# Abstracts of the Joint Annual Meeting of the International Society of Exposure Science and the International Society for Environmental Epidemiology 2025

**DOI:** 10.1038/s41370-025-00801-2

**Published:** 2025-08-19

**Authors:** José F. Cordero, Antonia M. Calafat, Gwen W. Collman, Yang Liu, Maria C. Mirabelli

**Affiliations:** 1 University of Georgia, Athens, GA USA; 2 National Center for Environmental Health, Centers for Disease Control and Prevention, Atlanta, GA USA; 3 National Institute of Environmental Health Sciences (retired), Research Triangle Park, NC USA; 4 Emory University, Atlanta, GA USA

The Joint Annual Meeting of the International Society of Exposure Science (ISES) and the International Society for Environmental Epidemiology (ISEE) took place in Atlanta, Georgia, USA, from August 17 to 20, 2025. The ISES/ISEE 2025 conference, themed “Global Environmental Health Equity across the Lifespan,” convened experts in exposure science, environmental epidemiology, and laboratory science, and offered a critical platform for sharing innovations and advancing interdisciplinary collaboration.

The conference attracted more than 1800 attendees from 68 countries, showcasing a strong international presence. Attendees were from North America (65%), Asia & Western Pacific (20%), Europe (7%), Latin America & Caribbean (4%), Africa (3%), and the Eastern Mediterranean (1%). Conference attendees included 225 ISES members, 738 ISEE members, and 91 participants who identified as members of both societies. Among the participants, 529 registered as students and 460 as early-career professionals, reflecting a significant engagement from the next generation of researchers.

Seven pre-meeting workshops provided training for attendees in important topics which covered a range of advanced methodologies and practical applications in environmental epidemiology, including Bayesian spatiotemporal modeling for environmental data and high-resolution mass spectrometry workflows to analyze the exposome. The emphasis on hands-on learning and interdisciplinary collaboration helped participants develop new skills and foster cross-sector partnerships for addressing complex environmental health issues. A dynamic series of six plenary presentations underscored the global and interdisciplinary nature of environmental health research. Two of the talks highlighted both scientific advancements and persistent translational gaps in addressing air pollution’s health burden across world regions. These discussions underscored the complexities of air pollution epidemiology, emphasizing the need for innovative solutions to overcome existing challenges. Another plenary lecture explored the potential and current limitations of integrating advanced analytics including artificial intelligence into environmental epidemiology. Meanwhile, two other plenaries underscored public engagement approaches to research, policy, and communication to many types of audiences on environmental science and health issues. A forward-looking plenary introduced novel biomonitoring methods using extracellular vesicles to showcase the emerging frontier of molecular epidemiology.

The scientific program featured 492 oral presentations distributed in 36 symposia, 42 traditional oral sessions, and 24 flash talk sessions, 1,614 poster displays presented at 6 poster viewing sessions, and over 100 late-breaking e-posters. Scientific sessions addressed the latest in air pollution research, including methods for assessing personal exposure and implications for public health interventions. Sessions on the exposome and biomarkers of exposure to chemical pollutants provided updated methodologies for detection and risk assessment. Sessions presented new data on specific topics such as per- and polyfluoroalkyl substances (PFAS), noise pollution, and community exposures focused on population-specific vulnerabilities and community-level impacts of a variety of co-occurring environmental exposures. The comprehensive scientific program provided a platform for in-depth discussions and networking among attendees, fostering cross-sector partnerships for addressing complex environmental health issues. Additional details about the scientific contributions are available in the abstract book of the ISES/ISEE 2025 conference in the Supplementary Material to this paper.

It is a unique opportunity when both the ISES and ISES come together and co-organize a scientific meeting, as each society has individual annual meetings. The 4-day meeting also prioritized community-building across the two societies and among its participants by creating opportunities to discuss innovative new methods to better integrate exposure science and environmental epidemiology in research, public health, policy and society. Dedicated gatherings for women in environmental health science celebrated achievements while tackling persistent challenges. Events geared toward students and new researchers provided mentorship opportunities, career development resources, and a platform for emerging voices in exposure science and environmental epidemiology. These meetings reinforced the conference’s commitment to cultivating an innovative future for global environmental health.

## Disclaimer

The findings and conclusions in this report are those of the authors and do not necessarily represent the official position of the Centers for Disease Control and Prevention.

## Supplementary information

Supplementary Material

