# Supplementary material for: Abstracts of the Joint Annual Meeting of the International Society of Exposure Science and the International Society for Environmental Epidemiology 2025
Source: J Expo Sci Environ Epidemiol. 2025 Aug 19;36(3):616–7. doi: 10.1038/s41370-025-00801-2 (PMC13143809; doi:10.1038/s41370-025-00801-2)
Supplement: Supplementary file 1 — Supplementary Material [file 41370_2025_801_MOESM1_ESM.pdf]

ISES '25 ATLANTA
ISEE GEORGIA, US
AUGUST 17–20

ABSTRACT BOOK

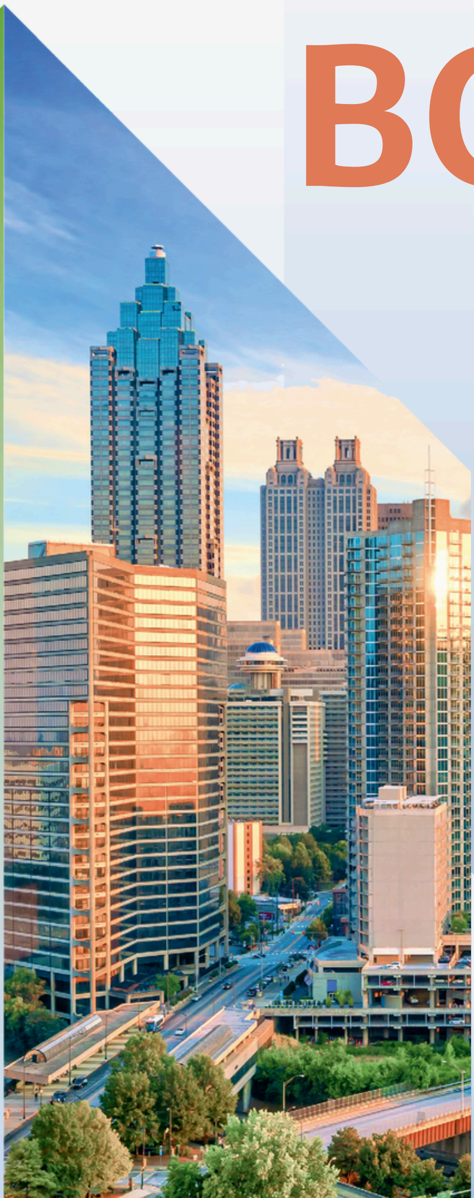

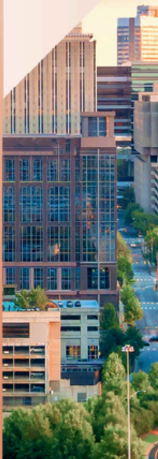

INTERNATIONAL SOCIETY OF EXPOSURE SCIENCE ISES

ISEE

Joint Annual Meeting
of the International Society of Exposure Science and the International Society for Environmental Epidemiology

Abstracts of the

Joint Annual Meeting of the International Society of Exposure Science and the International Society for Environmental Epidemiology 2025

ISES-ISEE 2025

Global Environmental Health Equity across the Lifespan

August 17 to 20, 2025

Contents

Plenary Speakers

PL01 | Air Pollution Epidemiology: Promises and Challenges

PL02 | Beyond the Buzzwords: Data Science, Machine Learning, and AI in Environmental Epidemiology

PL04 | Air Quality and Environmental Health in Africa: The Ghana Example

PL05 | Liquid Biopsies for Environmental Health – Leveraging Bloodborne Extracellular Vesicles to Assess Exposure and Risk

PL06 | Working with the media to share your exposure science

Pre-Meeting Workshops

WS01 | Bayesian spatiotemporal modelling for environmental data with NIMBLE

WS02 | High-resolution mass spectrometry workflows to study the exposome

WS04 | Health Assessment: Primer on Risk of Bias Evaluation for Epidemiological Studies in a Systematic Review

WS05 | Hypothetical interventions for exposure mixtures: Practical theory and applications for epidemiologists

WS06 | Resources and Tools to Support Integration of Geospatial and Environmental Exposure Data into Epidemiological and Clinical Health Research

WS07 | Science Communication for Environmental Epidemiologists and Exposure Scientists

WS09 | Wristbands 101: Harnessing Wearable Tech for Personalised Environmental Exposure Assessment

---

Symposia

S01 | Adapting Interventions for Complex Environmental Challenges in Community Settings: An Implementation Research Approach

S02 | Advancing Exposome Research Across the Lifespan: Biomarkers, Big Data, and Exposure Assessment

S03 | Joining Forces in the Face of Disaster: How Five Universities Address Exposure and Health Concerns Resulting from the East Palestine, Ohio Train Derailment

S04 | Micro- and nanoplastic and human health; where do we stand?

S05 | Bridging the Gap: Discovery of Novel and Uncommonly Measured PFAS in Human Exposure Studies

S06 | Household Air Pollution and Health: Recent evidence and the future of clean cooking research and policy

S07 | The Exposome in Critical Windows over the Lifespan and Multiple Generations: Prospective Multi-omics to Predict Breast Cancer Risk

S08 | Environmental Chemicals and Microbiota: Mechanisms of Interaction and Influence

S09 | Artificial Intelligence and Ethics: The Pros and Cons of Its Use in Environmental Health and Epidemiology

S10 | From Home, Preschool, and Beyond: Addressing Common Chemical Exposures Across Life Stages

S11 | New research on early life fluoride exposure and neurocognitive outcomes in infancy, childhood, adolescence and adulthood from United States and Canadian studies

S12 | Health Impacts of Firefighting-Related Chemical Exposures

S13 | Health-based interpretation and communication of human biomonitoring data

S14 | Prenatal Exposure to Endocrine Disrupting Chemicals and Maternal and Child Health in the U.S. Environmental Influences on Child Health Outcomes (ECHO) Cohort

S15 | The Red Hill Water Crisis and Steps to Address Community Concerns

S16 | Climate change and health across the lifespan in urban Latin America

S17 | Silencing an Environmental Injustice: How noise and health research, regulation, and policy differ in the US and around the world

S18 | From Data to Connection: Best Practices for Engaging Communities in Sharing Research Findings on Exposures

S19 | Advancing Pesticide Exposure Assessment and Understanding of Health Effects in Global Communities

S20 | Reducing Children's Lead Exposures in Sub-Saharan Africa – Exposure Science to Help Optimize the Balance Between Primary and Secondary Prevention

S22 | Global disparities in the exposome and human health

S23 | Extreme heat, air pollution & pregnancy: exploring interactions of environmental epidemiology and exposure science with health equity, focusing on vulnerable populations

S24 | Radon Exposure Across the Lifespan: Emerging Evidence Beyond Lung Cancer

S25 | Beyond the “impact factor:” environmental epidemiologists & exposure scientists in action across the United States

S26 | AI-Driven Early Warning Systems for Climate-Sensitive Infectious Diseases: Spatiotemporal Models and Big Data Integration

S27 | Milk Matters: Exploring Exposure, Impact, and Solutions for Endocrine Disrupting Chemicals in Human Milk

S28 | Unraveling the Links Between PFAS Exposure and Cancer Risk: Advances in Research and Mechanistic Insights

S29 | Bridging Mixture Exposure to Human Reproductive Health

S30 | Smoke and mirrors: reflecting on our understanding of connections between air pollution and infectious disease

S31 | Bridging Science and Policy: Shaping Global and Local Responses to Environmental and Climate Health Challenges

S32 | Environmental Exposures in CKDu epidemic regions: Updates from the field

S33 | Systems Biology Meets Environmental Health: Omics Approaches to Maternal and Child Health among Vulnerable Populations

S34 | Community Involvement in Environmental Health and Beyond: Lessons Learned and Upcoming Challenges

S35 | Wildfire Smoke Exposure and Health Effects in a Warming World: Epidemiological Insights, Health Equity, and Policy Responses

S36 | Addressing gaps in preconception environmental epidemiology

Traditional Oral Sessions

TO01 | Ambient air pollution and pediatric health: Impacts on growth, development, and disease

TO02 | Exposure assessment methods for environmental chemicals

TO03 | Omics technologies

TO04 | Environmental exposures and epigenetic aging

TO05 | Food contaminants

TO06 | Understanding and communicating elevated and occupational chemical exposures

TO07 | From cradle to cognitive decline: Air pollution and mental health across the life course

TO08 | Exposure to metals and elements in water

TO09 | Maternal exposure to climate extremes: Heat and wildfire smoke impacts on birth outcomes

TO10 | Chemical exposures and birth outcomes: Disparities in exposure and susceptibility to metals and endocrine-disrupting chemicals

TO11 | Chemical exposures, epigenetics, and molecular epidemiology

TO12 | Air pollution and extreme heat around the world

TO13 | Sounding the alarm: Monitoring and disentangling the health impacts of noise pollution

TO14 | Greenspace and health around the world

TO15 | Urban environment and brain health among adults

TO16 | Next-gen monitoring: Low-cost sensors, personal exposure, and advanced modeling

TO17 | Methodological approaches for improving accuracy in environmental epidemiology and exposure science

TO18 | Exposure assessment methods for PFAS

TO19 | Global risks of declining planetary health

TO20 | Advances in exposome research: Approaches, frameworks, and health outcomes

TO21 | Metal and element exposures and health outcomes

TO22 | Pesticide exposures: Trends, health, and mortality

TO23 | From data to justice: Air pollution, health burdens, and community empowerment

TO24 | Cancer risks from air pollution

TO25 | Strategies and approaches for improving the utility of environmental health data

TO26 | Heat, mind, and health: Exploring climate-driven mental and neurological risks

TO27 | Improving our understanding of the chemical exposome

TO28 | Links between exposure to PFAS and health outcomes in adults

TO29 | Air pollution and cardiometabolic outcomes: From glucose to heart disease

TO30 | Beyond the flames: The far-reaching health effects of wildfire smoke exposure

TO31 | Climate change and neurological health

TO32 | Environmental chemicals and reproductive outcomes

TO33 | Prioritizing and evaluating air pollution policy

TO34 | Air pollution, climate, and reproductive outcomes

TO35 | Innovations in exposure science: Modeling, monitoring, and mapping air pollution

TO36 | Improving our understanding of maternal, prenatal, and neonatal PFAS exposures

TO37 | Compounding climate hazards and health: Smoke, heat, storms, and vulnerable populations

TO38 | The heat-kidney connection: Risks, responses, and mechanisms across populations

TO39 | Heat in the city: Social and spatial dimensions of health risk and resilience

TO40 | Leveraging human biomonitoring to understand chemical exposures in populations

TO41 | A focus on environmental disparities related to personal care product chemicals

TO42 | From dust storms to urban pollution: Fine particulate matter and its role in respiratory dysfunction and disease

TO43 | Environmental chemical exposures during pregnancy and childhood

Flash Talk Sessions

FT01 | Chemical exposures: From PFAS to plasticizers to volatile organic compounds

FT02 | Health effects of air pollution components and mixtures: Global evidence and insight

FT03 | Exposure to metals and elements flash talks

FT04 | Biological signatures of air pollution exposure: From epigenetics to the metabolome

FT05 | Climate epidemiology in action: Tools, trends, and translational insights

FT06 | Global approaches to high-resolution air quality monitoring and exposure modeling

FT07 | Exposure to pesticides flash talks

FT08 | Air pollution and global mortality burden: Insights across borders, pollutants, and policies

FT09 | The air we regulate: Health effects of policy and behavioral interventions on pollution

FT10 | Built environment flash talks

FT11 | Exposure assessment methods for climate and urban environment

FT12 | Neurologic and mental health flash talks

FT13 | Cancer and cancer precursor flash talks

FT14 | Water pollution flash talks

FT15 | PFAS: Toxicology, modeling, exposures, and health

FT16 | Air pollution and systemic health outcomes across the life course

FT17 | Chemical exposures and pregnancy: From PFAS to extreme weather, new insights into birth outcomes and health trajectories

FT18 | Air pollution, pregnancy, and birth outcomes

FT19 | Climatic stressors and health: Modeling vulnerability, risk, and response across regions

FT20 | Vulnerability across the life course: Health impacts of heat and climate extremes

FT21 | Chemical exposures in mothers and children flash talks

FT22 | Air pollution: Exposures, methods, approaches, and health

FT23 | Environmental pollutants and chronic disease: Linking air quality to public health

FT24 | Chemical exposures and neurological health

Posters

P01 | Air Pollution: From Biomarkers to Population-Level Health Effects

P02 | Advances in Air Quality Monitoring and Modeling - PART I

P03 | Biomarkers of Environmental Exposures and Diseases

P04 | Environmental Chemical Exposures and Health Outcomes Across the Lifespan

P05 | Children's Environmental Health - PART I

P06 | Climate and Health: Exposures and Impacts

P07 | Community-Engaged Environmental Health Research

P08 | Data Analysis and Modeling Approaches in Environmental Health

P09 | Integrating Environmental Health into Public Health Practice and Education

P10 | Environmental and Occupational Health Policy

P11 | Environmental Exposures and Cancer - PART I

P12 | Environmental Determinants of Mental Health and Well-being

P13 | Environmental Chemical Exposures and Health

P14 | Climate and Health: Novel Methods in Climate Research

P15 | Environmental Exposures and Cardiovascular Health - PART I

P16 | Advancing Exposomics: Comprehensive Assessment of Environmental and Lifestyle Exposures - PART I

P17 | Environmental Exposure Assessment Methods

P18 | Fluoride Exposure and Health

P19 | Breathing Easier, Living Longer: The Power of Greenspace - PART I

P20 | From Policy to Practice: Global Efforts to Eradicate Lead Exposure - PART I

P21 | Occupational Exposures and Health - PART I

P22 | Multi-Omics Approaches to Environmental Health Research

P23 | Per- and Poly-fluoroalkyl Substances: Sources, Measurements, Trends, and Health Effects - PART I

P24 | Planetary Health and One Health

P25 | Reproductive Health

P26 | Air Pollution Exposure, Health Effects, and Mitigation Strategies Across Populations and Regions

P27 | Built Environment and Health

P28 | Chemical Exposures: Improving Measurements and Understanding Risks

P29 | Impact of Extreme Heat and Environmental Factors on Health Outcomes

P30 | Environmental Exposures and Aging - PART I

P31 | Environmental Exposures and Birth Outcomes - PART I

P32 | Advancing Environmental Justice through Research, Policy, and Community Engagement

P33 | Improving Measures of Environmental Exposure and Risk

P34 | Food Contamination, Food Safety, and Food Security

P35 | Environmental Exposures During Pregnancy and Maternal/Infant Health Outcomes - PART I

P36 | Beyond the Backyard: Understanding Indoor Environmental Health

P37 | Beyond the Usual Suspects in Exposure Science and Environmental Epidemiology

P38 | Environmental Exposures and Respiratory and Allergic Health

P39 | Water Quality and Health

P40 | Wildland Fire, Smoke, and Health - PART I

P41 | Air Pollution and Health Across the Lifespan

P42 | Advances in Air Quality Monitoring and Modeling - PART II

P43 | Children's Environmental Health - PART II

P44 | Climate and Health: Investiging Effects of Heatwaves and Hot Weather

P45 | Environmental Exposures and Aging - PART II

P46 | Environmental Exposures and Cancer - PART II

P47 | Advancing Exposomics: Comprehensive Assessment of Environmental and Lifestyle Exposures - PART II

P48 | Breathing Easier, Living Longer: The Power of Greenspace - PART II

P49 | Global Air Pollution: Sources, Trends, and Health Burdens

P50 | Climate and Health: Health Outcomes Across the Lifespan

P51 | Environmental Exposures and Birth Outcomes - PART II

P52 | Environmental Exposures and Cardiovascular Health - PART II

P53 | Environmental Exposures During Pregnancy and Maternal/Infant Health Outcomes - PART II

P54 | From Policy to Practice: Global Efforts to Eradicate Lead Exposure - PART II

P55 | Occupational Exposures and Health - PART II

P56 | Per- and Poly-fluoroalkyl Substances: Sources, Measurements, Trends, and Health Effects - PART II

P57 | Environmental Exposures and Sleep Health

P58 | Wildland Fire, Smoke, and Health - PART II

E-posters

EP01 | E-posters

EP02 | E-posters (late breaking abstracts)

PL01 | Air Pollution Epidemiology: Promises and Challenges

C. Arden Pope, III

Brigham Young University, United States of America

One promise of air pollution epidemiology is that it can provide essential information on the health effects of air pollution, supporting evidence-based public policies aimed at safeguarding air quality and public health. Over the past several decades, evidence has accumulated, indicating that the health impacts of fine particulate air pollution are greater than initially expected. Air pollution is now recognized as a leading risk factor for disease and death. However, the work of air pollution epidemiology is not yet complete. It faces at least two sets of ongoing challenges. The first set of challenges includes methodological issues such as difficulties in estimating human exposures and relying on observational data in real-world environments without experimental control. Advances, including improved exposure assessment, natural experiments, quasi-experimental approaches, and causal modeling methods, have significantly contributed to increasingly rigorous air pollution epidemiology. These advances have strengthened the evidence that air pollution impacts human health. The second set of challenges relates to efforts to cast doubt on scientific results that support public policies aimed at protecting air quality. The validity of environmental epidemiology is challenged by characterizing it as “junk science”, “secret science”, or “advocacy science”. Dan Greenbaum understood that the efficacy of using air pollution epidemiology to inform public policy depends on adeptly, collaboratively, and civilly addressing both sets of challenges.

PL02 | Beyond the Buzzwords: Data Science, Machine Learning, and AI in Environmental Epidemiology

Marianthi-Anna Kioumourtzoglou

Brown University, United States of America

The field of environmental epidemiology has evolved rapidly with the explosion of data, from terabytes characterizing natural, urban, and social environments to nationwide administrative records for outcome assessment and -omics datasets. Alongside these opportunities come important challenges, including the need for methods that can handle high-dimensional, complex data and algorithms that are computationally efficient. Enter the world of buzzwords: data science, machine learning, and artificial intelligence, which offer powerful tools to meet these needs. AI, in particular, holds promise for harmonizing disparate data sources, including—but not limited to—text and images not traditionally used for exposure assessment and health studies. Yet critical questions remain around the appropriateness, robustness, and generalizability of these methods in our field. This talk will explore both the promise and the pitfalls of these approaches and reflect on what lies beyond the buzzwords.

PL04 | Air Quality and Environmental Health in Africa: The Ghana Example

Kofi A. Amegah

University of Cape Coast, Ghana

The burden of disease (BOD) attributable to ambient air pollution exposure in Africa is growing and yet estimates of its magnitude and impact are possibly underestimated due to lack of air quality monitoring capacity and limited air pollution epidemiological studies. The integrated exposure-response (IER) function used to estimate the BOD attributable to air pollution is derived from long-term (cohort) studies conducted in USA and Europe, locations with low ambient $PM_{2.5}$ exposures. The IER function thus have limited applicability in Africa, a region where air pollution concentrations are very high (annual $PM_{2.5}$ average exceeds 100µg/m^3) and the relative contribution of specific sources of air pollution differ from those in North America and Europe. There is therefore an urgent need to bridge the huge air quality data gaps in Africa to enable the conduct of robust epidemiologic studies to inform the IER functions for better quantifying the BOD attributable to air pollution exposure on the continent and provide a solid evidence base for investments in air pollution control for public health protection. The proliferation of low-cost air quality sensors in recent times represents an excellent opportunity for bridging these data gaps and is gradually being leveraged in African countries. In this lecture, I will highlight the extent of the air pollution problem in Africa, identify the driving factors and diverse sources, pinpoint the thriving monitoring networks, and showcase the limited exposure health effects studies conducted in the region. I will proceed to outline how the Ghana Urban Air Quality and Breathe Accra Projects which have deployed low-cost sensors interspersed with reference grade monitors in metropolitan areas of Ghana is facilitating a data-driven solutions to the air pollution problem in Ghana as well as helping bridge the epidemiologic evidence gaps in the country.

PL05 | Liquid Biopsies for Environmental Health – Leveraging Bloodborne Extracellular Vesicles to Assess Exposure and Risk

Andrea Baccarelli

Harvard T.H. Chan School of Public Health, United States of America

Precision environmental health draws on epidemiological tools to focus on prevention, early risk identification, and interventions to advance public health. My research group developed wet lab and data analysis techniques that examine the biological “fingerprints” left behind by past exposures that serve as biomarkers of disease risk and epidemiological aging. Our novel investigations utilizing extracellular vesicles are enabling us to study target organ health in a non-invasive manner, expanding what we can analyze in a simple blood test. Combining these lab techniques with advances in high-throughput omics and exposome science enables us to take a holistic approach, analyzing multiple exposures and their synergistic effects. By using these tools to examine exposures and factors long before clinical symptoms appear, precision environmental health promises a future where predictive, preventive, and personalized health interventions become possible.

PL06 | Working with the media to share your exposure science

Brenda Goodman

CNN, United States of America

Working with media outlets is a mechanism to share scientific findings with the general public. Based on my two decades of reporting on science and health issues, I will share recommendations and tips for communicating with media to ensure that your research is shared with greatest meaning and impact.

WS01 | Bayesian spatiotemporal modelling for environmental data with NIMBLE

G. Konstantinoudis[1], R. Parks[2]

[1] *Imperial College London, Grantham Institute for Climate Change and the Environment, London, United Kingdom*
[2] *Columbia University, Department of Environmental Health Sciences, New York, New York, United States of America*

In environmental epidemiology, spatial and spatiotemporal methods are increasingly used with high-resolution environmental and health data, which allows detailed insights into associations between exposures and outcomes. In this context, Bayesian methods provide a natural setting to incorporate uncertainty and prior knowledge, borrow information between neighbouring units, and create hierarchical structures. However, appropriate usage of these methods requires careful consideration and knowledge of building technical models, which can be intimidating to the uninitiated user.

During this full-day course, our aim is to introduce the ideas of Bayesian inference and modelling for spatiotemporal environmental data. The workshop is designed to be as approachable and friendly as possible while still providing technical and practical know-how.

The objectives of the workshop are:

- An Introduction to Bayesian inference
- An Introduction to hierarchical model structures
- An Introduction to spatiotemporal priors
- Applications in real world environmental data and health outcomes

We will focus on three illustrative case studies.

In the first case study, we will evaluate the effect of long-term air-pollution exposure on COVID-19 mortality in England and examine the crucial role of spatial autocorrelation.

In the second case study, we will estimate the non-linear effect of temperature in all-cause mortality in Italy and examine how this effect has changed over time and varies in space using spatiotemporal Gaussian processes.

In the third case study, we will show how spatiotemporal models can be used to evaluate excess deaths due to events such as heatwaves in Switzerland and ways to propagate the different sources of uncertainty.

All analysis will be performed in R with the NIMBLE software and data and code will be made available for future studies.

By the end of this workshop, attendants shall develop an understanding of Bayesian spatiotemporal models and be able to write their first spatiotemporal model in NIMBLE.

WS02 | High-resolution mass spectrometry workflows to study the exposome

D. I. Walker[1], G. W. Miller[2], A. S. Young[1], D. Liang[1], X. Hu[1]

[1] *Emory University, Gangarosa Department of Environmental Health, Atlanta, Georgia, United States of America*
[2] *Columbia University, Department of Environmental Health, New York, New York, United States of America*

Untargeted high-resolution mass spectrometry (HRMS) has become a key platform for assessing the exposome, and its use is rapidly growing in environmental epidemiology and biomedical research for measurement of thousands of chemicals in human populations. While these methods provide a powerful strategy for comprehensively studying the role of environmental factors in human disease, the resulting data is complex, and proper interpretation and analysis requires an understanding of underlying instrumental methods and data pre-processing approaches. The objective of this full-day workshop is to provide participants with training in all aspects of the untargeted HRMS workflow for characterizing the exposome and will be taught by internationally recognized experts in the development and application of untargeted HRMS approaches for studying the exposome. Theoretical sessions will include the role of untargeted HRMS for exposome research and a detailed overview of the untargeted HRMS workflow, including instrumentation platforms, open-source methods for data pre-processing, and strategies for compound identification/annotation and reporting. Practical sessions will focus on the latest advances in data analysis methods for untargeted HRMS, including hands-on experience with single-variable and mixture-based methods, combining exposome-wide association studies with high-dimensional mediation analysis in a meet-in-the-middle framework for studying the intersection of exposures, biological response, and disease, and multi-omic approaches for integrating the exposome within biological networks. Upon completion of the workshop, participants will have developed expertise in the theoretical principles underlying the use of advanced analytical chemistry methods for measuring the exposome and gain hands-on experience in the application of cutting-edge data analysis methods that maximize information gained from untargeted HRMS measurements, empowering attendees to apply these methods to their own research projects.

WS04 | Health Assessment: Primer on Risk of Bias Evaluation for Epidemiological Studies in a Systematic Review

R. Nachman[1], Suril S. Mehta[2]

[1] *Washington DC, United States of America*
[2] *National Institute of Environmental Health Sciences, United States of America*

The identification and evaluation of information used in health assessments increasingly relies upon systematic review methods to ensure transparency, consistency, and reproducibility. Systematic reviews typically include several steps, such as scoping and problem formulation, literature search and screening, and study evaluation and data extraction. Components of study evaluation include risk of bias (RoB) and sensitivity. RoB assesses the internal validity of the study, whereas sensitivity is the ability of the study to detect a true association where one exists. There are multiple frameworks that may be used to structure a RoB evaluation, which have been developed to increase transparency and reduce subjectivity in reviews. Selection of a specific tool depends on the goal of the systematic review and available resources. This workshop will provide an overview of identifying the relevant body of evidence for a systematic review; evaluating RoB and sensitivity for observational epidemiology studies; briefly describe commonly encountered frameworks such as ROBINS-E, Navigation Guide, OHAT, the U.S. Environmental Protection Agency's (US EPA) Toxic Substances Control Act (TSCA) and Integrated Risk Information System (IRIS) frameworks; and discuss tailoring a framework to fit the needs of the systematic review. The workshop will also include a hands-on exercise tailoring and applying a RoB framework for a specific research or assessment need using the US EPA IRIS framework. This workshop is designed for epidemiologists and other researchers working with human data, to learn how their research may be evaluated during the systematic review process, as well as those directly engaged in conducting systematic reviews in the research or practice arena and/or using epidemiologic research in risk assessment.

WS05 | Hypothetical interventions for exposure mixtures: Practical theory and applications for epidemiologists

B. Welch[1], A. Keil[2], M. Spaur[2]

[1] *University of Nevada, Reno, School of Public Health, Reno, Nevada, United States of America*
[2] *National Cancer Institute, Occupational and Environmental Epidemiology Branch, Rockville, Maryland, United States of America*

Environmental epidemiologists are often interested in the health effects of chemicals that are, or may become, subject to policy and regulations aimed at reducing population-level exposures. Increasingly, the field of environmental epidemiology needs tools that can directly inform the public health impacts of future interventions. Further, there is an ongoing need for epidemiologic tools to address the complex reality of exposure to multiple contaminants in a way that informs decisions in an interpretable way. Causal inference methods, such as g-computation, are analytic approaches that can address both of these needs. In this workshop, we will provide an overview and practical advice on how to use g-computation, a methodological approach that leverages standard regression tools, to assess exposure-health relationships in terms of hypothetical interventions that can mimic potential public health actions. Hypothetical interventions can be crafted to address questions that may provide more insight than typical regression output in epidemiologic studies, such as:

- How would disease risk change if we could reduce all exposures within a mixture?
- Could health disparities be reduced or eliminated by ensuring that all groups receive similar exposures?

Using a combination of theory and worked examples, we will provide attendees the foundational tools to address such questions. Workshop material will cover theoretical topics such as: a basic introduction to the potential outcome framework of causal inference, and causal inference for exposure mixtures. Attendees will be given a synthetic dataset and sample R code for analysis. We will also consider practical issues such as: data management in causal analysis, sensitivity analyses to consider, and basic use of non-parametric bootstrapping to calculate confidence intervals. Deliverables for attendees include: a) an understanding of how to frame a hypothetical intervention; and b) experience using code to analyze a hypothetical intervention.

WS06 | Resources and Tools to Support Integration of Geospatial and Environmental Exposure Data into Epidemiological and Clinical Health Research

A. Miller[1], M. Conway[2], K. Messier[3], M. Shatz[2], A. Liu[1, 5], K. Lane[4]

[1] *National Institute of Environmental Health Sciences, Division of Extramural Research and Training, Bethesda, Maryland, United States of America*
[2] *National Institute of Environmental Health Sciences, Office of Data Science, Durham, North Carolina, United States of America*
[3] *National Institute of Environmental Health Sciences, Division of Translational Toxicology, Durham, North Carolina, United States of America*
[4] *Boston University School of Public Health, Environmental Health, Boston, Massachusetts, United States of America*
[5] *Kelly Government Solutions, Rockville, Maryland, United States of America*

Researchers investigating the health impacts of environmental exposures require access to data from multiple disciplines, ranging from earth and climate sciences to social determinants of health to clinical health. Though a wealth of data from numerous organizations exists, they often are generated for purposes other than scientific research and therefore reside in disparate locations, lack standardized ontologies and approaches for harmonization; require linkage; and are subject to inconsistent rules for access and use. This half-day workshop aims to introduce resources and tools developed by the NIH Climate and Health Outcomes Research Data Systems (CHORDS) project and the CAFÉ Research Coordinating Center to facilitate such health research using geospatial and environmental exposure data.

The outline of the workshop is as follows:

- Overview of the CHORDS data ecosystem and showcase of the curated data catalog and relevant resources, such as the climate change and human health literature portal and glossary
- Overview of the CAFÉ data management function, Harvard Dataverse repository, and research tools and educational resources
- Tutorial on the ‘amadeus’ software tool using a case example
- Tutorial of the processing and analysis guides available from CAFÉ
- Question and answer session
- Focus group discussion of climate and environmental data standards efforts

Following the completion of the workshop, attendants will have a better understanding of the diverse geospatial and environmental exposure data, tools, and resources; evolving approaches for linking and harmonizing data; and considerations for data standards in the context of epidemiologic and clinical research.

WS07 | Science Communication for Environmental Epidemiologists and Exposure Scientists

R. M. Shaffer[1], E. Copeland[2], A. Kostant[2], P. Pant[3]

[1] *Washington DC, United States of America*
[2] *Science Communication Network, Bethesda, Maryland, United States of America*
[3] *Health Effects Institute, Boston, Massachusetts, United States of America*

Effective communication of science concepts and scientific research is an essential skill for environmental epidemiologists and exposure scientists. Science communication makes our science more accessible, collaborative, and impactful. This workshop will provide theoretical and practical guidance to improve science communication across multiple platforms and across all career stages.

The first part of the workshop will include two trainings and one interactive breakout session. First, Science Communication Network (SCN) experts will provide “Media Training 101,” which will teach the nuances of communicating environmental health science to the media and other public audiences. An essential component of this training will be a focus on delivery of key research findings in an approachable manner while protecting scientific credibility. Following this presentation, participants will practice incorporating aspects of this training using ISEE/ISES-related examples in small breakout groups. Next, SCN experts will provide “Social Media for Scientists 101” training, which will include a discussion of best practices for scientists using social media in order to maximize their comfort and credibility using a variety of platforms.
In the second part of the workshop, two ISEE and ISES members will provide an overview of best practices based on real-world experiences in environmental health science communication, including in low and middle-income countries. The session will close with a discussion of opportunities and resources to advance skills in science communication and a Q&A with participants.

These trainings, coupled with the planned interactive break-out and discussion sessions, will provide essential guidance and practice for a world of increasing polarization and rapidly changing media and social media landscapes.

WS09 | Wristbands 101: Harnessing Wearable Tech for Personalised Environmental Exposure Assessment

K. Pollitt[1], E. Lin[1]

[1] *Yale School of Public Health, Environmental Health Sciences, New Haven, Connecticut, United States of America*

Capturing the diverse and dynamic environmental influences that we are exposed to is challenging. Wristbands have recently emerged as wearable tools that can provide personalized measures of chemical and biological exposures and track how these exposures change over time. The lightweight and lower cost design of wristbands have enabled exposures to be assessed in remote communities, vulnerable populations, and large-scale cohorts. This workshop will provide a comprehensive guide to using wristbands for exposure assessment, from the fundamentals of passive sampling to practical applications of the tool and an opportunity to work hands-on with real data. Participants will gain insight on study design and logistics, including available wristband technologies, best practices for deploying these wearable tools, optimal wear durations, and essential protocols for quality control and assurance. The session will also discuss mass spectrometry methods for targeted and non-targeted chemical analysis and participants will gain experience with data handling, analysis, and integration with various EPA databases, and interpretation. We welcome investigators at all career stages to attend. A basic understanding of R is recommended to maximize engagement in the workshop demonstrations.

S01 | Adapting Interventions for Complex Environmental Challenges in Community Settings: An Implementation Research Approach

L. M. Thompson[1], M. A. Handley[2]

[1] *Emory University, Atlanta, Georgia, United States of America*
[2] *University of California, San Francisco, San Francisco, United States of America*

The global environmental risk landscape is shifting rapidly in response to a changing climate. While public health interventions aim to address growing inequities in environmental risks, adaptation strategies are not being implemented effectively. Adapting these interventions and tailoring them to meet the needs of diverse communities is a critical step. Theory-informed approaches that adapt evidence-based interventions, addressing barriers and facilitators, and in response to local contexts, are key elements of implementation science. In this symposium we will: (1) describe key principles of implementation science relevant to adaptation of community-level interventions, (2) outline methods for engaging communities and other stakeholders to advance implementation strategies; and (3) present case studies incorporating implementation science approaches into broader environmental health initiatives to acheive global health equity.

S01-01 | Linking Implementation Science (ImS) and Participatory Research Methods for Adapting Interventions

Margaret Handley

University of California, San Francisco, San Francisco, USA

Implementation science can provide an essential set of methods to adapt interventions to local conditions in order to reduce the health risks from complex environmental challenges. Concurrent with the development of a more ‘local’ Implementation science approach is the need to foregrounding participatory methods across a range of implementation phases. Together, application of implementation science frameworks and participatory methods can promote well-tailored interventions that address ‘real world’ implementation challenges. We present a selection of applied methods relevant to intervention tailoring and implementation including: (1) *determinants and adaptation-specific frameworks* to guide community engaged research for designing interventions; (2) *rapid qualitative methods* to determine how local communities might experience barriers or enablers to new interventions focusing on behavior change at the individual and community level; and (3) *hybrid study designs* which incorporate context-relevant implementation outcomes into study design and planning processes. Examples for (1) will cover use of COM-B and the Behavior Change Wheel and Intervention Mapping. For (2) we will describe Rapid Ethnographic Assessment approaches, and for (3) we will cover fundamental approaches to type 1 hybrid designs and how to incorporate relevant implementation outcomes, such as acceptability, sustainability and reach.

S01-02 | Applied Implementation Research for Clean Cooking in Cambodia (AIR-C3): equity, exposure, costs, and benefits of induction stove interventions

Ajay Pillarisetti[1], Matthew Freeman[2]

[1] University of California, Environmental Health Sciences, School of Public Health, Berkeley, USA; [2] Emory University, Atlanta, USA

Background: Approximately 3 billion people use biomass fuels for cooking. Exposure to the resulting household air pollution (HAP) is attributed to 3 million deaths yearly. Interventions focused on burning these fuels more cleanly have failed to (a) reduce exposures to air pollution; (b) displace biomass fuel stove use, leading to mixed use of stoves and fuels; and (c) provide strong evidence of health benefits. Efforts have shifted to evaluating cleaner solutions as viable interventions.
Objectives: AIR-C3 is a four-arm, hybrid type II cluster randomized trial that will test intervention strategies to transition households from biomass toward sustained and exclusive use of induction cookstoves. Trial arms include direct sales, public sector promotion, and fuel and stove subsidies. The study aims to design and demonstrate the effectiveness of a scalable strategy for achieving equitable access that can be applied to other low- and middle-income settings.
Methods: In formative work we (a) identify determinants of stove purchase and use and biomass disuse, (b) develop messaging and intervention strategies to promote purchase and use; and (c) establish targeted subsidies to drive uptake. We will randomly allocate 62 villages to receive one of four interventions. We will use the RE-AIM framework

to interrogate and compare each strategy, with primary outcomes of induction stove uptake and use, and will estimate costs, benefits, and incremental cost-effectiveness of the stove promotion strategies.
Findings. We measure the impact of theory- and evidence-based strategies to increase uptake and sustained use of induction stoves to reduce biomass fuel use. We apply systematic approaches to implementation strategy development, establish plausible mechanisms of change for these strategies, and conduct economic evaluation. We provide strategies to achieve equitable access to induction stoves that may translate to other cooking interventions and highlight the value of implementation science for addressing persistent global environmental health challenges.

S01-03 | Ecolectivos trial: Implementing village-level strategies to adress plastic waste burning in household fires in rural Guatemala

Lisa Thompson

Emory University, Atlanta, USA

Background: Burning plastic waste is a major but unaddressed environmental and health hazard in countries that lack infrastructure to manage waste properly. Ecolectivos is implementing community working groups that aim to improve the health-related quality of life of women of reproductive age in Guatemala by reducing household-level plastic waste burning in indoor cooking and outdoor waste fires.
Aims: Using a type 1 hybrid-effectiveness-implementation study design, we randomized 16 rural villages and randomly selected 400 women 15-44 years old (25/village) who reported burning plastic as the primary method of waste disposal. In 8 intervention villages, we are currently conducting 12-week working groups, followed by an evaluation of community-initiated interventions that address plastic waste over 9 months. We use the Behavior Change Wheel and RE-AIM, two implementation science frameworks, to evaluate outcomes. At baseline, 4 and 12 months, we measure 24-hour filter-based personal exposures to fine particulate matter, black carbon, and 1,3,5-Triphenylbenzene, a known tracer of plastic incineration, and assess urinary biomarkers (e.g., bisphenols, phthalates, polycyclic aromatic hydrocarbons, and volatile organic compounds) to estimate exposures to air pollutants, including those from plastic burning. Participant-promotors are recruited from each intervention village to boost intervention activities, visiting intervention households monthly. Tree saplings (over 25,000) are provided to control villages as control compensation for their participation at the request of their village leaders.
Results: Of the 400 women screened at baseline, 365(91%) completed 4 months and 222(55%) completed 12 months (study to be completed December 2025). Fourteen promotoras have made 1,334 monthly visits to 200 intervention households. Baseline urine and filter-based measures will be presented.
Discussion: Fairs will be conducted June-July 2025 to disseminate intervention activities to control villages. Preliminary findings suggest that community clean-up and recycling interventions are demonstrating evidence of sustainment after 12 months in some communities.

S01-04 | Implementing School-based water conservation interventions in less developed countries

Wael Al-Delaimy

University of California, San Diego, San Diego, USA

Background: Jordan is one of the most water-deprived countries in the world; water is rationed to be piped once a week for the whole population. Interventions are needed to decrease water usage and increase water collection and storage. The Global Center on Climate Change and Water Energy, Food, and Health Systems is piloting community-based interventions in low-income agricultural communities in the Northern Mafraq Region.
Aim: We will describe several interventions funded by the center and evaluate the challenges and opportunities of establishing several water saving interventions within a school in a rural low-income area in Jordan.
Methods: We evaluated the efficacy of educational components through a quasi-experimental pre- and post-intervention surveys on student perceptions, knowledge, attitudes, and self-reported behaviors related to water conservation. The water saving posters, self-shutting water faucets, and rain harvesting efforts were implemented to determine the impact of these interventions on 219 high school children and their school's use of water. The control groups were students who did not receive specific targeted educational seminars.

Results and Discussion: There was a high response rate and participation by the schoolteachers and the students. We collected data from the students at two time points pre- and post-intervention. Results will be presented to demonstrate the measures of where the intervention had an impact and where it did not in terms of behavior change, decline in water usage, and attitudes and knowledge of students involved in the seminar education, compared to controls who only had access to posters within the school. Challenges of implementing these interventions, along with a description of the capacity building needed to increase the scientific rigor and quality of studies in an ethically responsible manner in less developed countries will be discussed.

S02 | Advancing Exposome Research Across the Lifespan: Biomarkers, Big Data, and Exposure Assessment

Y. Wang[1], C. Marsit[1]

[1] Emory University, Rollins School of Public Health, Atlanta, Georgia, United States of America

This symposium will highlight cutting-edge research on environmental exposures through the lens of the exposome. By integrating biomarkers, big data analytics, and omics technologies, speakers will explore how exposures during critical developmental windows at different points in life shape health trajectories across the lifespan. Presentations will focus on advances in environmental epidemiology, epigenetics, and statistical modeling to elucidate the mechanisms linking prenatal and early-life exposures to long-term health outcomes.

Key topics will include exposome-wide association studies (ExWAS) for identifying critical risk factors, the role of epigenetic modifications in mediating early-life exposures, and the use of multi-omics to uncover molecular pathways affected by environmental insults (adjust with the speakers' topics). The session will also address the challenges of applying exposome research to actionable public health interventions, emphasizing strategies to protect vulnerable populations and advance health equity.

Bringing together leading experts, this session aims to inspire new approaches to studying and mitigating environmental health risks while advancing the integration of exposome science into public health practice.

S02-01 | *Integrating Multi-Omics to Elucidate the Lifespan Impact of Prenatal Exposures within the Exposome Framework*

Yewei Wang

Emory University, Rollins School of Public Health, Atlanta, USA

Prenatal environmental exposures are critical determinants of lifelong health, yet their mechanistic impacts on early development remain incompletely understood. In this study, we integrated multi-omics approaches—including epigenomics, transcriptomics, and metabolomics—with comprehensive assessments of maternal pesticide exposures to elucidate mechanistic links between prenatal exposures, placental function, and early-life outcomes within an exposome framework.

We evaluated 248 mother–infant pairs from the SAWASDEE cohort, measuring maternal urinary biomarkers of organophosphate (OP) and pyrethroid (PY) pesticides repeatedly during gestation, profiling placental transcriptomes at birth, and conducting first-trimester maternal serum metabolomics alongside early neurodevelopmental screening. Our analyses reveal profound sex- and trimester-specific disruptions in placental gene co-expression networks. Averaged across pregnancy, male placentas exhibited significant up-regulation of cell cycle and invasion pathways - exemplified by increased E2F Targets and EMT - coupled with suppression of metabolic and DNA repair modules. In contrast, female placentas demonstrated down-regulation of developmental and immune-related modules (e.g., Myogenesis and Interferon Response) along with up-regulation of oxidative phosphorylation. With the sole exception of DNA Repair—which consistently correlated with reduced head circumference in both sexes—these transcriptomic responses were entirely divergent between male and female placentas. Trimester-specific analyses identified the first trimester as the most sensitive window for placental transcriptomic perturbations, particularly in males, while mid-gestational pesticide exposures were most strongly associated with increased birth size in male infants.

Metabolomic profiling implicated pathways related to oxidative stress and mitochondrial dysfunction, and neurodevelopmental screening suggested that these molecular perturbations may be linked to early neurobehavioral changes. Collectively, our integrated multi-omics approach provides novel mechanistic insights into how exposure timing and fetal sex interact to shape placental function and neonatal outcomes, highlighting critical windows for interventions to mitigate the adverse effects of prenatal pesticide exposures.

S02-02 | *Placental Epigenomics and its impact on Health*

Nora Fernández-Jiménez

University of the Basque Country (UPV/EHU) and Biobizkaia Health Research Institute, Department of Genetics, Physical Anthropology and Animal Physiology, Bilbao, Spain

Increasing evidence suggests that maternal exposures during pregnancy, including maternal smoking and psychosocial stress can leave molecular signatures in the placenta, potentially influencing fetal development and later life health. Additionally, it is known that the prenatal environment plays a crucial role in shaping early neurodevelopmental trajectories. In a previous work by our group, we demonstrated that placental DNA methylation patterns are associated with maternal body mass index at the beginning of the pregnancy, close to obesity-related genes and altogether, enriched in cancer and oxidative-stress pathways. These findings support the notion that the placenta can act as both a mediator and a biosensor of early-life environmental influences.

Expanding on this, our most recent study investigated the placenta as a potential conduit for genetic influences on neurodevelopmental risk. By integrating the placental DNA methylation under genetic control with large-scale psychiatric genome-wide association studies (GWAS), we identified genes whose placental expression may mediate the effects of polygenic liability for neurodevelopmental and psychiatric conditions. Using Mendelian Randomization and colocalization approaches, we provided evidence for a prenatal component to genetic risk, potentially channeled through pathways active in the placenta.

Together, these findings suggest that the placenta is not merely a passive intermediary but a dynamic interface where genetic and environmental factors converge to influence the developmental origins of health and disease. While specific mechanisms remain to be fully elucidated, immune regulation, neurodevelopmental processes such as dendritic spine formation, and oxidative stress pathways emerge as candidates warranting further investigation. In conclusion, placental DNA methylation may offer a valuable lens through which to study the complex interplay of genes and environment—helping us understand how prenatal life can shape vulnerability or resilience to later neuropsychiatric conditions.

S02-03 | *Exposome discovery approaches inform molecular and targeted Alzheimer's disease research*

Kelly Bakulski

Michigan Alzheimer's Disease Research Center, Epidemiology, Data Management and Statistical Core, Michigan, USA

Dementia, including Alzheimer’s disease, is a prevalent public health challenge. In 2024, the Lancet commission evaluated the full weight of evidence and determined dementia risk is partially attributed to air pollution. Building on the air pollution findings, it is likely many other chemical exposures contribute to dementia. To prioritize novel chemicals for investigation, we performed exposome-wide association studies of cognitive impairment and dementia using both human epidemiologic and computational toxicology approaches. These tandem and complementary study designs generate a rigorous discovery process. Select hits from the exposome-wide association studies were

followed up in animal and cell model systems to determine molecular pathways, tissues, and cell types linking exposures and dementia, and suggesting new biomarkers. Together, in the example of dementia, this pipeline successfully links exposome-wide screening methods with molecular mechanisms and biomarker development.

S02-04 | Predicting Long-Term Health Outcomes Using Early-Life Multi-Omic Data

Rebecca Fry

UNC-Chapel Hill, Department of Environmental Sciences and Engineering, Chapel Hill, USA

Children born preterm face a significantly higher risk of experiencing neurodevelopmental challenges, including Autism Spectrum Disorder (ASD). This association underscores the importance of understanding the biological mechanisms that may contribute to these outcomes. The placenta plays a crucial role in fetal neurodevelopment, acting as a regulator and mediator of developmental processes. However, the specific mechanisms by which the placenta influences neurodevelopment and how these mechanisms are disrupted in conditions like ASD remain poorly understood.
To shed light on this, we employed a multi-omic approach to investigate placental changes linked to ASD at age 10 in children born preterm. Our study utilized advanced data analytics to examine mRNA expression, CpG methylation patterns, and miRNA expression profiles in placental tissues from a cohort of 368 children. Through this comprehensive analysis, we identified 111 genes associated with ASD. Among these, key genes such as EWSR1 and BAZ2A emerged as predictors of ASD status, offering potential biomarkers for early identification and intervention.
Interestingly, some genes, including LRRFIP1, exhibited associations across multiple molecular layers, linking both CpG methylation and miRNA expression to ASD. These multi-layered connections suggest intricate regulatory networks that may influence neurodevelopmental outcomes. The integration of these findings highlights the complexity of placental biology and its potential role in shaping long-term neurodevelopment.

These discoveries hold promise for identifying novel intervention targets. By addressing specific molecular changes in the placenta, it may be possible to develop strategies to mitigate the risk of neurodevelopmental disorders, including ASD, in preterm children. Future research should build on these findings to explore therapeutic possibilities and further unravel the biological underpinnings of placental influence on neurodevelopment. This work represents a significant step toward understanding and addressing the challenges faced by preterm children at risk for ASD.

S03 | Joining Forces in the Face of Disaster: How Five Universities Address Exposure and Health Concerns Resulting from the East Palestine, Ohio Train Derailment

E. Haynes[1], M. Allison[2]

[1] University of Kentucky, Epidemiology and Environmental Health/College of Public Health, Lexington, Ohio, United States of America
[2] Columbiana Health Department, Lisbon, Ohio, United States of America

A Norfolk Southern train carrying 150 railcars derailed on the evening of February 3, 2024 in East Palestine, Ohio. Of the 52 cars that derailed, 11 were carrying hazardous chemicals. The derailment resulted in the explosion and burning of chemicals including vinyl chloride, ethylene glycol, ethylhexyl acrylate, isobutylene, benzene, and butyl acrylate. In response to community concerns about exposure and health effects, the National Institute of Environmental Health Sciences (NIEHS) funded six (6) time-sensitive R21s. Each project, led by five (5) universities, address a unique aspect of the environmental exposure and/or effects on public health. Through ongoing collaboration, the research teams have shared methodologies, coordinated community engagement efforts, and aligned their approaches to maximize impact. The collaboration among the institutions has resulted in greater impact for the community and improved communication pathways and messages. This session will highlight each university's research and how their collective efforts have addressed community concerns.

S03-01 | Integrated Environmental Monitoring and Health Risk Assessment Post-East Palestine Derailment

Natalie Johnson[1], Peng Gao[2]

[1] Texas A&M University School of Public Health, Environmental and Occupational Health, College Station, USA; [2] Harvard T.H. Chan School of Public Health, Environmental Health, Boston, USA

Objective: This presentation combines two complementary environmental monitoring initiatives following the February 2023 East Palestine train derailment.
Material and Methods: The research integrates mobile VOC air sampling with comprehensive soil, water, and sediment SVOC analysis to characterize the spatial and temporal distribution of contaminants. Using both targeted and non-targeted approaches, the studies examine airborne VOC concentrations and other matrices' SVOC concentrations while implementing novel health assessment tools.
Results: The combined findings provide critical insights into environmental fate and potential exposure patterns, establishing a template for disaster response research.
Conclusion: Our integrated approach emphasizes multi-matrix sampling for identifying (S)VOC hotspots and informing potential health risks.

S03-02 | University of Kentucky East Palestine Health Tracking Study

Erin Haynes[1], Timothy Hilbert[2], Christine Kim[1], Jay Christian[1], Candace Brancato[3]

[1] University of Kentucky, Department of Epidemiology and Environmental Health/College of Public Health, Lexington, USA; [2] University of Kentucky, Center for the Environment, Lexington, USA; [3] University of Kentucky, Department of Biostatistics/College of Public Health, Lexington, USA

On February 3, 2023, a 150 car Norfolk-Southern freight train derailed and 11 railcars containing hazardous chemicals caught fire in East Palestine, Ohio. About 402 tons of vinyl chloride were subsequently vented and burned. The University of Kentucky East Palestine Train Derailment Health Tracking Study launched in April 2023 to evaluate the long-term health effects. We screened for post-traumatic stress disorder (PTSD) and geocoded residential addresses. The online survey was completed by 379 participants within a few months, and 229 participants completed a follow up survey over a year later. In the months following the derailment, 38% of participants screened having PTSD, a result which declined only slightly to 34% over a year later. Notably, females screened for significantly more PTSD than males. Younger age and having school-aged children were independently associated with PTSD at both time points. Additionally, lower income was associated with PTSD in the early months following the disaster, but was not significant over a year later. About 75% reported health symptoms over a year later, including upper and lower respiratory symptoms and headaches. While the overall number of reported symptoms decreased over time, GI symptoms persisted, and a similar pattern was also observed in children of participants. In the months following the derailment, the rate of new upper respiratory symptoms was 81% among those living within one mile of the derailment, compared to 69.5% for those who lived between 1-5 miles, and 51.2% greater than five miles. There was a highly significant cluster ($p < 0.001$) of new upper respiratory symptoms and PTSD that align with plume projections of smoke following the derailment. We strongly recommend long-term follow-up for health outcomes for the community.

S03-03 | Healthy Futures Research Study: Linking somatic mutation rate with baseline exposure in East Palestine

Fredrick R. Schumacher[1,2], Erika S. Trapl[1,2], Maeve G. MacMurdo[3], Catherine C. Osborn[1], Anthony Orsino[1], Maura K. McCall[4,2], Lynn T. Singer[1]

[1] Case Western Reserve University, Department of Population and Quantitative Health/School of Medicine, Cleveland, USA; [2] Case Western Reserve University, Case Comprehensive Cancer Center, Cleveland, USA; [3] Cleveland Clinic, Respiratory Institute, Cleveland, USA; [4] Case Western Reserve University, Frances Payne Bolton School of Nursing, Cleveland, USA

Objective

On February 3, 2023, a Norfolk Southern train carrying hazardous chemicals, including vinyl chloride and butyl acrylate,derailed in the village of East Palestine, Ohio. A subsequent controlled combustion of five tankers was undertaken, resulting in the release of phosgene and hydrogen chloride into the atmosphere and contaminating two surface water streams. The subacute and long-term health impacts of this environmental and public health disaster remain unknown. Chemical exposure assessment is typically done via interrogation of specific chemical substrates in blood and urine. Our novel approach provides a snapshot of short-term chemical exposure by quantifying the somatic mutation rate (SMR) in DNA collected from study participants.

Material and Methods

Our research team developed and engaged participatory research in East Palestine and the greater region to establish a relationship with residents and engage in additional research inquiries. Our study utilizes a cross-sectional study design establishing the baseline impact of a subacute chemical exposure utilizing a genomic biomarker from saliva derived DNA as a surrogate measure for direct chemical concentration levels given the mixture of potential contaminants. This proposal will establish and develop a shared partnership with community residents, elucidate perceived experiences post-disaster with qualitative approaches, formally organize a Community Advisory Board among community members, and providing a baseline biomarker for chemical exposure thus serving as a baseline for longitudinal follow-up.

Results

Here, we will utilize a cross-sectional study to assess the correlation between the SMR, a genomic biomarker, with geographical proximity to the train derailment epicenter and the prevalence of self-reported symptoms at the time of the event.

Conclusions

Overall, this study establishes a cornerstone working within the community, health departments, and other research institutions across the region to fully comprehend the short- and long-term health impacts of an environmental disaster in the East Palestine community.

S03-04 | Incorporating Community Engagement into Studies of Environmental Exposure, Health, and Biobanking Following the East Palestine Derailment.

Erika Kinkead[1], James Fabisiak[2], Carl Ng[3], Laura Dietz[4], Firoz Abdoel Wahid[2], Samantha Totoni[2], **Maureen Lichtveld**[2], Juliane Beier[5]

[1] Community Partner, East Palestine, USA; [2] University of Pittsburgh, Environmental and Occupational Health, Pittsburgh, USA; [3] University of Pittsburgh, Civil and Environmental Engineering, Pittsburgh, USA; [4] University of Pittsburgh, Counseling and Behavioral Health/School of Health and Rehabilitation Sciences, Pittsburgh, USA; [5] University of Pittsburgh, Medicine/School of Medicine, Pittsburgh, USA

"East Palestine (EP) Community-Engaged Environmental Exposure, Health Data, and Biospecimen Bank" project, is one of 6 NIH-funded time-sensitive R21 grants awarded to assess human health and environmental impacts following the Norfolk Southern train derailment disaster and subsequent chemical release and fire on February 3, 2023. Since then, EP residents have faced significant risks given their exposure to both chemical and non-chemical stressors. Integrated into our methods is a community-driven approach guided by a Community Advisory Board (CAB) whose members are partners at every stage of our study – from planning and implementation to dissemination of findings and actionable recommendations. Of special note is that several EP residents, trained as Community-based Researchers (CbRs), collaborate in data acquisition activities: As envisioned in our first aim, we willmeasure chemical contaminants in indoor air and water in 100 EP homes at varying distances from the derailment. Partnering with the CbRs, we are conducting the following activities: a) real-time measurements of vinyl chloride in basement indoor air using colorimetric gas detection tubes, and b) grab sampling indoor air and tap water for analysis at University of Pittsburgh laboratories covering multiple chemicals of interest. Aim 2 focuses on recruiting a cohort of 300 EP residents as study participants and monitoring liver health by measuring biomarkers of early liver dysfunction, chemical exposure, and conducting an abdominal ultra-sound using Fibroscan to determine liver disease progression. We will also create a biospecimen repository to assess adverse liver outcomes over time. Our CAB has raised community concerns related to child health. refore, our third aim will characterize parents' concerns about their children's health and development following the derailment. Parents or guardians from our EP participant cohort will be invited to complete comprehensive surveys to help quantify the extent and nature of those concerns.

S03-05 | East Palestine, Ohio Train Derailment Health Effects: Echoes of Gulf War Illness

Janhavi Kulkarni[1,2], Jessica Y.F. Situ[1], Brinton K. Berg[1], Alexander L. Chen[1], Vincent Davidson[1], Marcella A. Evans[1], Jun Hee Han[1,3], Grace H. Hoang[1], Bruce J. Miller[1], Chayush H. Patel[1], Janis B. Ritchie[1], Beatrice A. Golomb[1]

[1] University of California- San Diego, Department of Medicine, La Jolla, USA; [2] Yale School of Public Health, Department of Environmental Health Sciences, New Haven, USA; [3] California State University, Fullerton, Fullerton, USA

Objective: The mixed toxicant exposure (**MTE**) from the Feb. 2023 train derailment in East Palestine, Ohio (**EP**) follows other MTEs that have left a legacy of health consequences – e.g. Gulf War illness (**GWI**), World Trade Center, burn pits. We assess if lessons from one MTE can apply to/accelerate understanding of others.

Material and Methods: We assessed whether findings in those reporting persistent health effects post-derailment in EP fit findings for GWI.

Results: 73% of 55 surveyed EP-exposed participants met MTE-illness (proxy Kansas GWI) criteria. For a GWI-severity instrument assessing 20 symptoms each present (prior 2-weeks) in ≥50% of a GWI sample, 19 were present in ≥45% of EP participants with MTE-illness (prior 2-weeks). 71% with MTE-illness cite symptoms of sleep-disordered-breathing (**SDB**), prior two weeks, supporting increased SDB – as in GWI. HsCRP, the most frequently abnormal routine-lab (increased) in GWI, was the most frequently abnormal routine-lab in EP. HsCRP related *inversely* to MTE-illness severity in EP as in GWI: β(SE)[95% CI]p= -4.00(1.81)[-7.74, -0.27]0.037. Membranes are altered in GWI: biopsies were not available for EP participants but membrane fatty acids powerfully predicted MTE-illness severity with β(SE)[95% CI]p= 669(171)[320, 1017]<0.001 for eicosadienoic acid and -104(29)[-165, -45]0.001 for nervonic acid. As for GWI, a marker tied to age-related macular degeneration (involving mitochondrial mechanisms documented in GWI) significantly predicted greater MTE-illness severity. Finally, conditions reportedly increased in GWI are now emerging in apparent excess in EP.

Conclusion: Symptoms, objective features, and their interrelationship in those experiencing persistent health effects in EP closely mirror findings in GWI. This suggests that lessons from one MTE may aid in accelerating understanding, post-exposure prophylaxis, and treatment for the next MTE, with prospects to mitigate suffering, disability and morbidity, and to extend life.

S04 | Micro- and nanoplastic and human health; where do we stand?

R. Vermeulen, **D. Walker**, R. D. Smith Jr.[1], M. Braselton[1], R. Zou, E. Lenssen, N. Sehgal[1], G. Read[1], K. Shao, A. Durkin, A. Robuck[2], N. Saenen[3], A. Durkin[4, 5], Y. Ni[6], B. Knox[7], M. Bustamante[7], T. Nawrot[3], M. Vrijheid[7], R. Vermeulen[8, 4], D. I. Walker[1]

[1] *Emory University, School of Public Health, Atlanta, Georgia, United States of America*
[2] *The University of Rhode Island, Graduate School of Oceanography, South Kingstown, Rhode Island, United States of America*
[3] *Hasselt University, Centre for Environmental Sciences, Diepenbeek, Belgium*
[4] *University Medical Center Utrecht, Julius Center, Utretch, Netherlands*
[5] *Utretch University, Institute for Risk Assessment Sciences, Utretch, Netherlands*
[6] *Boston University, School of Public Health, Boston, Massachusetts, United States of America*
[7] *ISGlobal, Barcelona Institute for Global Health, Barcelona, Spain*
[8] *Utretch University, Environmental Epidemiology, Utretch, Netherlands*

Micro- and nanoplastics (MNPs) have infiltrated every corner of our environment—from the air we breathe to the food we eat—raising pressing concerns about their impact on human health. Emerging research suggests that MNPs may contribute to a range of health issues, including respiratory problems, reproductive challenges, and disruptions to the gut microbiome. Despite ongoing studies, significant gaps persist in our understanding of MNPs' biological effects and exposure pathways.

The complexity of MNPs is multifaceted: they are composed of various polymers, contain numerous additives, and undergo aging or weathering due to environmental conditions, both externally (in water and air) and internally (within the respiratory or gastrointestinal tracts). These particles can also absorb environmental pollutants and microbes, further complicating their physicochemical properties such as size, shape, density, and surface chemistry. As MNPs degrade, their complexity increases, posing challenges for exposure assessment and hazard identification, which are crucial for effective risk characterization. In real-world scenarios, MNPs do not exist in isolation but as complex mixtures with other pollutants, adding another layer of complexity to their study. Laboratory analyses are also susceptible to artifacts, including contamination, making exposure assessment in epidemiological studies particularly challenging.

In this symposium, we will delve into research from human studies conducted within the CUSP cluster of five European projects and the Dutch MOMENTUM project. Our focus will be on innovative quantification methods for detecting MNPs in biological matrices. We will present and triangulate findings from experimental panel studies that explore varying exposure levels to traffic-related airborne MNPs and respiratory health effects, a cross-sectional study examining the presence of MNPs in blood and their potential immune and respiratory effects, and a prospective study investigating MNPs in the placenta and their implications for early life health.

S04-01 | Advancing the detection of micro- and nanoplastics in human tissues: a comprehensive analytical framework

Kuanliang Shao[1], Ronald Smith Jr.[1], Mattie Braselton[1], Neha Sehgal[1], Gwen Read[1], Anna Robuck[2], Nelly Saenen[3], Amanda Durkin[4,5], Yingyue Ni[6], Bethany Knox[7], Mariona Bustamante[7], Tim Nawrot[3], Martine Vrijheid[7], Roel Vermeulen[8,5], Douglas I. Walker[1]

[1] Emory University, Gangarosa Department of Environmental Health, Atlanta, USA; [2] The University of Rhode Island, Graduate School of Oceanography, South Kingstown, USA; [3] Hasselt University, Centre for Environmental Sciences, Hasselt, Belgium; [4] University Medical Center Utrecht, Julius Center, Utretch, Netherlands; [5] Utretch University, Institute for Risk Assessment Sciences, Utretch, Netherlands; [6] Boston University, School of Public Health, Boston, USA; [7] ISGlobal, Barcelona Institute for Global Health, Barcelona, Spain; [8] Utretch University, Environmental epidemiology, Utretch, Netherlands

Objectives

Micro- and nanoplastics (MNPs) are an emerging concern for human health that have been reported in a wide range of human tissues and biological fluids. Despite growing evidence of their exposure in human populations, standardized methods for detecting MNPs are still lacking. Existing detection strategies typically focus on a limited set of polymers or additives, failing to capture the full diversity of nanoplastics and associated compounds, and are limited by throughput and sensitivity for detecting a wide range of particle sizes.

Materials and Methods

In this work, we present a comprehensive analytical framework for MNP biomonitoring in human specimens that integrates sample pretreatment, microplastic separation and enrichment, Pyrolysis-GC/HRMS analysis with a hybrid analytical approach combining traditional targeted methods with state-of-the-art untargeted chemometric models for detection and quantification of MNPs. This framework enables a detailed and reliable characterization of polymers present in biological samples.

Results

Two major highlights of this work are: (1) the accurate and efficient detection and quantification of 12 types of MNPs, along with the qualitative identification of 26 additional polymer types; and the ability to achieve high-throughput analysis—with an average processing time of 80 samples per week, which makes the large-scale biomonitoring of MNPs feasible. (2) The integration of machine learning models enhances the precision of polymer fingerprint identification (e.g., polyethylene), significantly reducing false positives and increasing confidence in results.

Conclusions

This platform represents a significant advancement in the detection of MNPs and offers a scalable solution for assessing plastic exposure in human populations.

S04-02 | Human Exposure to Micro- and Nanoplastics: A Systematic Review of Evidence from Human Biological Matrices

Runyu Zou[1,2], Florian Meirer[3], Jane Muncke[4], Lisa Zimmermann[4], Barbara Scholz-Böttcher[5], Douglas Walker[6], Roel Vermeulen[1,2]

[1] University Medical Center Utrecht, Julius Center for Health Sciences and Primary Care, Utrecht, Netherlands; [2] Utrecht University, Institute for Risk Assessment Sciences, Utrecht, Netherlands; [3] Utrecht University, Institute for Sustainable and Circular Chemistry and Debye Institute for Nanomaterials Science, Utrecht, Netherlands; [4] Food Packaging Forum Foundation, Zürich, Switzerland; [5] Carl von Ossietzky University of Oldenburg, Institute for Chemistry and Biology of the Marine Environment, Oldenburg, Germany; [6] Emory University, Gangarosa Department of Environmental Health, Atlanta, USA

Micro- and nanoplastics (MNPs) are ubiquitously distributed throughout the ecosystem and raise emerging concerns about their impact on human health. Over the past five years, cumulative research has revealed the presence of MNPs in diverse human biological matrices, such as feces, urine, blood, placenta, breastmilk, lung, and semen, using microscopy-based (e.g., FTIR, LDIR, Raman) or mass-based (e.g., GC/MS) methods. However, small sample sizes and heterogeneities in study subjects, sample processing and treatment, and technical parameters substantially limit the comparability and generalizability of results. This systematic literature review aims to provide an updated overview of human internal exposure to MNPs, along with the mostly investigated determinants and health consequences. Using AI-assisted software for abstract screening, we identified 93 empirical studies published by the end of 2024 from PubMed, Scopus, Embase, and Google Scholar. China contributed nearly half (n=44, 48.4%) of the articles, followed by the EU countries (n=20, 22.0%) and the Unites States (n=5, 5.5%). Feces (n=19, 20.4%), blood (n=15, 16.1%), and placenta (n=11, 11.8%) were the most frequently examined matrices, and three studies collected samples from deceased individuals. Most of the research used microscopy-based methods, while an increasing trend toward employing mass-based techniques, or combining both approaches, is evident in the quantification of MNPs in human biological matrices. In addition, we integrated and applied QA/QC criteria focusing on efficiency of sample treatment, the reporting of technical parameters, and measures to minimize background or procedural contamination, which enabled us to synthesize and highlight evidence from a subset of studies with the highest methodological quality. These findings are not only of scientific interest but also offer valuable insights for policymaking and the development of intervention strategies.

S04-03 | Traffic-related micro- and nanoplastics and immunological and respiratory health effects in healthy adults

Esther Lenssen[1], Lorenzo Scibetta[2], Marja Lamoree[2], Tim Skrabanja[3], Nienke Vrisekoop[3], Annemijne van den Berg[1], Kas Adriaans[1], Laura Caiazzo[4], Roel Vermeulen[1], Raymond Pieters[1], Gerard Hoek[1]

[1] Utrecht University, Institute for Risk Assessment Sciences, Utrecht, Netherlands; [2] Vrije Universiteit, Amsterdam Institute for Life and Environment, Amsterdam, Netherlands; [3] University Medical Centre Utrecht, Dept. of Respiratory Medicine, Center of Translational Immunology, Utrecht, Netherlands; [4] ENEA, Environmental Chemistry Division, C.R. Casaccia, Roma, Italy

Micro- and nanoplastics (MNPs) are abundant in the environment, with traffic-related tyre wear contributing substantially to atmospheric levels. MNPs have been detected in human tissues; however, their health effects remain poorly understood. We aimed to assess changes in respiratory and immune responses following short-term exposure to traffic-related MNPs in healthy adults.

In a semi-controlled study, 23 healthy volunteers (23–38 years) participated in four-hour exposure sessions at three traffic locations: a stop-and-go high-traffic road, a highway, and an urban background location. Spirometry tests were conducted before and immediately after. Venous blood samples were collected at the same timepoints, and the following morning, to assess changes in whole blood MNP levels, and total and differential white blood cell counts. Cytokine expression levels were additionally assessed using an OLINK® Target 48 Immune Surveillance Bundle. During the sessions, atmospheric synthetic and natural rubber markers were measured using double-shot pyrolysis gas-chromatography mass-spectrometry, within PM10 fraction. Additional traffic-related pollutants, e.g. black carbon, ultrafine particles, trace metals, and polycyclic aromatic hydrocarbons, were quantified. Mixed model analyses were used to evaluate associations between air pollutants and pairwise differences in health endpoints (post-exposure minus baseline), adjusting for multiple covariates.

Compared to the urban park, rubber marker levels were 2–5 fold higher near major roads. Polymers were moderately to highly correlated with brake-wear trace metals and black carbon (r_p=0.36–0.77). We observed positive associations between tyre-wear rubber markers and monocytes (7.1–9.3%) immediately after exposure. Furthermore, the following morning positive associations were observed for monocytes (9·3 – 17·7%) and additionally neutrophils (7·4 – 14·0%). These associations remained consistent after adjusting for covariates and other traffic-related air pollutants. No associations were observed for lung function and symptoms. Future analyses will include cytokines expression results.

The associations between MNP levels and WBC subtypes, might indicate an increased inflammatory response in healthy adults.

S04-04 | MNP exposure, immune cell activation, and lung function in young adults: The PIAMA cohort

Amanda Durkin[1,2], Tim Skrabanja[3], Ulrike Gehring[2], Martin Brits[4,5], Runyu Zou[1,2], Virissa Lenters[4,1], Federica Nardella[4], Gerard Koppelman[6,7], Judith Vonk[7,8], Martin van Velzen[4], Nienke Vrisekoop[3], Marja Lamoree[4], Roel Vermeulen[2,1]

[1] University Medical Center Utrecht, Julius Center, Utrecht, Netherlands; [2] Utrecht University, Institute for Risk Assessment Sciences, Utrecht, Netherlands; [3] University Medical Center Utrecht, Department of Respiratory Medicine and Center for Translational Immunology, Utrecht, Netherlands; [4] Vrije Universiteit Amsterdam, Department Environment and Health, Amsterdam, Netherlands; [5] Grain Building-Agri Hub Office Park, The Southern African Grain Laboratory, Pretoria, South Africa; [6] University of Groningen, University Medical Center Groningen, Beatrix Children's Hospital, Pediatric Pulmonology and Pediatric Allergology, Groningen, Netherlands; [7] University of Groningen, University Medical Center Groningen, Groningen Research Insititute of Asthma and COPD, Groningen, Netherlands; [8] University of Groningen, University Medical Center Groningen, Department of Epidemiology, Groningen, Netherlands

Micro- and nanoplastics, an emerging public health concern, enter the human body through inhalation and ingestion. The potential for this exposure to induce a pro-inflammatory immune response or impair lung function remains unknown. This study aimed to estimate exposure to micro- and nanoplastics in young adults and investigate associations with immune activation and lung function. We performed a cross-sectional study among 100 participants of the Dutch PIAMA (Prevention and Incidence of Asthma and Mite Allergy) cohort during the 25-year follow-up. Micro- and nanoplastic concentrations in blood were quantified with pyrolysis gas chromatography mass spectroscopy. Innate immune cell activation was assessed with flow cytometry by measuring CD11b, CD62L, and CD10 expression on neutrophils as well as CD11b expression on monocytes and eosinophils. Lung function was measured by spirometry. Linear regression analysis was used to assess associations of micro- and nanoplastic exposure with selected immune markers and lung function parameters. Micro- and nanoplastics were detected in all samples with a median total concentration of 424.9 ng/mL. Higher total micro- and nanoplastic concentrations were associated with higher expression of neutrophil, eosinophil, and monocyte CD11b, and neutrophil CD10. Higher polyvinyl chloride concentrations were associated with higher expression of eosinophil CD11b. No associations were found between micro- and nanoplastic exposure and lung function. These findings suggest that micro- and nanoplastics may be associated with health perturbations in young adults. Further research is warranted to confirm these observations in larger, more diverse populations.

S04-05 | Micro- and nanoplastic exposure and early life health: The Aurora project.

Roel Vermeulen, The Aurora Consortium

Utrecht University, Environmental epidemiology, Utrecht, Netherlands

Micro- and nanoplastic (MNP) pollution is increasingly recognized as a global environmental concern, yet its potential impacts on human health—particularly during early-life stages—remain poorly understood. The AURORA project aims to deliver an actionable European roadmap for assessing early-life health risks of MNP exposure, to inform regulation of MNPs, the products and processes generating secondary MNPs, and the development of safer alternatives.

To address this, AURORA has advanced the exposure assessment methodologies by enhancing capabilities to detect and quantify MNPs and associated chemicals (e.g., additives) in tissues relevant to early development—such as placenta, and cord blood. Currently, over 800 placenta samples have been characterized on their plastic exposome and the effects on placenta function, biological effects and early life health are currently being researched. Using a novel tiered-testing strategy and by synthesizing traceable MNPs, AURORA has employed different in-vitro testing systems in varying complexity to screen for impacts on immune and inflammatory responses, oxidative stress, and endocrine disruption. These in-vitro results have shown selected biological responses at high levels of pristine MNPs. Further testing using more realistic MNPs (i.e weathered) are ongoing.

Launched in 2020, AURORA is a five-year initiative funded under the EU Horizon 2020 programme and is part of the European CUSP (Cluster to Understand the Health Impacts of Micro- and Nanoplastics) research cluster. The project brings together 11 partners from Europe and the United States.

S05 | Bridging the Gap: Discovery of Novel and Uncommonly Measured PFAS in Human Exposure Studies

J. P. Koelmel[1], C. McDonough[2], A. Salamova[3]

[1] *University of Kentucky, Epidemiology and Environmental Health/College of Public Health, Lexington, Ohio, United States of America*
[2] *Columbiana Health Department, Lisbon, Ohio, United States of America*

Objective:
Rapid advancements in non-targeted analysis have the potential to revolutionize our understanding of xenobiotic effects on human health; significantly expanding the coverage of chemicals detected at a fraction of the cost and time. We will discuss advancements in acquisition technologies for per- and polyfluoroalkyl substances (PFAS), including gas chromatography, ion mobility, and liquid chromatography. The pros, cons, and synergy of these platforms will be examined in terms of both expanding coverage and improving accuracy in PFAS non-targeted studies.

Materials and Methods:
We will exemplify the unique attributes of each technique via applications to air, water, soil, and blood measurements. We will also discuss major data-analytic and visualization approaches to mine through non-targeted data, with a specific lens on PFAS analytics using our software ecosystem FluoroMatch.

Results:
FluoroMatch can be used for rapidly screening tens of thousands of PFAS and for de novo identification of complete unknowns, and has implementations for ion mobility, liquid chromatography, and gas chromatography, with unique algorithms catered to each approach. Specifically, we will discuss about leveraging accurate mass data using Kaufmann plots, a novel 11-step formula prediction workflow aided by homologous series detection, mass defect filtering, and isotopic percentage filers, leveraging fragmentation data using massive rule-based suspect screening libraries, in-silico libraries, and fragment screening, and leveraging drift time for collision cross section matching and drift time region PFAS classification algorithms.

Conclusion:
The audience should be able to understand the broad methods used for annotating PFAS and the role of each technique in enhancing the space of PFAS coverage after this talk.

S05-01 | Understanding Indoor Air and Dust on PFAS Exposures in Homes: A Comprehensive Analysis Using LC- and GC-MS

Zhuoyue Zhang[1,2], Robin E. Dodson[3], Laurel Schaider[3], Minghao Kong[4], **Amina Salamova**[4]

[1] Moganshan Institute ZJUT, Ka,ngqian DistrictDeqing, China; [2] International Joint Research Center for Persistent Toxic Substances (IJRC-PTS), College of Environment, Zhejiang University of Technology, Hangzhou, China; [3] Silent Spring, Newton, USA; [4] Emory University, Gangarosa Department of Environmental Health, Rollins School of Public Health, Atlanta, USA

Residential PFAS exposure is poorly understood. To address this knowledge gap, we recruited 50 households from Massachusetts and collected indoor air and dust samples to analyze 93 PFAS, including 70 ionic and 23 neutral species—one of the most comprehensive analyses to date. Samples were extracted using solid phase extraction, analyzed by LC-MS/MS and GC/MS, and quantified via isotope dilution. Results reveal widespread PFAS in the indoor environment; ultra-short chain PFAS, PAPs and FTOHs were frequently detected in both dust and air. We also determined unique patterns in PFAS partitioning between air and dust.

S05-02 | The Forest for the Trees: What is PFAS Biomonitoring Missing?

Courtney Carignan

Michigan State University, Department of Food Science and Human Nutrition, Department of Pharmacology and Toxicology, East Lansing, USA

Biomonitoring of per- and polyfluoroalkyl substances (PFAS) in human serum is a good marker of long-term exposure to common and persistent PFAS (e.g., PFOA, PFOS, PFHxS). However, many PFAS currently used in products are not found in serum. As there is an urgent need to identify alternative approaches to assess exposure to these PFAS we compared mixtures identified in serum with modeled exposure for three vulnerable populations. Serum and environmental samples (water, food, etc.) were previously analyzed for extended panels of PFAS. Exposure was modeled using environmental measurements and exposure factors. Several current use PFAS with predominant modeled exposure were not identified in serum. We conclude that current serum biomonitoring approaches should be supplemented with alternative approaches such as exposure modeling to more comprehensively assess exposure to these mixtures.

S05-03 | Identifying and Prioritizing Bioaccumulative PFASs from Complex Mixtures with High-Resolution Mass Spectrometry

Carrie McDonough

Carnegie Mellon University, Department of Chemistry, Pittsburgh, USA

Objective: The objective of this work is to learn about toxicokinetics and bioaccumulation potential of poorly-studied per/polyfluoroalkyl substances (PFASs) and identify PFASs to prioritize for targeted biomonitoring efforts and toxicity studies.
Material and Methods: Complex exposure-relevant mixtures containing known and unknown PFASs (including aqueous film-forming foams; AFFFs) were analyzed to determine the bioaccumulation potential of poorly-studied components using biomimetic chromatography, *in vitro* assays, and ion mobility spectrometry coupled with quadrupole-time-of-flight mass spectrometry (IM-QTOF-MS). These results will be discussed in comparison with findings from previous *in vivo* studies.
Results: Poorly studied perfluoroalkyl sulfonamides (FASAs) exhibit chain-length-dependent glucuronidation, with long-chain (C8) FASAs accumulating to a greater extent in liver and kidney tissue of AFFF-dosed mice. No FASAs were detected in blood serum despite detection in kidney, liver, and urine. Preliminary results of biomimetic chromatography analyses suggest C2 bis-perfluoroalkyl sulfonimide (bis-FEtSI) hinds to human serum albumin (HAS) at two sites with affinity similar to or greater than PFOS despite low retention on C18 (a proxy for hydrophobicity).
Conclusion: The study of complex PFAS mixtures has highlighted several poorly-studied sulfonamide-based structures as having high bioaccumulation potential. More work on their biological fate, including the extent of conjugation, tissue distributions, and propensity for *in vivo* transformation is needed. We acknowledge funding from CDC NIOSH and AHA.

S05-04 | Acquisition Technology and Software for Expanded PFAS Coverage in Non-Targeted Analysis: a High-Level Overview

Jeremy Koelmel

Yale University, Yale School of Public Health, New Haven, USA

Rapid advancements in non-targeted analysis have the potential to revolutionize our understanding of xenobiotic effects on human health, significantly increasing the rate of dissemination and richness of information pertaining to the diversity of chemical exposures. We will discuss advancements in acquisition technologies for PFAS (gas chromatography, ion mobility, and liquid chromatography), and our software ecosystem FluoroMatch for rapidly screening tens of thousands of PFAS and for de novo identification of complete unknowns. Applications to air, water, soil, and blood measurements will be discussed.

S05-05 | Assessing PFAS Contamination in South Florida: Correlations Between Drinking Water, Human Serum, and Liver Cancer Mortality

Carolina Cuchimaque, Natalia Quinete

Florida International University, Institute of Environment, Miami, USA

Per- and polyfluoroalkyl substances (PFAS) are persistent environmental pollutants with widespread distribution and growing public health concern due to their association with various health effects, including certain cancers. Among the primary pathways of human exposure, drinking water is particularly significant. This study assessed PFAS contamination in tap water across Miami-Dade and Palm Beach counties in Florida, USA, with a focus on potential associations with liver cancer mortality counts. A total of 147 tap water samples were collected from 18 zip codes in Miami-Dade and 22 in Palm Beach, with sampling locations strategically selected based on liver cancer mortality quartiles from the Florida Health Charts Mapper. Human serum samples were obtained in collaboration with a local South Florida biospecimen repository. Cases and controls were selected based on zip code residency and matched for key factors including body mass index, age, gender, smoking history, ethnicity, and other limited but relevant health data. A rapid and sensitive method was developed and validated for the detection of PFAS in human serum, utilizing solid-phase extraction combined with liquid chromatography-tandem mass spectrometry (SPE-LC-MS/MS). Miami-Dade exhibited significantly higher PFAS concentrations (mean Sum of PFAS ∑16PFAS= 323.2 ng/L) compared to Palm Beach (mean ∑16PFAS= 22.2 ng/L), with contamination hotspots aligning with zip codes in higher liver cancer mortality quartiles. This study offers valuable insights into PFAS exposure patterns and concentrations within affected communities. However, due to the limited cohort size, the findings are not intended to establish a direct correlation between PFAS exposure via drinking water and bioaccumulation in human serum. Nonetheless, these results lay the groundwork for future research exploring such associations in greater depth and support efforts to address public health concerns related to PFAS exposure and potential links to liver cancer in South Florida populations.

S06 | Household Air Pollution and Health: Recent evidence and the future of clean cooking research and policy

T. Clasen[1]

[1] *Emory University, Rollins School of Public Health, Environmental Health, Atlanta, Georgia, United States of America*

More than 3 billion people cook on open fires and traditional stoves using solid fuels such as wood, dung, charcoal and agricultural waste. Exposure to the resulting household air pollution is a leading health risk in low- and middle-income countries, accounting for an estimated 2.3 million premature deaths annually from pneumonia, chronic lung disease, cardiovascular disease, cancer, hypertension and other health disorders. It also contributes to a heavy disease burden associated with low birthweight and impaired child development. Governments and non-governmental organizations have attempted to address this disease burden, first by promoting advanced biomass cook stoves to improve combustion efficiency and more recently cleaner cooking technologies such as liquefied petroleum gas, electricity, and ethanol. However, much of the epidemiological evidence supporting this disease burden was drawn from observational studies, in some cases of ambient air pollution or of smoking studies. Recently, randomized controlled trials, including the Ghana Randomized Air Pollution and Health Study (GRAPHS) trial and the multi-country Household Air Pollution Intervention Network (HAPIN) trial, have tested cleaner cooking interventions during pregnancy, infancy and early childhood on a range of health outcomes. In some cases, there are differences between the intention-to-treat and the exposure-response results. This symposium will begin with an introduction to household air pollution and health, the estimated disease burden, and the epidemiological evidence generated over the past decades. Leaders of the GRAPHS and HAPIN trials will then present the results of these trials and continued follow-up, both on intention-to-treat and exposure-response study designs. A panel researchers will then explore the implications of these results on future clean cooking research and policy.

S06-01 | Background on household air pollution and health: disease burden and evidence from observational studies and initial trials

Kalpana Balakrishna[1]

[1] Sri Ramachandra Institute For Higher Education and Research, Chennai, India

Lays the foundation for the other talks by providing updated information on disease burden from household air pollution. Focuses on results from observational studies in contrast to experimental study designs. Emphasizes how policy has been shaped by these study designs.

S06-02 | Effects of an LPG intervention on birthweight, pneumonia, stunting and blood pressure: an intention-to-treat analyses from the HAPIN trial

William Checkley

Johns Hopkins University, School of Public Health, International Health, Baltimore, USA

Presents results from the multi-country HAPIN trial on adherence, exposure and each of the primary outcomes. Focuses on intention-to-treat analyses which were primary in this randomized controlled trial. Compares results with the CHAPS trial in Peru. Contrasts these results with exposure response results. Points to policy implications.

S06-03 | Effects of an LPG intervention on birthweight, pneumonia, stunting and blood pressure: exposure-response analyses from the HAPIN trial

Kyle Steenland

Emory University, Rollins School of Public Health, Environmental Health, Atlanta, USA

Presents the exposure results on PM2.5, CO and Black Carbon from the multi-country HAPIN trial and the exposure-response associations on primary outcomes (birthweight, child stunting, child pneumonia, adult hypertension). Contrasts these with intention-to-treat results from HAPIN, but compares them with recent systematic review on clean fuels and health. Discusses policy implications.

S06-04 | Longer-term health effects from a clean cooking intervention: evidence from continued follow up of the GRAPHS cohort

Seyram Kaali

Kintampo Health Research Centre, Kintampo, Ghana

Presents the longer-term results from the GRAPHS household air pollution and clean cooking study in Ghana that suggest protective health benefits that emerge among children at ages 4 and above. Discusses the benefits and challenges of long term follow up of exposure and health endpoints. Suggests research and policy implications.

S06-05 | Panel Discussion: Josh Rosenthal (NIH), Kalpana Balakrishna (SRIHER, Chennai, India), KP Asante (Kintampo, Accra, Ghana) and Ajay Pillarisetti (UC Berkeley): What does recent evidence mean for the future of clean cooking policy

Darby Jack

Columbia University, Mailman School of Public Health, Environmental Health Sciences, New York, USA

Professor Jack (Columbia) and Professor Thomas Clasen (Emory) will facilatate a panel discussion with researchers from US, India and Ghana to discuss where current evidence on household air pollution and clean cooking lead in terms of future research and policy.

S07 | The Exposome in Critical Windows over the Lifespan and Multiple Generations: Prospective Multi-omics to Predict Breast Cancer Risk

B. Cohn[1], **P. Cirillo**[1], **A. White**[3], **C.-J. Chang**[3], **X. Hu**[2]

[1] *Public Health Institute, Child Health and Development Studies, Berkeley, California, United States of America*
[2] *Emory University, Environmental Health, Atlanta, Georgia, United States of America*
[3] *National Institutes of Health, NIEHS, Research Triangle Park, North Carolina, United States of America*

Breast cancer remains a leading cause of women's morbidity and mortality worldwide, with growing evidence pointing to environmental factors as a key driver of risk. The exposome captures the totality of an individual's cumulative, lifelong environmental exposures and the associated biological responses. Previous studies have highlighted the importance of considering both individual and combined chemical exposures in relation to breast cancer etiology, yet few studies have undertaken a comprehensive assessment of internal exposures to better characterize the breast cancer exposome. In addition, there is ample evidence that exposure during critical life stages, such as mammary gland development and pregnancy, impacts progression, initiation, and susceptibility to breast cancer. Discovery of molecular targets during this particularly sensitive and responsive period of maternal adaptation and fetal development holds great promise in developing mitigation and intervention strategies. Recent methodologic advances in exposomics, metabolomics, genomics, epigenomics, and computational modeling permits the elucidation of the complex, dynamic and multitudinous relationships between the environment and biological systems. In this session, we will present research findings from various large-scale cohort studies, share insights and lessons from applying multi-omics research in epidemiology, and examine the complex interplay between chemical exposures, reproductive or developmental transition periods, and key molecular mechanisms like metabolic reprogramming and epigenetic inheritance. By investigating the roles of widespread chemical exposures, including per- and polyfluoroalkyl substances (PFAS) or forever chemicals and emerging contaminants, as well as exposure during sensitive life periods in the life course, we aim to identify new opportunities for early detection, intervention and strategies to reduce the breast cancer burden at the population level.

S07-01 | Multigenerational and lifespan impacts of in utero and pregnancy exposures on breast cancer risk: Insights from CHDS

Barbara Cohn[1], Piera Cirillo[1], Nickilou Krigbaum[1], Sami Teeny[2], ViLinh Tran[2], Young-Mi Go[2], Dean Jones[2]

[1] Public Health Institute, Berkeley, USA; [2] Emory University, Medicine/Pulmonary, Atlanta, USA

Background: In the 1960’s ~15, 000 pregnant women and their partners enrolled in the Child Health and Development Studies (CHDS), generating two subsequent generations, ~19,000 offspring and ~18,000 grand offspring.

Methods: Assays of archived prenatal maternal and paternal serum collected during the founding pregnancies cover multiple windows of susceptibility that differ by generation: For founding mothers the window is the biological storm of pregnancy. For offspring the window is the development of organs during fetal life. For the grand offspring it is the germline forming at conception of the grandmother’s pregnancy and development of that germline during their grandmother’s pregnancy.

Results: CHDS revealed variable exposure and outcome windows for environmental determinants of breast cancer associated with maternal pregnancy exposures. We measure the maternal and paternal metabolome (endogenous chemicals that are a read out of gene by environment interaction), the exposome (thousands of external chemicals) and the epigenome in offspring and grand offspring (gene regulation that instructs the metabolome and might be inherited across generations). CHDS provides the only human data to address dramatic, short term secular trends in cancer and cancer risk factors across 3+ generations that may have origins in exposures in the founding maternal and the paternal lines. The increase in early onset of cancer seen globally is recapitulated over the CHDS generations. Determinants of this transition can be directly investigated controlled for shared family factors.

Conclusion: Environmental exposures link to breast cancer risk and depend on exposure and outcome windows that reflect biological susceptibility. These continuing studies resolve apparent paradoxes in the field. Recently, using multi-omics for discovery, we observe that endogenous predictors are driven by environmental exposures with impact over multiple generations and across maternal and paternal lines. Findings provide a clearer picture of etiology relevant to current generations with implications for personalized cancer prevention.

S07-02 | Metabolome Wide Association Studies (MWAS) of placental and pregnancy characteristics associated with maternal breast cancer

Piera Cirillo[1], Nickilou Krigbaum[1], Boris Minasenko[2], Sami Teeny[3], Young-Mi Go[3], Dean Jones[3], Xin Hu[2,3], Barbara Cohn[1]

[1] Public Health Institute, Berkeley, USA; [2] Emory University, Environmental Health, Atlanta, USA; [3] Emory University, Medicine/Pulmonary, Atlanta, USA

Objectives: Previously we found that maternal floor infarction (MFI) of the placenta, characterized by fibrin deposition on the maternal surface, protected against breast cancer in mothers and daughters in the Child Health and Development Studies (CHDS). We conducted a Metabolome Wide Association Study (MWAS) in trimester 2 (T2) and trimester 3 (T3) serum to compare MFI pregnancies with controls.

Methods: The study is based on an 8% random case-cohort sample of CHDS women with available placental information, pregnancy serum and cancer diagnoses through 1997: N=523 in T2 with 49 MFI cases; N= 566 in T3 with 43 MFI cases. Untargeted pathway and metabolite analyses were performed using Rodin (https://github.com/BM-Boris/rodin.git). HILIC ESI+ feature intensities were log2 transformed and tested using ANOVA to discover MFI-enriched pathways ($p<0.05$).

Results: MFI-enriched T2 pathways were biopterin, retinol, aspartate and asparagine, and glycerophospholipid metabolism. Notable up-regulated T2 compounds included tetrahydrobiopterin, 5-hydroxyeicosatetraenoate, arachidonic acid, and diacylglycerol (DAG), which may be directly linked to cancer supervision through their regulation of transient receptor potential (TRP) channels and inflammatory processes. MFI-enriched T3 pathways included serine, aspartate and asparagine, vitamin D3 metabolism and bile acid biosynthesis. Notable up-regulated compounds were N4-acetylaminobutanal, serine and 27-hydroxycholesterol (27-HC); down-regulated compounds were desmosterol and cholesterol.

Conclusion: Pathways and features identified for MFI in T2 during rapid placental development are consistent with its putative inflammatory-related etiology. 27-HC has been shown to be protective of breast cancer risk in the post-menopause, while lower cholesterol has been associated with lower risk. Up-regulation of serine, endowed with immunosuppressive potential, in T3 may be a response to heightened inflammatory activity in T2. These results support the possibility of multiple mechanisms contributing to the dramatic reduction in breast cancer risk associated with MFI. Next steps are feature confirmation and targeted validation using proportional hazards modeling fit to a case-cohort design.

S07-03 | Characterizing the breast cancer internal exposome: findings from the Sister Study

Alexandra White[1], Che-Jung Chang[1], Catherine Mullins[2], Jenna Weil[2], Katherine Pullella[2], Anna Young[2], Xin Hu[2], Douglas Walker[2,3], Dean Jones[3]

[1] National Institutes of Health, National Institute of Environmental Health Sciences / Epidemiology, Research Triangle Park, USA; [2] Emory University, Environmental Health, Atlanta, USA; [3] Emory University, Medicine/Pulmonary, Atlanta, USA

Objective. Individuals are exposed to a vast number of chemicals in daily life, yet, few have been thoroughly investigated for long-term health impacts. This study used exposomics technology to comprehensively characterize the internal blood exposome and evaluate associations between chemicals and breast cancer, the most common cancer diagnosed in women.
Materials and Methods. Sister Study participants with blood samples at baseline (2003-2009) were selected for a nested case-cohort study (N=1,992 cases and N=2,328 subcohort). Plasma samples were analyzed using both gas and liquid chromatography mass spectrometry to identify exogenous chemicals using both targeted and untargeted methods. Only chemicals with pairwise correlation >0.6 between fragments were retained. Chemical abundances were averaged across duplicates, log_2-transformed, and imputed with the minimal concentrations/sqrt(2) if below detection. Self-reported breast cancer diagnoses were validated using medical records. An exposome-wide association study was conducted for an interquartile range increase using Cox proportional hazards models to estimate hazard ratios (HRs) and 95% confidence intervals (CIs). We also conducted analyses limiting to chemicals identified by Silent Spring as potential breast carcinogens.
Results. We quantified >250 unique chemicals with confirmed identities. With an average of >7 years of follow-up, we found a higher incidence of breast cancer for PCB-28 (HR=1.08, 95% CI: 1.03-1.14, adjusted p=0.002) and possibly 3-hydroxycarbofuran (HR=1.11, 95% CI: 1.00-1.23, adjusted p=0.2). In the Silent Spring subset analysis, we observed that DDT was associated with a higher breast cancer incidence (HR=1.08, 95% CI: 0.98-1.18, adjusted p<0.001).
Conclusions. In this preliminary analysis, we observed that certain pesticides and industrial chemicals may contribute to breast cancer incidence. We will be extending our analysis to consider additional chemical features, tumor subtype information and to incorporate mixtures analyses.

S07-04 | Exposure to PFAS on an 'omics scale and breast cancer risk

Che-Jung Chang[1], Katherine Pullella[1], Catherine Mullins[2], Jenna Weil[2], Anna Young[2], Dean Jones[3], Xin Hu[2], Douglas Walker[2], Alexandra White[1]

[1] National Institutes of Health, National Institute of Environmental Health Sciences, Research Triangle Park, USA; [2] Emory University, Environmental Health, Atlanta, USA; [3] Emory University, Medicine/Pulmonary, Atlanta, USA

Objective: Per- and polyfluoroalkyl substances (PFAS) represent a large and expanding class of nearly 15,000 synthetic chemicals. While human exposure to PFAS is widespread, their potential role in breast cancer etiology remains poorly understood. This study leverages both targeted and untargeted analytical approaches to measure PFAS on an 'omics' scale in blood and examine their relationship with breast cancer incidence.
Methods: We conducted a case-cohort study within the Sister Study, including 1,992 incident breast cancer cases and 2,328 subcohort. Baseline blood samples (collected 2003−2009) were analyzed using liquid chromatography–high-resolution mass spectrometry. PFAS were measured through both targeted and untargeted approaches. For untargeted data, chemical features were extracted using apLCMS and xMSanalyzer, and annotated via xMSannotator using the EPA CompTox PFAS Master List. Values below detection were imputed as the minimum detected value divided by $\sqrt{2}$. Breast cancer diagnoses were self-reported and medically confirmed. Cox proportional hazards models estimated hazard ratios (HRs) and 95% confidence intervals (CIs) per interquartile range increase in PFAS abundance for chemicals detected in ≥60% of samples, adjusting for age, race, income, education, and menopausal status.
Results: At baseline, participants had a mean age of 55.8 years and were predominantly non-Hispanic White with relatively high socioeconomic status. In a subset of participants with completed measurements (418 cases, 490 subcohort), 16 of the 24 PFAS met the ≥60% detection threshold. With an average follow-up of 7.4 years, perfluoroheptanoic acid (PFHpA) was positively associated with overall breast cancer incidence (HR = 1.10, 95% CI: 1.03−1.19) and estrogen receptor-positive tumors (HR = 1.13, 95% CI: 1.04−1.22).
Conclusion: In this prospective analysis, PFHpA was associated with a higher breast cancer incidence, particularly for estrogen receptor-positive cases. Ongoing analyses will extend to the full case-cohort, include untargeted PFAS, and apply mixtures modeling.

S07-05 | Male and female grandparental transmission of metabolic and reproductive risks over three generations

Xin Hu[1,2], Dongxue Wang[1,3], Piera Cirillo[3], Nickilou Krigbaum[3], Boris Minasenko[1], ViLinh Tran[2], Mary Nellis[2], Young-Mi Go[2], Dean Jones[2], Barbara Cohn[3]

[1] Emory University, Environmental Health, Atlanta, USA; [2] Emory University, Medicine/Pulmonary, Atlanta, USA; [3] Public Health Institute, Berkeley, USA

Background: Many breast cancer risk factors, such as early menarche and obesity, have worsened across generations with unclear underlying mechanisms. Using samples from the Child Health and Development Studies (CHDS), we examined how prenatal metabolic profiles of both grandmothers and grandfathers influence health outcomes in their daughters and granddaughters.
Methods: High-resolution metabolomics was performed on serum samples collected from 250 F0 parental pairs between 1959 and 1967. We assessed associations with age at menarche and obesity in daughters (F1, n = 247) and granddaughters (F2, n = 139).
Results: While median age at menarche remained stable between F0 and F1, it declined by a full year in F2. Distinct (grand)maternal and (grand)paternal metabolic profiles associated with age at menarche, indicating differential contributions from the male and female lines. We observed key interactions between the male and female (grand)parental metabolite associations with age at menarche, particularly tryptophan, tyrosine, taurine, and citric acid cycle intermediates known to modify epigenetic regulations. These interactions were more pronounced for F2 than for F1.
Prenatal grandparental metabolism was also associated with obesity. Tyrosine metabolism in lean grandmothers was linked to obesity risk in their granddaughters, while inflammation-mediating metabolites (e.g. tryptophan and arachidonic acid) were implicated in overweight grandmothers. When both grandparents were lean, androgen/estrogen biosynthesis, carnitine shuttle, and caffeine metabolism predicted granddaughter obesity. Grandmaternal and grandpaternal mitochondrial metabolites interacted in association with obesity, including those in carnitine shuttle and branched-chain amino acid degradation. In contrast, discordance in BMI between lean grandmothers and overweight grandfathers shifted associations toward tyrosine and urea cycle metabolism.
Conclusion: These findings underscore the complex interplay between male and female metabolic environments and their generational influence on offspring endocrine and metabolic health. The observed generational amplification of health risk in F2 suggests epigenetic inheritance and highlights the underappreciated role of paternal exposures.

S08 | Environmental Chemicals and Microbiota: Mechanisms of Interaction and Influence

K. R. Dalton[1], **S. Eggers**[2]

[1] *University of Iowa College of Public Health, Occupational & Environmental Health Department, Iowa City, Iowa, United States of America*
[2] *University of Iowa College of Public Health, Epidemiology Department, Iowa City, Iowa, United States of America*

Emerging evidence suggests interactions between chemicals and the microbiome may affect host health. Exposure to environmental chemicals has been preliminarily shown to disrupt the microbiome. This resulting microbial dysbiosis could potentially impact host health and the development of diseases, thus mediating the pathway between environmental exposures and health. In addition, microbial dysbiosis can result in unique compositions of microbial enzymes, which can cause differential metabolism of chemicals, thereby modifying the potential toxicity to the host and associated health outcomes. However, the influence of chemicals on human microbial communities and their contributions to the progression of adverse disease conditions remain largely understudied. This lack of cohesive understanding inhibits identification of potential mechanisms by which environmental exposures may impact population health.

Within this symposium, we will explore current research looking at the complex relationship between exposure to environmental contaminants, like chemical toxins, and the human microbiome. This is a critical issue in environmental health as it can further our understanding of the biological mechanisms whereby environmental exposures lead to adverse health conditions, and shed light on novel intervention targets, namely the microbiome. Our proposed symposium is relevant to an environmental epidemiology and exposure science audience, as it will highlight the under-recognized microbial component within the growing field of exposomics. The goal of this symposium is to discuss current research on the interconnection between the human microbiome and chemical exposure and co-exposure to environmental microbiota and contaminants, and postulate avenues for future research.

S08-01 | Timing Matters: Windows of Susceptibility for Gut Microbiome Development

Hannah E. Laue

University of Massachusetts Amherst School of Public Health and Health Sciences, Department of Biostatistics and Epidemiology, Amherst, USA

Objective: As research into the gut microbiome's role in environmental epidemiology advances, understanding when in a person's life the microbiome is most susceptible to environmental exposures—and the duration of these effects—is critical.

Materials and Methods: We utilized longitudinal data from 167 participants in the Health Outcomes and Measures of the Environment (HOME) Study, a prospective birth cohort in Cincinnati, Ohio. Urinary concentrations of triclosan, an antimicrobial compound, were measured up to twice during gestation and up to eight times in childhood. Antibiotic use was recorded up to 12 times between 6 months and 12 years via caregiver-reported questionnaires. At the 12-year study visit (ages 11-14 years), participants provided stool samples on fecal occult blood test cards. We used metagenomic sequencing to determine diversity and composition (species, gene pathways) of the gut microbiome. Using generalized estimating equations and sequence analysis, we identified windows of exposure to triclosan and antibiotics associated with differences in the gut microbiome.

Results: We found that childhood triclosan concentrations, particularly ages 1 and 12 years, were more strongly associated with differences in the adolescent gut microbiome than gestational triclosan concentrations. In contrast, we found that antibiotic use around age 5 years had the strongest associations with the adolescent gut microbiome. Using sequence analysis, we identified 3 patterns of antibiotic use across childhood (no use, early use, persistent use). Those in the "persistent use" category had microbiomes less similar to those in the "no use" category compared to those with "early use."

Conclusion: We utilized generalized estimating equations and sequence analysis to identify windows of susceptibility in adolescent gut microbiome development. Using triclosan and antibiotics as illustrative examples, we highlight the importance of investigating sensitive developmental windows and the need for research into the long-term implications of early-life environmental exposures on the gut microbiome.

S08-02 | Interpretable machine learning models to identify microbial cliques

Vishal Midya[1], Shoshannah Eggers[2], Jamil Lane[1]

[1] Icahn School of Medicine at Mount Sinai, Environmental Medicine and Climate Science, New York City, USA; [2] University of Iowa College of Public Health, Iowa City, USA

Gut microbes interact with each other, and their interplay influences multiple health outcomes in humans. The collective impact of multiple bacterial species, called microbial cliques, may even surpass the effects of individuals. However, studying this multi-microbial interaction is challenging because of the high computational complexity. We introduce an interpretable machine-learning algorithm, the Microbial Co-occurrence Analysis (MiCA), to identify and study these non-linear interactions of microbial cliques on multiple health outcomes. Using a prospective longitudinal birth cohort in Mexico City, we demonstrate the utility of MiCA in understanding the relationship between microbial cliques, gut inflammation, and depressive symptoms in childhood. In another example from the Survey of the Health of Wisconsin and its ancillary Wisconsin Microbiome Study, using MiCA, we demonstrate that food insecurity may modify the relationship between the gut microbiome and the risk of cognitive impairment in older adults. Using a combination of weighted quantile sum regression and MiCA, we illustrate how to detect shape-based toxicologically mimicking interactions among gut microbes. We discuss the potential drawbacks of this method and discuss avenues for improvement.

S08-03 | Early-Life Metal Exposure and Childhood Cognition: The Role of the Oral Microbiome in Neurological Outcomes

Shoshannah Eggers[1], Jamil M. Lane[2], Libni A. Torres Olascoaga[3], Megan K. Horton[2], Martha M. Téllez Rojo[3], Robert O. Wright[2], Vishal Midya[2]

[1] University of Iowa College of Public Health, Epidemiology Department, Iowa City, USA; [2] Icahn School of Medicine at Mount Sinai, Environmental Medicine, New York, USA; [3] National Institute of Public Health, Cuernavaca, Mexico

Early-life metal exposures are associated with neurological health and alterations to the human microbiome, but their combined influence on cognitive outcomes remains unclear. The oral microbiome is an under-investigated player in cognitive health, linked through the oral-brain axis. This study examined the relationship between early-life metal exposure, the oral microbiome, and cognition in late childhood. Using data from the Programming Research in Obesity, Growth, Environment, and Social Stressors (PROGRESS) cohort, we assessed prenatal maternal blood metals, characterized salivary microbiome composition using 16S amplicon sequencing at 10-12 years old, and evaluated cognitive scores using the NIH Toolbox. We conducted a Microbial and Chemical Exposure Analysis (MiCxA), which identified and estimated joint associations of metal exposure signatures and microbial cliques that characterized subgroups of participants with better cognitive outcomes. All analyses were adjusted for relevant covariates. Our analysis of 61 children identified a metal signature of high levels of Selenium and Copper in the second trimester and an oral microbial clique of high abundance of *Atopobium spp*. and/or presence of *Prevotella shahii*. The combined metal signature and microbial clique was found in 43% of participants. Presence of either the metal signature ($B=0.02$, $p=0.02$) or the microbial clique ($B=0.02$, $p<0.001$) was associated with an increase in

crystalized cognition score. When both the metal signature and microbial clique were present, the positive association with crystalized cognition doubled (B=0.04, $p<0.001$). Associations between the metal signature and microbial clique with other NIH Toolbox domains of cognition were similar in magnitude, however, did not reach statistical significance. Our results highlight the importance of combined environmental and microbial influences on childhood cognition, demonstrating the potential influence of the oral microbiome and providing support for the development of future targeted probiotic interventions.

S08-04 | Does Human Milk PFAS Exposure Affect Infant Gut Microbes? Insights from a US Cohort

Devendra Paudel[1], Nathan D. Young[1], Elizabeth A. Holzhausen[1], Haonan Li[1], Roshonda Jones[2], Hailey Hampson[3,5], Michael I. Goran[4], Jesse A. Goodrich[5], Sandrah Eckel[5], Douglas I. Walker[6], Donghai Liang[6], David V. Conti[5], Lida Chatzi[5], Tanya L. Alderete[1]

[1] Johns Hopkins Bloomberg School of Public Health, Department of Environmental Health and Engineering, Baltimore, USA; [2] North Carolina Agricultural and Technical State University, Department of Biology, Greensboro, USA; [3] University of Washington, School of Medicine, Seattle, USA; [4] Children's Hospital, The Saban Research Institute, Los Angeles, USA; [5] University of Southern California, Department of Population and Public Health Sciences, Los Angeles, USA; [6] Emory University Rollins School of Public Health, Gangarosa Department of Environmental Health, Atlanta, USA

Objective: Human milk can be a source of early-life exposure to per- and polyfluoroalkyl substances (PFAS). This pilot study aims to examine the associations between human milk PFAS and the infant gut microbiome.
Materials and Methods: We analyzed 50 Latino mother-infant pairs from the Southern California Mother's Milk Study. Human milk PFAS at 1-month postpartum were characterized using untargeted high-resolution mass spectrometry, and the infant gut microbiome was characterized using metagenomics sequencing at 6 months. To examine relationships between individual PFAS, PFAS mixtures, and the gut microbiome, we applied a novel Bayesian Hierarchical Zero-Inflated Negative Binomial Regression model with G-Computation (BaH-ZING) that leverages the microbiome's taxonomic hierarchy to provide association estimates at each taxonomic level. Models were adjusted for birth weight, delivery mode, gestational age, socioeconomic status, and breastfeeding proportion.
Results: Seven PFAS were detected in human milk at 1-month postpartum, including one legacyandfour short-chain PFAS. At the genus level, individual PFAS were associated with 36 taxa, including some PFAS showing either positive or negative associations with pathogenic genera (e.g., *Enterococcus*, *Klebsiella*, *Enterobacter*, *Staphylococcus*). Meanwhile, individual PFAS exposure was negatively associated with 50 taxa, including a lower abundance of beneficial genera (e.g., *Bifidobacterium*, *Faecalibacterium*, *Blautia*, *Roseburia*, *Veillonella*). Similarly, exposure to the PFAS mixture was associated with a reduced abundance of beneficial genera, including *Bifidobacterium*, *Blautia*, and *Segatella*. At the species level, individual PFAS were associated with 57 unique microbial species and the PFAS mixture was associated with 14. Overall, 13 species were associated with both individual PFAS and the mixture.
Conclusion: Exposure to individual PFAS and PFAS mixtures through human milk is linked to infant gut microbial profiles. These microbial changes may have important implications for infant health. Further studies are urgently needed to understand how these early-life microbial disruptions might influence long-term child health outcomes.

S08-05 | Understanding the impact of PFAS mixtures on the gut microbiome using novel Bayesian hierarchical methods

Jesse Goodrich

Keck School of Medicine of the University of Southern California, Department of Population and Public Health Sciences, Los Angeles, USA

Methods for analyzing hierarchical microbiome data alongside correlated chemical mixture exposures remain limited. Moreover, strategies for integrating microbiome and environmental exposure data with other omics datasets to uncover drivers of disease are not well-established. Here, we introduce two innovative methods addressing these gaps. First, we present a novel Bayesian Hierarchical Zero-Inflated Negative Binomial Regression with G-Computation (BaHZING), designed to leverage the microbiome's inherent taxonomic hierarchy. Applying BaHZING to a multiethnic cohort of young adults at elevated risk for metabolic disease, we investigated associations between per- and polyfluoroalkyl substance (PFAS) mixtures and gut microbiome composition. Our findings revealed that higher exposure to PFAS mixtures was associated with lower abundances of Lachnospiraceae, a protective and highly abundant family of gut bacteria, along with elevated levels of bacterial taxa previously implicated in chronic disease development. Simulation studies demonstrated that BaHZING reduced Type I error rates, provided stabilized effect estimates, and yielded greater positive predictive values compared to existing methods, indicating improved estimation of true associations. Second, we introduce a high-dimensional multiomics mediation analysis that jointly incorporates microbiome and metabolomics data to understand how these omics layers link exposure to PFAS with risk of kidney disease. Applying this method to the same cohort, we examined how PFAS associated differences in the microbiome and metabolome are collectively associated with decreased kidney function. For example, this method identified that joint changes in the microbiome and metabolome, characterized by reduced Lachnospiraceae and 17bestradiol and increased succinate, retinoate and dodecanoic acid mediated 38% of the association between the PFAS burden score and estimated glomerular filtration rate. These two analytical approaches are both available through the R packages BaHZING and EnvirOmix and offer powerful new tools for elucidating how correlated chemical exposures affect microbiome composition and impact health outcomes.

S09 | Artificial Intelligence and Ethics: The Pros and Cons of Its Use in Environmental Health and Epidemiology

K. M. Zierold[1], **A. Sarkar**[2], M. G. Weisskopf[3], R. A. Etzel[4], C. Byrne[5], A. Mustapha[6], N. A.B. Islam[7]

[1] *University of Alabama at Birmingham, Environmental Health Sciences/Ryals School of Public Health, Birmingham, Alabama, United States of America*
[2] *Memorial University of Newfoundland, Population Health and Applied Sciences, St. John's, Newfoundland and Labrador, Canada*
[3] *Harvard University, Environmental Health/ Harvard T. H. Chan School of Public Health, Boston, Massachusetts, United States of America*
[4] *The George Washington University, Occupational & Environmental Health/Milken Institute School of Public Health, District of Columbia, United States of America*
[5] *Uniformed Services University of the Health Sciences, Preventive Medicine and Biostatistics/School of Medicine, Bethesda, Maryland, United States of America*
[6] *Nigerian Institute of Medical Research, Lagos, Nigeria*
[7] *Bangabandhu Sheikh Mujib Medical University, Preventive and Social Medicine, Dhaka, Bangladesh*

Artificial Intelligence (AI) has emerged to be at the forefront of science. AI technology uses computers and computer-controlled robots to think similar to and perform the tasks of human beings. Using created algorisms, AI technology uses large data to assess and predict outcomes related to the health and well-being of the environment and populations throughout the world.

The introduction of AI and its increased use has created challenging ethical struggles for scientists and educators in environmental health and epidemiology. As proponents of global health of the environment and populations, we must consider the harms and respectable use of AI. The ethics of AI use centers on accountability, transparency, and privacy. Questions surrounding (1) Who is responsible for both the programming of the algorisms and interpreting the results, (2) How is privacy maintained when data is being used without consent, (3) What are the stakes when this data is mishandled, (4) How is academic integrity maintained among students and researchers, and (5) What are the challenges of the lack of access to AI throughout the global health and environment. The ethics of AI use are particularly challenging for education and research for which harms to the health of populations and the environment are at stake.

This symposium will discuss the ethics of using AI in research and education with specific focus on environmental epidemiology and environmental health. Using global perspectives from researchers, educators, and journal editors, this symposium will bring light and lead to an in-depth discussion of the ethical use of AI

S09-01 | *AI and Environmental Epidemiology: Where are we going and how are we getting there?*

Marc Weisskopf

Harvard University, Environmental Health/ Harvard T. H. Chan School of Public Health, Boston, USA

The increasing availability of “big data” of many sorts holds tremendous potential, including for environmental health. Artificial intelligence (AI) can provide the means to wrangle such data to unlock this potential, and, indeed, is already being used widely in environmental epidemiology. AI techniques like machine learning and deep learning algorithms are used to generate, for example, air pollution exposure models from large complex datasets like those of satellite imaging and sensor network data. AI is used on google street view images to parse images into different exposure categories like trees, grass, or sidewalks. AI holds great potential on the outcome side as well, with possible uses of other large data like medical records, biometrics, and ‘omics from tissue samples to predict disease and disease states. The typically large scale of AI work, however, may mask, or make less likely that we explore, “what’s going on under the hood.” We need to understand the implications of using AI for environmental epidemiology.
In this presentation I will delve into several different issues related to AI in environmental epidemiology. First, there is the issue of predictive modeling vs. causal modeling. Understanding what the objective of the AI-related modeling is, is the first step in understanding potential pitfalls. Second, there are many ethical issues embedded both explicitly and implicitly in AI modeling. There are not only issues of data privacy, but also whose data is being used? And as importantly, whose data is not being used. How does this affect the outputs from AI models and who do the results apply to?
The potential for AI to revolutionize how we do our work is tantalizing. Deeply understanding what AI is doing and how, though, will be critical to appropriately interpreting results, and generating trust in the results, both by scientists and the general public.

S09-02 | *Ethical Issues with Using Artificial Intelligence for Peer Review of Manuscripts – An Editor’s Perspective*

Ruth A. Etzel

The George Washington University, Occupational & Environmental Health/Milken Institute School of Public Health, District of Columbia, USA

Peer review has been a foundation of scientific discourse for hundreds of years. With the massive increase in the number of journals and scientific publications during the past decade, scientists are receiving many more requests to conduct peer reviews than they previously received. Some reviewers may be tempted to consider the use of artificial intelligence (AI) to assist with this task. When invited to provide a peer review of a manuscript, it is usually because you have specific expertise and practical experience studying the topic; you probably have intimate familiarity with the challenges and methodological problems that can arise and you are, therefore, expected to be able to spot those problems during your review. A number of generative AI tools are now available to assist with peer review of manuscripts. Some journals explicitly prohibit their use, but other journals have remained silent on whether these tools may be used. The use of generative AI tools typically involves uploading the manuscript into the

generative AI tool. To do so, however, would breach the authors' confidentiality, and most journal editors do not consider it to be ethical because it risks possible disclosure of the study's findings before the date of publication. A second issue is that AI tools cannot take responsibility for the content of the peer review, and it is the editor's expectation that the author of a peer review should take responsibility for it. A third issue is that AI tools may bias against breakthroughs. Although AI tools may have the ability to assesses how a new study's findings fit within existing knowledge, AI is likely to miss scientific breakthroughs that result in paradigm shifts. This is a serious issue because journal editors and the scientific community at large place a high value on discovery of new scientific knowledge.

S09-03 | *When is AI "appropriate" for use by Epidemiology students or faculty?*

Celia Byrne

Uniformed Services University of the Health Sciences, Preventive Medicine and Biostatistics/School of Medicine, Bethesda, USA

Today AI creates models to analyze large datasets, provides an interpretation and writes the reports. How do we convince students they need to think through each step in a "DAG" when AI can do it without them having to think about steps in planning an analysis, never mind writing the final paper?

Many universities initial prohibited the use of AI, yet many now accept its use as the checkers are inadequate to always identify AI. Still students are often asked to sign they abided by an honor code in their work. Faculty must determine their own policies and practices. If work generated by AI is not the student's own should it be classified as plagiarism.

Consideration of a "DAG" implies one thinks through how exposures relate to each other and if each is independently related to the outcome of interest. It is that critical thinking process in planning, conducting and analyzing a study, as well as writing the report of the study that we hope to develop and promote in students as they learn the skills they will later apply in environmental epidemiology.

Various teaching methods are proposed to minimize the use of AI in courses. All writing assignments could be assigned in class. While practical for short answers, in epidemiology we draw on references and our data when writing so this does not develop the skills students will need. Some suggest students react and critique a piece generated by AI. How do we provide students with the tools to critically evaluate AI results and to know when it might be appropriate to use AI?

We will discuss these issues as we try to take Alexander Pope's advice from 1709 to "Be not the first by whom the new are tried, nor yet the last to lay the old aside."

S09-04 | *AI in Academic Research: Opportunities and Barriers for Bangladeshi Students*

Nabhira Islam

Bangabandhu Sheikh Mujib Medical University, Preventive and Social Medicine, Dhaka, Bangladesh

Background: Bangladesh has a rapidly growing economy with a large population of students. Artificial intelligence (AI) is increasingly used to assist students with activities like writing texts, literature reviews, and data analysis. Use of software may improve effectiveness but they lose their potential for growth and critical thinking.
Objective: This study explores the opportunities and barriers of using AI for Bangladeshi students in their academic research.
Methods: A narrative review was conducted analyzing national and international publications from January 2022 to January 2025, and a total of twenty-three articles were selected from databases like PubMed, Google Scholar and Scopus based on AI in academic research. The inclusion criteria for this review include articles published in English, with a focus on AI's impact on student research practices, higher education policies and plagiarism, while the exclusion criteria involve non-academic sources and unrelated studies. Thematic analysis was employed to categorize findings into opportunities and barriers.
Results: AI tools are helpful as they can significantly improve the quality and efficiency of academic work. However, overuse of AI may reduce students' ability to develop critical thinking skills. Plagiarism remains a major issue due to the unregulated use of AI-generated content. The key challenges include: (1) Lack of funding for AI infrastructure and training programs, (2) Shortage of skilled manpower, (3) Limited access to detect AI software, and (4) There is an absence of clear university policies and ethical guidelines.
The thematic analysis categorized findings into opportunities and barriers, highlighting the need for structured policies and investments to maximize AI’s benefits while safeguarding academic integrity.
Conclusion: Although AI has a huge potential in contributing to the improvement of academic research, a balanced approach is useful to maximize benefits while maintaining academic integrity.

S09-05 | *Opportunities, challenges and ethical considerations of artificial intelligence in environmental health: Perspectives from Africa*

Adetoun Mustapha

Nigerian Institute of Medical Research, Lagos, Nigeria

Nearly a quarter of all deaths in Africa are reportedly due to environmental causes. Therefore, Africa presents research challenges and opportunities for environmental health researchers. Many researchers predict that the introduction of artificial intelligence (AI) systems powered by big data will have a huge impact on research and public health (Sparrow and Hatherley, 2019). AI can help disease surveillance systems by analyzing large amounts of data from various sources, diseases diagnosis with greater accuracy, in the prediction of disease risk and the spread of diseases and provide more effective prevention measures and interventions, faster countermeasures and better health outcomes (Suarjana et al, 2023). However, AI algorithms can have inherent biases that affect clinical decisions and healthcare equity (Obermeyer et al, 2019). AI bias occurs when an algorithm’s output becomes prejudiced due

to false assumptions based on the data fed into it (Silberg and Manyika, 2019). Lack of datasets and the relevance of this data to African problems is a significant challenge to implementation AI in environmental health in Africa. Machine learning relies on vast amounts of data to train algorithms, and if this data is sparse and unrepresentative, the resulting algorithms will be less effective and could cause harm to the vulnerable populations (Okolo et al, 2023). AI-based apps may face the risk of exacerbating global injustices and create data colonialism (Ferryman 2021). There are challenges in integrating AI technologies into existing public health infrastructure, such as interoperability issues, data sharing protocols, data quality and workforce capacity (Suarjana et al, 2023). For responsible use of AI in environmental health, there is the need to evaluate the practicality of implementing AI-based systems in various healthcare settings and resource-constrained environments. Further, involvement of citizens in AI governance and policies that ensure equitable access and equitable health outcomes for all populations are vital.

S10 | From Home, Preschool, and Beyond: Addressing Common Chemical Exposures Across Life Stages

J. Hoponick Redmon[1], **N. DeLuca**[1], E. Norman[2], M. Napier[2], M. Del Rio[3], A. Alde[3], J. MacDonald Gibson[4], H. Long[4], A. Kondash[1], M. Lee[1]

[1] *RTI International, Durham, North Carolina, United States of America*
[2] *NC Department of Health and Human Services, Raleigh, North Carolina, United States of America*
[3] *Indiana University - School of Public Health, Bloomington, Indiana, United States of America*
[4] *NC State University - Dept. of Civil, Construction, and Environmental Engineering, Ra, North Carolina, United States of America*

Child care centers and schools are essential pillars of communities in the United States and beyond. The time that children and adults spend in these built environments and their households comprise a large part of one’s potential exposure to common everyday chemical hazards that affect human health. The risks of cumulative chemical exposures over time and across the exposome compound health risks. This symposium overviews statewide implementation programs and novel participatory research related to environmental exposures across built environments and life stages from infancy to adulthood; geographic scales from the US to Guatemala; chemical occurrence including lead, other metals, asbestos, flame retardants, cigarette smoke, and PFAS; media including drinking water, building materials, dust, soil, and beer; and tools to communicate and mitigate risks. We will highlight our methods to identify chemicals, work with communities, empower participatory scientists to take actions to improve environmental health outcomes, and advance laboratory analytical methods and predictive risk models that support data-driven policies and consumer choices. We encourage you to bring your preferred beverage and engage with our collaborative symposium presenters about strategies to advance environmental health equitably across space and time.

S10-01 | Clean Classrooms for Carolina Kids: Addressing environmental health hazards at child-occupied facilities through community engagement and partnerships

Ed Norman[1], **Melanie Napier**[1], Madison Lee[2]

[1] NC Department of Health and Human Services, Raleigh, USA; [2] RTI International, Durham, USA

Objective: Exposure to lead and asbestos can have lifelong health impacts, especially for children. The Clean Classrooms for Carolina Kids™ program aims to identify and eliminate exposure to lead in water, lead-based paint, and asbestos hazards at every child care center, family child care home, and public school across North Carolina.
Material and Methods: In partnership with the North Carolina Department of Health and Human Services (NC DHHS) and Division of Child Development and Early Education (NC DCDEE), child-occupied facilities have been notified to enroll in the program to help fulfill state requirements. Identification of lead in water is completed through participatory science, while identification of lead-based paint and asbestos hazards are completed through on-site assessments by accredited/certified professionals.
Results: To date, we identified lead in water above the 1 part per billion American Academy of Pediatrics' reference level and above 10 part per billion in 20% and 3% of samples, respectively. One or more taps at 52% of facilities exceeded 1 ppb and 12% exceeded 10 ppb. Among 2,564 facilities (24%) reviewed for lead-based paint and 2,563 facilities (35%) for asbestos required further inspection. Lead-based paint hazards were identified in 34% of inspections, significantly higher than asbestos hazards, which were identified in 6%.
Conclusion: A child's learning environment is commonly a potential lead and asbestos exposure source. Scientifically rigorous hazard testing programs, paired with infrastructure improvements, could eliminate early childhood exposure to lead and asbestos, and support school readiness, social-emotional development, and lifelong health.

S10-02 | The Preschool Environmental Experiences and Development Study - Evaluating how everyday chemical exposures and caregiving environment affect neurodevelopmental outcomes for preschoolers

Jennifer Hoponick Redmon

RTI International, Durham, USA

Objective: Children are exposed to many common chemicals in their day-to-day life. The first few years of life is a stage with very fast growth and brain development. Toddlers' growing brains are particularly sensitive to chemical exposures. The goal of this research is to understand how chemicals in children's everyday surroundings, such as their indoor living spaces, the water they drink, they dust and dirt on the floor they crawl through, and other factors might impact their early development.
Material and Methods: Characterizing the occurrence and effects of our target chemicals (metals, flame retardants, PFAS, cotinine) on children who are exposed to them can help families, child care providers, and early educators support healthy, thriving children. Our experimental approach was to recruit and enroll children attending licensed

childcare centers into the study and test their indoor environmental exposure pathways at both the home and childcare center, then pair that data with neurodevelopmental and caregiving environment evaluations.

Results: We have enrolled over 300 families, collected samples from 60 child care centers and 100 homes, completed 196 interviews, and 219 cognitive assessments. We characterized the occurrence of chemicals including metals, flame retardants, PFAS, and cotinine in both environments and with biological specimens. In this presentation, we'll overview preliminary results from the study along with the challenges and pathways to success enrolling participants.

Conclusion: Findings were translated to families, child care providers, and early educators to support healthy, thriving children.

S10-03 | PREDICTing Healthy Homes: Using multi and mixed modeling, participatory science testing, and mapping to identify households with high lead exposure risks

Alyson Alde[1], Michelle Del Rio[1], Jennifer Hoponick Redmon[2], Jacqueline MacDonald Gibson[3], Hana Long[3], Emmanuel ObengGyasi[4]

[1] Indiana University - School of Public Health, Bloomington, USA; [2] RTI International, Durham, USA; [3] NC State University, Raleigh, USA; [4] NC Agricultural and Technical State University, Greensboro, USA

Objective: This study developed an interactive map allowing users to see the percent chance a child will have a blood lead level (BLL) at or above 3.5 µg/dL. To do this, we (1) linked individual-BLL data with environmental and demographic factors; (2) used this individual level data to train predictive Machine-Learned Bayesian Network (BN) models; (3) collected environmental samples to validate the models.

Materials and Methods: Individual BLL records were linked with prior soil lead measurements and publicly available census block group (CBG) data, including demographics, socioeconomic indicators, and home characteristics. Once linked, BN models were trained. Participatory science was used to obtain household water, dust, and soil samples in Indiana and North Carolina. As a measure of total lead exposure for participant-provided environmental samples, we compared the BN model to total lead exposure in each household as estimated by the Environmental Protection Agency's (EPA) Integrated Exposure Uptake and Biokinetic (IEUBK) model. Finally, a risk mapper was created by applying the trained and validated model to publicly available census data at the block group level.

Results: Key predictors from the BN model included child's age, home construction year, BLL test year, specimen type, water source, home value, and proximity to known lead sources, along with CBG-level variables such as racial composition, poverty rate, and median income. Participant environmental sample BN-predicted risk was significantly correlated with IEUBK measured exposure from participant environmental samples (Spearman's $\rho = 0.519$, $p < 0.001$). A pilot mapper has been developed for selected counties in Indiana and North Carolina, estimating the probability that a child's BLL exceeds 3.5 µg/dL.**Conclusion:** This approach shifts from reaction to prevention for childhood lead exposure. By leveraging publicly available data and machine learning, we demonstrate a cost-effective and scalable method for identifying at-risk children without relying solely on blood testing.

S10-04 | Hold My Drink: Chemical occurrence from drinking water to beer across the globe

Nicole DeLuca, Jennifer Hoponick Redmon

RTI International, Durham, USA

Objective: We have conducted two studies on chemical occurrence in drinking beverages that will be discussed in this presentation. There are limited data on the occurrence and distribution of metals (including lead) and emerging contaminants such as per- and polyfluoroalkyl substances (PFAS) in urban Guatemalan tap water. Water is a main component of beer and the brewing process, therefore PFAS in drinking water systems could be a source of PFAS in beer.
Material and Methods: In the first study, 113 households served by a range of water providers across the Guatemala City metropolitan area were recruited as participatory scientists to collect tap water samples that were analyzed for 25 per- and polyfluoroalkyl substances (PFAS) and 20 metals. In the second study, 23 beers from various regions, brewery types, and water sources were analyzed for 17 PFAS using a modified EPA Method 533.
Results: In the Guatemala City study, at least one PFAS was detected in 19% of the tap water samples, and PFAS was associated with households' use of plastic water storage tanks. At least one metal exceeded Guatemalan Maximum Permissible Limits for drinking water in 63% of households. In the beer study, PFAS were detected in most of the beers tested, particularly from smaller scale breweries located near drinking water sources with known PFAS. Some beers had levels of PFOA and PFOS above the U.S. EPA's Maximum Contaminant Limits for drinking water. We found a county-level correlation between total PFAS, PFOA, and PFBS concentrations in municipal drinking water local to breweries and corresponding beers.
Conclusion: Chemical occurrence information from commonly consumed beverages like drinking water and beer can help to drive data-informed decisions by consumers, public health authorities, and policy makers.

S10-05 | Forever Chemicals in Well Water: A participatory science approach to evaluate, model, map, and communicate PFAS risk across the U.S.

Hana Long[1], **Jacqueline MacDonald Gibson**[1], Grace Kilroy[1], Krishna Mohan Ganta[1], Kelly Hoffman[2], Erica Wood[2], Rohit Warrier[2], Ted Lillys[2], Riley Mulhern[3], Jennifer Hoponick Redmon[2]

[1] NC State University - Dept. of Civil, Construction, and Environmental Engineering, Raleigh, USA; [2] RTI International, Durham, USA; [3] University of Colorado Boulder, Boulder, USA

Objective: Rural communities encounter exposure to per- and polyfluoroalkyl substances (PFAS) from agricultural operations, industrial applications, and water sources. Limited testing, monitoring, and funding in rural communities hinder efforts to assess and mitigate exposure risks. This research aims to enhance understanding of PFAS occurrence, fate, and transport in rural water sources and agricultural operations. Objectives include predicting source water risk of PFAS exposure in rural areas from industrial environmental point sources, wastewater, and agriculture; examining PFAS-soil interactions; and developing effective risk communication strategies.

Material and Methods: In four areas across the U.S. (North Carolina, Minnesota, Washington, and Indiana), 270 households collected tap water samples as participatory scientists. Soil and runoff samples were collected from agricultural sites in Robeson County, NC—near a known industrial GenX source—and a control site in Monroe County, IN. Comparative analysis of wastewater process samples and farmers' land use surveys aim to develop nationwide, web-based PFAS exposure risk maps for rural areas, supplemented by targeted risk communication materials for affected communities. Using study data and publicly available datasets, we developed a hybrid mechanistic groundwater fate and transport and machine-learned Bayesian Network (MLBN) model, and a geospatial risk mapper to better predict the likelihood of PFAS in wells.
Results: Overall, 63% of households had detectable PFAS, and 49% had PFAS above a state or federal guideline. Findings reveal how PFAS transport and transformation characteristics shift with land-applied biosolids. The hybrid mechanistic-MLBN model outperformed the mechanistic model in ranking wells in order of health risk.
Conclusions: PFAS are difficult and costly to measure and the accuracy of traditional modeling methods is uncertain. Data and modeling insights from this research will improve PFAS exposure prediction, particularly in rural areas where data are limited and private well owners are responsible for monitoring and managing their own risks.

S11 | New research on early life fluoride exposure and neurocognitive outcomes in infancy, childhood, adolescence and adulthood from United States and Canadian studies

A. J. Malin[1], **K. W. Taylor**[2]

[1] *University of Florida, Department of Epidemiology, College of Public Health and Health Professions; College of Medicine, Gainesville, Florida, United States of America*
[2] *National Institutes of Health (NIH), Division of Translational Toxicology, National Institute of Environmental Health Sciences, National Institutes of Health, Research Triangle Park, North Carolina, United States of America*

Fluoride exposure is ubiquitous in North America with 72% of United States (US) and 39% of Canadian communities on public water systems receiving fluoridated drinking water (recommended level of 0.7 mg/L) for dental caries prevention. Other sources of fluoride include foods and beverages reconstituted with fluoridated water, black tea, foods sprayed with fluoride-containing pesticides, some seafood, and certain pharmaceuticals. The potential for chronic low-level fluoride exposure to contribute to neurotoxicity, particularly during critical periods of brain development has received widespread attention recently. This awareness was spurred in part by the publication of the National Toxicology Program's (NTP's) Monograph in August 2024, followed later by an associated meta-analysis by Taylor et al. in January 2024. Both reported consistent inverse associations between fluoride exposure and children's IQ. The monograph concluded that these associations occurred at water fluoride levels above the World Health Organization's safe level of 1.5 mg/L; however, associations at lower water fluoride levels remain unclear. The monograph highlighted the immediate need for US-based studies, studies focused on lower exposure levels, and studies on neurocognitive effects in adults. One US-based study on prenatal fluoride exposure and child neurobehavior in Los Angeles, California, published by Malin et al. in May 2024, after the monograph's literature cut-off date, found that higher maternal urinary fluoride was associated with more neurobehavioral problems at age 3. Since then, other studies on chronic low-level fluoride exposure and neurocognitive outcomes in US and Canadian populations have been conducted. The following symposium presents preliminary data from the latest studies on this topic. These studies include various fluoride exposure measures such as maternal and child urinary fluoride, toenail fluoride, and dental fluorosis, with exposure windows spanning prenatal development and childhood. Outcomes include infant and child neurodevelopment, child sleep behaviors, adolescent neurological function assessed via neuroimaging and neurobehavior, and adult depression.

S11-01 | Prenatal fluoride exposure and infant motor development in Los Angeles, California

Durdana Khan[1], Sandrah P. Eckel[2], Ixel Hernandez-Castro[3], Howard Hu[2], Tingyu Yang[2], Shohreh F. Farzan[2], Carrie V. Breton[2], Theresa M. Bastain[2], Ashley J. Malin[1]

[1] University of Florida, Epidemiology; College of Public Health and Health Professions; College of Medicine, Gainesville, USA; [2] University of Southern California, Department of Population and Public Health Sciences, Keck School of Medicine, Los Angeles, USA; [3] Stanford University, Department of Epidemiology and Population Health, Stanford Medicine, Stanford, USA

Background: Chronic prenatal fluoride exposure, at United States (US) population relevant levels, has been associated with worse child neurodevelopment; however, to our knowledge, no US-based study has examined these associations in infants. This study investigated associations between maternal urinary fluoride during pregnancy and infants' motor developmental outcomes among mother-child pairs in Los Angeles, California.

Methods: Participants were from the Maternal and Developmental Risks from Environmental and Social Stressors (MADRES) cohort. MADRES is a prospective pregnancy and birth cohort of predominantly Hispanic women with low income living in urban Los Angeles. Specific gravity-adjusted maternal urinary fluoride (MUFsg) in the third trimester was used as a biomarker of fluoride exposure. Infant motor development at 6, 9, 12, and 18-months was assessed with the Ages and Stages Questionnaire-3 (ASQ). Fine and gross motor development scores were reverse coded, such that higher scores indicate worse performance. Longitudinal negative binomial regression examined associations between MUFsg and ASQ scores adjusted for maternal age, race, income, BMI, child sex and corrected age at ASQ assessment.

Results: Among 347 mother child pairs, the median (IQR) MUFsg was 0.79 (0.54) mg/L. In longitudinal models, higher third trimester MUFsg was marginally associated with higher gross motor score (IRR=1.13, 95% CI: 0.99, 1.29, p=0.06), and higher fine motor score (IRR=1.13, 95% CI: 1.00, 1.27, p=0.05), indicating worse performance. There was no evidence for associations with other ASQ scores [problem solving (IRR=0.99, 95% CI: 0.82,1.19), personal-social score (IRR=1.01, 95% CI: 0.86,1.19), communication score (IRR=0.94, 95% CI: 0.79,1.12)].

Conclusions: Prenatal fluoride exposure may be associated with developmental neurotoxicity manifesting in infancy. Additional studies are needed to further explore this association in different regions and across the nation.

S11-02 | Prenatal Fluoride Exposure and Neuropsychological Outcomes in the New Hampshire Birth Cohort Study

Carly V. Goodman[1], Margaret Karagas[2], Janet Peacock[2], Susan Korrick[3], David Flora[1], Bruce Lanphear[4], E. Angeles Martinez-Mier[5], Guillermo Tamayo-Cabeza[5], Gina Castiblanco[5], Frank Lippert[5], Christine Till[1]

[1] York University, Department of Psychology, Toronto, Canada; [2] Dartmouth College, Department of Epidemiology; Geisel School of Medicine, Hanover, USA; [3] Harvard University, Harvard T.H. Chan School of Public Health and Brigham Women's Hospital, Boston, USA; [4] Simon Fraser University, Faculty of Health, Burnacy, Canada; [5] Indiana University, Indiana University School of Dentistry, Indianapolis, USA

Background: Fluoride has been associated with deficits in intelligence, and increased externalizing and internalizing problems, but the neuropsychological domains most sensitive to fluoride remain unclear.

Objective: We examined whether prenatal fluoride exposure is associated with children's neuropsychological outcomes in the New Hampshire Birth Cohort Study (NHBCS).

Methods: Our analysis included 358-409 mother-child dyads from the NHBCS. Fluoride was measured in toenails collected at ~24-28 weeks' gestation (referred to as 24-week toenail fluoride; 24WTF) and ~6 weeks postpartum (6WPTF), reflecting exposures in early- to mid-pregnancy. Children's cognition, motor function, and behaviour were assessed at age 5 years. Generalized estimating equations were applied to estimate the association between toenail fluoride and neuropsychological outcomes, modeling behavioural measures with odds ratios for T-scores≥60. Covariates included child sex, maternal education, maternal age, and study site. Effect modification by child sex was evaluated.

Results: Median (IQR) µg/g fluoride concentration were 2.77 (1.90) for 24WTF and 2.13 (1.42) for 6WPTF.In a complete case analysis,24WTF was associated with higher odds of depression in girls (OR=1.82 [1.19, 2.79]), but not boys (p-for-interaction=0.007). 24WTF was associated with elevated odds of hyperactivity (OR=1.20 [1.01, 1.43]) and externalizing problems (OR=1.39 [1.15, 1.67]) in both sexes; a similar pattern was observed with 6WPTF. Associations differed between toenail sampling times and child sex for working memory (p-for-interaction=0.044); among girls, 24WTF was associated with decreased working memory (β=-1.24, [–2.40, –0.09]); no association was observed for boys or for 6WPTF in either boys or girls. A 6WPTF-by-sex interaction was observed for fine motor precision (p-for-interaction=0.032), with higher fluoride related to lower fine motor precision among boys (β=–0.35 [–0.70, 0.01]), but not girls.

Conclusions: Preliminary findings from this U.S. cohort suggest that prenatal fluoride exposure is associated with sex- and domain-specific effects on children's neuropsychological outcomes; however, further analyses are needed.

S11-03 | Prenatal fluoride exposure and child sleep behaviors in a nationally representative Canadian cohort

Zhilin Wang[1], Christine Till[2], Meaghan Hall[2], Jana El-Sabbagh[2], Gina Muckle[3], Jean Séguin[4], Linda Booij[5], Khrista Boylan[6], Constadina Panagiotopoulos[7], Katherine Morrison[8], Maryse Bouchard[9], Bruce Lanphear[10], Ashley J. Malin[1]

[1] University of Florida, Epidemiology; College of Public Health and Health Professions; College of Medicine, Gainesville, USA; [2] York University, Psychology; Faculty of Health, Toronto, Canada; [3] Université Laval, psychology; Faculty of Social Sciences, Québec, Canada; [4] Université de Montréal, psychiatrie et d'addictologie; Faculté de médecine, Montreal, Canada; [5] McGill University, Psychiatry; Faculty of Medicine and Health Sciences, Montreal, Canada; [6] Mcmaster University, Psychiatry & Behavioural Neurosciences; Faculty of Health Science, Hamilton, Canada; [7] University of British Columbia, Pediatrics; Faculty of Medicine, Vancouver, Canada; [8] McMaster University, Pediatrics; Faculty of Health Sciences, Hamilton, Canada; [9] Institut National de la recherche scientifique, Armand-Frappier Santé Biotechnologie Research Centre, Laval, Canada; [10] Simon Fraser University, Health Science; Faculty of Health Science, Burnaby, Canada

Objective: Fluoride accumulates in the pineal gland, which produces melatonin, the hormone that regulates sleep. We examined associations between maternal fluoride exposure during pregnancy and sleep patterns in Canadian children.

Materials and Methods:

Participants included mother-child pairs from the Maternal-Infant Research on Environmental Chemical (MIREC) cohort. We measured urinary fluoride concentrations (UFC; mg/L) in spot urine samples collected during each trimester and adjusted for specific gravity (n= 433). Water fluoride concentrations (WFC; mg/L) were ascertained from municipal water treatment reports (n=370). Covariate-adjusted linear and logistic regression analyses examined associations between fluoride exposures and sleep behaviors self-reported by the child (n=421 for UFC; n=361 for WFC) and accelerometer-derived sleep outcomes in a subsample (n=89 for UFC; n=74 for WFC). Covariates included mothers' education level, marital status, race, annual household income, age, urbanicity, and child's sex and age; we also included city for models using UFC as a predictor.

Results:

Children's ages ranged from 7 to 13 years. Median (interquartile range; IQR) UFC and WFC were 0.50 (0.39) mg/L and 0.52 (0.52) mg/L, respectively. Each 1-IQR increase in WFC was associated with a 10-minute later self-reported weekday bedtime (B = 10.40, 95% CI: 0.83, 19.97, p=0.04), and 1.73 times theodds of snoring compared to not snoring (OR = 1.73, 95% CI: 1.04, 2.88, p=0.04). Each IQR increase in UFC was associated with a 14-minute later bedtime (B=13.57,95%CI: 2.05, 25.09, p = 0.02), an 11-minute later child sleep midpoint (B=11.42,95%CI: 1.13, 21.72, p = 0.03), and a trend of later wake-up time (B=9.25,95%CI:-1.42, 19.92, p = 0.09) all assessed via accelerometry.

Conclusion:

In this Canadian cohort, children with higher prenatal fluoride exposure had later bedtimes, later sleep midpoint, and higher odds of snoring. Studies employing polysomnographic assessment of sleep are needed further to explore potential effects of fluoride on sleep patterns.

S11-04 | Urinary Fluoride Concentration is Associated with Structural Changes in the Adolescent Brain

Kim M. Cecil[2], Kelly J. Brunst[1]

[1] University of Cincinnati, Environmental Health; College of Medicine, Cincinnati, USA; [2] University of Cincinnati, Departments of Radiology, Pediatrics, Environmental and Public Health Sciences, Cincinnati, USA

Despite the emerging evidence of fluoride's adverse impact on neurobehavioral outcomes, the impact on brain structure is unclear. Using magnetic resonance imaging (MRI), we examined the relationship between childhood urinary fluoride and brain structure, featuring morphometrics of brain volume, cortical thickness and curvature, while also exploring sex effects.

Data from participants (n=329, median 12.1 years (range 11-14.7), 166 female) enrolled in the Cincinnati Combined Childhood Cohorts (C4) Study collected at the 12-year study visit were used for this study. Dilution-corrected childhood urinary fluoride (CUF) concentrations were determined, adjusted for specific gravity and log-transformed. High-resolution, three dimensional T1-weighted images were collected using a 3 Tesla MRI scanner and analyzed with FreeSurfer software to determine whole and subcortical brain volumes, with the cortex parcellated to determine volume, thickness and curvature measurements. Each regional volume, cortical thickness and curvature metric was compared to CUF using covariate-adjusted general linear models including participant sex, maternal age at delivery, household income, neighborhood deprivation index score, maternal depression score, blood lead, household smoking status and total intracranial volume. Sex-interaction and stratified models were developed. The models were adjusted for multiple comparisons.

CUF concentration was 1.12 $\pm$ 0.72 (range 0.31 – 7.78) mg/mL. Increases in CUF were associated with greater cortical thickness among several brain regions including the left inferior temporal gyrus, left pars orbitalis, right middle temporal gyrus and right fusiform gyrus in females. Associations of CUF and measures of cortical curvature were observed in males. However, no associations with CUF and volume metrics were observed.

Significant fluoride associated cortical thickness changes were observed in regions implicated in perception, language processing and social cognition, which may reflect delays in brain maturation. Understanding the impact on neurodevelopment is essential to protecting pediatric health and making informed decisions about appropriate fluoride exposure in public health settings.

S11-05 | Dental Fluorosis and Depression Among Young Adults in the United States

Angela M. Miles[1,2], Durdana Khan[2], Alexandra Mattia[2], Zhilin Wang[2], Ashley J. Malin[2]

[1] University of Florida, Rehabilitation Science; College of Public Health and Health Professions, Gainesville, USA; [2] University of Florida, Department of Epidemiology, College of Public Health and Health Professions, College of Medicine, Gainesville, USA

Objective

Fluoride exposure can contribute to depressive symptoms in animals, even at low levels; however, to our knowledge, no human studies have examined this association. We investigated associations between dental fluorosis (a proxy for excess fluoride exposure during tooth development) and depression in young adults in the United States (US).

Materials and Methods

The study included 886 adults aged 18-29 years from the National Health and Nutrition Examination Survey (NHANES) 2015-2016. Fluorosis was assessed using the Dean's Fluorosis Index (DFI). Participants with a DFI score ≥ 1.0 had fluorosis ranging from very mild to severe; we classified those with a DFI ≤ 0.5 (i.e., normal/questionable fluorosis) as not having fluorosis. Depression was measured using the Patient Health Questionnaire (PHQ-9). We classified participants as having major depression if they scored ≥10 and satisfied certain clinical criteria. We also classified depression severity as no depression (score < 5), minimal symptoms/mild depression (score 5-14), and moderately severe/severe depression (score >=15). Survey-weighted and covariate-adjusted binomial and multinomial logistic regression examined associations of dental fluorosis with presence and severity of depression, respectively. Covariates included age, gender, race/ethnicity, BMI, income, and serum cotinine.

Results

Participants were 24 years-old on average and males and females were evenly distributed. Approximately 70% of participants had dental fluorosis and 5% reported currently experiencing major depression. Participants with dental fluorosis were 1.8 times more likely to report current major depression compared to those without fluorosis (OR=1.78; 95% CI: 1.03, 3.07; *p*=0.04). Dental fluorosis was not associated with severity of depressive symptoms (OR=1.23, 95% CI: 0.56, 2.69; *p*=0.59 for minimal symptoms/mild depression; OR=1.03,95% CI: 0.27, 3.98; *p*=0.96 for moderately severe/severe depression, compared to no depressive symptoms).

Conclusion

Excess fluoride exposure during tooth development is associated with higher odds of major depression in adulthood. Prospective studies are needed to further examine this association

S12 | Health Impacts of Firefighting-Related Chemical Exposures

K. Hoffman[1], H. Stapleton[1], B. Ormond[2]

[1] *Duke University, Durham, North Carolina, United States of America*
[2] *North Carolina State University, Raleigh, North Carolina, United States of America*

Firefighting is a hazardous profession involving intense physical demands, disrupted sleep, cardiovascular stress, and exposure to high temperatures, all of which may impact health. Firefighters face elevated exposure to combustion byproducts like polycyclic aromatic hydrocarbons (PAHs) and synthetic chemicals in building materials, vehicles, and equipment. In addition, flame retardants and per- and polyfluoroalkyl substances (PFAS) are prevelent in turnout gear, firehouses and fire engines.

Assessing environmental mixtures is challenging and more complex for firefighters, whose chemical exposures vary across fire types and tasks. Conventional exposure tools, such as wearable air samplers, are impractical during firefighting. Innovative methods like passive silicone samplers (wristbands, dog tags) and gear wipes are emerging as alternatives to evaluate exposures.

The International Agency for Research on Cancer (IARC) recently classified firefighting as a carcinogenic occupational exposure (Class 1). Epidemiological studies link firefighting to cancers like mesothelioma and bladder cancer and suggest associations with colon, prostate, and testicular cancer. Research has also correlated firefighting exposures with reduced lung function, allergic airway sensitization, cardiovascular issues, and long-term diseases like cardiovascular disease. However, these findings are often based on cross-sectional studies with limited hazard characterization.

This symposium will highlight research on firefighters' exposure to chemical mixtures and related health outcomes. Presentations will address novel exposure assessment tools, explore occupational exposure levels, and discuss potential links to health impacts. Understanding these exposures is critical for protecting firefighters' health and developing strategies to mitigate occupational risks.

S12-01 | Effects of Intermittent Fasting and Moderate Physical Activity on Epigenetic Age in Firefighters: a Randomized Controlled Trial

James Hollister, Reagan Conner, Jefferey Burgess, Melissa Furlong

University of Arizona, Tucson, USA

Objective

There is increasing evidence supporting the benefits of intermittent fasting and low-intensity, moderate aerobic physical activity, also known as zone 2 activity. However, the effects of intermittent fasting and zone 2 activity on cellular aging rates are unclear. Accelerated cellular aging has previously been shown to be predictive of several adverse health outcomes, include an increase in all-cause mortality. Firefighters are a population of particular interest for accelerated cellular aging, as the occupational exposures of firefighting are classified as a group 1 carcinogen.

Materials and Methods

In this randomized controlled trial, we explored the effects of these activities on epigenetic aging. 310 and 127 active firefighters from 25 departments across Arizona were enrolled into the intermittent fasting arm and the zone 2 arm of the Firefighter Collaborative Research Project from 2023-2024. Once enrolled, participants collected a pre-study blood sample and were randomized into an intervention or control group in a 1:1 ratio. Individuals in the intermittent fasting intervention were asked to fast 14 to 16 hours/day for four days/week, for 4 months. Individuals in the zone 2 intervention group were asked to complete four 45-minute zone 2 workouts each week, for 4 months. All participants completed weekly surveys, and participants in the zone 2 arm wore a fitness tracking device. At the end of the 4-month study period, individuals collected a post-study blood sample. DNA methylation analysis using Illumina’s V2 chip was conducted on the pre- and post-study blood samples, and epigenetic clocks were calculated.

Results

Differences in the changes from pre- to post-study blood collection between the intervention and control groups were assessed.

Conclusions

The results from this trial will advance our knowledge on the effect of intermittent fasting and zone 2 physical activity as a method to reduce accelerated cellular aging.

S12-02 | PFAS Levels and Differential Proteomics in Firefighters and Other Front-Line Workers

Melissa Furlong, Tuo Liu, James Hollister, Shawn Beitel, Jefferey Burgess

University of Arizona, Tucson, USA

Objective Per- and Poly-fluoroalkyl substances are ubiquitous in our environment, with wide ranging health effects. Firefighters and other frontline workers experience increased occupational exposures to PFAS. Here, we adopt an untargeted proteomics analysis to evaluate biological and health effects of PFAS across the proteome in these workers.

Material and Methods From 2020-2022, 5,793 frontline workers in Arizona and other states were enrolled into the HEROES Study (Healthcare, Emergency Response, and Other Essential Workers). Of these, 1,084 enrollment samples were sent for PFAS (CDC-NCEH) and proteomics analysis (SOMAscan). We evaluated associations between 17 PFAS species, along with total PFOA and PFOS, using linear regression and adjusting for sex, occupation, smoking, marital status, education, age, COVID status, and geographic region, with a Bonferroni adjustment for multiple testing. We used gene set enrichment analysis and the KEGG and Human Phenotype Ontology (HPO) databases for pathway analysis.

Results: Participants were mostly female (64%), on average 45 years of age, highly educated (84% with a college degree), with good distribution across six states. We observed associations for several PFAS with 44 different proteins. One protein, ferritin light chain, was positively associated with 14 PFAS at Bonferroni significance, and another, hepcidin antimicrobial peptide, was positively associated at Bonferroni significance with 6 individual PFAS and with both total PFOA and total PFOS. 34 pathways were enriched, including several related to ferritin and iron transport, glycolysis and amino acid metabolism, insulin signaling, and xenobiotic metabolism. Most pathways (22) were enriched for PFHPA.

Conclusion: PFAS may disrupt several biological processes as reflected by the proteome, including ferritin and iron processes, immune response, and amino acid metabolism. Future analyses will use variable selection techniques to identify specific PFAS drivers and evaluate long-term outcomes.

S12-03 | Serum PFAS and Associations with Thyroid Hormones in Firefighters

Kate Hoffman, Mackenzie Laney, Sharon Zhang, Daniel Ehrlich, Candance Van Vleet, Eve Marion, Alexander Beste, Heather M. Stapleton

Duke University, Durham, USA

Per- and polyfluoroalkyl substance (PFAS) exposure in firefighters is a growing concern due to its potential impact on thyroid health. Firefighters face elevated PFAS exposure from turnout gear and aqueous film-forming foam, which may contribute to thyroid hormone disruption. In this study, we assessed current PFAS exposure and thyroid hormone levels among 145 firefighters enrolled in the North Carolina Firefighter Cancer Cohort Study (FFCCS).

Among the 52 PFAS analyzed in blood serum, 45 were detected in at least one sample, and 13 were detected in at least half of the samples. PFAS levels for commonly detected compounds were generally comparable to those in the

general U.S. population (NHANES), except for perfluorohexanesulfonic acid (PFHxS), which was slightly elevated in firefighters. Trifluoroacetic acid (TFA) was detected at the highest concentrations (median: 19.3 ng/mL); however, TFA has not been widely measured, limiting direct comparisons with other populations.
Thyroid stimulating hormone (TSH) and free thyroxine (FT4) were measured in serum samples. While most firefighters had levels within the normal range, higher levels of perfluorooctanoic acid (PFOA) and perfluorononanoic acid (PFNA) were associated with increased FT4. For example, firefighters in the highest PFNA exposure tertile had FT4 levels that were 0.08 ng/dL higher (95% CI: 0.02–0.14, p = 0.01) than those in the lowest tertile, suggesting a potential thyroid-disrupting effect.
These findings suggest a potential link between PFAS exposure and thyroid hormone disruption in firefighters, highlighting the need for further research on occupational PFAS exposure and its health effects.

S12-04 | Evaluating Fire Investigator PPE Effectiveness against PAH Exposure During Structure Fire Investigations

Mark Gaskill

North Carolina State University, Raleigh, USA

While modern firefighters have made strides in exposure mitigation through improved PPE and procedure, fire investigators remain at significant risk due to prolonged exposure at fire scenes and often ineffective PPE. This study aimed to measure fire investigators' PAH exposure during controlled structure fires using silicone samplers. Samplers were placed inside and outside of ensembles providing locational measures of PAH exposure and a determination of PPE efficacy. Six different ensembles were selected for evaluation, each with four replicate tests using experienced members of the fire service as test subjects. Each control burn was conducted using fully furnished ‘burn cells’, reusable structures that are burned to flashover to simulate a typical fire scene. Subjects performed three 30-minute investigation sessions inside the burn cells, with two separate 15-minute breaks. The silicone samples were extracted and analyzed using GC-HRMS, focusing mainly on PAH compounds. The internal and external exposures were used to identify the reduction in dermal exposure created by each ensemble. In turn, this was used to calculate the protective performance of each ensemble. All the data from this research will be used to provide fire investigators with insight into the effectiveness of currently fielded PPE and to help researchers and manufacturers understand future improvements in PPE that can be used to reduce fire investigator exposure at the fire scene.

S12-05 | Airborne contaminants encountered during electric vehicles fires and suppression operations

Richard Kesler

Fire Safety Research Institute, Urbana-Champaign, USA

This presentation examines the unique airborne hazards released during electric vehicle fires, focusing on the chemical contaminants generated around and above the vehicles. The study examines contamination throughout the fire development and during manual suppression operations by firefighters. Full scale fire tests were conducted with both electric and internal combustion engine vehicles utilizing both active and passive sampling methodologies to examine volatile organic compounds, polycyclic aromatic hydrocarbons, heavy metals, acid gases, and inhalable particulate. Strategies for exposure mitigation will also be discussed.

S12-06 | Occupational and Non-Occupational Predictors of PFAS Concentrations in Firefighters

Reagan Conner, James Hollister, Melissa Furlong, John Gulotta, Jefferey L. Burgess

University of Arizona, Tucson, USA

[BACKGROUND AND OBJECTIVE] Exposure to per- and polyfluoroalkyl substances (PFAS) is a widespread concern among the fire service. The general population is exposed to PFAS through the environment and consumer products, and firefighters experience additional exposure through class B aqueous film-forming foam (AFFF) and other occupational sources. This study aims to evaluate both occupational and non-occupational predictors of serum PFAS concentrations.
[METHODS] Between 2023-2024, we measured serum PFAS concentrations in 2,056 United States firefighters, as part of the Fire Fighter Cancer Cohort Study (FFCCS). At the time of sample collection, a survey was administered to each participant to gather information on potential predictor variables including demographics, lifestyle behaviors, career history, and occupational exposures. To assess the relationship between predictor variables and serum PFAS concentrations, we fit linear regression models for each predictor variable and each PFAS analyte, adjusting for age, gender, and department.
[RESULTS] Participants were mostly male, non-Hispanic white, with an average age of 40. Occupational characteristics associated with PFAS concentrations included current use of AFFF (11%; [2%, 21%]) for PFOA), performing on-scene decontamination after fires (-9% [-16%, -1%] for PFOA), and bottled water consumption while at work (-14% [-19%, -9%] for PFOS, -11% [-16%, -6%] for PFOA, and -21% [-27%, -15%] for PFHxS). Non-occupational characteristics included water source at home (well water: 19% [8%, 31%] for PFOS, bottled water: -8% [-13%, -2%] for PFOA), and history of blood or plasma donation (blood: -5% [-10%, 0%] for PFOS, plasma: -17% [-29%, -2%] for PFOA, -32% [-46%, -16%] for PFHxS, and -22% [-35%, -8%] for PFOS.
[CONCLUSION] These findings provide important insight into predictors of PFAS concentrations and identifies possible ways to reduce PFAS exposures.
This study was funded by the Arizona Board of Regents.

S13 | Health-based interpretation and communication of human biomonitoring data

T. Pollock[1], **S.F. Nakayama**[2]

[1] *Health Canada, Environmental Health Science and Research Bureau, Ottawa, Ontario, Canada*
[2] *National Institute for Environmental Studies, Japan Environment and Children's Study Programme Office, Tsukuba, Japan*

Human biomonitoring – the direct measurement of how much of a chemical or its metabolites or breakdown products is present in a person – is one of the most important instruments in exposure science. The health-based interpretation and communication of human biomonitoring data is paramount for its effective use in risk assessment, risk management, and public health policy, and to advance research on the associations between environmental chemical exposures and adverse health outcomes. The development of human biomonitoring health-based guidance values (HB2GVs) by regulatory agencies and researchers has facilitated the use of human biomonitoring data to understand the potential impacts of chemicals on human health. HB2GVs allow for more direct comparisons between environmental chemical levels measured in people and exposure thresholds derived from toxicological studies, such as Tolerable Daily Intakes (TDIs) and Reference Doses (RfDs).

Given the value of HB2GVs as a tool to assist with the health-based interpretation and communication of human biomonitoring data, the International Society of Exposure Science (ISES) International Human Biomonitoring Guidance Value (i-HBM) Working Group developed the Human Biomonitoring Health-Based Guidance Value (HB2GV) Dashboard (https://www.intlexposurescience.org/i-hbm/) as a platform to easily access available HB2GVs. The dashboard contains pertinent information for over 600 HB2GVs derived by regulatory agencies and researchers. In this symposium hosted by the i-HBM Working Group, we aim to highlight ongoing work that involves the derivation of novel HB2GVs and their use for interpreting HBM data in research, policy, and clinical contexts.

S13-01 | Derivation of health-based HBM guidance values for the UV filter homosalate

Daniel Bury, Katharina E. Ebert, Holger M. Koch

Ruhr University Bochum, Institute for Prevention and Occupational Medicine of the German Social Accident Insurance (IPA), Bochum, Germany

Homosalate (3,3,5-trimethylcyclohexyl salicylate, HMS) is a salicylic acid derivative and a technical mixture of *cis*- and *trans*-isomers (*cis*- and *trans*-homosalate). It is a UV filter used worldwide in sunscreens and other personal care products with maximum concentrations of 15% in the U.S. and, until recently, 10% in the EU. HMS use in the EU has now been restricted to face products, due to nephrotoxicity observed in an OECD guideline no. 422 study in rats (combined repeated oral dose toxicity study with reproduction/developmental toxicity screening test).
In our previous human toxicokinetic studies of HMS we identified specific exposure biomarkers for *cis*- and for *trans*-HMS, i.e., HMS-CA5 ((1*R*,3*S*,5*S*)-/(1*S*,3*R*,5*R*)-3-((2-hydroxybenzoyl)oxy)-1,5-dimethylcyclohexane-1-carboxylic acid) and tHMS-CA ((1*R*,5*S*)-/(1*S*,5*R*)-5-((2-hydroxybenzoyl)oxy)-3,3-dimethylcyclohexane-1-carboxylic acid), respectively. These biomarkers can be sensitively quantified in urine for several days after single sunscreen application. After oral dose, urinary excretion fractions (F_{UE}) were 0.0097% (0.0066-0.012%, n=4) for HMS-CA5 and 1.0% (0.76-1.3%, n=4) for tHMS-CA. Very similar metabolite ratios (including the parent HMS isomer and further metabolites) after oral dose and after sunscreen use demonstrated the applicability of these F_{UE}s for exposure assessment for both oral and dermal uptake.
In analogy to the assessment by the European Scientific Committee on Consumer Safety, we chose the LOAEL of 60 mg/(kg bw · d) from the OECD guideline study as point of departure (POD). With an overall assessment factor of 600 (factor 3 for NOAEL-to-LOAEL extrapolation, factor 2 for sub-chronic-to-chronic extrapolation, and default factors for intra- and interspecies differences of 10) we propose TDI-analogous HBM guidance values of 0.16 µg/L HMS-CA5 and 3.7 µg/L tHMS-CA in urine for the HBM-based risk assessment of HMS.

S13-02 | Selection of biological guidance values for occupational health purposes: Development of a decision diagram

Philippe Sarazin[1,2], Maude Pomerleau[1], Sabrina Gravel[1,2], Sébastien Gagné[1], Nolwenn Noisel[2]

[1] Institut de recherche Robert-Sauvé en santé et en Sécurité du travail (IRSST), Chemical and Biological Hazard Prevention, Montréal, Canada; [2] Université de Montréal, School of Public Health, Montréal, Canada

Background
Occupational health organizations propose biological guidance values (BGVs) for various substances, often resulting in several BGV candidate values for the same chemical.
Objectives
We aim to 1) synthesize the fundamental scientific and technical elements considered for BGV selection in occupational settings, and 2) to develop a decision diagram for a reproducible and evidence-based BGV selection approach for occupational biomonitoring.
Methods

Nine scientific and grey literature databases were consulted for this systematic review, using the concepts Derivation, Biological value, and Workplace. Renown occupational health and safety organizations' websites were also consulted. Studies and documents from high-income countries, focusing on the derivation of BGVs in workplaces, were included. Analytical methods validation, biomarker measurements or general population studies were excluded. Study selection was performed by two authors, and data extraction was done by one author with 25% validated by another. The compiled relevant BGV derivation criteria were analysed by a team of occupational toxicology experts for the development of the decision diagram.

Results

The search yielded 118 studies, of which 24 were retained, spanning from 1997 to 2023 and mostly originating from Europe (n=19). Twenty-one scientific and technical elements used for BGV derivation were documented and grouped into five categories. They include: 1) the biomarker appropriateness (e.g. specificity, sensitivity; n=21), 2) the analytical method availability (n=18), 3) the sampling conditions (e.g. timing of sample collection; n=17), 4) the approach used for BGV derivation (e.g. based on an external OEL value or a toxicological endpoint; n=16), and 5) a validation that the BGV is correlated with the legislated OEL (for BGV based on an external value; n=19). These categories were used to shape the diagram.

Conclusions

Next steps include testing the diagram with priority substances and validating with stakeholders. This diagram will facilitate further BGV updates and will be useful to other organizations.

S13-03 | Exceedance of health-based guidance values in the German adult population – results from the German Environmental Survey for Adults 2023–2024 (GerES VI)

Marike Kolossa-Gehring, Philipp Zimmermann, Petra Apel, Aline Murawski

German Environment Agency, Toxicology, Health-related Environmental Monitoring, Berlin, Germany

The German Environmental Survey (GerES) is a population-representative examination and interview survey repeatedly conducted since the mid-1980s. GerES VI (2023-2024) human biomonitoring (HBM) data were collected from a sample of the adult general population living in Germany. HBM of GerES VI comprises more than 100 exposure biomarkers already measured and a hundred more biomarkers are to be expected in the near future. Biomarkers were included because of their toxicological properties, to control success of regulatory measures, to verify expected non-occurrence of banned substances, and to identify further needs for actions to protect the whole population or sub-groups by targeted measures. Health impacts were interpreted by use of toxicologically and epidemiologically derived health-based Human Biomonitoring values (HBM values) indicating above which levels health impacts can no longer be excluded (HBM-I) or have to be expected (HBM-II). Currently for 24 biomarkers HBM values exist, additional ones equivalent to HBM-I are in preparation by the German Human Biomonitoring Commission and also in the European Partnership for the Risk Assessment of Chemicals (PARC). Exceedance of HBM values were observed for the plasticizers DEHTP and DiBP, the UV-filter EHS, cadmium, mercury, PFOS, PFOA, and the bisphenols A und S. Most exceedances ranged between 0.1 and 10% of the samples, for bisphenol A it was the vast majority of participants. Only PFOS levels exceeded the HBM-II level in 0.3% of the samples. Given the population-representativity, these exceedances concern extrapolated between some thousand and some million people in Germany. Different from former studies no exceedances were observed for the plasticizers DEHP, BBzP, DnBP, DINCH in a single substance assessment. Mixture risk assessments are under work.

GerES VI again proves health-relevant exposure of the general population. This underlines the need for a continuous HBM of the general population and for international cooperation to share burdens and promote progress.

S13-04 | Comparison of human biomonitoring data from the Canadian Health Measures Survey with health-based guidance values

Subramanian Karthikeyan, **Tyler Pollock**, Annie St-Amand

Health Canada, Environmental Health Science and Research Bureau, Ottawa, Canada

Objective: We conducted a direct comparison of the biomonitoring data collected as part the Canadian Health Measures Survey (CHMS) to human biomonitoring health-based guidance values (HB2GVs) for 43 chemicals including metals, per- and polyfluoroalkyl substances (PFAS), pesticides, plasticizers, flame-retardants, and volatile organic compounds.
Material and methods: We analyzed 15 chemicals for the first time using recently published HB2GVs such as zinc, lithium, some chlordane isomers, and toxaphene. Our previously reported assessment for 28 chemicals was updated with more recent data from individual or pooled samples collected as part of CHMS cycles 5 (2016-2017) and 6 (2018-2019). We calculated Hazard Quotients (HQs) as the ratio of the average concentrations [geometric mean (GM) or arithmetic mean (AM)] or upper concentrations [95th percentile (P95) or maximum] of a chemical to an HB2GV.
Results: HQs at P95 exceeded 1.0 for inorganic arsenic, 3-PBA, PFAS and xylenes in the general population, and for acrylamide, cadmium and benzene in smokers. HQs at the mean concentrations for the total population, by age group or by smoking behaviours were below 1.0 for all chemicals. For bisphenol A, diethyl phthalate, dioxins and furans, PFOS and PFOA, HQs calculated based on more recent CHMS data were lower than those reported earlier, consistent with a temporal decline in concentrations of these chemicals.
Conclusion: This screening analysis included for the first time data from pooled samples for many persistent organic compounds. It shows the importance of continued biomonitoring of chemicals in Canada to monitor for potential exceedances of HB2GVs over time and to identify disproportionately impacted populations.

S13-05 | Electronic PFAS blood test reporting in Maine: Considerations for public health oversight and case follow-up

Emily Pennoyer, Thomas Simones, Rebecca Lincoln, Andy Smith

Maine Center for Disease Control and Prevention, Toxicology and Environmental Epidemiology Program, Augusta, USA

Agricultural communities impacted by land spreading of biosolids can be exposed to high levels of PFAS through multiple exposure pathways. In Maine, statewide investigations have identified high soil concentrations of PFAS in farm fields with a history of biosolids application and contamination of nearby private wells, with some wells reporting PFAS levels in the hundreds to thousands of parts per trillion. Elevated PFAS levels have also been measured in freshwater fish, turkey, and deer near impacted fields, prompting consumption advisories for people eating sport-caught fish and game. Blood testing can provide information about potential exposures from these various pathways

and can help to inform health monitoring and interventions. Previously reported blood serum levels from a limited number of Maine residents with highly contaminated private wells showed PFOA and PFOS serum concentrations above 600 ng/mL and 2,000 ng/mL, respectively. In response to widespread PFAS contamination, the State of Maine will provide funding to support blood testing for individuals working on or living near contaminated farmland and establish public health oversight by making PFAS blood testing results a notifiable condition. For serum levels sufficiently above background, case follow-up can help identify possible exposure sources and inform strategies for exposure reduction. We will describe a framework for electronic PFAS blood test reporting and how toxicokinetic modeling based on PFAS concentrations measured in water, agricultural products, and fish and game can be used to identify possible exposures that could result in elevated serum levels. These serum levels will be compared to existing human biomonitoring (HBM) guidance values, including those used by the National Academies of Science, Engineering, and Medicine. Additional considerations for interpreting HBM data in this context include clinical education, supporting shared decision-making around blood testing, and helping individuals and providers understand and interpret results.

S14 | Prenatal Exposure to Endocrine Disrupting Chemicals and Maternal and Child Health in the U.S. Environmental Influences on Child Health Outcomes (ECHO) Cohort

L. Quiros-Alcala[3], J. Oh[4], J. P. Buckley[2], R. Strakovsky[1]

[1] *Michigan State University, Department of Food Science and Human Nutrition, East Lansing, Michigan, United States of America*
[2] *University of North Carolina at Chapel Hill, Department of Epidemiology, Chapel Hill, North Carolina, United States of America*
[3] *Johns Hopkins University, Department of Environmental Health and Engineering, Baltimore, Maryland, United States of America*
[4] *UC Davis, Department of Epidemiology, Davis, California, United States of America*

The Environmental influences on Child Health Outcomes (ECHO) Cohort Consortium, initiated in 2016 and funded by the U.S. National Institutes of Health, aims to understand the effects of early life environmental exposures on children's health and development. The ECHO Cohort has measured over 150 chemicals, many of which have endocrine disrupting potential during the prenatal period, a critical time for child development and maternal health. Both legacy and understudied chemicals spanning multiple chemical classes have been measured, including pesticides, flame retardants, preservatives, plasticizers, and perfluorinated and aromatic compounds. Many of these EDCs are widely detected among pregnant individuals worldwide and have been linked to adverse maternal and child health outcomes. Among pregnant ECHO participants across the U.S., widespread detection of these chemicals has been confirmed, with higher levels observed among Black and Latina participants, raising significant public health and health equity concerns. Given the pervasive exposure to these contaminants during a crucial period of fetal development, it is imperative to identify their impact on maternal and child health and associated disparities that could lead to health inequities to better understand and mitigate risks to maternal and child well-being.

The proposed symposium will discuss ongoing efforts in the nationwide ECHO Cohort to measure chemical exposures in pregnant individuals and determine their potential adverse effects on maternal and child health. Part 1 will address characterization of prenatal EDC exposures and maternal health outcomes, while Part 2 will focus on prenatal EDC exposures and associated child health outcomes. This symposium is relevant to exposure scientists, environmental epidemiologists, physicians, policymakers, and biomedical researchers with an interest in legacy and emerging chemical exposures and their impact on maternal and pediatric health and health equity. The session will foster discussions on future research directions aimed at protecting the health of vulnerable populations.

S14-01 | Variability of non-persistent chemicals in urine samples across multiple time-points during pregnancy in the ECHO Cohort

Giehae Choi[1], Xiaoshuang Xun[2], Deborah Bennett[3], John Meeker[4], Susan Schantz[5], Deborah Watkins[4], Edo Pellizzari[6], Wenlong Li[7], Kurunthachalam Kannan[7], Tracey J. Woodruff[8], Jessie P. Buckley[9], On behalf of the ECHO Cohort Consortium

[1] Johns Hopkins University, Department of Environmental Health and Engineering, Baltimore, USA; [2] Johns Hopkins University, Department of Epidemiology, Baltimore, USA; [3] University of California Davis, Department of Public Health Sciences, Davis, USA; [4] University of Michigan, Department of Environmental Health Sciences, Ann Arbor, USA; [5] University of Illinois Urbana-Champaign, Beckman Institute for Advanced Science and Technology and Department of Comparative Biosciences, Urbana, USA; [6] Research Triangle Institute, Fellows Program, Research Triangle Park, USA; [7] New York State Department of Health, Wadsworth Center, Albany, USA; [8] University of California San Francisco, Department of Obstetrics, Gynecology, and Reproductive Sciences and the Philip R. Lee Institute for Health Policy Studies, San Francisco, USA; [9] University of North Carolina at Chapel Hill, Department of Epidemiology, Chapel Hill, USA

Objective: Exposure assessment and epidemiologic studies of chemicals often use measurements from a single urine sample due to logistical and cost considerations; however, variability of biomarker levels across multiple pregnancy time-points are not well-characterized for many chemicals.

Methods: The current study comprised 30 participants each from three Environmental influences on Child Health Outcomes Cohort sites: IKIDS (Illinois), PROTECT (Puerto Rico), and NYU CHES (New York). We used urine samples collected at 2-4 pregnancy time-points to assess intra-individual variability of 156 analytes belonging to 14 chemical classes: synthetic pyrethroids (4), fungicides and herbicides (11), neonicotinoids (13), other insecticides (3), halogenated phenols (5), organophosphate esters (10), benzophenones (6), bisphenols (14), parabens (6), antimicrobials (2), phthalates/alternative plasticizers (32), PAHs(7), melamine and analogs (4), and aromatic amines (41). We modeled 49 analytes with ≥50% detection continuously and calculated intra-class correlation coefficients (ICCs); 25 analytes with 20-50% detection dichotomously (detected vs. not) and calculated kappa statistics. We conducted all analyses stratified by site.

Results: Across all 3 ECHO sites, we observed moderate-high ICCs for mono-benzyl phthalate (0.53-0.95) and 2-hydroxynaphthalene (0.33-0.43) but the results for other analytes varied substantially by site. We observed moderate-high ICCs (0.33-0.95) for most of the continuously modeled phthalates/alternative plasticizers, parabens, and antimicrobials in IKIDS and PROTECT; most aromatic amines and few phenols, phthalates/alternative plasticizers, and melamine analog in NYU CHES; while consistently low ICCs for 6-oxo-MPHP, MChP, and N-desmethyl acetamiprid. Kappa statistics for most dichotomously modeled analytes indicated high intra-individual variability. The large differences by ECHO site may reflect site-specific differences such as proximity to exposure sources and timing of urine sample collection.

Conclusion: The high intra-individual variability demonstrated in our study suggests the need to obtain serial urine samples to more reliably quantify exposures during pregnancy in future studies.

S14-02 | Diet quality and maternal pregnancy exposure to legacy and understudied endocrine disrupting chemicals in the Environmental influences on Child Health Outcomes Cohort

Diana C. Pacyga[1], Deborah H. Bennett[2], Michael S. Bloom[3], Joseph M. Braun[4], Courtney C. Carignan[5,6], Caitlin G. Howe[7], Margaret R. Karagas[7], Kristen Lyall[8], John D. Meeker[9], Melissa M. Melough[10], Wei Perng[11], Christy Porucznik[12], Lesliam Quiros-Alcala[13], Zorimar Rivera-Nunez[14], Rebecca J. Schmidt[2,15], Rita S. Strakovsky[5], Ana K. Rosen Vollmar[16], Yeyi Zhu[16], Tracey J. Woodruff[17], Jessie P. Buckley[1]

[1] University of North Carolina at Chapel Hill, Gillings School of Global Public Health, Department of Epidemiology, Chapel Hill, USA; [2] University of California Davis, Department of Public Health Science, School of Medicine, Davis, USA; [3] George Mason University, Department of Global and Community Health, College of Public Health, Fairfax, USA; [4] Brown University, Department of Epidemiology, Program in Public Health, Providence, USA; [5] Michigan State University, Department of Food Science and Human Nutrition, East Lansing, USA; [6] Michigan State University, Department of Pharmacology and Toxicology, East Lansing, USA; [7] Geisel School of Medicine at Dartmouth, Department of Epidemiology, Lebanon, USA; [8] Drexel University, AJ Drexel Autism Institute, Philadelphia, USA; [9] University of Michigan School of Public Health, Department of Environmental Health Sciences, Ann Arbor, USA; [10] University of Delaware, Department of Health Behavior and Nutrition Sciences, Newark, USA; [11] University of Colorado Anschutz Medical Campus, Department of Epidemiology and the Lifecourse Epidemiology of Adiposity and Diabetes (LEAD) Center, Colorado School of Public Health, Aurora, USA; [12] University of Utah, Department of Family and Preventive Medicine, Spencer Fox Eccles School of Medicine, Salt Lake City, USA; [13] Johns Hopkins University, Department of Environmental Health and Engineering, Bloomberg School of Public Health, Baltimore, USA; [14] Rutgers University, Department of Biostatistics and Epidemiology, Environmental and Occupational Health Sciences Institute, Piscataway, USA; [15] University of California Davis, The MIND (Medical Investigations of Neurodevelopmental Disorders) Institute, Sacramento, USA; [16] Kaiser Permanente, Kaiser Permanente North California Division of Research, Oakland, USA; [17] University of California San Francisco, Department of Obstetrics, Gynecology, and Reproductive Sciences and the Philip R. Lee Institute for Health Policy Studies, San Francisco, USA

Objective: Although diet is a source of endocrine disrupting chemicals (EDCs), Dietary Guidelines for Americans (DGA) do not consider chemicals in foods. We tested whether higher DGA adherence is associated with pregnancy EDC exposure.

Materials/Methods: Pregnant participants in the Environmental influences on Child Health Outcomes (ECHO) Cohort completed a dietary screener, food frequency questionnaire, or 24-hour recall. We calculated the Healthy Eating Index (HEI)-2015 to assess DGA adherence. We measured 113 chemical analytes in mid-gestation urines. We estimated overall, race/ethnicity-, and income-specific percent differences (β) and 95% confidence intervals (CIs) in analyte concentrations using linear mixed effects regression accounting for cohort (random effect), maternal age, educational attainment, pre-pregnancy body mass index, tobacco use, and specimen collection season and year.

Results: Most individuals (n=1487) self-identified as non-Hispanic White (40%) or Black (36%) and 50% had incomes ≥$50,000. Median (25th, 75th percentile) HEI-2015 was 61.6 points (52.8, 70.2; out of 100). Thirty-three chemical analytes were detectable in ≥70% of participants. Overall, each 10-point increase in the HEI-2015 (reflecting higher DGA adherence) was associated with lower bis(1,3-dichloro-2-propyl) phosphate (β: -8.2%; 95%CI: -15.3%, -1.1%), diphenyl phosphate (β: -9.5%; 95%CI: -15.9%, -2.9%), mono-benzyl phthalate (β: -9.7%; 95%CI: -16.8%, -2.5%), mono-isobutyl/mono-n-butyl phthalate (β: -7.4%; 95%CI: -12.5%, -2.2%), 2-hydroxynaphthalene (β: -7.5%; 95%CI: -13.5%, -1.4%), 1/9-hydroxyphenanthrene (β: -8.8%; 95%CI: -15.2%, -2.4%), and 1-hydroxypyrene (β: -6.4%; 95%CI: -11.2%, -1.5%) concentrations. Conversely, the HEI-2015 was positively associated with trans-3-2,2-dichlorovinyl)2,2-dimethylcyclopropane carboxylic acid (β: 8.2%; 95%CI: -0.5%, 17.0%), benzophenone-1 (β: 10.9%;

95%CI: 1.9%, 20.0%), benzophenone-3 (β: 18.3%; 95%CI: 8.4%, 28.3%), and ethylparaben (β: 12.9%; 95%CI: 2.8%, 23.0%) concentrations. Most associations were strongest in non-Hispanic White and higher-income individuals.
Conclusion: Higher pregnancy DGA adherence was associated with lower organophosphate ester, phthalate, and polycyclic aromatic hydrocarbon, and higher pyrethroid, benzophenone, and paraben concentrations with socioeconomic differences. Current guidelines support minimizing exposure to specific EDCs.

S14-03 | Investigating Disparities in Toxic Chemical Exposures with Neurodevelopmental Effects

Teresa Herrera[1], Kim N. Cajachagua Torres[1], Jiwon Oh[2], Lesliam Quirós-Alcalá[3], Andrea Titus[1], Jessie Buckley[4], Micheal Bloom[5], Aisha S. Dickerson[6], Deborah H. Bennet[2], John Meeker[7], Hana Sadeghi[8], Iván Díaz[1], Carol Duh-Leong[9], Leonardo Trasande[1,9], Akhgar Ghassabian[1,9]

[1] New York University Grossman School of Medicine, Department of Population Health, New York, USA; [2] UC Davis School of Medicine, Department of Public Health Sciences, Davis, USA; [3] Johns Hopkins University Bloomberg School of Public Health, Department of Environmental Health and Engineering, Baltimore, USA; [4] University of North Carolina at Chapel Hill, Department of Epidemiology, Chapel Hill, USA; [5] George Mason University, Department of Global and Community Health, Fairfax, USA; [6] Johns Hopkins University Bloomberg School of Public Health, Department of Epidemiology, Baltimore, USA; [7] University of Michigan, School of Public Health, Ann Arbor, USA; [8] Avera Research Institute, Avera McKennan Hospital & University Health Center, Sioux Falls, USA; [9] New York University Grossman School of Medicine, Department of Pediatrics Health, New York, USA

Objective: Disparities in modifiable environmental exposures may contribute to disparities in neurodevelopmental outcomes. Weaimed to identify disparities in exposure to endocrine-disrupting chemicals (EDCs) by race, ethnicity, and education while also exploring within-group differences.
Methods: We used data from 6,098 pregnant participants in the Environmental influences on Child Health Outcomes (ECHO) Cohort who provided urine samples for EDC analysis (2000-2023). We examined sociodemographic predictors of mean urinary levels of molar sums of seven EDC groups: High molecular weight (∑HMW) phthalate metabolites, low molecular weight (∑LMW) phthalate metabolites, LMW polycyclic aromatic hydrocarbons (∑PAH), ∑HMW PAH, ∑bisphenols, organophosphate esters (∑OPE), and organophosphate pesticides metabolites (∑OP). Multivariable linear regression models were used to assess differences in mean molar sum EDC concentrations between race/ethnicity and education levels and within-group differences adjusting for covariates.
Results: The mean molar sum urinary concentrations of bisphenols for participants with some college education was 18.7% higher (95%CI: 8.2, 30.3) than for those with a bachelor's degree. For LMW phthalates, higher mean molar sums were seen in participants with less education, especially those with some high school education (%difference=31%, 95%CI: 15.2, 49.0). Similar patterns were observed for HMW phthalates and all PAHs. Non-Hispanic White and Asian participants had lower HMW PAH concentrations compared to Hispanic participants. Non-Hispanic Black participants had higher levels of HMW phthalates (%difference=32.2%, 95% CI 21.0, 44.3) and LMW phthalates (%difference=74.2%, 95% CI 58.5, 91.4). All racial groups had higher OP exposure than Hispanics. Non-Hispanic Black and multiracial participants had higher OPE exposure. Among non-Hispanic White participants, differences in mean concentrations of all phthalates and LMW PAHs were observed by education level but not observed within other racial ethnic groups.
Conclusion: These findings illustrate the heterogeneity in exposure to non-persistent EDCs across social characteristics. Future research should consider whether elevated co-exposure to multiple neurotoxicants exacerbates disparities.

S14-04 | Pregnancy PFAS concentrations in association with maternal depression and perceived stress during pregnancy and postpartum

Susanna D. Mitro[1], Wei Yang[1], Assiamira Ferrara[1], Monique Hedderson[1], Max Aung[2], Himal Suthar[2], Radhika S. Raghunathan[3], Yeyi Zhu[1], Alicia Peterson[1], Morgan Reynolds[4], Kurunthachalam Kannan[4], Emily Oken[5], Margaret R. Karagas[6], Alison E. Hipwell[7], Thomas G. O'Connor[8], Courtney Carignan[9], Carrie V. Breton[2], Theresa M. Bastain[2], Lyndsay A. Avalos[1]

[1] Kaiser Permanente, Division of Research, Pleasanton, USA; [2] University of Southern California, Los Angeles, USA; [3] Johns Hopkins Bloomberg School of Public Health, Department of Mental Health, Baltimore, USA; [4] New York State Dept of Health, Wadsworth Center, Albany, USA; [5] Harvard Medical School, Department of Population Medicine, Boston, USA; [6] Dartmouth College, Geisel School of Medicine, Hanover, USA; [7] University of Pittsburgh, Department of Psychiatry, Psychology and Clinical & Translational Science, Pittsburgh, USA; [8] University of Rochester Medical Center, Departments of Psychiatry, Psychology, Neuroscience, and Obstetrics and Gynecology, Rochester, USA; [9] Michigan State University, Department of Food Science and Human Nutrition, Department of Pharmacology and Toxicology, East Lansing, USA

Background: Inconsistent evidence links per- and polyfluoroalkyl substances (PFAS) to depression with potential effect modification by social factors. We assessed associations of prenatal PFAS concentrations with prenatal and postpartum perceived stress and depression.

Methods: We analyzed concentrations of 7 PFAS in maternal plasma or serum in the Environmental influences on Child Health Outcomes (ECHO) cohort participants (n=4,403). We ascertained three outcomes: prenatal depression diagnosis (medical records or self-reported); prenatal and postpartum depressive symptom severity using a Patient-Reported Outcomes Measurement Information System Depression (PROMIS-D) t-score; and prenatal and postpartum perceived stress using a Perceived Stress Scale (PSS) t-score. Outcomes were ascertained after PFAS measurements. Linear and modified Poisson regression models assessed associations of $\log_2$-transformed PFAS concentrations with outcomes, adjusted for cohort, year, and demographic characteristics. Quantile g-computation assessed associations of the PFAS mixture with outcomes. We evaluated effect modification by nativity and Social Vulnerability Index.

Results: PFOS and NMeFOSAA were positively associated, while PFUnDA was inversely associated, with multiple outcomes. For example, each doubling of PFOS was associated with 0.13 (95%CI: -0.01, 0.28) units higher postpartum PROMIS-D t-scores and 0.28 (95%CI: -0.11, 0.67) units higher prenatal PSS t-scores. The highest (vs lowest) tertile of NMeFOSAA was associated with 1.43 (95%CI: 0.50, 2.35) units higher postpartum PROMIS-D t-scores, and 2.34 (95%CI: 1.73, 2.95) units higher postpartum PSS t-scores. In contrast, the highest (vs lowest) tertile of PFUnDA was associated with 0.91 (95%CI: -1.44, -0.37) units lower postpartum PROMIS-D t-scores and 1.98 (95%CI: -3.15, -0.81) units lower postpartum PSS t-scores. Social vulnerability and nativity appeared to modify some associations; for example, the association between PFOS and prenatal depressive symptoms appeared driven by non-U.S.-born participants.

Conclusion: Though associations were small with wide confidence intervals, results suggest that PFOS and NMeFOSAA may be associated with increased maternal depressive symptoms and perceived stress.

S14-05 | Joint effects of well-studied and understudied chemicals and psychosocial stressors on birth outcomes in the ECHO-wide cohort

Stephanie Eick[1], Patricia Brennan[2], Jessie Buckley[3], Donghai Liang[1], Xiuhong Li[4], Kayla Karvonen[5], Whitney Cowell[6], Yeyi Zhu[7], Emily Barrett[8], Alicia Peterson[7], Brennan Baker[9], Max Aung[10], Tracey Woodruff[11], MyDzung Chu[12], Christine Hockett[13], Courtney Carignan[14], Jiwon Oh[15], Anne Dunlop[16]

[1] Emory University, Gangarosa Department of Environmental Health, Atlanta, USA; [2] Emory University, Department of Psychology, Atlanta, USA; [3] Gillings School of Global Public Health, University of North Carolina at Chapel Hill, Department of Epidemiology, Chapel Hill, USA; [4] Bloomberg School of Public Health, Johns Hopkins University, Department of Epidemiology, Baltimore, USA; [5] University of California San Francisco, Department of Pediatrics, San Francisco, USA; [6] New York University Grossman School of Medicine, Departments of Pediatrics and Population Health, New York, USA; [7] Kaiser Permanente Northern California, Division of Research, Pleasanton, USA; [8] Rutgers School of Public Health, Department of Biostatistics and Epidemiology, Piscataway, USA; [9] University of Washington, Department of Environmental and Occupational Health Sciences, Seattle, USA; [10] Keck School of Medicine, University of Southern California, Department of Population and Public Health Sciences, Los Angeles, USA; [11] University of California San Francisco, Program on Reproductive Health and the Environment, Department of Obstetrics, Gynecology, and Reproductive Sciences, San Francisco, USA; [12] Tufts Medical Center, Institute for Clinical Research and Health Policy Studies, Boston, USA; [13] Avera Research Institute, Sioux Falls, USA; [14] Michigan State University, Department of Food Science and Human Nutrition, Department of Pharmacology and Toxicology, East Lansing, USA; [15] University of California Davis, Department of Public Health Sciences, Davis, USA; [16] Emory University, Department of Gynecology and Obstetrics, Atlanta, USA

Background: Psychosocial stressors may exacerbate observed associations between environmental toxicants and adverse birth outcomes. However, studies have primarily focused on well-known compounds rather than a broader range of chemicals of concern. Here we leveraged the Environmental influences on Child Health Outcomes (ECHO) cohort to assess interactions between emerging chemicals and psychosocial stressors on birth outcomes.

Methods: Pregnant women included in this analysis had a prenatal urine sample analyzed for an extensive panel of well-studied and understudied chemical analytes in 12 chemical classes, and at least one measure of prenatal psychosocial stress. Chemical analytes detected in $\geq$70% of samples were included in this analysis and were adjusted for specific gravity. Principal component (PC) analysis was used to identify clusters of exposure patterns. Linear regression was used to assess associations between individual PCs and molar sums of each chemical class with birthweight z-scores and gestational age, stratified by binary levels of adverse childhood experiences (ACEs) and prenatal perceived stress, depression, and anxiety.

Results: Our socioeconomically diverse study population comprised 1,613 participants from 12 ECHO study sites. In models stratified by ACEs, we observed that the PC reflecting high exposure to all analytes was negatively associated with birthweight z-scores only among those who experienced any ACE (e.g., β=-0.05, 95% CI=-0.10,0.00 for any ACE; β=-0.01, 95% CI=-0.06,0.03 for no ACEs). The PC reflecting high polycyclic aromatic hydrocarbons and organophosphate esters was associated with shorter gestational age at delivery only among those who experienced high levels of depression and anxiety (e.g., β=-0.18, 95% CI=-0.33, -0.03; β=-0.35, 95% CI=-0.58, -0.12, respectively). We observed no evidence of interaction by perceived stress in stratified models.

Discussion: Our findings provide some evidence that certain psychosocial stressors modify associations between chemicals and perinatal health outcomes.

S14-06 | Prenatal PFAS Exposure and Pubertal Development: Insights from the ECHO Consortium

Alicia K. Peterson[1], Xiaoshuang Xun[2], Stacey E. Alexeeff[1], Jennifer L. Ames[1], Jessie P. Buckley[3], Joseph Braun[4], Che-Jung Chang[5], Lisa A. Croen[1], Lauren B. Ellis[6], Monique M. Hedderson[1], Linda G. Kahn[7], Kurunthachalam Kannan[8], Mark Klebanoff[9], Thuy Lam[10], Donghai Liang[11], John D. Meeker[12], Susanna D. Mitro[1], Seonyoung Park[12], Lesliam Quiros-Alcala[13], Morgan Reynolds[8], Megan E. Romano[14], Ana K. Rosen Vollmar[1], Susan L. Schantz[15], Anne P. Starling[3], Julia Varshavsky[6], Yeyi Zhu[1], Assiamira Ferrara[1]

[1] Kaiser Permanente Northern California, Division of Research, Pleasanton, USA; [2] John Hopkins University, Environmental influences on Child Health Outcomes (ECHO) Data Analysis Center (DAC), Baltimore, USA; [3] University of North Carolina at Chapel Hill, Department of Epidemiology, Chapel Hill, USA; [4] Brown University, Department of Epidemiology, Providence, USA; [5] National Institutes of Environmental Health Sciences, Epidemiology Branch, Research Triangle Park, USA; [6] Northeastern University, Department of Public Health and Health Sciences, Boston, USA; [7] Grossman School of Medicine, New York University, New York, USA; [8] New York State Department of Health, Wadsworth Center, Albany, USA; [9] Nationwide Children's Hospital, Center for Perinatal Research, The Abigail Wexner Research Institute, Columbus, USA; [10] Brandeis University, Health: Science, Society, Policy, Waltham, USA; [11] Emory University Rollins School of Public Health, Gangarosa Department of Environmental Health, Atlanta, USA; [12] University of Michigan School of Public Health, Department of Environmental Health Sciences, Ann Arbor, USA; [13] Johns Hopkins University, Department of Environmental Health and Engineering, Baltimore, USA; [14] Dartmouth Geisel School of Medicine, Department of Epidemiology, Lebanon, USA; [15] University of Illinois Urbana-Champaign, Beckman Institute for Advanced Science and Technology, Urbana, USA

Introduction: Exposure to per-and polyfluoroalkyl substances (PFAS) is a concern for pubertal development due to their endocrine-disrupting properties. Few studies have explored the link between prenatal PFAS exposure, a critical period for reproductive programming, and offspring pubertal status.

Methods: We examined the relation of prenatal blood levels of eight PFAS (PFOS, PFOA, PFHxS, PFDA, MeFOSAA, EtFOSAA, PFUnDA, PFNA) with pubertal development in a subset of the Environmental influences on Child Health Outcomes (ECHO) cohort in the USA (N=1,646). Pubertal development was measured using the first available Pubertal Development Scale (PDS), dichotomized as prepubertal (1) vs. pubertal (>1). Associations between natural log-transformed PFAS concentrations and pubertal status were analyzed via Poisson regression with robust standard errors, stratified by sex, and adjusted for maternal age, education, pre-pregnancy BMI, prenatal smoking, parity, social vulnerability index, age at PDS assessment, and enrollment site.

Results: Mothers were 4% Asian, 22% Black, 11% Hispanic, 60% White, with mean age of 30.0±5.8 at delivery. Children (52% female) had a median age of 11.6 years (IQR: 9.6-17.1, minimum: 6.9) at first PDS assessment. Higher concentrations of PFOS, PFOA, PFHxS, MeFOSAA, and EtFOSAA were associated with a lower likelihood of pubertal development at assessment (RR[95%CI] in males per log unit increase: 0.96 [0.95, 0.97]; 0.97 [0.96, 0.98]; 0.98 [0.97, 0.99]; 0.97 [0.96, 0.99]; and 0.95 [0.94, 0.97], respectively). Higher concentrations of PFDA were associated with a higher likelihood of pubertal development (1.01 [1.00, 1.03]). Results were consistent in females. After stratifying by age at PDS assessment (6.9-10.0 vs. ≥11.0), associations remained consistent but were significant only in younger children. No associations were found for PFUnDA or PFNA.

Conclusion: Prenatal exposure to PFOS, PFOA, PFHxS, MeFOSAA, and EtFOSAA was linked to later puberty, whereas PFDA was associated with earlier puberty. Future research should confirm associations and evaluate long-term health impacts.

S15 | The Red Hill Water Crisis and Steps to Address Community Concerns

R. H. Weldon[1, 2], **C. M. Pirkle**[1]

[1] *University of Hawai‘i at Mānoa, Office of Public Health Studies, Honolulu, Hawaii, United States of America*
[2] *University of Hawai‘i System, Office of Strategic Health Initiatives, Honolulu, Hawaii, United States of America*

In November 2021 over 5,500 gallons of JP-5 jet fuel spilled from the United States Navy’s Red Hill Bulk Fuel Storage Facility and entered into the Navy water system serving Joint Base Pearl Harbor-Hickam and surrounding communities in O‘ahu, Hawai‘i. This unprecedented exposure through ingestion, dermal contact, and inhalation affected about 93,000 residents across 9,600 households, schools, and workplaces, including civilians, military personnel, and their families, who may now reside around the world. There is a need for increased research on the health effects of JP-5, but exposures to other hydrocarbons are better studied. Immediate health impacts were documented by surveys conducted by the Centers for Disease Control and Prevention’s Agency for Toxic Substances and Disease Registry and show widespread symptoms affecting multiple organ systems. Now the affected community is left to negotiate ongoing health issues with healthcare providers who may not be familiar with the exposure scenario. In response to community concerns, the National Academies of Sciences, Engineering, and Medicine (“National Academies”) is assembling a committee of experts to author a report which will review the health risks associated with JP-5 exposure, make recommendations regarding clinical follow-up for those exposed, and provide guidance on clinical actions that can be taken to monitor the health of community members exposed. In addition, the University of Hawai‘i (UH) is working to establish a Red Hill Registry with the goals of tracking the health of impacted Red Hill community members over time, connecting them to needed services, and providing educational programming for health providers and community members. Both the National Academies and UH are employing a community-driven approach by engaging impacted residents. By collecting comprehensive data on exposures, health outcomes, and service needs from participants, the Registry will enable future studies to examine exposure-outcome relationships.

S15-01 | The Red Hill Water Crisis And Known Health Effects of Jet Fuels and Other Hydrocarbons

Catherine Pirkle

University of Hawai‘i at Mānoa, Office of Public Health Studies, Honolulu, USA

Total petroleum hydrocarbons, or TPHs, are a mixture of hundreds of hydrocarbons found in crude oils. Most pollution from these compounds is a consequence of human activities from the extraction/use of fossil fuels. An estimated third of contaminated sites globally are a result of petroleum pollution.
In Hawai‘i, in the United States, there is a fuel storage facility that was built in secret during World War II; it is known as the Red Hill Bulk Fuel Storage Facility. It was declassified in 1995 and an unknown number of TPH spills have occurred since its construction. The facility is 100 feet (30 meters) above two aquifers that supply the majority of drinking water to the city of Honolulu. Since 2014, several spills at the site have been publicly reported, culminating in November 2021, when Jet Propellant 5 fuel, a type of TPH, entered the Navy's public water line, sickening tens of thousands. The 2021 spill eventually led to funding for a Registry to track the health outcomes of affected individuals over time.
This presentation will provide background on the Red Hill water crisis and summarize the currently documented health effects associated with the 2021 jet fuel spill. The presentation will then situate the Red Hill spill within the broader context of TPH spills/leaks both in the United States and globally, focusing on drinking water pollution. To contextualize the spill, the presentation will briefly introduce the audience to two other jet fuel spills resulting from military activities, one in Serbia and another in Guam. Finally, it will emphasize the need for interdisciplinary global collaborations regarding the environmental and health effects associated with TPH pollution in groundwater.

S15-02 | Public Health Investigations After Petroleum Contamination of a Drinking Water System — O‘ahu, Hawai‘i, November 2021-March 2023

Daniel Nguyen[1], Nicholas V. Titelbaum[2], Stacey Konkle[2]

[1] Centers for Disease Control and Prevention, Hawai‘i State Department of Health, Environmental Health Administration, Honolulu, United States of America; [2] Agency for Toxic Substances and Disease Registry, Atlanta, United States of America

BACKGROUND On November 20, 2021, petroleum fuel contaminated the Red Hill well, which provided water to ~93,000 persons on Oahu, Hawaii. The Hawaii State Department of Health and U.S. Department of Defense (DoD) independently requested U.S. Centers for Disease Control and Prevention (CDC)/Agency for Toxic Substances and Disease Registry (ATSDR) assistance evaluating health effects associated with the contamination event and understanding prevalence of conditions and symptoms.
METHODS CDC/ATSDR conducted initial and follow-up Assessment of Chemical Exposures (ACE) investigations in January and September 2022, respectively, and reviewed medical records in February 2023. *ACE*: Surveys were designed using the ACE toolkit to assess water use, exposure, and recent symptoms. We used convenience sampling from the affected areas and recruited participants ≥18 years to complete surveys online or by telephone; parents completed surveys on behalf of children. *Records review*: We reviewed a sample of electronic military health records from potentially exposed patients with: DoD outcomes of interest; relevant International Classification of

Diseases, Tenth Revision codes for toxic effects; or higher risk (i.e., children, pregnant women). Conditions or symptoms were categorized as preexisting worsening or new persistent.

RESULTS *ACE*: There were 2,289 participants representing 14% of households in the initial investigation, and 972 representing 8% of households in the follow-up. Most participants were military-affiliated (88% and 90%, respectively), and most reported experiencing symptoms associated with water contamination (86% and 79%, respectively). *Records review*: Of 653 records reviewed, 449 (69%) patients had documented self-reported exposure, and 357 (55%) had preexisting worsening or new persistent conditions or symptoms.

CONCLUSIONS Affected community members experienced symptoms attributed to the November 2021 water contamination exposure. Most patients had documented preexisting worsening or new persistent conditions or symptoms that might benefit from reinforced access to health care systems. Continued monitoring for long-term health outcomes was recommended.

S15-03 | Developing Clinical Guidance for Individuals Exposed to JP-5 Contamination in the Red Hill Bulk Fuel Storage Facility Incident - The National Academies of Sciences, Engineering, and Medicine

Elizabeth Boyle

National Academies of Sciences, Engineering, and Medicine, Health and Medicine Division, Board on Population Health and Public Health Practice, Washington, DC, USA

In 2021, approximately 20,000 gallons of Jet Propellant 5 (JP-5) leaked from the U.S. Navy's Red Hill Bulk Fuel Storage Facility into the Joint Base Pearl Harbor-Hickam water distribution system, affecting approximately 93,000 people. Many reported a range of acute symptoms and ongoing health concerns. In response, the U.S. Department of Defense and Department of Veterans Affairs asked the National Academies of Sciences, Engineering, and Medicine to convene an independent expert committee to assess the health risks of JP-5 exposure and provide recommendations for clinical follow-up.

The committee's charge includes reviewing current scientific evidence on the health effects of JP-5 and similar fuels; evaluating the strength of evidence for symptoms and conditions reported by those exposed; and examining existing clinical guidance, exposure assessments, and modeling efforts. The study will also identify key research needs to inform future clinical care and benefit-related decisions for people affected by the release.

The committee is conducting public information-gathering activities to inform its work, including virtual listening sessions and open meetings with impacted community members. These activities ensure that a broad range of experiences and concerns are considered.

This presentation will provide an overview of the committee's charge, the context for the study, and the approach to public engagement and evidence gathering to date. The final consensus report is expected in Spring 2026. It is intended to support federal and local agencies in developing responsive and evidence-informed clinical guidance for communities affected by JP-5 and similar fuel exposures.

S15-04 | The Red Hill Registry: A Community-Engaged Approach to Identifying and Addressing Community Needs from the 2021 Jet Fuel Spill at Red Hill

Rosana Weldon[1,2], Tara Sutton[2], Stephanie Wagner[3], Catherine Pirkle[1], Aimee Grace[2]

[1] University of Hawaiʻi at Mānoa, Department of Public Health Sciences, Honolulu, USA; [2] University of Hawaiʻi System, Office of Strategic Health Initiatives, Honolulu, USA; [3] University Health Partners of Hawaiʻi, Red Hill Registry, Honolulu, USA

Following the unprecedented Red Hill water crisis of 2021 in Oʻahu, Hawaiʻi, federal, state, and local stakeholders called for a registry to track the health of impacted Red Hill community members over time, connect them to needed medical or social services, and provide educational programming for healthcare providers and community members. Funded in June 2024 and facilitated by the University of Hawaiʻi, the Red Hill Registry employs a community-driven approach, engaging impacted residents in co-developing its priorities and protocols.

Community engagement activities have included numerous speaking engagements; focus groups with impacted water users, professionals and advocates, and concerned residents; online surveys; and the formation of a Community Advisory Workgroup. Community outreach has also included providing opportunities to connect with the Registry by signing up for a listserv to receive updates; completing a pre-enrollment survey to capture basic exposure, geography, and contact information; following the Registry on social media; and visiting the website. Community engagement specialists will assist participants by facilitating recruitment and enrollment, connecting participants to needed services, and delivering education materials. These educational materials will include fact sheets, podcasts, and videos on the potential health impacts of jet fuel for the community and health care providers.

Successful implementation of the Registry has also involved extensive scientific engagement. This has included forming a Scientific Oversight Workgroup, establishing relationships with leadership from other registries, performing a literature review of the impacts of jet fuel on health, submitting a report to Congress on the feasibility and necessity of conducting an epidemiological health outcomes study of individuals exposed at Red Hill, and developing all study instruments.

The Registry is slated to launch for formal enrollment in mid-2025 and will serve as a data repository for future epidemiological investigations of jet fuel exposure and outcomes, including long-term health consequences.

S16 | Climate change and health across the lifespan in urban Latin America

J. Kephart[1], S. Lopes de Moraes[2], C.-K. Hsu[3], K. Rangel Moreno[4], H. Jardel[1], N. Gouveia[2]

[1] *Drexel University, Urban Health Collaborative, Philadelphia, Pennsylvania, United States of America*
[2] *University of São Paulo, Faculty of Medicine, São Paulo, Brazil*
[3] *University of California Berkeley, Institute of Transportation Studies, Berkeley, California, United States of America*
[4] *National Institute of Public Health, Cuernavaca, Mexico*

Latin America has high urbanization (80% live in urban areas), a rapidly aging population, and substantial health impacts from anthropogenic climate change. However, there remains a dearth of health evidence to inform climate change mitigation and adaptation policies in this primarily middle-income region. The Climate Change and Urban Health in Latin America Project (SALURBAL-Climate) is an international scientific collaboration of researchers across Latin America and the United States that studies how climate change, urban environments, and urban policies impact the health of residents in nearly 400 cities across 11 Latin American countries. This symposium will bring together SALURBAL-Climate researchers from Latin America and the United States to present new findings on climate change, urban environments, and health in Latin America. We will place a particular focus on how climate-related health risks vary across the lifespan. A panel of early-career researchers and trainees based in Brazil, Mexico, and the U.S. will present findings on the impacts of heat and floods on health outcomes, including homicide mortality, diabetes mortality, and road traffic deaths from 10 Latin American countries. We will also discuss intra-urban disparities in heat-related mortality and how these disparities vary across four countries. The session will conclude with a moderated Q&A session co-chaired by SALURBAL investigators. Participants will leave with a deeper understanding of climate change and health challenges and the potential effectiveness of climate adaptation urban policies at local and regional levels across this understudied region.

S16-01 | The effect of temperature on homicide mortality in Latin American cities

Sara Lopes de Moraes[1], Nelson Gouveia[1], Brisa Sanchez[2], Josiah L. Kephart[2], Maryia Bakhtsiyarava[2], Iryna Dronova[3], Waleska T. Caiaffa[4], Leah H. Schinasi[2], Marie O'Neill[5], Karla Rangel-Moreno[6], Marcio Alazraqui[7], Amanda S. Magalhães[4], Amélia A.D.L. Friche[4], Daniel A. Rodriguez[3], Olga L. Sarmiento[8], Laura A. Rodriguez-Villamizar[9], Astrid B.H. López[8]

[1] University of São Paulo, Department of Preventive Medicine, São Paulo, Brazil; [2] Drexel University, Philadelphia, USA; [3] University of California Berkeley, California, USA; [4] Federal University of Minas Gerais, Belo Horizonte, Brazil; [5] University of Michigan, Michigan, USA; [6] Instituto Nacional de Salud Pública, Cuernavaca, Mexico; [7] Universidad Nacional de Lanús, Buenos Aires, Argentina; [8] Universidad de los Andes, Bogota, Colombia; [9] Universidad Industrial de Santander, Bogota, Colombia

Background and objective: Homicides is a public health concern worldwide and studies have suggested that ambient temperature is associated with increased risk of homicide. However, the knowledge about the effects of temperature on homicide deaths remains limited, especially in Latin America the most violent region in the world. Therefore, our aim was to estimate the association between homicide deaths and daily mean temperature in Latin American cities.

Material and Methods: A case time-series design combined with a distributed lag non-linear model was used to evaluate the temperature-homicide association among 1,057,993 homicide deaths across 272 cities in six countries from 2000 to 2019. We considered a lag period of 0-7 days and used the minimum temperature mortality as reference point to estimate the association.

Results: Our findings shows that higher temperatures were associated with an increased risk of homicide deaths. Specifically, for temperatures at the 90th percentile we found a relative risk of 1.126 (95% CI: 1.107-1.145), while extremely high temperatures at the 99th percentile showed a relative risk of 1.166 (95% CI: 1.143-1.189) compared to the minimum mortality temperature. We also found that heat is associated with a greater risk of homicide deaths across both sexes (female and male) and different age groups (0–24, 25–39, and ≥40 years old). The 25–39 years age group showed the greatest risk of homicide deaths across the temperature distribution compared to the other groups.

Conclusion: This study highlights the association between homicide with temperature, particularly high temperatures. These findings are essential for developing targeted interventions and public health policies to mitigate the effects of temperature on homicides a concern that is likely to increase due to climate change scenario.

S16-02 | Associations between Heat Exposure and Road Traffic Mortality in 272 Latin American Cities

Cheng-Kai Hsu

University of California Berkeley, Institute of Transportation Studies, Berkeley, USA

Latin America experiences both high road-traffic mortality and extreme heat, which have been shown elsewhere to be interrelated. However, evidence on temperature-related road-traffic mortality remains scarce in Latin America; even globally, few studies have examined multiple countries and cities, and even fewer considered diverse climates, urban conditions, and vulnerable road users. We examine this association and its variations across Latin America—one of the world's most urbanized and rapidly motorizing regions, with a high share of vulnerable road users, particularly motorcyclists. Using data from the SALURBAL study, we analyzed 272 cities across six countries—Brazil, Mexico, Chile, El Salvador, Panama, and Costa Rica—between 2000 and 2019. Road-traffic mortality was identified using ICD-10 codes, and temperature data were sourced from ERA5-Land reanalysis. We applied a time-stratified case-crossover design with a distributed lag non-linear model, pooling estimates across cities. Multiple imputation was used to redistribute ill-defined codes, and model results were combined using Rubin's rules across 100 imputations. Across 1,956,856 city-days, we estimated that the relative risks (RRs) of road-traffic mortality at the 95th and 99th temperature percentiles, compared to the minimum mortality temperature, were 1.16 [95% CI: 1.14–1.19] and 1.18 [1.15–1.21], respectively. The excess death fraction (EDF) for extreme heat (≥95th percentile) indicated that 0.74% [0.64%–0.84%] of road-traffic deaths were attributable to extreme heat—exceeding regional estimates for cardiovascular and respiratory mortality. Effects were more pronounced among younger individuals (≤19 years), males, motorcyclists, bicyclists, and residents of cities with hotter climates, longer street segments, and extended commute times. Policymakers in the tropical Global South should invest in built environments that promote sustainable transportation while protecting vulnerable road users—many of whom rely on non-climate-controlled informal transportation for long, heat-exposed commutes—from heat-induced road-traffic mortality.

S16-03 | Short-term diabetes mellitus mortality associated with hot temperatures in Latin American cities

Karla Rangel Moreno

National Institute of Public Health, Cuernavaca, Mexico

Objective: Latin America (LATAM) is one of the regions most affected by extreme heat, which has been associated with increased diabetes mellitus (DM) mortality in high-income countries. We assessed the association between heat and short-term diabetes mellitus mortality and its modification by individual variables and climate zones, using a case time series design in 294 LATAM cities.

Material and Methods: We conducted a case-time series design using data from 294 cities in eight countries included in the Climate Change and Urban Health in Latin America project (SALURBAL-Climate). We used percentiles of the population-weighted daily average temperature from ERA5-LAND and daily DM death counts in adults obtained from national statistical offices. We assessed the interest association using a conditional quasi-

Poisson with distributed-lag (0-21 lags) non-linear model, and added interaction terms in the models to evaluate effect modification by sub-group variables (age and sex) and climate zone.
Results: A total of 1,731,781 DM deaths were included in this analysis, most of them from Mexico (61.3%) and Brazil (32.6%). The minimum mortality temperature percentile (MMTp) corresponded to the 83nd percentile and the curve of its association showed an inverted J-shaped curve. Compared to the MMTp, the increase in DM mortality associated with heat ranged from a 1% increase at the 86th percentile (95% CI: 1%, 2%) to a 9% increase at the 99th percentile (95% CI: 5%, 13%). The DM mortality risk was higher for older adults and for women, and it was heterogeneous across countries and climate zones.
Conclusion: Our results show that heat exposure increased diabetes mortality in LATAM cities, with the risk being higher for elderly females. Cold temperatures were also an important concern regarding DM mortality. Given the high diabetes prevalence and temperature variability in LATAM, public health strategies are needed to protect the most vulnerable populations.

S16-04 | Flood-related mortality and modification by individual and urban features in Latin American cities

Hanna Jardel[1], **Josiah Kephart**[1], Juan Carlos Figueroa Morales[1], Ana Diez Roux[1], Olga L. Sarmiento[2], Laura B. Chiquillo[2], Waleska Caiaffa[3], Fatima Pina[4], Nelson Gouveia[5], Serena Perner[6], Luz M. Cardenas[7], Alex Quistberg[1], Usama Bilal[1], Jessica Uruchima[1], Steve Melly[1], Natalie Rodrigues[1]

[1] Drexel University, Urban Health Collaborative, Philadelphia, USA; [2] Universidad de los Andes, Bogota, Colombia; [3] Universidade Federal de Minas Geraisv, Belo Horizont, Brazil; [4] ICICT/FIOCRUZ, Rio de Janeiro, Brazil; [5] Universidade de São Paulo, São Paul, Brazil; [6] Universidad Nacional de Lanús, Instituto de Salud Colectiva, Remedios de Escalada, Argentina; [7] Instituto Nacional de Salud Pública, Cuernavaca, Mexico

Background and objective: Floods affect more people than other environmental hazards and little is known about how floods impact human health beyond immediate direct deaths. Urban populations, with high population density and reduced capacity to handle excess water, may be particularly impacted. We analyzed the impact of floods on mortality in 1181 sub-city areas within 317 Latin American cities and over 2000-2020.
Methods: We linked mortality, population, and environmental data from the SALURBAL project with daily flood data from the Dartmouth Flood Observatory to calculate incidence rate ratios (IRRs) for mortality (all-cause, injury-related, non-injury related) across lags of 0-60 days with distributed lag nonlinear models. We examined modification by individual level modifiers (age group and sex) and sub-city area-level modifiers (socioeconomic indicators, greenness, climate zone, and ocean adjacency).
Results: We observed an immediate increase in all-cause mortality at day 0 followed by a sharp decline in mortality day 1 after a flood event. Following this harvesting effect, mortality increased above baseline between days 6 to 16, with a peak at day 7 (IRR 1.001 95%CI 1.000, 1.003), before returning to baseline. The overall IRR point estimate was 1.005 (95% CI 0.983, 1.029). When stratified by sex, mortality among males shows higher magnitude extreme values and longer durations of above-null mortality and a higher cumulative IRR. Sub-city areas with the lowest education attainment had greater mortality impacts compared to sub-city areas with higher educational attainment.
Conclusion: All-cause mortality due to floods exhibited a temporal pattern indicating immediate direct-cause deaths as well as less immediate, indirect-cause deaths. This information should inform plans for resource allocation following floods to reduce mortality among those most at risk or death.

S16-05 | Heterogeneity in heat-related mortality by neighborhood in 5 cities of the Americas

Josiah Kephart[1], Maryia Bakhtsiyarava[1], Derek Weix[1], Ana Diez Roux[1], Brisa Sanchez[1], Nelson Gouveia[3], Mariana Lazo[1], Katherine Indvik[1], Cheng-Kai Hsu[2], Daniela DaCosta[4], Leah Schinasi[1], Daniel Rodriguez[2], M Fernanda Kroker-Lobos[4], Usama Bilal[1]

[1] Drexel University, Urban Health Collaborative, Philadelphia, USA; [2] University of California Berkeley, Institute for Transportation Studies, Berkeley, USA; [3] University of Sao Paulo, Department of Preventive Medicine, Sao Paulo, Brazil; [4] Instituto de Nutrición de Centroamérica y Panamá, Guatemala City, Guatemala

Background and Objective: Heat adaptation plans and heat-health research are commonly enacted at the city-level. However, there is strong intraurban variation in social, physical, and natural features that are understood to buffer or increase the health risks associated with heat. We studied five cities across the Americas, diverse in climate and urban environments, to examine how neighborhood-level social, physical, and natural environments modify the association between ambient heat and mortality.
Methods: We leveraged daily city-level ambient temperature (ERA5-Land) and daily neighborhood mortality (vital records) within Panama City, Panama (N=53 neighborhoods), Guatemala City, Guatemala (N=14), Philadelphia, USA (N=125), Rio de Janeiro, Brazil (N=332), and São Paulo, Brazil (N=621). We examined neighborhood-level variation in heat-related mortality by neighborhood educational attainment (social environment), population density (physical environment), and greenspace (natural environment), stratified by age at death (<65, 65+ years).
Results: We found a higher risk of heat-related mortality in one or both age groups in neighborhoods with lower greenspace in Rio de Janeiro, Panama City, and Philadelphia, but no evidence of modification by greenspace in São Paulo or Guatemala City. We observed higher risk in lower education neighborhoods in Philadelphia only. Finally, we found higher risk in neighborhoods with higher population density in Panama City and Philadelphia, but no evidence of modification in other cities.
Conclusion: Intraurban patterns of social, physical, and natural environments associated with heat-related mortality vary by context and interact to buffer or reinforce neighborhood-level disparities in heat-mortality distinctly within different cities. Locally derived studies are essential to understanding how neighborhood-level urban environments contribute to health disparities in specific contexts and to developing municipal heat adaptation plans that effectively protect public health.

S17 | Silencing an Environmental Injustice: How noise and health research, regulation, and policy differ in the US and around the world

C. Roscoe[1, 2], **S. Grady**[3], M. D. Willis[4], C. Nica[5], R. E. Arku[6], D. Vienneau[7], J. L. Peters[3]

[1] *Oregon Health & Science University OHSU-PSU School of Public Health, Environmental Systems and Human Health, Portland, Oregon, United States of America*
[2] *Knight Cancer Institute, Cancer Prevention and Control, Oregon Health & Science University, Portland, Oregon, United States of America*
[3] *Boston University School of Public Health, Environmental Health, Boston, Massachusetts, United States of America*
[4] *Boston University School of Public Health, Department of Epidemiology,, Boston, Massachusetts, United State of America*
[5] *Brown University, Community Noise Lab (Jackson, Mississippi Site), Providence, Rhode Island, United States of America*
[6] *University of Massachusetts School of Public Health and Health Sciences, Department of Environmental Health Sciences, Boston, Massachusetts, United States of America*
[7] *Swiss Tropical and Public Health Institute, Allschwil, Switzerland*

Health impacts of environmental noise have been primarily studied in Europe, which has led to the development of regional WHO guidelines and noise reduction directives. The guidelines, however, do not provide specific recommendations for vulnerable populations. Thus, even in Europe where exposure data are available, little has been done to quantify health disparities. Meanwhile, the US lags behind in environmental noise and health research, regulation, and policy, despite compelling neighborhood-level evidence of inequities by race/ethnicity and socioeconomic status (SES).

Historic US policies such as ‘Redlining’ and the ‘Federal Highways Act’ discriminated against or displaced majority-Black and Latine neighborhoods, and have a legacy of underprotection and underinvestment in racial/ethnic minority and low-SES neighborhoods, resulting in present day environmental noise inequities. Road traffic, rail, and airports are often situated in or near these neighborhoods, and novel industrial sources of noise (e.g., cryptocurrency mines) have more recently been located in or near underserved communities. Given the limited research funding and regulation for noise in the US and other countries around the world, including those in the Global South, underserved communities continue to suffer the health consequences of high noise exposure.

This symposium will compare noise research and policies in the United States, past and present, including environmental justice repercussions, with Europe and other regions around the world. We will hear from experts in noise and health inequities research, regulation, and those building sustainable infrastructure for community-led environmental justice, as they discuss advancing the field of noise and health, locally and globally.

S17-01 | The Sound of Bitcoin: Community Noise Concerns from Cryptocurrency Mining in the U.S.

Mary D. Willis[1], Brian Sousa[2], Fintan Mooney[3], Stephanie T. Grady[2], Joan A. Casey[4], Jonathan J. Buonocore[2]

[1] Boston University School of Public Health, Department of Epidemiology, Boston, USA; [2] Boston University School of Public Health, Department of Environmental Health, Boston, USA; [3] Columbia University Mailman School of Public Health, Department of Environmental Health Sciences, New York, USA; [4] University of Washington School of Public Health, Department of Environmental and Occupational Health Sciences, Seattle, USA

Objective: The cryptocurrency mining industry has rapidly expanded across the U.S. and around the world. The most popular currency, Bitcoin, uses an energy intensive “proof-of-work” algorithm – this process accounts for an estimated 2.3% of all U.S. electricity consumption. These operations are expected to increase in the U.S., with several state and federal programs aiming to incentivize the growth of proof-of-work cryptocurrency mining. Communities who host this industry have suffered from excessive noise from the digital mining operations. This novel source of noise pollution may adversely impact local communities, with residents’ reports of hearing impairment, headaches, stress, sleep disturbances, and hypertension. Here, we present preliminary conservative estimates of population-level exposures to this industry across the U.S., including evaluation of environmental justice concerns related to industry siting.

Methods: We identified the geocoded locations of proof-of-work cryptocurrency mines using an existing database, supplemented by updated temporal information on individual mines. We quantified the number of people who reside in close proximity to this industry using the National Structures Inventory and evaluated important environmental justice concerns among these populations at the block group level using proportional apportionment from the American Community Survey.

Results: Among the proof-of-work cryptocurrency mines that we identified in the U.S. (n = 151), preliminary results show that at least 45,000 people reside within 500 m. The states with the highest populations near this industry include New York, Washington, and Texas. These exposed populations are overwhelmingly in urban areas, with Black populations disproportionately residing in close proximity.

Conclusion: Current U.S. federal and state policies are promoting proof-of-work cryptocurrency. Estimates indicate that the industry is expected to skyrocket in the coming years, thus understanding this industry is highly relevant to affected communities. Planned future work includes a pilot sampling campaign to evaluate the spatial-temporal scope of noise near these mines.

S17-02 | Spatial modelling and inequalities of environmental noise in Accra, Ghana

Sierra N. Clark[1], Iris E. Fynn[2], Rachel Fowden-Hulme[1], Allison F. Hughes[4], Raphael E. Arku[5]

[1] City St. George's, University of London, School of Health and Medical Sciences, London, UK; [2] University of Ghana, Department of Geography and Resource Development, Legon, Accra, Ghana; [3] City St. George's, University of London, School of Health and Medical Sciences, London, UK; [4] University of Ghana, Department of Physics, Legon, Accra, Ghana; [5] University of Massachusetts Amherst, Department of Environmental Health Sciences, Amherst, Germany

Background: Noise pollution is a growing environmental health concern in rapidly urbanizing sub-Saharan African (SSA) cities, yet data limitations hinder policy and research. This study, the first of its kind in West Africa, measured and predicted noise levels across the Greater Accra Metropolis of Ghana.

Methods: For a 1-year period, we conducted large-scale measurements at 146 unique monitoring sites covering diverse land use features. The data were used to develop high-resolution land use regression models to estimate and predict metrics of environmental noise exposures ($LAeq_{24hr}$, L_{den}, L_{night}, L_{day}) at all locations in the city. The modelled predictions were subsequently linked to geocoded school and home addresses of a new cohort of 919 schoolchildren aged 8-12 years old (60% girls) from 90 (75% public) elementary schools in Accra.

Results: We found that environmental noise levels exceeded the World Health Organisation and national guidelines almost everywhere in the city. Children attending public schools, on average, had higher day-time noise exposures (64 dB L_{day}) than children attending private schools (60 dB L_{day}). This relationship remained for public vs private schoolchildren even for their exposures at home (55 vs 52 L_{night}). Furthermore, we observed negative linear correlations between the level of wealth in an area (based on median household consumption levels in each area) and environmental noise levels in those areas, with Pearson correlations of -0.63 for L_{day} at school locations and –0.38 for L_{den} at home locations.

Conclusion: These findings highlight socioeconomic inequalities in environmental noise exposures across a major SSA city but also for key microenvironments in which children spend most of their time. The results also provide a unique opportunity for epidemiological studies and policies to mitigate health risks and environmental disparities in the context of fast-growing SSA cities.

S17-03 | Transportation noise and health in vulnerable groups in Europe

Danielle Vienneau

Swiss Tropical and Public Health Institute, Allschwil, Switzerland

Objective: Research in Europe has largely focused on children as a key vulnerable group, though other groups may also be disproportionately affected by and susceptible to the impacts of transportation noise. Personal characteristics such as age (including elderly), gender, lifestyle and those with pre-existing health conditions may influence susceptibility. The impacts can be a particular concern in situations of environmental injustice, whereby certain vulnerable groups may experience higher exposures to noise levels.

Method: Ongoing work within the European Topic Centre on Human Health and the Environment focuses on gaining a broader understanding of whether there are injustices in transportation noise exposure, and if these exacerbate relevant health outcomes in such vulnerable groups. Given that information on potential vulnerabilities is typically included in studies as subset analyses, not always detectable within abstracts and by search terms, the project uses a scan of recent literature plus crowdsourcing within the noise-health community to identify relevant studies on the health effects of transportation noise.
Results: The findings are summarised in a narrative review, with most evidence for cardiovascular disease (CVD). For example, several large cohort studies investigating CVDs have reported potential effect modification by sex or age. The results are somewhat mixed; however, most report the relative risk, not absolute risk, which can influence interpretation. At least one study specifically studied the combination of high exposure and socioeconomic deprivation, showing an increased risk of major adverse CVD events in this vulnerable group. A study on recurrence of events in patients with acute coronary heart disease also showed stronger associations in relation to aircraft noise compared to studies in the general population.
Conclusion: The overarching question – are existing WHO guidelines adequate for protecting vulnerable groups? – will be discussed.

S17-04 | Research on Patterns of Residential Noise Exposure and Health in the United States

Junenette L. Peters[1], Ieza Arshad[1], Jean M. Costello[1], Matthew Bozigar[2], Stephanie T. Grady[1]

[1] Boston University School of Public Health, Environmental Health, Boston, USA; [2] Oregon State University, College of Health, Corvallis, USA

Objective: Noise has been a concern for many communities surrounding transportation and industrial sources. From the phase out of the United States (U.S.) Environmental Protection Agency's Office of Noise Abatement and Control in the early 1980's, there has been little research or policy update related to environmental noise in the U.S. Here, we provide an overview of the research on sociodemographic patterns of noise exposure in the U.S. that have been published over approximately the last 40 years, as well as an overview of the research on noise and health outcomes to date and future needs of noise research and policy in the U.S.
Methods: We conducted an informal review of the literature using PubMed to identify published studies conducted since the phase out in 1982 involving environmental noise and health. We excluded studies that were related to occupation, school, or outside the home, as our focus was on residential exposure to noise.
Results: Although there are variations in noise exposure patterns across neighborhoods, overall minoritized and low socioeconomic populations are disproportionately exposed. These groups are also more likely to experience disparities in health. Many of the U.S. studies to date use cohort studies that may not reflect the sociodemographic characteristics of the population. Additionally, although some epidemiologic studies have been conducted in the U.S., many have had challenges with obtaining spatiotemporally refined noise exposures, overcoming study biases (confounding and selection bias), and convincing others of the studies' generalizability.
Conclusions: Much work is needed to convince policymakers and other stakeholders in the U.S. of the importance of environmental noise as a public health issue. We will use this presentation as a starting point for dialogue with U.S. and non-U.S. colleagues on how to advance the field of noise and health to the forefront.

S18 | From Data to Connection: Best Practices for Engaging Communities in Sharing Research Findings on Exposures

R. Dodson[1], **E. Erdei**[5], **A. Rosofsky**[2], **A. Rule**[6], M. Ramírez-Andreotta[3], A. McIntyre[1], B. Youpee[4], J. Nelson[7], K. Korfmacher[8], T. Fingesi[3]

[1] *Silent Spring Institute, Newton, Massachusetts, United States of America*
[2] *Health Effects Institute, Boston, Massachusetts, United States of America*
[3] *University of Arizona, Tuscon, Arizona, United States of America*
[4] *Missouri Breaks Industries Research, Eagle Butte, South Dakota, United States of America*
[5] *University of New Mexico, Albuquerque, New Mexico, United States of America*
[6] *Johns Hopkins University, Baltimore, Maryland, United States of America*
[7] *Minnesota Department of Public Health, St Paul, Minnesota, United States of America*
[8] *University of Rochester, Rochester, New York, United States of America*

Sharing research results with study participants is essential for fostering trust, transparency, and community engagement. It acknowledges participants' Right-to-Know, has been shown to increase environmental health literacy, empowers participants to take action, and promotes health equity. Effective strategies for sharing research findings, especially measured or modeled chemical exposures, involve tailoring information to diverse audiences and considering literacy levels, cultural contexts, and language preferences. Researchers must also address ethical considerations, such as ensuring accuracy, contextualizing uncertainties, and supporting understanding. In this symposium, presenters will share detailed examples and effective approaches for sharing research results with study participants and their communities. They will highlight available tools, approaches for evaluation, and lessons learned from reporting back research results. We will follow each session with a discussion of best practices and future directions.

S18-01 | Co-created environmental health science: Centering community, culture, and design for shared learning, data engagement, and report back

Monica Ramírez-Andreotta

University of Arizona, Tuscon, USA

Objectives:
This study explores how participatory research approaches can address the social/political/cultural complexities present in environmental justice (EJ) communities. Specifically, it investigates how community scientists (CS) develop data/environmental health literacy (D/EHL), engage in sense-making, and build trust in science through co-created environmental monitoring and data report back efforts. The goal is to better understand how personal relevance and community engagement can drive learning and action in contexts of environmental pollution and (in)justice.

Methods:
The project centered community learning and prioritized participant experience in the practice of data report back. Data collection included surveys, focus groups, and semi-structured interviews focused on three key questions:

1. How do personal connections and local relevance drive sense-making?
2. How does data report back enhance visibility of pollution and (re)shape existing mental models?
3. How does co-creation of knowledge and data report back efforts build D/EHL, strengthen relationships with science of science, and trust in scientific processes?

Results:
The project framing facilitated CS to meaningfully engage with data through personal experience and relevance. Participants connected environmental data to their lived experiences and local histories, using that understanding to evaluate contaminant transport and environmental risks. This process supported their movement along the D/EHL continuum. CS adopted protective health measures based on the evidence they helped generate and began applying these skills to new contexts.

Conclusion:
Co-created science and data report back fostered a renewed relationship with science, built trust, enabled action, and promoted sustained engagement. This community-driven model of science and data report back not only supports individual learning but also lays the groundwork for social transformation and structural change in EJ communities. When report back materials are intentionally co-designed, they can serve as a boundary object; data maintains its integrity while being presented/used in different ways across multiple social spheres to support communication and action.

S18-02 | Approaches to returning personal exposure results: Lessons learned and future directions

Alina McIntyre[1], Katherine Franz[1], Katherine Boronow[1], Julia G. Brody[1], Carmen Milagros Vélez Vega[2], Phil Brown[3], Tamarra James-Todd[4], Jennifer Ohayon[1]

[1] Silent Spring Institute, Newton, USA; [2] University of Puerto Rico, San Juan, Puerto Rico; [3] Northeastern University, Boston, USA; [4] Harvard T. H. Chan School of Public Health, Boston, USA

Objective: Reporting back individual-level exposure results to study participants is vital for advancing community-engaged research. Drawing from over two decades of research and practice, we present case examples highlighting different report-back modalities: online report-back using the Digital Exposure Report-Back Interface (DERBI), clinician-facilitated report-back, and group report-back.
Methods: Engagement with report-back and impacts on participants' environmental health literacy have been evaluated in randomized trials and other research designs using surveys, semi-structured interviews, and online analytics across studies including the Child Health and Development Studies (CHDS), Green Housing Study, Puerto Rico Testsite for Exploring Contamination Threats (PROTECT), and Environmental Reproductive and Glucose Outcomes Study (ERGO).
Results: Participants in the CHDS who received personalized DERBI exposure reports spent about twice as much time engaging with their results compared to those given only aggregate data. All participants reported positive emotions like curiosity and feeling informed. Participants in PROTECT and ERGO who received clinician-facilitated report-back reported positive experiences overall and increased knowledge about reducing environmental exposures (e.g., replacing plastic with glass). In qualitative interviews, clinicians delivering the reports also reported learning about exposure sources. Our experience returning individual results in group settings—such as in PROTECT and the Green Housing Study—highlighted opportunities for participants to connect and identify shared sources of elevated exposure. Key barriers to clinician-facilitated and group report-back included clinician recruitment and logistical challenges for participants, such as childcare, transportation, and work obligations.
Conclusion: Findings highlight report-back as a tool for both information-sharing and building meaningful connections between researchers, clinicians, and communities. Personalized and participatory approaches can boost engagement, strengthen environmental health literacy, and support community agency. Ongoing evaluation in different settings is critical to advance methods and assess long-term impacts, including behavior change.

S18-03 | The Importance of Report-back Processes and Community Education in Tribal Settings

Brittany Youpee[1], Esther Erdei[2], Anna Tillery[3], Ana Rule[3], Lesliam Quiros-Alcala[3], Krystal Godri Pollitt[4], Rae O'Leary[1]

[1] Missouri Breaks Industries Research Inc.s Research, Eagle Butte, USA; [2] University of New Mexico, College of Pharmaceutical Sciences, Albuquerque, USA; [3] Johns Hopkins University, Department of Environmental Health and Engineering, Baltimore, USA; [4] Yale University, Department of Environmental Health Sciences, New Haven, USA

This presentation will highlight Tribal community processes to develop meaningful, informative, and educational report-back materials. We support participants who are exposed in their homes, Tribal workplaces, and schools. Specifically, this presentation will highlight current report-back material development, processes, and participant feedback for the indoor home environment study “Cheyenne River Sioux Tribe (CRST) COVID-19 Wayaktá He” that shared NO2 and Air Nicotine results and the “Women’s Volatile Organic Compound (VOC)” substudy that shared VOC results.

Our processes to the report-back and community engagements were based on data that directly generated from home indoor toxicant measurements. We found that as high as 75% of the Wayakta He study homes (N=298) had increased level of NO2 exposures over the USEPA suggested outdoor limits. Further survey analyses and home visits with visual inspections showed that these measurements were attributed to home cooking, gas oven use and had no seasonal variabilities that would indicate the influence of heating season on the indoor NO2 concentrations. This prompted then to develop flyers to the participating households and to enhance our Team’s educational opportunities on decreasing exposures during cooking. This has been a significant concern during the pandemic as home cooked meals were the standard due to quarantine conditions. Also, found that increased NO2 concentrations were predictors of the number of SARS-CoV-2 infections in the homes.

Examining our newly emerging chemicals in homes and among female participants (N=20), we find extremely high level of phthalates and VOCs as well as PAHs in the households. Our current messages considered graphical comparisons to other USA women exposure measures of the same chemicals and also adding frequent usage and exposure route considerations. These report backs are rooted in Lakota cultural imagery and applicable to further works on health effects that we are currently ongoing.

S18-04 | Improving Biomonitoring Results Return: Feedback from Minnesota Families

Jessica Nelson, Nicole Frederickson, Fathi Ahmed, Clara Lucero, Sheila Amenumey

Minnesota Department of Public Health, St Paul, USA

Objective. As the use of biomonitoring expands in public health practice, an essential question is how best to share individual results with participants in an accessible, informative, and culturally appropriate way. Interviews with a diverse group of Minnesota families provide important information about the experience of receiving biomonitoring results.

Methods. The Minnesota Department of Health's Healthy Kids Minnesota program assesses environmental exposures in preschool-age children across the state. Recruitment is conducted by partners at Early Childhood Screening Programs in school districts, local public health agencies, and tribal nations. Families who agree to participate complete an interview and help their child give a urine sample. Urine samples are analyzed for metals, pesticides, environmental phenols, phthalates, flame retardants, and air pollution chemicals. Full results are provided to families in multiple mailings along with information on reducing childhood exposures.

Based on previous feedback from families, the results packet was re-designed in 2024, relying on a more visual representation of results and contextual information. After implementing the new design and mailing results to families from the second program cycle (n=541), structured telephone interviews with approximately 40 families will be conducted to gather feedback. Families who speak languages other than English (including Hmong, Karen, and Spanish) will be prioritized for contact.

Results. Results of qualitative and semi-quantitative analyses will show whether the new results return approach is understandable and accessible to families, and how their experience of receiving results compares with the previous approach. Analyses will assess whether there were differences in families' understanding of their child's results based on preferred language, site of recruitment, caregiver's educational attainment, and child's biomonitoring results.

Conclusion. These findings will be used to improve accessibility of future results return materials and contribute to the growing body of research on best practices for biomonitoring results communication.

S18-05 | Sharing Home-Based Exposures: Lessons from the ROC HOME Study

Katrina Korfmacher[1], Rebecca Lauzon[1], Julia Udesky[2], Robin Dodson[2]

[1] University of Rochester, Rochester, USA; [2] Silent Spring Institute, Newton, USA

Objective: We sought to assess engagement with healthy homes education and action after report-back of personal study results among low-income homeowners.
Methods: The ROC HOME study evaluated the influence of lead hazard housing rehabilitation programs on levels of common contaminants in the home that affect children's health. We developed study results reports for participants using Silent Spring's Digital Exposure Report-Back Interface (DERBI) with feedback from local community partners. After participants received their reports, we interviewed a subset to learn about their report back experience.
Results: At an earlier study visit, 97% of participants (66 of 68) rated finding out what chemicals are in their house dust as an "extremely" (n=41) or "very" important (n=25) reason for participating; nearly half reported it as *the* most important reason for joining the study. After receiving their reports, nearly all participants interviewed agreed that receiving information from the study helped them keep their home healthier. About half of participants shared that receiving their results made them more worried about contaminants in their home, but many reported taking additional steps to reduce exposure after receiving their individual results. Participants' preferences on how the results were presented varied between individuals, including for receiving print or digital reports and for having their results compared to others in the study. Many participants were able to engage with their results, interpret them, and discuss potential exposure-reduction strategies. However, others were overwhelmed by the amount of information and challenged by the graphical data. Anecdotally, participants who struggled to interpret results on their own were able to better engage with them after brief in-person instruction by study staff.
Conclusion: Participants strongly desire individual-level environmental health results. However, report-back of complex study results may be overwhelming. Future research should explore effective approaches (e.g., in-person visit, video) to explain report-back materials.

S18-06 | Evaluating Per- and Polyfluoroalkyl Substances (PFAS) Report-Back Impact on Knowledge, Attitudes, and Behaviors

Tina Fingesi[1], Katie Boronow[4], Nathan Lothrop[1], Daniel Sullivan[2], Shawn Beitel[1], James Hollister[1], Pedro Flores Gallardo[1], Farshad Shirazi[3], Julia Brody[4], Karen Lutrick[1], Kate Ellingson[1], Jefferey Burgess[1], Paloma Beamer[1]

[1] University of Arizona, Mel and Enid Zuckerman College of Public Health, Tuscon, USA; [2] University of Arizona, College of Science, Tucson, USA; [3] Arizona Poison and Drug Information Center, Tucson, USA; [4] Silent Spring Institute, Newton, USA

Objective

Reporting individual exposure results collected as part of research studies is essential for meaningful community engagement. This study assessed participants' baseline PFAS knowledge, its demographic associations, and examined participants' concerns about PFAS exposure.

Methods

A pre-report survey was conducted before reporting individual PFAS exposure results to assess participants' understanding of PFAS health risks, contamination sources, and confidence in reducing exposure. Total knowledge index (TKI) was calculated by summing correct responses on a four-point Likert scale. Multiple linear regression was used to examine the relationship between demographic factors and TKI as a measure of baseline PFAS knowledge.

Results

Among 519 participants in the pre-report survey, 57% were familiar with PFAS. Most (93%) recognized PFAS in consumer products and drinking water, but 84% were unaware of declining blood concentrations of legacy PFOS and PFOA over the past two decades. Additionally, 60% were unaware that the U.S. EPA has established regulatory limits for PFAS in public water systems. TKI scores ranged from 0 to 10, with a mean of 5.52 (SD = 1.78). Participants earning $150,000+ had significantly higher TKI scores (0.41 points higher than those earning less than $50,000, p = 0.044). Males had higher TKI (0.47 points higher on average than females, p = 0.007), after adjusting for age, household income, ethnicity, and education. No significant associations were found between TKI scores and age or education. More participants were concerned about PFAS in drinking water (83%) than in food (78%). Despite 85% being concerned about the health effects of PFAS on their family, only 12% felt confident in reducing exposure, and 11% believed they had adequate resources to do so.

Conclusion

Findings indicate that demographic factors influence PFAS knowledge and concerns, highlighting the need for tailored communication and accessible exposure reporting to improve public health literacy and support informed decision-making.

S19 | Advancing Pesticide Exposure Assessment and Understanding of Health Effects in Global Communities

D. Martins Figueiredo[1], **A. R. Sherris**[2], A. Connolly[3], M. Rosa[4], C. Hyland[5], G. L. Kayser[6], C. L. Curl[7]

[1] *Utrecht University, Institute for Risk Assessment Sciences, Utrecht, Netherlands*
[2] *University of Washington, Department of Environmental & Occupational Health Sciences, Seattle, Washington, United States of America*
[3] *University College Dublin, UCD Centre for Safety and Health at Work, Dublin, Ireland*
[4] *Icahn School of Medicine at Mount Sinai, Department of Environmental Medicine and Public Health, New York, ´ New York, United States of America*
[5] *University of California, Berkeley, School of Public Health and UC Agriculture and Natural Resources, Berkeley, California, United States of America*
[6] *University of California, San Diego, Herbert Wertheim School of Public Health, San Diego, California, United States of America*
[7] *Boise State University, School of Public and Population Health, Boise, Idaho, United States of America*

Pesticide exposure is a longstanding global public and environmental health threat, disproportionately affecting farmworkers and agricultural communities. Exposure from environmental sources, such as pesticide residues in indoor dust and air, raises concerns—especially for structurally marginalized populations. These populations often face systemic barriers that compound chemical exposures and health risks, yet their contexts, priorities, and resilience are diverse. Addressing these challenges requires robust exposure science to inform policymaking, particularly within Integrated Pest Management (IPM) initiatives aimed at reducing pesticide use, as well as improved understanding of pesticide health risks. This symposium will bring together researchers studying pesticide exposure and health effects across geographically and demographically diverse populations, including communities in the U.S., Europe, Ecuador, Mexico, and Rwanda.

Cumulative and aggregate exposure assessment, considering variability across the lifespan and geographies, is essential to address health equity. Biomonitoring studies increasingly highlight the need to connect internal exposure metrics with external sources and routes of exposure. This is particularly important in low- and middle-income countries (LMICs), where resource limitations and unique environmental contexts demand adaptable and cost-effective exposure measurements. Additionally, exposure science must grapple with the real-life complexity of pesticide mixtures encountered by farmers and people living nearby fields. Measuring and predicting exposure and consequently health risks associated with these mixtures is therefore vital for prioritizing interventions. Presentations will explore key drivers of exposure to widely used pesticides, such as manganese-based fungicides, glyphosate, pyrethroids, neonicotinoids, and organophosphates. Speakers will discuss non-dietary pesticide exposure and health effects across the lifespan, ranging from acute poisoning events to child neurodevelopmental outcomes. By describing cutting-edge methodologies, challenges, and opportunities in exposure science and environmental epidemiology, we aim to support regulatory decision-making, sustainable agriculture, and improved health equity.

S19-01 | The ‘EIRE’ study investigating neonicotinoid insecticide exposures among workers, home users and the general public.

Alison Connolly[1], Sonja A. Wrobel[2], Heiko-Udo Käfferlein[2], Marike Kolossa-Gehring[3], Darragh M. Doherty[1], Sewon Lee[1], Holger M. Koch[2]

[1] University College Dublin, UCD Centre for Safety and Health at Work, Dublin, Ireland; [2] Institute of the Ruhr-University Bochum, Institute for Prevention and Occupational Medicine of the German Social Accident Insurance, Bochum, Germany; [3] German Environment Agency (Umweltbundesamt), Dessau-Roßlau, Germany

Neonicotinoid insecticides (NNIs) are pesticides widely used as plant protection products and as biocides in pet flea treatments. Many studies have investigated the adverse effects of NNIs on pollinators, but more data is needed on human exposures. Numerous international agencies and initiatives, including the Partnership for the Assessment of Risk of Chemicals, Health Canada, and the National Health and Nutrition Examination Survey, have identified NNIs as priority chemicals.
The EIRE project aimed to characterise NNI exposures among the general public, workers and users using human biomonitoring (HBM) sampling strategies. The study analysed urine samples for seven major NNIs and nine metabolites using online solid-phase extraction coupled to liquid chromatography-tandem mass spectrometry.
Irish families (i.e. both parents and at least one child) provided a first-morning void urine sample. Of the 227 urine samples analysed, widespread NNI exposure was found, with 76% of samples having at least one NNI or metabolite above the limit of quantification. The two most frequently detected were N-desmethyl acetamiprid (dme-ACE) and imidacloprid-olefin (IMI-olefin), which were found to have maximum urinary concentrations that corresponded to less than a third and less than 3% of the acceptable daily intakes (ADI) values, respectively.
Further investigations were conducted among pet owners who applied imidacloprid-based flea treatments. Each family was asked to provide the first morning void for 5 days; the first urine sample was given prior to the use of the IMI product. Among the 67 pet owners, a significant increase in the urinary levels of imidacloprid was observed following the application of flea treatments. Further investigations revealed possible exposure determinants, including the quantity of product used and the activities (e.g. sleeping with the pet, combing). Similarly, a smaller study among pet shelter workers using imidacloprid-based flea treatments showed increased urinary imidacloprid and imidacloprid olefin levels compared to the general population.

S19-02 | Household dust and urinary pesticide measures in Mexico City, Mexico

Maria Rosa[1], Cecilia Alcala[1], Hector Lamadrid-Figueroa[2], Adriana M. Garcia[3], Lisa Peterson[4], Carin Huset[5], Heather Stapleton[6], Kitrina M. Barry[5], Nicholas Herkert[6], Martha M. Tellez-Rojo[3], Rosalind J. Wright[1,7], Robert O. Wright[1,7]

[1] Icahn School of Medicine at Mount Sinai, Department of Environmental Medicine and Public Health, New York, USA; [2] National Institute of Public Health (INSP), Department of Perinatal Health, Center for Population Health Research, Morelos, Mexico; [3] National Institute of Public Health, Center for Nutrition and Health Research, Morelos, Mexico; [4] University of Minnesota, Masonic Cancer Center, Minneapolis, USA; [5] St Paul, Minnesota Department of Health, Minneapolis, USA; [6] Duke University, Nicholas School of the Environment, Durham, USA; [7] Icahn School of Medicine at Mount Sinai, Department of Public Health, New York, USA; [8] Icahn School of Medicine at Mount Sinai, Institute for Exposomic Research, New York, USA

Objective: Indoor environmental risk factors for the development and exacerbation of childhood respiratory disease in Latin America, including non-allergen exposures like pesticides, remain understudied. This project leveraged collected biospecimen and environmental samples from an ongoing birth cohort in Mexico City, in order to investigate pesticide metabolite concentrations and pesticide levels in household dust in childhood.

Materials and Methods: Study sample included 272 children enrolled in the Programming Research in Obesity, Growth, Environment, and Social Stressors study. Spot urine samples and household dust from the kitchen and child's bedroom floor were collected when the children were 8-10 years of age. A subset of 106 participants had sufficient dust mass for chemical analysis. Pesticide exposure assessment was conducted through the Human Health Exposure Analysis Resource. Concentrations for 16 current use pesticide metabolites were measured in urine using electrospray ionization high-performance liquid chromatography tandem mass spectrometry. Combined household dust from kitchen and bedroom floor, was sieved, extracted and then analyzed for 14 pesticides using a Q Exactive GC hybrid quadrupole-Orbitrap gas chromatography tandem mass spectrometry system.

Results: The most frequently detected urinary pesticide metabolites were 4-nitrophenol (PNP, 100% above limit of quantitation), 3,5,6-trichloro-2-pyridinol (TCP, 97%), 3-phenoxybenzoic acid (PBA, 84%), 2,4-dichlorophenoxyacetic acid (D24, 74%), trans-3-(2,2-dichlorovinyl)-2,2-dimethyl-cyclopropane-1-carboxylic acid (TDCCA, 71%) and 5-hydroxy-imidicloprid (IMI5OH, 51%). Cis- and trans-permethrin were detectable in >93% of dust samples and chlorpyrifos was detected in 44% of samples. Dust measures of trans-permethrin were strongly correlated with PBA and TDCCA metabolite concentrations (r=0.73).

Conclusions: Children living in an urban megacity had frequent detectable levels of organophosphate and pyrethroid insecticides and a commonly used herbicide. Indoor use of the pyrethroid permethrin may be an important source of exposure. Future work will examine other exposure sources and link exposure to childhood health outcomes.

S19-03 | Non-dietary personal pesticide exposure using silicone wristbands across 10 European countries

Daniel Martins Figueiredo[1], Hongyu Mu[2], Rima Osman[2], Vera Silva[2], Martien Graumans[3], Maurice van Dael[3], Souleiman El Balkhi[4], Hans Mol[5], Anke Huss[1], Roel Vermeulen[1], Jakub Hofman[6], Nelson Abrantes[7], Isabel Campos[7], Francisco Alcon[8], Josefa Contreras[8], Isabelle Baldi[9], Mathilde Bureau[9], Abdallah Alaoui[10], Florian Christ[10], Daniele Mandrioli[11], Daria Sgargi[11], Igor Paskovic[12], Marija P. Paskovic[12], Matjaz Glavan[13], Paula Harkes[2], Esperanza H. Lwanga[2], Lingtong Gai[2], Trine Norgaard[14], Violette Geissen[2], Vivi Schlunssen[15], Paul T. Scheepers[3]

[1] Utrecht University, Institute for Risk Assessment Sciences, Utrecht, Netherlands; [2] Wageningen University & Research, Soil Physics and Land Management Group, Wageningen, Netherlands; [3] Radboud University, Radboud Institute for Biological and Environmental Sciences, Nijmegen, Netherlands; [4] Inserm, Pharmacology & Toxicology, Limoges, France; [5] Wageningen University & Research, Wageningen Food Safety Research, Wageningen, Netherlands; [6] Masaryk University, RECETOX, Faculty of Science, Brno, Czech Republic; [7] University of Aveiro, CESAM and Department of Biology, Aveiro, Portugal; [8] Universidad Politécnica de Cartagena, Agricultural Engineering School, Cartagena, Spain; [9] Univ. Bordeaux, INSERM, Bordeaux, France; [10] University of Bern, Institute of Geography, Bern, Switzerland; [11] Ramazzini Institute, Cesare Maltoni Cancer Research Center, Bologna, Italy; [12] Institute of Agriculture and Tourism, Department of Agriculture and Nutrition, Porec, Croatia; [13] University of Ljubljana, Agronomy Department, Biotechnical Faculty, Ljubljana, Slovenia; [14] Aarhus University, Department of Agroecology, Aarhus, Denmark; [15] Aarhus University, Department of Public Health, Research Unit for Environment, Aarhus, Denmark

Introduction: Individuals are exposed to pesticides via dietary and non-dietary exposure routes. There is a growing need for aggregated exposure estimates across occupationally and non-occupationally exposed populations. This study investigated exposure to current-use and legacy pesticides using silicone wristbands worn by farmers, residents living close to treated fields (neighbors), and the general population (consumers).

Methods: In total, 641 participants across 10 European countries. Wristband extracts were analyzed for 193 pesticides by targeted Liquid and Gas Chromatography-Mass Spectrometry. Information on exposure predictors, such as frequency of cleaning, was collected by questionnaire. Logistic regressions and multivariable generalized least squares models were used to study predictors of pesticide occurrence and concentrations, respectively.

Results: We detected 173 out of 193 pesticides (89%) across all samples. Per wristband 9 – 74 (median of 36) pesticides were detected in conventional farmers (FC), 8 – 66 (median of 20) in organic farmers, 3 – 66 (median of 20) in neighbors and 2 – 54 (median of 17) in consumers. Insecticides, like permethrin and chlorpyrifos, were commonly detected across all groups (>85%). Concentrations varied significantly across groups, with the highest levels often observed in FC. We identified pesticide use and having pets as important predictors of personal non-dietary exposure. Mixture analysis revealed one major combination (in 72% of all samples) of three insecticides, namely permethrin, chlorpyrifos, DDE p,p', and the synergist piperonyl butoxide.

Conclusions: This study highlights that farm workers in conventional fields are a highly exposed group. Ownership of pets explained pesticides used as biocides to be found in wristbands. Banned pesticides are prominent and confirm persisting non-dietary occurrence. It is unclear what the identified prevalent mixtures mean for cumulative exposure and consequent health risks.

S19-04 | *Pesticide Exposure and Barriers to Protection among Pregnant Farmworkers in California*

Carly Hyland[1,2], Ellena Soule-Siler[1], Moncerrat Hernandez[1], Jimena Romano-Silva[1], Antonia M Calafat[3], Maria Ospina[3], Kim Harley[4]

[1] University of California, Berkeley, School of Public Health, Division of Environmental Health Sciences, Berkeley, USA; [2] University of California Agriculture and Natural Resources, Berkeley, USA; [3] Centers for Disease Control and Prevention, National Center for Environmental Health, Atlanta, USA; [4] University of California, Berkeley, School of Public Health, Division of Maternal, Child, and Adolescent Health, Berkeley, USA

Objective: Gestational pesticide exposure has consistently been associated with adverse maternal and child health outcomes, underscoring the importance of minimizing occupational exposure during pregnancy. Farmworkers in California are legally entitled to unique benefits during the prenatal period that require certification by a medical provider, including work accommodations and paid leave. The goal of this study was to examine urinary pesticide concentrations and the experiences of pregnant farmworkers, including barriers in adopting protective behaviors.
Materials and Methods: We conducted urinary biomonitoring (n=57) and administered surveys (n=128) and open-ended interviews (n=10) with pregnant farmworkers recruited at prenatal visits in California's Central and Salinas Valleys from May-August 2024. Spot urine samples were analyzed for biomarkers of herbicides and of organophosphate (OP) and pyrethroid insecticides.
Results: Urinary concentrations were typically lower than studies of other farmworkers. For analytes detected in at least 60% of participants, median specific gravity-adjusted concentrations ranged from 0.33-1.11 μg/L for biomarkers of OP and pyrethroid insecticides and 0.19-0.33 for the herbicides glyphosate and 2,4-dichlorophenoxyacetic acid. While 75% of participants knew farmworkers were eligible for additional paid leave during pregnancy, only 50% had been certified by a physician for this leave; the average gestational age at certification was around 22 weeks (range 8-36 weeks). Participants commonly reported changes in work tasks related to heavy lifting after becoming pregnant, but few received workplace modifications to reduce pesticide exposure.
Conclusion: Our study highlighted gaps in the utilization of prenatal benefits to avoid potential harmful effects of occupational pesticide exposure, including lack of awareness from medical providers and farmworkers, language discordance with medical providers, inconsistent access to prenatal care, and fear that employers would not accommodate their request. We found evidence of exposure to some pesticides among pregnant farmworkers, underscoring the importance of improving access to and understanding of these benefits.

S19-05 | *Children's Home Proximity to Pesticide Spray Sites and Neurobehavioral Performance in Ecuador*

Georgia Kayser[1], Jasen Zhang[1], Alehlí Calderón-Villareal[1], Briana Chronister[1], Jose Suarez-Torres[2], Harvey Checkoway[1], Franklin De la Cruz[2], Jose Ricardo Suarez-Lopez[1]

[1] University of California, San Diego, Herbert Wertheim School of Public Health, San Diego, USA; [2] CIMAS, QUITO, Ecuador

Background: Proximity to pesticide spray sites may affect neurodevelopment in children. Longitudinal studies assessing residential proximity to flower plantation and crops on children's neurobehavioral performance are needed to understand chronic effects of pesticide exposure and to inform policy on how far away residential homes and schools should be stationed from agricultural pesticide spray sites.

Methods: In this longitudinal analysis of 629 Ecuadorian children in the ESPINA study, home proximity to floricultural pesticide spray sites and neurobehavioral performance, using the NEPSY-II (A Developmental Neuropsychological Assessment), were studied over three time periods, between 2008 and 2016. A higher score on the NEPSY-II indicates better neurobehavioral performance. Child proximity to flower plantation was measured by distance between participant's households and the nearest flower plantation and total surface area of floricultural greenhouses around children's household. A generalize estimating equation with an exchangeable covariance structure was used to model each NEPSY II score with respect to various measure of pesticide exposure.

Results: We found most participants had floriculture plantations within a 500m area around their households in visit 1(62.6%), 2 (61.8%), and 3 (63.6%), respectively. Across the three visits, NEPSY-II scores mean (SD) ranged from 5.9 (3.0) to 10.7 (4.9). Doubling area of floriculture within 300m of children's homes decreased Attention/Inhibitory Control domain scores by -0.026 (CI: -0.045, -0.007; p = 0.0064). Increasing floriculture distance between children's homes and floriculture crop spray sites was associated with better performance on the subdomains related to Attention/Inhibitory Control, Language, and Memory/Learning performance.

Discussion: Our results suggest geographical proximity to pesticide spray sites may create exposures that increase risk of poorer neurobehavioral performance, specifically related to impulsivity and cognitive flexibility, understanding complex verbal instructions, and challenges with verbal-visual associative learning among adolescents.

S19-06 | *Understanding pesticide exposure in Rwanda: Identifying commonly used pesticides and their health effects*

Cynthia L. Curl[1], Concorde I. Rwibutso[2], Therese Nyirahabimana[3], Emmanuel Irimaso[4], Stephanie Mitchell[5], Liberata Mukamana[6], Dawn Zimmerman[7], Sabrina Sholts[8]

[1] Boise State University, School of Public and Population Health, Boise, USA; [2] Gorilla Doctors, Wildlife Medicine, Musanze, Rwanda; [3] Rwanda Biosolution, Waste Management, Kigali, Rwanda; [4] University of Rwanda, School of Veterinary Medicine, Nyagatare, Rwanda; [5] University of Washington, Center for One Health Research, Seattle, USA; [6] University of Rwanda, School of Economics, Kigali, Rwanda; [7] Veterinary Initiative for Endangered Wildlife, Wildlife Medicine, Bozeman, USA; [8] Smithsonian Institution, Anthropology, Washington, D.C., USA

Objective: The extent of pesticide exposure and the frequency of pesticide poisonings among Rwandan farmers is unknown, despite widespread anecdotal evidence indicating these issues are prevalent. We aimed to identify the main pesticide products used in agriculture in Rwanda, to determine their potential health effects and to compare these effects to those reported.
Methods: In December 2024, we visited the Rwamagana, Nyagatare, Kayonza, Kirehe, and Ngoma districts of Rwanda and conducted five focus groups with 31 sector agricultural officers (SAOs) and animal resource officers (SAROs). Focus group participants were asked to list the main pesticides used in their districts; these pesticides were recorded and any other pesticides mentioned in the discussion were extracted from focus group transcripts. We visited agrochemical stores in each district and documented those pesticides most commonly available for sale. We describe the potential health effects of these pesticides and compare with the most common health issues reported to affect Rwandan farmers.
Results: An initial list of 57 compounds was evaluated to distinguish names of active ingredients (n=24) from trade names/formulations (n=33). We then evaluated the relative frequency of use of each pesticide based on the number of regions in which it was reported and the frequency with which it was observed in agrochemical shops. We identify the six most common active ingredients: abamectin, cypermethrin, lambda-cyhalothrin, profenofos, mancozeb and metalaxyl. Health effects of exposures to these compounds were consistent with outcomes reported by SAOs and SAROs in this study.
Conclusions: Participants reported significant challenges in pesticide management among Rwandan farmers, including instances of pesticide application outside of recommended guidelines, limited access to personal protective equipment, and a need for more robust and consistent pesticide safety education and information. We identified the most commonly used pesticide products in Rwanda and confirmed their relationships to the health effects reported.

S20 | Reducing Children's Lead Exposures in Sub-Saharan Africa – Exposure Science to Help Optimize the Balance Between Primary and Secondary Prevention

A. M. Riederer[1], **A. McCartor**[2], E. A. Lumumba[3], A. J. Gilbert[1], F. H. Were[3], C. J. Karr[1, 4]

[1] *University of Washington, Environmental & Occupational Health Sciences, Seattle, Washington, United States of America*
[2] *Pure Earth, New York, New York, United States of America*
[3] *University of Nairobi, Chemistry, Nairobi, Kenya*
[4] *University of Washington, Pediatrics, Seattle, Washington, United States of America*

Lead (Pb) exposures in the prenatal period and early childhood are associated with adverse health outcomes across the lifespan. Efforts to reduce Pb exposures in sub-Saharan Africa (SSA) are urgently needed to close global environmental health equity gaps. Achieving these goals requires investments in both primary and secondary prevention through source reduction and exposure mitigation, and blood lead surveillance and screening, respectively, especially of pregnant women and young children. Current data on early-life lead levels and exposure sources in SSA communities are limited and programs focused on pediatric lead poisoning are lacking.

In four presentations, we share recent evidence from Ghana and Kenya on children's lead exposures. These are followed by a presentation on early efforts for pediatric blood lead screening and public health campaigns to address sources of concern using Kenya as a case study. Discussion with the audience will focus on: data needs to guide and promote progress on reducing early life lead exposure, transportability of child lead poisoning prevention programs to SSA based on experience in other settings, and opportunities to advance local exposure science capabilities for lead.

S20-01 | Potential lead exposure sources among children across 3 regions in Ghana

Emily Nash[1], Caroline M. Baylor[1], Blessing E. Gbadago[2], Wilson Baaku[2], Esmond W. Quansah[2], Ursula Berger[3], Stephan Böse-O'Reilly[4]

[1] Pure Earth, New York, USA; [2] Pure Earth Ghana, Accra, Ghana; [3] LMU Munich Faculty of Medicine, Institute for Medical Information Processing, Biometry and Epidemiology, Munich, Germany; [4] LMU Munich University Hospital, Institute and Clinic for Occupational, Social and Environmental Medicine, Munich, Germany

Objective: Lead exposure is well known to cause wide-ranging harmful effects especially in children. Data on lead exposure levels and sources are limited in most low- and middle-income countries. We followed a blood lead level (BLL) survey conducted by Ghana Health Service with household environmental assessments to better understand potential lead exposure sources in households across 9 communities in 3 regions of Ghana.
Methods: 293 households (hhs) were randomly selected for environmental assessments from 3,227 hhs where children aged 12–59 months had been recently tested for BLLs. In this subset, lead content was measured with a portable X-Ray Fluorescence (XRF) device of the following media: soil, dust, paint, toys, cosmetics, metal cookware, ceramic foodware, dried/powdered spices, and jewelry. Detection limits ranged from 0.2 μg/cm^2 (paint) to 20 ppm (metal cookware). Dust was collected using dust wipes, measured with the XRF, and sent for laboratory confirmation. Drinking water was collected for laboratory analysis. We used Additive Mixed Regression Models, factoring in age and gender, to determine associations between BLLs above 10 μg/dL and environmental exposure sources.
Results: Of 288 valid hh assessments, 31% of children had BLLs >10 μg/dL (60% >5 μg/dL). Environmental media showing low prevalence of elevated lead levels included drinking water, ceramic foodware, toys, spices, jewelry, and paint. Applying the Additive Mixed Regression Model on the remaining variables yielded the following factors of concern: age (with each additional month of age, chance of elevated BLL reduced by 6%), exposure to soil with lead levels >100 ppm (20x increased risk of elevated BLLs compared to soil lead levels <50 ppm), and traditional eyeliner use (40x increased risk of elevated BLLs).
Conclusion: Our findings suggest that contaminated soil and contaminated eyeliner use may be contributing to elevated blood lead levels among children in Ghana.

S20-02 | Rapid Market Screening of consumer products in Ghana

Aelita Sargsyan[1,2], Caroline M. Baylor[1], Emily Nash[1], Gordon Binkhorst[1], Jenna E. Forsyth[3], Barbara Jones[4], Gabriel S. Ibarra[1], Sarah Berg[1], Andrew McCartor[1], Richard Fuller[1], Stephan Böse-O'Reilly[1,5]

[1] Pure Earth, New York, USA; [2] University of Valencia, Faculty of Biological Sciences, Valencia, Spain; [3] Stanford University, Division of Infectious Diseases and Geographic Medicine, Stanford, USA; [4] Cardinal Resources Inc., Monroeville, USA; [5] LMU Munich University Hospital, Institute and Clinic for Occupational, Social and Environmental Medicine, Munich, Germany

Objective: Lead exposure causes wide-ranging harmful effects especially in children. Data on lead exposure sources are limited in most low- and middle-income countries. We conducted a Rapid Market Screening of consumer products in 25 countries, including Ghana, to better understand potential sources of lead.

Methods: Researchers visited markets in the Ghanaian cities of Cape Coast, Koforidua, and Kumasi and purchased items from the following categories: ceramic foodware, metallic cookware, plastic foodware, cosmetics, toys, paints, spices, and staple/dry foods. These items were tested for lead content with an X-Ray Fluorescence (XRF) analyzer with a subset of samples sent to a laboratory for confirmatory testing. Reference values were established for each category.

Results: Of 11 ceramic foodware samples, 18% were above the reference value of 100 ppm (max=6570 ppm). Of 22 metallic cookware samples, 55% were above the reference value of 100 ppm (max=24,100 ppm). Of 12 plastic foodware samples, 0% were above the reference value of 100 ppm (max=32 ppm). Of 28 cosmetics samples, 7% were above the reference value of 2 ppm (max=220 ppm). Of 22 toy samples, 14% were above the reference value of 100 ppm (max=1533 ppm). Of 4 paint samples, 0 were above the reference value of 90 ppm (max=non-detect). Of 47 spice samples, 0 were above the reference value of 2 ppm (max=non-detect). Of 29 staple/dry food samples, 0 were above the reference value of 0.2 ppm (max=non-detect).

Conclusion: Our findings suggest that contaminated metallic foodware may be a source of lead exposure in Ghana with ceramic foodware, toys, and cosmetics potential additional areas of concern. Funding: GiveWell, Effective Altruism Global Health and Development Fund, Open Philanthropy, Affinity Impact.

S20-03 | Household food insecurity and blood lead levels in 12-month-olds from Dandora, Nairobi, Kenya

Edith A. Lumumba[1], Anne M. Riederer[2], Faridah H. Were[1], Priscilla W. Edemba[3], Beatrice C. Mutai[3], Judith Adhiambo[3], Lewis Olweywe[3], Prestone Owiti[3], Barbra A. Richardson[4,5], Catherine J. Karr[2,6], Elizabeth Maleche-Obimbo[3], Sarah Benki-Nugent[4]

[1] University of Nairobi, Department of Chemistry, Nairobi, Kenya; [2] University of Washington, Department of Environmental and Occupational Health Sciences, Seattle, USA; [3] University of Nairobi, Department of Paediatrics and Child Health, Nairobi, Kenya; [4] University of Washington, Department of Global Health, Seattle, USA; [5] University of Washington, Department of Biostatistics, Seattle, USA; [6] University of Washington, Department of Pediatrics, Seattle, USA

Objective: Lead exposure affects neurodevelopment. We measured blood lead levels (BLLs) of 163 infants in an ongoing pregnancy cohort and explored associations with peeling/chipping paint in the home, household food insecurity, and breastfeeding frequency.
Methods: Mothers were interviewed on demographics and lead exposure factors at enrollment. At the 12-month visit, infant venous blood was drawn, iron status measured, and mothers were interviewed on household hunger in the past 30 days and frequency of breastmilk and other foods consumed by the infant in the past 24 hours. Blood was analyzed for Pb using ICP-MS, with accuracy evaluated using matrix spikes and certified reference materials. Results were blank corrected. Three observations below the detection limit (0.01 ug/dL) were replaced with 0.01 ug/dL. Generalized linear regression with robust standard errors was used to examine univariate associations between log-transformed BLLs and factors associated with children's BLLs in prior studies [maternal age, education, parity, infant hemoglobin, 24 h green leafy vegetable consumption, 24 h dairy consumption, water source, family member with potentially lead-exposed job, use of kohl on self or siblings, proximity (geospatial) to Dandora dumpsite (<=500 m, >500m), and proximity (self-reported) to battery/e-waste recycling or garages] and fit multivariable models.
Results: BLLs (N=161) ranged from 0.01-6.8 µg/dL [median (inter-quartile range) 1.9 (1.3-2.5) ug/dL]. 26% of homes had peeling/chipping paint at enrollment while 20% experienced hunger the past month. In the multivariable model, infants in households experiencing hunger had 29% (95% CI: 2%, 62%) higher BLLs; peeling/chipping paint and breastfeeding frequency were not associated with BLLs.**Conclusion:** Our study contributes novel data on infants' BLLs in Nairobi and demonstrates a link with household food insecurity, offering insight into potential interventions.

S20-04 | Characterizing neurotoxic metal exposures in early childhood using hair in a Nairobi, Kenya, birth cohort

Abigail Gilbert[1], Anne M. Riederer[1], Michael H. Paulsen[1], Shar Samy[1], Sarah Benki-Nugent[2], Wanini Edemba[3], Elizabeth Maleche-Obimbo[3], Christopher D. Simpson[1]

[1] University of Washington, Environmental & Occupational Health Sciences, Seattle, USA; [2] University of Washington, Global Health, Seattle, USA; [3] University of Nairobi, Pediatrics, Nairobi, Kenya

Objective: Our study determines concentrations and variability of five neurotoxic metals in hair samples from mother-infant pairs using laser ablation inductively coupled plasma mass spectrometry (LA-ICP-MS). LA-ICP-MS preserves the spatial distribution of metals along the hair shaft, allowing changes in metal deposition to be seen across time.

Methods: We collected hair from 40 mother-infant pairs in a birth cohort study in Nairobi, Kenya when infants were 6 or 12 months of age. Five hairs per participant were analyzed. The ICP-MS was tuned using NIST612 glass standard. We utilized gelatin standards to create calibration curves for the target metals and determined limits of detection (LODs). We averaged concentrations along the ablated length of hair then calculated a mean concentration across the 5 strands for each metal.

Results: LODs were: 1 µg/g (Mn), 0.1 µg/g (As), 0.05 µg/g (Cd), 0.1 µg/g (Hg), and 0.05 µg/g (Pb). Our Mn LOD is marginally higher than others reported using laser ablation calibration techniques for hair analysis (0.05 – 0.4 µg/g), while our other LODs are within or lower than the range of published LODs: 0.2-2.1 µg/g (As), 0.05-0.6 µg/g (Cd), 0.04-0.7 µg/g (Hg), 0.01-1.3 µg/g (Pb). Mean (± standard deviation) hair metal concentrations in the one mother-infant pair analyzed to date are above our LODs (except for Hg): 5.58±1.73 µg/g (Mn), 2.18±0.374 µg/g (As), 0.64±0.029 µg/g (Cd) and 6.62±3.66 µg/g (Pb). These hair metal concentrations fall within the ranges of previously published values when including environmentally and occupationally exposed populations.

Conclusions: Our method performance is comparable to previous laser ablation studies using hair. This relatively novel method is being applied to samples from the remaining 39 mother-infant pairs with a goal of using concentrations along the individual strands to provide a time-resolved picture of metals exposures during critical windows of neurodevelopment. Funding: R21ES033745 (U.S. NIEHS)

S22 | Global disparities in the exposome and human health

D. Sarigiannis[1, 2], K. K. Olympio[3], V. Vasiliou[4], T. Hartung[5]

[1] National Hellenic Research Foundation, Athens, Greece
[2] Aristotle University of Thessaloniki, HERACLES Research Center on Health and the Exposome, Thessaloniki, Greece
[3] University of Sao Paulo, Sao Paulo, Brazil
[4] Yale University, School of Public Health, Department of Environmental Health, New Haven, Connecticut, United States of America
[5] Johns Hopkins University, Bloomberg School of Public Health, Department of Environmental Health Engineering, Baltimore, Maryland, United States of America

Global disparities in the exposome and human health are increasingly recognized as critical factors influencing disease outcomes and public health. This ENVESOME workshop brings together experts to explore this complex interplay between environmental exposures, socioeconomic factors, and health disparities.

Kelly P. K. Olympio's research with the eXsat Group focuses on exposome studies in workers' health, particularly in informal and challenging workplace settings. In Latin America, exposome research faces unique obstacles, including economic constraints and the dual burden of infectious and non-communicable diseases. Despite these challenges, the group leverages health surveillance systems and administrative data to develop cost-effective exposome methodologies.
Vasilis Vasiliou's work highlights the exposome's role in Alzheimer's disease (AD) risk, emphasizing the interaction between genetics and social determinants of health. Using high-resolution mass spectrometry and machine learning, researchers analyze multi-scale data to understand how structural inequities shape exposure patterns and disease outcomes in AD.

Thomas Hartung introduces the concept of "Exposome Intelligence," which integrates AI, omics technologies, and biomonitoring to study environmental exposures systematically. This approach accounts for inequities and social disparities, utilizing deep learning for toxicity predictions and expanding into multi-modal deep learning and interpretable models.

Dimosthenis Sarigiannis explores how inequities affect children's exposure to pollutants of emerging concern. Using New Approach Methodologies and exposomics, this research investigates the roles of air, noise, and light pollution in respiratory disease, metabolic syndrome, and developmental neurotoxicity in children.

These diverse perspectives highlight the global nature of exposome research and the urgent need for targeted interventions to address health disparities and environmental exposures across different populations and regions.

S22-03 | Deciphering the human exposome considering the broad spectrum of population vulnerability and risks: the role of exposome intelligence

Thomas Hartung

Johns Hopkins University, Bloomberg School of Public Health, Department of Environmental Health Engineering, Baltimore, USA

The term “Exposome Intelligence” integrates AI, omics technologies, and biomonitoring to systematically study environmental exposures and their impacts on human health accounting explicitly for inequities and social and economic disparities.
Deep learning on chemical and bioassay data has shown successes for toxicity predictions and mechanisms. Active research is expanding into multi-modal deep learning, synthetic data generation, and interpretable models. Coupling AI with ongoing improvements in primary evidence generation and appraisal integrating exposomics data, toxicological and social determinants and modifiers of pathology is pivotal for it to robustly augment environmental health science. We show herein a viable roadmap towards achieving this.

S22-04 | Children exposure to pollutants of emerging concern – the environmental exposome and how it is affected by inequities

Dimosthenis Sarigiannis

National Hellenic Research Foundation, Athens, Greece

The impact of pollutants of emerging concern on children is explored through the lens of the environmental exposome, highlighting how inequities shape exposure and health outcomes. Using New Approach Methodologies and exposomics, this integrative strategy uncovers the roles of pollutants such as air, noise, and light pollution, as well as hazardous waste, in respiratory disease, metabolic syndrome, and developmental neurotoxicity in children. By identifying key sources of exposure and socio-political drivers of disease, scientific evidence is translated into actionable policy recommendations. These targeted interventions aim to address disparities and promote cost-effective public health strategies tailored to specific regions or communities.

S23 | Extreme heat, air pollution & pregnancy: exploring interactions of environmental epidemiology and exposure science with health equity, focusing on vulnerable populations

S. Bhatnagar[1], **J. Hirst**[4], **N. Wadhwa**[1], S. Dey[2], V. Venugopal[3], **R. Thiruvengadam**[5, 1], **P. Kshetrapal**[1], S. Sopory[1], B. K. Desiraju[1], D. Praveen[4], C. M. Kurian[4], R. Shanmugam[3], A. Ayushi[1], S. Raghavan[1], S. Singh[1], G. C. Flores[4], K. Kumar[4], T. S. Isaac[3]

[1] *Translational Health Science and Technology Institute, Maternal and Child Institute, Faridabad, India*
[2] *Indian Institute of Technology, Delhi, Centre for Atmospheric Sciences, Delhi, India*
[3] *Sri Ramachandra Institute of Higher Education and Research, Department of Environmental Health Engineering, Faculty of Public Health, Chennai, India*
[4] *The George Institute for Global Health, Maternal and Child Health, London, United Kingdom*
[5] *Pondicherry Institute of Medical Sciences, Biochemistry, Puducherry, India*

A recent significant threat that has emerged for maternal and child health is climate change, more specifically rising global temperatures and air pollution levels. These factors further compound the risks for adverse outcomes in socioeconomically disadvantaged regions, e.g., low and middle-income countries (LMICs). This symposium aims to outline a novel international, multi-institutional, transdisciplinary research venture investigating the impact of heat exposure, air pollution, and pregnancy outcomes—the Heat in Pregnancy-India (HiP-India) study, funded by the Wellcome Trust, UK. The team also has recently finished a systematic review on the associations between PM 2.5, heat and preterm birth focusing exclusively on low and middle income countries (LMICs) where greater vulnerabilities to these environmental stressors was obsereved in LMICs.

The session will outline, through a series of presentations, the findings from the Heat in Pregnancy (HiP) India Project. The project has an ambispective study design and envisages the assessment of associations of environmental exposures on maternal and fetal health. It also prospectively aims to focus on physiological outcomes and bio-signatures across distinct geographical settings within India. The presentations will cover the preliminary results from the retrospective analyses and discuss the plan of analysis to discover novel biomarkers associating high heat stress to adverse pregnancy outcomes. They will also detail the prospective cohort with specific focus on the insights and challenges in capturing individualized exposure data. The session will conclude with a summary of proposed qualitative assessment methods aimed to derive community insights towards heat stress linked with socioeconomic factors and adaptation strategies towards the same.

The symposium is proposed to finish with a moderated discussion on exposure science methodologies in clinical cohorts, with the ultimate deliverable being implementing equitable interventions in vulnerable populations to mitigate health risks.

S23-01 | Retrospective epidemiological Insights: Linking Heat and Air Pollution to Pregnancy Outcomes- Statistical methods used and results from the GARBH-INi cohort

Ayushi Ayushi[1], Sreevatsan Raghavan[1], Ramachandran Thiruvengadam[2,1], Bapu K. Desiraju[1], Nikhil Sharma[1], Nitya Wadhwa[1], Shinjini Bhatnagar[1]

[1] Translational Health Science and Technology Institute, Maternal and Child Institute, Faridabad, India; [2] Pondicherry Institute of Medical Sciences, Biochemistry, Puducherry, India

Robust methodological frameworks are critical to disentangling the effects of environmental exposures on adverse pregnancy outcomes just like any other epidemiological assessment. This presentation outlines a comprehensive Statistical Analysis Plan (SAP) developed to assess the independent effects of heat and air pollution exposure on outcomes such as preterm birth, low birth weight, and small for gestational age in the GARBH-Ini pregnancy cohort from India.

This presentation outlines a retrospective analysis of pregnancy data from the GARBH-Ini cohort with high-resolution (1 X 1 Km) daily and weekly ambient temperature and PM2.5 exposure data modelled by the HiP- India study team. One of the key analytical objectives is to identify vulnerable gestational exposure windows, specific periods during pregnancy when heat or air pollution may have the highest impact on outcomes. The analysis team explored a range of statistical modeling strategies to address the primary research aims: identifying critical exposure periods and quantifying their associations with adverse pregnancy outcomes such as preterm birth, low birth weight, and small-for-gestational age. The choice of models was guided by the data structure, temporal nature of exposure and potential for nonlinear and delayed effects. Covariate selection and possible data-generating processes were guided by directed acyclic graphs (DAGs) to minimize confounding effects and strengthen causal inference.

The presentation aims to establish a robust and reproducible analysis plan for conducting retrospective epidemiological analysis in maternal health using environmental data. The structured approach ensures clarity in model selection, exposure definition, and interpretation strategies.

This presentation will highlight a framework of high-quality, policy-relevant epidemiological research on climate-sensitive pregnancy outcomes. Its modular structure can be adapted to other cohorts and LMIC contexts, and it lays the foundation for valid, interpretable results that inform public health interventions.

S23-02 | Modelling in-utero exposure to air pollution and heat for individual participants of the Ganrbhini cohort in North India

Sagnik Dey, Arunangsu Das

Indian Institute of Technology, Delhi, Centre for Atmospheric Sciences, Delhi, India

Exposure to heat and air pollution during pregnancy has been identified as key environmental risk for adverse birth outcomes. While existing studies from India and other lower- and middle-income countries (LMICs) have explored the effect of ambient and household air pollution on birth outcomes, the impact of heat and how it modifies the air pollution-birth outcome association have not been studied. In this work, we developed air pollution and heat exposure data at a very high spatial scale to assign exposure to pregnant mothers at their household level, leveraging the ongoing and new birth cohorts across India.

For air pollution, we considered fine particulate matter ($PM_{2.5}$) and its composition at 1-km by 1-km spatial scale. We developed a machine learning-based framework to concert satellite-derived columnar aerosol optical depth to surface PM2.5. We trained machine learning models using mass fractions of PM2.5 species derived from chemical transport model outputs based on geospatial and meteorological predictor variables. We then multiplied the machine-learning predicted mass fractions with satellite-derived PM2.5 to predict PM2.5 species concentrations. For heat exposure, we processed temperature and humidity data from ERA5 reanalysis. We then interpolated the data to a finer spatial scale and bias-corrected it, utilising ground monitoring data from the Central Pollution Control Board and India Meteorological Department.

We extracted the exposure to PM2.5 and its composition during nine months of the subjects recruited in the cohorts to assign in-utero exposure to air pollution based on their household geocodes. Air pollution exposure has been estimated separately for the three trimesters to understand seasonal variations. We assigned exposure during the peak summer months for heat, inclduing exposure to extremes based on conditions exceeding 90th percentiles. The exposure data are being used for epidemiological analysis.

S23-03 | Discovering novel biological markers for adverse pregnancy outcomes associated with extreme heat

Divya Arya[1], Himadri Devvanshi[1], Katherine Dibbon[2], Study Team Garbhini[1], Manu Vatish[2], **Pallavi Kshetrapal**[1]

[1] Translational Health Science and Technology Institute, Maternal and Child Institute, Faridabad, India; [2] University of Oxford, United Kingdom, Nuffield Department of Women’s & Reproductive Health, Oxford, UK

Heat stress disrupts thermoregulation in pregnant women, leading to significant complications for both mother and fetus. Pregnant women face increased physiological strain when exposed to heat (extreme temperature), with effects varying across trimesters. Impaired thermoregulation can result in dehydration, heat exhaustion, and placental dysfunction, all of which could contribute to pregnancy complications and adverse outcomes. Multiomic studies in both in-vitro and in-vivo models have identified altered critical pathways (ROS signalling, immune homeostasis,

placental vascularization, and nutrient transport) through which heat stress leads to placental insufficiency and disruption. However, the translation of these findings to human pregnancy remains limited, especially in real-world settings with multiple stressors. Despite the increasing impact of climate change, mechanistic studies in human pregnancies remain scarce, particularly in LMICs, where environmental and socioeconomic challenges compound risks. Understanding how heat stress influences pregnancy outcomes in these settings is crucial for developing region-specific targeted interventions. We propose a retrospective longitudinal study leveraging well-phenotyped, high-quality samples from the GARBH-INi pregnancy cohort, archived at an ISO 20387:2018 accredited biorepository. This study will explore the effects of maternal heat exposure on the expression of inflammatory and placental angiogenesis-related biomarkers associated with pathological pregnancies. By analyzing serially collected maternal blood samples across pregnancy, we aim to identify critical gestational windows where heat exposure exerts the most significant effects on placental function. Additionally, we will compare the placental biomarkers circulating in maternal blood between the pregnancies with no extreme heat exposure to those exposed to high ambient temperatures. This comparative approach will help delineate the differential mechanistic pathways contributing to placental insufficiency under heat stress. The findings from this study will offer new insights into the biological pathways linking heat exposure to pregnancy complications and aid in development of targeted interventions to mitigate its adverse effects, ensuring better maternal and fetal health outcomes.

S23-04 | Individual Exposure assessment monitoring- Insights from the prospective cohorts for the HiP- India study

Vidhya Venugopal, Rekha Shanmugam, Tanya S. Isaac

Sri Ramachandra Institute of Higher Education and Research, Department of Environmental Health Engineering, Faculty of Public Health, Chennai, India

Maternal health is facing significant challenges due to the increasing temperatures associated with climate change. In our research, we utilised advanced, non-invasive wearable devices, specifically the iButton Hygrochron, integrated with a Fitbit smartwatch, to continuously monitor individual heat stress exposure. These devices provide real-time measurements of air and skin temperature, humidity, and heart rate, allowing individuals to engage in daily activities without disruption. Additionally, we incorporated a data logger to track continuous heat exposure, serving as an effective alternative to conventional environmental measurement methods. It became apparent that the wearable device was uncomfortable for some participants, resulting in instances of it being removed or misplaced. This observation prompted a targeted strategy to enhance participant adherence to device usage. Furthermore, we assessed environmental heat exposure within the home using the QuesTemp°34WBGT (wet bulb globe temperature) device, which accounts for all critical factors, including dry bulb temperature, wet bulb temperature, globe temperature, radiant heat, and humidity. These elements significantly influence an individual's heat exposure. The device's size and participants' privacy concerns sometimes impacted its placement, often stemming from misunderstandings with in-laws, neighbours, and landlords who misconstrued it as a surveillance camera. We measured physiological heat strain using infrared thermometers to determine core body temperature. This non-invasive, rapid, and portable method makes it well-suited for field evaluations. We also monitored hydration status utilising refractometers and dipsticks to ascertain urine specific gravity, allowing for reliable results. Despite encountering various challenges in the field, our findings provide essential insights into the heat exposure experienced by women. This investigation employs rigorous, internationally validated methodologies to collect precise and robust data regarding women’s heat exposure, and we present our findings on the effects of heat stress

on pregnancy while underscoring the necessity for personal exposure measurements, as well as the advantages and challenges experienced in the field.

S23-05 | Developing Adaptive Strategies: Socioeconomic and Policy Perspectives

Jane Hirst, Gabriela C. Flores, Karthika Kumar, Surya Surendran, Devaki Nambiar, Chris M. Kurian, Diana M. Thomas, Devarsetty Praveen, Yogesh Jain

The George Institute for Global Health, Maternal and Child Health, London, UK

Extreme heat has emerged as a critical public health crisis, disproportionately impacts pregnant women in low- and middle-income countries (LMICs), where physiological vulnerabilities intersect with deeply rooted gender inequities. The team’s scoping review on community-level heat adaptation interventions in LMICs revealed a troubling pattern: only one implemented gender- sensitive approaches. Analysis of 35 Indian Heat Action Plans and State Climate Change Action Plans substantiated this gap, revealing merely 40 strategies explicitly targeting pregnant and lactating women, compared to 174 instances where women were aggregated within broader vulnerability classifications. Further, the predominance of behavioral interventions reflects insufficient investment in healthcare infrastructure and policy mechanisms. Notably, 11 Indian states and territories demonstrated a complete absence of targeted interventions. This pattern not only indicates inadequate gender mainstreaming but also fundamental disconnection from women's lived experiences. While policy inconsistencies are evident in documentation, implementation efficacy remains largely unexamined.

To address this, the presentation will outline a qualitative study that explores how pregnant and lactating women experience and navigate extreme heat in India. Pregnant women (10-15) and lactating women (3-5) from backgrounds of social disadvantage will be sampled purposively in the three agro-ecologically diverse sites of the HiP-India Study. Key Informant interviews with community leaders (2-3), Focused Group Discussions with frontline workers (1-2), and family members (1-2) will help identify and locate existing practices, opportunities and barriers to adaptation embedded in different contexts. Furthermore, a community engagement strategy through Round Tables and Community Advisory Committees will foster the participation of key stakeholders to inform the study and facilitate knowledge exchange among participants and communities. By integrating critical policy analysis and empirical insights, this work aims to advance policy for an intersectional, equity-driven climate adaptation that centers the needs of pregnant and lactating women from marginalized communities, recognizing them as agents in building climate resilience.

S24 | Radon Exposure Across the Lifespan: Emerging Evidence Beyond Lung Cancer

L. Longxiang[1], A. Ruano Raviña[2], M. Bozigar[3], E. Whitsel[4], K. Kahe[5]

[1] Emory University, Rollins School of Public Health, Atlanta, Georgia, United States of America
[2] University of Santiago de Compostela, Preventive Medicine and Public Health, Santiago de Compostela, Spain
[3] Oregon State University, College of Health, Corvallis, Oregon, United States of America
[4] University of North Carolina, Department of Epidemiology/Gillings School of Public Health, Chapell Hill, North Carolina, United States of America
[5] Columbia University, Department of Epidemiology/Mailman School of Public Health, New York, New York, United States of America

Residential exposure to radon decay products is increasing due to more insulated home construction and even post-pandemic work-from-home trends. While radon decay products are a known cause of lung cancer in middle-aged and older adults, emerging evidence links even low ambient concentrations to a broader range of health effects across various age groups.

By synthesizing research across physics, atmospheric science, and toxicology, there is growing recognition that radon, an inert but unstable gas, decays into radioactive particles that rapidly attach to airborne particulates small enough to translocate throughout the body. These “attached fractions” emit highly toxic and energetic alpha radiation, which can damage DNA and disrupt signaling in numerous tissues. These mutagenic mechanisms might contribute to various disease processes, extending beyond lung cancer.

Given recent findings, it is urgent to reassess the health impacts of radon decay products, particularly in the context of climate change, evolving building practices, and critical periods of the lifespan. Buildings designed for energy efficiency often trap and concentrate indoor air pollutants, including radon decay products. While the health effects of radon exposure have been extensively studied in adult male miners, research on females, children, and other demographic groups remains very limited.

This symposium highlights the far-reaching health implications of radon decay product exposure, the largest global source of ionizing radiation both in residential settings and workplaces. Drawing from studies conducted worldwide and across the lifespan, we examine associations with respiratory diseases, cardiovascular conditions, non-lung cancers, and adverse maternal and child health outcomes. The symposium aims to advance understanding of radon-related risks, emphasizing the need for further research and policy action to mitigate exposures and protect public health.

S24-01 | Updated understanding of the spatiotemporal distributions of radon in the United States based on massive measurements during 2001-2021

Li Longxiang[1,2], Brent A. Coull[2], Petros Koutrakis[2]

[1] Emory University, Rollins School of Public Health, Atlanta, USA; [2] Harvard University, TH Chan School of Public Health, Boston, USA

Radon is the second leading cause of lung cancer in the United States, underscoring the need for precise and current data on its distribution to inform risk assessments and regulatory measures. Existing national radon maps lack the necessary resolution and temporal specificity to effectively guide such efforts. To address this gap, we compiled a comprehensive database of over six million radon measurements conducted by independent laboratories between 2001 and 2021. Employing a random forest model, we analyzed these data alongside approximately 200 geological, meteorological, architectural, and socio-economic variables to predict monthly community-level radon concentrations. Our high-resolution radon map reveals a slight decrease in average radon levels in historically high-radon regions over the study period. Despite this trend, an estimated 83 million individuals reside in areas where radon concentrations exceed the recommended action level of 148 Bq/m³, many of whom are in zones previously classified as low-risk. These findings highlight the critical need for comprehensive radon surveys and suggest that our refined mapping approach can significantly aid federal and local agencies in formulating and updating radon-related regulations. Furthermore, the model offers a valuable tool for assessing residential radon exposure, thereby enhancing our understanding of radon's health impacts.

S24-02 | Individual residential Radon exposure, COPD and brain tumors.

Alberto Ruano Raviña

University of Santiago de Compostela, Preventive Medicine and Public Health, Santiago de Compostela, Spain

Objective: To describe the design and results in brief of: 1) case-control study on radon and COPD performed exclusively in never-smokers and, 2) the preliminary results of a case-control study on radon and brain tumors.
Material and methods: Both studies have collected incident cases of COPD in never-smokers and brain tumors (mainly glioma and its variants and meningioma). Cases and controls were mainly recruited in a previously classified radon-prone area. Both studies are multicentric and hospital-based and individual radon measurements have been performed with alpha track devices (with at least 3 months placed at each dwelling).
Results: The study on radon and COPD in never smokers has included 300 cases and more than 400 never-smoking controls with a close to significant association with indoor radon exposure for those who have lived more than 40 years in the same dwelling and with COPD severity. The case-control study on radon and brain tumors is still recruiting cases and controls. To date, we have collected more than 200 cases and 150 controls. The results of the first 86 incident cases show that median radon concentration does not differ significantly from radon concentration of general population living in the same area. It is expected that this study will recruit more than 300 cases and 600 controls in the next 2 years.

Conclusions: radon exposure might be associated with other diseases different than lung cancer and particularly other respiratory diseases such as COPD. Case-control studies with individual radon measurements, especially in radon-prone areas, are needed to explore new potential associations, particularly for those diseases having stronger biological plausibility due to the ionizing radiation received through radon exposure.

S24-03 | Domestic radon exposure and risk of 20 cancers by age, sex, and smoking status in Massachusetts, United States, 2001-2020

Matthew Bozigar[1], Garyfallos Konstantinoudis[2], Ernani Choma[3], Carolina Zilli Vieira[3], Yazan Alwadi[3], Longxiang Li[4], Rena Jones[5], Petros Koutrakis[3,6]

[1] Oregon State University, School of Nutrition and Public Health, College of Health, Corvallis, USA; [2] Imperial College London, MRC Centre for Environment and Health, Department of Epidemiology and Biostatistics, School of Public Health, London, UK; [3] Harvard University, Department of Environmental Health, TH Chan School of Public Health, Boston, USA; [4] Emory University, Department of Environmental Health, Rollins School of Public Health, Atlanta, USA; [5] National Cancer Institute, Occupational and Environmental Epidemiology Branch, Division of Cancer Epidemiology and Genetics, Rockville, USA; [6] Harvard University, Exposure Epidemiology and Risk Program, TH Chan School of Public Health, Boston, USA

Objective:
Radon, a known lung carcinogen for lung cancer, may pose broader cancer risks. Leveraging fine-scale spatiotemporal data in Massachusetts (MA), we investigated associations between domestic radon exposure and the risk of 20 major cancers by smoking status, age, and sex.

Materials and Methods:
We merged monthly ZIP code tabulation area (ZCTA)-level basement radon estimates (2001-2021) from a cutting-edge national exposure model with annual cancer diagnoses (1995-2020) from the MA Department of Public Health. Due to frequent zero cancer counts, outcomes were aggregated into annual 5-year cumulative sums, matched to radon exposures averaged over the same periods within ZCTAs. We modeled each cancer separately, stratified by smoking status (never/ever), age (child/adult), and sex (male/female). Bayesian spatiotemporal models with zero-inflated Poisson distributions further addressed outcome zero-inflation. A directed acyclic graph identified a minimally-sufficient adjustment set, including age, sex, housing characteristics, socioeconomic status, smoking, and weather, accounted for via stratification or model adjustments. Models also incorporated structured and unstructured spatial effects at ZCTA and county levels, and annual temporal effects.

Results:
In 483 MA ZCTAs, a 50 Becquerel per cubic meter (Bq/m^3) radon increase was associated with higher relative risks (lower value of 95% credible interval >1.0) for breast, colorectal, and possibly brain and central nervous system (CNS), oral, and thyroid cancers in never-smoking adult females; and bladder, leukemia, and possibly esophageal, myeloma, prostate, and thyroid cancers in never-smoking males. Higher risks were also observed among adult ever-smokers for several cancers. Among children, higher risk of brain and CNS cancers and lower risk of Hodgkin lymphoma were noted in never-smoking males.

Conclusion:
Mounting evidence suggests broader carcinogenic impacts of radon exposure beyond lung cancer. There is a critical need for population-based and individual-level research to elucidate radon's role in non-lung cancers.

S24-04 | Radon exposure and stroke risk

Eric Whitsel

University of North Carolina, Department of Epidemiology/Gillings School of Public Health, Chapell Hill, USA

Stroke is a globally burdensome cerebrovascular disease characterized by the rapid onset of neurological symptoms related to the blockage or rupture of an artery that supplies blood to the brain. Little is known about the role of indoor air pollutants in incident stroke among older women in the U.S. However, this demographic group has historically spent much of the day at home and has been disproportionately burdened by this disease, thereby motivating investigation of residential factors that may contribute to its pathogenesis, epidemiology, and geographic variation. This presentation considers one such factor—radon—a radioactive and mutagenic gas that is ubiquitous in residential environments, drawing on recent results from the study of a prospective, longitudinal cohort of 161,808 postmenopausal women from forty U.S. clinical centers in the *Women's Health Initiative Radon RiskInvestigation* (*WHI Risk It?*). As previously reported (https://doi.org/10.1212/WNL.0000000000209143), 158,910 of the women (mean age: 63.2 years; 83% White) had no history of stroke at baseline (1993-1998), but among them, 6,979 had an incident, neurologist-reviewed, classified, and adjudicated stroke during follow-up (mean: 13.4 years). Incidence rates were 333, 343 and 349 strokes per 100,000 woman-years at radon concentrations of <2, 2–4, and >4 pCi/L. Compared with women at concentrations <2 pCi/L, those at 2–4 and >4 pCi/L had higher covariate-adjusted hazards of incident stroke: hazard ratio (95% confidence interval) 1.06 (0.99–1.13) and 1.14 (1.05–1.22). Using non-linear spline functions to model radon, stroke hazards were significantly elevated (p=0.0004) at concentrations as low as 2 pCi/L, i.e., one-half the U.S. EPA Radon Action Level for mitigation (4 pCi/L). Associations were stronger for ischemic than hemorrhagic stroke but otherwise robust in sensitivity analyses, collectively highlighting the potential public health impact of reducing the Radon Action Level on a common cerebrovascular disease not previously considered under the U.S. EPA National Radon Action Plan.

S24-05 | Associations between radon exposure and adverse pregnancy outcomes in the nuMoM2b cohort

Ka Kahe

Columbia University, Department of Obstetrics and Gynecology and Department of Epidemiology, New York, USA

Objective: To examine the associations between radon exposure and adverse pregnancy outcomes in a large, diverse cohort of American women.
Methods: This secondary analysis used data from the Nulliparous Pregnancy Outcomes Study: Monitoring Mothers-to-Be (nuMoM2b), which enrolled approximately 10,000 participants at baseline from eight US clinical centers. Radon exposure was estimated at county-level using data developed by the Lawrence Berkeley National Laboratory based on the Environmental Protection Agency's short- and long-term indoor home radon measurements. Exposure was categorized into three groups: <1, 1 to <2, and ≥2 pCi/L. Pregnancy outcomes were diagnosed by clinicians during routine prenatal care. Smoking status and fine particulate matter air pollutants (PM2.5) were considered as both potential confounders and effect modifiers.
Results: After adjusting for potential confounders, participants residing in counties with the highest radon levels (≥2 pCi/L) had significantly higher odds of developing gestational diabetes (GD) compared to those in counties with the

lowest radon levels (<1 pCi/L) (odds ratio [OR], 1.37; 95% CI, 1.02-1.84). This association remained after further adjustment for PM2.5, (OR, 1.36; 95% CI, 1.00-1.86). Although interaction tests between radon and smoking or PM2.5 were not statistically significant, elevated odds of GD were more pronounced among smokers living in areas with higher radon levels (OR, 2.09; 95% CI, 1.41-3.11) or higher PM2.5 exposure (OR, 1.93; 95% CI, 1.31-2.83). Additionally, radon exposure was associated with lower birth weight z-scores among women who smoked ≥20 cigarettes/d (β=-0.16; 95% CI, -0.33-0.01). No significant associations were observed with other adverse pregnancy outcomes.

Conclusions and relevance: Findings from this large cohort suggest that county-level radon exposure is associated with increased odds of GD and reduced BW, particularly among smokers and individuals exposed to higher levels of PM2.5. Further research using individual-level radon exposure data is warranted to validate these findings and explore the underlying biological mechanisms.

S25 | Beyond the “impact factor:” environmental epidemiologists & exposure scientists in action across the United States

R. M. Shaffer[1], J. Braun[2], E. Haynes[3], C. Frey[4], E. Coffman[5], A. Zota[6], **C. Hyland**[7]

[1] *Washington, DC, United States of America*
[2] *Brown University, Providence, Rhode Island, United States of America*
[3] *University of Kentucky, Lexington, Kentucky, United States of America*
[4] *North Carolina State University, Raleigh, North Carolina, United States of America*
[5] *U.S. Environmental Protection Agency (EPA), RTP, North Carolina, United States of America*
[6] *Columbia University, New York City, New York, United States of America*
[7] *University of California Berkeley, Berkeley, California, United States of America*

This session will feature environmental epidemiologists and exposure scientists across the United States and across diverse career stages applying their expertise outside of traditional academic models to translate science to legal, assessment, and policy contexts and to engage the power of communities. The aims of this session are to 1) provide guidance on making environmental health work more applicable outside academia, and 2) inspire others from the ISEE-ISES community to apply new or underutilized approaches to create tangible public health impact. Each panelist will discuss lessons learned from following avenues for engagement: legal (Dr. Joe Braun), community engagement (Dr. Erin Haynes), policy (Dr. Chris Frey and Mr. Evan Coffman), and science communication (Dr. Ami Zota). All presentations will include specific lessons and guidance that will help ISEE-ISES participants to use these approaches in their work.

S25-01 | Epidemiologists providing expert witness testimony to help those harmed by pollutants

Joseph Braun

Brown University, Providence, USA

The United States legal system provides an avenue for individuals to receive compensation for harms they experienced due to environmental pollutants. Epidemiologists and exposure scientists play a critical role in establishing (or not) the causal relations between pollutants and human health in toxic tort cases – lawsuits where the plaintiffs claim to be harmed or injured by a chemical or pollutant. This talk will provide an overview of my experiences as an expert witness in toxic tort cases related to per- and polyfluoroalkyl substances (PFAS). My objective is to help epidemiologists and exposure scientists understand how they can use their expertise to help those who have been harmed by pollutants and effectively apply their skills in the medico-legal context. Specifically, I will describe how members of ISEE and ISES can work with attorneys to interpret epidemiological and exposure science evidence under the standards of causation used in the medico-legal framework, communicate to diverse audiences, and be prepared to defend their opinions and expertise. While the decision to serve as an expert witness is a individual one, choosing to do so can be scientifically and personally rewarding, and most importantly provide justice to those harmed by pollutants.

S25-02 | Effectively engaging with the power of communities

Jill Johnston

University of California, Irvine, USA

Building effective community partnerships can be challenging. This presentation will review lessons learned from community-engaged research (CEnR) partnerships that were formed to address the community health and exposure concerns related to urban oil drilling in Los Angeles, CA. Community organizations found that changing regulations was hampered by the lack of local health studies or air monitoring data. Using community-driven research methods, we will describe collaborative strategies to quantify the impacts of living near urban oil drilling sites on lung function, blood pressure, stress and air quality; and how research informed influenced local and state policies for improved public health protections.

S25-03 | Federal air pollution advisory committees: Sticking to the science

Chris Frey

North Carolina State University, Raleigh, USA

Serving on Federal scientific advisory committees is an opportunity for external independent experts, including faculty and others, to provide high impact external professional service and to gain experience and perspectives regarding communication across disciplines and translation of science to inform decisions. There are scientific, legal, policy and process contexts to consider in developing and delivering reviews and advice that are useful to the Federal agency. Co-benefits of committee service include developing a network with other top experts and increased understanding of science needs of Federal agencies. Lessons learned from personal experience serving on numerous advisory committees will be shared. These lessons draw especially upon experience with the U.S. Environmental Protection Agency (EPA) Science Advisory Board (SAB) as a member, the EPA Clean Air Scientific Advisory Committee (CASAC) as a member and as chair, as a member of numerous ad hoc panels of both the SAB and CASAC, and as a reconvener of a CASAC Particulate Matter (PM) Review Panel as the Independent Particulate Matter Review Panel. The CASAC PM Review Panel was dismissed by EPA in October 2018 just as it was about to receive an EPA draft scientific assesment for review, and reconvened to deliver advice to the EPA in late 2019 as it would otherwise have if it had not been dismissed. The lessons are also based on experience as EPA Science Advisor in working with the SAB, CASAC, and the EPA Board of Scientific Counselors (BOSC) which advises EPA's Office of Research and Development regarding its research portfolio. Examples of key factors for advisory committee members to consider in developing and delivering advice include ethics requirements, situations for which recusal is appropriate, understanding the scope of the committee and its underlying charter and mandate, understanding agency charge questions, and understanding processes, procedures, and requirements for deliberations.

S25-05 | Science communication strategies for getting the message out beyond the ivory tower

Ami Zota

Columbia University, New York City, USA

Science communication to the public is an essential yet underappreciated work among the research community. This presentation will highlight the critical role of science communication in translating research into action, both at the individual and collective level. It will also discuss the ethical responsibilities of scientists to share their findings with the public and ways to support this work. The presenter will provide specific examples of science communication from their work on toxics with an emphasis on successes and challenges encountered. The session will include guidance on how to develop science communication strategies and factors to consider given the changing media ecosystem. Lastly, the session will highlight innovations in science communication training for early career scientists and scholars based on the Agents of Change training program at Columbia University.

S26 | AI-Driven Early Warning Systems for Climate-Sensitive Infectious Diseases: Spatiotemporal Models and Big Data Integration

W. Hu[1], **M. Bonsall**[2], **L. Yang**[3], **T. Zhang**[4], **K. Wangdi**[5]

[1] *Queensland University of Technology, School of Public Health and Social Work, Brisbane, Australia*
[2] *University of Oxford, Department of Biology, London, United Kingdom*
[3] *Hong Kong Polytechnic University, School of Nursing, Hong Kong, China, Hong Kong Special Administrative Region*
[4] *Chinese Academy of Medical Sciences & Peking Union Medical College, School of Population Medicine and Public Health, Beijing, China*
[5] *University of Canberra, HEAL Global Research Centre, Canberra, Australia*

Climate change exacerbates the spread and intensity of climate-sensitive infectious diseases, such as vector-borne and respiratory illnesses, with disproportionate impacts on vulnerable populations. Rising temperatures, extreme weather events, and shifting ecosystems heighten these risks. Effective early warning systems (EWS) are crucial for predicting and mitigating disease outbreaks, but current systems often rely on retrospective data, limiting their utility in real-time responses.

This symposium explores innovations in big data, spatiotemporal modelling, and artificial intelligence (AI) for developing climate-driven EWS. Real-time, multi-source data integration enhances outbreak forecasting, while spatiotemporal models provide insights into disease dynamics under diverse climate scenarios. AI-driven approaches, including machine learning and neural networks, improve predictive accuracy and enable tailored interventions in climate-sensitive regions, particularly for low-resource communities.

These advancements demonstrate how interdisciplinary research strengthens public health responses, supports timely decision-making, and reduces global disparities in disease prevention and control. By leveraging cutting-edge technologies, we can transform EWS into proactive tools for addressing the health impacts of climate change.

S26-01 | Big Data in Early Warning Systems for Climate-Sensitive Infectious Diseases

Wenbiao Hu

Queensland University of Technology, School of Public Health and Social Work, Brisbane, Australia

Background
The burden of climate-sensitive infectious diseases such as dengue is intensifying due to climate variability and environmental change. Traditional surveillance systems often lack the predictive capacity to support timely and effective interventions. Big data analytics and advanced modelling techniques are increasingly essential for strengthening early warning systems (EWS) to improve preparedness and response.
Methods
We conducted multi-country studies across China, Indonesia, Vietnam, Thailand, the Philippines, and Australia. These studies integrated diverse datasets, including high-resolution climate data, remote sensing, electronic health records, internet search trends, and human mobility data. Advanced modelling approaches such as machine learning, distributed lag non-linear models, and Bayesian spatiotemporal models were applied to capture delayed, nonlinear, and spatially heterogeneous climate–dengue relationships.
Results
The research demonstrated the high potential of big data and advanced analytics to enhance dengue EWS across different settings. To support consistent and scalable risk assessment, we developed a Suitable Conditions Index (SCI) based on temperature thresholds relevant to Aedes aegypti and Ae. albopictus mosquitoes for all six countries. SCI enabled the identification of high-risk periods and locations, providing a unified framework for forecasting under current and future climate conditions. Across countries, we identified varied but complementary patterns: from region-specific climate–disease dynamics in Thailand and China, to the impact of heatwaves in Vietnam, cross-border implications between Indonesia and Australia, and humidity-driven second seasonal peaks in the Philippines. In Australia, SCI was the most important predictor of future dengue risk, with projections indicating a southward expansion of outbreaks under business-as-usual climate scenarios.
Conclusion
Big data-driven EWS offer a powerful, scalable approach to manage climate-sensitive diseases. By integrating diverse data streams and advanced analytics, these systems enable improved risk prediction and early action. Strengthening data integration, cross-sector collaboration, and policy translation will be critical for building public health resilience in a changing climate.

S26-02 | Role of imported malaria on local transmission in a near elimination setting: a case study from Bhutan using Hawkes Processes

Kinley Wangdi

University of Canberra, HEAL Global Research Centre, Canberra, Australia

Objective

The aim of this study was to determine the role of imported malaria in driving local malaria transmission in Bhutan.

Materials and Methods

Laboratory-confirmed malaria cases between 2016 and 2020 was obtained from the Bhutan Vector-borne Disease Control Program. Information about the cases included the date of diagnosis and treatment, type of cases classified as indigenous or imported, and malaria species. A semi-mechanistic Hawkes Processes were used to study the role of imported malaria in local transmission in Bhutan. A 15-day delay was imposed in the model to account for a mosquito to become infectious.

Results

A total of 285 malaria cases were recorded during the study period and 58.6% (159) were imported malaria. Nearly two-third of imported cases (71.1%, 113) were *Plasmodium vivax,* and 73.6% (117) cases originated from India. The infectivity of *Plasmodium falciparum* and *P. vivax* were 8 and 19 days, respectively. The background intensity from imported malaria cases was much greater for *P. vivax* cases (maximum 0.17) resulting in more importations than *P. falciparum* cases (maximum 0.06). However, model fitting suggested that local *P. falciparum* transmission was mainly driven by importations but additional factors such as relapse played a role for *P. vivax*.

Conclusion

Imported malaria cases were key drivers of transmission in Bhutan. *P. falciparum* cases were mostly driven by imported cases while *P. vivax* transmission had other sources of infection in addition to imported *P. vivax*. Actively detection and treatment of imported *P. falciparum* cases can prevent local transmission while other sources of infection should be investigated for *P. vivax*.

S26-03 | AI-Driven Contact-Tracing and Outbreak Management System for Respiratory Infections in Vulnerable Communities

Jingbo Liang[1], **Lin Yang**[1], Yaojie Xie[1], Justina Liu[1], Man Sing Wong[2], David Shum[3]

[1] Hong Kong Polytechnic University, School of Nursing, Hong Kong, Hong Kong; [2] The Hong Kong Polytechnic University, Department of Land Surveying and Geo-Informatics, Hong Kong, Hong Kong; [3] The Hong Kong Polytechnic University, Department of Rehabilitation Sciences, Hong Kong, Hong Kong

Objective:
The COVID-19 pandemic underscored the need for advanced outbreak management systems tailored to high-risk congregate settings such as residential care homes (RCHs). This study developed and evaluated an artificial intelligence (AI)-powered indoor digital contact-tracing system to monitor transmission risks and optimize intervention strategies for respiratory infections.

Material and Methods:
We deployed a low-energy Bluetooth-based system to monitor contact networks among RCH residents, staff, and daycare participants. Effective contacts were defined as interactions within 2 meters for ≥20 minutes. Statistical analyses were conducted to compare contact patterns during and after the pandemic, while transmission dynamic models simulated outbreak sizes across various scenarios of social contact levels, vaccine coverage (10–90%), efficacy (30–80%), and pathogen transmissibility (R_0=1.2–8.5).

Results:
Post-pandemic network analysis demonstrated a significant increase in daily effective contacts across all groups: residents (from 2 to 5, p=0.04), staff (from 5 to 10, p=0.03), and daycare participants (from 8 to 15, p<0.001). Simulations demonstrated that combining high vaccine efficacy (80%) and coverage (≥70%) with strict social distancing contained outbreaks to <10% of the population across all R_0 levels (1.2–8.5). Conversely, uncontrolled social contacts with low-efficacy vaccines (30%) resulted in outbreaks exceeding 60% when R_0≥2.5, even with moderate vaccine coverage (50%). These findings underscore the importance of digital contact-tracing and implementing contact control measures in high-transmissibility and low-efficacy settings.

Conclusion:
The AI-driven system provided real-time contact network surveillance and predictive outbreak modelling. Integrating adaptive social contacts with vaccination strategies based on pathogen-specific parameters (R_0, vaccine profiles) offers a scalable framework for protecting vulnerable populations in RCHs and similar settings.

S27 | Milk Matters: Exploring Exposure, Impact, and Solutions for Endocrine Disrupting Chemicals in Human Milk

K. E. Manz[1], **M. E. Romano**[2], C. Howe[2], S. M. Tsunoda[3], N. Johnson[4], K. Dinh[4]

[1] *University of Michigan, Ann Arbor, Michigan, United States of America*
[2] *Dartmouth, Hanover, New Hampshire, United States of America*
[3] *University of California San Diego, San Diego, California, United States of America*
[4] *Texas A&M, College Station, Texas, United States of America*

Human milk is widely recognized as the optimal source of nutrition for infants, offering essential nutrients that support vital growth and developmental processes. However, recent concerns have emerged regarding the presence of chemicals, such as environmental pollutants and medications, in human milk, which may pose significant health risks to infants. Despite their pivotal role as the primary nutritional source during the first year of life, the concentration and impact of chemicals in human milk remain under-explored in scientific research. Some chemicals, such as per and poly-fluoroalkyl substances (PFAS), can accumulate in a mother's bloodstream following exposure from various environmental sources, including food, water, and air. During lactation, these chemicals may be mobilized from maternal tissues and consequently accumulate in human milk, leading to potential exposure for nursing infants. The impacts of these exposures on milk composition and infant health outcomes are largely unknown, and further investigation presents several scientific and methodological challenges. Emerging research is beginning to focus on strategies for removing contaminants from human milk, which is an important area of study to further enhance infant safety. This symposium aims to address these challenges by showcasing innovative exposure assessment studies and applied research that advance our understanding of the intersection of environmental exposures and human milk. The forum will also serve as a collaborative forum for researchers and healthcare professionals to discuss and identify both current obstacles and future research directions. By addressing these gaps, we can work towards improving the safety and health outcomes for infants globally.

S27-01 | Interactions between Per and poly-fluoroalkyl substances (PFAS) and Nutrients in Human Milk

Katherine Manz

University of Michigan, Ann Arbor, USA

Per- and polyfluoroalkyl substances (PFAS) exposure during infancy can occur through ingestion of contaminated human milk; and early-life exposure to PFAS is associated with adverse neurodevelopment, metabolic disruption, and diminished vaccine response. Adverse health outcomes associated with early-life PFAS exposure could be due to the indirect effects of PFAS on the nutritional components of human milk. However, limited information is available regarding the impact of PFAS on human milk quality. We will present initial results from a maternal-infant cohort that PFAS interact with micronutrients, such as vitamins and minerals, antioxidant enzymes, and immunoprotective components.

S27-02 | Metal Impacts on the miRNA Composition of Human Milk

Caitlin G. Howe

Dartmouth, Hanover, USA

MicroRNAs (miRNAs) are key epigenetic regulators abundant in human milk that increasingly have been associated with measures of infant growth and adiposity. Many of these miRNAs also play important roles in lactation. Milk-derived miRNAs may therefore be useful biomarkers of offspring health and breastfeeding outcomes. However, little is known about environmental impacts on the miRNA composition of human milk. In the New Hampshire Birth Cohort Study, we profiled miRNAs in mature human milk samples (n=54, collected at approximately 6 weeks postpartum) and evaluated relationships between biomarkers of five metals (arsenic, lead, manganese, mercury, selenium) reflecting exposures during periconception and pregnancy with the total abundance of miRNAs and the levels of specific miRNAs in milk. We found that arsenic exposure during both periconception and pregnany was associated with lower overall abundance of miRNAs in milk (PBonferroni<0.05) and also lower levels of specific miRNAs important for mammary gland development, intestinal development, and energy homeostasis (PBonferrroni<0.05). Similar patterns of association were observed for other metals including manganese and lead, although associations were not statistically significant after multiple testing correction. In addition to presenting these initial results, we will discuss our plan to expand this work to 400 human milk samples, which will allow us to assess how these metals, as a complex mixture, impact milk miRNA composition and downstream health outcomes. We will also discuss plans to investigate the potential mechanisms by which metal exposures during periconception and pregnancy alter human milk composition postpartum.

S27-03 | Impact of Drugs on Human Milk Composition and the Metabolome

Shirley Tsunoda, Nina Zhao

University of California San Diego, San Diego, USA

Objective: Pharmaceutical and personal care products (PPCPs) are ubiquitous in modern life, and infants may be unintentionally exposed to a wide range of these chemicals through breastfeeding. Documented risks of such exposures include low birth weight, developmental delays, reproductive issues, and immune dysfunction. Despite these concerns, our knowledge of PPCP-derived chemicals in human milk remains limited. A critical gap exists in identifying and characterizing these compounds, hindering our understanding of their health implications.
Methods: We analyzed 2,500 breast milk samples from the UCSD Human Milk Biorepository using untargeted liquid chromatography–high-resolution tandem mass spectrometry (LC-HRMS/MS). To support comprehensive annotation, we developed two mass spectrometry resources focused on PPCPs: (1) the GNPS Drug Library, an MS/MS spectral library of ~5,000 drugs, metabolites, and structural analogs mined from over 2 billion public MS/MS spectra; and (2) personalcareMASST, a rapid search tool to match acquired spectra against those from ~500 personal care product samples.
Results: After blank subtraction, 10,909 molecular features were detected. Of these, 662 (6%) were annotated using the GNPS Spectral Library. Cosmetic-related compounds such as Surfynol 104 and lauramidopropyl dimethylamine were found in >20% of samples, while the plasticizer acetyl tributyl citrate appeared in >10%. Using the GNPS Drug Library, we annotated 205 features as drugs or structural analogs-most commonly antibiotics, antidepressants, and antihistamines. Breast milk metabolomic profiles varied significantly with exposure to antibiotics and antidepressants. Key compounds driving this separation include dipeptides, tripeptides, and carnitines.
Conclusion: Our study presents a large-scale, systematic characterization of PPCP exposures in human milk, highlighting the need for deeper investigation into their potential impacts on infant health.

S27-04 | Novel Sorption Strategies to Reduce Per- and Polyfluoroalkyl Substances (PFAS) in Milk

Natalie Johnson, Kaylyn Dinh

Texas A&M University School of Public Health, Environmental and Occupational Health, College Station, USA

Objective: Given the growing concern regarding PFAS contamination in human milk, there is a need for mitigation strategies that do not impact milk composition. The objective of this work was to evaluate the efficacy of clay-based sorbents to reduce PFAS in a milk matrix and establish initial proof-of-concept as a mitigation strategy.

Material and Methods: Native and internal standard solutions of perfluorooctanoic acid (PFOA) and perfluorooctanesulfonic acid (PFOS) were spiked into bovine milk. Calcium montmorillonite (CM), CM-carnitine, CM-choline, and activated carbon (AC) were added into spiked milk individually at 0.05-1% w/v and shaken at various time intervals (5-120 min). Supernatants were subjected to QuEChERS extraction, dispersive solid phase extraction clean-up, and analyzed via LC-MS/MS. To compare to current filtration technologies, bovine milk was filtered through a commercially available water filter pitcher and sent to TX DHIA for nutrient analysis.

Results: CM, CM-carnitine, CM-choline, and AC, added at 0.2% w/v, were effective at reducing PFOA and PFOS. Notably, parent CM reduced PFOA and PFOS by 43% and 55%, CM-carnitine reduced PFOA and PFOS by 36% and 58%, and CM-choline reduced PFOA and PFOS by 51% and 31%. Reduction of PFOA and PFOS was variable for activated carbon which showed the least amount of reduction at 32% and 29%, respectively. The kinetic study at varying time intervals suggested stable adsorption of PFAS on all sorbents. Regarding nutrient composition, clay sorbents did not impact milk fat, protein, milk urea nitrogen, lactose, or solids-non-fats levels. However, the addition of AC significantly reduced protein levels. Following filtration of bovine milk through the water filter pitcher, nutrients were all significantly reduced.

Conclusion: Findings highlight clay-based sorption as a viable strategy for mitigating infant PFAS exposure.

S27-05 | Navigating the Unknown: Addressing challenges in human milk research at the intersection with environmental health

Megan Romano

Dartmouth, Hanover, USA

Following the presentations, Dr. Romano will lead an engaging panel discussion designed to delve further into this vital subject matter. This interactive forum aims to create a collaborative environment for attendees, encouraging the exchange of ideas and perspectives as they explore the multifaceted implications of contaminants present in the environment on breastfeeding and the well-being of infants. Participants will have the opportunity to share insights, raise questions, and highlight their own experiences in the field. Additionally, the discussion will focus on identifying key challenges that researchers and healthcare professionals face in understanding and mitigating these risks, as well as uncovering potential opportunities for future studies and innovations. By fostering dialogue and collaboration, this session seeks to contribute to the advancement of knowledge and strategies aimed at protecting human milk quality and ensuring the healthiest start for infants.

S28 | Unraveling the Links Between PFAS Exposure and Cancer Risk: Advances in Research and Mechanistic Insights

L. Chatzi[1], T. Alderate[2], R. Vermeulen[3], S. Fenton[4], D. Liang[5]

[1] University of Southern California, Department of Population and Public Health Sciences, Los Angeles, California, United States of America
[2] Johns Hopkins Bloomberg School of Public Health, Department of Environmental Health and Engineering, Baltimore, Maryland, United States of America
[3] Institute for Risk Assessment Sciences (IRAS), Department Population Health Sciences, Utrecht, Netherlands
[4] NC State University, Department of Biological Sciences, Raleigh, North Carolina, United States of America
[5] Emory University, Department of Environmental Health, Atlanta, Georgia, United States of America

The association between exposure to per- and polyfluoroalkyl substances (PFAS) and cancer risk has emerged as a critical public health concern, particularly given the widespread presence of these persistent environmental contaminants. Evaluating the state of the evidence linking PFAS to various cancers is essential to advancing our understanding of these associations, identifying vulnerable populations, and informing risk mitigation strategies.

This symposium will provide a comprehensive overview of the current evidence on PFAS exposure and cancer risk, synthesizing findings from epidemiological studies, toxicological research, and systems biology approaches. It will examine key gaps in knowledge, challenges in causal inference, and emerging opportunities for advancing the field. Cutting-edge omics technologies, including genomics, metabolomics, proteomics, and single-cell omics, will also be discussed as powerful tools for identifying biomarkers and elucidating mechanisms of PFAS-induced carcinogenesis.

The session will emphasize the critical role of synthesizing evidence from diverse research disciplines to evaluate and address the potential health impacts of PFAS exposure. By combining insights from population-level studies, experimental models, and omics-driven discoveries, the symposium aims to promote a deeper understanding of the mechanistic pathways linking PFAS to cancer and highlight opportunities for actionable interventions. The symposium format includes a brief introduction (3 minutes), five presentations (75 minutes), and a moderated discussion to foster interdisciplinary engagement and collaboration (12 minutes). This dialogue will provide a platform for advancing evidence-based strategies to address the public health challenges posed by PFAS exposure and its link to cancer risk.

This symposium will highlight innovative research on PFAS exposure, cancer risk, and emerging scientific methods, with an emphasis on evaluating evidence and addressing disparities in vulnerable populations.

S28-01 | *Integrating Human and In Vitro Studies to Assess PFAS and Liver Cancer: Insights and the Potential of Diet as a Mitigation Factor*

Lida Chatzi[1], Roselyn Tanghal[1], Ana Maretti[2], Sarah Rock[1], Adelynn Paik[1], Lucy Golden[2], Matthew Salomon[3], Jesse Goodrich[1], David Conti[1], Veronica Setiawan[1]

[1] University of Southern California, Department of Population and Public Health Sciences, LOS ANGELES, USA; [2] University of Southern California, Division of Gastrointestinal and Liver Diseases, Department of Medicine, Keck School of Medicine, LOS ANGELES, USA; [3] University of Southern California, Department of Cancer Biology, Keck School of Medicine, LOS ANGELES, USA

Objective
Hepatocellular carcinoma (HCC) is the fastest-growing cause of cancer deaths in the U.S., with rising incidence linked to metabolic dysfunction-associated steatotic liver disease (MASLD). Per- and polyfluoroalkyl substances (PFAS) are emerging environmental pollutants with hepatotoxic effects and underexplored role in HCC. This study integrates data from the Multiethnic Cohort (MEC) with 3D liver spheroids and single-cell transcriptomics to elucidate PFAS-induced cancer pathways and the potential protective role of the Planetary Health Diet (PHD).

Material and Methods
In a cohort of 2,800 participants (44% male, 56% female; 55% Japanese American; mean age 65), plasma PFAS levels (PFCAs and PFSAs) were measured using untargeted LC-MS. Dietary quality was assessed using the PHD Index (PHDI) based on food frequency questionnaires. A nested case-control study (240 HCC cases and 240 controls) further assessed PHDI association with HCC risk.

In vitro, multicellular human liver spheroids were exposed to 20μM of individual PFAS (PFCA: PFOA and PFNA; PFSA: PFOS and PFHxS) for 7 days and analyzed via scRNAseq and Nile red lipid staining.

Results
In vitro, PFAS exposures significantly altered hepatocyte transcriptional profiles, with PFNA showing the strongest oncogenic impact—marked by upregulation of cancer-associated pathways including cell cycle progression, DNA repair, and metabolic reprogramming. Both PFOS and PFNA suppressed anti-tumor signaling pathways in T and NK cells, suggesting immunosuppressive effects relevant to tumor initiation and progression. In the human study, higher adherence to the PHD was associated to lower PFCA and PFSA plasma levels. Each quintile increase in PHDI corresponded to a 36.6% PFCA decrease (β =-0.65, 95% CI=-1.13,-0.16, p=0.01) . Higher PHD adherence was also associated with reduced HCC risk (OR =0.77, 95% CI=0.64-0.91).

Conclusion
Our integrated human and in vitro data provide converging evidence that PFAS exposures may contribute to HCC development, while diet can be a mitigating factor.

S28-02 | A Scoping Review on Per- and poly-fluoroalkyl substances (PFAS) and Colorectal Cancer: Evidence from *In Vitro*, Animal, and Epidemiological Studies

Devendra Paudel[1], Haonan Li[1], Nathan Young[1], Elizabeth A. Platz[2], Douglas I. Walker[3], Donghai Liang[3], Max Aung[4], Jesse A. Goodrich[4], Veronica Wendy Setiawan[4], Loic L. Marchand[5], Brian Z. Huang[4], David Conti[4], Lida Chatzi[4], Tanya L. Alderete[1]

[1] Johns Hopkins Bloomberg School of Public Health, Department of Environmental Health and Engineering, Baltimore, USA; [2] Johns Hopkins Bloomberg School of Public Health, Department of Epidemiology, Baltimore, USA; [3] Emory University Rollins School of Public Health, Gangarosa Department of Environmental Health, Atlanta, USA; [4] University of Southern California, Keck School of Medicine, Department of Population and Public Health Sciences, Los Angeles, USA; [5] University of Hawaii Cancer Center, Epidemiology Program, Honolulu, USA

Objective: Per- and poly-fluoroalkyl substances (PFAS) are detected in most of the US population, raising concerns about potential health impacts. Our scoping review aimed to synthesize evidence from *in vitro*, animal, and epidemiological studies to assess the relationship between PFAS exposure and colorectal cancer (CRC) risk, identify methodological gaps, and propose future research directions.

Materials and Methods: A systematic scoping review was conducted by following PRISMA-ScR guidelines and a pre-registered protocol. A comprehensive literature search was conducted across multiple databases and studies were identified based on predefined inclusion criteria. Data from included studies were synthesized using the PECO framework. To assess the internal validity, a tiered quality appraisal was implemented, utilizing criteria adapted from the EPA's IRIS Handbook for human and animal studies. For *in vitro* studies, we used a customized criteria based on best practices in experimental design.

Results: Twenty-six studies were identified, including three *in vitro*, six animal, and twelve epidemiological studies. *In vitro* studies consistently demonstrated that PFAS exposure promotes CRC cell proliferation and migration, potentially through pathways like PI3K/Akt-NF-κB signaling, MMPs upregulation, and epithelial-mesenchymal transition. Animal studies showed mixed results, with some suggesting a protective effect of PFAS mixtures on CRC tumorigenesis, while others linked PFAS exposure to increased tumor development, depending on the PFAS type, dose, exposure window, and animal model. Epidemiological studies also presented inconsistent results, with positive, inverse, or null associations reported in occupation-based and community-based studies, likely due to variations in study design, exposure and outcome assessments, and population characteristics.

Conclusion: The existing evidence on the relationship between PFAS exposure and CRC risk remains limited and inconclusive. Our findings highlight the need for large-scale, prospective epidemiological studies with rigorous and pre-diagnostic exposure assessments, diverse populations, and comprehensive control for potential confounders to clarify the role of PFAS in CRC development and progression.

S28-03 | *Are prospective biobank studies on PFAS and cancer informative?*

Roel Vermeulen

Institute for Risk Assessment Sciences (IRAS), Department Population Health Sciences, Utrecht, Netherlands

The emergence of large biobanks and advancements in biomonitoring have opened new avenues for investigating associations between chemical exposures and health outcomes. A commonly used design in this context is the nested case-control study, where cases of a specific disease are matched to controls from within a cohort. Biological samples from these studies are analyzed using targeted, suspect screening, or untargeted (agnostic) exposomic approaches, each offering trade-offs between sensitivity and breadth of chemical detection.
Targeted analyses, while well-suited for hypothesis-driven research, may overlook less well-characterized compounds or complex interactions among co-occurring chemicals. In contrast, broader screening approaches can detect a wider range of compounds—including unknown metabolites—but may lack the sensitivity required for precise quantification and robust association analyses. These methodological choices can lead to variability in study findings.
Another critical source of variability stems from the timing of biospecimen collection relative to disease onset. Samples collected near the time of diagnosis may be susceptible to reverse causality or disease-related organ functions and metabolic changes, whereas samples collected long before diagnosis may not accurately reflect relevant cumulative exposures, particularly if PFAS levels change over time and are not repeatedly measured.
Greater attention to these methodological differences—including analytical approach and etiologically relevant exposure windows—is essential to interpreting inconsistencies across studies and advancing our understanding of chemical-related disease risk. Addressing these sources of heterogeneity, through approximation of organ functions, will improve the reliability of future research and help inform risk assessment and regulatory decisions.

S28-04 | *PFAS interference in platinum-based chemotherapy – mechanisms and mitigation*

Sue Fenton, Imran Rizvi, Brittany Rickard

NC State University, Department of Biological Sciences, Raleigh, USA

Certain PFAS have been linked to reduced fertility and increased risk of epithelial ovarian cancer (EOC), but the effect of PFAS on treatment response in EOC, and related mechanisms, remain poorly understood. Platinum resistance is a major barrier to the effective treatment of EOC, and external stressors, such as PFAS, may increase the likelihood of developing platinum-resistant disease. We have shown that certain PFAS induce platinum resistance in EOC and endometrial cancer models. We hypothesize the mechanism(s) underlying the effects of poorly characterized (emerging) PFAS on cancer cell aggressiveness and platinum resistance is mitochondrial in nature. We reported that emerging PFAS and their mixtures increase mitochondrial membrane potential and superoxide production in EOC cell lines and that photodynamic priming (PDP), a mitochondrial specific light-based treatment modality, abrogates chemoresistance in these EOC models. Leveraging these findings, we are trying to better understand how chronic PFAS exposure (more like what happens in humans) affects the

mitochondrial bioenergetics and abundance, and dysregulated lipidomic profiles. We are also working to identify the relationship between plasma PFAS and chemoresistance in EOC patient samples. Findings from these experiments will identify PFAS associated with platinum resistance in EOC patients as well as mechanisms underlying PFAS-induced platinum resistance in EOC models.

S28-05 | Unraveling the Link between Per- and Polyfluoroalkyl Substances (PFAS) and Breast Cancer among Participants in the American Cancer Society's Cancer Prevention Study-II

Shamim Shahi[1], W. Ryan Diver[1], James M. Hodge[1], Emily Deubler[1], Alpa V. Patel[1], Donghai Liang[2], Christina C. Newton[1], Lauren R. Teras[1]

[1] American Cancer Society, Population Science, Atlanta, USA; [2] Emory University, Gangarosa Department of Environmental Health, Atlanta, USA

Background and Objectives:
Per- and polyfluoroalkyl substances (PFAS) are hypothesized to increase breast cancer risk; findings from prior studies are inconsistent, with many not accounting for subtypes. We conducted this analysis to evaluate the association between serum PFAS concentrations and breast cancer incidence, overall and by selected characteristics, and explore the underlying mechanisms using high-resolution metabolomics.
Methods:
We conducted a case-cohort study among Cancer Prevention Study-II LifeLink Cohort female participants (mean age= 68) who developed breast cancer (n=781) during follow-up (1998–2015) or were in a randomly selected subcohort (n=498). Concentrations of 4 PFAS (PFOA, PFOS, PFHxS, and PFNA) were measured in participant blood samples collected at baseline (1998–2001). Each PFAS was modeled using a categorical (quartiles) and a continuous (log2-transformed concentrations) variable. Multivariable Cox proportional hazards models with Prentice weighting were used to estimate hazard ratios (HR) and 95% confidence intervals (CI) for associations between PFAS concentrations and breast cancer. An untargeted metabolome-wide association study was conducted to explore metabolic signatures associated with PFAS exposures among 667 study participants.
Results:
PFAS concentrations were not associated with breast cancer overall, but we observed suggestive positive associations in some subgroups including those with estrogen receptor negative (ER^-) tumors (e.g., for Quartile 4 vs Quartile 1: PFNA HR=2.01, 95% CI: 0.91–4.42; PFOS : HR=1.82, 95% CI:0.83–4.00), with a family history of breast cancer (PFHxS and PFOA), aged ≥70 years at blood collection (all PFAS except PFOS), and current estrogen replacement therapy (ERT) users.
Conclusion:
These results suggest a possible association between PFAS and breast cancer in specific subgroups and for ER- breast cancer in particular. The ongoing metabolomic study will help elucidate potential mechanisms underlying the observed differences in associations between PFAS levels and subgroups of breast cancer patients.

S29 | Bridging Mixture Exposure to Human Reproductive Health

B. Wang[1], **M. Bloom**[2], **A. Covaci**[3], **M. Ren**[4], **T. Wu**[1]

[1] *Peking University, School of Public Health, Beijing, China*
[2] *George Mason University, Department of Global and Community Health, Fairfax, Virginia, United States of America*
[3] *University of Antwerp, Toxicological Center, Antwerp, Georgia, Belgium*
[4] *Emory University, Rollins School of Public Health, Atlanta, Georgia, United States of America*

Environmental exposures play pivotal roles in affecting human health. The mixture-centered risk assessment strategy has been developed by integrating epidemiological and experimental evidence. As a new strategy aimed at linking environmental risk factors to various health outcomes, the exposome has become the focal point of next-generation environmental-health studies. In the assessment of human health risks, it takes all environmental exposures into account, from conception onwards throughout the lifespan. Exposomic studies typically integrate multi-dimensional data, ranging from a few to thousands of external environmental risk factors originating from physical, chemical, or biological sources, as well as those linked to social activities. However, despite the conceptual appeal of the exposome, its advancement has been hindered by challenges in study design, analytical capabilities, and the high cost of measuring such a vast array of risk factors. Thus, compared to the rapidly advancing fields of genomics, proteomics, and metabolomics, exposomics remains in its early stages, particularly in terms of characterization and analytical approaches. A standardized workflow for exposomic analysis is still in high demand. In this context, we discuss key applications of exposomics and outline strategies for conducting exposomic studies to establish relationships between the exposome and human reproductive health. To enhance the feasibility and efficiency, we propose the following topics for our symposium: (1) develop an exposome platform that meets these requirements using the statistical or experimental method; (2) combine both data- and hypothesis-driven exposomics to prioritize the risk of environmental factors; (3) develop reliable statistical analysis for high-dimensional data of external and internal exposure factors; (4) build causal relationships between exposome or exposure mixtures and human reproductive health outcomes; (5) summarize the strengths and limitations of the current non-target approaches in expsomics. Dr. Bin Wang and Dr. Michael S. Bloom worked as the co-chairs of this symposia.

S29-01 | ExposomeX: Development of An Integrative Exposomics Platform to Expedite Discovery of “Exposure-Biology-Disease” Nexus

Bin Wang

Peking University, School of Public Health, Beijing, China

BACKGROUND: Exposome has become the hotspot of next-generation health studies. To date, there is no available effective platform to standardize the analysis of exposomic data.
OBJECTIVES: In the present study, we aim to propose one new framework of exposomic analysis and establish one novel integrated platform “ExposomeX” to expediate the discovery of the “Exposure-Biology-Disease” nexus.
METHODS: We have developed six major functions including exposome database search (E-DB), biological link (E-BIO), statistical analysis (E-STAT), mass spectrometry data processing (E-MS), meta-analysis (E-META), and data visualization (E-VIZ), which are supported by a total of 15 analytical modules.
RESULTS: A total of 23 supporting databases were curated, encompassing exposome and diseases, along with their related biological pathways and molecules. The platform includes data on up to 119,247,057 unique exposures, 17,186 diseases, 153,780 proteins, 19,122 metabolites, 572,278 annotated pathways, and 43,324 annotated gene ontology IDs, along with their established links. Using ExposomeX, we can effectively analyze multidimensional exposomic data and investigate the "Exposure-Biology-Disease" nexus by exploring association strengths, understanding statistical and biological mechanisms, enhancing prediction performance, and automatically conducting meta-analyses based on high-quality literature databases and publications. The performance of ExposomeX has been well validated by re-analyzing three typical multi-omic datasets. The results revealed that ExposomeX can efficiently help discover new associations, as well as the relevant in-depth biological network analysis.
DISCUSSION: In sum, we have proposed a comprehensive new framework for comprehensive exposomic analysis, accessible both through R and an online interactive platform (http://www.exposomex.cn/).

S29-02 | Using Mixture Analysis to Uncover Differences in Chemical Impacts on Reproductive Outcomes

Michael S. Bloom[1], Meghana Varde[1], Roy R. Gerona[2], Abby G. Wenzel[3], Roger B. Newman[3]

[1] George Mason University, Department of Global and Community Health, Fairfax, USA; [2] University of California San Francisco, Department of Obstetrics, Gynecology, and Reproductive Sciences, San Francisco, USA; [3] Medical University of South Carolina, Department of Obstetrics and Gynecology, Charleston, USA

Objective: The exposome paradigm suggests that mixtures-based statistical analyses offer a more realistic exposure profile than analysis of individual chemicals. However, the optimal statistical approach to mixtures-based analysis for uncovering differences between populations of concern is unknown.
Materials and Methods: We measured concentrations of nine environmental phenols and eight phthalate metabolites in mid-gestation urine samples (18-22 weeks) from 310 pregnant women. We compared methods to estimate associations between urinary chemical mixtures and gestational age at delivery (GA), among 152 self-identified non-Hispanic Black (NHB) and 158 non-Hispanic White (NHW) women, adjusted for maternal urinary specific gravity, age, body mass index, education, cigarette smoking, and sampling season.

Results: Using principal component analysis with linear regression, GA was negatively associated with co-exposure to urinary MBP, MiBP, MBzP, MEP, and MMP (β=-0.13, 95%CI: -0.29, 0.03), and positively associated with MEHP, MEOHP, and MEHHP (β=0.12, 95%CI: -0.04, 0.29) among NHB, while coexposure to urinary triclosan, bisphenol A, and bisphenol S was positively associated with GA among NHW (β=0.13, 95%CI: 0.03, 0.22). Using quantile g computation (qgcomp), the mixture of 17 urinary chemicals was negatively associated with GA among NHB (ψ=-0.23 per mixture quartile; 95%CI: -1.00, 0.55), driven primarily by benzophenone-1 (BP-1) and BP-3. Among NHW, there was a positive association using qgcomp (ψ=0.39 per mixture quartile; 95%CI -0.18, 0.95), driven mainly by urinary MEOHP and MEHHP. Bayesian kernel machine regression suggested non-linear associations, with an earlier GA at lower EP concentrations among NHB, driven mostly by MEP, BP-1, and BP-3, but a null association among NHW.
Conclusion: Associations between urinary chemical mixtures and GA differed among NHB and NHW women, suggesting potential differences in their reproductive effects. However, different statistical methods produced different associations and different drivers of the associations. We recommend a multimethod approach to investigate associations that may indicate important reproductive health disparities.

S29-03 | What are the expectations and limitations of suspect and non-target screening for the characterization of the human chemical exposome?

Adrian Covaci

University of Antwerp, Toxicological Center, Antwerp, Belgium

Thecharacterization of thehuman chemicalexposome still relies on measurements of multiple classes of inorganic and organic contaminants through targeted analytical approaches that allows the standardization of resulting exposomic data. Suspect and non-target screening have been heavily promoted in the past 5 years and are possible contributors to the characterization of the chemical exposome. However, there are several hurdles which need to be properly addressed before their large-scale implementation in cohort studies. We summarize the expectations and limitations of exposomics data obtained through high-resolution mass spectrometric suspect and non-target screening approaches.

S29-04 | Predicting Early Pregnancy Loss Using Mixture Exposure Triggered Biological Knowledge-driven Machine Learning

Mengyuan Ren

Emory University, Rollins School of Public Health, Atlanta, USA

Background: Knowledge graph networks offer valuable advantages capturing the mixture effects of exposures and biological interpretation, while their application in epidemiological studies remains scarce. We developed a framework coupled with biological knowledge graph-based networks (BKGNs) and machine learning (ML) to predict early pregnancy loss (EPL).
Methods: We measured typical environmental exposures, including 41 metal(loid)s and 16 per- and polyfluoroalkyl substances (PFASs) among 116 women underwent *in vitro* fertilization and embryo transfer. EPL was confirmed by absence of gestational sac with a fetal heartbeat evident in ultrasonography after six weeks of gestation. BKGNs were constructed to link mixture exposure to EPL with biological pathways, including Gene Ontology (GO) and protein. Chemical-specific BKGNs were integrated with individual-level mixture exposures to build ML models with 5-fold cross-validation.
Results: Employing stack generalization approach, the GO-integrated model outperformed others even when sample size decreased to 60% of the total, demonstrating an area under the curve (AUC) value of 0.876 and overall improvement of 11%–15%. While no significant improvement in prediction was observed when using protein-integrated model. Beyond predicting EPL risk, this framework deciphers how mixture exposures link to EPL by featuring critical exposures (*e.g.*, serum selenium and chromium) and captures key biological perturbations on PRKCE and GO:0008285 pathway involving in apoptotic nuclear changes and negative regulation of cell proliferation.
Conclusion: Our proposed novel framework is both robust and cost-effective, offering a mechanistic lens for predicting exposure-associated health outcomes. BKGNs model reveals key roles of trace elements on EPL by triggering reduced cell proliferation and apoptotic processes.

S29-05 | Prompt Learning on Biological Knowledge Graphs for Few-Shot Prediction of Exposure-Protein Interactions

Tianxiang Wu

Peking University, School of Public Health, Beijing, China

Self-supervised learning (SSL) on biological networks offers new opportunities for predicting interactions between environmental exposures and proteins. However, the traditional "pretrain-and-finetune" paradigm often suffers from inefficiency and negative transfer, particularly in few-shot scenarios. Inspired by the success of prompt learning in natural language processing (NLP) for leveraging prior knowledge, we explore prompt learning for biological network graphs to bridge the gap between pretrained models and exposure-protein interaction tasks. We propose two prompt engineering strategies: graph-based prompts and token-based prompts, significantly improving model performance under limited data. Extensive experiments validate the superiority of our approach in enhancing predictive capabilities.

S30 | Smoke and mirrors: reflecting on our understanding of connections between air pollution and infectious disease

K. M. Rappazzo[1], **H. Chang**[2], S. Kannoth[3], J. Zhang[4], J. Markwiese[5]

[1] *U.S. Environmental Protection Agency, ORD/CPHEA/PHESD, Durham, North Carolina, United States of America*
[2] *Emory University, Rollins School of Public Health/Biostatistics and Bioinformatics and Environmental Health, Atlanta, United States of America*
[3] *Icahn School of Medicine at Mount Sinai, New York, New York, United States of America*
[4] *University of Copenhagen, Department of Public Health/global Health Section, Copenhagen, Denmark*
[5] *U.S. Environmental Protection Agency, ORD/CPHEA/PESD, Corvalis, Oregon, United States of America*

The influence of environment on infectious disease, or how infection can change susceptibility to environmental factors, has been understudied and is important in protecting public health in a changing environment. The health effects of air pollution have been extensively studied for cardiorespiratory morbidity and mortality. However, among respiratory outcomes, there has been less attention given specifically to infectious diseases in contrast to chronic conditions (e.g., asthma, COPD). This is despite a biologically plausible mechanism by which air pollution could contribute to infectious disease transmission, susceptibility, and severity. For example, wildfires produce concentrated air pollution exposures and can impact dispersion of infection vectors as well as individual behaviors that may increase contact with those vectors. Additionally, infection-related illness may place individuals at increased risk to air pollution due to weakened immune systems or damage to interfacing organ systems. The complex and interacting nature of these exposures and responses of both humans and disease vectors offers a wide variety of ways to study air pollution impacts on infectious disease.

In this symposium, presenters will cover a diverse array of research relating to air pollution and infectious disease. Howard Chang will present work on the synergistic effect of ambient air pollution and community-level influenza activity on emergency department visits; Sneha Kannoth will present work on how cardiovascular disease might mediate air pollutant associations with COVID-19 severity; Jiawei Zhang will present on modification of associations between long term air pollution pneumonia and acute lower respiratory infections by underlying health conditions and demographic factors; and Jim Markwiese will present work on wildfire smoke as a potential vector for fungal pathogens.

S30-01 | The synergistic effect of associations between ambient air pollution and community-level influenza activity on emergency department visits

Howard Chang, Fred Huang, Stefanie Ebelt

Emory University, Rollins School of Public Health/Biostatistics and Bioinformatics and Environmental Health, Atlanta, USA

Since the 2009 H1N1 influenza pandemic, considerable resources have been invested in the United States and worldwide to improve influenza surveillance. We leverage data from the Influenza Hospitalization Surveillance Network in 6 cities (Albany NY, Atlanta GA, Baltimore MD, Rochester NY, San Francisco CA, and Salt Lake City UT) to investigate effect modification of the short-term effects of ambient air pollution on emergency department visits. We report evidence of stronger air pollution effects during periods of higher community-level activity for seasonal influenza. We also explore effect modification stratified by patient age groups, respiratory disease outcomes and influenza types.

S30-02 | The role of cardiovascular morbidity in mediating the relationship between historical ambient air pollution exposure and COVID-19 severity

Sneha Kannoth[1], Pam Factor-Litvak[2], Stephen Morse[2], Earle Chambers[3], Kristen Rappazzo[4], Cong Zhang[2], Sandra Albrecht[2], Alexander Azan[5], Min Qian[2], Perry Sheffield[1], Azure Thompson[6], Jennifer Woo Baidal[7], Stephanie Lovinsky-Desir[8], Jeanette Stingone[2]

[1] Icahn School of Medicine at Mount Sinai, Department of Environmental Medicine, New York, USA; [2] Columbia University, Mailman School of Public Health, New York, USA; [3] Albert Einstein College of Medicine, Department of Family and Social Medicine, New York, USA; [4] Environmental Protection Agency, Office of Research and Development, Durham, USA; [5] New York University, Grossman School of Medicine, New York, USA; [6] SUNY Downstate Health Sciences University, School of Public Health, New York, USA; [7] Stanford University, School of Medicine, Stanford, USA; [8] Columbia University, Vagelos College of Physicians and Surgeons, New York, USA

Objective

Research suggests a relationship between particulate matter ($PM_{2.5}$) exposure and adverse COVID-19 outcomes, requiring exploration of underlying mechanisms. We hypothesize that cardiovascular morbidity, which is strongly associated with air pollution exposure, mediates the relationship between historical $PM_{2.5}$ and adverse COVID-19.

Material and Methods

We used harmonized electronic health records (INSIGHT Clinical Research Network) across New York City (NYC) healthcare systems, including NYC adults with COVID-19 hospitalizations (3/1/2020-2/28/2021; N=20118). We assigned historical $PM_{2.5}$ to residential ZIP Codes using the NYC Community Air Survey (2009-2019). Mediators included fifteen cardiovascular conditions before COVID-19 infection, including myocardial infarction, atrial fibrillation, heart failure, hypertension, diabetes, obesity, etc. Mediator models used logistic regression to estimate odds of cardiovascular morbidity. Outcome models used modified Poisson regression to estimate risk of adverse COVID-19 outcomes including acute respiratory distress syndrome (ARDS), pneumonia, ventilation, and dialysis, and Cox

proportional hazards regression to estimate hazard of COVID-19 fatality and discharge. We adjusted for age, sex, smoking, neighborhood environmental vulnerability, and exposure-mediator interaction when significant ($p<0.05$). We conducted mediation analyses to identify total, direct, indirect effects, and proportion mediated.

Results

In the initial pandemic phase, within hospital catchment (3/2020-6/2020; N=7461), total and direct effects suggest greater historical $PM_{2.5}$ increased risk of ARDS, pneumonia, and dialysis. Indirect effects yielded the largest magnitudes for atrial fibrillation (AF) and myocardial infarction (MI). Nearly 25% of the total effect of historical $PM_{2.5}$ on risk of ARDS, ventilation, and dialysis was mediated by AF or MI, and nearly 5-10% of the total effect of historical $PM_{2.5}$ on fatality risk was mediated by AF or MI.

Conclusion

Historical $PM_{2.5}$ exposure influences adverse COVID-19 risk, via onset of AF and MI, only in the initial pandemic phase. Findings suggest a causal relationship between historical $PM_{2.5}$ and adverse COVID-19. Thus, pollution mitigation measures can ensure future population-level pandemic preparedness.

S30-03 | Individual Variability to Air Pollution and Risk of Pneumonia: A Population-based Cohort Study

Jiawei Zhang

University of Copenhagen, Department of Public Health/global Health Section, Copenhagen, Denmark

Air pollution is a recognized risk factor for pneumonia, yet its impact may vary by age and pre-existing health conditions. In our study, we followed 3,083,227 Danish residents aged ≥30 years from 2000 to 2018, tracking first-ever hospital contacts for pneumonia. Annual concentrations of fine particulate matter ($PM_{2.5}$) were estimated using hybrid land-use regression models and assigned to residential addresses. We examined effect modification by age, sex, and 19 specific comorbidities, which included cardiovascular diseases (ischemic heart disease, congestive heart failure, atrial fibrillation, stroke, and post-stroke complications), respiratory diseases (COPD, asthma, lung cancer), metabolic disorders (type 2 diabetes), infectious conditions (HIV/AIDS, tuberculosis), neurological disorders (Parkinson's disease, dementia), renal conditions (chronic kidney disease, acute kidney injury), liver disease (cirrhosis), and psychiatric disorders (schizophrenia, bipolar disorder, depression). Stratified Cox models were used to assess heterogeneity, while Aalen additive models estimated absolute risks.

Our analyses showed that the hazard ratio (HR) of PM2.5-associated pneumonia was highest among individuals aged 45–60 years, whereas the absolute risk was greatest in the elderly. Hazard ratios were generally consistent across most disease groups and between sexes. However, absolute hazard estimates were markedly higher among patients with COPD, post-stroke complications, Parkinson's disease, dementia, and congestive heart failure.

These findings emphasize that while the relative increase in pneumonia risk due to air pollution is similar across various subgroups, the overall burden is particularly pronounced among older adults and patients with these high-risk conditions, underscoring the need for targeted public health interventions.

S31 | Bridging Science and Policy: Shaping Global and Local Responses to Environmental and Climate Health Challenges

M. Kogevinas[1, 2], **M.E. Quinteros Caceres**[3], R. Slama[4], K. Ebi[5], **P. Vineis**[6], E. Malmqvist[7]

[1] *ISGlobal, Barcelona, Spain*
[2] *CIBERESP, Madrid, Spain*
[3] *Universidad de Talca, Departamento de Salud Pública, Talca, Chile*
[4] *INSERM, Paris, France*
[5] *University of Washington, Centre for Health and the Global Environment, Seattle, Washington, United States of America*
[6] *Imperial College, London, MRC Centre for Environment and Health, London, United Kingdom*
[7] *Lund University, Lund, Sweden*

The role of scientists in shaping policies on environment, climate, and health has never been more critical, yet the pathways to influence remain fraught with challenges. This symposium explores how scientific evidence can effectively inform and influence both global and local policies in the face of pressing environmental and health crises.

The session begins by addressing the science-policy interface, highlighting opportunities for collaboration and the barriers that limit the impact of scientific associations and international organizations. It will delve into the debate on whether scientists should prioritize global frameworks or focus on localized interventions, using case studies to illustrate successes and lessons learned.

Discussions will also examine the role of international organizations and major research centers in driving policy change, exploring strategies to revitalize their influence and foster transdisciplinary collaborations. The symposium emphasizes the importance of grassroots movements and community engagement, showcasing how local voices and co-created solutions can drive meaningful change.

Finally, the session reflects on the evolving role of scientists in policy advocacy, debating whether they should remain neutral providers of evidence or take a more active stance in shaping policies. Ethical considerations, skills for effective communication, and actionable steps for bridging science and policy will be explored.

This symposium aims to provide a comprehensive and critical analysis of the scientist’s role in policy development, offering practical insights and a roadmap for future engagement. By bringing together experts from diverse fields, it seeks to foster dialogue and identify strategies for addressing the global and local dimensions of environmental and health challenges. The symposium will combine specific presentations and secure adequate time for discussion led by the co-chairs.

S31-01 | The Science-Policy Interface: How can scientific evidence influence policy?

Remy Slama

Inserm and ENS-PSL, PARSEC (Paris Research in Climate Environment and Health), IBENS, Paris, France

Science plays a critical role in informing actions to prevent adverse effects from the environment in the broad sense on human health and ecosystems. We discuss here the types of scientific outputs relevant for policy making and the requirements allowing science to play this role.
Scientific research can support environmental health policies by contributing in 5 key dimensions: 1) hazard identification (e.g., is benzene a carcinogen?), 2) exposure distribution (e.g., food contamination by metals), 3) health impacts (how many deaths are expected from climate change in 2050?) and ranking exposures in terms of societal burden; 4) identification and validation of efficient interventions allowing to reduce exposure or risk (e.g., expected mortality decrease if cars are banned from the city center?); 5) scientific knowledge synthesis and knowledge dissemination. While it may seem relevant to be able to rely on this full chain of scientific evidence, some interventions can be enacted based on only partial information.
Several factors can contribute to such information being generated by science. These include: strong funding schemes for environmental health science (with mechanisms making research support proportional to the needs, e.g., via a tax or fund related to the sales of newly marketed substances or to healthcare costs) and related agencies; protection against conflicts of interest among scientists, environmental health agencies and decision makers; strong support to education, scientific knowledge dissemination and possibly limitation of advertisement on harmful substances or relying on unproven scientific allegations (as done for tobacco products and alcohol in some countries); strong environmental and health laws with explicit risk management logics and science-based thresholds for acceptable hazards. In addition, a balanced engagement with stakeholders, ensuring decision-makers to equilibrate the time spent with the industry, scientists and patients and environmental organizations in the preparation of regulations may be relevant.

S31-02 | Global vs. Local Policy Priorities: Finding the Right Balance

Manolis Kogevinas

ISGlobal, Barcelona, Spain

Addressing environmental and climate health challenges demands coordinated action at both global and local levels. Scientists play a pivotal role in shaping policies, whether by influencing broad international frameworks such as COP agreements or WHO strategies, or by driving localized interventions tailored to community-specific needs. Striking the right balance ensures that scientific evidence translates into equitable and impactful solutions.
Global policies set goals, standards, and long-term commitments, guiding national and local actions. Their effectiveness often hinges on local adaptation, as illustrated by air pollution regulations. The WHO Air Quality Guidelines provide targets, but their real-world impact emerges through city-level measures like London's Ultra Low Emission Zone or Beijing's air quality strategies. Similarly, global recognition of endocrine-disrupting chemicals stemmed from European regulatory efforts that shaped international discourse. However, global policies often fail to

account for asymmetries in resources and priorities between the Global North and South, with local implementation facing constraints like funding shortages or cultural differences.
Conversely, local policies can scale to global influence when successful models are replicated regionally. Plastic bag bans, initially adopted in African and Asian cities, informed international agreements on plastic pollution. Community-based heat action plans from Ahmedabad, India, have influenced broader frameworks, yet scaling-up local innovations like WASH remains inefficient. Northern institutions often dominate the global recognition and funding of such initiatives.
Global frameworks provide direction, but their success relies on local implementation and rebalancing power dynamics in policy design. Scientists must engage across scales, ensuring locally generated evidence informs global frameworks and international commitments yield actionable solutions. Case studies will demonstrate how local and global policies intersect, advocating for a dynamic, bidirectional approach to environmental and climate health governance.

S31-03 | Engaging with International Organizations and Major Research Centers

Kristie L. Ebi

University of Washington, Department of Global Health, Seattle, USA

The Intergovernmental Panel on Climate Change (IPCC) and the Intergovernmental Science-Policy Platform on Biodiversity and Ecosystem Services (IPBES) conduct regular international assessments of the current impacts and future risks of global environmental change for health and well-being. The assessments focus on systems-based, transdisciplinary approaches and include official processes for engagement between scientists and policymakers to ensure policy-relevance and to facilitate uptake to benefit sustainability and resilience. Scientists can participate in these assessments through providing policy-relevant research, volunteering to be an author, and reviewing drafts; the processes for doing so are publicly available.
In addition are international collaborations to broaden understanding of the risks of and responses to global environmental change, such as Future Earth, an international research program to build knowledge about the environmental and human aspects of global change to increase the impact of scientific research on sustainable development; the Pathfinder Initiative that is collecting evidence of the health benefits of mitigation polices; and the Earth Commission that is exploring safe and just earth system boundaries.
Many philanthropy organizations and EU-funded projects are facilitating the networking of major research centers to advance the uptake of best practices and lessons learned in effectively managing the health risks of global change. Further, it is increasingly becoming standard practice for research projects to include decision-makers and boundary organizations to ensure impact is achieved.
These and other opportunities for researchers to contribute to furthering the understanding of global environmental challenges will be discussed.

S31-05 | The Future Role of Scientists in Policy Advocacy

Paolo Vineis

Imperial College, London, MRC Centre for Environment and Health, London, UK

Science in its current form has been developed, starting in the seventeenth century, together with other characteristics of liberal societies. The motto of the English Royal Society (1660) still is “Nullius in verba” , “trust no one's word”, expressing the Royal Society members' desire to oppose the rule of authority by resorting to facts, possibly determined by experiments. The reality today is quite different, since the rise of populism and the spread of social media has led to an inter-mingling of facts, fake news and opinions: ideology often drives statements and decisions that should be based instead on scientific evidence. Based on the liberal background (let us call it the “received view”), independence and neutrality of scientists were valued as a guarantee of sticking to “facts” and avoiding their mingling with opinions or ideology. Advocacy has been instead included traditionally in the realm of opinions and ideology. As a matter of fact, today we need to defend freedom of science and its peculiar nature of being a powerful, critical and self-critical way to produce knowledge without external conditioning. However, the received view reveals some weaknesses. First, a century of philosophical reflections on science has consistently shown that facts are conditioned by theoretical models, metaphors and assumptions, so that “objectivity” is impossible to attain. Second, biomedical and environmental sciences have a goal that is by its own nature “value-laden”, like curing patients, preventing diseases and preserving the environment. The difficulty if not impossibility of keeping separate science-making (independent, value-free) and advocacy (exerted in the name of overarching principles like the survival of humankind) has become manifest with climate change. The development of guidelines on how to be involved in advocacy while doing independent science that sticks to evidence could be an important achievement of ISEE.

S32 | Environmental Exposures in CKDu epidemic regions: Updates from the field

M. K. Scammell[1], **A. Navas-Acien**[2]

[1] *Boston University School of Public Health, Environmental Health, Boston, Massachusetts, United States of America*
[2] *Columbia Mailman School of Public Health, Environmental Health Sciences, New York, New York, United States of America*

Chronic kidney disease of uncertain origin (CKDu) may represent a single disease or a spectrum of conditions, and has emerged as a major public health problem, primarily affecting young adults in rural, agricultural communities in Mesoamerica and South Asia. It is also known as Mesoamerican Nephropathy (affecting people who live between Southern Mexico and Panamá, and primarily reported among men <45 years old), and Chronic Interstitial Nephritis of Agricultural Communities (CINAC, reflecting the predominant histologic findings on renal biopsy). The commonality shared among these CKDu "hotspots" is that traditional CKD risk factors (diabetes, age, and hypertension) are not observed among CKDu cases. While heat exposure is a known risk factor for CKDu in Central America, it does not fully explain the occurrence of CKDu in the region, and it is not consistently implicated worldwide. Other hypothesized contributors include exposure to trace metals, pesticides, fertilizers, and other airborne and biological contaminants.

This symposium will showcase findings from interdisciplinary studies conducted across affected regions globally among children and adults. Talks will cover exposure assessment of environmental contaminants in resource-constrained settings, addressing the rationale, methodologies, challenges, cultural considerations, and data. Additionally, talks will address epidemiological findings on CKDu and novel chemical exposure mixtures (e.g., pesticides, metals, air pollutants) across different populations, including at-risk children and workers in underserved areas. Efforts to report results back to participants and communities will also be highlighted in both. The symposium aims to engage exposure scientists, environmental epidemiologists, physicians, policymakers, and biomedical researchers interested in addressing renal health disparities caused by environmental and occupational exposures globally. By fostering collaboration among diverse stakeholders, this symposium seeks to advance strategies for mitigating CKDu's devastating impact on vulnerable populations.

S32-01 | Chemical exposures and kidney health among adolescents and young adults at risk of CKDu in Nicaragua

Jessica Leibler[1], Yirong Yuan[1], Samantha Hall[1], Selene Vences Brown[1], Juan Jose Amador Velasquez[3], Damaris Lopez Pilarte[3], Magaly Amador Sanchez[3], Madeleine Scammell[1], David Friedman[4,5], Daniel Brooks[2]

[1] Boston University School of Public Health, Environmental Health, Boston, USA; [2] Boston University School of Public Health, Epidemiology, Boston, USA; [3] Centro Medico del Pacifico (CENMED), Masaya, Nicaragua; [4] Beth Israel Deaconess Medical Center, Division of Nephrology, Brookline, USA; [5] Harvard Medical School, Boston, USA

Chronic kidney disease of uncertain etiology (CKDu) imposes significant mortality in Central America, primarily in agricultural communities. Chemical exposures in affected regions are understudied, particularly during early life when exposures and exposure mixtures may impact kidney development. Using silicone wristband passive samplers (n=80), we assessed exposure to >1500 chemicals among adolescents ages 13–23 years in Nicaragua not working in agriculture. We evaluated exposures descriptively and used Spearman's correlation to assess associations between chemical concentrations and estimated glomerular filtration rate (eGFR). K-means clustering was applied to scaled concentrations of 16 chemical classes to identify common exposure profiles. SHapley Additive exPlanations (SHAP) values were used to interpret feature contributions in eGFR prediction models adjusted for sex and region. In total, 130 chemicals were recovered, including 21 pesticides. Sixty-one chemicals, including seven pesticides, appeared on >5% of wristbands. Pyrethroid pesticides were more frequently detected in a sugarcane-intensive region (p=0.007). We identified three distinct exposure clusters. Cluster 1 (n=9) showed elevated min-max scaled concentrations of esters (mean: 0.38) and synthetic musks (0.20), suggesting personal care–associated exposures. Cluster 2 (n=7) had the highest ester and phthalate levels (0.62 each), representing a higher-intensity, multi-chemical profile. Cluster 3 (n=63) exhibited lower-intensity exposures with organophosphate (0.0066), pyrethroid (0.0120), and PAH (0.0020) contributions, suggestive of environmental agrochemical exposure. eGFR was negatively correlated with aldehyde concentrations (r = -0.3). SHAP analysis identified esters (mean SHAP = 0.551), organophosphates (0.527), and synthetic musks (0.514) as the strongest contributors to eGFR predictions. Pyrethroids consistently displayed negative associations, while other chemicals showed weak or inconsistent associations. The most common exposure profile reflected low-level but widespread agrochemical mixtures, with modest associations with kidney function. These findings support the need to evaluate chemicals, including pesticides and personal care products, and chemical mixtures in association with kidney health in a high-risk region for CKDu.

S32-02 | Pediatric kidney health in chronic kidney disease of unknown etiology (CKDu) endemic, emerging, and nonendemic regions in Sri Lanka

Nishad Jayasundara[1,5], Anna Strasma[2], Anisha Gerber[3], Sameera Gunasekera[4], Mercedes Bravo[5], Christina Wyatt[2], Mangala De Silva[4,5]

[1] Duke University, School of the Environment, Durham, USA; [2] Duke University, School of Medicine, Durham, USA; [3] University of Virgina, UVA Health, Charlottesville, USA; [4] University of Ruhuna, Department of Zoology, Matara, Sri Lanka; [5] Duke University, Duke Global Health Institute, Durham, USA

Environmental exposures – such as heat stress and chemical contaminants – can affect kidney health, even in childhood. Environmental exposures that are deleterious to kidney health are highlighted in the emergence of chronic kidney disease of unknown etiology (CKDu), which disproportionately affects young, primarily male, agricultural workers in Mesoamerica and South Asia who lack traditional risk factors for kidney disease. A critical gap in CKDu studies is our limited understanding of disease manifestation through childhood and the role of environmental drivers in disease initiation and progression. Here we leveraged a pediatric cohort (N= 3048) representing participants (age 10-17) from CKDu-impacted and unimpacted communities in Sri Lanka and used a cross sectional analysis stratified as CKDu endemic, emerging, and nonendemic based on the prevalence of CKDu, to determine (i) if pediatric kidney health outcomes are associated with CKDu-endemicity based on urinary levels of kidney injury molecule -1 (KIM-1), neutrophil gelatinase-associated lipocalin (NGAL), and albuminuria, and (ii) if there is a relationship between pediatric kidney health and environmental exposures, specifically heat and drinking water contaminants. Participants from CKDu-impacted regions had elevated levels of urinary KIM-1 ($p < 0.0001$; n=804) compared to those from the other regions. However, exposures to heat, fluoride, heavy metals, and water hardness did not strongly correlate with pediatric kidney injury markers. Notably, a follow-up systematic literature search from eight different countries with reported cases of CKDu indicates subclinical kidney damage in children living in areas where adults are impacted by CKDu. Overall, our findings and emerging data emphasize the importance of pediatric studies in CKDu-impacted communities and the need for improved exposure assessments for the community at large.

S32-03 | Elevated fluoride in drinking water and children's kidney function in Mexico

Marcela Tamayo-Ortiz[1], Leonora Rojas-Bracho[2], Elodia Rojas-Lima[4], Karla Rangel[2], Luz Del Razo[3], Olivier Barbier[3], Paulina Farías[2]

[1] Columbia University, New York, USA; [2] Instituto Nacional de Salud Pública, Mexico, Mexico; [3] Centro de Investigación y de Estudios Avanzados del Instituto Politécnico Nacional, Mexico, Mexico; [4] Instituto Mexicano del Seguro Social, Mexico, Mexico

Background Fluoride is a known nephrotoxicant. Communities in Central Mexico experience high levels of exposure to waterborne fluoride. Less is known on this exposure and children's kidney function.

Methods Our team has successfully carried out three pilot studies in different communities, with samples sizes of 75-80 children each, involving water and urine fluoride testing and dental fluorosis assessments. Kidney function markers (KIM-1, NGAL, B2MG, osteopontin, cystatin-C and creatinine) were measured in urine samples.

Results We found water fluoride levels (mg/L) in: well water=3.7–4.2, bottled water=1.3–2.1, rainwater=0.4–0.9. Mean urinary fluoride concentrations of children varied by drinking water source (mg/L): well water=4.4–5.0, bottled water=2.1–3.4, rainwater=1.3–2.9. For each 1 mg/L increase in water fluoride, urinary fluoride increased by 0.61–0.96 mg/L ($p < 0.001$). Dental fluorosis was detected in 78–82% of children, with moderate to severe in 37–60% of cases. Positive and significant associations of urinary fluoride levels with KIM-1, NGAL, and B2MG. Additionally, 43% of children were overweight or obese (25% excess fat, 18% obese) and family burden (parents/grandparents) of chronic diseases was 41% type 2 diabetes, 27% hypertension and 17% with CKD.

Conclusions Findings from pilot studies in children have confirmed: (1) a high prevalence of dental fluorosis (2) elevated urinary fluoride concentrations, particularly among children who drink well water; and (3) associations between urinary fluoride levels and renal function biomarkers. In these studies, we demonstrate the urgent need for larger epidemiologic studies to confirm these associations and examine other potential health risks. Our findings support larger studies to inform exposure mitigation interventions and raise awareness of associated health risks for this highly vulnerable and at-risk population.

S32-04 | *Associations of acute kidney injury biomarkers with exposure to polycyclic aromatic among Chilean agricultural workers in the Maule Cohort (MAUCO)*

Grant Tore[1], Melissa DeSantiago[1], Wassim Obeid[2], Chirag R. Parikh[2], Heather M. Stapleton[3], Margarita Valenzuela[4], Catterina Ferreccio[5,4], Sandra Cortes[4], Lesliam Quiros-Alcala[1]

[1] Johns Hopkins University, Department of Environmental Health and Engineering, Baltimore, USA; [2] Johns Hopkins University, Division of Nephrology, School of Medicine, Baltimore, USA; [3] Duke University, Nicholas School of the Environment, Durham, USA; [4] Pontificia Universidad Católica de Chile, Escuela de Salud Pública, Santiago, Chile; [5] Instituto de Salud Pública de Chile, Santiago, Chile

Polycyclic aromatic hydrocarbon (PAH) exposures from agricultural and household woodburning may adversely impact kidney health. Still, research remains limited, especially in Latin America, where these practices are prevalent. We aimed to examine associations of PAH exposures with kidney injury biomarkers among agricultural workers participating in a study on kidney disease in the Maule Cohort, Chile.

We conducted a cross-sectional study of 43 male agricultural workers following the Disadvantaged Populations eGFR Epidemiology (DEGREE) Study protocol. Participants reported demographic, household, and occupational characteristics via questionnaire, provided a urine sample, and wore a silicone wristband for 7-11 days. Urinary concentrations of five kidney injury biomarkers (IL-18, KIM-1, MCP-1, NGAL, YKL-40) and eight hydroxy-PAHs were quantified; we measured 23 parent PAHs in wristbands. Associations were modeled using linear regression adjusting for age, education, and agricultural work category; Spearman correlations were used to compare concentrations between exposure matrices.

All eight hydroxy-PAHs, representing exposure to four parent PAH compounds, were frequently detected (≥84%) in urine samples; 12 PAHs were detected in ≥90% of wristbands. Across exposure matrices, correlations were strongest between urinary concentrations of total 2&3-hydroxyphenanthrene (PHEN2&3: r=0.67), 1-hydroxyphenanthrene (PHEN1: r=0.66), and 2-hydroxyfluorene (FLUO2: r=0.63) with respective PAHs in wristbands. We observed consistent positive associations of IL-18 and KIM-1 with phenanthrene exposure. Per doubling of PHEN1 and PHEN2&3, we observed 61% and 35% increases in urinary IL-18, and 43% and 32% increases in KIM-1, respectively. IL-18 urinary concentrations were positively associated with wristband concentrations of four PAHs: phenanthrene, anthracene, pyrene, and perylene. Concentrations for three PAH biomarkers (PHEN1, PHEN2&3, PYR1) were associated with a composite subclinical kidney injury outcome.

Our findings suggest that select PAH exposures are associated with increased kidney injury among agricultural workers. Our comprehensive assessment addresses major knowledge gaps for this population and will inform future studies of kidney health in agricultural communities.

S33 | Systems Biology Meets Environmental Health: Omics Approaches to Maternal and Child Health among Vulnerable Populations

D. Liang[1], **T. Alderete**[2], N. Güil-Oumrait[3], K. Makris[4], A. Peters[5]

[1] *Emory University, Gangarosa Department of Environmental Health, Rollins School of Public Health, Atlanta, Georgia, United States of America*
[2] *Johns Hopkins University, Departmental of Environmental Health and Engineering, Johns Hopkins Bloomberg School of Public Health, Baltimore, Maryland, United States of America*
[3] *ISGLOBAL, Instituto de Salud Global de Barcelona, Barcelona, Spain*
[4] *Cyprus University of Technology, Cyprus International Institute for Environmental and Public Health, Limassol, Cyprus*
[5] *Helmholtz Munich, Institute of Epidemiology, Munich, Germany*

Maternal and child health disparities remain a pressing global challenge, particularly among underserved populations disproportionately burdened by environmental exposures. Leveraging systems biology through advanced omics technologies offers transformative potential to unravel the complex molecular mechanisms driving these health inequities. Omics approaches—encompassing genomics, epigenomics, metabolomics, lipidomics, microbiomics, and proteomics—have enabled high-resolution investigations of how environmental exposures impact human health across the lifespan. Recent innovations in single- and multi-omics profiling, coupled with integrative analytics, have revealed actionable biomarkers and pathways that underlie the link between environmental exposures and adverse maternal and child health outcomes.

This symposium will showcase cutting-edge research at the intersection of omics science and environmental maternal and child health, with a focus on vulnerable and underserved populations. Presentations will explore how various environmental exposures impact maternal and child health outcomes using multi-omics approaches. Specifically, we will showcase studies led by experts around the globe that investigate (1) endocrine-disrupting chemical mixtures and obesity risk in childhood and adolescence; (2) metabolomics insights into organic food interventions and pesticide exposure in school children; (3) epigenetic approaches to understanding environmental insults across life stages; (4) multi-omics analyses linking environmental pollutants to adverse birth outcomes among African American populations; and (5) gut microbiome-metabolome interactions in response to environmental exposures in infants.

This session will emphasize the critical role of omics technologics in advancing precision environmental health and promoting health equity by identifying biomarkers, elucidating pathways, and informing targeted interventions towards better maternal and child health. The symposium format includes a brief introduction (Liang/Alderete, 3 minutes), five presentations (75 minutes), and a moderated discussion to foster engagement and collaboration (12 minutes). This interdisciplinary dialogue aims to inspire novel ideas and catalyze progress in addressing maternal and child health disparities through cutting-edge omics applications.

S33-01 | Hallmarks of environmental insults in maternal and child health: Using epigenetics to understand health impacts of the external exposome

Annette Peters

Helmholtz Munich, Institute of Epidemiology, Munich, Germany

Omic approaches allow characterising the hallmarks of environmental insults including gene regulation in response to the external exposome. We find differences between methylation signatures between external exposures such as air pollution or greenspace and these patterns vary from early childhood to childbearing age. Recent developments building upon systems biology approaches and results from the European Expanse project will be discussed.

S33-02 | Integrating Stool Microbiome and Fecal Metabolome Data to Understand Environmental Influences on Infant Health

Elizabeth A. Holzhausen[1], Maximilian J. Bailey[2,1], Roshonda B. Jones[3], Michael I. Goran[4], Donghai Liang[5], Tanya L. Alderete[1]

[1] Johns Hopkins University, Departmental of Environmental Health and Engineering, Johns Hopkins Bloomberg School of Public Health, Baltimore, USA; [2] Stanford University School of Medicine, Stanford, USA; [3] North Carolina Agricultural and Technical State University, Greensboro, USA; [4] Children's Hospital Los Angeles, Los Angeles, USA; [5] Emory University, Atlanta, USA

Objective. The gut microbiome and its metabolic products play a critical role in early-life health and development. Previous work has shown that air pollution exposure is associated with the infant gut microbiome and fecal metabolome independently. This presentation builds on past work by employing novel, integrated multi-omics analyses to jointly assess the impact of air pollution exposure on the infant gut microbiome and fecal metabolome.
Materials and Methods. We examined the association between prenatal fine particulate matter exposure ($PM_{2.5}$) and the infant gut microbiome and fecal metabolome at 1-month, among infants in the Mother's Milk Study. First, multi-omics factor analysis (MOFA) was used to identify factors that are principal sources of variation among omics layers. Next, we used multivariable linear regression to assess the association between each factor and $PM_{2.5}$, adjusting for covariates. Finally, a permutational multi-omic network-based analysis, using Fastspar and SparCC, was used to generate two sets of co-occurrence networks between 1) $PM_{2.5}$ and gut microbes, and 2) $PM_{2.5}$, gut microbes, and fecal metabolites. Linear models were used to quantify multi-omic networks.
Results. MOFA identified two factors associated with $PM_{2.5}$ ($P_{BOTH}<0.05$). The top-weighted features for these factors were *Bacteroides caecimuris*, *Bacteroides ovatus*, *Bacteroides faecis*, and lysopc(18:0), lysopc(16:0), and urocanate. Infants with low $PM_{2.5}$ (below the population mean) had lower clustering (tendency of nodes to form groups) (t=-2.2, p=0.03) in microbiome-only networks and lower clustering (t=-1.5, p<0.05) and centrality (node relative importance) (t=-32.5, p<0.05) in microbiome-metabolome networks.
Conclusions. These findings highlight the impacts of $PM_{2.5}$ on the gut microbiome, fecal metabolome, and its intercorrelated network structure. *Bacteroides* is a commensal constituent of the human gut microbiome, which produces SCFAs and protects against pathogens. Lower centrality and clustering may indicate more robust networks.

These findings underscore the importance of multi-omics approaches in understanding the early-life effects of environmental exposures.

S33-03 | Per-and polyfluoroalkyl substance (PFAS) exposure, maternal and newborn omics perturbation, and adverse birth outcomes in the Atlanta African American Maternal Child Cohort

Youran Tan[1], Susan Hoffman[1], Kaitlin R. Taibl[1], CheJung Chang[1], Anne L. Dunlop[1], Dana B. Barr[1], Stephanie M. Eick[1], Todd Everson[1], Kristal Manner-Smith[1], P. Barry Ryan[1], Alicia Smith[1], Blake Rushing[2], Susan Sumner[2], Kurunthachalam Kannan[3], Carmen Marsit[1], Dean P. Jones[1], **Donghai Liang**[1]

[1] Emory University, Atlanta, USA; [2] University of North Carolina, Kannapolis, USA; [3] New York State Department of Health, Albany, USA

Background and Objectives:
Per- and polyfluoroalkyl substances (PFAS) are persistent environmental toxicants linked to adverse birth outcomes, particularly among Black and African American populations. However, the underlying biological mechanisms remain unclear. We examined maternal epigenetic, metabolomic, and lipidomic perturbations as potential mediators of PFAS-associated birth outcomes in the Atlanta African American Maternal Child Cohort.
Methods:
We analyzed maternal serum PFAS levels, DNA methylation, untargeted metabolomics, and untargeted lipidomics using blood samples collected during early and late pregnancy from 307 and 238 participants, respectively. Gestational age (GA), small-for-gestational-age (SGA), and birthweight Z-scores (BWZ) were abstracted from medical records. We conducted differential methylation region (DMR) analysis, metabolome-wide association studies (MWAS), and lipidome-wide association studies (LWAS) following a parallel mediation workflow of applying Meet-In-The-Middle (MITM) approach and High-dimensional Mediation Analysis (HIMA).
Results:
Four PFAS compounds were detected in >98% of participants. Increased maternal serum levels of perfluorooctanoic acid (PFOA) and perfluorononanoic acid (PFNA) were associated with higher risk of early GA and SGA. DNA methylation analysis identified 134 CpG sites associated with PFAS exposure, with six CpGs (e.g., EVA1A, FCRL1) also linked to reduced BWZ. Genes annotated to PFAS-associated CpG sites were involved in immune response and lipid homeostasis. Metabolomic signatures identified via MITM and HIMA implicated bile acid, purine, and steroid hormone metabolism as mediating pathways. Lipidomic analysis revealed that a one-quartile increase in PFAS mixture was associated with a 43% increase in SGA risk (odd ratios (OR) = 1.43; 95% confidence interval (CI): 1.02, 2.00). Overlapping lipid intermediates included phosphatidylinositol 18:1-20:4, phosphatidylcholine 32:0, and monoacylglycerol 18:0, enriched in glycerophospholipid metabolism, insulin resistance, and inflammatory pathways.
Conclusion:
Multi-omics integration identified maternal epigenetic, metabolic, and lipidomic disruptions as potential mediators of PFAS-associated adverse birth outcomes. These findings highlight molecular mechanisms underlying PFAS toxicity and may inform interventions to mitigate risks during pregnancy.

S33-04 | Prenatal and childhood exposure to mixtures of endocrine-disrupting chemicals in association with obesity in childhood and adolescence

Nuria Güil-Oumrait

ISGLOBAL, Instituto de Salud Global de Barcelona, Barcelona, Spain

Objective: Early-life exposure to endocrine-disrupting chemicals (EDCs) may increase obesity risk, but the effects of multiple EDCs as mixtures and the underlying biological mechanisms are not fully understood.
Material and methods: We used data from the Human Early Life Exposome (HELIX) project, a multi-cohort study across six countries spanning from northern to southern Europe (France, Greece, Lithuania, Norway, Spain, and the UK), to examine the associations between prenatal and childhood exposure to mixtures of 45 EDCs (measured in blood and urine) and obesity outcomes in 1,301 mother-child pairs. Using a multi-group LASSO-type Bayesian Weighted Quantile Sum Regression, we assessed associations between prenatal and childhood EDC mixtures and obesity markers in childhood and adolescence. We conducted mediation analyses to identify childhood protein pathways that may underlie the associations between prenatal EDC exposure and zBMI in adolescence.
Results: Our results demonstrate that prenatal exposures to metals, organochlorine pesticides, and perfluoroalkyl substances, along with childhood exposure to phenols and high-molecular-weight phthalates, were consistently associated with adverse obesity outcomes and that these associations persisted from childhood into adolescence. Mediation analysis suggests that pro-inflammatory pathways may underlie the association between prenatal metal exposure and obesity in adolescence, particularly through the C-type lectin, NOD-like receptor, MAPK, and NFκB pathways.
Conclusion: These findings underscore the importance of critical developmental windows in shaping obesity risk, while the identification of mediating pro-inflammatory pathways provides deeper insight into the biological mechanisms underlying the association between prenatal metal exposure and obesity in adolescence.

S33-05 | Use of metabolomics in refining the effect of an organic food intervention on biomarkers of exposure to pesticides and biomarkers of oxidative damage in primary school children in Cyprus: A cluster-randomized cross-over trial

Corina Konstantinou, Pantelis Charisiadis, **Konstantinos C. Makris**

Cyprus University of Technology, Cyprus International Institute for Environmental and Public Health, Limassol, Cyprus

Exposure to pesticides has been associated with oxidative stress in animals and humans. Previously, we showed that an organic food intervention reduced pesticide exposure and oxidative damage (OD) biomarkers over time; however associated metabolic changes are not fully understood yet. We assessed perturbations of the urine metabolome in response to an organic food intervention for children and its association with pesticides biomarkers [3-phenoxybenzoic acid (3-PBA) and 6-chloronicotinic acid (6-CN)]. We also evaluated the molecular signatures of metabolites associated with biomarkers of OD (8-iso-PGF2a and 8-OHdG). We used data from the ORGANIKO LIFE cluster-randomized cross-over trial conducted among primary school children in Cyprus. Participants ($n = 149$) were asked to follow an organic food intervention for 40 days and their usual food habits for another 40 days, providing up to six first morning urine samples (>850 samples in total). Untargeted GC-MS metabolomics analysis was performed. Metabolites with RSD ≤ 20% and D-ratio ≤ 50% were retained for analysis. Associations were examined using mixed-effect regression models and corrected for false-discovery rate of 0.05. Pathway analysis followed. Following strict quality checks, 156 features remained out of a total of 610. D-glucose was associated with the organic food intervention ($\beta = -0.23$, 95% CI: -0.37,-0.10), aminomalonic acid showed a time-dependent increase during the intervention period ($\beta_{int} = 0.012$; 95% CI:0.002, 0.022) and was associated with the two OD biomarkers ($\beta = -0.27$, 95% CI:-0.34,-0.20 for 8-iso-PGF2a and $\beta = 0.19$, 95% CI:0.11,0.28 for 8-OHdG) and uric acid with 8-OHdG ($\beta = 0.19$, 95% CI:0.11,0.26). Metabolites were involved in pathways such as the starch and sucrose metabolism and pentose and glucuronate interconversions. This is the first metabolomics study providing evidence of differential expression of metabolites by an organic food intervention, corroborating the reduction in biomarkers of OD.

S34 | Community Involvement in Environmental Health and Beyond: Lessons Learned and Upcoming Challenges

R. Wagoner[1], **J. Honan**[2], J. Wickliffe[2], A. Maybank[3], A. McIntyre[4], C. Stephens[2], C. Hyland[5, 6]

[1] University of Arizona Mel and Enid Zuckerman College of Public Health, Department of Community, Environment, and Policy, Tucson, Arizona, United States of America
[2] University of Alabama at Birmingham School of Public Health, Department of Environmental Health Sciences, Birmingham, Alabama, United States of America
[3] Yale School of Public Health, Department of Environmental Health Sciences, New Haven, Connecticut, United States of America
[4] Silent Spring Institute, Newton, Massachusetts, United States of America
[5] University of California, Berkeley School of Public Health, Division of Environmental Health Resources, Berkeley, California, United States of America
[6] University of California Agriculture and Natural Resources, Berkeley, California, United States of America

This symposium will highlight innovative environmental health research and exposure science projects that deeply involve community partnerships. Speakers will share their experiences navigating the complexities of collaborating with diverse populations, addressing community concerns such as air quality, chemical exposures, housing vulnerabilities, and heat stress.

The session will foster dialogue about the challenges researchers encounter in aligning community priorities with scientific goals, addressing difficult-to-answer questions posed by communities, and integrating principles of social justice into study designs. Topics will include how structural racism and other inequities shape environmental health outcomes, as well as the role of community outreach and engagement efforts in addressing these issues.

Attendees will gain insight into strategies for overcoming these challenges, balancing research and community needs, and preparing for emerging hurdles, such as shifting policies and climate impacts. By sharing lessons learned and identifying actionable paths forward, this session aims to inspire researchers, policymakers, and advocates to develop impactful, community-centered solutions to environmental health challenges.

S34-01 | Academic research and community partnering experiences in the southern United States

Jeffrey Wickliffe

University of Alabama at Birmingham, School of Public Health, Environmental Health Sciences, Birmingham, USA

Environmental health is, at its core, a community problem. Air and water quality, food safety, weather events, noise, the built environment, and social factors among other environmental factors directly affect communities and the health of those living in those communities. Academic researchers live in communities along with those that are not academic researchers. Researchers often have their own interests regarding environmental health issues in communities. These interests may not align with the interests of the communities. Communities in need often are looking for help in generating information that can be used for litigation purposes. Community partners are often small groups or individuals with a keen concern. Community organizations are often volunteer-driven, short on resources, non-profit, and focused on specific issues. Academic researchers, on the other hand, have a host of expectations as they navigate their careers that are not well known by those outside of the academic research community. From issues related to resource allocation among academic researchers and community partners to the slow and often unsuccessful process of acquiring funding to different objectives that academic researchers and community partners have for environmental health research, I hope to facilitate thoughts around the complexity and time-consuming challenge these partnerships represent. I'll share my experiences including air quality and industrial zoning problems in southeast Houston, Texas, oil spill work in southeast Louisiana, preliminary work with residents of Mossville, Louisiana, environmental quality work with public elementary schools in central Alabama, and recent water quality work in Wilcox County, Alabama.

S34-02 | Doctoral student involvement in community-based participatory research: Post graduate reflections

Alina McIntyre[1], Bianca N. Bowman[3], Barbara E. Barrera[3], M. Patricia Fabian[2], Madeleine K. Scammell[2]

[1] Silent Spring Institute, Newton, USA; [2] Boston University School of Public Health, Environmental Health, Boston, USA; [3] GreenRoots, Inc., Chelsea, USA

Urban environmental health challenges, such as air pollution and extreme heat exposure, disproportionately impact environmental justice (EJ) populations. Through a long-standing academic-community partnership, we employed a community-based, mixed methods approach to characterize local-level exposures in Chelsea and East Boston, two EJ communities in Massachusetts. Reflections and lessons learned aim to highlight doctoral student positionality and role in community-engaged research and partnerships.
Three projects in Chelsea and East Boston are presented as case examples that included doctoral student involvement at various stages of the research: 1) interviewing community leaders about extreme heat and air pollution, 2) piloting portable air cleaners to reduce particulate matter in resident homes, and 3) monitoring and mapping local heat variation. Each project highlights challenges and opportunities specific to exposure assessment and environmental health.

Important areas of training to develop student competencies for successful community-engaged environmental health research include community-based participatory research approaches, science communication for diverse audiences, and institutional support for relationship-building through research rotations. The value of applied coursework in qualitative and mixed methods research was realized through conducting interviews with community leaders. These skills were critical in piloting portable air cleaners, which presented challenges and opportunities for developing culturally responsive, bilingual approaches to communicating air pollution exposures and health impacts. This pilot revealed the need for protected time for shared learning (i.e., working on-site, outreach events, community meetings) and knowledge exchange. Local heat monitoring and mapping went beyond traditional GIS methods to consider local context and relevant heat adaptation policies.
This work highlights the key role of graduate students in community-academic collaborations and underscores the need for institutional commitment to building academic frameworks that prioritize training, mentorship, and sustainable community-engaged research in environmental health. Expanding these efforts within environmental health will better prepare researchers to work across disciplines and in partnership with communities.

S34-03 | Building Effective Community Air Monitoring Networks: Engagement, Accuracy, and Advocacy

Sarita Hudson[2], **Aline Maybank**[1], Krystal Pollitt[1]

[1] Yale School of Public Health, Environmental Health Sciences, New Haven, USA; [2] Public Health Institute of Western Massachusetts, Healthy Air Network, Springfield, USA

The Healthy Air Network is a community-led initiative addressing air pollution and environmental injustice in Springfield, Massachusetts. Springfield has some of the highest asthma rates in the U.S., shaped by I-95 corridor traffic, valley geography, and historical disinvestment. In partnership with the Pollitt Lab at the Yale School of Public Health, the Healthy Air Network builds local capacity to monitor ground-level air pollution and respond to air quality challenges through equitable, actionable, and science-informed approaches.

This presentation will highlight the network's collaborative efforts to deploy and maintain a community air sensor network using PurpleAir monitors. We will explain strategies for sensor installation in vulnerable areas, air quality data validation protocols, and the importance of ensuring the data is public, accessible, and valid. The Healthy Air Network empowers local residents with the tools and knowledge to protect their health and advocate for change through real-time air quality mapping, community workshops, and DIY air purifier demonstrations. Central to the Healthy Air Network's success is its commitment to equity and community ownership. Residents are involved in every step, from sensor placement to community education, ensuring that the research work reflects lived experiences and local priorities. This network can be an example of how hyperlocal air monitoring can support resilience, inform public health, and drive policy advocacy.

The Healthy Air Network transforms data into a catalyst for community-led environmental justice by bridging scientific research with grassroots action. Learn more at: healthyairnetwork.org

S34-04 | Exploring Environmental Health Knowledge and Attitudes in Urban High School Students: A Mixed Methods Approach

Crystal Stephens[1,2], Kevin Dsouza[2], Veena Antony[2], Jenna Honan[1], Jeffrey Wickliffe[1], Emmanuel Odame[1], Amy Chatham[1]

[1] University of Alabama at Birmingham, School of Public Health, Environmental Health Sciences, Birmingham, USA; [2] University of Alabama at Birmingham, Department of Medicine, Pulmonary, Allergy, and Critical Care Medicine, Birmingham, USA

Despite significant advancements in recent years, the issue of environmental health knowledge in vulnerable communities remains a critical challenge, particularly in regard to the gap in environmental health education in underrepresented students from environmental justice communities. Environmental exposures such as air pollution and neighborhood-level concerns can adversely impact human health. Adolescents may be more vulnerable to exposure risks and provide an opportunity to introduce environmental health education. With their power of peer association and determination, adolescents can influence the health and well-being of their community.

Using mixed methods, I explored the relationships between environmental exposures and associated health effects of high school students in North Birmingham, Alabama. We aimed to categorize attitudes, knowledge, and identify any perceived barriers and facilitators that influenced student's understanding of environmental health. While my research initially focused on traditional environmental health metrics like air quality, asthma outcomes, and awareness of a nearby superfund site, interview responses revealed a striking divergence.

Results from focus groups revealed five main themes. Emerging themes showed a hierarchy of needs that substantially interfere with students' ability to feel safe, secure, and healthy, overshadowing environmental concerns. Students primarily voiced environmental concerns centered less on air quality or pollution, but more on immediate safety threats like gun violence and crime.

These findings reveal underlying differences in how students define their environment, challenge conventional approaches to environmental health education, and highlight the need for more contextually relevant interventions. Students displayed increased interest in taking action on behalf of themselves and their community. By acknowledging student concerns and social stressors, this study informs decision makers about ways to engage and promote environmental health with community youth.

S34-05 | Moving Community-Engaged Research from Buzzword to Action: Case Studies with Farmworkers

Carly Hyland

University of California, Berkeley, School of Public Health, Division of Environmental Health Resources, Berkeley, California, USA

Our field has seen an explosion in the research deemed ‘community-based’ and ‘community-engaged’ in recent years. It is imperative to reflect on strategies to responsibly engage with community to ensure these are not simply buzz words slapped on grant proposals but result in meaningful partnerships with those impacted by the exposures and policies we aim to study. ‘Surface-level’ community engaged work, even by well-intended researchers and public health practitioners, often benefits researchers but inflicts greater harm on the communities we seek to serve. This presentation will highlight case studies of conducting research with farmworkers and farmworker advocates, including research examining the health impacts of wildfire smoke among agricultural workers and efforts to establish a farmworker resource center. The presentation will also highlight logistical challenges in academic-community partnerships, with the goal of facilitating dialogue and sharing strategies across institutions to move this work forward across our field.

S35 | Wildfire Smoke Exposure and Health Effects in a Warming World: Epidemiological Insights, Health Equity, and Policy Responses

Y. Li[1], M. Qiu[2, 3]

[1] The University of Texas at Arlington, Department of Earth and Environmental Sciences, Arlington, Texas, United States of America
[2] Stony Brook University, School of Marine and Atmospheric Sciences, Stony Brook, New York, United States of America
[3] Stony Brook University, Program in Public Health, Stony Brook, United States of America

Increasing wildfires and its impacts on air quality and human health have emerged as a global crisis, and are projected to get worse under a warming planet. Policymakers and researchers around the world have increasingly recognized the important challenge of wildfire smoke. However, key research gaps remain in both exposure estimation, health impacts of wildfire smoke, climate change influences, and equity-driven solutions to mitigate the health burden of wildfires. This symposium will bring together environmental epidemiologists, exposure scientists, climate scientists, and public health experts to present cutting-edge research on wildfire smoke exposures and their health consequences across diverse populations and settings. The presentation and moderated discussion at this symposium will focus on the following topics:

Health impacts of wildfire smoke: Quantifying both short- and long-term effects of wildfire smoke exposure, on a variety of health outcomes, with a focus on vulnerable populations (such as the elderly population, racial minority groups, etc.) and disparities in health outcomes.

- Exposure estimation methods: Highlighting advancements in modeling wildfire smoke effects on air quality from a variety of methods, ranging from machine learning approaches to field measurements; examining the differences across methods and implications for health effect assessment.
- Policy implications: Discussing the implications of the latest scientific evidence on policies to mitigate wildfire health risks, such as strategies to protect the vulnerable populations during smoke and residents who return to their smoke-influenced houses.

Climate change influences: Investigating how climate change will increase wildfire smoke risks around the globe, and the policy solutions to address this challenge in a warming world.
By bridging exposure science, health impact assessments, and equity-driven solutions, this session aims to catalyze collaborative research and policy actions to address the growing health burden of wildfires in a warming world.

S35-01 | Impact of long-term exposure to wildfire smoke $PM_{2.5}$ on cardiovascular hospital admissions among Medicare beneficiaries in the United States

Siqi Zhang[1], Yongfei Wang[2], Jing Wei[3], Tarik Benmarhnia[4], Yang Liu[5], Harlan M. Krumholz[2], Yuan Lu[2], **Kai Chen**[1]

[1] Yale University, School of Public Health, New Haven, USA; [2] Yale University, Yale School of Medicine, New Haven, USA; [3] University of Maryland, Earth System Science Interdisciplinary Center, College Park, USA; [4] University of California San Diego, Scripps Institution of Oceanography, La Jolla, USA; [5] Emory University, Rollins School of Public Health, Atlanta, USA

Objective: As wildfires intensify in a warming climate, prolonged exposure to smoke-derived fine particulate matter ($PM_{2.5}$) poses significant health risks. Our study aimed to investigate the association between long-term exposure to wildfire smoke $PM_{2.5}$ and first cardiovascular disease (CVD) hospitalizations among U.S. Medicare beneficiaries and to assess potential effect modification by sociodemographic factors.
Methods: This population-based cohort study analyzed 65.2 million Medicare Fee-For-Service or Medicare Advantage beneficiaries aged 65 years or older across the contiguous U.S. from 2017 to 2022. The primary outcome was the first hospitalization for overall CVD and major subtypes, including ischemic heart disease, cerebrovascular disease, heart failure, hypertension, and cardiac arrhythmias. Daily mean wildfire smoke $PM_{2.5}$ concentrations were estimated using a spatiotemporal model on a 10 × 10 km grid and were assigned to beneficiaries' residences at the ZIP code level. We applied quasi-Poisson regressions to estimate relative risks (RRs) and 95% confidence intervals (CIs) of CVD hospitalizations for each decile of wildfire smoke $PM_{2.5}$ concentrations, using exposures below the 1st percentile (0.10 μg/m³) as the reference.
Results: Higher three-year average wildfire smoke $PM_{2.5}$ exposure was significantly associated with increased CVD hospitalization risk. The exposure-response relationship was non-linear, with risk peaking at moderate exposure levels (e.g., RR: 1.02, 95% CI: 1.02–1.03 at 0.34–0.43 μg/m³ for overall CVD). Among CVD subtypes, heart failure exhibited the strongest association (RR: 1.26, 95% CI: 1.21–1.31 at 0.83–1.18 μg/m³). Beneficiaries aged 65–74 years and those of lower socioeconomic status were potentially more vulnerable.
Conclusion: Long-term exposure to wildfire smoke $PM_{2.5}$ is associated with an elevated risk of CVD hospitalizations, with variations by exposure level and individual characteristics. These findings underscore the need for targeted public health strategies and policies to mitigate cardiovascular risks among older adults in the face of increasing climate-driven wildfires.

S35-02 | From Wildfire Emissions to Public Decision-Making: Leveraging Health Research for Better Outcomes

Kevin Cromar

New York University, Department of Population Health, New York, USA

This talk will examine how research on the health impacts of wildland fires can inform public decision-making, focusing on risk communication and climate policy. It will explore how tailored messaging can address community-specific needs during wildfire events, such as assessing pollution levels for behavior modification or identifying smoke exposure risks. The presentation will also discuss how understanding the health impacts of climate change, including those mediated through wildfire activity, can enhance climate policy decisions. By quantifying the potential health benefits of reducing greenhouse gas emissions, this research provides critical insights to guide more effective and informed policy actions.

S35-03 | Perceptions of air quality and physical health symptoms among residents of smoke-damaged homes after a Wildland Urban Interface (WUI) fire

Colleen Reid[1,2], Jessica Finlay[1,2], Michael Hannigan[3], Emma Rieves[1,2], Hannah Walters[4], Courtney Welton-Mitchell[4], Christine Wiedinmyer[3,5], Joost de Gouw[5,6], Katherine Dickinson[4]

[1] University of Colorado Boulder, Department of Geography, Boulder, USA; [2] University of Colorado Boulder, Institute of Behavioral Science, Boulder, USA; [3] University of Colorado Boulder, Department of Mechanical Engineering, Boulder, USA; [4] Colorado School of Public Health, Department of Environmental and Occupational Health, Aurora, USA; [5] University of Colorado Boulder, Cooperative Institute for Research in Environmental Sciences, Boulder, USA; [6] University of Colorado Boulder, Department of Chemistry, Boulder, USA

WUI fires burn human-made materials such as buildings and vehicles. Incineration of human-made materials creates higher emissions factors of some metals, polycyclic aromatic hydrocarbons (PAHs), and volatile organic compounds (VOCs) than burnt biomass. While many people lose their homes in WUI fires, others return to smoke and ash damaged homes. The Marshall Fire was a wildland urban interface (WUI) fire that destroyed more than 1000 structures in two communities in Colorado. We aimed to understand whether smoke or ash damage to one’s home was associated with physical health impacts of the fire event for people living in and around the fire zone whose homes were not completely destroyed. We analyzed data collected from participants who responded to the Marshall Fire Unified Research Survey. We assessed whether self-reported exposure to smells and ash in their homes and spatial exposure measures based on proximity to destroyed structures were associated with reporting of symptoms after the fire. Reporting a headache was statistically significantly associated with all exposure metrics (self-reported and spatial proximity), and reporting a strange taste in one’s mouth was also significantly associated with having more destroyed buildings within 250 m of the home. To our knowledge, this is the first study to examine physical health symptoms associated with smoke-damage of homes after a wildfire. Our findings can inform response planning for future WUI fires to protect the health of residents of smoke-damaged homes.

S35-04 | Long-term exposure to wildfire smoke and mortality: heterogenous effects by exposure metric and vulnerable populations

Lara Schwarz[1], Timothy Frankland[2], Sara Tartof[3], Gina Lee[3], Yuqian Gu[3], Elizabeth R. Mayeda[4], David J. Gonzalez[1], Tarik Benmarhnia[5,6], **Joan A. Casey**[7,8], Chen Chen[5]

[1] University of California Berkeley, Division of Environmental Health Sciences, Berkeley, USA; [2] Kaiser Permanente Hawaii Center for Integrated Health Care Research, Honolulu, USA; [3] Kaiser Permanente Southern California, Department of Research & Evaluation, Pasadena, USA; [4] University of California Los Angeles, Department of Epidemiology, Los Angeles, USA; [5] University of California San Diego, Scripps Institution of Oceanography, La Jolla, USA; [6] University of Rennes, Irset Institut de Recherche en Santé, Rennes, France; [7] University of Washington, Department of Environmental and Occupational Health Sciences, Seattle, USA; [8] University of Washington, Department of Epidemiology, Seattle, USA

Wildfire smoke, once rare, has become a repeated exposure globally. Evidence of single acute health effects of wildfire particulate matter ($PM_{2.5}$) is growing, but less is known about its long-term effects related to repeated exposures or what exposure regime is the most harmful. Using a cohort of Kaiser Permanente Southern California members aged ≥60, we estimated the association between three-year exposure to five different census tract-level wildfire smoke metrics (mean daily wildfire-specific $PM_{2.5}$, mean daily wildfire-specific $PM_{2.5}$ during the peak wildfire week, number of days with daily wildfire-specific $PM_{2.5}$ above zero, number of weeks with average wildfire-specific $PM_{2.5}$ above five, and number of smoke wave days) and all-cause mortality, and evaluated differences by individual and community-level sociodemographics. We applied a discrete-time approach with pooled logistic regressions, adjusting for sex, age, race and ethnicity, marital status, smoking status, requiring an interpreter, calendar year, and census tract-level poverty and population density. Of the 1,250,083 KPSC members aged ≥60 included in the study population, 53% were women, and most identified as non-Hispanic White (49%) or Hispanic (26%). When comparing those highly exposed (95th percentile) to those minimally exposed (5th percentile), we found an increased odds of mortality across all five wildfire smoke metrics. We found that mean daily wildfire $PM_{2.5}$ is the most strongly associated with mortality (odds ratio: 1.07; 95% confidence interval: 1.05, 1.09). Those under age 75, identifying as Black or the Other race or ethnic group, or living in a census tract with higher poverty appeared to be more vulnerable to the long-term health effects of smoke. Understanding which pattern of exposure to long-term wildfire smoke is most harmful and which populations are most at risk will help us understand biological mechanisms and inform effective adaptation strategies to limit exposure to protect health in a changing climate.

S35-05 | Long-term Exposure to Fire Smoke Particulate Matter and Risk of Heart Failure

Yang Liu[1], **Hua Hao**[1], Ke Xu[1], Yanling Deng[1], Sadeer Al-Kindi[2], Varun Pattisapu[3], Kyle Steenland[1], Howard Chang[1], Alvaro Alonso[1]

[1] Emory University, Rollins School of Public Health, Atlanta, USA; [2] Houston Methodist DeBakey Heart & Vascular Center, Houston, USA; [3] Piedmont Hospital, Piedmont Heart Institute, Atlanta, USA

Fine particulate matter (PM2.5, particles <2.5 μm in diameter) is the most important environmental risk factor for global mortality. Wildfires have been increasing globally, posing a global health challenge. Fire smoke-specific PM2.5 is believed to have more cardiovascular toxicity compared with other PM2.5 sources, however, the impact of fire smoke PM2.5 and heart failure (HF) remains undefined. We sought to investigate the association between long-term exposure to fire smoke PM2.5 and HF and compare it with non-fire PM2.5 in a large national cohort. This retrospective cohort study analyzed data from Medicare beneficiaries between 2007 and 2018. We linked individuals to high-resolution exposure estimates of fire smoke-derived and non-fire smoke PM2.5 at a 1×1 km spatial resolution, based on their ZIP codes, and were followed for incident HF. Relationship between PM2.5 and incident HF were examined using Cox Proportional Hazard Models, adjusting for comorbidities and socioeconomic risk factors. The cohort included approximately 22 million enrollees, with a follow-up totaling 115 million person-years. Average fire smoke PM2.5 exposure was 0.51 μg/m³. The hazard ratio (HR) per 1 μg/m³ increase in smoke PM2.5 was 1.014 (95% CI: 1.007–1.020), which was notably higher than the corresponding HR for non-smoke PM2.5 (1.005 [95% CI: 1.003–1.006]). Additionally, the number of days exposed to smoke PM2.5 exceeding 1 μg/m³ and 2.5 μg/m³ over the past 2 years were both significantly associated with an elevated risk of HF. We additionally found that the association between smoke PM2.5 and HF was stronger in women, Medicaid eligible individuals, and those living in lower income areas, indicating higher susceptibility. In summary, long-term exposure to fire smoke-related PM2.5 was linked to a higher risk of HF compared to non-smoke PM2.5, with greater susceptibility in women and socially vulnerable populations.

S36 | Addressing gaps in preconception environmental epidemiology

J. Ashley-Martin[1], L. Wise[2], J. Braun[3], J. Buckley[4], C. Porucznik[6], N. Cohen[5]

[1] *Health Canada, Environmental Health Science and Research Bureau, Ottawa, Ontario, Canada*
[2] *Boston University, Epidemiology, Boston, Massachusetts, United States of America*
[3] *Brown University, Epidemiology, Providence, Rhode Island, United States of America*
[4] *University of North Carolina, Epidemiology, Chapel Hill, North Carolina, United States of America*
[5] *Mt Sinai School of Medicine, Environmental Medicine & Public Health, New York City, New York, United States of America*
[6] *University of Utah, Public Health, Salt Lake City, Utah, United States of America*

Environmental chemicals may adversely impact parental reproductive health - a vital sign for the overall health of parents and potential offspring. Despite the challenges of assessing exposure prior to conception, biomonitoring data collected during this window are essential to understanding potential environmental chemical-related contributions to critical endpoints such as fecundity and fetal loss. The few existing studies have rather homogenous study populations and subsequent limited ability to robustly answer critical questions about reproductive health. Specifically, male partners and individuals experiencing marginalization are underrepresented in the existing literature.

Both historic and contemporary perceptions of reproductive health create barriers to engaging fathers in reproductive health research. However, the limited data on environmental chemicals and fecundity report that male partners exposures are just as or more relevant to risks of diminished fecundity than exposures in female partners – underscoring the need to engage with and collect biomonitoring data from male partners.

Preconception study designs reliant on recruitment online or through fertility clinics has resulted in minimal sociodemographic diversity. Exposure and health disparities in racialized, newcomer and low-income populations are well established for many chemicals and health outcomes; however, knowledge of these disparities within the context of reproductive health is scarce. Novel study design and data collection methods are necessary to investigate patterns of exposure and health effects in the individuals who may be mostly likely to experience disproportionate exposure or susceptibility to environmental chemicals.

This symposium will highlight novel, contemporary research efforts to address these research gaps. Specifically, the panel will provide global perspectives on initiatives to recruit families prior to conception, engage with male partners and broaden the sociodemographic diversity of existing and future study populations. We will present findings from studies that have recruited from preconception and fertility clinics, web-based platforms, and the community.

S36-01 | Broadening diversity in an online preconception cohort

Lauren Wise, Martha Koenig, Mary Willis, Samantha Schildroth, Elizabeth Hatch, Amelia Wesselink

Boston University, Epidemiology, Boston, USA

Background: Preconception cohort studies can help advance science on environmental determinants of reproduction. While online research methods provide efficient and effective strategies to collect questionnaire-based data, few online studies incorporate biospecimen collection, which can enhance the validity of exposure assessment.
Methods: We evaluated the feasibility and cost-effectiveness of in-clinic and mail-based biospecimen collection protocols in Pregnancy Study Online (PRESTO), a North American web-based preconception cohort study. Couples residing in the contiguous U.S. with conception attempt times ≤3 months at enrollment were eligible to participate. We invited participants in Boston, MA and Detroit, MI to attend a study visit and provide urine and blood (hereafter "in-clinic protocol"). We invited all other participants to complete mail-based collection of urine and blood spots (hereafter "mail-based protocol"); starting 5/2024, we oversampled participants of color and randomly-sampled 25% of non-Hispanic White participants. We compared overall consent and protocol completion rates, demographic characteristics of those who consented and completed either protocol, and costs between mail-based and in-clinic protocols (urine collection only).
Results: During 1/2022-3/2025, 64% of female participants (1,303/2,037) and 39% of male participants (198/509) consented to participate in the mail-based protocol. Among those who consented, 68% of females (N=886) and males (N=136) provided urine and blood specimens. Consent rates for the in-clinic protocol were 39% for female participants (289/739 during 3/2014-7/2022) and 25% for male participants (40/157 during 3/2017-7/2022). Participants were generally of higher SES than non-participants. Oversampling by race and ethnicity increased the percent of mail-based participants of color from 15% to 32%. The cost per participant enrolled was similar across protocols (mail-based: $276.14 vs. in-clinic: $270.38).
Conclusions: Mail-based collection of biospecimens in a national preconception cohort study may create opportunities to recruit a larger, more geographically heterogeneous, and more racially and ethnically diverse participant population at a comparable cost-per-participant-enrolled to in-clinic methods.

S36-02 | Preconceptional Phthalate Exposures and Child Neurobehavior: The PEACE Study

Joseph Braun[1], Jordana Leader[2], Cecilie Skaarup Uldbjerg[3], Lidia Minguez-Alarcon[6], Jenn Ford[4], David Bellinger[5], Russ Hauser[4]

[1] Brown University, Epidemiology, Providence, USA; [2] Icahn School of Medicine at Mt. Sinai, Environmental Medicine and Public Health, New York, USA; [3] Rigshospitalet, Growth and Reproduction, Copenhagen, Denmark; [4] Harvard School of Public Health, Environmental Health, Boston, USA; [5] Boston Children's Hospital, Neurology, Boston, USA; [6] Brigham and Women's Hospital, Medicine, Boston, USA

Objectives: Experimental studies in animals suggest that preconceptional exposures, including to endocrine disrupting chemicals (EDCs) – may adversely affect offspring health via epigenetic programming. However, few studies have investigated whether preconceptional EDC exposures impact neurobehavioral outcomes in humans. Thus, we leveraged the Preconception Environmental exposure And Childhood health Effect (PEACE) Study to estimate the potential impact of paternal and maternal preconception phthalate exposure on child behaviors.
Methods: Since 2018, we have conducted follow-up on 273 children (age 6-11 years) whose parent(s) previously enrolled in the prospective preconception Environment and Reproductive Health (EARTH) Study (2005-2019). Before (mothers and fathers) and during (mothers) pregnancy we assessed exposure to eight phthalate dieters via urinary phthalate metabolites. Parents reported on children's problem, reciprocal social, and eating behaviors via the Behavioral Assessment System for Children-3, Social Responsiveness Scale-3, and Child Eating Behavior Questionnaire, respectively.
Results: Higher maternal preconception and pregnancy monobenzyl phthalate (MBzP) were associated with worse parent-reported child behavior, while higher maternal and paternal preconception mono-carboxyoctyl phthalate (MCOP) concentrations were related to lower BASC-3 scores. Urinary phthalate metabolite levels were not associated with higher SRS scores. Paternal and maternal preconception MBzP and maternal preconception mono-n-butyl phthalate (MnBP) concentrations were positively associated with scores on measures of emotional overeating, food responsiveness, and desire to drink in children. These associations remained present after adjusting for confounders and phthalate exposures biomarkers in other time periods or parent.
Conclusions: We found some evidence suggesting that maternal preconception urinary levels of MBzP were associated with more behavior problems and greater food approaching behaviors. Paternal phthalate levels were not as consistently associated with child behaviors. Future studies in the PEACE cohort could leverage biobanked samples and questionnaire data to identify potential modifiers or mediators of these associations or investigate other preconceptional exposures in relation to child health.

S36-03 | Characterizing preconception environmental exposures in the ECHO Cohort

Jessie Buckley

University of North Carolina, Epidemiology, Chapel Hill, USA

Objective: The National Institutes of Health's Environmental influences on Child Health Outcomes (ECHO) Cohort is the largest study of children's health in the U.S., with more than 50,000 children enrolled to date. ECHO's infrastructure provides a unique opportunity to collect preconception biospecimens and environmental exposure data for subsequent pregnancies.
Methods: This presentation will discuss ECHO's recent initiatives to collect biospecimens and measure environmental exposures during the preconception window in a subset of ~10,000 pregnancies.
Results: After their first ECHO pregnancy, participants and their partners are followed as a preconception cohort for subsequent pregnancies. ECHO enrolls current partners of previously pregnant participants and collects data and biospecimen from both biological mothers and their partners during the inter-pregnancy period. Biospecimen collection after the first ECHO birth may include maternal blood spots, urine, and hair as well as partner saliva, urine, and sperm, allowing for future analysis of chemical exposure biomarkers. Residential address history is collected to facilitate a variety of geospatial-based assignments, such as air pollution, greenspace, and drinking water contaminants. Questionnaires are administered to collect information on potential chemical exposure sources including household heating sources, pesticide use, and pets as well as maternal diet, food source, and preparation; maternal and paternal occupation; and breastfeeding practices. By collecting this detailed information, ECHO is prospectively assessing preconception environmental exposures that can be leveraged for future studies of pregnancy outcomes.
Conclusions: The ECHO Cohort represents an unparalleled and cost-effective data collection platform to characterize preconception environmental exposures, ultimately informing policies, practices, and programs to improve lifelong health.

S36-04 | Successful serial biospecimen collection in couples recruited from the community

Christy Porucznik

University of Utah, Public Health, Salt Lake City, USA

Dr. Porucznick, Principal Investigator of the Home Observation of Periconceptional Exposures (HOPE) study in Utah, will describe recruitment strategies and methods to invite both male and female partners to collect biospecimens at home during the windows of conception, implantation and early pregnancy. The HOPE study has leveraged this biospecimen collection to generate serial measurements of bisphenol A and investigate associations with time to pregnancy. Dr. Porucznick will describe HOPE's study successes in generating paternal data as well as the challenges of recruiting a demographically diverse population. 183 couples planning pregnancy enrolled in the study between 2011 and 2015. Both partners collected daily first-morning urine during the fertile window, with females continuing to the end of the menstrual cycle. We collected 6,118 urine samples from 284 menstrual cycles and 169

couples. Women collected at least one urine specimen within three days of ovulation in 97% of cycles, and men collected at least one in that time frame in 91%. Both partners collected 97% of the requested saliva samples, and men collected 89% of the requested semen samples. Data and samples from HOPE have been used to identify air pollution impacts on semen quality, temporal variability of urinary cadmium, develop analytical methods for bisphenol A analysis, the association between exposure to bisphenol A and semen quality, and daily perceived stress and time to pregnancy. Children conceived during HOPE have been enrolled into the Utah site of the national Environmental Influences on Child Health Outcomes cohort.

S36-05 | Examining modifiable determinants of fecundity in a preconception cohort

Nathan Cohen[1], Jordana Leader[1], Srinivasan Narasimham[1], Syam Andra[1], Meizhen Yao[1], Anil Meher[1], Manish Arora[1], Vishal Midya[1], Jerry K.Y. Chen[2,3], Shiao-Yng Chan[4,5], Lidia Minguez-Alarcon[10], Jonathan Huang[11], Robert Cabrera[8], Youssef Oulhote[1], Damaskini Valvi[1], See L. Loy[2,3], James C.Y. Chan[4,5], Richard Finnell[8,9]

*[1] Mt Sinai School of Medicine, Environmental Medicine & Climate Science, New York City, USA; [2] KK Women's and Children's Hospital, Department of Reproductive Medicine, Singapore, Singapore; [3] Duke NUS Medical School, Academic Clinical Program in Obstetrics and Gynaecology, Singapore, Singapore; [4] Agency for Science, Technology and Research, Institute for Human development and Potential, Singapore, Singapore; [5] National University of Singapore, Department of Obstetrics and Gynaecology, Singapore, Singapore; [6] Agency for Science, Technology and Research, A*STAR Skin Research Labs, Singapore, Singapore; [7] Agency for Science, Technology and Research, Singapore Institute of Food and Biotechnology Innovation, Singapore, Singapore; [8] Baylor College of Medicine, Department of Molecular and Cellular Biology, Houston, USA; [9] University of Texas at Austin, Department of Pediatrics, Austin, USA; [10] Harvard School of Public Health, Department of Environmental Health, Boston, USA; [11] University of Hawaii, Thompson School of Social Work and Public Health, Honolulu, USA*

Dr. Cohen, a postdoctoral research fellow at the Icahn School of Medicine at Mount Sinai, will discuss recent findings from the Singapore Preconception Study of Long-Term Maternal and Child Outcomes (S-PRESTO) study related to perfluoroalkyl substances and women’s infertility and the potential modifying effects of vitamin B12. Dr. Cohen will explain the importance of assessing chemical exposures at preconception as opposed to during pregnancy and the importance of including male partners in future studies.

TO01 | Ambient air pollution and pediatric health: Impacts on growth, development, and disease

TO01-01 | Ambient air pollution during pregnancy and offspring cerebral palsy

Haoran Zhuo[1,2], Beate Ritz[3,4], Jason Su[5], Joshua Warren[6], Zeyan Liew[1,2]

[1] Yale School of Public Health, Department of Environmental Health Sciences, New Haven, USA; [2] Yale School of Public Health, Yale Center for Perinatal, Pediatric, and Environmental Epidemiology, New Haven, USA; [3] UCLA, Department of Epidemiology, Fielding School of Public Health, Los Angeles, USA; [4] UCLA, Department of Neurology, Los Angeles, USA; [5] University of California, Berkeley, School of Public Health, Berkeley, USA; [6] Yale School of Public Health, Department of Biostatistics, New Haven, USA

Objective Ambient air pollution has been shown to have a detrimental effect on childhood neurodevelopment. Cerebral palsy (CP) is a severe neuromotor disorder with emerging evidence implicating environmental exposures in the disease etiology. This study investigated whether prenatal exposures to ambient air pollution was associated with childhood CP.

Methods We conducted a nested case-control study with all CP cases (N=4,657) identified from a statewide diagnostic system and randomly selected controls (N=872,684) representing 20% of all live births in California in 2007-2015. A high-resolution (100m) daily land-use regression spatiotemporal model based on a machine learning algorithm was used to estimate the prenatal exposure to nitrogen dioxide (NO_2), fine particulate matter ($PM_{2.5}$), and ozone (O_3) weekly averages that we also combined over the entire pregnancy period. We utilized logistic regression to estimate the odds ratio (OR) and 95% confidence interval (CIs) for CP risk with pregnancy-averaged exposures. A Bayesian distributed lag model was implemented to assess the existence of more susceptible windows of exposures.

Results A per interquartile-range increase in NO_2 over the pregnancy was associated with a 29% (95%CI: 1.22-1.35) increased risk of CP, adjusting for confounders and other air pollutants. Ozone exposure was also associated with CP but at a smaller magnitude (OR=1.12), while $PM_{2.5}$ was not associated with CP. We found gestational weeks 2-4 and weeks 22-24 to be most sensitive for NO_2 exposures, while no such windows noted for ozone and $PM_{2.5}$.

Conclusion Maternal exposures to NO_2 and Ozone from the ambient air, largely attributable to traffic-related emissions, were associated with increased risk of CP in California.

TO01-02 | Associations of Fine Particulate Matter Components with Neurodevelopment in Early Childhood: Evidence from Mixture Approaches

Haonan Li[1], Elizabeth A. Holzhausen[1], Nathan Young[1], Devendra Paudel[1], Jonatan Ottino-González[2], Frederick W. Lurmann[3], Douglas I. Walker[4], Howard H. Chang[5], Donghai Liang[4], Ravi Bansal[2], Michael I. Goran[2], Bradley S. Peterson[2], Tanya L. Alderete[1]

[1] Johns Hopkins University, Department of Environmental Health and Engineering, Bloomberg School of Public Health, Baltimore, USA; [2] University of Southern California, Department of Pediatrics, The Saban Research Institute, Children's Hospital Los Angeles, Los Angeles, USA; [3] Sonoma Technology, Inc., Petaluma, USA; [4] Emory University, Gangarosa Department of Environmental Health, Rollins School of Public Health, Atlanta, USA; [5] Emory University, Department of Biostatistics and Bioinformatics, Rollins School of Public Health, Atlanta, USA

Objective: Early-life exposure to ambient fine particulate matter ($PM_{2.5}$) is linked to adverse neurodevelopment in children, yet the specific components driving these associations remain uncertain. This study investigates the independent and joint effects of $PM_{2.5}$ components on neurodevelopment.
Methods: This study included 162 Latino mother-infant dyads from the Southern California Mother's Milk Study. Annual average concentrations of 15 chemical components of $PM_{2.5}$ [2 carbonaceous components: elemental carbon and organic carbon; 3 secondary inorganic salts: sulfate (SO_4^{2-}), nitrate (NO_3^-), and ammonium (NH_4^+); and 10 trace elements: bromine, calcium (Ca), copper (Cu), iron, potassium, nickel (Ni), lead (Pb), silicon, vanadium (V), and zinc (Zn)] were estimated in the calendar year of birth based on residential addresses. Neurodevelopment was assessed using the Bayley-III Scales of Infant and Toddler Development at 2 years. Linear regression models examined the associations between individual exposures with neurodevelopmental scores. Weighted quantile sum (WQS) regression and Bayesian kernel machine regression (BKMR) examined the mixture effects and individual contributions of these components.
Results: $PM_{2.5}$, SO_4^{2-}, NO_3^-, NH_4^+, Cu, Ni, Pb, and V were negatively associated with social-emotional and adaptive behavior scores, while Ca and Zn were positively associated. WQS regression showed that the $PM_{2.5}$ component mixture tended to be negatively associated with multiple adaptive behavior scores (*p*-value = 0.02–0.09). Based on BKMR, there were negative associations between the component mixture and adaptive behavior scores as well although these did not reach statistical significance. Both WQS and BKMR identified Ni as the strongest contributor to the effects, with SO_4^{2-} consistently ranking among the top contributors in both models.
Conclusion: These findings indicate that exposure to specific components of $PM_{2.5}$ mixtures may negatively impact infant neurodevelopment, particularly adaptive behavior skills, which are essential for navigating and managing everyday life demands. Notably, Ni and SO_4^{2-} appear to be key contributors to these effects.

TO01-03 | Long-term effects of cooking with liquefied petroleum gas or biomass on linear growth and neurodevelopmental outcomes in pre-school aged Peruvian children

Laura Nicolaou[1,2], Carolyn J. Reuland[1], Kendra N. Williams[1], Stella M. Hartinger[4], Marilu Chiang[5], William Checkley[1,3]

[1] Johns Hopkins University, Division of Pulmonary and Critical Care, Baltimore, USA; [2] Johns Hopkins University, Department of Environmental Health and Engineering, Baltimore, USA; [3] Johns Hopkins University, Department of International Health, Baltimore, USA; [4] Universidad Peruana Cayetano Heredia, Latin American Center of Excellence on Climate Change and Health, Lima, Peru; [5] A. B. PRISMA, Biomedical Research Unit, Lima, Peru

Objective: Household air pollution (HAP) is a leading environmental risk factor that may adversely affect child health. We recently completed an 18-month multi-center RCT of a liquefied petroleum gas (LPG) intervention during pregnancy and infancy. The intervention successfully lowered $PM_{2.5}$ exposures but failed to improve infant health outcomes. In this follow-up study in Puno, Peru, we evaluated the longer-term effects of reduced HAP exposures during a critical period of child development on linear growth and neurodevelopment.

Material and Methods: We measured height at age 2-4 years; and assessed neurodevelopment using the Bayley Scales of Infant and Toddler Development Third Edition (Bayley-III) in a subset at 2-3 years. We conducted intention-to-treat analyses of the intervention on height-for-age Z-scores (HAZ) and Bayley-III scores, and exposure-response analyses between prenatal/infant $PM_{2.5}$ exposures and both health outcomes. We also examined differences in the growth trajectories between birth and 2-4 years of age by study arm.

Results: We revisited 683 children for height measurements (mean age 34.0±6.6 months, 49.3% male, 52.3% in intervention), of which 301 also had Bayley-III testing. Mean HAZ was -0.92±0.83 SDs in intervention children and -1.00±0.80 SDs in controls (p=0·21). On intention-to-treat, adjusted differences between intervention and control were 0.08 SDs (95% CI -0.04 to 0.20) for HAZ, and -1.8 (98.3% CI -4.9 to 1.2), -2.9 (-6.0 to 0.1), and -1.4 (-5.1 to 2.2) for the composite cognitive, language, and motor scores. In longitudinal analysis, we did not find important associations between growth trajectories and study arm. There were no associations between prenatal/infant $PM_{2.5}$ exposures and HAZ or Bayley-III scores at age 2-4 years.

Conclusion: Linear growth or neurodevelopment at 2-4 years was not affected by an LPG intervention during pregnancy and infancy despite significant reductions in exposures; and were also not associated with either prenatal or infant $PM_{2·5}$ exposures.

TO01-04 | Spatial variability in the association of ambient $PM_{2.5}$ exposure with child undernutrition in Sub-Saharan Africa

A. Kofi Amegah[1], Patrick Attey-Yeboah[1], Paul K. Adjorlolo[2], Justice M. Aheto[2], John Molitor[3], Eric S. Coker[4]

[1] University of Cape Coast, Biomedical Sciences, Cape Coast, Ghana; [2] University of Ghana, School of Public Health, Accra, Ghana; [3] Oregon State University, College of Health, Corvalis, USA; [4] BC Centre for Disease Control, Vancouver, Canada

Objective: Studies have associated ambient $PM_{2.5}$ expsoure with child undernutrition. The expsoure-response relationships, however, vary significantly within and between countries and geographical regions, and yet has not been investigated. We therefore investigated spatial variability in the association of prenatal and postnatal $PM_{2.5}$ exposure with childhood undernutrition in sub-Saharan African (SSA) countries to better tailor intervention strategies.
Material and Methods: We integrated data on child undernutrition from Demographic and Health Surveys (DHS) conducted in countries between 1998 and 2022 with high resolution satellite-derived geospatial $PM_{2.5}$ exposures to create a dataset for our analysis. The dataset included 490,250 children under-five in 33 SSA countries. The exposure metrics was computed using weighted averages during the prenatal and postnatal periods with adjustment for geographic coordinate displacement via urban and rural buffers. We used a spatial random coefficient model set in a Bayesian framework (Integrated Nested Laplace Approximations [INLA] and Stochastic Partial Differential Equation [SPDE]) to model the spatially varying relationships controlling for individual- and area-level confounders assembled from the DHS data.
Results: Prenatal and postnatal $PM_{2.5}$ exposure was associated with 4.6% and 4.2% increased odds of stunting (AOR = 1.046, 95%CI: 1.039, 1.053 and 1.042, 95%CI: 1.035, 1.049, respectively), and 4.4% and 2.8% increased odds of wasting (AOR = 1.044, 95%CI: 1.0363, 1.052 and 1.028, 95%CI: 1.020, 1.036, respectively). Prenatal $PM_{2.5}$ exposure was associated with 3.1% increased odds of underweight (AOR = 1.031, 95%CI: 1.021, 1.0412). Postnatal $PM_{2.5}$ exposure showed no significant association with underweight. The observed associations varied significantly within and between the SSA sub-regions and countries, with West Africa recording the highest exposure health effects followed by East Africa.
Conclusion: The association of ambient $PM_{2.5}$ exposure with child undernutrition varied within and between SSA countries, and calls for targeted interventions to help address the pervasive child undernutrition problem in the SSA region.

TO01-05 | Fine particulate matter exposure above the new US National Ambient Air Quality Standard and risk of small-for-gestational-age: Evidence from a North American preconception cohort

Jonathan Y. Lee[1], Julie McDonald[2], Afroze Qutab[1], Owais Gilani[2], Laura Corlin[2], Mary Willis[3], Lauren Wise[3], Ameila Wesselink[3], Kipruto Kirwa[1]

[1] Boston University School of Public Health, Environmental Health, Boston, USA; [2] Tufts University School of Medicine, Public Health and Community Medicine, Boston, USA; [3] Boston University School of Public Health, Epidemiology, Boston, USA

Objective: In 2024, the US Environmental Protection Agency strengthened the health-based National Ambient Air Quality Standard (NAAQS) for fine particulate matter ($PM_{2.5}$) to 9 u g/m^3. Evidence linking $PM_{2.5}$ exposure to pregnancy and birth outcomes was, however, deemed inconsistent and insufficient to infer causality, given scant data in population subgroups and concerns about unmeasured confounding. We assessed the association between $PM_{2.5}$ and risk of small-for-gestational-age birth (SGA), a proxy for intrauterine growth restriction and predictor of infant morbidity, comparing participants exposed to $PM_{2.5}$ levels below and above the new NAAQS during pregnancy. We evaluated risk in strata of household income, region of residence, race/ethnicity, and educational attainment.

Methods: We analyzed data from Pregnancy Study Online (PRESTO), a preconception cohort of female pregnancy planners aged 21-45 years not using fertility treatment at baseline. We restricted analyses to 3,003 US participants who delivered singletons during 2013-2019. Participants reported sociodemographic, behavioral, and medical data on baseline and follow-up questionnaires, and birth outcomes on a postpartum questionnaire. We estimated weekly ambient residential concentrations of $PM_{2.5}$ using a well-validated fine-scale spatiotemporal model and calculated average residential exposures throughout pregnancy for each participant. We used multivariable logistic regression to estimate odds ratios (ORs) and 95% confidence intervals (CIs) for the association between $PM_{2.5}$ (≥9 *vs.* <9 u g/m^3) and risk of SGA (<10th percentile of birth weight for gestational age).

Results: Mean age at delivery was 30.7 years, 85.0% identified as non-Hispanic White, 47.1% had a graduate degree, and 13.8% had average $PM_{2.5}$ pregnancy exposures ≥9 u g/m^3. SGA prevalence was 6.0%. Exposure ≥9 u g/m^3 was associated with higher odds of SGA (OR=1.51, 95%CI: 0.99-2.27). The association persisted in strata of income, region, race/ethnicity, and education, with few exceptions where numbers were small.

Conclusion: Achieving population-wide $PM_{2.5}$ NAAQS may reduce risk of adverse pregnancy outcomes.

TO01-06 | Ambient air pollutant mixture and lung function among children in Fresno, California

Wenxin Lu[1], Ellen Eisen[1], Liza Lutzker[2], Elizabeth Noth[1], Tim Tyner[3], Fred Lurmann[4], S Katharine Hammond[1], Stephanie Holm[5,6], John Balmes[1,6]

[1] University of California, Berkeley, Division of Environmental Health Sciences, Berkeley, USA; [2] University of California, Berkeley, Division of Epidemiology, Berkeley, USA; [3] Central California Asthma Collaborative, Fresno, USA; [4] Sonoma Technology, Petaluma, USA; [5] University of California, San Francisco, Western States Pediatric Environmental Health Specialty Unit, San Francisco, USA; [6] University of California, San Francisco, Department of Medicine, San Francisco, USA

Objective

Ambient air pollutants such as $PM_{2.5}$, $PM_{10,}$ and NO_2 have been associated with lower lung function among children. However, the associations found could be due to correlation with other pollutants. We investigate the relationships between exposures to eight ambient air pollutants and children's lung function and apply mixture analysis to identify key risk factors.

Material and Methods

The Children's Health and Air Pollution Study (CHAPS) in Fresno, California, is a prospective cohort study that recruited 299 children and assessed their pulmonary function at two visits, at approximately 7 and 9 years of age. The children's forced expiratory volume in 1 second (FEV1), forced vital capacity (FVC), and FEV1/FVC ratio were standardized using the Global Lung Function Initiative (GLI) race-neutral calculators. We assessed the children's average residential exposures to eight ambient air pollutants, namely, $PM_{2.5}$, PM_{10}, NO_x, NO_2, ozone (O_3), carbon monoxide (CO), elemental carbon (EC), and polycyclic aromatic hydrocarbons (PAHs), during the 1-week, 1-month, 3-month, 6-month and 12-month periods before each visit, and the 2-year period between visits. We applied generalized estimating equations (GEE) and quantile-based g-computation (qgcomp) for statistical analysis.

Results

The children's exposures to the eight ambient air pollutants exhibited high intercorrelation: all pollutants were positively correlated except for O_3, which was negatively correlated with the other pollutants. PM_{10} was associated with lower FEV and FEV1/FVC ratio, and the association was the strongest for the 3-month exposure timeframe. O_3 exposure was negatively associated with the change in FEV1 and FEV1/FVC ratio between visits. CO exposure was negatively associated with the change in FVC between visits. Qgcomp identified the same key pollutants as single-pollutant models.

Conclusion

Among the eight ambient air pollutants, PM_{10}, O_3, and CO were the strongest risk factors for impaired lung function among children.

Funding: EPA RD83543501, NIH ES022849, and NIEHS R24 ES03088.

TO01-07 | Association between early-life exposure to multiple air pollutants and incident Kawasaki disease: A nested case-control study in Taiwan

Chau-Ren Jung[1,2], Lina Madaniyazi[3,4], Xerxes T. Seposo[5], Bo-Ting Chen[1,6], Bing-Fang Hwang[6]

[1] China Medical University, Department of Public Health, Taichung, Taiwan; [2] National Institute for Environmental Studies, Japan Environment and Children's Study Programme Office, Tsukuba, Japan; [3] Nagasaki University, Department of Pediatric Infectious Diseases, Nagasaki, Japan; [4] Nagasaki University, School of Tropical Medicine and Global Health, Nagasaki, Japan; [5] Hokkaido University, Department of Hygiene, Sapporo, Japan; [6] China Medical University, Department of Occupational Safety and Healt, Taichung, Taiwan

Objective: Several studies have explored the association between air pollutants and Kawasaki disease (KD); however, few have considered prenatal exposure and the combined effects of multiple air pollutants. This study aimed to investigate the associations between exposure to multiple air pollutants, including fine particles ($PM_{2.5}$), sulfur dioxide (SO_2), and total oxides (O_x), during pregnancy and the first five years of life, and KD.
Methods: This study employed a nested case-control design using data from the Taiwan Maternal and Child Health Database (TMCHD), which included 1,845,668 newborns between 2005 and 2015, followed for five years. Cases were identified based on the International Classification of Diseases, Ninth and Tenth revisions. A total of 8,857 cases were identified and matched to 88,570 controls by sex and birth date. Air pollution data were estimated using satellite-based models. Conditional logistic regression was used to assess associations between individual air pollutants and KD, expressed as odds ratios (ORs) with 95% confidence intervals (CIs). Additionally, quantile g-computation (QGC) was applied to assess the combined effects of multiple air pollutant exposures.
Results: The single-pollutant model showed that exposure to O_x and SO_2 during the first and second trimesters was associated with an increased risk of KD. Additionally, O_x exposure from the first year to fourth year of life significantly increased the risk of KD. The QGC model revealed that exposure to multiple air pollutants during all three trimesters of pregnancy and the first four years postnatally was significantly and positively associated with KD, with O_x and SO_2 contributing the most to this association.
Conclusion: Our findings suggest early-life exposure to O_x and SO_2 increased the risk of KD. The results of multiple exposure models further support this conclusion, mothers and infants should minimize exposure to oxides and consider implementing mitigation strategies to reduce air pollution-related health risks.

TO02 | Exposure assessment methods for environmental chemicals

TO02-01 | A Dynamic Two-Compartment Mass Balance Model for Probabilistic Assessment of Dermal Exposure to Insect Repellents

Noshin Anjum Kamal, Hyeong-Moo Shin

Baylor university, Environmental Science, Waco, USA

Background: Assessing dermal exposure to insect repellents for consumer use in both indoor and outdoor environments require models that account for chemical properties, environmental conditions, and user-specific application behaviors. Existing regulatory models, such as the U.S. EPA's Standard Operating Procedures (SOPs), provide a standardized framework for exposure estimation but overlook critical factors like environmental variability, reapplication patterns, and absorption dynamics. This study develops a probabilistic two-compartment mass balance model to enhance time-dependent dermal exposure predictions by systemically incorporating these factors.
Methods: Our model consists of two homogeneous, well-mixed compartments (skin surface and air/environmental interface) with rate-limiting transport processes. We conducted Monte Carlo uncertainty analysis to assess parameter uncertainty, incorporating variations in application rates, environmental factors (temperature, relative humidity) and absorption parameters. Model calibration was performed using experimental data from controlled chamber studies. Independent studies on DEET absorption under different environmental conditions were used for validation, and sensitivity analysis was used to identify key influential parameters.
Results: The model estimated dermal absorption rates of DEET across a range of percentages compared to existing models. Sensitivity analysis identified temperature and relative humidity as critical factors, contributing a substantial portion of overall exposure variability. Monte Carlo simulations quantified the uncertainty, demonstrating exposure variability under different environmental scenarios. Validation results confirmed alignment between simulated and observed DEET dermal absorption profiles, with acceptable error margins. Compared to the SOP model, our estimates reflected the impact of incorporating chemical-specific transport, environmental interactions, and absorption processes.
Conclusion: This study demonstrates the potential for advancing dermal exposure assessment and supporting regulatory assessments and product safety for consumer insect repellent use in indoor and outdoor settings. By integrating probabilistic methods with mechanistic dermal absorption models, we achieve a more comprehensive characterization of exposure dynamics. Continued experimental validation is necessary to support evidence-based decision-making.

TO02-02 | Evaluation of integrated, multimedia biomarkers of prenatal metals exposure in association with child neurodevelopment in Puerto Rico

Savannah M. Sturla Irizarry[1], Amber L. Cathey[1], Emily Zimmerman[2], Wei Hao[3], Yi-Ting Lin[3], Bhramar Mukherjee[4], Zaira Y. Rosario Pabón[5], Gredia Huerta Montañez[5], Carmen M. Vélez Vega[6], Akram N. Alshawabkeh[5], José F. Cordero[7], John D. Meeker[1], Deborah J. Watkins[1]

[1] University of Michigan, Environmental Health Sciences, Ann Arbor, USA; [2] Northeastern University, Communication Sciences and Disorders, Boston, USA; [3] University of Michigan, Biostatistics, Ann Arbor, USA; [4] Yale University, Biostatistics and Department of Chronic Disease Epidemiology, New Haven, USA; [5] Northeastern University, Civil and Environmental Engineering, Boston, USA; [6] University of Puerto Rico, Medical Sciences Campus, Department of Social Sciences, Doctoral Program in Social Determinants of Health, San Juan, Puerto Rico; [7] University of Georgia, Epidemiology and Biostatistics, Athens, USA

Objectives: Biomarkers can be measured in various media, including urine or blood, which represent different biological compartments and toxicokinetic pathways, resulting in biases when estimating the internal dose of an environmental exposure. Integrating measurements across multiple media as a multimedia biomarker (MMB) may better capture the effects of exposure on health outcomes. This study utilized concentrations of 13 metals in urine, blood, and MMBs, integrated using intraclass correlation coefficients and weighted quantile sum regression.

Materials and Methods: We investigated log-transformed geometric mean prenatal metal concentrations (urine, blood, MMBs) in association with repeatedly measured Child Behavior Checklist (CBCL) scores for ages 1.5 to 5 years, in 284 mother-child pairs in the PROTECT birth cohort in Puerto Rico. Summary scores were modeled as log(CBCL+1). Associations were assessed as single-pollutants and as a mixture, using linear mixed-effects models, adaptive elastic net, and Bayesian kernel machine regression, adjusted for sex, maternal education level, maternal age, and child age at assessment.

Results: In single-pollutant models, prenatal urinary As and Co; blood Cd and Mn; MMBICC Cd and Co; and MMBWQS Cd, Co, and Mn were associated with increased emotional and behavioral problems in children. For example, Cd was associated with a 0.24 (0.08, 0.41) increase in CBCL total problem scores per interquartile range (IQR) increase in blood, 0.19 (0.07, 0.31) per IQR increase in MMBICC, and 0.18 (0.05, 0.31) per IQR increase in MMBWQS. In mixture models, unexpectedly, combined effects of MMB metals were not more strongly associated with CBCL scores compared to blood media.

Conclusion: The results of this study can inform future research on optimizing biomarker assessment and reducing measurement error to assess the effect of metal mixtures on child neurodevelopment.

TO02-03 | Personalized exposure Factor-based Exposure assessment (PerFact-Expo) for Polycyclic Aromatic Hydrocarbons in Korean population

Suhyun Park[1], Jiaqi Zhang[2], Sangah Shin[2], Gowoon Lee[3], Sunmi Kim[4], Kyungho Choi[1]

[1] Seoul National University, Institute of Health and Environment, Seoul, South Korea; [2] Chung-Ang University, Department of Food and Nutrition, Gyeonggi-do, South Korea; [3] Korea National University of Transportation, Department of Safety Engineering, Chungcheongbuk-do, South Korea; [4] Korea Research Institute of Chemical Technology, Chemical Analysis Center, Daejeon, South Korea

Polycyclic aromatic hydrocarbons (PAHs) are generated by incomplete combustion such as wildfires, traffics, grilled meats, and smoking. Despite extensive monitoring, comprehensive exposure assessments integrating multiple pathways remain limited. We developed a novel personalized exposure assessment model, *PerFact-Expo*, to estimate aggregated PAH exposure in Koreans (n = 52,182). The model integrates individual dietary data from the Korea National Health and Nutrition Examination Survey (KNHANES) and total diet survey (TDS) with time-activity patterns from the Korean Statistical Information Service (KOSIS) to estimate personalized PAH exposure from diet, air, and dust. Target PAHs included BaA, BaP, BbF, BkF, BghiP, Chry, DBahA, and IcdP. Total daily PAH intake was estimated to be highest among children, followed by adolescents, adults, and elders. Dietary intake was the primary exposure route, on average, 93.5% of the total exposure, followed by environmental sources (6.5%). The major contributors to dietary intake were BbF (23.0%) followed by BaP (16.7%). For the environmental sources, BghiP (23.9%) and DBahA (19.9%) explained the most. Among high PAH exposure groups (>90th percentile, n = 5,218), dietary intake accounted for 96.8% of total exposure. In this group, grilled pork contributed 24.2% of the total dietary PAH intake, followed by fried chicken and stir-fried beef and pork, each exceeding 5%. These findings indicate that cooked meats, particularly those grilled or fried, are major contributors to PAH exposure. Additionally, major cancer risk drivers were identified as oral intake of BaP(65%), DBahA(12.1%), and BbF(6.6%).

This PerFact-Expo model characterized personalized PAH exposure across multiple pathways in Koreans, providing insights for mitigation and safety management. This approach was successfully utilized in association studies for endocrine related diseases employing national health databases.

This research was supported by Korea Ministry of Food and Drug Safety (20162MFDS112) and the National Research Foundation of Korea (NRF) grant funded by the Korea government (MSIT) (RS-2025-00513967).

TO02-04 | Mixed Metal Exposure and Adolescent Motor Function: A Multi-Media Biomarker Approach

Emily R. Leonard[1], Stefano Renzetti[2], Alexa Friedman[1,3], Xiruo Kou[4], Donatella Placidi[2], Megan Horton[5], Birgit Claus Henn[1]

[1] Boston University School of Public Health, Environmental Health, Boston, USA; [2] University of Brescia, Brescia, Italy, Department of Medical and Surgical Specialties, Radiological Sciences and Public Health, Brescia, Italy; [3] Environmental Working Group, Washington DC, USA; [4] University of Rovira i Virgili, Nutrition and Mental Health (NUTRISAM) Research Group, Reus, Spain; [5] Icahn School of Medicine at Mount Sinai, Department of Environmental Medicine and Public Health, New York, USA

Objectives: Metal exposures in epidemiologic studies are typically estimated using individual biological media (e.g., blood). However, there is no consensus on the most consistent and valid biomarker of exposure for many metals, and the uneven distribution of metals in the body limits each biospecimen's representation of total body burden. To address this, we derived multi-media biomarkers (MMBs) integrating multiple biomarkers to investigate the joint association of four metals—lead (Pb), manganese (Mn), copper (Cu), and chromium (Cr)—with adolescent motor function, an understudied neurodevelopmental domain.

Methods: We evaluated cross-sectional associations between a metal mixture and motor function among 554 adolescents (10-14 years old) from the Public Health Impact of Metals Exposure (PHIME) project. Metals concentrations were quantified in blood, hair, fingernails, urine, and saliva using ICP-MS. Motor function was assessed using visual reaction time (VRT), which measures response speed to a visual stimulus, with lower scores indicating better performance. Using weighted quantile sum (WQS) regression, we derived MMBs for Pb, Mn, Cu, and Cr using all five biomarkers. A subsequent WQS model estimated the joint effect of the MMBs on VRT, adjusted for sex, age, and socioeconomic status. Beta coefficients and 95% confidence intervals (CI) are reported as percent change in VRT per quartile increase in the metal mixture.

Results: A quartile increase in the metal mixture was associated with a 2.4% slower visual reaction time (95% CI = 0.5%, 4.3%), driven by Cr (weight = 0.67). Similarly, a quartile increase in the mixture was associated with greater variability between VRT responses (ß = 5.1%, 95% CI: -0.6%, 11.2%), also driven by Cr (weight=0.46).

Conclusion: Adverse associations between the metal mixture and psychomotor response and attention were driven by Cr. MMBs may improve exposure assessment, as previously published associations between individual biomarkers and these outcomes were weak and imprecise.

TO02-05 | Phthalates and Children, are we doing enough? Evaluating exposure patterns and assessing risk in the Modena Cohort Study

Lucia Palandri[1,2], Laura Lucaccioni[3], Lisa De Pasquale[1], Viola Trevisani[2,3], Camilla Lugli[1,2], Angela Ferrari[1], Lorenzo Iughetti[3], Elena Righi[1]

[1] University of Modena and Reggio Emilia, Department of Biomedical, Metabolic and Neural Sciences, Public Health Section, Modena, Italy; [2] University of Modena and Reggio Emilia, PhD Program in Clinical and Experimental Medicine, Modena, Italy; [3] University of Modena and Reggio Emilia, Department of Medical and Surgical Sciences of the Mother, Children and Adults, Pediatric Unit, Modena, Italy

Background-Safeguarding newborns' futures is paramount for pediatric epidemiologists, yet the environment may pose a threat. The European Union and governments worldwide have strictly regulated phthalates, known endocrine disruptors. Therefore, we wanted to evaluate levels of exposure, exposure patterns, and time trends during the first years of life in Italian newborns and children, and we used this data to perform risk assessment evaluations.

Methods-Between 2019 and 2020, we enrolled 197 mother-child couples in a prospective cohort study in the province of Modena (Italy). Urine samples were collected at birth, 3, 6, 15 and 36 months. For mothers we collected samples at delivery and at 36 months. 8 phthalates metabolites (MMP,MEP,MnBP,MBzP,MEHP,MEHHP,MEOHP,MCOP) of 6 phthalates were analysed. Descriptive statistics and overall time trends and interclass correlation were calculated. Agglomerative hierarchical clustering was performed to assess exposure patterns in phthalate excretion. Risk assessment (Risk Quotients and Hazard Indices) was performed.

Results-Investigated phthalates were detected in all samples showing an increased trend over time. MnBP and MBzP were higher in newborns and at 36-months, MEP at 15-months. Samples collected at 3-months showed overall lowest concentrations. At 36-months, concentrations were higher in children than mothers. Mothers and infants were interclass-correlated. Clustering analysis showed stricter clusters and specific time patterns with 3-and 6-months old infant. Risk assessment showed values below reference levels yet higher than threshold values were detected at each visit.

Conclusions-Phthalate exposure in Modena infants remains widespread, even for strictly regulated toxic compounds. Our data underscore patterns where specific mother-infant dyads consistently demonstrate elevated metabolite levels compared to others in the cohort. This suggests household-specific exposure patterns affecting all family members. Clustering analysis further indicates that increased environmental interactions may result into a loss in exposure protection. While legislation on childcare products has shown effectiveness, further efforts are needed.

TO02-06 | Impacts of Missing Data Imputation on Statistical Models for Environmental Mixtures

Yvonne Boafo[1,2], Emmanuel Obeng-Gyasi[1], Sayed Mostafa[2]

[1] North Carolina Agricultural and Technical State University, Department of Built Environment, Greensboro, USA; [2] North Carolina Agricultural and Technical State University, Department of Mathematics and Statistics, Greensboro, USA

Objective
Human exposure to chemical mixtures, including metals and PFAS, is linked to adverse health outcomes, yet missing data can bias estimates and reduce inferential validity. This simulation study evaluates the impact of missing data on multipollutant mixture models. It assesses the performance of four imputation methods—mean, median, Multivariate Imputation by Chained Equations (MICE), and Amelia—across six models: Weighted Quantile Sum (WQS), Bayesian WQS (BWQS), Quantile G-Computation (Q-Gcomp), Bayesian Kernel Machine Regression (BKMR), Elastic Net (Enet), and Least Absolute Shrinkage and Selection Operator (LASSO).
Methods
Monte Carlo simulations were conducted under Missing Completely at Random (MCAR) and Missing at Random (MAR) conditions. Data were simulated under both multivariate normal and multivariate T distributions to assess the robustness of imputation techniques. Missingness was introduced in exposures, outcomes, and both exposures and outcomes, and models were evaluated based on sensitivity, specificity, mean squared error (MSE), and false discovery rate (FDR).
Results
Multivariate imputation methods (MICE and Amelia) performed best under both distributional assumptions, closely approximating complete data estimates. However, performance varied under the multivariate T distribution, with increased false discovery rates when both exposures and outcomes were missing. Simple imputation methods (mean and median) resulted in biased estimates, particularly under MAR conditions.
Conclusion
Missing data significantly impacts multipollutant mixture models, with imputation performance influenced by missingness patterns and data distributions. Elastic Net and LASSO performed best for linear associations, while BKMR excelled in non-linear relationships. These findings highlight the need for appropriate imputation strategies to ensure valid environmental health inferences.

TO02-07 | Improving Design of Epidemiology Studies that Use Biomonitoring for Exposure Assessment: A SciPinion Delphi Panel Recommendation

Igor Burstyn[1], Tony Cox[2], Tyler Carneal[3], Sean Hays[3]

[1] *Drexel University, Philadelphia, USA;* [2] *Cox Associates, Denver, USA;* [3] *SciPinion, Bozeman, USA*

Background: Epidemiological studies that rely on biomarkers of exposure typically estimate each subject's exposure from measurements on that individual. If repeated measurements of biomarkers of exposure are obtained on an individual, they are typically averaged. This averaging helps to reduce error from within-person variability if average exposure is a better measure of biologically effective dose than instantaneous one. However, approaches that ignore variability in exposures within individuals through averaging can bias effect estimates and lead to inaccurate risk assessment. Because biomarkers typically follow log-normal distribution, epidemiologists face a choice of using biomarker levels either on the original scale or after logarithmic transformation. These choices imply different measurement error models and associated biases.
Objective: To develop software ("calculators") that help design studies of continuous and binary outcomes that rely on biomarkers of exposure.
Methods and Results: We present calculators designed to estimate sample sizes (number of individuals sampled and number of samples per individual), power and bias in epidemiological studies that use biomonitoring to assess each subject's exposure in the presence of classical additive and multiplicative measurement errors. Our web-based tools estimate the required sample sizes, number of repeated measurements, and the trade-offs between power and bias in simple linear and logistic regression models under classical additive and multiplicative measurement error models. Application of the calculators was illustrated in a case study of investigation of the association between urinary levels of bisphenols during pregnancy and fetal growth.
Significance: By providing user-friendly tools that account for two types of measurement error typically assumed in epidemiology, researchers can design more accurate and reliable studies, ultimately improving public health outcomes.

TO03 | Omics technologies

TO03-01 | Implications for the Mediating Role of Plasma Proteins in the Association between Air Pollution Exposure and Lung Cancer Risk

Ziyin Tang[1], Ying Wang[2], Jeremy A. Sarnat[3], W.Ryan Diver[4], Todd M. Everson[5], Sabrina S. Chow[6], Emily Deubler[7], Aparna H. Kesarwala[8], Michelle C. Turner[9], Mattias Johansson[10], Hilary A. Robbins[11], Carmen J. Marsit[12], Donghai Liang[13]

[1] Emory University, Gangarosa Department of Environmental Health, Rollins School of Public Health, Atlanta, USA; [2] American Cancer Society, Department of Population Science, Atlanta, USA; [3] Emory University, Gangarosa Department of Environmental Health, Rollins School of Public Health, Atlanta, USA; [4] American Cancer Society, Department of Population Science, Atlanta, USA; [5] Emory University, Gangarosa Department of Environmental Health, Rollins School of Public Health, Atlanta, USA; [6] Emory University, Gangarosa Department of Environmental Health, Rollins School of Public Health, Atlanta, USA; [7] American Cancer Society, Gangarosa Department of Environmental Health, Rollins School of Public Health, Atlanta, USA; [8] Emory University, Department of Radiation Oncology, School of Medicine, Atlanta, USA; [9] Barcelona Institute for Global Health (ISGlobal), Barcelona Institute for Global Health (ISGlobal), Barcelona, Spain; [10] International Agency for Research on Cancer, Genomic Epidemiology Branch, Lyon, France; [11] International Agency for Research on Cancer, Genomic Epidemiology Branch, Lyon, France; [12] Emory University, Gangarosa Department of Environmental Health, Rollins School of Public Health, Atlanta, USA; [13] Emory University, Gangarosa Department of Environmental Health, Rollins School of Public Health, Atlanta, USA

Objective: The biological mechanisms underlying air pollution-induced lung carcinogenesis remained underexplored. To identify key proteins and pathways involved in mediating this process, we assessed individual and joint effects of six criteria air pollutants on human proteome and their associations with lung cancer risk. **Methods:** We included 95 matched case-control pairs of elderly (N=190) with pre-diagnosis blood samples from the Cancer Prevention Study-II Nutrition Cohort (1998-2001). Plasma levels of 484 proteins were measured. Annual average exposures to fine and coarse particulate matter, nitrogen dioxide, ozone, sulfur dioxide, and carbon monoxide in the calendar year of blood draw were retrieved. We used linear regression for individual pollutant effects and quantile g-computation for joint effects on proteins, and conditional logistic regression for proteins and lung cancer risk, adjusting for confounders and multiple testing. Pathway analysis was conducted to identify overlapping proteins and pathways associated with both exposures and lung cancer risk, followed by an exploratory high-dimensional mediation analysis (HIMA) to investigate the potential mediation roles of proteins. **Results:** Fourteen proteins were associated with air pollutants and six with lung cancer risk (FDR <0.2). Pathway analysis revealed twelve pathways related to air pollution and two to lung cancer risk, closely linked to immunity, signaling, and cell activities. Eleven proteins were associated with both exposures and lung cancer risk, including CX3CL1, ADAM15, CD300E and LEPR. HIMA identified nine proteins as significant mediators of the association between air pollution and lung cancer risk, including IFN-gamma, several ILs, CD300E, and LEPR. **Conclusion:** Various immunity, signaling, and cell activities related proteins and pathways may play significant roles in the association between air pollution exposures and lung cancer. The findings provide novel insights into understanding the effects of air pollution exposures on lung carcinogenesis, contributing to future biomarker development for preventive and therapeutic strategies.

TO03-02 | Residential Proximity to Concentrated Animal Feeding Operations Associates with Increased Prevalence of Antimicrobial Resistance in Homes

Kathryn R. Dalton[1,2], Mikyeong Lee[2], Jared A. Fisher[3], Laura E. Beane Freeman[3], Rena R. Jones[3], Stephanie J. London[2]

[1] University of Iowa College of Public Health, Occupational & Environmental Health Department, Iowa City, USA; [2] NIH National Institute of Environmental Health Sciences, Epidemiology Branch, Durham, USA; [3] NIH National Cancer Institute, Division of Cancer Epidemiology & Genetics, Occupational and Environmental Epidemiology Branch, Bethesda, USA

Objective: Antimicrobial resistance (AMR) in environmental microorganisms can serve as a reservoir for AMR-infections in humans. Concentrated animal feeding operations (CAFOs) are a potential source of AMR genes to the surrounding community. We sought to examine the association between residential proximity to CAFOs and the prevalence of AMR genes in home environments.

Material and Methods: We detected AMR genes using whole genome shotgun sequencing in household dust samples from 781 participants in the Agricultural Lung Health Study of licensed pesticide applicators (mostly farmers) and their spouses in Iowa (N=534) and North Carolina (N=247), USA. We assessed the number of CAFOs (CAFO density) and the inverse distance-weighted number of livestock animals (animal density) within 10km of the residence. We used logistic regression, adjusted for demographic and farm exposures, to evaluate the association of CAFO exposures (in quartiles) with detection of AMR and multidrug resistance (MDR, resistant to 2 or more drug classes).

Results: Overall, 24% of dust samples had detectable AMR genes, with MDR in 14% of samples. In Iowa, we found increased odds of AMR genes detected in the highest quartile of CAFO density compared to the lowest (OR=1.98, 95% confidence interval [CI] 1.15-3.47), and increased odds for the highest animal density quartile (OR=1.94, CI=1.09-3.51). The associations were stronger for MDR detection for the highest CAFO density quartile (OR=2.14, CI=1.14-4.1) and the highest animal density quartile (OR=2.92, CI=1.44-6.25). In North Carolina, we observed a greater proportion of AMR and MDR detection in the highest quartiles, but the odds ratios were not significant.

Conclusion: We found that residential proximity to CAFOs was associated with increased odds of antimicrobial resistant genes, including multidrug resistant genes, in the homes of rural Iowa farmers, indicating a possible risk for resistant infections in inhabitants.

Supported by the Intramural Research Program of the NIH, NIEHS (ZO1ES102385)

TO03-03 | Differential DNA methylation in blood as potential mediator of the association between ambient $PM_{2.5}$ and cerebrospinal fluid biomarkers of Alzheimer's disease among a cognitively normal population-based cohort

Tszshan Ma[2], Jiaqi Liu[3], Donghai Liang[1,2], Stefanie Ebelt[1,2], Kyle Steenland[1,2], Allan Levey[7,8], James J. Lah[7,8], Aliza P. Wingo[9,3], Thomas S. Wingo[10,11], Anke Hüls[1,2]

[1] Emory University, Department of Epidemiology, Rollins School of Public Health, Atlanta, USA; [2] Emory University, Ganagarosa Department of Environmental Health, Rollins School of Public Health, Atlanta, USA; [3] University of California, Davis, Department of Psychiatry, Sacramento, USA; [4] The University of British Columbia, Centre for Molecular Medicine and Therapeutics, Vancouver, Canada; [5] BC Children's Hospital Research Institute, Vancouver, Canada; [6] Emory University, Department of Human Genetics, School of Medicine, Atlanta, USA; [7] Emory University, Goizueta Alzheimer's Disease Research Center, School of Medicine, Atlanta, USA; [8] Emory University, Department of Neurology, School of Medicine, Atlanta, USA; [9] Northern California VA, Division of Mental Health, Sacramento, USA; [10] University of California, Davis, Department of Neurology, Sacramento, USA; [11] University of California, Davis, Alzheimer's Disease Research Center, Sacramento, USA

Introduction: Fine particulate matter ($PM_{2.5}$) is a known risk factor for Alzheimer's disease (AD), with emerging evidence showing its effects detectable in the pre-clinical stage through cerebrospinal fluid (CSF) biomarkers of AD. While studies have linked $PM_{2.5}$ exposure and AD to DNA methylation (DNAm) alterations, the role of DNAm as potential mediator in the association between $PM_{2.5}$ and AD biomarkers in cognitively normal individuals remains largely unexplored, and formal mediation analyses addressing this question are scarce.

Methods: Genome-wide DNAm profiles (Illumina EPIC BeadChips) in whole blood and CSF $A\beta_{42}$ concentrations were assessed in 535 cognitively normal individuals from the Emory Healthy Brain Study (EHBS). Residential $PM_{2.5}$ exposure for the year preceding participants' blood collection was estimated. A multi-stage analytical pipeline was applied, incorporating single-mediator analysis, high-dimensional mediation analysis, and causal mediation analysis.

Results: Nine CpG sites were identified as noteworthy mediators of the relationship between $PM_{2.5}$ and decreased CSF $A\beta_{42}$ concentrations. Causal mediation analysis confirmed significant natural indirect effects (NIE) for eight CpGs, with the proportion mediated ranging from 14% to 43%. The joint NIE for all nine CpGs was -0.115 (95% CI: -0.215, -0.101) per 1 ug/m^3 increase in $PM_{2.5}$ exposure. Six CpGs are annotated to genes implicated in neuroinflammatory pathways.

Conclusion: Our findings suggest that differential DNAm, particularly in genes related to neuroinflammation, mediates the association between $PM_{2.5}$ exposure and CSF $A\beta_{42}$ concentrations. This highlights the utility of blood DNAm in detecting and studying biological pathways underlying $PM_{2.5}$ toxicity in the pre-clinical stages of AD.

TO03-04 | Harmonization and complementarity of Non-Targeted Screening Methods in the occupational exposure context: lessons learned from a large EU interlaboratory study

Baninia Habchi[1], Tarek Moufawad[2], Tina Kosjek[3,4], Guillaume Antoine[1], Rosalie Nijssen[5], Alkistis Kevrekidou[6,7], Aurelie Remy[1], Žan Rekar[3,4], Helena Plešnik[3,4], Catherine Gabriel[6,7], Dimosthenis Sarigiannis[6,7], Lucyna Kozlowska[8], Lidia Belova[9], Ardiana Kajtazi[9], Adrian Covaci[9], Katriina Sunnarborg[10,11], Olli Kärkkäinen[10], Herbert Oberacher[12], Ogier Hanser[1], Aikaterini Termentzi[13], Eirini Baira[13], Konstantinos Kasiotis[13], Anna Laura Lamiceli[14], Riccardo De Santo[14], Radu Corneliu Duca[15,16], An Van Nieuwenhuyse[15,16], Clément Chalet[17], François Fenaille[17], Annelaure Damont[17], Bruno Le Bizec[2], Gaud Dervilly[2], Andrea Rodriguez-Carrillo[18], Mariana F. Fernández[18,19], Marja Lamoree[20], Jean-Philippe Antignac[2], Sophie Ndaw[1]

[1] INRS (French National Research and Safety Institute for the Prevention of Occupational Accidents and Diseases), Department of Toxicology and Biomonitoring, Vandoeuvre-Les-Nancy, France; [2] Oniris, INRAE, LABERCA, Nantes, France; [3] Jožef Stefan Institute, Ljubljana, Slovenia; [4] Jožef Stefan International Postgraduate School, Ljubljana, Slovenia; [5] Wageningen Food Safety Research, part of Wageningen University and Research, Wageningen, Netherlands; [6] Aristotle University of Thessaloniki, Department of Chemical Engineering, Environmental Engineering Laboratory, University Campus, Thessaloniki 54124, Greece; [7] HERACLES Research Center on the Exposome and Health, Center for Interdisciplinary Research and Innovation, Balkan Center, Bldg. B, 10th km Thessaloniki – Thermi Road, 57001, Greece; [8] Warsaw University of Life Sciences, Nowoursynowska 166, 02-787 Warsaw, Poland; [9] Toxicological Centre, University of Antwerp, Antwerp, Belgium; [10] School of Pharmacy, University of Eastern Finland, Kuopio, Finland; [11] Finnish Institute of Occupational Health, Kuopio, Finland; [12] Medical University of Innsbruck, Institute of Legal Medicine and Core Facility Metabolomics, Austria, Austria; [13] Laboratory of Toxicological Control of Pesticides, Scientific Directorate of Pesticides' Control & Phytopharmacy, Benaki Phytopathological Institute, 14561 Kifissia, Germany; [14] Department of Environment and Health, Italian National Institute for Health (ISS), Rome, Italy; [15] Laboratoire national de santé, Department Health Protection, Dudelange, Luxembourg; [16] Environment and Health, Department of Public Health and Primary Care, KU Leuven, Leuven, Belgium; [17] Paris-Saclay University, CEA, INRAE, Département Médicaments et Technologies pour la Santé (DMTS), MetaboHUB.F-91191, Gif sur Yvette, France; [18] Instituto de Investigación Biosanitaria de Granada (ibs.GRANADA), 18016 Granada, Spain. & Spanish Consortium for Research on Epidemiology and Public Health (CIBERESP), Madrid, Spain; [19] Department of Legal Medicine, Toxicology, and Physical Anthropology, School of Medicine, University of Granada, 18016 Granada, Spain; [20] Vrije Universiteit Amsterdam, Amsterdam Institute for Life and Environment (A-LIFE), Section Chemistry for Environment and Health, De Boelelaan 1085, 1081 HV Amsterdam, Netherlands

Objectives: Occupational exposure to chemicals poses major challenges for risk assessment and regulatory decision-making. Innovative approaches based on suspect and non-targeted screening (SS/NTS) using high-resolution mass spectrometry (HRMS) have emerged as a promising solution. As part of the European Partnership for the Assessment of Risks from Chemicals (PARC), a project has been launched to conduct a proof of concept study of SS/NTS methods across multiple European laboratories. The first phase involved an interlaboratory study to assess comparability, coverage and complementarity between partners' methods. This activity will support further evaluation of workers' exposure in waste management and healthcare sectors under the PARC project.

Materials and Methods: A quality control mixture of 80 standards was developed to include chemicals commonly found in the environment and in humans working in the waste management and healthcare sectors. Urine (low, medium, high specific gravities) and plasma samples were spiked with the QA/QC mix at 0, 5, 20 and 100 ng/mL and analyzed by 12 partners using their own sample preparation and LC-HRMS and/or GC-HRMS methods.

Results: Comparison was based on the number of substances detected and the assigned identification levels. Preliminary findings suggest varying detection limits related to the substance polarity and volatility and the partners'

methods. Despite these variations, consistent detection of substances was achieved across multiple laboratories, highlighting the complementarity of their methods and validating the proof of concept of the NTS approach in detecting multiple exposures.

Conclusion: The study indicates that NTS is valid to screen large numbers of chemicals in workers and that SS/NTS methods are complementary across European laboratories, improving cross-comparability. Developing the most appropriate SS/NTS methods is essential to advancing occupational exposure assessment and evidence-based regulatory decision-making in Europe.**Acknowledgements:** This project has received funding from the European Union's Horizon Europe research and innovation programme under Grant Agreement No101057014

TO03-05 | Associations among prenatal exposure to per-and polyfluoroalkyl substances, maternal DNA methylation, and birth size in the Atlanta African American Maternal Child Cohort

Youran Tan[1], Anne L. Dunlop[2], Todd M. Everson[1], Alicia K. Smith[2], Stephanie M. Eick[1], Ziyin Tang[1], Dana B. Barr[1], Kurunthachalam Kannan[3], Peter B. Ryan[1], Carmen J. Marsit[1], Donghai Liang[1]

[1] Emory Univeristy, Gangarosa Department of Environmental Health, Atlanta, USA; [2] Emory University, Department of Gynecology and Obstetrics, School of Medicine, Atlanta, USA; [3] New York State Department of Health, Wadsworth Center, Albany, USA

Background: Exposures to per- and polyfluoroalkyl substances (PFAS) have been linked to reduced size at birth, although underlying mechanisms remain unclear. We aimed to investigate associations of prenatal PFAS exposures with maternal DNA methylation and assess whether methylation at selected CpG sites is associated with infant birth size.

Method: Among 296 mother-infant dyads in the Atlanta African American Maternal-Child Cohort (2016-2020), maternal blood samples collected in early pregnancy were processed to obtain serum for PFAS measurement and peripheral blood mononuclear cells for DNA methylation profiling using Illumina HumanMethylation EPIC BeadChip. Birth size was assessed using sex-specific birthweight Z-scores (BWZ, continuous) and small-for-gestational age (SGA, categorical). Robust generalized linear regression and quantile g-computation was used to estimate the effect of individual PFAS and PFAS mixture on DNA methylation, respectively. Differentially methylated regions (DMRs) analysis and gene ontology enrichment analysis were performed on PFAS-associated CpG sites. We also estimated associations between methylation of significant CpG sites and DMRs with BWZ and SGA.

Results: Multiple maternal serum PFAS levels were significantly associated with higher odds of SGA, including PFOA (OR=1.92, 95%CI: 1.01, 3.75), PFNA (OR=1.37, 95%CI:1.04, 1.84), and PFAS mixture (OR=1.30, 95%CI: 1.00-1.68). After adjusting for covariates, 134 CpG sites were significantly associated with at least one PFAS levels (FDR<0.05), 2 overlapped between PFOS and PFOA, with PFOS exhibiting the greatest effect and as the main driver within PFAS mixture. Specifically, 6 CpG sites annotated to genes *EVA1A, FCRL1,PRPSAP1, CTIF, ABHD12, WDYHV1*, were associated with both PFAS levels and birth size . We identified 2-9 DMRs for each PFAS and PFAS mixture. The PFAS-associated genes are involved in inflammatory response, immune activity, and lipid homeostasis.

Conclusion: Prenatal PFAS exposure could affect DNA methylation levels, suggesting potential associations with infant birth size. Future studies should examine mediation roles of these significant CpG sites.

TO03-06 | Metabolomics approach to unravel metabolites specific to high aggressive prostate cancer from cigarette smoking among African American and European American men

Amarnath Singh[1], Se-Ran Jun[2], Katherine Wallis[1], Reid D Landes[3], Edgar T Ellis[4], L. Joseph Su[5], Ping-Ching Hsu[1]

[1] University of Arkansas for Medical Sciences, College of Public Health, Little Rock, USA; [2] University of Arkansas for Medical Sciences, Department of Biomedical Informatics, Little Rock, USA; [3] University of Arkansas for Medical Sciences, Department of Biostatistics, Little Rock, USA; [4] Columbia University, Mailman School of Public Health, New York, USA; [5] UT Southwestern Medical Center, Peter O'Donnell Jr. School of Public Health, Dallas, USA

Cigarette smoking and its association with prostate cancer (PCa) aggressiveness remains unclear, but increasing evidence has shown positive associations between smoking and PCa incidence, aggressiveness, and mortality, particularly among African American (AA) men. This study explores smoking-related metabolomic patterns associated with PCa aggressiveness by race, aiming to understand racial health disparity in PCa through cigarette smoking. In this cross-sectional study, 479 PCa patients (239 AAs; 240 European Americans, EAs) from the North Carolina-Louisiana PCa project were included. Untargeted serum metabolomics were performed, and statistical and machine learning models were used to examine the metabolic signatures for PCa aggressiveness, stratified by race and smoking status. We compared current smokers to never smokers. Across all strata, 18 metabolites common to all were associated with tobacco, benzoate metabolism, and food. Venn diagrams revealed 15 distinct metabolites in AA with low aggressive PCa and 25 in AA with high aggressive PCa; and in EA, 8 distinct metabolites in low aggressive PCa and 38 in high aggressive PCa were revealed. Notably, key metabolites observed specific to AA high aggressive PCa smokers included metabolites in benzoate metabolism that might indicate potential involvement in gut microbiome; profiles specific to EA high aggressive PCa smokers were enriched with metabolites in the bile acid metabolism potentially impacting the balance of bile acids regulation. Metabolites specific to AA low aggressive PCa smokers were highly impacted on the fatty acid beta oxidation, whereas for EA low-aggressive PCa smokers, more metabolites affected were linked to fatty acid metabolism and diet. This study reveals racial differences and commonalities in PCa metabolism among smokers, and highlights unique metabolic signatures by PCa aggressiveness. Our data suggest that a dynamic interplay of multiple factors exists in PCa aggressiveness, highlighting the urgent need for a deeper understanding to improve PCa risk prediction models and prevention strategies.

TO03-07 | Brain tissue based multi-omics risk scores for Alzheimer's disease and their association with air pollution exposure

Terry Zhou[1], Timur Tug[1,2], Zhihao Jin[3], Marla Gearing[4,5], Allan I. Levey[5], James J. Lah[5], Aliza P. Wingo[6], Thomas S. Wingo[7,8], Katja Ickstadt[2], Donghai Liang[1,3], Anke Hüls[1,3]

[1] Rollins School of Public Health, Emory University, Department of Epidemiology, Atlanta, USA; [2] TU Dortmund University, Department of Statistics, Dortmund, Germany; [3] Rollins School of Public Health, Emory University, Gangarosa Department of Environmental Health, Atlanta, USA; [4] Emory University, Department of Pathology and Laboratory Medicine, Atlanta, USA; [5] Emory University School of Medicine, Department of Neurology, Atlanta, USA; [6] University of California, Davis, Department of Psychiatry, Sacramento, USA; [7] University of California, Davis, Department of Neurology, Sacramento, USA; [8] University of California, Davis, Alzheimer's Disease Research Center, Sacramento, USA

INTRODUCTION

Air pollution is a known risk factors for Alzheimer's disease (AD) and has been associated with AD-related neuropathology markers. Advances in omics technologies have provided novel insights into the biological mechanisms underlying AD. However, little is known about how these biological mechanisms relate to neurotoxicity of air pollutants.

METHODS

We analyzed 157 post-mortem samples from the Emory Goizueta Alzheimer's Disease Research Center brain bank. Residential exposure to fine particulate matter ($PM_{2.5}$), nitrogen oxides (NO_x) and carbon monoxide (CO) was estimated at a 250m resolution and averaged over a 5-year period before death. DNA methylation was measured in frontal cortex samples with the Infinium MethylationEPIC v1.0 array. DNA methylation risk scores (MRS) for AD pathology (Amyloid, Braak, CERAD) were calculated using the pruning and thresholding approach, followed by a pathway enrichment analysis for the included CpG sites. Associations between air pollution exposures and the MRS were estimated using linear regression models and weighted quantile sum (WQS) regression, adjusting for potential confounding variables.

RESULTS

The MRS showed a prediction R^2 of 0.17 for AD pathology. The pathway analyses identified lipid metabolism and signal transduction as key biological pathways among the CpG sites included in the MRS. While the associations between air pollution exposure and the MRS were positive for all pollutants (e.g., for one interquartile range increase in NO_x b=0.07 [95% confidence interval: -0.16, 0.31]), for increasing all exposures by one decile b=0.016 [-0.09, 0.13] (WQS, positive direction)), none of the associations were statistically significant.

DISCUSSION

We developed a MRS for AD pathology, which highlights the importance of lipid metabolism and signal transduction as key biological pathways for AD. Future large-scale research should further investigate how these changes in the brain epigenome relate to environmental exposures.

TO04 | Environmental exposures and epigenetic aging

TO04-01 | Glyphosate Exposure and Epigenetic Aging in Latino Adolescents and Young Adults from a California Agricultural Region

Saher Daredia[1], Dennis Khodasevich[2], Anne K. Bozack[2], Julianna Deardorff[1], Corinne A. Riddell[1], Kim G. Harley[1], Katherine R. Kogut[1], Nina Holland[1], Brenda Eskenazi[1], Ana M. Mora[1], Robert B. Gunier[1], Andres Cardenas[2]

[1] University of California, Berkeley, School of Public Health, Berkeley, USA; [2] Stanford University, Department of Epidemiology and Population Health, Stanford, USA

Objective: Glyphosate, a widely used herbicide, has been linked to adverse health outcomes, but biological mechanisms remain poorly understood. We leveraged data from CHAMACOS, a cohort of Latino families in California's agricultural Salinas Valley, to test associations between glyphosate exposure and DNA methylation (DNAm)-based biomarkers of aging.

Material and methods: Glyphosate and its degradation product, aminomethylphosphonic acid (AMPA), were measured in urine at ages 14 and 18. Glyphosate application within 1-kilometer of each participant's residence was estimated using California Pesticide Use Reporting data from birth to age 16. Blood DNAm was measured at ages 14 and 18 to estimate several epigenetic age acceleration (EAA) biomarkers. Multivariable linear regression models estimated associations between glyphosate exposure (as measured by urinary glyphosate, urinary AMPA, or nearby applications) and EAA at each time point, adjusting for maternal prenatal characteristics (occupation, socioeconomic status, smoking, alcohol consumption), pre-pregnancy BMI, and child sex.

Results: At age 14, a 1-SD increase in $\log_2$ urinary AMPA was associated with 0.82 (95% CI: 0.06, 1.59) years higher Horvath EAA (N=82). At age 18, a 1-SD increase in $\log_2$ urinary AMPA was associated with 0.75 (0.03, 1.46) and 0.87 (0.05, 1.70) years higher Horvath and Skin & Blood EAA, respectively (N=95). For residential proximity models, a 1-SD increase in $\log_{10}$ glyphosate application from birth to age 16 was associated with 0.51 (0.08, 0.93), 0.55 (0.06, 1.05), and 0.49 (0.12, 0.85) years higher Horvath, Skin & Blood, and intrinsic EAA at age 18, respectively (N=247).

Conclusion: Earlier and concurrent exposure to glyphosate was associated with accelerated epigenetic aging, specifically for biomarkers related to increased morbidity and mortality. Alongside our earlier finding that glyphosate was linked with higher risk of liver and metabolic dysfunction in this cohort, our result suggests one possible biological mechanism and strengthens the hypothesis that glyphosate harms human health.

TO04-02 | Mapping of CpG Methylation across North Carolina Identifies Epigenetic Age Acceleration Linked to Private Well Water Arsenic Contamination

Allison C. Spring[1,2], Kyle Roell[1,2], Farida Akhtari[3], Ellie Jiang[1,2], Lauren A. Eaves[1,2], Rebecca C. Fry[1,2], Alison Motsinger-Reif[3], Julia E. Rager[1,2]

[1] The University of North Carolina at Chapel Hill, Department of Environmental Sciences and Engineering, Chapel Hill, USA; [2] The University of North Carolina at Chapel Hill, The Institute for Environmental Health Solutions, Chapel Hill, USA; [3] National Institute of Environmental Health Sciences, Biostatistics and Computational Biology Branch, Research Triangle Park, USA

Objective: Inorganic arsenic (iAs) contamination is a health threat to private well users in the U.S., where private well water quality is unregulated. North Carolina (NC) has the highest population of residents on well systems in the U.S. and documented concerningly high levels of arsenic, ranging up to 800 ppb. Recent studies suggest that toxic metal exposure can lead to epigenomic changes, such as accelerated epigenetic aging, and mediate health outcomes, including cardiovascular disease and type two diabetes. This study aims to investigate the association between iAs in well water and epigenetic age acceleration (EAA) throughout NC.

Material and Methods: CpG methylation patterns were analyzed across blood samples from Personalized Environment and Genes Study participants. This study evaluated individuals who self-reported relying on private well water and having no occupational arsenic exposure (N=398). Participant addresses were geocoded to assign census tract-level, mean iAs well water concentrations using the NCWELL database. Epigenetic ages of participants were calculated and generalized linear models were employed to evaluate associations between iAs concentrations and EAA (the difference between epigenetic and chronological age), controlling for a sufficient set of confounders (body mass index, childhood socioeconomic status, sex, smoking, and water filter use).

Results: The difference in EAA comparing participants residing in tracts with elevated iAs concentration (defined as >50th percentile [2.78 ppb]) between participants with lower iAs concentrations (<50th percentile) averaged to 1-year [p-value =0.02]. Stratifying by whether participants reported water filtering in regions with elevated iAs demonstrated that filtering was associated with -1.9-year difference in EAA [p-value =0.03], suggesting potential protective influences of water filtering on EAA.

Conclusion: Findings suggest that geospatial variations in well water iAs may contribute to geospatial differences in biological vulnerability related to EAA, with water filtering potentially mitigating these associations.

Funding: NIH

TO04-03 | Associations between long-term exposure to $PM_{2.5}$ components and epigenetic age acceleration

Alexander Vervaet[1], Sarina Abrishamcar[1], Lina Dimitrov[1], Stefanie Ebelt[1,2], Donghai Liang[1,2], Allan Levey[3,4], James J. Lah[3,4], Aliza P. Wingo[5], Thomas S. Wingo[6,7], Anke Hüls[1,2]

[1] Emory University, Rollins School of Public Health, Department of Epidemiology, Atlanta, USA; [2] Emory University, Rollins School of Public Health, Gangarosa Department of Environmental Health, Atlanta, USA; [3] Emory University, School of Medicine, Department of Neurology, Atlanta, USA; [4] Emory University, School of Medicine, Goizueta Alzheimer's Disease Research Center, Atlanta, USA; [5] University of California, Davis, Department of Psychiatry, Sacramento, USA; [6] University of California, Davis, Department of Neurology, Sacramento, USA; [7] University of California, Davis, Alzheimer's Disease Research Center, Sacramento, USA

Objective

Epigenetic age acceleration (EAA) quantifies the difference between biological and chronological age and is linked to age-related diseases. While fine particulate matter ($PM_{2.5}$) has been associated with EAA, the impact of particular components remains unclear. We examined associations between long-term exposure to major $PM_{2.5}$ component mixtures and EAA in cognitively healthy adults from the Emory Healthy Brains Study (EHBS).

Material and Methods

We analyzed 620 adults (45-76 years) from the EHBS in Georgia, USA (2016-2018). Residential exposure to elemental carbon (EC), organic carbon (OC), ammonium (NH_4^+), nitrate (NO_3^-), and sulfate (SO_4^{2-}) was estimated at a 50m-1km resolution and averaged over 3-, 5-, 10-, and 15-year periods before blood collection. We measured whole blood DNA methylation using the Infinium MethylationEPIC v1.0 array and calculated EAA using four epigenetic clocks (Horvath, Horvath Skin and Blood, Hannum, GrimAge). Single pollutant linear regression models estimated associations between $PM_{2.5}$ components and EAA, adjusting for demographics and neighborhood deprivation. Environmental mixture methods—Bayesian Weighted Sums (BWS), Weighted Quantile Sums (WQS), and quantile g-computation (QGcomp)—assessed overall mixture effects and individual component contributions.

Results

NH_4^+ and SO_4^{2-} were associated with increased GrimAge (e.g., per 0.07 µg/m³ IQR increase in NH_4^+: 3-year: 0.20 year [95% confidence interval: 0.09-0.31]; 15-year: 0.14 [0.05-0.22]) and Hannum EAA (e.g., 3-year: 0.26 year [0.10-0.43]; 15-year: 0.14 year [0.02-0.27]). No significant associations were identified for EC, OC, and NO_3^-. BWS, WQS, and QGcomp identified significant overall mixture effects for the $PM_{2.5}$ components with GrimAge and Hannum EAA, with NH_4^+ and SO_4^{2-} contributing the most to the overall effect (e.g., NH_4^+ and SO_4^{2-} explained 59.3% of the GrimAge EAA effect for 3-year exposure).

Conclusion

$PM_{2.5}$ component mixtures were associated with EAA, particularly for shorter exposures and increased NH_4^+ and SO_4^{2-} levels. Reducing exposure to these pollutants may help mitigate long-term health impacts for aging-related diseases.

TO04-04 | Per- and Polyfluoroalkyl Substance Exposure is Associated with Sex-Specific Changes in Epigenetic Age in the National Health and Nutrition Examination Survey

Dennis Khodasevich[1], Anne K. Bozack[1], Belinda L. Needham[2], David H. Rehkopf[1], Andres Cardenas[1]

[1] Stanford University, Department of Epidemiology and Population Health, Palo Alto, USA; [2] University of Michigan, Department of Epidemiology, Ann Arbor, USA

Objective: Per-and polyfluoroalkyl substances (PFAS) are a large family of synthetic compounds with a wide range of reported health effects. Epigenetic clocks are DNA methylation-based predictors of chronological and biological age that have proven to be invaluable biomarkers for characterizing biological aging in humans. However, the extent to which PFAS exposure impacts epigenetic aging in the general US population remains poorly understood.
Materials and Methods: In the 1999-2000 National Health and Nutrition Examination Survey (NHANES) cycle (N = 262), eleven PFASs were measured in serum and DNA methylation was measured in blood with the EPICv1 array. Seven epigenetic clocks (Horvath panTissue, Hannum, Skin&Blood, PhenoAge, GrimAge2, DunedinPoAm, and DNAmTL) and their respective epigenetic age acceleration (EAA) measures were calculated. Survey-design weighted generalized linear regression models were used to model associations between individual $\log_2$-transformed PFAS concentrations and EAA measures, further adjusted for age, race/ethnicity, poverty-to-income ratio, BMI, and smoking status and stratified by sex.
Results: Among males, a doubling of PFNA exposure was consistently associated with greater EAA across several clocks including the Horvath clock (beta = 1.48, 95% CI: 0.35, 2.61), Skin&Blood clock (beta = 1.27, 95% CI: 0.21, 2.32), and PhenoAge (beta = 1.43, 95% CI: 0.41, 2.44), and doubling of PFOS exposure was associated with greater Skin&Blood acceleration (beta = 1.14, 95% CI: 0.04, 2.24). When utilizing cell-adjusted EAA measures, each of these associations among males remained significant, and PFOSA was further found to be associated with decreased PhenoAge acceleration (beta = -0.84, 95% CI: -1.49, -0.18) and GrimAge2 acceleration (beta = -0.81, 95% CI: -1.51, -0.11) among females.
Conclusions: We found evidence of sex-specific associations between PFAS exposure and epigenetic aging in a sample of older adult NHANES participants, indicating that PFAS exposure is influencing biological aging at levels of exposure observed within the general US population.

TO04-05 | The Joint Effects of Prenatal Pesticide Exposure and Psychosocial Factors on Epigenetic Age Acceleration Over Time in a South African Birth Cohort

Sarina Abrishamcar[1], Stephanie M. Eick[1,2], Todd Everson[1,2], Shakira F. Suglia[1], Robert O. Wright[3], Syam S. Andra[3], Nastassja Koen[4,5], Nadia Hoffman[4,6], Alexandre A. Lussier[7,8], Erin C. Dunn[9], Kristy Dever[10,11], Julie L. MacIsaac[10,11], Michael S. Kobor[10,11], Heather J. Zar[12], Dan J. Stein[4,5], Anke Hüls[1,2]

[1] Emory University, Department of Epidemiology, Rollins School of Public Health, Atlanta, USA; [2] Emory University, Gangarosa Department of Environmental Health, Rollins School of Public Health, Atlanta, USA; [3] Icahn School of Medicine at Mt. Sinai, Department of Environmental Medicine, New York, USA; [4] University of Cape Town, Neuroscience Institute, Cape Town, South Africa; [5] University of Cape Town, South African Medical Research Council Unit on Risk and Resilience in Mental Disorders, Cape Town, South Africa; [6] University of Cape Town, Department of Psychiatry and Mental Health, Cape Town, South Africa; [7] Massachusetts General Hospital, Center for Genomic Medicine, Boston, USA; [8] Harvard Medical School, Department of Psychiatry, Boston, USA; [9] Purdue University, Department of Sociology, College of Liberal Arts, West Lafayette, USA; [10] University of British Columbia, Department of Medical Genetics, Vancouver, Canada; [11] BC Children's Hospital Research Institute, Vancouver, Canada; [12] University of Cape Town, Department of Paediatrics and Child Health, SA-MRC Unit on Child & Adolescent Health, Red Cross War Memorial Children's Hospital, Cape Town, South Africa

Objective: Prenatal pesticide exposure and psychosocial factors have been separately implicated in adverse childhood development, often co-occurring in disadvantaged communities, like the South African Drakenstein Child Health Study (DCHS). Epigenetic aging is a potential biological mechanism linking these two exposures to poor health outcomes. We investigated the joint effects of prenatal pesticide exposure and psychosocial factors on epigenetic age acceleration (EAA) at 1, 3, and 5 years of age in the DCHS (N=643).
Methods: Eleven pesticide metabolites were measured in urine at the 2nd trimester of pregnancy. Concurrently, seven psychosocial factors were measured via questionnaires. DNA methylation (EPICv2.0 Beadchip)was measured in whole blood in children at 1, 3, and 5 years. We calculated the Horvath and Wu EAA as the residual of epigenetic age regressed on chronological age. We utilized 2 environmental mixture methods, self-organizing maps (SOM) and quantile-based g-computation (QGCOMP) to assess the joint effects of prenatal pesticides and psychosocial factors on EAA longitudinally using generalized estimating equations, adjusting for maternal age, ethnicity, HIV status, and child sex.
Results: With QGCOMP, we identified a 0.38 [95%-CI: 0.11,0.65] year increase in Wu EAA and a 0.43 [0.08,0.77] year increase in Horvath EAA per one quintile increase in the exposure mixture. For both clocks, food insecurity, substance use, neonicotinoid and pyrethroid pesticide metabolites, and interpersonal violence contributed most to the total effect. SOM identified four clusters of participants with distinct exposure profiles. Based on these exposure profiles, we note adverse psychosocial factors, with (β [95% CI] = 0.15 [0.02,0.28] and without high levels of pesticide exposure (β [95% CI] =0.19 [0.06,0.33]), are associated with Wu EAA, but not with Horvath EAA.
Conclusion: Prenatal exposure to co-occurring psychosocial factors and pesticides is associated with EAA longitudinally, providing insight into how environmental injustice gets under the skin and contributes to developmental disparities.

TO05 | Food contaminants

TO05-01 | Illness Associated with Diamond Shruumz^{TM} Products, and Other Mushroom-Containing Chocolate Products—United States, 2024

Andrea Winquist[1], Jelonia T. Rumph[1], Alyssa N. Troeschel[1], Skylar Pulver[1,2], Amy H. Schnall[1], Joseph Ebersole[1], Michael Yeh[1], Brooke Burt[1], Johnni Daniel[1], Arthur Chang[1]

[1] Centers for Disease Control and Prevention, National Center for Environmental Health, Atlanta, USA; [2] Oak Ridge Institute for Science Education, Oak Ridge, USA

Objective: On May 31, 2024, CDC was alerted to an outbreak of illness potentially associated with Diamond Shruumz^{TM} (DS) microdosing products (chocolate bars, gummies, and cones), marketed as containing a proprietary mushroom blend. CDC coordinated a nationwide call for cases. We analyzed data on reported cases to estimate the magnitude of the outbreak and to investigate relationships between consumption of specific products and severe health effects.

Material and Methods: A case was defined as an illness with moderate/major clinical effects (as defined by poison centers) in someone who consumed any DS product or another mushroom-containing chocolate product during January–October 2024. States identified cases and reported them to CDC using a standardized case investigation form. We conducted descriptive analyses of case characteristics, and assessed associations between product type (DS bars, DS gummies, DS cones, non-DS chocolates) and indicators of severe health effects [hospitalization, intensive care unit (ICU) admission, and intubation] using odds ratios (ORs), and 95% CIs; and assessed dose-response relationships between the amount of DS bars consumed and these indicators using the Cochran-Armitage test.

Results: In total, 180 cases were reported by 34 states; 73 involved hospitalization, 38 involved ICU admission, and 29 involved intubation. Those who consumed DS bars had higher odds than those who only consumed other products of experiencing hospitalization (OR: 2.3; 95% CI: 1.1-4.8), ICU admission (OR: 4.2; 95% CI: 1.5-12.0), and intubation (OR: 5.7; 95% CI: 1.6-20.1). Among those who consumed DS bars, the proportion experiencing hospitalization, ICU admission, and intubation increased with increasing amount consumed (P-Trend: 0.0237, 0.0035, and 0.008, respectively).

Conclusion: This outbreak affected nearly 200 people nationwide, suggesting that microdosing products marketed as containing mushrooms, particularly DS bars, may cause moderate/major health effects. Public health messaging about the risks of consuming these microdosing products should be provided to consumers.

TO05-02 | Prenatal exposure to acrylamide and cardiometabolic health at 20 years of age: a biomarker-based cohort study from Denmark.

Stephane Tuffier[1], Efstathios Vryonidis[2], Thorhallur I. Halldorsson[3,4], Anne A. Bjerregaard[3,5], Damian Chandia-Poblete[1], Dorte Rytter[6], Bodil H. Bech[6], Tine B. Henriksen[7], Thorkild I. Sørensen[1,8], Sjurdur F. Olsen[3,9], Margareta Törnqvist[2], **Marie Pedersen**[1]

[1] University of Copenhagen, Department of Public Health, Copenhagen, Denmark; [2] Stockholm University, Department of Environmental Science, Stockholm, Sweden; [3] Statens Serum Institut, Department of Epidemiology Research, Copenhagen, Denmark; [4] University of Iceland, Faculty of Food Science and Nutrition, Reykjavik, Iceland; [5] Copenhagen University Hospital - Bispebjerg and Frederiksberg Hospital, Centre for Clinical Research and Prevention, Copenhagen, Denmark; [6] University of Aarhus University, Department of Public Health, Aarhus, Denmark; [7] Aarhus University Hospital, Department of Paediatrics, Skejby, Denmark; [8] NNF CBMR, Novo Nordisk Foundation Center for Basic Metabolic Research, Genomic Physiology and Translation, Copenhagen, Denmark; [9] Centre for Childhood Health, Copenhagen, Denmark; [10] Harvard School of Public Health, Department of Nutrition, Boston, USA

Background Cardiometabolic health and development of obesity may be influenced by early-life exposure to dietary endocrine compounds such as acrylamide (AA).
AA forms in carbohydrate-containing foods, like potatoes and bread, during high temperature cooking. AA disrupts metabolic homeostasis and induces adiposity in offspring of mice. AA crosses the human placenta and has been associated with intrauterine growth restriction and childhood overweight/obesity. It is unknown if prenatal exposure to AA increases the risk of poor cardiometabolic health in young adults.
Objectives We examined associations between prenatal exposure to AA and cardio metabolic health at 20 years of age in singletons born in 1988–89 in Aarhus, Denmark.
Methods Hemoglobin adducts from AA (HbAA) were measured using a recent developed high-through method in blood from pregnant women collected in gestational week 30 with information on maternal health behaviour and socio-economic status. Offspring attended a clinical follow-up at 20 years of age with measurement of weight, height, waist circumference, blood pressure, blood glucose, insulin, leptin, adiponectin and lipid levels (n=638). We assessed associations using multivariate regression and natural spline models.
ResultsHbAA was detected in all women with a median level of 85 (range: 27-650) pmol/g Hb. For most of the outcomes examined before and after stratification by maternal smoking, there was no evidence of associations. Still, each 10 pmol/g HbAA increment was associated with larger waist circumference (0.13 cm (95% confidence interval (CI): 0.01; 0.25)) and with increased low-density lipoprotein (LDL) cholesterol (0.013 mmol/L (95% CI: 0; 0.03)).
DiscussionWhereas the study suggests an association of prenatal exposure to AA with increased waist circumference and LDL levels, we did not observe compelling evidence that prenatal exposure to AA is associated with deteriorated cardio-metabolic health in general of young adults.

TO05-03 | Associations of prenatal exposure to acrylamide and risk of neurodevelopmental and mental disorders: A longitudinal biomarker-based study from Denmark

Damian Chandia-Poblete[1], Stéphane Tuffier[1], Efstathios Vryonidis[2], Thorhallur I. Halldorsson[3,4], Anne A. Bjerregaard[3,5], Marin Strøm[3,6], Dorte Rytter[7], Bodil H. Bech[7], Tine B. Henriksen[8], Margareta Törnqvist[2], Sjurdur F. Olsen[3,9], **Marie Pedersen**[1]

[1] University of Copenhagen, Department of Public health, Copenhagen, Denmark; [2] Stockholm University, Department of Environmental Science, Stockholm, Sweden; [3] Statens Serum Institut, Department of Epidemiology Research, Copenhagen, Denmark; [4] University of Iceland, Faculty of Food Science and Nutrition, Reykjavik, Iceland; [5] Copenhagen University Hospital - Bispebjerg and Frederiksberg Hospital, Centre for Clinical Research and Prevention, Copenhagen, Denmark; [6] University of the Faroe Islands, Faculty of Health Sciences, Torshavn, Faroe Islands; [7] University of Aarhus, Department of Public Health, Aarhus, Denmark; [8] Aarhus University Hospital, Department of Paediatrics, Skejby, Denmark; [9] Harvard School of Public Health, Department of Nutrition, Boston, USA

Objective: Exposure to acrylamide (AA) from diet and tobacco smoke during prenatal development is of concern as AA is neurotoxic. We aimed to assess the associations of prenatal exposure to AA with the incidence rates of neurodevelopmental and mental health disorders.
Material and methods: Prenatal exposure to AA was quantified through measurement of the hemoglobin adducts from AA (HbAA) in maternal blood samples from a Danish pregnancy cohort (N=996). Poisson models were fitted with incidence of attention deficit hyperactivity disorder (ADHD) and depression using diagnosis and/or prescription for medication and diagnosis only for autism spectrum disorder (ASD), anxiety, and any mental health disorders.
Results: The median (IQR) HbAA concentration was 91 (78.5) pmol/g Hb. We identified 11 cases of ASD, 45 cases of ADHD, 59 cases of anxiety, 151 cases of depression, and 147 cases of any diagnosed mental disorders over a mean of 34 follow-up years. Higher AA was associated with increased risk of anxiety with an IRR of 1.03 (95%CI: 1.01,1.05) before and 1.02 (0.98, 1.05) per 10-pmol/g Hb increase in HbAA after adjustment for smoking, education, and income. Among offspring of smokers, the IRR of developing anxiety was 2.22 (1.07, 4.91) for the highest exposed offspring compared with those exposed to AA levels lower than the median level. An IRR of 1.02 (1.00,1.04) for any diagnosis of mental disorder(s) per 10-pmol/g Hb increase was observed before adjustment and attenuated to 1.00 (0.97,1.02) after adjustment. The corresponding IRR was 1.36 (1.01, 1.84) for the highest compared to the lowest exposure group. Higher prenatal exposure to AA were not found to be associated with increased risk of ASD, ADHD, and depression.
Conclusion: These preliminary findings suggest that prenatal exposure to AA increase the risk of developing anxiety, and any diagnosis of mental disorders in the offspring later in life.

TO06 | Understanding and communicating elevated and occupational chemical exposures

TO06-01 | Urinary Plasticizer Levels and Their Associations with Sex Hormones in Male Workers

Sohyeon Choi[1], Sohyeon Bang[1], Young-Jun Jeon[1], Jae-Hyun Kim[1], Younglim Kho[2], Gi Jeong Cheon[3], Jeongim Park[1]

[1] Soonchunhyang University, Dept. of Environmental Health Sciences, Asan, South Korea; [2] Eulji University, Seongnam-si, South Korea; [3] Seoul National University Hospital, Seoul, South Korea

Objective: Phthalates and alternative plasticizers, widely recognized as endocrine-disrupting chemicals, are commonly used in manufacturing as additives. However, research on plasticizer exposure and its endocrine effects among workers remains limited. This study aimed to assess urinary concentrations of 33 plasticizers in male workers and examine their relationships with sex hormone levels. **Material and Methods:** This study encompassed 490 male workers in automobile parts factories. At the end of the week, urine and serum samples were collected. LC/MS/MS was performed to examine concentrations of 33 metabolites of urinary plasticizers. Sex hormones were measured in serum, including total testosterone (TT), free testosterone (FT), estradiol (E2), luteinizing hormone (LH), follicle-stimulating hormone (FSH), and sex hormone binding globulin (SHBG). In addition, the free androgen index (FAI) and the ratio of TT to LH and LH to FSH were calculated. Generalized multiple linear regression and quantile g-computation analyses were performed to assess the associations of individual plasticizer and mixture levels with sex hormones while adjusting for age, BMI, urinary cotinine levels, alcohol consumption, income, education, and exercise using R software version 3.4.1. **Results:** A positive association was observed between urinary plasticizers—including MEP, MiBP, MnBP, MCPP, metabolites of DEHP, metabolites of DEHTP, and OH-MINCH—and E2. In contrast, 5OH-MEHTP (-0.03) and 5oxo-MEHTP (-0.04) were negatively associated with LH. For the calculated index, MEP (0.03), MiBP (0.04), 5oxo-MEHP (0.08), 2cx-MMHP (0.07), and DEHP metabolites (0.04~0.06) showed a positive association with TT/LH. Mixture analysis revealed a positive association between plasticizer mixture and E2(0.038). No associations were found with FSH, SHBG, FAI, or LH/FSH. **Conclusion:** This study identified significant associations between plasticizer exposure and sex hormones in male workers. Further research is needed to explore workplace exposure sources.

TO06-02 | Evolution of volatile organic compounds and their health risks following the opening of a newly built office building

Thiphanie P. Riveron, Rebecca L. Cordell, Anna L. Hansell

University of Leicester, Leicester, UK

Little is known about the time profile of volatile organic compounds (VOCs) concentrations in newly built office buildings. Several studies conducted in private buildings have shown that high concentrations of VOCs are present during and shortly after construction, which then decrease over the next few months to reach lower and more stable concentrations. In this study, VOCs were sampled in four rooms of a newly built office building over the 15 months following its opening. The samples were analysed by comprehensive two-dimensional gas chromatography with dual flame ionisation detection and mass spectrometry. The present study found high but fluctuating concentrations of VOCs, with a maximum TVOC concentration reaching 1492 μgm^{-3} in the one office one month after the opening. After six months, the TVOC concentration reduced by 60 to 76% and reached stable concentrations under 200 μgm^{-}

^{3} in all the rooms. VOC concentrations and composition between rooms were impacted by the number of occupants and the position of the offices inside the building. To determine health impacts, hazard quotients (HQ) and cancer risks (CR) were calculated using US Environmental Protection Agency (US.EPA.) reference values for inhalation exposures. At the end of the sampling period, all the HQs were under 1 suggesting that exposure to non-carcinogenic VOCs should not impact the health of the building's workers. CRs were between 1E-04 and 1E-06, which US.EPA. identifies as possible risks from long-term exposure. More studies are needed to characterise VOC exposures and their potential risks to the occupants of newly built public buildings.

TO06-03 | Trends of Types of Tobacco Products Used and Urinary Cotinine Levels among Korean Adults

Sohyun Kang[1], Kiyoung Lee[1,2]

[1] Seoul National University, Department of Environmental Health Sciences, Graduate School of Public Health, Seoul, South Korea; [2] Seoul National University, Institute of Health and Environment, Seoul, South Korea

The smoking habits of the Korean population have changed significantly following the introduction of alternative tobacco products (ATPs), such as electronic cigarettes (ECs) and heated tobacco products (HTPs), marketed as reduced-risk alternatives or aids for smoking cessation. However, ATPs pose health risks through exposure to toxic substances such as nicotine. This study examined the usage trends of tobacco products and compared nicotine exposure by tobacco product types in Korean adults. Trends in the use of conventional cigarettes (CCs) and ATPs among Korean adults were analyzed using the data from Korea National Health and Nutrition Examination Survey (KNHANES) between 2013 and 2022. Urinary cotinine levels by tobacco product types were compared using KNHANES data from 2014 to 2021. The types of tobacco products used were determined through self-reported surveys. The urinary cotinine levels were adjusted for the urinary creatinine levels. The overall tobacco use rate among Korean adults were 20.3% and 17.7% in 2013 and 2022, respectively. Although the use of CCs decreased, the use of ECs and HTPs increased during this period. Urinary cotinine levels remained consistently high among both conventional and alternative product users, with the average urinary cotinine levels being 1,020.36, 832.11, 887.88, and 0.85 ng/Cr mg in CC-only users, ATP users, dual users (both CCs and ATPs), and non-smokers, respectively. ATP users in Korea are exposed to a significant amount of nicotine. Although ATPs have gained popularity in Korea, they pose significant harm to consumers in terms of nicotine exposure.

TO06-04 | Polycyclic aromatic hydrocarbons in female firefighters post-fire response: concentrations and occupational risk factors

Jenni A. Shearston[1,2], Edythe S. Glazer[1,3], Jessica Trowbridge[4], Lauren Baehner[1], ShiZhong Wang[5], Josephine DeGuzman[8], Jianwen She[8], Ruthann A. Rudel[6], Heather Buren[7], Rachel Morello-Frosch[1]

[1] University of California Berkeley, Department of Environmental Science, Policy and Management and the School of Public Health, Berkeley, USA; [2] University of Colorado Boulder, Department of Integrative Physiology, Boulder, USA; [3] California Environmental Protection Agency, Department of Toxic Substances Control, Berkeley, USA; [4] University of California San Francisco School of Medicine, Department of Obstetrics Gynecology and Reproductive Sciences, San Francisco, USA; [5] California Department of Public Health, Biomonitoring California, Sacramento, USA; [6] Silent Spring Institute, Newton, USA; [7] United Fire Service Womoen, San Francisco, USA; [8] California Department of Public Health, Center for Laboratory Sciences, Richmond, USA

Objective: The International Agency for Research on Cancer classifies occupational exposure as a firefighter as carcinogenic to humans. Yet, few studies have evaluated exposure biomarkers in female firefighters, despite evidence that combustion byproducts, including polycyclic aromatic hydrocarbons (PAHs), increase breast cancer risk. We evaluated PAH concentrations in female firefighters after fire events and identified firefighting tasks associated with higher exposure.

Material and Methods: We collected urine samples and conducted an exposure interview with female firefighters from the Women Workers Biomonitoring Collaborative at two time points: 24-48 hours and 1-week post structural fire response (n = 18 participants). Eight PAH metabolites were analyzed by the California Department of Public Health using isotope dilution gas chromatography coupled with high resolution mass spectrometry operated in electron impact mode. We calculated summary statistics and used linear mixed-effect regression to measure temporal change and identify firefighting tasks associated with each PAH metabolite.

Results: Creatinine-adjusted PAH metabolites ranged from non-detection to a maximum of 56,488 pg/mL for 2-NAP. Median concentrations decreased between time points (range: 16%-56% decrease) for all analytes except 3-FLUO. Participating in salvage and overhaul non-significantly increased levels for most analytes: firefighters who participated had 283 pg/mL higher 1-PYR concentrations than those who did not (95% Confidence Interval: -1168-1735). Apparatus operators (elevated 3-FLUO, 2-NAP) and firefighters (elevated 1-PYR, 2-FLUO, 1-PHEN, 3-PHEN, 2-PHEN) had different patterns of PAH exposure, compared to officers.

Conclusion: Female firefighters who responded to a structure fire had elevated PAH metabolite concentrations compared to the general population. Concentrations varied by the firefighter’s role at the incident; for example, apparatus operators exhibited different patterns of PAH exposure than firefighters engaged directly in suppression efforts. This likely reflects variations in exposure sources, such as diesel exhaust versus fire smoke. PAH exposures from firefighting may have implications for breast cancer risk among female firefighters.

TO06-05 | Dealing with extreme values in exposure studies: setting priorities for reporting back

Franco Catuogno[1,2], Alba Iglesias-González[1], Brice M.R. Appenzeller[1]

[1] Luxembourg Institute Of Health, Human Biomonitoring Research Unit / Department of Precision Health, Luxembourg, Luxembourg; [2] University of Luxembourg, Esch-sur-Alzette, Luxembourg

Objectives:
In human biomonitoring studies, the presence of extremely high concentrations of chemicals in some samples can prompt reporting back to participants, with a view to suggest targeted interventions to decrease exposure. Resource limitation can however force to prioritize the participants to reach back. Based on toxicological consideration and chemical concentration, we developed approaches to rank chemicals and participants in the prioritization process.
Materials and Methods:
Results from the analysis of 153 biomarkers of exposure measured in hair samples of 218 children living in Luxembourg between October 2020 and April 2021 were used in this study.
Different approaches, by either filtration or ranking were tested. Filtration consisted in, first filtering chemicals based on their toxicity (carcinogenicity, mutagenicity, reprotoxicity and endocrine disruption), and second, filtering participants based on their levels of pollutants in hair. Ranking approaches were based on concentration, concentration/median-in-the-population (including and excluding non-detected values) ratios, and concentration/minimum-level-in-the-population ratio to build hierarchical scales, for both chemicals and participants. Subsequently, for each chemical, participants were sorted in increasing order of concentration to highlight population exposure profiles: flat profile (narrow concentration range showing no extreme values) vs steep profile (wide range with extreme values). Considering that steep profile chemicals would have more chances of impactful report-back, we used concentration profile as a gold standard to assess the relevance of the prioritization approaches.
Results:
The best approach to prioritize chemicals with steeper profiles over those with flatter profiles was using the ratio concentration/minimum-level-in-the-population. This hierarchization approach ranks participants based on their pollutant levels relative to the minimum detected value in the population for each pollutant.
Conclusion:
A system to rank participants based on the outlying levels for any targeted chemical was successfully developed allowing setting priorities for reporting back results of epidemiological studies when resources are limited.

TO07 | From cradle to cognitive decline: Air pollution and mental health across the life course

TO07-01 | Ambient exposure to hazardous air pollutants and associations with neurological function in the United States Gulf region

Christina Norris[1], Dale Sandler[2], Praful Dodda[3], Amelia Martin[1], Kate Christenbury[4], W. Braxton Jackson II[4], Emily Werder[2], Chi-Tsan Wang[5], Bok Haeng Baek[5], William Vizuete[3], Marc Serre[3], Fredric Gerr[6], Nora Abbott[3], Lawrence Engel[1,2]

[1] University of North Carolina at Chapel Hill, Department of Epidemiology, Gillings School of Global Public Health, Chapel Hill, USA; [2] National Institute of Environmental Health Sciences, Epidemiology Branch, Research Triangle Park, USA; [3] University of North Carolina at Chapel Hill, Department of Environmental Sciences & Engineering, Gillings School of Global Public Health, Chapel Hill, USA; [4] DLH, LLC, Bethesda, USA; [5] George Mason University, Center for Spatial Information Science and Systems (CSISS), Fairfax, USA; [6] University of Iowa College of Public Health, Department of Occupational and Environmental Health, Iowa City, USA

Objective: Styrene, benzene, toluene, ethylbenzene, and xylenes (SBTEX) are volatile organic compounds common in indoor and outdoor air in the United States. They affect the nervous system at occupational exposure levels, with some evidence of effects at lower levels. We leverage new geographically and temporally resolved estimates of individual-level ambient exposures to SBTEX over varying lags (0-6 day) of daily means to evaluate associations with performance on standardized tests of sensory and motor neurological function among 3,363 persons living in the Gulf States.

Materials and methods: We used linear regression to estimate differences in handgrip strength, vibrotactile threshold and postural sway, and log-binomial regression to evaluate failure to maintain a single leg stance, across quintiles of exposure. For visual contrast sensitivity we compared mean scores among high- vs. low-exposed groups. We adjusted for age, self-identified race, sex, smoking, alcohol consumption and educational attainment.

Results: Benzene exposure in the 0-4 days prior to testing was associated with less sensitivity to vibrotactile stimuli at all levels of exposure compared to the lowest quintile, although there was no clear exposure-response pattern, and not all associations were significant. There was some evidence of a detriment in grip strength associated with exposure to all pollutants except xylene for most lags, with some indication of an exposure-response, and with same-day exposure to xylene. Results were mixed for postural sway, with some evidence of more sway with eyes open among those exposed to xylene in the 1-4 days prior to testing. There was no compelling evidence of effects for the single leg stance or for visual contrast sensitivity.

Conclusion: We found some evidence of a detrimental effect of recent ambient SBTEX exposure on certain measures of sensory and motor nerve function, although results were mixed.

Funding: NIH/NIEHS R01ES031127, Intramural Research Program of NIH, NIEHS Z01 ES102945

TO07-02 | The role of the components of $PM_{2.5}$ in the incidence of Alzheimer's disease and related disorders

Haisu Zhang, Yifan Wang, Haomin Li, Qiao Zhu, Tszshan Ma, Yang Liu, Kyle Steenland

Emory University, Rollins School of Public Health, Atlanta, USA

Background: The associations of $PM_{2.5}$ mass and various adverse health outcomes have been widely investigated. However, fewer studies focused on the potential health impacts of $PM_{2.5}$ components, especially for dementia and Alzheimer's diseases (AD).
Methods: We constructed a nationwide population-based open cohort study among Medicare beneficiaries aged 65 or older during 2000-2018. This dataset was linked with the predicted levels of 15 $PM_{2.5}$ components, including 5 major mass contributors (EC, OC, NH_4^+, NO_3^-, SO_4^{2-}) and 10 trace elements (Br, Ca, Cu, Fe, K, Ni, Pb, Si, V, Zn) across contiguous US territory. Data were aggregated by ZIP code, calendar year and individual level demographics. Two mixture analysis methods, weighted quantile sum regression (WQS) and quantile g-computation (qgcomp), were used with quasi-Poisson models to analyze the health effects of the total mixture of $PM_{2.5}$ components on dementia and AD, as well as the relative contribution of individual components.
Results: Exposure to $PM_{2.5}$ components over the previous 5 years was significantly associated with increased risks of both dementia and AD. The cumulative associations in rate ratio (RR) was 1.027 (95% confidence interval (CI): 1.025-1.028) for dementia, and 1.041 (95% CI: 1.039-1.042) for AD in qgcomp models with 1 decile increase in all $PM_{2.5}$ components. WQS models showed similar but slightly stronger associations with RR = 1.029 (95% CI: 1.028-1.030) for dementia and RR = 1.048 (95% CI: 1.047-1.049) for AD. SO_4^{2-}, OC, Cu were identified as major contributors to the combined positive association of the mixture from both WQS and qgcomp models.

Conclusion: We found positive associations between the 15 $PM_{2.5}$ components and the incidence of dementia and AD. Our findings suggest that reducing $PM_{2.5}$ emissions from traffic and fossil fuel combustion could help mitigate the growing burden of dementia and Alzheimer's disease.

TO07-03 | Impact of Short-Term Air Pollution and Extreme Heat on Pediatric Psychiatric Exacerbations: A Case-Crossover Study

Erika R. Manning[1], Andrew Vancil[1], Patrick H. Ryan[1,2], Cole Brokamp[1,2]

[1] Cincinnati Children's Hospital Medical Center, Division of Biostatistics and Epidemiology, Cincinnati, USA; [2] University of Cincinnati, Department of Pediatrics, Cincinnati, USA

Objective

Evidence links environmental factors, particularly ambient air pollution and extreme heat, to psychiatric morbidity, a growing public health concern. This study investigates associations between short-term exposure to fine particulate matter (PM2.5) and extreme heat with psychiatric emergency department (ED) and urgent care (UC) visits among youth.

Materials and Methods

Patient addresses and visit dates from psychiatric ED/UC visits to Cincinnati Children's Hospital from July 1, 2016, to July 1, 2022 were geocoded and linked to a spatiotemporal model estimating daily PM2.5 levels. Extreme heat days were defined as those exceeding the 95th percentile of 30-year heat indices. We fit time-stratified case-crossover models to estimate odds ratios (OR) for psychiatric exacerbations associated with increased PM2.5 (adjusted for holiday effects and lagged temperature and humidity) and extreme heat exposure (adjusted for holiday effects and lagged PM2.5). To investigate susceptibility, we also fit models stratified by community deprivation.

Results

Among 12,767 psychiatric ED/UC encounters, a 10 µg/m³ increase in PM2.5 was associated with 6% increased odds for a psychiatric exacerbation two days later (OR 1.06, 95% CI: [0.99, 1.13]), driven by anxiety (1.34, [1.07, 1.68]), mood (1.17, [0.99, 1.38]), schizophrenia (1.43, [0.90, 2.28]), and intermittent explosive (1.18, [0.96, 1.44]) disorders. Extreme heat was linked to 11% increased odds for a psychiatric exacerbation one day later (OR 1.11, 95% CI: [1.02, 1.21]), driven by anxiety (1.5, [1.12, 2.01]), adjustment (1.18, [0.94, 1.49]), and intermittent explosive (1.27, [0.98, 1.65]) disorders. Children living in highly deprived communities were more susceptible to the two-day lagged increases in PM2.5 (OR 1.14, 95% CI: [1.01, 1.28] versus 1.03, [0.95, 1.11]), but less susceptible to one-day lagged extreme heat days (1.07, [0.92, 1.25] versus 1.13, [1.01, 1.26]).

Conclusion

Acute PM2.5 and extreme heat exposure are modifiable population-level influences that could be leveraged to reduce pediatric psychiatric morbidity.

TO07-04 | Long-term Wildfire Smoke Exposure and Risk of Dementia: A Nationwide US Cohort Study

Yanling Deng[1], Kyle Steenland[1], Danlu Zhang[2], Hua Hao[1], Ke Xu[1], Qiao Zhu[1], Yun Hang[3], Howard Chang[2], Sadeer Al-Kindi[4], Tarik Benmarhnia[5,6], Yang Liu[1]

[1] Emory university, Environmental health, Atlanta, USA; [2] Emory university, Department of Biostatistics and Bioinformatics, Atlanta, USA; [3] University of Texas Health Science Center at Houston, Department of Environmental and Occupational Health Sciences, Houston, USA; [4] Department of Cardiovascular Medicine, Houston Methodist DeBakey Heart and Vascular Center, Houston, USA; [5] Scripps Institution of Oceanography, University of California San Diego, La Jolla, USA; [6] Irset Institut de Recherche en Santé, University of Rennes, EHESP, France

Background: Although increasing evidence indicates that long-term exposure to fine particulate matter ($PM_{2.5}$) may increase the risk of dementia, little is known about the comparative significance of $PM_{2.5}$ from wildfire smoke.
Objectives: To investigate the associations of long-term exposure to wildfire smoke $PM_{2.5}$ with overall dementia and Alzheimer's Disease (AD) incidence and identify subpopulations that are particularly susceptible.
Material and Methods: In this nationwide population-based cohort study conducted from 2007 to 2018 using Medicare data,we used a two-stage, twin-model machine learning modeling framework to estimate wildfire smoke $PM_{2.5}$ and non-smoke $PM_{2.5}$ concentrations.Cox proportional hazard models were applied to estimate the associations of 5-year average exposure to wildfire smoke $PM_{2.5}$, non-smoke $PM_{2.5}$, and annual smoke days with incident dementia and AD. Stratified analyses by demographic characteristics and comorbidities were also conducted to evaluate the potential effect modifiers.
Results: We identified approximately 3.6 million incident dementia cases and 1.2 million AD cases. Wildfire smoke $PM_{2.5}$ was associated with an increased risk of both dementia and AD [per 1 μg/m^3 increase in the 5-year average exposure: HR=1.042; 95% CI: 1.032, 1.052 and HR=1.050; 95% CI: 1.034, 1.067, respectively], larger than the impact of non-smoke $PM_{2.5}$ exposure [HR: 1.002 (95% CI: 1.001, 1.003) for dementia and 1.007 (95% CI: 1.005, 1.009) for AD, respectively]. Both days with non-zero wildfire $PM_{2.5}$ concentrations and days with wildfire $PM_{2.5}$ concentrations ≥2.5 μg/m^3 were linked to an increased risk of dementia and AD. The risks from wildfire $PM_{2.5}$ were higher in those with higher income and no comorbidities, while non-smoke $PM_{2.5}$ had stronger associations with AD and dementia in individuals with comorbidities.**Conclusions:** Our study suggests that long-term exposure to wildfire smoke $PM_{2.5}$ may have greater effects on dementia than $PM_{2.5}$ from other sources, with varying risks among subpopulations.

TO07-05 | Long-term exposure to air pollution and road traffic noise in relation to dispensed antidepressant medications: A Swedish cohort study

Sai San Moon Lu[1,2], Erik Ekbäck[4,5], Johan Sommar[1], Sara Anderson[6], Henrik Olstrup[1], Sophia Harlid[2], Susanna Gustafsson[7], Kristoffer Mattisson[3], Leo Stockfelt[8,9], Theo Rein[10], Ivan Nalvarte[11], Joëlle Rüegg[12], Anna Oudin[1,3]

[1] Umeå University, Department of Epidemiology and Global Health, Umeå, Sweden; [2] Umeå University, Department of Diagnostics and Intervention, Umeå, Sweden; [3] Lund University, Division of Occupational and Environmental Medicine, Lund, Sweden; [4] Umeå University, Department of Public Health and Clinical Medicine, Umeå, Sweden; [5] Umeå University, Department of Clinical Science, Umeå University, Umeå, Sweden; [6] Region Västerbotten, Occupational and Environmental Medicine, Umeå, Sweden; [7] City of Malmö, Environmental Department, Malmö, Sweden; [8] University of Gothenburg, Occupational and Environmental Medicine, School of Public Health and Community Medicine, Institute of Medicine, Sahlgrenska Academy, Gothenburg, Sweden; [9] Sahlgrenska University Hospital, Department of Occupational and Environmental Medicine, Sweden, Sweden; [10] Max Planck Institute of Psychiatry, Project Group Signaling Pathways in Depression, Munich, Germany; [11] Karolinska Institutet, Department Neurobiology, Care Sciences and Society, Stockholm, Sweden; [12] Uppsala University, Department of Organismal Biology, Uppsala, Sweden

Objective: To investigate long-term residential exposure to air pollutants and road traffic noise in relation to depression.

Material and Methods: A cohort study was conducted using the Swedish Malmö Diet and Cancer Study cohort, linked to the National Prescribed Drug Register and the National Patient Register. The outcome was defined as a first dispensed antidepressant (ATC: N06A) and/or a first depression diagnosis (ICD-10: F32/F33) during follow-up (2007-2011). Exposure was assessed through modeled annual mean concentrations of particulate matter ($PM_{2.5}$, PM_{10}), nitrogen oxides (NO_x), and road traffic noise (L_{den}) level across two exposure periods: one year prior to event (lag1) and one to five years prior (lag1-5). Cox proportional hazards regression was applied, adjusted for potential confounders including sociodemographic and lifestyle factors.

Results: The study included 18 373 individuals, among whom 2 224 with the outcome (88% had dispensed antidepressant, 12% clinical diagnosis). Since most outcome events were identified from the National Prescribed Drug Register, the interpretation focused primarily on dispensed antidepressants. Long-term exposure to air pollutants was associated with increased dispensed antidepressants, exclusively in women. For road traffic noise, a tendency toward increased hazard ratios (HRs) was found. Conversely, neither air pollution nor road traffic noise was associated with dispensed antidepressants in men. For the lag1-5 in women, HRs per 10-unit increase were: $PM_{2.5}$, 1.29 (95% Confidence Intervals (CI), 1.09–1.52); PM_{10}, 1.10 (95% CI, 1.03–1.17); NO_x, 1.15 (95% CI, 1.04–1.26); and L_{den}, 1.06 (95% CI, 0.99–1.13). Similar association patterns were observed for the lag1 and in the two-pollutants models (air pollutants and road traffic noise).

Conclusions: Long-term exposure to air pollution was associated with antidepressant use among women, while no clear link was identified for road traffic noise. These findings suggest that air quality improvements may help reduce health burdens related to depressive symptoms.

TO07-06 | Short-term exposure to outdoor and indoor air pollution is linked to cognitive function in Mexico

Erika Meza[1], Joseph L. Saenz[2], Jennifer Weuve[3], Marcia P. Jimenez[3]

[1] Harvard University, Center for Population and Development Studies, Cambridge, USA; [2] Arizona State University, Edson College of Nursing and Health Innovation, Phoenix, USA; [3] Boston University, Department of Epidemiology, Boston, USA

Objective: Most evidence on the effect of air pollution on dementia risk has evaluated outdoor air pollution.We investigated the combined effects of outdoor and indoor air pollution on cognitive function in a cohort of older Mexican adults.

Material and Methods: We analyzed data on adults aged 50+ from the 2012 Mexican Health and Aging Study to evaluate the relationship between outdoor and indoor air pollution and cognitive function. Outdoor air pollution was assessed using 2012 PM2.5 levels (mean-centered), and indoor pollution was measured through participants' reported cooking fuel use (wood or coal versus gas). We computed a global cognitive function score based on each participant's averaged verbal learning, memory, fluency, visual scanning, and orientation assessment z-scores. We estimated the independent and then joint associations of outdoor and indoor air pollution with cognitive function using generalized linear models, adjusting for age, sex, education, and rurality.

Results: The 1,989 participants had a mean age of 64.6 years (SD: 9.3) and an average of 7 years of education (SD: 5); 59% were female. The average PM2.5 exposure was 21.54 μg/m³. In adjusted models, each 10-unit increase in PM2.5 exposure and the use of wood or coal for cooking were independently associated with lower global cognitive scores ($B_{PM2.5}$: -0.04 [95% CI: -0.12, 0.04] and $B_{Wood/Coal}$: -0.13 [95% CI: -0.43, 0.18]), although confidence intervals included the null. In adjusted models, we found evidence of a multiplicative interaction between outdoor and indoor air pollution, where higher PM2.5 exposure and use of wood or coal were associated with a lower global cognitive score (B: -0.67 [95% CI: -1.32, -0.02]).

Conclusion: Multiplicative interaction suggests that the combination of outdoor and indoor air pollution may worsen cognitive performance. Results emphasize the need to address air pollution exposure, particularly among doubly exposed populations.

TO07-07 | Associations between early-life exposure to air pollution and cognitive development: impacts on Intelligence Quotient (IQ) scores in children at 8 years of age

Yingxin Chen[1,2], Katie Eminson[1,3], Yutong S. Cai[1,4], Calvin Jephcote[1], John Gulliver[5], Samantha J. Johnson[6], Anna L. Hansell[1,2]

[1] University of Leicester, Centre for Environmental Health and Sustainability, Leicester, UK; [2] University of Leicester, The National Institute of Health Research (NIHR) Health Protection Research Unit (HPRU) in Environmental Exposure and Health, Leicester, UK; [3] University of Leicester, National Institute for Health Research (NIHR) Leicester Biomedical Research Centre (BRC), Leicester General Hospital, Leicester, UK, Leicester, UK; [4] Leicester General Hospital, NIHR Leicester Biomedical Research Centre, Leicester, UK; [5] St George's, University of London, Environmental and Exposure Sciences, Population Health Research Institute, London, UK; [6] University of Leicester, Department of Health Sciences, Leicester, UK

Objective: This study examines the impact of source-specific PM_{10}, and NO_2 on Intelligence Quotient (IQ) scores in children from birth to age 8 years in the Avon Longitudinal Study of Parents and Children (ALSPAC).

Material and Methods: ALSPAC recruited pregnant women in southwest England with expected delivery dates between April 1, 1991, and December 31, 1992. Source-specific PM_{10} exposure was assessed with dispersion models and local monitoring data, while NO_2 were estimated using Land Use Regression (LUR) models from the European ELAPSE project. Analyses used average air pollutant concentrations at ages 0–2, 2–4, and 4–8 years. IQ was assessed at 8.5 years using the Wechsler Intelligence Scale for Children 3rd Edition (WISC-III). Associations were evaluated using linear regression models adjusted for sex, preterm birth (<37 weeks), maternal education, maternal smoking, as well as maternal age, environmental tobacco smoke exposure, maternal body mass index (BMI), and road traffic noise

Results: A total of 7,154 children were included. NO_2 at age 2–4 years was associated with lower IQ at age 8.5 years (β = -0.51, 95% CI: -1.01 to -0.00) per 5.43 μg/m³ increase. No significant association was found for any of PM_{10} from traffic, PM_{10} from local sources and PM_{10} from total source. Subgroup analysis showed stronger effects among children with lower maternal education. PM_{10} from traffic exposure at ages 4–8 was associated with lower IQ (β = -0.74, 95% CI: -1.41 to -0.06). NO_2 exposure across all early-life periods (0–2, 2–4, and 4–8 years) also showed significant negative associations (β ranging from -0.97 to -1.23).

Conclusion: Early-life exposure to NO_2 was associated with lower cognitive performance at age 8. The effects were stronger in children from lower-educated backgrounds, highlighting potential socioeconomic disparities in pollution-related cognitive impacts.

TO08 | Exposure to metals and elements in water

TO08-01

This abstract has been withdrawn.

TO08-02 | Prenatal Exposure to Arsenic in Drinking Water and Type 1 Diabetes

Thoranna Gilbertsdottir[1], Louise Lindholdt[1], Birgitte Hansen[2], Denitza Voutchkova[2], Torben Sigsgaard[1,3], Jörg Schullehner[1,3]

[1] Aarhus University, Department of Public Health, Aarhus, Denmark; [2] Geological Survey of Denmark and Greenland (GEUS), Department of Geochemistry, Aarhus, Denmark; [3] Aarhus University, Big Data Centre for Environment and Health (BERTHA), Aarhus, Denmark

Objective: Exposure to inorganic arsenic during pregnancy has been linked to epigenetic changes in cord blood of newborns, resulting in dysregulation of immune responses and changes in proportions of leukocyte subpopulations. While biological evidence has provided insight into potential mechanisms of arsenic toxicity, there remains a need to link these findings to actual cases of disease in human populations. Therefore, this study explores the association between prenatal drinking water arsenic exposure and the autoimmune disease type 1 diabetes (T1D) in Danish children.

Material and methods: This follow-up study links prenatal arsenic exposure from household drinking water with information on T1D diagnoses from The Danish National Patient- and Prescription Registries. All singleton births from 2002-2012, located from The Danish Medical Birth Registry, and living in a household using a public water supply, were included. Cox proportional hazards models were used with age as underlying time scale and adjusted for calendar year, sex at birth, maternal age, smoking status, BMI, parental T1D status, education and income.

Results: In a total of 563,871 births, a positive association between prenatal arsenic drinking water exposure and T1D was observed. In fully adjusted models using <1 µg/L as reference, a monotonically increasing association was observed, HR (95% CI): 1.10 (0.98-1.24), 1.29 (0.88-1.87) and 2.04 (1.15-3.60) for 1-5, 5-10, and ≥ 10 µg/L, respectively. A higher risk was observed among children born above median compared with below median birth weight across all exposure categories.

Conclusion: This is the first study, to our knowledge, to assess the association of prenatal arsenic exposure on T1D. We found prenatal drinking water arsenic exposure to increase risk of T1D in public supply users at relatively low levels, even under the WHO's guidelines, demonstrating that arsenic may be a significant modifiable risk factor for T1D.

Funding: Independent Research Fund Denmark.

TO08-03 | Exposures to drinking water contaminants and risk of uterine cancer in the California Teachers Study cohort

Maya Spaur[1], Lydia Post[1], Laura Beane Freeman[1], Danielle Medgyesi[2], Jared Fisher[1], Jessica Madrigal[1], Samantha Ammons[1], Emma Spielfogel[3], Komal Bangia[4], Alexander Keil[1], James Lacey, Jr.[3], Mary Ward[1], Rena Jones[1]

[1] National Cancer Institute, Occupational and Environmental Epidemiology Branch, Division of Cancer Epidemiology and Genetics, Rockville, USA; [2] Columbia University Mailman School of Public Health, Department of Environmental Health Sciences, New York, USA; [3] Beckman Research Institute City of Hope, Division of Health Analytics, Department of Computational and Quantitative Medicine, Duarte, USA; [4] Office of Environmental Health Hazard Assessment, Community and Environmental Epidemiology Research Branch, Oakland, USA

Objective: Some drinking water contaminants including disinfection byproducts are known or suspected carcinogens with endocrine disrupting properties; limited epidemiologic studies have evaluated associations with uterine cancer.
Methods: We investigated multiple regulated contaminants and uterine cancer in the California Teachers Study, a prospective cohort of female California educators. Participants were cancer-free, without hysterectomy at enrollment (1995-1996), with addresses linked to a community water supply (CWS; N=81,094, 92%), with follow-up through 2020. Among women with a residential duration at enrollment $\geq$10 years (N=55,927, 70%), we computed long-term averages (1990-2005) of individual and total trihalomethanes (THMs), nitrate, arsenic, and uranium. We estimated hazard ratios and 95% confidence intervals (HRs, CIs) per continuous ($\log_{10}$) and quartiles/90th percentile increase for all uterine cancers (n=1,098), and for endometrioid (n=901) tumors, in Cox models adjusting for age, body mass index, and smoking status. We evaluated mixture effects of individual THMs, arsenic, uranium, and nitrate using quantile-based g-computation.
Results: Almost all women (>98%) had exposures below regulatory limits. Higher average concentrations of total THMs were associated with risk of uterine cancer ($HR_{perlog10}$=1.06, CI: 1.00-1.13) and endometrioid tumors specifically ($HR_{perlog10}$=1.08, 1.00-1.15). We observed similarly positive associations in continuous models for some individual THMs and at ≥90th percentile: bromodichloromethane ($HR_{perlog10}$=1.08, 1.00-1.17 and 1.11, 1.02-1.21 for uterine cancer and endometroid types, respectively), chloroform (HR_{90vsQ1}=1.33, 1.07-1.65 and 1.37, 1.08-1.74, p trends$\leq$0.05), and dibromochloromethane (HR_{90vsQ1}=1.19, 0.95-1.48 and 1.26, 0.99-1.61). We found no associations with nitrate, arsenic or uranium. HRs per interquartile range increase in the mixture were 1.17 (CI 0.93-1.47) and 1.27 (CI 0.99-1.64), for uterine and endometrioid types, respectively. Dibromochloromethane and chloroform were the major contributors.
Conclusions: Associations between drinking water THMs and uterine and endometrioid cancer are consistent with positive findings from limited prior studies and warrant further investigation into drinking water contaminants as modifiable risk factors for this gynecologic cancer.

TO08-04 | Progress toward modeling human exposures to drinking water contaminants in private wells: A predictive model for groundwater trace elements across the contiguous United States

Xindi C. Hu[1], Jennifer M. Sun[2], Jonas LaPier[2], Elsie M. Sunderland[2,3]

[1] George Washington University, Department of Environmental and Occupational Health, Washington, USA; [2] Harvard University, Department of Earth and Planetary Sciences, Cambridge, USA; [3] Harvard University, Department of Environmental Health, Boston, USA

Objective: Monitoring gaps in federally unregulated private well drinking water limit our understanding of health risks from drinking water contaminants, particularly in rural and remote communities facing environmental justice concerns. This study aims to develop machine learning models to predict trace element concentrations in groundwater across the contiguous United States.

Material and Methods: We employed XGBoost models trained on data from over 50,000 private wells, predicting concentrations of arsenic, cadmium, lithium, manganese, and strontium. We made predictions to 0.1-degree by 0.1-degree grid cells across the contiguous U.S. and quantified prediction uncertainty using bootstrapping methods. SHAP values identified key predictors influencing predictions, while a two-stage clustering approach (GeoSOM and K-means) characterized geographic and chemical co-occurrence patterns.

Results: The models effectively predict continuous concentrations of the trace elements, with R-squared values ranging from 0.44 (for manganese) to 0.68 (for lithium), which compared favorably to previous studies on more widely studied contaminants such as nitrate. Categorical evaluations, classifying concentrations above or below health-based thresholds, achieved accuracy ranging from 0.88 (for manganese) to 0.99 (for cadmium). SHAP values identify the most influential factors for each element, revealing variations across national and regional scales. Finally, GeoSOM and K-means clustering identified three main groups of co-occurring trace elements, each with distinct geographic patterns and chemical profiles.

Conclusion: This study demonstrates the utility of continuous machine learning models to predict chemical concentrations in groundwater on the national scale. Addressing the identified limitations to better capture localized contamination sources and allow geospatially varying relationships will further enhance the accuracy and applicability of these models, ultimately contributing to an improved understanding of human exposure to chemical mixtures in private wells and more effective risk mitigation strategies.

TO08-05 | Generating spatial prediction surfaces for groundwater arsenic in rural areas of the western United States using machine learning and GIS

Aaron Nuanez[1], Austin Martinez[2], Joseph Hoover[1]

[1] University of Arizona, Environmental Science, Tucson, USA; [2] California State University Dominguez Hills, Biology, Ecology, and Environmental Science, Carson, USA

Objectives. The objective of this project was to utilize machine learning and GIS to create models to spatially predict the occurrence of arsenic in the groundwater of the Colorado Plateau to highlight communities at risk of elevated arsenic in their groundwater. Rural and Tribal communities residing in geologically diverse areas, such as the Colorado Plateau, use groundwater that may contain arsenic derived from different geologic sources.
Materials. The modelling approach incorporated geochemical, geologic, soils, meteorological/climate, and water quality data. Water quality data were acquired from the Water Quality Portal (WQP), and a public data repository for wells in the Navajo Nation archived with the Environmental Data Initiative. Other public data were aquired from the US Geological Survey Oregeon State Univesity, and NOAA.
Methods. Data were converted to a common data format for compilation. Once geospatial data were harmonized our team developed predictive models using random forest and extreme gradient boosting. The models predicted the probability of arsenic exceeding relevant concentrations thresholds (5 ug/L or 10 ug/L). Models were fully tuned, and variables were selected to create the most parsimonious model while maintaining overall model accuracy.
Results. Notable variables retained in the final models included water pH, water Fe concentrations, depth to groundwater, average annual precipitation, and select soil trace metals and minerals. Distinct geographic patterns of arsenic occurrence were observed in spatial prediction surfaces. To explore why these variables may contribute to the model output we overlayed regional geology to explain potential arsenic sources across the Plateau.
Conclusions. Findings from this study will inform ongoing public health oriented investigations of groundwater arsenic exposure and gastric health. This research will also inform future community-based research that will refine knowledge of water use behavrious and risk factors for gastric cancer.

TO08-06 | Prenatal Public Drinking Water Arsenic Exposure and Hypertensive Disorders of Pregnancy in the ECHO Program

Sharon Kulali[1], Tessa Bloomquist[1], Tushara Rajeev[1], Amii Kress[2], Mohammad Burjak[2], Daniel Beene[2], Heather H. Burris[3], Whitney Cowell[4], Anne Dunlop[5], Caitlin G. Howe[6], Miranda Jones[2], Margaret Karagas[6], Zorimar Rivera-Nunez[7], Tiffany Sanchez[8], Allison Sherris[9], Pei-Wen Tung[1], Julia Varshavsky[10], Blair J. Wylie[11], Yeyi Zhu[12], Anne E. Nigra[1], Julie Herbstman[1], Maya Deyssenroth[1]

[1] Columbia University Mailman School of Public Health, Department of Environmental Health Sciences, New York, USA; [2] Johns Hopkins Bloomberg School of Public Health, Department of Epidemiology, Baltimore, USA; [3] The Children's Hospital of Philadelphia, Division of Neonatology, Department of Pediatrics, Philadelphia, USA; [4] New York University Grossman School of Medicine, Division of Environmental Pediatrics, Department of Pediatrics, New York, USA; [5] Emory University School of Medicine, Department of Gynecology and Obstetrics, Atlanta, USA; [6] Geisel School of Medicine at Dartmouth, Department of Epidemiology, Lebanon, USA; [7] Rutgers School of Public Health, Department of Biostatistics and Epidemiology, Piscataway, USA; [8] Colorado School of Public Health, Department of Epidemiology, Aurora, USA; [9] University of Washington, Department of Environmental and Occupational Health Sciences, Seattle, USA; [10] Northeastern University, Department of Health Sciences, Department of Civil and Environmental Engineering, Boston, USA; [11] Beth Israel Deaconess Medical Center, Department of Obstetrics, Gynecology, and Reproductive Biology, Boston, USA; [12] Kaiser Permanente Northern California, Division of Research, Pleasanton, USA

Objective: Arsenic is prevalent in public water systems and exposure to arsenic during pregnancy has been associated with hypertensive disorders of pregnancy (HDP). However, studies on public water arsenic below the current regulatory standard (10 µg/L) and HDP are limited in the United States. Leveraging the Environmental influences on Child Health Outcomes (ECHO) cohort consortium, we conducted one of the largest US-based studies to assess associations between prenatal, time-weighted average public water arsenic exposure and HDP outcomes.
Material and Methods: We included 14,802 pregnant individuals (birth years 2005-2019) from ECHO and generated individual, prenatal, time-weighted average public water arsenic estimates by leveraging >900k public water monitoring records (2006-2019) and linking census tract-level estimates to pregnancy residential address histories. HDP outcomes, including gestational hypertension (GHTN) and preeclampsia (PE), were ascertained from medical records and self-report. Missing covariate data were imputed. We used generalized estimating equation models to estimate relative risks (RRs) with arsenic exposure analyzed continuously, per log-doubling, and across categories of exposure. Models were adjusted for maternal age, maternal education, pre-pregnancy body mass index, prenatal tobacco use, HDP history, and population density.
Results: Prenatal public water arsenic estimates (mean: 0.905 µg/L; range: 0.15-37.6 µg/L) significantly declined over time (2005-2019). Among 1,586 HDP cases (GHTN: 563; PE: 774), the covariate-adjusted RR comparing the highest (>5 µg/L) to lowest (≤0.35 µg/L) exposure category was 0.74 (95% CI: 0.58, 0.95). All other results were not statistically significant.
Conclusion: We observed an inverse association between prenatal water arsenic exposure and HDP, though unmeasured confounding, co-exposures, or cohort-specific differences may explain this finding. Future studies will explore temporal trends, non-linear relationships, and cohort-specific differences, as well as stratifying by sociodemographic factors. These steps are critical to understand the true association and inform public health policies for arsenic in drinking water at low concentrations.

TO08-07 | Association of biomarker-derived heavy metal exposures with childhood anemia in Tumbes, Peru: A cross-sectional mixture analysis

Sofia I. Chapela-Lara[1], Seth E. O'Neal[1], Emily Henkle[1], William K.-Y. Pan[2], Percy M.Q. Vilchez-Barreto[3], Ricardo Gamboa-Moran[3], Charlotte Roscoe[1]

[1] Oregon Health & Science University - Portland State University, School of Public Health, Portland, USA; [2] Duke University, Global Health Institute and Nicholas School of Environment, Durham, USA; [3] Universidad Peruana Cayetano Heredia, Centro de Salud Global - Tumbes, Tumbes, Peru

Background: Heavy metal (HM) exposure is a global health concern, particularly in low- and middle-income countries affected by gold mining pollution. In underregulated regions, HM contamination of water and food threatens children's health, who are particularly vulnerable due to ongoing organ development. The Puyango-Tumbes River, Tumbes, Peru's main freshwater source, often exceeds the Environmental Protection Agency's thresholds for multiple HM, including lead, arsenic, cadmium, and manganese, potentially from upstream Ecuadorian gold mining. Lead disrupts hemoglobin synthesis and iron metabolism causing anemia. However, the combined impact of lead and other HM is understudied. This study examined the association of lead and other HM exposures from hair samples, both individually and as a mixture, with clinically assessed anemia in Tumbes' children.

Methods: We recruited 404 children (4-17 years) from 14 Tumbes villages (January-March 2023) and collected questionnaires, hair, and blood samples. Hair samples were analyzed using Inductively Coupled Plasma Mass Spectrometry to assess lead, arsenic, cadmium, and manganese exposure (~3-month window). Blood hemoglobin levels were analyzed to determine anemia status; iron, folic acid, and vitamin-B12 levels assessed anemia causes. Quantile G-computation models estimated anemia odds from lead and HM mixture exposure, adjusting for sociodemographic and behavioral confounders.

Results: The prevalence of anemia was 11.4%; all cases were due to iron deficiency. The adjusted odds of anemia for each quartile increase in the natural logarithm of lead (mg/kg) were 1.31 (95% CI: 0.96, 1.79), while for each quartile increase in all metals in the HM mixture, the odds of anemia assuming additivity were 1.46 (95% CI: 1.02, 2.08) or 1.51 (95% CI 0.98, 2.34) considering interactions within the mixture.

Conclusions: Lead exposure when combined with other HMs, showed a stronger association with childhood ferropenic anemia than lead exposure alone. Children with high simultaneous exposure to multiple HMs may face an increased anemia risk.

TO09 | Maternal exposure to climate extremes: Heat and wildfire smoke impacts on birth outcomes

TO09-01 | Exposure to extreme temperatures may increase the risk of preterm birth in dose response manner: Findings from a US cohort

Siddharth Rawat[1], Elizabeth DeVilbiss[2], Elizabeth Scholl[3], Taylor Petty[3], Brian Kidd[3], Deepak Mav[3], Shyamal Peddada[1], Jagteshwar Grewal[2], Neil Perkins[2]

[1] National Institute of Environmental Health Sciences, Biostatistics and Computational Biology Branch, Durham, USA; [2] Eunice Kennedy Shriver National Institute of Child Health and Human Development, Division of Population Health Research, Bethesda, USA; [3] Sciome, Durham, USA

OBJECTIVE: The frequency and severity of extreme temperatures have been increasing and are projected to grow in the coming decades. Relationships between chronic heat and cold temperatures and early deliveries are not well understood. The goal of this study is to investigate these relationships using the National Institute of Child Health and Human Development (NICHD) Consortium on Safe Labor (CSL) cohort (2002-2008) consisting of 203,691 pregnant women from 19 hospitals representing 11 states and one district across the United States.
MATERIAL AND METHODS: We investigated the effects of extreme heat and cold exposures on preterm birth categories defined by the gestational age (GA). A novel location-specific measure of exposure to extreme temperatures during pregnancy is introduced, along with a model-free statistical methodology to assess monotonic trends in preterm birth rates associated with extreme temperatures. We also performed cumulative logistic regression analyses to evaluate the effect of extreme temperatures on preterm birth while controlling for the month of conception and socioeconomic factors. Additionally, we conducted 1st and 2nd trimester-specific analyses with a fetuses-at-risk analysis to understand the effects of chronic heat and cold for different exposure windows.
RESULTS: We discovered a monotonically increasing trend in preterm birth rates with increased chronic heat and cold exposures (p-value < 0.0001). Additionally, when a woman is exposed to as few as 25% days of extreme heat or extreme cold temperatures, the probability of preterm birth rates exceeds CSL baseline preterm birth rates (p-value< 0.0001). Surprisingly, we discovered that pregnant women exposed to chronic cold temperatures were more likely to deliver early preterm (GA < 34 weeks) than those exposed to normal temperatures (Odds-ratio: 5.57, C.I.(5.19, 5.97)).**CONCLUSION**: Both extreme cold and heat were significantly associated with an increased risk of early deliveries, exhibiting a monotonic dose-response relationship.

TO09-02 | Associations between Wildfire Smoke and Pregnancy Health: A Nationwide Study in the United States from 2006 to 2023

Harshita Sharma[1], Muwu Xu[1], Danlu Zhang[2], Jing Nie[1], Tao Xue[3], Yang Liu[2], Meng Wang[1]

[1] The State University of New York at Buffalo, New York, Department of Epidemiology and Environmental Health, School of Public Health and Health Professions, Buffalo, USA; [2] Emory University, Atlanta, Georgia, Gangarosa Department of Environmental Health, Rollins School of Public Health, Atlanta, USA; [3] Peking University Health Science Center, Beijing, China, Institute of Reproductive and Child Health/Ministry of Health Key Laboratory of Reproductive Health and Department of Epidemiology and Biostatistics, Beijing, China

Objective: Wildfires have been increasing in frequency and intensity in the United States. However, the health effects of wildfire smoke during pregnancy have mostly been studied at a regional or local scale, over a long-term period. We aim to examine the associations between wildfire-sourced fine particulate matter (wildfire $PM_{2.5}$) and major adverse pregnancy and birth outcomes in the contiguous US.

Methods: Births records of 65,599,589 singleton deliveries from 2006 to 2023 were obtained across the US counties and were linked to wildfire $PM_{2.5}$ during the entire gestational period. Distributed lag non-linear models were used to examine the lag effects of wildlife exposures on pregnancy outcomes, including preterm birth, low birthweight, admission to neonatal intensive care unit (NICU) outcome, gestational hypertension (GHT) and gestational diabetes (GDM), for all exposure windows, adjusting for urban $PM_{2.5}$ and individual- and area-level confounders.

Results: Wildfire $PM_{2.5}$ contributed to 25% of daily $PM_{2.5}$ concentrations in the US. For preterm birth, NICU, and GDM, strongest associations with wildfire $PM_{2.5}$ (1 $\mu g/m^3$ increment) were observed during the second trimester (highest OR: 1.038, 95% CI: 1.019 - 1.058 for preterm birth, 1.002, 95% CI: 1.000 - 1.004 for NICU, and 1.003, 95% CI: 1.002 - 1.004 for GDM). For GHT, the strongest associations were observed during first trimester (highest OR: 1.002, 95% CI: 1.000 - 1.004). These associations strengthened when restricted to the fire-impacted areas, and among groups with lower education and greenness in the residential county, and high social vulnerability. These associations are stronger than those with urban $PM_{2.5}$.

Conclusion: As the largest study in the US that focused on wildfire $PM_{2.5}$ and pregnancy health, we found strong evidence of associations between wildfire $PM_{2.5}$ exposure and increased risks of preterm births, NICU admissions, GDM and GHT among a significant US population than the effects of urban $PM_{2.5}$.

TO09-04 | Disentangling Socioeconomic Inequality and Future Climate Impacts on Adverse Birth Outcomes in Greater Metropolitan Sydney

Tanya Singh[1,2], Geoffrey Morgan[3,4], Katrin Meissner[1,2], Donna L. Green[5], Edward Jegasothy[3]

[1] University of New South Wales, Climate Change Research Centre, Sydney, Australia; [2] University of New South Wales, ARC Centre of Excellence for Climate Extremes, Sydney, Australia; [3] The University of Sydney, Sydney School of Public Health, Sydney, Australia; [4] Health Environments and Lives (HEAL) National Research Network, Canberra, Australia; [5] University of New South Wales, School of Biological, Earth and Environmental Sciences, Sydney, Australia

Background: Preterm birth and stillbirth represent significant public health concerns, influenced by multifactorial pathways that include socioeconomic and environmental determinants. While climate warming is projected to exacerbate heat-related health risks globally, the relative importance of existing socioeconomic inequalities versus future temperature increases remains understudied for perinatal outcomes.

Methods: We analysed data on nearly 1.18 million live births, including over 62,500 preterm and 6,302 stillbirths in Sydney (2000–2019). Log-binomial regression quantified the relationship between area-level socioeconomic quintiles and birth outcomes relative to the least disadvantaged quintile. Temperature effects were modelled using distributed lag non-linear timeseries models, then applied to climate projections under moderate mitigation (RCP4.5) and minimal mitigation (RCP8.5) scenarios, assuming no changes in temperature related vulnerability and birth rates. Counterfactual scenarios enabled estimation of attributable fractions and numbers for socioeconomic inequality and future warming.

Results: Socioeconomic disadvantage explained up to 13.6% (95% CI:12.0%-15.0%) of all preterm births (8,477 excess cases) and 24.6% (95% CI: 20.2%-29.1%) of all stillbirths (1,518 excess cases) over two decades. In contrast, by 2080-2099, changes in temperature distributions under a high-emissions scenario were projected to produce a net excess of 446 preterm births and 205 stillbirths—substantially lower than the current burden attributable to disadvantage. Nevertheless, stronger climate mitigation (shifting from RCP8.5 to RCP4.5) could avert a meaningful fraction of that excess, particularly for stillbirths, which showed heightened vulnerability to heat. While stillbirths showed stronger relative associations with both determinants, the absolute impact was larger for preterm births due to their higher baseline prevalence.

Conclusions: The current burden from socioeconomic disadvantage substantially exceeds projected climate change impacts on adverse birth outcomes. Addressing persistent inequities now will confer near-term perinatal health benefits while increasing societal resilience to ongoing environmental challenges. However, synergistic policy approaches that both reduce longstanding socioeconomic disparities and mitigate climate change are vital as well.

TO09-05 | The short-term effect of multiple heat indicators on birth outcomes in four countries in Africa and Europe

Lebohang Radebe[1], Chloe Brimicombe[3], Michalis Koureas[4], Federica Nobile[5,6], Mian Bao[7], Paul Lokubal[8], Tobias Monthaler[3], Katharina Wieser [3], Georgios Charvalis[4], Varvara Mouchtouri[4], Christos Hadjichristodoulou[4], Massimo Stafoggia[5,6], Nathalie Roos [7], Cherie Part [9], Shobna Sawry[2], Sibusiso Mkwananzi[2], Ilona M. Otto [3], Debra Jackson[10,11], Stanley Luchters[12,13], Matthew Chersich[1,14], Jeroen de Bont[6], on behalf of the HIGH Horizons Study Group

[1] University of the Witwatersrand, Wits Planetary Health Research Division, Faculty of Health Sciences, Johannesburg, South Africa; [2] University of the Witwatersrand, Wits RHI, Faculty of Health Sciences, Johannesburg, South Africa; [3] University of Graz, Wegener Centre for Climate and Global Change, Graz, Austria; [4] University of Thessaly, Laboratory of Hygiene and Epidemiology, Faculty of Medicine, Larissa, Greece; [5] Lazio Region Health Service /ASL Roma 1, Department of Epidemiology, Rome, Italy; [6] Karolinska Institutet, Institute of Environmental Medicine, Stockholm, Sweden; [7] Karolinska Institutet, Clinical Epidemiology Division, Department of Medicine, Stockholm, Sweden; [8] London School of Hygiene and Tropical Medicine, Department of Infectious Disease Epidemiology and International Health, Faculty of Epidemiology and Population Health, London, UK; [9] London School of Hygiene and Tropical Medicine, Department of Public Health, Environments and Society, Faculty of Public Health and Policy, London, UK; [10] London School of Hygiene and Tropical Medicine, MARCH Centre, London, UK; [11] University of the Western Cape, School of Public Health, Cape Town, South Africa; [12] Centre for Sexual Health and HIV/AIDS Research, Harare, Zimbabwe; [13] Liverpool School of Tropical Medicine, Liverpool, UK; [14] Trinity College Dublin, Public Health and Primary Care, Dublin, Ireland

Objective: Comparing studies worldwide that evaluate the adverse effects of heat exposure on birth outcomes poses multiple challenges, including heterogeneity in study designs. In this study, we assessed the association between multiple heat indicators and various birth outcomes using large, high-quality neonatal data from Africa and Europe, applying a harmonized statistical approach.

Material and Methods: Using regional (Italy, South Africa) and national (Greece, Sweden) birth registry data, we employed a time-stratified case-crossover design to evaluate the association between short-term (lag 0-6 days) heat exposure and neonatal outcomes (preterm birth (PTB) and stillbirth (SB)). We generated country-specific effect estimates and meta-analyzed the results for mean ambient temperature. We further compared the association using four different heat metrics (temperature, WBGT, UTCI, and heat index), and considered socio-demographic factors potential effect modifiers.

Results: From 1999-2022, 3,923,639 births occurred across the four settings [range: 189,669 – 2,316,655]; with 7.8% [range: 5.0%-9.6%] PTBs and 0.4% [range: 0.1%-1.3%] SBs. In meta-analysis, compared to the 50th percentile of mean temperature, the 95th percentile was associated with a 10% increase in odds of PTB [95% CI: 1.04, 1.15; I^2=0%], with all country-specific estimates indicating elevated risk. Inconsistent associations, with both harmful (South Africa and Sweden) and protective (Italy and Greece) effects were observed for SB. All four heat exposure metrics were highly correlated, with similar effect estimates observed. Younger mothers (<25) and female infants were at greater risk of heat-associated adverse birth outcomes.

Conclusion: As one of the largest studies using harmonized statistical approaches, we provide robust estimates for the detrimental effects of heat on PTB across Africa and Europe. Consistent effects across countries and climate zones suggest its relevance as a potential indicator. Urgent action to reduce global greenhouse gas emissions is needed to mitigate the effects of heat on pregnant women.

TO09-06 | Heat Beyond Percentiles: Exploring Preterm Birth Risks in Santiago, Chile Using a Novel Metric

Estela Blanco[1,2], José D. Conejeros[1,3], Alvaro González-Reyes[4], Paola Rubilar[5], Pablo Sarricolea[6,2]

[1] Pontificia Universidad Católica de Chile, College UC y Escuela de Salud Pública, Santiago, Chile; [2] Centro de Ciencia del Clima y la Resiliencia (CR2), Santiago, Chile, Santiago, Chile; [3] Pontificia Universidad Católica de Chile, Escuela de Gobierno, Santiago, Chile; [4] Universidad Austral, Instituto de Ciencias de la Tierra, Facultad de Ciencias, Valdivia, Chile; [5] Universidad del Desarrollo, Centro de Epidemiología y Políticas de Salud, Facultad de Medicina Clínica Alemana, Santiago, Chile; [6] Universidad de Chile, Departamento de Geografía, Santiago, Chile

Objective: We used data from Santiago, Chile to evaluate the relationship between extreme heat in the week and month before birth and risk of preterm birth (<37 weeks' gestation) using exact and relative measures of heat and the excess heat factor (EHF), a metric which incorporates both historical temperature extremes and short-term acclimatization.

Material and Methods: We conducted a population-based cohort design using secondary information from birth records (1991-2020) and limited analysis to births in spring/summer months (n= 969,842). Minimum and maximum daily temperatures were obtained from a statistical model (CR2MET) derived from re-analyses of data from ERA6 and MODIS satellites for the 33 urban municipalities of Santiago. Temperature percentiles and EHF were calculated for each municipality. EHF considers high historical temperatures (>95th percentile) and acclimatization in the previous 30 days. We calculated 15 metrics of heatwaves: 2, 3, and 4 consecutive days of temperatures >= 30 degrees Celsius, >=90th, 95th, and 99th percentile, and EHF for each municipality and assigned exposure (Yes or No) for each metric in the month prior to delivery. Adjusted Cox proportional hazard models were estimated.

Results: Preterm birth occurred in 6% of births. Exposure temperatures >= 30 degrees Celsius were associated with decreased hazards. Exposure heat waves that used relative temperatures, did not relate to increased risk of preterm birth. Exposure to EHF for 2, 3, 4 days was associated with 1.02 (95%CI 1.00-1.03), 1.02 (95%CI 1.00-1.04), and 1.03 (95%CI 1.01-1.05), respectively.

Conclusion: We found evidence of increased risk of preterm birth, but only when evaluating exposure using EHF, potentially underscoring the importance of considering both heat intensity and acclimatization when assessing the impact of extreme temperatures on pregnancy outcomes. In temperate climates, EHF may capture risk of adverse birth outcomes better than absolute or relative measures of extreme temperature.

TO09-07 | Associations between maternal exposure to ambient temperature and adverse birth outcomes, and potential modifying effects of socioeconomic factors in Queensland.

Yohani Dalugoda[1], Dwan Vilcins[2], Darsy Darssan[3], Dung Phung[4]

[1] The University of Queensland, School of Public Health, Brisbane, Australia; [2] The University of Queensland, Child Health Research Centre, Brisbane, Australia; [3] The University of Queensland, School of Public Health, Brisbane, Australia; [4] The University of Queensland, School of Public Health, Brisbane, Australia

Objective: Queensland is the second largest state in Australia and typically experiences high temperatures and extreme heat events. Research on the effects of heat exposure on adverse birth outcomes in Queensland is limited and inconclusive. This study examined relationships between temperatures and adverse birth outcomes and evaluated the modifying effects of socioeconomic factors on temperature-adverse birth outcome relationships in Queensland.

Method: Data were collected from 82 statistical areas in Queensland. Binary logistic regression was applied to investigate relationships between high temperature and adverse birth outcomes, including preterm birth [PTB], low birth weight [LBW], stillbirth, congenital anomaly, and neonatal deaths. A random-effects meta-analysis was utilised to estimate the pooled risk of adverse birth outcomes associated with temperatures, while a random-effects meta-regression was applied to examine the modification effect of socioeconomic factors on the temperature-adverse birth outcome relationships.

Results: For a 1°C increase in average temperature, the risk of PTB increased by 0.6% (95% CI, 0.2–0.9) during the initial 0–8 weeks of gestation, by 0.7% (95% CI, 0.2–1.1) for LBW during the second trimester, by 0.5% (95% CI, 0.2–0.7) for congenital anomalies during the last week of pregnancy, and by 3.4% (95% CI, 0.0–6.8) for neonatal deaths during the third trimester. Stillbirth did not show any significant association with high temperatures for any exposure periods. Meta-regression showed that lower income, Indigenous status, lower education, and unemployment increased the risk of adverse birth outcomes related to high temperatures, while higher income reduced this risk.

Conclusion: Maternal exposure to high temperatures in Queensland significantly associated with the risk of PTB, LBW, stillbirth, congenital anomalies, and neonatal deaths. Our findings offer critical insights regarding the potential impacts of climate change on maternal and newborn populations. Heat health policies and adaptation strategies will require protecting Queensland mothers and newborns from extreme temperatures.

TO10 | Chemical exposures and birth outcomes: Disparities in exposure and susceptibility to metals and endocrine-disrupting chemicals

TO10-01 | Exposure to a large mixture of endocrine disrupting chemicals (EDCs) and anogenital distance across infancy

Maria E. Cinzori[1,2], Susan L. Schantz[3], Rita S. Strakovsky[1,2]

[1] Michigan State University, Department of Food Science and Human Nutrition, East Lansing, USA; [2] Michigan State University, Institute for Integrative Toxicology, East Lansing, USA; [3] University of Illinois Urbana-Champaign, Beckman Institute, Urbana, USA

Objective: Pregnant women are ubiquitously exposed to endocrine disrupting chemicals (EDCs). Anogenital distance (AGD) is a hormonally-mediated marker of reproductive development. Whether EDCs influence AGD across infancy is unclear. Therefore, we evaluated associations of gestational EDC exposure with AGD at birth, in infancy, and the change in AGD from birth to infancy.

Material and Methods: Pregnant Illinois women (n=563) provided up-to-five first-morning urines monthly across pregnancy, which we pooled to measure metabolites of 10 phthalates/replacements, 11 phenols (three bisphenols, four parabens, one benzophenone, two chlorinated phenols, triclosan), and triclocarban. We excluded bisphenol F, butylparaben, and triclocarban from analyses due to poor detection. In newborns and 5-month-olds, AGD_{short} (females: anus-to-fourchette; males: anus-to-scrotum) and AGD_{long} (females: anus-to-clitoris; males: anus-to-penis) were measured and normalized to body length (cm). Change in AGD across infancy was calculated as the difference between 5-month and newborn $AGD_{short-or-long}$. We evaluated associations of a 19-EDC mixture with AGD at birth and 5 months and change in AGD using covariate-adjusted quantile-based g-computation.

Results: The EDC mixture was associated with AGD only in female infants. In females at birth, each 10% increase in the EDC mixture was associated with 4% longer AGD_{short} (95%CI: -1%, 9%), driven by monocarboxynonyl phthalate (MCNP) and monoethyl phthalate. However, at median 5 months, each 10% increase in the mixture was associated with -4% shorter AGD_{short} (95%CI: -8%, 0%), driven by triclosan and sum of di(2-ethylhexyl) terephthalate metabolites; and with -2% shorter AGD_{long} (95%CI: -5%, 0%), driven by MCNP and methylparaben. Finally, each 10% increase in the mixture was associated with -12% smaller change in AGD_{long} from birth to median 5 months (-23%, 1.0%), with triclosan and MCNP driving the mixture effect.

Conclusion: Prenatal EDC exposure may sex-specifically disturb AGD trajectory across infancy. Future studies should investigate implications for child long-term reproductive health.

TO10-03 | High-Resolution Metabolomics of Maternal Polybrominated Diphenyl Ethers (PBDEs) Exposure and Preterm Birth in the Atlanta African American Maternal Child Cohort

Siddhi Desai[1], Anne Dunlop[2], Dana B. Barr[3], Susan S. Hoffman[1], ViLinh Tran[4], Youran Tan[3], Paula Nesbeth[3], Mengyuan Ren[3], Grace Lee[3], Parinya Panuwet[3], Stephanie Eick[3], P. Barry Ryan[3], Dean Jones[4], Donghai Liang[3]

[1] Emory University, Rollins School of Public Health, Department of Epidemiology, Atlanta, USA; [2] Emory University, School of Medicine, Department of Gynecology and Obstetrics, Atlanta, USA; [3] Emory University, Rollins School of Public Health, Gangarosa Department of Environmental Health, Atlanta, USA; [4] Emory University, Pulmonary Division, School of Medicine, Department of Medicine, Atlanta, USA

Objective: Black and African American women disproportionately experience high rates of preterm birth (PTB, <37 weeks gestation) and are at increased risk of exposure to environmental pollutants such as polybrominated diphenyl ethers (PBDEs). While recent studies have found links between exposure to PBDEs and PTB, the underlying biological mechanisms remain unclear. This study aimed to identify metabolomic signatures and pathways associated with PBDE exposure and PTB in a cohort of pregnant African Americans.

Methods: This study included 177 pregnant women from the Atlanta African American Maternal Child Cohort (2014-2019) who delivered either PTB or full-term birth. Six PBDE congeners (47, 99, 100, 85, 154, and 153) were measured via targeted analysis in maternal serum samples collected between 8-14 weeks of gestation. We conducted metabolome-wide association studies (MWAS), and the resulting features underwent pathway analyses and chemical annotation to identify potential metabolites and pathways linking PBDE exposure to PTB via a *meet-in-the-middle* approach.

Results: Among the study participants, 46 (35%) women experienced PTB delivery. Among the 25,516 metabolic features detected, 297 were associated with PTB and at least one of the six PBDEs ($p<0.05$). Pathway analyses revealed three pathways overlapping with PBDEs and PTB: androgen and estrogen metabolism, c21-steroid hormone biosynthesis and metabolism, and steroid hormone biosynthesis. We confirmed the association of 12 metabolites with Level 1 evidence with both PBDEs and PTB, including ω-hydroxydodecanoic acid, trans-cinnamate, thiamine, and decanoylcarnitine. Taken together, alterations in these metabolites and pathways suggest perturbations in hormonal homeostasis and energy metabolism.

Conclusion: This study provided important insights into the metabolic perturbations linking PBDE exposure to PTB in a prospective cohort of pregnant African Americans. Particularly, pathways involving steroid biosynthesis and metabolism may play an important role in mediating the association between PBDE exposure and PTB.

TO10-04 | Interactions Between Per- and Polyfluoroalkyl Substances, Folate Metabolism Gene Variants, and Birth Outcomes: Results from the Taiwan Birth Panel Study

Mei-Huei Chen[1,2], **Chih-Fu Wei**[3,4], Ching-Chun Lin[5], Tzu-Pin Lu[6], Chia-Yang Chen[5], Wu-Shiun Hsieh[2,7], Pau-Chung Chen[8,9]

[1] National Health Research Institutes, Institute of Population Health Sciences, Miaoli, Taiwan; [2] National Taiwan University College of Medicine and Hospital, Department of Pediatrics, Taipei, Taiwan; [3] National Taiwan University Hospital Yunlin Branch, Department of Environmental and Occupational Medicine, Yunlin, Taiwan; [4] National Taiwan University College of Medicine and Hospital, Department of Environmental and Occupational Medicine, Taipei, Taiwan; [5] National Taiwan University College of Public Health, Institute of Environmental and Occupational Health Sciences, Taipei, Taiwan; [6] National Taiwan University, Institute of Health Data Analytics and Statistics, Department of Public Health, College of Public Health, Taipei, Taiwan; [7] Cathay General Hospital, Department of Pediatrics, Taipei, Taiwan; [8] National Health Research Institutes, National Institute of Environmental Health Sciences, Miaoli, Taiwan; [9] National Taiwan University College of Public Health, Department of Public Health, Taipei, Taiwan

Objective

Per- and polyfluoroalkyl substances (PFAS) are associated with adverse birth outcomes, and folate metabolism is crucial for fetal development. However, no study tested the interaction between PFAS exposure and genetic variations in the folate pathway on birth outcomes. The study investigated whether folate metabolism genetic variants modify the association between prenatal PFAS exposure and birth outcomes.

Material and methods

Data from 341 mother-infant pairs in the Taiwan Birth Panel Study were analyzed. Four PFAS concentrations in cord blood were measured using ultra-performance liquid chromatography–tandem mass spectrometry, and folate metabolism-related single nucleotide polymorphisms (SNPs) were genotyped using Taiwan Biobank Array. Interactions were tested with linear regression and mixture models.

Results

Each standard deviation increase in PFOS concentration was associated with 82.5 g reduction. Additionally, PFUA and PFAS mixture exposure was also associated with lower birth weight in the study population. We also observed SNP and individual PFAS and the PFAS mixture interactions on birth weight. Individuals carrying *MTR* rs1805087 AG/GG genotypes showed larger PFOS-related birth weight reductions. The associations between PFAS exposure, birth weight and head circumference varied according to the included SNPs.

Conclusion

These findings suggest that genetic variations in folate metabolism influence the adverse effects of prenatal PFAS exposure on birth outcomes. Understanding these gene-environment interactions may guide targeted interventions to mitigate PFAS-related risks and improve child health outcomes.

TO10-05 | The role of environmental metal exposures on adverse birth outcomes in Jefferson County, Kentucky 2017-2021

Felicia Pugh[2,1], Seyed Karimi[2,1], Nick Peiper[2], Kira Taylor[2], Laura Schummers[3], Natalie DuPré[2]

[1] Louisville Metro Public Health and Wellness, Center for Health Equity, Louisville, USA; [2] University of Louisville, School of Public Health and Information Sciences, Louisville, USA; [3] University of British Columbia, Collaboration for Outcomes Research and Evaluation, Faculty of Pharmaceutical Sciences, Vancouver, Canada

Objective: Compare the incidence of preterm birth (PTB) and small for gestational age (SGA) by ambient heavy metal exposures in Jefferson County, KY.

Material and Methods: We examined birth certificate records from singleton births to self-identified Black or white birthing parents in Jefferson County, KY, from 2017-2021. We used year-specific quartiles of ambient arsenic, cadmium, chromium, and nickel exposure from the US EPA Air Toxics Screening Assessment 2017-2020 to derive a composite exposure score for the birthing parent's address at delivery. We categorized the composite as low (4-7), moderate (8-9), high (10-13), or very high (14-16). We used logistic regression to estimate adjusted odds ratios (ORs) with 95% confidence intervals (CI) for PTB and SGA, adjusting for maternal health variables and socioeconomic status. We assessed effect modification by race and the COVID-19 pandemic using likelihood ratio tests.

Results: In our sample of 30,216 births, we found significant associations between ambient heavy metal composite scores and risk of PTB and SGA. Compared with those with low exposure, those with very high exposure had increased risks of PTB (11.3% vs. 7.7%; OR 1.13, 95% CI 1.00-1.28) and SGA (12.9% vs. 6.3%; OR 1.25, 95% CI 1.10-1.41).

Additionally, we observed a significant interaction between race and exposure scores for PTB: among Black birthing parents, the OR for high exposure was 1.29 (95% CI 1.03-1.60) and 1.36 (95% CI 1.10-1.67) for very high exposure, compared with the low exposure reference group. Among white birthing parents, very high air pollution exposure increased SGA odds by 36% (95% CI 1.14-1.62) compared with the low exposure reference group.

Conclusion: This study found significant associations between ambient heavy metals and adverse birth outcomes. Further research should explore potential mechanisms underlying these associations and interventions that may reduce such exposures to promote equitable birth outcomes.

TO11 | Chemical exposures, epigenetics, and molecular epidemiology

TO11-02 | Prenatal per- and polyfluoroalkyl substances exposures and plasma folate levels on newborn mitochondrial DNA copy number in Boston Birth Cohort

Zeyu Li[1], Xueqi Qu[1], Giehae Choi[2], Jessie P. Buckley[3,2], Xiaobin Wang[1]

[1] Johns Hopkins Bloomberg School of Public Health, Center on the Early Life Origins of Disease, Department of Population, Family and Reproductive Health, Baltimore, USA; [2] Johns Hopkins Bloomberg School of Public Health, Department of Environmental Health and Engineering, Baltimore, USA; [3] University of North Carolina at Chapel Hill, Department of Epidemiology, Chapel Hill, USA

Objective: Mitochondrial DNA copy number (mtDNAcn) is a marker of mitochondrial dysfunction, a potential mechanistic pathway for health effects of prenatal per- and polyfluoroalkyl substances (PFAS) exposures. Folate, essential for DNA synthesis and repair, may have protective effects on PFAS-mtDNAcn relations. We estimated PFAS-mtDNAcn associations and assessed folate as a modifier.

Material and Methods: We included 572 mother-newborn dyads from the Boston Birth Cohort, a predominantly Black and Hispanic population. We measured eight PFAS in maternal plasma collected at delivery using HPLC-MS/MS and mtDNAcn in cord blood using a targeted sequencing approach (STAMP). We used adjusted linear regression and Bayesian kernel machine regression to estimate associations of individual PFAS and PFAS mixture with mtDNAcn Z-score, overall and stratified by newborn's sex and maternal plasma folate level (dichotomized by median).

Results: Participants were 54% non-Hispanic Black and 26% Hispanic. We observed inverted U-shaped relations of Me-PFOSA-AcOH, PFDeA, PFNA, and PFAS mixture with mtDNAcn. For example, compared to newborns with maternal PFDeA in Tertile(T)1, mtDNAcn was higher among those in T2 (β = 0.26, 95%CI: 0.06, 0.46) but not T3 (β = 0.10, 95%CI: -0.10, 0.30). When stratified by newborn's sex, the inverted U-shaped relations of PFDeA and PFNA remained only in males. When stratified by maternal folate, the inverted U-shaped relations of Me-PFOSA-AcOH and PFDeA remained only in newborns with low maternal folate levels. PFOS was monotonically associated with lower mtDNAcn (β_{T2} = -0.11, 95%CI: -0.31, 0.09; β_{T3} = -0.25, 95%CI: -0.45, -0.05), with stronger associations in females (β_{T3} = -0.35) and newborns with low maternal folate levels (β_{T3} = -0.48), consistent with the inverted U-shaped relations for other PFAS given PFOS had the highest levels.

Conclusion: Inverted U-shaped relations of certain PFAS and PFAS mixture with mtDNAcn differed by newborn's sex and were attenuated by adequate maternal folate levels.

TO11-03 | Leukocyte DNA methylation signatures of arsenic metabolites and metabolism efficiency: Epigenome-wide associations in the Strong Heart Study

Anne Bozack[1], Katarzyna E. Sokolowska[2], Arce Domingo-Relloso[3], Shelly Cole[4], Marcia O'Leary[5], Rae O'Leary[5], Tauqeer Ali[6], Ying Zhang[6], Jason Umans[7,8], Ronald Glabonjat[9], Walter Goessler[10], Brandon Pierce[11], John Witte[1], Andres Cardenas[1], Ana Navas-Acien[9]

[1] Stanford University, Department of Epidemiology and Population Health, Stanford, USA; [2] Pomeranian Medical University, Independent Clinical Epigenetics Laboratory, Szczecin, Poland; [3] Columbia University, Department of Biostatistics, Mailman School of Public Health, New York, USA; [4] Texas Biomedical Research Institute, San Antonio, USA; [5] Missouri Breaks Industries Research, Inc., Missouri Breaks Industries Research, Inc., Eagle Butte, USA; [6] University of Oklahoma Health Sciences, Department of Biostatistics & Epidemiology, Center for American Indian Health Research, Hudson College Public Health, Oklahoma City, USA; [7] MedStar Health Research Institute, Washington, DC, USA; [8] Georgetown/Howard Universities, Center for Clinical and Translational Sciences, Washington, DC, USA; [9] Columbia University, Department of Environmental Health Sciences, Mailman School of Public Health, New York, USA; [10] University of Graz, Institute of Chemistry, Graz, Austria; [11] Department of Public Health Sciences, University of Chicago, Chicago, USA

Objective: Liver methylation of inorganic arsenic (iAs) by arsenic 3 methyltransferase (AS3MT) decreases toxicity and facilitates urinary excretion, a process that generates monomethyl (MMA)- and dimethyl (DMA)-arsenic species. This study investigated epigenetic signatures of arsenic metabolite concentrations and metabolism efficiency.
Materials and Methods: The Strong Heart Study is a prospective cohort of American Indian adults with low-to-moderate drinking water arsenic exposure (N=2,133). Leukocyte DNA methylation was measured using the Illumina MethylationEPIC BeadChip; arsenic metabolite concentrations were measured using HPLC-ICP-MS. Arsenic metabolism efficiency was represented by the proportion of DMA (DMA%). We tested differentially methylated positions (DMPs) using robust linear models adjusted for total arsenic concentration, demographics, BMI, smoking, study center, cell-type proportions, genetic principal components, and estimated glomerular filtration rate.
Results: Concentrations of iAs, MMA, and DMA were associated with 8, 1, and 12 DMPs (p_{FDR}<0.05), respectively. DMA% was associated with 24 DMPs ($p_{Bonferroni}$<0.05), with 22 DMPs located within a 900kb region of chromosome 10 including *AS3MT*, *CNNM2*, *NT5C2*, *C10orf32*, *WBP1L*, *SUFU*, *C10orf95*, and *PITX3*. The top DMP was in the gene body of *AS3MT* and explained 8.20% of variation in DMA%. Three iAs-associated DMPs and 7 DMA-associated DMPs have previously been associated with total arsenic concentrations in the Strong Heart Study. However, 3 of the iAs-associated DMPs and the MMA-associated DMP were mapped to *AS3MT* and *CNNM2* and were also associated with DMA%.
Conclusion: DNA methylation in chromosome 10, including *AS3MT*, was associated with arsenic metabolite concentrations and arsenic metabolism, an important pathway for detoxification and excretion. This epigenetic signature may be used as a biomarker of arsenic metabolism efficiency in the absence of arsenic speciation as well as to estimate health risk. Future research will investigate persistence of these associations in other arsenic-exposed populations. This work was funded by the NIH grant K99ES035109 and grants listed under: https://strongheartstudy.org/Research/Papers-and-Abstracts/Funding-Acknowledgement.

TO11-04 | Glyphosate use and mosaic loss of chromosome Y: Investigations in the Agricultural Health Study

Vicky C. Chang[1], Mitchell J. Machiela[2], Weiyin Zhou[3], Lauren M. Hurwitz[1], Gabriella Andreotti[1], Christine G. Parks[4], Dale P. Sandler[4], Nathaniel Rothman[1], Stella Koutros[1], Laura E. Beane Freeman[1], Sonja I. Berndt[1], Jonathan N. Hofmann[1]

[1] National Cancer Institute, Division of Cancer Epidemiology and Genetics, Occupational and Environmental Epidemiology Branch, Rockville, USA; [2] National Cancer Institute, Division of Cancer Epidemiology and Genetics, Integrative Tumor Epidemiology Branch, Rockville, USA; [3] National Cancer Institute, Division of Cancer Epidemiology and Genetics, Cancer Genomics Research Laboratory, Rockville, USA; [4] National Institute of Environmental Health Sciences, Epidemiology Branch, Durham, USA

Objective: Glyphosate, the most widely applied herbicide worldwide, has been linked to certain hematologic malignancies; however, the underlying biological mechanisms remain unclear. To investigate the association between lifetime glyphosate use and mosaic loss of chromosome Y (mLOY), a potential biomarker of genomic damage or instability, we conducted two investigations in the Biomarkers of Exposure and Effect in Agriculture (BEEA) study and its parent study, the Agricultural Health Study (AHS), a large cohort of pesticide applicators in Iowa and North Carolina.
Material and Methods: Using blood samples from 1,606 male pesticide applicators from BEEA and buccal samples from 1,868 male pesticide applicators in the AHS (with no overlapping participants), we assessed the presence of mLOY (and fraction of affected cells) using genotyping array intensity data. Information on lifetime use of glyphosate prior to biospecimen collection was derived from questionnaires. Multivariable logistic regression was used to estimate associations of glyphosate use with any mLOY and high cell fraction mLOY, adjusted for potential confounders (e.g., age, smoking).
Results: Increasing lifetime use of glyphosate was associated with having high cell fraction mLOY in blood samples of BEEA participants (e.g., highest vs. lowest quartile of lifetime days of use: OR=1.75; 95%CI=1.00-3.07; P_{trend}=0.03). Similar results were observed in buccal samples of participants from the separate AHS study (OR=1.76; 95%CI=1.04-2.99; P_{trend}=0.06). In both investigations, the positive association between glyphosate use and high cell fraction mLOY was more apparent among older men (≥70 years) and never smokers; however, tests of interaction were not statistically significant.Conclusion: Results from these investigations in BEEA and the AHS using different sample types suggest that high lifetime use of glyphosate may be associated with mLOY affecting higher cell fractions (indicative of greater expansion of mutated clones). Together, these findings can help inform our understanding of the potential role of glyphosate in carcinogenesis.

Sources of funding: This work was supported by the Intramural Research Program of the National Institutes of Health, National Cancer Institute (Z01 CP 010119) and the National Institute of Environmental Health Sciences (Z01 ES 049030).

TO11-05 | Linking Occupational and Environmental Metal Exposure to Genotoxicity and Haematological Changes in Nigerian Bronze Casters: Biomarker Trends and Health Implications

Osaretin G. Igharo[1], Isoken H. Igbinosa[2], Bibiana O. Eiya[3], James O. Osakue[1], Edouwaye L. Igharo[1], Joan O. Osunbor[1], Iroghama A. Aruomaren[1], Christopher F. Harrington[8], Humphrey B. Osadolor[1], John I. Anetor[10], On behalf of BIOTOXCS Bronze Casters' Research Collaboration

[1] University of Benin, Medical Laboratory Science, Benin City, Nigeria; [2] University of Benin, Environmental Management and Toxicology, Benin City, Nigeria; [3] University of Benin, Physiology, Benin City, Nigeria; [4] University of Benin, Medical Laboratory Science, Benin City, Nigeria; [5] University of Benin, Medical Laboratory Science, Benin City, Nigeria; [6] University of Benin, Medical Laboratory Science, Benin City, Nigeria; [7] University of Benin, Medical Laboratory Science, Benin City, Nigeria; [8] Royal Surrey County Hospital NHS Trust, Pathology Services, Guildford, UK; [9] University of Benin, Medical Laboratory Science, Benin City, Nigeria; [10] University of Ibadan, Chemical Pathology, Ibadan, Nigeria

Nigerian bronze casters are routinely exposed to hazardous metals through traditional casting practices, particularly in underregulated environments, posing potential environmental and public health risk. While toxic metal exposure is widely recognized, its link to genotoxicity, immune suppression and haematological alterations in this population remains underexplored. This study investigates toxic metal exposure, associated biomarker changes, and potential health risks in in Nigerian bronze casters. A cross-sectional study was conducted with 200 participants from Benin City, Nigeria, comprising 100 Benin Bronze Casters (BBC), 40 environmentally exposed individuals (EC), and 60 unexposed participants (UP). Blood samples were analyzed for toxic metal levels using Inductively Coupled Plasma Mass Spectrometry (ICP-MS). Enzyme-Linked Immunosorbent Assay (ELISA) was employed to assess biomarkers such as mutant p53 (m-p53), 8-hydroxy-2'-deoxyguanosine (8-OHdG), DNA methyltransferase-1 (DNMT-1), and Siglec-3 (CD33), while haematological indices were analyzed with a 3-part differential analyzer. Bronze casters exhibited significantly elevated blood levels of cadmium, lead, mercury, and arsenic compared to controls. Genotoxic biomarker m-p53 was upregulated in BBC and EC, while DNA methyltransferase-1 (DNMT-1) activity was reduced. Elevated oxidative DNA damage marker 8-OHdG and altered haematological indices suggested potential haematotoxicity. Siglec-3 (CD33) expression was notably higher in BBC (340.20±19.35 pg/mL) compared to UP (233.00±14.63 pg/mL), alongside significantly reduced WBC and lymphocyte counts. These findings reveal a heightened body burden of toxic metals in Nigerian bronze casters, with evidence of genotoxic, epigenotoxic, and haematological risks. Of particular note is the elevated CD33 levels, potentially pointing to inflammatory stress and immune dysfunction risk. This study underscores the need for enhanced environmental and occupational safety measures, biomonitoring protocols, and public health interventions to mitigate these risks. This may enhance the sustainability and economic viability of the historic bronze casting vocation in Nigeria.

TO12 | Air pollution and extreme heat around the world

TO12-01 | Mitigating Acute Respiratory Infections in Children by Meeting World Health Organisation Nitrogen Dioxide Interim Targets in India

Neha Singh[1,2], Sagnik Dey[1,7], Joe V. Buskirk[3,4], Nimish Singh[5], Luke D. Knibbs[3,4]

[1] Indian Institute of Technology Delhi, Centre for Atmospheric Sciences, New Delhi, India; [2] Indian Institute of Technology Delhi, UQIDRA, New Delhi, India; [3] The University of Sydney, Faculty of Medicine and Health, School of Public Health, NSW, Australia; [4] Sydney Local Health District, Public Health Research Analytics and Methods for Evidence, Public Health Unit, NSW, Australia; [5] The Energy and Resource Institute, Earth Science & Climate Change Division, New Delhi, India; [6] Indian Institute of Technology Delhi, Arun Duggal Centre of Excellence for Research in Climate Change and Air Pollution, New Delhi, India; [7] Korea University, The Department of Health Policy and Management, Seoul, South Korea

Objective: While most air pollution epidemiological studies in India focus on fine particulate matter ($PM_{2.5}$), global evidence highlights significant health effects of nitrogen dioxide (NO_2), particularly in children. This study aimed to quantify the burden of acute respiratory infection (ARI) in children below five (B5) attributable to ambient NO_2 and sectoral oxides of nitrogen (NO_x) exposures. It also evaluated the potential health benefits of India achieving the World Health Organization (WHO) interim targets (ITs) and Air Quality Guidelines (AQG) for NO_2.
Methods: Outcome and confounder data were sourced from the Indian National Family Health Survey (NFHS-4, 2015–16). NO_2 concentrations were derived from a satellite-based land-use regression model. Sectoral contributions to annual NO_x were estimated using the subtraction method by comparing simulations with and without sector-specific emissions. Cross-sectional associations between annual NO_2 and sectoral NO_x with ARI in B5 children were analyzed using multivariate logistic regression, adjusting for maternal, child, and household covariates. Stratified analysis by place of residence was conducted for NO_2.
Results: ARI prevalence was 2.82%. A 10-μg m^{-3} increase in NO_2 was associated with greater odds of ARI (OR: 1.26; 95% CI: 1.19–1.33) with a stronger effect in rural (1.47; 1.4–1.6) than urban areas (1.08; 0.98–1.19). Residential NO_x had the highest association with a 23% (CI: 18%–28%) increase in ARI prevalence per interquartile range, followed by power, other, agricultural burning, road dust, and transportation sectors. The district-level analysis estimated a 7.1% reduction in national ARI prevalence if all districts met their nearest WHO ITs and 14.3% if they met WHO AQG.
Conclusion: Our results suggested achieving sector-specific emissions reductions is crucial, as the National Clean Air Programme (NCAP) for $PM_{2.5}$ alone is insufficient. These findings support efforts to meet Sustainable Development Goal (SDG) 3.2, which aims to eliminate preventable neonatal and B5 child mortality by 2030.

TO12-02 | Spatial Analysis of Heat Exposure, Cardiovascular Disease, and Social Determinants of Health in New Orleans

Sarah Yozzo, Martino Pepe, Marco Matteo Ciccone

Università degli studi di Bari Aldo Moro, Bari, Italy

Background: Urban heat islands (UHIs) exacerbate cardiovascular disease (CVD) risk, disproportionately affecting socioeconomically disadvantaged communities. This study investigates the spatial correlation between heat exposure, cardiovascular disease prevalence, and social determinants of health in New Orleans, a city with significant environmental and racial disparities.

Methods: Using spatial epidemiology and remote sensing, this study develops two novel indices: the Census Tract Heat Exposure Index (ctHEI) derived from Landsat 9 satellite imagery and the Cardiovascular Disease Prevalence Index (CVDPI) based on 2024 CDC PLACES data. Bivariate Local Indicators of Spatial Association (BiLISA) analysis identified geographic hotspots where high heat exposure and CVD burden overlap. Statistical comparisons using the Mann-Whitney U test and logistic regression assessed socioeconomic and health disparities between hotspot and non-hotspot census tracts.

Results: Analysis revealed 23 high-risk clusters where ctHEI and CVDPI were significantly elevated. Hotspot areas exhibited lower median income ($36,905 vs. $61,258, $p < 0.0001$), higher uninsured rates (14.66% vs. 9.39%, $p < 0.0001$), and greater prevalence of insufficient sleep, distress, and physical inactivity ($p < 0.01$ for all). African American residents were overrepresented in hotspot tracts (65.3% vs. 50.8%), though initial statistical significance was masked by flood-prone neighborhoods with paradoxically lower surface temperatures.

Conclusion: Findings highlight systemic environmental and health inequities, emphasizing the need for targeted urban planning and public health interventions to mitigate heat-related cardiovascular risks. Addressing these disparities requires a multidisciplinary approach integrating climate resilience, healthcare access, and social justice initiatives.

TO12-04 | The Impact of Geomagnetic Disturbances and Air Pollution on Mortality in South Korea

Ji Soo Kim[1], Carolina Leticia Zilli Vieira[2], Petros Koutrakis[2], Kiyoung Lee[1,3]

[1] Seoul National University, Department of Environmental Health Sciences, Graduate School of Public Health, Seoul, South Korea; [2] Harvard T. H. Chan School of Public Health, Department of Environmental Health, Boston, USA; [3] Seoul National University, Institute of Health and Environment, Seoul, South Korea

Geomagnetic disturbances (GMD), quantified by the Kp index, represent fluctuations in Earth's magnetic field caused by solar activity. Previous studies have identified GMD as a potential environmental factor associated with increased risks of cardiovascular diseases (CVD) and total mortality. This study aims to investigate the impact of GMD on total, CVD, stroke, and myocardial infarction (MI) mortality in Korea from 2001 to 2019. Daily mortality data from 237 districts were obtained from Statistics Korea; Kp index and sunspot number from NASA; PM_{10} and temperature from air quality monitoring stations and the Korea Meteorological Administration. In the first stage, a Generalized Additive Model (GAM) was applied using the daily Kp index to assess the impact of GMD on mortality, with additional analyses incorporating 3-day, 7-day, and 28-day moving averages to evaluate lagged effects. Temporal trends and temperature were included as confounders for adjustment. A sensitivity analysis examined whether PM_{10} modified the effect of the Kp index on daily mortality. Second, a mixed-effects meta-analysis estimated overall seasonal mortality risks, accounting for spatial heterogeneity across districts. A one standard deviation rise in the z-score Kp index was significantly associated with increased total mortality across all seasons, with the strongest effects in spring (0.33%) and autumn (0.32%), followed by winter (0.29%) and summer (0.25%). The results were consistently significant when replacing the daily Kp index with the moving averages. Results also remained stable after adjusting for PM_{10}. Based on the daily Kp index, CVD deaths significantly increased in all seasons except summer, stroke in all except winter, and MI only in winter. These seasonal effects might result from stronger GMD in spring and autumn, driven by Sun-Earth positional changes. This study determined the independent effect of GMD on mortality in Korea. The findings provide valuable insights into the public health implications of GMD.

TO12-05 | Compound Urban Infrastructure Deficiency and Extreme Heat in the Mediterranean Region

Tzu-Hsin Karen Chen[1,2], Joan Casey[2]

[1] University of Washington, Urban Design and Planning, Seattle, USA; [2] University of Washington, Environmental and Occupational Health Sciences, Seattle, USA

Objective

The Mediterranean coast is warming at a rate 25% faster than the global average. This rapid climate change has heightened heat exposure for local populations, particularly in areas experiencing urban expansion without adequate electricity infrastructure. This study identifies areas of increased heat and hotspots of electricity deficiencies across the Mediterranean region.

Material and Methods

Annual mean land surface temperature from June 15 to September 15 over 2012-2020, along with the annual duration of extreme heat—defined as the number of days exceeding the 90th percentile of historical local temperatures—was extracted for the Mediterranean region (N = 7,495,152 for 1 km × 1 km grid cells) using MODIS satellite imagery. Annual urban area and nighttime luminosity were measured using the Global Human Settlements Layer and Visible Infrared Imaging Radiometer Suite (VIIRS) composite, respectively. Urban electricity deficiency was identified by regressing urban area and nightlight signals on year for each cell; a significant increase in urban area without a corresponding increase in nightlight signals ($p < 0.05$) indicated electricity deficiency.

Results

The rate of temperature increase was predominantly higher in North Africa, Spain, Italy, and Jordan, ranging from 0.5°C to 1.5°C from 2012 to 2020. In contrast, temperature decreases were primarily detected in Turkey. The duration of extreme heat followed similar spatial patterns. Among urban areas experiencing significant increased duration of extreme heat, Egypt and Libya had the highest percentage of urban expansion with electricity deficiency, at 9% (46 km^2) and 8% (5 km^2), respectively, followed by Spain (1%) and other Mediterranean countries (below 1%).

Conclusions

Results show heterogeneity of compound risks from warming and urbanization across Mediterranean countries, with Northeast African nations experiencing the most extensive areas of combined risk. This multi-sensor satellite imagery analysis provides a novel and replicable method for identifying these risks and investigating regional disparities.

TO13 | Sounding the alarm: Monitoring and disentangling the health impacts of noise pollution

TO13-01 | Residential exposure to aircraft noise and depression risk in a US prospective cohort

Stephanie Grady[1], Unnati Mehta[2], Li Yi[2,3], Olivia I. Okereke[4], Jaime E. Hart[2], Francine Laden[5], Charlotte Roscoe[6], Jean Costello[1], Matthew Bozigar[7], Eric A. Whitsel[8], Joel D. Kaufman[9], Jonathan I. Levy[1], Junenette L. Peters[1]

[1] Boston University School of Public Health, Department of Environmental Health, Boston, USA; [2] Brigham and Women's Hospital and Harvard Medical School, Channing Division of Network Medicine, Department of Medicine; Harvard T.H. Chan School of Public Health, Department of Environmental Health, Boston, USA; [3] Harvard T.H. Chan School of Public Health, Department of Nutrition; Harvard Medical School and Harvard Pilgrim Health Care Institute, Department of Population Medicine, Boston, USA; [4] Brigham and Women's Hospital and Harvard Medical School, Channing Division of Network Medicine, Department of Medicine; Harvard T.H. Chan School of Public Health, Department of Epidemiology; Massachusetts General Hospital and Harvard Medical School, Department of Psychiatry, Boston, USA; [5] Brigham and Women's Hospital and Harvard Medical School, Channing Division of Network Medicine, Department of Medicine; Harvard T.H. Chan School of Public Health, Departments of Environmental Health & Epidemiology, Boston, USA; [6] Oregon Health and Science University–Portland State University School of Public Health, Department of Environmental Systems and Human Health, Portland, USA; [7] Oregon State University School of Nutrition and Public Health, College of Health, Corvallis, USA; [8] University of North Carolina at Chapel Hill, Gillings School of Global Public Health Department of Epidemiology, Department of Medicine, Chapel Hill, USA; [9] University of Washington, Departments of Environmental and Occupational Health Sciences, Epidemiology, and Medicine, Seattle, USA

OBJECTIVE: Evidence of associations between aircraft noise and mental health, such as depression, is mixed, with limited research in the US. Our objective was to investigate the association between residential exposure to aircraft noise and incident depression in a US cohort.

METHODS: We leveraged data from Nurses' Health Study II, a nationwide prospective cohort of female nurses. Participants were followed from 2001-2017 through biennial questionnaires on their health and medications. We defined incident depression as self-reported physician/clinician-diagnosed depression *or* self-reported regular use of anti-depressants. Given the distribution of noise exposure within our cohort, we dichotomized day-night average sound levels surrounding 90 airports ≥45 A-weighted decibels (dBA), for the nearest questionnaire year. We fit time-varying Cox proportional hazards models stratified by age and time-period and adjusted for a number of covariates, including race, marital status, spouse's education level, menopausal status, living arrangement, neighborhood socioeconomic status, region of residence, population density, and air pollution.

RESULTS: The sample included 18,705 participants free of prevalent depression at baseline and living within a 35.7 km-radius (maximum extent of noise contours) of an airport. The median age was 54 years (range 36.5-72.6 years), and 93% identified as white. The total person-time was 253,180 person-years, and depression incidence was 17%. Overall, 15% of participants were exposed to noise ≥45 dBA over follow-up. In models adjusting for age and time-period, the hazard ratio (HR) of developing depression was 1.00 (95%CI: 0.91, 1.10) comparing ≥45 dBA to <45 dBA. After further adjustment, the HR was 1.03 (95%CI: 0.93, 1.13).

CONCLUSION: In this sample of female nurses without depression at baseline, residential exposure to aircraft noise was not associated with incident depression. Further exploration of associations by age and co-exposure with green space could provide insight into complex relationships between noise and depression in mid-life and older populations.

TO13-02 | Health effects of exposure to railway noise and ground-borne vibration in the Netherlands

Jurriaan Hoekstra, Elise van Kempen, Sendrick Simon, Arnoud Kok, Irene van Kamp

Dutch National Institute for Public Health and the Environment (RIVM), Bilthoven, Netherlands

Objective: People living near railway lines are exposed to environmental stressors produced by railway traffic, especially noise and ground-borne vibration. The effects of these exposures are understudied and therefore there is limited information on their health impact. This study examined the associations between self-reported health outcomes and indicators of environmental exposure to railway traffic.

Material and Methods: Data was obtained from a cross-sectional survey study performed among people living within 300m of a railway line in The Netherlands in 2021. Three different exposure indicators of railway traffic were used: the maximal ground-borne vibration level (measured in Vmax), average nighttime noise of railway traffic (Lnight) and distance to the nearest railway line. The impact of these exposures on high sleep disturbance (HSD), sleep quality score and self-perceived general health (SPGH) was investigated using logistic regression models corrected for age, gender, education and level of urbanization.

Results: Within the final study population (n = 4.339), there was a strong association between all three exposure indicators and HSD. Similar results were seen for poor sleep quality. For SPGH, we found a significant association between vmax and poor SPGH in both the continuous (OR=1.13 per 1 unit of Vmax 95%-CI 1.03-1.23) and categorical logistic regression model. There was no effect of Lnight on SPGH status but persons living within 25m of railway line were more likely to have poor SPGH status (OR=1.40, 95%-CI 1.00-1.96) compared to persons living 100-300m. The continuous model for distance did not show a significant effect.

Conclusions: Environmental exposures from railway traffic are associated with multiple negative health outcomes and should be taken into account by decision-makers in the domain of public health and spatial planning. The outcomes also have implications in the context of environmental justice, as predominantly lower-income groups are exposed to railway noise and ground-borne vibrations.

TO13-03 | Improving noise pollution surveillance in the United States

Lauren M. Smith, **Abas Shkembi**, Richard L. Neitzel

University of Michigan School of Public Health, Environmental Health Sciences, Ann Arbor, USA

Objective:
Understanding of noise pollution and its distribution by source in the United States is limited. Our objective is to showcase improved noise exposure assessment across three domains: (i) personal, 24-hr, repeated noise exposures; (ii) geographically fine-scale transportation noise pollution; and (iii) noise pollution accumulated from both occupational and transportation sources.
Material and Methods:
First, we analyzed >30 million person-days of personal, 24-hr average LAeq noise measurements from >110,000 consented participants in the nationwide Apple Hearing Study (AHS), funded by Apple Inc. Second, we piloted new mobile noise monitoring methods in a local transportation study near a stretch of I-375 freeway in Detroit, Michigan, USA, funded by the University of Michigan School of Public Health IDEAs. Third (unfunded), we quantified cumulative noise exposure (occupational and transportation) at the census tract level using publicly-available, secondary data sources (Noise JEM and DOT National Transportation Noise Map).
Results:
A model trained on AHS data (currently under review for publication) estimated 34% of US adults (88 million; 95% CI: 81-95) are consistently overexposed to noise (24-hr LAeq >70 dB, per WHO). Mobile monitoring around I-375 showed median 10-second noise levels (equivalent to 45 meters) of 58 dBA, ranging 45-82 dBA. At the census-tract level an estimated 46.5 million (44.2-48.7) Americans are exposed to transportation-related noise (>55 dBA over 24 hours) and an estimated 21.8 million (20.0-23.7) are exposed to workplace-related noise pollution (>85 dBA over 8 hours). Higher proportions of racial and ethnic minority individuals were associated with significantly higher odds of combined transportation and workplace noise exposure.
Conclusion:
Our models estimate tens of millions of Americans are exposed to excessive noise pollution. There is substantial spatial, temporal variability and variability in noise source. Our results show that noise pollution is ubiquitous and improved methods to quantify and better understand noise are important.

TO13-04 | Associations between Aircraft Noise and Cardiovascular Inpatient Admissions and Deaths: Evidence from the UK Biobank Cohort near Four Major Airports

Kritika Anand[1,2], Xiangpu Gong[1], Katie Eminson[1,2], Constantin-Cristian Topriceanu[3,4], Garyfallos Konstantinoudis[5,6], Gabriella Captur[3,4], Yutong S. Cai[1,2], Calvin Jephcote[1], Kathryn Adams[1], John Gulliver[7], Marta Blangiardo[8], Anna L. Hansell[1,2]

[1] University of Leicester, Centre for Environmental Health and Sustainability, Leicester, UK; [2] Leicester General Hospital, National Institute for Health Research (NIHR) Leicester Biomedical Research Centre (BRC), Leicester, UK; [3] University College London, UCL Institute of Cardiovascular Science, London, UK; [4] University College London, UCL MRC Unit for Lifelong Health and Ageing, London, UK; [5] Imperial College London, Grantham Institute for Climate Change and the Environment, London, UK; [6] Imperial College London, UK Small Area Health Statistics Unit, Dept Epidemiology and Biostatistics, London, UK; [7] University of London, Population Health Research Institute, City St George's, London, UK; [8] Imperial College London, MRC Centre for Environment and Health, Department of Epidemiology and Biostatistics, London, UK

Objective: Aircraft noise has been associated with cardiovascular health, but evidence from population-based cohorts remains limited. This study explored associations of aircraft noise with all-cause, cardiovascular disease (CVD, ICD-10 I) and ischemic heart disease (IHD,ICD-10 I20-25) mortality and hospitalizations, in UK Biobank participants near four major UK airports: Heathrow, Gatwick, Manchester, and Birmingham.

Methodology: We used a subset of 105,751 cohort participants residing in one of 44 districts partially or wholly within Civil Authority Aviation (CAA) weighted 24-hour day-evening-night (L_{den}) airport noise contours. CAA aircraft noise data, estimated using the ANCON noise model, at residential postcodes for 2011 for L_{den} and night-time (L_{night}) were categorized into 5 dB bands, with <45 dB (L_{night}) and <50 dB (L_{den}) as reference categories. Cox regression models investigated associations with incident primary hospital admissions and deaths (2011-2021), controlling for individual and area-level confounders and clustering variance by airport.

Results: Participants had a mean recruitment age of 56 years [Standard Deviation (SD):8.2], with 54.3% female. A total of 1,404 (1.3%) and 4,153 (3.9%) participants were exposed to the highest noise category of L_{night} (>55 dB) and L_{den} (>60 dB), respectively. Those living in areas with highest level of aircraft noise had significant increased risks of all-cause mortality and hospital admissions for CVD and IHD compared to reference categories and positive but non-significant associations with CVD mortality. The strongest associations were observed for IHD mortality with L_{den} [Hazard Ratio (HR): 1.43, 95% Confidence Interval (CI): 1.18–1.73] and IHD hospital admissions with L_{night} [1.46 (1.41–1.51)].

Conclusion: Our study suggests a detrimental impact of aircraft noise on all-cause mortality and cardiovascular disease, offering insights from a large cohort study with individual-level data. Previous experimental and epidemiological research support a potential causal association, with biological plausibility via stress response, autonomic dysfunction, and vascular inflammation.

TO13-05 | Untargeted Metabolomics Reveals the Impact of Air and Noise Pollution on Chronic Disorders: Insights from the URBANOME – Greece Cohort

Nafsika Papaioannou[1,2], Anthoula Chatzimpaloglou[1,2], Catherine Gabriel[1,2], Thanasis Papageorgiou[1,2], Dayna Schulz[1,2], Fotini Nikiforou[1,2], Ilias Frydas[1,2], Marianthi Kermenidou[1,2], Eleni Feleki[1,2], Achilleas Karakoltzidis[1,2], Spyros Karakitsios[1,2], Dimosthenis Sarigiannis[1,2]

[1] Aristotle University of Thessaloniki, Department of Chemical Engineering, Environmental Engineering Laboratory, Thessaloniki, Greece; [2] Center for Interdisciplinary Research and Innovation, HERACLES Research Center on the Exposome and Health, Thessaloniki, Greece; [3] National Hellenic Research Foundation, Athens, Greece; [4] University School of Advanced Study IUSS, Pavia, Italy

Objectives

URBANOME aims to establish a unified EU framework for evaluating multi-sector urban policies that align with the WHO's "Health in All Policies" approach. Citizen involvement in co-creating the URBANOME prototype and data acquisition process led to a large-scale cohort, which will enable the identification of causal links between pollution exposure and vulnerability due to chronic conditions using high-dimensional biology approaches.

Materials and Methods

In total, 1200 adults from targeted areas of Thessaloniki metropolitan area were recruited during Phase I of the URBANOME cohort. Data was collected via standardized questionnaires and non-invasive respiratory-focused medical examinations. After data assessment in terms of risk, a group of participants (n=246) advanced to Phase II, which included individual interventions, physical exercising, medical examinations, exposure to air and noise pollution, repetition of standardized surveys and biological sample collection, pre and post urban interventions. Here we focus on untargeted metabolomics analysis of pre-intervention plasma samples using an an Agilent 6540 UHD Accurate-Mass QTOF LC–MS. A Reversed Phase column was used in both positive and negative ionization modes. Data preprocessing, batch effect correction, and metabolite annotation were performed in R.

Results

A total of 3,864 unique metabolites were identified, primarily glycerophospholipids (18.57%), glycerolipids (11.22%), fatty acyls (8.59%), carboxylic acids and derivatives (6.46%), and sphingolipids (5.76%). Key annotated biomarkers linked to oxidative stress, inflammation, and cardiometabolic disturbances exacerbated by air pollution and noise exposure included leukotrienes (A4, C5, D4, F4), citrate, and linoleic acid.

Conclusion

The identified metabolites are central to pathways that, when disrupted by environmental stressors like air pollution and noise, contribute to adverse health effects. Understanding and characterizing these associations is critical for developing strategies to mitigate health risks linked to urban environment.

Acknowledgments

The URBANOME Project has received funding from the European Union's Horizon 2020 programme under grant agreement No 945391.

TO14 | Greenspace and health around the world

TO14-01 | Associations between tree canopy configuration and under-5 mortality in 59 low-income and middle-income countries

Dengkai Chi[1], Daniel Richards[2], Jun Yang[3], Gabriele Manoli[4], Xing Zheng[5], Brenda Lin[6], Puay Yok Tan[7]

[1] Singapore-ETH Centre, Future Cities Laboratory Global, Singapore, Singapore; [2] Manaaki Whenua – Landcare Research, Lincoln, New Zealand; [3] Tsinghua University, Department of Earth System Science, Institute for Global Change Studies, Ministry of Education Ecological Field Station for East Asian Migratory Birds, Beijing, China; [4] École Polytechnique Fédérale de Lausanne, Laboratory of Urban and Environmental Systems, Lausanne, Switzerland; [5] City University of Hong Kong, Department of Architecture and Civil Engineering, HongKong, China; [6] CSIRO Environment, Dutton Park, QLD, Australia; [7] National University of Singapore, Department of Architecture, College of Design and Engineering, Singapore, Singapore

Objective: Beyond mere coverage, tree canopy configuration affects how effectively trees deliver ecosystem services and health benefits. There is limited evidence on the links between tree canopy configuration and children's health. We aimed to evaluate the association between tree canopy configuration and under-5 children mortality risks in low-income and middle-income countries (LMICs).
Material and Methods: We quantified five tree canopy configuration metrics within 2 km of each urban sampling cluster centre using land cover data with a resolution of 30 m. These metrics were linked to 402,110 children younger than 5 years in 59 LMICs, accounting for annual changes in tree canopies between 2000 and 2019. Time-varying Cox regression models were applied to estimate the association between each tree canopy configuration metric and mortality. We estimated the hazard ratios (HRs) and corresponding 95% confidence intervals (CIs) per unit increase in each configuration metric adjusting for a set of covariates including tree canopy cover, mother's sociodemographic status and fertility behaviour, and environmental factors. Effect modification by tree canopy cover, $PM_{2.5}$, age, and household wealth condition was explored.
Results: In the fully adjusted models, we observed robust negative associations between mortality risk and aggregation index (a metric quantifying the aggregation level of green patches). A 1% increase in the aggregation index was associated with a 0.048% (95% CI 0.012% - 0.084%) decrease in the risk of under-5 mortality. Analyses on modification effects suggested that aggregated tree patches may be more beneficial for areas with a higher $PM_{2.5}$ concentration.
Conclusion: When the tree canopy cover is constant, increasing the aggregation level of tree patches at the neighbourhood level may reduce the under-5 mortality risk. This study provided valuable insights into optimizing forested greenspaces and highlighted future directions for the planning and management of urban forests towards healthy and green cities.

TO14-02 | Investigating the Long-Term Residential Exposure to Air Pollution and Greenness in Relation to Cognitive Function in Older Chinese

Yanxi Zeng[1], Weiju Zhou[1], Yaojing Chen[2,3], He Li[3,4], Xiangwei Dai[3,4], Zhanjun Zhang[2,3], John S. Ji[1]

[1] Tsinghua University, Vanke School of Public Health, Beijing, China; [2] Beijing Normal University, State Key Laboratory of Cognitive Neuroscience and Learning, Beijing, China; [3] Beijing Normal University, Beijing Aging Brain Rejuvenation Initiative (BABRI) Centre, Beijing, China; [4] China Academy of Chinese Medical Sciences, Institute of Basic Research in Clinical Medicine, Beijing, China

Objective: This study investigates the longitudinal impact of year-to-year changes in greenspace and air pollution on cognitive performance among older adults in Beijing, China.

Material and Methods: We analyzed data from 6,706 participants aged over 50, enrolled in the ongoing Beijing Aging Brain Rejuvenation Initiative (BABRI) from 2009 to 2022. Standardized cognitive tests were used to measure participants' global cognition and five domains: memory, attention, execution, visuospatial function, and language.Individual exposure to greenspace was quantified using the Normalized Difference Vegetation Index (NDVI), while air pollution exposure was assessed based on annual average PM2.5 and PM10 levels. Longitudinal changes in environmental exposures over one-, three-, five-, and ten-year periods prior to cognitive assessment were examined using multivariable regression models, adjusted for key covariates.

Results: Increased NDVI over time were associated with significant improvements in executive function, attention, and memory, with stronger effects observed over longer exposure periods. For instance, the beta coefficient for Stroop-C (a measure of executive function) was 18.30 (95% CI [11.42,25.18]) for a one-year increase in NDVI, rising to 21.85 (95% CI [17.61,26.09]) for a ten-year increase in NDVI. Declines in PM2.5 and PM10 levels over time were associated with better global cognition enhanced performances across almost all cognitive domains assessed, but paradoxically, with lower memory scores (AVLT: β=-0.09 per unit decrease in PM2.5). Language showed insensitivity to variations in these environmental factors.

Conclusion: Long-term exposure to increased greenspace and reduced air pollution positively impacts cognitive health in older adults, particularly in executive function and attention. However, the observed negative association with memory suggests potential irreversible effects of prior high pollution exposure. These findings emphasize the need for urban planning strategies that enhance greenspace and improve air quality to support cognitive well-being in aging populations.

TO14-03 | Early-life Greenness Exposure and Childhood Asthma and Allergic Rhinitis: Findings from An Ontario Birth Cohort

Erjia Ge[1], Chengchun Yu[2,3], Eric Lavigne[4,2], Nicholas Grubic[1], Teresa To[5,6], Paul J. Villeneuve[7], Jeffrey Brook[1], Wendy Lou[1], Xin Liu[8], Lennon Li[9,1], Zihang Lu[10]

[1] University of Toronto, Dalla Lana School of Public Health, Toronto, Canada; [2] ICES uOttawa, Ottawa, Canada; [3] The Ottawa Hosptial, Ottawa Hospital Research Institute, Ottawa, Canada; [4] Health Canada, Health Environment & Consumer Safety Branch, Ottawa, Canada; [5] The Hospital for Sick Children, Child Health Evaluative Sciences, Toronto, Canada; [6] ICES UofT, Toronto, Canada; [7] Carleton University, Department of Neuroscience, Ottawa, Canada; [8] Southern Unviersity of Science and Technology, School of Public Health and Emergency Management, Shenzhen, China; [9] Public Health Ontario, Toronto, Canada; [10] Queen's Unviersity, Department of Public Health Sciences, Kingston, Canada

Objective: This study explores whether early-life exposure to greenness can mitigate the development of childhood asthma and allergic rhinitis.

Methods: Wefollowed a cohortof 1.4 million singleton live births in Ontario from 2006 to 2018, tracking outcomes until diagnosis of asthma or allergic rhinitis, loss to follow-up, or study end in 2019. The study assessed the impact of early-life greenness exposure (ages <3 and 3-5 years) on the development of asthma and allergic rhinitis. Exposure data included monthly averages of $PM_{2.5}$, NO_2, and O_3, 16-day $NDVI_{250m}$, and annual travel time to parks/forests, linked to children based on their maternal residential data at birth. Distributed lag non-linear models (DLNM) quantified lagged effects of exposures, and multivariate Cox regressions assessed associations, adjusting for potential confounders. Hazard ratios (HRs) with 95% confidence intervals (CIs) were calculated per interquartile range (IQR) changes in exposure.

Results: Of 1,440,360 children, 17.8% developed asthma, 23.3% allergic rhinitis, and 5.4% both. Early greenness exposure (< 3 years), measured by $NDVI_{250m}$, was linked to increased asthma [HR=1.141 (1.093-1.192)] and allergic rhinitis [HR=1.046 (1.019-1.073)]. Long-term exposure (3+ years) seemed to tune down the risk for asthma [HR=0.986 (0.953-1.021)] and allergic rhinitis [HR=0.945 (0.926-0.964)]. Longer travel times to forests between ages 3-5 years were associated with higher asthma risk [HR=1.012 (1.001-1.023)], but not allergic rhinitis [HR=0.979 (0.958-1.001)]. For children with early-life $PM_{2.5} \geq 7.5 ug/m^3$ (the cohort's median exposure level, higher than the WHO air quality guideline for health), shorter travel times to forests were linked to reduced asthma risk after age 5. No such reduction was observed for those with lower $PM_{2.5}$ or greater park exposure.

Conclusion: Early-life greenness exposure may help reduce allergy risks, but addressing air pollution remains crucial for protecting children's health.**Funding:** Canadian Institute of Health Research #DA4-170261

TO14-04 | Smartphone-based Greenness Exposure and Smartphone-assessed Physical Activity in an Intensive Longitudinal Cohort Continuously Measured over a Year

Li Yi[1,2], Jaime E. Hart[3,4], William Kessler[3], Jorge E. Chavarro[2,4], Francine Laden[3,4], Peter James[3,5]

[1] Harvard Medical School and Harvard Pilgrim Health Care Institute, Department of Population Medicine, Boston, USA; [2] Harvard T.H. Chan School of Public Health, Department of Nutrition, Boston, USA; [3] Harvard T.H. Chan School of Public Health, Department of Environmental Health, Boston, USA; [4] Brigham and Women's Hospital and Harvard Medical School, Channing Division of Network Medicine, Department of Medicine, Boston, USA; [5] University of California Davis School of Medicine, Department of Public Health Sciences, Davis, USA

Background: Growing evidence has linked greenspace to increased physical activity (PA). However, most studies measure residential greenspace, failing to capture personalized mobility-based exposure and potentially misclassifying greenspace, and use self-reported physical activity collected infrequently (e.g., every two years), which does not capture day-to-day variation and is subject to recall bias.

Methods: In a Substudy of the US-based Nurses' Health Study 3 (2021–2023), participants (N=2,127) provided smartphone global positioning system (GPS) and smartphone accelerometer-based PA continuously measured over a year. GPS data were gathered every 15 minutes and linked with 30-meter resolution Landsat normalized difference vegetation indices (NDVI), temporally matched by season. Average steps-per-minute for were calculated for each 15-minute period following each GPS observation. Generalized Additive Mixed Models were used to examine the associations of greenspace with PA, adjusting for participants' age, sex, race and ethnicity, marital status, education level, and neighborhood socioeconomic status.

Results: Our preliminary sample included 983 participants ($mean_{age}$= 39 years, n = 3,795,228 observations at 15-minute intervals; equivalent to 948,807 person-hours). Mean NDVI was 0.2 (SD=0.3) and mean steps-per-minute were 3.8 (SD=5.4) out of 7.2 months of average follow-up time. The association of NDVI with PA was non-linear. Specifically, each 0.1 increase in NDVI exposure was associated with a 0.25 steps-per-minute decrease (95% CI: -0.40, -0.12) where NDVI <0.6). Above NDVI 0.6, each 0.1 increase in NDVI was associated with 1.1 steps-per-minute increase (95%CI: 0.84, 1.34).

Conclusions: In this prospective cohort, momentary exposure to satellite-based greenness was inversely associated with PA in low-to-medium NDVI areas, but the association was positive in areas of higher NDVI. Future research should confirm these results in other populations, determine the specific types of nature underlying these associations, and explore the mechanisms explaining the associations between greenness and PA.

TO14-05 | Can increases in greenspace abate heat-related mortality in Latin American cities?

Maryia Bakhtsiyarava[1], Iryna Dronova[2], Daniel Rodriguez[2], Brisa Sanchez[1], Josiah Kephart[1], Leah Schinasi[1], Ana Diez-Roux[1], Sarav Arunachalam[3], Marie O'Neill[4], Nelson Gouveia[5], Waleska Caiaffa[6]

[1] Drexel University, Urban Health Collaborative, Dornsife School of Public Health, Philadelphia, USA; [2] University of California Berkeley, Berkeley, USA; [3] University of North Carolina, Chapel Hill, USA; [4] University of Michigan Ann Arbor, Ann Arbor, USA; [5] University of Sao Paulo, Sao Paulo, Brazil; [6] Federal University of Minas Gerais, Belo Horizonte, Brazil

Objective

As climate change intensifies, expanding greenspace is often proposed as a strategy to reduce heat-related mortality. However, its impact remains particularly understudied in the Global South. We assessed whether counterfactual projections of greenspace mitigate or exacerbate heat-related mortality in 323 Latin American cities.

Material and Methods

We used city-level daily mortality for 2002-2015, daily average temperature from ERA5-Land, and greenness (Normalized Difference Vegetation Index [NDVI]) for 323 cities in Latin America. First, we estimated the effect modification of heat-related mortality by the baseline amount of greenspace (NDVI) using distributed lag nonlinear models and a random effects meta-regression. Second, we generated counterfactual greenspace scenarios using Monte Carlo simulations separately for arid (n=79) and non-arid (tropical and temperate, n=244) cities. Third, we predicted effect modification of heat-related mortality by counterfactual greenspace. We then compared the baseline and counterfactual excess heat mortality to examine the impact of changes in greenspace on heat-related mortality.

Results

In arid cities, simulated increases in greenness are associated with a decrease in heat-related mortality from 0.68% of all deaths (95% CI 0.48, 0.89) to 0.29% of all deaths (95% CI 0.08, 0.50), which translates to a decrease from 17,908 heat-related deaths to 7,579 deaths over the 12-year period considered. In contrast, increasing greenspace in tropical and temperate cities is projected to raise heat-related mortality from 0.53% (95% CI 0.47, 0.59) to 0.62% (95% CI 0.56, 0.68), contributing approximately 10,000 additional heat-related deaths.

Conclusion

While greenspace generally reduces urban heat and may decrease heat-related mortality, its effectiveness depends on climate conditions. Greenspace in hot, humid regions can contribute to higher humidity and heat stress, exacerbating heat-related mortality instead of mitigating it. Understanding these dynamics in Latin America is crucial for designing region-specific climate adaptation policies and optimizing urban greenspace planning to mitigate heat-related health risks.

TO14-06 | Multidimensional Green Space and Its Associations with Lipid Health

Cui Guo

The University of Hong Kong, Department of Urban Planning and Design, Hong Kong, Hong Kong

Background

Green space is well-known for its significant health benefits for adults. However, most studies have relied on one- or two-dimensional metrics to evaluate green space, limiting the understanding of its full impact. This study aims to investigate the associations between multidimensional green space and lipid health among Chinese adults.

Methods

This is a longitudinal cohort study in Hong Kong. A total of 25,102 adults aged ≥18 years with 43,112 observations were included between 2008 and 2018. We assessed two-dimensional greenness (Normalized Difference Vegetation Index, Enhanced Vegetation Index, and street-view greenness) and three-dimensional greenness indicators (volume of greenness) within 500m and 1km buffers around residential addresses. Health outcomes included total cholesterol, triglycerides, high-density lipoprotein cholesterol, and the development of dyslipidaemia. Linear mixed-effects models and time-dependent Cox regression models were used to examine the impacts of greenness on lipid profiles and incident dyslipidaemia, respectively. Vulnerable populations were identified using stratified analyses. The potential pathways underlying their associations were examined using causal mediation analyses.

Findings

We found that long-term exposure to increased levels of multi-dimensional green space was consistently associated with an improved lipid profile. Multi-dimensional green space was also linked to a reduced risk of incident dyslipidaemia, with a risk reduction of 15% to 26%. Subgroup analyses revealed that males and individuals with sedentary lifestyles were generally more sensitive to the health benefits of green space. Physical activity was identified as a significant mediator in the greenness-lipid health relationship, accounting for 3.71% to 6.23% of the total health benefits.

Interpretation

This study addresses the health benefits of green space on lipid health from a multidimensional perspective. Both the area and volume of green space play a crucial role in improving lipid profiles and reducing the risk of dyslipidaemia in adults.

TO14-07 | Environmental risk factors and risk of early onset dementia in the Modena province, Northern Italy

Tommaso Filippini[1,2], Camilla Soncini[1], Annalisa Chiari[3], Sofia Costanzini[4], Francesca Despini[4], Niccolò Martini[4], Manuela Tondelli[1,3], Giovanna Zamboni[1,3], Sergio Teggi[4], Marco Vinceti[1,5]

[1] University of Modena and Reggio Emilia, Department of Biomedical, Metabolic and Neural Sciences, Modena, Italy; [2] University of California Berkeley, School of Public Health, Berkeley, USA; [3] University Hospital of Modena, Neurology Unit, Moden, Italy; [4] University of Modena and Reggio Emilia, Department of Engineering 'Enzo Ferrari', Modena, Italy; [5] Boston University School of Public Health, Department of Epidemiology, Boston, USA

Introduction: Dementia is a group of disorders characterized by the development of multiple cognitive deficits, including memory loss and dysfunction in at least one other cognitive domain. When the condition affects individuals under the age of 65, it is referred to as early-onset dementia. Risk factors include genetic predisposition, cardiovascular diseases, head injuries, unhealthy diet and lifestyles, and possibly environmental determinants. The aim of this study is to investigate the role of environmental factors, such as air pollution from motorized traffic, availability of green spaces (the so-called "greenness") and artificial light at night (ALAN) as possible risk factors for early-onset dementia.

Methods: We conducted a case-control study on a population from the province of Modena, Norther Italy, including 327 cases and 1949 sex and age-matched population controls. We georeferenced each subject's address of residence and estimated exposure data to benzene as marker of motorized traffic, the Normalized Difference Vegetation Index (NDVI) for assessment of greenness, and ALAN using Visible Infrared Imaging Radiometer Suite satellite data. We assessed EOD risk using conditional logistic regression model and computing odds ratio (OR) and 95% confidence interval, mutually adjusting for environmental factors.

Results: We found increased EOD risk for subjects exposure to high (≥1 μg/m3) of benzene from motorized traffic using those below <1 μg/m3 as reference. As regard NDVI, we noted a non-linear relation with higher EOD risk in the middle category of exposure, and lower risk at low and high NDVI values. ALAN showed lower EOD risk with increasing exposure. Stratified analyses by sex showed higher risk for males than females due to benzene exposure, while NDVI showed almost linear decrease in risk in females. ALAN did not demonstrate sex-differences.

Conclusions: Overall, our finding suggest that environmental factors may modify EOD risk, with also non-linear relations and sex-specific differences.

TO15 | Urban environment and brain health among adults

TO15-01 | Associations between long-term exposure to $PM_{2.5}$ components and brain MRI signs of Alzheimer's disease and vascular dementia among cognitively normal adults

Haomin Li[1], Lina Dimitrov[2], Stefanie Ebelt[1,2], Donghai Liang[1,2], Allan Levey[3,4], James J. Lah[3,4], Aliza P. Wingo[5], Zhihao Jin[1], Qixiang Lin[3], Deqiang Qiu[4,8], Thomas S. Wingo[6,7], Anke Huels[1,2]

[1] Emory University, Gangarosa Department of Environmental Health, Rollins School of Public Health, Atlanta, USA; [2] Emory University, Department of Epidemiology, Rollins School of Public Health, Atlanta, USA; [3] Emory University, Department of Neurology, School of Medicine, Atlanta, USA; [4] Emory University, Goizueta Alzheimer's Disease Research Center, Emory University School of Medicine, Atlanta, USA; [5] University of California, Davis, Department of Psychiatry, Sacramento, USA; [6] University of California, Davis, Department of Neurology, Sacramento, USA; [7] University of California, Davis, Alzheimer's Disease Research Center, Sacramento, USA; [8] Emory University, Department of Radiology and Imaging Sciences, Atlanta, USA; [9] Emory University and Georgia Institute of Technology, Joint Department of Biomedical Engineering, Atlanta, USA

Objective

Alzheimer's disease and vascular dementia are leading causes of morbidity and mortality worldwide, with progression often beginning long before clinical symptoms appear. Fine particulate matter ($PM_{2.5}$), a complex air pollution mixture, is a key modifiable risk factor for neurodegenerative diseases, yet the specific role of $PM_{2.5}$ components in subclinical brain changes remain unclear. To address this gap, we investigated the association between long-term exposure to major $PM_{2.5}$ components and early neurodegenerative changes estimated from MRI in cognitively normal adults.

Material and Methods

We analyzed 970 participants from the Emory Healthy Brain Study, who underwent standardized MRI scans to assess left and right hippocampal volumes, total gray matter volume, and white matter hyperintensity volume during 2019-2023. Residential exposure to 15 $PM_{2.5}$ components, including 5 major components and 10 trace elements, was estimated and averaged over 5-, 10-, and 15-year periods prior to MRI scan, accounting for residential history. We conducted single-pollutant models for each component and applied Weighted Quantile Sum (WQS) regression on the pollution mixture across these time windows to assess both individual and joint effects on brain volumetric measurements, adjusting for demographic and socioeconomic covariates.

Results

In single-pollutant models, zinc and calcium were associated with both left and right hippocampal volumes ($p < 0.05$) across all exposure windows. WQS analyses showed a significant association between the 15-year exposure mixture and left hippocampal volume ($\beta=-0.11$ SD per 1 quantile increase; 95% CI: -0.21, -0.01), with calcium as the major contributor. Overall, 15-year exposures showed stronger associations than 10- or 5-year exposures across all outcomes.

Conclusion

Our findings suggest a potential long-term cumulative effect of the $PM_{2.5}$ exposure mixture, particularly driven by calcium, on left hippocampal volume. Calcium may play important roles in hippocampal volume changes, highlighting the need for further research into component-specific effects on early neurodegenerative processes.

TO15-03 | Long-term exposure to air pollution and road traffic noise and incidence of psychiatric disorders in a Danish Nurse Cohort Study

Rina So[1], Marie Bergmann[1], Stephan Tuffier[1], Thomas Cole-Hunter[1], Jiawei Zhang[1], Zorana Andersen[1], Youn-Hee Lim[1]

[1] University of Copenhagen, Deperment of Publich Health, Copenhagen, Denmark; [2] University of Copenhagen, Deptartment of Clinical Medicine, Copenhagen, Denmark

Objective: Air pollution and road traffic noise have been linked to mental health, yet most epidemiological studies focus on single exposures and primarily on depression. This study investigated the association of long-term exposure to air pollution, road traffic noise, and the incidence of major psychiatric disorders.

Material and Methods: We followed 24,608 women from the Danish Nurse Cohort (>44 years), recruited in 1993/1999, until their first hospital contact for psychiatric disorders, or 31 December 2018, whichever came first. Residential annual mean concentrations of fine particulate matter ($PM_{2.5}$) and nitrogen dioxide (NO_2) since 1979 were estimated using the Danish DEHM/UBM/AirGIS dispersion model, while annual mean day-evening-night road traffic noise levels (L_{den}) were estimated since 1970 by the Nord2000 model. We applied time-varying Cox regression models to estimate the association of 14-year mean exposure to air pollutants and L_{den} with the incidence of mood (including depression), neurotic, and substance use disorders, after adjusting for potential confounders.

Results: Over 21-year mean follow-up, 982 nurses were diagnosed with mood disorders, 895 with depression, 640 with neurotic disorders, and 317 with substance use disorders. We observed positive associations between $PM_{2.5}$ and all outcomes, with hazard ratios (HR) (95% confidence interval) per interquartile range (IQR) (2.8 μg/m³) of 1.09 (0.93, 1.28) for mood, 1.08 (0.93, 1.25) for depressive, 1.18 (0.97, 1.45) for neurotic, and 1.19 (0.93, 1.51) for substance use disorders. Positive associations with L_{den} and NO_2 were only observed for substance use disorders [HR: 1.18 (1.01, 1.37) per IQR of 8.6 dB in L_{den}; 1.14 (0.97, 1.35) per IQR of 7.64 μg/m³ in NO_2].

Conclusion: Our findings suggests that long-term exposure to $PM_{2.5}$ may increase risk of psychiatric disorders, while exposure to traffic-related pollution, including traffic noise and NO_2, may elevate risk of substance use disorders.

The presented work is funded by LUNDBECKfonden (#R449-2023-1412).

TO15-04 | Long-term cumulative effects of annual air pollution exposure on hospitalization with Parkinson's Disease

Scott Delaney[1], Lauren Mock[2], Veronica Wang[1], Antonella Zanobetti[1], Danielle Braun[2,3], Daniel Mork[2]

[1] Harvard TH Chan School of Public Health, Department of Environmental Health, Boston, USA; [2] Harvard TH Chan School of Public Health, Department of Biostatistics, Boston, USA; [3] Dana-Farber Cancer Institute, Department of Data Science, Boston, USA

Objective

Parkinson's Disease (PD) poses substantial health challenges among an aging population, and underlying biological processes likely begin several years before diagnosis. Previous studies suggest increased same- or prior-year exposure to PM2.5 and NO2 air pollution increases PD morbidity. In this study, we examined 10-year lagged relationships between air pollution exposure and hospitalization with PD.

Material and Methods

We analyzed data from over 9 million Medicare fee-for-service beneficiaries aged 65 years and older in the contiguous United States in 2000-2016. We defined "hospitalization with PD" as the first inpatient hospitalization claim with any ICD-9 or -10 diagnosis code indicating PD. We calculated average exposures during the previous 10 years for annual PM2.5, annual NO2, and summer O3 exposure. We applied distributed lag models and estimated the lagged associations between air pollution and odds of first hospitalization with PD using a discrete time survival approach adjusted for several individual- and area-level confounders.

Results

Increased PM2.5 and NO2 (but not O3) exposure was associated with increased odds of hospitalization with PD at least 4 years prior to hospitalization. The 10-year cumulative odds ratio for hospitalization with PD comparing annual PM2.5 exposure to the 90th versus the 0.5th percentile (i.e., 11.88 ug/m3 versus 2.93 ug/m3) of the PM2.5 distribution was 1.771 (95% CI: 1.591, 1.972). The corresponding OR for NO2, comparing exposure to 31.36 ppb versus 3.73 ppb, was 1.530 (95% CI: 1.408, 1.663).

Conclusions

These findings suggest effects of air pollution on PD morbidity may last at least 4 years after initial exposure, and they highlight the long-lasting health impact of toxic air pollution. In turn, reducing air pollution exposure may confer long-lasting health benefits for adults with PD.

TO15-05 | Residential Greenness and Cognitive Dysfunction Among Older Adults in High-Density Urban Areas

Weiju Zhou[1], Peiyi Liu[2], Xingkang Chai[3], Yanhui Jia[1], Haida Tang[3], Jianjun Liu[2], Xifei Yang[2], John S. Ji[1]

[1] Tsinghua University, Vanke School of Public Health, Beijing, China; [2] Shenzhen Center for Disease Control and Prevention, Shenzhen Key Laboratory of Modern Toxicology, Shenzhen Medical Key Discipline of Health Toxicology, Shenzhen, China; [3] Shenzhen University, School of Architecture & Urban Planning, Shenzhen, China

Introduction: The increasing prevalence of cognitive dysfunction among older adults highlights the importance of investigating modifiable environmental determinants, such as residential greenness, particularly in high-density urban settings where exposure to green spaces may be limited.

Methods: This study included 4,737 registered individuals aged 60-85 years from the Luohu District of Shenzhen, a high-density urban area in China, as part of the Shenzhen Ageing-related Disorder Cohort. Participants underwent physical examinations and Mini-Cognitive Assessments conducted across 51 community health centers. Residential greenness was assessed using the satellite-derived Enhanced Vegetation Index (EVI) at 30m and 90m buffer sizes. Multivariable logistic regression models were applied to evaluate the association between residential greenness and cognitive dysfunction, including gender differences, with adjustments for socioeconomic factors, health behaviors, comorbidities, and building density.

Results: Our findings indicated that residential greenness was significantly associated with a reduced risk of cognitive dysfunction. Each interquintile range (IQR) increase in EVI at the 30m buffer was associated with a 12% reduction in the risk of cognitive dysfunction, with an odds ratio (OR) of 0.89 (95% CI: 0.80-0.99, $p = 0.028$) after adjustment for confounders. Gender-stratified analyses revealed a stronger protective association among males (0.85, 0.73-0.99, $p = 0.040$) compared to females (0.92, 0.80-1.06, $p = 0.273$). Similar trends were observed for the 90m buffer, though the associations approached but did not reach statistical significance.

Conclusion: Our study underscores the protective role of residential greenness in mitigating cognitive dysfunction among older adults in high-density urban areas. The observed gender differences suggest that public health and urban planning strategies should incorporate gender-specific approaches to maximize the cognitive health benefits of green spaces.

Funding: This work was supported by Sanming Project of Medicine in Shenzhen (SZSM202211010), Shenzhen Key Medical Discipline Construction Fund (SZXK069) and National Natural Science Foundation of China (NSFC) (42307535 and 82422064).

TO15-06 | The impacts of ambient particulate radioactivity on hospitalizations related to neurodegenerative disorders: a national retrospective cohort study in the USA

Shuxin Dong[1], Marc Weisskopf[1,2], Petros Koutrakis[1], Brent Coull[3,1], Ernani Choma[1], Longxiang Li[4], Antonella Zanobetti[1]

[1] Harvard T.H. Chan School of Public Health, Department of Environmental Health, Boston, USA; [2] Harvard T.H. Chan School of Public Health, Department of Epidemiology, Boston, USA; [3] Harvard T.H. Chan School of Public Health, Department of Biostatistics, Boston, USA; [4] Emory Rollins School of Public Health, Department of Environmental Health, Atlanta, USA

Objective: Neurodegenerative disorders are a major global public health challenge, with Alzheimer’s Disease (AD) and Parkinson’s Disease (PD) being the most prevalent. Identifying modifiable risk factors is critical, especially given the lack of effective treatments. Among the potential risk factors, we focused on ambient particulate radioactivity (PR) – whose primary source is radon gas and its decay products -- due to its promising biological pathways supported by current literature. In this study, we aim to estimate the long-term effects of PR on Alzheimer's disease and related dementias (ADRD) and PD.
Methods: We assembled a nationwide retrospective cohort of all Medicare fee-for-service beneficiaries aged ≥65 in the contiguous USA from 2000 to 2016, following individuals from enrollment until their first hospitalization with ADRD or PD. Annual average modeled gross beta-activity (a proxy for PR) and modeled $PM_{2.5}$ concentrations both from spatiotemporal models were estimated at each individual’s residential ZIP code. A Poisson survival analysis employing time-varying covariates was applied, with baseline hazards modeled by age and stratified by individual risk factors while adjusting for $PM_{2.5}$, socioeconomic status, seasonal temperature, relative humidity and census regions.
Results: The cohort comprised 68,640,730 individuals, with 7,108,045 (10.4%) hospitalized for ADRD and 1,019,099 (1.5%) for PD during the study period. Average follow-up was 8.10 years (SD 5.00) for ADRD and 8.26 years (SD 5.03) for PD hospitalizations. A significant association was observed between gross beta-activity and both outcomes; an interquartile range increase in gross b activity (0.0025 mBq/m³) corresponded to an incidence rate ratio of 1.033 (95% CI 1.031–1.034) for ADRD and 1.030 (95% CI 1.027–1.034) for PD.*Conclusions*: These findings provide novel evidence that long-term exposure to particulate radioactivity is linked with an increased risk of hospitalization with ADRD and PD, underscoring the importance of environmental factors in neurodegenerative disease risk.

TO15-07 | Childhood and adolescent air pollution exposure and young adult mental health

Laura McGuinn[1], Kathryn Keenan[2], Alison Hipwell[3]

[1] University of Chicago, Department of Family Medicine, Chicago, USA; [2] University of Chicago, Department of Psychiatry and Behavioral Neuroscience, Chicago, USA; [3] University of Pittsburgh, Department of Psychiatry, Pittsburgh, USA

Objective: This study examines the prospective association between childhood and adolescent nitrogen dioxide (NO_2) and fine particulate matter ($PM_{2.5}$) exposure and anxiety symptoms and psychological well-being in young adult women, addressing a gap in research on air pollution's impact on young adult outcomes and positive psychological measures.

Material and Methods: Participants included women from the longitudinal community-based Pittsburgh Girls Study. Anxiety symptoms were self-reported at age 21 using the DSM-IV based Adult Self-Report Inventory-4. Psychological well-being was assessed using the Flourishing Scale, a self-report measure of overall psychological functioning. Anxiety and flourishing symptoms were dichotomized at the 75th percentile. Daily NO_2 and $PM_{2.5}$ predictions were estimated using a 1km satellite-based model and averaged over the late childhood (ages 8-10), early (11-13), and late adolescent (14-16) developmental periods. Logistic regression was used to estimate associations between each interquartile range (IQR) increase in NO_2 and $PM_{2.5}$ for each period with odds of elevated anxiety and flourishing symptoms. Models were adjusted for race/ethnicity, socioeconomic status, age, and area deprivation. Analyses were stratified by race/ethnicity.

Results: Of the 1,836 women included, 53.1% were Black. NO_2 medians (IQR) ranged from 27.4 (4.1) to 36.0 (3.6) ppb and $PM_{2.5}$ medians from 12.7 (1.6) to 15.6 (0.9) μg/m³, decreasing from childhood to adolescence. Overall, a 4.1 ppb increase in NO_2 during late adolescence was associated with increased odds of elevated anxiety symptoms (OR: 1.32, 95% CI: 1.10, 1.59). In stratified analyses, increases in NO_2 (OR: 1.35, 95% CI: 1.03, 1.78) and $PM_{2.5}$ (OR: 1.31, 95% CI: 1.03, 1.66) were associated with elevated anxiety symptoms only in Black girls.

Conclusion: These findings suggest that exposure to air pollution exposure during late adolescence may have lasting effects on mental health in young adulthood, a relatively understudied period. Future analyses will expand exposure periods and assess trajectories over time.

TO16 | Next-gen monitoring: Low-cost sensors, personal exposure, and advanced modeling

TO16-01 | Detecting wood smoke with mobile monitoring

Jules Kerckhoffs, Gerard Hoek

Utrecht University, Institute for Risk Assessment Sciences, Utrecht, Netherlands

Objective: Due to the limited spatial coverage of monitoring stations that measure black carbon (BC), mobile monitoring can help in identifying spatial patterns and source attribution, especially for local sources, like residential wood burning. This study aimed to detect wood burning trough mobile monitoring and assess its contribution to the total BC and particulate matter (PM) concentration.

Material and Methods: We used a car with a AE33 (Magee Scientific; capable of measuring 7 different wavelengths) that measured two specific routes in Rotterdam in winter evening hours at least 20 times each. Routes were chosen based on municipal knowledge on neighborhoods with many woodstoves and paired with streets without wood stoves. Because the different channels relate to the type of BC, we calculated the contribution of different sources to the total black carbon concentration and subsequently to the total particulate matter concentration. We compared car measurements with a reference station before every drive.

Results: We found mean concentrations of 2.34 μg/m^3 (BC1) and 1.71 μg/m^3 (BC6), which means there is a substantial contribution of biomass burning to the total black carbon concentration. The average contribution across all street segments was 20% (IQR: 16-23%), with a standard error of 2.5% (IQR: 1.9-3.5%). Clear spatial patterns were observed between road types and neighborhoods. The biomass fraction was more than twice as high in neighborhoods with many wood stoves opposed to major roads and neighborhoods with high-rise buildings.

Conclusion: The contribution of residential wood burning to the total BC concentration in a winter season can reach 40% in certain neighborhoods in Rotterdam. Policymakers should consider this source in air pollution mitigation strategies.

TO16-02 | Distinguishing indoor- and outdoor-generated components of personal exposure to air pollutants at home for improved health metrics

Yilin Li[1], Lia Chatzidiakou[1], Anika Krause[1,2], Elizabeth Martin[1], Roderic L. Jones[1], Chiara Giorio[1], Frank Kelly[3], Tong Zhu[4]

[1] University of Cambridge, Yusuf Hamied Department of Chemistry, Cambridge, UK; [2] Airgradient Limited, Chiang Mai, Thailand; [3] Imperial College London, Environmental Research Group, MRC Centre for Environment and Health, London, UK; [4] Peking University, BIC-ESAT and SKL-ESPC, College of Environmental Sciences and Engineering, Center for Environment and Health, Beijing, China

Objective

Understanding the relative contribution of different air pollutants to overall personal exposure is essential for assessing the impacts of air quality on health. This study presents a methodological framework that differentiates the contribution of indoor and outdoor sources on overall household exposure using data collected with low-cost sensor technologies.

Methods

Data were collected with 60 portable air quality sensor platforms deployed among 250 participants during winter and summer in Beijing, China. We applied physical models and novel sensor network methodologies to disaggregate the indoor- and outdoor-generated exposures for carbon monoxide (CO), nitrogen dioxide (NO_2), and fine particulate matter ($PM_{2.5}$) at home, providing novel exposure metrics for studying exposure-health relationships.

Results

All households had high levels of indoor-generated air pollutants, primarily from cooking and biomass and coal burning for heating. However, personal exposure at home was found to be heavily influenced by and in some cases primarily from air pollutants originating from outdoor sources. Outdoor-generated NO_2 and $PM_{2.5}$ dominated household exposure, and CO was nearly evenly split between indoor and outdoor sources. CO and NO_2 can serve as proxies for co-emitted pollutants, while $PM_{2.5}$ varies in composition by source, indicating potential differences in pollutant mixtures' toxicity and health effects.

Conclusion

The methodology presented here has the potential to advance air pollution epidemiology by enabling the identification of source-related health effects using affordable and widespread sensor technologies. While this study focused on household exposures, the approach could be adapted to other microenvironments, offering a potential framework for exposure assessment in diverse settings. As mitigation measures for indoor and outdoor air pollution may be very different, this work will provide the important additional underpinning for effective environmental policy.

Funding: The National Natural Science Foundation of China (grant no. 81571130100) and the Natural Environment Research Council of UK (grant nos. NE/N007018/1 and NE/S006729/1).

TO16-03 | De potentia restitutiva of micro air sensors for democratizing air quality monitoring and mirroring air pollution at schools using citizen science.

Collins G. Hodoli[1], Victor D. Ablo[1], Francis Dugbartey[2], Francis-Xavier K. Sosu[3]

[1] Clean Air One Atmosphere, Accra, Ghana; [2] University of Northern Iowa, Cedar Falls, USA; [3] Parliament of Ghana, Parliament House, Accra, Ghana

In the African Union, children are exposed to $PM_{2.5}$ levels hundred times more than their peers in high income countries but the lack of air quality monitoring and meaningful data communication infrastructure hinders mitigation and children's health protection specifically at schools. We employed miniaturized air sensors using participatory science at a senior high school in Ghana to demonstrate the potentials of these devices for democratizing air quality monitoring for quantifying and tracking sources of $PM_{2.5}$ emissions. An appropriately calibrated air sensor was deployed at the West Africa Senior High School in the Greater Accra Region of Ghana as part of ongoing efforts for the "Schools for Earth" project. Based on 3,628 data points from June to December, 2024, monthly peaks were observed in December (34 μgm^{-3}) and the lowest in October (<15 μgm^{-3}). Weekly, low $PM_{2.5}$ levels were reported on Wednesdays (15 μgm^{-3}) in contrast to high levels on Saturdays (24 μgm^{-3}). Hourly observations were linked to the early morning rush hour; remained high throughout the day except at and around 20:00 hr. Higher $PM_{2.5}$ observations (45 μgm^{-3}) were identified from all wind sectors at low wind speed (2ms^{-1}) tied to background activities such as open waste burning, solid fuel use, vehicular emissions and windblown dust. A four-cluster shows contributions from all wind speed/direction intervals associated with high levels from the N direction pinpointing signals that are linked to anthropogenic activities and natural sources such as sea salt from the S. These preliminary findings and

recommendations were presented as a report to the Member of Parliament for Madina Constituency under whose jurisdiction the project was collaboratively carried out and the Ghana Environmental Protection Authority for urgent action to reduce emissions at source- vehicular, solid fuel, and develop a constitutional roadmap to a clean, healthy, and sustainable environment in Ghana.

TO16-04 | Implementation of a Low-Cost Sensor Network for $PM_{2.5}$ Monitoring in Peru: Progress, Challenges, and Lessons Learned

Luciana Blanco-Villafuerte[1], Qiang Pu[2], Stella Hartinger[1], Camila Llerena-Cayo[1], Solange Aznaran[1], Laura Nicolaou[3,5], William Checkley[3,6], Yang Liu[4], Kyle Steenland[4], On behalf of the Global Environmental and Occupational Health (GEOHealth) Hub in Peru

[1] Universidad Peruana Cayetano Heredia, Centro Latinoamericano de Excelencia en Cambio Climático y Salud, Lima, Peru; [2] Saint Louis University, College for Public Health and Social Justice, St. Louis, USA; [3] Johns Hopkins University, Division of Pulmonary and Critical Care, School of Medicine, Baltimore, USA; [4] Emory University, Rollins School of Public Health, Atlanta, USA; [5] Johns Hopkins University, Department of Environmental Health and Engineering, Bloomberg School of Public Health, Baltimore, USA; [6] Johns Hopkins University, Center for Global Non-Communicable Disease Research and Training, Baltimore, USA

Background and Aims

Ambient fine particulate matter ($PM_{2.5}$) pollution poses a health risk, especially in low- and middle-income countries where air quality monitoring is poor. Recent advances, like low-cost $PM_{2.5}$ sensors, improve pollution surveillance and data collection for environmental health studies. We will discuss the development of a national low-cost sensor (LCS) network for ambient $PM_{2.5}$ monitoring in Peru, designed to expand coverage in under-monitored regions and integrate into the country's air quality management (AQM) program while also highlighting key achievements, challenges, and lessons learned.

Methods

The monitoring network was designed to cover urban and rural areas across all 24 departments in Peru, selected through an algorithm considering geographical representativeness, altitude, and population size. To ensure data accuracy, sensor calibration was conducted in six regions of the country, covering different ecosystems: the Amazon rainforest, the coast, and the Andean highlands.

Results

To date, we have installed 120 of the 163 low-cost sensors across 24 departments, providing real-time air quality data in previously unmonitored areas despite ongoing challenges such as regulatory validation, infrastructure limitations, and logistical constraints. In the Amazonas department, where no AQM program previously existed, our monitoring network recorded a sharp increase in air pollution levels during the 2024 wildfires, with daily $PM_{2.5}$ averages reaching 180 µg/m³ in Pedro Ruiz, a town approximately 50 km from the nearest fires. This data offers real-time evidence of extreme pollution in Amazonas, showing the network's potential to enhance environmental monitoring and serve as an early warning system during such events.

Conclusion

Implementing a LCS network in Peru advances air pollution monitoring, covering areas without regulatory oversight and enabling environmental health studies. Despite challenges, our project shows that establishing a nationwide LCS network is feasible. Lessons from this project can guide similar initiatives in low—and middle-income countries, enhancing air quality monitoring capacity.

TO16-05 | Approximate Bayesian Kernel Machine Regression via Random Fourier Features for Estimating Joint Health Effects of Multiple Exposures

Danlu Zhang[1], Stephanie M. Eick[2], Howard H. Chang[1,2]

[1] Emory University, Department of Biostatistics and Bioinformatics, Atlanta, USA; [2] Emory University, Gangarosa Department of Environmental Health, Atlanta, USA

Objective: Bayesian Kernel Machine Regression (BKMR) is a popular approach to flexibly estimate the joint and nonlinear effects of multiple exposures. However, BKMR faces computation challenges for large datasets, as inverting the kernel repeatedly in Markov chain Monte Carlo (MCMC) algorithms can be time-consuming and often infeasible in practice. This study aimed to develop a computationally efficient alternative to BKMR to make it applicable to large datasets.

Material and Methods: We proposed a fast version of BKMR (Fast BKMR) using supervised random Fourier features to approximate the Gaussian process. We used periodic functions as basis functions and this approximation re-framed the kernel machine regression into a linear mixed-effect model that facilitates computationally efficient estimation and prediction. Bayesian inference was conducted using MCMC with Hamiltonian Monte Carlo algorithms. Simulation studies were conducted to evaluate the model performance. We also applied this approach to analyze over 270,000 birth records, examining associations between multiple ambient air pollutants and birthweight in Georgia. Analytic code for implementing Fast BKMR was developed for R.

Results: Simulation studies showed that Fast BKMR provides comparable estimates to the original Gaussian process while significantly reducing computation time by 29% to 99%, depending on the number of basis functions and sample sizes. The method also presented greater robustness to kernel misspecification in some scenarios. In the birthweight analysis, we identified stronger multi-pollutant associations compared to single-pollutant analysis, and evidence of nonlinear interactions.

Conclusion: Fast BKMR provides a computationally efficient alternative without sacrificing estimation accuracy. Its ability to handle large datasets makes it a valuable tool for environmental epidemiology and other fields analyzing high-dimensional exposure data.

Funding source: This work was supported by grants from the National Institute of Environmental Health Sciences under Award Numbers R01ES028346 and K01ES035082.

TO16-06 | Bayesian Nonparametric Ensemble (BNE) algorithm for predictions of high spatiotemporal $PM_{2.5}$ concentrations

Vijay Kumar[1], Jaime Benavides[1], Carlos Carrillo-Gallegos[1], John Paisley[2,3], Brent Coull[4], Arlene Fiore[5], Marianthi-Anna Kioumourtzoglou[1,3]

[1] Columbia University, Mailman School of Public Health, Environmental Health Sciences, New York, USA; [2] Columbia University, Department of Electrical Engineering, New York, USA; [3] Columbia University, Data Science Institute, New York, USA; [4] Harvard T.H. Chan School of Public Health, Department of Biostatistics, Boston, USA; [5] MIT-Massachusetts Institute of Technology, Department of Earth, Atmospheric and Planetary Sciences, Cambridge, USA

Accurate assessment of fine particle ($PM_{2.5}$) concentrations is a critical component of health studies and regulatory action. Although multiple prediction models exist, their accuracy varies in space and time. The Bayesian Nonparametric Ensemble (BNE) algorithm offers a structured framework for spatiotemporal prediction. BNE optimizes prediction by flexibly weighing existing models across space and time by their spatio-temporal accuracy and estimates spatiotemporal uncertainties associated with ensemble predictions.

We applied the BNE algorithm to estimate daily $PM_{2.5}$ concentrations at 1×1 km grid cells for the contiguous United States (2010-2016), leveraging a combination of four existing daily $PM_{2.5}$ datasets developed using observations, models, or in some cases blending both. BNE was trained on data from the US Environmental Protection Agency's Air Quality System database. The BNE-estimated concentration at a location and time was sampled as the mean and the uncertainty as the standard deviation of the posterior predictive distribution (PPD).

The mean BNE-predicted $PM_{2.5}$ concentration was 6.4 µg/m^3 (IQR: 5.3-7.2). The average uncertainty of these BNE-predictions, presented as standard deviation at each space and time point, was 0.13 µg/m^3 (IQR: 0.12-0.15). The BNE predictions achieved a daily 10-fold spatial cross-validated root mean square error (RMSE) of 2.24 µg/m^3 and an R^2 value of 0.86. We tested the robustness of BNE by evaluating various combinations of input models and found that BNE maintained consistent performance even when fewer models were used.

BNE robustness is crucial when data availability is limited, especially when some models have lower spatial coverage or missing data. Beyond robust spatiotemporal predictions, the uncertainties provided by BNE can help identify areas where additional monitoring sites may be needed. Additionally, BNE's predictions, along with associated uncertainties, can improve estimation of health effects. We are also evaluating BNE predictions on independent data for external validation to further assess its reliability.

TO16-07 | Enhancing Community Air Quality Monitoring: A Spatiotemporal PM2.5 Modeling Approach Using Multiple Data Sources

Eunhye Yoo[1], Jeongwon Lee[1], Yonghun Suh[2]

[1] University at Buffalo, SUNY, Geography, Buffalo, USA; [2] Seoul National University, Geography, Seoul, South Korea

Background: To improve the availability and resolution of air quality data ($PM_{2.5}$) in marginalized communities, we developed a low-cost air pollution sensor network using a community-based participatory approach. The New York State Department of Environmental Conservation also conducted mobile community air quality monitoring in Buffalo, NY, though in different years. Low-cost sensors provide high-frequency temporal data at fixed locations, while mobile monitoring captures fine-scale spatial variation that are not detected by EPA monitors or stationary low-cost sensors. However, little effort has been made to integrate these data sources to comprehensively assess the spatiotemporal variations of $PM_{2.5}$ exposure in marginalized communities.
Objectives: This study aims to develop spatiotemporal models of $PM_{2.5}$ by integrating data from the low-cost sensor network with annual average of $PM_{2.5}$ estimates derived from mobile monitoring.

Methods: We developed two models to predict daily $PM_{2.5}$ concentrations at 500 m resolution grid and evaluated their predictive performance. In the Bayesian hierarchical model, daily $PM_{2.5}$ measurements were integrated with annual $PM_{2.5}$ surfaces, which served a smooth spatial trend and were updated daily using the Integrated Nested Laplace Approximation to estimate spatiotemporal effects. In the machine learning approach, daily $PM_{2.5}$ concentrations were predicted with covariates, with residuals interpolated with Universal Kriging to refine spatiotemporal predictions.

Results: The machine learning model outperformed the Bayesian hierarchical model in the cross-validation (CV) R^2 of 0.05 - 0.09. The temporal variation of the predictions was influenced by weather variables, including windspeed and relative humidity. The predictions also revealed spatially resolved $PM_{2.5}$ hotspots associated with population, traffic, and other non-road emission sources (e.g., airport).

Conclusions: The integration of both low-cost sensor data and mobile monitoring data allows predictions to capture short-scale variations of $PM_{2.5}$.

TO17 | Methodological approaches for improving accuracy in environmental epidemiology and exposure science

TO17-01 | Improving Causal Inference with Measurement Errors in Exposures and Confounders: Application to Air Pollution Exposure Assessment and Epidemiology

Honghyok Kim

University of Illinois Chicago, Environmental and Occupational Health Sciences, Chicago, USA

Background: When exposure measurement error (EME), confounder measurement error (CME), or both are present, health effect estimates regarding environmental mixtures and critical exposure time-window may not represent the true effects. In air pollution epidemiologic studies, modelled estimates of multiple ambient air pollutants and meteorological factors may serve as surrogate exposure or confounder variables.
Objectives: I developed a two-stage causal effect modeling framework to estimate average exposure/treatment effects (AEE) by addressing both EME and CME and reanalyzed the association between air pollution and COVID-19 mortality in Cook County, Illinois, reported by Kim et al.(2022).
Methods: I identified conditions under which AEE is identifiable with minimal bias given linear or non-linear potential outcomes models and developed a new method, referred to as multi-dimensional regression calibration (MRC). The first stage of the framework estimates MRC models. The second stage estimates AEE by using g-computation with MR-Calibrated variables. Simulation analyses with non-linear outcome models (i.e., logistic, log-linear), including non-linear terms of variables, were conducted to evaluate the framework. I estimated the association between air pollution and COVID-19 mortality by reducing EME and CME from multiple sources (e.g., exposure/confounder prediction modeling, unmeasured individual mobility, differential error sources). Machine learning-based 500m-gridded daily estimates of air pollutants and meteorological factors were developed in a way that they are integrated with the framework for doubly EME&CME-corrected causal effect estimation.
Results: Simulations confirmed the bias-correction capabilities of the framework. An IQR (22.7ppb) increase in 3-week O_3 exposure below the US NAAQS was associated with an 135.3% (95% CI: 68.4, 233.0) increase in COVID-19 mortality risk, comparable to that for $PM_{2.5}$ exposure. This finding contradicts the previously reported no possitive association for 3-week O_3.
Conclusion: This new framework addresses EME and CME in causal effect estimation. At low levels, reducing $PM_{2.5}$ and O_3 may have prevented premature deaths from COVID-19.

TO17-02 | When exposure is a product but only factor is measured: implications for measurement error and the proximal/distal tradeoff

Thomas Webster

Boston University School of Public Health, Dept Environmental Health, Boston, USA

Objective: Proximal exposure measures tend to be more prone to confounding while distal measures tend to have greater measurement error. Epidemiologists and exposure scientists typically consider non-differential exposure measurement error as classical or Berkson where the error is additive. But exposure is often the product of two factors, e.g., exposure to PFAS in drinking water depends on the product of the water concentration and the amount of water consumed. Epidemiologists would often treat water concentration as the exposure.

Material and Methods: We review classical and Berkson error, using directed acyclic graphs (DAGs) and properties of the covariance and variance for an outcome (Y) linearly dependent (with coefficient beta) on a continuous exposure (X) with additive error. Now suppose that X=Cq where C is the media concentration of a compound and q is the exposure factor for that media. Treating C as the exposure variable has a DAG like the Berkson case, but the “error term” for X vs C is now multiplicative. We expand cov[Cq,C]. We then examine potential confounding by an unmeasured variable U that points into both C and Y.

Results: Assuming independence of q and C, the regression coefficient equals beta multiplied by the mean of q. This result is not biased but is rather what we would want. Uncontrolled confounding by U leads to bias magnification when C is the exposure variable instead of X, but with a somewhat more complicated expression compared to regular Berkson error.

Conclusion: Exposure is often a product of two factors, but often only one is measured. This can be thought of as a multiplicative form of Berkson error yielding unbiased results that contain the mean of the unmeasured factor. Bias magnification of uncontrolled confounding occurs, complicating the proximal-distal exposure variable tradeoff.

This work began under R01ES028800.

TO17-03 | Quantitative Bias Analysis in Environmental Epidemiology: Advancing the Science of Bias Assessment

David J. Miller

U.S. Environ. Prot. Agency (retired), Washington, D.C., USA

Objective
Quantitative Bias Analysis (QBA) is a tool used to quantify the impact of systematic biases in epidemiologic studies, providing estimates of the direction and magnitude of bias. By answering “what would have been” had biases been absent, QBA offers insights that extend beyond traditional confidence intervals and qualitative risk of bias (RoB) assessments. Despite its potential to improve research transparency and validity, QBA remains underutilized. This presentation will introduce QBA, its applications in environmental epidemiology, and its advantages in strengthening bias assessments.

Materials and Methods
This talk will explore QBA as an approach to quantitatively assess biases identified by qualitative RoB tools. Examples will illustrate how QBA can help evaluate the magnitude and direction of biases in published environmental epidemiology studies, particularly in hazard identification and regulatory risk assessment. The presentation will also highlight the integration of QBA with traditional epidemiologic methods, demonstrating its utility in reducing overconfidence in study findings.

Recent advancements, including IARC’s new publication *Bias Assessment in Case–Control and Cohort Studies for Hazard Identification*—part of the *Statistical Methods in Cancer Research* series—will be discussed. This volume, based on a 2022 workshop, provides researchers with explicit and quantitative methods for bias assessment and a practical toolkit for evaluating systematic errors in the literature.

Results
QBA enhances epidemiological assessments by addressing systematic errors in a structured and quantitative manner. By moving beyond confidence intervals and qualitative RoB determinations, QBA provides a transparent framework for evaluating uncertainty in effect size estimates.
Conclusion
QBA is gaining recognition in epidemiology, with increasing incorporation into graduate curricula and scientific discourse. As tools and educational resources improve, QBA has the potential to become a routine practice, enhancing the credibility, transparency, and utility of epidemiologic evidence in both academic and regulatory contexts.

TO17-04 | Investigating the Consequences of Measurement Error of Gradually More Sophisticated Long-Term Personal Exposure Models in Assessing Health Effects: The London Study (MELONS)

Dimitris Evangelopoulos[1,2], Dylan Wood[1,2], Benjamin Barratt[1,2], Hanbin Zhang[1,3], Barbara Butland[4], Evangelia Samoli[5], Heather Walton[1,2], Sean Beevers[1,2], Audrey de Nazelle[6], Vangelis Evangelou[7], Joel Schwartz[8,9], Klea Katsouyanni[1,5]

[1] Imperial College London, Environmental Research Group, MRC Centre for Environment and Health, London, UK; [2] Imperial College London, NIHR HPRU in Environmental Exposures and Health, London, UK; [3] University of Exeter, European Centre for Environment and Human Health, Exeter, UK; [4] City St George's, University of London, School of Health & Medical Sciences, London, UK; [5] National and Kapodistrian University of Athens, Department of Hygiene, Epidemiology and Medical Statistics, School of Medicine, Athens, Greece; [6] Imperial College London, Centre for Environmental Policy, London, UK; [7] Imperial College London, Department of Epidemiology and Biostatistics, School of Public Health, London, UK; [8] Harvard T.H. Chan School of Public Health, Department of Epidemiology, Boston, USA; [9] Harvard T.H. Chan School of Public Health, Department of Environmental Health, Boston, USA

Objective: Cohort studies estimate health effects of long-term exposure to ambient air pollution using surrogate exposure assessment methods such as models. The MELONS study evaluated whether individualised exposure estimates in large-scale studies can improve health effect estimation. Using personal exposure campaigns and London's dense monitoring network, we aimed to assess bias from measurement error (ME) in exposure-response associations.
Materials and methods: Total personal exposure to $PM_{2.5}$, NO_2, O_3 and Black Carbon (BC) from 344 participants across four campaigns (2015–2019) for 12,901 person-days were separated into exposures from outdoor and indoor sources and extrapolated to annual exposures. These personal exposures were compared with surrogate exposures from monitoring and modelling. MEs were calculated and used to quantify bias in epidemiological estimates. Two correction methods (SIMEX and regression calibration) were assessed using simulations. The UK Biobank's London cohort was used to investigate mortality and morbidity effect estimates before/after correction.
Results: Outdoor-generated pollution accounted for at least 50% of total personal exposure, even for participants staying mostly indoors. Large MEs were observed, with mean differences between personal exposures and surrogates up to 10.1μg/m^3, 40.0μg/m^3, 61.7μg/m^3 and 2.6μg/m^3 for $PM_{2.5}$, NO_2, O_3 and BC respectively. This led to substantial downward bias in health effect estimates, up to 100%. Larger total and classical ME caused higher health effect underestimation. SIMEX and regression calibration corrected ME bias effectively. Applying correction methods

to the UK Biobank data revealed ME corrected hazard ratios double or more in size compared with uncorrected estimates.

Conclusions: The MELONS study highlighted the difficulty in obtaining accurate estimates of people's true exposure to outdoor-generated pollution and the impact of exposure ME in underestimating epidemiological estimates. Designing appropriate validation studies and applying ME correction methods is important for air pollution epidemiology. This work was funded by the US Health Effects Institute (agreement #4974-RFA19-1/20-8-3).

TO17-05 | Does reference group matter in biomonitoring? National vs. State comparisons of Per- and Polyfluoroalkyl Substances (PFAS) prevalence and predictors among New Jersey volunteer firefighters.

Katherine A. Lubina[2], Robert J. Laumbach[1], Shou-En Lu[2], Zorimar Rivera-Nunez[2], Miriam M. Calkins[6], Tina Fan[3], Chang-Ho Yu[3], Brian Kubiel[4], Jefferey L. Burgess[5], Judith M. Graber[2]

[1] Rutgers, the State University of New Jersey, Environmental and Occupational Health and Justice, Piscataway, USA; [2] Rutgers, the State University of New Jersey, Biostatistics and Epidemiology, Piscataway, USA; [3] New Jersey Department of Health, Public Health and Environmental Laboratories, Trenton, USA; [4] Toms River Fire Department, District 1, Toms River, USA; [5] University of Arizona, Mel and Enid Zuckerman College of Public Health, Tucson, USA; [6] US Centers for Disease Control and Prevention, National Institute for Occupational Safety and Health, Cincinnati, USA

Objective: Volunteer firefighters are an understudied occupational group who may experience firefighting and community-related per- and polyfluoroalkyl substances (PFAS) exposures. The Firefighter Cancer Assessment and Prevention Study (CAPS) conducted PFAS biomonitoring of New Jersey (NJ) volunteer firefighters between 2020 and 2023. We assessed their PFAS serum profiles and predictors, and the utility of comparing them to state and national reference populations.

Methods: Volunteer firefighters were enrolled in NJCAPS using referral sampling. Serum profiles of 6 PFAS and associations with sociodemographic and firefighting characteristics collected by survey were assessed. Data from 2016-2018 NJ Biomonitoring Study (NJBM) and 2017-March 2020 National Health and Nutrition Examination Survey (NHANES) were used to construct demographically similar state and national reference populations following NHANES analytic guidelines. We calculated geometric means, detection frequencies, and 95% confidence intervals (CI) for serum PFAS in each population. For the NJCAPS-NJBM analyses, NJ was divided into five county-based regions to examine geographic differences in serum PFAS profiles and associations with predictors of interest.

Results: Among 212 male NJCAPS participants from five NJ county groups, the average PFHxS and PFNA concentrations were slightly higher among NJCAPS than NHANES non-Hispanic white male participants but were similar or lower than NJBM males (PFHxS GM (95%CI) ng/mL NJCAPS: 1.50 (1.36-1.65) NHANES: 1.04 (0.97-1.12) NJBM: 1.47 (1.32-1.65); PFNA GM(95%CI) ng/mL: NJCAPS: 0.59 (0.55-0.64) NHANES: 0.51 (0.47-0.56) NJBM: 0.94 (0.88-1.01)). Compared with NJBM, PFOA and PFHxS levels were higher among NJCAPS participants in northern-NJ regions but PFNA levels were similar or lower in all five regions. We also observed variation in magnitudes of association of several serum PFAS predictors (age, firefighting tenure, education) by regional group among NJCAPS.

Conclusions: When contextualizing exposures, different reference groups used in biomonitoring studies have unique advantages and limitations. These are important considerations when interpreting and comparing PFAS exposures across study populations.

TO18 | Exposure assessment methods for PFAS

TO18-01 | Upgrades to a Suite of One-Compartment Pharmacokinetic Models for Per- and polyfluoroalkyl substances (PFAS) and Implementation as a Batch Tool

Meghan T. Lynch[1], Rachel D. Rogers[2], Claire Lay[1], Sara Sokolinski[1], Weihsueh Chiu[3]

[1] Abt Global, Cambridge, USA; [2] Centers for Disease Control and Prevention (CDC)/Agency for Toxic Substances and Disease Registry (ATSDR), Atlanta, USA; [3] Texas A&M University, Department of Veterinary Physiology and Pharmacology, College of Veterinary Medicine and Biomedical Sciences, College Station, USA

Objective: The Agency for Toxic Substances and Disease Registry (ATSDR) previously created the PFAS Blood Level Estimation Tool to estimate perfluorooctanoic acid (PFOA), perfluorooctanesulfonic acid (PFOS), perfluorononanoic acid (PFNA), and perfluorohexanesulfonate (PFHxS) serum concentrations resulting from drinking PFAS contaminated water, calibrating model parameters for biological half-life (T½) and volume of distribution using Bayesian methods. Based on stakeholder feedback, we improved functionality to allow for batch processing, inclusion of multiple water concentrations and variable non-drinking water exposures through time, and changes in time-step calculations (mass vs. concentration). We also updated the breastfeeding pathway.
Methods: Using simulated exposures for individuals with known PFAS water and serum concentrations, we compared predicted serum PFAS concentrations resulting from each model change to both the measured and originally predicted serum levels. We then evaluated if these model changes, individually and combined, improved fit. For further evaluation of the changes to historic exposure assumptions, we will evaluate Area Under the Curve estimates of modeled levels.
Results: The original model accurately estimates serum levels for each PFAS for most adults, but somewhat overestimated serum concentrations for children. Results indicate that changes in moving PFAS mass rather than concentration to the next time-step improve model fit overall, particularly for children. Incorporating higher historic exposure, and changes to placental transfer assumptions had little change on overall prediction accuracy. Changing to an age independent volume of distribution resulted in slightly increased overestimates in children's serum levels.
Conclusions: Preliminary results indicate updates to the ATDSR tool modestly improve serum PFAS predictions. However, remaining sources of uncertainties include accuracy in historical water concentrations, exposure from non-drinking water sources, and life-history characteristics of individuals.
Disclaimer: The findings and conclusions in this report are those of the authors and do not necessarily represent the official position of Abt, CDC or ATSDR.

TO18-02 | Integrated GC- and LC-HRMS Non-Targeted Analysis Approaches to Characterize PFAS Exposures from Residential Floor Dust

Parker I. Chang[1], Jeremy P. Koelmel[2], Sheng Liu[2], Joseph M. Braun[3], Katherine E. Manz[4], Aimin Chen[5], Bruce P. Lanphear[6], Kimberly Yolton[7], Krystal J. Godri Pollitt[2]

[1] Yale University, Yale College Environmental Studies, New Haven, USA; [2] Yale School of Public Health, Department of Environmental Health Sciences, New Haven, USA; [3] Brown University School of Public Health, Department of Epidemiology, Providence, USA; [4] University of Michigan School of Public Health, Department of Environmental Health Sciences, Ann Arbor, USA; [5] University of Pennsylvania Perelman School of Medicine, Department of Biostatistics, Epidemiology & Informatics, Philadelphia, USA; [6] Simon Fraser University, Faculty of Health Sciences, Burnaby, Canada; [7] Cincinnati Children's Hospital Medical Center, Department of Pediatrics, Cincinnati, USA

Objective: Given the structural diversity of per- and polyfluoroalkyl substances (PFAS), non-targeted analytical techniques are needed to improve coverage of this chemical space. The objective of this study was to use non-targeted analyses to comprehensively detect PFAS in residential floor dust and to characterize the diversity of these exposures.

Materials and Methods: Settled dust was collected from 194 homes in the HOME Study from 2005-2008 to evaluate the influence of toxic chemicals on children's health. The HOME Study is a pregnancy and birth cohort study that enrolled pregnant mothers in the greater Cincinnati area. In each participant's home 24 months after birth, a high-volume surface sampler was used to collect dust from the mid-point of floors in the child's bedroom and main activity room. PFAS in settled dust samples were assessed using complementary gas chromatography high-resolution mass spectrometry (GC-HRMS) and liquid chromatography high-resolution mass spectrometry (LC-HRMS). GC-HRMS data was screened for PFAS through a trace analysis of eight common low-mass PFAS fragments, and LC-HRMS data was annotated using FluoroMatch 5.5.

Results: The number of PFAS detected in each sample varied greatly, ranging from 0 to 62 detected by GC-HRMS and 1 to 23 identified by LC-HRMS. The majority of PFAS detected by GC-HRMS were found in <10% of homes. However, two PFAS were found in over 50%, indicating that some PFAS exposures were common. Certain PFAS identified through LC-HRMS also had a high detection frequency; perfluorodecanesulfonic acid (PFDS), a PFAS used in floor polish, was found in 87% of homes.

Conclusions: Findings underscore that PFAS exposure profiles from floor dust have some common elements but can also vary significantly between homes, emphasizing the need for comprehensive analytical techniques that can capture the uniqueness of individual exposures and the diversity of PFAS in our environment.

TO18-03 | First quantification of ADVs, a novel class of PFAS in serum samples of residents living near a fluoropolymer plant in Northern Italy

Thomas Schettgen[1], Giuseppe Ungherese[2], Mirella Benazzo[3], Jens Rengelshausen[1], Andre Esser[1], Thomas Kraus[1]

[1] RWTH Aachen University, Medical Faculty, Institute for Occupational, Social and Environmental Medicine, Aachen, Germany; [2] Greenpeace Italy, Rome, Italy; [3] private, Vercelli, Italy

Background: Per- and polyfluorinated alkyl substances (PFAS) are highly persistent and bioaccumulative organic pollutants. Human exposure to PFAS has been associated with various health effects, including cancer. A former Solvay fluoropolymer plant in Northern Italy has contaminated the surrounding environment for decades. Besides the historic use of PFOA in the production of fluoropolymers, the plant also used a novel and previously completely unknown group of PFAS called “ADV” since 1996. The residents living in the vicinity of the plant were growingly concerned about their potential exposure to these PFAS.
Aims: A group of residents (n=36, 16 m, 20 f) demanded a human biomonitoring. Analytical standards for quantification of ADV have recently become available. We were aimed to include these standards in our routine analytical method and to quantify the PFAS-levels in serum of the residents.

Methods: Standards for 6 ADV-fractions with increasing molecular weight were obtained and included in our method. Anamnestic data were queried in a questionnaire and serum samples of the residents were drawn and shipped to our laboratory.
Results: The residents showed elevated levels of PFAS in serum. ADV could be determined in every serum sample with a median of 5.5 µg/L serum (max.: 40.1 µg/L serum) with even higher levels than PFOA (Median: 4.2 µg/L serum (max: 22.8 µg/L serum). The levels of ADV showed a highly significant correlation with PFOA in serum.
Conclusions: This is the first report on quantification of ADVs as a novel class of PFAS in serum of residents of a fluoropolymer plant. The good correlation of serum ADV-levels with PFOA prove the fluoropolymer plant as unique source of exposure. The health impact of ADV-levels in blood remains yet unknown.

TO18-04 | Evaluating a novel model for soil screening guidelines for farmworker exposure to per- and polyfluoroalkyl substances

Sara Lupolt[1], Thomas Simones[2], Emily Pennoyer[2], Andy E. Smith[2]

[1] Johns Hopkins Bloomberg School of Public Health, Department of Environmental Health and Engineering, Baltimore, USA; [2] Maine Center for Disease Control and Prevention, Toxicology & Environmental Epidemiology Program, Augusta, USA

Objective: Land application of biosolids is a global management strategy for wastewater sludge. Per- and polyfluoroalkyl substances (PFAS) have been detected in soils of Maine farmland with a history of biosolids application. Reported soil levels of perfluorooctane sulfonate (PFOS) range from < 10 ng/g to 1000 ng/g. There is need to assess PFAS exposure to farmworkers from contact with contaminated soils during their work activities.
Methods: Building on U.S. EPA's Regional Screening Levels (RSL) framework, we developed an exposure scenario representative of a typical farmworker's soil exposure for a small to moderate size vegetable farm in Maine. Key RSL modeling parameters were adjusted to better represent farmworkers, such as incidental soil ingestion rates, soil-to-skin adherence, inhalation of resuspended particulate matter, and time-activity patterns. Using this scenario and toxicity values from the EPA RSL database, we derived non-carcinogenic SSLs for four PFAS. We performed sensitivity analyses on key inputs to these SSLs. To evaluate the significance of these SSLs, we extracted data from a State of Maine ongoing investigation of PFAS soil levels on farms with a history of land application of biosolids.
Results: SSLs for PFOS, PFOA, PFNA, and PFDA were 15, 4.5, 450, 0.3 ng/g dw. These SSLs were largely driven by the incidental soil ingestion pathway, followed by the dermal contact pathway. Field soil averages from the testing of 712 Maine farms with a history of biosolids application exceeded SSLs for these four PFAS on 22%, 12%, 0%, and 58% of fields tested.
Conclusion: These results were sensitive to 2-fold changes in estimates of incidental soil ingestion and dermal absorption coefficients, which is well within the range of uncertainty in these key input values. These results demonstrate the need for improved exposure information and a planned biomonitoring and exposure assessment study of farmworkers.

TO18-05 | Evaluation of Historically Reconstructed PFAS Serum Levels in Communities Exposed to PFAS Contaminated Drinking Water in the Multi-Site Study

Laurel Schaider[1], Aaron Maruzzo[1], Scott Bartell[2,3], Claire Lay[4], Meghan Lynch[5], Rachel Rogers[6], Jason Sautner[7], John Adgate[8], Abigail Bline[9], Linda Brown[10], Weihsueh Chiu[11], Russell Detwiler[12], Timothy Fennell[10], Panos Georgopoulos[13], Ted Lillys[10], Steve Shost[14], Joost Van 't Erve[15], Brent Windebank[8], Meghan Weems[7], Marian Pavuk[7]

[1] Silent Spring Institute, Newton, USA; [2] University of California, Irvine, Joe C. Wen School of Population & Public Health, Department of Environmental and Occupational Health, Irvine, USA; [3] University of California, Irvine, Joe C. Wen School of Population & Public Health, Department of Epidemiology and Biostatistics, Irvine, USA; [4] Abt Global, Boulder, USA; [5] Abt Global, Worcester, USA; [6] Centers for Disease Control and Prevention, National Center for Environmental Health, Atlanta, USA; [7] Centers for Disease Control and Prevention, Agency for Toxic Substances and Disease Registry, Atlanta, USA; [8] Colorado School of Public Health, Department of Environmental and Occupational Health, Aurora, USA; [9] Harvard T.H. Chan School of Public Health, Department of Environmental Health, Boston, USA; [10] RTI International, Research Triangle Park, USA; [11] Texas A&M University, College of Veterinary Medicine and Biomedical Sciences, College Station, USA; [12] University of California, Irvine, Samueli School of Engineering, Department of Civil and Environmental Engineering, Irvine, USA; [13] Rutgers School of Public Health, Department of Environmental and Occupational Health and Justice, Piscataway, USA; [14] New York State Department of Health, Albany, USA; [15] Michigan Department of Health and Human Services, Lansing, USA

Objective: Epidemiological studies investigating potential links between contaminant exposures from drinking water and adverse outcomes often face challenges due to sparse water concentration data. For emerging contaminants like per- and polyfluoroalkyl substances (PFAS), drinking water measurements are rarely available for >5-10 years, limiting assessments of associations with health outcomes over time. Furthermore, reconstructing exposures among participants is complicated by varying residential histories and water-related behaviors. As part of the Agency for Toxic Substances and Disease Registry's Multi-Site PFAS Health Study (MSS), we reconstructed historical serum concentrations for individual study participants for perfluorooctanoate (PFOA), perfluorooctane sulfonic acid (PFOS), perfluorohexane sulfonic acid (PFHxS), and perfluorononanoate (PFNA).

Methods: The MSS is a cross-sectional epidemiological investigation that enrolled over 5,700 adults (ages 18+) and 700 children (ages 4-17) in communities with a history of PFAS drinking water contamination within eight U.S. states. Participants completed questionnaires (2019-2023) that captured residential histories back to 2000 and water-related behaviors (tap/bottled water, home filtration) and provided blood samples for serum PFAS analysis. As a longitudinal component, historical concentrations of PFAS in drinking water were estimated using hydrogeological and numerical modeling approaches and used to estimate monthly serum concentrations for each participant back to 2000 using a one-compartment pharmacokinetic model. To evaluate model performance, we compared measured concentrations to modeled serum concentrations (matched by month and year).

Results: Geometric mean serum concentrations varied 3- to 7-fold across sites, reflecting differential patterns in water contamination. Historically reconstructed serum concentrations were substantially higher than recent serum measurements, reflecting declining exposures following water remediation and population-level declines in background exposures.

Conclusion: Reconstructed serum levels provide insight into past exposures among study participants within and

across sites. Results will be used in ongoing MSS assessments of potential associations of health biomarkers and disease prevalence with chronic PFAS exposure from drinking water over time.

TO19 | Global risks of declining planetary health

TO19-01 | Spatiotemporal variation in risk of *Campylobacter* infection in childhood: a global risk mapping and prediction model using individual participant data

Josh M. Colston[1,2], Md Nasif Hossain[1,3], Benjamin F. Zaitchik[5], Venkataraman Lakshmi[4], Margaret N. Kosek[1,2]

[1] University of Virginia, Department of Medicine, Charlottesville, USA; [2] University of Virginia, Department of Public Health Sciences, Charlottesville, USA; [3] University of Virginia, Environmental Institute, Charlottesville, USA; [4] University of Virginia, Department of Civil and Environmental Engineering, Charlottesville, USA; [5] Johns Hopkins University, Department of Earth and Planetary Sciences, Baltimore, USA

Background: *Campylobacter bacterium* is a major cause of diarrheal illness in children globally, with transmission dynamics influenced by factors like sanitation and climate. This study aims to develop a risk mapping and prediction model of *Campylobacter* infection using individual participant data compiled by the Planetary Child Health and Enterics Observatory (Plan-EO) Initiative to analyze spatiotemporal variation across low- and middle-income countries (LMICs). **Methods:** Individual participant data for *Campylobacter* positivity in stool samples were sourced from multiple LMIC-based studies of children aged 0-59 months. Covariates included household-level and participant-level factors ascertained by study investigators and environmental and hydrometeorological variables extracted from various data products at georeferenced child locations. Multivariate models were fitted to obtain prevalence predictions by diarrheal syndrome and age stratum. **Results:** 30 studies from 44 countries (including locations in Central America and South America, sub-Saharan Africa, and south and southeast Asia) contributed over 90,000 sample results. The probability of *Campylobacter* infection increased exponentially when wind speed exceeded 8 m/s, precipitation and surface runoff surpassed 50% at 250 mm with a similar pattern, and specific humidity also exceeded 50% when above 20 g/kg. Piped, unimproved, and ground drinking water sources increased the odds of infection by 8% (OR = 1.08, 95% CI [1.04–1.12]), 13% (OR = 1.13, 95% CI [1.07–1.18]), and 22% (OR = 1.22, 95% CI [1.17–1.27]), respectively compared to surface water. On the other hand, compared to open defecation, improved sanitation reduced the odds of *Campylobacter* infection by 16% (odds ratio [OR] = 0.84, 95% CI [0.81–0.86]). **Conclusions:** The distribution of Campylobacter is strongly influenced by environmental factors, with higher infection risks associated with increased wind speed, precipitation, surface runoff, and specific humidity. These findings highlight the need for targeted interventions, such as adaptive public health strategies to mitigate environmental exposure and reduce infection risks.

TO19-03 | Global Mapping of Temperature-Related Mortality: A Health Impact Assessment Study in Cities

Huiqi Chen[1], Malcolm Mistry[1], Francesco Sera[2], Pierre Masselot[1], Antonio Gasparrini[1]

[1] London school of hygiene and tropical medicine, Department of Public Health, Environments and Society, LONDON, UK; [2] University of Florence, Department of Statistics, Computer Science and Applications "G Parenti", Florence, Italy

Background
Extreme temperatures, both heat and cold, are well-recognized environmental hazards that pose significant risks to human health. However, quantifying their global burden remains challenging due to the lack of available data on population-specific vulnerabilities in many countries. This study aims to provide a comprehensive assessment of temperature-related mortality at a global scale by integrating multiple datasets and applying advanced statistical modelling techniques.

Methods
We integrated daily time series data from 848 cities in the Multi-Country Multi-City (MCC) Network (2000–2019) to estimate city and age-specific exposure-response functions. We then combined them, with the Global Human Settlement Urban Center Database, to predict exposure-response functions covering in 13,232 urban areas worldwide. Environmental and socioeconomic factors, including population demographics, GDP, urban characteristics, and air pollution were incorporated as meta-predictors using partial least squares (PLS) regression to handle the large number of meta-predictors. Finally, we extrapolated the estimated risks to cities without mortality data, mapping global patterns of heat- and cold-related mortality.

Findings
Preliminary findings indicate that multiple socioeconomic and environmental factors influence the temperature-mortality relationship. We selected six PLS components that optimized model performance while minimizing heterogeneity. Five of the six components are statistically significant predictors of temperature-related mortality risks ($p < 0.01$). Across 13,232 urban areas worldwide, cold- and heat-related mortality risks at the 1st and 99th percentiles versus minimum mortality temperature (MMP) exhibited substantial spatial variability, with the interquartile range (IQR) of risks spanning 1.17–1.25 (IQR = 0.08) for cold-related mortality and 1.03–1.09 (IQR = 0.06) for heat-related mortality.
Interpretation
This study provides a global assessment of heat- and cold-related excess mortality using advanced predictive modelling and diverse data sources. Our approach improves understanding of spatial disparities in climate-related health risks and supports targeted adaptation strategies.

TO19-05 | Burden of infant mortality associated with flood in 37 African countries

Yixiang Zhu[1], Cheng He[1,2], Jovine Bachwenkizi[3], Zafar Fatmi[4], Lu Zhou[1], Jian Lei[1,5], Cong Liu[1], Haidong Kan[1], Renjie Chen[1]

[1] Fudan University, Shanghai, China; [2] Harvard University, Bostan, USA; [3] Muhimbili University of Health and Allied Sciences, Dar es Salaam, Tanzania; [4] Aga Khan University, Karachi, Pakistan; [5] Xi'an Jiaotong University, Xi'an, China

Objective: To investigate the associations between short-, mid-, and long-term flood exposure and infant mortality, and to estimate the burden of flood-related infant mortality in Africa.
Material and Methods: We conducted a sibling-matched case-control study using individual-level data from Demographic and Health Surveys in Africa during 1990–2020. Individual flood experience was determined by matching the residential coordinates with flood events from the Dartmouth Flood Observatory database. The conditional logistic regression was used to analyze the association between flood and infant mortality.
Results: Using data from 514,760 newborns, we found increased risks of infant mortality associated with flood exposure across multiple periods. The risk of infant death remained elevated for up to four years after the flood event, resulting in an increase of 9.69 infant deaths (95% confidence interval: 7.70, 11.69) per 1,000 births. These associations were particularly prominent among female infants, those born to mothers with lower education levels, or those residing in rural areas, houses with basic water or sanitation infrastructures. Overall, flood exposure caused 3.42 infant deaths per 1,000 births in Africa from 2000 to 2020, which was about 1.7 times the burden attributable to life-period flood exposure. We also revealed a general increase in the burden of infant mortality attributable to floods in Africa over the first two decades of the 21st century, with a sharp rise in the last 5 years.
Conclusion: This multi-country study in Africa provides novel and compelling evidence that flood events could have significant impacts on infant mortality risk and burden, even over 4 years after exposure.
Funding/Support: This study was supported by National Natural Science Foundation of China grant, National Key Research and Development Program grant, Shanghai International Science and Technology Partnership Project, and a grant from the Alexander von Humboldt Foundation for the Humboldt Research Fellowship.

TO20 | Advances in exposome research: Approaches, frameworks, and health outcomes

TO20-01 | The Bangladesh Longitudinal Child-Adolescent Development, Education, and Environment Study (BLADES): A Community-level Multipollutant Study on Mother-Child Health in Bangladesh

Juwel Rana[1,2], Rakib Islam[3,2], Dipak Mitra[4], Abdus Salam[5], Jay Kaufman[1]

[1] McGill University, Epidemiology, Biostatistics and Occupational Health, Montreal, Canada; [2] South Asian Institute for Social Transformation (SAIST), Research and Innovation Division, Dhaka, Bangladesh; [3] Monash University, School of Public Health and Preventive Medicine, Melbourne, Australia; [4] North South University, School of Health and Life Sciences, Dhaka, Bangladesh; [5] University of Dhaka, Department of Chemistry, Dhaka, Bangladesh

Concurrent exposures to environmental and climate-related factors, inadequate living conditions, poor nutrition, psychosocial stress, and structural inequities significantly impact child-adolescent development, academic attainment, and health outcomes. To understand their mechanism, the South Asian Institute for Social Transformation (SAIST) launched the Bangladesh Longitudinal Child-Adolescent Development, Education, and Environment Study (BLADES) in 2021 across the Dhaka and Dinajpur districts. Adopting a community-level exposomics framework, BLADES aims to examine the concurrent, prospective, and lifetime effects of environmental, climate-sensitive, psychosocial, lifestyle, and structural factors on child development, academic performance, and mother-child health. BLADES enrolled 2,455 households with children under five, school-going children (aged 0–18), and mothers/caregivers from 2022 to 2025, following them annually. Exposure measures include indoor $PM_{2.5}$ and outdoor air PM_1, $PM_{2.5}$, PM_{10}, SO_2, CO, NO_2, O_3, 11 heavy metals, and 10 PAHs, climate-sensitive exposures (e.g., greenness, temperature, floods), and socioeconomic factors. Outcomes span growth, early childhood development, school attendance, academic performance, blood pressure, lung function, mental health, functional difficulties, ASQ3, SDQ, and quality of life. At baseline, the median age was 24 years for mothers of under-five children and 35 years for mothers of school-going children. About 30% of participants are under five, 70% are school-going children, and 45% of households were in the poorest wealth quintile. The concentrations of air pollutants, heavy metals, and PAH were high in all the study areas, with higher concentrations in Dhaka and the Coal mine areas. For example, the median $PM_{2.5}$ was higher in Dhaka (104 μg/m³) than in Dinajpur (70 μg/m³); the lead ranged from 10 to 3802 ng/m3, arsenic ranged from 3.86 to 34 ng/m3 and benzo(a)fluo ranged 34 to 207 ng/m^3 in the ambient air. BLADES will produce critical evidence on how community-level exposomics affect child-adolescent development and long-term health outcomes for children and their mothers, guiding interventions for marginalized communities.

TO20-02 | Interaction between Environmental Exposures and Healthy Diet on Risk of Type 2 Diabetes in U.S. Women

Jie Chen[1,2], Jaime E. Hart[1,2], Melissa R. Fiffer[3], Peter James[1,4], Qi Sun[5,6], Francine Laden[1,2]

[1] Harvard T.H. Chan School of Public Health, Department of Environmental Health, Boston, USA; [2] Brigham and Women's Hospital and Harvard Medical School, Channing Division of Network Medicine, Department of Medicine, Boston, USA; [3] University of Illinois Chicago, Children's Environmental Health Initiative, Chicago, USA; [4] University of California Davis School of Medicine, Department of Public Health Sciences, Davis, USA; [5] Harvard T.H. Chan School of Public Health, Department of Nutrition, Boston, USA; [6] Harvard T.H. Chan School of Public Health, Department of Epidemiology, Boston, USA

Objective: Harmful environmental exposures may attenuate the benefits of a healthy diet. We examined the interaction between long-term exposure to environmental factors (air pollution, noise, and surrounding greenness) and the Mediterranean diet in association with type 2 diabetes (T2D) risk in a pooled cohort of two US nationwide prospective studies: the Nurses' Health Study (NHS) and Nurses' Health Study II (NHSII).
Materials and Methods: We followed 186,740 participants from June 1992 to T2D diagnosis, loss to follow-up, death, or through June 2019. We applied Cox proportional hazards models to assess the independent associations of 24-month moving average residential exposure to modeled fine particles ($PM_{2.5}$), nitrogen dioxide (NO_2), nighttime noise, satellite-based greenness, and questionnaire-based alternative Mediterranean diet (aMED) scores updated every 4 years and the multiplicative interaction between the environment and diet on T2D risk, adjusting for potential confounders.
Results: We observed 15,441 incident T2D cases over 27 years of follow-up. In fully adjusted models, exposures to air pollution, noise, and lower greenness were individually associated with an increased T2D risk, while adherence to the Mediterranean diet was associated with a reduced T2D risk. The benefits of Mediterranean diet adherence attenuated with higher exposure to environmental pollution and lower greenspace exposure. For example, the hazard ratios (HRs) and 95% confidence intervals (CIs) for high adherence (score 8-9) versus low (score 0-1) were 0.57 (95% CI: 0.45, 0.71) in the lowest $PM_{2.5}$ quintile (<8.9 μg/m^3) but attenuated to 0.80 (95% CI: 0.62, 1.04) in the highest $PM_{2.5}$ quintile (>14.9 μg/m^3). Similar patterns were found for NO_2, noise, and reduced greenspace.
Conclusion: Exposure to higher air pollution, noise, and lower greenness was individually associated with increased T2D risk, while adherence to the Mediterranean diet reduced T2D risk. Harmful environmental exposures may attenuate the benefits of a healthy diet on T2D risk.

TO20-03 | Global environmental measures through the Gateway to Global Aging Data Study allow for harmonized investigations of the exposome

Nia Clements[1], Adam Taggart[1], Carina Gronlund[1], Kayleigh Keller[2], Katherine Francois[1], Jinkook Lee[3], Sara Adar[1]

[1] University of Michigan, Ann Arbor, USA; [2] Colorado State University, Fort Collins, USA; [3] University of Southern California, Los Angeles, USA

Objectives. Environmental exposures are increasingly recognized as modifiable risk factors for disease. A lack of standardized exposures measures, however, poses challenges for cross-study comparisons. To address this limitation, we aimed to link consistent environmental measures to eight harmonized global cohorts. Here, we describe the patterning of these environmental measures across eight countries to understand the relationships of exposures across settings.

Material and Methods. As part of the Gateway to Global Aging Data project, we curated measures of outdoor air pollution ($PM_{2.5}$, NO_2, O_3) and natural space (greenspace, bluespace) for longitudinal cohorts on aging in Brazil, Chile, England, India, Ireland, Mexico, Northern Ireland, and the United States. We focused on global exposure measures derived from satellite data, empirical spatiotemporal prediction models, and physics-based deterministic models. We then linked these data to population density-weighted points in each country and evaluated spatiotemporal patterns within and between countries.

Results. Median concentrations ranged from 7.3-52.8 mg/m^3, 5 -12 ppb, and 31.3-68.9 ppb across countries for $PM_{2.5}$, NO_2, and O_3, respectively. Measures of greenspace and bluespace within a 1 km buffer of population points were similar across place (e.g., greenspace medians ranged from 0.5-0.8, with this measure being indexed from -1 to +1). Within-country variation was lower for $PM_{2.5}$ and O_3 and larger for natural space measures. Although temporal trends and correlations between exposures were often consistent across countries, some unique patterns emerged, such as how from 1990 to 2017, O_3 in India increased by 0.52 ppb/year whereas O_3 in Mexico decreased by 0.60 ppb/year.

Conclusions. Global harmonization of environmental data for research facilitates cross-country comparisons and shows variation within and between countries, enhancing the power to detect associations and identify sources of risk and resilience in environmental impacts on health.

This project is funded by National Institute on Aging, National Institutes of Health (R01 AG030153).

TO20-04 | Establishing an analytical framework to support large-scale exposome epidemiology

Douglas I. Walker, Catherine E. Mullins, Mattie Braselton, Anna S. Young, Karthikeyan Bagavathy Shanmugam, Maria E. Cardelino, Ronnie Smith, Parth Jariwala, Samyukta Sah

Emory University, Gangarosa Department of Environmental Health, Atlanta, USA

Objective: Over a lifetime, humans experience thousands of chemical exposures from multiple sources. A more complete estimate of environmental exposures across the lifespan would be a transformative research initiative. The use of high-resolution mass spectrometry (HRMS) provides a key platform for assessing the exposome; however, application in large populations has been limited due to challenges in sample throughput, instrument robustness, and data handling methods.
Material and Methods: To support large-scale population research for exposome epidemiology, we have established a high-throughput untargeted HRMS analytical framework combining parallel analysis by liquid (LC) and gas (GC) chromatography to enable robust analysis of up to 20,000 blood samples per year. Sample preparation is achieved using open-source automated liquid handlers allowing processing of extracts for LC and GC analysis from a single blood aliquot of 150µL to allow preparation of 80 samples in under 2.5 hours. Exposome profiles are measured using five different HRMS analytical configurations that include reverse phase and HILIC chromatography with both positive and negative ionization, and GC-HRMS to provide detection of 60,000-100,000 chemical signals. Resources for annotation include a standard library of over 7,000 compounds covering a wide range of environmental, drug and endogenous metabolites.
Results: We are applying these advanced untargeted LC- and GC-HRMS profiling approaches to identifying environmental drivers of cancer through analysis of over 10,000 pre-diagnostic blood samples collected from patients later diagnosed with cancer and matched controls. This effort will establish the foundation for a transformative Cancer Exposome Atlas initiative and provide a resource for assembling exposome-disease atlases.
Conclusion: Development of a robust and scalable analytical framework for large-scale exposome epidemiology is a critical first step to provide a robust foundation for exposome research and to facilitate development of a knowledge base of environmental chemicals, their products, distributions, and associated effects.

TO20-05 | From the environment to the heart: Joint effects of long-term external exposome on ischemic heart disease incidence in Europe

Huyen T.K. Nguyen[1], Regina Pickford[2], Massimo Stafoggia[3,1], Federica Nobile[3,1], Göran Pershagen[1], Kathrin Wolf[2], Evangelia Samoli[4], Konstantina Dimakopoulou[4], Klea Katsouyanni[4], Krista Fischer[5], Jaanika Kronberg[6], Mara Delesa-Velina[7], Danielle Vienneau[8], Sara Kress[9], Tamara Schikowski[9], Marta Manczuk[10], Cathryn Tonne[11,12], Fabian Coloma[11,12], Natalia Blay[13], Daniel Szabó[14], Andrea Dalecka[14], Olga Chervova[15], Christopher Åström[16], Marc Chadeau-Hyam[17], Ruben Colindres-Zuehlke[17], Tarik Benmarhnia[18,19], Erik Melén[1,20], Roel Vermeulen[21], Kees de Hoogh[8], Chris Thio[21], Jelle Vlaanderen[21], Rafael de Cid[13], Annette Petters[2,22], Jeroen de Bont[1]

[1] Karolinska Institutet, Stockholm, Institute of Environmental Medicine, Stockholm, Sweden; [2] Helmholtz Zentrum München – German Research Center for Environmental Health (GmbH), Neuherberg, Institute of Epidemiology, München, Germany; [3] Lazio Region Health Service /ASL Roma 1, Rome, Italy, Department of Epidemiology, Rome, Italy; [4] School of Medicine, National and Kapodistrian University of Athens, Department of Hygiene, Epidemiology and Medical Statistics, Athens, Greece; [5] University of Tartu, Institute of Mathematics and Statistics, Institute of Genomics, Tartu, Estonia; [6] University of Tartu, Estonian Genome Centre, Institute of Genomics, Tartu, Estonia; [7] University of Tartu, Institute of Mathematics and Statistics, Tartu, Estonia; [8] Swiss Tropical and Public Health Institute, Allschwil, Switzerland, Basel, Swaziland; [9] IUF – Leibniz Research Institute for Environmental Medicine, Düsseldorf, Germany, Düsseldorf, Germany; [10] Maria Sklodowska-Curie National Research Institute of Oncology, Department of Cancer Epidemiology and Primary Prevention, Warsaw, Poland; [11] ISGlobal, Barcelona, Spain; [12] Universitat Pompeu Fabra (UPF), Barcelona, Spain; [13] Germans Trias i Pujol Research Institute, Genomes for Life–GCAT laboratory, Barcelona, Spain; [14] Masaryk University, RECETOX, Faculty of Science, Kotlarska, Czech Republic; [15] University College London, UCL Research Department of Epidemiology & Public Health, London, UK; [16] Umeå University, Department of Public Health and Clinical Medicine, Umeå, Sweden; [17] Imperial College London, School of Public Health, London, UK; [18] University of California San Diego, Scripps Institution of Oceanography, Califonia, USA; [19] Irset Institut de Recherche en Santé, Environnement et Travail, Paris, France; [20] Karolinska Institutet, Department of Clinical Science and Education Södersjukhuset, Stockholm, Sweden; [21] Utrecht University, Institute for Risk Assessment Sciences, Utrecht, Netherlands; [22] Ludwig-Maximilians-Universität München, Chair of Epidemiology, Institute for Medical Information Processing, Biometry and Epidemiology, Medical Faculty, München, Germany

Introduction: The relationship between the external exposome and cardiovascular health is understudied. We evaluated the joint association between the long-term external exposome and ischemic heart disease (IHD) incidence across Europe within the EXPANSE project.

Methods: We followedeight adult cohorts (Czechia, Estonia, 2x Germany, Poland, Spain, Sweden, United Kingdom) and six administrative cohorts (nationwide and regionwide cohorts in Greece, Italia, Netherlands, 2x Spain, Sweden and Switzerland) from baseline until IHD incidence, death, migration, loss of follow-up, or study end (up to 2019). We measured annual external exposome at the individual residential addresses creating air pollution (NO_2, $PM_{2.5}$, PM_{10}) and built environment domains (vegetation indices, distance to nearest green/blue space, impervious surfaces, walkability). We used time-varying Cox proportional hazard models to evaluate single exposure associations and Quantile g-computation models to evaluate joint exposure associations between the external exposome and IHD. We performed random effects meta-analyses by cohort type.

Results: About 15 million participants were followed of which 5.2% developed IHD. In single-exposure models, higher air pollution increased IHD incidence in both cohort types whereas lower greenspace and higher impervious surfaces increased IHD incidence only in administrative cohorts. In joint-exposure models, a joint increment in the air pollution domain increased IHD incidence in both cohort types while a cumulative increment in the built environment domain increased IHD incidence only in administrative cohorts. In total, one quartile increment in the external exposome increased IHD incidence by 1.4% in adult cohorts (Hazard ratio (HR):1.014(0.918;1.121)) and by

4.1% in administrative cohorts (HR:1.041(1.024;1.058)). Air pollutants, lower greenspace and higher impervious surfaces were the main contributors to the joint effects of external exposome on IHD across cohorts.

Conclusion: Our findings suggest adverse effects of the external exposome on IHD and identify air pollutants, greenspace, and impervious surfaces as key external factors for promoting cardiovascular health.

TO21 | Metal and element exposures and health outcomes

TO21-01 | Relationship between Mammographic Density and Arsenic among a Rural Population

Shelbie D. Stahr[1], Eryn K. Matich[1], Edgar T. Ellis[2], Sean G. Young[3], Gwendolyn M. Bryant-Smith[4], L. Joseph Su[3], **Ping Ching Hsu**[1]

[1] University of Arkansas for Medical Sciences, College of Public Health/Environmental Health Sciences, Little Rock, USA; [2] Columbia University, Mailman School of Public Health, New York, USA; [3] UT Southwestern Medical Center, O'Donnell School of Public Health, Dallas, USA; [4] University of Arkansas for Medical Sciences, Winthrop P. Rockefeller Cancer Institute, Little Rock, USA

Objective. Mammographic density (MD) is both a risk factor and susceptibility marker of breast cancer. Although it is largely inherited, recent studies suggest that the exposure to environmental pollutants can significantly impact MD. This study aims to examine the relationship between arsenic exposure with MD in a rural state.

Methods. 233 participants recruited through collaboration with the mobile mammography unit were included as an ancillary study of Arkansas Rural Community Health Study. MD scores were dichotomized (fatty vs. dense), and serum, saliva, and creatinine-adjusted urine were analyzed for arsenic concentrations using inductively coupled plasma mass spectrometer. Census-tract level ambient air arsenic concentrations and soil arsenic concentrations were linked to participants' baseline addresses. Both environmental and biological arsenic measurements were categorized into respective tertiles. Unadjusted and multivariate logistic regression analysis was used to estimate potential associations between MD and various arsenic concentrations.

Results. Unadjusted models produced a significant increased risk for having dense breasts among medium (OR_{T2} = 3.39; 95% CI 1.18 – 9.72) and high urinary arsenic concentrations (OR_{T3} = 3.38; 95% CI 1.16 – 9.82). When adjusting for parturition, age at baseline, and BMI, the risk for having dense breasts is increased among medium (OR_{T2} = 2.83; 95% CI 0.95 – 8.40) and significantly increased among high urinary arsenic concentrations (OR_{T3} = 3.13; 95% CI 1.05 – 9.32). Both adjusted and unadjusted models produced a significant trend (P trend=0.03 and 0.04, respectively). No relationships were seen among MD and saliva, plasma, residential air or soil arsenic concentrations within this study.

Conclusion. Women with high urinary arsenic concentrations had increased odds of dense breasts. Investigation of arsenic exposure routes is needed to better understand the biological mechanism of arsenic and its effects on mammographic density.

Funding. Arkansas Breast Cancer Research Program.

TO21-02 | Associations of pre- and postnatal exposure to three heavy metals with visual impairment in 3-year-old children: Korean Children's Environmental Health Study

Dajeong Ham[1], Sanghyuk Bae[1,2], Group Ko-CHENS Study group[3,4]

[1] The Catholic University of Korea, Department of Preventive Medicine, College of Medicine, Seoul, South Korea; [2] Environmental Health Center, The Catholic University of Korea, Department of Preventive Medicine, College of Medicine, Seoul, South Korea; [3] National Institute of Environmental Research, Ministry of Environment, Environmental Health Research Division, Department of Environmental Health Research, Incheon, South Korea; [4] Ministry of Environment, Environmental Health Policy Division, Office of Environmental Health, Sejong, South Korea

Background: Visual impairment is a major public health problem affecting children. Heavy metals such as lead, mercury, and cadmium are known to have neurotoxicity and affect children’s development. However, evidence regarding the association between pre-and postnatal exposure to these metals and children’s visual health remains limited

Objectives: The present study aimed to investigate the association between pre- and postnatal exposure to cadmium, lead,and mercury on the risk of visual impairment in 3-year-old children. We also examined how the associations of heavy metal exposure varied across different exposure time points and how sex and environmental factors may modify these associations.

Methods: We used data from the Korean Children's Environmental Health Study. Among the cohort of 5,451, a total of 599 children were included in the analysis. Maternal blood lead, mercury, and cadmium concentrations were measured at early (<20 weeks) and late pregnancy (>30 weeks), and children’s blood concentrations at 24 and 36 months of age. Vision tests were conducted at 36 months follow-up. Visual impairment was defined as a score lower than 0.5 on Jin’s vision chart results, corresponding to 20/40 on the Snellen chart. The association between metal exposure and visual impairment was analyzed using inverse probability of treatment weighting with logistic regression and generalized estimating equations.

Results: Cadmium exposure during early pregnancy showed a stronger adverse risk for unilateral visual impairment (OR = 2.49, 95% CI: 2.01, 3.10, per 1 μg/L increase in blood cadmium levels). The magnitudes of associations were larger among girls and in groups with more screen time and less outdoor activity. However, we did not observe time-dependent variation in the associations.

Conclusion: We observed an increased risk of visual impairment in 3-year-old children with higher prenatal exposure to cadmium.

TO21-03 | Sex-specific urinary metal mixture effects on insulin resistance in Afro-Caribbean adults: A cross-sectional analysis in the Tobago Health Study

Natalie F. Price[1], Ryan Cvejkus[1], Victor Wheeler[2], Patrick J. Parsons[3], Chris Gennings[4], Aaron Barchowsky[5], Joseph Zmuda[1], Alison P. Sanders[5,1], Iva Miljkovic[1]

[1] University of Pittsburgh, Department of Epidemiology, Pittsburgh, USA; [2] Scarborough General Hospital, Scarbrorough, Trinidad & Tobago; [3] Wadsworth Center, New York State Department of Health, Division of Environmental Health Sciences, Albany, USA; [4] Icahn School of Medicine at Mt. Sinai, Department of Environmental Medicine and Climate Science, New York, USA; [5] University of Pittsburgh, Department of Environmental and Occupational Health, Pittsburgh, USA

Objective: Environmental exposures, including metal mixtures, may contribute to insulin resistance and the development of type 2 diabetes (T2D). However, populations of African ancestry, especially in the Caribbean, who experience a high T2D burden, remain understudied.
Methods: This cross-sectional analysis of the Tobago Health Study included 638 Afro-Caribbeans over the age of 50 without T2D (253 women, 385 men). Urinary concentrations of 11 metals and 1 metalloid (“metals” hereafter) were measured: arsenic (As), barium (Ba), cadmium (Cd), cobalt (Co), cesium (Cs), copper (Cu), molybdenum (Mo), lead (Pb), tin (Sn), thallium (Tl), uranium (U), and zinc (Zn). Insulin resistance was assessed using HOMA-IR. We used sex-stratified linear regression models to examine associations between individual metals and HOMA-IR. We applied weighted quantile sum regression (WQSR) to assess potential mixture effects and Bayesian Kernel Machine Regression (BKMR) to identify non-linearities and interactions among metals with HOMA-IR. Covariates included age, BMI, education, and urinary creatinine.
Results: Among women, each quintile increase of Cu and Sn was individually associated with 13.6% (95% CI: 2.3% - 26.1%) and 13.1% (95% CI: 3.5% - 23.5%) higher HOMA-IR, respectively, though effect estimates no longer met significance criteria after multiple comparisons adjustments. In WQSR models, the metal mixture was associated with 17.8% (95% CI: 3.1% - 32.6%) higher HOMA-IR, with Sn, Cu and Ba driving the mixture effect. BKMR results aligned with WQSR, but we observed no strong evidence of non-linear relationships or interaction effects. No significant associations were identified among men.
Conclusions: The observed mixture effect in women was greater than the effect of any metal alone, highlighting the importance of assessing the sex-specific effects of metal mixtures for increased insulin resistance. With T2D rising globally and evidence linking metals to increasing insulin resistance, future mechanistic and observational studies should account for potential mixture effects.

TO21-04 | Association between urinary metals concentrations in Gulf War veterans and Gulf War Illness and cognitive outcomes

Jean M. Costello[1], Kimberly A. Sullivan[1], Brian P. Jackson[3], Maxine H. Krengel[2], Junenette L. Peters[1]

[1] Boston University, Department of Environmental Health, Boston, USA; [2] Boston University, Chobanian & Avedisian School of Medicine, Boston, USA; [3] Dartmouth College, Trace Element Analysis Core, Dartmouth, USA

Introduction: Exposure to heavy metals is an occupational hazard for both military and civilian workers; such exposures have been linked to adverse health and cognitive decrements. Metal exposures have been proposed as a potential contributor to Gulf War Illness (GWI), a chronic and multisymptomatic condition affecting Gulf War (GW) veterans. Here, we measured urinary heavy metal levels in a cohort of 1990-1991 GW veterans and examined the relationships between metals, GWI and cognitive outcomes.
Methods: GW veteran urine samples (N=187) and corresponding health information were obtained from the Boston Biorepository, Recruitment and Integrative Network for Gulf War Illness (BBRAIN) repository; samples were collected between 2020-24. GWI was determined using the Kansas criteria. Our main cognitive outcome used was a measure of verbal learning and memory called the California Verbal Learning Test (CVLT-II). Triple quadrupole inductively coupled plasma mass spectrometry (ICP-MS) was used to determine concentrations of 32 metals. Concentrations were adjusted for creatinine and log-transformed. We used generalized additive models to test the relationships between each element (as a spline term) and the presence of GWI and CVLT-II (total of trials 1-5) scores, adjusting for age, sex, and education.
Results: Measurement detection rates varied, from 100% above the detection limit (arsenic), to 33% above the detection limit (chromium). No element was significantly associated with GWI. Increased levels of zinc were associated with an increased CVLT-II score (OR = 1.03, 95% CI = (1.01, 1.06)) and increased levels of aluminum were associated with a decreased CVLT-II score (OR = 0.95, 95% CI = (0.93, 0.97)).
Conclusion: In our cohort of GW veterans, we detected some associations between urinary metal levels and cognitive outcomes, but not with the presence of GWI. Further work should examine metal mixtures and blood markers to further elucidate these relationships.

TO21-05 | Trends in blood lead levels and cardiovascular disease mortality: the National Health and Nutrition Examination Survey (NHANES), 1976-2020.

Irene Martinez-Morata[1], Filippo Ravalli[2], Gervasio A. Lamas[3], Maria Tellez-Plaza[4], Bruce Lanphear[5], Ana Navas-Acien[1], Anne E. Nigra[1]

[1] Columbia University Mailman School of Public Health, Department of Environmental Health, NEW YORK, USA; [2] Columbia University, Vagelos College of Physicians and Surgeons, NEW YORK, USA; [3] Mount Sinai Medical Center, Department of Medicine, Miami, USA; [4] Instituto de Salud Carlos III, Department of Chronic Diseases Epidemiology., Madrid, Spain; [5] Simon Fraser University, Faculty of Health Sciences, Burnaby, Canada

Objective: Low-level lead poisoning is responsible for >5 million cardiovascular disease deaths annually worldwide. The National Health and Nutrition Examination Survey (NHANES) has been key to inform global estimates and public health interventions. This data has been reported separately for different periods and is not completely comparable to assess trends over time, as different analytical decisions were used across publications. We bring together 40 years of NHANES to examine trends in blood lead levels (BLLs) and their association with heart disease mortality.
Material and Methods: A total of 60,127 adults >20 years with BLLs available through 1976-2020 were included. We estimated the change in weighted adjusted geometric means (aGM) of BLLs at disaggregated time periods and by sociodemographic subgroups. Survey-weighted Cox proportional hazard models were used to estimate the hazard ratio (HR) of heart disease mortality (ischemic+hypertensive+heart failure+rheumatic origin) for ~10-year periods from 1988-2018 in 51,697 participants with follow-up data.
Results: From 1976-1980 to 2017-2020, BLL declined by 94% (aGM(95%CI) 147.9 *µ*g/L(147.2-148.6) vs. 8.5 *µg/L*(8.5-8.6)), with the largest decline (86.8%) between 1976-1980 vs.1999-2000. In models adjusted by sociodemographic+clinical factors, a doubling of the dose in baseline BLLs was associated with heart disease mortality risk in every decade (HRs (95%CI): 1.14 (1.06-1.23) in 1988-1994, 1.29 (1.18-1.41) in 1999-2008, and 1.25 (1.07-1.46) in 2009-2018)), with a largely linear dose-response relationship identified in restricted spline models. BLLs in 2009-2018 remained higher among populations with lower income (aGM(95%CI): 11.0*µ*g/L(10.9-11.1) vs. high income (9.9*µ*g/L(9.8-10)), men (10.8*µ*g/L(10.7-10.8)) vs. women (10.5*µ*g/L(10.4-10.5)), and people >60 (14.2*µ*g/L(14.1-14.3)) vs. 20-39 years (8.1*µ*g/L(8-8.2)).
Conclusion: Despite major declines, low-level lead poisoning is a risk factor for heart disease mortality in US adults with comparable associations across the past four decades and consistent with a log-linear dose-response. Public health interventions to further reduce lead exposure are urgently needed, particularly among disproportionally exposed populations.

TO21-06 | Early Childhood Blood Lead Concentrations and Depressive and Anxiety Symptoms in Adolescence: The HOME Study

Christian Hoover[1,6], George Papandonatos[10], Kim Cecil[2,7], Aimin Chen[3], Bruce Lanphear[4,9], Jeffrey R. Strawn[5], Kimberly Yolton[2], Joseph M. Braun[1]

[1] Brown University, Department of Epidemiology, Providence, USA; [2] Cincinnati Childrens Hospita Medical Center, Department of Pediatrics, Cincinnati, USA; [3] University of Pennsylvania Perelman School of Medicine, Department of Biostatistics, Epidemiology and Informatics, Philadelphia, USA; [4] Simon Fraser University, Faculty of Health Sciences, Burnaby, Canada; [5] University of Cincinnati, Department of Psychiatry and Behavioral Neuroscience, Cincinnati, USA; [6] Harvard University, Harvard Injury Control Research Center, Boston, USA; [7] University of Cincinnati College of Medicine, Department of Environmental and Public Health Sciences, Cincinnati, USA; [8] University of Cincinnati College of Medicine, Department of Radiology, Cincinnati, USA; [9] Simon Fraser University, BC Children's Hospital Research Institute, Burnaby, Canada; [10] Brown Univeristy, Department of Biostatistics, Providence, USA

Objective:
To examine associations between low-level, serial blood lead concentrations in childhood with the development of depression and anxiety in adolescence, and to identify critical periods of susceptibility.

Material and Methods:
We used data from a prospective cohort of 218 caregiver-child dyads (the HOME Study) in Cincinnati, Ohio. We followed children and their families from 2nd trimester to age 12 years. Serial blood lead concentrations in children were measured at ages 1, 2, 3, 4, 5, 8, and 12 years. We measured adolescent self- and caregiver-reported depressive and anxiety symptoms at age 12 years using the Behavioral Assessment System for Children–3 (BASC-3). We used linear and modified Poisson regression with generalized estimating equations to examine the association of average and age-specific (via multiple-informant models) childhood blood lead on adolescent mental health adjusting for covariates. This included testing for interactions, stratified analysis, and sensitivity analysis that included calculating E-values for unmeasured confounding.

Results:
Each doubling in average childhood blood lead concentrations was associated with 2.7-times the risk of elevated adolescent-reported depressive symptoms (95% CI:1.1-6.7) and 1.9-times the combined risk of self and caregiver-reported adolescent depressive symptoms (RR:1.9; 95% CI:1.1-3.4). We observed increasingly stronger associations between self-reported depression scores and blood lead concentrations from age 12 months (ß:1.1; 95% CI:-0.4, 2.7) to 144 months (ß:1.7; 95% CI:-0.1, 3.4). Adolescent sex and race did not modify these associations.

Conclusion:
Low-level childhood lead exposure was associated with elevated adolescent depressive symptoms, especially for exposures occurring in late childhood and early adolescence. Efforts to prevent and reduce lead exposure remain crucial for safeguarding adolescent mental health.

TO21-07 | Metal exposures and kidney function among adolescents in Nicaragua at risk of Chronic Kidney Disease of Uncertain Etiology (CKDu)

Samantha M. Hall[1], Birgit Claus Henn[1], Selene B. Vences[1], Juan José Amador Velasquez[2], Damaris López-Pilarte[2], Magaly R. Amador Sánchez[2], Stephanie Gonzalez Gill[1], Maria Argos[1], David Friedman[3], Madeleine Scammell[1], Daniel Brooks[4], Jessica Leibler[1]

[1] Boston University School of Public Health, Department of Environmental Health, Boston, USA; [2] Centro Médico del Pacífico (CENMED), Masaya, Nicaragua; [3] Beth Israel Deaconess Medical Center and Harvard Medical School, Division of Nephrology, Department of Medicine, Boston, USA; [4] Boston University School of Public Health, Department of Epidemiology, Boston, USA

OBJECTIVE: Chronic kidney disease of uncertain etiology (CKDu) is an ongoing epidemic with high mortality rates among laborers in rural settings, especially in Central America and South Asia. Data on nephrotoxic metal exposure in Central America is very limited, particularly among youth, a population with developmental susceptibility and elevated CKDu risk.

MATERIAL AND METHODS: We collected toenail specimens among a subset of participants in a prospective, community-based cohort study of kidney health among adolescents in Nicaragua (n=300, 50% male, 14-31 years). Toenail metal concentrations were assessed for 23 metals using inductively coupled plasma mass spectrometry (ICP-MS). We used generalized additive models and linear regressions to evaluate individual and pairwise-interactions of metal concentrations in association with estimated glomerular filtration rate (eGFR). Bayesian kernel machine regression (BKMR) was used to assess metal mixtures while adjusting for sex, age, region, water source, smoking, fish consumption, and occupation.

RESULTS: EGFR was notably lower among participants compared to a U.S. NHANES referent (median 102.6 ml/min/1.73m², IQR 20.3; prevalence of eGFR$\leq$75 ml/min/1.73m²: 7.7%). Twenty-two of 23 metals were detected above LOD in >90% of participants. Mixture analyses indicated higher-order interactions in a nephrotoxic metal mixture including As, Cd, Hg, Ni, Pb, and U. Nickel (median: 11.8 ug/g; IQR 20.9) and arsenic (0.19 ug/g; 0.13) concentrations were associated with low eGFR in all models, and relationships appeared non-linear. With nickel, the middle exposure tertile was associated with >3 times the odds of low eGFR (OR: 3.1; 95% CI 1.6, 6.1) in adjusted regressions.

CONCLUSIONS: Ni and As were consistently associated with low eGFR when accounting for confounding, metal co-exposures, and possible interactions. Our results suggest nephrotoxic metal exposures may be elevated among Central American youth and associated with kidney health.

TO22 | Pesticide exposures: Trends, health, and mortality

TO22-01 | A prospective analysis of urinary biomarkers of organophosphate insecticides and mortality among US adults

Emily Segal, Mingyang Song

Harvard T.H. Chan School of Public Health, Epidemiology, Boston, USA

Objective: To investigate the association between urinary concentrations of six dialkyl phosphate (DAP) metabolites of organophosphate insecticides (OPIs) and all-cause and cause-specific mortality.

Methods: DAPs are biomarkers quantifying OPI exposure within hours or days of sample collection. Participants ≥18 years-old enrolled in National Health and Nutrition Examination Survey (NHANES) between 1999-2018 with National Death Index (NDI) linkage eligibility, measured urinary DAP concentration (ng/mL), and ≥1 month follow-up were included. Adjusted hazard ratios (HRs) and confidence intervals (CIs) were estimated using survey-weighted Cox proportional hazard regression models with follow-up (months) as time-scale. We evaluated heterogeneity by smoking and birth cohort for dimethyl alkyl phosphates (DMAPs), a DAP subclass including dimethylphosphate (DMP), dimethylthiophosphate (DMTP), and dimethyldithiophosphate (DMDTP) metabolites. We assessed determinants of DMAP levels using logistic regression.

Results: Our study included 12,846 participants (mean age=44.6 years), of whom 1465 died during follow-up. Comparing quartiles 4 versus 1, DMP and DMDTP were associated with higher cancer-specific morality (HRadj=1.55 (95%CI: 1.07, 2.23); HRadj=1.45 (1.03, 2.04), respectively). Multiple DAPs had positive associations with borderline significance. Diethyldithiophosphate was excluded due to low detection. Comparing above versus below DAP-specific percentile cutoffs ensured no overlapping categories, and metabolites were positively associated with cancer-specific mortality (DMP 75th percentile cutoff: HRadj=1.41 (1.03, 1.93); DMDTP 80th percentile cutoff: HRadj=1.37 (1.01, 1.87)).There was potential heterogeneity by smoking comparing quartiles 4 versus 1 DMP with cancer-specific mortality (current smokers: HRadj=2.47 (1.16, 5.23); nonsmokers: HRadj=1.80 (0.91, 3.55)), and by birth cohort for DMTP and all-cause mortality (1910-1950: HRadj=1.38 (1.08, 1.77); 1951-2000: HRadj=0.91 (0.62, 1.34)). Smoking was inversely and older age was positively associated with quartile 4 DMAPs (i.e. DMP: adjusted odds ratio (ORadj)=0.74 (0.57, 0.95); ORadj=1.53 (1.27, 1.85), respectively).

Conclusions: Individual urinary DMAP concentrations are positively associated with mortality. Their association with smoking and age may reflect differences in OPI dietary consumption.

TO22-02 | Glycemic Status Modifies Organochlorine Pesticides Associated Liver Fibrosis Risk: Evidence from FIB-4 Biomarker Profiling

Taimeng Chen, Zhenxing Mao

Zhengzhou University, College of Public Health, Zhengzhou, China

Context: Little epidemiological evidence exists that human exposure to organochlorine pesticides (OCPs) in different glucose status is associated with liver fibrosis. We aimed to investigate the association of the risk of liver fibrosis with exposure to a single OCP and multiple OCPs.
Methods: A total of 2654 subjects were from the Henan Rural Cohort Study, including 871 patients with type 2 diabetes mellitus (T2DM), 892 patients with impaired fasting glucose (IFG), and 891 individuals with normal glucose tolerance (NGT). The liver fibrotic was assessed using fibrosis 4 score (FIB-4). Plasma OCPs levels were determined by gas chromatograph-mass spectrometry. We explored the relationship between single OCPs and multiple OCPs and the risk of liver fibrosis in different Glucose status using generalized linear modelling (GLM), quantile g-computation (QGC) and bayesian kernel machine regression (BKMR) models.
Results: High detection rates ranged from 75% to 100% for all OCPs. After adjusting for covariates, endrin, op-DDD, and pp-DDT were positively associated with FIB-4 in the IFG and T2DM groups. Notably, a positive correlation was found between op-DDD and high-risk fibrosis (HRF). The QGC analysis indicated that a significant positive association was identified between OCP mixtures and HRF. Finally, the BKMR analysis indicated that concurrent exposure to OCPs was associated with an increased risk of HRF in the NGT and IFG groups (all $P<0.05$).**Conclusion:** Our findings suggest that single or mixed OCPs is associated with increased liver fibrosis biomarkers and HRF, and abnormal glucose status changes the risk of liver fibrosis resulting from such exposures.

TO22-03 | Glyphosate Exposure and Mortality in the United States

Malia Cortez[1], Jiayu Lin[1], Seigi Karasaki[1], Caroline Nondin[1], Hiwot Y. Zewdie[2], Trang VoPham[1,2]

[1] Fred Hutchinson Cancer Center, Epidemiology Program, Seattle, USA; [2] University of Washington, Department of Epidemiology, Seattle, USA

OBJECTIVE
Glyphosate is the most widely used herbicide in agricultural and residential settings across the world. Glyphosate usage substantially increased after the introduction of glyphosate-resistant crops in 1996. Glyphosate is hypothesized to adversely impact health through mechanisms related to genotoxicity, oxidative stress, and metabolic dysregulation. Epidemiologic evidence on glyphosate exposure and health outcomes has been mixed. Further, the association between glyphosate exposure and non-cancer outcomes is understudied. The objective of this study was to examine the association between glyphosate exposure and all-cause mortality.
MATERIAL AND METHODS
Mortality and sociodemographic data from death certificates for all deaths occurring in the United States from 1997-2023 were obtained from the National Center for Health Statistics. Cumulative annual average glyphosate pesticide exposure was estimated by linking nationwide agricultural pesticide application data from the United States

Geological Survey with the year and county of residence at death. Glyphosate exposure was examined using quintiles and continuously per interquartile range [IQR] increase (2.64 kg/km^2). Quasi-Poisson regression with robust standard errors was used to estimate mortality rate ratios (MRRs) with 95% confidence intervals (CIs) for the association between glyphosate exposure and all-cause mortality risk adjusted for individual-level age, sex, race, ethnicity, year, marital status, and education level, and county-level socioeconomic status.

RESULTS

A total of 69,199,816 deaths were included in this study. Higher cumulative annual average glyphosate pesticide exposure was associated with a 66% increased risk for all-cause mortality (adjusted MRR highest quintile [>3.16 kg/km^2; 7,786,473 deaths] vs. lowest quintile [<=1.10 kg/km^2; 18,557,360 deaths]: 1.66, 95% CI 1.30-2.11; p trend<0.01). A positive association was observed when examining glyphosate exposure continuously (adjusted MRR per IQR increase: 1.09, 95% CI 1.02-1.17; p<0.01).

CONCLUSION

Higher agricultural glyphosate exposure was associated with increased all-cause mortality risk in the United States. Future research should examine the role of glyphosate exposure in cause-specific mortality.

TO22-04 | Determination of country-specific exposure patterns of neonicotinoids by human biomonitoring

Sonja A. Wrobel[1], Daniel Bury[1], Marike Kolossa-Gehring[2], Aline Murawski[2], Kyungho Choi[3], Joeun Jung[3], Jeongim Park[4], Ramadhan Tosepu[5], Khaled Hossain[6], Kraichat Tantrakarnapa[7], Mohamed Bedair[8], Alison Connolly[9], Darragh Doherty[9], Jochen Mueller[10], Danielle E. Que[10], Henrique C.S. Silveira[11], Paula Rohr[11], Thomas Brüning[1], Heiko U. Käfferlein[1], Holger M. Koch[1]

[1] Ruhr University Bochum, Germany, Institute for Prevention and Occupational Medicine of the German Social Accident Insurance (DGUV), Bochum, Germany; [2] German Environment Agency (UBA), Berlin, Germany; [3] Seoul National University, Department of Environmental Health Sciences, School of Public Health, Seoul, South Korea; [4] Soonchunhyang University, Department of Environmental Health Science, Asan, South Korea; [5] University of Halu Oleo, Department of Environmental Health, Faculty of Public Health, Kendari, Indonesia; [6] University of Rajshahi, Department of Biochemistry and Molecular Biology, Rajshah, Bangladesh; [7] Mahidol University, Department of Social and Environmental Medicine, Faculty of Tropical Medicine, Ratchathewi, Thailand; [8] National Research Centre, Food Contaminants Department, Cairo, Egypt; [9] University College Dublin, Dublin, Ireland; [10] The University of Queensland, Queensland Alliance for Environmental, Health Sciences (QAEHS), Brisbane, Australia; [11] Molecular Oncology Research Center, Barretos Cancer Hospital, Sao Paulo, Brazil

Neonicotinoids (NNI) are the most frequently applied insecticides worldwide and are used as active substances in plant protection products and veterinary medicines. Due to the different country-specific uses and regulations of NNIs, variances in human exposure are expected. Human biomonitoring (HBM) can be used as a tool for exposure assessment and provide evidence of global differences by analysing urine samples and quantifying NNIs concentrations across different continents.

We investigated exposures to acetamiprid, clothianidin, flupyradifurone, imidacloprid, sulfoxaflor, thiacloprid, and thiamethoxam in urine samples from nine different countries including Australia, countries in South America, Europe and Asia by HBM. Urine samples were analyzed for 17 biomarkers (parent NNI and/or NNI-specific metabolites) using an online-SPE-LC-MS/MS method with stable isotope dilution.

The main metabolite of acetamiprid, *N*-desmethyl-acetamiprid, was the most frequently detected biomarker in all countries (range <LOQ-44 µg/L, approximately 70% positive samples), followed by a major metabolite of imidacloprid, imidacloprid-olefin (range <LOQ-57 µg/L, approximately 50% positive samples). While NNI exposures

in the two European countries were similar, characteristic differences were observed between the Asian countries, i.e. frequent exposures to sulfoxaflor specifically in Korea.

In summary, detection rates and urinary NNI levels differed considerably between countries. Together with data from other national studies, our biomonitoring results help to characterize current environmental exposure patterns of NNIs on a global scale.

TO22-05 | Association between Pesticide Exposure and Impaired Glucose Regulation among Adolescents in the ESPINA Cohort in Ecuador

C. Ssemugabo[1], K. Yang[2], R. P. Parajuli[2], J. Suarez-Torres[3], D. Martinez[3], J. R. Suarez-Torres[2, 3]

[1] Makerere University School of Public Health, Disease Control and Environmental Health, Kampala, Uganda; [2] University of California San Diego, Herbert Wertheim School of Public Health & Human Longevity Science, San Diego, California, United States of America; [3] Fundación Cimas del Ecuador, Quito, Ecuador

Objective: Growing research shows that organophosphate insecticides, and to an extent pyrethroids, may increase glucose dysregulation and risk of pre-diabetes and diabetes. Other commonly used pesticides, such as neonicotinoids or herbicides have not been studied in this context and no research exists among adolescents. We tested the relationship between urinary pesticide biomarkers and impaired glucose regulation among adolescents and young adults living in agricultural communities in Ecuador
Methods: We include data from 499 participants (17 – 23 years) in 2022 who had measures of urinary pesticide biomarkers (organophosphate, pyrethroid, neonicotinoid, sulfoxaflor, and a phenoxy acid herbicide) and hemoglobin A1c% (HbA1c%) in whole blood. We assessed associations between imputed metabolite concentration and HbA1c levels using linear and logistic regression models, and modelled pesticide mixture analyses using quantile g-computation. We adjusted all modes for age, gender, BMI, and urinary specific gravity.
Results: The mean HbA1c% was 5.5% (SD= 0.3), 20% were prediabetic and none diabetic. Doubling the concentration of para-nitrophenol (PNP, organophosphate) and 3,5,6-Trichloro-2-pyridinol (TCPY, organophosphate) were associated with odd ratio (OR) for prediabetes of 1.37 (95%CI: 1.00, 1.88) and 1.35 (95%CI: 1.08, 1.69), respectively. Doubling of TCPY was positively associated with HbA1c% (β_{double}=0.02%, 95%CI:-0.00, 0.04), whereas sulfoxaflor (SLF)2 (β_{double}=-0.02%, 95%CI: -0.03, -0.00]) and SLF1 (borderline non-significant; β_{double}=-0.02, 95%CI: -0.04, 0.00]) were negatively associated. The mixture of all pesticides was negatively associated with HbA1c ($\beta_{mixture}$= -0.08, 95%CI: -0.15, -0.00), in which SLF2, SLF1, trans-3-(2,2-Dichlorovinyl)-2,2-dimethylcyclopropane carboxylic acid (TCC), and 2,4-D were the main contributors to the association.
Conclusion: Two urinary organophosphate metabolites were associated with increased odds of prediabetes in this population of adolescents and young adults in agricultural communities. Furthermore, TCPY was associated with higher HbA1c%, and sulfoxaflor metabolites were associated with lower HbA1C. Longitudinal analyses and replication in other populations are warranted.

TO22-06 | Organochlorine pesticides in serum of people in California, 2006-2017: Are hexachlorobenzene (HCB) concentrations decreasing?

Ian Tang[1], Dina Dobraca[1], Aalekhya Reddam[2], Meltem Musa[2], Kathleen Attfield[1], Martha Sandy[2], Stephanie Jarmul[2], June-Soo Park[3], Nerissa Wu[1]

[1] California Department of Public Health, Richmond, USA; [2] California Environmental Protection Agency, Office of Environmental Health Hazard Assessment, Oakland, USA; [3] California Environmental Protection Agency, Department of Toxic Substances Control, Berkeley, USA

Objective: Organochlorine pesticides (OCPs) are persistent, bioaccumulate, and have high toxicity. These synthetic chemicals are used as pesticides and in chemical industries. Many OCPs were restricted or banned in the USA during the 1970s-1980s, and globally in 2004. We evaluated trends of OCPs in serum among different populations in California from 2006-2017 as part of the statewide efforts of Biomonitoring California.

Methods: Biomonitoring California has conducted eight studies from 2006-2017. Populations for these studies include mothers of children with leukemia (2006-2007), female school employees (2011), mother and infant pairs (2010-2011), Kaiser Permanente Northern California members (2011-2012; 2013), and pregnant Californians (2012; 2015-2016; 2016-2017). In each study, restricted to females, lipid-normalized geometric means (GM) were calculated for six OCPs: beta-hexachlorocyclohexane (β-HCH), p,p′-dichlorodiphenyldichloroethylene (p,p′-DDE), p,p′-dichlorodiphenyltrichloroethane (p,p′-DDT), hexachlorobenzene (HCB), oxychlordane, and trans-nonachlor. We used linear regression to calculate the percent change of OCP GM by year. For analytes with a detection frequency of <65%, GM was not calculated and substituted with the level of detection.

Results: Among 1587 Californians, the average GM was 2.7, 162.7, 1.2, 9.6, 3.0, and 5.7 ng/g lipid for β-HCH, p,p′-DDE, p,p′-DDT, HCB, oxychlordane, and trans-nonachlor, respectively. The average percent change in GM per year ranged from -29.1% to -100.0% for all chemicals except for HCB (34.9%). Only the change in GM for p,p′-DDT was significant.

Conclusion: OCP concentrations appear to decline in different California sub-populations. In contrast, HCB concentrations did not show a downward trend over the decade. NHANES data, collected between 2005-2016, similarly do not indicate a large downward trend for HCB compared to other OCPs. The possible sources of HCB are unclear, but may persist through food, industrial byproducts, or re-emission from other sources. Biomonitoring data illustrates the need to continually monitor exposures after enacted bans so that other exposure sources can be identified and reduced.

TO22-07 | Trends in urinary glyphosate concentrations in the United States: Data from the National Health and Nutrition Examination Survey (2013–2018)

Maria Ospina, Andre Schuetze, Pilar Morales-Agudelo, Meghan Vidal, Lee-Yang Wong, Antonia M. Calafat

Centers For Disease Control And Prevention, National Center for Environmental Health, Atlanta, USA

Objective: Glyphosate is the most widely used herbicide in the United States. Glyphosate toxicity in animals is known, but human carcinogenicity is controversial; limited epidemiologic evidence suggests associations between exposure and respiratory diseases (e.g., asthma) and adverse child neurodevelopment. With public concern mounting over how glyphosate affects human and environmental health, understanding the extent of the general U.S. population exposure to glyphosate is important.

Material and Methods: We analyzed 7,067 urine samples collected from three cycles (2013–2014, 2015–2016, 2017–2018) of the National Health and Nutrition Examination Survey (NHANES) and determined glyphosate concentrations using on-line ion-chromatography isotope-dilution tandem mass spectrometry. We analyzed NHANES data using SAS and SUDAAN. We estimated the weighted detection frequency for the entire population sample per NHANES cycle. We calculated geometric means (GMs) and distribution percentiles for glyphosate urinary concentrations for the entire population and by sex, age, and race/ethnicity. We relied on estimated glyphosate agricultural use data from the U.S. Geological Survey.

Results: Most of the population (70%–81.7%, depending on cycle) was exposed, including children as young as 3 years of age. Glyphosate GM concentrations decreased 36% from 0.411 µg/L (2013–2014) to 0.263 µg/L (2017–2018) for the total population. Downward trends were also observed by age, sex and race/ethnicity. Children 6–11 years old had the highest GMs and smallest decrease (21%) in GM concentrations.

Conclusion: Exposure to glyphosate was prevalent in the U.S. general population during 2013-2018. Nevertheless, glyphosate urinary concentrations declined during this period suggesting a decline in exposure. The downward trend likely reflects changes in glyphosate use resulting, in part, from changes in agricultural practices, regulatory actions, and public awareness of glyphosate toxicity. Continuing glyphosate biomonitoring will help understand how changes in use and actions to restrict applications of this common herbicide affect human exposures.

TO23 | From data to justice: Air pollution, health burdens, and community empowerment

TO23-01 | Environmental Inequities in Adolescent Mental Health: The Role of Air Pollution and Neighborhood Context in Immigrant and US-Born Families

Carlos Cardenas-Iniguez, Hongxu Wang, Megan M. Herting, Max Aung, Claire Campbell, Hedyeh Ahmadi

Keck School of Medicine of the University of Southern California, Department of Population and Public Health Sciences, Los Angeles, USA

Objective: Adolescence is a critical period for mental health development, with depression and anxiety being prevalent concerns. Environmental factors, including air pollution and neighborhood conditions, may play a significant role in shaping mental health outcomes, particularly among immigrant populations. This study examines the relationship between air pollution exposure, neighborhood characteristics, and adolescent mental health, focusing on disparities between immigrant and US-born families.

Materials/Methods: We analyzed longitudinal data from the Adolescent Brain and Cognitive Development (ABCD) Study, including 11,880 participants aged 9–10 years. Geocoded environmental exposure metrics—fine particulate matter ($PM_{2.5}$), nitrogen dioxide (NO_2), and ozone (O_3)—were integrated with sociodemographic variables. Mental health outcomes were assessed using the Child Behavior Checklist. Statistical analyses employed zero-inflated negative binomial models to assess associations between environmental exposures and mental health outcomes in the overall sample and stratified by immigrant status.

Results: NO_2 exposure was linked to lower risk of withdrawn-depressed symptoms but increased rule-breaking behavior, mainly in children with US-born parents. O_3 showed lower risk of rule-breaking but increased withdrawn-depressed and social problems, with higher aggressive and externalizing behaviors in children with US-born parents. $PM_{2.5}$ showed lower risk of withdrawn-depressed and thought problems but increased rule-breaking, primarily in children with US-born parents. However, children with immigrant parents exhibited lower total and aggressive behavior problems. Children with immigrant parents demonstrated greater resilience in some domains but were disproportionately affected by neighborhood disadvantage, suggesting an interplay between social environment and pollution exposure.

Conclusion: This study highlights the complex interaction between environmental and social factors in adolescent mental health, emphasizing the heightened vulnerability of immigrant populations due to neighborhood disadvantages. Targeted public health interventions and policies are necessary to reduce environmental risks and improve neighborhood conditions, particularly in immigrant communities. Future research should further explore longitudinal trajectories and the role of acculturation stress in shaping mental health vulnerabilities.

TO23-03 | Prenatal Exposure to Nitrogen Dioxide and Neurodevelopmental Delays in Children Among Medicaid Recipients: A National Cohort Study

Wanyu Huang[1], Xinye Qiu[1], Krista Huybrechts[2], Sonia Hernandez-Diaz[3], Matthew Shupler[3], Hayon Michelle Choi[1], Michael Leung[1], Yaguang Wei[1,4], Joel Schwartz[1,3], Antonella Zanobetti[1], Marc Weisskopf[1,3], Stefania Papatheodorou[5]

[1] Harvard T.H. Chan School of Public Health, Department of Environmental Health, Boston, USA; [2] Brigham and Women's Hospital, Division of Pharmacoepidemiology and Pharmacoeconomics, Boston, USA; [3] Harvard T.H. Chan School of Public Health, Department of Epidemiology, Boston, USA; [4] Icahn School of Medicine at Mount Sinai, Department of Environmental Medicine and Climate Science, New York, USA; [5] Rutgers School of Public Health, Department of Biostatistics and Epidemiology, Piscataway, USA

Objective: Prenatal period is critical for brain formation and children's neurodevelopment, yet few epidemiologic studies have examined the effects of prenatal exposure to gaseous pollutants, compared to $PM_{2.5}$, particularly in underprivileged populations. Nitrogen dioxide (NO_2) is one of those gaseous pollutants, also deemed as a major traffic emission tracer.
Methods: We assigned ambient NO_2 concentrations at the residential zip code of mother-infant pairs in a longitudinal cohort from the Medicaid Analytic eXtract (MAX). Identification of autism spectrum disorder (ASD) and attention-deficit/hyperactivity disorder (ADHD) were based on ICD-9 codes. Weekly prenatal NO_2 levels from spatiotemporal models were calculated over the gestational period. We applied distributed lag models (DLM) coupled with stratified Cox models (by county, birth year, race), adjusting for temperature, relative humidity, maternal age, behavioral risk factors (e.g. smoking, alcohol, drug use), SES (area-level), as well as season of delivery.
Results: We included 1,544,986 births from 2001 to 2013, to assess the association between NO_2 level and neurodevelopmental delays. We observed a positive association between a 10 ppb increase of prenatal NO_2 exposure and the hazard of ASD during the gestational period (with a 0-to-37-week lag), with a cumulative HR of 1.08 (95% CI: 1.01, 1.15) as the overall effect. We observed no association between NO_2 exposure and ADHD (cumulative HR: 0.97, 95% CI: 0.94 – 1.00). The association between NO_2 and the hazard of ASD were stronger in females compared to males.
Conclusion: Among the Medicaid population, prenatal exposure to NO_2 was associated with increased risk of ASD but not ADHD. This is the first and largest study providing evidence on gaseous pollutants and neurodevelopment delays, among the US socioeconomically disadvantaged population.

TO23-04 | Access to interpretable data to assess cancer risks from industrial releases: EPA datasets and Latinx communities in Houston

Hannah Wheless[1], Lori Hoepner[2]

[1] New York University, Gallatin School of Individualized Study, New York, USA; [2] SUNY Downstate Health Sciences University, Department of Environmental and Occupational Health Sciences, School of Public Health, Brooklyn, USA

Latinx communities face disproportionate environmental injustices, targeted due to systematic economic and political inequities. This research evaluates the ease at which links between industrial releases and risk for adverse health effects such as cancer can be defined to influence policy change in Houston, TX. Data were obtained from the Environmental Protection Agency (EPA)'s Toxic Release Inventory (TRI) and its risk-based extension, the Risk Screening Environmental Indicators (RSEI) model. Using the free and open-source software QGIS, TRI and RSEI datasets for Houston in 2022 were assessed at the neighborhood level via Community Tabulation Areas (CTAs) to identify communities experiencing disproportionate industrial releases and consequent potential health risks. Additionally, a novel risk score was developed by integrating aggregated TRI emissions with PLACES cancer estimate data to assess the correspondence between RSEI cancer risk scores and actual cancer diagnosis estimates. Geospatial visualizations revealed Latinx communities in Houston are home to the most heavily polluting industrial facilities. These areas exhibited the highest cumulative RSEI cancer scores, reflecting elevated potential risk for developing cancer from exposures to multiple hazardous chemicals, particularly carcinogens 1,3-butadiene, benzene, and chromium. Moreover, the strong alignment between the RSEI cancer risk scores and the TRI/PLACES-derived risk score validates the use of RSEI cancer scores as a reliable predictor of cancer risk due to emissions exposure. This correlation was especially evident in Baytown, a Houston community with a 54% Latinx population and over 4 million pounds of industrial pollution released in 2022. The geospatial analysis of TRI, RSEI, and PLACES data elucidates the burden of gathering and analyzing chemical release data in a public health context – reflecting why change beginning at the local level can be difficult for under-resourced Latinx communities facing industrial pollution. Improving access and utility of these resources will provide a resource to advocate for data-driven policy change.

TO23-05 | Sector-Specific Energy Policy Impacts on Mortality Co-Benefits and Socioeconomic Disparities Across Three Midwestern States in the Year 2050

Rory K. Stewart[1], Kiarash Farzad[2], Yang Zhang[2], Kenneth T. Gillingham[1], Benjamin F. Hobbs[4], Daniel C. Esty[1,3], Michelle L. Bell[1,5]

[1] Yale University, School of the Environment, New Haven, USA; [2] Northeastern University, College of Engineering, Boston, USA; [3] Yale University, School of Law, New Haven, USA; [4] Johns Hopkins University, Department of Environmental Health and Engineering, Baltimore, USA; [5] Korea University, School of Health Policy and Management, Seoul, South Korea

Fine particulate matter ($PM_{2.5}$) is one of the leading causes of global disease burden and $PM_{2.5}$ exposure has been shown to be unequally distributed across populations within the United States. Thus, strategies to reduce greenhouse gas emissions, including $PM_{2.5}$, may provide differential distribution of health benefits through improved air quality. Understanding health co-benefits and who receives them can aid policymakers in prioritizing mitigation strategies to ensure the most impactful strategies are undertaken. We investigated three future energy scenarios (electric vehicles, natural gas, energy efficiency) and a business-as-usual scenario to determine how changes to $PM_{2.5}$ levels impact health in three midwestern states (Illinois, Indiana, Ohio) by race/ethnicity, urbanicity, income, residential isolation, and educational isolation. Health impact function analyses that utilize state-of-the-science econometric energy and air quality modeling to the 1km scale, census-tract level populations and projections, and numerous concentration-response functions are used to determine subpopulation-specific avoided health projections. Our preliminary projections estimate future avoided mortalities due to policy changes range from 6,010 (95% Confidence Interval: 4,093, 7,396) to 6,560 (4,469, 8,072) lives saved in 2050. We preliminarily found the highest benefit, by percent mortality change in 2050, for the Black population (34-41% relative benefit), those making under $75,000 (58-63% relative benefit), and the most educationally isolated populations (8-9% relative benefit). Our findings indicate energy policies transitioning from fossil fuel production in the U.S. provide substantial health benefits but vary by population, with some of the most burdened populations benefitting the most from decarbonization policy action.

TO23-06 | Associations between city-level socioeconomic status and urban air pollution-related disease burdens across 13,000 cities worldwide

Soo-Yeon Kim, Gaige H. Kerr, Susan C. Anenberg

George Washington University, Department of Environmental and Occupational Health, Washington, DC, USA

Objective: Area level socioeconomic status (SES) is associated with air pollution and related health impacts. However, studies characterizing this association are still limited to developed countries. We examine the associations between city-level SES and disease burdens attributable to fine particulate matter (PM2.5), nitrogen dioxide (NO2), and ozone (O3) from a global perspective, using state-of-the-science datasets covering 13,189 cities worldwide.
Material and Methods: All-cause mortality attributable to PM2.5, new-onset pediatric asthma attributable to NO2, and chronic respiratory disease mortality attributable to O3 were calculated at the city-level, using pollution data from

global gridded air pollution datasets and disease rates and concentration-response functions from the Global Burden of Disease (GBD) 2021 study. We used Bayesian multilevel Poisson regression to identify associations between these disease burdens and city-level SES, measured with the Human Development Index (HDI) which ranges from 0 (lowest development) to 1 (highest). We controlled for various city- and country-level sociodemographic and geographic characteristics, including total population, population density, total area, and climate.
Results: Globally, we found a significant negative association between city-level SES and disease burdens attributable to PM2.5 (0.29% decrease in PM2.5-attributable mortality rate per 0.01 HDI increase; 95% Credible Interval (CrI): -0.43, -0.15) and a significant positive association with disease burdens attributable to NO2 (6.81% increase in NO2-attributable asthma incidence rate per 0.01 HDI increase; 95% CrI: 6.40, 7.21). Disease burdens attributable to O3 did not show a significant association after adjusting other covariates (0.06% increase in O3-attributable mortality rate per 0.01 HDI increase; 95% CrI: -0.17, 0.29).
Conclusion: City-level SES was associated with PM2.5- and NO2-attributable disease burdens, but in opposite directions. Our findings shed light on disparities in disease burdens attributable to air pollution across cities with different development levels globally. This information can be helpful to improve environmental conditions in overburdened cities.

TO23-07 | Air pollution source impacts at fine scales for long-term regulatory accountability and environmental justice

Lucas R. Henneman, Ting Zhang, Sumaiya Hussein

George Mason University, Sid and Reba Dewberry Department of Civil, Environmental, and Infrastructure Engineering, Fairfax, USA

Background. A growing body of evidence has identified differing health impacts and inequalities associated with source-specific fine particulate matter ($PM_{2.5}$). Our objectives are to 1) create high resolution daily $PM_{2.5}$ source impacts from major source categories from 2011-2020, and 2) use the source impacts fields to quantify source-specific exposure differences among population groups.
Methods. We applied dispersion-normalized positive matrix factorization (DN-PMF) at over 300 monitoring locations across the contiguous United States. We identified five $PM_{2.5}$ source categories across most monitors: traffic tailpipe, secondary sulfate, secondary nitrate, biomass burning, and soil/dust. Next, we used the Community Multiscale Air Quality Model with the Integrated Source Apportionment Method (CMAQ-ISAM) to quantify source impacts on a 12 x 12 km^2 grid. We developed machine learning models to predict each $PM_{2.5}$ sources' daily concentrations from DN-PMF. The models included multiple spatial-temporal predictors, including CMAQ-ISAM $PM_{2.5}$ source impacts, meteorology, and land use. We then applied the machine learning models to predict $PM_{2.5}$ source impacts from each of the five sources at a fine spatial resolution on each day from 2011-2020 in the contiguous United States.
Results. We found changes in the dominant regional sources: secondary sulfate $PM_{2.5}$ decreased on average (by ~1.5%/year) while biomass burning $PM_{2.5}$ increased (by 1%/year). Local sources such as industrial sources and automobile tailpipe emissions show an increase in some locations. Machine learning models performed well, predicting DN-PMF $PM_{2.5}$ source impacts with mean bias of ±0.05 $\mu g\ m^{-3}$ and R^2 greater than 0.90 on average across spatial cross-validation holdout analyses for all sources.
Conclusions. Fine scale $PM_{2.5}$ source impacts reveal divergent trends in regional and local air pollution sources and opportunities for interventions to address elevated exposure and inequalities.

TO24 | Cancer risks from air pollution

TO24-01 | Outdoor $PM_{2.5}$ components and risk of lung cancer in a large prospective U.S. cohort

Lydia Marcus Post[1,2], Alex Keil[1], Jared A. Fisher[1], Joel D. Kaufman[3], Debra T. Silverman[1], Rena R. Jones[1]

[1] National Cancer Institute, Occupational and Environmental Epidemiology Branch, Division of Cancer Epidemiology and Genetics, Rockville, USA; [2] National Cancer Institute, Cancer Prevention Fellowship Program, Division of Cancer Prevention, Rockville, USA; [3] University of Washington, Department of Environmental and Occupational Health Sciences, Seattle, USA

Objective: Regional heterogeneity in the association between outdoor fine particulate matter ($PM_{2.5}$, <2.5μm) and lung cancer suggests that $PM_{2.5}$ chemical components may differentially influence risk. However, few studies have investigated these relationships.
Materials and Methods: We used the U.S. Environmental Protection Agency's Community Multiscale Air Quality model (12 x 12 km) to estimate historical concentrations of outdoor $PM_{2.5}$ components at residential enrollment (1995-1996) addresses of 486,101 NIH-AARP Diet and Health Study participants in eight states. Components included ammonium, nitrate, sulfate, elemental carbon (EC), organic carbon (OC), soil, and other. We used Cox models to estimate hazard ratios and 95% confidence intervals (HR [CI]) of associations between $PM_{2.5}$ components (per 1 μg/m^3) and lung cancer risk overall and by major histologic subtype (adenocarcinoma, squamous cell carcinoma, small cell carcinoma). We treated residential outdoor nitrogen dioxide (NO_2) as an independent exposure (per 10 ppb) given its known relationship with lung cancer. We fit single pollutant models and specified the multi-pollutant mixture using elastic net regularization to account for multicollinearity of components, adjusting for age, smoking, and other key confounders.
Results: 22,426 lung cancers were diagnosed over $\bar{x}$=18 years of prospective follow-up. In single-pollutant models, all $PM_{2.5}$ components were associated with increased risk of lung cancer overall, adenocarcinoma, and squamous cell carcinoma, with the strongest relationships observed for ammonium (HR=1.05 [1.01-1.09], HR=1.08 [1.01-1.15], and HR=1.11 [1.02-1.22], respectively) and nitrate (HR=1.03 [1.01-1.05], HR=1.05 [1.01-1.09], and HR=1.05 [1.01-1.09], respectively). Elastic net retained only OC in models of lung cancer overall (HR=1.02 [1.01-1.03]) and NO_2 for adenocarcinoma (HR=1.05 [1.02-1.08]). There were no clear associations with small cell or squamous cell carcinoma.
Conclusion: Our novel findings in this large cohort suggest that $PM_{2.5}$ chemical constituency may be an important consideration in studies of air pollution and lung cancer.

TO24-03 | Long-term exposure to ambient fine particulate matter and risk of renal cell cancer in the NIH-AARP Diet and Health Study

Jinhui Li[1], Zhengyi Deng[1], Minji Jung[1], John T. Leppert[1,2], Andres Cardenas[3], Marvin E. Langston[3], Benjamin I. Chung[1]

[1] STANFORD UNIVERSITY, Department of Urology, Palo Alto, USA; [2] Veterans Affairs Palo Alto Health Care System, Division of Urology, Palo Alto, USA; [3] STANFORD UNIVERSITY, Department of Epidemiology & Population Health, Palo Alto, USA

Background: Renal Cell Carcinoma (RCC) is the most common type of kidney cancer and a target organ of ambient pollution. However, few studies have tested the association between ambient fine particulate matter ($PM_{2.5}$) and renal cell cancer incidence. We aim to evaluate the association between ambient fine particulate matter exposure and the risk of RCC in a large, geographically diverse U.S. cohort. **Method:** The outdoor residential $PM_{2.5}$ concentrations (1980-2015) were estimated using a nationwide spatiotemporal model for the NIH-AARP Diet and Health Study in six states and two metropolitan areas. Participants were enrolled at the age of 62 years (1995-1996). We parameterized $PM_{2.5}$ exposures in 5-year moving averages that lagged 10 years prior to RCC diagnosis, and evaluated associations continuously (per IQR), and categorized them in quartiles. Time-varying Cox Models were used to estimate associations with RCC per IQR increase in estimated ambient $PM_{2.5}$ levels, adjusting for age, sex, race/ethnicity, education level, BMI, physical activity, alcohol use, smoking, caloric intake, and other dietary factors. We also evaluated $PM_{2.5}$ interactions with hypothesized effect modifiers. **Results**: Among 497,987 participants eligible for analysis, 3359 incident cases were identified. Per IQR (4.6 μg/m^3) increase in $PM_{2.5}$ was significantly associated with an 11% increase in RCC incidence (HR = 1.11, 95% CI = 1.04 to 1.18), which was similarly elevated across quartiles and greatest for participants in the highest exposure category (HR_{Q4vsQ1} = 1.30, 95% CI = 1.09 to 1.55). Although no significant effect modification was detected, a slightly higher HR was observed in males(HR = 1.11, 95% CI = 1.03 to 1.19), older age(HR = 1.14, 95% CI = 1.03 to 1.27), and non-Hispanic white participants (HR = 1.10, 95% CI = 1.01 to 1.21) subgroups. **Conclusion:** In this large US cohort with historical air pollutant exposure, $PM_{2.5}$ was associated with increased RCC risk

TO24-04 | Flash Talk: - Cancer Incidence and Residential Proximity to Coldwater Creek—The Health Legacy of the Atomic Bomb in St. Louis, Missouri

Michael Leung[1], Ian W. Tang[1], Joyce J. Lin[1], Lorelei Mucci[2], Justin G. Farmer[1], Kaleigh McAlaine[1], Joseph J. Mangano[3], Marc G. Weisskopf[1,2]

[1] Harvard T.H. Chan School of Public Health, Environmental Health, Boston, USA; [2] Harvard T.H. Chan School of Public Health, Epidemiology, Boston, USA; [3] Radiation and Public Health Project, Ocean City, USA

Objective: Beginning in the 1940s, radioactive waste from the effort to develop an atomic bomb was stored in the open near the St. Louis airport, and over several decades, contaminated the nearby Coldwater Creek. Here, we evaluated whether living near Coldwater Creek in childhood was associated with increased cancer incidence in the St. Louis Baby Tooth—Later Life Health Study (SLBT), a cohort centered in St. Louis, Missouri.
Material and Methods: We used data from 4,209 eligible SLBT participants. Self-reported cancer diagnoses were grouped into four composite cancer outcomes: any cancer, solid cancers (all except leukemias and lymphomas), known radiosensitive cancers (thyroid, breast, leukemia), and non-radiosensitive cancers (all except thyroid, breast, and leukemia). We estimated ORs and 95% CIs between our cancer outcomes and residential proximity to Coldwater Creek (≤1km, >1-5km and >5km) in childhood when participants donated their baby teeth, adjusting for sociodemographics and neighborhood median income. We also estimated the number of excess cases associated with living near Coldwater Creek.
Results: About 3% of participants lived ≤1km from Coldwater Creek. Compared to those living >5km from the Creek, the OR was 1.45 (95% CI: 0.97, 2.15) for any form of cancer, which translates to 749 (95% CI: -41, 1614) excess cases per 10,000 persons. We observed that associations with known radiosensitive cancers (OR=1.87 [95% CI: 0.96, 3.6]) was stronger than non-radiosensitive cancers (OR=1.32 [95% CI: 0.87, 2.01]) for participants living ≤1km versus >5km. Cancer risks were still elevated for those living in the >1-5km buffer. Despite imprecision in some of the site-specific estimates, we detected clear signals for radiosensitive organs like the thyroid (OR=4.89 [95% CI: 1.38, 17.41] for living ≤1km versus >5km.
Conclusion: Our study provides novel evidence of the long-term effects of living near Coldwater Creek in childhood and the risk of cancer.

TO24-05 | Flash Talk: - Air Pollution and Cardiovascular Risks in Testicular Cancer Survivors: A Case-Crossover Study

Judy Ou[1,3], Joemy Ramsay[2], Heydon Kaddas[3], James VanDerslice[4,3], Brock O'Neill[5,3], Sarah Garcia[3], Heidi Hanson[6], Anne Kirchhoff[1,3]

[1] University of Utah, Department of Pediatrics, Salt Lake City, USA; [2] University of Utah, Department of Surgery, Salt Lake City, USA; [3] Huntsman Cancer Institute, Salt Lake City, USA; [4] University of Utah, Division of Preventive Medicine and Public Health, Salt Lake City, USA; [5] University of Utah, Department of Surgery, Salt Lake City, USA; [6] Oak Ridge National Laboratory, Oak Ridge, USA

Aim: Chemotherapy for testicular cancer is highly effective but contributes to long-term risks for heart and lung problems. Air pollution leads to and worsens heart and lung conditions, but its impact on the health of testicular cancer survivors is unknown. We assessed associations between exposure to nitrogen dioxide (NO_2) and ozone (O_3) and health events with a cardiovascular or respiratory diagnosis among survivors of adolescent and young adult (AYA) testicular cancer.

Methods: This Utah-based case-crossover study included 385 survivors, diagnosed 2000-2016 at age 15-39 years and living 2 years after diagnosis, who had emergency department/urgent care [ED/UC] or inpatient admissions with a cardiovascular (n_{event}=257) and/or respiratory (n_{event}=685) diagnosis. Conditional logistic regression with robust standard errors estimated associations while controlling for temperature and humidity. Continuous and dichotomous (≥moderate Air Quality Index (AQI)) measures were included for NO_2 and O_3 for the 1-4 days before events (lag days). We conducted subgroup analysis according to cancer treatments.

Results: Exposure to NO_2 ≥moderate AQI on lag day 1 increased odds for any cardiovascular event (OR=1.97, 95% CI=1.08-3.59) and cardiovascular inpatient admission (OR=2.48, 95% CI=1.21-5.10). Survivors treated with chemotherapy and exposed to NO_2 ≥moderate AQI on lag day 1 had increased odds for cardiovascular inpatient admission (OR=3.00, 95% CI=1.29-7.00). O_3 ≥moderate AQI on lag day 4 also increased odds for respiratory ED/UC visit (OR=1.34, 95% CI=1.00-1.79). O_3 on lag day 4 was associated with any cardiovascular event (OR=1.02/1-ppb, 95% CI=1.00-1.03) and cardiovascular inpatient admission (OR=1.03/1-ppb, 95% CI=1.00-1.05).

Conclusions: Air pollution may worsen long-term outcomes among AYA testicular cancer survivors. This information can improve survivorship care plan for testicular cancer by addressing air pollution as a risk factor for exacerbation of these conditions.

TO25 | Strategies and approaches for improving the utility of environmental health data

TO25-02 | Development and Validation of a Neighborhood Psychosocial Index (NPSI) to Address Socioeconomic Disparities in Air Pollution-Related Respiratory Health

Adjani A. Peralta[1,2], Edgar Castro[2], Joel Schwartz[2]

[1] *Amsterdam University College, Amsterdam, Netherlands;* [2] *Harvard T.H. Chan School of Public Health, Boston, USA*

Introduction

Air pollution significantly affects respiratory health, with low socioeconomic status (SES) communities facing a disproportionate burden. Traditional SES indices often overlook complex stressors like psychological stressors leading to residual confounding in epidemiological analyses. This study introduces a Neighborhood Psychosocial Index (NPSI) that integrates conventional SES measures with additional factors such as crime statistics and access to resources to accurately capture the complexities of socioeconomic disparities affecting health outcomes.

Methods

The NPSI was developed using Independent Component Analysis (ICA) to integrate psychosocial stressors with traditional SES measures at the census tract level. Variables were regressed individually against the Social Vulnerability Index (SVI), with residuals undergoing ICA to identify key components contributing to the NPSI. This innovative approach highlights the multifaceted nature of socioeconomic stressors within communities.

Results

In Detroit, MI, using ICA identified three components capturing various psychosocial stressors. The NPSI conceptualization involves positively weighted components reflecting urban services and emergency needs, with a negatively weighted component for community support services. Component 1 (Urban Services and Community Segregation) is linked to an 18.73% increase in asthma hospital admissions (95% CI: 8.58-29.77%) per SD, highlighting urban areas with social challenges. Component 2 (Demographic and Community Support Services) correlates with an 11.79% decrease (95% CI: -17.51 to -5.61%) per SD, suggesting effective community support reduces admissions. Component 3 (Emergency Response and Community Needs) indicates a 15.54% increase (95% CI: 4.69-27.54%) per SD, denoting elevated needs in areas with emergency services. Additionally, NPSI is associated with a 15.17% increase in admissions (95% CI: 8.58-22.16%) per SD, reflecting the impact of psychosocial stress on asthma rates.

Conclusions

The NPSI captures psychosocial stressors beyond traditional SES indices, offering insights into air pollution's health impacts. Identifying often-overlooked at-risk populations enhances targeted interventions to reduce environmental health disparities and promote equitable outcomes.

TO25-03 | Increasing utility of environmental health data through Data Management and Sharing policies, FAIR principles, and harmonized language.

Maria Shatz

NIEHS/NIH, Office of Data Science, Durham, USA

The extensive generation of data and knowledge at the intersection of environmental epidemiology and exposure science is unlocking new opportunities to answer large-scale, complex questions in toxicology, public health, and environmental health sciences. However, a significant challenge remains around our ability to find, share, and reuse this data, thereby reducing utility of data and diminishing impact of scientific research investments. To address this challenge, the NIH issued an updated Data Management and Sharing (DMS) Policy, effective January 2023. This policy aligns with similar initiatives in Europe and other regions, aiming to streamline data sharing and enhance scientific progress globally. To facilitate data harmonization and reuse, NIH encourages development and use of data and metadata standards such as Common Data Elements (CDEs) to enable standardized and consistent use of data in research, especially translational and clinical research, and to facilitate efforts to replicate and validate findings. CDEs are data elements (or variables) that are defined and used the same way across multiple studies, to standardize the way data is collected. In this talk, we will discuss where to find CDEs and how to use them. Additionally, we will explore gaps in environmental health CDEs, identify areas of priorities for this community and strategies for filling the gaps.

TO25-04 | Developing a framework for collecting and reviewing strategies to address environmental public health problems

Ana Pomales, Kirsten Reed

CDC, NCEH/DEHSP/WFEHSB, Atlanta, USA

Objective
Information about effective environmental public health (EPH) practices is rarely published in scientific literature or available online. As a result, practitioners often spend valuable time searching for or recreating strategies to tackle common EPH problems. The National Center for Environmental Health’s Division of Environmental Health Science and Practice (DEHSP) developed a framework to identify and review information about EPH practices. The framework will provide actionable information to EPH practitioners through an online repository of EPH practices for addressing environmental hazards.
Material and Methods
DEHSP assembled a team with expertise in areas of EPH including asthma, lead, water, food, and evaluation. The team reviewed existing methods for evaluating evidence of effectiveness from the public health and medical fields by scanning the internet and scientific literature. They developed a framework for collecting information from EPH practitioners and assessing both research-based and practice-based evidence. The team tested the framework by assessing four EPH practices championed by EPH practitioners. These practices addressed private well repairs,

swimming pool operator outreach, digital tickborne disease education through dashboard and storyboards, and early outbreak interventions in food establishments.

Results

The pilot showed the submission process was effective for gathering information needed to review and share practices. Submitters found the submission process reasonable, appreciated development of a logic model, were excited for CDC to highlight their practice, and found summaries of practices helpful. Reviewers found the review rubrics helpful and provided suggestions for improving practices.

Conclusion

The proposed online repository for EPH practices will include guidance for implementation, highlight evidence gaps, and offer suggestions for strengthening the supporting evidence. Providing access to proven strategies will enhance public health practices and empower practitioners to implement effective solutions and evaluate them.

TO25-05 | Environmental Justice and Health Inequities Research in India: A Systematic Review

Rachit Sharma[1], Chelsea Lam[2], Ajay Pillarisetti[2]

[1] Drexel University, Dornsife School of Public Health, Philadelphia, USA; [2] University of California, School of Public Health, Berkeley, USA

Objective:

India's economic growth has come at a steep environmental cost, with industrial expansion, rapid urbanization, and climate change amplifying environmental risks. These burdens disproportionately impact structurally marginalized populations, reinforcing long-standing health inequities through exposure to polluted air, unsafe water, and extreme weather events, among others. We conducted a systematic review to evaluate the scope of environmental justice and health inequities research in India, with a focus on theoretical frameworks, key environmental exposures, affected populations, and methodological approaches.

Material and Methods:

A comprehensive search of four databases (Web of Science, Scopus, MEDLINE, and EMBASE) was conducted from their inception to September 1, 2024. Population-based, observational studies on environmental justice and studies assessing inequities in environmental exposures and associated health outcomes in India were included. Risk of bias was assessed using the ROBINS-E tool, and findings were synthesized narratively.

Results:

A total of 76 studies were identified. Air pollution (ambient and household), water contamination, and heat stress were the predominant environmental exposures studied. Health outcomes frequently examined included respiratory diseases, cardiovascular conditions, and child health indicators. Socioeconomic position, caste, and urban-rural disparities were commonly investigated, but limited research considered gender, disability, or intersectional vulnerabilities. Methodologically, cross-sectional designs dominated, with relatively few longitudinal studies. Many studies lacked explicit environmental justice frameworks, and institutional contributions were concentrated in select research institutions, highlighting regional disparities in academic engagement. Notably, few studies incorporated community-driven approaches or policy-relevant interventions.

Conclusion:

Despite growing recognition of environmental justice and health inequities in India, research remains methodologically limited and lacks intersectionality. Future studies should incorporate longitudinal analyses, expand

demographic representation, and adopt community-based approaches. Tailored frameworks reflecting India's diverse population and historical context should guide justice-related research. Strengthening interdisciplinary collaborations and integrating environmental justice principles into policy-driven research are essential to addressing environmental health inequities in India.

TO26 | Heat, mind, and health: Exploring climate-driven mental and neurological risks

TO26-01 | A Critical Gap in Addressing Mental Health in Heat Health Action Plans Worldwide

Allison Stewart-Ruano[1], Raenita Spriggs[1], Emma L. Lawrance[3], Alessandro Massazza[4], Alexandra Czerniewska[7], Alejandro S. Reale[6], Joy Shumake-Guillemot[6], Katherine M. Keyes[2], Yoshira O. Van Horne[5], Robbie M. Parks[1]

[1] Columbia University Mailman School of Public Health, Department of Environmental Health Sciences, New York, USA; [2] Columbia University Mailman School of Public Health, Department of Epidemiology, New York, USA; [3] Imperial College London, Climate Cares Centre, Institute of Global Health Innovation and Grantham Institute for Climate Change and the Environment, London, UK; [4] United for Global Mental Health, London, UK; [5] University of California Los Angeles Fielding School of Public Health, Department of Epidemiology, Los Angeles, USA; [6] World Health Organization, WHO/WMO Joint Climate and Health Office, Geneva, Switzerland; [7] Health Equity and Human Rights LLP, Princeton, USA

Objective: Extreme heat is associated with mental health conditions such as suicide, anxiety, and substance use disorders. However, the integration of mental health in heat health planning remains limited and no comprehensive assessment of its inclusion exists to date. This study aimed to: 1) identify and categorize mental health-related content in heat health action plans globally; and (2) analyze the gaps in included interventions related to extreme heat and mental health.

Material and methods: We analyzed 83 Heat Health Action Plans from 24 countries to evaluate mental health inclusion in extreme heat strategies. Using qualitative content analysis, we assessed mental health coverage and categorized interventions across socioecological levels (i.e., individual, relationship, community, societal). Specifically, we identified and analyzed references to (1) mental health impacts of extreme heat; (2) heat exposure risk factors related to mental health; (3) mechanisms linking heat exposure and mental health; and (4) relevant interventions. We analyzed the inclusion of mental health across countries, WHO region, World Bank income levels, and administrative level.

Results: Mental health was mentioned in 63 plans (75.9%), with 26 plans (31.3%) acknowledging the mental health impacts of extreme heat. Only 18 plans (21.7%) included specific interventions, primarily focused at the societal-level, with limited attention to individual or community-level support. Individuals with mental illness were often grouped with other vulnerable communities without addressing their unique challenges. Low-income countries were not represented, while 44 plans (53%) came from high- and upper-middle-income countries. Among lower-middle-income countries, most plans originated from India (35; 89.7%).

Conclusion: Our findings highlight a broader challenge within climate adaptation policies, where recognition of mental health risks is often not matched by necessary resources, planning, and interventions. This analysis highlights critical gaps in mental health inclusion and emphasizes the need for targeted strategies to address the mental health impacts of extreme heat.

TO26-02 | Assessing Filipino mental health professionals’ knowledge, attitudes, and practices on climate change and mental health

Alexis M. Cacioppo[1], Josefina Ly-Uson[2], Vincenzo Bollettino[3], Antonio Sison[2], Mario Lorenzo L. Bautista[2], Kate Burrows[4]

[1] The University of Chicago, Pritzker School of Medicine, Chicago, USA; [2] University of the Philippines, College of Medicine, Manila, Philippines; [3] Harvard University, Harvard Humanitarian Initiative, Cambridge, USA; [4] The University of Chicago, Public Health Sciences, Chicago, USA

Objective: Climate change exacerbates social and environmental risk factors for mental health problems, especially in countries that face challenges with geography, adaptive capacity, and infrastructure like the Philippines. The World Health Organization recommends mental health professionals take measures to understand and address climate-related mental health problems.

Material and Methods: Cross-sectional data was gathered from 216 licensed mental health professionals through an online, self-report, English-language survey disseminated by the Philippine Psychiatric Association and the Psychological Association of the Philippines (05/05-06/16/2024). Respondents’ knowledge, attitude, and practice(s) regarding climate change and mental health were assessed with items adapted from the 2019 Minnesota Survey of Climate and Mental Health. This study was approved by the University of the Philippines Manila Research Ethics Board.

Results: Respondents included psychometricians (36%), guidance counselors (18%), psychologists (13%), and psychiatrists (11%) with patients living in urban areas (79%). More than 9 in 10 respondents agreed or strongly agreed that they are “concerned about impacts [of climate change] on [patients’] mental health” (97%) and are “positioned to help patients cope with impacts [of climate change] on their mental health” (91%). Many respondents reported sometimes or often “see[ing] evidence of …mental health impacts in patients…influenced by…climate change,” including anxiety (73%), depression (68%), and chronic psychological distress (65%). Few respondents agreed or strongly agreed that they “feel well-prepared to discuss mental health impacts [of] climate change” (41%) or that they were “familiar with tools and resources” to “asses[s]” and “trea[t]” “patients suffer[ing] from impacts [of] climate change” (23% and 22%, respectively).

Conclusion: Filipino mental health professionals sampled demonstrate knowledge and attitudes favorable to the care of patients’ suffering from the mental health impacts of climate change. Our results underscore the need to bolster their capacity and preparedness to address and mitigate climate-related mental health problems.

TO26-03 | Prenatal and postnatal exposure to ambient temperature and risk of autism spectrum disorder and attention-deficit/hyperactivity disorder among Medicaid recipients

Matthew Shupler[1], Xinye Qiu[2], Krista Huybrechts[3], Sonia Hernandez-Diaz[1], Hayon Choi[2], Michael Leung[2], Wanyu Huang[2], Yaguang Wei[4], Joel Schwartz[1,2], Antonella Zanobetti[2], Marc Weisskopf[1,2], Stefania Papatheodorou[5]

[1] Harvard T.H. Chan School of Public Health, Department of Epidemiology, Boston, USA; [2] Harvard T.H. Chan School of Public Health, Department of Environmental Health, Boston, USA; [3] Brigham and Women's Hospital, Division of Pharmacoepidemiology and Pharmacoeconomics, Boston, USA; [4] Icahn School of Medicine at Mount Sinai, Department of Environmental Medicine and Climate Science, New York, USA; [5] Rutgers School of Public Health, Department of Biostatistics and Epidemiology, Piscataway, USA

Background: Studies on the association between ambient temperature and risk of autism spectrum disorder (ASD) and attention-deficit/hyperactivity disorder (ADHD) has been limited, especially among low-income populations who may be particularly vulnerable to environmental exposures.
Methods: The Medicaid Analytic eXtract (MAX) was used to generate a mother-infant longitudinal cohort from 2001-2013. Cases of ASD within the cohort were identified via ICD-9 codes. Ambient temperature exposure was assigned at the zip-code of residence.Cox proportional hazard models examined the association between both average zip-code level prenatal and postnatal ambient temperatureexposure with the risk of developing ASD and ADHD. Distributed lag non-linear models were used to characterize temperatureexposures (prenatal: 0-to-37-week lag; postnatal: 0-to-3-year lag). Cox models were stratified by county, birth year and race and adjusted for demographics, behavioral risk factors, ambient fine particulate matter concentration, season of delivery, and area-level socioeconomic status.
Results: A total of 1,548,303 births were included in the modeling.A 1-degree Celsius increase in average weekly prenatal temperature exposure was associated with 1.05 (95% CI: 1.00–1.11) and 0.99 (95%CI: 0.97-1.00) times the risk of ASD and ADHD, respectively, at the cumulative lag (0-37 weeks). A 1-degree Celsius increase in average annual postnatal temperature exposure was associated with 1.02 (95% CI: 0.96–1.08) and 1.02 (95% CI: 0.98–1.05) times the risk of ASD and ADHD, respectively, at the cumulative lag (0-3 years).
Conclusion: Among low-income children, prenatal temperatureexposure was associated with elevated risk of ASD but not ADHD risk. Although there were potentially sensitive windows of exposure in early childhood for ASD and ADHD, cumulative postnatal temperatureexposure was not associated with ASD and ADHD risk.

TO26-04 | Extreme Heat and Mortality from Neurodegenerative and Mental Health Disorders: Evidence from a Nationwide Climate-Health Analysis in Mexico

Ivan Gutierrez-Avila[1], Marlene Cortez-Lugo[2], Julio Landero[1], Elena Colicino[1,3], Liliana Gomez Flores-Ramos[4], Mike Z. He[1], Itai Kloog[1,3], Robert O. Wright[1,3], Allan C. Just[5,6]

[1] Icahn School of Medicine at Mount Sinai, Department of Environmental Medicine, NYC, USA; [2] National Institute of Public Health, Department of Environmental Health, Cuernavaca, Mexico; [3] Icahn School of Medicine at Mount Sinai, Institute for Exposomic Reseach, NYC, USA; [4] National Institute of Public Health, Center for Population Health Research, Cuernavaca, Mexico; [5] Brown School of Public Health, Department of Epidemiology, Providence, USA; [6] Brown University, Institute at Brown for Environment and Society, Providence, USA

Objective. Nervous system, mental, and behavioral disorders (NMBD) are increasing globally, and extreme temperatures can elevate mortality risks for affected individuals. This study explores the relationship between extreme temperatures and NMBD mortality across Mexico's diverse climate regions, addressing a gap in evidence for LMICs.

Methods. We used the novel case-time series study design to assess the impact of extreme temperatures on NMBD mortality in Mexico (2002–2019). Analyzing 292,844 adult death records, we stratified data by Köppen climate regions [Tropical (Af, Am, Aw), Arid (BSh, BSk, BWh), and Temperate (Cfa, Cfb, Cwa, Cwb)] and NMBD outcomes. ICD-10-coded deaths included mental disorders (F00-F99), nervous system diseases (G00-G99), and suicides (X60-X84), with specific codes for neurodegenerative diseases including, Alzheimer's, Parkinson's, and non-Alzheimer's dementias. Daily population-weighted exposures to mean ambient temperature were estimated using ERA5-Land reanalysis and the Gridded Population of the World. We assessed mortality risks from extreme heat (99th percentile) and extreme cold (1st percentile) relative to the region-specific 75th percentile using quasi-Poisson regressions with distributed-lag non-linear models, accounting for up to 21 days of lagged effects. Köppen-region associations were pooled using random-effects meta-analysis.

Results. Neurodegenerative mortality was predominantly observed in older adults (88.4%, ≥65 years-old), and primarily associated with extreme heat, with the strongest associations observed within two days post-exposure (lag_{0-2}) in tropical regions. Pooled relative risks (RR-lag_{0-2}) were 1.31 (95% CI: 1.19–1.43) for mental disorders, 1.24 (95% CI: 1.09–1.40) for nervous system diseases, and 1.24 (95% CI: 1.13–1.36) for suicides. The relative risk (RR-lag_{0-2}) for neurodegenerative-related mortality was 1.38 (95% CI: 1.22–1.57) overall, with specific estimates of 1.44 (95% CI: 1.14–1.82) for Alzheimer's, 1.40 (95% CI: 1.13–1.74) for Parkinson's, and 1.46 (95% CI: 1.24–1.72) for non-Alzheimer's dementias.

Conclusion. Extreme heat increases NMBD mortality risk, with the strongest associations consistently observed in tropical climate regions within the first two days post-exposure.

TO26-05 | Advancing mental and physical health in changing working environments: the EU INTERCAMBIO project

Michelle C. Turner[1], Xavier Basagaña[1], Karin Broberg[2], Alex Burdorf[3], Alexander Gorny[4], Irina Guseva Canu[5], Henrik Kolstad[4], Neil Pearce[6], Anjoeka Pronk[7], Jenny Selander[8], Ingrid Sivesind Mehlum[9], Sara Svensson[10], Maria Albin[8], Manosij Ghosh[11], Lode Godderis[11], Bernice Scholten[7], Kurt Straif[1], Susana Viegas[12], Jordi Julvez[13], Manolis Kogevinas[1]

[1] Barcelona Institute for Global Health (ISGlobal), Barcelona, Spain; [2] Lund University, Lund, Sweden; [3] Erasmus MC, Rotterdam, Netherlands; [4] Aarhus University, Aarhus, Denmark; [5] University of Lausanne, Unisanté, Lausanne, Switzerland; [6] London School of Hygiene and Tropical Medicine (LSHTM), London, UK; [7] Netherlands Organisation for Applied Scientific Research (TNO), Utrecht, Netherlands; [8] Karolinska Institute, Stockholm, Sweden; [9] Copenhagen University Hospital Bispebierg and Frederiksberg, Copenhagen, Denmark; [10] Halmstad University, Halmstad, Sweden; [11] KU Leuven, Leuven, Belgium; [12] NOVA National School of Public Health, Lisbon, Portugal; [13] Pere Virgili Institute for Health Research (IISPV), Reus, Spain

Objective: The EU INTERCAMBIO project will perform and evaluate interventions in strategic industries in Europe impacted by climate change and the transition towards the green and circular economy. We will also leverage upon large-scale European initiatives regarding occupational, industrial, and population-based cohorts, to advance climate-relevant occupational health research.

Material and Methods: INTERCAMBIO is structured by five main objectives, to 1) promote use of the most advanced research tools and methods, 2) evaluate potential short- and long-term mental and physical health effects of occupational heat, cold, and solar UV radiation exposures, 3) evaluate impacts of specific workplace interventions in five key industries relevant to climate change and the green transition, 4) model the socio-economic impacts of workplace interventions and develop a policy framework for social protection and decent green jobs; and 5) coordinate multi-level stakeholder engagement and develop a new health research agenda. INTERCAMBIO forms part of the WISEWORK-C (Workplace Innovation for Sustainable Well-Being Network) Cluster.

Results: Detailed workplace interventions are ongoing in outdoor construction (ISRCTN12209427), health care (ISRCTN25033513), public transit (NCT06688721), renewable energy (wind turbine manufacturing) (ISRCTN10031201), and waste management/recycling (ISRCTN19236285) industries. An international stakeholder survey was conducted to identify research gaps and ensure subsequent stakeholder consultation efforts to develop a new European health research agenda. Open online research and policy tools are being developed including protocols and training materials, a living systematic review, quantitative JEMs for occupational heat, cold, and solar UV radiation, and a policy tool kit.

Conclusion: INTERCAMBIO is the first large-scale initiative to systematically assess the health effects of climate change and green transition-related occupational exposures through intervention-based research across multiple industries. By integrating advanced research methodologies, economic modeling, and stakeholder-driven policy development, INTERCAMBIO will provide an evidence-based foundation for protecting workers' health in a changing climate and ensuring the sustainability of green jobs.

TO27 | Improving our understanding of the chemical exposome

TO27-01 | Advancing Chemical Exposomics with Multi-omics: An Integrative Framework for Precision Environmental Health

Peng Gao

Harvard T.H. Chan School of Public Health, Boston, USA

Objective: To develop an integrated exposomics-multi-omics framework for characterizing environmental exposure signatures and their biological impacts across respiratory, cardiovascular, and neurodegenerative conditions.
Material and Methods: We applied a comprehensive exposomics approach in three disease-specific cohorts: an asthma cohort, a lung cancer cohort, a cardiovascular disorder cohort, and a neurodegenerative disease cohort. Environmental exposures were assessed using targeted and untargeted high-resolution mass spectrometry of serum samples and passive sampling devices. Integration with proteomics and metabolomics data was conducted using correlation network analysis and pathway enrichment methods. Bioinformatic workflows were developed to identify statistically significant associations between xenobiotic exposures and altered molecular pathways.
Results: Disease-specific exposome profiles were identified across all conditions. Exposure to air pollutants was associated with increased respiratory disease severity ($p<0.05$) and altered inflammatory cytokine profiles. In the cardiovascular cohort, we identified 14 significantly up-regulated and 3 down-regulated compounds compared to controls. Network analysis revealed significant correlations between specific chemical classes and metabolic pathways, particularly those involved in amino acid metabolism, oxidative stress, and immune function ($|r|>0.9$; adjusted $p<0.05$). Both site-specific and non-site-specific variations in neurodegenerative-related exposome signatures were observed across multiple Florida locations.
Conclusion: Our integrative exposomics approach demonstrates the utility of combining chemical exposure assessment with multi-omics profiling to identify disease-relevant exposure signatures and affected biological pathways. This framework enhances our understanding of how environmental exposures contribute to complex disease development and progression, providing potential targets for intervention and precision environmental health strategies.

TO27-02 | The untargeted chemical exposome and metabolome in follicular fluid and associations with fertility outcomes

Anna Young[1], Audrey Gaskins[2,1], Chris Gennings[3], Donghai Liang[1,2], Jessica Spencer[4], Alicia Smith[4], Martha Braselton[1], Catherine Mullins[1], Parth Jariwala[1], Anna Knight[4], Douglas Walker[1]

[1] Emory Rollins School of Public Health, Gangarosa Department of Environmental Health, Atlanta, USA; [2] Emory Rollins School of Public Health, Department of Epidemiology, Atlanta, USA; [3] Icahn School of Medicine at Mount Sinai, Department of Environmental Medicine and Climate Science, New York, USA; [4] Emory School of Medicine, Department of Gynecology and Obstetrics, Atlanta, USA

Objective: Many endocrine-disrupting chemicals are reproductive toxicants that impair ovarian function and fertility. Most research has been limited to a small number of known chemicals measured in blood or urine. We aimed to characterize the complex chemical exposome in follicular fluid, a more toxicologically relevant ovarian tissue, and examine its associations with outcomes of controlled ovarian stimulation.

Materials and Methods: Follicular fluid was collected from 84 patients undergoing egg retrieval for assisted reproduction at the Emory Reproductive Center in Atlanta (2018–2020). These samples were analyzed for environmental chemicals and endogenous metabolites using untargeted high-resolution mass spectrometry (HRMS) with gas- (GC) and liquid- (LC) chromatography. We conducted single-chemical regression models as well as mixture models based on weighted quantile sum regression with random subsets (WQS_{RS}) to evaluate single and cumulative mixture effects on total number of oocytes retrieved, adjusted for age, race, smoking, and ovarian stimulation protocol.

Results: We detected ~70,000 untargeted features in follicular fluid, including confirmed plasticizers, pesticides, flame retardants, and per- and polyfluoroalkyl substances with detection rates >90%. In single-chemical models, ~3,000 features were negatively associated with oocyte yield (false discovery rate<20%). In WQS_{RS} models, 587 selected environmental chemicals detected by GC, for example, were cumulatively associated with a 21% lower expected oocyte yield (95% CI: -30%, -12%) per standard-deviation increase in the exposure mixture index, compared to average single-chemical effect estimates of -10%. In pathway enrichment analysis, 22 pathways, including methionine, cysteine, nicotinamide, glycine, serine, and histidine metabolism, were significantly enriched with the chemical mixture indices and oocyte yield (FDR<5%), indicating possible underlying mechanisms.

Conclusion: Complex chemical mixtures are detectable in the oocyte microenvironment and may impact ovarian reserve and response to gonadotropin stimulation. Discovery-based untargeted exposomics enabled identification of potential emerging exposures of concern and cumulative mixture effects larger than any single chemical effect alone.

TO27-03 | Exposure to Silent Spring potential breast carcinogens and association with breast cancer incidence in a diverse U.S cohort

Katherine Pullella[1], Che-Jung Chang[1], Sami Teeny[2], Jennifer E. Kay[3], Ruthann A. Rudel[3], Anna S. Young[4], Douglas I. Walker[4], Xin Hu[4], Dean P. Jones[2], Alexandra J. White[1]

[1] National Institute of Environmental Health Sciences, Epidemiology Branch, Research Triangle Park, USA; [2] Emory University, Division of Pulmonary, Allergy, Critical Care and Sleep Medicine, Department of Medicine, Atlanta, USA; [3] Silent Spring Institute, Newton, USA; [4] Emory University, Gangarosa Department of Environmental Health, Rollins School of Public Health, Atlanta, USA

Objectives: We investigated the risk of breast cancer associated with exposure to a priority list of 920 chemicals previously identified to be relevant for breast cancer, based on *in vivo, in vitro* and *in silico* research.

Materials and Methods: In a case-cohort of 1,437 invasive breast cancer cases and 2,411 controls from the Sister Study cohort, gas chromatography high-resolution mass spectrometry was used to quantify exogenous chemicals in baseline blood samples (2003–2009). Samples were run in duplicates, and data were extracted using XCMS. PeakWalk was used to annotate features with three in-house libraries (n=921 chemicals). Cox regression models were used to estimate adjusted hazard ratios (HR) and 95% confidence intervals (95%CI) between an interquartile range increase in individual chemical exposure intensity and breast cancer incidence with a false-discovery rate (FDR) correction, adjusting for age, education, household income and menopausal status. Models were stratified by self-reported race and ethnicity and estrogen receptor (ER) status.

Results: Women were followed for 7.35 years (mean). 55 priority chemicals were quantified and detected in over 60% of samples. Overall, there were no statistically significant associations after FDR correction. Among ER- tumors (n= 389), 11 chemicals were associated with a higher breast cancer incidence, with the strongest associations observed for industrial chemical nonylphenol (HR = 1.09, 95%CI 0.98-1.21) and herbicide bromacil (HR=1.06, 95%CI 0.99-1.16). In non-Hispanic Black women, associations varied by ER status, with the strongest association observed for benzo(a)pyrene and ER+ tumors (HR= 1.25, 95%CI 0.99-1.60).

Conclusion: In this US-wide cohort, we observed some evidence that these priority chemicals, previously identified based on experimental evidence for breast-carcinogenic activities, are associated with breast cancer incidence in women. Preliminary analyses suggest that associations may vary by ER tumor status and race and ethnicity. Future steps will include mixture analyses to evaluate associations with patterns of exposure.

TO27-04 | Characterizing untargeted chemical exposome changes among adolescents undergoing bariatric surgery

Shudi Pan[1], Zhenjiang Li[1], Douglas I. Walker[2], Mattie Braselton[2], Brittney O. Baumert[1], Qiran Jia[1], Elizabeth Costello[1], Jesse A. Goodrich[1], Hongxu Wang[1], Sarah Rock[1], Todd M. Jenkins[3], Stephanie Sisley[4], Scott M. Bartell[5], Thomas H. Inge[6], Stavra Xanthakos[7], Catherine E. Mullins[2], Max T. Aung[1], Erika Garcia[1], Sandrah P. Eckel[1], Rob McConnell[1], David V. Conti[1], Justin R. Ryder[6], Lida Chatzi[1]

[1] University of Southern California, Department of Population and Public Health Sciences/Keck School of Medicine, Los Angeles, USA; [2] Emory University, Gangarosa Department of Environmental Health, Atlanta, USA; [3] University of Cincinnati College of Medicine, Division of Biostatistics & Epidemiology, Cincinnati Children's Hospital Medical Center, Department of Pediatrics, Cincinnati, USA; [4] Baylor College of Medicine, USDA/ARS Children's Nutrition Research Center, Department of Pediatrics, Houston, USA; [5] University of California, Irvine, Department of Environmental and Occupational Health, Department of Epidemiology and Biostatistics, and Department of Statistics, Irvine, USA; [6] Northwestern University Feinberg School of Medicine and Ann & Robert H. Lurie Children's Hospital of Chicago, Department of Surgery, Chicago, USA; [7] University of Cincinnati College of Medicine, Division of Gastroenterology, Hepatology, Nutrition, Cincinnati Children's Hospital Medical Center, Department of Pediatrics, Cincinnati, USA

Objective:
Lipophilic chemicals stored in adipose tissue can be mobilized into circulation with weight loss. This study aimed to characterize chemical exposome changes among adolescents undergoing bariatric surgery and to identify key determinants modifying these changes.
Material and Methods:
We performed untargeted plasma chemical exposome analyses using gas and liquid chromatography with high-resolution mass spectrometry (LC-HRMS) from 149 adolescents in the Teen-Longitudinal Assessment of Bariatric Surgery (Teen-LABS) study. Samples were collected prior to surgery, and 6mo, 12mo and 36mo post-surgery. Based on PubChem, chemicals were grouped by shared chemical properties (e.g., lipophilicity) via k-means clustering. Changes in individual chemical concentrations at 6mo were examined using a linear mixed model to 1) determine whether factors such as sociodemographic, and clinical characteristics were associated with average post-surgery chemical changes at 6mo, and 2) assess if these associations were consistent across chemical groups.
Results:
Thirty-two chemicals in seven classes [polycyclic aromatic hydrocarbons (PAHs), nitro-PAHs, organochlorine pesticides, organophosphates, pyrethroid pesticides, per- and polyfluoroalkyl substances (PFAS) and phthalates] were identified and categorized into four groups. Most chemical increases were observed by 6mo post-surgery. For example, 80% of the 10 chemicals in group 3 (representing lipophilic chemicals) exhibited increases in concentrations which peaked at 6 months. After adjusting for study site, older age (p-value:0.01), race/ethnicity (p-value:0.04), type of surgical procedure (p-value:0.02) at baseline were significantly associated with average chemical changes at 6mo. We also found associations of parent's income and use of any weight loss medication with changes in chemical concentrations varied across chemical groups.
Conclusion:
This study characterized chemical exposome over time among adolescents undergoing bariatric surgery and found key social and behavioral factors contributing to rapid changes 6mo after bariatric surgery. These findings inform clinical recommendations aimed at minimizing potential adverse health effects related to the chemical release among adolescents preparing for obesity treatment.

TO28 | Links between exposure to PFAS and health outcomes in adults

TO28-03 | Longitudinal associations between per- and polyfluoroalkyl exposures and postpartum psychological dysfunction

Gary Joseph[1], Léa Maitre[2], Azzurra Invernizzi[1], Elena Colicino[1], Haibin Guan[1], Rosalind Wright[1,3], Robert Wright[1,4], Maria J. Rosa[1,4], Megan Horton[1,4], Veerle Bergink[6], Martha M. Téllez Rojo[5], Lauren M. Petrick[1,7]

[1] Icahn School of Medicine at Mount Sinai, Department of Environmental Medicine, New York, USA; [2] Instituto de Salud Global de Barcelona, Barcelona, Spain; [3] Icahn School of Medicine at Mount Sinai, Department of Public Health, New York, USA; [4] Icahn School of Medicine at Mount Sinai, Institute for Exposomics Research, New York, USA; [5] Instituto Nacional de Salud Pública, Centro de Investigación en Nutrición y Salud, Cuernavaca, Mexico; [6] Icahn School of Medicine at Mount Sinai, Department of Psychiatry, New York, USA; [7] Sheba Medical Center, The Bert Strassburger Metabolic Center, Tel-Hashomer, Israel

Background: Emerging evidence links perinatal per- and polyfluoroalkyl substances (PFAS) exposure to postpartum depression (PPD). To our knowledge, no previous study assessed this association using multiple time points of postpartum psychological functioning—including PPD and subscales of anhedonia, anxiety, and depression. Therefore, we assessed the association between PFAS at 1 month and mothers' postpartum psychological functioning from 1 to 24 months.

Methods: Eight serum PFAS were analyzed in 389 women at 1 month postpartum from Mexico City enrolled in the Programming Research in Obesity, GRowth, Environment and Social Stressors (PROGRESS) cohort using untargeted liquid chromatography-high resolution mass spectrometry. Postpartum psychological functioning was assessed with the Edinburgh Postnatal Depression Scale (EPDS) at 1, 12, and 24 months postpartum. Modified Poisson regression assessed the association between individual PFAS and PPD (EPDS≥13), while negative binomial regression assessed the EPDS subscales. Weighted Sum Quantile regression, constrained in the positive direction, evaluated PFAS mixtures' association with the outcomes.

Results: A doubling of PFDoA and PFDA was associated with higher PPD risk at 1 and 24 months postpartum, respectively. PFDoA was associated with anhedonia at 1 and 12 months and PFDA at 1 and 24 months, with larger effect sizes at later time. Both branched and linear PFOS were positively associated with anhedonia and depression at 1 month postpartum, with a stronger effect for anhedonia. Additionally, PFOA and PFNA were associated with anhedonia at 1 month. A higher PFAS mixture was associated with increased PPD at 1 month (RR: 1.51, 95% CI: 1.03-3.67), with anhedonia at 1 month (IRR: 1.38, 95% CI: 1.12-1.72), and with anhedonia at 24 months (IRR: 1.45, 95%CI: 1.08-1.95) postpartum.

Conclusion: These results suggest a persistent negative effect of early postpartum PFAS exposure on postpartum psychological functioning, in particular, anhedonia, and highlights the need to mitigate PFAS exposure to support maternal well-being.

TO28-04 | Associations between serum per- and polyfluoroalkyl substances and blood pressure outcomes among adults in the Multi-site Study

Yuting Wang[1], Abigail Bline[2], Rebecca DeVries[3], Mary B. Terry[1], Linda Brown[4], Naida Gavrelis[3], Philippe Grandjean[5], Emily Heckel[1], Yerin Jung[6], Robert Laumbach[7], Marian Pavuk[8], Celina Phillipson[6], Marlee Quinn[2], Patrick Rago[8], Ruthann Rudel[1], Adam Schaefer[9], Veronica Vieira[6], Tamarra James-Todd[2,10], Laurel Schaider[1]

[1] Silent Spring Institute, Newton, USA; [2] Harvard T.H. Chan School of Public Health, Department of Environmental Health, Boston, USA; [3] Eastern Research Group Inc., Concord, USA; [4] RTI International, Research Triangle Park, USA; [5] University of Southern Denmark, Copenhagen, Denmark; [6] University of California Irvine, Department of Environmental and Occupational Health, Irvine, USA; [7] The State University of New Jersey, Department of Environmental and Occupational Health and Justice, New Brunswick, USA; [8] Agency for Toxic Substances and Disease Registry, Centers for Disease Control and Prevention, Office of Community Health Hazards Assessment, Atlanta, USA; [9] Abt Global, Cambridge, USA; [10] Harvard T.H. Chan School of Public Health, Department of Epidemiology, Boston, USA

Objective: Exposure to per- and polyfluoroalkyl substances (PFAS) has been positively associated with blood pressure and hypertension, although prior findings were inconsistent. We examined associations between serum PFAS and blood pressure measurements among adults in a large, cross-sectional study in communities in eight U.S. states with historical PFAS drinking water contamination.
Methods: Adult participants (ages 18+) recruited in 2019-2023 completed questionnaires and blood sample collection (n=5,776). Serum was analyzed for perfluorooctanoate (PFOA), perfluorooctane sulfonic acid (PFOS), perfluorononanoate (PFNA), perfluorohexane sulfonic acid (PFHxS), perfluorodecanoate (PFDA), perfluoroundecanoate (PFUnDA) and 2-(N-Methyl-perfluorooctanesulfonamido)acetic acid (MeFOSAA). Resting blood pressure was measured in triplicate during a clinical visit and averaged. Linear regression was used to estimate associations between individual PFAS and systolic and diastolic blood pressure (SBP, DBP, respectively), after excluding pregnant women and people taking anti-hypertension medications (n=1,688). Models were adjusted for age, sex, race/ethnicity, income, education, body mass index (BMI), smoking, and alcohol consumption. Models were stratified by age, sex, and BMI to assess effect modification.
Results: Among 3,622 participants with complete data, 62% were female; the mean age was 52 years (SD: 15 years). Six serum PFAS were positively associated with SBP and/or DBP, with the strongest associations observed for PFOS and PFHxS. Each doubling of PFOS and PFHxS was associated with 0.5 (95% CI: 0.1, 0.9) and 0.5 (0.2, 0.7) mmHg increases in SBP, and 0.6 (0.3, 0.9) and 0.7 (0.5, 0.9) mmHg increases in DBP, respectively. Positive associations were stronger among participants who were under 50, male, and had BMI 25-29.9 kg/m^2.
Conclusions: We observed positive associations of serum PFAS with SBP and DBP. Our findings emphasize the importance of reducing PFAS exposure to improve cardiovascular health, especially in highly-exposed communities. Our future analyses will assess associations of PFAS mixtures and historically-reconstructed serum PFAS levels with hypertension and blood pressure.

TO28-05 | Exposure to Per- and Polyfluoroalkyl Substances and Osteoporosis among Sister Study Participants

Rebecca N. Noga[1], Che-Jung Chang[3], Douglas Walker[2], Catherine E. Mullins[2], Alexandra White[3], Jessie Buckley[1]

[1] University of North Carolina at Chapel Hill, Department of Epidemiology, Gillings School of Global Public Health, Chapel Hill, USA; [2] Emory University, Department of Environmental Health, Rollins School of Public Health, Atlanta, USA; [3] National Institute of Environmental Health Sciences, Epidemiology Branch, Research Triangle Park, USA

Objective: Exposure to per-and polyfluoroalkyl substances (PFAS) has been associated with poor bone health among adult women, although results are inconsistent. Using data from the Sister Study, a nationwide cohort of women whose sisters were diagnosed with breast cancer, we conducted a pilot study to examine associations of PFAS exposure with osteoporosis prevalence and incidence.
Material and Methods: Serum PFAS was assessed for 200 women in stored samples collected at baseline (2003-2009) using liquid chromatography-mass spectrometry. Women self-reported osteoporosis diagnoses and dates at baseline and throughout follow-up (2022). We used modified Poisson regression models to estimate prevalence ratios (PR) of baseline osteoporosis and Cox proportional hazard models to estimate hazard ratios (HR) for incident osteoporosis during follow-up in relation to $\log_2$-transformed serum PFAS concentrations, adjusted for baseline age, menopause duration, BMI, race/ethnicity, income, education, diet, and smoking status.
Results: Most participants were higher socioeconomic status and identified as non-Hispanic White. A total of 57 participants (34%) reported an osteoporosis diagnosis, including 21 (11%) diagnosed prior to baseline, 33 (17%) during follow-up, and 13 (7%) with unspecified timing. 17 PFAS were detected in >70% of the participants. In adjusted models, multiple PFAS were associated with higher osteoporosis prevalence at baseline (e.g., PFDoA PR: 1.67, 95%CI: 1.08, 2.57; PFTrDA PR: 1.47, 95%CI: 0.94, 2.31). However, associations of PFAS with incident osteoporosis were less consistent. Higher serum 8:2 FTS concentrations were positively associated with osteoporosis incidence (HR: 1.21, 95% CI: 0.82, 1.78), whereas higher PFBS concentrations were inversely associated (HR: 0.58, 95%CI: 0.23, 1.50).Conclusion: These preliminary data suggest that exposure to PFAS in adulthood may be associated with osteoporosis among older women, although larger prospective studies are needed. Given their ubiquitous nature and long half-life, reducing exposure to PFAS is potentially an important modifiable factor impacting women’s bone health later in life.

TO28-06 | Inverse associations between serum concentrations of per- and polyfluoroalkyl substances and adult obesity endpoints in the Multi-Site Study

Yerin Jung[1], Abigail Bline[2], Linda M. Brown[3], Robert J. Laumbach[4], Celina N. Phillipson[1], Anne P. Starling[5], Veronica M. Vieira[1], Carolina Villanueva[1], Yuting Wang[6], Scott M. Bartell[1]

[1] University of California, Irvine, Department of Environmental and Occupational Health, Joe C. Wen School of Population & Public Health, Irvine, USA; [2] Harvard University, Department of Environmental Health, Harvard T.H. Chan School of Public Health, Boston, USA; [3] RTI International, Research Triangle Park, USA; [4] Rutgers, The State University of New Jersey, Department of Environmental and Occupational Health and Justice, New Brunswick, USA; [5] University of North Carolina at Chapel Hill, Department of Epidemiology, Gillings School of Global Public Health, Chapel Hill, USA; [6] Silent Spring Institute, Newton, USA

Objective: While evidence suggests exposure to per- and polyfluoroalkyl substances (PFAS) may influence adipose tissue development, epidemiological studies have shown inconsistent results. This study investigates the cross-sectional association between serum PFAS concentrations and obesity-related measures among adults.

Material and Methods: This study analyzed 5,524 adults enrolled in the Multi-site Study across eight US sites (2019-2023); serum samples and body measurements were collected at the same clinical visit. Seven frequently detected PFAS compounds were measured in serum samples; each and their sum were investigated. PFAS concentrations were log-transformed, and PFAS with detection frequencies below 70% were categorized as binary variables. Endpoints included body mass index (BMI), waist circumference (WC), waist-to-hip ratio (WHR), and body roundness index (BRI). Prevalent obesity was determined using standardized BMI and WC cutoffs. Age, sex, race/ethnicity, income, and education were adjusted as potential confounders.

Results: Higher serum concentrations of all seven PFAS showed weak inverse associations with continuous obesity measurements. For example, each natural log increase in the sum of seven PFAS serum concentrations was associated with lower BMI (-0.3544 kg/m^2 [95% CI: -0.56, -0.14]), WC (-1.18 cm [-1.67, -0.68]), WHR (-0.0009 [-0.0034, 0.0015]), and BRI (-0.16 [-0.23, -0.1]). The largest effect size was observed for detection of perfluoroundecanoic acid (PFUnDA), which was associated with lower odds of prevalent obesity using BMI (OR: 0.91 [0.88, 0.94]) and WC (OR: 0.93 [0.90, 0.95]) cutoffs.

Conclusion: Higher serum concentrations of PFAS were associated with slightly lower obesity measures. Although our results do not support the hypothesis that these PFAS compounds are effective obesogens in this population, reverse causation cannot be ruled out in this cross-sectional study; more prospective studies are needed.

This study was funded by Agency for Toxic Substances and Disease Registry, Centers for Disease Control and Prevention.

TO28-07 | Serum per- and polyfluoroalkyl substances and prevalent heart disease in the Multi-Site Study.

Celina N. Phillipson[1], Marian Pavuk[2], Yerin Jung[1], Robert Laumbach[3], Linda M. Brown[4], Anne Starling[5], Veronica Vieira[1], Michael Lewin[2], Teresa Wang[2], Tamara James-Todd[6], Carolina Villanueva[1], Yuting Wang[7], Scott M. Bartell[1]

[1] University of California Irvine, Department of Environmental and Occupational Health, Irvine, USA; [2] Centers for Disease Control and Prevention, Agency for Toxic Substances and Disease Registry, Atlanta, USA; [3] Rutgers/The State University of New Jersey, Department of Environmental and Occupational Health and Justice, New Brunswick, USA; [4] RTI International, Research Triangle Park, USA; [5] Department of Epidemiology, University of North Carolina at Chapel Hill, Chapel Hill, USA; [6] Department of Environmental Health, Harvard T.H. Chan School of Public Health, Boston, USA; [7] Silent Spring Institute, Newton, USA

Objective: The objective of this study was to estimate associations between serum per- and polyfluoroalkyl substances PFAS and prevalent heart disease in a large, cross-sectional, multi-site study of U.S. adults residing in communities with historical drinking water contamination.

Materials and methods: Adult participants were recruited at eight sites across the U.S. (2019-2023), where they completed questionnaires and provided blood samples. Serum PFAS concentrations were measured for seven PFAS; PFAS detected in >75% of participants were treated as continuous variables - perfluorooctanoic acid (PFOA), perfluorooctanesulfonic acid (PFOS), perfluorohexanesulphonic acid (PFHxS), and perfluorononanoic acid (PFNA), while others were treated as binary at the limit of detection – perfluorodecanoic acid (PFDeA), perfluoroundecanoic acid (PFUnDA), and 2-(N-methyl-perfluorooctane sulfonamido) acetic acid (MeFOSAA). Logistic regression models estimated the odds ratios (ORs) and 95% confidence intervals (CIs) for the association between each PFAS and prevalent self-reported heart disease.

Results: A total of 5,761 participants were included in the analysis, with a mean age of 56 years and 53.4% female. The prevalence of self-reported heart disease was 9.2%. Covariate-adjusted analyses showed null associations between most PFAS and heart disease, except for PFUnDA, which was inversely associated. Detectable PFUnDA levels were associated with lower odds of self-reported heart disease (OR: 0.98, 95% CI: 0.96, 0.99) compared to non-detectable levels.

Conclusions: We observed no significant cross-sectional associations between most serum PFAS concentrations and self-reported heart disease, with the exception of PFUnDA, which was inversely associated. Despite finding no significant associations between most serum PFAS concentrations and self-reported heart disease, future analysis exploring potential joint effects of PFAS mixtures could lead to better understanding the impact of PFAS on heart disease. Funding for this study was provided by the Agency for Toxic Substances and Disease Registry, Centers for Disease Control and Prevention.

TO29 | Air pollution and cardiometabolic outcomes: From glucose to heart disease

TO29-01 | Source-Specific $PM_{2.5}$ Exposure and Atrial Fibrillation Risk in U.S. Older Adults: A Nationwide Cohort Study

Qiao Zhu, Hua Hao, Yanling Deng, Yang Liu

Emory University, Gangarosa Department of Environmental Health, Rollins School of Public Health, Atlanta, USA

Fine particulate matter ($PM_{2.5}$) is associated with an increased risk of atrial fibrillation (AF), but the impact of different $PM_{2.5}$ sources on AF remains unclear. In this study, we assessed the association between long-term exposure to source-specific $PM_{2.5}$ and incident AF among U.S. older adults using nationwide Medicare data from 2000 to 2019 with a 3-year clean period. $PM_{2.5}$ sources was resolved by using the Multilinear Engine model based on $PM_{2.5}$ components data from monitoring stations and assigned exposures for Medicare participants using an ordinary kriging model. We employed Cox proportional hazards models to estimate AF risk associated with interquartile range (IQR) increases in source-specific $PM_{2.5}$ concentrations, adjusting for demographic and socioeconomic factors as well as other environmental covariates. Among 34.1 million Medicare beneficiaries, 6.15 million AF cases were identified. $PM_{2.5}$ from vehicle emissions (HR: 1.038, 95% CI: 1.037–1.039), secondary sulfate (HR: 1.048, 95% CI: 1.046–1.051), secondary nitrate (HR: 1.043, 95% CI: 1.041–1.045), and heavy oil/coal combustion (HR: 1.058, 95% CI: 1.055–1.060) were significantly associated with increased AF risk. In contrast, biomass burning and secondary organic aerosols (SOA) did not show an association with AF. Further quartile-based analyses indicated a dose-response relationship for vehicle emissions, secondary sulfate, secondary nitrate, and heavy oil/coal combustion, with higher exposure groups experiencing greater AF risk. Our findings highlight the differential toxicity of $PM_{2.5}$ sources in AF risk and emphasize the need for targeted air quality regulations to reduce emissions particularly from traffic and industrial sources to mitigate AF incidence in older adults.

TO29-02 | Associations between Long-term Exposure to Ultrafine Particles and Type 2 Diabetes: A Large-scale, Multicenter Study in China

Tingting Yang[2], Yi Guo[1], **Cong Liu**[1], Feng Hong[2]

[1] Fudan University, School of Public Health, Shanghai, China; [2] Guizhou Medical University, School of Public Health, Guiyang, China

Objective: Few studies have examined the associations between long-term exposure to ultrafine particles (UFP) and type 2 diabetes (T2DM). We aimed to investigate the impact of long-term UFP exposure on diabetes prevalence, progression, and glycemic markers using a large multi-center cohort.
Material and Methods: This study was based on the China Multi-Ethnic Cohort (CMEC), with baseline data collected from 2017 to 2021. Health outcomes contained diabetes prevalence, progression (from normoglycemia to pre-diabetes and then to diabetes), and glycemic markers, including fasting blood glucose (FPG) and glycated hemoglobin (HbA1c). Three-year average UFP concentration prior to baseline was used as UFP exposure.
Results: This cross-sectional study included 93,990 participants for, with a diabetes prevalence of 10·97% (10,315 individuals). An interquartile range increase in UFP (6,326·35/cm^3) was significantly associated with diabetes prevalence and progression, with ORs of 1·20 (95% CI: 1·14, 1·26) and 1·11 (95% CI: 1·07, 1·44), respectively. For

transitions from normoglycemia to pre-diabetes and then to diabetes, the ORs were 1·01 (95% CI: 0·96, 1·04) and 1·24 (95% CI: 1·17, 1·31), respectively. Long-term UFP exposure was significantly associated with elevated levels of FPG (0·09 mmol/L, 95% CI: 0·06, 0.11) and HbA1c (0.03%, 95% CI: 0.02%, 0.05%). Additionally, the concentration-response curves of UFP-diabetes, UFP-FPG, and UFP-HbA1c relationships were monotonically increasing without obvious thresholds.
Conclusion: This study demonstrated positive association between long-term UFP exposure and various diabetes outcomes, with pre-diabetes population potentially vulnerable. The findings suggest long-term UFP exposure may be a potential risk factor for diabetes.

TO29-03 | Regional and Racial/ethnic Disparities in Cardiovascular Mortality Attributable to $PM_{2.5}$ Components in the Contiguous United States

Ying Hu[1,2], Lingzhi Chu[1,2], Stefano Renzetti[3], Emma Zhang[4], Ijeoma Opara[5], Yuan Lu[6,7], Erica S. Spatz[6,7], Harlan M. Krumholz[6,7], Kai Chen[1,2]

[1] Yale University, Department of Environmental Health Sciences, School of Public Health, New Haven, USA; [2] Yale University, Yale Center on Climate Change and Health, School of Public Health, New Haven, USA; [3] University of Brescia, Department of Medical and Surgical Specialties, Radiological Sciences, and Public Health, Brescia, Italy; [4] Yale University, Department of Sociology, New Haven, USA; [5] Yale University, Department of Social & Behavioral Sciences, School of Public Health, New Haven, USA; [6] Yale New Haven Hospital, Center for Outcomes Research and Evaluation, New Haven, USA; [7] Yale University, Section of Cardiovascular Medicine, Department of Medicine, School of Medicine, New Haven, USA; [8] Yale University, Department of Health Policy and Management, School of Public Health, New Haven, USA

Fine particulate matter ($PM_{2.5}$) exposure is a major risk factor for cardiovascular disease (CVD) mortality, but its components differ in sources, toxicity, and distribution despite regulations targeting total mass. Prior studies fail to address collinearity in large-scale spatiotemporal data, limiting understanding of component-specific contributions. This study examines $PM_{2.5}$ component-attributable CVD mortality and its disparities across the contiguous U.S. from 2001 to 2020.
We extended the generalized weighted quantile sum regression model by incorporating two-way fixed effects (gWQS-feglm) to estimate the health effects of six major $PM_{2.5}$ components: black carbon (BC), ammonium (NH_4^+), nitrate (NO_3^-), organic matter (OM), sulfate (SO_4^{2-}), and soil dust (DUST). We assessed the association between 12-month moving average $PM_{2.5}$ components exposure and monthly CVD mortality and estimated county-level attributable deaths.
A decile increases in all $PM_{2.5}$ components was associated with 1.75 (1.17, 2.39) additional CVD deaths per million individuals per month, with NH_4^+ (29%) and SO_4^{2-} (27%) as dominant contributors. $PM_{2.5}$-attributable CVD deaths declined from 42,200 (95% CI: 28,200, 57,600) in 2001 to 23,500 (15,700, 32,100) in 2020, mainly in association with NH_4^+ and SO_4^{2-} reductions. In 2020, high-attributable-mortality regions remained the Middle Atlantic, the South, the East North Central, and California, with NH_4^+ dominant in California and the East North Central, SO_4^{2-} in the South, and BC and SO_4^{2-} in the Middle Atlantic. Racial/ethnic disparities persisted, with non-Hispanic Blacks and Hispanics experiencing slower $PM_{2.5}$-attributable-mortality declines compared with non-Hispanic Whites.
Component-specific regulations are needed to reduce $PM_{2.5}$-attributable CVD mortality. Regional disparities require localized control, while racial inequities demand environmental justice. Strengthening air quality policies can reduce CVD mortality by targeting harmful $PM_{2.5}$ components and refining standards.

TO29-04 | Effect of long-term air pollution reduction on insulin resistance and fasting glucose in children: A causal analysis

Rajini Nagrani[1], Maike Wolters[1], Christoph Buck[1], Danielle Vienneau[2,3], Kees deHoogh[2,3], Stefaan De Henauw[4], Lauren Lissner[5], Dénes Molnár[6], Luis Moreno[7], Marika Dello Russo[8], Valeria Pala[9], Wolfgang Ahrens[10,1], Vanessa Didelez[10,1], Claudia Börnhorst[1]

[1] Leibniz Institute for Prevention Research and Epidemiology, Department of Epidemiological Methods and Etiological Research, bremen, Germany; [2] Swiss Tropical and Public Health Institute, Allscwil, Switzerland; [3] University of Basel, Basel, Switzerland; [4] Ghent University, Department of Public Health and Primary Care, Ghent, Belgium; [5] University of Gothenburg, School of Public Health and Community Medicine, Institute of Medicine, Gothenburg, Sweden; [6] University of Pécs, Department of Paediatrics, Medical School, Pécs, Hungary; [7] University of Zaragoza, GENUD (Growth, Exercise, Nutrition and Development) Research Group, Zaragoza, Spain; [8] National Research Council, Institute of Food Sciences, Avellino, Italy; [9] Fondazione IRCCS Istituto Nazionale Tumori, Milan, Italy; [10] University of Bremen, Institute of Statistics, Faculty of Mathematics and Computer Science, Bremen, Germany

Objective: Observational studies have suggested that air pollution is associated with impaired glucose metabolism; yet randomized controlled trials to confirm the causality of this association are ethically and practically unfeasible. We emulated a hypothetical trial to evaluate the effects of sustained reductions in ambient air pollutants on homeostasis model assessment for insulin resistance (HOMA-IR) and fasting glucose (FG) in children and adolescents.

Material and Methods: Combining target trial emulation with g-computation, we estimated the effects of sustained hypothetical reductions of air pollutants on HOMA-IR and FG considering interventions targeting lower standard levels as well as percental reductions, compared to no intervention (natural course). Our sample comprised 1,417 children aged 2–9 years at baseline (2007/2008) participating in the pan-European IDEFICS/I.Family cohort. Children were eligible if they provided fasting blood samples, had no insulin resistance/diabetes, were non-smokers, and participated in baseline, 2- and 6-year follow-ups. Ambient annual average levels of particulate matter<2.5µm ($PM_{2.5}$), black carbon (BC), and NO_2 were estimated at residential addresses using land use regression models.

Results: The median levels of $PM_{2.5}$, BC and NO_2 at baseline were 17.9µg/m^3, 1.5×10^{-5}/m and 25.3µg/m^3 respectively. We found a clear dose-response relationship between sustained reductions in $PM_{2.5}$ and BC and decreasing levels of HOMA-IR and FG. Hypothetically reducing $PM_{2.5}$ to WHO's recommended annual level of 5µg/m^3, the mean HOMA-IR z-score declined by -0.95(95%CI: -1.65; -0.44). Similarly, lowering BC to 0.8×10^{-5}/m reduced HOMA-IR by -0.36(95%CI: -0.61; -0.12) and FG by -0.28(95%CI: -0.50; -0.04), while there was no clear evidence for an effect on HOMA of reducing NO_2.

Conclusion: Despite low pollution levels, we observed a dose-response relationship, suggesting that even small reductions in $PM_{2.5}$ and BC can improve HOMA-IR and FG levels in children and adolescents. Our findings provide evidence for the development of BC recommendations for air quality guidelines.

TO29-06 | Differential effects of fine particulate matter constituents on acute coronary syndrome onset: A nationwide case-crossover study in China

Yixuan Jiang[1], Chuyuan Du[1], Renjie Chen[1], Jialu Hu[2], Xinlei Zhu[1], Xiaowei Xue[1], Qinglin He[1], Jun Lu[1], Junbo Ge[2], Yong Huo[3], Haidong Kan[1]

[1] Fudan University, School of Public Health, Shanghai, China; [2] Zhongshan Hospital, Fudan University, Department of Cardiology, Shanghai, China; [3] Peking University First Hospital, Department of Cardiology, Beijing, China

Objective

Short-term exposure to fine particulate matter ($PM_{2.5}$) has been linked with acute coronary syndrome (ACS). Nevertheless, the key constituents have not been elucidated. This study aimed to quantify the associations between different $PM_{2.5}$ constituents and ACS onset, and identify the critical contributors.

Material and Methods

A national time-stratified case-crossover study was conducted in China. Daily onset information of ACS between 2015 and 2021 were obtained from the Chinese Cardiovascular Association Database-Chest Pain Center. Daily concentrations of $PM_{2.5}$ constituents including organic matter, black carbon, nitrate, sulfate, and ammonium were obtained from satellite-based predicting models. Conditional logistic regression was applied to analyze individual effect of each constituent on ACS onset, and weighted quantile sum regression was used to investigate the joint effects and each constituent's contribution.

Results

A total of 2,113,728 ACS patients from 2,096 hospitals were included. All five constituents of $PM_{2.5}$ were observed to be significantly associated with onset of ACS as well as its subtypes. The magnitude of associations peaks on the concurrent day, attenuates thereafter, and becomes null at lag 2 day. The largest effects were found in organic matter and black carbon, followed by sulfate, nitrate, and ammonium. Each interquartile range increase in concurrent-day $PM_{2.5}$, organic matter, black carbon, nitrate, sulfate, and ammonium concentrations was associated with 2.00%, 2.15%, 2.03%, 1.54%, 1.57%, and 1.51% increase in ACS onset, respectively. Two carbonaceous components contributed the most to the joint effects, with 31% from organic matter and 22% from black carbon.

Conclusion

Carbonaceous components play crucial roles in the $PM_{2.5}$-related risk of ACS onset, highlighting tailored clinical management for patients and targeted control measures on $PM_{2.5}$ emissions.

Funding: National Key Research and Development Program of China (2022YFC3702701), Shanghai 3-year Public Health Action Plan (GWVI-11.1-39), Shanghai Committee of Science and Technology (21TQ015), and Shanghai Sailing Program (24YF2706700).

TO29-07 | The Association of Air Pollution Exposure with Glucose and Diabetes outcomes.

Helena Krasnov[1], Pablo Knobel[2], Leon Hsu Hsiao-Hsien[3], Susan L. Teitelbaum[4], Mary Ann McLaughlin[5], Allan Just[6], Itai Kloog[7], Maayan Yitshak Sade[8]

[1] Icahn School of Medicine at Mount Sinai, Department of Environmental Medicine, New York, USA; [2] Icahn School of Medicine at Mount Sinai, Department of Environmental Medicine, New York, USA; [3] Icahn School of Medicine at Mount Sinai, Department of Environmental Medicine, New York, USA; [4] Icahn School of Medicine at Mount Sinai, Department of Environmental Medicine, New York, USA; [5] Icahn School of Medicine at Mount Sinai, Department of Cardiology, New York, USA; [6] Brown University School of Public Health, Department of Epidemiology, Providence, USA; [7] Icahn School of Medicine at Mount Sinai, Department of Environmental Medicine, New York, USA; [8] Icahn School of Medicine at Mount Sinai, Department of Environmental Medicine, New York, USA

Objective: Residential exposure to fine particulate matter ($PM_{2.5}$) in the two decades following the 9/11 attack, was associated with increased cardiometabolic risk among the members of the World Trade Center Health Program General Responders Cohort (WTCHP GRC). However, current studies do not consider $PM_{2.5}$ chemical composition. We studied the effect of source-specific $PM_{2.5}$ exposures on diabetes risk and glycemic control among general responders.

Material and Methods: We included 41,776 members of the WTCHP GRC. We used non-negative matrix factorization to attribute 14 annual $PM_{2.5}$ component exposures to $PM_{2.5}$ sources and identified five sources: Biomass burning, oil combustion, metal industry, other industry, motor vehicle. We used multivariable mixed-effect models to estimate the simultaneous associations of the five source-apportioned $PM_{2.5}$ exposures with self-reported diabetes or repeated measurements of blood glucose (n = 141,926). Models were adjusted for age, sex, race, census tract-level socioeconomic variables, mean temperature, and year.

Results: We included 41,776 responders, of whom 14.6% reported diabetes and mean glucose level was 102.69 mmol/L. An interquartile range (IQR) incremental increase in metal industry (RR 1.071, 95% 1.005; 1.142) and motor vehicle-sources $PM_{2.5}$ (RR 1.056, 95% 1.003; 1.113) were associated with a higher diabetes risk. IQR increase in oil combustion apportioned $PM_{2.5}$ was associated with a 0.35% (0.02%, 0.69%) increase in glucose levels, and an IQR increase in metal industry apportioned $PM_{2.5}$ was associated with a 2.29% (1.37%, 3.22%) increase in glucose levels. The associations with metal industry, biomass burning, and motor vehicle-apportioned $PM_{2.5}$ were more pronounced among women while the association with oil combustion apportioned $PM_{2.5}$ was more pronounced among men.

Conclusion: Long-term $PM_{2.5}$ exposureattributed to oil combustion, metal industry, and motor vehicle sources is associated with increased cardiometabolic risk among WTC responders, especially among women. These findings can inform policy and interventions aimed at reducing cardiometabolic burden.

TO30 | Beyond the flames: The far-reaching health effects of wildfire smoke exposure

TO30-01 | Prenatal Exposure to Wildfire-Related $PM_{2.5}$ and Autism Spectrum Disorder in California, 2010–2019

Karl O'Sharkey[1], Sanjali Mitra[1], Ting Chow[1], Amanda Goodrich[2], Kathryn Conlon[2], Miriam Nuno[2], Deborah Bennett[2], Sean Raffuse[3], Michael Kleeman[4], Beate Ritz[1], Rebecca Schmidt[2]

[1] University of California, Los Angeles, Epidemiology, Los Angeles, USA; [2] University of California, Davis, Public Health Sciences, Davis, USA; [3] University of California, Davis, Air Quality Research Center, Davis, USA; [4] University of California, Davis, Civil and Environmental Engineering, Davis, USA

Background: Wildfires (WF) are increasing in frequency and intensity, leading to greater exposure to fine particulate matter ($PM_{2.5}$), a known environmental risk factor for adverse neurodevelopmental outcomes. This study examined the association betweenprenatal WF-related $PM_{2.5}$ exposure and autism spectrum disorder (ASD).
Methods: This study used 2010-2019 California (CA) birth records linked to ASD cases reported by the CA Department of Developmental Services up to the end of 2022 (N = 4,186,416; ASD = 70,782). Daily exposure to WF smoke was predicted with a chemical transport model adjusted for measured $PM_{2.5}$ concentrations using Random Forest Regression. WF $PM_{2.5}$ exposure during the whole of pregnancy was assessed usingcontinuous (per IQR increase), categorical (top 10%, 90th–50th percentile, <50th percentile), and episodic (distinct high-exposure events with 3- or 7-day unexposed washout periods) metrics, adjusting for non-WF $PM_{2.5}$ and key maternal and demographic covariates.
Results: A one-IQR (1.18 μg/m^3) increase in WF $PM_{2.5}$ was associated with a 4% increased risk of ASD (95% CI: 1.03, 1.05). Pregnancies exposed in the top 10% of exposure had a 17% increased risk (95% CI: 1.14,1.21), while those in the 90th–50th percentile had a modest 3% increased risk (95% CI: 1.01, 1.04), compared to those below the median exposure. The number of distinct high-exposure episodes also increased risk, with each additional episode of WF $PM_{2.5}$ ≥35 μg/m^3 after a 3-day washout period increasing risk by 10% (95% CI: 1.08, 1.12) and after a 7-day washout period by 12% (95% CI:1.09, 1.14). In contrast, lower-intensity WF exposure events (≥9 μg/m^3) showed no associations.
Conclusion: Higher WF $PM_{2.5}$ exposure levels and repeated high exposure episodes (≥35 μg/m^3) were associated with increased risk of ASD, even after accounting for non-WF $PM_{2.5}$. These findings highlight the potential for long-lasting neurodevelopmental disturbances associated with extreme and recurrent wildfire smoke exposure in pregnancy.

TO30-02 | Prenatal exposure to wildfire smoke and the risk of neurodevelopmental delays in children among Medicaid recipients: a national cohort study

<u>Stefania Papatheodorou</u>[1,2], Krista Huybrechts[4], Yang Liu[3], Danlu Zhang[3], Xinye Qiu[2], Sonia Hernandez-Diaz[2], Wanyu Huang[2], Matthew Shupler[2], Hayon Choi[2], Michael Leung[2], Brent Coull[2], Joel Schwartz[2], Yaguang Wei[2], Marc Weisskopf[2], Antonella Zanobetti[2]

[1] Rutgers School of Public Health, Piscataway, USA; [2] Harvard School of Public Health, BOSTON, USA; [3] Emory Rollins School of Public Health, Atlanta, USA; [4] Mass General Brigham, Boston, USA

Objective: Wildfires pose a threat to human health. Although past studies have shown that wildfire smoke (WFS) exposure may be associated with adverse birth outcomes, its association with children 's neurodevelopment has been understudied. We examined the association between prenatal WFS exposure and neurodevelopmental outcomes in a national birth cohort of Medicaid enrollees from 2007 to 2014.

Methods: We assigned WFS pregnancy average concentrations from a daily spatiotemporal model at the residential zip code of mother-infant pairs in a longitudinal cohort from the Medicaid Analytic eXtract (MAX) dataset from 2007 to 2014. Identification of autism spectrum disorder (ASD) and attention-deficit/hyperactivity disorder (ADHD) were based on ICD-9 codes. We applied stratified Cox models (by year and county), adjusting for non-wildfire $PM_{2.5}$, temperature, maternal age, race/ethnicity, behavioral risk factors (e.g., smoking, alcohol, drug use), and socioeconomic status (area-level). We examined effect modification by sex.

Results: We included 1,010,197 births from 2001 to 2014. The overall R^2 of the WFS model was 0.75, and the pregnancy average wildfire $PM_{2.5}$ ranged from 0 to 19 μg/m^3. A 1-μg/m^3 increase in prenatal exposure to WFS was associated with an increased risk of ASD (Overall HR:1.07, 95% CI: 1.01, 1.15, Males: HR:1.05, 95% CI: 1.00, 1.08, Females: HR:1.12, 95% CI: 1.03, 1.26) and ADHD (Overall: HR:1.13, 95% CI: 1.09, 1.17, Males: HR:1.11, 95% CI: 1.07, 1.15, Females: HR:1.18, 95% CI: 1.13, 1.23).

Conclusion: Our findings suggest that prenatal exposure to wildfire smoke is associated with an increased risk of ASD and ADHD among children. If causal, these results highlight the need for targeted public health interventions and policies to protect pregnant women and young children from wildfire-related air pollution. Further research is needed to explore underlying biological mechanisms for sex differences and long-term impacts, as well as sensitive windows of exposure.

TO30-03 | Associations between wildfire smoke exposure and risk of renal, ophthalmologic, and neurologic complications among type 1 and type 2 diabetes mellitus patients.

Trenton Honda[1], Trenton D. Henry[1], Jennifer Stowell[3], Christina A. Porucznik[2], James A. VanDerslice[2], Marcus Pezzolesi[2]

[1] Northeastern University, School of Clinical and Rehabilitation Sciences, Boston, USA; [2] University of Utah, Salt Lake City, USA; [3] Boston University School of Public Health, Department of Environmental Health, Boston, USA

Objective

Wildfires driven by climate change are increasingly contributing to air pollution across the US, with burgeoning information on the deleterious health impacts of wildfire smoke among particularly vulnerable individuals, such as those with type 1 (T1D) and type 2 diabetes mellitus (T2D). While the renal, ophthalmologic, and peripheral neurologic complications resulting from diabetes mellitus are well described, it remains unknown whether, and to what extent, wildfire smoke may contribute to the development of these complications in T1D and T2D populations.

Methods

We linked ambient 1-year moving average wildfire PM2.5 estimates from a well validated spatiotemporal model to the residential addresses of 16,793 T1D patients and 239,967 T2D patients residing in the U.S. state of Utah from 2006 to 2021. We used competing-risk Cox proportional hazards regression to explore associations between ambient wildfire PM2.5 exposure and incident diabetic nephropathy, ophthalmopathy, and neuropathy among T1D and T2D patients. All health effects models were adjusted for sex, race, age, BMI, and socioeconomic status.

Results

T1D and T2D follow-up times were 121,860 and 1,759,105 years, respectively. For T1D patients, an IQR increase in wildfire PM was associated with an 11.3% increased risk of peripheral neurologic complications (HR: 1.113, 95% CI: 1.045, 1.186). For T2 DM patients, an IQR increase in PM2.5 was associated with 4.6% increased risk of ophthalmologic complications (HR: 1.046, 95% CI: 1.021, 1.071) and 3.9% increased risk of peripheral neurologic complications (HR: 1.039, 95% CI: 1.024, 1.094). Associations were null for all renal outcomes in both T1D and T2D patients.

Conclusions

Our findings suggest that wildfire PM2.5 exposure may be an understudied risk factor for the development of microvascular complications in type 1 and type 2 diabetes mellitus.

TO30-04 | Quantifying the wildfire smoke influences on ambient ozone concentration: going beyond wildfire smoke PM2.5

Minghao Qiu[1], Yangmingkai Li[1], Xiaomeng Jin[2]

[1] Stony Brook University, School of Marine and Atmospheric Sciences; Program in Public Health, Stony Brook, USA; [2] Rutgers University, Department of Environmental Sciences, New Brunswick, USA

Ground-level ozone can negatively influence human health and lead to premature mortality. Wildfire activity can significantly influence ambient ozone concentration through complex pathways. Despite the emerging evidence on the adverse health effects of wildfire smoke, most prior studies focus on the impacts of wildfire fine particulate matter, while the potential ozone-related impacts are not well understood. Here, we quantify wildfire-driven changes in ambient ozone concentrations across the United States using surface air quality measurements, satellite-derived smoke information, and machine learning models. Our analysis reveals that ambient ozone concentrations significantly increase during smoke days by up to 10 ppb or 20\% relative to non-smoke days. Interestingly, stronger enhancements of ozone are observed in the eastern US compared to the western US -- a pattern notably different from wildfire smoke influences on PM2.5. We find that this notable difference in wildfire smoke ozone is largely driven by the different timing and associated meteorological conditions of smoke days across the eastern and western US. Using established dose-response functions, We estimate that wildfire smoke-related ozone leads to 3,600 premature deaths each year in the US during 2006-2023, which is about 30\% of the estimated deaths due to wildfire smoke PM2.5. However, estimated mortality due to smoke O3 is comparable to PM2.5-related deaths in 2022 and 2023 when the eastern US experienced intense wildfire activity and ozone enhancements. These findings underscore the importance of assessing ozone alongside PM2.5 to capture the full public health burden of wildfires, particularly as ozone and PM2.5 health effects do not necessarily coincide.

TO30-05 | The association between wildfire smoke-specific $PM_{2.5}$, COVID-19 infections, and infection severity in the population of British Columbia during the summers of 2020 and 2021.

Yanbo Li[1,2], Hind Sbihi[3,1], Agatha Jassem[4,5], Naman Paul[2], Kathleen McLean[2], Eric Coker[2,1], Sarah Henderson[2,1], Collaborating Institutions:UBC School of Population and Public HealthBC Centre for Disease Control Environmental Health Services, Provincial Health Services AuthorityBC Ministry of Health;Funded through BC Lung Health Foundation Grant

[1] University of British Columbia, School of Population and Public Health, Vancouver, Canada; [2] BC Centre for Disease Control, Environmental Health Services, Vancouver, Canada; [3] BC Centre for Disease Control, Data & Analytic Services, Vancouver, Canada; [4] BC Centre for Disease Control, Public Health Laboratory, Vancouver, Canada; [5] University of British Columbia, Pathology & Laboratory Medicine, Vancouver, Canada

Objectives: Wildfire smoke (WFS) is a growing public health challenge and may exacerbate risk of respiratory infections such as COVID-19. This study investigates the association between WFS-specific fine particulate matter ($PM_{2.5}$) exposure and COVID-19 tests and hospitalizations during the 2020 and 2021 wildfire seasons in British Columbia, Canada.

Methods and Materials: A test-negative design with a time-stratified case-crossover approach was used to assess the association between $PM_{2.5}$ exposure and COVID-19 test results, comparing effects between individuals with positive vs. negative tests. The same design was also used to examine COVID-19 hospitalizations, as an indicator of severe infections, Wildfire smoke $PM_{2.5}$ at the time of each COVID-19 test or hospitalization was compared with exposure during similar periods without these outcomes using conditional logistic regression. High-resolution $PM_{2.5}$ estimates were derived from the Canadian Optimized Statistical Smoke Exposure Model (CanOSSEM) and wildfire smoke presence from the Hazard Mapping System (HMS). Health outcomes were identified from linked provincial health registries.
Results: In 2020, each interquartile range (IQR) increase in daily wildfire smoke $PM_{2.5}$ was associated with an 11% increase in positive COVID-19 tests (OR: 1.11; 95% CI: 0.982–1.26), 5% decrease in negative tests (OR: 0.95; 95% CI: 0.94–0.97), and a 61% increase in hospitalizations (OR: 1.61; 95% CI: 1.04–2.48). In contrast, 2021 findings showed no significant effects as results on tests and hospitalizations were null.
Conclusion: Exposure to wildfire smoke $PM_{2.5}$ had a significant association with positive COVID-19 tests and hospital admissions in 2020, but not in 2021. The pandemic context was quite different in both summers, as were the SARS-CoV-2 variants. These findings suggest the effect of smoke exposure on COVID-19 infections may have changed over time in British Columbia.

TO30-06 | Impact of Short-Term Wildfire Smoke Fine Particulate Matter Exposure on Cardiovascular Hospitalization Risk

Yue Zhang[1], Feiyun Yan[1], A. Gannet Hallar[5], Timothy W. Collins[2], Kristina A. Fillman[4], Sara E. Grineski[3]

[1] University of Utah, Department of Internal Medicine, Salt Lake City, USA; [2] University of Utah, Society & Sustainability, Salt Lake City, USA; [3] University of Utah, Sociology, Salt Lake City, USA; [4] Oregon State University, Geography and Geospatial Science, Corvallis, USA; [5] University of Utah, Atmospheric Science, Salt Lake City, USA

Objective: This study examines the association between short-term wildfire smoke fine particulate matter ($PM_{2.5}$) exposure and cardiovascular hospitalization risk in Oregon from 2011–2020. With increasing wildfire events, understanding these acute health impacts is crucial for public health preparedness.
Methods: We analyzed 127,492 cardiovascular hospitalizations from the Oregon Health Authority. Outcomes included (1) any cardiovascular condition, (2) congestive heart failure, (3) ischemic heart disease, and (4) myocardial infarction. Wildfire smoke $PM_{2.5}$ exposure data from Stanford University's Environmental Change and Human Outcomes Lab were assessed using cumulative average $PM_{2.5}$ within two weeks before hospitalization, sustained exposure (≥2 days of $PM_{2.5}$ >15 μg/m³ in the first 4 lag days), and weekly exposure duration (**number of days with non-zero wildfire smoke** $PM_{2.5}$ within 2 weeks before index date). A unidirectional case-crossover design controlled for time-invariant confounders, and conditional logistic regression estimated odds ratios (ORs) and 95% confidence intervals (CIs).
Results: Short-term wildfire smoke $PM_{2.5}$ exposure was significantly associated with increased hospitalization risk across all outcomes. A 10 μg/m³ increase in cumulative $PM_{2.5}$ was linked to higher odds of hospitalization for any cardiovascular condition (OR = 1.30, 95% CI: 1.29–1.32), congestive heart failure (OR = 1.29, 95% CI: 1.25–1.32), ischemic heart disease (OR = 1.29, 95% CI: 1.26–1.31), and myocardial infarction (OR = 1.34, 95% CI: 1.30–1.39). Sustained exposure statistically significantly increased hospitalization risk (ORs: 1.67–1.75; all p-values<0.05). For

each additional day of wildfire smoke $PM_{2.5}$ exposure during the first 7-day lag period, the risk of hospitalization for all cardiovascular outcomes increased significantly, with odds ratio ranging from 1.31 to 1.32.

Conclusion: Wildfire smoke $PM_{2.5}$ exposure elevates cardiovascular hospitalization risk, highlighting the need for proactive public health interventions. Strengthening wildfire management, forecasting, and protective measures could reduce health impacts. Future research should investigate underlying biological mechanisms.

TO30-07 | Impact of wildfire smoke waves on long-term association between low-level fine particulate matter and hypertension hospitalizations in the United States

Siqi Zhang[1,2], Yongfei Wang[3,4], Jing Wei[5], Yuan Lu[3,4], Harlan M. Krumholz[3,4], Kai Chen[1,2]

[1] Yale School of Public Health, Department of Environmental Health Sciences, New Haven, USA; [2] Yale School of Public Health, Yale Center on Climate Change and Health, New Haven, USA; [3] Yale New Haven Hospital, Center for Outcomes Research and Evaluation, New Haven, USA; [4] Yale School of Medicine, Section of Cardiovascular Medicine, New Haven, USA; [5] University of Maryland, Department of Atmospheric and Oceanic Science, College Park, USA; [6] Yale School of Public Health, Department of Health Policy and Management, New Haven, USA

Objective: This study examined how wildfire smoke waves influence the association between first hypertension hospitalizations and long-term exposure to total fine particulate matter ($PM_{2.5}$) at concentrations below 9 μg/m^3 among U.S. Medicare beneficiaries.

Materials and Methods: We conducted a population-based cohort study of 26.8 million Medicare beneficiaries (enrolled in Fee-For-Service or Medicare Advantage program) aged ≥ 65 years across the contiguous U.S. from 2017 to 2022, all exposed to annual $PM_{2.5}$ concentrations below 9 μg/m^3 throughout the study period. The primary outcome was the first hospitalization for hypertension during the study period. Daily mean total $PM_{2.5}$ and wildfire smoke $PM_{2.5}$ concentrations were estimated using spatiotemporal models and assigned to beneficiaries' residences at the ZIP code level. Wildfire smoke waves were defined as periods with daily wildfire smoke $PM_{2.5}$ concentrations exceeding 15 μg/m^3 for at least two consecutive days. We applied quasi-Poisson regressions with an interaction term to estimate the relative risks (RR) and 95% confidence interval (CI) of hypertension hospitalizations associated with long-term $PM_{2.5}$ exposures stratified by wildfire smoke wave occurrence.

Results: At $PM_{2.5}$ exposure levels below 9 μg/m^3, each 1 μg/m^3 increase in annual mean $PM_{2.5}$ concentrations was associated with a 4.9% higher risk of hypertension hospitalizations (RR=1.049, 95% CI: 1.046-1.052). This association was significantly stronger with the occurrence of wildfire smoke waves in the same year (RR=1.065, 95% CI: 1.058-1.072 vs. RR=1.048, 95% CI: 1.045-1.051, *p*-value for interaction<0.002).

Conclusion: Long-term exposure to $PM_{2.5}$ is associated with a higher risk of hypertension hospitalizations even at levels below the current U.S. air quality standard, with wildfire smoke further amplifying this effect. These findings highlight the necessity for refined public health interventions and air quality regulation to protect vulnerable older adults from cardiovascular risks attributable to air pollution, particularly as climate change intensifies wildfire frequency and severity.

TO31 | Climate change and neurological health

TO31-01 | Temporal patterns in temperature-related psychiatric emergency department visits

Kate Burrows[1], Flannery Black-Ingersoll[2], Stephanie T. Grady[2], Katharine A. Teigen[3], Kaylin Vrkljan[4], Allan C. Just[9], Alison Duncan[5,6], Mary D. Willis[4], Rachel Oblath[5,6], Amruta Nori-Sarma[8]

[1] University of Chicago, Department of Public Health Sciences, Chicago, USA; [2] Boston University, Department of Environmental Health Sciences, Boston, USA; [3] University of Washington, Department of Environmental and Occupational Health Sciences, Seattle, USA; [4] Boston University, Department of Epidemiology, Boston, USA; [5] Boston University, Chobanian & Avedisian School of Medicine, Boston, USA; [6] Boston Medical Center, Department of Psychiatric, Boston, USA; [7] Boston University, Institute for Global Sustainability, Boston, USA; [8] Harvard University, Department of Environmental Health, Boston, USA; [9] Brown University, Department of Epidemiology, Providence, USA

Background: Previous studies have demonstrated associations between extreme heat and mental health; however, the temporal dynamics of this relationship, including diurnal variation and lag periods, remain poorly understood.

Methods: We conducted a time stratified case-crossover study to investigate the association between hourly ambient temperature exposure and psychiatric emergency department (ED) visits, evaluating the temporal variation in this relationship across different times of day and lag periods. Using data from the Boston Emergency Services Team (BEST), we identified psychiatric ED visits for a vulnerable population in the greater Boston area (2005-2019). We estimated hourly ambient temperature using a 1x1km geostatistical model for each individual's residential location for each of the 24 hours preceding ED visits. We estimated risk using a mixed effect conditional logistic regression model.

Results: Among 155,233 psychiatric ED visits, preliminary results show non-linear associations between temperature and psychiatric emergencies, with peak risk occurring between 20.5-23.8°C across different exposure windows. The risk increased with longer exposure periods, with 0-24 hours cumulative exposure showing the strongest association (risk ratio [RR]: 1.29 [95% CI: 1.27-1.30] at 20.5°C) compared to 1-hour immediate exposure (RR: 1.13 [95% CI: 1.12-1.14] at 23.8°C). Significant diurnal variation was observed, with the strongest temperature-psychiatric ED associations for encounters during morning hours (7h-11h, RR: 1.87 [95% CI: 1.79-1.94] at 30.8°C) and evening hours (18h-21h, RR: 1.75 [95% CI: 1.65-1.86] at 24.2°C), while risk was lower for encounters during afternoon (12h-17h, RR: 1.18 [95% CI: 1.15-1.21] at 30.8°C) and night (22h-6h, RR: 1.17 [95% CI: 1.14-1.19] at 15.9°C).

Discussion: These preliminary results indicate that heat impacts accumulate over the course of a day, with longer exposure windows showing progressively stronger associations with psychiatric emergencies. Findings also suggest the importance of considering temporal patterns when implementing heat-health interventions and provide insights for clinical resource allocation during periods of extreme heat.

TO31-02 | Association of cold spells with mental health among Chinese adolescents

Yaqi Wang, Jiajia Dang, Jianhui Guo, Di Shi, Xinyao Lian, Jing Li, Yi Song

Peking University, Institute of Child and Adolescent Health, Beijing, China

Objective: To investigate the impacts of cold spells defined with different intensities and durations on adolescent mental health across China, establish risk-driven thresholds for cold spells and identify vulnerable subpopulations.

Material and Methods: This cross-sectional study utilized data from the 2019 wave of Chinese National Surveillance on Students' Constitution and Health (CNSSCH), conducted between September and December 2019. A total of 149,697 adolescents aged 10-18 years from 30 provinces were included. Cold spell exposures were assessed using two metrics: frequency and cumulative intensity, calculated over the 12 months preceding each participant's survey date, based on 0.1° × 0.1° gridded apparent temperature data. Cold spells were defined using a series of cutoff temperature thresholds (1st–10th percentiles) and durations (≥2, 3, 4, or 5 consecutive days). Troubled symptoms, the primary mental health outcome, were measured using the Kessler Psychological Distress Scale (K10) and the Warwick-Edinburgh Mental Wellbeing Scale (WEMWBS). Generalized linear mixed models were used to estimate the associations, and risk-driven thresholds for cold spells were determined based on the distribution of risk estimates.

Results: Among participants, 10.9% reported troubled symptoms. Higher cold spell frequency and cumulative intensity were associated with increased odds of troubled symptoms (odds ratios [ORs]: 1.032-1.240). Risk-related thresholds were defined as a 5th-percentile daily mean apparent temperature lasting for ≥3 days (P5_3d) or a 2.5th-percentile lasting for ≥2 days (P2.5_2d). Avoiding cold spell exposure through early warnings could have potentially prevented 2.8% to 3.5% of troubled symptoms. Younger adolescents, girls, rural residents, and those in lower GDP regions were more vulnerable.

Conclusion: Cold spell exposure may elevate the odds of adverse mental health outcomes when exceeding specific thresholds, highlighting the need for development of targeted cold-health early warning systems for adolescents.

Funding: National Natural Science Foundation of China (82273654 and 42307133).

TO31-03 | Projections of temperature-related excess suicide mortality under climate change scenarios: a multi-country time-series study

Hyeyeong Ro[1], Yoonhee Kim[2], Masahiro Hashizume[3], Lina Madaniyazi[4], Yeonseung Chung[5], On behalf of the Multi-Country Multi-City (MCC) Collaborative Research Network

[1] Korea Advanced Institute of Science and Technology, Graduate School of Data Science, Daejeon, South Korea; [2] The University of Tokyo, Department of Global Environmental Health, Tokyo, Japan; [3] The University of Tokyo, Department of Global Health Policy, Tokyo, Japan; [4] Nagasaki University, School of Tropical Medicine and Global Health, Nagasaki, Japan; [5] Korea Advanced Institute of Science and Technology, Department of Mathematical Sciences, Daejeon, South Korea

Background
Research has demonstrated a link between temperature and suicide, raising concerns that climate change may amplify temperature-related suicide mortality. Yet, few studies have provided global projections of this impact.
Methods
We analyzed daily time series data on mean temperature and suicide counts from 751 locations in 26 countries (1971–2021) provided by the Multi-City Multi-Country Collaborative Research Network. Modeled temperature data for 2010–2059 under three climate change scenarios (SSP1-2.6, SSP2-4.5, and SSP5-8.5) were obtained from ISIMIP. Using a two-stage time series analysis, we first estimated location-specific temperature–suicide associations from the observed data. Then, for each scenario, we projected future excess suicide mortality associated with temperatures above and below the 50th percentile, assuming no changes in adaptation or population. The net change was defined as the difference in excess suicide mortality per decade relative to the 2010–2019 baseline.
Results
Projected increases in temperature-related excess suicide mortality were more pronounced under more extreme scenarios and varied by region. Warmer regions, including Central and South America, South-East Asia, and South Africa, are expected to experience substantial increases across all scenarios, with net changes by 2050–2059 ranging from 4.7% (95% eCI: 2.2, 7.9) in South America to 6.4% (95% eCI: 3.9, 10.5) in Central America under SSP5-8.5. In contrast, temperate or colder regions, such as North and Central Europe, North America, East Asia, and Australia, are projected to see moderate increases even under SSP5-8.5, with net changes ranging from 1.2% (95% eCI: 0.3, 2.8) in North Europe to 3% (95% eCI: 1.3, 6.0) in South Europe.
Conclusions
Our findings underscore an increasing burden of temperature-related suicide mortality under climate change, particularly in warmer regions. This highlights the urgent need for robust climate mitigation and adaptive interventions.

TO31-04 | The Association of Environmental Exposures with Changes in Depression and Anxiety in the German National Cohort

Yanding Wang[1,2], Susanne B. Busch[1,2], Kathrin Wolf[1], Fiona Niedermayer[1,2], Frank Padberg[3,4], Andrea Rachow[5], Marie T. Huemer[1], Annette Peters[1,2]

[1] Helmholtz Zentrum München, German Research Center for Environmental Health (GmbH), Institute of Epidemiology, Neuherberg, Germany; [2] The University of Munich (LMU), Faculty of Medicine, Pettenkofer School of Public Health, Institute for Medical Information Processing, Biometry, and Epidemiology (IBE), München, Germany; [3] LMU University Hospital Munich, Department of Psychiatry and Psychotherapy, München, Germany; [4] Partner Site Munich, German Center for Mental Health (DZPG), München, Germany; [5] Medical Centre of the University of Munich (LMU), Division of Infectious Diseases and Tropical Medicine, München, Germany

Objective: Environmental exposures such as air pollution, air temperature, and the built environment are assumed to influence the mental health of individuals. This study examines which environmental exposures are associated with changes in depression and anxiety in a large German prospective population-based cohort study.

Material and Methods: We analyzed data of more than 70,000 participants from the German National Cohort (NAKO) between 2014-2022. Environmental exposures assessed at baseline examination (2014-2019) and rescaled by an interquartile range (IQR) included air pollution from the German Environment Agency (PM_{10}, $PM_{2.5}$, NO_2) and ELAPSE (black carbon), meteorological factors (frost days, heat days, precipitation, humidity, annual mean temperature), and indicators of the built (imperviousness, greenness) and social (German index of socioeconomic deprivation, unemployment rate) environment. Depression and anxiety were assessed at three time points (baseline examination, 2020, and 2022) using the Patient Health Questionnaire-9 and the Generalized Anxiety Disorder 7-item, respectively. Linear mixed models were employed to assess associations for repeated measures adjusted for sociodemographic and lifestyle factors.

Results: The depression (N=72,227) and anxiety scores (N=72,909) increased on average from 3.59 at baseline to 4.06 in 2022 and from 2.95 at baseline to 3.27 in 2022, respectively. Air pollution was not associated with depression or anxiety. An IQR decrease of annual precipitation at baseline was associated with a 0.20 (*95% CI: 0.09-0.32*) increase in depression scores and a 0.13 (*0.03-0.24*) increase in anxiety scores. An IQR decrease in imperviousness density within 100m was associated with a 0.06 (*0.01-0.12*) increase in depression scores. Additionally, an IQR increase in mean annual air temperature was associated with a 4.08 (*1.10-7.06*) increase in depression scores.

Conclusions: Our findings highlight annual precipitation as an environmental risk factor associated with changes in depression and anxiety, while higher temperature and lower urban imperviousness were additional risk factors for increased depression levels.

TO31-06 | Independent and joint associations of ozone and wet-bulb globe temperature with suicide mortality: Evidence from a case-crossover study in Utah, USA

Dirga K. Lamichhane[1], James VanDerslice[2], Fred Lurmann[3], Douglas Tharp[4], Alina Peluso[5], Brandy Byrwa-Hill[1], Yue Zhang[6], Anna Docherty[1], Hilary Coon[1], Amanda Bakian[1]

[1] University of Utah School of Medicine, Department of Psychiatry & Huntsman Mental Health Institute, Salt Lake City, USA; [2] University of Utah School of Medicine, Department of Family & Preventive Medicine, Salt Lake City, USA; [3] Sonoma Technology, Inc., Petaluma, USA; [4] University of Utah, Department of Geography, Salt Lake City, USA; [5] Oak Ridge National Laboratory, Oak Ridge, USA; [6] University of Utah School of Medicine, Department of Internal Medicine, Salt Lake City, USA

Objective

Increasing ozone (O_3) concentrations and ambient heat exposure have been independently associated with suicide; however, their combined effects remain unknown. We aimed to estimate the independent and joint associations of short-term exposures to O_3 and wet-bulb globe temperature (WBGT), a measure of heat stress, with suicide mortality.

Materials and methods

We conducted a bidirectional time-stratified case-crossover study among 7,551 suicide cases in Utah, USA, from 2000 to 2016. Daily maximum 8-hour average O_3 (ppb) and daily maximum 1-hour average WBGT (°C) were obtained from high-resolution prediction models. We formulated conditional logistic regression models to measure the associations of these exposures (considering 0-6 day lags) with suicide. Models were stratified by season, and various O_3 and WBGTthresholds were applied to estimate their joint associations. Departures from additive joint associations were assessed using the relative excess risk due to interaction (RERI).

Results

For each 5°C increase in WBGT(adjusted by O_3), the odds of suicide increased by 5.3% (95% CI: 1.0%–9.9%) across the entire sample and by 13.1% (95% CI: 2.8%–24.3%) in the warm season. Each 10 ppb increase in the warm-season O_3 (adjusted by WBGT) was associated with a 13.7% (95% CI: 3.2%–25.3%) increase in suicide risk. When the joint associations of WBGT and O_3 were estimated by the 50th percentile threshold in the warm season, compared to the low-low exposure group, the odds of suicide increased by 15.3%, 29.6%, and 33.4% in the low-high, high-low, and high-high groups, respectively. The RERIs of WBGT and O_3 were insignificant for the entire sample but were less than additive (RERI <0) for the warm season.

Conclusion

Our findings indicate that WBGT and O_3 have significant independent and joint associations with suicide risk, and O_3-related risk may be especially heightened in areas experiencing lower temperatures in the summer.

TO31-07 | Association Between Heatwave and Length of Hospital Stay for Alzheimer's Disease and Related Dementias

Yongsoo Choi[1], Danielle Braun[2], Daniel Mork[2], Honghyok Kim[3], Garam Byun[1], Damien Foo[1], Yimeng Song[1], Michelle Audirac[2], Antonella Zanobetti[4], Thomas M. Gill[5], Tarik Benmarhnia[6], Scott W. Delaney[4], Michelle L. Bell[1,7]

[1] Yale University, School of the Environment, New Haven, USA; [2] Harvard T.H. Chan School of Public Health, Department of Biostatistics, Boston, USA; [3] The University of Illinois Chicago, Division of Environmental and Occupational Health Sciences, School of Public Health, Chicago, USA; [4] Harvard T.H. Chan School of Public Health, Department of Environmental Health, Boston, USA; [5] Yale School of Medicine, Department of Internal Medicine, New Haven, USA; [6] University of California, Scripps Institution of Oceanography, San Diego, USA; [7] Korea University, 7School of Health Policy and Management, College of Health Sciences, Seoul, South Korea

Extreme heat has been linked to many adverse morbidity outcomes among patients with Alzheimer's disease and related dementias (ADRD). However, its impact on length of hospital stay (LoS)—a commonly used metric reflecting disease severity and healthcare system burden—remains less studied.
This study analyzed all Medicare fee-for-service hospitalizations among beneficiaries aged 65 years and older residing in the contiguous United States who had at least one hospitalization with ADRD-related conditions, as identified by ICD codes, from May to September in the years 2016–2018. The heatwave-exposed group was identified based on residential address and admission date, and then matched to non-heatwave-exposed individuals using propensity score matching. LoS between the two groups was compared using linear regression.
Among 120,934 hospitalizations during the study period, 2,422 were identified as heatwave-exposed prior to hospitalization, and 9,688 matched controls were selected from the non-exposed group. Exposure to a heatwave one to two days before admission was associated with an increase of 0.56 days (95% CI, 0.15–0.95) in LoS, while any exposure within three days prior to admission was associated with an increase of 0.35 days (95% CI, 0.10–0.62) in LoS. Overall, an estimated 1,799 (95% CI: 468–3,188) additional inpatient days occurred due to heatwaves, resulting in an extra inpatient medical cost of $1,104,172 (95% CI: $286,975–$1,956,983) during the study period.
While effective strategies to prevent heat-related impacts exist, heatwave exposure is associated with longer LoS for ADRD or ADRD-related conditions, placing additional strain on individuals, families, and society in terms of resources and costs. This prolonged hospital stay may reflect more severe initial presentations among heatwave-exposed patients compared to those admitted during non-heatwave periods. These findings underscore the need for proactive measures and preventive strategies to protect the ADRD population during extreme heat events and to mitigate the additional burden on the healthcare system.

TO32 | Environmental chemicals and reproductive outcomes

TO32-01 | Associations of Phenols and Parabens with Anti-Müllerian Hormone During the Menopause Transition: The Study of Women's Health Across the Nation

Wenzhiyuan Zhang[1], John F. Randolph Jr.[3], Sioban Harlow[1], Ellen B. Gold[2], Sung Kyun Park[1,4]

[1] University of Michigan,School of Public Health, Department of Epidemiology, Ann Arbor, USA; [2] University of California, Davis School of Medicine, Department of Public Health Sciences, Davis, USA; [3] University of Michigan, School of Medicine, Department of Obstetrics and Gynecology, Ann Arbor, USA; [4] University of Michigan,School of Public Health, Department of Environmental Health Sciences, Ann Arbor, USA

Objective

Phenols, including bisphenols and parabens, are endocrine-disrupting chemicals that may influence ovarian aging and reproductive hormones. However, their relationship with anti-Müllerian hormone (AMH), a marker of ovarian reserve and aging, during the menopause transition remains unclear. We examined the associations between urinary phenols and AMH in midlife women.

Material and Methods

We included 636 women with 2,516 repeated AMH measurements from 10 to 0 years before the final menstrual period (FMP) over 6 years from the 3rd visit (1999-2000) to the 8th visit (2005-2006) in the Study of Women's Health Across the Nation. Serum AMH concentrations were measured using pico AMH ELISA. Urinary concentrations of ten phenols (bisphenol-A, bisphenol-F, 2,4-dichlorophenol, 2,5-dichlorophenol, triclosan, methylparaben, ethylparaben, propylparaben, butylparaben, benzophenone-3) were measured twice at the 3rd and 6th visit, three years apart, using online solid-phase extraction coupled to high-performance liquid chromatography-tandem mass spectrometry. Linear mixed-effects models were used to evaluate associations between time-varying urinary phenol (log2-transformed) and AMH levels (natural log-transformed), adjusting for urinary creatinine, race, site, age at FMP, education, smoking status, parity, body mass index, and time to the FMP.

Results

Higher concentrations of bisphenol-A, bisphenol-F, 2,4-dichlorophenol, and triclosan were associated with higher AMH levels per doubling of exposure: 5.99% (95% CI: 0.86%, 11.39%) for bisphenol-A, 4.71% (-0.41%, 10.10%) for bisphenol-F, 5.54% (1.88%, 9.33%) for 2,4-dichlorophenol, and 2.74% (-0.13%, 5.69%) for triclosan. By contrast, urinary ethylparaben was inversely associated with AMH levels (-2.72% per doubling of exposure [95% CI: -5.66%, 0.32%]). No associations were found for other phenols.

Conclusion

These findings suggest that various phenol compounds may affect ovarian reserve, possibly hastening ovarian aging during the menopausal transition, while parabens, particularly ethylparaben, might reduce ovarian reserve. Further epidemiologic studies are needed to confirm our findings.

TO32-02 | Maternal and Neonatal PFAS Concentrations in Twin Pregnancies: Transplacental Transfer and Association with Health Outcomes

Heeyeon Chae[1,2], Na-Youn Park[3], Ye Eun Seo[3], Inae Lee[4], Young Ah Lee[5], Jong Kwan Jun[6], Eun Na Kim[7], Gi Jeong Cheon[8], Young Joo Park[9,10], Younglim Kho[3], Sun Wook Cho[9], Kyungho Choi[1]

[1] Seoul National University, Graduate School of Public Health, Seoul, South Korea; [2] Seoul National University, Institute of Health & Environment, Seoul, South Korea; [3] Eulji University, Department of Health, Environment & Safety, Seongnam, South Korea; [4] Keimyung University, Department of Public Health, Daegu, South Korea; [5] Seoul National University Hospital and Seoul National University College of Medicine, Department of Public Health, Seoul, South Korea; [6] Seoul National University Hospital and Seoul National University College of Medicine, Department of Obstetrics and Gynecology, Seoul, South Korea; [7] Seoul National University Hospital, Department of Pathology, Seoul, South Korea; [8] Seoul National University College of Medicine, Department of Nuclear Medicine, Seoul, South Korea; [9] Seoul National University College of Medicine, Department of Internal Medicine, Seoul, South Korea; [10] Seoul National University, Department of Molecular Medicine and Biopharmaceutical Sciences, Graduate School of Convergence Science and Technology, Seoul, South Korea

In vitro fertilization with twin pregnancies has increased worldwide. However, the transplacental transfer (TPT) of per- and polyfluoroalkyl substances (PFASs) in twin pregnancies and their endocrine effects are not well understood. Employing 79 twin-pregnant women and 199 neonatal twins from the Ideal Breast Milk (IBM) cohort at Seoul National University Hospital (2016-2019), we assessed the TPT of PFASs and their association with thyroid hormones. PFASs and alternatives (n=33) were measured in maternal serum and neonatal cord serum using LC-MS/MS, and thyroid hormones (free T4, free T3, TSH) were assessed. Gestational diabetes mellitus (GDM) was diagnosed with the 50g oral glucose tolerance test (OGTT).

TPT ratio showed a U-shaped pattern by carbon chain length, with PFTrDA and PFHxS having median ratios above 1. This trend aligns with previous studies reported for a Chinese singleton-pregnancy population. PFAS exposure was strongly associated with GDM, with substances like PFOA positively correlating even within normal OGTT ranges (β=1.85, 95% CI: 0.41 to 3.28, p=0.02). Maternal PFAS exposure was linked to isolated hypothyroxinemia, a condition with unchanged TSH levels and decreased FT4. One notable compound, 9Cl-PF3ONS, showed significant associations with maternal thyroid hormones. PFOA was also positively correlated with placental malperfusion. In cord serum, PFOA was associated with increased FT4 (β=0.02, 95% CI: 0.00 to 0.03, p=0.03) and reduced birth weight (β=-0.05, 95% CI: -0.08 to -0.01, p=0.01), with the birth weight trend consistent with those reported for singleton pregnancies.

These findings suggest twin pregnancies may amplify the health impacts of PFAS exposure, especially regarding thyroid function, GDM, and fetal development. Minimizing PFAS exposure during pregnancy is crucial for protecting maternal and fetal health.

This work was supported by Korea Environmental Industry & Technology Institute (KEITI) through “Core Technology Development Project for Environmental Diseases Prevention and Management,” funded by Korea Ministry of Environment (MOE) (RS-2022-KE002173).

TO32-03 | Endocrine-disrupting Chemicals and Female Sexual Function: Investigating Phthalate and Phthalate Alternative Exposure in a Cohort of Pregnancy Planners

Samantha Schildroth[1], Julia Bond[2], Martha R. Koenig[1], Amelia K. Wesselink[1], Jasmine Abrams[3], Elizabeth E. Hatch[1], Lauren A. Wise[1]

[1] Boston University School of Public Health, Department of Epidemiology, Boston, USA; [2] Boston University School of Public Health, Department of Community Health Sciences, Boston, USA; [3] Yale University School of Public Health, Department of Social and Behavioral Sciences, New Haven, USA

Background: Sexual function, or the ability to engage in and enjoy sexual activity, is important for fertility, sexual expression, and general well-being. Endocrine-disrupting chemicals (EDCs) may affect sexual function by dysregulating hormonal systems supporting function (e.g., estrogen interference). However, EDCs, like phthalates, remain understudied in relation to sexual function.

Methods: We used cross-sectional preconception data from Pregnancy Study Online (PRESTO, n=347) to investigate associations between phthalate exposure and sexual function, measured using the Female Sexual Function Index-6 (FSFI-6). We summed FSFI-6 responses (range=2-30); lower scores reflected poorer function. We measured 16 phthalate or alternative metabolites in urine samples using online solid phase extraction coupled with high-performance liquid chromatography isotope dilution tandem mass spectrometry and creatinine-adjusted metabolite concentrations. Because phthalate metabolites were non-linearly associated with FSFI-6 scores, we categorized metabolite concentrations into tertiles. We used multivariable linear regression to estimate mean differences (β) with 95% confidence intervals (CIs) in FSFI-6 scores per tertile increase in concentrations, adjusting for confounders. In secondary analyses, we considered individual FSFI-6 items (interest/desire, arousal, lubrication, orgasm, sex life satisfaction, and pain; range=1-5) as outcome variables.

Results: Most metabolites were not appreciably associated with female sexual function. Mono-n-butyl phthalate concentrations were weakly and non-monotonically associated with lower summed FSFI-6 scores (β=-0.8, 95% CI=-1.8, 0.2) and orgasm scores (β=-0.3, 95% CI=-0.7, 0.1) at the 2nd (vs. 1st) tertile. Mono-2-ethyl-5- carboxypentyl terephthalate concentrations were modestly associated with poorer scores for orgasm, while other phthalate metabolites (e.g., mono-carboxyisononyl phthalate) were associated with higher scores for the summed FSFI-6 and FSFI-6 items.

Conclusion: Some phthalate metabolites were weakly associated with female sexual function, but the direction of association varied by metabolite, and most associations were non-monotonic. These findings suggest a complex relationship between EDC exposure and sexual health, warranting further investigation in longitudinal studies to clarify temporal associations and underlying mechanisms.

TO32-04 | Associations of gestational blood lead concentrations with adolescent reproductive hormones in a Cincinnati, Ohio pregnancy and birth cohort

Hannah S. Craig[1], Bruce Lanphear[2], Aimin Chen[3], Kim Cecil[4], Yingying Xu[5], Nicholas Newman[5], Heidi Kalkwarf[5], Kimberly Yolton[5], Joseph M. Braun[6], Jessie P. Buckley[1]

[1] University of North Carolina at Chapel Hill, Gillings School of Global Public Health, Department of Epidemiology, Chapel Hill, USA; [2] Simon Fraser University, Faculty of Health Sciences, Vancouver, Canada; [3] University of Pennsylvania Perelman School of Medicine, Epidemiology and Informatics, Department of Biostatistics, Philadelphia, USA; [4] Cincinnati Children's Hospital Medical Center, University of Cincinnati College of Medicine, Department of Radiology, Cincinnati, USA; [5] Cincinnati Children's Hospital Medical Center, University of Cincinnati College of Medicine, Department of Pediatrics, Cincinnati, USA; [6] Brown University, Department of Epidemiology, Providence, USA

Objective: Lead is a contaminant of global concern. In animal models, higher gestational exposure results in decreased concentrations of certain reproductive hormones and delayed pubertal development in offspring. Few epidemiologic studies have investigated associations in prospective cohorts or included both sexes. Therefore, we examined associations of gestational blood lead concentrations with reproductive hormones at age 12 years among males and females.

Material and Methods: We studied 187 mother-child pairs from the HOME Study, in Cincinnati, Ohio (enrolled: 2003–2006). We averaged blood lead concentrations measured twice in pregnancy. At age 12 years, we quantified serum concentrations of dehydroepiandrosterone sulfate, luteinizing hormone, and follicle-stimulating hormone in both sexes; estradiol in females; and testosterone in males. We estimated associations of averaged gestational lead and reproductive hormones using linear regression models among males (n=87) and females (n=100) separately. We adjusted for household income, parity, prenatal serum cotinine concentration, maternal age at birth of child, maternal pre-pregnancy body mass index, and child age at study visit.

Results: The median (25th–75th percentile) averaged gestational blood lead concentration was 6.7 µg/L (5.2–8.2 µg/L) in females and 6.7 µg/L (5.4–8.4 µg/L) in males. There were no clear patterns of association between lead and reproductive hormones in adjusted models, and many of the 95% confidence intervals were centered around the null. In models stratified by menarche status, estradiol was higher by a factor of 1.12 (12% higher) (95% CI: 1.01-1.24, or 1%–24% higher) per µg/L increase in gestational lead among menstruating females but null among non-menstruating females (interaction p=0.03), but the sample size was small (n=46).

Conclusion: Relatively low gestational blood lead concentrations were not associated with serum reproductive hormone concentrations in adolescent males or females. Future analyses will include repeated lead measures across childhood and assess associations with Tanner stages and age at menarche.

Supported by NIEHS grants.

TO32-05 | Maternal Perinatal Distress Symptoms in Relation to Exposure to Per- and Polyfluoroalkyl Substances (PFAS) in the Danish National Birth Cohort

Pengfei Guo[1], Onyebuchi Arah[2], Cecilia Ramlau-Hansen[3], Zeyan Liew[1]

[1] Yale School of Public Health, Department of Environmental Health Sciences, New Haven, Connecticut, USA; [2] UCLA Fielding School of Public Health, Department of Epidemiology, Los Angeles, USA; [3] Aarhus University, Department of Public Health, Aarhus, Denmark

Objective Per- and polyfluoroalkyl substances (PFAS), widespread endocrine-disrupting chemical pollutants, can dysregulate stress responses and affect the risk for depression. We evaluated whether PFAS exposure affects maternal distress, including depression and anxiety symptoms, during pregnancy and postpartum.

Methods Using data from 3759 pregnancies in the Danish National Birth Cohort (DNBC), we examined the associations between plasma concentrations of six PFAS (median: 8 weeks of gestation) and maternal distress. Maternal distress, and symptoms for depression and anxiety, were collected in self-reported Checklist-92 and the General Health Questionnaire, during 30 weeks of gestation and 6-month postpartum. We utilized log-binomial regression to estimate the risk ratios (RR) for the presence of high distress, depression, or anxiety (scores >90th) according to per doubling increase of plasma PFAS (ng/ml), adjusting for potential confounding and selection biases by applying inverse probability weights. We also estimated the joint effect of PFAS mixture using the quantile g-computation (QGC) method.

Results A doubling increase in plasma perfluorooctanoic acid (PFOA) was associated with high distress (RR=1.26, 95% CI: 1.12-1.41), as well as, the symptoms for depression (RR=1.33, 95% CI: 1.13-1.57) and anxiety (RR=1.45, 95% CI: 1.27-1.65) during pregnancy. Moreover, multiple PFAS exposures were also consistently associated with depressive symptoms at 6 months postpartum (e.g., PFOA: RR=1.12, 95% CI: 1.02-1.23; perfluoroheptanesulfonic acid (PFHpS): RR=1.15, 95% CI: 1.03-1.28). No apparent associations were found for PFAS mixture and maternal distress measures.

Discussion Gestational exposures to PFAS, particularly PFOA and PFHpS, were associated with worsened perinatal distress and depressive symptoms. These findings support that PFAS exposure may disrupt pregnancy mood/emotion regulation, with effects extending into the postpartum period.

TO33 | Prioritizing and evaluating air pollution policy

TO33-01 | Early evidence for spatiotemporal impacts of New York City's Congestion Relief Zone on PM2.5

Sarah McCuskee[1], Maida Galvez[2], Nicholas DeFelice[2]

[1] Icahn School of Medicine at Mount Sinai, Emergency Medicine, New York, USA; [2] Icahn School of Medicine at Mount Sinai, Environmental Medicine, New York, USA

New York City (NYC) implemented a congestion relief zone (CRZ) on January 5, 2025, charging tolls to vehicles entering southern Manhattan. Detailed evaluation of environmental impacts of this policy across wider NYC will take months to years. To provide an early indication of potential policy impacts, we investigated NYC PM2.5 levels in the first weeks after CRZ implementation.

This was a retrospective observational study. The outcome was PM2.5 measured by NYC Community Air Survey (NYCCAS) monitors, for the period January 5 to February 12 of years 2020, 2023, 2024, and 2025. Pandemic years were excluded. We stratified monitors into four zones: (1) within the CRZ, (2) upper Manhattan and the Bronx, (2) Brooklyn and Queens, and (4) Staten Island (no pre-implementation data). We compared daily PM2.5 by day of week between pre- and post-implementation years in each zone using Wilcoxon rank sum tests. Separately, we constructed linear mixed models for the number of vehicles entering the CRZ (Metropolitan Transportation Authority data). All analyses were conducted using R 4.2.

The 17 NYCCAS monitors active during the study period provided 243,063 hourly PM2.5 measurements. Median daily PM2.5 during the study period at monitors outside the CRZ was 7.16µg/m^3 (IQR 5.07-11.2), and inside the CRZ was 7.99µg/m^3 (5.93-11.8). After CRZ implementation, PM2.5 was significantly lower in each zone ($p<0.01$ for Upper Manhattan/Bronx and Brooklyn/Queens; $p<0.0001$ for CRZ). However, when stratified by day of week, the effect persisted only on Tuesdays, Wednesdays, and Thursdays in each zone. A daily median of 22,300 (IQR 14,400-25,800) vehicles entered the CRZ post-implementation. In longitudinal mixed models, daily CRZ vehicle entries were not significantly associated with PM2.5 ($p=0.08$).

Early findings suggest that PM2.5 may have lowered throughout NYC after CRZ implementation, particularly mid-week; however, possible seasonal effects necessitate longer-term evaluation to clarify impacts of this policy.

TO33-02 | Reflections on the 25th Anniversary of the HEI Reanalysis Project

Douglas W. Dockery[1], C. Arden Pope[2]

[1] Harvard TH Chan School of Public Health, Environmental Health, Boston, USA; [2] Brigham Young University, Departmetn of Economics, Provo, USA

The Harvard Six Cities (1993) and the American Cancer Society CPS-II (1995) prospective cohort studies linked $PM_{2.5}$ to decreased life expectancy. They provided crucial evidence for proposed new air quality standards for $PM_{2.5}$ in 1997. Industry groups and some lawmakers challenged these results and demanded access to the data of individual study participants. Harvard and ACS investigators declined to release these data due to legal, ethical, and confidentiality concerns. Instead, they agreed to independent review and reanalysis by the Health Effects Institute with assurances of rigorous review while maintaining the confidentiality of study participants. The HEI reanalysis, released on July 26, 2000, confirmed the data quality of both cohorts, verified the reproducibility of the original results,

and demonstrated the robustness of the results to alternative analytic approaches and extended control of confounders. Subsequently, independent analyses by researchers using different cohorts have corroborated the original Six Cities and ACS findings. The HEI Reanalysis Project prompted extended investigations of both the Six Cities and ACS cohorts, resulting in substantial advancements in understanding the health effects of air pollution. Nevertheless, critics have continued pressuring for access to the original individual data. The Shelby Amendment to the 1999 Omnibus Spending Bill mandated changes to the rules governing funding for universities and non-profits, making their research accessible under Freedom of Information requests. Proposed federal legislation (including the "Secret Science Reform" Acts of 2014, 2015, and the HONEST Act of 2017) and a 2018 proposed rule would have prohibited EPA from using epidemiologic studies in rulemaking unless the raw data was released publicly. These actions to undermine the use of environmental epidemiology studies in rulemaking were not implemented. They did motivate analyses of various public data sets (including Medicare, NHIS, and SEER data) and have considerably influenced the practice of environmental epidemiology.

TO33-03 | Risk-Based Prioritization of Air Toxics in Colorado: A Screening-Level Approach

Stephanie Pusker[1], Tara Webster[1], Rachel Burmeister[1], Kristy Richardson[1], Vinay Kumar[2], Heather Walsh[2], Patrick McKean[2], Miriam Hacker[2], Amanda Damweber[2], Rebecca Nagy[2]

[1] Colorado Department of Public Health and Environment, Division of Environmental Health and Sustainability, Denver, USA; [2] Colorado Department of Public Health and Environment, Air Pollution Control Division, Denver, USA

Objective: People are exposed to a multitude of toxic air contaminants from numerous sources and background levels in the ambient air that may act cumulatively to produce adverse impacts to public health. In response to growing concerns about air toxics, Colorado enacted House Bill 22-1244 (HB 22-1244), Public Protections from Toxic Air Contaminants, on June 2, 2022. This legislation mandates the Colorado Department of Public Health and Environment (CDPHE) to develop policies and regulations aimed at reducing public exposure to hazardous air pollutants. In January 2025, CDPHE completed its first rulemaking under HB 22-1244, identifying five priority air toxics: benzene, ethylene oxide, formaldehyde, hexavalent chromium, and hydrogen sulfide.
Methods: CDPHE developed a risk-based framework to guide the prioritization of air toxics. The prioritization process incorporated multiple data sources, including the EPA's AirToxScreen, CDPHE modeling, and monitoring data from both CDPHE and external sources. The data were analyzed using a screening-level risk assessment approach to evaluate potential health risks and inform regulatory decision-making.**Results and Conclusion:** This assessment's findings highlight the strengths and limitations of using screening-level analyses to prioritize air toxics. The outcome of regulatory decision-making provides a foundation for future risk assessment and policy development in Colorado, and the methodological approach presented here offers a framework for other jurisdictions seeking to implement data-driven strategies for air toxics management. Future efforts could focus on improving exposure characterization, enhancing community engagement in regulatory processes, and addressing data gaps to ensure equitable health protections.

TO33-04 | Mediated impacts of China's clean heating policy on blood pressure by indoor air pollution and indoor temperature

Talia Sternbach[1], Jill Baumgartner[1], Xiaoying Li[2], Collin Brehmer[2], Yuanxun Zhang[4,5], Wenlu Yuan[1], Shu Tao[3], Kaibing Xue[4], Kennedy Hirst[2], Guofeng Shen[3], Chris Barrington-Leigh[1], Xiang Zhang[1], Brian Robinson[1], Ellison Carter[2], Sam Harper[1]

[1] McGill University, Montreal, Canada; [2] Colorado State University, Fort Collins, USA; [3] Peking University, Beijing, China; [4] University of Chinese Academy of Sciences, Beijing, China; [5] Chinese Academy of Sciences, Xiamen, China

The Clean Heating Policy (CHP) was implemented in 2016 to transition millions of homes in northern China from coal- to electric-based heating. Household heating interventions may improve health, including blood pressure (BP), but the mechanisms through which this may happen are unclear. We leveraged the staggered rollout of the CHP across villages in rural Beijing and applied causal mediation analysis to understand how much of the CHP's impact on BP may be mediated through indoor temperature and air pollution ($PM_{2.5}$).
We enrolled 1,003 participants >40y from 50 villages unaffected by the CHP in winter 2018-19. Over the next 3 winters 11, 6, and 3 villages were treated, respectively We visited homes each winter to measure BP, administer questionnaires, and place indoor temperature and $PM_{2.5}$ sensors. Mediators were the indoor temperature measured 5-min before BP in all homes and the winter average indoor $PM_{2.5}$ measured in 25% of homes. We estimated the CHP's total effect on BP using a difference-in-differences design, then controlled for each mediator separately, and both together, in additional models to estimate controlled direct effects (CDE) of the CHP.The CHP reduced systolic BP by 1.4 mmHg (95%CI: -3.3, 0.5) and diastolic BP by 1.6 mmHg (95%CI: -2.9, -0.3). Holding indoor temperature and $PM_{2.5}$ at their baseline means (14˚C and 71 μg/m3, respectively), the CDEs for systolic and diastolic BP in the multiple mediation analysis were 0.3 mmHg (95%CI: -1.9, 2.5) and -0.6 mmHg (95%CI: -2.1, 0.9), respectively. Our analysis shows that CHP-driven improvements to indoor temperature and $PM_{2.5}$ contributed to modest reductions in BP. These results provide insight into *how* the CHP affected BP and may inform future clean heating policies.

TO33-05 | Environmental and health inequalities across Just Transition territories in Europe

Hans Orru, Taavi Tillmann, Marilin Poogen, Katrin Lang

University of Tartu, Institute of Family Medicine and Public Health, Tartu, Estonia

As a key initiative of the European Green Deal, the Just Transition Fund mechanism has been established to ensure that no individual or region is left behind in the shift to a climate-neutral economy. It includes altogether 104 territories, with different size, across European Union (EU). Mostly those are areas with extensive fossil fuel-based energy production as well as numerous industrial activities. The social impacts of the transition to climate neutrality are primarily tied to employment, directly affecting household livelihoods, contributing to social exclusion, and carrying significant gender implications. Current study aims to map the present environmental, and health inequalities and build the theoretical model to project the future changes. With-in this framework, air quality in terms of fine particle exposure, health status in terms of life expectancy at birth among males/females and income were mapped and compared to the national figures in 28 EU member states. We used EU wide air quality dispersion modelling, national

health statistics databases and we applied rapid-literature review methodology to build our theoretical model. In general, those areas except Northern-Europe, where Just Transition territories lie on more peripheral areas, with less industrial activities, are currently characterized with higher air pollution exposures. With industrial transition, air quality in those areas is expected to be improved, that would have positive effect on physical health; however, mental is expected to worsen due to increased economic uncertainties. In terms of life expectancy, those areas are characterized up to a five-year difference compared to the national average and the effect is more profound among males. Despite the extensive industrial activities, those areas also have lower income level per person that reinforce health inequities. These findings highlight the complex interplay between environmental improvements and socio-economic challenges in Just Transition territories, emphasizing the need for targeted policies that mitigate health inequalities.

TO34 | Air pollution, climate, and reproductive outcomes

TO34-01 | Fine Particulate Matter Exposures and Semen Quality in a U.S.-Based Cohort.

Sanjana Bhaskar[1], Makiko Mitsunami[2], Charles L. Bormann[3], Jorge E. Chavarro[4,5], Manoj K. Kanakasabapathy[6], Martin Kathrins[7], Hadi Shafiee[6,8], Li Yi[2,9], Rachel Nethery[10], Gary Adamkiewicz[1], Jaime E. Hart[1,5]

[1] Harvard T.H. Chan School of Public Health, Department of Environmental Health, Boston, USA; [2] Harvard T.H. Chan School of Public Health, Department of Nutrition, Boston, USA; [3] Massachusetts General Hospital, Harvard Medical School, Division of Reproductive Endocrinology and Infertility, Department of Obstetrics and Gynecology, Boston, USA; [4] Harvard T. H. Chan School of Public Health, Department of Epidemiology and Department of Nutrition, Boston, USA; [5] Brigham and Women's Hospital and Harvard Medical School, Channing Division of Network Medicine, Department of Medicine, Boston, USA; [6] Brigham and Women's Hospital and Harvard Medical School, Division of Engineering in Medicine, Department of Medicine, Boston, USA; [7] Brigham and Women's Hospital, Department of Urology, Boston, USA; [8] Harvard Medical School, Department of Medicine, Boston, USA; [9] Harvard Medical School and Harvard Pilgrim Health Care Institute, Department of Population Medicine, Boston, USA; [10] Harvard T.H. Chan School of Public Health, Department of Biostatistics, Boston, USA

Objective: We estimated the association between $PM_{2.5}$ exposure and semen quality across multiple exposure windows in a U.S.-based cohort. **Materials and Methods**: Participants in the Reproductive Effects of Chemicals and Air Pollution (RECAP) study collected semen samples remotely, used a smartphone-based application to assess sperm motility, and shipped samples to Massachusetts General Hospital for analysis of total sperm count and concentration. $PM_{2.5}$ exposure was estimated from the nearest EPA monitor to each participant's home address and averaged over five biologically relevant windows (0-9, 10-14, 15-69, 70-90, and 0-90 days before sample collection). Linear regression models were used to estimate unadjusted, age-adjusted, and fully adjusted associations controlling for age, abstinence duration, and recent exposure to a hot bath/sauna. **Results**: A total of 142 participants provided data on sperm count (Mean [SD]: 136.45 [121.08] million/ejaculate) and sperm concentration (Mean [SD]: 36.50 [31.91] million/mL), and 106 participants provided data on sperm motility (Mean [SD]: 39.45% [25.56%]). Mean $PM_{2.5}$ exposure in the 90 days before sample collection was 8.06 μg/m^3 (SD: 2.71). Higher $PM_{2.5}$ exposure was generally associated with lower semen quality. Exposure windows 0-9, 10-14, 15-69, and 0-90 days showed a pattern of lower sperm concentration (e.g., fully adjusted models showed that a 1 μg/m^3 increase in $PM_{2.5}$ during the 15-69-day window was associated with 6.4% lower concentration [95% CI: -12.2%, -0.3%], and similarly, 4.7% lower concentration [95% CI: -10.6%, 1.6%] for the 0-90-day window). $PM_{2.5}$ exposure during the 0-9 and 10-14-day windows was also associated with lower sperm count and motility (e.g., 0-9 days: fully adjusted models showed a 2.3% lower count [95% CI: -9.7%, 5.7%] and a 0.23 percentage-point lower motility [95% CI: -1.78, 1.32]). **Conclusion**: Data from this general population sample of men suggest that $PM_{2.5}$ exposure may contribute to lower sperm quality, particularly during key spermatogenic windows.

TO34-02 | Climate-related water contaminants and fetal loss in Northern California

Dana Goin[1], Sylvia Sudat[2], Jenny Rempel[3], Katherine Wolf[3], Amy Padula[4], Anne Nigra[1], Joan Casey[5], Komal Bangia[6], Rachel Morello-Frosch[3]

[1] Columbia University, Mailman School of Public Health, New York, USA; [2] Center for Health Systems Research, Sutter Health, Walnut Creek, USA; [3] University of California, Berkeley, School of Public Health, Berkeley, USA; [4] University of California, San Francisco, Department of Obstetrics, Gynecology, and Reproductive Sciences, San Francisco, USA; [5] University of Washington, School of Public Health, Seattle, USA; [6] Community and Environmental Epidemiology Research Branch, Office of Environmental Health Hazard Assessment, Oakland, USA

Background: Climate change is a threat to California's commitment to clean and safe drinking water for all. In particular, drought and wildfires can increase water contaminants in drinking water. The relationship between these water contaminants and fetal loss is not well known.

Objective: To estimate the effect of climate-related water contaminants during pregnancy on the risk of fetal loss using electronic health records (EHR).

Methods: We used Sutter Health EHR data to identify cases of fetal loss and match them to ongoing pregnancies using prenatal care visits. We linked pregnancies to water systems and levels of nitrate, benzene, arsenic, hexavalent chromium, manganese, uranium, and vanadium using geocoded addresses. We used Poisson regression to estimate the incidence rate ratio of fetal loss. We also estimated odds ratios using a continuation ratio logit discrete hazard model with gestational weeks as the time unit, to account for variable entry into prenatal care. We controlled for birthing parent age, race/ethnicity, primary language spoken, parity, and the year and season of conception.

Results: The overall rate of loss was about 0.5% for stillbirth and 4% for miscarriage. We observed a higher rate of fetal loss for a one-ug/L increase in prenatal arsenic exposure (IRR = 1.03, 95% CI = 1.02, 1.04). We also observed higher rates of fetal loss for a 0.1 ug/L increase in benzene (1.17, 95% CI 1.01, 1.32) and a 10 pCi/L increase in uranium (1.73, 95% CI 1.54, 1.94). Results from the discrete hazard model were similar.

Conclusions: Water contaminants, especially arsenic, benzene, and uranium, are associated with an increased risk for fetal loss.

TO34-04 | Warm season air pollution and Chinese couple fecundability: A nationwide preconception cohort study

Xinghou He[1,2], Hongbing Xu[1,2], Mengyao Li[1,2], **Wei Huang**[1,6]

[1] Peking University School of Public Health, Department of Occupational and Environmental Health, Beijing, China; [2] Peking University, Institute of Environmental Medicine, Beijing, China; [3] National Research Institute for Family Planning, Beijing, China; [4] National Human Genetic Resource Center, Beijing, China; [5] National Human Reproduction and Health Resource Center, Beijing, China; [6] Peking University Health Science Center, State Key Laboratory of Vascular Homeostasis and Remodeling, Beijing, China; [7] Peking University, Institute for Global Health and Development, Beijing, China

Objective

To assess the associations between warm season air pollution exposure and couple fecundability at nationwide population level.

Material and Methods

This cohort study used health data from the Chinese National Free Prepregnancy Checkups Project conducted across 31 mainland provinces in China, which included couples aged 20-49 years who planned to conceive between January 1, 2014, and December 31, 2019. Time to pregnancy (TTP) was used as the outcome measure indicative of couple's fecundability. Residence-specific concentrations of ambient particulate matter in diameter ≤10 μm and ≤2.5 μm (PM_{10} and $PM_{2.5}$), nitrogen dioxide (NO_2), sulfur dioxide (SO_2), carbon monoxide (CO), and 8-hour maximum ozone (O_3) were estimated using the Nested Air Quality Prediction Modeling System. Associations of air pollutants exposure and fecundability were characterized using Cox regression models, controlling for demographic and meteorologic parameters.

Results

Among the 14,029,184 Chinese couples included in this study, 4,285,222 (30.5%) females became pregnant within one year, resulting in 94,903,116 person-months of follow-ups. The warm season $PM_{2.5}$,PM_{10}, NO_2, SO_2, CO, and O_3 levels during the study period were 50.4 μg/m^3, 72.3 μg/m^3, 28.9μg/m^3, 20.0μg/m^3, 0.90 mg/m^3, and 118.5 μg/m^3, respectively. Higher exposure to O_3 was independently associated with reduced fecundability, with appropriately linear exposure-response relationships. Each interquartile range increase in O_3 concentrations was significantly associated with higher risk of delayed TTP, with a HR of 0.9270 (95%CI, 0.9256, 0.9285). The estimates were robust after adjustment for co-pollutants, and the risks were more pronounced among participants aged ≥40 years, farmers, those with low education levels, pregnancy history, and rural residents.

Conclusion

This cohort study showed that warm season exposure to O_3 was associated with heightened risk for reduced fecundability, suggesting that policies to control tropospheric O_3 pollution are urgently needed from the perspectives of managed environmental determinants of fecundability and improved couples' fertility.

TO34-05 | Ambient Temperature and Sperm DNA Methylation

Carrie J. Nobles[1], Timothy P. Canty[2], Pauline Mendola[3], Lindsey M. Russo[4], Kaniz Rabeya[1], Karen C. Schliep[5], Neil J. Perkins[6], Rachael Hemmert[5], May Shaaban[5], Akanksha Singh[2], Allison M. Ring[2], Matthew Peterson[7], Erica Johnstone[7], Richard Pilsner[8]

[1] University of Massachusetts Amherst, School of Public Health and Health Sciences, Amherst, USA; [2] University of Maryland College Park, Department of Oceanic and Atmospheric Chemistry, College Park, USA; [3] University at Buffalo, School of Public Health and Health Professions, Buffalo, USA; [4] Weill Cornell Medicine, Division of Internal Medicine, New York, USA; [5] University of Utah School of Medicine, Department of Family and Preventive Medicine, Salt Lake City, USA; [6] National Institutes of Health, Eunice Kennedy Shriver National Institute of Child Health and Human Development, Bethesda, USA; [7] University of Utah School of Medicine, Department of Obstetrics and Gynecology, Salt Lake City, USA; [8] Wayne State University School of Medicine, Department of Obstetrics and Gynecology, Detroit, USA

Objective: Spermatogenesis is uniquely sensitive to temperature, with heat stress associated with disruption of the blood-testes barrier and decrements in semen quality. Sperm DNA methylation may similarly be sensitive to heat stress with exposure during the cell division stages of spermatogenesis, with downstream implications for men's fertility.

Material and Methods: We evaluated ambient temperature and sperm DNA methylation among 1,192 men enrolled in the FAZST trial (2013-2018) in Utah who provided a semen sample 6 months after enrollment. Sperm DNA methylation was measured using the MethylationEPIC BeadChip array. Outdoor wet bulb temperature (relative temperature with 100% humidity) was abstracted from local weather monitoring stations and heat stress estimated as hours/day ≥95th and ≥98th percentiles. Exposure was averaged across spermatogenesis (0-74 days before ejaculation) and cell division stages of spermatogenesis (mitosis: 59-74 and meiosis: 35-58 days). Differences in methylation of CpG sites (M-values) for 1) change in temperature for warm versus cold season, and 2) heat stress thresholds were estimated using generalized linear models.

Results: In models evaluating temperature by season, few clear associations were observed in the warm season. However, in the cold season, increasing temperature was associated with 254 differentially methylated CpG sites with exposure during spermatogenesis (Benjamini Hochberg $p<0.05$) and 2572 and 193 sites during mitosis and meiosis, respectively. In threshold models estimating heat exposure, increasing proportion of time ≥95th (≥17.2°C) and ≥98th (≥17.8°C) percentiles of wet bulb temperature was associated with 151 and 465 differentially methylated CpG sites during mitosis and 1 and 17 sites during meiosis, respectively. For heat exposure, CpGs were most highly enriched in genes regulating inflammation, DNA repair, chromatid cohesion, and maintenance of stem cell populations.

Conclusions: Outdoor temperature exposure during early spermatogenesis was associated with sperm DNA methylation, with cell division during mitosis and meiosis a potential sensitive window.

TO35 | Innovations in exposure science: Modeling, monitoring, and mapping air pollution

TO35-01 | National-scale outdoor air pollution modelling and exposure assessment

Calvin Jephcote[1], John Gulliver[2]

[1] University of Leicester, School of Geography, Geology and the Environment, Leicester, UK; [2] City St George's, University of London, Population Health Research Institute, London, UK

Urban air pollution is traditionally estimated by land use regression (LUR) or dispersion modelling approaches. LUR applies proximity measures to indirectly quantify the influence of pollution sources and sinks, however, this approach can effectively account for terrain, street canyon effects, and long-range chemical transport. Dispersion models quantify the complex spatiotemporal relationship between emission inventories and airflows, however, these benefits come with increased data, computational and financial cost.
We developed a national-scale model of outdoor air pollution (Hybrid Air Dispersion Exposure System - HADES) for rapid production of concentration maps of nitrogen dioxide (NO_2) and ozone (O_3) at very high spatial resolution (10m). The model combines local dispersion modelling with satellite-derived estimates of background concentrations, land cover, and a 3-D representation of buildings, using open access datasets that are available in many countries. The model's statistical calibration parameters were constructed from 6,072 hourly-annual average concentrations, collected at 130 monitoring sites across England and Wales in 2018-19.
Concentration surfaces have been developed across England and Wales for 2018-2020. Modelling explained 77-79% for NO_2 and 87-89% for O_3 of the variability in hourly-annual average concentrations, and was robust to changes in NOx emissions during the COVID pandemic. In using these surfaces for exposure assessment, all residential locations, and neighbourhoods in urban areas, are unlikely to be below the 2021 World Health Organisation Air Quality Guidelines threshold for annual average NO_2 concentrations (10 µg/m^3). Rural and suburban areas are likely to exceed the peak-season 8-hour daily maximum O_3 threshold (60 µg/m^3). We suggest that our model output may be used for research and policy support, including health impact assessment, in the UK and elsewhere.

TO35-02 | Measuring differential air quality impacts of prescribed fires: An analysis in southeastern US

Jingting Huang[1], Jeffery Cannon[2], Scott Goodrick[3], Holly Nowell[4], Christina Fuller[1]

[1] University of Georgia, College of Engineering, School of Environmental, Civil, Agricultural and Mechanical (ECAM) Engineering, Athens, USA; [2] The Jones Center at Ichauway, Newton, USA; [3] USDA Forest Service, Center for Forest Disturbance Science, Southern Research Station, Athens, USA; [4] Tall Timbers Research Station, Tallahassee, USA

Objective: Prescribed fire is essential for southeastern US ecosystem management, with approximately six million acres treated annually to reduce wildfire risks and support biodiversity. While ecologically beneficial, these burns can also impact air quality in surrounding communities. Previous studies have shown that in some cases communities of color, particularly in rural areas, may experience disproportionate exposure to air pollution from fires. However, little is known about whether this pattern persists across the southeastern region.
Material and Methods: We integrate a well-developed prescribed fire permit database with atmospheric, emissions, and chemical transport models to estimate daily 24-hour average $PM_{2.5}$ attributable to prescribed burns during the

annual active burn season. We assess demographic and socioeconomic inequalities by census tract in prescribed fire smoke pollution across three southeastern states: Florida, Georgia, and South Carolina. We also evaluate their relationships using correlation and regression analyses stratified by two measures of racial segregation.
Results: Georgia exhibited the highest prescribed fire $PM_{2.5}$ concentrations(2.16±0.57 μg/m³) with frequent high-smoke days, while South Carolina (1.37±0.22 μg/m³) and Florida (1.2±0.52 μg/m³) experienced lower, more localized fire smokeburden. Exposure disparities varied by state: in Georgia, both low-income and predominantly African American communities faced higher exposure; South Carolina also demonstrated consistent prescribed fire pollution disparities in African American communities regardless of socioeconomic status; while Florida presented a contrasting pattern with white-dominant areas having higher fire-specific $PM_{2.5}$ levels, though wealthier areas were less affected.
Conclusion: Prescribed fire remains an important conservation tool in southeastern US forests, yet our results reveal distinct environmental inequalities across states. This research will inform future policies for the strategic use of prescribed burning that maximizes environmental benefits while reducing human exposure.

TO35-03 | Evaluating EPA's AirFuse Real-time Fusion-based Air Pollution Mapping Algorithm Using Computer Vision-based Techniques

__Meng Qi__[1], Ke Xu[1], Barron Henderson[2], Alqamah Sayeed[3], Pawan Gupta[3,4], Shobha Kondragunta[5], Yang Liu[1]

[1] Emory University, Gangarosa Department of Environmental Health, Atlanta, USA; [2] United States Environmental Protection Agency, Durham, USA; [3] Universities Space Research Association (USRA), Huntsville, USA; [4] NASA Marshall Space Flight Center, Huntsville, USA; [5] National Oceanic and Atmospheric Administration (NOAA), National Environmental Satellite, Data, and Information Service (NESDIS), Silver Spring, USA

Objective: There is a growing demand from the public to take protective actions against air pollution based on real-time air quality reading, especially during wildfire seasons. EPA proposed a data fusion framework named AirFuse which aims to integrate surface measurements and satellite data to generate national hourly $PM_{2.5}$ fusion surface. However, spatial anomalies exist due to the varied quality of source data, potentially undermining user confidence. Our study aims to develop a spatial anomaly detection algorithm to evaluate this issue.
Material and Methods: We investigated a representative case during the 2023 Canadian wildfire, where a small area exhibited significantly lower concentrations than its surroundings. We inspected fusion input sources and weights in the anomaly area. To automate anomaly detection, we developed a computer vision-based algorithm. Specifically, we applied a Canny edge detector to identify sharp transitions in model estimates and fusion weights, a fusion filter to locate regions with substantially discrepancies among fusion inputs, and image morphological processing to retrieve suspicious areas with a size larger than 100 pixels.
Results: Two conditions contributed to the representative anomaly: (1) large difference among fusion sources, and (2) abrupt changes in fusion weights. For example, in the anomaly area, satellite weight dramatically increased from near 0 to almost 1, while PurpleAir weight dropped from near 0.8 to nearly 0. Applying our anomaly detection pipeline to AirFuse maps in May-October 2020 revealed that anomalies peaked in the fire season, with a strong correlation with fire occurrences (r=0.989). We proposed a new fusion weighing scheme which significantly reduced detected anomalies by 42%. In contrast, traditional model evaluation metrics (e.g., RMSE) barely change.

Conclusion: Our method may serve as a diagnostic to systematically locate potential anomalies in AirFuse. The system improvements are expected to better inform the public and support decision-making for air pollution exposure mitigation.

TO35-05 | Gap-Filling AOD for Ethiopia between 2018-2023

Bryan N. Vu[1,2], Yinying Qian[1], Alexandra Shtein[1], Grace Chan[3,2], Joel Schwartz[1,3]

[1] Harvard University, Department of Environmental Health, Boston, USA; [2] Children's Hospital of Philadelphia, Clinical Futures, Philadelphia, USA; [3] Harvard University, Department of Epidemiology, Boston, USA

There is increasing evidence of adverse health affects from air pollution, particularly $PM_{2.5}$ (particulate matter with aerodynamic diameter of 2.5 μm or less). However, such studies are limited in low- and middle-income countries, such as Ethiopia, due to lack of consistent spatiotemporally resolved $PM_{2.5}$ estimates for the epidemiological studies. Aerosol optical depth (AOD) is a successful predictor of air pollution in geospatial models and its spatiotemporal coverage is crucial in $PM_{2.5}$ models with high performance. However, since AOD is a remotely sensed data product, its spatiotemporal coverage is hindered by cloud cover and algorithm limitations in processing satellite data. This study aims to gap-fill MODIS AOD over Ethiopia at 250m resolution from November 2018 to September 2023 using a Random Forest model that calibrates meteorological variables from the European Centre for Medium-range Weather Forecasts Reanalysis v5 (ERA5), Aster GDEM elevation, Gridded Population of the World v4 population count, land use, and light at night information. In total, we have almost 22,758,000 observations and the RF model achieved an R^2 of 0.89 (MSE= 0.0050). The most important predictors for AOD in this model were date, temperature, and relative humidity, indicating the importance of both spatial and also temporal predictors in the model. Predictions obtained of this model provides critical estimates for use in geospatial modeling of $PM_{2.5}$ for Ethiopia to use in future epidemiological studies.

TO36 | Improving our understanding of maternal, prenatal, and neonatal PFAS exposures

TO36-01 | Relationship between trimester-specific maternal serum PFAS distribution and placental and fetal cord serum levels

Kyle Campbell[1], Dana B. Barr[1], Andrew Morris[3], Volha Yakimavets[1], Parinya Panuwet[1], Stephanie Eick[1], Kartik Shankar[2], Clark Sims[3,5], Kevin Pearson[4], Aline Andres[3,5], Todd Everson[1,6]

[1] Emory University Rollins School of Public Health, Gangarosa Department of Environmental Health, Atlanta, USA; [2] USDA Agricultural Research Services, Responsive Agricultural Food Systems Research Unit, College Station, USA; [3] University of Arkansas for Medical Sciences, Arkansas Children's Nutrition Center, Little Rock, USA; [4] University of Kentucky College of Medicine, Department of Pharmacology & Nutritional Sciences, Lexington, USA; [5] University of Arkansas for Medical Sciences, Department of Pediatrics, Little Rock, USA; [6] Emory University Rollins School of Public Health, Department of Epidemiology, Atlanta, USA

OBJECTIVE: To characterize the relationship between trimester-specific maternal serum levels of prenatal per- and polyfluoroalkyl substances (PFAS) and paired placental and cord levels at term.

MATERIALS AND METHODS: Data came from Glowing, a prospective birth cohort (n=151). Seventeen PFAS were measured in maternal serum, cord serum, and pulverized flash-frozen villous placenta with liquid chromatography-tandem mass spectrometry. We analyzed 5 placental (PFOS, PFOA, PFHxS, PFNA, and PFDA) and 2 cord PFAS (PFOS and PFOA) with limit-of-detection rates >70%. Each analysis was stratified by PFAS, case-complete, and adjusted for maternal age and education, gestational age, and fetal sex with placental (n=104) or cord (n=67) PFAS as the outcome. Mixed effects models tested transplacental transfer efficiencies (TTEs) changes over pregnancy. Quantile g-computation tested the association between maternal and placental or cord PFAS levels. Cord models also included placental PFAS exposure.

RESULTS: Geometric means for PFAS-specific TTEs ranged from 27.9% in trimester one (T1) to 102.0% in trimester three (T3). TTEs increased linearly between 19% and 48.9% each trimester for all PFAS (p<0.001). A PFAS-specific tertile increase in maternal or maternal and placenta PFAS was associated with an 82.8% to 383.7% increase in placental or cord levels (p<0.001). Maternal T2 and T3 levels were usually the largest contributors to placental PFAS levels ranging from 47.4% for PFNA to 93.6% for PFOA. Placental PFOA was the most important contributor to cord levels at 47.6% whereas T1, T2, and Placenta PFOS contributed 28.6-33.9% each.

CONCLUSIONS: TTE increased throughout pregnancy, reflecting decreasing maternal serum PFAS concentrations across gestation. Our results suggest that mid-to-late pregnancy maternal PFAS levels may be particularly important biomarkers of placental levels at term. Placental PFAS levels were important contributors to cord levels for PFOS and PFOA, with varied effects for trimester maternal blood levels, suggesting heterogeneity in PFAS placental transfer or metabolism.

TO36-02 | Measuring Associations Between Per- and Poly-Fluoroalkyl Substances and Maternal Plasma and Cord Blood Bioactive Lipids in the Maternal and Developmental Risks from Environmental and Social Stressors (MADRES) Cohort

Himal Suthar[1], Helen Foley[1], Tingyu Yang[1], Sandrah P. Eckel[1], Rima Habre[1], Shohreh Farzan[1], Xinci Chen[1], Subramaniam Pennathur[2,3], Lixia Zeng[3], Carrie V. Breton[1], Theresa Bastain[1], Max Aung[1]

[1] University of Southern California, Department of Population and Public Health Sciences, Los Angeles, USA; [2] University of Michigan, Department of Internal Medicine-Nephrology, Ann Arbor, USA; [3] University of Michigan, Department of Molecular and Integrative Physiology, Ann Arbor, USA

Objective: We sought to evaluate associations between prenatal per- and poly-fluoroalkyl substances (PFAS) and bioactive lipids from three enzymatic pathways (cyclooxygenase, cytochrome p450, lipoxygenase) and their parent compounds.

Materials and Methods: 40 mother-infant dyads, an additional 58 pregnant women, and 5 newborns (N = 103) from a Los Angeles, CA based pregnancy cohort were included. We measured 50 bioactive lipids in prenatal plasma (N = 98; median 31 weeks' gestation) and newborn cord blood (N = 45; median 40 weeks' gestation). Of fourteen PFAS measured in prenatal serum (median 18 weeks' gestation), four were analyzed. Spearman correlations between maternal and newborn bioactive lipids were calculated, and associations between PFAS and bioactive lipids were quantified using linear regressions and quantile g-computation for pairwise and mixtures effects, respectively, adjusting for maternal age, educational attainment, parity, pre-pregnancy BMI, and gestational age at bioactive lipid sampling.

Results: 17.2% of pairwise plasma-cord bioactive lipid correlations were significant ($p \leq 0.05$; Range: [-0.71, 0.65]). Among 42 significant associations between individual PFAS congeners and prenatal bioactive lipids, most were negative, the strongest linking PFHxS and arachidonic acid (Doubling Effect: -40%; 95% CI: [-51%, -25%]). By contrast, associations in newborns were mostly positive, the strongest between PFHxS and 15-oxoETE (Doubling Effect: 59%; 95% CI: [6%, 138%]). PFAS mixtures revealed 6 negative and 1 positive signature in maternal plasma, the strongest linking a 1-SD increase in the PFAS mixture to decreased arachidonic acid levels, driven by PFHxS (Mixtures Effect: -39%; 95% CI: [-49%, -27%]). In newborn cord blood, 1 negative and 2 positive signatures were found, the strongest a 49% increase in 15-oxoETE driven by PFOA (95% CI: [10%, 101%]).

Conclusion: Bioactive lipid levels varied between prenatal maternal plasma and newborn cord blood. Their associations with PFAS may inform mechanisms linking PFAS to pregnancy outcomes.

TO36-03 | Prenatal per- andpolyfluoroalkyl substances, metal mixtures, and adiposity in childhood and adolescence

Ixel Hernandez-Castro[1], Sheryl L. Rifas-Shiman[2], Pi-I D. Lin[2], Izzuddin M. Aris[2], Abby F. Fleisch[3,4], Diane R. Gold[5,6], Mingyu Zhang[7], Brent Coull[8], Marie-France Hivert[2,9], Emily Oken[2], Andres Cardenas[1]

[1] Stanford University, Department of Epidemiology and Population Health, Stanford School of Medicine, Stanford, USA; [2] Harvard Medical School and Harvard Pilgrim Health Care Institute, Division of Chronic Disease Research Across the Lifecourse, Department of Population Medicine, Boston, USA; [3] Maine Medical Center, Pediatric Endocrinology and Diabetes, Portland, USA; [4] Maine Health Institute for Research, Center for Interdisciplinary Population and Health Research, Westbrook, USA; [5] Brigham and Women's Hospital and Harvard Medical School, Channing Division of Network Medicine, Boston, USA; [6] Harvard University, Department of Environmental Health, Harvard T.H. Chan School of Public Health, Boston, USA; [7] Harvard Medical School, Department of Medicine, Beth Israel Deaconess Medical Center, Boston, USA; [8] Harvard University, Department of Biostatistics, Harvard T.H. Chan School of Public Health, Boston, USA; [9] Massachusetts General Hospital, Diabetes Unit, Boston, USA

OBJECTIVE:
Per- and polyfluoroalkyl substances (PFAS) and non-essential metals are ubiquitous contaminants and have been individually linked to higher childhood adiposity. However, the potential interactive or cumulative effects of prenatal exposure on offspring adiposity remain understudied. We evaluated joint associations of prenatal PFAS and metals with adiposity in childhood and adolescence.

MATERIAL AND METHODS:
We measured first trimester PFAS (PFOA, PFOS, PFNA, PFHxS, EtFOSAA, and MeFOSAA) in plasma and non-essential (arsenic, barium, cadmium, cesium, mercury, lead) metals in red blood cells in the Project Viva cohort (n=845). We measured body mass index (BMI) *z*-scores and dual-energy X-ray absorptiometry (DXA) total- and truncal- fat mass index in mid-childhood (median= ~7.7y), early adolescence (median= ~13.0y), and late adolescence (median= ~17.5y). We used Bayesian kernel machine regression to evaluate joint associations of PFAS and metals with adiposity in childhood and adolescence, adjusting for key covariates.

RESULTS:
Mothers had a mean (SD) age of 32.7 (4.8) years and were primarily non-Hispanic white (72.9%). Higher levels of the prenatal PFAS and non-essential mixture were associated with higher BMI z-scores (75th vs 50th percentile: 0.17 [95% Credible Interval (CrI): 0.06, 0.28]) and DXA total (0.17 kg/m^2 [95% CrI: 0.05, 0.30]) and truncal fat mass index (0.20 kg/m^2 [95% CrI: 0.08, 0.32]) in mid-childhood. Similarly, higher levels of the prenatal PFAS and non-essential metal mixture were prospectively associated with BMI z-scores (75th vs 50th percentile: 0.14 [95% CrI: 0.02, 0.25]) and DXA total (0.20 kg/m^2 [95% CrI: 0.07, 0.32]) and truncal fat mass index (0.25 kg/m^2 [95% CrI: 0.11, 0.39]) in early adolescence. There were no associations of the PFAS and metal mixture with adiposity in late adolescence.

CONCLUSION:
First-trimester PFAS and metal mixtures were associated with higher childhood and early adolescent adiposity measures.

TO36-04 | Prenatal PFAS Exposure and Early Childhood Sleep Health: Longitudinal Analysis from the Environmental influence on Child Health Outcomes (ECHO) Study

Cai Zhang[1,2], Taylor Etzel[3], Susan Schantz[2,4], Sarah Geiger[1,2]

[1] The University of Illinois Urbana-Champaign, Department of Health and Kinesiology, Champaign, USA; [2] The University of Illinois Urbana-Champaign, Beckman Institute for Advanced Science and Technology, Urbana, USA; [3] Johns Hopkins University, Johns Hopkins Bloomberg School of Public Health, Baltimore, USA; [4] University of Illinois Urbana-Champaign, Department of Comparative Biosciences, Champaign, USA

Objective: Sleep quality in early childhood is critical for neurodevelopment and long-term health outcomes. Per- and polyfluoroalkyl substances (PFAS) are environmental contaminants that may influence child sleep, but evidence remains limited. We examined associations between prenatal PFAS exposure and child sleep health among children aged 1-5 years.

Material and Methods: We analyzed data from 2,811 mother-child pairs enrolled in the ECHO Cohort. Prenatal PFAS concentrations, including perfluorooctanoic acid (PFOA), perfluorooctane sulfonate (PFOS), perfluorononanoic acid (PFNA), and perfluorohexane sulfonate (PFHxS), were log2-transformed. Sleep outcomes were assessed using T-scores from the Child Behavior Checklist (CBCL) Sleep Problems Syndrome Scale. Linear mixed effects models examined associations between PFAS and child sleep problems, adjusting for cohort site, pre-pregnancy BMI, parental stress, alcohol and tobacco use, education, household income, employment during pregnancy, birth weight z-score, and child sex at birth. Missing data were handled using multiple imputation (MICE, 25 datasets).

Results: The study sample had a mean maternal age of 29.6 years and was racially and socioeconomically diverse, with 43% Non-Hispanic White, 24% Black, and 23% Hispanic. Education and income varied, with 27% holding an advanced degree and 36% earning less than $30,000. Prenatal PFAS exposure was not strongly associated with sleep disturbances in early childhood, as adjusted estimates for PFOA (β = 0.03 95% CI: -0.038, 0.096), PFOS (β = 0.06, 95% CI: -0.08, 0.19), PFNA (β = 0.02, 95% CI: -0.06, 0.10), and PFHxS (β = 0.03, 95% CI: -0.08, 0.13) were small and non-significant.

Conclusion: Although the associations were weak, they were consistently in the expected direction, with higher prenatal PFAS concentrations associated with more sleep problems. Future research should explore potential effect modification by socioeconomic or environmental stressors and alternative modeling approaches, including considering PFAS as a mixture, to better understand the impact of prenatal exposure on early childhood sleep health.

TO36-05 | Leveraging Non-Targeted PFAS Analysis of Neonatal Dried Blood Spots to Reveal Novel Exposures in Children

Sheng Liu[1], Jeremy P. Koelmel[1], James Pyke[2], David Schiessel[3], Elizabeth Z. Lin[1], Michael Kummer[3], David Godri[4], John Fortner[5], Libby Morimoto[6], Xiaomei Ma[7], Joseph L. Wiemels[8], Emma E. Rennie[2], Veronica M. Vieira[9], Catherine Metayer[6], **Krystal J. Godri Pollitt**[1,5]

[1] Yale University, Environmental Health Sciences, New Haven, USA; [2] Agilent Technologies, Santa Clara, USA; [3] Innovative Omics Inc., Sarasota, USA; [4] 3rd Floor Solutions, Toronto, Canada; [5] Yale University, Chemical and Environmental Engineering, New Haven, USA; [6] University of California, Berkeley, Epidemiology and Biostatistics, Berkeley, USA; [7] Yale University, Chronic Disease Epidemiology, New Haven, USA; [8] University of Southern California, Population and Public Health Sciences, Los Angeles, USA; [9] University of California, Irvine, Environmental and Occupational Health, Irvine, USA

Objective: Leukemia is the most common childhood cancer in the US, and its incidence has increased in the past 40 years, suggestive of environmental influences. Per- and polyfluoroalkyl substances (PFAS) are a growing class of over 14,000 chemicals that can cross the placenta and potentially modulate immunodevelopment and hormone levels. To comprehensively assess PFAS exposure during the fetal developmental period, we used discovery-based approach to identify neonatal exposure to previously unknown PFAS which will support future evaluation of the association of these novel exposures and childhood acute lymphoblastic leukemia risk.
Methods and Materials: Archived newborn dried blood spots (DBS) of 374 participants in the California Linkage Study of Early-Onset Cancers were analyzed for PFAS using liquid chromatography high-resolution mass spectrometry with targeted and non-targeted approaches. Paired card blanks were additionally analyzed for each neonatal DBS to assess potential contamination. Acquired DBS and card blank data were processed (Agilent Explorer) for peak picking and feature extraction. PFAS-like molecular features were identified using FluoroMatch Modular and subsequently confirmed based on retention time trends in homologous series, MS/MS fragmentation peaks, and isotopic patterns with FluoroMatch Visualizer.
Results: Of the 24 PFAS included in our targeted panel, two were found in >50% of neonatal DBS (PFOS, PFOA). Non-targeted analysis (NTA) revealed an additional 31 PFAS-like features that were detected in <1% to 85% of neonatal DBS after blank filtering, including PFSAs, ethers, H-substituted PFCAs and other carboxylic acids. Three PFAS identified by NTA were elevated in card blanks. Five of 31 PFAS detected via NTA were found in >50% of neonatal DBS, highlighting the need for discovery-based approaches.
Conclusions: Quantification of 24 known and 31 previously unknown PFAS strengthens are understanding of neonatal exposures and this comprehensive chemical analysis will support studying fetal exposure to PFAS and childhood cancer risk.

TO36-06 | Prenatal per- and polyfluoroalkyl substance exposure in relation to maternal prenatal and postpartum anxiety and depression

Claire Nurse[1], Sally Thurston[2], Kurunthachalam Kannan[3], Jessica Brunner[4], Susan W. Groth[5], Richard K. Miller[4], Thomas G. O'Connor[7], Emily S. Barrett[8]

[1] University of Rochester School of Medicine and Dentistry, Department of Biostatistics and Computational Biology, Rochester, USA; [2] University of Rochester School of Medicine and Dentistry, Department of Biostatistics and Computational Biology, Rochester, USA; [3] Wadsworth Center, Department of Health/Division of Environmental Health Sciences, Albany, USA; [4] University of Rochester Medical Center, Department of Obstetrics and Gynecology, Rochester, USA; [5] University of Rochester, School of Nursing, Rochester, USA; [6] University of Rochester School of Medicine and Dentistry, Department of Environmental Medicine, Rochester, USA; [7] University of Rochester School of Medicine and Dentistry, Department of Psychiatry, Rochester, USA; [8] Rutgers University School of Public Health, Department of Biostatistics and Epidemiology, Piscataway, USA

Objective: Per- and polyfluoroalkyl substances (PFAS), are ubiquitous, endocrine-disrupting synthetic chemicals. Considering the importance of hormones to mental health and the small literature linking PFAS exposure to depressive symptoms, we examined gestational PFAS exposure in relation to maternal anxiety and depression both pre- and postnatally.

Materials and Methods: Using LC-MS/MS, we measured five PFAS species in second trimester serum samples from participants in the UPSIDE-ECHO cohort (Rochester, NY; n=283). In each trimester and at 6 and 12 months postpartum, participants reported on symptoms of depression (Edinburgh Prenatal Depression Scale; EPDS) and anxiety (Penn State Worry Questionnaire; PSWQ), with higher scores indicating more affective symptoms. In covariate-adjusted linear regression models, we examined log-transformed PFAS concentrations in relation to average prenatal and average postnatal depression and anxiety scores, imputing missing data using MICE.

Results: Approximately 85% of PFAS in the sample were above the limit of detection (0.02 ng/mL). Median prenatal and postnatal scores for EPDS were 5.0 and 5.3 respectively, while PSWQ scores were 42 and 45 respectively. In our primary models, associations between PFAS and prenatal EPDS scores tended to be positive, with the strongest result observed for PFOS (β=0.22, 95% CI: 0.07, 0.38). No significant associations between PFAS and prenatal PSWQ scores were observed. In complete case models, PFNA was associated with lower postpartum EPDS (β=-0.133, 95% CI: -0.26, -0.004) and PSWQ scores (β= -0.071, 95% CI: -0.13, -0.015).

Conclusion: These preliminary findings suggest that PFAS exposures impact maternal perinatal affective symptoms. Further studies are needed to investigate potential causal mechanisms, including altered hormone profiles.

Funding provided by NIEHS T32ES007271 and P30ES001247; and P30ES05022, R01HD083369, UG3/UH3OD0223349, R01NR17602

TO36-07 | Maternal diet is associated with PFAS levels in human milk: implications for infant exposure

Nathan D. Young[1], Devendra Paudel[1], Elizabeth A. Holzhausen[1], Haonan Li[1], Douglas Walker[2], David Conti[3], Lida Chatzi[3], Sandrah P. Eckel[3], Michael I. Goran[4], Jesse Goodrich[3], Donghai Liang[2], Tanya L. Alderete[1]

[1] Johns Hopkins University, Department of Environmental Health and Engineering, Baltimore, USA; [2] Emory University, Rollins School of Public Health, Atlanta, USA; [3] University of Southern California, Department of Population and Public Health Sciences, Keck School of Medicine, Los Angeles, USA; [4] Children's Hospital Los Angeles, Department of Pediatrics, Los Angeles, USA

Background: Poly- and perfluoroalkyl substances (PFAS) are present in human milk—the primary source of infant nutrition during the first six months of life. However, the relationship between maternal diet and PFAS levels in human milk remains largely unexplored.

Methods: We utilized dietary and PFAS data from a pilot study of 63 Latino mothers in the Southern California Mother's Milk Study. Maternal diet data were assessed via 24-hour dietary recalls at 1-month postpartum. Human milk PFAS were characterized at 6-months postpartum using untargeted, high-resolution mass spectrometry. Statistical analyses utilized multivariable linear regression models to examine associations between 37 *a priori* dietary variables (e.g., macro/micronutrients, essential vitamins, etc.) and 8 human milk PFAS chemicals (PFHxA, PFECA, 8:2 FTSA, PFOS, Me-PFOSA-AcOH, Me-PFHxA, AHFP, KPFBS). Analyses adjusted for proportion of human milk feedings, socioeconomic status, and total kcal of energy consumed. Results were considered statistically significant based on $p<0.05$.

Results: Maternaldiet was associated with PFAS in human milk. For example, intake of fats and saturated fatty acids were positively associated with FTSA. Additionally, consumption of omega-3 and omega-6 fatty acids were positively associated with PFHxA. At the same time, higher intake of vitamins and minerals were associated with lower levels of human milk PFAS. As an example, higher vitamin E intake was associated with lower PFECA and FTSA. Higher intake of iron and zinc were also associated with lower PFOS and Me-PFHxA.

Conclusion: Our results suggest that maternal diet may influence levels of human milk PFAS. Given that human milk is a vital part of the infant diet, the presence of PFAS may have important implications for healthy growth and development. Future research with an expanded sample size will examine specific food consumption patterns to gain deeper insights into dietary sources of PFAS that may influence human milk.

TO37 | Compounding climate hazards and health: Smoke, heat, storms, and vulnerable populations

TO37-02 | Health impacts of $PM_{2.5}$ and O_3 from prescribed fire burning in 11 Southeastern US states.

Jennifer Stowell[1], Kamal Maji[2], Zongrun Li[2], Yongtao Hu[2], Ambarish Vaidyanathan[2], Chad Milando[1], Armistead Russell[2], Patrick Kinney[1], M. Talat Odman[2], Gregory Wellenius[1]

[1] Boston University School of Public Health, Department of Environmental Health, Boston, USA; [2] Georgia Institute of Technology, School of Civil and Environmental Engineering, Atlanta, USA

Prescribed burning is a tool used to mitigate wildfire spread. However, prescribed burning contributes to pollution, including $PM_{2.5}$ (particulate matter < =2.5µm) and O_3 (ozone). While the health impacts of wildfire smoke (WFS) are well-studied, less is known about prescribed fire smoke (PFS). Leveraging healthcare claims for residents of 11 Southeastern US states (2013-2021), we investigated health impacts from PFS using fusion-based models to estimate exposure and a time-stratified case-crossover design, including lags from 0-3 days. Models adjusted for non-fire $PM_{2.5}$ and O_3, temperature, humidity, and holidays. We also examined relative risk (RR) variations across subgroups. Comparing the 95th percentile of exposure to 0µg/m^3, PFS $PM_{2.5}$ was associated with higher RR of emergency department (ED) visits for non-external causes (1.2%), upper respiratory infections (URI, 3.9%), and ischemic heart disease (5.5%). PFS O_3 was associated with higher RR of ED visits for non-external causes (2.9%), URI (5.0%), total respiratory diseases (4.9%), and bronchitis (9.5%). RR varied across outcomes and population subgroups. For PFS-related $PM_{2.5}$, children and older adults showed higher RR for non-external visits, while those 35-64 years exhibited higher RR for ischemic heart disease and upper respiratory infections. For PFS-specific O_3, adults 35-64 years had a RR for total respiratory diseases and URI. Older adults exhibited higher RR for non-external causes and overall cardiovascular and respiratory diseases. A higher RR related to PFS-specific O_3 exposures was evident in females for overall respiratory disease, bronchitis, COPD, non-external causes, and dysrhythmia. A higher RR related to PFS-specific $PM_{2.5}$ was evident in females for non-external causes, bronchitis, overall cardiovascular disease, and dysrhythmia, with a higher RR of stroke in males. We found higher RR among socially vulnerable individuals for several outcomes, especially when evaluating minority status. Some findings differed from WFS literature, highlighting the need for comparisons of exposures to WFS versus PFS.

TO37-03 | Long-term exposure to wildfire smoke and breast cancer incidence among California women

Jennifer Ish[1], Tarik Benmarhnia[2], Scarlett L. Gomez[3], Dale Sandler[1], Alexandra White[1]

[1] National Institute of Environmental Health Sciences, Epidemiology Branch, Durham, USA; [2] University of California San Diego, Scripps Institution of Oceanography, La Jolla, USA; [3] University of California San Francisco, Department of Epidemiology and Biostatistics, San Francisco, USA

Objective: Wildfires have increased dramatically in frequency and intensity over the years leading to increased long-term population exposures. Wildfire smoke contains carcinogenic and endocrine disrupting compounds relevant to breast cancer etiology. Our objective was to evaluate the impact of long-term wildfire smoke exposure on breast cancer risk.

Methods: We included 4,412 participants from the Sister Study, a prospective cohort of women enrolled from 2003-2009, who lived in California. An ensemble machine learning approach incorporating monitoring, remote-sensing,

and smoke data was used to estimate daily mean concentrations of wildfire-specific fine particulate matter ($PM_{2.5}$). We linked zip code-level concentrations to participant enrollment and follow-up addresses. Breast cancer diagnoses were self-reported and verified using medical records. Using a discrete-time approach with pooled logistic regression, we evaluated the association between the odds of breast cancer and a 3-year rolling mean wildfire $PM_{2.5}$ exposure; other metrics of wildfire $PM_{2.5}$ characterizing peak levels and frequency of exposure were also considered. We separately evaluated associations with overall, estrogen receptor (ER)-positive and ER-negative breast cancer. Models were adjusted for calendar year and age at enrollment, race and ethnicity, income, and educational attainment.

Results: During a median of 11 years of follow-up, 267 breast cancer cases, including 231 ER-positive and 32 ER-negative cases, were diagnosed. A 1-μg/m^3 increase in 3-year mean wildfire $PM_{2.5}$ was associated with 2.6 times increased odds of ER-negative breast cancer (95% CI: 1.1-6.6). Null associations were observed for overall (OR=0.9, 95% CI: 0.5-1.4) and ER-positive breast cancer (OR=0.8, 95% CI: 0.4-1.4). Similar patterns were observed for all outcomes across metrics of exposure frequency and peak intensity.

Conclusions: In preliminary analyses of Sister Study participants in California, long-term exposure to wildfire $PM_{2.5}$ was associated with ER-negative breast cancer. Future work will extend the analysis to the entire nationwide cohort and examine potential socioeconomic disparities.

TO37-04 | Individual and compound exposure to wildfire smoke and extreme heat in California from 2008-2018: exploring regional and population vulnerability

Rachel Connolly[1], Miriam Marlier[2], Michael Jerrett[2], Joseph Wilkins[3], Michelle Wong[4], Joanna Wilkin[4], Catherine Carpenter[4]

[1] UCLA, Luskin Center for Innovation & Center for Healthy Climate Solutions, Los Angeles, USA; [2] UCLA, Department of Environmental Health Sciences, Los Angeles, USA; [3] Howard University, Department of Earth, Environment and Equity, Washington D.C., USA; [4] Tracking California, Oakland, USA

In California, wildfire-associated air pollution and extreme heat contribute to numerous adverse health outcomes. Certain communities are more vulnerable due to underlying health or socioeconomic characteristics, co-exposures, and/or lack of adaptive capacity. In this study, we integrated Earth science datasets on smoke pollution and extreme heat with sociodemographic data to assess vulnerability to climate-related exposures. Using Google Earth Engine, we combined eleven years (2008-2018) of simulated wildfire-associated fine particulate matter ($PM_{2.5}$) from the Community Multiscale Air Quality modeling system (CMAQ) with daily heat index (HI) data derived from temperature and relative humidity from the publicly available GridMET dataset. We estimated the frequency of days over the eleven-year period that each census tract in California was exposed to an extreme heat day, a wildfire smoke day, or both at the same time ("compound" exposures), using various exceedance thresholds. We define the heat threshold as the 85th percentile historical HI in each tract for the months of July and August (with absolute minimum and maximum thresholds to avoid "false positives" or "false negatives"), drawing from U.S. Environmental Protection Agency guidelines, and the smoke threshold as 15 μg/m^3 for daily smoke $PM_{2.5}$, drawing from health-based guidelines for $PM_{2.5}$ exposure from the World Health Organization. We then overlaid these exposures with population vulnerability characteristics from the California Office of Health Hazard Assessment's well-established CalEnviroScreen tool to assess climate vulnerability. In partnership with Tracking California, we are conducting

outreach to communities across the state to gain their perspectives on the most useful approaches to share these exposure and vulnerability datasets to best meet community needs.

TO37-05 | Assessing Tropical Cyclone Exposure and Mortality Among Older Adults in Socially Vulnerable Neighborhoods in NC

Melissa Fiffer[1], Aaron Lilienfeld[1], Ryan Zomorrodi[1], Joshua Tootoo[1], Rafiga Gasymova[1], Sue Anne Bell[2,3], Matthew Davis[2,3], Marie Lynn Miranda[1,4]

[1] University of Illinois Chicago, Children's Environmental Health Institute, Chicago, USA; [2] University of Michigan, School of Nursing, Department of Systems, Populations, and Leadership, Ann Arbor, USA; [3] University of Michigan, Institute for Healthcare Policy and Innovation, Ann Arbor, USA; [4] University of Illinois Chicago, Departments of Pediatrics and Mathematics, Statistics, and Computer Science, Chicago, USA

Objective

Older adults living in high social vulnerability index (SVI) communities may face greater tropical cyclone-related mortality risk due to healthcare services dependency and lower resilience. Prior studies assessed one severe weather exposure dimension. We evaluate multiple exposure types and mortality in the year following Hurricanes Matthew (2016) and Florence (2018).

Methods

For Census tracts within 500km of each hurricane at its closest approach, in states containing at least one FEMA disaster-declared county, we calculate sustained maximum wind speed and duration and distance to the central storm track from National Hurricane Center HURDAT2 best track estimates and mean cumulative precipitation from NCEI’s nClimGrid (https://ryanzomorrodi.github.io/exposur/). Combining EAGLE-I and EIA data, we estimate county-level percent out and average hours out. We perform Pearson correlations and Principal Components Analysis (PCA) to identify key exposure dimensions. Leveraging North Carolina death records for 33,225 adults over age 65 residing in 541 high SVI Census tracts, we use adjusted Cox proportional hazards models to assess associations between one standard deviation increase in individual exposures, PCA components, and overall mortality.

Results

Compared to Matthew, for Florence we find a weaker correlation between distance and each exposure dimension, as well as between wind and precipitation. For Matthew, we find the largest increased mortality risk associated with precipitation (HR: 1.26, 95%CI:1.24,1.28) in single-exposure models, and the PCA component with high precipitation loadings (HR:1.20, 95%CI:1.18,1.22). For Florence, we observe the largest associations for wind (HR: 1.15, 95% CI: 1.13,1.17), precipitation (HR: 1.16, 95% CI: 1.15, 1.18) and power outages (HR=1.13, 95% CI: 1.11,1.15) in single-exposure models, and for PCA components representing distance (HR=0.85, 95% CI: 0.83, 0.87) and power outages (HR=1.11, 95%CI: 1.10,1.13).

Conclusion

Mortality risk among older adults residing in high SVI areas differs by severe weather exposure dimension. Future studies should consider quantifying multiple local exposure metrics.

TO37-06 | Extreme weather events and dengue in Southeast Asia: a regionally-representative analysis of 291 locations from 1998 to 2021

Sophearen Ith[1,2], Xerxes Seposo[3,4], Vitou Phy[5], Kraichat Tantrakarnapa[6], Geminn Louis C. Apostol[4], Pandji Wibawa Dhewantara[7], Mohd Rohaizat Hassan[8,9], Hidayatulfathi Othman[10], Rozita Hod[8], Mazrura Sahani[10], Jue Tao Lim[11], Ha Hong Nhung[12], Nguyen Hai Tuan[12], Ngu Duy Nghia[12], Taichiro Takemura[13], Inthavong Nouhak[14], Felipe J Colón-González[16,17], Paul Lester Carlos Chua[1], Alex R Cook[15], Masahiro Hashizume[1,18]

[1] The Unviersity of Tokyo, Department of Global Health Policy, Tokyo, Japan; [2] National Institute of Infectious Diseases, Center for Surveillance, Immunization, and Epidemiologic Research, Tokyo, Japan; [3] Hokkaido University, Department of Hygiene, Graduate School of Medicine, Hokkaido, Japan; [4] Ateneo de Manila University-School of Medicine and Public Health, Ateneo Center for Research and Innovation, Pasig, Philippines; [5] Indeed Inc, Tokyo, Japan; [6] Mahidol University, Department of Social and Environmental Medicine, Faculty of Tropical Medicine, Bangkok, Thailand; [7] National Research and Innovation Agency (BRIN), Research Center for Public Health and Nutrition, Bogor, Indonesia; [8] Universiti Kebangsaan Malaysia, Department of Public Health Medicine, Faculty of Medicine, Kuala Lumpur, Malaysia; [9] University of Cyberjaya, Selangor, Malaysia; [10] Universiti Kebangsaan Malaysia, Centre for Toxicology and Health Risk Studies, Faculty of Health Sciences, Kuala Lumpur, Malaysia; [11] Nanyang Technological University, Lee Kong Chian School of Medicine, Singapore, Singapore; [12] National Institute of Hygiene and Epidemiology, Hanoi, Vietnam; [13] Nagasaki University, Institute of Tropical Medicine, Nagasaki, Japan; [14] Ministry of Health, Lao Tropical and Public Health Institute, Vientiane, Laos; [15] National University of Singapore and National University Health System, Saw Swee Hock School of Public Health, Singapore, Singapore; [16] Wellcome Trust, Data for Science and Health, London, UK; [17] London School of Hygiene & Tropical Medicine, Department of Infectious Disease Epidemiology, Faculty of Epidemiology and Population Health, London, UK; [18] Nagasaki University, Department of Global Health, School of Tropical Medicine and Global Health, Nagasaki, Japan

Background and Objective: Climate change, leading to more frequent and intense extreme weather events (EWEs), could significantly impact dengue transmission. However, the associations between EWEs and dengue remains underexplored in the Southeast Asia (SEA) region. We investigated the association between selected EWEs (i.e. heatwaves, extremely wet, and drought conditions) and dengue in the SEA region.
Material and Methods: Monthly dengue case reports were obtained from 291 locations across eight SEA countries between 1998 and 2021. Heatwaves are defined as days where temperatures exceed the 95th temperature percentile of the all-period distribution for at least two consecutive days. Droughts and extremely wet conditions are defined by a self-calibrating Palmer Drought Severity Index (scPDSI). We implemented a generalized additive mixed model coupled with a distributed lag non-linear model to estimate the association between each EWE and dengue.
Results: Months with fewer than 12 heatwave days increased dengue risk with delayed effect after two months lag, compared with months without any heatwave. Highest dengue risk is at 7 heatwave days (RR=1·28; 95%CI: 1·19,1·38). Compared to normal conditions (i.e. scPDSI=0), drought conditions (i.e. scPDSI=–4) were positively associated with dengue risk (RR=1·85; 95%CI: 1·73,1·99), while extremely wet conditions (i.e. scPDSI=4) have reduced dengue risk (RR=0·89; 95%CI: 0·87,0·91). Although the findings of this study are significant, its limitations arise from the inconsistency of dengue case reporting, which might complicate dengue risk estimation.
Conclusions: This study shows that the delayed effect of heatwaves and drought conditions magnifies the risk of dengue in the SEA region. The findings highlight the need for public health interventions to mitigate the potential dengue risks posed by EWEs in the context of climate change in SEA. Future research should investigate the factors influencing variations in the EWE-dengue association across the region to support the development of tailored, location-specific mitigation and prevention strategies.

TO37-07 | Interaction between Extreme Temperature Events and Fine Particulate Matter on Cardiometabolic Multimorbidity: Evidence from Four National Cohort Studies

Hao Xiang, Shouxin Peng, Zhaoyuan Li, Yuxuan Tan

Wuhan University, School of Public Health, Wuhan, China

Objective:
Accumulating evidence linked extreme temperature events ETEs and fine particulate matter ($PM_{2.5}$) to cardiometabolic multimorbidity (CMM); however, it remained unknown if and how ETEs and $PM_{2.5}$ interact to trigger CMM occurrence.
Material and Methods:
Participants'information was derived from four large-scale nationally representative surveys, involving China Health and Retirement Longitudinal Study (CHARLS), China Longitudinal Aging Social Survey (CLASS), China FamilyPanel Studies (CFPS), and Chinese Longitudinal Healthy Longevity Study (CLHLS). These national prospective cohorts covered 262 cities in 30 Chinese provinces. After merging the above four Chinese national cohorts, we collected 64,140 free-CMM adults as participants. Meteorological data was accessed from the National Center for Environmental Information. $PM_{2.5}$ concentration along with 5major chemical constituents, was derived from the Tracking Air Pollution in China dataset,34,35 with a 10km spatial resolution at a monthly level (2010−2020). Participants were identified as CMM if they experienced at least two concurrent conditions among heart disease, stroke, and diabetes.
Results:
Performing Cox hazards regression models along with additive interaction analyses, we found that the hazards ratio (HRs) of CMM occurrence associated with heatwave and cold spellwere1.006−1.019 and 1.063−1.091, respectively. Each 10 μg/m3 increment of $PM_{2.5}$ concentration was associated with17.9% (95% confidence interval: 13.9− 22.0%) increased risk of CMM. Similar adverse effects were also found among $PM_{2.5}$ constituents of nitrate, organic matter, sulfate, ammonium, and black carbon. We observed asynergetic interaction of heatwave and $PM_{2.5}$ pollution on CMM occurrence with relative excess risk due to the interaction of 0.999(0.663−1.334).
Conclusion:
Our study provides novel evidence that both ETEs and $PM_{2.5}$ exposure were positively associated with CMM occurrence, and the heatwave interacts synergistically with $PM_{2.5}$ to trigger CMM.
Funding:
This work was supported by the Bill & Melinda Gates Foundation, the National Natural Science Foundation of China, and the Funda mental Research Funds for the Central Universities in China.

TO38 | The heat-kidney connection: Risks, responses, and mechanisms across populations

TO38-01 | Evaluating Impacts of Heat on Body Temperature and Kidney Function Across the Sugarcane Harvest Season

Diana Jaramillo[1,2], Amy Dye-Robinson[1,2], Lyndsay Krisher[1,2], Miranda Dally[1,2], Jaime Butler-Dawson[1,2], Karely Villarreal Hernandez[1,2], Kevin Josey[3], Joshua Schaeffer[4], John Adgate[1,2], Lee Newman[1,2]

[1] Center for Health, Work, & Environment, Colorado School of Public Health, Aurora, USA; [2] Colorado School of Public Health, Department of Environmental and Occupational Health, Aurora, USA; [3] Colorado School of Public Health, Department of Biostatistics and Informatics, Aurora, USA; [4] Colorado State University, Department of Environmental and Radiological Health Sciences, Fort Collins, USA

Objective: Chronic Kidney Disease of unknown etiology (CKDu) is a growing public health concern, particularly in low-and middle-income countries where environmental and occupational risk factors remain understudied. Heat exposure is increasingly recognized as a key contributor to kidney dysfunction among agricultural workers who endure extreme temperatures and strenuous labor. This study investigates the relationship between occupational heat stress, core body temperature, and kidney function in male sugarcane harvesters in southwest Guatemala.

Methods: As part of the Chronic Kidney Disease, Heat, and Air Pollution Study (CHAP I), we conducted a longitudinal observational study during the 2021–2022 and 2022–2023 harvest seasons. Participants (n=145) were enrolled at the start of the season and followed during three work shifts over six-months.

Ambient heat and humidity data, clinical biomarkers, and core body temperature were measured to assess relationships between exposures and kidney function. Heat Index (HI) was used to quantify environmental heat stress, while telemetric temperature pills recorded core body temperature throughout work shifts. Kidney function was evaluated using estimated glomerular filtration rate (eGFR) based on creatinine. The relationships between heat index, core body temperature, and eGFR was analyzed using marginal structural models and longitudinal modifiable treatment policy implementations.

Results: The average daily HI and relative humidity was 37.4 °C (SD=2.0, Range=32.1, 40.6)) and 53.7% (SD=6.0, Range=39.7, 63.8) respectively. The workers had an average core body temperature of 37.3 °C (SD=0.37, Range=36.4, 38.8) and an average core body temperature increase of 1.16 °C (SD=0.48, Range=0.14, 2.70) during the workday. The change in eGFR - between the start and the end of the season was 0.59 ml/min/1.73m^2 (SD=16.5, Range=-63.7, 85.0). Analyses of the exposure-response relationship from marginal structural models and stochastic intervention evaluations are ongoing.

Conclusion: These findings highlight the significant occupational risks posed by heat exposure and reinforce the urgent need for workplace interventions.

TO38-03 | Occupational Heat and Kidney Function Decline

Kathryn M. Rodgers[1], Jocelyn Fimbres[1], Zoe Petropoulos[1], Juan José Amador Velazquez[2], Emmanuel Jarquin[3], Jessica H. Leibler[1], Ramon Garcia-Trabanino[4,5], Damaris López-Pilarte[2], Sinead A. Keogh[1], Samantha Hall[1], Daniel R. Brooks[6], David J. Friedman[7,8], Madeleine K. Scammell[1]

[1] Boston University School of Public Health, Environmental Health, Boston, USA; [2] Centro Medico del Pacifico (CENMED), Masaya, Nicaragua; [3] Agencia para el Desarrollo y la Salud Agropecuaria (AGDYSA), San Salvador, El Salvador; [4] Centro de Hemodialisis, San Salvador, El Salvador; [5] Emergency Social Fund for Health, Tierra Blanca, El Salvador; [6] Boston University School of Public Health, Department of Epidemiology, Boston, USA; [7] Beth Israel Deaconess Medical Center, Division of Nephrology, Boston, USA; [8] Harvard Medical School, Boston, USA; [9] Broad Institute of Massachusetts Institute of Technology and Harvard, Boston, USA

Objective: Chronic kidney disease of uncertain etiology (CKDu) is a growing public health crisis. It was first observed in young male outdoor workers in Central America, where it is also called Mesoamerican Nephropathy (MeN). It does not share the same risk factors as conventional CKD, such as diabetes, hypertension, and older age. Cross-sectional studies indicate heat exposure is a CKDu risk factor. The objective of this analysis was to evaluate longitudinal associations between occupational heat and change in kidney function over a three-year period.
Methods: In 2018, we measured continuous core body and wet bulb globe temperature (T_c and WBGT) for 569 participants in the Mesoamerican Nephropathy Study (MANOS) in El Salvador and Nicaragua. Participants were followed for three years to measure kidney function via estimated glomerular filtration rate (eGFR). We used linear mixed effect models to evaluate longitudinal associations between various T_c and WBGT measures during the workday taken at baseline and changes in eGFR over the course of the study, adjusting for participant's age, country, industry (sugarcane, corn, plantain, brickmaking, or road construction), job, self-reported hydration, and a random intercept for each participant.
Results: Percent of the workday with T_c exceeding 38°C was significantly associated with a reduction in eGFR over the study period. Drinking more water at work was associated with reduced eGFR, though not in a dose-dependent manner. There were no significant associations between continuous or categorical WBGT measures at baseline and eGFR over the study period.
Conclusion: T_c is a predictor of longitudinal kidney function decline, with exceedance of 38°C as an important metric. Hydration having an inverse and inconsistent association with kidney function may reflect more strenuous labor or exposure misclassification. Heat stress as measured by WBGT was not a predictor of kidney function decline.

TO38-04 | Variety of climates in Australian major cities and their impact on patients receiving kidney replacement therapy

Lijalem M. Tesfaw[1,2], Mark Tiong[3,4], Nicholas J Osborne[1,5], Gail Williams[6], Darsy Darssan[1]

[1] The University of Queensland, School of Public Health, Brisbane, Australia; [2] Bahir Dar University, Department of Statistics, Bahir Dar, Ethiopia; [3] The Royal Melbourne Hospital, Department of Nephrology, Melbourne, Australia; [4] University of Melbourne, Department of Medicine (RMH), Melbourne, Australia; [5] University of Exeter Medical School, Knowledge Spa, Royal Cornwall Hospital, European Centre for Environment and Human Health (ECEHH), Truro, Cornwall, UK; [6] The University of Queensland, Faculty of Medicine, Brisbane, Australia; [7] University of New South Wales, School of Population Health, Sydney, Australia

Objective: The impacts of climate on the incidence of patients receiving kidney replacement therapy (KRT) in Australian major cities.

Material and Methods: Data on patients receiving KRT data were sourced from the Australian and New Zealand Dialysis and Transplant (ANZDATA) registry for the period 2000-2020, while the temperature/humidity climate zones were obtained from the Australian Bureau of Metrology. Patients were located within the Statistical Area Level 3 (SA3), defined by the Australian Statistical Geography Standard, containing their usual residential address, with 157 regions considered where KRT patients are located. Spatial Bayesian modeling was employed to estimate the relative risk of climate zones within major cities.

Results: A total of 27,413 patients starting KRT were included from major cities. A large proportion of Melbourne (88.6%), Sydney (95.8%), Brisbane (74.2%), and Perth (67.2%) are located within the warm summer/cold winter zone. Adelaide and Darwin are the only cities with a single climate: warm summer/cold winter and hot humid summer, respectively. Darwin had the highest incidence of kidney failure with KRT incidence at 389 per 100,000 at risk (RR: 1.78, 95% CI: 1.17–2.96). In Melbourne and Sydney, regions with a warm summer/cold winter had a higher relative risk than those with a mild/warm summer or cold winter.

Conclusion: Considerable differences were noticed in the estimated relative risk of kidney failure across climate zones within and between major cities. Major cities in hot humid/dry summer zones have higher incidence of patients receiving KRT than those in warm summer/cold winter zones. This suggests that climate may play a role in the geographical distribution of kidney failure, highlighting the need for further investigation into environmental factors contributing to kidney failure incidence.

TO38-05 | Heat-Induced Acute Kidney Injury in Agricultural Workers: Mechanisms, Risk Factors, and Protective Adaptations

Hsiao-Yu Yang[1,4]

[1] National Taiwan University, Institute of Environmental and Occupational Health Sciences, Taipei, Taiwan; [2] National Taiwan University Hospital Yunlin Branch, Department of Community and Family Medicine, Yunlin, Taiwan; [3] National Taiwan University Hospital, Department of Environmental and Occupational Medicine, Taipei, Taiwan; [4] National Taiwan University, Population Health Research Center, Taipei, Taiwan

Background

There is a global epidemic of heat-induced kidney injury among agricultural workers. However, its underlying mechanism and risk factors remain unclear. This study aimed to investigate the mechanisms and risk factors of heat-induced kidney injury.

Methods

We used a repeated measures design to analyze changes in renal function and biomarkers before and after agricultural work to assess heat-induced incident acute kidney injury (AKI) and measure renal artery resistance to validate the mechanism of ischemic kidney injury. Mixed-effects linear regression model was used to estimate the associations of AKI adjusting for traditional risk factors (age, diabetes, hypertension, kidney stone) as well as with occupational risk factors (knowledge, adaptative behaviors).

Results

This study enrolled 119 agricultural workers exposed to high-heat environments between August 2023 and September 2024. The maximum personal heat index reached 54.6 (SD 9.9). After a work shift exposure, 24 participants (20.17%) developed AKI. There was a significant dose-response relationship between decline in kidney function and physiological strain index ($p = 0.047$). Among participants with AKI, renal artery resistance (RI) significantly increased after exposure ($p = 0.01$). Wet-bulb globe temperature (WBGT, $p = 0.00$), maximum core temperature ($p = 0.03$), and PSI ($p = 0.05$) significantly increased creatinine changes. Renal artery resistance (RI) significantly increased after exposure ($p = 0.032$). Risk factors for AKI included kidney stones ($p = 0.03$), hyperuricemia ($p = 0.02$), male sex ($p = 0.00$), and smoking ($p = 0.02$). Knowledge of heat stress impacts ($p = 0.02$) and adaptation behaviors, including shading ($p = 0.05$), wearing light clothing ($p = 0.02$), and eating during work shift ($p = 0.00$) reduced the risk of AKI.

Conclusion

Heat stress contributes to AKI in agricultural workers. Ischemic injury, occupational risks, and adaptive behaviors influence AKI risk.

TO38-06 | Heat Stress Exposure in Association with DNA Methylation and Kidney-related Health Outcomes

Wei-Yu Hsieh[1], Kuan-Chih Chiu[1], Hsiao-Yu Yang[1], Chen-Yu Liu[1,2]

[1] National Taiwan University, Institute of Environmental and Occupational Health Sciences, College of Public Health, Taipei, Taiwan; [2] National Taiwan University, Global Health Program, College of Public Health, Taipei, Taiwan

Objective:
Climate change has caused an increase in ambient temperatures over the past century, making farmers a vulnerable population. Animal studies suggest that extreme heat activates toll-like receptor 4 *(TLR4)* pathway could contribute to acute kidney injury (AKI), though relevant studies remain limited. Therefore, our study aimed to explore the relationships between heat stress exposure, DNA methylation changes, and kidney-related health outcomes among farmers in central Taiwan.

Material and Methods:
The study included 129 participants aged 28 to 82 between 2022 and 2024. Most participants were enrolled during the warm season (82.17%). The wet bulb globe temperature (WBGT) was measured using the TSI QUESTemp heat stress monitor during the participant's clinic appointment to assess short-term heat exposure. Based on the residential address of participants and weather monitoring data, universal kriging was used to estimate daily temperature exposure for the 52 weeks before the blood tests. Thus included calculation of the mean, minimum, maximum temperature, and relative humidity to estimate long-term exposure. The study participants' heat stress exposure was also estimated through a questionnaire that included daily farming time outdoors, pesticide spraying frequency, and water intake. Multivariable linear regression models and distributed lag non-linear models (DLNMs) were employed to evaluate the impact of ambient temperature and heat index (HI) exposure on health outcomes.

Results:
Each unit increase in WBGT was associated with a decrease of 0.0667 ($p = 0.81$) in the estimated glomerular filtration rate (eGFR). Exposure to high ambient temperature and HI were associated with changes in DNA methylation levels in *TLR4* pathway, and the associations were stronger in the prolonged exposure.

Conclusion:
Epigenetic regulation can alter health outcomes. Therefore, exposure to heat stress may lead to human *TLR4* hypomethylation, resulting in kidney diseases through inflammatory responses.

Taiwan's Ministry of Education and Ministry of Science and Technology supported this work.

TO38-07 | Ambient temperature and estimated glomerular filtration rate (eGFR) among US veterans with normal baseline kidney function

Abu Mohammed Naser[1], Xichen Mou[1], Chunrong Jia[1], Mohammad Abdullah Al Zubair Naim[2], Csaba P. Kovesdy[2]

[1] University of Memphis, Division of Epidemiology, Biostatistics, and Environmental Health, Memphis, USA; [2] University of Tennessee Health Science Centre, Division of Nephrology, Memphis, USA

Limited data are available on how temperature influences kidney function, as measured by estimated glomerular filtration rate (eGFR), in individuals with normal kidney function. We identified 57,603 U.S. veterans with normal baseline kidney function, defined as eGFR ≥60 ml/min/1.73m² and urine albumin-creatinine ratio <30 mg/g, from the Racial and Cardiovascular Risk Anomalies in Chronic Kidney Disease cohort.

We recorded 937,398 eGFR observations between 2004 and 2012. We modeled eGFR using linear random effects models for panel data, incorporating a natural spline function (4 degrees of freedom) for the temperature predictor. Models were adjusted for age, sex, race, baseline socioeconomic status, baseline comorbidities (hypertension, diabetes mellitus, coronary artery disease, and heart failure), dew-point temperature, and systolic and diastolic blood pressure. Separate models were run for same-day (lag 0) and 6-day lag (lag 6) temperatures. We plotted covariate-adjusted predicted eGFR and extracted values for -30°C, -10°C, 0°C, 25°C, and 40°C.

Among participants, 97.6% were male, 73.6% were White, and 15.7% were African American, with a median age of 63.7 years. We found an inverse J-shaped association between ambient temperature and predicted eGFR, indicating lower eGFR values at colder temperatures. For same-day temperature, the predicted mean eGFR was 71.67 ml/min/1.73m² (95% CI: 70.08, 73.26) at -30°C, 75.67 ml/min/1.73m² (95% CI: 74.64, 76.70) at -10°C, 77.87 ml/min/1.73m² (95% CI: 76.89, 78.87) at 0°C, 79.58 ml/min/1.73m² (95% CI: 78.58, 80.59) at 25°C, and 77.24 ml/min/1.73m² (95% CI: 75.90, 78.58) at 40°C. For the 6-day lag temperature, the predicted mean eGFR was 71.37 ml/min/1.73m² (95% CI: 70.37, 72.37) at -30°C, 75.18 ml/min/1.73m² (95% CI: 74.20, 76.17) at -10°C, 77.57 ml/min/1.73m² (95% CI: 76.58, 78.56) at 0°C, 79.66 ml/min/1.73m² (95% CI: 78.27, 81.04) at 25°C, and 77.96 ml/min/1.73m² (95% CI: 76.96, 78.96) at 40°C.Our findings suggest that lower ambient temperatures are associated with reduced kidney function.

TO39 | Heat in the city: Social and spatial dimensions of health risk and resilience

TO39-01 | Tree canopy cover and extreme heat-related asthma and adverse birth outcomes in the Bay Area, California (2011-2018): simulating the benefits of multiple hypothetical interventions

Andrew V. Nguyen[1], Tarik Benmarhnia[2], Amy Padula[3], Neeta Thakur[1]

[1] University of California, San Francisco, Division of Pulmonary and Critical Care Medicine, School of Medicine, San Francisco, USA; [2] University of California, San Diego, Scripps Institution of Oceanography, La Jolla, USA; [3] University of California, San Francisco, Department of Obstetrics, Gynecology and Reproductive Sciences, San Francisco, USA

Objective: Extreme heat (EH) events in California are increasing and are associated with exacerbating asthma outcomes and higher risk of adverse birth outcomes (ABOs). Improving tree canopy cover (TCC) can be an effective strategy to decrease ground temperatures. We investigate the effect of TCC on EH-related asthma emergency department visits (EDVs) and ABOs and simulate changes under different greening scenarios.
Material and Methods:We defined EH days as any census tract-day with daily minimum or maximum temperatures exceeding a percentile threshold. We estimated the rate of asthma EDVs and ABOs as a function of EH days using mixed-effects models across 1591 census tracts (CT) in the Bay Area 2011-2018 period, adjusting for $PM_{2.5}$ and poverty. We derived the CT-specific number of excess EH-related outcomes by differencing predicted outcomes under the scenario of no increased EH events from 2011 to 2018 and observed outcomes. We estimated associations between TCC and excess EH-related outcomes. We performed parametric G-Computation to predict the proportion of excess outcomes avoided under different scenarios of increased TCC.
Results: EH days were positively associated with asthma EDVs and ABOs. The increase in EH days over the study period was associated with 12% of asthma EDVs, 5% of term low birthweight births, and 4% of preterm births. CT-level TCC was associated with reduced EH-related outcomes. Targeting greening interventions using a vulnerability index led to the largest reductions in outcomes and improved equality of TCC distribution for the same cost as blanket population-wide increases.
Conclusion: To our knowledge, this is the first work to simulate benefits of greening for EH-related asthma and ABOs. Using a case study in the Bay Area, we found that increasing TCC may be a viable strategy to reduce the impacts of EH events, specifically via interventions delivered proportionally to a community’s existing infrastructure and susceptibility.

TO39-02 | Socio-economic factors affecting temperature and air pollution related mortality in Norway: a nationwide cohort-based study

Shilpa Rao-Skirbekk

Norwegian Institute of Public Health (NIPH), Oslo, Norway

Background: This time-stratified case-crossover study examined short-term associations of air temperature with cause-specific mortality and potential effect modification by daily mean air pollution concentrations , socio-economic factors and built environment in the Cohort of Norway (CONOR) cohort.
Methods: The CONOR cohort recruited ~173,000 participants from 1994 to 2003. Participants' vital status and cause of death were collected from the Cause of Death Registry of Norway until 2018. Daily mean air temperatures and concentrations of fine particulate matter (PM2.5) and ozone (O3) estimated by spatial-temporal models were assigned to participants' residences. We applied conditional logistic regression models with the distributed lag non-linear model approach to assess cold and heat effects on cause-specific mortality. The potential effect modification was analyzed by incorporating an interaction term between air temperature and the modifier in the regression model. The cold and heat effects were estimated for different subgroups of participants and at the low (5th percentile), medium (50th percentile), and high (95th percentile) levels of air pollution.
Results: During the follow-up period, we identified 40,040 cases of natural-cause deaths, including 14,457 and 3,699 cases of deaths from cardiovascular and respiratory diseases, respectively. This was accompanied by a trend for increased risks of cardiopulmonary, cardiovascular, ischemic heart disease, and cerebrovascular mortality associated with decreasing air temperature. The cold effect on natural-cause mortality was more pronounced among participants who were female, had lower socioeconomic status, lived in sparsely populated areas, and had no history of CVD. We did not find adverse heat effects on mortality.**Conclusion**: Our findings provide evidence for adverse short-term cold effects on mortality at the individual level in a Nordic country. Besides, we identify sub-populations who were likely to be more at risk of cold-related mortality. These findings could be important in developing integrated strategies for adaptation

TO39-03 | The diurnal dynamics of summer urban heat island intensity in heat vulnerable neighborhoods in New York City

P. L. Arab[1], A. Yuan[1], K. Lane[1], K. Ito[1], L. Smalls-Mantey[1]

New York City Department of Health and Mental Hygiene, Bureau of Environmental Surveillance and Policy, New York, USA

Objective
The diurnal pattern of air temperature in cities is complex and can vary depending on the surrounding environment. New York City (NYC) relies on temperature data from weather stations in non-residential environments (e.g., Central Park) for heat warnings and alerts, as well as the local new media. However, the actual air temperature in heat vulnerable neighborhood could differ. This temperature variation can potentially cause disparities in the distribution of heat-related outcomes across residents.
Material and Methods

We compared in-situ hourly summer (June-August) diurnal temperature variations, rate of change and relative humidity from three weather stations that we installed in heat-vulnerable neighborhoods in NYC from 2019-2022. The stations represent varying landscapes and geographical locations, including compact midrise residential and heavy industrial local climate zones. We compared average summer days and heat advisory days. The heat-vulnerable neighborhood stations were compared to airport weather stations at LaGuardia and John F. Kennedy (JFK). Central Park was used as a reference site.

Results

Relative to Central Park, weather stations in high heat-vulnerability neighborhoods experienced consistently higher temperatures than those at airport locations. LaGuardia exhibited the closest temperature patterns to neighborhood stations, with the highest temperature differences at 16:00. JFK, located along the coast, deviated from this pattern with peak differences after 18:00. The heavy industrial site in Hunts Point experienced the highest median temperature difference from Central Park of 5°F versus JFK with the lowest temperature difference at -3°F. JFK also experienced a greater rate of cooling during the day.

Conclusion

Our findings illuminate the differences between the temperature at weather stations used for National Weather Service heat warnings and messaging and in heat-vulnerable neighborhoods. Higher temperatures and varying diurnal patterns observed in heat-vulnerable neighborhoods need to be considered for prioritizing heat-risk messaging and local mitigation strategies.

TO39-04 | Risks of emergency hospitalizations associated with joint exposure to temperatures and air pollutants: a time-series study in Hong Kong, 2000-2019

Yi Tong Guo[1], Ka Hung Chan[2,3], Hong Qiu[1], Eliza Lai-yi Wong[1], Kin Fai Ho[1,4]

[1] The Chinese University of Hong Kong, JC School of Public Health and Primary Care, Hong Kong, Hong Kong; [2] University of Oxford, Clinical Trial Service Unit and Epidemiological Studies Unit, Nuffield Department of Population Health, Oxford, UK; [3] University of Oxford, Oxford British Heart Foundation Centre of Research Excellence, Oxford, UK; [4] The Chinese University of Hong Kong, Institute of Environment, Energy and Sustainability, Hong Kong, Hong Kong

Objective: Non-optimal temperatures and air pollution are well-documented risk factors for population mortality, yet the health risks associated with their joint exposure remain underexplored. This study investigates whether air pollutants modify and interact with the effects of temperature on emergency hospitalizations.

Material: We analyzed daily time series data on emergency hospitalizations, meteorological factors, and five criteria air pollutants (O_3, $PM_{2.5}$, NO_2, SO_2, and CO) from 2000 to 2019 in Hong Kong.

Methods: Utilizing generalized additive models integrated with distributed lag nonlinear models, we developed two comparative models to assess the potential joint effects between temperature and air pollution: (1) Model 1 included an interaction term between temperature and indicators for various pollutant scenarios; (2) Model 2 utilized dummy variables for temperature-pollutant joint scenarios to estimate the relative excess risk due to interaction (RERI).

Results: Over the 7,305 study days, more than 10 million hospitalizations for non-cancer, non-external causes were recorded. Model 1 revealed that the risks of cold-related hospitalizations increased significantly at higher pollutant levels by 2.4-3.3% for O_3, 3.0-4.2% for $PM_{2.5}$, 2.1-3.1% for NO_2, and 3.4-5.2% for SO_2. In contrast, differentiated heat-related risks were significant only for $PM_{2.5}$ and CO concerning non-cancer, non-external hospitalizations. Model

2 indicated significant synergistic effects between cold temperatures and $PM_{2.5}$, NO_2, and SO_2, while antagonistic effects were observed between heat and pollutants.
Conclusion: Notably, the results regarding cold temperatures and air pollutants were consistent across both models, reinforcing the significance of these interactions. These findings emphasize the need for public health strategies that address the joint effects of temperature and air pollution on health outcomes to inform effective mitigation policies.Acknowledgement: We would like to thank the Hong Kong Hospital Authority, the Hong Kong Observatory, and the Environmental Protection Department for providing the data for this study.

TO39-05 | Heat Exposure Risk of Urban Residents in Extreme Heat: A Case Study in Nanjing

Xinyuan Hao[1], Ziyu Tong[2]

[1] University of Washington, Department of Environmental & Occupational Health Sciences, Seattle, USA; [2] Nanjing University, School of Architecture and Urban Planning, Nanjing, China

Objective: To develop and apply a comprehensive framework for quantifying the heat exposure risk during extreme heat, addressing current methodological limitations in real-time temperature monitoring, population mobility tracking, and integrated risk assessment across vulnerable demographics.

Materials and Methods: We developed a novel heat exposure risk assessment framework based on Crichton Risk Triangle, incorporating three dimensions: hazard (quantified through urban heat island intensity and thermal comfort index), exposure (measured by population density during high-temperature periods), and vulnerability (assessed through elderly proportion, accessibility to emergency healthcare and public green space). We integrated meteorological simulation outputs, mobile signaling data, and infrastructure information for spatial statistics. The framework was applied to 2,601 blocks in central Nanjing City during extreme heat in 2022. Risk values were classified using 50th and 85th percentiles as thresholds.

Results: Neighborhoods with high exposure risk values (>5.5, 85th percentile), predominantly in southwestern districts, exhibited 5.2-fold higher risk than low-risk neighborhoods (<2.5, 50th percentile). We identified three significant high-risk clusters (Local Moran's I >0.65, $p<0.01$), where elderly proportion averaged 23.7%, higher than the citywide mean (14.1%, t=5.04, $p<0.05$). High-risk neighborhoods had lower accessibilities (mean=0.27) compared to central districts (0.85), with 47.3% negative spatial overlap ($\chi^2=8.76$, $p<0.01$). Floor area ratio (r=0.47, $p<0.001$) and block-level compactness (r=0.48, $p<0.05$) were positively correlated with thermal comfort index, while open space ratio (r=-0.72, $p<0.05$) and non-built area (r=-0.67, $p<0.05$) were negatively correlated.

Conclusion: We found significant disparities in heat exposure risk among elderly demographics across urban areas driven by disparities in hazard, exposure, and vulnerability factors. Study findings can inform public health policies and planning interventions crucial for protecting vulnerable populations during extreme heat. The assessment framework advances exposure science methodology by integrating environmental and socioeconomic vulnerability determinants and provides a reproducible tool for climate adaptation in built environments.

TO39-06 | Heat-related impacts on all-cause emergency hospitalization vary by area deprivation and urbanicity in Japan: A time-stratified case-crossover study

Hisaaki Nishimura[1,2], Nobutoshi Nawa[1,2], Tomoki Nakaya[3,4], Kiyohide Fushimi[2,5], Takeo Fujiwara[1,2]

[1] Institute of Science Tokyo, Department of Public Health, Tokyo, Japan; [2] Institute of Science Tokyo, Center for Well-being Research Advancement, Tokyo, Japan; [3] Tohoku University, Department of Frontier Science for Advanced Environment, Graduate School of Environmental Studies, Sendai, Japan; [4] Tohoku University, Department of Earth Science, Graduate School of Science, Sendai, Japan; [5] Institute of Science Tokyo, Department of Health Policy and Informatics, Tokyo, Japan

Objective: Heat-related health impacts have been a public health concern globally. Identifying vulnerable populations is crucial in developing adaptation strategies. We aimed to assess how heat-related impacts on all-cause emergency hospitalizations vary by area deprivation and urbanicity. Material and Methods: All-cause emergency hospitalizations were extracted from the Japanese nationwide administrative database during the warm season (June to September) between 2011 and 2019. A time-stratified case-crossover design was used to examine short-term associations between daily mean temperature and hospitalization. Heat exposure days were identified as days when the daily mean temperature exceeded the minimum morbidity temperature (i.e., temperature with the lowest relative risk between the 25th and the 75th percentiles of the daily mean temperature distribution). Analyses were stratified by area deprivation and urbanicity. Heat-related excess hospitalizations were estimated using the population attributable fraction (PAF), derived as a fraction of heat-attributable emergency hospitalizations to the total number of emergency hospitalizations for all study subjects or within specific subgroups. Results: We identified 5 914 084 hospitalizations. PAF for heat-related excess hospitalizations was 1.69% (95% CI 1.54% to 1.87%) among all study subjects. PAF for heat-related excess hospitalizations was more pronounced among those in the most deprived areas (1.87%, 95%CI: 1.68% to 2.06%) than people in the least deprived (1.19%, 95% CI 0.98% to 1.41%) and in urban residents (2.03%, 95%CI: 1.78% to 2.30%) than rural ones (1.42% (95%CI: 1.24% to 1.60%)). When stratified by area deprivation and urbanicity simultaneously, PAF for heat-related excess hospitalizations was most significant among urban residents living in the most deprived areas (2.62%, 95%CI: 2.26% to 3.03%). Conclusion: Individuals living in the most deprived areas in urban settings were disproportionately vulnerable to heat exposure. Adaptation strategies tailored to socioeconomic and geographical inequalities can potentially reduce heat-related health impacts. Funding: JSPS KAKENHI (JP23K19768 and 20H00040) and SATREPS (JPMJSA2402).

TO40 | Leveraging human biomonitoring to understand chemical exposures in populations

TO40-01 | Bisphenols (A/S/F) in urine samples of the German Environmental Specimen Bank from 2000 to 2024: time trends, exposure and risk assessment

Rebecca K. Moos[1], Till Weber[2], Monika Kasper-Sonnenberg[1], Daniel Bury[1], Thomas Brüning[1], Holger M. Koch[1], Marike Kolossa-Gehring[2]

[1] Ruhr University Bochum, Institute for Prevention and Occupational Medicine of the German Social Accident Insurance (DGUV), Bochum, Germany; [2] German Environment Agency (UBA), Berlin, Germany

Background: Bisphenol A (BPA), S (BPS) and F (BPF) are endocrine disruptors and reprotoxic chemicals classified as substances of very high concern in Europe. They have been used widely in consumer products and consumer-near applications. BPS and BPF are known substitutes for BPA.
Material and methods: We analyzed BPA, BPS and 4,4‘-BPF (total after hydrolysis of glucuronides) in 24h-urine samples of the German Environmental Specimen Bank (ESB). In continuation of the previous BPA measurement campaign covering the years 1995-2009, we have now analyzed samples collected between 2000 and 2024 for all three bisphenols (30 women and 30 men per year, total 480 samples).
Results: For BPA, a consistent decline in median concentrations was observed, decreasing from 1.73 µg/L in 2000 to 0.32 µg/L in 2024, alongside a reduction in detection frequency from 100% in 2000 to 68% in 2024. In contrast, an increasing trend was noted for BPS: the proportion of samples with concentrations above the limit of quantification (LOQ) rose from 21% in 2000 to 73% in 2024, with a median concentration of 0.19 µg/L. BPF, which has consistently been present to some degree (primarily from mustard as a source), appears to have experienced a slight increase in recent years, reaching a median concentration of 0.16 µg/L in 2024.
Conclusion: Despite the rapid decline, BPA concentrations in all samples (above the Limit of Quantification) surpassed the newly established Human Biomonitoring (HBM) guidance value of 11.5 ng/L (which is 20,000 times lower than previous assessments). This value was derived from the Tolerable Daily Intake of 0.2 ng/(kg bw*day) set by the European Food Safety Authority in 2024. For BPS, approximately 6% of the samples exceeded the HBM guidance value of 1 µg/L. Currently, no guidance value has been established for BPF.

TO40-02 | Diet and Everyday Product Use and Urinary Phthalate Metabolites Levels in Korean population: Findings from the Korean National Environmental Health Survey

Hyun-Wook Park, Bomi Park

Chung-Ang University, Dept. of Preventive Medicine, College of Medicine, Seoul, South Korea

[Objective] Phthalates are widely used plasticizers that easily leach from plastic products into the environment, contaminating food, water, air, dust, and personal care products. Given their potential health impacts, such as endocrine disruption, understanding exposure pathways is essential. This study aims to investigate how dietary intake and daily product usage patterns—closely tied to individual behavior and modifiable—affect phthalate exposure.

[Material and Methods] We analyzed data from 3,760 Korean adults aged 19 years or older, whose urinary concentrations of eight phthalate metabolites—mono-methyl phthalate, mono-ethyl phthalate, mono-n-butyl phthalate, mono-benzyl phthalate, mono-(2-ethyl-5-hydroxyhexyl) phthalate, mono-(2-ethyl-5-oxohexyl) phthalate, mono-(2-ethyl-5-carboxypentyl) phthalate, and mono-(3-carboxypropyl) phthalate—were measured using a nationally representative study population recruited during the fourth cycle (2018–2020) of the Korean National Environmental Health Survey. A total of 25 dietary factors and 16 products were assessed as potential exposure sources of phthalates. Sparse reduced rank regression (sRRR) was employed to identify patterns of exposure associated with urinary phthalate metabolite levels. Models were adjusted for age, sex, household income, education level, marital status, residential area, smoking status, drinking status, body mass index, daily indoor time, and recent renovation or repair work by exempting these variables from penalties during model processing.
[Results] ThesRRR analysis identified a pattern in which consuming groundwater, tap water, grains, beans, beverages in plastic pouches, beverages in PET bottles, and using air fresheners contributed to overall increased phthalates exposure. Among males, consuming groundwater, tap water, grains, and using non-stick cooker were associated with higher phthalate exposure. For females, consuming grains, beans, beverages in plastic pouches, and using makeup products were contributors to increased phthalate exposure.
[Conclusion] This study identified dietary and product use patterns, including drinking water sources, grains and beans consumption, use of plastic containers, non-stick cookware, makeup products, contribute to increased phthalate exposure. Interventions targeting these patterns are necessary to reduce phthalate exposure.

TO40-03 | Association of multi-pollutant mixtureexposure with oxidative stress and cardiovascular endpoints using environmental risk score: Findings from the Korean National Environmental Health Survey (KoNEHS)

Kyeongmin Kwak[1,2], Sung Kyun Park[2]

[1] *Korea University College of Medicine, Department of Occupational and Environmental Medicine, Seoul, South Korea;* [2] *University of Michigan School of Public Health, Department of Epidemiology, Ann Arbor, USA*

Background: Given the ubiquitous exposure to environmental pollutants, evaluating the health effects of mixtures, rather than individual exposures, is crucial.
Methods: We utilized the environmental risk score (ERS), a summary measure for assessing multi-pollutant exposure risks, to investigate the associations between pollutant mixtures and cardiovascular endpoints. Our analysis included 2,970 participants aged ≥20 years (mean age=52.5 [standard deviation=14.7]) from the fourth cycle of the Korean National Environmental Health Survey (KoNEHS). We examined 32 pollutant biomarkers from urine and blood, including 3 heavy metals, 4 polycyclic aromatic hydrocarbon metabolites, 8 phthalate and phthalate alternative metabolites, 5 per- and polyfluoroalkyl substances, 9 phenols, 2 volatile organic compound metabolites, and 1 insecticide metabolite. The data were randomly split into training and test sets in a 1:1 ratio. Gamma-glutamyl transferase (GGT), an oxidative stress marker linked to cardiovascular disease (CVD), was used to construct the ERS via quantile g-computation. Survey-weighted generalized linear models were used to assess associations between ERS and cardiovascular endpoints (HbA1c, systolic [SBP] and diastolic blood pressure [DBP]), adjusting for sex, age, smoking, alcohol consumption, exercise, income, urinary cotinine, and medical history of diabetes and hypertension.

Results: Important pollutants associated with GGT were Mono(2-ethyl-5-carboxypentyl) phthalate, urinary cadmium, propylparaben, perfluorooctane sulfonate, 2-hydroxyfluorene, and blood mercury. One quartile increase in all pollutants was associated with 6.16 mg/dL (95% CI: 3.24–9.41) higher GGT. A one-interquartile range increase in ERS was associated with higher levels of HbA1c by 0.13% (95% CI: 0.05–0.21), SBP by 4.08 mmHg (95% CI: 1.90–6.27), and DBP by 3.34 mmHg (95% CI: 1.23–5.45).
Conclusion: Our study suggests that exposure to multi-pollutant mixtures may increase the cardiovascular risks, such as diabetes and hypertension, through oxidative stress, a common pathway in the development of CVD. ERS construction for GGT could be a useful tool for identifying groups at high risk for CVD.

TO40-04 | A cumulative dietary pesticide exposure score based on pesticide residue and dietary questionnaire information is associated with urinary pesticide biomarkers in a national biomonitoring sample

Alexis Temkin[1], Varun Subramaniam[1], Elvira Fleury[2], Alexa Friedman[1], Dayna de Montagnac[1], Chris Campbell[1], David Andrews[1], Olga Naidenko[1]

[1] The Environmental Working Group, Washington, DC, USA; [2] Brown University School of Public Health, Department of Epidemiology, Providence, USA

Objective: This study aimed to develop and validate a dietary pesticide exposure score to assess the relationship between pesticide-contaminated food consumption and urinary pesticide metabolites.
Methods: A pesticide load index was developed to rank 44 fruits and vegetables based on residue data from the U.S. Department of Agriculture Pesticide Data Program (2013–2018). This index incorporated pesticide detection frequency, number, concentration, and toxicity. Using dietary questionnaire data from the 2015–2016 National Health and Nutrition Examination Survey, dietary pesticide exposure scores were calculated for 1,837 individuals. Covariate-adjusted weighted linear regression estimated the relationship between dietary pesticide exposure scores and cumulative urinary pesticide metabolite rank for 18 biomarkers.
Results: Pesticide residues corresponding to 175 unique parent pesticides were detected across 44 food commodities. The top and bottom ten commodities in the pesticide load index remained largely consistent across different toxicity indicators (NOAEL, RfD/ADI) or when toxicity was omitted. Higher dietary pesticide exposure scores were positively associated with urinary pesticide biomarker rank, with stronger associations when pesticides measured in urine were considered. Excluding potatoes from the dietary pesticide exposure score improved model precision.
Conclusion: Greater consumption of pesticide-contaminated produce was associated with higher urinary pesticide metabolite concentrations, including organophosphate, pyrethroid, and neonicotinoid insecticides. This validated dietary pesticide exposure score provides a tool for future research on dietary pesticide exposure and health outcomes.

TO40-05 | Federal Housing Assistance and Blood Lead Levels in Children Age 1-5 Years, NHANES 1999-2016

MyDzung T. Chu[1], Andrew Fenelon[2], Gary Adamkiewicz[3], Ami R. Zota[4]

[1] Tufts Medical Center, Institute for Clinical Research and Health Policy Studies, Boston, USA; [2] University of Minnesota, Division of Epidemiology and Community Health, Minneapolis, USA; [3] Harvard T.H. Chan School of Public Health, Department of Environmental Health, Boston, USA; [4] Columbia University, Department of Environmental Health Sciences, New York, USA

Objective: Lead is toxic for children's neurodevelopment, and low-income families face higher lead exposure. Federal housing assistance has been shown to decrease blood lead levels (BLLs) among low-income families, though its impact on children's BLLs is underexplored. We examined BLLs by housing assistance status among assistance-eligible children aged 1-5 years in the US, overall and by program type.

Materials and Methods: Using the 1999-2016 National Health and Nutrition Examination Survey (NHANES) and US Department of Housing and Urban Development (HUD)-linked data, we compared BLLs among children receiving HUD assistance (i.e., current recipients, n=557) versus those who will receive assistance within two years after NHANES (i.e., pseudo-waitlist recipients, n=317). We estimated geometric means (GM), standard errors (GSE), and adjusted odds ratios (aOR) for BLL ≥ 3.5 μg/dL - CDC's 97.5th blood lead reference value for children. We also examined effect modification by program type (i.e., project-based [public and multifamily income-restricted housing] and housing choice vouchers [HCV]).

Results: Current recipients had lower GM BLLs and prevalence of BLL ≥ 3.5 *μ*g/dL (1.58 [GSE: 1.05] μg/dL, 12.8%) than pseudo-waitlist recipients (2.10 [GSE: 1.06] μg/dL; 24.9%) (p≤0.0001). In adjusted models, HUD assistance was marginally protective of BLL ≥ 3.5 μg/dL (aOR: 0.70 [95% CI: 0.47, 1.03]). There was effect modification by program type ($p_{interaction}$=0.024), with the protective association of HUD assistance observed only among project-based housing (0.47 [0.25, 0.87]) and not HCV recipients (1.54 [0.76-3.11]).

Discussion: Children in low-income families continue to face high lead exposure. Receiving housing assistance, particularly project-based (i.e. public and multifamily-income restricted) housing, was protective against elevated BLLs in children nationally. Our findings suggest that differences in housing quality, management, or enforcement practices across programs could influence lead exposure. Comprehensive policy tools are needed to ensure all low-income families are safe from lead.

TO41 | A focus on environmental disparities related to personal care product chemicals

TO41-01 | Title: Hair Products and Health Inequities: Chemical Exposure Disparities Faced by Black Women

Sara Vaezafshar[1], Sabmiro Kitawi[1], Zoha Irfan[1], Vy K. Nguyen[2], Joseph Okeme[1]

[1] McMaster University, Chemistry & Chemical Biology, Hamilton, Canada; [2] Harvard University, Harvard Medical School, Boston, USA

Hair products, including relaxers, wigs, and extensions, often contain additives, some of which are classified as endocrine disruptors, such as phthalates (solvents and fixatives), parabens (preservatives), benzophenones (UV filters), among others. Many harmful additives detected in hair products are not disclosed on product labels, limiting individuals to reduce their exposure to chemicals of concern. Previous studies identified racial disparities in chemical exposure from hair products, with Black women experiencing disproportionately higher levels. However, there is few data on concentrations of chemicals of concern in hair products marketed for Black women with no data in Canada. Our primary goal was to investigate exposure disparities among Black women in Canada resulting from use of hair products. We identified and quantified chemicals in hair products through employing a multi-platform approach using both targeted and untargeted modes: Orbitrap Mass Spectrometry (Orbi-MS) and Quadrupole Time-of-Flight High-Performance Liquid Chromatography (Q-TOF-HPLC). We measured concentrations of targeted chemicals, including parabens (n=6), phthalates (n=6), UV filters (n=9), and bisphenol A, in 20 hair relaxers as well as 10 wigs and extensions. We assessed Black women exposures by integrating targeted analysis findings with biomarker data from the Canadian Health Measurement Survey (CHMS). Additionally, we identified chemical profiles in tested hair products through untargeted analysis and assessed lack of transparency in product labeling.

The findings of our study highlighted hair products as an exposure pathway of chemicals of concern for Black women and confirmed the existence of exposure disparities. We measured high concentrations of chemicals in tested hair products for those reported at elevated levels, such as parabens and phthalates, in biomarker data from CHMS. Targeted analysis results generate valuable data for further exposure risk assessment. Additionally, our untargeted analysis highlights the need for updates to existing labeling regulations, having detected broader chemical profiles not disclosed on product labels.

TO41-02 | Beauty on the Surface, Chemistry Beneath: Decoding Cosmetic Ingredients

Jieqiong Zhou, Sheng Liu, Elizabeth Lin, Krystal Godri Pollitt

Yale School of Public Health, Environmental Health Sciences, New Haven, USA

Objective: In the United States, women use an average of 12 personal care products every day, many of which are cosmetic products. While ingredients are listed for most cosmetics, unintentionally added chemicals, specific formulations of fragrances, and chemicals used in packaging materials are typically not detailed. The objective of this study was to comprehensively characterize chemicals found in a range of cosmetic products.

Materials and Methods: Fifty cosmetic products for the eyes, face and lips were purchased in North America and Asia. Products included green/natural and synthetic formulations and spanned a range of costs and brands.

Cosmetics were chemically analyzed using solid phase microextraction gas chromatography high-resolution mass spectrometry.

Results: Non-targeted analysis identified 118 organic chemicals in cosmetic products, including phthalates, siloxanes, alkylphenols, paraben, salicylates, and glycol ethers, many of which have endocrine disrupting properties, are reproductive toxicants, genotoxic and/or mutagenic. The primary functional uses of these chemicals were antimicrobials, catalysts, fragrances, flavoring agents, and, in some cases, humectant or adhesion promotors. Products applied to the eyes, face, or lips did not differ in chemical profiles; however, assessed eyeliners consistently had a greater number of detected chemicals, notably preservatives. Products marketed as natural/green were not found to contain fewer chemicals. One mascara marketed as vegan had the highest levels of preservatives (2-phenoxyethanol), and a lip gloss labelled as phthalate-free had the highest levels of butyl benzyl phthalate. Higher-cost cosmetics had slightly lower levels of detected chemicals.

Conclusions: The presence of endocrine disruptors, reproductive toxicants, and genotoxic or mutagenic chemicals in these widely used cosmetic products raises concerns about the long-term health impact of repeated use. Stricter regulations and great transparency in ingredient labeling are needed to help consumers make informed choices and minimize exposures to toxic compounds.

TO41-03 | Personal Care Product Use is Associated with Elevated Urinary Phthalate Metabolite Levels During Pregnancy in the Atlanta African American Maternal Child Cohort

J. A. Darville[1, 2], J. Bailey[1], M. McNamara[2], A. Dunlop[4], D. Barr[1], C. Hill[4], P. Brennan[3], Y. Tan[1], D. Liang[1], S. Eick[1]

[1] Emory University, Rollins School of Public Health, Atlanta, Georgia, United States of America; [2] Emory University, School of Medicine, Atlanta, Georgia, United States of America; [3] Emory University, Department of Psychology, Atlanta, Georgia, United States of America; [4] Emory University, Department of Gynecology and Obstetrics, Atlanta, Georgia, United States of America

Introduction: Personal care products (PCPs) are known to contain phthalates, a class of endocrine disrupting chemicals that have been linked to an increased risk of preterm birth. Few studies have evaluated if PCP use is associated with higher levels of phthalates of emerging concern.

Methods: Participants were a subset of the Atlanta African American Maternal-Child Cohort (N=122). Concentrations of nineteen individual phthalate metabolites were measured in urine samples collected during mid pregnancy. PCP use was obtained through a self-reported questionnaire. Linear regression was used to estimate adjusted associations between PCP use (e.g., cosmetics, hair products) and urinary concentrations of the sum of di(2-ethylhexyl) phthalate metabolites (ΣDEHP), di(isononyl) phthalate metabolites (ΣDINP), anti-androgenic phthalate metabolites (ΣAA), and individual phthalate metabolites.

Results: Associations between use of PCPs and phthalate metabolite concentrations were stronger among those who used these products more frequently. For example, monthly hair product use was associated with a statistically significant increase in urinary concentrations of MPCHP (β=0.82, 95% confidence interval [CI]=0.08, 1.57) compared to those who did not use hair products. The use of cosmetics was associated with a significant increase in individual metabolites levels, including MCIOP, MEP, and MHINP, compared to those who did not use cosmetics.

Conclusions/Implications: Among African American pregnant persons, more frequent use of hair products and cosmetics was associated with a significant increase in urinary concentration of phthalates. Elimination of phthalates in PCP formulations and patient counseling to reduce the use of these products can effectively reduce exposure.

TO41-04 | Self-reported personal care product usage and environmental chemical awareness in a digital US cohort

Zifan Wang[1], Elizabeth Peebles[1], Carrie Sarcione[1], Jukka-Pekka Onnela[1], Michelle A. Williams[1,2], Russ Hauser[1], Brent A. Coull[1], Shruthi Mahalingaiah[1], Funding: This study received funding from Apple Inc. The funding source provided platforms and software for data collection and participated in the review of the abstract. It played no role in the analysis and interpretation of data, or in the decision to submit.

[1] Harvard T.H. Chan School of Public Health, Boston, USA; [2] Stanford School of Medicine, Department of Epidemiology and Population Sciences, Stanford, USA

Objective: Certain personal care products (PCPs) are modifiable sources of environmental chemical exposures. We evaluated sociodemographic variations in PCP usage and chemical awareness in a digital US cohort.
Material and Methods: We included 4,753 Apple Women's Health Study (AWHS) participants who met AWHS eligibility criteria, consented, and responded to demographic and PCP-related questions. Participants were asked their PCP usage frequency during the past year and awareness-related questions such as reading or avoiding certain ingredients when buying PCPs and whether their sunscreen contained oxybenzone. We evaluated the distributions of these variables, stratified by age, race/ethnicity, education, and socioeconomic status (SES).
Results: Participants aged 39.8 years on average. Non-Hispanic (NH) Black and Hispanic participants were more likely to use lip makeup (NH Black: 47%, Hispanic: 39%, NH White: 33%) and fragrances daily (NH Black: 42%, Hispanic: 36%, NH White: 32%). Younger participants also used fragrances more frequently (daily use: 41% among age 18-19 vs. 15% among age 50+). Asian and NH Black participants used more skin lightener in the past year (NH Black: 11%, Asian: 10%, NH White: 4%). NH Black participants used hair oil, gel, perm/relaxer, and leave-in conditioner more frequently than others. Those with ≤high school degree or low SES were more likely to use fragrances daily (≤high school: 39%, graduate degree:17%; low SES: 33%, high SES: 18%). The most avoided ingredients were lead (52%), mercury (49%), formaldehyde (41%), and parabens (36%). Participants of older age or higher education/SES were more likely to avoid most of the ingredients surveyed. Hispanic participants were least likely to avoid ingredients such as fragrances, PFAS, or toluene, and were most likely to report "don't know" when asked about ingredient avoidance or whether their sunscreen contained oxybenzone.
Conclusion: PCP usage and awareness vary by sociodemographic characteristics, potentially contributing to environmental chemical exposures and health disparities.

TO41-05 | Disparities in the safety of personal care products and chemical exposures based on demographic marketing

Alexa Friedman[1], Hong Lin[1], Kristian Edwards[2], Kaley Beins[1]

[1] Environmental Working Group, Washington DC, USA; [2] BLK + GRN, Maryland, USA

Objective**:** Disparities in chemical exposures by racial and ethnic groups are well documented. Recent studies have reported that associations between personal care products and health outcomes are greater in magnitude among Black women. Investigate specific differences in ingredient exposures in products marketed towards Black women (PMBW) compared to products without demographic marketing.
Methods**:** Using the Environmental Working Group (EWG)'s Skin Deep® database, we analyzed over 110,000 products (~ 4,000 marketed to Black women) and examined differences in hazard scores and hazardous ingredients. Products are scored 1 to 10 using a hazard stack approach based on ingredient safety (hazard rating: low = 1-2; moderate = 3-6; high hazard 7-10). Products marketed to Black women were identified based on brand names, use instructions, and marketing language. This included brands like Africa's Best and Black Girl Sunscreen, mentions of specific hairstyles or curl patterns, and phrases like "for melanin-rich skin" or "natural hair."
Results:Seventy-nine percent of PMBW scored moderate to high hazard in Skin Deep compared to 74% of products with no demographic marketing. Hair relaxers were one of the most hazardous product categories and 26% of hair relaxers contained a formaldehyde-releasing preservative. The most common hazardous ingredients commonly found in PMBW included quaternary ammonium compounds, undisclosed fragrance blends, and harmful preservatives. Black women have fewer safe alternatives where 21% PMBW scored low hazard and 27% of products without demographic marketing scored low hazard.
Conclusion**:** Black women may have fewer safe options when it comes to personal care products. Personal care products may explain observed differences in chemical exposures by racial/ethnic groups in exposure studies and health outcomes in epidemiological studies.

TO42 | From dust storms to urban pollution: Fine particulate matter and its role in respiratory dysfunction and disease

TO42-01 | Ultrafine particle exposure during prenatal and infantile periods and childhood asthma: A birth cohort study in central Taiwan

Chia-Ching Wu[1], Li-Hao Young[2], Bing-Fang Hwang[2], Chau-Ren Jung[1,3]

[1] China Medical University, Public Health, Taichung, Taiwan; [2] China Medical University, Occupational Safety and Health, Taichung, Taiwan; [3] National Institute for Environmental Studies, Japan Environment and Children's Study Programme Office, Tsukuba, Japan

Objective: Ultrafine particles (UFPs) are particles with an aerodynamic diameter < 0.1 μm, can penetrate deep into the alveoli, inducing inflammation and oxidative stress. UFPs have strong oxidative capacity, potentially increasing the risk of childhood asthma. However, epidemiological evidence assessing the association between UFP and asthma remains limited. This study aimed to investigate the association between early-life UFP exposure and asthma.

Material and Methods: Data were sourced from the Taiwan Maternal and Child Health Database. Infants born in central Taiwan from 2006 to 2015 were recruited and followed for five years. Incident cases were defined using the International Classification of Diseases, Ninth and Tenth revisions. UFP number concentrations were estimated using a hybrid model. Cox proportional hazard models were applied to estimate hazard ratios (HR) and 95% confidence intervals (CI), adjusting for sex, birth season, birth weight, socioeconomic status, maternal age, and comorbidities. Additionally, two-pollutant models were employed to assess the associations between UFP exposure and asthma with consideration of other air pollutants.

Results: A total of 59,606 incident asthma cases were identified among 340,702 live births (incident rate = 32.6 per 1,000 person-year), with a higher incidence among boys. After adjusting for covariates, UFP exposure per interquartile range increase during infancy was positively associated with incident asthma (HR = 1.11, 95% CI: 1.09 - 1.12). In contrast, UFP exposure during pregnancy was negatively associated with asthma. A linear exposure-response relationship was observed, suggesting no safe level of UFP exposure. These associations remained statistically significant in the two-pollutant model (HR = 1.12, 95% CI: 1.11 - 1.14).

Conclusion: Exposure to UFP during infancy was significantly associated with childhood asthma, highlighting the adverse effects of early-life UFP exposure on respiratory health. These findings provide valuable insights for future policymaking and underscore the need for future research on UFP exposure mitigation strategies.

TO42-02 | The burden of acute respiratory infection attributable to fine particulate matter brought by dust storms among children under 5 years of age in low-and middle-income countries

Hong Lu[1], Mingkun Tong[1], Ruohan Wang[1], Ning Kang[1], Tianjia Guan[2], Tao Xue[1,3]

[1] Peking University, Institute of Reproductive and Child Health / National Health Commission Key Laboratory of Reproductive Health and Department of Epidemiology and Biostatistics / Ministry of Education Key Laboratory of Epidemiology of Major Diseases (PKU), School of Public, Beijing, China; [2] Chinese Academy of Medical Sciences and Peking Union Medical College, School of Health Policy and Management, Beijing, China; [3] Peking University, State Environmental Protection Key Laboratory of Atmospheric Exposure and Health Risk Management, Center for Environment and Health, Beijing, China

Background
Long-term exposure to fine particulate matter brought by dust storms (dust $PM_{2.5}$) poses a significant adverse impact on children's respiratory health and increases the risk of acute respiratory infection (ARI), particularly those in low- and middle-income countries (LMICs). However, the relationship used to evaluate the attributable burden of ARI is mainly based on the total $PM_{2.5}$ concentration, neglecting the specific effect of dust $PM_{2.5}$ distinct from other $PM_{2.5}$.
Method
We integrated the Demographic and Health Survey covering 53 LMICs with several well-recognized environmental datasets, and then associated dust $PM_{2.5}$ with ARI in children <5 years of age (U5-ARI). In addition to the linear association between annual average dust PM2.5 and U5-ARI, we developed a dust-specific $PM_{2.5}$ exposure response function (ERF). Based on the function, we estimated the burden of dust-associated U5-ARI across 100 LMICs and compared it with the results from two well-established functions of total $PM_{2.5}$ mass.
Results
The analysis of 1,223,118 children showed that a 10 μg/m^3 increase in dust $PM_{2.5}$ was associated with a 7.43% (95% confidence interval [CI]: 4.77–10.15%) increase in the prevalence of U5-ARI. The dust-specific ERF indicated that the risk of U5-ARI increased monotonically and linearly with dust $PM_{2.5}$ concentration with no evident effect threshold. In 2017, based on the dust-specific ERF, across 100 LMICs, about 159,000 (95% CI: 153,000–165,000) U5-ARI was attributable to dust $PM_{2.5}$, which was consistently higher than the estimates from the other two well-established ERFs based on total $PM_{2.5}$ mass. The geographic hotspots were the arid and populous areas of South Asia and Africa.
Conclusion
This study provided critical insight into the unique effect of dust $PM_{2.5}$, and highlighted the need for specific ERFs to distinguish the adverse effects of $PM_{2.5}$ from different sources. Personal protection during sand dust storms should be taken to safeguard children's respiratory health.

TO42-03 | Association Between Long-term Exposure to Fine Particulate Matter from Sand Dust Storms and Small Airway Dysfunction

Hailu Zhu[1], Ke Huang[2], Tianjia Guan[1], Ting Yang[2], Chen Wang[3,2]

[1] Chinese Academy of Medical Sciences and Peking Union Medical College, School of Health Policy and Management, Beijing, China; [2] China-Japan Friendship Hospital, National Center for Respiratory Diseases; National Clinical Research Center for Respiratory Diseases; Institute of Respiratory Medicine, Chinese Academy of Medical Sciences; Center of Respiratory Medicine, Beijing, China; [3] Chinese Academy of Medical Sciences and Peking Union Medical College, Beijing, China

Objective: Despite growing concern on the health effects of dust-sourced fine particulate matter (dust $PM_{2.5}$), especially in the context of climate change, but the respiratory health effect has not been well characterized. Investigating the impact of dust $PM_{2.5}$ on small airway dysfunction (SAD) is important for early prevention, since SAD represents an early stage of airway obstruction and it is potentially reversible. This study evaluated the dose-response associations and effect modifications of long-term exposure to dust $PM_{2.5}$ on the risk of SAD at a national scale.

Material and Methods: SAD were identified based on lung function data from the China Pulmonary Health Study (N = 50,326). Logistic regression and multivariable linear models were used to quantify the associations of long-term dust $PM_{2.5}$ with SAD and lung function parameters, respectively. Stratified analyses were performed based on demographic characteristics.

Results: An interquartile range (IQR) increment in long-term dust $PM_{2.5}$ concentration was associated with the increased risk of SAD (Odds ratio [OR]:1.18, 95% confidence interval [CI]:1.08, 1.28) and SAD without airflow obstruction (OR: 1.24, 95% CI: 1.10, 1.39). We also found that dust $PM_{2.5}$ exposure were significantly associated with decrease among pre- and post-bronchodilator lung function. Individuals with lower levels of education were found to be more vulnerable.

Conclusion: We found significant associations of long-term dust $PM_{2.5}$ exposure with higher risk of SAD and decreased lung function, which call for awareness on the harm of fine dust particle on early-stage lung impairment. Studies on the countermeasures of respiratory hazard caused by SDS are warranted.

This study is supported by Science & Technology Fundamental Resources Investigation Program (Grant No.2023FY100604) and Noncommunicable Chronic Diseases-National Science and Technology Major Project (2023ZD0506306). We also appreciated the continuous support, assistance, and cooperation of the hospitals and healthcare facilities in the China Pulmonary Health Study project.

TO42-05 | Associations of Short and Long Duration Exposure to Particulate Matter and Nitrogen Dioxide with Radiologic Sinus Inflammation in Pennsylvania, USA

Margaret M. Tomann[1], Karen Bandeen-Roche[2], Kirsten Koehler[1], Joan A. Casey[3], Jonathan S. Pollak[1], Joseph J. Dewalle[4], Elizabeth M. Blake[3], Ashton E. Lehmann[5], Annemarie G. Hirsch[4,1], Brian S. Schwartz[1,4]

[1] Johns Hopkins University Bloomberg School of Public Health, Environmental Health & Engineering, Baltimore, USA; [2] Johns Hopkins University Bloomberg School of Public Health, Biostatistics, Baltimore, USA; [3] University of Washington School of Public Health, Environmental and Occupational Health Sciences, Seattle, USA; [4] Geisinger Health System, Population Health Sciences, Danville, USA; [5] Geisinger Health System, Otolaryngology, Danville, USA

Objective. Chronic rhinosinusitis is a disease of the nasal and sinus mucosa with a risk of transition to serious lower airway diseases. We evaluated associations of short and long duration exposure to fine particulate matter (PM2.5) and nitrogen dioxide (NO2) with radiologic sinus inflammation (RSI) using electronic health record data from Geisinger in Pennsylvania.

Methods. In a nested case-control study, we included individuals with residence in a 38-county study region, aged 18–80 years from 2008–2018. We identified cases with RSI (n=2,382) using a validated text algorithm applied to sinus computed tomography scan reports. Controls (n=11,910) were frequency-matched 5:1 on age, sex, and year of encounter. Air pollutant metrics were assigned within a 1-kilometer residential buffer using short and long latency and duration windows. We evaluated single pollutant models using logistic regression with robust standard errors clustered on community to estimate odds ratios (OR) and 95% confidence intervals (CI), adjusting for potential confounders.

Results. Cases and controls had a mean (SD) age of 49.5 (15.3) years and were predominantly non-Hispanic White (96%). Mean air pollutant levels fell at or below National Ambient Air Quality Standards with expected variation across years, seasons, and geographies. The fourth quartile of NO2 (range: 19.0-37.3 parts per billion) was associated with increased odds of RSI across short (0 months latency, 2 months duration, OR=1.61 [95%CI: 1.31-1.99]) and long (3 months latency, 2 years duration, OR=1.49 [95%CI: 1.21-1.81]) exposure windows. We found null associations of PM2.5 with RSI across all latency and duration windows.

Conclusions. In this population-based study, we found that exposure to the highest quartile of NO2 was associated with increased odds of RSI across short and long exposure windows. Findings suggest that NO2 may contribute to the onset and exacerbation of sinus inflammation, even at levels below current regulatory standards.

TO43 | Environmental chemical exposures during pregnancy and childhood

TO43-01 | Phthalates metabolites and their association with Inflammatory Biomarkers in Children: The Hokkaido Study

Rahel Mesfin Ketema[1,2], Yu Ait Bamai[2], Houman Goudarzi[3], Takeshi Yamaguchi[2], Yi Zeng[1,2], Ayaka Yasuda[4,5], Megasari Marsela[1], Satoshi Konno[3], Reiko Kishi[2], Atsuko Ikeda[1,2]

[1] Faculty of Health Sciences, Hokkaido University, Sapporo, Japan; [2] Center for Environmental and Health Sciences, Hokkaido University, Sapporo, Japan; [3] Faculty of Medicine, Hokkaido University, Sapporo, Japan; [4] Graduate School of Pharmaceutical Sciences, Health Sciences University of Hokkaido, Sapporo, Japan; [5] Graduate School of Health Sciences, Sapporo, Japan

Background and objective: Inflammatory biomarkers of Type 2 (T2) and non-T2 serves as objective indicators of asthma phenotyping and allergic responses. Previous studies showed a positive association between phthalates and asthma/allergies. Thus, this study investigates the associations of individual and mixture phthalates exposure with T2 markers; fraction of exhaled nitric oxide (FeNO), blood eosinophil count, total immunoglobulin E (IgE), and non-T2 marker; absolute neutrophil count (ANC), in Japanese children.

Methods: This study included 421 children (ages 9–12) from a prospective Hokkaido Cohort. Ten urinary phthalate metabolites were quantified using UPLC-MS/MS. Inflammatory biomarkers were assessed in blood samples. Binary outcomes were defined as elevated levels as: FeNO ≥35 ppb, IgE > 170 IU/mL, eosinophil count ≥ 300 cells/μL, and ANC ≥ 4400 cells/μL. Logistic regression evaluates individual metabolites, while quantile g-computation (qg-comp) and Bayesian kernel machine regression (BKMR) assessed the mixture effects of phthalates on binary outcomes. Molar sums of same-parent metabolites were also analyzed.

Results: Individual analyses revealed mono-n-butyl phthalate (MnBP) and all metabolites of di(2-ethylhexyl) phthalate (DEHP) were positively associated with eosinophil count. Additionally, MnBP, sum of di-n-butyl phthalate, and mono-(hydroxyisononyl) phthalate were linked to a higher prevalence of FeNO level. Individual phthalate metabolites showed no significant associations with IgE or ANC. Qg-comp showed a mixture of phthalates was associated with a higher odds ratio (OR) for FeNO prevalence, with MnBP as main contributor. While BKMR revealed that the DEHP metabolites group was dominant in the higher prevalence of eosinophil count and ANC. Overall combined effect showed increasing trend in eosinophil and ANC when all metabolites exceeded their 55th percentile.

Conclusion: Our findings suggest phthalate, individually and as mixtures, may influence inflammation biomarkers linked to asthma phenotypes in children. These supports previous evidence and underscores the need for further research on the role of phthalates in immune modulation.

TO43-03 | Polybrominated diphenyl ether concentrations during pregnancy and bone mineral density at age 12 years

Katherine Marquess[1], Jordan Kuiper[2], Bruce Lanphear[3], Kim Cecil[4], Yingying Xu[5], Kimberly Yolton[5], Heidi Kalkwarf[5], Joseph Braun[6], Aimin Chen[7], Jessie Buckley[8]

[1] Johns Hopkins Bloomberg School of Public Health, Department of Environmental Health and Engineering, Baltimore, USA; [2] The George Washington University Milken Institute School of Public Health, Department of Environmental and Occupational Health, Washington, D.C., USA; [3] Simon Fraser University, Faculty of Health Sciences, Vancouver, Canada; [4] Department of Radiology, Cincinnati Children's Hospital Medical Center, University of Cincinnati College of Medicine, Cincinnati, USA; [5] Department of Pediatrics, Cincinnati Children's Hospital Medical Center, University of Cincinnati College of Medicine, Cincinnati, USA; [6] Department of Epidemiology, Brown University, Providence, USA; [7] Department of Biostatistics, Epidemiology and Informatics, University of Pennsylvania Perelman School of Medicine, Philadelphia, USA; [8] Department of Epidemiology, Gillings School of Global Public Health, University of North Carolina at Chapel Hill, Chapel Hill, USA

Objective: Animal and *in vitro* studies suggest that polybrominated diphenyl ether (PBDE) flame retardants impair bone accrual and strength, but no epidemiologic studies have examined gestational exposures and adolescent bone health. We measured serum PBDE levels in pregnant women and analyzed associations with their child's bone mineral density (BMD) in early adolescence. We also examined whether these associations differed by adolescent's sex.

Materials and Methods: Among 184 mother-adolescent pairs enrolled in the Health Outcomes and Measures of the Environment Study (Cincinnati, OH: 2003 - 2006), we quantified five PBDEs in maternal serum collected at 16- or 26- weeks gestation. At age 12 years, we conducted dual energy x-ray absorptiometry scans and calculated height-, age-, sex-, and race-specific BMD Z-scores for six skeletal sites. In multivariable linear regression models, we estimated covariate-adjusted associations per log10 increase in maternal serum PBDE concentrations. We assessed effect measure modification (EMM) by adolescent's sex.

Results: In adjusted models, higher maternal serum BDE-153 concentrations (median: 3.9 ng/g lipid; IQR: 4.9 ng/g lipid) were associated with lower BMD Z-scores at age 12 years for all skeletal sites. For example, each 10-fold increase in maternal serum BDE-153 was associated with 0.42 (95% CI: -0.79, -0.05) lower femoral neck BMD Z-score. Other PBDE associations were similar in direction but weaker. Adolescent's sex modified associations of BDEs -28, -47, and -100 with total hip and femoral neck BMD. For example, differences in total hip BMD Z-scores were -0.75 (95% CI: -1.50, 0.01) for males and 0.21 (95% CI: -0.31, 0.74) for females (EMM $p = 0.04$).

Conclusion: *In utero* BDE-153 exposure appears to negatively impact bone accrual and strength in early adolescence. For some PBDEs, associations with total hip and femoral neck BMD were sex-dependent.

Supported by NIEHS grants.

TO43-04 | Associations of Gestational and Childhood Polybrominated Diphenyl Ether (PBDE) Concentrations with Sleep Patterns in School-age Children

Kelli Williams[1], Jagadeesh Puvvula[1], John Holmes[1], Wei Yang[1], Sigrid Veasey[2], Jianghong Liu[3], Kimberly Yolton[4], Kim Cecil[4], Yingying Xu[4], Joseph Braun[5], Bruce Lanphear[6], Clara Sears[7], Ann Vuong[8], Andreas Sjödin[9], Aimin Chen[1]

[1] University of Pennsylvania, Department of Biostatistics, Epidemiology, and Informatics, Perelman School of Medicine, Philadelphia, USA; [2] University of Pennsylvania, Department of Medicine, Perelman School of Medicine, Philadelphia, USA; [3] University of Pennsylvania, Penn Nursing, Philadelphia, USA; [4] University of Cincinnati College of Medicine, Department of Pediatrics, Cincinnati Children's Hospital Medical Center, Cincinnati, USA; [5] Brown University, Department of Epidemiology, Providence, USA; [6] Simon Fraser University, Burnaby, Canada; [7] University of Louisville, Department of Medicine, Louisville, USA; [8] University of Nevada Las Vegas, Las Vegas, USA; [9] Center for Disease Control and Prevention, Division of Laboratory Services, Atlanta, USA

Gestational polybrominated diphenyl ethers (PBDEs) exposures have been associated with thyroid disruption in pregnant women and neurobehavioral problems in their children, but it is unknown if they interfere with children's sleep health. Our previous study found associations between gestational PBDEs with sleep irregularity and sleep disruption at ages 5 and 8 years, so exploring window-specific susceptibility would clarify whether the association can be explained by postnatal exposures. We assessed postnatal (at ages 5 and 8 years) PBDE exposures and child sleep patterns at age 8 years using 188 mother-child dyads in the Health Outcomes and Measures of the Environment (HOME) Study. Biomarkers of serum PBDEs (lipid-adjusted) include PBDE-153, PBDE-100, PBDE-99, PBDE-47, PBDE-28, and their arithmetic sum, SPBDEs. We measured child sleep patterns using an adapted version of the Children's Sleep Habits Questionnaire, measuring sleep irregularity, hypersomnolence, sleep disruption, and sleep duration. We used multiple informant models, a modified version of Generalized Estimating Equation (GEE) to assess the window of susceptibility for the PBDE exposure and sleep outcomes at age 8 years, adjusting for covariates. We found that PBDE exposure at 5 years, but not 8 years, was associated with higher sleep irregularity at 8 years. Each 10-fold increase in 5-year PBDE-28, PBDE-47, PBDE-99, PBDE-100, and SPBDEs was linked to lower sleep irregularity at 8 years. At age 5 years, PBDE-153 was linked to higher hypersomnolence at 8 years. At age 5 years, PBDE-28, -47, -100, and SPBDEs was linked to lower sleep disruption at 8 years. No notable associations were observed for sleep duration. Five year PBDEs were associated with lower year sleep irregularity and disruption at 8 years of age, underlining the importance of investigating periods of susceptibility. Future research should be conducted in larger cohorts to further explore and validate these findings.

TO43-05 | Gestational Phthalate Exposure Modifies Age-Specific DNA Methylation From Birth Through Adolescence in an Epigenome Wide Association Study

Ariana Haidari[1], Jaclyn Goodrich[1], Dana Dolinoy[1,2], Deborah Watkins[1], Adriana Mercado Garcia[3], Libni Torres Olascoaga[3], Zhenke Wu[4], Peter Song[4], Martha M. Tellez-Rojo[3], Karen Peterson[2]

[1] University of Michigan, Dept. of Environmental Health Sciences, Ann Arbor, USA; [2] University of Michigan, Dept. of Nutritional Sciences, Ann Arbor, USA; [3] National Institute of Public Health, Cuernavaca, Mexico; [4] University of Michigan, Dept. of Biostatistics, Ann Arbor, USA

Objective

We sought to investigate whether gestational exposure to phthalates, a class of plasticizer chemicals, modify the age-related rate of change in DNA methylation assessed at birth and two time points in childhood.

Methods

The analytical sample comprised of 70 participants in the Early Life Exposures in Mexico to Environmental Toxicants (ELEMENT) prospective cohort study. Phthalate metabolite concentrations measured in urine samples collected during pregnancy included monoethyl phthalate (MEP), mono-n-butyl phthalate (MBP), monobenzyl phthalate (MBzP), and mono(3-carboxypropyl) phthalate (MCPP). Exposure to di(2-ethylhexyl) phthalate (DEHP) was approximated by summing four metabolites of DEHP. DNA methylation was measured at 781,903 loci in infant cord blood as well as venous blood from childhood and adolescence via the Infinium EPIC array. Linear mixed effects models of the longitudinal DNA methylation measures were used, adjusting for confounders including cell type and batch, to identify loci associated with phthalate exposure, and loci with a significant interaction between phthalate concentration and age.

Results

Phthalate metabolites were significantly associated with repeated measures of DNA methylation at 13 CpG sites: 1 with MBP, 9 with MBzP, 2 with MEP, with 1 for DEHP (q value < 0.05). The interaction between gestational phthalate exposure and age on DNA methylation was significant at 21 CpG sites (q-value < 0.05): 1 site with DEHP, 2 with MEP, 2 with MBP, 11 with MBzP, and 2 with MCPP. These included sites in the genes B*AT3, WDR82, SCAND3, RAD51B, PDK3, GJC3, POMT1, TRPV1, CDH22, IGLL3P,* and *PTPRD.*

Conclusions

In this epigenome-wide association study, we demonstrate exposure to phthalates during gestation is associated with DNA methylation at multiple loci and also modifies the rate of age-related change in DNA methylation from birth to adolescence. This is one of the first human studies do so, illustrating how gestational exposures could impact health later in life.

FT01 | Chemical exposures: From PFAS to plasticizers to volatile organic compounds

FT01-01 | Drinking water predictors of serum per- and polyfluoroalkyl substances concentrations in select Orange County, CA communities

Carolina Villanueva[1], Briseida Miranda[2], Nicholas Cuvelier[3], Karen S. Valladares[1], Celina N. Phillipson[1], Ramandeep Kaur[1], Jayamalee DeSilva[1], Meghan Weems[4], Marian Pavuk[4], Veronica M. Vieira[1], Scott M. Bartell[1,2], Disclaimer: The findings and conclusions in this presentation have not been formally disseminated by the Centers for Disease Control and Prevention/the Agency for Toxic Substances and Disease Registry and should not be construed to represent any agency determination or policy.

[1] University of California, Irvine, Department of Environmental and Occupational Health/ Joe C. Wen School of Population & Public Health, Irvine, USA; [2] University of California, Irvine, Department of Epidemiology & Biostatistics/ Joe C. Wen School of Population & Public Health, Irvine, USA; [3] California Department of Public Health, Irvine, USA; [4] Centers for Disease Control and Prevention, Office of Community Health Hazards Assessment/ Agency for Toxic Substances and Disease Registry, Atlanta, USA

Objective: Cities in Orange County (OC), California have previously documented per-and polyfluoroalkyl substances (PFAS) in drinking water. This study aims to characterize serum PFAS levels in eligible OC cities, and examine predictors of serum PFAS levels.

Material and Methods: The OC study population was part of a cross-sectional multi-site study to examine the health effects of PFAS exposure. Participants resided in OC cities with at least one PFAS detected in their local water system between 2000-2019. We assessed differences in seven individual serum PFAS levels using Generalized Linear Models to calculate effect estimates and 95% confidence intervals (CI) for predictors. We categorized water/filter use as mostly unfiltered tap, mostly filtered tap, similar amounts unfiltered tap/ bottled, mostly bottled water (referent). PFAS detected in ≥70% of the population were modeled as continuous and log transformed, while PFAS detected in <70% were dichotomous at the level of detection, with adjustment for race/ethnicity, income, education, sex, age, and city.

Results: A total of 516 adults and 51 children completed the questionnaire and provided a serum sample. Predictors of higher serum PFAS levels were older age, Asian/Pacific Islander race, male sex, and city. After adjusting for water and filter use, city was no longer associated with serum PFAS levels. Except for perfluorononanoic acid (p=0.689), water filter use was an important determinant of serum PFAS concentrations among adults. Drinking unfiltered tap water (n=57) was associated with a 37.7% increase (95%CI: 13.9%, 68.2%) in log-transformed sum of seven PFAS levels compared to drinking mostly bottled water.**Conclusion:** In OC, consumption of unfiltered water was associated with higher individual and total serum PFAS levels. This is a modifiable risk factor with potentially significant public health impact. Funding for this study was provided by the Agency for Toxic Substances and Disease Registry, Centers for Disease Control and Prevention.

FT01-02 | Differences in Per- and Polyfluoroalkyl Substance Contamination Across Water Providers in Los Angeles

Elizabeth Costello[1], Shiwen Li[1], Tamar Galindo[1], Bianca Costa[2], Christelle Sawaya[2], Ana-Sofia Arena[1], Tomas Manea[1], Dominic Pak[1], Shudi Pan[1], Jiawen Carmen Chen[1], Jesse A. Goodrich[1], David V. Conti[1], Lucy Golden-Mason[4], Ana C. Maretti-Mira[4], Daniel L. McCurry[3], Amy E. Childress[2], Rob S. McConnell[1], Adam L. Smith[2], Max T. Aung[1], Lida Chatzi[1]

[1] University of Southern California, Population and Public Health Sciences, Los Angeles, USA; [2] University of Southern California, Sonny Astani Department of Civil and Environmental Engineering, Los Angeles, USA; [3] University of Southern California, Research Center for Liver Disease, Los Angeles, USA; [4] University of Southern California, Department of Medicine, Los Angeles, USA

The largest water provider in Los Angeles (LA) is the LA Department of Water and Power (LADWP), which sources water primarily from Central California or the Colorado River. However, many communities in East and Southeast LA are served by smaller providers which rely more on local groundwater. These communities are also more likely to be located near industrial PFAS sources, which poses a risk of groundwater contamination and PFAS-associated health risks. This study aimed to assess PFAS contamination across tap-water providers in Central, East, and Southeast LA.

Methods

From April-November 2024, 48 participants provided a tap-water sample and completed a questionnaire about their water use, health history, and demographics. Twenty-one PFAS were measured in the water samples using EPA Method 533. PFAS detections were compared between households served by LADWP and all small water systems (SWS) combined using Fisher's exact tests.

Results

Participants were primarily female (98%), Hispanic/Latino (94%), and in middle age (mean:51y, SD:11.3). Most (57%) reported having at least one metabolic condition previously linked to PFAS exposure (liver/kidney disease, diabetes, etc). LADWP (45% of samples) and 14 SWSs were represented. At least one PFAS was detected in 92.3% of SWS samples and in 9.5% of LADWP samples ($p<0.0001$). The mean sum concentration of all PFAS was 13.2ng/L (SD:14.5) in SWS samples and 0.54ng/L (SD:1.90) in LADWP samples. One LADWP sample exceeded the US EPA Maximum Contaminant Level (MCL; 4ppt for PFOS and PFOA) for PFOS. In SWS samples, 57.7% exceeded the MCL for PFOS and 15.4% exceeded the MCL for PFOA.

Conclusion

PFAS were detected more frequently and at higher concentrations in small water systems in LA. These differences may be related to ground water contamination or industrial PFAS sources. Households served by PFAS-contaminated water providers may be at higher risk for PFAS exposure and related health conditions.

FT01-04 | A comprehensive study on the full chain of household phthalate exposure

Yuxuan Zhao, Yuexia Sun

Tianjin University, Tianjin, China

Phthalates are ubiquitous pollutants in the indoor environment with potential negative impacts on children’s health. The aim of this study was to investigate the full chain of phthalate exposure in the home environment by examining the associations between sources of phthalates in the home environment, indoor environmental exposure, and asthma and allergies in children. We conducted home inspections for 398 children aged 0-8 years in Tianjin, China, collected dust samples from their bedrooms, measured environmental parameters, and administered questionnaires on household consumer products and childhood asthma and allergic symptoms. The primary phthalates detected in Tianjin households were DEHP, DnBP, and DiBP, with median concentrations of 126.94, 40.82, and 16.27 μg/g, respectively. We found a significant association between DiBP and diagnosed asthma (AOR: 2.26, 95% CI: 1.02-5.03), BBzP and current rhinitis (AOR: 2.48, 95% CI: 1.25-4.94), as well as DEHP and diagnosed asthma (AOR: 3.77, 95% CI: 1.00-14.15). Potential sources of these phthalates were identified: cosmetics were linked to DiBP, polyvinyl chloride (PVC) window frames to BBzP ($p<0.05$), and laminated wood floors to DEHP ($p<0.05$). Additionally, environmental parameters were found to influence phthalate concentrations, with ventilation having a dilution effect on low molecular weight phthalates, particularly DiBP. This study establishes a comprehensive chain of household phthalate exposure. Reducing the use of cosmetics, opting for green building materials, and ensuring continuous ventilation may help control indoor phthalate concentrations and protect children’s health.

FT01-05 | The Association between Volatile Organic Compounds and Sleep Health

Clara Sears[1], Daniel Riggs[1], Manjiri Kulkarni[1], Rachel Keith[1], Cameron Stopforth[1], Sanjay Srivastava[1], Pawel Lorkiewicz[1], Aruni Bhatnagar[1], Mathias Basner[2]

[1] University of Louisville, Christina Lee Brown Envirome Institute, Louisville, USA; [2] University of Pennsylvania Perelman School of Medicine, Unit for Experimental Psychiatry, Division of Sleep and Chronobiology, Department of Psychiatry, Philadelphia, USA

Objective: Emerging evidence suggests that exposure to ambient air pollutants is associated with poorer sleep health. However, the contribution of volatile organic compound (VOC) exposure to sleep disruption is not clear. Rigorous analsyes with objective sleep assessments could provide insights into the dimensions of sleep health that may be impacted by VOCs. We hypothesize that higher VOC metabolite concentrations will be associated with lower sleep efficiency and shorter total sleep time.

Methods: We evaluated the relation of urinary VOC metabolites with objective sleep measures in a cross-sectional study of 128 participants (ages 25-71) from Louisville, KY. Concentrations of 16 urinary metabolites corresponding to 12 VOCs were quantified by LC/MS. Sleep efficiency and total sleep time were estimated via ActiGraph accelerometer. Using adjusted linear regression, we estimated relations of VOC metabolites with sleep outcomes stratifying by weekday and weekend.

Results: The mean age of participants was 46 years with a majority being female (61%) and non-Hispanic White (89%). The mean total sleep time was 377.2 min for weekdays and 389.3 min for weekends. We found an interquartile increase in the 1,3-butadiene metabolite, was associated with -0.94 % (95%CI= -1.75%, -0.13%) lower weekday sleep efficiency. We observed significant sex differences in our associations, with males consistently showing stronger negative associations with VOC metabolites than females. Specifically, total sleep time and sleep efficiency were negatively associated with xylene metabolites in males. In males, we also observed negative associations between weekday sleep efficiency and metabolites of acrylonitrile (-0.99% per IQR; 95% CI: -1.93, -0.05), and propylene oxide (-1.92% per IQR; 95% CI: -3.21, -0.22). Among females, we did not find evidence that higher VOC metabolites were associated with poorer sleep.
Conclusion: Our results suggest that exposure to specific VOCs, such as 1,3-butadiene and xylene could contribute to inadequate sleep, particularly in males.

FT01-06 | Environmental PFAS Exposure and change in C reactive protein among US Population: A Mixture Method

Rezaul Karim Ripon[1,2], Narayana G Prasad[3], Jagdish Khubchandani[8], Satish Govind[9], Edima Ottoho[6], Rashmi Paudel[7], Shishir Gokhale[10], Mayra Volquez[5], Sujata Saunik[4], Srikanth Sola[11]

[1] Harvard T H Chan School of Public Health, Environmental Health, Boston, USA; [2] McHigher Centre For Health Research, Environmental Epidemiology, Dhaka, Bangladesh; [3] Public Health Literacy/BWH, Department of Cardiology, Miami, USA; [4] Harvard T H Chan School of Public Health, Takemi Fellow, Boston, USA; [5] Public Health Literacy/UT South Western, Preventive Cardiology, Dallas, USA; [6] Simmons University, Boston, USA; [7] Boston University, Boston, USA; [8] New Mexico State University, NM, USA; [9] Narayana Health, Department of Cardiology, Bengaluru, India; [10] Manipal College of Medical Sciences, Dean, Pokhara, Nepal; [11] Devic Earth, Founder, Bengaluru, India

Background: Per- and poly-fluoroalkyl substances (PFAS) are widespread environmental pollutants associated with inflammation, as indicated by C-reactive protein (CRP) levels. This study evaluates the link between PFAS exposure and CRP levels in the US population using the National Health and Nutrition Examination Survey 2015-2020 data.

Methods: The analysis included participants with serum PFAS levels, CRP levels, and covariates. Firth's logistic regression and generalized Weighted Quantile Sum (gWQS) regression assessed single and cumulative PFAS effects.

Results: The study includes 19,481 participants from 2015 to 2020. Regarding C-reactive protein, 8.5% of participants are above the detection limit. For PFAS levels, notable averages include 0.7 ng/mL for Perfluorononanoic acid and 4.73 ng/mL for n-perfluorooctane sulfonic acid. Significant associations were found between Perfluorononanoic acid (estimate = 0.2547, $p < 0.0001$) and 2-(N-methyl-PFOSA) acetic acid (estimate = 0.0919, $p < 0.00001$) with elevated CRP levels. The gWQS index had a significant cumulative effect estimate of 0.586 ($p < 0.001$). GWQS weights indicated that perfluorononanoic acid iso had the highest contribution (weight = 0.543), followed by n-perfluorooctane sulfonic acid (weight = 0.445).

Conclusions: PFAS exposure is significantly linked to increased CRP levels, suggesting a role in inflammation. These findings support regulatory efforts to curtail PFAS exposure and prompt further research into their health impacts.

FT01-07 | Assessing Infant PFAS Exposure: Contributions from Maternal Serum, Breast Milk, and Formula Mixing Water

Jiahe Tian[1], Rachel Bauer[2], Sarah S. Comstock[1], Amina Salamova[3], Jaclyn Goodrich[4], Michael Petriello[5], Doug Ruden[5], Jean Kerver[6], Courtney C. Carignan[1]

[1] Michigan State University, Department of Food Science and Human Nutrition, East Lansing, USA; [2] Michigan State University, Department of Pharmacology and Toxicology, East Lansing, USA; [3] Emory University, Department of Environmental Health, Atlanta, USA; [4] University of Michigan, Department of Environmental Health Sciences, Ann Arbor, USA; [5] Wayne State University, Institute of Environmental Health Sciences, Detroit, USA; [6] Michigan State University, Department of Epidemiology and Biostatistics, East Lansing, USA

Per- and polyfluoroalkyl substances (PFAS) are persistent and toxic chemicals that transfer from mother to infant via the placenta and breast milk. Despite being a sensitive developmental window, few studies have investigated infant exposure in early life. We estimated infant serum concentrations (CI) of four PFASs using median maternal serum concentrations (CM) (n=206) from the Michigan Archive on Child Health cohort and a toxicokinetic model for infants from birth to 24 months. Infants were assumed to consume exclusively breast milk or formula for the first year with 4 µg/L of each PFAS in drinking water and also predicted CM based on this exposure. Maternal-fetal (0.34–0.66) and maternal-breast milk (0.018–0.068) transfer factors were identified by literature review. Median CM for PFOA, PFOS, PFHxS and PFNA (0.46, 1.60, 0.53 and 0.18 µg/L) were similar to the general population. CI peaked at 12 months in breastfed infants and 2 months in formula-fed infants, followed by a steady decline (breastfed) or slight increase (formula-fed) through 24 months. Among breastfed infants, peak CI based on median CM were highest for PFOS followed by PFOA, PFHxS and PFNA (2.58, 1.78, 1.74 and 1.11 µg/L), and were 3.25–3.80 times higher when based on predicted CM. In formula-fed infants, peak CI based on median CM were highest for PFNA followed by PFHxS, PFOS and PFOA (1.12, 1.11, 0.75 and 0.70 µg/L), increasing 1–2.5 times with predicted CM. CI peaks were 1-5 times higher in breastfed compared to formula-fed infants, and notably exceeded benchmark doses for PFOA (0.93 µg/L) and PFOS (2.60 µg/L) among breastfed infants when based on 95th% and predicted CM. These findings underscore the role of maternal serum and drinking water as important infant PFAS exposure sources and highlight the need for reductions to protect infant health.

FT01-09 | Associations of prenatal polyfluoroalkyl substances (PFAS) and metals with childhood vaccine induced immunity

Anna R. Smith[1], Sheryl L. Rifas-Shiman[2], Joseph A. Lewnard[3], Alan Hubbard[4], Marie-France Hivert[2,5], Emily Oken[2], Diane R. Gold[6,7], Andres Cardenas[1]

[1] Stanford University, Department of Epidemiology and Population Health, Stanford, USA; [2] Harvard Medical School and Harvard Pilgrim Health Care Institute, Department of Population Medicine, Division of Chronic Disease Research Across the Lifecourse, Boston, USA; [3] University of California Berkeley, School of Public Health, Division of Epidemiology, Berkeley, USA; [4] University of California Berkeley, School of Public Health, Division of Biostatistics, Berkeley, USA; [5] Massachusetts General Hospital, Diabetes Unit, Boston, USA; [6] Harvard Medical School and Brigham and Women's Hospital, Department of Medicine, Channing Division of Network Medicine, Boston, USA; [7] Harvard T.H. Chan School of Public Health, Department of Environmental Health, Boston, USA

Objective: Per- and polyfluoroalkyl substances (PFAS) may induce immunosuppression and alter cell signaling involved in immune function, while nonessential metals may impair immune response to vaccination. Essential metals are necessary for immune cell growth and maintenance and could influence vaccine response. We evaluated prospective associations of prenatal PFAS and metal mixtures with childhood MMR (measles, mumps, rubella) and DTaP (diphtheria, tetanus, pertussis) antibody titers.

Material and Methods: We measured blood PFAS (PFOA, PFOS, EtFOSSA, PFHxS, MeFOSSA, PFNA), nonessential (arsenic, barium, cadmium, cesium, lead, mercury), and essential (magnesium, manganese, selenium, zinc) metals in first trimester blood from participants in the Project Viva cohort, recruited 1999-2000 in Massachusetts, USA. We measured childhood plasma MMR and DTaP titers (*n*=479-528), categorized as detect or borderline/nondetect. We used adjusted quantile g-computation models to estimate associations between the mixtures of PFAS, nonessential, or essential metals and each of the antibody titers.

Results: Median (interquartile range) age of participants at antibody titer measurement was 7.7 years (7.4, 8.4 years). Approximately 98%, 50%, 14%, and 4% of participants had borderline/nondetect pertussis, measles, rubella, and mumps titers. All participants had detectable diphtheria and tetanus titers. Risk of having nondetectable mumps titers was associated with prenatal PFAS [relative risk (RR)= 2.08, 95% confidence interval (CI): 1.05, 4.13 per quartile increase)] and nonessential metal mixture (RR=2.95, 95% CI: 1.04, 8.23). The nonessential metal mixture was also associated with a higher risk of having nondetectable measles titers (RR=1.20, 95% CI: 1.01, 1.44). There were no associations between the PFAS or metal mixtures and rubella, pertussis, diphtheria, or tetanus titers.

Conclusions: Prenatal PFAS were associated with lower mumps titers, while the nonessential metals were associated with lower mumps and measles titers. Future research will examine associations between postnatal mixtures of PFAS and metals and these antibody titers.

FT01-10 | Investigating disparities in exposure to PFAS and other industrial chemicals in US drinking water using nationwide monitoring data

Aaron Maruzzo[1], Jahred Liddie[2], Amanda Hernandez[2], Christopher Schwartz[3], Laurel Schaider[1]

[1] Silent Spring Institute, Boston, USA; [2] Harvard University, Harvard T.H. Chan School of Public Health, Department of Environmental Health, Boston, USA; [3] Stockholm Environment Institute, Somerville, USA

Objective: Prior studies have shown that communities of color and low-income communities can experience disproportionately high exposures to regulated drinking water contaminants, like lead and nitrate. However, few studies have examined associations between sociodemographic factors and exposures to unregulated drinking water contaminants, which are not subject to the same regulatory enforcement but may also pose health risks.
Methods: We compiled occurrence data for four industrial contaminants (1,4-dioxane, 1,1-dichloroethane, chlorodifluoromethane, and six per- and polyfluoroalkyl substances [PFAS]) from 4,815 public water systems (PWSs) in Environmental Protection Agency's (EPA) Third Unregulated Contaminant Monitoring Rule (UCMR, 2013-2015) and county-level race/ethnicity and socioeconomic data from the U.S. Census Bureau. We also compiled data on possible sources of these contaminants from known PFAS dischargers, relevant facilities in the Toxics Release Inventory, and wastewater treatment plants. We conducted multivariable logistic regression models to analyze associations between detections of target contaminants and sociodemographic variables.
Results: 27% of U.S. PWSs, serving >97 million people, detected at least one target contaminant. Average proportions of Hispanic/Latino and non-Hispanic Black residents were higher in counties with relevant industrial sources compared to counties without. In logistic models, odds of PWS-level detections were positively associated with proportions of Hispanic and Black residents. Detections were also positively associated with large system size, urbanized areas, and systems reliant on groundwater or mixed groundwater-surface water sources. Associations between Hispanic residents and contaminant detections persisted even after accounting for socioeconomic status and proximity to contamination sources.
Conclusions: PWSs in underserved communities can benefit from additional resources to protect drinking water sources from industrial contamination. Future work will analyze more recent results from U.S. EPA's 2023-2025 UCMR 5 monitoring for 29 PFAS and incorporate newly available service area boundaries for community water systems to assess ongoing disparities in exposures to both legacy and current-use PFAS from drinking water.

FT02 | Health effects of air pollution components and mixtures: Global evidence and insights

FT02-01 | Short-term effects of ultrafine particles on stroke events: An assessment using four different exposure metrics

Minqi Liao[1,2], Siqi Zhang[3], Maximilian Schwarz[1], Cheng He[1], Susanne Breitner-Busch[1,2], Josef Cyrys[1], Markus Naumann[4], Lino Braadt[4], Claudia Traidl-Hoffmann[5,6], Gertrud Hammel[7], Annette Peters[1,2], Michael Ertl[4], Alexandra Schneider[1]

[1] Helmholtz Zentrum München, Institute of Epidemiology, Munich, Germany; [2] Ludwig-Maximilians-Universität München, Institute for Medical Information Processing, Biometry, and Epidemiology (IBE), Munich, Germany; [3] Yale School of Public Health, Department of Environmental Health Sciences, New Haven, USA; [4] University Hospital Augsburg, Neurology and Clinical Neurophysiology, Augsburg, Germany; [5] University Augsburg, Environmental Medicine, Augsburg, Germany; [6] Center for Allergy and Research and Education, CK-CARE, Davos, Switzerland; [7] University of Augsburg, Institute for Social Sciences, Augsburg, Germany; [8] German Center for Cardiovascular Health, Munich Heart Alliance, Munich, Germany

Objective

Evidence on the effects of particles in the ultrafine range (10-100 nm in diameter, UFP) on stroke occurrence is scarce. We aimed to investigate the association between four different metrics of short-term exposure to submicron particulate air pollution and the occurrence of strokes and their subtypes.

Material and methods

We used 19,518 patient records of stroke events from the University Hospital Augsburg, Germany, between 2006 and 2020. Daily averages of four size-fractioned UFP metrics, including particle number (PNC), mass (PMC), length (PLC), and surface area concentrations (PSC), were collected from fixed urban background sites in Augsburg during the same period. We applied a time-stratified case-crossover study with conditional logistic regression to estimate the association between these four UFP metrics and stroke events. We further conducted subgroup analyses by stroke-related variables (subtypes, stroke-induced disability, and severity) and effect modification by sex, age, season, and 5-year periods.

Results

Four different UFP metrics were associated with elevated odds of stroke events. Each interquartile range (IQR) increase in the cumulative (lag 0-6 days) UFP exposure was associated with an increase in the odds of stroke events by 4.94% (1.22%; 8.81%) for PNC, 4.12% (1.04%; 7.29%) for PMC, 4.70% (1.25%; 8.26%) for PLC, and 4.29% (1.12%; 7.56%) for PSC. Particles in the Aitken mode (30-100 nm) showed more consistent associations than smaller particles in the nucleation mode (10-30 nm). The cumulative effects of UFPs were more pronounced for ischemic strokes and stroke patients with milder disability and severity.

Conclusion

Our research has shown that four different metrics of short-term UFP exposure were consistently associated with the occurrence of stroke events, particularly ischemic strokes. PLC and PSC may serve as promising alternative indicators for measuring UFP exposure. This finding underscores the need for effective approaches to reduce traffic-related air pollutants for stroke prevention.

FT02-02 | Patterns of elemental composition and sources of ambient fine particulate matter ($PM_{2.5}$) in Accra, Ghana

Carissa Lange[1], Aaron Specht[2], Abosede Alli[1], Sierra Clark[3], James Nimo[5,4], Allison Hughes[4], Majid Ezzati[6,7], Raphael Arku[1]

[1] University of Massachusetts Amherst, Department of Environmental Health Sciences, Amherst, USA; [2] Purdue University, School of Health Sciences, Lafayette, USA; [3] City St George's, University of London, School of Health and Medical Sciences, London, UK; [4] University of Ghana, Department of Physics, Accra, Ghana; [5] State University of New York, Department of Environmental & Sustainable Engineering, Albany, USA; [6] Imperial College London, Department of Epidemiology and Biostatistics, School of Public Health, London, UK; [7] University of Ghana, Regional Institute for Population Studies, Accra, Ghana

Objective: The ongoing economic development and urban growth in Sub-Saharan Africa (SSA) may be altering air pollution sources, emission patterns, and pollutant mixtures. While data on the levels and distribution of ambient fine particle matter ($PM_{2.5}$) are accumulating in the region, there remains a lack of empirical studies that identify and quantify major pollution sources and their contributions, which is relevant for effective air quality management in growing cities. Here, we provide detailed data on the composition and source-specific contributions to ambient $PM_{2.5}$ pollution in Accra, one of the fastest growing metropolises in SSA.
Material and Methods: Between April 2019 and June 2020, we collected >600 weekly gravimetric (filter-based) $PM_{2.5}$ samples from 146 sites covering a range of land-use and source influences. The filters were analyzed for 45 elements using energy dispersive x-ray fluorescence. We applied positive matrix factorization (PMF) to identify and quantify major sources.
Results: In preliminary analyses, elements linked to biomass burning (K, Br, As, Pb) and vehicle emissions (Ti, Fe, Cu, Zn, Ba) were highest in high-density residential and high-traffic areas, respectively. Elements associated with e-waste burning (Co, Cr) and aluminum production (Sc) were detected in relatively high abundance. Furthermore, our PMF models identified 5-7 major $PM_{2.5}$ sources across different sites: fresh and aged biomass burning (13–43%), road dust and traffic emissions (9–31%), solid and electronic waste combustion (13–26%), construction dust (3–29%), crustal dust (10–17%), and sea salt (8–46%).
Conclusion: Understanding $PM_{2.5}$ composition and sources in growing SSA cities is essential for developing effective, equitable air quality policies that protect public health. Our results will support air quality management plans and inform regulatory and non-regulatory approaches to reduce source contributions in SSA.

FT02-03 | Phthalates and Pediatric Infection Risk in Rural South Africa: Insights from the VHEMBE Birth Cohort

Soma Barsen[1], Elizabeth Lin[1], Sébastien Tchoubi[3], Emily Johnson[1], Jieqiong Zhou[1], Sheng Liu[1], Riana Bornman[2], Muvhulawa Obida[2], **Krystal Godri Pollitt**[1], Jonathan Chevrier[3]

[1] Yale School of Public Health, Environmental Health Sciences, New Haven, USA; [2] University of Pretoria, Institute for Sustainable Malaria Control and School of Health Systems and Public Health, Pretoria, South Africa; [3] McGill University, Epidemiology, Biostatistics and Occupational Health, Montreal, Canada

Objective: Environmental exposures to endocrine disrupting plastic additives may affect children's susceptibility to infections. In regions with inadequate waste management, plastics are commonly burned in open pits, leading to elevated exposure to phthalates and other plasticizers. This study aimed to 1) comprehensively characterize the children's personal exposure to plastics-related chemicals in rural South Africa and 2) evaluate the impact of these pediatric exposures on risk and frequency of infections—including ear, throat, and respiratory infections and persistent fevers.

Materials and Methods: Eight-year-old children (n=495) enrolled in the Venda Health Examination of Mothers, Babies, and their Environment (VHEMBE) birth cohort wore a wearable passive sampler (Fresh Air wristband) for three days. Wristbands were analyzed using gas chromatography high resolution mass spectrometry for a targeted panel of 20 phthalates. Non-targeted analysis was further used to screen for additional plastic additives, phthalates, and phthalate replacements. Residential refuse combustion events were characterized using similar passive sampling and chemical analysis approaches. Data on infection symptoms were collected via questionnaires administered to primary caregivers and associations were determined via Poisson regression with robust variance estimates.

Results: Exposure to the 20 assessed phthalates were detected. Diethyl phthalate, diisobutyl phthalate, dimethyl phthalate, and terephthalate were found in 99% of participants and observed to be characteristic of emissions released from waste burning events. The greatest exposure concentrations were measured for diisobutyl phthalate. Non-targeted analysis further revealed exposure to di(2-ethylhexyl) phthalate, and chemical additives, including diethylene glycol dibenzoate. Preliminary analyses suggest that a 10-fold increase in exposure to high-molecular weight phthalates was associated with 2.5-fold (95%CI=1.2, 5.1) elevated risk of ear infection among girls but a lower risk of colds among boys (RR=0.75; 95%CI=0.6, 0.9).

Conclusions: Exposure to phthalate was associated with risk of infections, the direction of which appears to differ based on biological sex.

FT02-04 | Long-term exposure to major constituents of fine particulate matter and sleep disorders among children and adolescents: A population-based survey in Guangdong province, China

Qing-Guo Zeng[1], Guang-Hui Dong[1,2], **Xiao-Wen Zeng**[1,2]

[1] Sun Yat-sen University, Department of Occupational and Environmental Health, Guangzhou, China; [2] Joint International Research Laboratory of Environment and Health, Ministry of Education, Guangzhou, China

Long-term exposure to PM2.5 is associated with sleep health in adults, but its impact on children and adolescents remains unclear. We conducted a cross-sectional study of 64,203 children and adolescents (6-18 years) from four cities in Guangdong, China, to assess the impact of PM2.5 and its major constituents [black carbon (BC), organic matter (OM), ammonium (NH4+), nitrate (NO3-), sulfate (SO42-), and chloride (Cl-)] on sleep disorders. Sleep disorders were assessed using the Sleep Disturbance Scale for Children. Generalized linear mixed models and weighted quantile sum regression were applied to assess the joint effects of PM2.5 constituents. Results show that PM2.5 and its constituents were positively associated with sleep disorders. For example, the odds of sleep disorder increased with per interquartile range (IQR) increase in PM2.5 concentrations [odds ratio (OR): 1.19, 95% confidential interval (CI):1.12, 1.26). BC and OM increase the odds by 26% (95% CI: 16, 36%) and 29% (95% CI: 18, 42%), respectively. Stratified analysis revealed that children and adolescents with limited outdoor activity (< 2 hours/day) were more vulnerable to PM2.5 exposure. Our findings suggest that long-term exposure to PM2.5 and its constituents, particularly combustion-related BC and OM, is linked to sleep disorders in children and adolescents.
ACKNOWLEDGMENTS: This work was supported by Natural Science Foundation of Guangdong Province (No. 2021B1515020015), National Key Research and Development Program of China (2018YFE0106900) and the Guangzhou Science and Technology Project (No. 2024A04J6476).

FT02-05 | Comparative health impacts of personal exposure to fine ($PM_{2.5}$) and coarse particulate matter (PM_{coarse}) in elderly residents of Hong Kong

Tianhang Zhang[1], Chung Ling Man[1], Ka Hei Lui[1], Steven Sai Hang Ho[2,3], Linli Qu[3], Siu Wai Choi[4], Timothy Chi Yui Kwok[5], Tony Ward[6], Kin Fai Ho[1]

[1] Chinese University of Hong Kong, The Jockey Club School of Public Health and Primary Care, Hong Kong, Hong Kong; [2] Desert Research Institute, Division of Atmosphere Sciences, Reno, USA; [3] Hong Kong Premium Services and Research Laboratory, Hong Kong, Hong Kong; [4] University of Hong Kong, Department of Orthopaedics & Traumatology, School of Clinical Medicine, Hong Kong, Hong Kong; [5] The Chinese University of Hong Kong, The Jockey Club Centre for Osteoporosis, Hong Kong, Hong Kong; [6] University of Montana, School of Public and Community Health Sciences, Missoula, USA

Objective: Fine particulate matter ($PM_{2.5}$) is documented for its adverse health effects, while the health effects of coarse particulate matter (PM_{coarse}) remain less understood due to measurement difficulties and spatial variability. The study aims to investigate the respiratory health impacts of $PM_{2.5}$ and PM_{coarse} in an elderly cohort in Hong Kong, with a novel focus on personal exposure and its chemical constituents. **Materia and Methods:** A panel study was conducted in Hong Kong with 48 healthy elderly residents aged 60 or above, each followed for a period of 6 days

during the campaign. Personal particulate matter samples were collected for PM constituents (organic carbon, elemental carbon, polycyclic aromatic hydrocarbon PAHs, water-soluble ions) analysis. Lung function parameters, including forced vital capacity (FVC), forced expiratory volume in 1 s (FEV1) and peak expiratory flow (PEF), were assessed and urine was collected for analysis of oxidative stress biomarkers 8-hydroxydeoxyguanosine and Malondialdehyde (8-OHdG and MDA) after 24-hour PM exposure. A mixed-effects model evaluated PM's impact on health outcomes adjusted for multiple covariates. **Results:** SO_4^{2-} in personal exposure to $PM_{2.5}$ was inversely associated with the lung function parameter FEV1/FVC ratio. Higher personal exposure to PM_{coarse} correlated with elevated oxidative stress biomarker 8-OHdG. Personal exposure to PAHs was linked to increased levels of the oxidative stress marker MDA, with PM_{coarse} having a greater impact. Mixture analysis identified NH_4^+ and Cl^- as key contributors to 8-OHdG levels from PM_{coarse} exposure. **Conclusion:** The findings highlight the need to include PM_{coarse} and its components in health impact assessments to develop more effective public health strategies.

Acknowledgments
This study was partially supported by the Theme-based Research Scheme Project of Research Grants Council of Hong Kong SAR, China (Ref. no. T24-508/22N) and the Health and Medical Research Fund of Food and Health Bureau of Hong Kong SAR, China (Grant No. HMRF17180401).

FT02-06 | High resolution ultrafine to ultra-coarse particulate matter concentrations:Factors affecting concentrations and size distributions near roads

Tian Xia, Austin Dymont, Sang-jin Lee, Stuart Batterman

University of Michigan, Environmental Health Sciences, Ann Arbor, USA

On-road and near-road exposure to pollutants including particulate matter (PM) are of concern given the elevated concentrations in these environments, called the roadway increment, and the large and often vulnerable populations who live near roads, especially in environmental justice communities. In this study, PM levels over a wide size range (10 nm – 20 μm) were measured in southwest Detroit with high spatial and temporal resolution using laboratory grade instruments installed in the Michigan Pollution Assessment Laboratory (MPAL), a mobile sampling platform. The study area experiences the highest PM levels in the state and will be designated as a non-attainment area for $PM_{2.5}$. Here we report on field data for about 150 sampling days between 2019 and 2025.

PM concentrations and size distributions were measured by an aerodynamic particle sizer (TSI 3321, 0.5–20 μm), an optical particle sizer (TSI 3330, 0.3–10 μm), and a scanning mobility particle sizer (TSI 3910) for ultrafine particles (UFPs, 10–422 nm). High resolution maps based on repeated measurements reflect long-term concentration patterns and suggest local sources. Elevated levels of UFPs and $PM_{2.5}$ near busy surface streets and highways demonstrated traffic emissions, and elevated PM_{10} levels near construction sites and scrap processing facilities indicated fugitive dust. We characterize roadway increments using estimates of background levels, contrast diurnal and seasonal patterns of PM size distributions, and provide a statistical analysis of meteorological, geographic and emission factors using generalized additive models that show non-linear relationships for several predictors.

The use of multiple instruments on a mobile platform that capture a full-size range of PM allows high-resolution mapping and insight into PM pollution sources and exposures. We conclude by discussing implications for exposure assessment, source apportionment, and the development of controls to improve air quality and reduce exposure.

FT02-08 | Associations between volatile organic compounds (VOCs) mixture and immune inflammation in US adults from the National Health and Nutrition Examination Survey (NHANES)

Nozomi Sasaki, Mahdieh D. Yazdi

Stony Brook Medicine, Program in Public Health, Stony Brook, USA

Objectives: Volatile organic compounds (VOCs) are ubiquitous, and exposure to multiple VOCs occurs in daily life. This study assessed NHANES data to examine the mixture effect of multiple VOCs on the systemic immune-inflammation index (SII) and systemic inflammation response index (SIRI). SII and SIRI are new inflammatory markers calculated from routine complete blood counts and have been used in environmental health studies.

Material and Methods: A cross-sectional study was conducted using NHANES data from 2011-2014 (unweighted n=2,371) to assess the combined effect of multiple VOCs exposure on the immune system. A mixture model employing Weighted Quantile Sum regression was adjusted for participant's sociodemographic characteristics, behaviors, and chronic disease status. 15 VOC metabolites were included in the mixture model: N-Acetyl-S-(2-carboxyethyl)-L-cysteine (CEMA), N-Acetyl-S-(3-hydroxypropyl)-L-cysteine (3-HPMA), N-Acetyl-S-(2-carbamoylethyl)-L-cysteine (AAMA), N-Acetyl-S-(2-cyanoethyl)-L-cysteine (CYMA), N-Acetyl-S-(3,4-dihydroxybutyl)-L-cysteine (DHBMA), N-Acetyl-S-(4-hydroxy-2-buten-l-yl)-L-cysteine (MHBMA3), N-Acetyl-S-(3-hydroxypropyl-1-methyl)-L-cysteine (HPMMA), 2-Amnothiazolne-4-carboxylic acid (ATCA), N-Acetyl-S-(N-methylcarbamoyl)-L-cysteine (AMCC), Phenylglyoxylic acid (PGA), N-Acetyl-S-(2-hydroxypropyl)-L-cysteine (2-HPMA), Mandelic acid (MA), N-Acetyl-S-(benzyl)-L-cysteine (BMA), 2-Methylhippuric acid (2-MHA), and 3-Methipurc acid and 4-Methipurc acid (3-MHA + 4-MHA). SII and SIRI were calculated from participant's complete blood counts data. All VOCs metabolites, SII, and SIRI were log-transformed due to skewed distributions.

Results: The study population was 48.8% female, with a median age of 48.0 years, and 12.6% had diabetes. Preliminary results showed a weak mixture effect of VOCs on SII and SIRI, was statistically significance only with SIRI (estimated risk difference=0.05, p=0.006) and close to significant with SII (estimated risk difference=0.03, p=0.08). Significant contributors to the mixture effect were HPMMA, MHBMA3, CYMA, CEMA, and MA, and their parent compounds were crotonaldehyde, 1,3-butadiene, acrylonitrile, acrolein, and styrene.**Conclusion:** Exposure to multiple VOCs may be associated with increased systemic immune inflammation. These findings suggested that exposures to tobacco smoke, vehicle exhaust, wood-burning smoke, and chemicals in flame resistant fabric may increase inflammation response in the immune system.

FT02-09 | Assessing oxidative potential of residential outdoor $PM_{2.5}$ and its potential drivers in four Indian cities

Priya Samdarshi[1], Rajdeep Singh[1], Vinayak Sahota[1], Chaitanya B. Shivakumar[1], Mahesh P. Anand[2], Harish C. Phuleria[1]

[1] Indian Institute of Technology Bombay, Environmental Science and Engineering Department, Mumbai, India; [2] JSS Academy of Higher Education and Research, Department of Pulmonary Medicines, Mysuru, India

Multiple studies have demonstrated that inhaling $PM_{2.5}$ components triggers oxidative stress, elevating the risk of cardio-pulmonary disorders. Oxidative potential (OP), indicating PM's ability to generate reactive oxygen species, is considered a more relevant biological metric than $PM_{2.5}$ mass. This study investigates the seasonal variation in OP of fine particulate matter at urban residential outdoor locations in India using dithiothreitol (DTT) assay. As a part of the APEAL (Longitudinal Effect of **A**ir **P**ollution **E**xposure on **A**dolescents' **L**ungs), a multi-centric cohort study, 24-hourly $PM_{2.5}$ was measured outside 60 systematically selected households each in four cities in India – Mumbai, Bangalore, Mysore, and Delhi – during summer and winter seasons using low volume air samplers. Water-extracted $PM_{2.5}$ samples were subjected to a widely used acellular DTT assay and its association was examined with various PM chemical components. Out of the total, 147 samples were analysed using DTT assay. Delhi exhibited the highest OP^{DTT} activity in both summer (3.85 ± 1.79 nmol $min^{-1}m^{-3}$) and winter (3.85 ± 1.79 nmol $min^{-1}m^{-3}$), exceeding Mumbai, Bengaluru, and Mysuru by 19%, 40%, and 16% in summer, and 30%, 39%, and 42% in winter. $PM_{2.5}$ concentrations varied significantly ($p < 0.05$) across cities and seasons, but OP values remained comparable ($p>0.05$). In summer, OP correlated with EC (traffic) in Mumbai, Mn (non-exhaust emissions), and NO_3^- (secondary transformation) in Delhi, and Ca^{2+} (crustal element) in Bengaluru. In winter, correlations included Na^+ (sea salt) and EC in Mumbai, Pb (traffic) in Delhi, NO_3^- and OC1 (Biogenic) in Bengaluru, and Pb and K^+ (crustal element) in Mysuru. This study highlights seasonal variations in OPDTT levels across urban residential settings in four Indian cities. Despite varying PM mass, OP levels remained consistent within and between the seasons, underscoring PM's epidemiological significance. Further analysis of remaining samples is in progress to enhance insights.

FT03 | Exposure to metals and elements flash talks

FT03-01 | Effect Modification by Cadmium on the Association between Polycyclic Aromatic Hydrocarbons and Oxidative Stress

Dahee Han[1], Yong Min Cho[1], Mijeong Kim[1], Haneul Kim[1], Kyung-Hwa Choi[2], Woo Jin Kim[3], Young-Seoub Hong[4]

[1] Seokyeong University, Institute of Environmental Health, Seoul, South Korea; [2] Dankook University College of Medicine, Department of Preventive Medicine, Cheonan, South Korea; [3] Kangwon National University, Department of Internal Medicine and Environmental Health Center, Chuncheon, South Korea; [4] Dong-A University College of Medicine, Department of Preventive Medicine, Busan, South Korea

Objective: Both cadmium and polycyclic aromatic hydrocarbons (PAHs) are well-known contributors to oxidative stress. We investigated whether cadmium modifies the effect of PAHS on oxidative stress, and association between oxidative stress and cancer.

Materials and Methods: A total of 1,025 urine samples were collected from polluted and control areas in Korea, from June 2021 to September 2023. Participants were interviewed and self-reported about their demographic information and diagnosed cancer history. Urinary cadmium was analyzed using inductively coupled plasma-mass spectrometer. Urinary PAHs metabolites were analyzed using gas chromatography-mass spectrometry. Malondialdehyde (MDA) and 8-hydroxy-2'deoxyguanosine (8-OHdG) were analyzed using high-performance liquid chromatography and Enzyme-linked immunosorbent assay, respectively. All biomarkers were natural log-transformed. Linear regression was conducted to examine the associations among cadmium, ΣPAHs, and oxidative stress, adjusting for age, sex, income, smoking, alcohol consumption, and occupational exposure. Additionally, samples were stratified into cadmium quartiles to investigate its effect modification on the association between PAHs and oxidative stress. Student's t-test was performed to examine association between oxidative stress and cancer.

Results: Cadmium and ΣPAHs were significantly associated with oxidative stress. After stratification by cadmium quartiles, the effect of ΣPAHs on MDA increased in higher quartile subgroups (Q2-Q4), which was not significant in Q1. Furthermore, MDA in cancer patients was significantly higher than in non-patients.

Conclusion: These findings suggest that cadmium may modify the effect of PAHs on oxidative stress, particularly in individuals with higher cadmium levels. Higher MDA levels in cancer patients emphasizes the potential health impact of these combined exposures. Our results highlight the need for strategies to reduce cadmium and PAH exposures, especially in high-risk populations.

Funding: This work was supported by Korea Environmental Industry & Technology Institute (KEITI) through Core Technology Development Project for Environmental Diseases Prevention and Management Program, funded by Korea Ministry of Environment (MOE)(2480000101/RS-2021-KE001378).

FT03-02 | The maternal urinary metabolome in the relationship between trace metals exposure and preterm birth

Sarahna A. Moyd[1], Maureen Kachman[2], David E. Cantonwine[3], Thomas F. McElrath[3], John D. Meeker[4], Kelly K. Ferguson[1]

[1] National Institute of Environmental Health Sciences, Epidemiology Branch / Division of Intramural Research, Research Triangle Park, USA; [2] University of Michigan, Medical School / Metabolomics Core / Biomedical Research Core Facilities, Ann Arbor, USA; [3] Harvard Medical School, Division of Maternal-Fetal Medicine / Brigham and Women's Hospital, Boston, USA; [4] University of Michigan, Department of Environmental Health Sciences / School of Public Health, Ann Arbor, USA

Objective:
While numerous environmental exposures may increase risk of preterm birth (PTB), investigation of mechanisms that underpin these relationships has been narrow in focus. The pregnancy metabolome may illuminate novel biological processes that have a role in these relationships. In this study, we applied targeted metabolomics to investigate urinary metabolites associated with PTB as well as trace metals exposure.

Material and Methods:
In a case-control study of PTB (n=99 cases with births <37 weeks gestation and 291 unmatched controls), maternal urine samples from median 26 weeks (range: 22,29 weeks) gestation were analyzed to quantify trace metals and targeted metabolomics. We examined associations between specific gravity (SG) adjusted urinary metabolites and PTB with logistic regression models. For metabolites associated with PTB ($p<0.05$), we examined associations with SG adjusted urinary trace metals via linear regression. Models were adjusted for maternal age, pre-pregnancy body mass index, race and ethnicity, education level, and gestational age at sample collection.

Results:
Of 119 urinary metabolites, 2 were inversely and 10 were positively associated with PTB. These included both exogenous and endogenous metabolites. We identified numerous associations between trace metals and the metabolites that were associated with PTB. For example, cholic acid, a bile acid related to liver function, was increased 2.36 (95%CI: 1.40, 7.82) times in association a natural-log increase in urinary copper and 1.13 (95% CI: 1.02, 3.20) times in association with PTB.

Conclusion:
We observed associations between several exogenous and endogenous urinary metabolites and PTB. The endogenous metabolites were also associated with several urinary trace metal concentrations. These data could provide information on biological mechanisms linking trace metals exposures to PTB.

FT03-03 | Interaction effects of social vulnerability and tungsten on cognition among older adults in the United States

Laura C. Arboleda Merino, Erika Walker, Kelly Bakulski, Sung Kyun Park

University of Michigan, School of Public Health, Ann Arbor, USA

Objective: A recent exposome-wide association study found tungsten as a risk factor for lower cognitive performance. We examined how social vulnerability influences the relationship between tungsten exposure and cognition in older adults.

Materials and Methods: We analyzed data from 1,470 participants 60+ years old from the US National Health and Nutrition Examination Survey (NHANES) 1999–2002 and 2011–2014 cross-sectional cycles. Cognition was assessed using the Digit Symbol Substitution Test (DSST). A modified Social Vulnerability Index (SVI) was constructed by summing seven individual-level risk indicators: income, education, health insurance, race/ethnicity, employment, housing tenure, and food security (range: 0 [least vulnerable] to 7 [most vulnerable]). Urinary tungsten was log_2-transformed after covariate-adjusted standardization. Survey-weighted linear regression models evaluated associations between SVI, tungsten, and their interaction on DSST scores, adjusting for age, sex, BMI, cotinine (log_2-transformed), NHANES cycle, and urinary creatinine (log_2-transformed).

Results: SVI and urinary tungsten were negatively associated with DSST scores, but their interaction was positive (β = 0.37, 95% CI: -0.006, 0.74), showing a stronger negative association in less socially vulnerable individuals and a weaker association in more vulnerable ones. For individuals with the mean SVI (1.80), mean DSST score was 51.13 (95% CI: 50.28, 51.99), decreasing by -0.64 points (95% CI: -1.22, -0.06) per doubling of tungsten. Those with lower SVI (0.34, mean -1SD) and higher SVI (3.26, mean +1SD) had mean DSST scores of 59.10 (57.90, 60.31) and 43.16 (42.18, 44.14), with slopes of -1.18 (-1.95, -0.41) and -0.10 (-0.90, 0.71), respectively. Tungsten exposure is not inconsequential for vulnerable populations. Instead, their lower baseline DSST scores suggest a floor effect, where cumulative stressors may have already impaired cognition, limiting tungsten's additional measurable impact.

Conclusions: Tungsten exposure and social vulnerability are independently associated with lower cognitive function in older adults. In highly vulnerable individuals, existing stressors may mask tungsten's impact.

FT03-05 | Early childhood metals mixtures and preadolescent metabolic parameters

Carolyn Gigot[1], Haotian Wu[1], Guadalupe Estrada-Gutierrez[2], Mari Cruz Tolentino[2], Alejandra Cantoral[3], Martha Tellez-Rojo[4], Julio Landero[5], Chitra Amarasiriwardena[5], Robert Wright[5], Allison Kupsco[1]

[1] Columbia University, Environmental Health Sciences, New York City, USA; [2] National Institute of Perinatology, Mexico City, Mexico; [3] Iberoamerican University, Health Department, Mexico City, Mexico; [4] National Institute of Public Health of Mexico (INSP), Center for Research in Nutrition and Health, Mexico City, Mexico; [5] Icahn School of Medicine at Mount Sinai, Environmental Medicine, New York City, USA

Objective
Exposures to several metals and metalloids (henceforth, metals) have been linked to impaired fasting glucose and insulin resistance that can develop into type 2 diabetes, which is increasing in prevalence among children and adolescents. However, the impacts of metals mixtures on children's metabolic parameters are less well characterized. Our objectives were to examine relationships between early childhood blood metals and preadolescent fasting plasma glucose (FPG); glycated hemoglobin (HbA1c), a longer-term marker of glucose levels; and homeostatic model assessment for insulin resistance (HOMA-IR).**Materials and Methods**
We measured 11 metals (essential: cobalt, copper, manganese, selenium, zinc; nonessential: arsenic, cadmium, chromium, cesium, lead, antimony) in whole blood among children 4-6 years of age enrolled in the Mexico City Programming Research in Obesity, Growth, Environment and Social Stressors (PROGRESS) cohort. We measured children's FPG (n=418), HbA1c (n=417), and HOMA-IR (n=216) at 8-12 years. We determined associations between metals mixtures and metabolic parameters using Bayesian Kernel Machine Regression (BKMR). We evaluated robustness using generalized additive models (GAMs), linear regression, and quantile-based g-computation (qgcomp). All models were adjusted for sex, socioeconomic status, maternal education, BMI, energy intake, age, and visit date.**Results**
Cobalt (Co) was associated with lower HbA1c but higher HOMA-IR in BKMR models, GAMs, and linear regressions. In linear regressions, a doubling of Co was associated with a 2.6% decrease in HbA1c (95% confidence interval [CI] 0.20 to 4.9%) but 42% increase in HOMA-IR (95% CI 7.0 to 91%). Lead (Pb) had a U-shaped association with HOMA-IR in BKMR models and GAMs. Higher levels of the overall mixture were associated with lower HOMA-IR in BKMR and qgcomp models.**Conclusion**
Overall metals mixture associations with HOMA-IR underline the importance of considering multiple exposures. Given inconsistent findings, more work is needed to understand the effects of essential and nonessential metals on children's metabolic health.

FT03-06 | Perinatal cadmium levels are associated with shifting distributions in extracellular vesicle tissue-type origin and microRNA profiles

Maya Deyssenroth, Allison Kupsco, Tessa Bloomquist, Melanie Caria, Kathrin Schilling, Julie Herbstman

Columbia University Mailman School of Public Health, Environmental Health Sciences, New York, USA

Objective: During the prenatal period, cadmium can pass through the placental barrier to enter fetal circulation and is implicated with impaired fetal growth at birth and neurodevelopment later in childhood. Cadmium-responsive biomarkers can reveal mechanistic insight and early signals foreshadowing altered postnatal health. Here, we evaluate cord blood cadmium concentrations in association with cord blood extracellular vesicle (EV) microRNA (miRNA) profiles.

Material and Methods: Cadmium concentrations and EVs were measured in a subset (n=48) of cord blood collected from participants enrolled in the New York City-based Sibling-Hermanos cohort. Cadmium in red blood cells was measured using inductively coupled mass spectroscopy (ICP-MS) and dichotomized above and below the limit of detection (LOD). Plasma EV miRNAs were profiled using small RNA sequencing. Tissue-type contributions of detected EVs were deconvoluted using the CIBERSORT R package. Differences in EV tissue-type contributions were assessed using compositional regression analysis. Individual EV-derived miRNAs associated with cadmium concentrations were assessed using the DESeq2 R package. All models were adjusted for gestational age and infant sex.

Results: Cord blood cadmium was detected in 38% of participants, with concentrations ranging from 0.06-0.65 µg/L. A total of 625 individual miRNAs were detected in cord blood EVs. Predominant tissue-types contributing to EVs in cord blood included peripheral blood mononuclear cells, red blood cells, liver, and lung. Cadmium exposure shifted tissue-type EV contributions, including increasing the proportion of placenta-derived EVs. Sixteen miRNAs were significantly associated with cadmium exposure (FDR<0.05). Placenta (miR-379-5p, miR-411-5p, miR-431-3p, miR-431-5p) and brain (miR-9-5p) were among tissues enriched for miRNAs significantly associated with cadmium concentrations.

Conclusion: We report associations between cord blood cadmium concentrations and EV miRNA profiles, with specific tissues (e.g., placenta) contributing to the altered signals in neonatal circulation. These results highlight the potential for EVs as early biomarkers for physiologic signals in response to prenatal cadmium exposure.

FT03-08 | Citizen Science Towards Environmental Health Equity: A Community-Based Assessment of Exposure and Health Risks from Arsenic in Groundwater Sources in a Volcanic Region in the Philippines

Geminn Louis C. Apostol[1,2], Augustyne Isabelle Adapon[3], Anne Margareth Chan[3], Mariane Nicole Gamboa[3], Miranda Ellen Meneses[3], Shannen Louise So[3], Marie Christine Zamora[3]

[1] Ateneo de Manila University - School of Medicine and Public Health (ASMPH), ASMPH Center for Research and Innovation, Pasig City, Philippines; [2] Philippine Inter-agency Committee on Environmental Health (IACEH), Manila, Philippines; [3] Ateneo de Manila University - School of Medicine and Public Health (ASMPH), Pasig City, Philippines

Objective: Arsenic contamination of drinking and household water sources poses disproportionate risks to vulnerable populations with limited access to safe water infrastructure. Leveraging citizen science, this study assessed health risks from arsenic exposure through household groundwater in six high-risk municipalities surrounding Taal Volcano, Philippines, where 90% of the population depends on groundwater for drinking and household water needs, with particular attention to socioeconomic disparities in exposure patterns and health effects.
Methods: We engaged 112 participants from six high risk municipalities, resident in these areas for at least five years. Water sample collection utilized a citizen science approach, with mothers, youth, and community health workers trained in sampling techniques following the Philippine National Standards for Drinking Water 2017. Arsenic levels were determined using electrothermal atomic absorption spectrometry. Health risk assessments included standardized arsenicosis evaluation forms documenting exposure history, medical history, and physical examinations. Multiple logistic regression analyses determined associations between arsenic levels and health outcomes.
Results: Significant associations emerged between elevated arsenic exposure (>10 ppb) and dermatological manifestations, particularly hyperkeratosis ($p=0.012$) and hypopigmentation ($p=0.017$). Physical examinations revealed 46.67% of individuals with high arsenic exposure exhibited abnormal skin findings compared to only 1.49% with normal exposure levels ($p<0.0001$). Educational attainment was significantly associated with hypopigmentation ($p=0.0002$), with less educated individuals showing greater vulnerability. Poorer communities relying exclusively on groundwater faced higher exposure risks due to limited water treatment infrastructure.
Conclusion: This assessment confirms arsenic exposure disproportionately affects socioeconomically disadvantaged populations in Batangas. Risk mitigation requires equitable interventions including accessible water testing, alternative water sources, and targeted health education. The citizen science approach demonstrated valuable community participation in environmental health research, building local capacity to understand exposure risks and advocate for remediation efforts, providing a transferable model for addressing environmental health inequities in resource-limited settings.

FT03-09 | Exploring the manufacturing process, supply chain dynamics and manufacturer's perceptions on lead in aluminum cookware in Bangladesh

Sanjida Adiba[1], Mahbubur Rahman[1], Shaikh Sharif Hasan[1], Jesmin Sultana[1], Asaduzzaman Hridoy[1], Soha Rafa[1], Supta Sarker[1], Musa Baker[1], Stephen P. Luby[2], Jenna E. Forsyth[2], Peter J. Winch[3]

[1] *icddr,b, Environmental Health and WASH, Health Systems and Population Studies Division, Dhaka, Bangladesh;* [2] *Stanford University, Woods Institute for the Environment, Stanford, USA;* [3] *John Hopkins University, Bloomberg School of Public Health, Baltimore, USA*

Background: Lead toxicity is a global crisis, and one potential exposure source of lead is leaded aluminum cookware, from which lead leaches into our daily food. This study examines the pathway of lead contamination in aluminum cookware and manufacturers' perceptions.

Methods: Between September 2024 and January 2025, 12 in-depth interviews were conducted with cookware manufacturers in Dhaka, Bangladesh, using a qualitative guideline and observation checklist. Due to many manufacturers' reluctance, convenience sampling was used. Data was analyzed using the deductive thematic analysis method, focusing on raw materials, manufacturing processes, quality control methods, and manufacturers' perceptions. Raw material samples were also collected for analysis.

Results: Cookware manufacturing occurs in two types of factories; those using pre-made aluminum discs and those making discs from the raw materials to make cookware. The steps for making cookware include sorting and melting raw materials into aluminum bars, rolling and cutting the bars into discs, and molding them into cookware. The key raw materials include imported aluminum ingot, aluminum sheets, electric wires, and aluminum scraps, including recycled utensils, food cans, medicine strips, and manufacturing byproducts. While most manufacturers deny intentional lead use, contamination may occur through recycled materials, as x-ray fluorescence (XRF) analysis of more than 25 unique raw materials shows that the recycled utensils and byproducts had the highest lead concentration (above 1000 ppm). Their quality control relies on a manual inspection for the shine and strength of the cookware rather than any chemical testing. There is a lack of awareness amongst the manufacturers about the presence of lead in cookware and the harmful effects on human health.

Conclusion: This study identifies recycled materials, particularly utensils and factory byproducts, as the primary sources of lead contamination in aluminum cookware. Improved sourcing, quality checks, and manufacturing guidelines are essential to reduce lead exposure in cookware.

FT03-10 | Analysis of metabolic and inflammatory biomarkers in human subjects with exposure to tungsten.

Katherine James[1], Charlotte McVeigh[2], Jennifer Tjung[2], Kevin Josey[1], Alicia Bolt[2]

[1] University of Colorado, Anschutz Medical Campus, Aurora, USA; [2] University of New Mexico, Albuquerque, USA

Tungsten is an emerging environmental toxicant due to increased human exposure and limited knowledge of health risks. Epidemiological and *in vivo* studies have shown that tungsten exposure is associated with increased risk for cardiometabolic diseases and cancer, however there limited understanding of the underlying molecular mechanisms. Previous publications have correlated urinary tungsten concentrations with risk for diabetes and CKD outcomes. A hypothesized mechanism is adipocyte dysfunction leading to a pro-inflammatory state. *In vivo* studies conducted by our lab have shown that tungsten enhances adipogenesis in the bone marrow niche of mice. By looking at pro-inflammatory cytokines/adipokines in the serum, we aim to provide some of the first evidence linking tungsten exposure to adipocyte dysfunction that could play a role in disease pathogenesis.
Utilizing human serum samples from the San Luis Valley Diabetes Study, we conducted a multi-analyte enzyme-linked immunosorbent assays using the multi-plex Luminex platform. Human serum samples were analyzed for adipokine/pro-inflammatory cytokines as well as several disease biomarkers. The analytes analyzed included: FABP4, Insulin, Leptin, Chemerin, OPN, Adiponectin, Adipsin, Resistin, PAI-1, IL-6, IL-1β, TNFα, MCP-1, IL-8, IL-18, VCAM-1, ICAM-1, VEGF-A, TFF3, MMP-9, MMP-1, CRP, Cystatin C, β 2-Microglobulin, Calbindin D, and NGF. Simple liner regression and Pearson Correlation analyses were used to correlate urinary tungsten concentration with analyte concentration in the serum.
There was a significant positive correlation between increasing urinary tungsten concentrations with increasing levels of serum analytes MMP-1, $p = 0.0004$; OPN, $p = 0.0448$; and VCAM-1, $p = 0.0644$. Negative correlations of increasing urinary tungsten concentrations with decreasing levels of analytes FABP4, $p = 0.0565$ and Chemerin, $p = 0.0818$.
This is the first study to correlate tungsten exposure to levels of adipokines/pro-inflammatory cytokines in human serum. This is promising early data suggesting that tungsten exposure could lead to adipocyte dysfunction.

FT04 | Biological signatures of air pollution exposure: From epigenetics to the metabolome

FT04-01 | The nasal microbiome is associated with short-to-medium term air pollution and environmental exposures in adolescents

Javier Perez-Garcia[1], Anne Bozack[1], Sheryl L. Rifas-Shiman[2], Brent Coull[3], Heike Gibson[3], Joel Schwartz[4], Marie-France Hivert[2], Emily Oken[2], Diane R. Gold[4,5], Andres Cardenas[2]

[1] Stanford University School of Medicine, Epidemiology and Population Health, Palo Alto, USA; [2] Harvard Medical School and Harvard Pilgrim Health Care Institute, Division of Chronic Disease Research Across the Lifecourse, Department of Population Medicine, Boston, USA; [3] Harvard T.H. Chan School of Public Health, Biostatistics, Boston, USA; [4] Harvard T.H. Chan School of Public Health, Environmental Health, Boston, USA; [5] Brigham and Women's Hospital, Harvard Medical School, Channing Division of Network Medicine, Boston, USA

Objectives: Climate change and air pollution are risk factors for respiratory diseases through multiple mechanisms, including airway epithelial barrier disruption. We aimed to identify nasal bacterial dysbiosis in adolescents associated with air pollution and meteorological conditions.

Methods: We analyzed the nasal microbiome using 16S rRNA sequencing in 416 adolescents from the Massachusetts-based Project Viva cohort. We measured particulate matter ($PM_{2.5}$), nitrogen dioxide (NO_2), ozone (O_3), ambient temperature, humidity, and greenness at exposure periods relative to nasal sampling, ranging from the past two days to the previous year. We tested associations between environmental exposures and alpha diversity (Richness and Shannon's Index), microbial clusters, and bacterial genera through regression models adjusted for age, sex, race and ethnicity, body mass index, season, and sequencing run. We adjusted for multiple comparisons using a false discovery rate (FDR)<5% and conducted sensitivity analyses further adjusting for socioeconomic factors.

Results: Adolescents had a mean (SD) age of 13.0 (0.7) years, 52% were female, and the mean $PM_{2.5}$ exposure in the prior year was 7.2 (0.8) μg/m^3. Alpha bacterial diversity was correlated with NO_2 exposure (r^2=0.20, FDR=1.2x10^{-4}) and temperature (r^2=-0.16, FDR=2.9x10^{-3}) in the previous month. Short-term exposure (past week) to O_3, temperature, and relative humidity were associated with nasal microbiome clusters (FDR<0.05). Participants with a microbiome dominated by *Moraxella* showed lower exposure to O_3 compared to those dominated by *Propionibacterium* (OR, 95% CI: 0.73, 0.62-0.86, FDR=0.002). 13 bacterial genera were associated with short-to-medium-term exposures to $PM_{2.5}$, NO_2, and/or temperature (FDR<0.05). The most significant association was for $PM_{2.5}$ and *Dialister* abundance (log_2fold-change=0.37, FDR=4.2x10^{-4}).

Conclusions: Several environmental exposures were associated with variations in the diversity and composition of the nasal microbiome. Future research should evaluate whether microbiome changes mediate the effect of air pollution and weather conditions on respiratory health.

Funding: US NIH Grants R01ES031259, P42ES004705, R01HD034568, R24ES030894, P30-ES000002.

FT04-03 | Pregnancy as a Susceptible Period to Ambient Air Pollution Exposure on the Maternal Postpartum Metabolome

Sandra India Aldana[1], Lauren Petrick[1], Megan M. Niedzwiecki[1], Damaskini Valvi[1], Allan C. Just[2], Ivan Gutierrez-Avila[1], Itai Kloog[1], Dinesh Barupal[1], Martha M. Téllez Rojo[3], Robert O. Wright[1], Andrea A. Baccarelli[4], Haotian Wu[5], Elena Colicino[1]

[1] Icahn School of Medicine at Mount Sinai, Department of Environmental Medicine and Climate Science, NEW YORK, USA; [2] Brown University, Institute at Brown for Environment and Society, Providence, USA; [3] National Institute of Public Health, Center for Nutrition and Health Research, Cuernavaca, Mexico; [4] Harvard T.H. Chan School of Public Health, Boston, USA; [5] Columbia University, Mailman School of Public Health, Department of Environmental Health Sciences, NEW YORK, USA

Objective

Pregnancy is a potential critical window to air pollution exposure for long-term maternal metabolic effects. Yet, little is known about potential early metabolic mechanisms linking air pollution to metabolic health. This study aims to examine whether both overall pregnancy exposure and critical exposure windows to airborne particulate matter with a diameter ≤2.5μm ($PM_{2.5}$) are associated with altered metabolites and pathways in women during postpartum.

Material/Methods

We included 544 pregnant Mexican women with both serum samples collected at 1-month postpartum and ambient (overall and monthly) $PM_{2.5}$ levels during pregnancy. Serum untargeted metabolomics was assessed using liquid chromatography high-resolution mass spectrometry. $PM_{2.5}$ exposure levels were estimated at each residential address using validated spatiotemporal satellite-based models. To examine associations between (overall and monthly) pregnancy $PM_{2.5}$ exposures and metabolite levels, we used covariate-adjusted robust linear models (RLMs). Pathway enrichment analyses (Reactome and MetaboAnalyst) and effect modification by fetal sex and folic acid intake were also evaluated.

Results

Mothers were exposed to an average (SD) $PM_{2.5}$ concentration of 22.8 (2.9) μg/m^3 throughout pregnancy. Higher $PM_{2.5}$ exposure throughout pregnancy was associated with lipid alterations. We observed consistent pregnancy $PM_{2.5}$ associations with higher levels of bile acids and amino acids, as well as dysregulated glycerophospholipids, and lower levels of fatty acyls (*FDR*<0.05). Potential critical windows of susceptibility to monthly $PM_{2.5}$ levels on metabolites were observed in early to mid-pregnancy (*FDR*<0.005). Main findings were consistent by strata of fetal sex and FA intake. Metabolic pathways corresponding to positive $PM_{2.5}$-metabolite associations indicated enriched bile acid, dietary lipid, and transmembrane transport metabolism, whereas for negative $PM_{2.5}$-metabolite associations we identified altered pathways involving adipocyte differentiation or adipogenesis, incretin peptide hormone, GLP-1, PPAR-alpha, and fatty acid receptors (*FDR*<0.05).

Conclusion$PM_{2.5}$ exposures during pregnancy, especially in early gestation, altered maternal postpartum lipid and amino acid metabolism.

FT04-04 | Air pollution exposure and microRNA content of neuronal extracellular vesicles in Major Depressive Disorder patients: Results from the FREEDOM Study

Paola Monti[1], Elisa Borroni[1], Laura M. Antonangeli[1], Rachele Matsagani[1], Eva Dariol[1], Laura Dioni[1], Davide Biganzoli[1], Simona Iodice[1], Francesca Legnani[2], Angela C. Pesatori[1,4], Valentina Bollati[1], Luca Ferrari[1], Massimiliano Buoli[2,3], **Michele Carugno**[1,4]

[1] University of Milan, Department of Clinical Sciences and Community Health, Milan, Italy; [2] University of Milan, Department of Pathophysiology and Transplantation, Milan, Italy; [3] Fondazione IRCCS Ca' Granda Ospedale Maggiore Policlinico, Department of Neurosciences and Mental Health, Milan, Italy; [4] Fondazione IRCCS Ca' Granda Ospedale Maggiore Policlinico, Epidemiology Unit, Milan, Italy

Objective

To evaluate the interplay between air pollution exposure, plasmatic microRNA (miRNA) content of neuron-derived extracellular vesicles (NdEVs), and severity of major depressive disorder (MDD) within the FREEDOM study.

Materials and Methods

Two hundred MDD patients were recruited at the Policlinico Hospital (Milan, Italy) in 2020-2022 and administered questionnaires on lifestyle, personal, and clinical characteristics. MDD severity was assessed using psychiatric rating scales. Particulate matter ≤2.5 μm (PM2.5) exposure was evaluated at patients' residential addresses by averaging concentrations of the day of recruitment with levels of each preceding day up to one month before. Plasmatic NdEVs were obtained by ultracentrifugation and immunocapture, and miRNAs were quantified by RT-qPCR. Associations between PM2.5 and miRNA expression, and between miRNA expression and MDD severity were evaluated by multivariate linear and ordinal regression models.

Results

Different PM2.5 exposure windows were associated with decreased miRNA expression, such as miR-320 (β=-0.70, 95% confidence interval (CI): -1.23; -0.18) for exposures of the three weeks before recruitment, miR-451 (β=-0.56, 95%CI: -1.08; -0.04) for the week before, miR-572 (β=-0.54, 95%CI: -1.05; -0.02) for the month before. Besides, decreasing levels of miR-320 (β=0.28, CI95%: 0.02; 0.54), miR-451 (β=0.24, 95% CI: 0.02, 0.46), and miR-572 (β=0.46, CI95%: 0.15; 0.78) were associated with increased MDD severity, similarly to what observed also in previous studies. These results seem supported also by plausible biological mechanisms: e.g., miR-451 exerts an anti-inflammatory action in the brain, while miR-320 and miR-572 are involved in the response to oxidative stress and in regulating numerous processes at neuronal level.

Conclusions

Our results suggest that air pollution exposure might trigger NdEV miRNAs expression, which might worsen MDD severity. NdEV miRNAs analysis could thus pave the way for identification of minimally invasive biomarkers for MDD prognosis.

Funding

CARIPLO Foundation (grant #2019-3354), Italian Ministry of University and Research (grant #2022WNW97F)

FT04-05 | PAH Carboxylic Acids as Novel Biomarkers of Coal Combustion Exposure and Associations With Urinary Mutagenicity and Lung Cancer Risk in Xuanwei Women

Xiangtian Wang[1], Yan Lin[1], Wei Hu[2], Nathaniel Rothman[2], George Downward[3], Qing Lan[2], Roel Vermeulen[3], Junfeng(Jim) Zhang[1]

[1] Duke University, Nicholas School of Environment, Durham, USA; [2] National Cancer Institute, Division of Cancer Epidemiology and Genetics, Bethesda, USA; [3] Utrecht University, The Institute for Risk Assessment Sciences, Utrecht, Netherlands

Lung cancer risks among non-smoking women in Xuanwei County, China, have historically been high due to indoor air pollution from burning smoky coal. Burning this bituminous coal emits carcinogenic polycyclic aromatic hydrocarbons (PAHs), such as 5-methylchrysene (5-MC) and benzo[a]pyrene (BaP). Previous studies showed that 5-MC, among all measured air pollutants, had the strongest association with both lung cancer risk and urinary mutagenicity, a potential contributor to lung cancer.
This study evaluates PAH metabolites as biomarkers of coal combustion exposure and examines their associations with external PAH exposures (5-MC and BaP) and urinary mutagenicity, respectively. We analyzed urinary metabolites, including hydroxylated PAHs (OH-PAHs), PAH carboxylic acids (PAH-CAs), and amino-PAHs, in 48 women exposed to four fuel types: smoky coal, smoky coal with a chimney, smokeless coal, and electricity.
Results show that 5-MC exposure had a Spearman correlation coefficient of 0.68 with 2-phenanthrene carboxylic acid (2-PHECA) and 0.66 with urinary 1-hydroxypyrene (1-OHP). Moreover, 1-OHP was not significantly associated with urinary mutagenic potency, whereas 2-PHECA showed a significant association. An interquartile range (IQR) increase of 31.4 ng/ml in 2-PHECA was associated with a 10.9% (95% CI: 0.55%, 21.3%) increase in mutagenic potency. Additionally, 2-PHECA concentrations were higher in individuals exposed to smoky coal with a chimney compared to those exposed to smokeless coal, aligning with 5-MC and BaP exposures, while OH-PAHs did not show this pattern.
Our findings suggest that urinary 2-PHECA may serve as a coal smoke exposure biomarker more relevant to lung cancer risk than OH-PAHs.

FT04-06 | Potential Mediating Role of Lipoprotein and Gut Microbiome in the Associations of Fine Particulate Matter and Ozone with Bone Health

Wenhao Qi[1], Mengmeng Kong[1], Xia Meng[2], Zhonghan Sun[1], Zhendong Mei[1], Yanni Pu[1], Xiaofeng Zhou[1], Qichu Wang[1], Jian-Ge Qiu[3], Bing-Hua Jiang[3], Jie Shen[4], Changzheng Yuan[4], John S. Ji[5], Xiaofeng Wang[1], Haidong Kan[2], Yan Zheng[1]

[1] Fudan University, Human Phenome Institute, School of LifeScience, Shanghai, China; [2] Fudan University, School of Public Health, Shanghai, China; [3] Zhengzhou University, The Academy of Medical Science, Zhengzhou, China; [4] Zhejiang University, School of Public Health, Hangzhou, China; [5] Tsinghua University, Vanke School of Public Health, Beijing, China

Objective

This study aimed to investigate how exposure to particulate matter with aerodynamic diameter ≤2.5 µm ($PM_{2.5}$) and ozone (O_3) influences bone health in older adults. Specifically, we explored whether circulating lipoproteins and gut microbiota act as intermediary mechanisms linking air pollution to bone metabolic disorders.

Materials and Methods

We integrated data from two prospective cohorts in China: the Rugao Longevity and Aging Study (RLAS; n=2,192) and the Central China Cohort (CCC; n=597). Daily $PM_{2.5}$ and O_3 concentrations were estimated based on participants' residential addresses, using geocoded national monitoring station data and meteorological information. Circulating lipoprotein profiles were quantified via nuclear magnetic resonance (NMR), and gut microbiota compositions were characterized by bacterial metagenomic sequencing. Associations between pollutant exposure, lipoprotein measures, microbial features, and bone outcomes (osteoporosis, bone fractures) were examined using multivariate regression models adjusted for demographic, lifestyle, and comorbidity factors.

Results

Elevated O_3 exposure was consistently associated with a higher risk of bone metabolic disorders across both cohorts (RLAS: OR=1.47, 95% CI: 1.19–1.81; CCC: OR=1.22, 95% CI: 1.00–1.50). High O_3 exposure was also linked to elevated NMR-based lipoprotein risk scores (P=0.038) and decreased abundances of potentially beneficial bacterial species. $PM_{2.5}$ showed suggestive but less consistent effects in both cohorts. Mediation analyses indicated that the lipoprotein risk score explained 6.4% and *Gordonibacter pamelaeae* accounted for 1.3% of the association between O_3 and bone disease probability.

Conclusion

Our findings suggest that $PM_{2.5}$ and O_3 contribute to bone health deterioration in older adults, partially through alterations in circulating lipoprotein profiles and gut microbiota. Targeting these mechanisms may offer novel strategies for mitigating air pollution–related skeletal risks, advancing understanding of environmental metabolic bone disorders and informing targeted intervention strategies against pollution-induced bone pathology.

FT04-07 | Effects of Exposure to Ambient Air Pollution on Human Metabolome in Patients with Coronary Artery Disease

Duan Wang[1], Chang Liu[2], Yi-An Ko[3], Howard H. Chang[3,1], Zhihao Jin[1], Youran Tan[1], ViLinh Tran[4], Dean P. Jones[4], Arshed A. Quyyumi[4], Donghai Liang[1,2]

[1] Emory University, Gangarosa Department of Environmental Health, Atlanta, USA; [2] Emory University, Department of Epidemiology, Atlanta, USA; [3] Emory University, Department of Biostatistics and Bioinformatics, Atlanta, USA; [4] Emory University, Department of Medicine, Atlanta, USA

Objective:Despite the established associations between air pollution and cardiovascular diseases (CVD), the biological mechanisms underlying the toxic impact of air pollution on CVD remain largely unknown. To identify molecular signatures and pathways altered upon exposure to ambient air pollutants among individuals living with CVD, we performed metabolomics analysis on plasma samples collected in patients with coronary artery disease with varying ambient air pollutant exposure over time.

Material and Methods: In 244 participants within the Emory Cardiovascular Biobank cohort, we estimated individual residential exposures to air pollutants carbon monoxide (CO), nitrogen oxides (NOx), and fine particulate matter ($PM_{2.5}$) across 4 exposure time windows (1-day, 1-week, 1-month, and 1-year) using a spatiotemporally-resolved model and conducted high-resolution metabolomics profiling on plasma samples to characterize specific metabolites and metabolic pathways associated with exposure to ambient air pollutants. An untargeted metabolome-wide association study framework with pathway enrichment analysis and chemical annotation was employed.

Results: We identified 9, 17, and 19 pathways associated with CO, NOx, and $PM_{2.5}$ exposures, respectively, which are indicative of perturbations in lipid, inflammatory, and vitamin metabolism. Thirty-eight unique metabolites were found associated with NOx and $PM_{2.5}$ under different exposure time windows, with 15 and 31 metabolites associated with long-term NOx exposure and various $PM_{2.5}$ exposure time windows, respectively. Specifically, protoporphyrin, thyroxine, methyl beta-d-galactoside, lauroylcarnitine, retinoate, bis(2-ethylhexyl)phthalate, biliverdin, and threonine were associated with multiple air pollutants, which were involved in lipid metabolism, energy production, and vitamin metabolism.

Conclusion: We identified numerous novel metabolites and pathways perturbed by air pollution under different exposure durations. These metabolic perturbations are closely involved in lipid metabolism, inflammation, vitamin metabolism, and energy production, revealing potential differential effects of air pollution exposure on human metabolome. These results provide important insights for potential biomarker development and future investigation of molecular mechanisms for air pollution effects on CVD.

FT04-08 | Untargeted serum metabolomics and air pollution in Parkinson's disease

Dayoon Kwon[1], Kimberly C. Paul[2], Yuyuan Lin[1], Jun Wu[3], Jason Su[4], Jeff M. Bronstein[2], Beate Ritz[1,2]

[1] University of California, Los Angeles, Department of Epidemiology, Los Angeles, USA; [2] University of California, Los Angeles, Department of Neurology, Los Agneles, USA; [3] University of California, Irvine, Department of Environmental and Occupational Health, Irvine, USA; [4] University of California, Berkeley, Division of Environmental Health Sciences, Berkeley, USA

Objective: Identify metabolic disturbances associated with air pollution in Parkinson's disease (PD) using untargeted metabolomics.

Material and methods: Serum samples were collected from 616 PD patients and 271 controls who were enrolled in a population-based case-control study of PD and resided in central California, a region known for high air pollution levels. Air pollution exposure (particulate matter < 2.5 mm [$PM_{2.5}$], carbon monoxide [CO], and nitrogen dioxide [NO_2]) was estimated for years prior to serum collection based on residential addresses and three modeling approaches (chemical transport, dispersion, and land use regression). A metabolome-wide association study was conducted using serum-based untargeted metabolomics data derived from liquid chromatography with high-resolution mass spectrometry (detecting 2,716 HILIC and 2,046 C18 metabolite features). Empirical Bayes linear regression was used to identify metabolite features associated with air pollution exposure in PD patients and controls, and pathway enrichment analysis was conducted using *mummichog*. Additionally, we compared our results with 22 previous air pollution metabolomics studies to assess external validity.

Results: We identified 26 annotatable metabolites and 23 pathways associated with air pollution exposure, particularly $PM_{2.5}$ and traffic-related air pollution (measured by CO and NO_2), in PD patients and controls. Metabolic profiles we observed in controls were consistent with findings from previous studies, supporting the validity our results. PD patients' metabolome also suggested disease-specific disruptions. In PD patients, air pollution exposure was associated with inflammation-related lipid metabolism, characterized by increased leukotrienes and decreased eicosatrienoic acid and docosahexaenoic acid. Additionally, several amino acids, including alanine, aspartate, and glutamate, were negatively associated with air pollution exposure in PD patients.**Conclusion**: We identified metabolomic features and pathways associated with $PM_{2.5}$ and traffic-related pollution. Our results suggested that these exposures may also contribute to PD through affecting inflammation, oxidative stress, and mitochondrial dysfunction pathways.

FT04-09 | Sex-Specific Changes in Placental DNA Methylation Associated with $PM_{2.5}$ Exposure During Early Gestation

Emily Craig[1], Yan Lin[1], Yihui Ge[1], Carole Grenier[2], Xiangtian Wang[1], Donald Harrington[3], Richard Miller[6,7], Sally Thurston[4], Philip Hopke[3,5], Emily Barrett[3,8], Thomas O'Connor[9,6], David Rich[3,7], Junfeng(Jim) Zhang[1], Susan Murphy[1,2]

[1] Duke University, Nicholas School, Durham, USA; [2] Duke University, Department of Obstetrics and Gynecology, Durham, USA; [3] University of Rochester Medical Center, Department of Public Health Sciences, Rochester, USA; [4] University of Rochester Medical Center, Department of Biostatistics and Computational Biology, Rochester, USA; [5] Clarkson University, Center for Air and Aquatic Resources Engineering and Sciences, Potsdam, USA; [6] University of Rochester Medical Center, Department of Obstetrics and Gynecology, Rochester, USA; [7] University of Rochester Medical Center, Department of Environmental Medicine, Rochester, USA; [8] Rutgers University School of Public Health, Department of Biostatistics and Epidemiology, Rochester, USA; [9] University of Rochester, Department of Psychology, Rochester, USA; [10] University of Rochester School of Medicine and Dentistry, Department of Psychiatry, Rochester, USA; [11] University of Rochester School of Medicine and Dentistry, Department of Neuroscience, Rochester, USA; [12] University of Rochester School of Medicine and Dentistry, Department of Medicine, Rochester, USA

Objective: Ambient air pollution, including $PM_{2.5}$, is implicated in adverse pregnancy outcomes, yet mechanisms remain poorly understood. $PM_{2.5}$ exposure is associated with altered DNA methylation of growth regulatory imprinted genes in the placenta. Epigenetic reprogramming involves removal and re-establishment of DNA methylation, occurring between gestational days 14 (fertilization) and 24 (implantation). Imprinted gene methylation, established in the gametes, resists reprogramming, potentially making this window susceptible to air pollution

Material and Methods: Using estimated residential, daily concentrations of ambient $PM_{2.5}$ concentrations in the UPSIDE cohort of 262 pregnant women, we examined covariate-adjusted associations between daily PM2.5 concentrations during gestational days (GD) 1 to 28 and DNA methylation of imprinted genes using sex-specific distributed lag nonlinear models with penalized splines.

Results: Each interquartile range increase (3.34 μg/m^3) in $PM_{2.5}$ on the specified GDs was associated with the following sex-specific DNA methylation differences: In females, *H19* decreased, GD 19–28 (-11.0%; 95% CI: -21.9%, -0.20%); *NNAT* increased, GD 25–28 (12.3%; 95% CI: 1.65%, 22.9%); *SNRPN* increased, GD 0–2 (10.8%; 95% CI: 4.64%, 1.71%) and decreased, GD 6–7 (-5.36%; 95% CI: -10.4%, -0.30%). In males, *IGF2* decreased, GD 13–18 (-2.32%; 95% CI: -4.39%, -0.27%); *MEG3* decreased, GD 0–6 (-9.22%; 95% CI: -18.1%, -0.29%); *MEST* decreased, GD 0–7 (-9.29%; 95% CI: -16.5%, -2.07%) but increased GD 16–21 (5.48%; 95% CI: 0.66%, 10.3%); and *PLAGL1* increased GD 18–29 (21.8%; 95% CI: 2.47%, 41.2%).

Conclusion: Appropriate imprinted gene expression is crucial for normal growth, development and energy homeostasis. These findings highlight sex-specific epigenetic sensitivity to $PM_{2.5}$ exposure during early pregnancy, including epigenetic reprogramming. Future studies should explore associations between changes to placental gene expression and mechanisms of putative placental impairments and their impact on fetal growth restriction and metabolism.

FT04-10 | Exploring the role of material hardship on the associations between PM2.5 exposure and cord blood DNA methylation in a New York City-based pregnancy cohort

Abigail Gaylord[1], Jia Guo[2], Shuang Wang[2], Frederica Perera[1,3], Julie Herbstman[1,3], Allison Kupsco[1]

[1] Columbia University Mailman School of Public Health, Department of Environmental Health Sciences, New York, USA; [2] Columbia University Mailman School of Public Health, Department of Biostatistics, New York, USA; [3] Columbia University Mailman School of Public Health, Columbia Center for Children's Environmental Health, New York, USA

Objective

Studies of prenatal air pollutant exposure and cord blood DNA methylation typically adjust for socioeconomic status. However, individuals from different socioeconomic backgrounds may experience air pollution differently, suggesting a potential for effect modification. Still, effect modification by economic stress remains unknown. We assessed whether economic stress, represented by material hardship, modifies the associations of prenatal exposure to particulate matter (PM2.5) on cord blood DNA methylation.

Material and Methods

We leveraged data from the Mothers and Newborns Study, a New York City-based prospective birth cohort (N=346). Prenatal PM2.5 exposure was estimated using spatiotemporal models and averaged over the entire pregnancy. Material hardship was defined as the presence of self-reported concerns over inability to afford food, gas/electricity, housing, or clothing. Using linear models, we tested the associations of log2-transformed PM2.5, adjusted for hardship, on CpG M-values at 387 CpG sites associated with PM2.5 in previous studies. We then examined associations with PM2.5 stratifying by hardship status or including an interaction between PM2.5 and hardship.

Results

Material hardship was experienced by 44% of participants and was not significantly associated with PM2.5. PM2.5 was associated with 152 of the 387 probes after adjustment for material hardship. Material hardship alone was not significantly associated with methylation at these sites. We observed effect modification and significant associations of PM2.5 with decreased methylation at 35 CpG sites among participants who reported prenatal material hardship compared to the entire study sample. Gene ontology analysis suggested that these sites map to genes key to B cell development and immune cell chemotaxis.

Conclusion

Our results suggest that economic stress does not have an independent effect on cord blood DNA methylation at sites associated with PM2.5 exposure. However, economic stress may modify the effect of prenatal PM2.5 at specific CpG sites related to cellular processes crucial to in-utero development.

FT05 | Climate epidemiology in action: Tools, trends, and translational insights

FT05-01 | Toward a cohort perspective of climate epidemiology: The case of examining intergenerational inequalities in susceptibility to non-optimal temperatures in Japan

Lei Yuan[1], Yasushi Honda[2,3], Lina Madaniyazi[3], Aurelio Tobias[4], Chris F.S. Ng[1,3], Masahiro Hashizume[1,3]

[1] The University of Tokyo, Department of Global Health Policy, Graduate School of Medicine, Tokyo, Japan; [2] National Institute for Environmental Studies, Center for Climate Change Adaptation, Tsukuba, Japan; [3] Nagasaki University, School of Tropical Medicine and Global Health, Nagasaki, Japan; [4] Spanish Council for Scientific Research (CSIC), 4Institute of Environmental Assessment and Water Research (IDAEA), Barcelona, Spain

BACKGROUND: Younger generations are projected to experience more severe climate exposure impacts during their lifetimes than older generations as global warming progresses. Despite the increasing evidence of the recent temporal changes in heat-related mortality risks,there remains a lack of research exploring this association from a cohort perspective.
OBJECTIVES: Our objective was to quantify the variation in susceptibility to short-term effects of non-optimal temperature on mortality, across generations and over the life course of specific generations,using a novel age–period–cohort approach.
METHODS: An extended two-stage analytical approach was applied to a nationwide mortality dataset covering individuals born from1866 to 2019 in all 47 prefectures in Japan. Daily mortality counts observed between 1972-2019 were aggregated into 5-year birth cohorts and corresponding age groups of the decedents. For each prefecture and birth cohort, the age-dependent association between ambient temperature and daily mortality was modeled using conditional quasi-Poisson regression. Then, the prefecture-specific associations were pooled across cohorts, separately for each age group, using a repeated-measure meta-regression. To model the intergenerational changes in risks, a nonlinear, continuous term for cohort was applied in the meta-analysis.
RESULTS: The relative risk (RR) of heat-related mortality decreased across generations for elder adults(65–89 years of age), for those born in1901–1905 to those born in1926–1930 (*p-trend* = 0.004). Similarly, the cold-related mortality risk also decreased across the same cohorts (*p-trend* < 0.001). The predicted continuous trends in heat- and cold-related mortality risks exhibited a nonlinear decline across generations. An inconsistent pattern was observed for middle-aged people(40–64 years of age) born between1930 and 1960, with a slight increase in risks for cold and heat over generations.
DISCUSSION: Our findings revealed disparities in susceptibility between generations, highlighting the importance for researchers and policymakers to consider cohort differences in efforts to promote future health advancements and reduce inequalities.

FT05-02 | Refining the Suitable Condition Index to Predict Dengue Fever Transmission in Bangladesh and Sri Lanka

Jahirul Islam[1], Xin Qi[2], Wenbiao Hu[1]

[1] Queensland University of Technology, School of Public Health and Social Work, Brisbane, Australia; [2] Xi'an Jiaotong University, School of Public Health, Xian, China

Objective: The Suitable Condition Index (SCI), based on optimal temperature ranges for *Aedes* vectors, was developed by our previous research to identify and predict risk areas in six Asian-Pacific countries. However, we aimed to refine the index using data from two dengue-endemic tropical countries, Bangladesh and Sri Lanka.

Material and Methods: We calculated daily SCI based on the temperature range of 17.05-34.61℃ for *Aedes aegypti* and 15.84-31.51℃ for *Aedes albopictus* and refined it by integrating waterbody areas. The generalized linear models were used to examine the association of dengue incidence with SCI and other covariates. We further classified the incidence rate (IR) into above- and-below-median IR and conducted a binomial logistic regression (BLR) to explore the model prediction accuracy.

Results: The refined SCI was positively associated with dengue incidence for both dengue vectors in Bangladesh ($\beta_{aegypti}$ = 0.03, p <0.001; $\beta_{albopictus}$= 0.06, p <0.001) and Sri Lanka ($\beta_{aegypti}$ = 0.33, p <0.001; $\beta_{albopictus}$= 0.41, p <0.001). The BLR model demonstrated statistical significance for Bangladesh ($\beta_{aegypti}$ = 0.26, p = 0.011; $\beta_{albopictus}$= 0.48, p <0.001), but showed no significance for Sri Lanka ($\beta_{aegypti}$ = 0.62, p = 0.625; $\beta_{albopictus}$= 0.47, p = 0.756).

Compared to the previously used precipitation-weighted SCI, which achieved a crude agreement rate (CAR) of 72% for *Aedes aegypti* and 64% for *Aedes albopictus*, our refined SCI achieved a CAR of 77% for *Aedes aegypti* (sensitivity: 71%, specificity: 81%) and 75% for *Aedes albopictus* (sensitivity: 67%, specificity: 81%). These findings highlight the higher predictive accuracy of refined SCI.

Conclusion: SCI is a valuable method for predicting vector suitability, but it must be refined with local climatic and environmental factors to improve predictive power. This is particularly crucial in regions with extreme climatic conditions, where region-specific adjustments to the index may enhance accuracy.

FT05-03 | Effect of Universal Thermal Climate Index on Mortality: analysis of longitudinal data from three Health and Demographic Surveillance Sites in Ghana - CLEAR Project

Stephaney Gyaase[1], Timothy Awine[3], Cascade Tuholske[6], Alison Lee[7], Darby Jack[9], Kwaku P. Asante[12], on behalf of Climate and Health Evaluation for Adaptive Resilience (CLEAR) PROJECT

[1] Kintampo Health Research Centre, Biostatistics, Kintampo, Ghana; [2] Kintampo Health Research Centre, Biostatistics, Kintampo, Ghana; [3] Navrongo Health Research Centre, Biostatistics, Navrongo, Ghana; [4] Navrongo Health Research Centre, Biostatistics, Navrongo, Ghana; [5] Montana State University, Department of Earth Sciences, Bozeman, USA; [6] Montana State University, Department of Earth Sciences, Bozeman, USA; [7] Mount Sinai Hospita, Mount Sinai, USA; [8] Mount Sinai Hospita, Mount Sinai, USA; [9] Columbia Mailman School of Public Health, School of Public Health, Columbia, USA; [10] Columbia Mailman School of Public Health, School of Public Health, Columbia, USA; [11] Kintampo Health Research Centre, Kintampo, Ghana; [12] Kintampo Health Research Centre, Kintampo, Ghana

Objective

To assess the impact of the Universal Thermal Climate Index (UTCI) on mortality using longitudinal data from three Health and Demographic Surveillance System (HDSS) sites in Ghana.

Material and Methods

Data were collected from three HDSS in Ghana. These sites have gathered mortality data over 20+ years, covering over 511,000 individuals. We harmonized data from these HDSS to create an analytic dataset that includes date of death, age, biological sex, and community residence. We performed a case-crossover analysis using a bidirectional, asymmetric time-stratified strategy to examine associations between the Universal Thermal Climate Index (UTCI) and all-cause mortality. We also explored subgroups' associations, specifically age (<5 and >60 years) and sex.

Results

We observed an association between an increase in daily UTCI levels and mortality. The effect was however high at UTCI levels above 30. Exploring the effect among sex, the average exposure among males was a little higher compared to females (28.59 in males versus 28.56 in females). The overall effect of UTCI on mortality among sex was similar, the odds of death increased as daily UTCI increased, the increase in both sexes was higher between 25°C and 30°C. The average UTCI among children under 5 years was 28.52 (15.81 - 36.85) compared to 28.58 (16.63 - 36.80) among adults. Again, the overall effect of UTCI on mortality was similar among age groups, with the effect being high between 25 and 30, especially amongst more vulnerable groups (e.g., those aged 60 years).

Conclusion

This study shows that higher UTCI levels increase mortality in Ghana. Variations across sites indicate regional climate sensitivity, underscoring the need for localized adaptation strategies. Findings highlight thermal stress's impact on health and the importance of integrating climate resilience into public health planning.

FT05-05 | Understanding historical population exposure to extreme ambient temperature indices in Peru at the national level

Yasna Palmeiro Silva[1], Stella Hartinger[1], William Checkley[2,4], Kyle Steenland[3]

[1] Universidad Peruana Cayetano Heredia, Centro Latino Americano de Excelencia en Cambio Climático y Salud, Lima, Peru; [2] Johns Hopkins University, Division of Pulmonary and Critical Care, School of Medicine, Baltimore, USA; [3] Emory University, Rollins School of Public Health, Atlanta, USA; [4] Johns Hopkins University, Center for Global Non-Communicable Disease Research and Training, School of Medicine, Baltimore, USA

Introduction: Under global warming, a new climate, and their consequences on population health, comprehensive assessments of population exposure to extreme ambient temperatures are a foundational step for health adaptation efforts. Comprehensive analyses in low- and middle-income settings remain limited. This study aims to understand historical population exposure to extreme ambient temperature indices in Peru.

Methods: Population data in Peru were analysed together with ERA5-Land daily temperature records for 2000–2024. We selected eight temperature indices (TNx, TXx, TNn, TXn, TN90p, TX90p, TN10p, TX10p) reflecting extreme heat and cold. Indices were computed monthly and aggregated by department using population-weighted methods. Analyses were conducted in R.

Results: From 2000 to 2009 and 2015 to 2024, cold indices—particularly the monthly number of cold nights and cold days—declined by around 30% (TN10p (-32.4%) and TX10p (-30%)). In contrast, the monthly number of hot nights and hot days (TN90p (+103.6%) and TX90p (+104.8%)) increased by slightly more than 100% over the same periods. Peruvians experienced rises in both monthly minimum and maximum temperatures: the monthly minimum of daily maximum and minimum temperatures increased by about 0.7°C, while the monthly maximum of daily maximum and minimum temperatures rose by around 0.8°C between the two intervals. Spatial maps show that warming is not uniform, with densely populated urban areas and certain Amazonian regions exhibiting greater increases.

Conclusion: This study represents the first nationwide analysis in Peru, revealing that people are experiencing increases in temperature extremes with potential health consequences, including heat-related morbidity, mortality, and other associated illnesses. This information underpins a comprehensive climate risk assessment for human health and provides critical data to enhance public health adaptation, including the prioritisation of resources and targeted interventions.

Funding: FIC, NIA, NIEHS, and under the Global Environmental and Occupational Health program awards U01TW010107 and U2RTW010114.

FT05-06 | Using electronic health record data for a rapid response inquiry into the 2025 Los Angeles Wildfires

Joan A. Casey[1,5], Yuqian M. Gu[2], Lara Schwarz[4], Timothy B. Frankland[3], Lauren B. Wilner[1], Heather McBrien[5], Nina M. Flores[5], Arnab K. Dey[6], Gina S. Lee[2], Chen Chen[6], Tarik Benmarhnia[6], Sara Y. Tartof[6,7]

[1] University of Washington, School of Public Health, Seattle, USA; [2] Kaiser Permanente Southern California, Research & Evaluation, Pasadena, USA; [3] Kaiser Permanente Hawaii, Integrated Healthcare Research, Honolulu, USA; [4] University of California, Berkeley, School of Public Health, Berkeley, USA; [5] Columbia University, Mailman School of Public Health, New York, USA; [6] UC San Diego, Scripps Institution of Oceanography, San Diego, USA; [7] University of Rennes, Irset Institut de Recherche en Santé, Rennes, France

Objective: January 2025 brought devastating wildfires to Los Angeles (LA) County, California, causing poor air quality, destroying homes and businesses, and displacing thousands of people. As part of ongoing work, we partnered with our collaborators at Kaiser Permanente Southern California (KPSC) to leverage their electronic health record (EHR) data to investigate acute health impacts.

Material and Methods: Our study population consisted of 3.7 million KPSC members of all ages, of whom 305,258 lived <20km from an LA Fire burn zone, accounting for nearly 13% of all LA residents living within that distance. In January-February 2025, we identified daily acute care visits across the exposure categories in two care settings (virtual, outpatient) and five disease categories (all-cause, cardiovascular, injury, neuropsychiatric, and respiratory). We conducted a novel 2-stage interrupted time-series analyses using machine-learning algorithms to determine if and by how much the 2025 LA Fires increased outpatient acute healthcare utilization.

Results: In the week after the January 7 LA Fires' ignition, KPSC saw an excess of 3,221 (+35%) respiratory and 2,424 (+35%) cardiovascular virtual visits from members living <20 km from a burn zone or in LA County. Injury virtual and neuropsychiatric outpatient visits were >20% higher than expected on January 7 for patients living <20km from burn zones. On January 7, outpatient injury visits were 18% and neuropsychiatric visits 26% above expected among members living <20 km from a burn zone.

Conclusion: We demonstrated that EHR data can rapidly inform healthcare delivery needs and population health impacts after such events. We identified substantial increases in acute care visits, driven primarily by virtual care-seeking, the role of which will likely grow in serving patients during disruptive climate events.

FT05-07 | Leveraging HeatRisk Forecasts to Enhance Public Health Strategies: A Comprehensive Assessment of Heat-Related Impacts Using Syndromic Surveillance Information

Ambarish Vaidyanathan[1], Abigail Gates[2], Michael Staudenmaier[3]

[1] Centers for Disease Control and Prevention (CDC), National Center for Environmental Health, Atlanta, USA; [2] Centers for Disease Control and Prevention (CDC), National Syndromic Surveillance Program, Atlanta, USA; [3] NOAA, National Weather Service, Salt Lake City, USA

Heat prevention and mitigation efforts, including communication and outreach, rely on real-time monitoring of weather conditions. HeatRisk is a multi-tiered, impact-based tool providing localized seven-day heat risk forecasts. For this assessment, an evaluation of HeatRisk tool was conducted using emergency department (ED) visits from CDC's National Syndromic Surveillance Program (NSSP).

County-level data on heat-related illness (HRI) and all-cause ED visits, occurring during summer months (May–September) of 2018–2023, were extracted for 12 states, located in different regions of the Continental United States. The primary exposure measure was the population-weighted average daily, county-level HeatRisk levels obtained from weather stations. A conditional quasi-Poisson regression with strata by county, year, month, and day of the week was used to estimate the relative risks (RRs) and 95% confidence interval (CI) of ED visits associated with HeatRisk levels. Furthermore, the modelling approach controlled for seasonality, holidays, and estimated overall cumulative RRs [CI] for HeatRisk categories over a 2-day lag period.

Daily HRI rates and HeatRisk levels were positively correlated with state-specific Spearman's rank-order correlation coefficients ranging between 0.56 and 0.85 (Median=0.78). During the summer months, any day with HeatRisk levels at or above minor risk was associated with a significant increase in risk of HRI ED visits (RR: 6.301; 95% CI: 5.972–6.652); the association between HeatRisk levels and total all-cause ED visits was significant but the magnitude of risk was smaller (RR: 1.031; 95% CI: 1.029–1.033). Regional differences in RRs were observed and individual factors, such as age and sex, influenced relationships between ED visits and HeatRisk levels.

Heat exposure continues to be a public health concern, with heat events becoming longer, more frequent, and more intense. Robust tools for forecasting heat exposure combined with mechanisms for tracking and reporting health effects are essential for protecting affected populations.

FT05-08 | Assessing drought metrics in climate exposure research: Insights from India

Grace M. Betts, Anoop Jain

Boston University School of Public Health, Department of Environmental Health, Boston, USA

Drought is an important predictor of numerous public health outcomes. However, drought variables often rely on previously established cutoffs that may not be context specific or sensitive. Using precipitation data and a new variable from the Demographic and Health Surveys (DHS) Program tracking water disruptions in India, we examine the robustness of this exposure and its associations with household water disruptions across India as a case study.

From DHS household surveys, we obtained 2019-21 data reflecting whether a household experienced a disruption to their water supply in the two weeks preceding the survey. Drought for each DHS cluster was calculated using Climate Hazards Group InfraRed Precipitation with Station rainfall data and reflects deviations from historic levels. By ranking annual rainfall since 1981 for each cluster and converting rankings to percentiles, current drought status was assigned as extreme if the percentile was under 10%, moderate for 10-30%, and no drought if over 30%. Using DHS Geospatial Covariates, we controlled for elevation and 2015 aridity and population density. Odds ratios estimated the association between drought and water disruptions at the cluster level across all states with sufficient variation in drought

Adjusted model results were heterogenous with negative and positive associations found across states. For example, in Sikkim, the odds of a household water disruption was 2.03 (95% CI: 1.20, 3.64) compared to households in areas with no drought. However, in Gujarat the odds of experiencing a water disruption in areas of extreme drought was 0.67 (95% CI: 0.51, 0.88) compared to areas with no drought.

Such heterogeneity across states suggests the need to examine drought measures more carefully to better understand why this association varies so greatly within the same country, and in what contexts, if any, this measure of drought is accurate and useful for predicting public health outcomes in India.

FT05-10 | Development and application of an index to capture meteorological and air pollution impacts on health in Alberta, Canada

Niklas J. Hayden[1,2], Tianqi Zhao[1], Urvi Rai[1], Samuel A. Lowe[1], Shelby S. Yamamoto[1,2], On behalf of the ACCLIMATES collaboration

[1] University of Alberta, School of Public Health, Edmonton, Canada; [2] University at Buffalo, School of Public Health and Health Professions, Buffalo, USA

Objective: Climate change is a critical issue impacting health. Socio-environmental and other determinants can predispose older adults, pregnant people, immigrants, infants, children and those living in rural areas to increased health risks, but the collective impacts of these exposures are still an emerging area of research. The objective of this study was to develop and apply a vulnerability index, comprising the domains of exposure, sensitivity, and adaptive capacity, to capture these impacts in urban areas of Alberta, Canada.
Material and Methods: Variables were identified through a process involving literature reviews, team discussions, and pilot study findings. Exposures included meteorological variables (temperature, precipitation) and criteria air pollutants. Sensitivity incorporated social determinants such as age, sex, visible minorities, education, and income. Adaptive capacity included variables related to climate adaptation (e.g., greenspace, healthcare facility access). Polychoric Principal Component Analysis was used to combine the variables into separate dimensions. An overall index was created by combining the dimensions. Negative binomial regression was used to evaluate associations between the separate dimensions, the overall index, and ambulatory and hospital visits for mental health, injuries, cardiovascular, pregnancy, and respiratory events between 2011 and 2021.
Results: The number of principal components that explained at least 70% of the variance was two for exposure, four for adaptive capacity, and eight for sensitivity. The vulnerability index was positively associated with mental health

(Incidence rate ratio (IRR) [95% CI], 1.028 [1.023, 1.033]), cardiovascular (1.030 [1.024, 1.035]), pregnancy (1.035 [1.031, 1.040]), and respiratory (1.019 [1.016, 1.022]) events.

Conclusion: Findings indicated that the index was associated with the occurrence of health events in urban areas. Next steps will include extending this to rural areas and developing a surveillance approach incorporating the index for health events across populations in Alberta.

FT06 | Global approaches to high-resolution air quality monitoring and exposure modeling

FT06-01 | Enhancing Hyperlocal Air Pollution Exposure Models with nuanced built environmental features and undocumented emission sources extracted from Street View Images

Zhendong Yuan[1], Jules Kerckhoffs[1], Debby P.-I. Lin[2], Esra Suel[3], Hao Li[4], Li Yi[2,5], Marcia P. Jimenez[6], Peter James[2,7], Kees D. Hoogh[8,9], Gerard Hoek[1], Roel Vermeulen[1,10]

[1] Utrecht University, Institute for Risk Assessment Sciences, Utrecht, Netherlands; [2] Harvard Medical School and Harvard Pilgrim Health Care Institute, Division of Chronic Disease Research Across the Lifecourse, Department of Population Medicine, Boston, USA; [3] University College London, The Bartlett Centre for Advanced Spatial Analysis (CASA), London, UK; [4] National University of Singapore, Department of Geography, Singapore, Singapore; [5] Harvard T.H. Chan School of Public Health, Department of Environmental Health, Boston, USA; [6] Boston University School of Public Health, Department of Epidemiology, Boston, USA; [7] University of California, Davis School of Medicine, Department of Public Health Sciences, Davis, USA; [8] Swiss Tropical and Public Health Institute, Allschwil, Switzerland; [9] University of Basel, Basel, Switzerland; [10] University Medical Centre, Utrecht University, Julius Centre for Health Sciences and Primary Care, Utrecht, Netherlands

BACKGROUND

Mobile monitoring campaigns combined with Land Use Regression (LUR) models effectively capture fine spatial variations in urban air pollution. However, traditional predictor variables often fail to capture the nuances of the built environment and undocumented emission sources.

METHODS

To address this, we developed a Visual LUR framework (VLUR) that enhances LUR models by integrating nuanced customizable object-level and segmentation-level features extracted from street-view images. We used OWL-ViT (Open-World Localization Vision Transformer) to count user-defined objects - users simply input names of desired objects by natural language. We considered twenty nuanced built environmental objects such as chimneys, windows, trucks and traffic lights. The segmentation module employed Mask2Former to calculate the pixel proportion of road, trees, grass, sky, etc.

Using 5.7 million mobile air pollution measurements (2019–2020) and 0.37 million street-view images (2008–2024), we mapped nitrogen dioxide (NO_2), black carbon (BC), and ultrafine particles (UFP) across 46,664 road segments in Amsterdam, The Netherlands.

RESULTS & CONCLUSION

We found that incorporating street-view images improved the performance of classic LUR models, increasing R^2 by 0.01–0.05 and reducing mean absolute errors by 0.7%–10.3%. Our VLUR approach identified influential but previously unrecognized object predictors, such as chimneys, traffic lights, and shops. Combined with segmentation-level predictors (e.g., walls, roads, and grass), street-view images contributed 8%–18% to the total predictive power. To our knowledge, this is the first study to incorporate chimneys as predictors in LUR models, capturing residential combustion emissions that traditional LUR models often overlook.

Beyond air pollution, our approach can be easily adapted to estimate other environmental exposures like noise and heat. User only needs to list the suspected objects according to prior knowledges. Moreover, as visual datasets—such as street-view, satellite, and crowdsourced imagery—become increasingly ubiquitous, our VLUR framework offers a scalable solution for large-scale urban environmental exposure assessments.

FT06-02 | Wildfire smoke $PM_{2.5}$and mortality rate in the contiguous United States: a causal modeling study

Min Zhang[1], Edgar Castro[2], Alexandra Shtein[2], Adjani A. Peralta[2], Mahdieh D. Yazdi[3,2], Xiao Wu[4], Joel D. Schwartz[5,2], Robert O. Wright[1], Yaguang Wei[1,2]

[1] Icahn School of Medicine at Mount, Environmental Medicine, New York, USA; [2] Harvard T.H. Chan School of Public Health, Environmental Health, boston, USA; [3] Renaissance School of Medicine at Stony Brook University, Program in Public Health, Department of Family, Population, and Preventive Medicine, New York, USA; [4] Columbia University, Biostatistics, New York, USA; [5] Harvard T.H. Chan School of Public Health, Epidemiology, boston, USA

Objective: Wildfire smoke $PM_{2.5}$ has emerged as a significant environmental hazard. However, the relationships between chronic exposure to wildfire smoke $PM_{2.5}$ and mortality remain poorly understood, with causal evidence being particularly scarce. We aimed to examine the causal effect of chronic exposure to wildfire smoke $PM_{2.5}$ on mortality across the contiguous US.

Methods: The annual mortality data for 3068 US counties from 2006 to 2020 were collected, covering all-cause and cause-specific deaths (e.g., circulatory, neurological, respiratory diseases). The concentrations of wildfire smoke $PM_{2.5}$ were estimated using spatio-temporal models at a 10km × 10km resolution, which were then aggregated to the county level. We used a doubly robust method, incorporating flexible generalized propensity score estimation strategies, to estimate additive effects of annual exposure to wildfire smoke $PM_{2.5}$ on all-cause and cause-specific mortality. Transport accident mortality was used as a negative outcome control to test the key assumption of no unmeasured confounding.

Results: A 0.1 mg/m^3 increase in annual wildfire smoke $PM_{2.5}$ concentrations was associated with increased all-cause mortality rate (rate difference: 1.904, 95% CI: 1.620 to 2.188 per 100,000 persons), as well as increased mortality rates for all cause-specific outcomes, with neurological diseases being the most susceptible. This translated to approximately 5,594 attributable deaths, along with 235 to 981 deaths from specific causes annually. The exposure-response curve for all-cause mortality increased monotonically, with no evidence of a “safe” threshold. Effects were greater in communities with a higher percentage of population under 65 years old, in rural areas, and during periods of lower temperatures. There was no evidence of the association between wildfire smoke $PM_{2.5}$ and the negative outcome control.

Conclusion: Our study provided robust evidence for the chronic effect of wildfire smoke $PM_{2.5}$ on mortality, underscoring the urgent need for targeted measures to mitigate the significant and escalating burden of wildfires.

FT06-03 | A Monitoring-Based Exposure Index to Delineate Petrochemical Emission Impact Zones: Preliminary Results from a Large Industrial Complex in Taiwan

Jui Huan Lee[1,2], Chang-Chuan Chan[1]

[1] National Taiwan University, Institute of Environmental and Occupational Health Sciences, Taipei City, Taiwan; [2] Industrial Technology Research Institute, Micropollutant Analysis Department, Hsinchu, Taiwan

Objective: We introduce a wind-weighted Exposure Index (E) to quantify petrochemical emissions, examining whether conventional 5–10 km buffer zones underestimate actual impact zones, particularly affecting communities facing environmental health inequities.

Methods: Hourly pollutant data for $PM_{2.5}$ (μg/m³), NOx, SO_2, and VOCs (ppb) were collected from regular and Special Industrial Parks (SIP) monitoring stations (1999–2024) surrounding a large petrochemical complex in southwestern Taiwan. The E retains pollutant units by multiplying concentrations with dimensionless wind-speed and wind-direction weights. An “exposure event” was defined as E>0. Rigorous quality assurance and sensitivity analyses were conducted. Resulting E-based zones were compared to traditional model-driven buffers.

Results: Mailiao (~5 km east, downwind) showed consistently elevated $PM_{2.5}$-E (max=459 μg/m³; mean E excluding zeros=14.48 μg/m³; ~9.5% non-zero event frequency), indicating significant persistent exposure. Taixi (~6 km south) recorded the highest $PM_{2.5}$-E (508 μg/m³), while Taixi and Dacheng showed elevated NOx-based E (max: 474 and 21 ppb, respectively). Special Industrial Parks (SIP) stations (operational since 2015), notably Dongshi (~10 km southeast), recorded elevated benzene, toluene, and ethylene E-values, confirming extended impacts beyond conventional model boundaries.

Conclusion: This study demonstrates that high-exposure zones extend beyond traditional Environmental Impact Assessments (EIA) buffers, highlighting the value of empirical monitoring combined with meteorological weighting. Aligning with ISES-ISEE 2025’s global health equity theme, our framework emphasizes the importance of integrated monitoring and environmental equity in future regulatory and community-level impact assessments.

FT06-06 | Familial Differences in Personal $PM_{2.5}$ Exposure Explained with Spatiotemporal Exposure Apportionment

Ky T. Tanner[1], Howard Chang[2], Maggie Clark[3], Vincent Cleveland[3], Egide Kalisa[4], Kayleigh Keller[5], Christian L'Orange[1], Theoneste Ntakirutimana[6], Casey Quinn[1], Rebecca Witinok-Huber[3], Bonnie Young[3], John Volckens[1,3]

[1] Colorado State University, Department of Mechanical Engineering, Fort Collins, USA; [2] Emory University, Department of Biostatistics and Bioinformatics, Atlanta, USA; [3] Colorado State University, Department of Environmental and Radiological Health Sciences, Fort Collins, USA; [4] Western University, Department of Epidemiology and Biostatistics, London, Canada; [5] Colorado State University, Department of Statistics, Fort Collins, USA; [6] University of Rwanda, College of Medicine and Health Sciences, Kigali, Rwanda

Exposure to fine particulate matter ($PM_{2.5}$) from solid fuel combustion is a major determinant of global morbidity and mortality. However, variations in exposure remain uncertain across many high-risk populations. This work describes personal $PM_{2.5}$ exposures among household members (adult men, adult women, and children) in rural sub-Saharan Africa, where biomass fuel is the primary household energy source. We assessed personal $PM_{2.5}$ exposure using wearable monitors that combined real-time sensing, time-integrated (gravimetric filter) sampling, and continuous location-activity tracking over 48-hr periods. A total of 1,280 samples were collected from 579 different homes over a 15-month period (in total: 304 men, 495 women, and 481 children). Linear mixed models, controlling for household-level effects, suggested that children were exposed to 14% (CI: 6, 22%) more $PM_{2.5}$ than their mothers and 100% (CI: 85, 117%) more than their fathers. Analysis of spatiotemporal exposure patterns, aggregated into various microenvironments (e.g., home, school, transit, agricultural fieldwork), reveal that children bore a disproportionate exposure burden from in-home cooking activities compared with their parents. Results from this work indicate that interventions for household energy systems, in conjunction with familial lifestyle and behavior modifications, are necessary to reduce household air pollution personal exposure in rural Rwanda, especially among children.

FT06-07 | Characterization and Risk Assessment of Atmospheric polycyclic aromatic hydrocarbons using Passive air sampler in six cities of East Africa

Egide Kalisa[1], Andrew Sudmant[2], Augustine Omodieke[1], Frank Kwabe[4], Gabriel Habiyaremye[5], Yves Ujeneza[6], Matthew Adams[7]

[1] University of Western Ontario, Department of Epidemiology and Biostatistics, Schulich School of Medicine & Dentistry, London, Canada; [2] The University of Edinburgh, Edinburgh Climate Change Institute, Edinburgh, UK; [3] University of Western Ontario, Department of Epidemiology and Biostatistics, Schulich School of Medicine & Dentistry, London, Canada; [4] Institut Supérieur Pédagogique de Bukavu, Department of Geography and Environmental Management, Bukavu, Congo - Kinshasa; [5] Carleton University, Department of Sociology and Anthropology, Ottawa, Canada; [6] HumeKaneza, Environmental Education, KIGALI, Rwanda; [7] University of Toronto Mississauga, Department of Geography,Geomatics and Environment, Mississauga, Canada

Background and Aims

Particulate matter air pollution exposure is suggested to affect every cell in the human body. These particulates contain several toxic organic species, including polycyclic aromatic hydrocarbons (PAHs), which are well known for

their carcinogenic and mutagenic properties. However, few studies have characterized PAH species in Africa due to a lack of funding and expensive research equipment. In this study, polyurethane foam disk passive air samplers (PUF-PAS), which are cost-effective, simple to use, and have electricity-free operation, were deployed for the first time in six East African cities.

Material and Methods

A total of 23 PAH compounds collected using PUF-PAS over 60 days were analyzed using gas chromatography-mass spectrometry.

Results

Results showed that the total PAH concentrations were significantly high in Bukavu and Goma sites in the Democratic Republic of Congo (DRC), followed by Bujumbura in Burundi, Kigali in Rwanda, Dar es Salaam in Tanzania, and Nairobi in Kenya. PAHs of 5 and 6 rings were dominant in the Dar es Salaam site compared to all other sites. Source analysis using PAHs diagnostic ratios showed that diesel vehicles and biomass burning are significant sources of PAHs in this region. Our study demonstrates that the lung cancer risk exceeds the World Health Organization threshold across all cities and can be classified as a definite risk in 3 cities (Goma, Bujumbura, and Bukavu).

Conclusion

We demonstrate that more affordable PUF-PAS can monitor atmospheric PAHs in low-resource settings without electricity in Africa, where emissions are high, and PAH data are lacking.

FT06-09 | Patterns of fine particulate matter ($PM_{2.5}$) and black carbon (BC) pollution in Kigali City.

Chantal Umutoni[1,2], Silas Mirau[1], Paterne Gahungu[2,7], James Nimo[3,4], Jean Remy Kubwimana[1,2], Pacifique Karekezi[1,2], Barbara Mottey[5], Claudette Nyinawumuntu[2], Samson Niyizurugero[2], Pie-Celestin Hakizimana[6], Isambi Mbalawata[2], Majid Ezzati[8], Allison F. Hughes[3], Raphael E. Arku[5]

[1] The Nelson Mandela Institution of Science and Technology, School of Computational and Communication Science and Engineering, Arusha, Tanzania; [2] African Institute for Mathematical Sciences, Research and Innovation centre, Kigali city, Rwanda; [3] University of Ghana, Physics, Accra, Ghana; [4] State University of New York, Environmental & Sustainable Engineering, Albany, USA; [5] University of Massachusetts, Environmental Health Sciences, Amherst, USA; [6] Rwanda Environment Management Authority, Kigali city, Rwanda; [7] Imperial College London, Department of Mathematics, Faculty of Natural Sciences, London, UK; [8] Imperial College London, Department of Epidemiology and Biostatistics, School of Public Health, London, UK

Background: As cities in sub-Saharan Africa continue to expand and densify, it is important for them to use planning and regulation to meet the associated services and infrastructure (e.g. housing, and mobility) needs while minimizing environmental impacts like air pollution. Yet, most cities lack baseline city-wide measurement data that capture the levels and variations in relation to land use to support policy formulation and evaluations to ensure that the ongoing urban change enhances health and wellbeing of residents.

Objective: We conducted large-scale measurement of fine particulate matter ($PM_{2.5}$) and black carbon (BC) in Kigali City to examine their space-time patterns.

Methods: Between November 2022 and December 2023, we measured weekly gravimetric (filter-based) and minute-by-minute $PM_{2.5}$ concentrations at 130 monitoring stations comprising of 10 year-long and 120 week-long monitoring sites that spans a variety of land-use and source impacts. The light absorbance of the filters was used as a substitute for BC pollution.

Results: The overall annual mean $PM_{2.5}$ concentration was 33 μg m^{-3}, with site-specific means ranging from 20 μg m^{-3} at a background site to 52 μg m^{-3} at a commercial, business, and industrial (CBI) site. Individual weekly samples were 2-15 times the WHO guideline of 5 μg m^{-3}, with 42% of all sites not meeting even least stringent target of 35 μg m^{-3}. BC levels showed clear and distinct increasing gradient across land-use patterns, from background sites to low/medium and high-density residential to CBI sites. $PM_{2.5}$ levels were on average 13-17μg m^{-3} higher in the dry than wet season, did not vary by days of the week nor weekend. From 6 PM to noon, levels exceeded the national standard35 μg m^{-3}.
Conclusion: Our results will support effective and equitable planning and regulations to minimize environmental exposures in the face of the ongoing urban growth.

FT06-10 | Traffic, Air Quality, and Health Impacts Resulting from Pricing Incentives and Commuter Programs using a Regional-Scale Agent-Based Transportation System Model

Cristian Poliziani[1], Ling Jin[1], Xiaodan Xu[1], Carlos Guirado[2], Nazanin Rezaei[3], Yuhan Wang[2], James Butler[2], Zachary Needell[1], Tom Wenzel[1], C. Anna Spurlock[1], Thomas W. Kirchstetter[1,2], Haitam Laarabi[1]

[1] Lawrence Berkeley National Laboratory, Energy Analysis & Environmental Impacts Division, Energy Technologies Area, Berkeley, USA; [2] University of California, Berkeley, Berkeley, USA; [3] University of California, Santa Cruz, Santa Cruz, USA

Background: Transportation system planning strategies that shift travel patterns may reduce Traffic-related Air Pollution (TrAP), improve air quality, and benefit public health. Berkeley Lab's comprehensive, open-source, agent-based transportation model integrating atmospheric dispersion enables detailed TrAP analysis at regional scale across various spatiotemporal scenarios.
Methods: We integrated BEAM CORE (beam.lbl.gov) with InMAP (inmap.run) to map vehicle emission changes and quantify population exposure and mortality outcomes across the San Francisco Bay Area. By pairing detailed 10-minute link-level vehicle movement with mode-specific emission factors from California's 2021 EMFAC model, we evaluated four scenarios: cordon pricing (up to $6.50 during peak hours), free transit, active mode incentives ($2/mile), and 60% telecommuting (varying by job sector). Annual changes in primary $PM_{2.5}$ and precursor emissions were then chemically dispersed using InMAP to generate high-resolution air quality maps and estimate associated health impacts.
Results: Calibration and validation confirm that the framework reliably replicates observed traffic and emission patterns. Across all scenarios, reductions in privately owned VMT occurred and lowered pollutant emissions. Telecommuting demonstrated the strongest impact, cutting VMT by ~11% and reducing $PM_{2.5}$ exposures by ~0.04 μg/m³, decreasing the mortality rate by 0.19 deaths/100k. Active mode incentives reduced VMT ~7% and increased bicycle usage fivefold, producing the second highest benefit: reducing $PM_{2.5}$ by 0.02 μg/m³ and mortality by 0.10 deaths/100k. Cordon pricing boosted biking 39% and transit ridership 19% as residents within the cordon zone experienced higher benefits (-0.58 deaths/100k) than those outside the zone (-0.4 deaths/100k)—45% difference underscores how location-specific interventions create spatial variations in health benefits. Free transit produced the smallest impact of the four incentives (-0.01 deaths/100k).**Conclusion:** This integrated framework addresses how micro-scale travel dynamics drive local air quality and health outcomes. Its versatile, study area–agnostic design supports advanced analysis and extension to other regions and inclusion of additional emissions and scenarios.

FT07 | Exposure to pesticides flash talks

FT07-01 | Longitudinal Associations of Urinary Pesticide Metabolites with Depression and Anxiety Symptoms from Adolescence to Young Adulthood

Briana N. Chronister[1], Dolores Lopes-Paredes[2], Harvey Checkoway[1], Jose Suarez-Torres[2], Sheila Gahagan[3], Danilo Martinez[2], Asa Bradman[5], Suzi Hong[1], Raeanne C. Moore[4], **Jose R. Suarez-Lopez**[1]

[1] University of California San Diego, Herbert Wertheim School of Public Health and Human Longevity Science, La Jolla, USA; [2] Fundación Cimas del Ecuador, Quito, Ecuador; [3] University of California San Diego, Department of Pediatrics, La Jolla, USA; [4] University of California San Diego, Department of Psychiatry, La Jolla, USA; [5] University of California Merced, Department of Public Health, School of Social Sciences, Humanities and Arts, Merced, USA

Introduction: Although growing evidence suggests exposure to pesticides, such as organophosphates, is a risk factor for mental health disorders, longitudinal studies remain limited. We assessed longitudinal associations between urinary pesticide biomarker concentrations with depression and anxiety symptoms in adolescent to young adulthood in the ESPINA study.

Material and Methods: We included 514 (12-17 years) and 485 (17-23 years) participants who participated Jul-Oct 2016 and Jul-Sep 2022 following a 2008 baseline visit; 375 individuals participated in both periods. Organophosphates, pyrethroid, neonicotinoid, herbicide and insect repellent urinary biomarkers (n=12) were measured using mass spectrometry methods. We used the Multidimensional Anxiety Scale for Children (MASC-2) and Children's Depression Inventory 2 (CDI-2) to measure anxiety and depression symptoms in 2016. In 2022, the age-appropriate Generalized Anxiety Disorder-7 (GAD-7) and Beck's Depression Inventory-II (BDI-II) were used. Generalized estimating equations (GEE) were used to test associations of pesticide exposure with percentile ranks of depression and anxiety scores, adjusting for socio-economic, demographic and anthropometric variables.

Results: We observed positive associations between higher para-nitrophenol (PNP; β=0.92 95%CI [0.0003, 1.84]), 3,5,6-Trichloro-2-pyridinol (TCPY; β=0.40 [0.05, 0.76]), 3-phenoxybenzoic acid (3-PBA; β=1.52 [0.21, 2.82]), trans-3-(2,2-Dichlorovinyl)-2,2-dimethylcyclopropane carboxylic acid (trans-DCCA; β=1.44 [0.59, 2.30]), 2,4-Dichlorophenoxyacetic acid (2,4-D; β=0.33 [0.08, 0.58]), and 3-(diethylcarbamoyl)benzoic acid (DCBA; β=0.002 [0.001, 0.002]) concentrations with depression percentiles over time. However, a unit increase in 2-isopropyl-4-methyl-6-hydroxypyrimidine (IMPY) was associated with a 3.43 95%CI:(-5.62, -1.23) decrease in depression percentile. Anxiety percentiles were inversely associated with PNP (β=-1.07 [-2.10, -0.04]) but positively associated with DCBA (β=0.002 [0.001, 0.002]).

Conclusion: Over time, higher urinary concentrations of PNP, TCPy, 3-PBA, 2,4-D, and DCBA were associated with higher depression symptoms, while higher DCBA concentrations were associated with greater anxiety symptoms. Some inverse associations were noted. These longitudinal findings highlight potential long-term mental health impacts of pesticide exposure. Research on mechanisms and public health intervention strategies is warranted.

FT07-02 | Associations of organophosphates, pyrethroids, and herbicide urinary biomarkers with Cognitive Performance in Adolescents and Young Adults in agricultural communities in Ecuador.

Rajendra P. Parajuli[1], Kun Yang[1], Harvey Checkoway[1,2], Jose Suarez-Torres[3], Danilo Martinez[3], Jose R. Suarez-Lopez[1,3]

[1] University of California San Diego (UCSD), Herbert Wertheim School of Public Health and Human Longevity Science, San Diego, USA; [2] University of California, San Diego (UCSD), Department of Neurosciences, School of Medicine, San Diego, USA; [3] Fundación Cimas del Ecuador (CIMAS), De los Olivos, Ecuador

Objective: Organophosphates (OPs), pyrethroids, and 2,4-D are widely used agricultural pesticides. While their cognitive effects have been studied, findings remain mixed, especially in adolescents and young adults. This study examined associations between urinary pesticide metabolites in adolescence and young adulthood in relation to cognition in young adulthood in participants from an agricultural region in Ecuador.
Methods: Data from 505 participants (mean age 19.8 [SD:1.84 years]) examined during follow-up year (FUY) 14 of the ESPINA study in 2022 were analyzed. Urinary concentrations of six insecticide biomarkers (i.e., 4 organophosphates and 2 pyrethroids) and one herbicide (2,4-dichlorophenoxyacetic acid [2,4-D]) were measured. Multivariate linear regression assessed associations of biomarkers with NIH Toolbox Cognitive scores, adjusting for covariates. We also assessed whether FUY-8 (2016, mean age 14.5y [SD:1.8]) pesticide biomarkers predicted FUY-14 cognitive performance; these models further adjusted for concurrent pesticide biomarkers (FUY-14).
Results: FUY-14 3,5,6-trichloro-2-pyridinol (TCPy) was significantly associated with lower cognition fluid composite (β = -4.40 [95% CI: -7.87, -0.94]), cognition total composite (β = -3.22 [95% CI: -6.28, -0.15]), dimensional change card sort (β = -3.94 [95% CI: -7.44, -0.44]), flanker inhibitory control and attention (β = -3.18 [95% CI: -6.25, -0.11]), and picture sequence memory (β = -3.58 [95% CI: -6.64, -0.51]). 2,4-D was linked to lower list sorting working memory (β = -2.63 [95% CI: -5.21, -0.06]). No associations were observed for FUY-14 concentrations of malathion dicarboxylic acid (MDA), 2-isopropyl-4-methyl-6-hydroxypyrimidine (OXY-2), para-nitrophenol (PNP), 3-phenoxybenzoic acid (3-PBA), trans-3-(2,2-dichlorovinyl)-2,2-dimethylcyclopropane carboxylic acid (TCC) with cognitive performance in FUY-14. FUY-8 urinary biomarkers of 2,4-D, TCPy and 3-PBA were associated with lower cognitive scores in FUY-14.
Conclusions: Higher urinary concentrations of TCPy and 2,4-D, and to a lesser extent 3-PBA, were linked to lower cognitive performance in young adulthood, with associations observed for both adolescent and young adult exposures.

FT07-03 | Glyphosate Exposure and Biomarkers of Oxidative Stress and Metabolic Function in Adolescents from the CHAMACOS Study

Ana Maria Mora, Robert Gunier, Stephen Rauch, Katherine Kogut, Kim Harley, Nina Holland, Brenda Eskenazi

University of California at Berkeley, Center for Environmental Research and Community Health (CERCH), School of Public Health, Berkeley, USA

Objective: Glyphosate (GLY), the world's most widely used herbicide, and its primary degradate, aminomethylphosphonic acid (AMPA), have been shown to induce oxidative stress and disrupt metabolic function in *in vitro* and animal studies. We previously identified an association between early-life GLY exposure and liver inflammation and metabolic disease in adolescents from the CHAMACOS cohort in California's Salinas Valley. In this analysis, we further investigate metabolic health by examining adipokine levels and assess whether oxidative stress, measured by urinary 8-isoprostane, may serve as a potential mechanism.

Materials and Methods: We estimated exposure using two approaches: (1) urinary concentrations of measured GLY and AMPA at age 14, and (2) the amount of agricultural GLY use within 1 km of participants' residences from birth to age 5, based on California Pesticide Use Reporting data. At age 14, we measured concentrations of urinary 8-isoprostane and plasma adipokines, including adiponectin and leptin. We fitted multivariable linear regression models to examine the associations between these exposure measures and biomarkers of oxidative stress and metabolic function, adjusting for sex, household poverty, and food security at age 14.

Results: Higher urinary AMPA concentrations at age 14 were associated with lower adiponectin concentrations at the same age (β per 2-fold increase=-2.7; 95% CI: -4.9, -0.5; n=74). Additionally, exposure to agricultural GLY applications during early childhood (birth to age 5) was linked to higher urinary 8-isoprostane concentrations at age 14 (β per 2-fold increase=1.2; 95% CI: 0.1, 2.2; n=236). Null associations were observed between exposure measures and plasma leptin concentrations.

Conclusion: Both early-life and concurrent GLY exposure were associated with oxidative stress and alterations in metabolic function biomarkers in adolescents. These findings contribute to the growing evidence of GLY's potential metabolic health effects and provide insight into a biological mechanism that may mediate these and other long-term health outcomes.

FT07-04 | Organophosphorus Pesticides, Glycemic Dysregulation, and Platelet Dysfunction: Synergistic Cardiovascular Risks in a Population-Based Study

Erbao Han, Taimeng Chen, Guozhen Han, Huilin Lou, Yumeng Yan, Zhenxing Mao

Zhengzhou University, College of Public Health, Zhengzhou, China

Organophosphorus pesticides (OPPs), widely linked to cardiovascular diseases (CVDs) through platelet dysfunction, were investigated for their effects on platelet activity across glycemic states. In 2,250 participants stratified by glycemic status [normal glucose tolerance (NGT), impaired fasting glucose (IFG), type 2 diabetes (T2DM)], plasma OPPs levels and platelet indices—platelet count (PLT), distribution width (PDW), mean platelet volume (MPV), MPV/PLT ratio (MPVP), platelet-large cell ratio (P-LCR), and plateletcrit (PCT)—were analyzed. Using generalized linear models (GLM), quantile g-computation (QGC), and weighted quantile sum (WQS) regression, we assessed individual and mixed OPPs exposures and their Joint effect with glycemic status. Key findings revealed that in NGT individuals, single OPPs (e.g., fenthion, cadusafos) significantly reduced MPV and P-LCR ($P < 0.05$), while mixed OPPs increased PLT and PCT but decreased PDW, MPV, P-LCR, and MPVP ($P < 0.05$). IFG groups exhibited similar trends, whereas T2DM showed attenuated effects. Strikingly, combined high OPPs exposure and dysglycemia (IFG/T2DM) synergistically amplified platelet dysfunction, with pronounced reductions in MPV, P-LCR, and MPVP ($P< 0.05$), except in low-OPPs + IFG subgroups. Conversely, PLT and PCT displayed positive correlations across most combined exposure groups. These results demonstrate that OPPs disproportionately impair platelet activity in NGT and IFG populations, with elevated OPPs concentrations and glycemic dysregulation jointly exacerbating CVD risk. Our study underscores the critical interplay between metabolic health and environmental toxicants, advocating for integrated regulatory strategies targeting pesticide mixtures and cardiometabolic comorbidities.

FT07-05 | Residential proximity to agricultural pesticide use and cardiometabolic outcomes at age 18 years in the CHAMACOS study

Cheng-Yang Hu[1], Ana M. Mora[2], Robert B. Gunier[2], Stephen Rauch[2], Katherine Kogut[2], Ayca Erkin-Cakmak[3], Brenda Eskenazi[2], Maria J. Rosa[1]

[1] Icahn School of Medicine at Mount Sinai, Department of Environmental Medicine, New York, USA; [2] University of California Berkeley, Center for Environmental Research and Community Health (CERCH), School of Public Health, Berkeley, USA; [3] University of California San Francisco, Department of Pediatrics, Division of Pediatric Endocrinology, San Francisco, USA

Objective:
To assess the association of prenatal and early childhood exposure to pesticide mixtures with cardiometabolic outcomes in young adults enrolled in the Center for the Health Assessment of Mothers and Children of Salinas (CHAMACOS) study (n=519).

Materials and Methods:
We estimated exposure from agricultural pesticide use within one km of each participant's residence during the prenatal and early childhood (birth to 5-y visit) periods using California's Pesticide Use Reporting data. We selected 12 pesticides for analysis based on their extensive use in Monterey County from 1999 to 2007. Body mass index,

waist circumference, glucose, insulin resistance, triglycerides, high-density lipoprotein (HDL), and metabolic syndrome were assessed at age 18 years. Associations for individual pesticides were evaluated using linear or logistic regression models; pesticide mixture associations were assessed through weighted quantile sum regression. All models were adjusted for potential confounders and stratified by sex.

Results:

Sex-stratified individual pesticide analyses indicated associations between early childhood acephate (OR=1.7; 95% CI: 1.1, 3.0), chlorpyrifos (OR=2.0; 95% CI: 1.3, 3.4), diazinon (OR=2.1; 95% CI: 1.3, 3.5), permethrin (OR=3.2; 95% CI: 1.6, 7.1), and neonicotinoid insecticides (OR=2.6; 95% CI: 1.4, 5.1) exposure and increased odds of metabolic syndrome in males but not in females. Higher prenatal exposure to the pesticide mixture was associated with lower HDL levels in males (β=-0.4; 95% CI: -1.0, 0.3) but not in females (β=0.7; 95% CI: 0, 1.2). Higher early childhood exposure to the pesticide mixture was associated with increased odds of metabolic syndrome (OR=1.8; 95% CI: 1.1, 2.1) in males but not in females (OR=0.9; 95% CI: 0.5, 1.3).

Conclusion:

We found associations between early-life exposure to pesticide mixtures and adverse cardiometabolic outcomes in young adult males. These findings suggest that exposure timing and sex differences play important roles in the association of pesticide mixtures with cardiometabolic health.

FT07-06 | Association between pesticide mixtures and pediatric cancer mortality in the United States

Jabeen Taiba[1], Yeongjin Gwon[2,5], Richard V. Remigio[3], Jesse E. Bell[1,5]

[1] University of Nebraska Medical Center, Department of Environmental, Agriculture, Occupational and Health, Omaha, USA; [2] University of Nebraska Medical Center, Department of Biostatistics, Omaha, USA; [3] National Cancer Institute, Occupational and Environmental Epidemiology Branch, Division of Cancer Epidemiology and Genetics, Rockville, USA; [4] University of Nebraska Medical Center, Department of Environmental, Agriculture, Occupational and Health, Omaha, USA; [5] University of Nebraska, Daugherty Water for Food Global Institute, Lincoln, USA; [6] University of Nebraska, School of Natural Resources, Lincoln, USA

Objective: We evaluated the joint association between a pesticide mixture and pediatric cancer mortality across counties in the conterminous United States (US).

Materials and Methods: County-level pesticide exposures for commonly applied pesticides (n=30) were quantified using the USGS Pesticide National Synthesis Project from 1992 to 2019. , We used restricted-use mortality data from the National Center for Health Statistics from 2003-2021 to evaluate the association between pesticide exposures and pediatric cancer mortality among children under 20 years of age (n=38,941 cases). We analyzed the associations between common pesticides and pediatric cancer mortality (including sub-types) at the county level using the Generalized Weighted Quantile Sum Regression (gWQS) model. The joint association model was fitted with a Poisson distribution and used pediatric population per county as an offset term. Additionally, a repeated holdout validation approach was used to assess variance in the joint effect estimates. We adjusted for social vulnerability factors based on socioeconomic status, household characteristics, racial and ethnic backgrounds, housing type, and transportation.

Results: We observed a statistically significant positive association between exposure to a mixture of 30 pesticides and pediatric cancer mortality. Associations were found with all-cause cancers (β=0.25, CI = (0.19, 0.31)). Notable cause-specific associations were found with lymphoid hematopoietic (β=0.29, CI = (0.22, 0.36)) and mesothelial and

soft tissue (β=0.27, CI = (0.17, 0.37)) subtypes. Fungicides, such as Mancozeb and Chlorothalonil, and the herbicide Triclopyr, largely contributed to the joint association between pesticide mixture and overall cancer.
Conclusion: Our hypothesis-generating findings suggest potential evidence of the association of pesticide mixture with pediatric cancer mortality rates at the national level.

Acknowledgments: This work was funded by the Water, Climate, and Health Program at the University of Nebraska Medical Center

FT07-07 | Early life exposure to pesticides and respiratory symptoms in young adults from the CHAMACOS cohort

Robert B. Gunier[1], Madelyn L. Eippert[1], Katherine Kogut[1], Stephanie M. Holm[1], John R. Balmes[1], Kim G. Harley[1], Asa Bradman[2], Brenda Eskenazi[1]

[1] University of California, Berkeley, School of Public Health, Berkeley, USA; [2] University of California, Merced, Public Health, Merced, USA

Objective: Evaluate the relationship of prenatal and early life pesticide exposure with respiratory symptoms, use of asthma medication, and asthma diagnosis at 9 and 18 years of age among participants living in an agricultural community.
Materials and methods: Participants included mother-child dyads (n = 469) enrolled in the Center for the Health Assessment of Mothers and Children of Salinas (CHAMACOS) birth cohort study. We used questionnaire data collected at ages 9 and 18 years for respiratory symptoms, asthma medication use, and doctor diagnosed asthma. We estimated pesticide exposure during the prenatal period and from birth to age 5 years using urinary biomarkers of organophosphates (OP) and residential proximity to agricultural use of OPs, Mn-based ethylene bisdithiocarbamates (Mn-EBDC), 1,3 – dichloropropene and sulfur. We calculated rate ratios (RR) and 95% confidence intervals (CI) using negative binomial models, adjusting for covariates.
Results: We observed a higher risk (RR=3.5; 95% CI: 1.2, 10.4) of ongoing respiratory symptoms or asthma medication use at 9 and 18 years of age with each 10-fold increase in early childhood (0 - 5 years of age) urinary biomarkers of OP exposure. We also observed associations between prenatal residential proximity to agricultural use of OPs (RR=1.5; 95% CI: 1.0, 2.3) and Mn-EBDCs (RR=1.5; 95% CI: 1.0, 2.2) with asthma diagnosis at 9 years of age or earlier. We observed significant interactions by sex for estimated exposure to OP pesticides and Mn-EBDC fungicides and asthma symptoms or medication use at 18 years of age, with males having higher risk estimates than females.**Conclusion:** Prenatal and childhood pesticide exposure was related to respiratory health at 9 and 18y of age; at 18y, relationships were stronger for males. Future analyses will incorporate exposure to air pollutants and follow up at adulthood.

FT07-09 | Organophosphate Pesticide Metabolites and Chronic obstructive pulmonary disease (COPD): A Multi-Method Study on Exposure Risk

Zhen Ma, Wenqian Huo, Qian Gao, Pengcheng Yuan, Xin Li, Qingqing Cao

Zhengzhou University, Department of Occupational and Environmental Health, College of Public Health, Zhengzhou University, Zhengzhou, Henan, PR China., Zhengzhou, China

Background and Aims
There is little epidemiological evidence linking the exposure of organophosphate pesticides (OPs) to lung function in the general population. This study aims to investigate the relationship of urinary OPs metabolites with Chronic obstructive pulmonary disease (COPD).
Materials and Methods
A total of 9291 participants (including 2598 COPD patients) from the National Health and Nutrition Examination Survey 1999-2012 and 2015-2017 were included. Weighted logistic regression, combining quantile g-computation (Qg) and weighted quantile sum (WQS) regression were used to evaluate the relationship between individual or mixed exposure of OPs and COPD.
Results
Dimethyl phosphate (DMPT) (*OR* = 1.15, 95% *CI*: 1.01, 1.22), diethylthiophosphate (DEPT) (*OR* = 1.29, 95% *CI*: 1.21, 1.38), dimethyldithiophosphate (DMDTP) (*OR* = 1.25, 95% *CI*: 1.20, 1.29) and diethyldithiophosphate (DEDTP) (*OR* = 1.54, 95% *CI*: 1.40, 1.70) were significantly associated with the increased risk of COPD. The Qg results indicated that the mixed OPs exposure significantly elevated the risk of COPD (*OR* = 1.58, 95% *CI*: 1.47, 1.70, $P < 0.001$), with DMPT, DEPT, DMDTP, DEDTP having relatively large weights. In the WQS model, co-exposure to OP mixtures was significantly associated with an increased risk of COPD. (*OR*: 1.8; 95% *CI*: 1.67, 1.94).
Conclusion
This study reveals that OPs exposure increases COPD risk through mixture effects, highlighting the significant impact of OPs and emphasizing the need to prioritize regulation of high-risk components such as DMPT, DEPT, DMDTP and DEDTP.

FT08 | Air pollution and global mortality burden: Insights across borders, pollutants, and policies

FT08-01 | Long-term exposure to outdoor ultrafine particles and black carbon and mortality from neurodegenerative diseases in Canada's two largest cities.

Emmanuelle Batisse[1], Marshall Lloyd[1], Christel Renoux[1], Arman Ganji[2], Junshi Xu[2], Marianne Hatzopoulou[2], Jill Baumgartner[1], Scott Weichenthal[1]

[1] McGill University, Epidemiology, Biostatistics and Occupational health, Montreal, Canada; [2] University of Toronto, Toronto, Canada

Background: In urban areas, transportation and industrial activities emit large quantities of air pollutants such as ultrafine particles (UFP, <0.1 um) and black carbon (BC). Due to their smaller size, UFP can reach vital organs including the central nervous system. Emerging evidence suggests that long-term exposures to UFP may increase the risk of mortality from neurodegenerative diseases, but studies are limited and none accounted for UFP size.

Objective: We evaluated associations between long-term exposures to outdoor UFP and BC and mortality from neurodegenerative diseases among members of the Canadian Census Health and Environment Cohorts living in Montreal and Toronto.

Methods: We followed 2.1 million adults between 2001 and 2019. Information on vital status and cause of death were from the Canadian Vital Statistics Database and high-resolution exposure data for UFP number concentrations, UFP size, and BC mass concentrations were assigned to residential locations using three-year moving averages, based on annual postal code entries in income tax records. We used Cox proportional hazards models to estimate associations between outdoor UFP/BC concentrations and mortality from dementia, Parkinson's disease and amyotrophic lateral sclerosis (ALS), adjusting for sociodemographic confounders and co-pollutants. In future analyses, we will use inverse-probability weighting to estimate the controlled direct effect of UFP/BC under the hypothetical scenario where competing events are eliminated.

Results: We includedover19 million person-years and 20,000 deaths. UFP were associated with increased neurodegenerative mortality (HR, 95% CI per 10,000 particles/cm³ UFP: 1.112, 1.074–1.152; per 500 ng/m³ BC: 0.993, 0.977–1.008). Further, UFP were linked to dementia mortality with stronger associations observed after controlling for UFP size (1.149, 1.107-1.192). BC was associated with mortality from Parkinson's disease (1.051, 1.002-1.103) and ALS (1.028, 0.947-1.116).

Conclusions: Our findings suggest that UFP and BC could be modifiable risk factors to target in efforts to reduce neurodegenerative mortality.

FT08-02 | Source-Specific $PM_{2.5}$ and Mortality in the Health and Retirement Study

Meredith Pedde[1], Jennifer Weuve[2], Kenneth Langa[3,4], Jennifer D'Souza[1], Adam Szpiro[7], Jessica Faul[3], Carlos Mendes de Leon[8], Joel Kaufman[9,10], Richard Hirth[12,13], Sara Adar[1]

[1] University of Michigan, Department of Epidemiology, Ann Arbor, USA; [2] Boston University, Department of Epidemiology, Boston, USA; [3] University of Michigan, Institute for Social Research, Ann Arbor, USA; [4] University of Michigan, Medical School, Ann Arbor, USA; [5] University of Michigan, Institute for Healthcare Policy and Innovation, Ann Arbor, USA; [6] US Veterans Affairs, Center for Clinical Management Research, Ann Arbor, USA; [7] University of Washington, Department of Biostatistics, Seattle, USA; [8] Georgetown University, Department of Global Health, Washington DC, USA; [9] University of Washington, Department of Epidemiology, Seattle, USA; [10] University of Washington, Department of Environmental and Occupational Health Sciences, Seattle, USA; [11] University of Washington, Department of Medicine, Seattle, USA; [12] University of Michigan, Department of Health Management and Policy, Ann Arbor, USA; [13] University of Michigan, Department of Internal Medicine, Ann Arbor, USA

OBJECTIVES: Existing research has found that both short- and long-term exposures to fine particulate matter ($PM_{2.5}$) are associated with increased overall mortality. There is less known, however, about the importance of $PM_{2.5}$ from specific emissions sources in these associations. We examined the association between source-specific long-term $PM_{2.5}$ exposure and increased mortality in older adults.

MATERIAL AND METHODS: We used biennial survey data from 1998 through 2016 for participants in the Health and Retirement Study (HRS), a nationally representative, population-based cohort study in the United States. We estimated time-varying 1-year average exposures to $PM_{2.5}$ from nine emissions source sectors at participants' residential addresses over their follow-up period. To estimate the associations with mortality, we used a Cox proportional hazards model, adjusted for individual- and area-level covariates, co-pollutants, and survey weights.

RESULTS: Among 32,550 participants, there were 10,219 deaths during the follow-up period. A one-interquartile range (IQR) increase in 1-year average $PM_{2.5}$ from agriculture was associated with an 8% increase in mortality (HR 1.08, 95% CI: 1.00-1.17). One-year average $PM_{2.5}$ from traffic, energy production, wildfires, and windblown dust was not associated with mortality, while $PM_{2.5}$ from coal production for industry and other industry emissions was associated with decreased mortality (industry coal: HR 0.97, 95% CI: 0.92-1.01; industry other: HR 0.95, 95% CI: 0.92-0.98).

CONCLUSION: Higher levels of $PM_{2.5}$ from agriculture, but not other source sectors, were associated with higher mortality rates in this large cohort study of older adults in the United States. These results suggest that considering sources may be beneficial when developing $PM_{2.5}$-lowering strategies, though further confirmatory research is needed.

FT08-04 | Global Mortality Attributable to Air Pollution: Findings from Global Burden of Disease 2023

Pallavi Pant[1], Aaron Cohen[1,2], Katrin Burkart[2], Nadim Hashmeh[2], Sandra Spearman[2], Elizabeth Marsh[2], Michael Brauer[2]

[1] Health Effects Institute, Boston, USA; [2] Institute for Health Metrics and Evaluation, Seattle, USA; [3] Boston University, School of Public Health, Boston, USA

Using the Global Burden of Disease (GBD) methodology, the mortality burden associated with exposure to air pollution was estimated for 1990 to 2023.
Mortality linked to air pollution was estimated based on the GBD methodology for more than 190 countries around the world. Population-weighted exposure to ambient $PM_{2.5}$ was estimated by combining ground monitoring data from 21,888 stations across 168 countries or territories with satellite-derived $PM_{2.5}$ datasets. For the first time, impacts of exposure to $PM_{2.5}$ on dementia were also included. Data from more than 200 epidemiological studies was used to develop concentration-response functions for ambient and household $PM_{2.5}$ while data from 3 cohorts was used to estimate the mortality linked with ozone. Strength of evidence for each risk-outcome pair was determined using the novel star rating system which considers both the reported magnitude of risk and consistency of findings between studies; one and five stars indicate the least and highest likelihood of adverse outcome respectively.
In 2023, exposure to ambient $PM_{2.5}$, household air pollution and ambient ozone contribute to 4.9 million (95% UI: 3.88-6.04), 2.77 (2.08-3.73), and 0.47 million (0.11-0.79) million deaths respectively. Between 2000 and 2023, the death rate linked to exposure to household air pollution due to cooking with solid fuels declined by 53%, while deaths due ambient $PM_{2.5}$ and ozone increased by 23% and 36% respectively. In addition to changes in exposure, changes in attributable mortality were impacted by changes in demographic structure of the population and health status. Strength of evidence for $PM_{2.5}$ varied across outcomes: COPD received 4 stars while stroke, lower respiratory infections, Type 2 diabetes and ischemic heart disease received 3 stars; dementia, received 2.
Air pollution is a leading risk factor for mortality. Exposure to particulate matter, including ambient and household $PM_{2.5}$, accounted for most deaths in 2023.

FT08-06 | Assessing the nonlinearity of PM2.5-related mortality associations across 309 cities

Devon Nenon, Antonio Gasparrini, Pierre Masselot

London School of Hygiene and Tropical Medicine, Environment & Health Modelling (EHM) Lab, Department of Public Health Environments and Society, London, UK

Objective: It has been suggested that the association between PM2.5 and mortality could be supralinear, especially at low-levels. The shape of the exposure-response relationship between PM2.5 and mortality, and how it varies between cities, can be difficult to characterize in many settings due to lack of statistical power. In this study, we aimed to characterize this shape and its heterogeneity using a large multilocation dataset and novel constrained methods that improve the precision of the analysis.
Material and methods: We estimated the exposure-response relationship between PM2.5 and daily mortality in 309 cities from 23 countries. In the first stage, we estimated city-specific exposure-response relationships through quasi-

Poisson regression with shape-constrained spline functions of PM2.5. Coefficients representing this association were then pooled into a second-stage multivariate meta-analysis model. We calculated the numbers and fractions of excess deaths attributable to PM2.5 exposure above 15 μg/m^3, the World Health Organization (WHO) 24-hour guideline limit. We also assessed the sensitivity of these results to including PM2.5 as linear or unconstrained spline functions.
Results: The average daily concentration of PM2.5 across all cities was 10.5 μg/m^3, with 27% of days exceeding WHO guideline. We observed a supralinear shape in the pooled exposure-response relationship with marked heterogeneity in both shape and magnitude between cities. We found that short-term exposure to PM2.5 above 15 μg/m^3 was associated with 2168 [95% confidence interval (CI): 339-3957] excess deaths per year. Using a linear exposure-response relationship instead, these figures would be 1995 [95% CI: -454-4437].
Conclusion: The results of this analysis are consistent with other studies suggesting a supralinear shape in the exposure-response relationship between PM2.5 and mortality. Exposure-response curves based on shape-constrained splines can improve statistical power in both pooled and location-specific analyses.
This work is supported by UKRI (MR/X029476/1) and done in collaboration with the MCC Collaborative Research Network.

FT08-07 | Urban-rural Differences in Long-term Effects of $PM_{2.5}$ on Mortality: Different Composition or Different Population?

Garam Byun[1], Yimeng Song[1], Nicole C. Deziel[2], Michelle L. Bell[1]

[1] Yale University, School of the Environment, New Haven, USA; [2] Yale University, School of Public Health, New Haven, USA

Objective: While previous studies have suggested that the health effects of $PM_{2.5}$ may vary between urban and rural areas, findings have been inconsistent. This study examines differences in mortality effects of long-term $PM_{2.5}$ exposure among U.S. older adults, considering variations in $PM_{2.5}$ composition and population characteristics.
Material and Methods: We analyzed a cohort of 73 million Medicare beneficiaries aged 65 and older in the contiguous U.S. (2000–2016). Annual $PM_{2.5}$ exposure was estimated by residential ZIP code. Given the lack of a standard urban-rural metric, urban-rural classifications were determined using five different indicators: population size, population density, Rural-Urban Commuting Area code, Census urban area population, and Census urban area coverage. Cox-equivalent Poisson regressions were used to estimate the effect of $PM_{2.5}$ on all-cause mortality in urban and rural areas. Additionally, the effects of 10 $PM_{2.5}$ components were assessed, and stratified analyses were conducted for individual and community-level demographic and financial characteristics.
Results: Preliminary findings indicated that average $PM_{2.5}$ levels were lower in rural than urban areas (9.2±3.2 μg/m^3 vs. 10.8±3.1 μg/m^3, using population density-based urban-rural classification). Across all urban-rural classification indicators, effects of $PM_{2.5}$ (per 10 μg/m^3) were higher in rural (relative risk [RR]: 1.086; 95% confidence interval [CI]: 1.082, 1.089, using population density) than in urban areas (RR: 1.035; 95% CI: 1.033, 1.038). Additionally, rural areas consistently exhibited higher effect estimates across different $PM_{2.5}$ components and population characteristics, including age, sex, Medicaid eligibility, and community-level variables.
Conclusion: Our findings suggest that the magnitude of mortality effects of $PM_{2.5}$ was higher in rural versus urban areas. Given that most previous research has focused on urban settings, the overall health impact of $PM_{2.5}$ may have been underestimated. Variations in $PM_{2.5}$ composition or population characteristics did not fully explain urban-rural differences in $PM_{2.5}$ effects, warranting further research.

Funding source: National Institutes of Health

FT08-08 | Trend of Fine Particulate Matter and Its Related Health Risks in China from 2013 to 2023

Fengchao Liang[1], Qian Li[1], Jiahao Duan[1], Huaiyue Xu[2]

[1] Southern University of Science and Technology, School of Public Health and Emergency Management, Shenzhen, China; [2] Wuhan University, School of Public Health, Wuhan, Germany

Background: Fine particulate matter ($PM_{2.5}$) pollution poses a significant threat to public health in China. Accurate predictions of $PM_{2.5}$ exposure and estimates of its health risks are important for disease prevention.
Methods: We developed a 1-km resolution $PM_{2.5}$ estimation machine learning model using satellite-based aerosol optical depth and predictor variables which were associated with the generation or transmission of $PM_{2.5}$, such as meteorological factors. A meta-analysis based on the evidence of different health outcomes caused by short-term $PM_{2.5}$ exposure was conducted to quantify the exposure-response associations, including all-cause mortality, cardiovascular diseases, and respiratory diseases. The Air Quality Health Index for $PM_{2.5}$ ($AQHI_{PM2.5}$) was developed to quantify health risks in different regions and time periods
Findings: The model established had high accuracy in estimating daily average $PM_{2.5}$ at 1-km resolution from 2013 to 2023. We observed a decrease in concentrations across China and various subregions from 2013 to 2019 and then a slightly increase after 2020. The meta-analysis found that each 10 μg/m³ increase in short-term $PM_{2.5}$ exposure was associated with excess risks of all-cause mortality (0.62, 95% CI: 0.49, 0.75), cardiovascular hospitalization (0.80, 95% CI: 0.49, 1.12), and respiratory hospitalization (1.10, 95% CI: 0.72, 1.49) among Chinese population. The result of $AQHI_{PM2.5}$ shows that Northern and Central China experience a longer high-risk periods than other area. The burden of respiratory hospitalization caused by high daily $PM_{2.5}$ concentration was more serious than other outcomes, with high-risk days covering a larger area.
Interpretation: This study highlights the persistent health risks posed by $PM_{2.5}$ in China, despite overall improvements in air quality. The integration of high-resolution $PM_{2.5}$ estimates with health risk assessments provides a robust framework for targeted air quality management and public health interventions.

FT08-09 | Evaluating time-varying and nonlinear short-term effects of PM2.5 on mortality: evidence from a 12-year study in Tuscany (Italy)

Chiara Marzi[1], Daniela Nuvolone[2], Michela Baccini[1]

[1] University of Florence, Department of Statistics, Computer Science, Applications "G. Parenti", Florence, Italy; [2] Regional Health Agency of Tuscany, Unit of Epidemiology, Florence, Italy

Objective

This study investigates the short-term effects of fine particulate matter with an aerodynamic diameter of less than 2.5 micrometers (PM2.5) on natural mortality over 12 years in an urbanized area of Tuscany, focusing on time-varying and nonlinear dose-response relationships.

Material and Methods

We analyzed daily mortality data for all natural causes from 2008 to 2019 for Florence and seven neighboring municipalities (597740 residents in 2019). Tuscany Regional Environmental Protection Agency provided daily PM2.5 concentrations, while Tuscany Regional Hydrological Service and Laboratory of Monitoring and Environmental Modelling for Sustainable Development acquired meteorological data. We handled missing data by multiple imputation by chained equations (MICE), using CART models to capture nonlinearities and interactions. We analyzed each imputed dataset separately and combined results using a multivariate version of Rubin's rule. We estimated the short-term effects of PM2.5 on natural mortality by applying Poisson regression models: (1) a time-invariant linear model, (2) a time-varying linear model with a combination of a linear coefficient and a natural regression spline on calendar days as the coefficient for PM2.5, and (3) nonlinear models using regression splines on PM2.5 concentrations. We adjusted all models for long-term trends, seasonality, day of the week, holidays, temperature, and relative humidity.

Results

Over the whole period, we found no significant association between PM2.5 and natural mortality (0.55% increase per 7.5 μg/m^3 PM2.5; 90% CI: -0.10, 1.21). However, time-varying analyses revealed notable fluctuations, with a peak effect in 2015 (1.57%; 90% CI: 0.44, 2.72). Nonlinear models suggested slightly stronger associations at higher PM2.5 concentrations (>50μg/m^3), but the 2015 peak occurred despite declining PM2.5 levels.

Conclusion

Our findings highlight the dynamic nature of PM2.5's impact on mortality, potentially influenced by changes in pollutant composition and contextual factors, such as temperature, alongside nonlinear dose-response effects.

FT08-10 | Estimating under-five mortality attributable to fine particulate matter brought by wind-blown dust in 100 low- and middle-income countries in 2000–2017

Ruohan Wang, Hong Lu, Ning Kang, Mingkun Tong, Tao Xue

Peking University, School of Public Health, Beijing, China

Objective

Wind-blown dust (dust $PM_{2.5}$) is a major contributor to fine particulate matter ($PM_{2.5}$) in low- and middle-income countries (LMICs), yet its impact on under-five mortality (U5M) remains underexplored. In particular, due to the lack of an exposure-response function (ERF) focusing on dust $PM_{2.5}$, the relevant burden is evaluated based on pre-established ERFs for total $PM_{2.5}$ mass. Our study aimed to evaluate the association between long-term dust $PM_{2.5}$ exposure and U5M, estimating the attributable mortality burden in LMICs.

Material and Methods

Using high-resolution $PM_{2.5}$ mass-maps, well-validated dust ratio data, and 125 Demographic and Health Surveys, we applied a fixed-effects Cox model to examine the association between life-course dust $PM_{2.5}$ exposure and the survival status of 1,411,851 children from 53 LMICs. A nonlinear ERF was developed by integrating the marginal effects of within-strata exposure variation. We then extrapolated the function to estimate the U5M burden attributable to dust $PM_{2.5}$ in 100 LMICs and compared the attributable fraction with those from two pre-established functions for total $PM_{2.5}$ mass.

Results

Each 10-μg/m^3 increment in dust $PM_{2.5}$ exposure raised U5M risk by 7.13% (95% confidence interval [CI]: 4.54-9.78%). The ERF showed no threshold effect at low concentrations and a steeper slope at higher levels. Based on this ERF, we estimated that dust $PM_{2.5}$ contributed to 1.74 million (95% CI: 0.24-3.00), 1.30 million (95% CI: 0.13-2.28), and 1.07 million (95% CI: 0.17-1.81) premature deaths in children under five in 2000, 2010, and 2017 across 100 LMICs, accounting for 19.5% (95% CI: 2.7-33.4%), 19.4% (95% CI: 1.9-33.8%), and 21.3% (95% CI: 3.3-35.6%) of the total U5Ms, respectively. These estimates were higher than those from total $PM_{2.5}$ mass ERFs in most countries.

Conclusion

Long-term dust $PM_{2.5}$ exposure significantly contributes to the U5M burden. Early warnings for dust $PM_{2.5}$ can efficiently protect child health.

FT09 | The air we regulate: Health effects of policy and behavioral interventions on pollution

FT09-01 | Room for improvement: National Air Quality Standards worldwide are far from WHO recommendations

Karla Cervantes-Martínez[1], Meltem Kutlar-Joss[2,3], Sigme Duman[2,3], Kerolyn Shairsingh[1], Pierpaolo Mudu[1], Sophie Gumy[1]

[1] World Health Organization, Environment Climate Change and Health Department, Geneva, Switzerland; [2] Swiss Tropical and Public Health Institute, Allschwil, Switzerland; [3] University of Basel, Basel, Switzerland

Background. WHO has set the Air Quality Guidelines (AQG) since 1987. The suggested levels (and interim targets [IT]) can be used as evidence-informed reference to guide decision-makers in setting legally binding standards for air quality management and public health protection. The aim of this work was to compile information on National Air Quality Standards (NAQS) setting worldwide and analyze its compliance and progress with a view to approaching WHO recommendations.
Methods. We searched and extracted information on short and long-term NAQS through a: 1) questionnaire shared with WHO regional offices, 2) review websites of national ministries and related websites, 3) approach with local experts/researchers in the field. 40% of the records were validated by peer review. Preference was given to the latest first-hand information (i.e., decrees, laws, norms, etc.) else, secondary sources were consulted. For NAQS compliance analysis, long-term PM2.5 standards were compared to annual population-weighted PM2.5 concentrations, derived from a Data Integration Model for Air Quality (DIMAQ).
Results. To date, 142 out of 251 countries around the world have set NAQS for at least one criteria pollutant. 94 of them have established an annual PM2.5 NAQS, only 2 (2%) are below the AQG of 5 µg/m3, while 42 (45%) are below the IT4 of 10 µg/m3, evidencing a commitment to drive policy changes. Nevertheless, based on national population-weighted concentrations, only 65% of countries comply with their own annual PM2.5 NAQS. From 6.7 million deaths attributed to air pollution worldwide, at least 10% occur in countries that have not set NAQS.
Conclusion. The setting of and compliance with NAQS should lead to global public health enhancement, even if their levels are higher than those recommended in WHO AQG. There is an urgent need to enforce NAQS where they already exist and promote its establishment where they are lacking.

FT09-03 | Cardiovascular benefits of reducing household air pollution: Results from a 26-month clean fuel trial in Bangladesh

Muhammad Ashique Haider Chowdhury[1,2], Shyfuddin Ahmed[8,2], Mohammad Hasan Shahriar[3,7], Shirmin Bintay Kader[2], Saira Tasmin[3], Evana Akhter[4], Md. Ahsanul Haq[4], Mahbub Eunus[7], Golam Sarwar[7], Bilkis Ara Begum[5], Faruque Parvez[6], Rubhana Raqib[4], Mohammad Yunus[9], Habibul Ahsan[3,1]

[1] University of Chicago, Public Health Sciences, Chicago, USA; [2] icddrb, Non-Communicable Diseases, Nutrition Research Division (NRD), Dhaka, Bangladesh; [3] University of Chicago, Institute of Population and Precision Health, Chicago, USA; [4] icddrb, Nutritional Biochemistry, Dhaka, Bangladesh; [5] Dhaka Atomic Energy center, Atomic Energy Commission, Dhaka, Bangladesh; [6] Columbia University, Mailman School of Public Health, New York, USA; [7] Uchicago Research Bangladesh, Dhaka, Bangladesh; [8] National Cancer Institute, Infections and Immunoepidemiology Branch, Maryland, USA; [9] icddrb, Maternal and Child Health Division, Dhaka, Bangladesh

Objective: Cardiovascular health benefits achievable through long-term reduction of personal exposure to air pollutants are less studied. We examined the changes in carotid artery wall thickness in response to reduced PM2.5 & black carbon (BC) exposure from exclusive liquid petroleum gas (LPG) used for cooking.

Materials and Methods: We conducted a clean fuel trial (NCT02824237) among 400 rural Bangladeshi women using biomass fuel. LPG stoves were installed in 200 households with 26 months of uninterrupted refill. Personal exposure to PM2.5 and black carbon (BC) was measured before and after the intervention. Carotid intima-media thickness (cIMT) was assessed using a SonoSite MicroMax ultrasound. We used multiple linear regression for the exposure-response analyses and mixed effect models to evaluate the intervention effect.

Findings: Exclusive LPG use reduced median 24-hour PM2.5 from 125.1 to 66.5 μg/m^3 (47%, p=<0.001) and BC from 6.4 to 4.2 μg/m^3 (35%, p=<0.001). Carotid wall thickness increased in both groups, as expected, but less in the intervention group (12.1 vs 15.4 μm, p=0.63). Exposure-response analyses showed detrimental effects of PM2.5 & BC on cIMT, though the PM2.5's effect was not significant. BC exposure increased cIMT by 4.27 μm (95%CI: 0.54, 7.9). Mixed effect models showed the intervention group had lower average cIMT compared to comparison group both in PM2.5 (-10.7 μm) and BC (-12.2 μm) models, but differences were not statistically significant. A significant time-PM2.5 interaction suggested PM2.5's effect on cIMT increases over time. Most variability in cIMT was within individuals (59.2%) with minimal clustering by village (2.6%).

Interpretations: Black carbon exposure increases carotid wall thickness. Intervention group experienced less increase in carotid wall thickness, but it was not statistically significant. Longer follow up with a larger population may yield significant results.

FT09-04 | Triggering of ST-elevation myocardial infarction by source-specific particle number concentrations: Before and after Tier 3 vehicle standard policy implementation

Fariha Hoque Rimu[1], Philip K. Hopke[1,2], Catherine Yount[1], Sally W. Thurston[4], Daniel Croft[5], Mark J. Utell[5], Frederick S. Ling[6], David Chalupa[3], David Q. Rich[1,3]

[1] University of Rochester Medical Center, Public Health Sciences, Rochester, USA; [2] Clarkson University, Institute for a Sustainable Environment, Potsdam, USA; [3] University of Rochester Medical Center, Environmental Medicine, Rochester, USA; [4] University of Rochester Medical Center, Biostatistics and Computational Biology, Rochester, USA; [5] University of Rochester Medical Center, Division of Pulmonary and Critical Care, Department of Medicine, Rochester, USA; [6] University of Rochester Medical Center, Division of Cardiology, Department of Medicine, Rochester, USA

Objective: To investigate the association between source-specific ultrafine particle (UFP) concentrations and the rate of ST-elevation myocardial infarction (STEMI) before and after the implementation of the Tier 3 vehicle emissions program (2014–2016 vs. 2017–2019).

Methods: Using a time-stratified case-crossover design, conditional logistic regression, and source-specific particle number concentrations (PNCs) estimated via positive matrix factorization and STEMI patients treated at the University of Rochester Medical Center, we estimated the rate of STEMI associated with increased source-specific PNC concentrations at multiple lag times within 120 hours. Models included residual PNC, temperature, and relative humidity at the same lag time and an interaction term between the period and PNC-source.

Results: In the 2014-2016 period, interquartile range (IQR) increases in traffic1-PNC (spark ignition vehicle emissions) were associated with an increased rate of STEMI (lag hour 0: OR = 1.18, 95% CI = 1.06-1.32; lag hours 0-119: OR = 1.27, 95% CI: 1.00- 1.60). However, in the 2017-2019 period, there was no association with traffic1-PNC concentrations in the previous hour (OR = 0.96, 95% CI: 0.82-1.11), while for lag hours 0-119 the increased rate of STEMI associated with traffic1-PNC concentrations remained (OR = 1.19; 95% CI: 0.93-1.51). There was a similar pattern for traffic2-PNC (diesel/aged traffic particles). In contrast, we observed an inverse association with O_3-rich-PNC concentrations in the previous 1 hour in 2014-2016 (OR = 0.84, 95% CI: 0.71-0.99), but not in 2017-2019. There were similar protective effects/patterns for aged SIA-PNC and fresh SIA-PNC concentrations.

Discussion: Our findings suggest that the associations observed with traffic-related sources during the 2014-2016 period weakened in the 2017-2019 period, potentially due to the reduced toxicity of these source-specific UFPs. However, the observed inverse association with O_3-rich-PNC in 2014-2016 is consistent with previous findings of inverse associations with ambient O_3 concentrations and requires further examination and explanation.

FT09-05 | The Role of Media in Raising Awareness about Air Quality Issues

Gifty Agyekum[1], Dina Adjei-Boadi[2]

[1] Maptech Systems, Research, Accra, Ghana; [2] University of Ghana, Department of Geography, Accra, Ghana

The rapid urbanization in Ghana has exacerbated air quality issues, posing significant health risks, particularly to vulnerable populations such as children. The media plays a crucial role in raising awareness and informing the public about the dangers of air pollution. This paper explores how newspapers, television, radio, and digital platforms in Ghana contribute to public awareness about air quality and solutions that advocate for cleaner air. It examines the strategies by media outlets to disseminate information, including investigative journalism, public service announcements, and collaborative campaigns with non-governmental organizations (NGOs). Through detailed case studies, the paper highlights successful media initiatives that have brought air quality issues to the forefront of public discourse. For instance, investigative reports in leading newspapers have uncovered sources of pollution and held industries accountable, while television and radio programs have educated the public on the health impacts of poor air quality. Social media has also emerged as a powerful tool, enabling real-time information sharing of some sources of air pollution and community engagement. However, challenges such as media censorship, funding constraints, and limited access to scientific data are identified as barriers to effective advocacy. The paper concludes with recommendations for enhancing the media's role in environmental health advocacy. These include training programs for journalists, increased collaboration between media and environmental scientists, and leveraging digital platforms to reach a wider audience. By strengthening these strategies, the media can continue to play a vital role in promoting cleaner air and protecting public health in Ghana.

FT09-06 | Will the air pollution health and economic co-benefits of UK Net Zero policy help justify future action?

Sean Beevers[1], Nosha Assareh[1], Andrew Beddows[1], Tuan Vu[1], Gregor Stewart[1], Mike Holland[2], Daniela Fecht[1], Bethan Davies[1], Dimitris Evangelopoulos[1], Dylan Wood[1], Christian Brand[3], Heather Walton[1], David Dajnak[1]

[1] Imperial, Environmental Research Group, School of Public Health, London, UK; [2] EMRC, Reading, UK; [3] University of Oxford, Oxford, UK

Background: The UK's Net Zero (NZ) targets are hugely challenging to achieve and with large associated costs. We have investigated whether the air pollution health and active travel co-benefits help justify NZ policies economically.

Method: We used European emissions, UK emissions and air pollution models to compare 2030/40, with and without NZ policies, and 2050, providing UK NO_X, NO_2, PM_{10}, $PM_{2.5}$ and O_3 exposure outdoors and $PM_{2.5}$ and NO_2 indoors. Lifetable analysis was used to quantify mortality and 13 morbidity impacts, including for stroke, ischemic heart disease (IHD), asthma in children and adults, dementia, and active travel. Economic analysis used UK Government methods and included capital and operating costs, GHG impacts, mortality and morbidity effects.

Results: NZ means more electric vehicles, increased active travel, more low carbon building heating, and reduced NO_2 and $PM_{2.5}$ concentrations. By 2040 the buildings sector was three times more effective at reducing $PM_{2.5}$ than road transport. To reduce inequalities, the NZ building transition was tailored toward those most in need, with air pollution exposure inequalities less pronounced by 2040. By 2050, air quality health benefits for the NZ buildings and transport sectors were £21.3 billion and £9.1 billion, respectively. The NZ building sector is difficult to justify economically. By adding air pollution health benefits, building-sector time to break-even improved by between 3 and 6 years. Removing gas cooking at home, for NZ, reduced $PM_{2.5}$ and NO_2 exposure by up to 3.6 mg m^{-3} and 21 mg m^{-3}, respectively, greater reductions than outdoor air pollution.
Conclusions: The switch to low carbon building heating, such as heat pumps, is challenging to justify economically and the health co-benefits difficult to assess methodologically. These challenges must be addressed as evidence presented here suggests that they will be important in justifying NZ policy in the coming decades.

FT09-07 | Potential health impacts of current and historical air contaminants in an industrial community in southern Illinois

Motria Caudill

Agency for Toxic Substances and Disease Registry, Region 5, Chicago, USA

Objective: In 2019, a federal elected official from Illinois asked the Agency for Toxic Substances and Disease Registry (ATSDR) to investigate whether the Veolia North America-Trade Waste Incineration (Veolia) facility was releasing contaminants into outdoor air and potentially harming people's health in the town of Sauget and Cahokia Heights.
Material and Methods: ATSDR evaluated concentrations of metals and volatile organic compounds (VOCs) in air in samples collected by the U.S. Environmental Protection Agency (EPA) between June 2021 and March 2022 on properties adjacent to Veolia. ATSDR also reviewed historical metals and VOC air data collected by EPA from 1990 to 1992 and sulfur dioxide (SO_2) data reported by the Illinois Environmental Protection Agency (IEPA) between 1986 and 2002 at a site about ½-mile downwind of Veolia and near industries with significant releases of metals at the time. The author followed ATSDR's public health assessment guidance, using a screening analysis and in-depth toxicological evaluation to determine the public health implications of exposure to air contaminants.
Results: ATSDR determined that the health of people in Sauget and Cahokia Heights could have potentially been harmed by breathing lead, VOCs, and SO_2 released into the air from 1990 to 1992 by industrial facilities in Sauget. People living in the area today are unlikely to be harmed by breathing metals at concentrations found in the air based on data from 2021 and 2022. ATSDR did not have adequate air monitoring data to determine whether people are currently being harmed by breathing VOCs in the air.
Conclusion: ATSDR recommended additional community-based air monitoring to better understand potential health effects of VOCs released by various local industries. ATSDR also offered residents recommendations on how to limit exposures to common air pollutants, as well as lead and mercury in local soils, housing, and waterways.

FT09-08 | Health benefits of source-specific air pollution reductions following emission regulations in New York City, 2005-2019

Masha Pitiranggon, Sarah Johnson, Rebecca Goldberg, Ariel Spira-Cohen, Kazuhiko Ito

New York City Department of Health and Mental Hygiene, Bureau of Environmental Surveillance and Policy, New York City, USA

Objective: We estimated the air quality and health benefits attributable to reductions in different source sectors.

Material and Methods: Air pollution, mortality, and morbidity data were obtained from US EPA's Air Quality System, NYC Department of Health and Mental Hygiene Vital Statistics, and New York Statewide Planning and Research Cooperative System, respectively. Changes between 2005-2019 (2008-2019 for nickel) were evaluated for effects of policy (e.g., 2015 residual oil boiler phaseout) and market trends (e.g., declining use of coal for power). Time-series models were developed to estimate short-term associations of NO_2 and $PM_{2.5}$ constituents with mortality, asthma, and cardiovascular outcomes. Particulate nickel and sulfate were analyzed as indicators of residual oil boilers and coal-fired power plants, respectively, and NO_2 as an indicator of traffic and building boilers. The health benefits of source-specific pollution reductionswere calculated using EPA's BenMAP.

Results: Sulfate fell 3 μg/m^3 (77%) with avoided excess morbidity and mortality estimated at 6% attributable fraction (AF) for both asthma ED visits and hospitalizations, 1% for both CVD ED visits and hospitalizations, and 2% for mortality. Nickel fell 0.005 μg/m^3 (86%) with avoided outcomes estimated at 3% AF for both asthma ED visits and hospitalizations, 1% for both CVD ED visits and hospitalizations, and 2% for mortality. NO_2 fell 15ppb (35%) with avoided outcomes estimated at 8% and 6% AF for asthma ED visits and hospitalizations, respectively, 0.5% and 1% for CVD ED visits and hospitalizations, respectively, and 0.5% for mortality.

Conclusion: Our findings suggest that substantial health benefits resulted from the declining use of coal-fired power plants and residual oil boilers, supporting continued reductions in fossil fuel use. NO_2 reductions exhibited comparable or greater health benefits than $PM_{2.5}$ constituent reductions, despite a smaller percentage reduction, underscoring the potential benefits of further NO_x mitigation, such as vehicle and building electrification.

FT09-09 | Air Quality Health Benefits of Transportation Solutions in Indore, India

Ananya Roy[1], Andrea Orfanoz-Cheuquelaf[5], Veronica Southerland[1], Abhinand Krishnashanka[1], Kaushik Hazarika[3], Saurabh Porwal[4], Ritesh Kumar[3], Sanjar Ali[3], Azra Khan[3], Vandana Tyagi[3], Prakash Doraiswami[3], Suvarna Tikle[5], Tammy Thompson[1], Idir Bourarar[2], Rajesh Kumar[5]

[1] Environmental Defense Fund, New York, USA; [2] Max Planck Institute for Meteorology, Hamburg, Germany; [3] World Resources Institute, New Delhi, India; [4] Vital Strategies, New York, USA; [5] National Center for Atmospheric Research, Boulder, USA

Objective:
Transportation is a major source of air pollution and climate emissions in Indian cities. This study evaluates the climate, air quality and health benefits of the proposed Clean Air Catalyst (CAC) solution in Indore, India. The intervention, developed through stakeholder consultations, targets city transportation emissions through six strategies, including expanding public transport, promoting low emission vehicles, restricting heavy-duty commercial vehicles, parking regulations, reducing road dust, and enforcing vehicle testing requirements.
Methods: $PM_{2.5}$ and NO_2 impacts for 2019 were modeled using WRF-Chem, based on a local fine-scale emission inventory. Air pollution-attributable mortality was estimated using census population data, epidemiologically derived concentration-response functions, and state-level disease rates.
Results: Transportation accounted fornearly half of the CO emissions and 78% of the black carbon emissions in the city. Indore population-weighted annual averages for $PM_{2.5}$ and NO_2 are 44 µg/m³ and 24 µg/m³ in 2019, respectively, with city emissions contributing 35% and 85%. The intervention is projected to cut 49% of city transportation CO emissions, reduce NO_2 by 12 µg/m³ (49%) and $PM_{2.5}$ by 2 µg/m³ (4%). In Indore 4,257 deaths/ year and 495 new cases of childhood asthma were attributable to $PM_{2.5}$ and NO_2 respectively, with 1,178 deaths linked to city emissions. The intervention would prevent 124 deaths (a 10% reduction in locally driven $PM_{2.5}$ mortality) and 405 fewer (81% of total) NO_2 attributable new cases of childhood asthma annually.
Conclusion:
The proposed intervention has the potential to improve air quality and avoid health risks in Indore. Local transportation interventions would have sizable NO_2 asthma benefits. City level interventions need to be supported with regional air quality strategies to further address severe particulate pollution health burdens in the city.

FT09-10 | NO_2 WHO guidelines threshold exceedances during pregnancy and risk of childhood asthma, rhinitis, and eczema.

Alan Ricardo Patlan Hernandez[1], Taos Benoussaïd[2,5], Christine Monfort[1], Etienne Audureau[3,5], Isabelle Coll[2,5], Adriana Coman[2,5], Ralph Epaud[4,5], Sophie Lanone[5], Charline Warembourg[1], Cécile Chevrier[1], Marine Savouré[6], Bénédicte Jacquemin[1]

[1] Univ Rennes, Inserm, EHESP, Irset (Institut de recherche en santé, environnement et travail) - UMR_S 1085, Rennes, France; [2] CNRS, Université Paris Cité, Laboratoire Interuniversitaire des Systèmes Atmosphériques (LISA), Paris, France; [3] Assistance Publique-Hôpitaux de Paris AP-HP, Hôpital Henri Mondor, Unité de Recherche Clinique (URC Mondor), Créteil, France; [4] Centre Hospitalier Intercommunal de Créteil, Centre des Maladies Respiratoires Rares (RESPIRARE), Service de Pédiatrie Générale, Créteil, France; [5] Univ Paris Est Créteil, INSERM, IMRB, Créteil, France; [6] Institute for Global Health, Universitat Pompeu Fabra, CIBER Epidemiología y Salud Pública (CIBERESP)., Department of Experimental and Health Sciences, Barcelona, Spain

Background

While air pollution levels have decreased, exceedances of the 2021 WHO Air Quality Guidelines remain frequent in many regions. Prenatal NO_2 exposure has been linked to childhood respiratory and allergic diseases, but no studies have examined threshold exceedance effects on these diseases. We aimed to evaluate the effects of such exceedances on asthma, rhinitis, and eczema prevalence.

Methods

We included 1299 children from the 6-year follow-up of the Pélagie mother-child cohort in Brittany, France. Asthma, rhinitis, and eczema were defined using validated questionnaires. Exposure was assessed at the residential address during pregnancy using 1) the number of days NO_2 levels exceeded the 2021 WHO threshold (25µg/m^3) and 2) the cumulative exceedance. We performed logistic regressions per increase of one interquartile range (IQR) adjusting on child's age and sex, maternal pregnancy smoking and education level, and area-level socioeconomic status, further stratifying by urbanization degree.

Results

Among included mothers, mean age was 30 years, 23% smoke during pregnancy, and 64% lived in rural areas. Median number of exceedance days was 42 (IQR=35); 68 in urban, and 36 in rural areas. Median cumulative exceedance was 246 µg/m^3 (IQR=290); 486 µg/m^3 in urban, and 184 µg/m^3 in rural areas. Asthma, rhinitis, and eczema prevalence were 12%, 20%, and 23% respectively. Non-stratified analyses shown increased odds of eczema associated with both the number of exceedance days 1.13 (CI:1.01–1.25), and the cumulative exceedance 1.07 (1.01–1.13). In urban areas, both indicators were statistically associated with eczema, but not in rural areas. No associations were found for asthma and rhinitis, with or without stratification.

Conclusion

The number of exceedance days and the cumulative exceedance were associated with eczema, reinforcing the need to comply with WHO guidelines. Further analyses, including using EU regulatory thresholds for 2030 are planned.

FT10 | Built environment flash talks

FT10-01 | Lessons learned from parcel datasets: an exploration of effect modification of building structures on extreme heat-related mortality

Futu Chen[1], Andre Comandon[2], Lu Zhang[1], Erika Garcia[1]

[1] University of Southern California, Department of Population and Public Health Sciences, Keck School of Medicine, Los Angeles, USA; [2] University of Southern California, METRANS Transportation Consortium, Sol Price School of Public Policy, Los Angeles, USA

Objective: The built environment is crucial to climate vulnerability, yet studies on how it modifies extreme heat effects are inconsistent, and use of fine spatial resolution datasets has been limited. We implemented a data-driven approach to understand how parcel-level data could be used to examine effect heterogeneity of extreme heat-related mortality.

Material and Methods: We conducted a time-stratified case-crossover study of 318,194 all-cause deaths in Los Angeles County from January 1, 2014–December 31, 2019. Daily maximum temperature, relative humidity, and mean particulate matter <2.5μm ($PM_{2.5}$) were assigned to decedents based on residential census tract. We used the within-census tract 90th, 95th, 97th, and 99th percentiles of daily maximum temperatures from 2014–2019 to define census tract-specific extreme temperatures. Geocoded residential addresses were matched to the nearest 2016 residential parcel within 20 meters. We used K-prototype to generate groups of building structures with parcel housing type, census tract median home value, and rental price. Conditional logistic regression was used with a product term to evaluate effect modification by parcel-level building variables and building structure groups. Sensitivity analyses were conducted among decedents aged >55 or >65 years and census tracts with low variability in home values and rental prices.

Results: Extreme heat was associated with elevated mortality. Three groups of building structures were identified: Lower-value multifamily, 20.8%; Higher-value single-family, 18.1%; and lower-value single-family, 61.2%. There was some evidence of lower heat-mortality risk among the lower-value single-family group compared with the higher-value single-family group. No effect modification observed for other parcel variables. Sensitivity analyses produced similar findings.

Conclusion: Parcel datasets provide detailed building location and attribute information; however, the data alone or combined with census tract values did not capture effect heterogeneity. To realize the high potential of parcel datasets, future research should focus on quality assurance and supplementation.

(Funding: NIH/NIEHS/NIMHD grants #P30ES07048-27, #P50MD015705)

FT10-02 | Understanding the Interpersonal Variability in Human Exhaled Droplet Ability

Yifan Li, Mengjie Duan, Weiqi Guo, Li Liu

Tsinghua University, Department of Building Science, 北京市, China

Objective: Respiratory infections, especially those caused by emerging viruses like SARS-CoV-2, have become a major global health concern. Superspreaders significantly accelerate outbreaks, particularly in high-density environments. However, understanding the mechanisms behind superspreading remains insufficient. This study aims to explore the release characteristics of exhaled respiratory infectious particles, their impact on respiratory exposure doses, and the role of dynamic interpersonal microenvironment changes in individual transmission capacity.
Materials and Methods: A decoupling analysis method was used to separate viral load and particle exhalation behavior. A total of 44 healthy volunteers were recruited, and food-grade fluorescent agent was used as a tracer for real exhaled particles. The particle airway dosage measurement platform, based on real human CT images, was employed to quantitatively measure the respiratory exposure doses of susceptible individuals inhaling exhaled particles. Additionally, the study evaluated the effect of interpersonal relative positioning on the stability of individual transmission capacity.
Results: The results showed that the positive exposure proportion in the respiratory model caused by subjects was 13.6%.Additionally, under horizontal conditions, the calculated difference in respiratory exposure doses was 42 times. Individual factors, such as respiratory morphology, lung function, and the distance and angle of interaction with susceptible individuals, were identified as dominant factors influencing transmission efficiency. The study also revealed that dynamic microenvironment changes significantly impact superspreading phenomena.
Conclusion: This study, combining experiments and numerical simulations, provides crucial data on particle transmission and deposition patterns in the respiratory tract. It offers scientific evidence for particle pollution control in indoor environments and highlights the importance of precise control strategies for infectious particles. The findings contribute to a deeper understanding of particle exposure characteristics and provide essential data for health risk assessments in complex environments.

FT10-03 | Exposure to Per- And Polyfluoroalkyl Substances (PFAS) in North Carolina Homes: Results from the Indoor PFAS Assessment (IPA) Campaign

Naomi Chang[1,4], Clara Eichler[1], Elaine Cohen Hubal[2], Jason Surratt[1,3], Glenn Morrison[1], Barbara Turpin[1]

[1] University of North Carolina at Chapel Hill, Department of Environmental Sciences and Engineering, Chapel Hill, USA; [2] US Environmental Protection Agency, Office of Research and Development, Research Triangle Park, USA; [3] University of North Carolina at Chapel Hill, Department of Chemistry, Chapel Hill, USA; [4] Colorado State University, Department of Systems Engineering, Fort Collins, USA

Objective: Indoor environments may be an important contributor to population exposure for per- and polyfluoroalkyl substances (PFAS). However, exposure assessments that consider indoor pathways are limited. We used measurements from the Indoor PFAS Assessment (IPA) Campaign to estimate potential exposure to PFAS in North Carolina (NC) homes for adults and young children via inhalation of indoor air, ingestion of dust and tap water, transdermal uptake from contact with dust, air, and surfaces, and, for children, mouthing of clothing.
Materials and Methods: During the IPA Campaign (2021-2022), we sampled air, dust, surfaces, clothing, and tap water in 10 homes in NC repeatedly over a period of up to nine months. We used gas chromatography-mass spectrometry (GC-MS) and liquid chromatography-tandem mass spectrometry (LC-MS/MS) for targeted analysis of 35 PFAS, followed by an indoor exposure assessment. Diet was not considered.
Results: We calculated average intake rates for sum of measured PFAS to be 12.4 ng/(kg d) (children) and 3.6 ng/(kg d) (adults). Inhalation contributed most to total PFAS exposure, i.e., 68% (children) and 87% (adults). Dust ingestion (25%) and mouthing of clothing (4.8%) contributed substantially to children's exposure to ∑(PFAS), while ingestion of tap water (7.3%) and dust (5.9%) were important exposure pathways to ∑(PFAS) for adults.
Conclusion: This research highlights that exposure to ∑(PFAS) from indoor air, dust, and clothing may be of similar or greater magnitude than exposure from tap water ingestion, especially for children. Similar indoor measurements in combination with biomonitoring are needed to characterize the contribution of indoor exposures to body burden.
Funding: This work was funded by the Alfred P. Sloan Foundation, NIOSH through NC OSHERC, NSF GRFP, and the NC Policy Collaboratory.
Disclaimer: The views expressed in this presentation are those of the authors and do not necessarily represent the views or policies of the US EPA.

FT10-04 | Initial Impacts of Personalized Environmental Report-Back and Household-Level Mitigation Strategies on In-home Air Pollution

Xiaoying Li[1], Olivia Cecil[1], Jessica Tryner[1], Mollie Phillips[1], Thomas Reilly[1], Kayleigh P. Keller[1], John Volckens[1], Ellison Carter[1]

[1] Colorado State University, Fort Collins, USA; [2] Access Sensor Technologies, Fort Collins, USA

Objectives: Reducing in-home exposure to air pollution is an understudied pathway for protecting public health. We hypothesize that reporting in-home pollution levels alongside personalized mitigation strategies may help households understand their indoor air quality (IAQ) and take actions to reduce harmful exposures. Methods: Households in Colorado were recruited for in-home air sampling with the Home Health Box (HHB), an innovative IAQ monitoring technology, which was used to quantify fine particulate matter (PM2.5), nitrogen dioxide (NO2), and carbon dioxide (CO2). To communicate the IAQ data, a Home Health Report (HHR) was developed, categorizing pollutant levels as 'low', 'medium', or 'high' based on USEPA standards and ASHRAE recommendations. The report also provided personalized, no-to-low-cost mitigation strategies, e.g., improving ventilation when hosting guests at home. Households randomly assigned to the treatment group received an HHR after each of three week-long measurements, while control homes received all three HHRs only after completing all measurements. Intake and several follow-up surveys were conducted to collect information on the actions participants took to improve their IAQ. A difference-in-differences design was used to evaluate the impact of the report-back intervention. Results: Nearly 50% of homes had at least one pollutant ranked 'high' at baseline. In treated homes, CO2 decreased between the first and second measurements, while PM2.5 and NO2 did not. As of March 2025, data collection was still ongoing, with one-third of treated homes reporting they took actions to improve IAQ, including increasing ventilation when hosting guests at home. Conclusion: Preliminary results suggest that the report-back treatment has potential to improve IAQ (e.g., CO2). However, it did not lead to significant reductions in PM2.5 and NO2, possibly due to the higher costs associated with mitigating these pollutants.

FT10-05 | Global Trends in Green Space Exposure, Inequality, and Mortality: A Multi-Country Analysis

Tian Ma, Michelle Bell, On behalf of the MCC Collaborative Research Network

YALE UNIVERSITY, School of Environment, NEW HAVEN, USA

Greenspace and its inequality have undergone significant changes in the 21st century due to rapid urbanization, climate change, and environmental policies. While greenspace is known to influence mortality, most studies are limited to single-country or regional analyses, and little is known about temporal variation in the health impacts. This study aims to examine the relationship between greenspace, greenspace inequality, and mortality on a global scale and to investigate how these associations have changed over time. We obtained all-cause mortality data for 906 locations across 52 countries from the Multi-Country Multi-City (MCC) Collaborative Research Network and retrieved Enhanced Vegetation Index (EVI) data for the period 2001–2020. EVI was used as a measure of greenspace, and the Gini index was used to assess the inequities in population exposure to greenspace. We first applied a negative

binomial generalized linear mixed-effects model to estimate country-specific associations between greenspace, greenspace inequality and mortality. We then pooled country-level estimates to generate global and regional estimates using a meta-regression model. Our preliminary analyses suggest that greenspace exposure has a protective effect on all-cause mortality, whereas greenspace inequality increase mortality risk. For instance, in Canada, a 0.1-unit increase in EVI is associated with a 10% (4%-15%) reduction in mortality risk. While a 0.1-unit increase in EVI inequality corresponds to a 20% (5% - 39%) increase in mortality risk. Besides, these associations varied over time, with the protective effect of EVI peaking in 2006–2010 and the adverse impact of inequality strongest in 2011–2015. This study highlights the protective effects of greenspace on mortality and the adverse impacts of greenspace inequality. The findings underscore the need for policies that not only expand urban green space but also ensure its equitable distribution to maximize health benefits.

FT10-07 | Associations of Urban Blue and Green Spaces with Coronary Artery Calcification in Black Individuals and Disadvantaged Neighborhoods

Kai Zhang[1], **Kyeezu Kim**[2], Brian Joyce[2], Yinan Zheng[2], Drew R. Nannini[2], Jun Wang[2], Penny Gordon-Larsen[3], Ankeet S. Bhatt[4], Kelley P. Gabriel[5], James M. Shikany[6], Ming Hu[7], Aimin Chen[8], Orna Reges[9], Mercedes R. Carnethon[2], Donald M. Lloyd-Jones[2], Lifang Hou[2]

[1] The University of North Texas Health Science Center at Fort Worth, Population and Community Health, Fort Worth, USA; [2] Northwestern University, Feinberg School of Medicine, Chicago, USA; [3] University of North Carolina at Chapel Hill, Gillings School of Global Public Health, Chapel Hill, USA; [4] Kaiser Permanente Northern California Division of Research, Kaiser Permanente San Francisco Medical Center, San Francisco, USA; [5] University of Alabama at Birmingham, Department of Epidemiology, Birmingham, USA; [6] University of Alabama at Birmingham, Heersink School of Medicine, Birmingham, USA; [7] University of Notre Dame, School of Architecture, South Bend, USA; [8] University of Pennsylvania, Perelman School of Medicine, Philadelphia, USA; [9] Ariel University, Department of Health Systems Management, Ariel, Israel

BACKGROUND: Proximity to urban blue and green spaces has been associated with improved cardiovascular health; however, few studies have examined the role of race and socioeconomic status in these associations.

METHODS: Data were from the CARDIA study (Coronary Artery Risk Development in Young Adults). We included longitudinal measurements (1985–1986 to 2010–2011) of blue and green spaces. Presence of coronary artery calcification (CAC) was measured with noncontrast cardiac computed tomography in 2010 to 2011. The associations of blue and green spaces with CAC were assessed with generalized estimating equation regression with adjustment for multiple potential confounders. We conducted stratified analyses by race and neighborhood deprivation score to investigate whether the association varied according to social determinants of health.

RESULTS: The analytic sample included 1365 Black and 1555 White participants with a mean±SD age of 50.1±3.6 years. Among Black participants, shorter distance to a river and greater green space cover were associated with lower odds of CAC (per interquartile range decrease [1.45 km] to the river: odds ratio [OR], 0.90 [95% CI, 0.84–0.96]; per 10 percentage-point increase of green space cover: OR, 0.85 [95% CI, 0.75–0.95]). Among participants in deprived neighborhoods, greater green space cover was associated with lower odds of CAC (per a 10 percentage-point increase: OR, 0.89 [95% CI, 0.80–0.99]),

whereas shorter distance to the park was associated with higher odds of CAC (per an interquartile range decrease [5.3 km]: OR, 1.07 [95% CI, 1.00–1.15]).

CONCLUSIONS: Longitudinally, shorter river distance and greater green space correlated with less CAC in Black participants and deprived neighborhoods, while shorter park distance increased CAC odds in deprived areas. Black participants in deprived neighborhoods had lower CAC odds with greater river and green space exposure.

FT10-08 | Development of a comprehensive index to measure the relationship between urban design and traffic

Sabah Usmani, Darby Jack, Jaime Benavides, Marianthi-Anna Kioumourtzoglou

Columbia University, Environmental Health Sciences, New York, USA

Objective: The urban built environment impacts health through various pathways, including its influence on traffic and traffic-related exposures (e.g., air pollution, noise). While prior research has examined individual built environment factors (BEFs) such as greenspace in relation to health, a comprehensive analysis of BEFs that impact traffic is lacking. We developed the Built Environment Traffic Solutions (BETS) Index, the first composite measure to characterize BEFs impact on traffic levels in New York City (NYC). By providing a standardized metric, the BETS Index can support health research and guide urban design policies for healthier, lower-traffic environments, offering actionable guidance for urban policy decisions.
Material and Methods: We selected variables representing BEFs based on their known associations with traffic and transportation choice using publicly available data (2019-2024) and used them as inputs in exploratory factor analysis. Factor scores were computed and summed across three factors to generate a composite index at the census tract level. Using annual traffic data from Streetlight, which provides crowdsourced traffic estimates, we will use a negative binomial model to assess the association between the BETS Index and traffic, controlling for relevant sociodemographic confounders.
Results: Preliminary results indicate that the index comprises three factors (with eigenvalues >1) capturing different dimensions of the urban built environment: (1) mixed-use/high-density development, (2) residential areas, and (3) factory/industrial areas, together explaining 49% of the data variance. The composite BETS Index shows high variability across census tracts in NYC, with higher values concentrated in Lower Manhattan and Downtown Brooklyn.
Conclusion: Our index provides a novel traffic-related built environment measure to inform health studies and guide urban design policies to reduce traffic exposure and related health risks. While the index was developed for NYC, the approach is generalizable and can be applied to other cities, subject to data availability.

FT10-10 | A Natural Experimental Evaluation of Cool Roof Installation and Heat-Related Pediatric Health Outcomes in Urban Heat-Vulnerable Communities

Alexander Azan[1,4], Yifan Xu[1], Elle Anastasiou Pesante[1], Andrea Titus[1], Mehdi Heris[2], Perry Sheffield[3], On behalf of the NYU Langone Collaborative Center in Children's Environmental Health Research & Translation

[1] New York University Grossman School of Medicine, Department of Population Health, New York, USA; [2] City University of New York, Urban Policy and Planning at Hunter College, New York, USA; [3] Icahn School of Medicine at Mount Sinai, Department of Environmental Medicine and Climate Science, New York, USA; [4] New York University Grossman School of Medicine, Division of General Internal Medicine and Clinical Innovation, New York, USA

Objective: To examine the impact of highly solar-reflective roof installations (termed "cool roofs") on trends in child heat-related health outcomes in heat-vulnerable New York City (NYC) communities.
Materials and Methods: Our sample included children aged 0-17 living in census blocks (CBs) with a high NYC Heat Vulnerability Index score. Cool roofs were defined as the CB-level percentage of roof surface area with a solar reflectivity index ≥ 65%. Heat-health outcomes were selected based on prior literature and included pediatric emergency department (ED) diagnosis codes derived from all-payer claims data: heat-specific illness, asthma, otitis media and externa, respiratory tract infections, digestive-related illness, constitutional symptoms, and physical injury. We estimated linear fixed-effects difference-in-differences (DiD) regression models with an auto-correlative structure to examine trends in monthly ED visit rates per 1000 children during summer (May-September) before (2016-2017) and after (2019-2020) cool roofs were installed in 2018 in response to local policy. Absolute (none to some cool roofs) (N=168) and relative (<50% to > 50% cool roofs) (N=378) treatment CBs were compared to control CBs (N=1135, N=6630, respectively).
Results: The DiD parallel trends assumption for monthly outcome rates pre-2018 was met only with relative treatment metrics. In these DiD models, we observed a significant reduction in a composite of child heat-health outcomes -2.58(-4.33, -0.84) and otitis media and externa -1.59(-3.12, -0.05) in CBs with cool roofs installed, compared to controls. Changes in other child health outcomes followed similar trends but were not statistically significant (p>0.05).
Conclusions: Our findings support further evaluation of cool roofs as a scalable urban heat mitigation and adaptation strategy for pediatric health. While trends toward improved heat-health outcomes were observed across multiple conditions at the CB-level, future research is needed to clarify the potential causal mechanisms linking building-level cool roof interventions to heat-health outcomes in children and other heat-vulnerable populations.

FT11 | Exposure assessment methods for climate and urban environment

FT11-01 | Predicting daily fine particulate matter in Mexico City: a hybrid spatiotemporal modeling approach

Mike Z. He[1], Ellen Ren[2], Iván Gutiérrez-Avila[1], Robert O. Wright[1], Itai Kloog[1]

[1] Icahn School of Medicine at Mount Sinai, Department of Environmental Medicine, New York, USA; [2] New York City Department of Buildings, Bureau of Sustainability, New York, USA

Background and Aim: In recent years, there has been growing interest in the development of air pollution prediction models, particularly in low- and middle-income countries that are disproportionately impacted by the effects of air pollution. Recent methodological advancements, particularly in machine learning, provide novel opportunities for modeling efforts.
Methods: We estimate daily ground-level fine particulate matter ($PM_{2.5}$) concentrations in the Mexico City Metropolitan Area at 1-km^2 grids from 2005 to 2023 using a multistage approach. Spatial and temporal predictor variables include data from the Moderate Resolution Imaging Spectroradiometer (MODIS), Copernicus Atmosphere Monitoring Service (CAMS), and additional meteorological and land use variables. We employed machine-learning based approaches (random forest and gradient boosting algorithms) to downscale satellite measurements and incorporate local sources, then utilized a generalized additive model (GAM) to geographically weight predictions from the initial models. Model performance was evaluated using 10-fold cross-validation.
Results: On average, the random forest, gradient boosting, and GAM models explained 75, 82, and 83% of variations measured in $PM_{2.5}$ concentrations. $PM_{2.5}$ levels were generally higher in densely populated urban centers and lower in suburban and rural areas. Important predictors of ground-level $PM_{2.5}$ included wind (both u and v components), 2-meter mean air temperature, elevation, and the normalized difference vegetation index (NDVI).
Conclusions: Using novel machine learning based approaches, we developed robust models with fine-scale spatial (1-km^2) and temporal (daily) variations of $PM_{2.5}$ in Mexico City from 2005 to 2023. The predicted $PM_{2.5}$ concentrations can further advance public health research on air pollution in Mexico City and beyond.

FT11-02 | Validation and Application of Ultrasonic Personal Air Samplers for Household and Personal Temperature Exposure Assessment Across 120 Communities in Eight Countries

Madalyn Nones, Perry Hystad

Oregon State University, College of Health / Public Health, Corvallis, USA

Background: Traditional methods for assessing temperature exposure in environmental epidemiology rely on meteorological station or remotely sensed data, which fail to capture individualized exposures. Personal heat exposure varies substantially based on indoor environments and microclimatic conditions. However, little information is available on personal temperature modification in low- and middle-income countries, despite these populations being the most susceptible to temperature-related health impacts.
Objective: To validate and apply Ultrasonic Personal Air Samplers (UPAS) for measuring household and personal temperature exposure in the Prospective Urban Rural Epidemiology (PURE) cohort.

Methods: Data were collected from 2,541 households and 998 individuals (442 men and 556 women) in 120 communities across eight countries as part of the PURE study. The UPAS monitors continuously recorded air temperature and collected monitor GPS data. To evaluate the accuracy of UPAS temperature readings, a calibration study was conducted using Kestrel temperature monitors as the gold standard. The evaluation accounted for environmental influences and air filter loading, which may affect sensor performance. We then applied adjusted UPAS temperature data to examine personal, household and ambient temperature differences.
Results: Across all temperature conditions, UPAS measurements were highly correlated with Kestrel monitor readings (average R-squared = 0.89). Systematic temperature offsets that remained relatively stable throughout the data collection period were observed, with UPAS monitors recording temperatures 3 – 9°C higher on average than Kestrel measurements. After applying corrections, significant differences were observed between household exposures, personal exposures, and ambient conditions. Next steps will focus on evaluating how housing characteristics, personal time-activity patterns, and community factors (e.g., greenspace) modify personal temperature exposures.
Conclusion: UPAS monitors provide reliable estimates of temperature exposure, but offset adjustments are necessary. The PURE temperature data presents new opportunities to examine temperature exposures across diverse communities and populations, enhancing our understanding of heat-related health risks.

FT11-03 | Data-Driven Metrics for Characterizing Episodic, Multiyear Wildfire Smoke Exposures for Epidemiologic Studies on Chronic Health Effects

Stephanie Cleland[1,2], Olivia Q. Hamilton[3], Sarah Henderson[3]

[1] Simon Fraser University, Faculty of Health Sciences, Burnaby, Canada; [2] Vancouver Coastal Health Research Institute, Centre for Lung Health, Vancouver, Canada; [3] British Columbia Centre for Disease Control, Environmental Health Services, Vancouver, Canada

Objective. Wildfires are becoming more frequent, widespread, and intense, increasing many populations' exposure to wildfire smoke (WFS). Despite increasing WFS, little is known about the chronic health effects of prolonged exposure. Previous studies on long-term air pollution exposure have relied on annual averages, which do not capture WFS' episodic and extreme nature. We aimed to develop data-driven exposure metrics that capture the frequency, intensity, and duration of WFS exposure over extended periods.
Material and Methods. We used estimates of daily average total fine particulate matter ($PM_{2.5}$) from the Canadian Optimized Statistical Smoke Exposure Model, available for all 6-digit postal codes in British Columbia (BC) for 2010-2022. We first generated estimates of weekly total $PM_{2.5}$ to identify the typical accumulation of $PM_{2.5}$ during weeks without WFS impacts. We then leveraged this information to extract estimates of weekly WFS-related $PM_{2.5}$, which were used to develop a suite of metrics that capture different aspects of episodic, multiyear WFS exposure.
Results. The metrics included cumulative exposure to WFS-related $PM_{2.5}$, exposure during WFS-impacted weeks, exposure during multiweek WFS episodes, and recovery from WFS exposure. In BC, some regions accumulated nearly 10,000 μg/m^3 of WFS-related $PM_{2.5}$, with >60 WFS-impacted weeks over 13 years. Correlation between the metrics was moderate to low, indicating their ability to capture different features of multiyear exposure. A combination of these metrics will be used in epidemiologic studies of chronic disease incidence in Canada, including endpoints such as asthma and cancer.

Conclusion. We demonstrated the application of a reusable, data-driven framework for characterizing episodic, multiyear exposure to WFS-related $PM_{2.5}$.The metrics are easily calculable using readily available $PM_{2.5}$ data and can be combined and accumulated over any period. These relevant, interpretable metrics allow for a comprehensive understanding of multiyear WFS exposure. This work was funded by the Canadian Institutes for Health Research.

FT11-05 | Application of Multi-Angle Spaceborne Observations for Characterizing Long-Term Particulate Organic Carbon Pollution in Poor Data Regions

Yun Hang[1,3], Qiang Pu[2,3], Yang Liu[3]

[1] University of Texas Health Science Center at Houston, School of Public Health, Houston, USA; [2] Saint Louis University, College for Public Health and Social Justice, St. Louis, USA; [3] Emory University, Rollins School of Public Health, Atlanta, USA

Ambient $PM_{2.5}$ pollution is a leading global environmental health risk, contributing to significant mortality and morbidity. In data-poor regions, such as low and middle-income countries (LMICs) and rural areas, while total $PM_{2.5}$ levels may be monitored, the chemical composition of $PM_{2.5}$ remains poorly characterized due to limited ground-based monitoring networks. Understanding the specific chemical components is crucial because each component has a different level of toxicity. This knowledge gap hinders effective public health interventions and environmental policy development, leading to persistent health inequities. To address this challenge, we developed a machine learning framework that combines multi-angle spaceborne observations from the NASA MISR instrument, chemistry transport model simulations, and land use data. Our approach accurately predicted daily organic carbon (OC) concentrations at a 10 km spatial resolution across China, a representative data-poor region, from 2003 to 2019. The model demonstrated high predictive accuracy, achieving a cross-validation R^2 of 0.84 and an RMSE of 4.9 $\mu g/m^3$, effectively capturing spatial and temporal variations in OC levels. To better understand pollution sources, we analyzed the OC to EC ratio. Higher ratios were observed in rural areas, indicating agricultural and biogenic emissions, while lower ratios in urban clusters pointed to primary sources such as traffic and industrial activities. Since 2013, significant decreases in the OC to EC ratio were recorded in the North China Plain, likely reflecting the impact of stringent biomass burning regulations. This study demonstrates the utility of multi-angle spaceborne observations and machine learning in characterizing long-term OC pollution in data-poor regions. Our findings provide valuable exposure estimates for assessing the health effects of OC and inform targeted policy interventions to reduce air pollution and promote environmental health equity. By enhancing the understanding of OC trends and source contributions, this research supports evidence-based strategies to address pollution-related health disparities in under-monitored regions globally.

FT11-06 | Advancing Exposure Assessment: A Comprehensive Review of Hybrid Modeling Approaches for Predicting Indoor Temperature and Humidity in Residential Buildings

Chima C. Hampo[1], Leah H. Schinasi[2], Simi Hoque[1]

[1] Drexel University, Civil, Architectural, and Environmental Engineering, Philadelphia, USA; [2] Drexel University, Environmental and Occupational Health, Dornsife School of Public Health, Philadelphia, USA

Objective: Accurate prediction of indoor temperature and humidity is crucial for assessing occupant exposure to thermal conditions. Traditional approaches rely on physics-based or data-driven models, each with inherent limitations in adaptability, computational cost, or interpretability. Hybrid modeling, which integrates physical and data-driven techniques, has emerged as a modern and more flexible approach in predicting indoor temperature, and humidity remains absent. This study aims to bridge this gap by reviewing hybrid modeling techniques, evaluating their effectiveness, and identifying key advancements and limitations.

Materials and Methods: A comprehensive literature review was conducted, analyzing studies that employ hybrid modeling techniques for predicting indoor thermal and humidity conditions in residential settings. The review categorizes methodologies based on model integration strategies, simulation tools, calibration techniques, and validation approaches. Comparative insights are drawn against conventional white-box and black-box methods to assess improvements in accuracy, computational efficiency, and real-time adaptability.

Results: The findings reveal that hybrid models significantly outperform traditional white-box or black-box approaches, with several studies reporting RMSE reductions of up to 75% and R^2 values exceeding 0.9. The integration of physics-based simulations with deep learning algorithms improves model generalizability while ensuring reliability in diverse residential settings. Moreover, co-simulation techniques have been shown to enhance the accuracy of humidity predictions, an area often neglected in conventional exposure assessments. Despite these advancements, the lack of standardized validation frameworks and the challenge of integrating real-time adaptation into hybrid models remain critical gaps in the field.

Conclusion: Hybrid modeling represents a significant advancement in exposure assessment for indoor thermal and humidity conditions, offering a more robust alternative to traditional methods. This review underscores the need for further research in refining hybrid frameworks, improving real-time applications, and integrating occupant behavior dynamics. Addressing these challenges will enhance predictive capabilities and support evidence-based strategies for sustainable residential environments.

FT11-07 | Improved $PM_{2.5}$ Mapping by Incorporating the Sensor Network in Beijing, China

Mei Zheng[1], Xiaomeng Liu[1], Xiaoliang Qin[2], Zhi Ning[2], Xiue Shen[3], Baoxian Liu[3], Tong Zhu[1]

[1] Peking University, College of Environmental Sciences and Engineering, Beijing, China; [2] The Hong Kong University of Science and Technology, Division of Environment and Sustainability, Hong Kong, Hong Kong; [3] Beijing Municipal Environmental Monitoring Center, Beijing Key Laboratory of Airborne Particulate Matter Monitoring Technology, Beijing, China

To combat severe haze pollution and protect public health, China introduced $PM_{2.5}$ into its national air quality standards for the first time in 2012, setting the annual average limit at 35 μg/m³. Recently, China has been working to tighten its $PM_{2.5}$ standards and improve air quality further. Accurate estimation of $PM_{2.5}$ exposure at the city level is crucial, especially in Beijing, a megacity with a population of nearly 22 million.
This study aims to develop a method for achieving more accurate estimates of $PM_{2.5}$ exposure, using Beijing as a case study. Although many sensor networks have been installed and operated in China, their applications remain limited. In this study, a spatiotemporal model for estimating $PM_{2.5}$ concentrations was developed using the land-use random forest (RF) method. The model utilized data from a 342-node $PM_{2.5}$ monitoring network with 1-hour resolution, collected during December 2018 and from August to December 2023. The model incorporated 39 variables, including aerosol optical depth (AOD), emission inventory, land use, and meteorological variables.
The $PM_{2.5}$ concentrations estimated by the newly developed RF method were compared with personal monitoring results and two commonly used datasets: TAP (Tracking Air Pollution) and CHAP (China High Air Pollutants). Our method showed better agreement with personal monitoring results, while TAP results differed by up to 13 μg/m³. Additionally, our results demonstrated that incorporating sensor network data could better capture $PM_{2.5}$ hotspots within the city. In summary, this study successfully explored the use of data from multiple sensor networks in a city and applied it to $PM_{2.5}$ exposure studies, setting a valuable example for other cities and aiding in the management of $PM_{2.5}$ pollution.

FT11-08 | Ultrafine particle mobile monitoring study designs for epidemiology: Cost and performance comparisons in various monitoring scenarios

Sun-Young Kim[1,2], Amanda J. Gassett[2], Magali Blanco[2], Lianne Sheppard[2,3]

[1] National Cancer Center Graduate School of Cancer Science and Policy, Department of Public Health & AI, Goyang, South Korea; [2] University of Washington, Department of Environmental and Occupational Sciences, Seattle, USA; [3] University of Washington, Department of Biostatistics, Seattle, USA

Objective: Given the difficulty of collecting ultrafine particle (UFP) measurements for individuals, researchers use mobile monitoring to develop models to predict long-term average exposure to UFP allowing the investigation of its association with human health. While recent mobile monitoring studies focused on predictive models' abilities to select optimal designs, cost is also an important feature. This study aims to compare costs to predictive model performance for different mobile monitoring designs in various monitoring scenarios.

Materials and methods: We used data on UFP mobile monitoring and associated costs collected by the Adult Changes in Thought Air Pollution study. We considered alternative designs by selecting subsets of fewer sites, visits, seasons, days of week, and hours of day in various monitoring scenarios. In addition to the single instrument and fixed staff cost scenario, we constructed extended scenarios using multiple instruments for UFP as well as gaseous pollutants and a temporally-varying shift premium for weekend and evening driving. We calculated cross-validation statistics of prediction models and estimated monetary costs for each alternative design in each monitoring scenario. Results: For designs with less visits per site, the costs were lower, and model performance also worsened, but with a slight decline above 12 visits per site. Designs with fewer sites showed a consistent reduction in cost and model performance. Restrictions on the number of seasons, days of week, and/or hours of the day adversely impacted model performance, but did not affect cost. Adding multiple instruments or a shift premium gave consistent findings. Conclusion: Our study provides practical guidance to future mobile monitoring campaigns that have the ultimate goal of assessing the health effect of long-term air pollution. Temporally-balanced designs with 12 visits per site are a cost-effective option that provide relatively good prediction accuracy with optimized costs regardless of instrumentation or shift premium.

FT11-09 | Are Volatile Organic Compounds (VOCs) Transported by Ambient Particulate Matter (PM)?Implications for Wildfire-related PM and VOC Exposures

Daniel J. Conklin, Lexiao Jin, Alexis Miller, Shweta Srivastava, Pawel Lorkiewicz

University of Louisville, Christina Lee Brown Envirome Institute, Louisville, USA

Objective: Effective VOC exposure assessment tools are needed for better VOC source apportionment as VOC exposures pose significant health risks. The contribution of particulate matter (PM) to VOC exposure assessment has not been adequately explored.

Materials and Methods: To test whether ambient PM and especially PM from wildfire smoke contributes directly to VOC exposure, we used a Versatile Aerosol Concentration Enrichment System (VACES) to separate and concentrate $PM_{2.5}$ from ambient Louisville, KY air during the Canadian wildfires of summer 2023. For PM-associated VOC assessment, we exposed male C57BL/6J (wild type, WT) mice to a single 6h episode of either filtered air (control) or of low (38 μg/m^3), medium (134 μg/m^3), or high (216 μg/m^3) level of $PM_{2.5}$, and then collected urine post-exposure for UPLC-MS/MS analysis of 23 VOC metabolites (VOCm). As both inhaled VOCs and $PM_{2.5}$ can be irritants that activate the transient receptor potential ankyrin-1 (TRPA1) and nocifensive reflexes (e.g., decreased breathing rate, coughing), we exposed mice deficient in TRPA1 (TRPA1-null). Thus, we tested dual hypotheses: 1) VOCs are present in $PM_{2.5}$; and, 2) that $PM_{2.5}$/VOC may activate TRPA1.

Results: Seven VOCm measured were below the limit of detection (LOD) in all exposures. Although acrolein metabolites (3-hydroxypropylmercapturic acid, 3HPMA; carboxyethylmercapturic acid, 2CoEMA) were detected in urine of all mice, these VOCm were not elevated by $PM_{2.5}$ exposures. In contrast, styrene-derived 1PHEMA levels were increased by $PM_{2.5}$ in both WT and TRPA1-null mice, whereas 2HEMA, a metabolite of acrylonitrile, vinyl chloride, or ethylene oxide, was increased in WT mice only.

Conclusion: These findings highlight both a complexity of VOC exposure assessment and a need for more research to better understand how $PM_{2.5}$ may influence VOCm especially with respect to wildfire exposures. This work supported by: NIH (P30ES030283, P42ES023716, S10OD026840), DoD (HT94252410596), and the Jewish Heritage Fund for Excellence.

FT12 | Neurologic and mental health flash talks

FT12-01 | Gene-Environment Interactions between Cumulative Lead Exposures and Genetic Risk Factors for Iron Burden on Parkinson's Disease

Kanghong Shao[1], Cynthia D. Kusters[1], Jeff Bronstein[2], Adrienne Keener[2,4], Danielle Thordarson[2], Kimberly C. Paul[2], Beate Ritz[1,3]

[1] UCLA Fielding School of Public Health, Epidemiology, Los Angeles, USA; [2] UCLA David Geffen School of Medicine, Neurology, Los Angeles, USA; [3] UCLA Fielding School of Public Health, Environmental Health Sciences, Los Angeles, USA; [4] VA Greater Los Angeles Healthcare System, Parkinson's Disease Research, Los Angeles, USA

Background

Parkinson's disease (PD) risk has been linked to cumulative lead exposure. Iron is known to accumulate in the substantia nigra and may contribute to PD etiology via oxidative stress, and may also interact with lead metabolism to contribute to PD development. Few studies have investigated this interaction.

Methods

We estimated tibia lead levels using DNA methylation biomarkers for 523 PD patients and 236 population controls enrolled in the Parkinson's Environment and Gene study, and created iron-handling polygenic risk scores (PRSs) (total iron binding capacity [TIBC] and transferrin saturation [TSAT]) based on iron-homeostasis related single-nucleotide polymorphisms (SNPs) in previous studies. Logistic regression analyses were conducted to estimate effects of lead biomarkers on PD stratified by iron-handling PRS percentiles. We also stratified by gender.

Results

A low TSAT PRS (<20th percentile), reflecting a genetic predisposition for iron deficiency, enhanced estimated tibia lead effects on PD (OR=1.86; 95% CI: 1.18, 3.04), while no association was found among those with a high TSAT PRS (>50th percentile). The effect modification of tibia lead on PD by TSAT PRS was most pronounced among and limited to women (OR=3.37; 95% CI: 1.53, 8.84). A genetic profile representing higher binding capacity for iron attenuated the estimated tibia lead effects on PD (TIBC PRS Q4 vs Q1-Q3: OR=1.11; 95% CI: 0.74, 1,66).

Conclusion

Genetic susceptibility to iron deficiency may exacerbate the effect of cumulative lead exposure on PD development, while genetic predilection to maintain higher iron loads and a higher total iron binding capacity reduces the estimated effects of bone lead on PD. We saw evidence for larger modifying effects from a genetic predisposition for iron deficiency among women but not for men. Our findings suggested a protective role of iron handling capacity and an increased risk with iron deficiency for the neurotoxicity of lead in PD.

FT12-02 | The Social Exposome and Default Mode Network Connectivity: Identifying Risk and Protective Factors in Adolescent Brain Development

Vida Rebello[1], Elza Rechtman[1], Stefano Renzetti[2], Kristie Oluyemi[1], Azzurra Invernizzi[1], Megan K. Horton[1]

[1] Icahn School of Medicine at Mount Sinai, Environmental Medicine, New York, USA; [2] University of Brescia, Medical and Surgical Specialities, Brescia, Italy; [3] Icahn School of Medicine at Mount Sinai, Environmental Medicine, New York, USA; [4] Icahn School of Medicine at Mount Sinai, Environmental Medicine, New York, USA; [5] Icahn School of Medicine at Mount Sinai, Environmental Medicine, New York, USA; [6] Icahn School of Medicine at Mount Sinai, Environmental Medicine, New York, USA

Background: The social exposome– the totality of neighborhood-level social and environmental factors- includes both protective (e.g., educational resources, green space) and risk (e.g., air pollution, housing instability) factors that shape neurodevelopment. Individual-level adverse childhood experiences (ACEs) may moderate the social exposome, promoting vulnerability or resilience. We examine the relationship between the social exposome and functional connectivity within the default mode network (DMN) and its connection with the amygdala (DMN-Amygdala), networks regulating thought and emotion, and assess effect modification by ACE.

Methods: We analyzed data from 6,359 children (9-11 years; 49% female) from the Adolescent Brain Cognitive Development Study, including resting-state MRI, three census-tract indices (Child Opportunity, Social Vulnerability, Area Deprivation), and individual ACE scores. Connectivity estimates were derived using Gordon parcellation. We computed a social exposome index using weighted quantile sum (WQS) regression to examine risk/protective factors associated with connectivity. We used stratified interaction WQS to determine whether associations differed by ACE (yes/no). We considered increased connectivity neuroprotective; decreased connectivity indicated neural vulnerability. Models adjusted for sex, age, MRI manufacturer, handedness, and site.

Results: The social exposome was associated with DMN and DMN-Amygdala connectivity (Protective: b_{DMN} = 0.01, 95% CI [0.006, 0.02]; $b_{DMN—Amyg}$= 0.01, 95% CI [0.008, 0.02]; Risk: b_{DMN} = -0.01, 95% CI [-0.02,- 0.006]; $b_{DMN—Amyg}$ = -0.01, 95% CI [-0.02, -0.006]). ACE exposure modified the association with the DMN-Amygdala ($b_{wqs*ACE}$ = -0.04, 95% CI [-0.06, -0.01]; among ACE-exposed children, the social exposome was more strongly associated with decreased connectivity. Protective factors included higher math/reading proficiency, income, and green space; risk factors included home crowding, school poverty, and teacher inexperience.

Conclusion: Our exposomic approach demonstrates the impact of neighborhood-level social factors on children's brain connectivity. Individual-level ACEs increase vulnerability. Findings guide resource allocation for child health policy.

FT12-04 | Prenatal critical windows of $PM_{2.5}$ exposure associated with structural brain changes in adolescents

Elza Rechtman[1], Lindsay Alexander[2], Allan Just[1], Robert Wright[1], Michael Milham[2], Chris Gennings[1], Megan Horton[1]

[1] Icahn School of Medicine at Mount Sinai, Department of Environmental Medicine, New York, USA; [2] Child Mind Institute, Healthy Brain Network, New York, USA

Objective. With the growing climate crisis, particulate matter (PM) air pollution increasingly impacts human health, especially children. Early-life $PM_{2.5}$ (< 2.5 μm) exposure is linked to adverse neurodevelopment, yet prenatal critical windows and underlying neural mechanisms remain unclear. This study examines prenatal critical windows of $PM_{2.5}$ exposure and structural brain changes during early adolescence in a cohort enriched for neurodevelopmental disorders.

Material and Methods. We analyzed structural magnetic resonance imaging (MRI) data from 238 children (ages 5–15; 90 females) enrolled in the Healthy Brain Network (HBN), a well-established behavioral and MRI phenotyping biobank. Using Freesurfer, we quantified 14 subcortical volumes from high-resolution T1-weighted MRI scans. Weekly prenatal $PM_{2.5}$ levels were estimated using a satellite-based gradient-boosting hybrid model at a 1 × 1 km spatial resolution. Treating the 14 brain regions as an aggregate, we used lagged weighted quantile sum (L-WQS) regression to assess time-varying associations between weekly prenatal $PM_{2.5}$ exposure (mean 12 μg/m^3) and brain structure, adjusting for age and sex.

Results. We identified two prenatal critical windows in which $PM_{2.5}$ exposure was negatively associated with subcortical brain volume, with the strongest associations observed at 20 weeks before birth (β = -0.11 [95% CI: -0.08, -0.14]) and 4 weeks before birth (β = -0.12 [95% CI: -0.09, -0.15]). These associations were primarily driven by reductions in the nucleus accumbens volume.

Conclusions. Our findings highlight two prenatal critical windows in which $PM_{2.5}$ exposure is associated with reduced subcortical brain volume, particularly in the nucleus accumbens, a key region involved in motivation, reward processing, impulsivity, and risk-taking behaviors. These results provide new insight into the neurodevelopmental vulnerability to air pollution and emphasize the importance of targeted interventions during pregnancy to mitigate long-term outcomes.

FT12-06 | Combined and synergistic effects of heat and fine particulate matter on hospitalization among patients with Alzheimer's disease and related dementias

Federica Spoto[1,2], Antonella Zanobetti[2], Scott W. Delaney[2], Thomas M. Gill[3], Michelle L. Bell[4,5], Francesca Dominici[1], Danielle Braun[1,6], Daniel Mork[1]

[1] Harvard T.H. Chan School of Public Health, Department of Biostatistics, Boston, USA; [2] Harvard T.H. Chan School of Public Health, Department of Environmental Health, Boston, USA; [3] Yale School of Medicine, Department of Internal Medicine, New Haven, USA; [4] Yale University, School of the Environment, New Haven, USA; [5] College of Health Sciences, Korea University, School of Health Policy and Management, Seoul, South Korea; [6] Dana Farber Cancer Institute, Department of Data Science, Boston, USA

Objective: Patients with Alzheimer's disease and related dementias (ADRD) are vulnerable to environmental stressors, including heat and air pollution. Both exposures have been independently associated with adverse health outcomes, but their combined effects remain less understood. Our goal was to quantify the joint impact of short-term exposure to extreme heat and fine particulate matter exposure ($PM_{2.5}$) on the risk of all-cause hospitalization among a cohort of Medicare beneficiaries with ADRD aged 65 years and older.

Material and Methods: We conducted a time-stratified case-crossover analysis using data from Medicare beneficiaries who had an ADRD-related hospitalization in the contiguous United States from 2000-2016. Daily heat index and $PM_{2.5}$ data were linked to each beneficiary's residential zip code. Conditional logistic regression models were applied to evaluate the effects of single and joint exposures, considering both linear and nonlinear effects and interactions to explore potential synergistic impacts.

Results: We found a linear trend between heat index and all-cause hospitalization with an odds ratio (OR) of 1.017 (95% CI: 1.004, 1.031) for extreme heat days (99th percentile) versus median. The $PM_{2.5}$-hospitalization relationship was nonlinear with a steeper slope at lower concentrations. The OR for hospitalization at 10μg/m^3 versus 5μg/m^3 $PM_{2.5}$ exposure was 1.010 (95% CI: 1.005, 1.015). A joint-exposure analysis confirmed these findings: the OR was 1.017 (95% CI: 1.002, 1.032) for hospitalization on extreme heat days compared to the median when simultaneously adjusting for the expected change in $PM_{2.5}$.

Conclusion: Findings underscore the importance of considering both heat and air pollution in assessing health risks for ADRD populations. Future research should explore extended lag effects and additional pollutants to refine our understanding and guide public health interventions for addressing extreme heat and air pollution events.

Acknowledgments: Work funded by NIH RF1AG080948, RF1AG074372, R01AG066793.

FT12-07 | Association of atmospheric pressure with deaths of despair among U.S. Veterans

Alina Peluso[1], Dirga Lamichhane[2], James VanDerslice[2], Jeremy Logan[1], Anuj Kapadia[1], Jodie Trafton[3], Amanda Bakian[2]

[1] Oak Ridge National Laboratory, Oak Ridge, USA; [2] University of Utah, Salt Lake City, USA; [3] VHA Office of Mental Health and Suicide Prevention, Palo Alto, USA

Objective: Atmospheric pressure may increase deaths of despair risk by reducing oxygen levels, leading to hypoxia and worsening mood and depressive symptoms. This study explores the link between atmospheric pressure exposure and deaths of despair in U.S. veterans, a vulnerable population due to unique risk factors.

Material and Methods: Using National Death Index data, we identified veterans who died in 2018-2019 by suicide (N=6,394), overdose (N=4,414), or opioid overdose (N=2,762). Average daily county-level sea level atmospheric pressure was estimated based on hourly data collected from ERA5. Potential confounders including daily average precipitation, maximum temperature, and average shortwave radiation, were sourced from NASA's ORNL Daymet; daily average $PM_{2.5}$ concentrations were processed from the CMAQ model. Veteran decedents were assigned exposures based on their county FIPS code of residence. Adjusted conditional logistic regression models were employed to estimate associations between daily average atmospheric pressure and cause of death over 0-6 lag days, utilizing a bidirectional time-stratified case-crossover design. Models were stratified by season.

Results: In *winter*, suicide, overdose, and opioid overdose risks increased following exposure to increasing atmospheric pressure on lag day 6 (aOR:2.18, 95% CI: [1.39-3.43]; 6 (1.71[1.19-2.46], 1.66[1.13-2.43], respectively). In *summer*, atmospheric pressure was similarly associated with increased suicide risk (lag day 6: 2.09[1.02-4.31]) and overdose risk (lag day 3: 1.94[1.04-3.64]). In contrast, in *fall*, increasing atmospheric pressure was associated with a decreasing risk of suicide on lag day 0 (0.52[0.34-0.81]), and with overdose and opioid overdose risks on cumulative lag days 0-6 (0.37[0.18-0.74]; 0.34[0.16-0.74], respectively). In *spring*, suicide and overdose risks were negatively associated with atmospheric pressure on lag day 6 (0.50[0.29-0.85]; 0.59[0.38-0.93], respectively).

Conclusion: These findings contribute to our understanding of how weather factors may be associated with deaths of despair, yet these relationships appear to be complex and vary based on other moderating effects such as season.

FT12-08 | How does air pollution affect the incident risk of Alzheimer's Disease? Findings from a prospective Italian population cohort

Antonietta Pepe[1], Simona Costanzo[1,2], Alfonso Di Costanzo[3], Maria I. De Bartolo[4,5], Sabatino Orlandi[1], Matteo Renzi[6], Americo Bonanni[1], Assuntina Cembalo[7], Francesco Martone[7], Gaetano Zazzaro[7], Giovanni Veronesi[2], Chiara Cerletti[1], Maria B. Donati[1], Francesco Gianfagna[2], Alfredo Berardelli[4,5], Giovanni de Gaetano[1], Licia Iacoviello[1,8], Alessandro Gialluisi[1,8]

[1] IRCCS NEUROMED, Research Unit of Epidemiology and Prevention, Pozzilli, Italy; [2] University of Insubria, Department of Medicine and Surgery, EPIMED Research Center, Varese, Italy; [3] University of Molise, Department of Medicine and Health Science, Campobasso, Italy; [4] IRCCS NEUROMED, Pozzilli, Italy; [5] Sapienza University of Rome, Department of Human Neurosciences, Rome, Italy; [6] Lazio Region Health Service/ASL Roma 1, Department of Epidemiology, Rome, Italy; [7] CIRA-Italian Aerospace Research Centre, Capua, Italy; [8] LUM University, Department of Medicine and Surgery, Casamassima, Italy

Objective: To clarify the link between air pollution and Alzheimer's Disease (AD) risk, particularly the joint influence of nitrogen dioxide (NO2) and particulate matter with aerodynamic diameter <10 µm (PM10).

Material and Methods: We built bi-pollutant survival models of incident AD risk vs exposure to NO2 and PM10, in an Italian population cohort, the Moli-sani study (baseline recruitment 2005-2010; N=24,325; ≥35 years; 51.9% women). Yearly exposure levels to pollutants were estimated applying Kriging interpolation algorithms to fourteen land boxes of the regional environmental agency (ARPA Molise, 2006-2018), while incident AD cases up to December 31st, 2022 were identified through interrogation of the main regional dementia centers' records, and validated by qualified neurologists based on neuroimaging, clinical and neuropsychological assessments.

Results: Over 16,814 participants with non-missing covariates and follow-up information and apparently free from prevalent dementia at baseline (216 incident cases, median (IQR) follow-up 15.4 (1.7) years), we observed significant independent associations for both PM10 and NO2, in Cox Proportional Hazard models incrementally adjusted for age, sex, education, lifestyles, quality of life, adiposity and prevalent health conditions. Participants above the median exposure levels for PM10 and NO2 (11.6 and 25.3 µg/m^3) showed a ~9.5x (95% Confidence Interval: 5.8-15.5) and ~2.1x (1.3-3.4) increase of AD risk, respectively, compared to those exposed to pollutants levels below the median. However, no significant interactive associations were detected. The fraction of incident AD risk explained by PM10 and NO2 represented more than 30% of the total risk explained by the most adjusted model (Nagelkerke R^2: 5.1% vs 15.7%).

Conclusion: This evidence suggests a notable influence of air pollution on incident AD, supporting a role of PM10, in addition to NO2. Further analyses are on the way to validate these findings through satellite data and in independent cohorts, and to identify potential mediation pathways in this link.

FT12-09 | Associations between pre-disease biomarkers of persistent organic pollutants (POPs) and amyotrophic lateral sclerosis (ALS) risk in Denmark

Aline Davias[1], Johnni Hansen[2], Marc Weisskopf[1]

[1] Harvard university, Departments of Environmental Health and Epidemiology, Boston, USA; [2] Danish Cancer Institute, Copenhagen, Denmark

Objective: Amyotrophic lateral sclerosis (ALS) is a neurodegenerative disease that is typically rapidly fatal. Exposure to persistent organic pollutants (POPs) like polychlorinated biphenyls (PCBs), polybrominated diphenyl ethers (PBDEs), and organochlorine pesticides (OCPs) has been suggested to increase risk of ALS, but there are no studies with biomarkers of exposure pre-disease. We assessed the association between POPs exposure and ALS using pre-disease plasma samples from the Danish Diet, Cancer, and Health cohort, established between December 1993 and May 1997.

Materials and Methods: We identified 167 incident ALS cases using the Danish National Patient Register (NPR) and selected 334 controls (two per case)—individuals with no record of ALS in the national register up to the date of diagnosis of the corresponding case—by individual matching on --age and sex. We assessed 13 PCB, 3 PBDE and 9 OCP concentrations in plasma samples collected at baseline. To assess the relationship between POP exposure and ALS, we employed conditional logistic regression models and generalized additive mixed models (GAMMs), adjusting for age, sex, smoking status, body mass index (BMI), total serum cholesterol, marital status, and education.

Results: Our results suggested generally inverse trends between several POPs and the predicted risk of ALS; especially for chlordane (logistic regression model comparing quartile 4 to quartile 1, OR: 0.3; 95%CI: 0.1, 0.7; p-value: 0.005). GAMMs results were consistent with the conditional logistic regression models, though they were not statistically significant. Additionally, for some POP groups (non-dioxin-like PCBs, chlordane, and DDT) there was the suggestion of higher ALS odds at the highest levels of exposure, but the data at these levels was sparse.

Conclusion: Our findings of inverse trends with POP exposures could suggest the presence of uncontrolled neuroprotective confounding factors, perhaps fish consumption as a potential route of exposure. Further examination of higher level exposures is needed.

FT12-10 | Residential public green space, traffic exposure and mental health-related consultations in primary care: a cross-sectional study.

Simon G. Beerten[1], Arne Janssens[1], Raf Aerts[2,3], Gijs Van Pottelbergh[1], Lidia Casas[4,5], Bert Vaes[1]

[1] KU Leuven, Department of Public Health and Primary Care, Leuven, Belgium; [2] Sciensano, Risk and Health Impact Assessment, Brussels, Belgium; [3] KU Leuven, Department of Biology, Leuven, Belgium; [4] University of Antwerp, Social Epidemiology and Health Policy, Department of Family Medicine and Population Health, Antwerp, Belgium; [5] University of Antwerp, Institute for Environment and Sustainable Development, Antwerp, Belgium; [6] University of Antwerp, Laboratory of Applied Microbiology and Biotechnology (LAMB), Department of Bioscience Engineering, Antwerp, Belgium

Objective: Green spaces have a beneficial effect on health, but the perspective from primary care is often missing. This study investigated the association between residential green space, traffic exposure, and anxiety and depression within primary care, often the first point of contact for mental health problems.

Methods: This cross-sectional study of adults was conducted in the urbanized region of Flanders, Belgium, based on consultations registered in the INTEGO primary care database from 2014 to 2023. Exposures were visible green, residential green space cover in different buffers (150m, 500m and 1500m) and access to green space. The outcomes were diagnoses of depression and anxiety, and prescriptions for antidepressants and benzodiazepines. Models were adjusted for traffic exposure, gender, age, socioeconomic status and living in a city and included a random effect of primary care practice, to account for spatial clustering of subjects.

Results: We identified 180,569 unique patients in the study period of which 16,659 (9.2%) had a diagnosis of depression or anxiety. Overall, we found inverse associations between diagnoses of depression or anxiety and residential total green cover (adjusted OR 0.95; 95%CI [0.93,0.98]) and high green cover (OR 0.97; [0.95,0.99]) within 150m. View on green was inversely associated with all outcomes (OR 0.89 [0.86,0.91]). There were no associations with green space accessibility. Associations remained robust when controlling for air pollution.

Conclusion: We found protective associations between mental health consultation outcomes in primary care and visible green and green space cover, but not accessible green. These results are important as a basis for non-pharmacological, preventive mental health care interventions, and as a call for urban policy to prioritize green in urban areas.

Funding: Supported by the Research Foundation Flanders (reference: G0B4820N).

FT13 | Cancer and cancer precursor flash talks

FT13-01 | Effect of Menthol Flavor on E-Cigarette Users' Toxicants Exposure: A Crossover Clinical Trial among US Young Adults

Sreshtha Chowdhury, Tarana Ferdous, Simanta Roy, Wasim Maziak

Florida International University, Department of public health, Epidemioogy, Miami, USA

Background: Limiting flavors to menthol and tobacco for e-cigarette “pod-mods” was one of the first major actions taken by the FDA to reduce the spread of e-cigarette among young people. However, menthol flavor can present a potential risk given its popularity among young people in the US and its puffing and nicotine-enhancing properties which increases toxicant exposure in cigarettes. This study examined the effect of menthol flavor on e-cigarette users' toxicant exposure.

Methods: In this 2X2 crossover clinical lab study, 67 current e-cigarette users aged 21-35 attended up to 60-minute ad libitum e-cigarette use sessions using 4th-generation e-cigarette, preceded by 12-hour nicotine abstinence. Participants used the same e-cigarette device in two separate sessions, differing by flavor (menthol/tobacco) in random order. Next, the puffing topography parameters recorded during each vaping session were replicated using a digital playback smoking machine for each participant to measure the toxicant emissions (14 aldehydes) at an Aerosol Research Laboratory.

Results: Majority of the participants (mean age 24.00 years) were male (56%) and employed (64.13%), and the majority were Hispanic (60.71%). Higher median values were observed for 10 among 14 aldehydes for sessions with menthol condition compared to the sessions with tobacco condition (i.e., formaldehyde: 0.56 vs. 0.50 µg/session, p=0.6154; acetaldehyde: 9.31 vs. 8.15 µg/session, p=0.4769; crotonaldehyde: 0.39 vs. 0.32µg/session, p=0.0518).

Conclusion: Although the differences were non-significant and smaller, using an acute lab based within-subject design and digital playback smoking method, the results of this study suggest that menthol flavor enhances e-cigarette users’ toxicant exposure. It highlights that limiting menthol flavor could play an important role in reducing e-cigarette harm and addictiveness among young people.

FT13-02 | A Risk Assessment of Aflatoxin B_1 in Five Mexican States with a High Mortality Rate of Liver Cancer

Paulina Farías[1], Karla Rangel-Moreno[1], Obed Solís-Martínez[2], Adriana Monge[1], Martín Romero-Martínez[3], Katherine A. McGlynn[5], John D. Groopman[4], Martín Lajous[1,6]

[1] National Institute of Public Health, Mexico, Center for Research on Population Health, Cuernavaca, Mexico; [2] National Institute of Public Health, Mexico, Center for Nutrition and Health Research, Cuernavaca, Mexico; [3] National Institute of Public Health, Mexico, Center for Research on Evaluation and Surveys, Cuernavaca, Mexico; [4] Bloomberg School of Public Health, Johns Hopkins University, Department of Environmental Health and Engineering, Baltimore, USA; [5] National Cancer Institute, Division of Cancer Epidemiology and Genetics, Rockville, USA; [6] Harvard T.H. Chan School of Public Health, Department of Global Health and Population, Boston, USA

Hepatocellular carcinoma (HCC) is a major public health concern in Mexico, particularly among individuals over 60 years old, where it is a leading cause of cancer mortality. Aflatoxin B1 (AFB_1), one of the most potent hepatic carcinogens, frequently contaminates staple foods such as maize and is a significant risk factor for HCC. This study aimed to assess the role of AFB_1 exposure in five Mexican states with high HCC mortality and exposure prevalence to AFB_1 (92%) —Campeche, Chiapas, Tamaulipas, Veracruz, and Yucatán—through a risk assessment approach.
Using serum AFB_1-lysine (AFB1-lys) biomarker data from 936 subjects recruited during the 2018 National Health and Nutrition Survey (ENSANUT), we estimated the daily intake of AFB_1. Overall, the average estimated daily intake (EDI) was 14.9 ng/kg/day and the 95th percentile of exposure reached 58.1 ng/kg/day. We calculated the local carcinogenic potency factor —central and upper bound— ($q^* = 0.018–0.052$) adjusting for hepatitis B virus prevalence, given the synergistic effect of these risk factors. The margin of exposure (MOE), the ratio of the benchmark dose to EDI values, for the mean (25) and the 95th percentile (6) were significantly lower than the safety threshold of 10,000 for genotoxic and carcinogenic substances, suggesting substantial health risks. The highest HCC (MOE) risk was observed in Veracruz (29) followed by Chiapas (42), Yucatan (55), Campeche (75), and Tamaulipas (181).
We estimate that AFB_1 exposure could be responsible for 27 to 279 HCC cases annually in the studied states, with over 60% occurring in Veracruz due to high exposure levels and population size. This study provides critical risk data for previously unexamined regions, enhances exposure estimation accuracy, and underscores the preventable nature of AFB_1-related HCC. Furthermore, climate change may expand high-risk areas by increasing temperature and humidity, highlighting the need for proactive food safety measures.

FT13-03 | The Michigan Cancer and Research on the Environment Study (MI-CARES): the First 10,000 Participants

Alison M. Mondul[1], Justin Colacino[1], Sailing Tang[1], Lilah Khoja[1], Bhramar Mukherjee[2], Dana Dolinoy[1], Leigh Pearce[1]

[1] University of Michigan, Ann Arbor, USA; [2] Yale University, New Haven, USA

Objective: Young-onset cancer is increasing, and environmental factors are the likely cause. Most cancer cohort studies recruited middle-aged and older adults and began decades ago. Therefore, we lack data on contemporary environmental exposures affecting younger populations. Thus, the National Cancer Institute and the National Institute of Environmental Health Sciences initiated the Cohorts for Environmental Exposures and Cancer Risks (CEECR) consortium. In 2022, the Michigan Cancer and Research on the Environment Study (MI-CARES) was launched within CEECR to understand the relationship between environmental exposures and intermediate cancer markers. Here, we present methods for establishing MI-CARES and characteristics of our first 10,000 participants.

Material and Methods: MI-CARES plans to recruit 100,000 18-49 year-olds residing in Michigan. Recruitment targets participants from across Michigan, focusing on metropolitan Detroit, Flint, Grand Rapids, Kalamazoo, Lansing, and Saginaw. These hubs were chosen for their population density and varying environmental exposures. Participation is entirely remote; the questionnaire is completed online. MI-CARES aims to collect dried blood spots (DBS) and saliva for participants living in the hubs listed above. Kits for biospecimen self-collection are mailed to participants.

Results: Nearly 10,000 participants have enrolled; 79% are female. Our racial/ethnic distribution is similar to that of Michigan overall. We have excellent representation from our recruitment hubs, as well as throughout the state yielding a wide range of environmental exposures in our study population. Pilot studies in 48 participants' biospecimens have yielded valid results for PFAS, DNA-methylation, metabolomics, and stress/inflammation markers. We detected 9 PFAS chemicals in participants' DBS (ranges in ng/1 punch: L-PFOA 0.007-0.023, L-PFOS 0.012-0.155).

Conclusion: The initial phase of recruitment for MI-CARES has been successful. Recruitment is ongoing and sampling frame considerations will be taken into account to ensure a representative sample.**Funding:** National Cancer Institute UH3 CA267907-01

FT13-04 | Epigenome-wide association study of leukocyte DNA methylation and lung cancer: A prospective study among never smokers.

Mohammad L. Rahman[1], Jason Y. Wong[2], Wei Zheng[3], Charles E. Breeze[1], Batel Blechter[1], Wei Hu[1], Qiuyin Cai[3], Gong Yang[3], Jirong Long[3], Yu-Tang Gao[4], Nathaniel Rothman[1], Xiao-Ou Shu[3], Qing Lan[1]

[1] National Cancer Institute, National Institutes of Health, Division of Cancer Epidemiology and Genetics, Rockville, USA; [2] National Heart Lung and Blood Institute, Epidemiology and Community Health, Bethesda, USA; [3] Vanderbilt University Medical Center, Division of Epidemiology, Department of Medicine, Nashville, USA; [4] Shanghai Cancer Institute, Shanghai, China

Objective: The etiology of lung cancer in never-smokers remains unclear, despite 53% of cases in women worldwide being unrelated to smoking. Epigenetic alterations, particularly DNA methylation (DNAm), can capture non-smoking environmental risk factors and may contribute to lung cancer development. Here, we aimed to investigate peripheral blood DNAm markers to identify etiologic signatures of lung cancer in never-smokers.

Methods: This nested case-control study within the prospective Shanghai Women's Health Study included 683 never-smoking women with incident lung cancer and 683 controls, matched 1:1 on date of birth (±2 years) and sample collection (±3 months). DNAm was assessed in pre-diagnostic samples using the MethylationEPIC v1.0 BeadChip. Robust linear regression models, adjusted for age, body mass index, sample plate, secondhand smoking, and estimated cell types, were used to identify differentially methylated positions (DMPs) and regions (DMRs) associated with lung cancer.

Results: We identified two significantly hypomethylated DMPs, cg09082427 (*NSMF*; $P=8.03\times10^{-09}$) and cg03459185 ($P=1.81\times10^{-08}$), associated with future lung cancer risk at the epigenome-wide significance level. Notably, cg09082427 has been previously linked to arsenic exposure, a known lung cancer risk factor. Additionally, five DMRs were identified near or within *RNG39, ZFP57, HOXA5*, *EHBP1L1*, and *PAX8-AS1,* with *RNG39, ZFP57,*and *HOXA5* significantly hypomethylated, while *EHBP1L1* and *PAX8-AS1* were significantly hypermethylated in association with lung cancer risk. Prior studies have linked differential methylation at *ZFP57* and *HOXA5* to lung cancer and air pollution exposure. Cell-type-specific integrative analysis of top DMPs showed significant enrichment in immune regulatory regions, while functional enrichment analyses indicated involvement in airway epithelial cell proliferation, immune regulation, and non-small cell lung cancer.

Conclusion: Our findings provide novel insights into epigenetic alterations associated with lung cancer development. The identified DNAm patterns suggest a potential role for non-smoking environmental exposures in lung cancer etiology among never-smokers in Shanghai.

FT13-05 | Residential radon exposure and risks of various cancers in adulthood in the CONSTANCES cohort

Afi M.S. Henyoh[1,2], Emeline Lequy[3,4], Olivier Laurent[1], Claire Gréau[5], Caroline Vignaud[5], Sophie Ancelet[1], Choisie Mukakalisa[1], Céline Ribet[3], Mireille Coeuret-Pellicer[3], Sophiane Kab[3], Corinne Mandin[1], Justine Sauce[1,2], Géraldine Ielsch[5], Marcel Goldberg[3], Marie Zins[3], Enora Cléro[1]

[1] Autorité de Sûreté Nucléaire et de Radioprotection (ASNR), PSE-SANTE/SESANE/LEPID, F-92260 Fontenay-aux-Roses, France; [2] Université Paris-Saclay, F-91190, Gif-sur-Yvette, France; [3] UMS 011 Inserm/UVSQ/U Paris Cité/U Paris Saclay, "Cohortes épidémiologiques en population", F-94800 Villejuif, France; [4] Université Paris-Saclay, UVSQ, Gustave Roussy, Inserm, Team "Exposome and Heredity", F-94800 Villejuif, France; [5] Autorité de Sûreté Nucléaire et de Radioprotection (ASNR), PSE-ENV/SERPEN/BERAD, F-92260 Fontenay-aux-Roses, France

Objective

Radon exposure is an established risk factor for lung cancer, but its role in other health outcomes remains unclear. A recent systematic review recommended more in-depth studies of specific diseases as potential long-term effects of radon exposure, especially during childhood. Here we investigated the association between childhood and lifelong residential radon exposure and the risks of various cancers in adulthood.

Material and Methods

Study participants were selected from the French population-based CONSTANCES cohort recruited at adult age over period 2012-2021. Radon exposure was reconstructed by linking lifelong residential histories with estimated indoor radon concentrations by municipality. We included participants with available radon exposure data covering at least 80% of their lifetime in 2022 (n = 62,519; 45% male). Some of the targeted cancers were lung, liver, colorectal, non-melanoma skin (NMS) cancers, melanoma, non-Hodgkin lymphoma and leukaemia. We used time-dependent Cox proportional hazard models adjusted for attained age (underlying time scale), region of residence, and birth cohort, to assess the hazard ratio (HR) and the 95% confidence interval (CI) per 1000 Bq.m^{-3}.years increase in lagged cumulative radon exposure over specific age periods and the entire follow-up time.

Results

Overall, 7260 cancer cases were identified. The median age at time of censoring was 55 years old (Q1-Q3 = 44-65). The 10-year lagged median radon exposure cumulated over lifetime was 2541 Bq/m^3.years. Preliminary results indicate statistically significant increased risk associated with cumulative radon exposure over 16 to 30 years old for colorectal cancer incidence among females (HR = 1.42; 95%CI: 1.08-1.87) and NMSC among males (HR = 1.23; 95%CI: 1.01-1.50); and over 51 to 82 for melanoma (HR = 1.34; 95%CI: 1.06-1.69). No significant association was observed with the lifelong exposure dynamic.

Discussion

Sensitivity analyses including additional adjustment for other risk factors (e.g. smoking) and analyses of potential effect modification are underway.

FT13-06 | Parental occupational automotive gasoline exposure and risk of pediatric cancers

Julia E. Heck[2,1], Yixin Chen[1], Beate Ritz[1], Johnni Hansen[3]

[1] UCLA, Epidemiology, Los Angeles, USA; [2] University of North Texas, Rehabilitation and Health Services, Denton, USA; [3] Danish Cancer Institute, Copenhagen, Denmark

Objective
The potential importance of automotive gasoline exposure to cancer risk is highlighted by its selection of a topic for a recent IARC Monograph. There is a limited literature on parental gasoline exposure and cancer risk in children. The goal of this study was to investigate the relation between parental occupational exposure to automotive gasoline and risk for pediatric cancers.
Material and Methods
We included all childhood cancers diagnosed in Denmark 1968-2013 from the Cancer Registry, and linked these to parental demographic, health, and occupational records, with controls selected from national registries. Parental occupations were taken from the Supplementary Pension Fund. We utilized job-exposure matrices developed for the Nordic Occupational Cancer Study to examine parental occupational gasoline exposure: for fathers, 3 months preconception to conception; and for mothers, exposure in pregnancy. The pregnancy period was ascertained by the Medical Births Registry. Conditional logistic regression was used to estimate the child's cancer risk in relation to parental automotive gasoline exposure.
Results
Participants included 10 442 cases and 261 050 controls. Maternal occupational gasoline exposure in pregnancy was imprecisely related to the child's risk for medulloblastoma (OR=1.92, 95% CI 0.94-3.90) and acute lymphoblastic leukemia (OR=1.13, 95% CI 0.80-1.60). Paternal pre-conception exposure was related to the child's risk for glioma (OR=1.21, 95% CI 0.97-1.50), and bone tumors (OR=1.48, 95% CI 1.04-2.10), particularly osteosarcoma (OR=1.63, 95% CI 0.99-2.67).
Conclusion
This study provides additional evidence of the carcinogenicity of automotive gasoline in children, when the parents are occupationally exposed in the perinatal period.

Acknowledgements
This study was funded by the US National Institutes of Health (T32CA009142, R03CA252788).

FT13-08 | Atrazine use and kidney function and nephrotoxicity markers in the Biomarkers of Exposure and Effect in Agriculture Study

Gabriella Andreotti[1], Joseph Shearer[2], Dale Sandler[3], Anna Lukkari[4], Richard Remigio[1], Christine Parks[3], Vicky Chang[1], Venkata Sabbisetti[5], Laura Beane Freeman[1], Amy Karger[4], **Jonathan Hofmann**[1]

[1] National Cancer Institute, Occupational and Environmental Epidemiology Branch, Division of Cancer Epidemiology and Genetics, Bethesda, USA; [2] National Heart, Lung, and Blood Institute, Heart Disease Phenomics Laboratory, Epidemiology and Community Health Branch, Bethesda, USA; [3] National Institute of Environmental Health Sciences, Epidemiology Branch, Research Triangle Park, USA; [4] University of Minnesota, Department of Laboratory Medicine & Pathology, Minneapolis, USA; [5] Brigham and Women's Hospital, Harvard School of Medicine, Boston, USA

Objective: Atrazine, an herbicide widely used in U.S. agriculture, has been associated with kidney cancer and non-malignant kidney disease in the Agricultural Health Study (AHS). However, the relationships between atrazine use and specific markers of kidney disease remain unclear. We evaluated associations between atrazine use and kidney function and nephrotoxicity biomarkers among male farmers in the Biomarkers of Exposure and Effect in Agriculture study, a substudy within the AHS.

Methods: Our investigation included 246 male farmers with: 1) both recent (last three months) and past atrazine use (n=83); 2) high lifetime atrazine use [top tertile of lifetime days) without recent use (n=88); and 3) no recent use and no/low lifetime use (<50 lifetime days; n=75). These groups were frequency-matched on age and state of residence (Iowa, North Carolina). We measured markers in serum (creatinine, cystatin C, urea nitrogen, uric acid) and urine (kidney injury molecule-1, albumin, creatinine). Estimated glomerular filtration rate (eGFR) was calculated using the 2021 CKD-EPI creatinine-cystatin C equation. We estimated the percent difference in each marker across groups using multivariable linear regression.

Results: Compared to farmers with no/low atrazine use, recent atrazine users had lower eGFR (-9.4%; 95%CI: -16.1%, -2.2%) and higher levels of serum creatinine (9.4%; 95%CI: 1.6%, 17.9%), cystatin C (10.8%; 95%CI: 2.0%, 20.4%), and uric acid (8.2%; 95%CI: -1.3%, 18.5%). Among the recent atrazine users, increasing lifetime use was associated with having a higher urine albumin-creatinine ratio (P_{trend}=0.03).

Conclusion: Our findings add to the evidence that atrazine use, in particular recent use, is associated with diminished kidney function. These findings support the biologic plausibility of observed associations with malignant and non-malignant kidney disease. Future mechanistic studies are needed to better understand how atrazine may contribute to kidney dysfunction.

Funding: NIH Intramural Research Program

FT13-09 | Examining the interaction between PAH-albumin adducts and underlying genetic susceptibility on breast cancer using a family-based cohort

Michelle L. Lui[1], Hui-Chen Wu[3], Yuyan Liao[1], Susan E. Lloyd[1], Jeanine Genkinger[1,2], Robert MacInnis[4,5], Irene Andrulis[6,7], Sarah Colonna[8], Mary Daly[9], Esther John[10,11], Melissa Southey[4,12], Regina M. Santella[3], Mary Beth Terry[1,2]

[1] Columbia University Mailman School of Public Health, Department of Epidemiology, New York, USA; [2] Columbia University Irving Medical Center, Herbert Irving Comprehensive Cancer Center, New York, USA; [3] Columbia University Mailman School of Public Health, Departments of Environmental Health Sciences, New York, USA; [4] University of Melbourne, Centre for Epidemiology and Biostatistics, Parkville, Australia; [5] Cancer Council Victoria, Cancer Epidemiology Division, Melbourne, Australia; [6] Sinai Health System, Lunenfeld-Tanenbaum Research Institute, Toronto, Canada; [7] University of Toronto, Departments of Molecular Genetics and Laboratory Medicine and Pathobiology, Toronto, Canada; [8] Huntsman Cancer Institute, Division of Medical Oncology, Salt Lake City, USA; [9] Fox Chase Cancer Center, Department of Clinical Genetics, Philadelphia, USA; [10] Stanford University School of Medicine, Department of Medicine (Oncology), Palo Alto, USA; [11] Stanford University School of Medicine, Department of Epidemiology & Population Health, Palo Alto, USA; [12] Monash University, School of Clinical Sciences at Monash Health, Clayton, Australia

Objective: Polycyclic aromatic hydrocarbons (PAH) are environmental contaminants with known carcinogenic mechanisms. Studies have found that PAH exposure may particularly increase breast cancer risk for women with higher underlying genetic susceptibility, making cohorts enriched for underlying genetic susceptibility important for studying this gene-environment interaction. In this study, we aimed to examine the interaction of PAH-albumin adducts in blood and underlying genetic susceptibility on breast cancer risk in a cohort enriched for genetic susceptibility to breast cancer.

Material and Methods: We examined the association between PAH-albumin adducts measured in baseline blood and breast cancer risk in a nested matched case-control study, using data from 6 sites of the Breast Cancer Family Registry (371 cases and 719 controls). We used the BOADICEA model to estimate the 1-year absolute breast cancer risk based on known pathogenic variants and family pedigree data and used conditional logistic regression to evaluate whether the increased risk from PAHs differed by BOADICEA score.

Results: We detected significant gene-environment interaction between exposure to PAH and BOADICEA risk score in our study sample. For each one-unit increase in BOADICEA score, women in the highest quartile of PAH exposure had 2.84 (95% CI: 1.06, 7.62) times the odds of breast cancer compared to women in the referent quartile of exposure. This association was stronger in women under age 50 (OR=10.56, 95% CI: 1.50 – 74.29) and weaker in women ages 50 and older (OR = 1.93, 95% CI: 0.34-10.91).

Conclusion: These results support the use of cohorts enriched for genetic susceptibility to breast cancer to detect gene-environment interactions. Furthermore, we demonstrated how cancer susceptibility from germline and somatic mutational burden changes across the life course, with stronger gene-environment interaction in earlier life and higher environmental burden in later life.

FT13-10 | A fine needle-shaped silica contaminant of food in the high esophageal cancer area of coastal South China

Haisheng Wu, Yang Li, Linwei Tian

University of Hong Kong, School of Public Health, Hong Kong, China

Objective: Emerging genomic evidence points to an important role of non-mutagenic environmental cancer promoters in driving esophageal squamous cell carcinoma (ESCC) incidence in the world. One such ESCC promoter suspect is crop-derived silica hairs in contaminated food in South Africa, Iran and North China. Repeated injuries and chronic inflammation of the esophagus epithelium caused by these silica needles can be analogous to microneedling for cosmetic purposes in dermatology. This study investigated needle-shaped diatom frustules (highly siliceous cell walls of diatom) in guts of small filter-feeding trash fish possibly underlying the ESCC endemic in coastal South China where there is a dietary culture allowing guts of trash fish into human food.

Materials and Methods: We collected the most common cooked and sun-dried trash fish from local wet markets, and undissolved substances during the fermentation process of fish sauce made from trash fish (aka, fish earth). A rat feeding experiment with 95% standard feed and 5% fish earth was conducted to explore the potential of food-borne diatom frustules embedding in rodent esophagus. The acid digestion-microscopy method was used for analyzing diatom frustules.

Results: Every gram of fish earth contained 0.33 million diatom frustules, over 70% of which are needle-shaped diatoms with a length of 10-50 μm and a diameter of 1-5 μm. Gut samples of cooked or dried trash fish show similar results. The diatom needle count in each gram of rat esophagus ranged from 115 to 687, significantly higher than the control group.

Conclusion: Abundant diatom needle contaminants are present in human food in coastal South China, which can lodge in esophagus tissue when ingested. We present a hypothesis for the involvement of diatom needles in the etiology of ESCC in coastal South China.

FT14 | Water pollution flash talks

FT14-01 | Public water disinfection byproducts and birth outcomes: the Environmental Influences on Child Health Outcomes Cohort

Tessa Bloomquist[1], Tushara Rajeev[1], Mohamad Burjak[2], Amii Kress[2], Zorimar Rivera-Nunez[3,4], Blair Wylie[5], Shuang Wang[6], Julie Herbstman[1], Allison Kupsco[1], Anne Nigra[1]

[1] Columbia University Mailman School of Public Health, Environmental Health Sciences, New York, USA; [2] Johns Hopkins Bloomberg School of Public Health, Epidemiology, Baltimore, USA; [3] Rutgers University, Environmental and Occupational Health Sciences Institute, Piscataway, USA; [4] Rutgers University, Department of Biostatistics and Epidemiology, Piscataway, USA; [5] Columbia University, Obstetrics and Gynecology, New York, USA; [6] Columbia University, Biostatistics, New York, USA

Objective: Disinfection byproducts are regulated in US public water systems as total trihalomethanes (TTHM) and haloacetic acids (HAA5). The US Environmental Protection Agency (USEPA) is reevaluating maximum contaminant levels for TTHM (currently 80 µg/L). Our objective was to evaluate the association between public water TTHM and HAA5 consumed prenatally and birth outcomes (birthweight, gestational age, birthweight-for-gestational-age z-score, preterm birth, and weight for gestational age) in the Environmental Influences on Child Health Outcomes (ECHO) Cohort.

Methods: We evaluated n=13,059 participants (n=26 cohort sites).We generated individual, prenatal, time-weighted average estimates of public water TTHM and HAA5 by leveraging >4 million public water monitoring records (2006-2019) and linking tract-level estimates to pregnancy residential address histories. We used generalized estimating equation models to evaluate the association between prenatal public water TTHM and HAA5 and birth outcomes per 20 µg/L (a value similar to the interquartile range) higher exposure, in flexible spline models, and across quantiles of exposure.

Results: Mean average prenatal TTHM and HAA5 were 32.0 and 21.3 µg/L. Per 20 µg/L higher TTHM, the risk ratio of preterm birth was 1.04 (95% CI 1.00, 1.08). We also observed a marginally significant relationship between higher TTHM and a lower mean difference in birthweight. In splines, we observed evidence of non-linear associations with preterm birth and birthweight, with higher risk ratios at the highest TTHM exposure levels. Similar linear associations of a stronger magnitude were observed for HAA5. Compared to those in the lowest quantile of HAA5 exposure (<10.1 µg/L), the fully adjusted mean difference in birthweight-for-gestational-age z-score for those in the highest quantile (>36.9 µg/L) was -0.11 (95% CI -0.20, -0.02).

Conclusion: Prenatal exposure to TTHM and HAA5, as measured in regulated public water systems, was associated with adverse birth outcomes at exposure levels below the current USEPA maximum contaminant level.

FT14-02 | Association between location, perfluoroalkyl substances in residential water supply, and childhood cancer in Orange and Los Angeles Counties, California (2000-2019)

Natalie R. Binczewski[1], Libby M. Morimoto[2], Joseph L. Wiemels[3], Xiaomei Ma[4], Catherine Metayer[2], Veronica M. Vieira[1]

[1] University of California, Irvine, Department of Environmental and Occupational Health, Joe C. Wen School of Population & Public Health, Irvine, USA; [2] University of California, Berkeley, Division of Epidemiology, School of Public Health, Berkeley, USA; [3] University of Southern California Keck School of Medicine, Center for Genetic Epidemiology, Department of Population and Public Health Sciences, Los Angeles, USA; [4] Yale School of Public Health, Department of Chronic Disease Epidemiology, New Haven, USA

Objective: We examined associations between residence at birth, perfluorooctane sulfonic acid (PFOS) and perfluorooctanoic acid (PFOA) in residential water supply, and childhood cancer (age 0-21 at diagnosis) in California.
Methods: Cases (n=6,448) and controls (n=13,044) born in Orange or Los Angeles Counties 2000-2019 were linked to water districts serving their residence at birth. Binary detection of PFOS/PFOA was derived from the third Unregulated Contaminant Monitoring Rule data. We used generalized additive models to predict geographic variation in odds of childhood cancer with detection of PFOS/PFOA in local water supply. Our reference model included a smoothing term for location, birth year, age at diagnosis or index year (for controls), and child sex. We compared spatial patterns from the reference model to models that only adjusted for PFOS/PFOA exposure and models that additionally adjusted for type of health insurance and maternal race and ethnicity, age, and education. We calculated odds ratios (ORs) and 95% confidence intervals (CIs) for PFOS/PFOA exposure.
Results: 2,515 (13%) children had their birth residence served by a water district with detectable PFOS or PFOA. Location was a significant predictor of increased childhood cancer risk in all models, ranging from OR=1.11 (95% CI: 1.00, 1.23) to OR=1.20 (95% CI: 1.08, 1.34). Children who had detectable PFOS in their residential water supply at birth had significantly higher odds of cancer diagnosis compared to unexposed children (OR=1.09; 95% CI: 1.00, 1.21). The OR for PFOA exposure was also elevated but not statistically significant (OR=1.03; 95% CI: 0.91, 1.16).
Conclusion: PFOS/PFOA contaminated residential water supply was associated with higher cancer risk, but exposure did not fully explain the observed geographic variation. This work can inform policies to reduce exposure to perfluoroalkyl substances and possible health impacts.
This work was supported by grant R01 ES032196 from the National Institutes of Health.

FT14-04 | The Well Water Index: Community-level characterization across the continental United States

Komal Peer; Angela Werner

US Centers for Disease Control and Prevention, Atlanta, USA

Objective: Private wells use groundwater as their source. Those reliant on private wells have the responsibility of ensuring that the water is safe to drink. Contamination exacerbated by weather hazards adds further layers of complexity for those relying on private wells. We sought to characterize community-level hazards and vulnerability for persons dependent on private wells in the continental United States (CONUS).
Material and Methods: We created the Well Water Index (WWI) by searching the literature and identifying nationally consistent, publicly available, sub-county data to build Overall, Drought, Flood, and Wildfire PWWCIIs at the national and state scales. We adapted the technical construction of this relative index from the California Communities Environmental Health Screening Tool (CalEnviroScreen 4.0).
Results: The distribution of impacted census tracts varied across CONUS by nationally-normed PWWCII type. Compared to the Southeast where the majority of the 2010 estimated U.S. private well water population lived, the estimated persons dependent upon private well water living in the West had an increased odds of living in higher impact census tracts for the Overall, Drought, and Wildfire WWIs across CONUS.
Conclusion: The WWI fills a gap as it provides a baseline understanding of potential impacts from contamination exacerbated by weather to communities reliant on private well water across CONUS.

FT14-05 | Nitrate in drinking water and colorectal cancer in the Danish Diet, Cancer and Health Cohort

Jörg Schullehner[1,5], **Dorit W. Erichsen**[2], Nicola P. Bondonno[2,3], Pratik Pohkarel[2,3], Kirsten Frederiksen[2], Susanne Rosthøj[2], Catherine P. Bondonno[3,4], Liezhou Zhong[3,9], Torben Sigsgaard[1,10], Peter F. Hendriksen[2], Frederik Dalgaard[6], Ole Raaschou-Nielsen[2,7], Jonathan M. Hodgson[3,4], Christina C. Dahm[1], Anja Olsen[2,1], Anne Tjønneland[2,8], Cecilie Kyrø[2]

[1] Aarhus University, Department of Public Health, Aarhus, Denmark; [2] The Danish Cancer Institute, Copenhagen, Denmark; [3] Edith Cowan University, Nutrition and Health Innovation Research Institute, School of Medical and Health Sciences, Perth, Australia; [4] Royal Perth Hospital, Medical School, The University of Western Australia, Perth, Australia; [5] Geological Survey of Denmark and Greenland, Department of Geochemistry, Aarhus, Denmark; [6] Herlev & Gentofte University Hospital, Department of Cardiology, Copenhagen, Denmark; [7] Aarhus University, Department of Environmental Science, Roskilde, Denmark; [8] University of Copenhagen, Department of Public Health, Copenhagen, Denmark; [9] The University of Western Australia, Institute of Agriculture, Perth, Australia; [10] Aarhus University, Danish Big Data Centre for Environment and Health (BERTHA), Aarhus, Denmark

Objective: Nitrate intake may increase risk of colorectal cancer due to endogenous formation of *N*-nitroso compounds (NOCs). In 2018, we published a widely-cited nationwide cohort study in the Danish population showing an association between nitrate in drinking water and colorectal cancer below regulatory levels. The study design was limited by a lack of data on factors governing NOC formation, especially related to diet and lifestyle. Here, we address these limitations by replicating the 2018 study in a cohort with well-defined diet and lifestyle information.

Material and Methods: Among 54,610 cancer-free participants of the Danish Diet, Cancer, and Health Cohort, we estimate source-specific intake of nitrate from food frequency questionnaires and the drinking water monitoring program. During a maximum of 27 years of follow-up, we identified 2245 colorectal cancer cases through health registries. Cox proportional hazards models were adjusted for sociodemographic, diet and lifestyle factors and stratified by factors hypothesized to promote (smoking, red meat) or inhibit (vitamin C/E, folate, flavonoids) NOC formation.

Results: When modelling exposure in the same categories as in the 2018 study, a higher rate of colon cancer was observed among participants exposed to nitrate concentrations ≥9.25 mg/L [1.52 (1.11, 2.07)] compared to <1.27 mg/L. This association was more pronounced among former or current smokers [1.77 (1.24, 2.44)], and in participants with a low dietary intake of folates [1.67 (1.03, 2.70)], and flavonoids [1.85 (1.16, 2.95)], or a high intake of red meat [1.58 (0.97, 2.57)]. No association was observed for rectal cancer, or when categorizing exposures as quintiles in this comparatively low-exposed population.

Conclusion: We confirmed the results of the 2018 study and showed that the association of nitrate in drinking water and colorectal cancer is especially pronounced in subpopulations with dietary and lifestyle factors that promote endogenous NOC formation.

FT14-06 | Microcystin-leucine-arginine Induces Liver Fibrosis and Intrahepatic Cholangiocarcinoma Progression

Chengxiang Su[1,2], Yudong Qiu[1,2]

[1] Nanjing University, Medical School, Nanjing, China; [2] Nanjing Drum Tower Hospital, Nanjing, China

Objective: Microcystin-leucine-arginine (MC-LR) is one of the most common biotoxins that can contaminate water bodies. Considering its potential carcinogenicity and hepatotoxicity, we aim to clarify the relationship between MC-LR exposure and the occurrence and development of liver/biliary tract tumors.
Material and Methods: Acute and chronic MC-LR-exposed models were established through 14-day intraperitoneal injections or 180-day water exposure. By Masson staining, immunofluorescence, and CCK8 assays, we confirmed liver fibrosis degree and the involvement of hepatic stellate cells. The role of Hedgehog pathway was assessed by inhibitors GANT58/61. Inflammation caused by MC-LR was examined with HE staining and qPCR. Through co-culture experiment of MC-LR, HiBEC, and THP-1 cells, MC-LR's effect on macrophage polarization and bile duct epithelial cells was showed. Multivariate and univariate analyses of clinical data confirmed MC-LR's impact on intrahepatic cholangiocarcinoma (ICC) prognosis, we identified its molecular mechanisms using RNA-seq and in vitro experiments.
Results: Acute MC-LR exposure for 14 days significantly enhanced liver fibrosis in mice by promoting hepatic stellate cell proliferation and activation via the Hedgehog pathway. Increased inflammatory factors and M2 macrophage infiltration in the intrahepatic bile duct area was observed after Chronic MC-LR exposure for 180 days. In vitro, we found that MC-LR can promote monocyte polarization into M2 macrophages and interact with intrahepatic bile duct epithelial cells, enhancing their proliferation and migration through IL-6/JAK2/STAT3, MEK/ERK, and PI3K/AKT pathways. Clinical data from ICC patients at Nanjing Drum Tower Hospital revealed MC-LR as an independent risk factor for worse overall survival (OS) and progression-free survival (PFS), since the activation of the ERK/MEK pathway via SET protein, which promotes ICC cell proliferation and inhibits its apoptosis.**Conclusion:** MC-LR exposure can induce the occurrence and development of liver fibrosis and ICC.

FT14-07 | Drinking Water Sources, Contaminants, and Associated Health Outcomes in Rural Virginia: Salivary Antibody Response Results from a Prospective Cohort Study

Alasdair Cohen[1], Andrew Mertens[2], Md Rasheduzzaman[3], Bethesda O'Connell[3], Andrey Egorov[4], Timothy Wade[4]

[1] Virginia Tech, Blacksburg, USA; [2] University of California, Berkeley, Berkeley, USA; [3] East Tennessee State University, Johnston City, USA; [4] Environmental Protection Agency, Chapel Hill, USA

OBJECTIVE: Relatively little is known about exposures to waterborne pathogens and the associated burden of disease in Central Appalachia, USA. Our objective was to assess associations between water sources, use, and health outcomes over time. MATERIAL AND METHODS: We conducted three rounds of data collection in two rural counties in southwest Virginia. At each round we administered surveys and collected water and saliva samples. First draw and five-minute flush water samples were analyzed for physicochemical parameters, total coliforms, *E. coli*,

and heavy metals; results were shared with households. Saliva samples were assayed for antibody responses to potentially waterborne infections. We calculated the incidence of infections at baseline, and across rounds by identifying antibody responses that increased 4-fold and were also above the 75th percentile. RESULTS: We enrolled 33 households (83 individuals), 82% (n = 27) with utility-supplied water and 18% with well or spring water (n = 6). Across rounds, total coliforms were detected in water samples from 27% (n = 20) of homes; *E. coli* was detected in 11% (n = 7), all with well-or-spring water. Diarrhea was reported for 14% of individuals (n = 27). At baseline, 34% (n = 15) of saliva samples had detectable antibody responses for *Cryptosporidium*, *Campylobacter*, and Hepatitis E. Three individuals had changes in antibody responses across rounds indicating additional post-baseline infections with *Cryptosporidium* and *Campylobacter*. After controlling for covariates and clustering, individuals in households without utility supplied water had significantly higher antibody responses for *Campylobacter* (and two norovirus genotypes), pooled across all three rounds. CONCLUSION: The incidence of reported diarrhea and measured enteric pathogen infections were both relatively high compared to other settings in the USA. More research is needed to better quantify waterborne pathogen exposures and infections for rural households will well and spring water in this region of Central Appalachia.

FT14-09 | Exposure to Disinfection By-Products in Drinking Water: A Spatial and Temporal Analysis of National Data in Israel and the impact of desalination

Nitzan Sagie[2,3], Ronnie Levin[1], Irit Hen[4], Atar Adout[4], Luda Groisman[4], Tamar Berman[4], Noa Cedar[4], Natalie DeFalco[2], Shimon Rachmilevitch[2], Lena Novack[2,3]

[1] Harvard TH Chan School of Public Health, Environmental Health, Boston, USA; [2] Ben Gurion University of the Negev, Beer Sheva, Israel; [3] Soroka Clinical Research Center, Beer Sheva, Israel; [4] Ministry of Health, Environment, Jerusalem, Israel

Introduction: Rising freshwater demand from population growth and climate change necessitates disinfection, mainly through chlorination, to prevent waterborne diseases. However, when chlorine interacts with organic matter it can produce disinfection by-products (DBPs), such as trihalomethanes (THMs) which are carcinogenic. To meet the demand for freshwater, desalination is increasingly used worldwide, especially in Israel. The water supply in the country varies by region, with the north relying more on groundwater, while the south depends primarily on desalinated water.

Objective: This study examined the distribution of THMs in Israel's drinking water by comparing different water sources, including desalination.

Materials and methods: The study utilized water quality data provided by the Ministry of Health covering all testing results between 2015-2024 as part of regulatory requirements. Geographic locations of sampling sites were determined by geocoding their locations, and the analysis focused on samples from the drinking water distribution system. We examined concentrations of DBPs, including bromoform, dibromochloromethane, bromodichloromethane, chloroform, and total trihalomethanes (TTHMs). THMs levels were compared across seven major regions in Israel using monthly and annual geometric means.

Results: The analysis of 19,367 TTHMs samples, most frequently tested in Haifa and the north regions, revealed elevated TTHMs levels, mainly due to Chloroform, in the Haifa region 0.035 mg/L (95%CI :0.034-0.036) and northern region 0.025 mg/L (95%CI: 0.024-0.025), although none of the annual averages exceeded the regulatory limit of 0.1 mg/L. A seasonal pattern was observed in the Haifa and northern regions, with higher TTHM concentrations recorded during summer (p-value<0.001).

Conclusions: THM levels are significantly affected by water sources. Our findings show that districts supplied by higher proportion of natural water sources exhibited elevated TTHM concentrations, and that incorporating desalinated water could serve as a potential regulatory measure to mitigate these levels.

FT14-10 | Causal mediation analysis to assess the effects of drinking water contamination and water storage on enteric infections in Beira, Mozambique

Rebecca S. Kann[1], Joshua Garn[3], Joan Casey[1], Rassul Nalá[4], Matthew Freeman[2], Karen Levy[1], on behalf of the PAASIM Authorship Group

[1] University of Washington, Department of Environmental and Occupational Health Sciences, Seattle, USA; [2] Emory University, Gangarosa Department of Environmental Health, Atlanta, USA; [3] University of Nevada-Reno, School of Public Health, Reno, USA; [4] Ministério de Saúde, Instituto Nacional de Saúde, Maputo, Mozambique

Objective: Interventions that deliver community-level piped drinking water infrastructure in low-income, urban settings have the potential to reduce exposure to enteric pathogens, but water intermittency and household water storage practices may reduce their effectiveness. We investigated the relationship between contamination in both point-of-collection (PoC) and stored drinking water on enteric pathogen infections among infants in a low-income, urban setting with access to an intermittent piped water supply.
Materials & methods: We used data from household surveys as well as drinking water and stool samples from 12-month-old children enrolled in the PAASIM study in Beira, Mozambique. We used a causal mediation analysis to assess (a) the direct effect of PoC water contamination and (b) the indirect effect mediated by water storage practices on enteric infections.
Results: PoC water contamination was associated with a 31% higher risk of stored water contamination [aRR: 1.31; 95% CI: (1.10, 1.57)] and a 9% higher risk of bacterial infections [1.09 (0.99, 1.20)]. Stored water contamination was associated with a 19% higher risk of protozoan infections [1.19 (0.94, 1.51)] and an 11% higher risk of co-infections [1.11 (0.97, 1.27)]. These results were supported by the causal mediation analysis, which showed a direct effect of PoC water contamination on bacterial infections [aRD: 0.063; 95% CI: (-0.02, 0.15)] and an indirect effect of stored water contamination on protozoan infections [0.008 (-0.00, 0.03)].
Conclusions: Our findings suggest that reducing water intermittency and improving storage practices are critical for mitigating waterborne disease risk in urban areas with piped water systems.

Funding: This work was supported by National Institute of Allergy and Infectious Diseases (NIAID) Grant #R01AI130163. Research was partially funded by the Future Rivers program at the University of Washington as part of an NSF National Research Traineeship award (DGE 1922004) as well as NIAID Training Grant #F31AI183829.

FT15 | PFAS: Toxicology, modeling, exposures, and health

FT15-01 | Toxicity Assessment of Fluorinated and Fluorine-free Firefighting Foams Using A Liver Cell Model

Fatema Tuj Jahura, R. Bryan Ormond, Jessica M. Gluck

North Carolina State University, Raleigh, USA

This study investigated the cytotoxicity of different firefighting foam formulations using human liver cell line HepG2. The foam formulations tested include a soy-based foam, three fluorinated aqueous film-forming foams (AFFF) (one C6-based AFFF, one C6-based alcohol resistant AFFF, and one C8-based alcohol resistant AFFF), and four fluorine-free foams (F3). The cells were exposed to different concentrations of foam samples (0.009%, 0.019%, 0.038%, 0.075%, and 0.15%) for up to 144 hours. After the exposure, cytotoxicity was assessed using LIVE/DEAD® and alamarBlue® assays. Later, the relative toxicity of these foam samples was determined by comparing the half-maximal inhibitory concentration (IC50) values. The oxidative stress induced by foam formulation was measured by dichloro-dihydro-fluorescein diacetate (DCFH-DA) assay. All foam formulations exhibited toxicity to the cells at concentrations below the recommended application level (3%), with varying degrees of cytotoxicity across foam types. Initially, fluorine-free foams (F3) exhibited higher toxicity than AFFF; however, AFFF became more toxic over time. In addition, all foam samples demonstrated the potential to induce oxidative stress through reactive oxygen species (ROS) production at higher concentrations. A moderate correlation between observed foam toxicity and their surface tension suggests that the chemical composition of the foam formulation may play a role in determining their toxic effects.

FT15-02 | Prenatal exposure to per- and polyfluoroalkyl substances, maternal metabolomics, and childhood neurodevelopment in a cohort with elevated familial likelihood of autism spectrum disorder

Jeong Weon Choi[1], Donghai Liang[2], Youran Tan[2], Rebecca J. Schmidt[3,4], Hyeong-Moo Shin[1]

[1] Baylor University, Department of Environmental Science, Waco, USA; [2] Emory University, Gangarosa Department of Environmental Health, Rollins School of Public Health, Atlanta, USA; [3] University of California, Davis, Department of Public Health Sciences, Davis, USA; [4] University of California, Davis, MIND Institute, Sacramento, USA

Background: Prenatal exposure to per- and polyfluoroalkyl substances (PFAS) has been linked to metabolic disturbances and neurodevelopmental outcomes in offspring separately, suggesting potential molecular pathways. However, studies examining the associations between maternal PFAS exposure, metabolomic alterations, and child neurodevelopmental outcomes remain limited.
Method: We analyzed 195 mother-child pairs from MARBLES (Markers of Autism Risk in Babies – Learning Early Signs), a cohort with an elevated familial likelihood of autism spectrum disorder (ASD). Maternal serum samples collected during pregnancy were analyzed for nine PFAS, and plasma metabolites were profiled using high-resolution metabolomics (HRM). At age 3 years, children were clinically assessed and classified as ASD, other non-typical development (non-TD), or typically developing (TD). Multiple linear regression was used for examining individual PFAS–metabolite associations and quantile-based g-computation for PFAS mixture. Partial least squares

discriminant analysis (PLS-DA) was used to identify metabolic differences in mothers of children with ASD or non-TD compared to TD.
Result: Among 16,251 metabolic features, 20 features were associated with individual PFAS or PFAS mixture (false discovery rate < 0.05). PLS-DA identified 622 and 850 metabolic features differentiating mothers of children with ASD vs. TD and non-TD vs. TD, respectively (variable importance in projection [VIP] score > 2). Among the Level 1 confirmed metabolites, taurine was associated with both maternal perfluorooctane sulfonate and ASD. Based on raw *p*-values and VIP scores, we identified 12 and 26 unique pathways associated with PFAS exposures and neurodevelopmental outcomes, respectively, with four overlapping pathways: protein digestion and absorption, purine metabolism, mineral absorption, and valine, leucine, and isoleucine degradation.
Conclusion: With HRM, we identified several novel metabolic pathways potentially linking prenatal PFAS exposure to neurodevelopmental outcomes. While our study population has an elevated familial likelihood of ASD, these findings enhance our understanding of metabolic alterations that could underly PFAS-induced neurodevelopmental effects.

FT15-03 | Associations between per- and polyfluoroalkyl substances (PFAS) and liver steatosis in children and young adults

Shiwen Li[1], Jiawen Carmen Chen[1], Elizabeth Costello[1], Douglas I. Walker[2], Jesse A. Goodrich[1], Tanya L. Alderete[3], Michael I. Goran[4], Damaskini Valvi[5], Brittney O. Baumert[1], Sarah Rock[1], Sandrah P. Eckel[1], David V. Conti[1], Zhanghua Chen[1], Frank D. Gilliland[1], Rob McConnell[1], Max Aung[1], Lida Chatzi[1]

[1] University of Southern California, Department of Population and Public Health Sciences, Los Angeles, USA; [2] Emory University, Gangarosa Department of Environmental Health, Atlanta, USA; [3] Johns Hopkins University, Department of Environmental Health and Engineering, Baltimore, USA; [4] Children's Hospital Los Angeles, Department of Pediatrics, Los Angeles, USA; [5] Icahn School of Medicine at Mount Sinai, Department of Environmental Medicine and Climate Science, New York, USA

Background
Longitudinal studies on associations between per- and polyfluoroalkyl substances (PFAS) exposure and liver steatosis are limited, particularly among children and young adults.
Method
We included participants from two cohorts to evaluate the effects of PFAS across several age ranges: 1) children (8-13 yrs with 8 yrs follow-up; n=74 with baseline data only and n=88 with follow-up data) from the Study of Latino Adolescents at Risk of Type 2 Diabetes (SOLAR) and 2) young adults (17-23 yrs with 4 yrs follow-up; n=122 with 75 longitudinally followed) from the Metabolic and Asthma Incidence Research (Meta-AIR) study. Liver outcomes included hepatic fat fraction (HFF), which was measured via magnetic resonance imaging scans at a minimum of one visit. Liver steatosis, which was defined as HFF ≥5.5%. Eight PFAS were quantified in plasma using liquid chromatography–high-resolution mass spectrometry. Linear/logistic regressions were used to assess associations between baseline PFAS and liver outcomes. We included an interaction term between baseline PFAS and baseline age.
Results
Cross-sectionally, although we did not find any main effect of baseline PFAS on HFF, we observed that PFNA in SOLAR and PFHxS in Meta-AIR were associated with higher baseline HFF at higher baseline ages (percent changes [95% CI]: $\beta_{\text{PFNA-SOLAR}}$ Age-Interaction = 33 [0, 76], and $\beta_{\text{PFHxS-Meta-Air}}$ Age-Interaction =5 [0, 10]). Longitudinally, in SOLAR, baseline PFHxS was associated with higher HFF at follow-up and PFHxS had greater effect on HFF at

higher baseline ages ($\beta_{PFHxS-SOLAR}$ Main-Effect =15 [0, 32] and Age-Interaction = 8 [2, 15]). Cross-sectionally, we did not observe any main effect between PFAS and liver steatosis but observed an interaction of baseline PFNA and PFOA with baseline age on baseline liver steatosis risk ($OR_{PFNA-SOLAR}$ Age-Interaction: 7.4 [1.2, 46.8] and $OR_{PFOA-SOLAR}$ Age-Interaction: 2.9 [1.1, 7.8]).

Conclusions

PFAS, especially PFHxS, were associated with HFF/liver steatosis at higher baseline ages.

FT15-05 | One Way Out: Source-to-outcome modeling methods and software to support environmental risk assessment

Kyle P. Messier, Skylar Marvel, Minoli Fernando, David M. Reif

National Institute of Environmental Health Sciences, Division of Translational Toxicology, Durham, USA

Objective

Source-to-outcome (S2O) modeling tracks chemicals from their release into the environment to determine human exposure levels and then uses biological pathways, toxicity mechanisms, dose-response modeling to predict potential adverse outcomes resulting from these exposures. S2O asserts that phenotypic outcomes caused by exogenous exposures at the individual or population level occur only after a sequence of source, exposure, and biological dynamics. GeoTox is an open-source, tested, and extensible software for S2O modeling. Our objective is to demonstrate novel methods and software in GeoTox for linking geospatial exposures with toxicological and mechanistic data to support risk mapping of chemical mixtures.

MATERIAL AND METHODS

New advancements in GeoTox allow the incorporation of multiple assay end-points into the geospatial risk mapping work-flow, which facilitate a more complete mechanistic risk mapping by incorporating multiple biological end-points or modes of action. First, we characterize the county-level, annual air toxic carcinogenic risk based on 200+ assays from the key characteristics of carcinogens (KCC). Second, we conduct multi-assay analysis of Per-and-polyfluoroalkyl substances (PFAS) at the county level, using age and obesity data from the North Carolina based Personalized Environment and Gene Study (PEGS) cohort.

RESULTS

The multi-assay GeoTox analysis for air toxics shows varying spatial patterns for assays representing genotoxicity and oxidative stress demonstrating a diversity in mechanisms for air toxics risk. Preliminary mapping results for PFAS in North Carolina implicated community water systems for heightened risk, with multi-assay hazard quotients exceeding the recommended safe level.

CONCLUSIONS

Our case study on mapping the total risk quantified by the KCC modes of action provides a straightforward approach to evaluate risk using NAMs that includes multiple routes or modes of toxicity. The PFAS and PEGS case study demonstrates how individual-level cohort data can be incorporated into S2O modeling with GeoTox to characterize risk to chemical mixtures.

FT15-06 | High Adherence to a Planetary Health Diet is Associated with Reduced PFAS Exposure

Roselyn B. Tanghal[1], Sarah Rock[1], Adelynn Paik[1], Hongxu Wang[1], Sihao Han[1], Ana Maretti-Mira[2], Lucy Golden-Mason[2], Jesse A. Goodrich[1], David V. Conti[1], Veronica W. Setiawan[1], Lida Chatzi[1]

[1] University of Southern California, Department of Population and Public Health Sciences, Los Angeles, USA; [2] University of Southern California, Department of Medicine, Division of Gastrointestinal and Liver Diseases, Los Angeles, USA

Objective

Per- and polyfluoroalkyl substances (PFAS) are persistent environmental chemicals linked to adverse health outcomes. While diet is a major source of PFAS exposure, its potential role in mitigating PFAS burden remains largely unexplored. The Planetary Health Diet (PHD) – developed by the EAT-Lancet Commission to promote health and environmental sustainability – may lower PFAS levels by limiting meat and emphasizing plant-based food. This study examines the association between PHD adherence and PFAS levels in a diverse population.

Material and Methods

We analyzed a subset of 2,800 participants from the Multiethnic Cohort, including those with liver diseases and controls. PFAS levels were measured in blood samples collected between 1998 and 2004 using untargeted liquid chromatography-mass spectrometry (LC-MS) and were $\log_2$-transformed for analysis. Planetary Health Diet Index (PHDI) scores were derived from baseline validated food frequency questionnaires and categorized into quintiles. We conducted linear regression analyses to assess the association between PHDI quintiles and PFAS levels.

Results

The study included male (44%) and female (56%) participants, with a mean age of 65 years. Japanese Americans comprised 55% of the cohort. PHDI scores ranged from 16 to 103. A higher PHDI score was associated with lower perfluorocarboxylic acids (PFCAs) and perfluorosulfonic acids (PFSAs) exposure. Each one-quintile increase in PHDI was linked to a 36.6% decrease in PFCA sum levels (β = -0.65, 95% CI: -1.13, -0.16, p = 0.01). A similar inverse association was observed for PFSA but was not statistically significant (β = -0.34, 95% CI: -0.77, 0.08, p = 0.11), corresponding to a 21.7% decrease in PFSA sum levels per quintile increase in PHDI.

Conclusion

Higher adherence to PHD is associated with lower PFAS exposure, particularly PFCAs. These findings suggest that dietary modifications aligned with PHD could serve as a potential strategy for reducing PFAS accumulation and mitigating health risks.

FT15-10 | Prenatal exposure to per- and polyfluoroalkyl substances (PFAS) and childhood asthma and allergies and vaccine antibodies: The Hokkaido Study

Yu Ait Bamai[1], Rehel M. Ketema[1], Atsuko Ikeda[2,1], Houman Goudarzi[3], Sachiko Ito[1], Takeshi Yamaguchi[1], Reiko Kishi[1]

[1] Hokkaido University, Center for Environmental and Health Sciences, Sapporo, Japan; [2] Hokkaido University, Faculty of Health Sciences, Sapporo, Japan; [3] Hokkaido University, the Department of Respiratory Medicine, Faculty of Medicine and Graduate School of Medicine, Sapporo, Japan

Concerns about their immunosuppressive effects of per- and polyfluoroalkyl substances (PFAS) have been raised, particularly at the population with high exposure levels, however evidence at general exposure levels remains scarce. Aim of this study is to examine the association between prenatal PFAS exposure and allergies, as well as vaccine antibody levels, in Japanese schoolchildren.

This study included 346 school children (9–12 years-old) who participated to face-to-face examinations of the Hokkaido Study and for whom the maternal blood from late pregnancy were available. Wheezing, allergic rhino-conjunctivitis, and eczema of children were assessed using the ISAAC questionnaire. Biomarkers for asthma and airway inflammation, eosinophil, Clara cell protein 16 (CC16) and fractional exhaled nitric oxide (FeNO), were measured, with FeNO ≥35 ppb indicating possible asthma. Vaccine antibody levels for diphtheria and tetanus were assessed. Concentrations of 13 PFAS in maternal blood were measured using LC-MS/MS. Logistic and multiple regression analyses were adjusted for confounders.

Higher prenatal PFDoDA, PFTrDA, and PFOS concentrations were associated with lower odds ratios (ORs) of rhino-conjunctivitis (PFDoDA: OR[95%CI] = 0.3 [0.1–0.72]; PFTrDA: 0.24 [0.07–0.67]) and eczema (PFDoDA: 0.51 [0.26–0.97]; PFOS: 0.23 [0.08–0.62]). PFOS levels were positively correlated with CC16 (β[95%CI] = 0.16 [0.01–0.30]). Eosinophil and FeNO were not associated with any PFAS. Among 10-year-old children who haven't taken booster at late-school-age, prenatal PFOS level was negatively correlated with tetanus antibody levels (β = -0.55 [-0.98, -0.12]).

Prenatal PFOS exposure may reduce the risk of allergies and suppress immune responses in school children, even at low exposure levels. However, the interpretations should be caution, as the potential effects of exposure to polychlorinated biphenyls (PCBs) and dioxins, which share common exposure sources with PFAS, have not been considered. Additionally, some associations were based on a small sample size and thus, further detailed evaluations are needed.

FT16 | Air pollution and systemic health outcomes across the life course

FT16-01 | Creating healthier homes for children with asthma: Exposure at home to indoor- and outdoor-generated $PM_{2.5}$ and their associations with asthma exacerbation using the West London Healthy Home and Environment (WellHome) study .

George Young, Dimitris Evangelopoulos, Klea Katsouyanni, Dylan Wood, Diana Varaden, David Greene, Benjamin Barratt

Imperial College London, Environmental Research Group, School of Public Health, Faculty of Medicine, London, UK

Background and Aim:
While associations between ambient air pollution and respiratory health in children with asthma are well documented, limited insight exists into the impact of personal and indoor $PM_{2.5}$ exposure. This project aims to combine indoor and outdoor $PM_{2.5}$ measurements and integrate these into refined metrics used in epidemiological models linking exposures to respiratory symptoms in children. This approach aims to clarify the contributions of indoor and outdoor sources to overall exposure and the subsequent health impacts.

Methods:
Indoor $PM_{2.5}$ (one-minute resolution) in three rooms and matched outdoor $PM_{2.5}$ (15-minute resolution) measurements were collected over two 28-day periods in 103 households with children with asthma. We estimated time spent in different microenvironments and respiratory symptom frequency via daily time-activity diaries. We combined home-specific infiltration factors with indoor and outdoor measurements to create multiple personal exposure metrics, including indoor- and outdoor-generated $PM_{2.5}$. Mixed-effects models will quantify associations between exposures (including appropriate lags) and health outcomes, adjusted for confounders identified via directed acyclic graphs (DAGs).

Results:
213 time-activity diaries were completed by 126 children. Preliminary results indicate that children spend 72.3% of their time at home and 90.1% indoors, underscoring the importance of considering indoor concentrations. Coughing and stuffy nose are the most common symptoms, reported on 27% of days. Averaging daily indoor $PM_{2.5}$ by household yielded an overall mean of 11.7 µg/m³ (intra-household SD: 13.5 µg/m³), with household means ranging from 2.06 to 89.9 µg/m³. Separate DAGs for the different exposure definitions have been constructed to account for their unique confounding structures.

Conclusion: This is one of the first extensive studies measuring $PM_{2.5}$ at homes of children with asthma. Observed indoor levels vary widely, with some households experiencing potentially hazardous exposures. Policy-makers, paediatricians, and families may consider strategies to reduce indoor and outdoor pollutant sources and mitigate asthma exacerbation risk.

FT16-03 | Prenatal Exposure to aerosol acidity and Preterm Birth, a birth cohort study in Beijing

Bin Han, Zhipeng Bai

Chinese Research Academy of Environmental Sciences, Beijing, China

As the major constituents of particulate matter, sulfate, nitrate, and ammonium (SNA) are associated with an elevated risk of preterm birth (PTB). However, epidemiological and toxicological studies show inconsistent health evidence caused by SNA, resulting in no direct biological mechanism that can explain the link between SNA and PTB. In this study, we proposed aerosol acidity as the intermediary factor mediating SNA impact on health outcomes. We investigated the association between prenatal exposure to aerosol acidity and the risk of PTB in a birth cohort from Beijing, China. Utilizing data from 2,520 singleton live births between November 1, 2022, and October 31, 2023, we assessed trimester-specific exposure to secondary inorganic aerosols (SNA) and aerosol acidity. In addition, we employed distributed lag models to identify critical exposure windows and estimate odds ratios (ORs) for PTB risk. Key findings revealed significant trimester-specific effects of aerosol acidity on PTB risk. A 1-unit decrease in aerosol pH during the first trimester was associated with a reduced PTB risk (OR = 0.52, 95% CI: 0.37-0.73), while third-trimester exposure significantly increased PTB risk (OR = 1.79, 95% CI: 1.27-2.52). Weekly-specific analysis identified the 21st to 27th gestational weeks as the susceptible window for aerosol acidity exposure, with elevated PTB risk observed during this period. This study highlights the critical role of aerosol acidity in influencing PTB risk, particularly during late pregnancy. It underscores the need for targeted interventions to mitigate the adverse effects of acid air pollutants on maternal and infant health.

FT16-05 | The Impact of War-Related PM2.5 Pollution on Public Health in Lebanon in 2024: A Case Study

Alicia Abi Nader[1,2], Nelly Ziade[4], Jowy Abi Hanna[1,2], George Mitri[5], Wehbeh Farah[6], Mira El Zarif[2], Emile Whaibeh[1,2], Jean Paule Joumaa[2], Elie Matta[3], Georges Abi Tayeh[7,8], Myriam Mrad[2,3]

[1] Saint-Joseph University of Beirut, Institut Supérieur de Santé Publique, Faculty of medicine, Beirut, Lebanon; [2] University of Balamand, Department of Public Health, Beirut, Lebanon; [3] Health and Environment Response Agency (HERA), Beirut, Lebanon; [4] Saint Joseph University and Hôtel-Dieu de France Hospital, Rheumatology Department, Beirut, Lebanon; [5] University of Balamand, Institute of the Environment, El Koura, Lebanon; [6] Saint-Joseph University of Beirut, Physics Department, Beirut, Lebanon; [7] Hôtel-Dieu de France Hospital, Obstetrics and Reproduction Department, Beirut, Lebanon; [8] Saint Joseph Fertility Center, Beirut, Lebanon

Objectives:
Armed conflicts hinder the quality of the air we breathe by generating particulate matter (PM2.5) resulting from explosions, and infrastructure destruction. This study addresses the health effects of PM2.5 pollution related to war consequences, focusing on skin and pulmonary effects in conflict-affected areas.
Material and Methods:
Data on ambiant PM2.5 levels prior to, during, and following conflict events were analyzed. Epidemiological studies were reviewed to establish associations between the exposure to PM2.5 and adverse health effects. PM2.5 levels were compared against WHO air quality guidelines to evaluate the extent of exceedance. Additionally, satellite data,

obtained from the Copernicus Atmosphere Monitoring Service (CAMS), were retrieved and compared with collected air quality data for the analysis. To estimate the relative risk (RR) for respiratory and skin diseases associated with increased exposure, RR functions obtained from the Beirut Air Pollution and Health Effects project were used. The attributable risk was calculated to estimate the proportion of disease cases in the population that could be attributed to the increased levels of PM2.5.

Results:

Compared to pre-war levels, PM2.5 concentrations increased drastically with an increase of 33.13%. Health risks also increased, with respiratory illness risk rising from 0.06 to 0.5 and skin disease risk from 0.06 to 0.6. These findings show the degradation of air quality resulting in health hazards during conflict, emphasizing the importance of monitoring and mitigation methods.

Conclusion:

This analysis provides insight into the public health impact of the observed air quality changes in Beirut following the war. The rise in PM2.5 causes an increased burden on public health, especially on individuals with pre-existing morbidities. To prevent the long-term health effects of war-related air pollution, immediate measures such as real-time air quality monitoring and emergency health interventions are required.

Funding: No Funding

FT16-06 | Spatial variability in the association of ambient $PM_{2.5}$ exposure with acute respiratory infections among children in Sub-Saharan Africa

Justice Moses Kwaku Aheto[1], Paul K. Adjorlolo[1], Patrick Attey-Yeboah[2], Kofi A. Amegah[2], John T. Molitor[3], Eric Coker[4]

[1] University of Ghana, Department of Biostatistics, School of Public Health, Legon, Accra, Ghana; [2] University of Cape Coast, Biomedical Sciences, Cape Coast, Ghana; [3] Oregon State University, College of Health, Corvalis, Oregon, USA; [4] BC Centre for Disease Control, Vancouver, British Columbia, Canada

Objective: Ambient $PM_{2.5}$ exposure was found to be associated with acute respiratory infections (ARIs). However, the effect of $PM_{2.5}$ on ARIs could vary substantially within and between countries, and over geographical locations, but not yet investigated. This study examined spatial heterogeneity in the association of $PM_{2.5}$ metrics on ARIs in sub-Saharan African (SSA) countries for targeted high-impact public health intervention strategies.**Material and Methods:** This modelling work utilized integrated data on child ARIs from the Demographic and Health Surveys (DHS) spanning from 1998 to 2022 across 33 SSA countries with high resolution (1km x 1km) satellite-derived geospatial $PM_{2.5}$ exposures to create a final dataset for this analysis. A total of 127,991 children <5 years across 21,924 clusters and 33 countries were included in the study. DHS GPS coordinates facilitated the extraction of $PM_{2.5}$ exposure metrics (i.e., prenatal and postnatal) from geospatial $PM_{2.5}$ data with a buffer of 2 km for urban residences and 5 km for rural residences to account for geographic coordinate displacement. Bayesian multilevel spatial logistic regression model was employed under the Integrated Nested Laplace Approximations (INLA) supported with interactive web-based mapping tools to model and map spatially varying associations while adjusting for suit of DHS assembled individual- and community-level confounders.

Results: The study found prenatal (*aOR* = 1.040, 95% CrI [1.024, 1.056]) and postnatal (*aOR* = 1.053, 95% CrI [1.036, 1.070]) $PM_{2.5}$ exposure metrics to be associated with 4.0% and 5.3% increased odds of ARIs, respectively.

The relationships found differ substantially within and between the SSA sub-regions and between countries with Uganda in East Africa recording the highest exposure health effects.

Conclusion: The association of ambient $PM_{2.5}$ exposure with child ARIs differ substantially within and between SSA sub-regions and between countries, requiring targeted high-impact interventions to help address the child ARIs problem in the SSA region.

FT16-07 | Kynurenine Pathways of Tryptophan Metabolism and Pro-inflammatory Effects of Air Pollution

Yan Lin[1], Xinchen Lu[4], Gajalakshmi Ramanathan[2], Fen Yin[2], Junfeng J. Zhang[1], Yifang Zhu[3], Jesus A. Araujo[2], Xinghua Qiu[4]

[1] Duke University, Nicholas School of Environment, Duhram, USA; [2] UCLA, David Geffen School of Medicine, Los Angeles, USA; [3] UCLA, Jonathan and Karin Fielding School of Public Health, Los Angeles, USA; [4] Peking University, College of Environmental Sciences and Engineering, Beijing, China

Objective: Mounting metabolomic studies including ours have associated exposure to air pollution with alterations in tryptophan metabolism. We aim to further understand the role of tryptophan metabolism in air pollution's health effects.

Material and Methods: We profiled tryptophan metabolomics in $ApoE^{-/-}$ mice with 2-week exposure to diesel exhaust (DE) or filtered air (FA) and in 26 healthy adults who traveled from Los Angeles (average $PM_{2.5}$ levels = 14.4 μg/m^3) to Beijing (average $PM_{2.5}$ levels = 67.6 μg/m^3) for 10 weeks. We previously reported that air pollution induced systemic lipid peroxidation consistently across the animal study and the travel study but induced pro-inflammatory effects only in the travelers. In this study, we compared the peripheral tryptophan metabolic alterations following air pollution in both studies and examined the associations of circulating and urinary tryptophan metabolites with biomarkers of inflammation and oxidative stress in the travelers.

Results: Air pollution induces different tryptophan metabolic alterations in mice versus humans. In mice, DE exposure activated indole pathways as evidenced by significant increases in tryptophan-normalized indole-3-lactic acid and indoele-3-propionic acids. In humans, traveling from Los Angeles to Beijing activated kynurenine pathways as evidenced by increased kynurenine to tryptophan ratio. The circulating kynurenine to tryptophan ratio is positively associated with circulating inflammatory biomarkers (i.e., CRP, IL-8, vWF, fibrinogen, and IL-10), but not with oxidative biomarkers (i.e., 8-isoprostane, 9- and 13-HODE, 5-, 12-, and 15-HETE). Circulating and urinary kynurenine to tryptophan ratios were positively correlated (Pearson r=0.44, p=0.0002).

Conclusion: Air pollution's pro-inflammatory effects may involve activated kynurenic pathways. Our results support the use of circulating or urinary kynurenine to tryptophan ratios to detect inflammatory responses to environmental changes.

FT16-08 | Air Pollution Predicts Healthcare Spending Among Older Adults in the United States

Sara D. Adar[1], Boya Zhang[1,2], Jennifer D'Souza[1], Adam Szpiro[3], Anjelica Gangaram[4], Jennifer Weuve[5], Carlos M. De Leon[6], Kayleigh Keller[7], Meredith Pedde[1], Jinkook Lee[8,9], Joel Kaufman[10,11], Jessica Faul[12], Kenneth Langa[12,13], Richard Hirth[4]

[1] University of Michigan School of Public Health, Epidemiology, Ann Arbor, USA; [2] Harvard TH Chan School of Public Health, Environmental Health, Boston, USA; [3] University of Washington School of Public Health, Biostatistics, Seattle, USA; [4] University of Michigan School of Public Health, Health Management and Policy, Ann Arbor, USA; [5] Boston University School of Public Health, Epidemiology, Boston, USA; [6] Georgetown University School of Health, Global Health, Washington DC, USA; [7] Colorado State University, Statistics, Fort Collins, USA; [8] University of Southern California, Center for Economic and Social Research, Los Angeles, USA; [9] University of Southern California, Economics, Los Angeles, USA; [10] University of Washington School of Public Health, Environmental and Occupational Health Sciences, Seattle, USA; [11] University of Washington School of Public Health, Medicine, Seattle, USA; [12] University of Michigan, Institute for Social Research, Ann Arbor, USA; [13] University of Michigan, Medicine, Ann Arbor, USA; [14] University of Michigan, Institute for Healthcare Policy and Innovation, Ann Arbor, USA

Objective: Air pollution is linked to poor health, including chronic disease, cognitive decline, and poor physical function. This likely leads to substantial healthcare spending, yet few studies have directly assessed these burdens using individual-level data and information on specific spending types. We investigated associations of air pollution with Medicare and out-of-pocket healthcare spending among older adults in the United States.

Material and Methods: We used Medicare-linked survey data from the nationally-representative Health and Retirement Study who were aged ＞67 years and enrolled in fee-for-service between 2006 and 2016. Using spatiotemporal models, we estimated 10-year average concentrations of $PM_{2.5}$, $PM_{10-2.5}$, NO_2, and O_3 at residential locations. We ran linear and quantile regressions with Medicare and out-of-pocket spending ($2016) for medical services, personal services, and prescription drugs per month, adjusting for confounding and accounting for survey design.

Results: Among 11,160 respondents (72±7yrs), the median annual total healthcare spending, including Medicare and out-of-pocket spending, was $9,130. Higher levels of $PM_{10-2.5}$ and NO_2 were associated with upward shifts in the distribution towards more spending. Interquartile range higher $PM_{10-2.5}$ and NO_2 concentrations were associated with $25 (95% CI: -19, 69) and $41 (95% CI: -35, 117) greater mean monthly total spending, respectively, whereas the 90th percentile of total spending increased by $51 (95% CI: 27, 75) for $PM_{10-2.5}$ and $87 (95% CI: 48, 126) for NO_2. O_3 was associated with lower mean total spending (-$66, 95% CI: -120, -12) and a lower 90th percentile (-$61, 95% CI: -85, -37), consistent with past findings for dementia and disability in this cohort. Medicare spending mainly drove the shifts for $PM_{10-2.5}$ and O_3 whereas NO_2 spending was split between Medicare and out-of-pocket spending.

Conclusions: Interventions to control key air pollutants may reduce societal and personal healthcare spending, particularly for individuals with substantial healthcare spending.

FT16-10 | Immune Marker Responses to Particulate Matter Exposure During Wildfire Events

Meredith Franklin[1], Mandy Yao[1], Marta Epeldegui[2], Michael Kleeman[3], Tarik Benmarhnia[5], Emiily Cauble[4], James Lacey[4], Sophia Wang[4]

[1] University of Toronto, Toronto, Canada; [2] UCLA, Los Angeles, USA; [3] UC Davis, Davis, USA; [4] Beckman Research Institute, Duarte, USA; [5] UCSD, San Diego, USA

Epidemiological studies investigating the short-term cardiovascular risks associated with fine particulate matter (PM2.5) exposure during wildfire events have yielded mixed results. Potential biological mechanisms underlying these effects include oxidative stress, systemic inflammation, and endothelial dysfunction, all of which can contribute to a heightened risk of cardiovascular events such as heart attacks and strokes.

A cross-section of 1900 female participants in the California Teachers Study cohort had 15 immune markers measured from serum collected in 2015. For the year leading up to serum collection, participant residences were geospatially linked daily PM2.5 concentrations from monitoring stations and wildfire smoke plumes. Outcomes were assessed for quantiles of the cytokine distributions. Individual and average lagged PM2.5 exposures were interacted with an indicator variable for the presence of wildfire smoke.

We observed statistically significant increased odds of elevated CCL2, a chemokine that plays a critical role in the recruitment of immune cells, associated with average and individual 6 to 8 day lagged PM2.5 on wildfire smoke-affected days. Other immune markers including CD14 and TNFR2, suggesting inflammatory response, showed similar lag patterns in increased odds associated with exposure. These associations suggest that immune activation and inflammatory pathways may play a key role in mediating cardiovascular health risks. This research helps to clarify the biological mechanisms through which wildfire smoke impacts cardiovascular health. As wildfire frequency and intensity continue to rise, understanding these pathways will be critical for mitigating air pollution-related health risks.

FT17 | Chemical exposures and pregnancy: From PFAS to extreme weather, new insights into birth outcomes and health trajectories

FT17-01 | Association of Maternal Bone Resorption with Urinary Fluoride Levels During Pregnancy- the ELEMENT Pregnancy and Birth Cohort

Howard Hu[1], Morteza Bashash[2], Karen Peterson[3], Angeles M. Mier[4], Martha M. Tellez-Rojo[5]

[1] University of Southern California, Keck School of Medicine, Population and Public Health Sciences, Los Angeles, USA; [2] University of Southern California, Keck School of Medicine, Population and Public Health Sciences, Los Angeles, USA; [3] University of Michigan School of Public Health, Nutrition, Ann Arbor, USA; [4] Indiana University School of Dentistry, Dental Public Health and Dental Informatics, Indianapolis, USA; [5] Instituto Nacional de Salud Publica, Mexico, Center for Nutrition and Health Research, Cuernavaca, Mexico

Objective

A growing body of evidence implicates fluoride, which is ingested in drinking water, salt, and various foods, as a neurodevelopmental toxicant. Fluoride accumulates in the skeleton, which may serve as an internal exposure source since maternal bone resorption accelerates during pregnancy to provide calcium to the growing fetal skeleton. We tested this hypothesis.

Material and Methods

We utilized data from 524 mothers participating in the Early Life Exposures in Mexico to Environmental Toxicants (ELEMENT) Birth Cohort project who contributed fasting morning urine samples that were analyzed for levels of fluoride and the N-terminal telopeptide of type I collagen (NTX-1; a specific biomarker of bone resorption). Generalized linear models (GLM) were used to evaluate the association between NTX-1 and MUF, adjusting for maternal age, parity (firstborn status), and neonatal birth weight. (Since both the exposure [NTX-1] and outcome [MUF] are urinary measures, correction for dilution was not necessary).

Results

Mean MUF levels were 0.84, 0.90, and 0.79 mg/L, and mean NTX-1 levels were 69.81, 98.36, and 133.56 nM BCE/mM during trimesters 1 (n=411), 2 (n=217), and 3 (n=236), respectively. GLM analysis showed a significant association between NTX-1 and MUF in all trimesters. The strongest relationship was observed in the second trimester, in which a 1 SD increase in NTX-1 was associated with an increase in MUF of 0.1325 mg/L (95%CI: 0.057, 0.208; $p<0.001$). Maternal age was significantly associated with higher MUF in trimester 1 ($p = 0.04$), while firstborn status was associated with higher MUF in trimester 2 ($p = 0.03$).

Conclusion

Maternal bone stores may represent a significant source of fetal fluoride exposure via maternal bone resorption. More research is needed to assess the contribution to fetal fluoride exposure of maternal bone resorption as well as potential mitigation strategies.

FT17-02 | Tropical Cyclone Exposure and Risk of Preterm Birth

Xuejuan Ning[1,2], Kai Chen[1], Zeyan Liew[1,2], Seulkee Heo[3], Michelle Bell[1,3], Nicole Deziel[1,2]

[1] Yale School of Public Health, Department of Environmental Health Sciences, New Haven, USA; [2] Yale School of Public Health, Yale Center for Perinatal, Pediatric and Environmental Epidemiology, New Haven, USA; [3] Yale School of Environment, New Haven, USA

Objective

Tropical cyclones have the highest mortality and property damage among U.S. environmental disasters. Evidence suggests an association between tropical cyclones and preterm birth, but findings have been inconsistent, potentially due to differences in storm metrics. This study evaluates the relationship between tropical cyclone exposure and preterm birth in Georgia counties from 2000-2018.

Materials and Methods

Preterm birth data (gestational age <37 weeks) was obtained from the Georgia Office of Vital Records. Tropical cyclones were defined using four metrics: maximum sustained wind speed, cumulative rainfall, distance to storm track, and flood events. Multiple thresholds were applied to these metrics to categorize counties as exposed or unexposed. Using a two-stage time series analysis, we first estimated county-level relative risks (RR) of preterm birth by comparing tropical cyclone weeks to non-tropical cyclone weeks using a zero-inflated Poisson model with cubic splines for time trend and temperature. Then, we pooled county-specific estimates in a meta-analysis.

Results

Our study included 56 storms (occurring within 250km of the study area during the storm seasons of May–November) and 1,444,551 births. Preliminary analyses found that higher sustained wind speeds were associated with increased risk of preterm birth with RR of 1.28 (95% CI: 1.10-1.49) and 1.83 (95% CI: 1.26-2.67) for wind speeds >17m/s and >25m/s, respectively. Proximity to storm track showed a higher risk for preterm birth within 5km (RR: 1.56, 95% CI: 1.17-2.08). Higher cumulative rainfall yielded increased preterm birth risk, with RR of 1.28 (95%CI: 1.10-1.49) and 1.52 (95% CI: 1.10-2.11) for rainfall of >125mm and >175mm. Flood events (any vs. none) were associated with an elevated RR for preterm birth (RR: 1.12, 95% CI: 0.99-1.25).

Conclusion

Tropical cyclone exposure, assessed using multiple metrics, was associated with increased risk of preterm birth, with higher intensity exposure levels linked to greater risk.

FT17-05 | Associations Between Maternal Semi-Volatile Organic Compound Exposure and Vaccine Responses in Pregnant Women

Elizabeth Boxer[1], Jillian Hurst[2,3], Rebecca Hoehn[1], Maria Dennis[4], Giny Fouda[4], Heather Stapleton[1], Kate Hoffman[1]

[1] Duke University, Nicholas School of the Environment, Durham, USA; [2] Duke University School of Medicine, Department of Pediatrics, Children's Health & Discovery Initiative, Durham, USA; [3] Duke University School of Medicine, Department of Pediatrics, Division of Infectious Diseases, Durham, USA; [4] Weill Cornell Medicine, Department of Pediatrics, New York, USA

Vaccinations are commonly administered to pregnant women to prevent neonatal infection and ensure that infants have protective antibodies prior to their own vaccination. Emerging research suggests that exposure to environmental chemicals may interfere with immune function and potentially alter the transplacental transfer of antibodies. We sought to investigate whether maternal exposure to a range of semi-volatile organic compounds (SVOCs) alter the immune response to Tdap (tetanus-diphtheria-acellular pertussis) vaccine administered during pregnancy.
SVOC exposures were evaluated in silicone wristbands, which were worn for 5 days in each trimester by participants in a pregnancy cohort in central North Carolina. Wristbands were extracted and analyzed via gas chromatography tandem mass spectrometry for SVOCs. IgG antibodies for tetanus, diphtheria, and pertussis vaccine antigens were measured in serum pre- and post-vaccination using a Luminex multiplex assay, reported in mean fluorescent intensity (MFI). Analysis is ongoing, but 78 wristbands from 33 pregnancies have been examined to date.
Of the 110 compounds analyzed in wristbands, twelve compounds were detected in all samples, and forty were detected in >60% of samples. Exposure to several SVOCs throughout pregnancy were associated with changes in maternal response to the Tdap vaccination. For example, doubling Di(2-ethylhexyl) adipate (DEHA) exposure on wristbands was associated with a 1963 increase in MFI value for pertussis antibodies (adjusted β=1963.5; 95%CI: 645.6– 3281.6). A doubling of 2-Ethylhexyl diphenyl phosphate (EHTBB) and pyrene on wristbands were associated with a decrease of 208.8 and 537.4 in MFI value respectively for tetanus at delivery (EHTBB adjusted β=-208.8 95%CI: =-408.6– -8.9; Pyrene adjusted β=-537.4; 95%CI: =-981.1– -95.6).
Preliminary findings suggest that SVOC exposure during pregnancy may alter maternal Tdap vaccine response. Understanding environmental influences on maternal immunity is crucial, as vaccination remains one of the most effective ways to protect newborns from life-threatening infections.

This work is funded by NIH NIAID.

FT17-06 | Diet as a modifier of the association between prenatal per- and polyfluoroalkyl substances (PFAS) exposure and adverse birth outcomes

Yunjie Huang[1], Donghai Liang[1], Anne Dunlop[2], Dana B. Barr[1], Kurunthachalam Kannan[3,4], P Barry Ryan[1], Parinya Panuwet[1], Volha Yakimavets[1], Youran Tan[1], Stephanie Eick[1]

[1] Emory University Rollins School of Public Health, Gangarosa Department of Environmental Health, Atlanta, USA; [2] Emory University School of Medicine, Department of Gynecology & Obstetrics, Atlanta, USA; [3] New York State Department of Health, Wadsworth Center, Albany, USA; [4] State University of New York at Albany, Department of Environmental Health Sciences, Albany, USA

Objective: Previous research has consistently linked prenatal PFAS exposure to adverse pregnancy and birth outcomes. However, little is known about potentially modifiable factors that may mitigate the harmful effects of PFAS. We assessed whether prenatal dietary supplements modified the inverse associations between prenatal PFAS exposure and gestational age at birth (GA) and birthweight for gestational age z-scores (BWZ). We hypothesized that PFAS would be associated with adverse birth outcomes among those who have low levels of folate and vitamin D, and high levels of dietary inflammation.

Material and Methods: Participants were a subset of the Atlanta African American Maternal-Child cohort (N=271). Serum samples collected between 8 and 14 weeks of gestation were analyzed for four PFAS (PFOA, PFOS, PFNA, PFHxS), as well as free and total 25(OH)D. A semi-quantitative food frequency questionnaire was administered during early pregnancy and was used to ascertain folate intake and dietary inflammation index (DII) over the last three months.We assessed whether diet measures (free and total Vitamin D, folate, and DII) modified the association between individual PFAS and birth outcomes using linear regression.We also examined individual diet measures as effect modifiers of a PFAS mixture using quantile g-computation and Bayesian kernel machine regression (BKMR).

Results: A natural log increase in PFOS and PFOA (analyzed separately) was associated with significant decreases in birthweight z-scores only among those whose diets were higher in inflammation (i.e., DII $\geq 75^{th}$ percentile) and lower in total 25(OH)D (i.e., total 25(OH)D $<75^{th}$ percentile).Associations were similar using quantile g-computation (e.g., Ψ= -0.50, 95% CI= -0.81, -0.19 for DII $\geq 75^{th}$ percentile, and Ψ= -0.10, 95% CI= -0.25, 0.06 for DII $<75^{th}$ percentile). No dietary measures modified associations between PFAS and gestational age.

Conclusion: Our findings suggested that diet may represent a possible intervention to mitigate harmful effects of PFAS exposure.

FT17-07 | Association of early PFAS exposure with maternal blood pressure and hypertension during pregnancy in the PROTECT cohort

Julia R. Varshavsky[1,2], Emily Zimmerman[1], Amber Cathey[4], Ram Siwakoti[4], Zaira Y. Rosario-Pabon[5], Carmen M. Vélez-Vega[5], Akram Alshawabkeh[2], José F. Cordero[6], John D. Meeker[4]

[1] Northeastern University, Department of Public Health and Health Sciences, Boston, USA; [2] Northeastern University, Department of Civil and Environmental Engineering, Boston, USA; [3] Northeastern University, Department of Communication Sciences & Disorders, Boston, USA; [4] University of Michigan School of Public Health, Department of Environmental Health Sciences, Ann Arbor, USA; [5] University of Puerto Rico Medical Sciences Campus, Department of Social Sciences, Graduate School of Public Health, San Juan, Puerto Rico; [6] University of Georgia, Department of Epidemiology and Biostatistics, College of Public Health, Athens, USA

Objective:
Per- and poly-fluoroalkyl substances (PFAS) are a large class of synthetic chemicals used as surfactants in stain and water-resistant products. They have been associated with an increased risk of hypertensive disorders of pregnancy, although findings across epidemiologic studies are inconsistent.
Material and Methods:
PFAS exposure was measured among 548 participants of the Puerto Rico PROTECT cohort study, at one time point during pregnancy (16–20 weeks) and blood pressure (BP) was measured at two time points during pregnancy (16–20 weeks, 24–28 weeks). We used multivariable linear regression to investigate associations between PFAS exposures and BP as continuous BP endpoints for diastolic (DBP) and systolic BP (SBP). We also examined hypertension during pregnancy (defined as stage 1 or 2 hypertensive blood pressure status) as a binary outcome in adjusted logistic regression models. Quantile g-computation was used to examine mixture effects.
Results:
Among four PFAS detected in more than 50% of the study population, which included perfluorooctanoic acid (PFOA), perfluorooctane sulfonic acid (PFOS), perfluorohexanesulfonic acid (PFHxS), and perfluorononanoic acid (PFNA), we found a consistent pattern of increased associations with continuous blood pressure measurements (DBP and SBP) at both pregnancy time points. We also found an association between exposure to all four individual PFAS and their mixture in early pregnancy (16–20 weeks) and increased odds of hypertension in later pregnancy (24–28 weeks), with an adjusted odds ratio (OR) = 1.61 and 95% confidence interval (CI) ranging from 1.17–2.21 for the PFAS mixture.
Conclusions:
Our findings indicate that 16–20 weeks' gestation may be a critical period of sensitivity for PFAS exposure with respect to maternal blood pressure and hypertension status during pregnancy. Further research is warranted in a larger sample testing PFAS and measuring BP. The role of PFAS-associated hypertension should be examined in relation to pre-eclampsia.

FT17-08 | Prenatal Exposure to PFAS, Associations with Child IQ and Modification by Maternal Vitamin D Status: The MABC Study

Xiaoyan Wu

Brown University, Epidemiology, Providence, USA

BACKGROUND: Prenatal PFAS exposure may adversely influence neurodevelopment. However, few studies have considered whether cumulative PFAS exposure over the entire course of pregnancy impacts child cognitive development and whether nutritional factors modify these associations.

OBJECTIVES: To investigate whether Vitamin D (VD) status modifies the association between maternal PFAS exposure and child cognitive function.

METHODS: This analysis used 728 mother-child pairs in the Ma'anshan Birth Cohort, a population-based Chinese birth cohort enrolled from 2013-2015. We measured serum PFAS and VD concentrations during the first, second, and third trimesters of pregnancy. Child intelligence quotient (IQ) was assessed at age 3.0-6.0 years. We used linear regression, Bayesian kernel machine regression, and quantile g-computation to estimate covariate adjusted associations of individual PFAS concentrations and their mixture with child cognitive scores. Analyses were stratified by sex to examine sex-specific associations, with further stratification by categorizing maternal VD levels into deficient (<20 ng/mL) and non-deficient (≥ 20 ng/mL) groups.

RESULTS: Overall, average br-PFOA, 8:2 Cl-PFESA and n-PFHxS concentrations were inversely associated with VCI, VSI and WMI scores, respectively. Generally, PFAS were associated with lower cognitive scores in boys, but not girls. Further, the PFAS mixture was negatively associated with boys' total cognitive scores (β = -1.94, 95%CI: -3.69, -0.19), with br-PFOA and n-PFHxS have the greatest weights. After stratifying by VD status, adverse associations were primarily observed in the deficient group among boys, whereas positive associations were present only in the deficient group among girls. Specifically, associations of cognitive scores with n-PFOA, br-PFOA, PFNA, n-PFHxS, and br-PFHxS were significant stronger among boys born to women with VD deficiency those with sufficient levels of VD.

DISCUSSION: We observed sex-specific adverse associations between prenatal PFAS and cognitive function. Our findings suggest that prenatal VD may modify these associations, potentially mitigating the neurotoxic effects of prenatal PFAS exposure.

FT17-10 | Associations between exposure to metals and trajectories of maternal blood pressure during pregnancy

Amber Cathey[1], Deborah Watkins[1], Thomas McElrath[2], Julia Varshavsky[3,4], Zaira R. Pabon[5], Carmen Vélez-Vega[5], Akram Alshawabkeh[3], José Cordero[6], John Meeker[1]

[1] University of Michigan School of Public Health, Department of Environmental Health Sciences, Ann Arbor, USA; [2] Brigham and Women's Hospital, Division of Maternal Fetal Medicine, Department of Obstetrics and Gynecology, Boston, USA; [3] Northeastern University College of Engineering, Department of Civil and Environmental Engineering, Boston, USA; [4] Northeastern University, Department of Public Health, Boston, USA; [5] University of Puerto Rico Graduate School of Public Health, Medical Sciences Campus, San Juan, Puerto Rico; [6] University of Georgia School of Public Health, Department of Epidemiology and Biostatistics, Athens, USA

Objective: Elevated blood pressure (BP) during pregnancy increases risk of early delivery and morbidity later in life. Environmental exposures may alter BP trends during pregnancy.

Materials and Methods: Utilizing the Puerto Rico PROTECT birth cohort, urine samples were collected at up to three time points (median 18, 22, and 26 weeks) for assessment of 14 toxic and essential metals, and BP was measured up to 17 times through gestation during ultrasound appointments. We calculated the geometric mean of specific gravity-corrected metals concentrations as a measure of overall pregnancy exposure. Women were excluded if they met criteria for pre-existing hypertension, giving a final sample size of 628. Latent class linear mixed models were used to group participants based on trajectories of BP change over pregnancy. Generalized linear models were then used to regress BP trajectory groups on metals exposure, adjusting for age, education, parity, and pre-pregnancy body mass index.

Results: Trajectory groups of diastolic BP (DBP) were “steady”, “falling” (starting low and decreasing), “rising” (steep increase from normal to very high), and “catch-up” (starting low, rising to normal). “Steady” and “rising” groups were also identified for systolic BP (SBP), plus a “high” group (starting high, rising to very high). An interquartile range (IQR) increase in toxic cadmium was associated with increased odds of “falling” DBP (OR: 1.80; 95%CI: 1.02, 3.16), while an increase in essential manganese (OR: 0.52; 95%CI: 0.38, 0.72) and tin (OR: 0.35; 95%CI: 0.16, 0.74) were associated with reduced odds of “falling” DBP relative to “steady”. Finally, increased mercury was associated with increased odds of “high” SBP (OR: 2.46; 95%CI: 1.18, 5.15), while tin was protective against “high” SBP (OR: 0.32; 95%CI: 0.12, 0.85).

Conclusions: Exposure to toxic metals during pregnancy is associated with adverse trajectories of BP over pregnancy, while essential metals are protective against these patterns.

FT18 | Air pollution, pregnancy, and birth outcomes

FT18-01 | Acute associations between ambient air pollution and risks of preterm and early-term births: results from 8 states in the United States

Xiaping Zheng[1], Amy Fitch[3], Matthew J. Strickland[3], Lyndsey A. Darrow[3], Joshua L. Warren[4], Andrew Newman[5], Howard H. Chang[1,2]

[1] Emory University, Department of Biostatistics and Bioinformatics, Atlanta, USA; [2] Emory University, Gangarosa Department of Environmental Health, Atlanta, USA; [3] University of Nevada, Reno, School of Public Health, Reno, USA; [4] Yale University, Department of Biostatistics, New Haven, USA; [5] NSF National Center for Atmospheric Research, Boulder, USA

Background: Exposure to higher levels of ambient air pollution during pregnancy has been linked to multiple adverse pregnancy outcomes. However, prior studies on acute exposures and reduced gestation length have reported inconsistent findings.
Objective:To examine the acute association between ambient air pollution and preterm (28-36 gestational weeks) or early-term (37-38 gestational weeks).
Methods: Daily concentrations of 12 air pollutants, based on bias-corrected numerical model outputs, were linked to vital records for singleton live preterm and early-term births in California, Florida, Georgia, Kansas, Nevada, New Jersey, North Carolina, and Oregon from 2005-2017. Under a time-stratified case-crossover design, we fitted conditional logistic regression with adjustment for at-risk ongoing pregnancies, meteorology, time trends and federal holiday. We estimated the 0-6 day cumulative associations using daily exposures and distributed lag models; shorter and longer lags were also examined. Risk estimates per interquartile range (IQR) increase in exposure were pooled across states using inverse-variance weighting.
Results: Our study included 1,085,162 preterm and 3,901,185 early-term births. We observed positive associations between weekly lagged exposure to several air pollutants and early-term births [e.g., odds ratio (OR) = 1.003 (95% confidence interval of 1.001, 1.005) per 7.9 *µg/m3* increase in PM_{10}, OR= 1.002 (95% CI: 1.000, 1.004) per 4.6 *µg/m3* increase in $PM_{2.5}$, OR= 1.002 (95% CI: 1.000, 1.004) per 1.7 *µg/m3* increase in organic carbon, OR =1.003 (95% CI: 1.000, 1.005) per 1.6 *µg/m3* increase in sulfate, and OR=1.002, (95% CI: 1.000, 1.003) per 0.26 *µg/m3* increase in elemental carbon]. Associations with preterm birth were mostly null.
Conclusion: We reported positive associations between short-term air pollution exposure, including PM and major $PM_{2.5}$ components, and higher odds of early-term birth.

FT18-02 | Prenatal Exposure to Fire-Related $PM_{2.5}$ and Birth Weight: Assessing the Impact of Prescribed Burns in Georgia, USA

Lei Li[1], Jennifer D. Stowell[2], Emily M. Gargulinski[3], Kamal Maji[4], Talat Odman[4], Hua Hao[1], Howard H. Chang[5], Danlu Zhang[5], Meng Qi[1], Amber Soja[6], Yang Liu[1]

[1] Emory University, Department of Environmental Health, Atlanta, USA; [2] Boston University, Department of Environmental Health, Boston, USA; [3] National Institute of Aerospace, Hampton, USA; [4] Georgia Institute of Technology, Atlanta, USA; [5] Emory University, Department of Biostatistics and Bioinformatics, Atlanta, USA; [6] NASA Langley Research Center, Hampton, USA

Objective: In the Southeastern United States, prescribed burning is a major but poorly characterized source of fine particulate matter ($PM_{2.5}$) emissions. While exposure to $PM_{2.5}$ from fires has been linked to adverse birth outcomes, the impact of prescribed fires on birth weight remains understudied, especially in the Southeast. This study investigates the association between $PM_{2.5}$ exposure from prescribed burns during pregnancy and birth weight in Georgia, USA.

Material and Methods: Term birth records for singleton live births (≥37 and ≤42 weeks of gestation) with an estimated gestational start date from January 1, 2013 to December 31, 2016 (n = 457,219), were obtained from Georgia Department of Public Health. Daily prescribed fire-related $PM_{2.5}$ concentrations at the ZIP code level were averaged for the first trimester (gestational weeks 1–13), second trimester (weeks 14–26), third trimester (weeks 27 to delivery), and the whole pregnancy. Linear regression estimated associations between birth weight and prescribed fire $PM_{2.5}$ exposure adjusting for residential county and maternal characteristics.

Results: In the first trimester, each interquartile range increase (0.75 μg/m^3) in $PM_{2.5}$ was associated with a 3.45g decrease in birth weight (95% confidence interval: 0.76g to 6.14g). No associations were observed in the second or third trimesters, or across the whole pregnancy.

Conclusion: Prescribed fire-related $PM_{2.5}$ exposure during pregnancy was associated with lower birth weight, particularly in the first trimester. These findings highlight the need for targeted public health interventions to reduce prenatal air pollution exposure and mitigate its adverse effects on birth outcomes.

FT18-03 | Association Of Gestational Exposure to Black Carbon and Birth Weight: Evidence From Denmark and Sweden

Zorana J. Andersen[1], Jiawei Zhang[1], Kajsa Pira[2], Marie Bergmann[1], Rina So[1], George M. Napolitano[1], Cale Lawlor[2], Tanya A. Nystedt[2], Youn-Hee Lim[1], Matthias Ketzel[3,4], Anna Oudin[2], Marie Pedersen[1], Ebba Malmqvist[2]

[1] University Of Copenhagen, Section of Environmental Health, Department of Public Health, Copenhagen, Denmark; [2] Lund University, Division of Occupational and Environmental Medicine, Lund, Sweden; [3] Aarhus University, Department of Environmental Science, Roskilde, Denmark; [4] University of Surrey, Global Centre for Clean Air Research (GCARE), Guildford, UK

Background: Air pollution has been linked to reduced birthweight, but the association with black carbon (BC) remains insufficiently explored. This study examined the association of gestational exposure BC with birth weight outcomes.
Method: We analyzed data from all singleton births in Denmark (DK, 1998–2016; n=1,058,906) and Scania, Sweden (SE, 2000–2009; n=43,676). BC and fine particulate matter ($PM_{2.5}$) were estimated by using high-resolution dispersion models based on maternal residential addresses during pregnancy. Linear, Logistic and Poisson regression models were employed to assess the associations of BC with birth weight, low birth weight (LBW), and small for gestational age (SGA), adjusting for spatiotemporal and individual-level covariates. Effect modification by gender, mothers' age and socio-economic status (SES) were examined.
Results: Gestational exposure to BC was associated with birth weight and SGA risk: interquartile range (0.34 μg/m^3 in DK, and 0.25 μg/m^3 in SE) increase in BC was associated with -6 g (95% CI: -7, -5.34) and -9.41 g (-17.05, -1.78) decrease in birth weight and a relative risk (RR) of 1.01 (1.00, 1.02) and 1.09 (1.02, 1.18) for SGA in DK and SE, respectively. No association was observed with LBW. After further adjustment for$PM_{2.5}$, the associations were attenuated but remained borderline significant with both for birth weight and SGA, in both cohorts. Associations of BC with birthweight in DK were stronger in boys, young mothers and mothers with low SES.
Conclusion: Our findings suggest that BC adversely affects birth weight, potentially independent of $PM_{2.5}$, and add important new evidence supporting regulation of BC in addition to $PM_{2.5}$.

FT18-05 | Fine particulate matter components and preterm birth: A hypothetical intervention study using parametric g-computation

Zilong Zhang[1], Yonggui Gao[1], Qingmei Lin[2], Hualiang Lin[1]

[1] Sun Yat-sen University, School of Public Health, Guangzhou, China; [2] Foshan Women and Children Hospital, Foshan, China

Objective
Fine particulate matter ($PM_{2.5}$) has been widely recognized as a risk factor for preterm birth (PTB), but the key components driving the $PM_{2.5}$-PTB association remain unclear. The present study was aimed at examining the associations between gestational exposure to $PM_{2.5}$ components and PTB using a hypothetical intervention approach.

Material and Methods

A birth cohort of 148,068 mother-child pairs from Foshan, China was constructed using health administrative database. Gestational exposure to $PM_{2.5}$ major components, including black carbon (BC), organic matter (OM), ammonium (NH_4^+), nitrate (NO_3^-), sulfate (SO_4^{2-}), was estimated using satellite-based spatial-temporal models at each participant's residence. Survival analyses incorporating parametric g-computation and distributed lag models were conducted to estimate the cumulative risks of PTB (gestation < 37 weeks) under hypothetical interventions of reducing the concentrations of $PM_{2.5}$ components in each gestational week. Potential benefits (number of preventable PTBs) were also calculated in comparison to the natural course with no interventions. The models were adjusted for a range of potential confounders (maternal age, socioeconomic status, etc.).

Results

A total of 9,757 (6.6%) PTBs were observed in the birth cohort. The hypothetical interventions of reducing all five $PM_{2.5}$ components changed the cumulative risks of PTB at week 36 (i.e., the end of preterm period). In particular, a 20% reduction in the concentrations of BC, OM, NH_4^+, NO_3^- and SO_4^{2-} was associated with a risk difference of -0.0012, -0.0019, -0.0024, -0.0025 and -0.0011, respectively. The corresponding numbers of preventable PTBs were 179, 286, 355, 376 and 163. No significant effect modification by maternal age or child sex was observed.

Conclusion

Findings of the present study indicate that reductions in $PM_{2.5}$ components, especially NH_4^+ and NO_3^-, were associated with lower risks of PTB, advocating the continuing efforts to mitigate $PM_{2.5}$ air pollution.

FT18-09 | Association between maternal exposure to particulate matters and birth defects: a retrospective cohort in Shanghai

Cheng Li[1], Si-Wei Zhang[1], Tao Li[2], Yan-Ting Wu[1], This contribution is not on behalf of any organization.

[1] Fudan University, Obstetrics and Gynecology Hospital, Institute of Reproduction and Development, Shanghai, China; [2] Shanghai Jiao Tong Univeristy, International Peace Maternity and Child Health Hospital, School of Medicine, Shanghai, China

Objective: The relationship between particulate matters of different diameters and birth defects exhibits significant heterogeneity across different regions and exposure time windows. This study aimed to estimate the effect of exposure to PM during different periods on the birth defects.

Methods: Pregnant women and their infants of this retrospective cohort were included in Shanghai between 2014 and 2020, with approval of the ethics committee of two obstetric hospitals. Individual exposure levels of PMs were estimated via satellite-based modeling methodology according to their residential geocoded addresses. The associations between different types of birth defects and exposure to PMs throughout different periods were estimated using generalized linear model.

Results: A total of 140,984 mother-infant pairs were included in the analysis. Logistic regression analyses showed that maternal exposure to $PM_{2.5}$ were associated with any birth defects during 3 months before conception (aOR=1.17, 95%CI=1.08-1.20), the first trimester (aOR=1.12, 95%CI=1.05-1.19), and the second trimester (aOR=1.17, 95%CI=1.09-1.25). Furthermore, maternal exposure to $PM_{2.5-10}$ were also linked to any birth defects during the first trimester (aOR=1.19, 95%CI=1.03-1.37). While PM_{10} exposures during the first trimester were found to have a borderline relationship with any birth defects (aOR=1.06, 95%CI=0.99-1.14). For isolated birth defect, we found that the risk of cardiovascular defects was increased after exposure to increased exposure of $PM_{2.5}$ during preconception period (aOR=1.23, 95%CI=1.09-1.38) and early pregnancy (aOR=1.19, 95%CI=1.05-1.34). We also

found that urogenital defects were associated with increased exposure of $PM_{2.5}$ (preconception: aOR=1.21, 95%CI=1.07-1.36; 1st trimester: aOR:1.20, 95%CI=1.05-1.37; 2nd trimester: aOR=1.24, 95%CI=1.07-1.44) and $PM_{2.5-10}$ (preconception: aOR=1.39, 95%CI=1.03-1.86; 1st trimester: aOR=1.36, 95%CI=0.99-1.85). Furthermore, chromosomal abnormalities were associated with $PM_{2.5-10}$ (aOR=1.57, 95%CI=1.03-2.40) and PM_{10} (aOR=1.48, 95%CI=1.05-2.09) during the second trimester.**Conclusions:** Maternal exposure to increased levels of PMs before and during pregnancy is associated with higher risk of birth defects, including cardiovascular defects, urogenital defects and chromosomal abnormalities in offspring.

FT18-10 | Association between PM2.5 exposures, extreme heat, and under-5 mortality (U5MR): A systematic review and meta-analysis

Sreevatsan Raghavan[1], Mudita Gosain[1], Divya Pillai[1], Nitya Wadhwa[1], Shinjini Bhatnagar[1], Ramachandran Thiruvengadam[2,1], Jane Hirst[3], Bapu K. Desiraju[1], Ayushi Ayushi[1]

[1] Translational Health Science and Technology Institute, Maternal and Child Health, Faridabad, India; [2] Pondicherry Institute of Medical Sciences, Biochemistry, Puducherry, India; [3] The George Institute for Global Health, Woman's Health, London, UK

Background: One of the major global health challenges is under-5 mortality (U5MR), which is more pronounced in low- and middle-income countries (LMICs). Limited evidence exists on the association between PM2.5, extreme heat, and U5MR, particularly in understanding differences in effect sizes between high-income countries (HICs) and LMICs.

Methods: Numerous databases (PubMed, EMBASE, Scopus, Web of Science, Global Health Library, Google Scholar, and grey literature) were systematically searched for peer-reviewed articles published between January 2010 and January 2025, with PM2.5 and ambient heat as exposures and U5MR as the outcome. After screening 2973 observational studies, 17 were included. The risk of bias assessment was done using the Non-randomized Studies of Exposures (ROBINS-E) tool. Random-effects meta-analyses using the DerSimonian and Laird method were done along with subgroup analysis by income level (HIC vs. LMIC). Egger's test, trim-and-fill method, and leave-one-out analysis were used to assess sensitivity.

Results: The pooled risk ratio (RR) for heat stress was 1.09 [95% CI: 0.95, 1.24], with higher and consistent risk in LMICs (1.07 [95% CI: 1.01, 1.13]). In contrast, estimates for HICs were inconclusive, with wide confidence intervals, crossing the null (1.11 [95% CI: 0.85, 1.44]. For PM 2.5 exposure, the pooled RR was 1.08 [95% CI: 1.03, 1.14], with LMICs exhibiting a higher risk (1.09 [95% CI: 1.03, 1.15]) compared to HICs (1.06 [95% CI: 1.01, 1.12]). Although high heterogeneity was observed for both heat and PM 2.5 studies [95-99%], sensitivity analyses validated the robustness of the model.

Conclusion: Postnatal high heat and PM 2.5 exposures are associated with increased risk of U5MR, particularly in LMICs where children are more vulnerable. The findings do underscore the need for large-scale harmonized studies in this domain along with targeted interventions aimed at mitigating environmental risks with the ultimate aim of bringing about change at the policy level.

FT19 | Climatic stressors and health: Modeling vulnerability, risk, and response across regions

FT19-01 | Heat Vulnerability Profiles Among Older Adults in Switzerland: A Case Time Series Analysis from 2019 to 2022

Sujung Lee[1,2], Ana M. Vicedo-Cabrera[1,2], This research is funded by the NCCS-Impacts programme (Swiss Federal Government), under the Project "Impacts of climate change on health, well-being and performance of humans and animals and on food safety in Switzerland"

[1] University of Bern, Institute of Social and Preventive Medicine, Bern, Switzerland; [2] University of Bern, Oeschger Centre for Climate Change Research, Bern, Switzerland

Objective: Older adults are more vulnerable to heat, yet our understanding of how heat-related health impacts vary across individual characteristics (e.g., comorbidities or levels of dependency) remains limited. To fill this knowledge gap, we assessed the risk of emergency hospital admission (EHA) related to heat among older adults included in homecare services in Switzerland from 2019 to 2022.
Methods: We collected detailed individual-level EHA data of older adults included in homecare services in Switzerland. For each admission, we utilized 1-km gridded temperature data to compute the daily maximum temperature percentile for the corresponding medical district of residence. We employed an individual-level case time series design to assess the association between EHA and heat using distributed lag non-linear models. We stratified the analysis by sex and various population subgroups, considering individual characteristics such as comorbidities, and daily activity capacity.
Results: We found higher heat-related EHA risks among younger males (aged 65-74 years) (RR:1.31; CI:1.04-1.66, vs. females (1.14; 0.88-1.48)). In contrast, within the oldest age group (85+), females faced greater risks than males. Regarding the main diagnosis at admission, females exhibited higher EHA risks due to genitourinary and skin diseases, while males faced increased EHA risks related to endocrine and circulatory diseases. Individuals with coronary heart disease, dementia/Alzheimer's disease, and cancer showed a higher risk of being admitted due to endocrine and mental disorders during heat days. People with diabetes showed a larger EHA risk due to mental disorders, neoplasms, and infectious or respiratory diseases. Older adults without assistance for daily tasks had a higher EHA risk than those receiving assistance.
Conclusion: Our findings show that older adults faced varying EHA risks during heat days depending on their health status and other characteristics. This highlights the need for targeted public health measures to specific subgroups of older adults with potentially higher vulnerability.

FT19-02 | Mortality risk and burden associated with non-optimum temperatures in Puerto Rico

Francisco Diaz-Collado[1,2], Lingzhi Chu[1,2], Daniel Carrión[1,2], Pablo Méndez-Lázaro[3], Kai Chen[1,2]

[1] Yale School of Public Health, Department of Environmental Health Sciences, New Haven, USA; [2] Yale School of Public Health, Yale Center on Climate Change and Health, New Haven, USA; [3] Environmental Health Department, Graduate School of Public Health, San Juan, Puerto Rico

The effects of a changing climate are already evident in Caribbean small island developing states (SIDS) like Puerto Rico (PR), where heat episodes have become more frequent. Despite reports of substantial increases in heat-related death rates, robust epidemiological evidence on the health impacts of heat, as well as the effects of cold temperatures, remains scarce, particularly outside of urban settlements in Caribbean SIDS. In this study, we conducted a case time series study on municipality-level mortality and temperature in PR from 2015 - 2023. We modeled the relationship between daily mortality count and mean temperature using a conditional quasi-Poisson regression, combined with a distributed lag non-linear model with a 21-day lag, adjusting for relative humidity, seasonality, and day of the week. We estimated the minimum mortality temperature (MMT)- the optimal temperature associated with the lowest mortality risk -and calculated the relative risk (RR) associated with extreme cold and heat, defined as the 2.5th and 97.5th percentiles of daily temperature. Additionally, we estimated the municipality- and island-level excess mortality fractions attributable to both cold and heat, relative to the MMT. Our findings indicate that exposure to non-optimum temperatures (both cold and heat) is significantly associated with increased mortality risk. Specifically, extreme cold was associated with 1.23 (95% CI: 1.07 - 1.40) times risk of all-cause mortality, while extreme heat was associated with 1.16 (95% CI: 1.05 - 1.27) times risk. We estimated that temperature-related mortality accounted for 3.88% of total 280,568 deaths (95% eCI 3.39% - 4.29%), with cold contributing 2.02% (95% eCI: 1.69% - 2.32%) and heat contributing 1.86% (95% eCI: 1.35% - 2.35%). Furthermore, we found substantial spatial variability in temperature-related mortality burdens across municipalities. Our study highlights the significant impact of non-optimal temperatures on mortality in PR, emphasizing the need for targeted localized climate adaptation and mitigation strategies.

FT19-03 | Acute temperature-related mortality in the Prospective Urban and Rural Epidemiology cohort: a case-crossover study in 713 communities and 24 countries

Kirstin R. Yeomans[1], John Paul Bigouette[1], Michael Brauer[3,4], Jeffrey W. Bethel[1], Adam Branscum[1], Justin Wettstein[2], David Wrathall[2], Sumathy Rangarajan[4], Salim Yusuf[4], Perry Hystad[1,4]

[1] Oregon State University, College of Health, Corvallis, USA; [2] Oregon State University, College of Earth, Ocean and Atmospheric Sciences, Corvallis, USA; [3] University of British Columbia, Vancouver, Canada; [4] Population Health Research Institute, Hamilton, Canada

Objective: The temperature-mortality relationship is not well quantified for low- and middle-income countries (LMICs), which may experience the largest future temperature extremes. We sought to quantify and compare the temperature-mortality relationship across country income statuses to better understand drivers of this association.

Materials and Methods: We leveraged the prospective urban and rural epidemiological (PURE) cohort, which includes over 200,000 adults in 780 urban and rural communities across 28 high, middle, and low-income countries. We extracted community-specific daily air and wet-bulb globe temperature (WBGT) estimates from the Global Land Data Assimilation System (v2.1). We used a novel method to estimate minimum mortality temperatures (MMTs) for each community and applied a bidirectional time-stratified case-crossover design to assess associations between acute temperature exposures and all-cause (n=17,539) and cardiovascular disease (CVD) mortality (n=5,905) for deaths occurring above and below the estimated MMTs.

Results: For deaths below the MMT, we estimated odds ratios (OR) for all-cause mortality of 1.06 (95% CI: 1.056, 1.073) and CVD mortality of 1.06 (95% CI: 1.050, 1.080) per 1 ^{o} C decrease in mean temperature on the day of death. The odds of mortality for deaths at temperatures above the MMT was 1.28 (95% CI: 1.259, 1.303) for all-cause mortality and 1.30 (95% CI: 1.260, 1.341) for CVD mortality per 1 ^{o} C increase in mean temperature. We observed stronger associations between cold temperature for LICs (1.18; 95%: CI: 1.153, 1.204) and MICs (1.05; 95% CI: 1.041, 1.062) compared to HICs (1.02; 95% CI: 0.997, 1.040), while associations for hot temperatures were larger for high-income countries.

Conclusion: Our study provides new information on temperature-mortality relationships in LMICs, highlighting the need for future adaptation strategies to both extreme cold and hot temperatures.

*This research is supported by a NIH grant (*R56ES035095-01A1).

FT19-04 | Evaluation of optimal heat stress metric for modelling heat-mortality relation across climatically distinct sub-regions of Southern California

C. Schimke[1], H. Schlaerth[2], J. Ko[2, 3], S. Silva[2, 4], S. P. Eckel[4], F. Chen[4], E. Garcia[4]

[1] University of Southern California, Infectious Diseases, Immunology, and Pathogenesis, Los Angeles, California, United States of America; [2] University of Southern California, Earth Sciences, Civil and Environmental Engineering, Los Angeles, California, United States of America; [3] Columbia University, Columbia Climate School, New York, New York, United States of America; [4] University of Southern California, Population and Public Health Sciences, Los Angeles, California, United States of America

Objective: Several metrics have been used to assess heat stress exposure, with the optimal heat stress metric for modelling the heat-mortality relation shown to vary by location. We evaluated how the optimal heat stress metric may vary even within geographically close, yet climatically distinct sub-regions of Southern California.
Material and Methods: The daily average of nine distinct heat stress metrics, including two definitions of apparent temperature, heat index, dry- and wet-bulb temperatures, and weighted averages of dry- and wet-bulb temperatures representing varying levels of evaporative cooling efficiency were derived from hourly predictions from the Weather Research and Forecasting model for 2015-2016 at a resolution of 2 km. Each metric was related to all-cause mortality in Southern California using a distributed lag nonlinear model with a time-stratified case-crossover design. To evaluate optimal heat stress metric by sub-region, models were stratified by: (i) Koppen-Geiger climate zone, (ii) National Land Cover Database landuse type, and (iii) an inland/coastal binary designation. The best fit metric for each sub-region was selected according to the Akaike Information Criterion. We obtained estimates of the overall mortality burden attributable to temperatures above the minimum mortality temperature for each fitted model using a supplemental function to the dlnm package.
Results: No single heat stress metric was the best fit metric for all sub-regions. Apparent temperature–derived from dry-bulb temperature, relative humidity, and wind speed–was the best fit metric for several sub-regions; however, dry-bulb temperature was the best fit metric for select other sub-regions. Estimates of the attributable fraction of deaths to heat stress varied significantly between metrics.
Conclusion: Local climate and built environmental factors may influence the optimal heat stress metric for quantifying heat-mortality risks. These factors should be considered when selecting metrics in heat-mortality studies.
Funding: NIEHS grants P30ES007048, T32ES013678

FT19-06 | Escalating Heat, Escalating Risk: Historical Associations of Heat and Mortality in Priority Health Diseases Across 82 Provinces in the Philippines with Future Projections Under Various Climate Scenarios

Percival Ethan Lao[1], Paul Lester Chua[2], Hazel Ann Fajardo[1], Sary Valenzuela[1,3], Geminn Louis Apostol[1,4]

[1] Ateneo de Manila University - School of Medicine and Public Health (ASMPH), ASMPH Center for Research and Innovation, Pasig City, Philippines; [2] The University of Tokyo, Department of Global Health Policy, Graduate School of Medicine, Bunkyo City, Japan; [3] Georgetown University, Department of Global Human Development, Washington D.C., USA; [4] Murdoch University, Harry Butler Institute, Perth, Australia

Objective: Rising global temperatures pose growing risks for heat-related mortality, especially in climate-vulnerable countries such as the Philippines, where frequent heat extremes compound existing public health vulnerabilities. This

study aimed to quantify historical heat-attributable mortality across six major disease categories and two age groups in the Philippines from 2006 to 2022 and to project future burdens under various climate scenarios to inform targeted public health adaptation strategies.
Materials and Methods: Daily mortality data (n=9,516,153 all-cause deaths) from 82 Philippine provinces were analyzed, categorized by cause (infectious, cancer, endocrine, circulatory, respiratory, and all-cause) according to ICD-10 classification, and by age groups (0–59 and ≥60 years). Historical population-weighted daily mean temperatures were derived from ERA5-Land datasets, while future temperature projections were obtained from bias-adjusted Inter-Sectoral Impact Model Intercomparison Project (ISIMIP) climate datasets under Shared Socioeconomic Pathway (SSP) scenarios (1-2.6, 3-7.0, 5-8.5). A two-stage modeling approach was employed: province-level conditional quasi-Poisson regression models estimated temperature–mortality associations across a 14-day lag period, and meta-regression provided nationwide heat-related mortality estimates and future province-specific projections.
Results: Between 2006 and 2022, heat contributed to approximately 200,964 all-cause deaths (2.11% of total mortality), including 30,264 deaths linked to extreme heat. Circulatory diseases had the highest heat-attributable fraction (3.00%), accounting for 99,614 deaths predominantly among individuals aged ≥60 years (84,061 deaths). Projected warming scenarios, especially under high-emissions conditions (SSP5-8.5), suggest substantial future increases in heat-related mortality, posing significant risks to public health infrastructure.
Conclusion: These findings emphasize the critical necessity of implementing localized heat-health action plans, strengthening disease surveillance systems, and developing climate-informed clinical guidelines. Enhancing healthcare workforce capacity through specialized climate training and integrating climate considerations into routine practices is imperative. Embedding climate adaptation measures into national health policies will further enhance preparedness and resilience, safeguarding vulnerable populations against intensifying heat-related health impacts.

FT19-07 | Projections of changes in dengue risk attributable to rising temperatures in Peru

Taehee Chang[1], Saebom Choi[2], Whanhee Lee[3], Sung-il Cho[2], Kevin S. Martel[4,5], Pierre J. Velásquez[4,5], César V. Munayco[5], Kyung-Duk Min[1], This research was supported by a National Research Foundation of Korea (NRF) grant funded by the Korea government (MSIT) (grant number: NRF-2021R1C1C2012611) and by a National Research Foundation of Korea (NRF) grant funded by the Korea government (MSIT) (grant number: RS-2025-00517731). We express our gratitude to the officials at the National Centre for Epidemiology, Disease Prevention, and Control (Peru CDC) under Peru's Ministry of Health for providing the data for this study.

[1] Chungbuk National University, College of Veterinary Medicine, Cheongju, South Korea; [2] Seoul National University, Graduate School of Public Health, Seoul, South Korea; [3] Pusan National University, College of Information and Biomedical Engineering, Busan, South Korea; [4] Universidad Peruana Cayetano Heredia, School of Public Health and Administration, Lima, Peru; [5] Prevención y Control de Enfermedades, Peruvian Ministry of Health, Centro Nacional de Epidemiología, Lima, Peru

Background

Climate change is expected to influence the transmission dynamics of mosquito-borne diseases. Dengue remains a significant public health challenge in Peru, imposing a considerable socioeconomic and health burden. However, there is still limited research on how climate change affects dengue risk. Therefore, this study aims to estimate the future impact of climate change on dengue cases associated with temperatures conducive to transmission (temperature-attributable dengue cases) in Peru.**Methods**

Meteorological data and dengue case records from 2016 to 2023 were used to analyze department-specific temperature-dengue relationships through a standard time series quasi-Poisson regression model combined with a distributed lag non-linear model. These associations were then applied to temperature projections from climate models covering the baseline period and the scenario period (2016–2050) to estimate temperature-attributable dengue cases. Data from five general circulation models (GCMs) were incorporated under three climate change scenarios (Shared Socioeconomic Pathways).**Results**

The overall cumulative temperature-dengue association in Peru showed that the relative risk (RR) peaked at 24.6°C, reaching 2.15 (95% CI: 1.28–3.62). Temperature-attributable dengue cases are projected to remain close to baseline levels in the 2020s but are expected to rise significantly between the 2030s and 2050. The degree, timing, and rate of increase in temperature-attributable dengue cases differed across departments. However, differences across SSP scenarios were not statistically significant.**Conclusion**

Alongside climate change mitigation, implementing proactive disease intervention policies appears essential to curb the projected increase in dengue transmission risk. This study provides actionable insights for public health policymakers to manage the growing threat of dengue.

FT19-09 | Enduring Impact of Extreme Temperature Events on Mortality: Decadal Insights from a Nationwide Cohort

Xu Gao

Peking University, Department of Occupational and Environmental Health Sciences, Beijing, China

Objective: The toll and destruction of heat waves and cold spells on health may last long due to underlying chronic pathological changes but rarely studied, it is imperative to investigate their enduring impacts on mortality.
Material and Methods: We conducted a prospective analysis in ChinaHEART, a national cohort of China, including 3.25 million participants with a median follow-up of 5.5 years. Individual decadal records of the temperature events were assessed using a percentile-based approach (heat waves: 95%, 97.5%, and 99% for 2/3 days; cold spells: 5%, 2.5%, and 1% for 2/3 days) based on ERA5-Land data. We primarily assessed the associations of events occurring within a decade prior to enrollment (y1 to y10) with all-cause and cause-specific mortality (stroke and IHD). Additionally, we examined the vulnerability of specific subpopulations by stratifying analyses.
Results: Extreme temperature events, particularly the most severe ones, had persistent but gradually diminishing effects on mortality. Specifically, the mortality risks per each exposure to a heat wave at the 99th percentile for 3 days were 1.5%, 1.3%, and 1.2% for y1, weighted y2–y5, and weighted y6–y10, respectively. Similar patterns were found for cold spells. Notably, heat waves were more strongly associated with IHD mortality, whereas cold spells exhibited a greater impact on stroke mortality. We observed substantial differences by sex, urbanization, geographic region, and ASCVD risk for all but the most extreme events. For the most intense events, subgroup differences were largely diminished.
Conclusion: Heat waves and cold spells exert prolonged effects on mortality which should be duly considered as the acute death burden. While the risks of less extreme events may be mitigated through targeted interventions tailored to specific demographic and cardiovascular risk groups, the most severe events pose a broadly uniform substantial threat across populations to warrant more comprehensive and stringent adaptation strategies.

FT19-10 | Effects of non-optimal temperature exposure on children and adolescents mortality risk in Brazil: small-area case time series approach

Ludmilla V. Jacobson[1,2], Jony A. Pinto Júnior[1], Mauricio Barreto[2], Rochelle Schneider[3,4]

[1] Fluminense Federal University, Department of Statistics, Niterói, Brazil; [2] Oswaldo Cruz Foundation, Center for Data and Knowledge Integration for Heath-Cidacs, Salvador, Brazil; [3] European Space Agency, Φ-lab, Frascati, Italy; [4] London School of Hygiene and Tropical Medicine, Faculty of Epidemiology and Population Heath, London, UK; [5] European Centre for Medium-Range Weather Forecast, Forecast Department, Reading, UK

The aim of this study is to quantify the non-linear and delayed effects of non-optimal temperature exposure on children and adolescents from 0-19 years of age with mortality risk in Brazil using a case-time series analysis. Methods: A small-area analysis was performed using data on all non-accidental causes of mortality and air temperature for the 5570 municipalities within 27 Federation Units (FU`s) across Brazil between Jan 1, 2000, and Dec 31, 2019. First, we applied the case-time series design, modeling multiple municipalities-specific series within

each of 27 FU and Regions through a conditional Poisson regression. Temperature–mortality associations were modeled through distributed lag non-linear models (lag: 0-7 days). Then, a meta-regression was used to pool FU-specific estimates. Results: The total non-optimal temperature-attributable fraction of death was estimated at 2.8% (CI95%: 1.53%; 4.08%), of which 2.35% (CI95%: 1.06%; 3.66%) was attributable to heat exposure. The pooled curve suggests an increase in relative risk for hot temperatures with a steeper increase for extreme temperatures when compared to mild heat. Region-specific shapes for the mortality risk pooled curve vary, e.g., in the south and southeast regions U-shape and J-shape were observed, respectively. The heat effects were higher in the first 0-3 days of exposure, although the cold effects were higher after 3 days (except for the Northeast region where there was no cold effect). The fractions of deaths attributable to heat exposure were significant for all FU`s from the Midwest, Southeast, and South regions, varying from 0.8% to 2.6%. In the North, Rondônia(1.39%), Acre(1.44%), Amapá(2.96%), and Tocantins(1.60%) had a significant heat-attributable fraction of death. In the Northeast, heat-attributable fractions were significant for Sergipe(0.99%), Piauí(2.81%), and Maranhão(1.34%). Cold-attributable fraction of deaths were notable for all FU`s in the south region (Paraná=2.54%; Rio Grande do Sul=1.47%; Santa Catarina=1.11%) and for São Paulo state (0.76%).

FT20 | Vulnerability across the life course: Health impacts of heat and climate extremes

FT20-01 | Tropical cyclones and psychoactive drug-related deaths in the United States

Raenita Spriggs[1], Victoria D. Lynch[1], Yuanyu Lu[2], Lincole Jiang[2], Wil Lieberman-Cribbin[1], G. Brooke Anderson[3], Katherine M. Keyes[4], Marianthi-Anna Kioumourtzoglou[1], Diana Hernandez[5], Anne E. Nigra[1], Robbie M. Parks[1]

[1] Columbia University Mailman School of Public Health, Department of Environmental Health Sciences, New York, USA; [2] Columbia University Mailman School of Public Health, Department of Biostatistics, New York, USA; [3] Colorado State University, Department of Environmental and Radiological Health Sciences, Fort Collins, USA; [4] Columbia University Mailman School of Public Health, Department of Epidemiology, New York, USA; [5] Columbia University Mailman School of Public Health, Department of Sociomedical Sciences, New York, USA

Objective: Existing evidence indicates that tropical cyclone exposure can exacerbate the mental health-related vulnerabilities of impacted communities, potentially leading to increased rates of psychoactive drug use. However, rigorous multi-decade modeling of the association between tropical cyclone exposure and psychoactive drug deaths is needed.

Material and Methods: We leveraged over 2 million deaths from the National Center for Health Statistics and a record of tropical cyclone occurrence in the United States during 1988-2019. We formulated a Bayesian conditional quasi-Poisson model to quantify the association between county-level tropical cyclone exposure (defined by sustained maximal wind speed and stratified by cyclone strength—all cyclones, gale to violent storms, and hurricanes) and monthly psychoactive drug-related death rates up to three months after exposure using unconstrained distributed lag terms. Analyses were further stratified by broad age group (0-64, 65+ years), sex (female, male), and county-level sociodemographics at their median (e.g., poverty, education).

Results: For all cyclones, there was a 4.2% (Credible Interval [CrI]: 2.1%, 6.2%) average increase in psychoactive drug-related death rates in the month of exposure, an overall positive association which persisted up to three months (2.6% (CrI: 0.6%, 4.6%)) post-exposure. This association was more pronounced for hurricanes (7.6% increase (CrI: 1.0%, 16.2%)) compared to gale to violent storms (3.5% increase (CrI: 1.4%, 5.6%)) in the month of exposure. When stratified by age group, sex, and sociodemographics, the results persisted across all variable dichotomies in the three months post-exposure for all cyclone categories.

Conclusion: We found a positive association for tropical cyclones and psychoactive drug-related deaths, with effect modification by cyclone strength. Our work provides a critical observational understanding for this understudied cause of death, demonstrating the need for targeted interventions and additional preparedness during and after tropical cyclone exposure, especially as cyclones are likely to increase in strength with global climate change.

FT20-02 | Burden of Enteric Infections Attributable to High temperature in Australia: Current estimates and future projections

Yohannes Tefera Damtew, Blesson Varghese, Olga Anikeeva, Peng Bi

University of Adelaide, School of Public Health, Adelaide, Australia

Background:
Salmonella and *Campylobacter* infections are leading causes of bacterial gastrointestinal infections, and their incidence is increasing worldwide. Climate change and rising temperatures are expected to further drive the transmission and prevalence of these enteric infections. Quantifying the current and future burden of *Salmonella* and *Campylobacter* infections in Australia attributable to increasing temperatures is crucial for developing effective prevention strategies. This study aims to evaluate the present and future burden of *Salmonella* and *Campylobacter* infections linked to rising temperatures in Australia.

Methods:
Disability-adjusted life years (DALYs) for Salmonella and Campylobacter infections (2003–2018) were obtained from the Australian Institute of Health and Welfare. A meta-regression model, using relative risks (RRs) from a prior meta-analysis, estimated infection risks per 1°C temperature increase. Climate-zone-specific exposure distributions were compared to a theoretical minimum risk exposure to assess attributable burdens. Future projections for the 2030s and 2050s were estimated under Representative Concentration Pathways (RCP4.5 and RCP8.5), considering population growth and adaptation scenarios.

Results:
From 2003–2018, rising temperatures contributed to 11.2% (41.8 ± 2.8 DALYs) and 8.5% (28.1 ± 1.8 DALYs) of the observed burden of Salmonella and Campylobacter, respectively. The highest burdens were in the tropical rainforest climate zone and in the Northern Territory (Salmonella) and Queensland (Campylobacter). Under RCP8.5, the annual burden by the 2050s could reach 100.6 ± 10.9 DALYs for Salmonella and 67.9 ± 7.4 DALYs for Campylobacter. A 10% adaptation measure could reduce these to 89.5 ± 8.3 and 61.8 ± 6.7 DALYs, respectively.

Conclusion: The findings indicate that 11.2% and 8.5% of *Salmonella* and *Campylobacter* infections in Australia are attributed to increasing mean temperatures, varying across states and territories, and climate zones. This warrants region-specific adaptive measures and policies to control climate-sensitive enteric infections.

FT20-03 | Association of cyclonic storms with traumatic injuries and diarrheal illness in Madagascar

Victoria D. Lynch[1], Marissa L. Childs[2], Lingke Jiang[1], Oladimeji Mudele[3], Giacomo De Nicola[3], G. Brooke Anderson[4], A.K. Symphonia Razafinimanana[5], Christopher D. Golden[3], Robbie M. Parks[1]

[1] Columbia University Mailman School of Public Health, Environmental Health Sciences, New York, USA; [2] University of Washington, Department of Environmental and Occupational Health Sciences, Seattle, USA; [3] Harvard T. H. Chan School of Public Health, Department of Nutrition, Boston, USA; [4] Colorado State University, Department of Environmental and Radiological Health Sciences, Fort Collins, USA; [5] Ministry of Public Health, Antananarivo, Madagascar

Introduction: Cyclonic storms are an urgent public health concern in Madagascar, which experiences an average of two cyclonic storms annually and is in a storm basin with increasing cyclonic storm severity. Madagascar is particularly vulnerable to the impacts of cyclonic storms as decades of food insecurity and inadequate sanitation have led to chronic malnutrition and persistent waterborne disease. However, the association of cyclonic storms with health outcomes has not been systematically assessed in Madagascar.

Methodology: We leveraged complete national public clinic and cyclonic storm records during 2010 – 2022 to evaluate the association of cyclonic storm exposure with specific health outcomes. We developed a Bayesian conditional quasi-Poisson model to estimate the association of cyclonic storm exposure with monthly district-level clinic diagnoses for diarrheal illness and traumatic injuries, including sub-cause analyses for injuries by suicide attempts, violence, road accidents, and workplace-related trauma, up to three months post cyclone. We conducted separate analyses for tropical storm-force (34 – 63 knots; n=19) and tropical cyclone-force (≥64 knot; n=7) winds and by age group, sex, and ecological region.

Results: There were 6,484,450 clinic diagnoses (49.7% female, 50.3% male) for included causes at 2,539 facilities in 104 districts. Each additional exposure day was associated with increased traumatic injury diagnosis rates (29.6%;95% Credible Interval [CrI]:22.2%, 37.1%) during tropical cyclone months; in particular, violent injury diagnosis rates increased for females (114.6%;95% CrI:83.9%, 145.3%) and males (60.7%;95%CrI:28.7%,92.7%). Tropical cyclones were associated with increased suicide attempt rates for females during the storm month (113.7%;95%CrI:34.1%,193.5%) and for males (32.2%;95%CrI:18.0%,46.5%) two months post-cyclone. Tropical cyclones were associated with increased diarrheal illness (11.9%;95%CrI:9.6%,14.2%) diagnosis rates during the storm month.

Conclusion: Climate change and population growth will lead to greater cyclonic storm exposure in Madagascar. Our results can inform preparedness efforts by identifying population groups and regions at risk of specific health outcomes.

FT20-04 | Associations between Ambient Temperature and Community Falls in Older Adults in Michigan, Ohio, and Pennsylvania, 2010-2016

Lisa Koh[1], Carina J. Gronlund[1,2], Marie S. O'Neill[1]

[1] University of Michigan, School of Public Health, Ann Arbor, USA; [2] University of Michigan, Institute for Social Research, Ann Arbor, USA

Objective: The goal of the study was to examine associations between ambient temperature and community falls among Medicare beneficiaries aged 65 and above in 30 counties in Michigan, Ohio, and Pennsylvania, U.S.A.--3 northern industrial states with aging housing and infrastructure.
Material and Methods: We obtained Medicare emergency department and urgent care (ED) billing records from the Centers for Medicare and Medicaid Services and temperatures and precipitation estimates from the PRISM Climate Group from 2010 through 2016. We calculated the daily number of fall-related ED visits, the number of Medicare beneficiaries who were not hospitalized at the start of the day, average daily maximum temperature, and average precipitation at a county level. We fit a distributed lag nonlinear model to estimate the association between extreme temperature (maximum ambient temperatures) and the cumulative incidence rate of fall-related ED visits, adjusting for daily precipitation. Random-effects meta-analysis was used to obtain an overall temperature-fall association.
Results: There were 961,549 (104 per 100,000 person years) fall-related ED visits. Cumulative associations between temperature and community falls followed a U-shaped relationship, with a small increased incidence rate of fall-related ED visits over the subsequent 14 days, at both high and low temperatures, compared to the minimum morbidity temperature, or nadir in the exposure-response relationship. Specifically, extreme cold (minimum observed temperature of -18 C) was associated with a 0.36% (95% CI = 0.06%, 0.66%) and extreme heat (maximum observed temperature of 39 C) was associated with a 0.34% (95% CI = 0.19%, 0.48%) increased rate of fall-related ED visits.Conclusion: Both extreme heat and extreme cold are associated with a small increase in community falls among older adults in this U.S. region.
Source of funding: This work was supported by the University of Michigan Injury Prevention Center, the Graham Sustainability Institute, and NIEHS grants R01ES032157 and P30ES017885.

FT20-05 | Ambient Temperature and COVID-19 Emergency Department Visits in Ontario, Canada: A Population-Based Case-Crossover Study with Distributed Lag Non-linear Models

Nicholas Grubic[1], Zhou Zang[2], Haley Golding[3], Michael Campitelli[3], Tariq Cisse[1], Maria Koh[3], Eric Lavigne[4,5], Brice Batomen[1], Xiaolin Wei[1], Erjia Ge[1]

[1] University of Toronto, Dalla Lana School of Public Health, Toronto, Canada; [2] University of Toronto, Department of Geography and Planning, Toronto, Canada; [3] ICES, Toronto, Canada; [4] Health Canada, Environmental Health Science and Research Bureau, Ottawa, Canada; [5] University of Ottawa, School of Epidemiology and Public Health, Ottawa, Canada

Objective: Extreme temperatures can influence the spread and severity of respiratory infections, but their impact on COVID-19-related emergency department (ED) visits remains unclear. This population-based study assessed the association between short-term ambient temperature variations and COVID-19 ED visits.

Material and Methods: We conducted a bidirectional time-stratified case-crossover study of 200,509 laboratory-confirmed COVID-19 ED visits in Ontario, Canada (January 2020-December 2023). Conditional logistic regression with distributed lag non-linear models was used to assess the effects of mean daily temperature (lag_{0-5}) on COVID-19 ED visits while adjusting for mean daily relative humidity and holidays. To quantify the cumulative effects of extreme hot and cold temperatures, we estimated odds ratios (OR) and 95% confidence intervals (CI) at the 5th and 95th percentiles of the temperature distribution, referenced to the minimum risk temperature (MRT). Stratified analyses were performed for the three most prevalent COVID-19 variants (alpha, delta, omicron).

Results: The cumulative exposure-response curve showed an M-shaped pattern, with temperatures between -22◦C to 0◦C and 2◦C to 25◦C significantly associated with COVID-19 ED visits, and peak effects at -15◦C and 15◦C. Extreme hot ($OR_{22°C}$: 1.38, 95% CI: 1.31-1.45) and cold temperatures ($OR_{-10°C}$: 2.33, 95% CI: 2.21-2.46) were associated with COVID-19 ED visits (MRT=1◦C), with peak effects observed at lag days 2 and 4 for hot temperatures and lag days 2 and 3 for cold temperatures. Cold effects were stronger than hot effects for delta (OR_{Cold}: 2.88 vs. OR_{Hot}: 1.21, $p<0.05$) and omicron variants (OR_{Cold}: 3.71 vs. OR_{Hot}: 1.72, $p<0.05$), however, for alpha variants, cold temperatures were weaker (OR_{Cold}: 1.00 vs. OR_{Hot}: 5.40, $p<0.05$).

Conclusion: Extreme temperature exposure is associated with a higher risk of COVID-19 ED visits across viral variants, highlighting the need for temperature-responsive public health strategies to reduce the burden of COVID-19 and other respiratory diseases.

Funding: Dalla Lana School of Public Health

FT20-07 | Upward air temperature shifts trigger acute cardiovascular events in individuals with pre-existing atherosclerotic cardiovascular disease

Katharina Lechner[1,2], Siqi Zhang[3], Nils Krüger[1,4], Johannes Krefting[1,5], Kathrin Wolf[2], Marco Dallavalle[2], Frank Offenborn[7], Fabian Starnecker[1,5], Moritz von Scheidt[1,5], Tobias Dreischulte[6], Kai Chen[3,8], Annette Peters[2,9], Susanne Breitner[2,10], Alexandra Schneider[2], Heribert Schunkert[1,5]

[1] Universitätsklinikum der Technischen Universität München, Department of Cardiology, Deutsches Herzzentrum München, Munich, Germany; [2] Helmholtz Zentrum München - German Research Center for Environmental Health (GmbH), Institute of Epidemiology, Munich, Germany; [3] Yale School of Public Health, Department of Environmental Health Sciences, New Haven, USA; [4] Division of Pharmacoepidemiology and Pharmacoeconomics, Department of Medicine, Brigham and Women's Hospital and Harvard Medical School, Boston, USA; [5] DZHK (German Centre for Cardiovascular Research), Partner site Munich, Munich Heart Alliance, Munich, Germany; [6] LMU Klinikum, Ludwig-Maximilians-Universität München, Institut für Allgemeinmedizin, Munich, Germany; [7] Allgemeine Ortskrankenkasse (AOK) Bayern, Munich, Germany; [8] Yale School of Public Health, Yale Center on Climate Change and Health, New Haven, USA; [9] Harvard T.H. Chan School of Public Health, Department of Environmental Health, Boston, USA; [10] Faculty of Medicine, LMU Munich, Institute for Medical Information Processing, Biometry, and Epidemiology, Munich, Germany

Objective

Recent research has addressed the health impact of heat, but the effects of temperature fluctuations are largely unknown. We analyzed the association between upward air temperature shifts (uTS) and acute cardiovascular events.

Material and methods

This time-stratified case-crossover study utilized German insurance claims data from 1,315,553 individuals diagnosed with atherosclerotic cardiovascular disease (ASCVD) between 2012 and 2020. Daily counts of hospitalizations due to acute coronary syndrome (ACS), stroke, heart failure, and all-cause mortality, as well as daily mean outdoor air temperatures estimated on a 1×1km-grid, were aggregated to the 3-digit postcode level. When the mean temperature of a day was higher than the average of the preceding seven days, an uTS was denoted. Acute and lagged impacts of uTS on hospitalization counts were analyzed by conditional Poisson regression adjusted for confounders in each postcode area. Area-specific estimates were pooled using multivariate meta-analysis.

Results

In the linear relationship, an increase in uTS was significantly associated with incident ACS (relative risk 1.028; 95%-confidence interval [1.018; 1.038], per 5°C increase in uTS), stroke (1.020 [1.010; 1.030]), heart failure (1.013 [1.006; 1.021]) and all-cause mortality (1.032 [1.024; 1.040]). Risks for ACS and stroke hospitalizations peaked on the same day of uTS; all-cause mortality and HF hospitalizations peaked one and three days after the uTS, respectively. The effect sizes of uTS on these outcomes were more pronounced in the warm season of the year. The average number of deaths attributable to uTS was 23 [18; 29] per 100,000 patients per year.

Conclusion

Independent of heat per se, uTS were associated with hospitalizations for ACS, stroke, heart failure, and all-cause mortality in individuals with ASCVD, particularly in the warm season. These findings are concerning, given the projected increase in temperature variability due to climate change.

FT20-09 | Impact of temperature on emergency department visits for individuals with intellectual and developmental disabilities in California

Sabrina S. Chow[1], Xiaping Zheng[2], Rebecca Zhang[2], Stefanie T. Ebelt[1], Noah C. Scovronick[1], Howard H. Chang[2,1]

[1] Emory University, Gangarosa Department of Environmental Health / Rollins School of Public Health, Atlanta, USA; [2] Emory University, Department of Biostatistics and Bioinformatics / Rollins School of Public Health, Atlanta, USA

Objective: Individuals’ risk of heat-related illness and other heat-related health outcomes is influenced by compounding social, environmental, and physiological factors. A growing body of research assesses heat-health disparities to identify at-risk populations. Despite approximately one in four Americans having a disability, little is known about the effect of temperature on emergency department (ED) visits for people with intellectual and developmental disabilities (IDD).
Methods: We examined the association between daily ambient temperature and ED visits for patients aged 18 years and older between 2005 and 2019 in California. IDD ED visits were identified using primary and secondary International Classification of Diseases diagnosis codes. Daily ZIP code-level temperatures were derived from 1-km gridded Daymet products. We used a time-stratified case-crossover design to assess associations between three-day cumulative average temperature and IDD ED visits.
Results: There were 386,997 IDD ED visits, with less than one percent ascertained from a disability-related primary diagnosis. Approximately five percent of visits were from people with both at least one intellectual and one developmental disability. For all visits, an increase in the three-day cumulative average temperature from the 50th to 95th percentile was associated with an odds ratio of 1.05 (95% CI: [1.03, 1.08]). The results were similar when restricted to only intellectual disability visits (1.06, 95% CI: [1.03, 1.10]) and only developmental disability visits (1.05, 95% CI: [1.01, 1.09]). We observed a higher odds ratio among ED visits with more severe ID, based on IQ. Results remained robust against temporal confounder adjustments.
Discussion: People with IDD are affected by higher temperature exposure, especially those with more severe or pervasive diagnoses. With increasing extreme heat events, there is a need to elucidate underlying biological pathways leading to heat-related ED visits. Additionally, improving health care systems and preparedness strategies for people with IDD can mitigate increased heat-related ED use.

FT20-10 | Associations of Prenatal and Childhood Exposures to Ambient Temperature with Menstrual Cycle Regularity across the Reproductive Lifespan

Boya Zhang[1], Makiko Mitsunami[1], Elizabeth Peebles[1], Brent A. Coull[1], Tamarra James-Todd[1], Jorge E. Chavarro[1,2], Jaime E. Hart[1,2], Francine Laden[1,2], Shruthi Mahalingaiah[1,3]

[1] Harvard T.H. Chan School of Public Health, Boston, USA; [2] Brigham and Women's Hospital and Harvard Medical School, Department of Medicine, Channing Division of Network Medicine, Boston, USA; [3] Massachusetts General Hospital, Department of Obstetrics and Gynecology, Division of Reproductive Endocrinology and Infertility, Boston, USA

Objective: Temperature exposure early in life may influence menstrual cycle characteristics later, increasing the risk of irregularities and fertility issues. We examined the association of ambient temperatures during gestation and childhood with the risk of menstrual cycle irregularity across the reproductive lifespan.
Material and Methods: We included female participants born after 1989 from a US nationwide study, Growing Up Today Study, who responded at least three times to the menstrual cycle length survey after menarche and who had exposure data during prenatal (N=2685) and childhood periods (N=2814). Monthly average temperatures from PRISM Spatial Climate Datasets were assigned to biennial-updated participants' residential addresses for prenatal (all trimesters and 12 months before birth) and childhood (birth to one year before menarche) time windows. Menstrual cycle regularity was categorized using latent class mixed-effect models with two (regular/irregular) and three (regular/transition to irregular around early 20s/transition to irregular around early 30s) classes. We estimated odds ratios (ORs) and 95% confidence intervals (CIs) for cycle irregularity per interquartile range increase in temperatures using logistic regression models.
Results: Approximately 3–4% of participants had irregular cycles when categorized into two to three classes. With two classes, higher ambient temperatures during the prenatal periods were suggestively associated with higher risk of irregularity (OR 1.57 per 4.8 ℃, 95%CI, 0.92, 2.68). Among three trimesters, only temperature during the first trimester was associated with higher risks of irregularity (OR 2.39 per 14.5 ℃, 95%CI, 1.10, 5.18). With three classes, similar associations were observed with irregularity defined as transiting to irregular around early 20s. No associations were observed with temperatures during childhood.
Conclusion: Higher ambient temperatures during the prenatal period, particularly in the first trimester, may increase the risk of menstrual cycle irregularity. This implies that lower ambient temperatures during early development may influence long-term reproductive health (funded by NIH R01ES035106).

FT21 | Chemical exposures in mothers and children flash talks

FT21-01 | Gestational Phthalates Exposure and High-Throughput Protein Profiles during Pregnancy

Meizhen Yao[1], David E. Cantonwine[2], Amber Cathey[1], Jennifer Fernandez[1], Kelly K. Ferguson[3], Thomas F. McElrath[2], John D. Meeker[1]

[1] University of Michigan, Environmental Health Sciences, Ann Arbor, USA; [2] Brigham and Women's Hospital, Harvard Medical School, Division of Maternal-Fetal Medicine, Boston, USA; [3] National Institute of Environmental Health Sciences, National Institutes of Health, Department of Health and Human Services, Epidemiology Branch, Division of Intramural Research, Research Triangle Park, USA

Objective: Gestational exposure to phthalates has been linked to adverse pregnancy outcomes, potentially through endocrine disruption, oxidative stress and inflammation. However, epidemiological studies leveraging high-throughput biomarkers to elucidate these mechanisms in pregnant women are limited. In this study, we investigated the association between gestational phthalates exposure and comprehensive protein profiles during pregnancy.
Material and Methods: We conducted a nested case-control study within the LIFECODES pregnancy cohort, selecting 86 women for proteome-wide analysis with a 1:1 case-control ratio (43 preterm birth cases and 43 controls). Nine urinary phthalate metabolites were measured across up to three pregnancy visits. Plasma samples were collected at gestation week 26 (Q1, Q3 = 25, 27), and 5,416 plasma proteins were quantified using Olink Explore HT Platform. To identify differentially expressed proteins associated with each phthalate exposure, we applied pairwise association-wide analysis using multivariable linear regression. Over-representation analysis (ORA) in Reactome pathways was performed to investigate alterations in functionally linked protein sets.
Results: We identified 5,602 significant phthalates-protein associations (FDR < 0.2) out of 48,735 tested associations. The frequently associated phthalate exposures included mono(2-ethyl-5-carboxypentyl) phthalate (MECPP), mono(2-ethyl-5-oxohexyl) phthalate (MEOHP), and mono(2-ethylhexyl) phthalate (MEHP), with the most significant protein changes observed in C-type lectin domain family 5 member A, Proline and serine-rich protein 3, and Interleukin-1 receptor type 2. Pathway analysis revealed enrichment in neutrophil degranulation and NF-kB activation, suggesting a potential role of immune activation in response to phthalate exposure. Additionally, pathways involving FGFR2 signaling, platelet function, and metabolic regulation are significantly enriched, highlighting potential disruptions in cellular signaling and coagulation processes.
Conclusion: In this exploratory study, gestational phthalate exposure was associated with widespread protein expression changes, potentially disrupting immune activation, inflammatory signaling, and metabolic regulation during pregnancy. These findings provide molecular insights into the mechanisms linking phthalate exposure to adverse pregnancy outcomes at the omics scale.

FT21-02 | Prenatal exposure to mixtures of non-persistent endocrine-disrupting chemicals and fetal growth, fetoplacental hemodynamics, and angiogenic biomarkers

Bethany Knox[1,2], Nuria Güil-Oumrait[1,2], Vishal Midya[3], Xavier Basagaña[1,2], Mariona Bustamante[1,2], Payam Dadvand[1,2], Maria Foraster[1], Mireia Gascon[1,9], Maria Dolores Gómez-Roig[4,7], Catherine Thomsen[5], Elisa Llurba[6,8], Sandra Márquez[1,2], Claire Philippat[10], Ioar Rivas[1,11], Zoraida García Ruiz[7], Amrit Sakhi[5], Jordi Sunyer[1,2], Jose Urquiza[1,2], Hana Vespalcová[1,2], Maria Julia Zanini[6,8], Martine Vrijheid[1,2]

[1] ISGlobal, Environment and Health over the Lifecourse, Barcelona, Spain; [2] Spanish Consortium for Research on Epidemiology and Public Health (CIBERESP), Instituto de Salud Carlos III, Madrid, Spain; [3] Icahn School of Medicine at Mount Sinai, Department of Environmental Medicine, New York, USA; [4] University of Barcelona, BCNatal, Fetal Medicine Research Center, Hospital Sant Joan de Déu and Hospital Clínic, Barcelona, Spain; [5] Norwegian Institute of Public Health (NIPH), Department of Food Safety, Oslo, Norway; [6] Institut d'Investigació Biomèdica Sant Pau - IIB Sant Pau. Hospital de la Santa Creu i Sant Pau, Department of Obstetrics and Gynaecology, Barcelona, Spain; [7] Primary Care Interventions to Prevent Maternal and Child Chronic Diseases of Perinatal and Developmental Origin Network (RICORS), RD21/0012/0003, Instituto de Salud Carlos III, Madrid, Spain; [8] Primary Care Interventions to Prevent Maternal and Child Chronic Diseases of Perinatal and Developmental Origin Network (RICORS),RD21/0012/0001, Instituto de Salud Carlos III, Madrid, Spain; [9] Unitat de Suport a la Recerca de la Catalunya Central, Fundació Institut Universitari per a la Recerca a l'Atenció Primària de Salut Jordi Gol i Gurina (IDIAPJGol), Manresa, Spain; [10] University Grenoble Alpes, Inserm U-1209, CNRS-UMR-5309, Environmental Epidemiology Applied to Reproduction and Respiratory Health Team, Institute for Advanced Biosciences, Grenoble, France; [11] Institute of Environmental Assessment and Water Research (IDAEA-CSIC), Barcelona, Spain

Background: Non-persistent endocrine-disrupting chemicals (EDC) exposure may alter fetal growth. However, evidence is lacking on EDC mixtures and clinical indicators of optimal placental function. This study aimed to examine associations between eight EDC mixtures and fetal growth, fetoplacental hemodynamics, and angiogenic markers.

Materials and Methods: We included 734 pregnant participants residing in Barcelona, Spain (2018-2021). Thirty-three EDCs were detected at 18- and 34-weeks gestation using pooled urine samples (≥10 voids collected over 6 days at 18- and 34-weeks): metabolites of low and high molecular-weight phthalates (LMWP, HMWP), DINCH, chlorinated or hydroxylated aromatic compounds, polycyclic aromatic hydrocarbons, organophosphates, and parent compounds phenols and parabens. Estimated fetal weight (EFW) (20-, 32-, 37-weeks) was assessed via ultrasound examination, and hospital birth weight obtained. Uterine artery (20- and 32 weeks) and cerebroplacental (CPR) (32 weeks) fetoplacental hemodynamics, and soluble fms-like tyrosine kinase receptor-1 (sFlt-1)/placental growth factor (PlGF) ratio (32 weeks) were assessed. Penalized LASSO-type multi-group Bayesian Weighted Quantile Sum Regression models were used to estimate associations between chemical mixtures and outcomes at single and repeated time points, controlled for relevant covariates.

Results: Single time-point analyses showed significant decreases in birth weight per quartile increase in the LMWP mixture at 18 weeks (β = -0.119; CrI, -0.224 to -0.008; monobutyl phthalate weight 0.53) and 34 weeks (β = -0.116; CrI, -0.217 to -0.016; monoisobutyl phthalate weight 0.60), which were consistent, but not significant in repeated time-point models. The LMWP mixture was associated with a decrease in CRP and an increase in the sflt-1/PlGF ratio. Exposure to mixtures of other EDC classes was inconsistent in single and repeated time-point models across outcomes.

Conclusion: This comprehensive study of multi-class non-persistent EDC mixtures suggests that exposure to LMWP mixtures is associated with decreased birth weight, and altered fetoplacental hemodynamics and angiogenic markers.

FT21-03 | Identifying Dietary Predictors of Per- and Polyfluoroalkyl Substances in Pregnancy Using Supervised Mixtures Methods

Heather M. Guetterman[1,2], George D. Papandonatos[3], Kelsi A. Morris[1,2], Sabrina Smith[4], June-Soo Park[5], Joseph M. Braun[6], Susan L. Schantz[7], Rita S. Strakovsky[1,2]

[1] Michigan State University, Department of Food Science and Human Nutrition, East Lansing, USA; [2] Michigan State University, Institute for Integrative Toxicology, East Lansing, USA; [3] Brown University, Department of Biostatistics, Providence, USA; [4] California Environmental Protection Agency, Environmental Chemistry Laboratory, Department of Toxic Substances, Berkeley, USA; [5] University of California, Department of Obstetrics, Gynecology and Reproductive Sciences, San Francisco, USA; [6] Brown University, Department of Epidemiology, Providence, USA; [7] University of Illinois, Department of Comparative Biosciences and Beckman Institute, Urbana-Champaign, USA

Objectives: Per- and polyfluoroalkyl substances (PFAS) are known toxicants, and diet is a major source of PFAS in the general population. Given the multicollinear and high-dimensional nature of dietary data, we evaluated associations of diet mixtures – using several supervised statistical methods – with PFAS concentrations in pregnant women, a vulnerable subgroup.

Material and Methods: Pregnant Illinois women (n=505, enrolled 2013-2018) provided fasted blood samples at median 17-weeks gestation to quantify serum perfluorooctane sulfonic (PFOS), perfluorooctanoic (PFOA), perfluorononanoic (PFNA), perfluorodecanoic (PFDeA), perfluoroundecanoic (PFUdA), 2-(N-methyl-perfluoro-octane sulfonamido) acetic, and perfluorohexane sulfonic acids. Women completed a three-month food frequency questionnaire at median 13-weeks gestation. Of 193 reported foods, we generated a list of 100 foods using prior literature and removing foods consumed by <15% of women. We evaluated associations of 100 foods in grams with PFAS using multivariable quantile g-computation (qGcomp), weighted quantile sum regression (WQS), and Bayesian kernel machine regression (BKMR) to estimate the overall mixture effect and identify primary dietary contributors.

Results: Median PFAS concentrations were similar to women in the U.S. at the same time. Using qGcomp, each quartile increase in the food mixture was associated with 96% higher PFOS (95%CI: 23-212), 101% higher PFOA (95%CI: 36-198), 116% higher PFNA (95%CI: 51-211), 334% higher PFDeA (95%CI: 93-879), and 400% higher PFUdA (95%CI: 54-1521); WQS and BKMR confirmed these findings. All methods identified shellfish as one of the main determinants of PFOS, PFNA, PFDeA, and PFUdA; fatty fish of PFUdA and PFDeA; tuna of PFUdA; and some non-animal foods, including breakfast/energy bars of PFOA and iced tea of PFOA, PFNA, and PFDeA.

Conclusions: By concurrently evaluating most foods consumed by this pregnant population, we identified fish/seafood, along with non-animal-sourced products, as predictors of several serum legacy PFAS. Future studies may consider mixtures methods to understand dietary predictors of other environmental contaminants.

FT21-04 | Associations between Halogenated Flame Retardants Exposure and Childhood Gut Microbiome Multi-omics Profiles in a Canadian Cohort

Yidan Zhang[1], Feng Gao[2,3], Peng Gao[4], Yihan Fan[2], Virginie Gillet[5], Jean-Philippe Bellenger[6], Andrea Baccarelli[7,5], Larissa Takser[5], Yike Shen[1]

[1] University of Texas at Arlington, Department of Earth and Environmental Sciences, Arlington, USA; [2] University of California Los Angeles, Department of Environmental Health Sciences, Fielding School of Public Health, Los Angeles, USA; [3] University of California Los Angeles, Department of Molecular and Medical Pharmacology, David Geffen School of Medicine, Los Angeles, USA; [4] Harvard University, Department of Environmental Health, Harvard T.H. Chan School of Public Health, Boston, USA; [5] Université de Sherbrooke, Département de Pédiatrie, Faculté de Médecine et des Sciences de la Santé, Sherbrooke, Canada; [6] Université de Sherbrooke, Département de Chimie, Sherbrooke, Canada; [7] Harvard University, Harvard T.H. Chan School of Public Health, Boston, USA

Objective: Imbalance in the childhood gut microbiota has been linked to long-term adverse health outcomes, including metabolic disorders, infections, and autoimmune diseases. Early-life exposure to halogenated flame retardants (HFRs) may alter the gut microbiota, but this association remains unexplored. This study examined the associations of HFRs in plasma and stool with gut microbiome and transcriptome profiles in children aged 8-12 years from the GESTE birth cohort in Sherbrooke, Quebec, Canada.

Material and Methods: We measured concentrations of 16 HFRs in plasma (N = 140) and stool (N = 182) samples from 8–12-year-old children in the GESTE cohort. Five plasma HFRs (BDE47, BDE99, BDE100, BDE153, BB153) and seven stool HFRs (BDE47, BDE99, BDE100, BDE153, BDE209, s-DP, t-DP) with detection rates >50% were included. Shotgun metagenomic and transcriptomic sequencing was used to profile gut microbiome species and functional pathways, respectively. Covariate-adjusted linear regression models assessed HFR-microbiome feature associations.

Results: After false discovery rate correction, plasma BDE99 was inversely associated with *Bacteroides dorei* (β = -1.36, 95%CI: -2.03 ~ -0.68). Stool BDE209 was negatively associated with *Monoglobus pectinilyticus* (β = -1.21, 95%CI: -1.75 ~ -0.67), and t-DP levels were negatively associated with *Eubacterium siraeum* (β = -0.93, 95%CI: -1.38 ~ -0.49). The stool BDE153 was linked to five functional pathways: 4-aminobutanoate degradation V, TCA cycle II, myo-, chiro-, and scyllo-inositol degradation, super-pathway of pyridoxal 5'-phosphate biosynthesis and salvage, and CDP-diacylglycerol biosynthesis III. Stool t-DP was positively associated with two pathways: 4-aminobutanoate degradation V and phosphatidylcholine acyl editing. No significant associations were observed between plasma HFR levels and gut microbiome functional pathways.

Conclusion: In children from the GESTE cohort, both plasma and stool HFR exposure were associated with alterations of gut microbiome, with stronger associations observed for stool HFRs. Future studies may investigate whether these microbiome changes are related to children's health outcomes.

FT21-05 | Predictors of organophosphate ester flame retardants in U.S. pregnant mothers in the ECHO cohort.

Thuy Lam[1], Lesliam Quiros-Alcala[2], Xuan Li[3], Jose F. Cordero[4], Megan E. Romano[5], Deborah J. Watkins[6], John Meeker[6], Emily S. Barrett[7], Tom O'Connor[8], Jessie P. Buckley[9], Rachel A. Morello-Frosch[10], Dana M. Dabelea[11], Assiamira Ferrara[12], Deborah H. Bennett[13], Donghai Liang[14], Tracy Bastain[15], Anne M. Riederer[16], Cindy T. McEvoy[17], Akhgar Ghassabian[18]

[1] Brandeis University, Health: Science, Society, and Policy Program, Waltham, USA; [2] Johns Hopkins Bloomberg School of Public Health, Department of Environmental Health and Engineering, Baltimore, USA; [3] Johns Hopkins Bloomberg School of Public Health, Department of Epidemiology, Baltimore, USA; [4] University of Georgia, Department of Epidemiology and Biostatistics, Athens, USA; [5] Dartmouth Geisel School of Medicine, Department of Epidemiology, Lebanon, USA; [6] University of Michigan School of Public Health, Department of Environmental Health Sciences, Ann Arbor, USA; [7] Rutgers School of Public Health, Department of Biostatistics and Epidemiology, Piscataway, USA; [8] University of Rochester Medical Center, Departments of Psychiatry, Psychology, Neuroscience, and Obstetrics and Gynecolog, Rochester, USA; [9] University of North Carolina Gillings School of Global Public Health, Department of Epidemiology, Chapel Hill, USA; [10] University of California, Berkeley School of Public Health, Department of Environmental Science, Policy and Management, Berkeley, USA; [11] Colorado School of Public Health, Department of Epidemiology, Aurora, USA; [12] Kaiser Permanente, Division of Research, Pleasanton, USA; [13] University of California, Davis School of Medicine, Department of Public Health Sciences, Davis, USA; [14] Emory University Rollins School of Public Health, Gangarosa Department of Environmental Health, Atlanta, USA; [15] University of Southern California, Department of Population and Public Health Sciences, Los Angeles, USA; [16] University of Washington, Department of Environmental and Occupational Health Sciences, Seattle, USA; [17] Oregon Health & Science University, Department of Pediatrics, Portland, USA; [18] New York University Grossman School of Medicine, Department of Pediatrics and Department of Population Health, New York, USA

Background: Increasing evidence suggests that exposures to organophosphate ester (OPE) flame retardants, chemical additives used as an alternative to the polybrominated diphenyl ethers, could impact health across the life course. Few studies have evaluated predictors of OPE exposure during gestation.

Objectives: We assessed predictors of OPE exposure among 420 pregnant individuals from 16 cohorts in the U.S. Environmental Influences on Child Health Outcomes (ECHO) cohort.

Methods: Nine OPE biomarkers were measured in prenatal urine samples collected between 10-41 gestational weeks. We used data on demographics, personal behaviors, household characteristics, and occupations captured in questionnaires to evaluate potential exposure determinants. General linear regression was used to identify determinants of $\log_2$-transformed biomarker concentrations modeled as continuous, categorical as high/low/non-detect, or dichotomous (detect/non-detect) depending on the detection frequency.

Results: Participants were primarily White (55%) and non-Hispanic (71%). The average age was 31.9 years (SD: 4.2 years). Women who had their rugs/carpets cleaned during pregnancy had 41% (95% CI: -7.53, 116.0) higher diphenyl phosphate (DPHP) concentrations compared to those who did not in adjusted models. Participants who worked in a hair salon had higher bis(1-chloro-2-propyl) phosphate (BCPP) concentrations than those who did not. Higher gravidity predicted lower concentrations of a composite of dibutyl phosphate and di-isobutyl phosphate (DBUP/IP) and bis(butoxyethyl) phosphate (BBOEP). Inverse associations were also observed for OPE biomarker concentrations and home maintenance or improvement activities, including installation of rugs/carpets (BBOEP), floor cleaning (BBOEP, dipropyl phosphate (DPRP)), rug/carpet cleaning (BCPP), home renovations (BBOEP, bis(2-ethylhexl) phosphate (BCETP), using air fresheners (BCPP), and working as a janitor/house cleaner (bis(1,3-dichloro-2-propyl) phosphate (bis(1,3-dichloro-2-propyl) phosphate (BDCPP).

Conclusion: Predictors of OPE concentrations in pregnancy may include factors related to household activities and occupation. Understanding predictors during this sensitive window will increase awareness and identify potential exposure mitigation strategies.

FT21-06 | Chemical flame retardants in breast milk: Assessing infant exposure in Lagos, Nigeria

Temilola Oluseyi[1], Oluwatoyin Olagoke[2], Kelechi Njoku[2], Stuart Harrad[3], Mohamed Abdallah[3]

[1] University of Lagos, Department of Chemstry, Lagos, Nigeria; [2] University of Lagos, 2Department of Cell Biology and Genetics, Lagos, Nigeria; [3] University of Birmingham, School of Geography, Earth and Environmental Sciences, Birmingham, UK

Halogenated flame retardants (HFRs) are chemicals used in consumer products to prevent flammability. They are persistent, toxic and have been reported to bioaccumulate in human milk. This report investigates the levels of polybrominated diphenyl ethers (PBDEs), novel brominated flame retardants (NBFRs) and dechlorane plus (DPs) in breast milk at three stages of lactation and associated health risks to nursing infants. Levels of PBDEs, NBFRs and DPs were determined at 3 stages of lactation via analysis of human milk samples provided by mothers. 126 breast milk samples were collected from 87 (primiparous and multiparous) mothers at colostrum, transitional and matured stages. HFRs were extracted by ultrasonication, cleaned up by solid-phase extraction and analysed using GC/MS. BDE99,183 and 209 were the dominant PBDEs with >80% detection frequency and median concentrations of 0.09, 0.02, and 0.03 ng/g lw, respectively. EH-TBB and DBDPE had the highest NBFR detection frequency of >90%, with median concentrations of 0.17 and 0.10 ng/g lw. DPS (syn and anti) were detected in >75% of the samples, with median concentrations of 0.05 and 0.07 ng/g lw, respectively. Colostrum had the highest concentration for most PBDEs, NBFRs and DP, with concentrations decreasing significantly ($p<0.05$) for BDE-183, 99, 153, EH-TBB, DBDPE, syn-DP and anti-DP across lactation stages. Primipara recorded higher median concentrations than multipara for most PBDES, NBFRs and DP. ΣNBFRs had the highest median estimated daily intake (EDI) of 13.26 ng/kg bw/day for breastfed infants. BDE-47, 153, 209, and DBDPE had hazard quotient (HQ) values less than the noncarcinogenic threshold of 1. Conversely, BDE-99 had EDI values above the EPA reference dose and HQ greater than 1 in five samples. These findings reveal that some infants are at risk of HFR exposure through breastfeeding in Lagos, Nigeria.

FT21-07 | Assessing Polychlorinated Biphenyls (PCB) Exposure in Lebanese Newborns' Cord Blood Two Decades After the Ban: Findings from the EELI Study

Emile Whaibeh[1,2], Jowy Abi Hanna[1,2], Sarkis Kerkemzian[2], Norma Aouad[4], Alicia Abi Nader[1,2], Cyrille Khalil[2,3], Georges Abi Tayeh[4,5], **Myriam Mrad**[2,3]

[1] Saint-Joseph University of Beirut, Institut Supérieur de Santé Publique, Faculty of medicine, Beirut, Lebanon; [2] University of Balamand, Department of Public Health, Beirut, Lebanon; [3] Health and Environment Response Agency (HERA), Beirut, Lebanon; [4] Hôtel-Dieu de France University Hospital, Beirut, Lebanon; [5] Saint Joseph Fertility Center, Beirut, Lebanon

Objective: Despite being banned in Lebanon since 1997, polychlorinated biphenyls (PCBs) remain a significant public health threat due to their persistence. Previous studies have detected PCBs at concerning levels in adults and pregnant women in Lebanon, yet data on fetal exposure via cord blood analysis remains limited. The current study aims to identify the presence of 13 PCB congeners in cord blood samples and to determine dietary, environmental, and sociodemographic predictors of exposure.
Materials and Methods: PCB concentrations were quantified using gas chromatography-mass spectrometry in cord blood samples (N=74) from the Environmental Exposures in Lebanese Infants (EELI) study. Descriptive statistics characterized PCB levels, while bivariate analyses explored associations between PCB concentrations and potential exposure predictors. Multivariate linear regression models identified significant predictors of PCB exposure.
Results: PCBs were detected in all cord blood samples, with total PCB (∑PCBs) concentrations ranging from 22.19 ng/L (median) to 373.16 ng/L (maximum). The predominant congeners were PCB-153 (median: 7.62 ng/L, 95th percentile: 23.5 ng/L), PCB-180 (median: 4.88 ng/L, 95th percentile: 21.9 ng/L), and PCB-138 (median: 4.29 ng/L, 95th percentile: 16.8 ng/L). Multivariate analyses identified significant associations between PCB levels and maternal dietary habits (higher vegetable and seafood intake, $p < 0.05$), environmental factors (proximity to industrial areas and agricultural land, $p < 0.01$), and sociodemographic characteristics (age and employment type, $p < 0.05$).**Conclusion:** The detection of PCBs in Lebanese newborns underscores ongoing prenatal exposure, despite their ban around two decades ago. Our findings highlight the need for continuous biomonitoring and targeted public health interventions addressing dietary sources, environmental contamination, and vulnerable sociodemographic groups.

Funding and Acknowledgments: The research is made possible by an intramural fund from the University of Balamand and partially by an NIH Fogarty International Center grant (Reference number: [1U01TW012237-01]). We thank RECETOX, the MoPH, and WHO for their invaluable assistance.

FT21-08 | Associations of trimester specific environmental chemical concentrations and first and third trimester total folate concentrations in Canadian pregnant women.

Jordana Leader[1], Josh Alampi[2], Joseph Braun[3], Amanda MacFarlane[4,5], Aimin Chen[6], Tye E. Arbuckle[7], Ana Maria Siega-Riz[8], Raji Balasubramanianh[8], Mandy Fisher[7], Jillian Ashley-Martin[7], Bruce P. Lanphear[2], Youssef Oulhote[1]

[1] ICAHN School of Medicine at Mount Sinai, Department of Environmental Medicine, New York, USA; [2] Simon Fraser University, Faculty of Health Sciences, Burnaby, Canada; [3] Brown University, Center for Climate, Environment and Health, Providence, USA; [4] Health Canada, Nutrition Research Division, Ottawa, Canada; [5] Carleton University, Department of Biology, Ottawa, Canada; [6] University of Pennsylvania, Department of Biostatistics, Epidemiology and Informatics, Perelman School of Medicine, Philidalphia, USA; [7] Health Canada, Environmental Health Science and Research Bureau, Healthy Environments and Consumer safety branch, Ottawa, Canada; [8] University of Massachusetts, Epidemiology and Biostatistics, School of Public Health and Health Sciences, Amherst, USA

Objective: Folate is essential for fetal development, but few studies have investigated the association between environmental chemicals and folate concentrations during pregnancy. We examined the associations of trimester specific gestational exposure to phthalates, organophosphate pesticides (OP) metabolites, and metal concentrations with total plasma folate concentrations in the 1st and 3rd trimester among pregnant Canadian women.
Method: Approximately 2000 women were enrolled in the Maternal-Infant Research on Environmental Chemicals (MIREC) study during the first trimester of pregnancy from 10 Canadian cities. We measured concentrations of 24 phthalates and six OP metabolites in first and third trimester urine samples. Arsenic, cadmium, lead, manganese and mercury and total folate concentrations were measured in first and third trimester blood samples. We used quantile g-computation to estimate covariate-adjusted joint associations between the mixture of each class of environmental chemicals with total folate in first and third trimesters separately.
Results: The sample size for this analysis ranged from 1604 to 1983 women. Each quartile higher first trimester metal mixture was associated with 10.6 nmol/L (95% CI: -19.4, -1.2) lower total folate concentrations in the third trimester only. This association was mainly driven by cadmium, manganese and mercury. Similarly, a one quartile increase in first trimester phthalate mixture was non-significantly associated with lower total folate concentrations in both first (b= -7.6 nmol/L, 95% CI: -16.8, 1.7) and third (b= -8.1, 95% CI -25.0, 8.9) trimesters. This relationship was driven by metabolites of di(2-ethylhexyl) phthalate. Finally, the first trimester OP mixture was non-significantly associated with lower total folate concentrations in the first (b= -21.9, 95% CI: -51.3, 7.5) trimester. This relationship was primary driven by diethyldithiophosphate.
Conclusion: Gestational exposure to some environmental chemicals, especially during the first trimester, was associated with reduced total plasma folate in both the first and third trimesters.

FT21-09 | Associations of prenatal maternal urinary concentrations of a mixture of parabens with cognition in 7.5-month-old infants

Nicholas Cragoe[1], Megan L. Woodbury[2], Sa Shen[3], Andrea Aguiar[1], Susan L. Schantz[1]

[1] University of Illinois Urbana-Champaign, Urbana, USA; [2] Northeastern University, Boston, USA; [3] Stanford University, Palo Alto, USA

Objective

Few studies have assessed the consequences of prenatal exposure to parabens, common chemical preservatives, for early neurodevelopment. We evaluated associations of prenatal exposure to three parabens with infant cognition at 7.5 months via performance on a visual recognition memory (VRM) task.

Materials and Methods

Methylparaben (MePB), ethylparaben (EtPB), and propylparaben (PrPB) were measured in pooled urine samples collected at five time points across pregnancy. Cognition was measured using a VRM task with infrared eye-tracking, where infants (n=310) were shown two identical photographs of human faces (familiarization), followed by the familiar face paired with a novel face (test trials). Covariate-adjusted generalized linear regression assessed associations of each individual paraben with time to familiarization (time to accrue 20 s of looking; attention), run duration (time looking before looking away; processing speed), novelty preference (time looking at the novel face; recognition memory), and shift rate (rate of gaze-shifts between stimuli; attention). Quantile g-computation assessed associations of the paraben mixture with VRM outcomes. Stratified models assessed modification by infant sex.

Results

Mothers were mostly white and college-educated with incomes >$50,000/yr. EtPB was associated with shorter time to familiarization in females (IQR-adjusted β:-0.015; 95% CI:-0.034;0.000) and longer in males (IQR-adjusted β:0.010; 95% CI 0.000;0.015), as well as longer run duration in females (IQR-adjusted β:0.015; 95% CI 0.000;0.029), and PrPB was associated with lower shift rate (IQR-adjusted β:-0.016; 95% CI -0.032;0.027), in females. The paraben mixture was associated with longer run duration (IQR-adjusted β:0.317; 95% CI -0.020;0.654), higher novelty preference (IQR-adjusted β:0.029, 95% CI 0.002; 0.056) and higher shift rate (IQR-adjusted β:0.027; 95% CI 0.000; 0.054) in males.

Conclusion

Paraben exposure is associated with changes in infant cognition, and the patterns of association differ markedly in their sex-specific and domain-specific effects when parabens were considered individually from than when they were considered as a mixture.

FT21-10 | What's in the Diaper? A Hybrid Targeted and Non-Targeted Analysis of Chemicals in Disposable Diapers Utilizing High-Performance Liquid Chromatography and High-Resolution Mass Spectrometry (HPLC-HRMS)

Elisia White[1], Trevor Johnson[2], Dimitri Abrahamsson[3], Linda G. Kahn[4]

[1] New York University Grossman School of Medicine, Department of Environmental Medicine, New York, USA; [2] New York University Grossman School of Medicine, Department of Pediatrics, New York, USA; [3] University of California San Francisco School of Medicine, Department of Obstetrics, Gynecology and Reproductive Sciences, San Francisco, USA; [4] New York University Grossman School of Medicine, Department of Population Health, New York, USA

Objective: Infancy is a critical developmental period for the reproductive system, which can be dysregulated by exposure to endocrine-disrupting chemicals (EDCs). Infants and toddlers are continuously exposed to exogenous chemicals through commercial baby products including bottles, pacifiers, creams, wipes, and diapers. Most parents in industrialized countries use disposable diapers, yet the diaper manufacturing industry remains unregulated. Research on the chemicals used in commercial diapers is limited, and manufacturers are not required to disclose this information, leaving a knowledge gap. Infants spend all day and night in diapers, which cover a large portion of their bodies. The combination of sweat and urine inside the diaper increases dermal absorption. Therefore, it is important to ascertain the chemical constituents of disposable diapers, as they may have adverse effects on infants' overall health and developing endocrine systems. **Method:** To investigate the chemical components of disposable diapers and identify potential novel compounds for future epidemiologic analyses, we performed a hybrid targeted and non-targeted analysis of 15 commercial diapers that included conventional, overnight, and eco-friendly types. Each diaper was carefully cut and weighed. Before analysis, the diaper samples were incubated and extracted in a 50:50 Methanol/Dichloromethane solution. The chemical composition of diapers was analyzed using High-Performance Liquid Chromatography coupled with High-Resolution Mass Spectrometry and Tandem Mass Spectrometry (MS/MS) in both positive and negative ionization modes. The chemical screening was performed using targeted analysis of known substances, coupled with non-targeted techniques to detect uncharacterized compounds. **Results:** Phthalates, Flame Retardants, Pesticides, Parabens, Plasticizers, and Polycyclic Aromatic Hydrocarbons were among the EDCs detected. Identities of phthalates were then confirmed and quantified using analytical standards. **Conclusion:** This study is an important first step in building an evidence base for regulating chemicals used to produce disposable diapers. This research was supported by funding from the NYU Langone Department of Pediatrics.

FT22 | Air pollution: Exposures, methods, approaches, and health

FT22-02 | A Mixed-Frequency Distributed Lag Non-Linear Model for Estimating Short-Term Environmental Exposure Risks

Nidhi Shukla[1,2], Anna L. Hansell[2], Suzanne E. Bartington[3], Tim CD. Lucas[1]

[1] University of Leicester, Department of Population Health Sciences, Leicester, UK; [2] University of Leicester, Centre for Environmental Health and Sustainability, Leicester, UK; [3] University of Birmingham, Department of Applied Health Sciences, Birmingham, UK

Background: Extreme environmental conditions significantly increase the risk of asthma exacerbation. Short-term changes in air pollutants, such as nitrogen dioxide (NO_2) from traffic emissions, are prevalent in urban areas. However, in environmental epidemiology, high-frequency health data are often unavailable. In the absence of high-frequency response data, researchers typically aggregate exposure data (e.g., from hourly to daily) to match the frequency of health data, leading to a loss of information. To address this, we developedthe mixed-frequency distributed lag non-linear model (mf-DLNM).
Method: We investigated the association between hourly NO_2 exposure and asthma-related emergency hospital visits to University Hospitals Birmingham NHS Foundation Trust. We developed mf-DLNM, a method inspired by the Mixed-Data Sampling (MIDAS) model from econometrics. The model employs the DLNM framework to estimate high-resolution risk using low-frequency response and high-frequency exposure data. We compared the predictive performance of mf-DLNM with standard DLNM, where mf-DLNM was fitted using hourly NO_2 and daily hospital visit data, while standard DLNM used same-frequency (sf) hourly data for both variables.
Results: The sf-DLNM showed a positive association between hourly NO_2 levels and daytime asthma-related emergency visits, with the most pronounced effects observed at 3 to 6-hour lags. Despite data limitations, mf-DLNM effectively predicted hourly effects. Using the sf-DLNM estimate as the gold standard, for the cumulative relative risk (RR) of mean NO2 over a 24-hour lag, mf-DLNM had a Mean Absolute Error (MAE) of 0.017 and an R-value of 0.97, relative to sf-DLNM. Additionally, mf-DLNM successfully captured 24-hour temporal variations, and showed a positive effect at 3 to 6-hour lag, consistent with sf-DLNM.
Conclusion: The mf-DLNM advances environmental epidemiology by enabling high-resolution health risk estimation despite data constraints. It offers researchers an alternative to aggregating high-frequency exposure data. By retaining high-frequency information, mf-DLNM enhances public health decision-making and strengthens preparedness during episodic environmental events.

FT22-04 | Associations between ambient air pollutant exposures and risk of obstructive sleep apnea in the Nurses' Health Study II

Jing Wang[1,2], Cindy R. Hu[1], Susan Redline[2], Jeff D. Yanosky[3], Francine Laden[1,4], Joel D. Kaufman[5], Charlotte Roscoe[6,7], Tianyi Huang[8], Jaime E. Hart[1,4]

[1] Harvard T.H. Chan School of Public Health, Department of Environmental Health, Boston, USA; [2] Brigham and Women's Hospital, Division of Sleep and Circadian Disorders, Boston, USA; [3] The Pennsylvania State University College of Medicine, Department of Public Health Sciences, Hershey, USA; [4] Brigham and Women's Hospital and Harvard Medical School, Channing Division of Network Medicine, Department of Medicine, Boston, USA; [5] University of Washington, Departments of Environmental and Occupational Health Sciences, Epidemiology, and Medicine, Seattle, USA; [6] Oregon Health & Science University Portland State University OHSU-PSU School of Public Health, Environmental Systems and Human Health, Portland, USA; [7] Division of Population Sciences, Dana Farber Cancer Institute, Boston, USA; [8] National Institute on Aging, Laboratory of Epidemiology and Population Sciences, Intramural Research Program, Baltimore, USA

Objective: Ambient air pollutant exposures may contribute to obstructive sleep apnea (OSA) development. However, existing epidemiological evidence is inconsistent and is limited to cross-sectional studies. This study aimed to prospectively assess the associations between ambient particulate matter ≤ 2.5 μm in aerodynamic diameter ($PM_{2.5}$) and nitrogen dioxide (NO_2) exposure with the risk of OSA.

Material and Methods: We followed 108,246 females from the US based Nurses' Health Study II from 2000 to 2017. Participants' time-varying 12-month average residential exposures to $PM_{2.5}$ and NO_2 preceding biennial questionnaires were estimated by spatiotemporal models. Clinical diagnosis of OSA was self-reported via a validated questionnaire in 2017. Cox proportional hazards models were used to estimate the hazard ratios (HRs) and 95% confidence intervals (CIs) for the associations. Primary models adjusted for age, calendar year, race and ethnicity, neighborhood socioeconomic status, and region. We also developed two-pollutant models and further adjusted for health behaviors, meteorological factors, and health conditions.

Results: We identified 5,924 OSA cases over 1,896,684 person-years of follow-up. The mean (SD) ambient $PM_{2.5}$ and NO_2 concentrations 2000-2017 were 10.3 (± 2.0) μg/m^3 and 8.9 (± 4.4) ppb. Each interquartile range (IQR) increase in $PM_{2.5}$ (2.60 μg/m^3) and NO_2 (4.74 ppb) were associated with a 17% (HR=1.17, 95% CI: 1.02, 1.33) and 0% (HR=1.00, 95% CI: 0.93, 1.07) change in OSA risk, respectively. The association between $PM_{2.5}$ exposure and OSA risk persisted in the two-pollutant model (HR=1.25, 95% CI: 1.07, 1.46) and strengthened in the model additionally adjusted for meteorological factors including temperature and relative humidity (HR=1.32, 95% CI: 1.15, 1.52).

Conclusion: We found increased ambient $PM_{2.5}$ exposure, but not NO_2, was associated with a higher risk of OSA in a nationwide longitudinal cohort of females. The findings support the need for consideration of ambient $PM_{2.5}$ mitigation strategies to reduce the risk of OSA.

FT22-06 | Prenatal household air pollution personal exposure and pulmonary function by oscillometry in 3-year-old Guatemalan children

Laura M. Grajeda[1], Anaite Diaz-Artiga[2], Maria R. Lopez[2], Keyla Castellanos[2], Tatiana Petrovick[3], Albino Barraza-Villarreal[4], Lisa M. Thompson[5], Shirin Jabbarzadeh[6], Jiantong Wang[6], Lance A. Waller[6], Maggie L. Clark[7], Amy E. Lovvorn[8], Christina M. Eckhardt[9], Nadia E. Hoekstra[10], Michael A. Johnson[11], Ajay Pillarisetti[12], Diddier Prada[13], Andrea A. Baccarelli[14], Ye Shen[15], Jessica Knight[15], Luke P. Naeher[16], Thomas Clasen[8], Jennifer Peel[7], Eric D. McCollum[17], John P. McCracken[1]

[1] University of Georgia, Global Health Institute, Department of Epidemiology and Biostatistics, Athens, USA; [2] Universidad del Valle de Guatemala, Centro de Estudios en Salud, Guatemala, Guatemala; [3] University of Massachusetts, UMass Chan Medical School, Worcester, USA; [4] National Institute of Public Health, Morelos, Mexico; [5] Emory University, Nell Hodgson Woodruff School of Nursing, Atlanta, USA; [6] Emory University, Department of Biostatistics and Bioinformatics, Rollins School of Public Health, Atlanta, USA; [7] Colorado State University, Department of Environmental and Radiological Health Sciences, Fort Collins, USA; [8] Emory University, Gangarosa Department of Environmental Health, Rollins School of Public Health, Atlanta, USA; [9] Columbia University, Columbia University Vagelos College of Physicians and Surgeons, New York, USA; [10] University of North Carolina, School of medicine, Chapel Hill, USA; [11] Berkeley Air Monitoring Group, Berkeley, USA; [12] University of California, Division of Environmental Health Sciences, Berkeley, USA; [13] Icahn School of Medicine at Mount Sinai, Institute for Health Equity Research, Department of Population Health and Policy and Department of Environmental Medicine and Public Health, New York, USA; [14] Harvard University, Department of Environmental Health, T.H. Chan School of Public Health, Boston, USA; [15] University of Georgia, Department of Epidemiology and Biostatistics, Athens, USA; [16] University of Georgia, Department of Environmental Health Science, Athens, USA; [17] Johns Hopkins University, Global Program in Pediatric Respiratory Sciences, Eudowood Division of Pediatric Respiratory Sciences, Baltimore, USA

Objective: Household air pollution is a risk factor for obstructive lung diseases. We assessed the exposure-response relationship between prenatal air pollution and lung function in preschool-aged children in Guatemala.

Materials and methods: The Household Air Pollution Intervention Network (HAPIN) trial randomized pregnant women (9-19 weeks gestation) to a free liquefied petroleum gas (LPG) cookstove and fuel intervention versus continued use of a biomass cookstove through their child's first year. We measured 24-hour personal exposure to particulate matter with aerodynamic diameter < 2.5 μm ($PM_{2.5}$) and black carbon (BC) at baseline and at 24-28 and 32-36 weeks gestation. We predicted the mothers' time-weighted average exposure between 9-40 weeks gestation by linear mixed-effects models conditioned on study arm, period, and day of year. We assessed lung function at age 3 by 7-41 Hertz multifrequency respiratory oscillometry (Tremoflo C-100, Thorasys). Endpoints were two-test averages of resistance and reactance at 7 Hertz, and resistance pooled over prime frequencies. We used linear mixed-effects and generalized additive mixed models to assess associations between log-transformed exposures and lung function adjusted for baseline sociodemographic variables and second-hand smoke exposure.

Results: Of 800 pregnant women, 524 children had valid exposure measurements and oscillometry tests. The median (interquartile range) $PM_{2.5}$ exposure was 67 (39-112) μg/m^3 and BC exposure was 8 (6-12) μg/m^3. We did not find evidence of exposure-response associations with lung resistance or reactance at 7 Hertz. However, an interquartile increase of 73 in $PM_{2.5}$ and 6 in BC exposures were associated with 0.35 (95% CI: 0.10, 0.60) and 0.31 (95% CI: 0.05, 0.57) cmH_20•s L^{-1} higher airway resistance.

Conclusion: In a population from a randomized control trial of an LPG cookstove intervention, higher prenatal exposure to $PM_{2.5}$ and BC was associated with higher resistance in 3-year-old children, suggesting increased air pollution exposure may lead to airway narrowing.

FT22-07 | Evaluating Personal Exposure to $PM_{2.5}$ and O_3 in Population Groups over Multiple Seasons in South Korea

Hyunwoo Jeon[1], Jiyoung Park[2], Kiyoung Lee[1,2]

[1] Seoul National University, Department of Environmental Health Science, Graduate School of Public Health, Seoul, South Korea; [2] Seoul National University, Institute of Health and Environment, Seoul, South Korea

Objective

Personal exposures to $PM_{2.5}$ and O_3 can be affected by time-location patterns and season. This study aimed to evaluate the impact of time-location patterns of various population groups and seasonal variations using the measured personal exposure to $PM_{2.5}$ and O_3 in South Korea.

Material and Methods

A longitudinal personal exposure measurement was conducted from March 2022 to November 2024 covering 886 person-days. Personal exposures to $PM_{2.5}$ and O_3 were measured using portable monitors carried by field technicians simulating daily activities based on predefined 24-hour time-location scenarios derived from Korea Statistics survey. Scenarios of ten distinct population groups (e.g., students, housewives, office workers) adjusted according to seasonal variations were applied. Analysis of variance and Bonferonni post-hoc tests were used to evaluate differences in exposure across population groups and seasons.

Results

The average personal exposures to $PM_{2.5}$ in ten population groups ranged from 13.58±9.26 μg/m³ to 23.7±18.6 μg/m³, with the highest exposure in night shift security workers and the lowest in office workers who visit café after work. The average personal exposure to O_3 in ten population groups ranged from 4.8±3.4 ppb to 7.0±8.8 ppb, with the highest exposure in senior citizen and the lowest in shopping mall night workers. Personal $PM_{2.5}$ and O_3 exposures showed significant difference by season. $PM_{2.5}$ exposure was the highest in spring with 19.86±18.04 μg/m³ and the lowest in summer with 15.8±13.39 μg/m³. O_3 exposure was the highest in spring with 8.2±7.69 ppb and the lowest in autumn with 4.07±2.82 ppb. Significant differences in personal $PM_{2.5}$ exposure across population groups were identified in summer, autumn, and winter. However, personal O_3 exposures were not different by population groups.

Conclusion

This study underscores the critical role of time-location patterns which vary across population groups and seasonal variability in determining personal exposure to $PM_{2.5}$ and O_3.

FT22-08 | Environmental noise and fine particulate matter ($PM_{2.5}$) and black carbon (BC) pollution in Holyoke, MA

In Kyu Park, Lily Juda, Sean O'Connor, Kultumie Tejan-Jalloh, Carissa Lange, Barbara Mottey, Jiayuan Wang, Raphael Arku

University of Massachusetts Amherst, Environmental Health Sciences, Amherst, USA

Introduction: Holyoke, a small city in Western Massachusetts (MA), has a predominantly Hispanic population, with one in four residents living below the poverty line. The city also has the highest prevalence of respiratory health problems in the state, including pediatric asthma and COPD. Studies consistently show that socioeconomically disadvantaged communities bear a disproportionate burden of environmental pollution and related health burden. Yet, environmental pollution in many smaller cities in Western MA has not been well-characterized.
Objective: We implemented a city-wide measurement to understand the space-time patterns in environmental noise and multiple air pollutants in Holyoke.
Methods: We conducted a one-year campaign from August 2021 to August 2022 and captured real-time sound level along with real-time and weekly integrated (filter-based) $PM_{2.5}$ concentrations at 35 sites, consisting of 5 year-long and 30 week-long sites. The light absorbance of the filters was used as a proxy for BC pollution.
Results: The overall median day-night average noise level (Ldn) was 64 dBA in high-density residential communities, compared to 57 dBA in low-density residential areas, both of which exceeded the EPA guideline of 55 dBA. The overall annual mean $PM_{2.5}$ concentration across the city was 6.2 µg/m3, ranging from 6.1 µg/m^3 in low-density residential areas to 8.3 µg/m^3 in high-density residential areas. The annual equivalent of the weekly samples ranged from 3.4 ug/m^3 to 31.7 ug/m^3. BC pollution followed the same land use pattern.
Conclusions: Although ambient $PM_{2.5}$ concentrations in Holyoke met the EPA standard of 9 µg/m^3, environmental noise levels were considerably high and exceeded EPA's recommended guidelines in most cases.

FT22-09 | Exploring the Relative Effects of Different Stove Types on Indoor Air Quality in Residential Kitchens

Dinela Dedic, Kailey Sipe, Alexander Jones, Megan Benka-Coker, **Wande Benka-Coker**

Dickinson College, Environmental Studies, Carlisle, USA

Gas stoves are a significant source of indoor air pollution, emitting nitrogen dioxide (NO_2) and other pollutants linked to adverse cardiorespiratory health effects. Biogas stoves offer a renewable alternative, yet their impact on indoor air quality (IAQ) remains understudied. This study quantifies NO_2 and fine particulate matter ($PM_{2.5}$) concentrations in kitchens using mainly natural gas and/or biogas stoves to assess their relative contributions to indoor pollution levels. Time-resolved kitchen area NO_2 and $PM_{2.5}$ concentrations were obtained over multiple 72-hour monitoring windows using Home Health Box aerosol samplers at one-minute intervals, with complementary time-integrated NO_2 samples from Ogawa passive samplers analyzed at RTI International. Outdoor pollutant concentrations and meteorological data were also monitored and recorded, and finally, questionnaires and event logs were used to obtain information on kitchen characteristics and stove usage. Linear mixed-effect regression models estimated the effect of cooking events on NO_2 and $PM_{2.5}$ levels, adjusting for house-level random effects, ventilation, and temperature.

Mean kitchen NO_2 concentrations were significantly higher in homes with natural gas stoves (6.87 ppb) compared to biogas stoves (3.42 ppb), though both remained below WHO's 1-hour guideline (13 ppb). $PM_{2.5}$ levels varied by cooking activity but showed no substantial difference between stove types. Regression models revealed similar patterns, suggesting a 95.4% increase in NO_2 exposure with gas stove use (-21.3%, 385.5%), though the confidence interval included the null.
Kitchens with natural gas stoves had higher NO_2 concentrations, though overall exposures were low. $PM_{2.5}$ levels fluctuated during cooking but did not differ significantly by stove type. These findings suggest that biogas could be a promising option for improving IAQ and reducing health risks associated with traditional cooking methods. However, further research is needed to understand the factors that influence pollutant levels, such as kitchen size, ventilation systems, and cooking patterns.

FT22-10 | Long-term $PM_{2.5}$ Composition Data for Climate and Air Quality Research in South Asia using Satellite observations

Shreya Srivastava[1], Sagnik Dey[1], Vasu Singh[1], Sudipan Saha[2], Chandra Venkataraman[3], Yang Liu[4]

[1] IIT Delhi, Centre for Atmospheric Sciences, New Delhi, India; [2] IIT Delhi, School of Artificial Intelligence, New Delhi, India; [3] IIT Bombay, Department of Chemical Engineering, Mumbai, India; [4] Emory University, Rollins School of Public Health, Atlanta, USA

Objective
Assessing aerosol composition is important for determining the detrimental aerosol impacts on climate, air quality, and human health. Conversely, South Asian countries lack systematic aerosol composition data, with only a few sites and limited time, and the extensive satellite data remains relatively unexplored.
Material and Methods
In this study, we use Multi-angle Imaging Spectro-Radiometer (MISR) Level 2 version 23 aerosol optical properties (spatial resolution = 4.4 km x 4.4 km) for the period 2000–2021. We calculated eight fractional AODs (f_{AODs}), which correspond to the eight aerosol models used in MISR's aerosol retrieval algorithm, as well as size-shape segregated AODs. The species-specific AODs and EDGAR emission data are used to train a Random Forest model, along with land use and meteorological variables. The model is trained on a quarter-degree grid covering South Asia, with the chemical model's simulated aerosol species mass fraction as the target variable.
Results
We train model to predict sulfate, nitrate, ammonium, black carbon, organic carbon, and dust aerosols. Using out-of-bag bootstrap sampling for cross-validation, Random Forest with 80% training and 20% testing yields R^2 values of 0.87, 0.92, 0.85, 0.89, 0.90, and 0.88 for sulfate, nitrate, ammonium, dust, OC, and BC, with RMSE values of 0.02, 0.03, 0.01, 0.001, 0.03 and 0.01 for test data. Predicted mass fraction and satellite-derived total $PM_{2.5}$ concentration estimate absolute mass concentration in a 1km grid. The model works well on all species except dust (overestimation) when validated against ground-based observations. The model estimates long-term aerosol composition climatology and trend from 2000 to 2021 over the study region.
Conclusion
Permutation feature importance analysis suggests that MISR's f_{AODs} have a significant influence on model predictions. The model anticipates the aerosol composition will improve climate and health impact assessments of aerosols by focusing on their differential effects in low-income South Asian countries.

FT23 | Environmental pollutants and chronic disease: Linking air quality to public health

FT23-02 | Fine particulate matter composition and breast cancer incidence in a United States-wide prospective cohort study

Meklit Daniel[1,2], Jennifer L. Ish[1], Jared A. Fisher[3], Christiana Kartsonaki[2], Kin Bong H. Lam[2], Rena R. Jones[3], Alexandra J. White[1]

[1] National Institute of Environmental Health Sciences, Epidemiology Branch, Division of Intramural Research and Training, Durham, USA; [2] University of Oxford, Clinical Trial Service Unit & Epidemiological Studies Unit, Nuffield Department of Population Health, Oxford, UK; [3] National Cancer Institute, Occupational & Environmental Epidemiology Branch, Division of Cancer Epidemiology & Genetics, Rockville, USA

Objective: Despite its carcinogenic properties, fine particulate matter ($PM_{2.5}$) has been inconsistently associated with breast cancer risk, which may be due in part to the heterogeneous chemical constituency of $PM_{2.5}$. We investigated the individual and joint effects of $PM_{2.5}$ components on breast cancer incidence.

Material and Methods: Using the Community Multiscale Air Quality modeling system (12-kilometer grid scale), we estimated the annual average outdoor concentrations (µg/m^3) of $PM_{2.5}$ and seven components (elemental carbon, ammonium, nitrate, organic carbon, sulfate, soil, and other) at the geocoded residential addresses of 45,574 Sister Study participants in the United States at baseline (2003–2009). We used Cox proportional hazards regression, with adjustments for sociodemographic and reproductive factors, to estimate hazard ratios (HR) and 95% confidence intervals (CI) for the association between 5-year mean concentrations of $PM_{2.5}$ components and breast cancer incidence overall and by tumor characteristics. We applied quantile g-computation to estimate the joint effects of $PM_{2.5}$ components on incident breast cancer.

Results: During follow-up (median = 14.4 years), 4,373 breast cancer cases were diagnosed. Associations for the individual and joint effects of $PM_{2.5}$ components were largely negligible. However, we observed a positive association between a 1-µg/m^3 increase in organic carbon and risk for estrogen receptor-positive (HR = 1.03, 95% CI = 0.98–1.08) but not estrogen receptor-negative breast cancer (HR = 0.92, 95% CI = 0.81–1.04). A 1-µg/m^3 increase across all $PM_{2.5}$ components was associated with slightly elevated incidence of ductal carcinoma *in situ* (HR = 1.07, 95% CI = 0.97–1.19) but not invasive (HR = 1.00, 95% CI = 0.95–1.06) breast cancer.

Conclusion: Our findings suggest that $PM_{2.5}$ components are not strongly related to overall breast cancer incidence although we observed suggestive evidence indicating that they may individually and jointly impact certain tumor types. Ongoing work will explore heterogeneity by geographic region and socioeconomic factors.

FT23-04 | Ambient air pollution exposure and risk of renal cell carcinoma in a large prospective U.S. cohort

Jared Fisher[1], Debra Silverman[1], Jessica Madrigal[1], Stella Koutros[1], Joel Kaufman[2], Jonathan Hofmann[1], Rena Jones[1]

[1] National Cancer Institute, Division of Cancer Epidemiology and Genetics, Rockville, USA; [2] University of Washington School of Public Health, Department of Environmental and Occupational Health Sciences, Seattle, USA

Objective: Prior studies examining associations between outdoor air pollution and renal cancer have had inconsistent findings and limited ability to evaluate heterogeneity by histologic subtype. We aimed to investigate these associations in a large U.S. cohort with well-characterized exposures and outcomes.

Methods: We estimated outdoor fine particulate matter ($PM_{2.5}$) and nitrogen dioxide (NO_2) concentrations at residential enrollment (1995-1996) addresses for 483,873 participants of the NIH-AARP Diet and Health Study, a prospective cohort in 6 states (California, Florida, Louisiana, New Jersey, North Carolina, and Pennsylvania) and 2 metropolitan areas (Atlanta, Georgia, and Detroit, Michigan). We used estimates from a national spatiotemporal model to assess exposures during a 5-year period prior to study enrollment (1990-1994) and Cox regression to estimate hazard ratios and 95% confidence intervals (HR[CI]) for associations (per IQR increase) with renal cell carcinoma (RCC), including clear cell (ccRCC) and papillary (pRCC) subtypes. Models were adjusted for participant demographics, smoking status and intensity, catchment state, and census-tract median household income. We evaluated mutual adjustment of $PM_{2.5}$ and NO_2 and interactions with smoking status.

Results: 4,216 RCC cases were diagnosed through 2018. We observed a small increased risk for RCC associated with both $PM_{2.5}$ (HR=1.03[0.98-1.09] per 4.4μg/m^3) and NO_2 (HR=1.02 [0.98-1.07] per 10.4ppb). Risk was elevated for ccRCC in association with both $PM_{2.5}$ (HR=1.11 [1.02-1.21] and NO_2 (HR=1.07 [1.00-1.15]) but not for pRCC (HR=0.98 [0.83-1.17] and HR=0.97 [0.84-1.12], respectively). Risk estimates for $PM_{2.5}$ were similar for RCC and ccRCC after additionally adjusting for NO_2 (HR=1.03 [0.95-1.12] and HR=1.10 [0.97-1.25], respectively), though associations with NO_2 were null after mutual adjustment. We found no evidence of effect modification by smoking status (*p*-interaction >0.05).

Conclusions: Our novel findings indicate that residential $PM_{2.5}$ concentrations may increase RCC risk, particularly the clear cell subtype. Replication of these results in other study populations will be important.

FT23-05 | Exposure to Fine Particulate Matter and Hypertension Progression: Insights from the Largest Taiwanese Cohort on Gene-Environment Interaction

Yu-Ting Lin[1,2], Yi-Ting Lin[3], Ping-Hsun Wu[3], Hung-Lin Chen[1,2], Hsiu-Yin Chiang[1,2], Ya-Chi Lin[1,2], Che-Chin Lin[1], Chau-Ren Jung[4], Chin-Chi Kuo[1,2]

[1] China Medical University Hospital, Big Data Center, Taichung, Taiwan; [2] China Medical University, Department of BioMedical Informatics, Taichung, Taiwan; [3] Kaohsiung Medical University Hospital, Department of Family Medicine, Taichung, Taiwan; [4] China Medical University, Department of Public Health, Taichung, Taiwan

Background

Although air pollution has been linked to elevated blood pressure, the long-term impact of fine particulate matter (PM2.5) on hypertension progression in patients with pre-existing hypertension remains unclear. This study investigates the association between long-term PM2.5 exposure and hypertension deterioration, with a particular focus on its interaction with genetic susceptibility.**Methods**

We conducted a retrospective cohort study involving 12,602 patients with hypertension in Taiwan from 2007 to 2018. Long-term average PM2.5 concentrations were estimated using satellite-based models at a 1 km resolution. Multivariable logistic regression was employed to calculate odds ratios (ORs) for hypertension deterioration, defined as worsening blood pressure classification over a 3-year period. Restricted cubic splines were applied to explore exposure–response relationships. Additionally, we developed a polygenic risk score (PRS) to assess whether genetic susceptibility modifies the effect of PM2.5 on blood pressure deterioration.**Results**

The study cohort included 12,602 adults with hypertension, with a median age of 61.1 years. A 10 μg/m³ increase in PM2.5 was associated with a 29% higher odds of hypertension deterioration (adjusted OR: 1.29, 95% CI: 1.20–1.39), following a linear exposure–response pattern. Interaction analysis revealed a significant sub-multiplicative interaction between genetic susceptibility and PM2.5 exposure (p for interaction = 0.009), indicating a greater adverse effect of PM2.5 in individuals with lower genetic risk.**Conclusions**

Our findings suggest that both PM2.5 exposure and genetic susceptibility independently contribute to hypertension progression, with a more pronounced impact of PM2.5 observed in individuals with lower genetic risk. These results highlight the importance of personalized intervention strategies to mitigate the effects of air pollution and underscore the need for further research to elucidate the underlying biological mechanisms.

FT23-07 | Associations between Long-term Exposure to Wildfire Smoke Carbonaceous $PM_{2.5}$ Anomalies and Incident Ischemic Heart Disease and Congestive Heart Failure: A Nationwide Cohort Study in the United States

Zhihao Jin, Hua Hao, Yang Liu

Emory University, Atlanta, USA

Background

Wildfire smoke is a major source of fine particulate matter ($PM_{2.5}$) and contains various chemical constituents that may differentially impact cardiovascular disease (CVD) risk, especially the carbonaceous matter. While chronic exposure to $PM_{2.5}$ has been linked to CVD, limited studies have specifically examined the long-term health effects of wildfire smoke PM2.5 and its chemical components.

Methods

We obtained hospital admission records from Medicare beneficiaries spanning 2002–2019 and applied a stratified Cox proportional hazards model to assess the association between long-term exposure to wildfire smoke carbonaceous $PM_{2.5}$ (i.e., organic carbon (OC) and elemental carbon (EC)) and CVD outcomes, specifically ischemic heart disease (IHD) and congestive heart failure (CHF). Exposure assessment included both background $PM_{2.5}$ and smoke-specific components. Exposure anomalies were defined as deviations from long-term mean concentrations to account for temporal variations. Stratification was performed based on sex, age, and Medicaid eligibility, and models were adjusted for potential confounders, including meteorological factors and socioeconomic variables.

Results

Our findings suggest that increased chronic exposure to wildfire smoke $PM_{2.5}$ OC was associated with a higher risk of both IHD (HR: 1.028, 95% CI: 1.016–1.040) and CHF (HR: 1.015, 95% CI: 1.004–1.025). Additionally, a 1 $\mu g/m^3$ increase in carbonaceous $PM_{2.5}$ (OC + EC) was associated with a 1.015-fold (95% CI: 1.004–1.025) and 1.007-fold (95% CI: 0.998–1.017) increased risk for IHD and CHF, respectively.

Conclusions

This study highlights the long-term health impacts of carbonaceous $PM_{2.5}$ from fire smoke on cardiovascular outcomes. Our findings emphasize the need for targeted air quality management strategies to mitigate wildfire smoke exposure and protect vulnerable populations.

FT23-08 | Duration versus intensity of radon exposure on the risk of lung cancer: a pooling study

Cristina Candal-Pedreira, Lucía Martín-Gisbert, Julia Rey-Brandariz, Mónica Pérez-Ríos, Guadalupe García, Carla Guerra-Tort, Ana Teijeiro-Teijeira, Alberto Ruano-Ravina

University of Santiago de Compostela, Preventive Medicine, Santiago de Compostela, Spain

Objective: To describe lung cancer risk based on both the duration and intensity of radon exposure and to analyze potential differences by sex.
Material and methods: Data were pooled from case-control studies on radon and lung cancer conducted in a radon-prone area of Spain. Exposure duration was measured as the number of years living in the measured dwelling, while intensity was considered in Bq/m³ (measured using 3 month-alpha track devices). Logistic regression models were used to estimate lung cancer risk based on exposure duration, intensity, and both combined. Generalized additive models (GAM) were also applied to assess the profile of lung cancer risk while considering both variables. All models were adjusted for tobacco consumption, and results were analyzed separately by sex.
Results: A total of 3,415 participants were included, 1,619 were lung cancer cases. Results suggest that both exposure duration and intensity play a similar role in lung cancer risk. A dose-response relationship was observed in the overall group and in men, regardless of years spent in the dwelling. This relationship was not observed in women. Overall, for those exposed less than 20 years, lung cancer risk rises with radon concentration. In individuals with 21-40 years of exposure, the odds ratio (OR) increases from 1.17(0-50Bq/m³) to 2.48(151-300Bq/m³), while in those with over 40 years of exposure, OR reaches 3.28 for radon levels above 300Bq/m³. GAM analysis confirms that, at radon concentrations below 100Bq/m³ the OR remains close to 1, but for concentrations above 100Bq/m³ risk increases with longer exposures.
Conclusion: This study suggests that both duration and intensity of exposure to residential radon significantly contribute to lung cancer risk. Exposure durations exceeding 40 years are particularly relevant when radon concentrations surpass 300 Bq/m³. Additionally, lung cancer risk due to residential radon exposure may differ between men and women

FT23-09 | Pro-thrombotic changes in response to ambient ozone exposure exacerbated by temperatures

Teng Wang[1], Xi Chen[2], Yuan Yao[3], Wu Chen[3], Haonan Li[3], Yifan Xu[3], Tianjia Guan[1], Jicheng Gong[3], Xinghua Qiu[3], Tong Zhu[3]

[1] Chinese Academy of Medical Sciences and Peking Union Medical College, School of Health Policy and Management, Beijing, China; [2] Shenzhen Institute of Building Research Co., Ltd, Hebei Technology Innovation Center of Human Settlement in Green Building, Xiongan, China; [3] Peking University, College of Environmental Sciences and Engineering, Beijing, China

Recent evidence links high temperatures to increased ozone-related cardiovascular mortality in a changing climate, but the underlying biological mechanisms remain unclear. We investigated the associations between short-term ozone exposure and pro-thrombosis, a key process in the pathophysiology of cardiovascular diseases across varying temperatures (5–30 ℃) in a longitudinal panel study of 135 participants in Beijing, China. Pro-thrombotic biomarkers and whole blood transcriptome data were measured repeatedly. Bayesian Kernal Machine Regression revealed that higher serum thromboxane (Tx)B_2 levels were associated with increasing levels of joint exposure to air pollutants over one week when ozone rather than other pollutants contributed most to the overall effect. Causal mediation analyses found 3233 transcripts associated with an increase in TxB_2 following ozone exposure, which were enriched in pathways including Ribosome, Thermogenesis, Oxidative phosphorylation, and Pathways of neurodegeneration. As the temperature increased, we observed a stronger association between ozone exposure and TxB_2 increase. The TxB_2 increments per interquartile range increase in the one-week average of ozone were 6.6, 13.2, 14.8, 16.6, and 18.4 units when the temperatures were at 6.5, 15.0, 17.6, 21.4, and 26.7℃, respectively. The number of mediating transcripts enriched in pathways related to translation, environmental adaptation, energy metabolism, and human diseases was also greater at higher temperatures than at lower ones. This study suggests that higher temperatures exacerbate ozone-related pro-thrombotic response, providing a biological basis for the increased risk of ozone-associated cardiovascular mortality at high temperatures. This study was supported by the National Natural Science Foundation of China (Grants 42293324, 42407583) and the Ministry of Science and Technology of the People's Republic of China (2015CB553401).

FT23-10 | Monthly oil and gas production associated with cardiovascular-related hospital visits

Arbor Quist[1], Yan Xu[2], Roxana Khalili[2], Elizabeth Kamai[2], James Lacey[3], Emma Spielfogel[3], Kristen Savage[3], Rima Habre[2], Jill Johnston[2]

[1] Ohio State University, Division of Epidemiology, Columbus, USA; [2] University of Southern California, Division of Environmental Health, Los Angeles, USA; [3] City of Hope, Department of Computational and Quantitative Medicine, Duarte, USA

Objective: Oil and gas production exposes nearby residents to numerous pollutants, including volatile organic compounds, particulate matter, and heavy metals. Over 7 million Californians live within 1600m of an oil or gas well. We sought to understand the association between oil and gas production and cardiovascular health in California.
Methods: Using monthly oil and gas production data and well locations from the California Geologic Energy Management Division, we applied kernel density estimation method to create a continuous, barrel of oil equivalent (BOE) production surface for each month. We linked this monthly production data to residential addresses of 114,833 California Teacher's Study participants. We split the monthly BOE production variable at the median (168 BOE) to categorize high and low oil or gas production, which we compared to no production. We used discrete time hazards models to examine the relationship between monthly BOE production levels and incident cardiovascular-related inpatient hospital visits and emergency department visits (1995-2020), adjusting for smoking status, alcohol, BMI, and age.
Results: The probability of a participant having an incident cardiovascular-related hospital visit given they survived the previous month increased 5% during a high production month (HR=1.05, 95% CI: 1.02, 1.08) and 3% during a low production month (HR=1.03, 95% CI:1.00, 1.06), compared to no oil or gas production. The association was slightly stronger for ischemic heart disease (HR=1.08, 95% CI: 1.02, 1.15) and slightly lower for heart failure (HR=1.04, 95% CI: 1.00, 1.08) hospital visits during high oil production months compared to no oil production months.
Conclusion: Oil and gas production occurs in various regions around the world and often affects the health of vulnerable populations. Monthly production volume and well distance from homes impacts the extent to which nearby residents are affected. High oil and gas production is associated with acute cardiovascular issues.
(Funding: NCI 3U01CA199277-09S1)

FT24 | Chemical exposures and neurological health

FT24-01 | Joint associations between gestational exposure to environmental chemical mixtures and parent-reported behaviors in preschool children

Jagadeesh Puvvula[1], Wei-Ting Hwang[1], Lawrence McCandless[2], Changchun Xie[3], Joseph M. Braun[4], Ann M. Vuong[5], Enrique F. Schisterman[1], Russell T. Shinohara[1], Kristin Linn[1], Linda Booij[7,8], Youssef Oulhote[9], Maryse F. Bouchard[11], Michael M. Borghese[11], Jean R. Seguin[12,8], Angelika Zidek[13], Christine Till[14], William Fraser[15], Kimberly Yolton[16,3], Kim M. Cecil[16,17], Jillian Ashley-Martin[11], Tye E. Arbuckle[11], Bruce Lanphear[2], Aimin Chen[1]

[1] University of Pennsylvania, Department of Biostatistics Epidemiology and Informatics, Philadelphia, USA; [2] Simon Fraser University, Faculty of Health Sciences, Burnaby, Canada; [3] University of Cincinnati, Department of Environmental and Public Health Sciences, Cincinnati, USA; [4] Brown University, Department of Epidemiology, Providence, USA; [5] University of Nevada Las Vegas, Department of Epidemiology and Biostatistics, Las Vegas, USA; [6] McGill University, Department of Psychiatry, Montreal, Canada; [7] Douglas Mental Health University Institute, Montreal West Island Integrated Health and Social Services Centre, Montreal, Canada; [8] CHU Sainte-Justine Research Centre, Montreal, Canada; [9] Icahn School of Medicine at Mount Sinai, Department of Environmental Medicine and Public Health, New York, USA; [10] Institut national de la recherche scientifique (INRS) - Centre Armand-Frappier Santé Biotechnologie, Laval, Canada; [11] Health Canada, Environmental Health Science and Research Bureau, Ottawa, Canada; [12] Université de Montréal, Department of Psychiatry and Addictology, Montreal, Canada; [13] Health Canada, Existing Substances and Risk Assessment Bureau, Ottawa, Canada; [14] York University, Psychology Department, Toronto, Canada; [15] University of Sherbrooke, Department of Obstetrics and Gynecology, Sherbrooke, Canada; [16] Cincinnati Children's Hospital Medical Center, Department of Pediatrics, Cincinnati, USA; [17] Cincinnati Children's Hospital Medical Center, Department of Radiology, Cincinnati, USA

OBJECTIVE: This study examined the joint associations between gestational chemical biomarkers and behaviors among preschool children.
METHODS: We pooled data from 695 mother-child dyads from two birth cohorts in North America (HOME & MIREC Studies). We examined 29 gestational biomarkers measured in both cohorts, including toxic metals, persistent organic pollutants (POPs), per- and polyfluoroalkyl substances (PFAS), organophosphate esters, parabens, phenols, phthalates, and organophosphate pesticides. Parents reported their child's behaviors and executive functions at age 3 years using the Behavioral Assessment System for Children (BASC-2) and Behavior Rating Inventory of Executive Function (BRIEF-P), respectively, with higher scores indicating adverse behavior. We examined joint associations using quantile g-computation, adjusted for maternal variables (cotinine, education, race, parity, age at delivery, depression), childhood variables (sex, HOME score), cohort, study site, and considered child sex as a modifier.
RESULTS: Overall, we observed null associations between gestational chemical biomarker mixture with child behavioral scores (BASC-2) such as the externalizing (beta:1.38 95%CI: -0.65, 3.41), internalizing (beta:1.34 95%CI: -0.97, 3.65), behavior symptom index (beta:0.84 95%CI: -0.89, 2.57), and executive function scores (BRIEF-P) plan/organize (beta:0.31 95%CI: -2.11, 2.73) and working memory (beta:-0.38 95%CI: -3.01, 2.25). There was evidence of sex-specific associations between the gestational biomarker mixture and BRIEF-P working memory scores (EMM p-value = 0.02), with a positive trend among boys (beta:3.04, 95% CI: -0.46, 6.55) and a negative trend among girls (beta:-3.40, 95% CI: -7.33, 0.53). In contrast, no such sex-specific association was observed for BASC-2 internalizing scores (EMM p-value = 0.69), though the gestational chemical biomarker mixture was associated with higher scores among boys (beta:3.59, 95% CI: 0.42, 6.75) but not girls (beta:-0.89, 95% CI: -4.07, 2.30).
CONCLUSION: Gestational exposure to a mixture of environmental chemicals was associated with greater anxiety, depression, withdrawal, and somatic behaviors among male children at age 3 years.

Funding:P01ES011261,P01R829389,R01ES014575,R01ES020349,R01ES027224,R01ES028277,R01ES033054, R01ES032552,R01ES033200; Canadian-Institute-of-Health-Research:(MOP-81825); Ontario-Ministry-of-the-Environment; Canada’s-Chemicals-Management-Plan.

FT24-02 | Early Life Lead Exposure, Sensitive Periods, and Long-Term Mental Health: Findings from the Saint Louis Baby Tooth – Later Life Health Study

Joyce Lin[1], Ruby Hickman[1], Kaleigh McAlaine[1], Justin Farmer[1], Ian Tang[1], Felicitas Bidlack[3], Tracy Punshon[5,6], Brian Jackson[5,6], Joseph Mangano[4], Marc Weisskopf[1,2]

[1] Harvard School of Public Health, Environmental Health, Boston, USA; [2] Harvard School of Public Health, Epidemiology, Boston, USA; [3] ADA-Forsyth Institute, Boston, USA; [4] Radiation and Public Health Project, New York, USA; [5] Dartmouth College, Biological Sciences, Hanover, USA; [6] Dartmouth College, Earth Sciences, Hanover, USA

Background: Early life lead exposure is associated with psychological concerns in later childhood and adolescence. However, limited research has examined sensitive periods of exposure and whether these mental health effects persist into adulthood.
Objective: This study examines the prospective association between early life lead exposure across prenatal and early postnatal periods and anxiety and depression in older adulthood among participants of the Saint Louis Baby Tooth – Later Life Health Study (SLBT) who donated deciduous (“baby”) teeth in childhood.
Methods: Baby tooth dentin lead levels were determined using laser-ablation inductively coupled plasma mass spectrometry in 718 SLBT participants (53% female). Tooth lead levels were evaluated in prenatal (2nd trimester to birth) and early postnatal periods (birth to ~6 months). Probable cases of anxiety and depression were ascertained using the PHQ-9 and GAD at the time of study re-enrollment at a mean age of 62 (standard deviation: 3.6). Associations of tooth lead with later-life anxiety and depression were examined using logistic regression models adjusted for key demographic characteristics and early life socio-economic variables.
Results: Median (25th – 75th percentile) tooth lead concentration across the early life period was 1.34 ppm (1.02 – 1.82). For every interquartile range increase in combined pre and postnatal tooth lead, we saw a nearly twofold increase in the odds of depression (1.86, 95% CI: 1.48, 2.36). We also found that the later prenatal period (roughly 3rd trimester) appeared to be a sensitive period for lead exposure on later life depression (OR=2.15, 95% CI: 1.47, 3.15). Meanwhile postnatal, but not prenatal, lead exposure was associated with 1.37 (95% CI: 0.98, 1.19) times greater odds of major anxiety in later life.
Conclusion: Lead exposures during specific periods in development likely have long-term consequences on depression and anxiety well into later adulthood.

FT24-03 | Effect of early-life cognitively stimulating activities on the association between prenatal lead exposure and later adulthood cognitive function in the St. Louis Baby Tooth Study

Ruby Hickman[1], Joyce Lin[1], Kaleigh McAlaine[1], Tracy Punshon[2], Brian P. Jackson[2], Felicitas B. Bidlack[3], Justin Farmer[1], Laura Germine[4,5], Scott M. Bartell[6,7], Joseph J. Mangano[8], Susan A. Korrick[1,9], Joel D. Schwartz[1], Rajarshi Mukherjee[10], Marc G. Weisskopf[1]

[1] Harvard T.H. Chan School of Public Health, Department of Environmental Health, Boston, USA; [2] Dartmouth College, Department of Biological Sciences, Hanover, USA; [3] The ADA-Forsyth Institute, Cambridge, USA; [4] Harvard Medical School, Department of Psychiatry, Boston, USA; [5] McLean Hospital, Institute for Technology in Psychiatry, Belmont, USA; [6] University of California, Irvine, Department of Environmental and Occupational Health, Irvine, USA; [7] University of California, Irvine, Department of Epidemiology and Biostatistics, Irvine, USA; [8] Radiation and Public Health Project, Ocean City, USA; [9] Brigham and Women's Hospital and Harvard Medical School, Channing Division of Network Medicine, Boston, USA; [10] Harvard T.H. Chan School of Public Health, Department of Biostatistics, Boston, USA

Objective: We previously reported that prenatal lead exposure is associated with poorer cognitive function in adulthood. Animal models suggest deleterious neurocognitive effects of lead exposure are ameliorated by the presence of a cognitively enriching environment. We tested the hypothesis that early-life cognitively-stimulating activities modify the association of prenatallead exposure with later adulthood cognitive function.

Materials and Methods: Participants in the St. Louis Baby Tooth – Later Life Health Study (SLBT) who donated shed deciduous teeth in 1959-1972 were recontacted and completed online cognitive testing in later adulthood (ages 55-75 years, mean 62). We assessed prenatal lead exposure using laser ablation inductively coupled plasma mass spectrometry in N=673 participants. Early-life cognitively-stimulating activities were assessed by averaging responses to the Rush Alzheimer's Disease Center Lifetime Cognitive Activity scales for activities at ages 6, 12, and 18. We used weighted generalized estimating equations to assess the impact of cognitively-stimulating activities on the lead-cognition association, adjusting for age, sex, race/ethnicity, mother's and father's education, maternal smoking during pregnancy, childhood urbanicity and financial status, and childhood area income and age of housing.

Results. The effect of prenatal lead on later adulthood cognition varied by amount of cognitively-stimulating activities (interaction p: 0.001). For those with low levels of cognitively-stimulating activities (<= 2.5 on 1-5 scale, N=162, 24%), a 1 ppm increase in prenatal tooth lead was associated with a 0.23 standard deviation decrement (95% CI: -0.35, -0.11) in cognition score, but there was no association among those with high levels of cognitively-stimulating activities (-0.01, 95% CI: -0.08, 0.05). Results were consistent when adjusting for additional early-life socioeconomic status variables (maternal/paternal employment, number of cars in home) and cognitive testing device.

Conclusions. Our results suggest that the association of prenatal lead exposure with later adulthood cognitive function is dependent on level of early-life cognitively-stimulating activities.

FT24-04 | Early-life metal mixture exposure is associated with neurocognitive mechanisms underlying adolescent risk-taking

Erik de Water[1], Elza Rechtman[1], **Kristie Oluyemi**[1,6], Azzurra Invernizzi[1], Christine Austin[1], Paul Curtin[1], Michelle A. Rodriguez[1], Vida Rebello[1], Libni A. Torres-Olascoaga[2,3], Luis F. Bautista-Arrendondo[2], Sandra Martínez-Medina[3], Rafael Lara-Estrada[4], Erika Proal[2], Viviana Villicaña-Muñoz[2], Chris Gennings[1], Abraham Reichenberg[7], Francisco X. Castellanos[8,9], Roberta White[10], Martha M. Tellez-Rojo[2], Robert O. Wright[1], Cheuk Y. Tang[5], Manish Arora[1], Megan K. Horton[1]

[1] Icahn School of Medicine at Mount Sinai, Environmental Medicine, New York, USA; [2] National Institute of Public Health of Mexico, Center for Nutrition and Health Research, Cuernavaca Morelos, Mexico; [3] National Institute of Perinatology of Mexico, Developmental Neurobiology, Mexico City, Mexico; [4] Universidad Autónoma Metropolitana, National Research Laboratory in Imaging and Medical Instrumentation, Mexico City, Mexico; [5] Icahn School of Medicine at Mount Sinai, Diagnostic, Molecular, and Interventional Radiology, New York, USA; [6] Icahn School of Medicine at Mount Sinai, Nash Family Department of Neuroscience; Friedman Brain Institute; Graduate School of Biomedical Sciences, New York, USA; [7] Icahn School of Medicine at Mount Sinai, Psychiatry, New York, USA; [8] NYU Langone Health, Child and Adolescent Psychiatry, New York, USA; [9] NYU Langone Health, Neuroscience and Physiology; Radiology, New York, USA; [10] Boston University School of Public Health, Environmental Health, Boston, USA

Objective: This study aimed to identify early-life critical windows when metal mixture exposure is associated with neurocognitive correlates of reward-based risky decision-making in adolescence.
Materials and Methods: We analyzed data from adolescents (8-14 years; 45% females) enrolled in the Programming Research in Obesity, Growth, Environment and Social Stressors (PROGRESS) cohort in Mexico City. Weekly concentrations (-19 prenatal weeks – 43 postnatal weeks) of nine metals (Mn, Pb, Cu, Li, Mg, Sn, Sr, Zn, Ba) were measured in deciduous teeth using laser ablation-inductively coupled plasma-mass spectrometry. Brain activation and behavior were assessed using a functional magnetic resonance imaging (fMRI) gambling paradigm. A generalized mixed-effects model was used to estimate risk and reward sensitivity. Functional regions of interest included brain regions involved in cognitive control (right angular gyrus/inferior parietal lobule (AG/IPL)) and reward processing (left insula/rolandic operculum (INS/ROL)). Time-varying associations between the metal mixture and neurocognitive correlates were assessed using lagged weighted quantile sum (LWQS) regression, adjusting for age, sex, and socioeconomic status.
Results: Postnatal metal mixture exposure (weeks 2–43) was associated with increased risk sensitivity (β = 0.18 [95% CI 0.042, 0.321]), driven by Pb, Sn, and Cu. Prenatal and postnatal exposure (weeks -19 to -16 and 22–41, respectively) were associated with increased reward sensitivity (β = 0.24 [95% CI 0.120, 0.355]), driven by Zn, Mg, and Sr. At 20-43 postnatal weeks, metal mixture exposure was associated with reduced right AG/IPL activation (β = -0.24 [95% CI -0.408, -0.067]), driven by Sr. Exposure at 27 – 43 postnatal weeks was associated with increased left INS/ROL activation (β = 0.28 [95% CI 0.094, 0.469]), driven by Mg, Cu, Ba, and Sn.Conclusion: Early-life metal exposure impacts neurocognitive mechanisms underlying adolescent risk-taking, emphasizing the importance of exposure timing in shaping neurodevelopmental outcomes.

FT24-06 | Neurobehavioral Effects of Prenatal Exposure to Phthalates in Early Childhood: Evidence from the PROTECT Cohort

Seonyoung Park[1], Amber Cathey[1], Wei Hao[2], Sung Kyun Park[1,3], Bhramar Mukherjee[4], Gredia H. Montañez[5], Zaira R. Pabón[5], Carmen M. Vélez-Vega[6], José F. Cordero[7], Akram Alshawabkeh[8], Deborah J. Watkins[1], John D. Meeker[1]

[1] University of Michigan School of Public Health, Department of Environmental Health Sciences, Ann Arbor, USA; [2] University of Michigan School of Public Health, Department of Biostatistics, Ann Arbor, USA; [3] University of Michigan School of Public Health, Department of Epidemiology, Ann Arbor, USA; [4] Yale University School of Public Health, Department of Biostatistics, New Haven, USA; [5] Northeastern University, Department of Electrical and Computer Engineering, Boston, USA; [6] University of Puerto Rico Graduate School of Public Health, Department of Social Sciences, San Juan, Puerto Rico; [7] University of Georgia, Department of Epidemiology and Biostatistics, Athens, USA; [8] Northeastern University, Department of Civil and Environmental Engineering, Boston, USA

Background and Aim

Phthalates are endocrine-disrupting chemicals widely used as plasticizers and solvents in consumer products, resulting in pervasive human exposure. Although previous epidemiological studies have investigated potential neurotoxic effects of prenatal phthalate exposure, findings remain inconsistent. Furthermore, few have examined newer replacement chemicals or multiple phthalates as a mixture. We aimed to address these gaps.

Methods

We included 306 mother–child dyads from the PROTECT birth cohort in Puerto Rico. Maternal urine samples were collected up to three times during pregnancy (median 18, 22, and 26 weeks) to measure 16 urinary metabolites of phthalates and the replacement chemical di(2-ethylhexyl) terephthalate (DEHTP). Child behavior and emotional problems at ages 1.5–3 years were assessed using the Child Behavioral Checklist (CBCL). Since CBCL scores are summed counts of behaviors and were overly dispersed, we employed negative binomial regression to examine associations with individual metabolites with CBCL scores. We additionally evaluated sex-specific associations and employed mixture analyses using adaptive elastic net and quantile g-computation.

Results

Several metabolites were positively associated with CBCL scores, indicating more frequent problematic behaviors with increased exposure. For example, a doubling of MHIBP was associated with a 13.23% increase in Internalizing scores (95% CI: 1.18, 26.72). The association between phthalates and CBCL scores varied by child sex, in general, showing stronger associations in males. Specifically, MIBP was associated with CBCL score among male children (17.38%/doubling, 95%CI: 3.31, 33.38), while not among female children (12.19 %/doubling, 95%CI: -2.45, 29.02). The adaptive elastic net identified MIBP and MHIBP as key contributors within the mixture; however, quantile g-computation did not detect additional combined effects.

ConclusionOur findings suggest prenatal phthalate exposure may adversely impact neurobehavioral outcomes, with certain metabolites driving these associations. Further research should explore underlying biological mechanisms of different phthalates and employ mixture analyses based on shared modes of action.

FT24-07 | Childhood Metals and Longitudinal Sleep Duration and Efficiency in a Mexico City Cohort

Allison Kupsco[1], Nia Mcrae[2], Mahmoud Awawda[2], Inessa Lopez[2], Libni Torres-Olascoaga[3], Julio Landero[2], María L. Pizano-Zárate[4, 7], Mariana Figueiro[2], Francheska Merced-Nieves[2], Emily Oken[5, 6], Rosalind Wright[2], Martha M. Téllez Rojo[3], Robert Wright[2], Dinesh Barupal[2]

[1] Columbia University Mailman School of Public Health, New York, New York, United States of America; [2] Icahn School of Medicine at Mount Sinai, New York, New York, United States of America; [3] National Institute of Public Health of Mexico, Cuernavaca, Mexico; [4] Instituto Nacional de Perinatología, Mexico City, Mexico [5] Harvard Medical School and Harvard Pilgrim Health Care Institute, Boston, Massachusetts, United States of America; [6] Harvard T. H. Chan School of Public Health, Boston, Massachusetts, United States of America; [7] Instituto Mexicano del Seguro Social, Mexico City, Mexico

Background: Sleep is essential to children's physiological, emotional, behavioral, and cognitive health. Ubiquitous exposures to essential and nonessential metals can adversely impact child neurodevelopment, a major determinant of sleep behaviors. However, the impact of metals exposure during early childhood on sleep remains underexplored.

Methods: We included 495 children from the Programming Research in Obesity, GRowth, Environment and Social Stress (PROGRESS) cohort from Mexico City. Inductively coupled mass spectrometry was used to quantify 16 elements (Al, As, Ba, Cd, Cr, Co, Cs, Cu, Mg, Mn, Mo, Pb, Sb, Se, V, Zn) in whole blood at 48 months of age. Sleep duration (hours) and efficiency (percent) were quantified with actigraphy over a seven-day period at 48 and 72 months of age. We used linear mixed effects models with a random intercept for subject to investigate assocations between individual log2 transformed metals and longitudinal sleep outcomes, adjusting for key sleep determinants, such as education, age, sex, BMI, season, and physical activity. Generalized additive mixed effects models were used to investigate nonlinear associations and quantile g-computation was used to investigate associations with the total metal mixture.

Results: Copper (Cu) was negatively associated with longitudinal sleep duration (-0.20 hours per doubling of Cu [95% confidence interval: -0.39, -0.014; p=0.034]). Vanadium (V) was associated with reduced sleep efficiency (-1.21% per doubling of V [95% CI: -2.34, -0.082; p=0.035]). Cadmium (Cd) was significantly nonlinearly associated with both sleep duration and efficiency, with Cd levels > 0.12µg/L associated with increased sleep disruption. Associations between the metal mixture and sleep outcomes were not significant.

Conclusions: Greater levels of known neurotoxic metals, such as Cd, and essential metals, such as Cu, which may reflect inflammation, in early childhood may influence child sleep health that could lead to long-term consequences. Future research will investigate circadian rhythms and longitudinal sleep trajectories.

FT24-08 | Pesticide exposure in childhood and adolescence: association with executive functions among adolescents from Montevideo, Uruguay

Danelly Rodríguez[1], Gabriel Barg[2], Elena I. Queirolo[2], Lai-Har Chi[1], James Olson[1], María I. Beledo[2], **Katarzyna Kordas**[1]

[1] University at Buffalo, Department of Epidemiology and Environmental Health, Buffalo, USA; [2] Catholic University of Uruguay, Department of Neuroscience and Learning, Montevideo, Uruguay

Objective: Numerous studies in agricultural settings have linked prenatal exposure to pyrethroids and organophosphates, including chlorpyrifos, with cognitive deficits. However, few have examined executive functions (EFs) during adolescence. This study investigates associations between chlorpyrifos and pyrethroid exposure and EFs in an urban cohort.

Methods: Data were analyzed from 332 participants (50% girls) in the Salud Ambiental Montevideo (SAM) cohort, with urinary metabolites of chlorpyrifos (TCPy) and pyrethroids (3-PBA) measured in childhood (mean ~7 years) and adolescence (mean ~12 years). EF domains (working memory, inhibitory control, cognitive flexibility) and the Fluid Cognition Composite were assessed using the NIH Cognitive Toolbox. Pesticide metabolites were measured in urine via GC/MS, adjusted for creatinine, and log-transformed for analysis. Multiple imputation addressed missing data. Multivariable linear regressions evaluated associations between each pesticide biomarker, the three EF domains and cognitive flexibility, adjusting for age, sex, BMI, neighborhood disadvantage, maternal education, and household possessions. Additional models tested the effect modification by age (<11.6 vs. ≥11.6) and sex.

Results: In childhood, median (IQR) TCPy and 3-PBA levels were 7.4 (4.4, 13.6) and 25.2 (8.4, 78.8) ng/mg creatinine, respectively. At adolescence, TCPy and 3-PBA levels were 2.8 (1.8, 4.9) and 11.0 (4.0, 30.2) ng/mg creatinine, suggesting marked reductions in exposure. In multivariable models, 3-PBA in adolescence was negatively associated with fluid cognition (β [95% CI]: -0.71[-1.2, -0.14]. Associations were stronger in younger (-1.22 [-1.9, -0.39] than older adolescents (-0.18 [-1.0, 0.66]). No associations were found for specific EF domains, with TCPy exposure or effect modification by sex.

Conclusion: Pyrethroid exposure was associated with lower fluid cognition but not individual EF domains in adolescence, particularly among younger adolescents. These findings suggest age-related susceptibility to pyrethroid exposure. Future research should incorporate repeated exposure and outcome measures to clarify pesticide exposure impacts on EF outcomes.

FT24-10 | Early-Life Metal Exposure and Adolescent Brain Age Maturation: Identifying Critical Windows of Neurodevelopmental Vulnerability

Azzurra Invernizzi[1], Elza Rechtman[1], Francesco La Rosa[2,3], Anna Sather[1], Avraham Reichenberg[4], Christine Austin[1], Michelle A. Rodriguez[1], Xavier Castellanos[5], Libni A. Torres-Olascoaga[6], Luis Bautista[6], Sandra Martínez-Medina[7], Rafael Lara-Estrada[8], Chris Gennings[1], Erin Beck[2], Martha M. Téllez-Rojo[6], Robert Wright[1], Roberta White[9,10], Manish Arora[1], Megan K. Horton[1]

[1] Icahn School of Medicine at Mount Sinai, Department of Environmental Medicine, New York, USA; [2] Icahn School of Medicine at Mount Sinai, Department of Neurology, New York, USA; [3] Icahn School of Medicine at Mount Sinai, Department of Artificial Intelligence and Human Health, New York, USA; [4] Icahn School of Medicine at Mount Sinai, Department of Psychiatry, New York, USA; [5] Department of Child and Adolescent Psychiatry, NYU Robert I. Grossman School of Medicine, New York, USA; [6] National Institute of Public Health, Center for Nutrition and Health Research, Cuernavaca, Mexico; [7] National Institute of Perinatology, Cuernavaca, Mexico; [8] Universidad Autónoma Metropolitana, National Research Laboratory in Imaging and Medical Instrumentation, Mexico City, Mexico; [9] Boston University School of Public Health, Department of Environmental Health, Boston, USA; [10] Boston University Medical School, Department of Neurology, Boston, USA

Background. Brain age, reflecting deviations in aging when compared to chronological age, is estimated using machine learning models applied to brain magnetic resonance imaging (MRI) data. Established as a biomarker for accelerated aging in adults, the interpretation of MRI-derived brain age in children and adolescents is limited due to the nonlinear nature of brain development. We used brain age estimates to examine the impact of perinatal metal exposure on neurodevelopment, providing new insights into the long-term neurodevelopmental consequences of early life metal exposures.

Methods. Among 215 adolescents (9-14 years, 49%females) enrolled in the PROGRESS cohort, we predicted brain age using BrainAgeNeXt, a convolutional neural network trained and validated on >11,000 MRI T1-weighted scans (mean squared error = 2.1 years). We calculated the brain age gap (BAG) by subtracting chronological age from predicted brain age. We estimated weekly exposure (14th-week gestation through one year of age) of 9 metals (Zinc (Zn), Manganese (Mn), Copper (Cu), Tin (Sn), Magnesium (Mg), Lead (Pb), Barium (Ba), Strontium (Sr), Lithium (Li)) in deciduous teeth using laser ablation-inductively coupled plasma-mass spectrometry and used lagged weighted quantile sum regression to examine time-varying associations between the metal mixture and BAG, adjusting for chronological age.

Results. BAG ranged from -3 to +4.98 years (mean= 0.18y; std= 1.44y). Postnatal (18th-41st postnatal weeks) metal mixture exposure was associated with a negative deviation from chronological brain age (i.e., maximum β =-0.15 (95% CI [-0.03; -0.27]), driven mainly by Ba, Li, Sn, Zn.

Conclusion. MRI-derived brain age reflects deviations from typical brain maturation in children and adolescents, serving as an early biomarker for neurodevelopmental outcomes. These findings emphasize exposure timing in shaping brain development and inform targeted public health interventions.

P01 | Air Pollution: From Biomarkers to Population-Level Health Effects

P01-01 | Air quality Indices, Public Health Risks and Biomarkers in Laboratory Mice Exposed to PM2.5 and CO at High-Density Traffic Location in Ojota, Lagos, Nigeria

Benjamin Suleiman, Sogbanmu Temitope, Alani Rose

University of Lagos, Department of zoology, Lagos, Nigeria

Traffic-related air pollution driven by fossil fuel combustion poses a global health threat worsened by climate change. This study aimed to evaluate the ambient air and noise quality, public health risks and biomarkers in laboratory mice (Mus musculus) exposed to air emissions at a high-density traffic location (Ojota) in Lagos, Nigeria over a period 90 days. The mice were housed in sex-segregated sectioned cages at test and control sites. Behavioural, oxidative stress and lung histological biomarkers were assessed at 30 d intervals during the study period. The average 24 h and 8 h Air Quality Index for PM2.5 and CO at the test site were 35.2 μg/m³ and 0.4 ppm respectively with PM2.5 levels exceeding the WHO guideline value of 15 μg/m³. Noise levels recorded ranged from 78.1 to 92 dB, surpassing the national set limit of 65 dB for outdoor commercial environments. Mice exposed to traffic air emissions exhibited elevated exposure duration-dependent aggression and 13.3% mortality (3 males and 1 female) after 45 days compared to control mice. After 90 days, superoxide dismutase (SOD) level was elevated (454.5 min/mg) in Traffic-exposed compared to the Ccontrol mice (312.5 min/mg); Malondialdehyde (MDA) demonstrated significant increase (0.488) contrasting the Control (0.252). Histological biomarkers in traffic-exposed mice showed distended vessel in the alveoli wall, many red blood stain and severe vascular congestion with edema in the lung, contrasting with normal features in the control. Statistical analysis indicated a cumulative weight increase of 38.46% for mice at the Control location and 25% at the Ojota Traffic spot. The results unveiled the potential respiratory health effects of exposure to traffic-related air emissions, emphasizing the need for further studies on chronic impacts, especially on the brain and liver. This study is crucial for policymaking aligned with the UN SDG 3 and 11./data/user/0/com.samsung.android.app.notes/files/clipdata/clipdata_bodytext_250213_160924_133.sdocx

P01-02 | Residential Location Shapes Exposure Risk: Spatial Variability in Air Pollution Exposure

Cheng-pin Kuo[1,2], Joshua S. Fu[2]

[1] Industrial Technology Research Institute, Hsinchu, Taiwan; [2] University of Tennessee Knoxville, Department of Civil and Environmental Engineering, Knoxville, USA; [3] University of Tennessee Knoxville, Department of Civil and Environmental Engineering, Knoxville, USA

Exposure to ambient fine particulate matter ($PM_{2.5}$) threatens public health and leads to premature death and cardiovascular diseases (CVD). However, the spatial variability of exposure risks associated with different land-use characteristics around residential locations remains underexplored.

This study analyzed 11-year emergency department (ED) visit data from a regional hospital in Tainan City, Taiwan to investigate $PM_{2.5}$-related risks of non-accidental death and cardiovascular diseases among urban, suburban, and rural areas. Air quality, meteorology, emission, and land-use pattern data around residential locations were also collected. We used a case-crossover study design and conditional logistic regressions to model the death and CVD

risks of $PM_{2.5}$ exposure. Further, we conducted a stratified analysis to retrieve the diverse risks among different land-use pattern variations, emission densities, and urbanization levels.
The results showed that living in residential locations with low land-use variability would face higher death risk and lower CVD risk, while those with high variability had lower death risk and high CVD risk. Meanwhile, living around medium-level emission density has higher death and CVD risks. Concerning spatial variability, rural residents had a higher death risk, while urban residents had a higher CVD risk. Living near main roads and highways had a lower death risk. The disparate risks emphasize different health impacts within the same $PM_{2.5}$ exposure and could be related to human-environment interactions, individual factors, and $PM_{2.5}$ toxicity.
In conclusion, this study highlights the spatial variability in $PM_{2.5}$ exposure risk of death and CVD in Tainan City. Understanding these disparities can help local governments allocate public and medical resources more efficiently. Integrating spatial risk information can also improve air quality management and ED preparedness.

P01-03 | The association between air pollution and lung function in southern Taiwan

Fang-Yu Kuo[1], Chia C. Wang[2], Ming-Lung Yu[3], Ya-Yun Cheng[1,2]

[1] National Sun Yat-sen University, College of Medicine, School Of Medicine, Kaohsiung, Taiwan; [2] National Sun Yat-sen University, Aerosol Science Research Center (ASRC), Kaohsiung, Taiwan; [3] National Sun Yat-sen University, Center of Excellence for Metabolic Associated Fatty Liver Disease, Kaohsiung, Taiwan

Objective: Air pollution has become a public health concern in recent years. It is a well-established fact that high levels of air pollution are detrimental to human health, particularly in developing lung diseases. However, concerns remain regarding the effects of low levels of pollution. This study focuses on the correlation between air pollution ($PM_{2.5}$, PM_{10}, CO, O_3, NO_2) and pulmonary function in a rural environment in southern Taiwan.

Methods: This cross-sectional study was conducted in Pingtung County, Taiwan, from 2023 to 2024. After excluding subjects with cancer, a total number of 243 subjects were enrolled in the study. Each participant was interviewed using a structured questionnaire, and pulmonary function tests were performed. An air pollution meter was placed to measure the ambient air pollution concentration. First, differences in characteristics between 2023 and 2024, and between the two countries in Pingtung, were compared. Then, the association of air pollutants with lung function and exhaled carbon monoxide was analyzed by the logistic regression. Finally, stratified analyses were conducted to explore whether the associations varied by year and region.
Results: Descriptive statistics showed that CO, O_3, PM_{10}, and $PM_{2.5}$ in 2023 were significantly higher than those in 2024; and CO, PM_{10}, $PM_{2.5}$, and NO_2 in Fangshan Township were significantly higher than those in Manzhou Township. All air pollutant concentrations were below the WHO standards. A significant negative correlation between CO, O_3, PM_{10}, $PM_{2.5}$, and lung function was observed in the logistic regression. In the stratified analysis by year, compared with 2024, PM in 2023 showed a significant negative correlation with FEV1(%), FVC(%), and FEV_1/FVC.
Conclusion: Pollution prevention policies should not only focus on high-pollution areas, but also establish appropriate protection measures for susceptible groups in low-pollution environments to reduce health risks.

P01-05 | Socioeconomic inequality and renewable energy adoption shape mortality risks and adaptation to short-term air pollution exposure in Europe

Zhao-yue Chen[1,2], Hicham Achebak[1], Wenzhong Huang[3], Blanca Paniello-Castillo[1,2], Hervé Petetin[4], Raúl F. Méndez Turrubiates[1], Carlos Pérez García-Pando[4,5], Joan Ballester[1]

[1] ISGlobal, Barcelona, Spain; [2] Universitat Pompeu Fabra (UPF), Barcelona, Spain; [3] Monash university, School of Public Health and Preventive Medicine, Melbourne, Australia; [4] Barcelona Supercomputing Center, Barcelona, Spain; [5] ICREA, Catalan Institution for Research and Advanced Studies, Barcelona, Spain

The spatial heterogeneity of population vulnerability to acute air pollution exposure and its driving factors remains poorly understood across large geographical contexts. Our study examines how socioeconomic factors and renewable energy adoption influence regional disparities in air pollution-associated mortality risk and adaptation patterns across Europe. Using a dataset of 88.8 million deaths from 653 contiguous regions in 31 European countries between 2003 and 2019. We identify a pronounced socioeconomic modification effect in vulnerability and adaptation to $PM_{2.5}$, PM_{coarse}, NO_2 and O_{3_8h} exposures. Regions with lower GDP per capita, higher poverty rates, limited educational attainment, and reduced life expectancy—predominantly in Eastern and Southern Europe—exhibit significantly higher mortality risks across all pollutants and weaker adaptation over time. In contrast, wealthier regions, more commonly in Northern and Western Europe, display lower baseline risks and greater adaptive capacity. Furthermore, regions at the forefront of renewable energy adoption, particularly in transport sectors, experience markedly lower mortality risks and greater adaptation. Our findings suggest that persistent socioeconomic inequalities and disparities in clean energy transition contribute to environmental health vulnerabilities in disadvantaged regions. Addressing these disparities through targeted interventions is essential for reducing health risks and enhancing resilience to air pollution exposure.

P01-06 | Does retinitis pigmentosa make a difference in air pollution-related cataract risk?

Juyeon Kang[1,2], Sera Kim[2], Jong-Tae Lee[1,2]

[1] School of Health Policy and Management, College of Health Sciences, Korea University, Seoul, South Korea; [2] Research and Management Center for Health Risk of Particulate Matter, Seoul, South Korea

Background and Aim

Retinitis pigmentosa (RP) is the most common inherited retinal disease, characterized by progressive vision loss without definitive cure and involving oxidative stress and inflammation. Cataracts, the most prevalent RP complication, share similar oxidative stress mechanisms, which are implicated in air pollution-related health effects. However, few studies have examined whether RP increases susceptibility to air pollution-related ocular damage. This study aims to investigate whether the association between long-term air pollution exposure and cataract incidence differs in individuals with or without RP.

Material and Methods

We conducted a national cohort study using the National Health Insurance Service–National Sample Cohort from 2007 to 2019. Air pollution exposure was assessed using a machine learning-based model with five-year mean concentrations of PM_{10}, $PM_{2.5}$, NO_2, CO, and O_3. Pooled logistic regression models were used to estimate associations between air pollution exposure and cataract incidence, with odds ratios (OR) and 95% confidence intervals (95% CI) for each interquartile range increase. Models were adjusted for age, sex, smoking status, household income, region, calendar year, and RP status. Stratified analyses were performed by RP status.

Results

There were 104,364 new cases of cataract during the study period. In the total population, NO_2 and CO were positively associated with cataract risk, while O_3 showed a negative association. The association between NO_2 and cataract incidence were stronger in RP patients (OR: 1.11, 95% CI: 0.73-1.68) compared to non-RP individuals (OR: 1.07, 95% 1.06-1.10). Similar patterns were observed with CO, PM_{10}, and $PM_{2.5}$, although interaction terms were not statistically significant.

Conclusions

This study found a positive association between long-term NO_2 and CO exposure and cataract incidence. Furthermore, RP patients showed potentially greater susceptibility to air pollution-related ocular damage. These findings imply the need to consider environmental exposures in RP management.

P01-07 | Childhood exposure to $PM_{2.5}$ in Nepal and Indonesia: A health impact assessment of respirator wearing

William Mueller[1], Fiona Carson[1], Claire Horwell[2], Megnath Dhimal[3], Muhammad Iqbal[4], Miranda Loh[1], Karen S. Galea[1]

[1] IOM, Edinburgh, UK; [2] Durham University, Durham, UK; [3] Nepal Health Research Council, Kathmandu, Nepal; [4] Institut Teknologi Bandung, Center for Climate Change, Bandung, Indonesia

Objective

Children's exposure to particulate air pollution in urban environments can vary greatly depending on the microenvironment. Using data from the Factors Affecting Childhood Exposures to Urban Particulates (FACE-UP) project, we conducted a health impact assessment (HIA) of childhood respirator wearing to reduce exposure to $PM_{2.5}$ in three microenvironments: school commute, school classroom, and outdoor play.

Material and methods

Our analysis applied to urban children aged 5-19 years living in Indonesia and Nepal and quantified the percent prevented of asthma hospital admissions, incident asthma cases, lost school days, and future premature mortality. HIA scenarios based on exposure microenvironment, uptake of respirators, and the fit factor of the respirator (function of respirator material and seal around wearer's face) were developed.

Results

Overall, the prevented cases associated with reduced $PM_{2.5}$ exposure from child respirator wearing were <20% of the annual baseline cases for each outcome. Results varied widely, with the highest contribution to reduced health impacts being associated with respirator wearing in the school classroom, coinciding with the longest duration of wear time. Fewer cases for all outcomes were prevented in the commute and outdoor play microenvironments, with

percentage reductions of similar magnitudes in each (<4%). Respirator wearing in Nepal during the highest 6-months of pollution in a year prevented around two thirds of the cases associated with annual use.

Conclusion

The child population health benefits of respirator wearing depend on many factors, especially uptake, quality and fit of the respirator, time spent wearing the respirator, and the surrounding air pollution concentrations. Our findings should be considered alongside evidence for other interventions to lower childhood exposures to air pollution while, at the same time, striving to curtail pollutant emissions.

Acknowledgments

FACE-UP is funded by a UKRI Collective Fund award under the Global Challenges Research Fund (UKRI GCRF grant reference: MR/T029897/1).

P01-08 | Low-grade long-term ambient particle exposure and chronic kidney disease

Karl Emritz Kilbo Edlund[1], Yiyi Xu[1], Eva Andersson[1,2], Peter Molnár[1,2], Annika Rosengren[3], Leo Stockfelt[1,2]

[1] University of Gothenburg, School of Public Health and Community Medicine, Gothenburg, Sweden; [2] Sahlgrenska University Hospital, Occupational and Environmental Health, Gothenburg, Sweden; [3] University of Gothenburg, Department of Molecular and Clinical Medicine, Gothenburg, Sweden

Objective

Emerging evidence indicates that long-term air pollution exposure is associated with a relatively strong increased risk of chronic kidney disease (CKD). However, this risk-outcome pair is not yet included in established health impact assessments of air pollution. One obstacle for this is the lack of large cohort studies at low exposure levels.

Material and Methods

We performed a retrospective cohort study in two population-based cohorts including 11 324 individuals in Gothenburg, Sweden. Both cohorts utilized validated high-resolution dispersion modelling of annual average air pollution concentration at the residential address. Participants were followed between 1990 and 2011. We retrieved dates of CKD diagnoses and start of renal replacement therapy (RRT) from national registers. In the main regression model, we adjusted for age, gender, area-level income, education, smoking, and physical activity.

Results

The median exposure to fine particles ($PM_{2.5}$) between 1990 and 2011 was 9.3 μg/m^3. Over this period, we recorded in total 268 cases of CKD and 27 cases of RRT. In the preliminary results, $PM_{2.5}$ exposure was associated with elevated hazards for CKD (aHR 1.20, 95% CI 1.03, 1.41, per 1 μg/m3) and, though not reaching statistical significance, RRT. The strongest signal was seen for $PM_{2.5}$ exposure lagged over 1 to 5 years.

Conclusion

Our results add to previous evidence that air pollution is a modifiable risk factor for CKD also at low exposure levels, motivating the inclusion of CKD in impact assessment of air pollution exposure and warranting further public health measures to reduce air pollution exposure.

This study is funded by the Swedish Research Council for Health, Working Life and Welfare.

P01-09 | Prenatal Exposure to Criteria Air Pollution and Traffic-Related Air Toxics and Risk of Autism Spectrum Disorder: A Population-Based Cohort Study of California Births (1990–2018)

Karl O'Sharkey[1], Sanjali Mitra[1], Ting Chow[1], Seung-a Paik[1], Laura Thompson[2], Jason Su[3], Myles Cockburn[2], Beate Ritz[1]

[1] University of California, Los Angeles, Epidemiology, Los Angeles, USA; [2] University of Southern California, Population and Public Health Sciences, Los Angeles, USA; [3] University of California, Berkeley, Environmental Health Sciences, Berkeley, USA

Background: Autism spectrum disorder (ASD) prevalence has risen steadily in California (CA) over the past three decades, with environmental factors like air pollution (AP) increasingly implicated. This study investigates the association between prenatal exposure to both criteria AP and traffic-related air toxics and ASD risk for 1990-2018 births.

Methods: Utilizing CA Department of Public Health birth registry data from 1990 to 2018, linked with ASD diagnoses from the CA Department of Developmental Services (n = 13,348,678 children; ASD cases = 135,232), we assessed prenatal exposure to $PM_{2.5}$, NO_2, O_3, and six traffic-related air toxics (benzene, 1,3-butadiene, chromium, lead, nickel, zinc) using machine learning-enhanced land-use regression models. Logistic regression estimated odds ratios (OR) for ASD per interquartile range (IQR) increase in pollutant levels across four time periods (1990–1997, 1998–2004, 2005–2011, 2012–2018). Analyses were stratified by race/ethnicity, neighborhood socioeconomic status (SES), and region within CA.

Results: Prenatal exposure to $PM_{2.5}$ (OR: 1.10; 95% CI: 1.04, 1.16) and NO_2 (OR: 1.25; 95% CI: 1.16, 1.35) were associated with increased ASD risk, with effect sizes declining over time. When mutually adjusting for NO_2, the association between $PM_{2.5}$ exposure and ASD risk was attenuated, whereas the association with NO_2 exposure remained largely unchanged. Among air toxics, benzene (OR: 1.55; 95% CI: 1.51, 1.59) and nickel (OR: 1.33; 95% CI: 1.21, 1.45) were strongly associated with ASD, persisting across time. Stratified analyses revealed that associations differed by race/ethnicity, SES, and region. Traffic-related air toxics consistently exhibited elevated ASD risks, highlighting persistent vulnerabilities despite reductions in traditional pollutants.**Conclusions:** Prenatal exposure to AP, especially traffic-related toxics, is linked to increased ASD risk with temporal and spatial variability. While reductions in NO_2 and $PM_{2.5}$ lessen ASD risk, persistent traffic-related pollutants like benzene and nickel highlight the need for targeted interventions.

P01-10 | Optimizing air purification strategies to reduce household $PM_{2.5}$ exposure in Uganda

Megan L. Benka-Coker[1], Edwin Nahabwe[2], Michael Johnson[3], Peter P. Moschovis[4,5]

[1] Gettysburg College, Department of Health Sciences, Gettysburg, USA; [2] World Alliance for Lung and Intensive Care Medicine, Kampala, Uganda; [3] Berkeley Air Monitoring Group, Berkeley, USA; [4] Massachusetts General Hospital, Divisions of Global Health and Pulmonary Medicine, Boston, USA; [5] Harvard Medical School, Department of Pediatrics, Boston, USA

Background: The health effects of air pollution are disproportionately experienced by people in low and middle-income countries (LMICs) who are exposed to substantial household and ambient sources. Although extensive research has demonstrated benefits associated with reducing particulate matter (PM) exposure, the multiple sources of air pollution in LMIC settings suggest that a single source-focused intervention (e.g., a clean cookstove) may not be sufficient. High-efficiency particulate air (HEPA) filter-based portable air cleaners (PACs) have demonstrated the ability to reduce PM exposure indoors regardless of the source, but there is limited evidence of their potential effectiveness in LMICs.

Methods: We tested the efficacy of a HEPA-filter based PAC on removing particulate matter in a typical home in Mbarara, Uganda. Particulate matter was introduced into the home via a kerosene wick lamp. When $PM_{2.5}$ concentrations reached 200-400 µg/m^3, we measured the reduction in $PM_{2.5}$ concentration under four conditions: PAC fan off, PAC fan on low, PAC fan on medium, and PAC fan on high. We calculated air changes per hour (ACH) equivalent rates based on particle clearance. We used the World Health Organization (WHO) Household Multiple Emissions Source (HOMES) Model to estimate how increasing ACH equivalents could impact daily $PM_{2.5}$ concentrations.

Results: The ACH equivalent rate was 1.0 per hour at baseline (PAC off), 1.1 for PAC on low, 2.9 for PAC on medium, and 3.3 for PAC on high. When ACH equivalents were input from the experiment into the HOMES model, modelled concentrations demonstrated that baseline concentrations could be reduced by 63% on on the high fan setting.

Conclusion: The PAC unit reduced the $PM_{2.5}$ levels with efficacy with to increasing fan speed. A HEPA-filter based PAC may be effective in reducing PM, especially when indoor $PM_{2.5}$ concentrations are high and ambient $PM_{2.5}$ is low.

P01-11 | Characterizing space-time patterns of nitrogen oxides (NO_2 and NO) pollution in Kigali, Rwanda.

Pacifique Karekezi[1,2], Kate Kyeremanteng[3,4], Barbara E. Mottey[3], Carissa Lange[3], Chantal Umutoni[1,2], Jean Remy Kubwimana[1,2], James Nimo[5,6], Jiayuan Wang[3], Paterne Gahungu[2,7], Silas S. Mirau[1], Claudette Nyinawumuntu[2], Samson Niyizurugero[2], Pie-Celestin Hakizimana[8], Isambi S. Mbalawata[2], Majid Ezzati[9], Allison F. Hughes[5], Raphael E. Arku[3]

[1] The Nelson Mandela Institution of Science and Technology, School of Computational and Communication Science and Engineering, Arusha, Tanzania; [2] African Institute for Mathematical Sciences, Research and Innovation Centre, Kigali, Rwanda; [3] University of Massachusetts, Department of Environmental Health Sciences, Amherst, USA; [4] University of Massachusetts, Department of Biostatistics and Epidemiology, Amherst, USA; [5] University of Ghana, Department of Physics, Accra, Ghana; [6] State University of New York, Department of Environmental & Sustainable Engineering, Albany, USA; [7] Imperial College London, Department of Mathematics, London, UK; [8] Rwanda Environment Management Authority, Kigali, Rwanda; [9] Imperial College London, MRC Centre for Environment and Health, London, Germany; [10] Imperial College London, Department of Epidemiology and Biostatistics, London, UK; [11] University of Ghana, Regional Institute for Population Studies, Accra, Ghana; [12] Imperial College London, Abdul Latif Jameel Institute for Disease and Emergency Analytics, London, UK; [13] 13Imperial Global Ghana Hub, Accra, Ghana

Background: In rapidly growing cities across sub-Saharan Africa (SSA), authorities are faced with the challenge of providing essential services to ever-growing population in a sustainable way that also minimizes exposure and health impacts from environmental pollution. Understanding air pollution emissions from combustion related sources in response to the increasing motorization, traffic congestion, and industrial (formal and informal) activities is critical for city planning and regulation.

Objective: We conducted a city-scale measurement to evaluate the patterns of nitrogen dioxide (NO_2) and nitric oxide (NO) pollution in relation to land use in Kigali, Rwanda.

Methods: Between November 2022 and December 2023, we deployed Ogawa passive samplers and measured weekly integrated NO_x (n= 556) and NO_2 (n= 556) concentrations at 130 (10 year-long and 120 week-long) locations of diverse land-use and source features.

Results: NO_2 concentrations ranged between < 2 - 62 μg/m^3 with an overall annual mean (SD) of 14.3 (12.11) μg/m^3. Across board, the levels were highest in densely populated communities and areas with commercial/business/industrial activities (CBI). While nearly all samples from background and sparsely populated communities were below the WHO guideline of 10 μg/m^3, over 65 % and 95% of the CBI and high-density residential samples exceeded this threshold. NO concentrations follow the same land use patterns. While NO concentrations were slightly elevated in the rainy season, NO_2 levels slightly increased in the dry season.

Conclusion: Residential areas in Kigali are experiencing elevated NO_2 pollution. Considered as a marker for traffic-related air pollution in cities, our city-wide data will guide land use planning and regulation in this city with unique physical layout of varying topography and greenery.

P01-12 | Long-term exposure to air pollution is associated with ever diagnosed periodontitis in the German National Cohort (NAKO)

Regina Pickford[1], Asma'a Alselwi[1], Fayed Mohammed[2], Susanne Breitner-Busch[1,2], Kathrin Wolf[1], Stefan Rupf[3], Thomas Kocher[4], Annette Peters[1,2], Alexandra Schneider[1], Birte Holtfreter[4]

[1] Helmholtz Zentrum München – German Research Center for Environmental Health (GmbH), Institute of Epidemiology, Neuherberg, Germany; [2] Ludwig-Maximilians-Universität München, Medical Faculty, Institute for Medical Information Processing, Biometry and Epidemiology, Munich, Germany; [3] Saarland University, Synoptic Dentistry, Homburg/Saar, Germany; [4] University Medicine Greifswald, Department of Restorative Dentistry, Periodontology, and Endodontology, Greifswald, Germany

Objective:
Periodontitis is a chronic inflammatory disease affecting the supporting structures of the teeth and a leading cause of tooth loss globally. Exposure to air pollution may play a critical role in the development and progression of periodontitis due to inflammation and oxidative stress. We assessed the relationship between long-term exposure to air pollution and ever diagnosed periodontitis in a large German cohort.
Material and Methods:
We included data of 159,420 participants from the German National Cohort (NAKO) collected between 2014 and 2019. Long-term exposure to $PM_{2.5}$ (particle diameter ≤ 2.5μm), NO_2 (nitrogen dioxide) and black carbon (BC) at the participants' home addresses was estimated for 2010 using land use regression models. Data were analyzed using logistic regression models adjusted for age, sex, study center, education, marital status and smoking. Results are reported as odds ratios (OR) with 95% confidence intervals (CI) per one interquartile range increase in the respective air pollutant.
Results:
Participants had a mean age of 49.6 years. About half of them were female, most had at least high school degree, 53% were current or former smokers, 58% were married living together. Median levels of $PM_{2.5}$, NO_2 and BC were 17.22 μg/m³, 26.98 μg/m³ and 1.63 10^{-5}/m, respectively. We found a clear association between air pollution and ever diagnosed periodontitis with an OR of 1.07 (95% CI: 1.04;1.11) per increase of 2.93 μg/m³ for $PM_{2.5}$, 1.05 (95% CI: 1.03;1.08) per increase of 10.37 μg/m³ for NO_2 and 1.04 (95% CI: 1.02;1.06) per increase of 0.48 10^{-5}/m for BC. Results remained stable in sensitivity analyses.
Conclusion:Untreated periodontitis can decrease the quality of life as it leads to tooth loss, affecting essential functions such as eating and speaking. Thus, more research is needed for this important public health issue regarding its association with air pollution.

P01-13 | India's sub-populations suffer from a disproportionate health burden attributable to ambient air pollution

Sagnik Dey[1,3], Debajit Sarkar[1], Santu Ghosh[2]

[1] Indian Institute of Technology Delhi, Atmospheric Sciences, New Delhi, India; [2] St. John's Medical College, Biostatistics, Bengaluru, India; [3] Korea University, Department of Health, Policy, Management, Seoul, South Korea

Background
Chronic exposure to ambient $PM_{2.5}$ represents the most significant environmental risk factor in India. However, it remains unclear whether its association with non-communicable diseases (NCDs) differs across demographic subgroups and whether its consequences lead to health disparities among them.

Material and Methods
We applied a hierarchical logistic mixed-effects model to evaluate the association between satellite-derived $PM_{2.5}$ exposure and the risks of diabetes, high blood pressure, and heart disease across gender, wealth, and caste subgroups while controlling for other covariates. We utilized an interaction model to analyze the combined impacts of $PM_{2.5}$ and unhealthy lifestyles, poor diets, and the use of solid fuels. In line with the Global Burden of Disease framework, we used the relative risk estimates obtained from our model to analyze the disparities in $PM_{2.5}$-related morbidity across these subgroups.

Results
In India, the link between ambient $PM_{2.5}$ levels and NCDs differed among various subgroups, with males being more affected than females and individuals from the Other Backward Classes (OBC) showing a higher impact than their demographic counterparts. The association of $PM_{2.5}$ with diabetes was found to be the weakest among the 'poorest' individuals (OR: 1.025, 95% UIs: 1.02-1.03) and strongest among the 'richest' (OR: 1.062, 1.056-1.068), while the relationships with the other two types of NCDs presented an opposite trend. We observed a larger combined effect of ambient $PM_{2.5}$ and other modifiable risk factors in males, OBC individuals, and lower-income groups compared to their counterparts. The excess morbidity burden of ambient $PM_{2.5}$ was significantly greater in males, OBC individuals, and deprived groups compared to their demographic counterparts, largely for diabetes and heart disease.

Conclusions
In India, air pollution affects males, OBCs, and deprived subgroups more. To achieve environmental and health equities in the future, other modifiable risk factors should be controlled simultaneously.

P01-15 | Hospital admissions attributable to reduced air pollution due to clean-air policies in China

Huimeng Liu[1], Jian Lei[1], Yuewei Liu[2], Tong Zhu[3], Kahung Chan[4], Xi Chen[5], Jing Wei[6], Furong Deng[7], Yunxing Jiang[1], Lijun Bai[1], Kai Wang[1], Juan Chen[1], Yang Lan[1], Xi Xia[1], Renjie Chen[8], Jicheng Gong[3], Xiaoli Duan[9], Kai Zhang[10], Haidong Kan[8], Xiaoming Shi[11], Xinbiao Guo[7], **Shaowei Wu**[1]

[1] Xi'an Jiaotong University Health Science Center, Department of Occupational and Environmental Health, School of Public Health, Xi'an, China; [2] Sun Yat-sen University, Department of Epidemiology, School of Public Health, Guangzhou, China; [3] Peking University, State Key Joint Laboratory for Environment Simulation and Pollution Control, College of Environmental Sciences and Engineering and Center for Environment and Health, Beijing, China; [4] University of Oxford, Clinical Trial Service Unit and Epidemiological Studies Unit, Nuffield Department of Population Health, Oxford, UK; [5] Chinese Center for Disease Control and Prevention, National Institute of Environmental Health, Beijing, China; [6] University of Maryland, Department of Atmospheric and Oceanic Science, Earth System Science Interdisciplinary Center, College Park, USA; [7] Peking University, Department of Occupational and Environmental Health Sciences, School of Public Health, Beijing, China; [8] Fudan University, School of Public Health, Key Lab of Public Health Safety of the Ministry of Education and NHC Key Lab of Health Technology Assessment, Shanghai Institute of Infectious Disease and Biosecurity, Shanghai, China; [9] University of Science and Technology Beijing, School of Energy and Environmental Engineering, Beijing, China; [10] University at Albany, State University of New York, Department of Environmental Health Sciences, School of Public Health, Rensselaer, China; [11] Chinese Center for Disease Control and Prevention, China CDC Key Laboratory of Environment and Population Health, National Institute of Environmental Health, Beijing, China

Objective: The Air Pollution Prevention and Control Action Plan (APPCAP) is considered to be the most stringent air pollution control policy in China implemented since 2013. This policy is a milestone in China to mitigate serious air pollution. However, health benefits attributable to reduced fine particulate air pollution after the implementation of the APPCAP have not been quantitatively estimated on a PM2.5 constituent-specific and morbidity cause-specific basis.

Material and Methods: A nationwide two-stage time-stratified case-crossover study using conditional logistic regression models was conducted based on a total of 48,623,468 cause-specific hospital admission records spanning major respiratory, cardiovascular, mental, neurological and kidney diseases in the urban population from 292 Chinese cities of prefecture-level or above during 2013-2017. The attributable fractions for major cause-specific hospital admissions were calculated based on the exposure-response relationships obtained from the above models.

Results: Compared with 2013, the annual average concentrations of PM2.5 and black carbon (BC) in 2017 decreased by 28.61% and 20.35%, respectively. As a result, the average relative reductions in annual attributable fractions of nine major cause-specific hospital admissions associated with PM2.5 and BC were 30.00% and 21.14%, respectively, among which annual attributable fraction for depression showed the largest reduction. Nationally, cities with higher reductions in PM2.5 and BC were found to have higher absolute reductions in annual hospital admission attributable fraction associated with PM2.5 and BC, while geographic inequality in health benefits still exists.

Conclusion: Our study highlights the substantial wide-ranging health benefits of reduced PM2.5 and BC levels following the nationwide implementation of the APPCAP in China.

Funding: This study was supported by the National Key Research and Development Programs of China (grant numbers 2022YFC3702600, 2022YFC3702700 and 2023YFE0117400), National Natural Science Foundation of China (grant numbers 82273587, 82073509 and 82203991), and Youth Top Talent Program of Xi'an Jiaotong University.

P01-16 | Verification of the Indoor Air Quality Improvement Effects of Particulate Matter and Gaseous Pollutants in Residential and Office Spaces Using Portable Air Purifiers

ShinYoung Park[1], JiYoon Chung[1], Kwang-Chul Noh[2], CheolMin Lee[1]

[1] Seokyeong university, environmental chemical engineering, Seoul, South Korea; [2] Airlab, Gwangju, South Korea

This study aims to evaluate the impact of air purifier usage on indoor air quality improvement in residential and office environments and to compare air purifier operation strategies for different spaces. Indoor air quality can be influenced by various sources such as cooking activities, cleaning, photocopiers, and outdoor air infiltration, which can significantly affect health and daily life. Methods for improving indoor air quality include natural ventilation, range hood operation, and the use of air purifiers, with recent focus on reducing particulate matter and gaseous pollutants through air purifiers. However, a fixed air purifier may struggle to uniformly improve indoor air quality in multi-room spaces, such as typical homes. In particular, its effectiveness in enhancing overall air quality may be limited in environments with multiple rooms. It is necessary to operate air purifiers in individual rooms or utilize portable air purifiers. In this study, three scenarios were set up in two locations each of a residential house and an office to analyze the indoor air quality improvement effects of different air purifier operation strategies. The first scenario involved no air purifier operation, the second involved operating a single fixed air purifier, and the third involved operating a portable air purifier based on IoT devices. The study measured the changes in particulate matter and gaseous pollutants in each scenario and compared the performance of portable air purifiers across different indoor spaces (residential and office). The results are expected to provide valuable foundational data for developing more effective air purifier operation strategies for indoor air quality improvement. The results will provide essential data for optimizing air purifier operation strategies and highlight the potential of IoT-based monitoring for future indoor air quality management and practical solutions.

P01-17 | Association between Air Pollution and the Persistency of Atopic Dermatitis in a Childhood Cohort in South Korea

Suhong Min[1,2], Sera Kim[2], Jong-Tae Lee[1,3]

[1] Interdisciplinary Program in Precision Public Health, Department of Public Health Sciences, Graduate School of Korea University, Seoul, South Korea; [2] Research and Management Center for Health Risk of Particulate Matter, Seoul, South Korea; [3] School of Health Policy and Management, College of Health Sciences, Korea University, Seoul, South Korea

Background

In South Korea, the prevalence of atopic dermatitis (AD) has decreased among infants but increased in older age groups, suggesting that AD is becoming persistent. While previous studies have shown a positive association between air pollution and the development of AD, few have explored its role in persistency. Therefore, this study aims to investigate the association between air pollution and persistency of AD in young children diagnosed with AD.

Methods

We conducted a retrospective cohort study using the National Health Insurance Service-National Sample Cohort from 2002 to 2019. The study included children aged 0 to 5 with AD-related diagnostic codes (ICD-10, L20). The

persistency of AD, defined by AD-related hospital revisits during a 3-year follow-up, was the outcome variable. The level of air pollutants, including particulate matter (PM_{10}, $PM_{2.5}$) and gaseous pollutants (NO_2, O_3, CO), provided by AiMS-CREATE team, was the independent variable of interest. Covariates included sex, age, household income, and the year of initial visit. We used logistic regression to estimate odds ratios (OR) and 95% confidence intervals (95% CI) for each 10-unit increase in pollutants.

Results

The data included 82,459 children diagnosed with AD, contributing 197,807 person-years. During the follow-up period, 37,662 children (45.8%) experienced AD-related hospital revisits. PM_{10} was positively associated with revisits (OR: 1.33, 95% CI: 1.31–1.36), as were $PM_{2.5}$ (OR: 1.63, 95% CI: 1.54–1.74), NO_2 (OR: 1.14, 95% CI: 1.11–1.16), and CO (OR: 1.49, 95% CI: 1.42–1.55). In contrast, O_3 was negatively associated with revisits (OR: 0.59, 95% CI: 0.57–0.62).

Conclusions

This study highlights the role of air pollution in the persistency of AD among young children in South Korea. Our findings suggest that reducing air pollution level is essential not only to prevent the AD onset but also to manage existing cases and reduce the disease persistency.

P01-18 | Associations of air pollutants, temperature, and road traffic noise with the incidence of chronic kidney disease in Taiwan elders: a retrospective cohort study

Ta-Yuan Chang[1], Ting-Yu Zhuang[1], Wan-Ju Cheng[2,3], Yun-Chieh Yang[2]

[1] China Medical University, Occupational Safety and Health, Taichung, Taiwan; [2] National Health Research Institute, National Center for Geriatrics and Welfare Research, Miaoli, Taiwan; [3] China Medical University, Public Health, Taichung, Taiwan

This study aimed to elucidate the associations between exposure to air pollutants, temperature, and road traffic noise with the incidence of chronic kidney disease (CKD) and to explore the interaction effects of multiple environmental pollutants on CKD in Taiwan. A total of 3,041 elders (aged ≥ 55 years) were recruited in 2009 and followed-up to 2019. Land use regression (LUR) was applied to estimate individual exposure levels of air pollutants ($PM_{2.5}$, NO_2, CO, SO_2, and O_3), temperature, and the 24-hour A-weighted equivalent sound pressure level ($L_{Aeq,24}$). Cox regression was used to examine the associations between exposure to these environmental pollutants and CKD incidence. In single-exposure models, a one-interquartile range (IQR) increase in $PM_{2.5}$ (7.8 μg/m^3) and temperature (2.3 ℃) were significantly associated with a higher risk of CKD incidence (relative risk [RR] = 1.14, 95% confidence interval [CI] = 1.00–1.25; RR = 1.10, 95% CI = 1.02–1.20), whereas other exposure variables did not show significant associations. In the multiple-exposure model, a one-IQR increase in $PM_{2.5}$ remained significantly associated with an increased risk of CKD (RR = 1.13, 95% CI = 1.03–1.25) after adjusted for temperature, NO_2, and $L_{Aeq,24}$. A synergy effect between PM2.5 (>=34.2 μg/m^3) and annual temperature (>=25.7 ℃) was observed (Rothman's synergy index: 3.18, 95% CI: 2.20-4.60). In conclusion, this study only found a significant and positive association between $PM_{2.5}$ exposure and the risk of CKD in the multiple-exposure model. An additive synergistic effect between $PM_{2.5}$ and annual temperature was also observed.

P01-20 | Association between nitrogen dioxide exposure and out-of-hospital cardiac arrest onset in China: A multi-center, time-stratified, case-crossover study

Pan Chang[1], **Xinyue Li**[2], Jiaqi Zheng[1], Ruixue Song[1], Ziyang Zhang[1], Renjie Chen[2], Haidong Kan[2], Feng Xu[1], Yuguo Chen[1], Xia Meng[2]

[1] Qilu Hospital of Shandong University, Shandong, China; [2] Fudan University, Shanghai, China

Background: Existing studies on the association between NO_2 exposure and out-of-hospital cardiac arrest (OHCA) are limited and present inconsistent results. In the context of global urbanization, a larger population have been being exposed to high levels of ambient NO_2 pollution, highlighting the need for further clarification of the health hazards posed by NO_2 pollution. Furthermore, Previous studies mostly applied exposure data from monitoring stations, with relatively few investigations examining acute effects using modeled data with high resolution.
Methods: To explore the association between NO_2 and the risk of cardiac OHCA onset, a time-stratified case-crossover study was conducted utilizing data from emergency medical service (EMS) systems across 23 provinces in China throughout 2020. Conditional logistic regression models were employed to investigate the potential association between NO_2 and OHCA onset. Additionally, individual-level NO_2 data obtained from both models and monitoring stations were analyzed separately to evaluate their comparability in practice.
Results: The analysis incorporated 76,263 EMS-attended cardiac OHCA onsets. The health estimates from NO_2 predictions and measurements were comparable without statistically difference, with each 10 μg/m³ increase associated with a 1.16% (95% confidence interval [CI]: 0.38-1.94%] and 1.03% (95% CI: 0.37-1.69%] increase in the risk of OHCA onset, respectively. Stratified analyses revealed heightened effect estimates in patients with respiratory diseases.
Conclusion: This nationwide multi-center study demonstrated adverse effects of NO_2 exposure on OHCA onset in a large population residing in the region with higher and variable NO_2 levels. These findings contribute robust epidemiological evidence to this field and offer new evidence to support global policy-making, particularly in developing countries. Additionally, modeled NO_2 predictions with high resolution and coverage can serve as effective alternatives to traditional monitoring station data in epidemiological studies.

P01-21 | Evaluating the energy transition in power generation: A cleaner alternative or a hidden health burden?

Xiaorong Shan, Lucas Henneman

George Mason University, Civil, Environmental, and Infrastructure Engineering, Fairfax, USA

Objective
The transition to cleaner fossil fuels in power generation led to widespread air quality improvements, yielding environmental and public health benefits. However, analyses of the EPA's National Emissions Inventory for Virginia from 2011 to 2020 suggest that increases in primary $PM_{2.5}$ emissions from natural gas electric generating units (EGUs) now exceed $PM_{2.5}$ emissions from coal EGUs. Motivated by potential nationwide shifts toward natural gas as a dominate emissions source of multiple air pollutions, this study aims to explore the temporal trends in fuel-specific EGU emissions and EGU-specific $PM_{2.5}$ exposure from 1940 to 2020.
Material and Methods
Present-day emissions data are obtained from the Clean Air Markets Program Data (CAMPD) datasets. For periods without direct measurements, we estimate SO_2, NOx, PM and CO_2 emissions using emission factors (EFs) derived from existing U.S. power plants that fuel type and consider presence or absence of emissions controls. We use the HYSPLIT average dispersion (HyADS) model, to quantify $PM_{2.5}$ concentrations attributable to power plant emissions.
Results
Power plant emissions of SO_2, NO_x, and PM increased by 86.1%, 41%, and 56.5% from 1940 to 1970, respectively, and were subsequently reduced by 95% by 2020 due to the fuel transition from coal to oil and natural gas. In contrast, CO_2 emissions remained relatively stable, with only a 30% difference compared to 1970 levels. Additionally, EGU-specific $PM_{2.5}$ exposure declined by 97.7% from 1970 to 2020, substantially mitigating associated health risks.
Conclusions
This comparison shows that shifting to alternative fuels has significantly reduced power plant emissions, though regional differences in control technologies can still lead to higher emissions in some areas. Since SO_2 from coal remains the primary source of EGU-specific $PM_{2.5}$, continued fuel transitions and improved emissions controls are essential for further mitigating environmental and public health risks.

P01-22 | *Exploring the interaction between ambient air pollution and road traffic noise on stroke incidence in ten Nordic cohorts*

Huyen T.K. Nguyen[1], Massimo Stafoggia[1,2], Mette Sørensen[4,5], Aslak H. Poulsen[4,5], Ole Raaschou-Nielsen[4,6], Jibran Khan[6,7], Jørgen Brandt[6], Anja Olsen[4,8], Zorana J Andersen[9], Mette Kildevaeld Simonsen[10], Youn Hee Lim[9], Jiawei Zhang[9], Tom Cole-Hunter[9], Göran Pershagen[1], Andrei Pyko[1], Agneta Åkesson[1], Leonard Stockfelt[11,12], Eva M Andersson[11,12], David Segersson[13], Annika Rosengren[14,12], Mikael Ögren[14,12], Anna Oudin[1,15], Maria Albin[1], Kristoffer Mattisson[15], Gunnar Engström[15], Debora Rizzuto[16], Patrik KE Magnusson[17], Hrafnhildur Gudjonsdottir[18], Karin Leander[1], Timo Lanki[20,19], Pekka Tiittanen[19], Tarja Yli-Tuomi[19], Pekka Jousilahti[19], Petter Ljungman[1,3], Jeroen de Bont[1]

[1] Karolinska Institutet, Stockholm, Institute of Environmental Medicine, Stockholm, Sweden; [2] Lazio Region Health Service, Department of Epidemiology, Rome, Italy; [3] Danderyd University Hospital, Department of Cardiology, Stockholm, Sweden; [4] Danish Cancer Institute, Copenhagen, Denmark, Copenhagen(CPH), Denmark; [5] Roskilde University, epartment of Natural Science and Environment, Roskilde, Denmark; [6] Aarhus University, Department of Environmental Science, Roskilde, Denmark; [7] Aarhus University, Danish Big Data Centre for Environment and Health (BERTHA), Roskilde, Denmark; [8] Aarhus University, Department of Public Health, Aarhus, Denmark; [9] University of Copenhagen, Department of Public Health, Copenhagen(CPH), Denmark; [10] Bispebjerg and Frederiksberg Hospital, Department of Neurology and the Parker Institute, Copenhagen, Denmark; [11] University of Gothenburg, Occupational and Environmental Medicine, School of Public Health and Community Medicine, Institute of Medicine, Sahlgrenska Academy, Gothenburg, Sweden; [12] Sahlgrenska University Hospita, Department of occupational and Environmental Medicine, Gothenburg, Sweden; [13] Swedish Meteorological and Hydrological Institute, Norrköping, Sweden; [14] University of Gothenburg, Department of Molecular and Clinical Medicine, Sahlgrenska Academy, Gothenburg, Sweden; [15] Lund University, Division of Occupational and Environmental Medicine, Lund, Sweden; [16] Karolinska Institutet, Aging Research Center, Department of Neurobiology, Care Science and Society, Stockholm, Sweden; [17] Karolinska Institutet, Department of Medical Epidemiology and Biostatistics, Stockholm, Sweden; [18] Karolinska Institutet, Department of Global Public Health, Stockholm, Sweden; [19] Finnish Institute for Health and Welfare, Department of Public Health, Kuopio, Finland; [20] University of Eastern Finland, Department of Environmental and Biological Sciences, Kuopio, Finland

Introduction: Ambient air pollution and road traffic noise are stroke risk factors, but evidence on their potential joint effects remains limited. This study investigated the independent and joint associations of air pollution and road traffic noise on stroke incidence using both multiplicative and additive scales.

Methods: We followed stroke incidence in ten cohorts in Sweden, Denmark, and Finland. We modelled annual average levels of outdoor particulate matter < 2.5 µm ($PM_{2.5}$), nitrogen dioxide (NO_2) and road traffic noise at residential addresses. We applied Cox proportional hazards regression to evaluate their single association. We assessed multiplicative interaction with interaction terms in Cox models and additive interaction using the Relative Excess Risk due to Interaction method.

Results: We followed 136,897 adults for 20 years, and 8.0% experienced stroke incidence. $PM_{2.5}$, NO_2 and road traffic noise were associated with higher stroke risk in single-exposure models. Multiplicative models showed higher HRs between $PM_{2.5}$ and stroke at higher levels of noiseand vice versa: HRs per 5 µg/m³ of $PM_{2.5}$ were 1.06 (95% CI:0.94–1.21) at 40 dB and 1.11 (95% CI:0.85–1.44) at 80 dB of road traffic noise; HRs per 12 dB of road traffic noise were 1.06 (95% CI:1.01–1.11) at 4 µg/m³ and 1.17 (95% CI:0.82–1.68) at 48 µg/m³ of $PM_{2.5}$. Additive models showed that the combined association of $PM_{2.5}$ and road traffic noise was 4% (RERI = 0.04 (95% CI:-0.05;1.12)) higher than the sum of their individual association.

Conclusion: $PM_{2.5}$ and road traffic noise showed a non-significant synergistic association on stroke incidence.

P01-23 | Short-term Associations between Ambient Air Pollution and Emergency Department Visits for Parkinson's Disease, Multiple Sclerosis, Migraine and Seizure in California, 2005 to 2018

Shumeng Chen[1], Danlu Zhang[1], Rebecca Zhang[1], Morgan Lane[2], Noah C. Scovronick[2], Stefanie T. Ebelt[2], Howard H. Chang[1,2]

[1] Emory University, Department of Biostatistics and Bioinformatics, Atlanta, USA; [2] Emory University, Gangarosa Department of Environmental Health, Atlanta, USA

Objective: Previous studies have reported associations between ambient air pollution and exacerbation of neurological conditions, but research in the United States is limited. This study aims to investigate short-term associations between emergency department (ED) visits for four disorders that affect the nervous system (Parkinson's disease, multiple sclerosis, migraine, and seizure) and three major air pollutants, fine particulate matter ($PM_{2.5}$), carbon monoxide (CO) and nitrogen dioxide (NO_2), in California for the period 2005 to 2018.
Materials and Methods: We acquired data on over 6 million ED visits for nervous system disorders. Daily estimated $PM_{2.5}$, NO_2, and CO concentrations at 12 km spatial resolution were aggregated to the ZIP code level as exposures. We estimated pollution-ED visit associations using a time-stratified case-crossover design with conditional Poisson regression. We also considered different exposure lags and stratified analysis by patient characteristics. Risk ratios (RR) are reported per interquartile range increase in exposure.
Results: For migraines, we observed positive associations with same-day (lag 0) NO_2 [RR = 1.009, 95% confidence interval (CI): 1.005, 1.015], CO (RR = 1.006, 95% CI: 1.003, 1.008) and $PM_{2.5}$ (RR = 1.003, 95% CI: 1.001, 1.005). For Parkinson's disease, CO showed the strongest short-term association at lag 0 (RR = 1.007, 95% CI: 1.003, 1.011). For seizures, the strongest association was observed for NO_2 at lag 0 (RR = 1.006, 95% CI: 1.003, 1.010). Compared to same-day exposure, associations for cumulative exposure effects over multiple days decreased for CO and NO_2 but remained similar for $PM_{2.5}$. For multiple sclerosis, associations with all three pollutants were null.
Conclusion: Same-day high ambient air pollution levels were associated with increased ED visits for migraine, Parkinson's disease, and seizures. This study adds to the growing epidemiological evidence that short-term exposure to ambient air pollution may affect the nervous system.
NIH R01ES027892 and P20ES036110.

P02 | Advances in Air Quality Monitoring and Modeling - PART I

P02-01 | High-Resolution AOD Reconstruction Using Deep Learning: A Foundation for Fine-Scale $PM_{2.5}$ Prediction Across the Contiguous United States (2000–2023)

Ali Sheidaei[1], Kimiya Gohari[1], Maayan Yitshak-Sade[1,2], Ryan Michael[3], Itai Kloog[1,2]

[1] Icahn School of Medicine at Mount Sinai, Department of Environmental Medicine, NEW YORK, USA; [2] Icahn School of Medicine at Mount Sinai, Institute for Exposomic Research, NEW YORK, USA; [3] Icahn School of Medicine at Mount Sinai, Department of Public Health, NEW YORK, USA

Objective: Accurate estimation of fine particulate matter ($PM_{2.5}$) at high spatial resolution is crucial for exposure assessment and environmental health research. MODIS AOD at 470 nm (AOD470) provides high-resolution data but suffers from missing values due to cloud cover, while the Copernicus Atmosphere Monitoring Service (CAMS) AOD dataset offers continuous coverage at a coarser resolution and tends to underestimate AOD in high-pollution regions. This study develops a deep learning-based data fusion approach to reconstruct MODIS AOD470 from CAMS AOD estimates (AOD1240, AOD469, AOD550, AOD670, AOD865) over the contiguous United States (CONUS) at a 100m daily resolution. The reconstructed dataset aims to improve air pollution exposure modeling for epidemiological studies.

Material and Methods: MODIS and CAMS datasets were spatially and temporally aligned, with resolution mismatches addressed using inverse-distance weighting. A hybrid Convolutional Neural Network and Recurrent Neural Network with Long Short-Term Memory (LSTM) units captured spatial and temporal dependencies, respectively. CNNs extracted spatial features across multiple scales, while LSTMs modeled long-range temporal variations to enhance prediction stability. The model was trained using an 80%-10%-10% data split and benchmarked against Random Forest, Gradient Boosting, and LightGBM.

Results: The deep learning model significantly surpassed conventional methods, delivering an R^2 exceeding 75-88% across the years. It successfully reconstructed MODIS AOD470, accurately capturing the spatial and temporal patterns of aerosols. The model addressed the underestimation of AOD by CAMS in regions with high aerosol levels, facilitating extrapolations at a 100m resolution that enhances fine-scale $PM_{2.5}$ exposure assessments. By adopting this method, the predictive capability for $PM_{2.5}$ models was markedly improved, leading to more precise estimates of air pollution exposure.

Conclusion: This study demonstrates the effectiveness of deep learning-based data fusion for high-resolution AOD reconstruction. Enhancing AOD coverage and resolution strengthens $PM_{2.5}$ exposure modeling, improving air pollution assessment for environmental epidemiology and public health research.

P02-02 | Enhancing 5-Day Particulate Matter (PM10) Forecasts in Morocco Using U-Net: A Deep Learning Approach

Anass Houdou[1,2], Kenza Khomsi[3], Luca D. Monache[4], Weiming Hu[5], Fayez Abdulla[6], Imad el Badisy[2,9], Wael K. Al-Delaimy[7], Mohamed Khalis[1,2]

[1] Mohammed VI University of Sciences and Health, Mohammed VI International School of Public Health, Casablanca, Morocco; [2] Mohammed VI Center for Research and Innovation, Department of Public Health and Clinical Research, Rabat, Morocco; [3] General Directorate of Meteorology, Casablanca, Morocco; [4] University of California San Diego, Center for Western Weather and Water Extremes, Scripps Institution of Oceanography, San Diego, USA; [5] James Madison University, School of Integrated Sciences, Harrisonburg, USA; [6] Jordan University of Science and Technology, Civil Engineering Department, Irbid, Jordan; [7] University of California San Diego, School of Public Health, San Diego, USA; [8] Ministry of Health and Social Protection, Higher Institute of Nursing Professions and Health Techniques, Rabat, Morocco; [9] Inserm UMR912 Sciences Economiques & Sociales de la Santé & Traitement de l'Information Médicale (SESSTIM), Marseille, France

Accurately predicting particulate matter is crucial for preventing health risks and protecting public health. This study improves the accuracy of particulate matter $d<10\ \mu m$ (*PM10*) forecasts over Morocco for the next five days using a U-Net-based deep learning model, marking the first work of its kind in the Middle East and North Africa (MENA) region. The U-Net model was used to post-process and improve *PM10* forecasts from the Copernicus Atmosphere Monitoring Service (CAMS), with reanalysis data from CAMS serving as a reference to guide the model's learning. The U-Net architecture was modified to predict outputs at a resolution different from the inputs, eliminating the need for interpolation and preserving critical spatial details. The results demonstrated significant improvements over two baselines—CAMS forecasts and the Analog Ensemble model (AnEn)—by enhancing metrics such as Mean Absolute Error (MAE), Root Mean Square Error (RMSE), Coefficient of Determination (*R2*), and biases, particularly in regions prone to dust storms. U-Net also proved effective in capturing particulate pollution, providing reliable predictions for values up to 500 μg/m³. These findings underscore U-Net's potential for operational forecasting, supporting accurate early warnings to mitigate the health and environmental impacts of *PM10* pollution.

P02-03 | Mapping Hourly Ozone Trends in Texas and the U.S.-Mexico Border using TEMPO Satellite Data: Insights into Spatial Patterns and Wintertime High-Ozone Episodes near Oil and Gas Fields

Aodong Mei, Yun Hang

University of Texas Health Science Center at Houston, School of Public Health, Houston, USA

Objective

Ground-level ozone is an essential air pollutant with significant impacts on public health and agriculture. The new state-of-the-art satellite platforms, Tropospheric Emissions: Monitoring of Pollution (TEMPO) could provide high-resolution, real-time air quality data, enabling a more accurate air pollutant prediction. This study aims to integrate satellite-driven air quality data and machine learning to predict ground-level ozone concentrations at an hourly scale in Texas the U.S.-Mexico border region.

Material and Methods

The study utilizes TEMPO's satellite data, Air Quality System (AQS) ground ozone observations, meteorological inputs from the NOAA High-Resolution Rapid Refresh (HRRR) model, population, and the distances to the main roads to capture the hourly variability in ozone levels within Texas and the U.S.-Mexico border region at 1 km spatial resolution. A hybrid machine learning model combining convolutional neural networks (CNNs), long short-term memory (LSTM) networks, and extreme gradient boosting (XGBoost) was developed, and ten-fold cross-validation was conducted to evaluate model performance.

Results

The hybrid CNN-LSTM-XGBoost model achieved a high predictive accuracy, with an R^2 of 0.88 and an RMSE of 0.0053 ppm. Spatial and seasonal variations in ozone levels were observed, with elevated concentrations in the U.S.-Mexico border region, the Edwards Plateau, and Texas metropolitan areas. A case study of wintertime high-ozone episodes near oil and gas fields highlighted the model's ability to identify ozone hotspots and capture hourly-scale variations.

Conclusion

This study demonstrates the potential of integrating TEMPO's satellite data with machine learning to enhance the understanding of transboundary pollution gradients, industrial emission impacts, and hourly ozone fluctuations. TEMPO could empower policymakers and environmental researchers to address environmental health equity and develop targeted strategies for mitigating ozone pollution. This work also lays a foundation for future advancements in ozone modeling and utilizing other geostationary instruments designed for air pollutants, such as the upcoming Sentinel-4 satellite in Europe.

P02-04 | Enhancing $PM_{2.5}$ Component Predictions Across the Continental U.S. Using Multivariate Machine Learning Frameworks

Kimiya Gohari[1], Ali Sheidaei[1], Maayan Yitshak-Sade[1,2], Elena Colicino[1,2], Itai Kloog[1,2]

[1] Icahn School of Medicine at Mount Sinai, Department of Environmental Medicine, New York, USA; [2] Icahn School of Medicine at Mount Sinai, Institute for Exposomic Research, New York, USA

Objective: Fine particulate matter ($PM_{2.5}$) is composed of various chemical constituents, including sulfate (SO_4^{2-}), nitrate (NO_3^-), ammonium (NH_4^+), organic carbon (OC), and elemental carbon (EC), all of which play a critical role in air pollution and public health. Accurate prediction of their spatial and temporal distributions is essential for regulatory policies, exposure assessments, and epidemiological studies. This study developed and evaluated multivariate machine learning models. We **greatly improved modeling efficiency across large spatiotemporal extents** by leveraging interdependencies among components to enhance accuracy and computational efficiency.

Materials and Methods: We utilized daily $PM_{2.5}$ chemical speciation data from 947 monitoring sites across the United States spanning 2000 to 2019, incorporating 50 predictor variables derived from different sources. We implemented machine-learning models, including Random Forest (RF) and Extreme Gradient Boosting (XGBoost) in univariate and multivariate frameworks (MRF and MXGBoost). We evaluated model performance using R^2, RMSE, spatial and temporal R^2. We assessed feature importance using Shapley Additive Explanations values to interpret model predictions.

Results: Multivariate models outperformed univariate approaches by effectively capturing interdependencies among $PM_{2.5}$ components. MXGBoost achieved the highest predictive performance, with R^2 values around 70% for key components, while spatial R^2 exceeded 90% and temporal R^2 reached 80% for some components. Key predictors influencing $PM_{2.5}$ component distributions included wind speed, relative humidity, and aerosol optical depth. The

multivariate approach demonstrated improved accuracy while reducing computational complexity across different geographic and temporal scales.

Conclusion: Our findings highlight the advantages of multivariate machine learning models in predicting $PM_{2.5}$ components by capturing interdependencies, leading to enhanced accuracy and efficiency compared to conventional univariate methods. This approach offers valuable applications in air quality monitoring, environmental risk assessment, and public health research. Future work should focus on refining multivariate modeling frameworks and extending their application to other air pollutants for more comprehensive exposure assessments.

P02-05 | Peak Ambient $PM_{2.5}$ Exposure in Peri-urban Delhi Exceeds Urban Core: Evidence from Community Air Sensor Networks

Mark J. Campmier[1], Amarendra Singh[2], Sofiya Rao[2], Saumya Singh[4], María T. Hernández[1], Ajay Pillarisetti[1], Kalpana Balakrishnan[3], Sagnik Dey[2], Joshua Apte[1]

[1] University of California, Berkeley, School of Public Health, Berkeley, USA; [2] Indian Institute of Technology, Delhi, Delhi, India; [3] Sri Ramachandra Institute of Higher Education and Research, Chennai, India; [4] Indian Institute of Forest Management, Bhopal, India

Fine particulate matter ($PM_{2.5}$) air pollution is the largest environmental risk factor for ill health and death in India. Delhi and the surrounding National Capital Region are considered the most polluted megacity in the world. Outside of the urban core, there is sparse ground-based monitoring in the populous, poorer non-urban surrounding communities. Previously, we demonstrated that the PurpleAir PA-II, a lower-cost monitor, could report high fidelity (R^2 = 0.8–0.9) sub-hourly $PM_{2.5}$ concentrations across India with proper calibration procedures. Subsequently, we partnered with communities across a 100-km transect spanning non-urban settlements northeast of Delhi (n=30 sites) through the urban core (n=17 sites) from July - December 2024. Monthly, we found that although non-urban and urban sites were well correlated, non-urban sites were persistently more polluted by about 20%. Leveraging the temporal resolving power of the PA-II, we found the higher campaign median and interquartile range at the non-urban site was driven by sharper peaks in the morning (05:00-08:00 LT) and evening (17:00-20:00 LT). We further decomposed the sub-hourly variability ("peak") using background subtraction. We found that the median morning peak $PM_{2.5}$ of the non-urban sites is more than double the urban concentration (35 ± 79 μg/m^3 and 15 ± 28 μg/m^{3,} respectively). Conversely, at midday (12:00 – 15:00 LT), we found urban sites featured higher peak $PM_{2.5}$. Since exposure assessment in India typically relies on satellite data corresponding to midday observations, we found that remote sensing products weakly predicted the morning and evening spatial gradients from our network. Therefore, exposure and health studies using satellite data in sparsely monitored regions likely underestimate household pollution and may incorrectly report spatial gradients of air pollution exposure. Monitoring in non-urban areas provides additional evidence of the role of household sources in impacting exposure and community-scale air quality.

Funding: Open Philanthropy, Global Health Emerging Scholars

P02-06 | Spatial variability in the association of ambient PM2.5 exposure with child anemia in Sub-Saharan Africa

Patrick Attey-Yeboah[1], A. Kofi Amegah[1], Paul K. Adjorlolo[2], Justice Moses Kwaku Aheto[2], John T. Molitor[3], Eric Coker[4]

[1] University of Cape Coast, Biomedical Sciences, Cape Coast, Ghana; [2] University of Ghana, School of Public Health, Accra, Ghana; [3] Oregon State University, College of Health, Corvalis, USA; [4] BC Centre for Disease Control, Vancouver, Canada

Objective: Emerging evidence suggests that prenatal and postnatal exposure to PM2.5 may contribute to adverse child health outcomes, including anemia. However, spatial variability in this association remains unexplored. We investigated the spatial heterogeneity in the relationship between prenatal and postnatal PM2.5 exposure and childhood anemia in Sub-Saharan Africa (SSA) to inform region-specific intervention strategies.
Material and Methods: We integrated data on child anemia from Demographic andHealth Surveys (DHS) conducted in countries between 1998 and 2022 with high resolution satellite-derived geospatial PM2.5 exposures to create a dataset for our analysis. The dataset included 260,244 children under-five in 33 SSA countries. The exposure metrics was computed using weighted averages during the prenatal and postnatal periods with adjustment for geographic coordinate displacement via urban and rural buffers. We used a spatial random coefficient model set in a Bayesian framework (Integrated Nested Laplace Approximations [INLA]and Stochastic Partial Differential Equation [SPDE]) to model the spatially varying relationships controlling for individual- and area-level confounders assembled from the DHS data.
Results: Prenatal PM2.5 exposure was associated with a 1.1% increased odds of anemia (AOR = 1.011, 95% CI [1.0026, 1.0195]). In contrast, postnatal PM2.5 exposure showed no significant association with anemia. The observed associations exhibited notable spatial variation both within and across SSA sub-regions and countries, with West Africa experiencing the greatest exposure-related health impacts, followed by East Africa
Conclusion: The association of ambient PM2.5 exposure with childhood anemia varies within and between SSA countries, and calls for region-specific intervention strategies and public health policies to address child anemia within the SSA region.

P02-08 | Machine learning models can predict poor housing conditions at building level resolution in Boston

Zichuan Li[1], Sophia S. Carryl[1], Diane Gold[1], Elizabeth A. Samuels[2], Marissa Hauptman[3], Dinah Foer[4,5], Adam L. Haber[1]

[1] Harvard T.H. Chan School of Public Health, Department of Environmental Health, Boston, USA; [2] David Geffen School of Medicine, University of California, Department of Emergency Medicine, Los Angeles, USA; [3] Boston Children's Hospital, Division of General Pediatrics, Boston, USA; [4] Brigham and Women's Hospital, Divisions of Allergy and Clinical Immunology and General Internal Medicine and Primary Care, Department of Medicine, Boston, USA; [5] Harvard Medical School, Boston, USA

Objective: Investigating the feasibility of using municipal data and electronic health records (EHR) to predict the risk of poor housing conditions.
Material and Methods: Tenant reports of poor housing conditions and tax assessor data were obtained from the Analyze Boston website. Adult asthma-related ED visits were obtained from the Massachusetts General Brigham EHR. Results of physical inspections in US Department of Housing and Urban Development (HUD) subsidized housing Feb 2013-Dec 2018 were obtained from the HUD website. We calculated population rate of asthma-relevant and non-asthma relevant tenant reports and asthma ED visits linked to the tax parcel of the property 24 months prior to each inspection. In addition to these three features, the models used the percent change of assessed value between 2013 and 2018. The binarized inspection result (pass or fail) was used as outcome. Inspections before Jan 2018 were used as training data and after were used as test data. Random forest and SuperLearner models in R were used.
Results: We included 580 inspections at 303 parcels in the full sample, consisting of 461 and 119 inspections in training and test data respectively. We also introduced a reduced sample that only included parcels with non-zero asthma ED patient, which included 274 inspections at 162 parcels, consisting of 219 and 55 inspections in training and test data. In the full sample, random forest yielded a result of AUC=0.670 in the test data, and SuperLearner showed similar results (AUC=0.674). Both random forest and SuperLearner performed better in the reduced sample, giving 0.753 and 0.761 of AUC, respectively.
Conclusion: By integrating municipal and EHR data and into machine learning models, we were able to predict failed HUD inspections. Our results on the restricted dataset showed that where the asthma ED visit data is available, the performance of the models improved.

P02-09 | Development of a predictive model for $PM_{2.5}$ over the greater Athens metropolitan area, Greece at 1 km x 1 km grid resolution using satellite measurements and machine learning methods.

Seyong Chang[1], Evangelia Samoli[2], Evangelina Diapouli[3], Petros Koutrakis[1]

[1] Harvard T. H. Chan School of Public Health, Department of Environmental Health, Boston, USA; [2] National and Kapodistrian University of Athens School of Medicine, Department of Hygiene, Epidemiology and Medical Statistics, Athens, Greece; [3] National Centre for Scientific Research Demokritos, Athens, Greece

Objective: Ambient $PM_{2.5}$ exposure is strongly associated with numerous adverse health effects, including all-cause mortality. However, the lack of monitoring networks globally necessitates a better understanding of the spatiotemporal distribution of near-surface $PM_{2.5}$ pollution to better assess risks to the general population and vulnerable groups. While previous studies in the Mediterranean have primarily used land use regression models, this study aims to demonstrate the effectiveness and feasibility of ensemble machine learning methods for estimating $PM_{2.5}$ at a high spatiotemporal resolution, using Athens, Greece (from 2007-2019) as a case study.
Material and Methods: Daily data from 15 pollution monitors were collected and combined with meteorological and satellite datasets, including MERRA-2, MODIS-Terra/Aqua, and the Copernicus Land Monitoring Service, spanning the years 2007–2019. The final model adjusted for a total of 53 variables related to aerosols, meteorology, and land use. To predict daily $PM_{2.5}$ concentrations, a stacked ensemble machine learning model was implemented, integrating distributed random forest, gradient boosting, and neural networks.
Results: Cross-validation indicated strong predictive performance, with a R^2 of 0.89 and an average error (RMSE) of 3.51 μg/m^3. The annual average $PM_{2.5}$ predictions was 22.3 μg/m^3, which is higher than current US and WHO advisory guidelines, with considerable spatiotemporal differences in $PM_{2.5}$ across Athens. The highest annual $PM_{2.5}$ concentrations were in 2007 (33.18 μg/m^3) and on average, $PM_{2.5}$ concentrations were highest during the months of November to February, which is consistent with seasonal variations in $PM_{2.5}$.
Conclusions: This is the first study to estimate $PM_{2.5}$ exposures in Athens at a high spatiotemporal resolution using diverse measurements and land use data that will enable the investigation of health effects, exposures, and the development of regional environmental policies and may be used in other regions with limited spatiotemporal monitoring networks. This study was supported by funding from the Harvard-Cyprus Endowment Program.

P02-10 | GPS-based mobility and associated personal exposure to fine particles

Goeun Jung[1], Won Kyung Kim[1], Soriul Kim[3], Miji Kim[4], Chol Shin[3], Seung-Hyun Cho[5], Sun-Young Kim[1]

[1] National Cancer Center, Public Health & AI, Goyang-si, South Korea; [2] National Cancer Center, Public Health & AI, Goyang-si, South Korea; [3] Korea University, Institute of Human Genomic Study, College of Medicine, Seoul-si, South Korea; [4] Kyung Hee University, Department of Biomedical Science and Technology, Graduate School, Seoul-si, South Korea; [5] RTI International, Durham, USA

Exposure to ambient fine particulate matter ($PM_{2.5}$) has been identified as a major risk factor of various health issues. Since individuals spend most time indoors and move across locations, outdoor concentrations may not reflect actual personal exposure. Global Positioning System (GPS) data can help capture mobility patterns of individuals which affect personal exposure to $PM_{2.5}$. This study aims to explore the variations in personal exposure based on individual mobility using two mobility metrics: Radius of Gyration (RoG) and Entropy.

We measured 10-second personal $PM_{2.5}$ concentrations from 52 elderly residents in South Korea who carried bags equipped with RTI MicroPEMs for approximately five days across four seasons. Concurrently, GPS devices attached to the same bags recorded movement trajectories and allowed us to compute RoG and Entropy. RoG represents the spatial dispersion of an individual's movement trajectory, calculated as the average distance between all locations visited and the central location, which is defined as the weighted mean of all visited locations. Entropy indicates the complexity of an individual's mobility pattern and is quantified as the sum of time fractions weighted by their log-transformed values across visited locations. Using daily average of $PM_{2.5}$, RoG, and Entropy, we explored the relationship between personal $PM_{2.5}$ concentrations and mobility patterns.

The averages of daily $PM_{2.5}$ concentrations, RoG, and Entropy were 13.3 (SD = 8.1) µg/m3, 2.5 (6.5) km, and 1.8 (1.1), respectively. $PM_{2.5}$ concentrations increased within a 1 km of RoG and flattened afterwards. Entropy showed no clear patterns with personal $PM_{2.5}$ concentrations.

This study suggests the positive association of mobility boundary and $PM_{2.5}$ concentrations within a few kilometers that possibly indicate the impact of daily-basis local pollution sources such as traffic. Future research should identify specific sources related to individual mobility patterns and exposure to air pollution.

P02-11 | Geostationary satellite meets deep learning techniques: Hourly Pollution Monitoring and Public Co-Exposure Assessment

Eunjin Kang[1], Sihun Jung[1], Jungho Im[1], Hyunyoung Choi[1], Soomin Hwang[1], Cheolhee Yoo[2], Julian Marshall[3], Deoksu Kim[4,5], Jhoon Kim[6], Sang-Min Kim[7]

[1] Ulsan National Institute of Science and Technology, Department of Civil Urban Earth and Environmental Engineering, Ulsan, South Korea; [2] The Hong Kong Polytechnic University, Department of Land Surveying and Geo-Informatics, Hung Hom, Hong Kong; [3] University of Washington, Department of Civil and Environmental Engineering, Seattle, USA; [4] Korea Institute of Ocean Science and Technology, Marine Natural Disaster Research Department, Busan, South Korea; [5] University of Science and Technology, Department of Ocean Science, Daejeon, South Korea; [6] Yonsei University, Department of Atmospheric Sciences, Seoul, South Korea; [7] National Institute of Environmental Research, Environmental Satellite Centre, Climate and Air Quality Research Department, Incheon, South Korea

Monitoring multiple air pollutants simultaneously is crucial for understanding their spatial distribution and combined health effects. However, accurately predicting multiple pollutants remains challenging due to limitations in spatial and temporal data availability. To address this, we developed the Multiple Air Pollutants Network (MAPNET), a deep learning framework that integrates multiple data sources through a multitasking, multi-branch structure and image-based modeling to enhance pollutant mapping at a 10-km resolution. MAPNET effectively captured transboundary pollution transport, including severe dust storms from 2021 to 2023, and outperformed global operational chemical transport model in tracking hourly particulate matter (PM) dispersion over marine areas. By analyzing both short- and long-term exposure, we found that an estimated 158 million, 158 million, and 191 million individuals in densely populated regions of East China are chronically exposed to PM10, PM2.5, and O3, respectively. Across East Asia, annual co-exposure to pollutant pairs such as PM10-PM2.5, PM-NO2, and PM-SO2 is widespread, with hotspots particularly concentrated near coal power plants, limestone quarries, and industrial sites. Exposure to four or more pollutants is notably prevalent in these high-emission regions. Our findings underscore the urgent need for targeted mitigation strategies to address multi-pollutant exposure and its potential health risks.

P03 | Biomarkers of Environmental Exposures and Diseases

P03-01 | Urinary metals trends in the Strong Heart Family Study: 2001-2024

Carolyn Gigot[1], Annie Nigra[1], Kathrin Schilling[1], Ronald Glabonjat[1], Anirban Basu[1], Whitney Collado[1], Walter Goessler[2], Ying Zhang[3], Jason Umans[4], Amanda Fretts[5], Shelley Cole[6], Ana Navas-Acien[1], Allison Kupsco[1]

[1] Columbia University, Environmental Health Sciences, New York, USA; [2] Universitat Graz, Institute of Chemistry, Graz, Austria; [3] University of Oklahoma Health Sciences Center, Center for American Indian Health Research, Department of Biostatistics and Epidemiology, Oklahoma City, USA; [4] MedStar Health Research Institute, Georgetown/Howard Universities, Center for Clinical and Translational Sciences, Washington, DC, USA; [5] University of Washington School of Public Health, Epidemiology, Seattle, USA; [6] Texas Biomedical Research Institute, Population Health, San Antonio, USA

Objective

While exposure to several metals has declined in recent years among the general US population, less is known about changes among American Indian populations. Our objectives were to estimate changes in urinary metals levels in Strong Heart Family Study (SHFS) participants over the past 20 years and identify subgroups with greater changes.**Material and Methods**

The SHFS has followed men and women from 12 American Indian tribes and communities across Arizona, Oklahoma, South Dakota, and North Dakota since the early 2000s. We measured 9 metals (essential: molybdenum, selenium, zinc; nonessential: arsenic, cadmium, lead, antimony, tungsten, uranium) in urine at SHFS visit 4 (2001-2003, n=2309), 5 (2006-2009, n=289), and 7 (2019-2024, n=894). We used linear mixed effects models to estimate percent change of each creatinine-adjusted urinary metal from visit 4 to 5 and 4 to 7 and to evaluate differences in changes by participant sex, age, site, smoking, BMI, and diabetes status.**Results**

Nonessential metals declined from visit 4 to 5 (from 21% for arsenic to 66% for lead) as well as 4 to 7 (from 29% for arsenic to 78% for lead), except for cadmium, which did not change from 4 to 5 and increased 21% from 4 to 7. Of essential metals, selenium increased; molybdenum did not change from 4 to 5 and increased from 4 to 7; and zinc decreased. Lead, antimony, and zinc decreases were greater for Oklahoma versus North and South Dakota participants. Arsenic and tungsten decreases from 4 to 7 were greater for Arizona versus North and South Dakota participants. Cadmium increases from 4 to 7 were greater for smokers.**Conclusion**

Except for cadmium, nonessential urinary metals concentrations have declined over the past 20 years in the SHFS. Essential metals have not experienced the same uniform changes, which is expected given their physiological regulation.

P03-02 | Sociodemographic and environmental predictors of urinary concentrations of metabolism-disrupting metals among pregnant women in the Healthy Start cohort

Celeste Connell[1], Dorothy Nakiwala[1], Fátima Pacheco Vásquez[1], Oniza Chaman[1], Jared Brown[2], Stephen Brindley[2], Dana Dabelea[1], Anne Starling[1,3]

[1] University of Colorado Anschutz Medical Campus, Lifecourse Epidemiology of Adiposity and Diabetes (LEAD) Center, Colorado School of Public Health, Aurora, USA; [2] University of Colorado Anschutz Medical Campus, Department of Pharmaceutical Sciences, Skaggs School of Pharmacy and Pharmaceutical Sciences, Aurora, USA; [3] University of North Carolina at Chapel Hill, Department of Epidemiology, Gillings School of Global Public Health, Chapel Hill, USA

Objective: Obesity, metabolic syndrome, and insulin resistance are increasingly prevalent and have been linked to environmental exposures, particularly metals that may disrupt metabolism. This study aims to identify predictors of exposure to several metabolism-disrupting metals in the Denver, Colorado-based Healthy Start pregnancy cohort.
Materials and Methods: Participants were 442 women who provided spot urine samples in mid-pregnancy (2009-2014). Metal concentrations (arsenic, cadmium, cesium, copper, and lead) in urine were quantified using Inductively Coupled Plasma Mass Spectrometry (ICP-MS) and adjusted for urinary creatinine. Metals with <15% of values below the limit of detection (arsenic, cesium, copper) were log-transformed and treated as continuous, while others were binary (at or above versus below the limit of detection). Unadjusted linear and logistic regression models assessed the relationship between sociodemographic and environmental factors and urinary metal concentrations.
Results: Higher maternal age, education, and household income were positively associated with cesium, while higher maternal pre-pregnancy body mass index was inversely related to cesium. Non-Hispanic white participants had 26% higher cesium concentrations than other racial and ethnic groups. Higher traffic density within a 50-meter and 100-meter radius of the residential address was positively associated with detectable cadmium concentrations. Urinary arsenic concentrations were highest in the group of participants who identified as Asian, American Indian, Alaska Native, Native Hawaiian, or Other Pacific Islander, with levels 69% higher than other racial and ethnic groups.
Conclusion: We identified sociodemographic and environmental factors that predict urinary concentrations of metabolism-disrupting metals in a US-based cohort of pregnant women, which may provide insight into preventable sources of exposure in this population. Future research will examine how these metals affect metabolic health in both pregnant women and their infants, emphasizing the need to consider environmental and socioeconomic contexts when assessing metal exposures during pregnancy.
Funding: National Institutes of Health (R01DK076648 and R01ES032213).

P03-03 | Effects of Exposure to Volatile Organic Compounds and Polycyclic Aromatic Hydrocarbons on high-sensitivity C-reactive protein Levels and Chronic Disease Risk

Haneul Kim[1], Yong Min Cho[1], Dahee Kim[1], Mijeong Choi[1], Kyung Hwa Choi[2], Woo Jin Kim[3], Young-Seoub Hong[4]

[1] Seokyeong University, Institute of Environmental Health, Seoul, South Korea; [2] Dankook University College of Medicine, Department of Preventive Medicine, Cheonan, South Korea; [3] Kangwon National University, Department of Internal Medicine and Environmental Health Center, Chuncheon, South Korea; [4] Dong-A University College of Medicine, Department of Preventive Medicine, Busan, South Korea

Objective: Exposure to volatile organic compounds (VOCs) and polycyclic aromatic hydrocarbons (PAHs) has been well-documented to increase the risk of inflammation and chronic diseases. This study aimed to investigate the association among organic compound exposure, high-sensitivity C-reactive protein (hs-CRP) levels, and chronic disease prevalence.

Materials and Methods: A total of 110 urine samples were collected from residents near a smelter in Korea in February 2022. Participants provided self-reported their disease prevalences. Four urinary metabolites of PAHs were analyzed using gas chromatography-mass spectrometry, and four metabolites of VOCs were analyzed using liquid chromatography-mass spectrometry. All variables were natural log-transformed, and covariates such as age, sex, income, occupational exposure, smoking, and alcohol consumption were adjusted. General linear regression analysis was conducted to examine the association between organic compounds and hs-CRP levels. Additionally, logistic regression analysis was conducted to assess the relationship between hs-CRP levels and chronic disease prevalence.

Results: Urinary concentrations of trans, trans-muconic acid and methylhippuric acid significantly elevated hs-CRP levels (β=0.286, p value=0.047; β=0.132, p value=0.031, respectively). Otherwise, urinary metabolites of PAHs did not show such associations. In logistic regression, the risk of cardiovascular disease (CVD) was significantly increased with hs-CRP (OR=1.908, 95% CI: 1.040, 3.501).

Conclusion: Exposure to organic compounds was associated with inflammation, which in turn increases the risk of CVD. Our findings underscore the urgent need for regulatory measures to mitigate exposure to organic compounds among residents in polluted areas, such as smelting regions.

Funding: This work was supported by Korea Environmental Industry & Technology Institute (KEITI) through Core Technology Development Project for Environmental Diseases Prevention and Management Program, funded by Korea Ministry of Environment (MOE)(2480000101/RS-2021-KE001378).

P03-04 | Investigating Astrocyte-Derived Extracellular Vesicles as Novel Biomarkers of Metal Exposure in Veterans with Amyotrophic Lateral Sclerosis (ALS)

Tanya H. Butt[1], Lina Marcinczyk[1], Grace Katz[1], Cherine Jaafar[1], Roheeni Saxena[1], Elijah Stommel[3], Brian Jackson[2], Marianthi-Anna Kioumourtzoglou[1], Diane Re[1]

[1] Columbia University, Environmental Health Sciences, New York, USA; [2] Dartmouth College, Earth Sciences, Chemistry, Hanover, USA; [3] Dartmouth Hitchcock Medical Center, Neurology, Lebanon, USA

Amyotrophic lateral sclerosis (ALS) is the most common adult-onset paralytic disorder worldwide. Multiple epidemiological studies have identified associations between environmental risk factors and ALS, although few have evaluated molecular biomarkers of exposures to persistent neurotoxicants, like metals. Currently, there are no accurate, non-invasive biomarkers of central nervous system (CNS) metal burden. Circulating extracellular vesicles, derived from astrocytes (ADEVs), may address this gap, due to the key role of astrocytes in metal homeostasis. We hypothesize that metal levels in ADEVs may be indicative of CNS metal burden in veterans with ALS.
The US Veterans Affairs Biorepository provided samples of motor cortex (n=145), lumbar spinal cord (n=145), and plasma (n=85) from veterans with ALS. ADEVs were isolated from plasma in metal-free conditions via immunoprecipitation using a biotinylated antibody against GLAST (Miltenyi Biotec, Cat#130-132-738). In ALS veterans (n=10) and age- and sex-matched veteran controls (n=10), motor cortex and spinal cord samples were screened for 53 elements using ICP-MS. 19 elements were subsequently screened in the remaining tissue and ADEV samples. Student's t-tests were used to compare mean metal levels in ALS vs. controls. Four linear regression models, adjusted for age, evaluated whether individual ADEV metal levels predicted cortex and spinal cord metal levels, and whether plasma metal levels predicted cortex and spinal cord metal levels.
Magnesium and zinc were increased, and cooper was decreased, in ALS cortex and spinal cord compared to controls (Mg: ALS 130.8±10.5, Control 119.71±19.5 PPM). Preliminary findings for magnesium and cooper show negative associations between ADEVs and cortex and spinal cord. The variation in ADEV metal levels also seems to account for more of the variation of CNS tissue metal levels compared to plasma.
This study can provide novel insight into the utility of neural EV-based methods for evaluating exposures and improving our understanding of metal metabolism in ALS.

P03-05 | Abdominal MRI adipose tissue depots and mitochondrial DNA copy number in adolescents

Pei Wen Tung[1], Dympna Gallagher[2], Wei Shen[2], Eliza Kinsey[3], Tessa Bloomquist[1], Andrea Baccarelli[1], Frederica Perera[1], Julie Herbstman[1], Andrew Rundle[4], Allison Kupsco[1]

[1] Columbia University, Department of Environmental Health Sciences, New York, USA; [2] Columbia University Irving Medical Center, Department of Medicine, New York, USA; [3] University of Pennsylvania, Department of Family Medicine and Community Health, Philadelphia, USA; [4] Columbia University, Department of Epidemiology, New York, USA

Objective: Increased abdominal adipose tissue (AT) is related to increased risk for obesity and metabolic diseases. A frequently used measure of weight status, body mass index, does not reflect fat quantity or fat distribution or capture

metabolic risk. Abdominal magnetic resonance imaging (MRI) is a non-invasive technique that provides high-resolution, cross-sectional images of abdominal AT distribution. Several environmental pollutants have been associated with increased obesity risk, and mitochondrial DNA copy number (mtDNAcn) may serve as a key mediator of these effects. mtDNAcn alternations are associated with mitochondrial dysfunction, which is implicated in various metabolic diseases. However, mtDNAcn relationship with AT quantity, especially in adolescents, remains understudied. This study aims to explore whether mtDNAcn levels are associate with abdominal AT distribution via MRI measurements in adolescents.
Material and Methods: Abdominal MRI measurements were obtained to quantify AT volume in visceral, subcutaneous, and intra- and inter-muscular adipose tissue depots from a predominantly African American and Dominican cohort in Northern Manhattan (mean = 17.3 years). mtDNAcn relative to nuclear DNA in blood was quantified through qRT-PCR. Sociodemographic and lifestyle (e.g., food insecurity and physical activity) information were included as covariates. We used multivariable linear regression models, with $\log_2$-transformed MRI measurements, to evaluate the association between mtDNAcn and AT volume (N=251).
Results: With every doubling in mtDNAcn, we observed a significant relative increase in abdominal intra- and inter-muscular AT volume by 69% (β=1.69, 95% CI: [1.11, 2.56], p=0.01) and visceral AT volume by 31% (β=1.31, [1.00, 1.72], p=0.05). No association was observed between mtDNAcn and abdominal subcutaneous AT.
Conclusion: By combining imaging-based body composition data with mtDNAcn, this research could contribute to a deeper understanding of obesity risk and potentially offer novel biomarkers for early metabolic disease diagnosis in adolescents.

P03-06 | Associations between individual and mixtures of urinary trace element concentrations with odds of uterine fibroid prevalence among African-Caribbean women in the Tobago Health Study

Alexis Kiyanda[1], Natalie Price[2], Ryan Cvejkus[2], Janet Catov[2], Victor Wheeler[2,3], Patrick J. Parsons[4,5], Aaron Barchowsky[1], Iva Miljkovic[2], Alison P. Sanders[1]

[1] University of Pittsburgh School of Public Health, Department of Environmental and Occupational Health, Pittsburgh, USA; [2] University of Pittsburgh School of Public Health, Department of Epidemiology, Pittsburgh, USA; [3] Tobago Health Studies Office, Scarborough, Trinidad & Tobago; [4] New York State Department of Health, Wadsworth Center, Division of Environmental Health Sciences, Albany, USA; [5] University of Albany School of Public Health, Department of Environmental Health Sciences, Albany, USA

Uterine fibroids are benign smooth muscle neoplasms of the uterine wall. While common risk factors include African Ancestry, family history, obesity and early onset menstruation, exposure to metals may pose additional unknown risk. The objective of this cross-sectional study, within the Tobago Women's Health Study, was to assess associations between individual urinary concentrations of trace elements and metal mixtures with uterine fibroid prevalence.
444 women had complete information on the history of uterine fibroid(s) and urinary trace element measurements collected between 2019-2023. We used logistic regression to estimate associations between concentrations of six prioritized elements (arsenic, cadmium, lead, copper, manganese and zinc) individually and as a mixture with odds of uterine fibroids. Models were adjusted for urinary creatinine, age, BMI and age of menarche. We assessed metal mixture models in negative and positive directions using weighted quantile sum (WQS) regression.
The mean age was 55.7 years; the mean BMI was 31.02 kg/m^2; and the mean age of menarche was 12.8 years. The prevalence of uterine fibroids was 48.4%. In adjusted models, each ten-fold increase in ng/mL urinary concentration

of cadmium (β:2.25; 95% CI: 1.01, 5.08) and arsenic (β:1.65; 95% CI: 1.07, 2.57) corresponded to higher odds of uterine fibroids. Urinary concentrations of lead, copper, manganese, and zinc were not associated with odds of uterine fibroids. The metal mixture model observed no significant associations between metal mixtures and odds of uterine fibroids (β_{nwqs}:0.99; 95% CI: 0.87, 1.14); (β_{pwqs}:1.10; 95% CI: 0.96, 1.26).
This study is important to better understand the relationship between essential and non-essential trace elements and women's health in a cohort of African-Caribbean women. Further research must confirm these findings and assess biological mechanisms of cadmium and arsenic in pathogenesis of uterine fibroid.
Acknowledgements: Supported by RustToResilience (R2R) Environmental-Chemical Research Center; NIDDK: R01-DK097084, R01-HL143793; NIH: U2CES026542, NIH U2CES026555.

P03-07 | Advanced Biomonitoring of Diisocyanate Exposures – Achieving Increased Sensitivity by Minimizing Background Contaminations

Oliver Lerch, Ina Hüsecken, Stephan Koslitz, Stefanie Kösling, Nina Reichelt, Thomas Brüning, Heiko U. Käfferlein

Ruhr University Bochum, Institute for Prevention and Occupational Medicine of the German Social Accident Insurance, Bochum, Germany

Objective
Human biomonitoring of diisocyanate exposures is carried out by analyzing diisocyanate-associated diamines in urine. Currently, due to limited sensitivities, biomonitoring methods are almost entirely restricted to workplace settings. Here, we present an improved method with increased sensitivities and the associated challenges and solutions we encountered during method development.
Material and Methods
Analysis of 6 diisocyanate-associated diamines (11 compounds/isomers in sum), among them hexanediamine (HDA), toluenediamine (TDA) and methylenedianiline (MDA), was carried out by GC-NCI-MS/MS. Urine samples were treated with hydrochloric acid under heating and the analytes were extracted by toluene and derivatized using heptafluorobutyric acid anhydride (HFBA). Focus was on minimizing sources of background contamination in the laboratory setting and on optimizing key MS parameters.
Results
Multiple background contamination sources in terms of diisocyanate-specific diamines in laboratory chemicals and materials were identified, e.g., in selected septum caps, vials, solvents and HFBA batches. Once eliminated (or reduced), the limits of quantification for the diamines were between 0.05 and 0.1 µg/L with upper calibration limits of 5 or 10 µg/L. The recoveries in spiked urine samples with different creatinine values were between 80 and 120%. The imprecisions were <10%. Ultimately, an external proficiency test was successfully passed.
Conclusion
Our results show ubiquitous and significant contamination sources of diisocyanate-specific diamines in laboratory settings that require individually tailored "search and eliminate" strategies to correctly assess diisocyanate exposures by biomonitoring at low exposure levels. The method presented here is suitable for routine use and is currently applied in a large cohort study of diisocyanate exposed workers and non-exposed controls.
Funding
European Trade Association for Producers of Diisocyanates and Polyols (ISOPA)

Federal Institute for Occupational Safety and Health (BAuA)
Several German Social Accident Insurances

P03-08 | Comprehensive Analysis of Environmental Toxic Metal Exposure and Biomarker Outcomes

Rezaul Karim Ripon[1,2], Narayana G Prasad[3], Mayra Volquez[3], Sujata Saunik[4], Satish Govind[5], Srikanth Sola[6], Rajesh Melaram[7]

[1] Harvard T H Chan School of Public Health, Environmental Health, Boston, USA; [2] McHigher Centre For Health Research, Environmental Epidemiology, Dhaka, Bangladesh; [3] Public Health Literacy/BWH, Department of Cardiology, Miami, USA; [4] Harvard T H Chan School of Public Health, Takemi Fellow, Boston, USA; [5] Narayana Institute of Cardiac Sciences, NH Health City, Department of Cardiology, Bengaluru, India; [6] Devic Earth, Founder, Bengaluru, India; [7] Texas A&M University, College of Nursing and Health sciences, Texas, USA

Background: Environmental exposure to lead, mercury, and cadmium poses significant adverse health effects, including cardiovascular diseases. This study examines the relationships between these metal exposures and biomarker outcomes.

Objectives: The aim of this study is to investigate the associations between blood levels of lead, mercury, and cadmium and biomarkers, including systolic and diastolic blood pressure, glucose, cholesterol, hs-CRP (high-sensitivity C-reactive protein), and ferritin levels.

Methods: Data from the National Health and Nutrition Examination Survey was utilized for this analysis. Structural Equation Modeling assessed the direct and indirect relationships between the metals and biomarkers outcomes.

Results: The study included males (49.11%) and females (50.89%), with a mean age of 44.13 years. The mean levels of exposure were as follows: lead (1.07453 µg/dL), mercury (1.13737 µg/L), and cadmium (0.43557 µg/L). Significant associations were identified: Lead was positively associated with systolic blood pressure ($\beta = 0.141$, $p < 0.001$) and cholesterol ($\beta = 0.063$, $p = 0.001$); Mercury was positively associated with cholesterol ($\beta = 0.112$, $p < 0.001$) and ferritin ($\beta = 0.073$, $p < 0.001$); Cadmium was associated with higher systolic ($\beta = 0.094$, $p < 0.001$) and diastolic blood pressure ($\beta = 0.079$, $p < 0.001$), as well as hs-CRP ($\beta = 0.064$, $p < 0.001$).

Conclusion: Based on this study, environmental metal exposures lead to change in multiple biomarkers such blood pressure, glucose, cholesterol, CRP and ferritin. Future research should focus on understanding these exposures' mechanistic pathways and long-term health impacts.

P03-09 | Exposure of the U.S. Population to Ethylene Oxide in the National Health and Nutrition Examination Survey 2013–2020

Enada Archibold, Liqun Wang, Alec Ricciuti, Khadija Omri, Tunde Frame, Hubert Vesper

Centers for Disease Control and Prevention, National Center for Enironmental Health/Division of Laboratory Sciences, Atlanta, USA

Objective: Ethylene oxide (EO) is an industrial and environmental chemical classified by the International Agency for Research on Cancer (IARC) as a human carcinogen (Group 1) and categorized as ‘known to be a human carcinogen’ by the National Toxicology Program (NTP). Exposure to EO is mainly via occupation, tobacco smoke, and endogenously. This study assessed continued human EO exposure in the U.S. general population.
Material and Methods: Specimen were obtained from the National Health and Nutrition Examination Survey (NHANES) 2013-2020 cycles, a nationally representative survey of the non-institutionalized civilian population of the U.S. Hemoglobin adducts of ethylene oxide (HbEO) were measured in hemolyzed erythrocytes samples from 8213 individuals ≥ 6years old using the CDC’s method for measurement of HbEO by ultra-high performance liquid chromatography-tandem mass spectrometry.

Results: Data obtained were stratified by smoking status. HbEO concentrations ranged from non-detectable (<LOD, 8.20 pmol/gHb) to 1460pmol/gHb. Exposure is elevated in smokers (bimodal frequency distribution), and the smoker and non-smoker distributions overlap (i.e., 5.8-41.5 pmol/gHb and 12.4-473.0 pmol/gHb in non-smokers and smokers, respectively). In non-smokers, HbEO levels in males were 6.8% higher than females, while it is 22% higher female smokers than male. Multiple regression models were developed with HbEO as the dependent variable and race/ethnicity, cotinine, BMI, alcohol consumption, sex, and A1c as independent variables which demonstrated nonsignificant-to-significant associations in both smokers and non-smokers.

Conclusions: This study demonstrates that smoking significantly contributes to higher HbEO levels. Sex, age, race/ethnicity, BMI, alcohol consumption, and A1c were also impacted HbEO levels. More investigations are needed to determine other factors that influence HbEO in the body (e.g., genetics) and additional endogenous and exogenous EO exposure routes. Understanding where exposures originate can lead to reduction in, or better management of, human exposure to EO, and thus potentially result in improvements to public safety and disease prevention.

P03-10 | Exposures to phthalate and phthalate replacements among women of childbearing age in a Great Plains Tribal community.

Lesliam Quiros-Alcala[1], Deborah Kanda[2], Rae O'Leary[3], Britanee Youpee[3], Elizabeth Z. Lin[4], Jean Zhou[4], Ana M. Rule[1], Krystal Godri Pollitt[4], Esther Erdei[5]

[1] Johns Hopkins University, Department of Environmental Health and Engineering, Baltimore, USA; [2] University of New Mexico, UNM Comprehensive Cancer Center, Albuquerque, Germany; [3] Missouri Breaks Industries Research Inc., Timber Lake, USA; [4] Yale University, Department of Environmental Health Sciences, New Haven, USA; [5] University of New Mexico, College of Pharmaceutical Sciences, Albuquerque, USA

Background: Endocrine disrupting chemicals (EDCs), like phthalates and their replacements, are ubiquitous in consumer products, including personal care products (PCPs). These EDCs may pose health risks across the life course, including reproductive health. Data on Tribal community women's exposure to these EDCs is scarce, hindering targeted interventions for this at-risk population.

Objective: We assessed exposures to phthalates and their replacements in residential air and urine samples from childbearing-age women in a Great Plains Tribal community and assessed exposure correlates.**Methods:** Twenty women between 18-45 years completed a questionnaire for information on demographics and personal behaviors (consumer product use, work, hobbies, and diet). Participants wore FreshAir badges to passively sample EDCs over one week and provided a urine sample on the last monitoring day. We estimated correlations between parent EDCs in badges and respective biomarkers and assessed exposure correlates using biomarker concentrations and questionnaire data in bivariate analyses.

Results: Detection frequencies for chemicals detected in air badges ranged between 5 and 100% and 15 and 100% in urine samples. Median airborne concentrations for DEP, BBzP, and DBP were 5-10 times higher in participants than U.S. women. Correlations between parent EDCs in air and respective biomarkers ranged between r=0.17 and r=0.33. Increased consumption days were associated with elevated concentrations of phthalates and/or replacements in women who consumed processed meats (MBzP/p=0.006), used air fresheners (CX-MINCH/p=0.04,OH-MINCH/p=0.004), burned medicinal plants (mBP=/p=0.03,MCNP/p=0.05,MCPP/p=0.02), and used gas or propane heaters (MNP/p=0.005,MCNP/p=0.03). PCP use was linked with elevated biomarker concentrations [e.g., hair spray (MECPP/p=0.03,CX-MINCH/p=0.04), hair sheen (CX-MINCH/p=0.01,MCOP/p=0.02), detangler (MEP=0.012,MiBP=0.02), hair dye (MECPTP,MCNP/p=0.04; MEP/p=0.01), facial cleanser (CX-MINCH/p=0.08, MECPTP/p=0.03,MEHHTP/p=0.02,CNP/p=0.002,MCOP/p<0.001,MCPP/p=0.01), shower gel (DEHP/p=0.03,MECPTP/p=0.05), tooth whitener (MBP,MBzP/p=0.01, tampons (DEHP/p=0.05,MCPP/p=0.04, MEP/p=0.007) and hand soap (OH-MINCH/p<0.001)].

Conclusions: Findings confirm widespread exposures to phthalates and their replacements, supporting larger studies that inform culturally sensitive interventions critical to improving Native American women's health across the life course.

P03-12 | Associations of individual and mixture exposure to volatile organic compounds with renal insufficiency in the Korean populations

Uhyeon Han[1], Heeseon Jang[2,3], Hyunah Son[1], Changsoo Kim[2,3]

[1] Graduate School, Yonsei University, Department of Public Health, Seoul, South Korea; [2] Yonsei University College of Medicine, Department of Preventive Medicine, Seoul, South Korea; [3] Yonsei University, Institute of Human Complexity and Systems Science, Incheon, South Korea

Background: Volatile organic compounds (VOCs), may contribute to renal dysfunction, but evidence in the general population is limited. Therefore, we aimed to investigate the association between exposure to VOCs and the risk of renal insufficiency in the general population.
Methods: This cross-sectional study included 1405 Korean adults without kidney disease selected from the Korea National Health and Nutrition Examination Survey conducted (2020–2022). Nine urinary VOCs metabolites were quantified using liquid chromatography with tandem mass spectrometry. Renal insufficiency was defined as an estimated glomerular filtration rate below 60mL/min/1.73m2 as determined by the Modification of Diet in Renal Disease equation, or Albumin-to-Creatinine Ratio exceeding 30mg/g. The associations between exposure to individual VOC and a mixture of VOCs and the risk of renal insufficiency were examined using multiple logistic regression and the Weighted Quantile Sum (WQS) model, respectively.
Results: Among 1405 participants, 117 individuals (8.33%) were identified to have renal insufficiency. A one-unit increase in the log-transformed 2-Methylhippuric acid (2-MHA) (Odds ratio [OR]=1.60, 95% confidence interval [CI]: 1.07–2.40), 3-and 4-Methylhippuric acid (3-MHA+4-MHA) (OR=1.62, 95% CI: 1.15, 2.29) and N-Acetyl-S-(3,4-dihydroxybutyl)-L-cysteine (DHBMA) (OR=3.10, 95% CI: 1.18, 8.17) metabolites were associated with an increased risk of renal insufficiency. The findings from the WQS analysis indicated that the urinary VOCs mixture was positively associated with an increased risk of renal insufficiency (OR=1.37, 95% CI: 1.15– 1.63). Ethylbenzene and styrene were determined to be the most prominent chemicals associated with renal insufficiency.
Conclusion: This study found that exposure to specific VOC or VOCs mixture is associated with an increased risk of renal dysfunction in the general population.
Acknowledgements*: This work was supported by Korea Environment Industry & Technology Institute(KEITI) through Core Technology Development Project for Environmental Diseases Prevention and Management, funded by Korea Ministry of Environment(MOE)(grant No.2022003310011), and by Smart HealthCare Program(www.kipot.or.kr) funded by the Korean National Police Agency(KNPA, Korea) [Project Name: Precision & Personalized Medicine for Predicting Cardiovascular Disease Risk in Police Officers (4Ps study) / Project Number: 220222M02].*

P03-13 | Respiratory Virus Season Surveillance in the United States Using Wastewater Metrics, 2023–2024

Elana M.G. Chan, Alexandria B. Boehm

Stanford University, Department of Civil and Environmental Engineering, Stanford, USA

Objective: Wastewater samples from a municipal treatment plant represent an entire contributing population, and measurements of nucleic acids from pathogens in wastewater samples can be available within 24 hours. We aimed to infer seasonal occurrence patterns for common respiratory viruses alongside transmission dynamics for SARS-CoV-2 across the USA using wastewater samples.
Material and Methods: We used wastewater RNA concentrations of influenza A and B (IAV/IBV), respiratory syncytial virus (RSV), human metapneumovirus (HMPV), and SARS-CoV-2 from 175 treatment plants (July 2023–June 2024).

For IAV, IBV, RSV, and HMPV, we determined epidemic onset, offset, peak, and duration at national and subnational scales. For SARS-CoV-2, we categorized wastewater measurements based on recent wastewater levels and trends. Results: Epidemic onset occurred in November for IAV and RSV which aligned with prepandemic norms. Onset occurred in January for IBV and April for HMPV which were later than expected according to historical data. Duration was longer for IAV and shorter for IBV, RSV, and HMPV than expected based on historical data. Epidemic peak dates were consistent with prepandemic norms for all viruses. Peak dates for influenza and RSV coincided with high, upward trending SARS-CoV-2 RNA concentrations.
Conclusion: Seasonal occurrence patterns for influenza, RSV, and HMPV have partially returned to prepandemic tendencies and influenza and RSV may co-occur with SARS-CoV-2 in the future. Wastewater measurements represent a complementary approach to infectious disease surveillance that can improve epidemiological understanding of infectious diseases. Enhanced knowledge about respiratory virus seasonality can improve population health outcomes (e.g., better-timed policies and interventions) and strengthen preparedness against public health threats (e.g., early warning signaling of abnormal virus activity).Acknowledgements: CDC Foundation and the Sergey Brin Family Foundation.

P03-14 | Exploring Causal Links Between Smoking Exposure, Epigenetic Modifications, Altered Gut Microbiota, and Obesity Risk: A Bidirectional Two-Sample, Two-Step Mendelian Randomization Study

Xueqi Wu[1,2], Ye Peng[1,3], Shilin Zhao[3,4], Xi Zhang[5], Hein M. Tun[1,3]

[1] The Chinese University of Hong Kong, The Jockey Club School of Public Health and Primary Care, Shatin, Hong Kong; [2] The Chinese University of Hong Kong, Li Ka Shing Institute of Health Sciences, Shatin, Hong Kong; [3] Microbiota I-Center (MagIC), Shatin, Hong Kong; [4] The Chinese University of Hong Kong, Department of Medicine and Therapeutics, Shatin, Hong Kong; [5] HKU-SIRI, Materials Innovation Institute for Life Sciences and Energy (MILES), Shenzhen, China

Objective
While smoking and obesity are epidemiologically linked, the causality and underlying mechanisms remain elusive. Here we investigated causal relationships and mediation roles of DNA methylation (DNAm) and gut microbiota between smoking and obesity with genetic variants.
Material and Methods
Published data from genome-wide association analysis studies of European ancestry were used. Cross-trait linkage disequilibrium score (LDSC) regression analysis assessed shared genetic architecture between smoking and obesity. Bidirectional two-sample Mendelian Randomization (MR) evaluated causation, adjusting for confounders via multivariable MR. A two-step MR followed to identify potential mediators among 2,084 smoking-related CpGs, 105 microbial species, and 205 microbial pathways. Mediation analysis quantified indirect effects and co-localization analysis probed shared causal variants.
Results
All traits related to smoking and obesity were heritable with significant genetic correlations (all P<0.05). Bidirectional MR revealed smoking initiation and lifetime smoking causally increase body mass index (BMI; b=0.08-0.062, $P<1.8\times10^{-6}$) , and BMI elevated smoking likelihood (OR=1.07-1.13, $P<1.5\times10^{-10}$), with robustness in sensitivity analyses across age/sex-stratified and body fat distribution data. Multivariable MR demonstrated independence from other smoking-related traits and alcohol use ($P<9.1\times10^{-8}$). Seven smoking-related CpGs [e.g. cg08548559 (PIK3IP1),

cg15963913 (ZFYVE21), cg01127300 (TMEM184B)], five microbial species (e.g., *Eubacterium siraeum*) and five microbial pathways (e.g., vitamin B6 biosynthesis), were found to be mediators between smoking and obesity. Notably, smoking may indirectly increase obesity risk by reducing the abundance of *E.siraeum*(proportion mediated: 7.23%), and methylation levels at cg08548559 (64.65%) (P<0.05). Co-localization identified shared causal variants (PPH4 >0.75) and a causal direction from DNAm to microbial alterations was established.

Conclusion

These findings suggest a dynamic, bidirectional crosstalk wherein smoking-induced epigenetic modifications and gut dysbiosis may jointly increase metabolic perturbations. Restoration of *E. siraeum* may thus be targeted to mitigate smoking-aggravated obesity.

Funding

Research Grants Council of Hong Kong SAR (Project No. 14113923 to Dr. Tun).

P03-15 | Exploring Epidemiological, Biomonitoring and Toxicological Data for Toxicity Assessment of Metal-based Chemicals

Custodio V. Muianga

ATSDR/OIA/Toxicology Section, ATSDR/OIA/Toxicology Section, Atlanta, USA

ATSDR/OIA/TS has developed and has regularly updated toxicological profiles (tox profiles) for approximately 400 hazardous substances found at National Priorities List (NPL) sites. Among these tox profiles, approximately 26 pertain to metals, metalloids, and related compounds, including metallic element, metal oxides, and metal sulfides. Most of these metals, metalloids and related compounds are carcinogens and legacy hazardous materials. Metals like nickel have a rich occupational epidemiology database and biomonitoring data; however, both the chronic epidemiological and toxicological data provide a clear noncancer dose-response curve at serious effect level, which does not allow the derivation of a protective minimal risk level (MRL). This research explores a combined use of environmental and occupational epidemiologic and biomonitoring data to innovatively derive health-based guidance values using one of the following methods: (i) biomonitoring guidance values dashboard; (ii) combination of biomonitoring data from the National Health and Nutrition Examination Survey (NHANES) and corresponding chemical specific pharmacokinetic and pharmacodynamic data (i.e., similar to the CDC blood lead reference values for children), and (iii) the combination of biological monitoring data such as the NHANES chemical specific biomonitoring data and existing validated physiologically based pharmacokinetic models (i.e., either forward or reverse dosimetry approaches). These approaches will increase our scientific understanding and awareness on the use of biomarkers of exposure, effect, and susceptibility along with physiologically based pharmacokinetics to derive toxicity values. These approaches have great potential in addressing data needs and gaps for multiple metal mixtures in emerging environmental health hazards such as microplastics and nanomaterials.

P03-16 | Exposure to Heavy Metals During Pregnancy in Residents of Communities Adjacent to Superfund Sites (Mining) in Colorado.

Francesca Macaluso[1], Naomi Perlman[1], Kathrin Schilling[2], Ana Navas-Acien[2], Marc Weisskopf[3], **Katherine James**[1]

[1] University of Colorado, Anschutz Medical Campus, Aurora, USA; [2] Columbia University, New York, USA; [3] Harvard University, Boston, USA

Background: Exposure to heavy metals increases the risk of miscarriage or pre-term birth in pregnant people and can also lead to neurocognitive impairment for infants and young children. The Metal Mixtures: Cognitive Aging and Exposure Sources Remediation Superfund Research Center's Community Engagement Core is conducting a community-engaged research study to characterize metal exposures in these populations in the San Luis Valley.

Appropriateness of Methods: We used community-engaged research methods to collect longitudinal environmental (water, soil) and biological (urine, fingernails) samples from pregnant persons and newborns living in communities adjacent to Superfund sites (predominantly mining) in Colorado. Biological sample collection over the course of pregnancy allows for a more in-depth characterization of the impact of maternal exposure on infant biological burden.

Presentation of Results: Our results strengthen the concerns that people residing in fence line areas of Superfund sites are exposed to elevated levels of metals through water, dust, aerosolized soil, and PICA (young children). Maternal participants had elevated levels of urinary inorganic arsenic, nickel, cadmium, and zinc. These elevated levels were associated with metals levels in newborn nails and young child urine.

Conclusions/Interpretations of Results: Preliminary results (n=12) suggest maternal urinary levels of inorganic arsenic, cadmium, nickel, and molybdenum are in exceedance of levels seen in similar cohorts in the United States. Metals concentrations in infant fingernails (n=10) contain elevated arsenic and nickel levels.

P04 | Environmental Chemical Exposures and Health Outcomes Across the Lifespan

P04-01 | Dermal absorption of fluorotelomers present in firefighter’s gear using porcine skin model

Anita Amir Labonno, Bryan R. Ormond

North Carolina State University, Wilson College of Textiles, Raleigh, USA

Firefighter’s protective clothing uses per- and polyfluoroalkyl substances (PFAS) finishes, including side-chain fluorotelomers to obtain water and oil repellency. 6:2 fluorotelomer alcohol (FTOH) and 6:2 fluorotelomer methacrylate (FTMAC) have been detected at high concentrations in firefighters’ gear. These compounds are volatile and may enter the body through inhalation or dermal absorption. While previous studies have investigated the dermal absorption of non-volatile PFASs, which showed limited absorption, there is limited information on volatile fluorotelomers. Since these compounds have high log K_{ow} values and reactive functional groups, they may have greater potential for dermal absorption. They can also biotransform inside the body after metabolism and persist for years. This study investigates the dermal absorption of 6:2 FTOH and 6:2 FTMAC using an in vitro with a flow-through diffusion system on an excised porcine skin model. A 5 µg dose of both 6:2 FTOH and 6:2 FTMAC in methanol as a vehicle was applied to porcine skin mounted in diffusion cells. Bovine serum albumin (BSA) media at pH ~7.4 was used as the receptor fluid and collected in the receptor vials at 0, 0.5, 4, and 8 hours after exposure. The collected media was further subjected to protein precipitation and liquid-liquid extraction using acetone and ethyl acetate, respectively. Residual doses on the skin were swabbed, and both swabs and skin samples were extracted via methanol and sonication. The extracted samples were analyzed using Orbitrap Exploris gas chromatography-mass spectrometry (GC-MS) to quantify fluorotelomer compounds. The findings from this study will help to understand the dermal bioavailability of volatile fluorotelomers from firefighter gear. Since firefighters may be exposed to these chemicals through multiple routes, the contribution of percutaneous absorption from gear is crucial. Overall, these results will form the foundation for the future in vivo studies of fluorotelomer metabolism and biotransformation.

P04-02 | A solute transport model to simulate groundwater flow and perfluorooctanoic acid transport in the Village of Hoosick Falls, New York.

Cindy Arthur[1,2], Erin Bell[1], Steve Shost[2]

[1] University at Albany State University of New York, Department of Environmental Health Sciences, Albany, USA; [2] New York State Department of Health, Center for Environmental Health, Albany, USA

Objective: As part of a larger effort to hindcast historical tap water perfluorooctanoic acid (PFOA) doses among Village of Hoosick Falls residents, develop a novel model that describes the transport of groundwater PFOA in the Village, and then calibrate the model employing groundwater monitoring well data.

Materials and Methods: Visual MODFLOW Flex software (Waterloo Hydrogeologic, Waterloo, ON) was used to develop and calibrate the PFOA transport model. Subsurface layers were the unconfined aquifer, aquitard, confined aquifer, and bedrock. Flow boundaries were defined by the Hoosic River, recharge areas, and the eastern-most monitoring wells. Input and calibration files incorporated monitoring well data obtained between 2016 and the present, characterizing hydraulic head and PFAS concentrations over about 326 acres that included documented PFOA

source areas and the Village wellfield. Calibration was facilitated by 3-D visualizations and was iterative, focusing on minimizing the Normalized Root Mean Square Error (NRMSE).

Results: Model calibration yielded a good model fit with NRMSE less than 0.2, while flow directions and PFOA groundwater iso-concentration contours were reasonable based upon Village wellfield PFOA concentrations and site investigation reports. The calibrated flow model indicates that shallow groundwater flows radially away from major PFOA source areas at a manufacturing facility located northwest of the wellfield, along the river. Conversely, deep groundwater flows southeast from the site into the Village municipal wellfield, and then into a deep groundwater transmissive zone, eventually flowing northward toward the Hoosic River. There is no evidence of a substantial eastward groundwater flow component.

Conclusion: Available groundwater monitoring well data supported the development and calibration of a groundwater PFOA transport model for the Village of Hoosick Falls. The groundwater PFOA transport model can now be employed for additional efforts to hindcast groundwater PFOA transport and ultimately evaluate historical tap water PFOA doses among Village of Hoosick Falls residents.

P04-03 | Vaping Induces Oxidative Stress-Driven DNA Damage in the Gingival Epithelium

Maureen Meister[1], Xiaojia He[1], Shaligram Sharma[1], Pam Cushenan[2], Jonathan Shannahan[3], Christa Wright[1]

[1] Chemical Insights Research Institute of UL Research Institutes, Marietta, USA; [2] Soft Smiles LLC, Marietta, USA; [3] Purdue University, School of Health Sciences, West Lafayette, USA

Objective: The health implications of the use of electronic nicotine delivery systems (ENDS), commonly referred to as vaping, has garnered significant public health attention. Given the concerns regarding the use of these devices and their impact on oral health, we sought to evaluate the potential links between vaping and the oral epithelium at the cellular level.

Methods: Young ENDS users (n=20) and respective non-user controls (n=15) were recruited for participation. Upon enrollment, a Registered Dental Hygienist performed Gingival Epithelial Swabs (GES) of the oral cavity. Following protein extraction of GES, malondialdehyde (MDA) concentrations and protein expression of DNA damage markers were evaluated.

Results: No significant differences in race, income or age existed between non-ENDS users and ENDS users. ENDS users had significantly higher levels of MDA compared to non-ENDS users ($p = 0.0014$). Further, protein expression of all assessed DNA damage markers (i.e., ATR, Chk1, Chk2, MDM2, p-p21, p-p53, H2A.X) were significantly higher in GES of ENDS users compared to non-ENDS users. When assessing the use of different flavored e-liquids, individuals who utilized menthol flavored e-liquids, had higher MDA levels compared to non-users ($p = 0.0001$) and to those ENDS users who used fruit flavored e-liquids ($p=0.0117$). Moreover, those ENDS users who preferred a menthol flavored e-liquid, had significantly elevated levels of protein expression of all DNA damage markers compared to non-users, this was not the case for those who utilized fruit flavored e-liquids or others such as tobacco or candy flavors.

Conclusion: Oxidative stress and DNA damage are often initiating events in oral health conditions. This evidence suggests that vaping, and potentially the type of e-liquid utilized, may increase the risk of developing such conditions. Further research will focus on the physical manifestations of oral health conditions and elucidating the role vaping plays in oral health disease.

P04-04 | Investigating the Mediating Effects of Urinary Phenols and Parabens on the Relationship Between Phthalate Exposure and Metabolic Changes in Adults

Rezaul Karim Ripon[1,2], Narayana G Prasad[4], Rashmi Paudel[3], Elizabeth Meda-Monzon[5], Mayra Volquez[6]

[1] Harvard T H Chan School of Public Health, Environmental Health, Boston, USA; [2] McHigher Centre For Health Research, Environmental Epidemiology, Dhaka, Bangladesh; [3] Boston University, School of Public Health, Boston, USA; [4] Public Health Literacy/BWH, Miami, USA; [5] Pharmamanagement EAS De RL, Mexico, Mexico; [6] Public Health Literacy/UT Southwestern, Dallas, USA

Background: Phthalates and other environmental contaminants, such as phenols and parabens, have been implicated in metabolic health disturbances. This study investigates the mediating effects of urinary phenols and parabens on the relationship between phthalate exposure and Metabolic Changes in adults.

Methods: Data from the National Health and Nutrition Examination Survey 2009-2010 based on exposure availability from this cycle encompassed 1669 adults. Phthalate exposure was measured via urinary Ethyl Paraben. BMI served as a proxy for metabolic health changes. The analysis employed mediation models to explore whether urinary phenols and parabens mediate the relationship between phthalate exposure and metabolic Changes, adjusting for covariates.

Results: The median age of participants was 46 years, with 51% males and 49% females. The mediation analysis revealed significant mediation effects for Benzophenone-3 and Propyl Paraben. For Benzophenone-3, the Average Causal Mediation Effect (ACME) was -0.0008 ($p < 0.001$), and the direct effect (ADE) was -0.004 ($p = 0.016$). Similarly, Propyl Paraben presented significant mediation with an ACME of -0.001 ($p = 0.020$), and ADE was -0.004 ($p = 0.036$).

Conclusion: Based on this finding, urinary phenols, and parabens significantly mediate the relationship between phthalate exposure and metabolic Changes, shedding light on the pathways through which environmental contaminants might influence metabolic health. The findings underscore the necessity for further research and regulatory measures to mitigate exposure to these chemicals and their impacts on metabolic health.

P04-05 | Associations between environmental phenols and anogenital distance among non-Hispanic White and non-Hispanic Black infants

Meghana Varde[1], Roy R. Gerona[2], Jenna R. Krall[1], Helen B. Chin[1], Roger B. Newman[3], Rebecca J. Wineland[3], Michael S. Bloom[1]

[1] George Mason University, Department of Global and Community Health, Fairfax, USA; [2] University of California at San Francisco, Department of Obstetrics, Gynecology and Reproductive Sciences, San Francisco, USA; [3] Medical University of South Carolina, Department of Obstetrics and Gynecology, Charleston, USA

Objective: Environmental phenols (EP), endocrine-disrupting chemicals found in food and beverage packaging as well as personal care products, may affect fetal genital development. We conducted a prospective birth cohort study to estimate the associations between maternal exposure to EPs and anogenital distance (AGD) among newborns from 153 non-Hispanic White and 139 non-Hispanic Black women.
Materials and Methods: Mid-gestational (18-22 weeks) urine samples were collected from pregnant women between 2011-2014. We measured methyl-, ethyl-, propyl-, and butyl-parabens, benzophenones-1 (BP-1) and 3 (BP-3), triclosan (TCS), and bisphenols (BPA, BPS) using liquid chromatography-tandem mass spectrometry. We collected two measures of AGD for males (anopenile and anoscrotal) and females (anofourchette and anoclitoral). Linear regression estimated associations between individual EPs (log-transformed) and infant AGD, adjusted for maternal urinary specific gravity and confounders, stratified by race. Bayesian kernel machine regression (BKMR) estimated the association between the mixture of nine EPs and AGDs in White and Black infants, allowing for non-linear and non-additive associations.
Results: Most EP concentrations were greater in White than Black women. Among males, shorter anopenile distance was associated with increased BP-1 (β=-0.56mm, 95%CI: -0.90, -0.22) and BP-3 (β=-1.85mm, 95%CI: -3.60, -0.11). However, longer anoscrotal distance was associated with increased BP-3 (β=1.00mm, 95%CI: -0.55, 2.56). We did not observe differences in male AGDs by race, but associations varied by race among females ($p<0.10$ modification). For Black infants, shorter anofourchette distance was associated with increased BP-3 (β=-1.50mm, 95%CI: -2.89, -0.11), TCS (β=-0.41mm, 95%CI: -0.79, -0.03), BPA (β=-0.34mm, 95%CI: -0.66, -0.02), and the total EP mixture using BKMR; however, associations for White infants were consistent with the null.
Conclusion: Associations between maternal exposure to individual EPs and AGDs varied by race in female but not male infants. These results showed a racial disparity between the associations between urinary EPs and anofourchette distance, but not in males.

P04-06 | Association between persistent organic pollutants and thyroid-related hormones and homeostasis parameters in postmenopausal women: The Hispanic Community Health Study/Study of Latinos (HCHS/SOL)

Chibuzor Abasilim[1,2], Victoria Persky[3], Robert M. Sargis[4,5], Tessa Day[3], Martha Daviglus[6], Jianwen Cai[7], Sally Freels[3], Konstantina Tsintsifas[3], Arielle Grieco[3], Brandilyn A. Peters[8], Carmen R. Isasi[8], Gregory A. Talavera[9], Bharat Thyagarajan[10], Mary E. Turyk[3]

[1] University of Wisconsin-Milwaukee, Epidemiology Program, Joseph J. Zilber College of Public Health, Milwaukee, USA; [2] University of Illinois Chicago, Division of Environmental and Occupational Health Sciences, School of Public Health, Chicago, USA; [3] University of Illinois Chicago, Division of Epidemiology and Biostatistics, School of Public Health, Chicago, USA; [4] University of Illinois Chicago, Division of Endocrinology, Diabetes, and Metabolism, Department of Medicine, Chicago, USA; [5] Jesse Brown VA Medical Center, Medical Service, Chicago, USA; [6] University of Illinois Chicago, Institute for Minority Health Research, Chicago, USA; [7] University of North Carolina at Chapel Hill, Department of Biostatistics, Chapel Hill, USA; [8] Albert Einstein College of Medicine, Department of Epidemiology and Population Health, New York, USA; [9] San Diego State University, Department of Psychology, San Diego, USA; [10] University of Minnesota, Department of Laboratory Medicine and Pathology, Twin Cities, USA

Background: Persistent organic pollutants have been implicated in endocrine disruption including thyroid dysregulation. However, previous studies have reported inconsistent findings, and few have specifically examined associations with thyroid homeostasis parameters or focused on postmenopausal women.

Methods: Using data collected at baseline examination between 2008 and 2011, we studied 716 postmenopausal women participating in an ancillary study of the HCHS/SOL. Survey-weighted multivariable linear regression models examined associations of organochlorine pesticides (OCPs), polychlorinated biphenyls (PCBs), polybrominated diphenyl ethers (PBDEs) and polybrominated biphenyl (PBB) 153 with free thyroxine (FT4), triiodothyronine (T3), thyroid stimulating hormone (TSH) and homeostasis parameters. Thyroid homeostasis parameters included T3 to FT4 and TSH to T3 ratios, TSH index (TSHI), thyroid feedback quantile-based index (TFQI), and parametric thyroid feedback quantile based index (PTFQI). POPs were analyzed in quartiles, except for mirex and p,p'-DDT which were categorized as non-detected (reference), ≤ median of detected values, and > median.

Results: Each quartile increase in PBB-153 was inversely associated with FT4 (β=-0.03ng/dL; 95% CI:-0.05,-0.01) and positively associated with ratio of T3 to FT4 (β=3.34, 95% CI:0.53,6.14) suggesting increased deiodination or peripheral thyroid hormone sensitivity. PBB-153 was also inversely associated with PTFQI (β=-0.04, 95% CI:-0.08,-0.001) and TFQI (β=-0.05, 95% CI:-0.09,-0.001) indicating greater pituitary or central sensitivity to FT4. Concentrations of p,p'-DDT above and below the median of detected values were also positively associated with T3 (β=10.9ng/dL, 95% CI:1.16,20.7). Inverse associations of borderline significance were noted between ∑PBDEs and TSH, ratio of TSH to T3, TFQI and TSHI.

Conclusion: Findings suggest that brominated flame retardants (PBB-153 and PBDEs) and p,p'-DDT rather than other OCPs and PCBs, may disrupt both central and peripheral thyroid hormone sensitivity in postmenopausal women. Given the persistence of these chemicals in the environment, understanding their effects on thyroid homeostasis pathways may be critical for diabetes prevention in postmenopausal women.

P04-07 | Prenatal exposure to endocrine-disrupting chemical mixture and maternal, newborn and childhood thyroid function

Yuchan Mou[1,2], Hanan El Marroun[1,3], Akhgar Ghassabian[4]

[1] Erasmus University Medical Center, Department of Child and Adolescent Psychiatry/Psychology, Rotterdam, Netherlands; [2] Erasmus University Medical Center, The Generation R Study Group, Rotterdam, Netherlands; [3] Erasmus University Rotterdam, Department of Psychology, Education and Child Studies, Erasmus School of Social and Behavioural Sciences, Rotterdam, Netherlands; [4] New York University Grossman School of Medicine, Departments of Pediatrics and Population Health, New York, USA

Objective:
While experimental data suggest thyroid disruption by exposures to chemicals, such as phthalates, bisphenol, and organophosphate (OP) pesticides, epidemiological evidence is inconsistent. We examined the association of prenatal exposure to a mixture of these chemicals with maternal, newborn, and early childhood thyroid function.

Methods:
We included participants from a birth cohort (Netherlands, 2002-2006), who had urinary levels of 10 phthalates, bisphenol A, and 5 non-specific OP pesticide metabolites measured three times during gestation. Serum thyroid stimulating hormones (TSH), free thyroxine (FT4) and total thyroxine (TT4) were measured in early pregnancy. Child TSH and FT4 were measured in cord blood and in childhood serum collected at age 6. Molar sum of chemical levels was averaged, log-transformed, and corrected for urinary dilution. Thyroid parameters were log-transformed. We applied multiple regression and Bayesian kernel machine regression (BKMR) to test associations.

Results:
The study population comprised 710 mothers, 486 newborns, and 493 children. In multiple regression models, higher ∑diethyl (DE) was associated with higher maternal FT4 (β=0.05, [95% CI: 0.01, 0.09]). An inverted U-shaped association was found between ∑DE and FT4 in newborns. No other associations were observed. In BKMR models, mixture concentrations above the 50th percentile were associated with lower TSH compared to the median (estimate of 75th percentile = -0.07, 95% CrI: -0.00, -0.14). Dimethyl phthalate (DMP) exposure was the main contributor. A lower concentration of the chemical mixture was associated with lower FT4 in newborns when compared to the median concentration, with ∑DE being the main contributor. However, estimates for this association were uncertain. There were no interactions among metabolites.

Conclusion:
Exposure to the chemical mixture in early pregnancy may interfere with thyroid function in the cord blood and in early childhood.

P04-08 | Sex-specific associations between persistent organic pollutants and changes in blood pressure traits among Hispanic/Latino adults: The Hispanic Community Health Study/Study of Latinos (HCHS/SOL)

Chibuzor Abasilim[1,2], Victoria Persky[3], Robert M. Sargis[4,5], Maria Argos[6,3], Martha Daviglus[7], Jianwen Cai[8], Sally Freels[3], Konstantina Tsintsifas[3], Arielle Grieco[3], Tessa Day[3], Brandilyn A. Peters[9], Carmen R. Isasi[9], Gregory A. Talavera[10], Mary E. Turyk[3]

[1] University of Wisconsin-Milwaukee, Epidemiology Program, Joseph J. Zilber College of Public Health, Milwaukee, USA; [2] University of Illinois Chicago, Division of Environmental and Occupational Health Sciences, School of Public Health, Chicago, USA; [3] University of Illinois Chicago, Division of Epidemiology and Biostatistics, School of Public Health, Chicago, USA; [4] University of Illinois Chicago, Division of Endocrinology, Diabetes, and Metabolism, Department of Medicine, Chicago, USA; [5] Jesse Brown VA Medical Center, Medical Service, Chicago, USA; [6] Boston University, Department of Environmental Health, School of Public Health, Boston, USA; [7] University of Illinois Chicago, Institute for Minority Health Research, Chicago, USA; [8] University of North Carolina at Chapel Hill, Department of Biostatistics, Chapel Hill, USA; [9] Albert Einstein College of Medicine, Department of Epidemiology and Population Health, New York, USA; [10] San Diego State University, Department of Psychology, San Diego, USA

Background: Persistent organic pollutants (POPs), historically used in agriculture and manufacturing, have been linked to cardiometabolic diseases. Understanding specific impacts of POPs on blood pressure (BP) traits is important for hypertension prevention but is not well understood. We investigated sex-specific associations between POPs and changes in BP traits among Hispanic/Latino adults with average follow-up of six years.

Methods: We studied 348 pre-and-perimenopausal women, 782 postmenopausal women and 1,134 men participating in an ancillary study of the HCHS/SOL. BP traits including systolic BP (SBP), diastolic BP (DBP), pulse pressure and mean arterial pressure (MAP) were analyzed. We adjusted SBP and DBP for antihypertensive medication use by adding 10 mmHg and 5 mmHg, respectively. Multivariable linear regression models evaluated sex-specific associations of organochlorine pesticides (OCPs), polychlorinated biphenyls (PCBs), polybrominated diphenyl ethers (PBDEs) and polybrominated biphenyl (PBB) 153 with changes in BP traits. Models accounted for the complex survey design of HCHS/SOL and adjusted for corresponding baseline BP trait, time elapsed between visits, and potential confounders. POPs were analyzed in quartiles, except for mirex and 1,1,1-trichloro-2,2-diphenylethane (p,p'-DDT) which were categorized as non-detected (reference), ≤ median, and > median of detected values.

Results: Among men, ∑PBDEs was associated with positive change in DBP (β=0.71mmHg,95% CI:0.10,1.33) and MAP (β=0.66mmHg,95% CI:0.03,1.30). In postmenopausal women, p,p'-DDT was associated with positive change in SBP (β=3.79mmHg, 95% CI:0.47,7.11). Among pre-and-perimenopausal women, p,p'-DDT was associated with positive change in DBP (β=2.70mmHg, 95% CI:0.02,5.39), MAP (β=2.57mmHg, 95% CI:0.03,5.11) and pulse pressure (β=3.93, 95% CI:0.27,7.59) while mirex was associated with positive change in pulse pressure (β=3.09, 95% CI:0.33,5.86).

Conclusion: Our findings suggest isolated and varied sex-specific associations between POPs and longitudinal changes in BP traits. While these chemicals have not been produced for decades, they may impact cardiovascular risk due to their environmental persistence and biological half-lives measured in years to decades.

P04-09 | Prenatal and postnatal exposure to endocrine-disrupting chemical mixture and cognition in adolescents

Yuchan Mou[1,2], Hanan El Marroun[1,3], Akhgar Ghassabian[4]

[1] Erasmus University Medical Center, Department of Child and Adolescent Psychiatry/Psychology, Rotterdam, Netherlands; [2] Erasmus University Medical Center, The Generation R Study Group, Rotterdam, Netherlands; [3] Erasmus University Rotterdam, Department of Psychology, Education and Child Studies, Erasmus School of Social and Behavioural Sciences, Rotterdam, Netherlands; [4] New York University Grossman School of Medicine, Departments of Pediatrics and Population Health, New York, USA

Objective:
The long-term impact of fetal and childhood exposures to endocrine-disrupting chemicals (EDCs) on cognition is unclear. We examined the extent to which exposure to a mixture of EDCs, i.e., phthalates, bisphenols, and organophosphate (OP) pesticides, during pregnancy and in childhood were associated with cognitive function in adolescents.
Methods:
We analyzed data from mother-child pairs participating in a birth cohort (Netherlands, 2002-2006), who had urinary levels of 10 phthalates, bisphenol A, and 5 non-specific OP pesticide metabolites measured three times during gestation (n = 565) or when children were 6 years (n = 539). Cognition was assessed using the Wechsler Intelligence Scale at 14 years, i.e., verbal, coding, digital span, and matrix reasoning subtests. Molar sum of chemicals was averaged, log-transformed, and corrected for urinary dilution. We applied multiple regression and hierarchical Bayesian kernel machine regression (hBKMR) to test associations of single EDC and in mixture with cognition.
Results:
In regression models, higher ∑di-2-ethylhexyl phthalate (∑DEHP) was inversely associated with matrix reasoning score, with sex differences (boys: β=-0.41 [95%CI: -0.79, -0.06]; girls: β=0.08 [95%CI: -0.24, 0.41]). No associations were observed with childhood exposure. In hBKMR models, there were inverse associations between EDC mixture during pregnancy and matrix reasoning and verbal test scores, but estimates for verbal test were uncertain. Compared to mixture level at the median, mixture level at the 25th percentile was associated with 0.43 increase in matrix reasoning scores (95%CrI: 0.03, 0.83), and 0.32 increase in verbal scores (95%CrI: -0.12, 0.75). There was no indication of interactions among EDCs. Mixture exposure at 5 years was generally not associated with cognition in adolescence, except for verbal test score.
Conclusion:
Higher prenatal EDC mixture was associated with lower matrix reasoning. The positive association between childhood mixture exposure and adolescent cognition may be explained by residual confounding and selection bias.

P04-10 | Home proximity to agricultural crops in relation internalizing symptoms in Ecuadorian adolescents and young adults

Briana N. Chronister[1], Jose Suarez-Torres[2], Danilo Martinez[2], Sheila Gahagan[1], Daniel Sousa[3], Humberto Parada Jr.[4], Penelope J. Quintana[4], Tommi Gaines[6], Raeanne C. Moore[5], Jose R. Suarez-Lopez[1]

[1] University of California San Diego, Herbert Wertheim School of Public Health and Human Longevity Science, La Jolla, USA; [2] Fundación Cimas del Ecuador, Quito, Ecuador; [3] San Diego State University, Department of Geography, San Diego State University, USA; [4] San Diego State University, School of Public Health, San Diego State University, USA; [5] University of California San Diego, Department of Psychiatry, La Jolla, USA; [6] University of California San Diego, Department of Medicine, Division of Infectious Diseases and Global Public Health, La Jolla, USA

Introduction: Depression and anxiety are among the leading contributors to Disability Adjusted Life Years in Ecuadorians. Elevated depression and anxiety have been observed in occupational populations with direct pesticide exposure suggesting that pesticide exposure may impact psychological well-being. In this study, we examined the associations between proximity to agricultural pesticide spray sites and depression and anxiety symptoms among adolescent and young adults in Ecuador.

Materials and Methods: This cross-sectional study included 484 participants (17-23 years) living in Pedro Moncayo, Ecuador in 2022. The Generalized Anxiety Disorder-7 (GAD-7) and Beck's Depression Inventory-II (BDI-2) measured anxiety and depression symptoms, respectively. Open-air agriculture was mapped using Spectral Mixture Analysis and Uniform Manifold Approximation and Projection on 2022 Sentinel-2 images. We used generalized estimating equations (GEE) and logistic regression to estimate the associations between home proximity to the nearest agricultural field (home-field proximity) and anxiety and depression symptoms. Geospatially weighted regression assessed variations in associations across the five parishes of Pedro Moncayo. GEE, logistic regression, and GWR models adjusted for demographic, socioeconomic, anthropometric, and alternative pesticide exposure pathway variables.

Results: The median home-field proximity was 16.39m (IQR 7.08m, 27.89m). A 10% increase in home-field proximity was associated with higher anxiety (3.04% 95% CI: [1.15%-4.93%]) and depression (6.26% [1.83%-10.69%]) scores. Living a standard deviation closer to fields (19.7 m) increased the odds of mild (OR=1.33 [1.08-1.67]) and moderate/severe (OR=1.45 [1.08-2.00]) anxiety symptoms, as well as moderate/severe depression symptoms (OR=1.49 [1.02-2.22]). GWR demonstrated that the association between home-field proximity and BDI-II was strongest in Malchinguí (central location) and weakened eastward. For anxiety symptoms, the association was strongest in Tupigachi (easternmost location) and weakened westward.

Conclusions: Living near open-air agricultural crops was associated with greater depression and anxiety symptoms in adolescents and young adults. Mitigating pesticide exposures for nearby residents may be important for preserving mental health.

P04-12 | Skin conditions associated with dermal exposure to oil spill chemicals among *Deepwater Horizon* disaster response and cleanup workers

Dazhe Chen[1], Kaitlyn G. Lawrence[1], Patricia A. Stewart[2], Melanie Gorman Ng[3,4], Mark R. Stenzel[5], John W. Cherrie[3,6], Kate E. Christenbury[7], William B. Jackson II[7], Lawrence S. Engel[8], Dale P. Sandler[1]

[1] Epidemiology Branch, National Institute of Environmental Health Sciences, Research Triangle Park, USA; [2] Stewart Exposure Assessments, LLC, Arlington, USA; [3] Centre for Human Exposure Science, Institute of Occupational Medicine, Edinburgh, UK; [4] School of Population and Public Health, University of British Columbia, Vancouver, Canada; [5] Exposure Assessment Applications, LLC, Arlington, USA; [6] Institute of Biological Chemistry, Biophysics and Bioengineering, Heriot-Watt University, Edinburgh, UK; [7] DLH, LLC, Bethesda, USA; [8] Department of Epidemiology, Gillings School of Global Public Health, University of North Carolina, Chapel Hill, USA

Objective: Previous studies have associated oil spill response and cleanup (OSRC) work with skin symptoms, but evidence is lacking on the specific exposures contributing to these effects. We investigated OSRC-related exposures and skin conditions among the *Deepwater Horizon (DWH)* OSRC workers enrolled in the GuLF Study.

Methods: At enrollment, workers reportedstart and end work dates, jobs performed, and skin contact with crude oil/tar, decontamination chemicals, and dispersants during *DWH* OSRC work. Cumulative dermal exposure to polycyclic aromatic hydrocarbons (PAHs) from oil/tar was estimated based on the “GuLF DREAM model”. We used adjusted modified Poisson regression to evaluate associations of exposures with prevalent skin conditions during spill cleanup and 1-3 years later among enrolled workers (N=23,833) and incident eczema diagnosed after the start of OSRC work among those with follow-up questionnaire data on eczema (N=14,305). We examined modification of associations by rubber/synthetic glove use.

Results: Duration of OSRC work was positively associated with skin conditions and eczema diagnoses (p-trend<0.01). Workers in operations, response, and decontamination jobs had higher skin condition prevalence (during cleanup: PR range=3.13-4.51; 1-3 years later: PR range=2.20-2.94) and eczema risk (RR range=1.44-1.89) compared to support workers. We found positive associations of skin conditions during cleanup with dermal exposure to oil/tar (PR=3.41, 95%CI: 3.14, 3.69), decontamination chemicals (PR=1.55, 95%CI: 1.46, 1.64), dispersants (PR=1.44, 95%CI: 1.33, 1.57), and PAHs (p-trend<0.01). These associations remained elevated 1-3 years later. Eczema was associated with oil/tar (RR=1.56, 95%CI: 1.20, 2.04) and PAH exposures (Tertile 3 vs. 1: PR=1.33, 95%CI: 0.86, 2.07). Effect estimates were, on average, 21% lower among workers who used rubber/synthetic gloves.

Conclusion: Duration of work, working in non-support jobs, and dermal exposure to oil/tar, dispersants, decontamination chemicals, and PAHs were associated with acute and longer-term skin effects among the *DWH* OSRC workers.

Funding: Intramural Research Program of the NIH (Z01ES102945).

P04-13 | Microplastic Particles in Human Blood, and Their Association With Coagulation Markers

Dong-Wook Lee[1], Jaehak Jung[2]

[1] Inha University, Department of Occupational and Environmental Medicine, Incheon, South Korea; [2] Korea Institute of Analytical Science and Technology, Seoul, South Korea

Background

Microplastics (MPs) are increasingly recognized as emerging contaminants in various ecosystems, including the human body. Recent findings of MPs in human blood raise concerns about their potential health effects. To enhance our understanding of the presence of MPs in blood and their possible health implications, it is crucial to develop and validate reliable analytical methods.

Materials and Methods

We recruited 36 healthy adults and collected whole blood samples, which were analyzed for MPs using Fourier-transform infrared (μ-FTIR) spectroscopy. Lifestyle factors potentially related to MP exposure, such as the use of plastic food containers, were also assessed. In addition, coagulation and inflammatory markers were measured, including C-reactive protein, prothrombin time, activated partial thromboplastin time (aPTT), antithrombin III, platelet count, erythrocyte sedimentation rate, and fibrinogen.

Furthermore, to compare detection and identification outcomes, 16 of the blood samples were analyzed using both μ-FTIR spectroscopy and Pyrolysis-Gas Chromatography/Mass Spectrometry (Py-GC/MS).

Results

MPs were detected in 88.9% of the participants, with a mean concentration of 4.2 MPs/mL. Polystyrene and polypropylene were the most commonly identified polymers. Participants reporting higher use of plastic food containers exhibited significantly greater MP levels in their blood. Moreover, a high MP load (≥3 MPs/mL) was significantly associated with elevated aPTT, C-reactive protein, and fibrinogen.

When both μ-FTIR and Py-GC/MS were applied to 16 samples, the correlation between microplastic counts per unit of blood (μ-FTIR) and mass (Py-GC/MS) was low; similarly, the correlation by monomer type was also weak.

Conclusion

Our findings confirm the presence of MPs in human blood and suggest an association with specific lifestyle factors and notable changes in coagulation markers. However, further research is needed to elucidate the relationships among different analytical methods and to develop standardized approaches for using microplastics in blood as a biological exposure index.

P04-14 | Association of Blood Arsenic Concentrations with Triglycerides, and Total and High-density Lipoprotein Cholesterol among Uruguayan Adolescents

Gauri Desai[1], Elena I. Queirolo[2], Teresa Quattrin[3], Patrick J. Parsons[4,5], Christopher D. Palmer[4,5], María Inés Beldo[2], Katarzyna Kordas[1]

[1] The State University of New York at Buffalo, Department of Epidemiology and Environmental Health, Buffalo, USA; [2] Catholic University of Uruguay, Department of Neuroscience and Learning, Montevideo, Uruguay; [3] The State University of New York at Buffalo, Departement of Pediatrics, Buffalo, USA; [4] Wadsworth Center, New York State Department of Health, Division of Environmental Health Sciences, Albany, USA; [5] University at Albany, Department of Environmental Health Sciences, College of Integrated Health Sciences, Albany, USA

Objective: Arsenic (As) exposure is associated with cardiovascular disease (CVD). However, the link of As exposure with precursors of CVD including lipid markers is understudied, particularly in adolescence. Our study examined the association between blood As and the levels of triglycerides (TGL), total cholesterol (TC), and high-density lipoprotein cholesterol (HDL-C) among Uruguayan adolescents.

Material and Methods: Adolescents (ages 9-20 years) participated in the Salud Ambiental Montevideo (SAM) cohort in 2021-23. Arsenic was measured in whole blood at two time points (T1 and T2) at mean ages 12 and 13 years, respectively. Lipid markers were measured in serum at T2. Blood As was averaged and log-transformed for analysis. Of the 337 participants (50.5% females) in the analysis, 325 had data on the exposure and outcomes. Missing covariate data for 12 participants were imputed. Separate linear regressions were built for TGL, TC, and HDL-C, adjusting for sex, age, body mass index, secondhand smoke exposure, physical activity score, and blood lead levels.

Results: Based on age-appropriate guidelines, 8% and 24% adolescents had borderline or high levels of TGL (>150 mg/dL) and TC (>170 mg/dL), respectively, and 32% had low levels of HDL-C (<45 mg/dL). Median (range)values for TGL, TC, and HDL-C were 70.0 (21.0, 291.0) mg/dL, 151.0 (81.0, 302.0) mg/dL, and 48.0 (21.0, 93.0) mg/dL, respectively, whereas those for blood As at T1 and T2 were 0.39 (0.18, 4.01) μg/L and 0.36 (0.14, 4.81) μg/L, respectively. Averaged blood As was positively associated with TGL levels, β (95% confidence interval) = 12.07 (0.81, 23.33). No associations were seen with TC or HDL-C.

Conclusion: Among Uruguayan adolescents in the study, blood As was positively associated with serum triglyceride levels. Further understanding of this association will help in identifying adolescents at risk of developing CVD outcomes as adults, thereby enabling early interventions for disease prevention.

P04-15 | Prenatal exposure to mixtures of endocrine disrupting chemicals and children's telomere length

Nuria Güil-Oumrait[1,2], Citlalli Osorio-Yáñez[3,4], Vishal Midya[5], Anne Lise Brantsæter[6], Leda Chatzi[7], David Conti[7], Jesse Goodrich[7], Regina Grazuleviciene[8], Barbara Heude[9,10], Marianna Karachaliou[11], Rosemary McEachan[12], Rémy Slama[13], Line Småstuen Haug[6], John Wright[12], Tim Nawrot[14,15], Damaskini Valvi[5], Martine Vrijheid[1,2]

[1] ISGlobal, Environment and Health over the Lifecourse, Barcelona, Spain; [2] Pompeu Fabra University, Barcelona, Spain; [3] Universidad Nacional Autónoma de México, Departamento de Medicina Genómica y Toxicología Ambiental, Instituto de Investigaciones Biomédicas, Mexico City, Mexico; [4] Universidad Nacional Autónoma de México and Instituto Nacional de Cardiología Ignacio Chávez, Laboratorio de Fisiología Cardiovascular y Trasplante Renal, Unidad de Investigación en Medicina Traslacional, Instituto de Investigaciones Biomédicas, Mexico City, Mexico; [5] Icahn School of Medicine at Mount Sinai, Department of Environmental Medicine and Climate Science, New York, USA; [6] Norwegian Institute of Public Health, Department of Food Safety, Oslo, Norway; [7] University of Southern California, Department of Population and Public Health Sciences, Keck School of Medicine, Los Angeles, USA; [8] Vytautas Magnus University, Department of Environmental Sciences, Kaunas, Lithuania; [9] National Institute of Health and Medical Research (INSERM), Paris, France; [10] National Institute for Agriculture, Food and the Environment (INRAE), Paris, France; [11] University of Crete, Clinic of Preventive and Social Medicine, Medical School, Crete, Greece; [12] Bradford Teaching Hospitals NHS Foundation Trust, Bradford Institute for Health Research, Bradford, UK; [13] Université Grenoble Alpes, Department of Prevention and Treatment of Chronic Diseases, Institute for Advanced Biosciences (IAB; INSERM U1209, CNRS UMR 5309), Grenoble, France; [14] Hasselt University, Centre for Environmental Sciences, Diepenbeek, Belgium; [15] Leuven University, Department of Public Health and Primary Care, Leuven, Belgium

Objective: Telomere attrition begins early in life and is associated with an increased risk of aging-related diseases in later life. Prenatal exposure to endocrine-disrupting chemicals (EDCs) may alter telomere length, but this hypothesis remains understudied in children. We assessed whether prenatal exposure to EDC mixtures is associated with leukocyte telomere length (LTL) in children.

Material and Methods: We examined 1062 mother-child pairsfrom the Human Early Life Exposome project, involving ongoing birth cohort studies across 6 European countries. We analyzed 9 metals, 3 organochlorine pesticides, 5 polychlorinated biphenyls, 2 polybrominated diphenyl ethers, 5 perfluoroalkyl substances, 10 phthalate metabolites, 3 phenols, 4 parabens, and 4 organophosphate pesticide metabolites measured in urine and/or blood samples collected at pregnancy**.** LTL was measured in children at age 8 years using real-time polymerase chain reaction. Associations between each EDC group and child LTL were estimated using 2 exposure-mixture methods: quantile g-computation and Bayesian Weighted Quantile Sum (BWQS) regression.

Results: Adjusted quantile g-computation indicated that prenatal exposure to phthalates was associated with a 3.55% decrease in LTL in children (95% CI: -5.56, -1.54, p = <0.001, per quartile exposure-mixture increase). Mono-oxo-isononyl phthalate (oxo-MiNP) was the phthalate with the highest contribution to the exposure-mixture associations (weight=0.32). No associations were observed for other EDC groups. BWQS showed consistent associations with those observed in quantile g-computation.

Conclusion: Combining two state-of-the-art exposure-mixture approaches, we found that prenatal exposure to phthalates may decrease telomere length in children, potentially contributing to the acceleration of biological aging from an early age. Future analyses will explore associations with mitochondrial DNA (mtDNA) content as a marker of oxidative stress and cellular aging, as well as potential modifying effects by sex.

P04-16 | Exposure hazards of volatile organic compound emissions from vat photopolymerization 3D printing

Qian Zhang[1], Mark Wilson[1], Cristi Bell-Huff[1], Ivan Sanchez[2], Mabel Maroun[2], Beth Welmaker[3]

[1] UL Research Institutes, Chemical Insights Research Institute, Marietta, USA; [2] Nova Southeastern University, Department of Prosthodontics, Digital Dentistry, College of Dental Medicine, Davie, USA; [3] Nova Southeastern University, Environmental Health & Safety Department, Davie, USA

3D printing, particularly vat photopolymerization technology, has become widely applied in the dental field, which can be used to create customized dental appliances like crowns and dentures. The photopolymer resins used for vat photopolymerization 3D printing contain various irritating and/or sensitizing compounds that may cause irritation or allergic reactions when in contact with eyes, skin, and the respiratory tract. In addition, resins may off gas at room conditions if not sealed. Therefore, inhalation exposure could happen during resin loading and handling, printing and post-printing processes. This study characterized volatile organic compound (VOC) emissions from various commonly used resins in dental applications. Exposure chambers were used to isolate emission sources and collect air samples. VOCs and aldehydes were collected on sorbent tubes and further analyzed using gas chromatography-mass spectrometry and high-performance liquid chromatography separately. Results showed that the most abundantly emitted VOCs were associated with the resin compositions, which included various methacrylates. Other emitted VOCs included esters, aldehydes, hydrocarbons, alcohols, and ketones. These VOCs were continuously emitted from the resins, no mater during 3D printing operation or at storage. An indoor exposure model was used to estimate personal exposure to emitted VOC mixtures and evaluate potential inhalation exposure hazards when handling resins. The results showed exposure to health-related VOCs such as methacrylates, 2,6-di-tert-butyl-4-methylphenol, cyclohexanone and 1-butanol could be concerning. Overall, 3D printing resins emit chemicals that may cause adverse health impacts at room conditions. Therefore, 3D printing resins are a source of both dermal and inhalation exposure hazards that users should be aware of during normal use.

P04-17 | Associations between pesticides in honeybees/hive products and adjacent land use in eastern Taiwan

Chien-Che Hung, Chung-Wen Hsueh, Wei-Cheng Chang, **Lih-Ming Yiin**

Tzu Chi University, Public Health, Hualien, Taiwan

Honeybees' contamination with pesticides could be related with land covers adjacent to the apiary. We conducted a field study to explore the associations between pesticides in honeybees/hove products and land use (i.e., upland field, orchard, forest) in estern Taiwan. A total of 12 apiaries collaborated with this study, providing bioindicating samples of bees (healthy, sick, dead) and hive products (beeswax, bee pollen), which were collected during the blooming season (May through September). Samples were analyzed for 14 pesticides (6 insecticides, 5 herbicides, 3 fungicides) using gas or liquid chromatography coupled with mass spectrometry. Geographic information system was applied to determine the proportions of land covers, which were used to relate to pesticides in the apiarian sample matrices. All sample matrics but healthy bees carried high levels of pesticides with mean concentrations above 270 ng/g in total. Only few significant correlations of apiarian pesticides ($\rho > 0.59$) with land covers are observed, such as those with upland fields within the buffer zone of 1500 m. Negative correlations ($\rho < -0.58$) are

present between fungicides in the sample matrices and forests in the buffer zone near the apiaries, suggesting no farming activity in the forest. Orchards appear to be irrelevant to pesticides in the apiarian sample matrices, despite negative correlations that may be confounded between apiarian pesticides and orchards within the long-distance buffer zones. We consider that the associations between pesticides carried by the apiarian bioindicators and adjacent land use are effective but may have been weakened by non-agricultural use of pesticides elsewhere.

P05 | Children's Environmental Health - PART I

P05-01 | Distance to the Salton Sea and Lung Function Development: A Cohort Study of Children Living Near a Drying Lake

Fangqi Guo[1], Elizabeth M. Kamai[1], Sandrah P. Eckel[1], Dayane D. Barahona[1], Luis Olmedo[2], Esther Bejarano[2], Christian Torres[2], Christopher Zuidema[3], Edmund Seto[3], Shohreh F. Farzan[1], Jill E. Johnston[1]

[1] University of Southern California, Population and Public Health Sciences, Los Angeles, USA; [2] Comite Civico del Valle, Brawley, USA; [3] Department of Environmental & Occupational Health Sciences, University of Washington, Seattle, Seattle, USA

Background: The Salton Sea, a drying terminal lake in California, has exposed large areas of desiccated lakebeds, leading to increased wind-blown dust and elevated levels of particulate matter (PM) air pollution. However, the long-term impact of these environmental changes on children's lung function growth remains unknown.

Objective: Investigate the impact of proximity to the Salton Sea and dust exposure on children's lung function development from ages 10 to 12.

Methods: Between 2017 and 2019, 369 participants were recruited from five elementary schools near the Salton Sea and followed until 2022. Lung function was assessed via spirometry (FEV1, FVC) at each visit. We used mixed-effects linear modeling on repeatedly measured lung function data to estimate individual growth slopes. The main exposure was the distance from each child's home to the edge of the Salton Sea. We also estimated exposure to particulate matter (PM) and dust storm hours (PM10 > 150 μg/m³) as secondary outcomes, using data from regulatory monitors. Demographics, height, BMI, baseline respiratory health and asthma status were adjusted as covariates.

Results: The baseline age of participants was 10.1±0.6 years. Each participant was followed for 2 years, with an average of 3 spirometry measurements. Annual growth in FVC and FEV1 averaged 318 ml/year and 249 ml/year, respectively. Results of fully adjusted mixed-effects linear models showed that for each 1km increase in distance from the Sea, we observed a 0.9 ml/year increase in FVC growth (95% CI: 0.4, 1.3; p<0.001) and a 0.5 ml/year increase in FEV1 growth (95% CI: -0.1, 1.2; p=0.092). PM exposure did not significantly affect lung function growth in the entire cohort but was associated with slower FVC growth in participants living closer to the Sea (<11 km).

Conclusion: Children living in closer proximity to the Salton Sea may have slower lung function growth in mid-to-late childhood.

P05-02 | Prenatal exposure to $PM_{2.5}$ and NO_2 and its association with child cortisol measures during the first year of life

Nafisa Nawal Islam[1], Sally W. Thurston[2,3], Donald Harrington[2], Yihui Ge[4], Xing Qiu[2], Qiuyi Wu[4], Meherab Hossain[2], Jessica Brunner[5], Yan Lin[4], Richard K. Miller[5,3], Emily S. Barrett[7,5], Junfeng Zhang[4], David Q. Rich[3,6], Thomas G. O'Connor[8,5]

[1] University of Rochester School of Medicine and Dentistry, Clinical and Translational Science Institute, Rochester, USA; [2] University of Rochester School of Medicine and Dentistry, Department of Biostatistics and Computational Biology, Rochester, USA; [3] University of Rochester School of Medicine and Dentistry, Department of Environmental Medicine, Rochester, USA; [4] Duke University, Durham, USA; [5] University of Rochester School of Medicine and Dentistry, Department of Obstetrics and Gynecology, Rochester, USA; [6] University of Rochester School of Medicine and Dentistry, Department of Public Health Sciences, Rochester, USA; [7] Rutgers University, Department of Biostatistics and Epidemiology, Rutgers University School of Public Health, Environmental and Occupational Health Sciences Institute, Piscataway, USA; [8] University of Rochester School of Medicine and Dentistry, Department of Psychiatry, Rochester, USA

Objective: We examined whether prenatal exposure to fine particulate matter ($PM_{2.5}$) and nitrogen dioxide (NO_2) is associated with hypothalamic-pituitary-adrenal (HPA) axis activity at ages 6 and 12 months, with implications for infant stress regulation and neurodevelopment.

Materials and Methods:We recruited 326 pregnant individuals at normal medical risk at <14 weeks gestation into a pregnancy cohort in Rochester, New York. Ambient $PM_{2.5}$ and NO_2 levels at participants' residences were estimated using spatial-temporal models. Six and twelve-month saliva swabs were collected pre-stressor, and 15- and 30-minutes post-stressor. We calculated total cortisol output or the area under the curve with respect to ground (AUCg) and increase (or change) (AUCi) based on infant cortisol data. Using distributed lag models, we estimated the increase/decrease in the five log-transformed cortisol measures associated with interquartile range increases in weekly mean $PM_{2.5}$ (3 µg/m^3) and NO_2 (9 ppb) concentrations, adjusting for covariates.

Results:$PM_{2.5}$ exposure during gestational weeks (GW) in late pregnancy was associated with lower cortisol levels. Specifically, three μg/m^3 increases in $PM_{2.5}$ concentrations were associated with lower baseline cortisol (GW 31–37: -0.329 ln[nmol/L]; 95% CI = -0.628, -0.031), 15-minutes post-stressor cortisol (GW 28–37: -0.446 ln[nmol/L]; 95% CI = -0.867, -0.026), 30-minutes post-stressor cortisol (GW 21–37: -0.700 ln[nmol/L]; 95% CI = -1.225, -0.175), and AUCg (GW 22–37: -0.858 ln[nmol/L]; 95% CI = -1.485, -0.230) at 6-months. However, we found no associations between weekly increases in $PM_{2.5}$ concentrations and month-12 cortisol measures, or between NO_2 concentrations and cortisol measures during both time points.

Conclusions:Prenatal $PM_{2.5}$ exposure, particularly in late gestation, may contribute to altered stress regulation in early life, with lower cortisol levels in 6-month-old infants indicating an adaptive downregulation of the HPA axis. Reducing maternal air pollution exposure could help mitigate the risk of early-life stress dysregulation, with implications for long-term neurodevelopmental outcomes.

P05-03 | Estimating the effect of reducing annual ambient fine particular matterconcentrations on allostatic load in children

Selene Banuelos[1], Alejandro Schuler[2], Austin D. Le[1], Morgan Ye[3], Andrew Nguyen[3], Dayna Long[4,5], Neeta Thakur[3], Rosemarie de la Rosa[2]

[1] University of California, Berkeley, Environmental Health Sciences, Berkeley, USA; [2] University of California, Berkeley, Biostatistics, Berkeley, USA; [3] University of California, San Francisco, Department of Medicine, Division of Pulmonary and Critical Care Medicine, San Francisco, USA; [4] University of California, San Francisco, Department of Pediatrics, San Francisco, USA; [5] UCSF Benioff Children's Hospital, Oakland, USA

Objective: Chronic exposure to environmental stressors may result in dysregulation across the body's adaptive stress-response systems, which can be conceptualized as allostatic load. Here, we estimated the effect of reducing annual average ambient fine particulate matter ($PM_{2.5}$) on allostatic load in children.

Material and Methods: Cross-sectional data were collected from 279 participants, 1-12 years old. Participants were recruited at the Benioff Children's Hospital in Oakland, California, during routine child wellness checks from March 2017-October 2018. Monthly ground-level ambient $PM_{2.5}$ concentrations were mapped to each participant's residential address and averaged over the year prior to biospecimen collection. Inflammatory, metabolic, neurological and cardiovascular plasma biomarkers related to hypothalamic-pituitary-adrenal (HPA) axis activation were measured to operationalize allostatic load. Targeted maximum likelihood estimation paired with the Super Learner machine learning algorithm were used to estimate the effect of hypothetically shifting the observed ambient $PM_{2.5}$ exposure distribution down by 3, 4, 5, 6 and 7 μg/m^3 on average allostatic load score.

Results: The observed average annual ambient $PM_{2.5}$ concentration was 10.93 μg/m^3 (SD: 1.86) and the observed average allostatic load score was 1.46 (SD: 1.24). A dose-response relationship was observed between exposure shift amounts and reduction in average allostatic load score. Shifting the exposure distribution down by 4 and 5 μg/m^3 was associated with a -0.41 (95% CI: -0.71, -0.12) and -0.6 (95% CI: -1, -0.17) point decrease in average allostatic load score, respectively.

Conclusion: Reducing ambient $PM_{2.5}$ concentrations may be effective in reducing allostatic load in children, prompting further consideration of the current $PM_{2.5}$ national ambient air quality standard.

Funding Sources: This work was supported in part by the TARA Health Foundation, Genentech Corporate Giving, and the California Initiative to Advance Precision Medicine.

P05-04 | Prenatal heavy metals and trace elements and prevalence of congenital limb abnormalities among newborns: the Japan Environment and Children's Study

Megasari Marsela[1], Chihiro Miyashita[2], Takeshi Yamaguchi[3], Yasuaki Saijo[4], Yoshiya Ito[5], Hiroyoshi Iwata[6], Sachiko Itoh[7], Mariko Itoh[8], Keiko Yamazaki[9], Naomi Tamura[10], Sumitaka Kobayashi[11], Reiko Kishi[12], Atsuko Ikeda[13]

[1] Hokkaido University, Faculty of Health Sciences, Sapporo, Japan; [2] Hokkaido University, Center for Environmental and Health Sciences, Sapporo, Japan; [3] Hokkaido University, Center for Environmental and Health Sciences, Sapporo, Japan; [4] Asahikawa Medical University, Department of Social Medicine, Asahikawa, Japan; [5] Japanese Red Cross Hokkaido College of Nursing, Faculty of Nursing, Kitami, Japan; [6] Hokkaido University, Center for Environmental and Health Sciences, Sapporo, Japan; [7] Hokkaido University, Center for Environmental and Health Sciences, Sapporo, Japan; [8] Hokkaido University, Center for Environmental and Health Sciences, Sapporo, Japan; [9] Hokkaido University, Center for Environmental and Health Sciences, Sapporo, Japan; [10] Hokkaido University, Center for Environmental and Health Sciences, Sapporo, Japan; [11] Hokkaido University, Center for Environmental and Health Sciences, Sapporo, Japan; [12] Hokkaido University, Center for Environmental and Health Sciences, Sapporo, Japan; [13] Hokkaido University, Faculty of Health Sciences, Sapporo, Japan

Background: Heavy metals such as lead (Pb) and cadmium (Cd) have been associated with adverse pregnancy and developmental outcomes, including congenital abnormalities. This study investigated the association between exposure to heavy metals and trace elements during fetal life and congenital limb abnormalities in infants.
Methods: This study is based on a prospective ongoing nationwide birth cohort from the Japan Environment and Children's Study (JECS). The concentrations of Cd, Pb, mercury (Hg), selenium (Se), manganese (Mn) were measured in maternal blood collected during the mid–late trimesters. The inclusion criteria were obtained from questionnaires completed during pregnancy, which included information about congenital limb abnormalities present at birth or at one month of age. Logistic regression models were applied after log transformation or division into quartiles of Cd, Pb, Hg, Se, Mn concentrations to examine their associations with limb anomalies and individual chemicals. To assess the associations with the heavy metals and trace elements mixture, quantile g-computation was employed. All models were adjusted for age, maternal smoking history, maternal alcohol intake, history of smoking, and infant sex.
Results: Data from 90,163 participants were included in the analysis, of whom 369 had congenital limb abnormalities in any of the collected information, and 89,794 had none. Among the 369 cases of congenital limb abnormalities, there were 185 and 142 cases of polydactyly and syndactyly, respectively. There were no associations between maternal blood concentrations of Pb, Cd, Hg, Se, Mn with congenital limb abnormalities. No significant association was observed between the mixture of heavy metals and trace elements and any congenital limb abnormalities. There was no association with any type of abnormality as a subdivision.
Conclusion: At the maternal exposure levels of Cd, Pb, Hg, Se, Mn assessed in the present study, no association was identified with the risk of developing congenital limb abnormalities in children.

P05-05 | Exposure to Phthalates and their Associations with Short-Chain Fatty Acids in Plasma among School Children: The Hokkaido Study

Ayaka Yasuda[1,2], Yu Ait Bamai[3], Rahel Mesfin Ketema[3,4], Takeshi Yamaguchi[3], Yi Zeng[3,4], Megasari Marsela[4], Yonghan Li[2], Siddabasave Gowda B. Gowda[4], Divyavani Gowda[4], Hitoshi Chiba[4,5], Shu-Ping Hui[4], Reiko Kishi[3], Hiroyuki Kojima[1], Atsuko Ikeda[3,4]

[1] Health Sciences University of Hokkaido, Graduate School of Pharmaceutical Sciences, Tobetsu, Japan; [2] Hokkaido University, Graduate School of Health Sciences, Sapporo, Japan; [3] Hokkaido University, Center for Environmental and Health Sciences, Sapporo, Japan; [4] Hokkaido University, Faculty of Health Sciences, Sapporo, Japan; [5] Sapporo University of Health Sciences, Department of Nutrition, Sapporo, Japan

Background and objective: Phthalates are widely used plasticizers and recent animal studies have suggested that they reduce short-chain fatty acids (SCFAs). SCFAs, produced by gut microbiota, help regulate immunity, prevent allergies and autoimmune diseases, and support energy metabolism and appetite control. Lower plasma SCFA levels have also been observed in individuals with ADHD. Additionally, hydroxy short-chain fatty acids (HSCFAs) may function as markers of metabolic and inflammatory response. This study aimed to elucidate the association between urinary phthalate levels and plasma SCFAs and HSCFAs levels in school children.

Methods: The participants were children of the Hokkaido Cohort aged 9-12 years who completed face-to-face surveys. Ten urinary metabolites of the four phthalates, five plasma SCFA, and four HSCFAs were measured using LC-MS/MS. The final analysis included a total of 335 participants. Associations between phthalates and SCFAs and HSCFAs levels were examined using multivariable regression, and quantile g-computation to find mixture exposure to multiple targeted chemicals.

Results: All phthalate metabolites were detected in > 87% of the samples. The highest phthalate concentration was MnBP (median 20.3 ng/mL), followed by MECPP (14.5 ng/mL). Within SCFAs and HSCFAs, acetic acid (188.37 pmol/μL) was the highest, followed by 3-Hydroxypropanoic acid (94.09 pmol/μL). A natural log unit increase in DBP was associated with decreased levels of acetic acid, classified as SCFAs [β (95%CI): -0.08 (-0.16, -0.00)]. Higher DBP [-0.09 (-0.17, -0.01)], BBzP [-0.04 (-0.06, -0.01)], and DEHP [-0.07 (-0.13, -0.01)] were associated with lower 3-Hydroxyhexanoic acid, classified as HSCFAs. Additionally, one quartile increase in phthalates mixture was negatively associated with 3-Hydroxyhexanoic acid [-0.04 (-0.13, -0.03)], and DBP contributed the most (71.5%).

Conclusion: This study suggests that exposure to phthalates in school children may reduce plasma SCFAs and HSCFAs levels.

Funding sources: The Environment Research and Technology Development Fund (JPMEERF20175053, JPMEERF20205002), JSPS KAKENHI A (JP21HT04843).

P05-06 | The Association Between Maternal Semi-Volatile Organic Compound Exposure And Acute Respiratory Infection In Children Under 1 Year Of Age

Corbin Hite[1], Elizabeth Boxer[1], Rebecca Hoehn[1], Jillian Hurst[2], Heather Stapleton[1], Kate Hoffman[1]

[1] Duke University, Nicholas School of the Environment, Durham, USA; [2] Duke University, School of Medicine, Durham, USA

Exposure to semi-volatile organic compounds (SVOCs) can impair immune function, potentially increasing disease risk among highly exposed populations. Because the prenatal period and early infancy are a critical window for immune development, we hypothesized that higher prenatal SVOC exposures could result in increased susceptibility to acute respiratory infections (ARIs) among infants, the most common infectious condition of childhood. To test this hypothesis, we used data from the ongoing HOPE 1000 longitudinal pregnancy cohort in central North Carolina to evaluate associations between prenatal SVOC exposure and infant respiratory infection before age 12 months (n=77 infants). Prenatal SVOC exposure was measured using silicone wristbands, which were worn by pregnant HOPE 1000 participants for 5 days in each trimester of enrollment. Forty SVOCs were detected in at least 60% of wristbands, with phthalates and organophosphate esters detected at the highest levels. Acute respiratory infections were identified from infant electronic health records (EHRs) using International Classification of Diseases Tenth Edition (ICD-10) codes. We identified medically attended ARIs for 32 of 77 infants (41.6%) in the first year of life. We observed an increased odds of ARI among infants whose birthing parent had higher prenatal exposure to several SVOCs including, tri-isobutyl phosphate (TiBP; Odds Ratio: 1.42; 95% confidence interval: 0.92 - 2.26), a common flame retardant in plastics; however, associations were not statistically significant, potentially due to our small sample size. Protective associations were also observed for some compounds (e.g., tris(2-ethylhexyl) trimellitate), though none reached statistical significance. Analysis of additional wristbands and EHR information is ongoing. Taken together, our results provide critical insights into the potential impacts of prenatal SVOC exposures on child health.

Funding for this work was provided by the National Institutes of Health (grant number 5R01-AI167850) and the National Institute of Environmental Health Sciences (grant number T32ES021432).

P05-07 | Using Time Activity Data to Study Children's Exposure to Chemical Hazards: A Scoping Review

Elizabeth A. Chatpar[1], Keeve E. Nachman[1,2], Sara N. Lupolt[1,2]

[1] Johns Hopkins Bloomberg School of Public Health, Department of Environmental Health & Engineering, Baltimore, USA; [2] Johns Hopkins Bloomberg School of Public Health, Risk Sciences and Public Policy Institute, Baltimore, USA

Objective: This scoping review describes the body of peer-reviewed environmental health literature that collects time activity data (TAD) to estimate children's exposures to chemical hazards.

Materials and Methods: We conducted our search in PubMed and Scopus, screening 5,597 articles for inclusion following a Population-Exposure-Method framework. Studies were included if they collected children's TAD for the purpose of estimating exposure to chemical hazards. All methods of TAD collection (e.g., videography, activity diaries, questionnaire/surveys) were eligible for inclusion. Extracted data were coded using an iteratively developed codebook, and analysis was performed using Microsoft Excel and Tableau.

Results. We identified 44 studies collecting TAD to estimate children's exposure to chemical hazards. Three common research objectives emerged: (1) quantifying inhalation exposures using macroactivity data, (2) assessing non-dietary ingestion from microactivity data, and (3) calculating dermal exposures associated with activities of varying scales. For children aged 6 months to 6 years, microactivities are well documented, although larger-scale activity patterns are understudied. Macroactivity patterns in older children (6+ years old) are well documented in the literature, but microactivity data on these lifestages are lacking. Very few studies reported participant sex, race, and/or socioeconomic status data. Five studies were conducted in low- and middle-income countries, whereas the rest were conducted in high income countries, most commonly the United States, Italy, and South Korea.

Conclusion. Incorporating individual activity data may produce more precise exposure estimates as compared to estimates relying on generalized information about population behaviors. Since activity patterns vary across sex, age, socioeconomic status, culture, and geography, including demographic information in TAD studies is essential for assessing generalizability. This review identifies significant gaps in the literature and highlights areas of potential improvement for TAD methodology, across childhood lifestages, locations, and activity types.

Funding. This work was supported by US Environmental Protection Agency STAR Grant No. R840200.

P05-08 | Plasticizer Exposure in Children of Four Asian Countries: Regional Variations and Potential Sources

Joeun Jung[1,2], Ah-Reum Jo[1], Yuna Kim[1], Jin-Yeong Heo[3], Na-Youn Park[3], Sonja A. Wrobel[4], Jeongim Park[5], Kraichat Tantrakarnapa[6], Ramadhan Tosepu[7], Khaled Hossain[8], Holger M. Koch[4], Younglim Kho[3], Kyungho Choi[1]

[1] Seoul National University, Department of Environmental Health Sciences, School of Public Health, Seoul, South Korea; [2] Seoul National University, Institute of Health and Environment, Seoul, South Korea; [3] Eulji University, Department of Health, Environment & Safety, Seongnam, South Korea; [4] Ruhr University Bochum, Institute for Prevention and Occupational Medicine of the German Social Accident Insurance (IPA), Bochum, Germany; [5] Soonchunhyang University, Department of Environmental Health Science, Asan, South Korea; [6] Mahidol University, Department of Social and Environmental Medicine, Faculty of Tropical Medicine, Bangkok, Thailand; [7] University of Halu Oleo, Department of Environmental Health, Faculty of Public Health, Kendari, Indonesia; [8] University of Rajshahi, Department of Biochemistry and Molecular Biology, Rajshahi, Bangladesh

Plasticizers may cause adverse health outcomes, with children being particularly vulnerable. However, the extent of exposure among children is not well understood in many Asian countries. We compared plasticizer exposure levels and risks among children in four Asian countries, evaluating major source contributions. First-morning urine samples were collected from children aged 6–14 in Korea (n=89), Thailand (n=111), Indonesia (n=100), and Bangladesh (n=100) in 2022–2023. For the same Indonesian population (n=76), a two-day intervention on behavior and plasticware was conducted, with urine samples collected pre- and post-intervention. Urinary concentrations of 37 plasticizer metabolites were analyzed, and estimated daily intake (EDI) values were calculated with risk assessment. Detection levels of plasticizers varied among countries. Indonesia and Bangladesh had higher legacy plasticizers but lower alternatives, while Thailand and Korea transitioned to alternatives. DEHP alternatives accounted for 95.8%, 93.5%, 94.1%, and 87.9% of total EDI for Korean, Thai, Bangladeshi, and Indonesian children, respectively. DEHA was the most significant alternative, contributing over 81% of total exposure in all four countries. Bangladeshi children had 1.5 to 2 times higher plasticizer exposure than others across all EDI quartiles. Risk drivers varied by country, but DEHP and DnBP were consistently identified as substances of concern across countries. The intervention in Indonesia significantly reduced DMP, DEP, and DEHA, but only modestly decreased overall exposure, suggesting the presence of unidentified sources of exposure. While further studies with a more representative sampling design are needed to confirm these findings, this study highlights regional differences in plasticizer exposure and a shift from legacy phthalates to alternatives in children of some Asian countries. Plasticizers of priority concern were also identified for this vulnerable population. This work was supported by the Research Grant from Seoul National University (900-20240102) and the National Research Foundation of Korea (NRF) grant funded by the Korea government (MSIT) (RS-2025-00513967).

P05-09 | Prenatal exposure to non-persistent chemicals and obesity throughout childhood: A meta-analysis across 11 multi-ethnic birth cohorts

Nuria Güil-Oumrait[1,2], Katherine Svensson[3,6], Damaskini Valvi[3], Syam S. Andra[3], Andrea Beneito[19,15], Sophia Blaauwendraad[4,5], Carl-Gustaf Bornehag[3,6], Anne Lise Brantsæter[7], Alejandra Cantoral[8], Tim Cadman[1,9], Nathalie Costet[10], Brenda Eskenazi[11,12], Lindsey English[13], Robert Gunier[11], Kim Harley[11,12], Amrit Kaur Sakhi[7], Barbara Heude[14], Nina Holland[11], Jesús Ibarluzea Maurolagoitia[16,15], Vincent V. Jaddoe[4,5], Marianna Karachaliou[5,17], Allison Kupsco[18], Aitana Lertxundi[16,15], Sabrina Llop[19,15], Sandra Márquez[1,15], Rosemary McEachan[20], John Meeker[13], Antonis Myridakis[21], Claire Philippat[22], Karen E. Peterson[13], Maria-Jose Lopez-Espinosa[19,27], Elke Rouxel[10], Stephen Rauch[11], Loreto S.M. Rodríguez[16,15], Euripides G. Stephanou[17], Morris Swertz[9], Leonardo Trasande[24,25], Martha M. Téllez-Rojo[26], Marcella Warner[11], Charline Warembourg[10], Marieke Welten[4,5], John Wright[20], Robert Wright[3], Sverre Wikström[6,28], Maribel Casas[1,15], Martine Vrijheid[1,2]

[1] ISGlobal, Environmental Health over the Lifecourse, Barcelona, Spain; [2] Pompeu Fabra University, Barcelona, Spain; [3] Icahn School of Medicine at Mount Sinai, Department of Environmental Medicine and Climate Science, New York, USA; [4] Erasmus MC, The Generation R Study Group, University Medical Center Rotterdam, Rotterdam, Netherlands; [5] Erasmus MC, Department of Pediatrics, University Medical Center Rotterdam, Rotterdam, Netherlands; [6] Karlstad University, Department of Health Sciences, Karlstad, Sweden; [7] Norwegian Institute of Public Health, Department of Food Safety, Oslo, Norway; [8] Iberoamerican University, Department of Health, Mexico City, Mexico; [9] University of Groningen, Department of Genetics, Genomics Coordination Center, University Medical Center Groningen, Groningen, Netherlands; [10] Univ Rennes, IRSET (Institut de recherche en santé, environnement et travail), Rennes, France; [11] University of California, Center for Environmental Research and Community Health School of Public Health, Berkeley, USA; [12] University of California, Department of Epidemiology, School of Public Health, Berkeley, USA; [13] University of Michigan, Department of Michigan School of Public Health, Ann Arbor, USA; [14] Université Paris Cité and Université Sorbonne Paris Nord, National Institute of Health and Medical Research (INSERM), Paris, France; [15] Spanish Consortium for Research on Epidemiology and Public Health (CIBERESP), Instituto de Salud Carlos III, Madrid, Spain; [16] BIOGIPUZKOA, Instituto de Investigación Sanitaria, Donostia-San Sebastián, Spain; [17] University of Crete, Clinic of Preventive and Social Medicine, Medical School, Heraklion, Greece; [18] Columbia University, Mailman School of Public Health, New York, USA; [19] FISABIO-Universitat Jaume I-Universitat de València, Epidemiology and Environmental Health Joint Research Unit, Valencia, Spain; [20] Bradford Teaching Hospitals NHS Foundation Trust, Bradford Institute for Health Research, Bradford, UK; [21] Brunel University London, Centre for Pollution Research and Policy, London, UK; [22] University Grenoble Alpes, Institute for Advanced Biosciences, Grenoble, France; [23] University of Crete, Department of Chemistry, Heraklion, Greece; [24] NYU Grossman School of Medicine, Department of Pediatrics, New York, USA; [25] NYU Grossman School of Medicine, Department of Population Health, New York, USA; [26] Instituto Nacional de Salud Pública, Centro de Investigación en Nutrición y Salud, Cuernavaca, Mexico; [27] University of Valencia, Faculty of Nursing and Chiropody, Valencia, Spain; [28] Örebro University, School of Medical Sciences, Örebro, Sweden

Objective: Phthalates and phenols are non-persistent endocrine-disrupting chemicals commonly found in consumer products. Experimental evidence suggests they may contribute to obesity, but findings from prospective birth cohort studies remain inconsistent —especially for less-studied phenols, such as parabens, oxybenzone, and triclosan, as well as chemical replacements. This study examined the relationship between prenatal exposure to phthalates and phenols, and childhood Body Mass Index (BMI) and overweight status.

Material and Methods: We performed a two-stage individual participant data meta-analysis, including 7506 mother-child pairs from 11 cohorts in Europe, North America, and Central America. Urinary concentrations of 8 phenols and 13 phthalate metabolites were measured during pregnancy. BMI z-scores were calculated using the WHO growth

reference at early (≥2–5 years), mid- (≥5–9 years), and late childhood (≥9–13 years). Overweight was classified as a BMI z-score ≥1. Binomial generalized linear mixed models estimated adjusted associations between prenatal exposure and childhood overweight, with results pooled using generic inverse variance meta-analysis.
Results: The prevalence of childhood overweight increased from 24% in early childhood to 36% in late childhood, with variation across cohorts. Random-effects meta-analysis indicated increased odds for overweight across the periods with a doubling increase of butylparaben (BUPA) and monoethyl phthalate (MEP) (OR [95%CI] = 1.12 (1.02, 1.22) and 1.01 (1.00, 1.02)). In males, methylparaben (MEPA) and propylparaben (PRPA) were associated with increased overweight [OR (95% CI) = 1.14 (1.00, 1.29) and 1.04 (1.00, 1.09), per doubling increase]. Also in males, diisononyl phthalate (DiNP) metabolites —ohMiNP and oxoMiNP—were associated with higher overweight odds [OR (95% CI) = 1.47 (1.05, 2.08) and 1.37 (1.04, 1.80)]. Heterogeneity across cohorts was low to moderate (I^2 = 1–34%).**Conclusion:** Our findings suggest that prenatal exposure to certain parabens and phthalates may contribute to childhood obesity, with potential sex-specific effects.

P05-10 | The associations between lifestyle factors and urinary phthalates exposure in children: The Hokkaido Study

Yi Zeng[1,2], Yu Ait Bamai[2,3], Rahel Mesfin Ketema[1,2], Takeshi Yamaguchi[2], Ayaka Yasuda[1,4], Megasari Marsela[1], Sachiko Itoh[2], Reiko Kishi[2], Atsuko Ikeda[1,2]

[1] Hokkaido University, Faculty of Health Sciences, Sapporo, Japan; [2] Hokkaido University, Center for Environmental and Health Sciences, Sapporo, Japan; [3] University of Antwerp, Toxicological Center, Antwerp, Belgium; [4] Health Sciences University of Hokkaido, Graduate School of Pharmaceutical Sciences, Toubetsu, Japan

Objective: Phthalates, commonly used plasticizers in plastic products, can leach out and cause endocrine-disrupting effects through human exposure. This study investigated the relationship between lifestyle factors and urinary phthalate metabolite concentrations in children, and identified the relative importance of lifestyle factors using an interpretable machine learning method.

Material and Methods: Data and urine samples were collected from 421 Japanese children aged 9 to 12 years between 2017 and 2020. Ten urinary phthalate metabolites were measured by UPLC-MS/MS. Children's lifestyle factors including usage frequency and heating frequency of plastic products, usage frequency of personal care products, household environment, and basic characteristics were reported by mothers through a questionnaire. The associations between lifestyle factors and urinary phthalate metabolite concentrations were examined using regression models. The relative importance of lifestyle factors was evaluated using gradient boosting decision trees, with SHapley Additive exPlanation (SHAP) method for interpretation.

Results: All phthalate metabolites were detected in > 87% of children. Usage of plastic cookware (≥once/week) was reported by >70% of participants, while microwave heating of plastic lunchboxes and single-use containers (≥once/week) was reported by 5.4% and 18.5%, respectively. ΣDEHP (di(2-ethylhexyl) phthalate) levels increased significantly with each additional time/week of heating lunchboxes (β: 0.07, 95%CI: 0.02-0.12) and single-use containers (β: 0.05, 95%CI: 0.00-0.10) per week. SHAP analysis identified plastic utensil usage and heating frequency as key predictors of internal exposure to DEHP. While usage frequency of plastic utensils showed no clear association, heating frequency of plastic utensils >4 times/week predicted elevated ΣDEHP concentrations.

Conclusion: These findings suggest that microwave heating of plastic utensils may enhance phthalate leaching to food, potentially increasing internal exposure of DEHP.

Funding sources: The Environment Research and Technology Development Fund (JPMEERF20175053, JPMEERF20205002), JSPS KAKENHI A (JP21H04843).

P05-11 | Nationwide analysis of temperature-related ambulance dispatches in Czechia: Children, adolescents, and young adults as the most vulnerable groups

Tomáš Janoš[1,2], Joan Ballester[2], Raúl F. Méndez Turrubiates[2], Pavel Čupr[1], Hicham Achebak[2,3]

[1] Masaryk University, RECETOX, Faculty of Science, Brno, Czech Republic; [2] ISGlobal, Barcelona, Spain; [3] Inserm, France Cohortes, Paris, France

Only little is known about the impact of ambient temperature on emergency medical services. Here, we conducted the first nationwide study on the association between temperature and ambulance dispatches in Europe, including a comprehensive description of age-specific risks for 10-year age groups to identify the most vulnerable population subgroups.
We collected daily time-series of ambulance dispatches by 10-year age groups, and population-weighted daily mean temperatures for each district of Czechia (n=77) from 2010 to 2019. We applied a quasi-Poisson regression with distributed lag non-linear models to estimate the relationship between temperature and ambulance dispatches. Then, we fitted a multilevel multivariate meta-analysis to derive average associations across the country and calculated the burden of ambulance dispatches attributable to non-optimum temperatures.
The risk of ambulance dispatches due to heat was highest among young people (<29 years) and then declined during the rest of life. Likewise, the cold-related risk was the highest in childhood and adolescence, declined in young adults before gradually increasing in older ages. High and low temperatures were responsible for 2.32% (95% confidence interval = 2.15-2.47) and 1.23% (1.16-1.30) of all ambulance dispatches, respectively. This difference in heat- and cold-attributable ambulance dispatches was even more pronounced in age groups below 59 years. For instance, the attributable fraction of ambulance dispatches due to heat in young adults (20-29 years) was more than 17 times greater than the cold-related attributable fraction (7.93% [7.28-8.50] vs. 0.45% [0.37-0.53]).Our findings have important implications for healthcare system preparedness and provide important evidence for better management of existing medical and emergency resources. The results also suggest that public health policies and adaptation strategies need to be extended and refocused to consider a wider range of vulnerable subgroups (children, adolescents, and young adults), which are often overlooked.

P05-12 | A Novel Method for Estimating Children's Soil and Dust Ingestion Rates Using Simultaneous Measurements of Multiple Metal Tracers

Yongcheng Li[1], Olufunke Dina[2], Yeongkwon Son[2], You Fu[1], Dingsheng Li[1], Patrick Hurbain[2], Li Li[1]

[1] *University of Nevada, Reno, School of Public Health, Reno, USA;* [2] *Desert Research Institute, The Organic Analytical Laboratory, Reno, USA*

Objective: Children are exposed to various chemical contaminants in the environment through hand-to-mouth activities. Accurately estimating the rate of dust and soil ingestion is crucial for effective exposure assessment and risk evaluation. However, current methods, which typically rely on a single tracer element, suffer from high uncertainty and low reproducibility. In this presentation, we introduce a novel approach that combines measurements of multiple metal elements with optimization techniques to estimate soil and dust ingestion rates in children.

Material and Methods: This study assessed daily ingestion rates of dust and soil particles in over 100 healthy children from Reno and Sparks, Nevada. A total of 32 metal tracer elements were selected to balance their intake doses (via ingestion of soil, dust, and additional exposure pathways) against elimination doses (through urination and fecal egestion), as these metals are primarily excreted through these pathways. An optimization approach, based on a mass balance framework, was applied to minimize contributions from extra exposure pathways (η) and deviations in urination and fecal excretion from the population average (∂), identifying the most accurate tracer combination for children's daily dust and soil ingestion rate estimation.

Results: Among all tested metal combinations derived from the 15 most frequently detected elements, the combination of Ce-140, Pb-208, Tl-205, La-139, and Nd-146 exhibited the highest detection rate and provided the most reliable ingestion estimates. Using this combination, the median daily dust ingestion rate was estimated at 29.6 mg/d (range: 0.14–168 mg/d), while the median daily soil ingestion rate was 7.9 mg/d (range: 0.04–110 mg/d). This combination also minimized contributions from extra exposure pathways ($\eta = 0.47$) and deviations in urination and fecal excretion ($\partial < 0.001$).

Conclusion: By leveraging data from multiple chemical tracers, our method overcomes the limitations of traditional approaches and thus improves accuracy and reliability.

P05-13 | Prenatal Urinary Metal Concentrations and Neurodevelopmental Outcomes in Early Childhood: The Healthy Start Study

Dorothy Nakiwala[1], Celeste Connell[1], Fátima Pacheco Vásquez[1], Oniza Chaman[1], Greta Wilkening[2], Jared Brown[3], Stephen Brindley[3], Dana Dabelea[1], Anne Starling[1,4]

[1] University of Colorado Anschutz Medical Campus, Lifecourse Epidemiology of Adiposity and Diabetes (LEAD) Center, Colorado School of Public Health, Aurora, USA; [2] University of Colorado Anschutz Medical Campus, Department of Pediatrics, Children's Hospital Colorado, Aurora, USA; [3] University of Colorado Anschutz Medical Campus, Department of Pharmaceutical Sciences, Skaggs School of Pharmacy and Pharmaceutical Sciences, Aurora, USA; [4] University of North Carolina at Chapel Hill, Chapel Hill, NC, USA, Department of Epidemiology, Gillings School of Global Public Health, Chapel Hill, USA

Background: Metals are widespread environmental contaminants with both essential and toxic properties. While copper, manganese, selenium, and zinc support neurodevelopment, excessive levels may be neurotoxic, cadmium, uranium, arsenic, and lead are known neurotoxicants. Prenatal exposure to these metals raises concerns due to potential adverse effects on fetal brain development.

Methods: We examined associations between prenatal urinary metal concentrations and cognitive, motor, and behavioral development in children aged 4–6 years. Participants were 252 mother-child dyads from the Healthy Start cohort, with mid-pregnancy urinary metal concentrations measured via inductively coupled plasma mass spectrometry and creatinine-adjusted. We assessed children's executive function (with subsets of the NIH Toolbox specifically, the Flanker and Picture Vocabulary tests), fine and gross motor skills (Ages and Stages Questionnaire-3), and internalizing and externalizing behavioral problems (Child Behavior Checklist 1.5-5). Linear and logistic regression models, adjusted for covariates, were used to estimate associations between log-transformed metal concentrations and the continuous or binary neurodevelopmental outcomes respectively.

Results: The median age of children at assessment was 4.8 years. Higher prenatal cadmium exposure was significantly associated with better inhibitory control (Flanker test: $\beta = 0.74$, $p = 0.042$) but also linked to greater internalizing behavioral problems ($\beta = 0.56$, $p = 0.033$). Uranium exposure was associated with lower odds of poor fine motor performance (OR = 0.80, $p = 0.017$). Higher zinc exposure was non-significantly associated with greater odds of poor fine motor function (OR = 1.11, $p = 0.082$), while higher manganese exposure showed a non-significant inverse association with total behavioral problems ($\beta = -0.41$, $p = 0.099$).

Conclusions: These findings highlight the complex relationship between prenatal metal exposures and neurodevelopment. Future research should explore mixtures and nonlinear associations to better understand combined effects and threshold-dependent toxicity.

Funding: This work was supported by the National Institutes of Health (R01DK076648, R01ES032213).

P05-14 | *Karenia brevis* Algal Blooms and Emergency Department Visits among Children Living in Coastal Southwest Florida

Erik Rizzo[1], Bianca Irimia[1], Justin Arnold[2,3], Barbara Kirkpatrick[5], Catherine Bulka[1], Sources of Funding: This work was supported by the National Institutes of Health (R03 ES036276) supporting JKA and CMB.Acknowledgements: Research reported in this publication was supported in part by the OneFlorida+ Clinical Research Network, funded by the Patient-Centered Outcomes Research Institute numbers CDRN-1501-26692, RI-CRN-2020-005 and RI-FLORIDA-01-PS1; in part by the University of Florida Clinical and Translational Science Institute, which is supported in part by the NIH National Center for Advancing Translational Sciences under award number UL1TR001427 and UL1TR000064. The content is solely the responsibility of the authors and does not necessarily represent the official views of the Patient-Centered Outcomes Research Institute (PCORI), its Board of Governors or Methodology, the OneFlorida+ Clinical Research Network, the UF-FSU Clinical and Translational Science Institute, or the National Institutes of Health.

[1] University of South Florida, Department of Epidemiology, College of Public Health, Tampa, USA; [2] University of South Florida, Department of Emergency Medicine, College of Medicine, Tampa, USA; [3] Florida Poison Information Center, Tampa, USA; [4] Tampa General Hospital, Tampa, USA; [5] Texas A&M University, Gulf of Mexico Coastal Ocean Observing System, Department of Oceanography, College Station, USA

Objective: Algal blooms of *Karenia brevis*, known colloquially as ‘red tide,’ produce toxins (brevetoxins) that contaminate seawater, bioaccumulate in shellfish, and are aerosolized by surf and wind, resulting in human exposures via ingestion and inhalation. Several epidemiologic studies have linked exposures to gastrointestinal and respiratory symptoms in adults. However, there are no studies on children, who may be especially vulnerable due to their smaller body sizes and increased respiratory rates. We performed a case-crossover study of children to investigate whether short-term environmental exposures to brevetoxins trigger emergency department visits, overall and by diagnoses.

Materials and Methods: We included 2,891 children aged 0-18 years who resided in coastal southwest Florida and visited an emergency department within the OneFlorida Clinical Research Network between 2012-2019. We spatiotemporally linked each visit to water concentrations of *Karenia brevis* and wind measurements based on residential zip code and date of admission. The case window was 30 days before each visit, with two control windows of 30 days each before and after. We fit conditional logistic regression models to compute odds ratios for elevated *Karenia brevis* concentrations, defined as >100,000 cells/Liter (a level known to predictably cause widespread mortality in marine wildlife), contrasting levels in the case window to the control windows. Average wind speed and direction were included as time-varying covariates.

Results: The association between elevated *Karenia brevis* concentrations and overall pediatric emergency department visits was near null (OR: 0.93, 95% CI: 0.73-1.20). However, suggestive positive associations were observed for gastrointestinal diseases (273 cases; OR: 1.89, 95% CI: 0.79-4.49) and asthma (70 cases; OR: 2.54, 95% CI: 0.46-14.2).

Conclusion:*Karenia brevis* blooms may contribute to increased emergency care utilization by children in coastal southwest Florida. Further studies with more refined methods for exposure assessment are needed to confirm these findings and identify common exposure pathways.

P05-15 | Prenatal Arsenic Exposure is Associated with Gut Microbiome Composition in Children

Maria J. Banas[1], Jamil Lane[2], Libni A. Torres-Olascoaga[3], Ryan W. Walker[2], Robert O. Wright[2], Manish Arora[2], Martha María Téllez-Rojo[3], Vishal Midya[2], Shoshannah Eggers[1]

[1] University of Iowa College of Public Health, Department of Epidemiology, Iowa City, USA; [2] Icahn School of Medicine at Mount Sinai, Department of Environmental Medicine, New York, USA; [3] National Institute of Public Health, Center for Research on Nutrition and Health, Cuernavaca, Mexico

Background

Gestational exposures can be associated with composition and function in the gut microbiome of children into adolescence. Arsenic (As) has strong interactions with the human gut microbiome; however it is unclear whether prenatal exposures to arsenic influence the gut microbiome in childhood and adolescence.

Objectives

We assessed associations between maternal As exposure during pregnancy and children's gut microbiome at 9-11 years old.

Methods

Data came from the Programming Research in Obesity, Growth, Environment and Social Stressors (PROGRESS) cohort and its microbiome sub-study, based in Mexico City, Mexico. As exposure data came from maternal whole blood samples taken during the second and third trimesters of pregnancy. Children's stool samples collected at 9-11 years old were used for metagenomic gut microbiome sequencing. Data analysis included generalized linear regression of alpha diversity (Shannon) and individual taxa, and permutational analysis of variance (PERMANOVA) of beta diversity (Bray-Curtis). All analyses were adjusted for relevant confounders and individual taxa p-values were FDR corrected for multiple comparisons.

Results

From 123 mother-child pairs, 74 children were males and 49 were females. Mean prenatal As exposure was 0.785 (SE= 0.038) µg/L and 0.915 (SE= 0.066) µg/L for mothers during their second and third trimester, respectively. There was no significant association between prenatal As exposure and alpha diversity. PERMANOVA analysis yielded R^2 value of 0.018 (p-value = 0.037) for the third trimester. Individual taxa regressions included associations between As exposure in the second trimester and Lachnospiraceae (FDR p-value = 0.05), and a trend in the third trimester for As exposure and Eggerthella sp. (FDR p-value = 0.11).

Discussion

Results suggest an association between prenatal As exposure and children's gut microbiome at 9-11 years old. Third trimester may be a particularly critical window of exposure, however further well-powered studies are needed to support this conclusion.

P05-16 | Ambient air pollution and cognitive functions in school-aged children in Accra, Ghana

Kate A. Kyeremateng[1], Sarah A. Abosede[2], James Nimo[3], Allison F. Hughes[4], Geofrey K. Kotoka[4], Lilly Adzrakor[4], Nancy Dery[4], Philomina Oppong[4], Elizabeth Bertone-Johnson[1], Hannah Laue[1], Majid Ezzati[5,7], Raphael Arku[2], Youssef Oulhote[9]

[1] University of Massachusetts Amherst, Department of Biostatistics and Epidemiology, Amherst, USA; [2] University of Massachusetts Amherst, Department of Environmental Health Sciences, Amherst, USA; [3] State University of New York, Albany, Department of Department of Environmental & Sustainable Engineering, Albany, USA; [4] University of Ghana, Department of Physics, Accra, Ghana; [5] Imperial College, Department of Epidemiology and Biostatistics, School of Public Health, London, UK; [6] University of Ghana, Regional Institute for Population Studies, Accra, Ghana; [7] Imperial College, Abdul Latif Jameel Institute for Disease and Emergency Analytics, London, UK; [8] Imperial College, MRC Centre for Environment and Health, School of Public Health, London, UK; [9] Icahn School of Medicine at Mount Sinai, Department of Environmental Medicine and Climate Sciences, New York, USA

Background: Emerging evidence has linked ambient air pollution to cognitive function in children, but evidence is lacking for Sub-Saharan Africa (SSA) where air pollution levels are rising in fast-growing cities.

Objective: We examined the associations between ambient air pollution exposure and cognitive function in schoolchildren in Accra, Ghana, one of the fastest growing metropolises in SSA.

Methods: Our analysis involved 862 children (ages 8–12) from 90 elementary schools (75% public) recruited within the Accra School Health and Environment Study (ASHES). Cognitive functions including executive function, attention, processing speed, working memory, and episodic memory were assessed using the NIH Toolbox cognition battery. Ambient $PM_{2.5}$, BC, and NO_2 exposures were estimated using land-use regression models linked with the children's home and school locations. We estimated covariate-adjusted associations of the individual exposures with the outcomes using linear mixed-effects models, Generalized Additive Models (GAM), and of mixtures using quantile G-computation.

Results: The mean scores of the different cognitive function measures were: Flanker (6.40, range: 0–9.50), Card Sort (5.94, range: 0–9.60), List Sort (13.00, range: 0–26.00), Pattern Comparison (33.75, range: 11.00–56.00), Picture Sequence (-1.13, range: -2.19–1.55), and Composite (78.2, range: 44-112). The annual exposures of $PM_{2.5}$, BC, and NO_2, ranged 28.6–39.5, 3.51–10.3, and 21.8–103.6, respectively. We observed small inverse but nonsignificant associations between each pollutant and cognitive measures in all models. The effect of NO_2 on Flanker was significant in males (p-interaction < 001), while $PM_{2.5}$ was significant in private schoolchildren (-0.085, 95% CI: -0.175, -0.001).

Conclusion: We found no evidence of associations between ambient air pollution and cognitive function in Ghanaian schoolchildren. Additional studies, particularly, prospective studies investigating specific windows of susceptibility are needed to understand its broader implications for public health.

P05-17 | Associations between blood PFAS concentrations and 25-hydroxyvitamin D levels in Hispanic children with overweight/obesity from Northern Virginia

Rooshna Mohsin[1], Margaret T. Jones[2], Daniel Tomer[3], Jenna R. Krall[1], Allison Mckay[4], Lameck Chiwaka[1], Roy R. Gerona[3], Michael S. Bloom[1]

[1] George Mason University, Department of Global and Community Health, College of Public Health, Fairfax, USA; [2] George Mason University, College of Education and Human Development, Sport, Recreation, and Tourism Management, Fairfax, USA; [3] University of California at San Francisco, Department of Obstetrics, Gynecology and Reproductive Sciences, San Francisco, Germany; [4] George Mason University, Department of Nutrition and Food Studies, College of Public Health, Fairfax, USA

Objectives

To quantify the association between blood perfluoroalkyl substances (PFAS) concentrations and 25-hydroxyvitamin D as a biomarker of vitamin D in Hispanic children.

Materials and Methods

Hispanic children who were overweight/obese (n=126) were enrolled in a weight loss intervention from 2017-2021. We measured children's anthropometry, and parents' completed questionnaires about diet, socioeconomic factors, and acculturation. Blood samples were collected from the children to analyze serum 25-hydroxyvitamin D. Twelve PFAS were measured in pre-intervention blood specimens using liquid chromatography-tandem mass spectrometry. Generalized linear regression was used to estimate associations between log-transformed pre-intervention PFAS and 25-hydroxyvitamin D measures, adjusted for age, sex, maternal education as a measure of socioeconomic status, year of enrollment, physical activity, dairy intake, beef intake, and fish intake. Principal component analysis (PCA) was used to characterize the mixture of multiple PFAS.

Results

Children were 4-11 years old (mean=7.85); 90% had obesity (BMI > 95th age/sex percentile), and 50% had severe obesity (BMI ≥ 120% above 95th age/sex percentile). The mean 25-hydroxyvitamin D was 20.72 ng/mL with a standard deviation of 10.99 (range 1.30-61.20); 50% of children met the Institute of Medicine's recommended 25-hydroxyvitamin D levels. Higher levels of 25-hydroxyvitamin D were associated with greater perfluorohexanesulfonic acid (PFHxS) (mean difference (MD): 6.79 ng/mL, 95%CI: 2.47, 11.10) and perfluorooctanesulfonic acid (PFOS) (MD: 4.56 ng/mL, 95%CI: 0.67, 9.78). Using PCA, a mixture of perfluoroheptanoic acid (PFHpA), perfluorooctanoic acid (PFOA), and perfluorononanoic acid (PFNA) was associated with lower levels of 25-hydroxyvitamin D (MD: -2.58 ng/mL, 95%CI: -5.57, -0.04), adjusted for other PFAS and covariates.

Conclusion

Higher blood PFHxS and PFOS were individually associated with higher 25-hydroxyvitamin D, while a higher PFHpA, PFOA, and PFNA mixture level was associated with lower 25-hydroxyvitamin D in Hispanic children with overweight/obesity. Future larger studies are needed to confirm these findings and identify their health implications.

P05-18 | Dental caries in childhood is associated with gestational and early life exposure to secondhand smoke

Sneha Patil[1], Andres Cardenas[2], Ajay Pillarisetti[3], Kyle Steenland[4], Smita Patil[8], Akash Navilebasappa[7], Santu Ghosh[5], Kalpana Balakrishnan[1], Lance Waller[4], Shirin Jabbarzadeh[4], Jennifer Peel[6], Thomas Clasen[4], Dana Barr[4], Naveen Puttaswamy[1]

[1] Sri Ramachandra Institute of Higher Education and Research, Chennai, India; [2] Stanford University, Stanford, USA; [3] University of California, Berkeley, Berkeley, USA; [4] Rollins School of Public Health, Emory University, Atlanta, USA; [5] St. John's Medical College, Bengaluru, India; [6] Colorado State University, Fort Collins, USA; [7] MetaOmics Laboratory Services, Bengaluru, India; [8] Private Practitioner, Pune, India

Objective: We evaluated associations of gestational and early postnatal secondhand smoke (SHS) exposure with early childhood caries (ECC) among preschoolers.

Methods: This study is mounted on a prospective birth cohort (HAPIN trial) conducted in southern India (2018–2020). Comprehensive data on exposure, lifestyle, and health were collected at multiple maternal and child time points. Between April and September 2023, dental caries and current exposure were assessed in children aged 3 to 5 years by a trained dentist using standardized, calibrated methods. SHS exposure was measured via questionnaires and urinary nicotine metabolites (cotinine, 3′ hydroxycotinine). Negative binomial hurdle models were used to assess associations between SHS exposure and ECC prevalence.

Results: Among 712 participants (mean age: 4.01 ± 0.45 years; 53.4% boys), 478 children (67.1%) were diagnosed with ECC. Children exposed to SHS during gestational period exhibited significantly higher prevalence of caries compared to their unexposed counterparts, with an incidence rate ratio (IRR) of 1.28 (95% CI, 1.17–1.39); second trimester was identified as critical window of vulnerability. Similarly, postnatal exposure increased ECC risk (IRR, 1.18; 95% CI, 1.08–1.30). Paternal smoking significantly elevated ECC prevalence, with children whose fathers smoked ≥11 cigarettes/day showing higher odds of ECC (odds ratio (OR), 1.90; 95% CI, 1.10–3.41). Urinary nicotine metabolites exhibited a dose-response relationship with ECC, showing the strongest association during pregnancy. Children classified as slow metabolizers were more vulnerable to third-trimester SHS exposure (OR, 1.74; 95% CI, 1.13–2.68).

Conclusion: SHS exposure from pregnancy through early childhood was linked to higher odds of developing dental caries in childhood. The findings underscore another critical health risk associated with SHS exposure and emphasize the need to reduce this exposure and mitigate the health and economic burdens of ECC.

P05-19 | Burden of infant mortality associated with flood in 37 African countries

Yixiang Zhu[1], Cheng He[1,2], Jovine Bachwenkizi[3], Zafar Fatmi[4], Lu Zhou[1], Jian Lei[5], Cong Liu[1], Haidong Kan[1], Renjie Chen[1]

[1] Fudan University, Shanghai, China; [2] Harvard University, Boston, USA; [3] Muhimbili University of Health and Allied Sciences, Dar es Salaam, Tanzania; [4] Aga Khan University, Karachi, Pakistan; [5] Xi'an Jiaotong University, Xi'an, China

Objective: To investigate the associations between short-, mid-, and long-term flood exposure and infant mortality, and to estimate the burden of flood-related infant mortality in Africa in 2000-2020.
Material and Methods: We conducted a sibling-matched case-control study using individual-level data from Demographic and Health Surveys in Africa during 1990–2020. Individual flood experience was determined by matching the residential coordinates with flood events from the Dartmouth Flood Observatory database. The conditional logistic regression was used to analyze the association between flood and infant mortality.
Results: Using data from 514,760 newborns, we found increased risks of infant mortality associated with flood exposure across multiple periods. The risk of infant death remained elevated for up to four years after the flood event, resulting in an increase of 9.69 infant deaths (95% confidence interval: 7.70, 11.69) per 1,000 births. These associations were particularly prominent among female infants, those born to mothers with lower education levels, or those residing in rural areas, houses with basic water or sanitation infrastructures. Overall, flood exposure caused 3.42 infant deaths per 1,000 births in Africa from 2000 to 2020, which was about 1.7 times the burden attributable to life-period flood exposure. We also revealed a general increase in the burden of infant mortality attributable to floods in Africa over the first two decades of the 21st century, with a sharp rise in the last 5 years.
Conclusion: This multi-country study in Africa provides novel and compelling evidence that flood events could have significant impacts on infant mortality risk and burden, even over 4 years after exposure.
Funding/Support: This study was supported by National Natural Science Foundation of China grant, National Key Research and Development Program grant, Shanghai International Science and Technology Partnership Project, and a grant from the Alexander von Humboldt Foundation for the Humboldt Research Fellowship.

P05-20 | Air pollution and pulmonary function and respiratory health in schoolchildren in Accra, Ghana

Jessica Kumah[1], Ruixue Dai[2], Nancy Derry[3], Abosede S. Alli[1], Kate A. Kyeremanteng[1,4], James Nimo[3], Prince Anku[5], Lilly Adzrakor[5], Geofrey K. Kotoka[5,6], Philomina Oppong[5], Adwoa Asante-Poku[7], Mavis Amoa-Dadzeasah[8], Shivani Jadav[2], Padmaja Subbarao[9], Theo Moraes[9], Majid Ezzati[10,11], Allison F. Hughes[3], Raphael E. Arku[1]

[1] University of Massachusetts, Department of Environmental Health Sciences, Amherst, USA; [2] The Hospital for Sick Children, Translational Medicine Program, Department of Pediatrics, Toronto, Canada; [3] University of Ghana, Legon, Department of Physics, Accra, Ghana; [4] University of Massachusetts, Department of Epidemiology and Biostatistics, Amherst, USA; [5] University of Ghana, Legon, Department of Physics, Accra, Ghana; [6] University of Ghana, Legon, School of Public Health, Accra, Ghana; [7] University of Ghana, Legon, Noguchi Memorial Institute for Medical Research, Accra, Ghana; [8] Concordia University, Department of Mathematics and Statistics, Montreal, Canada; [9] The Hospital for Sick Children, Department of Pediatrics, Division of Respiratory Medicine, Translational Medicine, PGCRL Research Institute, Toronto, Canada; [10] Imperial College London, Department of Epidemiology and Biostatistics, School of Public Health, London, UK; [11] Imperial College London, MRC Centre for Environment and Health, School of Public Health, London, UK

Background: While children living in sub-Saharan African (SSA) cities have overall health advantage over their rural counterparts, they are also at higher risk of exposure to urban environmental pollution. Air pollution exposure can affect children's respiratory health and harm their growing lungs.

Objective: We examine the effects of air pollution on chronic respiratory symptoms and lung function in Accra schoolchildren. We also characterize bacterial composition in their exhaled bioaerosols.

Methods: In the Accra School Health and Environment Study (ASHES) conducted in 1,037 schoolchildren (8-12 years) from 90 schools, parents reported on chronic respiratory conditions (cough, wheeze, and asthma) and other household factors. Lung function tests were performed in a subset of 709 participants using handheld EasyOne spirometer to measure forced vital capacity (FVC) and forced expiratory volume in 1 second (FEV_1). In a subset (n=237), we conducted microbial analysis of the exhaled air captured on the spirometry filters. Exposure to ambient $PM_{2.5}$, black carbon (BC), and NO_2, assessed using land use regression models at the children's home and school were regressed separately against each outcome.

Results: Chronic respiratory symptoms were reported in 46% of the children. Parent-reported use of biomass for cooking was associated with 40% [OR = 1.40 (95% CI: 1.05–1.87)] higher odds of children having respiratory symptoms compared to clean fuel. $PM_{2.5}$, NO_2 and BC were associated with decreased lung function. E.g. An IQR increase in BC was associated with 31.04 mL lower FEV_1 (95% CI: -61.82, -0.25). High prevalence of *Bacillus cereus* (86%), *E. coli* (30%), and *Streptococcus salivarius* (24%) were identified in the exhaled bioaerosols, and could be a relevant (mechanistic) biomarker of respiratory infection in schoolchildren.

Conclusion: Air pollution adversely influences overall respiratory health among children living in a major metropolitan area in SSA, highlighting the need for air quality interventions in SSA cities.

P05-21 | Early-Life Exposure to Phthalates and Bisphenols, and Allergies in Children: A Pooled Analysis of Cohort Studies

Thomas Boissiere-O'Neill[1], Nina Lazarevic[2], Peter Sly[1], Anne-Louise Ponsonby[3], Aimin Chen[4], Meghan Azad[5], Joseph Braun[6], Jeffrey Brook[7], David Burgner[8], Bruce Lanphear[9], Theo Moraes[10], Richard Saffery[8], Padmaja Subbarao[9], Stuart Turvey[11], Kimberly Yolton[12], Dwan Vilcins[1], On behalf of BIS and CHILD investigator groups

[1] The University of Queensland, Child Health Research Centre, The Children's Health and Environment Program, Brisbane, Australia; [2] Australian National University, National Centre for Epidemiology and Population Health, Canberra, Australia; [3] The University of Melbourne, Florey Institute of Neuroscience and Mental Health, Melbourne, Australia; [4] University of Pennsylvania, Department of Biostatistics, Epidemiology and Informatics, Perelman School of Medicine, Philadelphia, USA; [5] University of Manitoba, Children's Hospital Research Institute of Manitoba, Department of Pediatrics and Child Health, Winnipeg, Canada; [6] Brown University, Department of Epidemiology, Providence, USA; [7] University of Toronto, Dalla Lana School of Public Health, Toronto, Canada; [8] The University of Melbourne, Murdoch Children's Research Institute, Royal Children's Hospital, Melbourne, Australia; [9] Simon Fraser University, Faculty of Health Sciences, Burnaby, Canada; [10] University of Toronto, Department of Pediatrics, Hospital for Sick Children, Toronto, Canada; [11] University of British Columbia, Department of Pediatrics, Child and Family Research Institute, Vancouver, Canada; [12] University of Cincinnati, College of Medicine and Department of Pediatrics, Cincinnati Children's Hospital Medical Center, Cincinnati, USA

Objective

Exposure to plastic additives, such as phthalates and bisphenols, has been associated with an increased risk of asthma and allergies, but the evidence is inconsistent for children younger than five. We examined the association between exposures to pre- and postnatal phthalates and bisphenols and childhood allergies through a pooled analysis.

Material and Methods

We pooled data from the Barwon Infant Study (Australia), the Canadian Healthy Infant Longitudinal Development study (Canada), the Health Outcomes and Measures of the Environment (USA) and the Environmental influences on Child Health Outcomes–wide cohorts (USA). Urinary phthalates and bisphenols were measured during pregnancy and early childhood. We estimated daily intakes from urinary concentrations. Outcomes, including asthma, wheeze, eczema, and rhinitis, were assessed up to five years of age. We used generalised estimating equations for single compounds and quantile G-computation for the chemical mixtures.

Results

5,306 children were included. A two-fold increase in prenatal dibutyl phthalates (DBP; risk ratio [RR] = 1.08; 95% confidence interval [CI]: 1.00-1.16) and benzyl butyl phthalate (BBzP; RR=1.06; 95% CI: 1.00-1.12) increased the risk of asthma in children under five. Prenatal MCPP levels were associated with rhinitis (RR=1.05; 95%CI:1.01-1.09). Postnatal BBzP levels increased the risk of wheezing (RR=1.09; 95%CI 1.06-1.12), as well as di(2-ethylhexyl) phthalate (DEHP; RR=1.06; 95%CI: 1.01-1.11) and MCPP (RR=1.09; 95%CI: 1.04-1.14). These were also inversely associated with eczema: BBzP (RR=0.96; 95% CI: 0.94-0.98), DEHP (RR=0.95; 95% CI: 0.91-0.98), and MCPP (RR=0.95; 95%CI: 0.92-0.98). A one-quartile increase in the postnatal chemical mixture increased the risk of wheezing (RR=1.20; 95%CI: 1.09-1.33).

Conclusion

This pooled analysis suggests that phthalates increase the risk of asthma, wheezing, and rhinitis in young children. Most associations were consistent across cohorts for prenatal exposures, but postnatal exposure showed higher heterogeneity. These findings provide evidence of adverse effects from early-life exposure to chemicals found in plastics.

P05-22 | Prenatal metal and metalloid exposures and early childhood body mass index z-score in the Environmental Influences on Child Health Outcomes Cohort

Gyeyoon Yim[1], Mohamad Burjak[2], Megan Romano[1], Ahlam Abuawad[1], Meghan Muse[1], Shohreh Farzan[3], Patrick Parsons[4,5], Pi-I Lin[6], Anne Starling[7], Anat Sigal[8], Diane Gilbert-Diamond[1], Margaret Karagas[1], Miranda Jones[2], Debra MacKenzie[9], Caitlin Howe[1]

[1] Geisel School of Medicine at Dartmouth, Epidemiology, Lebanon, USA; [2] Johns Hopkins Bloomberg School of Public Health, Epidemiology, Baltimore, USA; [3] Keck School of Medicine at USC, Population and Public Health Sciences, Los Angeles, USA; [4] Wadsworth Center, New York State Department of Health, Environmental Health, Albany, USA; [5] University at Albany, State University of New York, Environmental Health Sciences, Albany, USA; [6] Harvard Pilgrim Health Care Institute, Boston, USA; [7] University of North Carolina, Chapel Hill, Epidemiology, Chapel Hill, USA; [8] Hackensack University Medical Center, Pediatrics, Hackensack, USA; [9] University of New Mexico Health Sciences Center, Pharmaceutical Sciences, Albuquerque, USA

Objective: Both essential and non-essential metals and metalloids (“metals”) influence biological processes important for energy homeostasis and growth. Increasingly such exposures have been associated with altered fetal growth, but less is known about impacts on postnatal growth. The current project assessed relationships between prenatal metal exposures and early childhood growth in the U.S. Environmental Influences on Child Health Outcomes (ECHO) cohort.

Material and Methods: Analyses included mother-child pairs from 10 sites participating in the ECHO Program who had maternal urinary metal concentrations measured in pregnancy, relevant covariate information, and child anthropometry data (weight and length/height) between birth and 18 months of age (n=2582) or between 18 months and 5 years of age (n=1591). Analyses focused on 11 widely detected metals (above limit of detection for >=70% of participants). Associations between the specific gravity-adjusted concentration of each metal and body mass index-for-age (BMI) z-score were assessed using separate covariate-adjusted linear mixed effects models with site modeled as a random intercept. In secondary analyses (1) quantile g-computation was used to evaluate metal concentrations simultaneously as a mixture and (2) primary models were stratified by child sex.

Results: Thallium and tin were associated with a smaller BMI z-score, while cesium was associated with a larger BMI z-score between birth and 18 months. Similar though not statistically significant patterns of association were observed for cesium and thallium and BMI z-score between 18 months and 5 years. Associations were largely consistent in leave-one-site out sensitivity analyses. Directions of association for each metal were consistent across single element and mixture analyses, and potential sex-dependent associations were identified for antimony, cesium, mercury, nickel, and tin.

Conclusion: Findings from this large demographically and geographically diverse U.S. cohort provide new insights into *in utero* exposure to metals and altered growth in early childhood, a predictor of future obesity risk.

P06 | Climate and Health: Exposures and Impacts

P06-01 | Estimating the Risk of Women Anemia Associated with Ozone ExposureAcross123 Low-and Middle- Income Countries: A Multicenter Epidemiological Study

Hao Xiang, Jiahui Tong, Shouxin Peng, Yuxuan Tan

Wuhan University, School of Public Health, Wuhan, China

Objective:
Anemia in women of reproductive age (WRA) presents a pressing global public health issue, particularly in low- and middle-income countries (LMICs). Yet, the potential impact of ozone (O_3) exposure on anemia remains uncertain.

Material and Methods:
The study population was collected from the Demographic and Health Surveys (DHS) program, which covers 93 developing countries. And this study included 1,467,887 eligible women from 83 surveys of 45 LMICs between 2004 to 2020. Monthly O_3 exposure was estimated using machine learning, with the year preceding the survey as the primary exposure window. Fixed-effects models evaluated the association between O_3 and anemia. An exposure-response function was constructed using a varying-coefficient regression model, and then extrapolated to estimate the anemia burden in relation to O_3 in 123 LMICs.

Results:
In the fully adjusted model, the odds ratio for the risk of anemia was 1.08 (95% CI: 1.07−1.09) and 1.05 (95% CI: 1.04−1.06), respectively. We investigated the nonlinear exposure-response curve connecting yearly O_3 exposure to the odds ratio of anemia in WRA, and found that O_3-related anemia exhibited a threshold effect, with the threshold concentration around 47.2 ppb. In 2020, more than 7.6 million anemic WRA (1.58%) in 123 LMICs were associated with O_3 exposure. The potentially attributable burden has generally decreased from 2004 to 2020, notably in South Asia.

Conclusion:
Integrating environmental exposures into the epidemiological framework of anemia underscores the urgent need for comprehensive, multicenter research to fully elucidate the impact of pollutants like O_3 on public health.
This work was supported by the National Natural Science Foundation of China, and the Fundamental Research Funds for the Central Universities.

P06-02 | A Framework for Examining Community Violence as a Climate-Related Environmental Exposure

Jane E. Clougherty[2], Ellen J. Kinnee[3], Perry E. Sheffield[1]

[1] Icahn School of Medicine at Mt Sinai, Dept of Environmental Medicine and Climate Science, New York, USA; [2] Drexel University Dornsife School of Public Health, Dept of Environmental and Occupational Health, Philadelphia, USA; [3] University of Pittsburgh, University Center for Social and Urban Research, Pittsburgh, USA

Introduction

Violent crime is increasing with climate change and ambient heat. Homicides are expected to increase 6% globally per ^{O}C increase in global annual mean temperature, with 2.3 - 3.2 M additional heat-attributable violent crimes in the U.S. by 2099. Greater increases are projected in communities already high in crime, or lacking resources for response, exacerbating inequities. Finally, exposure to community violence (ETCV), as a psychosocial stressor, may exacerbate impacts of climate-related extreme heat and pollution on health.

Methods

We have developed a framework to assess the multiple and complex pathways through which ETCV may impact population health in a warming climate. We are testing that framework using exhaustive data on mental health (MH)-related emergency department visits and hospitalizations among NYC children aged 6-17, for 2005-19, and case-crossover methods, comparing child's risk of MH event on days following violent crimes to their risk on other days.

Results

We are refining ETCV metrics by exploring nonlinearity in violence-MH relationships, to identify critical distances and time lags. We are testing the following key pathways in our framework:

1. Independent associations between ETCV and risk of child health event, adjusting for extreme heat and pollution.
2. Testing whether ETCV, as a stressor, exacerbates heat-health or pollution-health associations.
3. Testing whether some portion of heat-health associations may be mediated by (operate via) ETCV.

Discussion

This conceptual model and analysis will help to quantify the impact of community violence on child health, and reveal the multiple pathways through which violence may impact health in coming decades, separately and in combination with other climate-related exposures.

P06-04 | The Effect of Drought on Human Mortality in Zahedan, Southeastern Iran from 2006 to 2023

Neda Malek Mohammadi[1], Narges Khanjani[2,1], Bahram Bakhtiari[3], Yunes Jahani[1]

[1] Kerman University of Medical Sciences, Kerman, Iran; [2] Texas Tech Health El Paso, El Paso, USA; [3] Shahid Bahonar University of Kerman, Kerman, Iran

Introduction: Global climate change has led to severe weather shocks and natural disasters. One of these natural disasters is drought. The aim of the present study was to investigate the relation between drought and mortality in Zahedan (southeast Iran).
Method: The data used in this ecological study were from 2006 to 2023. The exposure variable was Standardized Precipitation Evapotranspiration Index (SPEI) at time scales of 1-3-6-9-12-24-36 and 48 months, and the outcome variable was different causes of mortality (total mortality, respiratory mortality, and cardiovascular mortality). Generalized linear models with negative binomial link were used to calculate the Relative Risks (RRs) of different causes of mortality. Analyses were conducted at lags of 0, 6, 12, and 24 months.
Result: The results of multivariate analysis in lags zero, 6 and 12 months showed that for total, cardiovascular and respiratory death, SPEI_36 and SPEI_48 remained as significant variables in the model, and with a one-unit decrease in SPEI_36 and SPEI_48 (more severity of drought), the relative risk of deaths increased. At 24 months lag, in total deaths, SPEI_9, SPEI_12, SPEI_24, SPEI_36 and SPEI_48 remained significant. For the association between cardiovascular deaths and drought, only SPEI_24, SPEI_36 and SPEI_48 remained significant. For the association between respiratory deaths and drought, SPEI_6, SPEI_9, SPEI_12, SPEI_24, SPEI_36, and SPEI_48 remained significant, and with a one-unit decrease in each of the above indicators, the relative risk of mortality increased.
Conclusion: The results of the present study showed that mortality rates increased with increased drought severity and decreased humidity. This study also found that drought has negative long-term effects on human health, since the SPEI-36 and SPEI-48 indices were significantly related to mortality.

P06-05 | Climate-Related Disaster Event Frequency Across Köppen-Geiger Climate Zones in the Australia and the Pacific Region

Nicholas J. Osborne[1,2], David J. Heslop[2], **Nathan George**[2]

[1] University of Queensland, School of Public Health, Herston, Australia; [2] University of New South Wales, School of Population Health, Kensington, Australia; [3] University of Exeter, European Centre for Enviroment and Human Health, Penryn, UK

Objective

We examined the frequency of climate-related disaster events across Köppen-Geiger climate zones in the Australia and the Pacific region to assess whether equatorial zones experience a disproportionate increase in disaster event frequency compared to arid and temperate zones.

Material and Methods

A retrospective analysis was conducted using disaster records from the Emergency Events Database (EM-DAT) over a 40-year period (1980–2020) classified by the Köppen-Geiger climate zone at the location of impact. Statistical comparisons of event frequency showed significant trends between geographical climate zones. Descriptive statistics and trend analyses were used to assess variations over time.

Results

Targeting events subject to climatic variation over a sample period of 43 years, n = 545 disaster events occurred within Australia and the Pacific. Analysis indicates a statistically significant increase in the frequency of disaster events in Köppen-Geiger A (tropical) zones. In comparison, only a moderate trend was observed in Köppen-Geiger C (temperate) zones, and no trend was observed in Köppen-Geiger B (arid) zones. The increase in equatorial Köppen-Geiger A (tropical) zones disaster event frequency has significant implications for Pacific Island Countries which are disproportionately affected by climate-related disasters.

Conclusion

Our study provides evidence of a disproportional increase in the frequency of climate-related disaster events in the equatorial climate zones of Australia and the Pacific region. Findings provide support for targeted climate adaptation and disaster preparedness strategies in vulnerable regions, especially the tropics where there are many low-to-middle income countries. Further research is necessary to assess whether the magnitude and duration of events is also increasing alongside frequency and and how these trends interact with broader socioeconomic and geopolitical factors.

P06-06 | Metrics of Rurality and Urbanicity in US-Based Epidemiologic Studies of Ambient Temperature and Health: A Scoping Review

Nicholaus P. Johnson[1], Alexandra Del Favero-Campbell[2], Amruta Nori-Sarma[3], Audrey Amezcua-Smith[4], Brandon Lewis[4], Chen Chen[5], Chengyi Lin[1], Damien Foo[4], Garam Byun[4], Hayon M. Choi[3], Honghyok Kim[6], Jesse Berman[7], Jiyoung Y. Son[4], Joshua L. Warren[8], Kai Chen[1], Kate Burrows[9], Kelvin Fong[10], Leo Goldsmith[4], Marie C. Meadows[7], Morrison Smith[11], Rory Stewart[4], Seulkee Heo[4], Shuqi Lin[8], Xuejuan Ning[1], Yongsoo Choi[4], Michelle L. Bell[4,12], Nicole C. Deziel[1]

[1] Yale School of Public Health, Department of Environmental Health Sciences, New Haven, USA; [2] Dalhousie University, Department of Earth and Environmental Sciences, Halifax, Canada; [3] Harvard T. H. Chan School of Public Health, Department of Environmental Health, Boston, USA; [4] Yale School of the Environment, New Haven, USA; [5] University of California San Diego, Scripps Institution of Oceanography, La Jolla, USA; [6] University of Illinois Chicago School of Public Health, Division of Environmental and Occupational Health Sciences, Chicago, USA; [7] University of Minnesota School of Public Health, Division of Environmental Health Sciences, Minneapolis, USA; [8] Yale School of Public Health, Department of Biostatistics, New Haven, USA; [9] University of Chicago, Department of Public Health Sciences, Chicago, USA; [10] George Washington University Milken Institute School of Public Health, Department of Environmental and Occupational Health, Washington D.C., USA; [11] The Pennsylvania State University, Computational and Spatial Analysis Core, Social Sciences Research Institute, State College, USA; [12] Korea University College of Health Sciences, School of Health Policy and Management, Seoul, South Korea

Objective: The impacts of environmental health risk factors, including temperature, vary across rural and urban areas. Application of different metrics of rurality and urbanicity can yield different risk characterizations. We aimed to identify, describe, and quantify how rural/urban metrics are used in epidemiologic studies of ambient temperature and health across the United States (US).

Material and Methods: Using PubMed and Scopus, we identified epidemiologic studies, published between January 1, 2010, to March 14, 2024, examining ambient temperature and health in the US that included defined, quantitative metric(s) of rurality/urbanicity. Titles, abstracts, and full texts were evaluated by two independent reviewers. Data from included studies were abstracted using a predetermined data extraction tool.

Results: Of the 10,338 studies resulting from our search, 30 were included. We identified 19 distinct metrics drawing from nine data sources. The most frequently used metrics were population density and size from the US Census (n=12). Other studies used metrics reflecting connectivity and proximity to metropolitan areas, such as the Census's Urban-Rural Classification (n=6), and US Department of Agriculture's Rural-Urban Commuting Area Codes (n=3), and Rural-Urban Continuum Codes (n=1). Additional metrics included features such as greenspace, economic integration, and road density. Many studies did not provide a rationale for metric selection.

Conclusion: Approaches have moved beyond traditional population size and density metrics to include other features (e.g., greenspace). Providing explicit rationale for choice of metric(s) or the differential vulnerability or adaptive capacity captured by the metric could bolster understandings of rural-urban differences in the impact of temperature on health.

P06-07 | Assessment of observed climate change in the Continental United States

Ray Yeager[1], Cascade Tuholske[3], Matthew H. Browning[2], Summer Olmsted[1], Alessandro Ossola[4], Dexter Locke[4]

[1] University of Louisville School of Medicine, Louisville, USA; [2] Clemson University, Clemson, USA; [3] Montana State University, Bozeman, USA; [4] United States Forest Service, Baltimore, USA; [5] University of California, Davis, Davis, USA

Objective: Holistically characterize long-term climate changes and population exposures across the Continental United States.
Material and Methods: We used Google Earth Engine to collect ERA5-Land climate reanalysis data of hourly and monthly climate metrics from 1970 through 2022, including measures of temperature, humidity, sunlight, wind, surface pressure, and rainfall. We also calculated heat index; annual, seasonal, daytime, and nighttime heat; heat waves and variability. We calculated climate anomalies as the difference between a 1970-1979 baseline mean and a 2013-2022 modern mean in all metrics. We aggregated these data to the U.S. NOAA climate division spatial scale to mitigate influences of localized landcover changes and assigned division-scale changes to nested tracts. We evaluated correlations between all metrics and conducted principal components analyses (PCA) to identify covarying changes in climate.
Results: We observed overall mean tract-level increases in temperature (1.05°C) and heat index (1.11°C) across the continental U.S., with extensive and inconsistent spatial variability of all anomaly metrics. Both temperature and heat index increase were greatest in the West. Relative humidity increased in the Southeast but there were much larger decreases in the West, with anomalies ranging from ranging from -5.26% to 1.92%. Precipitation anomalies were concentrated in the South-central U.S., surface pressure in the Northeast, and wind in the North and South-central areas of the country. Results of our PCA indicate climate changes that were highly intertwined, with no clear

driver of concurrent climate changes that explain 55% of total anomaly variation. However, principal components 2, 3, and 4 were clearly linked to respective sunlight, wind, and temperature anomalies.
Conclusion: The extent and nature of climate change varies greatly across the continental U.S. Future evaluation of long-term changes in a single measure will not capture the full extent of our changing climate.

P06-08 | Increasing zero-emissions vehicle adoption and reduced satellite-measured NO_2 air pollution in California

Sandrah Eckel[1], Futu Chen[1], Sam Silva[2], Daniel Goldberg[3], Jill Johnston[1], Lawrence Palinkas[4], Alberto Campos[5], Wilma Franco[5], Erika Garcia[1]

[1] University of Southern California, Department of Population and Public Health Sciences, Keck School of Medicine, Los Angeles, USA; [2] University of Southern California, Department of Earth Sciences, Dornsife College of Letters, Arts and Sciences, Los Angeles, USA; [3] The George Washington University, Department of Environmental and Occupational Health, Washington, D.C., USA; [4] University of California, San Diego, Herbert Wertheim School of Public Health and Human Longevity Science, San Diego, USA; [5] Southeast Los Angeles Collaborative (SELA), Bell Gardens, USA

Objective: Electrifying the transportation sector is a key climate change mitigation strategy. Related reductions in tailpipe emissions have anticipated air quality and public health co-benefits; yet, evidence is primarily based on projections. Using observed data in California, we evaluated whether reductions in tailpipe emissions from the transition to zero-emissions vehicles (ZEVs: battery electric, plug-in hybrid, and hydrogen fuel cell) were detectable using TROPOMI satellite measurements of tropospheric nitrogen dioxide (NO_2) air pollution.
Material and Methods: We combined data from 2019 to 2023 on annual light duty ZEVs registrations in 1692 zip code tabulation areas (ZCTAs; cross-walked from zip codes) in California with annual mean TROPOMI NO_2. Longitudinal linear mixed-effects models related within-ZCTA ZEV changes to within-ZCTA NO_2 changes, adjusting for temporal trends and time-varying potential confounding, or excluding the 2020 pandemic year. In positive control analyses, we related internal combustion engine vehicle registrations to NO_2. In ground-truth analyses, we related number of ZEVs to annual average NO_2 from 123 Environmental Protection Agency (EPA) monitors from 2012 to 2023.
Results: The median within-ZCTA increase in ZEVs from 2019 to 2023 in California was 270 (Q_1:17.0, Q_3:836.5). A within-ZCTA increase of 200 ZEVs was associated with a 1.11% decrease in annual average NO_2 (95%CI: -1.21%, -1.02%). Sensitivity, positive control, and ground-truth analyses supported main findings.**Conclusion**: Using the natural experiment of the transition to ZEVs in California, we found that within-ZCTA increases in ZEVs were associated with reductions in NO_2 air pollution measured by the TROPOMI satellite and replicated with ground-level EPA monitors. This work in California serves as a proof-of-principle for future work using satellite-measured NO_2 to evaluate impacts on combustion-related air pollution of climate change mitigation efforts within the U.S. and around the globe.

P06-09 | Temperature and emergency police calls in Phoenix, Arizona

Veronica A. Wang[1], Ernani Choma[1], Barrak Alahmad[1], Mary Wright[3,2], David Hondula[3], John D. Spengler[1], Antonella Zanobetti[1]

[1] Harvard TH Chan School of Public Health, Environmental Health, Boston, USA; [2] Arizona State University, School of Geographical Sciences and Urban Planning, Tempe, USA; [3] City of Phoenix, Office of Heat Response & Mitigation, Phoenix, USA

Objective: Despite mounting evidence of temperature effects on human health, few studies have quantified the impact on municipal emergency services. Even fewer have examined the potential burden on police departments. As part of the Smart Surfaces Coalition program and in cooperation with the City of Phoenix, we investigated whether hotter temperatures measured from weather stations increased police calls in the city of Phoenix, Arizona.
Material and Methods: Quasi-Poisson regression models, adjusted for seasonality and long-term trends, were fitted separately for the total number of calls. Using police calls data across eight years from 2016 to 2023, we examined calls for violent crimes, nonviolent crimes, car accidents, health, domestic emergencies, and sexual-based emergencies. We considered average day of and prior-day maximum and minimum temperature to gain insight into daytime and overnight conditions, respectively. We found a higher demand for police services during hotter periods.
Results: A 5˚C increase in maximum temperature was associated with a 1.1% (95% CI: 0.9%, 1.5%) increase in police calls overall. Higher temperatures were also associated with a greater number of calls related to violent crimes (2.6% [95% CI: 2.0, 3.2%]), health (4.2% [95% CI: 3.3%, 5.0%]), and domestic emergencies (3.1% [95% CI: 2.1%, 4.0%]). Results were similar and oftentimes stronger for minimum temperature, with the addition of elevated calls for nonviolent and sexual-based emergencies.
Conclusion: Even in Phoenix, where the climate is hot and dry and residents are presumably well adapted to heat, the number of police calls increased with temperature, and differentiating by call reasons allowed us to also gain insight into the potential drivers. Furthermore, our findings suggest that the inability to cool down overnight should be taken into consideration when allocating resources to emergency police services and for the well-being of the individuals who live, work, and play in Phoenix.

P06-10 | Effect of School-Based Educational Climate Change on Student's Knowledge, Attitude and Practice in Malaysia.

Zulkhairul Naim Bin Sidek Ahmad[1,2], Kurnia Ardiansyah Akhbar[3], TJ Robinson T. Moncatar[4], Roslinawati binti Mohd Roslan[5]

[1] Faculty of Medicine and Health Sciences, Universiti Malaysia Sabah, Department of Public Health Medicine, Kota Kinabalu, Malaysia; [2] Faculty of Medicine and Health Sciences, Universiti Malaysia Sabah, Department of Medical Education, Kota Kinabalu, Malaysia; [3] Faculty of Public Health, Jember University, Jember, Indonesia; [4] College of Public Health, University of the Philippines, Department of Health Policy and Administration, Manila, Philippines; [5] Universiti Brunei Darussalam, Sultan Hassanal Bolkiah Institute of Education, Brunei, Brunei

Introduction

Climate change represents an urgent global challenge, with its impacts particularly pronounced in lower- and middle-income nations such as Malaysia. In response, it is imperative to empower the younger generation with robust knowledge and proactive attitudes. Therefore, facilitating the implementation of climate change education in schools is crucial. Hence, this study evaluates the effect of a school-based climate change education program on students' knowledge, attitude, and practice (KAP) in Tenom district, Sabah, Malaysia.

Methods

A school-based pre- and post-test was conducted among primary school students in Tenom district, Sabah, Malaysia, in December 2024. The intervention comprised an educational training session using a climate change education module. The information collected was on the socio-demographic and KAP on climate change. The means of pre- and post-intervention scores were compared using a paired t-test. A p-value of <0.05 was considered statistically significant.

Results

A total of 117 participants were enrolled in this study with an average age of 11.10 ± 0.77 years. The results indicated a significant increase in knowledge scores, rising from 4.66 ± 1.85 pre-intervention to 5.80 ± 2.47 post-intervention (p-value < 0.001). Attitude scores also demonstrated a significant improvement, increasing from 35.78 ± 5.13 to 37.21 ± 6.99, pre- and post-intervention, respectively (p-value = 0.02). However, practice scores increased but not significantly from 37.34 ± 6.73 to 38.44 ± 7.24 (p-value = 0.07).

Conclusion

The study suggests that the school-based education program significantly enhanced students' knowledge and attitude on climate change. These findings provide valuable insights to conduct similar methods of the health education activities in schools with suitable cultural modifications.

P07 | Community-Engaged Environmental Health Research

P07-01 | Piloting the Incorporation of Air Quality within an Urban Healthcare System's Community Health Needs/ Assets Assessment

Alexander Azan[1,4], Cassandra Thiel[1], Jocelyn Gan[3], Oyinkansola Babayode[2], Jenna Agins[3]

[1] New York University Grossman School of Medicine, Department of Population Health, New York, USA; [2] New York University, School of Global Public Health, New York, USA; [3] New York University Langone Health, Real Estate, Development & Facilities, New York, USA; [4] New York University Grossman School of Medicine, Division of General Internal Medicine and Clinical Innovation, New York, USA

Objective: to pilot incorporating air quality health topics into an urban healthcare system's Community Health Needs/ Assets Assessment (CHNAA) to inform healthcare-based air pollution adaptation efforts.
Materials and Methods: We performed focus groups with three adult, community-based programs in Brooklyn, New York partnered with NYU Langone Health's Community Service Plan. Licensed translators facilitated focus groups for non-English-speaking participants. We administered Likert scale surveys examining participant understanding and prioritization of potential air pollution health impacts in their communities, and gauging participant interest in the healthcare system as an adaptation partner.
Results: Adult participants (N=86) had a mean age of 63 years, 76% female sex, 23% Hispanic ethnicity, 55% Asian and 15% white non-Hispanic race, and 35% less than a high school education. Primary languages included 24% English, 32% Cantonese, 19% Mandarin, 22% Spanish, and 3% Russian. Among participants, 68% agreed that the air quality in their community harms their health, 56% agreed that they knew how to access clean air spaces in their community during air pollution events, 35% agreed they use a home air filter/purifier, 26% agreed they use the Air Quality Index to guide their behaviors during air pollution events, 15% agreed they use a home air quality monitor. Overall, 93% of participants agreed that they want and trust their primary care physician (PCP) and clinic to help them stay safe during air pollution events and connect them to community resources.
Conclusions: Participants identified air pollution as a threat to their health and well-being. Most participants want and trust their PCP and clinic to help them improve their air pollution adaptive resiliency. Our pilot findings will be used to expand the integration of air quality within NYU Langone CHNAA efforts to develop a community-partnered, healthcare-based air pollution adaptation program.

P07-02 | Toxic Work, Toxic Treatment: Documenting Experiences of Black Aluminum Smelting Workers in Badin, North Carolina

Cherrel K. Manley[1], Elizabeth Teka[2], Pavithra Vasudevan[3], Elizabeth S. McClure[1]

[1] UNC-CH Gillings School of Global Public Health, Epidemiology, Chapel Hill, USA; [2] University of North Carolina at Chapel Hill, College of Arts and Science, Chapel Hill, USA; [3] University of Texas at Austin, African and African Diaspora Studies, Austin, USA

Objective: Between 1917-2007, Alcoa operated an aluminum smelting plant in Badin, North Carolina, resulting in hazardous occupational and environmental exposures in the surrounding community. Jobs and residences were racially segregated, with the plant and its dumping sites located in the West Badin, a predominantly Black community. We partnered with the Concerned Citizens of West Badin Community (CCWBC) to document experiences of residents who worked or had family members work at the plant.

Material and Methods: We used a community-based survey approach to summarize collective experiences of West Badin residents both on the job and in the community. The CCWBC guided the development of the survey, helped train interviewers, and assisted with recruitment efforts. Interviews were coded to identify recurring themes.
Results: A total of 14 participants were interviewed. Pot room work was the most frequently reported job (N=5). Occupational exposures included asbestos (N=13), extreme heat (N=10), inhalation of dust (N=10), and skin exposure to chemicals/toxins (N=9). Additional job-related stressors included take-home exposures (N=11), racism (N=10), layoffs (N=8), intimidation and threats (N=6 for each). Most participants (N=10) were also concerned about environmental contamination. Health conditions experienced included cancer and heart disease (N=11 for each), lung disease (N=10), death (N=9), carpal tunnel (N=7), asbestosis (N=6), and injuries (N=5). Participants further reported not knowing of potential hazards (N=7), justifying the work by compensation (N=5) and employee benefits (N=3), and benefiting from unionization (N=4). Additional emerging themes included strong community relations and resources, company patronage, distrust of Alcoa, abandonment, and loss of community.
Conclusion: West Badin residents experience the cumulative impacts of occupational and environmental hazards resulting from Alcoa's operations. This survey served as a tool for documenting their experiences, identifying ongoing environmental concerns, supporting community organizing, and highlighting the resilience of the West Badin community. This study will inform future community-based survey work.

P07-03 | Echoes of Resilience: Community Sound Monitoring in Baltimore After the Key Bridge Collapse

Salvatore Milletich[1], Ernest Billingy[4], Kenyetta Kendrick[4], Kris Chesky[2,3], Kirsten Koehler[1], Ana Rule[1]

[1] Johns Hopkins University, Department of Environmental Health and Engineering, Bloomberg School of Public Health, Baltimore, USA; [2] Johns Hopkins University, Department of Performing Arts and Health, Peabody Institute, Baltimore, USA; [3] Johns Hopkins University, Department of Physical Medicine and Rehabilitation, School of Medicine, Baltimore, USA; [4] Baltimore County Public Schools, Dundalk High School, Baltimore, USA

Background: Noise exposure is a near-universal experience, yet monitoring often requires expensive equipment, limiting accessibility. In Baltimore, the recent collapse of the Francis Scott Key Bridge has raised concerns about suspected increases in noise pollution likely driven by changes in transportation patterns, demolition, and construction activities.

Objective: To conduct a community-engaged project designed to evaluate noise exposure in Baltimore while validating multiple low-cost sound level meters (SLMs).

Methods: Partnering with community members and high school students, this initiative integrates hands-on research training focused on environmental justice. Students built Arduino-based SLMs, comparing results to a reference grade device. Performance was assessed indoors and outdoors and calibration techniques (i.e. Generalized Additive Models and Machine Learning approaches) were explored to enhance accuracy.

Results: This presentation will detail methodologies, preliminary findings, and the feasibility of low-cost SLMs in democratizing noise exposure assessment. Two of the four devices, including the Arduino-based SLM, demonstrated a ± 2 dB(A) mean difference in indoor testing, meeting class 1 performance standards. Calibration methods improved

all SLMs to class 1 performance. Full results, including accuracy and coefficients of variation, will be presented at the conference.

Conclusion: By embedding this study within the context of the Key Bridge Collapse, we highlight how community-driven research can address environmental justice concerns in real time. This work underscores the importance of equity in environmental exposure monitoring, engaging key stakeholders across multiple age groups, and provides an example of how scientific collaboration can increase both the awareness of an issue and the technical capacity of the community. Its adaptable framework offers a model for global implementation, particularly in underserved regions where participatory monitoring can enhance community resilience.

P07-04 | Mapping Air Pollution Microenvironments Among Adolescents Using Geospatial-Temporal Analysis and Community Engagement

Kyungwoon Yang[1], Cesunica Ivey[1], Elizabeth Noth[3,5], Yishu Zhu[2], Carlos Vera[3], Alma Andrade[4], Karina Barragan[3], Neeta Thakur[4], Rosemarie de la Rosa[3,4]

[1] University of California, Berkeley, Department of Civil and Environmental Engineering, Berkeley, USA; [2] University of California, Berkeley, Department of Earth and Planetary Science, Berkeley, USA; [3] University of California, Berkeley, Environmental Health Sciences Division, School of Public Health, Berkeley, USA; [4] University of California, San Francisco, Department of Medicine, Division of Pulmonary and Critical Care Medicine, San Fransico, USA; [5] Center for Occupational and Environmental Health, Berkeley, USA

Objective: Environmental justice communities in California, such as Richmond-San Pablo, experience disproportionate air pollution burdens. Adolescents, who spend more time outdoors than adults and young children, may face higher exposure risks. Identifying locations with the highest pollution exposure is essential for targeted mitigation strategies.

Methods: The study analyzed the mobility patterns of 54 adolescents (ages 8-16 years) from the Richmond Environmental and Community Health (REACH) study. Using carbon dioxide (CO_2) as a proxy for air pollution, we integrated high-resolution CO_2 data from the Berkeley Atmospheric CO_2 Observation Network (BEACO_2N) and WRF-Chem CO_2 tracer modeling with participant GPS data. Next, we applied cKDTree spatial analysis and GIS-based clustering to identify locations recorded a minimum of 30 times and obtained their corresponding CO_2 exposure levels. Lastly, we collaborated with a Youth Council in Richmond to identify and categorize clusters into microenvironments.

Results: Participants had an average of 9.91 days of GPS data and were exposed to a mean CO_2 concentration of 423.14 ppm (IQR: 403.35 – 427.97 ppm). The average CO_2 exposure per participant ranged from 409.70 to 448.77 ppm. A total of 301 location clusters were identified, with each participant averaging 5.59 clusters. CO_2 exposure was highest in residential areas (446.94 ppm), followed by schools (436.61 ppm) and indoor leisure spaces (436.12 ppm).

Conclusion: This study demonstrates an approach to identifying where adolescents spend time and their corresponding air pollution exposure. These findings can inform pollution mitigation strategies by prioritizing frequently visited locations with high exposure levels.

P07-05 | Technology development and adoption of an environmental message mapping dashboard for enhancing environmental health literacy

Amanda J. Llaneza[1], James Brooks[1], Muhammed Idris[3], Maya Korin[1], Faven Araya[2], Kristelle Pierre[2], Rocio Baez Rodriguez[1], Luz Claudio[1]

[1] Icahn School of Medicine at Mount Sinai, Environmental Medicine, New York, USA; [2] Arthur Ashe Institute for Urban Health, New York, USA; [3] Morehouse School of Medicine, Atlanta, USA

Objective:
Environmental health literacy is an important aspect of increasing awareness of health threats from climate change for New York City residents. Utilizing community-based participatory research (CBPR) fosters collaboration between researchers and community members to ensure culturally relevant, and ethically conducted research that may improve health literacy among communities in NYC. There is a need for documented examples facilitating successful implementation of CBPR. This study aims to apply CBPR to develop a message mapping dashboard with a coalition of researchers and community leaders in New York City.
Methods:
Collaboration between academic researchers and community leaders led to the development of a novel framework “lights, facts, and goals” for developing health messages. Data were also collected through key informant interviews, and a Health Information Technology Usability Scale (Health-ITUES) post-assessment survey to develop the technology-assisted health information campaign dashboard.
Results:
The collaboration resulted in the Community Health Information Equity Forum (CHIEF), an online platform to facilitate creation, distribution, and evaluation of environmental health messages to key health influencers in the community. This collaborative approach led to the co-development of culturally tailored educational messages on environmental health. Results of using the framework were:

- **Lights:** Implemented as text messaging campaigns.
- **Facts:** Provided relevant, concise and actionable scientific information.
- **Goals:** Outlined culturally relevant and achievable behavior changes for community members that could reduce environmental exposures.

Conclusion
This study demonstrates the feasibility and effectiveness of CBPR in addressing health literacy. Results provide insights on sustained community partnerships, bidirectional knowledge exchange, and shared decision-making. Co-development of a message mapping dashboard for community-based organizations to promote environmental health literacy and utilizing CBPR at each stage of the technology development lifecycle has practical implications for technology adoption in diverse communities.

P07-06 | Illness from Mixed Toxicant Exposures: Smoking and Alcohol as Potential Modifiable Risk Factors

Jessica Y.F. Situ[1], Janhavi Kulkarni[1,2], Brinton K. Berg[1], Vincent Davidson[1], Bruce J. Miller[1], Beatrice A. Golomb[1]

[1] University of California, San Diego, Department of Medicine, La Jolla, USA; [2] Yale School of Public Health, Department of Environmental Health Sciences, New Haven, USA

Objective: To identify modifiable risk factors associated with illness severity following a mixed toxicant exposure (**MTE**) event in East Palestine, Ohio (**EP**). Alcohol and smoking are candidates: each are tied to greater risk of some environmentally-promoted problems and lesser risk of others (e.g. smoking and Parkinson's Disease).

Methods: Adults ≤20-miles from the EP derailment completed surveys assessing pre- and post-derailment symptoms and habits. MTE-illness severity was evaluated by the "UCSD-20" – assessing 20 symptoms that are common post-MTE (prior 2-weeks).

Results: 27% of participants smoked (maximum 1-pack per day) while 60% drank alcohol (of whom 93% drank ≤10 beverages/week – i.e. not heavy drinkers). Alcohol (drinks per week) and smoking (cigarettes per day) each significantly predicted greater MTE-illness severity on age-sex adjusted regression (robust standard errors): drinks per week β(SE)[95% CI]p= 3.65(1.41)[0.81, 6.49]0.013; cigarettes per day β(SE)[95% CI]p= 1.75(0.78)[0.17, 3.32]0.031. Examining relationships to individual symptoms from the UCSD-20, alcohol related significantly ($p<0.05$) to energy problems, muscle weakness, fatigue with exertion and dry skin, with borderline significant relationships ($0.05<p<0.1$) to irritability and aches and pains. Besides the significant relationship to shortness of breath, cigarettes per day related significantly to individual UCSD-20 symptoms of muscle pain, temperature regulation problems and rash, and borderline significantly for sleep problems and energy problems. Sleep-disordered-breathing (**SDB**) – increased in MTE-illness – contributes to symptoms. Alcohol (not smoking) was a borderline predictor of "SDB" (logistic-regression, robust SEs): β(SE)[95% CI]p= 0.21(0.12)[-0.027, 0.46]0.083.

Conclusion: Even modest alcohol and tobacco use were tied to greater MTE-illness severity following the EP derailment and constitutes potential modifiable risk factors. This fits findings from MTE-illness in Gulf War veterans. Moreover, it coheres with the documented MTE-illness mechanism of mitochondrial impairment, to which both alcohol and smoking contribute.

P07-07 | Air quality in an agricultural community and pistachio harvests: A community-engaged research study

Bonnie N. Young[1], Jessica Tryner[2], Luis Hernandez Ramirez[1], Sherry WeMott[1], Grant Erlandson[1], Grace Kuiper[1], Daniel Dean[1], Xiaoying Li[3], Nayamin Martinez[4], Mollie Phillips[2], John Volckens[3], Sheryl Magzamen[1]

[1] Colorado State University, Environmental & Radiological Health Sciences, Fort Collins, USA; [2] Access Sensor Technologies, Fort Collins, USA; [3] Colorado State University, Mechanical Engineering, Fort Collins, USA; [4] Central California Environmental Justice Network, Fresno, USA

Objective: One of the United States' largest pistachio processing facilities operates in the San Joaquin Valley, California. Residents near this facility expressed concerns about potential environmental impacts. We collaborated with local residents to explore factors related to indoor and personal air pollution concentrations before and during the pistachio-harvest season. Methods: Thirty-five participants from 18 nearby households were enrolled via community leaders. We visited each participant up to three times during non-harvesting (December 2023, May 2024) and pistachio-harvesting seasons (September 2024) to sample 24-hour personal and indoor $PM_{2.5}$ and PM_{10} concentrations and survey household characteristics. We assessed associations between harvesting season and each log-transformed exposure separately in mixed effects linear regression models, adjusted for ambient $PM_{2.5}$ and PM_{10} levels from regulatory monitors and household characteristics, with a random effect for participant or household. Results: Participants were mostly female (69%), Latino/a (97%), and spoke Spanish as their primary language (60%). Most households had at least one person working in agriculture (72%). The median household distance from the pistachio plant silos was 633 m (Q1, Q3: 550, 1294). We found no evidence of associations between pistachio-harvest season and higher PM concentrations (e.g., indoor $PM_{2.5}$ n=31,-0.07 μg m-3; 95% CI: -0.47, 0.32; indoor PM_{10} n=34, -0.09 μg m-3; 95% CI: -0.60, 0.42). Anyone in the household working in agriculture was consistently associated with all PM concentrations except personal $PM_{2.5}$. Ambient air quality was associated with indoor $PM_{2.5}$ and suggestively personal $PM_{2.5}$ exposures. Conclusion: We found no observable impacts of pistachio-harvest season on indoor and personal $PM_{2.5}$ and PM_{10} concentrations. Indoor and personal PM levels were more strongly impacted by household members working in agriculture and, potentially, ambient air pollution.

P07-08 | Community-based Intervention Study for the Health Effects of Air Pollution on the Elderly: a Pilot Study in South Korea

Dong-Wook Lee, Hyungdoo Kim, SaeByeol Kwon, Hwan-Cheol Kim

Inha University, Department of Occupational and Environmental Medicine, Incheon, South Korea

Background

Particulate matter with an aerodynamic diameter of less than 2.5 μm ($PM_{2.5}$) is a growing environmental health concern. While $PM_{2.5}$ exposure is known to negatively impact respiratory function, there is limited evidence on effective community-based strategies to mitigate exposure among vulnerable populations, particularly older adults.

Methods

We conducted a pilot intervention in Incheon, South Korea, targeting older adults (≥65 years) enrolled in a home-visit

healthcare program operated by a public health center in Dong-gu, Incheon. Sixty participants were assigned to either an intervention group (n=30) or a control group (n=30) based on visit schedules. The intervention included PM-related health education, distribution of protective masks, and real-time indoor air quality monitoring. Participants completed surveys assessing PM awareness and preventive behaviors before and after the intervention. Indoor $PM_{2.5}$ levels in participants' homes were monitored using certified sensors for six weeks both before and after the intervention.

Results
Indoor $PM_{2.5}$ levels were significantly lower in the intervention group compared to the control group ($p<0.05$). The average indoor $PM_{2.5}$ concentration decreased from 20.1 to 13.0 μg/m³ in the intervention group ($p=0.038$). Awareness of the need to limit outdoor activities on high-PM days increased slightly in both groups, with the intervention group showing an improvement from 3.80 to 4.00 on a 4-point scale. The frequency of checking air quality reports showed a greater increase in the intervention group (from 2.83 to 3.60) compared to the control group (from 3.07 to 3.31), indicating enhanced engagement in preventive behaviors.

Conclusions
Our findings suggest that a community-based intervention delivered through home-visit healthcare services can effectively reduce indoor PM exposure among older adults. Strengthening such interventions may improve environmental health literacy and promote protective behaviors in vulnerable populations.

P07-09 | From Data to Dissemination: Creating Dashboards for Buffalo African-American Community-based Participatory Air Monitoring

Yonghun Suh[1], Eunhye Yoo[2]

[1] Seoul National University, Geography, seoul, South Korea; [2] University at Buffalo, SUNY, Geography, buffalo, USA

The African-American community on the east side of Buffalo, New York, experiences a disproportionate burden of chronic disease compared to neighboring regions. While poor air quality is a known contributor to adverse health outcomes, community-specific air pollution exposures have been difficult to assess the absence of localized air monitoring. To address this gap, we partnered with the Buffalo Center for Health Equity (BCHE), a local environmental justice organization, to construct a two-year air monitoring study using community-based participatory research (CBPR) methods. This study focuses on the co-design and evaluation of products develop to communicate study findings.
Prototypes of study results were developed to report fine particulate matter (PM2.5) and volatile organic compound (VOC) levels. An interactive data dashboard, hosted on the study website, was refined through virtual meetings with both the BCHE members and online surveys from the study participants.

The co-design process led to the development of community-tailored online dashboards summarizing air monitoring and study design. Key insights from working group emphasized the use of color, large font for key results, and a preference for maps over traditional plots to enhance accessibility and engagement.

Through CBPR, we co-designed and refined dissemination products from a ten-month community air monitoring campaign. Future dissemination efforts will include public education events and virtual meetings to share findings to the broader community.

P07-10 | Using Photovoice to Identify Solutions for Barriers to Safer Hair Product Purchasing and Use in the RESTYLE Study

Marissa Chan[1,2], Marlee Quinn[1], Derek Shyr[3,4], Jackeline Morales[5], Cynthia Jones[6], Leopoldo Cabassa[7,8], Gary Adamkiewicz[1], Tamarra James-Todd[1,9]

[1] Harvard TH Chan School of Public Health, Environmental Health, Boston, USA; [2] Columbia University Mailman School of Public Health, Environmental Health Sciences, New York, USA; [3] University of Alabama at Birmingham Heersink School of Medicine, Birmingham, USA; [4] Harvard TH Chan School of Public Health, Biostatistics, Boston, USA; [5] Boston University School of Public Health, Environmental Health, Boston, USA; [6] Union Capital Boston, Boston, USA; [7] Center for Mental Health Services Research, St. Louis, USA; [8] George Warren Brown School of Social Work, St. Louis, USA; [9] Harvard TH Chan School of Public Health, Epidemiology, Boston, USA

Objective: Racial and ethnic inequities in exposure to personal care product (PCP) endocrine disrupting chemicals (EDCs) and associated adverse health outcomes have been reported in the United States (US). A lack of community-based interventions exists, particularly as it relates to culturally-driven EDC exposures, such as hair products. Photovoice, a participatory qualitative method, was used to identify community priorities, key actors, and solutions to address barriers to safer hair product purchasing and use.

Methods:The Retail Environment and Hair Styling Exposure (RESTYLE) Study utilized photovoice to identify barriers and facilitators to safer hair product purchasing and use in the greater Boston, Massachusetts area. Two community art shows were hosted, with ~120 in attendance to view the photographs and complete a community survey developed to assess key actors and solutions identified from the photovoice project. Open-ended survey questions exploring reflections of the photographs and additional solutions were analyzed using pile sorting techniques. Descriptive statistics were used to identify the top three rank ordered key actors and solutions that the participants believed should be incorporated into future interventions.

Results: Participants completed the survey to share their reflections, attitudes, and selection of the top ranked key actors and solutions (n=102). Qualitative analysis revealed the respondents resonated with the theme of time invested in identifying safer products (n=93). Policymakers were the number one ranked key actor who should be involved in developing and implementing solutions (32.3%). The number one ranked solution was the US government enacting stronger PCP regulations (16.8%). The top ranked key actors and solutions differed by respondent sociodemographic characteristics.

Conclusions: Using photovoice, community members identified important key actors and solutions that may contribute to future interventions to reduce inequities in EDC exposure from hair products. This study was funded by the François-Xavier Bagnoud Center for Health and Human Rights, Harvard University.

P07-11 | State and Academic Partnerships: A qualitative assessment of community knowledge and preferred communication about toxic chemicals in personal care products.

Mom TatahMentan[1], Casper Voyles[2], Christabel Asare[2]

[1] *Minnesota Department of Health, Saint Paul, USA;* [2] *Hamline University, Saint Paul, USA*

Background: Toxic chemical exposures in personal care products have the potential to cause adverse health effects for the exposed individuals. Specifically, sensitive populations including women of reproductive age, pregnant people, and infants can be exposed to harmful chemicals from the use of personal care products. There is a need to understand community perspectives on chemicals in personal care products, their utilization of these products, and methods of communicating health information about chemicals and potential adverse effects. Methods: The Minnesota Department of Health in partnership with a public health seminar course at Hamline University, conducted 5 focus group discussions with women of reproductive age (18-44) with a total of 23 participants. The purpose was to understand current knowledge and attitudes about toxic chemical exposure in personal care products, product utilization, and strategies for health communication on this topic. Results: Findings indicated that women had knowledge of health-related effects related to chemical exposures from personal care products, yet their actions with this knowledge differed. Data showed that for college-aged women, knowledge about risk didn't deter their decision to use these products, whereas the risk perception of chemical exposures was more likely to influence older-aged women's decision on what products they used. Women of older reproductive age used a wider variety of trusted sources to seek out information about products, while college-aged women relied mostly on social media, even while acknowledging skepticism about the accuracy of claims. Conclusion: Our findings indicate a need for more evidence-based communication to promote awareness for college-aged women and older women on this topic. These findings are novel, because they provide critical insights and guidance on how to effectively implement health and risk communications for sensitive populations.

P07-12 | Better addressing chemical disasters: Independent science and community input from East Palestine, Ohio

Timothy H. Ciesielski[1], Jessica Conard[3,2]

[1] Case Western Reserve University School of Medicine, Population and Quantitative Health Sciences, Cleveland, USA; [2] Bennington College, Appalachia Director of Beyond Plastics, Bennington, USA; [3] The National Academies of Sciences, Engineering, and Medicine, Cumulative Impacts Liaison, Washington DC, USA; [4] University of Kentucky, Community Advisory Board Member for the East Palestine Train Derailment, Lexington, USA

Objective_ Environmental Health Scientists from academia and chemically-impacted community members can each offer critical perspectives on chemical disasters. However, opportunities for collaboration are limited, leaving gaps in immediate response efforts and long-term strategies. The 2023 East-Palestine-train-derailment underscored the urgent need for independent scientific inquiry alongside community engagement. This presentation explores key challenges and actionable solutions that emerged in conversations between these groups.

Material and Methods_ Following the disaster, academic scientists saw an immediate need for data collection and analysis. The barriers to rapid independent data collection led a group of academic researchers to analyze existing publicly available data for the community, and host a series of non-adversarial work-shop style meetings in East Palestine.

Results_ These conversations raised pressing questions. Why were these specific chemicals, locations, and timepoints chosen for measurement by the Environmental-Protection-Agency (EPA)? For example, Isobutylene, known to be on the train, was not included in air monitoring, and formaldehyde– likely to result from the degradation of chemicals such as acrolein– was also omitted. Should non-targeted exposure assessments(e.g. mass spectrometry) be a larger part of chemical disaster response? The EPA's CompTox interface proved crucial, but inconsistent units-of-measurement created barriers in interpreting the data. Should the Agency-for-Toxic-Substances-and-Disease-Registry have supported biomonitoring efforts? From the community resident perspective, the disconnect between agency-based assurances of safety, and scientific uncertainty regarding long-term health impacts created widespread distress. Residents faced acute health symptoms, place-disruption, and cultural upheaval, with no clear path to medical follow-up. Meanwhile, independent scientists positioned outside the regulatory framework were uniquely able to highlight unmet environmental research and healthcare needs.

Conclusion_ Bridging the gap between impacted communities and independent academic scientists is essential for improving chemical disaster response. Open communication between academic scientists and impacted community members yielded actionable insights for federal agencies, clinicians, exposure scientists, and epidemiologists that respond to chemical disasters.

P07-13 | Building public trust, improving exposure science, and achieving public health through community engagement infrastructure

Erin Lebow-Skelley[1], Melanie A. Pearson[1], Alina Merceron[1], Lynne Young[4], Columbus Ward[2], Yomi Noibi[3]

[1] Emory University, HERCULES Exposome Research Center, Atlanta, USA; [2] Peoplestown Revitalization Corporation, Atlanta, USA; [3] ECO Action, Atlanta, USA; [4] Pathways to Sustainability, Atlanta, USA

Objective: Public trust in science is imperative to advance exposure science and environmental health. A long-promoted and tested approach to build trust is for the research community to partner equitably with impacted communities. However, there are challenges to building trust when engagement only happens one research project at a time. Funding community engagement infrastructure can build sustained and trusting partnerships between academics and communities that outlast individual projects. The National Institute of Environmental Health Sciences funds this infrastructure as a core component of its extramural research centers. We describe the infrastructure for one of these centers, the HERCULES Exposome Research Center (HERCULES) at Emory University in Atlanta, Georgia.

Material and Methods: HERCULES includes five infrastructure cores that support environmental health and exposome research at Emory; one of those is the Community Engagement Core (CEC). The HERCULES CEC uses several approaches to 1) build multi-directional dialogue between the Center and local communities, 2) enhance the existing capacity of communities to address their environmental health concerns, and 3) support community-engaged research. These approaches are guided and driven by a Stakeholder Advisory Board (SAB) that consists of representatives from local communities, non-profits, government agencies, and other academic institutions who are interested in environmental health.

Results: The SAB has helped HERCULES develop programming that builds partnerships with Atlanta-area communities, provides a forum for HERCULES scientists to interact with community partners, and ensures that the Center remains committed to translational science that benefits communities. Altogether, this community engagement infrastructure, guided by the SAB, has resulted in several funded research projects, sustained and trusting community partnerships, and translational environmental health research.

Conclusion: For exposure science to improve the public’s health and well-being, sustained and trusting partnerships are necessary. These partnerships are how we re-build trust in science so that research leads to tangible public benefits.

P07-14 | Outcome Measures in Community-Driven Environmental Chemical Monitoring Studies

Esther Erdei

UNM HSC, Albuquerque, USA

Complex exposure mixtures do not exist in isolation and they can affect all physiological systems in humans. Probable health effects of emerging chemical exposures require both accurate quantification and toxicological investigations that are not always available readily yet. Immune system and other effect biomarkers can identify chronic exposures to VOCs, PFAS and metal mixtures. The CRST women VOC study (N=20 all childbearing age adults) will be presented as an example that focused on immune system evaluations and clinical measures of toxic exposure outcomes. In addition, metals and autoimmunity measures in two Tribal communities residing in the Western USA that are scattered with extant hard rock mines will also be presented. With documented drinking water contamination in both communities, *in vivo* levels of more than half of the 24 measured serum and urine metals differed between the two communities and were substantially contrasting from their national median values (NHANES). Serum autoantibodies associated with systemic autoimmune disease were at low-level, but antibodies to denatured (single-stranded) DNA and thyroid-specific autoantibodies were commonly elevated (> 27% prevalence), especially in women. This was the case with the VOC study too when more than 50% of women had autoimmunity outcomes. A three-tier statistical modeling process was carried out to examine individual metals exposure as predictors of autoantibody levels. For the most part only weak positive associations between individual metals and systemic autoantibodies were found, although univariate quantile regression analysis showed positive statistical associations of serum lead and antimony with anti-chromatin and anti-histone autoantibodies. Using age and gender-adjusted multivariable statistical models, metals did not predict anti-thyroglobulin or -thyroid peroxidase significantly but VOCs and PFAS were significant positive predictors. Results suggest that elevated levels of environmental metals and chemical mixtures in these communities may result in various serum autoantibodies associated with systemic autoimmune disease.

P07-15 | Community-Engaged Research on Water Quality and Metal Exposure in South Texas Colonias

Taehyun Roh[1], Nishat Tasnim Hasan[1], Lindsay Sansom[2], Garett Sansom[2]

[1] Texas A&M University, Department of Epidemiology and Biostatistics, College Station, USA; [2] Texas A&M University, Department of Environmental and Occupational Heealth, College Station, USA

BACKGROUND AND AIM: Colonias are impoverished communities in the U.S.-Mexico border area that lack infrastructure, including reliable water systems. Water infrastructure issues have been reported, and communities have concerns about water quality in this area. To address this, a community-engaged research study was conducted to assess water quality and exposure to select metals in the Alamo and Progreso colonias in South Texas. METHODS: Water was tested for arsenic, nickel, uranium, and lead, and biomonitoring was conducted for arsenic in urine and toenail samples. National Health and Nutrition Examination Survey (NHANES) data was utilized to estimate the national average. As an intervention, water arsenic filters were provided, and follow-up urine samples were

collected to assess exposure reduction. RESULTS: A total of 203 participants were recruited from the Alamo and Progreso colonias in South Texas. Arsenic, nickel, and uranium were found in 100% of households in both locations, with lead detected in 2% of Alamo households and 7% in Progreso. Maximum metal levels (4.2, 26.3, 18.1, and 11.5 µg/L) remained below the EPA MCL. In Alamo, arsenic was detected in all participants' toenails (0.01–0.57 µg/g) and urine (0.91–59.3 µg/g creatinine), with 27% exceeding the national average (NHANES). After the water filter intervention, urinary arsenic levels dropped to 1.72–31.2 µg/g, reducing the proportion above the national average to 15%. In Progreso, arsenic was detected in all participants' toenails (0.03–0.37 µg/g) and urine (0.21–47.2 µg/g creatinine), with 23% exceeding the national average. After intervention, urinary arsenic levels decreased to 0.29–43.4 µg/g, with only 13% exceeding the national average. CONCLUSIONS: Our study emphasizes the importance of active community engagement and communication, which are essential for raising awareness and implementing evidence-based interventions in vulnerable communities. Further efforts are needed to assess other environmental exposures and ensure continued improvements in water infrastructure.

P07-16 | Community, health, and environment: transdiscipline in the territory

Fiorella Iaquinta[1,2], Patricia Iribarne[3,2]

[1] Universidad de la República, Faculty of Chemistry, Montevideo, Uruguay; [2] Universidad de la República, Ecosalud Uruguay, Montevideo, Uruguay; [3] Universidad de la República, Faculty of Science, Montevideo, Uruguay

Malvin Norte, located in Montevideo, is a heterogeneous neighborhood, where different economic, social, and cultural realities coexist. This leads to certain sectors of the territory being more vulnerable, economically and environmentally, leading to the search for survival strategies, such as waste classification, and cable burning, among other informal activities. This territory has a background of environmental problems, such as generation of informal garbage dumps, exposure to lead, and high levels of parasitosis. For several years, we have been working in the territory, from different perspectives under the Ecohealth approach, where the community actors are given a space to express their concerns and participate in the search and implementation of solutions.
This work presents some of the challenges and opportunities of transdisciplinary work in Malvín Norte, identified over the last 6 years, concerning the environmental problems of the territory. Among the opportunities we can highlight are the possibility of contributing to a holistic understanding of the territory's environmental problems, their dimensions, and their link with health; the generation of knowledge from the integration of different visions and experiences; community empowerment; collective solutions and strategies such as the creation and maintenance of green spaces and community gardens; and the strengthening of the link between academia and government actors. On the other hand, the challenges are related to the time required, which does not always correspond to bureaucratic, political, social, or academic times. They are also concerned with the language and technicalities of the disciplines and communities and, finally, the lack of funding for the development of transdisciplinary processes.
However, it is important to point out that addressing environmental problems from a transdisciplinary perspective can lead to the development of collective, self-managed, and sustainable adaptation or mitigation strategies and solutions, but can also lead to the creation of new or better public policies.

P08 | Data Analysis and Modeling Approaches in Environmental Health

P08-01 | Independent and joint effects of time-varying $PM_{2.5}$ Compositions on mortality in Canada: A Novel Methodologic Approach

Juwel Rana[1,2], Alexander P. Keil[3], Hong Chen[4,5], Tarik Benmarhnia[6], Scott Weichenthal[1], Jay S. Kaufman[1]

[1] McGill University, Epidemiology, Biostatistics and Occupational Health, Montreal, Canada; [2] South Asian Institute for Social Transformation (SAIST), Research and Innovation Division, Dhaka, Bangladesh; [3] National Cancer Institute (NCI), Maryland, USA; [4] Environmental Health Science and Research Bureau, Health Canada, Ottawa, Canada; [5] University of Toronto, Toronto, Dalla Lana School of Public Health, Toronto, Canada; [6] University of California San Diego, Scripps Institution of Oceanography, San Diego, USA

Background and Objective: Numerous studies have established a link between total $PM_{2.5}$ mass exposure and mortality in Canada. However, no research has specifically investigated the joint effects of $PM_{2.5}$ constituents on mortality in the Canadian context. This study examines the independent and joint effects of time-varying $PM_{2.5}$ constituent mixtures on nonaccidental all-cause and cardiovascular disease (CVD)-related mortality in Canada.

Methods and Materials: We conducted a population-based cohort study involving approximately 3 million adults (25 to 89 years) from the Canadian Census Health and Environment Cohort (CanCHEC) 2006. For each participant, we estimated annual mean concentrations of black carbon (BC), sulfate (SO_4^{2-}), ammonium (NH_4^+), nitrate (NO_3^-), and organic matter (OM) using satellite observations in combination with a global atmospheric chemistry transport model. In total, 3,070,547 observations were followed across Canada from 2007 to 2019. We have developed a novel approach- time-varying quantile g-computation (tvcQGcomp) method that estimates the independent and joint effects of increasing each time-varying pollutant by a quantile at every time point.

Results and Discussion: Results from the simulation showed that tvcQGcomp produced unbiased independent and joint effect estimates for time-varying mixtures with reliable Confidence Interval (CI) coverage (~95%) and good power (5% to 7% type 1 error rate). The average concentrations of OM, SO_4^{2-}, and NO_3^- were higher in our CanCHEC cohort. The correlation among these five $PM_{2.5}$ constituents ranges from 0.20 to 0.91. The cohort comprised 48% females and 52% males. We are applying tvcQGcomp in the CanCHEC population to estimate the independent and joint effects of time-varying $PM_{2.5}$ constituents 13-years nonaccidental all-cause and CVD-related mortality in Canada. Our novel approach will estimate overall exposure-response for the mixture over time and the health effects of time-varying $PM_{2.5}$ compositions.

P08-02 | Small-Sample Learning for Next-Generation Human Health Risk Assessment: Harnessing AI, Exposome Data, and Systems Biology

Tianxiang Wu[1], Bin Wang[1], Mengyuan Ren[2]

[1] Peking University, School of Public Health, Beijing, China; [2] Emory University, Rollins School of Public Health, Atlanta, Georgia, USA

Human health risk assessment (HRA) in environmentally complex scenarios faces challenges due to the high dimensionality and heterogeneity of exposome data, integrating exogenous environmental factors (e.g., pollutants, metals) and endogenous multiomics profiles (e.g., metabolome, proteome). Traditional epidemiological approaches, constrained by small-sample limitations, necessitate innovative frameworks combining artificial intelligence (AI) and systems biology. Emerging strategies include optimized hybrid study designs (e.g., longitudinal multiomics cohorts with repeated measures) to strengthen causal inference in limited populations, as demonstrated by predictive models integrating metabolomic, proteomic, and immunomic data for labor timing prediction in small cohorts (n=63). Large-scale biomonitoring datasets (e.g., UK Biobank, NHANES) and in vitro-to-in vivo extrapolation (IVIVE) methods enable prior knowledge transfer, exemplified by proteomic age clocks validated across diverse populations (N=45,441). AI-driven advancements address data scarcity through generative models (e.g., diffusion models, GANs) for synthetic omics data generation, transfer learning frameworks adapting pretrained models (e.g., GPT-inspired architectures) to toxicity prediction, and multimodal integration of numerical, textual, and graph-based data (e.g., molecular/knowledge graphs) to map exposure-disease pathways. Sparse biological networks embedding adverse outcome pathways (AOPs) or gene regulatory mechanisms enhance interpretability, though balancing model accuracy with biological plausibility (e.g., via SHAP explanations) remains challenging. Persistent barriers include harmonizing spatiotemporal multimodal data, mitigating population biases in transfer learning, and validating AI-derived hypotheses experimentally. Future efforts should prioritize open exposome platforms for data sharing, active learning pipelines bridging computational predictions with targeted lab validations, and robust evaluation frameworks addressing reproducibility and ethical AI use. By synergizing AI, exposome analytics, and systems toxicology, this integrated paradigm advances precision HRA, offering scalable solutions for risk prediction in low-data, high-complexity environmental health contexts.

P08-03 | Environmental Contributions to Hypertension Prediction: A Novel Machine Learning Pipeline

Harris Jamal[1], Yi-Han Hu[1], Xinlei Deng[2], Kaitlyn G. Lawrence[2], Emily J. Werder[2], Lenore J. Launer[1], Dale P. Sandler[2]

[1] National Institute on Aging, Laboratory of Epidemiology and Population Sciences, Baltimore, USA; [2] National Institute of Environmental Health Sciences, Epidemiology Branch, Durham, USA

Objective: Environmental factors, from neighborhood level characteristics to ambient environmental exposures, are associated with increased hypertension risk. However, hypertension prediction models primarily incorporate traditional risk factors (age, sex, race, BMI). Leveraging multi-dimensional environmental exposure data, this study evaluates whether incorporating environmental exposures into a novel machine learning pipeline improves hypertension prediction.

Methods: Analyses included 10,491 participants from the Gulf Long-Term Follow-up (GuLF) Study (2011-2013; average age 43.6, 78.4% male, 35.1% Black). Hypertension was defined as systolic/diastolic blood pressure ≥140/90 mmHg or self-reported use of anti-hypertensive medication at an in-home enrollment exam. Environmental predictors were linked to residential geocodes, selected via variable selection approaches, and introduced to the prediction model in three stages: Stage 1 (demographics: age, race, sex, and BMI); Stage 2 (selected neighborhood characteristics: area deprivation index, social vulnerability, and community resilience score); Stage 3 (selected geospatial environmental components: vegetation index, $PM_{2.5}$, NO_2, and formaldehyde exposure). Variable selection consisted of a two-step approach in which boruta identified key predictors, followed by bootstrapping area under the precision-recall curve (AUPRC) to confirm their respective contributions. Extreme Gradient Boosting Models with a 70/30 train-test split were used. Performance was assessed via sensitivity, specificity, precision, F1 score, and AUPRC.

Results: Approximately 35% of participants had hypertension at the in-home enrollment exam. Sensitivity increased across the three stages (0.749, 0.767, 0.788), indicating improved detection of true positives and reduced rate of false negatives with the addition of neighborhood and ambient environmental variables. However, specificity and precision decreased across stages (specificity: 0.645, 0.620, 0.598; precision: 0.537, 0.527, 0.519), reflecting a higher false positive rate.

Conclusion: Our findings demonstrate that combining neighborhood characteristics and ambient environmental exposures with individual demographic factors can modestly improve the ability of a machine learning prediction model to correctly identify hypertension cases while minimizing missed cases.

Funding: NIH Intramural Research Program.

P08-04 | Surveillance of Rotavirus Epidemiology in the United States Using Wastewater Metrics: Relationship with Vaccination Coverage and Population Sociodemographics

Elana M.G. Chan, Alessandro Zulli, Alexandria B. Boehm

Stanford University, Department of Civil and Environmental Engineering, Stanford, USA

Objective: Surveillance of rotavirus is important to understand its epidemiology in the post-vaccination era and identify gaps in vaccination coverage. We longitudinally monitored rotavirus in wastewater across the USA to assess rotavirus occurrence patterns in relation to clinical surveillance metrics, vaccination coverage, and population sociodemographics.
Material and Methods: We used wastewater RNA concentrations of rotavirus from 185 treatment plants (September 2023–December 2024) to infer the timing of onset, offset, and duration of rotavirus epidemics at national and subnational scales. We assessed spatiotemporal occurrence patterns of wastewater rotavirus activity and compared occurrence patterns to clinical metrics of rotavirus infections, estimates of rotavirus vaccination coverage, and population sociodemographics associated with rotavirus vaccination coverage.
Results: Nationally, wastewater rotavirus RNA concentrations peaked in late winter/spring during the 2023–2024 season which is consistent with known patterns of rotavirus activity during odd numbered seasons in the post-vaccination era. Wastewater rotavirus RNA concentrations were temporally correlated with clinical metrics of rotavirus infections. Duration of elevated wastewater rotavirus activity was shorter among treatment plant service areas with high vaccination coverage. We also observed a longer duration of elevated wastewater rotavirus activity among some population sociodemographic groups associated with low rotavirus vaccination coverage.
Conclusion: Rotavirus infection still remains the leading cause of diarrheal deaths worldwide despite the licensure of rotavirus vaccines. Wastewater monitoring can augment clinical surveillance of rotavirus infections given that a wastewater sample represents an entire contributing population, irrespective of individuals' symptom presentation or test-seeking behaviors. Expanding wastewater monitoring of rotavirus globally can inform vaccine administration and enhance rotavirus surveillance to help reduce disease burden.Acknowledgements: CDC Foundation and the Sergey Brin Family Foundation.

P08-05 | Application of clustering algorithms for defining multiple vulnerabilities for association between heat and emergency visits among the elderly

Ayoung Kim[1], Jinah Park[1], Ho Kim[1], Whanhee Lee[2], This work was supported by Korea Environment Industry & Technology Institute(KEITI) through Climate Change R&D Project for New Climate Regime, funded by Korea Ministry of Environment(MOE)(grant number:RS-2022-KE002235)

[1] Seoul National University, Graduate School of Public Health, Seoul, South Korea; [2] Pusan National University, Biomedical Convergence Engineering, Pusan, South Korea

Background: Extreme heat poses significant health risks, leading to increased morbidity and mortality. While previous studies have examined heat vulnerability, they often focus on a limited number of factors, failing to capture the complexity of multidimensional susceptibility.
Methods: To overcome these limitations, we applied an unsupervised learning approach to identify personalized heat vulnerability profiles. Using Korean national health insurance data (NHID) from 2011 to 2019, we analyzed the total population aged 65 and older. We utilized 39 variables, including health examination data (e.g., lifestyle factors, blood test results) and socioeconomic data (e.g., income level, Medicaid eligibility, sex) and Charlson Comorbidity Index [CCI]), to cluster individuals using K-Means and Gaussian Mixture Model (GMM). We then assessed the short-term association between heat exposure and emergency department visits using a case-crossover design with conditional logistic regression, and calculated akaike information criteria (AIC) with each model. Finally, we examined risk patterns and cluster characteristics through heatmap visualization.
Results: Our findings suggest that increasing the number of clusters enhances model performance when sample sizes remain constant. However, as the sample size increases, the advantage of finer clustering diminishes. Additionally, models performed better in smaller subgroups when more variables were included. In terms of risk stratification, small vulnerable clusters were predominantly characterized by Medicaid eligibility, fasting blood sugar levels (FBS), kidney disease screening questionnaire (KDSQ) scores, diabetes mellitus (DM), and CCI scores. In contrast, larger clusters were primarily driven by age, Medicaid status, KDSQ scores, and hearing impairment.

Conclusion: Our study highlights the advantages of unsupervised learning in identifying heterogeneous vulnerability profiles to extreme heat.

P08-06 | Comparing the two-stage meta-analytic approaches with Bayesian hierarchical models in temperature-mortality association studies

Jiseop Jeong, Yeonseung Chung

Korea Advanced Institute of Science and Technology, Department of Mathematical Sciences, Daejeon, South Korea

Background: The two-stage meta-analytic approach is commonly used in environmental epidemiology to estimate the temperature-mortality association across multiple locations. However, this method is limited for small area estimation due to data sparsity. To address this limitation, Bayesian hierarchical models have been proposed as an alternative. Yet, studies that directly evaluated its performance compared to the two-stage method are lacking.
Methods: We conducted a simulation study to compare the two-stage method and a Bayesian hierarchical model in small area estimation setting. The two approaches were compared in terms of estimation performance for the location-specific association curves. In Bayesian hierarchical modeling, we explored three different priors with varying informativity and evaluated their impact on the results.
Results: In small area setting, when using the two-stage method, the estimated association curves from the first stage were highly variable, while those obtained through the second stage showed little variation, producing nearly identical results across locations. In contrast, the estimated association curves from the Bayesian model varied depending on the priors. With informative prior, the Bayesian model produced the estimates that were close to the true ones, showing better performance than the two-stage model did. Meanwhile, the estimates were highly variable when weakly informative or non-informative prior was assigned.
Conclusions: Our results highlighted that the Bayesian hierarchical model is flexible and has the potential to more accurately estimate the location-specific association when priors are appropriately specified, compared to the two-stage method for small area estimation in environmental epidemiological studies for the temperature-mortality association.

P08-07 | Effects of environment on the double and triple burdens of infections among under-five children across low-middle-income countries: Machine Learning Algorithms

Haile M. Fenta[1,3], A. Kofi Amegah[2], Aino K. Rantala[1], Inês Paciência[1], Jouni J.K. Jaakkola[1,4]

[1] University of Oulu, Center for Environmental and Respiratory Health Research (CERH), Research Unit of Population Health, Oulu, Finland; [2] University of Cape Coast, Public Health Research Group, Department of Biomedical Sciences, Cape Coast, Ghana; [3] Bahir Dar University, Statistics, Bahir Dar, Ethiopia; [4] Finnish Meteorological Institute, Helsinki, Finland

Objectives: The study aimed to examine the prevalence and associated factors of the double and triple burden of infection (D/TBID) symptoms among children under five in low- and middle-income countries (LMICs) using multilevel machine learning methods.

Methods: We used data on infection symptoms from the Demographic and Health Surveys (DHS) of 58 LMICs between 2000 and 2023. These data were merged with cluster-level particulate matter (PM2.5) from the National Aeronautics and Space Administration (NASA) and country-level data on political, social, and economic globalization from the World Bank report. We applied multilevel models to identify the most important predictors of D/TBID symptoms and employed machine learning (ML) algorithms to predict these symptoms among children across LMICs. We trained and validated the ML algorithms using 80%, 70%, and 60% of the data and tested them on the remaining 20%, 30%, and 40%, with 2, 5, and 10 cross-validation iterations.

Results: Of the 1,546,243 children under five, 19.2%, 20.5%, and 12.6% had fever, cough, and diarrhea, respectively, while the overall prevalence of double and triple burdens was 11. 9% and 3.67%, respectively. The results revealed that the double and triple burdens were influenced by the child's location, survey years, wealth index, family size, air pollutants, and environmental covariates. The estimated prevalence of both double and triple burdens of symptoms varied substantially across districts [intraclass correlation (ICC) = 13.3%] and countries (ICC = 8.8%). We found that the Random Forest algorithm provided the highest Area Under the Curve (AUC) of 94% and 99% for the double and triple burdens of infection, respectively.

Conclusion: The study found varying prevalences of double and triple burdens of infection among children under five, with significant influences from environmental and sociodemographic factors. It also demonstrated that the Random Forest algorithm performed best in predicting these burdens.

P08-09 | Prediction of Low Birth Weight Using Machine Learning-based Analysis on Environmental and Maternal Risk Factors: Korean CHildren's ENvironmental health Study (Ko-CHENS)

Seoyeon Cho[1,2], Jongmin Oh[1,3], Eunji Kim[4], Jin-Hong Kim[5], Hwan-Cheol Kim[6], KyoungSook Jeong[7], Eunhee Ha[1,2], Yi-Jun Kim[1], On behalf of Ko-CHENS Study group

[1] Ewha Womans University College of Medicine, Deparment of Environmental Medicine, Seoul, South Korea; [2] Ewha Womans University, System Health & Engineering Major in Graduate School (BK21 Plus Program), Seoul, South Korea; [3] Seoul National University College of Medicine, Department of Human Systems Medicine, Seoul, South Korea; [4] The University of Melbourne, Melbourne, Australia; [5] Seoul National University, Department of Biological Sciences, Seoul, South Korea; [6] Inha University Hospital, Inha University, Department of Occupational and Environmental Medicine, Incheon, South Korea; [7] Yonsei University College of Medicine, Wonju Severance Christian Hospital, Department of Occupational and Environmental Medicine, Wonju, South Korea

Maternal factors during fetal development affect postnatal health, and adverse birth outcomes increase the risk of metabolic disorders, hypertension, and other diseases. Previous studies on low birth weight have primarily focused on maternal characteristics such as age and health status, while research on environmental factors has been relatively limited. This study aimed to predict low birth weight by incorporating maternal environmental factors, including air pollution and housing conditions. Additionally, machine learning was employed to enhance predictive accuracy.

We utilized cohort data from the Korean Children's Environmental Health Study (Ko-CHENS), conducted from 2015 to 2021. Various machine learning models were applied, including Logistic Regression, Naïve Bayes, Random Forest, Support Vector Machine, Gradient Boosting Machine, Extreme Gradient Boosting, Lasso, Ridge, and Elastic Net. The best-performing model was selected based on AUC-ROC, AUC-PRC, F1 score, and recall score. As a result, Logistic Regression was the most effective model, with an AUC-ROC value of 0.902. And feature importance analysis was conducted to determine key risk factors for low birth weight. The important variables included NO2 and PM2.5.

Our study confirmed that machine learning techniques can be effectively utilized to predict low birth weight. Additionally, it highlights the impact of environmental factors on low birth weight. Our study may contribute to air pollution policies and low birth weight prevention measures.

P08-10 | Improving the rigor of bias assessments in systematic reviews: A case study of cancer epidemiology studies of woodsmoke exposure

Ruth M. Lunn[1], Whitney D. Arroyave[2], Suril S. Mehta[1]

[1] National Institute of Environmental Health Sciences, Division of Translational Toxicology, Research Triangle Park, USA; [2] inotivco, Durham, USA

Objectives
Systematic reviews typically conduct risk of bias assessments; however, the risk or potential for bias does not mean that the study findings were biased. The International Agency for Research on Cancer recently published statistical methods for assessing bias impact of published studies. To decrease uncertainty in cancer health assessments, we applied these methods and those in the Report on Carcinogens (RoC) Handbook to assess confounding bias in studies of wood smoke exposure and cancer risk.
Methods
We used a three-step approach to evaluate confounding bias in lung and nasopharyngeal (NPC) cancer studies. Using guidance in the RoC Handbook and the evaluation’s protocol, we first assessed individual studies to identify the potential for bias, including the predicted direction. For each potential confounder, we conducted quantitative bias analyses of individual studies using a structured approach. Lastly, we evaluate bias across studies using triangulation methods, including stratified meta-analyses or forest plots.
Results
Because the bias direction was expected to be away from the null for most confounders, we limited our assessment to positive studies, which could influence health hazard conclusions. For each potential confounder for lung cancer, concern for potential confounding was found in 11%- 37% of all studies and up to a third of positive studies. However, bias impact analysis (including those using very conservative methods) of individual studies and stratified meta-analyses showed that confounding would not explain all the excess risk. For NPC, a larger proportion of studies did not adjust for key confounders, and bias impact analyses were less clear.
ConclusionsLimitations of this approach include the presence of multiple biases in a study often in different or unknown directions, often incomplete reported information to conduct the assessment, and the use of overly conservative methods. However, overall, these assessments can greatly improve the uncertainty of bias in systematic reviews

P08-11 | Pointwise spatiotemporal machine-learning framework for virtually continuous air pollution exposure assessment

Arturo De La Cruz Libardi[1], Pierre Masselot[1], Emily Nightingale[1], Ai Milojevic[2,1], Rochelle Schneider[3,4], Malcolm Mistry[1,5], Antonio Gasparrini[1]

[1] London School of Hygiene and Tropical Medicine, London, UK; [2] UK Health Security Agency, London, UK; [3] Phi-lab, European Space Agency, Frascati, Italy; [4] European Centre for Medium-Range Weather Forecast, Reading, UK; [5] Ca' Foscari UNiversity of Venice, Venice, Italy

Objective: Accurate exposure assessment is critical for the study of health effects of air pollution , but current national and regional models rely on grid-based implementations that prevent making full use of highly spatially-resolved data. This contribution presents a pointwise machine-learning (ML)framework to model air pollution levels with high accuracy and at near-continuous resolution.
Material and Methods: As in grid-based approaches, a large set of atmospheric, land-use, sociodemographic, and satellite-derived datasets are gathered as model features. Contrastingly, the pointwise method avoids fixed-resolution grids and forced data resampling. Instead, buffers of increasing size are drawn around monitoring stations and intersected with raw data to preserve spatial contrasts for the model. Observations are characterized at multiple relevant scales (e.g., road density within meters, land-cover within 1km, atmospheric conditions within kms). The same technique applies at prediction points, allowing near-continuous estimates and optional area-level prediction. Performance is evaluated in the reconstruction of nitrogen dioxide (NO_2) and particulate matter ($PM_{2.5}$) maps across Great Britain , with internal monitor-grouped cross-validation.
Results: In 2003-2021 we use an average of 205,105 and 125,652 daily observations from 394 and 617 stations to train annual NO_2 and $PM_{2.5}$ ML-models. Results show high cross-validated overall R^2 of 0.72 and 0.85, respectively, and spatial R^2 of 0.73 for both pollutants; representing a 4% and 3% improvement in overall R^2 and approximately 10% improvement in spatial R^2, compared to the grid-based framework. Mapping pointwise estimates reveals sub-1km-scale pollution variations, especially for NO_2. The method maintains reliability across varying monitoring densities and site types.
Conclusion: A key limitation of large-scale grid-based geospatial exposure models is addressed by optimising spatial-signal extraction from features and avoiding over-smoothing predictor data. The resulting models are better able to capture and predict pollutant concentrations and their spatial heterogeneity improving exposure estimation at epidemiologically relevant points or areas.

P08-12 | Advancing outdoor noise exposure assessment by leveraging high resolution data: From land use regression to deep learning prediction models

Matthew Bozigar[1], Andrew Larkin[1], Madeline N. Spawn[2], Suhas V. Sumukh[2], Carson Mowrer[1], Charlotte Roscoe[3], Stephanie T. Grady[4], Brendon Haggerty[5], Junenette L. Peters[4], Perry Hystad[1]

[1] Oregon State University, School of Nutrition and Public Health, Corvallis, USA; [2] Oregon State University, School of Electrical Engineering and Computer Science, Corvallis, USA; [3] Oregon Health and Science University-Portland State University, School of Public Health, Portland, USA; [4] Boston University, School of Public Health, Boston, USA; [5] Multnomah County Health Department, Healthy Homes and Communities Program, Portland, USA

Objective:
Outdoor noise pollution disproportionately affects marginalized communities, yet exposure assessments remain hindered by sparse measurements and limited spatial resolution. We enhanced land use regression (LUR) models with deep learning and publicly available datasets to improve predictive accuracy and characterization of noise injustices.

Materials and Methods:
We developed a novel LUR model to predict A-weighted continuous equivalent sound levels (LAeq) using multiday (≥5 consecutive 24-hour) measurements with Class I microphones at 212 locations in Portland, Oregon, United States. Lasso penalization selected 598 candidate predictors, including traffic characteristics, green space indices, and infrastructure elements. A key innovation was incorporating two-dimensional building shielding effects for traffic noise. Using the final LUR model with shielding, LAeq (dBA) was estimated citywide on a 10m² grid to resolve within- and across-neighborhood exposure differences.

To address LUR limitations—such as reliance on predefined variables—we supplemented it with deep learning. This enhanced framework integrates diverse spatial datasets, including satellite imagery and digital surface models, allowing vision models to extract nuanced spatial patterns relevant to noise exposure. Model training and validation leverage the same measurement dataset as the LUR model for comparability.

Results:
The citywide LUR model improved with building shielding, reducing root mean squared error from 3.05 to 2.58 dBA and increasing variance explained from 84.2% to 89.0%. These refinements highlight the value of incorporating built environmental context into LUR frameworks. While deep learning model development is ongoing, its ability to capture fine-scale noise variability offers promising advantages for improving exposure estimates in vulnerable populations.

Conclusion: By integrating deep learning-based predictions with LUR modeling, we generated precise, equitable, and scalable noise exposure assessments to inform exposure science, urban planning, and public health policy. Advancing deep learning models may further benefit epidemiological research, supporting efforts to identify and mitigate health disparities across the lifespan.

P08-13 | Assessing statistical methods for metal mixture exposure and neuropsychological effects in children

Susana Diaz-Coto[1], Leyre Notario-Barandiaran[2], Manuel Lozano[3,4], Sabrina Llop[3,5], Adonina Tardón[12,5], Cristina Rodríguez-Dehli[12], Amaia Irizar[7,5], Nerea Lertxundi[6,7], Monica Guxens[8,5], Jordi Julvez[8,5], Pablo Martinez-Camblor[9,10], **Antonio J. Signes-Pastor**[11,5]

[1] Dartmouth College, Department of Orthopaedics, Lebanon, USA; [2] Dartmouth College, Department of Epidemiology, Lebanon, USA; [3] FISABIO−Universitat Jaume I−Universitat de València, Epidemiology and Environmental Health Joint Research Unit, Valencia, Spain; [4] Universitat de València, Preventive Medicine and Public Health, Valencia, Spain; [5] Instituto de Salud Carlos III, Spanish Consortium for Research on Epidemiology and Public Health (CIBERESP), Madrid, Spain; [6] University of the Basque Country (UPV/EHU), Faculty of Psychology, San Sebastián, Spain; [7] Environmental Epidemiology and Child Development Group, Biogipuzkoa Health Research Institute, San Sebastián, Spain; [8] ISGlobal, Barcelona, Spain; [9] Dartmouth Health. Geisel School of Medicine at Dartmouth, Department of Anesthesiology, Lebanon, USA; [10] Universidad Autonoma de Chile, Faculty of Health Sciences, Tamuco, Chile; [11] Unidad de Epidemiología de la Nutrición, Universidad Miguel Hernández, Alicante, Spain; [12] Universidad de Oviedo, Departamento de Medicina, Oviedo, Spain

Human exposure to metal mixtures occurs through air, food, and water, with potential adverse effects on neuropsychological development, particularly in early life. While advanced novel statistical methods such as Bayesian kernel machine regression (BKMR) are becoming increasingly popular, their complexity and interpretability challenges may limit practical application. In contrast, linear regression models (LRMs) offer a more accessible approach that, under certain conditions, may provide comparable findings. This study evaluates the feasibility, interpretability, and performance of LRMs and BKMR in assessing the effects of metal mixture exposure on neuropsychological development in early childhood. We analyzed data from 962 children in the INMA (*INfancia y Medio Ambiente*) cohort in Spain. Urine samples collected at ages 4–5 were analyzed for metal(loid) concentrations including Co, Cu, Pb, Se, Mo, Zn and As speciation using inductively coupled plasma mass spectrometry, and children's neuropsychological function was assessed with the McCarthy Scales of Children’s Abilities. Principal Components Analysis identified three neuropsychological domains: executive function, motor function, and visual/verbal functions. BKMR modeled metal mixture effects, while generalized linear regression models (LRMs) incorporated first-order interaction terms to account for effect modification. Higher lead exposure linked to lower neuropsychological scores, whereas molybdenum showed a positive association, and selenium had mixed effects. LRMs and BKMR produced highly correlated estimates ($r > 0.90$), suggesting that, despite BKMR's ability to capture higher-order interactions, LRMs effectively model key exposure-response associations. Moreover, interaction terms in LRMs improved alignment with BKMR estimates, indicating that, in many cases, LRMs offer a computationally efficient and interpretable alternative. Our findings suggest that LRMs provide a robust and practical approach for assessing metal mixture effects on neuropsychological development, offering comparable performance to BKMR while being easier to implement and interpret, particularly when exposure-response associations are predominantly linear. However, when non-linearity and complex interactions prevail, BKMR may provide added insight.

P08-14 | Predicting Acute Upper Respiratory Infections among the Taiwanese Elderly Population: A Machine Learning Approach to Environmental Health

Gerry Andhikaputra[1], William C. Loe[2], Wei-Hsiang Weng[3], Chun-Jen Tsai[3], Fung-Chang Sung[4], Yu-Chun Wang[3]

[1] University of Maryland, Department of Epidemiology and Biostatistics, College Park, USA; [2] University of Maryland, Department of Science Academy, College Park, USA; [3] Chung Yuan Christian University, Department of Environmental Engineering, Taoyuan, Taiwan; [4] China Medical University, Health Service Administration, Taichung, Taiwan

Introduction: Acute upper respiratory infections (AURIs) remain among the most prevalent illnesses worldwide, contributing to a significant economic burden. This study applies machine learning (ML) to predict AURI incidence in elderly population (65 years and above) in Tainan City, Taiwan, from 2016 to 2019, considering multiple environmental exposures.

Methods: Weekly outpatient AURI data were obtained from the Taiwan CDC, while exposure data—including meteorological factors, air pollutants, and the greenness index—were sourced from the Taiwan CWB, EPA, and the NASA. To develop predictive models for AURI, various ML algorithms were applied, including random forest regression (RF), gradient boosting (XGBoost), lasso regression (L1 penalty), ridge regression (L2 penalty), and elastic net. The model was trained using data from Week 1, 2016 to Week 11, 2019 and tested on data from Week 12 to 52, 2019. Model performance was assessed using the test dataset, reporting mean absolute error (MAE) and root mean square error (RMSE).

Results: We found that $PM_{2.5}$ and NO_2 had stronger coefficient correlations with the outcome compared to PM_{10} and NOx, respectively.Our results showed that average temperature, relative humidity, SO_2, O_3,$PM_{2.5}$,NO_2, and NDVI (250m)are significant predictors of AURI. These results suggest that regularized linear models were the most effective. Among the predictive models, ridge regression performed best, achieving the lowest RMSE (330.96) and MAE (245.98), followed by elastic net (RMSE: 332.49, MAE: 245.95) and XGBoost (RMSE: 333.47, MAE: 255.45). Lasso regression had slightly lower accuracy (RMSE: 335.28, MAE: 257.62), while RF underperformed, with an RMSE of 400.85 and MAE of 334.51. This finding highlights the importance of model selection in environmental health forecasting.

Conclusion: The proposed methods can support outbreak preparedness and assist public health practitioners during both stable transmission periods and times of structural breaks in data.

P08-15 | Watershed-Level U.S. Population Estimates Stratified by Age, Race, and Sex (2010 to 2019): A New, Publicly Available Resource for Environmental Epidemiologists and Water Quality Researchers

Ryan Gavin[1,2]

[1] University of Minnesota, Division of Environmental Health Sciences, Minneapolis, USA; [2] University of Minnesota, Division of Biostatistics and Health Data Science, Minneapolis, USA

Introduction: Environmental epidemiologists are often concerned with estimating the incidence or prevalence of a disease in a population as a function of some environmental exposure. These estimands usually require estimates of the size of the target population at risk in their denominators, but this information isn't always readily available for the true target population of interest, and investigators often rely on a convenience target population based on some politically or economically defined geography rather than a target population based on an ecologically relevant exposure area.

Methods: Here, we describe a dasymetric algorithm for population interpolation used in demography that leverages public infrastructure information and remotely sensed impervious surface data to appropriately apportion areal population estimates to secondary geographies. We then apply this algorithm and describe a new, publicly available dataset containing watershed-level population estimates for the continental United States between 2010 and 2019 stratified by age, sex, and race. Finally, to illustrate the importance of using an ecologically relevant target population, we estimate the incidence of the waterborne zoonosis sporadic cryptosporidiosis as a function of precipitation and cattle count in Minnesota using case data from 2010 to 2019 obtained from the Minnesota Department of Health and two target populations: one which is watershed based and another based on census geographies.

Results: This investigation resulted in the creation of a new, publicly available dataset containing watershed-level population estimates for the continental United States between 2010 and 2019 stratified by age, sex, and race, which will be of particular interest to environmental epidemiologists interested in studying water-related exposure disease relationships. We then used these data to analyse the effect of precipitation and cattle count on the incidence of sporadic cryptosporidiosis in Minnesota and obtained different estimates of effect compared to those resulting from census-based analyses, suggesting important public health and scientific implications.

P08-16 | Deciphering the Adverse Effects of Environmental Mixture Exposure on Cardiopulmonary Health by Constructing A Knowledge-Driven Framework

Bin Wang

Peking University, School of Public Health, Beijing, China

Exposure to vast mixtures of environmental pollutants can cause damage to human cardiopulmonary health. Still, it remains a challenge to conduct the risk assessment of these mixtures on the cardiopulmonary health in a specific exposure scenario due to the heterogeneity of the concerned toxins and health biomarkers. We therefore proposed a knowledge-driven framework integrating the results from the local epidemiological study and the constructed knowledge network to determine the key exposures and their related biological pathways associated with the endpoints of cardiopulmonary health. We constructed the knowledge network of "Exposures-Gene Ontology (GO)-Outcome" linking exposures to cardiopulmonary health-related endpoints by integrating information from multiple sources. To validate its efficiency, we further adopted a randomized crossover trial under a true or sham air purifier intervention among 15 healthy college students and collected their urine and serum samples. We measured a total of 34 toxic organic pollutants and 16 cardiopulmonary health-related endpoints in their biological samples, as well as the DNA methylation levels under sham conditions. We finally identified eight key exposures and a total of 130 GO pathways associated with their adverse effects on cardiopulmonary health. Among them, urinary mono-(2-ethylhexyl) phthalate (MEHP) was the most important exposure, and it may cause adverse effects on cardiopulmonary health through the key GO pathways of lipid metabolism processes, cell proliferation and migration. In addition, we identified the mediation effects of 8-isoPGF2α and FeNO in blood pressure and lung function. We concluded that our constructed knowledge-driven framework is effective in deciphering the adverse effects of environmental mixture exposure on cardiopulmonary health, and can be referred to conduct the health risk assessment for other scenarios with smaller population size and lower expenses.

P08-17 | Sample Size and Bias Approximations For Continuous Exposures Measured with Error

Honghyok Kim

University of Illinois Chicago, Division of Environmental and Occupational Health Sciences, Chicago, USA

Background. Measurement error is ubiquitous in environmental epidemiologic research, yet its impact on sample size determination and the accuracy and precision of estimators remains understudied in real-world scenarios. These scenarios include continuous (spatiotemporal) exposures with unequal variance, error-prone measurements, multiple exposure time points, and the use of calibrated exposure variables.
Methods. This research theoretically develops approximation equations for sample size calculations, estimator accuracy, and standard errors in research settings involving spatiotemporal exposures with unequal variance, distributed lags, and/or non-linear effects in the presence of exposure measurement error, and incorporating considerations of (external) exposure validation data.
Results. The developed framework accommodates non-differential, autocorrelated, and differential additive or

multiplicative measurement errors in distributed lag models for exposures with unequal variance, accounting for the presence or absence of exposure validation data. This also considers non-linear effect estimation using polynomials. Statistical simulation analyses confirmed the utility of the framework. An application of this framework is demonstrated in the design of a nested case-control birth cohort study examining air pollution exposures during pregnancy. **Conclusion.** The proposed theory and methods provide practical tools for efficient research design and a deeper understanding of measurement error impacts, while seamlessly integrating uncertainty analyses.

P08-18 | A machine learning framework for modeling the associations between environmental factors and health: an application in the German National Cohort

Nikolaos Nikolaou[1], Donatella Cea[2], Marco Dallavalle[1], Jeroen Staab[3,4], Mahyar Valizadeh[2], Marie Piraud[2], Annette Peters[1,5], Alexandra Schneider[1], Hannes Taubenböck[3,6], **Kathrin Wolf**[1]

[1] Helmholtz Zentrum München, German Research Center for Environmental Health, Institute of Epidemiology, Neuherberg, Germany; [2] Helmholtz Zentrum München, German Research Center for Environmental Health, Helmholtz AI, Neuherberg, Germany; [3] German Aerospace Center (DLR), German Remote Sensing Data Center, Geo-Risks and Civil Security, Oberpfaffenhofen, Germany; [4] Humboldt-University Berlin, Geography Department, Berlin, Germany; [5] LMU Munich, Pettenkofer School of Public Health, Institute for Medical Information Processing, Biometry and Epidemiology (IBE), Faculty of Medicine, Munich, Germany; [6] Julius-Maximilians-Universität Würzburg, Institute for Geography and Geology, Würzburg, Germany

Objective: Human health has been associated with individual characteristics, environmental exposures, socio-economic and neighborhood settings but their interplay is not adequately understood. We aimed to build a machine learning (ML) pipeline to identify the driving environmental, socio-economic and individual factors for health outcomes, using hypertension as a case study.
Material and Methods: The ML pipeline is based on three main pillars: data extract/transform/load (feature selection, imputation of missing values), modeling (hyperparameter optimization), and explainability (permutation feature importance). For our use case, we included health data from the baseline examination of the population-based German National Cohort (NAKO), conducted between 2014-19 in 16 study regions across Germany. We assigned environmental exposures (e.g., air pollution, air temperature, noise, greenness) and neighborhood factors (e.g., urbanization, deprivation) based on the participants' residential addresses. We compared traditional regression approaches (Logistic Regression) with multiple ML methods, such as neighbor-based methods (K-Nearest Neighbor), Statistical Learning (Support Vector Machine), Ensemble Learning (Random Forest, XGBoost) and Neural Networks, to identify the main drivers for hypertension.
Results: Of 204,752 participants included in our analysis, 41.2% were classified as hypertensive. Most models performed well with comparable accuracy ranging from 0.69 (K-Nearest Neighbour) to 0.73 (XGBoost) in our test set. The different approaches identified similar factors as the main drivers for hypertension with highest feature importance attributed to individual characteristics (age, body mass index, and sex). SHapley Additive exPlanations and sub-group analyses also identified environmental and neighborhood variables (minimum air temperature, noise and deprivation index), following the primary individual factors.
Conclusion: Our results indicate some variation in performance and that a guided application is needed if evidence shall be generated beyond major drivers of disease such as age and sex. The ML pipeline for binary health outcomes shall be openly accessible soon, but we also plan to expand it to continuous outcomes.

P08-19 | Revisiting the reversal paradox: why do regression coefficients change sign when adding variables?

Nate Wiecha[1], Ran Rotem[2], Marc Weisskopf[2]

[1] North Carolina State University, Department of Statistics, Raleigh, USA; [2] Harvard T.H. Chan School of Public Health, Department of Environmental Health, Boston, USA

Objective: The reversal paradox—where effect estimates for two highly correlated variables become more extreme or even reversed when modeled together—has been raised as a problem for the study of environmental exposure mixtures. Despite demonstration that, in the multi-exposure model, divergence in expected effect estimates arises from specific causal structures, the misconception that merely including correlated variables automatically 'pushes' expected estimates apart persists. This is perhaps because empirically investigators may seem to observe this occurring even when causal knowledge suggests it shouldn't. Thus, we set out to explore whether, under a defined causal structure, some statistical properties could contribute to the apparent divergence of correlated variables' effects in multi-exposure models.

Material and Methods: Base simulations were each run 1000 times, using 100 observations. Correlation between exposures (*rho*) ranged from 0 to 0.99, and the true causal effects on a continuous outcome were either large (b1=b2=1) or small (b1=b2=0.05).

Results: In a linear regression model with two exposure variables with correlation *rho*, the correlation of the two regression point estimates over repeated sampling will be -*rho*, regardless of the causal effects. As *rho* increases, the joint sampling distribution of the two point estimates becomes more stretched in the 2-d space around the two true causal effects. Thus, the distributions of point estimates are in closer opposition to each other than when *rho*=0. However, the overall expected estimates are unbiased and validity of hypothesis tests is unaffected. When more than two correlated variables are included in models, the anticorrelation of the point estimates for any pair is less than when fewer are in the model.

Conclusion: With highly correlated exposures, there is a higher chance that in any single sampled dataset, the two regression point estimates will vary in opposite directions. However, the overall validity of inference is preserved.

P08-20 | A High-Resolution Methodological Approach for Identifying Extreme Heat Events and Heatwave Trends in the Houston Metropolitan Statistical Area (1998–2018)

Omar Rosales[1], Elyse Burgess[1], Jeremy Schraw[2], Abiodun Oluyomi[1]

[1] Baylor College of Medicine, Department of Medicine, Houston, USA; [2] Baylor College of Medicine, Department of Pediatrics, Houston, USA

Objective
Accurate identification and characterization of extreme heat events are critical for assessing public health risks and informing climate adaptation strategies. This study presents a high-resolution methodological approach to quantify heatwaves and extreme heat exposure using spatially refined climate data across the Houston Metropolitan Statistical Area (MSA) from 1998 to 2018.

Methods
Daily maximum and minimum temperature data were sourced from the Daymet dataset, a 1 km × 1 km gridded climate product. Relative humidity was estimated using the August-Roche-Magnus approximation, and the heat index was calculated via the Rothfusz regression equation, which accounts for both temperature and humidity to assess apparent temperature. The study employed a custom-year framework to capture seasonal trends. Spring was defined as March through May, Summer as June through August, Fall as September through November, and Winter as December through February. To maintain seasonal continuity across calendar years, Winter was structured such that December was associated with the current year, while January and February were assigned to the subsequent year. Thresholds for extreme heat events were established using the 90th (top 10%) and 95th (top 5%) percentiles of the seasonal heat index distribution. Heatwaves were identified as two or more consecutive days exceeding these thresholds. A grid-based temporal analysis examined spatial variability, heatwave frequency, duration, and intensity across the study period.

Results
The methodological approach successfully captured spatiotemporal trends in extreme heat, demonstrating a statistically significant increase in heatwave frequency and intensity across urbanized areas of Houston. The urban heat island effect was evident, with sustained extreme heat exposure disproportionately affecting highly developed regions.

Conclusion
This high-resolution analytical framework provides a scalable methodology for assessing heat exposure trends, with applications for public health surveillance, urban climate adaptation, and extreme heat forecasting. The approach offers a reproducible model for integrating climate data into environmental epidemiology research.

P08-21 | *Environmental Public Health Tracking (EPHT) Program: Capacity building, applied environmental epidemiology, and environmental health surveillance through CDC's National Tracking Program*

Angela Werner

USCDC, Atlanta, USA

Objective
Over the past 20 years, the U.S. CDC's Environmental Public Health Tracking Program collected, integrated, and analyzed non-infectious disease and environmental data from a nationwide network of partners to help inform public health decision-making. These data empower people to make information-driven decisions that affect their health, including public health practitioners, community members, policymakers, and others.

Material and Methods
The Tracking Network is a network of people, including 33 funded recipients, and information systems that deliver a core set of health, exposure, and hazards data, and tools to enable analysis, visualization and reporting of insights drawn from data. These data include data developed in collaboration with other federal partners, including NASA, NOAA, and EPA, disseminating data in a usable, standardized format. Data are disseminated through the Tracking Program's Data Explorer, topic-specific dashboards, and other channels to empower users. Some examples include daily and forecast air quality and near real-time flooding measures on the Data Explorer as well as the Heat & Health Tracker dashboard.

Results
Recent examples of data usage include the 2024 heat season, where Emergency Departments in Phoenix utilized the Heat & Health Tracker to monitor near real-time data on heat-related illness, enabling facilities to anticipate patient surges during extreme heat events. For the 2023 Hawaii wildfires, 5-day PM2.5 forecast data were used for air quality monitoring, supporting situational awareness and allowing for timely public health messaging. Near real-time flooding data were also utilized to generate more detailed exposure maps and real-time tracking for the 2023 Hurricane response, allowing for groups across the agency to coordinate and align efforts.

Conclusions
The Tracking Network has a vast amount of information, including health, exposure, and hazards data, alongside valuable tools that can be used for timely, information-driven decision-making to improve the health of our communities.

P08-22 | Environmental health information system pilot development for the Kingdom of Tonga.

Matthew Ashworth

ESR, Auckland, New Zealand

Background: Tonga faces some of the highest environmental and climate-based disaster risk exposures in the world. Working in partnership with the Government of Tonga we have a multi-stakeholder environment and health information system. The system has been developed using water security and associated health risk exposures as the proof of design; but is conceived to be applicable to other environment and health related exposure pathways.
Methods: a multi-stakeholder collaborative process was undertaken to develop measures and indicators in the water security. Outcomes of the consultation were transformed to operationally and strategically useable information for decision making and direction of development activities.
Additionally, ESR's ALMA digital twin platform has been adapted to create a comprehensive virtual model of Tonga, generating a synthetic population that reflects the island nation's demographic characteristics. This synthetic Tongan population incorporates multiple attributes per individual, while preserving privacy through anonymised data. The platform integrates ground-truth air quality measurements with satellite remote sensing data to produce high-resolution temporal and spatial maps of air pollution across the islands.
Results: Tonga has a functioning environmental health surveillance prototype with a Water Security focus at present. Additionally, the digital twin correlates respiratory condition reports with the air quality maps, local weather patterns, and water quality surveillance data to simulate potential health impacts on different communities. This can provide decision-makers with powerful visualisation tools to identify vulnerable populations and test intervention strategies before implementation.
While this application focuses on respiratory health as a proof of concept, ALMA's sophisticated AI-driven simulation capabilities can be expanded to model numerous other scenarios, from disease outbreaks to climate change impacts and infrastructure planning, providing Tongan authorities with an unprecedented decision support system for addressing complex challenges.
Conclusions: development of environmental Health Information system and AI capability has provided increase information availability and is improving Health Security.

P09 | Integrating Environmental Health into Public Health Practice and Education

P09-01 | Impacts of clinician-guided versus self-guided report-back of biomonitoring results on study participants' environmental health literacy and other outcomes

Katherine Franz[1], Alina McIntyre[1], Katherine E. Boronow[1], Julia Green Brody[1], Phil Brown[2], Nobel O. Hernández Otero[4], Carmen M. Vélez Vega[4], Tamarra James-Todd[3], Jennifer Liss Ohayon[1]

[1] Silent Spring Institute, Newton, USA; [2] Northeastern University, Department of Sociology and Anthropology, Boston, USA; [3] Harvard University, T.H. Chan School of Public Health, Boston, USA; [4] University of Puerto Rico, Medical Sciences Campus, San Juan, USA

Objective:
We evaluated impacts of clinician-led versus self-guided approaches to reporting back individual-level environmental exposure measurements on study participants' emotional response, environmental health literacy, and satisfaction with report-back.
Methods:
In collaboration with two pregnancy-based cohort studies, Puerto Rico Testsite for Exploring Contamination Threats (PROTECT) and Environmental Reproductive and Glucose Outcomes (ERGO), we developed exposure reports for participants using the Digital Exposure Report-Back Interface (DERBI). Reports included personal biomonitoring results and information about chemical sources, links to health, and ways to reduce exposure. Participants (n = 165) were assigned to receive their report during a meeting with a clinician (trained to report back as part of this study) or as a self-guided online report. Pre- and post-surveys assessed feelings, self-efficacy, knowledge, and exposure-related behaviors before and after report-back, as well as satisfaction with report-back.
Results:
Clinician-guided and self-guided report-back groups were similar across many outcomes. For both intervention groups, participants felt high levels of respect and empowerment and mild to moderate levels of worry related to exposures before and after report-back. Correct answers for questions about exposure sources and scientific uncertainty increased, but misconceptions remained regarding chemical regulation in the U.S. and clinical implications of results. In both intervention groups, more than a third of participants reported changes in product choices related to the chemicals in the report following report-back, and participants were also more likely to avoid microwaving in plastic. Although satisfaction with the report-back experience was high across both interventions, overall satisfaction with study participation was higher in the clinician-guided group in PROTECT.
Conclusions:
Our findings support the value of report-back in environmental health research. Involvement of study-affiliated clinicians may increase study engagement and satisfaction. However, when clinicians are not available, self-guided report-back offers similar benefits to environmental health literacy.
Funding: NIEHS R21, NIEHS T-32 ES023769 2015-2026

P09-02 | HEI Summer Fellowship Program: Pathway to the Environmental Health Sciences for Undergraduate Students

Hlina Kiros, Samantha Miller, Allison Patton, **Elise Elliott**, Ellen Mantus

Health Effects Institute, Boston, USA

Objective: Hands-on experience in research is essential for undergraduate students to build skills and explore potential career pathways. The Health Effects Institute (HEI) runs an annual Summer Fellowship Program in partnership with the International Society of Exposure Science (ISES) and the International Society for Environmental Epidemiology (ISEE) to support unique, paid environmental health research opportunities for highly qualified undergraduate students who would otherwise not have an opportunity like this one.

Materials and Methods: U.S.-based undergraduate students are matched with volunteer faculty mentors at academic and nonprofit organizations for an immersive 10-week environmental health research project. During the summer, fellows in the Summer Fellowship Program cohort learn to develop a research proposal, present their work, and participate in professional development seminars. Fellowship applications are first reviewed by ISES and ISEE volunteers. Next, finalists are selected by a joint HEI-ISES-ISEE committee based on the applicant's motivation, skills, experience, and need for the opportunity. Finally, HEI matches the finalists and mentors for a tailored experience.

Results: The number of applications received by the fellowship program grew from 52 in its inaugural year in 2023 to 168 in 2025. The program supported cohorts of 7 fellows in 2023 and 8 fellows in 2024. We anticipate supporting a similar size cohort in 2025. Fellowship alumni reported that the program gave them well-rounded experience in their academic journeys by contributing to research projects and building new relationships with professionals and organizations.**Conclusions:** The continuous development of the Summer Fellowship Program and growing success reflects HEI's efforts to support academic and career development for highly qualified people in the field of environmental health science. HEI's Summer Fellowship Program is funded by the American Chemistry Council, Clinton Family Foundation, and individual donors.

P09-03 | Utilizing Communication Strategies to Increase Public Engagement on Air Pollution

John Commey

University of Arkansas at Little Rock, Information Science, Little Rock, USA

Abstract
Many of the activities that contribute to Air pollution are those that make our lives more comfortable, such as driving cars and using electricity generated from coal-burning power plants.
As these essential activities continue, increasing public awareness of air pollution issues, causes, and potential prevention strategies is crucial.
Air pollution is the release of harmful substances into the atmosphere, whether gases, solids, or liquid droplets.
Air pollution has serious health implications. It is one of the causes of premature death, mainly from the onset of cardiovascular diseases, cancer, diabetes, and infertility. The environment is also affected by the adverse effects of air pollution. Acid rain and depletion of the ozone layer are some of the main effects.
Understanding and addressing air pollution is a collective responsibility that requires active public participation.
The objective of the study was to investigate the impact of various communication strategies on public engagement regarding air pollution.
Three communication strategies, namely social media campaigns, traditional media and community outreach were tested on a sample of 500 individuals from urban areas with varying air pollution levels.It was concluded that communication strategies play a critical role in increasing public engagement on air pollution issues. Tailored approaches that combine social media, traditional media, and community outreach are most effective.

P09-04 | I have a radon problem at home, what now? A citizen science project in Galicia, Spain

Lucia Martin-Gisbert, Guadalupe Garcia, Cristina Candal-Pedreira, Julia Rey-Brandariz, Carla Guerra-Tort, Ana Tejeiro, Alberto Ruano Raviña

University of Santiago de Compostela, Preventive Medicine and Public Health, Santiago de Compostela, Spain

Objetive: To generate a community-based asset to support radon mitigation in Galicia (Spain)
Methodology: Four citizen scientists (CS) who had radon concentrations above 300 Bq/m3 (reference level in Spain) were recruited via phone from those who measured radon with the University of Santiago de Compostela (USC) within the last year. All of them lived within the area of Santiago de Compostela. The participation implied their commitment to mitigate radon in their homes within a year, sharing and reporting the whole mitigation process, attending periodic team meetings at the USC, and developing a radon mitigation asset. The USC team provided guidance,logistic and financial support throughout the process.
Results
The CS searched for radon mitigation providers and their proposed solutions, and 14 budgets were discussed. Three CS chose as solution a radon sump with a local provider due to its price and its better adequacy, and one CS chose to simply improve the ventilation habits. A post-mitigation measurement confirmed that radon mitigation was

successful for all CS. A video and an open event for dissemination about radon were created as an asset for the community. Through CS brainstorming the content of both was drafted. The onsite open event was held on November 8th, 2024, with a total of 101 attendants who rated its usefulness with an average of 9.5 (response rate 100%), and the video is available in YouTube with almost 600 views since its publication on November (https://www.youtube.com/watch?v=AU7EwRUIMZU).
The USC gained valuable knowledge about the current mitigation practices in the community.
Conclusions
Citizens who overcome a health risk can create accessible and original health assets for their community and for the academy. CS, and probably citizens in general, can easily become ambassadors for health promotion when they are provided with an active, well-informed role.

P09-05 | From the Cell to the Street: Report-Back in Large Studies with Multi-Level Data

Katherine E. Boronow[1], Rachel Morello-Frosch[2], Amy Padula[3], Sarah Geiger[4], Erin DeMicco[3], Katherine Franz[1], Mildred Cho[5], Phil Brown[6], Julia G. Brody[1]

[1] Silent Spring Institute, Newton, USA; [2] University of California Berkeley, Berkeley, USA; [3] University of California San Francisco, San Francisco, USA; [4] University of Illinois Urbana-Champaign, Urbana, USA; [5] Stanford University, Stanford, USA; [6] Northeastern University, Boston, USA

Objective: To understand effects of the exposome on human health, studies are integrating early-effect biomarkers, measurements of hundreds of chemical contaminants, and sociodemographic and geospatial measurements that capture environmental and social determinants of health. The Cell to the Street Study is developing methods for reporting back to participants on these types of multi-level data.

Material and Methods: We reported back on targeted chemical analysis in two pregnancy cohorts: Chemicals In Our Bodies (CIOB), based in the San Francisco Bay Area, and Illinois Kids Development Study (IKIDS), based in Urbana-Champaign. User-centered digital reports were developed using DERBI, the Digital Exposure Report-Back Interface. To develop expanded report-back for multi-level data, we held workshops with the Participant Ambassadors Council. Topics included their prior experience receiving reports on chemical measurements, their views on what types of geospatial indicators they wish to receive (for example, voter participation, social vulnerability index, green space), and whether they wish to receive results for early-effect markers (telomeres, oxidative stress), given scientific uncertainties about interpretation. We developed sample report-back content for the new data types.

Results: Participants typically spent enough time on their reports to read all their summary headlines and click to details about at least one chemical group. We developed a highly successful tutorial (<3 minutes) that supported understanding of results graphs, particularly among participants who do not have a college degree. At the workshops, participants shared perspectives on the new variables for multi-level report-back and gave feedback on sample content.

Conclusion: Digital methods can support personalized report back for multiple data types in exposome studies. Using participant-engaged methods ensures that content is understandable and responsive to their interests and concerns.

This work was supported by grants R01ES036234, R21ES030454, U2COD023375 (Opportunities and Infrastructure Fund), and UH3OD023272.

P09-06 | Knowlege, Attitude and Practices of Healthcare Providers (HCPs) regading Climate Change and Health in Karachi, Pakistan

Muhammad M.U. Siddiqui[1], Zafar Fatmi[1], Faiz Alam[2]

[1] Aga Khan University, Community health sciences, Karachi, Pakistan; [2] Emory university, Medical College, Atlanta, USA

Introduction: Climate change is a global challenge with profound implications for public health. Healthcare providers, as trusted figures and advocates for health, play a crucial role in addressing climate change's impact on well-being. This study aimed to assess the knowledge, attitudes, and practices of healthcare providers in Karachi, Pakistan, regarding climate change and its associated health effects.
Methodology: A cross-sectional survey was conducted among 490 healthcare providers from diverse backgrounds and healthcare settings in Karachi. The survey instrument included questions related to climate change knowledge, attitudes, and practices. Descriptive statistics, chi-square tests, and logistic regression analyses were employed to examine the data.
Results: The findings revealed a significant level of knowledge and awareness among healthcare providers regarding climate change and its health implications, with 53% demonstrating satisfactory knowledge. Moreover, a majority of respondents (67%) exhibited a positive attitude toward climate change, expressing a willingness to engage with climate-related issues. Encouragingly, 57% of participants engaged in good practices related to climate change, reflecting a commitment to sustainability. Factors such as education level influenced both attitudes and practices.
Discussion: The study underscores the potential of healthcare providers as advocates for climate action and role models for sustainable practices. While positive attitudes were prevalent, challenges, including time constraints and limited resources, may hinder the translation of knowledge and attitude into meaningful action.
Conclusion: Healthcare providers in Karachi, Pakistan, possess a considerable level of knowledge, positive attitudes, and engagement in good practices related to climate change. Harnessing this potential will require targeted training, integration of climate discussions into medical education, and institutional support. These findings contribute to the growing body of knowledge on the role of healthcare providers in addressing climate change and its health impacts.

P09-07 | Advancing Environmental Health Education for Midwives and Maternity Care and Pediatric Nurses in Lebanon: The PENSE Project

Emile Whaibeh[1,2], **Jowy Abi Hanna**[1,2], Ali Sherri[2], Norma Aouad[4], Lina Jaalouk[1,2], Georges Abi Tayeh[4,5], Myriam Mrad[2,3]

[1] Saint-Joseph University of Beirut, Institut Supérieur de Santé Publique, Faculty of medicine, Beirut, Lebanon; [2] University of Balamand, Public Health Department, Faculty of Health Sciences, Beirut, Lebanon; [3] Health and Environment Response Agency (HERA), Beirut, Lebanon; [4] Hôtel-Dieu de France University Hospital, Obstetrics and Reproduction Department, Beirut, Lebanon; [5] Saint Joseph Fertility Center, Beirut, Lebanon

Objective: The Petite Enfance et Santé Environnementale (PENSE) project aims to raise the knowledge and awareness of healthcare professionals in maternity care and pediatrics about perinatal environmental exposures and their impact on pregnant women and infants. This first-of-its-kind hospital-based intervention in Lebanon led to the development of a guide and capacity-building training sessions on perinatal environmental exposures, piloted in Hotel-Dieu de France University Hospital in Beirut.
Materials and Methods: The PENSE project employs an ecological approach to develop and implement training materials for healthcare professionals, focusing on environmental health and its effects on maternal and infant well-being. Using a mixed-methods design, the training was evaluated using qualitative interviews and pre- and post-training assessment questionnaires to measure the changes in knowledge and perceptions.
Results: Qualitative interviews with 10 nurses and midwives revealed broad but vague definitions of environmental health, with limited knowledge of specific hazards. Comparisons between pre- and post-training assessments (N=27) showed statistically significant improvements in knowledge and perception, particularly in recognizing product-related health impacts and discussing environmental risks with patients more confidently. Familiarity with the term "exposome" increased significantly ($p = 0.0003$), and awareness of product-related health risks—particularly for pregnant women—rose markedly ($p = 0.0013$). Confidence in discussing environmental exposures with patients improved across multiple domains, with the largest gains observed in topics related to furniture and home renovation materials ($p < 0.0001$), personal care products ($p = 0.0015$), and indoor air pollution ($p = 0.0049$). Notably, 96.3% of participants expressed willingness to integrate environmental health discussions into their patient interactions.
Conclusion: Targeted education on environmental health improves healthcare professionals' knowledge and confidence in addressing related risks. Increasing the capacity of healthcare personnel working closely with pregnant women and infants ensures the continuous and sustainable transmission of critical health information.

Funding: The project was conducted without external funding.

P09-08 | Bridging Planetary Health and Medical Education in a Quilombola Territory: Insights from the Planet&AR Project

Nelzair A. Vianna[1,2], Marcos D.A. Moura[4,3], All other authors contributed to conceptualization in the first draft, technical inputs, case study material, review and editing of the final abstract.

[1] Fiocruz, Molecular Epidemiology and Biostatistics Laboratory, Salvador, Brazil; [2] Faculdade Zarns, School of Medicine, Salvador, Brazil; [3] Federal University of Juiz de Fora, School of Medicine, Juiz de Fora, Brazil; [4] Faculdade de Ciencias Medicas e da Saúde de Juiz de Fora, SUPREMA, Juiz de Fora, Brazil

Introduction: Planetary health integrates systemic thinking involving human-political, economic, environmental, and social systems to guide the construction of new interdisciplinary policies that strengthen a new field of study. The PlanetAR Project was created to develop strategies to identify health and environmental vulnerabilities on Maré Island, which historically was occupied by indigenous people and is now home to a predominantly "Quilombola" population. This area is recognized for its vulnerability to environmental impacts caused by industrialization, and socioeconomic inequality, and by the advocacy of its residents, who have mobilized researchers to assess air and water pollution impacts and the lack of access to health services. **Objective:** To promote medical student immersion in this territory, and to expand knowledge of the health disease process through the planetary health lens. **Methods:** This is a report on the experience of applying planetary health concepts in medical education, where medical students, through immersion in the territory, are encouraged to recognize the social, environmental, economic, and cultural context in which this community lives and the possible health impacts. The students remained in the area for 3 days interacting with residents and workers. The planetary health lens was applied, encouraging a holistic view of this territory. **Results:** The students were able to observe the consequences of environmental pollution on health and the cultural strength that guides the way of life of the local population. Field immersion proved to be a tool to support new strategies in medical education, expanding the scope of perception on the relationship between health and the environment through the lens of Planetary Health and vulnerabilities as well as the need to incorporate these concepts into the curriculum of Brazilian medical schools to promote a new perspective in clinical practice to address the climate change.

P09-09 | Media representations of the impacts to Hawai‘i residents by the Jet Propellant-5 Spill at the Red Hill Bulk Fuel Storage Facility on O‘ahu, Hawai‘i

Zoe Smith[1], Madison Young[1], Amanda Boyd[2], Nicole Rosendaal[1], Catherine M. Pirkle[1]

[1] University of Hawaii at Manoa, Department of Public Health Studies, Honolulu, USA; [2] Washington State University, The Elson S. Floyd College of Medicine, Spokane, USA

Objective: On November 20th 2021, the Red Hill Bulk Fuel Storage Facility on O‘ahu, Hawai‘i inadvertently released Jet Propellant-5 (JP-5) resulting in 5,542 gallons of JP-5 entering the United States' Navy-managed water system that supplies Joint Base Pearl Harbor Hickam and neighboring communities (i.e. the Spill). Consequently, the Hawaii Department of Health issued a water advisory on November 29th that lasted through March 18th, 2022. During this time an estimated 93,000 individuals were affected. Media reports capture contemporaneous experiences of those

affected by this environmental hazard and effectively reflect the public perceptions of the event. Here, we describe the results of the first media analysis on the health and sensory experiences of those affected by the Spill.
Material and Methods: A media content analysis of a civilian, online, local news outlet The Honolulu Civil Beat (84 articles) and a daily, American military newspaper Stars and Stripes (51 articles) was conducted. All articles regarding the Spill published between the date of fuel release to the lifting of the advisory were included. Data was extracted by source type and included descriptions of key events, sensory and health experiences, and affective responses. Source types included: Government, Military, NGO/advocacy, affected individual, and other.
Results: Early reporting focused on health and sensory effects. Health effects included primarily skin issues, headaches, nose bleeds, stomach aches, nausea, vomiting, dizziness, and fatigue. Sensory experiences included smelling gas and seeing an oil sheen on the water. Impacts to families, livelihoods, the broader community, and the environment were also highlighted. Later reporting focused on how the incident would be resolved.Conclusion: This analysis captured the contemporaneous perspectives on the exposures and outcomes experienced by Hawaiʻi residents affected by the 2021 Spill at Red Hill, as reported by and compared across a civilian and military news outlet.

P09-10 | *Adopting and integrating environmental determinants of health into routine clinical education, trainings, and practice for frontline health professionals*

Sumi Mehta, Yi Lu, Daniel Kass, Audrey Pennington

Vital Strategies, New York CIty, USA

Although environmental determinants are an increasing public health challenge, active efforts to mainstream environmental health within public health practice are limited. At present, few health agencies are sufficiently equipped to track the leading risk factors, exposures, and health outcomes associated with threats in built and natural environments. Urgently needed are expanded efforts to guide better collection, integration, and use of data on environmental determinants of health. This presentation will share lessons learned from initial efforts to integrate a focus on air pollution, temperature, and lead exposure into 1) mandatory, routine training for clinicians and other frontline professionals and 2) enhanced surveillance activities. Applied examples from work to scale efforts in Indonesia, India, Peru and Ethiopia will be provided, including efforts to enable clinicians and other frontline health workers to provide patient sensitization and guidance, as well as work underway to increase the routine collection of key indicators and measures of environmental risk. This will include work to strengthen routine measurement of air pollution-related and heat-related illness in India, as well as ongoing efforts to establish guidance for health professionals to identify and assess population level lead exposure among children in India, Peru and Indonesia. Efforts to prioritize and accelerate policies to support better integration of environmental determinants of health to prevent noncommunicable disease will also be described, with a focus on leading sources of ambient and household air pollution in Ethiopia.

P09-11 | Racial/Ethnic and Gender Disparities in Scientific Authorship in Public Health

Yimeng Song[1], Nabarun Dasgupta[2], Michelle L. Bell[1]

[1] Yale University, School of the Environment, New Haven, USA; [2] University of North Carolina at Chapel Hill, Gillings School of Global Public Health, Chapel Hill, USA

BACKGROUND: Racial/ethnic and gender disparities persist in scientific authorship, yet their extent and impact on manuscript acceptance rates remain understudied in public health. Understanding these disparities is crucial for promoting equity in academic publishing and ensuring diverse perspectives in scientific discourse. Machine learning algorithms provide a novel approach to estimating these disparities by predicting race/ethnicity and gender based on name-derived probabilities, though their inherent biases and limitations must be carefully considered.

METHODS: We analyzed 17,667 manuscript submissions from the United States between 2013 and 2022 to the American Journal of Public Health, using different machine learning algorithms to predict corresponding authors' race/ethnicity (Asian, Black, Hispanic, White) and gender categories, both based on name-derived probabilities. We compared the predictive performance of the different algorithms and examined their impact on disparity analysis. To account for uncertainties in classification, we employed a multiple imputation approach to probabilistically assign race/ethnicity and gender, ensuring robustness in our estimates.

RESULTS: Predicted White authors dominated submissions and had the highest acceptance rates (21.1%), while predicted Asian authors, despite contributing a substantial proportion of submissions (22.4%), had the lowest acceptance rates (14.9%). Predicted Black and Hispanic authors had intermediate acceptance rates of 18.1% and 17.4%, respectively. Gender disparities were also evident: predicted women, though forming the majority of submissions (57.4%), had lower acceptance rates (17.9%) than men (20.5%), a pattern consistent across most racial/ethnic groups. Differences in algorithmic predictions influenced the absolute estimates but consistently revealed these disparities.

CONCLUSION: Manuscript acceptance rates reveal persistent disparities by race/ethnicity and gender, with predicted White and male authors receiving higher acceptance rates. While machine learning algorithms are valuable for identifying such patterns, their limitations necessitate highlight the need for self-identified demographic data. Addressing these inequities requires continued efforts to promote diversity and inclusion in academic publishing.

P10 | Environmental and Occupational Health Policy

P10-01 | Analysing State-Level Heat Action Plans in India: Strategies for Protecting Pregnant, Lactating Women, and Women with Young Children

Gabriela De Jesus Cipriano Flores[1,2], Surya Surendran[3], Jane Hirst[1,2], Devaki Nambiar[3], Karthika Kumar[3]

[1] The George Institute for Global Health UK, London, UK; [2] Imperial College, School of Public Health, London, UK; [3] The George Institute for Global Health India, Hyderabad, India

1. Objective
To assess India’s heat adaptation and State Action Plans for Climate Change for strategies protecting pregnant, lactating women, and women with young children from extreme heat.

2. Material and Methods
We reviewed 20 Heat Action Plans (HAPs) and, where unavailable, analysed 14 State Action Plans for Climate Change (SAPCCs) using public sources and research networks. Two researchers identified direct strategies for pregnant, lactating women, and women with young children, as well as indirect strategies addressing vulnerable groups, within which they were included. Interventions were categorised using Chersich et al.’s (2022) framework and coded by action verbs (e.g., protect, prioritise). Non-English documents were translated. Independent coding was conducted by two researchers, with a third resolving discrepancies.

3. Results
We identified 214 strategies (Direct = 40). Most focused on behavioural changes (98), followed by health system interventions (42), structural and policy interventions (44), and built environment modifications (25), with five classified as other. There was an emphasis on communication (59) and identification of vulnerable populations and heat risk areas (41). 12 States/Union Territories yielded no relevant interventions. Direct strategies mainly involved heat-risk education and alert communication, often implemented by the Women and Child Development Department and the Integrated Child Development Services (ICDS). Some HAPs prioritised women in worker protection strategies, while indirect strategies included access to resources, emergency health services, and NGO outreach.

4. Conclusion
While numerous plans acknowledge heat-related risks, interventions specifically addressing the needs of women remain limited. Only strategies 40 explicitly target this group, and interventions remain largely communication-based, with limited structural or policy measures. Furthermore, 12 states and union territories yielded no relevant measures, underscoring critical gaps in adaptation planning that address women's needs. To effectively mitigate heat-related health risks for this group, it is imperative to strengthen targeted policies and implement concrete, action-oriented adaptation measures.

P10-02 | Public Perception of Human Biomonitoring and Pesticide Exposure in Latvia: Implications for Policy and Risk Communication

Lāsma Akūlova[1], Linda Matisāne[2], Ivars Vanadziņš[2]

[1] Rīga Stradiņš university, Laboratory of Hygiene and Occupational Diseases, Rīga, Latvia; [2] Rīga Stradiņš university, Institute of Occupational Safety and Environmental Health, Rīga, Latvia

Public trust in environmental health policies and exposure monitoring is critical for effective risk communication and regulatory decision-making. However, awareness of human biomonitoring and pesticide exposure remains limited, often shaped by media advocacy efforts.

This study examines public perceptions, concerns, and awareness of pesticide exposure and human biomonitoring in Latvia, highlighting implications for policy and public engagement.

Three focus group discussions were conducted between August 2021 and June 2024 with a diverse representation of Latvian citizens, exploring their understanding of human biomonitoring, chemical exposure concerns, and trust in governmental monitoring. Additionally, online content analysis was used to assess how pesticide-related topics are framed in public discourse by media and non-governmental organizations.

Awareness of human biomonitoring was generally low, with many participants mistakenly associating it with medical diagnostics rather than recognizing it as a tool for chemical exposure assessment. Public concerns primarily revolved around food additives and household chemicals, while pesticides received comparatively less attention. Among pesticides, glyphosate emerged as the most widely discussed, largely driven by non-governmental organisation advocacy and media influence, whereas other pesticides like acetamiprid are rarely mentioned despite being detected in 1-in-3 people. Trust in government-led monitoring initiatives was mixed, with concerns about political influence, industry affiliations, and data privacy contributing to public scepticisms. Notwithstanding these reservations, participants expressed strong support for further research, emphasizing the importance of locally tailored biomonitoring studies to inform policy and enhance public understanding.

Findings underscore the importance of public trust in scientific and regulatory institutions and the need for transparent communication on exposure risks. Addressing misconceptions about human biomonitoring, strengthening policy-driven risk communication strategies, and increasing public engagement are essential steps for advancing the chemical exposure, including pesticide, monitoring framework.

This work was done under the State Research Program “Development of Human Biomonitoring Program for Latvia - HBM4LV”, project No. VPP-VM-Sabiedrības_Veselība-2023/4-0001.

P10-03 | Regulatory Power Over Ethylene Oxide Emissions: Case Study of Salinas, Puerto Rico

Melanie Diaz-Bonet[1,2], Polaris Torres-Rodríguez[1], Ana P. Ortiz[1], Leticia Nogueira[3], Nancy Cardona-Cordero[1]

[1] UPR Comprehensive Cancer Center, Division of Cancer Control and Population Sciences, San Juan, Puerto Rico; [2] University of Puerto Rico Medical Sciences Campus, Environmental Health Department, San Juan, Puerto Rico; [3] American Cancer Society, San Juan, Puerto Rico

Background: A warning for residents of 23 states and Puerto Rico about the risk of cancer and other diseases due to high emissions of Ethylene Oxide (EtO) by sterilization facilities was issued by the Environmental Protection Agency in 2022. Steri-Tech, in Salinas, Puerto Rico has been operating since 1986 using only EtO for sterilization of medical equipment and materials. EtO exposure increases the risk of being diagnosed with breast cancer, leukemia, and lymphoma. Residents have issued a Class Action Complaint for injuries and damage as a result of exposure to this toxic chemical. **Objective:** Conduct a systematic review of regulations applicable in Puerto Rico to Ethylene Oxide (EtO) Emissions by sterilization facilities.

Material and Methods: Publicly available databases such as Google, Google Scholar, LexisNexis were used to access Puerto Rico's legal documents, and the Federal laws, including powers to the Environmental Protection Agency. Documents were organized by Puerto Rico's normative hierarchy and/or agencies with jurisdictional authority. Scientific studies and news reports were also included for context and to support the claim of environmental injustice and community outcry. Tables and diagrams were constructed to enhance communication and understanding.

Results: Using the term "Ethylene Oxide" 23,100 and 27,000 federal and state laws and regulations, respectively were identified through Google. Using Lexis Nexis, 25 archives and documents were identified. Considering the inclusion and quality criteria, 20 documents were included in our analysis.

Conclusions: Ethylene Oxide emissions is a critical issue that requires the participation of all sectors. For this, the creation of an interagency committee is recommended to address the emissions of carcinogens such as EtO holistically and the health of those exposed. Underserved communities are at most risk due to cultural, societal and economic factors that limit mobility.

P10-04 | Impact of China’s rural clean heating policy on indoor temperature

Talia Sternbach[1], Sam Harper[1], Xiaoying Li[2], Collin Brehmer[2], Yuanxun Zhang[4,5], Wenlu Yuan[1], Shu Tao[3], Kaibing Xue[4], Kennedy Hirst[2], Guofeng Shen[3], Chris Barrington-Leigh[1], Xiang Zhang[1], Brian Robinson[1], Ellison Carter[2], Jill Baumgartner[1]

[1] McGill University, Montreal, Canada; [2] Colorado State University, Fort Collins, USA; [3] Peking University, Beijing, China; [4] University of Chinese Academy of Sciences, Beijing, China; [5] Chinese Academy of Sciences, Xiamen, China

The Rural Clean Heating Policy (CHP) was introduced in 2016 to transition up to 70% of homes from coal- to electric-based space heating in northern China. Most rural homes relied on solid fuel space heating before the policy, which can be inefficient and lead to cold indoor temperatures that are inadequate to meet thermal comfort needs and harm health. We therefore leveraged the CHP’s staggered implementation across villages to evaluate its impact on indoor temperature.

We enrolled 977 households from 50 villages that were eligible for but not yet treated by the CHP in winter 2018-19. Over the next 3 winters, 20 villages were treated by the CHP. Thermochron iButton data-loggers taped to an interior home wall measured indoor temperature for ~5 months in a ~50% subsample. We estimated the CHP’s impacts on the mean winter and winter minimum indoor temperature using a difference-in-differences design. Standard errors were clustered at the village level, and models were adjusted for the number of rooms and wintertime occupants in the household, age of the primary respondent, and a composite wealth index.

The mean winter and minimum indoor temperatures at baseline were 14˚C and 7˚C, respectively, and 90% had average indoor temperatures below the WHO health-motivated guideline (18˚C). On average, the CHP increased the mean winter indoor temperature by 1.8˚C (95%CI: 0.9, 2.7) and the minimum indoor temperature by 4.2˚C (95%CI: 2.4, 6.0).

Our results show that the CHP increased rural homes’ indoor temperature in northern China, underscoring the importance of improved heating systems to maintain household warmth in colder climates. However, 68% of homes in treated villages still had average indoor temperatures <18˚C after follow-up. Additional measures, including insulation and training on new heating equipment, may be necessary to improve indoor temperature further.

P10-05 | Development of a Socio-Demographic Vulnerability Index to Improve the Effectiveness of Air Pollution Policy in South Korea

Solbit Hong[1,2], Sera Kim[2], Jong-Tae Lee[1,2]

[1] School of Health Policy and Management, College of Health Sciences, Korea University, Seoul, South Korea; [2] Research and Management Center for Health Risk of Particulate Matter, Seoul, South Korea

Objective

Public efforts have focused on reducing exposure in high-concentration areas to mitigate air pollution's health risks. However, since the health outcomes are composite results from pollutant exposure and population vulnerability, lowering concentration levels alone may not sufficiently improve public health.

This study develops a Socio-Demographic Vulnerability Index (SDVI) by integrating air pollution-related contextual indicators as a supplementary tool for public decision-making and examining their utilities.

Material and Methods

Key indicators reflect individual vulnerability (age-related, chronic disease) and neighborhood-level factors (socioeconomic status, healthcare accessibility, absolute poverty, medical accessibility index, and greenness). All data was collected at the Si-Gun-Gu level (city/county equivalent) and covered the whole country in 2020.

All indicators were standardized using Z-scores and compiled into SDVI-Mean and SDVI-Percentile. The indexes graphically examined regional distributions at both Si-Do (state equivalent) and Si-Gun-Gu levels.

Results

At the Si-Do level, the highest $PM_{2.5}$ concentrations appeared in central industrial regions (e.g., Gyeonggi, Chungcheongbuk-do) and urbanized areas, with over 90% of regions exceeding the annual standard of 15μg/m³. However, high SDVI scores emerged in the southern part (e.g., Jeollanam-do, Gyeongsangnam-do), where $PM_{2.5}$ levels were moderate, yet populations were older, had higher chronic disease prevalence, and had lower healthcare accessibility. We also observed clear variations in SDVI scores at the Si-Gun-Gu level even within the same Si-Do regions.

Conclusion

SDVI offers a new approach to identifying vulnerable areas beyond conventional pollution-based assessments. The highly urbanized cities, such as Seoul, showed lower SDVI scores despite higher pollution levels, reflecting secure social infrastructure. Furthermore, disparities in social privileges existed within the same Si-Do.

Since socio-demographic vulnerabilities vary independently from pollution levels, $PM_{2.5}$-related health impacts can be exacerbated even at moderate concentrations. Policy interventions must address these disparities to ensure targeted, equitable air pollution adaptation strategies, promoting environmental justice.

P10-06 | Effects of $PM_{2.5}$ Exposure and Air Quality Improvements on Colorectal Cancer Survival in Taiwan: A Nationwide Population-Based Study

Chi-Hsin S. Chen[1,2], Chia-Yi Lu[1], Wei-Chi Lin[1], Ruei-Hao Shie[3], Chun-Ju Chiang[4,5], Wen-Chung Lee[6,5], Chang-Chuan Chan[1,2]

[1] National Taiwan University, Institute of Environmental and Occupational Health Sciences, Taipei, Taiwan; [2] National Taiwan University, Global Health Program, Taipei, Taiwan; [3] Industrial Technology Research Institute, Green Energy and Environment Research Laboratories, Hsinchu, Taiwan; [4] National Taiwan University, Institute of Epidemiology and Preventive Medicine, Taipei, Taiwan; [5] Ministry of Health and Welfare, Taiwan Cancer Registry, Taipei, Taiwan; [6] National Taiwan University, Institute of Health Data Analytics and Statistics, Taipei, Taiwan

Background: Air pollution may influence cancer progression and survival, yet the impact of fine particulate matter ($PM_{2.5}$) on colorectal cancer (CRC) outcomes remains unclear. Taiwan's extensive air quality monitoring and nationwide cancer surveillance provide a unique setting to explore this association.

Objectives: This study evaluates the effects of $PM_{2.5}$ exposure on CRC survival in Taiwan over two decades, considering the regional implementation of CRC screening in 2004 and its nationwide expansion in 2010.

Methods: We linked Taiwan's national cancer registry with daily $PM_{2.5}$ concentrations from air monitoring stations for CRC cases diagnosed between 1997 and 2018. Cox proportional hazard models were used to assess associations between $PM_{2.5}$ exposure and CRC-specific mortality as well as all-cause mortality, adjusting for age, sex, cancer stage, tumor site, treatment, smoking, alcohol intake, and obstruction. Stratified analyses examined the modifying effects of CRC screening.

Results: From 1997 to 2018, 460,590 CRC patients were registered. The five-year CRC survival rate remained at 50% from 1997 to 2003 but gradually increased to 62% by 2018. Concurrently, average $PM_{2.5}$ concentrations declined from 30.9 μg/m³ in 1997 to 26.4 μg/m³ in 2011, reaching 15.0 μg/m³ by 2023. A 6.3 μg/m³ reduction in $PM_{2.5}$ was associated with a hazard ratio (HR) of 0.70 (95% CI: 0.69–0.71) for CRC-specific mortality and 0.70 (95% CI: 0.69–0.70) for all-cause mortality between 2011 to 2018, translating to a 30% survival improvement for both outcomes.

Conclusions: Our findings highlight the impact of air quality improvements on CRC survival and emphasize the synergistic benefits of environmental policies and public health interventions. This study provides critical evidence to inform future cancer prevention and control strategies.

P10-07 | Evaluating Environmental Policy Effectiveness by Urinary Heavy Metal Levels and Biochemical Indicators of Residents near a Petrochemical Complex in Taiwan

Tzu-Hsuen Yuan[1], Chih-Yun Li[1], Chang-Chuan Chan[2]

[1] University of Taipei, Department of Health and Welfare, Taipei, Taiwan; [2] National Taiwan University, nstitute of Environmental and Occupational Health Sciences, Taipei, Taiwan

Objective: Given the lack of research on the impact of environmental policies on residents' exposure and health near the big industry, this study examined how national environmental policies influence urinary metal concentrations and health indicators in affected populations in the vicinity of a petrochemical complex. **Materials and Methods:** The study was conducted in ten townships within 0–30 km of a petrochemical complex in central Taiwan, with high-exposure areas defined as within 10 km. A total of 414 residents were recruited from 2009 to 2011 and tracked through 2014–2015. Urinary heavy metals were analyzed by ICP-MS, and biochemical indicators were assessed by medical examination. The effectiveness of the 2011 national air pollution control policy by Taiwan was evaluated using the linear mixed model to analyze changes in urinary metal concentrations and biochemical indicators among study participants. **Results:** After policy intervention, urinary arsenic and mercury concentrations significantly decreased by 4.93% and 23.11%, respectively, while lead significantly increased by 38.82%. In high-exposure areas, chromium and arsenic significantly declined. For cardiovascular indicators, high- and low-density lipoprotein significantly decreased by 4.79%, and 4.88%, respectively, while diastolic blood pressure, pulse rate, and total cholesterol significantly increased by 12.45%, 8.97%, and 7.13%. For renal function, serum creatinine significantly decreased by 14.74%, whereas blood urea nitrogen significantly increased by 9.3%. For liver function, albumin, globulin, and platelet levels significantly declined by 2.43%, 7.57%, and 4.78%. Moreover, urinary heavy metal concentrations were significantly associated with cardiovascular, liver, and kidney indicators. **Conclusion:** Despite the reduction in certain heavy metal levels, some biochemical indicators related to cardiovascular diseases, kidney, and liver function still showed adverse trends. Future research will explore possible delayed health effects of long-term pollution exposure. We sincerely appreciate the financial support by the National Science and Technology Council, Taiwan.

P10-08 | Persistent Organic Pollutants: Impact on General Human Health, The Gut Microbiota, and Environmental Policy

Vaidehi Patel

University of Guelph, Population Medicine, Guelph, Canada

Objective: Persistent Organic Pollutants (POPs) are globally distributed toxic compounds that resist environmental degradation, bioaccumulate in organisms, and pose significant threats to human health. This study examines the links between POP exposure, gut microbiome dysbiosis, and systemic health effects, including endocrine disruption, immune suppression, metabolic dysfunction, and carcinogenicity. Additionally, it highlights gaps in multilateral environmental agreements and the necessity for strengthened global monitoring strategies.

Materials and Methods: A comprehensive literature review was conducted to assess the environmental presence and health impacts of five key POPs: Bisphenol A (BPA), Polychlorinated Biphenyls (PCBs), Phthalates, Dioxins, and Heavy Metals bound to organic ligands. Studies on microbiome alterations, policy frameworks, and monitoring techniques were analyzed, emphasizing the interplay between gut microbial changes and chronic health risks. Comparative analysis of regulatory mechanisms was also included to assess effectiveness.
Results: POPs significantly alter gut microbial composition, reducing bacterial diversity and increasing inflammation-related species, contributing to chronic diseases. Sex-specific microbiome alterations suggest differential susceptibility to POP exposure. Despite international agreements like the Stockholm Convention, enforcement gaps persist, particularly in remote and developing regions. Inadequate monitoring and underregulated industrial emissions exacerbate exposure risks, highlighting the urgent need for improved environmental governance.
Conclusion: Strengthened policy measures, enhanced microbiome stewardship, and globally coordinated POP monitoring efforts are essential to mitigate health risks. Future research must integrate microbiome health within regulatory frameworks to ensure sustainable exposure reduction strategies. Increased international cooperation and equitable policy enforcement are critical for protecting vulnerable populations from persistent pollutant exposure.

P10-09 | Persistent Organic Pollutant Regulation in Canada: Policies, Compliance, and Challenges

Vaidehi Patel

University of Guelph, Population Medicine, Guelph, Canada

Objective: Persistent Organic Pollutants (POPs) persist in the environment, bioaccumulate in living organisms, and pose severe risks to human health and ecosystems. This study evaluates Canada's past and current commitments to reducing POP emissions, compliance monitoring mechanisms, and the barriers to implementing safer chemical alternatives.
Materials and Methods: A review of regulatory frameworks and international agreements was conducted, including the Stockholm Convention, the North American Agreement on Environmental Cooperation, and the Great Lakes Water Quality Agreement. Canadian policies such as the Chemicals Management Plan and the Canadian Environmental Protection Act were assessed for their effectiveness. Data from government reports and scientific literature were analyzed to identify trends in compliance and enforcement.
Results: Canada has enacted multiple legislative measures to regulate POPs, yet industrial sources such as waste incineration, biomass burning, and transportation remain major contributors. Compliance monitoring programs like the National Pollutant Release Inventory provide valuable data but are limited by self-reporting issues. The Chemical Alternative Assessment process presents a viable solution for reducing POP reliance, yet regulatory and economic challenges hinder widespread adoption.
Conclusion: Canada's leadership in POP regulation is evident through its active participation in international treaties and domestic policies. However, enforcement inconsistencies and industrial emissions continue to pose challenges. Strengthening compliance mechanisms and enhancing the adoption of safer chemical alternatives are essential to mitigating POP exposure. A multidisciplinary approach, integrating environmental governance, policy, and public health, is necessary for future improvements.

P10-10 | Evaluating the Impact of Heat Adaptation Policies on Public Health

Ejin Kim[1], Insung Song[2], Ho Kim[1,2], This work was supported by Korea Environment Industry & Technology Institute(KEITI) through Climate Change R&D Project for New Climate Regime, funded by Korea Ministry of Environment(MOE)(grant number:RS-2022-KE002235)

[1] Seoul National University, Institute of Health and Environment, Graduate School of Public Health, Seoul, South Korea; [2] Seoul National University, Graduate School of Public Health, Seoul, South Korea

Objectives

This research aims to evaluate the effectiveness of different heat adaptation strategies in minimizing health risks during extreme heat events. It also explores how regional differences shape policy outcomes.

Methods

Health outcome data, including disease incidence and mortality rates, were obtained from Statistics Korea and the National Health Insurance Service. Environmental and regional characteristics were analyzed using data from the Korea Disease Control and Prevention Agency's Community Health Impact Survey. A two-stage distributed lag nonlinear model (DLNM) was applied to compare the effectiveness of adaptation policies across regions with different environmental and demographic conditions.

Findings

Preliminary findings show that areas with more policy initiatives have less health risks from excessive heat. In places with less green space, for example, the relative risk (RR) of heat-related mortality at the 95th percentile temperature compared to the minimum mortality temperature (MMT) was 1.21 (95% CI: 1.12–1.30), but in areas with more green space the RR was smaller at 1.15 (95% CI: 1.07–1.23). These results imply that, especially in susceptible groups like elderly persons, adaption techniques include boosting urban greenery could help lower heat-related mortality.

Conclusion

This study highlights the role of heat adaptation policies in reducing health risks from extreme heat. Regions with more adaptation measures tend to have lower mortality rates, though effectiveness varies by local conditions.

P10-11 | Expanse Urban Labs – Facilitating translation of exposure-disease associations to effective urban planning and policy interventions

Ayoung Jeong[1,2], Taymara Abreu[3], Joline Beulens[3], Kees de Hoogh[1,2], Benjamin Flückiger[1,2], Anke Huss[4], Medea Imboden[1,2], Magdalena Janc[5], Maria-Iosifina Kasdagli[6], Kalliopi Kyriakou[3], Joreintje Mackenbach[3], Maria G. Matias de Pinho[4], Erik Melén[7], Evangelia Samoli[6], Noreen Siddiqui[3], Melissa Witzig[1,2], Klea Katsouyanni[6], Kinga Polanska[5], Cathryn Tonne[8], Jelle J. Vlaanderen[4], Roel C. Vermeulen[4], Nicole Probst-Hensch[1,2]

[1] Swiss Tropical and Public Health Institute, Epidemiology and Public Health, Allschwil, Switzerland; [2] University of Basel, Department of Public Health, Basel, Switzerland; [3] Vrije Universiteit Amsterdam, Amsterdam UMC, Amsterdam, Netherlands; [4] Utrecht University, Utrecht, Netherlands; [5] Nofer Institute of Occupational Medicine, Department of Environmental and Occupational Health Hazards, Łódź, Poland; [6] National and Kapodistrian University of Athens, Department of Hygiene, Epidemiology and Medical Statistics, Athens, Greece; [7] Karolinska Institutet, Department of Clinical Science and Education Södersjukhuset, Stockholm, Sweden; [8] ISGlobal, Barcelona, Spain

Objective: Environmental factors influence the health and wellbeing of urban dwellers. The Urban Labs study aims (1) to obtain individual exposure assessments to facilitate the measurement error assessment and (2) to understand how urban dwellers use and interact with their environment in spatio-temporal contexts and how this influences the association between exposure and intermediate health parameters such as subjective wellbeing, obesity, and sleep quality.

Material and Methods: The study was conducted simultaneously in five urban areas: Basel, Switzerland (CH), Barcelona, Spain (ES), Athens, Greece (GR), the Netherlands (NL), and Lodz, Poland (PL). The participants filled in online questionnaire at recruitment. Time-varying information was repeatedly collected via a smartphone application and bimonthly online questionnaires. The participants were invited for two-week measurement campaigns. The participants carried a GPS tracker and a silicone wristband for the two weeks, and provided self-administered dried blood sample at the end of the two weeks. A subset of the participants received a pair of air pollution trackers, one to carry and the other to install at home for the two weeks. Exposure to ambient air pollution, green space, blue space, gray space, temperature, and population density was estimated at the participants' home address at recruitment.

Results: We assessed 3782 participants: 731 in CH, 978 in ES, 377 in GR, 1055 in NL, and 641 in PL. 1786 out of the 3782 participants completed the two-week measurement campaign: 665 (91%) in CH, 326 (33%) in ES, 229 (61%), 458 (43%) in NL, and 108 (17%) in PL. Exposure to the environmental factors and their correlation pattern varied between the centers.

Conclusion: The study collected novel data on the participants' exposure to and use and perception of the urban environment in the spatio-temporal context, which will refine urban planning towards healthier urbanization.

P10-12 | Piloting A Methodology for Health Impact-based Heat Threshold in the Caribbean: Leveraging the Benefits in the Region

Lindonne Telesford[1], Sally Edwards[2], Karen Polson[3], Cédric Van Meerbeeck[4], Hunter Jones[5], Shawn Charles[6]

[1] St. George's University, St. George's, Grenada; [2] Pan American Health Organization/World Health Organization, Panama City, Panama; [3] Pan American Health Organization/World Health Organization, Bridgetown, Barbados; [4] Caribbean Institute for Meteorology and Hydrology, Bridgetown, Barbados; [5] National Oceanic and Atmospheric Administration, Washington, USA; [6] Ministry of Health, Wellness, and Religious Affairs, St. George's, Grenada

Caribbean countries are making steady progress in identifying the impacts of climate change on the environment. The region, however, still lacks understanding of the “true” impact of rising temperatures on population health and healthcare systems. To address this challenge, PAHO commissioned a pilot study in Grenada to explore the development of health impact-based heat thresholds as a blueprint for methodology to support the development of evidence-based heat warning systems. Mortality and morbidity were used as proxies in distributed lag non-linear models to determine changes in the relative risk of emergency room visits, hospital admissions, and all-cause mortality corresponding to meteorological values from the 90th percentile. The results support the relevance of both mortality and morbidity data to generate heat-health relationships and to guide the development of effective thresholds. The findings also indicate the urgent need to strengthen heat early warning systems for health protection. At the regional level, four aspects need to be further explored to maximize the benefits of this study at the level of the wider Caribbean: Application of science to support evidence-building in small island states; climate change knowledge sharing; opportunity for local and regional responses to strengthen resilience; and public health protection. It is understood that despite the disproportionate magnitude of adverse impacts on small island states, resources are limited for research and development related to climate change. Extrapolation of the work in Grenada to other countries can be a crucial step in building climate change research capacity and strengthening health sector resilience at local and regional levels. This pilot study provides an opportunity to leverage regionally generated knowledge to advance climate change research methodologies. The regional health systems can benefit tremendously from efficiency in acquiring climate change knowledge that directly targets the sector.

P10-13 | Strengthening the Environmental Dimensions of AMR Policy and Implementation in the Philippines: A One Health Perspective

Percival Ethan Lao[1], Miguel Antonio S. Salazar[2], Sary Valenzuela[1,3], Lea Conda[1], Anna Beatrice Enriquez[1], Geminn Louis Apostol[1,4]

[1] Ateneo de Manila University - School of Medicine and Public Health (ASMPH), ASMPH Center for Research and Innovation (ACRI), Pasig City, Philippines; [2] Alliance for Improving Health Outcomes (AIHO) Inc., Quezon City, Philippines; [3] Georgetown University, Department of Global Human Development, Washington D.C., USA; [4] Murdoch University, Harry Butler Institute, Perth, Australia

Objective: Antimicrobial resistance (AMR) extends beyond healthcare settings, significantly involving environmental pathways. This study assessed the extent of the Department of Environment and Natural Resources' (DENR) involvement in Philippine AMR policies, identified gaps in environmental-based AMR initiatives—including wastewater surveillance—and proposed strategies to enhance environmental engagement through a strengthened One Health approach.
Materials and Methods: A qualitative policy analysis was conducted on key national documents (1990–2023), including policies on hazardous waste, water quality, food safety, and AMR-specific legislation. In-depth stakeholder interviews from governmental bodies, academia, and international organizations provided further context. Policies were categorized based on environmental integration, institutional mechanisms, financing, and clarity of mandates. Special focus was given to the WHO Quadripartite Tricycle protocol for environmental AMR surveillance.
Results: Analysis identified extensive Philippine regulations on water and waste disposal but revealed limited explicit reference to AMR mitigation. While policies such as the Clean Water Act implicitly cover contaminants, there are currently no specific budgetary or institutional provisions ensuring comprehensive surveillance and mitigation of antibiotic-resistant pathogens. The Department of Environment and Natural Resources (DENR), despite robust chemical monitoring capabilities, lacks a formal mandate and institutional funding streams specifically for AMR initiatives. Furthermore, formal DENR integration in the Inter-Agency Committee on Antimicrobial Resistance (ICAMR) remains minimal. Stakeholder interviews emphasized opportunities for greater integration of DENR expertise into One Health structures, particularly in municipal wastewater management and pharmaceutical runoff surveillance.
Conclusion: Strengthening DENR’s integration into national AMR governance frameworks, formalizing institutional mandates, and securing dedicated funding are essential. Embracing a “One Health in all policies” approach can significantly bolster the Philippines’ capacity to address environmental drivers of AMR, thereby safeguarding public health.

P10-14 | Bridging Policy Gaps for Enhanced Health Security Preparedness from Threats of Emerging Infectious Diseases and Environmental Hazards: A Systematic Mapping of National Health Security Policies in the Philippines

Percival Ethan Lao[1], Sary Valenzuela[1,3], Jenica Sy[1], Gabriella Corrigan[2], Nisha Marles[2], Noemi Bautista[2], Geminn Louis Apostol[1]

[1] Ateneo de Manila University - School of Medicine and Public Health (ASMPH), ASMPH Center for Research and Innovation (ACRI), Pasig City, Philippines; [2] Research Triangle Institute (RTI), Global Health Division, Research Triangle Park, USA; [3] Georgetown University, Department of Global Human Development, Washington D.C., USA

Objective: Responsive health security policies are critical in addressing global threats from emerging infectious diseases and environmental hazards, particularly in resource-constrained settings. The Philippines, a country frequently challenged by public health threats, provides a unique context for examining policy coherence in health security. This study aimed to map and analyze national health security policies in the Philippines to identify policy coherence, surveillance capabilities, risk assessment practices, and environmental exposure considerations influencing preparedness for and response to public health threats.
Materials and Methods: We conducted extensive thematic and content analysis of 438 national health security policy documents, applying frameworks from the International Health Regulations and the Global Health Security Agenda. The study employed qualitative approaches, including detailed policy content analysis and structured key informant interviews with stakeholders from governmental and non-governmental sectors involved in health security. Participants from health, environment, agriculture, and disaster response sectors provided insights into the practical challenges and gaps in policy implementation.
Results: The analysis uncovered significant fragmentation across policy domains, characterized by overlapping mandates and unclear accountability structures, leading to substantial implementation barriers. Notable gaps included limited environmental exposure surveillance capacities, inadequate mechanisms for chemical and zoonotic threat preparedness, workforce constraints, and unsustainable funding arrangements. The decentralized governance structure of the Philippines exacerbated these challenges, resulting in marked regional disparities in adapting international health security standards locally. Additionally, ineffective intersectoral communication and lack of formal data-sharing practices significantly hampered efficient and timely public health responses during crises.
Conclusion: To effectively address these policy gaps requires an integrated One Health strategy, strengthening surveillance infrastructures, and enhancing monitoring and evaluation systems. Importantly, environmental health hazards must be explicitly recognized and systematically integrated into our health security policies. These findings offer critical insights and actionable guidance for policymakers, and implementers, aiming to build resilience against emerging health security threats.

P10-15 | Addressing research gaps in environmental health: The need for human biomonitoring programs in sub-Saharan Africa

Hamid Y. Hassen[1,2], Jean-Pierre Van geertruyden[2], Jurgen Buekers[1], Liese Gilles[1], Eva Govarts[1]

[1] Flemish Institute for Technological Research (VITO), Environmental Intelligence, Mol, Belgium; [2] University of Antwerp, Family Medicine and Population Health, Antwerp, Belgium

Nearly a quarter of the total global burden of disease is attributable to an unhealthy environment with higher burden in low and middle-income countries (LMICs), mainly sub-Saharan Africa (SSA) (1). Environmental health research is crucial for understanding the impact of environmental stressors on human health, ultimately aiming to improve public health. In recent decades, there has been remarkable progress in environmental health research, marked by better methodologies, analytical techniques, and interdisciplinary collaborations.

Human biomonitoring (HBM) involves measurement of environmental pollutants or their metabolites in human tissues or fluids to assess internal human exposure levels, providing essential data to investigate exposure pathways, toxicological mechanisms, and potential health effects. This approach enables more accurate risk assessment and informed policy decisions. Recently, HBM has become the benchmark in environmental health research, leading to ongoing large-scale studies worldwide. To our knowledge, HBM efforts in most SSA are limited despite the region's environmental health challenges. Thus, it is imperative to effectively address these issues through increased research and comprehensive HBM programs.

There are significant disparities in the distribution of research efforts globally, with SSA often receiving less attention. Despite the disproportionately higher burden of diseases attributable to environmental pollutants, HBM programs are scarce in LMICs, particularly SSA. The region lacks research infrastructure and human capacity to run HBM programs. Comprehensive and continuous biomonitoring programs are necessary to provide reliable data for research, implement effective public health interventions, and guide policy decisions.

This abstract underscore the pressing need for increased research efforts and resources dedicated to HBM in SSA, highlighting the potential benefits for improving environmental health outcomes in the region. Our aim is to emphasize the need for HBM in SSA, support relevant authorities in establishing HBM, to raise public awareness about chemical pollution, to build research capacity in HBM, and propose preventive measures

P10-16 | Drafting the First Comprehensive Environmental Health Act for the Philippines: Advancing Beyond the 1975 Sanitation Code

Hazel Ann R. Fajardo, Percival Ethan Lao, Anna Beatrice B. Enriquez, Noelle Anne S. Cubacub, Marianne K. Bongcac, Lea Elora A. Conda, Jose Carlos Alexis C. Bairan, Geminn Louis C. Apostol

Ateneo School of Medicine and Public Health, Ateneo Center for Research and Innovation - Environmental Health and Global Health Security Flagship Program, Pasig, Philippines

Objective: To develop the first Environmental Health Act for the Philippines that modernizes the outdated 1975 Sanitation Code (PD 856), addresses emerging environmental health threats, and harmonizes fragmented policies through the One Health framework, anchored on the National Environmental Health Action Plan (NEHAP 2023-2030).

Materials and Methods: This policy development project utilized a mixed-methods approach conducted in two phases. Phase 1 (2023) involved policy mapping through systematic review of 150+ existing environmental health-related laws and administrative issuances. Key informant interviews (n=12) were conducted with government officials, sanitation engineers, and environmental health experts. Phase 2 (2024) employed stakeholder consultations (n=35) to develop an integrated framework aligned with NEHAP priorities. Gap analysis identified policy inconsistencies and emerging threats not covered in existing legislation. International benchmarking against ASEAN and global environmental health laws informed best practices.

Results: The draft Environmental Health Act contains nine chapters addressing: institutional frameworks for environmental health governance; standards for water, air, food safety, waste management, and chemical safety; provisions for climate change and antimicrobial resistance; specific considerations for vulnerable populations including persons deprived of liberty; monitoring systems; capacity-building measures; and financing mechanisms. The legislation enhances local government unit responsibilities, establishes an Environmental Health Bureau, and creates intersectoral coordination mechanisms. The One Health approach is integrated throughout to facilitate collaboration between human, animal, and environmental health sectors.

Conclusion: The Philippine Environmental Health Act represents a significant policy advancement that transforms fragmented environmental health regulations into a cohesive framework. By incorporating One Health principles, NEHAP priorities, and equity considerations, the legislation provides a forward-looking foundation for protecting public health from environmental hazards. The participatory approach ensured broad stakeholder input and strengthened prospects for implementation.

P10-17 | Assessing Heat Action Planning in Arizona Through Hierarchy of Controls and Socio-ecological Model Lens

Imran Hossain Mithu[1], Paloma Beamer[1], Maia Ingram[2], Mona Arora[1]

[1] Mel and Enid Zuckerman College of Public Health, University of Arizona, Community, Environment & Policy Department, Tucson, USA; [2] Mel and Enid Zuckerman College of Public Health, University of Arizona, Health Promotion Sciences Department, Tucson, USA

Objective: This study analyzes state and county-level heat action plans and prevention strategies in Arizona using the Hierarchy of Controls (HOCs) and Socioecological Model (SEM) frameworks to understand how well these plans address health inequities and national safety recommendations.

Materials and Methods: A structured search of heat action plans in Arizona was conducted using online and gray literature sources. Data were thematically analyzed based on the Hierarchy of Controls and the Socioecological Model. Intercoder reliability (ICR) was used to test the consistency and validity of extracted data.

Results: Of the 5 Arizona heat action plans we analyzed, 4 are at the county level and 1 at the state level. Our findings reveal that most heat actions rely on administrative controls, such as creating education campaigns and increasing public awareness efforts. Higher-order controls, such as the elimination or substitution of heat risks, are largely absent from current plans. Engineering controls, including shading and shelters, are implemented inconsistently across jurisdictions. Analysis within the SEM framework indicates that community-level interventions, such as collaboration with communities, are more common, while individual and policy-level responses are insufficient.

Discussion & Conclusion: The absence of higher-order HOC and inconsistent implementation of engineering controls, along with insufficient policy-level responses, undermine public health and exacerbate inequities by failing to address systemic barriers and vulnerabilities. These weaknesses in state and county planning disproportionately impact marginalized and low-income populations who are least equipped to manage heat risks. This study highlights the need for a comprehensive approach incorporating elimination and substitution strategies while strengthening policy and individual-level interventions. We conclude by recommending multi-level interventions and cross-sector collaboration to improve heat resilience and address the public health challenges posed by extreme heat in Arizona, which can also be applied to other states and countries vulnerable to extreme heat.

Funding: NIEHS P20ES036112 and P30ES006694

P10-18 | The Environmental Health Language Collaborative (EHLC): Harmonizing Toxicology Data Use and Sharing

Maria Shatz

NIEHS/NIH, Office of Data Science, Durham, USA

The extensive generation of data is rapidly advancing opportunities to answer large-scale, complex questions in toxicology and environmental health sciences. However, a significant challenge remains around the development and use of a harmonized language to allow for streamlined workflows to find, share, and reuse this data. The Environmental Health Language Collaborative (EHLC) was created to facilitate a community-driven effort to advance the development and adoption of harmonized language approaches within toxicology and environmental health science fields. This presentation describes EHLC activities and data harmonization and sharing opportunities for toxicologists.

EHLC has grown into a robust community that is led by an Executive committee made up of transdisciplinary subject matter experts from multiple institutions. The collaborative currently supports three active use case working groups focusing on Data Discovery, Data Harmonization, and Biomarkers and Biological Processes of Exposure. Additionally, EHLC hosts webinars and workshops to share relevant data harmonization information and methods with the scientific community.EHLC has planned and hosted two major workshops and numerous webinars collectively attended by ~300 participants. All three use case workgroups are currently developing manuscripts focusing on pain points, potential solutions, and existing best practices in the corresponding fields. To encourage engagement within the community, EHLC shares activity updates, opportunities, and key resources via the EHLC Community Listserv and the comprehensive EHLC website, which also has foundational information describing the mission of the collaborative.

P10-19 | Beyond exposure assessment: a method to convey worker harm at the point of purchase

Mary O'Reilly

University at Albany School of Public Health and Workplace Health Without Borders-US (WHWB-US), Department of Environmental Health Sciences, Manlius, USA

Objective:

This research promotes the development of a new metric for occupational health and safety by building on the work of the environmentalists who developed the carbon footprint as a measure of environmental harm associated with an activity or product. The new metric allows a measure of worker injury and fatality to be included with a product at the point of purchase and supplies consumers with an index to assess the harm that workers globally have suffered while producing the item they are purchasing.

Methods

Both the IMF and the World Bank require countries to keep economic input/output tables that represent their economies. Spurred on by the ISO 14001 and its promotion of life-cycle analysis, environmental scientists

substituted the carbon dioxide output of each sector in place of dollars used in the economic input/output tables which created the concept of carbon footprint.

Results
Substituting worker injury, including illness and death, associated with each sector in economic input/output tables is a way to convey the impact of a product on the occupational health of the people who made it. Although not every consumer would care, worker injury footprints provide a way for consumers to assess the impact of their purchase on the workers who made it. For example, consumer concern about environmental and health effects of per- and poly fluorinated alkyl substances is driving change in at least some of the 300 uses of this family of chemicals.

ConclusionConsumers are an untapped resource for promoting occupational health and safety. Consumers have power to influence companies and have changed business practices through their purchasing power. A worker injury footprint can improve workplace health and safety by making consumers partners with occupational health and safety practitioners.

P10-20 | Health Impact Assessment of Smoke-free Legislation in the Capital City of the World's Largest Tobacco Consumption Country

Yunting Zheng[1,2], Chun Chang[3]

[1] Fujian Medical University, School of Health Management, Fuzhou, China; [2] London School of Economics and Political Science, Department of Health, London, UK; [3] Peking University, School of Public Health, Beijing, China

Objective: Beijing, the capital city of the world's largest consumption country, implemented a smoke-free law in most compliance with the WHO FCTC in the country and nearly 20 cities followed Beijing's step. This study aimed to conduct a comprehensive health impact assessment of the smoke-free law in Beijing.
Materials and Methods: A theoretical framework on health impact assessment of smoke-free legislation was developed based on the Driving forces, Pressure, State, Exposure, Effect, Action Model, and the Determinants of Health model. Quantitative data including more than 13.6 million samples was collected from monitoring systems or public sources and analyzed using descriptive statistics, interrupted time series, slope inequity index, and relative inequity index.
Results: After the implementation of the smoke-free law, significant declines were observed in cigarette sales (a per capita reduction of 1388.2 cigarettes over 30 months), current smoking prevalence (a decrease of 3.1% over 4 years), hospital admissions for smoking-related diseases (stroke: 26.7% averted, chronic obstructive pulmonary disease: 10.7% averted), and medical costs associated with these diseases (stroke: 194 million yuan averted, chronic obstructive pulmonary disease: 46 million yuan averted). It also identified challenges including the overall upward trend of tobacco affordability, the proportion of "three noes" in public places first increased and then decreased, second-hand smoke exposure in some places first decreased and then rebounded.
Conclusion: The implementation of the smoke-free law in Beijing has yielded a series of positive health impacts. However, it still faces challenges in policy implementation. This study suggests conducting further research on health impact assessment of smoke-free legislation to accumulate evidence for implementing similar laws in additional districts.

P11 | Environmental Exposures and Cancer - PART I

P11-01 | Integrated meta-analyses of genome-wide effects of $PM_{2.5}$ in human cells identifies dysregulation of genes and pathways associated with cancer progression.

Anadi Bisht[1,4], Sagnik Dey[2,3], Ritu Kulshreshtha[4]

[1] Indian Institute of Technology Delhi, School of Interdisciplinary Sciences, New Delhi, India; [2] Indian Institute of Technology Delhi, Centre for Atmospheric Sciences, New Delhi, India; [3] Indian Institute of Technology Delhi, Centre of Excellence for Research on Clean Air, New Delhi, India; [4] Indian Institute of Technology Delhi, Department of Biochemical Engineering and Biotechnology, New Delhi, India

BACKGROUND AND AIM: Epidemiological studies have consistently shown a positive association between exposure to ambient $PM_{2.5}$, a major component of air pollution, and various types of cancer. Previous biological research has primarily focused on the association between $PM_{2.5}$ and lung cancer, with limited investigation into other cancers. **METHOD:** High-throughput transcriptomic data for $PM_{2.5}$ exposure was obtained from Gene Expression Omnibus (GEO) database. A meta-analysis was performed on multiple $PM_{2.5}$-treated human cell lines to identify differentially expressed genes (DEGs) and dysregulated pathways associated with $PM_{2.5}$ exposure along with validation using qRT-PCR in breast cancer cell lines. Oncogenes, tumor suppressor genes, fragile site genes, and immune-related genes were identified among $PM_{2.5}$-associated DEGs to study its carcinogenic potential. **RESULTS:** Our analysis revealed 310 common DEGs that exhibited significant dysregulation upon exposure to $PM_{2.5}$. These $PM_{2.5}$ associated DEGs spanned key functional categories, including oncogenes, tumor suppressor genes, and immune-related genes, which collectively contribute to $PM_{2.5}$-induced carcinogenesis. Pathway enrichment analysis revealed upregulation of pathways associated with HIF, VEGF, and MAPK signalling, all of which have been implicated in various cancers. Notably, the upregulation of HIF pathway genes (*HIF1α, HIF2α, VEGFA, BNIP3, EPO, and PGK1*) was validated in breast cancer cell lines upon $PM_{2.5}$ treatment. Furthermore, the construction of a protein-protein interaction (PPI) network identified $PM_{2.5}$-associated hub genes, including *NQO1* and *PDGFRB*, which are known to be dysregulated and significantly correlated with overall survival in lung and breast cancer patients, underscoring their potential clinical relevance. **CONCLUSION:** This study provides a comprehensive insight into $PM_{2.5}$-mediated dysregulation of oncogenes, tumor suppressor genes and immune related genes across various human cell lines, highlighting their potential roles in $PM_{2.5}$-induced carcinogenesis. Further exploration of these dysregulated molecular targets may improve our understanding of the biological effects of $PM_{2.5}$ and facilitate the development of preventive strategies and targeted therapies for $PM_{2.5}$-associated cancers.

P11-02 | Exposure to oil and gas development and childhood Ewing sarcoma risk in California

Cassandra J. Clark[1,2], Nicholaus Johnson[2], Rong Wang[3], Joseph L. Wiemels[4], Catherine Metayer[5], Nicole C. Deziel[2,6], Xiaomei Ma[3,6]

[1] University of Minnesota School of Medicine, Department of Pediatrics, Minneapolis, USA; [2] Yale School of Public Health, Department of Environmental Health Sciences, New Haven, USA; [3] Yale School of Public Health, Department of Chronic Disease Epidemiology, New Haven, USA; [4] University of Southern California, Department of Population and Public Health Sciences, Los Angeles, USA; [5] University of California Berkeley, Division of Epidemiology, Berkeley, USA; [6] Yale School of Medicine, Yale Comprehensive Cancer Center, New Haven, USA

Objective: Oil and gas development (OGD) has been linked to increased pediatric cancer risk, but the literature to date is limited and focuses on hematologic malignancies. The emergence of suspected clusters of other cancers such as the rare Ewing sarcoma in children living near OGD warrants investigation.

Materials/Methods: The study included 556 children born in California (1982-2015) and diagnosed with Ewing sarcoma at age 0-19 years (1988-2015), and 27,800 cancer-free controls frequency-matched to cases on birth year (50:1 ratio). We used birth address to assign OGD exposure using inverse distance-squared weighted (ID^{2}W) well counts at 2, 5, and 10 km buffer sizes for the exposure window from three months prior to conception to birth. We estimated odds ratios (ORs) and 95% confidence intervals (CIs) for the association between prenatal OGDexposure and Ewing sarcoma risk using multivariate logistic regression. We also evaluated exposure distribution by Hispanic ethnicity to identify potential disparities.

Results: There were associations between prenatal exposure to active (OR:0.92, 95% CI:0.70-1.20) or abandoned (OR:1.12, 95% CI:0.84-1.50) OGD and Ewing sarcoma risk. However, Hispanic cases were significantly more likely to be exposed to any active OGD within 10 km (15% vs. 8%, p=0.01), compared to controls, and Hispanic children overall were more likely to be exposed to both active and abandoned OGD wells within 10 km (17% vs. 11%, 10% vs. 9%, respectively; both p<0.01) as compared to non-Hispanic children.

Conclusion: In this registry-based case-control study, we did not identify any significant associations between OGD exposure and pediatric Ewing sarcoma risk in California. However, Hispanic children were significantly more likely to be exposed to any OGD activity than non-Hispanic children, which could have implications for other health outcomes including childhood cancers. Replication in different populations and regions with diverse patterns of OGD activity is necessary to draw concrete conclusions.

P11-03 | Associations between air pollution and cancer survivorship in the U.S: the NIH-AARP Diet and Health study

Jeonggyo Yoon[1], Rena R. Jones[2], Jared A. Fisher[2], George D. Thurston[3], Chris C. Lim[1]

[1] University of Arizona, Department of Community, Environment, and Policy, Mel and Enid Zuckerman College of Public Health, Tucson, USA; [2] National Cancer Institute, Occupational and Environmental Epidemiology Branch, Division of Cancer Epidemiology and Genetics, Rockville, USA; [3] New York University School of Medicine, Department of Environmental Medicine, New York, USA

Background: Ambient particulate matter ($PM_{2.5}$, <2.5μm) is a known human carcinogen, but research on associations with cancer survival remains limited.

Objectives: We sought to investigate the long-term effects of air pollution exposure on post-diagnosis cancer survival, utilizing data from the NIH-AARP Diet and Health Study.

Methods: Annual average concentrations of $PM_{2.5}$ (ug/m^3), nitrogen dioxide (NO_2, ppb), and ozone (O_3, ppb) were estimated from published models based on individual residential addresses. We analyzed associations with survival of all cancers combined (N=92,818), and lung (N=11,213), colon (N=8,389), prostate (N=25,774), and breast cancer (N=10,977), separately.Cox proportional hazards models with time-varying exposures adjusted for demographic, socioeconomic, and behavioral factors (age, sex, race and ethnicity, education level, body mass index, diet, alcohol, and smoking history). Different lag structures (1 to 10 years) were examined to assess both cumulative and delayed effects on survival outcomes.

Results: Among prostate cancer patients, a 10 ppb increase in NO_2 was associated with an increased risk of prostate cancer death (Hazard ratio [HR]=1.09 [0.99–1.21]) in the 5-year lag model. In contrast, lung cancer patients showed decreased HRs with exposure to $PM_{2.5}$ (per 10 ug/m^3; HR=0.86 [0.77–0.96]), and NO_2 (per 10 ppb; HR=0.92 [0.88–0.96]) in the 5-year lag model. We found no evidence of associations for either other cancer types or different lag models.

Conclusions: These findings suggest a role for air pollution in cancer survivorship. Although some associations were statistically significant, the effect sizes were small and may not be clinically meaningful, highlighting the need for further research to confirm these findings and explore underlying mechanisms.

P11-04 | Prenatal and Postnatal Exposure to Traffic-Related Air Pollution (TRAP) and Childhood Cancer: A Systematic Review and Meta-Analysis

Jongmin Oh[1,2], Surabhi Shah[1], Jieun Min[1], Sohee Wang[1], Ji Hui An[3], Ji Hyen Lee[4], Yi Jun Kim[1], Byungmi Kim[5,6], Bohyun Park[7], Eun Mi Jung[8], Logan G. Spector[8,9], Philip J. Landrigan[10], Kurt Straif[11,12], Eun Sun Yoo[4], Eunhee Ha[1,13]

[1] Ewha Womans University College of Medicine, Department of Environmental Medicine, Seoul, South Korea; [2] Seoul National University College of Medicine, Department of Human Systems Medicine, Seoul, South Korea; [3] Ewha Womans University College of Medicine, Institute of Ewha Medical Research, College of Medicine, Seoul, South Korea; [4] Ewha Womans University College of Medicine, Department of Pediatrics, Seoul, South Korea; [5] National Cancer Center, Division of Cancer Prevention, National Cancer Control Institute, Goyang, South Korea; [6] National Cancer Center, Department of Cancer Control and Population Health, Graduate School of Cancer Science and PolicyDepartment of Cancer Control and Population Health, Graduate School of Cancer Science and Policy, Goyang, South Korea; [7] National Cancer Center, Division of Cancer Control and Policy, National Cancer Control Institute, Goyang, South Korea; [8] University of Minnesota, Division of Epidemiology and Clinical Research, Department of Pediatrics, Minnesota, USA; [9] University of Minnesota, Masonic Cancer Center, Minnesota, USA; [10] Boston College, Program for Global Public Health and the Common Good, Global Observatory on Pollution and Health, Boston, USA; [11] Boston College, Global Observatory on Planetary Health at Boston College, Boston, USA; [12] Boston College, Schiller Institute for Integrated Science and Society, Connell School of Nursing, Boston, USA; [13] Ewha Womans University, System Health & Engineering Major in Graduate School (BK21 Plus Program), Seoul, South Korea

Background and Aims

Previous research has indicated potential associations between traffic-related air pollution (TRAP) and the risk of childhood cancers; however, corresponding evidence remains inconsistent. This study aimed to evaluate the association between TRAP exposure and the risks of childhood cancer through a systematic review and meta-analysis.

Methods

This systematic review focused on studies examining the association between prenatal and postnatal exposure to TRAP—specifically, fine particulate matter ($PM_{2.5}$), nitrogen dioxide (NO_2), and benzene—and the risk of all childhood cancers, including leukemia and central nervous system (CNS) tumors. Peer-reviewed manuscripts were searched in PubMed (n=1,116), Web of Science (n=578), and Cochrane Library (n=163) up to June 21, 2024. Five reviewers independently screened titles, abstracts, and full texts to identify eligible articles. A meta-analysis was conducted, with subgroup analyses based on exposure period (prenatal vs. postnatal) and age group. The meta-analysis results are presented as odds ratios (ORs) and 95% confidence intervals (CIs) per 10 μg/m^3 increase in exposure.

Results

Of 1,632 studies screened after duplicate removal, 25 were included in the analysis. The findings indicated an increased risk of leukemia (i.e., acute lymphoblastic leukemia) in children exposed to TRAP, specifically $PM_{2.5}$ and benzene ($PM_{2.5}$: OR=1.29, 95% CI: 1.01–1.63; benzene: OR=2.99, 95% CI: 1.19–7.49). An association was also observed between $PM_{2.5}$ exposure and an increased risk of retinoblastoma (OR=1.68, 95% CI: 1.16–2.43). Subgroup analysis revealed a stronger association between postnatal TRAP exposure ($PM_{2.5}$ and NO_2) and leukemia risk compared with prenatal exposure.

Conclusions

This study provides evidence of an increased risk of childhood cancer associated with TRAP exposure, particularly in the postnatal period. Additional research is needed to validate these findings.

P11-05 | Associations between exposure to a mixture of perfluoroalkyl substances (PFAS) and breast cancer development in the Athena Health Network: a pilot study

Kimberly Badal[1], Doug Walker[2], Catherine Mullins[2], Peggy Reynolds[3], Diane Heditsian[1], Vivian Lee[1], Susie Brain[1], Laura Esserman[1], Laura van 't Veer[4]

[1] University of California San Francisco, Department of Surgery, San Francisco, USA; [2] Emory University, Rollins School of Public Health, Atlanta, USA; [3] University of California San Francisco, Department of Epidemiology and Biostatistics, San Francisco, USA; [4] University of California San Francisco, Department of Laboratory Medicine, Atlanta, USA; [5] University of California San Francisco, Breast Science Advocacy Core, San Francisco, USA

Objective

To determine whether exposure to a mixture perfluoroalkyl substances (PFAS) is associated with breast cancer development in the University of California Athena Health Network.

Materials and Methods

A pilot set of 106 serum samples (39 cases: 67 controls) were selected and analyzed using non-targeted analysis with gas chromatography high resolution mass spectrometry. Samples from cases were collected at least one year before diagnosis and matched with controls by age. Identified PFAS detected in all samples were evaluated for association with future breast cancer development. Peak areas, which is a measure of abundance, were batch-corrected and log transformed. Correlation between PFAS pairs were estimated using Spearman's rank correlation. Differences in the abundances of each PFAS between cases and controls was compared using linear regression with an unadjusted $p<0.5$. Weighted quantile sum (WQS) regression was used to estimate PFAS mixture effects, adjusted for age, with 100 bootstraps and a 20% holdout validation set.

Results

Samples from cases were collected an average 4.84 years (1.05-9.24 years) before diagnosis. The mean age was 56 years (35-73 years). Twenty-two PFAS were selected for analysis. There was a weak overall average correlation between PFAS pairs ($\rho = 0.28$). In the univariate analysis, two PFAS were associated with breast cancer ($p<0.5$). In the mixtures analysis with 22 PFAS, a one quartile increase in the PFAS weighted quantile sum index was associated with a marginally significant increase in breast cancer odds (β: 2.23; OR: 9.3; $p = 0.09$). The three most important PFAS in the mixture were N-Methylperfluorooctanesulfonamidoacetic acid, perfluorohexanoic acid, and perfluoroheptanoic acid.

Conclusion

Preliminary analyses suggests that there could be a strong positive association between exposure to PFAS mixtures and breast cancer development. Ongoing research is increasing the sample size to 300 cases and 300 controls, and applying additional mixtures methods to refine the analyses.

P11-06 | Low-dose CT screening for lung cancer among high-risk smoky coal-exposed residents in Xuan Wei: a prospective cohort study

Lijun Liu[1], Hongli Lin[1], Zheshen Han[1], Renyue Ji[1], Haisheng Wu[1], Chao Jiang[1], Jialin Xu[1], Shijie Zhu[1], Bofu Ning[3], Yunchao Huang[2], Linwei Tian[1]

[1] University of Hong Kong, School of Public Health, Hong Kong, Hong Kong; [2] The Third Affiliated Hospital of Kunming Medical University, Department of Thoracic Surgery I, Kunming, China; [3] Xuanwei Center of Diseases Control, Xuan Wei, China

Objective: Indoor smoky coal emissions cause extremely high female lung cancer mortality in Xuan Wei, China. We build a prospective cohort to explore how coal emissions impact the early stages of lung cancer and further guide the design of effective interventions. Herein we report the baseline results and one-year follow-up until September 30, 2024.

Material and Methods: From April 2023 to September 2024, 3,303 individuals aged 25-75 years with ≥10-year residency in Xuan Wei underwent baseline low-dose CT (LDCT) screening. People who had prior lung cancer or other cancer diagnoses (n=66), or incomplete records/repeat participation/insufficient residential history (n=111) were excluded from further follow-up. The confirmation of lung cancer was based on CT scans and linkage to hospital records. Individuals with a positive scan, defined as a non-calcified nodule or mass larger than 6mm in average diameter, or a previous surgery manifestation were checked for hospital records to confirm lung cancer diagnosis. Information on demographic characteristics and lifetime coal usage was collected through face-to-face interviews.

Results: The cohort included 3126 participants (1897 females). At baseline, 97.0% (3032/3126) of participants had ever used Xuan Wei coal, with 95.5% (2986/3126) exclusively using Xuan Wei coal. The CT scans showed that 36.9% (1154/3126) had lung nodules (mean diameter:4.7mm), with 13.9% (432/3105) being positive. Among 2,073 participants who completed a one-year follow-up, 29 new lung cancers were identified (incidence rate: 1.4%; 15 females). The average age at diagnosis was 57 years for men and 58.1 years for women.

Conclusion: This is the first prospective lung cancer cohort involving the general population with an early recruitment age in high-risk areas in Xuan Wei. Continued follow-up will clarify longitudinal progression patterns.

This work was funded by the Research Grants Council of Hong Kong- General Research Fund (17613819) and the National Natural Science Foundation of China (NSFC-82173469).

P11-07 | Individual radon exposure among brain cancer cases: preliminary results from the RadonBrain Study

Lucia Martin-Gisbert[1], Guadalupe Garcia[1], Cristina Candal-Pedreira[1], Ana Collazo Lorduy[3], Raquel Gutierrez[3], Paula Roman-Pena[5], Isaura Pérez-Fernandez[7], Alejandro Rivero de Aguilar[9], Victor Sacristán-Santos[11], Ignacio Fernandez-Sobrado[13], Paula Sampedro-Domarco[15], Alberto Garrido-Fernandez[9], Santiago Aguín[5], Asunción Díaz-Serrano[18], Rocío Uriel[19], Luis Ruiz de Almirón[8], Alberto Ruano-Raviña[1]

[1] University of Santiago de Compostela, Preventive Medicine and Public Health, Santiago de Compostela, Spain; [2] University of Santiago de Compostela, Preventive Medicine and Public Health, Santiago de Compostela, Spain; [3] Hospital Puerta de Hierro, Majadahonda, Spain; [4] Hospital Puerta de Hierro, Majadahonda, Spain; [5] Hospital de Santiago, Santiago de Compostela, Spain; [6] Hospital de Santiago, Santiago de Compostela, Spain; [7] Hospital de Vigo, Vigo, Spain; [8] Hospital de Vigo, Vigo, Spain; [9] Hospital de Pontevedra, Pontevedra, Spain; [10] Hospital de Pontevedra, Pontevedra, Spain; [11] Hospital de A Coruña, A Coruña, Spain; [12] Hospital de A Coruña, A Coruña, Spain; [13] Hospital de Lugo, Lugo, Spain; [14] Hospital de Lugo, Lugo, Spain; [15] Hospital de Ourense, Ourense, Spain; [16] Hospital de Ourense, Ourense, Spain; [17] Hospital de Zamora, Zamora, Spain; [18] Hospital de Zamora, Zamora, Spain; [19] Hospital de Salamanca, Salamanca, Spain; [20] Hospital de Salamanca, Salamanca, Spain

Objective: To assess radon exposure among incident cases of brain tumors (gliomas and meningiomas).
Material and methods: Series of incident cases of brain tumors from an ongoing multicentric hospital-based case-control study in Spain were included, mostly recruited in a radon prone-area. Eight public hospitals from 3 regions (Galicia, Madrid and Castilla y León) took part, recruiting consecutive patients with a confirm diagnosis of a brain tumor from January to November 2024. Each participant was interviewed by the physician with a structured questionnaire, and was provided with an alpha track radon detector to be placed in each participant's dwelling for three months (in the master bedroom). Participants' radon concentration was described by sex and brain tumor type.
Results: A total of 86 incident cases, 66 (31 women) of glioma and 20 (14 women) of meningioma were included. Median age and range of age (in years) sorted by tumor type was 60 (22-80) for glioma and 65 (37-84) for meningioma; and sorted by sex 62 (36-84) for women and 61 (22-81) for men. Median radon concentration and inter-quartile range (IQR) was 93 Bq/m3 (IQR 46-163) for glioma and 74 Bq/m3 (IQR 43-129) for meningioma, and 74 Bq/m3 (IQR 37-139) for women and 115 Bq/m3 (IQR 58-192) for men. There were no significant differences on radon concentration found on glioma and meningioma cases, nor significant differences by sex. Furthermore, radon levels from 60 of the cases recruited in the region of Galicia were not higher than those found in the residential radon map of that same region.

Conclusions: There are no significant differences on radon concentrations found among women and men cases nor among glioma and meningioma cases. Average radon concentration for brain tumour cases seems to be similar to that of the general population.

P11-08 | Hormonal Risks of Early Adolescence Makeup Usage: Preliminary Results from HERSAFE

Sofía González[1,2], Brenda González[2], Imar Mansilla[3], Ana Ortiz[2], Nancy Cardona[1,2]

[1] University of Puerto Rico at Aguadilla, Department of Natural Sciences, Aguadilla, Puerto Rico; [2] University of Puerto Rico Comprehensive Cancer Center, Division of Cancer Control and Population Sciences, San Juan, Puerto Rico; [3] University of Puerto Rico Medical Sciences Campus, Division of Environmental Health, San Juan, Puerto Rico

Introduction. Cosmetic products, particularly makeup-related, are widely used for aesthetic enhancement, yet their prolonged contact with the skin raises concerns about potential health risks. Many foundations, mascara, eyeshadow, lipstick, among other products, contain ingredients that have been linked to endocrine disruption and possible carcinogenic effects.

Purpose. This study aims to assess the use of makeup products between 10-13 years of age and early age at first menstrual period associated with.

Materials and Methods. The ongoing "HERSAFE" case-control study examines the relationship between beauty product use and breast cancer among Hispanic women in Puerto Rico.Participants were recruited from Comprehensive Cancer Hospital and Radiotherapy Center. Data collection included a 45-60-minute survey on sociodemographics, lifestyle, maternal health, and beauty product use at: 10-13 years and in the past 12 months. Data was stored in RedCap. STATA was used for demographics and logistic regression analysis (95%CI).

Results. Participants (n=41) mean age was 52.9 (SD±10.5). Only 22.8% of participants had a household income <$30,000 and 82% had at least one child. Around 32.4% of the participants reported a darker skin tone, compared to the lightest color. Over half of the participants (51.4%) used makeup in early adolescence (10-13 years). Participants who used makeup in early adolescence were: (1) less likely with darker self-reported skin tone (AOR:-0.09, 95CI:-0.48-0.29) and (2) more likely to have a menstrual preiod at a younger age (<12years) (AOR:0.12, 95%CI:-0.31-0.57), when compared to their counterparts, respectively. However, none of these trends are statistically significant.

Conclusion. By analyzing existing literature and examining sociodemographic factors that may influence product use, this research aims to enhance awareness of cosmetic safety, encourage informed consumer choices, and advocate for stricter regulations on harmful ingredients in personal care products. Further research with larger samples and additional variables is needed to better understand potential influences on pubertal timing.

P11-09 | Teen Oral Hygiene and Breast Cancer Risks in Puerto Rico: A pilot study from HERSAFE

Yavier Morales[1,2], Brenda J. Gonzalez[2], Imar Mansilla[3], Ana P. Ortiz[2], Nancy R. Cardona-Cordero[1,2]

[1] University of Puerto Rico Aguadilla, Department of Natural Sciences, Aguadilla, Puerto Rico; [2] University of Puerto Rico Comprehensive Cancer Center, Division of Cancer Control and Population Sciences, San Juan, Puerto Rico; [3] University of Puerto Rico Recinto de Ciencias Medicas, Departamento de Salud Ambiental, San Juan, Puerto Rico

Introduction. Hormonal imbalances can contribute to cancer development. Mouthwashes are a care product recommended for oral hygiene. Ingredients like methyl- ethyl- and propyl-paraben, which have been classified as endocrine disrupting chemicals (EDCs) can be listed in mouthwashes. Additionally, alcohol-containing mouthwash and alcohol consumption can increase the permeability of the oral mucous membrane which can lead to absorption of EDCs.

Purpose. To examine the association between the use of mouthwash at 10-13 years old and the risk of breast cancer in Puerto Rican women.

Materials and Methods. The ongoing "HERSAFE" case-control study aims to assess the relationship between usage of care and beauty products and breast cancer in Puerto Rico. Currently 41 participants have been recruited since January 2025 at the Comprehensive Cancer Center Hospital. One-time questionnaire is administered to collect participants' socio-demographic, medical history and care products usage patterns between 10-13 years and the past 12 months. To assess the association, odds ratios (OR) with 95% confidence intervals (95%CI) were performed using STATA.

Results. The participants' mean age was 52.9 years (SD±10.5). Most participants were currently or previously married (65.8%), had a bachelor's degree (80%), consumed alcohol at least once in their lifetime (88%) from which 67% ingested alcohol in the last month. Mouthwash use between 10-13 years was reported by 46.8%. Observed trends among mouthwash users between 10-13 years who were more likely to have breast cancer when compared to those who did not use mouthwash at that age (OR: 2.72, 95%CI:0.42-17.6) and alcohol consumption can increase this risk (OR: 2.91, 95%CI:0.42-20.1).

Conclusion. The study sample size is a limitation. However, observed trends suggest that mouthwash usage between ages 10-13 can increse breast cancer risk, and alcohol consumption can contribute to an elevated risk. Mouthwash could be a modifiable cancer risk factor. Further studies can help develop targeted interventions.

P11-10 | Association between arsenic exposure and non-melanoma skin cancer prevalence among National Health and Nutrition Examination Survey adult participants (2003-2018)

Yingyue Ni[1], Lauren Giacobbe[2], Yu-Hsuan Shih[2], Maria Argos[1]

[1] *Boston University, School of Public Health, Department of Environmental Health, Boston, USA;* [2] *University of Illinois Chicago, School of Public Health, Division of Epidemiology and Biostatistics, Chicago, USA*

Objective: Arsenic is a well-established human carcinogen contributing to multiple cancers. This analysis aimed to evaluate the association between arsenic exposure and the prevalence of non-melanoma skin cancer (NMSC) in a general population sample in the United States (U.S.).
Material and Methods: We leveraged data on 13,690 adult participants from the National Health and Nutrition Examination Survey (NHANES; survey cycles 2003-2004 to 2017-2018). Measured urinary total arsenic (tAs) and dimethylarsinic acid (DMA) were corrected by arsenobetaine using regression calibration to represent non-seafood-derived arsenic exposure. The associations between estimated urinary tAs and DMA tertiles with NMSC and overall cancer prevalence were modeled using multivariate logistic regression to estimate adjusted odds ratios (aOR) and their 95% confidence intervals (CIs). Confounders were adjusted based on prior knowledge. We additionally explored associations stratified by folate status based on red blood cell concentrations.
Results: A dose-response relationship was observed for both estimated tAs and DMA with NMSC prevalence in U.S. adults (p for trend=0.09 and 0.005, respectively). Estimated tAs exposure was associated with higher NMSC prevalence (tertile 2 aOR=1.32, 95% CI=0.89-1.97 and tertile 3 aOR=1.59, 95% CI=0.93-2.72) in the highest two tertiles compared to the lowest exposure tertile. Similar associations were observed with DMA, although the magnitude of association was stronger (tertile 2 aOR=1.57, 95% CI: 1.03-2.39 and tertile 3 aOR=1.92, 95% CI: 1.23-3.02). No associations were observed for overall cancer prevalence. Stratified analyses showed that among participants with red blood cell folate above the median concentration, the slope of the dose-response relationship between arsenic exposure and NMSC prevalence was slightly weaker.
Conclusion: Longitudinal studies are needed to better understand the association of low-level arsenic exposure on cancer risk and the potential interactions with folate status.

P11-11 | A systematic review and meta-analysis on radon exposure and potential health effects other than lung cancer

Afi M.S. Henyoh[1,2], Olivier Laurent[1], Corinne Mandin[1], Enora Cléro[1]

[1] *Autorité de Sûreté Nucléaire et de Radioprotection (ASNR), PSE-SANTE/SESANE/LEPID, F-92260 Fontenay-aux-Roses, France;* [2] *2. Université Paris-Saclay, F-91190, Gif-sur-Yvette, France*

Objective: To date, lung cancer is the only well-established health effect associated with radon exposure in humans. To summarize available evidence on other potential health effects of radon exposure, we performed a comprehensive qualitative and quantitative synthesis of the available literature on radon exposure and health effects other than lung cancer, in both occupational and general populations.

Material and Methods: Eligible studies published from January 1990 to March 2023, in English and French languages, were identified in PubMed, ScienceDirect, Scopus, ScieLo and HAL. In the meta-analysis, we estimated average weighted standardized incidence ratios (metaSIR), standardized mortality ratios (metaSMR), and risk ratio (metaRR) per 100 unit (Bq/m^3 or Working Level Month) increase in radon exposure by combining estimates from the eligible studies using the random-effect inverse variance method.
Results: A total of 129 studies were included in the systematic review and 40 distinct studies in the meta-analysis. For most health outcomes, the results of the meta-analyses showed no statistically significant association, and heterogeneity was only present among occupational studies, especially between those included in the metaSIR or metaSMR analyses. However, the estimated exposure-risk associations were positive and close to the statistical significance threshold for: lymphohematological cancer incidence in children (metaRR = 1.01; 95%CI: 1.00–1.03; $p = 0.08$); malignant melanoma mortality among adults in the general population (metaRR = 1.10; 95%CI: 0.99–1.21; $p = 0.07$); liver cancer mortality (metaRR = 1.04; 95%CI: 1.00–1.10; $p = 0.06$), and intestine and rectal cancer mortality combined (metaRR = 1.02; 95%CI: 1.00–1.04; $p = 0.06$), among mine workers.
Conclusion: Although none of the exposure-risk associations estimated in the meta-analyses reached statistical significance, the hypothesis that radon may have other health effects apart from lung cancer could not be ruled-out and call for additional research. Larger and well-designed studies are needed to further investigate this question.

P11-12 | Nonlung Cancer Risks Linked to Silica Dust Exposure

David F. Goldsmith

George Washington University, Environmental & Occupational Health, Washington DC, USA

Objective: The International Agency for Researech on Cancer (IARC) has reviewed the cancer risk from workplace silica dust exposure three times--1986, 1997 and 2012. The primary focus was on pulmonary cancer in mining, construction and stone work. Other than lung cancer, what other tumor sites have been linked with exposure to crystalline silica?
Materials and Methods: The author consulted the PubMed database and sought cancer research studies published since 1998 (after the second IARC assessment), focusing on epidemiology findings that linked silica exposure to nonlung cancers. In particular, attention was paid to research on gastrointestinal (stomach), kidney, skin, laryngeal and esophageal malignancies. There was a desire to know the risks sfter diagnosis of silicosis and whether there was any association with smoking.
Results: There were excess odds ratios or relative risks for stomach cancer among Parent et al. (1998), Krstev et al. (2005) and Walsh et al (2025). Kidney cancer or renal cell carcinoma were elevated among granite workers (Atfield and Costello, 2004) and brick workers (Karami et al, 2011). Esophageal cancers demonstrated elevated risks for silica exposure in studies by Santibanez et al. (2008) and by Yu et al (2005) among Hong Kong silicotics. Excess laryngeal cancers were detected by Chen and Tse (2012) and by Elci and co-workers (2009). Although there were early concerns showing an increased risk for skin cancer by Partenan et al (1994), there did nor appear to be excess melanoma skin cancer asasociated with silica exposure in the past 2.5 decades.
Conclusions: Since 1998, there is published epidemiology evidence supporting links between silica exposed jobs, silicosis and the following cancers: stomach, kidney, larynx, and esophagus from a variety of dusty SiO_2 exposures.Clearly, continued and renewed prevention efforts are needed to lower the risks of nonlung cancer risks as well as pulmonary cancers.

P12 | Environmental Determinants of Mental Health and Well-being

P12-01 | The Psychological Impacts of Extreme Heat in Chicago's South Side

Ani Gogineni[1], Sarah Walton[2], Kristy Wei[1], Kimberley Mbayiwa[1], Ilaria Tare[1], Laura McGuinn[1], Kate E. Keenan[2], Kate Burrows[3]

[1] University of Chicago, Biological Sciences Division, Chicago, USA; [2] University of Chicago, Department of Psychiatry and Behavioral Neuroscience, Chicago, USA; [3] University of Chicago, Department of Public Health Sciences, Chicago, USA

Objective
Extreme heat events are intensifying due to climate change, disproportionately impacting marginalized populations in urban environments. This qualitative study investigates the mental health effects of extreme heat on black mothers residing in Chicago's South Side. Specifically, it explores how heat exposure interacts with existing psychosocial stressors, like economic stability and childcare, influencing emotional well-being, stress levels, and coping strategies.**Materials and Methods**
This study employs a qualitative, interview-based design. Semi-structured interviews were conducted with 15 participants from the Nutrition and Pregnancy Study (NAPS), an ongoing study examining stress and health disparities among black women and children living on Chicago's South Side. Participants were recruited based on prior involvement in NAPS and willingness to participate. Interviews explored emotional responses, behavioral adaptations, and systemic barriers to heat resilience. Thematic analysis was performed using a combined deductive-inductive coding approach to identify recurrent themes and patterns in participants' narratives.**Results**
Preliminary findings suggest that participants experienced heightened psychological distress during extreme heat, including increased irritability, fatigue, and social withdrawal. Many described significant disruptions to daily routines, difficulties sleeping, and exacerbation of pre-existing stressors, such as financial strain and childcare burdens. Limited access to cooling resources compounded these effects, particularly for those lacking a proper air conditioning system. Social and environmental inequities, including the urban heat island effect, further intensified vulnerability. Coping strategies varied but were often constrained by economic barriers, underscoring the need for systemic interventions.**Conclusion**
Extreme heat presents a significant, underrecognized public health challenge in marginalized urban communities. Beyond its physiological dangers, heat exacerbates mental health burdens, amplifying stress and diminishing quality of life. Addressing these disparities requires expanding access to cooling resources and heat-adaptive infrastructure. Community-informed strategies must be prioritized to mitigate the psychological toll of rising temperatures and to make cooling resources more accessible to high-risk populations.

P12-02 | The impact of temperature on mental health during spring and summer in a highly urbanized area: A case-crossover study.

Simon G. Beerten[1], Bert Vaes[1], Katrien De Troeyer[2], Endale A. Ali[1], Lidia Casas[2,3], Gijs Van Pottelbergh[1]

[1] KU Leuven, Department of Public Health and Primary Care, Leuven, Belgium; [2] University of Antwerp, Department Family Medicine and Population Health, Antwerp, Belgium; [3] University of Antwerp, Department of Bioscience Engineering, Antwerp, Belgium

Objective: Common mood disorders account for a large part of worldwide morbidity. The role of the urban environment, and specifically temperature, in depression and anxiety has been poorly studied in primary care.
Methods: In this time-stratified case-crossover study set in Flanders, Belgium, we used consultation data from primary care database Intego from 2012 to 2021. Mental health was defined as a first registration of depression, anxiety, or a first antidepressant or benzodiazepine prescription during the study period. We considered outcomes in vulnerable and non-vulnerable adults, with and without a two-year history of mental health problems, respectively. Daily maximum temperature from April to September was the exposure. The association with mental health was modeled using a conditional Poisson regression combined with distributed lag non-linear models, adjusted for weekday, month, year and relative humidity. Risks were cumulated over 21 days. We evaluated cold (P1,P5) as well as heat effects (P95,P99) relative to the median temperature.
Results: We identified 34,166 events in the vulnerable and 133,866 events in the non-vulnerable population. Median maximum temperature was 20.6 °C. In the non-vulnerable group, we found an overall effect of extreme cold on depression (IRR 2.42, 95% CI [1.14,5.15]). Protective effects of heat were found for prescriptions in this group. In the vulnerable group, we found cold effects at 14-day lags for depression (P1: IRR 1.85 [1.06,3.25]). Stratified analyses revealed a heat effect on any event for the low socioeconomic status (SES) category of the vulnerable group (P99: IRR 2.58 [1.03,6.47]).*Conclusion*: In addition to a cold effect for depression in a vulnerable population, we found that unusually high temperatures increased the risk of any mental health event in the low SES group of this population. These are important findings for both healthcare providers and policymakers.

Funding: Supported by the Research Foundation Flanders (reference: G0B4820N).

P12-03 | Nighttime Heat is Associated with An Elevated Risk ofEmergency Department Visits for Mental Health Disorders

Qingyang Zhu[1], Binyu Yang[1], Danielle A. Wallace[2,3], Qiang Pu[1], Ke Xu[1], Rebecca Zhang[4], Rohan R. D'Souza[4], Wenhao Wang[1], Benjamin G. Druss[5], Kyle Steenland[1], Noah C. Scovronick[1], Donald L. Bliwise[6], Stefanie Ebelt[1], Howard H. Chang[4], Yang Liu[1]

[1] Emory University, Gangarosa Department of Environmental Health, Rollins School of Public Health, Atlanta, USA; [2] Havard Medical School, Division of Sleep Medicine, Boston, USA; [3] Brigham and Women's Hospital, Division of Sleep and Circadian Disorders, Departments of Medicine and Neurology, Boston, USA; [4] Emory University, Department of Biostatistics and Bioinformatics, Rollins School of Public Health, Atlanta, USA; [5] Emory University, Department of Health Policy and Management, Rollins School of Public Health, Atlanta, USA; [6] Emory University, Department of Neurology, Sleep Center, School of Medicine, Atlanta, USA

Objectives: This study aimed to evaluate the effects of nighttime heat exposure on ED visits for anxiety and mood disorders in the United States, as well as the possible role that diurnal temperature range (DTR) plays in this association.

Methods: We analyzed ED visit data from ten US states. To distinguish between different heat scenarios, hot nights (HN) and hot days (HD) were defined as only daily minimum (Tmin), or maximum (Tmax) temperatures exceeded the 90th percentile of 30-year historical warm season temperature records from 1990-2019; The combined hot day and night (HDN) scenario was established when both Tmin and Tmax exceeded the 90th percentile threshold. We employed a case-crossover design using conditional logistic regression to model the association between heat exposure and ED visits. DTR was explored as a potential factor that underlies the heat-mental health relationship.

Results: The study included 4,484,913 ED visits for anxiety and mood disorders from 2005 to 2019. A HN was positively associated with ED visits for anxiety (OR and 95% CI: 1.026 [1.020-1.032]) and mood disorders (OR and 95% CI: 1.017 [1.011-1.024]). These effects were comparable to the effects of a HD, while a HDN showed the strongest associations. Days with less temperature cooling from Tmax of the previous day to Tmin were linked to higher odds of ED visits, while days with greater temperature heating from Tmin to Tmax showed weaker associations.

Conclusion: Our study highlights the significant, but underexplored, impact of nighttime heat on mental health. This association may be explained by a low temperature cooling, which could deteriorate sleep quality and mental health. These findings highlight the urgent need for targeted public health interventions designed to address the mental health emergencies intensified by the increasing frequency of heat events under global climate change.

P12-04 | Exploring Potential Associations between Mental Health/Indicators of Psychological Distress and Estimated Exposures of Ambient/Outdoor Air Quality Among Secondary School Teachers in New Jersey, 2022-2024

Maryanne L. Campbell[1], Juhi Aggarwal[1], Quincy W. Hunter[1], Midhat Rehman[1], **Derek G. Shendell**[2,3]

[1] Rutgers School of Public Health (Rutgers Health/Biomedical and Health Sciences), NJ Safe Schools Program, Piscataway, USA; [2] Rutgers School of Public Health (Rutgers Health/Biomedical and Health Sciences), Environmental and Occupational Health and Justice / NJ Safe Schools Program, Piscataway, USA; [3] Rutgers Health/Biomedical and Health Sciences, Environmental and Occupational Health Sciences Instititute, Piscataway, USA

*Background:*TheNew Jersey Safe Schools Program (NJSS) provides trainings, surveillance, and technical assistance to NJ secondary schools. NJSS has various training cohorts consisting of NJ secondary school teachers. Moreover, cross-sectional surveys of psychological distress were conducted amongst these cohorts starting winter of the 2021-22 school year (SY) through the end of the 2023-24 SY. Utilizing the Kessler-6 tool (K6+), NJSS assessed symptoms associated with general mental health. Utilizing data gathered from the NJ Department of Environmental Protection (NJDEP)/U.S. Environmental Protection Agency (EPA) Air Quality Monitoring Stations (AQMS), a database relating to estimated exposures to ambient/outdoor air quality was constructed.

Methods: This was an initial environmental epidemiological study with an ecological design. NJSS cohorts for the 2021-2024 SY were broken down by school district (SD) and paired with AQMS based on approximate geographic proximity for each SD's school utilizing NJDEP's NJ-GeoWeb after school addresses were retrieved utilizing the NJ School Performance Reports database. Once addresses were reported and associated with at least two AQMS, associated reviewed daily criteria pollutant data were retrieved from EPA AQMS. Only agency reviewed data were included, excluding preliminary data, from 2021-2023, for particulate matter (PM, as PM10 and PM2.5) and ozone; 2024 data are currently preliminary, i.e., pending agency verification. Data downloaded were refined to include those associated with twelve NJSS virtual training date-ranges related to specified teacher cohorts, including two live session trainings per date-range consisting of 30 calendar days prior to its date to relate to the K6+ questions. Data were averaged for relevant stations and included in NJSS database.

Results: We analyze 2021-2024 SY data for K6+ and selected criteria pollutants by NJ SD.

Conclusions: NJSS will present analyses of 2021-2024 data to spark discussion and inform future collaborative work with other researchers interested in mental health and effects of environmental factors.

P12-05 | Air pollution and postpartum psychological dysfunction symptoms: the interplay with prenatal stress

Gary Joseph[1], Megan Niedzwiecki[1], Itai Kloog[1], Allan C. Just[2], Rosalind J. Wright[1,4], Ivan G. Avila[1], Elena Colicino[1], Martha María T. Rojo[3], Robert O. Wright[1,5], Lauren M. Petrick[1,6]

[1] Icahn School of Medicine at Mount Sinai, Environmental Medicine and Climate Science, New York, USA; [2] Brown University School of Public Health, Department of Epidemiology, Providence, USA; [3] National Institute of Public Health, Center for Nutrition and Health Research, Cuernavaca, Mexico; [4] Icahn School of Medicine at Mount Sinai, Department of Public Health, New York, USA; [5] Icahn School of Medicine at Mount Sinai, Institute for Climate Change, Environmental Health and Exposomics, New York, USA; [6] Sheba Medical Center, The Bert Strassburger Metabolic Center, Tel-Hashomer, Israel

Objective: To examine the modifying role of prenatal stress in the association between ambient air pollution ($PM_{2.5}$) and psychological dysfunction in a cohort of Hispanic women in Mexico.

Materials and methods: This study utilized data from mothers in the PROGRESS cohort with available information on prenatal $PM_{2.5}$exposure and psychological dysfunction (PPD, anhedonia, anxiety, and depression) at 6 months postpartum (n = 457). Daily residence level $PM_{2.5}$ estimates were generated from our hybrid satellite-based machine learning model, and averaged over pregnancy. Psychological dysfunction was assessed using the Edinburgh Postnatal Depression Scale (EPDS) at 6 months postpartum. Modified Poisson regression evaluated the association between $PM_{2.5}$ and PPD (EPDS≥13), stratified by negative life events (NLEs) and perceived stress score (PSS), dichotomized around the median (low/high). Negative binomial regression was used for EPDS subscales (anhedonia, anxiety, and depression).

Results: A 5-μg/m^3 increase in average prenatal $PM_{2.5}$ exposure was associated with an increased risk of overall PPD (RR: 1.48, 95%CI: 1.02-2.17), and new onset of PPD (RR: 2.55, 95%CI: 1.27-5.12) at 6 months. Stratifying PPD risk by NLE and PSS scores showed increased overall PPD risk per unit increase in $PM_{2.5}$ in low-PSS subgroups (RR: 2.18, 95%CI: 1.11-4.28) and new-onset PPD in high-NLE (RR: 4.04, 95%CI: 1.47-11.12) and low-PSS subgroups (RR: 4.04, 95%CI: 1.60-10.22). $PM_{2.5}$ was positively associated with EPDS subscales of anhedonia and depression, with greater risk in low-PSS subgroups.

Conclusion: Prenatal $PM_{2.5}$ exposure is associated with an increased risk of postpartum psychological dysfunction symptoms, especially in those with low-PSS or high-NLE scores. These findings highlight the need for interventions addressing both air pollution and prenatal stress to prevent adverse postpartum mental health outcomes.

P12-06 | The impact of indoor air quality on mental health: A comparative study in three Northern-European countries

Milena Podžigun[1], **Hans Orru**[1,2], Anna-Sara Claeson[3], Ene Indermitte[1], Kalle Kipper[1], Einar Eidstø[4], Anniina Salmela[4,5], Juha Pekkanen[4]

[1] University of Tartu, Institute of Family Medicine and Public Health, Tartu, Estonia; [2] Umea University, Sustainable Health, Umea, Sweden; [3] Umea University, Department of Psychology, Umea, Sweden; [4] University of Helsinki, Department of Public Health, Helsinki, Finland; [5] Finnish Institute for Health and Welfare, Department of Health Security, Kuopio, Finland

Poor indoor air quality (IAQ) is an environmental risk factor, contributing to health burdens and reduced quality of life. Emerging research suggests that IAQ may play a role in mental health disorders. This study investigates the potential link between perceived IAQ and mental health outcomes, examining key indoor air and climate factors in three Northern-European countries.

The study utilizes data from national surveys involving randomly selected participants aged 25–64. The final sample included 1,614 participants from Finland, 1,023 from Estonia, and 930 from Sweden. Logistic regression analysis was applied to estimate odds ratios (OR) and confidence intervals (CI) with Bonferroni correction to determine associations between perceived IAQ and mental health indicators.

The results highlight the difference in exposures, symptoms and associations across countries. In Finland, stuffy indoor air was associated with depression, increasing odds by 3.7 times (99.6% CI: 1.5–8.3). In Estonia, exposure to indoor air allergens elevated depression risk by 3.5 times (99.6% CI: 1.7–6.9), while in Sweden, in the presence of cigarette smoke odor at home, the risk was raised fivefold. Chronic fatigue was associated with indoor allergens in Estonia (OR = 6.1; 99.6% CI: 1.8–17.2) and dry air and dust in Sweden. Burnout was associated with poor air exchange (OR = 3.3; 99.5% CI: 1.9–5.6) and cigarette smoke odor (OR = 3.3; 99.5% CI: 1.8–5.8) in Estonia, whereas in Finland, cigarette smoke odor was linked to burnout (OR = 4.0; 99.5% CI: 1.9–8.0). In Sweden, drafty workplaces were connected to a higher risk of burnout (OR = 4.4; 99.5% CI: 1.5–11.2). Additionally, in Estonia, frequent exposure to too hot temperatures was associated with up to 80% reduction in work ability.

These findings might suggest a negative impact of perceived IAQ on mental health, with variations observed across countries.

P12-07 | Heat Exposure and Depression-Related Hospitalizations: A Nationwide Time-stratified Case-Crossover Study in Taiwan

Chi-Jen Chen[1,2], Wei-Che Chiu[3,4], Ching-Chun Lin[2], **Pau-Chung Chen**[2,5]

[1] National Taiwan University, Institute of Epidemiology and Preventive Medicine, Taipei, Taiwan; [2] National Taiwan University, Institute of Environmental and Occupational Health Sciences, Taipei, Taiwan; [3] Cathay General Hospital, Department of Psychiatry, Taipei, Taiwan; [4] Fu Jen Catholic University, School of Medicine, New Taipei, Taiwan; [5] National Health Research Institutes, National Institute of Environmental Health Sciences, Miaoli, Taiwan

Background

Climate change has resulted in the increased frequency and intensity of heat waves, posing significant public health challenges. While the physiological effects of heat exposure are well-documented, its mental health impacts,

particularly on depression, remain underexplored. This study investigates the association between acute heat exposure and depression-related hospitalizations in Taiwan.

Methods

A time-stratified case-crossover design was employed to evaluate the short-term associations between heat exposure and depression-related hospitalizations. Nationwide inpatient records from Taiwan's National Health Insurance system, with ICD codes identifying depression-related admissions. High-resolution temperature data from meteorological stations were linked to patients' residential areas. Key exposure variables included daily mean, maximum, and minimum temperatures, diurnal temperature range, and day-to-day temperature fluctuations. Conditional logistic regression models were used to calculate odds ratios (ORs) for hospitalization. Distributed Lag Nonlinear Models (DLNMs) were also utilized to examine the exposure-lag-response effects between daily temperature parameters and depression-related hospitalizations.

Results

In the overall analysis, increases in daily mean, maximum, and minimum temperatures are consistently linked to higher odds of hospitalization, with winter showing the strongest associations (e.g., OR = 1.010 for mean and minimum temperatures, $p < .0001$). Diurnal temperature range (DTR) is also significantly associated overall (OR = 1.003, $p = 0.0015$), with the strongest impact observed in summer (OR = 1.009, $p = 0.0001$). Sudden day-to-day temperature increases significantly increase hospitalization risks in summer (OR = 1.010, $p < .0001$), while sudden decreases show no significant association across any season.

Conclusions

This study highlights a significant association between acute heat exposure and depression-related hospitalizations. Addressing the mental health consequences of climate change should be a public health priority.

P12-08 | Impact of Ambient Temperature on Mental Health-Related Emergency Room Visits in Chicago: A Time-Series Analysis

Saira Tasmin[1], Danielle Landron[2], Julie Johnson[2], Habibul Ahsan[1]

[1] The University of Chicago, Institute of Population and Precision Health, Chicago, USA; [2] The University of Chicago, Center for Research Informatics, Biological Sciences Division, Chicago, USA

Objective: Mental health conditions affect nearly 20% of U.S. adults, posing a significant public health challenge. Emerging evidence suggests that environmental factors, including temperature, may influence mental health outcomes. This study examines the association between short-term ambient temperature exposure and mental health-related emergency room (ER) visits at UChicago Medicine (UCM) hospital from 2012 to 2023.

Methods:

Using electronic health record data, we conducted a time-series analysis of ER visits with a primary mental health diagnosis at UCM. Daily meteorological data were obtained from the National Center for Environmental Information Climate Data Online system, specifically from the Global Historical Climatology Network (GHCN) for the study region (IL), including daily temperature, precipitation, and snowfall. Generalized linear models were used to assess the association between ambient temperature and ER visits, adjusting for seasonality, long-term trends, day of the week, and public holidays. Stratified analyses explored effect modification by age, sex, race, and diagnosis.

Results:

Between 2012 and 2023, 85,374 mental health-related ER visits were recorded. A 1°F increase in mean daily temperature at lag 0–1 was associated with a 4.1% increase in mental health-related ER visits (RR: 1.041, 95% CI:

1.040–1.0421). Using EHR data for mental health-related ED visits, we observed increased healthcare utilization for mental health diagnoses associated with high temperature, highlighting the need for climate-adaptive public health strategies to protect vulnerable populations.

P12-09 | Prenatal Exposure to Heat Stress and Maternal Depressive Symptoms at 12 Months Postpartum

Yuhong Hu[1], Noelle Pardo[1], Xiaoran Yang[1], Tingyu Yang[1], Yan Xu[1], Roxana Khalili[1], Aviva Wolf-Jacobs[1], Xinci Chen[1], Zhongzheng Niu[1,2], Brendan Grubbs[3], Laila Al-Marayati[3], Nathana Lurvey[4], Claudia Toledo-Corral[1,5], Jill Johnston[1], Genevieve F. Dunton[1], Sandrah P. Eckel[1], Shohreh F. Farzan[1], Carrie Breton[1], Rima Habre[1], Theresa Bastain[1]

[1] University of Southern California, Department of Population and Public Health Sciences, Keck School of Medicine, Los Angeles, USA; [2] University at Buffalo, Department of Epidemiology and Environmental Health, School of Public Health and Health Professions, Buffalo, USA; [3] University of Southern California, Department of Obstetrics and Gynecology, Keck School of Medicine, Los Angeles, USA; [4] Eisner Health, Los Angeles, USA; [5] California State University, Northridge, Department of Health Sciences, Northridge, USA

Objective

While extreme temperature has been linked to multiple mental disorders, the impact of prenatal heat stress on postpartum depression remains understudied. We aimed to evaluate the association between prenatal heat stress exposure and depressive symptoms one year postpartum, identify susceptible gestational windows, and assess whether neighborhood climate vulnerability modifies maternal susceptibility to heat stress.

Methods

We followed 275 predominantly low-income Hispanic/Latina participants with full-term pregnancies in the MADRES cohort (2015-2023) through 12 months postpartum. Daily residential wet bulb globe temperature (WBGT) was estimated throughout pregnancy using meteorological data from the Abatzoglou 4x4km gridMET model, incorporating temperature, humidity, winds, and solar insolation. Depressive symptoms were assessed using the Center for Epidemiologic Studies-Depression (CES-D) scale. A neighborhood-level Climate Vulnerability Index (CVI) was calculated to capture comprehensive factors affecting climate change vulnerability based on prenatal residence. We used multivariable linear regression to estimate associations between trimester-specific heat stress and postpartum depressive symptoms (log-transformed CES-D scores). We examined effect modification by CVI (≥ 75th percentile vs. < 75th percentile) for each trimester-specific effect. Distributed lag models (DLM) were used to identify susceptible exposure windows over 37 gestational weeks.

Results

Each interquartile range increase in first-trimester WBGT (1.98°C) was associated with a 5.35% (95% CI: 0.01%–11.06%) increase in 12-month CES-D scores. DLM analysis confirmed positive associations during gestational weeks 0-14. We observed significant effect modification by CVI (p-interaction = 0.05) in the second trimester, with stronger WBGT effects on postpartum depression among mothers in high-CVI neighborhoods (β = 8.96%; 95% CI: -3.89%–23.54%) compared to lower-CVI neighborhoods (β = -5.61%; 95% CI: -11.89%–1.11%).

Conclusion

Early-pregnancy exposure to heat stress is associated with increased postpartum depressive symptoms. Moreover, mothers living in highly climate-vulnerable neighborhoods show greater effects of heat stress during mid-pregnancy on postpartum depression compared to those in less vulnerable areas.

P12-10 | Early-Pregnancy Exposure to Ambient Air Pollution and Trajectories of Depressive Symptoms during Pregnancy

Yuhong Hu[1], Tingyu Yang[1], Sandrah P. Eckel[1], Xinci Chen[1], Zhongzheng Niu[1,2], Claudia M. Toledo-Corral[1,3], Nathan Pavlovic[4], Fred Lurmann[4], Erika Garcia[1], Santiago Morales[5], Jill Johnston[1], Genevieve F. Dunton[1], Rima Habre[1], Shohreh F. Farzan[1], Carrie Breton[1], Theresa M. Bastain[1]

[1] University of Southern California, Department of Population and Public Health Sciences, Keck School of Medicine, Los Angeles, USA; [2] University at Buffalo, Department of Epidemiology and Environmental Health, School of Public Health and Health Professions, Buffalo, USA; [3] California State University, Northridge, Department of Health Sciences, Northridge, USA; [4] Sonoma Technology, Inc, Petaluma, USA; [5] University of Southern California, Department of Psychology, Los Angeles, USA

Objective

Depression during pregnancy shows diverse patterns in severity and progression. While air pollution has been linked to depression risk, its impact on the course of prenatal depression remains understudied. We first aimed to identify distinct trajectories of depressive symptoms across pregnancy and then evaluate whether early pregnancy air pollution exposure impacts membership in trajectory groups.

Methods

This study followed 542 predominantly low-income Hispanic/Latina participants in the MADRES cohort who completed at least 2 depression assessments during pregnancy. We assessed depressive symptoms using the Center for Epidemiologic Studies-Depression (CES-D) scale at 1st, 2nd, and 3rd trimester visits. Daily residential concentrations of $PM_{2.5}$, PM_{10}, NO_2, and O_3 were estimated using inverse-distance squared spatial interpolation from monitoring data. We performed latent growth mixture modeling (LGMM) to identify clusters of individuals with similar progressions of continuous prenatal CES-D scores. Multinomial logistic regression models were further conducted to estimate the relative risk ratios (RRRs) of membership in each trajectory group relative to the lowest group with averaged air pollution levels over early pregnancy (gestational weeks 0-6).

Results

We identified four distinct trajectories: moderate-to-high symptoms (8.2%), consistently-moderate symptoms (17.3%), moderate-to-low symptoms (36.6%), and consistently-low symptoms (38.0%). Each interquartile range increase in early-pregnancy NO_2 (3.89 ppb) was associated with 66% higher relative risk (95% CI: 17%–137%) of following the moderate-to-high versus consistently low symptoms trajectory. Similar elevated risks were observed for $PM_{2.5}$ (RRR = 1.32; 95%CI: 1.04–1.66) and PM_{10} (RRR = 1.74; 95% CI: 1.01–3.00). No significant associations were found between air pollution with trajectories of consistently moderate or moderate-to-low symptoms versus consistently low symptoms.

Conclusion

Early pregnancy exposure to ambient air pollution is associated with increased risk of having moderate-to-high depressive symptoms across pregnancy. This finding suggests air pollution may interfere with early-pregnancy adaptations in maternal physiology, potentially altering mood regulation pathways.

P12-11 | Pesticide Exposure and Depression in Rural and Urban Workers of Maule, Chile

Boris A. Lucero[1,4], Maria T. Muñoz-Quezada[2], Andres C. Johnson[1,4], Cynthia Carrasco[1], Maria V. Rodriguez[1], Tannelle Reid[4], Pablo Mendez[3], Jandy Adonis[2], Tristan Bekinschtein[4]

[1] Universidad Católica del Maule, CINPSI Neurocog/Faculty of Health Sciences, Talca, Chile; [2] Universidad de Chile, School of Public Health/Faculty of Medicine, Santiago, Chile; [3] Universidad Católica del Maule, Departament of Psychology/Faculty of Health Sciences, Talca, Chile; [4] University of Cambridge, Consciousness and Cognition Laboratory/, Cambridge, UK

Exposure to pesticides has been increasingly identified as a contributor to adverse health outcomes, including depressive symptoms. This longitudinal study investigated the association between chronic exposure to organophosphate (OP) pesticides and depressive symptoms among agricultural workers in Chile's Maule region, an area characterized by intensive agricultural pesticide use. We assessed pesticide exposure in 78 participants—comprising agricultural workers occupationally exposed to pesticides and urban residents without direct occupational exposure—by quantifying six dialkyl phosphate (DAP) metabolites in four repeated urine samples collected over two years. Depressive symptoms were measured using the CES-D scale, while affect was evaluated with the PANAS scale. Generalized Estimating Equations (GEE) analyses, adjusting for sociodemographic, health-related, and other exposure variables, revealed that cumulative exposure to diethyl alkyl phosphate (DEAP) metabolites was significantly associated with increased depressive symptoms (CI = 0.0003–0.0127, $p = 0.040$). Although no direct differences in urinary DAP metabolites emerged between rural and urban groups, rural participants reported increased positive affect over time, whereas urban workers showed significantly reduced negative affect by the study's second year. These findings highlight that pesticide exposure occurs beyond occupational settings, potentially through environmental contamination or dietary sources, as indicated by comparable exposure levels across rural and urban populations. Our results emphasize the importance of biomarkers to assess cumulative pesticide exposure accurately. Effective public health strategies, tighter regulatory measures, and increased public awareness are crucial to mitigating mental health risks associated with chronic pesticide exposure. Further studies should explore genetic and environmental determinants of individual susceptibility to pesticide-related mental health outcomes.

P12-12 | DSM-5 Mental Disorder in Ohio, Pennsylvania, and West Virginia 9 Months after the East Palestine Train Derailment

Cameron P. Pugach[1], **Aaron Reuben**[2], Angela D. Moreland[1], Alex O. Rothbaum[3], John Boyle[4], Michael G. Schmidt[1], James Dayton[4], Rachel Kinder[4], Sandro Galea[5], Salma Abdalla[5], Mohammed Abba-Aji[5], Dean G. Kilpatrick[1]

[1] Medical University of South Carolina, Charleston, USA; [2] University of Virginia, Charlottesville, USA; [3] Emory University, Atlanta, USA; [4] ICF International, Reston, USA; [5] Boston University, Boston, USA

Objective: The February 3, 2023, Norfolk Southern train derailment in East Palestine, Ohio, released tens of thousands of hazardous compounds into the air, water, and soil. Such chemical disasters are potentially traumatic events (PTEs) capable of onsetting or exacerbating mental disorders. Study sought to determine the prevalence and correlates of DSM-5 PTSD and Major Depression among potentially exposed residents of Ohio, Pennsylvania, and West Virginia nine months after the accident.
Material and Methods: Rapid, low-cost, non-probability sampling of 1,000 adults living within 65 miles of the derailment was conducted via existing market-research panels. Participants completed a high-resolution, self-administered clinical survey interview assessing derailment-related experiences, physical health symptoms, diagnosablePTSD and Depression, and preexisting psychiatric risk factors. Analyses were demographically weighted to yield estimates representative of the 65-mile-radius population.
Results: Most respondents were concerned about potential toxic substance exposure (73.9%) and associated health problems (92.1%), and half did not fully trust information provided about the event by public officials (52.1%). Half of all respondents (49.7%) also reported at least one new or worsening physical health symptom since the derailment, primarily respiratory symptoms such as coughing or trouble breathing. Nine months after the derailment, diagnosable PTSD (15.4%), Major Depression (13.3%), and their comorbidity (8.7%) were prevalent in the area surrounding the derailment. Notably, distance from the derailment site was associated with greater endorsement of negative event-related beliefs and experiences (Cohen's d = .21-22), but not with actual risk for mental disorder.
Conclusion: Chemical disasters may have psychological consequences even in the absence of documented exposures. Communities in Ohio, Pennsylvania, and West Virginia may benefit from increased mental health services, as may other communities post technological disaster.

P12-14 | Community severance and mental health-related hospital visits in New York City

Jaime Benavides[1], Jeff Goldsmith[2], Marianthi-Anna Kioumourtzoglou[1]

[1] Columbia University, Department of Environmental Health Sciences, Mailman School of Public Health, New York, USA; [2] Columbia University, Department of Biostatistics, Columbia University Mailman School of Public Health, New York, USA

Objective: Mental health disorders tend to accumulate in cities; for instance, urban living reportedly has significant influence on anxiety, mood, and schizophrenia disorders. There is growing evidence demonstrating that traffic-related exposures, such as air pollution and noise, have a detrimental impact on mental health. However, the influence of road infrastructure and traffic on mental health through the pathway of severely separating communities and breaking down the social fabric has not been studied. We aim to investigate the association between mental health hospitalizations and community severance in New York City (NYC).
Material and Methods: We conducted analyses at the ZIP code-level for 2014 across NYC. We used daily mental health hospitalizations from the New York Department of Health Statewide Planning and Research Cooperative System (SPARCS), which we aggregated into annual ZIP code counts. We focused on four categories of mental health disorders: mood, anxiety, adjustment and schizophrenia. We used a Community Severance Index (CSI) developed to characterize severance in NYC using geospatial data and pattern recognition. We used generalized additive models to estimate the association between CSI and mental health, adjusting for ZIP code-level socioeconomic status and green space. We allowed for nonlinear exposure–outcome relationships using natural splines and conducted sensitivity analyses.
Results: In adjusted analyses, preliminary results indicate that increasing CSI levels were nonlinearly associated with schizophrenia disorders in NYC. For mood and anxiety disorders, high values of CSI were non-significantly associated with increases in hospitalizations. We found no association between CSI and adjustment disorders. The estimated associations were fairly robust in sensitivity analyses.
Conclusion: Our preliminary results suggest that community severance is associated with mental health hospitalizations for schizophrenia.

P12-15 | Associations of urinary phthalate metabolites with cognitive performance and mood in post-menopausal women with and without major depression

Jessica A. Harder[1], Katherine E. Burdick[2], Katharine Burns[3], Brent A. Coull[4], Russ Hauser[5]

[1] Massachusetts General Brigham and Harvard Medical School, Department of Psychiatry / Division of Neuropsychiatry, Boston, USA; [2] Massachusetts General Brigham and Harvard Medical School, Department of Psychiatry, Boston, USA; [3] Massachusetts General Brigham and Harvard Medical School, Department of Psychiatry, Boston, USA; [4] Harvard T.H. Chan School of Public Health, Departments of Biostatistics and Environmental Health, Boston, USA; [5] Harvard T.H. Chan School of Public Health, Departments of Environmental Health and Epidemiology, Boston, USA

Objective: Early life phthalate exposure adversely affects cognition and risk for affective disturbance, with sex-specific effects. There is limited data on the relationship of phthalates with cognition and mood in adults. Recurrent major depressive disorder (MDD) increases risk for cognitive and recurrent mood symptoms. This study aimed to assess associations between phthalate exposure biomarkers and mood and cognitive performance in midlife women with and without a history of MDD.

Material/Methods: Forty post-menopausal women (N=20 with MDD, recurrent; N=20 healthy controls) underwent mood assessment with the Hamilton Depression Rating Scale (HDRS) an average of 2.23 days (range 0 to 31 days) before comprehensive neuropsychological assessment with the modified MATRICS Consensus Cognitive Battery (MCCB) and single spot urine collection for urinary phthalate metabolite (PM) assays. Outcomes were regressed on PMs, adjusting for urine specific gravity and MDD status.

Results: Participants’ mean age was 59.8 years (SD6.5). Higher urinary miBP levels were associated with higher HDRS scores (β=0.17 point change in score per ng/ml change in PM, 95%CI:0.08,0.26) and lower MCCB composite scores (β=-0.18, 95%CI:–0.32,–0.04), Working Memory (β=-0.28, 95%CI: -0.47,-0.09), Attention/Vigilance (β=-0.21, 95%CI: –0.40,–0.03), and Visual Learning (β=-0.27, 95%CI:–0.53,–0.01) subscale scores. Higher levels of DEHP metabolites (summed across metabolites) were associated with higher Reasoning/Problem Solving (β=38.63, 95%CI: 9.63,67.62) and Working Memory (β=31.82, 95%CI: 6.89,56.75), with some evidence of association with MCCB composite (β=17.41, 95%CI:–0.69,–35.50) and Speed of Processing (β=27.91, 95%CI:–0.19,56.00) subscale scores.

Conclusion: In this pilot study of postmenopausal women with and without recurrent MDD, higher urinary miBP levels were associated with poorer cognitive performance and worse mood. In contrast, higher levels of DEHP metabolites were associated with better cognitive performance. These findings provide support for potential effects of phthalate exposure on neuropsychiatric outcome measures outside of critical prenatal and childhood windows.

JH support: NIH K23ES034472, BWH FCDA, Harvard-Chan NIEHS Pilot Project (P30ES000002).

P12-16 | Momentary Effects of Air Pollutants and Temperature on Depression: A Pilot Study Using Ecological Momentary Assessment and Portable Sensors

John E. Roberts[1], Dakota Hughes[1], Eunhye Yoo[2]

[1] University at Buffalo, Psychology, Buffalo, USA; [2] University at Buffalo, Geography, Buffalo, USA

OBJECTIVE Population-level studies suggest that exposure to environmental stressors, including heat and air pollution, contribute to mental health outcomes such as emergency department visits, but the extant literature provides limited information concerning the momentary effects of these environmental stressors on individuals. This pilot study aims to determine the feasibility of an individual-level study designed to test the impact of within-day exposures to environmental stressors on depression-related psychological and behavioral processes.

METHODS For 30 consecutive days participants carry portable sensors and complete an ecological momentary assessment (EMA) protocol. The portable sensors measure temperature and air pollutants ($PM_{2.5}$ and VOC) continuously throughout the day, whereas the EMA protocol measures the location of survey, psychological (e.g., fatigue, mood) and behavioral variables (e.g., physical and social activity) that may be early indicators of the onset of depressive episodes. EMAs are prompted on three random occasions each day. This pilot study consists of a sample of 30 individuals who meet criteria for Major Depressive Disorder in remission and data collection is ongoing.

RESULTS Preliminary results based on the first 14 participants indicate excellent EMA compliance with 84.6% completed within 30 minutes of the prompt. Each EMA took 101.8 seconds on average to complete and there has been no attrition from the protocol. Multilevel modeling results indicate that momentary elevations in VOCs predicted increased fatigue, lower self-worth, and less physical activity, and most effects were stronger when participants were at home. $PM_{2.5}$ did not significantly predict any outcome variables, whereas higher temperatures predicted lower self-worth and less physical activity.

CONCLUSIONS These initial data support the feasibility of a larger scale study examining the daily processes by which exposure to environmental stressors, such as fluctuations in temperatures and air pollutants, impact functioning among individuals with Major Depressive Disorder.

P12-17 | Residential proximity to oil and gas development and prevalence of postpartum depression in a North American preconception cohort study

Kaylin A. Vrkljan[1], Amelia K. Wesselink[1], Erin J. Campbell[2,1], Martha R. Koenig[1], Dmitrii Krivorotko[1], Jordan R. Kuiper[2], Nicole C. Deziel[3], Jonathan J. Buonocore[4], Yael L. Nillni[5], Lauren A. Wise[1], Mary D. Willis[1]

[1] Boston University School of Public Health, Epidemiology, Boston, USA; [2] The George Washington University Milken Institute School of Public Health, Environmental and Occupational Health, Washington D.C., USA; [3] Yale School of Public Health, Environmental Health Science, New Haven, USA; [4] Boston University School of Public Health, Environmental Health, Boston, USA; [5] Boston University Chobanian & Avedisian School of Medicine, Psychiatry, Boston, USA

Objective: As oil and gas production expands, a growing body of literature shows that communities residing near oil and gas development (OGD), compared to those living far, have worse mental health outcomes. Postpartum depression (PPD) affects ~15% of birthing people and may be influenced by environmental exposures. Our objective is to estimate the effect of OGD on PPD.

Material and Methods: We used data from U.S. participants between 2017-2024 in Pregnancy Study Online (PRESTO), an internet-based prospective preconception cohort study. At 6 months postpartum, participants who reported a birth were invited to complete a questionnaire, including a validated scale for symptoms of PPD (Edinburgh Postnatal Depression Scale, EPDS). We calculated the distance between a participant's geocoded address at the time of the postpartum questionnaire and the nearest active OGD site and restricted our analytic sample to PRESTO participants within 50 km of an active OGD site to ensure comparability between exposed and unexposed groups. We examined the relation between residential proximity to an active OGD site and prevalence of PPD (EPDS >13). We estimated prevalence ratios (PRs) and 95% CIs using log-binomial models, adjusting for maternal age, geographic region, year, and season.

Results: Among 1,021 participants, the prevalence of PPD was 11.6%. Residence within 5 km of an active OGD site was associated with a higher prevalence of PPD (PR=1.3, 95% CI: 0.9, 2.0) compared with residences 20-50 km away. This association attenuated among participants who resided at further distances (e.g., 5-10 km [PR=1.1, 95% CI: 0.7, 1.9], 10-15 km [PR=0.9, 95% CI: 0.5, 1.5], 15-20 km [PR=0.7, 95% CI: 0.3, 1.5]).

Conclusion: Preliminary results suggest that living within 5 km of an active OGD site may be associated with slightly higher prevalence of PPD.

P12-18 | Identifying sensitive windows of temperature exposure associated with childhood anxiety and depression

Kelly Brunst[1], Erika Rasnick Manning[2], Jeff Burkle[3], Melinda MacDougall[3], Yingying Xu[3], Kimberly Yolton[3,1], Kim Cecil[4,1], Cole Brokamp[2], Patrick Ryan[2,1]

[1] University of Cincinnati, Environmental and Public Health Sciences, Cincinnati, USA; [2] Cincinnati Children's Hospital Medical Center, Biostatistics and Epidemiology, Cincinnati, USA; [3] Cincinnati Children's Hospital Medical Center, Pediatrics, Cincinnati, USA; [4] Cincinnati Children's Hospital Medical Center, Radiology, Cincinnati, USA

Purpose: Exposure to elevated temperatures can adversely impact mental health. However, the critical windows for exposure to extreme ambient temperature and its impact on mental health remain poorly understood.

Materials and Methods: We estimated monthly temperature exposures among 544 children enrolled in the Cincinnati Combined Childhood Cohort (C4) by overlapping geocoded residential addresses with daily 12 km x 12 km temperature estimates from the North American Regional Reanalysis (NARR). We assessed depression and anxiety at age 12 years by child report with the Child Depression Inventory and the Spence Children's Anxiety Scale. Distributed lag models were used to estimate the associations of temperature exposure with depression and anxiety outcomes while adjusting for child sex, race, maternal education, community-level deprivation, maternal depression, parent child relationship factors, and cohort; secondary analyses controlled for blood Pb.

Results: Overall, exposure to higher temperature was associated with greater symptoms of generalized anxiety and anxiety related to panic/agoraphobia, separation, and social phobia ($p<0.05$). We identified sensitive windows for temperature exposure during early childhood associated with greater generalized anxiety (birth to age 2) and social phobia (birth to age 5); exposure to higher temperatures between ages 5 and 6 were associated with greater symptoms of panic/agoraphobia and separation anxiety. Analyses remained significant after controlling for blood Pb, with the exception of social anxiety that suggested more social anxiety with exposure to lower temperatures. No overall effects or sensitive windows were identified for depression.

Conclusions: Early life exposure to elevated temperatures may have an impact on adolescent anxiety symptoms, particularly generalized anxiety, panic/agoraphobia and separation anxiety. Our findings with social anxiety suggest bothlow and high temperatures might be important drivers of certain types of anxiety warranting further research.

P12-19 | Access to Social Support and Ancestral Medicine as Predictors of Mental Health in Adolescents and Young Adults in an Agricultural Region of Ecuador

Kristina Brandveen[1,2], Rajendra P. Parajuli[2], Briana N. C. Chronister[2], Dolores López-Paredes[3], Jose R. Suárez-López[2]

[1] San Diego State University, School of Public Health, San Diego, USA; [2] University of California, San Diego, Herbert Wertheim School of Public Health, La Jolla, USA; [3] Fundación CIMAS del Ecuador, Quito, Ecuador

Background:
Ancestral medicine (AM) and social support (SS) may improve mental health by alleviating anxiety and depression. However, conflicting findings on the effects of AM on mental health exist. This study explores their associations with mental health outcomes among adolescents and young adults in the agricultural region of Pedro Moncayo, Ecuador.

Methods and Materials:
Participants aged 17–23 years (n = 488) in the 2022 ESPINA assessment were included. Access to SS (1 = Never, 2=Rarely, 3=Sometimes, 4=Frequently; 5 = Always) and AM use (0 = No Use, 1 = Some Use, 2 = Always) were reported via survey. The Generalized Anxiety Disorder-7 (GAD-7) and Beck Depression Inventory-II (BDI-II) were used to measure anxiety and depression symptoms. Linear regression examined the associations between SS and AM with raw GAD-7 and BDI-II scores. Logistic regression estimated the odds ratios (ORs) of having mild, moderate or severe anxiety (GAD-7 score ≥ 5) and depression symptoms (BDI-II score ≥14). All models were adjusted for age, sex, race, education, and monthly household income.

Results:
AM use (vs not) was associated with greater anxiety scores (0.95, 95% CI: [0.25, 1.65]) and an elevated OR of 1.97 (1.34, 2.91) for mild-to-severe anxiety symptoms. SS was inversely associated with anxiety (-0.37 [-0.67, -0.07]) and depression (-2.08 [-2.74, -1.42]) symptoms. Compared with individuals who "rarely, never or sometimes" have SS, individuals who "always or frequently" use SS had lower odds of experiencing mild or greater depression (OR = 0.52 [0.34 to 0.78]) and anxiety (OR = 0.66 [0.45, 0.97]) symptoms. **Conclusion:**
AM use was associated with increased anxiety symptoms, highlighting a possible connection between experiencing anxiety and seeking traditional care in this population. Additionally, our findings highlight the benefits of SS in reducing anxiety and depression symptoms, suggesting that its integration into care practices could improve mental health.

P12-20 | Influence of social and built environment factors on the mental health of women with infertility

Lina Jaalouk[1,2], Myriam Mrad[2,3], Emile Whaibeh[1,2], Kassandra Fares[1,5], Georges Abi Tayeh[4,5]

[1] Saint-Joseph University of Beirut, Institut Supérieur de Santé Publique, Faculty of medicine, Beirut, Lebanon; [2] University of Balamand, Public Health Department, Faculty of Health Sciences, Beirut, Lebanon; [3] Health and Environment Response Agency (HERA), Beirut, Lebanon; [4] Hôtel-Dieu de France University Hospital, Obstetrics and Reproduction, Beirut, Lebanon; [5] Saint Joseph Fertility Center, Beirut, Lebanon

Objective

Infertility is a major worldwide multifactorial health issue that poses considerable mental and emotional challenges. This study aims at exploring the influence of the social and built environment factors on anxiety and depression levels among women with infertility.

Material and Methods

This study included 48 women with infertility issues seeking repeated medical assisted treatment at Saint Joseph Fertility Center in Lebanon. A validated questionnaire was used to record demographic, environmental, and socio-economic data, the General Anxiety Disorder-7 (GAD-7) to quantify anxiety and the Patient Health Questionnare-9 (PHQ-9) to quantify depression. Data was analyzed using STATA. Categorical data are displayed as frequencies and percentages, followed by Chi-Square or Fisher's exact test. Quantitative variables are reported as mean, standard deviation, and median. Significance was set at $p<0.05$.

Results

Participant age at study inclusion was 36.1±5.9 years (median=36). Average GAD-7 score was 8.1±5.2 (median=6) and women were distributed as follows: 14 (29.1 %) had minimal anxiety, 18 (37.5 %) mild anxiety, 9 (18.7%) moderate anxiety and 7 (14.5%) severe anxiety. Average PHQ-9 score was 7.5±5.5 (median=6) and women were distributed as follows: 17 (35.4%) had none-minimal depression, 18 (37.5%) mild depression, 7 (14.5%) moderate depression, 4 (8.3%) moderate-severe depression and 2 (4.1%) severe depression. There was a significant difference in anxiety levels when comparing living conditions (proximity to agricultural areas or to side roads with heavy traffic). Depression levels were also associated with proximity to heavy traffic.

Conclusion

Early findings show that women with infertility experience different levels of depression and anxiety related to surrounding social and built environment factors. In a country with high rates of psychological difficulties, integrating mental health promotion in the comprehensive medical care would greatly benefit women with infertility.

This work is funded by the National Institutes of Health Fogarty International Center (NIH/FIC) Grant #5U2RTW012228 and #U01TW012237

P12-22 | Mixed methods assessment of mental health among adolescents at high risk for CKD of non-traditional origin in Central America

Selene Vences Brown[1], Damaris López-Pilarte[2], Juan José Amador Velasquez[2,3], Samantha M. Hall[1], Magaly R. Amador Sanchez[2], Madeleine K. Scammell[1], Daniel R. Brooks[3], Jessica H. Leibler[1]

[1] Boston University, School of Public Health Department of Environmental Health, Boston, USA; [2] Centro Médico del Pacífico (CENMED), Masaya, Nicaragua; [3] Boston University, School of Public Health Department of Epidemiology, Boston, USA

Chronic kidney disease of uncertain origin (CKDu) is a leading cause of mortality in Central America and is widely considered a climate-associated disease. Known risk factors include occupational heat exposure, although the full disease etiology remains unclear. Research on the mental health burden of the CKDu epidemic among affected communities, including high-risk agricultural communities for whom employment and educational opportunities may be limited, is scant. We assessed frequency of experiencing depressive symptoms within an ongoing prospective cohort study of adolescents living in high-risk regions for CKDu in Nicaragua (n=486) using the Patient Health Questionnaire (PHQ-2). We used open-ended questions to explore concerns about CKDu, occupational health, and employment. Among participants, 14.8% (n=72) scored ≥3 on the PHQ-2, indicating high risk for depression. This finding is consistent with U.S. based estimates for Hispanic young adults. Most participants reported "always" worrying about kidney health of family and friends (n=298; 61.6%) while 28.9% (n=140) expressed concern that their daily activities harmed their kidneys. Nearly 20% (19.8%; n=96) indicated that the epidemic affected their employment decisions, while 9.6% (n=45) noted that the CKD epidemic disrupted their educational plans. Using a systematic inductive thematic qualitative analysis, we identified major themes around employment disruption, including: fear of developing CKDu, inability to work due to disease, and avoiding jobs that may harm the kidneys. Minor themes included limiting physical exertion at jobs with environmental heat exposure and concerns related to mandated occupational health screenings. Themes around educational decisions included concerns for career preparation, financial stability, and negative psychological and emotional impacts. Our findings suggest that the CKDu epidemic affects employment and educational opportunities for young people in affected regions, with implications for financial stability and mental health.

P12-23 | The Effect of Depression on Care: Revisiting the Health Access Resource Behavior Observation and Use Research (HARBOUR I) Cohort Project in the Eastern Caribbean

Steve D. Whittaker[1,2], Darnel Allen[2], Gabrielle Myers[2,1], Deitra Wharton[2,1], Jodie Lyn Watson[2,1], Tyon Slader[2,1], Shania Bidder[2,1]

[1] Clarence Fitzroy Bryant College, Arts Sciences and General Studies, Basseterre, St. Kitts & Nevis; [2] Science Technology Research and Work Study, NA, Basseterre, St. Kitts & Nevis

The World Health Organization (WHO) identifies depression as a leading cause of disability and death. However, despite this observation, health-seeking behaviors, and access to treatment tend to be relatively low among those affected. This study aimed to evaluate how persons who report depressive symptoms in the Eastern Caribbean nation of St. Kitts-Nevis (SKN) engage with the healthcare system, care products, and environmental factors. Surveys were administered to adult SKN residents between 2018 and 2020 with post-hoc open-ended discussions in 2023 to 2025. Of 396 respondents, majority Black (98%), female (57%), single (85%) and under 30 years old (62%), approximately 1 in 4 reported being depressed; less than 1% fully adhering to prescribed treatments. When seeking hospital care, relative to those not depressed, depressed persons were more likely to engage in physical activity when injured (OR 4.86; 95%CI: 2.76 - 9.12) and take medication on an empty stomach (OR: 2.40; 95%CI:1.48 - 4.00). These individuals were also more concerned about privacy (OR: 3.67; 95%CI: 2.16 - 6.50), cleanliness (OR: 2.11; 95%CI: 1.30 - 3.52), the threat of a disease outbreak (OR: 4.45; 95%CI: 2.49 - 8.50) and uncertainties in the aftermath of a storm (OR: 2.58; 95%CI: 1.56 - 4.41). Among 20 post-hoc focus group members, these results proved "surprising", 90% failing to rank depression in the top three of a list of common morbidities. Findings suggest a unique interaction between depressed individuals and health-protective aspects. Novel programs as well as policies need to be implemented to reduce risks, avoid adverse outcomes and improve effective engagement in and around this vulnerable subpopulation.

P12-24 | Associations between asthma and the risk of mental and behavioural problems in males

Nicholas J. Osborne[1,3], Darsy Darssan[1], Christopher Warren[2], **Diana Garcia-Sanchez**[1]

[1] University of Queensland, School of Public Health, Herston, Australia; [2] Northwestern University, Department of Preventive Medicine, Chicago, USA; [3] University of New South Wales, School of Population Health, Kensington, Australia

Objective To investigate the association between asthma and the risk of developing anxiety, depression and hazardous alcohol consumption in Australian men. This study also aims to assess how age influences this association.

Materials and Methods: The study included 16,021 men who participated in *Ten to Men:* The Australian Longitudinal Study on Male Health between 2012 to 2022 (Wave 1- Wave 4). Parental or self-report of lifetime, physicians-diagnosed asthma and the presence of asthma symptoms in the last 12 months defined asthma cases. Logistic regression models and generalized estimating equations (GEE) examined relationships between asthma and

anxiety, depression and hazardous alcohol consumption. Stratified models explored the impact of age on this association, dividing participants into three groups: boys aged 10 to 17 years, young adults aged 18 to 30 years and adults aged 31 to 57 years.

Results: Men with asthma had a higher risk of developing anxiety and depression compared to those without asthma in adjusted GEE models (OR 1.68 (95% CI 1.38, 1.96); OR (1.39 (95% CI 1.11, 1.73), respectively). For those with asthma, the increased risk of anxiety was highest in young adult group (OR 2.15 (95% CI 1.56, 2.96). For adults with asthma there was an increased risk of depression (OR 1.33 (95% CI 1.02, 1.72). Asthma was not associated with hazardous alcohol consumption (OR 0.96 (95% CI 0.84, 1.08).

Conclusion

These population-based survey findings improve understanding of sex-specific impacts of asthma on mental health in the Australian context. Similar to findings in children, Australian adults with asthma were at increased risk of anxiety and depression diagnosis. It might be particular important for Australian males aged 17 years old or older.

P12-25 | Noise Pollution, Sleep Quality and Mental Health of Individuals Living in Communities around Religious Centres in Ibadan, Nigeria

Oyewale M. Morakinyo[1], Martins Jejeola[2]

[1] University of Ibadan, Department of Environmental Health Sciences, Faculty of Public Health, Ibadan, Nigeria; [2] University of Ibadan, Department of Environmental Health Sciences, Faculty of Public Health, Ibadan, Nigeria

Noise pollution is an environmental stressor, particularly in neighborhoods surrounding religious institutions where noise levels may adversely affect mental health and sleep quality. This study determines the effect of noise pollution on mental health and sleep quality among residents living around religious centres in Ibadan, Nigeria.
The study involved noise measurements at selected religious centres using a sound level meter over three days. Measurements were taken at source within the religious centres and at 10 and 20 meters away. A semi-structured questionnaire was administered to 248 randomly selected individuals from neighborhoods with religious centres (Group A) and those without (Group B). Mental health was assessed using the General Health Questionnaire (GHQ-12), while sleep quality was assessed with the Pittsburgh Sleep Quality Index (PSQI). Data were analysed using descriptive statistics, logistic regression, Chi-square and t-test at $\alpha_{0.05}$.
Mean noise levels at source in Group A were significantly higher than those in Group B during daytime (98.7±1.7 dB(A) vs 41.6±0.31 dB(A)) and evening (96.6±3.1 dB(A) vs 41.6±0.51 dB(A)), p<0.001). In Group A, noise level at 10m (97.5±1.11 dB(A)) daytime; 95.6±1.31 dB(A) evening) and 20m (85.7±2.01 dB(A) daytime; 84.3±1.11 dB(A) evening) exceeded the WHO and NESREA standards of 60d dB(A). The GHQ scores were higher in Group A (11.90±5.71) than in Group B (4.08±3.83, p<0.001). Similarly, PSQI scores were worse in Group A (8.6±3.1) than in Group B (5.4±2.6, p<0.001). Higher noise levels (Leq 96.6 dBA, 94.8 dBA) were associated with poor mental health (OR = 2.54, 95% CI: 1.72-3.76, p<0.001) and poor sleep quality (OR = 3.21, 95% CI: 2.13-4.83, p<0.001).
Exposure to elevated levels of noise from religious centres was associated with mental health distress and sleep disruption. Implementing targeted interventions to reduce noise and raising awareness of its detrimental effects would promote good mental well-being and sleep quality.

P13 | Environmental Chemical Exposures and Health

P13-01 | Characterizing Polycyclic Aromatic Hydrocarbon and Chemical Exposure Mixtures for Individuals using Solid Fuels in Low- and Middle- Income Countries

Sigride Asseko[1], Sarah Rothenberg[2], Molly Kile[2], Adam Branscum[2], Nordica MacCarty[5], Nicole Ngo[6], Perry Hystad[7]

[1] California State University-Sacramento, Department of Environmental Studies, Sacramento, USA; [2] Oregon State University, College of Health, Corvallis, USA; [3] Oregon State University, College of Health, Corvallis, USA; [4] Oregon State University, College of Health, Corvallis, USA; [5] Oregon State University, College of Engineering, Corvallis, USA; [6] University of Oregon, School of Planning, Public Policy and Management, Eugene, USA; [7] Oregon State University, College of Health, Corvallis, USA

Background: Over 2.8 billion people rely on solid fuels for cooking and heating, resulting in high levels of air pollution exposure. This exposure has been well quantified in terms of fine particulate matter ($PM_{2.5}$) concentrations, but not as a complex chemical mixture.
Objective: To characterize chemical exposures from cooking (solid and clean fuels) in low- and middle- income countries using silicone wristbands.
Materials and Methods: This pilot study asked 40 people to wear a silicone wristband for 48 hours. They were located in five distinct rural regions in 4 low- and middle- income countries (China, Colombia, India, Zimbabwe). Wristbands were analyzed for 62 polycyclic aromatic hydrocarbons (PAHs) and screened for 1,500 organic chemicals (OCs). We characterized the number and type of PAHs and OCs detected and examined correlations between PAHs, OCs, and monitor-based $PM_{2.5}$. Linear regression models examined differences by cooking fuel type, region, and sex.
Results: On average, 45 PAHs and 79 OCs were detected in the wristbands worn by individuals using solid fuels for cooking compared to 44 PAHs and 76 OCs for individuals cooking with clean fuels. PAH concentrations were highest for solid fuel users, especially for 5- and 6- ring PAHS: 44 and 8 ng/g for solid fuels and 17 and 3 ng/g for clean fuels, respectively. Individuals cooking with solid fuels had higher exposure to dioxins/furans, flame retardants, pesticides, and pharmaceutical related chemicals compared to those using clean fuels. Differences in PAHs and OCs were observed by geographic region and sex.
Conclusion: Passive silicone wristbands are useful for characterizing complex chemical mixtures associated with cooking and could distinguish between solid fuels and clean fuels. Individuals who use solid fuels for cooking have higher exposures to PAHs, but exposure to OCs varied by sources.

P13-02 | Prenatal exposure to environmental chemicals and academic achievement during adolescence

Dean A. Marengi[1], Marc G. Weisskopf[1,2], Brent A. Coull[1,3], Maitreyi Mazumdar[1,4], Susan A. Korrick[1,5]

[1] Harvard T.H. Chan School of Public Health, Department of Environmental Health, Boston, USA; [2] Harvard T.H. Chan School of Public Health, Department of Epidemiology, Boston, USA; [3] Harvard T.H. Chan School of Public Health, Department of Biostatistics, Boston, USA; [4] Boston Children's Hospital, Department of Neurology, Boston, USA; [5] Brigham and Women's Hospital, Harvard Medical School, Channing Division of Network Medicine, Boston, USA

Objective: Academic achievement is a strong predictor of future well-being. Prenatal metal and organochlorine exposures have been associated with adverse neurodevelopment, but few studies directly examine associations with academic achievement. We assessed the relationships of prenatal metal and organochlorine exposures with academic achievement and potential effect modification by sex and socioeconomic disadvantage.

Materials and Methods: Participants were 371 New Bedford Cohort (NBC) adolescents born (1993-1998) to mothers residing in towns surrounding a Superfund Site in Massachusetts and who underwent periodic neuropsychological evaluations. Cord blood biomarkers of prenatal exposure to metals [manganese (Mn), lead (Pb)] and organochlorines [dichlorodiphenyldichloroethylene, hexachlorobenzene, polychlorinated biphenyls] were analyzed. Academic achievement was assessed using the Wide Range Achievement Test, 4th edition (WRAT-4) with scores standardized to a mean (SD) of 100 (15). A prenatal social disadvantage index was derived using maternal age and marital status, parental education, and household income. We used covariate-adjusted linear regression models, including all exposures, to examine associations with math and reading scores and interaction terms to assess effect modification.

Results: Mean (SD) age at testing was 15.5 (0.6) years with 52% female, 71% identified as non-Hispanic white, and 30% born in low-income households. Exposure biomarker levels were similar to the general U.S. population. An interquartile range increase in cord blood Mn was associated with a 1.41-point decrease in math scores (95% CI: -2.86, 0.05). We observed a similar, although weaker, association between Mn and reading. There were no adverse associations of Pb or organochlorines with WRAT-4 scores. Effect modification analyses demonstrated an adverse Mn-math association among females (β= -3.03; 95% CI: -5.09, -0.97) but not males (0.29; 95% CI: -1.83, 2.41). Associations between Mn and lower achievement were, paradoxically, stronger among adolescents with less prenatal disadvantage.

Conclusion: Our findings support the potential for prenatal Mn exposure to adversely impact adolescent academic achievement.

P13-03 | *In vitro* bioaccessibility assessment of metals in soil as a tool for the evaluation of the exposure in children

Lucía Falchi[1,2], Fiorella Iaquinta[1,2], Carolina de los Santos[3]

[1] Universidad de la República, Analytical Chemistry Area, Chemistry Faculty, Montevideo, Uruguay; [2] Universidad de la República, Analytical Chemistry Environmental Group, Chemistry Faculty, Montevideo, Uruguay; [3] Universidad de la República, Physicochemistry Area, Chemistry Faculty, Montevideo, Uruguay

Understanding the values of potentially toxic metals in soils is of utmost relevance, especially in areas where children have direct contact, such as educational centers or public parks. Even though the total concentration of these elements provides relevant information, *in vitro* bioaccessibility assays allow the evaluation of the ability of organisms to extract metals from the soil in the gastrointestinal tract. Therefore, these assays provide complementary information to the total concentrations in environmental exposure studies. In the present work, an *in vitro* bioaccessibility assay of potentially toxic metals was carried out on soils from an educational center for children aged 3 to 5 years. For this task, simulated gastric and intestinal solutions were prepared and added to 1 g of soil sample. The solution was incubated for 4 hours at 37°C with constant agitation, following an adapted INFOGEST protocol.

The bioaccessible fractions obtained were 1.50 - 28.38% for lead, 0.85 - 13.23% copper, 27.75 - 38.75% manganese and 0.33 - 2.61% chromium. It is interesting to highlight that, of the pool of samples evaluated, the variation in manganese was not significant, meanwhile for the other three metals, the samples with a higher bioaccessible fraction showed this tendency simultaneously. Therefore, a thermogravimetric analysis was carried out, which revealed a difference in the relative percentage of the phases present in the samples. This suggests that these phase amounts may be affecting the bioaccessibility of chromium, lead, and copper. These findings are promising as they present a potential mitigation strategy for soil exposure by adding organic matter, such as compost or soil, which could act as a metal binder in soils.

P13-04 | A dynamic toxicokinetic model for plasma p,p'-dichlorodiphenyldichloroethylene concentrations in obese adults

Yerin Jung[1], Adrian Covaci[2], Philippe G. Jorens[3], Scott M. Bartell[1]

[1] University of California, Irvine, Department of Environmental and Occupational Health, Joe C. Wen School of Population & Public Health, Irvine, USA; [2] University of Antwerp, Toxicological Centre, Antwerp, Belgium; [3] Antwerp University Hospital, Department of Intensive Care Medicine and Clinical Pharmacology, Edegem, Belgium

Objective: p,p'-dichlorodiphenyldichloroethylene (p,p'-DDE), a potential obesogen, may impede weight loss efforts in people with obesity through its mobilization from adipose tissue into plasma. We developed and validated a simple physiology-based toxicokinetic (TK) model to predict organs and plasma p,p'-DDE concentrations in obese adults during weight loss.

Materials and Methods: Our dynamic TK model for DDE incorporates six compartments: liver, subcutaneous adipose tissue, visceral adipose tissue, richly-perfused tissues, poorly-perfused tissues, and blood. The model assumed that weight loss primarily affects visceral adipose tissue volume, which releases stored p,p'-DDE into the blood. Model validation used clinical data from obese individuals at a weight management clinic (baseline, n=151),

with follow-up measurements at three (n=69), six (n=46) and twelve months (n=36). We ran the TK model incorporating the body-weight changes of this cohort over 12 months period. We back-calculated individual daily p,p'-DDE intake rates (normalized to body weight) from baseline plasma levels and assumed that they were constant throughout the simulation period.

Results: Median body-mass index (BMI) decreased from 38.5 to 31.1 kg/m^2 over the 12 months period, resulting in increased predicted and measured p,p'-DDE concentrations across all six compartments. Reconstructed p,p'-DDE intake rates ranged from 0.0008 to 0.75 µg/kg-bw/day (median = 0.010 µg/kg-b.w./day). Spearman correlation coefficients between predicted and measured plasma p,p'-DDE levels and levels are 0.74, 0.75, and 0.71 at three, six, and twelve months, with model predictions within two-fold of measured values for 97%, 98%, and 86% of participants, respectively.

Conclusion: The model effectively approximates plasma p,p'-DDE concentrations for obese individuals during weight loss. Incorporating individual-level adipose tissue p,p'-DDE measurements, dietary information, and body fat composition data could improve model fits even further.

This study was funded by National Institutes of Health (NIH) and the University of Antwerp.

P13-05 | Integrated Risk Assessment of heavy metals (Pb, Hg, Cd) for KoreansIntegrated Risk Assessment of heavy metals (Pb, Hg, Cd) for Koreans

Daehwan Kim, Gihong Min, Youngtae Choe, Sanghoon Lee, Wonho Yang

daegu catholic university, Department of Health and Safety, daegu, South Korea

Introduction Heavy metals existing in the environment are exposed through various exposure routes such as oral, inhalation, and skin contact, and can cause health effects by accumulate in the human body. This study aims to assess the exposure and risk of the entire population, including children and the elderly who are more vulnerable to heavy metal toxicity. It seeks to present the risk and contribution rate based on major exposure routes.

Material and Methods This study collected toxicity values and exposure factors for three heavy metals across all age groups through a literature review. Exposure assessments were conducted using Monte-Carlo simulation, considering the mean, standard deviation, and distribution model of exposure factors for each exposure route (inhalation, dermal, and ingestion) by age group and exposure algorithm. The risk assessment applied toxicity reference values to the exposure assessment results to calculate the hazard index (HI) and cancer risk (CR) for each exposure route and to determine the contribution rate of risk.

Results The carcinogenic risk of lead was 0.26% in 0-2 year olds and 21.76%-27.77% in the adult age group, while cadmium is a carcinogenic risk in all age groups. The health risk of mercury was 3.53% in 0-2 year olds. The contribution of the three heavy metals was mostly due to food intake. Lead and mercury showed a high risk contribution mainly from the consumption of agricultural and seafood products, while approximately 80% of cadmium exposure was attributed to seafood consumption.

Conclusion Lead exposure from soil and settled indoor dust contributed to some extent in the children's age group. Smoking rates should be reduced through anti-smoking campaigns to reduce exposure to lead and cadmium. Dietary

mitigation measures might reduce exposure to heavy metals by reducing the consumption of large fish and removing the guts from cephalopods.

P13-06 | Risk Assessment of Environmental Pollution and Its Impact on Health near a Mixed Waste Landfill

Youngtae Choe, Gihong Min, Sanghoon Lee, Daehwan Kim, Wonho Yang

Daegu catholic university, Changwon-si, South Korea

Objective :Landfills contribute to environmental pollution by contaminating soil, water, and air. While BTEX (benzene, toluene, ethylbenzene, and xylene) exposure in urban and industrial settings is well-documented, studies on BTEX levels and health risks from landfill remain limited. Nevertheless, research on contamination in surrounding communities remains insufficient. This study assesses pollutants from landfill leachate and their potential health risks in adjacent residential areas.
Methods :Environmental sampling was conducted at six landfill-affected and two control sites across three seasons (winter, spring, and summer). BTEX and heavy metals in water, soil, and sediment were sampled seasonally, while odor compounds were analyzed in spring and summer. BTEX and odor compounds were collected during the day (14:00–16:00) and night (20:00–22:00) with 18 samples per site. Water, soil, and sediment samples were collected once per season. Health risks were assessed by calculating excess lifetime cancer risk (ELCR) and hazard quotient (HQ).
Results :Mean BTEX concentrations showed no significant difference, but peak levels were higher in landfill-affected areas. BTEX posed a low non-carcinogenic risk (HQ < 1), but benzene and ethylbenzene exceeded the ELCR threshold of 1.0E-06. HQ values for ammonia (25.04) and hydrogen sulfide (20.88) significantly exceeded the acceptable threshold of 1, suggesting potential non-carcinogenic health risks. Water samples from landfill-affected areas contained significantly higher sodium (Na) and total organic carbon (TOC) levels than those from control sites, indicating substantial water contamination.
Conclusion : Based on average concentrations, the ELCR for benzene and ethylbenzene was 3 and 6 cases per million. In the worst-case scenario with 95th percentile concentrations, the risks increased to 13 and 28 cases per million. Odor compound concentrations exceeded regulatory thresholds in both landfill and residential areas, suggesting potential health concerns. Elevated Na and TOC levels indicate significant water contamination, underscoring the need for continued monitoring and mitigation efforts.

P13-07 | Cannabis-dependent associations of prenatal tobacco and insecticide metabolites with child neurobehaviors among African Americans

Neha Sehgal[1], Patricia Brennan[2], Anne Dunlop[3], Donghai Liang[1], Elizabeth Corwin[4], Youran Tan[1], Todd Everson[1,5], William M. Caudle[1], Laura Stroud[6], Parinya Panuwet[1], Priya D'Souza[1], Volha Yakimavets[1], Grace Lee[1], Dana B. Barr[1], Stephanie Eick[1,5]

[1] Emory University, Gangarosa Department of Environmental Health, Rollins School of Public Health, Atlanta, USA; [2] Emory University, Department of Psychology, Atlanta, USA; [3] Emory University, Department of Gynecology and Obstetrics, School of Medicine, Atlanta, USA; [4] Columbia University, School of Nursing, New York City, USA; [5] Emory University, Department of Epidemiology, Rollins School of Public Health, Atlanta, USA; [6] Brown University, Providence, USA

Objective: Cannabis and tobacco are widely used during pregnancy in the U.S. but are contaminated with insecticides, raising concerns for co-exposures and compounded neurodevelopmental vulnerabilities. However, the cumulative effect remains unexplored. We examined cumulative associations of prenatal urinary metabolites of cannabis, tobacco, pyrethroid, and organophosphate insecticides with neurobehavior in early childhood.

Methods: During 2014-2022, among 197 African American mother-child pairs in Atlanta, Georgia, levels of cannabis (THCOOH), tobacco (COT and 3OH-COT), pyrethroid (3PBA), and organophosphate (TCPY) metabolites were quantified in maternal urine sampled at 8-14- and 24-30-weeks gestation. Infant arousal and attention were evaluated using the NICU Network Neurobehavioral Assessment Scale 2 weeks postnatally. Externalizing and internalizing behaviors were assessed using the Child Behavior Checklist and averaged across ages 1 to 5. We estimated individual effects using linear regressions and cumulative effects using quantile g-computation and Bayesian kernel machine regression. Further, we examined tobacco and insecticide's effect modification by sex and cannabis exposure.

Results: Prenatal urinary insecticide metabolite levels were strongly individually associated with child neurobehaviors. Specifically, 3PBA was positively related to internalizing behaviors (β= 18.10%; 95% CI= 0.00%, 39.48%), and TCPY was negatively related to externalizing behaviors (β= -12.94%; 95% CI= -27.80%, 4.98%). These associations were both modified by prenatal THCOOH levels and were stronger among females. TCPY was more strongly negatively associated with externalizing behaviors among those exposed with detectable THCOOH (β= -26.8%; 95% CI= -42.96%, -6.05%) compared to those without (β= -4.07%; 95% CI= -30.74%, 31.96%). Similarly, the mixture of tobacco and insecticide metabolite levels was negatively associated with externalizing behaviors among females with detectable prenatal THCOOH levels (quantile g-computation β= -46.78%; 95% CI= -70.47%, -4.08%).

Conclusion: Prenatally, 3PBA and TCPY were associated with neurobehaviors in a sex-specific manner and differed among those co-exposed to cannabis, as measured by THCOOH.

P13-08 | The Reproductive Toxicity of Benzyl Alkyl Dimethyl Ammonium Chlorides and Didecyl Dimethyl Ammonium Chloride: A Systematic Review

Abena BakenRa[1], Emily Lasher[1], Nicholas Chartres[2], Rashmi Joglekar[1], Tracey Woodruff[1]

[1] University of California, San Francisco, Program on Reproductive Health and the Environment, San Francisco, USA; [2] University of Sydney, Sydney, Australia

Background

Quaternary ammonium compounds (QACs) are a chemical class comprised of hundreds of chemicals and mixtures that primarily function as antimicrobials. Two QACs, benzyl alkyl dimethyl ammonium chlorides (BACs) and didecyl dimethyl ammonium chloride (DDAC), are often researched together due to their combined use in antimicrobial products and similar hazard profiles. Their use is widespread and has been linked to several reproductive toxicity effects. To date, no systematic reviews have evaluated the evidence using best practices combining data from multiple evidence streams.

Objective

To fill this gap, this systematic review evaluates the reproductive toxicity of BACs and DDAC and will be a proof of concept for a framework to integrate new approach methodologies (NAMs) into hazard assessment.

Methods

We pre-defined our methods in a published protocol. We searched PubMed, Embase, Biosis Previews, Web of Science, pre-print servers, and grey literature for human, animal, and NAMs evidence evaluating the reproductive toxicity of exposure to BACs and/or DDAC. We will conduct a risk of bias assessment. We will then perform a meta-analysis for sufficiently homogenous data. Additionally, we will perform a dose-response assessment and rate the quality and strength of the evidence. Lastly, we will integrate the evidence streams to produce a final bottom-line statement regarding human reproductive toxicity to BACs and DDAC.

Results

Our literature search identified 4,986 studies. Our systematic review will quantify the effect of BACs and DDAC on reproductive toxicity through meta-analyses and dose-response analysis and rate the quality and strength of the evidence.

Conclusion

The findings of this systematic review establish what is known about the reproductive effects of exposure to BACs and DDAC and act as a proof of concept for a framework for integrating NAMs as an evidence stream into hazard assessment.

This work is supported by California's Office of Environmental Health Hazard Assessment.

P13-09 | DDT and DDE associated with executive function and frontal cortical thickening in middle-age CHAMACOS Latinas

Brenda Eskenazi[1], Yishu Chao[2], Lucia Calderone[3,1], Stephen Rauch[1], Katherine Kogut[1], Robert Gunier[1], Nina Holland[1], Marcella Warner[1], Weihong Guo[1], Hector Gonzalez[4], Julianna Deardorff[1], William Jagust[2], Jacqueline Torres[3,1]

[1] UC Berkeley, Public Health /Center for Environmental Research and Community Health (CERCH), Berkeley, USA; [2] UC Berkeley, Helen Willis Neurosciences Institute, Berkeley, USA; [3] UC San Francisco, Public Health/ Division of Epidemiology and Biostatistics, San Francisco, USA; [4] UC San Diego, School of Medicine, La Jolla, USA

Objective: To examine the relationship of p,p´-dichlorodiphenyltrichloroethane (DDT) and p,p´-dichlorodiphenyldichloroethylene (DDE) serum levels with cognitive functioning and brain magnetic resonance imaging (MRI) measures in middle-aged Latina women. DDT/E has been associated with poorer neurodevelopment in children and with Alzheimer’s disease (AD), possibly by impacting the amyloid pathway.

Material and methods: Women (N=472) participating in the CHAMACOS Maternal Cognition Study completed a neurocognitive battery (SOL-INCA2) assessing immediate and delayed verbal recall, attention, working memory, and executive functioning. DDT and DDE concentrations were measured in blood collected 13 years previously. T1-weighted structural brain MRIs were completed on a subset (n=96), and cortical thickness was calculated in FreeSurfer-defined regions of interest. We assessed associations between log10 DDT/DDE concentrations and cognitive measures and MRI cortical thickness using linear regression models, controlling for Apo-E genotype and other covariates.

Results: Women averaged 48.9 (±5.5) years old; most were Mexican born; 19.5% carried an e4 Apo-E allele. DDT and
DDE were detected in 97% and 100%, respectively. Each 10-fold increase in lipid-adjusted DDT and DDE concentrations was associated with a 0.10 standard deviation (95% CI=-0.18, -0.02) and a 0.09 standard deviation (95% CI=-0.19, 0.00) lower executive function score, respectively. We observed no associations with other measures of cognitive functioning. DDT/DDE were significantly associated with increasing age-adjusted thickening of the bilateral frontal cortex, specifically in the medial orbitofrontal cortex and the pars orbitalis (r=0.2 to 0.4, p=0.001 to 0.05). Medial orbitofrontal thickening was also associated with poorer executive function (r=-0.21, p=0.03).

Conclusion: DDT, a legacy pesticide with a long half-life, may impact neuropsychological functioning and brain structure among middle-aged women decades after exposure. Cortical thickening could reflect developmental effects of pesticide exposure.

P13-10 | Multi-statistical approach unraveling impact of prenatal metal mixtures on birth outcomes

Surabhi Shah[1], Jongmin Oh[1,2], Hwan-Cheol Kim[3], Kyoung Sook Jeong[4], Dong-Wook Lee[3], Kyung A Lee[5], Yun-Chul Hong[10,6], Eunhee Ha[1,7], Ko-CHENS Study group[8,9]

[1] Department of Environmental Medicine, College of Medicine, Ewha Womans University, Seoul, South Korea; [2] Department of Human Systems Medicine, College of Medicine, Seoul National University, Seoul, South Korea; [3] Department of Occupational and Environmental Medicine, College of Medicine, Inha University Hospital, Inha University, Incheon, South Korea; [4] Department of Occupational and Environmental Medicine, College of Medicine, Wonju Severance Christian Hospital, Yonsei University, Wonju, South Korea; [5] Department of Obstetrics and Gynecology, Ewha Womans University College of Medicine, Seoul, South Korea; [6] Institute of Environmental Medicine, Medical Research Center, Seoul National University, Seoul, South Korea; [7] System Health & Engineering Major in Graduate School (BK21 Plus Program), Ewha Womans University, Seoul, South Korea; [8] Core Center, Incheon, Sejong, South Korea; [9] Supportive Center, Seoul, South Korea; [10] Seoul National University Bundang Hospital, Seongnam-Si, South Korea

Objective:
Developing fetus is more susceptible to environmental pollutants like heavy metals. Adverse birth outcome like low birth weight has been considered as indicator of growth restriction for fetuses and adverse health outcomes in long term. In this study, we evaluate the exposure to mixture of heavy metals on birth outcome comparing different advance mixture analysis methods.

Material and Methods:
We analyzed 4,382 pregnant women in the Korean CHildren's ENvironmental health Study (Ko-CHENS), a prospective birth cohort that recruited pregnant women from 2015–2019 and has been following up their children . Early pregnancy, late pregnancy and cord blood heavy metals: lead, mercury and cadmium were examined after log transformation. Birth outcomes: birth weight was considered. Generalized linear model, Bayesian kernel machine regression and weighted quantile sum regression were applied to evaluate associations between mixture of prenatal heavy metals exposure and birth outcomes.

Results:
Linear models showed a negative association between late pregnancy lead (β= -52.97, 95% CI: -82.31 to -23.62) and cadmium (β= -37.59, 95% CI: -57.72 to 10.50) on birth weight. Weighted quantile sum regression confirmed a negative association between prenatal heavy metal exposure and birth weight, with late pregnancy lead as the primary contributor (β= -0.03, 95% C.I. -0.04, -0.01) followed by late pregnancy cadmium (β=-0.02, 95% C.I. -0.03, -0.08). Bayesian kernel machine regression revealed a non-linear and negative association between prenatal heavy metal mixtures and birth weight with late pregnancy lead having the highest posterior inclusion probability (0.99).

Conclusion:
Our multi statistical approach showed that prenatal exposure to mixture of heavy metal exposure, particularly lead and cadmium during late pregnancy period affects birth weight.

Acknowledgement: This work supported by a grant from the National Institute of Environmental Research (NIER-2019-03-01-001), funded by the Ministry of Environment (MOE), Republic of Korea.

P13-11 | Associations Between Bisphenol Exposure and Thyroid Function: A Cross-Sectional Study in Adults

Yen-Ting Hsu[1], Po-Chin Huang[2], Yuan-Ting C. Lo[1], **Han-Bin Huang**[1]

[1] National Defense Medical Center, School of Public Health, Taipei, Taiwan; [2] National Health Research Institutes, National Institute of Environmental Health Sciences, Miaoli, Taiwan

Background
Bisphenol A (BPA) has been regulated in many countries, leading to the development of substitutes such as bisphenol S (BPS) and bisphenol F (BPF). However, there have been several concerns about the health effects of BPS and BPF on thyroid function in adults. This study aims to evaluate the associations between urinary concentrations of BPA, BPS, and BPF and markers of thyroid function.

Methods
This cross-sectional study utilized data from the Taiwan Environmental Toxins Database (N= 262 adults). Thyroid function markers, including triiodothyronine (T3), thyroxine (T4), and free thyroxine (FT4), were measured in blood samples, while urinary concentrations of BPA, BPS, and BPF were quantified. Daily intake (DI) of these bisphenols was estimated. Generalized linear models (GLMs) were employed to assess the associations between bisphenol concentrations, DI, and thyroid function markers. Additionally, a quantile g-computation (qgcomp) model was applied to evaluate the combined effects of bisphenol exposure on thyroid function.

Results
BPA concentrations were positively associated with FT4 (β = 0.027, 95% CI: 0.002, 0.053) and SPINA-GT (β = 0.079, 95% CI: 0.009, 0.148). Both DI_{BPA} and DI_{BPS} exhibited a positive association with T_4 (β = 0.054, 95% CI: 0.016, 0.091 for BPA; β = 0.039, 95% CI: 0.006, 0.072 for BPS), whereas they were negatively associated with the T_3/T_4 ratio (β = -0.047, 95% CI: -0.092, -0.001 for BPA; β = -0.052, 95% CI: -0.097, -0.007 for BPS). Additionally, DI_{BPA} was inversely associated with the TSH/T_4 ratio (β = -0.144, 95% CI: -0.265, -0.024) but positively associated with SPINA-GT (β = 0.115, 95% CI: 0.033, 0.198). In the qgcomp model, combined exposure to BPA, BPS, and BPF was positively associated with T_4 (ψ = 0.025, 95% CI: 0.001, 0.050).

ConclusionThe findings indicate that environmental exposure to BPA, BPS, and BPF may affect thyroid hormone homeostasis in adults.

P13-12 | Variability in urinary biomarkers of oxidative stress among adolescents: implications for research in environmental epidemiology

Katarzyna Kordas[1], Matthew R. Bonner[1], Elena I. Queirolo[2], Diala Ghazal[1], Patrick J. Parsons[4,5], Christopher D. Palmer[4,5], María I. Beledo[2], Richard W. Browne[3]

[1] University at Buffalo, Department of Epidemiology and Environmental Health, Buffalo, USA; [2] Catholic University of Uruguay, Department of Neuroscience and Learning, Montevideo, Uruguay; [3] University at Buffalo, Department of Biotechnical and Clinical Laboratory Sciences, Buffalo, USA; [4] New York State Department of Health, Division of Environmental Health Sciences, Wadsworth Center, Albany, USA; [5] University at Albany, Department of Environmental Health Sciences, College of Integrated Health Sciences, Albany, USA

Objective: Despite interest in oxidative stress (OS) as the underlying mechanism linking environmental exposures and chronic diseases, it is unclear how OS variability affects the relationship between environmental factors and OS. This study characterized urinary F2-isoprostanes and 8-OHdG levels measured 10 times across 1 year and explored the potential implications of OS marker variability on inferences in environmental epidemiology studies.
Materials and Methods: Samples were taken from 100 adolescents from Montevideo, Uruguay every 6 weeks. F2-isoprostanes were measured in duplicate following solid phase extraction (SPE), using ELISA kits. 8-OHdG were analyzed without prior SPE, in triplicate, utilizing ELISA kits. Final sample included 80 children (siblings excluded) with 789 observations across 10 waves. We calculated the median [IQR] concentrations across and Pearson inter-correlations among study waves. Blood lead (BLL) was measured using ICP-MS in samples collected ~6 weeks prior to first urine sample. We selected six predictors often applied in statistical models as time-unvarying covariates (sex, maternal education, SES, neighborhood sociability, overweight status, BLL [correlation between years is >0.8 in our study]). A series of regressions tested association of each predictor with specific gravity-adjusted, log-transformed OS biomarker at each wave, adjusting for month of sampling.
Results: Study participants (54% girls) were ~11.1 years old. F2-isoprostane and 8-OHdG concentrations were highly variable overall and by sex. Pearson correlations ranged -0.09-0.55 for F2-isoprostanes and -0.05-0.44 for 8-OHdG. Sex and SES were consistent (³80% of models) in the direction of their associations with OS biomarkers. Higher BLLs were negatively associated with OS markers in ³70% of models. For the remaining predictors, the direction of associations was mixed. Magnitude of these associations varied highly.
Conclusions: OS markers in adolescents vary considerably across a year of study, possibly influencing the direction of observed associations with predictors, and inferences made in epidemiological studies.

P13-13 | Human biomonitoring of the UV filters octocrylene, 2-ethylhexyl salicylate and homosalate in Australian pooled urine samples.

Katharina E. Ebert[1], Danielle E. Que[2], Thomas Brüning[1], Holger M. Koch[1], Jochen F. Müller[2,3], Daniel Bury[1]

[1] Ruhr University Bochum, Germany, Institute for Prevention and Occupational Medicine of the German Social Accident Insurance (IPA), Bochum, Germany; [2] The University of Queensland, Queensland Alliance for Environmental Health Sciences (QAEHS), Brisbane, Australia; [3] Minderoo Centre – Plastics and Human Health, Brisbane, Australia

Ultraviolet (UV) radiation is commonly considered the main cause for skin cancer. In the light of changing climatic conditions, the importance of sufficient protection against the harmful effects of UV radiation is increasing further. Sunscreens are an important tool enabling the protection of areas not easily covered by clothing, such as the face and neck. However, potential health effects caused by several commonly used UV filters are currently being discussed. As sunscreens are required to be safe, this necessitates accurate exposure and risk assessments. Human biomonitoring (HBM) is the preferred method for exposure assessments of substances with predominantly dermal exposures, and both analytical methods and health-based HBM guidance values are available for several UV filters. For several decades, the Australian population has been sensibilized to the dangers of UV radiation, and regular sunscreen application has been encouraged. In this study, we investigated the concentrations of specific metabolites of the UV filters octocrylene, 2-ethylhexyl salicylate, and homosalate in Australian pooled urine samples stratified by age, gender, and sample collection year (187 pools representing 18,700 individuals). The results are compared to the results of previous population studies and available health-based HBM guidance values (German HBM-I values). Furthermore, differences between the different pools are statistically analysed.

P13-14 | Effect of prenatal arsenic exposure on childhood body mass index: a mediation analysis of birth weight.

María P. Muñoz[1], Verónica Iglesias[2], Paola Rubilar[3], Paulina Pino[2], Claudia López[4]

[1] Universidad de Chile, Programa de Doctorado en Salud Pública, Escuela de Salud Pública, Facultad de Medicina, Santiago, Chile; [2] Universidad de Chile, Programa de Epidemiología, Escuela de Salud Pública, Facultad de Medicina, Santiago, Chile; [3] Universidad del Desarrollo, Centro de Epidemiología y Políticas de Salud, Facultad de Medicina Clínica Alemana, Santiago, Chile; [4] Universidad de Chile, Escuela de Salud Pública, Facultad de Medicina, Santiago, Chile

Background and aim: Prenatal arsenic exposure has been linked to adverse effects on child growth, including changes in body mass index (BMI). However, whether the birth weight z-score for gestational age mediates this relationship is unclear. This study aims to estimate the total effect of prenatal arsenic exposure on children's BMI z-score and assess the potential mediating effect of birth weight z-score for gestational age.
Methods: A retrospective cohort study was conducted using secondary data from two previous studies. The first included 1,644 pregnant women who gave birth at the regional hospital of Arica between 2013 and 2016, providing information on urinary inorganic arsenic concentration, birth weight, gestational age, and covariates. The second

study evaluated their children (n=451), collecting BMI z-score data. A causal mediation analysis was performed to decompose the total effect into direct and indirect components. The log-transformed prenatal urinary inorganic arsenic concentration was the exposure, BMI z-score was the outcome, and the z-score of birth weight for gestational age was the mediator. Linear regression models were adjusted for confounders (maternal age, education, pre-pregnancy BMI, and ethnicity).
Results: The median age of the children was 8 years (IQR: 7-8), and the median prenatal arsenic concentration was 16 µg/L (IQR: 10-23). The median birth weight was 3,470.0 g (IQR: 3,130.0-3,740.0), and the gestational age was 39.0 weeks (IQR: 38.0-40.0). A total effect of arsenic on BMI z-score was observed ($\beta=-0.218$; 95% CI: -0.39 to -0.06), with a significant direct effect ($\beta=-0.199$; 95% CI: -0.37 to -0.04). However, the indirect effect through birth weight z-score for gestational age was not significant ($\beta=-0.019$; 95% CI: -0.06 to 0.02).
Conclusion: Prenatal arsenic exposure negatively affects child BMI primarily through direct effects, independent of birth weight. These findings highlight the importance of early interventions to reduce exposure and mitigate adverse effects on growth.

P13-15 | E-Waste and Flame Retardant Exposure: A Comparative Risk Assessment in Lagos, Nigeria, and Birmingham, UK

Temilola Oluseyi[1], Moyofoluwa Ogunyemi[2], Aderonke Oyeyiola[3], Mohamed Abdallah[4], Stuart Harrad[5]

[1] University of Lagos, Department of Chemistry, Lagos, Nigeria; [2] University of Lagos, Department of Chemistry, Lagos, Nigeria; [3] University of Lagos, Department of Chemistry, Lagos, Nigeria; [4] University of Birmingham, School of Geography, Earth and Environmental Sciences, Birmingham, UK; [5] University of Birmingham, School of Geography, Earth and Environmental Sciences, Birmingham, UK

In developing countries, informal processing of electronic waste (e-waste) leads to the release of persistent organic pollutants (POPs), posing environmental and health risks. E-waste serves as a major source of hazardous chemicals, necessitating urgent regulatory measures. This study assessed contamination levels of 8 polybrominated diphenyl ethers (PBDEs) and 8 organophosphate esters (OPEs) in 96 soil, dust, and air samples from the Alaba e-waste dumpsite in Lagos, Nigeria, and a multi-waste site in Birmingham, UK. Samples underwent ultrasonication extraction, solid-phase purification, and quantification using ISQ and Agilent GC/MS. Findings showed significantly higher contamination in Alaba than Birmingham. Mean $\sum_8$ PBDEs and $\sum_8$ OPEs in Alaba were 23,500 ng/g and 156,000 ng/g, respectively, compared to 772 ng/g and 18,700 ng/g in Birmingham. In Alaba, mean $\sum_8$ PBDEs in soil, dust, and air were 1,600 ng/g, 3,500 ng/g, and 19 ng/m³, respectively, while $\sum_8$ OPEs measured 1,300 ng/g, 9,800 ng/g, and 15 ng/m³. Dust had the highest contaminant levels, marking it as a key reservoir. BDE-209 was the dominant congener across samples. Soil from burning sites had higher FR levels than dismantling sites. In air, PBDEs (518.26 ng/m³) exceeded OPEs (298.40 ng/m³), with indoor concentrations exceeding outdoor levels. Despite high contamination in Alaba, levels were within the global range. Ingestion was the primary exposure pathway. Hazard quotient values for ingestion, inhalation, and dermal contact were below 1, suggesting low non-carcinogenic risk. These findings emphasize the need for stricter regulations and improved e-waste management to mitigate health and environmental hazards.

P13-16 | Associations of volatile organic compounds exposures with the risk of obstructive sleep apnea in Korean adults

Hyunah Son[1], Heeseon Jang[2], Hyunji Park[1], Changsoo Kim[2,3]

[1] Yonsei University College of Medicine, Department of Public Health, Seoul, South Korea; [2] Yonsei University College of Medicine, Department of Preventive Medicine, Seoul, South Korea; [3] Yonsei University College of Medicine, Institute for Environmental Research, Seoul, South Korea

BACKGROUND AND AIM: Evidence suggests that volatile organic compounds (VOCs) exposure has adverse effects on pulmonary health. However, no studies have examined the effect of VOC exposures on obstructive sleep apnea (OSA). Therefore, this study investigated the association between VOCs exposures and OSA risk in adults.
METHOD: A total of 916 adults (aged 40 years and over) were included from the 2020−2021 Korea National Health and Nutrition Examination Survey. The OSA risk was determined using the STOP-Bang questionnaire and with participants categorized into low-risk and high-risk groups. The STOP-Bang score was classified as high risk for OSA if answered with a 'yes' response to two or more of the 'STOP' with factors including male sex or a body mass index (BMI) ≥ 30 kg/m², or if a participant answered "yes" to five or more of the questions. Nine urinary VOC metabolites were analyzed from the urine samples. The VOC metabolites were log-transformed due to the skewed distribution. Logistic regression models were performed after adjusting potential confounders. Sampling weights were utilized in analyses to account for the complex survey design inherent in the KNHANES dataset.
RESULTS: Of the participants, 802 were defined as low-risk OSA (mean age: 56.30 years), and 114 as high-risk OSA (mean age: 58.17 years). A one-unit increase in log-transformed urinary BMA (Odds ratio [OR]: 1.80, 95% confidence interval [CI]:1.16−2.80), MA (OR: 1.62, 95% CI: 1.00−2.63) was associated with increased risk of high-risk OSA.
CONCLUSIONS: These findings suggests that VOC exposures is an environmental risk factor for sleep disorders.

***ACKNOWLEDGEMENT**: This work was supported by Korea Environment Industry & Technology Institute (KEITI) through Core Technology Development Project for Environmental Diseases Prevention and Management, funded by Korea Ministry of Environment (MOE) (grant No. 2022003310011).*

P13-17 | UPDATE ON THE IMPLEMENTATION OF PESTICIDE NON-DIETARY RISK ASSESSMENT IN BRAZIL

Fabiana C. Palma[1], Adriana T. de Sousa[2], Marcela Giachini[1], Daniele Lautenschalaeger[1]

[1] Instituto ProHuma de Estudos Cientificos, Technical Institute, Jundiai, Brazil; [2] Brazilian Health Regulatory Agency - ANVISA, General Office of Toxicology, Brasilia, Brazil

The implementation of Exposure Risk Assessment for Operators, Reentry workers, Residents and Bystanders in Brazil has been discussed for a few years and a regulation along with a Technical Guidance are to be published in 2025 by the Brazilian Health Surveillance Agency – ANVISA. In this scenario of technical discussion, the ProHuma Institute, a non-profit scientific entity, was created with the main objective of developing a generic database of pesticide handlers exposure representative of the Brazilian agricultural scenario, providing technical information and tools fostering scientific based decisions, to support this implementation. A Technical Cooperation Agreement

(ACT) between the National Health Surveillance Agency – ANVISA and the ProHuma Institute, was issued in 2020 and will be extended for another 5 years (2025 -2030) which enables a collaborative arrangement and increases local knowledge.
Over the past four years, the ACT Task Force has made great progress on key projects. Some of these key projects have focused on generating specified local data and developing a Brazil-specific Occupational Exposure Database. The results achieved in its first phase have been incorporated into the Technical Guidance, this also includes mapping agricultural scenarios, proposing transfer coefficients, and integrating international methodologies to enhance the assessment of exposure for rural workers, residents and bystanders. A standardized and easy-to-use exposure calculator in its first version has been developed to further support exposure assessment. The main outcome of the upcoming regulation will be the enhancement in human health safety and overall society benefit, through an assessment of the risk of exposure that is more representative of the Brazilian reality.

P13-18 | Associations of persistent organic pollutants with hearing loss in Hispanic/Latino adults: The Hispanic Community Health Study/Study of Latinos (HCHS/SOL)

Mariam Oladosu[1], Victoria Persky[1], Robert M. Sargis[2,9], Martha Daviglus[3], Jianwen Cai[4], Chibuzor Abasilim[1], Robert Kaplan[5], Gregory A. Talavera[6], Humberto Parada Jr[7], Sally Freels[1], Laura Coco[8], Mary E. Turyk[1]

[1] University of Illinois, Chicago, Division of Epidemiology and Biostatistics, School of Public Health, Chicago, USA; [2] University of Illinois, Chicago, Division of Endocrinology, Diabetes, and Metabolism, Department of Medicine, Chicago, USA; [3] University of Illinois, Chicago, Institute for Minority Health Research, Chicago, USA; [4] University of North Carolina, Department of Biostatistics, Chapel Hill, USA; [5] Albert Einstein College of Medicine, Department of Epidemiology and Population Health, Bronx, USA; [6] San Diego State University, San Diego, Division of Health Promotion and Behavioral Science, Graduate School of Public Health, San Diego, USA; [7] San Diego State University, San Diego, Division of Epidemiology and Biostatistics, School of Public Health, San Diego, USA; [8] San Diego State University, San Diego, SDSU/UC San Diego Joint PhD Program in Language and Communicative Disorders, San Diego, USA; [9] Jesse Brown VA Medical Center, Chicago and Medical Service, Chicago, USA

Background: Hearing loss is a common chronic condition in the United States, with exposure to persistent organic pollutants (POPs) identified as a potential risk factor. While some human and animal studies suggest associations between POPs and hearing loss, research in this area remains limited.
Methods: This study utilized baseline data (2008–2011) from a sub-cohort of the HCHS/SOL, a multi-site prospective cohort study of diverse Hispanic/Latino adults in the U.S. We studied 2,188 participants to investigate associations between serum concentrations of POPs, including organochlorine pesticides, brominated flame retardants, and polychlorinated biphenyls (PCBs), and hearing loss. Hearing loss was defined as a pure tone average (PTA) greater than 25 dB based on audiometric measurements at 0.5, 1, 2, and 4 kilohertz (kHz). Survey-weighted multivariable logistic regression models assessed associations between serum POPs concentrations (continuous and quartiles) and hearing loss. Effect modification by sex and body mass index (BMI) was also explored.
Results: In the full cohort, higher ∑PBDE concentration (Quartile 4 vs. Quartile 1: OR: 2.16, 95% CI: 1.08–4.33) and PCB-phenobarbital-type inducers concentration (Q3 vs. Q1: OR: 2.04, 95% CI: 1.05–3.95) were both significantly associated with increased odds of hearing loss. Significant linear associations were identified in subgroups. Among men, 2-fold increases in sum of polybrominated diphenyl ethers (∑PBDE) concentration were significantly associated with increased odds of hearing loss (OR: 1.39, 95% CI: 1.15–1.68). Among obese individuals, 2-fold increases in

polybrominated biphenyl-153 (PBB-153) concentration were associated with higher odds of hearing loss (OR: 1.35, 95% CI: 1.06–1.73).

Conclusion: These findings suggest that brominated flame retardants (PBDEs and PBB-153) and some PCBs may be associated with prevalent hearing loss. Further studies are warranted to confirm these associations and elucidate underlying biological mechanisms.

.

P14 | Climate and Health: Novel Methods in Climate Research

P14-01 | Factors Influencing Access to Non-Communicable Disease-Related Healthcare Services for Women in Coastal Bangladesh: A Community-Based Study

Anika Tasnim, Khandakar Fatema, Md. Maruf H. Khan, Marium Salwa, M Atiqul Haque

Bangabandhu Sheikh Mujib Medical University, Department of Public Health and Informatics, Dhaka, Bangladesh

Objective:
Access to non-communicable disease (NCD)-related healthcare is a significant challenge in coastal areas of Bangladesh, where socio-economic, geographical, and environmental factors exacerbate healthcare disparities. Coastal populations, particularly women, face heightened vulnerability to NCDs due to limited healthcare infrastructure. This study explores the determinants affecting access to NCD-related healthcare services among women in coastal areas of Bangladesh.
Material and Methods:
A community-based cross-sectional study was conducted among 576 women aged 18 years and above from three coastal districts of Bangladesh (Shatkhira, Cox's Bazar, and Patuakhali). Participants were selected using multistage systematic random sampling. Data on socio-demographics, NCD risk factors, and healthcare access were collected through structured face-to-face interviews and health screenings. Health literacy was assessed using the European Health Literacy Questionnaire.
Results:
The health literacy distribution showed that 43.6% of participants had inadequate health literacy, 41.0% had problematic health literacy, and 15.5% had sufficient health literacy. Logistic regression analysis identified several significant factors influencing healthcare access: inadequate health literacy (B = -2.615, Exp(B) = 0.073, $p < 0.001$), problematic health literacy (B = -0.742, Exp(B) = 0.476, p = 0.002), no formal education (B = -0.707, Exp(B) = 0.493, p = 0.019), primary education (B = -1.169, Exp(B) = 0.311, p = 0.008), and smokeless tobacco use (B = -0.454, Exp(B) = 0.635, p = 0.044). Other factors, including fruit and vegetable consumption, physical activity, BMI, hypertension, socioeconomic status, and age, did not show significant associations with healthcare access.
Conclusion:
Health literacy and education are key determinants of NCD healthcare access for women in coastal Bangladesh, highlighting systemic inequities in environmentally stressed, low-resource settings. Interventions must integrate climate-resilient healthcare strategies to address environmental and health challenges, advancing global environmental health equity. Empowering communities with knowledge and resources fosters sustainable, equitable healthcare access, enabling them to overcome environmental and health disparities.

P14-02 | The impact of heavy rainfall on ambulance response time in Japan: A threshold analysis

Athicha Uttajug[1,2], Xerses Seposo[2], Muhammad Abdul Basit A. Tajudin[2], Kayo Ueda[2]

[1] *Institute of Science Tokyo, Tokyo, Japan;* [2] *Hokkaido University, Sapporo, Japan*

Introduction: Ambulance response time (ART) is critical for delivering timely medical care during emergencies, significantly improving survival and recovery outcome. However, the impact of extreme weather events, such as heavy rainfall, on ART remains poorly understood.
Objective: To investigate the association between hourly rainfall and ART and identify a potential rainfall threshold beyond which ART is significantly delayed.
Methodology: We employed a linear threshold model to determine the hourly rainfall threshold and assess ART delays across 15 cities in the Kyushu region, Japan. The model was adjusted for temperature, public holiday, day of week, and random effect terms for the city. The analysis focused on data during rainy season (June to September) between 2012 to 2018. The stratification analysis was performed for case severity.
Results: A total of 170,195 ambulance dispatch records with a median ART of 8 minutes were included in this study. We found that hourly rainfall exceeding 34 mm was significantly associated with increased ART, with an additional 1 mm of rainfall beyond this threshold leading to a 0.84-minute delay (95% CI: 0.48, 1.25). The impact of heavy rainfall on ART varied by case severity, with a threshold of 10 mm and estimated delayed of 0.33 minutes (95%CI: 0.33, 0.64) for severe cases, whereas mild cases had a higher threshold of 35 mm with a longer delay of 1.63 minutes (95%CI: 0.64, 2.32).
Conclusion: Our findings revealed that heavy rainfall disrupted emergency medical services, especially for more vulnerable patients who are sensitive to weather conditions. This highlights the importance of incorporating weather-related risks into emergency service planning.

P14-03 | Knowledge and Implementation behavior towards mitigation initiatives of climate change: Community settings approach of Bangladesh context

Bilkis Banu[1], Nasrin Akter[2], Nusrat H. Sheba[2], Sujana H. Chowdhury[2]

[1] *Independent University, Bangladesh, Public Health, Dhaka, Bangladesh;* [2] *Northern University Bangladesh, Public Health, Dhaka, Bangladesh*

Bangladesh experiences different types of natural disasters almost every year which affect adverse human health. It is very essential to identify knowledge and implementation behavior as mitigation initiatives towards climate change in community settings of Bangladesh. This study was designed to explore this issue. It was an analytical type of cross-sectional study which was conducted among 450 adult people residing in Barisal district of Bangladesh. Data were collected through face-to-face interviews using semi-structured questionnaire included socio-demographic information, knowledge and implementation behavior regarding mitigation initiatives towards climate change. Bivariate and multivariate techniques were adopted to analyze the data. The outcome reflected that a large proportion of the respondents had poor knowledge (55.1%) and poor implementation behavior (52.0%) on mitigation initiatives

towards climate change. Poor knowledge was significantly more prominent among the people who were male (AOR=1.56), Muslim (AOR =2.55), respondents with larger family members (AOR =1.91) and 3 or more children (AOR=1.64) showed higher odds of poor knowledge. Poor implementation behavior was found significantly more leading among the female (AOR=2.91), service-holder (AOR =1.92) participants having higher monthly family incomes (AOR =2.91), who had only one child (AOR =2.70) belonging a smaller number of family members (AOR =30.09). An alarming proportion of poor knowledge and implementation behavior were found regarding mitigation initiatives towards climate change in community settings of Bangladesh. Concerning demographic major predictors, it is essential to plan and implement sustainable and comprehensive health educational program on climate change mitigation throughout the country.

P14-04 | Developing a Climate Threat Index (CTI) to characterize exposure to co-occurring climate threats

Carlos Carrillo-Gallegos[1], Jeff Goldsmith[2], Marianthi-Anna Kioumourtzoglou[1], Xiao Wu[2]

[1] Columbia University Mailman School of Public Health, Environmental Health Sciences, New York, USA; [2] Columbia University Mailman School of Public Health, Biostatistics, New York, USA

Several climate threats, such as heat waves, have been linked to adverse health outcomes, including mortality, cardiorespiratory disease, and neurodegeneration. As climate change increases the frequency and intensity of climate threats, it is important to understand when these events co-occur and identify those populations that are most vulnerable to specific combinations of exposures. To address this, we develop a Climate Threat Index (CTI) to quantify the co-occurrence of 10 climate threats (temperature, tropical cyclones, relative humidity, wildfires, nor'easters, flooding, droughts, tornadoes, extreme precipitation, and air pollution) in the continental United States daily from 2000-2020. We employ a Kernel Principal Component Analysis using the climate-related exposures as inputs to develop region-specific, ZIP code-level indices of co-occurrence. In secondary analyses, we train and evaluate the use of a deep autoencoder to estimate the CTI and incorporate zero-inflated factor analysis to account for the presence of zero climate threat values on certain days and ZIP codes. Additionally, we use feature importance ranking and SHAP (SHapley Additive exPlanations) values to rank the contribution of each climate threat to the region-specific CTIs, providing a useful qualitative measure of importance for each climate threat. In future analyses, the CTI will provide an overall exposure estimation to identify links between co-occurring climate threats and health outcomes such as Alzheimer's and Parkinson's diseases.

P14-06 | Hurricane Dashboard: A Tool for Emergency Response and Preparedness

Fatima Abdirizak

Centers for Disease Control and Prevention, National Center for Environmental Health, Atlanta, USA

Objective:
The impact of hurricanes can have lasting effects on communities including exposure to environmental toxins and reduced access to critical resources due to infrastructure damage. To address these challenges, the National Environmental Public Health Tracking Program (Tracking Program) developed a Hurricane Dashboard (HD), an interactive tool integrating near real-time data to assist public health professionals in accessing the needs of hurricane-impacted communities and supporting emergency response and recovery efforts.
Material and Methods: Over 100 data sources were evaluated to determine which sources where applicable in understanding the magnitude and severity of specific hurricanes impact on population and environmental health as well as the timeliness of the data. The HD uses data available on the Tracking Program's Data Explorer and incorporates dynamic infographics, text, maps, and charts. Content on the HD was informed by collaboration between the NCEH/ATSDR Emergency Management Unit and NCEH/ATSDR Data Analytics and Visualization Task Force.
Results:
The HD provides near real-time tracking of active and recently active hurricanes through detailed maps displaying storm information, including maximum category, maximum wind speeds, and storm paths. For each hurricane, users can explore three distinct maps with information on population characteristics, health, and environmental characteristics, along with optional layers such as hospital and emergency medical service locations. For granular insight, all data is available at the county or census tract level.
Conclusions: The HD consolidates near real-time data from multiple sources into a single, interactive tool to support emergency preparedness, response, and recovery efforts. The HD enhances public health professionals' ability to address immediate and long-term needs during hurricane events. The HD can be used to assess a community's preparedness and the potential impact of a hurricane which helps in implementing necessary measures to reduce harm and understand the factors that may influence environmental exposures and human health.

P14-07 | Integrating Gender, Disability, and Social Inclusion in Developing the First Philippine Health and Climate Change Roadmap

Hazel Ann R. Fajardo[1], Percival Ethan Lao[1], Anna Beatrice Enriquez[1], Giancarlo Pocholo Enriquez[1], Rafael Deo Estanislao[1], Jemar Anne Sigua[1], Noelle Anne Cubacub[1], Xerxes Seposo[1,2], Geminn Louis Apostol[1]

[1] Ateneo School of Medicine and Public Health, Ateneo Center for Research and Innovation Environmental Health and Global Health Security Flagship Program, Pasig, Philippines; [2] Hokkaido University, Graduate School of Medicine Department of Hygiene, Sapporo, Japan

Objective: To develop the first national health and climate change roadmap that integrates gender equity and disability inclusion considerations to address different climate impacts across population groups.

Materials and Methods: We conducted a mixed-methods approach to develop the first Philippine Health and Climate Change Roadmap 2025-2050. This included: (1) literature review across academic databases, grey literature, and government repositories; (2) key informant interviews (n=8) with Department of Health officials and partners; and (3) multi-stakeholder consultation workshops with government agencies, development partners, and civil society organizations. Key informant interviews assessed existing gender and equity provisions in climate-health policies and programs, while consultation workshops included representatives from the National Commission on Indigenous Peoples and disability advocacy groups. The roadmap development was guided by the WHO Operational Framework for Building Climate Resilient Health Systems and aligned with national policies.

Results: The Philippine Health and Climate Change Roadmap 2025-2050 established a framework structured around seven strategic outcomes using a SMARTER approach: Strengthened workforce capacity, Multilevel data integration, Accessible financing, Resilient service delivery, Transformative infrastructure, Engaged communities, and Responsive governance. Key informant interviews revealed a lack of explicit gender and disability provisions in existing climate-related health policies. To address this gap, the roadmap includes specific provisions for indigenous communities, persons with disabilities, women, children, and the elderly through targeted early warning systems, adaptive health service delivery, and participatory action planning integrated with Universal Health Care implementation.

Conclusion: The Philippine Health and Climate Change Roadmap establishes a foundation for equitable climate action in the health sector. By explicitly incorporating gender equity and disability inclusion considerations, the roadmap addresses different climate vulnerabilities while ensuring no populations are left behind in climate response. This framework provides a template for countries seeking to develop inclusive climate-health strategies that advance global environmental health equity.

P14-08 | CARBOMICA: A Financial Resource-Allocation Tool for Carbon Mitigation in Healthcare Facilities - A Case Study from Kenya

Jetina Juliet J. Tsvaki[1], Craig Parker[2], Farah Houdroge[3], Romesh Abeysuriya[3], Eloisa Perez-Bennetts[3], Thabani Muronzie[1], Aquinius Mung'atia[4], Progress Chiworeka[1], Jaspar Maguma[1], Josh Rusike[1], Nick Scott[3,5], Fortunate Machingura[1,8], Stanley Luchters[1,7], for the HIGH Horizons study group.

[1] Ceshhar Zimbabwe, Climate Environment & Health, Harare, Zimbabwe; [2] University of the Witwatersrand, Witswatersrand, South Africa; [3] Burnet Institute, Monash, Australia; [4] Aga Khan Hospital Mombasa, Mombasa, Kenya; [5] Monash University, Department of Epidemiology and Preventive Medicine, Monash, Australia; [6] Optima Consortium for Decision Science, Monash, Australia; [7] Ghent University, Department of Public Health and Primary Care, Ghent, Belgium; [8] Liverpool School of Tropical Medicine, London, UK

Objective:
The healthcare sector contributes 4-8% of global greenhouse gas (GHG) emissions through energy use, medical supply chains, and waste. Mitigation is critical for public health and cost reduction, yet funding gaps persist, especially in low- and middle-income countries (LMICs). CARBOMICA, a data science tool developed by the HIGH Horizons study group, optimizes financial resource allocation for carbon mitigation in healthcare facilities. This study demonstrates its application at Aga Khan Hospital, Mombasa, Kenya.
Material and Methods:
CARBOMICA, built on the 'Atomica' framework, was used to model carbon mitigation interventions at Aga Khan Hospital. The hospital's 2023 carbon footprint (758,445 kg CO_2) was measured using the Aga Khan Development Network (AKDN) carbon management tool, covering Scope 1, 2, and 3 emissions. Stakeholders identified context-specific interventions, including solar systems, energy-efficient appliances, biogas digesters, and tree planting. Costs, carbon savings, and co-benefits (e.g., health improvements, cost savings) were assessed. Three budget scenarios ($50,000,$125,000, $250,000) were analyzed for emission reductions and cost-effectiveness.
Results: With a $50,000 budget, emissions decrease by 19% through LED lights, energy-efficient ACs, biogas digesters, and a sustainability policy, leaving a $1,835 surplus. A $125,000 budget achieves a 29% reduction, while a $250,000 budget maximizes reductions at 34%, leaving surpluses of $715 and $576, respectively allocating funds to optimal mitigation interventions.
Conclusion:
CARBOMICA effectively guides financial resource allocation for carbon mitigation in healthcare facilities, balancing cost-efficiency and emission reductions. Stakeholder engagement was critical for aligning interventions with facility needs. Investments in renewable energy and energy-efficient technologies are recommended, with optimal spending capped at $125,000 due to diminishing returns at higher budgets.

Funding: This work was supported by the HIGH Horizons project.
Keywords: Carbon mitigation, resource allocation, healthcare sustainability, LMICs, data science tool.

P14-09 | Evaluation of CMIP6 Climate Models for Precipitation Projections in Jordan Under SSP4.5 and SSP8.5 Scenarios

Moawiah O. Hussine, Fayez Abdulla

Jordan university of science and technology, Civil Engineering / Water resources and environmental engineering, Irbid, Jordan

Objective: This study evaluated the performance of seven CMIP6 climate models to project future precipitation patterns across Jordan's diverse hydrological basins under SSP4.5 and SSP8.5 scenarios, employing a basin-specific approach to address spatial heterogeneity in precipitation patterns.
Material and Methods: We utilized high-resolution climate projections from RICCAR for the period 1961-2070. Models were validated against historical observations (1971-2014) using five statistical metrics: correlation coefficient, Nash-Sutcliffe Efficiency, Percent Bias, RMSE, and MAE. For each basin, the three best-performing models were selected, and ensemble approaches were implemented to reduce individual model uncertainties. Changes in precipitation were analyzed for near-future (2021-2040) and mid-future (2041-2060) periods relative to the reference period (1995-2014), with particular focus on seasonal variations.
Results: Performance evaluation revealed regional variability in model accuracy, with CMCC-CM2-SR5 performing optimally in five basins, while MPI-ESM1-2-LR, CNRM-ESM2-1, and NorESM2-MM showed regional strengths in other basins. MPI-ESM1-2-LR demonstrated the most balanced overall performance with high correlation consistency (0.85-0.95), favorable NSE distribution, and compact bias range. Precipitation projections showed distinct spatial patterns, with western highlands demonstrating greater sensitivity to changes, while eastern regions exhibited more stable patterns under SSP4.5.
Conclusion: Our basin-specific approach identified significant spatial variability in future precipitation patterns across Jordan, with implications for regional water resource management. The findings highlight the importance of high-resolution, localized climate projections for developing effective adaptation strategies in water-scarce regions.
Funding: This research was supported by the GeoHealth Hub.
Keywords: climate change, precipitation projections, CMIP6, Jordan, water resources

P14-11 | The Changing Flow: Rivers and Communities in Bangladesh

Nabhira Aftabi Binte Islam

Bangabandhu Sheikh Mujib Medical University (bsmmu, Preventive and Social Medicine, Dhaka, Bangladesh

Abstract
Background:
In Bangladesh, the main rivers like the Padma, Meghna, and Jamuna play immense roles in the country's economy, culture, and way of life. But since the past decade, these rivers have felt large changes because of a combination of manmade and natural factors.
Objective:
The evolving situations of Bangladesh's significant rivers follow the dynamic forms of landscape, people, and ecosystems. This study aimed to shed light on the causes of such transformation as well as prescribe remedies.
Methods:

A photocollectivist approach was adopted toward imaging visually changing patterns of rivers along with populations dwelling alongside them. Photos were taken from various locations along the Padma, Meghna, and Jamuna rivers, tracing changes in riverbanks, water levels, and human settlements. National database statistics and research articles were utilized to build a complete picture of such changes. Local community interviews through electronic media to learn about their experience and perception of these changes were also utilized.
Results:
Early findings present an appreciable decrease in the quality of water, an increase in siltation, transformation in river runoff patterns, and encroachment due to urbanization. All these have hampered regional livelihoods, agriculture, and transportation infrastructure. River barrage construction and conservation efforts have also produced unequal impacts on the rivers' natural behavior.
Conclusion:
The transformation of Bangladesh's rivers is influenced by multi-dimensional factors like climate change, urbanization, industrialization, and conservation efforts. This photo walk emphasizes the need for an integrated approach to river management to balance development and environmental protection.

P14-12 | Building a climate cohort to monitor the health impacts of climate change in Ghana: aims, methodology, and expected outcomes—CLEAR PROJECT.

Solomon Nyame[1], Alison Lee[2], Frank Atuguba[3], Japhet Anim[1], Patrick Ansah[4], Leonard Amekudzi[5], Cascade Tuholske[6], Darby Jack[7], Kwaku Poku Asante[1], on behalf of Climate and Health Evaluation for Adaptive Resilience (CLEAR) Project

[1] Kintampo Health Research Centre, Environmental Department, Kintampo, Ghana; [2] Mount Sinai Hospital, New York, NY, USA; [3] Dodowa Health Research Centre, Dodowa, Ghana; [4] Navrongo Health Research Centre, Navrongo, Ghana; [5] Kwame Nkrumah University of Science and Technology, Department of Meteorology and Climate Science, Kumasi, Ghana; [6] Montana University, Department of Earth Sciences, Montana State University, Bozeman, MT, USA., Montana, USA; [7] Columbia University, Columbia Mailman School of Public Health, New York, NY, USA

Objective
We aim to examine: (1) the impact of climate change on morbidity and mortality using our robust HDSS; and (2) public engagement activities to communicate the mpact of climate change on morbidity and mortality
Methods
Data collection and analysis utilise quantitative and qualitative approaches, relying on collaboration among seven institutions. We have partnered with community and government stakeholders to leverage longitudinal data from our health and demographic surveillance system (HDSS) and cohort studies. These HDSS sites have systematically collected mortality data over the past 20+ years, covering over 511,000 individuals. We harmonized data from the three HDSS and derived an analytic dataset including date of death, age at death, biological sex, and community of residence. We used satellite-derived climate variability and exposure metrics. We will apply statistical modeling techniques such as case-crossover and distributed lag non-linear models (DLNMs) to analyze the associations between climate parameters and health outcomes.
Results
The study analyzes health and demographic data from 2004 to 2022. By 2022, there were 61,550 deaths (12.0%), with males at 54.4%. Children under five and adults comprised 13.8% and 44.7%, respectively. The average UTCI,

maximum temperature, and heat index were 28.6 (27.8 - 29.2), 32.8 (28.9 - 34.6), and 40.2 (36.9 - 51.6). Stakeholder engagement emphasized using longitudinal data to generate evidence for targeted adaptation strategies and early warning systems, identifying vulnerable groups and risk factors. Stakeholders also highlighted the need for a robust climate-health surveillance system to inform public health policies.

Conclusion

This initiative underscores the urgency of integrating climate science with public health research to mitigate the adverse effects of climate change. Through interdisciplinary collaboration, this study aims to provide actionable evidence for policymakers, healthcare providers, and communities to safeguard health in a changing climate.

P14-14 | Characterizing global tropical cyclone events of 2024

Lingke Jiang[1], G. Brooke Anderson[3], Victoria D. Lynch[2], Xiao Wu[1], Robbie M. Parks[2]

[1] Columbia University, Department of Biostatistics, New York, USA; [2] Columbia University, Department of Environmental Health Sciences, New York, USA; [3] Colorado State University, Department of Environmental and Radiological Health Sciences, Fort Collins, USA

Objective: Tropical cyclones have a devastating effect on society, yet a comprehensive global sociodemographic exposure assessment is lacking. Our aim was to evaluate the exposure and sociodemographic characteristics of regions impacted by tropical cyclones during the 2024 calendar year and place in a recent historical context.

Methods: For tropical storms that occurred during 2024, we used tracking data from International Best Track Archive for Climate Stewardship (IBTrACS) to model local windfields and determine exposure to tropical cyclone- (≥17.1 m/s) and hurricane-force (≥33.1 m/s) sustained wind speed at each second-level administrative unit (ADM2) globally. We used gridded population estimates from Global Human Settlement Layer to generate person-days of exposure. We compared the 2024 exposures to 44 prior-year exposures since 1980. We analyzed the relationship between person-days and the Global Gridded Relative Deprivation Index (GRDI) (0 low, 100 high). We identified and ranked the most impacted ADM2s based on GRDI and tropical cyclone and hurricane person-days.

Results: Globally during 2024, there were 93 uniquely identified tropical storms recorded by IBTrACS, of which 35 contributed to 3,922 local tropical cyclone exposures in 2,951 ADM2s, with 15 contributed to 720 local hurricane exposures in 711 ADM2s. The Western Pacific (42,839,326 person-days) had the highest WHO region-level person-day exposure, followed by the Americas (19,576,238 person-days). The ADM2 with the highest person-event tropical cyclone exposures were Longzhouxian, China (8,264,775 person-days, GRDI 67), Trang Dinh, Vietnam (593,6385 person-days, GRDI 68), and Ningmingxian, China (2,764,006 person-days, GRDI 58). In context, 2024 ranked 12th in total tropical cyclone person-day exposures since 1980.

Conclusion: In 2024, tropical cyclones impacted regions in Western Pacific and the Americas the most, in relatively deprived areas. Our findings highlight the importance of further understanding the population characteristics of communities and places most impacted by tropical cyclones to better guide and accelerate future research efforts.

P14-15 | Enabling environmental exposure studies withtranscriptomic endpointsin hard-to-reach demographics and extreme climates

Filip Stefanovic[1], Lauren Brown[1], James MacDonald[1], Theo Bammler[1], Darawan Rinchai[1], Damien Chuassabel[2], Amanda Haack[1], Sanitta Thongpang[1], Erwin Berthier[1], **Ashleigh Theberge**[1]

[1] University of Washington, Chemistry, Seattle, USA; [2] The Jackson Laboratory, Computer sciences, Farmington, USA

Environmental exposures that have significant impact on health and disease are often found in hard-to-reach areas where climate or external conditions have limited the development and logistic solutions available. Areas experiencing arid, polluted, or extreme climate conditions both in the USA or internationally can display toxicants or pathogens in the air that are typically more impactful on human health. Climate change has further impacted these areas disproportionately through the expansion of pathogenic bacterial, fungal, smoke, and other types of dust and toxicants that have been shown to be harmful.

Accessing populations to perform exposome studies in these hard-to-reach areas present multiple challenges, particularly for studies that require blood-based endpoints. A first barrier to these studies is the ability to collect a blood sample in a manner that preserves the high quality results for the biomarkers. A second barrier, is accessing these locations given the limited logistical solutions and typically out-of-standard-range climates they experience.

We have developed homeRNA, a technology that enables the collection of a pristine blood sample for transcriptomic analysis from anywhere. The kit enables a reliable and painless collection of over 500 uL of blood that is immediately integrated to an RNA preservation buffer at the point of collection. We demonstrate that this system also preserves samples at elevated temperatures such as those found in Qatar or Thailand. Specifically, we demonstrate RINs of over 7.0 for samples that have been subjected to real-world shipping conditions ranging from 25 degrees Celsius up to 37 degrees Celsius. With the ability to ensure robust, simple, and cost-effective sample logistics, homeRNA has the potential to enable broad world-wide cohort studies and precision medicine applications while allowing access to any patient or participant.

P14-16 | Evaluating Citizen Science Approaches for Mosquito Surveillance: A Systematic Review

Syamala Buragadda[1], Chinenye L. Onyenwe[1], Rebecca Davis[2], Negar Elmieh[3], Wendy Pons[4], Atanu Sarkar[1]

[1] Memorial University of Newfoundland, Division of Population Health and Applied Health Sciences, St. John's, Canada; [2] London School of Hygiene and Tropical Medicine, London, UK; [3] National Collaborating Centre for Environmental Health, Vancouver, Canada; [4] Conestoga College Institute of Technology and Advanced Learning, School of Health & Life Sciences, Kitchener, Canada

Objective: Citizen science has emerged as a valuable approach for mosquito surveillance, engaging the public in data collection and expanding research capacity in regions with limited resources. This global review assesses the effectiveness, sustainability, and educational impact of citizen science-based mosquito surveillance methods in improving public awareness and disease risk communication.

Material and Methods: A comprehensive search was conducted across databases, including MEDLINE, Embase, Scopus, and Web of Science, with no publication year restrictions. Studies involving citizen-led mosquito surveillance were included, while those focused on non-mosquito vectors or control efforts without a surveillance component were excluded. Study selection, data extraction, and synthesis followed the PRISMA method for a systematic review using Covidence. The review protocol was registered on PROSPERO.

Results: The initial search identified 2219 studies, with 64 articles meeting the inclusion criteria. The review highlights the effectiveness of citizen science in mosquito surveillance, integrating smartphone apps (e.g., Mosquito Alert) and traps (e.g., BG-GAT) to enhance data accuracy. Global initiatives, including those in Germany, Spain, and Paraguay, demonstrate the value of community engagement in data collection. Training and multilingual outreach efforts in Africa and India led to increased participation. While citizen science expands surveillance coverage and improves intervention timeliness, challenges remain in data accuracy. Expert validation and machine learning can enhance data reliability. Compared to traditional surveillance, citizen-driven approaches are cost-effective and scalable, reinforcing their role in public health strategies for mosquito-borne disease prevention and control.

Conclusion: Our review findings underscore the critical role of citizen science in enhancing mosquito surveillance through technology, education, and community participation. For effective surveillance, sustained training, accessible tools, and strategic engagement are essential. Integrating citizen science with traditional surveillance may improve data quality. Future research should assess long-term impacts on mosquito control and disease prevention to optimize public health outcomes.

P14-17 | Diversifying Climate Response: Incorporating Gender, Disability, and Social Inclusion in the development of the first Philippine Roadmap for Health and Climate Change

Anna Beatrice Enriquez, Hazel Ann Fajardo, Percival Ethan Lao, Noelle Anne Cubacub, Giancarlo Pocholo Enriquez, Geminn Louis Apostol, Xerxes Seposo, Jemar Anne Sigua, Rafael Deo Estanislao

Ateneo School of Medicine and Public Health, Center for Research and Innovation Environmental Health and Global Health Security Flagship Program, Pasig City, Philippines

As one of the world's most climate-vulnerable nations, the Philippines faces severe health impacts that disproportionately affect marginalized populations, necessitating inclusive climate-health frameworks.

Objectives: The project aimed to develop a comprehensive Strategic Plan (2025-2050) and Operational Plan (2025-2030) on climate change and health by: (1) establishing appropriate institutional mechanisms within the Department of Health; (2) outlining strategic activities aligned with national and international commitments; and (3) creating an implementable operational plan for the current administration.

Methods: The development process employed an output-based methodological approach across three components: long-term Strategic Plan, near-term Operational Plan, and Organizational Set-up. Methods included systematic literature review, key informant interviews with health officials, and multi-sectoral consultation workshops with representatives from indigenous peoples' commissions and women's organizations.

Results: The Roadmap establishes climate-smart, gender-responsive strategies for 2025-2050. Six of seven strategic objectives integrate GEDSI components, including: gender-responsive capacity-building for health workers; climate emergency protocols for vulnerable groups; participatory vulnerability mapping with indigenous knowledge; universally accessible health facilities; and representation of women and marginalized groups in climate-health leadership. Implementation includes dedicated budget allocations and monitoring frameworks with disaggregated indicators to track outcomes across vulnerable populations.

Conclusion: The Philippine Roadmap for Health and Climate Change (2025-2030) is the first comprehensive health-climate framework with substantive GEDSI integration by systematically integrating social dimensions into national policy. By centering historically excluded voices, the plan addresses structural inequities while strengthening health system resilience, offering valuable lessons for other climate-vulnerable regions and advancing global commitments to health equity in climate adaptation.

P15 | Environmental Exposures and Cardiovascular Health - PART I

P15-01 | Effect of Seasonal Firefighting on Cardiovascular Health in Wildland Firefighters

Chieh-Ming Wu[1], Hilary Stroud[2], Anthony Akande[1], David Bahner[2], Travis Sharkey-Toppen[2], Olorunfemi Adetona[1]

[1] The Ohio State University, College of Public Health, Division of Environmental Health Sciences, Columbus, USA;
[2] The Ohio State University, College of Medicine, Department of Emergency Medicine, Columbus, USA

Objective: Wildland firefighters are repeatedly exposed to fire smoke, which can negatively affect cardiovascular health. To evaluate the effect of seasonal firefighting on their cardiovascular function, pre- and post-season measures of hemodynamic response, systemic arterial stiffness, endothelial function and vascular structure were taken among wildland firefighters.

Material and Methods: Brachial blood pressure (including systolic blood pressure [SBP], diastolic blood pressure [DBP], and pulse pressure [PP]), central augmentation index (cAIx), and pulse wave velocity (PWV) were measured using the Sphygmocor XCEL device. Carotid intima-media thickness (CIMT) and flow-mediated dilation (FMD) were determined using the GE VIVID ultrasound machine. Linear mixed model was used to determine cross-seasonal changes in cardiovascular function of wildland firefighters.

Results: Fifty-three wildland firefighters (aged 34.9±8.9 years, 92% males, 27.0±3.9 kg/m^2 BMI, 5 current smokers) from federal forestry agencies and a state forest service participated in the study. On average, wildland firefighters responded to 9 wildfires and participated in 24 prescribed burns during the study period. Wildland firefighters' SBP, DBP, and PP significantly decreased across wildfire season (−9.5±3.3 mmHg, p<0.01; −4.0±1.8 mmHg, p=0.04; −5.6±2.4 mmHg, p=0.03; respectively), while FMD significantly increased after the fire season (52.1±23.0%, p=0.04). Although the changes were non-significant, PWV and CIMT (left side) increased in post-season measurements (0.25±0.27 m/s, p=0.35 and 0.02±0.02 mm p=0.35, respectively). Insignificantly decreased cAIx was observed in wildland firefighters (−1.4±4.4%, p=0.75).

Conclusion: Wildland fire smoke exposure, high physical demand, and working in hot environments might be associated with cardiovascular responses observed in this study. Seasonal firefighting could also potentially increase arterial stiffness and thickness and impair endothelial function. Our results suggest that firefighting in forest areas can induce distinct cardiovascular responses in wildland firefighters. This study is supported by the National Institute for Occupational Safety and Health (R21OH011271) and is approved by The Ohio State University Institutional Review Board (2020H0102).

P15-02 | Cross-sectional association between household air pollution exposure and blood pressure amongst adults in rural Rwanda, East Africa

Christian Sewor[1], Ky Tanner[2], Vincent Cleveland[1], Bonnie N. Young[1], Christian L'Orange[2], Rebecca Witinok-Huber[1], John Balmes[3,4], Egide Kalisa[5], Lindsay Zurba[6], Theoneste Ntakirutimana[7], Kayleigh P. Keller[8], Howard H. Chang[9], John Volckens[2], Maggie L. Clark[1]

[1] Colorado State University, Environmental and Radiological Health Sciences, Fort Collins, USA; [2] Colorado State University, Department of Mechanical Engineering, Fort Collins, USA; [3] University of California, School of Public Health, Berkeley, USA; [4] University of California, Department of Medicine, San Francisco, USA; [5] Western University, Department of Epidemiology and Biostatistics, London, Canada; [6] Education for Health Africa, Durban, South Africa; [7] University of Rwanda, College of Medicine and Health Sciences (CMHS), School of Public Health, Department of Environmental Health Sciences, Kigali, Rwanda; [8] Colorado State University, Department of Statistics, Fort Collins, USA; [9] Emory University, Rollins School of Public Health, Department of Biostatistics and Bioinformatics, Atlanta, USA

Objective

Household air pollution (HAP) from biomass fuel combustion is a key contributor to cardiovascular disease (CVD) burden through mechanisms such as oxidative stress, systemic inflammation, and autonomic regulation. In Africa, where HAP exposure is high and CVD rates are rising, limited research has examined the impact of HAP on CVD risk. We analyzed baseline cross-sectional data from an ongoing trial to evaluate the association between fine particulate matter ($PM_{2.5}$) exposure and adult systolic and diastolic blood pressure (SBP and DBP, respectively) in rural Rwanda.

Material and Methods

We enrolled 622 households, recruiting up to two adult participants per household using traditional biomass cookstoves in Eastern Rwanda. Participants wore Ultrasonic Personal Air Samplers (UPAS) for 48 hours to collect gravimetric $PM_{2.5}$ samples. BP was measured up to four times per participant, following established guidelines. Linear mixed models were used to assess the associations between $PM_{2.5}$ exposure and BP, adjusting for age, sex, weighted asset index, and BP measurement time; effect modification by age and sex was evaluated.

Results

Of the 960 participants, 361 were men and 599 were women. The median 48-hour $PM_{2.5}$ exposure level was 138.2 μg/m^3 (25th percentile=81.6, 75th percentile=239.5); women were exposed to higher levels than men. The average SBP was 122 mmHg (standard deviation = 16.4). We did not observe evidence of an association between $PM_{2.5}$ exposure and SBP (-0.21mmHg BP [95% confidence interval: -1.32, 0.88] per interquartile range [157.9 μg/m^3] higher $PM_{2.5}$ exposure). Similarly, no evidence of associations was observed when DBP was the outcome or for any of the effect modification results.

Conclusion

Given the results observed in our robust, yet cross-sectional study, further prospective studies are needed to examine the impact of HAP on BP, particularly through interventions aimed at reducing exposures to health-relevant standards in low-resource settings.

Funding: NIH(R01ES029995)

P15-03 | Joint exposure to ozone and temperature and acute myocardial infarction among adults aged 18-64 years in the United States

Lingzhi Chu[1], Rong Wang[1], Cary P. Gross[1], Jing Wei[2], Yuan Lu[1,3], Harlan M. Krumholz[1,3], Xiaomei Ma[1], Kai Chen[1]

[1] Yale University, New Haven, USA; [2] University of Maryland, College Park, USA; [3] Yale New Haven Hospital, New Haven, USA

Objective: Both ambient temperature and ground-level ozone may lead to adverse health impacts, while effects of the joint exposure are less conclusive. This study aimed to evaluate the associations between joint exposure to ozone and temperature and risk of acute myocardial infarction (AMI) among younger adults aged 18-64 years, which population was relatively understudied in previous cardiovascular studies.

Methods: We included eligible young policyholders who experienced AMI, covered by a nationwide private insurance company in the U.S. (2016-2020). We used a time-stratified case-crossover design and adopted a two-stage model with climate-zone-specific conditional logistic regressions at lag 0 day in the first stage and multivariate meta-analysis in the second stage. To assess the interactive association of ozone and temperature, we included a statistical interaction term between these two exposures, with both specified as natural cubic splines to ensure entire-range evaluation and flexibility.

Results: In total, 270,123 adults with AMI were included. We found higher odds of AMI associated with joint exposure to ground-level ozone and temperature. We used the joint exposure to ozone at 35 ppb and temperature at the 1st percentile as the reference scenario. When both ozone and temperature were high (ozone: 60 ppb; temperature: the 95th percentile), the odds ratio was estimated to be 1.33 (95% confidence interval [CI]: 1.16, 1.51) at lag 0 day. Joint exposure to moderate ozone and temperature (ozone: 50 ppb; temperature: the median) was associated with an OR of 1.15 (95% CI: 1.04, 1.28).

Conclusion: Joint exposure to ground-level ozone and temperature adds to the risk of AMI among young people. Furthermore, not only extremely high exposures but also moderate joint exposures should be taken into account in public health management for AMI.

Acknowledgments: The National Heart, Lung, and Blood Institute of the National Institutes of Health (R01 HL169171).

P15-04 | Short-term association between ambient air pollution and heart rate variability: Unexpected findings from the population-based KORA S4 and FF4 studies

Yujiao Li[1,2], Susanne Breitner-Busch[1,2], Wayne E. Cascio[3], Siqi Zhang[4], Kathrin Wolf[1], Ina-Maria Rückert-Eheberg[1], Stefan Kääb[5,6], Georg Schmidt[5,7], Alexander Strom[8,9], Annette Peters[1,2], Alexandra Schneider[1]

[1] Helmholtz Zentrum München GmbH, German Research Center for Environmental Health, Institute of Epidemiology, Neuherberg, Germany; [2] LMU Munich, Institute for Medical Information Processing, Biometry, and Epidemiology, Pettenkofer School of Public Health, Munich, Germany; [3] US Environmental Protection Agency, Center for Public Health and Environmental Assessment, Office of Research and Development, Durham, USA; [4] Yale School of Public Health, Department of Environmental Health Sciences, New Haven, USA; [5] German Research Center for Cardiovascular Disease (DZHK), Partner site Munich Heart Alliance, Munich, Germany; [6] Medical Policlinic and University Clinic I, Department of Cardiology, Munich, Germany; [7] TUM University Hospital, Technical University of Munich, Department of Internal Medicine I, Ismaninger Str. 22, 81675, Munich, Germany; [8] German Diabetes Center, Leibniz Center for Diabetes Research at Heinrich Heine University Düsseldorf, Institute for Clinical Diabetology, Düsseldorf, Germany; [9] German Center for Diabetes Research (DZD), Partner Düsseldorf, Munich, Germany; [10] German Center for Cardiovascular Health (DZHK e.V., partner-site Munich), Munich Heart Alliance, Munich, Germany

Objective: This study investigates the associations between short-term exposure to particulate matter ($PM_{2.5}$), nitrogen dioxide (NO_2), and heart rate variability (HRV) indices, including SDNN, RMSSD, pNN50, LF, HF, LF/HF ratio, and heart rate (HR), across two waves of the KORA study conducted 14 years apart. Additionally, the study explores effect modification by individual characteristics, including age, smoking status, and medication use.

Material and Methods: Data from 4,032 participants in KORA S4 (1999–2001) and 1,912 participants in KORA FF4 (2013–2014) were analyzed. Air pollution data were obtained from fixed monitoring stations. HRV indices were derived from 5-minute electrocardiogram recordings. Generalized additive models were applied to assess short-term associations of air pollution with HRV and HR. Effect modification by age, smoking status, and other individual characteristics was explored using interaction terms.

Results: In KORA S4, each IQR increase in 14-day moving average $PM_{2.5}$ was associated with a -2.32% (95% CI: -4.41, -0.19) decrease in SDNN and a -0.74% (95% CI: -1.34, -0.13) decrease in pNN50. An IQR increase 14-day moving average NO_2 was associated with a HR increase of 1.2% (95% CI: 0.16, 2.26). By contrast, KORA FF4 showed the opposite results with positive associations for all HRV indices (SDNN, pNN50, RMSSD, HF, LF) for both pollutants. Effect modification by age and smoking status showed smaller HRV responses for older smokers.

Conclusion: Short-term exposure to $PM_{2.5}$ and NO_2 impacts cardiac autonomic function negatively, with delayed effects being observed. However, the direction of the associations differed between the two study waves, potentially due to aging, smoking, medication use, and lower pollution levels in FF4 (29.87% lower $PM_{2.5}$; 24.66% lower NO_2). These findings highlight the importance of reducing air pollution exposure and considering individual susceptibility factors in cardiovascular risk assessments.

P15-05 | Neighborhood Physical Disinvestment and Incident Diabetes between visits 1 and 2 of the Hispanic Community Health Study/Study of Latinos (HCHS/SOL)

Cara M. Smith[1], Elizabeth Spalt[1], Linda Gallo[2], Jordan Carlson[3], Bharat Thyagarajan[4], Earle Chambers[5], Matthew Allison[2], Amber Pirzada[6], Martha Daviglus[6], Christina Cordero[7], Qibin Qi[10], Elena Austin[1], Amanda Fretts[8], India Ornelas[9], Robert Kaplan[10], Joel Kaufman[1,8], Stephen Mooney[8]

[1] University of Washington, Department of Environmental and Occupational Health, Seattle, USA; [2] San Diego State University, Department of Psychology, San Diego, USA; [3] Children's Mercy Hospital and University of Missouri, Department of Pediatrics, Kansas City, USA; [4] University of Minnesota, Department of Laboratory Medicine and Pathology, Minneapolis, USA; [5] Albert Einstein College of Medicine, Department of Family and Social Medicine, Bronx, USA; [6] University of Illinois at Chicago, Institute for Minority Health Research, Chicago, USA; [7] University of Miami, Department of Psychology and Behavioral Medicine, Miami, USA; [8] University of Washington, Department of Epidemiology, Seattle, USA; [9] University of Washington, Department of Health Systems and Population Health, Seattle, USA; [10] Albert Einstein College of Medicine, Department of Epidemiology and Population Health, Bronx, USA

Objective: Neighborhood-scale environmental factors, including disinvestment in infrastructure, may impact cardiometabolic disease risk. To our knowledge, no prior studies have investigated the association between neighborhood disinvestment and incident diabetes.

Material and methods: We used a virtual street audit of Google Street View Imagery to generate a neighborhood disinvestment score for participants' residential addresses in the Hispanic Community Health Study/Study of Latinos (HCHS/SOL). An item response theory model was fit to indicators (litter, graffiti, under-maintained buildings, bars on windows, and abandoned buildings) to form a scale measuring a latent level of disinvestment. Ordinary kriging was used to estimate levels for each residential address within the HCHS/SOL census tracts via spatial interpolation. The HCHS/SOL is a longitudinal cohort study of self-identified Hispanic/Latino adults in the Bronx, Chicago, Miami, and San Diego. Using covariate-adjusted and survey-weighted Poisson regression models with data from 9120 participants free of diabetes at baseline (2008-2011), we investigated the association between neighborhood disinvestment z-score and incident diabetes at visit 2 (2014-2017). A sensitivity analysis included only those who did not move during the follow-up period.

Results: A one-standard deviation increase in neighborhood disinvestment score was associated with a 13% (95% CI: 1%-23%) lower risk of incident diabetes when adjusting for age, sex, education, income, study center/heritage, years in the US, family history, and a neighborhood socioeconomic index. Our sensitivity analysis yielded qualitatively similar results with lower precision.

Conclusion: Overall, our analysis does not support the hypothesis that neighborhood physical disinvestment is associated with incident diabetes in this Hispanic/Latino population.

P15-06 | Associations Between Repeated Exposure to Phthalate, Phenol, and Paraben Mixtures and Metabolic Syndrome in Mexican Midlife Women

Lindsey English[1], Wei Hao[2], Erica C. Jansen[1], Jaclyn M. Goodrich[3], Deborah J. Watkins[3], Libni A. Torres Olascoaga[4], John D. Meeker[3], Martha M. Tellez-Rojo[4], Karen E. Peterson[1,3]

[1] University of Michigan School of Public Health, Department of Nutritional Sciences, Ann Arbor, USA; [2] University of Michigan School of Public Health, Department of Biostatistics, Ann Arbor, USA; [3] University of Michigan School of Public Health, Department of Environmental Health Sciences, Ann Arbor, USA; [4] National Institute of Public Health, Cuernavaca, Morelos, Mexico

Objective: Using repeated urinary measures of phthalate, phenol, and paraben exposure during adulthood in Mexican women, we examined time-specific effects of exposure to endocrine disrupting chemical (EDC) mixtures on cardiometabolic risk in midlife.
Materials and Methods: The analytic sample comprised 422 women from the Early Life Exposures in Mexico to ENvironmental Toxicants cohort. Urinary concentrations of phthalate metabolites, phenols, and parabens were measured in 2008 and 2019, then log-transformed and specific gravity-corrected. Metabolic Syndrome (MetS) and its components (hypertriglyceridemia, hypertension, abdominal obesity, cholesterolemia, hyperglycemia) were assessed in 2019 and set as binary outcomes. For mixtures analyses, we examined the joint effect of chemical mixtures on MetS using quantile g-computation. We examined time-specific effects by examining longitudinal and cross-sectional associations along with cumulative exposure for three mixtures classes (all EDCs, phthalates-only, phenols-only). Models were adjusted for baseline age, smoking status, education, and BMI.
Results: In 2019, participants were 48.5 (SD 6.0) years of age, and 51.2% had MetS. Those with MetS were more likely to be older, postmenopausal, and have a higher BMI. In adjusted models, a one quartile increase in 2019 phthalate mixture was associated with 1.97 (95% CI:1.04, 3.73) higher odds of abdominal obesity and 0.61 (95% CI:0.38, 0.97) lower odds of hypertension. A one quartile increase in the cumulative phthalate mixture resulted in a stronger association than 2019 exposure alone with a 0.50 (95% CI:0.26, 0.94) lower odds of hypertension. A one quartile increase in the phenol mixture from 2019 had 1.40 greater odds of hyperglycemia (95% CI:1.03,1.89). There were no associations of mixtures from 2008 only and MetS.
Conclusion: Increased exposure to different EDC mixtures had different effects on MetS components, with stronger associations for concurrent exposure. Further research is needed to understand the observed negative association of phthalate mixtures on hypertension.

P15-07 | Effect of smoking on mortality in patients with ST-segment elevation myocardial infarction after primary percutaneous coronary intervention

Man-Ju Ting

National Taiwan University, Institute of Environmental and Occupational Health Sciences, Taipei, Taiwan

Background
The "smoker's paradox" suggests better outcomes for smokers following ST-segment elevation myocardial infarction (STEMI) treated with primary percutaneous coronary intervention (PCI); however, evidence remains conflicting. This study aimed to examine the association between smoking and mortality in STEMI patients and evaluate the prognostic utility of a novel triage index, the hypoxia-age-shock index (HASI).
Methods
A case-control study analyzed 404 STEMI patients (272 smokers and 132 non-smokers) treated with primary PCI. Patient characteristics, clinical outcomes, and laboratory findings were compared. The predictive power of the shock index (SI), age shock index (ASI), and HASI for adverse outcomes was assessed using receiver operating characteristic (ROC) curve analysis. DeLong's method was used to compare the ROCs.
Results
Smokers were younger (56.8 ± 11.1 vs. 63.2 ± 13.8 years, $p < 0.001$) and predominantly male (95.2% vs. 68.2%, $p < 0.001$). No significant differences were observed between smokers and non-smokers in intubation rates, ICU stay, hospital length of stay, or in-hospital mortality. HASI demonstrated superior prognostic accuracy for in-hospital mortality (AUC = 0.75, $p = 0.012$) compared to SI and ASI. Smokers exhibited elevated leukocyte counts and hemoglobin levels.
Conclusions
This study challenges the smoker's paradox in STEMI patients undergoing primary PCI and highlights HASI as an effective triage tool for early risk stratification. These findings emphasize the importance of tailored risk assessment and aggressive smoking cessation strategies in this high-risk population.

P15-08 | Investigating the Interplay of Toxic Metals and Essential Elements in Cardiovascular Disease

Aderonke G. Adetunji, Emmanuel Obeng-Gyasi

North Carolina A&T State University, Built Environment, Greensboro, USA

Cardiovascular diseases (CVDs) are the world's leading cause of death, accounting for approximately one-third of all deaths. Exposure to toxic metals presents significant cardiovascular health risks, contributing to the onset of CVDs. Essential elements are vital for maintaining proper cardiovascular function, but their imbalances or deficiencies can heighten the risk and progression of CVDs. Understanding how toxic metals interact with essential elements is key to comprehending their effect on cardiovascular health. This study aims to examine the individual and combined effects of toxic metals—lead (Pb), cadmium (Cd), and mercury (Hg)—along with essential elements—manganese (Mn), iron (Fe), and selenium (Se)—on CVDs. We analyzed National Health and Nutrition Examination

Survey (NHANES, 2017–2018) data to assess the individual and combined effect of toxic metals and essential elements on key cardiovascular biomarkers, using descriptive analyses and advanced methods including Bayesian Kernel Machine Regression (BKMR), Weighted Quantile Sum Regression (WQSR), and Quantile g-computation. The results revealed distinct relationships between the toxic metals and essential elements. Spearman correlation revealed a stronger association between toxic metals than essential elements. BKMR, posterior inclusion probability (PIP) analysis identified lead, mercury, iron, and selenium as key contributors to CVD risk. Univariate and bivariate analyses confirmed lead and mercury's strong associations with triglycerides and blood pressure, while lead, selenium, and iron were linked to different cholesterol outcomes. Single-variable analysis revealed an interaction between individual chemicals with combined exposures. The overall exposure revealed a steady positive association with CVD markers increasing from the 0.65 quantile. Quantile g-computation and WQSR confirmed lead's consistent dominant role in a mixture across all CVD markers, with variations among other toxic metals and essential elements. In conclusion, our study suggests that toxic metals and essential elements are important factors in CVD, with different metals and elements associated with variations in specific biomarkers.

P15-09 | Association of PFAS and Metals with Cardiovascular Disease Risk: Exploring the Mediating Effect of Diet

Augustina O. Odediran, Emmanuel Obeng-Gyasi

North Carolina Agricultural and Technical State Un, Applied Engineering Technology/ Built Environment, Greensboro, USA

Cardiovascular disease (CVD) is a major global health burden, influenced by genetic, behavioral, and environmental factors. Exposure to per- and polyfluoroalkyl substances (PFAS) and toxic metals has been increasingly implicated in adverse cardiovascular outcomes. However, the mediating role of dietary inflammation in these associations remains unclear.

This study investigates the relationship between PFAS, metal exposures, and CVD risk, focusing on the potential mediating role of diet, operationalized through the Dietary Inflammatory Index (DII). Additionally, the study examines age as an effect modifier.

Utilizing data from the National Health and Nutrition Examination Survey (NHANES) 2017-2018 cycle (n = 660), we assessed environmental exposures (lead, cadmium, mercury, PFOA, PFOS), dietary inflammatory potential (DII), and cardiovascular markers (blood pressure, lipid profile, C-reactive protein). Statistical analyses included linear regression and Bayesian Kernel Machine Regression-Causal Mediation Analysis (BKMR-CMA) to estimate direct, indirect, and total effects of exposure on CVD risk biomarkers while accounting for age-related modifications.

Linear regression revealed significant associations between mercury and reduced systolic blood pressure (SBP) ($p = 0.017$) and cadmium with increased C-reactive protein (CRP) ($p = 0.006$), suggesting an inflammatory response. Additionally, mercury was associated with higher HDL ($p = 0.001$), while PFOA showed a negative association with HDL ($p = 0.022$). BKMR-CMA indicated dietary inflammation (DII) mediated part of the relationship between PFAS/metals and cardiovascular markers, with stronger mediation in younger individuals. However, mediation effects were inconsistent across age groups, with certain biomarkers exhibiting negative mediation in older populations, suggesting complex age-related modifications.

This study provides evidence that PFAS and metal exposures contribute to CVD risk. While diet mediates some associations, its role is influenced by age, with younger individuals demonstrating greater susceptibility. These

findings highlight the need for targeted public health interventions integrating dietary modifications and exposure reduction strategies to mitigate CVD risk, particularly in vulnerable populations.

P15-10 | Combined Effects of Metals, PCBs and Dioxins on Cardiovascular Dysfunction

Bolanle Akinyemi, Emmanuel Obeng-Gyasi

North Carolina Agricultural and Technical State University, Applied Science and Technology / Built Environment, Greensboro, USA

Exposure to environmental pollutants such as heavy metals (lead, cadmium, and mercury), polychlorinated biphenyls (PCBs), dioxins, and furans has been individually associated with adverse effects on various physiological systems. However, limited research has examined the combined impact of these contaminants on cardiovascular dysfunction. This study investigates the joint effects of these pollutants on cardiovascular health using data from the National Health and Nutrition Examination Survey (NHANES) 2003–2004.
We assessed associations between exposure to metals, PCBs, dioxins, furans, and their derivatives with six cardiovascular-related biomarkers: diastolic blood pressure (DBP), systolic blood pressure (SBP), low-density lipoprotein (LDL) cholesterol, high-density lipoprotein (HDL) cholesterol, total cholesterol, triglycerides, and the Framingham Risk Score (FRS). A combination of statistical approaches, including descriptive statistics, Spearman correlation, linear regression, and Bayesian Kernel Machine Regression (BKMR), was used to evaluate potential non-linear relationships and interactions among exposures.
Findings from posterior inclusion probabilities highlighted differential pollutant-specific contributions to cardiovascular biomarkers. Metals exhibited a predominant association with DBP and HDL cholesterol, while PCBs were primarily linked to SBP and FRS. Furans demonstrated significant associations with total cholesterol, LDL cholesterol, and triglycerides. Univariate and bivariate exposure-response analyses revealed complex, multi-pollutant interaction patterns. Single-variable interaction analyses further confirmed significant interactions among pollutants for several cardiovascular-related biomarkers, underscoring the intricate nature of pollutant exposure and cardiovascular risk. These findings emphasize the need for a multi-pollutant approach in environmental health research and highlight the importance of considering pollutant interactions when assessing cardiovascular disease risk.

P15-11 | The AssociationBetween Vitamin D Deficiency and Hypertension in YoungAdults in Taiwan: Exploring Copper as a Mediator

Ching Way Chen[1], Ta-chen Su[2]

[1] National Taiwan University Hospital Yunlin Branch, Cardiology, Douliu, Taiwan; [2] Tungs' Taichung MetroHarbor Hospital, Cardiology, Taichung, Taiwan

Background: The relationship between vitamin D levels and blood pressure is of interest in cardiovascular research. This study investigates the association between serum vitamin D [25(OH)D] levels and systolic blood pressure (SBP) in young adults in Taiwan.
Methods: We conducted a prospective cohort study from 2017 to 2019 on 1,034 young adults, using cross-sectional analyses of data collected at enrollment. SBP was measured using an automated sphygmomanometer after participants rested for five minutes. Linear regression analyses assessed the relationship between vitamin D levels and SBP, adjusting for confounders such as age, sex, BMI, renal function, smoking, and physical activity.The nonlinear effects of vitamin D levels on hypertension risk were further examined using restricted cubic splines model.
Results: The mean age was 33.45 years, and 41.9% were male. The median 25(OH)D level was 19.20 ng/mL, with 54.06% of participants having levels below 20 ng/mL. Multivariable linear regression showed that lower 25(OH)D levels were significantly associated with higher SBP (β = -0.08, p = 0.04), DBP (β = -0.09, p = 0.03), and MBP (β = -0.10, p = 0.03). Restricted cubic spline analysis revealed a U-shaped relationship between vitamin D levels and hypertension risk, with an optimal level of 20 ng/mL.Subgroup analyses indicated no significant interaction between vitamin D levels and either age or gender.
Conclusion
A U-shaped relationship exists between vitamin D levels and blood pressure in young adults in Taiwan, with optimal levels around 20 ng/mL. Maintaining adequate vitamin D may be crucial for cardiovascular health, highlighting the need for further longitudinal studies to determine causality.

P15-12 | The Role of Coronary Artery Calcification in Metal-Related Cardiovascular Disease

Arce Domingo Relloso[1], Katlyn McGraw[1], Irene Martinez-Morata[1], Yuchen Zhang[1], Kathrin Schilling[1], Ronald Glabonjat[1], Ziqing Wang[1], Kiros Berhane[1], Brent Coull[2], Miranda Jones[3], Wendy Post[3], Joel Kaufman[4], Tiffany Sanchez[5], Maria Tellez-Plaza[6], Graham Barr[7], Steven Shea[7], Ana Navas-Acien[1], Linda Valeri[1]

[1] Columbia University, Mailman School of Public Health, New York, USA; [2] Harvard University, TH Chan School of Public Health, Boston, USA; [3] Johns Hopkins University, Bloomberg School of Public Health, Baltimore, USA; [4] University of Washington, Department of Medicine, Seattle, USA; [5] University of Colorado, Anschutz Medical Center, Aurora, USA; [6] Carlos III Health Institute, National Center for Epidemiology, Madrid, Spain; [7] Columbia University, Departments of Medicine and Epidemiology, New York, USA

Metals are associated with cardiovascular disease (CVD), but the underlying pathways remain largely unclear. We evaluated the potential intermediate role of coronary artery calcification (CAC) trajectory on the association between urinary metals and incident CVD, accounting for competing risks by death from other causes. We used data from 6,527 participants of the Multi-Ethnic Study of Atherosclerosis (MESA). CAC was measured longitudinally using the spatially weighted calcium score in five exams, starting in 2000. Participants were followed for CVD events through 2019. Cadmium, cobalt, copper, uranium, tungsten, and zinc were measured in urine at the baseline visit (2000-2002). We used a causal inference algorithm with a path-specific effects approach for longitudinal mediation analysis to evaluate the intermediate role of CAC on the association between metals and incident CVD. During follow-up, 1,140 participants had a CVD event and 1,147 died. The association with incident CVD mediated through the CAC trajectory was statistically significant for all metals except for uranium. The number of CVD cases (95% CI) per 100,000 person-years attributable to an interquartile range (IQR) increase in metal levels through the longitudinal trajectory of CAC was 46 (22, 73) for cadmium, 19 (2, 36) for cobalt, 21 (4, 40) for copper, 21 (3, 40) for tungsten, and 41 (24, 60) for zinc. The results were substantially attenuated for copper and zinc after adjusting for CVD risk factors, but not for the other metals, which supports differences in additionally relevant pathways across metals. This study supports that part of the association between urinary metals and CVD is attributable to changes in CAC over time. In particular, half of the association between urinary cadmium and CVD might be mediated by longitudinal changes in CAC. This study could inform strategies for early detection and prevention of CVD based on CAC measurements.

P15-13 | Heat and Cold effects of Ambient temperature on Heart Failure Hospitalization Risk: A systematic review and meta-analysis.

Hoimonty Mazumder[1], Md. Monir H. Shimul[2], John L. Jefferies[1], Abu M. Naser[1]

[1] The University of Memphis, Division of Epidemiology, Biostatistics, and Environmental Health, Memphis, USA; [2] Daffodil International University, Department of Public Health, Dhaka, Bangladesh

Background: Ambient heat has been identified as a risk factor of adverse cardiovascular outcomes, including stroke, myocardial infarction, and ischemic attacks. While studies have highlighted temperature-related impact of heart failure, a comprehensive synthesis of evidence on the relationship between ambient heat and heart failure is lacking.

Methods: We adhered to the Preferred Reporting Items for Systematic Reviews and Meta-analysis (PRISMA) for this review. A rigorous literature search was performed in leading electronic databases that include PubMed, CINAHL complete, Scopus, Web of Sciences and additional sources using a structured search strategy. We developed eligibility criteria a priori using the PECO (Population-Exposure-Comparators-Outcomes) framework. Finally, we employed the random-effects meta-analysis to estimate pooled effect.

Results: A total of seven articles were identified from the primarily retrieved literature that reported ambient heat induced risk of hospitalization among heart failure patients. Four articles reported cold temperature induced heart failure hospitalization. The meta-analysis showed a 1.1% higher risk of hospitalization among heart failure patients for a 1ºC higher ambient heat [Pooled RR: 1.011 (95% CI: 1.005, 1.017); I^2: 80.79%; p-value <0.001]. For a 1 ºC temperature decrement, the pooled RR of heart failure hospitalization was 1.058 [(95% CI: 1.025, 1.091); I^2: 95.60%; p-value <0.001].

Conclusion: This study reveals ambient temperature related to increased risk of hospitalization among heart failure patients, suggesting that heat and cold, both ambient temperature exposure may exacerbate cardiovascular outcomes.

P15-14 | Association between $PM_{2.5}$ and Daily Hospital Admissions in Heart Failure: A Case-Crossover Analysis in India

Taruna Singh[1], Sagnik Dey[2], Santu Ghosh[3], Sivadasanpillai Harikrishnan[4], Ajay Bahl[5], Animesh Mishra[6], Jayesh Prajapati[7], C N Manjunath Manjunath[8], Rishi Sethi[9], Santanu Guha[10], Santhosh Satheesh[11], Jeemon Panniyammakal[4], Ambuj Roy[12]

[1] Indian Institute of Technology Delhi, School of Interdisciplinary Research, Delhi, India; [2] Indian Institute of Technology Delhi, Centre for Atmospheric Sciences, Delhi, India; [3] St. John Medical College Bangalore, Department of Biostatistics, Bangalore, India; [4] Sree Chitra Tirunal Institute for Medical Sciences and Technology (SCTIMST), Trivandrum, India, Department of Cardiology, Trivandrum, India; [5] Postgraduate Institute of Medical Education and Research (PGIMER), Department of Cardiology, Chandigarh, India; [6] North-Eastern Indira Gandhi Regional Institute of Health and Medical Sciences (NEIGRIHMS), Department of Cardiology, Shillong, India; [7] U N Mehta Institute of Cardiology and Research Centre (UNMICRC), Ahmedabad, India; [8] Sri Jayadeva Institute of Cardiovascular Sciences and Research (SJICR), Bangalore, India; [9] King George's Medical University (KGMU), Lucknow, India; [10] Medical College Hospital (MCH), Department of Cardiology, Kolkata, India; [11] Jawaharlal Institute of Postgraduate Medical Education and Research (JIPMER), Pondicherry, India; [12] All India Institute of Medical Sciences Delhi, Department of Cardiology, Delhi, India

Introduction: Numerous epidemiological studies have shown that short-term changes in ambient particulate matter ($PM_{2.5}$) increase the risk of unplanned cardiovascular hospital admissions. However, there have been few studies from developing countries that have substantially higher levels of $PM_{2.5}$ exposure. This study examines the association between short-term $PM_{2.5}$ exposure and daily hospital admissions among patients with heart failure in India.

Methodology: We analyzedhospital admissions among a cohort of heart failure patients from the National Heart Failure Registry from January 2019 to December 2020. A satellite-based $PM_{2.5}$ dataset (1 km resolution) was used for exposure assessment (Katoch et al., 2023). This dataset was used due to its high spatial resolution and comprehensive coverage of India, ensuring accurate exposure estimation. The association between $PM_{2.5}$ exposure and hospitalizations was estimated using a case-crossover approach, controlling for the same day of the week, month, and year, and considering the running mean exposure over 0–7 days for cases and controls.

Results: During the study period, 10850 hospital admissions for heart failure (HF) were noted. Using conditional logistic regression, the effect of $PM_{2.5}$ on hospital admissions was most robust for the two-day running mean exposures, with an odds ratio of 1.038 (95% CI: 1.019–1.056) for every 10 μg/m³ increase in $PM_{2.5}$. We also found that older patients (≥ 65 years) were more susceptible to $PM_{2.5}$, with an odds ratio of 1.052 (95% CI: 1.021-1.083) for two-day running mean exposure.
Conclusion: In patients with heart failure, increased ambient air pollution was associated with higher hospital admissions. This is the first study from India on hospital admissions and calls for urgent interventions to control air pollution to reduce morbidity due to cardiovascular diseases.

P15-15 | Spatial analysis of socioeconomic variables and mortality due to cardiovascular diseases in São Paulo, Brazil.

Priscilla V. Ikefuti

State University of Maranhao, Department of Geography, São Luís, Brazil

With the epidemiological transition in Brazil, chronic diseases are now responsible for more deaths between men and women. Among the components that define the state of human health, some geographical context, as socioeconomic factors appear to influence the mortality from circulatory diseases, such as stroke. The main objective of this study was to investigate the influence of the geographical context in mortality from cardiovascular diseases (stroke) in São Paulo, Brazil, between 2002 to 2011. In order to analyze the association of mortality with socioeconomic variables we used spatial regression models Ordinary Least Squares (OLS) and Geographically Weighted Regression (GWR). As a result, we found that the highest values of relative risk (RR) for stroke mortality were concentrated in the outskirts of the city of São Paulo, which coincided with areas of lower per capita income and vegetation and higher percentage of black population. In conclusion, our research has shown that both environmental and socioeconomic variables influence on mortality from some diseases of the circulatory system. In view of the urgent care for stroke cases can avoid serious sequelae and deaths, a better understanding of the importance of geographic context may allow the development of warning systems at the urgent care services and targeting campaigns for the prevention of avoidable risk factors.

P16 | Advancing Exposomics: Comprehensive Assessment of Environmental and Lifestyle Exposures - PART I

P16-01 | Ambient temperature and endocrine metabolic disruption during pregnancy in two Spanish prospective cohorts

Adina Cazacu-De Luca[1,2], Esmée Essers[1,3], Mònica Guxens[1,4], Yu Zhao[1], Emily Laveriano[1,4], Estelle Renard-Dausset[1], Jordi Sunyer[1,4], Payam Dadvand[1,4], Martine Vrijheid[1,4], Óscar J. Pozo[5], Léa Maitre[1,4]

[1] ISGlobal, Barcelona, Spain; [2] Harvard T.H. Chan School of Public Health, Department of Environmental Health, Boston, USA; [3] Erasmus University Medical Center, Department of Child and Adolescent Psychiatry/Psychology, Rotterdam, Netherlands; [4] Universitat Pompeu Fabra, Barcelona, Spain; [5] Hospital del Mar Research Insititute, Barcelona, Spain

Objective: Exposure to higher temperature during pregnancy has been linked to adverse maternal and child health outcomes, but endocrine-disrupting effects remain unclear. We examined the association between temperature during pregnancy and sex steroid hormone metabolites (including progestogens, androgens, estrogens, corticosteroids) in 1,118 pregnant women from two Spanish birth cohorts: INMA Sabadell and BiSC.

Methods: Women from Barcelona province were recruited during pregnancy from 2004 to 2006 (INMA Sabadell) or 2018-2021 (BiSC). Daily temperature exposures at 100 and 250 m resolution during pregnancy were estimated using validated spatiotemporal models. We applied generalized linear models (GLMs) and distributed lag nonlinear models (DLNMs) to assess relationships between temperature and urine-collected steroid metabolites measured via targeted LC-MS/MS at 32 weeks of gestation. Analyses included 50 individual metabolites and 38 metabolic indicators. We adjusted for maternal age, parity, education, diet, BMI, physical activity, and season of urine sampling. Cohorts were analyzed separately. GLMs examined mean and maximum temperatures over various periods before third-trimester urine collection. DLNMs incorporated 1-week and 1-month lags.

Results: Weekly average temperatures ranged from 1 to 30°C. After multiple testing correction, the glucuronide-sulfate ratio for progesterone, progestogens, and androgens increased with temperature across time periods. For example, in INMA, 30-day maximum temperature was associated with a 1.05 [95% CI 1.03; 1.06] higher progesterone ratio, 1.03 [1.02; 1.05] for progestogens, and 1.02 [1.01; 1.03] for androgens. In both cohorts, DLNMs showed significant cumulative effects over monthly and weekly lag periods on five glucuronide-sulfate ratios (androgens, cortisol, estradiol, estrogens, testosterone).

Conclusions: The glucuronide and sulfate forms of steroid hormones differ in bioavailability, with the former more easily excreted. We found increases in the glucuronide forms of progesterone, progestogens, and androgens with increased temperature. These findings provide novel evidence of temperature-related endocrine disruption with potential maternal and child health implications.

P16-02 | Evaluating Sex-Mediated Transplacental Transfer of Environmental Chemicals in the Human Placenta and Fetal Growth

Michelle Misselwitz, Shaza Gaballah, Kate Hoffman, Heather M. Stapleton

Duke University, Durham, USA

Environmental exposures during pregnancy can impact fetal growth, increasing the risk of long-term adverse health outcomes. Given the crucial role of the *placenta*, the analysis of environmental chemicals in placental tissue provides novel insights into fetal exposure and adverse birth outcomes. While sex-disparate health outcomes have been observed, the mechanism for this modulation is still unknown.
To address this data gap, we analyzed 200 placental tissues collected as part of a prospective pregnancy cohort recruited in central North Carolina, USA. Maternal and fetal placental sections were dissected for separate analysis to evaluate the transfer of chemicals across the placental barrier. A suite of environmental chemicals (e.g., flame retardants, polychlorinated biphenyls (PCBs), pesticides, polycyclic aromatic hydrocarbons (PAHs), plasticizers, and industrial chemicals) were quantified via gas chromatography – high resolution mass spectrometry (GC-HRMS).
Out of 120 total chemicals evaluated, between 3 to 43 chemicals were detected in each of the first 100 placenta tissues. There were 9 chemicals that were detected in at least 50% of all samples. Chemical concentrations were, on average, higher on the fetal side of the placenta compared to the maternal side for 6 out of the 9 highly detected chemicals. Data were stratified by maternal and fetal placenta tissue and infant sex to evaluate associations with fetal growth indicators. Using multiple linear regression and adjusting for maternal age, concentrations of tris(2-butoxyethyl)phosphate (TBoEP) in female fetal placenta tissue was inversely correlated with birthweight for gestational age z-score (β = -0.50, p = 0.03). This association was stronger using the fetal placenta tissue concentrations compared to maternal tissue (β = -0.31, p = 0.25). In addition to single chemical evaluations, the effects of chemical mixtures will be evaluated. This study is funded by the National Institutes of Health NIH R01 grant.

P16-03 | Sex differences in daytime light exposure among the general US population

Danielle Wallace[1,2]

[1] Rollins School of Public Health, Emory University, Gangarosa Department of Environmental Health, Atlanta, USA; [2] Harvard Medical School, Division of Sleep Medicine, Boston, USA; [3] Brigham and Women's Hospital, Division of Sleep and Circadian Disorders, Departments of Medicine and Neurology, Boston, USA

Light is fundamentally important for biological rhythms, physiology, and health. Light is a complex, multi-dimensional (intensity, duration, timing, wavelength, exposure history) environmental exposure and component of the exposome, but it is relatively understudied in public health research. In addition to intensity and duration, one of the most important aspects of light exposure is timing of exposure. Here, we utilize objective personal (rather than satellite) measures of light from wrist-worn wearables in the US-based National Health and Nutrition Examination Survey (NHANES, 2011-2014). Building on our prior work characterizing light exposure patterns in the general population, we hypothesize that the amount and timing of 24-hour light exposure significantly differs by sex. To test this, we analyzed data from more than 11,000 participants ages 3-80+ years old. Using functional data analysis (FDA) methods with linear Function-on-Scalar Regression (FoSR) models, timeseries light data were modelled as the

outcome and sex as the predictor in models adjusted for age, season of measurement, income, sleep duration, and activity. Sensitivity analyses also evaluated the role of occupation and time spent outside. Results of the adjusted FoSR models show females have significantly lower light exposure compared to males, particularly from 7AM (β= -7.0 lux; 95%CI: -9.2, -4.7) to 7PM (β= -8 lux; 95%CI: -11, -5 lux), with the greatest difference at noon (β= -94 lux; 95%CI: -104, -83 lux). These findings support large sex difference in daytime light exposure, but not nighttime light, with females getting less daytime light compared to males. Results may be at least partly explained by sex differences in occupational factors and time spent outdoors. These results highlight the need for future work to consider the role of sex differences in light exposure on health and the incorporation of light measurement in exposome research.

P16-04 | Joint Effects of Lifestyle Habits and Heavy Metals Exposure on Chronic Stress Among U.S. Adults: Insights from NHANES 2017–2018

Esther O. Ogundipe[1], Emmanuel Obeng-Gyasi[2]

[1] North Carolina A&T State University, Applied Science & Technology / Built Environment, Greensboro, USA; [2] North Carolina A&T State University, Applied Science & Technology / Built Environment, Greensboro, USA

Chronic stress, characterized by sustained activation of physiological stress response systems, is a risk factor for numerous health conditions. Allostatic load (AL), a biomarker of cumulative physiological stress, offers a quantitative measure of this burden. Lifestyle habits such as alcohol consumption and smoking, alongside environmental exposures to toxic metals like lead, cadmium, and mercury, are implicated in increasing AL. However, the combined impact of these lifestyle habits and environmental factors remains underexplored. This study aims to investigate the joint effects of lifestyle habits and environmental factors on AL, using data from the NHANES 2017–2018 cycle.
Descriptive statistics were calculated to summarize key indicators with multivariate linear regression performed to assess associations between exposures and AL. BKMR was employed to estimate exposure–response relationships and posterior inclusion probabilities (PIPs).
Descriptive analysis indicated that the mean levels of lead, cadmium, and mercury were 1.23 μg/dL, 0.49 μg/dL, and 1.37 μg/L, respectively. The mean AL was 3.57. Linear regression indicated that alcohol consumption was significantly associated with increased AL (β = 0.0933; 95% CI [0.0369, 0.1497]; p = 0.001). Other exposures, including lead (β = −0.1056; 95% CI [−0.2518 to 0.0408]; p = 0.157), cadmium (β = −0.0001, 95% CI [−0.2037 to 0.2036], p = 0.999), mercury (β = −0.0149; 95% CI [−0.1175 to 0.0877]; p = 0.773), and smoking (β = 0.0129; 95% CI [−0.0086 to 0.0345]; p = 0.508), were not significant. BKMR analysis confirmed alcohol's strong importance for AL, with a PIP of 0.9996. The interaction between alcohol and cadmium showed a stronger effect on AL at higher exposure levels. In contrast, lead, mercury, and smoking demonstrated minimal effects on AL.
In conclusion, alcohol consumption and cadmium exposure are key contributors to increased AL. These findings emphasize the need to consider lifestyle and environmental factors in physiological stress assessment.

P16-05 | Bayesian Network Analysis of Environmental, Physiological, Lifestyle, Socioeconomic, and Demographic Factors in Cardiometabolic Risk

Hope Nyavor, Emmanuel Obeng-Gyasi

North Carolina Agricultural and Technical State University, Greensboro, USA

Abstract

Environmental exposures, including heavy metals (lead, cadmium, mercury) and per- and polyfluoroalkyl substances (PFAS: PFOA, PFOS), may contribute to cardiometabolic health risks. However, their interactions with biological, lifestyle, socioeconomic, and demographic factors remain underexplored. This study models these relationships using Bayesian network analysis to uncover dependencies among cardiometabolic risk factors.

This study utilized NHANES 2017-2018 data (n = 9,254) with 22 selected variables, including blood pressure, cholesterol, triglycerides, BMI, CRP, environmental pollutants, and lifestyle behaviors. Spearman's correlation analysis was performed to identify preliminary associations. Bayesian networks were constructed using constraint-based algorithms (Grow-Shrink, IAMB, Fast-IAMB) and score-based algorithms (Hill-Climbing) to infer relationships and assess structural differences.

Correlation analysis showed a strong positive correlation between total cholesterol and LDL cholesterol (0.90) and a moderate correlation between systolic and diastolic blood pressure (0.48). BMI and CRP exhibited a weak to moderate correlation (0.38), reinforcing a potential link between obesity and inflammation. HDL cholesterol showed an inverse correlation with triglycerides (-0.49) and allostatic load (-0.41), suggesting a protective cardiometabolic effect.

Among environmental exposures, cadmium demonstrated the strongest positive correlation with smoking (0.56) and lead was moderately correlated with income (-0.34), indicating disproportionate exposure among lower-income groups. PFOS exhibited a moderate correlation with gender (0.61), while PFOA was weakly correlated with income (0.25).

Bayesian network analysis revealed PFAS, heavy metals, and demographic factors as key influencers of cardiometabolic outcomes. The constraint-based Bayesian network identified broad associations, while the Hill-Climbing network captured specific pathways linking environmental exposures to cardiometabolic risk via BMI and inflammation (CRP). Constraint-based models were more conservative, whereas the Hill-Climbing algorithm identified more conditional dependencies.

This study identifies cardiometabolic risk relationships and highlights the impact of environmental pollutants. Bayesian networks reveal BMI and CRP as key mediators, emphasizing the need for further research on causal pathways to inform public health interventions.

P16-06 | An Exposome-Wide Association Study of Chronic Pain using the National Health and Nutrition Examination Survey 1999-2004

Jiaxin Wu[1], Erika Walker[1], Kelly M. Bakulski[1], Sung Kyun Park[1,2]

[1] University of Michigan, Department of Epidemiology, Ann Arbor, USA; [2] University of Michigan, Department of Environmental Health Sciences, Ann Arbor, USA

Objective

Chronic pain, affecting a significant portion of the population, is a major health issue with complex underlying mechanisms. Limited studies have explored the associations between environmental chemicals and chronic pain. We conducted an Exposome-Wide Association Study (ExWAS) to identify potential environmental risk factors for chronic pain.

Material and Methods

This cross-sectional study included 10,262 participants aged 20 and over from the United States National Health and Nutrition Examination Survey (NHANES), 1999-2004. Chronic pain was self-reported as having pain in any region persisting for more than 12 weeks. Biomarker levels for 131 environmental chemicals from 14 classes were $\log_2$ transformed. The relationships between chemicals and chronic pain were adjusted for age, sex, race/ethnicity, poverty income ratio, education, alcohol consumption, body mass index, and smoking status using separate survey-weighted modified Poisson regressions, where prevalence ratios (PRs), 95% confidence intervals (CIs), and false discovery rate (FDR) corrected p-values were computed.

Results

The prevalence of chronic pain was 18.2%. Three chemicals were associated with chronic pain (FDR corrected p-value < 0.05), including 3,5,6−trichloropyridinol (PR = 0.80, 95% CI: 0.72-0.88), thallium (PR = 0.87, 95% CI: 0.81-0.93), and polychlorinated biphenyl 99 (PR = 0.89, 95% CI: 0.83-0.95). Two additional chemicals were associated (p-value < 0.01) with chronic pain, including polychlorinated biphenyl 28 (PR = 0.83, 95% CI: 0.74-0.93) and m−/p−Xylene (PR = 1.16, 95% CI: 1.04-1.28).

Conclusion

As the first ExWAS of prevalent chronic pain, our findings indicated that exposure to different chemical families are associated with chronic pain in different directions: volatile organic compounds associated with higher prevalence of chronic pain, while metals, pesticides, and polychlorinated biphenyls associated with lower prevalence. Chemicals may impact chronic pain through inducing inflammation, oxidative stress, and disrupting pain signaling pathways. Future longitudinal studies are necessary to explore environmental chemicals’ specific roles in chronic pain development and progression.

P16-07 | Community Contexts of Health: A Comparative Study of Health Risk Factors and Outcomes Across 774 Communities in 25 Countries in the PURE Study

Madalyn Nones, Perry Hystad

Oregon State University, College of Health / Public Health, Corvallis, USA

Background: Communities play a critical role in shaping health-related behaviors and outcomes, reflecting the collective impact of environmental, social, and structural factors. Understanding variation in health across and within communities may provide insight into the "total" influence of community contexts to health. Assessing communities on a global scale provides insight on how the magnitude of community-dependent variation shifts based on place.
Objective: Use epidemiologic methods to estimate variations in health outcomes due to community specific contexts.
Methods: We leveraged the Prospective Urban and Rural Epidemiology (PURE) study, which included ~200,000 individuals from 774 communities across 25 countries. Individual data on community perceptions, health behaviors, physiological measures, health outcomes, and biomarkers were examined. Community intraclass correlation coefficients (ICCs) were calculated using linear, logistic, and Cox proportional hazards models adjusted for age, sex, education, and household wealth.
Results: Substantial between-community variation was observed across all outcomes. For example, 39.5% of crime perceptions, 18.5% of total METs, 28.9% of BMI, 8.5% of hypertension, 16.7% of severe CVD events, and 5.3% of leptin biomarker levels. Models controlling for country showed slightly attenuated ICCs (e.g., 13% of total METs, 9.1% for CVD events, 5.5% for hypertension).
Discussion: These findings demonstrate that a large portion of health-related behaviors and outcomes is shaped by "total" community context. Studies examining single environmental, social, or structural factors may fail to capture the complex, integrated influence of community environments on health. Future work intends to combine this modeling approach with modeling techniques for individual-level community factors to better estimate how community variables such as the built environment shape a variety of health outcomes.

P16-08 | Association of Combined Effect of Metals Exposure and Behavioral Factors on Depressive Symptoms in Women

Olamide Ogundare, Emmanuel Obeng-Gyasi

North Carolina A&T State University, Built Environment, Greensboro, USA

This study investigates the combined effects of environmental pollutants (lead, cadmium, total mercury) and behavioral factors (alcohol consumption, smoking) on depressive symptoms in women. Data from the National Health and Nutrition Examination Survey (NHANES) 2017–2018 cycle was used in this study. The analysis of these data included the application of descriptive statistics, linear regression, and Bayesian Kernel Machine Regression (BKMR) to explore associations between environmental exposures, behavioral factors, and depression. The PHQ-9, a well-validated tool that assesses nine items for depressive symptoms, was used to evaluate depression severity. Exposure levels of heavy metals were measured in blood samples. BKMR was used to estimate the exposure–response relationship, while posterior inclusion probability (PIP) in BKMR was used to quantify the likelihood that a

given exposure was included in the model, reflecting its relative importance in explaining depression. A descriptive analysis showed mean total levels of lead, cadmium, and total mercury at 1.21 µg/dL, 1.47 µg/L, and 0.80 µg/L, respectively, with a mean PHQ-9 score of 5.94, which corresponds to mild depressive symptoms. Linear regression indicated positive associations between depression and lead as well as cadmium. Alcohol and smoking were also positively associated with depression. BKMR posterior inclusion probability (PIP) analysis revealed alcohol and cadmium as significant contributors to depressive symptoms. Univariate and bivariate analyses revealed lead and total mercury's strong relationship with depression, with cadmium showing a complex pattern in the bivariate analysis. A cumulative exposure analysis of all metals and behavioral factors concurrently demonstrated that higher quantile levels of combined exposures were associated with increased depression. Finally, a single variable-effects analysis in BKMR revealed lead, cadmium, and alcohol had a strong impact on depression. Overall, the study findings suggest that exposure to environmental and behavioral factors in a mixture contribute to depressive symptoms.

P16-10 | Integrating Human Exposome Data into Next-Generation Risk Assessment: A Systematic Framework to Infer Real-Life Exposure Levels and Prioritize Chemical Compounds

Yifeng Dai[1,2], Bob van de Water[3], Jaione Telleria[4], Laura I. Furlong[4], Roel C. Vermeulen[1,2], Jelle J. Vlaanderen[2]

[1] Utrecht University Medical Center, Department of Global Public Health and Bioethics, Utrecht, Netherlands; [2] Utrecht University, Institute of Risk Assessment Sciences, Utrecht, Netherlands; [3] Leiden University, Division of Drug Discovery and Safety, Leiden, Netherlands; [4] MedBioinformatics Solutions SL, Barcelona, Spain

Background: Exposome research and next-generation risk assessment (NGRA) are parallel but complementary fields that could benefit from the two-way exchange of data and insights. However, specific implementation strategies for integrating human exposome data into NGRA have yet to be identified.

Objective: To develop a comprehensive framework to inform NGRA about real-life human chemical exposure levels and prioritize chemicals by integrating exposure, toxicity, and bioactivity data.

Material and Methods: We developed a framework consisting of three modules: a curated human chemical exposure database, model-predicted human chemical exposure estimates, and chemical-related information incorporating chemical properties, toxicity, and bioactivity data. Then, we validated the effectiveness of the constructed framework using two case studies. Case study #1 focused on organ-specific carcinogens, selecting potential hepatocarcinogens as the target candidate compounds. Case study #2 employed an exposure-driven approach, emphasizing compounds of concern commonly identified in biomonitoring programs across different human populations. We integrated exposure, toxicity, bioactivity, and risk estimate data to assess the utility of our approach for NGRA.

Results: The application of our framework in the two case studies yielded actual concentration value ranges of the compounds in different biological samples, predicted blood concentrations, and in vitro exposure intake values. These concentration values provide reference ranges for human-relevant exposure to be included in NGRA approaches and can be compared with existing in vitro cell toxicity AC50 values. We also demonstrated that our approach can be used to prioritize compounds in NGRAs. Notably, the prioritization of compounds shifts significantly when the human exposure data are factored in. However, the limited availability of human exposure data for chemicals remains a critical constraint, highlighting the need for enhanced data collection and integration in future risk assessment efforts.

Conclusion: This flexible framework provides a comprehensive approach to leveraging exposome data to generate human-relevant compound exposure insights to inform NGRAs.

P16-11 | Exposome-wide associations and polyexposure risks of workplace chemical exposures for adult asthma

Yujing Chen[1], Farida S. Akhtari[2], Chirag J. Patel[3], Alison Motsinger-Reif[2], Ming Kei Chung[1,4]

[1] The Chinese University of Hong Kong, The Jockey Club School of Public Health and Primary Care, Hong Kong, Hong Kong; [2] National Institute of Environmental Health Sciences, Biostatistics and Computational Biology Branch, Division of Intramural Research, Durham, USA; [3] Harvard University, Department of Biomedical Informatics, Harvard Medical School, Boston, USA; [4] The Chinese University of Hong Kong, Institute of Environment, Energy and Sustainability, Hong Kong, Hong Kong; [5] The Chinese University of Hong Kong, Li Ka Shing Institute of Health Sciences, Hong Kong, Hong Kong

Objective: Many potential occupational chemical asthmagens and their co-exposure risk remain underestimated. We employed an exposome-wide association study (ExWAS) to systematically explore occupational chemical exposures and estimate their combined risk for asthma and related multimorbidity.

Materials and Methods: This cross-sectional study included 3148 adults from the Personalized Environment and Genes Study (PEGS), a North Carolina-based cohort with questionnaire-based health and exposure data and genomic data. Standardized questionnaires investigated occupational exposure to 93 chemical agents across 18 groups and the use of personal protective equipment (PPE). Current adult asthma was defined as self-reported doctor-diagnosed asthma with recent attacks in the past year. Respiratory multimorbidity was ascertained if asthma coexists with allergic rhinitis or chronic obstructive pulmonary disease. Logistic regression-based ExWAS analyzed associations for individual chemicals. Weighted standardized polyexposure scores (PXSs) were developed for predefined known asthmagens (PXS_{known}) and suspected or novel risk agents ($PXS_{\text{suspected or novel}}$), selected through a regularized selection procedure.

Results: Approximately 9.7% of adults had current asthma. In ExWAS analysis, ever exposure to cleaning liquids, heavy metals, dust, and combustion emissions, was positively associated with asthma [adjusted odds ratio (aOR): 1.50-2.10; false discovery rate (FDR)< 0.05)], particularly when exposure occurred weekly or lasting over 1 year. PXS_{known} (four agents) and $PXS_{\text{suspected or novel}}$ (six agents) showed positive associations with asthma and multimorbidity (aOR: 1.22-1.94 per 0.1 increment). Additionally, participants using gloves and protective clothing had reduced risks of asthma and respiratory multimorbidity in relation to $PXS_{\text{suspected or novel}}$ ($P_{\text{interaction}}< 0.05$).

Conclusion: This data-driven study identified potential suspected and novel risk chemical agents not yet recognized as occupational asthmagens. PXS summarized joint exposures and their effects on asthma and respiratory multimorbidity, supporting occupational risk assessment in public health settings.

Supported by a grant from the CUHK (code 4937217)

P16-12 | Precision in a Pinprick: Biomonitoring from a Dried Blood Strip

Meghan M. Dillon[1], Alina M. Hamilton[2], Shobhan Gaddameedhi[1,2], Andrew J. Morris[3], Jonathan E. Katz[4,5], Terra D. Vincent-Hall[6], Sue E. Fenton[2,7]

[1] NC State University, Toxicology Program, Department of Biological Sciences, Raleigh, USA; [2] NC State University, Center for Human Health and the Environment, Office of Research and Innovation, Raleigh, USA; [3] Central Arkansas Veterans Affairs Healthcare System, Little Rock, USA; [4] Ellison Medical Institute, Los Angeles, USA; [5] Keck School of Medicine of the University of Southern California, Department of Medicine/Oncology, Los Angeles, USA; [6] United States Department of Veterans Affairs, Exposure Science Program, Health Outcomes Military Exposures, Washington, D.C., USA; [7] Uniformed Services University of the Health Sciences, Murtha Cancer Center Research Program, Bethesda, USA

OBJECTIVE: Per and poly-fluoroalkyl substances (PFAS) exposure has been associated with numerous human health effects, including adverse developmental and reproductive outcomes, altered immune, liver, and thyroid function, and various cancers. Firefighters risk PFAS exposure regularly due to their presence in turnout gear, aqueous film-forming foams (AFFFs), and the fumes and dust generated from a fire. The International Agency for Research on Cancer classifies firefighting as a Group 1 carcinogen, and we aim to discover if dried blood strips (DBS) can be used to quantitate carcinogenic biomarkers associated with PFAS exposures.

MATERIAL AND METHODS: In this pilot project, a minimally invasive Drawbridge Health OneDraw™ device was used to collect DBS from a convenience sample of 10 anonymous individuals who simultaneously donated a vial of venous blood (VB; 6 mL EDTA). VB was used for evaluation of DNA damage (comet assay and long PCR comparison), DNA hydroxymethylation analysis (bisulfite sequencing), miRNA profiling (cancer-focused PCR panels), and oxidative lipid characterization (LC-MS). Remaining VB was spun to plasma. DBS were extracted to prepare DNA, RNA, and lipids, and will be compared to outcomes in matched VB samples using similar methodological approaches. Biomarkers (regardless of original source) will be evaluated for associations with PFAS measured via UHPLC-coupled mass spectrometry.

RESULTS: Our work has optimized extraction of the DBS for measurement of approximately 27 PFAS, with 10 of those PFAS strongly associated with levels in VB. Ongoing research will further optimize DBS protocols for DNA damage assays, epigenetic/omic, and oxidative lipid profiling.

CONCLUSION: This research will 1) develop cellular and health biomarkers indicative of PFAS exposure using non-invasive and easy to store DBS and 2) further characterize DBS-based biomarkers of occupational exposure and enable longitudinal health and exposure monitoring of military and civilian firefighters. This work was funded by USUHS, NIEHS, VA, and NC State.

P16-13 | Are We Measuring the Same Thing? A Scoping Review & Framework for Harmonizing Measurement and Reporting of Nano- and Microplastics in Indoor Environments to Advance Human Exposure Research.

Soma Barsen, Elizabeth Z. Lin, Jean Zhou, **Krystal J. Godri Pollitt**

Yale School of Public Health, Environmental Health Sciences, New Haven, USA

Objective: Indoor environments contain numerous sources of nano- and microplastics (NMPs) that may lead to exposure through inhalation (air) and ingestion (settled dust). While the literature describing NMPs in indoor spaces is growing, diverse methods are used, presenting challenges when synthesizing evidence. The objectives of this study were (i) to conduct a scoping review on NMPs found in indoor environments; and (ii) to develop a framework for harmonization of methods for evaluating NMPs in air and settled dust to better understand their impact on health.
Materials and Method: A search of PubMed, Scopus, and Web of Science for “indoor,” “nanoplastic,” “microplastic”, “air,” and “dust” resulted in 169 publications between January 2017 and August 2024. Detailed review was conducted for 51 research publications, including 24 active, 13 passive, and 14 settled dust sampling techniques for NMPs in indoor spaces.
Results: Reviewed publications evaluated residential, transportation, medical, and childcare settings. Inconsistent terminology, heterogeneous sampling approaches, dissimilar analytical methods with varied reporting metrics hindered a comparative assessment to 22 studies. Compared to studies that used spectroscopic imaging (FTIR, Raman, LDIR), primarily reporting NMP characteristics for larger particles (>10 μm), studies that evaluated composition with pyrolysis coupled with high resolution mass spectrometry identified NMPs at the nanoscale (~<0.050 μm). The variety of activities and occupancy in indoor microenvironment influenced the magnitude and polymer composition of detected NMPs. While PET(S), PP, and PA were abundant indoors, PET and acrylic were more prevalent in childcare facilities and nail salons than in homes.
Conclusions: NMPs have been evaluated in a range of indoor environments, however, as a nascent field, rigorous meta-analysis of reported exposures requires implementation of standardized analytical approaches. Harmonization of measurement methods, controls and reporting metrics will facilitate advancement of NMP exposure assessment and our understanding of the associated health impacts.

P16-15 | Association between environmental chemicals and sex-steroid hormones in the U.S. general population: An exposome-wide association study and evaluation of complex mixture effects

Jiyun Lee, Hyuna Jang, Hyeong-Moo Shin

Baylor University, Department of Environmental Science, Waco, USA

Background: Studies investigated associations between environmental chemicals and sex-steroid hormones; however, most focused on specific chemical classes, and assessments of chemical mixtures remain limited. This study addressed that gap by employing an exposome-wide association study (ExWAS) approach with nationally representative U.S. data.

Methods: We analyzed data from the National Health and Nutrition Examination Survey (NHANES) two cycles (2013-2014 and 2015-2016) which included measurements of sex-steroid hormones (total testosterone [tT], estradiol [E2], and sex hormone-binding globulin [SHBG]) and chemical biomarkers from 9,172 participants. Analyses were stratified into six subgroups to capture sex- and age-related differences in sex-steroid hormone levels: adolescent boys (12-19 years), younger adult men (20-44 years), older adult men (45+ years), adolescent girls (12-19 years), pre-menopausal women (20-44 years), and post-menopausal women (45+ years). Robust regression, which accounts for outliers and survey weights, was used to identify biomarkers associated with hormone levels. Quantile g-computation was applied to assess chemical mixture effects using biomarkers identified in ExWAS and ≥70% detection frequency.

Results: After adjusting for multiple comparisons, we identified 7 to 36 biomarkers associated with at least one hormone across subgroups. Notably, acrylamide was consistently associated with changes in hormone levels across all male subgroups. Overall, biomarkers were identified 2.8 times more in men than in women. In adolescent boys, higher exposure to a mixture was associated with lower levels of tT (β = -0.41, 95% CI: -0.56, -0.26) and E2 (β = -0.36, 95% CI: -0.52, -0.19). In other subgroups, although the overall mixture effects were not significant, copper, manganese, and five other biomarkers were associated with higher or lower levels of at least one hormone.

Conclusion: Our study showed that chemical exposures, both individually and as mixtures, are associated with disruptions in sex-steroid hormone levels in the U.S. population and that adolescent boys are sensitive to chemical exposures.

P16-16 | Incorporating metabolomics and genome to understand the effects of prenatal phenol exposure on the first 1000-day growth in an exposome-based study

Wen Jiang[1,2], Jun Zhang[1,2]

[1] Shanghai Jiao Tong University, Ministry of Education-Shanghai Key Laboratory of Children's Environmental Health, Shanghai, China; [2] Shanghai Jiao Tong University, School of Public Health, Shanghai, China

Objectives: To screen for prenatal environmental factors that continuously affect first 1000-day growth and further explore the underlying bio-mechanisms and genetically susceptible populations.
Material and Methods: Within 1,944 mother-child pairs from the Shanghai Birth Cohort, neonatal sex-specific z-scores of weight-for-age (WAZ), length-for-age (LAZ), and weight-for-length (WLZ) were used as the fetal growth outcomes. The growth trajectories of WAZ, LAZ, and WLZ before 2 years of age were used as the child growth outcomes. Multivariable regression (MR), ENET, sPLS, XGBOOST, and random forest were applied jointly to determine important factors associated with both fetal and child growth outcomes among 70 prenatal environmental exposures covering outdoor and living environments, chemicals, lifestyles, and psychosocial status. Meet-in-the-middle, mediation, and enrichment analyses were integrated with early-pregnancy untargeted metabolomics to explore the biological pathways linking concerned exposures and fetal growth. MR included a product term of environmental variables and maternal dichotomous polygenetic risk scores (PRS) of the biomechanism-related phenotypes to explore the gene-environment interaction.
Results: Phenols were negatively associated with neonatal WAZ, LAZ, and WLZ and positively associated with the risks of WAZ's slow growth trajectory, LAZ's slow growth trajectory, and WLZ's rapid growth trajectory in children, suggesting the chemicals continuously affect fetal and child growth. 71 out of 413 metabolites were found to mediate the phenols-fetal growth associations, which were enriched in glucose and lipid metabolism-related pathways. The associations of phenols with fetal and child growth outcomes were significantly more pronounced in mothers with high type 2 diabetes, fasting glucose, 2-hour oral glucose tolerance test, and HbA1c PRS levels, and low fasting insulin, triglyceride, and cholesterols PRS levels.
Conclusion: Our study suggests that prenatal phenol exposure is an environmental factor that continuously affects offspring's first-1000-day growth by disturbing maternal glucose and lipid metabolisms. Mothers with high glucose and low lipid genetic predispositions were more susceptible.

P17 | Environmental Exposure Assessment Methods

P17-01 | Utility of low-cost sensor measurement for predicting ambient $PM_{2.5}$ concentrations: Evidence from a monitoring network in Accra, Ghana

Kelvin Yeboah[1], Patrick A. Yeboah[1], Christian Afful[1], Carl H. Korkpoe[2], Eric Coker[3], R(Subu) Subramanian[4], **Adeladza K. Amegah**[1]

[1] University of Cape Coast, Public Health Research Group, Department of Biomedical Sciences, Cape Coast, Ghana; [2] University of Cape Coast, Department of Finance, School of Business, Cape Coast, Ghana; [3] British Columbia Center for Disease Control, Vancouver, Canada; [4] Center for Study of Science, Technology and Policy, Karnataka, India

Objective Particulate matter ($PM_{2.5}$) is one of the most critically monitored pollutants due to its devastating health effects. Air quality (AQ) monitoring is very limited in sub-Saharan Africa (SSA). Satellite remote sensing has helped address significant AQ data gaps in countries, but measurements have not been validated against ground-level measurements in these countries. We evaluated the efficiency of low-cost sensors in estimating $PM_{2.5}$ concentrations in an African city through comparison of low-cost sensor data with satellite aerosol optical depth (AOD) data leveraging complex machine learning (ML) methods. **Material and Methods** Low-cost sensor data was collected from the Breathe Accra monitoring network in Accra. AOD measurements were extracted from the MODIS MCD19A2v061 dataset and processed using the Multi-Angle Implementation of Atmospheric Correction (MAIAC) algorithm. OLS regression, Random Forest, Extra Trees, Boosted Decision Trees, and XGBoost were used to establish the relationship between AOD and the low-cost sensor $PM_{2.5}$ measurements incorporating meteorological data. **Results** We observed significant positive relationships for two low-cost sensors deployed on the network (Clarity Node S and Airnote). The R^2 values were, however, small (0.16 to 0.23) with corrected Clarity Node S data recording the highest R^2. The ML models which integrated temperature and humidity improved the R^2 values. Boosted Decision Tree showed best predictive capability. Seasonal variability played a significant role in the model performances, as dry seasons performed significantly better compared to wet seasons. Consistent with other studies, AOD explained only a small proportion of ground-level $PM_{2.5}$ variations. **Conclusion** Our findings suggests that AOD predicts ground-level $PM_{2.5}$ measured with low-cost sensors similar to conventional air monitoring instrumentation. However, for low-cost sensors to be deemed a good substitute for satellite AOD, it will require correction of the data with complex algorithms developed in the same research location.

P17-02 | Characterizing Environmental Influences Impacting Children's Health in Rural South Africa: Application of Wearable Technologies to Measure the Exposome in the VHEMBE Birth Cohort

Inumidun Oyebode[1], Elizabeth Lin[1], Emily Johnson[1], Jieqiong Zhou[1], Jeremy Koelmel[1], Riana Bornman[2], Muvhulawa Obida[2], Jonathan Chevrier[3], Krystal Godri Pollitt[1]

[1] Yale School of Public Health, Environmental Health Sciences, New Haven, USA; [2] University of Pretoria, Institute for Sustainable Malaria Control and School of Health Systems and Public Health, Pretoria, South Africa; [3] McGill University, Epidemiology, Biostatistics and Occupational Health, Montreal, Canada

Objective: Children are especially vulnerable to environmental factors due to their developing airways, immature immune systems, and higher respiratory rates. In rural South Africa, children are exposed to a diverse mixture of pollutants from various sources such as cooking, waste management, and malaria control interventions. The objective of this study was to comprehensively characterize the exposome of children in low-income, malaria endemic areas of sub-Saharan Africa.

Material and Methods: We evaluated the chemical exposures of children, aged 8 years (n=495), enrolled in the Venda Health Examination of Mothers, Babies, and their Environment (VHEMBE) birth cohort using a wearable passive sampler (Fresh Air wristband) that was worn for three days. Wristbands were analyzed using thermal desorption gas chromatography high-resolution mass spectrometry to evaluate of the occurrence, distribution, and magnitude of expected environmental contaminants of concern (targeted analysis) and screen for thousands of unexpected chemical exposures (non-targeted analysis).

Results: We quantified personal exposure to 59 chemicals. Non-targeted approaches were further used to characterize the exposome of these children which was found to be dominated by combustion products (polycyclic aromatic hydrocarbons) from solid fuel combustion from cooking, plasticizers (phthalates and replacements) from open waste combustion and consumer products, and organochlorine pesticides from indoor residual spraying for malaria control. Exposure to a range of dyes (e.g., indigo dye, quinaldines, quinolines), fragrances (e.g., musk ketone, galaxolide), and organophosphates were also detected. Seasonal changes in behaviour were found to drive exposure differences that were either higher in the wet (e.g., propoxur, p,p'-DDT, DEET) or dry seasons (e.g., longipinene, D-limonene, and 3-carene). Cooking and refuse combustion exposures were elevated regardless of season.

Conclusion: Understanding the exposome of children in rural South Africa will offer insights into how the environment shapes the health and presents opportunities to support healthier communities.

P17-03 | Effectiveness of SPF 10–12 Sunscreen in UV Spot Reduction: An Epidemiological Assessment Referencing the Double Plate Method

Fu-Yu Chan[1], Chun-Tung Kuo[2,3]

[1] UNIYU BIOTECH CORPORATION, SKIN HEALTH PROMOTION RESEARCH CENTRE, Taipei City, Taiwan; [2] National Taiwan University, Institute of Health Behaviors and Community Sciences, College of Public Health, Taipei City, Taiwan; [3] Academia Sinica, Center for Survey Research, Research Center for Humanities and Social Sciences, Taipei City, Taiwan

Background: In vitro Sun Protection Factor (SPF) testing methods provide a reproducible alternative to in vivo assessments, reducing reliance on human subjects. The Double Plate Method (DPM), an ISO-recognized standard for SPF evaluation, remains limited in real-world applications. This study evaluates the effectiveness of an SPF10–12 physical day cream in reducing skin UV spots among Taiwanese residents using in vitro SPF assessment and skin imaging analysis.

Methods: A cross-sectional study was conducted in 2024, enrolling 292 participants (aged 10–70 years) in Taiwan. Among them, 87 regularly used an SPF10–12 physical day cream containing Titanium Dioxide (TiO_2), a commonly used physical UV filter, while 205 did not. The Margin of Safety (MoS) was calculated as 238.75 for children (6–10 years, 28 kg) and 511.6 for adults (60 kg), confirming product safety. Skin UV spots were assessed using the VISIA 7 Imaging System (UV lighting 365 nm, Canfield, NJ, USA) and facial image analysis software. Application was standardized at 2 mg/cm². Independent t-tests were performed to examine the association between SPF10–12 use and skin UV spots.

Results: Participants were distributed across age groups: 10-20 years (13.4%), 21-39 years (45.5%), 40-55 years (18.8%), 56-64 years (5.8%), and ≥65 years (16.4%). Regular SPF10–12 users exhibited significantly fewer UV spots than non-users ($p = 0.001$, 95% CI = 22.65–24.09). The SPF10–12 formulation effectively reduced direct UV exposure, forming a protective barrier.

Conclusion: The findings support the preventive role of SPF10–12 physical sunscreen in mitigating UV-induced skin UV Spot features. Findings support in vitro SPF testing methodologies and contribute to the development of alternatives to animal testing. Additionally, results highlight the feasibility of low-SPF, non-chemical filter formulations as viable daily sun protection options.

Keywords: Sun Protection Factor (SPF); Double Plate Method (DPM); Skin UV spots; Titanium dioxide; Physical sunscreen; In vitro testing

P17-04 | Predicting indoor PM2.5 concentrations from household and behavioural features: a tool for neurodevelopment epidemiology in low resource settings

Hallah Kassem[1], Edmund Seto[1], Vincent Kipter[2], Anne Riederer[1], Michael Gatari[3], Faridah H. Were[4], Chris Zuidema[1], Elizabeth M. Obimbo[5], Catherine Karr[1], Sarah Benki-Nugent[1]

[1] University of Washington, Department of Environmental and Occupational Health Sciences, Seattle, USA; [2] University of Auburn, Department of Chemistry, Auburn, USA; [3] University of Nairobi, Department of Engineering, Nairobi, Kenya; [4] University of Nairobi, Department of Chemistry, Nairobi, Kenya; [5] University of Nairobi, Department of Paediatrics and Child Health, Nairobi, Kenya

Background

In high income countries (HICs), early life exposure to air pollutants, particularly from combustion sources, are associated with neurodevelopmental disorders (NDDs).Clinical and exposure data constraints have limited research on air pollution and neurodevelopment in sub-Saharan Africa (SSA). New predictive machine learning methods present an opportunity to facilitate studies needed to inform policy and programs by making use of limited data.

Methods

The Nairobi based Air Pollutant Exposures and Brain Development in Children (ABC) cohort includes 400 woman-child dyads who completed Bayley Scales of Infant and Toddler Development (BSID) and household and neighborhood characteristic questionnaires. 39 participants also provided 24 hour PM2.5 indoor and outdoor residential sampling. We used these data in SuperLearner machine learning models of household features to predict indoor and outdoor PM2.5. We used a simple baseline mean (SL.mean) as a comparative benchmark for predictive power of four regressions: generalized linear (SL.glm), Elastic Net (SL.glmnet), and Random Forest (SL.randomForest).

Results

Household factors regarding ventilation, building materials, cooking fuel and other indoor combustion were highly homogeneous in the 39 sampled households. This precluded the machine learning model prediction of indoor PM2.5 beyond the baseline mean model**.** Reported ambient proximal sources included being near a factory (n=1, 2.6%), battery recycling facility (n=1, 2.6%), industry burning (n=1, 2.6%), charcoal burning (n=1, 2.6%), garage (n=5, 12.8%), waste recycling (n=6, 15.4%), dumpsite (n=11, 28.2%), welding (n=18, 46.1%), rubbish burning (n=28, 71.8%), and vehicle emissions (n=38, 97.4%). The SuperLearner model of these factors explained 43.4% of the variability in outdoor PM2.5.

Conclusion

This study informs understanding of strengths and limitations of machine learning models and their use with limited data in low-resource settings. Future work will apply the outdoor model to estimate PM2.5 in the ABC cohort and epidemiological analysis of infant neurodevelopment.

P17-05 | Space-time analysis of environmental noise pollution in Kigali, Rwanda

Jean Remy Kubwimana[1,2], Sierra N. Clark[3], James Nimo[4,5], Chantal Umutoni[1,2], Pacifique Karekezi[1,2], Barbara E. Mottey[6], Claudette Nyinawumuntu[2], Samson Niyizurugero[2], Silas S. Mirau[1], Pie-Celestin Hakizimana[7], Isambi S. Mbalawata[2], Paterne Gahungu[2,8], Majid Ezzati[9], Allison F. Hughes[4], Raphael E. Arku[6]

[1] The Nelson Mandela African Institution of Science and Technology, School of Computational and Communication Science and Engineering, Arusha, Tanzania; [2] African Institute for Mathematical Sciences, Research and Innovation Centre, Kigali, Rwanda; [3] University of London, School of Health and Medical Sciences, City St George's, London, UK; [4] University of Ghana, Department of Physics, Accra, Ghana; [5] State University of New York, Department of Environmental & Sustainable Engineering, Albany, USA; [6] University of Massachusetts, Department of Environmental Health Sciences, Amherst, USA; [7] Rwanda Environment Management Authority, Kigali, Rwanda; [8] Imperial College London, Department of Mathematics, Faculty of Natural Sciences, London, UK; [9] Imperial College London, Department of Epidemiology and Biostatistics, School of Public Health, London, UK; [10] University of Ghana, Regional Institute for Population Studies, Accra, Ghana; [11] Imperial College London, Abdul Latif Jameel Institute for Disease and Emergency Analytics, London, UK; [12] Imperial College London, MRC Centre for Environment and Health, School of Public Health, London, UK; [13] Imperial Global Ghana Hub, Accra, Ghana

Background: As Sub-Saharan African cities become more crowded, noise pollution is also emerging as an important environmental concern, after air pollution. Yet, unlike air pollution, which is enjoying a relatively more public attention, there is limited measurement data and policy efforts on environmental noise pollution. A recent large-scale study in Accra found elevated noise levels of public health concern, and the levels patterned diverse sources, some of which were unique to SSA.

Objective: We implemented the Accra protocol in Kigali City to understand environmental noise patterns in a contrasting city with very different topography and regulatory system than Accra to inform policy.

Methods: Between November 2022 and December 2023, we established 10 year-long and 120 week-long monitoring sites to capture the space-time patterns in Kigali's soundscape. The monitoring sites covered a variety of geographic and land-use factors across diverse neighborhoods and sources. We computed several noise metrics, including daily ($LAeq_{24hr}$), day-time (L_{day}), and night-time (L_{night}).

Results: Daily noise levels ranged between 38-85 dBA, and were patterned by land-use, with the highest pollution in CBI and HD residential communities. Overall, there was ~8 dBA difference between daytime and nighttime levels. Within land-use category, BG sites experienced the highest day- and night-time variability of ~10 dBA, compared to ~6.0 dBA at other land-use types. Daytime and nighttime levels, respectively, exceeded the Rwandan standards for residential areas by ~72% and 82% at CBI and ~57% and 66% at HD areas. Diurnal patterns were similar throughout the city, rising from ~5am, peaking at about 8am, and plateauing until 6pm before falling to their lowest by midnight. The levels did not vary across weekdays vs weekends or dry vs wet seasons.

Conclusion: The findings will support city-wide planning and regulations to minimize environmental exposures in the face of the ongoing urban growth and densification.

P17-06 | Evaluating survey-based indoor air quality data for Predicting exposure level

Kiya K. Abagero[1], Eva K. Salomonsson[2], Christina Isaxon[2], Rawleigh Howe[1], Yamlak Bereket[1], Ebba Malmqvist[2], This is a joint study conducted by Lund University and Armauer Hansen Research Institute

[1] Armauer Hansen Research Institute, Addis Ababa, Ethiopia; [2] Lund University, Lund, Sweden; [3] Lund University, Lund, Sweden; [4] Armauer Hansen Research Institute, Addis Ababa, Ethiopia; [5] Armauer Hansen Research Institute, Addis Ababa, Ethiopia; [6] Lund University, Lund, Sweden

Background: Indoor air quality greatly affects human health as most people spend their time in home. According to the report from WHO, Household air pollution was responsible for an estimated 3.2 million deaths per year in 2020, including over 237 000 deaths of children under the age of 5. Women and children are highly affected as they are responsible for household tasks such as cooking; using polluting fuels and traditional cooking technologies. In low- and middle-income countries like Ethiopia, because of technological and resource difficulties survey-based data have been used to assess indoor air quality data. However, the accuracy of such surveys has been questioned without objective validation.

Aim: This study aimed to validate responses from survey questionnaires on behaviour and environmental factors, such as fuel type and smoking habits and comparing them with objective data collected from monitoring campaigns in the households.

Method: A cross-sectional observational study was conducted involving 40 households located in three different parts of Addis Ababa, Ethiopia. A questionnaire/survey was collected from pregnant women during their prenatal care visit and subsequently indoor air quality was monitored for 48 hours using ground monitor installed in the selected household.

Result: The survey-based data has revealed that most of the households were to be categorised as low and/or medium exposure whereas the data from real time monitor instead indicated high level of exposure among the majority of the household. High degree of subjectivity was also reflected during the scoring of exposure status as there is no standard guideline.

Conclusion: Although we found no perfect consistency between survey responses and actual air quality measurements, surveys can still be an effective tool for identifying potential indoor air quality issues. Survey-Based assessment tend to identify general trends rather than pinpoint exact pollution sources or concentrations.

P17-07 | Evaluating methods to estimate the impacts of multiple environmental co-exposures on health outcomes

Kritika Anand[1,2], Tim CD. Lucas[3], Anna L. Hansell[1,2], Yutong S. Cai[1,2]

[1] University of Leicester, Centre for Environmental Health and Sustainability, Leicester, UK; [2] Leicester General Hospital, NIHR Leicester Biomedical Research Centre, Leicester, UK; [3] University of Leicester, Department of Population Health Sciences, Leicester, UK; [4] University of Leicester, The National Institute of Health Research (NIHR) Health Protection Research Unit (HPRU) in Environmental Exposure and Health, Leicester, UK; [5] University of Leicester, Glenfield Hospital, Leicester British Heart Foundation Centre of Research Excellence,Cardiovascular Research Centre, Leicester, UK

Objective: Environmental exposures such as temperature, noise, air pollution, and green space, have been known to affect health, yet their interactions remain complex and inconsistently assessed. This review examines methodological approaches to co-exposures analysis in environmental health research, highlighting key interactions, theoretical and mechanistic underpinnings, statistical considerations, and implications for interpreting health associations.

Methodology: To identify all relevant studies, we searched for combinations of exposure and did not specify health outcomes. We searched Scopus for original English-language studies and included all studies published up to 17th February, 2025. Screening was conducted using Rayyan. Data extraction focused on exposure assessment, statistical designs and biological mechanisms. We assessed interaction rationales mentioned in directed acyclic graphs of the studies and examined whether exposures were treated independently, as mediators, modifiers, confounders, or composite indices, along with statistical frameworks used for handling simultaneous exposures.

Results: The initial search yielded 5,706 articles, with 241 selected for full-text review. Studies fell into three categories: (1) independent assessment of exposures, (2) interaction, confounding or mediation analyses, and (3) mixture or composite indices. Noise, greenspace, and air pollution were commonly studied together, while temperature was rarely analysed with noise except in exposome studies. Mediation analysis was common when greenspace was the primary exposure. Only a few studies utilized composite indices or mixture models, and even fewer examined all four exposures together. Most treated exposures separately or adjusted for them rather than analyzing them as mixtures. Statistical approaches for examining these co-exposures included dimension reduction, mixture models, multivariate regression, Bayesian spatiotemporal modelling, and machine learning.

Conclusion: The integration of co-exposures in environmental health research remains an evolving field, with methodological differences impacting comparability across studies. The findings will inform future research on the health impacts of these commonly co-existing urban exposures.s

P17-08 | Improving Chemical Mixture Exposure Estimates for Chemical Disasters using Monte Carlo Methods

Philip Steenstra

University of Michigan, Department of Environmental Health Sciences, Ann Arbor, USA

Communities worldwide suffer from chemical accidents that adversely affect human and environmental health. Understanding the impacts of these accidents requires accurate assessment of chemical exposures and their health and environmental effects over both short and long time-periods. Estimating exposures for these accidents is challenging and in practice highly simplified, often considering only averaged exposure data and neglecting effects from chemical mixtures. These issues are particularly important for industrial disasters that release complex chemical mixtures over extended periods, as considerable uncertainty surrounds release, transport, and transformation of pollutants and the ultimate exposures. To obtain valid results, exposure assessments should address the complexity, variability, and uncertainty of these factors, and health and risk assessments should consider chemical mixtures.

This research utilizes a massive chemical warehouse fire in Cornubia, South Africa in 2021 as a case study to compare current deterministic modeling techniques against a novel use of Monte Carlo methods that accounts for uncertainty and variability. The Monte Carlo methods utilize the CALPUFF air dispersion model to create exposure probability distributions (EPD), as well as estimates and risks associated with chemical mixtures for the affected community, expressed as risk probability distributions (RPD).

Study results identify key variables and show that uncertainty in exposure modeling increases the variability of results and can increase highest exposure and risk values. Both EPDs for single chemicals and chemical mixtures are affected by factors such as the release rate of chemicals, meteorological variability, and population characteristics – including mobility –which also alter the areas and individuals affected.

Accounting for variability and uncertainty in the key inputs for exposure modeling can provide unexpected results which should be considered during response and disaster analysis. Modifications to current assessment methods could be applied to other types of chemical accidents to improve the realism of exposure estimates.

P17-09 | A fresh look at exposure profiles: utilizing z-scores to assess predictors of exposure to semi-volatile organic contaminants in wristbands

Rebecca M. Hoehn, Elizabeth Boxer, Kate Hoffman, Heather Stapleton

Duke University, Nicholas School of the Environment, Durham, USA

In a world full of complex chemical mixtures, a chemical-by-chemical approach is not sufficient to identify key determinants of exposure, necessitating new methods. We analyzed 209 silicone wristbands from 75 mothers in Durham, North Carolina, to measure exposure to ~130 semi-volatile organic contaminants (SVOCs), including flame retardants, phthalates, pesticides, and polycyclic aromatic hydrocarbons. Many of these chemicals are considered endocrine disruptors or neurotoxic, raising concerns during pregnancy.

Summing raw concentrations of these compounds is problematic due to vast differences in wristband concentrations. For example, the median concentration of Di-(2-ethylhexyl) terephthalate (DEHT) (87,700ng/g) is orders of magnitude

higher than that of Acenaphthene (5.29ng/g), potentially skewing exposure estimates. To address this, we used z-scores to standardize concentrations and summed these to assess total exposure.
This approach helps identify individuals who may otherwise be overlooked in a chemical-by-chemical analysis. For instance, among the three individuals with the highest total exposure, their exposure to individual chemicals was below the 75th percentile 36% of the time and below the median 11% of the time. If only single exposures were assessed, it would be more challenging to identify these individuals.
Comparing sum z-scores across the cohort also revealed socio-economic trends in exposure, with lower household income and government insurance status associated with higher total exposure to SVOCs. Mothers earning less than $49,999/year had sum z-scores 0.61 standard deviations higher than those earning above $150,000/year. Similarly, mothers with government insurance had sum z-scores 0.5 standard deviations higher than those with private insurance. Post-hoc analysis by individual chemicals revealed flame retardants and phthalates were major contributors to these trends.
These results demonstrate the value of z-scores in assessing total exposure and suggest that total exposure to SVOCs during pregnancy is associated with socio-demographic variables. Further exposure predictors will be explored as additional data become available.
Funded by NIH.

P17-10 | Assessment of $PM_{2.5}$ Inhalation Exposure and Contribution Rates Using Time-Activity Patterns from the Time-Use-Survey in Daegu, South Korea

Sanghoon Lee[1], Jihun Shin[2], Gihong Min[1], Youngtae Choe[1], Daehwan Kim[1], Wonho Yang[1]

[1] Daegu Catholic University, Department of Health and Safety, Gyeongsan-si, South Korea; [2] Songwon University, Department of Health and Safety Management, Gwangju, South Korea

Objective: The purpose of this study is to evaluate $PM_{2.5}$ exposure levels and derive contribution rates based on cl uster-specific behavioral patterns using Korean Time-Use Survey data.

Material and Methods: This study analyzed the weekday time-specific activity-patterns of 1,718 Daegu residents using data from the 2019 Time-Use Survey by Statistics Korea. Clustering analysis using location and behavior data (collected every 10 minutes) was followed by sociodemographic frequency analysis to classify occupational groups. Then, exposure scenarios were constructed to conduct an active simulation. Calculating $PM_{2.5}$ exposure and contribution rates, the Korean Exposure Factors Handbook was used to account for inhalation rates.

Result: Weekday participant analysis using K-means clustering identified 12 clusters via the silhouette score. Active simulation followed exposure scenarios over 24 hours for each specialized cluster. The average percentage of time spent in microenvironments was highest for manufacturing work (39.58%), followed by sleep (35.47%), office work (34.38%), security work (33.33%), and cafe work (29.52%). The average concentrations were lowest for security work (4.99 μg/m³) and school meals (5.05 μg/m³) but highest for barbecue restaurant work (350.46 μg/m³) and meal preparation (54.74 μg/m³). Contribution rates were highest for barbecue restaurant work (68.75%), followed by meal preparation (32.01%), manufacturing work (21.33%), drinking (16.62%), and restaurant dining (15.22%).

Conclusion: Although exposure durations were short, work (barbecue restaurant) and meal preparation had the hi

ghest contribution rates due to high concentrations, with rates increasing significantly as concentration levels rose. In contrast, cleaning was expected to have a high contribution rate but had minimal impact. This study helps identif y key behaviors and establish priorities for reducing $PM_{2.5}$ concentrations.

Acknowledgements: This study was supported by the Korea Environmental Industry & Technology Institute (KEITI) through the Core Technology Development Project for Environmental Diseases Prevention and Management Project, funded by the Korea Ministry of Environment (MOE) (grant number: RS-2021-KE001349).

P17-11 | Creation of a protocol to quantify and identify indoor pollutants

Thiphanie P. Riveron, Rebecca L. Cordell, Anna L. Hansell

University of Leicester, Leicester, UK

People spend around 90% of their time indoors, including 74% in their homes during winter. This can lead to long-term exposure to chemical and biological pollutants and have negative effects on human health. An indoor air sampling protocol was developed to investigate the composition and concentration of indoor pollutants in dwellings. This protocol was tested in 60 dwellings of different types and age in the winter period. The pollutants targeted included volatile organic compounds (VOCs), fine particulate matter ($PM_{2.5}$) and fungi. During a one-week period, air pollutant sensors were installed in the living room and bedroom of the participants' homes. In addition, a house questionnaire was created to help identifying pollutant sources.
The VOC data showed no significant difference between the total VOC concentration measured in the living rooms and bedrooms for all houses. Fungal samples showed that air samples were more representative of the current fungal spore concentration in the dwellings, while, dust samples gave indication of the long-term spore concentration and diversity. A combination of air and dust samples is therefore recommended to fully characterise fungi in dwellings. Regarding $PM_{2.5}$, the limit of detection of the optic sensor did not allow quantification in 19% of the dwellings, so an optical sensor-based PM sampler was switched to a gravimetric sampler. The protocol was generally well tolerated by participants, however, there were some complaints about the noise from the fungi samplers. To combat this, a passive fungal sampler is under development to be able to identify the airborne fungal spore diversity in the dwellings. The protocol developed effectively identifies and quantifies several indoor pollutants, and is suitable for application to larger scale studies. The data collected can help improve our knowledge of indoor pollutants in dwellings and their potential impact on human health.

P17-12 | Predicting the Variability of Measured Short-term Noise Levels in Four U.S. Metropolitan Areas

Tingfan Jin, Meredith Pedde, Sara D. Adar

University of Michigan School of Public Health, Department of Epidemiology, Ann Arbor, USA

Objective: To evaluate our ability to predict and explain the variation in measured short-term noise levels in four U.S. metropolitan areas using publicly available noise models and other features.
Material and Methods: We collected 1,160 short-term (mostly 5 minutes) noise samples in Chicago, Illinois (N=580), Seattle, Washington (N=234), Los Angeles (LA), California (N=206), and Riverside, California (N=140) between 2005-2024 at 1,017 unique sites (N=456, 230, 194, 137, respectively). Temporal predictors included time of day, day of week, month, year, and daily meteorological factors. Long-term spatial predictors were generated at and within buffers of the sampling sites as: variables derived from annual average U.S. Department of Transportation (DOT) National Transportation Noise Map estimates, long-term average U.S. National Park Service Sound Map estimates, and measures of annual average traffic flow, roads, railroads, subway lines, greenspace, population, and land cover. Extreme Gradient Boosting was fit using 5-fold cross validation to model the measured noise levels for each city with data split into training and testing sets by sampling sites at 70:30 ratio.
Results: Temporal-predictors-only-model explained a modest extent of variation in Chicago (R^2=0.29) but virtually nothing in other cities (R^2=0.003~0.06). Upon it, including DOT variables was more predictive than NPS variables, with best performance in LA (R^2=0.66), then Seattle (R^2=0.53) and Riverside (R^2=0.48), and modestly improved Chicago model (R^2=0.49). No additional improvement was seen when including both DOT and NPS variables simultaneously. Including all predictors only significantly improved the Chicago model to R^2=0.64, with slight improvement in Seattle (R^2=0.56) and no improvement in LA and Riverside.
Conclusion: We found that the DOT noise models were the strongest predictor of measured noise levels, explained approximately half of the variation in three out of four U.S. cities. Additional place and time variables only improved Chicago model, suggesting that localized models may be needed.

P17-13 | Enhancing Exposure Assessment: A Hybrid Within-Person Biospecimen Pooling Approach to Reduce Biomarker Variability and Cost

Yajnaseni Chakraborti[1], Enrique F. Schisterman[1,2], Sunni L. Mumford[1,2], Aijun Ye[3], Stefanie N. Hinkle[1,2]

[1] Perelman School of Medicine, University of Pennsylvania, Department of Biostatistics, Epidemiology and Informatics, Philadelphia, USA; [2] Perelman School of Medicine, University of Pennsylvania, Department of Obstetrics and Gynecology, Philadelphia, USA; [3] Horizon Analytics Consulting LLC, North Potomac, USA

Objective: Biomarkers are crucial for exposure assessment in environmental research, but single biospecimen measurements can be unreliable due to high intra-individual variability (IIV). Repeated within-person sampling improves reliability but is costly and impractical. The hybrid pooled-unpooled design, which pools biospecimens from individuals with varying pooled sizes, offers a cost-effective alternative but fails when pooling error (PE) exceeds IIV. We developed a regression calibration method to correct exposure effect estimates (βs) if post-experiment variance

components reveal excessive PE.

Material and Methods: This study, motivated on phenol measurements in the EAGeR trial where PE > IIV. A calibration factor was developed to correct for both IIV and PE, ensuring accurate β estimation and its 95% confidence intervals (CI). A simulation (1000 runs, n=1000, 10 repeated samples) was conducted with varying pooling sizes and three error scenarios (PE>IIV, PE<IIV, PE=IIV), assuming the normally distributed errors are independent with constant variance. We assessed the design's robustness by comparing bias and other metrics for uncalibrated versus calibrated β estimates in a binary outcome model, with 95% CI coverage.

Results: Uncalibrated estimates of β, were consistently underestimated across all scenarios, with percent bias ranging from 51.3-66.8%. Bias was most severe when PE > IIV or when both were similar (PE ≈ IIV). After applying calibration, bias improved substantially, reducing to a range of 7.8%-20.7%, with the highest residual bias observed when PE ≈ IIV. CI coverage was within acceptable limits, close to 95%.

Conclusion: Hybrid within-person biospecimen pooling with regression calibration is a cost-effective method for estimating β when biomarkers are variable and costly. Its accuracy depends on the relationship between PE and IIV—unknown before pooling. If PE is excessive, bias can occur, but our method provides a post-experiment correction, making it ideal for studies with high biomarker variability, like environmental phenols.

P17-14 | Development of a Mobile Application Utilizing Artificial Intelligence to Support the Key Indicator Method and Prevent Ergonomic Hazards

Yuju Chou[1], Tzyychyang Lu[2], Yutse Tsan[1], Kexian Kuo[2], On behalf of Devision of Occupation Medicine, Department Of Emergency Medicine, Taichung Veterans General Hospital

[1] Taichung Veterans General Hospital, Devision of Occupation Medicine, Department Of Emergency Medicine, Taichung, Taiwan; [2] Providence University, Department of Computer Science and Communication Engineering, Taichung, Taiwan

Objective: To develop a mobile application for assessing posture-related risks in the workplace, reducing musculoskeletal injuries and enhancing productivity. The app uses a 3D artificial intelligence(AI) recognition model to provide stable posture risk assessments, eliminating subjective errors.
Materials and Methods: The application integrates the Key Indicator Methods(KIM) checklist with a 3D posture tracking model. Users record work postures via smartphone, upload them for AI analysis, and receive risk reports with personalised improvement suggestions. The AI model uses MediaPipe Solutions, BlazeFace and BlazePose to perform complex posture analysis and motion status classification. The system architecture involves a smartphone app as the Key Indicator Method for assessing Lifting , Holding and Carrying(KIM-LHC) checklist carrier, coupled with BlazePose to estimate 33 human body key points. The app uses Flutter as the client-side development framework, with Flask used as the server-side framework for model determination and data transmission.
Results: The app accurately identifies posture changes, calculates risk levels, and offers tailored recommendations. It incorporates an algorithm to filter noise and address inaccuracies from model-based judgments. The app has a feature for additional scoring items such as trunk twisting, arm lifting. Testing of the app showed 100% accuracy when compared to professional assessment of different posture types.

Conclusion: The mobile app provides a rapid and convenient solution for assessing posture risks, promoting workplace safety and health management. The combination of the KIM checklist and AI-driven posture analysis demonstrates the potential of digital technology in occupational health.
Acknowledgements: This research was supported by the Rong Jing Cooperation Research Project, grant number TCVGH-PU1138105.

P17-15 | Synthesis of results across exposure scales in environmental epidemiology systematic reviews and meta-analyses: methods in use and an evaluation

Emma Rosen[1], Matthew Longnecker[2]

[1] Ramboll, Princeton, USA; [2] Ramboll, Raleigh, USA

Background: Suboptimal methods in environmental epidemiology systematic reviews (SR) and meta-analyses (MA) are well-documented, but how studies synthesize results across different exposure scales has received relatively little attention. Variation in exposure scales (e.g., log transformed and absolute, study-specific high vs. low categories) presents methodological challenges in SR/MA data synthesis, affecting informativeness and utility for risk assessment. The non-combinability of log and absolute scale results was recently reported. Furthermore, recent advances in Synthesis Without Meta-analysis (SWiM) may increase the efficiency of using available data. However, the extent to which these issues are applicable in environmental health research remains unclear.
Methods: We conducted a PubMed search targeting SRs and MAs of six types of environmental chemicals in six key environmental epidemiology journals published between 2022-2023. Studies were categorized into non-exclusive groups based on how they handled synthesis of results across different exposure scales.
Results: Of the 10 SRs that did not perform MA, only one adhered to recommended SWiM methods. Of the 18 studies conducting qualitative meta-analyses, only two applied optimal methodologies, while the remaining 16 relied on high-low exposure contrasts. Among the 10 studies using quantitative meta-analyses, only two employed methodologically sound approaches. The rest either excluded studies due to exposure scale differences, combined exposure scales inappropriately, or re-expressed results using questionable methods.
Discussion: Our review highlights widespread methodological weaknesses in how SRs and MAs synthesize results across exposure scales in environmental epidemiology. The limited adoption of SWiM suggests reduced bias and greater efficiency are possible. High-low exposure contrasts use data inefficiently and could easily be made quantitatively informative. SWiM could be used to combine results across scale results. A few specific improvements in methods of synthesis of results across exposure scales would enhance the rigor, reproducibility, and interpretability of SR and MA findings in environmental health research.

P17-16 | Smart-NanoEHS: Strategic Machine Learning and Artificial Intelligence Use for NanoEHS Discovery

Anjali Kumari[1], Rhema Bjorkland[2], Kristen Dellinger[1], Holly Mortensen[4]

[1] Joint School of Nanoscience and Nanoengineering, Greensboro, USA; [2] National Nanotechnology Coordination Office (Contractor), Washington, D.C., USA; [3] Centers for Disease Control and Prevention, National Center for Environmental Health, Atlanta, USA; [4] U.S. EPA, Office of Research & Development Center for Public Health and Environmental Assessment, Research Triangle Park, USA

The coordinated nanotechnology research and development efforts in the United States have and continue to make substantial investments in the responsible development of nanotechnology. We discuss potential advances, opportunities, challenges, and limitations of the use of artificial intelligence (AI) and machine learning (ML) tools to retrospectively analyze the nanotechnology environmental health and safety (nanoEHS) data generated since the early 2000s. We also explore the potential for organized crowdsourced solutions to use AI to tackle the problem of distinct data types, nanomaterials, and formats of nanoEHS data and to expand the availability of data for robust decision-making.

Leveraging existing data to extract novel findings and insights could advance our understanding of the factors that drive uncertainty in nanoEHS measurements, support and improve rigorous data quality assessment and extraction, and boost predictive risk assessment, pattern recognition, and the development of probabilistic models. These efforts may accelerate the development of data-driven minimum characterization standards, upgrade prevention-by-design tools for new materials, and spur greater use of new approach methodologies for risk reduction, dosage determination, process refinement, and replacement of current testing models, when feasible.

P17-17 | Assessing airborne PFAS personal exposure profiles using wearable and stationary passive sampling technology and a novel GC-HRMS workflow

Jeremy Koelmel[1], Elizabeth Z. Lin[1], Parker Chang[1], Wan-Ling Tseng[2], Zeyan Liew[1], Krystal G. Godri Pollitt[1]

[1] Yale University, Yale School of Public Health, Environmental Health Sciences, New Haven, USA; [2] Yale University, Yale Child Study Center, New Haven, USA

While exposure to per- and polyfluoroalkyl substances (PFAS) poses a major health concern due to PFAS toxicity, bioaccumulation, persistence, and widespread distribution, airborne exposure routes remain poorly characterized, hindering accurate risk assessment. This study investigates indoor PFAS emissions and personal PFAS exposure profiles using stationary and wearable Fresh Air passive sampling technology and gas chromatography high-resolution mass spectrometry (GC-HRMS). For personal exposure profiling, passive samplers (polydimethylsiloxane coated tubes embedded in a 3D printed silicon wristband) were worn by 48 children and adolescents (age 7 to 17 years) in New Haven, Connecticut, for 7 days. Samplers were deployed between 2020 and 2023. For stationary sampling passive and active samplers were placed in a single carpeted office space and measurements were taken at intervals from 1 to 15 days. Personal exposure to volatile and semi-volatile PFAS was found to vary significantly across individuals. In the cohort of 48 children and adolescents, GC-HRMS fragment screening revealed that 46%

of 40 unique airborne PFAS detected only occurred in a single child. In the office space, a unique fluorotelomer alcohol (FTOH) profile was monitored using passive and active air sampling (FTOH 8:2, 10:2 (two isomers), 12:2 (two isomers), 14:2, and 16:2), which were not detected in any other samples; certain FTOHs monitored have been linked to adverse health effects. In both studies, detected PFAS were not those either amenable to LC or commonly monitored using LC. These findings underscore the critical need for personal monitoring combined with non-targeted GC-HRMS for comprehensive PFAS exposure assessment, with LC alone not providing full coverage of PFAS exposures. This approach, leveraging wearable and stationary passive sampling, enhances our understanding of human health risks associated with airborne PFAS, ultimately informing effective regulatory policies and public health interventions.

P17-18 | Characterizing the Exposome Using Chemical Profiles of Tear Fluid Measured from Hydrogel Samplers

Anya Guo, Norah H.B. Beach-Diplock, Parshawn Amini, Joseph O. Okeme

McMaster University, Department of Chemistry & Chemical Biology, Hamilton, Canada

Objective: Novel methods for characterizing the chemical exposome are important for studying relationships between chemical exposures and health. Few sampling and analytical methods simultaneously characterize the internal exposome and the external exposome for longitudinal population-level studies. Computational workflows are also needed to process big data generated from exposome studies. We hypothesize that tear fluid, which is exposed to both the internal and the external environments, can be an accessible matrix to simultaneously characterize both the internal and external exposome. This study aims to assess variability in chemical exposure profile in the tear film captured in hydrogels as a function of lifestyle and demographic characteristics.
Material & Methods: We recruited 22 participants, who self-collected and provided worn hydrogels for three consecutive days at two different time points three weeks apart. We solvent extracted the samples and analyzed the extracts using a multi-platform approach with non-targeted LC-MS/MS and GC-HRMS. We processed the high-resolution data with an automated computational workflow. Our statistical analysis assessed temporal variability in measured compounds and their relationship with the behavior and demographic characteristics of participants.
Results: Non-targeted analysis identified over 1,000 unique exogenous and endogenous features with a 100% m/z match in the hydrogels. Examples of compound classes measured in the hydrogels include organophosphate esters, pesticide metabolites, amino acids, and steroid hormones. The abundance of the chemical features varied widely between and within participants and across sampling periods. This variability likely reflects differences in factors influencing exposure including physical environment, lifestyle, diet.
Conclusion: Our results demonstrate that hydrogels combined with a computational approach is promising for characterizing diverse compounds in the tear film.
Funding is acknowledged from the Natural Sciences and Engineering Research Council of Canada and the Canada Foundation for Innovation.

P17-19 | Validation of residential histories reconstructed through LexisNexis in a pediatric cancer population

Cassandra J. Clark[1], Yueqi Li[1], Helen Parsons[2], Jenny N. Pointer[1], Logan G. Spector[1], Erin L. Marcotte[1]

[1] University of Minnesota School of Medicine, Division of Pediatric and Clinical Epidemiology, Minneapolis, USA; [2] University of Minnesota School of Public Health, Division of Health Policy and Management, Minneapolis, USA

Background: Construction of accurate residential history is a common challenge in epidemiologic studies. LexisNexis Accurint (LN) is a database that uses multiple sources (e.g., bankruptcy filings, court filings, jury verdicts/settlements, real estate records, motor vehicle/driver's license records, professional licenses, voter registration) to obtain residential information and establish residential histories from subject names, but little is known about its accuracy or its role as a potential source of bias.
Methods: We compared 165 self-reported and LexisNexis-derived residential histories from osteosarcoma cases diagnosed ages 6-19 (2007-2010). Self-reported residential histories were collected as part of the Genetics of Osteosarcoma (GO) study at the time of interview; these also participated in the Children's Oncology Group Childhood Cancer Research Network (CCRN) protocol, which collected name and address at the time of diagnosis for the case and up to two parents/guardians. Name/address from CCRN enrollment was linked with LN in March 2024 to derive residential history. LN addresses associated with the parent/guardian were compared with GO self-reported addresses to determine accuracy of LN results. GO and LN results were considered matching only when results were an exact match on all elements (house/building number, street, city, state, zip code).
Results: Of 161 children with a primary parent/guardian with GO residential data, LN returned results for 146 (90.7%). Of these, 95.9% had a match with at least one self-reported residence from the GO data, 84.9% matched all available self-reported residences, and 1.4% did not match any self-reported residences.
Conclusion: Residential histories derived from LN show a high degree of accuracy with self-reported residential histories. Our results indicate that LN is a reliable source of residential information even given limited subject information. Its use could improve exposure assessment methods for studies of life-course area-based exposures, diseases with long latency periods, or study designs where subjects are not typically contacted.

P17-20 | Using Explainable Artificial Intelligence to Explore Factors Affecting the Spatial Variation of Water-soluble Ions in $PM_{2.5}$

Kang Lo[1], Yee-Lin Wu[2], Tang-Huang Lin[3], Chang-fu Wu[1]

[1] National Taiwan University, Institute of Environmental and Occupational Health Sciences, Taipei, Taiwan; [2] National Cheng Kung University, Department of Environmental Engineering, Tainan, Taiwan; [3] National Central University, Center for Space and Remote Sensing Research, Taoyuan, Taiwan

Objective: This study explore the non-linear relationships between environmental factors and the spatial variation of water-soluble ions (WSI) in fine particulate matter ($PM_{2.5}$), based on the explainable artificial intelligence (XAI) technique.
Material and Methods: Monthly average concentrations of $PM_{2.5}$ WSIs (SO_4^{2-}, NO_3^-, NH_4^+) were obtained from 28 air quality monitoring stations operated by the Taiwan Ministry of Environment from 2019 to 2021. Meteorological data and satellite-derived information (including land use, aerosol characteristics, greenness, and insolation) were

collected as model predictors. Extreme Gradient Boosting (XGBoost) algorithm was applied for model building to estimate the spatial variations of ionic $PM_{2.5}$. Shapley additive explanations (SHAP), an attributive XAI, were further calculated to quantify the contribution of each feature to the model prediction.
Results: The models demonstrated moderate performances for SO_4^{2-}, NO_3^-, NH_4^+, with an independent test set showing adjusted R^2 values of 0.85–0.87. Temporal and meteorological related variables accounted for 65%–75% of the overall variations. Among the land use variables, vegetation was identified as the major contributor to variations in $PM_{2.5}$ ions. However, different vegetation types, including natural greenness, half-natural greenness, and non-natural greenness, may have opposing effects.
Conclusion: This study demonstrates the utility of XAI for understanding the non-linear impact of environmental factors on water-soluble ions of $PM_{2.5}$.

P17-21 | Impact of exposure factor selection on deterministic dermal exposure assessment of personal care products

Gayoung Lee[1], Kiyoung Lee[1,2]

[1] Seoul National University, Department of Environmental Health Sciences, Graduate School of Public Health, Seoul, South Korea; [2] Seoul National University, Institute of Health and Environment, Seoul, South Korea

The selection of exposure factor in deterministic dermal exposure assessments is critical for accurate exposure assessment. The purpose of this study was to compare dermal exposure estimated by specific percentiles in deterministic assessment with population exposure. Ten personal care products were analyzed, including three skincare products, sunscreen, hair care product, body lotion, hand cream, facial cleanser, facial foundation, and men's skincare lotion. The population exposures were individually calculated by two exposure factors (frequency of use, amount of use) from 1,000 participants representing the national population. Deterministic exposure assessment was conducted according to various percentiles of exposure factors. For each product, the input percentile that closely aligned with the 95th and 99th percentiles of parent population exposure were identified. The 99thpercentiles of population exposures were 1.5 to 2.6 times higher than the 95th percentiles except for hair care product (1.2 times) and men's skincare lotion (1 times). Deterministic dermal assessments should use input percentiles above the 87th for most personal care products to estimate the 95th percentiles of population exposure, except for the liquid-based skincare product (78th) and facial foundation (80th). Exposure factors in deterministic assessment should be greater than the 94th percentiles to estimate 99th percentiles of population exposure. This study determined exposure factors in deterministic dermal assessment of 10 personal care products for more accurate exposure estimation of at-risk group.

P17-22 | Hazard Minimization vs. Risk Assessment Approaches to Assessing Product Safety for Mineral Actives in Powder Sunscreens

Jacob Persky, Frank Pagone

RHP Risk Management Inc., Chicago, USA

Objective

To evaluate inhalation exposures to titanium dioxide (TiO_2) and zinc oxide (ZnO) for three powder sunscreen formulations under realistic use scenarios and compare lifetime use acceptability conclusions following two approaches: 1) a particle-size dependent hazard minimization approach and 2) a toxicological human health risk assessment approach.

Material

Testing was conducted in a clean-room isolation chamber with a volunteer human subject using three retail powder-form sunscreen products with different concentrations of mineral active ingredients: Product A (15% ZnO and 10% TiO_2), Product B (22% ZnO and 7% TiO_2), Product C (23% ZnO and 0% TiO_2).

Methods

Exposure scenarios were evaluated in triplicate for each of the three products yielding nine assessments total. During each assessment, both real-time direct read instrumentation and filter-based time-integrated air sampling was conducted in the personal breathing zone of the subject. Real-time data was size-fractioned using laser light-scattering instrumentation and recorded on both mass-concentration and numerical count-concentration basis. Filter cassettes were analyzed by an analytical chemistry laboratory for both elemental analysis and electron microscopy for visualization of particle size and agglomeration characteristics.

Results

Particle size distributions within the breathing zone during application contained 14% respirable sized particles which does not meet current EU criteria for mineral actives in cosmetics following a hazard minimization approach which requires that no more than 1% of the particle size distribution relate to particles less than 10 microns. However, a toxicological human health risk assessment approach considering particle size influenced potency factors and electron microscopy evidence of particle agglomeration and coating/binding characteristics found that single application exposures were 1,500 times below estimates of a derived no-effect level for ZnO and 800 times below a currently proposed No Significant Risk Level (NSRL) for TiO_2.

Conclusion

These findings underscore the importance of a risk-based evaluation over hazard-minimization criteria in assessing product safety.

P17-23 | The Rise of Drones in Agriculture and Impact on Human Exposure to Pesticides

Jennifer Thomasen, Richard Anderson

Bayer, Human Safety, St. Louis, USA

Unmanned aerial vehicles (UAV), or drones, are increasingly being adopted globally as a pesticide application method and have the potential to provide numerous benefits to the environment, human health, and improve operational efficiency of the farm. The use of drones for pesticide application is advancing rapidly and in a short period, the technology has improved significantly, making drones a promising new tool for growers. A risk-based approach to characterize human safety for pesticide applications is critical to ensure that operators are adequately protected from the potential health risks associated with pesticide exposure. As this is a new technology and rapidly evolving, there are limited human exposure studies and data on operator exposure resulting from the use of drones.

A survey was conducted with a targeted audience to gain insights and information on drone applications of pesticides in agriculture. A variety of questions were asked to facilitate the characterization of pesticide exposure potential. Information related to use of personal protective equipment (PPE), workflow/job steps, volume of pesticide handled, post application activities, etc. were collected. A total of 70 respondents indicated that they use drones to apply pesticides to agricultural crops and were the focus of analysis.

Using this survey data, existing agricultural operator exposure models/data, and recent drone operator exposure studies, an exposure and risk assessment framework for drone pesticide application was established. Results indicate that potential pesticide exposure to drone operators may be significantly reduced or no higher than operators using traditional application methods. Survey results and risk assessment framework will be presented.

P17-24 | Development of a Novel Monitoring Method for Exposure Assessment of Nitrogen-Containing Organic Compounds

Kanae Ito-Bekki, Shigehisa Uchiyama, Yohei Inaba, Shoichi Kusunose, Akira Ushiyama

National Institute of Public Health, Environmental Science, Saitama, Japan

Objective: Isocyanates are known as widespread hazardous environmental pollutants emitted from various combustion products in our lives because they cause health effects to the nervous system and respiratory systems through sensitization. In this study, to obtain data on the concentration and composition of isocyanates in indoor and outdoor air, we developed a novel passive sampler (PSG-DBA) for monitoring gaseous and particulate isocyanates; e.g. isocyanic acid (ICA), methyl isocyanate (MIC), ethyl isocyanate (EIC), and propyl isocyanate (PIC), and acquired basic data of isocyanates on the behavior in general households.
Material and Methods: The passive sampler (PSG-DBA) developed in this study consists of a glass fiber filter impregnated with DBA (Dibutyl amine) and a stainless steel mesh (7 mesh/cm) installed in the specially designed holder. The sampling was conducted for one week in indoor and outdoor air of various houses. After collection, the isocyanate-DBA derivatives in the filter were extracted through liquid-liquid extraction, performed twice using a

methanol: sulfuric acid: toluene (3:3:4) mixture. This solution was subsequently analyzed by LC-MS/MS. Results: This proposed method allows for continuous sampling over a week. From the results of this monitoring, in particular, ICA and MIC were frequently detected in ambient air. In summer, indoor concentrations were typically higher than those outdoors in most houses, e.g., mean concentrations; ICA; 0.21 ppb (summer), 011 ppb (winter); MIC; 0.0052 ppb (summer), 0.0038 ppb (winter). PSG-DBA can monitor a wide range of environments to further investigate the environmental dynamics of isocyanates in the future. Conclusion: A novel passive sampler developed in this study made it possible to measure isocyanates in indoor and outdoor air in a wide range of locations such as school, office, work environment, etc. This enables simplified conduct of epidemiological investigations requiring multiple samples and long-term continuous surveys necessary to examine seasonal variations and environmental dynamics.

P17-25 | PopExp: An open-source Python package to quickly and efficiently estimate populations exposed to environmental hazards

Lauren B. Wilner[1], Heather McBrien[3], Nina M. Flores[3], Joan A. Casey[2,1]

[1] University of Washington, Department of Epidemiology, School of Public Health, Seattle, USA; [2] University of Washington, Department of Environmental Health and Occupational Health Sciences, School of Public Health, Seattle, USA; [3] Columbia University, Department of Environmental Health Sciences, New York, USA

Exposure assessment is a cornerstone of environmental epidemiology. The study of exposure-outcome relationships relies on accurate assignment of exposures and outcomes among study populations. Estimating population-level exposure to environmental hazards is challenging because it requires large, spatially-resolved datasets. Manipulation of such datasets is technically demanding, computationally slow and memory intensive, and difficult to optimize with existing geospatial analysis libraries in R. Individual research teams generally develop independent algorithms to complete these tasks, which risks inconsistent assumptions and methods between teams, potentially resulting in disparate analytic results and findings.

We developed an open-source Python package, PopExp, which quickly, efficiently, and consistently finds the number of people living near environmental hazards. The inputs to PopExp are (1) environmental hazard geospatial datasets, (2) a gridded population raster, and (3) optional additional administrative geographies such as census tracts or counties. PopExp determines the Universal Transverse Mercator (UTM) zone based on hazard location and uses these inputs and the UTM zone to estimate the number of people exposed to either (a) each hazard (e.g., the number of people exposed to each individual wildfire in 2018 in California, by wildfire incident) or (b) the number of people exposed to the cumulative set of hazards provided (e.g., the number of people exposed to all wildfires in 2018 in California). Additionally, PopExp can optionally determine the number of people residing within a buffer distance of each environmental hazard by administrative geography (e.g., the number of people residing within 10 km of wildfire burn zones in 2018 by census tract). The PopExp package contains the software and an example case study using wildfire burn zone disasters.

PopExp is a tool for population health researchers to ensure precise and reproducible exposure assessment of environmental hazards. Employing standard and consistent algorithms, such as PopExp, facilitates effective, efficient, and reproducible research.

P17-27 | Quantifying long-term exposures to fine particulate matter ($PM_{2.5}$) using real-time low-cost sensors to assess the impact of air pollution on birthweight in two rural-urban cohorts in southern India

Naveen Puttaswamy[1], Sudhakar R. Saidam[1], Saritha Senthil[1], Divya Jayakumar[1], Sneha S. Patil[1], Sreekanth Vackacherla[1], Santu Ghosh[2], Ajay Pillarisetti[3], Kalpana Balakrishnan[1]

[1] Sri Ramachandra Institute of Higher Education and Research, Faculty of Public Health, Chennai, India; [2] St John's Medical College, Biostatistics, BENGALURU, India; [3] University of California, Berkeley School of Public Health, Berkeley, USA

Objective: To estimate long-term exposures to $PM_{2.5}$ among pregnant women in the Tamil Nadu Air Pollution and Health Effects-II (TAPHE-II) and Reproductive effects from Exposure to Airborne Chemicals in urban Homes (REACH) cohorts using low-cost sensors (LCS).

Methods: Real-time battery-operated LCS equipped with PMS7003™ (Plantower Inc., China) were used to monitor living-room $PM_{2.5}$ levels for a period of 21-days and 7-months in the TAPHE-II (n=80) and REACH (n=15) cohort homes, respectively. LCS recorded PM, temperature, and relative humidity at 1-minute time intervals and transmitted data in real-time to the cloud. Sensors were collocated with pump-filter set-up for a period of 24-h on three consecutive days in 25 homes to develop indoor-specific calibration equations. In addition, all sensors were collocated with a reference-grade monitor pre- and post-monitoring periods. Linear models were fit to derive ambient calibration coefficients.

Results: Continuous PM levels were monitored on average (s.d.) for 21 (03) and 160 (30) days in the TAPHE-II and REACH cohort homes, respectively. Data availability ranged between 97 to 100% across rural and urban homes. Precision across all sensors was satisfactory with a standard deviation of 2.6 $\mu g/m^3$ and a coefficient of variation of 15.6%. The normalized root mean square error (NRMSE) for indoor and ambient collocation was 31.6% (r=0.86) and 43.2% (r=0.80), respectively. Long-term averages of indoor $PM_{2.5}$ levels were high on weekends (19.2 to 89.9 $\mu g/m^3$) compared to weekdays (13.4 to 48.6 $\mu g/m^3$). Long-term averages (min. max.) of indoor $PM_{2.5}$ levels were high in biomass users (49.8 $\mu g/m^3$ (35.8 – 80.6)) followed by mixed-fuel (28.7 $\mu g/m^3$ (29.4 – 61.9)) and liquefied petroleum gas (LPG) (25.7 $\mu g/m^3$ (21.3 – 39.5)) users.

Conclusion: We demonstrate the applicability of LCS for long-term indoor PM monitoring to assess health risks associated with indoor air pollution in mother-child cohort studies.

P17-28 | A hybrid geospatial modeling approach for $PM_{2.5}$ exposure estimation in low-resource settings: A case study from Peru

Qiang Pu[1], Luciana Villafuerte[2], Stella Hartinger[2], Camila Cayo[2], Solange Aznaran[2], Laura Nicolaou[3,4], William Checkley[3,5], Yang Liu[7], Kyle Steenland[7]

[1] Saint Louis University, Behavioral Science and Health Equity, St. Louis, USA; [2] Universidad Peruana Cayetano Heredia, Centro Latino Americano de Excelencia en Cambio Climático y Salud, Lima, Peru; [3] Johns Hopkins University, Division of Pulmonary and Critical Care, Baltimore, USA; [4] Johns Hopkins University, Department of Environmental Health and Engineering, Baltimore, USA; [5] Johns Hopkins University, Center for Global Non-Communicable Disease Research and Training, Baltimore, USA; [6] Johns Hopkins University, Program in Global Disease Epidemiology and Control, Baltimore, USA; [7] Emory University, Gangarosa Department of Environmental Health, Atlanta, USA

Background and Aim:
Exposure to fine particulate matter, $PM_{2.5}$, contributes to millions of premature deaths annually worldwide. Epidemiological studies increasingly rely on geospatial technologies, such as satellite remote sensing, low-cost sensors, and chemical transport models, to quantify air pollution exposure. However, in many low- and middle-income countries (LMICs), sparse air quality monitoring introduces sampling bias, leading to inaccuracies in exposure estimates. This study aims to develop a hybrid modeling approach that integrates multiple geospatial technologies and novel machine learning to address sampling bias and improve $PM_{2.5}$ predictions in resource-limited settings.

Methods:
We conducted the study in Lima, Peru using multi-sourced geospatial data, including ground $PM_{2.5}$ observations from both regulatory (10 monitors) and low-cost PurpleAir networks (22 monitors), satellite-retrieved aerosol optical depth, simulations from Weather Research and Forecasting model coupled with Chemistry, and various pollution-related variables (e.g., meteorological fields, land covers, etc.). Our hybrid modeling approach consists of two stages. Stage-1 applies transfer learning (TL) to mitigate sampling bias due to nonexistent rural monitoring. We transfer source data and knowledge from data-rich regions (e.g., U.S.) to data-poor target regions (e.g., Lima), and supplement training samples for rural areas via TL using TrAdaBoostR2. Stage-2 develops a sample-importance-weighted random forest (RF) exposure model that incorporates the transferred samples to estimated daily $PM_{2.5}$ levels from 2010 to 2023.

Results:
The hybrid model demonstrated robust performance with a cross-validated R^2 of 0.76. Notably, it alleviated overprediction in mountainous regions caused by a lack of observations (e.g., average $PM_{2.5}$ in mountainous regions reduced from 34.19 to 29.23 μg/m^3). The proposed approach enables $PM_{2.5}$ exposure modeling across entire Peru.

Conclusion:
Our hybrid approach provides a scalable solution to address sampling bias and enables high-resolution $PM_{2.5}$ exposure modeling in LMICs. Our results are expected to support robust epidemiological studies and inform public health planning.

P17-29 | Identification of Factors Affecting PM2.5 Exposure in Adults with Allergic Diseases: A Personal Exposure Study in the Seoul Metropolitan Area, South Korea

ShinYoung Park, JiYoon Chung, CheolMin Lee

Seokyeong university, environmental chemical engineering, Seoul, South Korea

It has been reported that approximately 6.7 million people worldwide die each year due to air pollution. Among various pollutants, particulate matter less than 2.5 μm in aerodynamic diameter (PM2.5) has a large surface area, making it more likely to adsorb harmful substances such as heavy metals. Long-term or short-term exposure to PM2.5 is known to cause respiratory diseases such as asthma, chronic obstructive pulmonary disease (COPD), and lung cancer. Consequently, numerous studies have been conducted to clarify the correlation between PM2.5 exposure and the occurrence of these diseases, typically using outdoor PM2.5 concentrations, personal PM2.5 measurements, or modeling data as indicators of exposure. However, previous studies have found a low correlation between outdoor PM2.5 concentrations and personal exposure levels, which is presumed to be influenced by the high proportion of time spent indoors in modern lifestyles. Therefore, to accurately assess the health impacts of PM2.5 exposure, it is essential to measure individuals' actual exposure levels. Furthermore, to accurately evaluate and reduce individual exposure in the future, it is crucial to identify the factors influencing personal exposure concentrations. This study aims to assess the factors affecting personal PM2.5 exposure among adults with allergic diseases (asthma, rhinitis, and conjunctivitis) in Seoul, South Korea. Over the course of one year, real-time PM2.5 measurements were taken, along with GPS coordinates, time-activity diaries (TADs), and daily surveys to analyze the levels and influencing factors of PM2.5 exposure across microenvironments. Ultimately, the study aims to identify potential interventions to reduce exposure for adults living in urban areas.

P17-30 | An environmental exposure indicator for selecting schools for environmental and health monitoring in Italian contaminated sites

Sofia Tagliaferro[1,2], Giuseppe Sarno[2], Sara Maio[2], Sandra Baldacci[2], Ilaria Stanisci[2,3], Elisa Bustaffa[2], Anna Bonomolo[4], Stefania La Grutta[4], Velia Malizia[4], Elisabetta Pace[4], Alessandra Pandolfo[3,4], Pietro Alfano[4], Giovanni Viegi[2,4], Fabrizio Minichilli[2]

[1] University of Verona, Unit of Epidemiology and Medical Statistics, Department of Diagnostics and Public Health, Verona, Italy; [2] National Research Council, Institute of Clinical Physiology, Pisa, Italy; [3] University of Palermo, Department of Economics, Business and Statistics, Palermo, Italy; [4] National Research Council, Institute of Translational Pharmacology, Palermo, Italy

Objective:

WHO recognizes contaminated sites (CSs) as a key environmental health priority. The SIN-SCUOLE study will evaluate air pollution exposure and respiratory health effects among students/parents/teachers from schools near 5 CSs (2 in Tuscany, 3 in Sicily).

To facilitate school selection, an exposure indicator (IND) integrating proximity to CSs and air pollution was developed.

Material and methods:
Schools in CSs municipalities were georeferenced, and their shortest distance (DIST) to the nearest CSs multi-centroid was calculated. $PM_{2.5}$ concentration values (proxy for exposure to air pollution from multiple sources, including industrial ones) were obtained through machine-learning-models and assigned to each school (year: 2022, resolution: $1km^2$). For each CS, IND was developed, prioritizing proximity to CS over air pollution: DIST + 0.5 * $PM_{2.5}$. Schools were classified at different levels of exposure based on IND quartiles for random sampling.
Results:
We identified 38 schools in Tuscany and 53 in Sicily. DIST ranged from 846–5912 m (Tuscany) and 306–7393 m (Sicily). Mean annual $PM_{2.5}$ concentrations ranged from 9.2 to 12.2 μg/m³ in Tuscany and 9.5 to 13.4 μg/m³ in Sicily, exceeding 2021 WHO's Air Quality Guideline of 5 μg/m³ almost twofold. Across all schools, mean (±SD) DIST and $PM_{2.5}$ in the first and fourth IND quartiles were 3854 m (±999) and 11.1 μg/m³ (±1.0) vs. 1338 m (±293) and 11.8 μg/m³ (±0.3), respectively. The inverse correlation between DIST and $PM_{2.5}$ (Pearson coefficient ranging from -0.28 to -0.47) was not statistically significant, indicating no collinearity and supporting their combined use in IND.
Conclusions:
By integrating both proximity and air pollution exposure, the indicator could be an appropriate basis tool for school selection and exposure assessment in contaminated sites. Its validity will be assessed against environmental monitoring data scheduled in the study.

Funding: The SIN-SCUOLE study was funded by the Italian Ministry of Health.

P17-31 | Optimization of illuminance and color-temperature conditions for railway passengers' comfort based on oxygenated hemoglobin saturation in the brain

Min-kyeong Kim[1], Duckshin Park[2]

[1] Korea Railroad Research Institute, Railroad Test & Certification Division, Uiwang-si, South Korea; [2] Korea Railroad Research Institute, New Transporation Innovation Research Center, Uiwang-si, South Korea

As railways are an eco-friendly means of transportation, various activities are carried out and the length of stay is increasing, so we aimed to examine the effect of lighting as a major factor affecting passenger comfort. In particular, since the lighting devices in railway vehicle cabins are installed in two rows of LED lights based on KSR 9159 and are presented only at 500lx or higher, we aimed to derive objective and quantitative values according to changes in cerebral blood flow. In this study, we measured the activation of the prefrontal cortex using fNIRS in 29 male and female college students, and examined brain activation under conditions of illuminance and color temperature warm white , neutral white, daylight color through ANOVA analysis. We confirmed that there was a significant difference in channel 38 out of 48 channels and in the Right DLPFC in the Brodmann area under illuminance and color temperature conditions. Oxy-Hb concentrations had negative values overall, and when examined by condition according to the one-sample t-test results, it was confirmed that the degree of activation was different in the channel and Brodmann areas. These results provide scientific basic data for designing comfort in railway vehicles, and ultimately can contribute to providing information for improving passenger satisfaction and enhancing the quality of railway services.
* KEY WORDS: fNIST; brain activation; light and color temperature; GLM

P17-32 | Content of elements in consumer products made of recycled plastics in Japan: A screening assessment of consumer exposure

Tomoko Oguri, Naoya Kojima, Naohide Shinohara, Isamu Ogura, Kyoko Ono, Masashi Gamo

National Institute of Advanced Industrial Science and Technology (AIST), Research Institute of Science for Safety and Sustainability, Tsukuba, Japan

Introduction
The global interest in using recycled plastics has been increasing. However, the safety of recycled plastics remains a concern. To better understand the non-intentionally added substances of the plastic recycling stream by regulated and other chemical substances, we are examining the current situation of chemical substance in materials and products made of recycled plastics.
Methods
A total of 138 general consumer products made of recycled plastics (e.g., stationery, calculators, carpets, sundries, detergent containers, sanitary supplies, food containers, garden supplies) were purchased in stores in Japan between March and December 2024. The collected products were labelled as containing recycled plastic. Each plastic product was dismantled, and 150 plastic component samples were analyzed for metal concentration with handheld X-ray fluorescence spectrometry.
Results and Discussion
The four elements regulated by the RoHS were detected: chromium (Cr) in 14% of samples (range: <N.D. to 96 mg/kg), cadmium (Cd) in 5% of samples (range: <N.D. to 72 mg/kg), mercury (Hg) in 5% of samples (range: <N.D. to 22 mg/kg), and lead (Pb) in 54% of samples (range: <N.D. to 152 mg/kg). None of the samples exceeded the maximum permitted concentration defined in the RoHS Directive. Bromine (Br) which might indicate the presence of brominated flame retardants, was detected in 17% of samples (range: <N.D. to 5468 mg/kg). Based on these results, we plan to carry out a consumer health risk assessment due to oral exposure to elements in recycled plastics.
Keywords: Recycled plastic, Commercial consumer products, Elements, XRF analysis

P17-33 | Automating Sensor Metadata Extraction for Exposure Health Research Using Large Language Models

Fatemeh Shah-Mohammadi[1], Sunho Im[2], Julio C. Facelli[1,3], Ramkiran Gouripeddi[1,3], Mollie R. Cummins[2,3]

[1] University of Utah, Department of BioMedical Informatics, Salt Lake City, USA; [2] University of Utah, College of Nursing, Salt Lake City, USA; [3] University of Utah, Center of Excellence for Exposure Health Informatics, Salt Lake City, USA

The rapid evolution of sensor technologies, driven by advancements on the Internet of Things (IoT), has led to a proliferation of diverse sensors with varied specifications and capabilities. However, the lack of standardized sensor metadata is a serious barrier for environmental health studies. Within the Sensors and Metadata for Analytics and Research in Exposure Health (SMARTER) project, our team is developing methods for consistent managing sensor

metadata that can be leveraged in informatics infrastructure such as the Exposure Health Informatics Ecosystem and elsewhere. Nevertheless, the extraction of the necessary metadata and data needed to populate the metadata model presents significant challenges. This still require costly and non-scalable manual curation. Additionally, sensor specifications are provided in non-computable and inconsistent formats, such as PDF datasheets, word documents, web pages, and catalog entries, creating barriers to efficient data management. On the other hand, Large Language Models (LLMs) have demonstrated remarkable potential for extraction of information, particularly in processing unstructured data, identifying relevant entities, and structuring information for various applications. In this study we present an LLM-based approach to automatically extract and harmonize sensor specifications, deployment settings, and operational characteristics, to enhance real-world contextualization of sensor metadata for scalable population of a metadata repository. The proposed framework addresses a critical bottleneck in environmental health research by enabling efficient and scalable metadata extraction, reducing manual effort, and ensuring accurate, comprehensive, and harmonized sensor information for FAIR exposure assessment. In this study, we focus on evaluating the sensor metadata extraction towards two goals: (1) Quality: accuracy of sensor metadata documents with metadata curated by exposure health experts, and (2) Quantity: depth of sensor metadata extracted from exposure health literature. As a future direction, we aim to expand our approach to encompass other common sources such as web pages, manufacturer catalogs, and other unstructured data sources.

P17-34 | Influence of enzymatic deconjugation on urinary metabolites of commonly used organophosphate esters

Nayana Jayatilaka, Paula Restrepo, Antonia Calafat, Maria Ospina

Centers for Disease Control and Prevention, National Center for Environmental Health/Division of Laboratory Sciences, Atlanta, USA

Objective: Some polybrominated diphenyl ethers (PBDEs) were phased out in the U.S. by 2013 due to health concerns, and chlorinated alkyl and non-chlorinated aryl organophosphate esters (OPEs) replaced them. OPEs are widely used in consumer products like furniture foam, electronics, and household items as flame retardants or plasticizers. OPEs are less persistent than PBDEs, but their metabolic transformation and health risks, including endocrine disruption, neurodevelopmental effects, and reproductive toxicity, remain a major concern. Research suggest widespread human exposure to mixtures of OPEs. Studies have shown that OPEs undergo various metabolic transformations in the body, including hydrolysis, O-dealkylation, hydroxylation, carboxylation, oxidative dehalogenation, and conjugation. These transformations lead to the formation of OPE metabolites, which are then excreted in urine, and are used as OPEs exposure biomarkers.
Material and Methods: We developed a highly sensitive method to quantify 15 urinary OPE metabolites reflecting about ten commonly used OPEs. The method uses 200 µL of urine, enzymatic hydrolysis, automated solid-phase extraction, reversed-phase high-performance liquid chromatography, and isotope dilution tandem mass spectrometry. We evaluated the performance of six commercial enzymes, two β-Glucuronidases and four β-Glucuronidase/sulfatases, to quantify these OPE metabolites.
Results: Of the 15 metabolites included in the method, only four out of six hydroxylated metabolites were fully conjugated. Two other hydroxylated metabolites and three none-hydroxylated metabolites were partially conjugated. Enzymes containing sulfatase yielded higher concentrations of metabolites, but some also suppressed the signal for certain non-conjugated metabolites. Optimization identified that 10µL of β-Glucuronidase/Arylsulfatase from *Helix pomatia* (liquid) incubated overnight at 37°C provided the best results.

Conclusion: These findings highlight the importance of enzyme selection and optimization for accurate, efficient biomonitoring methods. Additionally, developing multianalyte methods that can concurrently measure multiple biomarkers of OPEs from just a few drops of urine is crucial for assessing human exposure to OPE mixtures.

P17-35 | d-Band Center of Rare Earth Oxides Determines Biotransformation-Induced Cell Membrane Damage

Tianxiang Wu, Bin Wang

Peking University, School of Public Health, Beijing, China

Biotransformation of rare earth oxide (REO) nanoparticles on biological membranes may trigger a series of adverse health effects in biosystems. However, the physicochemical mechanism of the complicated biotransformation behavior remains elusive. By investigating the distinctly different biotransformation behavior of two typical REOs (Gd_2O_3 and CeO_2) on erythrocyte membranes, we demonstrate that dephosphorylation by stripping phosphate from phospholipids correlates highly with the membrane destructive effects of REOs. Density functional theory calculations decode the decisive role of the d-band center in dephosphorylation. Furthermore, using the d-band center as an electronic descriptor, we unravel a universal structure–activity relationship of the membrane-damaging capability of 13 REOs (R^2 = 0.82). The effect of ion release on dephosphorylation and physical damage to cell membranes by Gd_2O_3 are largely excluded. Our findings depict a clear physicochemical microscopic picture of the biotransformation of REOs on the nano-bio interface, providing a theoretical basis for safe application of REOs.

P17-36 | Navigating data linkages in environmental health research with CHORDS: Connecting Health Outcomes Research and Data Systems

Aubrey Miller[1], Adam Burkholder[2], Trisha Castranio[5], David Fargo[2], Ann Liu[1,7], Kyle Messier[4], Alison Motsinger-Reif[6], David Reif[4], Charles Schmitt[3]

[1] National Institute of Environmental Health Sciences, Division of Extramural Research and Training, Bethesda, USA; [2] National Institute of Environmental Health Sciences, Office of Environmental Science Cyberinfrastructure, Durham, USA; [3] National Institute of Environmental Health Sciences, Office of Data Science, Durham, USA; [4] National Institute of Environmental Health Sciences, Division of Translational Toxicology, Durham, USA; [5] National Institute of Environmental Health Sciences, Division of Extramural Research and Training, Durham, USA; [6] National Institute of Environmental Health Sciences, Division of Intramural Research, Durham, USA; [7] Kelly Government Solutions, Rockville, USA

Objective: Environmental determinants of health, such as poor air quality, extreme heat, and natural disasters, have a wide range of impacts on human health. To advance health research on these topics, comprehensive and robust data are needed. These complex environmental and health data are often collected for reasons other than health research and require much effort to be accessed, harmonized, and linked. *Material and Methods*: The NIH has developed a data ecosystem for the CHORDS project, with funding from the Office of the Secretary Patient-Centered Outcomes Trust Fund, aimed at addressing these challenges. The suite of CHORDS resources includes a curated data catalog, software tools and tutorials tailored to users of different levels of expertise, and standardized, linked

datasets. *Results*: We will showcase the data resources and provide short overviews of the software and educational materials that the research community, policymakers, educators, and the general public can use to examine environmental health questions. Additionally, we will present a wildfire use case to demonstrate a practical application of these resources. *Conclusion*: With these tools and tutorials available from the CHORDS data ecosystem, we hope clinicians, environmental health specialists, exposure science researchers, and earth and space scientists find their data access, harmonization, and integration processes easier to navigate and achieve.

P18 | Fluoride Exposure and Health

P18-01 | Evaluation of Fluoride-Containing Groundwater and Its Impact on Human, Livestock, and Environmental Health in the U.S. Southwest

Chrisa Whitmore[1], Jani Ingram[2], Anna Rondon[3], Joseph Hoover[1]

[1] The University of Arizona, Environmental Science, Tucson, USA; [2] Northern Arizona University, Chemistry and Biochemistry, Flagstaff, USA; [3] New Mexico Social Justice and Equity Institute, Gallup, USA

Objective: This research aims to model the spatial distribution and health risks of fluoride, a geogenic groundwater contaminant, in unregulated water sources. By integrating geospatial analysis with environmental health risk assessment, the study seeks to identify areas and populations with potential for elevated groundwater fluoride exposure and to understand impacts on individual organisms.
Materials: Publicly available datafor water quality, geology, land use, climate, hydrology, and more were compiled from federal and state sources. Risk assessments incorporate rural water hauling data, community demographics, and geospatial predictions of groundwater fluoride concentrations to evaluate the impact of varying fluoride levels in drinking water on both humans and livestock, which are vital to the local economy.
Methods: GIS spatial analysis methods are being employed to map and predict groundwater fluoride concentrations. A health risk assessment for humans and livestock will be conducted to evaluate the health risks of fluoride exposure using Monte Carlo simulation in Crystal Ball.
Results: Data from 20,437 water sources were compiled and mapped.Preliminary findings indicate spatial heterogeneity in fluoride levels throughout the region, which may be impacting both human populations and livestock. We observed that 10.4% of tested sources produced water with fluoride concentrations exceeding drinking water standards for people, and 23.4% exceeded the water standard for livestock water. Risk assessment using these data is underway, but preliminary outcomes suggest fluoride toxicity in livestock are associated with long term exposure in areas with elevated fluoride concentrations.
Conclusion: This study focuses on fluoride, a geogenic contaminant that is prevalent in groundwater in the southwest US. By combining geospatial analysis with health risk assessment, this research provides supporting data for public health interventions, policy development, and sustainable solutions to mitigate fluoride exposure and uphold the health of communities at risk for exposure and its agricultural systems.

P18-02 | Fluoride exposure and cardiometabolic risk in children: role of microRNAs.

Elihu A. Hernández-Rodríguez[1,2], Yazmín Godínez-Solís[1,2], Mónica I. Jiménez-Córdova[3,4], Sandra L. Romero-Cordoba[1,5], Marco Sánchez-Guerra[6], José E. Márquez-García[7], Angel Barrera[3], Luz M. Del Razo-Jiménez[3], Citlalli Osorio-Yáñez[1,2]

[1] Universidad Nacional Autónoma de México, Instituto de Investigaciones Biomédicas, Ciudad de México, Mexico; [2] Instituto Nacional de Cardiologia Ignacio Chavez, Unidad de Investigación UNAM-INC, Ciudad de México, Mexico; [3] Instituto Politécnico Nacional, Departamento de Toxicología-Centro de Investigación y de Estudios Avanzados, Ciudad de México, Mexico; [4] Programa de Investigadoras e Investigadores por México-SECIHTI, Ciudad de México, Mexico; [5] Instituto Nacional de Ciencias Médicas y Nutrición Salvador Zubirán, Departamento de Bioquímica Avanzada, Ciudad de México, Mexico; [6] Instituto Nacional de Perinatología Isidro Espinosa de los Reyes, Ciudad de México, Mexico; [7] Instituto Nacional de Enfermedades Respiratorias Ismael Cosío Villegas, Ciudad de México, Mexico

Background and Aim. In Mexico, 6.5 million children are exposed to fluoride, which may raise cardiometabolic risk (CMR). However, no previous studies have proposed microRNAs as possible circulating biomarkers linking F- and cardiometabolic outcomes. Therefore, the aim of this study was to evaluate the associations between urinary F-, CMR and specific microRNAs. **Methods**. A cross-sectional study was conducted in Chihuahua, Mexico with 235 children (5-12 years). A CMR score was constructed, and serum microRNAs -24, -122 and -222 were analyzed using TaqMan Advanced Probes by Real Time PCR. **Results**. Overall, the 45.1% of the children had CMR and no significant association was found between CMR and urinary F-. Looking at individual variables of CMR, urinary F- was associated with a decrease in HDL (-2.98 mg/dL; 95% CI:-6.05, -1.54; p=0.003) and insulin (-17.9; 95% CI:-37.17, 1.38; p=0.071) in girls and boys, respectively. Regarding microRNAs, urinary F- was significantly associated only with a decrease in miRNA-24 (-0.069; 95% CI:-0.122, -0.016; p=0.012). No significant associations were found between urinary F- and miRNA-122 or -222. On the other hand, sex-specific associations were observed between miRNAs -122 and -222 and CMR variables. In boys, miRNA-222 was associated with increased waist circumference (1.36 cm; 95% CI:0.48, 2.24; p=0.003), BMI z-score (0.35; 95% CI:0.03, 0.67; p=0.033), body fat percentage (0.72; 95% CI:0.10, 1.34; p=0.024), triglycerides (5.77 mg/dL; 95% CI:0.94, 10.61; p=0.02), and cholesterol (2.65 mg/dL; 95% CI:0.07-5.24; p=0.045). In girls, both miRNAs -122 and -222 were associated with lower insulin levels. **Conclusion**. We observed associations between urinary F- and some variables of CMR such as HDL and insulin, but not with CMR score. Additionally, we found no evidence that F- increases CMR through miRNAs. However, miRNA-122 and -222 could serve as CMR biomarkers in children, regardless of F- exposure. This study was funded by UNAM-PAPIIT-IN209323.

P18-03 | Causal Effect of Plasma Fluoride on Blood Pressure, Lipid Profiles, Renal Function, Metabolic Markers, Sex Hormones, and Serum Electrolytes: An Instrumental Variable Analysis Using NHANES Data

Shaheryar Shafqat, Hongmei Zhang, Chunrong Jia, Marian Levy, Xichen Mao, Xinhua Yu, Abu Mohammed N. Titu

University of Memphis, School of Public Health, Memphis, USA

Plasma fluoride concentrations may have multi-system effects; however, the association between plasma fluoride levels and systemic health effects may be confounded by demographic, socioeconomic, and unmeasured factors, limiting causal inference. To estimate plasma fluoride's causal effect on various organ function biomarkers, we conducted instrumental variable analyses using household drinking water fluoride levels as an instrument. A two-stage, survey-weighted regression model was implemented, adjusting for age, sex, BMI, income, income-to-poverty ratio, health insurance, food security, history of food assistance, maternal age at birth, and birth weight, utilizing data from the NHANES 2013–2016 surveys.
Each 0.25 µmol/L (250 nmol/L) increase in plasma fluoride was associated with a -1.97 mmHg (95% CI: -3.71, -0.23) change in systolic blood pressure (SBP) and a 0.41 mmHg (95% CI: -2.70, 3.53) change in diastolic blood pressure (DBP). Additionally, we observed changes of 2.01 mg/dL (95% CI: -19.78, 23.79) in triglycerides, -0.19 mg/dL (95% CI: -4.19, 3.79) in total cholesterol, 0.51 mg/dL (95% CI: -1.44, 2.47) in HDL cholesterol, -2.07 mL/min/1.73m² (95% CI: -4.05, -0.10) in estimated glomerular filtration rate (eGFR), and a 0.21 mg/dL (95% CI: -0.02, 0.43) increase in serum uric acid.
Further analyses revealed changes in HbA1c (-0.05; 95% CI: -0.14, 0.04), fasting blood glucose (-7.03 mg/dL; 95% CI: -18.31, 4.24), serum potassium (-0.04 mmol/L; 95% CI: -0.12, 0.04), serum chloride (0.69 mmol/L; 95% CI: -0.39, 1.77), serum sodium (0.82 mmol/L; 95% CI: 0.27, 1.36), and serum calcium (0.01 mmol/L; 95% CI: -0.08, 0.10) per 0.25 µmol/L increase in plasma fluoride. Moreover, plasma fluoride levels were causally linked to a -19.32 ng/dL (95% CI: -34.97, -3.67) change in testosterone and a 1.76 pg/mL (95% CI: -5.78, 9.30) change in estradiol levels.
Our findings suggest a potential causal relationship between plasma fluoride and various physiological biomarkers, including lower SBP, eGFR, and serum testosterone.

P18-04 | Fluoride exposure among the US children aged ≤ 19 years old: findings from NHANES surveys 2013-2016

Shaheryar Shafqat, Bikram Adhikari, Xinhua Yu, Xichen Mou, Marian Levy, Hongmei Zhang, Chunrong Jia, Abu Mohammed N. Titu

University of Memphis, School of Public Health, Memphis, USA

Evaluating fluoride levels in biological and environmental matrices helps understand fluoride exposure among U.S. children and adolescents.
We analyzed plasma and drinking water fluoride levels in children aged 6–19 using NHANES data from 2013–2016. Water fluoride was categorized based on USEPA's non-enforceable (≤2 mg/L) and enforceable (≤4 mg/L) standards. Plasma fluoride was dichotomized using Mayo Clinic's reference of 1–4 µmol/L. We assessed Pearson correlation between water and plasma fluoride and used survey-weighted linear regression to examine the association between water and plasma fluoride.
The median **water fluoride** was **0.474 mg/L** (IQR: 0.296–0.651) for males and **0.416 mg/L** (IQR: 0.224–0.609) for females. Age-group wise, **water fluoride**was **0.492 mg/L** (IQR: 0.343–0.641) for ≤6 years, **0.528 mg/L** (IQR: 0.404–0.652) for >6 to ≤10 years, **0.450 mg/L**(IQR: 0.289–0.610) for >10 to ≤12 years, and **0.495 mg/L** (IQR: 0.347–0.643) for >12 to ≤19 years. The median **plasma fluoride** was **0.348 µmol/L** (IQR: 0.328–0.368) for males and **0.327 µmol/L** (IQR: 0.304–0.350) for females.
Of participants, 0.156% (77/19,357) had >2 mg/L, and 0.009% (3/19,357) had >4 mg/L. Plasma fluoride was <1 µmol/L in 99.617% (19,257/19,353) participants. The Pearson correlation coefficient between water and plasma fluoride was 0.22. A 250 µgm/L (0.25 mg/L) increase in water fluoride was associated with a 0.054 (95% CI: 0.041, 0.067, R^2=0.15) µmol/L increase in plasma fluoride after adjusting for age, sex, race, BMI, income, health insurance presence, income-to-poverty ratio, food stamp receipt, mother's age at birth, U.S.-born status, and birthweight.
More than 99% participants had water fluoride below USEPA's non-enforceable level and plasma fluoride below lower range of Mayo Clinic's standard. Only 15% of variation in plasma fluoride was explained by the urine fluoride in the model.

P18-05 | Re-analysis of bone fluoride's association with bone strength, controlling for confounders, based on bone samples from Canadian hip-replacement-surgery patients

Chris Neurath[1], Hardy Limeback[2], Michael Connett[3]

[1] American Environmental Health Studies Project (AEHSP), North Sutton, USA; [2] Professor Emeritus, University of Toronto, Faculty of Dentistry, Toronto, Canada; [3] Siri & Glimstad LLP, Los Angeles, USA

Objective: Recent studies of US children and Swedish postmenopausal women found associations between commonly experienced fluoride exposures (≤1mg/L) and increased risk of bone fractures [Lindsay et al 2023, Helte et al 2021]. While these studies estimated exposures from drinking water or urine fluoride concentrations, bone fluoride is a more direct measure of long-term exposure to the target tissue. We therefore re-analyzed published data on bone fluoride and bone strength from a study of bone samples obtained from 92 patients undergoing hip

replacement surgery in fluoridated Toronto and non-fluoridated Montreal, adding control for age, sex, and city which the original study did not control for [Chachra et al 2010, Chachra 2001].
Material and Methods: Data was extracted from published scatterplots using digitizing software, and matched across variables common to two or more scatterplots to determine variable values for each patient. Our re-analysis assessed bone strength (Ultimate Compressive Stress or UCS) as a function of bone fluoride concentration, controlling for age, sex, and city using Generalized Linear Models.
Results: Higher bone fluoride had a large and highly significant association with reduced bone strength measured as UCS when controlling for age, sex, and city. The model predicted a decrease in UCS from 10.9 to 3.0 MegaPascals (MPa) as bone fluoride increased from 200 to 2,000 ppm. In the multivariable model, age had no effect on bone strength; Toronto's mean UCS was 2.2 MPa greater than Montreal's; and females averaged 1.3 MPa lower than males.
Conclusion: Bone fluoride had a large and significant adverse association with bone strength after controlling for age, sex, and city. This adverse association found in human bone samples is consistent with the increased risk of bone fractures found in both children and postmenopausal women in two recent epidemiological studies where the main exposure source was also water fluoride ≤1mg/L.

P18-06 | The sugar industry's efforts to manipulate research on fluoride effectiveness and toxicity: A ninety-year history

Chris Neurath

American Environmental Health Studies Project (AEHSP), North Sutton, USA

Objective: Scholars have recently started exploring efforts of the sugar industry to manipulate science to downplay sugar's role in obesity, diabetes, cardiovascular disease, and tooth decay. The sugar industry's tactics are like those in the better-known tobacco industry campaigns. Archival records show sugar-industry-funded scientists criticized evidence linking sugar to harms and tried to deflect attention to other risk factors. For tooth decay specifically, available documents allow investigation of whether the sugar industry turned to promoting fluoride as the solution, to avoid calls for reduced sugar consumption. Furthermore, as concerns for fluoride's own harmful side effects arise – most recently with developmental neurotoxicity – there is a continuing need to investigate whether the sugar industry is still manipulating science, now to defend fluoride.
Materials and Methods: Internal documents from sugar and dental organizations were reviewed and compared to the published scientific literature. The main source of records was the online Industries Documents collection at the University of California, San Francisco.
Results: Records dating back to the 1930s show the sugar industry, sometimes in cooperation with dental interests, exaggerated fluoride's effectiveness and downplayed safety concerns. This manipulation campaign predated the tobacco industry's defense of cigarettes. Key figures in the sugar industry's campaign later helped jumpstart the tobacco industry's campaign, bringing tactics and conflicted scientists along with them. An important current fluoride safety concern is developmental neurotoxicity. Researchers with undisclosed conflicts of interest with sugar and allied industries have recently produced biased reviews minimizing this risk.
Conclusion: Newly available records reveal a long history of the sugar industry distorting fluoride science. Many tactics originated by the sugar industry were later adopted in campaigns by industries involved with tobacco, asbestos, lead, pesticides, climate change denial, etc. Researchers and policymakers should be aware of the distorted scientific record on fluoride's effectiveness and toxicity.

P18-07 | XRF applications in Fluoride and PFAS assessment in biological samples

Abu Sayed Mohammed Sayam[1], Laimar Garmo[2], Joyce Lin[3], Michael Petriello[2], Izabela Podgorski[2], Frank Lippert[4], Esperanza Angeles Martinez Mier[4], Marc Weisskopf[3], Aaron J. Specht[1,3]

[1] Purdue University, School of Health Sciences, West Lafayette, USA; [2] Wayne State University, School of Medicine, Detroit, USA; [3] Harvard University, Harvard T.H. Chan School of Public Health, Boston, USA; [4] Indiana University, School of Dentistry, Indianapolis, USA

X-ray fluorescence (XRF) is widely used as a non-destructive tool for assessing environmental and biological exposures, with its effectiveness depending on the application, X-ray source, detector capability, and sample type. This study explores the potential of a lab-based micro-XRF system for spatial elemental mapping with micrometer-level resolution, including the quantification of fluorine and its derivatives like fluoride and per- and polyfluoroalkyl substances (PFAS). Fluoride exists in various forms in nature and has been associated with dental and skeletal fluorosis, neurodevelopmental issues, pulmonary edema, skin burns, and laryngeal spasms when chronically or acutely exposed above a threshold. On the other hand, PFAS chemicals are linked to liver damage, immune dysfunction, and reproductive harm. This study contains three distinctive parts. The first part involved a dose-response experiment on fluoride-treated tooth enamel embedded in acrylic rods. Three sets of six teeth were treated with 0, 250, and 1100 ppm fluoride, and measurements showed significant agreement by the treatment group ($r = 0.94$). Second, fourteen sliced baby teeth embedded in epoxy were measured in micro-XRF—seven of these teeth dated before fluoride's introduction to community water supplies. Upon analysis, we found that teeth samples dated after water fluoridation had 28% higher fluoride content in tooth dentin ($p = 0.006$) compared to the pre-fluoridation. In the final part, raw bone samples from euthanized mice, with three groups exposed to different PFAS chemicals (PFHxS, GenX, Mixtures) and one untreated, were examined. The fluorine(F) content in these bones was quantified and compared with calcium (Ca), a basic structural element in the bone. The subjects treated with GenX showed maximum F/Ca ratio. Based on the outcome of this study, Micro-XRF could be established as a tool to assess exposure related to fluorine derivatives and ultimately could pave the way for developing portable XRF tools for similar purposes.

P18-08 | Prenatal fluoride exposure and child IQ: Is tea a key source of fluoride?

Meaghan Hall[1], Richard Hornung[2], David Flora[1], Bruce Lanphear[3], Christine Till[1]

[1] York University, Psychology, Toronto, Canada; [2] Retired; consultant to York University, Psychology, Toronto, Canada; [3] Simon Fraser University, Health Sciences, Burnaby, Canada

Objective: Prenatal fluoride exposure has been associated with reduced child intelligence, but whether fluoride exposure from tea negatively affects children's IQ remains unclear. We examined the association of fluoride exposure from tea consumption in pregnancy with child IQ scores among mother-child pairs in the Canadian Maternal-Infant Research on Environmental Chemicals cohort.

Methods: We recorded thefrequency of tea, water, and coffee consumption through self-report questionnaires during the first and third trimesters. We estimated daily fluoride intake from tea (mg/day) by summing the fluoride content in a standard cup of green (0.260mg) and black tea (0.326mg) with the fluoride content of each 200mL cup of the participant's tap water. We derived drinking-water fluoride concentrations from water treatment plant reports. We assessed Full-Scale IQ (FSIQ) in 470 preschool-aged children. We quantified the association between maternal fluoride intake from tea (mg/day) and child FSIQ using linear regression, adjusting for relevant covariates. We then tested the interaction between tea-drinking status (yes/no) and total maternal fluoride intake (all beverages) to assess the exposure-response trend across tea- and non-tea-drinkers.

Results: Most participants (78%) reported tea consumption during pregnancy; 11% reported daily consumption.Mean (SD) maternal fluoride intake from tea was 0.12 (0.21) mg/day. We observed a significant negative association between fluoride intake from tea and child FSIQ. A 0.38mg/day increase in fluoride intake from tea (approximate difference between non-tea-drinkers and daily tea-drinkers) was associated with a 2.61-point decrease (95%CI:-4.66,-0.56) in child FSIQ. The association between total maternal fluoride intake and child FSIQ did not differ by tea drinking status (p-interaction-term=.92). Fluoride intake from non-tea beverages alone was not significantly associated with child FSIQ.

Conclusion: Fluoride from tea consumption contributes to reduced child IQ over-and-above fluoride from other beverages. As tea may be a significant source of fluoride exposure, pregnant women may choose to limit their intake.

P18-09 | Does fluoride exposure affect the risk of fractures and bone density? A dose-response meta-analysis

Riccardo Mazzoli[1], Inga Iamandii[1], Lisa De Pasquale[1], Linda S. Birnbaum[2], Tommaso Filippini[1,3], Marco Vinceti[1,4]

[1] University of Modena and Reggio Emilia, Environmental, Genetic and Nutritional Epidemiology Research Center (CREAGEN), Section of Public Health, Department of Biomedical, Metabolic and Neural Sciences, Modena, Italy; [2] Duke University, Nicholas School of the Environment, Durham, USA; [3] University of California Berkeley, School of Public Health, Berkeley, USA; [4] Boston University School of Public Health, Department of Epidemiology, Boston, USA

Background: Fluoride exposure and its effects on human health are controversial and highly debated. While beneficial in preventing dental caries, fluoride has been linked to skeletal and dental fluorosis at high levels, as well

as other adverse health endpoints. However, the safe range of exposure for bone health remains poorly defined. Our goal was to quantify the dose-response relation between fluoride exposure and bone health, focusing on fracture risk and bone mineral density (BMD).
Methods: We conducted a systematic review and dose-response meta-analysis (PROSPERO Registration no. CRD42022321899), by searching literature in online databases (PubMed, Embase and Web of Science) up to 20th November, 2024, and by pooling results of studies examining fluoride exposure and its associations with bone health outcomes, namely fracture risk and BMD. We also carried out stratified analyses by sex, age, and fracture site.
Results: We included 35 studies published between 1945-2024, 9 pertaining to bone density only; 18 on bone fractures only; and 8 on both outcomes. We found a non-linear, positive relation between fluoride exposure and fracture risk, with a threshold around 1.5 mg/L of fluoride in drinking water and increased fracture risk above that concentration, especially in females and for femoral fractures. Conversely, the association between fluoride and BMD was inconsistent, showing opposite trends depending on bone site (hip and spine) and sex. Overall, females and individuals aged over 50 years exhibited stronger associations of fluoride exposure with fracture risk and spine BMD.
Conclusion: This study suggests a potential higher risk of fractures at drinking water fluoride exposure>1.5 mg/L. It also indicates the impact of fluoride on BMD, depending on level of exposure and specific bone site. These findings provide additional evidence for the risk assessment of fluoride, including individuals more vulnerable to fracture risk, such as postmenopausal females.

P18-10 | FLUORIDE EXPOSURE AND CHILDREN'S IQ: A SYSTEMATIC REIVEW AND META-ANALYSIS OF HIGH-QUALITY STUDIES

Kyla W. Taylor[1], Sorina E. Eftim[2], Christopher A. Sibrizzi[2], Robyn B. Blain[2], Kristen Manguson[2], Pamela A. Hartman[2], John R. Bucher[1], Andrew A. Rooney[1]

[1] National Institute of Environmental Health Sciences, Division of Translational Toxiciology, Durham, USA; [2] ICF, Reston, USA

OBJECTIVE Conduct a systematic review and meta-analysis of epidemiological studies investigating children's IQ scores and pre- or post-natal fluoride exposure, primarily urinary and water fluoride.
METHODS AND MATERIALS Following OHAT methodology, literature searches were conducted in 8 databases through October 2023. Studies reporting children's IQ scores, fluoride exposure, and effect sizes were included. Study quality was evaluated and pooled standardized mean differences and regression coefficients were estimated with random-effects models. More recent data will be incorporated to evaluate its potential impact.
RESULTS Out of 74 total relevant studies from 10 countries, 22 were rated high quality, of which 19 reported inverse associations between fluoride exposure and children's IQ. Among high-quality studies, analyses of studies with group-level and individual-level fluoride measures found statistically significant decreases in IQ score. Associations were robust to stratification by study quality, sex, age, outcome assessment type, country, exposure timing, and exposure matrix. The dose-response meta-analysis considered linear, quadratic, and cubic splines.Associations remained inverse when exposed groups were restricted to <1.5 mg/L fluoride in water or urine. The linear model provided the best fit in all dose-response analyses with statistically significant associations. When estimating fluoride exposure solely from drinking water, results were not statistically significant at <1.5 mg/L, and there was no evidence to support a threshold.
CONCLUSIONS The meta-analysis suggests that early life exposure to total ingested fluoride is associated with lower childhood IQ. These results and updated analyses that incorporate more recent data inform ongoing

discussions on the level of fluoride exposure at which neurodevelopmental effects begin to occur. Confidence in the inverse association is based on the clear and consistent association across study quality, sex, age, outcome assessment type, country, exposure timing, and exposure matrix. This comprehensive meta-analysis may inform future risk-benefit assessments of the use of fluoride in children's oral health.

P18-11 | Fluoride and Arsenic Exposure from Groundwater and Kidney Function in Children:Assessment and Mitigation

Paulina Farías[1], Marcela Tamayo-Ortiz[5], Katherine James[6], Elodia Rojas-Lima[4], Leonora Rojas-Bracho[7], Karla Rangel-Moreno[1], Matthieu Carrière[3], Manolo Ortega-Romero[2], Olivier Barbier[2], Luz M. Del Razo-Jiménez[2]

[1] National Institute of Public Health, Mexico, Center for Research on Population Health, Cuernavaca, Mexico; [2] Center for Research and Advanced Studies of the National Polytechnic Institute, Mexico City, Mexico; [3] Caminos de Agua, San Miguel de Allende, Mexico; [4] Mexican Institute of Social Security, Mexico City, Mexico; [5] University of Columbia, New York, USA; [6] Colorado University, Center for Health, Work & Environment, Boulder, USA; [7] Living in a Chemical World, Mexico City, Mexico

Chronic exposure to fluoride and arsenic in groundwater poses significant public health risks, particularly in rural communities. This study evaluated fluoride and arsenic exposure and its potential impact on early kidney function in children from Guanajuato, Mexico. Our objective was to assess fluoride levels in drinking water, urinary fluoride concentrations, and their association with early renal function markers in schoolchildren. Seventy-five children (6–12 years old) were recruited with parental consent. Data collection included household drinking water samples, first-morning urine samples, anthropometric measurements, blood pressure assessments, and oral health evaluations. Sociodemographic and exposure questionnaires were administered to parents. Fluoride and arsenic concentrations in water and urine were measured, and kidney function biomarkers were analyzed. Nearly 50% of household drinking water samples exceeded the national fluoride limit (1.5 mg/L), with the highest concentrations in well water (average 3.7 mg/L). Urinary fluoride levels were elevated in 85% of children, indicating excessive exposure. Children drinking well water or eating food cooked with it had significantly higher urinary fluoride levels. Fluorosis was present in 78.1% of participants, indicating a chronic exposure starting in utero. A positive association was observed between urinary fluoride and most early renal function markers, particularly beta-2-microglobulin (B2MG) and cystatin C (controlling for age, sex, body mass index, and dental fluorosis as a proxy of chronic fluoride exposure). The risk of developing chronic kidney disease in these children waoutreach
s enhanced by the coexistence of other risk factors: 39% of them were overweight or obese, and a high prevalence of family history of diabetes (41%), hypertension (27%), and chronic kidney disease (17%) was reported. In conclusion, prolonged fluoride exposure, primarily from well water, may pose risks for early kidney dysfunction. Public health efforts should prioritize community education, water monitoring, and alternative safe water sources to reduce exposure and prevent long-term health consequences.

P18-12 | Knowledge, Attitudes, and Behaviors Related to Fluoride Among Canadian and American Adults

Christine Till[1], Jana El-Sabbagh[1], Mikel Subiza[2], Kyla W. Taylor[4], Bruce Lanphear[3]

[1] York University, Faculty of Health, Toronto, Canada; [2] University of the Basque Country, Department of Clinical and Health Psychology and Research Methods, Donostia-San Sebastián, Spain; [3] Simon Fraser University, Faculty of Health Sciences, Vancouver, Canada; [4] National Institutes of Health, National Institute of Environmental Health Sciences, Division of Translational Toxicology, Research Triangle Park, USA

Objective: The evolving science on fluoride neurotoxicity has triggered renewed debate over community water fluoridation (CWF). We conducted a survey to understand what people know about fluoride use and explore opinions around fluoride in the public water supply.
Methods: We surveyed 2,000 adults from Canada and 6,011 from the United States via CloudResearch's Prime Panels, an online platform that ensures data quality standards and country-specific census gender and age quotas are met.
Results: Sixty percent of respondents knew why fluoride is added to drinking water, whereas 48% knew whether fluoride is added to their tap water. Older age, females, and higher income and education were more likely to know more about CWF. Forty-eight percent of respondents support CWF while 27% opposed and 25% were neutral. People aged >65 years (73%) and identifying as politically-left (74%) were more likely to endorse strongly/somewhat support CWF compared with people <45 years (57%) and politically right-of-centre (56%). Reasons for supporting CWF mainly relate to beliefs that the benefits outweigh potential risks (81%), that CWF reduces the cost of dental care (71%), and it is safe (85%). Reasons for opposing CWF mainly relate to skepticism about its effectiveness (45%), the view that CWF imposes on personal rights or freedoms (59%), and concerns that it is not safe (46%) or unsure (32%). Among supporters, 85% trust the information they receive from health authorities about CWF compared with only 40% among non-supporters. Concern about potential health risks of ingesting too much fluoride was endorsed among 73% of people who do not support CWF versus 44% who do.
Conclusion: CWF is a polarized issue based on public perceptions. Differing beliefs about its effect in preventing tooth decay, its potential for adverse health effects, and trust in health authorities have contributed to mixed perceptions of this public health practice.

P18-13 | Fluoride Disrupts Calcium Metabolism and Affects the Expression of Klotho-Related Calcium Metabolism Proteins

Yang Liu, Yuting Chen, Yuchen Zhang, Xiaona Liu, Yanmei Yang, Yanhui Gao

Harbin Medical University, Center for Endemic Disease Control and Prevention, Harbin, China

Abstract

Background & Objective: Our previous population-based study showed that serum Klotho levels differed in individuals with varying degrees of fluorosis. To further investigate the effect of fluoride exposure on calcium metabolism-related proteins of the kidney and to clarify the mechanisms of fluorosis, we conducted this mechanistic study using an animal model.

Methods: A total of 45 male Wistar rats were randomly divided into 3 groups. The control group received deionized water, and the fluoride - exposed groups were given 50 mg/L and 100 mg/L NaF solutions for 12 weeks. After 12 weeks, urine, kidney and blood samples were collected. Urinary fluoride was measured by the ion-selective electrode method, and calcium ions in kidney, urine and serum by the MTB method. Western blot was used analyze protein expression in kidneys, and ELISA was used to measure serum Klotho levels.

Results: Compared with the control group, urinary fluoride levels were significantly elevated in the fluoride exposure groups. Urinary calcium ion concentrations were significantly higher in both fluoride-exposed groups. The 100 mg/L fluoride exposure group also showed a significant increase in renal calcium ion concentration and a notable decrease in serum calcium ion concentration. In rat kidneys, Klotho and TRPV5 expression levels were significantly upregulated in the 100 mg/L fluoride exposure group. Conversely, NCX1 and PMCA1 expression levels were significantly downregulated in the same group. CB-D28K expression levels were significantly elevated in fluoride exposure groups compared with the control group. ELISA results indicated that serum Klotho concentrations were significantly increased in the 100 mg/L fluoride exposure group.

Conclusion: Fluoride exposure may induce calcium metabolism disorders by modulating the expression of Klotho and other calcium metabolism-related proteins in the kidney.**Funding**

This work was supported by National Key R&D Program of China (2022YFC2503000)

P19 | Breathing Easier, Living Longer: The Power of Greenspace - PART I

P19-01 | Exposure to Neighborhood Greenness and Reduced Biological Aging in Adults: A Longitudinal Study in Taiwan

Yiqian Zeng, Ting Xiao, Weiyi Chen, Dezhong Chen, Yiyue Yin, Xiang Qian Lao

City University of Hong Kong, Biomedical Sciences, Hong Kong, China

Objective

The beneficial effects of neighborhood greenness in promoting cardiovascular health are increasingly acknowledged. However, the association between neighborhood greenness and acceleration of aging (AA) is not well understood. This study aims to investigate the association between long-term exposure to neighborhood greenness and AA among adults in Taiwan.

Material and Methods

We included 302,158 adults (≥18 years of age) with 655,935 medical observations in Taiwan during 2001-2016. Neighborhood greenness was assessed using the normalized difference vegetation index (NDVI) within a 500m buffer around participants' residences. The biological age was estimated using Klemera−Doubal method (KDM) algorithms based on clinical biomarkers. AA was determined by the difference between the Klemera–Doubal biological age and chronological age. A linear mixed model (LMM) was used to examine the association between neighborhood greenness and AA.

Results

Compared with living in areas within the first tertile of neighborhood greenness, living in areas within the second and third tertiles of neighborhood greenness was found to be associated with reduced AA, with coefficients and 95% confidence intervals (CIs) of -0.075 (95% CI: -0.115, -0.034) and -0.129 (95% CI: -0.168, -0.09), respectively. Each 0.1-unit increase in the NDVI was associated with a -0.938 (95% CI: -1.082, -0.793) year reduction in AA.

Conclusion

Residing in a higher level of neighborhood greenness was associated with reduced biological aging in Taiwanese adults. The finding highlights the importance of neighborhood greenness to improve healthy aging.
This study is supported by the RGC-General Research Fund of University Grant Committee of Hong Kong (14600522) and the New Research Initiatives/Infrastructure Support from the City University of Hong Kong (9610613).

P19-02 | Exposure to urban greenness and severity of Major Depressive Disorder: Results from the FREEEDOM Study

Elisa Borroni[1], Giovanni Sanesi[2], Vincenzo Giannico[2], Paola Monti[1], Massimiliano Buoli[3,4], Valentina Bollati[1], Guido Nosari[3,4], Luca Ferrari[1], Laura M. Antonangeli[1], Angela C. Pesatori[1,5], Michele Carugno[1,5]

[1] University of Milan, Department of Clinical Sciences and Community Health, Milan, Italy; [2] University of Bari Aldo Moro, Department of Agricultural and Environmental Sciences, Bari, Italy; [3] University of Milan, Department of Pathophysiology and Transplantation, Milan, Italy; [4] Fondazione IRCCS Ca' Granda Ospedale Maggiore Policlinico, Department of Neurosciences and Mental Health, Milan, Italy; [5] Fondazione IRCCS Ca' Granda Ospedale Maggiore Policlinico, Epidemiology Unit, Milan, Italy

Objective

To evaluate effects of exposure to urban greenness, alone and in combination with air pollution, on Major Depressive Disorder (MDD) severity within the FREEDOM study.

Materials and Methods

Three hundred and ninety-six MDD patients were recruited at the Policlinico Hospital (Milan, Italy) and administered questionnaires on lifestyle, personal, and clinical characteristics. MDD severity was assessed using five psychiatric rating scales. Exposure to urban greenness was estimated using the Normalized Difference Vegetation Index (NDVI), in a 100-m radius around patients' residence. Particulate matter ≤2.5 µm (PM2.5) and nitrogen dioxide (NO_2) average exposure in the year before recruitment was also evaluated. Multivariate linear or ordinal logistic regression models (adjusted by age, sex, education, occupation, month and year of recruitment) were applied to assess the association between NDVI and MDD severity, examining also the possible modifying role of PM2.5 and NO_2. Results are expressed as β with corresponding 95% confidence intervals (CI) per 0.1-unit increase in NDVI levels.

Results

NDVI levels were associated with decreasing scores of MDD severity scales: MADRS (focuses on melancholic symptoms: -1.23, 95% confidence interval (CI): -2.32; -0.13); HAMD (focuses on anxiety and somatization symptoms: -1.20, 95%CI: -2.27; -0.14); CGI (assesses global severity of illness: -0.21, 95%CI: -0.38; -0.04); GAF (evaluates patient's global functioning and is interpreted in a mirror-like manner to the previous ones: 1.61, 95% CI: 0.24; 2.97). The inverse association between NDVI and MDD severity was confirmed when annual PM2.5 values were below the average, while no effect was observed when values were above the average.

Conclusions

Green spaces have a protective role on MDD severity, especially when PM2.5 levels are low. Evaluation of greenness "three-dimensional" indicators (also capable of capturing volume of green spaces) will allow us to strengthen our results.

Funding

CARIPLO Foundation (grant #2019-3354), Italian Ministry of University and Research (grant #2022WNW97F)

P19-03 | Associations between ambient air pollution and temperature, green spaces, and adverse pregnancy outcomes in Bangladesh

Nandia Shirchindorj, Roman Pabayo, Joseph Syer, Shelby Yamamoto

University of Alberta, School of Public Health, Edmonton, Canada

Objective: Climate change and air pollution are significant public health challenges in Bangladesh. Both climate change and air pollution have been linked to adverse birth outcomes such as low birth weight and pre-term birth, while exposure to green spaces has been suggested as a potential adaptation strategy to mitigate the harmful effects of climate change and air pollution. The objective of this study is to investigate associations between air pollution and temperature exposures and the risk of preterm birth (PTB) and low birth weight (LBW), in Bangladesh. Additionally, this study will explore whether exposure to green spaces modifies these associations.

Material and Methods: The study will be completed using birth data obtained from the 2022 Bangladesh Demographic and Health Survey (DHS). Exposure variables that will be analysed include temperature (average monthly mean, maximum, minimum, and diurnal temperatures), air pollution (particulate matter 2.5), and green space (Normalized Difference Vegetation Index (NDVI)) variables. The planned analyses include multilevel logistic regression to estimate associations between PM 2.5, temperature, and birth outcomes, adjusting for relevant maternal and sociodemographic confounders. Effect modification will be used to explore whether NDVI modifies these associations.

Results: It is hypothesized that higher temperatures and PM 2.5 concentrations are associated with increased odds of PTB and LBW, while NDVI will weaken this association, suggesting a potential adaptive effect.

Conclusion: Bangladesh is among the most polluted countries worldwide, however, research concerning climate change and air pollution adaptation in Bangladesh is limited. This study will analyse the relationship between temperature and PM 2.5 variables, green spaces, and adverse birth outcomes. Evidence in this area is necessary to protect the health of highly vulnerable populations, pregnant women and newborns, from the impacts of air pollution and inform future adaptation policy, urban planning, and research.

P19-04 | Associations between changes in neighborhood tree canopy cover and $PM_{2.5}$ levels by gentrification status in ten Rust-Belt metropolitan areas, 2011-2018.

Daniel Wiese[1], Jordan Baeker Bispo[1], Qinran Liu[1], Kieran Kelly[1], Donghai Liang[2], Farhad Islami[1]

[1] American Cancer Society, Surveillance and Health Equity Science Department, Atlanta, USA; [2] Emory University, Gangarosa Department of Environmental Health, Atlanta, USA

Objective

Gentrification–a neighborhood socio-demographic transformation process that typically occurs alongside profit-driven housing modernization in historically disinvested communities–reshapes urban landscapes across the US, especially in the Rust-Belt cities. Besides changes in area socio-economic status, this process may improve street greening and greenspaces, reducing environmental pollution exposure. In this study, we aimed to investigate associations between census tract gentrification and changes in tree canopy cover and levels of fine particulate matter ($PM_{2.5}$) between 2011-2018 in ten Rust-Belt metropolitan areas, including Baltimore, Boston, Buffalo, Chicago, Cincinnati, Cleveland, Detroit, New York, Philadelphia, and Pittsburgh.

Material and Methods

Relative change in tree canopy cover was estimated using the USGS National Landcover Database. Relative change in the average $PM_{2.5}$ was estimated using NASA's Earthdata. Gentrification status was defined using US Census data, and tracts were categorized as ineligible for gentrification, earlier-gentrified (2000-2010), recently-gentrified (2011-2018), or non-gentrified. Associations between gentrification and changes in tree canopy cover or $PM_{2.5}$ level were estimated using linear regression overall and stratified by metropolitan areas.

Results

Overall, tree canopy cover and $PM_{2.5}$ level declined over time across all metropolitan areas and all gentrification categories. However, the magnitude of decline varied by gentrification status: for all metropolitan areas combined, compared to tracts ineligible for gentrification, earlier-gentrified tracts had a higher decline in tree canopy cover (coefficient=−4.23; 95%confidence interval[CI]=−5.29, −3.18) but an increase in $PM_{2.5}$ level (0.56; 95%CI=0.01, 1.11). Stratified by metropolitan areas, these associations varied in magnitude and significance level.

Conclusion

Our findings indicate that changes in tree canopy cover and $PM_{2.5}$ level differ by area gentrification. However, these associations vary across metropolitan areas, likely reflecting differences in local policies, and traffic/road patterns. Further studies should examine the role of change in tree canopy cover and other contextual neighborhood factors as potential mediators between gentrification and changes in $PM_{2.5}$ level.

P19-05 | Residential Green Space and Noise Annoyance in a Chicago-Based Cohort

Hannah Li, Laura McGuinn, Andrew Craver, Habibul Ahsan, Briseis Aschebrook-Kilfoy, Patrice Nolen

The University of Chicago, Institute for Population and Precision Health, Biological Sciences Division, Chicago, USA

Objective: We sought to characterize the distribution of noise annoyance and sensitivity within a Chicago-based cohort, examining how these factors vary across different demographic groups. We also explored the role of residential green space as a potential mitigating factor for noise annoyance, hypothesizing that greater access to green space may reduce the perceived impact of environmental noise.
Material and Methods: We deployed a community noise survey to assess perceptions of environmental noise within a subset of participants from the Chicago Multiethnic Prevention and Surveillance Study (COMPASS) cohort. The survey included the International Commission on Biological Effects of Noise (ICBEN) Noise Annoyance Scale, a widely used instrument to measure subjective noise annoyance across a variety of contexts. We also administered the Weinstein Noise Sensitivity Scale, a psychological assessment tool that measures individual sensitivity to noise in different contexts. We surveyed participants' subjective green space access, and residential addresses were linked to green space measures, including NatureScore, which scores the quantity and quality of natural elements in participants' neighborhoods. We generated descriptive statistics to summarize demographic characteristics of the study population and their survey responses. We compared noise annoyance and green space exposure by demographic factors using chi-square tests and regression analyses.
Results: Of the 210 participants in this pilot study, most participants were Black/African American (70%), female (63.8%), and had an annual household income less than $50,000 per year (66.7%). The mean NatureScore was 41.61 (Nature Adequate). Noise annoyance from road traffic and bars/restaurants was associated with NatureScore categories (p=0.06319, p=0.04936). Mean NatureScore was lowest for participants "Slightly" and "Extremely" annoyed (34.60, 36.55) and highest for participants "Not at all" annoyed by road traffic (47.92).
Conclusion: Residential greenspace may be associated with noise annoyance from some sources of urban noise.

P19-06 | Urban Heat Disparities in Boston's Chinatown: Measuring Wet Bulb Globe Temperature Across Open Spaces

MyDzung T. Chu[1,2], John Durant[4], Jenny Huang[3], Ponnapa Prakkamakul[5], Kiran Kui[2], Yanqing Huang[6], Aoife Schmitt[4], Isabelle Berman[4]

[1] Tufts Medical Center, Institute for Clinical Research and Health Policy Studies, Boston, USA; [2] Tufts Clinical and Translational Science Institute, Boston, USA; [3] Asian Community Development Corporation, Boston, USA; [4] Tufts University, Department of Civil and Environmental Engineering, Medford, USA; [5] Sasaki Associates, Boston, USA; [6] Tufts University, Department of Community Health, Medford, USA

Objective: Boston's Chinatown experiences extreme urban heat island effects due to high-density development, limited green space, and extensive impervious surfaces. While air pollution has been well-documented in the neighborhood, wet bulb globe temperature (WBGT), a critical indicator of heat stress, remains understudied. The *Chinatown HEROS (Heat Equity and Resilience in Open Spaces)* study applied a community-based participatory

research approach to assess WBGT disparities and their environmental determinants across 12 open spaces, identifying priority areas for climate adaptation interventions.

Materials and Methods: From July to August 2023, continuous WBGT and $PM_{2.5}$ data were collected at 12 sites using low-cost Kestrel DROP and PurpleAir sensors. Meteorological conditions were recorded from a rooftop weather station, while surface characteristics, vegetation cover, and site usage were documented through structured daily visual assessments. $PM_{2.5}$ measurements were corrected using U.S. EPA factors. Linear models estimated WBGT for each site, adjusting for time, meteorological conditions, relative humidity, and $PM_{2.5}$ levels.

Results: Median WBGTs were 67.8°F in July and 65.5°F in August, with multiple hourly exceedances of the National Weather Service's 80°F heat stress threshold (maximum 88.0°F). WBGT varied across sites (0.30–1.8°F difference), peaking between 9 AM and 2 PM. The hottest sites were surrounded by tall brick buildings and concrete pavement. WBGT was positively associated with $PM_{2.5}$ (+0.31°F) and relative humidity (+0.14°F), and negatively associated with wind speed (-0.09°F).

Conclusion: WBGT disparities across Chinatown's open spaces highlight the role of urban design in shaping localized heat exposure. These findings underscore the need for targeted infrastructural investments to enhance climate resilience in urban heat island communities.

P19-07 | What makes a park cool? A spatiotemporal analysis of urban heat and vegetation volume

Aviva R. Wolf-Jacobs[1], Yan Xu[2], Rima Habre[2], John P. Wilson[1]

[1] University of Southern California, Spatial Sciences Institute, Los Angeles, USA; [2] University of Southern California, Department of Population and Public Health Sciences, Keck School of Medicine, Los Angeles, USA

Extreme heat causes more deaths and hospitalizations than any other climate hazard in Los Angeles. Parks and urban vegetation are vital cooling resources during heatwaves. Trees, in particular, are powerful assets for mitigating urban heat. Evidence suggests that the park cooling effect (PCE), which measures the difference in temperature between a park and its surrounding area, varies greatly for parks both within and across cities due to a variety of factors, including park vegetation. Current research on PCE is limited by 2D assessments of vegetation and constrained spatiotemporal extents. These limitations hinder efficacy of assessments of high vegetation (trees, tall shrubs, etc.) volume's influence on PCE. This study uses a geographically weighted regression to explore the association between high vegetation volume and PCE for hundreds of public parks in Los Angeles City. Light Detecting and Ranging (LiDAR) 3D point cloud imagery and Landsat 8-9 30 m resolution thermal imagery are analyzed for all available days between 2018 and 2024. Results will inform vegetation-based urban planning efforts aimed at protecting the public from the dangers of extreme heat in Los Angeles and beyond.

P19-08 | Differences in Historical Redlining Measure Classifications and Their Associations with Contemporary Green Space Across the United States

Charlie H. Nguyễn[1], Jaime Madrigano[1,2], Carlos Calvo-Hernandez[2], Sarah E. Edgington[2], Beth Ann Griffin[2], Genee S. Smith[1], Scott R. Stephenson[2], Regina Shih[3,2]

[1] Johns Hopkins Bloomberg School of Public Health, Department of Environmental Health and Engineering, Baltimore, USA; [2] The RAND Corporation, Santa Monica, USA; [3] Emory University, Department of Epidemiology, Atlanta, USA

Objective: Historical redlining is increasingly recognized as an important contributor to disparities in green space and health outcomes, but approaches vary in how to operationalize it. We aimed to compare the use of two measures derived from 1930s Home Owners' Loan Corporation (HOLC) perceived credit risk maps in assessing the association between historical redlining and green space at the census tract level.

Methods: We used HOLC map scans from the Mapping Inequality project (Nelson et al., 2023) to compute proportions of the area of each present-day census tract covered by "A", "B", "C", and "D" credit risk grades. Using these proportions, we produced two redlining measures: a categorical variable incorporating the grades covering the largest and second-largest areas of each tract (as described by Noelke et al., 2022) and a continuous area-weighted variable. We compared descriptive demographic characteristics by redlining category and by continuous variable quartiles. We then estimated adjusted regression models assessing the association between historical redlining and proportion of census tracts covered by green space using 2010 National Land Cover Database data. We also explored how these analyses differed when including unrated tract area.

Results: Tracts with higher proportions of high-risk HOLC grades tended to have lower educational attainment, higher unemployment, and higher poverty rates. Regression models showed a negative association between historical redlining and green space when using either measure. Estimates did not change meaningfully when unrated area was included in the weighted continuous variable.

Conclusion: Despite consistency in the direction of association between historical redlining and green space using either measure, each measure has differing implications for predictive validity and interpretation. Studies linking redlining, green space and health outcomes should consider conducting sensitivity analyses to assess the impact of using different measures of redlining and the treatment of unrated tract area.

Funding: NIH NIA (R01AG077935)

P19-09 | Inequalities in road traffic noise exposure levels in greenspaces in Greater London

Kathryn Adams[1,2], Calvin Jephcote[1,2], John Gulliver[3]

[1] University of Leicester, Centre for Environmental Health and Sustainability, Leicester, UK; [2] University of Leicester, School of Geography, Geology and the Environment, Leicester, UK; [3] City St George's, University of London, Population Health Research Institute, London, UK

Background and Objective : Exposure to road traffic noise in residential settings has been associated with detrimental effects on health including annoyance, sleep disturbance, cardiometabolic outcomes, mental health, and cognition. Conversely, exposure to natural sounds improves cognitive performance and aids in stress recovery in humans. Environmental exposure studies have shown that the distribution of noise exposures is often not equitable across cities, but evidence related to noise in greenspaces is missing. This study provides an analysis of noise variability and inequalities in noise levels for greenspaces in Greater London related to residential addresses.
Materials and Method: Noise levels from major and minor roads were modelled across 2,532 greenspaces for the daytime and evening period in accordance with the European Commission Common Framework for Noise Assessment (CNOSSOS-EU) methods from Environment Noise Directive 2002/49/EC and the inverse square law of sound attenuation.
Results: Using modelled road traffic noise estimates, we found that 28% of greenspaces exceed adopted noise threshold levels during daytime and evening periods. Greenspaces in Central London are more likely to have noise levels that exceed the WHO noise guidelines compared to greenspaces in Outer London. Distance-based proximity analyses showed that populations in Central London are more likely to be affected by high noise levels in greenspace areas nearest to their residential address. As distance travelled from residential locations increases, the distribution of high greenspace noise levels becomes more dispersed across Greater London. However, no inequality gradient was observed between high greenspace noise levels and different deprivation groups, except for the least deprived communities', who experienced noise levels that were 2 dB lower within greenspace areas within 5km radius.

P19-10 | Greenspace Morphology, Air Pollution, and Cancer Risk: A Prospective Cohort Study

Fatemeh Yousefian[1], Erika Rees-Punia[2], Kierstin Faw[2], Ke Xu[1], Meng Qi[1], Yang Liu[1]

[1] Gangarosa Department of Environmental Health, Rollins School of Public Health, Emory university, Atlanta, USA; [2] Department of Population Science, American Cancer Society, Kennesaw, USA

Greenspace exposure had been associated with numerous health benefits, including increased physical activity, reduced stress, improved mental well-being, and lower risks of cardiovascular disease and mortality. However, its relationship with cancer risk remained unclear, as previous studies produced inconsistent findings. Some evidence suggested that greenspace might lower cancer risk by reducing air pollution exposure, mitigating chronic stress, and enhancing immune function, but quantitative assessments had yet to establish a definitive link.
We conducted a prospective cohort study using data from the Cancer Prevention Study-3 (CPS-3), which included approximately 300,105 cancer-free adults aged 30–65 years across 34 U.S. states. Greenspace morphology indicators—including connectivity, fragmentation, and spatial configuration—were analyzed alongside air quality

metrics, such as annual mean daily maximum 8-hour ozone concentration and annual mean $PM_{2.5}$ concentration. Cox proportional hazards regression models were used to estimate hazard ratios (HRs) and 95% confidence intervals (CIs) per interquartile range (IQR) increase in greenspace exposure. Models were adjusted for individual-level cancer risk factors, including age, sex, race, education, marital status, body mass index, smoking status, diet score, alcohol use, and comorbidity score. Overally, the percentage of green space dropped about 2% (78.5 to 76.8) over 35 the U.S. After applying inclusion and exclusion criteria, 244,434 participants were included in the final analysis. Among them, 5,223incident cancer cases were identified for the three most common cancer types. The study provided novel insights into the relationship between greenspace morphology, air pollution, and cancer incidence. By evaluating greenspace exposure in a large, diverse U.S. cohort, it aimed to clarify its role in cancer prevention and disease risk reduction. The findings could inform public health policies and urban planning strategies designed to mitigate cancer risk through environmental interventions. Identifying potential protective effects of greenspace supported the development of healthier, more sustainable urban environments that promoted long-term disease prevention.

P19-11 | Bird richness as a mediator between greenspace and mental health relationships

Huaqing Wang[1], **Sihao Chen**[2], Wenyan Xu[1]

[1] Utah State University, Landscape Architecture and Environmental Planning, Logan, USA; [2] University of Liverpool, Earth, Ocean and Ecological Sciences, Liverpool, UK

Neighborhood greenspace is widely known to benefit bird diversity and human mental health. However, whether bird richness mediates the relationship between greenspace and mental health is unknown. We ascertain such mediation effects in 294 census tracts in Los Angeles City. We obtained greenspace data from one-meter resolution satellite imagery, bird species data from eBird citizen science datasets, and prevalence of poor mental health from the US Centers for Disease Control and Prevention. Mediation analysis combined with spatial error models were used to assess the mediating effects while controlling for geographic, demographic, and socio-economic factors. We found that higher greenspace percentage was associated with increased bird richness ($\beta = 0.188$, $p < 0.001$) and such bird richness was associated with reduced prevalence of poor mental health ($\beta = -0.020$, $p = 0.010$). The richness of bird richness partially mediated the relationship between greenspace percentage and the prevalence of poor mental health, as evidenced by a decrease in coefficient efficiency from −0.020 to −0.017. Our findings underscore the need for public health policymakers, landscape architects, and greenspace managers to consider biodiversity-friendly strategies in the design of urban greenspaces to enhance both wildlife habitats and human mental health.

P19-12 | Longitudinal associations of long-term exposure to air pollution, temperature and greenness with type 2 diabetes subphenotypes: results from KORA cohort

Yue Xi[1,2], Susanne Breitner-Busch[1,2], Qiuling Dong[1,2], Kathrin Wolf[1], Marco Dallavalle[1,2], Nikolaos Nikolaou[1], Lars Schwettmann[3,4], Wolfgang Rathmann[5,6], Wolfgang Koenig[7,8], Barbara Thorand[1,2], Reiner J.V. Schwartzenberg[9,10], Annette Peters[1,2]

[1] Helmholtz Zentrum München - German Research Center for Environmental Health, Institute of Epidemiology, Neuherberg, Germany; [2] Ludwig-Maximilians-Universität München (LMU Munich), Chair of Epidemiology, Institute for Medical Information Processing, Biometry, and Epidemiology, Faculty of Medicine, Munich, Germany; [3] Helmholtz Zentrum München, German Research Center for Environmental Health, Institute of Health Economics and Health Care Management, Neuherberg, Germany; [4] Carl von Ossietzky Universität Oldenburg, Department of Health Services Research, School of Medicine and Health Sciences, Oldenburg, Germany; [5] German Center for Diabetes Research (DZD), Neuherberg, Germany; [6] German Diabetes Center, Leibniz Center for Diabetes Research at Heinrich Heine University Düsseldorf, Institute for Biometrics and Epidemiology, Düsseldorf, Germany; [7] German Research Center for Cardiovascular Disease (DZHK), Partner site Munich Heart Alliance, Munich, Germany; [8] Deutsches Herzzentrum München, Technische Universität München, Munich, Germany; [9] Eberhard Karls University Tübingen, Department of Internal Medicine IV, Division of Diabetology, Endocrinology and Nephrology, Tübingen, Germany; [10] Helmholtz Center Munich, Eberhard Karls University Tübingen, Institute for Diabetes Research and Metabolic Diseases, Tübingen, Germany

Objective: To examine the longitudinal associations between long-term exposure to environmental exposures and data-driven type 2 diabetes (T2D) subphenotypes.

Material and Methods: We categorized T2D participants from the KORA F4 (2006–08) and FF4 (2013–14) study waves in Germany into three clusters using k-means clustering: Cluster A (F4: 22 (6.9%), FF4: 28 (9.3%)) was characterized by insulin deficiency; Cluster B (F4:199 (62.8%), FF4:182 (60.5%)) was defined as age-related diabetes; and in Cluster C (F4: 96 (30.3%), FF4: 91 (30.2%)) participants showed higher levels of insulin resistance. Environmental exposures, including annual averages of fine particulate matter ($PM_{2.5}$) and $PM_{2.5}$ absorbance ($PM_{2.5abs}$), annual standard deviations of air temperature (T_{SD}), annual average of air temperature (T_M) and surrounding greenness (NDVI), were assessed at participants' residences. Covariate-adjusted mixed multinominal logistic regression models were fitted to examine the effects of per Interquartile Range (IQR) change in environmental exposures on diabetes subphenotypes, compared with individuals without diabetes. We further calculated joint Odds Ratios (ORs) to estimate the combined additive effects of single exposures using the Cumulative Risk Index.

Results: IQR increases in $PM_{2.5}$ (OR = 1.29, 95% confidence interval [CI]: 1.01,1.64) and $PM_{2.5abs}$ (OR = 1.30, 95% CI: 1.01,1.67) were positively associated with Cluster C risk, compared with non-diabetes individuals. Furthermore, we found higher joint effects of co-exposures than the single exposure estimates: per IQR increase in $PM_{2.5}$ and T_{SD} but decrease in NDVI and T_M elevated Cluster B (joint OR = 1.41, 95% CI: 1.03,1.93) and Cluster C (joint OR = 1.55, 95% CI: 1.02,2.36) risks, while the combination of $PM_{2.5abs}$ with other exposures increased the risk of Cluster C (joint OR = 1.54, 95% CI: 1.01,2.33).

Conclusions: Our study extends the knowledge about associations between environmental exposures and diabetes to specific diabetes subphenotypes, indicating increased risks for age-related and insulin-resistant diabetes.

P20 | From Policy to Practice: Global Efforts to Eradicate Lead Exposure - PART I

P20-01 | Lead, From Wood to the Air We Breathe…

Patricia Henegan[1,2], Maggie Stewart-Smith[3], Nora Traviss[5,4], Richard Peltier[1,2]

[1] University of Massachusetts-Amherst, Environmental Health Sciences, Amherst, USA; [2] University of Massachusetts-Amherst, ELEVATE, Amherst, USA; [3] University of Massachusetts-Amherst, Chemistry & Biochemistry, Amherst, USA; [4] Keene State College, Environmental & Sustainability, Keene, USA; [5] NESCAUM, Northeast States Coordinated Air Use Management, Boston, USA

Objective
To investigate wood fuel emissions as a source of trace lead exposure via ambient air in the northern regions of the United States.
Materials & Methods
Federal Reference Methods filters (n=182) were obtained from the state of Vermont's Department of Environmental Conservation (n=97) and New Hampshire Department of Environmental Services (n=85) from Rutland, Vermont and Keene, NH respectively. Filter samples were collected during the winters of 2011-2016 and analyzed by Energy Dispersive X-ray Fluorescence. Chemical speciation data was also compiled from 24 sites via the Interagency Monitoring of Protected Visual Environments and EPA Chemical Speciation Network. They were included to compare to the northeastern US region's association of lead as a tracer for wood fuel burning.
Results
Our evidence indicates a link between wood burning emissions with increases in particle-bound lead. These emissions may increase trace lead exposures populations where wood burning is common, which are often lower income and rural communities. Linear regressions of lead and potassium concentrations of samples collected in Keene, NH (R^2=0.438) and Rutland, VT (R^2=0.341) were our first indication that this is an association we should further explore whether this relationship could be seen in other regions. On average, lead concentrations were lower than EPA standards, ranging from 0.86 ng/m³ in the Rocky Mountains Region to 1.70 ng/m³ in the Southeast Region. Across the US, we observed varying agreement between lead and potassium linear regressions, with slight-to-modest agreement (R^2=0.09-0.47) at most sites; the strongest associations were on the Pacific Coast, where R2 >0.40. This suggests that the relationship between potassium and lead is strong in several locations nationwide.
Conclusion
Wood burning is shown in these results to be an anthropogenic source of lead that was introduced into ambient air throughout the northern portion of the US in the winters of 2011-2016.

P20-02 | Identifying Lead Soil Contribution to Dust Loading in Homes Built Before and After 1978 at a Potential Superfund Site

Sean Stratton[1,2], Adrienne Ettinger[1,2], Zorimar Rivera-Núñez[1,2], Shereyl Snider[3], Brian Buckley[1]

[1] Rutgers University, Environmental and Occupational Health Sciences Institute, Piscataway, USA; [2] Rutgers University, School of Public Health, Piscataway, USA; [3] East Trenton Collaborative, Trenton, USA

East Trenton, a neighborhood in the Trenton North Ward, is an environmental justice community burdened by lead (Pb). It is home to 4,000 residents (>85% African-American or Hispanic) in approximately 900 households. According to the U.S. EPA EJ Screen tool, it is in the 95th percentile nationally for Pb paint, low income, and demographic index. East Trenton Collaborative (ETC), a local community organization was created to revitalize their neighborhood and empower their community. ETC partnered with Rutgers Environmental and Occupational Health Sciences Institute (EOHSI) to reduce Pb exposure to their residents. ETC was specifically interested in capacitating and educating residents to develop mitigation strategies for Pb exposure. Using a community-based participatory research approach, EOHSI trained 6 residents living in the East Trenton neighborhood as community scientists to conduct soil sampling throughout the neighborhood.

In August of 2023, the 6 community scientists collected 242 soil samples from 121 homes in East Trenton. The mean Pb soil level was 620.8 ppm with a median Pb soil level of 478.8 ppm. 95% of homes had at least one sample above the EPA's Residential Lead Hazard Pb for Soil (200 ppm).

172 dust wipes were collected from 38 homes. Of the homes built prior to 1978 with lead-based paint, 65 floor dust samples were collected with a mean dust loading of 139 µg/ft^2. Of the homes with no lead-based paint, 20 samples were collected from floors with a mean dust loading of 263 µg/ft^2. A Wilcoxon rank-sum test compared indoor dust loadings from homes built before 1978 and homes built or fully renovated after 1978. For dust loading (W statistic = 732, $p = 0.39$) no difference between groups were observed. High Pb dust loadings were observed in homes with lead-based paint and without, indicating homes-built post 1978 are not necessarily Pb dust free.

P20-03 | The Effect of Physical Activity on Combined Cadmium, Lead, and Mercury Exposure

Akua K. Marfo[1], Emmanuel Obeng-Gyasi[2]

[1] North Carolina A&T State University, Built Environment, Greensboro, USA; [2] North Carolina A&T State University, Built Environment, Greensboro, USA

Environmental exposures, such as heavy metals, can significantly affect physical activity, an important determinant of health. This study explores the effect of physical activity on combined exposure to cadmium, lead, and mercury, using data from the 2013–2014 National Health and Nutrition Examination Survey (NHANES). Physical activity was measured with ActiGraph GT3X+ devices worn continuously for 7 days, while blood samples were analyzed for metal content using inductively coupled plasma mass spectrometry. Descriptive statistics and multivariable linear regression were used to assess the impact of multi-metal exposure on physical activity. Additionally, Bayesian Kernel Machine Regression (BKMR) was applied to explore nonlinear and interactive effects of metal exposures on physical activity. Posterior Inclusion Probabilities (PIPs) were calculated to quantify the relative importance of each metal. The linear regression analysis revealed positive associations between cadmium and lead exposure and physical activity.

PIP analysis identified lead as the most influential metal in predicting physical activity, followed by cadmium and mercury. The study also uncovered complex relationships between metal exposures and physical activity. In univariate BKMR exposure-response analysis, lead and cadmium showed positive associations with physical activity, while mercury exhibited a slightly negative relationship. Bivariate exposure-response analysis illustrated how the impact of one metal could be influenced by the presence and levels of another, confirming the trends observed in univariate analyses while also demonstrating the complexity varying doses of two metals can have on physical activity. Additionally, the overall exposure effect analysis across different quantiles revealed that higher levels of combined metal exposures were associated with increased physical activity. This study demonstrated the combined effects of multiple metals on physical activity. The findings underscore the necessity of using advanced methods such as BKMR to capture the complex dynamics of environmental exposures and their impact on human behavior and health outcomes.

P20-04 | The Combined Effects of Urine Zinc, Cadmium, Mercury, Lead, and Copper on Endometrial Cancer Staging

Issah Haruna[1], Emmanuel Obeng-Gyasi[2]

[1] North Carolina A & T State University, Applied Science and Technology/Built Environment, Greensboro, USA; [2] North Carolina A & T State University, Applied Science and Technology/Built Environment, Greensboro, USA

Endometrial cancer (EC) is a growing public health concern. This secondary data study of a case series leveraged existing samples and data to explore the potential link between exposure to heavy metals/essential elements and stage of EC. We analyzed urine samples from women with EC, measuring levels of toxic metals (cadmium, mercury, and lead) and essential elements (zinc and copper). Our findings revealed that higher levels of mercury, cadmium, and lead are associated with more advanced EC stages. Conversely, zinc showed a protective effect, potentially mitigating EC stage progression. Copper levels did not show a clear association with EC stage. These results highlight the potential impact of environmental exposures on EC stage and the crucial need for advanced statistical methods to understand the combined effects of these pollutants on health and the need for public health interventions. Further research is needed to explore the mechanisms by which these metals influence EC stage and long-term outcomes.

P20-05 | Feasibility of a novel optically filtered Monochromatic portable X-ray Fluorescence (MXRF) to quantify lead in bone and soft tissues

Kolawole E. Adesina[1], James A. DiNatale[1], Thomas R. Grier[1], Aaron J. Specht[1,2]

[1] Purdue University, School of Health Sciences, West Lafayette, USA; [2] Harvard University, T.H. Chan School of Public Health, Boston, USA

Lead (Pb) exposure threatens both wildlife and humans, causing neurological, cardiovascular, and renal damage. Traditional Pb analysis depends on costly, lab-based techniques like mass spectrometry. Monochromatic portable X-ray fluorescence (MXRF) now enables rapid, non-destructive, and field-deployable Pb detection for environmental and biomedical applications with a lower detection limit than any previous portable XRF measurement system. This study evaluated the feasibility of MXRF, developed by X-ray Optical Systems (XOS, East Greenbush, NY), for Pb quantification in bone using black vulture bones and soft tissues. Integrating monochromatic micro-focus XRF with doubly curved crystal (DCC) optics, MXRF enhances sensitivity, compactness, and energy efficiency for real-time Pb analysis. Pb concentrations in 37 black vulture bones (femur and tarsometatarsus (TMT)) were measured using MXRF and validated against inductively coupled plasma-mass spectrometry (ICP-MS). Soft tissue-equivalent layers were combined with Pb-doped bone phantoms to quantify attenuation effects and develop a correction model. MXRF signal attenuation followed a quadratic decrease with increasing soft tissue thickness, with reliable Pb quantification of collected samples. Calibration with Pb-bone standards demonstrated strong linearity between standard and measured Pb concentrations ($R^2 = 0.994$) and between Pb levels and MXRF counts ($R^2 = 0.996$). In comparison with ICP-MS, Pb bone samples showed high ICC values (0.933 for femur, 0.953 for TMT) and minimal bias in Bland-Altman analysis (-1.73 µg/g for femur, -0.42 µg/g for TMT, -1.07 µg/g overall) confirmed measurement consistency. Strong Pearson correlations ($r = 0.959$-0.962, $p < 0.0001$) and linear regression ($R^2 = 0.905$-0.926) further supported measurement reliability in bone samples. Passing-Bablok regression also indicated minimal proportional and constant bias (slopes: 0.921-0.961). The calibration-derived MDL was 0.03 µg/g for a 3-minute measurement. These findings validate MXRF as a rapid, non-destructive tool for Pb quantification, supporting its use in wildlife and human biological monitoring, further advancing portable XRF applications in environmental health research.

P20-06 | Determination of Lead Use in Buried Water Service Lines

Thomas Grier[1], Chase Hadu[1], Drew Novak[1], Yuchen Zhang[1], David Goodman[2], Aaron Specht[1]

[1] Purdue University, Health Sciences, West Lafayette, USA; [2] H3D Inc, Ann Arbor, USA

Legacy lead service lines pose a significant public health risk, yet many communities lack the records or tools needed to locate them efficiently. With the recent push for lead pipe replacement under federal infrastructure initiatives, water service providers are faced with the challenge of identifying buried or inaccessible lead pipes. Current detection methods are often incapable of detection without digging resulting in overly time-consuming and ineffective replacement strategies for lead pipes.

We present a high-energy X-ray fluorescence (XRF) technique capable of detecting lead service lines through at least 12 in (30.5 cm) of soil with a theoretical max depth of closer to 5 ft (1.5 m) in soil. This approach adapts traditional low-power lead detection methods to a 140 kV X-ray tube that enables deeper penetration and stronger signal strength. Our experimental setup utilized an H3D M400 CdZnTe detector and a Moxtek 140 kV X-ray source to

measure lead signal with lead sources buried at varying depths. We stacked bags of topsoil over lead to simulate buried infrastructure and made 10-minute measurements at 140 kV and 6 uA. Signal strength was modeled using a two-term power function and demonstrated a clear correlation between depth and detection efficiency (adj $R^2 = 0.98$ and RMSE = 10.8 counts, 0.0015% of the mean observed signal).
Our results confirm the feasibility of lead pipe identification at practical depths with further potential for optimization through higher-output X-ray sources and improved shielding. We also explore detection limits for real-world buried pipes and shorter measurement times with additional instrument optimization. This technology offers a scalable solution for municipalities and water service providers to efficiently locate and replace hazardous lead infrastructure, reducing long-term exposure risks for communities.

P20-07 | Children's 2023 Lead Exposure in SENDSS-Assessed and Unassessed Homes in North Central Health District, Georgia, United States.

Caroline Obi[1,2], Atin Adhikari[1]

[1] Georgia Southern University, Statesboro, USA; [2] Georgia Department of Public Health, North Central Health District, Macon, USA

Objective: Children are especially at risk for lead poisoning. In 2022, Georgia, in the United States- lowered the blood lead reference value from 5 µg/dL to 3.5 µg/dL. This adjustment established a new standard for initiating interventions in children identified with elevated lead levels through the State Entry National Database Surveillance System (SENDSS). The study aims to compare average blood lead levels in children from assessed versus non-assessed homes and evaluate significant differences in their follow-up results.
Materials and Methods: Blood lead data for children aged six and under was collected from SENDSS across 13 counties in Georgia under the North Central Health District (NCHD). Additional variables included assessment status, elevated blood lead levels, and follow-up status. Data analysis conducted using SPSS compared average blood lead levels between assessed and unassessed homes, while the Chi-Square Test of Independence examined the relationship between categorical variables and lead levels.
Results: Preliminary findings indicated that 30 children with elevated blood lead levels were recorded in Districts 5-2. Nineteen homes received assessments, while eleven did not. An independent samples t-test found revealed that the mean lead level in assessed homes was 1.4737 ± 0.51299 µg/dL, compared to 1.1818 ± 0.40452 µg/dL in unassessed homes, with no statistically significant difference ($p = 0.097$). However, a significant association was noted between assessment status and follow-up (X^2 (1) ≥ 4.966, $p = 0.0206$, $\alpha = 0.05$).
Conclusion: Both assessed and unassessed homes, particularly those built before 1978, show elevated blood lead levels in children. This suggests potential underreporting or a lack of awareness about lead exposure risks in assessed homes.

P20-08 | Characterizing relationships among biomarkers of arsenic, cadmium and lead exposure in US women

YiTing Hana Lee[1], Susan Korrick[2,3], Francine Laden[1,2]

[1] Harvard T.H. Chan School of Public Health, Department of Epidemiology, Boston, USA; [2] Harvard T.H. Chan School of Public Health, Department of Environmental Health, Boston, USA; [3] Brigham and Women's Hospital and Harvard Medical School, Channing Division of Network Medicine, Department of Medicine, Boston, USA

Objective:
Cohorts with previously collected biospecimens may provide a resource for studying the associations of chronic exposure to arsenic (As), cadmium (Cd) and lead (Pb) with aging. However, the utility of convenience samples depends on the metabolism, distribution, and half-life of metals in a biospecimen. In a sub-group of US-based Nurses' Health Study participants, toenail clippings were collected in 1984, and blood was collected in 1989/90 and 2000. We assessed the correlation of As, Cd, and Pb levels in these two biospecimens to understand their utility in epidemiological studies.
Material and Methods:
We selected 25 participants aged 38 to 63 in 1984 who provided biospecimens and did not move 1984-2000. We measured As, Cd and Pb in toenails and red blood cells (RBCs) and extracted information on smoking and menopause status from biennial questionnaires closest to sample collection. We calculated Spearman correlation coefficients of concentration between toenail and RBC (1990) overall and in subsets of women with no changes in menopausal (n=20) or smoking status (n=23) 1984-1990.
Results:
Median (IQR) concentrations (ng/g) in toenails were 70.4 (53.8), 79.8 (142.9), and 1083 (1407) for As, Cd and Pb, respectively. Toenail and RBC (1990) As and Pb were moderately correlated (respective r = 0.44 and 0.29), but Cd was inversely correlated (r=-0.43). RBC in 1990 and 2000 were correlated for all metals (0.65-0.91). Overall, the correlations were the same or attenuated in the subsets, and the inverse association seen for Cd went away among those who did not change smoking status.
Conclusion: Metals in toenails and RBCs represent different exposure periods (approximately 6-18 months versus days-months, respectively). In this pilot study, correlations were only modest, suggesting the biomarkers are not exchangeable. However, a single RBC measurement may be a reasonable metal exposure biomarker in this general population sample of women.

P20-09 | Childhood Lead Exposure's Contribution to Mental Disorder in the US Population over the Past Century

Michael J. McFarland[2], **Aaron Reuben**[1], Matthew Hauer[2]

[1] University of Virginia, Charlottesville, USA; [2] Florida State University, Tallahassee, USA

Objective. More than half of the current US population were exposed to adverse lead-levels in childhood as a result of lead's use in gasoline over the past century. We sought to estimate the total contribution of this exposure to mental disorder in the population.

Material and Methods. We combined serial, cross-sectional blood–lead level (BLL) data from National Health and Nutrition Examination Surveys (NHANES) with historic leaded-gasoline data to estimate US childhood BLLs from 1940 to 2015 and calculate population mental-health symptom elevations from known lead-psychopathology associations. We utilized five outcomes: 1) General Psychopathology "points," reflecting an individual's liability to mental disorder, scaled to match IQ scores (M=100, SD=15); 2) Symptoms of Internalizing disorders (anxiety and depression) and Attention-deficit/Hyperactivity Disorder (AD/HD), both z-scored (M=0, SD=1); and 3) Differences in the personality traits of Neuroticism and Conscientiousness (M=0, SD=1).

Results. Assuming that published lead-psychopathology associations are causal and not purely correlational: We estimate that by 2015, the US population had gained 602-million General Psychopathology factor points because of exposure arising from leaded gasoline, reflecting a 0.13-standard-deviation increase in overall liability to mental illness in the population and an estimated 151 million excess mental disorders attributable to lead exposure. Investigation of specific disorder-domain symptoms identified a 0.64-standard-deviation increase in population-level Internalizing symptoms and a 0.42-standard-deviation increase in AD/HD symptoms. Population-level Neuroticism increased by 0.14 standard deviations and Conscientiousness decreased by 0.20 standard deviations. Lead-associated mental health and personality differences were most pronounced for cohorts born from 1966 through 1986 (Generation X).

Conclusion. A significant burden of mental illness symptomatology and disadvantageous personality differences can be attributed to US children's exposure to lead over the past century. Lead's potential contribution to psychiatry, medicine, and children's health may be larger than previously assumed.

P20-10 | Arsenic, Lead, and Mood: Assessing Group Susceptibility in Nova Scotia, Canada

Alexandra Del Favero-Campbell[1], Ellen Sweeney[2], W. Shane Journeay[3], Christopher S. Greene[4], Daniel Rainham[5], Kelvin C. Fong[6]

[1] Dalhousie University, Department of Earth and Environmental Sciences, Halifax, Canada; [2] Dalhousie University, Atlantic PATH, Faculty of Medicine, Halifax, Canada; [3] Dalhousie Medicine New Brunswick & Dalhousie University, Departments of Medicine and Community Health & Epidemiology, Saint John, Canada; [4] Dalhousie University, Department of Earth and Environmental Sciences, Halifax, Canada; [5] Dalhousie University, School of Health and Human Performance, Faculty of Health, Halifax, Canada; [6] George Washington University, Department of Environmental and Occupational Health, Washington DC, USA

Objective: Lead (Pb) and arsenic (As) contamination in drinking water is associated with increased risks for cardiometabolic and neurological diseases. We hypothesize that higher than national average prevalence of depression and anxiety may be associated with concentrations of Pb and As in Nova Scotia. Our study characterized the association between metal concentrations in drinking water and mental health outcomes, including depression and anxiety.

Materials and Methods: The Atlantic Partnership for Tomorrow's Health (PATH) is a prospective cohort study designed to investigate chronic disease risk. The analytic dataset includes adults (35-79 years old) who have completed validated questionnaires to assess depressive disorder (Patient Health Questionnaire-9) and anxiety (Generalized Anxiety Disorder-7). As and Pb concentrations in drinking water were obtained from 4669 well water samples across Nova Scotia. Logistic regressions and generalized additive models analyzed associations between these metals and poor mental health, examining how demographic factors (e.g., gender, age, household income) influence susceptibility.

Results: Fifty-four (54%) percent of participants were classified as having major depression (PHQ-9 ≥10), 83% with generalized anxiety disorder (GAD-7 ≥8), and 38% as having both disorders (i.e., poorest mental health). Men (21% vs. 79% women) and lower income participants were significantly more likely to report poorer mental health ($p<0.05$). As and Pb concentrations were not significantly associated with the poorest mental health. However, an interaction between arsenic and age ($p<0.05$) showed younger participants (ages 40-50) had lower probabilities of poor mental health, even with high arsenic levels, whereas older participants exhibited increased probability at higher arsenic levels.

Conclusion: This study highlights the complex relationship between metals in drinking water and mental health, suggesting that older populations may be at a greater risk of neuropsychiatric effects from metal exposures. Identifying susceptible populations is essential for targeted public health interventions, particularly in regions with elevated arsenic and lead levels.

P20-11 | The Combined Effect of Lead and Non-chemical Stressors on Infant and Child Neurodevelopment: A Systematic Review Using the Navigation Guide Methodology

Jessica Trowbridge[1], Nicholas Chartres[2], Emily Lasher[1], Abena BakenRa[1], Rashmi Joglekar[1], Tracey Woodruff[1]

[1] University of California, San Francisco, Program on Reproductive Health and the Enviornment, San Francisco, USA; [2] University of Sydney, Sydeney, Australia

Background: The impact of non-chemical stressors (e.g., food insecurity, poverty, discrimination, housing insecurity, exposure to violence, perceived stress, among others) is rarely considered in risk assessment when evaluating the risk posed by environmental chemicals. This is partly due to a lack of understanding and methods for incorporating the additional risk posed by non-chemical stressors in risk assessment methodologies. A recent overview of reviews identified 18 systematic and scoping reviews evaluating the combined risks of non-chemical stressors and environmental exposures on adverse health outcomes. However, the quality of these systematic reviews and the exposures and outcomes studied precluded a quantitative evaluation of the impact of non-chemical stressors.

Objective: To fill these evidence gaps, we conducted a new systematic review evaluating the combined effect of non-chemical stressors and lead on child neurodevelopment.

Methods: We developed and pre-published a protocol to apply the Navigation Guide systematic review method. We searched PubMed and Web of Science for human studies (population) evaluating the combined impact of lead and non-chemical stressors, such as socioeconomic status (SES) (exposure), on child neurodevelopment (outcome). We will conduct a risk of bias assessment. We will perform a meta-analysis or other quantitative synthesis for sufficiently homogenous data. Additionally, we will rate the quality and strength of the evidence.

Results: Our literature search identified 4,232 studies. We identified 64 studies from a previous scoping review that meet our inclusion criteria. Our systematic review and evaluation will identify additional studies, describe the risk of bias, and quantify the effect of lead and SES on child neurodevelopment through meta-analyses.

Conclusions: Our review provides a rigorous evaluation of the combined impacts of lead and social stressors on child neurodevelopment, leading to improved decision-making on identifying and protecting all populations from lead impacts.

P20-12 | Lead exposure and school readiness among low-income urban children in Bihar, India

Aditi Roy[1,2], Aisha Yousafzai[3], Ashok Ghosh[4], Siddhartha Mandal[1,2], Madhavi Maganti[5], Poornima Prabhakaran[1,2]

[1] Centre for Chronic Disease Control, New Delhi, India; [2] Ashoka University, Centre for Health Analytics Research and Trend, Trivedi School of Biosciences, Sonipat, India; [3] Harvard T. H. Chan School of Public Health, Department of Global Health and Population, Boston, USA; [4] Mahavir Cancer Sansthan and Research Center, Patna, India; [5] Krea University, Sri City, India

OBJECTIVE:
High lead (Pb) exposure continues to be a global public health concern. Although the impact of Pb on cognition is known, whether Pb exposure is associated with school readiness (emergent academic skills and socioemotional development) is understudied. We examined the association between concurrent Pb exposure and school readiness in children.

MATERIAL AND METHODS:
In a longitudinal study in Patna, Bihar, India, we collected baseline data from 501 children aged 4.5 (0.6) years, attending public (68%) or private (32%) preschools in neighbourhoods with a history of Pb exposure from battery recycling. Capillary blood Pb concentration (BPb) was measured using the LeadCare Analyzer. School readiness, an outcome measure of early years covering multiple dimensions of development (emergent literacy and numeracy, fine motor, and socioemotional development) was assessed using the International Development and Early Learning Assessment (IDELA) tool. Hemoglobin (Hb), height, weight, and sociodemographic factors were also assessed.

RESULTS:
Median (IQR) child BPb was 11.1 (7.7) µg/dL. Half of the children (53%) had anemia (Hb<11.4 mg/dL), 69% were stunted (Height-for-age z scores<-2 SD) and 35% were underweight (Weight-for-age scores<-2 SD). There was an inverse cross-sectional association between BPb and standardized total IDELA score (IDELA total) [β = -0.002; 95% CI: -0.01, 0.008] adjusting for child age, sex, HAZ, WAZ, Hb, mother's education, household crowding, and preschool type. We found an interaction between BPb and Hb on IDELA total (p= 0.03). Furthermore, with a 1 µg/dL increase in BPb, there was a 0.02 SD decline in IDELA total [95% CI: -0.02, 0.004; p=0.1] among anemic children.

CONCLUSION:
Lead exposure may be inversely associated with school readiness among low-income urban Indian children and the association may be modified by nutritional status signifying the importance of holistic interventions. Reducing lead exposure to prevent adverse effects on human capital should remain a global public health priority.

P21 | Occupational Exposures and Health - PART I

P21-01 | Association of Occupational Injury and Cognitive Status among U.S. Aging Adults: A Cross-Sectional Study

Marielynn Herrera[1], Scarlet Cockell[1], Alexis J. Handal[1], Erin B. Ware[2], Kelly M. Bakulski[1]

[1] University of Michigan, Department of Epidemiology, Ann Arbor, USA; [2] University of Michigan, Institute for Social Research, Survey Research Center, Ann Arbor, USA

Objective:
Occupational environments can make injuries more likely. Injurious falls can lead to declines in functional status and loss of independence, and we hypothesize may serve as a risk factor for dementia among older adults. We aimed to assess the association of injuries in the workplace on cognitive status among older U.S. adults.
Methods:
In the 2010 cross-sectional sample of the nationally representative Health and Retirement Study, participants self-reported an impairment that limits their paid work activities caused by the nature of their work. Based on neuropsychiatric testing, cognitive status was classified as normal cognition, cognitive impairment non-dementia (CIND), and dementia. To test the association between workplace injury and cognitive status, we used multivariable logistic regression, adjusting for age, smoking status, parental educational attainment, race/ethnicity, sex, years of schooling, and drinks per week.
Results:
Our analysis included 3,831 adults (mean 55 years, SD: 3.7) comprising Non-Hispanic White (42%), Non-Hispanic Black (31%), Hispanic (22%), and 50.5% female participants. In the analytic sample, 9% (n=344) had experienced work injury, and these participants were predominantly Non-Hispanic Black (42%) and male (52%). Twenty-two percent of participants with a work injury had CIND compared to 13.3% of work injury-free participants ($p<0.01$). Participants with work injuries had 1.83 times higher odds of CIND (95% CI: 1.34 - 2.50) than normal cognition, compared to work injury-free participants. Three percent of participants with a work injury impairment had dementia compared to 2% of impairment free participants. Participants with work injuries had 1.92 times higher odds of dementia (95% CI: 0.84 – 4.39) than normal cognition, compared to work injury-free participants.
Conclusion:
Individuals with work-related injury have a higher probability of cognitive impairment than non-work injured adults. Exposures in the workplace, such as injuries, may be important contributors to dementia risk.

P21-02 | Occupational Exposure and Respiratory Health in Firefighters: A Baseline Comparison of Wildland and Structural Firefighters

Anthony O. Akande[1], Chieh-Ming Wu[1], Erick E. Mollinedo[2], Luke P. Naeher[2], Christopher Simpson[3], Rufus Edwards[4], Olorunfemi Adetona[1]

[1] The Ohio State University, Division of Environmental Health Sciences, College of Public Health, Columbus, USA; [2] University of Georgia, College of Public Health, Division of Environmental Health Science, Athens, USA; [3] University of Washington, Department of Environmental and Occupational Health Sciences, Seattle, USA; [4] University of California, Department of Environmental and Occupational Health, School of Population and Public Health, Irvine, USA

Objective: Occupational exposure to smoke could potentiate respiratory impairment in wildland firefighters. We conducted; i) a cross-sectional comparison of baseline pulmonary measurements between non-smoking wildland firefighters (WFFs, n=47) and structural firefighters/emergency medical technicians (SFFs/EMTs, n=43) as controls, and ii) changes in the pulmonary measurements across a wildland fire season among the WFFs.
Material and Methods: Lung health was assessed using the NDD EasyOne Air Spirometer and performed according to guidelines published by the American Thoracic Society. Testing at baseline included forced vital capacity (FVC), forced expiratory volume in one second (FEV1), peak expiratory flow (PEF), forced mid-expiratory flow (FEF25-75), the percentage of predicted values for these metrics, and FEV1/FVC after standardized pre-screening and exclusionary questionnaires were completed. Smokers were excluded at baseline and cross-seasons. Analysis of Covariance (ANCOVA) was used to compare baseline raw spirometry parameters between WFFs and SFFs while controlling age, sex, height, race, and weight. ANCOVA for the percentage of predicted values included BMI as a covariate. The cross-seasonal changes in the measures among non-smoking WFFs with both pre- and post-season measurements (n=26) were analyzed using paired t-test.

Results: EMTs had significantly higher weight ($p = 0.010$) and BMI ($p = 0.019$) than WFFs at baseline. WFFs had a lower percentage of predicted PEF (76.28 ± 37.36 vs. 97.95 ± 19.24, $p < 0.001$). No other spirometry measure was different between the two groups at baseline. All the spirometry measures were reduced post-season among WFFs but none of the cross-season declines was significant.
Conclusion: These preliminary findings suggest an adverse effect of cumulative wildland firefighting exposures on respiratory function. This research is continuing to recruit participants and will follow them across a three- year period.
This study is supported by the National Institute for Occupational Safety and Health (R010H012224), approved by The Ohio State University Institutional Review Board (2022H0330).

P21-03 | Continuous assessment of occupational exposures to fine particulate matter ($PM_{2.5}$) in U.S. wildland firefighters

Erick E. Mollinedo[1], Olorunfemi Adetona[2], Chieh-Ming Wu[2], Ryan Chartier[3], Luke P. Naeher[1]

[1] University of Georgia, Environmental Health Sciences, Athens, USA; [2] The Ohio State University, Environmental Health Sciences, Columbus, USA; [3] RTI International, Research Triangle Park, USA

Background/Objective: Wildland firefighters (WFFs) are exposed to fine particulate matter ($PM_{2.5}$) during fire control activities. Personal exposure assessment with air quality sensors is more accurate than relying on questionnaires. We conducted this pilot study that aimed to measure the continuous exposures to $PM_{2.5}$ in WFFs deployed for two weeks of wildfire control during the summer of 2021 in California.

Methods: MicroPEM® sensors were loaded with polytetrafluoroethylene filters, calibrated, and programmed for sampling. The sensors were sent to the WFFs, and they were instructed to manually turn on the MicroPEMs on-site when starting the fire control activity. The MicroPEMs were set operating at a duty cycle of 30 second On – 90 seconds Off to extend the sampling period. The mean $PM_{2.5}$ concentrations for the total sampling period were estimated gravimetrically by the filter samples. The raw real-time $PM_{2.5}$ concentrations for the total period and 24-hour averages were calculated based on the MicroPEM's nephelometric readings and were compared to the World Health's Organization (WHO) air quality guidelines (AQGs).

Results: We estimated the occupational $PM_{2.5}$ exposures in six WFFs. The MicroPEMs measured $PM_{2.5}$ in a span of 6.1 days on average (min= 2.95 days and max= 8.4 days). The mean gravimetric concentration was 79.53 μg/m^3 against the raw mean real-time concentration (314.3 μg/m^3). The raw 24-hour $PM_{2.5}$ real-time concentrations ranged from 13 μg/m^3 to 908.8 μg/m^3 (Mean= 261.3 μg/m^3, SD= 233.9 μg/m^3), compared to the WHO AQG of 15 μg/m^3.

Conclusion/Future directions: The mean 24-hour $PM_{2.5}$ levels from occupational exposure in WFFs while working in the fire line are above the WHO AQGs. We will conduct additional measurements in 2025 with WFFs from the Southeast U.S., modifying the MicroPEM sample settings to extend the sampling time, to improve the generalizability of our findings and to adjust the real-time concentrations.

Funding: NIOSH R21OH011271

P21-04 | Mapping Environmental Risks at Houston's Day Labor Corners: Air Pollution, Heat, and Worker Exposure

Juan Aguilera, Cesar Pinzon Gomez, Maria E. Fernandez-Esquer

UTHealth Houston School of Public Health, El Paso, USA

Objective: Across Houston, Latino day laborers gather at informal hiring sites, known as "corners," where they wait for work—often for hours under the open sky. While these sites provide economic opportunity, they expose workers to a hazardous mix of environmental threats. Surrounded by dense traffic, industrial activity, and urban heat islands, day laborers face prolonged exposure to harmful air pollutants, including fine particulate matter ($PM_{2.5}$), nitrogen dioxide (NO_2), and ozone.

Material and Methods: To understand the environmental burden at corners, we mapped 84 hiring sites across Houston between 2019 and 2023. Using geospatial analysis, we overlaid air pollution data from EPA monitoring

stations, traffic density from the Annual Average Daily Traffic dataset, and heat mapping data from community-driven campaigns.

Results: The corners are consistently located in areas with heavy traffic and high concentrations of $PM_{2.5}$ and NO_2. Additionally, elevated ozone levels at these locations pose further risks to cardiorespiratory health. Most sites fall within urban heat islands, amplifying the dangers of extreme heat. Statistical analysis shows a strong link between $PM_{2.5}$ exposure and traffic density (R=0.70, p<0.01), reinforcing the reality that day laborers are stationed in high-risk environments.

Conclusion: These environmental exposures are not incidental—they reflect broader inequities in urban planning and worker protections. Addressing this issue requires community-driven solutions: shade structures, air quality alerts, hydration stations, and policy changes to prioritize vulnerable workers. Public health must move beyond documenting these risks to actively mitigating them.

Funding: This research was supported by the National Institute on Minority Health and Health Disparities (NIH grant SR24MD007975-02) through the VALES+Tú project.

P21-05 | Biomonitoring and Environmental Assessment of Occupational Exposure to Potentially Toxic Substances in Greek Waste Recycling Facilities

Alkistis Kevrekidou[1,2], Catherine Gabriel[1,2], Marianthi Kermenidou[1,2], Anthoula Chatzimpaloglou[1,2], Nafsika Papaioannou[1,2], Tiina Santonen[3], Susana Viegas[4], Kukka Aimonen[3], Spyros Karakitsios[1,2], Dimosthenis Sarigiannis[1,2]

[1] Aristotle University of Thessaloniki, Department of Chemical Engineering, Environmental Engineering Laboratory, University Campus, Thessaloniki 54124, Greece; [2] Center for Interdisciplinary Research and Innovation, Balkan Center, HERACLES Research Center on the Exposome and Health, Bldg. B, 10th km Thessaloniki – Thermi Road, 57001, Greece; [3] Finnish Institute of Occupational Health (FIOH), P.O. Box 40, FI-00032, Työterveyslaitos, Finland; [4] NOVA University Lisbon, NOVA National School of Public Health, Public Health Research Centre, Comprehensive Health Research Center, CHRC, REAL, CCAL, Lisbon, Portugal

Objectives: Workers in recycling facilities processing plastic and electronic waste may be exposed to hazardous chemicals such as metals, flame retardants, plasticizers, per-/polyfluoroalkyl substances, and polycyclic aromatic hydrocarbons. Long-term exposure to these substances could pose significant health risks, yet comprehensive biomonitoring data remain scarce. This study, conducted as part of the European Partnership for the Assessment of Risks from Chemicals (PARC), aims to evaluate occupational exposure in Greece through biological and environmental sampling while recommending safety measures to mitigate risks.

Materials and Methods: The study target groups include both workers directly exposed to hazardous chemicals and a control group consisting of office workers. A total of 55 workers participated in the study, representing four recycling facilities. Among them, 35 were exposed workers involved in various tasks including raw material operators, welders, sorters, production managers, process engineers, track and forklift drivers, QC technicians and other related roles. Biological samples, including blood, urine, and buccal cells, were collected alongside personal exposure samples (hand wipes, inhalable and respirable fraction of air, and silicone wristbands) from recycling plant workers. Additionally, environmental samples such as air, settled dust, and soil were taken from workplace areas. Chemical analyses focus on detecting hazardous substances, while biomarker assessments explore potential links between exposure levels and health effects.

Results: The analysis is currently underway to establish connections between environmental contamination and biological markers of exposure. Initial findings suggest higher concentrations of certain hazardous substances in workers compared to unexposed individuals.

Conclusion: Preliminary results emphasize the urgent need for enhanced occupational safety measures in the recycling industries. However, further research is essential to determine long-term health impacts and develop strategies to reduce workers' exposure to harmful chemicals.

Acknowledgements: This project has received funding from the European Union’s Horizon Europe research and innovation programme under Grant Agreement No 101057014

P21-06 | Analysis of per/polyfluoroalkyl substance (PFAS) in serum and urine from firefighters: Baseline and post-fire event monitoring

N.L.Dilani Perera[1], Kiran Ali[1], Shawn Beitel[3], Liu Yiwen[2], Jefferey Burgess[2], Carrie McDonough[1]

[1] Carnegie Mellon University, Chemistry, Pittsburgh, USA; [2] University of Arizona, College of Public health, Tucson, USA; [3] University of Arizona, Community Environment and Policy, Tucson, USA

Firefighters are regularly exposed to per/polyfluoroalkyl substances (PFAS) from various sources, which may result in elevated PFAS levels and distinct exposure profiles in their blood and urine. This study analyzed serum and urine samples from municipal firefighters within 24 hours of responding to a fire or another potential exposure event. The results were compared to baseline samples from the same individuals to assess PFAS accumulation before the exposure and monitor any changes afterward. High-resolution mass spectrometry with liquid chromatography (LC-HRMS) was used to quantify known PFAS and screen for emerging ones. Eight targeted PFAS, including perfluoroalkyl acids (C7–C11 PFCAs, C5–C7 PFSAs), perfluoroalkane sulfonamides (e.g., FOSA), and perfluoroethylcyclohexane sulfonate (PFEtCHxS), were commonly found in serum. Through non-targeted analysis, two upregulated features (m/z 166.9865 Da @ 13.94 min and 262.0518 Da @ 5.62 min) and one downregulated feature (m/z 438.0625 Da @ 6.12 min) were detected in post-fire serum samples relative to baseline. In urine, eight targeted PFAS were measured, including FASAs (FHxSA, FOSA), PFCAs, and PFSAs. Some PFAS that were not detected in serum (e.g., PFO2HxA) were detected in urine. Additionally, FOSA was detected only in urine, likely due to its efficient renal excretion. The similarity in serum PFAS levels between baseline and post-exposure groups suggests that the presence of PFAS in serum is not solely due to short-term firefighting activities but reflects continuous environmental or occupational exposure. Here, similarities and differences between PFAS profiles in samples collected from post-fire and baseline monitoring will be discussed further. Overall, the widespread occurrence of these substances underscores the significant presence of PFAS in firefighter populations.

P21-07 | The effects of ambient temperature on occupational accidents in Santiago, Chile, from 2012 to 2019.

Rolando Vilasau[2,1], Rodrigo Puentes[3], Karla Yohannessen[1]

[1] Universidad de Chile, Escuela de Salud Pública, Santiago, Chile; [2] Instituto de Salud Pública de Chile, Departamento de Salud Ocupacional, Santiago, Chile; [3] Emory University, Gangarosa Department of Environmental Health, Rollins School of Public Health, Atlanta, USA

OBJECTIVE: To evaluate the association between daily ambient temperature and occupational accidents in Santiago, Chile, between 2012 and 2019.
MATERIALS AND METHODS: We fitted a time-series quasi-Poisson regression model controlling for time-varying confounders to analyze the association between daily ambient temperature and occupational accidents. A distributed lag non-linear framework was used to estimate the temperature-accident association, considering lagged effects. Subgroup analyses were conducted by industry, specifically in construction and transport.
RESULTS: 605,138 occupational accidents were recorded during the study period. There were 90,082 accidents in the construction sector and 55,918 in the transportation sector. Workers aged 25 to 34 accounted for approximately 26% of occupational accidents, followed by those aged 35 to 44, who accounted for 21%. Model results indicated an increased risk of accidents attributable to cold and hot temperatures compared to Minimum Morbidity Temperature (MMT, 8.8°C). For the 2.5th temperature percentile (4.8°C), the relative risk (RR) for all accidents was 1.12 (95% CI: 1.06–1.18), while for the 97.5th percentile, the RR increased to 1.42 (95% CI: 1.26–1.60). In the construction sector, the RR was 1.12 (95% CI: 1.04–1.20) at the 2.5th percentile and 1.45 (95% CI: 1.24–1.71) at the 97.5th percentile (23.4°C). In transportation, the RR was 1.11 (95% CI: 1.03–1.19) at lower temperatures and 1.47 (95% CI: 1.25–1.72) at higher temperatures.
CONCLUSION: The results of this study suggest that non-optimal temperature exposure (both cold and heat) is associated with an increased risk of occupational accidents in Santiago, particularly in sectors such as construction and transportation. Younger workers, especially those aged 25 to 34 years, may be the most vulnerable. These findings highlight the need to implement control measures, with a particular focus on younger workers in temperature-exposed industries.

P21-08 | Swiss bus drivers' workosome

Viviane F.M. Remy, Thomas C. Charreau, Irina Guseva Canu

Unisanté / Université de Lausanne, environmental and occupational health departement, Lausanne, Switzerland

Objective: To assess the occupational exposome of Swiss bus drivers and to classify them into different risk profiles.
Material and Methods: The Swiss Transport Personnel Health Cohort study (TRAPHEAC) was launched in 2024. Participants completed a comprehensive inclusion questionnaire encompassing their occupational history and the history of buses driven. The later was cross-referenced with the Bus-Exposure Matrix (BEM) to retrospectively assess the bus drivers' occupational exposure. The BEM contains quantitative exposure estimates for noise, high and low-frequency electric fields, low-frequency magnetic field, whole-body and floor vibration, air exchange rate, and global ergonomics score (with higher score meaning less constrains) used to define the latent profiles. Nine Latent Profile

Analysis (LPA) models with different numbers of profiles were compared using statistical fit indices (AIC, BIC, entropy). To characterize the profiles, we examined the mean scores on the indicators of the best model.
Results: Study sample comprised 508 bus drivers (16% female; mean age 48.8 (SD=10.5) years and the average length of employment as bus drivers 12.1 (SD=11.4) years). Daily occupational exposure to equivalent noise was 70.7 dB(C) (SD=2.2), whole-body vibration 0.26 m/s^2 (SD=0.02), floor vibration 0.25 m/s^2 (SD=0.06), high-frequency electric field 0.42 V/m (SD=0.20), low-frequency electric field 0.48 V/m (SD=0.20), magnetic field 0.17 µT (SD=0.09), and global ergonomic score 75.8 (SD=6.5) in average. Five latent profiles were identified: profiles 1 and 2 being the two extremes. Profile 1 drivers (N=188) have low physical-chemical exposures but high ergonomic constrains, while profile 2 drivers (N=44) have high physical-chemical exposures but few ergonomic constrains. Drivers in other groups differed mainly by the level of their exposure to noise, vibration and ergonomic constrains.
Conclusion: These results help understand differences between bus driver groups based on observed workosome and will enable predicting health outcomes based on profile membership, and targeting prevention on the most at-risk profiles.

P21-09 | Do Dry-Cleaning Shop Owners' Awareness and Knowledge of Microplastics Influence Their Occupational Protective Practices to Reduce Inhalation Exposures?

Chanmi Lee[1], Grant Kassan[1], Caesar Belchez[2], Heli Patel[1], Jin Young Seo[3], Inkyu Han[1]

[1] Temple University, Department of Epidemiology and Biostatistics, Philadelphia, USA; [2] Temple University, Department of Health Services Administration and Policy, Philadelphia, USA; [3] Hunter College - CUNY, School of Nursing, New York, USA

Objective: As part of an ongoing research project on microplastics exposure, this study assessed the awareness and knowledge of self-employed dry-cleaning shop owners regarding microplastic exposure and their workplace practices in Philadelphia. Data were collected through indoor air monitoring and surveys; here, we present the survey findings.
Methodology: In-person surveys were conducted with 34 dry-cleaning shop owners in Philadelphia during 2024. Data were collected on demographics, knowledge and awareness of microplastic exposure, and workplace practices. Using a comprehensive scoring index, we assessed participants' ability to define microplastics, identify sources of microplastics, and recognize the presence of microplastics in both the environment and the workplace.
Results: Participants had an average age of 64.3±7.9 years. Ninety-one percent were Korean, and 53% were female. About 80% operated their businesses six days a week, working an average of 9.3 hours per day and handling 145 clothing items daily. Less than half (40%) had heard of microplastics. The high-awareness group (those who had heard of microplastics) scored 8.1±2.3 on average, while the low-awareness group (those who had never heard of microplastics) scored 3.5±1.9 (p=0.002). Specifically, the high-awareness group had higher scores for: Definition of microplastics (high=0.70 vs. low=0.20, p=0.002), Identification of sources (high=3.46 vs. low=2.05, p=0.003), and Recognition of microplastics in the environment (high=1.61 vs. low=1.31, p=0.002). The high-awareness group showed a greater recognition score for microplastics in the workplace (high=0.70 vs. low=0.35), but the difference was not statistically significant (p=0.093). These findings may explain the limited use of protective practices (e.g., wearing surgical masks at work) in both groups (high=38% vs. low=45%, p=0.941).
Conclusion: Despite concerns about exposure and health risks, awareness and understanding of airborne microplastics were limited. Given their unique occupational environments, future educational interventions may help increase awareness of microplastics exposure and promote healthier workplace practices.

P21-10 | *Association Between PFAS Levels in Firefighter Serum and Drinking Water in Orange County, California*

Jesselle M. Legaspi[1], Jefferey L. Burgess[2], Derek Biering[3], Veronica Vieira[1]

[1] University of California-Irvine, Department of Environmental and Occupational Health, Joe C. Wen School of Population & Public Health, Irvine, USA; [2] The University of Arizona, Department of Community, Environment and Policy, Mel & Enid Zuckerman College of Public Health, Tucson, USA; [3] Orange County Fire Authority (OCFA), Irvine, USA

Objective:

Per- and polyfluoroalkyl substances (PFAS) are persistent environmental contaminants found in Orange County (OC), California drinking water and linked to adverse health outcomes. Filtered and bottled drinking water are recommended for reducing PFAS exposure from contaminated public drinking water. This analysis investigated whether perfluorooctanoic acid (PFOA) and perfluorooctane sulfonic acid (PFOS) concentrations in public drinking water systems are a predictor of OC firefighter serum concentrations.

Materials and Methods:

Participants included Orange County Fire Authority (OCFA) firefighters (n=176) enrolled in the Fire Fighter Cancer Cohort Study (FFCCS) and residing in Orange County. The U.S. Environmental Protection Agency's Unregulated Contaminant Monitoring Rule 3 (UCMR3) dataset was used to determine OC cities with detectable PFAS concentrations in their public drinking water. We compared serum PFOS and PFOA levels of firefighters residing in cities with and without UCMR3 detected PFAS in their public drinking water. Additionally, univariate linear regression models examined associations between PFOS and PFOA concentrations in water and firefighter serum.

Results:

Among the 176 OC residents, 87 (49%) provided serum samples. T-tests showed no statistically significant differences in serum PFOA and PFOS levels between firefighters in cities with (n=25) and without (n=62) detectable PFAS in their drinking water systems (PFOA: p=0.223; PFOS: p=0.796). Similarly, linear regression analyses showed no significant associations between serum PFOA (β=-0.12 μg/L; 95% CI=-0.31,0.07) or PFOS (β=0.02 μg/L; 95% CI=-0.31,0.34) per 0.01 μg/L increase in drinking water concentrations.

Conclusion:

This analysis found no significant association between PFAS levels in public water systems and firefighter serum PFAS levels. This is likely due to the low proportion of OC firefighters self-reporting drinking unfiltered tap water. Future analyses will incorporate covariates, including those from the drinking water survey. This work was supported by the Southern California NIOSH Education Research Center (SCERC). FFCCS thanks OCFA firefighters for assisting their community daily.

P21-11 | Beyond the Fields: A Farmworker-Centered Approach to Heat and Chemical Exposure Prevention

Brandon R. Toji Ruiz[1], Michelle Solorio[1], Rietta Wagoner[1], Isaiah Casarez[1], Linda Lara-Jacobo[2], Ana Solorio[3], Briana Toji[3,2], Ofelia Cruz[1], Eduardo Arreola[1], Laura Magaña[1], Alejandra Early[1], Benjamin Aceves[1], Nicolas Lopez-Galvez[1]

[1] San Diego State University, School of Public Health, San Diego, USA; [2] San Diego State University Imperial Valley, San Diego State University Imperial Valley, Brawley, USA; [3] Organización en California de Lideres Campesinas, INC., Lideres Campesinas: Valle Imperial, Imperial Valley, USA

Background and Objective: Farmworkers are at increased risk for exposure to extreme temperatures and chemicals on a daily basis. We implemented a community-based intervention toassess and reduce heat and chemical exposure among farmworkers and their families living in Imperial County, California.
Materials and Methods: We designed and implemented a heat and chemical exposure intervention for farmworkers, incorporating education, personal protective equipment, household modifications, and hydration support. Community health workers administered a tailored intervention to each participant. We measured individual heat stress and household temperature, along with indoor air quality indicators, including CO_2, $PM_{1.0}$, $PM_{2.5}$, PM_{10}, and total volatile organic compounds (VOCs), both before and after the intervention. Pre- and post-intervention levels were then compared to evaluate the intervention's impact.
Results: The cohort consisted entirely of Latino participants (n=27), with 60% identifying as male. Half had a secondary education or less, and 46% worked as field laborers. Additionally, 63% reported a monthly income of $2,500 or less. Following the intervention, we observed significant reductions in indoor air pollutants and temperature in participant homes, with decreases in CO_2 (44% of homes), $PM_{1.0}$ (30%), $PM_{2.5}$ (30%), PM_{10} (30%), total VOCs (48%), and temperature (37%). $PM_{1.0}$ levels were significantly lower after the intervention, with a geometric mean of 2.59μg/m^3 (SD:5.32) pre-intervention to 2.10 μg/m^3 (SD:6.16) post-intervention. $PM_{2.5}$ decreased from 4.36 μg/m^3 (SD:4.75) to 3.18 μg/m^3 (SD:6.05), and PM_{10} decreased from 5.04 μg/m^3 (SD:4.52) to 3.62 μg/m^3 (SD:5.70).
Conclusion
As extreme temperatures and chemical exposures continue to disproportionately affect migrant and seasonal farmworkers, it is crucial to collaborate with these communities to develop interventions that align with their priorities, address barriers to care and education, and promote health equity. Additionally, evaluating the effectiveness of these interventions is essential to ensuring meaningful and sustainable improvements.

P21-13 | Assessment of Plasticizer Contamination in Workplaces and Its Association with Urinary Metabolites

Sohyeon Bang[1], Sohyeon Choi[1], Yougjun Jeon[1], Jaehyun Kim[1], Younglim Kho[2], Nayoun Park[2], Jeongim Park[1]

[1] Soonchunhyang University, Department of Environmental Health Sciences, Asan, South Korea; [2] Eulji University, Department of Environmental Health and Safety, Seongnam, South Korea

Objective: Phthalates and alternative plasticizers are endocrine-disrupting chemicals linked to thyroid hormone disruption and anti-androgenic effects. However, occupational exposure data remain limited. This study assessed workplace contamination and urinary metabolites of plasticizers in workers and examined their association. **Material and Methods:** Surface samples were collected from 37 locations (production: 23; non-production: 14) using gauze pre-soaked in 70% isopropanol. Samples were analyzed for 25 plasticizers via GC–MS. End-of-work-week urine samples from 121 male workers (molding: 64, painting: 13, assembly: 32, office: 12) were analyzed for 33 metabolites of 17 plasticizers using LC-MS/MS. **Results:** DEHP, DiNP, and DEHTP were detected in over 50% of surface samples, with median concentrations of 4.45, 4.23, and 7.78 ng/cm², respectively. Molding areas had the highest DiNP (8.10 ng/cm²) and DEHTP (68.0 ng/cm²) levels ($p < 0.05$). Urinary metabolite concentrations did not significantly differ by job category. DiNP levels were highest in office workers, while DEHTP was highest in assembly workers. No correlation was found between surface contamination and urinary metabolites. The unexpected urinary metabolite patterns suggest non-occupational sources or alternative exposure pathways. **Conclusion:** DiNP and DEHTP were prevalent in workplace environments, yet no direct association with urinary metabolites was observed. This indicates the need to consider multiple exposure routes, such as dermal absorption, ingestion, or external sources, in occupational plasticizer exposure assessment. **Acknowledgments:** This research was supported by Basic Science Research Program through the National Research Foundation of Korea(NRF) funded by the Ministry of Education(No. 2022R1A2C2011685). This work was supported by the National Research Foundation of Korea(NRF) grant funded by the Korea government(MSIT) (RS-2022-KJE002055).

P21-14 | Characterizing occupational exposures and respiratory symptoms during cannabis processing activities

Callan Krevanko, Coralynn Sack, Christopher D. Simpson

University of Washington, Department of Environmental and Occupational Health, Seattle, USA

Objective: During cannabis processing activities, workers are exposed to airborne agents that can cause allergic or respiratory health symptoms. This study aims to characterize exposure to potential respiratory hazards during cannabis processing and determine whether these exposures are associated with respiratory and allergic health measurements in cannabis workers.

Methods: We collected a series of exposure and health measurements from workers (N=11) at a Washington indoor cannabis cultivation and processing facility. Workers wore personal air sampling pumps equipped to measure inhalable particle mass (PM), beta-glucan, endotoxin, and volatile organic compound terpenes. Participants completed a daily health symptom questionnaire with information on job activities and health symptoms experienced during the workday. Cross-shift and cross-week changes in pulmonary function and airway inflammation were

assessed using pre- and post-shift spirometry and fractional exhaled nitric oxide measurements. Cannabis sensitization was assessed through skin prick testing to a hemp slurry. Continued sampling at additional cannabis facilities is currently underway.
Results: Our preliminary findings demonstrated that total terpene air concentrations were highest during cannabis bucking, trimming, and sifting activities. Terpenes detected in all samples collected included Terpinolene, beta-Pinene, alpha-Pinene, beta-Myrcene, and D-limonene. The highest inhalable PM concentrations were observed for workers engaged in trimming and joint packing activities. Inhalable PM concentrations were generally lower during packaging- and office work-related activities. Workers reported the onset of respiratory symptoms, including chest tightness (27%), wheeze (9%), shortness of breath (18%), and cough (9%), during cannabis bucking, trimming, and rolling activities.
Conclusions: Our preliminary findings suggest that cannabis processing workers are exposed to inhalable PM, terpenes, and bioaerosols at varying levels depending on their job tasks. Continued sampling at additional facilities will improve the generalizability of exposure and health data. Study findings may inform exposure control strategies to protect cannabis industry workers from potential respiratory hazards.
Funding: UW PNASH Center

P21-15 | Western Construction and Museum Field Work Must Reckon with Valley Fever

David F. Goldsmith[1], Holly Cusack-McVeigh[2]

[1] George Washington University, Environmental & Occupational Health, Washington DC, USA; [2] University of Indiana, Department of Anthropology, Indianaoplis, USA

Introduction: Valley Fever (VF) or Coccidioidomycosis is a hazard found when museuem field work disturbs dry sandy desert soils, especially in Southwestern states and Northern Mexico. VF arises from ground disturbances common in construction, military maneuvers, other desert activities (like racing), dust storms, and in museum or archaeology digs. VF arises when spores of Coccidioides immitis, a soil fungus endemic to southwestern U.S. is made airborne. Outbreaks of VF have been reported in SE California, Arizona, New Mexico, Utah, Nevada, and in Northern Mexico. Symptoms include cough, shortness of breath, chest pain, myalgias, headache/sore throat; confirming clinical studies should include positive chest X-ray, + skin test, and + antibody test to C. immitis; average 10 days from initial exposure to symptoms (range 9-15 days).
Literature: Reviewing the published literature shows that southwestern museum field trips are the source of at least four case studies. Some modest epidemiology research suggests the risk is rising, though it is unknown whether this is associated with climate change or simply more worker incursions onto previously undisturbed desert lands.
Synthesis: Because the symptoms of VF can be severe, it suggests the need for better occupational epidemiology studies, particularly for construction workers and for volunteers and museum employees. Prevention steps include the use of wet methods, minimize soil disturbance, and wearing N95 respirators when digging into desert surfaces. Furthermore, there is a suggestion that fieldwork occur *after* rainfall events. Higher risks are suggested for pregnant women, subjects with weakened immune system, diabetics, and Africans & Philippinos. Smoking and silica dust exposure should be considered as possible co-factors.
Conclusion: Because the data suggests a rising risk for VF, there is a need for more health research, greater focus on prevention and the role of climate change, with deepened concern for Western museum field staff.

P22 | Multi-Omics Approaches to Environmental Health Research

P22-01 | Associations of maternal blood biomarkers of prenatal acetaminophen exposure with placental gene expression and child attention deficit hyperactivity disorder (ADHD)

Brennan H. Baker[1,2], Theo Bammler[1], Emily Barrett[3], Nicole Bush[4], Brent Collett[1,2], Karen Derefinko[5], Daniel Enquobahrie[1], Catherine Karr[1], Kaja LeWinn[4], Jiawang Liu[5], Christine Loftus[1], James MacDonald[1], Shanna Swan[6], Qi Zhao[5], Alison Paquette[1,2], Sheela Sathyanarayana[1,2], First published in Nature Mental Health (2025) by Springer Nature. https://doi.org/10.1038/s44220-025-00387-6. This research was conducted using specimens and data collected from the Conditions Affecting Neurocognitive Development and Learning in Early Childhood (CANDLE) study which was funded by the Urban Child Institute. Data for this study were collected with additional support from ECHO PATHWAYS, funded by the National Institutes of Health (NIH UG3OD023271 and UH3OD03271), and from additional NIH grants (NCATS of NIH UL1TR002319 and NIH R01HL132338). The generation of RNA-sequencing data for this study was supported by the University of Washington Interdisciplinary Center for Exposures, Diseases, Genomics, and Environment funded by the National Institute of Environmental Health Sciences (NIEHS P30ES007033). BHB was supported by the UW NIEHS sponsored Biostatistics, Epidemiologic and Bioinformatic Training in Environmental Health (BEBTEH) Training Grant (NIEHS T32ES015459). The authors would like to thank the study staff, data teams, and co-investigators involved in the CANDLE cohort as well as the ECHO-PATHWAYS consortium for their invaluable contributions. We are also grateful to the study participants who generously volunteered their time for this study. The content is solely the responsibility of the authors and does not necessarily represent the official views of the National Institutes of Health.

[1] University of Washington, Seattle, USA; [2] Seattle Children's Research Institute, Seattle, USA; [3] Rutgers School of Public Health, Piscataway, USA; [4] University of California San Francisco, San Francisco, USA; [5] University of Tennessee Health Science Center, Memphis, USA; [6] Icahn School of Medicine at Mount Sinai, New York, USA

Objective

Over 50% of individuals are prenatally exposed to acetaminophen in most populations. Randomized clinical trials have only shown safety of developmental acetaminophen exposure for short-term liver outcomes, while neurodevelopment has never been assessed. Observational studies indicate links between prenatal acetaminophen and adverse neurodevelopment, but are limited by reliance on maternal self-reported drug use. Mechanisms linking prenatal acetaminophen with adverse neurodevelopment are unknown. We addressed these gaps by examining associations of maternal plasma acetaminophen metabolites during pregnancy with child ADHD, and evaluating differential gene expression and transcriptomic pathway perturbations in the placenta.

Material and Methods

We conducted metabolomics analysis of acetaminophen in second-trimester plasma and assessed ADHD outcomes at age 8-10 years for 307 mother-child pairs from the Conditions Affecting Neurocognitive Development and Learning in Early Childhood study. Associations of prenatal acetaminophen exposure with ADHD-related outcomes were modeled while covarying for potential confounders including sociodemographics and indications for acetaminophen use. For 174 placentas among our study sample, we performed RNA-sequencing, differential gene expression analysis, and gene set enrichment analyses for KEGG pathways. We additionally assessed effect modification by sex.

Results

Detection of acetaminophen in second trimester plasma was associated with higher odds for child ADHD (Odds Ratio (OR)=3.15 [95%CI: 1.20-8.29]). In sex-specific analysis, prenatal acetaminophen exposure was associated with

higher odds for child ADHD in females (OR = 6.16 [1.58-24.05]) while there was a positive, though non-significant association in males (OR = 1.7 [0.44 - 6.61]; interaction P = 0.18). Prenatal acetaminophen exposure and ADHD were associated with placental upregulation of immune system pathways and downregulation of oxidative phosphorylation in both sexes. In females only, prenatal acetaminophen was associated with 5.22% higher odds (0.0456%-13.1%) of ADHD mediated through increased immunoglobulin heavy constant gamma 1 expression.

Conclusion

Results highlight placental molecular mechanisms that may underlie developmental toxicity of prenatal acetaminophen exposure.

P22-02 | Chronic arsenic exposure, serum folate levels, and DNA methylation in Bandadeshi women

Amy M. Inkster[1], Gwen Tindula[2], Anne K. Bozack[1], Afifah Farooque[6], Kian P. Koh[4], Richard H. Finnell[4], Xingyan Wang[5], Liming Liang[5], Maitreyi Mazumdar[3,6], Andres Cardenas[1]

[1] Stanford University, Department of Epidemiology and Population Health, Palo Alto, USA; [2] University of California Berkeley, School of Public Health, Berkeley, USA; [3] Harvard University, Department of Environmental Health, Harvard T.H. Chan School of Public Health, Boston, USA; [4] Baylor College of Medicine, Department of Molecular and Cellular Biology, Center for Precision Environmental Health, Houston, USA; [5] Harvard University, Department of Epidemiology, Harvard T.H. Chan School of Public Health, Boston, USA; [6] Boston Children's Hospital, Department of Neurology, Harvard Medical School, Boston, USA

Objectives

In Bangladesh, 17% of the population is exposed to high levels of arsenic in drinking water, which is associated with several adverse outcomes including spina bifida. Maternal folate status is protective and reduces neural tube defect risk, while folic acid supplementation enhances arsenic detoxification *in vivo* and contributes to DNA methylation (DNAme) maintenance. Here, we hypothesize that associations between arsenic exposure and leukocyte DNA methylation (DNAme) may be modified by folate status.

Materials and Methods

We analyzed leukocyte DNA methylation (DNAme) in 374 Bangladeshi women (246 with a history of spina bifida-affected pregnancies) using the Illumina Infinium MethylationEPIC v2.0 array. Measurements of serum folate (ng/mL) and chronic arsenic exposure (toenail, µg/g) were obtained. DNAme at 830,263 CpGs was evaluated for associations with arsenic, folate, and their interaction using linear models. All models were adjusted for age, BMI, education, offspring case/control status, and leukocyte cell composition. Multiple comparisons were corrected using the Benjamini-Hochberg method.

Results

While folate was not found to be associated with leukocyte DNAme, toenail arsenic was negatively associated with DNAme at one CpG in the *C5* gene body (cg20363688, $\Delta\beta = -0.01$). The arsenic*folate interaction was associated with DNAme at 64 CpGs, seven of which had absolute effect sizes >0.02 ($\Delta\beta$), such as CpGs in the TSS1500 of *DCAF10* and the first exon of *LINC01714*. For these seven CpGs, arsenic concentration (main effect) was associated with increased DNAme, while the interaction effect was associated with decreasing DNAme, suggesting that folate levels may protect against arsenic-associated DNAme changes at these loci.

Conclusions

Our work suggests that folate modifies the association between arsenic exposure and leukocyte DNAme among Bangladeshi women chronically exposed to high levels of arsenic. Future work will examine DNAme variation between mothers of spina bifida cases and controls, and in the tissues of affected children.

P22-03 | Ocular Biomarkers as Mediators between Environmental Exposures and Systemic Health Outcomes—Review of Current Evidence and Directions for Future Research

Haoran Cheng[1], Jeremy Sarnat[2], Douglas I. Walker[2], Anant Madabhushi[3,4], Amritpal Singh[3,5], Rohan Dhamdhere[3,5], Jodhbir S. Mehta[1], Tien Yin Wong[1,6], John S. Ji[7], Carmen Marsit[2], Dean Jones[8], Daniel Shu Wei Ting[1,9], Darren Shu Jeng Ting[1,10], Donghai Liang[2]

[1] Duke-NUS Medical School, Singapore National Eye Center, Singapore Eye Research Insitute, Singapore, Singapore; [2] Emory University Rollins School of Public Health, Gangarosa Department of Environmental Health, Atlanta, USA; [3] Emory University and Georgia Institute of Technology, Wallace H. Coulter Department of Biomedical Engineering, Atlanta, USA; [4] Emory Empathetic AI for Health Institute, Atlanta, USA; [5] Emory University School of Medicine, Department of Radiology and Imaging Sciences, Atlanta, USA; [6] Tsinghua University, Tsinghua Medicine, Beijing Tsinghua Changgung Hospital School of Clinical Medicine, Beijing Visual Science and Translational Eye Research Institute, Beijing, China; [7] Tsinghua University Vanke School of Public Health, Beijing, China; [8] Emory University School of Medicine, Department of Medicine, Atlanta, USA; [9] Stanford University, Byers Eye Insitute, Stanford, USA; [10] University of Birmingham College of Medicine and Health, Department of Inflammation and Ageing, Sandwell and West Birmingham NHS Trust, Birmingham and Midland Eye Centre, Birmingham, UK

Objective
The field of environmental epidemiology aims to identify biomarkers that provide early, accessible insights into the influence of exposures on disease risk. While multi-omics technologies offer valuable molecular insights, their high cost and invasiveness limit population-wide applications. The human eye provides a promising alternative for biomarker discovery through non-invasive imaging techniques, such as color fundus photography (CFP) and optical coherence tomography (OCT), a field known as oculomics. However, a systematic approach to link environmental exposures with systemic health using ocular biomarkers remains underexplored.
Method
We summarized current literature that examines ocular biomarkers associated with (1) exposome, including air pollution, psychosocial stress, socioeconomic status, and physical activity, and (2) systemic health outcomes, particularly cardiovascular and neurodegenerative diseases. Gaps in the literature were identified, and future research directions proposed.
Results
Particulate matter exposure is linked to narrower arterioles, wider venules, and retinal neurodegeneration, particularly in the retinal nerve fiber layer (RNFL). Psychosocial stress and adverse life events correlate with larger venules and smaller arterioles, while socioeconomic disadvantages accelerate retinal atrophy in the ganglion cell and inner plexiform layers. Conversely, higher physical activity levels are associated with narrower venules and wider arterioles, reflecting protective microvascular effects. Narrower arterioles and wider venules are linked to hypertension, diabetes, and cardiovascular disease, while retinal and choroidal thinning correlates with Alzheimer's, Parkinson's, and chronic kidney disease. Reduced vessel density and enlarged foveal avascular zones indicate ischemic damage in diabetes and hypertension.
Conclusion

Ocular biomarkers bridge exposome and systemic health, offering unique opportunities for precision health. Future research will benefit from new methodologies, such as exposome-wide association studies and high-dimensional mediation analysis for a more holistic insight. Addressing challenges such as data standardization and ethical considerations will unlock the potential of oculomics in advancing precision health.

P22-04 | Postpartum metabolomics profile predicts 8-years postdelivery women's subclinical atherosclerosis measures

Katherine Svensson[1], Marco Sanchez-Guerra[2], Vishal Midya[1], Haibin Guan[1], Megan Niedzwiecki[1], Georgia Dolios[1], Citlalli Osorio-Yáñez[3,4], Luz M. Del Razo[5], Julio C. Ayllon-Vergara[6], Guadalupe Estrada-Gutierrez[7], Maria Luisa Pizano-Zárate[8,9], Lauren Petrick[1], Mara M. Tellez-Rojo[10], Robert O. Wright[1], Elena Colicino[1]

[1] Icahn School of Medicine at Mount Sinai, Department of Environmental Medicine, New York, USA; [2] National Institute of Perinatology, Department of Developmental Neurobiology, Mexico City, Mexico; [3] Instituto de Investigaciones Biomedicas, Departamento de Medicina Genomica y Toxicologia Ambiental, Mexico City, Mexico; [4] Instituto de Investigaciones Biomedicas & Instituto Nacional de Cardiologia Ignacio Chavez, Unidad de Investigacion en Medicina Traslacional, Mexico City, Mexico; [5] Centro de Investigacion y de Estudios Avanzados del Instituto Politecnico Nacional, Departamento de Toxicologia, Mexico City, Mexico; [6] Servicio de Cardiologia del Hospital General de Mexico, Mexico City, Mexico; [7] National Institute of Perinatology, Department of Immuobiochemistry, Mexico City, Mexico; [8] National Institute of Perinatology, Nutrition and Bioprogramming Coordination, Mexico City, Mexico; [9] Mexican Social Security Institute (IMSS), UMF 4, South Delegation of the Federal District, Mexico City, Mexico; [10] National Institute of Public Health, Center for Nutrition and Health Research, Cuernavaca, Mexico

Background: Women experience significant physiological changes, which may contribute to long-term cardiovascular risk. Metabolomics profiling has the potential to aid in early risk identification. **Objective:** This study evaluated the prospective association between metabolomics profiles in early postpartum and 8-year later subclinical atherosclerosis. **Methods:** The PROGRESS study has followed women from the time of pregnancy and currently up to 16 years postdelivery. Pregnant women with less than 20 weeks´ gestation and living in Mexico City, Mexico, were recruited during the years 2007-2011. Serum untargeted metabolomics at 1-month postpartum was analyzed with two analytical modes of chromatography high-resolution mass spectrometry (LC-HRMS) to identify both polar and non-polar or semi-polar metabolites. Carotid intima media thickness (CIMT) was measured 8-years postdelivery using B-mode ultrasound on the right side of the neck area. A metabolome-wide association study consisting of linear regression models was conducted to identify metabolites associated with CIMT measures. Models were adjusted for age, education, estimated pre-pregnancy BMI, and smoking exposure. Significant metabolites (FDR-adjusted p-values <0.10) were analyzed using class-and pathway-based enrichment analysis for further biological interpretation. **Results**: Several metabolites of the superclasses organic acids, oxygen compounds, benzenoids and organoheterocyclic compounds were significantly associated with CIMT. Most of the significant polar metabolites were associated with a thinner CIMT, whereas among non-polar or semi-polar metabolites, significant associations were found with both thinner and thicker CIMT levels. Enriched pathway analysis identified related biological pathways including regulation of white adipocyte differentiation, PPARalpha activation of gene expression and regulation of lipid metabolism. **Conclusion:** We found several metabolites at 1-month postpartum associated with 8-years postdelivery CIMT. Associated biological pathways were connected to adipocyte and lipid metabolism, and PPARalpha regulated pathways. This study highlights the potential of measuring metabolomics as early markers associated with subclinical atherosclerosis for long-term follow-up of women´s cardiovascular health.

P22-05 | Association Between Urinary Cadmium Levels and EV-miRNA Expression in the Strong Heart Study

Stephanie Calluori[1], Arce Domingo-Relloso[1], Haotian Wu[1], Walter Goessler[2], Ying Zhang[3], Jason G. Umans[4,5], Maria Tellez-Plaza[6], Lyle G. Best[7], Shelley A. Cole[8], Abbas Hakim[9], Louise C. Laurent[9], Peter DeHoff[9], Andrea Baccarelli[10], Tiffany Sanchez[11], Ana Navas-Acien[1], Allison Kupsco[1]

[1] Columbia University Mailman School of Public Health, Department of Environmental Health Sciences, New York, USA; [2] Universität Graz, Institute of Chemistry, Graz, Austria; [3] The University of Oklahoma Health Sciences Center, Center for American Indian Health Research, Department of Biostatistics and Epidemiology, Oklahoma City, USA; [4] Georgetown-Howard Universities, Center for Clinical and Translational Sciences, Washington, DC, USA; [5] MedStar Health Research Institute, Washington, DC, USA; [6] Instituto de Salud Carlos III, Integrative Epidemiology Group, Department of Chronic Diseases Epidemiology, National Center for Epidemiology, Madrid, Spain; [7] Missouri Breaks Industries Research, Eagle Butte, USA; [8] Texas Biomedical Research Institute, Population Health, San Antonio, USA; [9] University of California San Diego, Department of Obstetrics, Gynecology, & Reproductive Sciences, La Jolla, USA; [10] Harvard T.H. Chan School of Public Health, Harvard University, Department of Environmental Health, Boston, USA; [11] Colorado School of Public Health, University of Colorado Anschutz, Department of Epidemiology, Aurora, USA

Objective: American Indians experience higher exposure to cadmium (Cd), a toxic metal associated with many diseases (e.g., cancers, cardiovascular disease), than the general U.S. population. Mechanisms of Cd toxicity are unknown, but studies suggest oxidative stress and epigenetics may play a role. MicroRNAs (miRNAs) are important epigenetic regulators; however, impact of Cd on extracellular vesicle (EV)-miRNA expression has not been investigated. We examined the association between urinary Cd levels and plasma EV-miRNA expression in an American Indian population.

Methods: The Strong Heart Study is a cohort of 12 American Indians tribes and communities across Arizona, Oklahoma, and South/North Dakota. Urine was collected from participants at Visit 1 (1989-1991) and plasma at Visit 2 (1993-1994). Urinary Cd was quantified using inductively coupled plasma mass spectrometry (N=842). Plasma EV-miRNA sequencing detected 182 distinct miRNAs in ≥70% of the study population. Linear regression was used to estimate associations between urinary Cd and individual EV-miRNAs (FDR≤0.05). To reduce data dimensionality and account for total EV-miRNA variability, we generated sparse principal components (sPCs) and examined associations between urinary Cd and each sPC using linear regression (p≤0.05). Pathway analysis was performed using DIANA MirPATH v4.

Results: Median urinary Cd was 0.95 μg/g creatinine [IQR: 0.13, 1.77]. No significant associations were observed between urinary Cd and individual EV-miRNAs after FDR correction. However, Cd was significantly associated with sPC1 ($\beta 1$= -0.59, p = 0.02, [95% CI: -1.10, -0.08]). sPC1 explained 41.5% of total EV-miRNA variability and selected 62 EV-miRNAs with non-zero loadings. Pathway analysis indicated that EV-miRNAs in sPC1 may be related to cancer and neurological disease pathways.

Conclusion: Urinary Cd levels were associated with EV-miRNA variability captured by sPCs. Further research is needed to assess EV-miRNAs as a biomarker of chronic Cd exposure and related disease.

Funding: NIEHS T32 ES007322, R01ES027747, P42ES010349. NHLBI 75N92019D00027, 75N92019D00028, 75N92019D00029, 75N92019D00030SHS.

P22-06 | Childhood Gut Microbial Multi-omics Profile and Neurodevelopmental Outcomes in a Canadian Birth Cohort

Yidan Zhang[1], Feng Gao[2,3], Anthony Gagnon[4], Yihan Fan[2], Virginie Gillet[4], Andrew Michael[5], Jonathan Posner[6], Jean-Philippe Bellenger[7], Andrea Baccarelli[8,4], Larissa Takser[4], Yike Shen[1]

[1] University of Texas at Arlington, Department of Earth and Environmental Sciences, Arlington, USA; [2] University of California, Los Angeles, Department of Environmental Health Sciences, Fielding School of Public Health, Los Angeles, USA; [3] University of California, Los Angeles, Department of Molecular and Medical Pharmacology, David Geffen School of Medicine, Los Angeles, USA; [4] Université de Sherbrooke, Département de Pédiatrie, Faculté de Médecine et des Sciences de la Santé, Sherbrooke, Canada; [5] Duke University, Duke Institute for Brain Sciences, Durham, USA; [6] Duke University, Department of Psychiatry and Behavioral Health, Durham, USA; [7] Université de Sherbrooke, Département de Chimie, Sherbrooke, Canada; [8] Harvard University, Harvard T.H. Chan School of Public Health, Boston, USA

Objective: The gut microbiome plays a crucial role in neurodevelopment through the gut-brain axis, and its imbalance has been linked to various neurodevelopmental impairments. This study aims to explore the associations of gut microbiome and transcriptome profiles with multiple neurodevelopmental outcomes in children aged 8-12 years from the GESTation and Environment (GESTE) birth cohort in Sherbrooke, Quebec, Canada.
Material and Methods: We included 251 children from the GESTE cohort. Stool samples were collected at the 8-12-year follow-up and analyzed using shotgun metagenomic and metatranscriptomic sequencing to profile microbial species and functional pathways. Neurodevelopmental outcomes were assessed using the Vanderbilt Rating Scale, Behavior Assessment System for Children, Third Edition (BASC-3), Continuous Performance Test, Third Edition (CPT-3), Wechsler Intelligence Scale for Children, Fifth Edition (WISC-V), and Neuropsychological Assessment, Second Edition (NEPSY-II). Associations of gut microbial species and functional pathways with neurodevelopmental test scores were evaluated using linear regression models, adjusted for potential confounders. False discovery rate correction was applied.
Results: Higher relative abundance of Streptococcus sanguinis was associated with increased hyperactivity (β = 0.26, 95%CI: 0.14-0.26) and ADHD symptom scores (β = 0.24, 95%CI: 0.12-0.36). Roseburia sp. 57 (β = 0.26, 95%CI: 0.14-0.26) was associated with more repetitive responses in CPT-3 test, while Eubacterium eligens (β =0.23, 95%CI: 0.11-0.36) was positively associated with full-scale IQ on WISC-V. Functional pathways including “L-lysine fermentation to acetate and butanoate” and L-arginine biosynthesis III were associated with higher hyperactivity scores and variabilities in completing CPT-3 test, respectively. Additionally, 80 pathways, such as folate transformations III and glycolysis III, were associated with less error incompleting NEPSY-II test.
Conclusion: Our study reveals significant associations between the gut microbiome and neurodevelopmental outcomes in children, with specific microbial species and functional pathways linked to behavior problems and cognitive functions. These findings underscore the potential influence of gut microbiota on neurodevelopment.

P22-07 | Microplastic Exposure Could Affect Immune Responses in Humans: A Pilot Study in Taiwan

Kuan-Chih Chiu[1], Yu-Cheng Chen[2], Pei-Chen Lin[1], Yin-Yi Chen[1], Tsun-Jen Cheng[1]

[1] National Taiwan University, Institute of Environmental and Occupational Health Sciences, Taipei, Taiwan; [2] National Health Research Institutes, National Institute of Environmental Health Sciences, Miaoli, Taiwan

Objective

Microplastics (MPs) are ubiquitous in the environment and have been detected in various human organs and biological samples. While animal studies suggest that MP exposure can negatively impact the immune system, relevant epidemiological studies in humans remains limited. Therefore, we aimed to investigate the associations between MP exposure and biomarkers in humans, and to explore the metabolic pathways potentially affected by MP exposure.

Material and Methods

A total of 50 participants, aged between 20 and 34 years, were recruited from northern Taiwan in 2022. Differential leukocyte counts were performed on whole blood. Serum cytokines were measured through enzyme-linked immunosorbent assay (ELISA). Serum samples were also analyzed for metabolic profiles using ultra-high performance liquid chromatography coupled with time-of-flight mass spectrometer (UHPLC-ToF-MS). MPs were filtered from digested stool samples, and MP particle counts were determined by micro-Fourier transform infrared spectrometry (micro-FTIR). We used generalized additive models (GAMs) to investigate the associations between MP exposure and leukocyte counts, cytokine levels, and metabolic profiles. Kyoto Encyclopedia of Genes and Genomes (KEGG) pathway analysis of candidate metabolites was conducted to explore the underlying mechanisms of MP exposure in humans.

Results

The median MP concentration was 7.48 particles/g stool (interquartile range (IQR) = 8.32), with higher concentrations observed in males. After adjusting for sex in GAMs, high MP exposure was associated with decreased lymphocyte and monocyte counts, and increased levels of interleukin 1β (IL-1β) and IL-6. Metabolome analysis revealed that 23 out of 97 metabolites fluctuated in association with MP exposure. The citrate cycle (TCA cycle) was identified as the most significantly affected pathway.

Conclusion

Metabolites in the TCA cycle play a role in regulating immune responses. Therefore, MP exposure may perturbate the TCA cycle and further influence inflammatory processes in humans.This study was supported by National Science and Technology Council, Taiwan (NSTC 112-2621-M-002-014-MY3).

P22-08 | Human metabolism and urinary excretion of Imidaclothiz and Cycloxaprid after a single oral dose in a human volunteer

Sonja A. Wrobel[1], Danielle E. Que[2,1], Holger M. Koch[1], Thomas Brüning[1], Heiko U. Käfferlein[1]

[1] Ruhr University Bochum, Institute for Prevention and Occupational Medicine of the German Social Accident Insurance (DGUV), Bochum, Germany; [2] The University of Queensland, Queensland Alliance for Environmental, Health Sciences (QAEHS), Brisbane, Australia

Neonicotinoids are the most commonly used insecticides worldwide and two of them, imidaclothiz and cycloxaprid, have been recently launched on the market. Both compounds are structurally similar to imidacloprid. However, no human biomarkers of exposure have been reported yet.

Here, we studied the human metabolism and excretion of imidaclothiz and cycloxaprid after a single oral dose at the current acceptable daily intake in a single male volunteer. Consecutive post-dose urine samples were collected up to 48 hours and a suspect screening of tentative metabolites was carried out using liquid chromatography coupled to high-resolution mass spectrometry (LC-HRMS). Screening hits were identified based on their accurate mass, isotope signal masses and ratios, product ion spectra, and excretion kinetics.

For imidaclothiz, extensive metabolization occurred. Similar to the metabolism of imidacloprid, a hydroxylated and olefinic metabolite were found. In addition, the parent compound also was excreted. Interestingly, cycloxaprid was hydrolyzed to nitromethyleneimidazole although hydroxylated and dealkylated metabolites could also be identified at low sensitivities. No peak at the retention time of the neat standard and therefore parent itself could be found.

The study provides first in vivo data on the human metabolism of imidaclothiz and cycloxaprid, a basic requirement to develop reliable human biomonitoring methods for these two compounds in future.

P22-09 | Deciphering the chemical exposome and metabolome of endometriosis using high-resolution mass spectrometry and bioinformatic tools: Preliminary results from the EndoxOmics project – A case-cohort study

German Cano-Sancho[1], Sabine Naudin[2], Meije Mathe[3], Axel Raux[1], Oceane Mohamed[2], Yann Guitton[1], Thérèse Truong[2], Ronan Pommereuil[1], Bruno Le Bizec[1], Francesca Romana Mancini[2], Clement Frainay[3], Jean-Philippe Antignac[1], Fabien Jourdan[3,4], Marina Kvaskoff[2]

[1] Oniris, INRAE, LABERCA, Nantes, France; [2] National Institute of Health and Medical Research (Inserm), Exposome and Heredity team, Centre for Research in Epidemiology and Population Health (CESP), Villejuif, France; [3] Université de Toulouse, INRAE, ENVT, INP-Purpan, UP, Toxalim (Research Centre in Food Toxicology), Toulouse, France; [4] MetaToul-MetaboHUB, National Infrastructure of Metabolomics and Fluxomics, Toulouse, France

A growing list of endocrine and metabolic disrupting chemicals such as persistent organic pollutants (POPs) are suspected to be involved in the development of endometriosis. However, little is known about the role of the metabolism on the link between chemical pollutants and endometriosis.

To address this gap, we conducted a metabolome-wide case-cohort study involving blood samples from 120 surgically-confirmed endometriosis cases and 462 randomly sampled non-cases from the French E3N prospective cohort study. Blood levels of 42 POPs were measured by liquid or gas chromatography coupled to mass spectrometry. We used liquid chromatography coupled to high-resolution mass spectrometry to measure the concentrations of 417 metabolites and lipids, and to conduct a non-targeted analysis (NTA) of endogenous and exogenous compounds. Weighted Cox models were used to calculate endometriosis hazard ratios (HR) and 95% confidence intervals (CI) for the associations between POPs, metabolites, and endometriosis diagnosis. In order to support the chemical annotation and hypothesis generation processes, we built a knowledge-mining computational method resulting in a knowledge network linking 295 concepts and 3,795 compounds associated to endometriosis based on MeSH concepts and ChEBI ontologies.
The preliminary results showed positive associations between four per- and polyfluoro alkylated substances (PFAS) and endometriosis diagnosis risk (p.e. N-MeFOSAA [HRQ4vs.Q1= 4.14, 95%CI= 1.25, 13.74], PFUnDA [HRQ4vs.Q1= 3.25, 95%CI= 1.13, 8.74]). In turn, twelve metabolites and lipids were associated with endometriosis risk (false discovery rate <0.10), including two bile acids that showed inverse associations with endometriosis risk: glycochenodeoxycholic acid (HR=0.47, 95%CI: 0.32, 0.69) and taurodeoxycholic acid (HR=0.51, 95%CI: 0.32, 0.79). The knowledge-mining approach enabled the identification of compound-concept relationships, simplifying hypothesis generation and adverse outcome pathway building, deepening our understanding of POPs mixtures' role in endometriosis. Preliminary results from this project support a role of the metabolome in the association between exposure to POPs and endometriosis.

P23 | Per- and Poly-fluoroalkyl Substances: Sources, Measurements, Trends, and Health Effects - PART I

P23-01 | Per- and polyfluoroalkyl substances and incident metabolic syndrome: The Study of Women's Health Across the Nation Multi-Pollutant Study

Benjamin R. Kessler[1], Victoria Zhang[1], Carrie Karvonen-Gutierrez[1], William H. Herman[1,2], Bhramar Mukherjee[3], Sung Kyun Park[1,4]

[1] University of Michigan, Department of Epidemiology, Ann Arbor, USA; [2] University of Michigan, Department of Internal Medicine, Ann Arbor, USA; [3] Yale University, Department of Biostatistics, New Haven, USA; [4] University of Michigan, Department of Environmental Health Sciences, Ann Arbor, USA

Objective: Per- and polyfluoroalkyl substances (PFAS) contribute to lipid and insulin dysregulation, central mechanisms of metabolic syndrome (MetS). However, cross-sectional studies of MetS have conflicting findings. We therefore examined associations between PFAS, incident MetS, and longitudinal changes in its components.
Material and Methods: This study included participants from the Study of Women Across the Nation Multi-Pollutant Study with follow-up visits from 1999-2000 to 2015-2016. Baseline serum PFAS concentrations were quantified using online solid-phase extraction coupled to high-performance liquid chromatography-tandem mass spectrometry. MetS was defined as having three or more of the following: race-specific abdominal obesity, impaired fasting glucose or antihyperglycemic use, hypertension or antihypertensive use, low HDL cholesterol, high triglycerides. Cox proportional hazard models (n=995) estimated hazard ratios (HR) and 95% confidence intervals. Linear mixed effect models (n=1,376) were used to estimate change in the continuous components of MetS. PFAS mixtures were assessed using quantile g-computation.
Results: There were 238 incident cases of MetS over 13,623 person-years. Those in the highest exposure tertile relative to the lowest had adjusted HRs of 1.59 (1.03-2.46, p-trend=0.04) for linear perfluorooctanoic acid (PFOA) and 1.67 (1.08-2.6, p-trend=0.02) for total PFOA. The same contrast was associated with a fasting glucose increase of 3.35% (1.43-5.3, p-trend<0.01) for total perfluorooctane sulfonic acid (PFOS) and 2.16% (0.24-4.11, p-trend=0.02) for linear PFOA as well as a waist circumference increase of 3.79% (1.66-5.97, p-trend<0.01) for total PFOA, 4.24% (2.19-6.32, p-trend<0.01) for total PFOS, and 3.57% (1.46-5.73, p-trend<0.01) for linear PFOA. A two-tertile increase in a PFAS mixture was associated with a 2.77% (0.53-5.06) increase in fasting glucose and a 4.55% increase in waist circumference (1.66-7.52).**Conclusion:** PFOA and PFOS consistently contributed to incident MetS through impaired fasting glucose and central adiposity.

P23-03 | Assessing the Role of Psychosocial Health on Associations between a Per- and Polyfluoroalkyl Substance Mixture and Perinatal Outcomes

Samantha Schildroth[1], Birgit Claus Henn[2], Amelia K. Wesselink[1], Victoria Fruh[2], Donna D. Baird[3], Ganesa Wegienka[4], Lauren A. Wise[1]

[1] Boston University School of Public Health, Department of Epidemiology, Boston, USA; [2] Boston University School of Public Health, Department of Environmental Health, Boston, USA; [3] National Institute of Environmental Health Sciences, Durham, USA; [4] Henry Ford Health System, Detroit, USA

Objective: Per- and polyfluoroalkyl substances (PFAS) are endocrine-disrupting chemicals that can dysregulate reproductive hormones that support healthy pregnancy and have been associated with adverse perinatal outcomes. Less is known about the combined effects of multiple PFAS, or the modifying role of psychosocial factors, which may affect perinatal outcomes through similar mechanisms as PFAS (e.g., hormonal dysregulation).

Methods: We leveraged data from the Study of Environment, Lifestyle, and Fibroids, a prospective cohort study of 1,693 reproductive-aged Black participants recruited in 2010-2012. We evaluated associations between a PFAS mixture and perinatal outcomes reported by 321 participants with live births during ~60 months of follow-up. We quantified concentrations of six PFAS in baseline plasma samples using online solid-phase extraction-liquid chromatography-isotope dilution tandem mass spectrometry. Participants reported social support (via questionnaire) and perceived stress (Perceived Stress Scale-4) at baseline. We used Bayesian Kernel Machine Regression (BKMR) to estimate mean differences (β) in birthweight (grams) and gestational age at birth (weeks) with 95% credible intervals (95% CrIs) across percentiles of the PFAS mixture, adjusting for hypothesized confounders. We evaluated effect measure modification of the PFAS mixture by social support and perceived stress by including these variables in the BKMR kernel function.

Results: The 75th percentile of the PFAS mixture (versus the 50th percentile) showed little association with birthweight (β=-8.8; 95% CrI=-95.3, 77.8) or gestational age (β=-0.1; 95% CrI=-0.2, 0.1). Several individual PFAS, including PFDA and PFHxS, were weakly associated with perinatal outcomes in BKMR univariate exposure response profiles, but directions of association varied by PFAS. There was no evidence of interaction between the PFAS or effect measure modification by social support or perceived stress.

Conclusion: Although some PFAS were individually associated with perinatal outcomes, there was no evidence of combined associations of the mixture, and associations were not modified by social support or perceived stress.

P23-04 | Exposure to PFBS (replacement PFAS) in a New York City-based pregnancy cohort

Eleanor A. Medley[1], Duong Q. Nguyen[2], Anne Nigra[3], Amy Padula[4], Vittorio Albergamo[2], Akhgar Ghassabian[1,2], Linda G. Kahn[1,2], Leonardo Trasande[1,2], Whitney Cowell[1,2]

[1] NYU School of Medicine, Department of Population Health, New York, USA; [2] NYU School of Medicine, Department of Pediatrics, New York, USA; [3] Columbia University Mailman School of Public Health, Department of Environmental Health Sciences, New York, USA; [4] University of California San Francisco, Department of Obstetrics, Gynecology, and Reproductive Sciences, San Francisco, USA

Objective: Perfluorobutane sulfonic acid (PFBS) is a short-chain per- or polyfluoroalkyl substance (PFAS) manufactured as a replacement for the long-chain PFAS, perfluorooctane sulfonic acid (PFOS). Few epidemiologic studies in the US have measured PFBS and relatively little is known about its health implications. We measured serum concentrations of PFBS and 12 other PFAS in the New York University Children's Health and the Environment Study (NYU CHES).

Methods: PFAS concentrations were measured in serum collected from 500 pregnant participants 2017-2020 (median gestational age: 24.7 weeks, range: 12.6-37.7 weeks). We included 7 PFAS with detection rates >50% in analyses. We calculated descriptive statistics for the distributions of each PFAS and Spearman's rank correlation coefficients. We investigated geospatial distributions by mapping PFAS concentrations to geocoded residential addresses.

Results: PFBS was detected at concentrations higher than the limit of quantification (0.025 ng/mL) in 95.8% of the sample. The median PFBS concentration was 0.40 ng/mL and the maximum was 2.10 ng/mL. This is significantly higher than serum PFBS concentrations reported in 11 other US birth cohorts which collectively had a detection rate of 4.4% (n=3339). PFBS concentrations were not correlated with any other PFAS (ρ = 0.03 PFOS, -0.05 PFOA, -0.01 PFDA, -0.05 PFNA, -0.02 PFUnDA, 0.04 PFHxS), while these other PFAS were moderately-to-highly correlated with each other (ρ = 0.38-0.83). There was no evidence for spatial clustering of PFBS across New York City (Moran's I = -0.009, p = 0.36). PFBS has been reported in one of the city's drinking water sources at a concentration of 2.6-3 ng/L in 2023, similar levels to the more regulated compounds PFOS (2.3-2.7 ng/L) and PFOA (2.8-3.3 ng/L).

Conclusion: These results suggest there is rising exposure to short-chain replacement PFAS, exemplified by serum PFBS concentrations in a population of pregnant individuals in New York City.

Funding: NIH UG3/UH3OD023305

P23-05 | Prenatal exposure to per- and polyfluoroalkyl substances and maternal thyroid function in a cohort with high familial likelihood of autism spectrum disorder

HyunA Jang[1], Deborah H. Bennett[2], Andrew N. Hoofnagle[3], Elizabeth N. Pearce[4], Daniel J. Tancredi[5], Rebecca J. Schmidt[2,6], Hyeong-Moo Shin[1]

[1] Baylor University, Department of Environmental Science, Waco, USA; [2] University of California, Department of Public Health Sciences, Davis, USA; [3] University of Washington, Department of Laboratory Medicine and Pathology, Seattle, USA; [4] Boston University Chobanian & Avedisian School of Medicine, Section of Endocrinology, Diabetes, and Nutrition, Department of Medicine, Boston, USA; [5] University of California Davis, Department of Pediatrics, Sacramento, USA; [6] University of California Davis, MIND Institute, Sacramento, USA

Background: Maternal thyroid hormones (THs) are essential for fetal brain development but known to be disrupted by per- and polyfluoroalkyl substances (PFAS). As abnormal gestational thyroid function is a known risk factor for child autism spectrum disorder (ASD), this study examined the effects of prenatal PFAS exposure on thyroid function in pregnant women of MARBLES (Markers of Autism Risk in Babies – Learning Early Signs), who are at elevated likelihood of having a child with ASD.

Methods: We selected 152 MARBLES pregnant women who provided multiple blood samples (n = 214) during pregnancy and who delivered a child with a final diagnosis (ASD, typical development [TD], non-TD). In maternal serum, we quantified nine PFAS, three THs (total triiodothyronine [TT3], total thyroxine [TT4], and free T4 [FT4]) and thyroid-stimulating hormone (TSH). For five PFAS with a detection rate of >80%, we used robust regression to assess the co-exposure effects of PFAS and principal component analysis and the generalized additive model to evaluate PFAS mixture effects. We also conducted sensitivity analyses by excluding samples from mothers who tested positive for thyroid antibodies and by combining ASD and non-TD groups.

Results: In the non-TD group, excluding antibody positive samples, higher perfluorodecanoic acid (PFDA) exposure was associated with overactive thyroid (β = 0.14, 95% CI: 0.04, 0.23 for FT4; β = -0.97, 95% CI: -1.83, -0.10 for TSH). In combined ASD and non-TD groups, higher perfluorooctanoic acid (PFOA) exposure was associated with underactive thyroid (β = -0.09, 95% CI: -0.16, -0.01 for FT4; β = 0.36, 95% CI: 0.11, 0.61 for TSH). Additionally, in the TD group, PFAS mixture showed bell-shaped associations with TT3 levels, while higher PFAS mixture was associated with higher TT4 levels.

Conclusion: Our findings suggest that maternal PFAS exposure is associated with thyroid function in children with neurodevelopmental concerns.

P23-06 | Assessing Human Exposure to Per- and Polyfluoroalkyl Substances (PFAS) Through Seafood Consumption From the Delaware Bay

Jacqueline Carroll, Mi-Ling Li

University of Delaware, School of Marine Science and Policy, Newark, USA

Per- and polyfluoroalkyl substances (PFAS) are a group of highly persistent, man-made chemicals known to cause adverse ecological and human health effects. Seafood consumption has been identified as an important dietary source of PFAS for human populations. It is observed that populations of recreational anglers consume more seafood than the general population, making them particularly susceptible to high levels of contaminant exposures from fish consumption. Prior research revealed that recreational fish species in the Delaware Bay generally exhibit higher PFAS concentrations than those in the US commercial seafood market. This study aims to assess human exposures to PFAS in recreational anglers through the consumption of self-caught fish from the Delaware Bay. We conduct seasonal fish consumption surveys that include fish consumption patterns and demographic information for recreational anglers who consume wild-caught fish from the Delaware Bay. We calculate exposure dose based on the self-reported fish consumption results and corresponding tissue concentrations of recreational fish species. Monte Carlo simulations are conducted to develop the current likely exposure distribution. We explore how changes in parameters like consumption frequency, portion size, fish species and socioeconomic factors affect the exposure distribution. These models reflect hypothetical scenarios that represent PFAS exposure reduction strategies in marine anglers. We anticipate the knowledge learned in this study can be extended to marine anglers in other regions and inform regulatory guidelines related to fish consumption advisories for mitigating human PFAS exposure.

P23-07 | Sociodemographic predictors of PFAS exposure among a pooled sample of U.S. pregnant women: an Environmental Influences on Child Health Outcomes (ECHO) cohort analysis

Jessie A. Gleason[1], Jerald Fagliano[1], Kristen Lyall[1], Lucy Robinson[1], Gloria Post[2], Anneclaire De Roos[1]

[1] Drexel University, Environmental and Occupational Health, Philadelphia, USA; [2] Retired NJ Department of Environmental Protection, Trenton, USA

Per- and polyfluoroalkyl substances (PFAS) are chemicals widely used in the manufacturing of commercial and industrial products since the 1940s; and associated with developmental and immune effects at levels observed in the general population. **Objective:** To evaluate exposure differences to 14 PFAS by sociodemographic factors among U.S. pregnant women. **Materials and Methods:** We pooled maternal PFAS measurements from twelve pediatric cohorts drawn from the Environmental influences on Child Health Outcomes (ECHO) consortium (n=3,236). Geometric means were estimated by race, ethnicity, maternal education, and cohort. We used multivariable linear regression to estimate percent changes in PFAS levels and logistic regression to assess odds of detectable PFAS levels by sociodemographic factors. **Results:** Mothers reporting Black race had lower levels of PFOA (-25% change; 95% CI -31%, -18%), PFOS (-10%; 95% CI -17%, -3%), PFHxS (-22%; 95% CI -29%, -14%), EtFOSAA, PFDoDA, and PFPeA compared with White mothers, with the exception of higher PFBS and PFHxA levels. Asian mothers had

higher levels of PFNA (+30%; 95% CI 16%, 45%), PFDA (+69%; 95% CI 40%, 104%), PFUnDA, PFDoDA, and PFPeA compared with White mothers. Greater weekly fish consumption was found to be a significant predictor of PFAS concentrations and did not generally attenuate associations with race (e.g. weekly fish meals >1.69 compared with less than 0.23 for PFNA: +17% change; 95% CI 5%, 29%; for PFDA: +34%; 95% CI 1%, 78%). Hispanic ethnicity was associated with lower levels of most of the PFAS analytes studied, compared with non-Hispanic ethnicity. Higher levels of maternal education were associated with increased levels of most PFAS analytes. **Conclusions:** In data drawn from this large US consortium, our work supports continued widespread PFAS exposure, with higher levels noted for certain racial groups, non-Hispanic ethnicity, higher educational attainment, and according to fish intake. **Funding:** NIH Award number 1R36OD037668-01

P23-08 | Analysis of ultra-short and short-chain per/polyfluoroalkyl substances (PFAS) in human urine

Kiran Ali, N.L.Dilani Perera, Carrie McDonough

Carnegie Mellon University, Chemistry, Pittsburgh, USA

Per- and polyfluoroalkyl substances (PFAS) are widespread in the environment and biota due to their persistence, aquatic mobility, and bioaccumulation potential. Due to their shorter biological residence times and lower bioaccumulation potential, short-chain (SC) and ultra-short chain (USC) PFAS were introduced as alternatives to longer-chain PFAS. However, studies have reported their presence in environmental matrices and humans. Due to their solubility, SC and USC PFAS do not accumulate to the same extent as longer-chain PFAS in blood, but they are excreted fairly readily in urine. This suggests urine may be a useful biomonitoring matrix for SC and USC PFAS. This study aims to optimize sample preparation and liquid chromatography high resolution mass spectrometry (LC-HRMS) analysis for SC and USC PFAS in human urine using a polar embedded analytical column. Sample preparation via liquid-liquid extraction (LLE) and solid-phase extraction (SPE) were compared for optimal recoveries and detection limits. Thus far, average recoveries of 100 ± 30% were obtained for 42 (out of 60) analytes in LLE experiments and 22 analytes in SPE experiments. Detection limits for LLE-prepared samples ranged from 0.01 ng/mL to 14 ng/mL, whereas detection limits for SPE ranged from 0.02 ng/mL to 20.3 ng/mL. Here, these methods will be compared to solid-phase microextraction (SPME) strategies and analysis of real samples from occupationally-exposed individuals will be discussed.

P23-09 | Gestational Per- and Polyfluoroalkyl Substance (PFAS) Exposure and Infant Non-Nutritive Suck in Two Birth Cohorts

Lauren B. Ellis[1], Emily Zimmerman[2], Max Aung[3], Megan Woodbury[4], Andréa Aguiar[5], Susan Schantz[5], John Meeker[6], Akram Alshwabakeh[4], José F. Cordero[7], Julia Varshavsky[1,4]

[1] Northeastern University, Department of Public Health and Health Sciences, Boston, USA; [2] Northeastern University, Department of Communication Sciences and Disorders, Boston, USA; [3] University of Southern California, Keck School of Medicine, Department of Population and Public Health Sciences, Los Angeles, USA; [4] Northeastern University, Department of Civil and Environmental Engineering, Boston, USA; [5] University of Illinois Urbana-Champaign, Beckman Institute for Advanced Science and Technology and Department of Comparative Biosciences, Urbana, USA; [6] University of Michigan School of Public Health, Department of Environmental Health Sciences, Ann Arbor, USA; [7] University of Georgia, Department of Epidemiology and Biostatistics, Athens, USA

Objective: Per- and polyfluoroalkyl substances (PFAS) have been associated with adverse childhood neurodevelopment, but no studies have explored their association with non-nutritive suck (NNS)—an early measure of neurofunction.We examined the relationship between gestational PFAS exposure and infant NNS in a sample of 191 mother-infant pairs enrolled in the Puerto Rico Testsite for Exploring Contamination Threats (PROTECT, n=119) and Illinois Kids Development Study (IKIDS, n=72) prospective birth cohorts.

Materials and Methods: Nine PFAS were measured in maternal serum during pregnancy and six NNS outcomes (duration, frequency, amplitude, cycles/burst, bursts/minute, and cycles/minute) were measured in 1- to 8-week-old infants using a custom research pacifier. We used linear regression and quantile g-computation to assess associations between PFAS (individually and as a mixture) and NNS outcomes in the separate and combined cohorts, adjusting for maternal age and education.

Results: Some individual PFAS (particularly PFOS, PFOA, and PFNA) were consistently associated with longer NNS duration (seconds) and more NNS cycles/burst and cycles/minute. For example, ln-transformed PFOS was significantly associated with longer ln-transformed NNS duration (adjusted $\beta_{\text{ln-PFOS}}$ [95%CI] = 0.25 [0.12, 0.38]), more NNS cycles/minute (10.53 [5.14, 15.92]), and more ln-transformed NNS cycles/burst (0.28 [0.14, 0.41]) in the combined cohort. The PFAS mixture (which included PFOA, PFOS, PFNA, and PFHxS; >50% detection frequency) was similarly associated with longer ln-transformed NNS duration (adjusted β_{Mixture} [95%CI] = 0.11 [0.02, 0.20]) and more ln-transformed NNS cycles/burst (0.13 [0.04, 0.22]) in the combined cohort.

Conclusion: We found associations between gestational PFAS exposure and altered NNS patterns during early infancy. Given research linking longer NNS duration and more NNS cycles/minute and cycles/burst at three months to developmental delays at 12 months, our findings may indicate potential adverse neurodevelopmental effects later in childhood. Further research is needed to better understand this relationship.

Funding: US NIH/NIEHS (P42ES017198, R01ES032203, P30ES007048, OD023272).

P23-10 | "Mechanisms driving Per- and Polyfluoroalkyl Substances (PFAS) Disposition in Human Breast Milk: Roles of Efflux Transporters, Permeability, and Binding"

Liam N. Geyer[1], Grayson Norman[1], Šimon Vojta[2], Jitka Becanova[2], Roberta King[1], Fabian C. Fischer[1]

[1] University of Rhode Island, College of Pharmacy, Kingston, USA; [2] University of Rhode Island, Graduate School of Oceanography, Kingston, USA

Objective: Per- and polyfluoroalkyl substances (PFAS) are widespread environmental pollutants linked to neurodevelopmental disorders in children. Maternal transfer of PFAS through breastfeeding represents a critical exposure pathway during early life. This study investigated the toxicokinetic mechanisms driving PFAS transfer into breast milk by evaluating interactions with the efflux transporter Breast Cancer Resistance Protein (BCRP), passive membrane permeability, and PFAS binding in human serum and breast milk.

Materials/Methods: PFAS binding in human serum and breast milk was quantified using solid-phase microextraction with C18 fibers. Passive permeability and BCRP-mediated transport were assessed in trans-well *in vitro* assays using transporter knock-out and BCRP knock-in cell lines and the results were compared to molecular docking simulations using the Molecular Operating Environment (MOE) software.

Results: PFAS binding in breast milk increased linearly with molecular weight for sulfonic acid and sulfonamide groups. In contrast, carboxyl and fluorotelomer PFAS followed this trend only up to 500 g/mol, after which binding declined. BCRP-mediated transport increased with molecular weight, with efflux ratios ranging from 10- to 50-fold higher in BCRP-expressing cells compared to inhibited controls, agreeing with molecular docking simulations. Similarly, passive permeability increased with molecular weight, with the highest permeability observed for PFAS above 500 g/mol.

Conclusions: Our preliminary results show that molecular weight strongly influences PFAS transfer into breast milk, with higher molecular weight compounds exhibiting increased binding, greater passive permeability, and enhanced BCRP-mediated transport. The mechanistic data will be integrated into a physiologically based toxicokinetic model to simulate PFAS transfer into breast milk under human physiological conditions, accounting for inter-individual variability such as BCRP polymorphisms and hypoalbuminemia as potential risk factors for disproportionate transfer of PFAS to the breastfeeding infant during lactation.

Acknowledgment: All reported research was supported by the University of Rhode Island, under Fund 5U54GM115677-09

P23-11 | PFAS-Associated Metabolic Disruptions After Weight Loss: A Longitudinal Metabolomics Analysis

Zhenjiang Li[1], Brittney O. Baumert[1], Elizabeth Costello[1], Shudi Pan[1], Hongxu Wang[1], Sarah Rock[1], Justin R. Ryder[2,3], Todd Jenkins[4,5], Stephanie Sisley[6], Xiangping Lin[7], Scott M. Bartell[8], Thomas H. Inge[2,3], Stavra Xanthakos[9], Brooklynn NcNeil[10], Anna R. Robuck[11], Michele A. La Merrill[12], Sandrah P. Eckel[1], Rob McConnell[1], David V. Conti[1], Douglas I. Walker[13], Lida Chatzi[1]

[1] University of Southern California, Department of Population and Public Health Sciences, Los Angeles, USA; [2] Northwestern University, Department of Surgery, Chicago, USA; [3] Ann & Robert H. Lurie Children's Hospital of Chicago, Chicago, USA; [4] Cincinnati Children's Hospital Medical Center, Division of Biostatistics & Epidemiology, Cincinnati, USA; [5] University of Cincinnati, Department of Pediatrics, Cincinnati, USA; [6] Baylor College of Medicine, Department of Pediatrics, Houston, USA; [7] Stanford University, Department of Genetics, Stanford, USA; [8] University of California, Irvine, Irvine, USA; [9] Cincinnati Children's Hospital Medical Center, Division of Gastroenterology, Hepatology, Nutrition, Cincinnati, USA; [10] Columbia University, Irving Institute for Clinical and Translational Research, New York, USA; [11] University of Rhode Island, Graduate School of Oceanography, Narragansett, USA; [12] University of California, Davis, Department of Environmental Toxicology, Davis, USA; [13] Emory University, Gangarosa Department of Environmental Health, Atlanta, USA

Objective: Per- and polyfluoroalkyl substances (PFAS) are ubiquitous environmental contaminants linked to adverse health effects. Prior studies have explored PFAS-associated metabolic disruptions, but longitudinal assessments of metabolic alterations following significant physiological changes, such as weight loss interventions, remain limited. This study leverages untargeted metabolomics to investigate PFAS-associated metabolic perturbations in adolescents undergoing bariatric surgery.

Material and Methods: We conducted a metabolome-wide association study (MWAS) using plasma samples from 214 participants in the Teen-Longitudinal Assessment of Bariatric Surgery (Teen-LABS) study. PFAS concentrations, including PFOS, PFHxS, PFHpS, PFOA, PFNA, PFDA, PFHpA, and PFUnDA, were measured pre-surgery, and metabolomic measurements were assessed from samples at surgery, and 0.5, 1, and 3 years post-surgery. We developed a linear mixed-effect model with random intercepts by participants to evaluate the associations between PFAS exposure and longitudinal changes in metabolic measures, with a product term between PFAS and time since baseline. . We performed pathway enrichment analysis to identify biologically relevant metabolic perturbations.

Results: Our findings reveal distinct PFAS-associated metabolic features over time, with key metabolites such as aminoisobutanoate, creatinine, and hydroxymyristic acid exhibiting significant associations with specific PFAS. Among them, hydroxymyristic acid showed the broadest association, being linked to PFOS (one-unit increase associated with -7.2% in hydroxymyristic acid), PFHxS (-11.3%), and PFHpS (-89.0%). Pathway enrichment analyses identified associated pathways primarily in lipid metabolism, amino acid metabolism, and xenobiotic processing pathways. Notably, PFOA was linked to the greatest number of metabolic pathways (16), predominantly involved in lipid and amino acid metabolism, suggesting a key role in metabolic dysregulation. Additionally, linoleate metabolism emerged as a central pathway, associated with PFOS, PFOA, PFHpS, and PFDA, indicating a potential shared mechanism of metabolic disruption across different PFAS compounds.

Conclusion: PFAS-perturbed metabolic pathways highlight the need for further research on PFAS-associated biological mechanisms and weight-loss interventions.

Funding: NIEHS and NIDDK, US.

P23-12 | Combined Effect of Urinary Metals, Per- and Polyfluoroalkyl Substances, Polycyclic Aromatic Hydrocarbons, Phthalates, and Plasticizer Metabolites on Cardiovascular Disease Risk

Doreen N. Jehu-Appiah, Emmanuel Obeng-Gyasi

North Carolina A&T State University, Greensboro, USA

This study investigated the combined effects of urinary metals, per- and polyfluoroalkyl substances (PFAS), polycyclic aromatic hydrocarbons (PAHs), phthalates, and plasticizer metabolites on cardiovascular biomarkers using National Health and Nutrition Examination Survey (NHANES) 2013–2014 data. The analysis included 15 metals, 10 PFAS, 14 phthalates/plasticizer metabolites, and 7 PAHs. Descriptive statistics, linear regression, Spearman correlation, and Bayesian Kernel Machine Regression (BKMR) were used to examine associations with systolic blood pressure (SBP), high-density lipoprotein (HDL), low-density lipoprotein (LDL), total cholesterol, and triglycerides.
Spearman correlation analysis indicated that metals, PFAS, PAHs, phthalates, and plasticizer metabolites were highly correlated. BKMR posterior inclusion probability (PIP) analysis indicated that phthalates and plasticizer metabolites had the highest PIP for HDL, metals and PFAS for LDL, and PAHs for SBP, while phthalates and PFAS influenced total cholesterol.
Triglycerides were mainly affected by phthalates and plasticizer metabolites. Univariate exposure-response analysis showed complex nonlinear associations, with PFAS demonstrating the most variability across cardiovascular markers.
The overall exposure effect, which assessed the combined exposure to all pollutants on cardiovascular disease (CVD) markers, revealed that increasing pollutant exposure consistently decreased HDL levels while increasing LDL and SBP, heightening cardiovascular risk. Total cholesterol followed a U-shaped trend, and triglycerides displayed a quantile-dependent effect, where lower exposures were associated with higher triglycerides, but higher exposures showed a decline, potentially due to metabolic saturation.
Single-variable interaction analysis suggested PAHs most strongly interacted with other pollutants for HDL, while PFAS and metals did so for LDL and SBP. Phthalates, plasticizer metabolites, and PFAS were key for total cholesterol and triglycerides. These findings underscore the complexity of multi-pollutant exposure effects on cardiovascular health, highlighting the intricate interactions among contaminants and emphasizing the need for comprehensive mixture analysis to better understand their combined impact.

P23-13 | Association of Per- and Polyfluoroalkyl Substances with Pan-Cancers Associated with Sex Hormones

Elizabeth O. Olarewaju, Emmanuel Obeng-Gyasi

North Carolina A&T State University, Built Environment, GREENSBORO, USA

Per- and polyfluoroalkyl substances (PFAS) are ubiquitous environmental contaminants with potential endocrine-disrupting properties. This study examines the association between exposure to multiple PFAS and pan-cancers associated with sex hormones (PCSH).

We analyzed data from the National Health and Nutrition Examination Survey (NHANES) spanning 2-year cycles from 1999 to 2012, including 14,373 participants. Serum concentrations of six PFAS (PFOA, PFOS, PFHxS, PFDE, PFNA, and PFUA) were assessed for their relationship with PCSH. Statistical analyses included descriptive statistics, Spearman and Pearson correlation analyses, and both linear and logistic regression models. Additionally, Bayesian Kernel Machine Regression (BKMR) was applied to capture potential non-linear relationships and interactions.

Initial t-tests showed a statistically significant difference in PFOS levels between individuals with and without PCSH (p=0.0022), with higher mean PFOS levels in the PCSH group. Chi-square tests revealed a significant association between ethnicity and PCSH (p<0.001). Linear and logistic regression analyses revealed signficant associations for PFOS. BKMR analysis identified PFOA as having the highest posterior inclusion probability, indicating its importance in explaining PCSH risk. Univariate exposure-response analysis revealed limited individual PFAS effects. However, bivariate analysis indicated a complex U-shaped interaction pattern among many joint PFAS assessments. Overall exposure effect analysis suggested that the combined impact of all PFAS was more strongly associated with PCSH at exposure levels below the 0.5 quantile compared to higher levels. Single-variable interaction analyses highlighted PFOA and PFOS as the most interactive PFAS when testing their interaction with combined exposure to all other PFAS.

In summary, while initial findings suggested a positive association between PFOS and PCSH, BKMR analysis revealed complex non-linear relationships and interactions among PFAS. These findings emphasize the importance of evaluating PFAS as a mixture rather than as individual chemicals and using techniques that can capture non-linear relationships and interactions.

P23-14 | A Hybrid Targeted and Untargeted Analytical Framework for Assessing Per- and Polyfluoroalkyl Substances (PFAS) in Human Milk

Aryan D. Patel[1], Catherine E. Mullins[1], Donghai Liang[1], David Conti[2], Lida Chatzi[2], Michael Goran[3], Tanya L. Alderete[4], Douglas I. Walker[1]

[1] Emory University, Gangarosa Department of Environmental Health, Rollins School of Public Health, Atlanta, USA; [2] University of Southern California, Department of Population and Public Health Sciences, Keck School of Medicine, Los Angeles, USA; [3] Saban Research Institute, Department of Pediatrics, Children's Hospital Los Angeles, Los Angeles, USA; [4] Johns Hopkins University, Department of Environmental Health and Engineering, Johns Hopkins Bloomberg School of Public Health, Baltimore, USA

Objective: For this study, we developed a hybrid targeted and untargeted liquid-chromatography high-resolution mass spectrometry (LC-HRMS) method to quantify 24 per- and polyfluoroalkyl substances (PFAS) in human milk while also enabling screening for novel and emerging PFAS-related compounds.
Material and Methods: Human milk samples were collected at 1- and 6-months from lactating mothers enrolled in the Southern California Mother's Milk Study. PFAS were extracted and prepared for analysis using an Opentrons Flex automated liquid handling system and 96-well plate format designed to facilitate high-throughput sample preparation requiring minimal volumes of human milk (<500 µL). Samples were analyzed using C18 chromatography interfaced to a Thermo Exploris 120 HRMS. Quality control measures included the use of 13C isotopically labeled internal standards and repeat analysis of NIST 1954. Novel PFAS were identified by comparison to a library of 150 PFAS standards and 13,054 PFAS compounds present in the EPA CompTox Chemical Dashboard.
Results: A semi-automated, targeted and untargeted LC-HRMS method was developed for quantification of known PFAS while enabling screening for novel and emerging PFAS. Targeted PFAS compounds were detected in a significant proportion of the human milk including perfluorooctanesulfonic acid (PFOS) and perfluorooctanoic acid (PFOA) that were frequently present at 1- and 6-months postpartum. Comparison to a PFAS library and databases identified additional novel PFAS, which were found to vary across the two time points.
Conclusions: Our methods can detect the presence of legacy and emerging PFAS in human milk using low sample volumes (<500 µL). While the detected concentrations are generally low, cumulative exposures to PFAS may be important early life. Thus, further research is needed to assess the health outcomes associated with early-life exposure to both legacy and emerging PFAS compounds.

P23-15 | Competitive Inhibition of Cytochrome P450 Enzymes by Per- and Polyfluoroalkyl Substances (PFAS) in Human Liver Microsomes

Nick Ferguson[1], Kimberly Lapham[2], Fabian Fischer[1]

[1] University of Rhode Island, College of Pharmacy, Kingston, USA; [2] Pfizer, Pharmacokinetic and Drug Metabolism, Groton, USA

Per- and polyfluoroalkyl substances (PFAS) are environmental contaminants with widespread human exposure. Studies using immortalized hepatic cells suggested that PFAS can interact with cytochrome P450 (CYP) enzymes, potentially altering drug metabolism, affecting treatment efficacy, and increasing the risk of drug toxicity. We aimed to provide physiologically relevant insights into the impact of PFAS exposures on hepatic metabolism by measuring the half-maximal inhibitory concentrations (IC50) for thirteen legacy and emerging PFAS on CYP1A1, CYP2B6, CYP2C8, CYP2C9, CYP2C19, CYP2D6, and CYP3A4 enzyme activities using pooled human liver microsomes (HLM).

PFAS were incubated at concentrations ranging from 0.4 to 400 µM in mixtures with HLM at protein levels between 0.01 and 0.1 mg/mL and selected CYP substrates dosed at concentrations near their Michaelis-Menten constant. Marker metabolite formation was quantified using liquid chromatography-mass spectrometry, normalized to solvent controls, and fitted to IC50 curves.

The tested PFAS exhibited a wide range of CYP inhibition, from no inhibition (IC50 > 400 µM) to potent inhibition (IC50 < 1 µM). EtFOSA was the most potent inhibitor, particularly for CYP2C19 (IC50 = 0.74 µM). Long-chain perfluorocarboxylates (PFUnDA, PFDoDA, and PFDA) showed strong inhibition of CYP2C8 (1.02–1.7 µM), while PFOA and PFHxS also inhibited CYP2C8 (10.5 µM and 7.75 µM, respectively). Moderate inhibition was observed for PFNA (CYP2C8, 2.56 µM), FOSA (CYP2C19, 7.37 µM), PFOS (CYP2C19, 34.7 µM), MeFOSA (CYP2C19, 28.2 µM), 8:2 FTS (CYP2C8, 12.6 µM), and PFDoDA (CYP2C19, 44.4 µM).

This study demonstrates that PFAS inhibit drug-metabolizing CYP enzymes. EtFOSA, the most potent inhibitor and a PFOS precursor, is found in human livers, suggesting interference with drug metabolism at relevant exposure levels. Integrating these findings into physiologically based toxicokinetic models will evaluate PFAS-drug interactions affecting drug efficacy and toxicity, while accounting for interindividual variability in CYP enzyme activity due to genetic polymorphisms and underlying health conditions.

P23-16 | Per- and Poly-fluoroalkyl Substances (PFAS) and Diabetes: A Systematic Review and Meta-Analysis of Epidemiological Studies

Sandra India Aldana[1], Meizhen Yao[1], Xin Yu[1], Nathan Cohen[1], Stephanie Eick[2], Vishal Midya[1], Anne P. Starling[3], Dinesh Barupal[1], Douglas I. Walker[2], Chris Gennings[1], Ruth J.F. Loos[4,5], Leda Chatzi[6], Veronica W. Setiawan[4], Ryan Walker[1], Elena Colicino[1], Damaskini Valvi[1]

[1] Icahn School of Medicine at Mount Sinai, Department of Environmental Medicine, New York, USA; [2] Emory University, Rollins School of Public Health, Atlanta, USA; [3] University of North Carolina Chapel Hill, Gillings School of Global Public Health, Chapel Hill, USA; [4] Icahn School of Medicine at Mount Sinai, Charles Bronfman Institute for Personalized Medicine, New York, USA; [5] University of Copenhagen, Novo Nordisk Foundation Center for Basic Metabolic Research, Faculty of Health and Medical Sciences, Copenhagen, Denmark; [6] University of Southern California, Keck School of Medicine, Los Angeles, USA

Objective

We conducted a comprehensive systematic review and meta-analysis of epidemiological studies to characterize the associations of exposures to per- and polyfluoroalkyl substances (PFAS) with diabetes and markers of insulin resistance and glucose homeostasis.

Material/Methods

A systematic search of publications through 01/28/2025 was conducted by two researchers in PubMed/MEDLINE and Ovid/EMBASE following PRISMA guidelines (PROSPERO-ID:CRD42022369711). Risk of bias was evaluated using the Navigation Guide. Random-effects meta-analyses stratified by study design were performed to estimate PFAS (per-doubling) associations with diabetes outcomes, including gestational diabetes mellitus (GDM), type 2 diabetes (T2D), and continuous HOMA-IR, HOMA-β, fasting insulin, fasting glucose, and HbA1c.

Results

We identified 114 eligible studies, most of which focused on at least one PFAS and diabetes outcome, with only three studies focusing on T1D. In meta-analyses of prospective studies, we found higher GDM odds [OR (95%CI)] for PFOA [n=7, 1.09 (0.97, 1.22)], PFOS [n=7, 1.12 (1.01, 1.25)], and PFHxS [n=6, 1.10 (1.00, 1.23)], among others. Associations with T2D odds were inconsistent, with a positive association observed only for PFHpS [n=4, 1.58 (1.07, 2.33)]. We also found positive associations with HOMA-IR [prospective studies β (95% CI)] for PFOS [n=5, 0.07 (0.01, 0.13)], PFOA [n=6, 0.05 (-0.01, 0.12)], and PFNA [n=4, 0.12 (-0.03, 0.26)]. HOMA-β was further associated [cross-sectional studies β (95% CI)] with PFOA [n=6, 5.80 (0.04, 11.6)], PFOS [n=6, 5.93 (1.72, 10.2)], and PFNA [n=4, 3.35 (0.12, 6.58)]. Fasting insulin was positively associated [prospective studies β (95% CI)] with PFOA [n=5, 0.29 (0.11, 0.48)], PFOS [n=4, 0.22 (0.04, 0.39)], and PFNA [n=3, 0.29 (0.08, 0.50)]. Associations with fasting glucose and HbA1c were mostly null.

Conclusion

Findings support an association between PFAS exposures and higher GDM odds, and with markers of insulin resistance and pancreatic β-cell function. Emerging PFAS are understudied, while PFAS-T1D research remains scarce.

P23-17 | Serum Per- and Polyfluoroalkyl Substances (PFAS) and High Cholesterol: Examining Effect Modification by Environmental and Social Factors in Orange County, CA Using the CDC/ATSDR Environmental Justice Index (EJI)

Karen S. Valladares, Jesselle M. Legaspi, Veronica M. Vieira, Scott M. Bartell

University of California, Irvine, Department of Environmental and Occupational Health, Joe C. Wen School of Population & Public Health, Irvine, USA

OBJECTIVE This study examines whether associations between PFAS exposure and self-reported high cholesterol in Orange County, CA, vary by CDC/ATSDR Environmental Justice Index (EJI) scores.
MATERIAL AND METHODS We used data from the California site of the PFAS Multi-Site Study, recruiting participants from 2021 to 2023 based on residence in eligible cities from 2000 to 2019. Participants completed a questionnaire and provided blood samples. Serum concentrations of seven PFAS compounds were measured, with those detected in <70% of samples treated as binary variables. The outcome was self-reported high cholesterol, dichotomized as a yes/no response based on a prior diagnosis by a healthcare provider. EJI modules, including social vulnerability (SVM), environmental burden (EBM), and a combined social-environmental ranking (SER), were analyzed as continuous variables and tertiles, assigned at the census tract level based on current residence. Logistic regression models adjusted for age, sex, race/ethnicity, body roundness index, education, income, estimated glomerular filtration rate, smoking, and alcohol consumption, with interaction tests for effect modification by EJI rankings. Complete-case analysis and multiple imputation were used to account for missing data.
RESULTS Among 392 adults (154 cases, 237 controls), significant effect modification was observed for PFOSA (dichotomous) with high cholesterol, by EBM (continuous) ($p = 0.01$), with a stronger effect of PFOSA at lower EBM values. Additionally, significant effect modification was found for PFOSA (dichotomous) with high cholesterol, by EBM (tertiles) ($p = 0.04$), as well as for perfluoroundecanoic acid (PFUA, dichotomous) with high cholesterol, by EBM (tertiles) ($p = 0.03$). The effects of dichotomous PFOSA and PFUA were strongest in the lowest EBM tertile.**CONCLUSION** EJI modified PFAS-cholesterol associations. These findings suggest environmental and social factors may influence susceptibility to PFAS-related health effects. This study was funded by CDC/ATSDR.

P23-18 | Follicular Fluid Per- and Poly-fluoroalkyl Substances (PFAS), Metabolomics, and IVF Outcomes: A Pilot Study

Elvira S.C. Fleury[1], Emily Silva[1], Elizabeth Peebles[1], Anna Young[3], Douglas Walker[3], Zifan Wang[1], Daniel Cramer[2], Allison Vitonis[2], Kathryn Terry[2], Shruthi Mahalingaiah[1]

[1] Harvard T.H. Chan School of Public Health, Department of Environmental Health, Boston, USA; [2] Brigham and Women's Hospital, Obstetrics and Gynecology Epidemiology Center, Boston, USA; [3] Rollins School of Public Health, Gangarosa Department of Environmental Health, Atlanta, USA

Objective

To characterize associations between follicular fluid (FF) per- and poly-fluoroalkyl substances (PFAS) and in vitro fertilization (IVF) outcomes and explore FF metabolic features and pathways associated with PFAS and IVF outcomes in a pilot study.

Methods

FF was collected from the first aspirated follicle of 36 women undergoing IVF treatment (16 resulting in a live birth) at three Massachusetts centers (1999–2002). We quantified eight targeted PFAS (N-EtFOSAA, N-MeFOSAA, PFBS, PFHps, PFHxS, PFNA, PFOA, and PFOS) and untargeted metabolic features in FF using liquid chromatography high-resolution mass spectrometry. We used linear regression to estimate age and infertility-type-adjusted associations between FF PFAS concentrations and metabolic feature intensities and logistic regression for associations with live birth. We also used logistic regression to estimate the age and infertility-type-adjusted association between metabolic feature intensities and live birth. We subsequently conducted pathway enrichment analyses. Finally, we used a meet-in-the-middle approach to screen for overlapping metabolic pathways associated with FF PFAS and live birth.

Results

Higher FF PFHps was associated with lower live birth odds (OR=0.42 [95% CI: 0.15-0.97]). All other PFAS, except PFBS, were non-significantly negatively associated with live birth. We detected 27,903 FF metabolic features in more than 25% of participants with acceptable measurement variability. Eleven pathways were significantly enriched ($p<0.05$) and associated with six or more PFAS. Forty metabolic pathways were significantly associated with live birth odds ($p<0.05$). Four pathways—ascorbate and aldarate metabolism, c5-branched dibasic acid metabolism, glyoxylate and dicarboxylate metabolism, and pentose and glucuronate interconversions—were significantly associated with both ≥ 6 PFAS and live birth.

Conclusions

Our pilot study suggests FF PFAS are negatively associated with live birth following IVF. Metabolic pathways associated with both PFAS concentrations and IVF outcomes may involve oxidative stress regulation and carbohydrate and amino acid metabolism. A larger sample size is needed to confirm these findings.

P23-19 | Prenatal exposure to per- and polyfluoroalkyl substances and metabolic changes in maternal serum and placenta associated with neurodevelopment in children

Jeong Weon Choi[1], Mariana Parenti[2], Carolyn M. Slupsky[2,3], Rebecca J. Schmidt[4,5], Hyeong-Moo Shin[1]

[1] Baylor University, Department of Environmental Science, Waco, USA; [2] University of California, Davis, Department of Nutrition, Davis, USA; [3] University of California, Davis, Department of Food Science and Technology, Davis, USA; [4] University of California, Davis, Department of Public Health Sciences, Davis, USA; [5] University of California, Davis, MIND Institute, Sacramento, USA

Background: Prenatal exposure to PFAS has been associated with changes in metabolism and child neurodevelopment; however, limited studies have integrated these associations. We investigated associations between prenatal PFAS exposure, maternal metabolism, and child neurodevelopment.
Methods: We included 172 mother-child pairs (n = 129 for maternal serum, n = 152 for placental metabolome data) from the MARBLES (Markers of Autism Risk in Babies–Learning Early Signs) cohort. Nine PFAS were measured in maternal serum during pregnancy, and metabolites were quantified in 3rd-trimester serum and placenta using ^{1}H-NMR spectroscopy. At age 3 years, children were diagnosed and classified as autism spectrum disorder (ASD), other non-typical development (non-TD), or typical development (TD). Multiple linear regression was used to investigate the association between individual PFAS and maternal serum or placental metabolites, while quantile-based g-computation examined the impact of PFAS mixtures. Principal component analysis (PCA) was used to explore associations between serum and placental metabolites, maternal serum PFAS and offspring ASD/non-TD risk.
Result: Higher maternal perfluorooctane sulfonate (PFOS) was associated with 2-hydroxybutyrate and 3-methyl-2-oxo-butanoic acid (FDR < 0.05). PFAS mixtures and some PFAS compounds were also associated with 2-hydroxybutyrate and glucose, suggesting endocrine-disrupting effects; however, these associations did not remain after FDR correction. No significant associations were observed between PFAS and placental metabolites. PCA showed that higher perfluorooctanoate, PFOS and PFAS mixtures were associated with some serum metabolite components. Certain metabolite components in serum and placenta were associated with increased ASD/non-TD risk compared to TD, but no clear link was found between PFAS and neurodevelopmental outcomes.**Conclusion:** Prenatal PFAS exposure may contribute to endocrine disruption; however, its impact on neurodevelopment through disruption of maternal metabolism remains unclear. The impact of PFAS on maternal metabolism appears to be subtle, and thus a more sensitive non-targeted method may capture metabolic perturbations from low-level exposure to endocrine disrupting chemicals.

P23-20 | PFAS concentration at birth and body mass index and blood pressure: The Upstate KIDS Study

Anonna Saha[1], Erin M. Bell[2]

[1] University at Albany, Department of Environmental Health Sciences, Rensselaer, USA; [2] University at Albany, Department of Environmental Health Sciences & Epidemiology and Biostatistics, Rensselaer, USA

Objectives: Per and Poly-fluoroalkyl substances (PFAS) are a group of persistent, synthetic chemicals ubiquitous in the environment and consumer products. PFAS can readily cross the placental barrier and enter the fetus's bloodstream. They have been associated with metabolic diseases later in life. This study examines the association between PFOA and PFOS concentrations in newborn dried blood spots (NDBS) and maternal-reported body mass index (BMI) and blood pressure (BP) at age 7-9 years.
Material and Methods: The Upstate KIDS study is a longitudinal, prospective birth cohort of live births occurring in New York State, excluding New York City, between September 2008 and December 2010. PFOA and PFOS concentrations were measured in NDBS using liquid chromatography-tandem mass spectrometry. Maternal reports of BMI and BP at ages 7-9 were examined, and covariates were selected using directed acyclic graphs. Due to the data's nested structure and non-independence, a linear generalized estimating equations model is used.
Results: More than 99% of PFOA and PFOS in NDBS were above the limit of detection with mean PFOA 1.27 ng/ml and PFOS 1.96 ng/ml. The mean maternal-reported child BMI at age 7-9 was 17 kg/m2, the mean systolic BP was 98 mmHg, and the mean diastolic BP was 61 mmHg. Adjusted log-transformed PFOA (β= 0.0078 (95% CI: -0.5153, 0.5309)) and PFOS (β= -0.1364 (95% CI: -0.6184,0.3456)) were not associated with an increase in BMI. Also, no association was found with BP after adjusting for covariates. Inverse probability weighting will be used to account for missing confounders, and stratified analysis by sex will be performed.
Conclusion: The results indicate that PFOA and PFOS at birth are not associated with increased BMI or BP at age 7-9.
Funding: Intramural Research Program, Eunice Kennedy Shriver National Institute of Child Health and Human Development, National Institutes of Health (contracts # HHSN267200700019C; HHSN275201200005C)

P23-21 | PFAS Multi-site Study in New York: Community level serum PFAS results

J. Moore[1], M. Liu[1], Y. Liu[1], S. Forand[1]

[1] *New York State Department of Health, Center for Environmental Health/Bureau of Environmental and Occupational Epidemiology, Albany, New York, United States of America*

Objective

To describe serum PFAS results from the New York site of the Multi-site PFAS Health study, a federally funded study of communities exposed to PFAS in drinking water. The New York site has three exposure groups including residents of the City of Newburgh and residents of the Village of Hoosick Falls, both served by public water systems; and residents in the Hoosick Falls area served by private wells. PFAS contamination originated from a nearby National Guard base in Newburgh and from industry in Hoosick Falls. PFAS contamination was mitigated in 2016.

Materials and Methods

Study invitation packets were mailed to eligible study area residents. Eligibility was based on residence in the study areas between 2005 and 2016. Participants completed an exposure survey and between 2021 and 2023 and 496 people provided blood serum which was analyzed for PFAS.

Results

The geometric mean (GM) serum PFOA levels were notably elevated in adult participants served by the Village of Hoosick Falls water system (19.84 ng/mL; n = 128) and those using private wells in the Hoosick Falls area (4.62 ng/mL; n=109) as compared to levels in the general US adult population (2017-18 NHANES 1.45 ng/mL). Children from the Village of Hoosick Falls had a GM serum PFOA level of 5.33 ng/mL (n = 11) as compared to 2017-18 NHANES (1.18 ng/mL). In the Newburgh study cohort, the GM for serum PFOS and PFHxS levels among adults were 7.95 and 6.14 ng/ml (n = 215) compared to 4.50 and 1.11 ng/ml in the general population, respectively.

Conclusion

Serum PFAS levels among residents of communities with documented exposure are higher than those in the general US population. The results highlight differences in PFAS exposure in the exposure groups. Further study of the associated health effects is underway.

P24 | Planetary Health and One Health

P24-01 | Integrating One Health Perspectives in Digital Health Education: Evaluating an IPC MOOC in Ecuador during the COVID-19 Pandemic.

Bernarda C. Espinoza Castro[1,2], Verónica Encina[1], Astrid Garrido[1], Fausto Ignatov[1,2], Juan Piedra[3], Ximena Garzón[4], Katja Radon[1,2]

[1] LMU University Hospital Munich, Center for International Health, Munich, Germany; [2] LMU University Hospital Munich, Institute and Clinic for Occupational, Social and Environmental Medicine, Munich, Germany; [3] Universidad de las Américas, Occupational Safety and Health, Quito, Ecuador; [4] Universidad San Francisco de Quito, School of Public Health and Nutrition, Quito, Ecuador

The COVID-19 pandemic has underscored the interconnections between human, animal, and environmental health, demonstrating the need for a One Health approach to emerging infectious diseases. In Ecuador, where environmental challenges and zoonotic risks increase public health vulnerabilities, a Massive Open Online Course (MOOC) titled "Infection, Prevention, and Control (IPC) of Acute Respiratory Infections for Healthcare Workers in Low- and Middle-Income Countries (IPC MOOC)" was developed and implemented. This study evaluates the IPC MOOC's impact by examining whether course completion varied by sociodemographic factors or occupational roles (manual vs. intellectual) and whether satisfaction and perceived learning outcomes differed among these groups.

The IPC MOOC was developed by an interdisciplinary team involving public and occupational health experts, environmental scientists, and sociologists. It integrated problem-based learning and interactive scenarios to teach IPC principles within a One Health framework. The course was offered to all healthcare workers between August and December 2021 and enrolled 3,498 participants. Data collected via registration and post-course surveys were analyzed using Chi-squared and Mann-Whitney U tests to assess the influence of demographic and occupational factors on completion rates, satisfaction, and learning outcomes and to compare perceived learning outcomes between manual and intellectual workers.

Seventy-five percent of participants completed the course, with no significant differences in completion rates based on gender, region, or occupation. Among the 809 respondents to the post-course survey, 80% reported high satisfaction. Although perceived knowledge differed between manual and intellectual workers before the course (3.41 vs. 3.57 on a 5-point scale; $p=0.02$), post-course evaluations showed similar perceived knowledge for both groups (4.08 vs. 4.14; $p=0.41$).

By integrating digital education with One Health principles, the IPC MOOC effectively bridged knowledge gaps across diverse healthcare roles. It promoted equitable access to IPC education, demonstrating its potential as a scalable intervention during health crises.

P24-02 | Impact of Veterinary Feed Directive Rule Changes on Antimicrobial Resistance of *Salmonella Typhimurium* isolated from Human Stool in the United States

Shamim Sarkar, Gary Vroegindewey

Lincoln Memorial University, College of Veterinary Medicine, Harrogate, USA

Objective: In 2017, the FDA implemented Veterinary Feed Directive (VFD) rules in the U.S., requiring a veterinarian's prescription or VFD for using medically important antimicrobials in feed or water, and prohibiting their use for growth promotion in food-producing animals. This study aimed to test the hypothesis that the implementation of the VFD rules would reduce the resistance of *Salmonella typhimurium* isolates from humans to antimicrobials used in food-producing animals, but not to those used primarily in human medicine.

Material and Methods: *Salmonella typhimurium* isolates from human stool, collected between 1996 and 2023, were obtained from the National Antimicrobial Resistance Monitoring System (NARMS) in the U.S. Variables of interest included antimicrobial susceptibility testing results, year, age group, specimen source, and region. Isolates were grouped by specimen collection year into pre-VFD (1996-2016) and post-VFD (2018-2023) periods. The outcome variable was binary (resistant vs. susceptible). Mixed-effects logistic regression models were fitted to assess the relationship between VFD implementation and resistance outcomes.

Results: Analyzed tetracycline (n = 6,949), ampicillin (n=7,083), gentamicin (n=7,083), ciprofloxacin (n= 7,083), trimethoprim-sulfamethoxazole (n=7,083), and ceftriaxone (n=7,083) susceptibility results for *Salmonella typhimurium* isolated from human stool. Following implementation of the VFD, the odds of detecting resistance to tetracycline (OR = 0.33, 95% CI: 0.28, 0.40), ampicillin (OR = 0.36, 95% CI: 0.30, 0.43), and gentamicin (OR= 0.52, 95% CI: 0.31, 0.90) in *Salmonella typhimurium* isolates significantly decreased compared to the pre-VFD period. In contrast, the odds of detecting ciprofloxacin resistance significantly increased (OR = 2.70, 95% CI: 1.87, 3.92) and no significant changes were observed in the odds of detecting resistance to trimethoprim-sulfamethoxazole and ceftriaxone during the post-VFD period.

Conclusion: VFD implementationlikely contributed to reduce resistance to antimicrobials (tetracycline, ampicillin, and gentamicin) commonly used in food animals but did not affect those primarily used in humans (ciprofloxacin and ceftriaxone).

P24-03 | Relative Humidity and Insomnia: A Nationwide Social Media Study

Qian Li, Jiahao Duan, Fengchao Liang

Southern University of Science and Technology, School of Public Health and Emergency Management, Shenzhen, China

Background: Relative humidity has been shown to affect population health. It may be harmful to insomnia and circadian rhythms by changing environmental comfort and melatonin secretion.
Methods: This ecological study used social media data from the platform Weibo (Sina) during the study period from May 2022 to April 2023. The study covered 336 cities in mainland China and included insomnia-related posts from platform users, representing exposure to different levels of nighttime heat exposure. Relative humidity exposure was determined by observation from satellite and transfer to 1km resolution through inverse distance weighted method. Associations were analyzed by linear regression based on time-series design.
Findings: Data from 1,147,583 social media posts about insomnia were included in the research. The relative humidity ranged from 7.96 to 99.84%. For each 10% increase in relative humidity exposure, the incidence of insomnia increased by 0.060% (95% CI: 0.140 to 0.178%). The effect is significantly different in different regions, with the effect in the south being 0.091% (95% CI: 0.058 to 0.125%, mean relative humidity: 71.57%) and the effect in the north being -0.158% (95% CI: -0.183 to -0.133%, mean relative humidity: 55.82%). The exposure-response curves showed a nonlinear trend, indicating that either excessively low or excessively high relative humidity may cause an increase in insomnia incidence rates. Subgroup analysis suggested that the effect of relative humidity on insomnia may be further amplified under high temperature and high outdoor artificial light exposure.
Interpretation: This study emphasizes that excessive exposure to both high or low relative humidity could lead to an increase in insomnia in the population, which provides evidence for targeted health prevention for the population.

P24-04 | Patterns in pesticide use among farmers across eastern Rwanda: Insights from agricultural and animal resource officers

Concorde I. Rwibutso[1], Therese Nyirahabimana[2], Emmanuel Irimaso[3], Cynthia Curl[4], Stephanie Mitchell[5], Liberata Mukamana[6], Dawn Zimmerman[7], Sabrina Sholts[8]

[1] Gorilla Doctors, Wildlife medicine, Musanze, Rwanda; [2] Rwanda Biosolution, Waste Management, Kigali, Rwanda; [3] University of Rwanda, School of veterinary medicine, Nyagatare, Rwanda; [4] Boise State University, School of Public and population Health, Boise, USA; [5] University of Washington, Center of One health, Washington, USA; [6] University of Rwanda, School of social sciences, Kigali, Rwanda; [7] Veterinary initiative for Endangered Species, Wildlife Medicine, California, USA; [8] Smithsonian Institution, Anthropology, Washington, USA

Background In Rwanda, more than 75% of the population works in agriculture, but the potential health risks of growing pesticide use on humans, domestic animals, and wildlife are understudied. This study aims to identify common themes in how farmers utilize and interact with pesticides across Rwanda’s Eastern Province within a One Health framework.
Materials and Methods

We conducted focus group discussions with sector agricultural officers (SAOs) and animal resources officers (SAROs) on pesticide use from an administrative perspective. We held five discussions in December 2024 with a total of 31 SAOs and SAROs from Rwamagana, Nyagatare, Kayonza, Kirehe, and Ngoma districts, using an interview guide that covered topics such as changes in pesticide use over time, sources of pesticide information, human and animal exposure to pesticides, and gender differences related to pesticides. Voice recordings of the discussions were transcribed, translated from Kinyarwanda into English, and subjected to deductive thematic analysis using Dedoose software.

Results

We identified key themes of climate change, pest resistance, and population growth as drivers of increased pesticide use, which were reported consistently in all focus groups. Each group shared numerous examples of pesticide exposure and acute poisoning events among humans and animals or via animal products. They also described similarities in how farmers acquire pesticides and often misuse them due to lack of information or understanding about safe practices, including a widespread lack of personal protective equipment. However, there were between-group differences in how women were perceived to know or care about the health risks of pesticide exposure compared to men.

ConclusionMost farmers in the Eastern Province of Rwanda have insufficient information, training, oversight, resources, and/or motivation for safe pesticide use. More research is needed to improve communications and support changes that can decrease shared health risks for humans, animals, and the environment.

P24-05 | Environmental Health Equity Through One Health: An Implementation Analysis of Antimicrobial Resistance Governance in the Philippines

Geminn Louis C. Apostol[1,4], Sary Valenzuela[1,5], Anna Beatrice Enriquez[1], Lea Elora Conda[1], Lystra Zyrill Dayapera[3,1], Percival Ethan Lao[1], Elaine Joy Diaz[1], Luh Rai Maduretno Asvinigita[1]

[1] Ateneo de Manila University - School of Medicine and Public Health (ASMPH), ASMPH Center for Research and Innovation, Pasig City, Philippines; [2] Philippine Inter-agency Committee on Environmental Health (IACEH), Manila, Philippines; [3] Ripple Innovative Science Consulting LLC, Austin, USA; [4] Murdoch University, Harry Butler Institute, Perth, Australia; [5] Georgetown University, Department of Global Human Development, Washington DC, USA

Objective: Antimicrobial resistance threatens global health security with disproportionate impacts on environmentally vulnerable communities lacking adequate water, sanitation, and healthcare infrastructure. This study evaluates implementation progress of the Philippine National Action Plan to Combat Antimicrobial Resistance (PNAP AMR) 2019-2023 through an environmental health equity lens, identifying gaps that disproportionately affect vulnerable populations.

Material and Methods: We conducted a mixed-methods implementation review using the traffic light assessment system across seven key strategies of the PNAP. Data collection involved comprehensive desk review of peer-reviewed literature, government documents, and policies from multiple agencies (DOH, DENR, DA, DILG, among others). We conducted over 50 key informant interviews with implementing agencies and stakeholders, and facilitated technical consultations with government and non-governmental participants to validate findings and build consensus on implementation challenges.

Results: Key Strategy 5 (infection prevention measures) showed the highest full implementation rate (26.7%), while surveillance and laboratory capacity (KS2) and rational antimicrobial use (KS4) had the highest "not started" ratings

(38.5% and 31.3% respectively). Environmental sector engagement remained limited, with inadequate regulatory frameworks and surveillance systems to monitor environmental dimensions of AMR. Implementation disparities were most pronounced at local government levels, where resource constraints and technical capacity gaps created inequitable protection against AMR threats. Sanitation, hygiene, and infection control activities in community settings revealed significant geographical and socioeconomic disparities.

Conclusion: Environmental health inequities significantly undermine Philippines' AMR response effectiveness. While multisectoral engagement through the Inter-Agency Committee on AMR has progressed, environmental monitoring and equity considerations require strengthening. Recommendations include integrating environmental justice metrics into surveillance systems, building local-level implementation capacity, and explicitly addressing socioeconomic disparities in AMR vulnerability to ensure equitable protection across all communities and life stages.

P24-06 | Unpacking the epidemiological determinants of COVID-19 preventive behaviours: evidence from Ghana’s urban informal settlements

Richmond Amponsah, Marie V. Miller, Nigel McConnell, Aaron Lawson

Ulster University, Belfast School of Architecture and the Built Environment, Belfast, UK

The COVID-19 pandemic highlighted significant disparities in the uptake of preventive health behaviours across various socio-economic groups, particularly within informal settlements. Achieving environmental health equity in low-resource settings has become an urgent issue, as these populations often face limited access to infrastructure (e.g., healthcare, water, and sanitation), accurate information, and various socio-economic inequalities. Addressing this knowledge gap aids the development of context-specific public health policies in advancing global health equity. However, little is known about the various factors influencing the uptake of COVID-19 prevention behaviours, particularly in sub-Saharan Africa, a region with numerous pockets of informal settlements. To this end, we applied the Information-Motivation-Behavioural (IMB) Skills Model of Pandemic Risk and Prevention to explore the pathways through which COVID-19 information and knowledge, personal and social motivation, and behavioural skills influenced the uptake of preventive practices in three informal settlements (Old Fadama, Nima, and Alogboshie) in Ghana. Drawing on a survey of 496 participants and employing Structural Equation Modelling (SEM), our results reveal a concerning knowledge gap, with only 30.6% of participants responding accurately to all COVID-19 knowledge questions. Further, our findings indicate that information provision and knowledge of COVID-19, personal and social motivations, improve behavioural skills and these in turn foster uptake of preventive behaviours. Despite the crucial role of knowledge and behavioural skills, majority of respondents (about 70%) had limited knowledge of COVID-19. Therefore, pandemic control efforts should prioritise awareness, skills development and self-efficacy in accessing and managing preventive behaviours. This could be done through relevant message framing, using appropriate communication channels and providing context-specific skills development training and resources to improve behavioural skills. Given that, low-income groups have peculiar characteristics, and their circumstances may differ across different contexts, further research may be needed on what constitutes the most appropriate message framing and information dissemination channels across various contexts.

P24-07 | Regional and Local Perceptions of Environmental change and its impacts on health and wellbeing in Amazonia

Beth J. Feingold[1], Paola A. Torres-Slimming[2], Carlos L. del Cairo Silva[3], Hortensia Caballero-Arias[4], Daniel S. Bustos-Echeverry[5], Danny Pinedo[6]

[1] University at Albany - State University of New York, Albany, USA; [2] Universidad Peruana de Ciencias Aplicadas, Lima, Peru; [3] Pontificia Universidad Javeriana, Bogota, Colombia; [4] Instituto Venezolano de Investigaciones Científicas, Caracas, Venezuela; [5] Noname SAS, Bogota, Colombia; [6] Universidad Nacional Mayor de San Marcos, Lima, Peru

Environmental changes produced by human activities have increased the negative impacts on the fragile health of the Amazonian ecosystem and the diverse populations whose livelihoods depend on it. The overall goal of this transboundary visual and participatory project was to highlight the voices and images of local communities on the perceived health-related effects of climate change and to generate spaces for collective dialogue for policy and decision-making. Using the qualitative research method of Photovoice, 33 individuals from 11 unique peasant and Indigenous localities in the Colombian, Venezuelan and Peruvian Amazon were invited to answer the question - through photographs and accompanying narratives- of how they perceive, experience, and adapt to the socio-environmental changes that are occurring in the Amazon. Common themes that emerged across at least two of the sites included community health concerns about: land conflict, fire, road access, loss of local craft, presence of illicit economies, food security, water security and availability, pollution and urbanization. Other themes that emerged at individual locations included drought, trash, deforestation, cattle ranching, migration, and loss of territorial lands. Some of the localities chose to exhibit their photos and narratives in their communities to spark discussion with leaders and other community members about risks and opportunities regarding community health impacts of climate and anthropogenic change. This project is significant because it highlights transboundary perspectives on the lived experiences of local communities, who have deep knowledge of their environment, and how they can offer important insights into finding alternatives to the impacts of climate change on their livelihoods and health. Taking such experiences into account is paramount when considering health impacts of climate and anthropogenic change in and across the Amazon.

P24-08 | Impact of Temperature on Diarrhoeal Diseases in Rohingya Refugee Camps, Cox's Bazar, Bangladesh

Md Nasif Hossain[1,2], Josh M. Colston[2]

[1] University of Virginia, Environmental Institute, Charlottesville, USA; [2] University of Virginia, Department of Medicine, Charlottesville, USA

Background and Aim: Diarrhoeal diseases remain a significant public health concern in refugee camps, where overcrowding, poor sanitation, and limited healthcare exacerbate disease transmission. This study investigates the impact of temperature on diarrhoeal disease incidence in the Rohingya refugee camps of Cox's Bazar, Bangladesh. ***Method:*** Daily time series data based on facility-based case reports were collected from several health services provider clinics from September 2017 to December 2023. Daily temperature data in the same period were downloaded from the ERA5-Land. We fitted a time series regression model to examine the relationship of daily diarrhoeal cases with daily ambient temperature for the last 7 days using a generalized linear model with distributed lag nonlinear functions. ***Results:*** A total of 2,795,618 diarrhoea cases were included in the analysis. During the study period, daily mean temperatures ranged from 16.9°C to 31.6°C. The relationship between temperature and diarrhoeal incidence followed a U-shaped curve, with the minimum risk temperature (MRT) at 21°C. Compared to the MRT, the relative risk (RR) of diarrhoea at the 1st percentile temperature (18.6°C) was 1.24 (95% confidence interval [CI]: 1.09–1.42), while at the 95th percentile temperature (29.8°C), the RR was 1.28 (95% CI: 1.07–1.53). A significant positive association was observed between diarrhoeal infections and high temperatures (31°C) on the same day, while low temperatures (17–18°C) exhibited a delayed effect, increasing diarrhoeal risk at lags of 6–7 days. ***Conclusion:*** Our findings highlight the critical role of extreme temperatures, both high and low, in increasing diarrhoeal disease incidence in the Rohingya refugee camps. These results underscore the need for climate-adaptive public health interventions to mitigate temperature-related diarrhoeal risks in vulnerable populations.

P24-09 | Associations of pathogen-specific diarrhoea with temperature and precipitation: a multi-country study in Asia and Africa of the Global Enteric Multicenter Study (GEMS)

Md Nasif Hossain[1,2], Lina Madaniyazi[2], Chris Fook Sheng Ng[3,2], Dilruba Nasrin[4], Josh M. Colston[5], Abu Sayed Golam Faruque[6], Masahiro Hashizume[3,2]

[1] University of Virginia, Environmental Institute, Charlottesville, USA; [2] Nagasaki University, Department of Global Health, School of Tropical Medicine and Global Health, Nagasaki, Japan; [3] The University of Tokyo, Department of Global Health Policy, School of International Health, Tokyo, Japan; [4] University of Maryland School of Medicine, Center for Vaccine Development and Global Health, Baltimore, USA; [5] University of Virginia, Department of Medicine, Division of Infectious Disease and International Health, Charlottesville, USA; [6] International Centre for Diarrhoeal Disease Research, Bangladesh, Nutrition Research Division, Dhaka, Bangladesh

Background: Enteric infections causing moderate to severe diarrhea (MSD) remain a major public health concern in low- and middle-income countries. However, the relationship between weather patterns and MSD risk in resource-limited settings is not well understood. This study investigates the association between weather variables and pathogenic MSD in children across seven countries. **Methods:** Data were obtained from the Global Enteric

Multicenter Study (December 2007–March 2011) across seven sites in The Gambia, Mali, Mozambique, Kenya, India, Bangladesh, and Pakistan. Daily weather data from ERA5-Land were used to assess associations between MSD and temperature and precipitation. A case-crossover model was applied to analyze the relationship between daily mean temperature, 21-day moving average precipitation, and MSD risk. **Results:** A total of 9,439 MSD cases were analyzed. Daily mean temperatures ranged from 15.2°C to 37.4°C. The risk of viral MSD increased with lower temperatures, with significant relative risks (RRs) in Pakistan [7.66 (95% CI, 1.36–43.28)] and India [29.21 (95% CI, 5.55–153.65)]. Conversely, bacterial MSD risk increased with higher temperatures, particularly in Bangladesh [RR: 4.64 (95% CI, 1.30–16.51)]. Protozoal MSD showed varied patterns; The Gambia exhibited a J-shaped association, with RR of 6.65 (95% CI, 1.59–27.74) at the 95th percentile temperature, whereas Bangladesh showed a reversed U-shaped pattern. High precipitation elevated protozoal MSD risk in Mali [RR: 3.34 (95% CI, 1.17–9.56)] and The Gambia [RR: 13.85 (95% CI, 1.46–131)]. **Conclusions:** The study found that enteric infection risks vary with temperature and precipitation. Viral infections increase in colder conditions, bacterial infections rise with warmer temperatures, and protozoal infections shift at different temperature ranges. High precipitation amplifies protozoal risks in sub-Saharan Africa.

P25 | Reproductive Health

P25-01 | Associations Between Monthly Fine Particulate Matter, Ozone, and Postneonatal Infant Mortality in the United States, 2005-2017

Sanjida Mowla[1], Alison Krajewski[2], Adrien Wilkie[2], Kristen Foley[3], Kristen Rappazzo[2], Thomas Luben[2]

[1] Oak Ridge Institute of Science and Education (ORISE) Research participant at the United States Environmental Protection Agency, Research Triangle Park, USA; [2] United States Environmental Protection Agency, Office of Research and Development, Center for Public Health & Environmental Assessment, Research Triangle Park, USA; [3] United States Environmental Protection Agency, Office of Research and Development, Center for Environmental Measurement and Modeling, Research Triangle Park, USA

We evaluated the association between air pollutants and postneonatal infant mortality in a US national cohort.
Using National Center for Health Statistics birth and death records, we estimated associations between monthly mean fine particulate matter ($PM_{2.5}$) or ozone (O_3) concentrations and postneonatal infant mortality (all-cause and cause-specific) among infants born between 2005-2017 in US counties with >250,000 residents. Air pollution concentration estimates from US EPA’s Air Quality Time Series (EQUATES) fused downscaler model were spatially weighted to each county and linked to birth and death records. We estimated incidence rate ratios (IRRs) and 95% confidence intervals (95%CI) using two-staged overdispersed Poisson regression, adjusted for temperature, by climate zone (arid, equatorial, snow, warm temperate) and pooling across zones for an overall estimate using random-effects meta-analysis.
There were 35,922,771 births and 72,359 postneonatal deaths across 261 counties. Congenital anomalies (19.8%) and sudden infant death syndrome (SIDS;16.8%) were leading causes of mortality. Mean $PM_{2.5}$ concentration was 9.77 µg/m³ (SD: 3.19) and O_3 was 38.73 ppb (SD: 9.78). A 10 µg/m³ increase in monthly $PM_{2.5}$ exposure was associated with increased infant mortality for all causes (IRR: 1.11 [1.05, 1.17]), respiratory causes (aIRR: 1.90 [1.60, 2.25]) and SIDS (IRR: 2.62 [2.14, 3.22]). Similarly, a 10-ppb increase in O_3 exposure was associated with increased mortality for respiratory causes (IRR: 1.89 [1.60, 2.25]) and SIDS (IRR: 2.62 [2.13, 3.22]). Geographically stratified analyses showed associations between $PM_{2.5}$ and all-cause mortality were strongest in warm temperate zones (IRR: 1.17 [1.13, 1.21]) and the Southeast (IRR: 1.18 [1.11, 1.25]). For O_3, associations were highest for all-cause infant mortality in the West (IRR: 1.10 [1.06, 1.13]).
Mean monthly $PM_{2.5}$ and O_3 exposure is associated with increased respiratory and SIDS-related postneonatal infant mortality. All-cause infant mortality risk was strongest among counties in the Southeast and the West regions.

P25-02 | Prenatal polycyclic aromatic hydrocarbon exposure and adverse birth outcomes in a pregnancy cohort in Nairobi, Kenya

Allison R. Sherris[1], Priscillah W. Edemba[2], Anne M. Riederer[1], Judy Adhiambo[3], Lewis Olweywe[2], Catherine J. Karr[1,4], John Kinuthia[5], Barbra Richardson[6], Christopher D. Simpson[1], Sarah Benki-Nugent[5], Elizabeth Maleche-Obimbo[3]

[1] University of Washington, Department of Environmental & Occupational Health Sciences, Seattle, USA; [2] Kenyatta National Hospital, Nairobi, Kenya; [3] University of Nairobi, Department of Paediatrics and Child Health, Nairobi, Kenya; [4] University of Washington, Department of Pediatrics, Seattle, USA; [5] University of Washington, Department of Global Health, Seattle, USA; [6] University of Washington, Department of Biostatistics, Seattle, USA

Objective: Prenatal exposure to polycyclic aromatic hydrocarbons (PAHs) has been linked to lower birth weight and shorter gestational age. Most research has focused on populations in high-income countries, leaving low- and middle-income countries (LMICs) understudied. We examined associations between pregnancy urinary PAH metabolites and birth outcomes in a prospective cohort in Nairobi, Kenya.
Methods: Analyses included women and their newborn babies enrolled in a pregnancy cohort (N=400) in Nairobi, Kenya. Mid-pregnancy urinary mono-hydroxylated metabolites (OH-PAH) were measured, and infant birth weight, gestational age, and birthweight-for-gestational-age z-scores were ascertained using medical record abstraction and self-report. Modified Poisson regression was used to estimate relative risk (RR) of birth outcomes associated with five OH-PAHs, adjusting for urine specific gravity, infant sex, maternal characteristics, and prenatal smoking. We evaluated effect modification by infant sex and assessed mixture effects with Weighted Quantile Sum regression.
Results: Among n=354 mother-infant pairs, the median infant birth weight was 3.2 kg and the prevalence of preterm birth was 10.7%. Mothers had a median age of 27 years at delivery and 72% completed secondary school. Individual OH-PAH concentrations were not associated with birth weight or birthweight-for-gestational-age z-scores. One metabolite, 2-hydroxyphenanthrene, was associated with gestational age (RR = -1.6 day per doubling in concentration, 95% confidence interval: -3.1, -0.1). This association was attenuated after adjusting for self-reported exposure to household fuel and outdoor combustion and was strengthened among female infants, but not male infants, in sex-stratified analyses. The OH-PAH mixture was not associated with birth outcomes.
Conclusion: Our findings suggest that mid-pregnancy exposure to 2-hydroxyphenanthrene may modestly reduce gestational age, with potential sex-specific differences in association. No associations were observed for markers of fetal growth. These results highlight need for further studies on sex-specific vulnerabilities and the role of environmental co-exposures in impacting birth outcomes in LMICs. Funding: R01ES032153 (U.S. NIEHS).

P25-03 | Maternal Demographic Predictors of Agricultural Pesticide Exposures in the Arizona Pregnant Women's Environmental and Reproductive Outcomes Study (2006-2020)

Audrey R. Yang, Rietta S. Wagoner, Melissa A. Furlong

University of Arizona, Department of Community, Environment & Policy, Tucson, USA

Objective
About 3.7 million tons of pesticides are used each year. Pesticide exposures are associated with a range of developmental health effects, and certain populations face an increased risk of exposure. We aimed to evaluate the association between demographics of pregnant women and carbamate, organophosphate, and pyrethroid pesticide exposures in Arizona from 2006-2020.

Material and Methods
We used data from Arizona's pesticide use registries and the Arizona Pregnant Women's Environmental and Reproductive Outcomes Study (Az-PEARS). We excluded study subjects missing covariate data. Exposure was defined as having a residential address within 500m of a carbamate, organophosphate, or pyrethroid agricultural pesticide application. We performed logistic regressions to evaluate maternal demographic predictors of exposure to agricultural pesticide applications during preconception and/or pregnancy.

Results
We included 1,168,284 pregnant women. 71,827 (6.15%) women were exposed to a carbamate, organophosphate, or pyrethroid pesticide during preconception and/or pregnancy. The majority identified as Hispanic (50.20%), were 20-35 years old (82.07%), graduated high school as their highest level of education (29.64%), were married (62.83%), and resided in Maricopa County (47.82%).
Demographic characteristics associated with higher odds of exposure included a Hispanic ethnicity (OR: 1.51, 95% CI: 1.48-1.54), an American Indian/Alaska Native race (OR: 1.26, 95% CI: 1.20, 1.33), and an age less than 20 years (OR: 1.24, 95% CI: 1.20, 1.28). Graduating high school (OR: 1.43, 95% CI: 1.39, 1.46), attending some college (OR 1.47, 95% CI: 1.43, 1.51) or graduating college or higher (OR: 1.39, 95% CI: 1.36, 1.43) as one's highest level of education were all associated with increased odds of exposure, along with residing in an agricultural county (OR: 20.35, 95% CI: 19.29, 21.47). Trends remained consisted when evaluating exposures to individual pesticide classes.

Conclusion
Our results may demonstrate the demographics of agricultural communities. These results could inform interventions aimed at mitigating pesticide exposures.

P25-04 | Urinary metal concentrations and their association with gynecological diseases in premenopausal women: A cross-sectional analysis

Aram Lee[1], Gyuyeon Choi[2], Hyo-Bang Moon[3], Kyungho Choi[4], Sungkyoon Kim[4], Jeongim Park[1]

[1] Soonchunhyang University, Department of Environmental Health Sciences, Asan, South Korea; [2] Soonchunhyang University, College of Medicine, Seoul, South Korea; [3] Hanyang University, Department of Marine Sciences and Convergent Technology, Ansan, South Korea; [4] Seoul National University, Department of Environmental Health Sciences, Seoul, South Korea

Objectives: This study aimed to examine urinary concentrations of toxic heavy metals and trace elements in premenopausal women and assess their associations with gynecological diseases.

Material and Methods: A total of 281 premenopausal women aged 20–49 years (non-pregnant) were recruited between 2015 and 2016. Gynecological conditions, including uterine leiomyomas (UL), adenomyosis (AM), endometrial polyps (EP), and ovarian benign tumors (OT) were diagnosed via ultrasonography. Urinary concentrations of five toxic heavy metals (As, Cd, Hg, Ni, Pb) and 11 trace elements (Ba, Co, Mn, Mo, Sb, Sn, Sr, Tl, U, W) were quantified using inductively coupled plasma mass spectrometry (ICP-MS). Metal concentrations were compared between cases and controls. Associations between single and mixture metal exposures and gynecological diseases were evaluated using logistic regression and g-computation analysis. Linear trends in disease prevalence across exposure levels were assessed using the Cochran-Armitage trend test.

Results: Among the participants, 198 were controls, and 83 were diagnosed with gynecological diseases (UL 53; AM 16; EP 12; OT 25). Detection rates exceeded 70% for all elements except Hg and U. No significant differences in urinary metal concentrations were observed between the control and disease groups. Logistic regression and mixture analysis did not indicate significant associations between metal exposure and the four gynecological diseases. However, trend analysis revealed a significant positive association between increasing urinary Ni and Sr and AM prevalence (p=0.01 for Ni,0.03 for Sr).

Conclusion: While no direct associations were observed between urinary metal concentrations and gynecological diseases, the significant trend between Ni and Sr exposure and adenomyosis prevalence suggests a potential role of these elements in disease progression. These findings highlight the need for exposure management and preventive measures against reproductive toxicants to protect women's reproductive health.

P25-05 | Relation between chemicals found in personal care products and prevalence of pelvic inflammatory disease

Gulan Pari, Robert B. Hood

The Ohio State University, College of Public Health, Columbus, USA

Objective: Pelvic inflammatory disease (PID) is a syndrome of upper genital tract and is associated with several adverse reproductive health outcomes. The role of endocrine disrupting chemicals (EDCs) on PID risk is currently unknown. Our study aimed to examine the potential association between chemicals found in personal care products and PID in a representative sample of US women.
Material and Methods: We examined the association between twelve phenols and parabens exposure and PID outcomes in the National Health and Nutrition Examination Survey (NHANES). We utilized two waves collected during the 2013-2014 and 2015-2016 cycles. Phenols and parabens were measured using solid phase extraction coupled with liquid chromatography and tandem mass spectrometry. Adult female participants were asked if they had ever been treated for a pelvic infection or PID. We restricted our sample to those with complete exposure and outcome data (n=1196). We conducted unadjusted and adjusted single-pollutant logistic regression models accounting for survey weights. Models were adjusted for age and ratio of family income-to-poverty.
Result: Among the twelve chemicals explored, methyl paraben, benzophenone-3 and propyl paraben had the highest median values, 87.3 ng/mL, 23.6 ng/mL, and 16.6 ng/mL, respectively. Among 1,196 participants, 55 (4.5%) had ever had a pelvic infection or PID. In both our crude and adjusted regression models, we observed limited evidence for a relation between these chemicals and PID. Interestingly, one chemical in our adjusted regression models, urinary triclocarban was inversely associated with the prevalence of PID (Odds Ratio=0.90), but this association was not statistically significant (95% Confidence Interval: 0.79, 1.02).
Conclusion: Overall, we did not observe evidence to suggest a relation between chemicals commonly found in personal care products and the prevalence of PID. Our study is limited due to its sample size and cross-sectional nature and should be repeated in a larger cohort study.

P25-06 | Volatile Organic Compound Mixtures on Liver and Mammary Gland Toxicity in Male and Female Rats

Savannah E. Jacobs[1,2], Adam J. Filgo[3], Alina M. Hamilton[1], Suzanne E. Fenton[1,2]

[1] North Carolina State University, Center for Human Health and the Environment, Office of Research and Innovation, Raleigh, USA; [2] North Carolina State University, Toxicology Graduate Program, Department of Biological Sciences, Raleigh, USA; [3] Zoetis, Inc., Kalamazoo, USA

Objective: Volatile organic compounds (VOCs) are ubiquitous anthropogenic chemicals linked to numerous cancers. At Camp Lejeune, North Carolina (NC; 1953-1987), United States Marine families exposed to VOC-contaminated drinking water developed adverse outcomes including male and female breast cancer. While this exposure resulted in the largest male breast cancer cluster to-date, mechanisms driving this relationship are unknown. Thus, we evaluated the effects of a VOC mixture in male and female rat offspring following exposure during pregnancy and adolescence.

Material and Methods: Timed-pregnant Sprague Dawley rats were exposed from gestational day 13 (GD13) through postnatal day 48 (PND48) to drinking water containing a VOC mixture mirroring that measured at Camp Lejeune. Upon necropsy at pre/post-pubertal timepoints (PNDs 23, 30, and 48), mammary gland (MG) and livers from male and female offspring were analyzed for RNA expression and morphology, and VOC concentrations and half-lives were quantified.
Results: Early-life VOC exposure resulted in decreased body and liver weights in both sexes at PNDs 30 and 48, and decreased liver P450 enzyme-related RNA expression at PND 48. Further, in both sexes, we observed a dose-dependent increase in MG developmental scores at PNDs 30 and 48. Following VOC mixture exposure, blood and MG tissue contained elevated benzene levels with extended half-lives relative to other VOCs in the mixture. However, an experiment restricted to benzene-only exposure revealed no effects on aforementioned endpoints.
Conclusion: Using a rat model, these data suggest early-life VOC mixture exposure increases risk for liver toxicity and advanced MG growth in males and females.
Funding: National Institute of Environmental Health Science P30ES025128 to the Center for Human Health and the Environment; Molecular Pathways to Pathogenesis in Toxicology T32ES007046-41 to NC State University.
Acknowledgements: The authors thank Dr. Ben Blout, Center for Disease Control and Prevention, for his role in developing mixtures for this study.

P25-07 | Exposure to organophosphate esters and gestational weight gain: New insights from a large prospective cohort

Belle Martin[1], David E. Cantonwine[2], John D. Meeker[3], Thomas F. McElrath[2], Kelly K. Ferguson[4], Barrett M. Welch[1]

[1] University of Nevada, Reno, School of Public Health, Reno, USA; [2] Brigham and Women's Hospital, Harvard Medical School, Division of Maternal-Fetal Medicine, Boston, USA; [3] University of Michigan, School of Public Health, Department of Environmental Health Sciences, Ann Arbor, USA; [4] National Institute of Environmental Health Sciences, Division of Intramural Research, Epidemiology Branch, Durham, USA

Objective. Organophosphate esters (OPEs) are flame retardants and plasticizers used in consumer products. Exposure during pregnancy may disrupt maternal metabolic function, which is dynamic during pregnancy. We aimed to determine association between maternal OPE exposures and gestational weight gain (GWG), an important indicator of metabolic health in pregnancy.
Materials and Methods. Five OPE biomarkers were measured and averaged across repeated urine samples in the LIFECODES Fetal Growth Study (n=901, 2008-2018). Maternal GWG was determined from extensive repeated weight measures (median:14 per/participant). Delivery weight was predicted by mixed-effects modeling. Linear regression was used to determine associations between individual biomarkers and total GWG. Group-based trajectory models identified three trajectories of GWG (expected, low-catch up, and constant-high). The expected trajectory had minimal first-trimester weight gain, followed by steadily increasing gains in following trimesters.
Results. We found mixed associations between OPE biomarkers and total GWG. For example, a doubling in bis(2-chloroethyl) phosphate (BCEP) was associated with 0.14 kg lower (95% confidence interval [CI]: -.26, .01) GWG, whereas a doubling in diphenyl phosphate (DPHP) was associated with 0.16 kg greater (95% CI: -.08, .46) GWG. Additionally, certain OPE biomarkers were associated with GWG trajectories. For example, compared to the expected trajectory group, a doubling in DPHP was associated with higher odds of being in the low-catch up (odds

ratio: 1.28, 95% CI: 0.98, 1.69) group. The low-catch up group was also most likely to have weight gain that exceeded clinical guidelines.
Conclusion. Our results in a large pregnancy cohort, with uniquely strong characterization of OPE biomarkers and GWG, show certain OPE exposures may have a range of impacts on maternal weight gain. We also provide novel evidence that OPE biomarkers are associated with trajectories of GWG that are outside of clinical guidelines. Thus, our study suggests gestational OPE exposures may impact maternal metabolism.

P25-08 | Using the Comparative Toxicogenomics Database (CTD) to explore air pollution-associated adverse pregnancy outcomes by integrating exposure data with toxicological mechanisms

Carolyn Mattingly, Brent Wyatt, Thomas Wiegers, Jolene Wiegers, Daniela Sciaky, Fern Barkalow, Melissa Strong, Sakib Abrar, Alan Davis

NC State University, Biological Sciences, Raleigh, USA

Objective: Chemicals are important factors that affect human health, yet the specific molecular mechanisms connecting an exposure to an adverse outcome often remain unknown, reflecting knowledge gaps. Here, we demonstrate how the publicly available Comparative Toxicogenomics Database (CTD; https://ctdbase.org) can be leveraged to discover molecular mechanisms linking air pollution to adverse pregnancy and birth outcomes to develop testable frameworks for environmental health.
Material and Methods: CTD is an innovative, publicly available resource that centralizes cross-species data for chemical exposures and includes over 4 million interactions that interrelate chemicals, genes, phenotypes, anatomical terms, diseases, and human exposure information reporting real-world biomarker measurements and health outcomes. Data are manually curated from more than 150,000 peer-reviewed scientific articles. Importantly, this content is integrated to generate novel inferences that help inform mechanistic knowledge gaps to connect and complement real-world exposure data with lab-based toxicology results.
Results: We evaluated articles in CTD that correlate air pollution exposure with pregnancy complications or adverse birth outcomes studied in more than 25 countries. Integrating additional toxicological mechanistic data with exposure findings identifies numerous air pollutants (e.g., ozone, particulate matter, vehicle emissions, soot) that affect specific genes (e.g., TNF, IL6, PTGS2, SOD2, MMP9), and biological phenotypes (e.g., cytokine production, apoptosis, blood vessel development, inflammatory responses) that can be connected to adverse pregnancy/birth outcomes (e.g., premature birth, fetal growth retardation, pre-eclampsia, spontaneous abortion).
Conclusion: We constructed specific, testable pathways for air pollution-induced adverse birth outcomes by leveraging the integrated content from CTD. Importantly, this methodology can be used to study any chemical or disease in public CTD, allowing researchers to survey the curated data-landscape and design molecular frameworks for their environmental health area of interest.

P25-09 | The link between prenatal exposure to a chemical mixture, cord blood hormones, and birth weight

Eva Govarts[1], Bianca Cox[1], Lützen Portengen[2], Andrea Rodriguez-Carrillo[3], Madeline Carsique[4], Adrian Covaci[5], Elly Den Hond[6], Stefaan De Henauw[7], Tim Nawrot[8], Amélie Crépet[4], Jacob Van Klaveren[9], Roel Vermeulen[2], Greet Schoeters[5]

[1] VITO, Mol, Belgium; [2] IRAS, Utrecht, Netherlands; [3] UGR, Granada, Spain; [4] ANSES, Paris, France; [5] UA, Antwerp, Belgium; [6] PIH, Antwerp, Belgium; [7] UGhent, Ghent, Belgium; [8] UH, Hasselt, Belgium; [9] RIVM, Bilthoven, Netherlands

Objective: Prenatal chemical exposure has frequently been associated with fetal growth, although the underlying molecular mechanisms remain unclear. This study aims to explore the potential mediating role of hormones in the association between prenatal chemical mixture exposure and birth weight.
Material and methods: We used data of 432 newborns from two Flemish birth cohorts. The common set of available and detectable exposure biomarkers and hormones analyzed in cord plasma are: 6 metals/trace elements, 3 polychlorinated biphenyl (PCB) congeners, hexachlorobenzene, dichlorodiphenyldichloroethylene and 2 perfluoroalkyl substances; and 3 thyroid, 3 reproductive and 2 metabolic hormones. Mixtures analyses were performed to assess each of the bilateral associations in the path exposures-hormones-birth weight, including mediation analysis.
Results: Combining all exposures, we found an inverse association between PCB 180 and birth weight. PCB 180 was positively associated with sex hormone-binding globulin (SHBG) and negatively associated with leptin and insulin. Similarly, thallium was positively associated with testosterone, estradiol, and SHBG, and negatively with insulin. Lead was positively associated with insulin. Higher free thyroxine (FT4), insulin, and leptin were associated with higher birth weight, whereas higher SHBG was associated with lower birth weight. Mediation analysis for PCB 180 indicated that 94% of the effect of this exposure on birth weight is mediated by FT4, SHBG, leptin, and insulin.
Conclusion: Assessing the health risk of chemical mixture exposure reflects better real-world situations, thereby allowing more effective risk assessment. Our results suggest that hormonal markers are on the causal path in the association between environmental exposure and birth weight, adding interesting insights for mechanistic research.

Acknowledgements/funding: PARC (Grant Agreement No 101057014); NAPED by FPS Health, Food Chain Safety and Environment, DG Environment; FLEHS studies by the Ministry of the Flemish Community (Department of Economics, Science and Innovation; Agency for Care and Health; and Department of Environment, Nature and Energy).

P25-10 | Association of urinary nitrophenol concentrations during pregnancy with preterm birth, low birth weight, and small-for-gestational-age: The Japan Environment and Children's Study (JECS)

Michihiro Kamijima[1], Kayo Kaneko[1], Yuki Ito[1], Takeshi Ebara[1,2], Hiroshi Yatsuya[3], Mayumi Sugiura-Ogasawara[4], Shinji Saitoh[5], Makiko Sekiyama[6], Tomohiko Isobe[6]

[1] Nagoya City University Graduate School of Medical Sciences, Dept of Occupational and Environmental Health, Nagoya, Japan; [2] University of Occupational and Environmental Health, Dept of Ergonomics, Institute of Industrial Ecological Sciences, Kitakyushu, Japan; [3] Nagoya University Graduate School of Medicine, Dept of Public Health and Health Systems, Nagoya, Japan; [4] Nagoya City University Graduate School of Medical Sciences, Dept of Obstetrics and Gynecology, Nagoya, Japan; [5] Nagoya City University Graduate School of Medical Sciences, Dept of Pediatrics and Neonatology, Nagoya, Japan; [6] National Institute for Environmental Studies, Japan Environment and Children's Study Programme Office, Tsukuba, Japan

Objective: Nitrophenols are widely detected in human urine, and whether their concentrations during pregnancy are associated with adverse birth outcomes remains inconclusive. The aim of this study was to clarify the association of 4-nitrophenol (4NP) and 3-methyl-4-nitrophenol (3M4NP) concentrations in maternal urine in mid-pregnancy with preterm birth, low birth weight (<2500 g), and small-for-gestational-age (SGA).
Methods: A total of 3650 non-hypertensive mothers with singleton births were enrolled from JECS. High 4NP (≥0.41 µg/L) and 3M4NP (≥0.29 µg/L) were defined as ≥lowest concentration minimum reporting level. Four groups were created using these dichotomized 4NP and 3M4NP concentrations: 'both low', 'either high/low', and 'both high'. Multivariable logistic regression models were used to estimate the adjusted odds ratios (aOR) and population attributable fraction (PAF).
Results: For 4NP and 3M4NP, 68.4% and 19.0% of participants had 'high' urinary concentrations, respectively. Compared to 'both low', the overall analysis showed no significant associations between 'both high' and any of the outcomes. However, the stratified analysis showed that the aOR (95% confidence interval [CI]) and PAF (95% CI) for 'both high' regarding preterm birth (<37 weeks' gestation) were 2.7 (1.3, 5.7) and 16.3% (2.5%, 28.1%), respectively, in male offspring. Among female offspring, the aOR and PAF for the 'both high' regarding SGA—defined as weight-for-gestational age <10 percentile—were 1.7 (1.02, 2.8) and 10.6% (-0.2%, 20.2%), respectively. Urinary 4NP and 3M4NP concentrations showed no association with low birth weight.
Conclusion: Higher concentrations of nitrophenols in maternal urine were associated with preterm births and SGA infants. The exposure source should be identified.
Acknowledgements: The authors thank all the study participants and the collaborative researchers/staffs/organizations. JECS is funded by the Ministry of the Environment, Japan (MOE), and the conclusions of this presentation are solely the responsibility of the authors and do not represent the official views of MOE.

P25-11 | Exposure risk assessment of bisphenol A and its substitute in relation to thyroid homeostasis in Taiwanese pregnant women nearby a Science Park

Po Chin Huang[1], Ching Chun Huang[2], Pao Ling Torng[3]

[1] National Health Research Institutes, National Institute of Environmental Health Sciences, Zhunan, Taiwan; [2] National Taiwan University Hospital Hsin-Chu Branch, Environmental and Occupational Medicine, Hsin-Chu, Taiwan; [3] National Taiwan University Hospital Hsin-Chu Branch, Department of Obstetrics and Gynecology, Hsin-Chu, Taiwan

Bisphenols, particularly bisphenol A (BPA) and its substitutes like BPS, BPF, and BPAF, are chemicals commonly used in the production of plastics, resins, and other industrial products. This study investigates the concentrations of bisphenol A (BPA) and its substitutes (BPS, BPF, BPB, BPAF) in the urine of 94 Taiwanese pregnant women living near a Science Park in Northern Taiwan from 2022 to 2024. It also evaluates their daily intake (DI) and potential health risks. The results show that the median concentration of BPF in urine was the highest (9.78 µg/L), followed by BPA (3.55 µg/L), with lower detection rates for other bisphenol compounds. After adjusting for urine creatinine, the median concentrations of BPF and BPA were 12.9 and 4.50 µg/g creatinine, respectively. The median daily intake (DI) for BPA and BPF were 1.28 and 3.48 ng/kg/day, respectively. Based on the 2023 evaluation by the European Food Safety Authority (EFSA), the acceptable daily intake (TDI) for bisphenols is 0.2 ng/kg/day. The study found that 72.2% of pregnant women exceeded the acceptable daily intake for BPA, and all (100%) exceeded the exposure limit for BPF. Hazard quotient (HQ) analysis revealed that the median hazard indices for BPA, BPS, and BPF were 18, 0.16, and 17.4, respectively, with BPF posing a greater risk to maternal health than BPA and BPS. Additionally, an analysis of the association between bisphenol exposure and thyroid hormone (T_4) concentrations showed a significant negative correlation between the second quartile of BPA concentration and T4 ($p = 0.045$). Furthermore, the second quartile of the combined molar concentrations of BPA and BPF (ΣBPs) was significantly negatively correlated with Free T_4 ($p = 0.029$). Exposure to bisphenol compounds may potentially affect thyroid function, emphasizing the need for enhanced monitoring and risk assessment of bisphenol exposure to protect maternal and fetal health.

P25-12 | Examining the Contribution of Air Pollution to Disparities in Preeclampsia

Jasmine Lee[1], Kelsey R. Bilsback[1], Yanelli Nunez[1], Jonathan J. Buonocore[2], Mary Willis[2], Sebastian Rowland[1]

[1] PSE Healthy Energy, Oakland, USA; [2] Boston University, Department of Environmental Health, Boston, USA

Vulnerable populations face persistent disparities in maternal health in the United States. For example, the prevalence of pre-eclampsia, a pregnancy complication marked by high blood pressure that impacts 3-6% of US pregnancies, is nearly 50% higher in Black patients than White patients. Exposure to air pollution may contribute to these disparities, given the inequitable exposure in environmental justice communities and air pollution's harmful effects on pregnancy and hypertension. Quantifying the role of air pollution can help communities and policymakers identify effective mitigation opportunities and priorities.
Here, we assess the contribution of fine particulate matter (PM2.5) and ozone exposure to Black-White disparities in preeclampsia in the United States.
The study population includes singleton pregnancies within the nationwide catchment area of Truveta, an electronic health records database, from 2019—2022. Pregnancies, including preeclampsia cases, have been identified via ICD-9, ICD-10, and treatment codes. We will examine gestational-week-specific exposure to PM2.5 and ozone estimated from a model developed by the U.S. Environmental Protection Agency (EPA) that fuses Community Multiscale Air Quality (CMAQ) model output with ground monitoring data to generate gridded estimates of air pollution exposures. We will apply logistic regression using ZIP-code average exposure and adjusting for meteorological and neighborhood-level risk factors as confounders associated with ambient air pollution.
Of the 1.4 million identified pregnancies, 5% experienced preeclampsia, with a 5.7% rate among White pregnancies and a 6.0% rate among Black pregnancies. In 2021, ZIP-code daily average PM2.5 concentrations averaged 8.4 µg/m3 (SD: 5.3 µg/m3) and 8-hour ozone daily maximum averaged 38.3 ppb (SD: 11.4 ppb). To generate actionable estimates, we quantify the impact as increases in preeclampsia prevalence and counts via additive associations from g-computation.
We would like to acknowledge funding support from the Robert Woods Johnson Foundation.

P25-13 | The seasonal factor in studies of temperature and pregnancy outcomes: A confounder, a collider, or a helpful source for realistic exposure contrasts?

Raanan Raz[1], Selin Girgin[1], Daniel Nevo[2]

[1] The Hebrew University of Jerusalem, Braun School of Public Health, Jerusalem, Israel; [2] Tel Aviv University, Tel Aviv, Israel

Objective
Temperature and pregnancy outcomes studies typically adjust for seasonality to prevent confounding by factors that change seasonally. However, actual confounding is not always evaluated. Additionally, some studies define seasonality based on birth dates, while others use the last menstrual period (LMP). For certain outcomes, such as

preterm birth, the birth date functions as a post-exposure collider, and its use may induce collider bias. Seasonal factors may also be included as time-varying covariates when associations are analyzed using a survival model. Finally, in some climates, seasonal changes account for most of the variability in the temperature exposure variable. This allows for using seasonal factors to illustrate the implications of different realistic exposure scenarios on risk.
This study aims to evaluate the various implications of methodological approaches to seasonality on confounding, collider bias, and risk quantification in a study of temperature and preterm birth in Israel.
Material and Methods
We employed a historical cohort design, including 131,599 singleton live births in Israel. We analyzed the risk of preterm birth using a time-varying Cox proportional hazards regression, modeling weekly temperatures with distributed lag models. Seasonality was represented by the month of birth, the month of LMP, a weekly time-varying covariate, or not at all. Additionally, seasonal exposure trajectories were utilized to demonstrate the predicted risk through survival curves.

Results
When using a time-varying seasonality covariate, we found no evidence of confounding by seasonality. In contrast, employing a fixed seasonal term based on LMP created a seasonal association pattern in some socioeconomic strata. Furthermore, adjusting for the birth month introduced a strong collider bias. The predicted relative risk by LMP month varied between 1.0 and 1.17.

Conclusion
The need for adjustment for seasonality should be assessed for each specific study. Researchers should avoid using post-exposure seasonal factors for adjustment.

P25-14 | Vitamin D and fertility: leveraging menstrual effluent to examine circulating levels of 25-hydroxyvitamin D (25OHD) and environmental effects on endometrial decidualization

Christine N. Metz[2], Stephanie Busch[2], Prodyot K. Chatterjee[2], Sophia Tarasenko[2], Peter K. Gregersen[2], Anne Z. Steiner[3], **Anne Marie Z. Jukic**[1]

[1] NIEHS, Epidemiology Branch, Durham, USA; [2] The Feinstein Institutes for Medical Research at Northwell Health, Manahasset, USA; [3] University of North Carolina School of Medicine, Division of Reproductive Endocrinology and Infertility, Chapel Hill, USA

Objective: The prevalence of infertility is increasing. Inadequate vitamin D may both exacerbate effects of endocrine disrupting chemicals, and directly affect uterine decidualization, a process essential for human embryo implantation. We investigated effects of vitamin D on decidualization using menstrual effluent (ME) derived endometrial stromal cells (eSCs).
Material and Methods: We used ME-eSCs from the Research Outsmarts Endometriosis (ROSE) study (NY) and the Investigation of Vitamin D and Menstrual Cycles Trial (inVitD) (Michigan & North Carolina). Both studies included healthy participants, ages 19-40, and not using hormones. ME was collected during peak flow (menses days 1-3) using a menstrual cup. ME-eSCs were isolated, cultured, and cryopreserved. First, we examined the effect of pre-treatment with active vitamin D (calcitriol, 10^{-7}M) on ME-eSC decidualization (vs vehicle). Second, we compared decidualization using inVitD eSCs collected before and after *in vivo* cholecalciferol supplementation. Decidualization

was stimulated *in vitro* with 8-bromoadenosine 3′,5′-cyclic monophosphate sodium salt (cAMP, 0.5mM) and medroxyprogesterone acetate (MPA [$1x10^{-7}$M], an analogue of progesterone). After 48hrs, supernatants were assayed for insulin growth factor-binding protein 1 (IGFBP1) and prolactin (PRL) protein, biomarkers of decidualization. Third, we compared ME-serum levels of 25-hydroxyvitamin D (25OHD) with peripheral serum levels. Results: In ROSE and inVitD eSCs, vitamin D significantly increased IGFBP1 expression 3-fold (vs vehicle, p<0.0001) and prolactin expression 1.5-fold (p<0.0001). There was a slight, though non-significant, increase in decidualization capacity after supplementation. ME-serum 25OHD was correlated with peripheral serum (r = 0.61, p=0.05).Conclusion: Vitamin D enhanced eSC decidualization. Supplementation may make *in vivo* eSCs more responsive to vitamin D. These effects on decidualization could explain observed associations with fertility. The correlation between ME and circulating 25OHD levels suggests utility of ME for measuring environmental exposures. ME can elucidate biological pathways affected by environmental exposures, providing an important link between epidemiologic and molecular research. Funding: NIEHS

P25-15 | Heavy metals and their associations with preterm birth: Study using GAPPS neonatal urine samples

Harpriya Kaur[1], Kelsey Brady[3], Prinn Panuwet[1], Jeffrey Jarrett[2], Cynthia Ward[2], Elizabeth Irvin[1]

[1] ATSDR, OCHHA, Atlanta, USA; [2] CDC, NCEH/DLS, Atlanta, USA; [3] ATSDR, OIA, Atlanta, USA

Background: Preterm birth (PTB) (<37 weeks) is one of the leading causes of neonatal mortality and morbidity in US. From 2014 to 2022, the PTB rate rose more than 10%. The objective of this study is to evaluate whether *in-utero* exposure to heavy metals are related to preterm births.
Methods: We analyzed 15 metal(loids) in 399 neonatal urine samples from the Global Alliance to Prevent Prematurity and Stillbirth (GAPPS) cohort. GAPPS research coordinators collected urine using diaper sponges at delivery. The urinary metal(loids) were analyzed using inductively coupled plasma dynamic reaction cell mass spectrometry. Using SAS, we performed bivariate and elastic net regression analyses to examine the associations between PTB and non-normalized urinary metal concentrations while controlling for maternal factors (including maternal age, obesity, anemia, ethnicity, smoking status, gestational diabetes and hypertension, and antepartum hemorrhage) and multicollinearity among mixture analytes.
Results: Mothers with PTB deliveries were older than mothers delivering at ≥ 37 weeks (mean age 31.6 vs 29.5 years) and had higher rates of obesity (45%), gestational diabetes (15.4%), antepartum hemorrhage (18%), and twin pregnancies (33.7%). These differences were statistically significant. After adjustment for confounders, higher neonatal tin levels were associated with increased odds of PTB (OR 3.75, 95% CI 2.12-6.62), while increased neonatal molybdenum decreased PTB odds (OR 0.17, 95% CI 0.10-0.30).
Conclusion: Our findings suggest that neonatal tin exposure may increases PTB risk while molybdenum exposure shows protective effects. Though tin and PTB association remains imprecise, future studies should investigate the underlying mechanisms of *in-utero* metal exposure effects on PTB.
Disclaimer: The findings and conclusions in this presentation have not been formally disseminated by the Centers for Disease Control and Prevention/the Agency for Toxic Substances and Disease Registry and should not be construed to represent any agency determination or policy.

P26 | Air Pollution Exposure, Health Effects, and Mitigation Strategies Across Populations and Regions

P26-01 | Concentrations of polycyclic aromatic hydrocarbons (PAHs) in personal air samplers and resulting inhalation exposure of Rwandan cooks

Clara Eichler[1], Gina Hidalgo Diaz[1], Joseph Pedit[2], Zachary G. Robbins[1], Ipsita Das[3], Karin Yeatts[4], Pamela Jagger[5], Sudhanshu Handa[2,6], Leena Nylander-French[1]

[1] University of North Carolina at Chapel Hill, Department of Environmental Sciences and Engineering, Chapel Hill, USA; [2] University of North Carolina at Chapel Hill, Carolina Population Center, Chapel Hill, USA; [3] Duke University, Sanford School of Public Policy, Durham, USA; [4] University of North Carolina at Chapel Hill, Department of Epidemiology, Chapel Hill, USA; [5] University of Michigan, School for Environment and Sustainability, Ann Arbor, USA; [6] University of North Carolina at Chapel Hill, Department of Public Policy, Chapel Hill, USA

Objective: Polycyclic aromatic hydrocarbons (PAHs) are products of incomplete combustion associated with the burning of biomass fuels, including fuelwood and charcoal commonly used for household cooking in low-income countries. PAHs are major contributors to household air pollution (HAP) and associated with multiple adverse health outcomes, including lung cancer. Here, we assessed inhalation exposure of Rwandan cooks to PAHs using personal air samplers and evaluated potential contributing factors.
Material and Methods: We measured concentrations of 16 priority PAHs in personal air samplers worn by the primary cooks of 300 households in Gisenyi, Rwanda between 2015 and 2019. Air samples were collected during four sampling rounds in which approximately 170 of the 300 households participated, with several households participating in some rounds but not in others. Information on stove and fuel types, as well as cooking environment, were collected. Extracted air samples were analyzed by gas chromatography-mass spectrometry (GC-MS) and resulting PAH concentrations were used to estimate the daily intake (DI) by the cooks via inhalation.
Results: The concentrations of the sum of the 16 PAHs, ∑(PAHs), in the air samples ranged from 0.061 μg/m³ to 69 μg/m³ with an overall mean of 2.4 μg/m³, and were dominated by naphthalene. Over the course of the study, we observed a modest transition towards cleaner stoves associated with a reduction in extremely high ∑(PAHs) levels. The average DI of ∑(PAHs) via inhalation was 0.65 μg/d/kg BW. PAH concentrations in personal air samples and estimated DIs were significantly influenced by stove and fuel type.
Conclusion: Burning biomass fuels was associated with high levels of personal PAH inhalation exposure. A shift to cleaner cooking technologies reduced PAH exposure, especially for household cooks.
Funding: This work was funded by NIOSH (T42OH008673), NIH/NIEHS (R01ES023861), and the United Nations Foundation (UNF-15-708).

P26-02 | Personal $PM_{2.5}$ Exposure During Commuting – A Comparison of Commuting Modes and Measurement Devices

Denise Feurer[1], Dolores Catelan[1], Annibale Biggeri[1], Francesco Pirotti[2], Daniele Grechi[3], Francesco Sera[4]

[1] University of Padova, Unit of Biostatistics, Epidemiology and Public Health, Padova, Italy; [2] University of Padova, Interdepartmental Research Center of Geomatics (CIRGEO), Padova, Italy; [3] Epidemiologia & Prevenzione, Social Enterprise, Milano, Italy; [4] University of Florence, Department of Statistics, Computer Science and Applications, Florence, Italy

Introduction

Air pollution is a major global health threat, causing over 4 million premature deaths annually. This study evaluates $PM_{2.5}$ exposure of commuters across different transportation modes and compares measurements from personal devices with low-cost citizen science sensors.

Methods

Our study was conducted in metropolitan Florence, Italy, measuring $PM_{2.5}$ exposure during commutes over 16 weeks in January-February 2019 and 2020. Each week, two participants wore personal monitors, recording exposure, coordinates and time each minute during their commute. Concurrently, 20 low-cost sensors and two local authority monitors measured $PM_{2.5}$.

We linked personal $PM_{2.5}$ measurements to the nearest low-cost sensor with readings within 60 minutes to analyze differences across commuting modes. Validity checks - including Pearson correlation, Lin's concordance correlation coefficient, and the correlation between differences and the mean - were used to compare personal monitors, low-cost sensors, and local authority monitors.

Results

A total of 4384 minutes of commute was recorded. Personal monitors recorded an average $PM_{2.5}$ exposure of 18 μg/m^3 (95% CI: 18, 18), which was comparable to the nearest low-cost sensors (18 μg/m^3, 95% CI: 18, 19). Overall, the validity test indicated strong correlations between measurement devices (Pearson's r = 0.81 to 0.97).

The highest personal exposure was observed in tram commutes (36 μg/m^3), followed by walking (25 μg/m^3), cycling and bus (22 μg/m^3), while scooters (17 μg/m^3) and cars (15 μg/m^3) measured the lowest exposure. The largest discrepancy was in cycling, where personal monitors recorded a 10 μg/m^3 higher exposure than low-cost sensors, followed by trams, with an 8 μg/m^3 lower exposure.

Discussion

$PM_{2.5}$ exposure varied significantly between commuting modes, with trams recording the highest and cars/scooters the lowest personal exposure. Discrepancies between monitoring devices highlight the differences in direct exposure, particularly for cyclists. These findings could help inform policies to improve transportation infrastructure and reduce exposure risk.

P26-03 | The effect of air pollution and seasonality on the respiratory microbiome of healthy office workers

Giulia Solazzo[1,2], Sabrina Rovelli[3], Simona Iodice[2], Valentina Bollati[2,4], Luca Ferrari[2,4], Elodie Ghedin[1]

[1] National Institutes of Health, Systems Genomics Section, National Institute of Allergy and Infectious Diseases (NIAID), Bethesda, USA; [2] University of Milan, Department of Clinical Science and Community Health, Milan, Italy; [3] University of Como, Department of Science and High Technology, Como, Italy; [4] Fondazione IRCSS Ca' Granda Ospedale Maggiore Policlinico, Occupational Health Unit, Milan, Italy

Objective: This study investigated the effects of seasonality and exposure to air pollutants, including microbes carried by air particles, on the nasal microbiome of health office workers. Given the direct exposure of the upper airways to the environment, studying the respiratory microbiome and its interactions with environmental factors is key to understanding its complexity.

Material and Methods: Nasal swabs were collected weekly from volunteers over a period of three weeks in the winter and three weeks in the summer. Extracted DNA was subjected to both 16S ribosomal RNA gene sequencing and whole genome shotgun sequencing. Samples of total suspended particulate (TSP) were collected weekly inside offices and outside office buildings. The TSP microbiome was analyzed by shotgun sequencing. Concentrations of PM_{10}, $PM_{2.5}$, benzene, carbon monoxide, and black carbon were obtained from the Italian Agency for Environmental Protection.

Results: While outdoor air pollution concentrations were higher in the winter, the indoor TSP concentrations were not different between seasons, likely due to the similarity in office activities across seasons. As for TSP concentrations, the microbiome on the outdoor TSP changed across the seasons and differed significantly from the indoor TSP microbiome. Indoor samples had a higher relative abundance of bacteria associated with opportunistic infections, such as *Paracoccus* sp. and *Klebsiella pneumoniae*. Additionally, TSP samples contained broad-spectrum antibiotic resistance genes including against tetracyclines. The nasal microbiome was stable over a short time-period (e.g., weeks), but it changed between seasons. Microbial diversity was higher in the summer, while winter was characterized by a higher prevalence of gram-negative bacteria like *Moraxella*. Finally, in both seasons, exposure to air pollution was associated with a decrease in the relative abundance of commensal bacteria such as *Corynebacterium* sp.**Conclusion:** All together these findings indicate a strong influence of seasonality and air pollution exposure on shaping the respiratory microbiome.

P26-04 | Adjustment Criteria for Air Quality Standards based on altitude: A Scoping Review

Lenin V. Rueda-Torres[1], Julio L. Warthon-Ascarza[2]

[1] Universidad Nacional Agraria La Molina, PhD program in Engineering and Environmental Sciences, Lima, Peru; [2] Universidad Nacional San Antonio de Abad del Cusco, Academic Department of Physics, Cusco, Peru

Air quality standards (AQS) are essential for protecting public health by regulating pollutant thresholds. However, high-altitude (HA) environments have unique atmospheric conditions, such as reduced pressure and lower oxygen levels, which may affect pollutant concentrations and human susceptibility. This raises a critical knowledge gap on whether altitude-specific AQS adjustments are needed to ensure adequate protection for HA populations. This study systematically assesses the literature to address this issue.

Methods: This review followed PRISMA-P guidelines, focusing on studies examining AQS adjustment approaches based on altitude. The Population/Concept/Context (PCC) framework was used to define search terms: Population (AQS), Concept (Air Pollutants), and Context (Altitude), with equivalents from PubMed MeSH and PAHO DeCS. Literature was retrieved from MEDLINE, SCOPUS, Web of Science, and SciELO. The protocol is available on OSF: https://osf.io/c3fnh/.

Results: 2,964 articles were identified, with 2,093 remaining after duplicate removal. After title and abstract screening, 2,075 papers were excluded, leaving 18 for full-text evaluation. Finally, six studies met the eligibility criteria (Table 1). Three studies focused on adjustment models based on atmospheric conditions, such as temperature and pressure changes, while the other three examined human physiological responses, particularly the effects of low oxygen levels and increased inhaled air volume.

The review highlights the necessity for tailored AQS studies focused on AH populations.

Table 1. AQS adjustments proposals identified.

Author, country, year		Basis of adjustment
W.B. Goddard	USA-1979	AQS correction by change of pressure and temperature.
H. Bravo, A. 1981	Mexico-	Hyperventilation at altitude increases the risk of pollutant exposure
Clarence R. Collier USA-1983		Less oxygen partial pressure at altitude increases risk of carboxyhemoglobin
Leizel Madueño 2020	Germany-	Hyperventilation at altitude increases the risk of pollutant exposure
H. Bravo Alvarez Mexico-2013		General gas law. Volume concentration change at different pressure and temperatures
Guanfeng Yan	China-2021	Low oxygen influences the vehicle pollutant emissions

P26-05 | Assessing Air Pollution Dispersion and Population Exposure from Unconventional Oil and Gas Development in Texas

Mohammadreza Bohloul, Lucas Henneman

George Mason University, 1Sid and Reba Dewberry Department of Civil, Environmental, and Infrastructure Engineering, Fairfax, USA

The rapid growth of UOGD in the U.S. has increased emissions of methane, ethane, propane, and VOCs, exacerbating environmental and health concerns. We aim to quantify UOGD processes in Karnes County, Texas, using different modeling systems, including Inverse Distance Weighting (IDW), Inverse Distance Weighting with wind speed and direction (IDWmet), Gaussian Model, and AERMOD. Our analyses indicate that meteorological variations have a greater impact on pollutant concentration fluctuations than emissions variability. Among emission sources, tank flash contributes the most (44%) to pollutant concentrations. Additionally, sources within a 15 km radius account for the majority (85%) of pollutant concentrations. Our spatial results show that pollutant concentrations form a southwest-to-northeast band, while the southeastern region of the study area exhibited lower levels. This spatial pattern is driven by the combination of emission source density and prevailing winds originating from the southeast. The modeled daily concentrations of all pollutants exhibit an average correlation (R) of 0.9 for IDWmet, 0.7 for AERMOD, and 0.5 for Gaussian with observations. Additionally, analysis of daytime and nighttime concentrations reveals that average nighttime pollutant concentrations are higher than daytime levels, primarily due to meteorological differences, while spatial concentration patterns remain consistent across both periods. Our results reveal that UOGD-related emissions lead to substantial spatial variability in pollutant concentrations, with nighttime exposures consistently higher than daytime levels. Population-weighted exposure estimates indicate pronounced disparities across racial, ethnic, and income groups. Hispanic and White populations experience the highest exposure levels, while Native American and Asian communities face comparatively lower concentrations, largely due to geographic distribution relative to emission sources. Income-based analyses show that both lower- and higher-income groups experience greater exposure than middle-income groups, suggesting complex socio-spatial factors influencing pollutant distribution.

P26-06 | Cross-Border Air Quality Monitoring: Los Dos Laredos Network for $PM_{2.5}$

Sofia M. Osuna Uribe[1], Anjala Pyakurel[2], Tricia Cortez[2], Richard Peltier[1]

[1] University of Massachusetts, Environmental Health Sciences, Amherst, USA; [2] Rio Grande International Science Center, Laredo, USA

Objectives
To address transnational pollution, the University of Massachusetts and the Rio Grande International Science Center have established Los Dos Laredos Network, which is guided by the first Binational Air Council in Laredo, TX, and Nuevo Laredo, México. This network investigates air quality by leveraging a new, binational network of low cost particle sensors. The network seeks to characterize $PM_{2.5}$ in moderately sized cities on each side of the border.

Methods
Launched in October 2024, the network consists of 19 low-cost scattering nephelometer nodes, generating approximately 10,000 hourly $PM_{2.5}$ estimates per month. The network also includes collocated sensors with the region’s only EPA reference monitor for PM2.5. The monitoring locations were identified by community-based partners in this participatory research approach and include neighborhoods near the World Trade Bridge and the Juarez-Lincon Bridge II, which receive substantial cross-border traffic.

Results
Monthly, bilingual meetings are convened with members of the public, regulators, activists, and academics to discuss network operations and develop broader knowledge on air quality. Data are displayed through a public-facing, bilingual dashboard, featuring data visualizations and descriptions aimed at a public audience. Though a nascent network, evidence suggests clear ‘hot spots’ of pollution arising from specific geographic regions and likely linked to specific economic sectors. Responding to recommendations from the binational air council, we continue to iterate complex scientific data towards broader understanding through improved engagement activities.

Conclusions
In a community with a paucity of monitoring infrastructure, community-based initiatives such as Los Dos Laredos Network builds long-term, community-based research capacity and knowledge creation that is essential for cleaner air in this binational region.

Funding
The North American Development Bank Border 2025 program.

P26-07 | Scalable Bayesian Geostatistical Regression Model for Bias-Correcting Large-Scale Daily Satellite-Retrieved AOD and CTM Simulations

Wyatt G. Madden[1], Yang Liu[2], Howard H. Chang[1]

[1] Emory University, Department of Biostatistics and Bioinformatics, Atlanta, USA; [2] Emory University, Department of Environmental Health, Atlanta, USA

Objective
Ambient fine particulate matter (PM2.5) is linked to adverse health outcomes such as respiratory and cardiovascular disease, low birth weight, and premature mortality. Accurate PM2.5 prediction and uncertainty estimation at fine resolution is crucial for health analyses. We introduce a scalable Bayesian spatial-temporal geostatistical model that can incorporate Chemical Transport Model (CTM) outputs or satellite-based Aerosol Optical Depth (AOD), and additional spatiotemporal covariates, to improve large-scale PM2.5 predictions. We demonstrate the high predictive performance and accurate uncertainty quantification of the model via two case studies in 2019 and provide open-source R software for environmental health researchers.

Material and Methods
In the first case study, daily PM2.5 measurements from 158 Air Quality System (AQS) monitors across California were used to bias-correct 1km AOD data with additional meteorology, and land-use covariates. In the second case study, daily PM2.5 from 533 AQS monitors over the contiguous United States was used to bias-correct 12km CTM. We also predicted PM2.5 for all 1km grid cells in California and all 12km grid cells in the U.S. Our framework employs a Nearest-Neighbor Gaussian Process (NNGP) for scalable inference of spatiotemporal random effects, enabling us to handle large datasets efficiently. Additional covariance function specifications were implemented to improve computational efficiency and model fit.

Results
For the 12km California data, our model performs similar to Random Forest (RMSE = 8.16 μg/m³) while maintaining accurate predictive uncertainty quantification (interval coverage = 0.96). For the 1km U.S. data, it also achieves high predictive performance (RMSE 4.42 μg/m³) and accurate uncertainty quantification (coverage 0.97).

Conclusion
Bayesian geostatistical regression models have been less utilized in large-scale PM2.5 prediction problems due to computational challenges. Our scalable approach produces high predictive accuracy and reliable uncertainty estimates, and with the creation of R software can facilitate its application in future air quality research.

P26-08 | Air Pollution and Psychiatric Diseases Among the Medicare Population Using the Generalized Weighted Quantile Sum Regression

Yichen Wang[1,2], Heresh Amini[3,4], Mahdieh Danesh Yazdi[5], Yaguang Wei[1,3], Michelle Bell[2], Joel Schwartz[1,6]

[1] Harvard T.H. Chan School of Public Health, Department of Environmental Health, Boston, USA; [2] Yale University, School of Environment, New Haven, USA; [3] Icahn School of Medicine at Mount Sinai, Department of Environmental Medicine and Climate Science, New York, USA; [4] Icahn School of Medicine at Mount Sinai, Institute for Climate Change, Environmental Health, and Exposomics, New York, USA; [5] Stony Brook University, Program in Public Health, Department of Family, Population, & Preventive Medicine, Stony Brook, USA; [6] Harvard T.H. Chan School of Public Health, Department of Epidemiology, Boston, USA

Background: The relationship between air pollution and psychiatric outcomes remains poorly understood. The cumulative effects of multiple-component pollution mixture and the most harmful components need to be clarified.
Objective: To estimate the mixture effect of $PM_{2.5}$ components, NO_2, and warm-season O_3 on hospitalization risks for psychiatric diseases and to analyze the relative contribution of each pollutant.
Methods: We derived annual and ZIP code-level exposure data for NO_2, warm-season O_3, and 15 $PM_{2.5}$ components (SO_4^{2-}, NO_3^-, NH_4^+, OC, EC, Br, Ca, Cu, Fe, K, Ni, Pb, Si, V, Zn, and Other) from high-resolution spatiotemporal models across the contiguous US from 2000 to 2016. We obtained the hospitalization data for depression, bipolar disorder, and schizophrenia among Medicare beneficiaries as three outcome measures. The Weighted Quantile Sum Regression was applied to estimate the mixture effects of all pollutant components and their weights. Separate analyses were conducted by socio-demographic groups to assess potential effect modification.
Results: We observed positive associations between the air pollution mixture with hospitalizations for depression, bipolar disorder, and schizophrenia. The relative risks were 1.07 (95% CI: 1.07, 1.08), 1.08 (95% CI: 1.07, 1.09), and 1.16 (95% CI: 1.15, 1.17), respectively, per decile increase in long-term exposure to the mixture. Ca and SO_4^{2-} were the largest contributors to the mixture effect on depression. SO_4^{2-} and NO_2 were the most influential components for bipolar disorder, while SO_4^{2-} and Zn contributed the most to schizophrenia. Blacks appeared more susceptible to the mixture-associated risk of all outcomes, while non-Medicaid people and those living in low-poverty areas were at greater risk of depression and schizophrenia.
Conclusions: Long-term exposure to the air pollution mixture could increase the hospitalization risk of psychiatric disease in the US elderly. Controlling emissions of NO_2 and certain $PM_{2.5}$ components such as SO_4^{2-} could effectively mitigate the related psychiatric disease burden.

P26-09 | Association Between Ambient Styrene, Benzene, Toluene, Ethylbenzene, and Xylenes (SBTEX) Exposure and Hospitalizations in the US Gulf Region

Yongsoo Choi[1], Beiming Tang[2,3], Joshua L. Warren[4,5], Lawrence S. Engle[6], Bok Haeng Baek[2], Michelle L. Bell[1,7]

[1] Yale University, School of the Environment, New Haven, USA; [2] George Mason University, Center for Spatial Information Science and Systems (CSISS), Fairfax, USA; [3] National Oceanic and Atmospheric Administration (NOAA), Air Resources Laboratory (ARL), College Park, USA; [4] Yale School of Public Health, Public Health Modeling Unit, New Haven, USA; [5] Yale School of Public Health, Department of Biostatistics, New Haven, USA; [6] University of North Carolina, Department of Epidemiology, Chapel Hill, USA; [7] Korea University, School of Health Policy and Management, Seoul, South Korea

Styrene, benzene, toluene, ethylbenzene, and xylenes (SBTEX) are volatile organic compounds known to pose risks to human health. However, relatively little is known about how ambient (non-occupational) concentrations of these compounds are distributed across different communities or socioeconomic groups, or how such lower-level exposures translate into health risks. We aimed to estimate the short-term effects of ambient SBTEX exposure on cardiovascular and respiratory hospitalizations among individuals aged 65 years and older in the US Gulf region from 2011–2016. We analyzed all Medicare fee-for-service hospitalizations in this population using a time-stratified case-crossover design. Conditional logistic regression was used to assess associations between daily ambient SBTEX concentrations and hospital admissions. Our preliminary results indicate that higher daily SBTEX concentrations were associated with increased risks of both cardiovascular and respiratory hospitalizations. Comparing the 75th versus 25th percentile of daily concentrations, we estimated changes in cardiovascular hospitalization risk of -0.01% (95% confidence interval [CI]: -0.09, 0.08) for styrene; 0.33% (CI: 0.02, 0.64) for benzene; 0.48% (CI: 0.14, 0.82) for toluene; 0.40% (CI: 0.13, 0.67) for ethylbenzene; and 0.41% (CI: 0.10, 0.73) for xylenes. The corresponding increases in respiratory hospitalizations were 0.03% (CI: -0.07, 0.13) for styrene; 1.05% (CI: 0.67, 1.44) for benzene; 1.11% (CI: 0.70, 1.53) for toluene; 0.91% (CI: 0.57, 1.24) for ethylbenzene; and 0.99% (CI: 0.61, 1.37) for xylenes. These findings provide evidence that even relatively low ambient SBTEX levels encountered in daily life can contribute to higher hospitalization rates.

P26-10 | Residential Wood Burning, Macrophage Carbon Load, and Health Effects: A Multi-Site Pilot Study in the Mountain West

Yue Feng[1], Yiliang Zhu[1], Xiaozhong Yu[1], Chelin J. Hu[1], Micah Hahn[2], Zeina Jaffar[3], Kristen McHenry[4], Akshay Sood[1], Paula Meek[5], Shuguang Leng[1]

[1] University of New Mexico, Albuquerque, USA; [2] University of Alaska Anchorage, Valdez, USA; [3] University of Montana, Missoula, USA; [4] Boise State University, Boise, USA; [5] University of Utah, Salt Lake City, USA

Objective: Residential wood smoke exposure is a critical public health issue in many communities in the United States. A knowledge gap exists regarding the lung deposition of black carbon from residential wood burning and its dose-response to health outcomes.

Maethods: A multi-site panel study was conducted on people who use wood stoves for winter heating in New Mexico (NM), Montana/Idaho, and Alaska. A 12-item questionnaire assessing indoor wood burning, St. George's Respiratory Questionnaire (SGRQ), and spontaneously expectorated sputum were collected during the peak (January and February) and shoulder (two weeks before or after peak) heating seasons across all sites. Additional biospecimens were collected during summer (blood and sputum) and peak (blood) surveys in NM. Scoring of macrophage carbon load (MaCL) was conducted manually (number of particles [NoP]) or using our cutting-edge artificial intelligence (AI) algorithm (NoP and area of particles [AoP]).

Results: Among 103 subjects enrolled, a total of 71 subjects had MaCL measures in at least one survey. Majority (>98%) of the black pitched particles detected in macrophages had diameters <1 μm, with median diameters at 0.66 μm. Among 12 questions assessing indoor wood burning, starting or attending the fire and leaving stove/fireplace doors open during burning were associated with higher MaCL, particularly for larger (diameter >0.66 μm) particles. Specially, always leaving stove/fireplace doors open (compared to never) was associated with increase in AoP by 0.78 μm^2. Additionally, each 1μm^2 increase in AoP was significantly associated with higher SGRQ score in activity domain (β=5.01, with a value of 4 indicating clinical significance). In New Mexico, we also found higher systemic inflammation response index (SIRI, 0.28) associated with 1μm^2 increase in AoP with larger particles demonstrating stronger pro-inflammatory potency.

Conclusions: Residential wood burning increased sputum MaCL. Increasing MaCL was associated with worse respiratory quality of life and higher systemic inflammation.

P26-11 | Evaluation of hazardous airborne volatile organic compounds and CO_2during work and non-work hours within Philadelphia dry cleaners

Grant Kassan[1], Heli Patel[1], Chanmi Lee[1], Caesar Belchez[1], Jin Y. Seo[2], Inkyu Han[1]

[1] Temple University, College of Public Health, Philadelphia, USA; [2] Hunter College, School of Nursing, New York, USA

Objective:
The objective of this study was to compare airborne total volatile organic compounds (TVOCs) and CO_2, inside dry cleaners in Philadelphia during work and non-work hours post 2014 local perchloroethylene (PERC) regulation implementation in Philadelphia.

Methods/Material:
Thirty-four dry cleaning, majority Korean owned, shops were recruited via canvassing in the Philadelphia area in 2024. Air quality was assessed using Air Assure sensors to measure TVOCs and CO_2, levels over at least 48 hours. Working hours were defined as Monday-Friday, from 7 AM - 7PM, aligning with typical operational schedules. Dry cleaning owners' workload, cleaning activities, and maintenance efforts (i.e., frequency of equipment cleaning) were reported via surveys.

Results:
TVOC levels were influenced by the cleaner's workload, cleaning activities, and maintenance efforts. Nonetheless, there was no significant difference in TVOC levels between work and non-work hours (median TVOCs: 412.21µg/m^3 vs. 403.71µg/m^3; p= 0.0516). In contrast, a significant difference was observed in CO_2 levels between work and non-work hours (median CO_2: 665.28 ppm vs. 598.83 ppm; p< 0.0001).

Conclusions:
Regulations enacted in Philadelphia in 2014 regarding PERC and n-Propyl Bromide (n-PB) pollution led many dry cleaners in the Philadelphia area to transition to off-site cleaning facilities, commonly known as "factories". This shift has reduced in-store air contamination.

The U.S. lacks indoor air quality standards for TVOCs and CO_2, with no universal TVOC exposure limits internationally, though CO_2 guidelines exist. In this study, measured concentrations remained below the recommended thresholds of 500 µg/m^3 for TVOC set in LEEDV4 and 1000 ppm for CO_2.

P26-12 | Characterizing a highway corridor's effect on the ambient air quality of America's oldest majority Black neighborhood

Brian W. Lucas, David Luglio, **Mostafijur Rahman**

Tulane University, Global Environmental Health Sciences, New Orleans, USA

Objective: The purpose of this study is to characterize the average concentration of ultrafine particles (pt/cc), PM2.5 (µg/m^3), and black carbon (µg/m^3) in proximity to the I-95 overpass in New Orleans' Tremé Neighborhood. Methods: Prior to sampling, a grid was mapped over the Tremé neighborhood to identify a coordinate scheme of evenly spaced points along each road. 9 evenly spaced street corners were sampled along streets parallel to the Tremé section of the I-95 overpass. Data was collected using a microAeth AE51, Personal DataRAM™ pDR-1500 Aerosol Monitor, and TSI P-Trak Ultrafine Particle Counter 8525. Samples were collected approximately 4 feet above the ground in 5-minute intervals to simulate the height at which children inhale ambient air. Street corners iso-distant to the overpass were aggregated and averaged to characterize the air quality as a function of proximity. Individual streets were sampled regularly on a random schedule from September 2024 to February 2025. Results: For three of the closest streets parallel to the highway, the average PM2.5 concentration for each street was found to be 13.59, 6.99, 5.81 µg/m^3 (in order of increasing distance from the highway). An exception was noted for a high-traffic road which had an average concentration of 9.77 µg/m3. In order of distance from the highway, carbon concentrations were found to be higher near the highway with concentrations 0.75, 0.46, and 0.47 µg/m^3 respectively. Conclusions: Overall, the study suggests that proximity to the highway could be a contributing factor towards poor air quality in the oldest majority Black neighborhood in the United States. While further study is needed, these initial findings corroborate complaints made by lifelong residents of the Claiborne Avenue Highway Corridor.

P26-13 | The role of FISE subsidies to accelerate LPG use in Peru

Carlos Carrillo-Gallegos[1], Annelise Gill-Wiehl[1], Karin Troncoso[2], Ajay Pillarisetti[3], Daivie Puccla[4], Juan Carlos Buleje[4], Isabel Atamara[4], Johnny Gonzales[4], Darby Jack[1]

[1] Columbia University Mailman School of Public Health, Environmental Health Sciences, New York, USA; [2] World Bank, Washington D.C, USA; [3] University of California, Berkeley School of Public Health, Environmental Health Sciences, Berkeley, USA; [4] Peru Ministerio de Energía y Minas, Fondo de Inclusión Social Energético, Lima, Peru

Introduction: Exclusive use of clean cooking fuels is necessary to reduce household air pollution exposure and associated negative health outcomes. Currently, over 2 billion people, mainly in sub-Saharan Africa, Asia, and Latin America, rely on solid cooking fuels. Cleaner alternatives, such as gas or electric, are unaffordable or unavailable. Liquefied Petroleum Gas (LPG) has been a broadly promoted clean fuel because of its transportability, availability across a global supply chain, and scalability. National government programs, including Peru's Fondo de Inclusión Social Energético (FISE), have pursued subsidies for recurring LPG costs, but their effectiveness remains understudied.

Objectives: Combining nationally representative data from Peru's 2 million FISE enrollees from 2014 to 2024, we characterize coverage and uptake of the program, describe participant voucher redeeming behaviour, and investigate drivers of program uptake.

Methods: We employ an ordinary least squares regression with cluster-robust standard errors to investigate the relationship between uptake and district-level characteristics from 2017 census data.
Results: We find that, per year, 38% of active participants redeem monthly, while 45% “bank” two vouchers together every two months. 17% redeem infrequently or not at all. These categories are largely stable overtime, with the exception of during the COVID-19 pandemic, where we see a large decrease in monthly redemptions. Conversely, a temporary subsidy increase in 2023 shifted more users towards monthly redemption. We find that incidence of dirt floors or adobe walls in homes, established LPG use, and stove ownership positively influence FISE uptake and LPG use, while incidence of households with 6 or more people, high school completion, and rurality hinder FISE uptake and LPG use.
Conclusion: We discuss the role of subsides and policy implications that can encourage continual use of clean fuels. Additionally, district-level respiratory disease outcomes provide further opportunity to study the impact of these subsidies on human health outcomes.

P26-14 | Associations between short-term exposure to ultrafine air pollution and mortality in two UK metropolitan areas

Devon Nenon[1], Pierre Masselot[1], Antonio Gasparrini[1], Gary Fuller[2]

[1] London School of Hygiene and Tropical Medicine, Environment & Health Modelling (EHM) Lab, Department of Public Health Environments and Society, London, UK; [2] Imperial College London, MRC Centre for Environment and Health, School of Public Health, London, UK

Objective: Ultrafine particulate matter (UFP) represents particles smaller than 100 nanometers in diameter and has been hypothesized to be more harmful than fine (PM2.5) and coarse (PM10) particulate matter components. In this study, we aimed to estimate the associations between UFP and daily mortality in the United Kingdom, as well as assess possible effect modification of the simultanous introduction of "sulfur free" diesel fuel and the London Low Emission Zone. These traffic policies, implemented in 2007, are known to have affected local UFP levels and could impact their toxicity.
Materials and methods: We estimated the associations between particle number concentration (PNC), a proxy of UFP, and daily mortality from non-external, cardiovascular, and respiratory causes in the urban areas of Greater London and the West Midlands from 2003 to 2018. We used quasi-Poisson time series regressions with a 0-1 day moving average of PNC, adjusting for seasonality, long-term trend, and temperature. We then included an interaction term to assess effect modification of the traffic policies.
Results: The average PNC in both cities was 15,257 particle/cm^3 during the study period. For a 10,000 particle/cm^3 increase in PNC we estimated risk ratios ranging from 0.9965 to 1.0002, with 95% confidence intervals largely including the null, providing no evidence suggestive of an association between UFP and mortality. We also found no evidence suggesting that the traffic policies have a modifying effect on this relationship.
Conclusions: In this study, we failed to find evidence of an association between UFP and mortality. This is consistent with previous studies finding mixed results for this association. However, this study was limited by a large amount of missing data, so results should be interpreted with caution.

P26-15 | Examining the social distributions in neighborhood black carbon and ultrafine particles in Montreal and Toronto, Canada.

Emmanuelle Batisse[1], Marshall Lloyd[1], Alicia Cavanaugh[2], Arman Ganji[3], Junshi Xu[3], Marianne Hatzopoulou[3], Jill Baumgartner[1], Scott Weichenthal[1]

[1] McGill University, Epidemiology, Biostatistics and Occupational Health, Montreal, Canada; [2] Scientific Consulting Group, Gaithersburg, USA; [3] University of Toronto, Toronto, Canada

Objective: We investigated socioeconomic inequities in outdoor ultrafine particles (UFP) and black carbon (BC) exposure in Canada, focusing on Montreal and Toronto, two metropoles with distinct sociodemographic diversity and urban characteristics compared to U.S. cities..

Methods: We collected vulnerability indicators, including social, economic, household composition, and immigration status, at the dissemination area level for Montreal and Toronto using data from the 2006 and 2021 Canadian Census of Population. Areas were classified as disadvantaged, intermediate, or advantaged following K-means clustering analysis. We aggregated and calculated population-weighted average concentrations of BC, UFP, and UFP sizes at the dissemination area and cluster levels using high-resolution exposure surfaces, derived from year-long mobile monitoring campaigns conducted in each city during 2020-2021. Final exposure surfaces were generated by integrating predictions from land-use regression models and deep convolutional neural network models.

Results: We observed high within-city variations in aggregated air pollutant levels, with higher outdoor BC and UFP concentrations and smaller UFP sizes in areas near local sources such as major roads, railways, airports, and densely populated regions. Advantaged areas experienced the lowest median UFP concentrations in both Montreal (10,707 pt/cm^3) and Toronto (10,988 pt/cm^3), as well as the lowest BC concentrations (650 ng/m^3) in Montreal. The highest median UFP concentrations were observed in intermediate areas in Montreal (15,709 pt/cm^3) and disadvantaged areas Toronto (12,228 pt/cm^3). Conversely, the highest BC concentrations were observed in disadvantaged and intermediate areas in Montreal (805-811 ng/m^3), and disadvantaged and advantaged areas in Toronto (1,228-1,252 ng/m^3). Notably, high priority areas for the double burden of high BC and UFP concentrations and vulnerability were located near air pollutants local emission sources.

Conclusions: Our findings highlight the importance of prioritizing exposure mitigation for populations residing near local sources and to understand contextual factors influencing inequities across cities and pollutants.

P26-16 | Prenatal exposure to air pollution and emotional and behavioral outcomes in early childhood: Environmental influences on Child Health Outcomes (ECHO) Cohort

Jiwon Oh[1], Akhgar Ghassabian[2,3], Judy Aschner[4], Catrina A. Calub[5], Yueh-Hsiu M. Chiu[6], Lisa A. Croen[7], Aisha S. Dickerson[8], Teresa Herrera[3], Lijun Li[9], Christine Loftus[10], Irva Hertz-Picciotto[1,11], Alicia K. Peterson[7], Rebecca J. Schmidt[1,11], Julie B. Schweitzer[5,11], Annemarie Stroustrup[12], Kirin N. Suri[4], Li Yi[13], Heather Volk[14], Deborah H. Bennett[1]

[1] University of California Davis, Department of Public Health Sciences, Davis, USA; [2] New York University Grossman School of Medicine, Department of Pediatrics, New York, USA; [3] New York University Grossman School of Medicine, Department of Population Health, New York, USA; [4] Hackensack Meridian School of Medicine, Department of Pediatrics, Nutley, USA; [5] University of California Davis, Department of Psychiatry and Behavioral Sciences, Sacramento, USA; [6] Icahn School of Medicine at Mount Sinai, Departments of Public Health and Environmental Medicine, New York, USA; [7] Kaiser Permanente Northern California, Division of Research, Oakland, Pleasanton, USA; [8] Johns Hopkins Bloomberg School of Public Health, Department of Epidemiology, Baltimore, USA; [9] Pennsylvania State University, Department of Psychology, University Park, USA; [10] University of Washington, Department of Occupational and Environmental Health, Seattle, USA; [11] University of California Davis, MIND (Medical Investigations of Neurodevelopmental Disorders) Institute, Sacramento, USA; [12] Northwell Health Cohen Children's Medical Center, Division of Neonatology, New Hyde Park, USA; [13] Harvard Medical School, Department of Population Medicine, Boston, USA; [14] Johns Hopkins Bloomberg School of Public Health, Department of Mental Health, Baltimore, USA

Objective: A growing body of evidence links prenatal exposure to ambient air pollution with emotional and behavioral outcomes in young children. However, few studies have been conducted in the U.S. Leveraging a geographically and sociodemographically diverse U.S. sample, this study investigated the extent to which prenatal exposure to air pollution were associated with child emotional and behavioral outcomes.
Materials and Methods: Participants were 8,405 mother-child dyads from 28 study sites within the Environmental influences on Child Health Outcomes (ECHO) Cohort. Prenatal air pollution exposure, specifically particulate matter ($PM_{2.5}$), nitrogen dioxide (NO_2), and ozone, from 2001−2020 were estimated based on residential addresses, and averaged across pregnancy and by trimester. Child internalizing and externalizing behaviors were assessed using the Child Behavior Checklist for Ages 1.5−5 years (CBCL/1.5−5). Covariate-adjusted linear mixed-effects models with cohort as a random effect were used to estimate associations between individual air pollutants and square-root-transformed CBCL/1.5−5 T-scores. Analyses were stratified by child opportunity index (COI) to assess whether associations vary by neighborhood factors.
Results: Higher pregnancy-average $PM_{2.5}$ exposure was associated with higher externalizing scores (β=0.013, 95% confidence interval [CI]: 0.004, 0.023). Similar associations were observed for first- and second-trimester exposures but not for third-trimester exposure. Higher pregnancy-average NO_2 exposure was associated with higher externalizing scores (β=0.003, 95% CI: 0.000, 0.006), with associations observed only in the third trimester. When stratified by COI, higher pregnancy-average $PM_{2.5}$ and NO_2 exposures were associated with higher externalizing and internalizing scores in neighborhoods with the lowest child opportunity.
Conclusions: We found that prenatal exposure to $PM_{2.5}$ and NO_2 may be associated with small but significant differences in externalizing behaviors in young children. Future studies are needed to further investigate the biological relevance.
This research was supported by the ECHO Program, Office of the Director, National Institutes of Health.

P26-17 | Ambient fine particulate matter concentration temporal trends in Grenada between 2009 and 2022 - a MERRA-2 atmospheric reanalysis

Natalie Kuo Hsuan Yang[1], Kerry Mitchell[1], Martin Forde[1], Sabrina Compton[1], Nicholas Dirienzo[2], Paul Villenueve[3], Andrea Sealy[4]

[1] St. George's University, Department of Public Health and Preventive Medicine, True Blue, Grenada; [2] Carleton University, Department of Health Sciences, Ottawa, Canada; [3] Carleton University, CHAIM Research Centre, Ottawa, Canada; [4] Caribbean Institute for Meteorology and Hydrology, Bridgetown, Barbados

To assess the trends in air quality in Grenada, an island often impacted by Saharan dust storms, a historical analysis was conducted using the Modern-Era Retrospective Analysis for Research and Applications, version 2 (MERRA-2), as a proxy for the absence of ground monitoring stations. A time series decomposition of daily mean concentrations and a density ridgeline plot of their monthly distribution were created for trend analysis. Although the mean concentrations of PM2.5 remained relatively stable, significant fluctuations were noted. Notably, concentrations increased consistently and significantly during the months typically associated with Saharan dust events, despite annual variability. Considering that PM2.5 exposure is linked to various adverse health effects, the findings from this study can serve as a basis for establishing more comprehensive monitoring networks. This can help inform air quality management decisions tailored to the region and protect public health. Furthermore, the lessons learned from this study can enhance research capabilities and support the implementation of air quality management initiatives in Grenada and other small island developing states in the region and beyond.

P26-19 | New Air Pollution Satellite Data Reveals Diurnal Nitrogen Dioxide Variation and Hotspots in Eastern Canada

Tsz Kin Siu[1], Dan L. Goldberg[2], Gaige H. Kerr[2], M. Omar Nawaz[2,3], Rachel Y. Chang[4], Kelvin C. Fong[1,2]

[1] Dalhousie University, Department of Earth and Environmental Sciences, Halifax, Canada; [2] George Washington University, Department of Environmental and Occupational Health, Milken Institute School of Public Health, Washington, USA; [3] Cardiff University, School of Earth and Environmental Sciences, Cardiff, UK; [4] Dalhousie University, Department of Physics and Atmospheric Science, Halifax, Canada

Objective:
Tropospheric Emissions: Monitoring of Pollution (TEMPO) is a new geostationary satellite that measures atmospheric trace gas pollution in North America during daytime hours. We investigated tropospheric nitrogen dioxide (NO_2) spatiotemporal patterns in eastern Canada from September 2023 to August 2024.

Material and Methods:
We obtained and extracted high-quality TEMPO tropospheric NO_2 measurements at high resolution (2 km x 2 km). We selected eight highly polluted municipalities, including Toronto, Montreal, Quebec City, Windsor, Sarnia, Halifax, Saint John, and Cape Breton. For each municipality, we visualized the population-weighted hourly averages of NO_2 and found the hours with peaked NO_2 level accounting for seasonal and weekday-weekend differences. Comparing

annual means of TEMPO NO_2 vertical column density with measurements from a sun-synchronous satellite instrument that only provides daily observations, we identified previously unrecognized spatial NO_2 hotspots.

Results:
On weekdays, NO_2 peaked in Atlantic and Quebec municipalities in the early morning (between 7 a.m. and 9 a.m.), while municipalities in Ontario had a slightly delayed peak (10 a.m.). After these morning peaks, NO_2 levels gradually decreased and flattened during the afternoon and evening, except for Sarnia, which maintained high levels. We observed seasonal variations. Elevated noontime levels occurred between 12 p.m. and 2 p.m. in Nova Scotia's port cities in autumn and in Toronto on spring weekends. The border cities (Windsor and Sarnia) had an afternoon-to-evening rise in NO_2 after 12 p.m. in summer. Uncertainty in measurements limited our understanding of winter pollution. Using TEMPO, we identified new hotspots along the north shores of the Gulf of St. Lawrence and Chaleur Bay in Quebec.

Conclusion:
Our work revealed new spatiotemporal patterns in tropospheric column NO_2 variabilities in eastern Canada. This motivates future research on NO_2 and its connections with emissions, atmospheric chemistry, transport, and health, through leveraging TEMPO observations over multiple years.

P26-20 | Synergistic Effects of Multiple Air Pollutants on Non-Suicidal Self-Injury Among Chinese Adolescents: A 7-Province Cross-Sectional Study

Runyu Wei[1], Weiju Zhou[2], Xingkang Chai[3], Haida Tang[3], Yuhui Wan[1]

[1] Anhui Medical University, School of Public Health, Hefei, China; [2] Tsinghua University, Vanke School of Public Health, Beijing, China; [3] Shenzhen University, School of Architecture & Urban Planning, Shenzhen, China

Objective: This study aimed to investigate the association between exposure to multiple air pollutants and non-suicidal self-injury (NSSI), focusing on synergistic effects of co-exposure, among Chinese adolescents.
Methods: A cross-sectional study was conducted with 11,167 adolescents (mean age: 14.9 years) from 26 schools across seven provinces in China. NSSI was assessed using a validated questionnaire, and annual concentrations of five air pollutants ($PM_{2.5}$, PM_{10}, O_3, SO_2, and CO) were estimated around schools. Multivariable logistic regression models assessed single- and two-pollutant associations, with subgroup analyses for urban-rural differences, while the quantile-based g-computation (qgcomp) model evaluated combined pollutant effects.
Results: In single-pollutant models, a 10 μg/m³ increase in $PM_{2.5}$, PM_{10}, O_3, SO_2, and CO was associated with elevated NSSI risk, with odds ratios (95% confidence intervals) of 1.015 (1.009, 1.021), 1.006 (1.004, 1.007), 1.010 (1.005, 1.014), and 1.001 (1.000, 1.001), respectively. In two-pollutant models, PM_{10} and SO_2 remained significantly associated with NSSI risk after adjusting for co-pollutants. Subgroup analyses showed urban adolescents were more susceptible to PM_{10}, $PM_{2.5}$, and CO exposure, while rural associations were non-significant. Multi-pollutant models revealed synergistic interactions among pollutants, identifying $PM_{2.5}$ as the primary driver of these associations.
Conclusion: Exposure to multiple air pollutants increases NSSI risk among Chinese adolescents, highlighting the mental health impacts of air pollution. Integrated air quality management and targeted mitigation of $PM_{2.5}$ are essential to improve public mental health in polluted regions.

Keywords: Air pollution, Non-suicidal self-injury, Adolescents, Synergistic effects, Mental health, China

P26-21 | Fine Particulate Matter Acidity, Source, and Mortality in the Six Cities Study Cohort

Wuyue Yu[1], George D. Thurston[1], Francine Laden[2]

[1] New York University Grossman School of Medicine, Department of Medicine, Division of Environmental Medicine, New York, USA; [2] Harvard T.H. Chan School of Public Health, Department of Environmental Health, Boston, USA

Objective: The association between ambient air pollution fine particulate matter ($PM_{2.5}$) and health has been well documented since the landmark Harvard Six Cities Study, in which a cohort from six U.S. cities were followed to assess the effect of air pollution on mortality. Despite its strengths in design and critical role in air pollution epidemiology, there remained gaps in the investigation of correlations between mortality and aerosol acidity and elemental constituents in this dataset, due to the differing PM characteristics' sampling time periods across PM components and study sites.
Material and Methods: We analyzed the PM constituent measurements in the Six Cities Study. Factor analysis and absolute principal component analysis source apportionment were applied across cities to estimate the source-specific $PM_{2.5}$ concentration for Coal, Mobile, Metal, Crustal, and Oil source categories in each city. We considered and compared the correlation between mortality risk and the mean measured H^+ vs. an overall study-sulfate-sampling-period (i.e. 1979-1984) adjusted H^+ employing the H^+/SO_4^{2-} ratio from summers when both H+ and sulfate levels were available.
Results: We found the highest mean level of coal- and metals-related source-specific PM at Steubenville (12.5±0.3 ug/m^3 and 4.3±0.1 ug/m^3), and the highest levels of fuel oil- and mobile-related source-specific PM at Watertown (3.1±0.1 ug/m^3 and 3.6±0.1 ug/m^3). Highest estimated overall PM acidity was found at Steubenville (37.2 nMol/m^3), followed closely by Kingston-Harriman (35.1 nMol/m^3). The reported all-cause mortality hazard ratios found for $PM_{2.5}$ were highly correlated with PM from Coal (R^2 = 0.85) and Metal (R^2 = 0.83), but not other sources. Sampling-period-adjusted H^+ is more highly correlated with all-cause mortality risk than measured H^+ from the original sampling period alone (R^2 = 0.64 vs. R^2 = 0.36).
Conclusion: The mortality impacts of long-term $PM_{2.5}$ exposure in the Harvard Six Cities Study vary with particle source and composition.

P26-22 | Trends in Exposure to On-road Diesel Truck emissions in Illinois Before and After COVID-19

Yi-Ling Cheng[1], **Jane Lin**[2]

[1] University of Illinois Chicago, Division of Environmental and Occupational Health Sciences, Chicago, USA; [2] University of Illinois Chicago, Department of Civil, Materials, and Environmental Engineering, Chicago, USA

During and after the COVID-19 pandemic, truck traffic has in fact gone up from the pre-pandeic level in many US cities including Chicago. The objectives of this research are (1) to assemble and analyze commercial vehicle emission measurement data, (2) classify the effects of vehicle characteristics and traffic activities on diesel truck emissions, (3) estimate diesel truck emissions statewide in Illinois, and (4) assess the exposure risks of commercial vehicle pollutants. The study finds that statewide Average Annual Daily Truck Traffic (AADTT) increased multi-fold from 2019 to 2021 and greater increases were observed outside of the Chicago Metropolitan region. In contrast, statewide truck vehicle miles of travel (VMT) increased only slightly between 2019 and 2021, Multi-unit truck VMT actually decreased. Such changes in truck traffic patterns suggest a lot more short-distance truck traffic in urban and local areas after the pandemic due to rapid increase in e-commerce/delivery demand. As a result, althgough all pollutant emissions (CO, NOx, PM10, PM2.5, CO2) have a downward trend from 2019 to 2021 in Illinois, greater health exposure risks of diesel truck emissions are concentrated in local environmental justice communities in Illinois and Chicago with the increased local truck traffic after the pandemic. Those adverse trends should warrant more scrutinized local adn regional studies and proper policy actions.

P26-23 | Exploring multilevel patterns in energy use and ambient PM2.5 concentrations using low-cost sensors in rural Tamil Nadu, India

Rashmi Krishnan[1], Ajay Pillarisetti[1], Manish Desai[2], Naveen Puttaswamy[3], Wenlu Ye[4], Mayank Bhushan[5], Saritha Sendhil[3], Adithi R. Upadhya[6], Arul Selvan[3], Rengaraj Ramaswamy[3], Durairaj Natesan[3], Sankar Sambandam[3], Sreekanth Vakacherla[7], Kalpana Balakrishnan[3]

[1] University of California, Environmental Health Sciences, Berkeley, USA; [2] University of Pennsylvania, Department of Policy and Ethics, Philadelphia, USA; [3] Sri Ramachandra Institute of Higher Education and Research, Faculty of Public Health, Chennai, India; [4] World Health Organization, Geneva, Switzerland; [5] University of California, School of Public Policy, Berkeley, USA; [6] University of Liverpool, Liverpool, UK; [7] World Resources Institute, Delhi, India

Objective: Despite advances in policies promoting clean cooking fuels and air quality monitoring, few bottom-up efforts link residential fuel use with rural ambient air quality. Our research uses a low-cost sensor network to establish this relationship in rural South India, where biomass burning is high and LPG consumption is low.

Material and methods: We deployed 80 air quality sensors and 160 stove use monitors (SUMs) in 8 small villages from July 2022 to September 2023 across two districts in Tamil Nadu. Each habitation of ~100 households received 10 outdoor PM2.5 monitors and 20 SUMs (tracking stove temperatures). We used multilevel linear models to partition within- and between-habitation variability and multivariate autoregressive models to examine stove-use as a driver of outdoor air quality.

Results: The mean [s.d.] biomass stove use duration per day was 179.20 minutes [123.81] in Kallakurichi (40 households) and 103.24 minutes [11.51] in Nagapattinam (39 households). Daily LPG use was 24.07 minutes [48.13] in Kallakurichi and 90.62 [18.05] minutes in Nagapattinam. On average, a household in Kallakurichi used LPG and biomass stoves 0.83 [1.66] and 2.86 [1.97] times a day, respectively, while in Nagapattinam these numbers were 4.34 [0.64] (LPG) and 2.61 [0.51] (biomass). Daily mean outdoor PM2.5 concentrations, with a coefficient of variation of 68.11%, were consistently higher in Kallakurichi (37.72 μg/m3 [21.18]) than Nagapattinam (26.76 μg/m3 [21.46]).

Conclusion: We noted strong diurnal peaks in PM2.5 levels and stove usage, potentially indicative of biomass combustion for cooking. Although LPG users stacked fuels, mean outdoor PM2.5 levels were lower in areas with higher LPG prevalence. Additional work is needed to disentangle household-derived ambient air pollution from other local sources.

P26-24 | Overlooked Health Risks in Rural Settings: $PM_{2.5}$ Exposure, Premature Mortality, and Economic Burden Assessment in Punjab and Haryana, India

Amrendra K. Singh[1], Ashutosh K. Pathak[2], Gaurav Saini[1]

[1] Netaji Subhas University of Technology, Civil Engineering Department, New Delhi, India; [2] AARC Engineers and Consultants India Private Limited, Noida, India

Particulate matter pollution is known to have significant health impacts, including respiratory, cardiovascular, neurological, and reproductive diseases. This study investigates premature mortality and economic burden associated with fine particulate matter ($PM_{2.5}$) in the agricultural heartland states of Punjab and Haryana, India using Integrated Exposure Response (IER) and Global Exposure Mortality Model (GEMM) Models.

The $PM_{2.5}$ associated premature mortality was the maximum for IHD (Ischemic Heart Disease). IER model estimated total premature mortality associated with $PM_{2.5}$ for five specific diseases is 20,259[95% CI: 15,558-25,562] for Haryana and 27,677[95% CI: 21,447-34,248] for Punjab whereas the GEMM model estimated total premature mortality for same five diseases are 28,059[95% CI: 21,523-35,208] for Haryana and 28,030[95% CI: 21,713-34,875] for Punjab.

Combined economic burden of this premature mortality for both states was found to be 74841.78 million USD [95% CI: 57710.54 – 93601.93] (IER), 90991.69 million USD [95% CI: 70057.87-113811.89] (GEMM-5 COD), 125374.72 million USD [95% CI: 104304.69 – 146839.27] (GEMM-NCD+LRI).

The importance of strict regulations, pollution management, and rural $PM_{2.5}$ health awareness may be shown in the possibility that many lives could have been saved and a large economic burden would have been released if air quality standards (NAAQS, WHO-2005 & 2021) had been followed.

P26-25 | Short-term impact of air pollution exposure on emergency room and hospital admissions in the Veneto Region, Northern Italy (2008-2022)

Anaam Arif[1], Francesca Locatelli[1], Sofia Tagliaferro[1,2], Lorenzo Di Spazio[1], Francesco Domenichini[3], Ugo Fedeli[4], Nicola Gennaro[4], Pierpaolo Marchetti[1], **Alessandro Marcon**[1]

[1] University of Verona, Unit of Epidemiology and Medical Statistics, Department of Diagnostics and Public Health, Verona, Italy; [2] National Research Council, Institute of Clinical Physiology, Pisa, Italy; [3] Environmental Protection Agency of Veneto (ARPAV), Teolo, Italy; [4] Azienda Zero, Veneto Region, Epidemiological Department, Padova, Italy

Objective:
Exposure to air pollutants is associated with increased emergency room (ER) visits and hospital admissions. We investigated these associations in the Veneto Region, Northern Italy, over 2008–2022.

Material and Methods:
We obtained daily air pollutant concentrations, hospital admissions for respiratory diagnoses, and ER visits for natural causes across all age groups. A space- and time-stratified case-crossover study design was used, with stratification by area, year, month, and weekday. Associations at lag 0–6 days were analyzed using Quasi-Poisson regression models, adjusting for temperature, precipitation, summer holidays, influenza rates, and age.

Results:
Among 2,470,101 individuals, 7,712,804 ER visits and 220,528 hospital admissions were analyzed. Air quality guidelines by WHO (2021) were frequently exceeded: $PM_{2.5}$ (57.3% of days >15 μg/m³), PM_{10} (19.3% >45 μg/m³), and NO_2 (43.9% >25 μg/m³). Relative changes in ER visits (95% CI) per standard deviation increase in pollutant levels were: PM_{10} +0.9% (0.7–1.2% per 23.4 μg/m³), $PM_{2.5}$ +1.0% (0.7–1.3% per 19.3 μg/m³), NO_2 +0.3% (0.0–0.7% per 15.3 μg/m³), SO_2 +0.5% (0.1–0.9% per 1.3 μg/m³), and summer O_3 +1.6% (0.8–2.4% per 29.2 μg/m³). Corresponding increases in hospital admissions were: PM_{10} +2.2% (1.3–3.2%), $PM_{2.5}$ +3.3% (2.1–4.5%), SO_2 +1.7% (0.1–3.4%), and summer O_3 +0.8% (-1.3–2.9%); NO_2 showed no association −0.6% (−2.0%–0.7%).

Conclusion:
$PM_{2.5}$ and PM_{10} had the strongest impact on healthcare utilization, emphasizing the urgent need for air quality policies to reduce particulate pollution in the Veneto Region.

Funding:
ESF REACT-EU Green and Innovation and NextGenerationEu programmes (Italian Ministry of University and Research, Decrees 1061/2021, 737/2021, and 118/2023).

P26-26 | Spatial and Temporal Patterns of Latino/Non-Latino White Disparity in Mortgage Lending and Traffic-Related Air Pollutants in Sacramento, California

Anjali D. Kumar[1], Thomas J. Luben[1], Arrianna M. Planey[2], Jessie P. Buckley[1], Allison E. Aiello[3], Chantel L. Martin[1]

[1] University of North Carolina Gillings School of Global Public Health, Department of Epidemiology, Chapel Hill, USA; [2] University of North Carolina Gillings School of Global Public Health, Department of Health Policy and Management, Chapel Hill, USA; [3] Columbia University, Robert N Butler Columbia Aging Center, Department of Epidemiology, New York, USA

Objective: Racial and ethnic residential segregation and mortgage discrimination have shaped neighborhoods and disproportionate environmental exposures, yet patterns of contemporary mortgage lending and environmental disparities in Latino populations are understudied. We examined the spatial and temporal patterns of traffic-related air pollutants (TRAPs) and Latino/Non-Latino White disparity in mortgage lending in the Combined Statistical Area (CSA) of Sacramento, California.

Material and Methods: Using the Home Mortgage Disclosure Act, we derived Latino/Non-Latino White disparity in mortgage lending, a predictive odds measure of mortgage denial for census tracts based on Latino ethnicity (compared to non-Latino white individuals). Annual census tract level estimates of nitrogen dioxide (NO_2), fine particulate matter ($PM_{2.5}$), and carbon monoxide (CO) represented TRAP exposures. We assessed spatial autocorrelation and clustering of Latino/Non-Latino White Disparity in mortgage lending and TRAPs through Global Moran's I and Local Indicators of Spatial Autocorrelation.

Results: Latino/Non-Latino White disparity in mortgage lending and TRAPs were significantly spatially clustered in the CSA when examined individually ($p<0.05$). NO_2 and CO were spatially autocorrelated with Latino/Non-Latino White disparity in mortgage lending when assessing joint clustering ($p<0.05$). Spearman correlations indicated Latino/Non-Latino White disparity in mortgage lending and TRAPs were moderately correlated with census tract level neighborhood characteristics such as Latino population, Latino homeownership, housing cost burden (mortgage lending: ρ= 0.375-0.430; TRAPS: ρ= 0.421-0.684) as well as median family income (mortgage lending: ρ= -0.659; TRAPs: ρ= -0.388 to -0.458).

Conclusion: These findings indicate that the patterns of mortgage lending and traffic-related exposures are intricately connected, requiring further research on the neighborhood environment to comprehend this complex relationship.

P26-27

This abstract has been withdrawn.

P26-28 | Contribution of Ship Emissions to Air Pollution in the Port of Los Angeles

Florence Lee, Fei Liu, Rob McConnell

University of Southern California, Population and Public Health Sciences, Los Angeles, USA

Ports are a large contributor to air pollution in surrounding urban areas. The contribution of emissions from ships burning low grade fuel to exposures in near-port communities is not well studied. To address this knowledge gap, this study examined the impact of ship activity in the Port of Los Angeles and in its harbor on $PM_{2.5}$ and black carbon measurements at an adjacent monitoring station. Measured pollutant concentrations on 330 days during 2019-2020 were modeled as a function of number of ships in the harbor, ships at berth for unloading, and ships departing the harbor, lagged by one day prior to pollutant measurements, and adjusted for confounders including a spline of date, day of week, daily truck counts out of the port, PM2.5 concentrations at a regional downtown LA monitor, and wind speed and direction. Concentrations of black carbon (but not PM2.5) were associated with number of ships at anchor (87ng/m^3/ship; 95% CI 30-145) and number of ships departing (37 ng/m^3/ship; 95% CI 11-63). Estimated effects were little changed in models co-adjusted for ships in harbor and ships departing, suggesting that these effects were independent. Approximately 20% of estimated black carbon concentrations at the near-port monitoring station could be attributed to the combined effects of these two ship activities during the study period.

P26-29 | Short-Term Air Pollution Increase from Large-Scale Firework Festivals in Korean Megacities: Insights from an Exposure-Crossover Analysis

Habyeong Kang[1], Eunjin Oh[2], Yoon-Hyeong Choi[3]

[1] Hanyang University, Department of Preventive Medicine, Seoul, South Korea; [2] Ajou University, Department of Preventive Medicine, Suwon, South Korea; [3] Korea University, School of Health and Environmental Science, Seoul, South Korea

Objective: Large-scale firework festivals are popular in Korean megacities, but few studies have investigated their contributions to ambient air pollution. This study aimed to evaluate the short-term impact of firework festivals on ambient $PM_{2.5}$, PM_{10}, and SO_2 concentrations in Seoul and Busan in 2023.
Material and Methods: A self-matched exposure-crossover design was applied to compare air pollution levels on festival days with reference days (one week before and after each festival day). Spatiotemporal dispersion patterns were visualized using data from multiple air quality monitoring stations.
Results: During the festival periods, $PM_{2.5}$ and PM_{10} concentrations dramatically increased, peaking at 320 and 371 μg/m^3 in Seoul and 241 and 253 μg/m^3 in Busan, respectively. These concentrations on the festival days exceeded those on the corresponding reference days by 7.4–12.2 times. While SO_2 levels also increased, the magnitude was small in both cities. Spatiotemporal analysis demonstrated that firework-induced $PM_{2.5}$ and PM_{10} dispersion was driven by wind direction.
Conclusion: This study highlights the significant contribution of large-scale firework festivals to ambient air pollution, especially regarding $PM_{2.5}$ and PM_{10} in Korean megacities. Given the dense populations in these areas and the large

number of festival attendees, the short-term air pollution spikes associated with large-scale firework festivals raise public health concerns, emphasizing the need for mitigation strategies.

P26-30 | Association between ozone and the emergency department visit in people with disabilities in South Korea: a nationwide case-crossover study

Jinah Park[1], Whanhee Lee[2], Ho Kim[1], On behalf of AiMS-CREATE team.

[1] Seoul National University, Department of Public Health Sciences, Graduate School of Public health, Seoul, South Korea; [2] Pusan National University, School of Biomedical Convergence Engineering, College of Information and Biomedical Engineering, Pusan, South Korea

Objective: Ozone is known to have detrimental impacts on health, and its concentration is projected to increase with global warming. However, limited evidence exists regarding its impact on the health of people with disabilities. This study aimed to estimate the association between ozone exposure and emergency department (ED) visits by disability status and type.
Material and Methods: We collected claims data spanning seven years (2015–2019, 2022–2023), excluding 2020 and 2021 due to the COVID-19 pandemic, during warm seasons (April to September) from the Korean National Health Insurance Service. A time-stratified case-crossover design was applied, and conditional logistic regression was used to estimate the association between ozone and ED visits.
Results: A total of 2,344,708 ED visits were recorded during the study period. The odds ratio (OR) for ED visits per 10 ppb increase in ozone was 1.075 (95% confidence interval (CI): 1.056–1.094). It was prominent on early warm season (April to June) than late warm season (July to September) (OR: 1.151 (1.119–1.184) vs. OR: 1.045 (1.021–1.070)). Furthermore, developmental and mental disability, and ED visit due to respiratory diseases showed the strongest association between ozone and ED visits.
Conclusion: This study quantified the risk of ED visits attributable to ozone exposure across various disability types at a national level. The findings highlight significant variations in risk by disability types, the cause of ED visits, and across the early and late warm seasons. These results underscore the importance of considering both disability types and visit causes when addressing the health risks of ozone exposure.
Acknowledgement: JP was supported by the National Research Foundation of Korea (NRF) grant funded by the Korean Government (RS-2024-00412028).

P26-31 | Prenatal and postnatal exposure to ambient fine particulate matter ($PM_{2.5}$) and risk of autism spectrum disorder and attention-deficit/hyperactivity disorder among Medicaid recipients

Matthew Shupler[1], Xinye Qiu[1], Krista Huybrechts[3], Sonia Hernandez-Diaz[1], Hayon Choi[2], Michael Leung[2], Wanyu Huang[2], Yaguang Wei[4], Joel Schwartz[1,2], Antonella Zanobetti[2], Marc Weisskopf[1,2], Stefania Papatheodorou[5]

[1] Harvard T.H. Chan School of Public Health, Department of Epidemiology, Boston, USA; [2] Harvard T.H. Chan School of Public Health, Department of Environmental Health, Boston, USA; [3] Brigham and Women's Hospital, Division of Pharmacoepidemiology and Pharmacoeconomics, Boston, USA; [4] Icahn School of Medicine at Mount Sinai, Department of Environmental Medicine and Climate Science, New York, USA; [5] Rutgers School of Public Health, Department of Biostatistics and Epidemiology, Piscataway, USA

Background: Evidence on the association between prenatal and early life exposure to fine particulate matter ($PM_{2.5}$) and risk of autism spectrum disorder (ASD) and attention-deficit/hyperactivity disorder (ADHD) is conflicting. In particular, studies in low socioeconomic status populations are lacking.

Methods: We created a linked mother-infant longitudinal cohort from the Medicaid Analytic eXtract (MAX) from 2001-2013, with cases of ASD and ADHD identified via ICD-9 codes. Ambient prenatal and postnatal exposure to $PM_{2.5}$ was assigned via a spatiotemporal pollutant prediction model at the mother's residential ZIP code. Distributed lag non-linear models were used to characterize $PM_{2.5}$ exposures (prenatal: 0-to-37-week lag; postnatal: 0-to-3-year lag). Cox models were stratified by county and birth year and adjusted for demographics, behavioral risk factors, ambient temperature, season of conception and area-level socioeconomic status (SES).

Results: A total of 1,548,303 births were included.A 10 μg/m^3 increase in average weekly prenatal $PM_{2.5}$ exposure was associated with 1.09 (95% CI: 0.95–1.25) and 1.24 (95% CI: 1.18–1.30) hazard of ASD and ADHD, respectively, at the cumulative lag (0-37 weeks). A 10 μg/m^3 increase in average annual postnatal $PM_{2.5}$ exposure was associated with 1.03 (95% CI: 0.76–1.40) and 0.87 (95% CI: 0.74–1.02) times the risk of ASD and ADHD, respectively, at the cumulative lag (0-3 years).

Conclusion: Low-income children in the US may have higher ADHD risk due to increased prenatal $PM_{2.5}$ exposure. Although there is a potentially sensitive window of exposure in late pregnancy for ASD, cumulative prenatal and postnatal $PM_{2.5}$ exposure was not associated with ASD risk.

P26-32 | Estimation of size fractionated lung deposition dose from low-cost sensor measured particulate matter in rural homes of Southern India

Sai Ganesan Karthiga Devi[1], Naveen Puttaswamy[1], Saritha Sendhil[1], Rengaraj Ramasami[1], Durairaj Natesan[1], Manish Desai[3], Ajay Pillarisetti[4], Sreekanth Vakacherla[5], Rashmi Krishnan[3], Sankar Sambandam[1], Padmavathi Ramaswamy[2], Kalpana Balakrishnan[1]

[1] Sri Ramachandra Institute of Higher Education and Research, Department of Environmental Health Engineering, Chennai, India; [2] Sri Ramachandra Institute of Higher Education and Research, Department of Physiology, Chennai, India; [3] University of California, Berkeley, Berkeley Air Monitoring Group, Berkeley, USA; [4] University of California, Berkeley, School of Public Health, Berkeley, USA; [5] World Resources Institute, India, Consultant, New Delhi, India

Background and Objective

Exposures to fine and ultra-fine particles (i.e., PM2.5 and PM1) affect human health. Most research relies on $PM_{2.5}$ as the 'reference' exposure metric to assess health risks. Size-segregated particulate matter assessment of exposures and effects are limited. Objective of the study is to estimate lung deposition dose for size-fractionated particulate matter measured using low-cost sensors.

Methods

We have utilized the data obtained from an ongoing study conducted in South Indian villages where biomass is the primary cooking fuel, and LPG consumption is low. Ambient PM measurement over a period of 1 year, using real-time low-cost PM sensors, was carried out in 80 rural households in southern India. Indoor sampling was carried out in a subset of households. The low-cost sensors measured PM concentrations in three size fractions (PM_1, $PM_{2.5,}$ and PM_{10}). Mean daily and monthly concentrations were determined from the real-time PM sensors.

Results

A mathematical model using the non-linear least squares method was developed to transform the measured PM concentrations into a continuous size distribution. Respiratory deposition doses were estimated by feeding the size distribution to a computational model of the lung designed to solve the spatial and temporal distribution of particles within the human respiratory system, incorporating various deposition models. The estimates of deposition doses ranged from ~0.2μg/min to ~1cg/min in the total lung. The coarse particles contributed to about 20% of the total lung dose, whereas the remaining 80% of the respiratory dose was predominantly of ultrafine size.

Conclusions

The study has estimated the size fractioned doses using 3 bin data obtained from low-cost sensors. Owing to the availability of low-cost sensor technology in air pollution monitoring, this study will serve as a benchmark for future work on utilizing such data to obtain size-fractioned dose-relevant implications for health.

P26-33 | Exposure-response relationships of wintertime outdoor and household air pollution with biomarkers of inflammation and oxidative stress in rural Beijing adults

Wenlu Yuan[1], Jill Baumgartner[1,2], Sam Harper[1,2], Chris Barrington-Leigh[3,2], Collin Brehmer[5], Ellison Carter[5], Kennedy Hirst[5], Xiaoying Li[6], Brian Robinson[4], Talia Sternbach[1], Kaibing Xue[10,11], Xiang Zhang[4], Guofeng Shen[7,8], Yuanxun Zhang[10,11], Shu Tao[7,8]

[1] McGill University, Department of Epidemiology, Biostatistics and Occupational Health, Montreal, Canada; [2] McGill University, Department of Equity, Ethics, and Policy, Montreal, Canada; [3] McGill Univeristy, Bieler School of Environment, Montreal, Canada; [4] McGill University, Department of Geography, Montreal, Canada; [5] Colorado State University, Department of Civil and Environmental Engineering, Fort Collins, USA; [6] Colorado State University, Department of Mechanical Engineering, Fort Collins, USA; [7] Peking University, College of Urban and Environmental Sciences, Beijing, China; [8] Peking University, Laboratory for Earth Surface Processes, Beijing, China; [9] Peking University, Sino-French Institute for Earth System Science, Beijing, China; [10] University of Chinese Academy of Sciences, College of Resources and Environment, Beijing, China; [11] University of Chinese Academy of Sciences, Beijing Yanshan Earth Critical Zone National Research Station, Beijing, China

Solid fuel combustion, common for cooking and heating in rural northern China, releases high levels of pollutants, resulting in disproportionate air pollution exposure among rural populations. Household and outdoor air pollution are established risk factors for cardiorespiratory diseases and certain cancers, potentially through chronic inflammation and oxidative stress. This study uses longitudinal measurements of air pollution and blood biomarkers of inflammation (IL-6, TNF-α, CRP) and oxidative stress (MDA) in middle-aged and older adults in rural Beijing, where solid fuel combustion is prevalent, to assess the impact of wintertime (Jan 15 to March 15) household and outdoor $PM_{2.5}$ exposure on inflammation and oxidative stress.

We enrolled participants ≥40 yrs from 50 villages in rural Beijing in winter 2018-19 and conducted follow-up visits in winter 2019-20. Trained field staff collected sociodemographic data via household surveys, and trained nurses collected blood samples in village clinics during both study waves. IL-6, TNF-α, and CRP concentrations were measured using enzyme-linked immunosorbent assay, and MDA concentration was measured with high-performance liquid chromatography. Wintertime outdoor $PM_{2.5}$ was continuously measured in each village. Household $PM_{2.5}$ was measured in a random ~30% subsample of participant homes. Missing data were addressed using multiple imputation. Multivariable mixed-effect models were used to estimate the effects of wintertime household and outdoor $PM_{2.5}$ on blood biomarkers.

A total of 1,064 participants were included. Wintertime outdoor and household $PM_{2.5}$ levels ranged from 19.8-100.1 and 11.7-431 μg/m^3, respectively. In multivariable models, we observed 1.9% (95%CI: 0.2, 3.6%) increase in TNF-a concentration per 10 μg/m^3 increase in outdoor $PM_{2.5}$. We did not observe effects of outdoor $PM_{2.5}$ on other biomarkers, nor did we observe effects of household $PM_{2.5}$ on any biomarkers. Our longitudinal study contributes to the understanding of $PM_{2.5}$ on inflammation in rural populations living in settings of solid fuel burning using a methodological robust design.

P26-34 | Power Plant Emission Trend from 1940 to 2020 in the United States

Xiaorong Shan, Lucas Henneman

George Mason University, Civil, Environmental, and Infrastructure Engineering, Fairfax, USA

Objectives
Historically, power plants have been major contributors to poor air quality in the United States and greenhouse gas emission. Power plant emissions have increased and decreased since the early 1900s because of factors such as environmental legislation, electricity demand, and available fuels. The objective of this project is to quantify and characterize the variation in emissions and exposure from power plants in the United States from 1940 to 2020.
Materials & Methods
For periods without direct measurements, we estimate SO_2, NOx, PM, and CO_2 emissions using emission factors derived from existing US power plants. These estimates consider operating capacity and installed emissions control technologies at individual electricity generating units. We use reduced complexity atmospheric models, the HyADS model, to quantify coal $PM_{2.5}$ concentrations.
Results
SO_2 emissions, 90% from coal power plants in the Midwest and West, surged 545% from 1940 to 1970, then declined 10% by 1990 and 93% between 1995–2020, with the steepest drop from 2005–2012. Similarly, NO_x emissions rose 1117% from 1940 to 1990, then fell 87% from 1995–2020, following a steady decline during 2005–2012. In contrast, CO_2 emissions saw a more gradual decrease of 22% during this period, highlighting the ongoing need for targeted efforts to mitigate greenhouse gas emissions from the 20th to the 21st century.
Conclusions
These reductions can be attributed to key regulations aimed at reducing air pollution and costs of fuel. Programs implemented for SO_2 and NOx, utilizing various control technologies and fuel switches significantly decreased emissions. However, the modest 22% reduction in CO_2 emissions underscores the continued need for focused regulatory efforts and initiatives to achieve further reductions in greenhouse gas emissions.

P26-35 | Sex Differences in Cardiorespiratory Responses to Urban Air Pollution Exposure During a Two-hour City Walk

Yihui Ge[1], Yan Lin[1], Xiaodong Liu[4], Ruoxue Chen[1], Kian Fan Chung[3], Junfeng Zhang[1,2]

[1] Duke University, 1. Nicholas School of the Environment, Durham, USA; [2] Duke Univeristy, 2. Duke Global Health Institute, Durham, USA; [3] Imperial College London, 3. National Heart & Lung Institute, London, UK; [4] The First Affiliated Hospital of Guangzhou Medical University, 4. State Key Laboratory of Respiratory Diseases, Guangzhou Institute of Respiratory Health, National Center for Respiratory Medicine, Guangzhou, China

Rationale: Animal studies documented clear sex differences in respiratory responses to air pollution. However, comparable evidence in humans, mostly derived from epidemiological studies, is mixed.
Objectives: This randomized crossover study examined sex differences in cardiorespiratory responses to air pollution and explored the potential role of lipid mediators derived from polyunsaturated fatty acids.
Methods: 40 healthy (F=21, M=19) and 40 COPD (F=21, M=19) adults aged > 60 years underwent a 2-hour walk either in the more-polluted Oxford Street or a less-polluted Hyde Park. Baseline and changes of lung function, airway resistance, and arterial stiffness were measured before and at multiple time points after exposure. Sputum was collected 24 hours after exposure for the measurement of inflammatory biomarkers. Sex differences were examined in associations between health outcomes and air pollution exposure during walks.
Results: Walking in the less-polluted Hyde Park consistently improved lung function (FEV1, FVC) and reduced airway resistance (R5, R20) among female but not among male participants. These benefits among female participants were significantly attenuated when walking on the more-polluted Oxford Street. Furthermore, increasing concentrations of ultrafine particles, black carbon, and NO_2 during walk were significantly associated with decreasing lung function and increasing airway resistance among women, whereas no significant associations were observed among men. In addition, females but not males exhibited significant increases in 15-LOX- and COX-derived lipid mediators (e.g., 17-HDHA, TXB_2) after walking in Oxford Street. In contrast, cardiovascular responses (arterial stiffness, blood pressure, and heart rate) showed similar post-exercise improvements in both sexes, with no sex-specific differences.**Conclusions:** There are sex differences in respiratory but not cardiovascular responses to air pollution. The sex difference in respiratory effects may be related to PUFA-derived lipid mediators. Our results provide mechanistic evidence in humans to support that women are more susceptible than men in respiratory effects of air pollution.

P26-36 | Association between long-term ambient $PM_{2.5}$ exposure and low bone mineral density: a longitudinal study in Taiwan and UK

Yiyue Yin[1], Dezhong Chen[1], Timothy Chi Yui Kwok[2], K. L. Kingston Mak[1], Yu Huang[1], G. Neil Thomas[3], Xiang Q. Lao[1]

[1] City University of Hong Kong, Department of Biomedical Sciences, Kowloon Tong, Hong Kong; [2] The Chinese University of Hong Kong, Department of Medicine and Therapeutics, Shatin, New Territories, Hong Kong; [3] University of Birmingham, Department of Public Health, Epidemiology and Biostatistics, Birmingham, UK

Objective:
The global health impact of low bone mineral density (BMD) is intensifying, yet the relationship between $PM_{2.5}$ air pollution and BMD remains underexplored. This study investigates the association between long-term $PM_{2.5}$ exposure and the incidence of low BMD in populations from Taiwan and the UK.
Materials and Methods:
We analyzed data from 104,781 participants in the MJ cohort in Taiwan and 10,219 participants in the UK Biobank, all of whom had normal BMD at baseline and underwent at least two medical examinations. $PM_{2.5}$ exposure was quantified using a Spatio-Temporal Model with a 0.01° × 0.01° resolution. We employed Cox proportional hazards regression models, adjusted for time-dependent covariates and incorporating random effects, and performed random-effects meta-analyses to pool estimates from both cohorts.
Results:
Each 1 μg/m³ increase in $PM_{2.5}$ concentration was associated with a 7% higher risk of developing low BMD in the MJ cohort (HR 1.07, 95% CI 1.06 to 1.08) and a 15% higher risk in the UK Biobank cohort (HR 1.15, 95% CI 1.10 to 1.19). The pooled hazard ratio, combining results from both cohorts, was 1.09 (95% CI 1.03 to 1.17).
Conclusion:
Our findings indicate that higher ambient $PM_{2.5}$ exposure is linked to an increased risk of low BMD, highlighting the critical importance of air quality. Improving ambient air quality could significantly reduce the disease burden associated with low BMD.
StartFragmentThis study was supported by the New Research Initiatives/Infrastructure Support from the City University of Hong Kong (9610613) and the RGC-Theme-based Research Scheme (T24-508/22-N).EndFragment

P26-37 | Elevated Fine Particulate Matter Levels at Electric Vehicle Fast Charging Stations

Yuan Yao[1], Muchuan Niu[1], Haoxuan Chen[1], Qiao Yu[1], Qingyang Wu[1], Yuhang Li[2], Yijie Zhang[2], Aydogan Ozcan[2], Michael Jerrett[1], Yifang Zhu[1]

[1] University of California, Los Angeles, Department of Environmental Health Sciences, Los Angeles, USA; [2] University of California, Los Angeles, Electrical and Computer Engineering Department, Los Angeles, USA

The global shift to electric vehicles necessitates the expansion of Direct Current Fast Charging (DCFC) stations, yet the related environmental and public health impacts remain unclear. Here, we report that the power cabinet at DCFC stations emit fine particulate matter ($PM_{2.5}$). We collected integrated filter samples from 50 DCFC stations across 47 cities in Los Angeles County, California. The daily $PM_{2.5}$ concentrations were between 7.3 and 39.0 μg m^{-3}, significantly higher than urban background sites ($p = 0.02$) and the nearest U.S. EPA monitoring stations ($p < 0.0001$). To understand the emission mechanism of these particles, we measured real-time $PM_{2.5}$ mass concentration, particle size distribution, and other pollutants, as well as characterized particle chemical compositions on integrated filter samples. Our results indicate that these particles, primarily in the sub-micrometer range (0.5–1.0 μm), are likely due to particle resuspension from the power cabinets. $PM_{2.5}$ samples from power cabinets showed higher levels of brake and tire wear tracers (Ba, Cu, Zn) and dust tracers (Ca, Al, Fe) compared to samples from nearby chargers and background sites. With no current emission standards for DCFC, managing particle resuspension is crucial for improving air quality and protecting public health as transportation electrification advances.

P26-38 | The effect of outdoor air pollution on healthcare utilization among people living with sickle cell disease: a scoping review

Zachary Smith[1,2], Kirsten Koehler[1,2], Jaime Madrigano[1], Louis Fazen[1,2], Christy Sadreameli[3]

[1] Johns Hopkins University, Bloomberg School of Public Health, Department of Environmental Health and Engineering, Baltimore, USA; [2] Johns Hopkins University, Education and Research Center for Occupational Safety and Health, Baltimore, USA; [3] Johns Hopkins University, School of Medicine, Baltimore, USA

Objective. People with sickle cell disease (SCD) may be vulnerable and susceptible to pain crises and other serious health exacerbations associated with outdoor air pollution. In this scoping review, we summarized evidence about the association between outdoor air pollutants and health among people with SCD. **Material and Methods.** PubMed and Embase databases were queried for articles published between January 1, 1946 and December 4, 2024. We used four terms for SCD and 23 for air pollution. The inclusion criteria were English-language peer-reviewed articles available online. We excluded commentaries, conference abstracts, reviews, and articles discussing indoor air pollutants. After screening articles, we abstracted relevant studies into a custom template. We used Covidence (Veritas Health Innovation, Melbourne, Australia) to organize the review. **Results.** We identified 1058 articles and screened 801 abstracts, after removing duplicates. Of 32 full-texts reviewed, we excluded 21, primarily conference abstracts (n=7) and those discussing primary or secondary tobacco smoke (n=6). Eleven articles met inclusion criteria. Nine studies were published after 2010, most were set in the United States (n=5), and seven focused exclusively on children. Various study designs were used and most focused on healthcare utilization outcomes (n=9), like emergency department visits (n=6). Studies generally supported positive

associations between at least one pollutant and healthcare utilization among people with SCD (n=8). All studies used area-level exposures, with nitric oxides (n=9), $PM_{2.5}$ (n=8), and ozone (n=8) the most commonly studied pollutants. No studies attributed exposure to specific sources (e.g., traffic, wildfires). **Conclusions.** We identified few studies investigating the role of outdoor air pollution on health among people with SCD. Heterogeneity of findings could be due to varying outcome and exposure assessment methodologies, study populations, and study designs. Further research is needed to examine air pollution as a contributor to health exacerbations among people with SCD.

P26-39 | Who’s at risk? Estimating population exposure to oil and gas methane super-emitters

Jasmine Lee[1], Angélica Ruiz[1], Quintin Munoz[1], Sofia Bisogno[1], Jeremy K. Domen[1], Christos Efstathiou[1], Nick Heath[1], Lee Ann L. Hill[1], Chowdhury G. Moniruzzaman[1], Sebastian T. Rowland[1], Ali Snell[1], Seth B. Shonkoff[1,2], Kelsey R. Bilsback[1], Yanelli Nunez[1,3]

[1] PSE Health Energy, Oakland, USA; [2] University of California, Berkeley, Department of Environmental Science, Policy and Management, Berkeley, USA; [3] Columbia University, Department of Environmental Health Sciences, Mailman School of Public Health, New York, USA

Methane (CH_4) detecting satellites and aerial surveys have identified thousands of methane super-emitter events (MSEEs) (>100 kg CH_4 hr^{-1}) across the U.S. oil and gas industry. These super-emitters can pose health risks to nearby populations due to the co-emission of non-methane volatile organic compounds with methane. Here, we assess who is at risk of exposure to MSEEs.

We compiled oil and gas MSEE data from available aerial and satellite surveys from 2016 to 2024. We characterized populations within a 1-mile and 3-mile radius of a detected MSEE. We used data from the U.S. Census, the Center for Disease Control and Prevention’s (CDC) PLACES, the CDC’s Environmental Justice Index (EJI), and the Homeland Infrastructure Foundation-Level Data. We estimated the total population and race/ethnicity breakdown near MSEEs. We also calculated the number of children, older adults, people living in poverty, people with asthma, cancer, chronic obstructive pulmonary disorder, stroke, or coronary heart disease and the number of sensitive receptors at risk of exposure, including childcare centers, schools, hospitals, nursing homes, and prisons. Lastly, we estimated the environmental justice burden within the 1-mile and 3-mile radius.

In our preliminary analysis, we found 2,059 MSEEs located across 10 states, the majority of which were in Texas, New Mexico, and California. Most events occurred in sparsely populated areas, but ~3% were in dense regions. The average population in the 1-mile radius was 118 (min: 0, max: 14,570). 793 MSEEs have a surrounding population with an EJI rank over the 50th percentile, indicating higher than average environmental and social burdens in some nearby communities. We anticipate our analysis will provide insight into who is impacted by MSEEs and help develop mitigation strategies to protect public health.

We acknowledge funding support from Global Methane Hub and the Waverly Street Foundation.

P26-40 | Morphological characterization & toxicity evaluation of Size Segregated Aerosols in Urban Households: Interaction between particulate pollution and Socio-economic status

Himanshi Rohra[1,2], Pg Satsangi[1], Ajay Taneja[2]

[1] Savitribai Phule Pune University, Pune, India; [2] Dr. Bhimrao AMbedkar University, Agra, India

Air pollution disproportionately affects marginalized populations of lower socioeconomic status (SES). As frequently reported, households with poor financial background are the ones that are vastly exposed to pollutant concentration. The study aims to explore the relationship between socio-economic disparity and indoor particulate exposure through morphometric and toxicity analysis at household level in Agra, India. Sampling of size-fractionated PM (10-0.25μm) was conducted using 5-staged Cascade Impactor in residential homes separated by SES; Low (LIG: <5K INR/month), Middle (MIG:5-60K INR/month and High (HIG: >70K INR/month) income groups.

Bimodal distribution of PM with primary mode in 0.25-0.5μm and high mass loading (28-39%) in LIG than higher waged groups was noted. Morphometric analysis using electron microscopy (SEM-EDX) revealed different kinds of particles like soot, tar balls, dust particles. Shape descriptor parameters revealed particles to be non-uniform (circularity=0.6-0.7 and Aspect Ratio (AR): 1.5-1.9) while more spherical particles (soot and TB) were found in HIG than LIG and MIG. EDX spectra revealed fewer concentration of crustal elements in ultrafine particles in LIG, indicating anthropogenicity. Correlation analysis revealed AR and circularity to decrease with increase of AED in LIG while in case of RN the trend was reversed. Toxicity evaluation (through USEPA (2009) health risk assessment model) suggested LIG residents (children>adults) to be highly susceptible to health hazard. Highest value of non-carcinogenic risk was posed by metals (Mn, Ni) in PM0.25 while highest carcinogenic risk was posed maximally by metals (Cr(VI)) in PM2.5-10. Overall, 231 adults and 613 children out of 1 million are likely to develop carcinogenic effects if exposed to such concentration levels in LIG households. These findings highlight the need to consider the role of air pollution in exacerbating the consequences of socio-economic disadvantages in India. Results will aid policy makers to design intervention policies for betterment of air quality in residential environments.

P27 | Built Environment and Health

P27-01 | The association of socio-economic and built environment characteristics with smoking behavior in a cohort of Greek Cypriot adults

Benjamin Weitz[1], Panayiotis Kouis[2], Carolina Z. Vieira[1], Panayiotis K. Yiallouros[2], Marina Neophytou[3], Andreas Protopapas[2], Petros Koutrakis[1], Petros Mouzourides[3], Angelos P. Kassianos[4], On behalf of the Cyprus Harvard Endowment Program for the Environment and Public Health - Master of Science Fellowship Program.

[1] Harvard T.H. Chan School of Public Health, Harvard University, Boston, USA; [2] Medical School, University of Cyprus, Nicosia, Cyprus; [3] Laboratory - Isle of Excellence of Environmental Fluid Mechanics, Department of Civil and Environmental Engineering, University of Cyprus, Nicosia, Cyprus; [4] Department of Nursing, School of Health Sciences, Cyprus University of Technology, Limassol, Cyprus

Background: Previous studies from Cyprus and elsewhere have demonstrated consistent associations between individual level characteristics and smoking behaviors in adults. Nevertheless, the association of urban built environment characteristics with smoking behavior as well as how individual level characteristics interact with built environment characteristics to modify smoking behavior remain unclear and have never been investigated in Cyprus and the region.

Objectives: This study aimed to explore associations between individual socio-economic characteristics and urban built environment characteristics with smoking behavior in Greek Cypriot adults.

Methods: Data on individual level characteristics and smoking outcomes (active smoking and whether participants had ever tried quitting) were obtained from the 2023 cross-sectional survey from the Cyprus National Addictions Authority. Information for built environment characteristics (building height, green urban area, sports and leisure facilities, and trees at the street level) were obtained from the Copernicus Land Monitoring Service, aggregated at the postal code level.

Results: From a total of 2643 adults ($\geq$18 years old), 49.2% were males (mean age = 35.74). Of them, 44.6% were active smokers, and individual factors were associated with smoking outcomes consistent with the literature. Building height was associated with increased odds of active smoking, especially among city center participants [1.21 (1.01, 1.46); p=0.036], and the presence of sports and leisure facilities was associated with decreased odds of active smoking [0.77 (0.63, 0.96); p=0.020]. The presence of green urban areas was associated with increased odds of having ever tried to quit [1.48 (1.07, 2.05); p=0.020], and attenuated the effects of building height.

Conclusions: Building height was associated with smoking behaviors and the opposite effects were observed for sports and leisure facilities and green urban area in adults. These results indicate the contrasting effects of urban built environment characteristics on health behavior.

P27-02 | Integrating Hyperlocal Environmental, Social, and Healthcare Data to Create Address-Level Risk for Child Hospitalization (ARCH) Scores

Carson S. Hartlage[1,2], Qing Duan[2], Erika R. Manning[2], Joseph Michael[2], Judith Dexheimer[1,2], Andrew F. Beck[1,2], Cole Brokamp[1,2]

[1] University of Cincinnati, College of Medicine, Cincinnati, USA; [2] Cincinnati Children's Hospital and Medical Center, Division of Biomedical Informatics, Cincinnati, USA

Objective: Traditional population health approaches that rely on area-level data may obscure critical spatiotemporal heterogeneity. More precise spatiotemporal exposure assessment and multi-scale data integration could provide more nuanced understanding of exposure-response relationships and inform more effective, targeted intervention strategies. Here, we aimed to use hyperlocal residence- and neighborhood-specific social and environmental data linked to population-wide healthcare data to characterize address-level risk for pediatric hospitalization.

Materials and Methods: Residential addresses associated with Cincinnati Children's hospital admissions (2016-2022) were linked to parcel-specific housing characteristics from the Hamilton County Auditor and enforced housing infractions from the Cincinnati Department of Buildings & Inspections. Addresses were geocoded to estimate hyperlocal crime using Cincinnati Police Department data and to link census tract-level American Community Survey measures and eviction filings. We trained a generalized random forest model to predict hospitalization totals for every residential address in Cincinnati. Predictions were calibrated using a ranked percentile-based approach and characterized using area under the receiver operating curve (AUC), sensitivity, specificity, and positive predictive value (PPV).

Results: Among 77,077 total residential addresses, 5,704 (7.4%) were linked with at least one of 10,085 pediatric hospital admissions and 1,850 (2.4%) were linked with multiple. Percentile-based predictions from the model trained on hyperlocal environmental characteristics were defined as the Address-Level Risk for Child Hospitalization (ARCH) scores. When calibrated to identify the 5% of residential addresses with the highest number of pediatric hospitalizations, ARCH scores were highly predictive (AUC: 0.957; 95% CI: [0.952, 0.963]), sensitive (0.972), specific (0.943), and had a fair PPV (0.447). Variable importance analysis indicated that residence-specific variables, including housing infractions, violent crime, and market value were important for risk stratification. Visual comparisons revealed within-tract heterogeneity in hospitalization risk at the address level.

Conclusion: ARCH scores can pinpoint addresses with higher predicted healthcare needs, guiding hyperlocal interventions while preserving individual-level privacy.

P27-03 | Factors associated with airborne fungal abundance in the homes of older adults in Northern Taiwan

Nicholas T. Minahan[1], Chi-Hsien Chen[2], Yu-Chen Chuang[2], Chih-Cheng Hsu[3,4], Yue Leon Guo[1,2]

[1] National Health Research Institutes, National Institute of Environmental Health Sciences, Zhunan, Taiwan; [2] National Taiwan University (NTU) College of Medicine and NTU Hospital, Department of Environmental and Occupational Medicine, Taipei, Taiwan; [3] National Health Research Institutes, Institute of Population Health Sciences, Zhunan, Taiwan; [4] National Health Research Institutes, National Center for Geriatrics and Welfare Research, Yunlin, Taiwan

Objective: Exposure to airborne fungi indoors is associated with allergic respiratory diseases. Taiwan has a humid climate conducive to fungal growth and has a rapidly aging population for whom the home environment is increasingly important. However, the factors shaping fungal exposure remain poorly characterized. This study aimed to identify environmental factors associated with airborne fungal abundance in the homes of older adults.

Material and Methods: A cross-sectional study was conducted in 192 homes in Yang-Mei District, Taoyuan, Taiwan, from March to October 2024. Airborne fungi were passively sampled in bedrooms for one week, and temperature and relative humidity (RH) were monitored. Building characteristics, mold indicators, and ventilation were investigated. Meteorological variables and land use areas were estimated within a 500-m buffer. Fungal genus abundance was quantified using high-throughput sequencing and quantitative PCR targeting fungal ITS2. Generalized additive models and mediation analysis were used.

Results: Among 1438 genera, 66 were commonly detected in ≥90% of homes, including allergenic genera *Cladosporium*, *Alternaria*, *Fusarium*, *Aspergillus*, and *Penicillium*. Higher ambient temperature and RH were associated with increased abundance of 55 and 46 common genera, respectively, after adjusting for indoor factors. Natural ventilation was associated with increased *Cladosporium*, *Alternaria*, *Fusarium*, *Penicillium*, and 42 other common genera. A larger paddy field area was associated with increased *Cladosporium*, *Alternaria*, *Fusarium*, and 11 other common genera. *Aspergillus*, *Wallemia*, and *Sterigmatomyces* were more abundant in ground-floor bedrooms than on higher floors and associated with visible mold. This floor-abundance relationship was partially mediated by the indoor-to-outdoor RH ratio.

Conclusion: Both outdoor and indoor environmental factors contribute to airborne fungal abundance in homes. Elevated indoor RH, particularly on ground floors, contributes to a higher abundance of specific genera. Targeted interventions, including air purification and dehumidification, are recommended to mitigate exposure. This work received support from the National Health Research Institutes (CG-113-GP-12).

P27-04 | The Association Between Nightlife Districts and Pedestrian Fatality: A Location-Based Case-Control Analysis

Nicole G. Itzkowitz[1], James R. Quinn[1], Kathryn M. Neckerman[2], Eliza W. Kinsey[3], Stephen J. Mooney[4], Michael D. Bader[5], Gina S. Lovasi[6], Andrew G. Rundle[1]

[1] Columbia University, Department of Epidemiology, New York, USA; [2] Columbia University, Columbia Population Research Center, New York, USA; [3] University of Pennsylvania, Department of Family Medicine and Community Health, Philadelphia, USA; [4] University of Washington, Department of Epidemiology, Seattle, USA; [5] Johns Hopkins University, Department of Sociology, Baltimore, USA; [6] Drexel University, Department of Epidemiology, Philadelphia, USA

Objective: Cities across the United States are developing and promoting nightlife districts consisting of high concentrations of bars, restaurants, and entertainment establishments to encourage economic growth. This clustering also groups intoxicated pedestrians in these districts, which may elevated injury risk. We evaluated the association between nightlife districts and risk of pedestrian fatality in the US.

Material and Methods: Using 2018 data from the National Establishment Time Series business database, we used SaTScan to identify clusters of nightlife establishments (e.g. bars, clubs, restaurants) in each metro area in the USA and identified the Census blocks that comprise each identified nightlife district. We used the 2017 and 2018 Fatality Analysis Reporting System database to identify the locations of 10,587 pedestrian fatalities. Using incidence density sampling, we selected 21,174 matched control locations, matching on county, quintile of Tract-level population density and roadway type (intersection, segment or ramp). We estimated covariate adjusted odd ratios for case and control locations being in a nightlife district Census block compared to being in a Census block outside of a nightlife district.

Results: Overall, case locations were more likely to be in nightlife districts compared to control locations (adjusted OR = 2.13, 95% CI 1.87-2.42). The odds ratio for being in a nightlife district was 2.33 (95% CI 1.85-2.94) for case locations involving a driver or pedestrian who was alcohol-impaired and 2.02 (95% CI 1.71-2.38) for case locations without alcohol involvement.

Conclusion: These results suggest pedestrian fatalities are more likely to occur in nightlife districts that outside of these areas, although the associations with nightlife districts was similar for alcohol involved and uninvolved fatalities. Understanding how the built environment interacts with alcohol use behaviors in the context of pedestrian fatalities can inform urban planning policies that can help mitigate the potential health risks posed by these spaces.

P27-05 | Turning Waste into Resource: Rethinking Waste Management through Architecture in Santurce, Puerto Rico

Nildalice Berrios Negrón

University of Puerto Rico Rio Piedras Campus, School of Architecture, San Juan, Puerto Rico

Waste management is a global challenge with significant environmental, economic, and public health implications. Abandoned buildings and unmanaged waste in Santurce, Puerto Rico, increase flood risk due to clogged sewers and potential vector-borne disease outbreaks such as dengue, chikungunya, and leptospirosis. This study examines current barriers to ecological behavior and effective waste management by exploring how architectural interventions can integrate waste as a resource in design.

A four-week cartographic analysis documented Santurce's current physical conditions and identified spatial patterns and prevalent waste disposal practices. Christian A. Klöckner and Anke Blöbaum's Comprehensive Action Determination Model (CADM) was applied theoretically to design a survey assessing the ecological behavior of residents, workers, and visitors. Additionally, semi-structured interviews of residents and workers provided qualitative insights into awareness levels, additional situational hurdles, and potential solutions.

The results indicate that plastic bags (45%), plastic trash cans (21%), and exposed domestic waste (18%) are the predominant methods of waste disposal concentrated on commercial properties (restaurants, cafes, bars), residences (occupied or abandoned), and sewers located in Santurce's main avenues. Situational influences such as perceived behavioral control and access to waste management services, were direct obstacles to proper waste disposal across all groups. At the same time, habitual processes, social norms, and personal norms may be indirect obstacles to ecological behavior, specifically for workers and visitors. Despite these challenges, local communities lead initiatives to improve waste management and rehabilitate abandoned buildings, though they remain vulnerable to environmental and health risks.

The findings suggest that architectural interventions, such as a community-based collection and educational center, could improve waste management accessibility and promote environmentally responsible practices. Future research should explore demographic variations in waste perceptions (e.g. children) and innovative architectural strategies for incorporating discarded materials into construction and urban design.

P27-06 | Building age-friendly and environmentally sustainable cities and communities in China: A Scoping Review

Yanhui Jia, Jiuxuan Gao, Weiju Zhou, John S. Ji

Tsinghua University, Vanke School of Public Health, Beijing, China

Climate change, population ageing, and rapid urbanization pose interconnected threats and challenges to public health in China, especially the vulnerable elderly. This scoping review synthesizes national policy documents that contribute to age-friendly and environmental sustainable cities and communities, alongwith sub-national innovations as support. 26 websites of government ministries affiliated with the Chinese State Council were searched from Jan 1st, 2020 to Dec 20th, 2024. Total of 126 documents were included after retrieving and screening process. National strategies including National Strategy for Climate Change Adaptation 2035, Healthy China 2030 initiative, Healthy Cities and Carbon Neutrality plans lay a comprehensive foundation in China. However, age-friendly and sustainable city initiatives operate as distinct policy domains managed by separate government departments. Age-friendly urban development prioritizes healthcare system optimization, accessibility improvements, and social equity measures, while sustainable urbanization emphasizes ecological restoration, pollution reduction and decarbonization, and climate-resilient infrastructure. Key innovations including 15-minute community life circles, comprehensive communities, sponge cities development, urban renewal actively promotes climate resilience and age-friendliness at community and city level, through enhancing infrastructure accessibility and inclusivity, managing rainstorm and flooding, and optimizing layout of green spaces. Large-scale ecological restoration such as Three-North Shelter Forest Program also contributes to elderly well-being by combating desertification and purifying air. However, institutional coordination remains limited, with climate adaptation strategies rarely incorporating targeted measures for elderly populations, and age-friendly programs seldom aligning explicitly with sustainability goals such as carbon reduction or disaster preparedness. Implementation varies greatly across regions due to vast geographic, economic, and administrative diversity. Though China has made significant progress in advancing age-friendly and sustainable urbanization, huge challenges persist in fragmented governance and implementation gaps. Future efforts should prioritize the inter-ministerial coordination, integrated urban projects for co-benefits, and promote elderly engagement in climate adaptation to jointly advance age-friendly, sustainable cities and maximize public benefits.

P27-07 | Neighborhood-level factors associated with gentrification and agreement of gentrification measures in two United States cities

Erin E. Bennett[1], Sarah Edgington[2], Charlie H. Nguyễn[1], Beth Ann Griffin[2], Huda Bashir[3], Genee Smith[1], Regina A. Shih[3,2], Jaime Madrigano[1,2]

[1] Johns Hopkins University Bloomberg School of Public Health, Department of Environmental Health and Engineering, Baltimore, USA; [2] The RAND Corporation, Santa Monica, USA; [3] Emory University Rollins School of Public Health, Department of Epidemiology, Atlanta, USA

Objective: Measuring gentrification, an emerging exposure of interest for health studies, remains challenging because the variables and reference geographies used to define it significantly affect which neighborhoods are considered gentrifying. Understanding factors that influence both gentrification and gentrification measure agreement is important for assessing potential impacts of confounding and exposure misclassification. We assessed whether tract-level characteristics are associated with (1) gentrification defined using four different measures and (2) agreement of all four gentrification measures. We use Baltimore, Maryland and Pittsburgh, Pennsylvania as case examples because previous work showed that agreement of gentrification measures varied substantially between these two Northeastern, mid-size cities.

Methods: We used 2008-2012 (proxy for 2010) and 2013-2017 (proxy for 2015) American Community Survey data to identify census tracts that gentrified between 2010 and 2015. We used two definitions (Freeman, 2005 and Ding et al., 2016) and two reference geographies (county and metropolitan statistical area) to create four gentrification measures. We used logistic regression models to determine whether tract-level resident race/ethnicity, age, and nativity status, housing unit density, median income, and income inequality were associated with each gentrification measure and measure agreement.

Results: Gentrification measure agreement was higher in Pittsburgh. Tract-level housing density, median income, and income inequality were associated with gentrification measures in both cities. Race was more consistently associated with gentrification in Pittsburgh. In Pittsburgh, race, housing density, and median income were significantly associated with gentrification measure agreement. No variables were associated with measure agreement in Baltimore.

Conclusion: Choice of gentrification measure may affect associations between gentrification and health. Investigators studying gentrification should conduct sensitivity analysis on the type of gentrification measure used, explore effect heterogeneity by urban center in multi-site analyses, and consider adjustment for factors known to influence gentrification measure agreement, which may make findings across studies using different measures more comparable.

P27-08 | The School Environment Modifies Pediatric Susceptibility to Air Pollution in New York State

Lisa Frueh[1], Rachit Sharma[1], Ellen J. Kinnee[2], Perry E. Sheffield[3], Jane E. Clougherty[1]

[1] Drexel University Dornsife School of Public Health, Environmental and Occupational Health, Philadelphia, USA; [2] University of Pittsburgh, Center for Social & Urban Research, Pittsburgh, USA; [3] Icahn School of Medicine at Mount Sinai, Environmental Medicine and Pediatrics, New York, USA

Objective: In the US, most children attend public schools, comprising both a social and physical environment and key predictor of pediatric health. Given the extant literature on how the neighborhood social and built environment modifies susceptibility to environmental hazards, we hypothesize that aspects of public school built and social environments may shape pediatric susceptibility to ambient air pollution.

Methods: To explore school environment variables as effect modifiers of air pollution and child health, we employed a time-stratified, case-crossover design. We estimated associations between residential ambient $PM_{2.5}$ exposure and risk of emergency department (ED) visit among children aged 5-17 in NY State between 2005-2019 using data from the NY Statewide Planning and Research Cooperative System (SPARCS), stratifying models by school quality indicators using publicly-available data and census sociodemographic characteristics.

Results: In preliminary analyses (N = 5,592,018) excluding NYC, $PM_{2.5}$ conferred a modestly-increased risk of all-cause ED visit [1.17% increased lag0 risk (95% CI: 1.03, 1.29)]. Risk decreased across increasing quintiles of teacher-to-pupil ratio [e.g., quintile 1: 1.26% (95%CI: 1.06, 1.46) vs. quintile 4: 0.84% (0.51, 1.17)]. Risk increased across increasing quintiles of school violent incident rate [quintile 2: 0.56% (95%CI -0.25, 1.38) vs. quintile 4: 1.77% (1.55, 2.01)] and proportion of classes taught by highly-qualified teachers (counter-to-hypothesis), though less so in the lowest-income areas. There was no evidence of effect modification by building quality indicators in single-modifier models, however, within tracts with the highest proportion of people of color, children in schools with better space adequacy ratings had lower risks per unit $PM_{2.5}$.

Conclusion: We find some evidence that children assigned to schools with a higher-quality educational environment showed weaker associations between acute $PM_{2.5}$ exposure and ED visit for any cause. Evidence of school-related effect modification varied across neighborhood sociodemographic characteristics.

Funding: US EPA (EPA STAR R840472)

P27-09 | The Spatial Distribution of School Environment Indicators in New York State and Co-Patterning with Sociodemographic Characteristics

Lisa Frueh[1], Ellen J. Kinnee[2], Perry E. Sheffield[3], Jane E. Clougherty[1]

[1] Drexel University Dornsife School of Public Health, Environmental and Occupational Health, Philadelphia, USA; [2] University of Pittsburgh, Center for Social & Urban Research, Pittsburgh, USA; [3] Icahn School of Medicine and Mount Sinai, Environmental Medicine and Pediatrics, New York, USA

Objective: Children aged 5-18 in the United States spend around 20% of their time in school, and school context is a key determinant of child health. We compiled data on public school educational, social, and building environment in New York State (NYS), to investigate spatial clustering across these domains, and co-patterning with neighborhood sociodemographic characteristics.

Methods: A suite of school educational, social, and physical indicators were compiled for public schools across 2005-2019 in NYS. Spatial regression models were used to estimate bivariate spatial associations between census tract-level sociodemographic characteristics and school quality indicators.

Results: Results suggest that schools with better educational and physical environments are more often found in census tracts with larger non-Hispanic White populations and higher socioeconomic position. For example, in preliminary non-spatial models in 2015, a 1-percent increase in census tract population of color was associated with 3% lower odds that $\geq$ 90% classes in the school are taught by a highly-qualified teacher [OR = 0.97 (95%CI: 0.96, 0.97)]. Similarly, a 1-percent increase in the rent-burdened population (spending $\geq$ 30% of income on rent) was associated with 2% lower odds that $\geq$ 90% classes in the school are taught by a highly-qualified teacher [OR = 0.98 (0.97, 0.99)]. Building quality indicators showed comparable patterns—for example, higher rent burdened population was associated with higher odds of receiving negative ratings for space adequacy, ventilation, overall building rating, and evidence of vermin.

Conclusion: Schools with poorer building quality, more crowding, and lower teacher qualifications are more likely to be found in census tracts of lower socioeconomic position and a greater proportion of people of color. These results highlight racial, ethnic, and socioeconomic disparities in access to high-quality public schools in NYS.

This work is funded by the Environmental Protection Agency (EPA STAR R840472).

P27-10 | Development of a Context-Specific Environmental Fall Risk Assessment Tool for Older Adults in Kerala, India

Aravind C. Balachandran[1], Ravi P. Varma[2], Rekha R. Ravindran[3]

[1] State Health Systems Resource Centre Kerala, Ministry of Health and Family Welfare Government of Kerala, Thiruvananthapuram, India; [2] Achutha Menon Centre for Health Science Studies, Sree Chitra Tirunal Institute for Medical Sciences & Technology, Public Health, Sree Chitra Tirunal Institute for Medical Sciences & Technology, Thiruvanathapuram, India; [3] Directorate of Health Service, Department of Health Services, Government of Kerala., Thiruvanathapuram, India

Falls are a significant global health concern, particularly among older adults, leading to injury, disability, and mortality.In India, Kerala has the highest proportion of older adults, comprising 12.6% of the population (Census of India, 2021), underscoring the urgent need for targeted public health interventions. Despite the high risk, no context-specific tools exist in India to assess environmental fall hazards, especially in Kerala. This study aimed to develop and validate the first environmental fall risk assessment tool tailored to the local setting. The objective was to create simple screening tool for Accredited Social Health Activists to identify home-based fall hazards in and around the built environment of older adults. The tool was designed to detect common hazards and improve safety. Methods included reviewing international fall risk assessment tools, adapting items to the local context, and conducting literature reviews and expert interviews to generate a list of home hazards. Content validity was ensured through expert evaluation and feedback from older adults. The tool was piloted in older adults' homes by trained health workers under researcher supervision to assess feasibility and accuracy. Results from screening 77 older adults revealed that 41% had low fall risk, 30% moderate risk, and 29% high risk. Common hazards included wet or slippery floors (44.2%), tripping hazards (37.7%), and wet sink areas (36.4%). Lack of handrails (31.2%) and poorly constructed handrails (27.3%) were also identified. Risk levels were categorized using percentile scores: ≤0.1020 (low), 0.1021–0.1837 (moderate), and >0.1837 (high). Older adults demonstrated awareness of hazards, with some using walking sticks or holding onto pipes for support. Carers reported advising precautions such as using bleaching powder on slippery floors. The study successfully developed an environmental fall risk assessment tool to identify home hazards, supporting Kerala's efforts to prevent falls and protect older adults from injuries and death. Funding National-Health-Mission-India.

P27-11 | Estimating Community Severance in Four Major US Cities

Atharv Jayprakash[1,2], Jaime Benavides[1], Marianthi-Anna Kioumourtzoglou[1]

[1] Columbia University, Mailman School of Public Health, Environmental Health Sciences, NYC, USA; [2] Rutgers University, School of Arts and Sciences, New Brunswick, USA

Background and aims

Traffic-related exposures including air pollution and noise pose substantial health risks, particularly in urban environments. However, a crucial gap remains in understanding community severance, which refers to the physical barriers created by road infrastructure and heavy traffic that restrict access to essential services, social interactions, and economic opportunities. These physical disruptions can weaken social cohesion and may have negative health implications. This study aims to create a community severance index in four major US cities: New York City (NYC), NY, Los Angeles (LA), CA, Seattle, WA, and Chicago, IL.**Methods**

We used geospatial location data and dimensionality reduction techniques to capture community severance variation across the four cities. We employed principal component pursuit along with factor analysis to estimate the Community Severance Index. We used public data for 2019 at the census block group (CBG) level on road infrastructure, road traffic activity, and pedestrian infrastructure.

Results

We identified one multidimensional factor related to community severance explaining 49% of the data variation across the four cities. We estimated the highest levels of community severance in LA compared to NYC, Seattle, and Chicago. However, for the development of a robust multi-city index, a one-size-fits-all approach may not be optimal. Smaller towns may require a different framework to define community severance effectively.

Conclusion

We developed a highly resolved Community Severance Index for four major US cities using publicly available nationwide data. By estimating community severance indices across multiple cities, we now have an objective metric to compare community severance across different urban centers. This is a step toward a generalizable model for community severance across all US cities. This tool may be used to inform urban policy aiming to minimize severance and improve urban health outcomes.

P27-12 | Limiting Microbial Exposures from Sink Drains in the Healthcare Environment

Hannah G. Healy[1], Kate Bowie[2], Irvan Luhung[2], Richard Martinello[3], Jordan Peccia[2]

[1] Harvard, Environmental Health, Harvard T.H. Chan School of Public Health, Boston, USA; [2] Yale, Chemical and Environmental Engineering, New Haven, USA; [3] Yale, Yale School of Medicine, New Haven, USA

Hospital sink drains are known reservoirs for many pathogens that cause healthcare-associated infections (HAIs). Bacteria from drains can migrate up to the drain cover and sink basin and then spread to surrounding surfaces and patients through droplet dispersal during sink use. Across several sampling campaigns, we sampled drain pipes, drain covers, sink basin surfaces, drinking water, and p-trap liquid from hospital sinks to elucidate factors that influence pathogen abundance in sink microbial communities. To limit transmission between drains and patients, we also investigated two potential intervention points: (i) cleaning sink surfaces and (ii) cleaning drains. Bacterial abundance was assessed via culture and ddPCR for the 16S rRNA gene, and bacterial taxonomy was assessed through shotgun metagenomics and 16S rRNA gene amplicon sequencing. Microbial communities of drains and tap water stayed relatively consistent over time at each sink, while surface communities were more stochastic. Bacterial abundance on sink surfaces and in drain biofilms were correlated. Many genera that contain overt and opportunistic pathogens were identified; notably, Legionella spp. was detected in drains but not tap water (Figure 1). Hallway sinks, which were used more frequently than sinks in patient rooms, had significantly higher bacterial abundance. Surface cleaning reduced bacterial gene copies, but median gene copies on drain covers rebounded to over 80% of pre-cleaning levels within 24 hours. After drain cleaning, both total bacteria and carbapenem-resistant bacteria had regrown to exceed initial values after one week, which remained elevated for an additional week before starting to decline. These results emphasize the need for strategies beyond disinfection for controlling microbial communities in the built environment.

P27-13 | The Effects of Early Life Social Environment on Systemic Lupus Erythematosus Risk in Adulthood

Jennifer M. Woo, Dale P. Sandler

NIEHS, Epidemiology Branch, Research Triangle Park, USA

Objective: Systemic lupus erythematosus (SLE) is a chronic autoimmune disease that predominantly affects women and is associated with notable socioeconomic position (SEP)-related health disparities. Early life social environment, including trauma (ELT) and neighborhood deprivation (ND), has been associated with immune dysfunction and inflammation. While stress and low SEP in adulthood have been independently associated with SLE risk, the intersecting impact of ELT and ND on adult SLE risk is unknown. Using a life course approach, I examined the effects of early life social environment on adult SLE risk.

Methods: Data are from The Sister Study, a prospective cohort of US women (ages 35-74; enrollment: 2003-2009). Eligible participants (N=23,839) were SLE-free at ELT recall and had US Census tract data for age 10 residence. Incident SLE was self-reported through 2021 (n=94). ELT measures were based on 20 trauma types (before age

18): 1) number of ELTs and 2) six patterns of co-occurring ELT latent classes. A continuous latent measure of early life ND was estimated from Census data using confirmatory factor analysis. Cox proportional hazards regression was used to calculate SLE hazard ratios (HR) and 95% confidence intervals (CI).

Results: Approximately 52% of participants reported at least one ELT. Increasing number of ELTs was associated with elevated SLE hazard (HR=1.24[1.11,1.38]). Compared to the low ELT latent class, greater SLE hazards were observed for high ELT (HR=3.71[1.56,8.83]), moderate ELT (HR=2.77[1.49,5.17]), and sexual trauma with household dysfunction (HR=1.59[0.79,3.21]), but not family health issues nor high betrayal trauma. The highest quartile of early life ND had 1.29 times greater SLE hazard (CI: 0.69,2.41) compared to the lowest quartile. In models stratified by ND quartiles, the highest ND quartile had the largest SLE hazard associated with ELT count (HR=1.40[1.20-1.63]).

Conclusion: Adverse early life social environment represents a potentially critical driver of disparities in SLE risk.

P27-14 | Urban Compactness, Neighborhood Proximity, Environmental Exposures, and Health Impacts in Spanish Cities

Natalie Mueller[1], **Avi Patel**[2], Silia Gomez[1], Jaime Orrego[3], Federica Montana[1], Marta Cirach[1], Tamara Iungman[1], Sasha Khomenko[1], Mark Nieuwenhuijsen[1], Oriol Marquet[3]

[1] Barcelona Institute for Global Health (ISGlobal), Barcelona, Spain; [2] Yale University, New Haven, USA; [3] Autonomous University of Barcelona (UAB), Barcelona, Spain

Background: Compact urban designs can reduce carbon emissions by decreasing car dependency and improving service access. However, they often face challenges such as high pollution and limited green space. European compact cities report persistent motorized traffic, elevated pollution levels, and increased premature mortality. This study examines the relationship between urban proximity, environmental quality, and health in Barcelona, Granada, Palma de Mallorca, and Valencia, Spain.

Methods: A spatial proximity index was developed at a 100m grid resolution, incorporating walkability, cyclability, urban vitality, and the 15-minute city concept. Environmental exposure data (PM2.5, NO2, NDVI) and baseline mortality rates were analyzed across proximity quartiles (Q1: least proximate, Q4: most proximate). A health impact assessment estimated mortality burdens attributable to non-compliance with recommended environmental exposure levels.

Results: Annual average PM2.5, NO2, and NDVI levels ranged from 7.5–17 µg/m³, 10–50 µg/m³, and 0.20–0.45 NDVI, respectively. The most proximate areas (Q4) had the highest air pollution levels and lowest NDVI, while the least proximate areas (Q1) had lower pollution and more green space. Non-compliance with PM2.5, NO2, and NDVI recommendations significantly contributed to mortality burdens, with the highest burdens in Q4. In Barcelona, PM2.5-related deaths were 529 in Q1 vs. 22 in Q4, NO2-related deaths were 1,445 in Q1 vs. 57 in Q4, and NDVI-related deaths were 365 in Q4 vs. 36 in Q1. Similar patterns, though less pronounced, were estimated for Granada, Palma, and Valencia. High proximity was strongly linked to increased pollution and reduced green space, primarily due to dense motorized traffic.

Conclusions: Despite their sustainability potential, compact Spanish cities face significant environmental and health challenges. Reducing motorized traffic should be a priority to improve air quality, expand green spaces, and align urban proximity policies with better health outcomes.

P27-15 | Traffic Exposure: A Public Health Comparative Analysis of Population-Based and Traffic Density Metrics in Minnesota

Nicole Frederickson[1,2], Tess Konen[1], Jessie Carr[1]

[1] Minnesota Department of Health, St. Paul, USA; [2] Council of State and Territorial Epidemiologists, Applied Epidemiology Fellowship Program, Atlanta, USA

OBJECTIVE: The built environment influences traffic density, which adversely affects health and disproportionately burdens low-income communities. This study evaluates population-based and traffic density indicators as a proxy for exposure to determine the most suitable metric for ongoing geospatial surveillance and environmental justice screening tools in Minnesota.
MATERIAL AND METHODS: Qualitative and quantitative analyses assessed the quality, strengths, and limitations of two population-based metrics-Council of State and Territorial Epidemiologists (CSTE) and CDC traffic indicators-and traffic density. Proximity buffer and “busy roads” definitions were compared. ArcGIS Pro was used to map traffic density and roadway buffers. The indicators were compared using summary statistics and correlation analyses in RStudio. Traffic data were sourced from the Minnesota Department of Transportation. Population and spatial boundary estimates were obtained from the 2018-2022 American Community Survey and IPUMS National Historical Geographic Information System.
RESULTS: The metrics varied in defining proximity buffer and classifying "busy roads". CSTE’s proximity buffer is 300 meters, whereas CDC’s buffer is 150 meters. The CSTE indicator classifies busy roadways based on annual average daily traffic, while CDC’s indicator uses highway functional classifications. Summary statistics suggest that the CSTE indicator captures a greater population in areas of high traffic exposure than the CDC indicator. A moderate correlation was found between the two metrics. Traffic indicator variation was further explored in relation to area-level poverty, with the CSTE metric including a higher proportion of exposed populations in disadvantaged areas.
CONCLUSION: The CSTE metric defines busy roads more broadly, incorporating inner-city roads. Consequently, it captures a greater number of neighborhoods affected by traffic density and exposure, particularly those in high-poverty areas. By integrating a health equity perspective, the CSTE metric provides a comprehensive view of traffic-related disparities. Mapping traffic exposure proxies is crucial for understanding health equity, informing ecological studies, and enhancing environmental justice screening tools.

P27-16 | Geographic distribution, regional trends, and city-level modifiers of floods in Latin American cities: the Salud Urbana en America Latina (SALURBAL) study

Hanna Jardel[1], Ana Diez-Roux[1], Juan C. Figueroa[1], Usama Bilal[1], Jinwen Xu[2], Marcio Alazraqui[3], Nelson Gouveia[4], Olga L. Sarmiento[6], Salvador Ayala[5], Juan P. Rodriguez[6], Karla Rangel[7], Josiah Kephart[1]

[1] Drexel University, Urban Health Collaborative, Philadelphia, USA; [2] University of California, Berkeley, Institute of Transportation Studies, Berkeley, USA; [3] Universidad Nacional de Lanús, Instituto de Salud Colectiva, Remedios de Escalada, Argentina; [4] Universidad de São Paulo, Departamento de Medicina Preventiva, São Paulo, Brazil; [5] Universidad de Chile, Centro de Epidemiología y Políticas de Salud, Santiago, Chile; [6] Universidad de los Andes, Escuela de Medicina, Bogotá, Colombia; [7] Instituto Nacional de Salud Pública, Cuernavaca, Mexico

Objective: Floods affect more people than any other natural hazard and they are likely to affect more people with increasing population density. Little is known about flood distribution in Latin America, which is highly urbanized. We examined the spatial distribution of recent floods and flood trends among cities in Latin America.

Materials and methods: We linked the Dartmouth Flood Observatory flood data, which collects information on all floods reported in news media, to SALURBAL data on Latin American cities. We calculated descriptive statistics for cumulative (total flood duration over 22 years) and annual urban flood duration per city, stratified by Köppen climate group, country, elevation, and coastal status. We will examine factors associated with cumulative flood duration and modifiers of annual flood trends, including climate and features of the urban population and built environment using multilevel random-intercept, random-slope Poisson models.

Results: We observed 326 floods in 366 cities across 10 countries and 22 years (2000-2021). The median of cumulative flood days was highest for tropical cities and lowest for arid cities. Inland cities generally had more flood days than coastal cities. Colombian cities experienced by far more flooded days on average than other cities and Argentinian cities experienced the broadest variability in flood days. Annual duration flooded varied most over the years for tropical cities, inland cities, cities above sea level, Colombian cities, and Peruvian cities. Coastal cities experienced fewer flooded days each year than inland cities and for some years arid cities experienced more flooding than did tropical and temperate cities.

Conclusion: Both cumulative flood duration and annual flood duration trends varied widely across Latin American cities. We need to understand what modifies urban flood exposure to support widely applicable flood adaptation efforts that promote health equity across Latin America.

P27-17 | Exploring potential associations between environmental factors and reported injuries in career-technical education programs in New Jersey 2012-2024

Derek G. Shendell[1,3], Juhi Aggarwal[2], Quincy W. Hunter[2], Midhat Rehman[2], Maryanne L. Campbell[2]

[1] Rutgers School of Public Health (Rutgers Health/Biomedical and Health Sciences), Environmental and Occupational Health and Justice / NJ Safe Schools Program, Piscataway, USA; [2] Rutgers School of Public Health (Rutgers Health/Biomedical and Health Sciences), NJ Safe Schools Program, Piscataway, USA; [3] Rutgers Health/Biomedical and Health Sciences, Environmental and Occupational Health Sciences Institute, Piscataway, USA

Introduction:
The State of New Jersey (NJ) administrative code requires reporting incidents or injuries to NJ Safe Schools Program (NJSS) to inform future training interventions. Students or teachers sustained injuries from participating in career and technical education programs, on- or off-campus, and required treatment by a licensed medical doctor.
Methods:
In 2012 into 2013, NJSS transitioned physical reporting forms to an online platform, fully implemented by 2014. For 2012-2024, 495 reported injuries were analyzed. Ambient/outdoor environmental data (minimum, maximum, and average temperature, relative humidity, and maximum and average wind speed and direction) were obtained from public online archives (NJ Office of the State Climatologist, Rutgers School of Environmental and Biological Sciences/NJ Agricultural Experiment Station). We analyzed 166 of 495 injuries with environmental data: 28 outdoors, 35 semi-enclosed, 103 possibly semi-enclosed (construction/architecture, welding, etc.). Other injuries (329) were excluded since they occurred indoors (162), in-transit/travel (6) or location undetermined per incident report descriptions (161). Data were managed and analyzed in Microsoft Excel then SAS 9.4.
Results:
Most reported injuries involved body parts: hands, fingers, wrists, followed by face/head; type of reported injury as skin injuries, which included abrasions, cuts/lacerations and scratches; and were in Architecture and Construction, Agriculture, Food and Natural Resources, and Transportation, Distribution and Logistics Career Clusters. Males had more reported injuries than females. Potential associations with higher outdoor air temperature on the day of injury (average, maximum) and average outdoor relative humidity on the day of injury were noted.
Conclusion:
Understanding environmental factors contributing to reported injuries among susceptible, vulnerable young workers can inform future policies to reduce identified physical exposures or weather-related risk factors and develop new or enhance existing workplace safety trainings.

P28 | Chemical Exposures: Improving Measurements and Understanding Risks

P28-01 | Characterizing Human Exposure Sources in Human Hair using a Thermal Desorption Vocus Proton Transfer Reaction Time of Flight Mass Spectrometry

Anna C. Neville[1], David A. Jarma[2], Kerry A. Kinney[3], Pawel K. Misztal[4]

[1] University of Texas at Austin, Austin, USA; [2] University of Texas at Austin, Austin, USA; [3] University of Texas at Austin, Austin, USA; [4] University of Texas at Austin, Austin, USA

Objective

Air pollution significantly impacts human health, yet current exposure assessment methods are often limited to outdoor measurements, are time intensive, or are unable to accurately measure certain compounds. This study introduces human hair as a non-invasive alternative for monitoring pollution exposure.

We developed a novel and non-targeted method of hair analysis involving chemical extraction using thermal desorption and detection using a Vocus 2R proton transfer reaction time-of-flight mass spectrometer (Vocus-PTR-TOF-MS). This method enables the rapid and sensitive detection of a wide spectrum of toxicologically relevant compounds in human hair, providing a more efficient and comprehensive alternative to the traditional use of liquid extraction and GC/MS or LC/MS.

Materials and Methods

Hair samples from Austin, TX were rinsed with methanol and then subjected to a temperature ramp (60 °C, 90 °C, and 120 °C) using a Gerstel C200 controller. The Vocus measured the resulting emissions in real time. Hierarchical clustering was used to create chemical fingerprints to help identify potential exposure sources.

Results

Over 1,000 compounds were detected, including a broad range of exogenous volatile and semi-volatile organic compounds. Notable compounds relevant to human health included benzyl butyl phthalate and dibutyl phthalate, toluene, benzene, and oxybenzone. The hair samples also contained monobutyl phthalate, a metabolite of dibutyl phthalate and a secondary metabolite of benzyl butyl phthalate, indicating that hair can capture environmental exposure and subsequent metabolic processes.

Conclusion

This study highlights the potential of hair analysis as a non-invasive and time-efficient method for assessing human exposure. In combining thermal desorption with the Vocus PTR-TOF-MS, we detected a diverse range of contaminants, including exogenous compounds and metabolites. This method has promising applications in exposomics, especially for large datasets involving solid biological samples. This study was funded by the NSF CAREER award (no. 2146347).

P28-02 | Comparing Nanoparticle and Lung-Deposited Surface Area Concentrations Released from Burning Synthetic and Natural Candles in an Enclosed Residential Environment

Dalton Crunkelton[1], Dorothy Seybold[2], Elena Ortez[1], Megan Nicol[1], Kenya Watson[1], Jhy-Charm Soo[2], Atin Adhikari[2]

[1] Georgia Southern University, Department of Biology, College of Science and Mathematics, Statesboro, USA; [2] Georgia Southern University, Jiann-Ping Hsu College of Public Health, Statesboro, USA

Objective: Candles are burned by billions every day at home for religion, therapy, and more. However, candles emit various air pollutants, including particulate matter and nanoparticles. Nanoparticles can have several harmful effects on the human body, including respiratory diseases, cardiovascular diseases, neurological disorders, and cancers. Different types of candles may release varying amounts of nanoparticles during combustion, depending on their material composition. However, little is known about nanoparticles released from synthetic versus natural candle burning. This study intends to measure the nanoparticle emissions from artificial and natural candles in a residential environment through a series of experiments. We also measured Lung-Deposited Surface Area (LDSA) concentration, which is the surface area of nanoparticles deposited in the lungs and a key predictor of health outcomes.

Materials and Methods: Candles were burned for one hour in an enclosed and unventilated 4.42m × 2.77m room, with number concentration ($C_{\#}$; size range: 10-300 nm) and LDSA concentration (C_{LDSA}) measured using a Testo DiSCmini device at distances of 15 cm, and 1 and 2 meters from the candles repeated two times for 1-minute durations at three different time intervals: 15, 35, and 55 minutes.

Results: The means ±SD of nanoparticle $C_{\#}$ (numbers/cm^3) across all distances were: (a) *15-minute*: natural: 629,546±55,534; synthetic: 32,564±3,937; (b) *35-minute*: natural: 493,125±32,825; synthetic: 31,000±1,540; (c) *55-minute*: natural: 393,699±45,539; synthetic: 22,352±711. The ranges of nanoparticle C_{LDSA} (μm^2/cm^3) across all distances were: (a) *15-minute*: natural 170.2; synthetic: 141.85; (b) *35-minute*: natural: 63.77; synthetic: 123.1; (c) *55-minute*: natural: 122.3; synthetic: 111.8.**Conclusion**: Preliminary findings suggest that, on average, natural candles release significantly more nanoparticles over a defined duration than synthetic candles. This is likely attributed to the organic material present in natural candles, as the incomplete combustion of these materials generates a substantial quantity of nanoparticles.

P28-03 | Associations between prenatal glyphosate concentrations and early childhood behavioral measures in northern Puerto Rico

Haley Jenkins[1], Emily Zimmerman[2], Jennifer Fernandez[1], Zaira Rosario Pabón[3], Carmen Vélez Vega[4], José Cordero[5], Akram Alshawabkeh[6], John Meeker[1], Deborah Watkins[1]

[1] University of Michigan School of Public Health, Department of Environmental Health Sciences, Ann Arbor, USA; [2] Northeastern University, Department of Communication Sciences and Disorders, Boston, USA; [3] Northeastern University, Department of Electrical and Computer Engineering, Boston, USA; [4] University of Puerto Rico Graduate School of Public Health, Department of Social Sciences, San Juan, Puerto Rico; [5] University of Georgia, Department of Epidemiology and Biostatistics, Athens, USA; [6] Northeastern University, Department of Civil and Environmental Engineering, Boston, USA

Objective: Glyphosate, the most commonly-used herbicide worldwide, has been detected in ~80% of prenatal urine samples collected from participants in the PROTECT cohort in Puerto Rico. In previous research, we reported significant associations between prenatal glyphosate concentrations and poorer Battelle Developmental Inventory scores at 6-24 months. This study builds on these findings by investigating associations between prenatal glyphosate and a measure of behavioral and emotional development, the Child Behavior Checklist (CBCL) at 1.5-5 years. We hypothesized that prenatal glyphosate exposure would be associated with higher CBCL scores, indicating more problem behaviors.

Material and Methods: Mother-child pairs from the PROTECT cohort with prenatal glyphosate measures and ≥1 CBCL score were included. Glyphosate (GLY) and its degradant, aminomethylphosophonic acid (AMPA), were measured in maternal urine up to three times in pregnancy; concentrations were adjusted for specific gravity and geometric means were taken across visits to represent total exposure per participant. CBCL was administered at ages 1.5, 2, 2.5, 3, 4, and 5 years; Internalizing, Externalizing, and Total Problems scores represent outcomes. Negative binomial mixed-effects models were selected to account for over-dispersed CBCL scores, adjusting for maternal education, child age, birthweight, and sex.

Results: This analysis included 254 pairs, with 414 total observations. Statistical analysis revealed no statistically significant associations between urinary glyphosate or AMPA concentrations and CBCL scores. Overall, trends indicated marginally lower CBCL scores linked to glyphosate exposure and slightly higher scores associated with AMPA.

Conclusion: These preliminary results suggest that early child behavioral and emotional development may not be significantly associated with prenatal glyphosate exposure. Additional analyses will assess sex interactions, as well as glyphosate/AMPA concentrations at individual prenatal visits to understand whether exposure at specific timepoints *in utero* has a stronger influence on development. Assessment of CBCL subscales will be considered to more closely examine specific domains of development.

P28-04 | Vaginal absorption of surfactants and chemicals: a narrative review

Hamsika V. Thumu[1], **Jenni A. Shearston**[2]

[1] Illinois Mathematics and Science Academy, Aurora, USA; [2] University of Colorado Boulder, Department of Integrative Physiology, Boulder, USA

Objective: Vaginally used products can contain toxic chemicals such as per- and polyfluoroalkyl substances, surfactants, metals, and others. Chronic dermal exposure to such chemicals can lead to adverse health effects, however the effects of vaginal exposure are unknown. The objective of this review is to evaluate if surfactants and other chemicals applied intravaginally can be absorbed systemically or negatively impact vaginal cells.

Material and Methods: We conducted a narrative review using the PubMed database and the search terms "vagina*" or "intravaginal" or "vaginal administration" and "permeation" or "toxicity" and "surfactant". We included studies about vaginally administered or mucosally administered drugs or chemicals in human, animal, or cell models representing vaginal exposure. We excluded reviews and studies that only evaluated microbiome impacts or ecosystem toxicity.

Results: Our primary search yielded 3,131 articles and 13 papers met our inclusion criteria. We reviewed the absorption potential of 24 surfactants, chemicals, and metals, including lubricants of varying osmolarity, lauryl glucoside, nanosilver particles, TSST-1, alpha hemolysin, endotoxin lipopolysaccharide, p-Coumaric acid, surfactants with varying ionic structures, tenofovir, natural rubber FLG, purasomes, nonoxynol-9, octoxynol-9, DSE-36, and DSE-27. We found a total of 6 human cell, 10 animal cell, and 2 animal experiments. All of the studies tested for absorption or effect on vaginal cells, and one evaluated systemic absorption. All of the surfactants reviewed were absorbed into vaginal cells, but effects were dependent on concentration and polarity. Nonoxynol-9 consistently inhibited cell growth, was cytotoxic to vaginal cells, and had a high irritation potency.

Conclusion: Many reviewed surfactants were found in commercial personal lubricants, including the cytotoxic surfactant nonoxynol-9. Given its cytotoxicity and irritation potential, we recommend against use of nonoxynol-9 in vaginally applied formulations. Only one study evaluated systemic absorption of surfactants and other chemicals, highlighting a large gap in research of vaginally applied formulations.

P28-05 | Health Risks of Inhaling Metal Aerosols from Electronic Cigarettes

Jinho Lee[1], Wei-Chung Su[2], Inkyu Han[3]

[1] Boston University School of Public Health, Department of Environmental Health, Boston, USA; [2] Baylor University College of Arts & Sciences, Department of Environmental Science, Waco, USA; [3] Temple University College of Public Health, Department of Epidemiology and Biostatistics, Philadelphia, USA

Several studies suggest electronic cigarettes (e-cigs) might be less harmful than conventional tobacco products due to their non-combustible mechanism and fewer emitted aerosols. However, concerns remain regarding potential health risks associated with their use, particularly due to the presence of potentially harmful metals in e-cig aerosols. Furthermore, although studies have estimated inhalation doses of these metals, the influence of aerosol particle size and atomizing power of e-cigs on metal inhalation doses remains underexplored. A thorough understanding of these determinants is crucial to accurately assess health risks from inhaling e-cig aerosols containing potentially harmful metals.

The aim of this study is to quantify the potentially harmful metals in e-cig aerosols, and their inhalation doses, by different particle sizes. Furthermore, the association between the generated four metals and the various atomizing powers and six flavors of e-cig was investigated.

Size-segregated e-cig aerosols were generated in an exposure chamber, and collected using an 11-stage cascade impactor (0.056 – 18 μm) for the analyses of aerosol mass and metal mass. The metal deposition dose in the human respiratory tract was calculated using a mathematical respiratory deposition estimation model and aerosol size-specific metal mass concentration.

The results showed that neither an increase in atomizing power (from 5 to 20 W) nor six e-cig flavors was associated with a significant increase in the total metal deposition in the respiratory tracts. Additionally, the concentrations of metals were not consistent across the size of the aerosol, challenging the common assumption used in e-cig inhalation health risk assessments. Although the factors did not significantly affect the calculated respiratory deposition of harmful metals under typical e-cig usage condition, the estimated average hazard index exceeded 1.0 in this study.

These findings highlight the potential dangers of inhaling metal aerosols from e-cigs, emphasizing the need for further research and regulation.

P28-06 | Living near industrial facilities linked to gynecological cancer risk in Puerto Rico: CARIB-CARES Study

Paola N. Rodriguez-Montoyo[1,2], Ana P. Ortiz[6,1], Carlos Torres[3], Karen Ortiz-Ortiz[3], Noreen Michael[4], Pablo Mendez[1,2], Marievelisse Soto-Salgado[5,2], Nancy Cardona-Cordero[5,2]

[1] University of Puerto Rico Medical Sciences Campus, School of Public Health / Department of Biostatistics and Epidemiology, San Juan, Puerto Rico; [2] UPR Comprehensive Cancer Center, San Juan, Puerto Rico; [3] Puerto Rico Cancer Registry, San Juan, Puerto Rico; [4] University of the Virgin Islands, St. Thomas, U.S. Virgin Islands; [5] Division of Population Sciences, San Juan, Puerto Rico; [6] UPR Comprehensive Cancer Center, Division of Population Sciences, San Juan, Puerto Rico

Purpose. Cancer is the second leading cause of death in Puerto Rico, with differences across counties. In 2006, 86 million pounds in total releases of production-related waste managed, including 170 types of carcinogenic substances were reported to the Environmental Protection Agency (EPA). Major contributors included hazardous waste, chemical industries, and electric utilities, collectively releasing over 1,000,000 pounds. This study aimed to assess age-adjusted incidence rate ratios for gynecological cancers near high-risk sites in Puerto Rico compared to low-risk sites.
Methods. Data from the Puerto Rico Central Cancer Registry (2016-2021 period) was used to analyze cancer incidence by type, using the SEER*Stat v8.4.4. For this study, high-risk sites were defined as counties in Puerto Rico with the presence of at least one industry that reported chemical emissions to the EPA Toxic Release Inventory (TRI) Program and low-risk sites were defined as counties in Puerto Rico without the presence of industries that reported chemical emissions to the EPA TRI Program.
Results. An increase of 24% incidence rate ratio (IRR=1.24, 95%CI: 1.04-1.49) of ovary cancer for populations living in a high-risk site compared to those living in a low-risk site. Similar increasing patterns were observed for cervix uteri (IRR=1.02, 95%CI: 0.88-1.20) and corpus uterus (IRR=1.08, 95%CI: 0.99-1.17) with no significant differences observed ($p<0.05$).
Conclusions. These findings suggest a link between location of facilities emitting toxic chemicals and increased cancer risk for a specific gynecological cancer type, emphasizing the need for further research into environmental contributors to these disparities. Integration of targeted objectives through the state cancer control plans can serve as a basis to improve environmental health awareness of cancer risk factors.

P28-07 | Perfluorooctanoic acid (PFOA) exposure perturbs the molecular clock and metabolic homeostasis in mouse liver cells

Rosanna Jiang[1,2], Lisa Bottalico[1,2], Aalim Weljie[1,2]

[1] University of Pennsylvania, Systems Pharmacology and Translational Therapeutics, Philadelphia, USA; [2] Perelman School of Medicine, Center of Excellence in Environmental Toxicology, Philadelphia, USA

Objective: Per- and polyfluoroalkyl substances (PFAS) are endocrine-disrupting chemicals that act as obesogens and interfere with hormone action. In parallel, the circadian clock regulates metabolic homeostasis and hormone signaling. However, the effects of PFAS on circadian-endocrine crosstalk and physiology remain unclear. We assessed circadian and biochemical impacts of perfluorooctanoic acid (PFOA) on the liver clock using a systems-based cell model and mass spectrometry-based metabolomics.
Materials and Methods: We monitored circadian metabolic disruptions across three facets of the liver system via in vitro studies of met-murine hepatocytes (MMH-D3) exposed to PFOA (0.01–10 μg/ml). : 1) Perturbations to the clock gene *PER2* expression rhythm using real-time bioluminescence tracing; 2) metabolism from cell metabolites collected every 2 hours over 24 hours and analyzed with liquid chromatography-mass spectrometry; and 3) transcription of 23 molecular clock and nuclear receptors (NR) genes via quantitative real-time PCR.
Results: The amplitude of *PER2* oscillation increased across all PFOA doses. Twenty-two metabolites involved in amino acid metabolism, TCA cycle, and lipid metabolism, including proline, succinate, acetylcarnitine, and phosphocholine, exhibited altered cycling (RAIN Q < 0.3). PFOA increased *PPARα/γ* transcription, which regulates fatty acid β-oxidation and lipid synthesis pathways. Transcription changes in clock genes (*CLK, BMAL1, CRY, PER, REV-erb, ROR*) and 11 NR genes were observed 4–12 hours post-exposure.
Conclusion: PFOA exposure induces perturbations in oscillating liver metabolism, including energy homeostasis, inflammation, and xenobiotic degradation. Given that PFOA is recognized as an obesogen—chemicals that disrupt lipid metabolism and promote fat accumulation—the interaction of circadian metabolic disruption on glucose and lipid metabolism warrants further investigation on PFOA's mode of action in the development of obesity and metabolic disorders.
This work was supported by the NIH/NIEHS P30ES013508. We would like to thank the University of Pennsylvania and the Center of Excellence in Environmental Toxicology for their support.

P28-08 | Chemical components of indoor $PM_{2.5}$ and associated household characteristics in a susceptible population

Seokmin Ji[1], KyuMin Kim[2], Miyoun Shin[1], Young Shin Park[1], Seung-Hyun Cho[3], Hee-Young Yoon[4], Jin Woo Song[5], Sun-Young Kim[1]

[1] National Cancer Center, Gyeonggi Goyang, South Korea; [2] Gyeonggi University of Science and Technology, Gyeonggi Siheung, South Korea; [3] Research Triangle Institute, North Carolina, USA; [4] Soonchonhyang University, Seoul, South Korea; [5] Asan Medical Center, Seoul, South Korea

[Background]

Indoor fine particulate matter ($PM_{2.5}$) was associated with health risks particularly for susceptible populations who spend most of their time indoors. $PM_{2.5}$ chemical components measured in residences can help identify potential sources of indoor $PM_{2.5}$. This study examines household characteristics associated with $PM_{2.5}$ chemical components at residences of individuals with idiopathic pulmonary fibrosis (IPF).

[Methods]

We conducted an indoor air monitoring campaign at homes of 130 participants with IPF in South Korea from September 2021 to August 2023. Our monitoring campaign collected 5-day samples of $PM_{2.5}$ and 35 chemical components at four seasonal visits by using RTI MicroPEMs placed in participants' living rooms or bedrooms. We quantified black carbon (BC) and brown carbon using an integrating sphere method, while X-ray fluorescence was used to determine 33 inorganic elements. In addition, using household questionnaires, we obtained obtain demographics, home environments, heating/cooking, and lifestyles. Then, we applied stepwise regression to identify the household characteristics associated with each of $PM_{2.5}$ component concentrations, adjusting for season and age.

[Results]

The average $PM_{2.5}$ concentration of 287 5-day samples from 130 participants across four seasons was 14.9 (standard deviation=21.6) $\mu g/m^3$. Our participants were mostly males (82.5%), aged over 65 years (81.4%), and used natural gas for cooking and heating (73.0 and 94.7%, respectively). In our regression analyses, building age, ventilation during cooking, and the use of air cleaners were associated with most inorganic elements, as opposed to $PM_{2.5}$ that showed weaker associations with fewer characteristics. In particular, barium, iron, and titanium were higher with buildings aged more than 10 years than new buildings. BC and sulfur showed no associations with any of household characteristics.

[Conclusion]

Our study suggested the benefit of using $PM_{2.5}$ components to identify residential pollution sources of $PM_{2.5}$. Future research for season specific analyses or different populations should confirm our findings.

P28-09 | Environmental Chemicals and Maternal Perinatal Depression: A Scoping Review

Yunyue Shi[1,2], Pengfei Guo[1,2], Cindy Nguyen[2,3], Haoran Zhuo[1,2], Zeyan Liew[1,2]

[1] Yale School of Public Health, Yale Center for Perinatal, Pediatric, and Environmental Epidemiology, New Haven, USA; [2] Yale School of Public Health, Department of Environmental Health Sciences, New Haven, USA; [3] Yale College, Department of Cellular, Molecular, and Developmental Biology, New Haven, USA

Objective Perinatal depression (PND) affects 10–20% of women during pregnancy and postpartum. We conducted a scoping review of epidemiological evidence regarding maternal exposure to environmental chemicals and PND.
Methods We searched PubMed, Embase, Web of Science, Dimensions, and Scopus through August 16, 2024 and identified 66 original research articles that examined chemical exposures and maternal PND.
Results Over two-thirds of the 66 studies were cohort design, published between 2021-2024, with outcome assessment based on self-rated symptoms. Nineteen studies have evaluated ambient air pollution exposure, including PM2.5, NO2, PM10, and O3, and twelve studies reported an exposure association with antenatal and postpartum depression symptoms. Sixteen studies that examined environmental tobacco exposures, and three studies of indoor air pollution, were reported to be associated with maternal PND. Eighteen studies that examined endocrine disrupting chemicals in blood or urine, such as phthalates (n=10), per- and polyfluoroalkyl substances (PFAS, n=8), polybrominated diphenyl ethers (PBDE, n=6), organophosphate esters (OPE, n=3), and pesticides (n=2), reported mixed findings. Metals were examined in nine studies with blood lead and cadmium most strongly reported to be associated with PND.
Conclusion Multiple environmental exposures, including airborne pollutants and those from contaminated household products and food sources, may be contributing to the risk of maternal depression. Environmental risk reduction should be considered in primary prevention of maternal depression.

P28-10 | Associating Individual and Environmental Exposures to PAH sources based on Isotopic Carbon Ratios collected with Passive Sampling Devices

Alison Clark, Richard Scott, Kim Anderson

Oregon State University, Department of Environmental and Molecular Toxicology, Corvallis, USA

Forensically identifying the sources of airborne exposure from chemical characteristics can be difficult environmental samples can degrade or weather over time. One technique that avoids this problem is isotopic abundance. Stable isotope ratios have been used in other fields such as food authenticity to provide origins of a contaminant. We are creating an isotopic source library for carbon isotope ratio data from archived samples from known sources as well as common sources of PAHs such as natural gas exhaust and superfund sites. We hypothesize that carbon isotope ratios collected from known sources will be unique, and that we can identify sources of exposure in collected passive samplers based on those identifiers. Low-density polyethylene (LDPE) and silicone wristband (WB) passive sampler data from archived studies as well as available standard reference materials were first analyzed for present PAHs via GC/MS/MS. PAH Detections were used to create a standard for further analysis via GC/IRMS for carbon isotope

ratios. Collections of additional PAH source material are currently ongoing with passive sampling devices and will be analyzed after extraction. Preliminary results from the analysis of NIST SRM 1991, a mixed petroleum and coal tar creosote standard, and a collected sample from creosote-impacted surface water in Northern Oregon near the Portland Harbor Superfund site show respective isotopic signatures within 1% of each other. A variance of 5% in isotopic ratio may be expected between samples from of the same origin. As a large contamination source of the surface water is expected to be creosote, the alignment of the two signatures suggests that identifying contaminant type from environmental samples and their isotopic ratios is feasible. Continued analysis of samples from the same source are ongoing to assess precision. Funding was provided by the National Institute of Environmental Health Sciences with grant numbers T32ES007060, P42ES016465, and P30ES030287.

P28-11 | Toxic Threads: Public Health Implications of Environmental Chemicals in Textile

Alexa Friedman[1], **Samantha Schildroth**[2]

[1] Environmental Working Group, Washington DC, USA; [2] Boston University, Department of Epidemiology, Boston, USA

Objective: We aimed to provide a high-level commentary on the current knowledge of environmental chemicals in textiles (e.g., clothing), evaluate the implications for human health, and highlight research needs for exposure science and epidemiology.
Methods: We reviewed peer-reviewed literature assessing 1) environmental chemical concentrations in textiles, 2) biomarker concentrations of environmental chemicals following textile use, and 3) health effects from textile (e.g., uniforms) use in occupational and epidemiological studies. We summarized the findings and identified future research needs.
Results: Several studies reported detectable concentrations of environmental chemicals (e.g., heavy metals, azobenzene dyes, and multiple classes of endocrine-disrupting chemicals [EDCs]) in textile materials, suggesting that textiles (e.g., clothing or upholstery) are important sources of exposure to environmental chemicals. A paucity of studies observed associations between textile use and increased biomarker concentrations of environmental chemicals. Finally, a handful of occupational and epidemiological studies reported a greater risk of adverse health outcomes (e.g., adverse skin reactions, difficulty breathing, headaches, and fatigue) associated with use of specific textile items (e.g., airline uniforms). However, studies characterizing human exposure to environmental chemicals from textile use or evaluating the potential health effects of these exposures are limited.
Conclusion: Environmental chemicals have been detected in textiles, and textile use has been associated with higher biomarker concentrations of environmental chemicals and adverse health effects in a small number of studies. Additional research is needed to 1) fully characterize the extent of human exposure, including potential inequities in exposure, from textile use and 2) the reproductive, perinatal, and development health effects associated with environmental chemicals from textile use.

P28-12 | Characterizing Community Exposure to Tetrachloroethylene (PCE): A Breath Analysis Study with Mixed-Effects Models to Evaluate Variability

Marwan Alajlouni, Sa Liu

Purdue University, West Lafayette, USA

Background and Purpose: This study evaluates community exposure to tetrachloroethylene (PCE), a volatile organic compound (VOC), probable human carcinogen, and known neurotoxin among residents near contaminated groundwater plumes. Chronic PCE exposure, particularly in environmental settings, raises substantial concerns about community exposure levels. This work advances breath analysis as a non-invasive tool for environmental health monitoring and lays the groundwork for data-driven public health strategies. We use exhaled breath analysis to assess personal exposure and will integrate mixed-effects models to quantify inter- and intra-personal variability in repeated measurements.

Methods: Breath samples were collected from 173 participants in a Midwestern community with four PCE plumes. Proton Transfer Reaction Mass Spectrometry (PTR-MS) provided rapid, sensitive quantification of PCE in exhaled breath via a mobile lab. A total of 44 repeated measurements were gathered, which will be used in mixed-effects model analyses to unravel intra-personal and inter-personal variability.

Results: This poster reports a preliminary analysis of 217 exhaled breath samples from 153 participants, revealing PCE concentrations ranging from 0.08 to 14.43 ppb (mean = 0.38 ppb, SD = 1.24). Individuals residing *within* plume & buffer areas (24 participants) exhibited average PCE (0.48 ppb, SD = 0.84) compared to *outside* plumes (0.37 ppb, SD = 1.30). While spatial proximity was not significantly higher (p = 0.138), observed variability across individuals and repeated measurements from mixed-effects model analyses for inter- and intra-personal variability will be finalized for the conference poster.

Conclusion: These interim findings highlight complexity of low-level PCE exposure in communities near contamination sites. The observed variability underscores the importance of upcoming mixed-effects model analyses, refining exposure assessments by quantifying intra- and inter-personal factors (e.g., temporal shifts, behavioral habits). The final results, to be presented at the conference, will help untangle some of the complexity of environmental community exposure for people living at or near contamination sites.

P28-13 | Longitudinal Associations between Persistent Organic Pollutant Exposure and Neurodegenerative Biomarkers in Postmenopausal Women

Wu Chen[1], Chenyu Qiu[1], Zhenchun Yang[1], Frank D. Gilliland[1], Wendy J. Mack[1,2], Howard N. Hodis[1,2], Roksana Karim[1,2], Zhanghua Chen[1]

[1] University of Southern California, 1Department of Population and Public Health Sciences, Keck School of Medicine, Los Angeles, USA; [2] University of Southern California, 2Atherosclerosis Research Unit, Keck School of Medicine, Los Angeles, USA; [3] University of Southern California, Department of Medicine, Keck School of Medicine, Los Angeles, USA

Objective: Exposure to persistent organic pollutants (POPs) has been linked to an increased risk of neurodegenerative disorders including Alzheimer's disease. Postmenopausal women face an elevated risk of neurodegeneration compared to premenopausal women and age-matched men. This study investigates the longitudinal associations between POP exposure and neurodegenerative biomarkers in postmenopausal women.

Material and Methods: Plasma concentrations of 24 polychlorinated biphenyls (PCBs), 7 organochlorine pesticides, 5 polybrominated diphenyl ethers (PBDEs), and 2,2',4,4',5,5'-hexabromobienyl were measured at a pre-randomization visit using gas chromatography isotope dilution high-resolution mass spectrometry in 72 women (mean age: 59.6 ± 7.2 years) from the Early versus Late Intervention Trial with Estradiol. Neurodegenerative biomarkers, including phosphorylated tau 181 (p-tau181), glial fibrillary acidic protein (GFAP), neurofilament light chain (NfL), and amyloid β-proteins (Aβ40, Aβ42, and Aβ42/40 ratio), were quantified from plasma samples at baseline and 30-month follow-up using SIMOA technology. Linear mixed-effects models were used to assess cross-sectional and longitudinal associations between plasma POP concentrations and neurodegenerative biomarkers adjusted for age and ethnicity.

Results: P-tau181, GFAP, and NfL concentrations significantly increased with age. Higher baseline concentrations of CB28 and CB66 were associated with increased p-tau181 levels, and 2,2-bis(4-chlorophenyl)-1,1,1-trichloroethane was associated with increased Aβ42 levels at baseline. BDE28 and BDE47 were negatively associated with GFAP, and BDE47 was negatively associated with Aβ40 at baseline. Over the 30-month follow-up, p-tau181 increased more with higher baseline concentrations of CB167 and hexachlorobenzene. GFAP showed a larger increase with several PCBs (congeners 28, 66, 74, 167, and 206) and hexachlorobenzene, and NfL increased more with CB167.

Conclusion: To our knowledge, this is the first study to demonstrate that POP exposure may contribute to longitudinal increases in neurodegenerative biomarkers, uncovering potential mechanisms underlying POP-related neurodegeneration and identifying key POP compounds for targeted prevention.

Fundings: National Institute on Aging (R01AG024154); National Institute of Environmental Health Sciences (R01ES033705, R01ES032247).

P28-14 | Exposure to Metals and Osteocalcin levels in Midlife Women

Marcela Tamayo-Ortiz[1], Gerard Karsenty[2], Kathrin Schilling[1], Mary B. Terry[3], Kendall Krutchen[1], Julie Herbstman[1]

[1] Columbia University, Department of Environmental Health, Mailman School of Public Health, NY, USA; [2] Columbia University, Department Of Genetics and Development, Biomedical Engineering, NY, USA; [3] Columbia University, Herbert Irving Comprehensive Cancer Center, Mailman School of Public Health, NY, USA

Objective Midlife hormonal changes affect the acceleration of bone loss in women. Several toxic metals (e.g., cadmium and lead) have long half-lives because they accumulate and are released from bone. However, the endocrine role of bone itself has not been sufficiently studied. In a cohort of midlife women, we analyzed the association between metals and undercarboxylated osteocalcin, a hormone produced by osteoblasts that plays critical role in bone mineralization.

Methods We analyzed urine and serum samples collected in midlife (~20 years after pregnancy) from 47 women in the Mothers and Newborns cohort from the Columbia Center for Children’s Environmental Health. Osteocalcin was measured in serum using enzyme-linked immunosorbent assay. A panel of 10 metals was measured in urine by inductively-coupled plasma mass spectrometry and corrected for specific gravity. We used linear models adjusted by age and body mass index (BMI) to evaluate the association between each log-metal separately and a model mutually adjusted for arsenic, cadmium, chromium, lead, manganese and strontium.

Results. Median osteocalcin concentrations were 3.69 ng/mL (min 0.64, max 9.83 ng/mL). Mean metal concentrations (μg/L) were: arsenic 68.1±149; cadmium 0.39±0.23; chromium 0.53±0.48; lead 1.29±1.57; manganese 1.62±8.49 and strontium 170±131. Although not statistically significant, increased arsenic, lead, manganese and strontium showed an overall inverse association with osteocalcin, whereas cadmium and chromium showed a positive association. The direction of these associations did not change depending on individual or aggregated metal models.

Conclusions In this pilot study, we were able to collect incipient evidence that exposure to toxic metals, such as lead, can act as an endocrine disruptor in midlife women. We will expand our sample size with the ongoing Pregnancy to Aging Through Health and Wellbeing Assessment across the Years (PATHWAYS) initiative to further investigate environmental exposures and the endocrine role of bone in midlife women.

P28-15 | How polluting is plastic burning? A pilot study of women's exposure to filter-based 1,3,5-Triphenylbenzene (TPB) in rural Guatemala

Lucia Buscemi[1], Eri Saikawa[1,2], Maria Renee Lopez[3], Alex Ramirez[3], John P. McCracken[3,4], Josie Welker[5], Elizabeth A. Stone[5], **Lisa M. Thompson**[2,6]

[1] Emory University, Department of Environmental Sciences, Emory College of Arts and Sciences, Atlanta, USA; [2] Emory University, Gangarosa Department of Environmental Health, Rollins School of Public Health, Atlanta, USA; [3] Universidad del Valle de Guatemala, Center for Health Studies, Guatemala City, Guatemala; [4] University of Georgia, Department of Environmental Health Science, College of Public Health, Athens, USA; [5] University of Iowa, Department of Chemistry, Iowa City, USA; [6] Emory University, Nell Hodgson Woodruff School of Nursing, Atlanta, USA

Objective: Plastic waste is a critical problem globally; without proper waste management infrastructure, rural households resort to burning plastic in trash fires outdoors and in cooking fires indoors. Women are particularly prone to the dangerous health effects of burning plastic, as they are typically responsible for domestic tasks. This is the first study to directly assess women's personal exposures to a unique chemical tracer generated from the burning of plastic waste.

Material and Methods: We conducted a pilot study among 12 non-pregnant women of reproductive age (mean age 27.1 years, SD 7.6 years), in rural Guatemala, where 70% of households burn household waste as a primary means of disposal. We assessed 24-hour personal exposures and kitchen concentrations of 1,3,5-Triphenylbenzene (TPB), a polycyclic aromatic hydrocarbon generated from burning plastic. Filter-based measurements using quartz fiber filters were assessed twice, 2 months apart. We also measured 1-hour concentrations at a 1-meter distance from six outdoor trash fires.

Results: The median concentration of TPB was 155.13 ng/m^3 (153.89 IQR) at the outdoor trash fire location, 1.10 ng/m^3 (3.79 IQR) at the kitchen location, and 1.40 ng/m^3 (2.52 IQR) for personal exposure. All households stated that plastic was burned during the monitoring period.

Conclusion: Although several studies have quantified TPB as a tracer of plastic burning in urban settings, ours is the first to focus specifically on women's exposures to compounds generated from plastic waste burning. This study informs a more extensive study, Ecolectivos, that is implementing strategies to reduce plastic waste burning in rural communities in Guatemala. [Funding National Institutes of Health, Award Number R01ES032009]

P28-16 | Tracing the UV filter DHHB and its contaminant Di-n-hexylphthalate across time and age groups – results from two cycles of the German Environmental Survey (GerES)

Aline Murawski[1], Holger M. Koch[2], Philipp Zimmermann[1], Sonja A. Wrobel[2], Nora Lemke[1], Nikola Pluym[3], Max Scherer[3], Marike Kolossa-Gehring[1]

[1] German Environment Agency (UBA), Toxicology, Health-related Environmental Monitoring, Berlin, Germany; [2] Institute for Prevention and Occupational Medicine of the German Social Accident Insurance, Institute of the Ruhr-University Bochum (IPA), Bochum, Germany; [3] ABF Analytisch-Biologisches Forschungslabor GmbH, Planegg, Germany

The German Environmental Survey (GerES) revealed widespread exposure of the general population to the reprotoxic phthalate Di-n-hexylphthalate (DnHexP) that is forbidden in cosmetics (EC Regulation 1223/2009). It may only be contained in traces if technically not avoidable. Preliminary analyses of questionnaire data indicated sunscreen use as the likely exposure source. Patents of the UV filter diethylamino hydroxybenzoyl hexyl benzoate (DHHB), which is approved in the EU in sunscreen and other cosmetics, indicated a likely contamination of the UV filter with DnHexP. Product analyses by federal authorities found DnHexP in DHHB-containing products like sunscreen up to 44 ppm. In the human biomonitoring (HBM) program of GerES, biomarkers of both – the UV filter DHHB and the contaminant DnHexP – were analysed and clearly showed broad and concurrent exposure of the general population and significant association with self-reported sunscreen use, thus confirming the exposure source. Data from two cycles of GerES (GerES VI on adults, 2023–2024 and GerES V on children and adolescents, 2014–2017) allow tracing the exposure to contaminated sunscreen across a broad range of age groups (3–79 years) and a time span covering major shifts in the UV filter market.This example emphasises the need of including even regulated substances and biomarkers deemed unimportant in HBM as a measure to monitor compliance with existing regulation. As a consequence, the Scientific Committee on Consumer Safety (SCCS) of the EU re-evaluated the safety of DHHB regarding the contamination with DnHexP. In a preliminary opinion, the SCCS concluded that 1 ppm is the maximal level of DnHexP as an unavoidable trace impurity in DHHB. Given that this limit will soon be adopted into EU cosmetic regulation and cosmetic products will furtheron adhere to it, HBM can claim to have effectively made consumer products safer by revealing the exposure and correctly identifying the source.

P28-17 | Cigarette and cigar smoking are major sources of benzene exposure among the U.S. population: NHANES 2017- March 2020

Brandon M. Kenwood, Can Zhang, David Chambers, Wanzhe Zhu, Benjamin Blount, Deepak Bhandari

Centers for Disease Control and Prevention, Tobacco and Volatiles Branch, Division of Laboratory Sciences, National Center for Environmental Health, Atlanta, USA

Disclaimer: The findings and conclusions in this report do not necessarily represent the official position of the CDC.
Objectives:
We characterized the association between smoke and fossil fuel exposure and concentrations of the benzene exposure biomarkers blood benzene, urinary phenyl mercapturic acid (PhMA), and urinary muconic acid (MUCA) among a representative sample of the US population.
Methods:
We measured blood benzene, PhMA, and MUCA concentrations as part of the National Health and Nutrition Examination Survey (NHANES 2017-March 2020). We excluded participants with missing data leaving 3,623 blood specimens and 2,195 urine specimens among participants ages ≥12, and we determined smoking status using serum cotinine concentrations and questionnaires. We created nationally representative statistics by accounting for the complex survey design of NHANES (e.g., examination weights) using SAS 9.4. We fitted multiple logistic regression models and multiple linear regression models using the odds of detecting benzene exposure biomarkers and log-transformed benzene exposure biomarker concentrations, respectively, as dependent variables and pumping gas, demographics, diet, and exclusively smoking cigarettes, cigars/little cigarettes/cigarillos, or marijuana as independent variables. We calculated multilinearity using a variance inflation factor.
Results:
People who smoked tobacco had ≥20-fold higher odds of detecting blood benzene and PhMA compared to participants not exposed to tobacco smoke ($p < 0.0001$). Participants who smoked 10-19 cigarettes per day (CPD) or >19 CPD had 129-168% higher blood benzene or PhMA concentrations compared to participants who smoked 1-9 CPD ($p < 0.0001$). Participants who smoked cigarettes or cigars had 104-168% higher MUCA compared to nonexposed participants ($p < 0.0001$). Pumping gas and smoking marijuana were associated with 1.74-fold higher ($p = 0.0014$) and 7.24-fold higher ($p = 0.0030$) odds of detecting blood benzene.
Conclusions:
Smoking cigarettes and cigars are sources of benzene exposure among the U.S. population. Pumping gas and smoking marijuana are other potential sources of benzene exposure.

P28-18 | Simultaneous measurement of 24 VOC exposure biomarkers using UPLC-MS/MS

Kyle Duplessis, Deepak Bhandari, Lanqing Wang, Brandon Kenwood

Centers for Disease Control and Prevention, Tobacco and Volatiles Branch, Division of Laboratory Sciences, National Center for Environmental Health, Atlanta, USA

Disclaimer: The findings and conclusions in this report do not necessarily represent the official position of the CDC.

Objectives:
We aim to develop an LC-MS/MS method that measures 24 urinary biomarkers of VOC exposure, including exposure biomarkers of crotonaldehyde, acrolein, methacrolein, glycidol, methyl vinyl ketone, acrylonitrile, benzene, and ethylene oxide.
Materials and Methods:
We stored all materials at -70 °C. Prior to analysis, we thawed the urine specimens and added 50 µL of urine and 25 µL of isotopically labeled internal standards to 425 µL sample preparation buffer (6.25 mM ammonium formate, pH 2.9). We detected VOC exposure biomarkers using liquid chromatography electrospray ionization tandem mass spectrometry (LC-ESI-MS/MS) in positive and negative ion mode. We separated the analytes using a Waters™ I-Class Plus UPLC equipped with a reversed phase column. We eluted the analytes with a Solvent A of 0.1% formic acid and a Solvent B of 90/10 methanol/acetonitrile with a flow rate of 600 µL/min. The gradient started at 0.5% B for 1 min, increased linearly to 5% B for 0.1 min, increased linearly to 10% B for 3.3 minutes, increased linearly to 40% B for 0.1 min, increased to 50% B with a curve of 4 for 2.1 min, decreased linearly to 0.5% B for 0.1 min before re-equilibrating. We detected analytes using a Sciex™ 5500 mass spectrometer. We validated the method according to Clinical Laboratory Improvement Amendments (CLIA) guidelines. This included calculating accuracy, calculating precision, and matrix validation.
Results:
We detected analytes in the urine of anonymous donors who smoked cigarettes. The method's accuracy was between 96.5% and 108%, precision between 1.74% and 8.05%, and urine-water slope differences between 0.143% and 2.73%.
Conclusions:
This method may be a valuable tool for population-based assessments of VOC exposure, particularly among participants exposed to tobacco smoke or e-cigarette aerosol.

P28-19 | Associations between prenatal phthalate exposures and the placental proteome

Brennan H. Baker[1,2], Samantha Lapehn[2], James MacDonald[1], Theo Bammler[1], Chaini Konwar[3], Michael Kobor[3], Kurunthachalam Kannan[4], Rachel Miller[5], Nicole Bush[6], Kaja LeWinn[6], Qi Zhao[7], Bhagwat Prasad[8], Alison Paquette[1,2], Sheela Sathyanarayana[1,2]

[1] University of Washington, Seattle, USA; [2] Seattle Children's Research Institute, Seattle, USA; [3] University of British Columbia, Vancouver, Canada; [4] New York State Department of Health, Wadsworth Center, Albany, USA; [5] Icahn School of Medicine at Mount Sinai, New York, USA; [6] University of California San Francisco, San Francisco, USA; [7] University of Tennessee Health Science Center, Memphis, USA; [8] Washington State University, Spokane, USA

Objective

Model associations between second and third trimester phthalate exposures and the placental proteome in the Conditions Affecting Neurocognitive Development and Learning in Early Childhood (CANDLE) study.

Material and Methods

We included 17 urinary phthalate metabolites in the second trimester and 14 in the third trimester that were detected in over 70% of participants. Untargeted, label-free placental proteomics measures were on 1,221 proteins from 99 participants. We fit a series of linear models to assess associations between each phthalate metabolite and the abundance of each individual protein, and analyzed mixtures using quantile g-computation. Models were adjusted for sociodemographics, genetic ancestry, and pregnancy-related variables. We performed rotational gene set testing (ROAST) to identify associations between phthalates and KEGG pathways. Proteins and KEGG pathways were considered statistically significant with a false discovery rate <0.05.

Results

Second trimester monobenzyl phthalate (MBZP) exposure was associated with higher placental Haptoglobin(HP) expression, and third trimester mono(2-carboxymethylhexyl) phthalate (MCMHP) exposure was associated with lower placental Coactosin Like F-Actin Binding Protein 1 (COLT1) expression. Phthalate mixtures in the second and third trimesters were not associated with the placental proteome. In ROAST analyses, third trimester monocarboxyisooctyl phthalate (MCIOP) exposure was associated with downregulation of the adrenergic signaling in cardiomyocytes pathway, and monoisobutyl phthalate (MIBP) was associated with upregulation of the axon guidance pathway.

Conclusion

HP binds to free hemoglobin, and higher HP expression may signal elevated red blood cell damage and oxidative stress. COLT1 regulates the actin cytoskeleton. Altered COLT1 expression could indicate disruptions to placental cytoskeletal rearrangement, a critical process for cell fusion and formation of placental syncytiotrophoblasts. Alterations in the adrenergic signaling in cardiomyocytes and axon guidance pathways could signal disruptions to placental vascularization. Findings highlight the need for additional research exploring whether prenatal phthalate exposures may promote adverse health outcomes through these mechanisms.

P28-20 | Assessing biomarkers of exposure to benzotriazoles, benzothiazoles, and benzotriazole UV-stabilizers in an Australian general population

Danielle E. Que[1], Xianyu Wang[1,2], Ian Zammit[1,2], Sandra Nilsson[1], Ryan G. Shiels[1], Geoff Eaglesham[1], Pritesh Prasad[1], Yuya Cheng[1], Leisa-Maree L. Toms[3], Peter Hobson[4], Cassandra Rauert[1,2], Derek C. Muir[1,5], Jochen F. Mueller[1,2]

[1] The University of Queensland, Queensland Alliance for Environmental Health Sciences (QAEHS), Woolloongabba, Australia; [2] The University of Queensland, Minderoo Centre – Plastics and Human Health, Woolloongabba, Australia; [3] Queensland University of Technology, School of Public Health and Social Work, Faculty of Health, Kelvin Grove, Australia; [4] Sullivan Nicolaides Pathology, Fortitude Valley, Australia; [5] University of Guelph, School of Environmental Sciences, Guelph, Canada

Benzotriazoles (BZTs), benzothiazoles (BTs) and benzotriazole ultraviolet (UV)-stabilizers (BUVs) are classes of high production volume industrial compounds. BZTs and BTs are mainly metal corrosion inhibitors and vulcanization accelerators for rubber production. BUVs are added in plastic products to protect them from UV-induced degradation. Extensive use of these chemicals has contributed to their ubiquitous presence in the environment and as a result, has raised concerns regarding human exposure and their potential adverse health effects. Human biomonitoring is the gold standard in assessing exposure to these chemicals as it incorporates all exposure pathways. Currently, there is limited understanding regarding human exposure to these compounds, particularly in Australia.

The aim of this study is to measure the concentrations of these chemicals in the Australian general population using deidentified surplus pathology urine samples that we have been pooling and stratifying based on sex (male and female) and age (0−5, 6−15, 16−30, 31−45, 46−60 and >60 years old) since 2012/2013 and 2002/2003 for serum. Concentrations of 4 BZTs, 5 BTs, and UV-P (a polar BUV) were measured in pooled urine using dilute and shoot tandem LC-MS/MS while the other 5 BUVs were quantified in pooled serum using liquid-liquid extraction coupled with Orbitrap GC-MS. Two BZTs: Tolyltriazole (TTri) and 5,6-dimethylbenzotriazole (DMBZT) and 3 BTs: 1,3-benzothiazole (BTH), 2-hydroxybenzothiazole (2-OH-BTH) and 2-aminobenzothiazole (2-amino-BTH) frequently detected in the urine samples (>60%) were assessed for age-, sex-, and temporal-related trends. Concentrations of DMBZT, BTH (females), 2-OH-BTH, and 2-amino-BTH (females) increased with age. In all sampling years, DMBZT levels consistently showed gender differences (females > males) while only in some for TTri (males > females: >45 years old), BTH (females > males), and 2-amino-BTH (males > females). A temporal increase in concentration was observed for BTH, 2-OH-BTH and 2-amino-BTH. Findings on BUVs in urine and serum will be presented and discussed.

P28-21 | Hand-to-Mouth Contact and Dermal Absorption Dominate Quaternary Ammonium Compounds (QACs) Exposure in Chinese Urban Homes: Evidence from Silicone Wristband Passive Sampling and Urinary Biomonitoring

Min Hu, Guomao Zheng

Southern University of Science and Technology, Shenzhen, China

BACKGROUND: The widespread use of quaternary ammonium compounds (QACs) in indoor environments leads to frequent human exposure. Given their potential adverse health effects, especially in children, it is crucial to identify the sources and pathways of QAC exposure.

OBJECTIVES: This study aims to comprehensively assess multiple QAC exposure pathways within a cohort in Shenzhen and to determine the relative contributions of each pathway to overall QAC exposure using advanced modeling and experimental design.

METHODS: 109 adults 20–34 years of age who reported to use disinfectants were recruited for this study conducted in 2022. First, we identified six major urinary exposure biomarkers of QACs using *in silico* and *in vitro* techniques. We then measured QACs and metabolites in matched dust, PDMS, hand wipes, and silicone wristband samples as well as urine samples. Finally, back-calculation from the internal dose is used to estimate the relative source contribution of different exposure pathways.

RESULTS: Twenty-three QACs, including 6 BACs, 6 DADMACs, 6 ATMACs, and 5 emerging QACs were ubiquitously detected in multiple environmental matrices, including dust, air, hand wipes, and silicone wristbands with median ∑QAC concentrations of 39.6 μg/g, 112 pg/m^3, 1420 ng/person and 225 ng/g, respectively. A significant correlation was observed between QAC levels in wristbands and the aggregate QAC mass from dust, air, and hand wipes (R^2=0.7061, $p<0.05$). Urinary biomarkers for specific BAC metabolites were validated and showed strong correlations with external BAC concentrations. Hand-to-mouth ingestion and dermal contact emerged as primary exposure routes for C12-BAC, contributing up to 90%.

CONCLUSIONS: The findings from this study propose suitable urinary exposure biomarkers and wristbands as valuable indicators for accurate internal and external exposure assessment, respectively, and demonstrate that hand-to-mouth contact and dermal absorption represent the major sources of QAC exposure in humans.

P28-22 | Occurrence of Current-Use Pesticides in Paired Indoor Dust, Drinking Water and Urine Samples from the United States: Risk Prioritization and Health Implications

Yichun Xie[1], Juying Li[2], Amina Salamova[3], Guomao Zheng[1]

[1] Southern University of Science and Technology, Shenzhen, China; [2] Shenzhen University, Shenzhen, China; [3] Emory University, Atlanta, USA

Despite being regarded as safer alternatives to legacy pesticides, current-use pesticides (CUPs) are now identified as emerging contaminants with growing evidence of their toxicity to wildlife and humans. In this study, we collected matched samples of dust, drinking water, and urine from 81 households in Indiana, United States, and analyzed these samples for 82 CUPs, including 48 insecticides, 25 herbicides, and 9 fungicides. Of these, 37 CUPs were identified across samples of dust, drinking water, and urine with median total CUP (∑CUP) concentrations of 18,300 ng/g, 101 ng/L, and 2.93 ng/mL, respectively. Notably, concentrations of neonicotinoids (NEOs) in dust were higher than those reported in other studies, indicating significant NEOs contamination in Indiana homes. Herbicides were the most abundant CUPs detected in drinking water, constituting 55% of the ∑CUP concentrations. The most abundant CUPs detected in urine were insecticides with a median total concentration of 2.30 ng/mL, followed by herbicides (median: 0.409 ng/mL) and fungicides (median: 0.0531 ng/mL). The highest estimated daily intake (EDI) from drinking water and dust ingestion was found for imidacloprid, with a median value of 1.00 ng/kg bw/day. Our results show that dust ingestion is a significant exposure pathway for most insecticides and fungicides, while herbicides mainly ingested by drinking water consumption. In addition, the toxicity equivalent factor model incorporated with data retrieved from the ToxCast database indicated that imidacloprid poses the greatest health risk based on its exposure levels through multiple routes and toxicity. This study underscores the importance of monitoring CUPs and sheds light on the potential health risks of CUPs, particularly for residents of agricultural regions.

P28-23 | A critical analysis of the evidence of the toxicity of chemical substances regulated under the EU Ambient Air Quality and Drinking Water Directives: Implications for health impact assessments

Hans Orru[1,2], Wasif Raza[1], Francesco Forastiere[3,4], Joseph Spadaro[5], Henrik Olstrup[1], Nataša Dragic[6], Ivana Radic[6], Sanja Harhaji[7], Sanja Bijelovic[6], Lars Modig[1], Mike Holland[8], Sandrine Andres[9], Adrien Troise[9], Antoine Guion[9], Alicia Gressent[9], Jörg Schullehner[10], Johan Nilsson Sommar[1]

[1] Umea University, Sustainable Health, Umea, Sweden; [2] University of Tartu, Institute of Family Medicine and Public Health, Tartu, Estonia; [3] IFT, National Research Council, Palermo, Italy; [4] Imperial College, Environmental Research Group, London, UK; [5] Spadaro Environmental Research Consultants, Philadelphia, USA; [6] University of Novi Sad, Faculty of Medicine, Novi Sad, Serbia; [7] Institute of Public Health of Vojvodina, Novi Sad, Serbia; [8] Ecometrics Research and Consulting, Reading, UK; [9] INERIS, Verneuil-en-Halatte, France; [10] Aarhus University, Department of Public Health, Aarhus, Denmark

The European Union (EU) Ambient Air Quality Directive (AAQD) and Drinking Water Directive (DWD) aim to enhance air quality and improve high drinking water standards across member states. Both directives regulate altogether, 35 chemical parameters, encompassing individual substances or groups of substances, many of which have established health effects. Although air pollution alone causes millions of deaths annually, many other chemicals have not been adequately assessed, potentially masking a significant portion of the environmental burden of disease. Several of the substances are also classified as carcinogenic or potentially carcinogenic based on causal associations.

To assess the health burden of chemical substances covered under the AAQD and DWD, it is essential to have data on population exposure, baseline mortality and morbidity rates, and exposure-response functions (ERFs) or unit-risk (UR) values derived from epidemiological studies. In the VALESOR (Valuation of Environmental Stressors) study we have evaluated the availability of ERFs and URs and their applicability in health impact assessments (HIAs).

From an HIA perspective, ERFs—expressed through relative risk (RR), or UR values—were available for most analyzed substances. After a critical approach, we have identified the appropriate ERFs/URs for a majority of the analyzed chemical substances; however, no established risk measures were identified for certain substances, including acrylamide, antimony, boron, chlorate, chlorite, copper, microcystin-LR (MC-LR), and selenium. If adequate exposure data and baseline health statistics were available, the health impact of a considerable number of chemical substances could be quantified.

Currently, HIAs primarily focus on major ambient air pollutants, such as particulate matter (PM_{10}, $PM_{2.5}$), nitrogen oxides (NOX), and ozone (O_3). However, the health risks associated with chemical exposure are much more complex, requiring a broader and more comprehensive assessment of their health burdens.

P28-24 | Parabens in urine samples of the German Environmental Specimen Bank from 1995 to 2021: new insights into exposure trends

Rebecca K. Moos[1], Monika Kasper-Sonnenberg[1], Petra Apel[2], Till Weber[2], Thomas Brüning[1], Holger M. Koch[1], Marike Kolossa-Gehring[2]

[1] Ruhr University Bochum, Institute for Prevention and Occupational Medicine of the German Social Accident Insurance (IPA), Bochum, Germany; [2] German Environment Agency (UBA), Berlin, Germany

Background: Parabens are widely used as antimicrobial preservatives in cosmetics, pharmaceuticals, and food since the 1920s. Exposure to parabens has become of concern because of evidence of their potency as endocrine disruptors.

Materials and methods: We measured nine parabens (methyl-, ethyl-, *n*- and *iso*-propyl-, *n*- and *iso*-butyl-, benzyl-, pentyl-, and heptyl paraben) in 24-hour urine samples collected from the German Environmental Specimen Bank between 2011 and 2021 (30 women and 30 men per year, total 420 samples). The data were integrated with measurements from a previous campaign covering the period from 1995 to 2012. This enables us to present insights into exposure trends, daily intake (DI), and risk assessments over a span of 26 years.

Results: We observed a significant decline in the levels of all parabens in recent years. Between 2011 and 2021, the median concentrations of the three most prevalent parabens - methyl, ethyl, and *n*-propyl paraben - decreased by 87-93%, reaching 1.7, <0.5, and <0.5 µg/L, respectively. Additionally, the median DI values for these parabens dropped by 97%, 99%, and 98% (0.27; 0.01; 0.01 µg/kg bw/day). For *n*-butyl paraben, the 95th percentile of DI decreased from 5.7 µg/kg bw/day in 2012 to 0.01 µg/kg bw/day, representing a reduction of 99.8%. Other parabens were increasingly infrequently detected, with no detections recorded in 2021. Since 2019 even the maximum values of the Hazard Index (HI) fell below 1 indicating to exposure levels of negligible concern.

Conclusions: Since 2014, the maximum allowable concentration of *n*-propyl and *n*-butyl paraben in cosmetic products in Europe has been reduced, while *iso*-propyl paraben and *iso*-butyl paraben have been entirely banned. The effectiveness of these regulatory measures is evidenced by the rapid decline in paraben levels observed in the ESB samples.

P28-26 | The characterization of phthalate concentrations in artificial turf and turf-air exchange

Kelly O'Malley, Alison Clark, Lane Tidwell, Kim Anderson

Oregon State University, Environmental and Molecular Toxicology, Corvallis, USA

Objective:

Artificial turf field users may be exposed to hazardous chemicals that emit into the air from some of the components used in artificial turf fields. Phthalates have been detected in crumb rubber in previous studies and are associated with having reproductive and developmental toxicity following exposure. It is unknown how phthalates move between the artificial turf and the overlying air. Low-density polyethylene (LDPE) passive samplers have been used extensively to measure a variety of chemical classes but have not been previously used to measure phthalates. The objectives

are to use LDPE to measure phthalates and to investigate the artificial turf – air exchange of phthalates. We hypothesized there will be greater volatilization of phthalates into the air above the artificial turf fields that have been recently refreshed with crumb rubber.

Materials and Methods:
Paired air and turf pore air LDPE passive samplers were deployed concurrently at indoor and outdoor artificial fields for 27 days during the summer. LDPE samplers were analyzed for 31 phthalates and alternatives using a gas chromatography mass spectrometer method.
Results:
Seven phthalates were detected among all air and turf pore air, including three recognized as reproductive toxicants by REACH (bis(2-ethylhexyl) phthalate, di-n-butyl phthalate, and diisobutyl phthalate). Bis(2-ethylhexyl) phthalate was detected in all artificial turf samples and generally had greater concentrations in the air than its paired turf pore air sample. Results showed the oldest field had the overall highest sum phthalate concentrations, and the newest field had the lowest.
Conclusion:
Results showed diisobutyl phthalate volatilizing at most sites, and bis(2-ethylhexyl) phthalate and bis(2-ethylhexyl) adipate depositing to turf at all sites. This may indicate the presence of other sources of phthalates in turf indoor and outdoor field environments releasing into the air that go on to deposit to the turf.

P42ES016465, P30ES000210, T32ES007060

P28-27 | Urinary biomarkers of exposure to UV filters

Lei Meng, Xiaoliu Zhou, Kyle Smith, Antonia Calafat, Julianne C. Botelho

Centers for Disease Control and Prevention, Atlanta, USA

Objective: Ultraviolet (UV) filters including avobenzone, homosalate, benzophenone-3, octocrylene, octisalate, and octinoxate are widely used as over the counter products in the USA. Recent data suggest emerging safety concerns for some of these UV filters, as a result, better understanding of the extent of exposure is needed. These UV filters are typically used in sunscreen products which may be used more frequently by certain populations and during specific times of the year. Yet, except for benzophenone-3, exposure data for these UV filters are limited.

Material and Methods: We developed a sensitive mass spectrometry method to measure metabolites of UV filters analytes, including octocrylene, and octisalate in 100 µL urine. We applied the method to 90 convenient urine samples collected in summer 2023 from anonymous adult male and female volunteers.

Results: The limit of quantitation for these biomarkers ranged from 0.1 to 0.5 ng/mL. The detection frequency ranged from 26.7 to 48.9%, with mean concentration ranging from 0.48 to 1.30 ng/mL. Concentration varied greatly from sample to sample, however, concentrations were higher in women compared to men for all the UV filter biomarkers. This trend is consistent with the benzophenone-3 concentration pattern previously reported in the general U.S. population.

Conclusion: We developed a sensitive and robust biomonitoring method suitable for epidemiological studies to estimate the prevalence of human exposure to four UV filters commonly used in sunscreen and other personal care products in human urine samples, including octocrylene and octisalate for the first time. Even in a small set of convenient samples, exposure to these UV filters is higher in women compared to men and show high variability between samples suggesting differences in product use and timing of exposure should be further explored in biomonitoring studies.

P28-28 | Harmonizing Human Biomonitoring Across Europe: The PARC Aligned Studies

Liese Gilles[1], Susana Pedraza-Diaz[3], Marta Esteban-Lopez[3], Marike Kolossa-Gehring[2], Aline Murawski[2], Sebastien Denys[4], Loïc Rambaud[4], Eva Govarts[1], This contribution is developed as part of the Partnership for the Assessment of Risks from Chemicals (PARC), human biomonitoring task.

[1] Flemish Institute for Technological Research (VITO), Health, Mol, Belgium; [2] German Environment Agency (UBA), Berlin, Germany; [3] Instituto de Salud Carlos III (ISCIII), National Centre for Environmental Health, Madrid, Spain; [4] Santé publique France (SPF), Department of Environmental and Occupational Health, Paris, France

As part of the Partnership for the Assessment of Risks from Chemicals (PARC), a human biomonitoring (HBM) survey is being conducted across 26 European countries. Known as the PARC Aligned Studies, this survey leverages existing HBM capacity in Europe by aligning national and regional HBM studies to obtain comparable HBM data across EU regions, to calculate European Exposure values for international comparison, and to identify exposure determinants.

The survey targets three age groups: (i) children (6-11 years), (ii) teenagers (12-17 years), and (iii) young adults (18-39 years), aiming to recruit and sample approximately 10,800 participants (around 3,400 children, 2,800 teenagers, and 4,600 young adults). Recruitment and sampling occur from May 2023 to April 2026.

Samples collected include urine and hair for children, and urine and blood for teenagers and adults. Depending on the age group, the survey assesses internal exposure to various chemicals, including phthalates and their alternatives, per-/poly-fluorinated compounds, metals, bisphenols, and pesticides (such as organophosphates, pyrethroids, neonicotinoids, and glyphosate). Supporting materials, such as questionnaires and best practice documents for sampling, have been developed centrally and are applied across the participating countries. Data from the different countries will be centralized for harmonized data processing and statistical evaluation.

The PARC Aligned Studies framework also addresses additional research questions by incorporating optional components into the studies. These components include the analysis of effect biomarkers, application of non-targeted screening (NTS), and investigating associations between concentrations observed in personal environmental samples of HBM participants, such as tap water, indoor air, dust samples and internal concentrations observed with HBM.

The primary goal of the program is to obtain quality-controlled and comparable HBM data on chemical exposure, prioritized under PARC, with comprehensive European coverage. This data will inform the development of environmental and health policies in Europe.

P28-29 | Individual, mutually adjusted, and joint associations between 10-year average pesticide exposures and cardiometabolic markers among women in an agricultural region

Lucia E. Calderon[1], Robert Gunier[2], Marcella Warner[2], Katherine Kogut[2], Stephen Rauch[2], Julianna Deardorff[2], Brenda Eskenazi[2], Jacqueline M. Torres[1]

[1] University of California, San Francisco, Department of Epidemiology and Biostatistics, San Francisco, USA; [2] University of California, Berkeley, Center for Environmental Research and Community Health, Berkeley, USA

Objective: We examined associations between chronic exposure to twelve pesticides and cardiometabolic risk factors among women living in an agricultural region.

Material and Methods: Women participating in the longstanding Center for the Health Assessment of Mothers and Children of Salinas study completed anthropometric measurements, blood collection, and a personal interview in 2022-2024. We used participant-reported addresses and California's Pesticide Use Reporting database to estimate average use (kg/year) of agricultural pesticides within 1 km of residences in the ten years preceding outcome assessment. We used generalized linear models and quantile G-computation to examine the effect of one-quartile increase in individual, mutually adjusted, and joint pesticide exposures on a suite of cardiometabolic risk factors, controlling for confounders.

Results: Participants (n=413) were 48.4 ± 5.4 years old at outcome assessment. Increases in a number of individual pesticides were associated with higher risk of metabolic syndrome and hypertension, higher blood pressure and c-reactive protein, and lower HDL cholesterol. In models mutually adjusted for pesticide coexposures, dimethoate was associated with higher risk of metabolic syndrome, naled was associated with higher blood pressure and risk of hypertension, and glyphosate was associated with lower HDL cholesterol and c-reactive protein. In the joint exposure model, we observed associations between a one-quartile increase in the entire pesticide mixture and blood pressure (systolic blood pressure: β=2.50 mm Hg, 95% CI=0.41-4.60; pulse pressure: β=2.15 mm Hg, 95% CI=0.65-3.64; hypertension: RR=1.22, 95% CI=1.08-1.39) and HDL cholesterol (β=-1.87 mg/dL, 95% CI=-3.60--0.13; <50 mg/dL or Rx treatment: RR=1.17, 95% CI=1.05-1.29).

Conclusion: Among women in an agricultural region, we observed associations between chronic exposure to pesticide mixtures and adverse blood pressure and cholesterol outcomes.

Funding and Acknowledgements: National Institutes of Health/National Institute on Aging, R01AG069090; National Institute of Environmental Health Sciences, P01ES009605, R01ES017054, R01ES02699, R24ES028529, U24ES028529-06; Environmental Protection Agency, RD83451301.

P28-30 | GSH/GSSG as a potential effect biomarker for risk assessment of workers exposed to real-life mixtures

Maria Torres Toda[1], Maria-Mirela Ani[1], Matteo Creta[1], Horatiu Moldovan[2], Lenard Farczadi[3], An Van Nieuwenhuyse[1], **Radu Duca**[1]

[1] Department of Health Protection, Laboratoire National de Santé (LNS), Dudelange, Luxembourg; [2] Occupational Medicine Department, George Emil Palade University of Medicine, Pharmacy, Science and Technology of Targu Mures, Târgu Mureș, Romania; [3] Center for Advanced Medical and Pharmaceutical Research, George Emil Palade University of Medicine, Pharmacy, Science and Technology of Targu Mures, Târgu Mureș, Romania

Glutathione redox ratio (GSH/GSSG) plays a crucial role in different detoxification pathways and regulation of oxidative stress. Exposure to metals and volatile organic compounds (VOCs) has been shown to disrupt this balance, potentially triggering future adverse health effects, particularly in workers exposed to high concentrations. In this study, the ratio of GSH/GSSG concentrations was evaluated as a potential tool for risk assessment of workers exposed to complex mixtures. To this aim, workers from mines (n=135) and industries with solvent exposure (n=49), as well as control groups (n=62), were recruited in Romania (recruitment is not finished, data from further recruitments will be available soon). Blood samples were collected to measure GSH/GSSG ratio, while exposure was assessed by quantifying metals in blood and VOC metabolites in pre-shift and post-shift urine. The quantification of exposure biomarkers in blood and urine highlighted a higher exposure to metals for mineworkers and a higher exposure to VOCs for workers involved in the industries. VOCs concentrations were higher in the post-shift samples indicating occupational exposure. Negative correlations were observed between GSH/GSSG ratio and the blood concentrations of Pb (rspearman=-0.49), Cd (rspearman=-0.35) and Cr (rspearman=-0.25) for the entire studied population. VOCs exposure also showed negative correlations with GSH/GSSG ratio. Predictive linear regression models were developed considering relevant covariates such as gender, age, smoking status, and years worked in the company. A significant depletion of GSH/GSSG ratio was observed between workers and controls. These findings might confirm the involvement of GSH/GSSG in several detoxification pathways following mixture exposure, strengthening the approach of effect-biomarkers determination for risk-assessment purposes. Definitive results are not yet available.

P28-31 | Endocrine-disrupting chemicals in breast milk and early life exposure for infants in the United States

Marnie Hazlehurst[1], Chenglin Liu[3], Guomao Zheng[3], Meghan Koch[4,5], Erika Schreder[6], Amina Salamova[2], Sheela Sathyanarayana[7,8]

[1] Seattle Children's Research Institute, Center for Child Health, Behavior, and Development, Seattle, USA; [2] Emory University, Gangarosa Department of Environmental Health, Rollins School of Public Health, Atlanta, USA; [3] Southern University of Science and Technology, School of Environmental Science and Engineering, Shenzhen, China; [4] Fred Hutch Cancer Center, Basic Sciences Division, Seattle, USA; [5] University of Washington, Department of Immunology, Seattle, USA; [6] Toxic Free Future, Seattle, USA; [7] Seattle Children's Research Institute, Center for Child Health, Behavior, and Development, Seattle, USA; [8] University of Washington, Department of Pediatrics, Seattle, USA

Objective

In a novel study of endocrine-disrupting chemicals (EDCs) in breast milk from US mothers, we (1) reported levels of melamine and melamine analogues including cyanuric acid, Bisphenol A (BPA) and BPA substitutes including 4-hydroxyphenyl sulfone (BPS), and triclosan; (2) described associations between participant demographic characteristics and each of these chemicals; and (3) calculated estimated daily intakes (EDIs) of these compounds through breast milk for infants.

Methods

We analyzed several EDCs in breast milk samples collected from 50 women in Seattle, Washington during 2019. We examined associations of maternal and infant age at time of sample collection, maternal education, neighborhood income, and maternal body mass index, with log10-transformed chemical concentrations using linear regression. EDIs of each chemical through breast milk was calculated for infants age <1, 1-3, 3-6, and 6-12 months using measured median chemical concentrations.

Results

This study frequently detected melamine, cyanuric acid, BPA, BPS, and triclosan (62%-92% of samples) in breast milk. Median concentrations were 0.48 ng/mL melamine, 0.59 ng/mL cyanuric acid, 0.311 ng/mL BPA, 0.012 ng/mL BPS, and 0.072 ng/mL triclosan. Older infant age at the time of sample collection (≥6 versus <6 months) was associated with lower melamine concentrations (β=-0.41, 95% CI: -0.80, -0.01). Maternal obesity or overweight was associated with higher concentrations of BPA (β=0.68, 95% CI: 0.14,1.23) and triclosan (β=0.43, 95% CI: 0.06, 0.80), respectively, compared to normal weight. Other associations with participant characteristics were suggestive but not statistically significant. EDIs for infants ranged by infant age from 40.3-72.8 and 86.5-156 ng/kg bodyweight/day for melamine and BPA, respectively.

Conclusions

We frequently detected melamine, cyanuric acid, BPA, BPS, and/or triclosan in breast milk. More work is needed to understand potential health effects of chronic exposures of infants to even low levels of these chemicals through breast milk.

P28-32 | Silicone Hydrogels as a Method for Characterizing the Neuroexposome

Norah H.B. Beach-Diplock, Anya Guo, Parshawn Amini, Joseph O. Okeme

McMaster University, Department of Chemistry and Chemical Biology, Hamilton, Canada

Objective: Exposure to environmental neurotoxins has been shown to significantly increase a person's risk for Parkinson's Disease, Alzheimer's Disease, and other neurodegenerative diseases. These exposures, and diseases are then reflected in changes to typical biomarker levels. Currently, we lack a method to adequately capture data from the internal and external neuroexposome, to comprehensively assess risks for neurodegenerative diseases. Our work aims to show silicone hydrogel samplers as a tool for sampling tear fluid, to characterize the neuroexposome.

Materials and Methods: We recruited 22 undergraduate participants to collect tear fluid samples through silicone hydrogel samplers, across two periods of 3 consecutive days, spaced 3 weeks apart. Each participant self-collected worn hydrogel samplers for all 3 days, as well as an unworn hydrogel. The silicone hydrogels were solvent extracted and analyzed on LC-MS/MS and GC-HRMS. Temporal changes were analyzed within participants, as well as correlations to the participants daily activities and demographic information.

Results: We have identified several environmental neurotoxins in the participant samples. These include polychlorinated biphenyls, organophosphate esters, phthalates and other prevalent pesticides. The risks for Parkinson's and Alzheimer's disease are escalated by these compound classes, which can be reflected in neurological biomarkers levels. Dopamine, cortisol, and other implicated biomarkers have been detected in the tear fluid samples, establishing silicone hydrogel samplers as a method for capturing endogenous and exogenous neurological information.

Conclusion: Overall, our work demonstrates that silicone hydrogel samplers can utilize tear fluid to characterize the neuroexposome. The combination of internal and external data captured by the hydrogels allows for a complete risk assessment for neurodegenerative diseases.

P28-33 | Using Web-Based Technology to Uncover Hazardous Ingredient Trends and Target Market Differences in Personal Care Products

Paula I. Johnson[1], Amy M. Le[1], Tamar Golovenzitz[2], Oren Agiv[2], Amit Rosner[2]

[1] California Department of Public Health, California Safe Cosmetics Program, Richmond, USA; [2] Clearya, Goshen, USA

Objective: Hazardous chemicals, including carcinogens, reproductive and developmental toxicants, neurotoxicants, endocrine disruptors, allergens, and others are used as ingredients in personal care products, yet ingredient information is often lacking, particularly for products sold online.
Material and Methods: We utilized a web-based tool, Clearya Insights, to automate identifying select ingredients of concern in personal care products marketed by several large retailers. We quantified the number of products with ingredients on hazard lists identified as reportable to the state of California. We examined ingredients in products of brands identified as "target-market" brands that are marketed to and/or used by women of color. We compared the number of products containing ingredients of concern between the target-market and the nontarget-market brands.
Results: Of 42,013 personal care products, we found over 100,000 instances of ingredient matches with chemical hazard lists, representing over a quarter of all products marketed online by the major retailers. We identified over 1,000 products noncompliant with California's hazardous ingredient disclosure laws. Across all product types, the target-market brands had higher proportions of products containing fragrance allergens and Lilial, a fragrance ingredient that is a reproductive toxicant. Compared to the nontarget-market brands, a greater proportion of makeup products used by Latinas contained talc.
Conclusion: Products marketed to and/or used by women of color can contain a higher number of hazardous ingredients. Using web-based technology can efficiently screen for hazardous ingredients in products sold online, help identify noncompliance with disclosure laws, contribute to more comprehensive product safety awareness, and inform targeted interventions to reduce potentially harmful exposures.

P28-34 | Exploring Trends in Evaluated Chemicals: Content Analysis of Public Health Assessments And Health Consultations

Ridwan A. Mahbub

Agency for Toxic Substances and Disease Registry (ATSDR), Central Section, Office of Community Health Hazard Assessment, Atlanta, USA

Objective: The Agency for Toxic Substances and Disease Registry (ATSDR) is a federal public health agency charged with enforcing the health-related provisions of the Superfund Act by performing public health assessment (PHA) evaluations of exposure to toxic substances. This study explores the frequency of chemicals identified as a public health hazard and describes the language and context used to communicate potential chemical hazards in PHA and health consultation HC reports released in the past five years (2020–2024).
Material and Methods: We analyzed reports published on ATSDR's website from 2020–2024 to identify evaluated chemicals of health concern and organize them into ATSDR's chemical classifications. We then performed text-

mining analysis on these reports to identify the language used to report the health hazard of these chemical classifications to the public.

Results: Preliminary results show that the majority of reports were HCs; however, the ratio of PHAs to HCs increased.Additionally, the results show that ATSDR published more reports investigating the health impacts of exposure to volatile organic compounds (VOCs) followed by metals (11 and 4 reports in 2024, respectively) compared to other chemical classes. Text-mining and content analysis identified language attributes that described public health implications of exposure to these hazardous chemicals.

Conclusion: The increase in the ratio of PHAs to HCs reflects a rise in the complexity of assessments. The higher number of reports addressing air public health hazards, often from vapor intrusion assessments, suggests that inhalation has become a more significant exposure pathway assessed to determine public health hazards associated with environmental exposures. This study provides a critical review of the structure and content of public health reports released to the public by ATSDR.

P28-35 | Identifying the known and unknown health hazard information from the East Palestine, Ohio train derailment: A phased scoping review

Ruth M. Lunn[1], Meredith Clemons[3], Robyn Blain[3], Somdat Mahabir[2], Andrew A. Rooney[1], Anisha Singh[1], Stephanie Smith-Roe[1], Kyla W. Taylor[1], Wren Tracy[3], Maricruz Zarco[3], Suzanne E. Fenton[4,1], Suril S. Mehta[1]

[1] National Institute of Environmental Health Sciences, Division of Translational Toxicology, Research Triangle Park, USA; [2] National Cancer Institute, Division of Cancer Control and Population Sciences, Bethesda, USA; [3] ICF, Reston, USA; [4] North Carolina State University, Center for Human Health and the Environment, Raleigh, USA

Objective

In February 2023, people residing in the village of East Palestine (EP, Ohio, USA), and surrounding areas were exposed to toxic chemicals from a Norfolk Southern Railway train derailment and subsequent burn and vent. Many hazardous chemicals, including volatile organic chemicals used in industrial manufacturing, were released. Our aim was to identify known health hazards and evidence gaps from these chemicals to inform the disaster-related response.

Methods

We used an iterative two-phase literature scoping review to identify health information. Phase one consisted of extracting data from 8 authoritative sources reporting on ~15 health outcomes for 22 chemicals potentially related to the train derailment and response. Phase 2 consisted of searching PubMed for studies on prioritized exposure-outcome pairs, summarizing and characterizing the evidence, and proposing next steps. Prioritization was based on the potential for human exposure, the recency of the authoritative review, and suggestive evidence for a given outcome.

Results

Based on our Phase One scoping of authoritative health assessments, all eight higher-priority chemicals (all high-production industrial chemicals) were classified as skin and eye irritants, and seven of these were respiratory irritants. Five of these chemicals were human or animal carcinogens; two may cause adverse immunological or neurological effects, and one may cause damage to reproductive organs or the developing fetus. Vinyl chloride had the most recent hazard assessment. We identified 14 chemical-outcome pairs (7 different chemicals) with suggestive data for

PubMed literature searches; however, these updated literature searches only found new data suggesting the need for two systematic reviews. Primary research gaps remain for the other 12 exposure-outcome pairs.
Conclusion: Our review aims to inform future research on chemical disaster-related health effects, facilitate communication with the affected EP community, and inform global disaster-response-related research, as these chemicals are commercially important and present in other chemical release incidents.

P28-36 | Washing Away the Risk? Chemical Profiles of Textiles Before and After Washing

Soma Barsen, Elizabeth Z. Lin, Allison Su, **Krystal J. Godri Pollitt**

Yale School of Public Health, Environmental Health Sciences, New Haven, USA

Objective: Many of the desirable functional properties of fibers and textiles in apparel are achieved through chemical additives, such as dyes and stain- or water-repellent treatments, enhancing the fabric's performance. The objective of this study was (i) to identify the chemical signatures of textiles, (ii) examine the impact of washing on reducing the exposure burden, and (iii) evaluate the toxicological profile of detected textile exposures.
Materials & Methods: Ten textiles were evaluated (plain cotton, flannel, denim, microfleece, and polyester) in various colours. Each fabric type underwent three different treatments: (i) a control condition, where each swatch was enclosed in a sealed container with a polydimethylsiloxane sorbent bar to passive sample volatilised chemicals; (ii) a series of cold wash cycles; and (iii) a series of hot wash cycles. Following the third and sixth wash cycles, swatches were place into sealed vials for passive sampling. Passive samplers were chemically analysed using non-targeted gas chromatography high resolution mass spectrometry.
Results: Polycyclic aromatic hydrocarbons (PAHs) and chlorinated aromatic hydrocarbons were identified in most fabrics, including two-dye related compounds, five dye solvents, and three environmental contaminants. Unwashed fabrics contributed to higher overall chemical exposures, though triple-washed indigo denim emitted more PAHs than unwashed polyester. Cold washes were less effective than hot washes in reducing chemical burden. Unwashed red polyester had nearly twice the chemical load of other colours and showed the smallest reduction after multiple washes. Identified chemicals included 1,3-dichlorobenzene, 2-methylnaphthalene, and hexachloroethane, all linked to potential respiratory and dermal irritation, carcinogenicity and neurotoxicity.
Conclusion: Given their pervasive presence in daily life, textiles are an important component of the exposome—the dynamic complex of environmental influences during the life-course. Identifying their chemical signatures is a critical first step in comprehensively characterizing their contribution to exposome, assessing potential health impacts, and developing strategies to mitigate risk.

P28-37 | The Agency for Toxic Substances and Disease Registry's Cancer Cluster Inquiry and Regional Activity Tracker (CCARAT)

Teresa Wang[1], Timothy Turner[1], Kelsey Brady[2], Brittany Luke[2], Chris Poulet[1], Elizabeth Irvin[1]

[1] Agency for Toxic Substances and Disease Registry (ATSDR), Office of Community Health Hazard Assessment, Atlanta, USA; [2] Agency for Toxic Substances and Disease Registry (ATSDR), Office of Innovation and Analytics, Atlanta, USA

Objective: At any given time, the Agency for Toxic Substances and Disease Registry (ATSDR) conducts multiple health activities to protect communities from harmful environmental exposures. The Cancer Cluster Inquiry and Regional Activity Tracker (CCARAT) platform aims to geospatially track cancer cluster inquiries and ATSDR's site-specific public health activities.

Materials and Methods: Within ATSDR, the Office of Community Health Hazard Assessment actively collaborates with the Geospatial Research, Analysis, and Services Program for its geospatial expertise and maintenance of the CCARAT platform. Built using ArcGIS Online technology, CCARAT utilizes geospatial tools and data visualization techniques to consolidate diverse datasets and display them in a centralized location. CCARAT is constructed on Enterprise Sites, enhancing its functionality through Dashboards and Experience Builders. CCARAT also integrates with Survey123, allowing users to submit, update, and visualize public health activities in real-time. Furthermore, CCARAT integrates information from various sources, incorporating boundary layers such as the Environmental Protection Agency's Superfund sites and Brownfield sites.

Results: CCARAT has resulted in two primary dashboards: (1) The CCARAT Dashboard, which provides a retrospective geospatial view of completed public health assessment activities, including published public health assessments, health consultations, and cancer cluster inquiries across ATSDR's 10 regions; and (2) The Site Activity Dashboard, which tracks ongoing general site activities and health education efforts. CCARAT's visual, geospatial, and data linkage components provide ATSDR staff with added visibility and site reference tools for responding to community concerns and accomplishing the agency's mission to protect communities from harmful health effects related to environmental exposures.

Conclusions: CCARAT enhances operational efficiency by aggregating and displaying a subset of ATSDR's diverse public health activities. An additional dashboard for tracking regional office-specific activities and requests is also underway. Others may learn from this adaptable framework to enhance data-driven decision-making and situational awareness in public health and environmental response efforts.

P28-38 | A Systematic Review of Human Studies Assessing the Health Effects of Kerosene Based Jet Fuels and Products Across Diverse Populations and Settings

Veronica Carvajal, Brandon Ng, Nicole Rosendaal, Catherine Pirkle

University of Hawaii at Manoa, Department of Public Health Sciences, Honolulu, USA

Objective:

In November 2021, the kerosene-based jet fuel, Jet Propellant-5 (JP-5), leaked from the U.S. Navy's Red Hill Bulk Fuel Storage Facility on Oʻahu, Hawaiʻi, contaminating the drinking water system and affecting approximately 93,000 individuals. In response, a registry is being developed to track and investigate potential health impacts of the spill. Previous reviews of jet fuel–the latest in 2017–focus on occupational settings, men, as well as dermal and inhalation exposures. Given the diverse population affected by the contaminated drinking water, there is a need to review recent literature to identify any health effects associated with oral, non-occupational exposures.

Material and Methods

With the assistance of a public health librarian, a search strategy, search terms, and eligibility criteria were developed following PRISMA guidelines. PubMed and Web of Science databases were searched for eligible literature published between 2017 and 2024. Rayyan was used for data management. Data extraction included topics such as study design, participant characteristics, exposure and health outcomes. An adapted version of the Modified Downs and Black framework was used for quality assessment.

Results

2,216 results were obtained, with 536 duplicates, resulting in 1,680 articles to screen for eligibility. Preliminary results indicate few studies assess the health effects of jet fuel in non-occupational settings and among vulnerable populations, such as children and pregnant women. Additionally, studies assessing health effects of kerosene have been primarily focused on inhalation of post-combustion byproducts.

Conclusion

This review contributes to the knowledge on the health effects of jet fuel and other kerosene-based products in order to inform the ongoing work of the Red Hill Registry. It confirms few studies outside of occupational settings, highlighting the need for research from the Red Hill Registry.

P28-39 | Follow-up Values, Health Limits, and Reference Ranges for the NJHANES Study - Results Reporting and Subject Matter Expert Calls

Eric Bind, Izabella Saini

New Jersey Department of Health, Public Health & Environmental Laboratories - Environmental & Chemical Laboratory Services, Trenton, USA

The NJ Department of Health (NJDOH) launched the NJ Health and Nutrition Examination Survey (NJHANES) in 2021 as its first statewide population biomonitoring surveillance study. NJHANES examines the health, nutrition, and chemical body burden of NJ residents using questionnaires and biospecimen analysis for over 120 chemicals including toxic metals, PFAS, and others. All 505 NJHANES participants opted into receiving their exposure reports.

Most chemicals do not have established health limits, follow-up values, or reference ranges so determining when exposures are high enough to merit immediate notification and a call with a subject matter expert (SME) was an important step. We established these values based on NJ-legislated reportable levels for arsenic, cadmium, lead, and mercury and 3X the CDC's NHANES 95th percentile for other chemicals while balancing concern for participants and program capacity to conduct calls.
For the 400 participants whose specimens were analyzed to date, NJHANES held 55 SME calls for 46 participants; 9 participants had multiple calls for multiple chemicals, while most had multiple chemicals explained in one call. Results above the follow-up value included 30 for toxic metals including arsenic, barium cadmium, lead, mercury, thallium, and uranium, 3 for PAH metabolites, 2 for nicotine metabolites, 5 for PFAS, and 34 for volatile organic compound metabolites. A participant satisfaction survey indicates the calls are well received with 79% of respondents who received a call stating they were very satisfied with the experience and the remaining 21% being satisfied with 0 unsatisfied. Twenty-one participants were reported to the NJDOH Consumer, Environmental, & Occupational Health Services for follow up case management and one case of mercury and thallium exposure was forwarded to NJ Poison Control. Follow up testing in this case following the initial SME call indicated an 87% reduction in blood mercury levels.

P28-40 | Analysis of Personal Care Product Ingredients Associated With Reproductive And Developmental Health Harms Utilizing U.S. Market Data From EWG's Skin Deep

Hong Lin, Alexa Friedman, Kaley Beins, David Andrews

Environmental Working Group (EWG), Washington, DC, USA

Objective: The potential reproductive and developmental health harms associated with personal care product use are a significant concern due to the widespread use of these products by persons of childbearing age and increasing use in children.
Methods: The Environmental Working Group (EWG)'s Skin Deep® database, which includes over 120,000 products with over 80,000 ingredients provides a unique opportunity to characterize ingredient use patterns and identify potential hazards within the U.S. cosmetics market. Using the aggregated toxicity datasets within Skin Deep, ingredients with reproductive and developmental health harms were identified. With this list of target ingredients we analyzed their usage frequency generally and across product categories.
Results: Of the 80,000+ ingredients in Skin Deep®, 1,533 had reproductive and developmental health hazard data from sources such as the European Chemical Agency, EPA, Environment Canada, and open scientific literature. The most frequently used ingredients with reproductive and developmental health hazards were fragrances, skin conditioning agents, and colorants.
Conclusion: Our findings highlight the ingredients contributing to potential consumer exposure to reproductive and developmental toxicants. While Skin Deep® primarily reflects the U.S. market and may have data gaps, it serves as a valuable screening tool to prioritize exposure reduction efforts. Our results demonstrate the importance of seeking alternative chemicals for less hazardous formulations. This exposure data is valuable for prioritizing chemical exposure studies in exposure and epidemiology studies.

P28-41 | Association between essential and toxic elements in semen and sperm quality in Japanese men

Miyuki Iwai-Shimada[1], Kenta Iwai[1], Nozomi Tatsuta[1], Tomohiko Isobe[1], Mai Takagi[1], Yayoi Kobayashi[1], Shoji F. Nakayama[1], Yukiyo Kumazawa[4], Kazumasa Takahashi[4], Takashi Tanaka[5], Yukihiro Terada[4], Kyoko Nomura[3], Hiroshi Okada[5], Akiko Tamakoshi[2], Eri Maeda[2]

[1] National Institute for Environmental Studies, Health and Environmental Risk Division, Tsukuba, Japan; [2] Hokkaido University, Faculty of Medicine and Graduate School of Medicine, Department of Public Health, Sappro, Japan; [3] Akita University Graduate School of Medicine, Department of Environmental Health Science and Public Health, Akita, Japan; [4] Akita University Graduate School of Medicine, Department of Obstetrics and Gynecology, Akita, Japan; [5] Dokkyo Medical University Saitama Medical Center, International Center for Reproductive Medicine, Saitama, Japan

Objective:
Male preconception health is important for improving family planning and pregnancy outcomes. In 50% of couples, infertility primarily lies on the male side, mostly attributable to semen abnormalities. The essential elements have been found to relate with male fertility, affecting proper spermatogenesis, sperm maturation and motility. We aimed to investigate the relationship between 13 essential and toxic elements in semen and sperm quality.
Material and Methods:
In total, 379 healthy male adults, aged between 20 and 39 years were asked to submit a semen sample to the hospital using sterile plastic vessels within 2 hours of collection after a 2-5-day abstinence period. Semen quality was assessed by trained physicians. Levels of 13 elements (magnesium (Mg), manganese (Mn), calcium (Ca), iron (Fe), cobalt (Co), copper (Cu), zinc (Zn), selenium (Se), molybdenum (Mo), Iodine (I), mercury (Hg), lead (Pb) and cadmium (Cd)) were assessed by inductively coupled plasma mass spectrometry (ICP-MS).
Results:
The median concentrations for Mg, Mn, Ca, Fe, Zn, Se, Mo, I, Hg, Pb and Cd in semen were 1.02×10^5, 7.0, 3.40×10^5, 179, 1.16×10^5, 69, 2.8, 3.6, 0.18, <0.025, and 0.30 ng g−1, respectively. Motile sperm counts and Fe and Se correlated strongly with each other (Spearman's $\rho = 0.619$ and 0.469). The k-means algorithm identified three clusters. One cluster (14% participants) had higher performance (motile sperm counts and motility) and higher essential (Mg, Mn, Fe, Co, Cu, Zn, Se and Mo) elements.
Conclusion:
Essential elements in semen (especially Fe and Se) had significant effects on sperm quality.

P28-42 | Chemical and Oxidative Properties of Indoor PM: Discerning the Influence of Residential Characteristics

P. S. Ganesh Subramanian[1], Alice Oloo[2], Vinh Vo[2], Brenda Koester[3], Vishal Verma[1], Sheena Martenies[2,4]

[1] University of Illinois Urbana-Champaign, Department of Civil and Environmental Engineering, Urbana, USA; [2] University of Illinois Urbana-Champaign, Department of Health and Kinesiology, Urbana, USA; [3] University of Illinois Urbana-Champaign, Center for Social and Behavioral Sciences, Urbana, USA; [4] University of Illinois Urbana-Champaign, Division of Nutritional Sciences, Urbana, USA

Objective: Oxidative potential (*OP*) is a metric that quantifies the ability of particulate matter (PM) to induce oxidative stress in humans and cause health outcomes. Although people spend the largest proportion of their time in residences, our understanding of exposure to *OP* and PM constituents in this critical environment is limited. Here, we attempt to address this by quantifying the *OP* and chemical composition of PM collected from diverse residential environments and evaluating the effects of residential characteristics in discerning residential exposures.
Methods: Here, we collected PM samples from 22 homes in the Champaign-Urbana region (CUR) and characterized their composition and *OP* using the dithiothreitol (DTT) assay. Residential characteristics were collected using questionnaires from each participant.
Results: The median *OP* of residential PM was 185.90 pmol/(min.m^3), comparable with the values reported for ambient $PM_{2.5}$ in CUR. Water soluble organic carbon, Cu, and Mn were mainly driving OP, aligning with past findings on ambient $PM_{2.5}$. Both particle mass concentrations (*PMC*) and *OP* consistently increased with increasing window-opening frequency or decreasing floor area. The median *PMC* and *OP* of residences using gas stoves were 72% and 42% higher than those using electric stoves, respectively. Similarly, scented candle usage also resulted in a 71% and 34% increase in the median *PMC* and *OP*, respectively, signifying the importance of indoor-origin PM in determining residential exposure.
Conclusion: Collectively, these findings reveal that both residential characteristics (floor area, gas vs electric stove) and individualized behavior (window-opening frequency, scented candle use) play a significant role in determining *PMC* and *OP* exposure indoors and emphasize the need for future studies to reliably estimate residential exposures to toxic PM, as a function of building characteristics and individualized activity patterns.
Funding for this research was provided in part by the Personalized Nutrition Initiative seed grant at the University of Illinois.

P28-43 | State-of-the-science review of micro- and nanoplastic in human tissues and fluids

Timothy Hua, Yulia Carroll

Centers for Disease Control and Prevention (CDC), National Center for Environmental Health (NCEH), Division of Environmental Health Science and Practice, Atlanta, USA

Background and Aim: Micro- and nanoplastics (MNPs), particles smaller than 5 mm and 100 nm, respectively, have become a global health concern due to increasing plastic waste. These particles can enter the human body through inhalation, ingestion, and dermal contact, travel through bodily fluids, and accumulate in tissues, posing potential health risks. Through this review we are (i) investigating the current knowledge on MNPs' uptake, distribution, and accumulation in human tissues and fluids, and their potential health risks; and (ii) evaluating how the findings can inform accurate determinations of exposure routes.
Methods: We conducted a literature search across nine electronic databases. Keywords included combinations of "microplastics and/or nanoplastics" with human tissues or fluids. We selected Covidence, an electronic platform to facilitate the conduct of transparent and unbiased reviews in three steps, (i) title and abstract screening, (ii) full-text review, and (iii) data extraction. We developed a rigorous quality assessment template to use for data extraction and select studies that would inform the scientific community. Our quality assessment focuses on study designs, methods used for reporting findings of MNPs in human tissues and fluids, and the connections between findings and potential routes of MNP human exposure in the environment.
Results: Our preliminary findings show that the presence, accumulation, and distribution of MNPs was more often reported in 11 human tissues (8) and fluids (3). Also, < 50% of the papers informed on the potential routes of exposure to MNPs in the environment. Our preliminary assessment also show no direct association between human exposure to MNPs and potential adverse health effects.
Disclaimer: The findings and conclusions in this report are those of the authors and do not necessarily represent the official position of the Centers for Disease Control and Prevention.

P28-44 | Micro- and nanoplastics in the environment: An emerging environmental health challenge

Yulia Carroll, Max A. Zarate-Bermudez

Centers for Disease Control and Prevention (CDC), National Center for Environmental Health (NCEH), Division of Environmental Health Science and Practice, Atlanta, USA

Objective:
Environmental micro- and nanoplastics (MNPs) are found in the environment from sources like microbeads or as result of the degradation of larger pieces of plastic waste. The aim of this presentation is to describe the challenges environmental health scientists face today to better understand human exposure to MNPs in the environment. **Materials and methods:** We planned and conducted state-of-the-science and systematic-evidence map reviews focused on MNPs in the environment. We registered our protocols in PROSPERO and used Covidence to conduct the reviews following the stages required. Also, we implemented a rigorous quality assessment process to make the final selection of papers to extract data from. We organized and analyzed the data to draft manuscripts.

Results:
Findings of our state-of-the-science and systematic-evidence map reviews revealed an exponential growth in the number of publications focused on MNPs in the environment. The number of publications on MNPs present in the human body is also starting to grow swiftly. However, knowledge gaps, regarding hazard identification, dose-response relationship, exposure, and risk characterization and assessment, still exist and complicate the determination of the potential impact of MNPs on human health. We also found that our scientific knowledge regarding MNPs in the environment and the human body ought to be enhanced via accurate characterization of MNPs in both environmental and biological samples.
Conclusion:
Environmental exposure to MNPs could potentially cause adverse health effects in humans. Implementing rigorous monitoring of MNPs in both the environment and the human body could assist in estimating health risk. Therefore, developing standard methods for characterizing MNPs in both environmental and biological samples is of upmost importance.
Disclaimer: The findings and conclusions in this report are those of the authors and do not necessarily represent the official position of the U.S. Centers for Disease Control and Prevention.

P28-45 | Environmental exposure to agrochemicals and health conditions in rural communities of the Maule Region, Chile (FONDECYT 1240899)

Maria Teresa Muñoz Quezada[1], Cristian Valdes[2], Liliana Zuñiga[2], Benjamín Castillo[2], Natalia Landeros[3], Boris Lucero[4], Juan Pablo Gutierrez[2], Alexis Castillo[2], Ramón Castillo[5], Cynthia Carrasco[4], Jandy Adonis[6], Maria Victoria Rodriguez[7], Andres Canales[8], Tristan Bekinschtein[8]

[1] Universidad de Chile, School of Public Health/Faculty of Medicine, Santiago, Chile; [2] Universidad Católica del Maule, Centro de Investigación de Estudios Avanzados del Maule, Talca, Chile; [3] Universidad Católica del Maule, Centro Oncológico/Facultad de Medicina, Talca, Chile; [4] Universidad Católica del Maule, The Neuropsychology and Cognitive Neurosciences Research Center CINPSI Neurocog, Talca, Chile; [5] Universidad de Talca, Centro de Investigación en Ciencias Cognitivas/Facultad de Psicologia, Talca, Chile; [6] Universidad de Chile, Magister en Salud Pública, Santiago, Chile; [7] Universidad Católica del Maule, Doctorado en Psicología, Talca, Chile; [8] University of Cambridge, Consciousness and Cognition Laboratory, Cambridge, UK

Background: Agrochemical use in intensive farming raises concerns about environmental contamination and health effects. Rural communities, particularly small-scale farmers, are highly exposed. This study evaluates pesticide contamination in deep well water and household soil and its association with physical, mental, and neurobehavioral health. It is the first to assess pesticide contamination in underground aquifers used for community water consumption.
Methods: A cross-sectional study was conducted with 95 adults from rural Maule (Chile). Health surveys were administered, and environmental samples (water, soil, and flies) were analyzed for cypermethrin, lambda-cyhalothrin, chlorpyrifos, diazinon, and pirimiphos-methyl. A 95% confidence level and a 5% margin of error were used. Statistical analyses included Spearman correlations and Mann-Whitney U tests.
Results:

- **Pesticide residues:** Chlorpyrifos, diazinon, and pirimiphos-methyl were detected. In soil (µg/kg), mean concentrations were 5.36 (chlorpyrifos), 0.42 (diazinon), 0.11 (pirimiphos-methyl), 0.23 (cypermethrin), and 0.26

(lambda-cyhalothrin). In deep well water (ppb), mean levels were 8.05 (chlorpyrifos), 0.40 (diazinon), 1.61 (cypermethrin), and 0.41 (lambda-cyhalothrin).
- **Proximity to agriculture:** Pesticide concentrations were inversely correlated with distance from agricultural enterprises. Chlorpyrifos in soil negatively correlated with vineyards (r=-0.29, p<0.01), while diazinon decreased near pig farms (r=-0.44, p=0.006). Chlorpyrifos, pirimiphos-methyl, and cypermethrin in water were associated with vineyards (r=-0.45, p=0.011; r=-0.379, p=0.035) and forestry plantations (r=-0.45, p=0.011).
- **Health outcomes:** Diazinon in soil was linked to lower physical health (r=-0.248, p=0.029). Cypermethrin in water correlated with poorer mental health (r=-0.254, p=0.025), increased depression (r=0.228, p=0.04), and reduced cognitive function (r=-0.233, p=0.040). Higher chlorpyrifos concentrations in soil were associated with migraines (median=6.02 μg/kg in affected individuals).

Conclusion: Rural communities in Maule (Chile) face pesticide contamination in soil and drinking water, with potential health risks. Strengthening environmental policies, monitoring health impacts, and ensuring community participation in decision-making are necessary steps toward addressing pesticide exposure.

P28-46 | Co-exposure to Organophosphate and Pyrethroid Pesticides and Human Brain Health: A Systematic Review

Maria Teresa Muñoz Quezada[1], Rocio Hojas[2], Joaquin Toro[2], Jose Norambuena[2], Boris Lucero[3], Tristan Bekinschtein[4], Liliana Zuñiga[5], Cristian Valdes[5], Andres Canales[4], Ramon Castillo[6], Juan Pablo Gutierrez[5], Benjamin Castillo[5], Natalia Landeros[7], Cynthia Carrasco[3], Jandy Adonis[8], Patricio Olguin[9]

[1] Universidad de Chile, Epidemiology/School of Public Health/Faculty of Medicine, Santiago, Chile; [2] Universidad de Chile, School of Medicine/Faculty of Medicine, Santiago, Chile; [3] Universidad Catolica del Maule, The Neuropsychology and Cognitive Neurosciences Research Center CINPSI Neurocog, Talca, Chile; [4] University of Cambridge, Consciousness and Cognition Lab/Department of Psychology, Cambridge, UK; [5] Universidad Catolica del Maule, Centro de Investigación de Estudios Avanzados del Maule, Talca, Chile; [6] Universidad de Talca, Centro de Investigación en Ciencias Cognitivas/Facultad de Psicología, Talca, Chile; [7] Universidad Católica del Maule, Centro oncológico/Faculty of Medicine, Talca, Chile; [8] Universidad de Chile, Magister en Salud Pública/Escuela de Salud Pública, Santiago, Chile; [9] Universidad de Chile, Departamento de Neurociencias/Facultad de Medicina, Santiago, Chile

Background

Organophosphate (OP) and pyrethroid (PYR) pesticides are widely used, yet little is known about their simultaneous exposure and effects on brain health. This systematic review synthesizes cohort study findings on the association between OP and PYR coexposure and neurological outcomes.

Methods

A systematic search was conducted in PubMed and Web of Science (2014–2024) following PRISMA guidelines and summarized with PECO framewok (1). Cohort studies assessing OP and PYR coexposure with validated methods and reporting neurological or psychiatric outcomes were included. The risk of bias was evaluated using ROBINS-E (2)

Results

Among 34 included studies, 62% found associations, and nine were cohort studies (four with low risk of bias):

- Autism Spectrum Disorder (ASD): A birth cohort study found no significant association between simultaneous OP and PYR exposure and ASD risk (3).
- Attention-Deficit/Hyperactivity Disorder (ADHD): One study found an association between OP and PYR coexposure and ADHD symptoms (4), while another found an association only with PYR exposure (5).
- Neurobehavioral Performance (NB): One study found an association between OP and PYR coexposure and poorer neurobehavioral performance (6), while two studies reported associations only with OP exposure (7, 8). One study found no association (9).
- Neurodegenerative Phenotypes (NDP): One study found an association between OP exposure and neurodegenerative outcomes (9), while another found no significant association with OP or PYR exposure (11).**Conclusions**

  Cohort studies suggest that coexposure to OP and PYR may be linked to ADHD symptoms and neurobehavioral deficits, while findings on neurodegeneration and ASD remain inconsistent. ROBINS-E requires adaptation for observational studies. Further longitudinal research is needed to clarify these risks and guide public health policies.

References

1.Morgan et al.,2018,https://doi.org/10.1016/j.envint.2018.07.015
2.Higgins et al.,2024,https://doi.org/10.1016/j.envint.2024.108602
3.Joyce et al.,2022,https://doi.org/10.1002/aur.2698
4.Dalsager et al., 2019,https://doi.org/10.1016/j.envres.2019.108533
5.Eadeh et al., 2023,https://doi.org/10.1016/j.neuro.2023.01.001
6.Fluegge et al.,2022,https://doi.org/10.5281/zenodo.218417
7.Hyland et al.,2021,https://doi.org/10.1016/j.envres.2021.111908
8.Hyland et al.,2022,https://doi.org/10.1097/EE9.0000000000000150
9.Shrestha et al.,2020a,https://doi.org/10.1136/oemed-2020-106818
10.Shrestha et al.,2020b,https://doi.org/10.1016/j.envres.2020.110186

P28-47 | Evaluation of heavy metals in cosmetic products in Nigeria and health implications

Blessing E. Edogbo[1], Shehu U. Adamu[2], Oluremi A. Saliu[1], Suwaiba Nasir[1], Salima S. Adamu[1], Idowu A. Oluwafemi[1], Tijjani Habibu[1], Frank Ebhodaghe[1], Chukwunonso Eya[1], Eunice O. Olukoba[3], Samira A. Bashir[2], Chidiebere Uchendu[4]

[1] National Open University of Nigeria, Environmental Health Science, Abuja, Nigeria; [2] National Open University of Nigeria, Public Health, Abuja, Nigeria; [3] National Open University of Nigeria, Nursing Science, Abuja, Nigeria; [4] University of Jos, Veterinary Physiology, Biochemistry and Pharmacology, Jos, Nigeria

Objectives: Heavy metals contamination in cosmetic products poses a significant global concern, including in Nigeria. The study aimed to evaluate the concentrations of heavy metals in various brands of cosmetic products and assess the associated health risks. Additionally, Consumer awareness was evaluated to determine the extent of knowledge regarding the presence of heavy metals in cosmetic products.

Materials and Methods: Five heavy metals, including cadmium (Cd), chromium (Cr), mercury (Hg), lead (Pb) and arsenic (As) were quantified in the different brands of skincare products, makeup, hair products, nail care products,

fragrances and personal hygiene commonly used in Nigeria. A total of 270 samples were digested and analysed for heavy metals using with Atomic Absorption Spectrophotometry. Risk assessment was carried out using Hazard quotient (HQ), hazard quotient (HQ) and lifetime cancer risk (LCR). Consumer awareness of heavy metals in cosmetic products was evaluated through well-structured questionnaires.
Results: Heavy metals were detected in the cosmetic products in varying concentrations. Notably, High levels of Pb (6.72 ±0.05 mg/kg) were found in nail polish. The majority of cosmetic product types used by participants were personal hygiene items (75.2%) followed by fragrances (71.3%) and skincare products (69.9%). The least used products included items like lip gloss, baby lotion and Vaseline, accounting for 0.3%/**Conclusion**: Regular use of certain cosmetic products can cause serious health threats, particularly the risk of skin cancer from long-term exposure. Continuous monitoring of cosmetic products, specifically adulterated ones, is important to ensure human safety.

P28-48 | Countering the cumulative health impacts of corporations to ensure health equity across the lifespan

Nicholas Chartres[1]

[1] The University of Sydney, School of Pharmacy, Faculty of Medicine & Health, Sydney, Australia; [2] University of California, San Francisco, Program on Reproductive Health and the Environment, Department of Obstetrics, Gynecology and Reproductive Sciences, San Francisco, USA

The World Health Organization estimates that approximately one quarter or 13.7 million global deaths each year are linked to modifiable environmental factors, including chemicals and pollution. The cumulative human health effects of these exposures have fallen disproportionately on low-wealth populations and communities of colour, leading to health inequities and environmental injustices, e.g. 89% of ambient air pollution-related deaths occur in LMICs. Chemical pollution is driven by extraction, production, and use of fossil fuels and fossil fuels are also the primary driver of climate change. Fossil-fuel consumption and petrochemical production are >15 time higher than in the 1950s, and global plastic production is set triple in the next 30 years as the fossil-fuel corporations increase production of plastics and other petrochemicals. As many of the chemicals used in plastics production including PFAS, are persistent, bio accumulative and toxic (PBTs), these chemicals take decades to degrade in the environment and therefore cause intergenerational harms. Additionally, chemicals used in ultra-processed foods targeted at children and minority populations has exploded since the 1980's when the tobacco industry acquired the world's largest food companies. The health effects of these chemicals include endocrine disruption and cancer, and thus cause harm across the lifespan. Addressing the role of corporations in the spread of these diseases is therefore essential to improve global health equity.
A primary mechanism by which corporations act to spread their products is through strategies designed to undermine the science and regulatory process, which delays regulation. It is critical to understand and accelerate research on industry strategies to identify and prevent corporations from continuing to spread disease. This symposium will discuss empirical case studies of individual and shared strategies used by corporations to proliferate chemical production and

pollution, along with other health harming products, and actionable solutions to safeguard scientific integrity and the regulatory process.

P28-49 | Identification of Highly Hazardous Pesticides Responsible for Poisoning and Death in Uganda

C. Ssemugabo[1, 2], J. Agenonga[1], E. Kigozi[1], L. Utyasheva[2, 3], J.C. Ssempebwa[1], M. Eddleston[3, 2]

[1] Makerere University, School of Public Health, Kampala, Uganda; [2] University of Edinburgh, Center for Pesticide Suicide Prevention, Edinburgh, United Kingdom; [3] University of Edinburgh, Centre for Cardiovascular Science, Edinburgh, United Kingdom

Background: Pesticide poisoning has increased in Uganda due to the increased use of pesticides in agriculture. Some pesticides responsible for the poisoning are highly hazardous.
Objective: We identified highly hazardous pesticides (HHPs) responsible for poisoning and death from 2018 to 2022.
Methods: We reviewed records from 56 police stations and 5 regional referral hospitals. Uganda's pesticide registry was reviewed to identify HHPs. HHPs were identified using the Joint Meeting on Pesticide Management (JMPM) Criteria 1 – Pesticide formulations that meet the criteria of classes Ia or Ib of the WHO Recommended Classification of Pesticides by Hazard, and Pesticide Action Network HHPs list.
Results: 120 pesticide-active ingredients (a.i.) are registered for agricultural use in Uganda, out of which 41.6% (50/120) are HHPs. Police and hospital record reviews between 2018 and 2022 revealed 482 and 397 pesticide poisoning cases respectively. Most of the pesticide poisoning cases were intentional with 249 and 338 from police and hospital respectively. Police data showed that 33-pesticide a.i. were associated with fatal pesticide poisonings, with 14 belonging to the organophosphorus group. Carbofuran, chlorfenviphos, chlorpyrifos, diazinon, and dichlorvos were associated with high numbers of deaths reported to police. However, pesticides such as chlorfenviphos and diazinon are not registered for agricultural use in Uganda. Pesticide a.i was not identified in 10 of the cases. From the hospital records, 25 pesticide a.i were responsible for 397 poisonings, of which 15% (59/397) victims eventually succumbed to the poison. Warfarin (37), Brodifacoum (20), and Cypermethrin (18) were responsible for the majority of non-fatal pesticide poisonings. Paraquat (5), Glyphosate (2), and Dicamba (2) caused fatal poisonings.
Conclusion: We identified HHPs responsible for poisoning and deaths in Uganda. Discussion with the national pesticide registration board will explore ways of enforcing current bans and removing these toxic licensed pesticides from agriculture in Uganda.

P29 | Impact of Extreme Heat and Environmental Factors on Health Outcomes

P29-01 | Assessing the potential reduction in heat-related illnesses ambulance transport through temperature exposure mitigation in Japan

Lisa Yamasaki[1,2], Chris F.S. Ng[1], Tatsuki Uemura[2], Ryo Sasaki[2], Masahiro Hashizume[1]

[1] The University of Tokyo, Department of Global Health Policy, Tokyo, Japan; [2] Center Hospital of the National Center for Global Health and Medicine, Department of Emergency Medicine and Critical Care, Tokyo, Japan

BACKGROUND AND AIM
With global warming, the frequency of extremely hot days is increasing. Given that older individuals are more susceptible to heat stress and nearly one-third of Japan's population is aged 65 and over, inadequate heatstroke prevention measures place additional strain on emergency healthcare systems. This study aims to evaluate the impact of reducing heat exposure on heat-related illness ambulance transport (HIAT) cases and identifies effective intervention targets.

METHODS
We analyzed HIAT data in Tokyo from June to September between 2015 and 2023, excluding 2020 and 2021 due to the COVID-19 pandemic. The data were categorized into two groups: individuals under 65 years old and those aged 65 and older. Daily maximum temperature data were obtained from the Japan Meteorological Agency. We estimated the relative risk of HIAT at different temperatures compared to a reference temperature. Counterfactual scenarios were then constructed, assuming reductions in population-level heat exposure. Based on these scenarios, we estimated the number of potentially preventable HIAT cases over the past seven years to quantitatively assess the impact of interventions.

RESULTS
A total of 44,509 HIAT cases were recorded, with a nearly equal proportion of cases among individuals under 65 and those 65 and older. When hypothetical temperature exposure was reduced by 50%, approximately 15,746 cases among younger individuals and 16,234 cases among older adults could have been prevented over eight years. Considering the higher incidence and severity of heat-related illness among older individuals, targeted interventions for this age group are expected to be a more effective strategy for reducing the healthcare burden associated with heatstroke.

CONCLUSIONS
Urban areas face significant challenges in implementing effective measures to reduce heatstroke-related healthcare burdens. While prioritizing older individuals, optimizing intervention strategies for both younger and older residents is essential to enhance resource allocation efficiency and maximize public health benefits.

P29-02 | Spatial heterogeneity in synergistic effects of extreme heat and NO_2 exposures on cardiorespiratory hospitalizations in California

Yiqun Ma[1], Chen Chen[1], Rosana Aguilera[1], Alexander Gershunov[1], Michael Jerrett[2], Rachel Connolly[2,3], Miriam E. Marlier[2], Tarik Benmarhnia[1,4]

[1] University of California San Diego, Scripps Institution of Oceanography, La Jolla, USA; [2] University of California Los Angeles, Department of Environmental Health Sciences, Fielding School of Public Health, Los Angeles, USA; [3] University of California Los Angeles, Luskin Center for Innovation, Los Angeles, USA; [4] University of Rennes, Irset Institut de Recherche en Santé, Rennes, France

Objective: In contrast to the overall decrease in ambient air pollution in the U.S. in recent years, extreme heat events have become increasingly frequent under climate change. Growing evidence suggests that extreme heat and high air pollution levels may co-occur and act synergistically, exacerbating adverse health outcomes. Synergistic health effects of extreme heat and NO_2, as well as the fine-scale spatial heterogeneity in such joint effects, remain unclear. In this study, we aim to (1) investigate the state-level additive synergistic effect of extreme heat and extreme NO_2 exposures on cardiorespiratory hospitalizations; (2) estimate the ZIP Code Tabulation Area (ZCTA)-specific synergistic effects of these two exposures; and (3) explore how community characteristics modify these synergistic effects in California.

Material and Methods: Using ZCTA-level daily time-series data in California from 2000 to 2019, we investigated the state-level synergistic effects of extreme heat and extreme NO_2 exposures on cardiorespiratory hospitalizations with a state-level case-crossover analysis. We then estimated the ZCTA-specific synergistic effects of these two exposures using a within-community matched design combined with a spatial BHM. Finally, we explored how community characteristics might modify these effects using meta-regressions.

Results: When considering California overall, we found mild synergistic effects of extreme heat and NO_2 exposures on cardiorespiratory hospitalizations, with a relative excess risk due to interaction (RERI) of 0.004 (95% confidence interval: -0.002, 0.011). Yet, great spatial heterogeneity was observed in the synergistic effects, with ZCTA-specific RERIs ranging from -1.082 to 2.218. Higher synergistic effects were observed in communities with lower socioeconomic status, higher population density, reduced green space, greater proportions of racial and ethnic minority residents, and higher historical temperatures.

Conclusion: Our findings underscore the urgent need for integrated adaptation policies that account for compound exposures to heat and air pollution and inform the development of targeted intervention strategies to protect vulnerable communities.

P29-03 | Association between Heat and Air Pollution ($PM_{2.5}$ and Black Carbon) exposure in Pregnancy and Preterm Birth in Low and Middle-Income Countries: A Systematic Review & Meta-Analysis

Sreevatsan Raghavan[2], **Tanya S. Isaac**[1], Divya Arya[2], Rekha Shanmugam[1], Ayushi Na[3], Bapu K. Desiraju[2], Vidhya Venugopal[1], Ramachandran Thiruvengadam[4], Gabriela C. Flores[5], Pallavi Kshetrapal[2], Nitya Wadhwa[2], Laura Downey[5], Jane Hirst[5], Shinjini Bhatnagar[2]

[1] Sri Ramachandra Institute of Higher Education and Research, Department of Environmental Health Engineering, Chennai, India; [2] Translational Health Science and Technology Institute, Centre of Maternal and Child Health, Faridabad, India; [3] Translational Health Science and Technology Institute, Clinical Development Services Agency, Faridabad, India; [4] Pondicherry Institute of Medical Sciences (A Unit of Madras Medical Mission), Biochemistry Department, Pondicherry, India; [5] Imperial College London, The George Institute for Global Health, London, UK

Background: Preterm birth (PTB) remains a major global health concern, contributing significantly to neonatal morbidity and mortality. However, limited research has examined the association between prenatal exposure to ambient heat and air pollution—particularly $PM_{2.5}$ and black carbon—in low- and middle-income countries (LMICs), where vulnerabilities to climate change and environmental hazards are more pronounced. This review integrates existing evidence on these exposures and their impact on PTB risk in LMICs.

Methods: A systematic search of PubMed, Embase, Scopus, and grey literature was conducted for peer-reviewed studies published between 2010 and 2024. Eligible studies assessed PTB in relation to $PM_{2.5}$, black carbon, and ambient heat exposure. After screening 4,524 studies, 32 met the inclusion criteria, with exposure assessments covering various pregnancy windows. Risk of bias was evaluated using the ROBINS-E tool. Random-effects meta-analyses (DerSimonian and Laird method) were performed when at least three studies were available; otherwise, a fixed-effects model was applied model was used to estimate the pooled effect sizes.

Results: Heat exposure significantly increased PTB risk, particularly in the first and third trimesters (OR = 1.29 and 1.39, respectively). $PM_{2.5}$ was associated with a modest but consistent increase in PTB risk across all trimesters (OR = 1.09). Black carbon exposure showed the strongest association in the third trimester (OR = 2.74).

Conclusion: The results point towards a consistent adverse effect on the exposures studied ($PM_{2.5}$, black carbon, and heat). There is also a dearth of representative data from LMICs where vulnerabilities to climate change, specifically for maternal and child health, are more pronounced. Furthermore, few studies have investigated the impact of combined exposures, highlighting a critical gap in understanding the synergistic effects of these environmental factors. This emphasizes the need for more geographically diverse and representative studies to permit policy framing aiming to reduce PTB incidence mediated by environmental factors.

P29-04 | Characterizing volatile organic compound (VOC) emissions in vehicles parked in extreme heat

Rafi Uddin, Inusng Kang

University of Texas at Arlington, Civil Engineering, Arlington, USA

According to the Texas Department of State Health Services (DSHS), the interior of vehicles can experience the greenhouse effect, with in-cabin temperatures exceeding 140°F when the outdoor temperature reaches 101°F. This study aims to characterize the emission rates of volatile organic compounds (VOCs) and other potential pollutants, including ozone (O_3) and ultrafine particles (UFPs), in vehicles parked during the summer season. Our preliminary study utilized low-cost air quality sensors to measure both indoor and outdoor pollutant concentrations continuously, as well as meteorological parameters such as temperature, humidity, and solar radiation. Air exchange rate was measured to be ~0.25 h^{-1} (with windows closed) via a CO_2 tracer gas method. Average TVOC emission rates during midday testing were approximately 5.6×10^2 µg/h when cabin air temperatures exceeded 125 °F and ambient temperature was around 96°F. We also found a significant correlation between in-cabin temperature and TVOC concentrations ($\rho = 0.80$, $p < 0.001$) using Spearman's correlation analysis. Additionally, we will utilize gas chromatography–mass spectrometry (GC-MS) between May and July 2025 to identify specific VOC types and quantify their precise emission rates.The study provides crucial insights about the emission pattern of VOC under extreme weather condition and how it may impact interior built environment of a car.

P29-05 | Assessing the Impact of Risk Perceptions on Extreme Heat Mitigation Behaviors among U.S. Adults

Bibiana Martinez, Jo Kay Ghosh

Heluna Health, Department of Research and Evaluation, City of Industry, USA

Objective: Extreme heat presents increasing hazards to human health; individual-level mitigation behaviors can help ameliorate such impacts. Our goal is to explore the effect of risk perceptions on likelihood of engaging in heat mitigation behaviors among U.S. adults.
Materials and Methods: Survey panel data were collected online during May 2024 to assess perceptions of heat risk and heat mitigation behaviors (e.g., seeking shade, staying cool, wearing loose-fitting clothes) among a representative sample of U.S. adults. A dichotomous outcome representing good vs. poor mitigation behaviors was developed by summing responses about likelihood to engage in each mitigation behavior; scores above the median value were considered good mitigation behaviors. We multiplied responses for questions regarding perceived frequency and personal impact of extreme heat events to calculate a heat risk perception score (range 1-25). Weighted logistic regression was used to model the impact of perceived risk on good mitigation behaviors, controlling for age, sex, political affiliation, race/ethnicity, education, and household income.
Results: A total of 6095 housed individuals responded to our online survey, and 2740 (45%) had scores classified as good mitigation behaviors. Each one unit increase in heat risk perception was associated with approximately 4% (95% CI: 1.03-1.04) increased odds of having good mitigation behaviors in our adjusted model. Younger age groups (vs. ages 65+), politically independent (vs. democrat), non-white (vs. non-Hispanic White), male (vs. female), and

having a household income <$55,000 (vs. $100,000+) were less likely to have good heat mitigation behaviors in our multivariable model.
Conclusion: Risk perception is a significant factor impacting the likelihood of engaging in heat mitigation behaviors. While other factors likely also play an important role, understanding the effect of risk perception on heat mitigation behaviors is paramount to designing effective communication strategies and interventions to protect the public against the health hazards of extreme heat.

P29-06 | Rising Extreme Heat Exposure in Gulf States of the United States

Chowdhury Arafat Hossain, David Luglio, Melissa Gonzales, Md Mostafijur Rahman

Tulane University Celia Scott Weatherhead School of Public Health and Tropical Medicine, Department of Environmental Health Sciences, New Orleans, USA

Background: Climate change is escalating extreme heat events globally, and the Gulf South of the United States is among the regions at highest risk.
Objective: We analyzed trends in summertime daytime and nighttime temperatures, as well as extreme hotter days and extreme hotter nights, in five Gulf States (e.g., Texas, Louisiana, Mississippi, Alabama, and Florida), from 2000 to 2023. We conducted the analysis at the county-level to identify the most vulnerable places due to extreme heat.
Method: We utilized the gridMET meteorological and NASA's grided population data to calculate geography and population-weighted county resolution daily maximum and minimum temperature. Increase in minimum temperature serves a proxy for nighttime temperature. An extreme day or night, in terms of temperature or heat index, is defined as a day or night when the temperature or heat index in a county exceeds its 90th percentile. We used the non-parametric Mann-Kendall test to identify monotonic trends and a generalized least squares (GLS) model with restricted maximum likelihood (REML) for trend estimation, adjusting for first-order autocorrelation.
Results: Our findings indicate a significant increasing trend for both daytime and nighttime temperatures during summer. However, the increase in nighttime temperature was observed in more counties (342 out of 534 counties), compared to 29 counties for daytime temperatures. The mean nighttime temperature increase across those counties was 1.23°C. Our analysis of extreme hotter days and nights revealed consistent results, with a widespread increase in extreme hotter nights across the Gulf States(370 counties compared to 48 counties for hotter days). On average, there were 34 additional extreme hotter days and nights during the study period.

Conclusion: This study underscores the increasing trend of nighttime heat in most counties of the Gulf-South, highlighting the need for targeted public health interventions and adaptive strategies to mitigate the health impacts of extreme heat.

P29-07 | Climate resilience adaptation and psychiatric emergency services utilization during extreme heat in Boston, MA: a difference-in-differences analysis

Flannery Black-Ingersoll[1], Mary D. Willis[2], Stephanie T. Grady[1], Kaylin Vrkljan[2], Alison Duncan[3,4], Kate Burrows[5], Rachel Oblath[3,4], Amruta Nori-Sarma[6]

[1] Boston University School of Public Health, Department of Environmental Health, Boston, USA; [2] Boston University School of Public Health, Department of Epidemiology, Boston, USA; [3] Boston University Chobanian & Avedisian School of Medicine, Boston, USA; [4] Boston Medical Center, Department of Psychiatry, Boston, USA; [5] University of Chicago, Department of Public Health Sciences, Chicago, USA; [6] Harvard T.H. Chan School of Public Health, Department of Public Health, Boston, USA

Objective: Emerging research shows that extreme heat events increase the risk of adverse mental health outcomes. Research on heat alert warning systems (developed to warn the public ahead of extreme heat events) has found limited effectiveness at preventing physical health outcomes. Additionally, the effect in vulnerable low-income populations has been challenging to assess and potential effects on mental health outcomes have not been examined. The objective of this project is to investigate the effect of a heat warning system on use of psychiatric emergency services during extreme heat.

Material and Methods: Using a quasi-experimental study design, we implemented a difference-in-differences analysis to examine associations between extreme heat and psychiatric emergency services (PES) use before and following the 2016 implementation of Boston's city-wide heat warning system as part of climate resilience adaptations. We calculated daily total PES encounters for 2012-2019 using the Boston Emergency Services Team (BEST) electronic health records, a program run by a safety net hospital designed to serve vulnerable uninsured and publicly insured persons. Using the National Weather Service temperature data, we flagged each day for extreme heat (i.e., maximum temperature ≥*90 °F*). We estimated risk ratios and 95% CIs using Poisson regression, adjusting for temporal variation.

Results: We identified 63,293 encounters within the BEST program (23.7% unhoused, 53.3% nonwhite, 84.7% publicly insured). Contrary to our hypothesis, extreme heat events occurring after the implementation of the heat warning system were associated with increased PES use among BEST patients compared with events occurring prior to the heat warning system implementation (RR=1.07, 95% CI: 1.01, 1.13).

Conclusion: Our findings show that the implementation of a heat warning system does not appear to yield the expected reductions in the direction or magnitude of association between extreme heat and PES use in this highly vulnerable study population.

P29-08 | The effect of ice cooling vests on thermoregulation and thermal sensation among users of personal protective equipment when exercising

Hung-Pang Hsieh, Yi-Chun Lin, Victor E.-W. Lo, **Chen-Peng Chen**

China Medical University, Department of Occupational Safety and Health, Taichung, Taiwan

Objective:
Personal protective equipment (PPE) may cause heat stress among the wearers when in use. To alleviate the stress from heat exposure, ice cooling vests have been suggested to be an effective strategy. This study evaluated the impact of wearing Level C PPE and ice cooling vest together on changes in thermoregulation and thermal sensation during exercise under simulated solar irradiation.
Material and Methods:
This study recruited 10 male and 10 female participants aged 20 to 40. Four outfit selections were simulated: Standard clothing with and without donning an ice cooling vest and Level C PPE (including chemical protective clothing, half-facepiece respirator, goggles, gloves, and safety boots) with and without a cooling vest. The experiments were conducted in a controlled microclimatic chamber (28 °C; 60 % relative humidity), where participants performed exercises corresponding to a moderate metabolic rate (235-360 W). Thermoregulatory and subjective perception indicators were observed to assess their changes.
Results:
The results indicated that differences in clothing/equipment (Level C PPE: 3.9 kg; ice cooling vest: 2.6 kg) were the key factors influencing change in thermoregulation and subjective perception. In the experiments donning Level C PPE, the heart rate of participants recovered slowly and failed to return to the baseline 40 min post-exercise, whereas, in those donning standard clothing, the heart rate recovered within 20 minutes. The medians of skin capillary blood flow and thermal sensation ratings were lower in the Level C PPE + Ice Cooling Vest group (39.3 PU; 2) than their counterparts in the Level C PPE group (53.6 PU; 3).
Conclusions:
The heat stress from PPE use should be considered a safety concern. Cooling garments such as ice cooling vests could be used to mitigate the heat stress.
This study was financially supported by National Science and Technology Council (NSTC), Taiwan (NSTC 112-2221-E-039-007-MY3).

P29-09 | Association between heat exposure and short-term kidney disease mortality in Latin American cities.

Karla Rangel Moreno[1], Horacio Riojas-Rodríguez[2], Magali Hurtado-Díaz[3], Josiah L. Kephart[4], Tonatiuh Barrientos Gutiérrez[5]

[1] National Institute of Public Health, Cuernavaca, Mexico; [2] National Institute of Public Health, Cuernavaca, Mexico; [3] National Institute of Public Health, Cuernavaca, Mexico; [4] Drexel Dornsife School of Public Health, Urban Health Collaborative, Philadelphia, USA; [5] National Institute of Public Health, Cuernavaca, Mexico

Objective: Kidney disease (KD) mortality is among the top ten causes of death in Latin America (LATAM), and it has been associated with heat exposure in high-income countries. We aimed to assess the association between heat exposure and short-term KD mortality in 294 LATAM cities from 2000 to 2019, and its modification due to sub-group and city-level variables.

Material and Methods: We conducted a case-time series design using data from 294 cities in eight countries included in the Climate Change and Urban Health in Latin America project (SALURBAL-Climate). We used percentiles of the population-weighted daily average temperature from ERA5-LAND and daily KD death counts in adults obtained from national statistical offices. We assessed the interest association using a conditional quasi-Poisson with distributed-lag (0-6 lags) non-linear model, and added interaction terms in the models to evaluate effect modification by sub-group variables (age and sex), and city variables (country and climate zone).

Results: A total of 592,810 KD deaths were recorded, with the majority occurring in Brazil (52.5%) and Mexico (35.6%). The minimum mortality temperature percentile corresponded to the 42nd percentile. An increase in the relative risk of KD mortality was observed from 1% at the 64th percentile (95% CI: 1%, 2%) to 20% at the 99th percentile (95% CI: 18%, 22%). The risk was higher for elderly people and females, and it was heterogenous across the countries and climate zones.

Conclusion: Exposure to heat temperatures was associated with an increase in KD mortality in LATAM cities. The results may contribute to the development of adaptation programs and public policy strategies to mitigate the health impacts of global warming, particularly for older adults and individuals with chronic diseases.

P29-10 | Heat Health Warning Systems and Emergency Department Visits: Investigating the Impact on Heat-Related Illnesses in San Diego, California

Oghenetanure Ryan Enaworu[1], Leire Luque García[2,3], Wael K. Al-Delaimy[4]

[1] University of California Irvine, Cancer Research Center, Irvine, USA; [2] University of the Basque Country (UPV/EHU), Department of Preventive Medicine and Public Health, Leioa, Spain; [3] Biogipuzkoa Health Research Institute, Environmental Epidemiology and Child Development Group, Donostia-San Sebastián, Spain; [4] University of California San Diego, Herbert Wertheim School of Public Health and Human Longevity Science, La Jolla, USA

Background: Heat Health Warning Systems are critical to mitigate the impact of extreme heat events. However, the evidence for the associated health implications remains underexplored, particularly regarding emergency department visits. The present study aims to investigate the association between Heat Health Warning System alerts and emergency department visits for heat-related illness in San Diego from 2012 to 2023, focusing on heat-related renal, cardiovascular, respiratory, metabolic, and mental health disorders.

Methods: Emergency department visit data from UCSD Health were analyzed alongside heat alert data from the Iowa Environmental Mesonet. Heat alert days were matched to comparable non-alert days. Initial statistical models assessed the association with maximum temperature and humidity, while further analyses adjusted for confounders such as age, gender, ethnicity, and Health and Human Services Agency region. Stratified analyses explored variations across demographic groups.

Results: Heat alert days did not significantly reduce overall emergency department visits, including those for heat-related renal, cardiovascular, respiratory, metabolic, or mental health disorders. However, stratified analyses identified variations across age, gender, and ethnicity, with some subgroups experiencing increases or decreases in specific health outcomes. No significant differences were observed across Health and Human Services Agency regions.

Conclusion: This study highlights the complexity of heat health warning systems and shows limited overall effectiveness in reducing overall heat-related emergency department visits in San Diego. However, the variations observed among demographic groups suggest that specific populations may benefit more than others. These findings highlight the need for targeted public health interventions that address specific demographic vulnerabilities by improving the effectiveness of heat warning systems in mitigating heat-related health risks.

P29-11 | Heat Exposure and Epigenetic Age Acceleration

Marina Sweeney[1], Kaitlyn Lawrence[3], Ian Buller[1], Nathaniel MacNell[1], Anna Jones[1], Zongli Xu[4], Dale Sandler[2]

[1] DLH, LLC, Data Sciences, Epidemiology, & Biostatistics, Bethesda, USA; [2] National Institutes of Health, Epidemiology Branch, National Institute of Environmental Health Sciences, Durham, USA; [3] National Institutes of Health, Division of Extramural Research and Training, National Institute of Environmental Health Sciences, Durham, USA; [4] National Institutes of Health, Biostatistics and Computational Biology Branch, National Institute of Environmental Health Sciences, Durham, USA

Objective. To investigate the association between heat exposure and markers of epigenetic aging.
Material and methods. Epigenome-wide DNA methylation was measured in peripheral blood samples collected from N=4,626 participants in the U.S.-wide Sister Study cohort (2003-2009). Heat exposure was determined using data from the Oregon State University PRISM Climate Group (1993-2009). Residential exposure to high heat was defined as the count of ‘hot days’ where the daily maximum apparent temperature (i.e., heat index or ‘feels like’ temperature; accounts for daily mean relative humidity) exceeded 85° Fahrenheit within the 4km-by-4km gridded location of each participant’s residential address for the 365-day exposure window prior to the participant’s blood draw date. We used sample-weighted linear regression models to evaluate associations between hot day count (quartiles and per 10-day increase) and three epigenetic clocks: PhenoAge, GrimAge, and DunedinPACE. Models were adjusted for race, alcohol consumption, pack-years of smoking, census region, and DNA methylation platform.
Results. Participants were exposed to a median of 74 hot days (interquartile range: 45-116). Those in the highest quartile (117-261 days) experienced hot days for 32-72% of the year prior to the blood draw. Compared to those in the lowest quartile of hot days (0-45 days), those in the highest quartile experienced greater GrimAge acceleration (beta=0.31, 95% CI: 0.06, 0.57) and suggestively greater PhenoAge acceleration (beta=0.41, 95% CI: -0.11, 0.92). DunedinPACE was also associated with the count of hot days (Q4vsQ1: beta=0.010, 95% CI: 0.002, 0.017) but to a lesser extent. Associations per 10-day increase in hot days were consistent.
Conclusion. Participants exposed to the greatest number of hot days over the year before their blood draw had elevated epigenetic age acceleration measures, regardless of the clock metric. Persistent high heat exposure may increase biological age relative to chronological age.
Funding: The Intramural Research Program of the NIH (Z01 ES044005).

P29-12 | Assessing the Impact of Heatwaves and Cold Snaps on Child Malnutrition: A Multilevel and Spatial Analysis of Burkina Faso and Kenya

Tavis C. Mansfield

University of Maryland, College Park, Geographical Sciences, College Park, USA

Objective
This study investigates the relationship between heatwaves and child malnutrition—specifically stunting and wasting—by comparing Burkina Faso and Kenya. These two countries, with differing levels of economic development and infrastructure, provide an opportunity to assess whether electricity access and other household factors mitigate the adverse effects of extreme temperatures on child nutrition.

Materials and Methods
This study utilizes Demographic and Health Survey (DHS) data combined with remotely sensed climate variables to analyze child nutrition outcomes in relation to heatwave exposure. A multilevel modeling approach is employed to assess the influence of household- and community-level factors on height-for-age (HAZ) and weight-for-height (WHZ) z-scores. Key variables examined include household income, food security, access to electricity, and parental education. The comparison between Burkina Faso and Kenya allows for an evaluation of how economic and infrastructural disparities shape the resilience of populations to heat stress.

Results
Preliminary analysis suggests that heatwaves are associated with declines in both HAZ and WHZ z-scores, indicating increased vulnerability to stunting and wasting. Households with limited access to electricity and lower socioeconomic status appear to be at higher risk. The findings also suggest that Kenya, with its higher electrification rate and economic capacity, may exhibit greater resilience to heatwave-related malnutrition compared to Burkina Faso.

Conclusion
This study highlights the urgent need for targeted interventions to mitigate the impact of extreme heat on child nutrition. Improving access to electricity, strengthening food security, and enhancing climate adaptation strategies are critical to reducing the adverse effects of heatwaves on vulnerable populations in sub-Saharan Africa.

P29-13 | Contrasting patterns of heat-related mortality and morbidity in Prague, Czech Republic

Tugba Dogan[1], Aleš Urban[1,2]

[1] Czech University of Life Sciences Prague, Faculty of Environmental Sciences, Prague, Czech Republic; [2] Czech Academy of Sciences, Institute of Atmospheric Physics, Prague, Czech Republic

Objective
Episodes of extremely high temperatures pose significant health risks to vulnerable populations in urban areas. While the associations between extreme temperatures and mortality in the Czech Republic have been documented by many studies, the impacts of heat on morbidity are less understood. In this study, we employed a unique individual database of ambulance callouts in Prague, Czech Republic, from 2014 to 2019, to compare heat-related morbidity and mortality patterns for selected population groups and diagnoses.

Material and Methods
A two-stage time-series design was employed to assess temperature-related health outcomes at both the district and city-wide levels. High-resolution (300 x 300 m) UrbClim model data were used to obtain daily temperature estimates for each of the districts of Prague. In the first stage, a district-specific distributed lag non-linear model quantified the non-linear and delayed associations between temperature and health outcomes, adjusting for temporal trends and potential confounders. In the second stage, meta-analytic techniques were applied to pool district-level estimates, evaluate effect modifiers, and derive population-wide exposure-response relationships. The relative risk at the 99th percentile of mean temperature compared to the median was considered as the heat effect.

Results
Analyses of morbidity and mortality outcomes consistently indicate an elevated risk associated with high temperatures, with similar trends observed across both sexes, respiratory cases, and all-cause outcomes. While morbidity indicated the largest risk among younger populations (0–29 years), moderate among middle-aged adults (30–64 years), and negligible among older adults (65+ years), mortality showed the opposite pattern. In addition, disease-specific analyses revealed a decline in cardiovascular morbidity at higher temperatures, while mortality risks increased, highlighting differing trends in cardiovascular outcomes during heat exposure.

Conclusion
This research highlights the contrasting patterns between heat-related mortality and morbidity in Prague, Czech Republic, which have important implications for the public health sector and heat adaptation strategies.

P29-14 | Assessing the Impact of Heat Alerts on Heatstroke Cases in Japan between 2020 and 2023

Youki Kiyomoto[1,2], Kazutaka Oka[3], Chris Fook Sheng Ng[4], Vera L.H. Phung[4], Satbyul Estella Kim[5], Aurelio Tobias[6], Yasushi Honda[3], Masahiro Hashizume[4], Lina Madaniyazi[2]

[1] National Center for Global Health and Medicine, Tokyo, Japan; [2] Nagasaki University, School of Tropical Medicine and Global Health, Nagasaki, Japan; [3] Center for Climate Change Adaptation, National Institute for Environmental Studies, Tsukuba, Japan; [4] Department of Global Health Policy, Graduate School of Medicine, The University of Tokyo, Tokyo, Japan; [5] Institute of Health and Sport Sciences, University of Tsukuba, Tsukuba, Japan; [6] Institute of Environmental Assessment and Water Research (IDAEA), Spanish Council for Scientific Research (CSIC), Barcelona, Spain

Objectives: Japan has seen a rise in heatstroke cases in recent years, prompting the implementation of a heat alert system to prevent health risks. This study evaluates the impact of Japan's heat alert system on emergency ambulance dispatches for heatstroke from 2020 to 2023.

Study design: A matched case-control study using conditional Poisson regression models.

Methods: Daily time series data on heatstroke ambulance dispatches and Wet Bulb Globe Temperature (WBGT) were collected for 47 prefectures from May to September 2020–2023. Heatstroke cases on heat alert days were compared to matched non-heat alert days with similar WBGT levels. Relative risks (RR) estimated the association between alert issuance and heatstroke cases. Subgroup analyses were conducted by age group, severity, and region.

Results: Of 2715 heat alert days reported, 1310 had matched non-heat alert counterparts, yielding 2620 total days and 63,753 cases for the analysis. Ambulance dispatches for heatstroke increased by 33.8% (95% confidence interval (CI): 24.1, 44.2) on heat alert days compared to matched non-heat alert days. This increase was more pronounced among individuals aged ≥ 65 years, sever heatstroke cases, and in southern Japan, though some 95%CIs overlapped.

Conclusions: Heat alerts in Japan were associated with higher ambulance dispatches for heatstroke, potentially due to increased awareness, reclassification of cases, alert fatigue, or behavioral responses. These findings should be interpreted with caution. Further research should consider other heat-related illnesses and assess public awareness and behavioral changes in response to heat alerts.

P29-15 | The impacts of carrying and transporting weights by hands or shoulder-backpacking on thermoregulation and muscle fatigue when walking on stairs

Yu-Chen Lai, Yi-Chun Lin, **Chen-Peng Chen**

China Medical University, Department of Occupational Safety and Health, Taichung, Taiwan

Objective:
Transporting heavy weights physically in warm environments poses physiological challenges, affecting both thermoregulation and muscle functions. This study aimed to examine how the magnitude of weight and the ways the transporter carried the weight influenced the development of heat strain and muscle fatigue as well as the kinetics involved in recovery.
Material and Methods:
Ten female and ten male participants performed moderate metabolic activity (235-360 W) under five experimental conditions, combining two load-carrying methods (bilateral hand-carrying and double-shoulder backpacking) with three load magnitudes (0, 5, and 10 kg). The experiment was conducted in a controlled microclimatic chamber simulating warm environment (28 °C, 60 % relative humidity). Participants performed step-walking exercises while core physiological and skin thermoregulatory parameters were continuously monitored.
Results:
Heart rate and metabolic rate increased significantly as the participants exercised, by 42 % and 186 %, respectively, after 20 minutes of step-walking. Skin moisture and skin capillary blood flow showed a notable increase under load conditions, with the effect being more pronounced in the bilateral hand-carrying group (skin moisture at 10 kg: 1,337.6 ± 980.6 μS for hand-carrying vs. 1,000.1 ± 582.5 μS for backpack-carrying). Deep tissue oxygenation was declined the most in the 10 kg hand-carrying condition (from 59.7 % to 49.44 %, a 17.18 % reduction), followed by the unloaded condition (8.39 %) and backpack-carrying condition (3.06 %), indicating a greater muscle fatigue manifested when hand-carrying the weight.
Conclusions:
Carrying weights with a double-shoulder backpack keeps the weight close to the body and reduces physiological stress and fatigue compared to bilateral hand-carrying. To alleviate the synergistic impacts of load carriage and environmental heat stress, appropriate work-rest cycles should be integrated into task planning.
This study was financially supported by National Science and Technology Council, Taiwan (NSTC 112-2221-E-039-007-MY3).

P29-16 | No Relief: Impact of Two-day Sustained Exposure To Elevated Minimum Temperature and Elevated NO_2 On Suicide In Utah, USA

Douglas S. Tharp[1], Jim VanDerslice[2], Fred Lurmann[3], Dirga Lamichhane[4], Yue Zhang[5], Brandy Hill[6], Amanda Bakian[7]

[1] University of Utah, SLC, USA; [2] University of Utah, Salt Lake City, USA; [3] Sonoma Technology, Petaluma, USA; [4] University of Utah, Salt Lake City, USA; [5] University of Utah, Salt Lake City, USA; [6] University of Utah, Salt Lake City, USA; [7] University of Utah, Salt Lake City, USA

Objective

To determine whether sustained exposure to high wet bulb global temperature (WBGT) and nitrogen dioxide (NO_2) is associated with an increased risk of suicide in Utah during the warm season.

Material and Methods

Utah's suicides (N=7,551) were ascertained through the Office of the Medical Examiner from 2000 to 2016 and geolocated to a 7x7km grid. Daily one-hour WBGT (℃) was combined with daily maximum NO_2 data. A unidirectional, time-stratified case-crossover design covering the warm season was employed, selecting death and control days. A binary 'no heat relief' measure was based on minimum WBGT exceeding 16℃ for the two days prior to death or control days. Similarly, a binary "no NO_2 relief" measure was created from the two lag days exceeding 20 ppb. A continuous "heat relief" variable was calculated as a one-day lag difference between maximum and minimum WBGT. We fit conditional logistic regression models to estimate the association between no heat relief" and "no NO_2 relief". Due to decliningNO$_2$ levels over time, models were stratified into three-year increments.

Results

"No NO_2 relief" was associated with an increase in suicide risk with a pattern suggesting a dose-response relationship across time with suicide risk most recently of 60.1% (1.29-1.91). Sustained "no heat relief" was associated with a 4.4% (1.03-1.06) increase in suicide risk. Increasing "Heat relief" showed a protective relationship with suicide risk (0.92,0.89-0.94).

Conclusion

Exposure to "no heat relief" and "no NO2 relief" was found to increase suicide risk in Utah during the warm season. In contrast, "heat relief" where there is a larger break in temperatures over the day seem protective against suicide. Despite improvements in air quality over time, continued efforts toward reducing sustained NO_2 exposures are needed. This also suggests the need for providing relief from sustained extreme heat.

P29-17 | Change in susceptibility to heat on suicide mortality within summer in Japan: a two-stage time-series analysis from 1973 to 2020

Nana Misawa[1], Jinah Park[2], Masahiro Hashizume[3], Yoonhee Kim[1]

[1] The University of Tokyo, Department of Global Environmental Health, Graduate School of Medicine, Tokyo, Japan; [2] Seoul National University, Department of Public Health Sciences, Graduate School of Public Health, Seoul, South Korea; [3] The University of Tokyo, Department of Global Health Policy, Graduate School of Medicine, Tokyo, Japan

Objective: The association between heat and suicide has been widely studied. However, evidence on how this risk varies within a season or between daytime and nighttime temperatures remains limited. This study aims to examine changes in the risk of heat-associated suicide during summer in Japan from 1973 to 2020.

Methods: A two-stage time-series analysis was used separately for maximum, mean, and minimum temperatures. In the first stage, we performed prefecture-specific analyses using a distributed lag non-linear model (DLNM) to estimate the exposure–lag–response association between temperature and suicide mortality and their variations within summer. In the second stage, we used a multivariate meta-regression to combine the temperature-suicide associations across 47 prefectures in Japan and estimated relative risks (RRs) for the 90th percentile of temperature compared to the minimum temperature value.

Results: A total of 383,115 suicides were included in this study. The RRs of suicide for the 90th percentile of maximum, mean, and minimum temperatures for whole summer were 1.23 (95% CI 1.14-1.32), 1.18 (95% CI 1.10-1.27), and 1.10 (95% CI 1.03-1.17), respectively. When comparing early and late summer, the RRs for maximum temperatures did not differ between early and late summer (1.24 [95% CI 1.16–1.33] and 1.24 [95% CI 1.10–1.40], respectively), while the RRs for minimum temperatures was higher for early summer (1.14, 95% CI 1.07–1.22) and lower for late summer (1.05, 95% CI 0.94–1.17).

Conclusions: We found a modest decline in susceptibility to heat-associated suicide for minimum temperatures in the late summer, suggesting potential adaptation to nighttime heat while, the risk associated with maximum temperatures, representing daytime heat, remained unchanged within the season.

Acknowledgement: This research was performed by the Environment Research and Technology Development Fund (JPMEERF20231007) of the Environmental Restoration and Conservation Agency provided by Ministry of the Environment of Japan.

P29-18 | Do heat warning alerts influence behavior? A study of the effectiveness of existing heat warning systems on limiting population mobility during periods of extreme heat in California

Jennifer Bailey[1,2], Chen Chen[3], Anna Dimitrova[3], Anaïs Teyton[1,2], Tarik Benmarhnia[3,4], Josephine Borghi[5,6], Roman Hoffman[6]

[1] University of California San Diego, Herbert Wertheim School of Public Health and Human Longevity Science, San Diego, USA; [2] San Diego State University, School of Public Health, San Diego, USA; [3] University of California San Diego, Scripps Institution of Oceanography, La Jolla, USA; [4] University of Rennes, Irset Institut de Recherche en Santé, Environnement et Travail (IRSET), Rennes, France; [5] The London School of Hygiene & Tropical Medicine, Department of Global Health and Development, London, UK; [6] The International Institute for Applied Systems Analysis (IIASA), Laxenburg, Austria

Climate change is expected to increase the frequency and intensity of extreme heat events (EHEs), which currently pose the deadliest weather hazard in the U.S. each year. Heat alerts issued by the National Weather Service aim to reduce exposure by encouraging avoidance behavior. The effectiveness of heat alerts is rarely evaluated, hindering the development of effective adaptation policies that protect public health, especially vulnerable populations. This study fills this gap by analyzing the observed relationship between heat alerts and mobility across California during the warm months of 2019 and 2020. Capitalizing on anonymized cellphone data inferred population mobility flow counts and high-resolution gridded temperature data from gridMET, we explored the influence of various extreme heat metrics and heat alerts on normalized population outflow at the daily level using panel regression models with fixed effects for census tracts and time. We found a consistent decrease in normalized population outflow across exposures to heat alerts and extreme heat for most definitions of EHEs. Across relative thresholds, a heat alert during a period of extreme heat reduced mobility more than the reduction when no heat alert was issued but temperatures still constituted an EHE. Effect modification at the neighborhood level was identified by vehicle access, income, and proportion of white residents and older residents. This research comprehensively assesses the impact of heat alerts on mobility and is an initial effort to evaluate existing heat warning systems in the context of climate change in California. Findings indicate that heat alerts may be effective in further reducing population flow beyond behavioural response to heat alone. This study uses a generalizable framework that can be used in other states or regions using high-resolution movement datasets to understand the usefulness and impact of early warning systems on people’s behaviour, with clear implications for climate change adaptation policies.

P29-19 | Air pollution, temperature, and HbA1c levels among children in Mexico City, Mexico.

Jeanne Wu[1], Pablo Knobel[1], Mike Z. He[1], Itai Kloog[1,2], Allan C. Just[1], Ivan Gutierrez-Avila[1], Elena Colicino[1], Martha M. Téllez-Rojo[3], Maria L. Pizano-Zárate[4,5], Marcela Tamayo-Ortiz[6], Alejandra Cantoral[7], Diana C. Soria-Contreras[8], Andrea Baccarelli[9], Robert O. Wright[1], Maayan Yitshak Sade[1]

[1] Icahn School of Medicine at Mount Sinai, Department of Environmental Medicine, New York, USA; [2] Ben-Gurion University of the Negev, Department of Geography and Environmental Development, Beer Sheva, Israel; [3] National Institute of Public Health, Center for Nutrition and Health Research, Cuernavaca, Mexico; [4] Nutrition and Bioprogramming Coordination, National Institute of Perinatology, Mexico City, Mexico; [5] UMF 4, South Delegation of the Federal District, Mexican Social Security Institute, Mexico City, Mexico; [6] Occupational Health Research Unit, Mexican Social Security Institute, Mexico City, Mexico; [7] Department of Health, Universidad Ibeoamericana, Mexico City, Mexico; [8] Diabetes Research Center, Massachusetts General Hospital, Boston, USA; [9] Department of Environmental Health Sciences, Columbia University Mailman School of Public Health, New York, USA

Objective: Glycated hemoglobin (HbA1c), a strong indicator of cardiovascular risk, is affected by air pollution and temperature exposures. However, studies examining the association between HbA1c, temperature, fine particulate matter ($PM_{2.5}$), and nitrogen dioxide (NO_2) among children are scarce, especially looking at all exposures simultaneously. We investigated the associations between these exposures and HbA1c among children, known to be a highly susceptible group.
Material and Methods: We included children enrolled in the Programming Research in Obesity, Growth, Environment, and Social Stressors (PROGRESS) cohort based in Mexico City (2013-2019). We obtained exposures from spatiotemporal models. HbA1c levels were measured at 4-5 years, 6-7 years, and 8-10 years post-birth. We used multivariable linear mixed-effects models to assess the simultaneous associations of three-month averages of $PM_{2.5}$, temperature, and NO_2 exposures with natural log-transformed HbA1c levels. We fitted stratified models by sex and age group. We included 1,186 HbA1c measurements from 503 children. **Results:** In multi-exposures models 1-μg/m³ increase in $PM_{2.5}$ was associated with 0.311% (95% CI:0.159, 0.464) relative increase in HbA1c and 1-degree Celsius increase in average temperature was associated with a -0.626% (95% CI: -0.977, -0.274) relative decrease. We did not find an association with a 1-ppb increase in NO_2 (-0.010%. 95% CI: -0.126, 0.106). The stratified analysis found slightly different associations by sex and age.
Conclusion: This study adds evidence of HbA1c increases associated with lower temperatures and higher $PM_{2.5}$ exposure in an area with high air pollution and moderate temperature fluctuation. These glycemic changes may translate into increased cardiovascular risk in later life.

P29-20 | The Impact of Ambient Environmental Stressors on Neurodevelopmental Disorders: Interactive Systematic Evidence Maps to Inform Decisions

Anisha Singh[1], Stefania Papatheodorou[2], Helena Hogberg-Durdock[3], Kyla Taylor[1], Vickie Walker[1], Chris Sibrizzi[4], Jenna Sprowles[4], Jeremy Frye[4], Johanna Rochester[4], Robyn Blain[4], Sam Goodman[4], Andrew A. Rooney[1]

[1] National Institute of Environmental Health Sciences, Integrative Health Assessments Branch, Durham, USA; [2] Rutger, The State University of New Jersey, Piscataway, USA; [3] National Institute of Environmental Health Sciences, Predictive Toxicology Branch, Durham, USA; [4] ICF, Reston, USA

Objective: Evidence on the association between prenatal and early-life exposure to ambient environmental stressors (e.g., air pollutants, weather related variables, residential greenness), and the risk of neurodevelopmental disorders (NDDs) suggests these exposures have adverse effects; however, data remain inconsistent. Epidemiologic and animal studies often yield mixed findings regarding impact of pollutants, critical windows of exposure, and potential interactions between ambient environmental stressors. This project aims to systematically identify, describe, and characterize literature on prenatal and early childhood exposure to ambient environmental stressors in relation to NDDs using a Systematic Evidence Map (SEM) approach. The SEM will make published research findable and accessible in an interactive format that will enable users to highlight research gaps as potential targets of future research, identify knowledge clusters that may support meaningful synthesis, and explore specific exposure combinations that may be of interest.

Materials and Methods: SEM applies systematic review methodologies to categorize and visualize data, enhancing synthesis and supporting evidence-based decisions. Our workflow integrates Artificial Intelligence (AI) in two key steps: (1) prioritizing references for screening to reduce manual workload and (2) extracting study details (e.g., exposures, models, NDD-related outcomes).

Results: We systematically searched PubMed, Scopus, and Web of Science with no date or language restrictions. The search identified 150,325 studies, with 117,347 remaining after removal of duplicates. Initial screening focused on 64,952 PubMed studies. After title-abstract and full-text screening in duplicate using DistillerSR, 720 studies met the inclusion criteria. Data extraction was performed using Dextr, and findings were displayed in interactive evidence maps via Tableau Public.

Conclusions: This study provides a comprehensive synthesis of evidence on the associations between ambient environmental stressors and NDDs, facilitating a better understanding of exposure risks and identifying key areas for future research.

P29-21 | Impact of tropical cyclones on child marriage rates in 23 affected countries

Anna Y. Palmer, Alissa Koski, Jill Baumgartner

McGill University, Epidemiology, Biostatistics, and Occupational Health, Montreal, Canada

Objective: Tropical cyclones (TCs) can disrupt livelihoods, displace communities, and curtail access to education—factors that may influence the rate of child marriage. We estimated the impact of TCs on child marriage rates across 23 cyclone-affected countries.
Material and Methods: We analyzed nationally representative child marriage data from the Demographic and Health Surveys and Multiple Indicator Cluster Surveys conducted between 1980 and 2015. These data were integrated with modelled subnational cyclone wind speed estimates based on data from the International Best Track Archive for Climate Stewardship. A distributed lag model was employed to estimate the effect of TC exposure on child marriage rates in each country for up to three years following landfall.
Results: In most countries, child marriage rates remained stable in the aftermath of TCs. We found no consistent evidence that TCs disproportionately affect rural populations or regions with higher wind speed exposure. Although minor variations emerged in a few settings, these differences were not robust across the overall sample.
Conclusion: Our results challenge the prevailing narrative that extreme weather events systematically drive increases in child marriage. Instead, the impact of TCs appears to be context-specific and limited, suggesting that deeply entrenched social and cultural norms play a more decisive role in marriage practices than short-term economic disruptions.

P29-22 | Increasing Susceptibility to Cold Spell but Not Heat Wave in Hong Kong over 1996–2015

Fang Guo[1,2], Mengdi Chai[3], Haisheng Wu[1], Vivian Do[4], Linwei Tian[1]

[1] The University of Hong Kong, School of Public Health, Hong Kong, Hong Kong; [2] Hong Kong Polytechnic University, Department of Civil and Environmental Engineering, Hong Kong, Hong Kong; [3] Harvard University, Harvard T.H. Chan School of Public Health, Boston, USA; [4] Columbia University, Mailman School of Public Health, New York, USA

Abstract
Background: A rise in both the frequency and intensity of extreme temperature events under climate change presents significant public health challenges, particularly in subtropical regions like Hong Kong. While prior studies have linked extreme temperatures to increased mortality, long-term trends in population susceptibility to heat waves (HWs) and cold spells (CSs) remain underexplored.
Objective: This study investigates temporal trends in mortality risks associated with HWs and CSs in Hong Kong from 1996 to 2015, focusing on all-cause, cardiovascular, and respiratory mortality.
Methods: Daily mortality data and weather records were collected from local bureaus. HWs and CSs were defined using fixed and dynamic thresholds assuming different scenarios of acclimatization. A distributed lag non-linear model (DLNM) was employed to evaluate the delayed effects of extreme temperature events on mortality, accounting

for humidity, seasonality, and long-term trends. A set of sensitivity analyses were performed to test the robustness of our results.
Results: The analyses revealed a generally declining trend in all-cause, cardiovascular, and respiratory (in particular) mortality risks associated with HWs during the study period. By contrast, CSs-related all-cause mortality risk exhibited a significantly increasing trend. Regarding cause-specific causalities, the relationship of CSs with deaths of respiratory conditions showed a pronounced upward trend over the years, while the risk estimates for cardiovascular mortality remained relatively unchanged. The findings are robust to different exposure metrics and model parameterizations.
Conclusion: These findings underscore that while preparedness and/or adaptation to hot extreme weather appears effective in Hong Kong, the escalating risks from CSs necessitate enhanced public health interventions, especially for vulnerable subpopulations. Continuous monitoring and tailored adaptation strategies are critical to mitigate health impacts from both HWs and CSs amid ongoing climate change. Future research avenues include exploring potential environmental health disparities in adapting to extreme temperatures in the long term.

P29-23 | A Perspective of Childhood Diarrhea Risk and Climate Dynamics from East Java Province, Indonesia

Gerry Andhikaputra[1], Bima S.S. Wibawa[2], Aussie T. Maharani[3], Fung-Chang Sung[4], Yu-Chun Wang[2], Amir Sapkota[1]

[1] University of Maryland, Department of Epidemiology and Biostatistics, College Park, USA; [2] Chung Yuan Christian University, Department of Environmental Engineering, Taoyuan, Taiwan; [3] Airlangga University, Institute of Tropical Disease, Surabaya, Indonesia; [4] China Medical University, Health Service Administration, Taichung, Taiwan

INTRODUCTION: Improved sanitation and hygiene along with rotavirus vaccination has resulted in declining burden of diarrheal disease in recent decades; however, increase in extreme weather events related to climate change has the potential to undermine this progress. In this study, we investigated the association between meteorological factors (maximum temperature and precipitation) and diarrhea incidence among under-five and all-age populations residing in three cities in Eastern Java, Indonesia: Surabaya City, Sidoarjo Regency, and Pasuruan Regency.
METHOD: We collected monthly diarrheal disease records and weather data from the East Java Provincial Health Office and the Indonesian Agency for Meteorological, Climatological, and Geophysics (BMKG), respectively. We extracted information of monthly Oceanic Niño Index (ONI) values from NOAA. We employed negative binomial generalized estimating equation models to assess the impacts of temperature and precipitation on diarrhea incidence, adjusting for ONI values and population.
RESULTS: The effects of temperature and precipitation on diarrhea varied by lag time and region. The association between maximum temperature and diarrhea was highest among children under five at lag of 2 months in Pasuruan Regency (IRR: 1.105, 95% CI: 1.037, 1.178), while the highest effect among all-age group was observed in Surabaya City (IRR: 1.081, 95% CI: 1.033, 1.131). Precipitation had an immediate effect (lag 0 months) in Surabaya City with a stronger impact among under-five (IRR: 1.017, 95% CI: 1.009, 1.025) and all-age group (IRR: 1.016, 95% CI: 1.009, 1.023) compared to Sidoarjo and Pasuruan Regency. However, no significant negative effects of temperature and precipitation were observed in the suburban area of Sidoarjo Regency.
CONCLUSION: We observed positive associations between meteorological factors and diarrheal disease, with a higher risk observed among younger children.

P29-24 | Power outages and cardiovascular and respiratory hospitalizations among US older adults

Heather McBrien[1], Daniel Mork[3], Vivian Do[1], Marianthi-Anna Kioumourtzoglou[1], Joan A. Casey[2]

[1] Columbia Mailman School of Public Health, Department of Environmental Health Sciences, New York, USA; [2] University of Washington, Department of Environmental and Occupational Health Sciences, Seattle, USA; [3] Harvard T. H. Chan School of Public Health, Department of Biostatistics, Cambridge, USA

Background: In the United States, already-prevalent power outages are increasing in frequency and duration with climate change. Studies from New York State show that power outages may increase hospitalizations for cardiovascular (CVD) and respiratory disease in vulnerable populations such as older adults, but exposure data limitations have constrained nationwide studies of power outages and health.

Question: Are power outages associated with emergency CVD and respiratory disease-related hospitalizations among older adults in the United States?

Methods: We developed a national dataset of power outage exposure and identified county-days with ≥1% of customers exposed to 8+ hour power outages in 2018. We leveraged data on 23 million Medicare Fee-For-Service beneficiaries aged 65+ to estimate daily county-level rates of emergency CVD- and respiratory-related hospitalizations. We applied a case-crossover design with a conditional Poisson model to estimate the lagged association (up to 1 week) between daily county-level power outage exposure and cause-specific hospitalization rates. Models controlled for daily temperature, precipitation, and wind speed.

Results: Power outages were associated with increased emergency CVD and respiratory hospitalizations. The association between power outage and CVD hospitalizations was strongest the day after power outage exposure (rate ratio [RR]=1.02, 95% CI: 1.01, 1.03), while the association between outage and respiratory disease was strongest the day of power outage exposure (RR=1.03, 95% CI: 1.01, 1.04).

Conclusion: Power outages increase risk of CVD and respiratory hospitalizations among US older adults. Improving electricity reliability could support community health and protect older adults from CVD and respiratory disease exacerbations.

P29-25 | Environment and Injury Outcome Tracker (EIOT):Visualizing Connections Between Environmental Factors and Injury Mortality

Jaemin Jeon[1], Douglas J. Wiebe[2,4], Jason Goldstick[2,4], Nichole Burnside[2], Peter S. Larson[2,3]

[1] University of Michigan, College of Engineering, Ann Arbor, USA; [2] University of Michigan, Injury Prevention Center, Ann Arbor, USA; [3] University of Michigan, Department of Epidemiology, School of Public Health, Ann Arbor, USA; [4] University of Michigan, Medical School, Ann Arbor, USA

Background: Injury-related outcomes, including drowning, violence including firearm-related incidents, and self-harm/suicide, are thought to have associations with environmental factors such as precipitation, extreme temperature and air pollution. Despite growing evidence of these associations, there are limited resources that explore the relationship between injury-related outcomes and environmental factors through an interactive interface.
Objective: This study will develop and test an interactive Environment and Injury Outcome Tracker (EIOT) to allow users to explore possible links between environmental factors and injury. This interactive tool will help to inform research and policy initiatives on environmental health and injury. EIOT will help to expand knowledge of how the ambient environment influences risk for important injury outcomes.**Methods**: EIOT will be developed using R Shiny, a web based tool for creating interactive data visualizations. This tool will visualize mortality from select injury related categories using data from the US Centers for Disease Control and other sources. For environmental data, we will use temperature and precipitation data from the PRISM Climate Group (Oregon State University) along with pollution data from the Environmental Protection Agency. This dashboard will aggregate and visualize state- and county-level injury data across multiple demographics, including gender, age, and race, over different time periods and provide an interactive U.S. map where users can explore injury trends at various geographic levels, allowing for comparative analysis between states and counties. Dashboard functionality will include summary statistics, normalization by population for all variables but will also include regression based tests for association between county level environmental exposures and mortality counts. The tool will visualize prominent clusters of hot and cold spots of injury measures using spatial clustering method (Getis Ord Gi*). This tool will also show where injury outcomes are significantly higher or lower than those predicted by environmental variables, accounting for neighbor effects.

P29-26 | Mental Health Disparities in the Displaced LGBTQ+ Population After Disasters in the United States

Leo Goldsmith, Jiyoung Son, Michelle Bell

Yale School of the Environment, New Haven, USA

Little is known about the mental health impacts of disaster displacement on the LGBTQ+ population in the United States (US), despite known social disparities before, during, and after disasters. The US Census Bureau Household Pulse Survey (HPS) is the first survey to collect national-level data at multiple scales (e.g. region, state, metropolitan statistical area) on disaster experiences and sexual orientation and gender identity demographic data. Using the HPS from January – July 2024, disparities regarding LGBTQ+ status compared to non-LGBTQ+ status in higher mental health burden outcomes among displaced populations up to one month after a disaster are assessed. The LGBTQ+ population has a 3.08 times higher odds of high mental health burden compared to the non-LGBTQ+ population. These results are driven by the significantly worse mental health outcomes of the transgender population who has

22.49 times the odd of high mental health burden. In addition, LGBTQ+ people with intersecting marginalized identities along axes of race/ethnicity, age, income, and disability have higher odds of high mental health burden after disaster displacement. This study is significant, because it is one of the first studies, if not the first, on mental health disparities among the displaced LGBTQ+ population and demonstrates significant health disparities in the US. This research indicates a need for policy change and increased resources for LGBTQ+ people, especially transgender people, and those with intersecting marginalized identities.

P29-27 | Contrasting patterns of temperature-related mortality and morbidity in Santiago, Chile, from 2011 to 2019.

Rodrigo Puentes[1,2], Luis Cifuentes[3], Sandra Cortés[4]

[1] Emory University, Gangarosa Department of Environmental Health, Rollins School of Public Health, Atlanta, USA; [2] Pontificia Universidad Católica de Chile, Escuela de Salud Pública, Programa de Epidemiología, Santiago, Chile; [3] Pontificia Universidad Católica de Chile, Facultad de Ingeniería y Centro de Cambio Global UC, Santiago, Chile; [4] Pontificia Universidad Católica de Chile, Escuela de Salud Pública, Advanced Center for Chronic Diseases (ACCDiS), Santiago, Chile

Objective: To conduct a comparative analysis of the associations between daily ambient temperature and mortality, ED visits, and hospital admissions by cardiovascular and respiratory diseases for the city of Santiago (32°55'S-34°19'S latitude and 69°47'W-71°43'W longitude) between 2011 and 2019.
Material and Methods: We fitted a time-series quasi-Poisson regression model controlling for time-varying confounders and estimated the temperature-mortality and temperature-morbidity associations considering a distributed lag non-linear framework. We evaluated non-optimal temperature effects at percentiles commonly used in literature. Associations were assessed by causes (all diseases, cardiovascular, and respiratory).
Results: We analyzed 296,136 deaths, 10,141,193 ED visits, and 4,493,183 hospital admissions in the study period. Temperature patterns showed contrasting effects on mortality and morbidity for different health endpoints. All-cause cold-related risk at 1st, 2.5th, and 5th temperature percentiles was highest for mortality with a relative risk (RR) of 1.30 (95% CI: 1.20-1.42), 1.23 (95% CI: 1.14-1.32), and 1.17 (95% CI: 1.10-1.26), respectively, compared to morbidity outcomes. Heat-related risk at 95th and 97.5th temperature percentiles was highest for ED visits with an RR of 1.13 (95% CI: 1.08-1.20) and 1.14 (95% CI: 1.08-1.20), respectively, but surpassed by mortality at the 99th percentile. For cardiovascular diseases, acute effects of heat were similar for mortality and ED visits. Mortality risk from respiratory diseases due to non-optimal cold and heat was higher than the risk of ED visits and hospital admissions.**Conclusion:** Cold temperatures had a greater effect on all-cause mortality than morbidity, while extreme heat increased ED visits until the 99th percentile, where mortality risk became higher. Heat-related cardiovascular morbidity should be monitored closely, as these results may suggest an acute burden on the healthcare system. Adaptation measures aimed at reducing temperature-related mortality trends should also evaluate health system utilization by addressing population susceptibility to temperature extremes.

P29-28 | Impact of weather on dementia-related hospital and ambulatory visits among older adults in Alberta, Canada: An ecological study

Sachit Gurung[1], C. Allyson Jones[1], Joey Syer[1], Sammy Lowe[1], Shelby Yamamoto[2,1]

[1] University of Alberta, School of Public health, Edmonton, Canada; [2] University at Buffalo, School of Public Health and Health Professions, Buffalo, USA

Introduction

The frequency and intensity of extreme events such as heatwaves, wildfires, are increasing with climate change. Older adults living with dementia are particularly vulnerable to climate change due to cognitive impairments, comorbidities, mobility issues, and weakened immune systems.

Objectives

To conduct an ecological study to investigate the effects of weather on dementia-related visits among older adults ≥65 years in Alberta, Canada.

Methods

A sample of counts of monthly hospital and ambulatory visits by urban dissemination areas in Alberta related to dementia between January 1, 2011, to December 31, 2021, from the Discharge Abstract Database and National Ambulatory Care Reporting System were analysed. Environmental exposures, including temperature, precipitation, and fine particulate matter ($PM_{2.5}$) were obtained from the Canadian Urban Environmental Health Research Consortium database, linked to counts of health outcomes, and analysed using negative binominal regression. Analyses were stratified by season.

Results

During the winter season, precipitation (prevalence risk ratio (PRR) = 1.061, C.I =1.004-1.122) and $PM_{2.5}$ (PRR= 1.017, C.I = 1.007-1.028) were associated with increased risks of dementia-related healthcare visits among older adults at the dissemination area level. An increase in the diurnal temperatures range was associated with decrease in dementia visit among older adults at the dissemination area level (PRR = 0.9706, C.I = 0.954-0.987) and (PRR= 0.986, C.I = 0.973-0.999) respectively during both the winter and summer. However, no significant associations were observed with minimum, average and maximum temperatures.

Significance of the research

Higher levels of $PM_{2.5}$ and precipitation in the winter were associated with an increase in dementia-related visits among older adults at the dissemination area level. Winter exposures may be a vulnerable time for older adults with dementia. Further exploration of weather and air pollution exposures focusing on older adults with dementia are still needed.

P29-29 | Building Climate-Informed Forecasting Capacity to Predict Near- and Long-Term Coccidioidomycosis Incidence in California

Simon Camponuri[1], Alexandra Heaney[2], Gail Sondermeyer-Cooksey[3], Duc Vugia[3], Seema Jain[4], Daniel Swain[5,6], John Balmes[8], Justin Remais[1], Jennifer Head[9,10]

[1] University of California, Berkeley, Division of Environmental Health Sciences, School of Public Health, Berkeley, USA; [2] University of California, San Diego, Herbert Wertheim School of Public Health and Human Longevity, San Diego, USA; [3] California Department of Public Health, Infectious Diseases Branch, Richmond, USA; [4] San Francisco Department of Public Health, Population Health Division, San Francisco, USA; [5] University of California Agriculture and Natural Resources, California Institute for Water Resources, Davis, USA; [6] University of California, Los Angeles, Institute of the Environment and Sustainability, Los Angeles, USA; [7] NSF National Center for Atmospheric Research, Capacity Center for Climate and Weather Extremes, Boulder, USA; [8] University of California, San Francisco, Department of Medicine, San Francisco, USA; [9] University of Michigan, Department of Epidemiology, School of Public Health, Ann Arbor, USA; [10] University of Michigan, Institute of Global Change Biology, Ann Arbor, USA

Coccidioidomycosis, or Valley fever, is an emerging infectious disease in the southwestern United States caused by inhalation of airborne *Coccidioides* spp. fungi released from soils via wind erosion or anthropogenic disturbance. Incidence in California is highly sensitive to climate variability, increasing nearly two-fold following wet winters that occur one and two years after drought. The most recent California drought (2020-2022) was followed by heavy precipitation during the 2022-2023 winter, prompting California Department of Public Health (CDPH) to issue public health alerts warning of potential increases in coccidioidomycosis cases in fall 2023. Indeed, the state saw a near record number of cases in 2023 (9,054 cases reported). Continued above average precipitation during the 2023-2024 California wet season yielded concerns about continued high coccidioidomycosis risk and motivated our development of a prediction modeling framework for coccidioidomycosis incidence in the state. We developed an ensemble prediction model consisting of five candidate prediction algorithms relating monthly reported cases per census tract in California to climatological, environmental, and other predictors. The model predicted a total of 11,846 cases (90% PI: 11,224–12,456) in California between April 1, 2023, and March 31, 2024, closely aligning with the preliminary state report of 10,519. For the current transmission year (April 1, 2024 – March 31, 2025), the model predicts 12,244 cases (90% PI: 11,638–12,917) statewide— a 62% increase over the cases reported during the same period two years prior, and on par with the high incidence seen in 2023. The forecasts predict pronounced seasonality in incidence, with cases peaking in November at 1,411 (90% PI: 1,267–1,587) statewide. We report on how climate-informed prediction of coccidioidomycosis incidence is supporting effective prevention efforts in California, and leverage the same climate sensitivities for long-term climate projections, finding that rising temperatures and increasingly volatile precipitation patterns may further impact coccidioidomycosis transmission in California.

P29-30 | Tracking Coccidioidomycosis in Arizona: Regional Trends and Seasonal Cycles of a Climate Sensitive Disease

Wenxin Chen[1], Sophia E. Kruger[2], Jennifer R. Head[2], Alexandra K. Heaney[1]

[1] University of California San Diego, San Diego, USA; [2] University of Michigan, Ann Arbor, USA

Objective: Coccidioidomycosis, a fungal disease caused by inhalation of spores of *Coccidioides* species, has increased across the southwestern US in association with increasing aridity, warming temperatures, and precipitation volatility. In Arizona, changes in incidence within distinct climatological regions remain to be characterized. Here, we investigate regional trends in and seasonal patterns of coccidioidomycosis incidence over time.
Methods: Using surveillance data reported to Arizona Department of Health Services since 2005, we calculated incidence rate ratios (IRRs) to describe increases in incidence within six ecoregions. To identify region-specific periodicity in incidence, we performed wavelet analyses on regional monthly incidence rates normalized in each year.
Results: Between 2005 and 2022, the annual incidence rate (IR) more than doubled in Arizona, with the greatest median annual IR in the southern Sonoran Desert ecoregion (112.13 per 100,000) and the lowest in the northern Plateaus ecoregion (19.91). From 2005-2007 to 2020-2022, the two northernmost regions – the Plateaus and the Mojave Desert – reported the highest relative increases in incidence rate, with the IR in 2020-2022 6.6 (95% CI: 4.0, 10.9) and 4.5 (95% CI: 3.3, 6.1) times that in 2005-2007, respectively. All other regions reported IRRs between 1.44-3.32. IRs also increased between 2011-2013 to 2020-2022 within all regions, with the greatest IRR observed in the Plateaus region (IRR: 1.81, 95% CI: 1.33, 2.45). Wavelet analyses revealed consistent annual and intermittent semi-annual cycles within the Sonoran Desert region, with bi-annual periodicity occurring in years with wetter Monsoon seasons.
Conclusion: Overall, while the periodicity of coccidioidomycosis incidence is most pronounced within the highly endemic Sonoran Desert region, incidence is increasing most rapidly within northern regions, including those considered less suitable (i.e., cooler, wetter) for *Coccidioides* species, necessitating targeted public health messaging in these regions.

P29-31 | Suicide and Homicide Mortality and Ambient Temperature in Brazil and Mexico: Two Nationwide Time-Stratified Case-Crossover Analyses

Zhou Yang, Hao Neng Huang, Tao Pan, Chun Quan Ou

Southern Medical University, State Key Laboratory of Organ Failure Research, Department of Biostatistics, Guangdong Provincial Key Laboratory of Tropical Disease Research, School of Public Health, Guangzhou, China

Objective: Deaths from intentional injuries, especially those resulting from interpersonal violence, constitute a major public health crisis in two tropical countries, Brazil and Mexico. Understanding the exposure-lag-response relationship between ambient temperatures and suicide and homicide may assist in identifying preventive approaches.
Material and Methods: By integrating 1,243,596 suicide and homicide records extracted from two national mortality information systems and daily weather data from ERA5 between 2000 and 2019, the relationship between short-term

exposure to increased temperatures and the risk of suicide and homicide mortality was investigated using a time-stratified case-crossover design. Odds ratios (ORs) and 95% confidence intervals (CIs) of temperature-associated mortality were estimated using the conditional logistic regression model combined with the distributed lag nonlinear model. Province-specific associations were estimated first, and then a random-effect meta-analysis was employed to obtain nation-level pooled estimates. The national fractions of intentional injury deaths attributable to temperatures were calculated.

Results: The risk of both suicide and homicide mortality rose almost monotonically with increasing temperatures. Pooled estimates suggested each 1°C increase in ambient mean temperature was associated with a 2.2% (95% CI:1.7–2.6%) and 1.8% (95% CI:1.3–2.3%) increase in homicide over lag 0–5 days in Brazil and Mexico, and the estimates for suicide were 2.8% (95% CI: 2.2–3.4%) and 3.8% (95% CI: 3.0–4.5%), respectively. The association seemed stronger in males, the mixed race, during the weekend, and summer–autumn. Throughout the study period, 12.56% of suicide deaths in Brazil and 22.55% in Mexico, along with 9.74% of homicide deaths in Brazil and 14.38% in Mexico, could be attributed to temperatures exceeding province-specific medians.

Conclusion: There are consistent positive associations between suicide/homicide and elevated ambient temperatures in Brazil and Mexico. Our findings emphasize the significance of heat adaptation strategies almost throughout the year to mitigate self-harm and interpersonal violence.

P29-32 | Climate Change Impacts on the Mental Well-being of Refugees: A Qualitative Analysis

Raghad Aljenabi[1], Nathan Gonzalez[2], Wael Al-Delaimy[1]

[1] University of California San Diego, Herbert Wertheim School of Public Health and Human Longevity Science, San Diego, USA; [2] University of California San Diego, 2. Center for Research and Evaluation, Division of Extended Studies, San Diego, USA

Introduction: Displaced populations are rising at alarming rates due to the climate crisis. This is a pressing issue that will only worsen as the climate crisis progresses. The Middle Eastern North African region is at the frontline of the consequences of climate change on mental health. The aim of this study is to explore the perspectives of resettled Syrian refugees about climate change and its impacts on their mental health. Method: Syrian refugees that have resettled in the U.S. less than two years ago were recruited in San Diego. They participated in semi-structured qualitative focus groups to address their beliefs, attitudes, and perceptions about climate change and their mental well-being. Interviews were translated and transcribed from Arabic to English. Themes were coded using ATLAS.ti software. Results: There were 12 total Syrian refugee participants in two semi-structured qualitative focus groups. Focus groups revealed perspectives on extreme weather and climate change that were attributed mostly to God. Participants reported severe weather conditions in their transition countries exacerbated stress and impacted their mental well-being. Pre-existing health conditions, income, unemployment, and discrimination were also made worse by these weather events during their transition compared to their native Syria. Participants reported that their mental and physical well-being is impacted by severe climate change. More notably, participants' recollection of their refugee experiences highlights the complex determinants of mental health within this population. These findings suggest the need for ongoing mixed method analysis to develop culturally adequate mental health interventions specific to the experiences and beliefs common to refugees who are impacted by climate change events.

P29-33 | The Effects of Atmospheric Temperature on Outpatient Admissions for Hand - Foot - and - Mouth Disease: A Time - Series Study in Beijing, China

Xuancheng Hu, Enmin Ding, Weizhong Yang

Peking Union Medical College, School of Population Medicine and Public Health, Beijing, China

Background: Currently, the evidence regarding the impact of atmospheric temperature on the risk of hand - foot - and - mouth disease (HFMD) remains limited. This study was designed to explore the influence of atmospheric temperature on HFMD outpatient admissions.

Methods: Daily HFMD case data was retrieved from the hospital admission registration system in Beijing, China (July 2015-December 2019). Daily meteorological data, including temperature and relative humidity, were sourced from the National Meteorological Information Center. Machine learning-based imputation algorithms preprocessed the data. At county level, a distributed lag non-linear model (DLNM) evaluated the lag-exposure-response relationship between daily mean temperature and HFMD outpatient admissions. Pooled effect estimates were derived from multivariate meta-regression model analysis. The model controlled for humidity, long-term and seasonal trends, day of week, and public holidays as covariates.

Results: From July 2015 to December 2019 in Beijing, a total of 128,137 HFMD cases were recorded. The results indicated positive non - linear relationships, presenting as J - shaped curves, between ambient temperature and HFMD. The maximum cumulative relative risk (RR) reached 3.47 (95% confidence interval (CI): 1.76 - 6.8), peaking at 31.9 °C with a 14 - day lag. Moreover, extreme high temperature (the 90th percentile of temperature) had significant effects at a lag of 0 - 1 days. Specifically, for every 1 °C increase in ambient temperature, the risk of HFMD increased by 1 - 4% (RR = 1.01 - 1.04).

Conclusions: This study demonstrates that high temperature is associated with an elevated burden of HFMD. In the context of future global warming, these findings highlight the significance of strengthening resilience to climate change as a preventive measure against HFMD.

P29-34 | Emerging Risk of Japanese Encephalitis Virus in Australia: Climate Drivers and Public Health Implications

Caroline K. Dowsett[1], Xuxia Wang[2], Wenbiao Hu[1]

[1] Queensland University of Technology, Department of Health, School of Public Health & Social Work, Brisbane, Australia; [2] Gansu Centre for Disease Control and Prevention, Gansu, China

OBJECTIVE

Japanese Encephalitis Virus (JEV) has emerged as a growing public health threat in Australia. The 2022–2023 outbreak of Japanese Encephalitis (JE) in Australia, followed by a re-emergence in 2024-2025, marked a significant shift in the JE epidemiology, with cases now occurring in temperate regions previously considered low risk. Once thought to be confined to tropical regions, the virus's expansion highlights an urgent need to investigate the environmental and climatic factors driving its transmission, particularly in the context of climate change. This study aims to assess JEV transmission risk across Australia using a One Health approach.

MATERIAL & METHODS
The study integrated human case data from the Australian National Notifiable Disease Surveillance System Dashboard with social factors, meteorological data, and agricultural determinants to enhance understanding of JEV dynamics in Australia. Geographic Information System and spatial analysis were used to identify the potential risk factors and estimate future outbreaks of JEV.
RESULTS
During the 2021-2022 outbreak, Australia reported 45 JE cases, primarily in New South Wales and Victoria, with peak transmission occurring between January and February 2022. After no reported cases in 2023, JEV re-emerged in late 2024, in six human cases and one fatality in the 2024–2025 season. Transmission was identified in 31 unique Local Government Areas (LGAs), all situated in regional Australia. Most affected LGAs experienced significantly above average rainfall, particularly during La Nina-driven extreme flooding events, creating favourable conditions for both mosquito vectors and reservoir hosts.
CONCLUSION
The spread of JEV into temperate regions underscores how climate change, rising temperature, altered rainfall patterns, extreme weather events, and expanded mosquito habitats are reshaping the epidemiology of JEV. As climate-driven environmental shifts become more frequent, understanding these factors is crucial for improving surveillance, refining predictive models, and guiding targeted public health interventions to mitigate future JEV risks.

P29-35 | Study on AI prediction model of severe fever with thrombocytopenia syndrome based on climate and natural environment factors--2009-2023,China

Jinzhao Cui, Jie Qian, Ting Zhang

Chinese Academy of Medical Sciences & Peking Union Medical College, School of Population Medicine and Public Health, Beijing, China

[Background] The case fatality rate of Severe Fever with Thrombocytopenia Syndrome (SFTS) has remained high. SFTS is mainly prevalent in five countries in Asia. The transmission dynamics of the virus are closely coupled with the ecology of tick vectors and are significantly regulated by environmental factors.
[Methods] Chinese SFTS cases from 2009 to 2023 as the research object. Through multiple rounds of feature selection , core predictive factors such as temperature, precipitation, and vegetation index were identified from the 14 environmental and social factors. Spatiotemporal aggregation techniques were employed to capture the spatiotemporal heterogeneity of SFTS transmission. The multiplicative seasonal model of the Holt-Winters exponential smoothing algorithm was used to capture the monthly fluctuation patterns, while XGBoost ensemble learning was employed to analyze the nonlinear interactions of environmental factors.Stacking architecture was used to input the predictions of the two models as meta-features into a second-layer XGBoost. Dynamic learning rate optimization was applied to weight allocation to complete the prediction.
[Results] The distribution of SFTS cases showed high aggregation, highly overlapping with the active areas of the tick Haemaphysalis longicornis. The monthly fluctuations were strongly positively correlated with temperature and NDVI, with a significant increase in incidence during the rainy season compared to the dry season, consistent with the ecological characteristics of tick vectors. After the COVID-19 pandemic, the spatiotemporal spread of SFTS accelerated.

[Conclusion] This study shown that if error is set to be less than or equal to 5 cases, the accuracy of the model reaches 90%, with an accuracy improvement of over 15% compared to a single model. COVID-19 pandemic may have accelerated the spread of SFTS. This alerts that other regions globally with similar natural environments suitable for the survival of Haemaphysalis longicornis are at high risk of transmission, especially those along the migratory routes of birds.

P29-36 | Age- and Cause-Specific Minimum Mortality Temperatures: A Multi-Country Observational Study (1990–2019)

Seunghyun Eom[1], Masahiro Hashizume[2], Aurelio Tobias[3], Yeonseung Chung[4], on behalf of the Multi-Country Multi-City (MCC) Collaborative Research Network

[1] Korea Advanced Institute of Science and Technology, Graduate School of Data Science, Daejeon, South Korea; [2] University of Tokyo, Department of Global Environmental Health, Tokyo, Japan; [3] Institute of Environmental Assessment and Water Research, Spanish Council for Scientific Research, Barcelona, Spain; [4] Korea Advanced Institute of Science and Technology, Department of Mathematical Sciences, Daejeon, South Korea

Background
The minimum mortality temperature (MMT) serves as a key indicator of how populations adapt to their local climates. Although MMT may vary across different age groups and causes of death, no study to date has systematically compared these differences.
Methods
Daily time-series data were obtained on mean temperature and mortality from 508 communities in 35 countries. Mortality was categorized by age group (elderly vs. nonelderly) and by cause (cardiovascular, respiratory, and non-cardiorespiratory). We used a two-stage meta-analytic approach to estimate MMT. In the first stage, Quasi-poisson regression was applied to estimate the temperature-mortality association curve for each age group and cause of death in each community. In the second stage, mixed-effects meta-regressions were used to pool these associations across communities for the entire study population as well as by climate zone, geographical region, and country. The MMT was then derived from the pooled association curves.
Results
For the overall population, MMT was highest for cardiovascular causes (23.8°C, 95% CI: 23.4–24.2°C), followed by non-cardiorespiratory (23.3°C, 95% CI: 22.3–23.6°C) and respiratory causes (23.1°C, 95% CI: 21.4–23.6°C). Similar trends were observed in the elderly group (22.7°C, 22.3–23.1°C; 22.3°C, 21.7–22.7°C; 22.1°C, 21.0–22.5°C). In the non-elderly group, MMT was highest for cardiovascular causes (23.1°C, 95% CI: 22.1–23.5°C), followed by respiratory (21.0°C, 95% CI: 17.9–30.1°C) with large uncertainty, and non-cardiorespiratory causes (19.8°C, 95% CI: 17.7–22.1°C). These patterns remained consistent across climate zones, regions, and countries.
ConclusionMMT varies by cause of death and age group, indicating that temperature adaptation differs across populations. These findings underscore the need for age- and cause-specific public health strategies to mitigate temperature-related mortality.

P29-37 | Association between ambient temperature and anovulation: a comparative analysis of the BioCycle and EAGeR studies

Kaniz Rabeya[1], Pauline Mendola[2], Lindsey M. Russo[3], Timothy P. Canty[4], Karen C. Schliep[5], Nidhi Manchikanti[1], Carrie J. Nobles[1]

[1] University of Massachusetts Amherst, Department of Environmental Health Sciences, School of Public Health & Health Sciences, Amherst, USA; [2] University at Buffalo, Department of Epidemiology and Environmental Health, School of Public Health and Health Professions, Buffalo, USA; [3] Weill Cornell Medicine, Division of Internal Medicine, New York, USA; [4] University of Maryland, Department of Atmospheric & Oceanic Science, College Park, USA; [5] University of Utah, Department of Family and Preventative Medicine, Salt Lake City, USA; [6] University of Massachusetts Amherst, Department of Environmental Health Sciences, School of Public Health & Health Sciences, Amherst, USA; [7] University of Massachusetts Amherst, Department of Environmental Health Sciences, School of Public Health & Health Sciences, Amherst, USA

Objectives: Rising temperatures due to climate change have been linked to adverse reproductive health outcomes, yet evidence of their impact on ovulatory function remains unclear. This study examines the association between ambient temperature, anovulation, and hormones around ovulation in two distinct cohorts, the BioCycle Study and EAGeR trial.

Material and Methods: BioCycle (2005–2007) enrolled 250 regularly menstruating women (18–44 years) from Buffalo, NY, while EAGeR (2006–2012) included 1,228 women attempting pregnancy (18–40 years) from Salt Lake City, Scranton, Denver, and Buffalo. Daily temperature was averaged across early-to-mid follicular (1-10 days after menses) and ovulation (11–16 days) phases. Anovulation was determined using absence of a luteinizing hormone (LH) peak and low luteal-phase progesterone in both studies. Log-binomial models estimated anovulation risk comparing high versus low temperature (≥22°C vs. <22°C in BioCycle; ≥25°C vs. <25°C in EAGeR) and using natural cubic splines to assess threshold and non-linear associations, adjusting for age, season, and study site.

Results: In BioCycle, higher anovulation was observed with higher temperature (≥22°C) in the early-to-mid follicular (RR = 1.66, 95% CI: 0.46, 5.34) and ovulation (RR = 1.06, 95% CI: 0.24, 3.49) phases, although estimates were imprecise. Similarly in EAGeR, higher anovulation was observed with higher temperature (≥25°C) in the early-to-mid follicular (RR = 1.45, 95% CI: 0.83, 2.37) and ovulation (RR = 1.26, 95% CI: 0.67, 2.13) phases. Spline models indicated a greater relative risk of anovulation above 25°C. Higher temperature was also associated with lower estradiol in Biocycle and lower follicle-stimulating hormone and LH in EAGeR leading up to ovulation, which correlates with increased risk of anovulation.

Conclusion: Findings suggest a potential association between higher ambient temperatures and anovulation, with impacts on hormonal patterns further supporting a relationship between temperature and ovulatory function.

P29-38 | Environmental Temperature and Their Effects on Child Growth and Mortality in South Asia

Vinod Joseph Kannankeril Joseph[1,2]

[1] Max Planck Institute for Demographic Research, Digital and Computational Demography, Rostock, Germany; [2] University of Bologna, Department of Statistics, Bologna, Italy

Temperature extremes are projected to become more frequent and intense as climate change continues and will be a tremendous public health concern in the coming years.
Objectives:
To assess whether exposure to high temperatures in South Asia is associated with child growth faltering.
To examine the association between environmental temperature and child survival in South Asia.
Methods:
This study uses cross-sectional data from the most recent DHS surveys from five south Asian countries. Gridded synoptic mean monthly daytime land surface temperature is being used as the indicator of environmental temperature in the study.
Multilevel regression models are used to determine the odds of stunting, wasting and being underweight relative to land surface temperature. The risk of under-five death is estimated using cox regression models, Kaplan-Meier survival plots, and log-rank test (Mantel test).
Key Outcomes:
Identifying environmental hazards prevalent in various settings for children's lives is essential for setting-based interventions.

P29-39 | Climate-Specific Patterns of Asthma Hospitalisations in Queensland: Seasonal and Environmental Impacts Across Diverse Climate Zones

Jialu Wang[1], Javier Cortes-Ramirez[1], Janet Davies[2], Wenbiao Hu[1]

[1] Queensland University of Technology, School of Public Health and Social Work, Brisbane, Australia; [2] Queensland University of Technology, School of Biomedical Sciences, Brisbane, Australia

This study examined patterns of asthma hospitalisations among children and adolescents (0–19 years) across five Queensland cities, each representing distinct climate zones. It assessed demographic characteristics, seasonal variations, and climate-specific effects of temperature, humidity, and $PM_{2.5}$ on asthma risk.

Asthma hospitalisation data (2016–2019) were analysed at the SA2 level on a weekly basis across Brisbane (Cfa – humid subtropical), Townsville (Aw – tropical savanna), Cairns (Am/Af – tropical monsoon/equatorial rainforest), Mackay (Cwa – subtropical highland), and Mount Isa (BSh – hot semi-arid). Environmental data were sourced from NASA and the Copernicus Climate Data Store. Age-standardised prevalence rates (ASPR) were calculated using the Australian Standard Population. Long-term trends were examined using time-series regression models, while multivariable distributed lag nonlinear models (DLNM) assessed delayed environmental effects (up to four weeks).

Seasonal variations were explored via time-series decomposition and heatmaps.

A total of 4,059 asthma hospitalisations were recorded across five regions from 2016 to 2019. Asthma hospitalisations were higher in Cfa and Am/Af, with 5–9-year-olds most affected in Cfa (25.24%) and 0–4-year-olds in BSh (25.37%). Adolescents (15–19 years) had the lowest rates, particularly in BSh (1.49%). Hospitalisations peaked during winter (June–August) and autumn (March–May), with the highest seasonal peak observed in BSh (35.29%). A higher increased asthma risk found in BSh (>25°C), while Cwa showed a U-shaped risk pattern. $PM_{2.5}$ increased risk in Cwa (<2 μg/m³) and Aw (<4 μg/m³). Humidity was associated with increased asthma risk in Am/Af, Cwa, and BSh at extreme levels. Lag effects varied, with immediate or delayed effects depending on the climate zone.

The impact of environmental factors on asthma risk differs across climate zones, highlighting the importance of tailored intervention strategies. Future research will incorporate spatiotemporal modelling to develop a climate-driven early warning system for asthma, enabling proactive public health responses and improved management strategies.

P29-40 | Mortality Dynamics of West Nile and Lyme Diseases in the US: Temporal, Spatial, and Socioeconomic Dimensions (2003–2021)

Raheleh Mohammadi[1], Yeongjin Gwon[4,6], Kelli Gribben[1], Babak JFard[1], Kristina Kintziger[1], Jocelyn Herstein[1], Jesse Bell[1,2]

[1] University Nebraska Medical Center, Department of Environmental, Agricultural, and Occupational Health; College of Public Health, Omaha, USA; [2] University Nebraska Medical Center, Executive Director of the Water, Climate and Health Program; College of Public Health, Omaha, USA; [3] University Nebraska Medical Center, Claire M. Hubbard Professor of Water, Climate and Health; College of Public Health, Omaha, USA; [4] University Nebraska Medical Center, Department of Biostatistics; College of Public Health, Omaha, USA; [5] Rollins College, Health Professions Department; Master of Public Health Program, Winter Park, USA; [6] University Nebraska Medical Center, Director of Biostatistics in Water, Climate, and Health Program (WCHP), Omaha, USA

Objective: Vector-borne diseases, particularly West Nile Virus (WNV) and Lyme disease, pose a growing public health threat in the United States (U.S.). This study aims to analyze the spatial and temporal mortality trends of WNV and Lyme disease in the U.S. and assess their associations with health insurance status and urban versus rural residency. **Methods**: We utilized National Center for Health Statistics (NCHS) data to identify the WNV and Lyme-related deaths (2003-2021) including demographics and the decedent's county. County-level insurance status was sourced from the Census Bureau, and urbanicity was defined using the NCHS 2013 classification. We applied negative binomial regression models to identify associations of demographics, insurance, and urbanicity with deaths. Spatial visualization was conducted in ArcGIS Pro and analyses in SAS 9.4. **Result**: Our analysis of 3,694 deaths (WNV: 2,628 [71.2%]; Lyme: 1,066 [28.8%]) revealed WNV peaks in 2003 (246 deaths, 9.4%) and 2012 (281 deaths, 10.7%), and a Lyme peak in 2021 (103 deaths, 9.7%). Overall, deaths were highest in 2012 (343, 9.3%) and lowest in 2009 (105, 2.6%). Age ≥65 (RR=1.45, p<0.0001), male sex (RR=1.16, p=0.007), and White race (RR=1.73, p<0.0001) increased mortality, while rural residency (RR=0.07, p<0.0001) decreased it; insurance status was not significant (RR=1.008, p=0.488) in predicting the mortality. Spatial analysis indicated high WNV mortality in California, Colorado, Arizona, and Texas, with midwestern (along the Mississippi River) hotspots. In contrast, Lyme mortality hotspots were concentrated in the northeastern U.S. (New York, New Jersey, Pennsylvania) and the upper Midwest (Minnesota and Wisconsin). **Conclusion:** Our results suggest that age, sex, race, and urbanicity, are important

factors influencing mortality from WNV and Lyme disease. More research is needed to link exposures and incidence to mortality of WNV and Lyme to better understand the drivers of regional variation, given the spatial and temporal variability.

P29-41 | Assessing tropical cyclone exposure definitions and diarrheal disease risk: A study of two urban contexts Tokyo and Taipei

Szu Yu Lin[1], Paul L.C. Chua[1], Tarik Benmarhnia[2,3], Masahiro Hashizume[1]

[1] The University of Tokyo, Department of Global Health Policy, Tokyo, Japan; [2] UC San Diego, Scripps Institution of Oceanography, San Diego, USA; [3] University of Rennes, EHESP, Irset Institut de Recherche en Santé, Environnement et Travail, Inserm, Rennes, France

Background: Climate change is intensifying tropical cyclones (TC), leading to stronger winds, heavier rainfall, and increasing threats to public health. Among these risks, the heightened incidence of climate-sensitive diarrheal diseases is of particular concern. However, existing research lacks consistency in defining TC exposure—some studies focus on wind speed, others on rainfall, and some use a combination—making cross-study comparisons challenging.

Objective: This study aims to evaluate multiple TC exposure metrics in two distinct urban contexts—Tokyo and Taipei—to provide insights for future research and improve the selection of TC definitions.

Methods: We first assessed the relationship between three TC wind intensity categories and diarrheal diseases, following prior literature. We then examined associations using absolute wind speed and rainfall separately. Based on the findings, we refined our categorical approach for wind intensity and tested different combinations of wind and rainfall to determine how exposure definitions influence diarrheal disease outcomes.

Findings: Our results highlight the importance of incorporating multiple TC-related metrics when defining exposure to better capture the impact of TC on diarrheal disease. These insights can help guide future epidemiological research and policy decisions in climate-sensitive health studies.

P29-42 | Exposure to tropical cyclones in 643 Latin American urban municipalities from 2002-2020.

Ana Peralta-Garcia[1], Jinwen Xu[2], Josiah L. Kephart[3,4], Waleska T. Caiaffa[5], Monica Mazariegos[6], Alex Quistberg[3,4], Yenisei Ramirez Toscano[7], Juan P. Rodriguez[8], Brisa Sanchez[1], Olga L. Sarmientos[9], Ana Diez Roux[1,4], Usama Bilal[1,4], Mariana Lazo[10,4]

[1] Drexel Dornsife School of Public Health, Department of Epidemiology and Biostatistics, Philadelphia, USA; [2] University of California at Berkeley, Institute of Transportation Studies, Berkeley, USA; [3] Drexel Dornsife School of Public Health, Department of Environmental and Occupational Health, Philadelphia, USA; [4] Drexel Dornsife School of Public Health, Urban Health Collaborative, Philadelphia, USA; [5] Universidade Federal de Minas Gerais, Observatório de Saúde Urbana de Belo Horizonte, Belo Horizonte, Brazil; [6] Instituto de Nutrición de Centroamérica y Panamá, Centro de Investigación del INCAP para la Prevención de las Enfermedades Crónicas, Guatemala City, Guatemala; [7] Universidad Autónoma de Nuevo León, Departmento de Medicina Preventiva y Salud Pública, Monterrey, Mexico; [8] Universidad de los Andes, Department of Civil and Environmental Engineering, Bogotá, Colombia; [9] Universidad de los Andes, School of Medicine, Bogotá, Colombia; [10] Drexel Dornsife School of Public Health, Department of Community Health and Prevention, Philadelphia, USA

Objective: To characterize and compare the exposure to tropical cyclones (TC) in urban municipalities across 6 Latin American countries over time (2002-2020).

Material and methods: We used population estimates from the Salud Urbana en América Latina (SALURBAL) project and TC data from the International Best Track Archive for Climate Stewardship (IBTrACS). The sample included 137 cities and 643 urban municipalities (or equivalent) across Mexico (MX), Guatemala (GT), El Salvador (SV), Costa Rica (CR), Panama (PA), and Colombia (CO). Yearly exposure was defined using (1) distance-based metrics (exposed if boundaries within ≤100km. or ≤250km. of a storm's central track), and (2) wind-based metrics (exposed if boundaries overlapped with TC wind fields at ≥34 [all TCs] , ≥50, or ≥64 knots [hurricanes]).

Results: Between 2002-2020, 370 urban municipalities (57.5%) were within 100 km. of storm tracks and 197 (30.6%) experienced at least one TC with winds ≥34-knots. Using the 100km. distance metric, exposed populations increased over time in CR, GT, MX, and SV. These changes ranged from 11.2 to 13.7 million people in MX and 44,526 to 552,097 people in SV (1.0% and 20.2% point increase, respectively) between 2002-2011 and 2012-2020. For the 34-knot wind metric, SV experienced the highest increase in exposed population from 22,374 people (1.01%) to 243,500 (9.8%); similarly, MX's exposed population increased from 6.6 million (9.39%) to 7.9 million (9.79%), while GT's exposure decreased by 8.7% points (268,879 fewer people exposed). MX was uniquely exposed to hurricane-strength winds, with the affected population decreasing from 2.74 (3.86%) to 2.67 million (3.27%) between 2002-2011 and 2012-2020.

Conclusion: Our analysis reveals important variations in TC exposure across urban municipalities in Latin America from 2002-2020, highlighting the importance of describing differential exposure across and within countries at the municipality level.

Funded by the Wellcome Trust and the National Institutes of Health.

P29-43 | Climate Disasters and Human Vulnerability in the US Caribbean: A 30-Year Trend Analysis

Ignacio Reinosa Mateo[2], Ana P. Ortíz[1], Pablo Méndez[3], Noreen Michael[4], Polaris Torres[1], Lenulisy Rosado Estrada[1], Marievellisse Soto Salgado[1], Nancy R. Cardona[1], This work was supported by the National Cancer Institute (Grant #1P20CA294096-01) and the American Heart Association. This work represents only the views of the authors and does not represent the official view of the National Cancer Institute or the American Heart Association.

[1] University of Puerto Rico Comprehensive Cancer Center, Division of Cancer Control and Population Sciences, San Juan, Puerto Rico; [2] University of Puerto Rico, Rio Piedras Campus, Department of Natural Sciences, San Juan, Puerto Rico; [3] University of Puerto Rico, Medical Sciences Campus, Department of Environmental Health, San Juan, Puerto Rico; [4] University of the Virgin Islands, Charlotte Amalie West, U.S. Virgin Islands

During the past three decades, Puerto Rico (PR) and the US Virgin Islands (USVI) have suffered a disproportionate burden from climate disasters. Hurricanes, floods, and other climate events, exacerbated by socioeconomic inequities, have caused economic losses and fatalities. This study integrates NOAA's Weather and Climate Disasters database and the Fifth National Climate Assessment (NCA5) to evaluate impacts and vulnerabilities.
We used NOAA's database from 1995 to 2024 for PR and USVI, capturing inflation adjusted losses from severe weather, adhering to standards and incorporating more than a dozen public and private sources (e.g., FEMA, National Weather Service, utility companies). Trend analyses were performed using STATA and the FNCA was consulted for additional context.
From 1995 to 2024, events with at least one death increased: 35 from 1995–2004, 32 from 2005-2014, and 112 from 2015–2024. Following an upward trend, climate events rose sharply in 2005 (in part due to Hurricane Katrina) and peaked in 2024 with 763 incidents—11.91% of all events recorded since 1995. Floods, thunderstorm winds, and rip currents stand out among hazards. Hurricanes Irma and Maria (2017) resulted in more than $218 billion in losses and 3,190 fatalities. Recent storms like Fiona (2022) underscore persistent vulnerability. Disrupted infrastructure and reduced food imports, worsening food insecurity, while healthcare failures threatened patients who needed ongoing treatment for chronic conditions. Non-hurricane added losses of events were underestimated by 10–15%.
Communities with limited disaster preparedness, unenforced building codes, and socioeconomic marginalization face high health risks. The surge in deadly climate events emphasizes targeted adaptation, including early warning systems, resilient construction standards, and investment in local preparedness and, as climate change intensifies, prioritization of food security and access to healthcare is crucial. Addressing structural inequities and implementing evidence-based interventions in the US Caribbean can reduce losses, protect public health, and increase resilience.

P29-44 | Are Indigenous North American Populations at Increased Risk from Extreme Temperature Exposures? A systematic review and meta-analysis

Marie-Claire Meadows, Alexandra R. Willis, Thuy N. Kim, Andrea L. Grzybowski, William Finical, Jesse D. Berman

University of Minnesota School of Public Health, Division of Environmental Health Sciences, Minneapolis, USA

Objective: The effects of extreme temperatures on acute and long-term health are well documented. However, less is known about the impacts of temperature extremes on the health of high-vulnerability and low-resource Indigenous populations. We have compiled available literature and reviewed them for relevance.
Material & Methods: A systematic literature review was conducted using both PubMed and SCOPUS databases. Studies for full text review contained an ambient temperature exposure (heat, cold, or generally focused), any cardiovascular, respiratory, mental health, kidney, heat- or cold-related illness, or mortality outcome, and included participants identifying as Indigenous in Canada, Mexico, or the United States. Published quantitative or mixed methods literature from 1990 to 2024 were included for review.
Results: Of the 4,676 studies identified from the databases, 4,606 were excluded in title/abstract screening, and seventy for duplication. Twenty-six total studies received a full text review. Each paper was reviewed by two independent reviewers with an interrater reliability of 99.25%. Of the twenty-six full-text studies, 11 were included for data extraction, which is ongoing. Of the included studies, five are focused on heat, one on cold, and four on the effect of both. Additionally, eight of these studies took place in the United States, two in Canada and one in Mexico. Finally, three studies include multiple health outcomes, two each focus on mortality, mental health, and heat-related illnesses, and one focuses on respiratory outcomes.**Conclusion:** The limited availability of studies on this topic indicates a need for further research, particularly in places with larger Indigenous populations. As the climate changes, it will be necessary to understand how different temperature extremes affect the most vulnerable populations, so that funding and mitigation practices can be allocated to specific high-risk groups to reduce potential disparities.

P29-45 | Association between ambient temperature and blood concentrations of three heavy metals: Korea National Health and Nutrition Examination Survey

Soonsu Shin[1], Dajeong Ham[1], Minkyo Song[2], Sanghyuk Bae[1,3]

[1] The Catholic University of Korea, Department of Preventive Medicine, Seoul, South Korea; [2] National Institute on Aging, Laboratory of Epidemiology and Population Sciences, Baltimore, USA; [3] The Catholic University of Korea, Environmental Health Center, Seoul, South Korea

Objective: Climate change may alter human exposure profiles to environmental pollutants. Warmer sea and air temperatures have been associated with increased heavy metal levels in fish and crops. However, the association between ambient temperature and human blood levels of heavy metal concentrations remains unclear. This study explores the association between ambient temperature exposure across different timeframes and blood concentrations of cadmium (Cd), mercury (Hg), and lead (Pb) in a nationally representative population-based study.

Material and Methods: We used data from the Korea National Health and Nutrition Examination Survey. Ambient temperature exposure was estimated using the Community Multiscale Air Quality model, linked to participants' residential addresses. Ambient temperature was categorized as short-term (0 to 14 days moving average), mid-term (30 to 180 days moving average), and long-term (1-year moving average). Blood Cd and Pb were measured using graphite furnace atomic absorption spectrometry, while Hg was analyzed using a direct mercury analyzer. Heavy metal concentrations were log-transformed. Survey-weighted multivariable linear regression models assessed associations between temperature and blood heavy metal concentrations.

Results: A total of 13,768 participants were included. A 1°C increase in Day 0 ambient temperature was associated with a −0.39% (95% confidence interval [CI]: −0.52, −0.26) decrease in ln(Cd), a −0.20% (95% CI: −0.35, −0.04) decrease in ln(Hg), and a −0.19% (95% CI: −0.28, −0.10) decrease in ln(Pb). These negative associations remained consistent over the mid-term exposure period. However, a 1°C increase in the 1-year moving average temperature was associated with a 2.37% (95% CI: 1.17, 3.57) increase in ln(Cd) and a 6.99% (95% CI: 5.58, 8.41) increase in ln(Hg).

Conclusions: Short- and mid-term temperature exposure was associated with decreased blood heavy metal levels, while long-term exposure was associated with increased Cd and Hg concentrations in the Korean population.

P29-46 | Examining the distribution of power outages after tropical cyclones in the United States, 2014-2018

Vivian Do[1], Lauren Wilner[2], Marianthi-Anna Kioumourtzoglou[3], Tarik Benmarhnia[1], Joan Casey[2]

[1] University of California, San Diego, San Diego, USA; [2] University of Washington, Seattle, USA; [3] Brown University, Providence, USA

Objective: Tropical cyclones (TCs) often cause power outages, which can negatively impact health. After TCs, outages can cause psychosocial stress by hindering crucial services like communications and transportation. Additionally, outages can exacerbate health conditions by preventing the use of electricity-dependent durable medical equipment (DME). Previous work on TCs and outages focused on single TC events or were based on simulations. Our goal was to investigate county-level outages 14 days after all TCs that occurred in the United States from 2014-2018 using historical outage data while considering vulnerability.

Material and Methods: Using the *hurricaneexposure* R package, we defined TC exposure as when individuals lived in a county where peak sustained wind in the population center was ≥34 knots on the day when the TC was closest. Leveraging EAGLE-I data, we measured outage exposure as the (a) percent of county customers without power and (b) outage hours where ≥5% of county customers were without power. Counties were vulnerable if their social vulnerability index score from the Centers for Disease Control was in the top tertile and the percentage of older adult DME users from emPOWER was >5.4%, the median percentage among study counties. We describe outage trends using means.

Results: From 2014-2018, 15 TCs impacted 418 counties, of which 168 (40.2%) were vulnerable. While TC Michael in 2018 affected the most counties (259, 62.0%), the greatest county average percentage of customers without power (53.8%) and average outage hours (23.5) during any one day was after TC Irma in 2017. Throughout the 14-day period post-TC, the average percentage of customers without power was consistently higher in vulnerable counties compared to non-vulnerable counties. Similarly, the average outage hours tended to be greater among vulnerable counties.

Conclusion: Counties that were both socially and medically vulnerable faced more power outages after a TC than their counterparts.

P29-47 | Kidney healthcare access for kidney failure patients across remote areas with diverse climates in Australia.

Lijalem M. Tesfaw[1,2], Mark Tiong[3,4], Nicholas J Osborne[1,5], Gail Williams[6], Darsy Darssan[1]

[1] The University of Queensland, School of Population Health, Brisbane, Australia; [2] Bahir Dar University, Department of Statistics, Bahir Dar, Ethiopia; [3] University of Melbourne, Department of Medicine (RMH), Melbourne, Australia; [4] The Royal Melbourne Hospital, Department of Nephrology, Melbourne, Australia; [5] University of Exeter Medical School, Knowledge Spa, Royal Cornwall Hospital, European Centre for Environment and Human Health (ECEHH), Truro, Cornwall, UK; [6] The University of Queensland, Faculty of Medicine, Brisbane, Australia

Objective: Investigate the accessibility of healthcare centers for individuals starting kidney replacement therapy (KRT) across remote areas with diverse climates.

Methods: The catchment area of each kidney healthcare center was assessed using a multiple-ring buffer analysis with 100, and 200 km radii. Data from the Australian and New Zealand Dialysis and Transplant Registry (2000-2020) were used to locate KRT patients within one of the 326 Australian Statistical Geography Standard (ASGS) Statistical Area Level 3(SA3) regions containing their residential addresses. Remote areas including remote and very remote regions were defined using the Accessibility/Remoteness Index of Australia Plus (ARIA+) based on service access, with corresponding climate zones obtained from the Australian Bureau of Meteorology.

Results: A total of 5,514 patients starting KRT in 30 kidney healthcare centres across remote areas of Australia were considered. Large proportion of remote (82.5%) and very remote (87.4%) areas are in the hot dry summer, cold/mild winter. In remote areas, 648 cases are within 100km, while in very remote areas 407 cases are within 200 km of kidney healthcare centers. On average, 64,601 people in remote areas travel up to 100 km, and 24,151 in very remote areas travel up to 200 km for KRT. The estimated kidney failure risk in remote (RR = 1.25, 95% CI: 1.00–1.61) and very remote areas (RR = 1.48, 95% CI: 1.37–1.72) was 25% and 48% higher than what would typically be expected for their populations, respectively.

Conclusion: Kidney healthcare centers were less accessible in remote areas, with higher kidney failure needs, predominantly characterized by a hot dry summer and cold/mild winter climate. This suggests that limited access may accelerate kidney failure progression, as distance barriers and climate factors limit regular healthcare visits.

P29-48 | The vulnerability of people with disabilities in hospital admission and emergency room visit due to extreme temperature-related injuries

Eunjin Oh[1], Hyungryul Lim[1], Jonghyuk Choi[2,3], Ho-Jang Kwon[2,3], Kyung-Hwa Choi[2,3]

[1] Ajou University School of Medicine, Department of Preventive Medicine and Public Health, Suwon, South Korea; [2] Dankook University College of Medicine, Department of Preventive Medicine, Cheonan, South Korea; [3] Dankook University College of Medicine, Research Institute of Healthcare Bigdata, Cheonan, South Korea

Objective: People with disabilities (PWD) may be more vulnerable to injury accidents during extreme weather than non-disabled (ND). This study evaluates whether extreme temperatures increase injury risk among PWD relative to ND.

Methods: We used the National Sample Cohort, a systematically sampled and nationally representative database of 1 million beneficiaries from the Korean National Health Insurance Service, from January 1, 2002, to December 31, 2019. We divided the cohort participants into PWD and ND, outcomes included all-cause and external cause including injury (ICD-10: S00-T98) hospitalizations and emergency room visits. Daily mean temperatures were obtained from ERA5-Land via Google Earth Engine with a 9 km resolution at the district level. We performed conditional logistic regression with a distributed lag non-linear model and 21-day cumulative effect using a time-stratified case-crossover design, adjusting for relative humidity and public holidays.

Results: We followed 790,655 person-years (PY) in PWD and 17,890,657 PY in ND. Incidence rates (/1,000 PY) for all-cause hospitalization and ER visits were 334.3 and 20.3 in PWD, and 134.5 and 10.6 in ND, respectively. Those for injury-related causes were 49.4 and 45.3 in PWD, and 17.9 and 28.6 in ND, respectively.

Conclusion: The incidence for all-cause hospitalization and ER visits in PWD were higher than ND. We are performing further analysis to estimate the risk of extreme temperatures relative to the lowest incidence temperature and evaluate whether disability increases vulnerability through effect modifiers such as gender, socioeconomic status, and regional deprivation.

Funding: This work was supported by the National Research Foundation of Korea grant funded by the Korea government (No. 2023R1A2C1002801).

P29-49 | Bayesian spatiotemporal analysis of heat-related emergency hospital admissions in Switzerland: a nationwide case-crossover study

Sujung Lee[1,2], Garyfallos Konstantinoudis[3]

[1] University of Bern, Institute of Social and Preventive Medicine, Bern, Switzerland; [2] University of Bern, Oeschger Centre for Climate Change Research, Bern, Switzerland; [3] Imperial College London, Grantham Institute for Climate Change and the Environment, London, UK

Background: Although several studies indicate that heat exposure increases the risk of hospital admissions in Switzerland, understanding how these effects vary across different spatial and temporal scales is limited. This ongoing project develops a Bayesian spatiotemporal model to assess spatial variability in sensitivity to heat using various temperature metrics and emergency hospital admissions (EHA) in Switzerland.
Methods: Data on individual-level all-cause EHA were collected from medical districts (Medstat regions), which consist of 706 districts across Switzerland. We computed the population-weighted daily maximum temperatures using 1-km gridded temperature data from MeteoSwiss. We employed a case-crossover study design within a Bayesian framework, utilizing non-linear functions to capture non-linear associations and the Besag, York, and Mollie (BYM2) model to account for spatial autocorrelation. We adjusted for national holidays and evaluated effect modification by areal-level factors (e.g., green space, language, urbanicity, and socio-economic status). In this ongoing project, we will conduct a stratified analysis based on the primary diagnosis of EHA.
Results: We will present heat-related EHA risks categorized by the primary diagnosis, including all-cause EHA and EHA due to mental disorders, respiratory diseases, genitourinary diseases, circulatory diseases, and infectious and parasitic diseases. To aid interpretation, we will report the results using epidemiological metrics, such as heat-related excess EHA and minimum hospital admission temperature (MHT). Furthermore, we will show how spatial effect modifiers contribute to disparities in EHA risks during heat episodes.
Conclusion: Our research will quantify heat-related EHA risks and identify the factors driving their spatial variations. These insights can improve the allocation of healthcare resources and promote more spatially targeted adaptation strategies that address community-level vulnerability factors.
This research was supported by the UniBE Doc.Mobility program of the University of Bern.

P29-50 | Associations between Weather, Wild Bird Abundance, and HPAI Spillover Events in Commercial and Backyard Poultry Flocks: A Case-Crossover Study in Minnesota

Fintan A. Mooney[1], Rishi Kowalski[2], Joshua Warren[3], Nicholas DeFelice[2], Sen Pei[1]

[1] Columbia University Mailman School of Public Health, Department of Environmental Health Sciences, New York, USA; [2] Mount Sinai Icahn School of Medicine, Department of Environmental Medicine and Climate Science, New York, USA; [3] Yale School of Public Health, Department of Biostatistics, New Haven, USA

Objective: Highly pathogenetic avian influenza (HPAI) H5N1 was first identified in the United States in 2021 and rapidly spread nationwide, spilling over from wild birds into backyard and commercial poultry flocks. Here, we evaluate the association between meteorological conditions, wild bird abundance, and HPAI spillover risk in Minnesota during 2022. Material and Methods: We conducted a time-stratified case-crossover study with a distributed nonlinear lag model on weekly aggregated data within 0.125-degree grid cells across Minnesota. Cases were defined as grid cells with a positive for HPAI, while controls were selected from the same cells within five weeks before and after each outbreak. Environmental variables included precipitation, soil moisture, temperature, and specific humidity extracted from the North American Land Data Assimilation System (NLDAS) Forcing 2.0 dataset. Wild bird abundance data were obtained from eBird for three species most commonly confirmed as HPAI positive (Mallards, Canada Geese, and Green-Winged Teal) in USDA wild bird surveillance. Results: A total of 109 spillover events occurred across 100 unique case cells, with 952 matched controls over the 52 weeks included in the model. The probability of HPAI outbreaks in Minnesota peaked in late March to early April (64.2% of all positive cases). Preliminary findings suggest that decreased soil moisture (OR: 0.42, 95% CI: 0.30 – 0.59) and decreased specific humidity (OR: 0.69, 95% CI: 0.49 – 0.97) two weeks prior were associated with a lower risk of spillover. Conversely, increased Canada Geese abundance (OR: 1.06, 95% CI: 1.01 – 1.12) three weeks prior was associated with a higher risk of wild bird to poultry flock spillover events. Conclusion: Weather variability and wild bird abundance may increase the risk of HPAI transmission from wild birds to farms. Understanding these transmission pathways is the first step in developing an environmentally focused risk assessment.

P30 | Environmental Exposures and Aging - PART I

P30-01 | Urinary Cadmium and Grip Strength in Older Women from the Study of Osteoporotic Fractures

Lissa F. Soares[1], Joseph Kawuki[1], Shamim Sarkar[1], James Harrington[2], Dylan Smith[1], Deborah M. Kado[3,4], Eric Orwoll[5], Li-Yung Lui[6], Peggy M. Cawthon[6,7], Jaymie Meliker[1]

[1] Stony Brook Medicine, Program in Public Health, Stony Brook, USA; [2] Research Triangle Institute, Analytical Science Division, Durham, USA; [3] Stanford University, Department of Medicine, Stanford, USA; [4] U.S. Department of Veterans Affairs, Geriatric Research Education and Clinical Center, Palo Alto, USA; [5] Oregon Health & Science University School of Medicine, Division of Endocrinology, Diabetes and Clinical Nutrition, Portland, USA; [6] California Pacific Medical Center, Research Institute, San Francisco, USA; [7] University of San Francisco, San Francisco, USA

Objective: To test the hypothesis that a high level of urinary cadmium (Cd) is associated with a faster decline in grip strength in older women.

Material and Methods: We used individual growth models in a random subcohort sample of 1325 women aged 65 years or older enrolled in the Study of Osteoporotic Fractures (SOF) to assess the association between urinary-Cd and repeated measures of grip strength assessed at baseline in 1997-1998, in 1999, and in 2002-2004. Cd and creatinine concentrations were measured in urine samples collected in 1997-1998. Covariates including factors that were associated with both Cd and grip strength were collected by questionnaire and clinical measurements.

Results: At baseline, women were an average age of 79 (range 65-96) with a creatinine-corrected urine-Cd median (IQR) concentration of 0.67 (0.43-1.01) µg/g. The average grip strength was 19.5 Kg (95% CI: 19.2; 19.7), meeting grip strength criteria for sarcopenia (<20 Kg). The average rate of grip strength decline was -0.28 Kg/year (95% CI: -0.32; -0.24). In mixed-models adjusted for age, urine creatinine, smoking status, pack-years, race, clinic site, body mass index, education, estimated glomerular filtration rate, parity, physical activity, self-rated health, diabetes, rheumatoid arthritis, and relevant medication use, compared to women in the referent (lowest) urinary-Cd quartile, those with the highest urinary-Cd had faster rate of decline in grip strength (Q4 vs Q1: β= -0.17 kg/year, p=0.03; Q3 vs Q1: p=0.05; Q2 vs Q1: p=0.1).

Conclusion: These results suggest that higher Cd exposures may have detrimental effects on muscle strength over time, with further confirmatory studies needed.

The Study of Osteoporotic Fractures (SOF) is supported by National Institutes of Health funding. The National Institute on Aging (NIA) provides support for SOF under several awards. The National Institute of Arthritis and Musculoskeletal and Skin Diseases (NIAMS) provides support for this study (R01 AR081125).

P30-02 | Urinary phthalate metabolite concentrations and vasomotor symptoms in midlife women: A Cross-sectional analysis in the Study of Women's Health Across the Nation

Rachael A. Brooks[1], Jiaxin Wu[1], Wenzhiyuan (. Zhang[1], Carrie Karvonen-Gutierrez[1], Bhramar Mukherjee[2], John Randolph Jr.[3], Ellen B. Gold[4], Siobán Harlow[1], Sung Kyun Park[1,5]

[1] Department of Epidemiology, University of Michigan, School of Public Health, Ann Arbor, USA; [2] Department of Biostatistics, Yale University, New Haven, USA; [3] Department of Obstetrics and Gynecology, University of Michigan, School of Medicine, Ann Arbor, USA; [4] Department of Public Health Sciences, University of California, Davis School of Medicine, Davis, USA; [5] Department of Environmental Health Sciences, University of Michigan, School of Public Health, Ann Arbor, USA

Objective. Vasomotor symptoms (VMS) are the most frequent symptoms of menopause. Phthalate exposure is associated with disrupted reproductive function, but few studies have examined the relation of phthalate exposures to VMS. We investigated associations between phthalates and prevalent VMS and their frequency in midlife women.
Materials and Methods. We included 1285 women from the Study of Women's Health Across the Nation (SWAN) in 1999-2000. Outcomes included VMS (none/any VMS in the last two weeks) and VMS frequency (none/moderate (1-5 days)/frequent (≥6 days). Eleven phthalate metabolites were measured in spot urine samples in 1999-2000. Covariates included age, race/ethnicity, site, education, BMI, physical activity, smoking status, secondary smoking exposure, menstrual status, prior anxiety, symptom sensitivity, and premenstrual symptoms. Logistic regression was used to examine associations between phthalates and VMS, while multinomial logistic regression was used to examine VMS frequency. Phthalate mixture effects were evaluated using quantile g-computation. Effect modification by race/ethnicity was examined using interaction terms.
Results. Overall, 48.1% of women reported VMS. Compared to women who did not report VMS, a doubling of di(2-ethylhexyl) phthalate (DEHP) metabolite concentrations were associated with non-significantly higher odds of reporting VMS (∑DEHP; OR: 1.08, 95% CI: 0.96, 1.22) and with moderate frequency of VMS (∑DEHP; OR: 1.09, 95% CI: 0.97, 1.23). Phthalate metabolite mixtures revealed similar non-significant results. Effect modification by race/ethnicity was observed. Among White women, a doubling of DEHP phthalate concentrations were associated with higher odds of VMS (∑DEHP; OR: 1.10, 95% CI: 1.01, 1.21) and moderate frequency of VMS (∑DEHP; OR: 1.12, 95% CI: 1.02, 1.23). Phthalates were not associated with VMS in Black or Asian women.*Conclusion.* Phthalate metabolites may increase the odds of VMS in midlife women, but associations vary across racial/ethnic groups. Further longitudinal analyses are needed to examine if phthalates are associated with duration of or incident VMS.

P30-03 | The influence of extreme weather on life expectancy in the contiguous United States

Yanni Cao, Jianyong Wu

The Ohio State University, Division of Environmental Health Sciences, College of Public Health, Columbus, USA

Climate change poses significant threats to human health, including its impact on life expectancy and mortality rates. However, the relationship between extreme climate and life expectancy remains insufficiently understood. This study investigates the influence of extreme weather on life expectancy in the contiguous United States using 2011 census tract-level data. Multi-level linear regression models were employed to analyze the effects of extreme weather events, elevation, and sociodemographic factors on life expectancy, which was derived from modeled predictions. Results indicate that extreme rainfall events (e.g., daily precipitation > 50 mm) and drought conditions (daily precipitation = 0 mm) negatively impact life expectancy, while higher maximum precipitation and Standardized Precipitation Index (SPI) values are positively associated with longevity. Extreme high-temperature days, particularly those exceeding 40°C, were linked to reduced life expectancy, whereas extreme low temperatures showed a significant positive association. Additionally, extreme weather affects life expectancy differently between urban and rural areas. Extreme rainfall events and drought conditions have significant negative impacts on life expectancy in urban areas, but not significant in rural areas. These findings highlight the complexity and disparity in the impact of extreme weather on life expectancy, emphasizing the need for targeted strategies. Key measures include enhancing drainage systems, improving water management, and expanding urban greening to mitigate the effects of heavy rainfall, drought, and extreme heat. Addressing resource disparities is critical, focusing on improving flood management and waste handling in rural areas and advancing sustainable planning in urban regions. Finally, leveraging the benefits of high elevations in rural areas can enhance resilience and health outcomes across populations.

P30-04 | Examining the role of cardiovascular disease as a potential mediator on dementia outcomes in the UK Biobank

Aksayaa Perinpakumar[1], Dylan Wood[1,2], Klea Katsouyanni[1,3], Dimitris Evangelopoulos[1,2]

[1] MRC Centre for Environment and Health, Environmental Research Group, Imperial College London, London, UK; [2] NIHR HPRU in Environmental Exposures and Health, Imperial College London, London, UK; [3] Department of Hygiene, Epidemiology and Medical Statistics, School of Medicine, National and Kapodistrian University of Athens, Athens, Greece

Exposure to air pollution (AP) has been identified as a potential risk factor for dementia with possible indirect pathways through cardiovascular disease (CVD). This study aims to investigate the role of CVD as a mediator and estimate the proportion of the association explained.
We utilised the UK Biobank cohort study and identified dementia incidence cases among people with pre-existing CVD. Participants with pre-existing major psychiatric conditions, as well as incomplete data, were excluded. Exposure to $PM_{2.5}$ and NO_2 was assessed at participants' residential addresses using hybrid exposure models that combined land-use variables, dispersion modelling, and satellite imagery. Mediation analysis was performed to decompose the total effect (TE) of AP on dementia into direct (ADE) and indirect effects (ACME) through CVD. Incident CVD, including myocardial infarction (MI), non-ST elevated MI, ST elevated MI and stroke, was ascertained

as a composite variable. Proportion mediated (ACME/TE) was calculated to estimate the extent to which the association between air pollution and dementia operates via CVD.
Preliminary analysis on London (n=61,797) showed no significant direct or indirect effects on hazard of dementia via CVD (ACME HR: 0.98 [0.94,1.01], ADE HR: 0.99 [0.94, 1.03]) per 1 μg/m³ increase in $PM_{2.5}$. A high proportion of hazard mediated was seen with variability (69% [-158.7%, 296.7%]). In contrast, NO_2 exposure showed a small but statistically significant mediation effect through CVD for each 1 μg/m³ increase (ACME HR: 0.99 [0.99, 1.00]) and a significant direct effect (ADE HR: 1.01 [1.00, 1.01]). Despite wide confidence intervals, the potential for mediation exists and will be explored further in the full UK Biobank cohort.
This study explores the mechanistic role of CVD as a mediator quantifying the proportion of dementia risk attributable to CVD pathways, which could provide valuable insights into midlife intervention strategies, potentially helping reduce dementia incidence and overall mortality.

P30-05 | Exposure to $PM_{2.5}$, Its Metal Constituents, and Cognitive Decline: Findings from the Adult Changes in Thought Study

Ting Zhang[1], Ya Y. Zhu[1], Paola Sebastiani[2], Paul Crane[3], Trenton Honda[4], Helen Suh[1]

[1] Tufts University, Department of Civil and Environmental Engineering, Medford, USA; [2] Tufts University, School of Medicine, Boston, USA; [3] University of Washington, Department of Medicine, Seattle, USA; [4] Northeastern University, School of Clinical and Rehabilitation Science, Boston, USA

Objective:Epidemiological evidence suggests $PM_{2.5}$ exposure contributes to cognitive decline, but findings remain inconsistent across studies and cognitive domains. Few studies have examined the role of $PM_{2.5}$-associated metal constituents, despite their potential neurotoxic effects. This study investigates the associations between long-term exposure to $PM_{2.5}$ and its metal constituents with cognitive decline, considering effect modification by APOE-ε4 and lifestyle factors, in the Adult Changes in Thought (ACT) cohort.
Methods:We analyzed 5,673 ACT participants (aged ≥65) from this longitudinal study (1994–2022) in the Greater Seattle area. $PM_{2.5}$ and metal exposure estimates (5-, 10-, and 20-year moving averages) were obtained from spatio-temporal models and linked to residential history. Cognitive function was assessed using the Cognitive Abilities Screening Instrument (CASI), providing domain-specific and overall cognitive scores. Linear mixed models with random intercepts and slopes were applied, using age as the analytical time scale and adjusting for key covariates. Effect modification by APOE-ε4 genotype and lifestyle factors was examined through interaction terms.
Results:Preliminary findings show baseline mean (SD) total cognitive score of 93.84 (4.49) (range: 0–100) and bifactor score of 0.18 (1.00) (standardized). Baseline domain-specific scores ranged from 7.64 (0.68) for attention to 17.80 (0.65) for orientation. Mean (SD) 10-year average $PM_{2.5}$ exposure through September 2018 among participants with non-missing APOE status was 10.1 (2.9) μg/m³. Our spatiotemporal model indicates that in Washington state, the 2022 mean predicted values for sulfur, lead, and silicon are 0.3572 μg/m³, 0.0012 μg/m³, and 0.0525 μg/m³, with cross-validation R^2 values of 0.84, 0.83, and 0.74. With 1,444 participants diagnosed with dementia (1994 – 2022), the bifactor cognitive score decreased by 0.0088 ± 0.0016 units per year of aging ($p < 0.001$), adjusting for baseline age, gender, education, race/ethnicity, and random effects.

Conclusion: Findings would clarify air pollution’s impact on cognitive aging, guiding future research and policy.

P30-06 | Lead exposure signatures overlap with cell composition, transcriptomic, and functional pathway changes in the hippocampus in Alzheimer's disease

Herong Wang[1], John Dou[1], Evelyn Matei[2], Justin Colacino[2], Kelly Bakulski[1]

[1] University of Michigan, Epidemiology, Ann Arbor, USA; [2] University of Michigan, Environmental Health Sciences, Ann Arbor, USA

Objective: Lead (Pb) is a neurotoxicant and risk factor for Alzheimer's disease (AD), but the potential mechanisms linking Pb to AD pathology remain ambiguous. In hippocampal transcriptomes from AD and cognitively normal human postmortem samples, this study estimated cell composition, identified transcriptomic and biological process enrichment in AD, and tested for overlap with perinatal Pb exposure signatures.
Materials and Methods: We pre-processed RNA expression data from five human postmortem hippocampus studies. We deconvoluted proportions of oligodendrocytes, astrocytes, microglia, endothelial and neurons. Beta regression tested for differences in cell proportions between AD and normal cognition. To identify AD differentially expressed genes, we performed linear regression, adjusted for age, sex, and cell type, and tested for enrichment of gene ontology biological processes. We meta-analyzed results across datasets. Pb exposure hippocampal response genes were identified from our published mouse study. Overlapping response genes and biological processes between Pb and AD were identified.
Results: Our study included 90 AD and 106 cognitively normal samples. AD samples had fewer neurons (-2.46%, 95% CI: -3.04, -1.88) and higher proportions of other cell types, including microglia (0.42% higher, 95% CI: 0.22, 0.63), astrocytes (0.31% higher, 95% CI: 0.12, 0.51), oligodendrocytes (0.46% higher, 95% CI: 0.27, 0.62), and endothelial cells (0.95% higher, 95% CI: 0.63, 1.27), relative to cognitively normal samples. AD enriched biological processes (p-adjusted<0.05) included cellular energy production and metabolism. AD signature genes were enriched for Pb exposure response genes (OR: 1.08, 95% CI: 1.02, 1.15) with 128 common transcripts differentially expressed (p-adjusted<0.05) in both AD and Pb including genes ATN1 and GRIN2B. The top AD and Pb overlapping processes were "synaptic vesicle maturation" and "vesicle docking". Conclusion: Our study sheds light on overlapping gene expression and pathways between AD and Pb, which reveals potential roles of Pb dysregulation in AD brains.

P30-07 | Lithium in drinking water and dementia risk: A nationwide county-level analysis in the U.S.

SeHyun Hwang, Hyeong-Moo Shin

Baylor university, Environmental Science, Waco, USA

Background: Lithium is a naturally occurring trace element found in food, widely used in various industries, and prescribed for bipolar disorder. Although its potential role in dementia has been investigated, findings remain inconclusive. In the U.S., lithium is widely present in groundwater, a major drinking water source. This study aimed to examine county-level associations between groundwater lithium exposure and dementia risk in the U.S. population.

Methods: County-level groundwater lithium exposure was estimated using 2023-2024 data from the U.S. Environmental Protection Agency's Fifth Unregulated Contaminant Monitoring Rule (UCMR 5), which monitors lithium in public water systems (PWSs). Lithium concentrations were population-weighted based on the population sizes served by each PWS. County-level dementia prevalence, incidence, and mortality data were obtained from the U.S. Dementia Data Hub. Our analysis, covering 2,123 counties (approximately 80% of U.S. Medicare beneficiaries), employed a Quasi-Poisson regression model and a generalized additive model (GAM) with sex-stratified analyses.

Results: Higher groundwater lithium levels were associated with a significant decrease in dementia prevalence compared to levels below 6.4 μg/L. Specifically, for lithium levels between 6.4 and 12.7 μg/L, the prevalence ratio (PR) was 0.979 (95% confidence interval [CI]: 0.966–0.992), and for levels above 12.7 μg/L, PR was 0.965 (95% CI: 0.949–0.981). Dementia incidence and mortality rates were also significantly lower at lithium levels between 6.4 and 12.7 μg/L, compared to levels below 6.4 μg/L. However, at levels exceeding 12.7 μg/L, an increased risk of dementia and mortality was observed. Sex-stratified analyses showed a continuous decline in dementia prevalence among males, while mortality rates steadily increased with rising lithium levels, as visualized in the GAM model.

Conclusion: Our findings indicate a non-linear relationship between groundwater lithium exposure and dementia risk, suggesting that moderate lithium levels may offer a protective effect, while higher exposures could pose adverse risks.

P30-08 | Indoor temperatures predict cognitive function among older adults in India: Findings from the LASI-DAD study

Ekta Chaudhary[1], Jinkook Lee[2,3], Aparajit B. Dey[4], Pranali Khobragade[5], Joyita Banerjee[6], Anushikha Dhankhar[2], Mark Davey[1], Sarah Petrosyan[2], Sandy Chien[2], Alden Gross[7], Emma Nichols[2], Albert Weerman[2], Meredith Pedde[1], Boya Zhang[8], Sharmistha Dey[6], Sara D. Adar[1]

[1] University of Michigan, Department of Epidemiology, Ann Arbor, USA; [2] University of Southern California, Center for Economic and Social Research, Los Angeles, USA; [3] University of Southern California, Department of Economics, Department of Economics, USA; [4] Venu Eye Institute, Department of Geriatric Medicine, New Delhi, India; [5] Maze Research, Mumbai, India; [6] All India Institute of Medical Sciences, New Delhi, India; [7] Johns Hopkins Bloomberg School of Public Health, Department of Epidemiology, Baltimore, USA; [8] Harvard T. H. Chan School of Public Health, Department of Environmental Health, Boston, USA

Objective: High temperatures present health risks, particularly in low and middle-income countries (LMICs) where many do not have access to cooled spaces. While the effects of outdoor heat exposure are well-explored, the influence of indoor temperature on cognition is understudied. This study examines the association of indoor temperature on cognitive function among older adults in India.

Methods: We used indoor environment data from the 2022-2024 wave of the Harmonized Diagnostic Assessment of Dementia for the Longitudinal Aging Study in India (LASI-DAD). We assessed objective cognitive functioning using a comprehensive neuropsychologic battery and measured indoor temperature over 24 hours using the ARANET4 device. We estimated cross-sectional associations of indoor temperature with the measures of general and domain-specific cognitive functioning based on confirmatory factor analysis of the available cognitive tests. We used linear regression accounting for survey weights and adjusting for risk factors including demographics, socioeconomic status, and other temporally varying factors like relative humidity, solar radiation, and holidays.

Results: The 558 participants (mean age: 73 (±7) years, 55% female) had indoor temperatures ranging from 10 to 35°C. After covariate adjustments, higher temperatures were linked to worse cognitive function (-0.063 per 10°C, 95% CI: -0.21, 0.09). This association remained consistent across cognitive domains, including memory (-0.07, 95% CI: -0.25, 0.1), executive functioning (-0.08, 95% CI: -0.24, 0.07), language (-0.01, 95% CI: -0.18, 0.15), and visuospatial ability (-0.02, 95% CI: -0.18, 0.14). Subgroup analyses revealed older adults, males, and people with dementia exhibited lower cognitive function at higher temperature levels.

Conclusion: Higher indoor temperatures were suggestively associated with poorer cognitive function in older adults, suggesting that mitigating indoor heat exposure may be important for promoting cognitive health in aging LMIC populations.

P30-09 | Association of Urinary Metal Mixtures and Serum Lipids in African Caribbean Men: The Tobago Health Study

Ruel E. Beresford[1], Natalie F. Price[1], Aaron Barchowsky[2], Alison P. Sanders[2], Ryan K. Cvejkus[1], Patrick J. Parsons[3], Joseph M. Zmuda[1], Allison L. Kuipers[4], Victor Wheeler[5], Iva Miljkovic[1]

[1] University of Pittsburgh School of Public Health, Department of Epidemiology, Pittsburgh, USA; [2] University of Pittsburgh School of Public Health, Department of Environmental and Occupational Health, Pittsburgh, USA; [3] New York State Department of Health, Division of Environmental Health Studies, Albany, USA; [4] Michigan State University College of Human Medicine, Department of Medicine, Grand Rapids, USA; [5] Tobago Health Studies Clinic, Scarsborough, Trinidad & Tobago

Objective
Exposure to toxic metals has been linked to changes in lipid metabolism and the development of atherosclerosis. However, these relationships remain understudied among populations of African ancestry, especially in the Caribbean. Thus, we assessed associations between a mixture of urinary arsenic, barium, cadmium, copper, and lead and circulating HDL-C, LDL-C, triglycerides, and cholesterol, in middle-aged and older African Caribbean men.
Material and Methods
The Tobago Health Study is a population-based observational study of Afro Tobagonian Men aged 40+. Metals were measured from spot urine samples collected between 2014-2016 that were analyzed by inductively coupled plasma mass spectrometry. Fasting serum lipid profiles, including HDL-C, LDL-C, triglycerides, and total cholesterol, were measured in 189 men not on lipid-lowering medication. Weighted Quantile Sum (WQS) regression was used to assess the relationship between the metal mixture and serum lipids, adjusted for age, BMI, energy intake, fish consumption, creatinine, and physical activity.
Results
We observed a significant association between the metal mixture index and higher LDL levels (β:13.14; 95%CI:2.26,24.01). Barium, copper, and arsenic contributed the greatest weights to the index. We observed no significant evidence of association between the metal mixture and HDL, triglycerides, or total cholesterol.
Conclusion
Our results suggest metal exposure is associated with a more atherogenic lipid profile, which can lead to atherosclerosis. Further research is needed to determine if the relationship between selected trace elements and cardiovascular disease is mediated by lipoprotein levels among African Caribbeans. Caribbean populations are often overlooked, due to their geographic localization, racialization, and economic position. Research like this ensures that there is a more equitable understanding of determinants of cardiovascular health, which can inform public health policy to promote healthy aging.

P30-10 | Short-Term $PM_{2.5}$ Exposure and Hospital Readmissions in Medicare Beneficiaries with Alzheimer's Disease and Related Dementias

Garam Byun[1], Scott W. Delaney[2], Daniel Mork[3], Danielle Braun[3], Jenny J. Lee[4], Mahima Kaur[3], Thomas M. Gill[5], Michelle L. Bell[1]

[1] Yale University, School of the Environment, New Haven, USA; [2] Harvard T.H. Chan School of Public Health, Department of Environmental Health, Boston, USA; [3] Harvard T.H. Chan School of Public Health, Department of Biostatistics, Boston, USA; [4] Ewha Womans University, Department of Data Science, Seoul, South Korea; [5] Yale School of Medicine, Department of Internal Medicine, New Haven, USA

Objective: Individuals with Alzheimer's disease and related dementias (ADRD) may experience higher rates of unplanned hospital readmissions than the general older population. This study investigates the impact of short-term exposure to fine particulate matter ($PM_{2.5}$) on 30-day hospital readmissions among older adults with ADRD.
Material and Methods: We utilized hospital admission data from Medicare fee-for-service beneficiaries aged 65 and older in the contiguous U.S. from 2000 to 2016. Among individuals with prior hospitalization with ADRD, all-cause readmissions occurring within 30 days following all-cause index admissions were identified. Daily ambient $PM_{2.5}$ concentrations were estimated at the ZIP code level. A time-stratified case-crossover design with conditional logistic regression was employed to assess the association between 3-day moving average (lag 0-2 days) of $PM_{2.5}$ exposure and 30-day all-cause readmissions.
Results: Among 6,131,018 individuals with prior hospitalization with ADRD, 1,641,758 (26.8%) had at least one 30-day all-cause readmission, totaling 2,767,674 readmissions. Preliminary results indicated that a 10 $\mu g/m^3$ increase in 3-day moving average of $PM_{2.5}$ was associated with increased odds of 30-day all-cause readmissions (odds ratio [OR]: 1.017; 95% confidence interval [CI]: 1.014, 1.020). The ORs for early (1–7 days) and late (8–30 days) all-cause readmissions were 1.011 (95% CI: 1.005, 1.016) and 1.020 (95% CI: 1.016, 1.024), respectively. The association remained robust when index admissions and readmissions were restricted to ADRD-related hospitalizations.
Conclusion: Short-term exposure to $PM_{2.5}$ was associated with increased risk of 30-day all-cause hospital readmissions in older adults with ADRD. Reducing $PM_{2.5}$ exposure may help reduce preventable readmissions in this vulnerable population, easing the burden on both ADRD patients and their caregivers.
Funding source: National Institutes of Health

P30-11 | The Association Between Air Pollution and Neighborhood-level Social Factors and Memory Performance in a Cognitively Healthy Mid-life Population

Lina V. Dimitrov[2], Donghai Liang[1,2], Zhihao Jin[1], Aliza Wingo[3], James Lah[4], Allan Levy[4], Stefanie Ebelt[1,2], Thomas Wingo[6], Anke Huels[2,5]

[1] Emory University, Environmental Health, Atlanta, USA; [2] Emory University, Epidemiology, Atlanta, USA; [3] UC Davis, Psychiatry and Behavioral Science, Davis, USA; [4] Emory University, Neurology, Atlanta, USA; [5] Emory University, Biostatistics and Bioinformatics, Atlanta, USA; [6] UC Davis, Neurology, Davis, USA

Objective: Air pollution and neighborhood-level social factors are linked to Alzheimer's disease, but they are often examined in isolation, oversimplifying real-world simultaneous co-exposures. We aimed to examine the combined effect of air pollution and neighborhood-level social factors on memory performance.

Methods: We studied 1,911 cognitively healthy participants from the Emory Healthy Brain Study in Atlanta, Georgia. Memory performance was assessed from 2016-2023 using the delayed recall and recognition tests (higher scores mean better performance). We estimated residential 10-year exposure averages (accounting for residential history) of 5 fine particulate matter ($PM_{2.5}$) components, including ammonium, nitrate, sulfate, organic carbon (OC), and elemental carbon (EC), from the Socio-economic Data and Application Center and 9 variables from the Social Vulnerability Index. We used a negative constraint Weighted Quantile Sum (WQS) regression with 10 quantiles to estimate the total mixture effect and the contribution of each mixture component, adjusting for age, gender, education level, race, and ethnicity. To assess effect measure modification (EMM) by individual education level, we ran stratified WQS models.

Results: The total mixture was associated with lower delayed recall z-scores (beta: -0.06, or a 0.06 standard deviation decrease per decile increase in mixture [95%-confidence interval: -0.10,-0.02]) and recognition z-scores (-0.04 [-0.07,-0.0005]). For the delayed recall scores, ammonium was the mixture component driving the association, closely followed by neighborhood-level low education levels, high unemployment, and households without a vehicle; for the recognition scores, it was low education levels, closely followed by OC. In the EMM analyses, associations were the strongest among the low education group (e.g., for delayed recall: less than college degree: beta:-0.12 [95%-CI:-0.20, -0.04]; college degree: -0.06 [-0.14, 0.01]; master’s or higher degree: -0.04 [-0.10, 0.01]).

Conclusions: Both $PM_{2.5}$ components and neighborhood-level social factors were associated with lower memory performance. More formal education may reduce vulnerability to these detrimental effects.

P30-12 | Glycemic Indices Modify the Associations of Metals with Depression and Cognitive Performance

Yi Wang[1], **Wending Li**[2]

[1] Albert Einstein College of Medicine, Department of Epidemiology and Population Health, Bronx, USA; [2] Columbia University, Mailman School of Public Health, Department of Environmental Health Sciences, New York, USA

Objective:
We investigated whether glycemic indices modify the associations between essential and toxic metal exposures with depression symptoms and cognitive performance. Understanding these interactions may help identify at-risk populations and inform prevention strategies.
Materials and Methods:
We analyzed data from the 2005–2018 waves of the National Health and Nutrition Examination Survey (NHANES). Metal exposures included serum (copper, selenium, zinc), blood (lead, cadmium, mercury, manganese, iron), and urinary (creatinine-adjusted arsenic) measures. Depression symptoms were assessed using the Patient Health Questionnaire-9 (PHQ-9), and cognitive performance was measured using the Immediate Recall Test (IRT), Animal Fluency Test (AFT), and Digit Symbol Substitution Test (DSST). Survey-weighted regression models adjusted for sex, age, race/ethnicity, education, and smoking status. Effect modification by glycemic indices—including physician-diagnosed diabetes, Homeostatic Model Assessment of Insulin Resistance (HOMA-IR), and hemoglobin A1c (HbA1c)—was tested using likelihood ratio tests.
Results:
Higher blood manganese levels were associated with lower AFT scores, while mercury, lead, iron, and arsenic were linked to lower PHQ-9 scores. We confirmed previous findings of a positive association between cadmium and depression, but this relationship became non-significant after adjusting for smoking. Effect modification analysis revealed that diabetes status modified the associations of mercury and cadmium with cognitive performance, and these relationships were also reflected in their associations with HbA1c. For depression, both diabetes status and HbA1c modified the relationship between manganese and PHQ-9, while HOMA-IR modified the association between cadmium and PHQ-9.
Conclusion:
Our findings suggest that glycemic status may alter the effects of metal exposures on brain health, influencing both depression symptoms and cognitive performance. These results emphasize the importance of considering glucose metabolism when assessing environmental risk factors for neuropsychiatric outcomes, highlighting the need for targeted interventions to reduce metal exposure in high-risk populations.

P30-13 | Persistent Organic Pollutants and Alzheimer's Disease and Related Dementias: A Systematic Review of Epidemiologic and Neuropathological Evidence in Humans

Julia Anglen Bauer[1], Zachary Sievers[1], Marc Weisskopf[2], Orly Lazarov[3], Robert Sargis[4], Jose Farfel[5], David Bennett[5], Mary Turyk[1]

[1] University of Illinois Chicago School of Public Health, Division of Epidemiology and Biostatistics, Chicago, USA; [2] Harvard T.H. Chan School of Public Health, Department of Environmental Health, Boston, USA; [3] University of Illinois Chicago College of Medicine, Department of Anatomy and Cell Biology, Chicago, USA; [4] University of Illinois Chicago College of Medicine, Division of Endocrinology, Diabetes, and Metabolism, Chicago, USA; [5] Rush University Medical Center, Rush Alzheimer's Disease Center, Chicago, USA

Background: There is an urgent need to identify early risk factors of dementia. Persistent organic pollutants (POPs), such as organochlorine pesticides (OCPs), polychlorinated biphenyls (PCBs), and per- and polyfluoroalkyl substances (PFAS), are ubiquitous environmental contaminants that accumulate in human tissues and have neurotoxic potential.
Methods: We performed a comprehensive PubMed search of human studies written in English that measured OCP, PCB, or PFAS in biomatrices in relation to dementia outcomes. MeSH and Supplementary Concept terms for specific POPs (e.g., polychlorinated biphenyls) and neurodegenerative outcomes (e.g., Alzheimer's disease) were combined using "AND." Studies were screened for relevance, categorized by exposure type, and synthesized.
Results: Our analysis included twelve unique studies. Three studies examined sex differences in which two found stronger harmful associations in females. OCPs: Medehouenou et al. (2014) found no association between plasma OCPs and dementia. Raffetti et al. (2020) reported a dose-response relationship between serum OCPs and dementia (HR = 1.45, 95%CI: 1.12–1.87). Postmortem studies identified elevated dieldrin and β-hexachlorocyclohexane (β-HCH) in Parkinson's disease brains as compared to non-diseased brains(Corrigan et al., 1999; Richardson et al., 2011). PCBs: Weisskopf et al. (2012) reported an inverse association between serum PCBs and Parkinson's disease, while Raffetti et al. (2020) found higher PCB exposure was associated with increased dementia risk (HR = 1.62, 95%CI: 1.28–2.04); finding discrepancies may be due to differences in PCBs measured. Postmortem studies found PCB accumulation in Parkinson's disease brains (Hatcher-Martin et al., 2013), and Bohler et al. (2019) found sex-specific gene expression responses to PCB exposure. PFAS: Delcourt et al. (2024) found PFAS in cerebrospinal fluid correlated with Alzheimer's biomarkers.
Conclusion: Current evidence suggests POPs may contribute to dementia risk, but inconsistencies across studies highlight the need for standardized exposure assessment and further research on susceptible populations.

P31 | Environmental Exposures and Birth Outcomes - PART I

P31-01 | Mediating role of placental transcriptome and metabolome in the association between early pregnancy fine particulate matter exposure and spontaneous preterm birth

Jagadeesh Puvvula[1], Aimin Chen[1,2], Rebecca Simmons[3,4], Rita Leite[5], Yu-Chin Lien[2,5]

[1] University of Pennsylvania, Department of Biostatistics Epidemiology and Informatics, Philadelphia, USA; [2] University of Pennsylvania, Center for Excellence in Environmental Toxicology, Philadelphia, USA; [3] University of Pennsylvania, Department of Pediatrics, Philadelphia, USA; [4] Children's Hospital of Philadelphia, Division of Neonatology, Philadelphia, USA; [5] University of Pennsylvania, Center for Women's Health and Reproductive Medicine, Philadelphia, USA

OBJECTIVE: This study aims to evaluate potential mediation effects of transcriptome/metabolome on the association between gestational exposure to fine particulate matter during early gestation and spontaneous preterm birth (sPTB; <32 weeks' gestation). METHODS: We analyzed data from 72 participants (46% sPTB) in the CellulaR Injury and Preterm Birth Study. Daily exposure to fine particulate matter (daily mean $PM_{2.5}$ and black carbon) during the first 20 weeks of pregnancy was assessed using spatiotemporally weighted deep forest model output at 1-kilometer resolution linked to maternal residential address at delivery. The transcriptome (24,205 genes upon pre-processing) and metabolome (866 named metabolites) were profiled from placenta tissue. sPTB (outcome) and covariates were collected from medical records. Transcriptome-wide associations with daily particulate matter exposures were assessed using gene-wise negative binomial generalized linear models, while metabolome-wide associations were analyzed using multiple linear regression, adjusting for covariates. Genes significantly associated with $PM_{2.5}$ exposures ($q<0.05$) in at least seven exposure windows were included in Ingenuity Pathway Analysis. Additionally, high-dimensional mediation analyses (without covariate adjustment) were performed using genes/metabolites with at least one significant hit ($q<0.05$). RESULTS: There are 214 genes associated with black carbon and 234 genes associated with $PM_{2.5}$, with at least 7 hits. These genes are enriched in pathways related to cellular immune response, cellular stress and injury, cellular growth, proliferation and development. We observed 36 metabolites associated with black carbon and 81 metabolites with particulate matter exposure during early pregnancy, linked to lipid, amino acid, and peptide metabolism. We observed nine genes (*WBP1L, CACNG4, AHSA2P, SERTAD4, VAC14-AS1, ANKHD1, PARP14, EIF2AK2, LSMEM1)* and two metabolites (N6-methyladenosine, 1-(1-enyl-palmitoyl)-2-linoleoyl-GPE(P-16:0/18:2)) mediated associations (*pmax<0.001*) between fine particulate matter (at varying gestational exposure windows) and increased odds of sPTB. CONCLUSION: Associations between gestational air pollution exposure and sPTB are mediated by altered placental gene expression and metabolite intensity.

P31-02 | Mixtures-based analysis of prenatal exposure to phthalates, parabens, and phenols and birth outcomes.

Alana J. Ferris[1], Kylie W. Riley[1,2], Lehyla Calero[2], Darrell Holmes[2], Catherine Tobon[2], Matthew Guttierez[2], Maya A. Deyssenroth[1], Julie B. Herbstman[1,2]

[1] Columbia University Mailman School of Public Health, Environmental Health Sciences, New York City, USA; [2] Columbia University Mailman School of Public Health, Columbia Center for Children's Environmental health, New York City, USA

Objective: Exposure to phthalates, parabens, and phenols is ubiquitous and a suspected risk factor for adverse pregnancy outcomes. Individuals are frequently exposed to these chemicals in mixtures, yet few studies have investigated their effect as a mixture on birth outcomes. Thus, we investigated the prospective association between mixtures of urinary biomarkers of phthalates, parabens, and phenols during pregnancy and birth outcomes.

Materials and Methods: We leveraged an ongoing longitudinal birth cohort study at the Columbia Center for Children's Environmental Health (n=639). Spot urine samples were collected in the third trimester of pregnancy and analyzed for 26 phthalates, 6 parabens, and 17 phenols by high performance liquid chromatography-tandem mass spectrometry and were specific gravity-adjusted to account for urine dilution. Metabolites detected in ≤70% of samples were excluded from statistical analyses. Concentrations below the limit of detection (LOD) were substituted with LOD/sqrt(2) and were natural-log-transformed to account for non-normal distributions. We estimated linear associations of each chemical with gestational age at birth and sex-specific birthweight-for-gestational age z-scores. Quantile g-computation and Bayesian Kernel Machine Regression were implemented to assess associations with the chemicals evaluated as mixtures. All models were adjusted for maternal age and sex of the child. P-values in single chemical analyses were adjusted for multiple comparisons using the Benjamini-Hochberg procedure.

Results: Out of the 49 chemicals measured in urine, 11 phthalates, 3 parabens, and 5 phenols were detected in >70% of samples. After adjustment for possible confounders, no significant individual or mixture effects were observed between the chemicals and gestational age or sex-specific birthweight-for-gestational age. We also observed no interactions between chemicals.

Conclusions: No significant associations were identified between exposure to phthalates, parabens, and phenols during pregnancy and gestational age at birth or sex-specific birthweight-for-gestational age z-scores when chemicals were analyzed individually or as a mixture.

Funding: T32 ES007322; UG3OD023290

P31-03 | Prenatal glyphosate or aminomethylphosphonic acid (AMPA) exposure and birth outcomes in a pilot study of women from the Midwestern U.S.

Kelsi Morris[1,2], Maria Cinzori[1,2], Bradley Ryva[3,4], Khyatiben Pathak[5], Patrick Pirrotte[5], Brooke Lovell[5], Susan Schantz[6,7], Rita Strakovsky[1,2]

[1] Michigan State University, Department of Food Science and Human Nutrition, East Lansing, USA; [2] Michigan State University, Institute for Integrative Toxicology, East Lansing, USA; [3] Michigan State University, College of Osteopathic Medicine, East Lansing, USA; [4] Michigan State University, Department of Pharmacology and Toxicology, East Lansing, USA; [5] Collaborative Center for Translational Mass Spectrometry, Translational Genomics Research Institute, Phoenix, USA; [6] University of Illinois, Department of Comparative Biosciences, Urbana-Champaign, USA; [7] University of Illinois, Beckman Institute, Urbana-Champaign, USA

Background: Pregnant women are ubiquitously exposed to the herbicide glyphosate and its major environmental metabolite aminomethylphosphonic acid (AMPA), which are potential endocrine disrupting chemicals. As consequences of their exposure in pregnancy are poorly understood, we examined associations of glyphosate and AMPA with birth outcomes.

Methods: Pregnant Illinois women (n=300) provided first-morning urine samples at median 13-weeks gestation for glyphosate and AMPA measurement. We calculated gestational age at birth using the last menstrual period after ultrasound confirmation. We abstracted birth weight from electronic medical records and calculated sex-specific birthweight-for-gestational-age z-scores (BWz). We measured birth length (cm) and anogenital distance (AGD, mm) within 24-hours of birth and normalized AGD to birth length (m). We evaluated associations of specific gravity-adjusted glyphosate or AMPA with birth outcomes using multivariable linear regression accounting for sociodemographic and lifestyle factors, and considered differences in associations by fetal sex. In secondary analyses, we excluded preterm infants (n=16).

Results: Women had high socioeconomic status and glyphosate concentrations lower than same-age U.S. women. Overall, glyphosate was not associated with birth outcomes, whereas each 2-fold increase in AMPA concentration was associated with -0.1 cm shorter birth length (95%CI: -0.2, 0.0). In male newborns, each 2-fold increase in glyphosate was associated with -0.1 lower BWz (95% CI: -0.1, 0.0), -0.1 cm shorter birth length (95%CI: -0.3, 0.0), and 0.6 mm/m longer AGD_{long} (95%CI: -0.2, 1.4). In females, each two-fold increase in glyphosate was associated with 0.5 mm/m longer AGD_{long} (95%CI: -0.2, 1.3). AMPA was associated with 0.6 mm/m (95%CI: 0.0, 1.2) and 0.5 mm/m (95%CI: 0.0, 0.9) longer AGD_{long} in males and females, respectively. Findings were consistent after removing preterm infants.

Conclusion: In low-exposed U.S. women, glyphosate or AMPA were associated with smaller birth size and longer AGD_{long}, with evidence of sexual-dimorphism. Future studies could evaluate implications for child lifelong health.

P31-04 | Preconceptional bisphenol and phthalate exposure, fetal growth, and adverse birth outcomes

Kim Nail Cajachagua Torres[1], Linda Khan[1,2], Leonardo Trasande[1,2], Akhgar Ghassabian[1,2]

[1] New York University Grossman School of Medicine, Department of Pediatrics, New York, USA; [2] New York University Grossman School of Medicine, Department of Population Health, New York, USA

Background: Data from couples seeking infertility treatment suggest that preconceptional bisphenol and phthalate exposure may impact fetal growth, but evidence outside of subfertile populations remains limited. We used data from repeat pregnancies in a birth cohort to examine the extent to which exposure to chemicals before conception were associated with fetal growth and birth outcomes.

Methods: In the NYU Children's Environmental Health Study, we used maternal urinary bisphenol and phthalate metabolite concentrations from participants' first pregnancy as preconceptional exposures for their second (interpregnancy interval median=2.5 years, 90% range=0.99-4.9) and examined associations with fetal biometry measures (n=139) and birth outcomes (n=181), including birthweight and gestational age. We averaged three pregnancy measurements of two bisphenols and fourteen phthalate metabolites with detection rate >50%, adjusted for urinary dilution, grouped into molar sums, and natural log-transformed. We obtained repeated fetal biometry measures from clinical ultrasounds and predicted measures at 20, 30, and 36 weeks using linear mixed-effects models assuming nonlinear effects of gestational age. We used mixed models to examine associations of preconceptional chemical exposure with repeated fetal growth estimations, and regression models for birth outcomes. Models were adjusted for sociodemographic, lifestyle, and health-related factors, and interactions with sex were explored before performing stratified analysis. False Discovery Rate was used to correct for multiple comparisons.

Results: The nteraction term between preconceptional chemical exposures and sex was statistically significant. Higher exposure to bisphenols and phthalates was mostly negatively associated with fetal growth measures in females; e.g., β for low molecular weight phthalates (LMWP) associated with biparietal diameter (BPD)=-0.42, 95%CI: -0.57, -0.27. The associations in males were mostly positive, e.g., β for LMWP with BPD=0.58, 95%CI: 0.47, 0.69. There were no associations between preconceptional chemical exposures and birth outcomes.

Conclusion: Preconceptional exposure to phthalates and bisphenols may influence fetal growth, with males and females affected differently.

P31-05 | Metabolic Pathways and Signatures Linking Pre- and Peri-conceptional Traffic-Related Air Pollution Exposure to Preterm Birth Risk

Mengyuan Ren[1], Anne L. Dunlop[2], Jeremy A. Sarnat[1], Youran Tan[1], Zhihao Jin[1], Paula-Dene C. Nesbeth[1], Stephanie M. Eick[1,3], Christine C. Ekenga[1,4], Alicia K. Smith[2], Dean P. Jones[5], Howard H. Chang[6], Carmen J. Marsit[1], Donghai Liang[1,3]

[1] Emory University, Gangarosa Department of Environmental Health, Rollins School of Public Health, Atlanta, USA; [2] Emory University, Gynecology and Obstetrics, School of Medicine, Atlanta, USA; [3] Emory University, Epidemiology, Rollins School of Public Health, Atlanta, USA; [4] Emory University, Behavioral, Social, and Health Education Sciences, Rollins School of Public Health, Atlanta, USA; [5] Emory University, Department of Medicine, Pulmonary Division, School of Medicine, Atlanta, USA; [6] Emory University, Biostatistics and Bioinformatics, Rollins School of Public Health, Atlanta, USA

BACKGROUND: African Americans (AA) experience disproportionately high traffic-related air pollution (TRAP) exposures and bear the highest burden of preterm birth (PTB). Exposure to TRAP has been linked to an increased risk of PTB. However, the molecular mechanisms underlying TRAP-PTB associations remain unclear. We investigated metabolic signatures and pathways linking pre- and peri-conceptional TRAP exposure to PTB among AAs.

METHOD: We included 326 participants recruited between 2014-2019 with singleton pregnancies from the prospective Atlanta AA Maternal-Child Cohort. TRAP exposure, including fine particulate matter ($PM_{2.5}$), carbon monoxide (CO), and nitrogen oxides (NO_x), were estimated using spatiotemporally resolved model with 250×250m resolution for a maximum of 1-year, 6, 3 and 1-months prior to biomonitoring. Prenatal longitudinal metabolic serum profiles in early (8-14 gestational weeks) and late-pregnancy (24-32 weeks) were characterized with untargeted high-resolution metabolomics. Generalized linear models were utilized to evaluate the associations between TRAP, metabolomic signatures and pathways, with completed gestational weeks and PTB using a *meet-in-the-middle approach*.

RESULTS: We identified 16 and 43 metabolic signatures during early-pregnancy, and 16 and 74 during late-pregnancy associated with PTB and completed gestational weeks, respectively. Betaine metabolism, 2-oxocarboxylic acid metabolism, and plasmalogen synthesis were the overlapping pathways linked with both TRAP exposure and PTB. We confirmed the identity of several metabolites with Level-1 evidence, including 4-hydroxy-3-methoxyphenylglycol, N-acetyl-D-galactosamine and endogenous phthalate metabolites. 1-year exposure window prior to biomonitoring at early pregnancy was the most sensitive time window with 2084, 19, and 11 metabolomic features significantly associated with $PM_{2.5}$, CO, and NO_x (FDR $q<0.05$), respectively.

CONCLUSIONS: Amino acid and lipid metabolism pathways and metabolic signatures centering around neurotransmission and glycoconjugate biosynthesis were associated with both longitudinal TRAP exposure and PTB in this AA cohort. These findings reveal novel mechanistic insights underlying the effects of prenatal TRAP exposure on PTB risk.

P31-06 | Noise at Work, Risks at Birth: A Review of Occupational Noise Exposure and Birth Outcomes

Mislael Valentin-Cortes, Miatta Buxton, Marie O'Neill, Alexis Handal

University of Michigan, Ann Arbor, USA

Objective: Occupational noise is one of the most common work-related exposures, affecting millions of workers globally, including pregnant individuals. Despite increasing workforce participation among women, particularly in industrial and service sectors where noise exposure is prevalent, the impact of occupational noise on pregnancy remains understudied. Noise is a known physiological stressor that can activate the hypothalamic-pituitary-adrenal (HPA) axis, leading to elevated maternal stress hormone levels, vascular changes, and fetal growth restriction. This review examines the current epidemiologic evidence on the relationship between occupational noise exposure and adverse birth outcomes, including low birth weight (LBW), small-for-gestational-age (SGA), intrauterine growth restriction (IUGR), and preterm birth (PTB).
Materials and Methods: A systematic search of peer-reviewed epidemiologic studies published between 1984 and 2024 was conducted. Studies assessing the relationship between occupational noise exposure and adverse birth outcomes among pregnant workers globally were included. Key findings were extracted and summarized.
Results: Eighteen studies met the inclusion criteria. Associations between occupational noise exposure and outcomes such as LBW, SGA, and IUGR were observed in some studies, while others reported null or inconsistent findings. No strong evidence supported an association with PTB. Notably, no studies focused on low- and middle-income countries (LMICs), despite the high prevalence of noise exposure in these settings.
Conclusion: The current evidence on occupational noise exposure and adverse birth outcomes is limited and inconsistent. Further research is needed to refine exposure assessment methodologies, investigate critical exposure windows during pregnancy, and address gaps in LMIC populations. Strengthening this evidence base is essential to inform workplace policies that better protect pregnant workers and their children.

P31-07 | Estimated Risk of Preterm Birth Based on Realistic Temperature Exposure Trajectories Throughout Pregnancy

Selin Girgin[1], Offer Erez[2,3], Daniel Nevo[4], Iaroslav Youssim[1], Itai Kloog[5], Raanan Raz[1]

[1] The Hebrew University of Jerusalem, Braun School of Public Health, Jerusalem, Israel; [2] Soroka University Medical Center, Division of Obstetrics and Gynecology, Beer Sheva, Israel; [3] Wayne State University, Department of Obstetrics and Gynecology, Detroit, USA; [4] Tel Aviv University, Department of Statistics and Operations Research, Tel Aviv, Israel; [5] Ben-Gurion University of the Negev, Department of Geography and Environmental Development, Beer Sheva, Israel

Objective: We aimed to examine the associations of cumulative exposure to ambient temperature throughout pregnancy with preterm birth using realistic exposure trajectories.
Material and Methods: We carried out a historical cohort study involving 131,599 singleton live births in Southern Israel, 2005-2019, utilizing electronic health records. Weekly mean temperatures were assessed based on residential address and a spatiotemporal model. We examined weekly associations using a Cox model with time-dependent covariates integrated with distributed lag non-linear models, adjusted for ethnicity, time-varying seasonality, and

socioeconomic status. We estimated the cumulative risk of preterm birth based on this model for each pregnancy using their 37-week exposure trajectories. Then, we calculated the average estimated risk for the pregnancies with the same month of the last menstrual period (LMP). 95% CIs were calculated by bootstrapping.
Results: High temperatures in weeks 29-37 were associated with preterm birth. The strongest weekly hazard ratio was 1.04 (95% CI: 1.01-1.07) for exposure to 31°C (99th percentile) in week 37 versus the minimum risk temperature (16°C). Weekly temperature exposures followed seasonal trajectories based on the month of LMP, where the highest temperatures in weeks 33-37 were observed for the pregnancies with LMP in the fall season. The estimated risk of preterm birth also followed a seasonal pattern; the risk was higher for the pregnancies whose LMP was in the fall season. The average estimated risk by LMP month varied between 6.5–7.6% for pregnancies with LMP in March and October, respectively, translated to a relative risk of 1.17 (95% CI: 1.06-1.25).**Conclusion:** In Southern Israel, pregnancies starting in the fall season follow a temperature exposure trajectory predicting a higher risk of preterm birth.

Funding: This research was partially funded by a research grant from Israel Higher Education Council to Prof. Raz through Hebrew University Center for Interdisciplinary Data Science Research.

P31-08 | Brominated Flame Retardants and Thyroid Hormone Levels in Fetal and Maternal Placental Tissues and their Associations with Birth Outcomes.

Shaza Gaballah, Michelle Misselwitz, Kate Hoffman, Heather M. Stapleton

Duke University, Integrated Toxicology and Environmental Health Program, Nicholas School of the Environment, Durham, USA

In utero exposure to several legacy and replacement brominated flame retardants (BFRs) has been linked to changes in maternal thyroid hormone (TH) levels, along with adverse pregnancy and birth outcomes. Despite evidence that some BFRs, including polybrominated diphenyl ethers (PBDEs), can differentially accumulate in the maternally- and fetally-derived placental tissues, no studies have assessed associations between maternal and fetal placental THs with legacy and replacement BFRs and birth outcomes. To address this, we micro-dissected human placentas (n=200) collected at delivery into the maternal and fetal layers and analyzed them using mass spectrometry for T_3, rT_3, and T_4, 10 PBDEs, and several replacement BFRs, including decabromodiphenyl ethane (DBDPE), 2-ethylhexyl-2,3,4,5-tetrabromobenzoate (EH-TBB) and bis(2-ethylhexyl)tetrabromophthalate (BEH-TBP). BDE-47 was detected most frequently (59% of samples), followed by BDE-99, -153, and -209 and DBDPE was the most frequently detected replacement BFR (20% detection frequency). rT_3 and T_4 were detected in all samples, while T_3 was above detection limits in 43% of tissues. BDE-47 and rT_3 concentrations were higher in the fetal placenta than maternal placenta ($p<0.0001$). Birth-weight-for-gestational age z-scores (BW) significantly differed by BDE-153 detection in the maternal ($p=0.007$) and fetal tissues ($p=0.019$). Similarly, gestational age was significantly different by BDE-153 detection in the maternal placenta ($p=0.0051$). Several sex-specific associations were also observed. For example, while maternal BDE-47 was weakly but positively correlated with T_4 ($r_s=0.25$) within male-associated placentas, the opposite trend was observed with rT_3 in female-associated placentas ($r_s=-0.16$). Linear regression analyses also revealed that rT_3 was significantly associated with BW in male-associated placental tissues ($\beta=0.6-0.8$, confidence interval=0.1-1.2). These data highlight associations between gestational exposure to legacy and replacement BFRs

and placental TH levels, including sex-specific differences with fetal birth outcomes, and may be indicative of adverse impacts on placental TH regulation and fetal health.

P31-09 | Metabolic Signatures and Pathways Linking Maternal PFAS Exposure to Gestational Age at Birth: A High-Dimensional Mediation Analysis

Susan S. Hoffman[1], Anne L. Dunlop[2], Dana B. Barr[3], Blake Rushing[4,5], Audrey J. Gaskins[1], Todd Everson[3], Ashley Naimi[1], Stephanie M. Eick[3], Youran Tan[3], Kaitlin R. Taibl[3], Susan McRitchie[5], Susan Sumner[4,5], Kurunthachalam Kannan[6], Michele Marcus[1,3], Dean P. Jones[7], Donghai Liang[3,1]

[1] Emory University, Department of Epidemiology, Atlanta, USA; [2] Emory University, Department of Gynecology and Obstetrics, Atlanta, USA; [3] Emory University, Gangarosa Department of Environmental Health, Atlanta, USA; [4] University of North Carolina at Chapel Hill, Department of Nutrition, Chapel Hill, USA; [5] University of North Carolina at Chapel Hill, Nutrition Research Institute, Chapel Hill, USA; [6] University at Albany, Department of Environmental Health Sciences, Albany, USA; [7] Emory University, School of Medicine, Atlanta, USA

Background: Per- and polyfluoroalkyl substances (PFAS) are persistent environmental toxicants. Epidemiological studies have linked PFAS exposure to reduced gestational age at birth (GA), which disproportionally affects Black and African American populations. Maternal metabolic perturbations may mediate this association. To address this, we conducted high-dimensional mediation analyses to identify metabolic pathways linking PFAS exposure to GA.
Methods: We used serum samples collected from participants in early (8-14 weeks; n=307) and late (24-30 weeks; n=238) pregnancy in women from the Atlanta African American Maternal Child Cohort (2014-2018) to measure PFAS chemicals and untargeted metabolomics. GA (in completed weeks and categorized into preterm and early term birth) was abstracted from medical records, and we applied parallel mediation methodologies to identify the metabolomic signature of the PFAS-GA associations, including the Meet-In-The-Middle (MITM) and High-dimensional Mediation Analysis (HIMA) workflows. MITM identifies overlapping metabolites by independently assessing their associations with PFAS exposure and GA categories, while HIMA applies a penalization-based regression approach to select mediators in a high-dimensional mediation setting.
Results: Four PFAS chemicals were detected in ≥ 98% of participants during pregnancy. Increased serum perfluorooctanoic acid (PFOA) and perfluorononanoic acid (PFNA) concentrations were associated with an increased risk of an early GA. In the MITM workflow, we confirmed the identities of 47 metabolites at early pregnancy and 26 metabolites at late pregnancy as potential mediators. In the HIMA workflow, we confirmed one metabolite (Palmitoleic acid) at early pregnancy and two metabolites (Cortisol; N-Acetyl-D-galactosamine) at late pregnancy as mediators, which were either the same or biologically linked to those identified in MITM.

Conclusions: Metabolite mediators of the PFAS-GA association were closely involved in the tricarboxylic acid (TCA) cycle, bile acid biosynthesis, steroid hormone biosynthesis, and purine metabolism. While MITM provides a broad biological signature, HIMA applies a more conservative approach that increases confidence in findings.

P31-10 | Prenatal Exposure to Nitrites and Nitrates from Drinking Water and Associated Birth Outcomes

Tushara Rajeev[1], Tessa R. Bloomquist[1], Mohamad Burjak[2], Amii M. Kress[2], Julie Herbstman[1], Margaret R. Karagas[3], Judy L. Aschner[4], Yeyi Zhu[5], Alicia K. Peterson[5], John Meeker[6], Monique M. Hedderson[5], Heather H. Burris[7], Allison R. Sherris[8], Yoshira O. Van Horne[9], Dana Goin[10], Anne E. Nigra[1]

[1] Columbia University Mailman School of Public Health, Department of Environmental Health Sciences, New York, USA; [2] Johns Hopkins Bloomberg School of Public Health, Department of Epidemiology, Baltimore, USA; [3] Geisel School of Medicine at Dartmouth, Department of Epidemiology, Hanover, USA; [4] Hackensack Meridian School of Medicine, Center for Discovery and Innovation, Department of Pediatrics, Nutley, USA; [5] Kaiser Permanente Northern California, Division of Research, Pleasanton, USA; [6] University of Michigan School of Public Health, Department of Environmental Health Sciences, Ann Arbor, USA; [7] University of Pennsylvania Perelman School of Medicine, Department of Pediatrics, Philadelphia, USA; [8] University of Washington, Department of Environmental & Occupational Health Sciences, Seattle, USA; [9] University of California Los Angeles, Department of Environmental Health Sciences, Los Angeles, USA; [10] Columbia University Mailman School of Public Health, Department of Epidemiology, New York, USA

Objective

The U.S. regulatory limits for nitrites (1000 mg/L) and nitrates (10,000 mg/L) in public water systems were established to protect infants against methemoglobinemia. Other health outcomes, including adverse birth outcomes, may be associated with exposure at sub-regulatory levels. We evaluated the association between prenatal public water nitrate/nitrite exposure and birth outcomes in the Environmental Influences on Child Health Outcomes (ECHO) Cohort.

Methods

We included n=15,390 participants from 56 cohort study sites, with birth years from 2006-2019. To estimate exposure, we leveraged >4.9 million public water monitoring records and linked tract-level summed nitrate and nitrite concentrations to pregnancy residential address histories. We modeled individual, prenatal, time-weighted average estimates of public water nitrate/nitrite (range: <1-12,490 mg/L; mean= 579 mg/L). We used generalized estimating equation models to calculate adjusted risk ratios (RR) of preterm birth, low birthweight (LBW), and small-for-gestational age (SGA), as well as mean differences in birthweight-for-gestational age Z-scores and birthweight, and the geometric mean ratio of gestational age at birth. We modeled nitrate/nitrite continuously, in flexible spline models, and across quantiles. We explored potential effect measure modification by maternal education, race and ethnicity, and area-level socioeconomic vulnerability index.

Results

Overall, we observed no consistent associations between prenatal public water nitrate/nitrite and birth outcomes. Per 100 mg/L higher nitrate/nitrite, the RR of preterm birth was 1.00 (95% CI 1.00, 1.00). Similarly, associations with LBW and SGA were null (RR 1.00, 95% CI 1.00, 1.00). We did not observe heterogeneity across subgroups in stratified analyses.

Conclusion

We did not observe associations between prenatal public water nitrate/nitrite and birth outcomes in the ECHO consortium. Because prior studies have identified maternal vitamin C intake as a critical effect measure modifier of these associations, we will further evaluate potential modification by vitamin C intake. Null results should be interpreted cautiously pending these additional analyses.

P31-11 | Prenatal exposure to the mixture of cotinine, polycyclic aromatic hydrocarbons, and metal(loid)s on birth weight in the Houston MIEHR study

Wei-Jen Chen[1,2], Elaine Symanski[3,4], Iman Moussa[4], Patrick J. Parsons[5,6], Morgan Reynolds[5], Kurunthachalam Kannan[5,6], Peter Chutcharaven[5], Kristina W. Whitworth[3,4]

[1] Taipei Medical University, Department of Public Health, School of Medicine, Taipei City, Taiwan; [2] Taipei Medical University, School of Public Health, College of Public Health, Taipei City, Taiwan; [3] Baylor College of Medicine, Department of Medicine, Section of Epidemiology and Population Sciences, Houston, USA; [4] Baylor College of Medicine, Center for Precision Environmental Health, Houston, USA; [5] New York State Department of Health, Division of Environmental Health Sciences, Wadsworth Center, Albany, USA; [6] University at Albany, State University of New York, Department of Environmental Health Sciences, College of Integrated Health Sciences, Rensselaer, USA

Objective: Exposure to polycyclic aromatic hydrocarbons (PAHs) and metals/metalloids (collectively termed 'metals') often occurs in mixtures, potentially affecting fetal development through shared mechanisms. Few studies have investigated associations between exposure to PAHs as a mixture and birth weight, particularly in conjunction with exposure to metals. Our study aimed to address this research gap.

Material and Methods: This study included 544 mothers from the Maternal and Infant Environmental Health Riskscape (MIEHR) study in Houston, Texas. Concentrations of cotinine, 6 hydroxylated PAHs (OH-PAHs), and 17 metals were measured in spot urine samples collected at delivery. Infant birth weight was abstracted from medical records and converted into z-scores, standardized by gestational age and sex. Linear regression models were conducted to examine the association between individual chemical constituents and birth weight z-scores, adjusted for relevant covariates. Bayesian kernel machine regression (BKMR) was employed to examine the chemical constituents as a mixture for their single and joint effects on birth weight z-scores.

Results: Both linear regression and BKMR analyses indicated inverse associations of 2-hydroxynaphthalene, 2/3/9-hydroxyfluorene, and molybdenum with birth weight z-scores, while zinc was positively associated with birth weight. No overall mixture effect was observed, but a potential antagonistic interaction between zinc and molybdenum on change in birth weight z-scores was identified. Although inverse associations of cotinine, 1-hydroxynaphthalene, 1-hydroxypyrene, lithium, and strontium, as well as the positive association of chromium on birth weight, were observed in linear regression models, these associations were attenuated when accounting for effects from other chemical constituents within the mixture in BKMR model.

Conclusion: Our study provides evidence that prenatal exposure to naphthalene and fluorene (as indicated by their urinary metabolites 2-hydroxynaphthalene and 2/3/9-hydroxyfluorene) and molybdenum is associated with decreased birth weight, while the essential trace element zinc was associated with increased birth weight.

P31-12 | Prenatal Transcriptomic and Metabolomic Signatures of Infant Birth Outcomes: A Multi-Omics Investigation in an Agricultural Cohort

Yewei Wang[1], Donghai Liang[1], Duan Wang[1], Karen Hermetz[1], Amber Burt[1], Corina Lesseur[2], Parinya Panuwet[1], Nancy Fiedler[3], Tippawan Prapamontol[4], Panrapee Suttiwan[5], Pimjuta Nimmapirat[5], Supattra Sittiwang[5], Warangkana Naksen[6], Volha Yakimavets[1], Dana Barr[1], Ke Hao[7], Jia Chen[2], Carmen Marsit[1]

[1] Emory University, Rollins School of Public Health/the Gangarosa Department of Environmental Health, Atlanta, USA; [2] Icahn School of Medicine at Mount Sinai, Icahn School of Medicine at Mount Sinai, New York, USA; [3] Rutgers University School of Public Health, Environmental and Occupational Health Sciences Institute, Piscataway, USA; [4] Chiang Mai University, Research Institute for Health Sciences, Chiang Mai, Thailand; [5] Chulalongkorn University, Life Di Center, Faculty of Psychology, Bangkok, Thailand; [6] Chiang Mai University, Faculty of Public Health, Chiang Mai, Thailand; [7] Icahn School of Medicine at Mount Sinai, Department of Environmental Medicine, New York, USA

Objective

Prenatal environmental exposures influence fetal growth and may be mediated through maternal metabolomic features, although how these features influence birth outcomes warrant further mechanistic investigation. The placenta given its key functions could be influenced by those features to impact fetal development. This study aimed to identify common molecular pathways representing both endogenous and exogenous exposures that influence neonatal birth outcomes through the placenta.

Materials and Methods

We leveraged data from an established cohort of pregnant farmworkers and their offspring in agricultural regions of Thailand (N = 40). From placental transcriptomic data, gene networks were identified using weighted gene co-expression network analysis, and the eigengenes of those networks were examined for associations with neonatal birth outcomes (birth weight, length, and head circumference) using linear regression. Based on the matching maternal serum metabolomics data, we conducted a metabolome-wide association study (MWAS) to identify metabolic features associated with the same birth outcomes and placental eigengenes. Pathway enrichment analyses were undertaken on those metabolites common between these analyses.

Results

A significant association was found between a placental gene expression module representing myogenesis function and birth weight (estimate = -0.11, $p = 0.03$). MWAS identified 17 unique metabolites associated with both the myogenesis module and with birth weight. A distinct phospholipid cluster (cytidine diphosphate-diacylglycerol, phosphatidic acid, phosphatidylglycerol, phosphatidylcholine, and sphingomyelin) related to membrane remodeling and oxidative stress pathways were identified amongst those metabolites, as well as exogenous metabolites representing polycyclic aromatic hydrocarbons and sulfanylbenzoic acid.

Conclusion

By integrating placental transcriptome and maternal serum metabolome data from the same individual, we discovered novel converging pathways involving phospholipid metabolism and oxidative stress response associated with neonatal growth. These results provide a foundation for future research investigating how placental molecular mechanisms contribute to long-term health outcomes in children exposed to environmental pollutants.

P32 | Advancing Environmental Justice through Research, Policy, and Community Engagement

P32-01 | Are Clean Air Equally Shared? A Gini-Based Approach to $PM_{2.5}$Inequality

Yifan Chao, Chris C. Lim

The University of Arizona, Tucson, USA

Background: Air pollution inequality is an emerging concern in environmental justice and public health. While Gross Domestic Product (GDP) inequality has been extensively studied, the relationship between economic disparities and air pollution distribution has not received as much of attention. This study explores the relationship between $PM_{2.5}$-based Gini coefficients, which measure air pollution inequality, and GDP per Capita Gini coefficients at the subnational level, accounting for economic and demographic factors.
Methods: Using the extracted remote-sensing global annual $PM_{2.5}$ data, we computed $PM_{2.5}$-basedGinito quantify air pollution inequality as well as GDP per Capita Gini for multiple years in 10 selected countries. The study controlled for economic and demographic variations, such as GDP and population size. We first calculated the Pearson correlation between $PM_{2.5}$-based Gini and GDP per Capita Gini. Then, linear mixed-effects models (LMMs) were used to assess how much GDP per Capita inequality predicts air pollution inequality while controlling for economic and demographic factors. The Akaike Information Criterion (AIC) and R^2 were used to assess model performance.
Results: The significant ($p < 0.05$) Pearson correlation suggested a positive association between GDP per Capita inequality and air pollution disparities. The best-fit LMM model, with the lowest AIC and greatest R^2, suggested that GDP per Capita Gini explains a substantial part of the variation in $PM_{2.5}$-basedGini, after accounting for economic and demographic factors.
Conclusion: Our findings demonstrated that economic disparity adds another layer in assessing and understanding air pollution distribution and exposure disparities. Regions with higher GDP per Capita inequality tend to experience greater unequal air pollution distribution, although variations were observed across regions and countries. These findings underscore the need for integrated plans that concurrently address economic, environmental, and public health issues.

P32-02 | Unmeasured rural populations: assessing equity in the distribution of $PM_{2.5}$ monitors

Zoe Haskell-Craig[1], Kevin P. Josey[2], Patrick L. Kinney[3], Priyanka deSouza[4]

[1] New York University School of Global Public Health, Department of Biostatistics, New York, USA; [2] Colorado School of Public Health, Department of Biostatistics and Informatics, Aurora, USA; [3] Boston University School of Public Health, Department of Environmental Health, Boston, USA; [4] University of Colorado Denver, Urban and Regional Planning Department, Denver, USA

US EPA regulatory monitors comprise the largest network of high-quality ground-based $PM_{2.5}$ data. This data is routinely used in epidemiological studies, and to calibrate high-resolution estimates of national pollutant concentrations. Monitors are also beneficial for neighborhoods, leading to the enforcement of air quality standards and decreased pollution levels. Yet monitor site selection is complex, with some evidence that agencies avoid placing monitors in pollution 'hotspots' in low-income communities. However to date, relatively little Environmental Justice (EJ) research has focused on equity in access to monitoring. We assess how siting tendencies impact the regulatory

network as a whole, using hierarchical (mixed-effect) models with county and state -level random intercepts to investigate whether census tract EJ attributes (%American Indian/Alaskan Native, %Asian, %Black, %Hispanic, %White, %living below the poverty line) are associated with neighborhood proximity to 2022 $PM_{2.5}$ monitor sites. Separate models are fit for each attribute, stratified by urbanicity and controlling for population density and annual average $PM_{2.5}$ concentration.

Our results show that most EJ attributes exhibit negative or weak associations with monitor proximity, suggesting that these EJ populations are well-represented in regulatory monitoring datasets. The exception is in rural areas: where higher poverty levels are significantly linked to greater monitor distances (β = 0.6, 95%CI = [0.49, 0.71]). This suggests that pollution concentrations experienced by low-income rural populations are systematically underrepresented in datasets, a critical omission at a time when increasing wildfires are driving changing $PM_{2.5}$ exposure patterns. We present a discussion of the challenges in defining equity in monitoring, and the risk of sampling-bias inducing measurement error for lower-income rural populations.

P32-03 | Environmental Justice Index Components and Ovarian Cancer Risk and Survival

Rachel Pomazal[1], Amy Trentham-Dietz[1,2], John Hampton[1,2]

[1] University of Wisconsin-Madison, Department of Population Health Sciences, Madison, USA; [2] University of Wisconsin-Madison, Carbone Cancer Center, Madison, USA

Objective: Ovarian cancer is the deadliest gynecological cancer. Previous studies suggest that geographic location and social deprivation are associated with ovarian cancer diagnosis and survival. However, opportunities remain to examine these associations from an environmental justice lens and to disentangle the aspects of disadvantage that are contributing to worse ovarian cancer outcomes. The aim of this research is to evaluate the association between the Environmental Justice Index (EJI) component indicators and ovarian cancer risk and survival.
Materials and Methods: Data are from the Wisconsin Women's Health Study, a case-control study of 592 ovarian cancer cases and 3490 controls conducted 1992-2001. Participants were identified using statewide cancer registry and population lists. Risk factor data were collected through telephone questionnaires. Date and cause of death were ascertained for cases by linkages with the National Death Index. Participants were mapped to EJI indicators for census years 1990 and 2000 based on their residence at the time of study enrollment. Logistic regression and Cox proportional hazards will be used to analyze associations between EJI components with ovarian cancer risk and survival.
Results: Among cases, 46% were aged ≥60, 32% lived in rural areas, and 55% had a high school degree or less education. Among controls, 55% were aged ≥60, 34% lived in rural areas, and 58% had a high school degree or less. 42% of cases were diagnosed with metastatic disease. Mean survival among cases was 9.9 years (range 0.9-25.3). In age-adjusted analyses, cases were 2.9% (95%CI: 0.5-5.5%) more likely to live within 1 mile of a railway and 9.4% (95%CI: 1.1-18.5%) more likely to have a 1-day increase in the mean number of ozone days/year.
Conclusion: These preliminary results suggest EJI components are associated with ovarian cancer outcomes. On-going analysis will include spatial, logistic regression, and Cox proportional hazards analyses.

P32-04 | Environmental Justice Dimensions of Orphaned Oil and Gas Wells in the United States

Grace M. Hauser[1,2], Nicole C. Deziel[1], Kyle Ferrar[2], Joshua L. Warren[1], Cullen Smith[2]

[1] Yale School of Public Health, New Haven, USA; [2] FracTracker Alliance, Pittsburgh, USA

Objective
Orphaned oil and gas (O&G) wells – non-operational and without a financially-responsible party – can emit methane and air toxics, accelerating climate change and posing a health risk to the estimated 14 million Americans living within one mile of an orphaned well. We evaluated the distribution of orphaned wells across the United States (US) in 2024 in relation to community environmental justice (EJ) characteristics.

Material and Methods
We constructed a national orphaned O&G wells dataset, current as of November 2024, using state databases and the publicly accessible FracTracker Alliance database. An EJ dataset was created using the 2017-2021 US Census American Community Survey and EPA's EJScreen tool v.2.2. Variables (e.g., poverty status, educational attainment, and PM2.5 concentration) captured socioeconomic and environmental disparities. Wells and EJ data were assigned to Census Block Groups (CBGs). We compared orphaned well counts to a 2022 US Geological Survey dataset and used logistic regression to examine EJ indicators' association with CBGs containing orphaned wells.

Results
We identified 111,848 orphaned wells across 27 states, mainly in Utah (29,406), Oklahoma (18,459), and Kentucky (15,367). Between June 2022 to November 2024, the number of documented orphaned wells increased by 52,981, mainly in Utah (+29,369), Texas (+5,391), and Oklahoma (+5,460). During the same period, only 4,994 wells were plugged, mainly in Texas (1,776), Louisiana (880), and Oklahoma (462). Ten states plugged no wells at all. Preliminary analyses reveal statistically significant associations between EJ variables and counts of orphaned wells (e.g., % mobile homes, % housing with incomplete plumbing), with some variation across states.

Conclusion
State-level variability in EJ indicators highlights the need for localized analyses and suggests an urban-rural divide. Effective policies should integrate EJ principles to ensure impacted communities receive necessary resources to address orphaned well hazards.

P32-05 | Elevated serum copper levels in a bariatric population in Southeastern Louisiana: Investigating distributions and racial disparities

Katherine McKeon[1], Michael Cook[2], John Baker[2], Kyle LaPenna[2], Jake Doiron[2], Felicia Rabito[1]

[1] Tulane University, Department of Epidemiology/ Celia Scott Weatherhead School of Public Health and Tropical Medicine, New Orleans, USA; [2] Louisiana State University, Health Sciences Center, New Orleans, USA

Objective: Copper (Cu) is essential for many physiological functions, but in excess can be harmful to health. Increased serum Cu is associated with obesity, however data on Cu levels in people with obesity is limited. The objective of this cross-sectional analysis was to quantify the prevalence of elevated (≥155 mcg/dl) serum Cu levels in pre-bariatric surgery patients and to explore racial disparities in Cu levels.

Methods: Data were obtained from electronic health records of adults presenting to the Center for Weight Loss and Bariatric Surgery at University Medical Center Hospital in New Orleans, LA between 2018-2023. Patients did not have to proceed with surgery to be included. Covariates including hormonal contraception and inflammatory markers were evaluated for adjustment. Self-reported race was categorized as Black, white, and other. Elevated serum Cu was defined as Cu ≥ 155 mcg/dl and analyzed using logistic regression.
Results: Of the n=1763 study participants, 70.7% were Black, 24.9% were white, and 4.4% fell into the other category. The median age was 40 years old, and 92.1% of participants were female. Overall, the median serum Cu was 141 mcg/dl; 31.8% of participants had elevated Cu. Black race was associated with elevated Cu in both the crude model (OR = 1.97 (95% CI: 1.54-2.51), and in the adjusted model (OR=1.94 (95%CI: 1.34-2.77). Female sex was associated with elevated Cu in both crude and adjusted models (OR= 6.73 95 % CI: 3.48, 12.79; OR=14.37 95%CI: 4.37-47.21, respectively).

Conclusion: This study found a high prevalence of elevated Cu levels with significant disparities by Black race and female sex, even after adjustment for inflammatory markers. These findings are higher compared to previous studies on Cu level in obese cohorts. Further research is needed to investigate Cu levels in high-risk populations and to identify potential environmental sources of elevated Cu.

P32-06 | Proximity to concentrated animal farming operations and the risk of vaginitis

Lesley Thompson[1], **Bo Park**[1], Pauline Lim[1], Ruofan Yao[2], Cristina Connolly[3], Shannon Roback[4]

[1] California State University, Fullerton, Department of Health and Human Development, Fullerton, USA; [2] Loma Linda University, Loma Linda, USA; [3] University of Connecticut, Mansfield, USA; [4] California State University, Dominguez Hills, Carson, USA

Objective:
To evaluate the risk of vaginitis associated with close proximity to concentrated animal farming operations (CAFO).
Methods:
This was a retrospective cohort analysis using the California Department of Health Care Access and Information (HCAI) linked birth file between 2007-2012 combined with the Concentrated Animal Farming Operations (CAFO) database linked by zip code. Maternal and neonatal diagnoses and procedure codes were based on the International Classification of Diseases, 9th Revision, Clinical Modification (ICD-9-CM). Close proximity to CAFO was defined as having a primary dwelling zip code within a 5 mile radius of CAFO. The outcome of interest was vaginitis. Multivariate logistic regression analysis was used to estimate the effect of CAFO exposure on vaginitis.
Result:
3,098,578 women were included in analyses, of which 343,781 live within a 5 mile radius of a designated CAFO. When compared to individuals living remote from CAFO, those who live within close proximity to CAFO were at higher risk of vaginitis (aOR 3.47 [3.15 – 3.83])after adjusting for potential confounders.
Conclusion:
Living within close proximity to CAFO increases the risk of vaginitis. The underlying etiology requires further investigation.

P32-07 | Socioeconomic Inequities Continue to be Drivers of Malaria in India: Mapping Districts between 2019-2023

Avik K. Sam[1], Neha Keshri[1], Harish C. Phuleria[1,2]

[1] Indian Institute of Technology Bombay, Environmental Science and Engineering Department, Mumbai, India; [2] Indian Institute of Technology Bombay, Inter-Disciplinary Program in Climate Studies, Mumbai, India

Introduction:
Malaria continues to be a significant contributor to the disease burden in India, bearing a substantial economic and policy mandate. The National Center for Vector-Borne Disease Control Programme (NCVBDC), an umbrella body under the Federal Ministry of Health and Family Welfare, has targeted 2027, two years from now, as the year of achieving zero Indigenous cases. Here, we map the malaria cases across the Indian districts between 2019 and 2023 using a digital dashboard and assess the potential drivers of malaria transmission.
Methods:
We collated the annual district-wise data on the Annual Parasite (API) and Falciparum Incidence (AFI) from the NCVBDC. Socioeconomic inequities data was obtained from the Demographic and Health Survey, while land-use land-cover and meteorological data were collated from the satellite reanalysis data platforms. We use an ensemble

of statistical models to identify the associated covariates responsible for malaria transmission and Getis-Ord Gi* statistics to identify the clusters.

Results:

India reported 0.33 million cases in 2019, which reduced to 0.18 million in 2020 when the COVID-19 pandemic occurred. The cases further decreased by 13.3% the following year, but then it again increased in 2022 and 2023 when 0.23 million cases were reported. The *P. falciparum-infested* regions are mainly prevalent in Northeastern and Central India, as the districts reported both API and AFI > 10. This is also evident from the spatially clustered hotspots, defined by the significant positive Getis-Ord G* statistics. However, while the clusters in Central India weakened, the districts in Northeast India experienced intensified clustering over the years. The ensemble models suggest that the marginalised socio-economic communities residing in forested regions witnessing high rainfall remain the most vulnerable to Malaria. Our dashboard will help the stakeholders and the general public to gain insights into malaria transition across Indian districts and will aid in better awareness.

P32-08 | Health Benefits from the Rapid Reduction in Ambient Exposure to Air Pollutants after China's Clean Air Actions: Progress in Efficacy and Geographic Equality

Ruohan Wang, Hong Lu, Tao Xue

Peking University, School of Public Health, Beijing, China

Objective

Clean air actions (CAAs) in China have been linked to considerable health benefits by enhancing air quality and potentially preventing premature mortality caused by air pollution. However, whether the beneficial effects of CAAs are equally distributed geographically is unknown. This study aimed to track spatiotemporal changes in health impacts from, and geographic inequality embedded in the reduced exposures to fine particulate matter [$PM_{2.5}$] and ozone [O_3] between 2013 and 2020.

Material and Methods

Using high-resolution maps of the distributions of air pollutants and population, we utilized the metric of attributable deaths and years of life lost (YLL) to measure the sum of health impact from air pollution among different subgroups, categorized by sex, age, residence, and spatiotemporal coordinate. We then calculated gains in life expectancy (GLE) from reduced $PM_{2.5}$ and O_3 exposure and assessed geographic inequality using the Gini Index (GI).

Results

In the first stage of CAAs (2013 to 2017), the population-weighted concentration of $PM_{2.5}$ decreased significantly, dropping from 68.98 μg/m^3 to 47.13 μg/m^3, and further to 35.77 μg/m^3 after the second stage (2017 to 2020). Conversely, O_3 concentrations showed an increase from 111.4 μg/m^3 to 121.1 μg/m^3 during the first stage and a slight decrease to 114.9 μg/m^3 after the second stage. The mean GLE was 1.87 months in the first stage, with half of the sum of the GLE was disproportionally distributed in about one quarter of the population exposed (GI 0.44). During the second stage of CAAs, the mean GLE increased to 3.94 months and geographic inequality decreased (GI 0.18).

Conclusions

CAAs were enhanced from the first to second stages, in terms of not only preventing premature mortality but also ameliorating health inequalities. The enhancements were related to increased sensitivity to the health effects of air pollution and synergic control of $PM_{2.5}$ and O_3 levels.

P32-09 | Increasing Decent Living Standards at the district level in India and its impact on climate mitigation and adaptation

Amrutha G.s.[1], Omkar Patange[2], Michael Kuhn[2], Arup Jana[1], Shilpa Rao[3]

[1] International Institute for Population Sciences (IIPS), Mumbai, India; [2] International Institute for Applied System Analysis (IIASA), Laxenburg, Austria; [3] Norwegian Institute of Public Health (NIPH), Oslo, Norway

Decent Living standards (DLS) are based on material requisites for human well-being. They are intrinsically linked to climate mitigation and adaptation. This study analyses district-level panel data constructed using the 4th (2015-16) and 5th (2019-21) rounds of National Family Health Surveys (NFHS). It constructs a DLS Index to assess well-being across the districts in both time periods. This study further links the DLS to air pollutant- particulate matter 2.5 (PM2.5) and greenhouse gas emission data. We find that a unit increase in DLS decreases pollutant (PM2.5) concentration by 0.6 units (µg/m3), controlling for urbanisation and age. This shows that increasing DLS is not necessarily associated with higher pollution and may, in addition, improve climate adaptation.

P32-10 | An Investigation of the Impact of Concentrated Animal Feeding Operations on the Historical Trends of Agricultural Communities

Brandon Lewis, Michelle Bell

Yale University, School of the Environment, New Haven, USA

Background: Concentrated Animal Feeding Operations (CAFOs) are large industrious animal farms that can impact nearby communities and the environment through waste pollution. Understanding how these farms are constructed, their history, and the relationship between farm exposure, the community, and the environment are essential for making CAFOs sustainable and for the local communities in which they reside.
Objectives: We investigate how the construction of concentrated animal feeding operations (CAFOs), specifically hog CAFOs, has impacted communities in North Carolina (NC) historically, socially, and economically, as compared to non-hog CAFO communities in North Carolina.
Methods: We identified the construction year of each hog CAFO using satellite imagery and established remote sensing techniques (i.e., changepoint detection) and additional publicly available data. We then investigated the association between the demographic and economic trends from 1986 to 2012 of hog CAFO counties. We compare trends of CAFO to non-CAFO counties during the increase in the development of hog CAFOs.
Results: Preliminary results indicate that the percentage of persons of color tended to decrease over time for counties with hog construction while increasing for counties without hog construction. The reverse is seen from trends in the white population. Counties without hog construction have seen a significant increase in average personal income (in thousands, $450,700) compared to counties without hog construction (in thousands, $296,800),

over time. Additionally, the number of wage and salary jobs tended to increase significantly in counties without hog construction compared to counties with hog construction during this period.
Discussion: These techniques allow for the first assessment, to our knowledge, of the temporal relationship between the construction of CAFOs and their potential impact on the community over time which will provide more insight into how industrial livestock production operates as a tool of sustainable local development and health in rural communities.

P32-11 | Comparative Analysis of Air Quality Data for Environmental Justice Assessment Considerations

Chloe Chung, Annette Rohr

EPRI, Palo Alto, USA

Recent advancements in environmental justice (EJ) screening and mapping tools have enhanced our ability to identify and address the disproportionate environmental burdens faced by various communities. These tools often utilize air pollution data, such as $PM_{2.5}$ and ozone levels, as key indicators due to their well-documented health impacts. However, the reliance on outdated air quality data can compromise the accuracy of these assessments.
In this study, we analyzed publicly available $PM_{2.5}$ and ozone monitoring data from the U.S. Environmental Protection Agency (EPA) for the years 2019, 2022, and 2023. Our objective was to assess the potential consequences of using outdated data in EJ evaluations. We observed significant variability in pollutant concentrations over time, with site-level differences ranging from a decrease of 5.25 μg/m³ to an increase of 9.29 μg/m³ for $PM_{2.5}$, and from a decrease of 20.7 ppb to an increase of 22.6 ppb for ozone between 2019 and 2023.
These findings highlight the dynamic nature of air quality and underscore the importance of using current data in EJ assessments. The observed variability is likely influenced by changes in emission sources, driven by local infrastructure developments, policy changes, regulatory measures, and technological advancements. While EJ tools are designed for preliminary screening, their outputs, often visualized as heat maps, can imply a level of precision that may not be warranted without up-to-date data.
To enhance the reliability of EJ assessments, it is crucial to provide comprehensive education and training for users, ensuring they understand the tools' capabilities and limitations. Continued efforts in refining and updating EJ screening tools are essential to better support overburdened communities and to inform effective policy and intervention strategies.

P32-12 | Disparities in Air Pollution Emissions Across Home and Workplace Environments in the United States

Jenny J. Lee[1], Priyanka deSouza[2,3]

[1] Ewha Womans University, Department of Data Science, Seoul, South Korea; [2] University of Colorado Denver, Department of Urban and Regional Planning, Denver, USA; [3] University of Colorado Boulder, CU Population Center, Boulder, USA

Objective

Human mobility plays a critical role in determining exposure to air pollution and associated health risks. However, most existing research has focused on exposure based on residential location alone, potentially overlooking variations related to workplace environments. This study quantifies disparities in emission sources that contribute to exposures experienced at residential and workplace locations for multiple air pollution emissions from different source sectors.

Materials and Methods

We leverage a database comprising the residential and workplace counties of of ∼128−144 million workers in the United States between the years 2009 and 2017. We evaluate county-level emissions from multiple pollution sources—industry (SO2), energy (SO2, NOx), agriculture (NH3), commercial (NOx), and on-road transportation (NOx)—for the same time period. We assess disparities in emissions changes, focusing on racial/ethnic, socioeconomic, and demographic factors.

Results

Exposure disparities between home and workplace were observed for all pollutants except SO2 from energy. Emissions were generally higher at workplaces than residences, except NH3. Workers with higher income and education, as well as those in sectors like real estate and finance, had the highest exposures. Racial/ethnic disparities were significant, with Asian workers experiencing the highest exposure, and white and Native Hawaiian/Pacific Islander workers the lowest. In the 9 climate regions, workers in the Northeast experienced the highest emissions at both locations for all pollutants, except NH3 and SO2 from energy.

Conclusion

Our study is the first nationwide, multiyear analysis that incorporates multiple emission pollutants and sectors based on both home and workplace addresses. This study emphasizes how accounting for workplace exposures can influence disparities in air pollution exposure and underscores the importance of considering mobility to fully capture environmental disparities.

P32-13 | Community Severance and Environmental Inequities in New Haven: A Geospatial Analysis

Aline Maybank[1], Pargoal Arab[1], Thomas Commander[2], Annie Lin[2], Andrei Harwell[3], Daniel Carríon[1]

[1] Yale School of Public Health, Environmental Health Sciences, New Haven, USA; [2] Yale College, New Haven, USA; [3] Yale School of Architecture, New Haven, USA

Objective: Community severance, the physical and social fragmentation caused by road infrastructure and motorized traffic, can limit access to essential services, social networks, and green spaces, potentially exacerbating environmental health disparities. While previous studies have quantified community severance in urban settings, little research has examined its relationship with environmental conditions, such as air pollution and urban heat. This entirely student-led study aims to quantify community severance in New Haven, Connecticut, and investigate its associations with land surface temperature, vegetation cover, and nitrogen dioxide (NO_2) concentrations at the census block group level.
Method: To investigate community severance in New Haven, we replicated the Community Severance Index (CSI) framework developed for New York City by Benavides et al. at Columbia University by integrating geospatial datasets on road infrastructure, pedestrian connectivity, and socioeconomic factors. CSI values were calculated using factor analysis, incorporating road network density, traffic volume, and pedestrian barriers. Also, environmental exposure variables, including land surface temperature (LST), tree cover, Normalized Difference Vegetation Index (NDVI) datasets were shared from the Seto lab at Yale University and NO_2 concentrations were retrieved from Sentinel-5P TROPOMI observations. Linear and spatial regression models were used to assess associations between community severance and environmental conditions.
Results: Preliminary analyses indicate spatial heterogeneity in CSI values across New Haven, with higher severance observed in historically marginalized neighborhoods. We hypothesize that census block groups with high CSI values will exhibit elevated LST, lower NDVI, reduced tree cover, and increased NO_2 concentrations, reflecting disparities in environmental exposures linked to transportation infrastructure and historical urban planning decisions.
Conclusion: By integrating geospatial analysis with environmental and demographic data, this study will provide novel insights into how community severance intersects with environmental health risks. These findings will inform equitable urban planning policies aimed at mitigating transportation-related barriers and improving environmental conditions for vulnerable communities.

P32-15 | Social inequalities in the distribution of health-related ecosystem services in the Italian population

Sofia Tagliaferro[1,2], Pierpaolo Marchetti[1], Linda Avesani[3], Chiara Badaloni[4], Nicola Caranci[5], Riccardo Greco[3], Francesca Locatelli[1], Maura Loi[6], Mauro Masiero[7], Silvia Pogliaghi[6], Alessandro Marcon[1]

[1] University of Verona, Unit of Epidemiology and Medical Statistics, Department of Diagnostics and Public Health, Verona, Italy; [2] National Research Council, Institute of Clinical Physiology, Pisa, Italy; [3] University of Verona, Department of Biotechnology, Verona, Italy; [4] Lazio Region Health Service/ASL Roma 1, Department of Epidemiology, Rome, Italy; [5] Emilia-Romagna Region, Department of innovation in healthcare and social services, Bologna, Italy; [6] University of Verona, Department of Neurosciences, Biomedicine and Movement Sciences, Verona, Italy; [7] University of Padua, Department of Land, Environment, Agriculture and Forestry, Padua, Italy

Objective:
The distribution of ecosystem services (ES) and their relationship with socioeconomic factors is underexplored. This study examines social inequalities in the supply of health-related ES in the Italian population.

Material and Methods:
We intersected 2018 Corine Land Cover data with 2021 census-tract limits and population data for Italy, then assigned ES supply to each census-tract portion using the Burkhard matrix (2012). We devised a score summing selected health-related regulation and cultural ES, ranging from 0 (no supply) to 35 (maximum supply). Socioeconomic disadvantage was defined as a municipality-level proportion of people with low education ≥ 80th percentile. We used logistic regression with population frequency weights to estimate adjusted proportions.

Results:
The score was ≤1 for 77% of Italy's 59 million population (median score: 1, 5th-95th percentile: 0-16). High ES supply (score ≥ 9, the 90th percentile) was more common in Central (19%) and Southern Italy (17%) than in the North (13%), and in rural areas (33%) than in towns (17%) and cities (6%). People living in disadvantaged areas had a greater supply of ES (18%) than those in less disadvantaged areas (15%). However, this was due to the uneven distribution of socioeconomic disadvantage across the country, with the disadvantaged population concentrated in Southern Italy (38% vs. 10% in Central/Northern Italy) and rural areas (35% vs. 22% in towns and 9% in cities). When adjusting for geographic zone and urbanization, high ES supply was more common in less disadvantaged areas (14%) than in disadvantaged areas (11%).

Conclusion:
This preliminary analysis highlighted the limited provision of ES in the country and possible social inequalities in its distribution. Urbanization emerged as the primary factor influencing ES supply in Italy.

Funding: Cariverona Foundation, call ‘Capitale Naturale 2023’

P32-16 | Quantifying the spatial inequalities of the effect of heat on health

Xinyi Chen, Marta Blangiardo, Connor Gasgoigne, **Garyfallos Konstantinoudis**

Imperial College London, London, UK

Exposure to high ambient temperatures is a significant driver of preventable mortality, with non-linear health effects and elevated risks in specific regions. To capture this complexity and account for spatial dependencies across small areas, we propose a Bayesian framework that integrates non-linear functions with the Besag, York, and Mollie (BYM2) model. Applying this framework to all-cause mortality data in Switzerland, we quantified spatial inequalities in heat-related mortality. We retrieved daily all-cause mortality at small areas (2,145 municipalities) for people older than 65 years from the Swiss Federal Office of Public Health and daily mean temperature at 1km×1km grid from the Swiss Federal Office of Meteorology. By fully propagating uncertainties, we derived key epidemiological metrics, including heat-related excess mortality and minimum mortality temperature (MMT). Heat-related excess mortality rates were higher in northern Switzerland, while lower MMTs were observed in mountainous regions. Further, we explored the role of the proportion of individuals older than 85 years, green space, average temperature, deprivation, urbanicity, and language regions in explaining these discrepancies. We found that spatial disparities in heat-related excess mortality were primarily driven by population age distribution, green space, and vulnerabilities associated with elevated temperature exposure.

P32-17 | Climate justice in the U.S.-Mexico borderlands: differential exposure to extreme heat by sociodemographics in three binational conurbations

Lara Schwarz[1], Joan A. Casey[2], David J. González[1]

[1] University of California, Berkeley, Environmental Health Sciences, School of Public Health, Berkeley, USA; [2] University of Washington, Department of Environmental and Occupational Health Sciences, School of Public Health, Seattle, USA

Objective: The U.S.-Mexico borderlands are highly affected by extreme heat, yet the majority of health equity research has been conducted on the U.S. side of the border. We evaluated extreme heat exposure in three cross-border conurbations (San Diego-Tijuana, El Paso-Ciudad Juárez and Brownsville-Matamoros), and explore exposure disparities by factors related to susceptibility and socioeconomic marginalization. The three conurbations were collectively home to 14.1 million people.

Methods: We obtained sociodemographic data from 2010 national census datasets for census tracts (CTs, U.S.) and Áreas Geoestadísticas Básicas (AGEBs, Mexico). Daily temperature estimates were obtained from Daymet. We defined extreme heat thresholds as the 99th percentile of maximum (daytime) and minimum (nighttime) temperature during a historical period (1990-1999) for each CT/AGEB. We estimated local relative warming as the number of days above this threshold from 2006–2014. We estimated relative exposure risks (RR) for people over age 65, with a high school degree or more, and unemployed. To estimate RRs, we compared the proportion of residents in each

subpopulation in CTs/AGEBs with the number of extreme heat days in the highest quartile to the lowest quartile of each sociodemographic in each conurbation and country.
Results: On average, Brownsville-Matamoros showed the highest exposure to daytime extreme heat (103.4 days) and El Paso-Ciudad Juárez to nighttime extreme heat (123.7 days) from 2006 to 2014. Mexican AGEBs had a higher exposure to extreme heat than U.S. CTs across all regions, up to 70%. Higher daytime extreme heat exposure was observed for older, less educated communities. No consistent differences were found for unemployment. With respect to education, the greatest observed disparities were in Brownsville (RR: 1.18), U.S., and Tijuana (RR: 1.16), Mexico.
Conclusion: Identifying subpopulations most affected by extreme heat is critical to inform effective cross-border adaptation, particularly as temperatures continue to trend hotter than historical averages.

P32-19 | Particulate matter and black carbon monitoring in urban environmental justice communities using a network of conventional and low-cost sensors

Tian Xia, Stuart Batterman

University of Michigan, Environmental Health Sciences, Ann Arbor, USA

Low-cost sensors for monitoring air quality are increasingly being used to supplement the generally spatially sparse network of regulatory air quality monitoring sites operated by states and the US EPA. These sensors can provide consistent and localized measurements of particulate matter (PM) concentrations, but performance can vary significantly from the federal reference methods (FRM) used by EPA, sensor performance has not been well documented across a broad range of environmental conditions, and few gases or PM constituents (like black carbon) are typically monitored. Our objectives in this study were to assess the long-term performance of low-cost black carbon (BC) and PM sensors, and then use these data to characterize temporal trends and major sources of PM pollution in Detroit, Michigan, a moderately polluted urban/industrial area. Seven DSTech ObservAir sensors were deployed across the study area for two years (2023 – 2025) to measure PM, NO_2, black carbon (BC), CO and weather parameters, including three which were collocated at one FRM site to test long-term performance and sensor variation. The sensor data was also compared to FRM measurements at four other sites in the study area. After initial calibration, BC and $PM_{2.5}$ agreement with FRM instruments was moderately high (R^2 = 0.45 and 0.83 respectively). After two years, several of the PM and gas sensors malfunctioned, and data completeness was lowered by power outages, poor Wi-Fi/LTE connections, and microSD failures. The collected data clearly showed diurnal, monthly and day-of-week trends for each pollutant, and the striking impact of the Canadian wildfire smoke events in June 2023. Pollution roses with nearby airport meteorological data indicated traffic emissions as the major BC and $PM_{2.5}$ source in this area. Advantages, disadvantages, precautions and lessons learned for low-cost sensors are discussed.

P32-20 | Heterogeneity in approaches in characterizing community exposures to animal feeding operations: implications for evidence synthesis and meta-analysis

Elizabeth A. Chatpar[1,2], Brent F. Kim[1,2], Bashiru C. Bakin[1,2], Sara N. Lupolt[1,2], Juleen Lam[3], Joan A. Casey[4], Keeve E. Nachman[1,2]

[1] Johns Hopkins Bloomberg School of Public Health, Department of Environmental Health & Engineering, Baltimore, USA; [2] Johns Hopkins Bloomberg School of Public Health, Center for a Livable Future, Baltimore, USA; [3] California State University, East Bay, Department of Public Health, Hayward, USA; [4] University of Washington, Department of Environmental & Occupational Health Sciences, Seattle, USA

Objective. This research summarizes the heterogeneity in exposure assessment approaches across epidemiologic studies on animal feeding operations and proposes ways to address this heterogeneity for more meaningful evidence synthesis.
Materials & Methods. This research is part of an ongoing systematic review of health impacts associated with community exposure to animal feeding operations. Studies were identified using a predetermined search strategy run in August 2024 and screened using a Population-Exposure-Comparator-Outcome-Study Design (PECOS) framework. Screening and data extraction were each conducted independently by two reviewers using DistillerSR. Extracted data included the exposure metric, its unit, type of animal exposure (e.g., species), transformations of exposures (e.g., continuous to categorical variables), and health outcome evaluated.
Extracted data were categorized by variable type (e.g., ordinal, binary, continuous) and exposure metric type (e.g., density, distance, indicator contaminant). Findings were summarized using narrative analyses.
Results. Our search returned approximately 20,000 results, of which approximately 160 were included in this analysis. Existing literature uses a range of metrics to assess exposure, including animal density, buffer distances, indicator contaminants (e.g., hydrogen sulfide), and participant-derived measures (e.g., perceived odor). Most exposures are modeled as categorical variables, which are limited in their use for evidence synthesis since categories are often population- or study-specific (e.g., quantiles of animal density in a specific region). Exposure measurement heterogeneity in this body of literature challenges evidence synthesis and meta-analysis of exposure-outcome relationships by limiting the ability to derive meaningful pooled estimates, due to the variation of metrics, units, and measurement techniques used.**Conclusion.** To improve comparability between studies, future research should prioritize the use of standardized (e.g., animal equivalent units per hectare) and continuous exposure variables. Harmonizing exposure assessment approaches across studies could enhance the ability to synthesize evidence and better understand the health impacts of animal feeding operations on nearby communities.

P32-21 | Association of deprivation and sleep disturbance related to road traffic noise exposure

Kathryn Adams[1,2], Calvin Jephcote[1,2], Tess Osborne[2,3], John Gulliver[4]

[1] University of Leicester, Centre for Environmental Health and Sustainability, Leicester, UK; [2] University of Leicester, School of Geography, Geology and the Environment, Leicester, UK; [3] University of Birmingham, School of Geography, Earth and Environmental Science, Birmingham, UK; [4] City St George's, University of London, Population Health Research Institute, London, UK

Background and Objective: Road traffic noise has been associated with negative health outcomes related to sleep disturbance. Environmental exposure studies have noted that the distribution of road traffic noise is not equitable across socioeconomic groups. Exposures to high noise levels may compromise the restorative benefits of sleep. It is of importance to identify which communities, based on deprivation level, are most at risk of sleep disturbances due to elevated road traffic noise levels, as this may exacerbate health inequalities across and within cities. This study analysed the socioenvironmental inequalities in night-time (L_{night}) road traffic noise exposures and sleep disturbance at residential locations in Greater Manchester, England.
Materials and Method: Noise levels from major and minor roads were modelled for residential locations at the neighbourhood (small area) level (N=8,966), in accordance with the European Commission Common Framework for Noise Assessment (CNOSSOS-EU) methods from Environment Noise Directive 2002/49/EC. Traffic-related noise exposures and high sleep disturbance (HSD%), the standard procedure for estimating HSD from the WHO 2018 systematic reviews, was assessed across deprivation groups defined by the Index of Multiple Deprivation – the official measure of relative deprivation in the UK.
Results: In Greater Manchester, at least 60% of residential locations in 2021 exceeded the current World Health Organisation noise guidelines of 45dB(A) for the night-time period (23:00 to 07:00). Noise exposures are unevenly distributed among deprivation groups, with most deprived communities experiencing higher residential exposures by 1.4dB(A). Furthermore, 5.6% of Greater Manchester's population was estimated to be highly sleep-disturbed. Populations in the most deprived areas had a 12.5% higher risk of HSD than the least deprived communities.
Conclusion: This study demonstrated that road traffic noise exposure may contribute to health inequalities.

P32-22 | Toxic layering and compound extremes: Contaminant exposures in rural, environmental justice mining communities

Monica D. Ramirez-Andreotta[1,2], Zain A. Alqattan[1], God'sgift N. Chukwuonye[1], Miriam Jones[1]

[1] University of Arizona, Department of Environmental Science, Tucson, USA; [2] University of Arizona, Division of Community, Environment & Policy, Mel and Enid Zuckerman College of Public Health, Tucson, USA

Wildfires/flash floods heavily impacted select AZ rural, underserved communities near active/legacy mines, worsening contaminant exposures and environmental health disparities. Mining operations are linked to environmental contamination, with many waste sites left unrehabilitated. Fire can alter the physical/chemical/biogeochemical properties of the soil/surface materials. The extent of contamination in Arizona's mining areas post-disasters remains unassessed and community members raised concerns about contaminant exposure and origin. This study analyzes sediments, soil, and dust samples for metal(loid)s, PAHs, dioxins, and/or

PFAS to assess contaminant release during climate extremes, focusing on exposure pathways and climate-related geohazards in environmental justice communities.

P32-24 | In Their Own Words: Assessing Impacts of Military Aviation from Noise Complaint Data

Shirley C.-H. Huang[1], Lauren M. Kuehne[2], Giordano Jacuzzi[3], Julian D. Olden[3], Edmund Seto[1]

[1] University of Washington, Department of Environmental and Occupational Health Sciences, School of Public Health, Seattle, USA; [2] Omfishient Consulting, Bremerton, USA; [3] University of Washington, School of Aquatic and Fishery Sciences, College of the Environment, Seattle, USA

Military aviation training noise, despite its far-reaching effects on urban, rural, and wilderness communities, remains an underexplored issue. The continuous low-frequency noise and repetitive nature of training exercises contribute to widespread noise pollution, yet existing studies often overlook the full spectrum of its health and quality-of-life implications. In this research, we examined two datasets of noise complaints related to Whidbey Island Naval Air Station: U.S. Navy records from 2017 to 2020 and data from the Quiet Skies over San Juan County initiative spanning 2021 to 2023. We applied sentiment analysis to chart spatial patterns of negative sentiment in relation to modeled annual noise levels, developed a typology to categorize the impacts, and utilized random effects modeling to identify the environmental and operational factors that influence complaint frequency.

Our analysis uncovered extensive negative sentiment and anger that frequently extended beyond the boundaries predicted by noise models, indicating that current annual noise assessments may underestimate community impacts. Common complaints included sleep disturbances, hearing and health issues, and disruptions in home life caused by shaking, vibration, and routine disturbances. Additionally, residents reported significant interruptions to their social, recreational, and work activities, alongside feelings of fear, helplessness, and concerns for their children's well-being. The correlation between the number of complaints and training schedules was strong, with late-night sessions emerging as the most significant predictor, and a noticeable lag in responses suggesting that residents accumulate a threshold of frustration before formally complaining.

Overall, our findings demonstrate the persistent negative sentiment and diverse adverse impacts of military aviation noise, highlighting the need for improved noise modeling and operational adjuetments to mitigate these disruptions.

P32-25 | Evaluating objective and subjective measures of neighborhood stress and vulnerability in relation to placental metals concentrations in a U.S. pregnancy cohort.

Megan C. Hansel[1], Sean A. Stratton[2,3], Alyssa Juenke[2], Anushka Pande[4], Carolyn W. Kinkade[1], Catherine S. Yount[5], Pamela Ohman-Strickland[1], Cathleen Doherty[6], Brian Buckley[2], Jessica Brunner[7], Richard K. Miller[7], Thomas G. O'Connor[7,8], Emily S. Barrett[1,2], Zorimar Rivera-Núñez[1,2]

[1] Rutgers School of Public Health, Department of Biostatistics and Epidemiology, Piscataway, USA; [2] Rutgers University, Environmental and Occupational Health Sciences Institute, Piscataway, USA; [3] Rutgers School of Public Health, Department of Environmental and Occupational Health and Justice, Piscataway, USA; [4] Rutgers University, Robert Wood Johnson Medical School, Piscataway, USA; [5] University of Rochester Medical Center, Department of Public Health Sciences, Rochester, USA; [6] Rutgers University, Department of Earth and Planetary Sciences, Piscataway, USA; [7] University of Rochester Medical Center, Departments of Obstetrics and Gynecology, Pediatrics, Pathology, Environmental Medicine, Rochester, USA; [8] University of Rochester Medical Center, Departments of Psychiatry, Psychology, Neuroscience, Rochester, USA

Objective: Given thatsocial and environmental exposures may co-occur and impact perinatal outcomes, we examinedneighborhood stress and social vulnerability in relation to placental metals concentrations.

Material and Methods: The U.S. UPSIDE study recruitedpregnant participants (n=242). In the third trimester, participants reported on neighborhood stressors using the City Stress Inventory which has two subscales: neighborhood disorder (e.g., vacant buildings) and violence (personal experiences of violence). Neighborhood disorder was considered as quartiles (Q) and exposure to violence was considered binary (any/none). Participant addresses were linked to the CDC's Social Vulnerability Index (SVI), which contains four themes (socioeconomic status, household composition and disability, minority status and language, housing type and transportation) and overall SVI. Each tract receives a U.S.-wide percentile ranking (expressed as a fraction from 0-1, with higher values indicating greater vulnerability). SVI measures were categorized as low, medium [M], and high. Placental lead, arsenic, cadmium, chromium, and manganese at delivery were analyzed by ICP-MS. We fitted adjusted linear regression models examining neighborhood stress and SVI measures in relation to each log-transformed metal. Results are reported as percentage difference (%Δ) in metals concentrations.

Results: Most participants (73%) reported ≥1 exposure to neighborhood disorder, 24% reported any exposure to violence, and the mean overall SVI was 0.4±0.3. Compared to the lowest neighborhood disorder category, higher neighborhood disorder was associated with higher cadmium (Q2:%Δ= 25.5, 95%CI: 0.3, 57.1). Positive associations were observed for minority status and language with cadmium (M:%Δ= 26.4, 95%CI: 2.9,55.1) as well as housing type and transportation with chromium (M:%Δ= 27.1, 95%CI: 1.3,59.6) compared to the lowest tertile.

Conclusion: Aspects ofneighborhood stress and social vulnerability are associated with placental metals concentrations with implications for perinatal health. Additional research is needed to examine other factors associated to neighborhood such as access to prenatal care and healthy food.

P32-27 | Environmental Justice Before the Courts: The Impact of Federal Financial Settlements on Communities

Yoshira O. Van Horne[1], Dylan Morgan[2], Madeleine Killough[3], Sanika Parasnis[2], Robbie M. Parks[2], Anne E. Nigra[2], Paris B. Adkins-Jackson[4]

[1] University of California Los Angeles, Department of Environmental Health Sciences, Los Angeles, USA; [2] Columbia University Mailman School of Public Health, Department of Environmental Health Sciences, New York, USA; [3] The New School University, Tishman Environment and Design Center, New York, USA; [4] Columbia University Mailman School of Public Health, Departments of Epidemiology and Sociomedical Sciences, New York, USA

Introduction

When communities challenge environmental polluters (e.g., chemical or mining companies) they typically experience very limited restorative justice, with the most successful cases resulting in financial settlements through federal court acts. Little is known about the short and long-term impact of settlements. We evaluated the association between the location of facilities implicated in federal settlements and social vulnerability and environmental justice indices.

Methods

We merged federal settlement data from the US Environmental Protection Agency (EPA) with the Social Vulnerability (SVI) and Environmental Justice (EJI) Indices at the county-level. We used non-parametric tests to assess the association between the quartile ranks of SVI and EJI scores and the total number of settlement facilities.

Results

A total of 109,223 unique settlements (from 85,891 unique facilities in all 50 US states) occurred from 1972-2023. A majority of these settlements were due to violations of the Clean Water (25.3%) and Clean Air Acts (16.5%). Most settlements occurred in EPA regions 2, 4, and 6 (US States: NY, NYJ, GA, KY, TN, NC, SC, AL, MS, FL, NM, TX, OK, AR, LA). We identified statistically significant differences in facility location by SVI and EJI quartiles. In counties where at least one settlement facility was located between 1972-2023. Of these, 11,046 facilities were in the least vulnerable SVI quartile 1 (SVI-qt1), 17676 were in SVI-t2, 21223 were in SVI-qt3, and 23587 were in the most vulnerable (SVI-qt4) ($P<0.001$). Similarly, 8348 facilities were in the lowest EJI quartile 1 (EJI-qt1), 13589 were in EJI-t2, 15406 were in EJI-qt3, and 18809 were in the most environmentally burdened EJI-qt4 ($p<0.0001$).

Conclusion

Most facilities implicated in federal settlements were located in areas of high social vulnerability and high environmental burdened areas. Policies aimed at bringing polluting facilities into compliance and strengthening enforcement practices are warranted.

P33 | Improving Measures of Environmental Exposure and Risk

P33-01 | Health Risks of Antibiotic Contamination in Rural Punjab: Exposure Pathways and Childhood Vulnerabilities

Abid Ali[1], Munnaza Khalid[2], Zeeshan Nawaz[3], Habib Ullah[4], Audil Rashid[5]

[1] PMAS Arid Agriculture University, Rawalpindi, Pakistan, Institute of Soil and Environmental Sciences, Rawalpindi, Pakistan; [2] Rawalpindi Medical University, Department of Obstetrics and Gynaecology (Unit-1), Rawalpindi, Pakistan; [3] SABIC Technology & Innovation, SABIC, Jubail Industrial City, Saudi Arabia, Olefins Platform, Technology & Innovation, Jubail Industrial City, Saudi Arabia; [4] Zhejiang University, Department of Environmental Science, Hangzhou, China; [5] University of Gujrat, Department of Botany, Gujrat, Pakistan

The widespread and unregulated use of antimicrobials in both human and veterinary applications has led to significant environmental contamination, particularly in rural areas, exposing populations to potential health risks. Despite the severity of the issue, research on the cumulative impact of antibiotic exposure through multiple pathways remains limited, especially for children, who are particularly vulnerable. This study investigates the correlation between antibiotic residues in soil and water with the exposure frequency of children in rural Punjab, aiming to assess potential health risks.

Our findings indicate that soil contamination primarily stems from human antibiotic use, with erythromycin (32.61 μg kg^{-1}), doxycycline (20.89 μg kg^{-1}), and ciprofloxacin (15.52 μg kg^{-1}) being the most prevalent. Water contamination, however, results from a combination of human, poultry, and veterinary antibiotic use. Notably, dust ingestion contributed to over 70% of the total daily antibiotic exposure dose. Health assessments revealed that children exposed to contaminated environments had significantly higher risks of allergic symptoms (OR 3.14, 95% CI 1.43-6.75) and atopic dermatitis (OR 2.21, 95% CI 1.13-5.77). Moreover, children frequently in contact with contaminated water were found to be 30 times more susceptible to allergic rhino-conjunctivitis compared to those living farther from stagnant water sources.

These findings underscore the urgent need for comprehensive exposure assessments to better understand the health risks associated with antibiotic contamination. Additionally, stricter regulations on antibiotic use are essential to mitigate their environmental and health impacts, particularly for vulnerable populations in rural communities.

P33-02 | Chemical exposure assessments in firefighters using silicone wristbands: associations with fire response history

Mackenzie Laney, Heather Stapleton, Nicholas Herkert, Duncan Hay, Sharon Zhang, Kate Hoffman

Duke University, Durham, USA

Firefighters face multiple occupational stressors, including extreme heat, physical and mental demands, shiftwork, and exposure to hazardous chemicals such as flame retardants and combustion byproducts, all of which may be related to higher risks for disease among firefighters. This study sought to assess firefighters' chemical exposure over several months using a wearable exposure monitor, the silicone wristband. Twenty-three firefighters enrolled in the study and wore a wristband over a continuous 9-day period, during which they alternated 24 hours on-duty and 24 hours off-duty. They then had 5 days off-duty where no wristbands were worn, and the entire process was repeated up to 8 times over a 4 to 5 months period. Firefighters used an App on their phone to record information on their time spent at a fire call, the type of fire, and the number of fire calls while on-duty. Wristbands were analyzed

for a suite of 130 different chemicals, including combustion byproducts, flame retardants and plasticizers. In addition, a composite wristband sample was prepared and analyzed for each firefighter to compare with individual levels measured over the 4–5 month period. Preliminary findings suggest the ng/g/day concentrations of flame retardant decabromodiphenyl ethane, along with combustion byproducts phenanthrene and pyrene significantly increased by a factor of two with the number of fire calls. The cumulative length of time spent at a fire was also associated with concentrations of BDE-209, phenanthrene and pyrene. Levels of almost all chemicals measured in the composite samples were significantly and highly correlated ($p<0.01$) with average levels in all wristbands worn by a firefighter, suggesting one composite sample could provide a good assessment of exposure over 4-5 months. This study provides critical insight into the utility of wristbands for monitoring firefighter's exposures over time.

P33-03 | Exposure to Commonly Used Disinfectants in Assisted Living Facilities: Implications for Older Adults

Minghao Kong[1], Tret Burdette[2], Raghu S. Kumar[1], Claire Dempsey[1], Parinya Panuwet[1], Amina Salamova[1]

[1] Emory University, Gangarosa Department of Environmental Health, Rollins School of Public Health, Atlanta, USA;
[2] University of Georgia, Center for Applied Isotope Studies, Athens, USA

Quaternary ammonium compounds (QACs) are a class of chemicals that are commonly used in disinfecting and personal care products, for their antimicrobial, surfactant, and preservative properties. This study provides the first comprehensive assessment of QACs in assisted living facilities, through the analysis of 19 QACs from three different QAC subgroups in indoor dust and air samples collected from three assisted living facilities in Indiana, United States, as well as in wristbands worn by the residents and staff of these facilities. The medians of the total QAC concentration (the sum of 19 [AS1] QACs) were 150,599 ng/g in dust, 2.85 ng/m^3 in air, and 2,118 ng/g in wristbands, respectively. Benzylalkyldimethyl ammonium compounds (BACs) were the most abundant QAC group in all three matrices and contributed 58-85% to the total QAC concentrations. Spearman correlation analysis demonstrated that C12-, C14-, and C16-BACs exhibited strong positive correlations among concentrations within and between dust, wristbands, and indoor air. The QAC distribution patterns found in dust, air, and wristbands were similar to those reported for disinfecting products, suggesting these products could be an important indoor source in assisted living. Levels in wristbands worn by staff during their work shift were significantly higher than those in wristbands worn by residents ($p < 0.05$). In addition, the levels found in dust from assisted living were several times higher than those previously reported in US residential households. The elevated QAC concentrations in assisted living facilities suggest stronger sources of QACs in non-residential public settings indicating high exposure to QACs in senior care facilities and health risk effects on older adults.

P33-04 | Examining the inter-rater reliability of a self-administered occupational history questionnaire

Olivia Cooper[1], Katherine Leggatt-Barr[1], Peggy Reynolds[2], Robert Harrison[3], Diane Heditsian[5], Susie Brain[5], Vivian Lee[5], Allison Stover-Fiscalini[1], Laura J. Esserman[1], Laura Van 't Veer[4], Kimberly Badal[1]

[1] UCSF, Department of Surgery, San Francisco, USA; [2] UCSF, Department of Epidemiology and Biostatistics, San Francisco, USA; [3] UCSF, Department of Occupational Medicine, San Francisco, USA; [4] UCSF, Department of Laboratory Medicine, San Francisco, USA; [5] UCSF, Breast Science Advocacy Core, San Francisco, USA

Objective

To test the inter-rater reliability of an occupational history questionnaire in the national Women Informed to treat Depending On Measures of risk (WISDOM) Study.

Material & Methods

An occupational history questionnaire was designed to capture participants' occupations from 18 years and their mother's occupation during pregnancy. Recently active WISDOM participants (n=131) randomly selected based on geography, race and ethnicity, age, and education were invited to the study. Interested participants (n=28) had seven days to complete the questionnaire. Two weeks later, participants were interviewed by an Occupational Health Expert (OHE). Participant and OHE occupations and industries were translated into Standardized Occupational Classification (SOC) codes using NIOSH's (National Institute for Occupational Safety and Health) NIOCCS (NIOSH Industry and Occupation Computerized Coding System) software. Agreement between SOC codes from participant and OHE entries was assessed using Krippendorff's alpha. Participants were recontacted to determine the most accurate SOC code.

Results

To date, 86% (n=24/28) of participants completed the study; 75% were White (n=18), 13% Hispanic (n=3), 4% Asian (n=1), and 8% Black (n=2). The average age was 59 years (range: 42–77 years), and 92% were college graduates. Geographically, 75% were from California (n=18), 13% (n=3) from the South and East Coast respectively. There was poor average agreement between SOC codes generated from participant entries and OHE entries, indicated by a Krippendorff's alpha of 0.47 (range: 0.36-0.59). There was poor average agreement between the job start and end dates from participant and OHE entries with an alpha of 0.66 (range 0.47-0.77). Of participants recontacted to date, 88% (n=14/16) indicated that the SOC code generated from their entry was more accurate than the SOC code from OHE entry.

Conclusion

Most participants found the SOC code generated for their entry was more accurate, suggesting self-reported questionnaires better capture occupational histories than OHE interviews.

P33-05 | Comparing mobility-driven and home-based particulate matter exposure estimates in the United States

Sanjana Bhaskar[1], Li Yi[2,3], Rachel Nethery[4], Jaime E. Hart[1,5], Gary Adamkiewicz[1]

[1] Harvard T.H. Chan School of Public Health, Department of Environmental Health, Boston, USA; [2] Harvard T.H. Chan School of Public Health, Department of Nutrition, Boston, USA; [3] Harvard Medical School and Harvard Pilgrim Health Care Institute, Department of Population Medicine, Boston, USA; [4] Harvard T.H. Chan School of Public Health, Department of Biostatistics, Boston, USA; [5] Brigham and Women's Hospital and Harvard Medical School, Channing Division of Network Medicine, Department of Medicine, Boston, USA

Objective: Air pollution studies frequently assess exposures at a participant's home address, without accounting for daily mobility patterns. This study evaluates whether incorporating GPS-based location information when assigning $PM_{2.5}$ exposures alters exposure estimates compared to a home-based assessment. **Material and Methods**: A total of 161 participants from the Growing Up Today Study provided 23,663 days of minute-level GPS data. The mobility-based $PM_{2.5}$ exposure metric was estimated by averaging the daily $PM_{2.5}$ values from the EPA monitors nearest to each GPS location, while the home-based metric was sourced from the daily $PM_{2.5}$ value at the monitor nearest to the participant's home address. Daily $PM_{2.5}$ averages were compared across both metrics, and absolute differences were calculated and stratified by urban-rural classification, and U.S. Census regions. **Results**: Overall, the two metrics were similar (Mean [SD]: 8.19 [8.34] μg/m³ for the mobility-based metric vs. 8.36 [6.84] μg/m³ for the home-based metric). However, the absolute difference between the two metrics increased with greater daily travel distance, as indicated by a positive Spearman correlation (0.59). These differences were more pronounced in urban (0.83 [2.59]) vs. rural areas (0.34 [1.31]), and in the West (0.99 [2.65]) and Northeast (0.94 [2.67]) compared to the South (0.62 [2.63]) and Midwest (0.47 [1.52]). Even when participants were not at home, their mobility-based and home-based exposure values were sourced from the same monitoring site more frequently in low-density areas, rural areas, and in the Midwest. **Conclusion**: While GPS-based mobility data can capture greater variation in $PM_{2.5}$ exposure, particularly for highly mobile individuals and those in urban areas, overall differences between home-based and mobility-based nearest monitor estimates were minor. This likely reflects the differences in the number and placement of regulatory monitors in the U.S. but suggests that exposure predictions using only residential addresses may have little bias.

P33-06 | Predicting missing data for estimating endotoxin exposure using ordinal classification trees in the Agricultural Health Study

Shuai Xie, Pabitra R. Josse, Gabriella Andreotti, Alexander P. Keil, Jonathan N. Hofmann, Laura E. Beane Freeman, Melissa C. Friesen

National Cancer Institute, Division of Cancer Epidemiology and Genetics, Occupational and Environmental Epidemiology Branch, Bethesda, USA

Objective: Epidemiologic studies often collect questionnaire information in a staged approach, creating a missing data problem for those who do not complete all questionnaires. The prospective Agricultural Health Study (AHS) cohort used two stages: participants enrolled in 1993-1997 completed a questionnaire while attending a pesticide training session, followed by a take-home questionnaire. To assess endotoxin exposure, we needed the self-reported frequency of three agricultural tasks (grinding animal feed, harvesting grain, milking cows) queried only in the take-home questionnaire. We evaluated the use of ordinal classification tree models to predict these frequencies to impute the missing values assuming missingness at random.

Material and Methods: The take-home questionnaire was completed by 47% of the 52,394 AHS private applicators. We used ordinal classification tree models (rpartScore) and farm characteristics and task information from the enrollment questionnaire to predict frequency (3-category scale). The data for those with a non-zero frequency was split into training (70%), test (15%), and validation (15%) sets separately for each task (n=22,919, 17,413, and 1,588 applicators for grinding feed, harvesting grain, and milking cows, respectively). We evaluated agreement between the predictions and responses in the validation set by calculating Somers' d, quadratically-weighted kappa (κ_w), and sensitivity.

Results: We observed moderately-high agreement for grind animal feed (Somers' d=0.65; κ_w=0.66), with moderate to high category-specific sensitivities (56-94%). For harvest grain and milk cows, we observed poor overall agreement (Somers' d=0.15-0.25; κ_w=0.20-0.27). However, category-specific sensitivity was high for the most reported category (harvesting grain=87%; milking cows=74%). Sensitivities were generally lower for the other reported categories (harvesting grain=15-23%; milking cows=27-38%).

Conclusion: The ordinal classification models had a good ability to predict the most reported frequency category for all three endotoxin-related tasks, and all categories for grinding animal feed. However, we were less successful in predicting the frequency of harvesting grain and milking cows.

P33-07 | Sampling and analysis of polycyclic aromatic hydrocarbons using pine needles: A ‘green’ approach to monitoring air pollutants.

Adriana C. Torres-Moreno, Sung H. Seo, Stuart A. Batterman

University of Michigan, Environmental Health Sciences, Ann Arbor, USA

Polycyclic aromatic hydrocarbons (PAHs) are a large group of compounds distinguished by their many chemical structures, multiple sources, ubiquitousness, toxicity, and difficulty and expense in monitoring. These lipophilic and semi-volatile organic compounds (SVOCs) are adsorbed into the waxes and oils of pine needles, which can serve as passive samplers, potentially providing a cost-effective and flexible approach for monitoring and for showing spatial and temporal trends. This sampling also allows long-term monitoring and applications in remote locations where the deployment of conventional samplers is challenging.

This study aims to detect and quantify 27 PAHs in pine needles from *Pinus strobus,* a native species to eastern North America, using a ‘green’ analytical approach that reduces solvent use and disposable materials. Sample preparation included freeze drying and sonication; cleanup included sorbents to remove waxes and pigments; and analysis used gas chromatography/mass spectrometry (QqQ) with 16 deuterated internal standards. Method optimization to address separation, recovery, and detection limits included tests of various extraction times, different adsorbents for cleanup (e.g., florisil, alumina), and other parameters. During method development, several compounds tended to volatize (e.g., naphthalene); others (e.g., benzo[e]pyrene, benzo[a]pyrene, and some dibenzopyrenes) were degraded or retained with Florisil cartridges. Ultimately, we were able to quantify 27 PAHs using alumina cartridges with solvent use reduced several folds from previously published methods. The method was applied to late-winter samples collected at 27 sites in rural, urban, and industrial sites in two consecutive years. Elevated concentrations were noted at locations where high levels of other persistent organic pollutants had been measured, showing the influence of both local and regional sources.

The use of natural passive samplers and the minimum use of disposable material and solvents make this approach an excellent tool for green analytical chemistry for the detection of air contaminants such as PAHs.

P33-08 | Literature Review of the Reliability of Personal Air Sensors for Air Pollution Epidemiological Studies

Alexa Gallagher[1], Ryan Drover[2], Lizzie Pinegar[2], James Dennison[3], **Linda Dell**[1]

[1] Ramboll Americas Engineering Solutions, Inc., Amherst, USA; [2] Ramboll Americas Engineering Solutions, Inc., Salt Lake City, USA; [3] Ramboll Americas Engineering Solutions, Inc., Seattle, USA

Objective. Recommendations for air pollution epidemiological studies include improving exposure estimates through monitoring technology and wearable devices. Personal air sensors offer real-time, individualized exposure data, but concerns remain regarding their reliability when used under a range of real-world conditions and over extended durations. We evaluated the reliability and validity of personal air sensors available for estimating personal exposure to particulate matter (PM) and ozone in epidemiological studies.

Materials and methods. We searched PubMed for epidemiological studies that used personal air sensors to measure PM or ozone. We extracted data on sensor manufacturer, testing duration, performance (sensor precision,

detection limits, response times, susceptibility to environmental factors like humidity and temperature), and usability (data collection, power requirements, ease of deployment, availability, cost, long-term operational durability). We also searched authoritative and regulatory agencies, conference proceedings, and academic research groups to identify sensors not yet used in published epidemiological research.
Results. There were relatively few different personal air sensors used to estimate PM and ozone in epidemiological studies. We extended the review to include approximately 30 personal air sensors that are on the market or in use by academic groups to fully evaluate the characteristics most suitable for use in epidemiological research and describe gaps or issues in performance or availability. While many sensors perform adequately for basic air quality monitoring, significant challenges remain in their ability to meet epidemiological research requirements, especially regarding long-term accuracy and reliability. Currently, personal air sensors used in epidemiological studies are most often limited to smaller studies or studies of shorter duration.
Conclusion. We recommend enhanced sensor validation, performance metrics, and practical deployment solutions to better support epidemiological studies. In cases where current technologies or certification options fall short, we provide recommendations for sensor improvements, standardization, and formal validation criteria.
Acknowledgments. The American Petroleum Institute sponsored this study.

P33-09 | *Performance of Low-Cost $PM_{2.5}$ Sensors Under West African Weather: Insights from the Air Sensor Evaluation and Training Facility for West Africa*

James Nimo[1,6], Mathias A. Borketey[6], Emmanuel K-E. Appoh[6], Morrison Abena Kyerewaa[6], Yussif Ibrahim-Anyass[6], Abraham Amankwah[2], Audrey Owusu Tawiah[6], Joanna A. Modupeh Hodasi[2], Selina Amoah[4], Esi Nerquaye-Tetteh[4], Nana Ama B. Klutse[4,2], Victoria Owusu-Tawiah[3,6], Raphael E. Arku[5], Allison Hughes[2,6]

[1] University at Albany, Department of Environmental and Sustainable Engineering, Albany, USA; [2] University of Ghana, Department of Physics, Accra, Ghana; [3] Carnegie Mellon University Africa, Kigali Collaborative Research Centre, Kigali, Rwanda; [4] Ghana Environmental Protection Authority, Accra, Ghana; [5] University of Massachusetts, Department of Environmental Health Sciences, Amherst, USA; [6] University of Ghana, The Air Sensor Evaluation and Training Facility for West Africa, Accra, Ghana

Background: Fine particulate matter ($PM_{2.5}$) pollution is a growing public health concern in sub-Saharan Africa (SSA). However, access to reference-grade air quality monitors remain limited, hindering effective assessment and control. Low-cost sensors (LCS) offer a promising alternative for air quality monitoring, supporting both policy and civic engagement. Despite their increasing use, there is limited research on their performance under SSA's unique meteorological conditions.
Objective: We evaluated the accuracy, reliability, and responsiveness of eight commercially available LCS for $PM_{2.5}$ monitoring and assessed their performance and suitability across different seasons.
Methods: We collocated 8 LCS that contained Plantower PMS, Alphasense OPC-N3, and AVO-Sensor technologies along with a Federal Equivalent Method (FEM) Teledyne T640 monitor over six months. Sensor performance was assessed during wet and dry seasons, focusing on intra- and inter-sensor variability, correlation with the reference monitor, and error metrics such as mean absolute error (MAE) and root mean squared error (RMSE).
Results: The LCS exhibited low intra-sensor variability across seasons, but high inter-sensor variability compared to the Teledyne T640. A strong correlation was observed between LCS and the T640, with an average R^2 of 0.7 (range: 0.5–0.9) in the wet period and 0.8 (0.64–0.97) in the dry period. However, larger errors were recorded during the dry

season, where MAE and RMSE were 4.5 and 5.3 times higher, respectively. Uncertainties were also noted in LCS data when $PM_{2.5}$ levels were high during the wet season, a condition more typical of the dry season due to long-range transport dust of varying pollution sources. **Conclusion**: LCS performance vary significantly by season, requiring routine maintenance and calibration, particularly in the dry season. To enhance the effectiveness of LCS-based air quality monitoring in SSA, strong collaboration among governments, industries, and civil society is essential to develop an effective framework for their use in SSA.

P33-10 | Indoor Exposure Measurements in Vulnerable Communities using Dust and Silicone Wristbands as Passive Samplers

Anna C. Neville, Evelyn Deveraux, Chou-Hsien Lin, Daniel Sung, Kerry A. Kinney, Pawel K. Misztal

University of Texas at Austin, Austin, USA

Objective

Exposure to volatile organic compounds and semi-volatile organic compounds impacts human health, but current methods of evaluating exposure are challenged by detection limits and time response. This study presents a non-invasive method of chemical exposure monitoring, combining novel approaches (dust and silicone wristbands) for passive sampling with thermal desorption and comprehensive chemical analysis using Vocus 2R Proton Transfer Reaction Time of Flight Mass Spectrometry (Vocus PTR-TOF-MS). This new approach reveals a unique profile of more than 1,000 chemicals adsorbed from the environment which can be quantitatively related to human exposure.

Materials and Methods

This study focuses on the Beaumont-Port Arthur region of Southeast Texas, a highly polluted industrial area. Silicone bracelets were distributed to Beaumont-Port Arthur residents. Subjects continuously wore the bracelets for 7 days. Dust was collected from air filters inside each home. Each bracelet and dust sample was subjected to a temperature ramp (60 °C, 90 °C, and 120 °C) using a Gerstel C200 controller. The Vocus measured the resulting emissions during desorption. The analysis included hierarchical clustering to create chemical fingerprints and identify potential exposure sources.

Results

Over 1,000 compounds were detected across samples. Dust contained contaminants including organophosphates, phthalates, and brominated flame retardants. Similar compounds were extracted from the silicone wristbands, including endocrine disrupting compounds such as dibutyl phthalate and di(2-ethylhexyl) phthalate (DEHP), along with siloxanes from personal care products. The similarities between the chemical composition of the bracelets and dust samples point to indoor exposure sources.

Conclusion

This combination of silicone bracelets and household dust with comprehensive PTR-TOF-MS analysis represents a rapid, untargeted, and widely implementable method for assessing indoor exposure. Insights gained from passive sampling could be used to identify trends in chemical exposure and human health. This study was funded by the NSF CAREER award (no. 2146347).

P33-11 | Interpretation of Spot Urine Biomonitoring: Insights from 24-hour Sampling Around a Known Exposure Event

Carrie Fleming[1], Xiaoyi Sopko[2], Elizabeth Sweeney[3], Alexa Neumann[3]

[1] Corteva Agriscience, Louisville, USA; [2] Corteva Agriscience, Wilmington, USA; [3] Corteva Agriscience, Indianapolis, USA

Objective: Most biomonitoring programs today collect spot urine samples from their participants, requiring extrapolation to estimate average daily exposure. Our objective was to use urine samples collected surrounding a known exposure event to understand the impact of assumptions in biomonitoring studies employing spot sampling.
Material and Methods: 24-hour urine was collected from 19 participants for 3 consecutive days surrounding a pesticide lawn application, with each urine void analyzed separately. Urine sample weight, specific gravity, creatinine, and pesticide concentrations were measured. Participants' age, sex, body weights, and heights were also collected. Research followed an Institutional Review Board-approved protocol. The ability to predict daily exposures (24-h) and average daily exposures across 72-h from individual urine voids was determined using extrapolation based on: expected urinary flow; body-weight adjusted expected urinary flow; and expected creatinine excretion.
Results: Prediction of 24-h or 72-h average daily exposure from spot samples was generally similar between extrapolation methods, with creatinine performing slightly better than other methods. Predictions were up to 86-fold different from measured exposures, with most predictions within 10-fold of measured. More outliers in fold-difference were observed on the day of the application, demonstrating the impact of exposure events on ability to predict longer term exposures.
Conclusions: Biomonitoring spot sampling may result in erroneous prediction of daily exposure. This effect may be more pronounced when an exposure event occurs during sampling. Significant departures of extrapolated vs. observed daily exposures indicate that there is a significant risk of misclassification when using concentrations of a biomarker in spot samples to classify participants into high vs. low exposure categories, as is sometimes done in epidemiological studies. A better understanding of inherent void-to-void variability may help in designing sampling procedures that balance convenience and expense with the quality of the generated data. Research was funded by Corteva Agriscience.

P33-12 | Exposures to Pesticides in a Residential Setting: Approaches to Highly-Refined Personalized Exposure Modeling

Carrie Fleming

Corteva Agriscience, Louisville, USA

Objective: Exposure modeling often accounts for uncertainty with conservative assumptions that overestimate exposure. Under certain circumstances, it may be desirable to more accurately estimate individual exposures. This work aimed to develop methodologies to accurately model pesticide exposures from different potential sources in a residential setting, replacing conventionally used conservative assumptions with more realistic modeled or measured values.

Material and Methods: Models were developed to estimate, as accurately as possible, individual exposures from pesticide residues found in residential settings, including residues in indoor and outdoor air, house dust, and home lawns. Starting with commonly used algorithms for estimating exposure from these sources, conservatisms within the algorithms and the constants within them were identified. Wherever possible, these conservative assumptions were replaced with less conservative assumptions. We also incorporated individual survey data to account for behavioral or physical exposure determinants.
Results: Disparities were found in the degree to which assumptions about exposure from the various residues (indoor air, outdoor air, house dust, or lawn residues) could be refined to result in more realistic individual exposure estimates. Inhalation exposures were refined substantially using survey information on individual daily activities and typical breathing rates for those activities. However, information on contact factors for exposures from lawn residues was limited to the highest contact activities, resulting in limited opportunities for refinement. This poster will present the algorithms and data used to refine exposure modeling, highlight limitations in the degree of refinement possible, and suggest ways to fill the identified gaps.
Conclusion: Existing data support highly refined individualized exposure modeling from some sources of exposure, while for other sources, additional information would be needed to remove conservatisms in commonly used models. Filling these gaps would support more refined and accurate individualized exposure modeling. This work was supported by Corteva Agriscience.

P33-13 | Qualitative and Mixed Methods in Exposure Science: Strategies and Recommendations

Christine C. Ekenga[1], Ashby Lavelle Sachs[2,3], Denise Moreno Ramirez[4]

[1] Emory University Rollins School of Public Health, Gangarosa Department of Environmental Health, Atlanta, USA; [2] Barcelona Institute for Global Health (ISGlobal), Barcelona, Spain; [3] CIBER Epidemiología y Salud Pública, Madrid, Spain; [4] University of Arizona Mel and Enid Zuckerman College of Public Health, Department of Community, Environment and Policy, Tucson, USA

Objective: Qualitative and mixed methods research offers valuable insights into the sociocultural factors that influence people's relationships with the environment and health-related behaviors. The objective of this project was to create a set of recommendations for qualitative and mixed-methods research in exposure science.
Material and Methods: We conducted a content analysis of research articles published in the Journal of Exposure Science and Environmental Epidemiology (JESEE) from 2003 to 2023. We extend prior work by proposing recommendations for qualitative and mixed-methods studies.
Results: Over the past 20 years, less than 1% of JESEE articles have employed qualitative or mixed methods. These studies concentrated on evaluating environmental exposures, elucidating exposure-related behaviors, assessing the impacts of regulatory interventions, and informing the design of exposure reduction interventions, all within historically marginalized or understudied populations. Key recommendations for researchers include selecting an appropriate qualitative or mixed-methods approach aligned with their research goals and the phenomenon studied. Additionally, researchers should develop a well-documented sampling strategy, explaining how participants are selected and when data collection is deemed complete, such as achieving saturation. Reflexivity is important, as researchers must acknowledge their own biases and preconceptions, which can influence data collection and interpretation. Assembling a skilled, interdisciplinary team with social scientists or qualitative/mixed methods experts can enhance methodological rigor and foster collaborative research.

Conclusion: Qualitative and mixed method approaches in exposure science offer invaluable insights into environmental health challenges by revealing sociocultural dynamics and nuanced community perspectives. Despite being underutilized, these methods can improve exposure assessment, risk perception, and intervention design.

P33-15 | An Improved High-Throughput Sample Preparation Method for Measuring Urinary Nicotine Metabolites.

Honest Achilihu, Patrick Bendik, June Feng, Lanqing Wang, Rayaj Ahamed

Centers for Disease Control and Prevention, National Center foe Environmental Health/Division of Laboratory Science, Atlanta, USA

Objective: Nicotine is the primary tobacco-specific alkaloid in tobacco plants and tobacco smoke. Assessing populational exposure to tobacco use via biomonitoring requires the analysis of large number of samples using high-throughput automation method. We present here an improved automated sample preparation method on a Hamilton® Vantage™ system.

Materials and Methods: We measure total urinary nicotine, six of its metabolites (*trans-3'*-hydroxycotinine, cotinine, 4-hydroxy-4-(3-pyridyl)-butanoic acid, cotinine N-oxide, nicotine N'-oxide, and nornicotine), and two minor tobacco alkaloids, anabasine and anatabine. Recently, we improved our sample preparation automation from a PerkinElmer Staccato® system to a Hamilton® Vantage™ system. On the new system, we simplified our sample preparation method, which was previously established on a much more customized and expensive system with many peripheral equipment and parts.

Results: For this new method, sample identifications are tracked automatically throughout the process to prevent human error. The sample order on the injection plate is in line with LC-MS/MS analysis sequence, making sample tracking and troubleshooting easier. The current method improved the accuracy (100.5%-115.4%) and precision (1.6 % - 5.1%) and maintained limit of detection for all analytes. Critical features on the Vantage™ include air displacement pipetting and Monitored Multi-Flow Positive Pressure Evaporative Extraction which has improved the method performance and affordability.

Conclusion: In summary, our improved automated sample-preparation method is robust, accurate, precise, and highly efficient. The new method saves time, is cost-effective, and has resulted in cleaner samples. These changes have also resulted in elimination of exposure to organic solvents (acetone and ethylene glycol) comparing to our previous method. Our updated method on the Hamilton® Vantage™ system uses less peripheral equipment, requires less customization than the previous automation system, and is easily transferrable to other laboratories that conduct similar analyses.

P33-16 | Optical Coherence Tomography to Investigate the Morphological and Physiological Changes in Human Skin after Standard Simulant Exposure and Tape-Stripping

Ian Choi[1], Alejandra Jaramillo[1], Nancy C. Urbano[1], Glenn Morrison[1], Leena Nylander-French[1], Rebecca Weed[2], Jeffrey Enders[2,3], Bryan Ormond[4], Ronald Baynes[5], Howard Maibach[6], Tim Burgin[7], Jonathan Kaufman[7]

[1] University of North Carolina at Chapel Hill, Gillings School of Global Public Health, Chapel Hill, USA; [2] North Carolina State University, Molecular Education, Technology and Research Innovation Centre (METRIC), Raleigh, USA; [3] North Carolina State University, Biological Sciences, Raleigh, USA; [4] North Carolina State University, Wilson College of Textiles, Raleigh, USA; [5] North Carolina State University, College of Veterinary Medicine, Raleigh, USA; [6] University of California San Francisco, Department of Dermatology, San Francisco, USA; [7] Joint Research and Development (JRAD), Stafford, USA

Objective: Optical Coherence Tomography (OCT) is a non-invasive 3-D imaging technology that generates real-time cross-sectional images of tissue using near-infrared laser light around the wavelength of 1300 nm. OCT images can obtain skin structural information down to 1 mm with a resolution of approximately 5 µm, sufficient to capture blood vessels and the dermal-epidermal junction. We investigated morphological and physiological changes before and after heavy-labeled methyl salicylate ($^{13}C_6$-MeS) exposure and tape-stripping to the skin.
Material and Methods: We recruited 21 healthy male (8) and female (13) participants to scan real-time images of volar forearm skin using a VivoSight Dx System (Michelson Diagnostics, UK). A treatment consists of 1-hour exposure to a filter dosed with ~1 mg of $^{13}C_6$-MeS combined with removal of the upper layers of the stratum corneum using tape-stripping. A repeated exposure and measures were conducted on 4 males and 4 females to provide intra-personal variation. A paired two-tailed t-test was employed to analyze inter-and intra-personal changes. Complementary Skin Surface Area Ratios (SSAR) were calculated with MeshLab software from exported DICOM files.
Results: Most notably, there were statistically significant differences ($p < 0.05$) in the Attenuation Coefficient, Plexus Depth, and SSAR before and after the treatments. There was no statistical significance before and after the treatments in several quantitative skin parameters computed by the OCT system: Mean Skin Roughness, Peak-Trough Difference, and Epidermal Thickness. There were no statistically significant intra-personal changes between the 1st and 2nd visit.
Conclusion: The OCT system, with complementary SSAR calculation, effectively quantifies morphological and physiological changes in human skin without altering fine skin texture following $^{13}C_6$-MeS exposure and tape-stripping. In tandem with human biomarker studies, this method will improve the underlying mechanistic understanding of skin absorption, enhancing human skin exposure assessment.
Funding: NIOSH (T42OH008673) and the United States Department of Defense (DoD).

P33-17 | Comparison between Cotinine, 2-CyEMA, and NNAL as Predictors for Levels of Ethylene Oxide Hemoglobin Adducts in Individuals of 6 Years and Older

James Pyon[1], Lanqing Wang[2], Michelle Zeager[1], Hubert Vesper[2], Enada Archibold[2]

[1] Centers for Disease Control and Prevention, Agency for Toxic Substances and Disease Registry, Office of Community Health Hazard Assessment, Atlanta, USA; [2] Centers for Disease Control and Prevention, National Center for Environmental Health, Division of Laboratory Sciences, Atlanta, USA

Objective

Ethylene oxide hemoglobin adducts (HbEO) are used as biomarkers to evaluate occupational exposure to ethylene oxide (EtO); however, using it to assess environmental EtO exposure is difficult because of possible confounding, such as tobacco smoking. In a previous analysis, a combination of two smoking biomarkers, cotinine and 2-CyEMA, were effective in predicting HbEO. Urinary NNAL is a major metabolite of tobacco specific nitrosamine NNK, with comparable half-life to HbEO. This analysis explores NNAL as an additional smoking biomarker to cotinine and 2-CyEMA for predicting HbEO levels.

Materials and Methods

A subsample of 2,291 participants (age six and older) in NHANES (2013–2014) had their whole blood analyzed for HbEO. Jackknife replicate weights were used with linear modeling to estimate model R-squared (R^2) with standard error. LASSO variable selection was applied to a full model. Correlation was calculated for cotinine and NNAL.

Results

The *R2* value of the model with cotinine and NNAL was greater than that of the model with either smoking biomarker. The R^2 value of the full model with cotinine, NNAL, and 2-CyEMA was slightly greater than that with cotinine and 2-CYEMA. Cotinine and NNAL were positively correlated; cotinine was selected over NNAL during LASSO variable selection.

Conclusion

A combination of cotinine and 2-CyEMA reduces the uncertainty from smoking in evaluating environmental EtO exposure.

The findings and conclusions in this presentation have not been formally disseminated by the Agency for Toxic Substances and Disease Registry and should not be construed to represent any agency determination or policy.

P33-18 | A natural nexus: Exposure and risk assessments can validate assumptions in, and establish meaningful context for, epidemiology studies on pesticide exposures

Jennifer Reed

Bayer Crop Science, Research & Development, Chesterfield, USA

Pesticide exposure classifications in epidemiology studies are often assessed indirectly; example approaches include those based on proximity, recall, and job or crop exposure matrices. Implicit in these methodologies are assumptions, which, if appropriately validated, can help investigators to accurately classify exposure. If an assumption is not sufficiently validated, however, there might be a degree of uncertainty regarding the extent to which an assessment tool's classification matches the true exposure of a study participant. Such uncertainty raises the need to pressure test assumptions to establish confidence in resulting exposure classifications. Subsequent to exposure assessment is the need to interpret the assessed exposure as it is classified in a study. Regulatory reference values like acceptable daily intake (ADI) and reference dose (RfD) are numeric values defined by regulatory agencies in the EU and US, respectively, which are derived from risk assessments, and represent the amount of residue that can be ingested every day over the course of a lifetime without an unreasonable risk to health. Urinary concentrations of an analyte can be translated mathematically to exposures that can be compared to the ADI or RfD if basic information is known about the distribution and metabolism of the active ingredient or its metabolite(s). If a study's participants report exposures less than the established safe limits, then this supports that the pesticide is being used in a safe manner. This presentation will 1. explore ways in which insights from exposure assessment can be used to assess and potentially strengthen the validity of example assumptions implicit in the classification methods used in some epidemiology studies which aim to investigate possible associations between pesticide exposures and health outcomes of interest, and 2. discuss how reference values can provide context for real world pesticide exposures in reference to levels which are considered permissible by regulatory agencies.

P33-20 | Influence of observed mortality on the estimation of attributable mortality to secondhand smoke exposure

Julia Rey-Brandariz, Carla Guerra-Tort, Cristina Candal-Pedreira, Guadalupe García, Lucía Martín-Gisbert, Ana Teijeiro, Alberto Ruano-Ravina, Bibiana Wandarlei-Flores, Mónica Pérez-Ríos

University of Santiago de Compostela, Department of Preventive Medicine and Public Health, Santiago de Compostela, Spain

Objective: To analyze differences in estimates of attributable mortality (AM) to secondhand smoke (SHS) exposure for lung cancer in a population aged ≥16 years, based on different observed mortality (OM) calculation methods.
Material and methods: AM was estimated as AM = OM × PAF, applying the different OM methods while keeping the population attributable fraction (PAF) constant. The different calculation methods where OM varied, were identified from a previous systematic review (López-Medina et al. 2024) and applied to data from Spain. An Excel table was designed to include: 1) prevalence of tobacco use (smokers, non-smokers, never smokers) and SHS exposure (exposed, not exposed) according to smoking habit (non-smokers, never smokers) and exposure setting (indoor,

home, work), obtained from a representative survey of the Spanish population ≥16 years carried out in 2024; 2) relative risks for lung cancer associated with tobacco use and SHS exposure, obtained from the Surgeon General Report, 2014 and Flor et al. Nat Med, 2024, respectively ; and 3) OM for lung cancer (ICD-10: C33-C34) in 2023, obtained from the National Institute of Statistics. Ratios were calculated to compare the AM estimates.
Results: Five OM calculation methods were identified. Two methods focused on the calculation of OM in never smokers (referred to as M1, M2, respectively), two in non-smokers (M3, M4) and one in both groups (M5). AM estimates varied according to the OM method used. In never smokers and considering all exposure settings, the M5 method estimated 2.5 times more SHS-attributed deaths than M2. In non-smokers, the largest difference in all exposure settings was seen with M3, estimating 5.8 times more deaths than M5.
Conclusions: Estimates of AM to SHS exposure may vary substantially based on the OM calculation method. This variability should be considered when assessing SHS-attributable mortality.

P33-21 | Silicone Ankle Bands as A Tool to Assess Infant Exposures to Semi-Volatile Organic Chemicals

Catherine Wise[1,2], Elizabeth Boxer[1], Rebecca Hoehn[1], Duncan Hay[1], Nicholas Herkert[1], Jillian Hurst[3], Heather Stapleton[1,2], Kate Hoffman[1,2]

[1] Duke University, Nicholas School of the Environment, Durham, USA; [2] Duke University, Duke Cancer Institute, Durham, USA; [3] Duke University, Duke University School of Medicine, Department of Pediatrics, Durham, USA

Exposure to semi-volatile organic chemicals (SVOCs) is of concern due to their potential health impacts. Exposure to SVOCs varies widely in the general population, but data on exposures in infants and young children is limited. Silicone wristbands have been shown to capture relevant SVOC exposures in adults and older children, with demonstrated correlations to biomarkers of exposure. However, their use and validation in infants remain unexplored. This study utilized data and samples from HOPE 1000, an ongoing prospective pregnancy cohort based in North Carolina, USA, investigating early-life exposures and maternal/infant health outcomes. Infants aged 6 to 18 months wore a silicone band on their ankle for three consecutive days. We analyzed the bands for 122 SVOCs across various classes. We also assessed whether the levels of parent organophosphate esters (OPEs) on ankle bands reflect internal exposure by comparing them to urinary biomarkers of OPE exposure, measured in spot urine samples collected while wearing the bands (n = 21 infants).
In total, 49 compounds were detected in over 50% of ankle bands, including 11 polycyclic aromatic hydrocarbon (PAHs), 3 pesticides, 2 phenols, lilial, 10 phthalates, 7 brominated flame retardants (BFRs), and 15 OPEs. Concentrations of individual SVOCs ranged across several orders of magnitude, with phthalates found at the greatest abundance, followed by OPEs. Urinary OPE biomarkers were significantly correlated with parent compounds on ankle bands ($r_s = 0.44–0.73$, $p < 0.05$). These findings suggest that silicone ankle bands effectively capture infants' internal OPE exposure, consistent with findings from older children and adults.
Overall, this study supports the use of silicone ankle bands as a valuable tool for assessing OPE and potentially other SVOC exposures in infants, offering a non-invasive approach for environmental health research.
Funded for this work was provided by NIH (NIAID 5R01AI167850).

P33-22 | Practical guidance on mobile monitoring design for epidemiology: A case study

Lianne Sheppard[1,2], Magali Blanco[1]

[1] University of Washington, Environmental & Occupational Health Sciences, Seattle, USA; [2] University of Washington, Biostatistics, Seattle, USA

Objective: Little is known about optimal mobile monitoring exposure assessment designs for health inference. We leveraged an extensive mobile monitoring campaign to advance our understanding of exposure assessment study design for estimating associations between long-term average air pollution exposure and cognitive function.
Materials and methods: We focused on ultrafine particle (UFP) measurements from a year-long mobile monitoring campaign with ~29 repeat visits to 309 stationary roadside locations in the Adult Changes in Thought Air Pollution study. We evaluated the impact of monitoring design by subsampling UFP measurements following reduced sampling approaches, evaluating the resulting exposure prediction performances, and assessing the resulting health inferences in cross-sectional analyses of UFP and cognitive function.
Results: We found mobile campaigns with 12 visits per site gave exposure prediction performances that were similar to the all-data reference campaign. Performance was poorest for temporally-restricted designs during weekday rush or business hours, and somewhat attenuated with an unbalanced number of visits per site. Inference about cognitive function was generally similar to the all-data reference campaign, with the largest differences occurring when temporally-restricted designs were considered.
Conclusion: Thoughtful monitoring design can enhance exposure assessment, and while potentially less critical in some settings, can support epidemiologic analyses. To maximize exposure and health inference accuracy, we recommend extending sampling beyond typical weekday business or rush hours, collecting data over at least two seasons if the goal is to estimate an annual average, collecting a balanced (fixed) number of visits across locations, and collecting more visits per location. Analyses in other contexts would provide further insights and complement this work.

P33-23 | Characterization of breath biomarkers in e-cigarette users, smokers, and non-users using a novel breath biopsy method

Mohamed J Ruzmyn Vilcassim, Samuel Stowe, Aakriti Chadha, Jefferey Wickliffe

University of Alabama at Birmingham, Department of Environmental Health Sciences, Birmingham, USA

Objectives: Electronic cigarette (e-cigarette) use has risen significantly, with U.S. adult prevalence increasing from 3.7% (12.2 million) in 2020 to 6.5% (21.8 million) in 2023. While e-cigarette vapor is considered less toxic than cigarette smoke, studies have detected harmful compounds and linked vaping to respiratory issues. Identifying biomarkers associated with vaping is crucial to understanding its health impacts. Breath biopsies, which analyze exhaled compounds, offer a novel, non-invasive method to distinguish vapers from smokers and non-users. Previous research has identified distinct breath biomarkers in cigarette smokers, but limited data exist for vapers. This study aims to characterize breath biomarkers in vapers, smokers, and non-users using a breath biomarker device

developed by Owlstone Medical. Identifying these biomarkers may improve differentiation between vapers and smokers, addressing a key research gap and facilitating early detection of health risks.
Methods: We recruited 24 male participants (ages 21-35): 8 vapers, 8 smokers, and 8 non-users. Exhaled carbon monoxide (eCO) and urinary cotinine tests confirmed nicotine presence/absence before enrollment. Each participant provided at least one breath biopsy sample using a ReCIVA breath sampler developed by Owlstone Medical, UK. Vapers provided two samples—one immediately post-vaping and another 24 hours later. Samples were analyzed for volatile organic compounds (VOCs) and their expression patterns.
Results: The mean participant age was 26, with 20 of 24 identifying as Asian. Smokers had higher eCO levels than vapers and non-users. All eCO and urinary cotinine results matched self-reported tobacco use. VOCs and their expression patterns for each tobacco use category are currently being analyzed.
Conclusions: This study has the potential to help identify vaping-specific biomarkers in exhaled breath, enabling non-invasive identification of exclusive vapers and early health risk detection.
This study is funded by the UAB School of Public Health Accelerator Award, which was awarded to Dr. Vilcassim.

P33-24 | Stability of Urinary Anabasine and Anatabine Measurements across 2012-2024

Patrick Bendik, June Feng, Lanqing Wang

Centers for Disease Control and Prevention, National Center of Environmental Health/Division of Laboratory Sciences/Tobacco and Volatiles Branch, Atlanta, USA

Objective: Anabasine and anatabine are minor alkaloids found in tobacco, and measured exposure of these compounds can distinguish recent tobacco product use from nicotine-replacement therapy product use. We have measured total urinary anabasine and anatabine for over a decade, and we must ensure that our measurements remain consistent as we continuously update our systems and methodology by routinely measuring testing materials.

Material and Methods: We established our method that measures total urinary anabasine and anatabine using liquid chromatography-tandem mass spectrometry (LC-MS/MS) in 2012. We completed sample preparation manually until mid-2013 when we automated the method, and we upgraded our LC-MS/MS systems to newer models. Proficiency testing (PT) occurred twice a year to evaluate our method's stability by measuring quality control materials (QCs), PT samples, and standard reference material (SRM) 3671 from the National Institute of Standards and Technology (NIST).

Results: We compared QC measurements from each PT cycle to limits characterized in 2013, and all except for two measurements of the high QC for anabasine were within those limits. PT sample measurements were within 25% of known concentrations among three levels for both anabasine and anatabine across all cycles from 2014 to 2024. Means of the low and high NIST SRM for anabasine were 1.1 ng/mL and 10 ng/mL while means for anatabine were 1.7 ng/mL and 17 ng/mL, respectively.

Conclusion: Our analysis showed that the method maintained high accuracy and precision throughout upgrades to instrumentation. We also provided data that could help with developing reference values for anabasine and anatabine in NIST SRM 3671. Our results showed that anabasine and anatabine were stable within our samples for at least 10 years, indicating that our method and sample storage techniques were excellent for long-term monitoring.

P33-25 | Evaluating CARES NG for Dietary Risk Assessments of Pesticides

Sheila Flack, Richard Anderson

Bayer Crop Science, Chesterfield, USA

The Cumulative and Aggregate Risk Evaluation System Next Generation (CARES NG) is a cloud-based model built upon publicly available data and developed by Crème Global with the support of the non-profit CARES NG organization to conduct aggregate and cumulative risks from pesticide use. This is an alternative tool to the Dietary Exposure Evaluation Model (DEEM) currently used by EPA and PMRA for performing dietary risk assessments for pesticide residues in food and drinking water. CARES NG can be used to conduct both deterministic and probabilistic risk assessments, with the latter used to refine acute dietary risk assessments using distributions of pesticide residues. In addition to aggregating risks from multiple exposure sources, such as air, water and food, these assessments may also include pesticide residue monitoring data available from USDA's Pesticide Data Program (PDP) to refine exposure estimates from foods, particularly for infants and children. This data combined with EPA's What We Eat in America-Food Commodity Intake Database (WWEIA-FCID) used to calculate food commodities consumed per day, allows the estimation of dietary exposure and risk associated with pesticide use. The use of CARES NG to conduct and refine dietary risk assessments was evaluated through several pesticide use case studies. The application of CARES NG in dietary risk assessments would allow pesticide registrants and regulatory agencies to make informed decisions about pesticide use and ensure that pesticide residues in food and drinking water are safe for consumers.

P33-26 | Occupational and Personal Exposures to Multiple Chemical Classes in Black Hairstylists using Silicone Wristbands: A Pilot Study

Anna Z. Pollack[1], Heather Stapleton[2], Melissa DeSantiago[3], Grant Tore[3], Magdalena Fandino Del Rio[3], Katrina Randolph[4], Sarrah Davis[5], Walkiria Pool[6], Julia Mandeville[1], Joanna Marroquin[1], Olivia Thornton[1], Paloma Beamer[8], Kelly Palmer[8], Jodi Flaws[7], Lesliam Quiros-Alcala[3]

[1] George Mason University, Global and Community Health Department, College of Public Health, Fairfax, USA; [2] Duke University, Durham, USA; [3] Johns Hopkins University, Environmental Health and Engineering, Baltimore, USA; [4] Tre Shadez, Capitol Heights, USA; [5] Taino Incorporated, Baltimore, USA; [6] Centro de Apoyo Familiar, Hyattsville, USA; [7] University of Illinois, Urbana-Champaign, USA; [8] University of Arizona, Tucson, USA

Objective: Despite growing concern over exposures to chemicals in personal care and beauty products, little is known about exposures among hairstylists. We sought to quantify occupational and personal exposure to polycyclic aromatic hydrocarbons (PAHs), phthalates, and organophosphate esters (OPEs) among Black hairstylists using silicone wristbands as personal passive samplers.
Methods: We conducted a pilot study to assess exposures via wristbands among 8 Black hairstylists in salons serving Black women. Hairstylists wore the wristbands for a mean of 6.9 days (minimum 5.8, maximum 7.1 days). Targeted analyses measured 10 phthalates and phthalate replacements, 23 PAHs, and 34 OPEs using GC-HRMS. Chemical selection was based on known or suspected presence in hair products and salon processes.

Results: Most (80%) phthalates but fewer PAHs (26%) and OPEs (32%) were detected in all wristbands. All phthalates, 39% of PAHs, and 50% of OPEs were detected in at least 75% of wristbands. The highest geometric mean concentrations for phthalates were bis (2-ethylhexyl) terephthalate (DEHT) 32,171 ng/g/day, di-isononyl phthalate (DINP) 8,412 ng/g/day, and trioctylmetallitate 1892 ng/g/day. Dibutyl phthalate (GM 166.3 ng/g/day) and diethyl phthalate (GM 451.7 ng/g/day), commonly used in personal care and beauty products, were detected in all wristbands. The highest geometric mean PAHs were phenanthrene 6.20 ng/g/day, fluorene 1.15 ng/g/day, and fluoranthene 1.04 ng/g/day. The highest geometric mean OPEs were tris (1-chloro-isopropyl) phosphate (TCPP1) 29.0 ng/g/day, tri-(2-butoxyethyl)-phosphate (TBOEP) 28.0 ng/g/day, and Tris (2,4-dichloro-isopropyl) phosphate (TDCPP) 24.2 ng/g/day.
Conclusion: These findings underscore the importance of conducting larger-scale studies that encompass hairstylists at the highest risk of exposure and the utility of using noninvasive wristband methods. Such research can inform targeted interventions and policies to protect worker health.

P33-27 | Developing Pollen Prediction Models: A High Spatial and Temporal Resolution Approach

Xueying Zhang[1,2], Yohei Saburi[3], Wenhao Wang[3], Zhihao Jin[3], **Yang Liu**[3]

[1] Baylor College of Medicine, Center for Precision Environmental Health, Houston, USA; [2] Baylor College of Medicine, Department of Medicine, Houston, USA; [3] Emory University Rollins School of Public Health, Gangarosa Department of Environment Health, Atlanta, USA

Background: Pollen is a significant cause of respiratory conditions; current monitoring data do not provide spatially resolved pollen concentrations.
Objective: To develop a model predicting daily pollen concentrations at a 1 km spatial resolution.
Methods: We collected daily speciated pollen counts in Atlanta, GA, and Houston, TX, from a total of ten sampling sites. Nine sites in Atlanta utilized automated pollen sensors, while the Houston site followed the National Allergy Bureau's protocol, employing a pollen trap machine and manual counting. Pollen counts were continuously collected from early spring to fall in 2020, 2021 and 2024. Fourteen tree species were selected for model development. The Research LINE-source dispersion model (RLINE) was used to estimate daily pollen concentrations. Pollen sources used for RLINE were estimated based on live tree species basal area data from the United States Department of Agriculture. Meteorological data for RLINE were collected at major airports in both cities. RLINE provided hourly deposited concentrations of species-specific pollen, converted to daily averages and adjusted using a daily growth rate estimated from measured pollen counts and season. Predicted daily pollen concentrations were compared to measured data using simple linear regression models, whose performance were evaluated using coefficient of determination (R^2).
Results: Modeling performance varied by tree species. Oak showed consistently better performance, with R^2 values ranging from 0.46 to 0.71 across sites and study years. Fagus in Atlanta and Sweet Gum in Houston had the highest R^2 values, with models achieving R^2 values of 0.81 in 2024 and 0.78 for 2021, respectively.
Conclusion: We developed a novel approach to predict daily pollen concentrations at high spatial and temporal resolutions. Utilizing the high resolution of the RLINE model, we can generate predictions at a 1 km resolution. Prediction performance is expected to be enhanced by incorporating additional meteorological and land use predictors.

P33-28 | Old tools to address persistent problems: assessing drivers of health differences with attributable fractions

Alexander Keil[1], Maria Kamenetsky[1], Maya Spaur[1], Jessica Madrigal[1], Whitney Robinson[2]

[1] National Cancer Institute, Occupational and Environmental Epidemiology Branch, Division of Cancer Epidemiology and Genetics, Rockville, USA; [2] Duke University, Duke-Margolis Institute for Health, Duke Cancer Institute, Durham, USA

Understanding differences in health across strata within populations is key to improving the overall health of populations. One focus of health research in epidemiologic studies has been the differential impact of individual exposures across groups with substantial differences in health outcomes. Current tools for assessing and understanding these differences in epidemiologic data include evaluation of effect measure modification (EMM, does the impact of an additional unit of exposure vary across groups on some scale?) and decomposition analysis (to what extent is the difference across groups mediated by the association between group membership and the exposure?). Despite common usage, these tools are limited. EMM evaluation fails to account for how the differential distribution of exposure across groups may impact overall health differences, while the decomposition analysis focuses directly on effects of group membership, which carries possibly untenable assumptions for making causal inferences and may not represent a modifiable factor.

Here, we make a modest proposal to adopt an existing, underutilized tool for assessing how exposures of interest drive differences in health: attributable fractions. Attributable fractions estimate the impact of eliminating exposure in a population, making them useful for studies of modifiable exposures that may or may not be evenly distributed across population groups. Using synthetic examples, we demonstrate that attributable fractions can identify exposures that drive health differences in settings in which there is no EMM. We demonstrate how attributable fractions can be extended to environmental settings, using tools like g-computation, to assess multiple exposures and to settings where eliminating exposure is not feasible. This work demonstrates a set of tools for studying health differences that focus on modifiable factors and thus better contribute to public health decision makers' efforts to improve public health.

P33-29 | Development of a new Method for Micro-Sampling Based Analysis of Trace Elements in Whole Blood

Julio A. Landero[1], Lucas Schmidt[1], Kayla Peterson[1], Malgorzata Knap[1], Lauren Petrick[1], Martha Koenig[2], Andrea Kuriyama[2], Samantha Schildroth[2], Amelia Wesselink[2], Lauren Wise[2]

[1] Icahn School of Medicine at Mount Sinai, Environmental Medicine, NYC, USA; [2] Boston University School of Public Health, Epidemiology, Boston, USA

Objective

To develop and validate a method to quantify metals and metalloids in 30µl of capillary blood collected in volumetric absorptive micro samplers (VAMS), using inductively-coupled plasma mass spectrometry (ICP-MS), for population-based studies.

Material and Methods

We developed a microwave-assisted method to extract metals from the samples collected in VAMS, and adapted the ICP-MS to analyze seventeen elements. To validate the method, we used certified reference materials and proficiency testing. We analyzed 360 samples from females who enrolled in Pregnancy Study Online (PRESTO), a North American preconception cohort study, and provided blood specimens via mail-based self-collection VAMS kits during 2022-2023. The median age (IQR) was 31 years (22-43 years) and participants resided across 47 states. More than 9% identified as Hispanic, 4% as Non-Hispanic Black, 6% as Non-Hispanic Asian, 5% as Non-Hispanic Multiracial, and 76% as Non-Hispanic White. Iron, sulfur and calcium levels indicated proper volumetric blood loading of the samplers.

Results

The digestion protocol extracted the elements of interest while eliminating >95% of the matrix. We validated the quantitative analysis of As, Be, Ca, Cd, Cs, Cu, Fe, Mg, P, Pb, Hg, S, Sb, Se, Tl, V, and U for measurement accuracy and reproducibility; Al, Ti, Cr, Mn, Co, Ni, Sn, Mo and Ba showed background contamination from the micro-samplers, indicating unreliable analysis. The samples showed >90% proper blood loading and blood levels of metals within the reference ranges of US populations for participants, with a lower analysis cost than the phlebotomy-based method.

Conclusion

We developed and validated a new method for the measurement of trace elements in capillary blood, based on the analysis of micro-sampled self-collected blood in PRESTO. This method is convenient for large population-based studies and can decrease costs associated with sample collection, storage, and transport, while maintaining high standards of data quality.

P33-30 | Development of a Novel Environmental Exposure Study Reporting Guideline

Nicole Deziel[1], Martin O'Connor[2], Mark Musen[2], Elaine Cohen Hubal[3], Kei-Hoi Cheung[4]

[1] Yale School of Public Health, New Haven, USA; [2] Stanford School of Medicine, Stanford, USA; [3] US Environmental Protection Agency, Raleigh, USA; [4] Yale School of Medicine, New Haven, USA

Objective

Characterizing environmental exposures is critical to advancing understanding of health throughout the lifespan. However, exposure-related data are not readily findable, accessible, interoperable, or reusable (FAIR), limiting optimal data usage and scientific reproducibility. We aimed to create an environmental exposure data reporting guideline and to encode a machine-readable metadata template to facilitate adherence to FAIR principles.

Material and Methods

We established a workflow using Google Sheets that enables domain experts to describe (1) a metadata specification (including data elements such as study design, study population, exposure assessment method, sampled media), and (2) value sets that contain ontology-based controlled terms for individual data elements in this template. These spreadsheets were then converted to (1) a machine-readable metadata template accessible through the Center for Expanded Data Annotation and Retrieval (CEDAR) Workbench, a platform designed to help researchers create, manage, and share structured metadata, and (2) a BioPortal value set. This value set was designed to fill gaps in available exposure-related ontologies.

Results

We completed development of a new exposure assessment template, which is now publicly available to scientists to submit annotated datasets to appropriate online data repositories. For approximately 40% of template questions, responseswere matched to existing BioPortal ontologies, while for 60%, we created new value sets. These value sets were primarily built using existing ontology terms, though, in a few cases, we created custom terms.

Conclusion

We developed a reporting guideline to provide a structured framework for researchers to describe and identify environmental exposure studies with rich metadata to facilitate scientific reuse and reproducibility. Planned community engagement is key to adoption of our guideline and will enable connection to the wider environmental health science community.

P33-31 | Development of a Method for Evaluating Skin Permeability of Phosphorus Flame Retardants Using a Franz Diffusion Cell and Pig Skin

Qi Wang[1], Megumi Ono[1], Makiko Iwase[1], Shuhan Yang[2], Yuichi Miyake[2], Masahiro Tokumura[3], Yukie Yanagiba[1]

[1] National Institute of Occupational Safety and Health, Japan (JNIOSH), Research Center for Chemical Information and Management, Kawasaki, Japan; [2] Yokohama National University, Yokohama, Japan; [3] University of Shizuoka, Shizuoka, Japan

With the ban on certain brominated flame retardants, phosphorus flame retardants (PFRs) are increasingly used as alternatives. Since some PFRs have been linked to adverse health effects, understanding their exposure pathways is crucial. While oral ingestion and inhalation are the major routes of exposure, recent studies highlight dermal absorption as a significant exposure pathway. This study developed a method using a Franz diffusion cell and pig skin to evaluate the skin permeability of PFRs.
To optimize the receptor solution for permeability testing, phosphate buffer solution (PBS), PBS with 5% bovine serum albumin (BSA), and PBS with 6% polyethylene glycol 20 oleyl ether (PEG) were assessed. Eighteen PFRs were introduced into each solution, and recoveries were measured using liquid chromatography-tandem mass spectrometry (LC-MS/MS). PBS with BSA demonstrated the most consistent recoveries (80–105%), while PEG led to unreliable results due to ion suppression or enhancement.
Three PFRs, triethyl phosphate (TEP), tris(2-chloroethyl) phosphate (TCEP), and tricresyl phosphate (TCsP), were used as model compounds in skin permeability tests. After 24 hours of exposure, TEP and TCsP showed low permeability (<55.6 ng cm^{-2}), while TCEP exhibited significantly higher permeability (8160 ± 649 ng cm^{-2}). This result aligns with previous studies (Shindo M. et al. (2024), Chemosphere, 353, 141555), confirming the validity of the developed method for assessing the skin permeability of PFRs.

P33-32 | Method Updates in the Measurement of Heterocyclic Aromatic Amines in Urine using Hamilton Starlet Automated Liquid Handling Platform and LC-ESI-MS/MS

Shrila Mazumder, Lanqing Wang

Centers for Disease Control and Prevention, Tobacco and Volatiles Branch / Division of Laboratory Sciences / National Center for Environmental Health, Atlanta, USA

Objective: Heterocyclic aromatic amines (HCAAs), many of which are probable/possible human carcinogens (IARC group 2A/2B), are formed during smoking of cigarettes and high temperature cooking of meats. Exposure to HCAAs such as 2-amino-9H-pyrido[2,3-b]indole (AAC); 2-amino-3-methyl-9H-pyrido[2,3-b]indole (MAAC); 3-amino-1,4-dimethyl-5H-pyrido[4,3-b]indole (TRP1); 3-amino-1-methyl-5H-pyrido[4,3-b]indole (TRP2); 2-amino-6-methyldipyrido[1,2-a:3',2'-D]imidazole (GLP1); 2-aminodipyrido[1,2-a:3',2-D]imidazole (GLP2); 2-amino-3-methyl-3H-imidazo[4,5-f]quinolone (IQU); 2-amino-1-methyl-6-phenylimidazo[4,5-b]pyridine (PHIP); harman (HMU); and norharman (NHM) can be assessed by measuring their concentrations in urine. Measurement of HCAAs in urine is

challenging because of their low abundance and labor-intensive sample preparation. Updates to an existing, automated sample preparation method, LC method and sample data flow process were made to more efficiently quantify and report “total” (free + conjugated) HCAAs in human urine samples.
Method: Sample aliquot, clean-up and reconstitution are all completed on a single Hamilton STARLET™ workstation. Subsequently, HCAAs are measured by isotope-dilution high-performance liquid chromatography coupled with electrospray ionization tandem mass spectrometry (ID-HPLC-ESI-MS/MS) in positive mode ionization.
Results: This new automated method increased sample throughput by reducing sample clean-up time from 6.5 hours to 5 hours while maintaining high precision (inter-run CV <9%) and accuracy (±10%). Updates to the LC method reduced the number of study samples needing repeat analyses (decreased from approximately 12% to 3%), while increasing the overall robustness of the LC method. Other updates made to this method also increased the data review throughput by using Indigo ASCENT™ program (Indigo BioAutomation, Inc.) for peak integration, quantification, sample QA evaluations, and result report generation. Furthermore, a streamlined data system was created in parallel to the automated method, in which, sample results can be tracked from initial analysis and repeat analysis to the final report results in laboratory information management system with minimal human intervention.
Conclusion: A sensitive and high-throughput automated method with an improved sample data flow is developed and used to quantify “total” HCAAs in human urine samples.

P33-33 | Advancing transparency, systematic review methods, and evidence integration approaches for identifying cancer hazards: The Updated Report on Carcinogens (RoC) Handbook

Whitney D. Arroyave[1], Suril Mehta[2], Neela Guha[3], Pamela Schwingl[4], Elizabeth Hodgson[4], Elizabeth Marder[3], Ruth Lunn[2]

[1] Integrated Laboratory Systems an Inotiv Company, RTP, USA; [2] National Institute of Environmental Health Sciences, RTP, USA; [3] Office of Environmental Health Hazard Assessment (OEHHA) (formerly), Sacramento, USA; [4] Integrated Laboratory Systems an Inotiv Company (Retired), RTP, USA

Objective:
Systematic review methods for evaluating health hazards from observational environmental epidemiology studies have been adapted from clinical medicine relatively recently and face additional challenges. We developed systematic review methods to address these challenges, applied them to several evaluations, and compiled them in the 2015 RoC Handbook. Since then, we and others have advanced approaches, including identifying and addressing research gaps for assessing human mechanistic studies. The need to create new and incorporate emerging methods and provide additional transparency prompted a RoC Handbook update.
Methods:
To update the Handbook, we built upon successful application and modification to several RoC evaluations, researched best practices, and developed new tools. The night shift work cancer hazard assessment was especially informative for the updated Handbook development.
Results:
New features in the updated Handbook apply to all parts of our four-step evaluation: scoping activities to construct the evaluation framework, evaluation of study informativeness (bias potential and study sensitivity), and integration of study evidence within and across evidence streams (human cancer, animal cancer, and mechanisms). To inform framework development, we now use interactive systematic maps to identify literature with an adequate database for

the assessment and key issues. A major advancement is the development of a new tool kit for all study informativeness domains for assessing human mechanistic studies. Building on revised questions for assessing exposure misclassification, we developed resources and guidance specific to common exposure measurements. Evidence integration enhancements include triangulation and systematic approaches to explore the impact of biases and confounding, effect modifiers, exposure metrics, and other sources of heterogeneity**.** Our presentation uses case studies from ongoing and past evaluations using these methods.

Conclusions:

The revised RoC Handbook utilizes robust and transparent methods for cancer hazard evaluations. We believe it will serve as a valuable resource in appraising the environmental epidemiology literature.

P33-34 | Environmental health webtool development

Yike Shen[1], Dhruvilkumar A. Chodvadiya[1], Shashwat Dhayade[1], Feng Gao[2]

[1] University of Texas at Arlington, Arlington, USA; [2] University of California, Los Angeles, Los Angeles, USA

Objective: The interdisciplinary field of environmental health sciences is rapidly evolving. Cohort studies have generated rich knowledge on exposure, omics, and disease relationships. Efforts have been made to build static knowledge graphs to summarize and visualize findings. However, users still need to manually search the edges in the dataset to extract the corresponding publications. An interactive web tool linking knowledge graph edges to publications can improve knowledge sharing efficiency. Additionally, machine learning (ML) models are widely applied to predict chemical toxicity, but users often lack Python expertise to develop, train, and validate ML models. A web-based ML tool that provides direct predictions will enhance accessibility. We aim to develop web tools for knowledge sharing and toxicity prediction in environmental health.

Materials and Methods: We developed two interactive webtools using Visual Studio Code for backend coding and React for the frontend development, connected through GitHub, and deployed them via Vercel with domains registered through GoDaddy. The knowledge graph tool, built with Cytoscape and custom web scraping scripts, links edges to publications via DOIs. The ML-based tool leverages AWS (API Gateway, Lambda, S3) to process user-uploaded data, applying a pre-trained model to compute ecotoxicity values and returning results via an API Gateway. We registered our websites using MIT license.

Results: We have launched our developed webtools: 1) ecotoxicity.org, a ML-based tool for predicting chemical ecotoxicity; 2) cohortnetwork.org, an interactive multi-layer knowledge graph linking exposures, outcomes, and their connections in environmental health cohorts. Both websites include landing pages with background information and relevant publications. For backend development, the interactive knowledge graph highlights interactive visualization, while the ecotoxicy tool highlights the ML-based prediction.

Conclusion: Our developed webtools provide a valuable approach for environmental health science community, including researchers and regulatory agencies, for more efficient knowledge sharing and more accurate toxicity prediction.

P33-35 | A Novel Air Sampler Evaluation for Drone Optimization: Using Drones as Tools for Environmental Air Sampling

Peter Kessinger[1], Marina Nieto-Caballero[2], Thomas Hill[3], Sonia Kreidenweis[4], Sheryl Magzamen[1]

[1] Colorado State University, Environmental and Radiological Health Sciences, Fort Collins, USA; [2] Colorado State University, Atmospheric Science, Fort Collins, USA; [3] Colorado State University, Atmospheric Science, Fort Collins, USA; [4] Colorado State University, Atmospheric Science, Fort Collins, USA; [5] Colorado State University, Environmental and Radiological Health Sciences, Fort Collins, USA

OBJECTIVE: Drones are an innovative exposure assessment tool that can be used to carry air samplers to different altitudes. However, drones are limited by their flight time, especially considering payload weight. Our goal is to evaluate the sampling efficiency of a novel lightweight air sampler in comparison to the well-established SASS 3100 bioaerosol collector, based on performance capability of the two.
MATERIALS AND METHODS: The novel air sampler was evaluated side-by-side with the SASS 3100 in two different approaches: 1) ground-based air sampling and 2) an elevated air sampling (12 m AGL). The ground-based air sampling approach placed the novel air sampler and the SASS 3100 a meter above the ground and collected bioaerosols for an hour in triplicate. The elevated air sampling approach included mounting the novel air sampler on two different drones and hovering at 12 meters AGL, while simultaneously the SASS 3100 was mounted on a boom lift at the same height. Samplers ran simultaneously in triplicate to assess any propeller effects on sampler efficiency. Samples were then processed to extract DNA and ran through Qubit software for DNA quantification.
RESULTS: Data is inconclusive but indicates that the novel air sampler performs similarly to the SASS 3100 at altitude when the novel air sampler is mounted on a drone (DJI M350). However, ground performance and performance when mounted on a hexacopter drone (Aurelia X2 PRO V6) do not show agreement between the novel air sampler and SASS 3100.
CONCLUSION: There were no conclusive findings from our data. However, there does appear that the novel air sampler can perform similarly to the SASS 3100 while cutting down on weight and increasing drone flight time. Further study is required to determine a trend and if the novel air sampler is comparable to the SASS 3100 in air sampling performance.

P34 | Food Contamination, Food Safety, and Food Security

P34-01 | Food Security and Phthalate Exposure: A Cross-Sectional NHANES (2001-2018) Analysis

Marshae Nickelberry[1], Emma V. Preston[1], Marlee R. Quinn[1], Kimberly L. Parra[1], Izzuddin M. Aris[3], Michele R. Hacker[4,5], Emily Oken[3,2], Tamarra James-Todd[1,5]

[1] Harvard T.H Chan School of Public Health, Department of Environmental Health, Boston, USA; [2] Harvard T.H Chan School of Public Health, Department of Nutrition, Boston, USA; [3] Harvard Medical School and Harvard Pilgrim Health Care Institute, Division of Chronic Disease Research Across the Lifecourse, Department of Population Medicine, Boston, USA; [4] Beth Israel Deaconess Medical Center and Harvard Medical School, Department of Obstetrics and Gynecology, Boston, USA; [5] Harvard T.H Chan School of Public Health, Department of Epidemiology, Boston, USA

Objectives: Insufficient food security may result in greater exposure to nonpersistent endocrine-disrupting chemicals. For example, individuals with lower food security often consume more processed food with packaging that contains plasticizers such as phthalates. We hypothesized that lower food security is associated with higher processed food-associated urinary phthalate metabolite concentrations and is modified by sex.

Methods: Using National Health and Nutrition Examination Survey (NHANES) data, we examined the association between food security status and urinary phthalate metabolite concentrations among 20,798 participants aged 6 and older (2001–2018). NHANES classified food security via the U.S. Household Food Security Survey Module as high food security (reference), marginal, low, or very low food security. We used multivariable linear regression to estimate associations between food security and ln-transformed urinary phthalate metabolite concentrations, adjusting for age, sex, education, creatinine, and cycle year, with survey weights incorporated. Secondary models also adjusted for race and ethnicity, as well as poverty-income ratio. Effect modification was assessed by sex.

Results: Lower food security was associated with higher mono-benzyl butyl phthalate (MBzP) concentrations, with significant percent differences in mean concentrations: [% difference [95% CI]: marginal: 7.89% [0.89-15.38], low: 15.37% [7.23-24.13], very low: 33.44% [17.05-52.12]. After adjusting for race/ethnicity and poverty-income ratio results were similar, though estimates in the most food secure categories were attenuated. Associations were generally stronger among females [low: 10.76% [1.13-21.30], very low: 22.84% [6.13-42.18].

Conclusions: Lower food security was associated with higher urinary phthalate metabolite concentrations, particularly MBzP, with stronger associations in females. These findings suggest a higher burden of chemical exposures in vulnerable populations. Further, longitudinal research is warranted to evaluate associations between food security and phthalate metabolite concentrations to identify those factors that may place individuals at greatest risk of exposure.

P34-02 | Food frequency patterns and hair metals among Chinese and Korean women of childbearing age in Chicago

Rose Hefferon[1], Mary Turyk[1], Jing Zhang[2], Hong Liu[2], Kyeezu Kim[3], Noel Chávez[4], Brian Jackson[5], Susan Buchanan[6], Julia Anglen Bauer[1]

[1] University of Illinois Chicago, Division of Epidemiology and Biostatistics, Chicago, USA; [2] Midwest Asian Health Association, Chicago, USA; [3] Sungkyunkwan University School of Medicine, Department of Social and Preventive Medicine, Seoul, South Korea; [4] University of Illinois Chicago, Division of Community Health Sciences, Chicago, USA; [5] Dartmouth College, Department of Earth Sciences, Hanover, USA; [6] University of Illinois Chicago, Division of Environmental Occupational Health Sciences, Chicago, USA

Objectives: Environmental elements are ubiquitous and can be essential for life, toxic, or both, depending on the dose.Our study aimed to characterize hair elements associated with diet among Asian women of childbearing age in Chicago.

Methods: From 2018 to 2020, Asian women were recruited for a 6-month intervention trial from community-based organizations (CBOs) serving Southeast Asian and Pacific Rim residents in Chicago through various community events sponsored by the CBOs and by word-of-mouth (Chinese(n=125), Korean(n=100)). This paper focuses on data collected at enrollment. Surveys assessed dietary patterns, fish consumption, lifestyle, and demographics. Hair samples were analyzed at Dartmouth Trace Elements Laboratory using ICP-MS. We used ln-transformed elements in adjusted multivariable linear regression models to estimate percent differences (95%CI) in hair element concentrations per unit increase in food frequency. We explored differences across Asian ethnicities using stratified models (Chinese or Korean).

Results: Fourteen metals and metalloids were analyzed in hair, including essential (Cu, Co, Cr, Fe, Mn, Se, Zn, V) and non-essential (As, Al, Ni, Sn, Pb, Hg) elements. Geometric mean(gsd) hair element concentrations (µg/g) that differed between Chinese and Koreans include: Al (Chinese: 4.0(2.1), Korean: 5.3(2.3)); Cu (Chinese: 13.6(1.7), Korean: 15.3(1.8)); and Hg (Chinese: 0.7(2.5), Korean: 0.5(2.7)). Daily to weekly consumption of various foods differed by ethnicity, for example, kimchi (Chinese: 4.4% vs. Korean: 85.2%) and mushrooms (Chinese: 46.3% vs. Korean: 70.1%). In adjusted models, the frequency of consuming the following foods was associated with increased hair elements: white rice and As (all participants: 26.1% (2.3, 55.3)), kimchi and Ni (Chinese: 66.5% (6.3, 160.9); Korean: 46.8% (9.6, 96.5)), and mushrooms and Sn (Chinese: 41.4% (-4.5, 109.3); Korean: 66.6% (4.5, 165.7)).

Conclusions: Kimchi, white rice, and mushroom consumption frequency were associated with metal exposures among Chinese and Korean women in Chicago. Asian ethnicity should be considered in exposure assessment.

P34-03 | Microplastics Exposure in Association with DNA Methylation and Hepatic Inflammatory Biomarkers

Yu-Chieh Chen[1], Kuan-Chih Chiu[1], Yu-Cheng Chen[2], Tsun-Jen Cheng[1], Chen-Yu Liu[1,3]

[1] National Taiwan University, Institute of Environmental and Occupational Health Sciences, College of Public Health, Taipei, Taiwan; [2] National Health Research Institutes, National Institute of Environmental Health Sciences, Miaoli, Taiwan; [3] National Taiwan University, Global Health Program, College of Public Health, Taipei, Taiwan

Objective:
Microplastics (MPs), ubiquitous environmental pollutants, have been detected throughout the human body. Evidence from animal studies suggests that MP exposure can activate the toll-like receptor 4 (TLR4) pathway, leading to hepatic injury and inflammation via the gut-liver axis. However, studies in humans remain limited. Therefore, we aimed to investigate the associations between MP exposure, DNA methylation patterns, and inflammatory and hepatic biomarkers in humans.

Material and Methods:
A total of 50 participants with a mean age of 24.35 years were recruited from northern Taiwan in 2022. MPs in fecal samples were detected using a micro-Fourier transform infrared spectrometer (micro-FTIR). DNA methylation levels of targeted genes were measured in whole blood samples via pyrosequencing. Serum inflammatory cytokines were measured using enzyme-linked immunosorbent assay (ELISA), while hepatic biomarkers were measured using Roche Cobas c702 analyzer. Linear regression models were used to examine the associations between MP exposure and DNA methylation levels, hepatic biomarkers, and inflammatory cytokines levels.

Results:
In univariable analyses, each unit increase in MPs exposure was associated with elevated levels of alanine transaminase (ALT), gamma-glutamyl transferase (γ-GT), and interleukin-1β (IL-1β) levels. After adjusting for potential confounders, the association between MPs exposure and IL-1β levels remained significant. Additionally, MPs exposure was linked to changes in DNA methylation levels within the TLR4 pathway, which may serve as a potential mechanism connecting MPs exposure to inflammatory response and hepatic injury.

Conclusion:
Epigenetics provides a potential explanation of the effect of environmental exposure on health outcomes. MP exposure may induce methylation changes in genes within the TLR4 pathway, leading to inflammation and hepatic damage.

This study was supported by the National Science and Technology Council, Taiwan (NSTC).

P34-04 | Socio-demographic, lifestyle, and dietary determinants of omega-3 status in Nunavimmiut (Inuit of Nunavik, Quebec)

Pauley Tedoff[1], Matthew Little[1], Mélanie Lemire[2], Pierre Ayotte[2], Tiff-Annie Kenny[3], Amira Aker[4], Federico Andrade-Rivas[1,5], Zeinab Zoveidadianpour[1]

[1] University of Victoria, Faculty of Human and Social Development, School of Public Health and Social Policy, Victoria, Canada; [2] Laval University, Faculty of Medicine, Department of Social and Preventive Medicine, Quebec City, Canada; [3] Université de Montréal, Faculty of Medicine, Department of Nutrition, Montreal, Canada; [4] Boston University, School of Public Health, Department of Epidemiology, Boston, USA; [5] University of Colorado Boulder, College of Art and Sciences, Department of Geography, Boulder, USA

Objective: Traditional Inuit foods, particularly those rich in omega-3 fatty acids from marine mammals and fish, play a crucial role in maintaining health. Reductions in traditional food consumption over time, along with environmental and lifestyle factors, have been associated with a higher incidence of cardiovascular disease in Inuit populations. Our objective was threefold: (1) to compare mean omega-3 levels of Nunavimmiut (Inuit from Nunavik, Quebec) between 1992, 2004, and 2017; and (2) estimate the association between socio-demographic, lifestyle, and dietary factors and omega-3 status (PUFA index); and (3) estimate the association between socio-demographic, lifestyle, and dietary factors and omega-3-related coronary heart disease risk.

Methods: We used data from Qanuilirpitaa? 2017 Nunavik Inuit Health Survey, the 1992 Santé Québec Survey and the 2004 Qanuipitaa? Health Survey to compare mean omega-3 levels over time. For the remainder of our objectives, we estimated linear and logistic regression models using data from Qanuilirpitaa? 2017. Our sample included 1,133 participants for whom complete food frequency questionnaire (FFQ) and blood sample data were available.

Results: We observed a steady decline in all omega-3 measures over time. Additionally, omega-3-related coronary heart disease risk was higher in 2017 compared to 2004. Our analysis of Qanuilirpitaa? 2017 data revealed that marine food consumption and food security were positively associated with omega-3 status. Further, high monthly marine country food consumption was associated with a lower likelihood of being in the high-risk category for coronary heart disease.

Conclusions: Higher omega-3 status is protective against coronary heart disease risk, yet omega-3 levels among Nunavimmiut are on the decline. Our findings underscore the importance of ensuring that Nunavimmiut have ongoing and enduring accessibility to omega-3 rich foods, particularly marine country foods, which are essential not only for physical health, but also for the preservation of traditional Inuit practices and livelihoods.

P34-05 | Assessing Fish Consumption Among High-Risk Lake Michigan Angler Communities

Susan Buchanan[1], Danielle Westnedge[1], Stephanie Sloane[2], Sonia Saxon[1], Drina Holliday[1], Kristen Malecki[1], Brenda Koester[2]

[1] University of Illinois at Chicago, Environmental and Occupational Health Sciences, Chicago, USA; [2] University of Illinois Urbana-Champaign, Center for Social and Behavioral Science, Urbana Champaign, USA

Objective: Subsistence and recreational fish consumption are common sources of PFAS and other contaminant exposures. There is a general lack of public awareness of the potential risks of exposure to these contaminants, especially among communities of color. We conducted a survey aimed at characterizing fishing habits, consumption practices, knowledge of PFAS and other contaminants, and trusted and preferred sources of health information in two populations on opposite sides of Lake Michigan.
Material and Methods: Using a mixed-methods approach we conducted intercept surveys conducted among recreational anglers in Chicago, Illinois and Benton Harbor, Michigan, two frontline communities facing numerous chemical and non-chemical stressors including high prevalence of food insecurity.
Results: A total of 85 recreational anglers aged 20-89yo completed surveys (50 males, 32 females, 1 other, 2 declined). The majority of participants (79%) identified as African American. Over 20% had yearly household income less than $10,000, and 40% were below the poverty threshold. Year-round fishing was common, with 25% reporting fishing at least 2-3 times per month in the winter. Over 60% noted that they fish to feed themselves or their families. While the majority reported fishing for recreation, the majority reported eating the fish and sharing with family and friends. Knowledge about fish consumption advisories and contaminants was limited. Family/friends was the most commonly reported source of information followed by social media platforms (e.g. Facebook). Over 50% of participants lacked trust in all sources of information including local paper, TV/radio news, healthcare providers, social media, fishing magazines, family/friends, and government websites (CDC, DNR).
Conclusion: Recreational fishing populations around Lake Michigan are high consumers of fish but have limited knowledge regarding health risks. Trusted sources of health information are limited and future messaging and risk assessment needs to consider vulnerable populations at high risk of PFAS exposure.

P34-06 | Health risk assessment of dietary cadmium exposure based on cadmium bioavailability in food: Opportunities and challenges

Di Zhao[1], Albert L. Juhasz[2], Lena Q. Ma[3]

[1] Nanjing Agricultural University, College of Resources and Environmental Sciences, Nanjing, China; [2] University of South Australia, Future Industries Institute, South Australia, Australia; [3] Zhejiang University, College of Environmental and Resource Sciences, Hangzhou, China

Long-term exposure to toxic cadmium (Cd) through contaminated food can result in multiple adverse health effects. While previous studies have addressed global food Cd concentrations and dietary Cd exposures across various populations, growing concerns have emerged about the adequacy of current food Cd safety standards in protecting public health. Additionally, the relative bioavailability (RBA) of Cd in food has been identified as a critical factor for improving health risk assessments, yet the factors influencing Cd-RBA have not been systematically addressed,

limiting its application in risk assessment. This study aims to provide an overview of Cd contents in food, highlight concerns regarding international food Cd concentration standards, explore factors influencing food Cd bioavailability, and identify both opportunities and challenges in refining differences between dietary Cd intakes and body burdens. Our findings suggest that existing safety standards may be insufficient for safeguarding human health, as they primarily focus on kidney damage as the protective endpoint, neglecting global and regional variations in food consumption patterns and the temporal changes in dietary habits. Key factors such as crop cultivars and food compositions play a significant role in determining food Cd-RBA. To improve the accuracy of Cd health risk assessment, food Cd-RBA, sociodemographic characteristics, nutritional status, and incidental Cd exposure should be incorporated. This information is essential in informing future bioavailability investigations, health risk assessment, and safety standard development.

P34-07 | Toxic metals in rice across Asian countries: A review of occurrence and potential human health risks

Hien T.T. Ngo[1], Hang T.T. Nguyen[2], Cuong X. Nguyen[3], Ngoc T.M. Nguyen[4], Duc D. Nguyen[5]

[1] Phenikaa University, Faculty of Public Health, Ha Noi, Vietnam; [2] Vietnam National University Ho Chi Minh City/University of Science, Department of Environmental Management and Informatics, Faculty of Environment, Ho Chi Minh, Vietnam; [3] Duy Tan University, Institute of Research and Development /Faculty of Environmental Chemical Engineering, Da Nang, Vietnam; [4] Haiphong University of Medicine and Pharmacy, Faculty of Public Health, Hai Phong, Vietnam; [5] Kyonggi University, Department of Environmental Energy Engineering, Suwon, South Korea

Background: Heavy metals such as cadmium (Cd), arsenic (As), and lead (Pb) pose significant health risks, especially in Asia, where rice is a staple food for nearly three billion people. Despite the known hazards and widespread environmental presence of these metals, there is a lack of comprehensive studies on their concentrations in rice across various regions and the related health risks.

Objective: This review aims to systematically explore the presence of Pb, Cd, and As contamination in rice from Asian countries and the potential ecological and health hazards associated with consuming rice containing these toxic metals.

Material and Methods: An extensive literature review was carried out utilizing multiple databases, including Science Direct, Web of Sciences, Springer, and PubMed up until May 2021. The search used the keywords “rice, toxic metals or As, Cd, Pb analysis methods, detection, environmental health risks” along with terms such as “food,” “staple food,” and “food crops” to ensure all relevant publications were included. Additional studies were identified by reviewing the reference lists from the selected research. The geographical areas concerning heavy metal presence in rice covered Asian countries like Vietnam, China, India, Malaysia, Iran, Korea, and Bangladesh.

Results: Data from seven countries indicated mean concentrations of heavy metals in the descending order of Pb>As>Cd, with levels of 0.255±0.013, 0.236±0.317, and 0.136±0.150 mg/kg, respectively. Uncertainty analysis revealed considerable variation, particularly for Cd, with a 95% confidence interval ranging from 0.220 to 0.992 mg/kg. The typical daily intake of heavy metals through rice consumption, in the order of As>Cd> Pb, often exceeded safe limits. Additionally, children were found to be more vulnerable to heavy metal exposure through rice consumption than adults.

Conclusion: This review is fundamental for ongoing monitoring, future research, and the development of strategies to mitigate heavy metal contamination in rice.

P34-08 | Mercury (Hg), Perfluoroalkyl Substances (PFAS), and Fish Consumption in the Great Lakes Fish Consumer Study

Meghan Cerpa[1], Saria Awadalla[1], Robert Sargis[2], Henry Anderson[3], Mary Turyk[1]

[1] University Illinois Chicago, Epidemiology and Biostatistics, Chicago, USA; [2] University Illinois Chicago, Department of Endocrinology, Diabetes and Metabolism, Chicago, USA; [3] University of Madison Wisconsin, Department of Population Health Sciences, Madison, USA

Objective: To examine associations of blood serum Hg and PFAS with fish meals in frequent and infrequent Great Lakes (GL) fish consumers.

Methods: The GL Fish Consumer Study recruited GL charter boat captains, non-GL fish consumers from the same geographic areas (referents), and anglers who fished other inland Wisconsin lakes (anglers) in the early 1990s. In 2004-2005, participants reported the number of fish meals eaten in the past year by source. Sera was analyzed for Hg and 12 PFAS. Rates of GL, inland lake and purchased fish meals consumed in the past year were regressed simultaneously on log_2-transformed serum biomarkers, adjusting for age, BMI, sex, and education.

Results: Median age was 58.0 years, BMI was 28.9kg/m^2, and 70% of participants were male (n=468). Captains had significantly higher geometric mean serum concentrations for the majority of PFAS, but not Hg, compared to anglers and referents. Participants ate a geometric mean of 6 GL sportfish, 10 inland lake sportfish, and 12 purchased fish meals over the past year. An increase of 5 GL sportfish meals/year was significantly associated with higher serum concentrations of several PFAS, ranging from 1.0% (PFHxS) to 4.1% (PFUnDA); smaller effects were noted for an increase of 5 purchased [0.6% (n-PFOS) to 1.0% (PFOA)] and 5 inland lake [-1.5% (FOSA) to 2.2% (n-PFOS)] fish meals/year. An increase of 5 GL meals/year and 5 inland lake meals/year were associated with a 3.3% and 7.2% higher serum Hg concentration, respectively, while purchased fish meals were not associated with serum Hg levels.

Conclusions: GL sportfish consumption was associated with higher serum PFAS concentrations than inland lake sportfish and purchased fish consumption. GL and other inland lake sportfish consumption were associated with significantly higher total Hg serum concentrations.

P34-09 | PFAS in Surface Water and Edible Fish Tissue from Freshwater Rivers and Lakes in Massachusetts

Rebecca DeVries[1], Anna Stanley Lee[1], Richard Chase[2], Richard Carey[2]

[1] Eastern Research Group, Concord, USA; [2] Massachusetts Department of Environmental Protection, Watershed Planning Program, Worcester, USA

Objective: PFAS pose a significant threat to human health through various exposure routes, including consumption of wild-caught fish and recreational swimming. This study aimed to characterize PFAS contamination in freshwater fish and surface water throughout Massachusetts.
Materials and Methods: In 2022, we collected co-located surface water and fish tissue samples at 52 freshwater rivers and lakes, prioritizing sites near known or suspected sources of PFAS contamination and waterbodies with high fishing pressure, while ensuring an appropriate geographic distribution. We collected 66 surface water samples and 242 composite fish samples (comprised of 948 individual boneless/skinless fish filets), which were analyzed for 40 PFAS analytes with EPA Method 1633. We summarized results for individual PFAS analytes and the sum of all detected analytes (ΣPFAS40), and explored patterns by media and species as well as bioaccumulation in fish. We also compared results to draft health-based action levels (AL) derived by the Massachusetts Department of Public Health and to EPA's aquatic life criteria recommendations, both of which are applicable to specific PFAS.
Results: All surface water samples and all but one fish sample had detectable levels of at least one PFAS analyte. The median ΣPFAS40 was 23.7 ng/L in surface water and 9.0 ng/g in fish tissue. Six of the 52 waterbodies had at least one PFAS concentration in surface water exceeding the draft AL of 20 ng/L for public beaches in Massachusetts. All but one waterbody had at least one fish sample with a PFAS measurement above the draft AL of 0.22 ng/g. All PFOS and PFOA detections were below EPA's aquatic life criteria recommendations.
Conclusion: PFAS are widespread in waterbodies across Massachusetts and in many cases are present at levels that could harm human health.
This work was funded by the Watershed Planning Program within the Massachusetts Department of Environmental Protection.

P34-10 | A Systematic Review of Seafood Consumption Rates for use in Human Health Risk Assessments in the Southeast Region

Shanna T. Alexander

U.S. Environmental Protection Agency, Region 4, Atlanta, USA

The finfish and shellfish (collectively referred to as seafood) consumption rate is a key limiting exposure factor in the chronic daily intake equation in human health risk assessments conducted at Superfund sites. Site-specific seafood consumption rates for vulnerable, high-consuming populations are lacking in most studies contributing to risk assessments and national fish consumption rate guidelines. Currently, there are no standardized or reproducible methods for selecting reliable seafood consumption surveys for use in risk assessments, which may lead to potential inclusion of studies with survey and response biases. In addition, conducting a reliable consumption survey can be cost and time prohibitive, taking years to complete. In the absence of representative seafood consumption rates, per

capita estimates protective of the general population or the default value provided by EPA's Office of Water for recreational fishers is often broadly applied in risk assessments. This can result in underestimation or overestimation of consumption risks for certain populations, including recreational and subsistence fish consumers. This study attempts to address the need for defensible, location, and population specific seafood consumption rates that are derived in a transparent, timely, and cost-efficient manner. Closing these information gaps will assist risk assessment practitioners in refining exposure and health risk outcomes used to inform risk management decisions at hazardous sites. Finally, this work may be used to further advance the practice of systematic review in exposure science and shed light on the need for additional seafood consumption studies.

P34-11 | Role of income in the associations of food consumption and handling practices with exposure to endocrine-disrupting chemicals in pregnant Taiwanese women.

Alexander Waits[1], Chia-Huang Chang[2], Yu-Fang Huang[3], Ming-Song Tsai[4], Pei-Wei Wang[5], Chih-Yao Chen[6], Chia-Jung Hsieh[7], Ming-Tsang Wu[8], Shu-Li Wang[9], **Mei-Lien Chen**[3]

[1] National Yang Ming Chiao Tung University, International Healt Program, Taipei, Taiwan; [2] Taipei Medical University, School of Public Health, Taipei, Taiwan; [3] National Yang Ming Chiao Tung University, Institute of Environmental and Occupational Health Sciences, Taipei, Taiwan; [4] Cathay General Hospital, Department of OBS & GYN, Taipei, Taiwan; [5] Taipei City Hospital, Heping Fuyou Branch, Department of Pediatrics, Taipei, Taiwan; [6] Taipei Veterans General Hospital, Division of Obstetrics and High-Risk Pregnancy, Taipei, Taiwan; [7] Tzu Chi University, Department of Public Health, Hualien, Taiwan; [8] Kaohsiung Medical University, Research Center for Environmental Medicine, Kaohsiung, Taiwan; [9] National Health Research Institutes, Division of Environmental Health & Occupational Medicine, Miaoli, Taiwan

This study explored the role of income in associations of food consumption and handling with exposure to endocrine-disrupting chemicals in pregnant women from Taiwan Maternal and Infant Cohort Study. The associations were estimated with linear regression adjusted for age, education, body-mass index, working status, and geographical region and expressed as percentage changes in urinary concentrations of bisphenol A (BPA) and parabens. The results were stratified by a cut-off point of two minimal salaries into the lowest and above-lowest income groups. Mediation analysis was performed to test the environmental justice hypothesis of the path from income via food consumption/handling to BPA and parabens exposure.

In the sample of 472 pregnant women in third trimester, higher parabens concentrations were associated with the consumption of low-fat milk (ethylparaben, 127.2 %, 95%CI = 24.0%, 316.3%) and peeled fruit (methylparaben, 143.8%, 95% CI = 24.5%, 377.6%, butylparaben, 212.3%, 95%CI = 11.2%, 776.8%), and with reusing bottles for refrigerating or heating food (methylparaben, 76.8%, 95% CI = 5.2%, 197.3%). Higher butylparaben concentrations were associated with meat/fish consumption (12.4%, 95%CI = 1.9%, 23.9%) and covering food for refrigerating/heating (462.5 %, 95%CI = 34.9%, 2246%) in the lowest-income group. Reusing bottles for refrigerating/heating food was associated with higher concentrations of methylparaben and propylparaben in the above-lowest income group. No significant mediation was detected.

While certain food consumption/handling was associated with higher paraben levels, further investigation of the association between paraben exposure and adverse health outcomes is needed, especially in disadvantaged populations. Reducing the use of bottles for heating/refrigerating food may reduce paraben exposure.

P34-14 | Outbreak investigation on mushroom poisoning by *Exsudoporus ruber cf.* and *Boletus*species in the Himalayan Region of Nepal

Aayush Adhikari[1], Rajesh Sharma[2], Rakesh Ghimire[2], Rabina K. Thagunna[3], Shiva Devkota[4], Sanjeeb Bhandari[5], Baburam Acharya[6]

[1] Manang Hospital, General Emergency, Chame, Nepal; [2] Poison Information Center, Drug and Toxicology Center, Tribhuvan University Teaching Hospital, Toxicology, Kathmandu, Nepal; [3] Health Office, Public Health, Chame, Nepal; [4] Gandaki Province Academy of Science & Technology, Pokhara, Nepal, Research, Pokhara, Nepal; [5] University of Pittsburgh Medical Center Western Maryland Hospital, Emergency Medicine, Cumberland, USA; [6] Deakin University, Health Economics, Burwood, Australia

Objectives

To find out the demographic, clinical and seasonal profile of mushroom poisoning events in Manang, Nepal from 2022 to 2024.

Materials and Methods

We conducted a retrospective descriptive analysis to investigate events in the Himalayan district of Manang from August 2022 to August 2024. Medical records of patients visiting Manang Hospital, Nepal in this time frame after consuming wild mushrooms were evaluated. We consulted with forestry officials to identify the wild mushroom. Data were analyzed for descriptive characteristics including age, gender, incubation period, clinical features, and outcomes.

Results

A total of 25 migrant workers ate wild mountainous mushrooms. Attack rate was 100%. Outbreaks mostly occurred from May to August. Mean age was 32.5 years (Range: 14-58 years). 80% of cases were males. Median incubation period was 51.6 minutes (range: 45 minutes- 2.5 hours). Common manifestations involved gastrointestinal (96%), nervous (64%), musculoskeletal (60%), cardiovascular (40%), and visual (20%) symptoms. Mean systolic blood pressure was 104.4 ± 17.57 mmHg and mean diastolic blood pressure was 68.8 ± 13.32 mmHg. Renal Function Tests showed mean urea concentrations of 35.22 ±14.61 mg/dl and mean creatinine concentration of 0.98 ± 0.23 mg/dl. Mean pulse rate was 97.52 ± 22.17 bpm. All patients were hospitalized for 16 to 48 hours. The mushroom was identified as *Exsudoporus ruber cf*. in 18 cases and *Boletus* species in rest cases. There was no associated mortality. The mushroom was unknowingly picked in all cases.

Conclusion

The poisoning event was due to consumption of wild mushrooms of genus *Exsudoporus* and *Boletus*. Our study adds valuable insight into the impact of poisoning events and highlights the need for further studies on classifying poisonous wild mushrooms of Himalayan regions to prevent such incidents in future.

Funding: Not involved

Acknowledgements:

1. Dr. Shiva Devkota (Mycologist)
2. Health Office Manang

P34-16 | Should Pregnant Women and Children Eat Fish? A Risk-Benefit Analysis – A Scoping Review

Nuria Jiménez-Redondo[1], Isabel Casanova-Martínez[1], Marina Cano-Lamadrid[1], Esther Sendra[1], **Antonio J. Signes-Pastor**[2,3]

[1] Universidad Miguel Hernández, Centro de Investigación e Innovación Agroalimentaria y Agroambiental (CIAGRO-UMH), Orihuela, Spain; [2] Universidad Miguel Hernández, Unidad de Epidemiología de la Nutrición, Departamento de Salud Pública, Historia de la Ciencia y Ginecología, San Joan d'Alacant, Spain; [3] Instituto de Salud Carlos III, CIBER Epidemiología y Salud Pública (CIBERESP), Madrid, Spain

Fish is a valuable source of long-chain omega-3 polyunsaturated fatty acids (LCPUFAs), which are essential for neurodevelopment and overall health, particularly during pregnancy and childhood. However, concerns persist regarding exposure to heavy metals, particularly methylmercury (MeHg), which is neurotoxic. This scoping review synthesizes the available literature to assess the risk-benefit balance of fish consumption in these vulnerable populations and provide evidence-based dietary recommendations. A systematic search was conducted across four databases, identifying 48 eligible studies after full-text screening. The selected studies were categorized based on observed health effects: beneficial (n = 20), harmful (n = 14), no effect (n = 12), and mixed effects (n = 2). Findings indicate that regular fish consumption supports fetal brain development, reduces the risk of preterm birth, and provides essential micronutrients, yet also raises concerns due to heavy metal accumulation, particularly in large predatory fish. To maximize benefits while minimizing risks, this review recommends prioritizing the consumption of small-sized oily fish, which are rich in n-3 LCPUFAs and have lower mercury concentrations, while limiting the intake of high-mercury species. These findings align with existing dietary guidelines but underscore the need for improved consumer awareness and further research on heavy metal mitigation strategies.

P34-17 | Evolution of Merchantability during storage of cowpea triple bagged containing Biopesticides (*Lippia multiflora*)

Constant K. Konan[1], Kouamé Kouadio[1], Gilles Léonce Niamketchi[2], Henri G. Biego[3]

[1] Institut Pasteur of Côte d'Ivoire, Environmental and Health Department, Abidjan, Côte d'Ivoire; [2] National Center for Agricultural Research, Programme Palmier, Station de Recherche Irho La Mé, Abidjan, Côte d'Ivoire; [3] University Félix Houphouët-Boigny of Abidjan, Biochemistry and Food Science Laboratory, Abidjan, Côte d'Ivoire

In rural areas, the preservation of the quality of cowpea grains stored in the presence of plants with biopesticide properties that are not harmful to health and the environment deserves to be promoted in order to limit producers' dependence on the use of synthetic pesticides. The storage technique used is triple bagging containing *Lippia multiflora* leaves. The marketable quality parameters (humidity, damage and weight loss) of cowpea grains were evaluated for 8 months of storage. The variance analysis on the marketable quality parameters of stored cowpea showed a significant influence between the type of packaging and the storage duration. Thus, the moisture content (from 10.03 ± 0.21% to 12.10 ± 0.10%), damage (from 3.25 ± 0.07% to 11.18 ± 0.01%), weight loss (from 0.28 ± 0.08% to 4.03 ± 0.27%) of cowpea stored in triple-bagged bags with different proportions of biopesticide were significantly lower than those recorded in the polypropylene woven control bag and in the triple-bagged control bag during the storage period. The results obtained seem to indicate a stability in the quality of cowpea grains stored for

8 months in triple-bagged bags containing different proportions of *Lippia multiflora* leaves. A proportion of at least 1.26% of *Lippia multiflora* leaves in triple-bagged bags is recommended for better preservation of the marketable quality of stored cowpea grains.

P34-18 | Modeling PFOS in Corn Stover and Beef to Assess Human Dietary Exposure

Jiahe Tian, Ankita Bhattacharya, Courtney Carignan

Michigan State University, Food Science and Human Nutrition, East Lansing, USA

Per- and polyfluoroalkyl substances (PFAS) in biosolid-amended soils can transfer and accumulate in crops, livestock, and humans. Given the persistence of PFAS, there is a critical need for modeling tools to estimate PFAS transfer to crops and livestock and associated human dietary exposures. This study aims to develop and apply modeling frameworks to quantify perfluorooctane sulfonic acid (PFOS) in crops and cattle from soil and estimate human dietary intake from beef consumption. A literature review was conducted to compile bioaccumulation factors (BAFs) from soil to corn stover. Median and maximum BAFs and PFOS concentrations from biosolid-amended soils were used to estimate PFOS in stover. A dynamic pharmacokinetic model was applied to predict PFOS in cattle muscle over two years. The predicted PFOS concentrations in cattle muscle were multiplied by body weight-adjusted consumption rates (BW-CR) for beef to estimate daily exposure for both children and adults. Median estimated PFOS exposure via beef consumption was notably higher for children compared to adults due to higher BW-CR. PFOS concentrations were estimated for beef, stover and soil that result in exposures of 0.1 ng/kg-bw/day (U.S. EPA reference dose). Exposure estimates increase with incorporation of upper bound BAF and BW-CR, cattle placental transfer, background PFOS exposure and mixtures of PFASs. This study presents a quantitative approach for estimating PFOS accumulation from crops to livestock along with human dietary exposure to inform intervention strategies.

P34-20 | Antibiotic Resistance of Bacteria Isolated from Medicinal Plant Powders Sold in Abidjan, Côte d'Ivoire

Kouassi B. Kouame[1], N'dri S. Vakou[2], Kouadio F. Konan[3], Kalpy J. Coulibaly[2], Mamidou W. Kone[4]

[1] Institut Pasteur Côte d'Ivoire, Environnement Santé, Unité de Botanique et Médecine traditionnelle, Abidjan, Côte d'Ivoire; [2] Institut Pasteur de Côte d'Ivoire, Environnement Santé, Unité de Chimie et Microbiologie Environnementale, Abidjan, Côte d'Ivoire; [3] Institut Pasteur de Côte d'Ivoire, Unité des Antibiotiques, de substances Naturelles et de la surveillance de la résistance des Microorganismes aux Anti-Infectieux, Abidjan, Côte d'Ivoire; [4] University Nangui Abrogoua Côte d'Ivoire, UFR Sciences de la Nature, Botanique, Biologie végétale, phytochime, Abidjan, Côte d'Ivoire

Objective: Medicinal plant powders are widely used in Côte d'Ivoire for treating various ailments and are often marketed without rigorous health controls. This lack of regulation increases the risk of contamination by pathogenic bacteria, particularly antibiotic-resistant enterobacteria and *Staphylococcus*, which poses a significant challenge for infection control.

Material and Methods: This study aimed to evaluate the antibiotic susceptibility of enterobacteria and *Staphylococcus* isolated from 100 samples of medicinal powders sold in different markets in Abidjan. Bacterial identification was performed using biochemical and proteomic methods (MALDI-TOF). Antibiotic susceptibility testing was conducted using the agar diffusion method, following the EUCAST-CASFM guidelines (2024_V1.0). **Results :** A total of 48 enterobacterial strains were isolated, including *Enterobacter hormaechei* (21), *Escherichia coli* (18), *Citrobacter freundii* (2), *Enterobacter asburiae* (2), *Klebsiella pneumoniae* (2), *Enterobacter cloacae* (1), *Enterobacter bugandensis* (1), and *Cronobacter sakazakii* (1) and 4 *Staphylococcus* strains, including *Staphylococcus sciuri*(3)*Staphylococcus cohnii* (1) . Antibiotic susceptibility testing revealed that 54.14% of the strains were resistant to at least one antibiotic, with the highest resistance rates observed forpefloxacin (33.34%) and ertapenem (8.35%). Resistance to ertapenem, a carbapenem frequently used as a last-resort treatment, is particularly concerning. **Conclusions** : The presence of enterobacteria in these samples suggests fecal contamination, reflecting poor hygienic conditions. These findings highlight the potential risk posed by medicinal plant powders as vectors of antibiotic-resistant bacterial strains. Implementing strict quality control measures and enhanced surveillance of antibiotic resistance is crucial to reducing their impact on public health.

P34-21 | Perceptions and awareness of the harmful effects of plastic packaging on food preservation on human health

Shahana Sultana, Tanjela Bushra, Md. Atiqul Haque

Bangabandhu Sheikh Mujib Medical University, Department of Public Health and Informatics, Dhaka, Bangladesh

Plastics are widely used in households for food storage and packaging because of their availability, cost-effectiveness, durability, and convenience. However, public awareness of the risks associated with plastic use is limited. This study aims to assess the public's perception and understanding of the toxicological dangers linked to using plastics for food preservation and packaging.

This cross-sectional study included 547 participants from two areas, the old part of Dhaka City (Lakshmi Bazar) and Mirpur. Data collection was conducted through face-to-face interviews, which gathered demographic information, knowledge, and perceptions regarding plastic use, waste disposal practices, and health effects.

Approximately 52% of respondents were female, 85% were married, and the average age was 35.2 years (SD = 11.7). Print media, social media, and friends were the main sources of information about the health risks of plastics. While 90% acknowledged these hazards, only 27% believed the public is aware of them. In terms of usage, 58% reused plastic products daily, and 30% did so 1 to 4 days a week. For daily use, 57% used plastics for drinking water, and 50% consumed frozen food from plastic containers. A significant 72% did not segregate plastic waste, with disposal methods mainly involving public dustbins or selling to vendors. While 60% supported banning plastic products, 80% noted that alternatives are costly. Additionally, 33% believed reusing plastic was beneficial for health. Only 45% thought white plastic was safer than colored, and 81% considered microwave use of plastic safe. However, 82% recognized health risks from using plastic to wrap frozen food, and 63% deemed this practice inappropriate.

This study highlights the gap between personal awareness and public knowledge of the health risks of plastic use, stressing the need for educational initiatives to promote safer handling and disposal practices.

P34-22 | Effects of different coffee roasting processes on the formation of diacetyl and 2,3-pentanedione

Tzu-Chieh Su, Yeh-Chung Chien

Yunlin University of Science and Technology, Douliu, Taiwan

Aims: This study investigated the formation of diacetyl (butanedione) and 2,3-pentanedione, known respiratory irritants, in the air during various coffee roasting processes.
Methods: A factorial experiment [roast degree (light, medium, dark) x process (roasting, packaging, grinding)] was conducted, supplemented by independent tests examining the effects of grinding fineness, bean type (origin), sampling distance, grind quantity and aging (days post-roasting). All tests were performed within a single coffee establishment to minimize variability. Real-time measurements of total volatile organic compounds (TVOC) and $PM_{2.5}$ were also performed where feasible.
Results: The factorial experiment revealed significant differences ($p<0.05$) in diacetyl and 2,3-pentanedione concentrations across processes, with grinding producing the highest levels, followed by roasting and then packaging. Diacetyl and 2,3-pentanedione concentrations were strongly correlated with TVOC levels during grinding ($r=0.827$) and roasting ($r=0.848$). Bean type significantly influenced diacetyl and 2,3-pentanedione concentrations, and the concentrations decreased with increasing sampling distance. Aging significantly affected 2,3-pentanedione concentrations, with the highest levels observed on the first day after roasting. However, grind size (coarse vs. fine) and grind quantity (0.5 kg vs. 2 kg) did not significantly impact the concentrations of either ketone.
Conclusions: This study demonstrates that coffee roasting operations, particularly grinding and roasting, significantly impact airborne diacetyl and 2,3-pentanedione concentrations. Coffee processing establishments should implement appropriate control measures to minimize exposure risks for both workers and consumers.

P34-23 | Testing for Paralytic Shellfish Poisoning Toxins in Community-Provided Samples in Southeast Alaska

Shannon Cellan[1], Hugh B. Roland[2], Esther G. Kennedy[3], Kari Lanphier[4], John R. Harley[3], Christopher Whitehead[1], **Matthew O. Gribble**[5]

[1] Sitka Tribe of Alaska, Resource Protection Division, Sitka, USA; [2] University of Alabama at Birmingham, Health Policy & Organization, Birmingham, USA; [3] University of Alaska, Southeast, Alaska Coastal Rainforest Center, Juneau, USA; [4] Southeast Alaska Tribal Ocean Research Consortium, Sitka, USA; [5] University of California, San Francisco, Division of Occupational, Environmental, and Climate Medicine, San Francisco, USA

Objective: Southeast Alaska is home to many communities with dietary traditions of seafood consumption including subsistence harvest of shellfish. Southeast Alaska is also a place where harmful algae that make the toxins causing paralytic shellfish poisoning are endemic. To reduce the risk of eating shellfish contaminated with these toxins, Sitka Tribe of Alaska Environmental Research Laboratory (STAERL) has been providing a testing service for shellfish (e.g., clam, cockle, mussel) samples provided by people living in the region.
Material and Methods: Samples were collected at tribally-designated harvest sites using hand collection techniques and shipped to STERL for analysis, with intention to report results back to community members within 48 hours whenever possible. Shellfish samples were then shucked and homogenized for testing as per FDA regulatory

protocols. Paralytic shellfish toxins were then extracted from the shellfish homogenates in a scaled version of the FDA-approved mouse bioassay protocol. The toxicity of these extracts was determined using a receptor binding assay and results were reported back to individual community members in an emailed report, with access to salient educational resources and guidelines. Community members who were unable to receive emailed reports were provided information via phone call or through their tribal representative.
Results: There have been >2,000 samples tested through this service since 2020. Although the food safety limit for commercial shellfish is 80μg toxin-equivalents/100g shellfish, in community-provided samples there were levels observed as high as 2,447μg/100g, which would be lethal. There were >450 community-provided samples found to have levels at or exceeding the FDA regulatory threshold. Despite the evidently high potential for dangerous exposures in the absence of testing, there have been no fatal cases of paralytic shellfish poisoning in the served communities since this food safety monitoring program started.
Conclusion: There has been significant utilization of this service, possibly saving lives.

P34-24 | One health challenge from heavy metal exposure risk in a coastal area of Northern Vietnam

Nguyen Thi Minh Ngoc[1], Nguyen Thi Thu Trang[4], Pham Van Han[2], Nguyen Van Ba[3], Ho Anh Son[3], Nguyen Bao Tran[6], Elizabeth V Wattenberg[7], Hiroyuki Nakamura[8], Pham Van Thuc[9], Nguyen Van Chuyen[10]

[1] Haiphong University of Medicine and Pharmacy, Environmental Health/Public Health, Haiphong, Vietnam; [2] Haiphong University of Medicine and Pharmacy, Public Health, Haiphong, Vietnam; [3] Vietnam Academy Medical University, Haiphong, Vietnam; [4] Vietnam Academy Medical University, Military hygiene, Hanoi, Vietnam; [5] Vietnam Academy Medical University, Hanoi, Vietnam; [6] Haiphong University of Medicine and Pharmacy, Anatomy, Haiphong, Vietnam; [7] University of Minnesota, School of Public Health, Minesota, USA; [8] Kanazawa University, Department of Environmental and Preventive Medicine, Kanazawa, Japan; [9] Haiphong University of Medicine and Pharmacy, Haiphong, Vietnam; [10] Vietnam Academy Medical University, Military hygiene, Vietnam, Vietnam

Objective: Heavy metal contamination of water sources has emerged as a major global environmental concern, which threatens to disrupt aquatic ecosystems, contaminate vegetables, and harm human health. The heavy metals Chromium (Cr VI), Cadmium (Cd), Lead (Pb), and Arsenic (As) have been recognized as toxic environmental contaminants in many parts of the world. A cross-sectional study was carried out to estimate the risk of human health effects from heavy metal-contaminated water and food consumption among residents of a northern coastal area of Vietnam.
Material and Methods: Cr, Cd, Pb, and As concentrations in 222 well-water, 135 vegetable, and 40 seafood samples were examined. A human health risk assessment was then conducted by following the guidelines from the US Environmental Protection Agency.
Results: 70%-90% of seafood and vegetable samples had Pb, Cd, and Cr concentrations higher than the permitted standards. 50%-60% of the water samples exceeded the TDI (Tolerable Daily Intake) for As and Pb. The estimated average cancer risk levels for adults consuming well water for drinking purposes and food were determined to be higher than acceptable for As, Cr, and Cd. The estimated cancer risk in the population due to consumption of food contaminated with heavy metals decreases gradually from As > Cr > Pb > Cd.
Conclusion: These results indicate that there is an urgent need to develop surveillance and interventions in order to improve drinking water quality and food safety, and to minimize the health risks to the local population, and contribute to a One Health vision for a better future and sustainable development.

P34-25 | Heavy Metal Accumulation in Fish from Mining and Non-Mining Areas: Health Risks and Implications

Serwaa Akoto Bawua, Katrine Enimil Armah

University of Ghana School of Public Health, Biological, Environmental and Occupational Health, Accra, Ghana

Abstract

Objective

This study assesses the concentrations of heavy metals (Hg, As, Pb, Cr, Mn, and Cd) in fish from mining and non-mining communities, evaluating human exposure risks through dietary consumption. It aims to determine the impact of mining activities on heavy metal accumulation in fish and the potential health risks to consumers.

Materials and Methods

An analytical cross-sectional study was conducted in three mining communities in Ghana, along the Ankobra River (Dominase, Prestea, Wassa Akropong) and three non-mining communities along the Volta River (Yeji, Akateng, Kpando). Fish species were selected based on trophic level, feeding habits, and dietary importance. Heavy metal concentrations were measured using inductively coupled plasma mass spectrometry (ICP-MS) and atomic absorption spectrometry (AAS). Bioaccumulation factors (BAFs) were calculated, and dietary exposure was assessed using a 24-hour dietary recall questionnaire from 384 fish consumers. Risk assessment models, including tolerable weekly intake (TWI) and hazard quotient (HQ), were used to evaluate health risks. Statistical analyses, including ANOVA and regression models, assessed the impact of mining on heavy metal concentrations.

Results

Fish from mining areas exhibited significantly higher concentrations of Hg, Pb, and As ($p < 0.05$), with Clarias sp. and Chrysichthys showing the highest levels. Pomadasys sp. exceeded WHO limits for As, with an estimated exposure of 0.43 mg/week among the 20–29 age group. Risk assessment confirmed that Hg and As levels in some species exceeded safety thresholds, posing potential chronic health risks. Mining activities were significant predictors of heavy metal accumulation ($p < 0.01$).

Conclusion

Mining significantly increases heavy metal contamination in fish, elevating health risks for consumers. Strengthened environmental monitoring, enforcement of mining regulations, and public health interventions are essential. Sustainable mining policies and community awareness campaigns are critical to reducing exposure and protecting aquatic ecosystems.

P34-26 | Environmental contamination associated with biofuel production involving pesticide-coated seed corn as feedstock: A survey of community environmental and health impacts

Kelli Gribben[1], Kylie Johnson[1], Pierce Greenberg[3], Ruth Mencia[1], Jabeen Taiba[1], Kristina Kintziger[1], Kaleb Michaud[4], Eleanor Rogan[1], Terra Uhing[5], Jesse Bell[1,2]

[1] University of Nebraska Medical Center, Department of Environmental, Agricultural and Occupational Health, Omaha, USA; [2] University of Nebraska Lincoln, Daugherty Water for Food Global Institute, Lincoln, USA; [3] Clemson University, Department of Sociology, Anthropology and Criminal Justice, Clemson, USA; [4] University of Nebraska Medical Center, Department of Internal Medicine, Omaha, USA; [5] Three Rivers Public Health Department, Fremont, USA

Objective: A United States ethanol company used pesticide-coated seed corn for fuel ethanol production, resulting in highly contaminated byproducts. Detectable levels of neonicotinoids have been found in soil, water, and air samples, raising questions about the potential health impacts. We evaluated the community's perceived physical and mental health impacts and needs following the contamination event.
Material and Methods: A 54-question survey was administered to households located within a 10-mile radius of the facility. Respondents could complete the paper survey and return it by mail or electronically. The general topics were household members' awareness, concerns, and perceptions of environmental and health impacts. Quantitative data were presented as frequencies and percentages, while qualitative data were grouped into themes based on keywords and summarized as counts.
Results: A total of 459 respondents completed the survey, a 38% response rate. The average household size was 2.7 (SD=1.4). Responding households were primarily single-family homes (88%) that were owned (85%), and were long-time residents (mean=18.4 years, SD=15.5). A total of 36% of households included children aged 18 or younger. Respondents were concerned about contaminants affecting drinking water (82%), soil (79%), and air (72%) quality. Most respondents (75%) felt at least 'some stress' related to potentially compromised health; however, 51% did not believe they had health symptoms resulting from the contamination. The most common self-reported symptoms among primary respondents were sinus (n=32), respiratory (n=30), cognitive/neurological symptoms (n=30), and allergies (n=28). Depression and anxiety were the primary mental health symptoms reported with 40 mentions. The top community need was wanting the environment cleaned-up, including proper removal of waste and land restoration.**Conclusion**: This study evaluated community-level perceived impacts of environmental contamination from an ethanol plant's improper handling of production waste containing pesticides. Findings can support community action and serve as a baseline for future health and environmental monitoring.

P35 | Environmental Exposures During Pregnancy and Maternal/Infant Health Outcomes - PART I

P35-01 | Effect of liquified petroleum gas cookstove with clean fuel intervention during gestation and infancy on stunting at ages 2-5 years: findings from the multi-country household air pollution intervention network trial.

Md Abul Kalam[1], Sheela Sinharoy[2], Kyle Steenland[3], Usha Ramakrishnan[2], Lance Waller[4], Christopher Sudfeld[11], Kalpana Balakrishnan[6], Lisa M Thompson[8], Anaite Diaz[7], Ghislaine Rosa[9], Ajay Pillarisetti[5], Shirin Jabbarzadeh[4], Jennifer Peel[10], Thomas Clasen[3]

[1] Emory University, Global Health and Development Program, Laney Graduate School, Atlanta, USA; [2] Emory University, Hubert Department of Global Health, Rollins School of Public Health, Atlanta, USA; [3] Emory University, Gangarosa Department of Environmental Health, Rollins School of Public Health, Atlanta, USA; [4] Emory University, Department of Biostatistics and Bioinformatics, Rollins School of Public Health, Atlanta, USA; [5] University of California, Environmental Health Sciences, School of Public Health, Berkeley, USA; [6] Sri Ramachandra Institute for Higher Education and Research (Deemed University), Department of Environmental Health Engineering, ICMR Center for Advanced Research on Air Quality, Climate and Health, Chennai, India; [7] Universidad del Valle de Guatemala, Center for Health Studies, Guatemala City, Guatemala; [8] Emory University, Nell Hodgson Woodruff School of Nursing, Atlanta, USA; [9] London School of Hygiene & Tropical Medicine, Department of Infectious and Tropical Diseases, London, UK; [10] Colorado State University, Department of Environmental and Radiological Health Sciences, Fort Collins, USA; [11] Harvard T.H. Chan School of Public Health, Global Health and Population, Boston, USA

Objective

Evidence on relationships between household air pollution (HAP) and child growth over the first five years of life is lacking. The Household Air Pollution Intervention Network (HAPIN) is a randomized controlled trial that enrolled ~3200 pregnant women across Guatemala, Peru, India, and Rwanda. Women in the intervention group received a liquefied petroleum gas (LPG) cookstove and fuel until the infant reached 12 months of age. We examined the effect of the HAPIN intervention on childhood stunting at ages 2-5 years to understand possible long-term effects of the intervention.

Materials and method

We continued following children in Guatemala, Rwanda, and India (N=2260) and measured child height at ages 2, 3, 4 and 5 years. We calculated height-for-age Z-score (HAZ) and created a binary variable for stunting (defined as HAZ<2.0). We will conduct an intention-to-treat analysis to evaluate the effect of the intervention on stunting at each time point (ages 2, 3, 4 and 5 years separately). We will also conduct interaction analyses to assess treatment effects by intervention country, maternal height, child sex and birthweight.

Results

We will present the results showing the risk of stunting in intervention and control arms at each time point, and the risk difference between the arms. We will place these results in the context of the primary fuel use patterns during and after the 18-month intervention period. We will also discuss the results in the context of other findings from HAPIN and explore potential reasons thereof. Results are anticipated prior to the ISEE 2025 meeting.

Conclusion

This study will contribute important new evidence on the potential for an LPG cookstove and free fuel intervention during gestation and infancy to reduce stunting risk at ages 2–5 years. Results can inform policy decisions for scaling interventions in low-resource settings to combat childhood stunting.

P35-02 | Gestational $PM_{2.5}$, Maternal Folate and Vitamin B12, and liver steatosis in offspring

Xin Yu[1], Sabiha Nasrin[2], Tarik Benmarhnia[3], Sambasivam Sendhil Velan[4], Katherine Reeves[2], Navin Michael[4], Suresh A. Sadananthan[4], Raji Balasubramanian[2], Yung Seng Lee[5,6], Fabian Yap[7], Yap Seng Chong[8], Jonathan Huang[9], Youssef Oulhote[1], Damaskini Valvi[1]

[1] Icahn School of Medicine at Mount Sinai, Department of Environmental Medicine, New York, USA; [2] University of Massachusetts, Department of Biostatistics and Epidemiology, Amherst, USA; [3] University of California San Diego, Scripps Institute, San Diego, USA; [4] Agency for Science Technology and Research, Institute for Human Development and Potential (IHDP), Singapore, Singapore; [5] National University of Singapore, Department of Paediatrics, Yong Loo Lin School of Medicine, Singapore, Singapore; [6] National University Health System, Division of Paediatric Endocrinology, Department of Paediatrics, Khoo Teck Puat-National University Children's Medical Institute, Singapore, Singapore; [7] KK Women's and Children's Hospital, Department of Paediatrics, Endocrinology Service, Singapore, Singapore; [8] National University of Singapore, Department of Obstetrics and Gynaecology, Singapore, Singapore; [9] University of Hawai'i at Manoa, Office of Public Health Studies, Honolulu, USA

Objective:
B vitamins such as folate (B9) and cobalamin (B12) may attenuate associations between prenatal air pollution exposure and liver outcomes in children, but population studies evaluating this hypothesis are scarce. We examined prenatal folate and cobalamin levels as potential effect modifiers of the association between PM2.5 and childhood liver steatosis.
Material and Methods:
We analyzed 525 mother-child pairs from the Growing Up in Singapore Towards Healthy Outcomes (GUSTO) cohort. Liver fat fraction (%) at age 6–7.5 was measured using proton magnetic resonance spectroscopy. Pregnancy and trimester-specific PM2.5 exposures were spatially interpolated from monitoring stations via inverse distance-squared weighting. Maternal plasma folate and cobalamin levels were measured around week 26 of gestation. We estimated covariate-adjusted associations between gestational PM2.5 and liver fat fraction, and effect modification by maternal folate and cobalamin concentrations, and sex, using generalized linear regression with a log-link function and gamma-distributed outcome. Associations were expressed as %change in outcome mean per 1 µg/m³ increase in PM2.5.
Results:
Median liver fat fraction in children was 0.51% (IQR: 0.31%–0.90%). PM2.5 during pregnancy, particularly in trimester 1, was associated with increased liver steatosis in girls [%change = 7.65% (95% CI: 2.72%, 12.80%)] but not in boys [%change = -0.90% (95% CI: -7.19%, 5.82%); p-interaction=0.03]. We observed a negative interaction between maternal plasma cobalamin and trimester 1 PM2.5 in girls (β = -0.05; p-interaction=0.03, per 1 µg/m³ increase in PM2.5 and per IQR=110.4 pg/ml in cobalamin). The positive association between gestational PM2.5 and liver fat fraction was only observed in girls whose mothers had inadequate plasma cobalamin (<300pg/ml) and attenuated at higher levels. No interactions were found for maternal folate levels.
Conclusion:Gestational PM2.5 exposure may increase liver steatosis risk, particularly among girls whose mothers had inadequate cobalamin in pregnancy, highlighting the potential of nutritional interventions to promote offspring liver health.

P35-03 | Associations of urinary polycyclic aromatic hydrocarbon (PAH) metabolites with serum biomarkers of inflammation during pregnancy in the PROTECT cohort

Amaleah F. Mirti[1], Amber L. Cathey[1], Deborah J. Watkins[1], Zaira Y. Rosario-Pabón[3], Carmen M. Vélez-Vega[2], Akram N. Alshawabkeh[3], José F. Cordero[4], John D. Meeker[1]

[1] University of Michigan, Department of Environmental Health Sciences, Ann Arbor, USA; [2] University of Puerto Rico, Department of Social Sciences, San Juan, Puerto Rico; [3] Northeastern University, Department of Civil and Environmental Engineering, Boston, USA; [4] University of Georgia, Department of Epidemiology and Biostatistics, Athens, USA

Objective:

Polycyclic aromatic hydrocarbons (PAH) are byproducts of incomplete combustion that are widespread in the environment. Several studies have demonstrated adverse effects of PAH exposure on pregnancy. Inflammation has been proposed as a potential mechanism through which PAH can adversely impact birth outcomes. While there is evidence that PAH may cause inflammation during pregnancy, studies have been limited in sample size and the range of inflammation biomarkers assessed.

Material and Methods:

This study included 1,643 pregnant women in the PROTECT birth cohort in Puerto Rico and aimed to explore the relationship between PAH exposure and serum concentrations of inflammation biomarkers during pregnancy. Eight urinary PAH metabolites were measured at three timepoints (18, 22, and 26 weeks), while six serum inflammation biomarkers were measured at the first and third timepoints. Linear mixed-effects models were constructed to examine the association between PAH metabolites and inflammation biomarkers while adjusting for maternal age, education, pre-pregnancy body mass index, income, smoking status, and specific gravity.

Results:

Several PAH were associated with significantly lower vascular cellular adhesion molecule 1 (VCAM), and intercellular adhesion molecule 1 (ICAM), but higher matrix metalloproteinase 9 (MMP9) and/or MMP2 concentrations. For example, a doubling in urinary 1-hydroxyphenanthrene concentration was associated with 6.7% lower VCAM1 (95%CI -10.0%, -3.4%), 4.5% lower ICAM (-7.9%, -0.9%), and 4.8% higher MMP9 (1.2%, 8.6%). Similar patterns were observed for 2-hydroxynaphthalene, 2-hydroxyfluorene, 2,3-hydroxyphenanthrene, 4-hydroxyphenanthrene, and 9-hydroxyphenanthrene.

Conclusion:

These findings increase our understanding of how PAH exposure may adversely impact pregnancy. While associations with MMPs were in the hypothesized direction, the associations with VCAM and ICAM highlight a need for further research on whether PAH exposure affects the signaling pathway for synthesizing these proteins.

P35-04 | Maternal environmental exposures during gestation and child's epigenetic age at birth

Anmol Anand Pardeshi[1], Alice Kang[2], Libby Morimoto[2], Xiaomei Ma[3], Adam de Smith[1], Catherine Metayer[2], Joseph L Wiemels[1], Charlie Zhong[4,1]

[1] University of Southern California, Population & Public Health Science, Keck School of Medicine, Los Angeles, USA; [2] University of California, Berkeley, School of Public Health, Berkeley, USA; [3] Yale University, Department of Chronic Disease Epidemiology, Yale School of Public Health, New Haven, USA; [4] Epidemiology Research, American Cancer Society, Atlanta, USA

An individual's biological age, when accelerated compared to calendar age, may provide insight into why some people may be more susceptible to diseases. We previously identified a significant association between childhood leukemia subtype and epigenetic gestational age acceleration (EGAA) using the Bohlin and Knight clocks. In this analysis, we investigated possible association between maternal exposure to two environmental factors (normalized difference vegetation index [NDVI], and ambient fine particulate matter [PM2.5]) and child's EGAA at birth. We included cases and controls from the California Childhood Leukemia Study, who were born in California. We assigned mean values for NDVI and PM2.5 for the 9 months prior to birth based on geocoded maternal residential address at the time of birth. Dried blood spots, taken at birth, were retrieved from the California Biobank and assayed for genome-wide DNA methylation using Illumina Infinium 450K or EPIC arrays. We computed Epigenetic Gestational Age [EGA] based on the Bohlin and Knight clocks, and EGAA was calculated as the residuals by regressing (chronological) gestational age against EGA for both clocks. We used linear regression to assess the relation of NDVI and PM2.5 level to EGAA, adjusting for household income and smoking (based on the *AHRR* CpG cg05575921). Of 1210 participants, the mean NDVI was 0.40 and the mean PM2.5 level was 13.80 µg/m3. A 0.1 unit increase in NDVI was associated with a non-statistically significant 0.52 (95% confidence interval [CI]: -0.33,1.39) week increase in EGAA based on the Bohlin clock and 0.71 (95%CI: -0.54,1.98) week increase in EGAA based on the Knight clock. There were no associations with PM2.5 (p-value = 0.95 for the Bohlin clock, p-value = 0.99 for the Knight clock). In our study, maternal exposure to greenspace or ambient fine particulate matter during pregnancy did not appear to influence child's epigenetic age at birth

P35-05 | Red Tides and Risk of Gestational Hypertension in Coastal Southwest Florida

Bianca Irimia[1], Rain E. Freeman[1], Sandy Haaf[2], Jason L. Salemi[1], Jean Paul Tanner[1], Catherine M. Bulka[1]

[1] University of South Florida, Department of Epidemiology, College of Public Health, Tampa, USA; [2] Zev Ross Spatial Analysis, Ithaca, USA

Background: The algae *K. brevis*, responsible for red tides in the Gulf waters of Southwest Florida, produces toxins that induce severe hypertension in animals. These toxins bioaccumulate in shellfish and are aerosolized and transported inland by wind, resulting in potential human exposures via ingestion and inhalation. However, the human health impacts, especially during pregnancy, remain understudied. This study investigated residential exposures to red tide early in pregnancy and the risk of new-onset hypertension during the latter half of pregnancy.

Methods: The study population included 37,619 Southwest Floridians who lived near the coastline, were hypertension-free before pregnancy, and delivered a live or stillborn singleton between 2006 and 2019. Birth certificate records were spatiotemporally linked to monthly estimates *of K. brevis* water concentrations from a published report. We constructed individual exposure histories by calculating time-weighted averages of *K. brevis* concentrations during the first and second trimesters, dichotomizing at >100,000 cells/Liter, a level known to predictably cause widespread mortality in marine wildlife. Generalized additive models adjusted for demographic factors, season of conception, wind patterns, and spatial clustering were fit to evaluate associations of trimester-specific exposure levels with gestational hypertension.
Results: Approximately 5% of the study population developed gestational hypertension. After adjustment, the relative risk for exposure to >100,000 cells/Liter was 1.23 (95% CI: 1.07–1.41) for the first trimester and 1.12 (95% CI: 0.96–1.31) for the second trimester. The relative risk for exposure to elevated levels sustained over both trimesters was 1.23 (95% CI: 1.00-1.50).
Conclusions: Our findings suggest that exposure to red tides early in pregnancy may increase the risk of gestational hypertension. Further epidemiologic studies with more refined exposure assessment methods are needed to confirm these results. If substantiated, additional research should explore the underlying biological mechanisms and evaluate public health interventions to protect pregnant coastal residents.

P35-06 | Per- and polyfluoroalkyl substances serum concentrations during pregnancy and gestational weight gain in the Healthy Start Study

Julia Kaplan[1], Dana Dabelea[2], John Adgate[3], Anne Starling[1,2]

[1] University of North Carolina at Chapel Hill, Department of Epidemiology, Gillings School of Global Public Health, Chapel Hill, USA; [2] University of Colorado Anschutz Medical Campus, Lifecourse Epidemiology of Adiposity and Diabetes (LEAD) Center, Aurora, USA; [3] Colorado School of Public Health, University of Colorado Anschutz Medical Campus, Department of Environmental and Occupational Health, Aurora, USA

Objective: Per- and polyfluoroalkyl substances (PFAS) are widespread and persistent contaminants detected in most humans. PFAS exposure has been linked to adverse health conditions, including excessive gestational weight gain (GWG), though results have been inconsistent. We were interested in understanding the association between GWG and PFAS in participants of the Healthy Start Study (HSS).
Material and Methods: Participants (N=618) were enrolled in the HSS, a Colorado-based cohort. Eleven PFAS were measured in serum collected during pregnancy (2009-2014). GWG was quantified in four ways: absolute weight change during pregnancy; adjusted for pregnancy duration (z-score); 3rd trimester rate of weight gain; and categories of recommended GWG according to Institute of Medicine guidelines. PFAS detectable in >60% of samples (perfluorohexane sulfonate [PFHxS], perfluorooctane sulfonate [PFOS], perfluorooctanoate [PFOA], perfluorononanoate [PFNA], and perfluorodecanoate [PFDA]), were included in this analysis. We fit covariate-adjusted general linear models for continuous outcomes and multinomial logistic regression for the categories of recommended gain and evaluated effect modification by pre-pregnancy body mass index (BMI) categories. Results: PFHxS, PFOS, PFOA, and PFNA were not significantly associated with GWG. Each doubling of PFDA was associated with a lower GWG z-score (-0.11 SD units; 95% CI: -0.21, -0.00). Some positive associations with GWG were only present among women with BMI classified as overweight, and the negative association between PFDA and GWG only persisted among women with BMI in the normal or underweight category.

Conclusion: In a diverse population of women with PFAS serum concentrations typical of the US general population, GWG was not positively associated with PFAS concentrations during pregnancy. The influence of PFAS may differ by women's BMI entering pregnancy.

Funding Sources: This study was supported by National Institutes of Health grants (R01ES032213, R01ES022934, R01DK076648, P30DK056350, UH3OD023248, T32ES007018 [Training Grant that supported J. Kaplan]).

P35-07 | Adherence to the Mediterranean and Planetary Diets During Pregnancy and Their Impact on Gestational and Neonatal Outcomes: Findings from a U.S. Rural Cohort

Leyre Notario, Juliette C. Madan, Margaret R. Karagas

Dartmouth College, Epidemiology Department, Lebanon, USA

Introduction: The Mediterranean diet prioritizes plant-based foods, healthy fats, and proteins, offering metabolic and cardiovascular benefits. More recently, the Planetary Diet has been developed to improve both human and planetary health. However, the impact of these diets on gestational and neonatal outcomes is not well-understood.

Aim: To evaluate adherence to the Mediterranean and Planetary diets during pregnancy and their impact on gestational weight gain (GWG) and infant birth outcomes in a rural U.S. pregnancy cohort.

Methods: This preliminary analysis included 1,359 pregnant participants from the ongoing New Hampshire Birth Cohort Study. We calculated the relative Mediterranean diet score (rMED) and the Planetary Healthy Diet Index (PHDI) from data provided through a validated food frequency questionnaire. The difference between pre-pregnancy weight and the last recorded weight before delivery was used to calculate GWG. Gestational age and birth outcomes were obtained from medical records. Multivariable regression models examined associations between rMED and PHDI and gestational and birth outcomes, adjusting for maternal age, pre-pregnancy BMI, smoking during pregnancy, education level, parity, and infant sex.

Results: Higher adherence to the Mediterranean diet (tertile 3 vs. tertile 1) was associated with a 0.23-week increase in gestational age ($\beta = 0.23$, 95% Confidence Interval (CI): 0.01, 0.45) and a 2.33 lb. decrease in GWG ($\beta = -2.33$, 95% CI: -4.19, -0.46). Similarly, higher adherence to the PHDI was associated with a 0.27-week increase in gestational age ($\beta = 0.27$, 95% CI: 0.02, 0.51), though no significant association was observed with GWG.

Conclusion: These preliminary findings suggest that adherence to the Mediterranean and Planetary diets during pregnancy may positively influence gestational age, while greater adherence to the Mediterranean diet may support healthy gestational weight management. Further research is needed to explore potential modifying factors and underlying mechanisms.

P35-08 | The Association between COVID-19 and Incident Gestational Diabetes: A Case-Control Study using the National Health Insurance Research Database in Taiwan

Liang-Yu Lin[1,2], Chi-Jen Chen[3], Mei-Huei Chen[4], Chun-Wei Chen[5], Pau-Chung Chen[1,6]

[1] National Taiwan University, Institute of Environmental and Occupational Health Science, Taipei, Taiwan; [2] National Taiwan University Hospital, Department of Environmental and Occupational Medicine, Taipei, Taiwan; [3] National Taiwan University, Institute of Epidemiology and Preventive Medicine, Taipei, Taiwan; [4] National Health Research Institutes, Institute of Population Health Science, Miaoli County, Taiwan; [5] National Taiwan University Hospital Hsin-Chu Branch, Department of Obstetrics & Gynecology, Hsinchu County, Taiwan; [6] National Health Research Institutes, National Institute of Environmental Health Sciences, Miaoli County, Taiwan

Objective: This study investigated the association between COVID-19 infections and incident gestational diabetes (GDM) in pregnant women in Taiwan.
Material and Methods: We conducted a population-based case-control study using data from the National Health Insurance Research Database (NHIRD) in Taiwan from 2020 to 2022. Adult pregnant women exceeding 23 weeks of gestation were included. Cases with GDM outcomes were identified using ICD-10 codes between gestational weeks 24 and 28, and each case was matched with four non-GDM controls by age, region of residence, and gestational weeks. COVID-19 exposure was determined from linked testing data (including PCR, rapid antigen tests, and clinical diagnosis). We investigated the association between COVID-19 and GDM using logistic regression models adjusting for covariates, and various sensitivity analyses were performed.
Results: We identified 24,194 individuals with GDM, and 96,776 matched non-GDM comparators with an average age of 32. The GDM group had a higher proportion of polycystic ovarian syndrome (3.79%) and morbid obesity (0.59%) than the control group. The control group had slightly more people with a history of COVID-19 infection (GDM group: 4.98%; Control group: 5.64%), while the GDM case group had more unvaccinated people (GDM group: 69.24%; control group: 66.91%). After adjusting for matching factors and covariates, we found no evidence of an association between COVID-19 infection history and incident GDM in the primary (adjusted odds ratio: 0.94; 95% confidence intervals: 0.88—1.00) and subsequent sensitivity analyses.
Conclusion: Our study found no evidence of an association between COVID-19 infections and incident GDM among pregnant women in Taiwan. Different prevalent variants, testing strategies, and possible competing risks of SARS0-CoV-2 during pregnancy may influence the results. Further studies regarding the potential adverse pregnancy outcomes due to COVID-19 are needed. We acknowledge the National Taiwan University for funding this study.

P35-09 | Residential Herbicide Exposure and Eclampsia Risk During Pregnancy

Rietta Wagoner, Audrey Yang, Rebecca Ramos, Tuo Liu, Melissa Furlong

University of Arizona, Mel and Enid Zuckerman College of Public Health, Tucson, USA

Background and Objective:
Hypertensive disorders of pregnancy (HDP), including eclampsia, increasematernal and offspring morbidity and long-term cardiovascular risks. While environmental factors such as pesticides and extreme temperatures have been implicated in HDP, exposures to herbicides and their interactions with temperature are not well understood. This study examines associations between herbicide exposure and eclampsia in Arizona and assesses potential interactions with extreme temperature.

Materials and Methods:
We linked Arizona’s Pesticide Use Registry data (2011–2020) with maternal eclampsia cases and covariates from birth certificates and assessed interactions with extreme temperaturefrom NOAA’S Daymet. Herbicide exposure was defined as any application within 500m of the maternal residence at birth during two preconception periods, each trimester, and the full pregnancy. A negative control exposure model with log-binomial regression was used to obtain adjusted risk ratios (aRR). We controlled for maternal race/ethnicity, education, and age, number of prenatal visits, gestational estimate, conception year, conception season, and residence in an agricultural area. Pregnancies with preexisting hypertension or maternal age >51 years were excluded.

Results:
Among 764,211 pregnancies and 22 herbicides analyzed, several were positively associated with eclampsia. Metribuzin was positively associated with eclampsia in the first (aRR=12.39, 95%CI: 3.14, 48.90) and third trimesters (aRR=8.17, 95%CI: 1.16, 57.62), and across pregnancy (aRR=4.80, 95%CI: 1.79, 12.82). Dimethenamide-P was positively associated 180–90 days preconception (aRR=9.76, 95%CI: 1.40, 68.11) and at 90 days preconception (aRR=7.08, 95% CI: 1.01, 49.61). 2,4-D (aRR=2.64, 95%CI: 1.18, 5.91), flumioxazin (aRR=14.24, 95%CI: 2.04, 99.59), and metolachlor (aRR=2.18, 95%CI: 1.03, 4.62) were also positively associated. Interactions with heat are under investigation.

Conclusion:
Exposure to some herbicides was positively associated with eclampsia during pregnancy in Arizona, though the estimates were imprecise with wide confidence intervals, warranting further investigation. As herbicide usage rises, understanding the health impacts and potential interactions with extreme temperatures is crucial.

P35-10 | A preliminary investigation of the perinatal effects of electronic cigarette use during pregnancy.

Samuel Stowe[1], Akila Subramaniam[2], Rachel Sinkey[2], Ruzmyn Vilcassim[1]

[1] The University of Alabama at Birmingham, Department of Environmental Health Sciences, Birmingham, USA; [2] The University of Alabama at Birmingham, Department of Obstetrics and Gynecology, Birmingham, USA

Electronic cigarette (e-cigarette) use during pregnancy is rising, which has led to concerns about potential perinatal health risks. Pregnant women may be more susceptible to e-cigarette use (vaping) due to their appealing flavors and perceived safety compared to traditional tobacco products. Additionally, hormonal and physiological changes during pregnancy may heighten vulnerability to e-cigarettes effects. This study aims to assess the prevalence and health effects of vaping during pregnancy in a cohort of pregnant patients at the University of Alabama at Birmingham (UAB).
Participants were recruited from UAB prenatal clinics to complete a survey on tobacco product use during pregnancy. Those who consented provided contact information to participate in subsequent aims. For Aim 1, 100 participants will complete exhaled carbon monoxide and urinary cotinine tests to biologically validate self-reported tobacco use. This will be completed prior to participating in Aims 2 and 3. For Aim 2, 60 pregnant vapers and 60 pregnant non-users will provide blood and urine samples for exposure and inflammatory biomarker analysis. For Aim 3, 20 vapers and 20 non-users from Aim 2 will provide umbilical cord blood samples for biomarker comparison.
Thus far, 249 participants have completed the survey, with 192 answering questions on tobacco use. Among them, 14 (7.3%) reported exclusive vaping during pregnancy. The mean age was 29, while vapers had a mean age of 28. The sample was predominantly Black (47.42%) and White (42.78%), while vapers were primarily White (64.29%). Most vapers reported using disposable e-cigarettes daily, taking 1-10 puffs per day, exclusively using flavored e-liquids, and having switched from cigarettes.
Vaping during pregnancy is concerning due to potential health risks. Our preliminary data shows 7.3% of participants exclusively vape during pregnancy. We are collecting samples for further analysis and will share our updated findings at the conference.
Funded by the Harvard JPB Fellowship.

P35-11 | Maternal health conditions during pregnancy and the Risk of Childhood Cancer: A Nationwide Cohort Study

Sohee Wang[1,2], Jieun Min[1,2], Jongmin Oh[1,3], Eun Mi Jung[5], Eun Sun Yoo[6], Kyung A Lee[7], Minho Kim[8], Ji Hyen Lee[4,6], Hae Ji Han[1], Logan G Spector[5,9], Eunhee Ha[1,4]

[1] Ewha Womans University, Department of Environmental Medicine, College of Medicine, Seoul, South Korea; [2] Ewha Womans University, Graduate Program in System Health Science and Engineering, College of Medicine, Seoul, South Korea; [3] Seoul National University, Department of Human System Medicine, College of Medicine, Seoul, South Korea; [4] Ewha Womans University, Institute of Ewha-SCL for Environmental Health (IESEH), College of Medicine, Seoul, South Korea; [5] University of Minnesota, Division of Epidemiology and Clinical Research, Department of Pediatrics, Minneapolis, USA; [6] Ewha Womans University, Department of Pediatrics, College of Medicine, Seoul, South Korea; [7] Ewha Womans University, Department of Obstetrics and Gynecology, College of Medicine, Seoul, South Korea; [8] Ewha Womans University Seoul Hospital, Information Department, Seoul, South Korea; [9] University of Minnesota, Masonic Cancer Center, Minneapolis, USA

Objectives

Maternal health conditions during pregnancy may affect childhood cancer risk, but comprehensive evidence is limited. This nationwide cohort study in South Korea evaluated associations between ten common maternal diseases during pregnancy and childhood cancer in offspring.

Methods

We conducted a retrospective cohort study using data from the Korea National Health Information Database (NHID) on children born between 2008 and 2021 and their mothers. Childhood cancer was defined as the first hospital visit before age 5 with a special calculation code for reduced cost-sharing and an ICD-10 ‘C’ code. Cox proportional hazards models estimated associations between maternal diseases during pregnancy and childhood cancer, adjusting for potential confounders.

Results

Among 4,405,953 mother-child pairs, 4,537 childhood cancer cases were identified. Anemia during pregnancy was associated with increased risk of non-Hodgkin lymphoma (HR [95% CI]: 1.16 [0.98, 1.37]) and acute myeloid leukemia (1.31 [0.99, 1.73]). Gestational infection was linked to higher risks of overall childhood cancer (1.09 [1.01, 1.17]), leukemia (1.13 [1.00, 1.27]), and acute lymphoblastic leukemia (1.18 [1.02, 1.36]). Hyperemesis gravidarum (1.09 [1.00, 1.20]) and thyroid disorders (1.12 [1.00, 1.25]) also increased risk of overall childhood cancer risk.

Conclusion

Our findings suggest anemia, genitourinary infections, hyperemesis gravidarum, and thyroid disorders during pregnancy are associated with increased childhood cancer risk. Further studies are needed to clarify underlying mechanisms and inform early screening strategies.

Acknowledgement

This study was supported by a grant from the National R&D Program for Cancer Control, Ministry of Health and We lfare, Republic of Korea (RS-2024-00332838).

This research was supported by the BK21 FOUR (Fostering Outstanding Universities for Research) funded by the Ministry of Education (MOE, Korea) and National Research Foundation of Korea (NRF-5199990614253, Education Research Center for 4IR-Based Health Care).

P35-12 | Indoor exposure to phthalates during pregnancy and its association with reproductive development in male infants

Yuxuan Zhao, Mengfang Wan, Yuexia Sun

Tianjin university, Tianjin, China

Phthalates have attracted considerable attention due to their ubiquity in indoor environments and endocrine-disrupting properties. These compounds can cross the placental barrier, leading to fetal exposure and adverse health outcomes. The objective of this study was to investigate household phthalate concentrations during pregnancy, assess non-dietary indoor intake among pregnant women, and examined associations between prenatal phthalate exposure and male infant reproductive development. Utilizing data from the CHemical Exposure and Children's Health (CHECH) cohort in Tianjin, China, we collected 978 bedroom dust samples during pregnancy and analyzed concentrations of six phthalates [diethyl phthalate (DEP), di-isobutyl phthalate (DiBP), di-n-butyl phthalate (DnBP), benzyl butyl phthalate (BBzP), di (2-ethylhexyl) phthalate (DEHP) and di-isononyl phthalate (DiNP)] and one alternative plasticizer [Di-2-ethylhexyl terephthalate (DEHT)]. The median concentrations of DEP, DiBP, DnBP, BBzP, DEHP, DiNP and DEHT (μg/g) were 0.00, 18.07, 41.00, 6.12, 208.59, 16.17 and 46.42 μg/g. Indoor phthalate intakes were assessed, with DnBP and DiBP exhibiting the highest daily intake estimates, at 3150.40 ng/kg/day and 2910.87 ng/kg/day, respectively. Notably, 5.9%-7.6% of pregnant women exceeded the tolerable daily intakes for DiBP and DnBP. In a subgroup of 78 male infants aged 7-12 months, we observed a significant inverse association between prenatal DiBP exposure and anogenital distance (AGD). These findings highlight the critical need for monitoring phthalate exposure in residential environments to safeguard children's health.

P35-13 | Prenatal Heat Stress and Risk of Hypertensive Disorders of Pregnancy

Noelle Pardo[1], Xiaoran Yang[1], Yuhong Hu[1], Yan Xu[1], Roxana Khalili[1], Aviva Wolf-Jacobs[1], Sandrah P. Eckel[1], Zhongzheng Niu[1,2], Tingyu Yang[1], Xinci Chen[1], Mario Vigil[1], Brendan Grubbs[3], Laila Al-Marayati[3], Nathana Lurvey[4], Jill Johnston[1], Genevieve F. Dunton[1], Carrie Breton[1], Theresa M. Bastain[1], Claudia M. Toledo-Corral[1,5], Rima Habre[1], Shohreh F. Farzan[1]

[1] University of Southern California, Department of Population and Public Health Sciences, Keck School of Medicine, Los Angeles, USA; [2] University at Buffalo, Department of Epidemiology and Environmental Health, School of Public Health and Health Professions, Buffalo, USA; [3] University of Southern California, Department of Obstetrics and Gynecology, Keck School of Medicine, Los Angeles, USA; [4] Eisner Health, Los Angeles, USA; [5] California State University, Northridge, Department of Health Sciences, Northridge, USA

Hypertensive disorders of pregnancy (HDPs), such as gestational hypertension (GHTN), are a leading cause of maternal mortality and morbidity. Recent evidence suggests that heat stress may be an important factor in the development of HDPs. As climate change leads to increased temperatures and shifts in weather, wet bulb globe temperature (WBGT) is thought to estimate heat stress more precisely by incorporating temperature, relative humidity, solar radiation, and wind speed. We aimed to examine associations between preconception and prenatal WBGT with GHTN risk.

We ascertained GHTN cases among a primarily low-income Hispanic pregnancy cohort in Los Angeles using physician diagnosis and blood pressure measurements from maternal electronic medical records. Average WBGT was evaluated in different exposure periods: 12 weeks preconception, the first trimester (0-13 weeks), and the second trimester (14-20 weeks) truncated at 20 gestation weeks, given that HDPs are diagnosed in the second half of pregnancy. Logistic regression models, adjusted for maternal age at consent, pre-pregnancy BMI, family income, maternal education, race/ethnicity, and conception season, were used to examine preconception and trimester-specific associations.

Among 661 participants, 43 (6.5%) had GHTN. The average WBGT in the 12 weeks preconception was 16.9°C [SD: 3.5], 17.0°C [SD: 3.5] during the first trimester, and 17.2°C [SD: 3.8] during the second trimester. Overall, we did not see evidence of an association of WBGT with GHTN risk across preconception, the first or second trimester (OR: 1.00, 95%CI: 0.84 – 1.20, p=0.98; OR: 1.03, 95%CI: 0.87 – 1.25, p=0.75; OR: 1.12, 95%CI: 0.93 – 1.37, p=0.25, respectively).

We did not observe an increased risk of GHTN related to preconception and trimester average prenatal heat stress exposure. However, we may have been limited in our ability to observe an association due to limited exposure variability and relatively low levels of preconception and prenatal heat stress in this region.

P35-14 | Maternal Heat Shock Protein as a Potential Biomarker of Adaptation to Heat Stress during Pregnancy

Xiaoran Yang[1], Yuhong Hu[1], Noelle Pardo[1], Yan Xu[1], Roxana Khalili[1], Aviva Wolf-Jacobs[1], Sandrah P. Eckel[1], Zhongzheng Niu[1,2], Tingyu Yang[1], Xinci Chen[1], Mario Vigil[1], Helen Foley[1], Danilo M. Guardado[1], Brendan Grubbs[3], Laila Al-Marayati[3], Claudia M. Toledo-Corral[4], Jill Johnston[1], Genevieve F. Dunton[1], Shohreh F. Farzan[1], Theresa M. Bastain[1], Rima Habre[1], Carrie Breton[1]

[1] University of Southern California, Department of Population and Public Health Sciences, Keck School of Medicine, Los Angeles, USA; [2] University at Buffalo, Department of Epidemiology and Environmental Health, School of Public Health and Health Professions, Buffalo, USA; [3] University of Southern California, Department of Obstetrics and Gynecology, Keck School of Medicine, Los Angeles, USA; [4] California State University, Department of Health Sciences, Northridge, USA

Heat shock proteins (HSPs) are a class of signaling molecules providing cellular resiliency for stressors. We evaluated whether HSP70 responded to heat stress during pregnancy and whether timing affected this association. To our knowledge, this is the first study evaluating HSPs among pregnant women.
The study population (N=228) comprised predominantly Latina mothers (81.3%) from the MADRES cohort. Serum HSP70 was measured during early and late pregnancy using ELISA assays. Daily heat stress in the 30 days prior to HSP70 measurement was computed as (1) Daily Maximum Heat Index (DMHI) and (2) Wet Bulb Globe Temperature (WBGT). Distributed lag models (DLMs) were used to identify sensitive windows of exposure to heat stress separately for early and late pregnancy. Heat stress variability (HSV) was defined as standard deviation of heat stress for a cumulative period prior to HSP70 measurement. Relationship between log-HSP70 and HSV was evaluated by linear regression. All models were adjusted for race/ethnicity, pre-pregnancy BMI, education, maternal age, parity, gestational age at serum collection, season at conception; trimester specific: AC use, physical activity, hours spent outdoors weekly.

HSP70 concentrations (geometric mean±SD) during early and late pregnancy were 3.0±3.1 and 3.3±2.2 ng/ml, respectively. Higher daily DMHI and WBGT (each ℃) were associated with higher HSP70 during early pregnancy, with days 1-5 and 1-3 showing significant effects with day 1 (7.2% [95% CI: 2.1, 12.5]; 9.8% [95% CI: 0.02, 20.6]) showing strongest effect from DLMs. No associations were observed during late pregnancy. Higher HSV (1-unit) in 1-3 days prior to HSP70 measurement was significantly associated with higher HSP70 (55.1% [95% CI: 10.5, 117.7]) only during late pregnancy.
HSP70 might be a biomarker for acute heat stress during early pregnancy. Further studies are needed to confirm specificity of HSP70 response to heat stress and possibility of adaptation across later pregnancy.

P35-15 | Ambient temperature during pregnancy and offspring cerebral palsy: A population-based study in California

Haoran Zhuo[1,2], Joshua Warren[3], Giselle Bellia[1,2], Pin Wang[4], Kai Chen[1,5], Zeyan Liew[1,2], Tormod Rogne[2,6]

[1] Yale School of Public Health, Department of Environmental Health Sciences, New Haven, USA; [2] Yale School of Public Health, Yale Center for Perinatal, Pediatric, and Environmental Epidemiology, New Haven, USA; [3] Yale School of Public Health, Department of Biostatistics, New Haven, USA; [4] University of Maryland, Department of Global, Environmental, and Occupational Health, College Park, USA; [5] Yale School of Public Health, Yale Center on Climate Change and Health, New Haven, USA; [6] University of Oslo, Department of Community Medicine and Global Health, Oslo, Norway

Objective Current evidence on early life exposure to ambient temperature and childhood neurodevelopment is sparse. Cerebral palsy (CP) is the most common neuromotor disorder in childhood with growing concerns on environmental etiological factors. We investigated whether prenatal exposure to ambient temperature was associated with offspring CP.
Methods We conducted a nested case-control study that included all CP cases (N=5,938) identified from a statewide system and randomly selected 20% of all live births without CP as controls (N=1,092,313) in California during 2005-2015. Gestational weekly average temperatures were calculated from a high resolution (1km) daily mean temperature data. Extreme heats were defined as days with mean temperatures above the 90th percentile of the distribution at each location during the study period. We implemented a Bayesian distributed lag model with logistic regression to estimate the associations between ambient temperatures at per 5°C increase, extreme heat and risk of CP. We then examined the effects of exposures in late pregnancy by estimating the cumulative effects in the last seven weeks before birth. Finally, we performed a sibling comparison design in sensitivity analyses to address uncontrolled confounding bias.
Results Per 5 °C increase in ambient temperature in gestational week0 to week3 was associated with a 2% increased risk in CP (odds ratio (OR)=1.02, 95% credible interval: 1.01-1.05) and slightly stronger effects were estimated for extreme heat (ORs between 1.03 and 1.05). We found a cumulative effect of high temperatures, but not extreme heat, in the last seven weeks before birth. Similar trend of increased risk of CP in early trimester was observed in sibling analyses.
Conclusion Early-pregnancy exposure to high temperatures was associated with higher risk of childhood CP. While the estimated effect size was small, our data suggested that CP risk should be monitored in the population within the context of climate change.

P35-16 | Prenatal mycoestrogen exposure and placental corticotropin releasing hormone (pCRH) in a U.S. pregnancy cohort

Alyssa Juenke[1,2], Carolyn Kinkade[3], Megan Hansel[3], Anita Brinker[1], Brian Buckley[1], Jessica Brunner[4,5], Maria Bowman[6], Roger Smith[6], Richard Miller[4], Tom G. O'Connor[5], Lauren Aleksunes[1,2], Zorimar Rivera-Núñez[1,3], Emily S. Barrett[1,3]

[1] Rutgers University, Environmental and Occupational Health Sciences Institute, Piscataway, USA; [2] Rutgers University, Department of Pharmacology and Toxicology, Piscataway, USA; [3] Rutgers University, School of Public Health, Piscataway, USA; [4] Rochester University, Obstetrics and Gynecology, Rochester, USA; [5] Rochester University, Psychiatry, Rochester, USA; [6] University of Newcastle, School of Medicine and Public Health, New Castle, Australia

Objective: Zearalenone (ZEN) is a mycotoxin that widely contaminates human food supplies. In animal models, ZEN and its metabolites have been associated with endocrine disruption and poor pregnancy outcomes. Here we translate that research to humans by examining gestational exposure to ZEN in relation to corticotropin releasing hormone (CRH), a hormone believed to regulate the timing of parturition.

Materials and Methods: Utilizing data from a medically normal risk pregnancy cohort (UPSIDE; Rochester, NY; n=273), we investigated gestational exposure to ZEN and its metabolites in relation to pCRH across pregnancy. In each trimester, ZEN and five of its metabolites were measured in urine using LC-MS, and pCRH was measured in serum using radioimmunoassay. We fitted trimester-specific, adjusted linear regression models examining log-transformed pCRH in relation to three log-transformed, specific gravity-adjusted urinary mycoestrogen measures: (1) ZEN; (2) its metabolite, α-zearalenol (AZOL); and (3) the sum of ZEN and its total metabolites (∑mycoestrogens).

Results: Mycoestrogens (ZEN and/or its metabolites) were detected in 94, 91, and 90% of urine samples in the first, second, and third trimesters, respectively. No statistically significant associations between mycoestrogens and pCRH were observed in the first and second trimesters. However, in third trimester models, mycoestrogens were positively associated with pCRH concentrations: ZEN (β: 0.06 pg/mL; 95%CI: -0.02, 0.14); aZOL (β: 0.08 pg/mL, 95%CI: 0.002, 0.16), ∑mycoestrogens (β: 0.06 pg/mL, 95%CI: -0.02, 0.14).

Conclusion: Our findings suggest that mycoestrogen exposure may be associated with higher pCRH in late pregnancy, a risk factor for preterm birth. Further modeling to examine associations between mycoestrogens and gestational age at birth is underway.

Supported by P30ES005022, UG3OD035527, T32E5007148, R01ES029275-02S1, R01HD083369, R212ES032047, UG3/UH3OD0223349, T32ES019854, F31ES034269.

P35-17 | Prenatal melamine, aromatic amine, and psychosocial stress exposures and their association with gestational diabetes in a San Francisco pregnancy cohort

Emily Lasher[1,2], Jessica Trowbridge[1], Alison Gemmill[2], Rachel Morello-Frosch[3], Erin DeMicco[1], Kurunthachalam Kannan[4], Jessie Buckley[5], Tracey Woodruff[1]

[1] University of California, San Francisco, Department of Obstetrics, Gynecology and Reproductive Sciences, Program on Reproductive Health and the Environment, San Francisco, USA; [2] Johns Hopkins Bloomberg School of Public Health, Department of Population, Family, and Reproductive Health, Baltimore, USA; [3] University of California, Berkeley, School of Public Health and Department of Environmental Science, Policy, and Management, Berkeley, USA; [4] New York State Department of Health, Wadsworth Center, Albany, USA; [5] Gillings School of Global Public Health, University of North Carolina at Chapel Hill, Department of Epidemiology, Chapel Hill, USA

Objective: Research suggests exposure to chemical and non-chemical stressors may increase the risk of pregnancy complications, including gestational diabetes mellitus (GDM). Exposure to melamine and aromatic amines (AAs) is ubiquitous among pregnant people. However, studies investigating the maternal and fetal health effects of prenatal exposure are limited. Thus, this cross-sectional study aimed to (1) evaluate relationships between exposure to aromatic amines, melamine, and its derivatives, and gestational diabetes in a pregnancy cohort in San Francisco, California, USA, (2) explore if non-chemical stressors modify these relationships, and (3) assess fetal sex differences using stratification.

Methods: We measured 36 AAs, melamine, and three of its derivatives in second-trimester urine samples (n=607). Psychosocial stress was assessed using self-reported questionnaires. GDM status was abstracted from medical records. We used unadjusted and adjusted logistic regression models to calculate the odds of GDM associated with an interquartile range increase in urinary concentrations of melamine and AAs or higher levels of non-chemical stress, overall and stratified by infant sex. Interaction terms between each chemical and non-chemical stressor were used to test for effect modification.

Results: Eight analytes were detected in >65% of participants, with 100% detection of melamine and cyanuric acid. Among male infants, summed urinary concentrations of melamine and its analogs and o-anisidine were associated with increased odds of GDM (OR: 1.08 [1.00, 1.17], OR: 1.18 [1.03, 1.36], respectively). Higher levels of perceived stress and discrimination were also associated with increased odds of GDM (OR: 1.41 [0.73, 2.70], OR: 2.33 [1.16, 4.67], respectively). We found limited evidence of interaction between chemical and non-chemical stressors.

Conclusion: Prenatal exposure to certain chemicals, including melamine, its analogs, and some AAs, as well as non-chemical stressors, may increase the risk of GDM. Future research should investigate these relationships in other cohorts.

Funding:
NIH U2CES026542, UG3OD023272, P30ES030284
JPB Foundation U24OD023382

P35-18

This abstract has been withdrawn.

P35-19 | Associations between Gestational Exposure to Brownfield Sites and Early Life Blood Pressure

Sam J. Neally[1,2], Erik Slawsky[1,2], Celeste Butts-Jackson[3], Cathrine Hoyo[4], Chantel L. Martin[2], Kristen M. Rappazzo[5]

[1] US EPA, Oak Ridge Institute for Science and Education, Oak Ridge, USA; [2] UNC Gillings School of Public Health, Department of Epidemiology, Chapel Hill, USA; [3] North Carolina Agricultural and Technical State University, Department of Health Services Management, Greensboro, USA; [4] North Carolina State University, Department of Biological Sciences and Center for Human Health and the Environment, Raleigh, USA; [5] US EPA, Office of Research and Development, Research Triangle Park, USA

Objectives: Childhood cardiovascular health (CVH) consistently associates with lifecourse cardiovascular morbidity and mortality. Blood pressure (BP) is a key indicator of childhood CVH. Brownfields are abandoned and disused sites potentially containing hazardous wastes. Closer proximity to brownfields during gestation has been associated with adverse birth outcomes related to CVH, including preterm birth, low birthweight, and overall and cardiovascular birth defects.We examined associations between brownfields during gestation and early childhood BP trajectories in a North Carolina (NC) birth cohort. We hypothesized that gestational brownfield exposure would associate with increased odds of abnormal childhood BP trajectories.

Methods: The Newborn Epigenetics Study (NEST) cohort includes 2,681 children recruited from prenatal visits at obstetrics care facilities in Durham County, NC (2005-2011). This study included participants with at least two clinical BP measurements from 3 to 9 years (n=1,040). Exposure was binary, specified as having prenatal residence within 2,000m of at least one brownfield. Outcome was systolic BP percentile trajectories, using group-based trajectory modeling (low stable, low increasing, high stable, high decreasing). Logistic regression models estimated odds ratios (OR (95%CI)) for relationships between having or not having brownfield exposure and BP trajectory groups (ref=low stable), adjusting for age, race, marital status, and education. Additional models stratified by race.

Results: Prior to covariate adjustment, children with gestational exposure to brownfields had greater odds of high stable (1.05 (1.01,1.09)) and low increasing trajectories (1.03 (1.00,1.07)). Associations were attenuated after adjustment but remained positive (high stable: 1.02 (0.98,1.06); low increasing: 1.02, (0.97,1.06)). In stratified adjusted models, Black children with brownfield exposure had greater odds of high stable (1.08 (1.01,1.15)) and low increasing (1.07 (1.00,1.14)) trajectories.

Conclusions: Brownfield exposure may associate with abnormal blood pressure trajectories during early childhood. Analyses of specific contaminants and brownfield sites may reveal additional associations.

P35-20 | Association of Urinary Phthalate Concentrations with Maternal Blood Pressure Measurements in the PROTECT Cohort

Julia Logan[1], John Meeker[2], Lauren Ellis[1], Emily Zimmerman[4], José Cordero[3], Akram Alshawabkeh[5], Julia Varshavsky[1]

[1] Northeastern University, Department of Public Health and Health Sciences, Boston, USA; [2] University of Michigan, Department of Environmental Health Sciences, Ann Arbor, USA; [3] University of Georgia, Department of Epidemiology and Biostatistics, Athens, USA; [4] Northeastern University, Department of Communication Sciences and Disorders, Boston, USA; [5] Northeastern University, Department of Civil and Environmental Engineering, Boston, USA

Objective:
The increasing incidence of gestational hypertension merits an exploration of environmental risk factors. Phthalates, a class of synthetic chemicals found in many consumer products, have been associated with an increased risk of hypertension during pregnancy in several studies, but more research is needed to understand the relationship in susceptible and highly exposed populations.

Material and Methods:
We examined associations between maternal urinary concentrations of 19 phthalate or phthalate replacement metabolites and maternal blood pressure measurements during pregnancy among n= 1,570 Puerto Rico PROTECT study participants. Blood pressure outcomes were modeled continuously as systolic and diastolic blood pressure (SBP and DBP) and dichotomously as hypertension status during pregnancy (defined as stage 1 or 2 hypertensive blood pressure levels). Associations were analyzed cross-sectionally at two clinic visits during pregnancy (16-20 and 24-28 weeks' gestation) using linear and logistic regression models.

Results:
Lower SBP and higher DBP were associated with individual metabolites after adjusting for maternal age, education, and pre-pregnancy BMI, with the strongest associations between MCOP ($\beta = 1.42$; 95%CI: 1.15, 1.70) and increased DBP at 24-28 weeks, and between MBZP ($\beta = -0.91$; 95%CI: -1.21, -0.61) and decreased SBP at 16-20 weeks. A significant association between MCOCH and increased odds of hypertension was found at 16-20 weeks (OR = 1.28, 95%CI: 1.06, 1.53). We found modest associations with imprecise effect estimates between multiple metabolites and increased odds of hypertension at 24-28 weeks, including MECPTP (OR: 1.04, 95%CI: 0.92, 1.18). Significant associations between multiple metabolites and reduced odds of hypertension were found at 16-20 weeks, including MECPP (OR: 0.65, 95%CI: 0.56, 0.75).

Conclusions:
Our findings indicate that maternal phthalate exposures may increase the risk of high blood pressure during pregnancy, but more research is needed to better understand the implications of these associations on pregnant people's cardiovascular health.

P36 | Beyond the Backyard: Understanding Indoor Environmental Health

P36-01 | The indoor air quality benefits of gas stove replacements in low-income rental housing: A university-community partnership

Niouma Semega[1], Breanna van Loenen[1], Tori Hass-Mitchell[4], Joel Wool[3], Gail Latimore[2], Jonathan I. Levy[1]

[1] Boston University School of Public Health, Environmental Health, Boston, USA; [2] Codman Square Neighborhood Development Corporation, Boston, USA; [3] Boston Housing Authority, Boston, USA; [4] City of Boston, Environment Department, Boston, USA

Objective: Building electrification is a critical strategy for achieving decarbonization, and the City of Boston has committed to electrifying at least 80 percent of existing buildings by 2050. Swapping out gas stoves for induction stoves is a crucial component of the electrification strategy, both because gas stoves are considered "gateway appliances" and because indoor air pollution from gas stoves is a well-documented public health concern. The goal of this study is to evaluate the indoor air quality and health implications of replacing gas stoves with induction stoves in low-income rental housing, as well as to determine the associated logistical issues to inform future decarbonization plans.
Method and Materials: Eighty households in two housing developments in the Dorchester neighborhood of Boston were selected to have their gas stoves replaced with induction stoves. Pre-monitoring activities include detailed building assessments, resident meetings, and cooking demonstrations with induction stoves. Recruited households have multiple pollutants (e.g., nitrogen dioxide, particulate matter) monitored continuously for one week before and after stove replacement with the MODULAIR, with continuous measurements of carbon dioxide (CO2) used to characterize ventilation. Residents fill out daily activity logs indicating cooking behaviors, other source contributions, and ventilation patterns.
Results: Our recruitment strategy coupled with a partnership between researchers and community organizations to conduct data collection led to robust data capture. In baseline monitoring, NO2 concentrations varied significantly over time and across households, with utilization of the gas stove in combination with inadequate ventilation leading to elevated indoor pollutant concentrations.Conclusion: A partnership among government agencies, housing authorities, and university researchers led to the replacement of 80 gas stoves with induction stoves, with indoor air quality monitors deployed to quantify the resulting indoor air quality improvements. Findings from this study will inform municipal policy decisions regarding appliance electrification in residential housing developments.

P36-02 | Oxidative Potential of Particulate Matter Emitted from Common Indoor Sources

P. S. Ganesh Subramanian[1], Zhuying Dai[1], Saman Haratian[2], Mohammad Heidarinejad[2], Brent Stephens[2], Vishal Verma[1]

[1] University of Illinois Urbana-Champaign, Department of Civil and Environmental Engineering, Urbana, USA; [2] Illinois Institute of Technology, Department of Civil, Architectural and Environmental Engineering, CHICAGO, USA; [3] University of Illinois Urbana-Champaign, Department of Civil and Environmental Engineering, Urbana, USA

Objective: The ability of particulate matter (PM) to induce oxidative stress in humans and cause adverse health outcomes can be quantified using oxidative potential (OP). Although people spend most of their time indoors, the OP and composition of PM emitted from indoor sources is largely unknown. Here, we address this by quantifying the chemical composition, OP, and emission rates of PM (*PER*) originating from 11 commonly used indoor sources (e.g., candles, incense, cigarettes, humidifier with tap water, toaster, air fryer, etc.).

Methods: PM from different sources were collected through controlled-chamber experiments. OP of collected PM was measured using three OP assays: dithiothreitol (DTT) consumption, glutathione (GSH) consumption, and hydroxyl radical (·OH) generation.

Results: The *PER* was the highest for toaster emissions (3,500 µg/min), followed by cigarettes (1257 µg/min). The mass-normalized OP (OP^m) of several residential sources were comparable (0.75-1.25×) or exceeded (1-6×) that of typical ambient $PM_{2.5}$, indicating the detrimental role they could play in human health. [if gte msEquation 12]><m:oMath><i style='mso-bidi-font-style:normal'><m:r>O</m:r></i><m:sSup><m:sSupPr><m:ctrlPr></m:ctrlPr></m:sSupPr><m:e><i style='mso-bidi-font-style: normal'><m:r>P</m:r></i></m:e><m:sup><i style='mso-bidi-font-style:normal'><m:r>m</m:r></i></m:sup></m:sSup></m:oMath><![endif][if gte vml 1]><v:shapetype id="_x0000_t75" coordsize="21600,21600" o:spt="75" o:preferrelative="t" path="m@4@5l@4@11@9@11@9@5xe" filled="f" stroked="f"> <v:stroke joinstyle="miter"/> <v:formulas> <v:f eqn="if lineDrawn pixelLineWidth 0"/> <v:f eqn="sum @0 1 0"/> <v:f eqn="sum 0 0 @1"/> <v:f eqn="prod @2 1 2"/> <v:f eqn="prod @3 21600 pixelWidth"/> <v:f eqn="prod @3 21600 pixelHeight"/> <v:f eqn="sum @0 0 1"/> <v:f eqn="prod @6 1 2"/> <v:f eqn="prod @7 21600 pixelWidth"/> <v:f eqn="sum @8 21600 0"/> <v:f eqn="prod @7 21600 pixelHeight"/> <v:f eqn="sum @10 21600 0"/> </v:formulas> <v:path o:extrusionok="f" gradientshapeok="t" o:connecttype="rect"/> <o:lock v:ext="edit"

aspectratio="t"/></v:shapetype><v:shape id="_x0000_i1025" type="#_x0000_t75" style='width:24.6pt; height:15pt'> <v:imagedata src="file:///C:/Users/psgs1/AppData/Local/Temp/msohtmlclip1/01/clip_image001.png" o:title="" chromakey="white"/></v:shape><![endif] was mainly driven by elemental carbon (EC) for PM emitted from sources undergoing incomplete combustion, whereas for others (i.e. based on water-droplet evaporation, heating, and complete combustion), metals were the most important drivers. A comparative exploratory exposure assessment in a hypothetical 100 m^3 apartment revealed operating certain sources (e.g., incense, cigarettes, toasters, and air-fryers) for just one hour could result in occupants being exposed to higher PM and oxidative burden than they would be exposed to via inhaling typical ambient $PM_{2.5}$ in the U.S. over an entire day.

Conclusion: Collectively, these results highlight the key role that PM emanating from indoor sources could play in human health and emphasize the need for a comprehensive toxicological and health impact assessment of PM emanating from common indoor sources.

Funding for this work was provided by the National Science Foundation under Grant No: CBET 2241332.

P36-03 | Sex-specific association between indoor total volatile organic compound and urinary VOC metabolites

Saerom Kim[1,2], Seung-Ah Choe[1]

[1] Deportment of Preventive Medicine, Korea University, College of Medicine, Seoul, South Korea; [2] Soonchunhyang University Seoul Hospital, Department of Laboratory Medicine, Seoul, South Korea

Objective: This study investigates the association between time spent at home and indoor total volatile organic compound (TVOC) concentrations and urinary VOC metabolites, and potential effect modification by sex.

Materials and Methods: This cross-sectional study utilized data from the Korean National Health and Nutrition Examination Survey (KHANES) from July 2020 to August 2021. A total of 1,859 Korean adults were analyzed. Linear regression models, including biological and socioeconomic variables were fitted for urinary concentration of nine VOC metabolites (SPMA, BMA, PGA, MA, 2-MHA, 3,4-MHA, 3-HPMA, BPMA, and DHBMA). We assessed effect modification by sex for pairs presenting positive associations. Linear coefficients and their 95% confidence intervals were calculated per interquartile range increase of indoor TVOC.

Results: Mandelic acid (MA; β=0.03, 95% confidence interval [CI]: 0.00, 0.06), 2-methylhippuric acid (2-MHA; 0.06, 95% CI: 0.03, 0.09), and 3,4-methylhippuric acid (3,4-MHA; 0.06, 95% CI: 0.03, 0.09) were positively associated with indoor TVOC concentrations. When stratified by sex, the association was not different for all three metabolites, regardless of model specification.

Conclusion: We identified MA, 2-MHA, and MHA as potential biomarkers for indoor TVOC exposure, demonstrating a consistent association across both sexes.

P36-04 | Indoor and outdoor fine particulate matter, black carbon, and carbon monoxide concentrations in homes of infants in Nairobi, Kenya

Vincent K. Kipter[1], Faridah H. Were[1], Michael J. Gatari[2], Christopher Zuidema[3], Edmund Y. Seto[3], Orly D. Stampfer[3], Bernard Makau[4], Barbra A. Richardson[5,8], Julian D. Marshall[6], Timothy V. Larson[3,6], Catherine J. Karr[9,3], Elizabeth Maleche-Obimbo[7], Sarah Benki-Nugent[5], Anne M. Riederer[3]

[1] University of Nairobi, Departments of Chemistry, Faculty of Science & Technology, Nairobi, Kenya; [2] University of Nairobi, Institute of Nuclear Science & Technology, Faculty of Engineering, Nairobi, Kenya; [3] University of Washington, Department of Environmental & Occupational Health Sciences, Seattle, USA; [4] Kenyatta National Hospital, Nairobi, Kenya; [5] University of Washington, Departments of Global Health, Seattle, USA; [6] University of Washington, Department of Civil & Environmental Engineering, Seattle, USA; [7] University of Nairobi, Department of Paediatrics & Child Health, Nairobi, Kenya; [8] University of Washington, Department of Biostatistics, Seattle, USA; [9] University of Washington, Department of Pediatrics, Seattle, USA

Introduction: Early life air pollution exposure is associated with adverse health outcomes. However, few studies have investigated children's exposure in urban sub-Saharan Africa (SSA).

Objective We measured fine particulate matter ($PM_{2.5}$), black carbon (BC), and carbon monoxide (CO) in a subset of homes with infants in Nairobi, Kenya and explored exposure factors as part of a longitudinal investigation of air pollution and neurodevelopment.

Methods: Indoor and outdoor 24-hour (24h) samples of $PM_{2.5}$ were collected using standard gravimetric procedures and collocated with CO sensors. BC was measured on two-thirds of $PM_{2.5}$ filters using multi-wavelength absorption method. Household characteristics and self-reported air pollution exposures were captured using questionnaires.

Results Forty-eight homes (median infant age of 11.5 months (range 0.8-26.2 months) were sampled. Mothers reported using liquefied petroleum gas (LPG) (66.7%), ethanol (18.8%), electricity (10.4%), and/or kerosene (10.4%). Median indoor and outdoor 24h $PM_{2.5}$ (n=39) were 39.9 ug/m^3 (range, 12.8-519.6 ug/m^3) and 23.3 ug/m^3 (2.6-68.2 μg/m^3), respectively. Most $PM_{2.5}$ concentrations (97% indoor; 79% outdoor) exceeded the World Health Organization (WHO) 24-hour air quality guideline (AQG) (15 ug/m^3). Median BC was 46 ug/m^3 (10-1,800 ug/m^3) indoors (n=25) and 34 ug/m^3 (14-280 ug/m^3) outdoors (n=24); median indoor BC in two kerosene-using homes was 10 times higher than non-kerosene homes. Median indoor (n=47) and outdoor (n=41) 24 h mean CO were 0.7 ppm (0.0-33.9 ppm) and 0.0 ppm (0.0-1.0 ppm). CO exceeded the WHO 24h AQG (6.2 ppm) in 9% of homes.

Conclusion: Indoor and outdoor $PM_{2.5}$, and CO in these Nairobi infant homes often exceeded WHO AQGs despite use of clean cooking fuels. Expanded studies of children's air pollution exposures in urban SSA are needed to build awareness and inform policy.

This research was supported by the U.S. National Institutes of Environmental Health Sciences (R01ES032153).

P36-05 | Evaluating PurpleAir Sensors and Low-Cost Household Clean Air Interventions: A Multi-year Participatory Science Study in King County, Washington

Katharine Teigen[1,2], Hannah McKinley[1,2], Mariana Cortes Espinosa[1,2], Addison Houston[2], Tania Busch Isaksen[1]

[1] University of Washington, Environmental and Occupational Health Sciences, Seattle, USA; [2] Public Health Seattle & King County, Environmental Health, Seattle, USA

Background: Indoor air quality (IAQ) has largely been understudied and under characterized. Limited research efforts have investigated how access to geographically-proximal air quality data and the use of portable air filtration systems can reduce wildfire smoke (WFS) exposure at the household level. The growing popularity of low-cost air sensors that measure fine particulate matter (PM2.5) provides an opportunity for individuals to monitor their own IAQ and make informed decisions on personal air quality improvement strategies.

Objective: Public Health–Seattle & King County (PHSKC) and the University of Washington (UW) employed participatory community science to evaluate the efficacy of low-cost public health interventions aimed at monitoring IAQ, reducing exposure, and safeguarding public health during WFS episodes.

Methods: King County households were recruited to monitor their indoor air quality throughout the summer months of 2023 and 2024 with PurpleAir Touch Indoor Air Quality Monitors. Participants completed surveys throughout the study period to assess building characteristics, personal decision making, and the performance of air filtration devices. PurpleAir data and survey responses were analyzed and summarized across the study periods.

Results: A total of 13 households in 2023 and 25 households in 2024 completed the study. In 2023, average indoor PM2.5 concentrations were higher during a WFS event (19.0 μg/m3 ∓ 27.3 μg/m3) than non-WFS events (10.7 μg/m3 ∓ 24.1 μg/m3). There were no WFS events during the 2024 study. Household-level knowledge of risks and health impacts varied amongst the two-year cohorts, showing variability in the relationship between information uptake and individual perceptions in informing behavioral changes in reducing exposure to indoor IAQ.

Conclusion: This community-based approach provides evidence of how IAQ education, risk communication, and IAQ monitoring can be used to protect the public's health in residential environments, both during ambient periods and WFS events.

P36-06 | The association between exposure to a mixture of indoor allergens on pediatric asthma control

Derek W. Werthmann, Felicia Rabito

Tulane University SPHTM, Epidemiology, New Orleans, USA

Background: Exposure to indoor allergens exacerbates asthma, with prior studies focused primarily on the association between a single allergen and asthma outcomes. However, indoor allergens typically occur as a mixture, making children's exposure to indoor allergens more complex. The objective of this study is to evaluate the association between the mixture of common indoor allergens and pediatric asthma control.
Methods: The study population is children (age 5-17 years) participating in The Allergen Reduction and Child Health Study (ARCHS). Children were recruited from the New Orleans, Louisiana area from 2020-2024. Vacuum dust samples were obtained from the bed or kitchen floor and analyzed for nine indoor allergens; cockroach (*Bla g 1*, *Bla g 2*, *Per a 7*), dust mite (*Der p 1*, *Der f 1*), dog (*Can f 1*), cat (*Fel d 1*), mouse (*Mus m 1*), and mold (*Alt a 1*). Asthma control was assessed via the Asthma Control Test (ACT) or Childhood Asthma Control Test (cACT). The association between the indoor allergen mixture adjusting for socio-demographic factors (age, sex, race, medication use, atopy) and asthma control was evaluated using generalized weighted quantile sum (qWQS) regression with repeated holdout validation.
Results: We found a negative association between the allergen mixture and asthma control in unadjusted (Effect: -0.17; 97.5% CI: -1.07-0.72) and adjusted (Effect: -0.14; 97.5% CI: -0.90-0.62) models. When evaluating the contribution of each allergen to asthma control, we found the greatest contribution from cockroach *Bla g 1* (29%) followed by dust mite Der p 1 (28%) in adjusted models.
Conclusion: The results suggest an association between the overall allergen mixture and pediatric asthma control. Among the nine individual indoor allergens investigated, cockroach (*Bla g 1*) and dust mite (*Der p 1*) contributed the greatest weight to the mixture and may be driving the reduction in asthma control.

P36-07 | Assessing Indoor Air Pollution in the Accra Metropolis: Household Exposure and Characteristics

Brandon Lewis[1], Raphael Arku[2], Michelle Bell[1]

[1] Yale University, School of the Environment, New Haven, USA; [2] University of Massachusetts Amherst, School of Public Health and Health Science, Amherst, USA

Background: Understanding the issue of indoor air pollution and its contributing factors in urban households in fast-growing sub-Saharan African cities is vital to implementing effective air quality management to mitigate air pollution and protect the most vulnerable populations.
Objectives: We measured household fine particulate matter ($PM_{2.5}$) and nitrogen dioxide (NO_2) and investigate the factors that drive increased exposure to indoor $PM_{2.5}$ and NO_2 in the homes of pregnant women and breastfeeding mothers in Accra, Ghana.
Methods: We recruited 55 households through women participating in an ongoing Accra Birth cohort study (ABC). We collected weekly average $PM_{2.5}$ and NO_2 concentrations in the kitchen and living areas of each home.

Questionnaires inquired about household socio-demographics, building characteristics, and residential activities. Descriptive and statistical methods are used to evaluate the main household factors that could be associated with increased concentrations of indoor $PM_{2.5}$ and NO_2.

Results: Most of the survey respondents are females (n=52) and primary cooks (n=53). The mean (max) $PM_{2.5}$ and NO_2 concentrations for all household areas are 56.8 ug/m^3 (428.7 ug/m^3) and 2.24 ppb (11.20 ppb), respectively. Homes using wood on average had the highest $PM_{2.5}$ and NO_2 concentrations followed by gas (for $PM_{2.5}$) and charcoal (for NO_2). Preliminary results show both $PM_{2.5}$ and NO_2 concentrations tend to increase as the number of reported cooking events increases and decreases with more open kitchen layouts. Homes that have fans, drapes, and access to air conditioning tend to have less $PM_{2.5}$ exposure on average than homes without these amenities.

Discussion: To the best of our knowledge, this project is the most comprehensive and first study addressing multiple indoor air pollutants in Accra, Ghana. Further research can provide guidance to substantially managing and reducing indoor air pollution, increase awareness, and empower local people and communities to further progress towards a cleaner environment.

P36-08 | Analysis of Spatial and Temporal Variations in Indoor $PM_{2.5}$ Levels in Urban Residential communities in four Indian cities

Chaitanya B. Shivakumar, Rajdeep Singh, Akshay Kumar, Vinayak Sahota, Harish C. Phuleria

Indian Institute of Technology, Environment science and Engineering Department, Bombay, India

Previous studies have shown the indoor concentrations, mainly in lower and middle income countries have higher indoor pollution levels as compare to outdoor and people spend 87% of their time indoors, emphasizing indoor air quality's impact on health. Therefore, this study examines housing factors affecting indoor pollution, seasonal $PM_{2.5}$ variations, and contributions from indoor and outdoor sources.This study is part of APEAL (Longitudinal effects of Air Pollution on Adolescents Lungs), a prospective cohort study, in four Indian cities, Mumbai, Mysuru, Bengaluru and Delhi

24-hour $PM_{2.5}$ measurements are done using low volume samplers for mass concentration and Atmos™ low-cost real-time sensor- used for diurnal variations in strategically selected homes followed by a 24-hour recall questionnaire. Indoor source contributions were estimated by an algorithm that separates real-time PM measurements into a fast variable signal(activity-related fluctuations) and smooth signals (persistent sources); while successive moving average subtraction method distinguished local and regional outdoor sources, enhancing source identification

Winter indoor $PM_{2.5}$ levels were highest in Delhi (274.9 ± 90.0 μg/m³), followed by Mumbai (120.6 ± 39.3 μg/m³), Bengaluru (89.8 ± 54.1 μg/m³), and Mysore (52.9 ± 14.7 μg/m³). Outdoor levels followed a similar trend. Indoor concentrations in Mumbai and Bengaluru were significantly (p-value <0.05) higher in winter than summer. The I/O ratio varied seasonally, with greater winter fluctuations in Bengaluru (0.6-3.3) and Mysore (0.9-3.2). Cooking emissions, incense burning, and mosquito coil combustion were key indoor $PM_{2.5}$ sources, contributing 32% in Delhi, 23% in Mumbai, 20% in Bengaluru, and 15% in Mysore. Outdoor sources like traffic and construction added 13% in Delhi, 12% in Bengaluru, and ~12% in Mumbai and Mysore

The findings provide critical insights into the dynamics of indoor and outdoor $PM_{2.5}$ interactions in diverse Indian urban settings, emphasizing that while outdoor pollution significantly influences indoor air quality, substantial contributions from household activities.

P36-09 | A Study on the Improvement Effect of Indoor Air Quality(IAQ) of Mobile Air Purifiers Using Health Risk Assessment

JiYoon Chung[1], ShinYoung Park[1], CheolMin Lee[1], KwangChul Noh[2]

[1] Seokyeong University, Chemical and Environmental Engineering, Seoul, South Korea; [2] AIR LAB, Gwangju, South Korea

Modern people are investigated to spend more than 80-90% of the day indoors, meaning that Indoor Air Quality (IAQ) is very closely related to human health. The importance of air purifiers is increasing as a way to manage indoor air quality, and interest in mobile air purifiers that can move throughout the room and manage air quality is increasing. Therefore, this study conducted a health risk assessment on indoor Volatile Organic Compounds (VOCs) as part of the evaluation of the indoor pollutant removal efficiency of air purifiers in indoor spaces. VOCs were measured at a total of five measurement points (main room, living room, kitchen, room 1, room 2) for a house. In order to evaluate the VOCs removal efficiency of the air purifier, it was measured by dividing it into a total of three conditions when there is no air purifier, when a fixed air purifier is installed, and when a mobile air purifier is installed. Health risk assessment was performed using the concentration of VOCs measured under each condition. The subjects of health risk assessment were conducted on adult men and women and children, and it was assumed that they were exposed to VOCs for 16 hours a day by referring to the average residence time of Koreans. After the exposure to inhalation exposure due to breathing was calculated, the health risk was calculated using carcinogenic and non-cancer toxicity values. By comparing the hazard calculated under each condition and evaluating the VOCs removal efficiency of the mobile air purifier, we tried to confirm the necessity of the mobile air purifier.

P36-10 | Health Risk Assessment for Exposure to Indoor Pollutants in Automobile for the Management of Indoor Air Quality in Automobile

JiYoon Chung[1], ShinYoung Park[1], InJi Park[2], HyunWoo Lee[2], CheolMin Lee[1]

[1] Seokyeong University, Chemical and Environmental Engineering, Seoul, South Korea; [2] Korea Automobile Testing & Research Institute, Gyeonggi-do, South Korea

It has been reported that exposure to pollutants indoors can actually cause more health hazards than exposure to outdoor air pollution, and indoor air pollution of newly manufactured automobiles among indoor air pollution has recently attracted attention. Exposure to Volatile Organic Compounds (VOCs) and carbonyl compounds emitted from interior materials in vehicles is known to have a harmful effect on the human body. Accordingly, as part of the development of automobile indoor air quality environmental friendliness rating, a health risk assessment was conducted on the exposure of automobile indoor pollutants. In this study, eight pollutants (Formaldehyde, Benzene, Toluene, Xylene, Ethylbenzene, Styrene, Acrolein, and Acetaldehyde) subject to indoor air quality management of newly manufactured vehicles were selected. The concentration of the target substance was measured in the driving mode for each newly manufactured vehicle, and the results of a survey of drivers such as weekly usage time and frequency of weekly use were used as exposure factors. In the case of automobile, the Average Daily Dose (ADD) was calculated by focusing on non carcinogens because the duration of stay is not as long as in the residential

environment, so the effect of carcinogens is not significant. The Hazard Quotient (HQ) was calculated by dividing the calculated ADD by the Reference Dose (RfD). If the calculated HQ exceeds the reference value of 1, it was judged that there is a possibility of health effects. By comparing the results of the health risk assessment on exposure to indoor pollutants in automobiles, it was intended to identify indoor pollutants that need to be managed first and come up with measures such as weighting when developing automobile rating.

P36-11 | Impact of Cooking Activities and Ventilation on $PM_{2.5}$ Concentrations in Semi-Open Restaurants in Vietnam

JuShin Oh[1], Kiyoung Lee[2]

[1] Seoul National University, Graduate School of Public Health, Seoul, South Korea; [2] Seoul National University, Seoul, South Korea

The expansion of food delivery services in Vietnam after the COVID-19 pandemic has led to a rise in small-scale restaurants, many of which operate in semi-open kitchens—structures with three enclosed walls and one open side. These kitchens rely on natural ventilation and often lack dedicated exhaust systems. Limited regulatory oversight on restaurant air quality management underscores the need for indoor air quality (IAQ) assessment in these environments. This study examined $PM_{2.5}$ concentrations in 30 semi-open restaurant kitchens in Ho Chi Minh City, Vietnam, focusing on variations by kitchen type, fuel type, cooking activity, and ventilation. $PM_{2.5}$ levels were measured using a real-time monitor during operating hours, alongside observational data collected at 10-minute intervals. The average $PM_{2.5}$ concentration in semi-open kitchens ranged from 12.1 $\mu g/m^3$ to 130.5 $\mu g/m^3$. Among the 30 monitored kitchens, 14 locations (46.7%) exceeded the Korean 24-hour $PM_{2.5}$ standard of 35 $\mu g/m^3$. Kitchen type and fuel type played a significant role, with stir-fry/wok kitchens and charcoal use associated with higher $PM_{2.5}$ levels. Cooking activities significantly influenced $PM_{2.5}$ levels, with steaming, frying, and stir-frying identified as major contributors. High-temperature and oil-based cooking processes were found to be key sources of $PM_{2.5}$ emissions in semi-open restaurant kitchens. These findings provide fundamental data on IAQ in semi-open restaurant kitchens in Ho Chi Minh City and highlight the impact of specific cooking activities and kitchen characteristics on $PM_{2.5}$ concentrations.

P36-12 | Indoor air quality and children's cognitive and academic outcomes in India

Jyothi S. Menon[1,2], Aditi Roy[1,2], Shweta Dixit[1], Debarati Mukherjee[3,4], Deepa Ravi[3,4], Smita Todkar[5,6], Astha Yadav[5], Chaman Lal[5], Amit Prakash[5], Suganya Rajendran[3], Giridhar R. Babu[7], Siddhartha Mandal[1,2], Poornima Prabhakaran[1,2], On behalf of COINCIDE consortium

[1] Ashoka University, Centre for Health Analytics Research and Trends, Trivedi School of Biosciences, Sonipat, Haryana, India; [2] Center for Chronic Disease Control, New Delhi, India; [3] Indian Institute of Public Health-Bengaluru, Public Health Foundation of India, Bengaluru, Karnataka, India; [4] Public Health Foundation of India, Centre for Developmental and Lifecourse Research, Bengaluru, Karnataka, India; [5] Sangath, Child Development Group, New Delhi, India; [6] Manipal University, Centre for Doctoral Studies, Manipal Academy of Higher Education, Karnataka, India; [7] Qatar University, Department of Population Medicine, College of Medicine, QU Health, Qatar

Objectives: Poor indoor air quality can affect children's cognitive development, although evidence from India is sparse. This study aims to assess the relationship between exposure to adverse indoor air quality conditions and cognitive and academic outcomes among 3-9 year-old children in two diverse Indian sites.

Material and Methods: This study is part of an ongoing prospective cohort study of 1,591 mother-child dyads from urban (Bengaluru, Karnataka) and rural (Rewari, Haryana) India. Data on household indoor air quality (fuel use (clean/unclean), smoking (yes/no) and dampness (yes/no)) were collected using parent-report questionnaires. Cognitive ability was assessed using Raven's Coloured Progressive Matrices (RCPM); early language and numeracy skills were measured using the Annual Survey of Education Report (ASER). Linear regression was used to assess the association, controlling for factors such as SES, child age, gender, mother's education and crowding index of households.

Results: In the rural cohort (n=601), 79% of households used Liquefied Petroleum Gas as their primary cooking fuel, compared to 100% in urban households (n=990). Rural households also used multiple fuel types for cooking, including solid fuels. Dampness was reported in 86% and 14% of rural and urban households, respectively. Second-hand smoke was higher in rural areas (55%) compared to urban areas (22%). Compared to children who were not exposed to dampness, children exposed to dampness had lower RCPM scores (β = -3.01(-5.85, -0.16), n=940) and early language scores (β = -0.35(-0.61, -0.08), n=906) in the urban cohort. Children exposed to unclean cooking fuels exhibited lower early language scores (β = -0.6(-0.94, -0.27), n=601) in the rural cohort. No significant association with numeracy outcomes was observed in both cohorts.

Conclusion: Children in both cohorts exposed to conditions such as dampness and unclean cooking fuels had lower cognitive and early language scores, highlighting the importance of maintaining good indoor home conditions.

P36-13 | Residential Indoor $PM_{2.5}$ exposure using low-cost sensors in Washington, Oregon and California: Source contributions and sociodemographic disparities

Ningrui Liu[1], Xuyuan Kang[3], Ching-Hsuan Huang[1], Enjia Zhang[4], Timothy Larson[1,2], Elena Austin[1], Da Yan[3], Edmund Seto[1]

[1] University of Washington, Department of Environmental and Occupational Health Sciences, Seattle, USA; [2] University of Washington, Department of Civil and Environmental Engineering, Seattle, USA; [3] Tsinghua University, Department of Building Science, Beijing, China; [4] Beijing University of Technology, College of Architecture and Urban Plannning, Beijing, China

Air pollution is one of the leading contributors to the burden of disease. Many studies have revealed large sociodemographic differences in ambient $PM_{2.5}$ exposure and the growing impact of wildfire events across US. Although people spend over 80% of their lifetime indoors, little is known about the indoor $PM_{2.5}$ exposure. Therefore, this study is aimed to use crowdsourced sensor data to estimate the attributable health burden of residential $PM_{2.5}$ exposure in US West Coast. We first collected daily average indoor $PM_{2.5}$ concentrations in 2021 from low-cost PurpleAir sensors in Washington, Oregon, and California. After calibrating the sensor data, we trained a two-layer hierarchical machine learning model based on XGBoost algorithm and predicted the annual average indoor $PM_{2.5}$ concentrations for all residential buildings across the three states. We further employed the random component superposition analysis and wildfire-related $PM_{2.5}$ identification approach to apportion the above attributable health burden into outdoor wildfire, non-wildfire, and indoor source contributions. Currently, a total of 4553 indoor PurpleAir sensors have been collected with over 1 million daily average concentration data. Data from about 50% of these sensors cover over 60% of days in 2021. The mean (SD) of the annual average residential $PM_{2.5}$ exposure levels across these sensors was 3.45 (2.83) μg/m^3 in 2021. Modeled predictions of indoor concentrations will be used to assess health burdens and disparities in burden by neighborhood sociodemographic factors, which is still in progress. The findings of this study can help target the sources, locations, and subpopulation to effectively control residential air pollution control and improve population health in the future.

P36-14 | A state-of-the-art review and performance evaluation of novel do-it-yourself (DIY) air cleaner designs

Rafi Uddin, Insung Kang

University of Texas at Arlington, Civil Engineering, Arlington, USA

Frequent exposure to wildfire smoke and respiratory pathogens, such as SARS-CoV-2, emphasizes the urgent need for affordable and accessible indoor air filtration solutions. Although high-efficiency particulate air (HEPA) purifiers are available on the market, their high cost and variable performance limit widespread use. On the other hand, do-it-yourself (DIY) air cleaners, which use box fans and furnace air filters, have become popular recently due to their low cost and simple assembly. However, there is limited knowledge regarding the effectiveness of different DIY air cleaner designs, which can vary significantly in terms of filter and fan choices, resulting in varying performance outcomes. This study aims to review the existing knowledge on the efficiency of DIY air cleaners and present performance testing results for various configurations, with a focus on Clean Air Delivery Rate (CADR), filtration

efficiency and noise levels.The best performing DIY air cleaner design will be deployed in the classrooms of the University of the Texas at Arlington, in the second phase. It is anticipated that the novel DIY design deployed in the classroom will enhance indoor air quality to levels comparable to that of commercial HEPA purifiers. The findings will also offer guidance for best practices in low-cost IAQ management, yielding better health for occupants in built environment.

P36-15 | Assessing Efficacy of Vehicle Air Cleaners to Improve In-Cabin Air Quality and Health Outcome

Rafi Uddin, Insung Kang

University of Texas at Arlington, Civil Engineering, Arlington, USA

Significant health concerns are associated with traffic-related air pollution (TRAP), especially during car commutes when passengers are exposed to high levels of particulate matter (PM), nitrogen dioxide (NO_2), carbon monoxide (CO), and volatile organic compounds (VOCs). Even though existing car filtration systems offer some protection, standalone vehicle air cleaners present a viable way to reduce in-cabin TRAP exposure even further. Therefore, we propose a two-phase approach to evaluate the effectiveness of vehicle air cleaners in reducing in-cabin exposure to TRAP. First, we will assess the pollutant removal efficiency of vehicle air cleaners currently available in the U.S. market (as of February 2025) in the laboratory. Following that, we will deploy the best-performing air cleaners in vehicles to conduct field testing under real-world driving conditions. The field testing will involve 60 controlled commutes with crossover design to measure pollutant concentrations with and without the air cleaners activated. Air pollutant sampling for both laboratory and field tests will utilize portable, research-grade air quality monitoring equipment. Additionally, we will monitor drivers' physiological responses through wearable sensors to assess potential health impacts such as blood pressure, heart rate, oxygen saturation levels, stress, and electrocardiogram.

P36-16 | The Impact of Cooking Fume Exposure on Lung Cancer in Non-smokers of Different Ethnicities.

Bría McAllister[1], Samuel Cai[1], Sam Khan[1,2], Karen Brown[1,2]

[1] University of Leicester, Leicester, UK; [2] University Hospitals Leicester, Leicester, UK

Objective

Lung cancer is increasingly prevalent in the non-smoking population, with Household Air Pollution (HAP) recognised as a major contributor, and some evidence that women of Asian backgrounds are at greater risk than other demographics.

Our systematic review showed that the majority of studies have been carried out in low-, and middle-income countries, with a clear research gap regarding the impact of cooking-related HAP on non-smoking lung cancer in high-income countries.

Building on this evidence, this project explores whether South-Asian households are exposed to higher levels of cooking-related HAP compared to households of White British ethnicity in a high-income country.

Material and Methods
Twenty households have been recruited, with White British, and South-Asian families equally represented among participants. Kitchen air quality is monitored for a variety of pollutants, including PM and VOCs, using active and passive methods. Week-long air sampling is conducted once in winter and once in summer to account for seasonal variation. Participants complete a daily diary noting activities related to cooking, cleaning and ventilation.
This study is ongoing, with the first round of data collection complete. This data includes accurate, real time air quality measurements allowing for direct comparison with the daily activity diaries to assess the impact of cooking on kitchen air quality.
Results
Initial findings have shown overall higher pollution levels in South-Asian households compared with White households. Exposure levels rise significantly during cooking events, frequently breaching WHO recommended levels in all households, and take several hours to return to baseline levels.
Conclusion
To the best of our knowledge, this is the first UK study to directly explore how cooking practices affect kitchen air quality, with a focus on ethnicity and cultural practices. It will provide valuable data on how household air quality affects the risks of non-smoking lung cancer in a high-income country.

P36-17 | Impacts of household cooling methods on indoor PM10 and PM2.5 concentrations in rural California agricultural communities

Sherry WeMott[1], Luis Hernandez Ramirez[1], Jessica Tryner[2], Xiaoying Li[3], Bonnie N. Young[1], Mollie Phillips[2], Grant Erlandson[1], Celine Campos[1], Daniel Dean[1], Nayamin Martinez[5], Grace Kuiper[1], John Volckens[3], Sheryl Magzamen[1]

[1] Colorado State University, Environmental and Radiological Health Sciences, Fort Collins, USA; [2] Access Sensor Technolgies, Fort Collins, USA; [3] Colorado State University, Mechanical Engineering, Fort Collins, USA; [4] California Environmental Justice Network, Fresno, USA; [5] California Environmental Justice Network, Fresno, USA

Background: Particulate matter (PM) can infiltrate homes from outdoors and be generated by indoor sources, worsening indoor air quality and affecting residents' health. Indoor PM concentrations may vary based on building characteristics, home appliances, and residents' activities. This study aimed to assess how home cooling methods impacted indoor air quality
Methods: The study, which took place from November 2023 - September 2024 recruited participants aged 18 or older from two agricultural towns in California's San Joaquin Valley. Each household had two Ultrasonic Personal Air Samplers placed in a common living space for 24 hours to measure fine ($PM_{2.5}$) and thoracic (PM_{10}) particulate matter concentrations. Researchers administered a survey to assess participants' exposures to pesticides and dust, home cooling methods, as well as other household characteristics and activities that could affect PM concentrations. Wilcoxon tests and linear regression models were applied to determine how various cooling methods affected in-home $PM_{2.5}$ and PM_{10} concentrations.
Results: Of the 127 participants, the majority identified as Hispanic/Latino. There were 59 observations for indoor $PM_{2.5}$ concentrations and 69 for PM_{10} concentrations. Central air, swamp coolers, fans, and opening windows were used as cooling methods in 51%, 38%, 81%, and 69% of homes, respectively. Households using fans showed a suggestive association with increased PM_{10} concentrations ($p = 0.06$). After adjusting for the number of children and workers exposed to dust in the home, fan use was associated with a 1.40 μg/m³ increase in indoor PM_{10} (95% CI:

0.98, 1.98). No cooling methods demonstrated a significant association with indoor $PM_{2.5}$ concentrations.

Conclusion: These results suggest that using fans to cool homes may be assoicated with increased indoor PM concentration, particularly PM_{10}.

This work was funded by the National Institute of Environmental Health Sciences under grant R44ES024041.

P36-18 | Indoor Air Quality in Disadvantaged Communities: The Role of Ventilation, Household Behaviors, and Perception Gaps

Tianjun Lu, Yisi Liu

University of Kentucky, Department of Epidemiology and Environmental Health, Lexington, USA

Fine particulate matter ($PM_{2.5}$, particles ≤ 2.5 μm) is linked to adverse respiratory and cardiovascular effects, yet indoor determinants remain understudied, especially in disadvantaged communities. While outdoor $PM_{2.5}$ can infiltrate indoors, household behaviors, ventilation, and air conditioning use further influence levels. Additionally, residents may misperceive their air quality, influencing risk awareness and mitigation behaviors.

We conducted a real-time air quality monitoring campaign in 11 households across disadvantaged communities in South Los Angeles from June to September 2022. We collected 22,836 hours of paired indoor and outdoor $PM_{2.5}$ measurements using low-cost PurpleAir sensors. Household residents participated in five bi-weekly surveys as well as pre-monitoring and post-monitoring surveys, capturing indoor pollution sources, ventilation behaviors, perceived air quality, and socio-demographic information. We analyzed indoor infiltration patterns using I/O ratios, identified key predictors of indoor $PM_{2.5}$ concentrations using generalized linear models, and assessed discrepancies between perceived and measured air quality through logistic regression.

On average, indoor hourly $PM_{2.5}$ (13.5 μg/m^3) levels were 18% higher than outdoor levels (Mean I/O ratio = 1.34). Frequent window opening (β = - 0.184, $p < 0.001$) and air cleaner use (β = - 0.142, $p < 0.001$) were associated with lower indoor $PM_{2.5}$, while cooking frequency (β = 0.118, $p < 0.001$) and cooking style (e.g., frying) significantly increased indoor pollution levels. Surprisingly, both exhaust fan use (β = 0.182, $p < 0.001$) and air conditioning use (β = 0.316, $p < 0.001$) were associated with higher indoor $PM_{2.5}$, suggesting ineffective pollutant removal or recirculation. Perceived and measured air quality were weakly correlated (ρ = 0.075, $p < 2.2e-16$), indicating that residents may underestimate exposures.

Household behaviors influence indoor $PM_{2.5}$ and assumed protective measures may not always be effective. Findings warrant improved public health messaging, ventilation strategies, and targeted interventions to reduce indoor pollution disparities in vulnerable communities.

P36-20 | Health Effects of Indoor Air Pollution: Research Needs and Pathways

Danny Malashock[1,2], Vito Ilacqua[1]

[1] U.S. Environmental Protection Agency, Indoor Environments Division, Washington, DC, USA; [2] U.S. Public Health Service Commissioned Corps, Washington, DC, USA

Exposure to air pollution in the indoor environment, where people spend roughly 90 percent of their time, plays an important role in shaping public health worldwide. Decades of research have improved our understanding of the health impacts of indoor air pollution exposure, yet gaps in knowledge remain. By filling existing research gaps and leveraging emerging technologies and approaches, we can improve our understanding of the health impacts of indoor air pollution and develop effective interventions to protect public health. This presentation will provide an overview of indoor air pollution research needs at the intersection of exposure science and epidemiology, and potential pathways to address them. The presentation will summarize indoor air pollution exposure and its multifaceted health effects, including a brief discussion of emerging issues and contaminants of concern in the indoor environment. Exposure science research needs and innovative methodologies for assessing indoor air pollution exposure will then be addressed, including advanced monitoring techniques (e.g., low continuous monitoring, occupancy sensing), personal exposure assessments, modeling, and the importance of integrating building characteristics and occupant data. Epidemiologic research needs will be discussed, including the need for long-term population-based studies of the health effects of indoor air pollution exposure, including on multiple organs and systems (in addition to cardiopulmonary), and more robust, adequately powered interventional studies examining health outcomes (i.e., exposure reduction). Finally, the implications of new research in each of these fields on risk assessment and public health interventions will be discussed, including how these efforts could allow for development of new estimates and refinement of the few existing estimates of the burden of indoor air pollution.

P36-21 | From agricultural fields to bedrooms: Investigating pesticides in house dust

Assif Friedman[1], Parisa Falakdin[1], Amitabha Govande[1], Geert Gomers[3], Yacine Ryckebusch[3], Lieven Bevoerts[4], Kai Wang[5], Samuel Fuhrimann[7], Vera Silva[2], Wim de Meyer[6], Anke Huss[1], Roel Vermeulen[1], Daniel Martins Figueiredo[1]

[1] Utrecht University, Institute for Risk Assessment Sciences, Utrecht, Netherlands; [2] Wageningen University & Research, Soil Physics and Land Management Group, Wageningen, Netherlands; [3] Velt vzw Vereniging voor Ecologisch Leven en Tuinieren, Antwerpen, Belgium; [4] University of Antwerp, ECOSPHERE, Wilrijk, Belgium; [5] China Agricultural University, College of Resources and Environmental Sciences, Beijing, China; [6] Primoris Belgium, Ghent, Belgium; [7] Swiss Tropical and Public Health Institute (Swiss TPH), Department of Epidemiology and Public Health, Arlesheim, Switzerland

Indoor dust in bedrooms, where individuals typically spend considerable time, can be a reservoir for pesticide residues. Nevertheless, the contamination levels and associated health risks remain poorly characterized. This study examined floor dust samples from 112 bedrooms across Belgium and the Netherlands to evaluate pesticide contamination levels in indoor dust and identify contributing factors. A total of 494 pesticides were analyzed via liquid

and gas chromatography-mass spectrometry. A total of 122 pesticides, comprised of 61 approved and 59 unapproved substances, were detected. Over 98% of the homes contained at least one pesticide, and 95% of the dust samples contained five or more different compounds. The most frequent mixture consisted of five pesticides—Dicloran, Permethrin, 2-phenylphenol, DEET (Diethyltoluamide), and Propiconazole—which co-occurred in 33% of all samples. Key factors included the proximity to agricultural fields and pet ownership, which were significantly linked to a higher presence of both approved and banned pesticides. Cumulative risk assessment revealed that while most organ systems had hazard index values below 1—indicating negligible risk—the risk of neurotoxicity exceeded the threshold (HI = 1.45) in worst-case scenario, primarily due to exposure to fipronil, also authorized as a biocide. Despite regulatory bans, the persistence of some legacy pesticides underscores the challenges of mitigating long-term indoor contamination. Our study highlights the incidence of pesticide mixtures in indoor environments and the necessity for comprehensive exposure assessments.

P36-22 | Performance Evaluation of Portable Indoor Air Cleaners: Particles and Organic Compounds Removal and Possible Byproduct Generation

Dong Gao[1], Elizabeth Lin[1], Sheng Liu[2], Krystal Pollitt[1]

[1] Yale University, School of Public Health, New Haven, USA; [2] Yale University, Department of Chemistry, New Haven, USA

Objective: Portable air cleaners are widely used to mitigate health risks associated with airborne contaminants in indoor environments. However, their effectiveness in removing different pollutants, especially organic compounds, remain uncertain. This study aims to assess the efficiency of four air cleaner types in removing particles, volatile and semi-volatile organic compounds (VOCs and SVOCs), while also examining the potential formation of byproducts such as ozone, providing insights into their overall impact on indoor air quality. ***MaterialsandMethods***: Four types of portable air cleaners were tested, including a commercially available high-efficiency particulate air cleaner, an electronic air cleaner, and two lower-cost, do-it-yourself (DIY) alternatives built from box fans – one coupled with a MERV-13 filter and the other with activated carbon filters, to cover a wide range of applications and affordability. The air cleaners were tested in a 3.5 m^3 Teflon chamber spiked with wood smoke. Particle and ozone concentrations were monitored continuously using an optical particle counter and a portable ozone monitor, respectively. Organic compounds were collected via passive sampling at different operational time intervals, analyzed by thermal desorption gas chromatography high resolution mass spectrometry, and identified through targeted analysis for known compounds and suspect screening for contaminants of emerging concern. ***Results***: All tested air cleaners excelled in particle removal, eliminating more than 95% of particles within 10 minutes of operation. The DIY activated carbon air cleaner outperformed the other cleaner types, maintaining low compound levels in the chamber. However, the electronic air cleaner with a charging system posed a risk of generating harmful oxidation byproducts, such as ozone or oxidized organic compounds, after extended operation (>20 hours). ***Conclusion***: The findings provide valuable insights regarding the selection and implementation of effective air cleaning solutions to promote healthier indoor environments.

P36-23 | The Influence of Cultural Background and Personal Behaviors on Sick Building Syndrome in College Students

Jingting Huang[1], **Linyan Li**[1,2]

[1] City University of Hong Kong, Department of Data Science, Hong Kong, Hong Kong; [2] City University of Hong Kong, Department of Infectious Diseases and Public Health, Hong Kong, Hong Kong

Objective

Indoor environments significantly impact human health and well-being. This population-based study examined the relationship between student origins, behaviors, and the prevalence of Sick Building Syndrome (SBS) symptoms among college students in Hong Kong. It explored how cultural backgrounds influence the use of air treatment devices, cleaning products, and fresheners, and how these behaviors mediate the association between SBS symptoms and student origins.

Materials and Methods

A questionnaire was administered to 1,076 City University of Hong Kong students from November 2022 to May 2023, with additional data collected from 1,072 students from November 2023 to May 2024. Data included demographics, student behaviors, and SBS symptoms. Statistical analyses (ANOVA, linear regression, multivariate logistic regression, and mediation analysis) were conducted using Python, R, and Jamovi, with significance set at $p < 0.05$.

Results

Frequent candle use was significantly associated with higher scores for general ($\beta_1 = 0.22$), mucosal ($\beta_2 = 0.20$), and skin ($\beta_3 = 0.16$) SBS symptoms. Incense use was linked to higher mucosal ($\beta_2 = 0.15$) and skin ($\beta_3 = 0.14$) symptom scores. SBS symptoms were more prevalent among Hong Kong students compared to mainland Chinese and international students. Student behaviors, particularly the use of air treatment and freshening products, mediated the relationship between student origins and SBS symptoms.

Conclusion

This study highlights cultural disparities in SBS symptoms and the role of student behaviors in shaping health outcomes. Cultural backgrounds significantly influence behaviors and vulnerability to SBS, emphasizing the need for cross-cultural understanding in public health initiatives.

Funding

This work was supported by a grant from the Research Grants Council of the Hong Kong Special Administrative Region, China (Project No. 21607223).

P36-24 | AERLIFT: *A*ssessing *E*xposure to *R*esidential Air Pollution for *L*ow-*I*ncome *F*amilies with Gas and Electric Cooking *T*echnologies– Pilot Results

Michael Johnson[1], Dan Bertoldi[2], Helen Mejia[2], Katherine Kearns[1], Mafe Aragon[1], Caleb Witzaney[3], Dana Charron[1], Laura Forni[7], Nicholas Lam[4], Peter Moschovis[5], Ajay Pillarisetti[6]

[1] Berkeley Air Monitoring Group, Berkeley, USA; [2] Ava Community Energy, Oakland, USA; [3] Franklin Energy, Port Washington, USA; [4] California State University, East Bay, Hayward, USA; [5] Harvard University, Cambridge, USA; [6] University of California, Berkeley, Berkeley, USA; [7] Stockholm Environment Institute, Davis, USA

Background: Electrification programs have the potential to improve indoor air quality and health, but limited research has quantified their programmatic benefits. Ava Community Energy's Health-e Communities program provides Ava customers with gas-to-induction stove replacements at no cost to the customer. The program's goal is to distribute 2,000-3,000 induction stoves, electrical upgrades, and compatible cookware to health-impacted and lower-income households.

Objectives: AERLIFT will quantify 48-hour personal exposures to $PM_{2.5}$, NO_2, CO, CO_2, black carbon, and the elemental composition of $PM_{2.5}$, as well as indoor concentrations of these pollutants and BTEX, in 300 homes before and after transitioning from gas to induction cooking. The study will also assess changes in energy costs, avoided emissions, and the social cost of carbon resulting from the electric stove transition. Additionally, AERLIFT will evaluate the impact of induction stove adoption on respiratory symptoms, health care utilization, and exacerbation frequency in a high-risk population.

Methods: We will present results from an ongoing 200-household pilot phase of AERLIFT, in which low-cost $PM_{2.5}$, NO_2, CO_2, and CO monitors collect approximately one month of continuous data before and after introducing an induction stove. This data is also being used to generate individualized air quality feedback reports for participating households.**Results and Conclusions:** The median amount of time per day NO_2 concentrations exceeded 100 ppb (USEPA's 1-hr standard) were reduced from 16.1 to 0.1 minutes in the first set of pilot homes (n=11). Given the constraints on funding for electrification programs, allocating resources strategically to maximize health and social benefits is critical. Evaluating targeted electrification programs like Ava Energy's is essential for understanding both their effectiveness and how to design impactful policies and programs.

P36-25 | Improving Classroom Indoor Air Quality with DIY Corsi-Rosenthal Boxes

Misti L. Zamora[1], Sharmin Akter[2], William William Gasparrini[5], Marina Creed[3], Jessica Hollenbach[4], Kristina Wagstrom[5]

[1] UConn Health, Public Health Sciences, Farmington, USA; [2] University of Connecticut, Department of Civil and Environmental Engineering, Storrs, USA; [3] UConn Health, Department of Neurology, Farmington, USA; [4] Connecticut Children's Medical Center, Asthma Center, Hartford, USA; [5] University of Connecticut, Department of Chemical and Biomolecular Engineering, Storrs, USA

Poor classroom ventilation creates an environment where particles can accumulate, leading to reduced indoor air quality (IAQ), heightened risks of infectious disease transmission, and exacerbating asthma symptoms. Children are particularly vulnerable to the health consequences of poor classroom ventilation due to a combination of physiological factors, such as developing respiratory and immune systems and higher breathing rates, and the significant amount of time they spend in school environments. Therefore, practical and effective air purification strategies are crucial for mitigating these risks in classrooms. Corsi-Rosenthal boxes (C-R boxes), low-cost DIY purifiers using box fans and MERV-13 filters, represent a practical and accessible intervention. This study, conducted by the UConn Indoor Air Quality Initiative, assessed C-R box effectiveness and practicality in two occupied classrooms without mechanical ventilation. We measured PM1, PM2.5, and PM10 mass and number concentrations with the C-R boxes on and off. They achieved an average particle reduction of 60% for both mass and number concentrations. Spatially resolved measurements showed consistent particle reduction throughout classrooms, which is key to ensuring all students are benefiting from the filter. These results demonstrate that C-R boxes can substantially and rapidly improve classroom IAQ by reducing particle exposures. Additional measurements during a severe smoke event revealed that the C-R box demonstrated rapid removal efficacy, achieving a sustained 95% reduction of particulate matter within minutes of operation. This accessible, DIY intervention, offers a highly effective strategy to lower children's airborne particle exposure in schools, potentially mitigating respiratory health risks and improving public health in school environments.

P36-26 | Assessment of Indoor Air Quality in a University Computer Room: A Case Study of a University in Thailand

Saowanee Norkaew[1,2], Monthawan Panket[1], Napasthamon Khan-audta[1], Teeraphun Kaewdok[1,2], Nontiya Homkham[1], Katiya Ivanovitch[1]

[1] Thammasat University (Rangsit Campus), Faculty of Public Health, Pathumthani, Thailand; [2] Thammasat University, Thammasat University Research Unit in Occupational Ergonomics, Pathumthani, Thailand

The majority of urban populations spend most of their daily time indoors. Indoor environments may be influenced by pollution sources originating both within and outside the building, which can potentially impact the health of occupants and building users. This study aims to evaluate the indoor air quality of a computer room at a university in Thailand. Air quality data were collected by measuring nine parameters: temperature, relative humidity, carbon monoxide, carbon dioxide, total particulate matter, particulate matter with diameters of ≤10 micrometers (PM10) and ≤2.5 micrometers (PM2.5), airborne microorganisms, fungi, and bacteria. The results indicated that all measured

parameters met the relevant air quality standards. However, despite compliance with the standards, the study recommends implementing measures to maintain and improve indoor air quality. These measures include regular maintenance of air conditioning systems and other equipment within the room, the installation of mechanical ventilation systems, and the use of exhaust fans to enhance air circulation and reduce the accumulation of pollutants. Additionally, further research on the health effects associated with indoor air quality is suggested.

P36-27 | Measurement of Toxic Substances in Environmental Tobacco Smoke from Heated Cigarettes and E-Cigarettes for Exposure Assessment in the Home

Satoshi Nakai[1], Ryoya Matsubara[1], Atsuhiro Motomiya[1], Izumi Takino[1], Yoshika Sekine[2], Masaki Kawaguchi[2], Yoshihiro Suzuki[3]

[1] Yokohama National University, Graduate School of Environment and Information Sciences, Yokohama, Japan; [2] Tokai University, Isehara, Japan; [3] SIBATA Scientific Technology LTD., Souka, Japan

Objective
In recent years, interest in heated tobacco and e-cigarettes as an alternative to conventional tobacco has increased in Japan. However, in addition to a lack of knowledge on the health effects and health risks of heated tobacco and e-cigarette smoking, there is also a lack of information on the effects of exhalation smoke on the surrounding environment and the health effects of passive smoking. The purpose of this study was to obtain data on the concentration of toxic substances when smoking heated cigarettes in an actual residential environment.
Material and Methods
In one room of a monthly apartment, six volunteers were asked to conduct a smoking experiment according to the established smoking protocol. Six brands of heated tobacco, two brands of e-cigarettes, and one brand of conventional cigarette sold in Japan were measured for PM2.5, nicotine, VOCs, and aldehydes in environmental tobacco smoke.
Results
For nicotine concentrations, indoor concentrations of heated tobacco ranged from 0.23 μg/m3 to 0.45 μg/m3, which were 0.056 to 0.11 times higher than those of conventional tobacco.
The concentrations of VOCs and formaldehyde were lower for all brands of heated tobacco than for conventional tobacco. Concentrations of acetaldehyde from some brands of heated tobacco and e-cigarettes were higher than those of conventional tobacco.
Conclusion
The contribution of heated tobacco and e-cigarettes to the concentration of toxic substances in the indoor environment is smaller than that of conventional tobacco, suggesting that the health risk of passive smoking due to each substance alone is small. However, since the results were obtained under limited conditions, further studies are needed, such as examining combined exposure.

P36-28 | Heat Action Plan (HAP) Design and Implementation: A state-of-the-science review evaluating published evidence using the WHO heat-health action plan guidelines

Tanya S. Isaac[1], Perumal K. Latha[1], Vidhya Venugopal[1], Laura Downey[2,3]

[1] Sri Ramachandra Institute of Higher Education and Research, Department of Environmental Health Engineering, Chennai, India; [2] University of New South Wales, The George Institute for Global Health, Sydney, Australia; [3] Imperial College London, The George Institute for Global Health, London, UK

Introduction: With global temperatures rising due to climate change, heatwaves are becoming increasingly common and intense, posing severe health risks to vulnerable populations. Heat Action Plans (HAPs) have been developed globally to mitigate these effects; however, the effectiveness of their implementation depends on several factors. This state of the science review synthesizes evidence on HAP design and implementation, emphasizing key facilitators and challenges in effective deployment.

Methods: We conducted this state-of-the-science review by comprehensively searching PubMed, Scopus, EMBASE, and Google Scholar for original articles describing HAP implementation as a single policy instrument, focusing on enablers and barriers to effective deployment. Searches identified 3,137 potential studies, of which a total of 11 eligible articles were included.

Results: The 11 included studies described HAP implementation across diverse geographical settings in sub-national and/or national contexts. Key enablers identified included established leadership for coordination, effective alert systems, communication strategies, and stakeholder collaboration. Key challenges identified were inadequate funding, limited capacity in health systems, insufficient training, and variable access to cooling resources. Public-private partnerships and strengthened cross-sectoral collaborations emerged as potentially promising models for overcoming resource constraints, especially in low-and middle-income countries.

Discussion: Our findings underscore the importance of systemic readiness, stakeholder coordination, and long-term planning in successful HAP implementation. Regions with well-defined leadership and robust health and social systems demonstrated greater resilience to heat. However, many HAPs focus on short-term responses, often neglecting preventative measures such as urban greening and infrastructural adaptation.

Conclusion: Effective HAP implementation requires a multi-sectoral approach, sustainable financing, and adaptive policies for local contexts. Enhancing HAPs with preventative strategies and equity-focused measures will be crucial to safeguarding public health in an era of escalating heat.

P36-29 | Post-trial Liquefied petroleum gas refill pattern among the beneficiaries of the household air pollution intervention network study from India site

Vigneswari Aravindalochanan[1], PadmaPriya Rajendran[2], Saritha Sendhil[1], Durairaj Natesan[1], Rengaraj Ramasamy[1], Sankar Sambandam[1], Kalpana Balakrishnan[1]

[1] Sri Ramachandra Institute of Higher Education and Research, Environmental Health of Engineering & Faculty of Public Health, Chennai, India; [2] St. Johns Research Institute, Bengaluru, India

Objectives

To assess the Liquefied petroleum gas (LPG) consumption among the rural households of south India who were part of a multicountry randomized controlled free LPG trial using their LPG refilling pattern between free refill phase (FRP) and post-free refill phases (PFRP) and to perform the price elasticity analyses between the phases

Methods

This was a follow-up study of the households from India sites of household air pollution intervention network (HAPIN). All these households had the free LPG stove, connection, and refills for being part of the trial during study period (intervention arm) or after the study period (control arm) as control compensation. The LPG refills availed by these households PFRP for one year were collected from their respective LPG agencies. The change in the LPG price for PFRP period was also documented to perform Arc price elasticity analyses between FRP and PFRP, and also comparing within PFRP as two periods- PFRP-1 (0 to6 months) and PFRP-2 (7 to 12 months) from FPR.

Results

The FRP ranged between May-2018 and Jul-2021 for the intervention houses and between Oct-2019 and Oct-2022 for the control houses, respectively and that of PFRP period was between May-2021 and Jul-2022 for intervention and between Jun-2022 and Oct-2023 for control houses, respectively. The average monthly LPG consumption during FRP, PFRP-1 and PFRP-2 of the intervention households were 0.66, 0.09, and 0.06, respectively and that of control households were 0.86, 0.09 and 0.06, respectively. Overall, a price inelasticity was observed between all the compared intervals except for the control group in one of the Indian sites between FPR-1 and FRP-2.

Conclusion

A decreasing trend in LPG consumption with price inelasticity pattern can be due to LPG being an essential commodity and increased awareness on the health benefits of the cleaner fuel use among these trial beneficiaries.

P36-30 | Indoor radon and COPD risk and severity in never-smokers. A case-control study in a radon priority area with individual measurements.

Alberto Ruano Raviña[1], Carlota Rodriguez García[2], Cristina Represas Represas[4], Rafael Golpe[3], Cristina Candal-Pedreira[1], Guadalupe García[1], Monica Pérez-Ríos[1], Lucía Martín-Gisbert[1]

[1] University of Santiago de Compostela, Preventive Medicine and Public Health, Santiago de Compostela, Spain; [2] Hospital Universitario de Ferrol, Neumology, Ferrol, Spain; [3] Hospital Universitario Lucus Augusti, Neumology, Lugo, Spain; [4] Hospital Universitario Álvaro Cunqueiro, Neumology, Vigo, Spain

Objective: To explore whether indoor radon exposure is associated with the risk of COPD or its severity in never-smokers.

Material and methods: Multicentric, hospital-based, case-control study, located in Northwestern Spain. Cases had a diagnosis of COPD, confirmed by spirometry, and most of them were incident cases. Controls were recruited from the same hospitals as cases. Controls had a sex and age frequency matching according to cases. All participants were never-smokers, defined as an individual who smoked less than 100 cigarettes in their lifetime or less than one daily cigarette during six months. All participants placed an alpha-track radon detector at home for three months (www.radon.gal). A logistic regression was fitted using case-control status as the dependent variable and indoor radon exposure as the main independent variable. We performed a sub-analysis considering participants who lived in the same dwelling for 20, 30 or more than 40 years, respectively. We also correlated radon exposure with COPD severity (GOLD status) and number of hospitalizations in the last 3 years.

Results: 300 cases and 462 controls. Indoor radon exposure was similar in cases and controls, but radon exposure had a marginally significant association with COPD when duration of residence in the same dwelling was >40 years, with an OR of 2.24 (95% CI 0.93-5.39) for those with the highest (>300 Bq/m^3) vs the lowest radon exposure (<100 Bq/m^3). Average radon exposure was 107, 114, 148 and 333 Bq/m^3 for GOLD status I, II, III and IV, respectively (p< 0,05). Patients with the highest number of hospitalizations in the last 3 years had also higher radon concentrations.

Conclusion: There is the suggestion of an association with COPD for those with higher exposures who lived for longer periods in the same dwelling. In cases, higher radon exposure was associated with more severe airflow limitations.

P36-31 | Effect of household environments on allergic diseases in preschoolers – A cross-sectional study in Greater Taipei

Hsing J. Chao[1], Ssu-Yin Chen[1], Ya-Wen Yang[1], Ming-Lun Zou[1], Yi Hua Chen[1], Ling-Chu Chien[1]

[1] Taipei Medical University, School of Public Health, Taipei, Taiwan; [2] Taipei Medical University, School of Public Health, Taipei, Taiwan

The prevalence of children's allergic diseases has been increasing for the past decades. In addition to genetics, environmental, social, and lifestyle factors are crucial determinants of the development and exacerbation of allergic diseases. We conducted a cross-sectional study in Greater Taipei to assess the relationships between multiple environmental factors and allergic diseases in preschoolers in Greater Taipei. A total of 120 preschool children were recruited. The participants' sociodemographic data, lifestyle factors, residential characteristics, and allergic diseases were collected using structured questionnaires. Indoor and outdoor household pollutants were measured in living rooms, children's bedrooms, and outdoor/balconies, including particulates, formaldehyde, environmental microbiome, and dust mite allergens. Land use characteristics (e.g., roads, airports, greenness) surrounding children's homes were evaluated using buffer analysis in Geographic Information System and land use regressions. We also collected children's nasal and fecal samples to analyze microbiota. The dominant gut bacterial genera were *Bacteroides* and *Bifidobacterium*. The main bacterial genera in the dust on children's beds were *Staphylococcus* and *Streptococcus*, while the dominant fungi were *Aspergillus* and *Malassezia*. The prevalent nostril bacteria were *Corynebacterium* and *Moraxella*. The prevalent nostril fungi were *Aspergillus* and *Aureobasidium*. According to the multivariable regression analyses, increased airborne fungal spores and living near schools or gas stations were correlated with asthma in children; outdoor O_3 significantly increased the risk of allergic rhinitis; dustborne fungal spores in the child's bed, smoking at home and the presence of gas stations near the home were significantly associated with atopic dermatitis. Our study results indicate that residential environments are closely related to allergic diseases in preschoolers. Regular household cleaning, proper indoor ventilation, and judicious urban planning are crucial to children's health.

P37 | Beyond the Usual Suspects in Exposure Science and Environmental Epidemiology

P37-01 | Heavy Metal Leaching in Colorado Market Cannabis Vape Devices

Francesca S. Macaluso[1], Katherine A. James[1], Stephen Goldman[3], Mike Van Dyke[2]

[1] University of Colorado Anschutz Medical Campus, Aurora, USA; [2] Colorado State University, Fort Collins, USA; [3] Kaycha Labs, Denver, USA

Objectives: There is a paucity of research into the potential health risks associated with cannabis-based vaping products. Heavy metal contaminants are of particular concern due to the cannabis plant's tendency to hyperaccumulate metals from the growing environment. Additionally, there is increasing evidence that the internal heating components of vaping devices leach additional heavy metals into vape oils, increasing overall heavy metal exposure from use. The goal of this study was to assess how factors such as vape device design and age, cannabis oil terpene content, and storage conditions may impact heavy metal leaching.
Materials and Methods: Several Colorado market cannabis vape devices from low, medium, and high price points were selected for testing based on popularity. Vape devices were filled with lab-generated cannabis vape oil concentrates and then stored under various scenarios that combined different vape oil terpene contents, new/used devices, lower/higher temperatures, and shorter/longer storage times. Post-storage samples were analyzed for 20 heavy metals using ICP-MS.
Results: Preliminary analyses revealed some heavy metals that were not present at detectable levels in cannabis vape oil cartridges prior to storage in post-storage oils. Lead (Pb) was one of the primary metals of contamination post-storage. There was considerable variability in the levels of metals detected in post-storage cannabis vape oils for some of the tested devices. Even mid-range quality vaping devices leached heavy metals under various storage conditions. Final results within this presentation will include an expanded analysis of additional heavy metals of concern under different storage scenarios and comparisons with this research group's prior work characterizing the distribution of 20 different heavy metals across a random sample of Colorado market cannabis vape oil concentrates.
Conclusion: Pre-market testing of heavy metals levels in cannabis vape oil concentrates may not sufficiently protect Colorado vape product consumers from heavy metals-related health impacts.

P37-02 | Ubiquitous, critical, and invisible: The role of maintenance and repair in sustaining health interventions

Naomi Chang, Tami Bond, Marilee Long

Colorado State University, Fort Collin, USA

Objective:
Despite studies demonstrating the efficacy of interventions in reducing exposures to pollutants, sustained use remains a challenge. Ignoring causes of cessation may allow intervention promoters to claim unrealistic health benefits and limit opportunities to address long-term adoption challenges. An important factor in cessation is the need for maintenance and repair (M&R), which often remain invisible to practitioners despite their ubiquity and importance for sustained effectiveness. Our objective is to provide recommendations for researchers to enable users to achieve sustained use.

Materials and Methods:
We reviewed literature across robotics, electronics, infrastructure, communications, and sustainability and synthesize definitions of M&R to conceptualize their relationship to sustained use. We identify factors that affect sustained use and draw from other behavioral theories and frameworks to extend the Technology Acceptance Model (TAM) to include sustained use.
Results:
We identify factors that affect M&R, which broadly fall under motivation and accessibility. The perceived necessity of the intervention to daily life and user self-efficacy affect motivation, while the availability and affordability of replacement parts/services influence accessiblity. We include these factors in the widely-used TAM to extend its application to include sustained use, which is critical to reduce exposures. By applying this model to various health interventions, we also provide recommendations for researchers to include M&R in their study design and implementation.
Conclusion:
The role of M&R in sustained use has been underexplored. By extending the TAM to include sustained use, we offer concrete recommendations to account for M&R in intervention design, implementation, and evaluation. These considerations are especially relevant to health interventions in extending their lifetime, thus reducing exposure to harmful pollutants.
Funding:
This research was supported by NSF Award #2153042, “LEAP-HI: Systemic, Anthro-centric Packages for Protecting Households In Realistic Exposure Situations (SAPPHIRES)” and the Walter Scott Jr. College of Engineering.

P37-03 | Long-term residential extremely low-frequency magnetic field exposure and mortality from neurodegenerative diseases: an 18-year nationwide cohort study in Switzerland

__Nekane Sandoval-Diez__[1,2], Anke Huss[3], Martin Röösli[1,2], Danielle Vienneau[1,2]

[1] Swiss Tropical and Public Health Institute, Epidemiology and Public Health, Allschwil, Switzerland; [2] University of Basel, Basel, Switzerland; [3] Utrecht University, Institute for Risk Assessment Sciences (IRAS), Utrecht, Netherlands

Objectives: Extremely low-frequency magnetic field (ELF-MF) exposure has been hypothesised to increase the risk of neurodegenerative diseases, but epidemiological evidence remains inconclusive. This study investigates the relationship between long-term ELF-MF exposure and mortality from neurodegenerative diseases in adults living in Switzerland.
Material and methods: We conducted a nationwide cohort study using the Swiss National Cohort (SNC), covering an 18-year follow-up (2001–2018) and including 3.5 million individuals. ELF-MF exposure was estimated using proximity models based on residential distance to high-voltage power and railway lines. Residential histories were used to account for mobility during the follow-up, and cumulative ELF-MF exposure was calculated in μT-years. Mortality outcomes included Alzheimer’s disease, other dementias (including vascular dementia and dementia in other diseases), amyotrophic lateral sclerosis (ALS), Parkinson’s disease, and multiple sclerosis. Associations were assessed using Cox proportional hazards models with age as the underlying time scale, stratified by sex, and adjusted for demographic, socioeconomic, and environmental factors.

Results: Over follow-up, over 128,000 deaths from neurodegenerative diseases were recorded. Cumulative ELF-MF exposure was low (95th percentile: 0.97 μT-years for power lines, 3.27 μT-years for railway lines). No increase in mortality risk was observed for Alzheimer´s disease, ALS, Parkinson's disease, and multiple sclerosis. A slight increase in risk for other dementias was observed with power line exposure (HR for one μT-year increase: 1.010, 95% CI: 1.002–1.019), though residual confounding cannot be ruled out.
Conclusions: In this large, nationwide population-based cohort study, preliminary analysis found no evidence linking long-term ELF-MF exposure to neurodegenerative disease mortality. The findings align with previous studies, which have not established a consistent link between residential ELF-MF and neurodegenerative outcomes.

Funding and Acknowledgements: NLEON-EMF has received funding from the Swiss Federal Office for the Environment (FOEN) under grant agreement No. 21.0121.PJ/01EAE8F65.

P37-04 | CKD Predictions Using Environmental Factors in South Korea

Jiwoo Park[1], Yejin Kim[1], Whanhee Lee[2], Youngrin Kwag[4], Duk-Hee Kang[6], Eunhee Ha[4,8]

[1] Pusan National University, Department of Information Convergence Engineering, Pusan, South Korea; [2] Pusan National University, School of Biomedical Convergence Engineering, Pusan, South Korea; [3] Ewha Womans University, Department of Environmental Medicine, College of Medicine, Seoul, South Korea; [4] Ewha Womans University, Department of Environmental Medicine, College of Medicine, Seoul, South Korea; [5] Ewha Womans University School of Medicine, Ewha Medical Research Center, Division of Nephrology, Department of Internal Medicine, Seoul, South Korea; [6] Ewha Womans University School of Medicine, Ewha Medical Research Center, Division of Nephrology, Department of Internal Medicine, Seoul, South Korea; [7] Ewha Womans University, Graduate Program in System Health Science and Engineering, College of Medicine, Seoul, South Korea; [8] Ewha Womans University, Graduate Program in System Health Science and Engineering, College of Medicine, Seoul, South Korea; [9] Ewha Womans University College of Medicine, Institute of Ewha-SCL for Environmental Health (IESEH), Seoul, South Korea; [10] Ewha Womans University College of Medicine, Institute of Ewha-SCL for Environmental Health (IESEH), Seoul, South Korea; [11] Ewha Womans University School of Medicine and Ewha Medical Research Institute, Department of Medical Science, Seoul, South Korea; [12] Ewha Womans University School of Medicine and Ewha Medical Research Institute, Department of Medical Science, Seoul, South Korea

Introduction
In the Kidney research area, CKD, Chronic Kidney Disease, is an important disease, and recent studies are uncovering the environmental factors' role in CKD. Therefore, we've constructed the study about quantifying the environmental factors' effect on CKD by predicting CKD.
Method
We've collected a dataset about CKD using the National Health Insurance Service National Sample Cohort (NHIS-NSC) in South Korea.
We've gathered the Health Examination Data from the same data source. And other data about regional factors are gathered using the Community Health Survey managed by the Korea Center for Disease Control and Prevention. Weather factors are collected using the Korea Meteorological Administration for temperature, related humidity, and modeling data using station data for O3, PM2.5.
Extreme Gradient Boosting, and Light Gradient-Boosting model was used for prediction.
Result
Using environmental factors, the prediction score showed more improvement and showed its effects on the CKD occurrence. Specificity showed around 0.90 and the Area Under Curve showed about 0.70 for both modeling

methods. Feature importance showed highly relying on eGFR(estimated glomerular filtration rate), but also regional factors showed the 0.40 importance to the CKD prediction. Weathers also showed the importance of 0.35.

P37-05 | Relationship between Environmental factor and Injuries in South Korea

Jiwoo Park[1], Jieun Oh[2], Whanhee Lee[3]

[1] Pusan National University, Department of Information Convergence Engineering, Pusan, South Korea; [2] Seoul National University, Department of Public Health Sciences, Graduate School of Public Health, Seoul, South Korea; [3] Pusan National University, School of Biomedical Convergence Engineering, Pusan, South Korea

Both climate change and intentional injuries are recognized as crucial factors that affect human health. Previous studies revealed the association between ambient temperatures and air pollution, and deaths due to intentional injuries, but mostly about the consequences of severe events. Our study aimed to examine the relationship between violence incidence, including mild and moderate cases, and environmental factors.
We collected violence cases nationally of 9,742 cases from the Korea National Hospital Discharge In-depth Injury Survey from 2005 to 2019. We performed a two-stage analysis with a time-stratified case-crossover design to evaluate the associations of outdoor temperatures with violence using historical data. Based on the historical period, we projected the excess violence attributable to ambient temperatures in the future (2020-2099) using the three shared socioeconomic pathway (SSP) scenarios. With air pollutant, we've collected 2,867 violence cases from 2015 to 2019 in the same data source. We evaluated the national association between short-term exposure to $PM_{2.5}$ and violence cases with a time-stratified case-crossover design.
We found positive linear associations between outdoor violence and environmental factors. The excess risk of violence attributable to temperatures in the historical period was around 3.5%, and it was also estimated to increase in the future (up to 7.92%). Short-term exposure to $PM_{2.5}$ and an increased risk of violence showed an estimated odd ratio per 10 μg/m^3 of $PM_{2.5}$ of 1.07 with 95% CI: 1.02–1.12 with estimated excess fraction of 14.53% (95% CI: 4.54%–22.92%).
The study shows the relationship between temperature and $PM_{2.5}$ is positively linear. Especially for the temperature, its relationship gets higher as the time passes. Our findings might provide informative evidence for relevant action plans against climate change and intentional injuries, as well as evidence for the need to establish stricter air quality guidelines to reduce the hazardous impacts of $PM_{2.5}$ in South Korea.

P37-06 | Assessment of quality of life and its determinants in type-2 diabetes patients using the Asian Diabetes Quality of Life - AsianDQOL instrument in Vietnam

Dat Ha Van[4], Hai Nguyen Thanh[5], Elizabeth V Wattenberg[3], Hiroyuki Nakamura[2], **Ngoc Nguyen Thi Minh**[1]

[1] Haiphong University of Medicine and Pharmacy, Dept of Environmental Health/ Faculty of Public Health, Haiphong, Vietnam; [2] Kanazawa University, Department of Environmental and Preventive Medicine, Kanazawa, Japan; [3] University of Minnesota, School of Public Health, Minnesota, USA; [4] Cam Giang district health center, Haiduong, Vietnam; [5] Haiphong University of Medicine and Pharmacy, Dept of Occupational Health/Faculty of Public Health, Haiphong, Vietnam

Objective: Quality of life (QoL) is a good indicator of lifespan, especially for individuals who are suffering from a particular illness, such as diabetes mellitus (DM), which is one of the most significant non-communicable diseases (NCDs) globally. This study aimed to assess QoL and identify factors associated with a good QoL among type-2 DM patients (T2DM) in Northern Vietnam.

Material and Methods: A cross-sectional study was carried out among individuals aged at or above 18 years with T2DM, who attending a rural district health center in the Hai Duong province, Northern Vietnam. A pretested structured questionnaire including the 21-item Asian Diabetes Quality of Life (AsianDQOL) questionnaire in 5 dimensions was used for face-to-face interviews. T-test, One-way ANOVA, Wilcoxon-Mann Whitney and Kruskal-Wallis tests were used to compare mean QoL scores at a significance level of $\alpha = 0.05$.

Results: Among 295 enrolled participants: 63.4% were female, 71.9% were aged ≥ 60 years, 56.2% graduated from secondary school and under, 68.7% had an annual income ≤ 2,500 USD, and 5.1% had no income. The overall QoL average score (± SD) was 50.51 ± 3.58. The highest and lowest domain scores were in the finance and the relationship domain, respectively. More than 90% of patients had a poor QOL, and only approximately 7% had a good QoL. Some variables that were seen significantly associated with poor QOL scores included aged over 60 years, female, single status, living alone, having co-morbidities, fasting blood glucose not achieving treatment target, and having complications of diabetes ($p< 0.05$).**Conclusion:** Most of the T2DM patients had a poor average score of overall quality of life. Health authorities and health workers should improve counseling and communication measures and educate patients about health, self-monitor, and early-detection to prevent complications, especially among the elderly, females, those who have comorbidities with diabetes.

P37-07 | Phthalate exposure in Chinese homes and its contribution to total intake

Yuexia Sun[1], Yeganeh Ataei[1], Gabriel Beko[2], Yuxuan Zhao[1]

[1] Tianjin University, Tianjin, China; [2] Technical University of Denmark, Lygnby, Denmark

Phthalate exposure may affect human health, with particular concerns for children. We measured phthalate metabolite concentrations in the urine of 324 Chinese children aged 0-8 years, and parent phthalate mass fractions in settled dust collected from the children's bedrooms. Concentrations of phthalates in the air (particulate and gas phase) were calculated from phthalate levels in the dust using equilibrium partitioning. The daily intakes of four phthalates, namely di-2-ethylhexyl phthalate (DEHP), diethyl phthalate (DEP), di-n-butyl phthalate (DnBP), and diisobutyl phthalate (DiBP), were estimated from concentrations of urine metabolites. The intakes attributable to residential indoor environmental exposure via air and dust were calculated for three exposure routes, namely dermal absorption, inhalation and ingestion. The primary residential indoor intake routes for lower molecular weight phthalates DnBP, DiBP and DEP were dermal absorption from air (median 0.12, 0.09, and 0.01 μg/d/kg-bw, respectively), and inhalation (median 0.13, 0.09, and 0.02 μg/d/kg-bw, respectively). For DEHP, a high molecular weight phthalate, dust ingestion was the predominant exposure route (median 0.10 μg/d/kg-bw). The findings emphasize the significant contribution of residential indoor intake from air and dust to total DnBP and DiBP exposure (with median values of 40.2% and 19.6%, respectively) and possibly to the consequent health effects.

P37-08 | Long-term field evaluation of a low-cost electrochemical formaldehyde sensor

Zheyuan Pei, William F. Kelly, Kerry E. Kelly

University of Utah, Department of Chemical Engineering, Salt Lake City, USA

Formaldehyde is the biggest driver of cancer risk in the United States among hazardous air pollutants. The Occupational Safety and Health Administration sets a permissible exposure limit for formaldehyde at 750 parts per billion (ppb) averaged over an 8-hour workday. However, indoor formaldehyde measurements are rarely collected because they are generally labor-intensive and/or costly. In recent years, numerous studies have highlighted the use of low-cost sensors for air quality monitoring. These sensors can provide a more affordable alternative as well as real-time measurements. We systematically evaluated the laboratory performance of a low-cost electrochemical formaldehyde sensor (Sensirion SFA30) over a formaldehyde concentration range of 0-326 ppb. The sensors exhibited good linearity of response, low limit of detection and good sensor accuracy to formaldehyde. Moreover, these sensors demonstrated limited (less than 5%) cross sensitivity to methanol and isopropyl alcohol. In this study, we deployed five SFA30 sensors in the anatomy laboratories on the University of Utah campus from February 2024 to November 2024. Although the median formaldehyde signal of each sensor was estimated to be below 1 ppb, we observed hourly signal increases exceeding 250-fold during dissection activities in the anatomy laboratories. We also compared the sensor formaldehyde signals with passive sampler measurements to develop an appropriate calibration relationship and estimate formaldehyde concentrations in these indoor environments. Our results indicate that the SFA30 sensors can be a useful tool for indoor formaldehyde measurements if properly calibrated.

P37-09 | Rebuilding Trust in Human Biomonitoring: Lessons from Slovenia's Post-COVID Experience

Milena Horvat[1,2], Janja Snoj Tratnik[1], Darja Mazej[1], David Kocman[1], Ingrid Falnoga[1], Marta Jagodic Hudobivnik[1], Polona Klemenčič[1], Janko Stankič[1,2], Adna Alilovič[1,2], Neža Palir[1,2]

[1] Jožef Stefan Institute, Department of Environmental Sciences, Ljubljana, Slovenia; [2] International Posgraduate School Jožef Stefan, Ljubljana, Slovenia

Objective: This abstract presents an overview of the Slovenian national human biomonitoring (HBM) programme, legally defined under the Act on Chemicals, with a focus on challenges encountered after the COVID-19 pandemic and lessons learned for improving future implementation.

Material and methods: Slovenia has conducted two national HBM cycles (2008–2014 and 2018–2024), targeting different population groups to assess exposure to selected environmental pollutants. These efforts relied on a combination of questionnaire-based surveys and chemical analysis of biological samples (e.g. blood, urine, hair). While the earlier cycle progressed steadily, the second cycle—launched before the pandemic—faced major disruptions due to COVID-19 and struggled with participant recruitment after restrictions were lifted. Low post-pandemic participation particularly affected the socio-economic representativeness and extended the duration of fieldwork.

Results: Post-pandemic hesitancy, reduced public trust in research activities, and increased reluctance to engage in health studies were identified as key factors affecting participation. Feedback from the field highlighted the need for stronger communication strategies and a more participant-centred approach. Transparent engagement with communities, including clear messaging about the purpose and benefits of HBM, was seen as critical. Integrating HBM activities into regular preventive healthcare services could also enhance future participation and resilience against disruptions. Institutional flexibility and cross-sectoral collaboration proved essential for maintaining momentum.

Conclusion: The Slovenian experience underscores the importance of adapting human biomonitoring programmes to societal changes in the post-pandemic context. A participatory, trust-building approach and improved integration into health systems are key to strengthening the relevance and resilience of HBM.

Acknowledgments: The programme is supported by the Ministry of Health of the Republic of Slovenia and implemented by the Jožef Stefan Institute in collaboration with the National Institute of Public Health and other partners.

P37-12 | Conducting scoping reviews for environmental epidemiology: Lessons learned from examining the health impacts of September 11th

Alyson B. Harding[1], Joachim O. Hero[2], Thomas W. Concannon[2]

[1] RAND, Pittsburgh, USA; [2] RAND, Boston, USA

Objective: Scoping reviews examine the nature and extent of literature on a given topic, and, as a result involve a heterogeneous array of literature. Environmental epidemiology topics encompass diverse study designs, objectives,

and disciplines, posing challenges for scoping reviews. This presentation will discuss lessons learned from an ongoing scoping review of literature evaluating the health impacts of the September 11th attacks.
Materials and methods: In collaboration with the World Trade Center Health Program, our team is conducting a scoping review on the health consequences of the September 11th attacks. The review currently encompasses 1,186 papers, representing a highly heterogeneous collection of peer-reviewed articles.
Results: A primary challenge of scoping reviews is developing a data collection instrument without full knowledge of the articles and data to be extracted. The instrument may need to be updated after data collection has begun. We recommend beginning with a broad data collection instrument and implementing a process to assess the quality of extracted data and determine if changes to the data collection instrument are required.
The data collection instrument should include multiple frameworks to capture various dimensions of the literature and should include free text response options to allow for flexibility. Additionally, the instrument should be designed to accommodate the fact that not all questions will be applicable to every article. After data collection has begun, researchers should assess data for missingness and examine free text responses. The data collection instrument should be evaluated to ensure that questions are appropriate for a scoping review and are returning high quality data.
Conclusion: When conducting a scoping review, the data collection instrument must accommodate a wide range of references. We recommend intentionally examining extracted data and the data collection instrument, and updating the instrument as needed, to ensure quality data are collected.

P37-13 | Improving efficiency and sustainability in science.

Lorena Tran[1], Alexandra Loren[2], Shakayla Lamer[2], Katarina Kulhankova[2], Mitchell Riley[1], Linda Powers[1], Ian M. Thornell[1], Ashley Cooney[2], Sarah Ersnt[1], **Emma M. Stapleton**[1]

[1] University of Iowa, Internal Medicine, Division of Pulmonary, Critical Care and Occupational Medicine, Iowa City, USA; [2] University of Iowa, Stead Family Department of Pediatrics, Iowa City, USA

Laboratories use more energy than any other sector aside from data centers—10x more than office buildings. Resource use is intensive; for example, labs account for ~2% of global plastic waste, and biohazardous waste is shipped out of state and incinerated or autoclaved. Because science seeks to solve complex problems, our Lung Biology Group formed a taskforce to improve sustainability in energy use and the waste-stream. Methods: Energy demand was measured (Suraielec watt monitors). For 220V devices, data were interpolated from comparable equipment and publicly available data. Reduced energy use was encouraged via device power-downs and removal of redundant, inefficient devices. Cold storage audits included removal of inefficient units. Temperatures were increased on ultralow temperature (ULT) freezers from -80°C to -70°C. Biohazardous waste use was audited over 1-year. A targeted biohazardous waste experiment measured baseline, intervention, and post-intervention (4-weeks each) waste in a common-use room. Outcomes included photo audit, bin weight and number, and user feedback. Cultural changes were invited through meeting presentations, weekly newsletters, signage, and “turn-off” stickers and positive incentives provided. Results: Annual energy savings were ~61,000 kWh (~$8,000), roughly the equivalent of 6 US homes’ energy usage. A pipette tip-box recycling pilot saved the equivalent of 200 lbs CO_2 emissions. The rate of biohazard bin use floorwide is trending down ($p=0.09$). Our 1-month biohazardous waste intervention significantly reduced waste (63 lbs, $p<0.001$) and the number of bins used ($n=3$). If scaled University-wide this would save ~$800,000/year. Supply-side improvements transpired at a systems-level by coordination with the local biochemistry store and supply representative. Conclusions: Easy measures, such as increasing the

temperature of ULT freezers from -80°C to -70°C and turning off biosafety cabinets can save energy and indirect costs. Supporting a culture of sustainability in science requires effort but has important financial co-benefits.

P37-14 | The impact of land cover and land use change on coccidioidomycosis incidence in California: a population-based longitudinal study

Alexandra K. Heaney[1], Isabel J. Jones[2], Simon K. Camponuri[3], Jennifer R. Head[4,5], Nicole Keeney[3], Erika Lee[3], Whitney Mgbara[3], Amanda K. Weaver[3], Philip A. Collender[3], Robert Wagner[6], Bo Zhou[7], Gail Sondermeyer-Cooksey[8], Seema Jain[8], Duc J. Vugia[8], John Balmes[3], Ellen Eisen[3], Dennis Lettenmaier[7], Gregory Okin[7], Adeyemi Adebiyi[9], John Taylor[6], Abinash Bhattachan[10], Justin V. Remais[3]

[1] University of California San Diego, Herbert Wertheim School of Public Health and Human Longevity Science, San Diego, USA; [2] US Environmental Protection Agency, San Francisco, USA; [3] University of California Berkeley, Division of Environmental Health Sciences, Berkeley, USA; [4] University of Michigan, Department of Epidemiology, Ann Arbor, USA; [5] University of Michigan, Institute for Global Change, Ann Arbor, USA; [6] Univerity of California Berkeley, Department of Plant and Microbial Biology, Berkeley, USA; [7] University of California Los Angeles, Department of Geography, Los Angeles, USA; [8] Califoria Department of Public Health, Infectious Disease Branch, Richmond, USA; [9] University of California Merced, Department of Life and Environmental Sciences, Merced, USA; [10] Texas Tech University, Department of Geosciences, Lubbock, USA

Coccidioidomycosis is an emerging infectious disease caused by inhalation of spores from soil-dwelling fungi of the *Coccidioides* genus. The San Joaquin Valley, a highly productive agricultural region in California, has both a high burden of coccidioidomycosis and is in transition: large regions of irrigated arable land have been or will likely be permanently fallowed (i.e., no longer cultivated) in the next decade to conserve groundwater resources. Such land cover changes may influence disease risk through hypothesized links to sporegrowth in soils and dispersal via fugitive dust emissions. To estimate associations between coccidioidomycosis incidence and changes in natural, agricultural, and fallow land covers in California, we analyzed surveillance data on 67,735 confirmed coccidioidomycosis cases reported to the California Department of Public Health between April 2008 and March 2021 using generalized mixed effects models. Natural land covers were positively associated with coccidioidomycosis incidence (shrubland: incidence rate ratio (IRR) per standard deviation (SD) increase in area = 1.62, 95% CI:1.36,1.92; barren land: IRR = 1.34 [1.20,1.49]; grassland: IRR = 1.18 [1.07,1.30]). Some agricultural land covers were positively associated with incidence (e.g., grain or hay (IRR = 1.09 [1.06,1.12]), field crops (IRR = 1.09 [1.07,1.12])), while others were negatively associated (e.g., deciduous fruits and nuts (IRR = 0.86 [0.82,0.90]), truck crops or berries (IRR = 0.96 [0.93,0.99])). Notably, fallow land area was positively associated with incidence (IRR = 1.03 [1.02,1.05]), especially when land had mixed agricultural land use in the three years prior to fallowing (IRR = 1.08 [1.06,1.10]). Although more research is needed to elucidate the mechanisms that underpin our findings, these results suggest that agricultural and natural land cover change, including fallowing of cultivated lands, could impact coccidioidomycosis risk in endemic regions. Expected future fallowing of agricultural land may thus exacerbate growing coccidioidomycosis risks in California.

P37-15 | Education-based oral health promotion interventions during pregnancy: A systematic review

Nabhira Aftabi Binte Isalam

Bangabandhu Sheikh Mujib Medical University (bsmmu, Preventive and Social Medicine, Dhaka, Bangladesh

Background
Physiological changes during pregnancy increase the risk of oral diseases such as gingivitis, periodontitis, and dental caries. If left untreated, these conditions can lead to adverse pregnancy outcomes. This systematic review evaluates the effectiveness of education-based oral health promotion interventions during pregnancy.Objectives
To evaluate the impact of education-based oral health promotion interventions during pregnancy and to identify existing gaps in this area of research.Methods
This systematic review focused on randomized controlled trials (RCTs) and non-randomized studies of interventions.Article searches were conducted in MEDLINE, Cochrane Library, Embase, Scopus, and CINAHL, covering studies published from 2014 to January 14, 2024. The review included studies evaluating education-based oral health promotion interventions among pregnant women aged 15–45 years. Non-educational interventions, secondary research, case reports, non-peer-reviewed papers, and studies with irrelevant or inadequate data were excluded.The risk of bias was assessed using Cochrane Risk of Bias 2.0 for RCTs and the McMaster tool for non-RCTs. Data were synthesized narratively.Results
Primarily, 2120 studies were identified, but after rigorous screening, only 14 articles met the inclusion criteria. The interventions studied included structured education (e.g., booklets, text messages, face-to-face sessions) and group discussions. Outcomes assessed included improved oral hygiene practices, reduced plaque levels, and enhanced oral health knowledge. Some concerns were noted regarding randomization processes and outcome measurements, particularly in non-RCTs. Overall, education-based interventions significantly improved oral health behaviours, such as brushing duration and oral hygiene practices.Conclusion
This systematic review confirms that education-based oral health interventions during pregnancy can significantly improve oral health knowledge, attitudes, and behaviours.

P38 | Environmental Exposures and Respiratory and Allergic Health

P38-01 | From air to hair: Biomonitoring of environmental metal levels and respiratory outcomes in an opencast coal mining community in India

Kamlika Gupta[1,2], Victor W.-C. Chang[1], Mohan Yellishetty[1], Harish C. Phuleria[2]

[1] Monash University, Civil Engineering, Melbourne, Australia; [2] Indian Institute of Technology-Bombay, Environmental Science and Engineering Department, Mumbai, India; [3] Indian Institute of Technology-Bombay, Centre for Climate Studies, Mumbai, India

Background

Communities around opencast coal mines are more vulnerable to fine particulate matter ($PM_{2.5}$) linked trace metals exposures. Biomonitoring of human hair and nail can be instrumental in studying the toxicological effects of such trace metals, due to ease in sampling and their non-invasive nature.

Method

Hair samples (N=40) were collected from consenting adult male and females (18-65 years) from communities around an opencast coal mining region in Eastern Maharashtra, India. Using ICPMS, metals such as Lithium, Chromium, Magnesium, Calcium, Manganese, Iron, Cobalt, Nickel, Copper, Zinc, Arsenic, Strontium, Selenium Cadmium, Antimony, Arsenic and Lead were quantified in biosamples and $PM_{2.5}$ after acid-assisted microwave digestion. Structured questionnaire was used to collect information on demography, occupation, and doctor diagnosed respiratory outcomes.

Results and conclusion

Out of the trace metals studied, Cu emerged as the dominant metal in biosamples (15.8 ± 9.6 ng/g), followed by Sr, Pb, Ni and Cr which showed 5-10 folds higher concentration than other metals ($p<0.05$). Based on occupation, higher Pb (36.23%), Cd (10.41%) and Ni (6.54%) was observed in truck driver sand crane operators when compared to street vendors and housewives indicating higher exposure to exhaust origin metals. Further, Cr (20 ± 2.3 μg/m3), Ni (5.6 ± 0.3 μg/m3), Cd (7.2 ± 1.5 μg/m3), As (2.8 ± 1.6 μg/m3), and Pb (3.7 ± 0.9 μg/m3) emerged as important carcinogenic metals across the sites likely attributable to coal combustion and vehicular exhaust. The current study underscores the need of considering both environmental and tissue metal levels to safeguard public health. It identifies, for the first time, the enrichment of PM bound metals in biosamples of coal mining community in India while also examining the relationship between metal levels and doctor diagnosed respiratory outcomes.

P38-02 | Effect of Hydration Levels on Respiratory Aerosol Emissions

Mahender Singh Rawat[1], Dinushani Senarathna[2], Byron D. Erath[3], Sumona Mondal[4], Sarah K. Sirsat[5], Andrea R. Ferro[1]

[1] Clarkson University, Civil and Environmental Engineering, Potsdam, USA; [2] SUNY Oswego, Mathematics, Oswego, USA; [3] Rochester Institute of Technology, Mechanical Engineering, Rochester, USA; [4] Clarkson University, Mathematics, Potsdam, USA; [5] SUNY Potsdam, Biology, Potsdam, USA

Previous literature indicates that emission rates of respiratory aerosols from speaking, shouting, singing, coughing, and breathing vary widely due to several factors, including loudness, frequency, and age. However, these factors alone do not fully account for variations in emission rates. Hydration levels may also significantly influence the rate of particle production during sustained vocalization activities, although the magnitude of the effect of hydration has not been quantified. This study hypothesizes that well-hydrated (Hy) individuals produce more respiratory droplets during vocalizations than dehydrated (Dhy) individuals, as dehydration reduces saliva and respiratory tract lining fluid production and alters the rheology of these fluids.

To evaluate this hypothesis, we conducted respiratory aerosol emission experiments with participants in both well-hydrated and dehydrated conditions. Participants phonated into a funnel connected to an aerodynamic particle sizer (APS) to measure aerosol emission rates and size distribution. Additionally, participants phonated into a well-sealed, half mask connected to an iWorx (Dover, NH) TA Model 220 spirometer to quantify expiratory flow rates.

The vocal exercises performed included the open vowel /a/, forced breathing at a similar flow rate as the open vowel /a/, running speech (Rainbow passage), and normal breathing, conducted in random order.

Our preliminary results, based on a limited sample size of nine participants (five females and four males), indicate that hydrated participants emitted 15% more particles than dehydrated participants during vocal activities, both in terms of particle number emission rate and particle number concentration for particles with diameters from 0.5 to 20 microns in size.

This study is ongoing, and we are currently recruiting more participants to investigate the relationship between hydration and aerosol emission rates during vocalization.

P38-03 | Childhood Pesticide Exposure and Lung Function in Mexico City: Findings from the PROGRESS Cohort

Cecilia S. Alcala[1], Hector Lamadrid-Figueroa[2], Marcela Tamayo-Ortiz[3], Adriana Mercado Garcia[4], Elena Colicino[1], Lisa Peterson[5], Kitrina Barry[6], Carin Huset[6], Syam Andra[1], Jamil M. Lane[1], Martha M. Tellez-Rojo[4], Robert O. Wright[1,8], Rosalind J. Wright[1,9], Kecia N. Carroll[1,7], Maria José Rosa[1]

[1] Icahn School of Medicine at Mount Sinai, Department of Environmental Medicine, New York, USA; [2] National Institute of Public Health (INSP), Center for Population Health Research, Cuernavaca, Mexico; [3] Columbia University Mailman School of Public Health, Department of Environmental Health Sciences, New York, USA; [4] National Institute of Public Health, Center for Nutrition and Health Research, Cuernavaca, Mexico; [5] University of Minnesota Masonic Cancer Center, Minneapolis, USA; [6] Minnesota Department of Health, St. Paul, USA; [7] Icahn School of Medicine at Mount Sinai, Jack and Lucy Department of Pediatrics, New York, USA; [8] Icahn School of Medicine at Mount Sinai, Institute for Exposomic Research, New York, USA; [9] Icahn School of Medicine at Mount Sinai, Department of Public Health, New York, USA

Objective: Childhood exposure to pesticides may impact lung function, yet research on this association remains limited, particularly among children in urban settings. This analysis examines the association between childhood exposure to organophosphate and pyrethroid pesticides and lung function. Materials and Methods: The study sample included 233 children enrolled in theProgramming Research in Obesity, Growth, Environment, and Social Stressors (PROGRESS) birth cohort. Urinary pesticide metabolite concentrations were measured at8 years of age as biomarkers of pesticide exposure. Lung function was assessed at ages8-11 years, with results standardized as age-, height-, and sex-adjusted z-scores. Associations between pesticide exposure and lung function were evaluated using generalized linear models, adjusting for key covariates, includingurinary creatinine, maternal education, maternal smoking, and child sex. Potential effect modification by child sex was examined through inclusion of product terms and stratification. Results: Higher urinary concentrations of 3-phenoxybenzoic acid (PBA), a pyrethroid metabolite, were associated with lower forced expiratory volume in 1 second (FEV1) z-scores (β: -0.06, 95% CI [-0.11, -0.01]). Similarly, higher urinary concentrations of trans-DCCA (TDCCA), another pyrethroid metabolite, were linked to lower FEV1 z-scores (β: -0.05, 95% CI [-0.09, -0.01]). Furthermore, increased PBA and TDCCA concentrations were also associated with lower forced expiratory flow (FEF)z-scores (β: -0.048, 95% CI [-0.094, -0.002]; β: -0.042, 95% CI [-0.079, -0.005], respectively).We did not find evidence of effect modification by child sex. Conclusion: Higher childhood pyrethroid metabolite concentrations were associated with lower lung function measures, suggesting a potential adverse impact of exposure to this pesticide on respiratory health. These findings underscore the need for research on environmental exposures impacting of lung function, to inform public health interventions to minimize early-life pesticide exposure.

P38-04 | Modifying Effects Of Inhaled Corticosteroids On Air Pollution-Asthma Associations In Philadelphia: A Retrospective Case-Crossover Analysis Of Electronic Health Records

Lizbeth F. Gomez[1,2], Anneclaire J. De Roos[3,4], Jane E. Clougherty[3,4]

[1] University of Pennsylvania, Perelman School of Medicine, Department of Biostatistics, Epidemiology and Informatics, Philadelphia, USA; [2] University of Pennsylvania, Perelman School of Medicine, Palliative and Advanced Illness (PAIR) Center, Philadelphia, USA; [3] Drexel University, Dornsife School of Public Health, Department of Environmental and Occupational Health, Philadelphia, USA; [4] Drexel University, Dornsife School of Public Health, Urban Health Collaborative, Philadelphia, USA

OBJECTIVE: While air pollution is well known to adversely affect childhood asthma, the potential for asthma medications to ameliorate these impacts is largely unexplored. Further, while inhaled corticosteroids (ICS) yield variable effectiveness in susceptible populations, few studies have examined how ICS prescription may alter associations between air pollutant exposures and pediatric asthma. This study examined potential modification of air pollution effects on asthma exacerbation in children by prescribed inhaled corticosteroids (ICS).

MATERIAL AND METHODS: We conducted a retrospective EHR-based cohort study using electronic health record (EHR) data from Children's Hospital of Philadelphia (CHOP), years 2005-2019. Patients were followed from their first visit within the CHOP health system during infancy (<6 months of age) through their remaining visits during childhood (max age 17.4 years; N=17,929). Daily census-tract-level $PM_{2.5}$ and O_3 were obtained from EPA's Downscaler model, and temperature data from NOAA nClim databases. We used a time-stratified case-crossover design and conditional logistic regression to examine associations between daily pollutant census tract exposures and exacerbation (i.e., healthcare encounter with asthma ICD-10 codes and an oral corticosteroid [OCS] prescription). We tested whether associations were modified by medication use in the six months prior to exacerbation (i.e., no medication, ICS alone, ICS + long-acting beta agonist [LABA]).

RESULTS: A one-unit increase in $PM_{2.5}$ was associated with increased odds of asthma exacerbation among children on ICS alone (OR: 1.011, 95% CI: 1.003, 1.018), whereas there was no association among children receiving ICS+LABA (OR: 1.00, 95% CI: 0.999, 1.008). O_3–asthma associations were not modified by the ICS medication regimens.

CONCLUSION: Our findings suggest a potential protective effect of ICS+LABA against air pollution exposures, indicating that ICS prescription may attenuate some impacts of $PM_{2.5}$ on pediatric asthma exacerbations. Future research should explore biological mechanisms underlying these interactions. Pilot study funded by NIEHS P2CES033428 award.

P38-05 | Asthma status and sex difference in association with short-term fine particulate matter exposure and respiratory health biomarkers

Shiyu Zhang[1], Yan Lin[1], Amy Herring[2], Hisham Abubakar-Waziri[3], Sharon Mumby[3], Junfeng Zhang[1], Susan Murphy[4], Pankaj Bhavsar[3], Ian Adcock[3], Kian Fan Chung[3]

[1] DUKE UNIVERSITY, Duke Global Health Institute & Nicholas School of the Environment, Durham, USA; [2] DUKE UNIVERSITY, Department of Statistical Science, Global Health, Biostatistics & Bioinformatics, Durham, USA; [3] Imperial College London, Airway Disease, National Heart & Lung Institute, London, UK; [4] Duke University Medical Center, Department of Obstetrics and Gynecology, Durham, USA

Background: There is substantial evidence that exposure to $PM_{2.5}$ increases asthma exacerbation risk and may lead to sex-specific differences in respiratory responses that disproportionately affect women. It remains unclear whether asthmatic individuals and whether females are more susceptible to acute respiratory responses.
Methods: A panel study was conducted in London, UK, involving 59 adult participants (36 women and 23 men; 26 with and 33 without asthma). Each participant was assessed twice in summer and twice in winter for airway mechanics, lung function, and biomarkers of systemic inflammation and oxidative stress. Hourly $PM_{2.5}$ exposure during the 48 hours prior to outcome measurement was estimated using an inverse distance weighting (IDW) method. Mixed linear models combined with distributed lag models (DLM), incorporating an interaction of $PM_{2.5}$ with sex or asthma status, were used to estimate the health effect differences by sex and asthma status, respectively.
Results: In asthmatic participants, significant associations were observed between increased airway resistance (R5, R5-R20, and R20) and 0–11 hours $PM_{2.5}$ exposure window, between decreased lung function (FEV_1 and FEF_{25-75}) and 12–31 hours exposure window, and between urinary MDA levels and 32–35 hours exposure window. In healthy participants, however, associations were only observed between R5 and R20 and 12–31 hours exposure window. In women participants, significant associations were found between R20 and 0-7 hour exposure window, between lung function and 8-15 hour exposure window, between MDA and 28-39 hour exposure window. In contrast, no significant associations were observed among male participants.
Conclusions: Women (versus men) and individuals with asthma (versus those without asthma) showed greater susceptibility and a more rapid response to the respiratory effects of short-term $PM_{2.5}$ exposure in a sample of urban residents. The order of biomarker changes, from earliest to latest, was airway resistance, lung function, and inflammatory reactions.

P38-06 | Short-term associations of pollen exposure with utilization of healthcare services in Northern Italy (2008-2022)

Pierpaolo Marchetti[1], Francesca Locatelli[1], Sofia Tagliaferro[1,2], Lorenzo Di Spazio[1], Francesco Domenichini[3], Nicola Gennaro[4], Morena Nicolis[5], Damaris Selle[6], Ugo Fedeli[4], Alessandro Marcon[1]

[1] University of Verona, Unit of Epidemiology and Medical Statistics, Department of Diagnostics and Public Health, Verona, Italy; [2] National Research Council, Institute of Clinical Physiology, Pisa, Italy; [3] Environmental Protection Agency of Veneto (ARPAV), Teolo, Italy; [4] Azienda Zero, Veneto Region, Epidemiological Department, Padova, Italy; [5] University of Verona, Section of Hygiene and Preventive, Environmental and Occupational Medicine, Department of Diagnostics and Public Health, Verona, Italy; [6] Environmental Protection Agency of Veneto (ARPAV), Belluno, Italy

Objective

We analysed the short-term associations between exposure to nine pollen taxa and utilization of healthcare services in the Veneto region, Northern Italy, between 2008 and 2022.

Material and Methods

In the MEETOUT study, we collected daily data on Emergency Room (ER) visits for natural causes and hospital admissions for respiratory diseases. The population included approximately 500,000 children aged 0-17 years residing within 10 km of 12 pollen monitoring stations. We designed a case-crossover study stratified by area and weekday/month/year. We estimated associations at lags 0-2 days using quasi-Poisson regression adjusted for holidays, daily mean temperature, and precipitation. The analysis for each pollen type was restricted to periods corresponding to its typical season.

Results

The dataset included approximately 1.5 million paediatric ER admissions and 30,000 hospital admissions. Across all areas combined, the 99th percentile of daily pollen concentrations during the season ranged from 19 p/m^3 for Ambrosia to 378 p/m^3 for Corylaceae. We primarily observed immediate effects of pollen exposure on both outcomes. At lag 0-2 days, the most consistent associations across outcomes (relative change with 95%CI per 100 p/m^3) were observed for Artemisia, with increases of 4.3% (1.2;7.5%) for ER visits and 15.5% (-0.13;34.7%) for hospital admissions; Betula, 1.2% (-0.1;2.5%) and 10.9% (3.8;18.3%), respectively; Cupressaceae, 0.5% (0.0;1.8%) and 2.1% (0.0-4.3%), respectively. In addition, Gramineae, 3.8% (2.5;5.2%) and Oleaceae, 2.9% (1.5;4.4%) were associated with ER admissions.

Conclusion

Short-term exposure to Artemisia, Betula and Cupressaceae pollen showed the strongest associations with increased utilization of paediatric healthcare services in the Veneto region. Targeted public health interventions, including early warning systems and careful urban planning, may be valuable public health strategies.

Funding

ESF REACT-EU Green and Innovation and NextGenerationEu programmes (Italian Ministry of University and Research, Decrees 1061/2021 and 737/2021).

P38-07 | Environmental and Population Health Factors Associated with County-Level Influenza-Attributable Emergency Department Visit Rates in the United States

Xucheng Huang[1], Stefanie Ebelt[1], Howard H. Chang[2,1]

[1] Emory University, Gangarosa Department of Environmental Health Science, Atlanta, USA; [2] Emory University, Department of Biostatistics and Bioinformatics, Atlanta, USA

Objective

Seasonal influenza is a major public health concern, yet its burden on emergency department (ED) visits is poorly quantified due to under-ascertainment challenges. This study first estimates county-level respiratory ED visits attributable to influenza using a newly developed time-series model, followed by an examination of spatial heterogeneity in influenza burden and its associations with spatial covariates.

Material and Methods

We used daily hospital discharge records as a measure of community-level influenza activity in California (2005-2018), Georgia (2010-2018), and New York (2005-2018), United States. County-level respiratory ED visit rates attributable to influenza were estimated by quasi-Poisson time-series models, adjusting for air pollution and meteorological factors. Bayesian spatial models were used to examine associations between respiratory ED visit rates and spatial covariates, including socioeconomic status (SES), environmental exposures, and prevalences of chronic health conditions.

Results

Poverty and uninsured rates were positively associated with influenza burden: 160 (95% CI: 127, 196) and 217 (95% CI: 168, 265) additional ED visits per 100,000 population per 10% increase in poverty or uninsured rate, respectively. Long-term $PM_{2.5}$, humidity, and ambient temperature also exhibited positive associations, with humidity having the strongest association (222/100,000; 95% CI: 183, 260 ED visits per IQR of 3.05g/kg increase). Chronic health conditions were strongly associated with ED visit rates: 1,476/100,000 (95% CI: 1,167, 1,778), 588/100,000 (95% CI: 400, 747), and 488/100,000 (95% CI: 402, 574) per 10% increase in stroke, chronic obstructive pulmonary disease, and diabetes prevalence, respectively. Associations with chronic disease prevalence generally decreased after adjustment for socioeconomic variables.

Conclusion

County-level respiratory ED visit rates exhibit significant spatial heterogeneity that can be explained by differences in socioeconomic measures, environmental exposures, and prevalences of chronic health conditions. These findings underscore the potential benefits of improving healthcare accessibility, reducing environmental risks, and enhancing chronic disease management to reduce influenza severity.

P38-08 | Who is Most Susceptible to Air Pollution Related Pneumonia

Jiawei Zhang[1], Stéphane Tuffier[1], Zhebin Yu[2], Rina So[1], Thomas Scheike[3], George Napolitano[1], Steffen Loft[1], Youn-Hee Lim[1], Erik Melén[4], Zorana J. Andersen[1]

[1] University Of Copenhagen, Section of Environmental Health, Department of Public Health, Copenhagen, Denmark; [2] Karolinska Institutet, Institute of Environmental Medicine, Stockholm, Sweden; [3] University Of Copenhagen, Section of Biostatistics, Department of Public Health, Copenhagen, Denmark; [4] Karolinska Institutet, Department of Clinical Sciences and Education, Södersjukhuset, Stockholm, Sweden

Background:
Air pollution is a known risk factor for pneumonia; however, evidence on individual susceptibility remains limited. We aimed to examine whether pre-existing comorbidities modify the association between long-term air pollution exposure and pneumonia incidence.
Methods:
We followed 3,083,227 Danish residents aged ≥ 30 years from 2000 to 2018 for first-ever hospital contact for pneumonia. Annual concentrations of fine particulate matter ($PM_{2.5}$) and nitrogen dioxide (NO_2) were estimated via hybrid land-use regression models and assigned to residential addresses. We identified 12 major comorbidities [cardiac, vascular, cancer, respiratory, metabolic, immunosuppressive, neurological, chronic infections, autoimmune/inflammatory, kidney, liver, and mental health disorders] and 19 specific diseases from prior hospital contacts. Stratified Cox models were used to assess heterogeneity in air pollution-pneumonia associations across comorbidities. Aalen additive models were used to quantify absolute risks attributable to air pollution. Results were reported as per 2 μg/m³ for $PM_{2.5}$ and 10 μg/m³ for NO_2.
Results:
During a mean follow-up of 16 years, 309,092 pneumonia cases were identified. Individuals with comorbidities exhibited higher (38% to 165%) pneumonia incidence than the general cohort. We found associations of air pollution and pneumonia among most comorbidity groups, with hazard ratios ranging from 1.02 (autoinflammatory) to 1.08 (mental health) for $PM_{2.5}$ and from 1.04 (kidney) to 1.11 (cancer) for NO_2, except among patients with immunosuppressive conditions and kidney diseases. Notably, patients with chronic obstructive pulmonary disease (COPD), post-stroke complications, Parkinson's disease, and dementia showed the highest susceptibility, with hazard differences of 424, 412, 341 and 321 cases per 100,000 person-years for $PM_{2.5}$ and 579, 577, 619 and 549 cases for NO_2, respectively.
Conclusion:
The association between air pollution and pneumonia risk varies by comorbidity status, with COPD, post-stroke complications, Parkinson's disease, and dementia patients being the most vulnerable.

P38-09 | Long-Term Exposure to Air Pollution and Incident Asthma and Chronic Obstructive Pulmonary Disease in adults: The Danish Nurse Cohort

Jiawei Zhang[1], Rina So[1], Marie Bergmann[1], Stéphane Tuffier[1], Cale Lawlor[2], Kajsa Pira[2], George Napolitano[1], Ebba Malmqvist[2], Anna Oudin[2], Youn-Hee Lim[1], Zorana J. Andersen[1]

[1] University Of Copenhagen, Section of Environmental Health, Department of Public Health, Copenhagen, Denmark; [2] Lund University, Division of Occupational and Environmental Medicine, Lund, Sweden

Background: Air pollution is a known risk factor for chronic respiratory diseases, including asthma and chronic obstructive pulmonary disease (COPD). However, evidence regarding the role of black carbon (BC) remains mixed and sparse. This study aimed to examine the association of long-term exposure to BC with incident asthma and COPD in adults.
Method: We followed 28,731 female nurses from the Danish Nurse Cohort from baseline (1993/1999) until the end of 2018, tracking their first-ever hospital contact (inpatient, outpatient, or emergency visit) for asthma or COPD. Residential annual mean concentrations of BC and fine particulate matter ($PM_{2.5}$) were modeled from 1980 onward using the Danish AirGIS modeling system at nurse's residential addresses. Time-varying Cox models were used to assess the association between 3-year running mean air pollution and incident asthma and COPD, adjusting for individual and area-level covariates.
Results: Over an average follow-up of 23 years, 633 nurses developed asthma, and 1,145 developed COPD. We found consistent associations between BC exposure and increased risks of both asthma and COPD, with hazard ratios (HR) per 0.34 μg/m³ (interquartile range) increase in BC of 1.06 (95% confidence interval [CI]: 1.00-1.13) for asthma and 1.04 (95% CI: 0.98-1.11) for COPD. These associations persisted and even strengthened after controlling for $PM_{2.5}$, with HR of 1.05 (0.95, 1.16) and 1.09 (1.02, 1.16) separately.
Conclusion: We showed that BC may be an independent risk factor for developing chronic respiratory diseases, such as asthma and COPD, and suggested BC may play a significant role with $PM_{2.5}$. This underscored the need for targeted air quality regulations to reduce BC emissions, which may have a greater impact on mitigating the burden of chronic respiratory diseases than focusing solely on $PM_{2.5}$.

P38-10 | Integrating epidemiological and toxicological approaches for assessing sulphur dioxide respiratory effects: results from the ASHES study

Cristina Candal-Pedreira[1,2], David Díaz-Pérez[2], María Carmen González-Caballero[3], Ana Fernández Agudo[3], Noelia Dominguez-Morueco[3], Valle Velasco[2], Ciro Casanova[2], Esther BArreiro[2], Ana Cañas[2], María Jesús Cruz Carmona[2], Xavier Muñoz[2], Francisco Javier Sánchez-Íñigo[3,2], Alberto Ruano-Ravina[1,2], José Vicente Tarazona[3]

[1] University of Santiago de Compostela, Preventive Medicine, Santiago de Compostela, Spain; [2] Scientific committee of the ASHES project, Spanish Society of Neumology and Thoracic Surgery (SEPAR), Madrid, Spain; [3] Health Institute Carlos III, National Centre of Environmental Health, Madrid, Spain

Objective: To evaluate the respiratory effects of SO_2 exposure during the three-month volcanic eruption in 2021 on La Palma (Canary Islands, Spain) by integrating epidemiological and toxicological approaches through Health Impact Pathways (HIPs).

Methods: ASHES is a cohort study assessing the health impact of the La Palma eruption. We analyzed the adult population, divided into high, moderate and low/no exposure groups. Participants underwent spirometry, and results were graded using predefined criteria. Ambient air SO_2 concentrations were measured at 11 different stations using gas analyzers grounded on UV fluorescence. Based on studies in humans, the correlation between short-term SO_2 exposure and respiratory effects was established, forming the basis for exposure thresholds in healthy and asthmatic individuals. First, we estimated the expected probabilities of respiratory effects for each group based on the previously established theoretical correlations. Second, the observed proportions of respiratory effects in our sample were calculated and compared to the expected ones. Lastly, respiratory health parameters were analyzed using the Respiratory Adversity Index (RAI), measuring the relative increase in frequency of respiratory effects based on exposure, and the Respiratory Severity Index (RSI), integrating frequency and severity of respiratory effects across groups.

Results: Among the 474 participants included, expected and observed probabilities of respiratory effects closely matched across groups. SO_2 exposure was linked to the increased proportion of respiratory effects, with the highest percentages seen in the exposed groups. The RAI confirmed more frequent altered respiratory parameters in highly and moderately exposed individuals compared to the low/no exposure group. The RSI indicated greater severity, particularly in municipalities with higher SO_2 concentrations.

Conclusion: SO_2 exposure increased respiratory effects, with HIP analysis revealing asthma-like effects in healthy individuals, increasing their vulnerability in future exposure episodes. These findings highlight the need for targeted health interventions in high-exposure areas to mitigate respiratory risks.

P38-11 | Short-term combined effects of ambient air pollutants and pollen exposure on asthma hospitalizations: a nationwide time-series analysis in Korea

Hyeinn Lim[1], Eunjin Oh[2], Hyungryul Lim[2], Jonghyuk Choi[3], Ho-Jang Kwon[3]

[1] Ajou University School of Medicine, Department of Medicine, Suwon, South Korea; [2] Ajou University School of Medicine, Department of Preventive Medicine and Public Health, Suwon, South Korea; [3] Dankook University College of Medicine, Department of Preventive Medicine, Cheonan, South Korea

Objectives: Previous studies suggest air pollutants and pollen grains have positive associations with asthma hospitalization. This study aims to investigate the short-term combined effects of air pollutants—fine particulate matter ($PM_{2.5}$) and ozone (O_3)—and pollen grains (pine, oak, and weed) on asthma hospitalization across South Korea.

Methods: We analyzed data from 15 South Korean provinces during tree (April–June) and weed (August–October) seasons from 2017 to 2019. We obtained province-specific daily counts of asthma hospitalizations from the National Health Insurance Service and linked them with daily $PM_{2.5}$ and O_3 concentration as well as the Pollen Concentration Risk Index. Distributed lag models (DLMs) and distributed lag non-linear models (DLNMs) using quasi-Poisson regression were employed to assess the effects of $PM_{2.5}$ and O_3 on asthma hospitalizations, sequentially evaluating the effect modification by pollen exposure. Pooled results across provinces were analyzed using multivariate random effects meta-analyses.

Results: O_3 exposure during the tree season (April–June) was significantly associated with increased asthma hospitalizations [Relative risk (RR): 1.012 per 10 ppb increase, 95% CI: 1.004–1.021] in the DLM analysis. In the DLNM analyses, pollen significantly modified these associations. The effect sizes of $PM_{2.5}$ was higher on pine pollen-affected days, while the effect of O_3 was higher on weed pollen-affected days. Furthermore, the interaction effect was stronger in those under 10 and over 65, as well as in higher socioeconomic status group.

Conclusions: Our research suggests that interactions between air pollutants and pollen may contribute to increased asthma hospitalization.

P38-12 | Community exposures to sulfur compounds from industrial sources and the potential for environmental odors and acute respiratory effects.

Aaron Young, James Durant, Debra Gable

ATSDR, Office of Community Health Hazard Assessment, Atlanta, USA

Abstract

Objective

Environmental odors are an ongoing concern for communities near industries that emit sulfur compounds. The Agency for Toxic Substances and Disease Registry (ATSDR) measured sulfur compounds in a community near a paper pulp mill to determine the potential for environmental odors and adverse health effects. Air monitoring data were used to substantiate community concerns of environmental odors and the potential for health effects from exposure to a mixture of sulfur compounds.

Materials and Methods

To address community concerns of strong sulfur-like odors, ATSDR conducted an exposure investigation (EI) that measured several sulfur compounds in addition to hydrogen sulfide and sulfur dioxide measured using standard methods. During the EI, sulfur compounds were measured using the $_{TRS}$MEDOR® ppb method, which individually measures several sulfur compounds (including hydrogen sulfide and sulfur dioxide) simultaneously and continuously. Concentrations of each sulfur compound were compared to health-based screening levels and odor thresholds where available.

Results

The $_{TRS}$MEDOR® ppb method measured 14 additional sulfur compounds in air that would not be captured with conventional sampling methods. The concentrations of the sulfur compounds were positively correlated, and their combined exposure could cause acute respiratory effects (irritation of the respiratory tract, shortness of breath, or breathing discomfort).

Conclusion

The air in this community contained several sulfur compounds that can combine to increase the potential for environmental odors and adverse health effects. Sampling for multiple sulfur compounds showed the presence of contaminants other than hydrogen sulfide and sulfur dioxide that can result in a combined toxic effect greater than what would be predicted by traditional methods.

P38-13 | Associations Between Airborne Bacterial Communities and Symptomatic Responses in Asian Dust-Affected Coastal Areas of Japan

Kazunari Onishi[1], Masanori Nojima[2], Atsushi Shimizu[3], Joseph Ching[4], Yasunori Kurosaki[5], Teruya Maki[6]

[1] St. Luke's International University, Division of Environmental Health, Graduate School of Public Health, Chuo-ku, Japan; [2] The University of Tokyo, Center for Translational Research, The Institute of Medical Science Hospital, Minato-ku, Japan; [3] National Institute for Environmental Studies, Tsukuba, Japan; [4] The Education University of Hong Kong, Department of Science and Environmental Studies, Hong Kong, Hong Kong; [5] Tottori University, Arid Land Research Center, Tottori city, Japan; [6] Kindai University, Department of Life Science, Faculty of Science and Engineering, Higashiosaka, Japan

Objective:
This study aimed to investigate variations in airborne bacterial communities in areas affected by Asian dust and haze events and their potential health effects.

Materials and Methods:
From March to June 2015, bioaerosol samples were collected during Asian dust and haze events. Simultaneously, a questionnaire survey was conducted among residents of Yonago, a region with minimal local pollution sources, to assess self-reported symptoms, including ocular and respiratory symptoms as well as physical discomfort, such as stiffness, shoulder discomfort, and lumbalgia. Asian dust levels were monitored using LIDAR. Bacterial compositions were analyzed using high-throughput DNA sequencing targeting 16S rRNA genes, and their associations with symptom severity were evaluated using a logistic mixed model.

Results:
The relative abundances of certain bacterial groups exhibited positive correlations with symptom severity. Actinobacteria, primarily soil-derived bacteria, were significantly associated with ocular symptoms ($OR = 1.032$, $p < 0.05$). Firmicutes (Clostridia) were linked to both ocular ($OR = 1.026$, $p < 0.05$) and respiratory symptoms ($OR = 1.027$, $p < 0.05$). Deltaproteobacteria and several other bacterial groups were positively correlated with physical discomfort ($OR = 1.023$, $p < 0.05$; $OR = 1.027$, $p < 0.05$). Conversely, other bacterial groups were negatively correlated with symptom severity, suggesting a possible dilution effect of harmful bacteria. Acidobacteria (Chloracidobacteria, Solibacteres) moderately correlated with the LIDAR dust extinction coefficient.

Conclusion:
Airborne bacteria, which increase during Asian dust events, are thought to impact various aspects of human health, contributing to ocular inflammation, airway damage, and physical discomfort. Further research is required to clarify the pathogenicity influenced by other categories of airborne bacteria.

P38-14 | Long-term exposure to ambient nitrogen dioxide and chronic obstructive pulmonary disease: a systematic literature review and quantitative synthesis

Tessa Haverkate[1], Periklis Charalampous[1,2], Elia Gonzato[1], Susanne Breitner-Busch (neé Breitner)[3,4], Juanita Haagsma[1]

[1] Erasmus MC, University Medical Center Rotterdam, Department of Public Health, Rotterdam, Netherlands; [2] Sciensano, Department of Epidemiology and Public Health, Brussels, Belgium; [3] LMU Munich, Faculty of Medicine, Institute for Medical Information Processing, Biometry, and Epidemiology, Neuherberg, Germany; [4] Helmholtz Munich, Institute of Epidemiology, Neuherberg, Germany

Objective: Epidemiological studies have suggested a link between long-term exposure to ambient nitrogen dioxide (NO_2) and chronic obstructive pulmonary disease (COPD). However, the extent to which NO_2 contributes to the burden of COPD has not been precisely quantified. Exposure-response functions (ERFs) are essential for this quantification, with region-specific ERFs needed for accurate burden estimates for specific populations. This study aimed to 1) systematically review the association between long-term exposure to ambient NO_2 and COPD; 2) derive and compare ERFs for the NO_2-COPD association using different meta-analytical approaches; and 3) derive global- and region-specific exposure-response curves.
Material and Methods: We searched for epidemiological studies published from inception until June 2024 in several electronic databases and enginges. Epidemiological studies were included if they reported on the general population, assessed long-term exposure to NO_2, investigated COPD incidence, prevalence, or mortality, and reported a quantifiable measure of association. Risk of bias of the included studies was assessed based on standardized critical appraisal tools. Individual study estimates were meta-analyzed using a conventional random-effects model, meta-regression, and the Meta Regression-Bayesian Regularized Trimmed (MR-BRT) approach.
Results: We included 24 studies for the quantitative synthesis; seven reported estimates for ambient NO_2 and COPD incidence, five provided estimates for prevalence, eleven for mortality, and one for both incidence and mortality. The pooled random-effects relative risk (RR) indicated a positive association between NO_2 and combined COPD incidence and mortality, with higher risks in Europe (RR=1.08, 95%CI:1.01-1.15), compared to all study regions (RR=1.04, 95%CI:1.00-1.09). Conventional meta-regression yielded a fluctuating ERF, while MR-BRT suggested a saturation effect at higher exposure levels globally.
Conclusion: This systematic review and quantitative synthesis showed an association between long-term exposure to ambient NO_2 and COPD. Tailored regional ERFs and standardized exposure definitions are essential for more accurate estimates of the disease burden attributable to ambient NO_2 exposure.

P38-15 | Long-term exposure to ultrafine particles and its association with cardiometabolic diseases, chronic obstructive pulmonary disease, and cardiometabolic biomarkers: a cross-sectional analysis of the German National Cohort (NAKO)

Susanne Breitner-Busch (neé Breitner)[1,2], Kathrin Wolf[2], Josef Cyrys[2], Alexandra Schneider[2], Regina Pickford[2], Marco Dallavalle[2], Susanne Sues[2,3], Beate Fischer[4], Michael Leitzmann[4], Jens Soentgen[3], Annette Peters[1,2]

[1] LMU Munich, Faculty of Medicine, Institute for Medical Information Processing, Biometry, and Epidemiology, Neuherberg, Germany; [2] Helmholtz Munich, Institute of Epidemiology, Neuherberg, Germany; [3] University of Augsburg, Environmental Science Center, Augsburg, Germany; [4] University of Regensburg, Department of Epidemiology and Preventive Medicine, Regensburg, Germany

Objective: Long-term exposure to ambient particulate air pollution has been associated with various adverse health effects. However, there is limited epidemiological evidence regarding ultrafine particles (UFP; particles with an aerodynamic diameter of 10-100 nm). This study investigated whether long-term exposure to total particle number concentration (PNC), used as a surrogate for UFP, is associated with cardiometabolic diseases, chronic obstructive pulmonary disease (COPD), and cardiometabolic biomarkers.

Material and Methods: Our cross-sectional study included 27,113 participants of the NAKO study centers in Augsburg and Regensburg in Southern Germany. Health outcomes included physician-diagnosed hypertension, myocardial infarction, stroke, diabetes, and COPD, alongside blood biomarkers such as glucose or hemoglobin A1c. Annual PNC averages for 2014 were estimated using supervised land use regression models and linked to participants' home addresses. We also obtained annual averages of further pollutants (e.g., particulate matter ≤2.5 µm ($PM_{2.5}$) and nitrogen dioxide (NO_2)) for 2010. We applied covariate-adjusted logistic and linear regression models to examine associations between PNC and health outcomes. Additionally, we assessed interdependencies between pollutants using two-pollutant models.

Results: Long-term exposure to PNC was associated with increased odds of hypertension, myocardial infarction, and COPD, as well as elevated glucose levels. For example, the odds ratio for hypertension was 1.05 (95% confidence interval: 1.02;1.08) for each increase in PNC by 1000 particles/cm³. Adding $PM_{2.5}$ or NO_2 in two-pollutant models did not substantially change the results for PNC but led to slightly wider confidence intervals. **Conclusions:** Our study suggests that long-term exposure to PNC, as a surrogate for UFP, contributes to the risk of hypertension, myocardial infarction, COPD, and elevated blood glucose levels in adults. These results highlight the role of UFP within the broader mixture of ambient air pollution and underscore the need for strategies to reduce UFP exposure to prevent adverse cardiometabolic and pulmonary health effects.

P38-16 | The Joint Impact of Ambient Air Pollution and Extreme Temperatures on Asthma-Related Emergency Department Visit in Kentucky.

Yisi Liu, Marissa English, Tianjun Lu

University of Kentucky, Department of Epidemiology and Environmental Health, Lexington, USA

Objective: Kentucky ranks 5th for the highest prevalence of asthma among adults in the US. This research aims to understand the joint impact of ambient air pollution and extreme temperature on asthma-related emergency department (ED) visit in Kentucky.
Material and Methods: The annual age-adjusted asthma ED visit rate (per 10,000 population) and annual average of 6 air pollutants (i.e. land-use regression modelled SO_2, NO_2, CO, O_3, $PM_{2.5}$, PM_{10} concentrations) was collected across 120 counties in Kentucky from 2008 to 2019. Daily meteorological data from gridMET were used to calculate the annual county-level temperature, relative humidity and wet bulb globe temperature (WBGT) for each county between 2008 to 2019. WBGT was categorized as extreme cold (<25th percentile), medium (25th–75th percentile), or extreme warm (>75th percentile). Linear regression models stratified on WBGT assessed the impact of air pollution on asthma ED visit rate after adjusting for regional demographical, socioeconomical, built environment, and behavioral factors.
Results: The average asthma ED visit rate decreased from 53.99 (2014) to 31.21 (2019) per 10,000 population, while WBGT increased from 11.32°C (2000) to 12.70°C (2019). All pollutants were independently associated with asthma ED visit, with the strongest effect found for O_3. SO_2 had a greater impact on asthma ED visit in extreme cold years than medium WBGT years (per SD increased in SO_2, asthma ED visit rate increased 3.32 [1.33, 5.31] vs. 0.82 [-0.86, 2.50] per 10,000 population). However, NO_2 had a stronger impact in extreme warm years than medium WBGT year (1.31 [-0.71, 3.33] vs. -2.23 [-3.82, -0.63]).Conclusion: These ecological study highlights significant interactions between ambient air pollution and extreme temperatures on asthma ED visit, emphasizing the need for climate-adaptive interventions

P38-17 | Housing Features Related to Asthma Quality of Life in Older Adults

Barbara Polivka[1], Luz Huntington-Moskos[2], Demetra Antimisiaris[3], Rodney Folz[4]

[1] University of Kansas Medical Center, School of Nursing, Kansas City, USA; [2] University of Louisville, School of Nursing, Louisville, USA; [3] University of Louisville, School of Public Health, Louisville, USA; [4] Houston Methodist Hospital, Division of Pulmonary, Critical Care and Sleep Medicine, Houston, USA

Objective: The Housing Typologies Framework (Howard, et al., 2023) proposes that location features, dwelling features, and occupancy features contribute to asthma quality of life. The objective of this study was to determine components of the framework specifically applicable to poor asthma quality of life in older adults with asthma.
Materials and Methods: Data are from our prospective study of older adults with asthma (N=185). Participants were non-smokers aged ≥60 with asthma. Baseline data included Mini Asthma Quality of Life (QoL) Questionnaire, 24-hour residential environmental sensor monitoring of volatile organic compounds (VOCs) and particulates ($PM_{2.5}$), and

home environmental assessments by a trained data collector. Indoors, a six-liter pre-evacuated steel canister with flow controls and the Dylos DC1100 Air Quality monitor were placed 1 meter off the flow in the room the participant spent most of their time (i.e., bedroom) for VOC and $PM_{2.5}$ sampling, respectively. The outdoor canister was near the residence, away from trees, >2 meters from a building, and not near other potential VOC sources. Data were analyzed descriptively and with inferential statistics.**Results:** Most participants were female (73%), white (77%), had at least a high school education (82%); the average age was 68±6 years. Poor asthma quality of life in older adults was significantly related to: (a) location feature of higher levels of outdoor VOCs (p=.03); (b) dwelling features included not having an attached garage (p=.04) and having higher level of indoor $PM_{2.5}$ (p=.01); and (c) occupancy features of home renting (p=.<.001) and having high levels of dust in the home (p=.03).
Conclusion: The Housing Typology Framework provides a comprehensive guide for evaluating housing features contributing to poor asthma quality of life in older adults. Findings have implications for interventions at multiple levels, including policies related to environmental health.

P38-18 | Self-Reported Symptoms and Exposure to Cyanobacteria Blooms in Florida

John Reif[1], Adam Schaefer[2], MIchael Parsons[3], Malcolm McFarland[4], Rebecca Koszalinski[5]

[1] Colorado State University, School of Public Health, Fort Collins, USA; [2] ABT Global, Rockville, USA; [3] Florida Gulf Coast University, The Water School, Fort Myers, USA; [4] Florida Atlantic University, Harbor Branch Oceanographic Inst, Fort Pierce, USA; [5] University of Central Florida, College of Nursing, Orlando, USA

Objective: Harmful algal blooms frequently impact coastal Florida and have raised concern about both acute and chronic health impacts among residents. Between 2016 and 2018, large scale blooms of blue green algae resulted in exposure to multiple cyanotoxins. We initiated a long-term study to incorporate environmental measures of cyanotoxin exposure and reported symptoms among Florida residents.
Material and Methods: Participants were recruited during an active algal bloom during summer, 2018 and non-bloom periods in subsequent years and completed a questionnaire, Nasal swabs, and blood specimens were collected and paired with water and air samples to assess exposure. The questionnaire queried the location, frequency, duration, and types of interaction with bloom impacted waterways. Self-reported outcomes included respiratory, gastrointestinal, ocular, and dermal symptoms.
Results: 125 individuals were sampled during the 2018 bloom event in southeastern Florida. Microcystin was detected in 95% of samples from the nasal mucosa. Respiratory symptoms were reported by 74%, ocular symptoms by 62%, and gastrointestinal symptoms by 35% of respondents. Those most reported included rhinorrhea, sneezing, headache, sore throat and dry cough. Residential and recreational exposures were associated with increased risks of respiratory, gastrointestinal, and ocular symptoms in univariate and multivariable analyses. Residential exposure was significantly associated with multiple respiratory symptoms (OR =2.45, 95%CI 1.05–5.74) including dry cough, dyspnea, and wheezy respirations. Notably, direct contact with waterways was not a necessary risk factor for respiratory effects, whereas recreational exposure was significantly associated with sore throat and eye irritation.
Conclusions: The results demonstrate that exposure to cyanobacteria algal blooms are associated with multiple symptoms that likely represent an inflammatory response to toxins. Inhalation appears to represent an important exposure pathway for beachgoers and persons living in proximity to impacted waterways. Analysis of symptom profiles during non-bloom periods will permit estimation of potential reporting bias.

P38-19 | The Impact of Alder, Birch, and Olive Pollen on Negative Sentiments: An Analysis of 400 Million Tweets Across 11 European Countries

Tareq Al-Ahdal

Heidelberg University Hospital, Heidelberg Institute of Global Health, Heidelberg, Germany

This study exploratory investigates the impact of alder, birch, and olive pollen levels on negative sentiments expressed on social media across 11 European countries. Analyzing over 400 million tweets from 2015 to 2022, we employed advanced natural language processing (NLP) tools, such as NLTK and LIWC22, to extract sentiment data. These tweets were aggregated weekly at the NUTS2 level, representing various regions in Europe. To assess the relationship between pollen levels and negative sentiments, we applied a Poisson generalized additive model (GAM) with integrated nested place approximation (INLA). This model was specifically chosen for its ability to account for both spatial and temporal dependencies within the data. To further improve the model's accuracy, we incorporated fused lasso regularization, which helps capture fluctuations in sentiment over time while preventing overfitting by penalizing large variations in the coefficients. The fused lasso method is particularly effective in modeling complex relationships where data points are correlated, as is the case with social media data that shows temporal trends and regional variations. Our findings revealed a significant association between higher pollen counts, particularly alder pollen levels above 135 grains/m³, and an increase in negative sentiment risk. This suggests that pollen exposure, especially from alder, birch, and olive, may contribute to heightened anxiety and poor emotional well-being in affected populations. Interestingly, while temporal and spatial variations in sentiment were observed, This study demonstrates the potential of using large-scale social media data as a proxy for monitoring public mental health and highlights the considerable influence of pollen exposure on emotional states, emphasizing the need to consider climate-related factors when assessing mental well-being across populations. Further interventional research is needed with causal inference models to establish the causal association between pollens exposure and public mood.

P38-20 | Investigation of Residential Tree Canopy Coverage in Relation to Development of Asthma during Early Childhood

Anneclaire J. De Roos[1,2], Leah H. Schinasi[1,2], Steven Melly[2], David A. Hill[3], Chen C. Kenyon[4]

[1] Drexel University, Department of Environmental and Occupational Health, Dornsife School of Public Health, Philadelphia, USA; [2] Drexel University, Urban Health Collaborative, Dornsife School of Public Health, Philadelphia, USA; [3] Children's Hospital of Philadelphia, Division of Allergy and Immunology, Philadelphia, USA; [4] Children's Hospital of Philadelphia, Division of General Pediatrics, Philadelphia, USA

Objective. To investigate the hypothesis that tree canopy coverage in residential neighborhoods is protective against development of asthma in early childhood.

Material and Methods. This study was funded by NIH (R21ES032963). We assembled a longitudinal cohort of children living in the Philadelphia metropolitan region using electronic health records (EHR) from the Children's Hospital of Philadelphia, followed from infancy until age 5, between 2005-2021. Tree canopy exposure, derived from high-resolution spatial data, was quantified as the percent coverage within circular buffer areas around children's homes (50-2000m) during two periods: 1) early-life: first six months after birth, and 2) cumulative: from birth until age

5. Low-lying vegetation (grasses/shrubs) was coded similarly. Poisson regression with robust standard errors was used to estimate associations (relative risk [RR] and 95% confidence interval [CI]), with adjustment for potential confounders at the individual and neighborhood levels.
Results. Greater tree canopy coverage surrounding children's homes was associated with lower risk of asthma by age 5. This association was observed with both early-life and cumulative exposures, and within all buffer area sizes. For example, 28% greater early-life tree canopy within 250m was associated with 5% lower risk of asthma by age 5 (16,555 cases, RR=0.95; 95% CI: 0.93-0.98), after adjustment for individual child's birth year, sex, race, Hispanic ethnicity, Medicaid/SCHIP coverage, and Philadelphia residence, as well as neighborhood population density, household income, education level, and traffic density. Low-lying vegetation was not associated with asthma (RR=1.02, 95% CI: 0.99-1.04 for interquartile range increase within 250m). These relationships were similar when tree canopy and low-lying vegetation were modeled together.
Conclusion. Our results suggest that living in areas with tree canopy coverage protects against development of asthma during childhood. Proposed mechanisms involve the contribution of natural landscapes to robust immune development, and removal of asthma-causing air pollution by tree canopy.

P38-21 | The association between wet bulb temperature and lung function in a cohort of elderly German women.

Ashtyn T. Areal[1], Claudia Wigmann[1], Tamara Schikowski[1,2]

[1] IUF-Leibniz Research Institute for Environmental Medicine, Epidemiology, Düsseldorf, Germany; [2] University of Bielefeld, Faculty of Public Health, Bielefeld, Germany

Objective: At-risk populations, such as the elderly, are at increased risk of adverse respiratory outcomes with exposure to climate-related factors such as temperature, humidity, and air pollution. This study investigated the association between wet bulb temperature and lung function in elderly women.
Materials & Methods: We used complete lung function data from the second follow-up of the prospective cohort study, SALIA. Exposure to wet bulb temperature was calculated from maximum temperature and relative humidity using an empirical arctangent-based approximation; we calculated the 5-day moving average to account for long-term trends. The primary outcomes were forced expiratory volume in 1 second (FEV_1), forced vital capacity (FVC), and the ratio FEV_1/FVC. We used multivariable linear regression adjusted for particulate matter less than 2.5μm diameter, the day of week (e.g. Monday, Tuesday etc.), smoking status, history of chronic respiratory disease, education status, and location. GLI z-scores were used to standardise the lung function outcomes and adjusted for age, height, sex, and ethnicity.
Results: The analysis included 735 women with an average age of 73.46 years with complete lung function data. The mean wet bulb temperature was 13.69 degrees Celsius. There was an increase in FEV_1/FVC with an increase in wet bulb temperature (b=0.012: 95% confidence interval (95%CI): 0.001-0.023). We found that FEV_1 tended to stay unchanged (b= -0.0002. 95%CI: -0.013 to 0.013) while FVC decreased (b= -0.008. 95%CI: -0.020 to 0.004) with increasing wet bulb temperature.
Conclusion: As climate change continues to affect health, it is important to assess how climate-related exposures affect health. The difference in direction for FEV_1/FVC, compared to FVC, is naturally explained by its calculation as a ratio. This study found that wet bulb temperature was associated with different lung function parameters: however, future studies are imperative to understand potential associations between wet bulb temperature and lung function.

P38-22 | ENSO and Respiratory Diseases: Analyzing the Relationship Between Socioenvironmental Vulnerability and Acute Respiratory Infections in Colombia (2010-2019)

Carlos A. Reina-Bolaños, Sonia Guzman, Camilo Blanco

Andean Region University Foundation, Health, Bogotá DC, Colombia

Objective

The El Niño-Southern Oscillation (ENSO) phenomenon drives global climate variability and is increasingly linked to adverse health outcomes, particularly in low- and middle-income countries (LMICs) with limited adaptation capacity. This study examines the impact of ENSO and socioenvironmental inequalities on the burden of Acute Respiratory Infections (ARI) in Colombia, an LMIC highly vulnerable to climate extremes, from 2010 to 2019.**Material and Methods**

A random-effects mixed model was employed to estimate the influence of ENSO, air pollution from particulate matter (PM10, PM2.5), precipitation, and multidimensional poverty on ARI rates. Additionally, Machine Learning techniques, including Decision Trees and Classification Boundaries, were applied to explore complex interactions and identify high-risk municipalities.**Results**

Severe El Niño and La Niña events were significantly associated with increased ARI rates, particularly in municipalities with higher socioenvironmental vulnerability. While PM2.5 and precipitation influenced ARI incidence, their effects varied by climatic classification. Notably, municipalities classified as high climate risk did not always present the highest ARI rates, suggesting that structural health disparities, access to care, and local mitigation strategies may buffer or amplify climate-related health impacts.**Conclusion**

These findings highlight the urgent need for climate-adaptive public health policies in LMICs, where health systems remain under-resourced to address climate-driven respiratory health risks. Strengthening epidemiological surveillance, integrating climate resilience into health systems, and advancing interdisciplinary climate-health research are critical to mitigating ENSO-related health burdens in vulnerable populations.

Funding: This study was conducted using publicly available government databases, with research efforts supported by the investigators’ independent work. No external funding was received.

P38-23 | Longitudinal effects of an urban factory fire incident on asthma development: A marginal structural model analysis

Changwoo Han[1,2], Jaeyoung Yun[1], Tarik Benmarhnia[2,3]

[1] Chungnam National University College of Medicine, Department of Preventive Medicine, Daejeon, South Korea; [2] University of California, San Diego, Scripps Institution of Oceanography, San Diego, USA; [3] Université de Rennes, Inserm, EHESP, Irset (Institut de recherche en santé, environnement et travail) - UMR_S 1085, F-35000, Rennes, France

Introduction

Studies on the chronic health effects of urban fire disasters on exposed residents are limited. This study utilizes the three-day tire factory fire that occurred in Daejeon, South Korea, on March 12, 2023, as a natural experiment to causally estimate the incident's long-term effect on asthma development among nearby residents.

Methods

We selected individuals of Daejeon with no prior history of chronic lower respiratory disease diagnosis between January 1, 2022, and March 12, 2023, from the nationwide health insurance data covering the entire Korean population. Based on a previous study, residents of three administrative districts near the factory were classified as the exposed (n=34,568), while residents of the remaining 79 districts were classified as the control (n=1,286,803). Using logistic regression and inverse probability weighting to adjust for differences in baseline characteristics and comorbidities, we constructed a marginal structural model to estimate the risk of asthma development until the end of 2023. A survival curves were used to estimate weekly time-varying hazards.

Results

Residents living close to the factory (exposed) had an increased risk for asthma development over the nine-month follow-up period [Odds Ratio (95% CI): 3.15 (2.93, 3.38)]. This increase was most pronounced among individuals aged 0–19 years [5.53 (5.06, 6.06)] compared to other age groups [20-64 years: 2.25 (2.00, 2.53); 65+ years: 1.25 (0.99, 1.58)]. The difference in cumulative disease-free probability between exposed and controls was 2% for the entire subjects, but 9% for the 0–19 years group.

Conclusion

An urban tire factory fire was associated with an increased risk of asthma development in nearby residents, particularly among children and adolescents. Even a few days of exposure to high concentration of pollutants during fire disasters necessitates respiratory health monitoring for residents in affected areas.

P38-24 | Vulnerability to pollen-related asthma hospital admissions in the UK Biobank: a case-crossover study

Elaine Fuertes[1], Garyfallos Konstantinoudis[2], Diana Van der Plaat[1], Adam Koczoski[1], Mikhail Sofiev[3], Paul Agnew[4], Lucy Neal[4], Debbie Jarvis[1]

[1] Imperial College London, National Heart and Lung Institute, London, UK; [2] Imperial College London, Grantham Institute for Climate Change and the Environment, London, UK; [3] Finnish Meteorological Institute, Helsinki, Finland; [4] Met Office, Exeter, UK

Background: Pollen worsens symptoms in people with allergic respiratory disease, but less is known regarding severe respiratory outcomes. Using individual-level data, we examined short-term associations between grass and tree pollen and emergency asthma hospital admissions among UK Biobank adult participants, and whether associations are stronger when air pollution levels are high.

Methods: Asthma hospital admissions were defined as any 493 ICD9 or J45/J46 ICD10 code. Daily concentrations of alder, birch, grass, oak and nettle were obtained from the UK Met Office pollen model (0.05^{o} x 0.05^{o} resolution, 2011-2022), dichotomized as low vs high ($\geq$ 30 grains/m^3 for all pollens except birch and nettle, for which it was $\geq$ 40 grains/m^3) and assigned to the home address of participants. Associations were assessed using bidirectional time-stratified case-crossover models adjusted for daily mean temperature, precipitation, relative humidity, wind speed and weekend/public holidays. Confounding and effect modification by short- and long-term air pollution (nitrogen dioxide (NO_2), particulate matter $\leq$ 2.5 μm in diameter *(*$PM_{2.5}$) and ozone) was assessed.

Results: There were 1,893 asthma hospital admissions among 1,489 adults from January to September, 2011-2022 (mean age: 57.7 years). Days of high vs. low alder and grass pollen were associated with higher asthma hospital admissions (1.51, 95% confidence interval: [1.10, 2.09] and 1.31 [1.00, 1.70], respectively). For most pollen types, associations were greater on days with high daily NO_2 and $PM_{2.5}$ mass concentrations (p-interaction < 0.05).

Conclusions: Days with high alder and grass pollen concentrations were associated with increased asthma hospital admissions. Associations with several pollen types were greater on days with high air pollution, particularly NO_2 and $PM_{2.5}$ mass.

P38-25 | Residential fungal β-(1,3)-D-glucan exposure correlates with increased pulmonary severity in a cohort of midwestern sarcoidosis patients.

Emma M. Stapleton[1], Nervana Metwali[2], Michael Shlossman[1], Alejandro C. Pezzulo[1], Alejandro P. Comellas[1], Peter S. Thorne[2], Alicia K. Gerke[1]

[1] University of Iowa, Internal Medicine, Division of Pulmonary, Critical Care, and Occupational Medicine, Iowa City, USA; [2] University of Iowa, College of Public Health, Department of Occupational and Environmental Health, Iowa City, USA

Background: Sarcoidosis is a systemic granulomatous inflammatory disease typically affecting the lungs. Presentation can range from mild resolving disease to severe end-stage progressive organ (heart/lung) fibrotic disease, which is more frequent in African Americans. While genetics increases disease risk, environmental antigens have long been suggested to play a role in incidence and outcomes. Pulmonary-specific disease risk, for example, is increased in people exposed to organic aerosol. **Methods:** Two-week residential sampling was carried out using electrostatic dust collectors (EDC) to collect residential fungal and bacterial cell wall material, assessed by Glucatell® (β-(1,3)-D-glucan, BDG), and kinetic chromogenic Limulus amebocyte lysate assay (endotoxin); participants with pulmonary sarcoidosis, n=59; participants without sarcoidosis, n=25. Sarcoidosis pulmonary severity was assessed by clinical pre-bronchodilator lung function, significant fibrosis by clinical chest CT. Serum biomarkers were assessed by multiplex (Luminex xMAP). Surveys were deployed to determine bioaerosol source and disease severity risk-factors. **Results:** Residential bioaerosol concentrations were significantly greater in homes of participants with sarcoidosis than controls. Disease severity (Wasfi, VAS) was greater in participants with significant pulmonary fibrosis (identified by chest CT). Lung function was also decreased in these participants. Because participants with fibrosis were more likely to own houseplants, we compared lung function to number of plants owned, and found an inverse association for % predicted FVC and FEV_1 and plant number (p=0.05, p=0.02). Residential BDG inversely correlated with FVC in patients with fibrosis, but not in those without (p=0.02 vs. p=0.82). Eight chemokines and cytokines correlated with BDG exposure in non-fibrotic participants (6CKine, IL-9, IL-17F, IL-21, IL-28A, I-309, MIP-1β, TARC), while only two did in those with fibrosis (eotaxin-3, M-CSF), likely indicative of immune dysfunction/anergy in fibrotic disease. **Discussion:** Fungal exposure likely increases severity in susceptible patients.

P38-26 | Strain-Dependent Phenotypic Variation and Genetic Influences on Sensitization Potentials Following Skin Exposure to 1,6-Hexamethylene Diisocyanate Oligomer and Monomer

Ian Choi[1], Jayne Boyer[1], Qing Shi[1], John E. French[2,3], Leena A. Nylander-French[1]

[1] University of North Carolina at Chapel Hill, Department of Environmental Sciences & Engineering, Chapel Hill, USA; [2] University of North Carolina at Chapel Hill, Department of Nutrition, Chapel Hill, USA; [3] University of North Carolina at Chapel Hill, Nutrition Research Institute, Kannapolis, USA

Objective: 1,6-Hexamethylene diisocyanate (HDI) and its oligomers are widely used in the automotive and aircraft refinishing paints and coatings. With the known potential for these compounds to cause occupational asthma, understanding the sensitization potential through skin exposure and the underlying mechanisms of these low-molecular-weight allergen-induced immune responses are crucial. We investigated phenotypic and genetic variations concerning skin sensitization in genetically diverse mouse strains following HDI oligomer (HDI isocyanurate, HDI-IC) and HDI monomer (HDI-M) skin exposure and airway challenge.
Material and Methods: Topical applications of HDI-IC and HDI-M at varying equimolar doses [0, 0.001, 0.01, 0.1, 1.0% v/v 4:1 ethyl acetate(EA):olive oil (OO) vehicle] followed by oropharyngeal challenge (0.1% v/v 1:4 EA:OO) was performed in ten Collaborative-Cross Recombinant Inbred Lines (CCRILs) as well as in C57BL/6 and BALB/cJ mice for reference. Bronchoalveolar lavage fluid (BALF) cell counts and plasma immunoglobulins IgE and IgG were measured for phenotypic biomarkers of exposure. Lung gene expression with selected human equivalent genes was explored. Group differences were evaluated using one-way analysis of variance (ANOVA), with $p < 0.05$.
Results: CCRILs exhibited more diverse phenotypic variations to HDI-IC in BALF cell counts and plasma IgE or IgG levels than those of C57BL/6 and BALB/cJ. Additionally, CCRILs displayed elevated cell responses and plasma IgE and IgG levels at baseline doses, compared to those of the reference strains. Dose-dependent immune responses were not observed. Notably, C57BL/6 exhibited compromised eosinophils at all doses of HDI-IC.
Conclusion: Genetic background influences HDI-IC sensitization potential, highlighting the importance of using genetically diverse models to understand and predict human responses to chemical allergens like isocyanates in genetically diverse worker populations. The study advances our understanding of HDI-induced asthma susceptibility based on genetic makeup and could provide scientific evidence to improve occupational safety measures accordingly.Funding: NIOSH/CDC R21OH011562

P38-27 | Spatiotemporal Patterns and Trends in Paediatric Asthma Hospitalisations in Queensland, Australia: A 20-Year Analysis Across Climate Zones

Jialu Wang[1], Javier Cortes-Ramirez[1], Janet Davies[2], Wenbiao Hu[1]

[1] Queensland University of Technology, School of Public Health and Social Work, Brisbane, Australia; [2] Queensland University of Technology, School of Biomedical Sciences, Brisbane, Australia

To examine the spatiotemporal trends of asthma hospitalisations among children and adolescents in Queensland from 2000 to 2019, focusing on variations in relative risks across different climate zones.
This retrospective, population-based study utilised asthma hospitalisation data from Queensland Health. Geographic patterns were assessed using Bayesian smoothing and Kriging-interpolated maps. Relative risks were calculated across age groups (0–4, 5–9, 10–14, and 15–19 years), sex, and distinct climate zones based on the Köppen climate classification, accounting for temporal shifts in risk distribution.

Children aged 0–4 years consistently had the highest proportion of hospitalisations, peaking at 55.04% in 2010 before declining to 23.19% by 2019. Adolescents aged 15–19 years had the lowest proportion (~10%). Males exhibited higher hospitalisation rates in younger age groups, whereas females had higher rates in the 15–19 age group. Distinct geographic and climate zone variations were observed. Initially, high-risk areas were found in southeastern Brisbane (2000–2006), shifting to central and western Queensland by 2010–2011 and southwestern Queensland by 2018–2019. Climate zone analysis revealed consistently higher risks in arid and subtropical regions, with fluctuations over time. The highest asthma burden was observed in drier inland regions, while coastal areas exhibited more variable trends. In recent years, risk patterns in tropical zones have also become more pronounced.

Significant variations in asthma hospitalisation risks across climate zones highlight the potential influence of climatic and environmental factors. The shifting spatiotemporal patterns suggest evolving risk dynamics, particularly in arid and subtropical zones, which may be linked to changes in temperature extremes, air quality, and humidity levels. These findings underscore the need for climate zone-specific asthma prevention strategies, including early warning systems and tailored public health interventions. Future research should integrate climatic and air pollution data to enhance risk prediction and inform targeted mitigation efforts.

P38-28 | Temperature inversions and emergency department visits for asthma in Salt Lake and Cache Couties, Utah, 1996-2017

John D. Beard[1], Carson T. Telford[1,2], Douglas G. Shumway[1], Cole G. Jensen[1,3], Maren M. Ball[1], Celeste Beck[4], Randall Graham[5], James D. Johnston[1], Chantel D. Sloan-Aagard[1]

[1] Brigham Young University, Public Health, Provo, USA; [2] University of North Carolina at Chapel Hill, Epidemiology, Chapel Hill, USA; [3] Yale University, Computational Biology and Biomedical Informatics, New Haven, USA; [4] Heluna Health, Research and Evaluation, City of Industry, USA; [5] National Weather Service, Kansas City, USA

Objective: Temperature inversions, which occur when cold air near the earth's surface is trapped by warmer air above, often lead to increased air pollution concentrations. Previous studies of inversions and emergency department (ED) visits for asthma have been limited in size and scope and did not consider features of inversions. We aimed to estimate associations between occurrence and features of inversions and ED visits for asthma.
Material and Methods: We conducted two time-stratified, case-crossover studies of 35,856 and 1,847 ED visits for asthma in Salt Lake and Cache Counties, respectively, in Utah from 1996-2017. We studied occurrence and features of inversions: height of bottom and top above ground level, depth, temperature at bottom and top, temperature differential, multiple inversions, number of inversion days during the lag period, day of inversion, inversion length. Inversions data were derived using weather balloons in Salt Lake County and ground-based monitors in Cache County. We used conditional logistic regression models and constrained quadratic polynomial distributed lag models to estimate odds ratios (OR) and 95% confidence intervals (CI) for associations between occurrence and features of inversions and ED visits for asthma during the 0-7 day-lag.
Results: In Salt Lake County, ED visits for asthma were positively associated with occurrence of inversions during the 0-7 day-lag (OR: 1.13; 95% CI: 1.07, 1.21). All features of inversions were positively associated with ED visits for asthma. In Cache County, ED visits for asthma were positively associated with occurrence of inversions at lag one (OR: 1.17; 95% CI: 1.01, 1.35), but inversely associated at lag six (OR: 0.85; 95% CI: 0.73, 0.98). Day of inversion and inversion length were positively associated with ED visits for asthma.
Conclusion: Occurrence and features of inversions were positively associated with ED visits for asthma in Salt Lake and Cache Counties in Utah.

P38-29 | Increased risk of respiratory disease during pollen season in Melbourne, Australia

Nicholas J. Osborne[1,2], Darsy Darssan[1], Sagnik Dey[3], Ed Lampugnani[4,5], **Arzoo Dhankhar**[1,3]

[1] University of Queensland, School of Public Health, Herston, Australia; [2] University of New South Wales, School of Population Health, Kensington, Australia; [3] Indian Institute of Technology Delhi, Centre for Atmospheric Sciences, Delhi, India; [4] University of Melbourne, Melbourne, Australia; [5] AirHealth, Melbourne, Australia

Objective

The purpose of this study is to use time series regression in Melbourne (population 5.21 million) to evaluate the short-term effects of pollen exposure on hospital visits and admissions related to respiratory and cardiovascular conditions.

Material and Methods

The Victorian Emergency Admitted Database and Victorian Emergency Minimum Dataset supplied hospital admission data for Greater Melbourne in the State of Victoria and we examined congestive heart failure, acute URI, asthma, ischemic heart diseases, COPD, Influenza and pneumonia, other acute LRI, other UR tract diseases, all cardiovascular diseases, all respiratory diseases. Patient information included age group and sex. Daily pollen counts (2003-2023) were used from a site in central Melbourne and were measured as grass pollen and total pollen. Relationships between total pollen and grass pollen levels and hospital visits was assessed for a variety of health outcomes using time series analysis. Models were adjusted for a range of other variables and potential confounders such as air pollution, age, sex and meteorological conditions.

Results

During October-December each year pollen ranged from 0-466/m^3 (grass) and 0-5312/m^3 (total pollen), with 95th percentiles 146/m^3 and 526/m^3 (grass and total pollen, respectively). Across Melbourne during October-December there was an average of 38.1 asthma visits and 23.6 ischaemic heart disease emergency visits (Minimum Dataset). A relationship was found between grass pollen and asthma hospital visits (RR 1.12 (95% CI 1.030, 1.23)) at 95th percentile grass pollen for lag day 1) but no relationship was found between grass pollen and ischaemic heart disease (RR 1.00, (95% CI 0.97, 1.03)) at 95th percentile grass pollen for lag day 1.

Conclusion

In Melbourne, Australia pollen is a risk factor for patients with asthma visiting emergency departments in hospitals. There was little evidence of increased the risk of emergency department visits for heart disease related diagnoses.

P38-30 | A novel prospective case time series evaluation of pollen levels and asthma and rhinitis symptoms in Melbourne, Australia

Rachel Tham[1,2], Hannah Murray[1], Prasanth Konduru[1], Jo Douglass[1,3]

[1] The University of Melbourne, Department of Medicine, Parkville, Australia; [2] The University of Melbourne, Allergy and Lung Health Unit, Parkville, Australia; [3] Royal Melbourne Hospital, Allergy and Immunology, Melbourne, Australia

Objective: Seasonal allergic rhinitis and allergic asthma are key risk factors for thunderstorm asthma, but there is limited prospective evidence on pollen exposure and allergic respiratory symptoms during the pollen season.
Methods: The TAISAR (Thunderstorm Asthma in Seasonal Allergic Rhinitis) study prospectively monitored self-reported asthma and rhinitis symptoms of 228 adults at risk for thunderstorm asthma over the 2018-2019 spring pollen seasons (1 October-31 December) in Melbourne, Australia using a smartphone (AirRater) app which recorded geolocated symptoms linked to the pollen and air quality conditions at the time and location of the report. The associations between 50 grains/m^3 increases in local pollen levels and symptoms were analysed using a distributed lag non-linear model adjusted for weather and air pollutants using a novel case-time series methodology. The associations were examined on the day of exposure and up to three days lag.
Results: Over 34,500 symptom reports were categorised into no symptoms, asthma and rhinitis. Of the 228 participants, 221 (96.1%) reported a history of seasonal allergic rhinitis and 140 (61%) a history of asthma. Increased grass pollen levels (50 grains/m^3) tended to be associated with asthma symptoms immediately (RR=1.02 95% CI 0.86-1.21) and three days after exposure (RR=1.15, 95%CI 0.98-1.32). Increased risk of allergic rhinitis symptoms occurred on the same day (RR=1.17, 95%CI 1.07-1.29) and one (RR=1.11, 95%CI 1.03-1.19) to two (RR=1.07, 95%CI 1.00-1.14) days after exposure.
Conclusion: Both immediate and delayed allergic respiratory symptoms occurred following exposure to elevated pollen counts. This should be considered in public health forecasting and managing allergic respiratory conditions.

P38-31 | Small-area assessment of pollen-related asthma hospitalisation for England: a case time series analysis.

Rebecca Cole[1], Leire Luque García[2,5], Arturo de la Cruz Libardi[1], Gillian Flower[1,4], Mikhail Sofiev[3], Antonio Gasparrini[1]

[1] London School of Hygiene and Tropical Medicine, Public Health and Policy, London, UK; [2] University of the Basque Country, Department of Preventive Medicine and Public Health, Bilbao, Spain; [3] Finnish Meteorological Institute, Atmospheric Composition Research, Helsinki, Finland; [4] UK Government, Department for Health and Social Care, London, UK; [5] Biogipuzkoa Health Research Institute, Environmental Epidemiology and Child Development Group, Donostia-San Sebastián, Spain

Objective: Existing evidence on the role of pollen exposure on asthma exacerbations is limited and heterogeneous, with inconsistent results for pollen type and age group. Here, we assess the association between pollen concentrations and asthma-related hospital admissions at small-area level across England.

Materials: Age-specific daily emergency asthma admissions were sourced from the Hospital Episode Statistics dataset and aggregated by 32,844 lower super output areas (LSOAs) in England. Daily pollen data (alder and birch) over a 10km grid were produced by the SILAM model and linked to LSOAs using area-weighted interpolation.

Analysis: We applied a two-stage design to account for spatial variations in risks, restricting the analysis to the pollen period (March-August) and analysing both species-specific and total pollen counts. In the first stage, we performed a case time series analysis through conditional Poisson regression of LSOA-specific data within 200 spatial clusters. Log-transformed pollen counts were modelled as linear with a 0-1 lag. Results were pooled in a second-stage meta-analysis. A secondary analysis coded pollen counts as low (<80), high (80-200) and very high (>200).

Results: Overall, asthma admissions increase by 0.7% (CI: 0.4%-0.9%) per doubling of pollen exposure. Statistically significant results are observed for ages 0-4 (1.5% CI: 0.7%-2.3%), 5-14 (1.1% CI: 0.5%-1.8%) and 15-29 (1.3% CI: 0.5%-2.1%), consistent with greater susceptibility to asthma exacerbation among younger individuals. High pollen was associated with a 1.029 (CI: 1.01-1.05) fold increase in hospital admissions, with no further increase for very high levels (0.988 CI:0.96-1.01) when compared with high. This is consistent with the log-transformed model, where risk increases more rapidly at lower exposure levels.

Conclusion: We find a significant effect of pollen levels on asthma-related hospital admissions for children and young adults, with the bulk of this effect concentrated at lower exposure levels. Further work aims to assess group and individual susceptibility.

P38-32 | Innovative approaches to asthma care: Home visits in Illinois

Evan Gustafson[1,2], Arlene Keddie[3], Cassandra Johnson[2], Heather Graham[2], Maddie Lamphear[2], M. Omar Khaium[2], Sofia Logacho[2], Jerrica V. Ampadu[4], Felicia Fuller[5], Julie Kuhn[6], Sarah D. Geiger[2]

[1] Southern Illinois University, School of Medicine, Springfield, USA; [2] University of Illinois at Urbana-Champaign, Department of Health and Kinesiology, Champaign, USA; [3] Northern Illinois University, Department of Health Studies, DeKalb, USA; [4] Southern Illinois University Edwardsville, School of Nursing, Edwardsville, USA; [5] American Lung Association, Chicago, USA; [6] Sinai Urban Health Institute, Chicago, USA

Objective: Asthma can significantly impact an individual's quality of life and create a substantial burden on individuals, families, and communities. Environmental factors, combined with limited awareness of effective asthma management strategies, can exacerbate inequities in care and health outcomes. Home visits are an impactful community-based interventiontoaddress these issues.The analysis aimed to explore predictors of asthma management in Illinois home visiting programs.

Material andMethods: We conducted cross-sectional analyses of baseline data among four home visiting programs. Visits were conducted both virtually and in person according to participant preference. Participants were classified into two groups: those with managed and unmanaged asthma, based on their scores from the Asthma Control Test (ACT). We compared these groups on various factors hypothesized to be associated with asthma control status.

Results: Mean age in our sample (N=271) was 23.4 years; 64.9% were female. Results of logistic regression analyses showed that for each additional asthma trigger, the odds of having well-managed asthma decreased by a factor of 0.86 (OR 0.86; $p < 0.05$). Nonsmokers had 9.49 times the odds of having managed asthma compared to smokers (OR = 9.49; $p < 0.05$). Participants without pests in their homes were more than 2.5 times as likely to have managed asthma compared to those with pests (OR = 2.66; $p < 0.05$).

Conclusions These results indicate a connection between health literacy, asthma education, and asthma management. Specifically, a greater understanding of asthma contributes to higher odds of effectively managing the condition. The data suggest that each increase in asthma knowledge enhances the likelihood of well-management asthma, underscoring the importance of asthma education as a key factor in successful asthma management. These analyses highlight the complex needs of different families and provide valuable insights that can guide programs to improve asthma management.

P38-33 | Mediation and Gene-Environment Interaction Analysis of Prenatal Phthalates and Bisphenols on Oxidative Stress with Childhood Allergic Diseases and Lung Function

Thomas Boissiere-O'Neill[1], Nina Lazarevic[2], Anne-Louise Ponsonby[3], Peter Sly[1], Aimin Chen[4], Tamara Blake[1], Cassidy Du Berry[5,7], Louise King[5,7], Cindy Pham[6,8], Samuel Tanner[3], Sarah Thomson[3], Dwan Vilcins[1], on behalf of the Barwon Infant Study Investigator Group

[1] The University of Queensland, Child Health Research Centre, The Children's Health and Environment Program, Brisbane, Australia; [2] Australian National University, National Centre for Epidemiology and Population Health, Canberra, Australia; [3] The University of Melbourne, Florey Institute of Neuroscience and Mental Health, Melbourne, Australia; [4] University of Pennsylvania, Department of Biostatistics, Epidemiology and Informatics, Perelman School of Medicine, Philadelphia, USA; [5] The Royal Children's Hospital, Department of Respiratory and Sleep Medicine, Melbourne, Australia; [6] The University of Melbourne, Department of Paediatrics, Melbourne, Australia; [7] Murdoch Children's Research Institute, Respiratory Group, Melbourne, Australia; [8] King's College London, Department of Forensic and Neurodevelopmental Sciences, Institute of Psychiatry, Psychology and Neuroscience, London, UK

Objective

Phthalates and bisphenols are plastic additives that may affect childhood allergies through oxidative stress. However, this area remains understudied. We aimed to examine whether the oxidative stress pathway during pregnancy mediated the association between prenatal phthalate and bisphenol exposure, childhood allergies, and lung function. Additionally, we examined whether genetic susceptibility to oxidative stress modifies these associations.

Material and Methods

Maternal urinary phthalates, bisphenols, and 8-hydroxydeoxyguanosine (8-OHdG) at 36 weeks gestation were measured in the Barwon Infant Study. Allergic outcomes included food allergy (oral food challenge, 1 year), atopy (skin prick tests, 4 years), and parent-reported asthma, wheeze, eczema, and rhinitis (4 years). Lung function (4 years) was determined by lung clearance index ($LCI_{2.5}$). Predisposition to oxidative stress was evaluated with a genetic pathway function score. Confounder-adjusted associations were analysed using generalised linear models for single compounds. We used a weighted quantile sum regression within a counterfactual framework for the mediation analysis of the exposure mixture and quantile G-computation for gene-environment interactions.

Results

833 children were included. While the total effects included the null, 10% of the increased risk of non-atopic rhinitis associated with the exposure mixture was mediated by 8-OHdG. Children genetically predisposed to oxidative stress had a higher likelihood of asthma (OR=4.47; 95% CI: 1.07-18.69) and increased $LCI_{2.5}$ (β = 1.06 SD; 95% CI: 0.06-2.07) associated with one quartile increase in the exposure mixture, compared to those not predisposed (asthma OR = 1.01; 95% CI: 0.60-1.70 and $LCI_{2.5}$ β = -0.04 SD; 95% CI: -0.38-0.30)

Conclusion

Prenatal exposure to phthalates and bisphenols may increase the risk of non-atopic rhinitis and impair childhood lung function through the oxidative stress pathway. Additionally, children with genetic susceptibility to oxidative stress may be at a greater risk of plastic chemical-related asthma.

P38-34 | The Impact of Farming Exposure on New Onset Asthma-Like Symptoms

Jesper S. Frandsen[1,2], Vivi Schlünssen[1], Ioannis Basinas[3], Anneli Bolund[4], Grethe Elholm[1,2], Signe Timm[5,6], Øyvind Omland[7], Torben Sigsgaard[1,2]

[1] Aarhus University, Public Health, Environment, Work and Health, Aarhus, Denmark; [2] Aarhus University, Danish Big Data Centre for Environment and Health (BERTHA), Aarhus, Denmark; [3] The University of Manchester, Health Services Research & Primary Care, Manchester, UK; [4] Aarhus University Hospital, Department of Clinical Genetics, Aarhus, Denmark; [5] University Hospital of Southern Denmark, Department of Oncolog, Vejle, Denmark; [6] University of Southern Denmark, Department of Regional Health Research, Vejle, Denmark; [7] Aalborg University Hospital, Department of Occupational Medicine, Aalborg, Denmark

Objectives: To examine the connection between adult-onset asthma like symptoms and exposure to farming. Methods: A 15-year follow up study of 917 Danish farming students and rural controls. Phenotypes and clinical examination were conducted at baseline and follow-up. Multiple Log regression assessed associations with adult-onset asthma. Asthma was defined as "definite or probable" according to the ECHRS algorithm previously employed in the baseline study and the ECRHS study.
Results: Almost 70% of 155 adult-onset probable asthma cases were non-atopic at baseline. Controlling for smoking, atopy, and bronchial responsiveness, upbringing at dairy farm reduced asthma risk; OR (95% CI) 0.5(0.3–0.9), while swine farm upbringing showed no association with new onset asthma-like symptoms. Working in dairy barn did not increase asthma risk, but working in swine confinements did; OR (95% CI) 1.8 (1.0–3.0).
Conclusion: The prevailing asthma type is in this adult cohort was non-atopic. Childhood exposure to dairy farming lowered asthma risk in adulthood, while occupational exposure to swine farming increased the risk of new onset asthma-like symptoms.

P38-35 | Genetic susceptibility to adult asthma interacts with occupational cleaning agent exposure in the Personalized Environment and Gene Study

Yujing Chen[1], Farida S. Akhtari[2], Chirag J. Patel[3], Alison Motsinger-Reif[2], Ming Kei Chung[1,4]

[1] The Chinese University of Hong Kong, The Jockey Club School of Public Health and Primary Care, Hong Kong, Hong Kong; [2] National Institute of Environmental Health Sciences, Biostatistics and Computational Biology Branch, Division of Intramural Research, Durham, USA; [3] Harvard University, Department of Biomedical Informatics, Harvard Medical School, Boston, USA; [4] The Chinese University of Hong Kong, Institute of Environment, Energy and Sustainability, Hong Kong, Hong Kong; [5] The Chinese University of Hong Kong, Li Ka Shing Institute of Health Sciences, Hong Kong, Hong Kong

Objective: To investigate the multiplicative and additive interaction between occupational cleaning agents and genetic risks on adult asthma using genome-wide and pathway-based approaches.

Materials and Methods: This study included 2615 adults (87.19% White individuals) from the Personalized Environment and Genes Study (PEGS) in North Carolina, with questionnaire-based health and exposure data and genomic data. Standardized questionnaires assessed ever exposure to any cleaning agent, ammonia, and chlorine bleach over 15 minutes/week in any job. Current adult asthma was defined as self-reported doctor-diagnosed asthma with an attack in the past year. Whole-genome sequencing was performed on blood samples by the Broad Institute. Genome-wide and pathway-specific polygenic risk scores (PRSs) were separately developed with external summary statistics and curated gene sets of oxidative stress and type 2 immune response pathways. Logistic regression analyzed interactions between PRS and exposure, and linear principal-component regression examined gene-based interactions.

Results: Current asthma was reported by 250 adults (9.56%). Ever exposure to any cleaning agent, particularly ammonia and bleach, was associated with increased asthma risk [adjusted odds ratio (aOR): 1.56-2.24]. Genome-wide and pathway-specific PRSs were positively associated with asthma. We identified multiplicative interaction between genome-wide PRS and any cleaning agent or bleach ($P_{multiplicativeinteraction}$=0.029 and 0.039). When genome-wide or pathway-specific PRSs were dichotomized into high and low genetic risk (median split), additive interactions between cleaning agents and high genetic risk were observed, with the highest asthma risk in individuals with both exposure and high genetic risk. Eight oxidative stress genes significantlyinteractedwith cleaning agents on asthma (Bonferroni-corrected $P_{interaction}$ < 0.05/29 genes).

Conclusion: This study suggests that cleaning agents may modify and synergistically interact with genetic risks of asthma. Precision prevention and intervention for occupational asthma should prioritize individuals with genetic susceptibilities.

Supported by a grant from the CUHK (code 4937217).

P38-36 | Tenant reports of in-home asthma triggers and neighborhood adult emergency department asthma use

Zichuan Li[1], Sophia S. Carryl[1], Elizabeth A. Samuels[2], Dinah Foer[3,4], Adam L. Haber[1]

[1] Harvard T.H. Chan School of Public Health, Department of Environmental Health, Boston, USA; [2] David Geffen School of Medicine, University of California, Department of Emergency Medicine, Los Angeles, USA; [3] Brigham and Women's Hospital, Divisions of Allergy and Clinical Immunology and General Internal Medicine and Primary Care, Department of Medicine, Boston, USA; [4] Harvard Medical School, Boston, USA

Objective: To test the association between tenant reports of in-home asthma triggers and the population-based incidence rate of asthma emergency department (ED) visits in Boston, Massachusetts.
Material and Methods: In this retrospective cross-sectional study, tenant reports of asthma triggers, i.e., asthma-related requests for inspections by a code enforcement officer for the City of Boston were obtained from the Analyze Boston website. Adult asthma ED visits for patients residing within Boston were obtained from a large health system's electronic health record. Tenant reports and ED data from 1/1/2014-12/31/2018 were aggregated at the block group level. Neighborhood demographics at the block group level were obtained from corresponding 5-year estimates of American Community Survey (2014-2018), and traffic associated air pollution data using the Traffic Proximity and Volume indicator (PTRAF) was obtained from the EPA's EJScreen tool. Associations were tested using a Bayesian generalized linear mixed model to account for spatial autocorrelation, block group demographics, household income, and PTRAF.
Results: 4,082 asthma ED visits from 2,753 unique patients and 14,752 tenant reports linked to 538 residential block groups were analyzed. The median (interquartile range) population-based incidence rate of tenant reports at the block group level was 6.49 (7.12) per 1,000 tenant-years, and of adult asthma ED visits was 9.32 (18.32) per 10,000 person-years. In adjusted models, for each increase of a tenant report per 1,000 tenant-years, there was a 3.0% (95% CI: 2.1-3.9%) increase in asthma ED visit rates.
Conclusion: Tenant-reported asthma triggers are substantially associated with adult asthma ED visits after adjusting for neighborhood-level confounders and spatial autocorrelation. These findings underscore the urgent need for policies to improve housing conditions to reduce neighborhood asthma burden.

P39 | Water Quality and Health

P39-01 | Residential well water and serum PFAS concentrations among individuals impacted by contamination from agricultural biosolids

Lisa B. Rokoff[1], Katherine Senechal[1], Anne M. Mullin[1,2], Julianne Botelho[3], Antonia M. Calafat[3], Rachel Criswell[4,5], Abby F. Fleisch[1,6]

[1] MaineHealth Institute for Research, Center for Interdisciplinary Population & Health Research, Westbrook, USA; [2] Tufts University School of Medicine, Boston, USA; [3] Centers for Disease Control and Prevention, Division of Laboratory Sciences, National Center for Environmental Health, Atlanta, USA; [4] Redington-Fairview General Hospital, Skowhegan Family Medicine, Skowhegan, USA; [5] Dartmouth College, Department of Epidemiology, Geisel School of Medicine, Lebanon, USA; [6] Maine Medical Center, Pediatric Endocrinology and Diabetes, Portland, USA

Objective: To describe residential well water and serum per- and polyfluoroalkyl substance (PFAS) concentrations among adults residing in central Maine where PFAS-contaminated biosolids were historically applied to farmland as fertilizer.

Methods: From September-December 2023, we collected serum from 145 Maine Biosolids Study participants who had PFAS measured in their private well water within the three prior years. We measured concentrations of seven PFAS in serum, and five of these were also assessed in water (PFOS, PFOA, PFHxS, PFNA, PFDA). We calculated geometric mean(standard deviation) [GM(SD)] and Spearman correlations of PFAS in water and serum. We used published serum half-lives to estimate peak PFOS and PFOA serum concentrations among participants whose water PFAS exceeded the state health advisory (∑PFOS, PFOA, PFHpA, PFNA, PFDA, PFHxS >20ng/L), and thus had a water filter placed by the Maine Department of Environment Protection.

Results: Participants were mean(SD) 62(13)years, 58% female, and 58% had water PFAS ≥20ng/L. In water, concentrations were highest for PFOA [13.6(20.1)ng/L], PFHpA [6.6(21.0)ng/L], and PFOS [3.6(14.6)ng/L]. Compared to the U.S. general population (i.e., NHANES ’17-18), GM(SD) serum PFOA [6.1(4.9)ng/mL)] was ~4x higher; PFOS [8.3(4.1)ng/mL], PFNA [1.3(3.8)ng/mL], and PFDA [0.4(4.1)ng/mL] were >1.5x higher. Of PFAS analyzed in both serum and water, PFOA had the strongest inter-matrix correlation (0.72); other PFAS had moderate inter-matrix correlations (0.32-0.48). Among participants with water PFAS >20ng/L, GM of estimated peak serum PFOA was 17.6ng/mL and PFOS was 14.2ng/mL.

Conclusions: Individuals exposed to PFAS-contaminated biosolids had highly prevalent and correlated concentrations of PFOA in their water and serum. Serum PFOS, PFNA, and PFDA were also elevated but more moderately correlated with drinking water concentrations, suggesting potential exposure to these PFAS via other pathways (e.g., occupation, diet). In future work, we will explore other non-water exposure pathways, and the psychosocial impact of living in a community impacted by PFAS-contaminated biosolids.

P39-02 | Association between Metal Exposure and Oxidative Stress Based on Drinking Water Sources Among Residents Living Near the Smelter

Mijeong Kim[1], Yong Min Cho[1], Dahee Han[1], Haneul Kim[1], Kyung-Hwa Choi[2], Woo Jin Kim[3], Young-Seoub Hong[4]

[1] Seokyeong University, Institute of Environmental Health, Seoul, South Korea; [2] Dankook University College of Medicine, Department of Preventive Medicine, Cheonan, South Korea; [3] Kangwon National University, Department of Internal Medicine and Environmental Health Center, Chuncheon, South Korea; [4] Dong-A University College of Medicine, Department of Preventive Medicine, Busan, South Korea

Objective: Metals are well known for its environmental and health impacts, particularly in areas with active industrial activities. This study aims to assess metal exposure through drinking water among residents living near a smelter and analyze its association with oxidative stress.

Materials and Methods: Urine samples were collected from 110 residents living near a smelter, and surveys were conducted on general characteristics, and drinking water. Nine metals (mercury, vanadium [V], chromium [Cr], manganese, nickel [Ni], molybdenum [Mo], cadmium [Cd], antimony [Sb], and lead [Pb]) were analyzed using inductively coupled plasma-mass spectrometry. Malondialdehyde (MDA) and 8-hydroxy-2'deoxyguanosine (8-OHdG) were analyzed using high-performance liquid chromatography and Enzyme-linked immunosorbent assay, respectively. Metal concentrations were compared with those from the Korea National Environmental Health Survey (KoNEHs). Student's t-test was performed to compare urinary metal concentrations based on drinking water (local-based or non-local-based water). General linear regression was conducted to examine the association between oxidative stress and urinary metal concentrations, adjusting for age, sex, income, smoking, alcohol consumption, and occupational exposure.

Results: Participants hadhigher concentrations of urinary metals than the general population in Korea, with V, Cr, Mo, Cd, Sb, and Pb showing significantly elevated levels. Residents consuming local-based water showed higher levels of all nine metals than those consuming non-local-based water, with only V displaying statistically significant differences (p-value=0.012). Among the metals, Mo, Cd, and Ni significantly increased 8-OHdG. MDA levels were significantly increased with V and Mo.

Conclusion: These findings suggest drinking water sources may influence metal exposure and consequently affect oxidative stress. Improving drinking water monitoring near smelters is essential for reducing metal exposure and health risks.**Funding: This work was supported by Korea Environmental Industry & Technology Institute (KEITI) through Core Technology Development Project for Environmental Diseases Prevention and Management Program, funded by Korea Ministry of Environment (MOE) (2480000101/RS-2021-KE001378).**

P39-03 | Perception of Drinking Water Quality and Detection of Per- and Polyfluoroalkyl Substances in Tap Water: Evidence from Two Community-Based Studies in Los Angeles

Shiwen Li[1], Elizabeth Costello[1], Tamar Galindo[1], Bianca Costa[2], Ana-Sofia Arena[1], Rebecca Schielzeth[2], Connie Valencia[1], Tomas Manea[1], Christelle Sawaya[2], Dominic Pak[1], Carmen Jiawen Chen[1], Shudi Pan[1], Jesse A. Goodrich[1], David V. Conti[1], Lucy Golden-Mason[3], Ana C. Maretti-Mira[3], Daniel L. McCurry[2], Amy E. Childress[2], Rob McConnell[1], Adam L. Smith[2], Lida Chatzi[1], Max Aung[1]

[1] University of Southern California, Department of Population and Public Health Sciences, Los Angeles, USA; [2] University of Southern California, Sonny Astani Department of Civil and Environmental Engineering, Los Angeles, USA; [3] University of Southern California, 3. Department of Medicine, Los Angeles, USA

OBJECTIVE

Per- and polyfluoroalkyl substances (PFAS) contamination in tap water poses potential health risks, but its alignment with public trust in water quality remains unclear. This study examines the association between PFAS detection in water and perceptions of drinking water safety in two community-based studies in Los Angeles (LA).

METHODS

We included participants from two studies in LA: PFAS ACT Study (n=48) and Focus Group Study (n=29). In both studies, participants provided tap/bottled water samples and completed a questionnaire assessing their perceptions of water safety. PFAS concentrations were quantified using liquid chromatography-mass spectrometry. In the Focus Group study, we conducted semi-structured interviews/focus groups on water safety.

RESULTS

In the PFAS ACT study, 80% of participants drank alternative water sources where perfluorobutane sulfonic acid (PFBS) (80%) and 6:2 fluorotelomer sulfonate (FTS) (50%) were commonly detected. People with higher level of knowledge of where to find information about their water quality had higher detection of PFAS in their tap water (Spearman's $\rho=0.37$, $p=0.01$). In the second study, PFAS were detected in 50% of tap water samples from small water systems but none in large systems. Participants from small water systems reported lower confidence in water quality ($\chi^2=4.39$, $p=0.04$). Qualitative data analysis suggested most participants preferred not to drink tap water although there's skepticism about the safety of bottled water. Participants suggested actions to build trust, such as regular community reports on water quality, visible indicators (i.e., tags with test dates) on water infrastructure, and educational resources to help individuals assess water safety at home.

CONCLUSION

These studies showed that PFAS were detected in tap/bottled water. Detection of PFAS was correlated with lower confidence in water safety and higher knowledge of water quality. Transparent reporting, visible infrastructure indicators, and educational resources may help empower communities to make informed decisions about drinking water.

P39-04 | Benefits of drinking water treatment to remove contaminants co-occurring with per- and polyfluoroalkyl substances in the United States

Sydney Evans, Varun Subramaniam, Anna Cullen, Tasha Stoiber, Chris Campbell, Olga Naidenko, David Andrews

Environmental Working Group, Washington DC, USA

Objective

Public drinking water quality is a critical environmental health equity issue. Understanding which contaminants co-occur in public drinking water systems and whether they can be concurrently removed or reduced through filtration is essential for cost-effective public health protection, particularly for communities most exposed.

Materials and Methods

We analyzed contaminant co-occurrence patterns in public drinking water systems across the United States, subsequently focusing on systems with high levels of PFAS. Data on advanced filtration technologies, including reverse osmosis (RO), granular activated carbon (GAC), and ion exchange (IX), as well as 12 reported treatment objectives were also assessed across different system sizes.

Results

Co-occurring contamination is widespread in U.S. drinking water systems. Over 40% (*n*=631) of systems with PFAS detections (*n*=1,439) also reported detecting contaminants from four other groups: metals, disinfection byproducts, nitrate/nitrite, and organic contaminants. On average, systems with PFAS detections also reported detecting contaminants belonging to three other groups. The implementation of advanced filtration technologies varies significantly by system size. Among very large systems (serving >100,000 people, *n*=417), 28% (*n*=116) implemented RO, GAC, or IX, compared to only 7% (*n*=2,050) of small systems (serving <3,300 people, *n*=28,759).**Conclusion**

Current drinking water regulations in the United States established through the Safe Water Drinking Act Amendments of 1996 require a cost benefit analysis to show net public benefit. Since 1996, no new drinking water regulations have yet been finalized for an unregulated contaminant using this regulatory process. Approaching drinking water regulation through a lens of treatment standards for co-occurring contaminants provides an opportunity to more accurately capture the health benefits that come with additional filtration. This approach also highlights how smaller systems inadequately address common contaminant mixtures, potentially posing compounded health risks for exposed, rural populations.

Funding

Research for this study was supported by donations from members of the public.

P39-05 | Health Impacts of Tijuana River Pollution: Preliminary Results from A Community-Based Survey on Environmental and Public Health Challenges

Yu Ni, Penelope J. Quintana, Eyal Oren, Eunha Hoh, Nicolas Lopez-Galvez, Miguel A.Z. Perez, Linda Lara-Jacobo, Kai-Chung Cheng, Jade Johnson, Yaritza Benitez, Elora Shakoor, Laura Magaña, Shannon McBride, Lauren Bonneval, Paula S. Granados

San Diego State University, College of Health and Human Services, San Diego, USA

Background: Raw sewage and industrial pollution from Tijuana, Mexico flow north via the Tijuana River into the estuary in southern San Diego County, CA, creating a longstanding transboundary pollution crisis. The situation has significantly worsened in 2024, with residents reporting unbearable odors and major health concerns.
Methods: A bilingual (Spanish/English) rapid response survey was launched online to evaluate the pollution's impact on community wellbeing. Individuals living and working near the estuary were recruited through outreach to community organizations, flyer distribution in targeted locations, and social media. The survey captured environmental perceptions and health symptoms across several domains. We fitted Poisson regressions to estimate the pollution-health symptom associations, controlling for sociodemographic and lifestyle factors.
Results: Since Oct 2024, 304 participants (40% Hispanic) have completed the survey. Three-quarters reported upper respiratory symptoms in the past two weeks, and 47% experienced diarrhea. Other common symptoms included allergic reactions (69%), headaches (77%), and trouble sleeping (64%). Most participants (78%) rated air quality as unhealthy, very unhealthy, or hazardous, and 23% reported severe odors daily. Compared to those perceiving neighborhood air quality as good or moderate, participants who rated it as unhealthy, very unhealthy, or hazardous were more likely to report various health issues – prevalence ratios (PR) of 1.2-1.5 for upper respiratory symptoms, 1.5-1.8 for allergic reactions, 2.6-3.0 for diarrhea, and 3.4-3.9 for trouble sleeping. Strong odors were associated with headaches (PR: 1.4–1.6) and loss of appetite (PR: 2.4–4.7).
Conclusion: The survey results underscore the severity of the sewage crisis. Follow-up surveys aiming to track changes over time are underway. Immediate action is needed to reduce sewage flows and improve living environment for affected environmental justice communities.

P39-06 | Evaluating the Prevalence of Antibiotics and Pesticides in Agricultural Irrigation Water in Mbeya, Tanzania

Ibiyinka M. Amokeodo[1], Amy Sapkota[1], Paul Turner[1], Eliezer Mwakalapa[2], Lillyanna House[1]

[1] University of Maryland, Global, Environmental and Occupational Health, College Park, USA; [2] Mbeya University of Science and Technology, Mbeya, Tanzania

Objective:
Water used for irrigation is at risk of chemical contamination from pesticides via runoff and from antibiotics via the inappropriate disposal of household wastewater. These chemicals are both a risk to the farmers and the consumers of food produced. To date, there are no data on the frequency or levels of these contaminants in irrigation waters from farms in Mbeya, Tanzania. In this study, the associations between contaminant concentrations and key water quality and environmental parameters were examined.

Materials and Methods:
Groundwater and surface water sources for agricultural irrigation were sampled at 10 farms in Mbeya between July 2024 and February 2025. Water quality (e.g., pH, turbidity) and environmental parameters (e.g., temperature) were measured at each sampling site. All water samples (n = 109 total) are being analyzed for target chemical contaminants using high-performance liquid chromatography-tandem mass spectrometry (LC-MS/MS). Regression analysis will be used to evaluate associations between contaminant levels and environmental factors.

Results:
We anticipate frequent detection of diverse pesticides and antibiotics across multiple irrigation water sources. Variability in contaminant concentrations is expected between surface and groundwater sources, with potential associations between contaminant levels and key water quality parameters.

Conclusion:
Our findings will be the first of their kind from this area of Tanzania and will provide critical insights into the prevalence and distribution of chemical contaminants in irrigation water, informing future agricultural water management strategies and public health risk assessments. Understanding the associations between contaminants and environmental parameters may support the development of targeted interventions to mitigate risks associated with agricultural irrigation water in Tanzania.

Funding & Acknowledgements:
This research is supported by the UMD Global FEWture Alliance, funded by the University of Maryland Grand Challenges Program. We acknowledge Mbeya Farmers and lab assistants at Mbeya University of Science and Technology for supporting sample collection and analysis.

P39-07 | Residential history and PFAS exposures from contaminated drinking water: A comparison of two Massachusetts communities

Emily Heckel[1], Rebecca DeVries[2], Abigail Bline[3], Yuting Wang[1], Marlee Quinn[3], Naida Gavrelis[2], Ruthann Rudel[1], Tamarra James-Todd[3,4], Laurel Schaider[1]

[1] Silent Spring Institute, Newton, USA; [2] Eastern Research Group, Concord, USA; [3] Harvard T.H. Chan School of Public Health, Department of Environmental Health, Boston, USA; [4] Harvard T.H. Chan School of Public Health, Department of Epidemiology, Boston, USA

Objective: Drinking water is considered the main source of per- and polyfluoroalkyl substance (PFAS) exposure for communities impacted by water contamination. Even after installation of water treatment, residents may continue to have elevated blood PFAS levels. However, the duration of elevated body burdens and influence of water-related behaviors (e.g., bottled water consumption, home filtration) are poorly characterized.

Methods: The MA PFAS & Your Health Study is part of the PFAS Multi-Site Study (MSS), a cross-sectional epidemiological study in 8 US states. We collected blood samples and questionnaire data from adults and children (2021-2023) in two Massachusetts communities (Hyannis, Ayer) where municipal supplies were contaminated by aqueous film-forming foam (AFFF). We analyzed residential histories of adults (n=399), ages 18-89, and calculated time spent in the study area between 2000 and the start of municipal water treatment (Hyannis: 2016, Ayer: 2018). We used multivariable linear regressions to examine relationships between years in the study area and serum perfluorooctane sulfonic acid (PFOS), perfluorohexane sulfonic acid (PFHxS), perfluorooctanoate (PFOA), and perfluorononanoate (PFNA).

Results: In both communities, time in the study area was associated with higher serum PFOS, PFHxS, and PFOA. Median PFOS and PFHxS were 2-2.5 times higher in Hyannis than Ayer, consistent with higher AFFF contamination in Hyannis. Long-term Hyannis residents (>15 years) had 1.9 times higher PFOS and 3.9 times higher PFHxS than long-term Ayer residents. In Hyannis, residential history and home filtration were stronger predictors of serum PFAS compared with Ayer.

Conclusion: Our findings demonstrate that long-chain PFAS serum levels can remain elevated for years after installation of municipal water treatment and exposure reductions from home filtration and bottled water consumption are reflected in long-term serum levels. Our findings will inform MSS analyses of potential associations between PFAS exposures from drinking water and health effects.

Funding was provided by ATSDR/CDC.

P39-08 | Per- and polyfluoroalkyl substances (PFAS) serum concentrations and half-lives among residents exposed to contaminated drinking water in Newburgh, New York

Yichun Liu, Ming Liu, June Moore, Steven P. Forand

New York State Department of Health, Bureau of Environmental & Occupational Epidemiology, Albany, USA

Objective:
City of Newburgh in upstate New York, USA, is near a military installation that used firefighting foams containing PFAS. Elevated PFAS were detected in the City's drinking water supply and by May 2016 the city was able to switch to an alternate water source. The New York State Department of Health (NYSDOH) conducted two rounds of PFAS biomonitoring in the Newburgh area between 2016 and 2024. In this study, we aimed to evaluate expected declines in serum PFAS levels and to estimate PFAS half-lives in this population.

Material andMethods:
The NYSDOH tested serum for six PFAS between 2016 and 2017 and 11 PFAS in 2024. Approximately 1,900 people served by Newburgh public water participated in serum testing by 2017. In 2024, 126 people participated in serum testing again. A questionnaire was conducted in both rounds to collect demographic and exposure information.

Results:
For the 122 adult participants served by Newburgh public water who participated in both rounds of serum testing, the results in 2024 showed that the highest PFAS detected was PFOS (geometric mean [GM] = 7.96 micrograms per liter [mcg/L], 95% confidence interval [CI]: 6.86-9.24), followed by PFHxS (GM = 6.42 mcg/L, CI: 5.35-7.71) and PFOA (GM = 1.34 mcg/L, CI: 1.18-1.52), with median declines of 62.3% (interquartile range[IQR]: 49.8%-72.1%), 47.9% (IQR: 38.4%-59.3%) and 61.3% (IQR: 48.5%-70.5%), respectively, from the initial testing. The estimated GM half-life was 5.4 years for PFOS, 6.9 years for PFHxS and 5.0 years for PFOA.

Conclusion: The results from two rounds of biomonitoring show the expected declines of serum PFAS levels after end of exposure to contaminated drinking water. Further research can focus on identifying important factors associated with serum half-lives of PFAS.

P39-09 | Effect of Seasonal Variability and Climatic Factors on Enteric and Respiratory Pathogens: Exploring Wastewater Surveillance in Dhaka City

Farjana Jahan, Rezaul Hasan, Mizanul Nasim, Afroza J. Suchana, Sk.Kamrul Bashar, Mahbubur Rahman

icddr,b, Environmental Health and WASH, Dhaka, Bangladesh

Background: Seasonal meteorological variations influence the spread of infectious diseases. Wastewater surveillance helps understanding pathogen transmission dynamics, particularly in urban areas of climate-vulnerable countries like Bangladesh.

Methods: We analyzed 52 weeks of wastewater surveillance, clinical surveillance, and meteorological data from Dhaka, Bangladesh. Samples from 11 sites were tested for *Vibrio cholerae (V. cholerae)*, SARS-CoV-2, *Salmonella enterica* subspecies *enterica* serovar Typhi (*S.* Typhi), and Group A rotavirus. Cholera case data were sourced from icddr,b, and meteorological data from the Bangladesh Meteorological Department. Regression models adjusted for site variations were used for statistical analysis.

Results: Cholera cases were highest during monsoon (adj. coef: 1.63; 95% CI: 1.21 to 2.05; $p < 0.001$). *V. cholerae* log10 concentrations in wastewater were positively associated with pre-monsoon (adj. coef: 0.92; 95% CI: 0.4 to 1.45; $p = 0.001$), while SARS-CoV-2 peaked during monsoon (adj. coef: 1.83; 95% CI: 1.34 to 2.32; $p < 0.001$). *S.* Typhi and rotavirus log10 concentrations showed negative associations with pre-monsoon (adj. coef: -0.96; 95% CI: -1.52 to -0.39; $p = 0.001$, and -0.84; 95% CI: -1.16 to -0.52; $p < 0.001$, respectively). Temperature positively influenced log10 concentrations of *V. cholerae* (adj. coef: 0.09; 95% CI: 0.04 to 0.13; $p < 0.001$) and SARS-CoV-2 (adj. coef: 0.17; 95% CI: 0.12 to 0.21; $p < 0.001$), but negatively associated with rotavirus (adj. coef: -0.06; 95% CI: -0.09 to -0.03; $p < 0.001$). Similar associations were found between pathogen-positive samples and temperature.

Conclusion: Our study shows that seasonal, and meteorological factors (particularly temperature) influence the patterns and abundance of pathogens in wastewater and help in understanding disease transmission across different weather patterns.

P39-10 | Data needs on micro- and nanoplastics in water to determine exposure and potential human health effects

Max A. Zarate-Bermudez, Yulia Carroll

Centers for Disease Control and Prevention (CDC), National Center for Environmental Health (NCEH), Division of Environmental Health Science and Practice, Atlanta, USA

Objective:
Human exposure to micro- and nanoplastics (MNPs) in water and their potential health effects are a serious public health concern nowadays. Following an extensive review of the scientific literature, we present the data needs identified to better understand human exposure to MNPs in water.
Materials and methods:
We selected 106 out of 6,472 papers via a rigorous selection process using Covidence. After screening titles and abstracts and reviewing full texts, the papers selected passed our quality assessment for data extraction. We organized findings by water matrices, methods used to characterize MNPs, and efforts conducted to control their mobility to minimize exposures.
Results:
Findings of the selected studies (2018 – 2021) showed the lack of standard methods for sampling, sample preparation, and characterization of MNPs in water. Authors used a diversity of methods, thus findings reported need a careful review for accurate interpretation. The latter is crucial to conduct exposure assessment studies. Authors identified ~40 polymers and used mostly a type of Fourier Transform Infrared spectroscopy (61%) to chemically characterize MNPs followed by a type of Raman spectroscopy (24%); pyrolysis gas chromatography-mass spectrometry was not yet widely used. Also, we found microscopic techniques like scanning or transmission electron microscopy could provide useful structural insights to describe MPs' physical characteristics.
Conclusion:
The lack of standard methods represents a barrier to assess potential associations of human exposure to MNPs in water and harmful health effects. However, understanding how MNPs enter drinking water sources and how they reach the tap is vital to minimize exposures. Thus, developing standard methods for sampling and characterizing MNPs is urgent to advance exposure and risk assessments.
Desclaimer:
The findings and conclusions in this document are those of the authors and do not necessarily represent the official position of the U.S. Centers for Disease Control and Prevention.

P39-11 | The Spatial, Temporal, and Demographic Distribution of Sporadic Cryptosporidiosis in Minnesota, USA: 2010 – 2019

Ryan Gavin[1,2], Bruce Alexander[1], Jeff Bender[1], Harrison Quick[2]

[1] University of Minnesota, Division of Environmental Health Sciences, Minneapolis, USA; [2] University of Minnesota, Division of Biostatistics and Health Data Science, Minneapolis, USA

Objective: Cryptosporidiosis is a common cause of waterborne illnesses in the United States. While the risk factors associated with outbreak-related cryptosporidiosis are well described, less is known about the epidemiology of sporadic cryptosporidiosis, which constitutes the majority of cases. Here, we describe the spatial, temporal, and demographic distribution of sporadic cryptosporidiosis by etiologic species (*C. parvum* and *C. hominis*) in Minnesota, USA between 2010 and 2019.

Methods: We obtained information on sporadic cases from the Minnesota Department of Health. After screening for eligibility, we fit Poisson regression models to each species' case data to estimate the 1) 10-year incidence rate by county, 2) monthly 10-year incidence rate by demographic group, and 3) annual incidence rate by demographic group for each species type. Finally, we fit binomial models to estimate the demographic distribution of cases by species type and compared these distributions to the average demographic distribution of Minnesota between 2010 and 2019.

Results: Our analytical sample contained 1,362 *C. parvum* cases and 339 *C. hominis* cases. The annual incidence of *C. parvum* cases is increasing over time amongst rural individuals, white individuals, and young individuals. The 10-year incidence of *C. parvum* cases followed seasonal and spatial patterns, with incidence peaking in August and being greater in west-central and southeastern Minnesota than in northern Minnesota or the 7-county twin-cities metropolitan area. *C. hominis* case occurrence followed a seasonal pattern, with cases peaking in September, but we saw no other clear spatial, temporal, or demographic trends.

Conclusion: The incidence of sporadic cryptosporidiosis in Minnesota varies spatially, temporally, and by demographic group. The overall incidence of *C. parvum* cases in Minnesota appears to be increasing, especially amongst rural individuals, suggesting specific environmental determinants of disease occurrence like climate, land use, and agriculture merit further investigation.

P39-12 | Biosynthesis of *Pseudomonas aeruginosa*-Mediated Silver Nanoparticles for Remediation of Crude Oil-Contaminated Water

Agatha A. Ikhumetse[1], Olabisi P. Abioye[2], Abdulsalami S. Kovo[3], Udeme J. Ijah[4], Sources of fundingThe study was not funded by any organization, the authors contributed their financial resources for the study.

[1] Federal University of Technology, Microbiology, Minna, Nigeria; [2] Federal University of Technology, Public Health, Minna, Nigeria; [3] Federal University of Technology, Chemical Engineering, Minna, Nigeria; [4] Federal University of Technology, Microbiology, Minna, Nigeria

Objective

This study aimed to synthesize bacterial silver nanoparticles (AgNPs) for the remediation of crude oil contamination in aquatic environments. The research utilized a novel bacterium, *Pseudomonas aeruginosa* strain AgA, isolated from crude oil-contaminated water samples from the Jones Creek in the Niger-Delta region of Nigeria.

Material and Methods

The isolate was identified at the molecular level, facilitating the synthesis of AgNP, which was characterized using UV–visible spectroscopy, dynamic light scattering (DLS), X-Ray diffraction (XRD), transmission electron microscopy (TEM) and energy dispersive spectroscopy (EDS) techniques. The Total Petroleum Hydrocarbons (TPH) in the water samples were adsorbed using the biosynthesized AgNP. The TPH response was designed and optimized using response surface methodology (RSM).

Results

The 16S rDNA sequencing showed that the microbe is related to *Pseudomonas aeruginosa*. The isolate was registered in the NCBI Bank and called *Pseudomonas aeruginosa* strain AgA with accession number ON600888. The optical properties of the AgNP showed a peak at 425 nm, was spherical with an average particle size distribution of 34.60±13.04 nm, and a significant signal in the silver region, as ascertained by TEM, DLS and EDS investigations. The AgNP's crystalline structure was revealed by the XRD patterns, with average mean crystallite size of 18.01504±4.03 nm. The RSM for design and optimization showed a good fit with the 2FI regression model for the AgNP with a coefficient of determination (R^2) of 0.9190. The maximum expected TPH removal was 99.98% with optimum contact time (22.5 min/100 mL), stirring speed (1620 rpm/100 mL), dosage (0.3206 g) and temperature (67.5 ^oC).

ConclusionThis proved that the bacterium was useful in synthesizing AgNPs for the efficient removal of contaminants from water, and that the model developed (2FI) using the RSM technique was useful in determining the ideal conditions for TPH removal in the Niger-Delta aquatic environment.

P39-13 | Bacteriological and Physicochemical Analyses of Crude Oil-Contaminated Surface Water from the Jones Creek Oil Field, Niger-Delta, Nigeria

Agatha A. Ikhumetse[1], Olabisi P. Abioye[2], Abdulsalami S. Kovo[3], Udeme J. Ijah[4], Sources of fundingThe study was not funded by any organization, the authors contributed their financial resources for the study.

[1] Federal University of Technology, Department of Microbiology, Minna, Nigeria; [2] Federal University of Technology, Department of Public Health, Minna, Nigeria; [3] Federal University of Technology, Department of Chemical Engineering, Minna, Nigeria; [4] Federal University of Technology, Department of Microbiology, Minna, Nigeria

Objective

This research focused on determining the bacteriological and physicochemical assessments of crude oil-contaminated surface water from the Jones Creek Oil Field, Niger-Delta, Nigeria

Material and Methods

Water samples from three communities (Akpata Ekpemu, Omadenor and Okerenkoko) in the Jones Creek Oil Field in Nigeria's Niger Delta were evaluated for bacteriological quality using standard protocols. The physicochemical properties of the water samples were determined using established techniques. Gas Chromatography/Mass Spectroscopy (GC/MS) was used to measure the amount of total petroleum hydrocarbons (TPH) in the samples after the TPH was extracted using a conventional solvent extraction procedure.

Results

The total heterotrophic bacterial counts and total hydrocarbon utilizing bacterial counts ranged from 16.67±2.73 X 10^5 - 120.33±0.88 X 10^5 cfu/mL and 2.33±0.88 X 10^3 - 23.33±2.96 X 10^3 cfu/mL respectively. Bacterial isolates from the water samples included *Pseudomonas aeruginosa, Pseudochrobactrum asaccharolyticum, Bacillus megaterium, Micrococcus luteus, Bacillus subtilis, Escherichia coli, Bacillus cereus, Macrococcus caseolyticus,* and *Bacillus thuringiensis.Pseudomonas aeruginosa, Pseudochrobactrum asaccharolyticum* and *Macrococcus caseolyticus,* were the most frequently isolated bacteria based on their frequency of occurrence (20.49%, 16.39%, and 13.93%, respectively).The results revealed that pH, turbidity, concentrations of potassium, calcium, BOD_5, nitrates, sulphates and phosphates in all water samples were below the WHO limits while the mean concentrations of electrical conductivity, total hardness, total dissolved solids, dissolved oxygen, chemical oxygen demand, chlorides and TPH (10750.00±50.42 - 13000.00±10.65 μs/cm, 372.00±28.28 - 1132.00±28.56 mg/L, 5350.00±50.11- 6450.00±50.62 mg/L, 14.10±1.25 - 17.75±0.15 mg/L, 540.00±55.27 - 860.00±60.00. mg/L, 167.43±2.22 - 466.40±3.12 mg/L, and 9.00±1.60 - 380.85±0.55 mg/L respectively) in all samples were above the permissible limits for surface water.

Conclusion

The study's conclusions showed that microbes and organic matter had contaminated the watercourse. As a result, the water does not meet WHO requirements for surface water and it is an effective way to spread waterborne diseases.

P39-14 | Development of a Poly-γ-Glutamic Acid Flocculation Method for Enhanced Virus Recovery from Wastewater

Cathleen Horng, Minola M. De Silva, Nicola K. Beck, John S. Meschke

University of Washington, Department of Environmental & Occupational Health Science, Seattle, USA

Environmental surveillance plays a critical role in monitoring viral prevalence and assessing disease burden, particularly for waterborne pathogens transmitted through fecal contamination. Current virus recovery methods, such as the Bag-Mediated Filtration System (BMFS), ultrafiltration, and skimmed milk flocculation, are often time-consuming, costly, or inefficient, potentially underestimating viral prevalence. This study investigates the potential of Poly-γ-Glutamic Acid (PGA), a flocculant used in wastewater treatment, for enhancing virus recovery from aquatic environments.

PolyGlu (PGL), a PGA-based product, was evaluated for its ability to recover nonenveloped bacteriophage MS2, a surrogate for human viruses, under varying conditions, including processed volumes (5 mL vs. 10 mL), sample types (pellet vs. supernatant), groups (silica powder alone vs. silica with PGL), and water sources (lake water vs. wastewater). Recovery efficiency (RE) was calculated to assess PGL's performance.

Results showed no significant effect of processed volume on MS2 concentration or RE, though sediment buildup suggested potential sample loss. Supernatant samples exhibited higher RE than pellets. PGL significantly improved virus recovery in lake water compared to silica powder alone, but its effect was less pronounced in wastewater. Generalized Linear Model (GLM) analysis indicated that treatment groups and sample types significantly influenced MS2 concentration and RE. Although PGL enhanced virus recovery in lake water samples (13.06%), further optimization is required. Adjustments to factors such as cation presence, pH levels, stirring conditions, temperature, and PGA dosage could further improve recovery efficiency.This study highlights the potential of PGL as a rapid, cost-effective method for virus recovery. However, further refinements are necessary to improve its reliability for environmental surveillance and public health applications. Further research is needed to optimize PGL-based virus concentration methods for broader implementation.

P39-15 | Method development and quantification of ultra-short to ultra-long chain PFASs in agricultural soil, runoff, and wastewater.

David Dukes[1], Grace Kilroy[2], Jacqueline MacDonald Gibson[2], Amina Salamova[1]

[1] Emory University, Gangarosa Department of Environmental Health, Atlanta, USA; [2] North Carolina State University, Department of Civil, Construction, and Environmental Engineering, Raleigh, USA

Per and polyfluoroalkyl substances (PFAS), often called forever-chemicals, are a group of thousands of different compounds typically used to make products oil and water-repellant. The characteristic carbon-fluorine chain that defines PFAS gives these compounds high thermal and chemical stability making them incredibly persistent in the environment. Additionally, PFAS have been linked to a variety of negative health outcomes ranging from multiple forms of cancer to high cholesterol. Common routes of PFAS exposure include ingestion of PFAS-contaminated drinking water and food and inhalation of contaminated indoor air and dust. Recently, agricultural products such as grains, vegetables, and fruits have been found to readily uptake some PFAS (particularly short-chain perfluoroalkyl

carboxylates). PFAS and biotransformation of perfluoroalkyl acid precursors (pre-PFAAs) in soil can act as a persistent long-term source of contamination to agricultural plants. Common sources of PFAS in agriculture include irrigation with contaminated water, pesticides, sewage sludge/biosolids, and industrial/municipal compost. Further, contamination of surface water and groundwater from the runoff or leachate of contaminated soil is possible. To evaluate and mitigate risks to our food chain as well as source and irrigation waters a comprehensive, quantitative analytical method for ultra-short to ultra-long chain PFAS and pre-PFAAs is needed. Here we developed and utilized a liquid chromatography-triple quadrupole-mass spectrometry method with a C18 and Raptor Polar X column to quantify 64 different PFAS from agricultural soil, runoff, and wastewater. Some of the PFAS included in the method are C2–C18 perfluoroalkyl carboxylates, C1–C10 perfluoroalkyl sulfonates, C1-C10 perfluoroalkyl phosphates, and pre-PFAAs such as perfluoroalkyl sulfonamides, perfluoroalkyl sulfonamido acetic acids, mono and di polyfluoroalkyl phosphates, fluorotelomer sulfonates, and fluorotelomer carboxylic acids. This presentation will discuss the details of the analytical methodology and results obtained from analyzing wastewater, runoff water, and soil collected from agricultural sites in Indiana and North Carolina.

P39-16 | Quantification and risk distribution of selected antibiotics in water, sediments, and fish from two rivers in Taiwan: assessment of ecological and human health risks

Hsiu-Ling Chen, Ching-Chang Lee

National Cheng Kung University, Department of Food Safety/Hygiene and Risk Management, Tainan, Taiwan

Antibiotics are compounds with bactericidal or bacteriostatic effects, they can be applied in livestock industries as growth promoters to enhance feed efficiency. Most antibiotics cannot be metabolized and absorbed within animals, resulting in antibiotic excretion to rivers, and these antibiotic residues can cause contamination of aquatic environments. This study aims to investigate the antibiotic concentration in two rivers in Taiwan, and to assess the potential ecological risks and human health risk.

Samples were collected from Jhuoshuei and Beigang River during dry season (March to April) and wet season (July to August). Antibiotic levels were analyzed using ultra performance liquid chromatography-tandem mass spectrometry. Ecological and human health risks were assessed based on antibiotic levels and predicted no-effect concentrations, finally, we presented the distribution of ecological risks and human health risk in the study region by using ToxPi GIS.

The four antibiotics analyzed were sulfamethoxazole (SMX), tylosin (TYL), chlortetracycline (CTC), and doxycycline (DOX). Based on the results of two years, the concentration range of four antibiotics in water samples was N.D.-0.970 µg/L, with SMX and CTC showing higher detection rates. In sediment samples, the concentration range was N.D.~2934 µg/kg dw, with CTC and DOX exhibiting higher detection rates. In fish samples, the concentration range was N.D.~2.50 µg/kg ww, with CTC and DOX being the antibiotics most frequently detected.

The ecological risks in water samples were assessed using risk quotients (RQ), with algae was selected as the indicator. The RQs for SMX ranged 0-1.64, TYL 0-42.08, CTC 0-6.11, and DOX 0-2.28. Results of ToxPi indicated that the ecological risks were higher in downstream of each sampling sites along the Beigang River. For the Zhuoshui River, the downstream of JS5 and JS6 exhibited higher risks. The combined results over the two years suggested that these two rivers are important regions of ecological risk.

P39-17 | Pinpointing pollutant pathways: Emerging concerns about residential drinking water quality in Wilcox County, Alabama

Jenna K. Honan, Amy Hutson Chatham, Jeffrey K. Wickliffe

University of Alabama at Birmingham School of Public Health, Department of Environmental Health Sciences, Birmingham, USA

In response to growing community concerns about water quality in Alabama's Black Belt region, we conducted a drinking water quality assessment across 30 households in Wilcox County. We leveraged existing community partnerships to quantify concentrations of aluminum, arsenic, calcium, copper, iron, magnesium, non-radioactive strontium, zinc, and total coliforms in both well water and municipal (“tap”) drinking water sources. Community volunteers collected samples from kitchen faucets after external disinfection via heat or bleach. Results of the laboratory analysis revealed an unexpected presence of arsenic (n=10/30, detected range: 7.27-11.3 µg/L), with two homes surpassing the maximum contaminant level of 10µg/L and 70% of detected samples above 8 µg/L. Arsenic is considered toxic at any non-zero concentration. This finding is particularly significant as arsenic is not historically documented as naturally occurring in Alabama’s geologic formations. The detection of arsenic in recent water quality reports, combined with our findings, suggests a potential emerging environmental health concern that warrants further investigation. Additionally, we identified the presence of coliform bacteria in some samples, indicating possible water system vulnerabilities. Our team is currently developing a follow-up investigation to increase the number of homes tested, incorporate biomarkers, and create more detailed mapping to identify potential arsenic sources. We will also evaluate residents’ habits related to frequency of exposure, such as how often they drink bottled water or whether they use in-home filtration systems. This next phase aims to determine exposure pathways and inform targeted intervention strategies. Our participatory approach, which we will continue to employ while scaling up our testing efforts, not only facilitated data collection but also strengthened community trust and participation in environmental health research. These findings highlight the importance of continued water quality monitoring in underserved regions and demonstrate the value of community-engaged research in identifying emerging environmental health challenges.

P39-18 | Pollutants in the Ozama and Isabela Rivers: Assessing Potential Human Health Risks

Kristy M. Polanco[1], Kelly R. Peña[2], Sayira Mueses[4], Jenny L. Cepeda[3]

[1] Universidad Iberoamericana, Medical Sciences Faculty, Santo Domingo, Dominican Republic; [2] Universidad Iberoamericana, Medical Sciences Faculty, Santo Domingo, Dominican Republic; [3] Universidad Iberoamericana, Research Department, Santo Domingo, Dominican Republic; [4] Universidad Iberoamericana, Research Department, Santo Domingo, Dominican Republic

Background and Objective:
The Ozama and Isabela Rivers play a crucial role in the socio-economic and environmental framework of Santo Domingo, Dominican Republic, supporting a diverse fish ecosystem. However, pollution from industrial and agricultural activities, population growth, and poor urban planning have significantly compromised water quality, raising concerns about the potential health risks of consuming water or fish from these rivers. This study aimed to

assess the possible health risks associated with the consumption of fish by analyzing mercury (Hg) levels and toxic organic pollutants, such as organochlorine pesticides, organophosphates, and polychlorinated biphenyls (PCBs), in both water and fish samples.

Materials and Methods:

Water samples and a total of twenty-one muscle tissue samples from four commonly consumed fish species were collected from three areas along the Ozama and Isabela Rivers. Mercury (Hg) levels in both sample types were measured using Inductively Coupled Plasma Atomic Emission Spectrometry, while toxic organic pollutants—including organochlorine pesticides, organophosphates, and PCBs—were analyzed using High-Resolution Gas Chromatography/High-Resolution Mass Spectrometry.

Results:

Analysis of the water and fish samples from the three locations along the Ozama and Isabela Rivers revealed that mercury (Hg) and all the analyzed organic pollutants were undetectable in both types of samples.

Conclusions:

This is the first study in the Dominican Republic to establish a potential link between water pollution and health risks from fish consumption. Based on the analyzed pollutants, the findings from our samples suggest no immediate health risk from mercury or organic contaminants in fish from the Ozama and Isabela Rivers. However, further studies are recommended to assess other potential contaminants, such as other heavy metals, which could pose a similar or greater risk to human health, as well to evaluate river soil sediments for the presence of these pollutants, and including a larger sample size.

P39-19 | Evaluating 1,4-Dioxane Exposure in Long Island, New York: Comparison of Environmental, Biological, and Survey-Based Measures

Nicholaus P. Johnson[1], Sheng Liu[1], Domenica Berardi[1], Cassandra J. Clark[1,2], Clare Loughlin[1], Keli M. Sorrentino[1], Xuejuan Ning[1], Joshua L. Warren[3], Zeyan Liew[1], Caroline H. Johnson[1], Krystal J. Godri Pollitt[1], Nicole C. Deziel[1]

[1] Yale School of Public Health, Environmental Health Sciences, New Haven, USA; [2] University of Minnesota, Department of Pediatrics, Division of Epidemiology and Clinical Research, Minneapolis, USA; [3] Yale School of Public Health, Department of Biostatistics, New Haven, USA

Objective: 1,4-Dioxane (1,4-DX), an industrial chemical in solvents and personal care products, is an emerging, potentially carcinogenic drinking water contaminant. Prior studies identified high 1,4-DX concentrations in Long Island, New York. This study (1) compared 1,4-DX concentrations in drinking water and biological samples, (2) evaluated exposure determinants, and (3) analyzed metabolomic profiles in relation to exposures.

Material and Methods: We analyzed tap water and blood samples from 92 participants for 1,4-DX using a newly optimized gas chromatography high-resolution mass-spectrometry method. We interviewed participants about their water consumption patterns and consumer product usage. We assessed correlations between 1,4-DX in water and blood and used chi-squared tests and logistic regression to evaluate exposure determinants. We compared metabolomic profiles among those with and without detectable 1,4-DX in water and blood using untargeted metabolomics-based mass spectrometry.

Results: 1,4-DX was detected in 32% of water and 24% of blood samples, with 24% of water samples exceeding

New York's Maximum Contaminant Level of 1 ppb. No correlation was found between 1,4-DX in water and blood (r=-0.39, p=0.38), even among those primarily consuming tap (versus bottled) water (Spearman r=-0.30, p=0.62). Homes served by public water systems with reported MCL exceedances were more likely to have detectable 1,4-DX in tap water (44% vs. 28%, p=0.2). Water consumption and personal care product use were not associated with blood 1,4-DX. Metabolomics analyses revealed small but significant differences between participants with and without detectable 1,4-DX in water (Fold Change <20%, false-discovery-rate-corrected p<0.05).

Conclusion: This study presents new exposure measurements of 1,4-DX in tap water and biospecimens. The lack of correlation between water and blood samples suggests contributions from other sources, differences in time windows captured by sample types, and/or sample size limitations. Further investigation into exposure determinants and metabolomic profiles in a larger population would clarify exposure sources and metabolic effects.

P39-20 | Desalination and mineral composition: Assessing water quality variations across sources

Nitzan Sagie[1,2], Atar Adout Hawlena[3], Irit Hen[3], Ronnie Levin[4], Luda Groisman[3], Tamar Berman[3], Noa Cedar[3], Natalie De Falco[2], Shimon Rachmilevitch[2], Lena Novack[1,2]

[1] Soroka Clinical Research Center, Negev Environmental Health Research Institute, Be'er Sheva, Israel; [2] Ben-Gurion University of the Negev, Be'er Sheva, Israel; [3] Ministry of Health, Jerusalem, Israel; [4] Harvard University, Cambridge, USA

Objective: Due to limited freshwater resources, Israel increasingly relies on desalination for drinking water. However, this process reduces essential minerals like magnesium (Mg), calcium (Ca), and fluorine (F), potentially posing health risks. In this study we compare the concentrations of these minerals across different water sources.
Materials and Methods: This analysis presents a cross-sectional analysis of Mg, Ca, and F concentrations in Israel from 2021 to 2024 based on a survey by the Israeli Ministry of Health for Mg and Ca levels and regulatory testing for F throughout this period. Geometric means of mineral concentrations were compared across the main water sources - desalinated water, groundwater, and mixed sources (a variable blend of desalinated water, groundwater, and possibly other sources, with unknown proportions). This classification reflects the country's geo-climatic patterns, with arid regions more reliant on desalinated water.
Results: The analysis utilizes 2,227, 2,735, and 3,375 individual measurements for Mg, Ca, and F, respectively. Desalinated water exhibited the lowest Mg (mean=4mg/L, 95%CI: 2-8mg/L), followed by mixed sources (mean=9mg/L, 95%CI: 8-11mg/L) and groundwater presenting with the highest levels (mean=37mg/L, 95%CI: 29-47mg/L). A similar pattern was recorded for Ca concentrations 36mg/L (95%CI: 30-43mg/L), 50mg/L (95%CI: 47-53mg/L), and 73mg/L (95%CI: 59-91mg/L) for desalinated, mixed and groundwater, respectively. F levels were similar between desalinated water (mean=0.15mg/L, 95%CI: 0.11-0.20mg/L) and mixed sources (mean=0.17mg/L, 95%CI: 0.16-0.18mg/L), whereas groundwater exhibited higher levels (mean=0.24mg/L, 95%CI: 0.22-0.27mg/L).
Conclusion: Desalinated water contains significantly lower Ca and Mg levels than groundwater or mixed water sources. F levels varied between water sources and were significantly low compared to 0.7–1.0 mg/L maintained in the country before mandatory fluoridation was ended in 2014. The findings highlight potential public health concerns. Further research is needed to determine minimum mineral levels in drinking water to guide re-mineralization strategies that meet the population's nutritional needs.

P39-21 | Metal Pollution and Bioaccumulation in Lualaba Aquatic Ecosystems, DR Congo

Paulin K. Kalomo Kitikimo

Université de Lubumbashi, École de santé publique, Lubumbashi, Congo - Kinshasa

Background:
Mining activities may contribute to metal pollution of aquatic ecosystems. This study assessed metal concentrations in surface water,sediments, and fish collected from selected locations in Kolwezi and Fungurume.
Methods:
Samples of water, sediment, and fish were collected between January and March 2024 from four water bodies in Kolwezi (60) and two in Fungurume (30). pH and metal concentrations (ICP-MS) were measured and bioconcentration factors (BCF) were calculated as metal concentration in fish/metal concentration in water (Cfish/Cwater) to assess the extent of metal accumulation in fish.
Results:
Water samples from Kolwezi (n=60) had higher metal concentrations than those from Fungurume (n=30), with median [interquartile range] concentrations (µg/L) as follows: Mn: 122 [19.6–3801] vs 53 [6.49–428], p = 0.04; Cd: 0.09 [0.01–0.59] vs 0.02 [0.01–0.03], p = 0.01; and Co: 0.55 [0.28–1.81] vs 0.17 [0.10–0.37], p < 0.001. Median pH was 6.72 (6.22–7.26) in Kolwezi and 6.54 (6.32–7.18) in Fungurume. In contrast, sediment samples from Fungurume (n=22) had significantly higher metal concentrations (µg/Kg) for Mn, Ni, and W compared to Kolwezi (n=24). Spearman correlations between water and sediment concentrations were positive for Cu (r = 0.5, p = 0.002) and Co (r = 0.3, p = 0.05).Substantial metal accumulation was found in fish, with median BCF values as follows: Cd: 1654, As: 1000, Co: 387, and U: 90 for Lualaba stream (n=5) and Cd: 2966, As: 458, Co: 1753, and U: 8.39 for Nzilo lake (n=6).
Conclusion
We found substantial metal contamination in surface water and sediments, with distinct spatial variation and evidence of bioaccumulation in fish. These findings highlight the need for continuous environmental monitoring and targeted mitigation strategies to reduce metal pollution and potential health impacts.

P39-22 | Simultaneous Removal of Co-occurring Contaminants Reduces Drinking Water-Attributed Cancer Risk: A United States Case Study

Tasha Stoiber, Sydney Evans, Chris Campbell, David Andrews, Olga Naidenko

Environmental Working Group, Washington DC, USA

Objective: Installation of drinking water treatment technologies capable of removing multiple contaminants can produce significantly greater potential health and economic benefits compared to the benefits estimates for removal of one contaminant at a time. Here we carry out a case study for the United States to calculate a decrease in attributable cancer risk that could be realized from a reduction in hexavalent chromium concentrations together with simultaneous reduction in co-occurring arsenic concentrations in drinking water.

Methods: Using a comprehensive dataset for drinking water contaminants in community water systems in the United States, we analyze water quality improvement scenarios for the subsets of community water systems where inorganic anionic forms of arsenic and Cr(VI) occur simultaneously. We estimate the reduction of theoretically attributable cancer cases associated with contaminant removal.

Results: For the entire United States, up to 7410 and up to 43,418 lifetime cancer cases could be avoided if Cr(VI) and As concentrations in drinking water, respectively, were decreased to the one-in-million cancer risk level in systems with Cr(VI) detections. For hypothetical maximum contaminant levels of Cr(VI) at 10 and 5 µg/L, estimated avoidable lifetime cancer cases for the entire United States are up to 575 and 1320, respectively. For these two water quality scenarios, a simultaneous reduction of 42% and 28% of arsenic, respectively, would double the number of lifetime cases avoided.

Discussion: Analysis of decreased cancer risk associated with the simultaneous reduction of co-occurring contaminants provides information for establishing new frameworks for mitigation of drinking water contaminants and protection of public health from exposure to pollution. Consideration of the comprehensive health and economic benefits associated with removal of co-occurring contaminants can document the value of environmental protection regulations and support the development of more efficient approaches for drinking water treatment.

Study funded in part by the Park Foundation.

P39-23 | Advancing Public Access to Drinking Water Contaminant Data: The EWG Tap Water Database

Tasha Stoiber, Sydney Evans, Chris Campbell, David Andrews, Olga Naidenko

Environmental Working Group, Washington, D.C., USA

Objective: Access to reliable, transparent data on drinking water quality is essential for public health protection and informed decision-making. The Environmental Working Group (EWG) developed the Tap Water Database, the most comprehensive publicly available resource on drinking water contaminants in the United States.
Methods: Here we will highlight the methodology behind the database, including data collection, interpretation of health risks, and comparison of contaminant levels to both federal legal standards and health-based standards. The database examines the gaps in current drinking water regulations and emphasizes the need for stronger health-protective limits and increased transparency in water quality reporting.
Results: The database compiles water quality data from nearly 50,000 utilities nationwide, assessing contaminants against health-based benchmarks and documenting their prevalence and potential health risks from long-term exposure over a lifetime. EWG has published 112 health-based standards for both regulated and unregulated contaminants, including 17 PFAS. The database includes information on 324 contaminants, including 231 that are unregulated, detailing their sources, detection in utilities, associated health concerns and what filtration technology is effective for reduction.
Discussion: The EWG Tap Water Database serves as a critical resource for researchers, policymakers, and the public to identify disparities in water quality and advocate for improved regulatory safeguards. By democratizing

access to drinking water data, this initiative empowers communities to demand cleaner, safer water and supports the push for science-driven policy reforms. The database is a tool for leveraging data for environmental advocacy, risk communication, and regulatory improvements in drinking water standards.

P39-24 | Effect of Water Type on Educational Achievements: A Cross-Sectional Study with National Representativeness in Colombia.

Victor Florez-Garcia[1,2], Javith Torres- Saballeth[2], Rafael Tuesca-Molina[2], Jorge Acosta-Reyes[2], Edwin Guevara-Romero[3], Natalia Nohora[4,5], Esmeralda Santacruz-Salas[6], Tania Acosta-Vergara[2]

[1] University of Illinois Chicago, Division of Epidemiology and Biostatistics, School of Public Health, Chicago, USA; [2] Universidad del Norte, Department of Public Health, Barranquilla, Colombia; [3] University of Wisconsin Milwaukee, Joseph J. Zilber College of Public Health, Milwaukee, USA; [4] University of São Paulo, Ribeirão Preto Medical School, São Paulo, Brazil; [5] University of Groningen, Department of Obstetrics and Gynecology, University Medical Center Groningen, Groningen, Netherlands; [6] Universidad de Castilla La Mancha, Faculty of Physiotherapy and Nursing of Toledo, Toledo, Spain

Aim: Our study investigates the relationship between water type and academic achievement in Colombian adults. **Material and Methods:** We conducted a cross-sectional analysis using data from the 2015 National Survey of Nutritional Situation in Colombia (ENSIN -2015). Our analytical sample was comprised of subjects of legal age (>18 years) who had no mental or physical disability, had available information on educational achievements and the type of water source consumed (for drinking), and had lived at least 5 years in the location where the survey was conducted. Educational achievements were evaluated by the staff as less than complete primary school (0-4 years), between complete primary and incomplete secondary (5-10 years), between complete secondary and incomplete higher education (11-15 years), and complete higher education and more (16-24 years). We employed ordinal logistic regression to estimate betas (β's) coefficients and Odds Ratios (OR) with 95% confidence intervals (95%CI) in cured and adjusted models. All estimations and models were kept weighted as recommended for prevalence studies with national representativeness. Models were adjusted by age (categorical), ethnicity (categorical), marital status (categorical), area of residence (categorical), and wealth quartile (categorical). **Results**: We included 17709 subjects, most 18 to 45 years old (66%), women (51%), and the first and second wealth quartiles (68%). Adjusted analyses revealed a negative association between untreated water consumption and educational attainment. Those consuming surface water [β: -0.625; 95%CI: -0.629, -0.620] or groundwater [β: -0.368; 95%CI: -0.372, -0.364] had lower chances of reaching higher education levels compared to individuals with access to treated water. These findings were consistent across all regions of Colombia. **Conclusion**: Our findings suggest that lower educational levels are linked to the consumption of untreated water, emphasizing the need for improved access to safe drinking water in Colombia.

P39-25 | Household Water Inadequacy in Ghana: A National-Level Analysis of Probability and Contributing Factors

Dominic B. Boyetey[1], Christian A. Hesse[2], Felix K. Akporsuer[1]

[1] University Of Environment And Sustainable Development . Ghana, Department of Built Environment, Accra, Ghana, Ghana; [2] University of Professional Studies, Department of Economics and Actuarial Science, Accra, Ghana, Ghana

Objective

Access to safe and sufficient water is a fundamental human right, yet inadequate water, sanitation, and hygiene contribute to an estimated 3.5 million deaths annually (UN, 2024). Despite the Earth's abundance of water, only 2.5% is freshwater, making sustainable access to clean water a critical Sustainable Development Goal. With the WHO recommending 50-100 liters per person per day for basic needs (UN Water, 2024), and a projected increase in household water demand due to population growth, this study investigates the probability of household water inadequacy in Ghana.

Material and Methods

Utilizing a nationwide survey dataset of 14,009 households, sampled through a multi-stage process, we analyzed water consumption, household size, location, and regional variations using logistic, probit, and linear probability regression models.

Results

Consistent across all models, we found an odds ratio of approximately 0.7, indicating a significant probability of water inadequacy for households with five members across all regions of Ghana.

Conclusion

The result highlights the pervasive nature of household water scarcity in the country, necessitating urgent policy interventions to ensure equitable and sustainable water access. Consequently, we recommend to the government and donors to invest robust and resilient water infrastructure, including pipelines, reservoirs and water treatment facilities, particularly in underserved regions.

Keywords: water inadequacy, probit regression, probability, households

P40 | Wildland Fire, Smoke, and Health - PART I

P40-01 | Exploring the Chemistry of Wildfire Smoke: Leveraging Non-Targeted High-Resolution Mass Spectrometry Approaches to Detect Chemical Signatures

Aline Maybank[1], Liddy Boland[1], Elizabeth Lin[1], Jean Zhou[1], Scott Weichenthal[2], Krystal J. Godri[1], Eric Coker[3]

[1] Yale School of Public Health, Environmental Health Sciences, New Haven, USA; [2] McGill University, Epidemiology, Biostatistics, and Occupational Health, Montreal, Canada; [3] BCCDC, Vancouver, Canada

Objective: Wildfire smoke is a growing public health concern. While research has focused on fine particulate matter (PM2.5), wildfire smoke contains a complex chemical mixture of volatile and semi-volatile organic compounds (VOCs and SVOCs), including carcinogens and endocrine disruptors, whose health impacts remain underexplored. The objective of this study was to quantify and characterize the chemical composition of air pollutants associated with wildfire smoke compared to non-wildfire smoke periods in British Columbia, Canada.
Method and Materials: Stationary passive samplers (Fresh Air Clips) were longitudinally deployed from March to September 2024 in Kamloops and Kelowna to collect chemical samples before, during and after wildfire season. Samples were analyzed by thermal desorption gas chromatography-high resolution mass spectrometry to identify and quantify VOCs and SVOCs. Wildfire smoke days were classified using NOAA's Hazard Mapping System plume data and a calculated wildfire smoke threshold.
Results: Sixteen wildfire smoke days occurred in Kamloops and 24 in Kelowna in 2024. Cluster analysis identified three distinct pollutant signatures, with cluster 3 strongly linked to wildfire-affected days. This cluster exhibited increased polycyclic aromatic hydrocarbons (PAH) concentrations, suggesting a wildfire-specific chemical fingerprint. The mean concentration of 23 chemical pollutants were elevated during these wildfire events, including six volatile organic compounds, four phthalates, and three low molecular weight PAHs. Non-targeted analysis of the stationary passive samplers further revealed 174 chemicals across the 2024 assessment period, many of which were determined to have potential genotoxic mutagenicity, endocrine disruption, and developmental effects.Conclusion: This study enhances our understanding of wildfire smoke's complex chemical composition by identifying carcinogenic PAHs, endocrine-disrupting phthalates, and toxic VOCs, which provides a foundation for developing exposure models, informing public health policies, and guiding interdisciplinary collaborations with local health authorities to mitigate wildfire-related health risks.

P40-02 | Examining associations between wildfire smoke exposure and hospitalizations among people who are incarcerated in California (2010-2019)

Kristen Cowan[1], Shabbar Ranapurwala[1], Sara Levintow[1], Lauren Brinkley-Rubinstein[3], Paul Delamater[2], Lawrence Engel[1]

[1] University of North Carolina at Chapel Hill, Epidemiology, Chapel Hill, USA; [2] University of North Carolina at Chapel Hill, Geography, Chapel Hill, USA; [3] Duke University, Population Health Sciences, Durham, USA

Objective: Short-term exposure to wildfire smoke has been shown to adversely impact population health. Individuals who are incarcerated lack agency to protect themselves from exposure and/or to relocate during a wildfire. The objective of this study was to examine the association between wildfire smoke exposure and hospitalizations among people who are incarcerated in California.

Material and Methods: Patient discharge data from all hospitalizations in 2010-2019 at California-licensed hospitals were obtained through the California Department of Health Care Access and Information. Incarcerated individuals were identified via ICD-9/10 codes for incarceration, Zip-code of residence and mode of entry into the hospital. A case-crossover design was employed, assigning up to four control dates on the same days of the week in the same month as each case. Using the patient's zip-code, hospitalizations were linked to daily wildfire PM2.5. data. Exposure was defined as ≥ 1 day of moderate to high average wildfire smoke (≥ 9mg/m^3) in the preceding 5 days. Conditional logistic regression was used to estimate odds ratios (OR) and 95% confidence interval (CI) for the associations between wildfire smoke exposure and all-cause, cardiovascular, respiratory and injury hospitalizations.

Results: Compared to 0 days in the past five,1-2 days with wildfire PM2.5 ≥9mg/m^3 was associated with an increased odds of hospitalization for respiratory causes (OR (95% CI): 1.18 (0.92, 1.51) and cardiovascular causes (OR (95% CI): 1.23 (1.02, 1.48), and decreased odds of hospitalizations for injury causes (OR (95% CI): 0.92 (0.75 1.13). 3-5 days were associated with decreased odds for all causes.

Conclusion: Incarcerated individuals may be at increased risk of hospitalization from wildfire smoke during moderate wildfire events. Further research should assess protective measures taken in carceral facilities and the extent of wildfire smoke exposure within prison buildings. Measures should be taken to protect incarcerated individuals from these exposures.

P40-03 | Wildfire-Attributed PM2.5 Exposure During Pregnancy: A National Perspectives

Menglu Liang[1], Hao He[2], Samyam Aryal[1], Nicole E. Sieck[1], Amir Sapkota[1], Michel Boudreaux[3]

[1] University of Maryland, Epidemiology and biostatistics, college park, USA; [2] university of maryland, Atmospheric & Oceanic Science, college park, USA; [3] university of maryland, Health Policy and Management, college park, USA

Objective: To characterize spatiotemporal patterns in prenatal exposure to wildfire-attributed fine particulate matter (PM2.5) among live-born infants in the United States from 2013 to 2019.
Material and Methods: We simulated U.S. air quality using the EPA's Air Quality Time Series platform and conducted a Community Multiscale Air Quality experiment by removing fire-related emissions to estimate wildfire-attributable PM2.5. Daily county-scale wildfire PM2.5 estimates were linked to county-level birth registry data. Statistical analyses included percentage change calculations, contribution analysis, and seasonal decomposition.
Results: Nationally, overall PM2.5 during pregnancy decreased by 0.8% to 14.1%; however, this decline would have been more pronounced (7.9-23.3%) without wildfires. Wildfire-attributable PM2.5 increased by 0.37 μg/m³ (68.5%) over the study period, with the Northwest region experiencing the highest increase of 1.21 μg/m³ (102.5%). The proportion of total prenatal PM2.5 attributable to wildfires rose from 8.2% to 14.3% nationwide. Substantial spatial heterogeneity existed, with 10% of counties accounting for 42.3% of the total increase. Peak fire season concentrations in Northwest and West regions reached 4.32 μg/m³ and 3.87 μg/m³ respectively by 2019. States with initially low exposure showed relative increases exceeding 200%, while western states showed more moderate relative increases despite larger absolute changes.**Conclusion:** While we observed overall declines in PM2.5 exposure during pregnancy, exposure to wildfire-attributable PM2.5 is increasing. Given the increasing frequency and intensity of wildfires supercharged by global change and the potentially higher toxicity of wildfire PM2.5, there is a need to investigate impacts on adverse birth outcomes, particularly among disproportionately exposed communities.

P40-04 | Differential impacts of wildfire, agriculture fire, and prescribed fire smoke PM2.5 on emergency department visits in California

Minghao Qiu[1], Makoto Kelp[2], Sam Heft-Neal[2]

[1] Stony Brook University, School of Marine and Atmospheric Sciences & Program in Public Health, Stony Brook, USA; [2] Stanford University, Doerr School of Sustainability, Stanford, USA

Increasingly frequent fires pose serious challenges to air quality and public health in the US and the globe. While it is widely recognized that air pollution originating from fires can negatively impact human health outcomes such as respiratory and cardiovascular conditions, limited research has examined how these impacts vary by fire type. Smoke pollution originating from different fire types could have different health impacts given potential differences in their chemical composition, influenced populations, and induced behavioral responses. Our study addresses this gap by focusing on fine particulate matter (PM2.5) from three major fire sources -- wildfires, agricultural burns, and prescribed fires -- across California from 2014 to 2017. We estimate daily smoke PM2.5 originated from three fire types by combining simulations from the atmospheric chemical transport model and satellite-derived machine learning-based estimates. We then combine these smoke PM2.5 estimates with geolocated data covering all emergency department

(ED) visits to non-federal hospitals in California from 2014 to 2017 to quantify how smoke originating from each fire type affects ED visits. Consistent with previous findings, we find that wildfire smoke PM2.5 increases overall ED visits and specifically visits related to respiratory conditions such as asthma. Interestingly, we find agricultural fire smoke PM2.5 has even stronger impacts on ED visits (all-cause and asthma), while no health effects are detected for prescribed fire smoke PM2.5. Our results underscore the need for refined and differentiated regulatory approaches and targeted protections for at-risk groups, including agricultural workers. Our results can also inform the design of management practices such as the use of prescribed fires to mitigate impacts from future wildfires while limiting the health impacts of prescribed fires themselves.

P40-05 | Impacts of Wildfire on Cardiorespiratory Hospitalizations in New Mexico in 2022

Courtney Maichak[1], Olivia Sablan[2], Bonne Ford[2], Chris Uejio[3], Chelsea Langer[4], Stephanie Moraga-McHaley[4], Emily Fischer[2], Jeffrey Pierce[2], Sheryl Magzamen[1]

[1] Colorado State University, Department of Environmental and Radiological Health Sciences, Fort Collins, USA; [2] Colorado State University, Department of Atmospheric Sciences, Fort Collins, USA; [3] Florida State University, Department of Geography, Tallahassee, USA; [4] New Mexico Department of Health, Environmental Health Epidemiology Bureau, Santa Fe, USA

Climate change has resulted in the increased frequency and intensity of many natural disasters, including wildfires. These wildfire events have resulted in growing concerns regarding unhealthy air quality and public health. Specifically, the western United States has heightened wildfire activity. In 2022, New Mexico experienced the state's largest wildfire, the Calf Canyon/Hermits Peak wildfire, that consumed nearly 1,400 km^2 of land in the northeast part of the state. New Mexico wildfires have major ecological, social, and economic consequences, however, associated health impacts from wildfire smoke have not been extensively documented with modern exposure assessment methods. We investigated the impacts of wildfire smoke (WFS) fine particulate matter ($PM_{2.5}$) on cardiorespiratory morbidity in New Mexico during the 2022 wildfire season. To estimate WFS exposure, we used daily population-weighted county averages of $PM_{2.5}$ from ground-based EPA monitors and satellite-based smoke plume estimate from the NOAA Hazard Mapping System. We used time-stratified case-crossover analyses, with distributed lag models to estimate the effect of WFS $PM_{2.5}$ on cardiorespiratory hospitalization health records. Exposure to WFS $PM_{2.5}$, there were increased odds of arrythmia on Lag Day 2 (OR = 1.529, 95% CI = 1.001, 2.336) and Lag Day 3 (OR = 1.569, 95% CI = 1.030, 2.390). For all respiratory diagnoses there were significantly lower odds on day of exposure (OR = 0.707, 95% CI = 0.504, 0.993) and Lag Day 1 (OR = 0.804, 95% CI = 0.679, 0.951). As the frequency and intensity of New Mexico wildfires continue to increase due to climate change, understanding the health impacts will be imperative. Being aware of the effects of WFS on specific geographic areas can help facilitate public health interventions and fire management protocols that address the adverse effects.

P40-06 | Effectiveness of Portable HEPA Filtration in Reducing Residential Indoor $PM_{2.5}$ Levels During the Palisades/Eaton Fire in Los Angeles

Ruoxue Chen[1], Jiawen Liao[2], Yan Lin[1], Chenyu Qiu[2], Wu Chen[2], Yihui Ge[1], Zhenchun Yang[2], Frank Gilliland[2], Mike Bergin[3], Marilyn Black[4], Zhanghua Chen[2], Junfeng Zhang[1]

[1] Duke University, Nicholas School of the Environment, Durham, USA; [2] University of Southern California, Department of Population and Public Health Sciences, Keck School of Medicine, Los Angeles, USA; [3] Duke University, Department of Civil and Environmental Engineering, Durham, USA; [4] Underwriters Laboratories Inc, Chemical Insights Research Institute (CIRI), Marietta, USA

Objectives: The Palisades/Eaton Fire broke out on January 7th, 2025, in Los Angeles (LA) County, California, USA, resulting in a significant wildfire smoke event. This study investigated residential indoor $PM_{2.5}$ trends and assessed the effectiveness of portable High Efficiency Particulate Air (HEPA) purifiers in reducing indoor $PM_{2.5}$ concentrations in 27 participant homes located in LA during baseline and wildfire phases.
Material and Methods: The study continuously monitored residential indoor and outdoor $PM_{2.5}$ using low-cost sensors from 3 days before fire (Jan 4-6), at the early stage of fire (Jan 7-9), to late fire (Jan 10-21). Among the 27 households, 11 had HEPA purifiers installed throughout the study, while 16 had air purifiers without a HEPA filter (non-HEPA homes). Generalized additive models (GAM) were conducted to compare $PM_{2.5}$ levels and determine HEPA effectiveness, with covariates controlled.
Results: The wildfire event elevated outdoor $PM_{2.5}$ levels (mean±SD) on the most severe smoke days (non-HEPA homes: from 10.69±6.61 to 68.68±92.35ug/m^3; HEPA homes: from 10.29±7.32ug/m^3 to 60.72±80.18ug/m^3). Indoor $PM_{2.5}$ concentrations significantly increased after the fire broke out (non-HEPA homes: from 7.03±4.36 to 26.76±29.75ug/m^3; HEPA homes: from 5.70±3.86ug/m^3 to 21.23±18.78ug/m^3), and decreased during late fire days (non-HEPA homes: 7.51±4.53ug/m^3; HEPA homes: 7.01±4.52ug/m^3). Indoor $PM_{2.5}$ levels were consistently lower in HEPA homes compared with non-HEPA homes. After adjusting for within-individual correlations and room volumes, HEPA filtration reduced indoor $PM_{2.5}$ concentrations by 6.46 µg/m³ (95% CI: 3.52, 9.39), in HEPA homes compared to non-HEPA during the early fire stage.
Conclusion: Portable HEPA purifiers reduced residential indoor $PM_{2.5}$ concentrations during the Palisades/Eaton Fire event. The findings highlight the importance of HEPA filtration as an effective and timely mitigation strategy to improve residential air quality during wildfire events.

Funding acknowledgement: This work is supported by Underwriters Laboratories Inc., NIEHS, and National Center for Advancing Translational Science of U.S. NIH.

P40-07 | Joint effects of wildfire smoke and extreme heat on preterm births in California (2018-2022)

Logan C. Dearborn[1], Heather K. McBrien[2], Allison R. Sherris[1], Lauren B. Wilner[3], Marianthi-Anna Kioumourtzoglou[2], Vivian Do[2], Rachel A. Morello-Frosch[4], Tarik Benmarhnia[5], Catherine J. Karr[1,6], Christine T. Loftus[1], Joan A. Casey[1,2]

[1] University of Washington, Department of Environmental and Occupational Health Sciences, Seattle, USA; [2] Columbia University Mailman School of Public Health, Environmental Health Sciences, New York City, USA; [3] University of Washington, Department of Epidemiology, Seattle, USA; [4] University of California, Berkeley, Environmental Health Sciences, Berkeley, USA; [5] University of California, San Diego, Scripps Institution of Oceanography, La Jolla, USA; [6] University of Washington, Department of Pediatrics, Seattle, USA

Introduction

While prenatal exposure to wildfire smoke (WFS) and extreme heat have separately been associated with preterm birth, limited research explores joint effects. We assessed the interaction of pregnancy-wide WFS and extreme heat on preterm birth and explored effect modification by neighborhood poverty.

Methods

We included singleton births born between 2018-2022 identified via California birth records. Daily exposure to WFS and heat were assessed at the census tract. We considered a census tract-day exposed if it experienced mean WFS $PM_{2.5}$ ≥ 10 μg/m^3 or mean temperature ≥ 95th percentile for the prior 11-year period (2007-2017). The cumulative number of pregnancy-wide exposure days was summed independently to the weekly level. We used pooled logistic regression with robust standard errors to estimate the independent association of WFS and the multiplicative interaction of WFS and extreme heat after adjusting for sociodemographic and maternal factors. We used three-way multiplicative interaction models to assess effect modification by neighborhood poverty, comparing pregnancies in the highest quartile of census tract-level poverty to the lower three quartiles.

Results

Of 1,458,702 included births, 8.9% were preterm. Median days of exposure were 2 (interquartile range [IQR]: 11) for WFS and 14 (IQR: 16) for extreme heat. Pregnancies exposed to 10 additional days of WFS had 1.1% (95% confidence interval [CI]: 0.9-0.13%) higher odds of preterm birth. Above and beyond WFS alone, we observed an additional 0.6% (95% CI: 0.4-0.7%) higher odds of preterm birth per 10 additional days of extreme heat exposure. The joint association of WFS and heat with preterm birth was higher among pregnancies in census tracts with more poverty (0.9%, 95% CI: 0.7-0.11%) versus less (0.4%, 95% CI: 0.2-0.6%).

Conclusion

Addressing co-exposures to WFS and extreme heat is important for reducing the burden of preterm birth, especially for those living in higher-poverty communities.

P40-08 | Chemical Composition of Wildfire Smoke and Public Health Impacts: Air Quality Trends in Butte County, California (2020)

Kathryn Phung[1,2], Yun Hang[2]

[1] Rice University, Department of Earth, Environmental and Planetary Sciences, Houston, USA; [2] The University of Texas Health Science Center at Houston, Department of Environmental & Occupational Health Sciences, Houston, USA

Objective: Wildfire smoke contains a complex mixture of particulate matter ($PM_{2.5}$), volatile organic compounds (VOCs), and hazardous air pollutants (HAPs), posing significant health risks. While $PM_{2.5}$ is widely used as a proxy for wildfire-related air pollution, the specific chemical composition of wildfire smoke and its disproportionate health burdens remain underexplored. Butte County, a wildfire-prone area in northern California, faces heightened exposure risks, particularly for low-income and marginalized communities. This study examines the chemical constituents of wildfire emissions in Butte County in 2020, assessing wildfire-related air pollution variability across monitoring sites with a focus on environmental health disparities.

Material and Methods: We analyzed daily air quality data from the EPA Air Quality System and Chemical Speciation Network to assess concentrations of heavy metals (lead, mercury, arsenic), VOCs (benzene, formaldehyde), and combustion-related HAPs. Wildfire activity data from the National Interagency Fire Center were used to determine high-exposure periods. Correlations between pollutant concentrations and wildfire events were evaluated across monitoring sites in Butte County.

Results: Pollutant levels varied significantly with wildfire activity, particularly during the North Complex Fire (August-December 2020). Benzene and formaldehyde concentrations exhibited clear spikes during wildfire periods, consistent with biomass combustion as a primary source, while heavy metals showed intermittent peaks, likely from structural and vehicular combustion. Notably, data from nearly all recorded days revealed concentrations of one or more combustion-related air pollutants exceeding EPA thresholds, with benzene reaching 1.6 ppm and formaldehyde at 3.4 ppm. These exceedances pose serious public health risks, particularly for low-income communities who are often the least equipped to mitigate exposure.

Conclusion: This study highlights additional details regarding the distinct chemical signature of wildfire smoke to better assess its public health risks. These findings emphasize the need for targeted air quality interventions to reduce exposure, particularly in low-income, wildfire-prone communities.

P40-09 | Stroke Incidence and Long-term Exposure to Wildfire Smoke Particulate Matter: A National Cohort Study in the United States

Hua Hao[1], Ke Xu[1], Danlu Zhang[4], Yanling Deng[1], Tarik Benmarhnia[2], Varun Pattisapu[3], Kyle Steenland[1], Howard H. Chang[4], Alvaro Alonso[5], Yang Liu[1]

[1] Emory University, Gangarosa Department of Environmental Health, Atlanta, USA; [2] University of California San Diego, Scripps Institution of Oceanography Department, San Diego, USA; [3] Piedmont Heart Institute, Piedmont Hospital, Atlanta, USA; [4] Emory University, Department of Biostatistics and Bioinformatics, Atlanta, USA; [5] Emory University, Department of Epidemiology, Atlanta, USA

Introduction

Wildfire smoke exposure has emerged as a growing environmental health concern due to its high concentrations of fine particulate matter ($PM_{2.5}$). While short-term cardiovascular effects of wildfire smoke are well-documented, the long-term impacts on cerebrovascular health, particularly stroke incidence, remain understudied. Given the increasing frequency and intensity of wildfires, it is critical to assess their long-term health consequences. This study investigates the association between chronic exposure to wildfire smoke $PM_{2.5}$ and stroke incidence among U.S. older adults.**Methods**

We conducted a national, population-based cohort study of Medicare Fee-for-Service beneficiaries aged ≥65 years from 2007 to 2018. Wildfire smoke $PM_{2.5}$ exposure was estimated at a 1 km² resolution using a two-stage machine learning model that distinguished smoke-specific $PM_{2.5}$ from other ambient sources. Stroke cases were identified using validated diagnostic codes from Medicare claims data. We applied stratified Cox proportional hazards models with generalized estimating equations to estimate hazard ratios (HRs) for stroke incidence per 1 μg/m³ increase in wildfire smoke $PM_{2.5}$, adjusting for non-smoke $PM_{2.5}$, meteorological conditions, and socioeconomic variables.**Results**

Among ~25 million Medicare beneficiaries, ~2.9 million stroke events were recorded. Long-term exposure to wildfire smoke $PM_{2.5}$ was associated with an increased risk of stroke, with stronger effects observed for longer exposure windows (HR per 1 μg/m³ increase: 1.009 for past 2 years; 1.013 for past 3 years). The association was more pronounced for wildfire smoke $PM_{2.5}$ compared to non-smoke $PM_{2.5}$. Sensitivity analyses confirmed the robustness of these findings.**Conclusion**

This study provides the first national-scale evidence linking long-term wildfire smoke $PM_{2.5}$ exposure to increased stroke risk. Given the rising frequency of wildfires due to climate change, targeted mitigation strategies are needed to reduce air pollution exposure and prevent adverse health outcomes in vulnerable populations.

P40-10 | The impacts of heat and air pollution on heat illness and renal diseases in the U.S. Midwest

Lillian Arp, Peter S. Thorne

University of Iowa, Department of Occupational and Environmental Health, Iowa City, USA

Objective: The Midwestern United States is experiencing more extreme weather due to climate change, producing exposures to wildfire smoke and heat waves, yet few studies have investigated the health impacts in this population. We explored the impacts of exposure to daily heat, PM2.5, ozone, and wildfire smoke on renal illnesses in Iowa and South Dakota.

Methods: Data was from a deidentified, commercial insurance data repository. Individuals residing in an Iowa or South Dakota ZIP-Code during summer of 2022 or 2023 were included (n= 1,030,368). A case control design was used, comparing the initial claims for a health outcome of interest (e.g., acute kidney disease) with ankle fractures. Exposure variables were determined per day for each ZIP-Code. Meteorological variables were maximum and minimum temperature, heat index, and relative humidity, ascertained from PRISM. PM2.5 and ozone were modeled from the EPA's AirNow daily data, and wildfire smoke plumes were from NOAA's Hazard Mapping System. Logistic regression was used to determine the relationship between exposure and outcome, adjusted for potential confounders.

Results: Maximum and minimum temperature, ozone, and minimum relative humidity were significant factors for heat-related illness with ORs (95% confidence intervals) of 1.20 (1.14-1.26), 1.12 (1.07-1.18), 1.02 (1.01-1.03), and 1.02 (1.00-1.03), respectively. Additionally, risk rose during the weekend for women but was protective for men. Minimum temperature was a significant risk factor for acute kidney injury 1.02 (1.01-1.04). Urolithiasis risk in women increased with smoke plume density (p-trend 0.0057) and in men increased with higher minimum temperature (OR 1.03 (1.02-1.05)). Risk of UTI increased in men with higher minimum temperature (OR 1.02 (1.01-1.04)) and increased in women with minimum humidity and maximum temperature (ORs 1.004 (1.000-1.007), 1.013 (1.003-1.023)).

Conclusion: These early results point to an impact of weather extremes and air pollution on renal health in the Midwest with potential sex-based differences.

P40-11 | Barriers and Facilitators to Community Wildfire Smoke Response in San Francisco Bay Area

Rebecca Sugrue, Andersen Yang, Arianne Teherani, Sheri Weiser, Neeta Thakur

University of California, San Francisco, Department of Medicine, San Francisco, USA

Objective. While the health impacts of wildfire smoke are well-documented, less is known about the programs communities implement to prepare for, respond to, and recover from smoke events. In California, despite numerous statewide guidelines aimed at reducing wildfire smoke exposure, the extent of their adoption, effectiveness, and equity remains uncertain.

Material and Methods. We surveyed community-based organizations and government agencies addressing climate change and health across the nine counties of the San Francisco Bay Area. The survey focused on: (1) target locations and populations served; (2) wildfire smoke mitigation activities such as advocacy, education, training, or

emergency response, and their status (in use, planned, or aspirational); and (3) implementation barriers and facilitators, guided by the APEASE criteria (affordability, practicality, effectiveness, acceptability, suitability, and equity), an implementation framework to facilitate intervention selection and prioritization.

Results. To date, 57 responses have been analyzed, including 20 from community-based organizations and 19 from government agencies. "Building public awareness and notification" (reported by 48 organizations) and "Strengthening community services and response" (reported by 36 organizations) were the most common activity areas. The criteria of "practicality" and "equity" were cited 96 and 95 times, respectively, by organizations engaged in one or more of the eight listed strategies for "Building public awareness and notification." Equity was a major consideration across all 35 listed strategies, whether currently implemented, planned, or aspirational. Cost, cited 193 times, was the primary barrier to implementation, mentioned over four times more frequently than any other barrier.

Conclusion. Although many government-recommended strategies are being implemented by organizations in the SF Bay Area, large portions of the population still lack access to these actions. Further research will explore how specific strategies highlight barriers and facilitators in various settings. County-level comparisons may reveal population and area-based differences in climate response strategies.

P40-12 | From Plywood to Laundry: Deriving a Mixture of Combustible Materials in American Homes at the Wildland-Urban Interface for Health Studies

Chloe K Chou[1], Amara L Holder[2], Adam Nored[2], M Ian Gilmour[2], Yong Ho Kim[2], Julia E Rager[1]

[1] University of North Carolina at Chapel Hill, Chapel Hill, USA; [2] U.S. EPA, Research Triangle Park, USA

Objective: Approximately 39% of homes in the U.S. are now located in the Wildland-Urban Interface (WUI) and at elevated risk of burning during wildfires. WUI fires emit a cocktail of chemicals from the combustion of anthropogenic materials in affected homes, including compounds that may be absent in biomass-only wildfires. There is currently limited knowledge on the mixture of combustible materials in WUI homes. If better defined, such mixtures could be used to characterize exposure chemistries and toxicological impacts of WUI-relevant smoke exposures, which can inform the actions needed to reduce exposures and protect public health. To address this rate-limiting step, this study aims to integrate all combustible materials in an average American WUI home to derive what we are referring to as the ‘Burning ExposHome’.

Materials and Methods: Items such as structural materials, plumbing, furnishings, and appliances are included in the Burning ExposHome. Calculations were based on the average American household, a 2,016 sq. ft. single family home of four bedrooms, using materials typical to California, due to the high incidence of WUI fires in that geographic region. All materials were then sorted and summed by type of base material such as wood materials, plastics, textiles, and metals.

Results: In summary, the total combustible mass of an average American home was around 38,000 kg, including 80% wood materials, 7% plastics, and 2% metals.

Conclusion: This list of materials can serve as a foundational mixture of home materials to address exposure characterization, health effects, and mechanistic toxicology related to wildfires occurring at the growing WUI. (This abstract does not reflect EPA policy.)

Funding: National Institutes of Health and a Cooperative Agreement with the US Environmental Protection Agency. Additional support was provided by the Institute for Environmental Health Solutions at the Gillings School of Global Public Health.

P40-13 | Spatiotemporal Distribution of Wildfire Exposure in Dialysis Clinics (2010-2024)

Samyam Aryal[1], Hao He[2], Menglu Liang[1], Evan Ellicott[3], Nicole E. Sieck[1], Amir Sapkota[1]

[1] University of Maryalnd College Park, Department of Epidemiology and Biostatistics, College Park, USA; [2] University of Maryalnd College Park, Department of Atmospheric and Oceanic Sciences, College Park, USA; [3] University of Maryalnd College Park, Department of Geographical Sciences, College Park, USA

Objective: To characterize the spatiotemporal distribution of wildfire events in 2551 Fresenius Kidney Care (FKC) dialysis clinics in the contiguous United States from June 2010 to December 2024.

Materials and Methods: We overlaid individual FMC clinic location information with the Hazard Mapping System Smoke (HMS-Smoke) database. We then extracted clinic-level exposure to wildfire smoke plumes (medium and heavy smoke density) for each day between 2010 and 2024. We then used statistical methods, including trends, percentage change, and seasonal decomposition, to evaluate changes in spatial and temporal patterns of wildfires and wildfire-related PM2.5.

Results: Nationally, an increasing trend ($\beta = 0.0004$, $p < .01$) was seen in the monthly wildfire rate over the study period. The largest increase year-over-year was seen between 2022 and 2023, with a 188.5% increase. The increases in rates were more pronounced in summer ($\beta = 0.0039$, $p < .01$) than in winter. In addition, all states showed an increasing trend in monthly wildfire rates. The largest increase during the study period was seen in Minnesota ($\beta =0.0007$, $p < .01$) followed by Iowa ($\beta =0.0007$, $p < .01$). The smallest increase was seen in Florida ($\beta =0.00008$, $p=.013$). Initial analyses using the Community Multiscale Air Quality (CMAQ) show that while the total PM2.5 is decreasing nationally, the wildfire-related PM2.5 is increasing. Additional analyses involving environmental variables including seasonality, precipitation, and drought index along with rural-urban classification, latitude bands, and climate regions will be analyzed to characterize how these factors influence clinic level exposure to wildfire smoke plume.

Conclusion: Dialysis clinics across the country are experiencing increases in wildfire-related smoke plumes. Future studies are needed to understand how such increases in wildfire smoke may impact the health of end-stage kidney disease patients living in the vicinity and receiving treatment in these facilities.

P40-14 | Hotwash for the Los Angeles Fire: How Social Media Reveals the Many Faces of Humanity

Sulong Zhou[1], Yun Hang[2]

[1] Texas A&M University, Urban AI Lab, College Station, USA; [2] The University of Texas Health Science Center at Houston, Houston, USA

This study investigates the immediate disaster response during the 2025 Los Angeles wildfires by examining how online discourse captured community experiences and illuminated the multifaceted nature of human behavior during crises. Social media (Reddit) posts and comments spanning from the onset of the wildfire to its full containment were collected and analyzed using state-of-the-art topic modeling techniques. To address variations in data volume and linguistic structure, we employed a dual-strategy methodology: Latent Dirichlet Allocation (LDA) combined with ChatGPT prompting and human-in-the-loop refinement for post-level annotation, and BERTopic for comment-level clustering. A hierarchical classification framework was developed, comprising two primary categories. The first, Situational Awareness, includes fire progression and suppression, public safety, emergency resources, recovery, influential figures, and loss and damage. The second, Crisis Narratives, covers emotional and social responses such as blame, renewal, victimhood, and heroism. The final dataset consisted of 385 posts and 114,879 comments, with 85.6% retained after preprocessing. Topic trends closely aligned with real-world developments, peaking within the first 2–5 days as the fire reached its peak burn extent. Notably, 2,466 comments were deleted, which indicates the perishable nature of social media data and the importance of timely collection. Within the Situational Awareness category, air quality, insurance, and community-based mutual aid emerged as persistent topics sustained throughout the event. In contrast, other topics were short-lived, driven by real-time incidents. The Crisis Narratives frequently conveyed collective expressions of grief and helplessness, revealing the emotional distress shared across affected communities. These findings highlight Reddit as a valuable platform for in-depth crisis communication. While situational awareness content provides actionable insights for emergency coordination, crisis narratives reveal the emotional and psychological dimensions of public experience. This study contributes a novel, annotated social media dataset and offers analytical insights to support more resilient and empathetic disaster response and coping strategies.

P40-15 | How Wildfire Displacement Policy Shapes Diabetes Outcomes and Management: Stories from Maui Residents

Whitney Denary, Danya Keene

Yale University, School of Public Health, New Haven, USA

Objective: Housing displacement disrupts the management of chronic health conditions. Diabetes is one disease particularly vulnerable to housing instability due to its complex and resource-intensive daily management.
The residents of Maui experienced three devastating fires in August 2023, resulting in over 10,000 people displaced. During this time, landmark housing-based resources took shape, including temporary rental assistance, eviction moratoriums, and freezes on rental price increases. This study interviews individuals with diabetes to better

understand how housing displacement, and subsequent housing policy protections, impacted the health and well-being of those displaced.
Material: Individuals with diabetes displaced following the Maui 2023 wildfires participated in one-time semi-structured individual interviews.
Results: Those displaced shared a sense of insecurity as they moved between different hotels every few weeks. Once housed, they felt constrained in their ability to enforce and benefit from protections provided by emergency housing policies. Key themes involved reductions in food security and autonomy, alterations in healthcare and medication accessibility, and the effects of community and governmental aid.
Conclusion: In a world where climate displacement of this magnitude is a matter of where, not if, it is critical to understand the housing needs of our most vulnerable. These findings highlight the chronic disease challenges during displacement and the coping mechanisms, strategies, and supportive resources that can be targeted in clinical and community interventions.
Funding: Stipends for this research were provided by the Yale Housing and Health Equity Lab and the Yale Center for the Study of Race, Indigeneity, and Transnational Migration (RITM) Research Grant.

P40-16 | The Impact of Wildland Fire Smoke $PM_{2.5}$ on Birth Weight in California

Xing Gao[1], Sally Picciotto[2], Dana Goin[3], Fred Lurmann[4], Nathan Pavlovic[4], ShihMing Huang[4], Tracey J. Woodruff[1], Rachel Morello-Frosch[5,6], Amy Padula[1]

[1] University of California San Francisco, Department of Obstetrics, Gynecology & Reproductive Sciences, San Francisco, USA; [2] University of California Berkeley, Division of Environmental Health Sciences, Berkeley, USA; [3] Columbia University, Department of Epidemiology, New York City, USA; [4] Sonoma Technology Inc, Petaluma, USA; [5] University of California Berkeley, Department of Environmental Science, Policy and Management, Berkeley, USA; [6] University of California Berkeley, Division of Community Health Sciences, Berkeley, USA

Background: The frequency, duration, and intensity of wildland fires have increased, making them a significant source of air pollution in California that can impact perinatal outcomes. Fine particulate matter <2.5 microns ($PM_{2.5}$) attributable to wildfires during pregnancy may impact fetal growth and birth weight.
Objectives: This study assessed associations between average daily exposure to wildland fire $PM_{2.5}$ and risk of adverse birth weight outcomes among singleton term births in California between 2007-2018.
Methods: Exposure over the entire pregnancy and each trimester was assessed using the United States Environmental Protection Agency's bias-corrected Community Multiscale Air Quality Model, linked to the geocoded residences of birthing people at the time of delivery. We used logistic and linear regression models to assess associations between wildfire $PM_{2.5}$ and low birth weight, small/large for gestational age, and in birth weight differences (in grams and z-score), adjusting for individual-level sociodemographic covariates and seasonality. We conducted race/ethnicity-stratified analyses to assess whether wildfire $PM_{2.5}$ impacted racially marginalized populations differentially.
Results: In a cohort of 4,537,418 births,we found that 1 μg/m^3 increase of average wildfire $PM_{2.5}$ during pregnancy was associated with increased odds of large for gestational age (OR=1.06, 95% CI: 1.05-1.06) and higher term birth weight (estimated difference=11.31, 95% CI: 10.78, 11.84), and associated with moderately decreased odds of low birth weight (OR=0.98, 95% CI: 0.97-0.99) and small for gestational age (OR=0.97, 95% CI: 0.96-0.97). Race/ethnicity-stratified results showed that these associations were more pronounced among Hispanic and Other groups. Among American Indian and Alaskan Native births, exposure to wildfire $PM_{2.5}$ was associated with decreased

odds of large for gestational age.
Conclusion: Findings demonstrate the importance of understanding how wildfire $PM_{2.5}$ impacts birth outcomes, especially among marginalized groups.

P40-17 | Wildfire Impacts on Drinking Water Quality in the Western United States (2000-2022)

Beth Haley[1], Wes Austin[2], Charlotte Wachter[3], Jordan Suter[3], Jesse Burkhardt[3], Siyu Pan[4], Bryan Parthum[2], Sonja Kolstoe[5]

[1] Oak Ridge Institute for Science and Education (ORISE), Pacific Northwest Research Station, USDA Forest Service, Portland, USA; [2] US Environmental Protection Agency, National Center for Environmental Economics, Washington, USA; [3] Colorado State University, Department of Agricultural and Resource Economics, Fort Collins, USA; [4] Northern Arizona University, Department of Economics, Finance, and Accounting, Flagstaff, USA; [5] USDA Forest Service, Pacific Northwest Research Station, Portland, USA

Wildfires can threaten the provision of safe drinking water by altering source water characteristics or directly damaging drinking water infrastructure. Wildfire-induced changes to source waters can disrupt drinking water treatment practices, especially in systems with limited operational capacity or flexibility, and have lasted as long as fifteen years in some locations. Recent work has shown that wildfires are associated with increases in the concentrations of certain drinking water contaminants as well as the frequency of Safe Drinking Water Act violations. However, previous studies have focused on a small subset of contaminants and geographic areas over a shorter time horizon that excludes years of exceptional wildfire frequency after 2016.
We study the impacts of wildfires on drinking water quality from 2000-2022 by linking a rich set of information on wildfire burn locations and severity, precise drinking water system intake locations, contaminant concentrations in drinking water, and Safe Drinking Water Act violations in all public drinking water systems in the Western United States. Using geospatial techniques to characterize the extent of wildfire impacts within intake catchment areas, we assess how the presence of an upstream wildfire affects drinking water quality delivered by downstream public water systems at different distances, burn severities, and time periods post-fire. Our analysis focuses on two types of outcomes related to drinking water quality: Safe Drinking Water Act violations and drinking water contaminant concentration levels. We include a diverse set of violation types as well as numerous drinking water contaminants such as disinfectant byproducts, nutrients, volatile organic chemicals, and metals. We test for heterogeneous impacts across ecoregions, and all models include a set of controls for water system, temporal, and state variation. Our research builds on a growing literature aiming to better understand the impacts of climate change on public drinking water systems.

P40-18 | Associations between ultrafine particulate matter from wildfires and late-life cognitive function in the KHANDLE and STAR cohorts by race and ethnicity

Kathryn C. Conlon[1], Amanda J. Goodrich[1], Stacey E. Alexeeff[2], Ai-Lin Tsai[2], Paola Gilsanz[2], Michael Kleeman[3], Rachel Whitmer[1]

[1] University of California, Davis, Department of Public Health Sciences, Davis, USA; [2] Kaiser Permanente, Division of Research, Pleasanton, USA; [3] University of California, Davis, Department of Civil and Environmental Engineering, Davis, USA

Objective: Substantial research has linked fine particulate matter (PM2.5) to dementia, however, the role of ultrafine PM (PM0.1), which is more readily absorbed, remains underexplored, especially in diverse populations.
Methods: The study included n=1648 adults aged ≥65 in the ethnically diverse Kaiser Healthy Aging and Diverse Life Experiences (KHANDLE) study, and n=746 African Americans in the Study of Healthy Aging in African-Americans (STAR). Three domains of cognitive outcomes (semantic memory, verbal episodic memory, and executive function) were measured using the Spanish and English Neuropsychological Assessment Scales and z-scored. Total PM0.1, and the fraction of PM0.1 from two sources (biomass combustion and wildland urban interface (WUI)) during 2000-2020 were generated using a chemical transport model and linked to geocoded residential addresses. We used linear regression to assess the association between long-term PM0.1 exposure (5-, 10-, and 17-year averages) and domain-specific cognitive function adjusting for confounders, remaining fraction of PM2.5, NO2, and O3, separately by study. Effect modification by race/ethnicity was assessed in KHANDLE.
Results: 10-year average total PM0.1 was associated with decreased verbal episodic memory in STAR (β (95% CI): -0.18 (-0.36, -0.01), but not KHANDLE. Effect modification by race/ethnicity was observed in KHANDLE with 5- and 10-year WUI PM0.1 exposures for verbal episodic memory ($P < 0.05$). Among non-Latino white participants, an IQR increase in 5-year average WUI PM0.1 was associated with decreased verbal episodic memory and executive function (-0.34 (-0.49, -0.19) and -0.17 (-0.33, -0.01), respectively). Similar decreases were observed among Latino participants for verbal episodic memory and semantic memory (-0.12 (-0.21, -0.03) and -0.10 (-0.20, -0.01), respectively). Associations were attenuated or null for 10- and 17-year exposures.
Conclusion: Long-term WUI PM0.1 exposure was associated with decreased cognitive function among non-Latino white participants. Wildfires are an increasing threat, and more research on their impact on cognition is needed.

P41 | Air Pollution and Health Across the Lifespan

P41-01 | Leveraging Low-Cost Sensors for Monitoring Air Quality in Lagos, Nigeria: Insights and Lessons Learnt

Adetoun Mustapha[1], Adesola Z. Musa[1], Abdul-Ganiyu Usman[2], Prince Ikonwa[1], Babatunde Salako[1], Deo Okure[2], Engineer Bainomugisha[3], Eric Coker[4]

[1] Nigeria Institute of Medical Research, Lagos, Nigeria; [2] AirQo, Kampala, Uganda; [3] Makerere University, Department of Computer Science, Kampala, Uganda; [4] N/A, Vancouver, Canada

Air pollution is a growing public health concern in Sub-Saharan Africa (SSA), yet many mega-cities in the region lack robust air quality monitoring systems. Lagos, Nigeria, one of Africa's most populous cities, faces significant challenges in understanding and mitigating the population health impacts of air pollution due to limited air monitoring infrastructure. This study addresses this gap by deploying four low-cost sensors manufactured by AirQo, an African-based organization, to measure fine particulate matter ($PM_{2.5}$) across Lagos between July 2023 and December 2024. Strategically deployed to capture variations in population socioeconomic status and emission sources, including traffic-related air pollution (TRAP), the sensors provided critical insights into spatiotemporal air quality patterns. The 24-hour overall median $PM_{2.5}$ concentration was 20.6 μg/m^3 (IQR=16.3, 29.8), indicating poor air quality throughout the city. Results revealed significant variability in $PM_{2.5}$ concentrations across the city, with levels lowest in the more affluent community of Eti Osa Island. Notably, dramatic seasonal fluctuations were observed. $PM_{2.5}$ levels were significantly elevated in December (mean=43.1 μg/m^3), January (mean=62.1 μg/m^3), and February (mean= 68.3 μg/m^3) at all study sites.

Despite these insights, challenges emerged during deployment, including optimizing sensor placement to accurately capture ground-level TRAP and navigating the region's absence of regulatory air quality standards. Collaborative efforts with local stakeholders were essential for sensor placement and data collection.

This study underscores the critical role of low-cost sensor networks in addressing air quality monitoring gaps in Sub-Saharan Africa. The dramatic elevations in $PM_{2.5}$ levels from December to February highlight the unique challenges of air pollution exposure research in SSA, where distinct $PM_{2.5}$ sources and meteorological conditions shape seasonal variations. These findings also contrast sharply with patterns observed in higher-income countries. This study's marked spatial and temporal variability underscores the need for tailored, context-specific approaches to air pollution monitoring and exposure assessment in SSA.

P41-02 | Short-term Effects of Ambient Particulate Matter on Mortality in Lebanon from 2017 to 2019: A Time-series Analysis

Alicia Abi Nader[1,2], Heba Safi[4], Nelly Ziade[5], Joyce Haddad[6], Georges Abi Tayeh[7,8], Myriam Mrad[2,3], Massimo Stafoggia[9]

[1] Saint-Joseph University of Beirut, Institut Supérieur de Santé Publique, Faculty of medicine, Beirut, Lebanon; [2] University of Balamand, Department of Public Health, Beirut, Lebanon; [3] Health and Environment Response Agency (HERA), Beirut, Lebanon; [4] World Health Organization, Regional Office of the Eastern Mediterranean, Healthier Populations Department, Amman, Jordan; [5] Saint Joseph University and Hôtel-Dieu de France Hospital, Rheumatology Department, Beirut, Lebanon; [6] Ministry of Public Health, Directorate of Preventive Health Care, Beirut, Lebanon; [7] Hôtel-Dieu de France Hospital, Obstetrics and Reproduction Department, Beirut, Lebanon; [8] Saint Joseph Fertility Center, Beirut, Lebanon; [9] Lazio Region Health Service / ASL Roma 1, Department of Epidemiology, Rome, Italy

Objective: Lebanon suffers from high concentrations of outdoor air pollutants recorded in the past decade, yet it is still unclear how these pollutants affect health. This study, as part of the Beirut Air Pollution and Health Effects (BAPHE) project initiated in 2010, evaluates the impact of short-term fluctuations in ambient particulate matter (PM10 and PM2.5) concentrations on general mortality in Lebanon from June 2017 to May 2019.

Material and Methods: This study was conducted in collaboration with the Ministry of Public Health (MOPH) and the World Health Organization Eastern Mediterranean Regional Office (WHO EMRO) following a time-series analysis approach. Mortality data was collected from the MOPH, while the ambient air pollution data were collected from the Ministry of Environment's air quality monitoring stations. Temperature was obtained using the Copernicus satellite data. Data on potential confounders such as influenza were not available due to the poor diagnostic infrastructure in Lebanon.

A quasi-Poisson regression model was applied to account for the overdispersion of daily mortality events. The study examined associations between daily mortality and independent variables, including pollutant concentrations, temperature and day of the week, following a Generalized Additive Model (GAM) and a distributed lag model framework. The model fit was evaluated using the Akaike Information Criterion (AIC), and sensitivity analyses were conducted to validate the results. Various spline functions were applied to reduce residual autocorrelations.

Results: Preliminary findings suggest a potential significant association between the increase in PM10 and PM2.5 levels and daily mortality, with temperature influencing the effect estimates. Sensitivity analyses are used to confirm the robustness of the results.

Conclusion: This study provides an understanding of air pollution's health impacts in Lebanon and shows evidence to inform decision-makers and public health interventions aimed at reducing air quality-related health risks.

Funding: No Funding

P41-03 | Residential mobility, changes in $PM_{2.5}$ exposure, and all-cause mortality among U.S. Veterans (2009-2019)

Andrea R. Titus[1], Tarik Benmarhnia[2], Rania Kanchi[1], Byoungjun Kim[3,1], Lorna E. Thorpe[1]

[1] NYU Grossman School of Medicine, Department of Population Health, New York, USA; [2] University of California, San Diego, Scripps Institution of Oceanography, San Diego, USA; [3] NYU Grossman School of Medicine, Department of Surgery, New York, USA

Objective: Residential mobility can be leveraged as a quasi-experiment to explore health outcomes associated with changes in exposures to environmental conditions. We examined whether moving to areas with different levels of ambient $PM_{2.5}$ was associated with differences in mortality risk among U.S. Veterans.

Material and Methods: We constructed an electronic health record-based dynamic cohort of U.S. Veterans (spanning 2009-2019) and identified Veterans who moved between zip codes exactly once over the study period, based on quarterly residential address information (N=594,035). We incorporated high-resolution $PM_{2.5}$ estimates, developed using information from satellite data, ground monitors, and chemical transport modeling. We assigned annual average $PM_{2.5}$ exposure for each residential census tract. We used discrete-time logistic regression to estimate associations between all cause-mortality and moving to areas where cumulative average $PM_{2.5}$ exposure was 1) more than one standard deviation (SD) lower than the pre-move area (“low” exposure), 2) more than one SD higher than the pre-move area (“high” exposure), and 3) within one SD of the pre-move area (“comparable” exposure). Models incorporated inverse probability weights to address confounding by individual-level demographic and health characteristics; area-level demographic, economic, and meteorological characteristics; destination state; and year of move. We plotted adjusted survival curves with bootstrapped confidence intervals.

Results: Average pre-move $PM_{2.5}$ exposure was 8.8 ug/m3 (SD = 1.8 ug/m3). Preliminary results from adjusted models suggest that Veterans moving to “low” exposure areas had a higher cumulative survival probability after 5 years post-move (probability (Pr)=75.0%, 95% CI = 74.7%-75.3%), compared to Veterans moving to “comparable” exposure areas (Pr=73.8%, 95% CI = 73.7%-74.0%), and Veterans moving to “high” exposure areas (Pr=73.2%, 95% CI = 72.5%-73.8%).

Conclusion: Moving to areas with lower $PM_{2.5}$ levels was associated with a higher survival probability. Leveraging residential history information can shed light on the impacts of changing environmental conditions on health outcomes.

P41-04 | Land use regression model for heavy metals in fine particulate matter across the Denver Metro Area

Anne Mielnik[1], Grace Kuiper[1], Sherry WeMott[1], Sheena Martenies[2], William Allhouse[4], Christian L'Orange[3], Anne Starling[5], John Adgate[4], Dana Dabelea[5], Sheryl Magzamen[1]

[1] Colorado State University, Department of Environmental and Radiological Health Sciences, Fort Collins, USA; [2] University of Illinois at Urbana-Champaign, Department of Health and Kinesiology, Champaign, USA; [3] Colorado State University, Department of Mechanical Engineering, Fort Collins, USA; [4] Colorado School of Public Health, Department of Environmental and Occupational Health, Denver, USA; [5] Colorado School of Public Health, Department of Epidemiology, Denver, USA

Heavy metals in fine particulate matter ($PM_{2.5}$) are associated with adverse health outcomes, even at low concentrations found in ambient air, yet pose significant challenges for risk assessment, as biological responses vary by metal species, and exposure quantification remains uncertain. Thus, to estimate heavy metal concentrations in airacross the Denver Metro Area, we created a land use regression (LUR) model to evaluate Iron (Fe), Copper (Cu), and Zinc (Zn) detected in $PM_{2.5}$ as part of the Environmental influences on Child Health Outcomes (ECHO) study. From May 2018 through March 2019, we collected filter-based $PM_{2.5}$ using personal air samplers at 67 locations within the study area. $PM_{2.5}$ mass was quantified gravimetrically. X-ray fluorescence was used for the determination of heavy metals. 10-fold cross-validation was used to assess model performance, which varied by metal species (i.e., long-term R^2 = 0.343 (Cu), 0.383 (Fe), and 0.201 (Zn)) and by meteorological season (e.g., Cu R^2 = 0.167 (Spring) to 0.562 (Fall); Fe R^2 = 0.337 (Winter) to 0.626 (Fall); and Zn R^2 = 0.106 (Summer) and 0.437 (Winter)). The final model used 83 predictors, with major road and railway length, traffic, and point source emissions within 500 meters being the strongest. As a result, our LUR model is the first of its kind to predict ambient heavy metal concentrations in Denver and can be used for risk assessment and health studies. However, model performance varies by season, likely due to the influence of atmospheric transport and other meteorological factors on $PM_{2.5}$ concentrations and may be improved by the integration of high-resolution $PM_{2.5}$ and meteorological data in the future. This work is supported by Colorado State University Grant Number T32GM144856 and National Institutes of Health (NIH) UG3/UH3OD023248. Contents are the author's sole responsibility and do not necessarily represent official NIH views.

P41-06 | Epidemiological Study on Human Health Risk Assessment of Exposure to Particulate Matter bound Toxic Metals in South-western India

Himanshi Rohra, Pg Satsangi

Savitribai Phule Pune University, Pune, India

The epidemiological research on the increasing mortality risks from inhalation of particulate matter has a wide range of results regarding impacts on people's health. This study aims to provide a comprehensive suite of information on exposure to Particulate Matter (PM) by assessing the particle size effect connected to human health risk. Sampling of size-fractionated PM (9-0.7µm) was conducted using a multi-orifice 8-staged Non-Viable Anderson Cascade Impactor in residential sites of southwestern (Pune) region of the Indian subcontinent. Bimodal distributions of PM mass concentrations in coarse fraction (MMAD±GSD=4.32±1.27) were noted while accumulation of elements was found higher in fine (0.43–2.1µm) mode particles. High mass median aerodynamic diameter (MMAD) (≥1.8) and geometric standard deviation (GSD) (>1.2) of all elements pointed towards the significance of coarse particles. PCA suggested crustal origin, resuspended road dust along with vehicular traffic and industrial emissions as major sources. Particle deposition in the human respiratory tract using Multiple Path Dosimetry Model (MPPD) was estimated to understand particle inhalation's health effects. Results showed that 8-year-old children recorded the maximum size-segregated PM deposition. Coarse particles (PM_{10}) were primarily deposited in the head (62-91%) and tracheobronchial (15–56%) regions, whereas fine particles ($PM_{2.5}$ and PM_1) depositions were observed maximum in the head (38–72%) and pulmonary (22–64%) regions. Also, maximum deposition (~70%) was found in lower lung lobes than middle and upper lobes while the clearance rate of deposited PM was high in the tracheobronchial region. Furthermore, the health risks of PM exceeded the safer limits (HQ=1) and ELCR≥10^{-6}, while Ni and Cr(VI) pose the highest hazard quotient (HQ) and Excess Lifetime Cancer Risk (ELCR) respectively within the respirable size range (1.0-2.1µm). When coupled with different environmental exposure scenarios, the obtained information can improve human risk assessment to enrich knowledge of particle effects and determine the best risk-control strategies.

P41-07 | Human Exposure to Size-Fractionated Particulate Matter in South-Western India: Potential Human Health Risk Assessment

Himanshi Rohra, Pg Satsangi

Savitribai Phule Pune University, Pune, India

Related research in recent years has now shifted from single specific particle fractions to varied aerosol modal classes giving deeper insight into particle size distribution. This study aims to provide a comprehensive suite of information on exposure to varied size fractions of Particulate Matter (PM) in residential microenvironments by assessing particle size effect on elemental mass concentration and, evaluating the human health risk of particle size-segregated metal constituents.

Sampling of size-fractionated PM (9-0.7 µm) was conducted using a multi-orifice 8-staged Non-Viable Anderson Cascade Impactor (TE-20-800 TISCH) on the terrace of a commercial building (~10m height) in the southwestern

(Pune) region of Indian subcontinent. Samples were analysed by ICP-MS to determine the elemental concentration. The computational analysis included lognormal particle size distribution statistics, source identification using Principal Component Analysis (PCA), and health risk assessment using the USEPA (2009) model.

Bimodal distributions of PM mass concentrations in coarse fraction (MMAD± GSD=4.32 ± 1.27) were noted while accumulation of elements was found higher in fine (0.43–2.1 μm) mode particles. High mass median aerodynamic diameter (MMAD) (≥1.8) and geometric standard deviation (GSD) (>1.2) of all elements pointed towards the significance of coarse particles in this study region. Ni and Cr(VI) via inhalation exposure pathway pose the highest hazard quotient (HQ) and Excess Lifetime Cancer Risk (ELCR) respectively within the respirable size range (2.1–3.3μm) while children were subject to potential health risks than adults mainly through ingestion mode of exposure. PCA suggested crustal origin, resuspended road dust along with vehicular traffic and industrial emissions as major sources. This study embarks upon the inclusion of an assessment of particle size dynamics in making environmental policy decisions. When coupled with different environmental exposure scenarios, the obtained information can improve human risk assessment to enrich knowledge of particle effects and determine the best control methods.

P41-08 | Heterogeneity scores for $PM_{2.5}$ sources and constituents using national datasets

Jenna R. Krall[1], Matthew J. Strickland[2], Lyndsey A. Darrow[2], Heather A. Holmes[3], Lucas R.F. Henneman[4]

[1] George Mason University, Department of Global and Community Health, Fairfax, USA; [2] University of Nevada, Reno, Department of Epidemiology, Biostatistics and Environmental Health, Reno, USA; [3] University of Utah, Department of Chemical Engineering, Salt Lake City, USA; [4] George Mason University, Department of Civil, Environmental, and Infrastructure Engineering, Fairfax, USA

Objective: Total mixture indicators such as fine particulate matter air pollution ($PM_{2.5}$) are convenient for epidemiologic studies, but these approaches obscure variations in chemical composition that may impact health. We develop an approach to quantify variability in composition for total mixture indicators and use it to compare heterogeneity between sources and constituents of $PM_{2.5}$.

Material and Methods: To quantify variability in $PM_{2.5}$ source mixtures, we used two datasets from the US Environmental Protection Agency: the National Emissions Inventory (NEI) for 2011-2020 and SPECIATE version 5.3. Our NEI data contained source emissions in tons for Virginia counties every three years. SPECIATE contains measured chemical compositions of $PM_{2.5}$ sources. For our heterogeneity scores, we used the scaled determinant from Principal Component Analysis (PCA), which characterizes the amount of variability across the mixture. Heterogeneity scores were applied to each source and constituent to mixtures from NEI and SPECIATE.

Results: We identified 398 $PM_{2.5}$ source profiles from SPECIATE that had detailed chemical composition data and represented sources commonly utilized in epidemiologic studies of $PM_{2.5}$ including biomass burning, dust, oil combustion, and motor vehicles. Heterogeneity scores were largest for residual oil combustion, residential wood burning, and soil dust. There was less heterogeneity in source profiles corresponding to motor vehicles and road dust. Using NEI, the sources associated with $PM_{2.5}$ sulfate varied most across Virginia counties and years, with large heterogeneity also observed for sources of $PM_{2.5}$ strontium, phosphorus, elemental carbon, and potassium.Conclusion: Even within major source categories and constituents such as dust and sulfate, heterogeneity exists in their $PM_{2.5}$ mixtures. Heterogeneity scores provide a summary necessary to compare relative variability in a mixture, for example between sources of $PM_{2.5}$.

Acknowledgements: This work was partially supported by NIH R01-ES032810. The content is the responsibility of the authors and does not represent official NIH views.

P41-10 | Association between long-term air pollution exposure and risk of autoimmune inflammatory rheumatic diseases in South Korea: effect modification by sex and age

Jiwon Choi[1,2], Sera Kim[2], Jong-Tae Lee[1,3]

[1] Graduate School of Korea University, Interdisciplinary Program in Precision Public Health, Department of Public Health Sciences, Seoul, South Korea; [2] Research and Management Center for Health Risk of Particulate Matter, Seoul, South Korea; [3] Graduate School of Korea University, School of Health Policy and Management, College of Health Sciences, Seoul, South Korea

The prevalence of autoimmune inflammatory rheumatic diseases (AIRDs) is increasing worldwide, posing significant public health burdens. However, only a few studies have investigated the association between air pollution and AIRDs. This study aims to investigate the association between long-term air pollution exposure and the risk of AIRDs in South Korea and to examine whether sex and age modify these associations.

We conducted a national retrospective cohort study using the National Health Insurance Service-National Sample Cohort in South Korea from 2006 to 2019. Air pollution data, including particulate matter (PM_{10}, $PM_{2.5}$) and gaseous pollutants (NO_2, O_3), were modeled using a machine learning-based technique. Time-varying Cox proportional-hazard models were used to estimate the association between the annual mean concentration of air pollutants and the incident AIRDs (rheumatoid arthritis, ankylosing spondylitis and systemic lupus erythematosus). We conducted stratified analyses by sex and age.

The study population at baseline was 721,853, and there were 29,308 new cases of AIRDs during the study period. In one-pollutant models, PM_{10}, $PM_{2.5}$, O_3 was positively associated with AIRDs (PM_{10}: HR = 1.07 [95% CI = 1.04, 1.10]; $PM_{2.5}$: HR = 1.29 [95% CI = 1.22, 1.36]; O_3: HR = 1.08 [95% CI = 1.04, 1.12]). The results from the two-pollutant models showed that the association between $PM_{2.5}$, O_3 and AIRDs incidences remained robust. These associations varied by sex ($PM_{2.5}$: HR in men = 1.25 [95% CI = 1.13, 1.38], HR in women = 1.30 [95% CI = 1.22, 1.39]; O_3: HR in men = 1.10 [95% CI = 1.03, 1.18], HR in women = 1.06 [95% CI = 1.02, 1.11]).

This study found positive associations between long-term exposure to $PM_{2.5}$ and O_3 and incident AIRDs, with differential susceptibility by sex. These findings suggest efforts to reduce air pollution levels may help reduce the public health burden from AIRDs.

P41-11 | Variation of Air Quality at Philadelphia Subway Stations

Kabindra M. Shakya[1], Jack Schelin[2], Aimee L. Eggler[3], Michael A. Posner[2]

[1] Villanova University, Geography and the Environment, Villanova, USA; [2] Villanova University, Mathematics and Statistics, Villanova, USA; [3] Villanova University, Chemistry, Villanova, USA

Subways offer an efficient and sustainable way to travel that reduces air pollution emission from cars on roadways. Underground subways are also known to have higher air pollution levels than outdoor levels. We present the air quality in Philadelphia subways by comparing matched belowground and aboveground locations and comparing these with suburban air quality. $PM_{2.5}$, black carbon (BC), and ultrafine particles – lung-deposited surface area (UFP-LDSA), and heavy metal concentrations were measured from three subways in Philadelphia, Pennsylvania. Measurements were conducted during the summers of 2022, 2023, and 2024 and the spring of 2024 with about five replicate measurements at each of the stations. We found higher concentrations of the pollutant concentrations in underground subways compared to their matched aboveground locations, but they were also highly variable depending on the subway stations. The ratio of belowground to aboveground BC was much higher compared to $PM_{2.5}$ and UFP-LDSA. We present the yearly and seasonal variation, variation among the subways, and the differences in pollutants between the aboveground and belowground for each of the pollutants.

P41-12 | The effect of temperature on particulate matter related incident hypertension in women: a cohort analysis

Karla M. Valdez-Trejo[1], Karla Cervantes-Martinez[2], Marlene Cortez-Lugo[1], Jose M. Salazar-Montiel[3], Alejandro Molina-Villegas[3], Pablo Lopez-Ramirez[3], Dalia Stern-Solodkin[1], Adrian Cortes-Valencia[1], Martin Lajous-Loaeza[1], Horacio Riojas-Rodriguez[1]

[1] National Institute of Public Health, Center for Population Health Research, Cuernavaca, Mexico; [2] World Health Organization, Department of Environment, Climate Change and Health, Geneva, Switzerland; [3] Geospatial Information Sciences Research Center, Mexico, Mexico

Background: Air pollution and temperature extremes are significant environmental risk factors for cardiovascular disease. However, longitudinal studies investigating the combined impact of these factors on hypertension are limited.
Objective: This study aimed to estimate the modifying effect of temperature on the association between $PM_{2.5}$ exposure and incident hypertension among women in the Mexican Teachers' Cohort (MTC).
Methods: The sub-sample included 25,949 female teachers from the MTC residing in the metropolitan areas of Mexico City, Guadalajara, Monterrey, and Toluca, with follow-up from 2006 to 2024 or until failure, censoring, or death. Participants reported in follow-up questionnaires if they had been diagnosed with hypertension by a physician and if they were receiving pharmacological treatment. Using a model that integrates satellite data and ground measurements, annual average exposure to $PM_{2.5}$ was estimated, considering both home and workplace. Time-varying Cox proportional hazards models were employed to evaluate the interaction between $PM_{2.5}$ and temperature with incident hypertension, considering the clustered nature of the data within each metropolitan area, and adjusting for confounding variables that were identified using a causal model.

Results: Significant interactions were observed between $PM_{2.5}$ and temperature for the annual average exposure. For each 1°C increase in temperature, the risk of hypertension increased by 5% for every 10 μg/m³ increase in PM2.5 (HR = 1.05, 95% CI: 1.01, 1.09, p = 0.014).
Conclusion: The interaction analysis suggests that the effect of $PM_{2.5}$ on the risk of hypertension may be modified by temperature. These findings underscore the urgent need to address both air pollution and climate change, as their combined effects may exacerbate public health problems such as hypertension.

Supported by: The National Council of Science and Technology #**316370**.

P41-13 | Disparities in Immigrants' Exposure to Nitrogen Dioxide in the United States

Kelvin Fong, Corinne Bassett, Daniel Goldberg, Gaige Kerr, Susan Anenberg

George Washington University, Department of Environmental and Occupational Health, Milken Institute School of Public Health, Washington DC, USA

Objective:
Immigrants in the United States (US) total over 41 million or 14% of the population. Are they exposed to higher levels of nitrogen dioxide (NO_2), an air pollutant linked to increased risks for asthma attacks and respiratory dysfunction? This study leverages satellite remote sensing to assess NO_2 disparities between immigrants and the US-born, as well as among immigrant groups by time since immigration and region of origin.
Material and Methods:
We obtained counts of immigrants residing in each US Census tract using the American Community Survey 5-year estimates (2019-2023). Each tract was classified as metropolitan or non-metropolitan using rural-urban continuum codes. We characterized tract-level annual average NO_2 levels in 2021 using satellite measurements by the Tropospheric Monitoring Instrument (TROPOMI). We calculated the population-weighted NO_2 mean for the US-born, immigrants, and immigrant groups by year of immigration and world region of origin.
Results:
On average, immigrants live in Census tracts with 33% higher NO_2 than the population average. We observed this disparity only in metropolitan areas. Among immigrants in metropolitan areas, those who immigrated earlier lived in tracts with higher NO_2. Those who immigrated before 1990 lived in tracts with 42% higher NO_2 compared to the overall population-weighted NO_2 mean; those who immigrated 1990-1999 37% higher, 2000-2009 29% higher, and 2010-2019 25% higher. Those who immigrated from Asia or Latin America lived in tracts with 36% higher NO_2 than the population mean; from Europe 25% higher, Africa 19% higher, and Oceania 17% higher.
Conclusion:
In the US, NO_2 disparities exist between immigrants and the US-born, as well as among immigrant groups by time since immigration and region of origin. Future research should assess the extent to which poor air quality contributes to immigrant health disparities and inform context-dependent healthcare and environmental policy.

P41-14 | Air pollution (PM 2.5) may induce cardiorespiratory effects on health of the population of the city of Korhogo in Cote d'Ivoire

Kouame Kouadio[1], Constant K. Konan[1], Sylvain N.K. Gnamien[3], Veronique Yoboue[4]

[1] Institut Pasteur of Côte d'Ivoire, Environmental science and health/ Eco epidemiology Unit, Abidjan, Côte d'Ivoire; [2] Institut Pasteur of Côte d'Ivoire, Environmental science and health Department/ Eco epidemiology Unit, Abidjan, Côte d'Ivoire; [3] Felix Houphouet Boigny University, Atmospheric Sciences Laboratory, Abidjan, Côte d'Ivoire; [4] Felix Houphouet Boigny University, Atmospheric Sciences Laboratory, Abidjan, Côte d'Ivoire

BACKGROUND AND AIM: Air quality and it impacts capture more attention of governments and public opinion. Indeed, air pollution has consequences on the health of populations. Epidemiological studies contributed much to the understanding of the eff ects. In order to understand this, an epidemiological study was done in the regional hospital of Korhogo in regards to the PM 2.5 concentration levels in the city.
The objective of this study was to assess the impact of PM2.5 concentration on health in Korhogo, with special attention to cardio-respiratory.
METHODS: All subjects coming to consultation in the regional hospital of Korhogo were selected in relation to the lungs and heart symptoms for 3 months beginning from December 2018 to March 2019. Patients to include were identified by a Medical Doctor through a questionnaire after their informed consent. The relationship between exposure to air pollution and health was done through time series. The main outcomes of the study were the incidence of cardiorespiratory (95% CI) and the indicator of health impact. An existence of a significant relationship between exposure to air pollution and health outcomes was done.
RESULTS:The results showed that 3410 patients consulted a physician during the 3 months visits in the regional hosptal ofKorhogo. Within those patients, 527 (17.46%) were recruted for pollution issues. More than 95% of the patients had cardiopulmonary diseases in the city of Korhogo. The average of PM2.5 concentration was higher than the daily recommended by WHO. A strong relationship was seen to be associated to cardiopulmonary disease in that regional hospital;CONCLUSIONS: The average of PM2.5 concentration was higher than the daily recommended by WHO. A strong relationship was seen to be associated to cardiopulmonary disease in that regional hospital Where a special attention should be taken for patie

P41-15 | Lung cancer mortality attributable to residential radon exposure, an estimation at small areas level in a radon-prone region.

Lucia Martin-Gisbert[1], Maria Isolina Santiago-Perez[2], Guadalupe Garcia[1], Cristina Candal-Pedreira[1], Julia Rey-Brandariz[1], Carla Guerra-Tort[1], Monica Perez-Ríos[1], Alberto Ruano-Raviña[1]

[1] University of Santiago de Compostela, Preventive Medicine and Public Health, Santiago de Compostela, Spain; [2] Goverment of Galicia, Public health Directorate, Santiago de Compostela, Spain

Objective:
To estimate lung cancer mortality attributable to residential radon exposure at local level (at municipality and/or shire level)
Material and methods:
Estimation was performed for Galicia, a European radon-prone region, located in Northwestern Spain for the 5-year period 2019-2023. Radon exposure prevalence was obtained from a total of 6,080 three-month measurements carried out with alpha track detectors at participant homes by an accredited radon laboratory. Data related to lung cancer observed mortality, demographics and smoking prevalence were obtained from the regional mortality registry (using CIE-10 codes C33-C34), the Galician Institute of Statistics, the census and the Galician Information System for Risk Behaviors.
Population Attributable Fractions (PAF) were calculated using the prevalence dependent method to estimate the radon attributable mortality (radon AM). The estimations were performed globally, by sex, and by smoking habit.
Results:
Prevalence of exposure to radon > 100 Bq/m3 ranged from 19% to 94% across a total of 7 municipalities (cities) and 53 shires, with a median of 63%. Average prevalence of never smoker females and males was 73% (ranging from 59 to 86.8%) and 38% (ranging from 28 to 52%), respectively.
PAF and radon AM were estimated for female and male population and sorted by smoking status (never smokers, ex-smokers and current smokers). PAF was highly variable depending on the shire, ranging from 6% to 24%. Radon AM was 311 deaths for woman and 927 deaths among men in the 5 year-period analyzed, from which never smokers women and men accounted for 40 deaths each.
Conclusions:
Radon attributable mortality varies widely between small areas such as municipalities or counties located in the same region. Calculations at small areas level is possible with adequate data sources and provide insightful estimations at community level to design health policies aimed to reduce the burden of lung cancer

P41-16 | Roads to health: The impact of new roadway development on traffic related air pollution

Maeve G. MacMurdo[1], Monica Harkey[3], Yanjun Wu[2], Xiaofeng Wang[2], Daniel A. Culver[1]

[1] Cleveland Clinic, Integrated Hospital Care Institute/Pulmonary Department, Cleveland, USA; [2] Cleveland Clinic Learner Research Institute, Quantitative Health Sciences, Cleveland, USA; [3] University of Wisconsin-Madison, Center for Sustainability and the Global Environment, Madison, USA

Objective: In 2019 a 3-mile, high traffic roadway running through historically redlined black neighborhoods was opened in the City of Cleveland. The impact of the roadway opening on community health is unknown. Utilizing satellite derived models of ambient nitrogen dioxide (NO2) and fine particulate matter (PM2.5) we aim to quantify the impact of roadway opening on traffic related air pollution and asthma outcomes.
Methods: Patients with a diagnosis of asthma who actively received healthcare between 2012-2022 were identified, and address history geocoded. Mean exposure to NO2 and PM2.5 were modelled prior to roadway opening (2016) the year of roadway opening (2019) and two years after roadway opening (2021). Tropospheric NO2 column amounts were generated at 1km x 1km resolution using OMI and TROPOMI data. Satellite derived estimates of PM2.5 were obtained from estimates generated by the Washington St. Louis Atmospheric Composition analysis group. The impact of roadway opening on concentrations of PM2.5 and NO2 were modeled.
Results: Annual concentrations of ambient NO2 decreased for the City of Cleveland over the study period, though this decrease was less pronounced for patients within 5km of the roadway (-0.810^15 molecules/cm2 versus –0.5 10^15 molecules/cm2., P <0.001). Conversely, ambient concentrations of PM2.5 increased for all participants, from 7.9µg/m3 in 2016 to 8.315µg/m3 in 2021.
Conclusion: Traffic related air pollution may have significant implications for community health. There is a pressing need to consider the impact of new infrastructure on local level air quality.

P41-17 | Methods for strong causal evidence in air pollution studies of metabolomics using data from a randomized crossover trial of air purifiers

Nicholas Pellegrino[1], Douglas Brugge[1], Misha Eliasziw[2]

[1] University of Connecticut Health Center, Department of Public Health Sciences, Farmington, USA; [2] Tufts University, Department of Public Health and Community Medicine, Boston, USA

Objective: Exposure to particulate matter has been associated with adverse health outcomes such as cardiovascular and respiratory illnesses, as well as mortality. There is a growing field of metabolomic research studying the biological mechanisms by which air pollution affects health. However, there is limited research on reducing air filtration on the metabolome.
Materials and Methods: We have plasma samples from 156 participants in the Home Air Filtration for Traffic-Related Air Pollution (HAFTRAP) study, which is a randomized cross-over study in Somerville, MA, USA. Participants were randomized to receive either a high efficiency particulate arrestance (HEPA) air purifier for one month, a washout period of one month, and then a sham unit for one month or vice-versa. Blood samples were collected at the start and end of each intervention period for a total of four samples per participant. We will use high resolution

metabolomics to identify approximately 8,000 metabolites. Metabolomic data will be normalized and batch corrected to limit variability between individual plasma samples and between batches of plasma that were not analyzed together. Data will be transformed and linear mixed models will be used to determine which metabolites are significantly different between intervention groups at all four time points. This will also account for within-person correlations and can be adjusted for other factors such as sex, age, etc. Finally, pathway enrichment will be employed to identify the biological function and pathways of the significant metabolites.

Results: The HAFTRAP trial has concluded and all blood samples are currently in storage. They will be analyzed in the coming months by the Emory metabolomics lab.

Conclusion: Our randomized crossover study design has the potential to generate strong causal evidence of effects of particulate matter exposure on the metabolome since it effectively controls for time invariant confounders.

P41-18 | Characterization of Sources and Sinks of the Oxidative Potential of Particulate Matter Emitted from Household Sources.

P. S. Ganesh Subramanian[1], Saman Haratian[2], Brent Stephens[2], Mohammad Heidarinejad[2], Vishal Verma[1]

[1] University of Illinois Urbana-Champaign, Department of Civil and Environmental Engineering, Urbana, USA; [2] Illinois Institute of Technology, Department of Civil, Architectural and Environmental Engineering, CHICAGO, USA

Objective: Although indoor exposure to particulate matter (*PM*) dominates in comparison to outdoor exposure, the sources of indoor PM and their sinks are not well characterized. Moreover, the oxidative potential (OP) of PM has emerged as a more representative metric to represent the toxicity of aerosols than particle mass concentrations (*PMC*). Our objective here is to parametrize the OP sources and sinks for PM emitted from household sources to develop an OPemission rate (*OER*) inventory.

Methods: Time-resolved PM samples (TRS; 10 samples per source) of emissions from six sources (candles in the presence of a wind-draft, two types of incense, cigarettes, ultrasonic humidifiers, and toasters) were collected using a manifold filter-holder configuration in a controlled-environmental-chamber. The mass-normalized OP (OP^m) was characterized using dithiothreitol depletion ($OP_{DTT}{}^m$) and glutathione depletion ($OP_{GSH}{}^m$) assays.

Results: The $OP^m$of TRS showed little to no temporal variation for any of the sources. Therefore, the temporal variations in the volume-normalized OP [OP^v; product of OP^m and *PMC*], were predominantly driven by variations in *PMC*. This is further substantiated by the loss coefficients of OP^v and *PMC* being almost equal in the post-emission phase. Both these losses followed first-order decay kinetics. Even though the $OP_{DTT}{}^m$ was highest for candles in the presence of a wind-draft [55.72 pmol/(min.µg)], its OER_{DTT} was relatively lower (4,871 pmol/min^2) than that of toaster emissions (50,970 pmol/min^2), due to relatively much higher particle mass emission rates (PER_m) of toasters (3520 µg/min vs. 86.8 µg/min).

Conclusion: Limited variation in the time-resolved OP^m indicates that the chemical composition of the PM emitted during these emissions remains largely similar. Thus, the oxidative burden of the emissions from tested sources is predominantly driven by the PER_m.

Funding for this work was provided by the National Science Foundation under Grant No: CBET 2241332.

P41-19 | Traffic impact on air quality at the Otay Mesa Commercial Truck Port of Entry

Yeonseung Cheong[1], Jeffry H. Shirai[1], Edmund Seto[1], Elena Austin[1], Alejandro Amador[3], Janie Paz[3], Penelope J. Quintana[2], Erica S. Hernandez[2], Jonas Contreras[2], Carina V. Zarate[2]

[1] University of Washington, Department of Environmental & Occupational Health Sciences, Seattle, USA; [2] San Diego State University, School of Public Health, San Diego, USA; [3] Casa Familiar, Department of Environmental Justice, San Ysidro, USA

Objective: The Caltrans-funded Otay Mesa study expanded a community air monitoring network along the U.S.-Mexico border to evaluate the impact of truck traffic at the Otay Mesa commercial port of entry (POE), the busiest commercial vehicle crossing in California, which is soon to be expanded.

Material and Methods: Led by Casa Familiar (community partner) in San Ysidro, CA, in partnership with San Diego State University and the University of Washington, low-cost sensors were deployed across 11 community-selected sites near border to measure traffic-related particles ($PM_{2.5}$, black carbon) and NO_2. Measurements from April 2022 to June 2024 were calibrated against collocated reference instruments. Linear regression was used to assess the associations between the dependent variable community-monitored pollutant concentrations and independent variables regional $PM_{2.5}$, wind direction, and truck traffic along freeway corridors (I-5, I-905, I-805). Findings were shared with the community for feedback.

Results: Elevated concentrations of $PM_{2.5}$, black carbon, and NO_2 were observed at sites near the Otay Mesa POE, with distinct morning peaks aligning with peak truck traffic hours. Black carbon levels near the Otay Mesa POE were notably higher than at sites less impacted by traffic, suggesting significant traffic influence and the need for community driven mitigation strategies. Linear regression models indicated regional $PM_{2.5}$ and wind direction influenced pollutant concentrations measured at community sites. $PM_{2.5}$ was moderately associated with traffic flow, particularly for the monitoring site at the California Highway Patrol truck facility, which is near to high truck-traffic. However, black carbon showed weaker associations with truck flow, suggesting additional sources in nearby Tijuana, Mexico.

Conclusion:

This community-engaged monitoring network provided valuable insights into local and transboundary air pollution near the U.S.-Mexico border. Continued monitoring will be critical in assessing changes as the border expansion progresses.

P41-20 | Preliminary Observations on Exposure Risk to Aerosolized Cyanotoxins in Florida

Michael L. Parsons[1], Malcolm McFarland[2], Adam M. Schaefer[3], Rebecca S. Koszalinski[4], John S. Reif[5]

[1] Florida Gulf Coast University, The Water School, Fort Myers, USA; [2] Florida Atlantic University, Harbor Branch Oceanographic Institute, Fort Pierce, USA; [3] Abt Global, Rockville, USA; [4] University of Central Florida, College of Nursing, Orlando, USA; [5] Colorado State University, Fort Collins, USA

Objective: Public health concerns arose in Florida following large blooms of the cyanobacterium, *Microcystis aeruginosa*, in 2016 and 2018. A subsequent study was initiated to measure the air-borne concentrations of the cyanotoxins produced by *Microcystis* (microcystins) present during bloom and non-bloom conditions in Florida.
Material and Methods: Anderson-style air impactors were deployed near Cape Coral and Stuart between 2018 and 2024, areas which experienced prior cyanobacteria blooms. Microcystin concentrations were measured using Abraxis ELISA kits. Overall, 72 samplers were deployed and 648 filters extracted in 22 separate sampling events.
Results: The 2018 sampling effort in Cape Coral captured a declining bloom of *Microcystis* in an affected canal adjacent to residences. Microcystin concentrations (ng m^{-3}) were up to 100-fold higher than during non-bloom concentrations (~1 vs 0.01 ng m^{-3}) and were comparable to levels measured elsewhere during blooms. Additionally, during the 2018 bloom event, “elevated” concentrations of microcystins were measured up to 10 miles (16 km) from the bloom in Cape Coral, suggesting that airborne toxins may affect an area beyond the immediate vicinity of the bloom.
In a health assessment conducted during the 2018 bloom, water samples were collected from locations adjacent to human exposure sites. Microcystin concentrations ranged from undetectable to > 5000 ppb, with approximately 40% exceeding the EPA limit for human recreational exposure. Microcystin was detected in the nasal passages of 95% of 121 human participants. Residential and recreational exposures were associated with an increased risk of reporting respiratory symptoms including cough, dyspnea and wheeze.
Conclusions: These results demonstrate the feasibility of measuring microcystin concentrations in air and support the conclusion that inhalation of cyanobacterial toxins represents an important pathway of human exposure.

Funding source: Florida Department of Health

P41-21 | Impact of Commute Timing on Personal Exposure: Findings from the ETCH Study

Misti L. Zamora, Abhay Anand

UConn Health, Public Health Sciences, Farmington, USA

There is increasing evidence that traffic-related air pollutants inflict a broad range of deleterious health effects in both health-compromised and healthy individuals, and it has been reported that traffic pollutants may cause up to half of all air pollution-related mortalities. The Effects of Transportation Choices on Commuter Health (ETCH) study investigates the impact of commute timing on exposure to traffic-related air pollutants (TRAPs) and associated acute health effects. Participants are being recruited (n = 65) to complete two 48-hour periods of comprehensive personal monitoring while modifying commute times (rush hour vs. non-rush hour). This includes backpacks equipped with

real-time sensors for PM2.5, CO, NO2, O3, volatile organic compounds (VOCs), temperature, relative humidity, noise levels, and GPS, complemented by traditional badge samplers for validation. Health assessments include spirometry (FEV1, FVC), fractional exhaled nitric oxide (FeNO), blood pressure (BP), and urinary biomarkers of PAH/nPAH exposure, oxidative DNA damage, and benzene exposure. Preliminary personal monitoring results revealed higher air pollution concentrations during rush hour commuting compared to non-rush hour commuting. Specifically, PM2.5 and particle number concentrations during commutes increased with decreasing driving speed ($p<0.01$), indicating the influence of traffic congestion. The average FeNO for our study participants was 20.3 ± 13.7 ppb. Overall, FeNO levels were found to be impacted by a variety of factors including PM2.5 concentrations, seasonal allergies, pollen levels, and respiratory cold symptoms, and FeNO increased after the rush hour commuting scenario. This talk will focus on characterizing the exposures during the distinct commuting periods, and preliminary relationships with the health endpoints. By integrating detailed personal monitoring with health assessments, the ETCH study provides a framework for evaluating commute timing as a TRAP mitigation strategy and informing public health interventions.

P41-22 | Different health impacts derived by different data sources of particulate matter air pollution

Miyoun Shin[1], Yoon-Hee Kang[2], Soontae Kim[3], Sun-Young Kim[1]

[1] National Cancer Center, Department of Public Health & AI, Ilsan, South Korea; [2] AJOU University, Environmental Institute, Suwon, South Korea; [3] AJOU University, Department of Environmental and Safety Engineering, Suwon, South Korea; [4] National Cancer Center, Department of Public Health & AI, Ilsan, South Korea

Objective: Ambient fine particulate matter ($PM_{2.5}$) is a major environmental risk of human health and associated burdens were widely reported. However, findings could vary depending on the PM data sources. We aimed to compare premature deaths attributable to $PM_{2.5}$ using four commonly-applied PM data across ~250 districts in South Korea for 2016-2019.
Material and Methods: We assessed district-specific annual-average $PM_{2.5}$ concentrations using 1) regulatory monitoring data (local measurements: LM), and three sets of predictions derived by 2) local geostatistical model (local estimates: LE-GS), 3) regional chemical transport model (regional estimates: RE-CTM), and 4) global chemical transport model (global estimates: GE-CTM). Given the district-specific population, age-specific mortality rates, and hazard ratios (HRs) from a South Korean cohort study, we estimated the number of premature deaths attributable to $PM_{2.5}$.
Results: The mean $PM_{2.5}$ concentrations for 2016-2019 were 24.09 (standard deviation=3.46), 23.59 (1.99), 23.39 (2.52), and 23.26 (2.70) μg/m³ for LM, LE-GS, RE-CTM, and GE-CTM, respectively. Across the four data sources, local measurements or estimates showed higher correlation between each other (Pearson correlation coefficients in 2019=0.76-0.85) than those with global estimates (0.56-0.68). In 2016, LM reported the largest death count (12,647), followed by LE-GS (12,565), RE-CTM (11,910), and GE-CTM (11,115). Over four years through the year 2019, LM, LE-GS, and RE-CTM showed slight increases by 4-10% (death counts= 13,257, 13,074, and 13,145, respectively). In contrast, GE-CTM exhibited the most notable increase by 23% (13,657 death counts).
Conclusion: Our study highlights the discrepancy of $PM_{2.5}$-related health burdens depending on the $PM_{2.5}$ data sources, particularly for exposure estimates from global models. These findings emphasize the need for careful selection of exposure models in local health impact assessments.

P41-23 | The role of particulate radioactivity on the impacts of $PM_{2.5}$ on hospitalizations related to neurodegenerative disorders with causal mediation analysis

Shuxin Dong[1], Marc Weisskopf[1,2], Petros Koutrakis[1], Brent Coull[3,1], Ernani Choma[1], Longxiang Li[4], Antonella Zanobetti[1]

[1] Harvard T.H. Chan School of Public Health, Department of Environmental Health, Boston, USA; [2] Harvard T.H. Chan School of Public Health, Department of Epidemiology, Boston, USA; [3] Harvard T.H. Chan School of Public Health, Department of Biostatistics, Boston, USA; [4] Emory Rollins School of Public Health, Department of Environmental Health, Atlanta, USA

Objective: Neurodegenerative disorders—especially Alzheimer's disease (AD) and Parkinson's disease (PD)—pose major global health challenges. With few effective treatments available, mitigating modifiable risk factors such as $PM_{2.5}$ is essential. However, the toxic properties of $PM_{2.5}$ remain poorly understood. We propose that particulate radioactivity (PR), a radiometric measure of $PM_{2.5}$, may be key to its harmful effects on neurodegenerative disorders.
Methods: We performed a nationwide cohort study using Medicare fee-for-service data for beneficiaries aged ≥65 in the contiguous USA from 2000 to 2016. Participants were followed from enrollment until their first hospitalization for Alzheimer's disease and related dementias (ADRD) or PD. Annual average levels of modeled gross beta-activity (a proxy for PR) and modeled $PM_{2.5}$ were estimated based on residential ZIP codes. A causal mediation framework was employed. First, we used a Poisson survival analysis employing time-varying covariates with PR and $PM_{2.5}$, adjusting for age, socioeconomic status, seasonal temperature, and relative humidity. Next, we fitted a linear mediator model. Natural direct and indirect (via PR) effects of $PM_{2.5}$ were then calculated and reported as incidence rate ratios (IRRs) with bootstrap-derived standard errors.
Results: The cohort comprised 68,640,730 individuals. During the study period, 7,108,045 (10.4%) were hospitalized with ADRD and 1,019,099 (1.5%) with PD. For an interquartile range increase in $PM_{2.5}$ (3.8 $\mu g/m^3$), the ADRD analysis yielded a natural direct effect IRR of 1.034 (95% CI: 1.032–1.035) and an indirect effect IRR of 1.008 (95% CI: 1.006–1.035), indicating that 19.7% of the effect was mediated by PR. In the PD analysis, the natural direct effect was 1.050 (95% CI: 1.048–1.052) and the natural indirect effect 1.005 (95% CI: 1.002–1.008), with 9.7% mediation.
Conclusion: Our findings suggest that PR significantly contributes to $PM_{2.5}$ toxicity, exerting a greater impact on ADRD hospitalizations compared to PD.

P41-25 | Impact of long-term exposure to air pollution on childhood cancer and endocrine disorders: A National Cohort Study from the National Health Insurance Service, Korea

Jongmin Oh[1,2], Eun Sun Yoo[3], Ji Hyen Lee[3], Yi Jun Kim[1], Do Kyeong Song[4], Kyung Hee Kim[3], Eunji Mun[3], Soontae Kim[5], Hae Soon Kim[3], Jieun Min[1], Sohee Wang[1], Ji Hui An[6], Byungmi Kim[7,8], Bohyun Park[9], Sanghyuk Bae[10], Eun Mi Jung[11], Logan G. Spector[12,13], Eunhee Ha[1,14]

[1] Ewha Womans University College of Medicine, Department of Environmental Medicine, Seoul, South Korea; [2] Seoul National University College of Medicine, Department of Human Systems Medicine, Seoul, South Korea; [3] Ewha Womans University College of Medicine, Department of Pediatrics, Seoul, South Korea; [4] Ewha Womans University College of Medicine, Department of Internal Medicine, Seoul, South Korea; [5] Ajou University, Department of Environmental and Safety Engineering, Suwon, South Korea; [6] Ewha Womans University College of Medicine, Institute of Ewha Medical Research, Seoul, South Korea; [7] National Cancer Center, Division of Cancer Prevention, National Cancer Control Institute, Goyang, South Korea; [8] National Cancer Center, Department of Cancer Control and Population Health, Graduate School of Cancer Science and Policy, Goyang, South Korea; [9] National Cancer Center, Division of Cancer Control and Policy, National Cancer Control Institute, Goyang, South Korea; [10] The Catholic University of Korea College of Medicine, Department of Preventive Medicine, Seoul, South Korea; [11] National Cancer Institute, Division of Cancer Epidemiology & Genetics, Maryland, USA; [12] University of Minnesota, Division of Epidemiology and Clinical Research, Department of Pediatrics, Minnesota, USA; [13] University of Minnesota, Masonic Cancer Center, Minnesota, Germany; [14] Ewha Womans University, System Health & Engineering Major in Graduate School (BK21 Plus Program), Seoul, Germany

Recent studies have highlighted air pollution as a significant environmental hazard affecting children's health, particularly in relation to both childhood cancer and endocrine disorders. However, due to the relatively lower incidence of these conditions, epidemiological research has been limited. Our team established the Childhood and Adolescent Cancer consortium between Korea and USA (CACKU) to identify risk factors and prediction models for childhood and adolescent cancer (5.3 million children) based on Korea-US consortium. In addition, we employed big data to investigate the association between long-term exposure to air pollution and the risk of endocrine disorders, including precocious puberty (PP, 1.2 million children) and autoimmune thyroid diseases (AITD, 1.4 million children). We analyzed the National Health Insurance Database from the National Health Insurance Service from 2007 to 2022 and found a significant association between air pollution exposure and the incidence of childhood cancers, particularly leukemia. Furthermore we found that exposure to these pollutants, especially fine particulate matter and sulfur dioxide, is associated with an earlier onset of PP in girls (2007–2019). Additionally, these pollutants are linked to an increased risk of AITD, which can lead to lifelong health complications (2002–2023). Notably, the effects of air pollution on these conditions are more pronounced in girls, with both early puberty and thyroid diseases showing a stronger association in girls compared to boys.

These findings underscore the growing recognition of air pollution as a significant environmental threat to pediatric health. As the incidence of both childhood cancer and endocrine disorders continues to rise, reducing exposure to these harmful pollutants is critical for mitigating these risks. Public health interventions aimed at limiting children’s exposure to air pollution are urgently needed. Further research into the mechanisms by which air pollution influences cancer development and endocrine function is essential to inform effective prevention and treatment strategies.

P41-26 | $PM_{2.5}$ constituents associated with childhood obesity and larger BMI growth trajectory: A 14-year longitudinal study

Yaqi Wang[1], Yanhui Dong[1], Lijuan Wu[2], Jing Li[1]

[1] Peking University, Institute of Child and Adolescent Health, Beijing, China; [2] Capital Medical University, Department of Epidemiology and Health Statistics, School of Public Health, Beijing, China

Objective: To investigate the associations of $PM_{2.5}$ constituents with incident childhood overweight/obesity (OWOB) risk and evaluate long-term $PM_{2.5}$ impacts on BMI trajectories in Chinese children.

Material and Methods: We conducted a longitudinal study of 1,450,830 Chinese children aged 6–19 years from Beijing and Zhongshan during 2005–2018. $PM_{2.5}$ mass and five major components were extracted from the Tracking Air Pollution in China (TAP) dataset. Cox proportional hazards models were applied to quantify exposure–response associations. We further performed principal component analysis (PCA) to handle the multi-collinearity and used quantile g-computation (QGC) approach to analyze the impacts of exposure mixtures. Additionally, we selected 125,863 children with at least 8 physical examination measurements and combined group-based trajectory models (GBTM) with multinomial logistic regression models to explore the impacts of exposure to $PM_{2.5}$ mass and five constituents on BMI and BMI Z-score trajectories during 6–19 years.

Results: Each interquartile range increment in $PM_{2.5}$ exposure was significantly associated with a 5.1 % increase in the risk of incident OWOB (95 % confidence Interval [CI]: 1.036–1.066). We also found black carbon, sulfate, organic matter, often linked to fossil combustion, had comparable or larger estimates of the effect (HR = 1.139–1.153) than $PM_{2.5}$. Furthermore, Exposure to $PM_{2.5}$ mass, sulfate, nitrate, ammonium, organic matter and black carbon was significantly associated with an increased odds of being in a larger BMI trajectory and being assigned to persistent OWOB trajectory.

Conclusion: Our findings provide evidence that the constituents mainly from fossil fuel combustion may have a perceptible influence on increased OWOB risk associated with $PM_{2.5}$ exposure in China. Moreover, long-term exposure to $PM_{2.5}$ contributes to an increased odds of being in a lager BMI and a persistent OWOB trajectories.

Funding National Natural Science Foundation of China (42307133).

P42 | Advances in Air Quality Monitoring and Modeling - PART II

P42-01 | Machine Learning-Based PM2.5 Exposure Modeling in Kampala and Accra: Integrating Mobile Air Monitoring for Sub-Saharan Africa

Eric S. Coker[1], Solomon Nsumba[2], Wabinai F. Raja[3], Joel T. Muhanguzi[2], Adeladza K. Amegah[4], Ernest Mwebaze[2], Engineer Bainomugisha[3,2]

[1] N/A, Vancouver, Canada; [2] Sunbird AI, Kampala, Uganda; [3] Makerere University, Department of Computer Science, Kampala, Uganda; [4] University of Cape Coast, Cape Coast, Ghana

Air pollution exposure modeling remains in its early stages in sub-Saharan Africa, limiting the ability to assess health risks and understand mitigation strategies. This study develops a novel fine particulate matter (PM2.5) exposure model for Kampala, Uganda, and Accra, Ghana, applying machine learning (ML) techniques and integrating mobile air monitoring data. The model addresses critical gaps in exposure assessment by incorporating diverse data sources, including satellite-derived air quality estimates, land-use variables, meteorological factors, and ground-monitored measurements from a high-resolution network of low-cost sensor networks.
A key innovation of this study is integrating more than a year of daily mobile air monitoring data, which can potentially reduce biases associated with static monitoring stations. We evaluate the model's predictive performance in Kampala and assess its generalizability to Accra, testing its adaptability across complex urban environments. This cross-city validation is crucial for understanding how well ML-based exposure models translate across regions.
Preliminary modeling results indicate strong prediction performance for Kampala, with XGBoost achieving the lowest RMSE (14.7 μg/m^3) and highest R^2 (0.71). LightGBM and CatBoost also performed well, with RMSE values of 14.22 μg/m^3 and 14.65 μg/m^3, and R^2 of 0.73 and 0.71, respectively. In contrast, MLP and LSTM showed lower predictive performance, with RMSE values above 19 and R-squared values below 0.5. Ongoing work involves further model development and validation using the mobile monitoring data and extending the analysis to Accra.
This research advances data-driven exposure assessment methodologies and contributes to the growing field of air pollution and health impact modeling in Africa. The findings will support local policymakers, public health practitioners, and researchers in developing evidence-informed air quality health research and interventions. The study highlights the potential of machine learning and mobile monitoring to improve exposure estimates in resource-constrained settings, ultimately enhancing public health protection in rapidly urbanizing African cities.

P42-02 | Moderating Effects of Meteorological Factors in Agricultural Straw Open-field Burning and Air Pollution

Haoneng Huang, Zhou Yang, Li Li, Chun-Quan Ou

Southern Medical University, Department of biostatistics, Guangzhou, China

Background: Straw burning remains prevalent in China despite stringent burning-ban policies. By identifying meteorological windows conducive to pollutant dispersion, prescribed burning is possible to help farmers alleviate the burden of straw disposal while avoiding heavy pollution.

Objective: To explore the moderating effects of meteorological factors on the relationship between straw burning and air pollution and identify combinations of meteorological conditions that favor pollutant dispersion when burning.

Methods: Multi-source remote-sensing monitored thermal anomalies were overlaid on cultivated land raster to identify straw-burning points. In the first stage, we employed a distributed-lag-nonlinear model to quantify the impact of surrounding fire points on pollutant levels at each of the 621 monitoring stations during 2015-2020. In the second stage, a meta-analysis was conducted to aggregate effects nationally. Interactive analyses between fire point counts and meteorological variables were conducted to investigate the differences in pollutant effects under varying meteorological conditions.

Results: Wind speed (WS), temperature, relative humidity (RH), and planetary boundary layer height (PBLH) were lower during straw-burning periods with higher AQI days. For $PM_{2.5}$, the cumulative effect from every 10-increase in upwind fire points over a lag of 0-3 days was twice as high as that from downwind fire points (14.62 [12.00,17.23] vs. 7.33[6.07, 8.59] $\mu g/m^3$). Interaction analysis indicated that the impact of fire points on particulate matter was strongest when daily mean temperatures were between 9-20°C, while the effect on O_3 increased with rising temperatures. The cumulative effects of pollutants other than O_3 primarily decreased with lower RH, increased WS, and higher PBLH. In contrast, the impact on O_3 was most severe at 60-75% RH levels, diminishing with increased WS and PBLH.

Conclusion: Suitable meteorological windows can significantly reduce the impact of straw burning on urban air quality. These findings provide a reference for enabling prescribed burning under specific conditions.

P42-03 | Unveiling Localized Air Quality Dynamics in Washington D.C. Using Fine-Scale CMAQ Modeling

Hoda Hallaji, Lucas Henneman

George Mason University, Department of Civil, Environmental and Infrastructure Engineering, Fairfax, USA

Abstract:
The Washington D.C. metropolitan area experiences complex air pollution patterns due to vehicular and industrial emissions. To investigate spatial and temporal variations in pollution levels, we employ the EPA's Community Multiscale Air Quality model (CMAQ v5.4+) with a nested domain configuration: 12 km² for the contiguous U.S., 4 km² for the eastern U.S., and a high-resolution 1 km² domain covering approximately 1,500 km² of the metropolitan area. This fine-scale modeling resolves meteorological and chemical processes at the neighborhood scale, providing improved accuracy over previous efforts.
Meteorological inputs are derived from the Weather Research and Forecasting (WRF v4.2.1) model, and emissions are processed using the Sparse Matrix Operator Kernel Emissions (SMOKE v4.7) model. We incorporate point-source emissions from the 2017 National Emissions Inventory (NEI2017) and gridded emissions from the Neighborhood Emission Mapping Operation (NEMO) dataset for localized precision.
By integrating air quality modeling with epidemiological analysis, we assess health burdens associated with traffic-related air pollution (TRAP). In Washington, D.C., on-road emissions contribute to over 50% of NO_2-related asthma cases and 23% of air pollution-attributable premature deaths, disproportionately affecting historically marginalized communities. Our analysis quantifies neighborhood-level exposure disparities and evaluates potential regulatory impacts on air quality and health outcomes.
Model evaluation is conducted using ground-based sensor data and satellite observations. Initial results show agreement with observed spatial patterns, reinforcing the model's utility for policy applications. By linking air pollution estimates with public health data, our study supports evidence-based policies aimed at mitigating environmental health disparities and advancing environmental justice.

Funding & Acknowledgments:
This research is supported by the NIH-funded REACH Center initiative. We acknowledge collaborations with Greater Greater Washington (GGWash) and George Washington University for their contributions to community-driven policy applications.

P42-04 | Reliable Air Quality Forecasting: An Integrated Approach Using the Choquet Integral and Möbius Representation

Houria Bouzghiba[1], Najiya Omar[2], Amine Ajdour[3], Abderrahmane Mendyl[5], Kenza Khomsi[6], Radouane Leghrib[3], Gábor Géczi[4]

[1] Hungarian University of Agriculture and Life Sciences, Doctoral School of Environmental Sciences, Gödöllő, Hungary; [2] Dalhousie University, Electrical & Computer Engineering Department, Halifax, Canada; [3] Ibn Zohr University, Laboratory of Materials, Signals, Systems and Physical Modeling, Physics Department, Faculty of Sciences, Agadir, Morocco; [4] Hungarian University of Agriculture and Life Sciences, Institute of Environmental Sciences, Gödöllő, Hungary; [5] Eötvös Loránd University, Institute of Geography and Earth Sciences, Budapest, Hungary; [6] General Directorate of Meteorology, Casablanca, Morocco

Poor air quality is one of the major environmental challenges that urban areas face, requiring robust planning and management. Particulate matter (PM) is a key pollutant in urban atmospheres and is the most harmful to human health. Therefore, accurate forecasting of this pollutant is essential. In this paper, we present a new aggregation function that combines the chemical transport model (CTM), specifically CHIMERE, with machine learning models, namely Univariate LSTM and Multivariate LSTM. The Choquet Integral function is employed as an aggregation operator to merge interrelated PM_{10} concentration outputs from different models. The results demonstrate high efficiency, with the R^2 reaching 0.98 for the aggregation function across all stations in Budapest, regardless of seasonal variations. Although PM_{10} levels in Budapest are not influenced by other pollutants - making the Univariate LSTM model more effective - the aggregation function significantly enhances the forecasting capabilities. Additionally, the use of the Möbius representation played a key role in this study by streamlining the process, reducing computational load, and dynamically adjusting weights. This enabled the model to prioritize the most relevant inputs without unnecessarily complicating the prediction process.

P42-05 | Rapid PM2.5-Induced Health Impact Assessment: A Novel Approach Using Conditional U-Net CMAQ Surrogate Model

Jong-Hyeon Lee[1], Jung-Hyun Park[1], Jinseok Kim[2], Jung-Hun Woo[2]

[1] EH R&C Co., Research Institute of Environmental Health and Safety, Incheon, South Korea; [2] Seoul National University, Environmental Planning Istitute, Seoul, South Korea

There is a pressing need for tools that can rapidly predict PM2.5 concentrations and assess health impacts under various emission scenarios, aiding in the selection of optimal mitigation strategies. Traditional chemical transport models (CTMs) like CMAQ are accurate but computationally intensive, limiting practical scenario analysis. To address this, we propose a novel method integrating a conditional U-Net surrogate model with health impact assessments, enabling swift estimation of PM2.5 concentrations and related health effects. The U-Net model was trained with 2019 South Korean PM2.5 data, including precursor emissions and boundary conditions. Our model showed high accuracy and significant efficiency, reducing processing times while maintaining reliability. The surrogate model, trained using annual PM2.5 data from South Korea, demonstrated impressive performance with an MAE of 0.221 μg/m3, NMAE of 1.762%, and an r^2 of 0.996, significantly reducing computational time compared to traditional CMAQ simulations. This model allows rapid predictions and assessments of health impacts under various

emission scenarios. By combining this surrogate model with the EPA's BenMAP-CE tool, we estimated potential premature deaths under various emission reduction scenarios in South Korea, extending projections to 2050 to account for demographic changes. Additionally, we assessed the required PM2.5 emission reductions needed to counteract the increase in premature deaths due to an aging population. This integrated framework offers an efficient, user-friendly tool that bridges complex air quality modeling with practical policy evaluation, supporting the development of effective strategies to reduce PM2.5-related health risks and estimate economic benefits.

P42-06 | Modelling of traffic-related air pollution and exposure assessment in Heredia City, Costa Rica.

Jose F. Rojas Marin

Universidad Nacional de Costa Rica, Escuela de Ciencias Ambientales, Heredia, Costa Rica

Heredia City is one of the most populated cities in the Metropolitan Area of Costa Rica, which holds around 60% of the country's population. Currently, the city suffers mobility problems for private and public transportation and pedestrians since there are limited areas for safe traveling (walking or cycling). This issue is related to a city's poor design and uncontrolled growth for many years, as for obsolete infrastructure for transportation. This problem causes public health issues for everyone who needs to enter the city for several activities: work, study, or recreation. To asses this issue, a project was developed to model the air pollution caused by the cars that moves through the city and determine the possible hotspots. CALROAD and AERMOD were the models used for this purpose. As a complementary objective, air pollution pedestrian exposure was measured using low-cost portable sensors. This includes people using several transportation modes (walking, cycling, and public transportation). The results show an important particulate matter exposure, mainly for people walking near hotspots located around a big farmers market, which also has several bus stations nearby. Also, the main road that enters the city has an important impact on air quality and the people who walk around every day.
Using modelling tools and air pollution sensor results, maps were generated for the local municipality for better city growth planning and mobility improvement. The results are going to be used to support the "Boulevard Project" that the municipality plans to start this year, which looks to make important changes in transportation and pedestrian mobility within the city to promote more visiting and a healthy environment.

P42-08 | Using Low-Cost Monitoring Devices and Random Forest to Investigate the Impact of Size-Fractionated Particulate Matter, Carbon Dioxide, and Heat Stress Exposure on Respiratory Health Indicators in Suburban Elementary School Children

Li-Te Chang[1], Chin-Sheng Tang[2], Tzu-Ying Lee[2], Hsiao-Yun Huang[3], Shih-Chun C. Lung[4]

[1] Feng Chia Univeristy, Dept. of Environmental Engineering and Science, Taichung, Taiwan; [2] Fu Jen Catholic University, Dept. of Public Health, New Taipei City, Taiwan; [3] Fu Jen Catholic Univeristy, Dept. of Statistics and Information Science, New Taipei City, Taiwan; [4] Academia Sinica, Research Center for Environmental Changes, Taipei, Taiwan

Objective

Airborne particulate matter (PM) is linked to respiratory and cardiovascular diseases, with potential interactions with temperature affecting health. This study examines the effects of size-fractionated PM, carbon dioxide (CO_2), and thermal environment indicators on respiratory health in elementary school children in a downwind suburban area in central Taiwan.

Materials and Methods

Fifty children (27 boys, 23 girls) participated in this panel study from July 2017 to December 2018. Each child wore personal monitors (AS-LUNG and HOBO) for five days to measure size-fractionated PM, CO_2, temperature, humidity, and illuminance. The wet bulb globe temperature (WBGT) was calculated for heat exposure assessment. Respiratory indicators, including fractional exhaled nitric oxide (FeNO), peak expiratory flow rate (PEFR), and forced expiratory volume in one second (FEV_1), were measured daily. Using the Random Forest method, we analyzed the cumulative effects of air pollution and thermal exposures over different time windows (1–48 hours) and their interactions.

Results

Short-term exposure (1–8 hours) showed demographic factors had a stronger impact on FeNO and PEFR, while longer exposure (24–48 hours) highlighted the importance of CO_2, thermal indicators, $PM_{1-2.5}$, and $PM_{2.5-10}$. PM exposure had cumulative effects on FeNO and PEFR but not FEV_1. CO_2 influenced FeNO but not PEFR or FEV_1. Furthermore, CO_2 and thermal environment indicators had a greater influence on respiratory health than PM, with temperature and relative humidity ranked higher than WBGT.

Conclusion

Air pollution and thermal exposure interact non-linearly with respiratory health. Synergistic effects were observed for FeNO, while PEFR and FEV_1 showed both synergistic and protective responses. Using a non-parametric Random Forest approach, this study highlights the significance, dose-response relationships, and interactions of environmental exposures, offering insights for future research on air pollution and climate change.

Acknowledgement

This study was funded by the Ministry of Science and Technology of Taiwan (108-2111-M-035-001).

P42-09 | High-Resolution Air Pollution Modeling Incorporating Intensive Mobile Monitoring datain Beijing, China

Muwu J. Xu[1], Xin Meng[2], Joel Kaufman[3], Yang Liu[4], Jicheng Gong[2], Meng Wang[1]

[1] University at Buffalo, Department of Epidemiology and Environmental Health, School of Public and Health Professions, Buffalo, USA; [2] Peking University, SKL-ESPC & SEPKL-AERM, College of Environmental Sciences and Engineering, and Center for Environment and Health, Beijing, China; [3] University of Washington, Department of Environmental and Occupational Health Sciences, Seattle, USA; [4] Emory University, Gangarosa Department of Environmental Health, Rollins School of Public Health, Atlanta, USA

Objective:
Exposure modeling for criteria air pollutants in metropolitan cities, such as fine particulate matter (PM2.5), oxides of nitrogen (NOx), and ozone (O3), has largely depended on monitoring data from regulatory stations, which often fail to represent residential exposure accurately. Our study examined whether integrating high-density mobile monitoring data into a spatiotemporal model enhances the ability to predict exposure to criteria air pollutants in Beijing, China, compared to models that rely solely on regulatory monitoring data.
Material and Methods:
Short-term mobile monitoring data for PM2.5, NO2, and O3 were collected at 164 sites in Beijing, with each site sampled 12 times from 2022 to 2023. A spatiotemporal modeling framework was developed by integrating routine (from 2014 to 2024) and mobile monitoring data with high-resolution predictor datasets, including geographical variables, satellite remote sensing, and chemical transport model predictions. Model performance was assessed through cross-validation and compared to models excluding mobile monitoring data. Finally, 100m-resolution maps of the three pollutants were generated to evaluate spatiotemporal variability across Beijing.
Results:
For mean (SD) for the $PM_{2.5}$, NO_2, and O_3, were 34.37 (12.19) ug/m^3, 41.70 (13.51) ug/m^3 and 67.23 (21.39) ug/m^3, respectively. The model exhibited good performance with an overall cross-validation R^2 of 0.81, 0.76 and 0.78, and root mean squared error of 9.17, 10.51, and 26.13 ug/m^3. The model was dominated by source-specific predictors such as population density, land use, factory, gas stations and traffic density. The best models greatly outperformed the models with routine monitoring data alone. Our spatio-temporal map shows a clear variation of the three criterion pollutants over the Beijing residential area.
Conclusion:
We successfully developed spatiotemporal model for criterion pollutants in Beijing that utilize the mobile monitoring data that capture a large spatiotemporal variability. These models will be great benefits for future environmental epidemiology studies in Beijing.

P42-10 | Wildfire Smoke Exposure at the Neighborhood Scale with Fusion of Models, Satellite, and Observations

Nathan Pavlovic, Daniel King, Fred Lurmann

Sonoma Technology, Petaluma, USA

Objective
Increasing wildfire activity in recent years and an expanding wildland urban interface have led to unprecedented wildfire smoke exposure events. These exposures have been linked to a broad range of health outcomes, including cardiometabolic health, mortality, and pregnancy outcomes. Minimizing exposure misclassification is critical for advancing the understanding of health outcomes in light of extreme spatiotemporal variability of wildland fire smoke concentration and composition. We developed and evaluated exposure estimates for wildland fire smoke events in which wildland fires proximate to dense urban areas resulted in neighborhood-scale exposure variability.
Materials and Methods
We used data fusion and machine learning methods to fuse modeled wildland fire smoke $PM_{2.5}$ concentrations, satellite observations, and in-situ observations of $PM_{2.5}$. Wildland fire smoke $PM_{2.5}$ concentrations were modeled using emissions developed from a merged satellite data record of fire activity with HYSPLIT dispersion. $PM_{2.5}$ measurements from regulatory monitors and low-cost sensors, adjusted to address measurement bias in wildfire smoke conditions, were also used to develop exposures with bilinear interpolation. Model results were validated using measurements and performance was compared to other common exposure estimates for smoke $PM_{2.5}$.
Results
We developed neighborhood-scale wildfire smoke $PM_{2.5}$ exposure for several wildfire smoke events that impacted local urban areas. Modeled smoke indicates good agreement with in-situ observations. The local exposure estimates we developed here compared favorably to other commonly-used methods, including satellite data.
Conclusion
Health studies examining the impact of extreme smoke events in urban areas due to fires in close proximity can benefit from neighborhood-scale estimation of wildfire smoke exposure. Our work demonstrates a technique and validation approach for smoke $PM_{2.5}$ exposure estimation to meet this need.

P42-11 | The Community of Practice for Air Quality Systems (COMPASS) Open Data Management System for Air Pollution Measurements

Nathan Pavlovic[1], Jennifer DeWinter[1], Chris Hagerbaumer[2], Russ Biggs[2], Sarah Elkotbeid[3], Matthew Tejada[3]

[1] Sonoma Technology, Petaluma, USA; [2] OpenAQ, Washington, USA; [3] Natural Resources Defense Council, New York, USA

Objective
Air pollution is a critical environmental exposure contributing to a wide range of adverse health outcomes. Air pollution concentration data requires a Data Management System (DMS) for data collection and storage, quality assurance, and analysis. Currently, organizations that measure air pollution must purchase a commercial system or develop a custom DMS, leading to substantial duplication and barriers to efficient collection and use of air quality data for research, public health, and regulatory purposes. The Community of Practice for Air Quality Systems (COMPASS) Open Data Management System project seeks to create a collaboratively developed, open-source DMS to address these challenges.
Materials and Methods
A more systematic and scalable approach to air quality data management is needed, one that reduces reliance on proprietary systems and avoids the need to build bespoke systems from scratch. The COMPASS project convenes core stakeholders in a collaborative, consensus-based process to develop an open-source DMS that meets the needs of organizations that collect air data or aim to do so. This customizable DMS will be built to handle core functions, such as data ingestion, aggregation, harmonization, storage, quality control, validation, the ability to share data, and more. DMS development is guided by extensive global stakeholder engagement efforts.
Results
The COMPASS has been convened by key stakeholders, including the National Resource Defense Council, OpenAQ, and Sonoma Technology, and has engaged with core COMPASS members and stakeholders. COMPASS participants indicate the significant capabilities and requirements of the DMS. In addition, the state of existing systems has been surveyed. Preparations are underway to develop and validate the prototype DMS system.
Conclusion
The collaborative, open-source COMPASS DMS process provides an impactful, sustainable tool for practitioners and managers who need to efficiently collect air pollution measurements.

P42-12 | Nationwide High-Resolution Ultrafine Particle Exposure Modeling in Switzerland: Advancing Environmental Epidemiological Research

Eric P. Twomey[1,2], Marloes Eeftens[1,2], Martin Röösli[1,2]

[1] University of Basel, Faculty of Science - Epidemiology, Basel, Switzerland; [2] Swiss Tropical and Public Health Institute, Environmental Epidemiology, Allschwil, Switzerland

Introduction
Air pollution is a major global health burden, contributing to an estimated 8 million premature deaths annually. While particulate matter (PM10, PM2.5) is regulated, ultrafine particles (UFPs; <100 nm) remain largely unregulated despite mounting evidence of their adverse health effects. The lack of large-scale, high-resolution UFP exposure models limits epidemiological assessments and policy interventions.

Objective
This study aims to develop a nationwide, high-resolution daily and annual UFP exposure model for Switzerland, integrating mobile and indoor-outdoor measurements, environmental predictors using machine-learning approaches. The model will support future epidemiological studies on UFP-related health risks and inform regulatory strategies.

Methods
Between July 2025 and June 2026, within the MARKOPOLO (Markers of Pollution) project, a nationwide measurement campaign will be conducted in Switzerland, covering large and intermediate cities, small towns, as well as rural areas across three seasons. UFP particle number concentration will be measured using mobile monitoring units, alongside co-pollutants (PM2.5, NO_2, black carbon, brown carbon). Additionally, an indoor-outdoor penetration model will be developed based on measurements in 60 households selected according to heating type, ventilation, and building characteristics. The model will be built using random forest classifiers, integrating spatial and temporal predictors such as land-use, altitude, meteorological, and traffic data. Model performance will be assessed through 5-fold hold-out cross-validation.
Exposure surfaces will be generated at 25m x 25m resolution, and back-extrapolated using long-term monitoring at five network sites in Switzerland from 2010 onwards, as well as previous UFP measurement campaigns. This will enable nationwide retrospective UFP exposure estimation at individual-level for epidemiological studies linking UFP exposure to the Swiss National Cohort to study mortality from cardiovascular disease, and tumors of the central nervous system.[m1]

Conclusion
This study will provide the first comprehensive, high-resolution UFP exposure model for Switzerland, forming the foundation for future epidemiological research, health impact assessments and regulatory recommendations.

P42-13 | Spatiotemporal modeling of mobile black carbon measurements using machine learning and integrated multisource data

Jia Xu[1], Ziqi Liu[2], Wen Yang[1], Bin Han[1], Zhipeng Bai[1]

[1] Chinese Research Academy of Environmental Sciences, State Key Laboratory of Environmental Criteria and Risk Assessment, Beijing, China; [2] University of Washington, Department of Environmental & Occupational Health Sciences, Seattle, USA

Few studies have investigated the link between particulate matter component exposure and human health in epidemiological research because of a lack of routine ambient monitoring and the challenge of using an unbalanced dataset of measurements obtained from mobile monitoring campaigns. In this study, equivalent black carbon (eBC) was monitored on a mobile platform in four seasonal campaigns from 2023 to 2024 in the ambient environment around the residential area in urban Beijing. A spatially rich and temporal sparse hourly dataset was obtained and adjusted by a XGBoost model to fill in the missing data at the time points. This model has integrated multiple open-source data, including NO_2 measurements at adjacent municipal air pollutant monitoring sites, meteorological observations, satellite retrieval data and publicly available online dispersion model predictions, to represent the meteorological condition and real-time traffic volume. The adjusted spatiotemporal dataset of eBC was then used to develop a data-driven hierarchy geostatistical model based on intrinsic temporal trends and a large array of geographic covariates. The prediction ability of the exposure model was determined using 10-fold cross-validation (CV). Good performance of the spatiotemporal model was achieved, with the 10-fold CV R^2 of 0.89 and the root mean squared error (RMSE) of 181.81 ng/m^3. An external validation was also performed at a central urban monitoring site during the Spring and Summer of 2023. This study developed a multi-step approach using hourly short-term measurements from a high-efficiency mobile campaign to develop a daily spatiotemporal exposure model for black carbon. The temporal adjustment approach can be used for spatially rich and temporal sparse mobile monitored datasets for traffic-related air pollutants.

P42-14 | So Fresh, So FAIR: Updateable and FAIR Geostatistical Air Pollution Exposure Models

Mitchell Manware[1], Insang Song[3], Eva Marques[1], Mariana A. Kassien[1], Anisha Singh[1], Lara Clark[1], Cavin Ward-Caviness[2], **Kyle P. Messier**[1]

[1] National Institute of Environmental Health Sciences, Division of Translational Toxicology, Durham, USA; [2] US Environmental Protection Agency, Clinical Research Branch, Chapel Hill, USA; [3] Seoul National University, Department of Geography, Seoul, South Korea

BACKGROUND AND AIM

Electronic health records and modern epidemiological cohorts offer opportunities to conduct large-scale epidemiological studies focused on spatiotemporal exposures in recent years. However, the lack of high-resolution environmental exposure data at recent temporal scales often precludes this opportunity. Continued efficacy of environment and health studies will require geospatial exposure models that are rapidly (< 1 year) updatable in a transparent, flexible, and timely manner. This study develops a new, regularly updating (So Fresh), open source (So

FAIR), high-resolution, daily model for air pollution, which meets the timeliness, reproducibility, and extensibility needs for the next generation of environmental health studies.

MATERIAL AND METHODS

The model utilizes air pollution data from the Environmental Protection Agency's air quality monitoring network, and includes atmospheric, meteorological, land cover, and emissions covariates from public data sources. Our open-source amadeus R-package facilitates reproducible downloading, processing, and covariate calculation of these data. Random subsets train 100 base learners from elastic-net, lightGBM, and feed-forward neural networks using space-time cross-validation. Base learner output is input into M elastic-net meta-learner models with 50 percent column drop-out and space-time cross-validation. The model's workflow was developed in a test-driven, reproducible pipeline that will be automated for regular model updates as new monitoring data and covariates become available.

RESULTS

Space-time cross-validation results are comparable to state-of-the-art daily $PM_{2.5}$ models; however, ours are available at multiple space and time aggregations, cover recent time ranges, and include uncertainty quantification. Total human and computational time were dramatically reduced for updating the model with an additional one year of data.

CONCLUSIONS

The proposed model expands both the spatial and temporal range of high-quality, publicly available, environmental exposure data, enabling novel environmental epidemiology studies that can take advantage of larger and most recent health outcomes datasets.

P42-16 | An Ensemble Machine Learning Model to Enhance Extrapolation Ability of Predicting Coarse Particulate Matter with High Resolutions in China

Su Shi, **Xia Meng**

Fudan University, School of Public Health, Shanghai, China

Accurate exposure assessment is important for conducting $PM_{10-2.5}$-related epidemiological studies, which have been limited thus far. In this study, we aimed to develop an ensemble machine learning method to estimate $PM_{10-2.5}$ concentrations in mainland China during 2013–2020. The study was conducted in two stages. In the first stage, we developed two methods: the indirect method refers to developing models for $PM_{2.5}$ and PM_{10} separately and subsequently calculating $PM_{10-2.5}$ as the difference between them; and the direct method refers to establishing a model between $PM_{10-2.5}$ measurements and relevant predictors directly. In the second stage, we employed an ensemble method by integrating predictions from both indirect and direct methods. Internal and external cross-validation (CV) were performed to validate the extrapolation capacity of models. The ensemble method demonstrated enhanced extrapolation accuracy in both internal and external CV compared to indirect and direct methods. The predictions produced by the ensemble method captured the spatiotemporal pattern of $PM_{10-2.5}$, even in the sand and dust storm seasons. Our study introduces an ensemble strategy leveraging the strengths of both indirect and direct methods to estimate $PM_{10-2.5}$ concentrations, which holds significant potential to support future epidemiological studies to address knowledge gaps in understanding the health effects of $PM_{10-2.5}$.

P42-17 | Design and application of residential indoor air monitoring to assess long-term exposure to air pollution in susceptible population for epidemiology

Sun-Young Kim[1], Seokmin Ji[1], Miyoun Shin[1], Hyunji Kim[1], Seung-Hyun Cho[2], Hee-Young Yoon[3], Jin Woo Song[4]

[1] National Cancer Center, Department of Public Health & AI, Goyang, South Korea; [2] Research Triangle Institute, Department of Center for Exposure and Protection, RTP, USA; [3] Soonchunhyang University Hospital, Department of Pulmonary and Allergy, Seoul, South Korea; [4] Asan Medical Center, Department of Pulmonary and Critical Care Medicine, Seoul, South Korea

While there has been accumulating evidence of the association between long-term exposure to air pollution and various health endpoints, most epidemiological studies relied on ambient air pollution which showed substantial deviation from indoor. This difference could lead to biased or imprecise health effects, particularly for the susceptible population who spends most time indoors. Although a few studies focused on indoor air pollution, their monitoring was limited to one-time short-term periods. We aimed to design and apply an indoor monitoring campaign to assess long-term exposure to residential indoor $PM_{2.5}$ for epidemiological application, leveraging a well-established cohort of older adults with idiopathic pulmonary fibrosis. Specifically, our design focused on 1) representation of long-term exposure, 2) identification of pollution sources, 3) assessment of infiltration, and 4) implementation of non-contact monitoring. Using RTI MicroPEMs, we measured concentrations of indoor $PM_{2.5}$ and chemical components for five days including weekdays and weekends in four seasons as well as household information. For a subset of homes, we collected matched outdoor samples to assess infiltration. We also planned non-contact monitoring using postal mails for repeat visits. We applied this campaign to 130 participants in the Seoul Metropolitan Area, South Korea, for 2021-2023. Our analyses for $PM_{2.5}$ and component measurements showed hourly, weekly, and seasonal patterns. Indoor concentrations were lower than outdoor with consistent seasonal patterns but contrasted hourly patterns. Average $PM_{2.5}$ concentrations tended to be high at households with humidifiers, pets, and smokers. Our indoor monitoring approach provides a practical guidance for future epidemiological studies of $PM_{2.5}$.

P42-18 | Post-Wildfire Geospatial Data Collection in Los Angeles: Advancing Public Health Research

Elena Austin, Jacqueline Zdebski, Karen Dedinsky, Jeffrey Berman, Joseph Wartman

University of Washington, Seattle, USA

Following the devastating wildfires in Los Angeles in early 2025 the NHERI RAPID Facility assisted several interdisciplinary research teams with data collection to document and quantify the damage and provide information on the environmental health impacts. The RAPID Facility is a shared use facility supported by the National Science Foundation and the National Institutes of Health that provides tools and support services for researchers to collect perishable data following natural hazards. This presentation will detail the data sets that have been collected and made openly available and will propose applications of the data in public health research. The data includes: (i) high-

resolution imagery collected via uncrewed aerial systems over all of Altadena and much of the Palisades burn areas, (ii) streetview imagery for both entire areas affected by the wildfires, (iii) multi-spectral imagery obtained from uncrewed aerial systems, and (iv) hyperspectral data for selected portions of the burn area. The UAS camera imagery has been processed into 2D flat orthomosiacs and is available as geotiffs and also into 3D point cloud models using structure from motion. Potential uses for the data in public health include enabling novel high-resolution exposure assessments by integrating multi-spectral and hyperspectral imagery with geospatial air quality data to identify localized health risks. This unique dataset provides an unprecedented opportunity to link structural damage, pollutant dispersion, and health outcomes, supporting innovative epidemiologic studies and targeted public health interventions in wildfire-affected communities.

P42-19 | GPS-based kernel matching for estimating the average dose-response curve: An analysis of the short-term effects of $PM_{2.5}$ on mortality in the Florence area (Italy)

Giulio Biscardi[1], Chiara Marzi[1], Alessandra Mattei[1], Daniela Nuvolone[2], **Michela Baccini**[1]

[1] University of Florence, Florence, Italy; [2] Regional Health Agency, Florence, Italy

Objective

The generalized propensity score (GPS) is used to account for confounding when exposure is continuous and the objective is to estimate an average dose-response function (aDRF). With the aim to estimate the relationship between $PM_{2.5}$ and mortality in the Florence area (Italy) from 2008 to 2019, we propose a novel method that combines machine learning techniques with a local kernel matching (KM) procedure based on GPS to estimate the aDRF.

Material and methods

For each day, we define a set of Potential Outcomes (PO) for mortality, interpreted as the number of deaths that would be observed on that day at different levels of PM2.5. Since only the PO associated with the actual exposure is observed each day, missing POs are imputed using a KM procedure, under local ignorability assumptions. After dividing the exposure range into quantile-based intervals and calculating the median point for each interval, we estimate, for each day, the GPS value corresponding to each median point. Then, for each day, the PO associated with each median point is imputed as the weighted average of observed deaths on days with an actual exposure within the interval. The weights are defined to be inversely proportional to the distance between GPS values and the distance between $PM_{2.5}$ levels. Finally, the aDRF is estimated by specifying a regression spline on the imputed POs. Confidence bands are calculated via non-parametric bootstrap.

Results

The causal effect of $PM_{2.5}$ on mortality is evident. The curves for natural, cardiovascular, and respiratory mortality show an increasing trend, with a steeper slope for lower pollutant concentrations.

Conclusion

The proposed method has the advantage of not requiring the specification of an outcome model. Compared to 1:1 matching, it ensures greater precision in estimates and can be used even when the number of daily events is relatively low.

P43 | Children's Environmental Health - PART II

P43-01 | Exposure assessment of personal $PM_{2.5}$ for children and the association with chronic kidney disease

Ching Chang Lee, Tzu Huai Wei, Quang Oai Lu

National Cheng Kung University, Department of Environmental and Occupational Health, Tainan, Taiwan

The objective of the study is to investigate the association between $PM_{2.5}$ exposure and chronic kidney disease(CKD) in children. There are 58 CKD children and 34 controls were recruited and their parents signed the informed consent and accepted to collect serum and urine in this study. Most of CKD children in this study were stated early stage of CKD, and 89.7% of them were diagnosed with CKD due to structural abnormalities. In CKD children and controls, increased $PM_{2.5}$ exposure was associated with the onset of inflammatory responses, leading to elevated concentrations of TNF-R1 and TNF-R2, showing a significant positive correlation ($P<0.05$). In the control group, a significant positive correlation was found between $PM_{2.5}$ exposure and GFR (P=0.015), along with a significant negative correlation with serum creatinine (P=0.017). This may be due to the acute response to short-term higher $PM_{2.5}$ exposure, which may temporarily elevate the glomerular filtration rate and decrease serum creatinine levels, while also increasing ANG-II concentrations. In CKD children, the concentration of KIM-1 in urine showed a significant positive correlation with most of the single-lag-day and all multi-lag-day of $PM_{2.5}$ exposure ($P<0.005$, $P<0.001$). In the controls, a significant negative correlation was observed between $PM_{2.5}$ exposure and EGF concentrations (P=0.003). When $PM_{2.5}$ exposure increased, kidney damage occurred, leading to a decrease in EGF levels.

P43-03 | Effects of web-based behavioral intervention on fine particulate matter, pulmonaryfunction, and airway inflammation in children: The COCOA randomized controlled trial

Hwan-Cheol Kim[1], Dirga K. Lamichhane[2]

[1] Inha University School of Medicine, Department of Occupational and Environmental Medicine, Incheon, South Korea; [2] University of Utah School of Medicine, Department of Psychiatry, Salt Lake City, USA

Background: Although web-based interventions have been shown to be effective in changing health behavior, evidence about their effectiveness in relation to air pollution and respiratory health in children is missing. We assessed the impact of web-based behavioral intervention on fine particulate matter (PM ≤2.5μm in diameter [PM2.5]), lung function, and airway inflammation in children.
Methods: We randomized 80 mother-child pairs into intervention or control groups (two arms, 1:1 allocation). Personal and indoor PM2.5 concentrations over 24-hour sampling periods on up to four occasions during the study period were measured in participant homes. We used linear mixed models to assess the intervention effects on PM2.5 concentrations and lung function parameters including forced vital capacity (FVC), forced expiratory volume in one second (FEV1), FEV1/FVC, and forced expiratory flow at 25–75% (FEF25%–75%) and fractional exhaled nitric oxide (FeNO), and the association between PM2.5 and lung function and FeNO. Quantile regression was also used to examine the effects of PM2.5 at various locations of the outcomes.
Results: Compared with the control, the intervention reduced average indoor and personal PM2.5

concentrations by 20.5 % (95% confidence interval [CI]: –30.7, –8.7) and 12.9 % (95% CI: –20.1, –5.1), respectively. The lung function parameters including FVC, FEV1, and FEF25%–75% were higher in the intervention group, with evidence of larger benefits for children at the lower end of these parameters. Higher levels of outdoor and personal PM2.5 (≥ 90th percentile) were negatively associated with the lung function parameters at the lower quantiles, whereas the same level of outdoor PM2.5 was positively associated with FeNO at the lower quantiles. *Conclusions:* Reducing PM2.5 concentrations in the homes have impact on lung function parameters and the marker of airway inflammation in children, particularly at the lower quantiles.

P43-04 | Prenatal and postnatal exposure to air pollution and the child lipid levels at 24 and 36 months: The Korean CHildren's ENvironmental health Study (Ko-CHENS)

Jongmin Oh[1,2], Hwan-Cheol Kim[3], Kyung Sook Jeong[4], Ji Hyen Lee[5], Dong-Wook Lee[3], Yun-Chul Hong[2,6], Eunhee Ha[1,7], On behalf of Ko-CHENS Study group

[1] Ewha Womans University College of Medicine, Department of Environmental Medicine, Seoul, South Korea; [2] Seoul National University College of Medicine, Department of Human Systems Medicine, Seoul, South Korea; [3] Inha University Hospital, Inha University, Department of Occupational and Environmental Medicine, Incheon, South Korea; [4] Wonju Severance Christian Hospital, Yonsei University College of Medicine, Department of Occupational and Environmental Medicine, Wonju, South Korea; [5] Ewha Womans University College of Medicine, Department of Pediatrics, Seoul, South Korea; [6] Seoul National University Medical Research Center, Institute of Environmental Medicine, Seoul, South Korea; [7] Ewha Womans University, System Health & Engineering Major in Graduate School (BK21 Plus Program), Seoul, South Korea

Background

Early life exposure to air pollution has been linked to adverse health outcomes, including changes in lipid levels. However, studies on the impact of prenatal and postnatal air pollution exposure on child lipid profiles have yielded inconsistent results. We aimed to evaluate the association between prenatal and postnatal exposure to air pollution and child lipid levels at 24- and 36-month.

Material and methods

We used data from the Korean CHildren's ENvironmental health Study (Ko-CHENS) Core cohort, which tracked children born between 2015 and 2020 until they reached 36 months. Blood lipid profiles [total cholesterol (TCHOL), triglyceride (TG), fat free acid (FFA), and phospholipid] were assessed at 24 and 36 months, and all lipid profiles were log-transformed for analysis. Prenatal and postnatal air pollution exposures were assessed using land use random Forest (LURF) modeling. Linear mixed models were used to analyze associations, adjusting for maternal age, child sex, multiple births, birth season, birth weight, gestational age, and child height and weight. Effect sizes were expressed as the percentage change for each 1 μg/m^3 increase in $PM_{2.5}$ and PM_{10}, and each 1 ppb increase in SO_2, NO_2, CO, and O_3.

Results

A total of 880 children from the Ko-CHENS were included in this analysis. We observed that increases in $PM_{2.5}$ and CO were associated with higher FFA levels, while increases in O_3 were linked to elevated TG levels and reduced FFA levels. No significant associations were found for other pollutants.

Conclusion

We observed that increases in $PM_{2.5}$ and CO were associated with higher levels of FFA, while increases in O_3 were linked to elevated TG levels and decreased FFA levels. These findings highlight the need to monitor air pollution exposure during critical developmental periods due to its potential lasting effects on children's metabolic health.

P43-05 | A narrative review of the association between Particulate Matter exposure and children's internalizing disorders

Joselyn B. Dionisio, Yuki Ito, Sayaka Kato, Michihiro Kamijima

Nagoya City University, Department of Occupational and Environmental Health, Nagoya, Japan

Objective
Childhood internalizing disorders have a short-term impact on peer relationships and academic success, but also severe long-term consequences. Environmental exposures should be important in the etiology of these disorders, but the role of chemical exposures is not as well established compared with social or psychological exposures. We aim to assess the current knowledge about the association between particulate matter (PM) and early-life internalizing disorders.
Material and Methods
Literature search on 3 databases, using keywords related to children (≤18 years by time of outcome measurement), mental health (depression, anxiety, suicidality or self-harm, internalizing symptoms, other indicators) and environmental chemical exposures (1274 results). Duplicates were removed and the results filtered out according to selected exclusion criteria (78 results). Of these, we selected articles where the exposure measured included PM.
Results
We identified 19 articles with measurements of $PM_{2.5}$ (n=15), PM_{10} (6) and/or $PM_{0.1}$ (1), and one where the exposure was coal ash detected in household PM10 filters. Of the articles identified, eight followed a cohort design and five had a cross-sectional design. Ten studies found significant associations between exposure to PM and increased odds of internalizing disorder events or score, one study found an association only in a group after stratified analysis, and two found associations between the exposure and stress or reactivity.
Conclusion
This review describes the growing evidence of positive association between PM exposure and pediatric internalizing disorders, however, more research is needed to fully understand the mechanism of how PM affects pediatric mental health.
This study was financed by the Grant-in-Aid for Promotion on Co-Creative Urban Development in Nagoya City University Grant Number 2408234, and JSPS KAKENHI Grant Number 22H00495. The authors have no conflict of interest concerning to disclose concerning the presentation.

P43-06 | Assessment of personal $PM_{2.5}$ exposure of children living in Bandung, Indonesia and Kathmandu, Nepal

Fiona Carson[1], Claire Horwell[2], Mohamad Iqbal[3], Kusum Shahi[4], Rabindra Bhandari[4], Asep Sofyan[3], Djoko Santoso[3], Megnath Dhimal[4], Katharina Oginawati[3], Will Mueller[1], Miranda Loh[1], Karen S. Galea[1]

[1] IOM, Edinburgh, UK; [2] Durham University, Institute of Hazard, Risk & Resilience, Department of Earth Sciences, Durham, UK; [3] Institut Teknologi Bandung, Center for Climate Change, Bandung, Indonesia; [4] Nepal Health Research Council, Kathmandu, Nepal

Objective

The Factors Affecting Childhood Exposures to Urban Particulates (FACE-UP) project aims to reduce lifetime vulnerability of urban children, particularly in Indonesia and Nepal, to noncommunicable diseases by reducing exposure to particulate air pollution through suitable personal interventions. Children's exposure to $PM_{2.5}$ was investigated over a week-long period to investigate: 1) The exposure levels for children living in Bandung, Indonesia and Kathmandu, Nepal; 2) The environments/activities lead to their highest exposures; and 3) The similarities/differences in exposure between the two study populations.

Material and Methods

Low-cost $PM_{2.5}$ sensors (PurpleAir) and GPS devices were carried by participants, aged 6-13 years old, in custom designed backpacks for one week. Questionnaires were deployed to the children's carers and teachers to gather information about the household and school environment, as well as the child's daily routine. Sixty-one children were recruited from four schools and PurpleAir sensors were also installed outdoors at each school to measure ambient $PM_{2.5}$ concentrations.

Results

Personal exposure levels in the home were found to be high but no specific source driving these elevated concentrations was identified. Travelling by scooter or walking exposes children to the highest $PM_{2.5}$ levels while commuting. Car or bus commutes expose children to lower concentrations, but the longer duration of these commutes might offset this benefit. The air quality in school classrooms was found to be better than the other microenvironments but $PM_{2.5}$ concentrations were still high compared to WHO guidelines.

Conclusions

Our study suggests children in urban areas of Nepal and Indonesia are regularly exceeding WHO recommended levels of $PM_{2.5}$. Our results emphasise the urgent need for effective interventions to protect children from high pollution levels.

Acknowledgments

Thanks to our study participants and schools. FACE-UP is funded by a UKRI Collective Fund award under the Global Challenges Research Fund (UKRI GCRF grant reference: MR/T029897/1).

P43-07 | Assessment of the fit and wearability of commercially available KN95 respirators for children in Indonesia and Nepal

Anne Sleeuwenhoek[1], Claire Horwell[2], Kusum Shahi[3], Jihan Nur Azizah[4], Rabindra Bhandari[3], William Mueller[1], Muhammad Iqbal[4], Megnath Dhimal[3], Miranda Loh[1], Karen S. Galea[1]

[1] IOM, Edinburgh, UK; [2] Durham University, Institute of Hazard, Risk & Resilience, Department of Earth Sciences, Durham, UK; [3] Nepal Health Research Council, Kathmandu, Nepal; [4] Institut Teknologi Bandung, Center for Climate Change, Bandung, Indonesia

Objective

Availability and use of facemasks to protect against outdoor particulate pollution has increased, however there are few published studies on respirator fit and wearability relating to children. Our study aimed to determine how well KN95 respirators, marketed for children, fit the faces of 6-13 years olds in two urban sites with elevated levels of air pollution: Kathmandu, Nepal and Bandung, Indonesia. Wearability of the respirators and the children's style preferences were assessed.

Material and Methods

Thirty children in each country were fit tested with three different ear loop respirators in two combinations (with and without additional adjustable ear loop clip worn around head). The fit factor (FF) for each respirator was determined using the Occupational Safety and Health Administration approved modified fit test protocol using a TSI PortaCount™ Respirator Fit Tester. Facial dimensions were measured, and wearability of the respirators and children's style preferences were assessed through questionnaires administered after the fit tests.

Results

Most FFs were less than 10, i.e. less than 90% reduction in exposure. Using an additional ear loop clip was associated with increases in FF of 42 and 50% for Indonesian and Nepalese respirators, respectively. There were no significant differences among the respirators for any of these perceptions: comfort, feeling hot, breathability, fit, embarrassment or appearance for either country. The appearance of the respirator was important to the children.

Conclusions

Although the use of an additional ear loop clip improved the fit, the respirators were generally too large for the children's faces to achieve a good fit. Respirators marketed for children should be better designed to suit their facial dimensions.

Acknowledgments

Thanks to our study participants and schools. Factors Affecting Childhood Exposures to Urban Particulates (FACE-UP) is funded by a UKRI Collective Fund award under the Global Challenges Research Fund (UKRI GCRF grant reference: MR/T029897/1).

P43-08 | Short-term, lagged association of airway inflammation, lung function, and asthma symptom score with $PM_{2.5}$exposure among schoolchildren within a high air pollution region in South Africa

Minenhle S. Buthelezi[1], Graciela Mentz[2], Caradee Wright[3,4], Shumani Phaswana[1], Rebecca Garland[4,5], Rajen N. Naidoo[1]

[1] University of KwaZulu-Natal, Occupational and Environmental Health, Durban, South Africa; [2] University of Michigan, Anesthesiology Department, Ann Arbor, USA; [3] South African Medical Research Council, Environment and Health Research Unit, Pretoria, South Africa; [4] University of Pretoria, Geography, Geoinformatics and Meteorology, Pretoria, South Africa; [5] Council forScientific and Industrial Research, Smart Places, Pretoria, South Africa

Background: Asthma affects millions of people globally, and high levels of air pollution aggravate asthma. This study aimed to determine the association between short-term lagged $PM_{2.5}$ exposure and airway inflammation, lung function, and asthma symptom scores among schoolchildren in the Highveld high-pollution region in South Africa.

Methods: This is a cross-sectional study among schoolchildren aged 9–14 years in the Highveld region in South Africa, between October 2018 and February 2019. A NIOX 200 instrument measured fractional exhaled nitric oxide (FeNO). Lung function indices (FEV_1; FVC and FEV_1/FVC) were collected using spirometry and the percent of predicted of these was based on the reference equations from the Global Lung Initiative, without ethnic correction. Asthma symptoms were used to create the asthma symptom score. Daily averages of $PM_{2.5}$ data for the nearest monitoring station located in each community, were collected from the South African Air Quality Information System and created short-term 5-day lag $PM_{2.5}$ concentrations.

Results: Lag 1 (odds ratio [OR]: 1.01; 95% confidence interval [CI]: 1.00, 1.02, P = 0.039) and 5-day average lagged PM2.5 (OR: 1.02; 95% CI: 0.99, 1.04, P = 0.050) showed increased odds of the FeNO > 35 ppb. Lung function parameters (FEV_1 < lower limit of normal [LLN] [OR: 1.02, 95% CI: 1.00, 1.03, P = 0.018], and FEV1 / FVC < LLN [OR: 1.01; 95% CI: 1.00, 1.02, P < 0.001]) and asthma symptom score ≥ 2 (OR: 1.02; 95% CI: 1.00, 1.04, P = 0.039) also showed significant associations with lag 2, lag 4 and lag 1 of $PM_{2.5}$, respectively.

Conclusion: Lagged $PM_{2.5}$ exposure was associated with increased odds of airway inflammation and increased odds of lung function parameters below the LLN particularly for the later lags, but a significant dose-response relationship across the entire sample was not consistent.

P43-09 | Associations between the oxidative potential of indoor particulate matter and salivary biomarkers of inflammation and oxidative stress in school-aged children

Vinh Vo[1], P. S. Ganesh Subramanian[2], Alice Oloo[1], Brenda Koester[1], Vishal Verma[2], Jenna Riis[1], Naiman Khan[1], Sheena Martenies[1]

[1] University of Illinois Urbana-Champaign, Department of Health and Kinesiology, Urbana, USA; [2] University of Illinois Urbana-Champaign, Department of Civil and Environmental Engineering, Urbana, USA

BACKGROUND: Indoor air quality (IAQ) can negatively impact health in childhood, potentially through oxidative stress and inflammation pathways. Quantifying the oxidative potential (OP) of indoor particulate matter (PM) will provide further insights into the potential health effects of indoor air.

OBJECTIVE: This study aims to understand the association between the OP of indoor PM and salivary biomarkers of inflammation in children ages 6-11 years.

METHODS: Indoor PM samples (no size selection) were collected over a 7-day period during which the study team collected three passive drool saliva samples from participants. Salivary C-reactive protein (CRP) was measured in duplicate using a commercially available assay kit. PM samples were assessed for 28 metals using inductively coupled plasma mass spectrometry and OP was assessed using a dithiothreitol (DTT) assay. We examined correlations between OP, total metal concentrations, and salivary CRP concentrations.

RESULTS: We had complete PM and CRP data for n = 22 (mean age 7.9 ± 2.1) participants. OP was strongly correlated with transition metals such as Cu, Mn, and Fe. The correlations between OP and total metal concentrations were higher in the winter (n = 8, Pearson ρ =0.70) than in the summer (n = 21, Pearson ρ =0.17). CRP was inversely correlated with OP during winter months (Spearman ρ = -0.75; n =8). During the summer months, a similar trend was observed, although the correlation was weaker, with a Spearman ρ = -0.34 (n = 9).

CONCLUSION: During winter months, higher OP was correlated with higher total metal concentrations in PM samples. Contrary to our initial hypothesis, OP was also moderately negatively correlated with salivary CRP concentrations in children ages 6-11. Future work will further investigate the relationship between OP and inflammatory biomarkers.

Funding was provided by the Personalized Nutrition Initiative at the University of Illinois at Urbana-Champaign

P43-10 | Association of exposure to fine particulate matter components during pregnancy and early childhood with asthma and allergies in school children

Yoshiko Yoda[1,2], **Kazue Ojima**[2], Shin Araki[3], Hikari Shimadera[3], Narumi Tokuda[4], Yasuhiro Takeshima[2,4], Masayuki Shima[4,5]

[1] Kansai University of Welfare Sciences, Faculty of Health Sciences for Welfare, Kashiwara, Japan; [2] Hyogo Medical University, School of Medicine, Nishinomiya, Japan; [3] Osaka University, Graduate School of Engineering, Suita, Japan; [4] Hyogo Medical University, Hyogo Regional Center for the Japan Environment and Children's Study, Nishinomiya, Japan; [5] Hyogo Medical University, School of Nursing, Kobe, Japan

Objective: Exposure to fine particulate matter ($PM_{2.5}$) has been associated with asthma and allergies. However, information about the effects of specific $PM_{2.5}$ components is limited. This study aimed to investigate the relationship of exposure to chemical components of $PM_{2.5}$ during pregnancy and early childhood with the development of asthma, allergies, and sensitization in school-age children.
Methods: This study included 2,408 children in the second grade of elementary school. Questionnaire surveys of respiratory/allergic symptoms and measurements of specific IgE levels to house dust mite (HDM) and animal proteins were conducted. Exposures to ambient sulfate (SO_4^{2-}), nitrate (NO_3^-), ammonium (NH_4^+), elemental carbon (EC), and organic carbon (OC) of $PM_{2.5}$ in participants' residences from conception to age six were estimated using predictive models. Multiple logistic regression model was used to analyze the association of respiratory/allergic symptoms and allergen sensitization with estimated exposure concentrations, after adjustment for covariates.
Results: No significant association was found between $PM_{2.5}$ and its component concentrations and asthma. Wheezing significantly increased with mean NO_3^- concentrations during pregnancy (odds ratio of 1.68 [95% confidence interval: 1.08, 2.63] for an interquartile range increase). Significant associations were also found between EC in the second trimester of pregnancy and NO_3^-, EC, and OC concentrations in early childhood. Higher SO_4^- and NH_4^+ concentrations during the second trimester increased the risk of rhinitis. Sensitizations to HDM and animal proteins were significantly associated with exposure to components such as SO_4^{2-} and NH_4^+ during pregnancy but not with postnatal exposure.
Conclusions: Exposures to NO_3^-, EC, and OC during pregnancy and early childhood were associated with wheezing. SO_4^{2-} and NH_4^+ exposures during pregnancy were associated with sensitization.
Acknowledgement: This work was supported by JSPS KAKENHI [Grant Numbers JP18H03060, JP21H03205, JP24K02717] and the Environment Research and Technology Development Fund [JPMEERF20185002, JPMEERF20195055] by the Environmental Restoration and Conservation Agency of Japan.

P43-11 | Association between long-term exposure to PM2.5 and incident obesity in children: a prospective cohort study in Taiwan

Yu-Chuan Chen[1], Chung-Chin Lee[1], Yu-Ting Lin[2,3], Chau-Ren Jung[4], Bing-Fang Hwang[1]

[1] China Medical University, College of Public Health, Department of Occupational Safety and Health, Taichung, Taiwan; [2] China Medical University, College of Medicine, Department of Biomedical Informatics, Taichung, Taiwan; [3] China Medical University, China Medical University Hospital, Big Data Center, Taichung, Taiwan; [4] China Medical University, College of Public Health,Taiwan, Department of Public Health, Taichung, Taiwan

Objective:
This study explores the link between long-term exposure to fine particulate matter (PM2.5) and childhood obesity in Taiwan. Utilizing data from the iHi Data Platform of China Medical University Hospital, the research analyzed a cohort of 340,054 children under 18 years old in central Taiwan from 2004 to 2016. The follow-up period lasted up to four years, with obesity diagnoses based on specific ICD-9-CM codes (278.00 and 278.01) and ICD-10-CM codes (E66.0 and E66.01).
Materials and Methods:
Air pollution exposure was estimated using a satellite-based model to determine PM2.5 concentrations at a high resolution (1 km) and correlated with participants' residential addresses. A Cox proportional hazards model was employed to assess the association, with results expressed as hazard ratios (HR) and 95% confidence intervals (CI).
Results:
During the study, 327 cases of childhood obesity were identified. Monthly average PM2.5 levels ranged from 3.70 to 90.48 μg/m³. The analysis revealed that a 10 μg/m³ increase in PM2.5 concentration was associated with a significantly higher risk of childhood obesity (HR = 1.322; 95% CI: 1.125–1.554).
Conclusion:
The findings suggest a causal relationship between higher PM2.5 exposure and increased obesity risk in children, emphasizing the need for public health measures to mitigate air pollution's impact on younger populations.
Acknowledgments:
This research was supported by Taiwan's National Science and Technology Council (NSTC 113-2813-C-039-089-B) and acknowledges contributions from the iHi Data Platform team for data and analytical support.

P43-12 | Structural equation modelling of the associations between socioeconomic status, maternal exposure to endocrine-disrupting chemicals and children behavior

Alexander Waits[1], Chia-Huang Chang[2], Yu-Fang Huang[3], Ming-Song Tsai[4], Pei-Wei Wang[5], Chih-Yao Chen[6], Chia-Jung Hsieh[7], Ming-Tsang Wu[8], Shu-Li Wang[9], **Mei-Lien Chen**[3]

[1] National Yang Ming Chiao Tung University, International Health Program, Taipei, Taiwan; [2] Taipei Medical University, School of Public Health, Taipei, Taiwan; [3] National Yang Ming Chiao Tung University, Institute of Environmental and Occupational Health Sciences, Taipei, Taiwan; [4] Cathay General Hospital, Department of Obstetrics and Gynecology, Taipei, Taiwan; [5] Taipei City Hospital, Heping Fuyou Branch, Department of Pediatrics, Taipei, Taiwan; [6] Taipei Veterans General Hospital, Department of Obstetrics and Gynecology, Taipei, Taiwan; [7] Tzu Chi University, Department of Public Health, Hualien, Taiwan; [8] Kaohsiung Medical University, Research Center for Environmental Medicine, Kaohsiung, Taiwan; [9] National Health Research Institutes, Division of Environmental Health & Occupational Medicine, Miaoli, Taiwan

This study examined the environmental justice hypothesis regarding maternal exposure to endocrine-disrupting chemicals (EDCs) and child behavior. Using data from the Taiwan Maternal and Infant Cohort Study, we assessed urinary concentrations of eight phthalates, four parabens, nonylphenol, and bisphenol A in pregnant women as potential mediators linking socioeconomic status (SES) to child behavior. Child Behavior Checklist (CBCL) for ages 1.5–5 was reported by mothers to provide T-scores for internalizing/externalizing, affective, anxiety, autism spectrum, attention deficit/hyperactivity, and oppositional defiant problems. SES was measured at both individual and area levels. The associations were estimated using linear regression and structural equations models (SEM).

In the sample of 178 pregnant women in the third trimester, few EDCs were associated with lower maternal SES and higher CBCL T-scores of their children. CBCL T-scores were negatively associated with interquartile increases in area average household income and positively associated with the percentage of households using indigenous languages. Model fit indices indicated an acceptable fit (RMSEA = 0.077, CFI =0.791). SES included maternal education (β = 0.51), household income (β = 0.62), area average household income (β = 0.66), unemployment rates (β = -0.33), and indigenous language use (β = -0.39). SES was negatively associated with CBCL (β = -0.24, 95% CI = -0.43, -0.06), but no mediation was observed for EDCs.

Although this study did not confirm the environmental justice hypothesis, further research on broader EDC mixtures is recommended. The observed associations between SES and child behavior highlight the need for targeted interventions to protect vulnerable populations from differential environmental exposures.

P43-13 | The cytokine profile in 3-year-old children and its association with prenatal and postnatal inorganic arsenic exposure

Shu-Li Wang[1,2], Hui-Ju Wen[1], Chien-Wen Sun[1], Shih-Fen Tsai[1], Tsung-Lin Tsai[3], Chih-Hung Lee[4], On behalf of TMICS group.

[1] National Health Research Institutes, National Institute of Environmental Health Sciences, Miaoli, Taiwan; [2] National Defense Medical Centre, Department of Public Health, Taipei, Taiwan; [3] China Medical University, Department of Public Health, Taichung, Germany; [4] Chang Gung University College of Medicine, Department of Dermatology, Kaohsiung, Taiwan

Objective: Inorganic arsenic (iAs) exposure induces endocrine disruption, carcinogenesis, and immune dysregulation. We reported that iAs exposure results in metabolic syndrome, bladder cancer, and atopic diseases, likely through immune dysregulations. However, how the early life, iAs human exposure affects the early immune development remained unclear. We investigated various blood cytokines in 3-year-old children with prenatal and postnatal iAs exposure with a maternal-child cohort.
Methods: We analyzed data from 215 mother-child pairs participating in a birth cohort study conducted in central Taiwan since 2012. Maternal iAs exposure was estimated using urinary arsenic species measured by inductively coupled plasma mass spectrometry. We employed multiplex ELISA to assess serum levels of 65 analytes representing various immune pathways in children. To evaluate the associations, we used Spearman's correlation and multivariable regression analyses.
Results: Maternal urinary total iAs levels (in µg/g creatinine), which include arsenite, arsenate, monomethylarsonic acid (MMA), and dimethylarsinic acid (DMA), were positively associated with serum cytokine levels in 3-year-old children, In boys, a doubling of maternal urinary iAs concentration was significantly associated (p-value < 0.01) with increased levels (pg/mL) of BAFF (β [95% CI] = 20.8 [5.13, 36.59]), IL-10 (β [95% CI] = 20.87 [6.67, 35.08]), LIF (β [95% CI] = 18.40 [5.23, 31.57]), and TNF-α (β [95% CI] = 23.62 [7.52, 39.71]), even after adjusting for maternal age at childbirth, gestational week, child's BMI at age three. Postnatal iAs exposure generally showed a negative association with cytokine levels in both sexes and significant for IL-17A, IL-20, G-CSF, and MIF (macrophage inflammatory protein) specifically in boys.
Conclusion: Prenatal and postnatal exposure to iAs was associated with altered cytokine levels in young children, including inflammatory, growth factor, and regulatory immune pathways, particularly in boys. Further follow-up studies are warranted to assess the long-term implications for pathogenesis of diseases related to immune regulations.

P43-15 | Associations between exposure to plastic additives and cytokine levels among school-aged children: The Hokkaido Study

Atsuko Ikeda[1,2], Yi Zeng[1,3], Takeshi Yamaguchi[2,4], Rahel M. Ketema[1,2], Yu Ait Bamai[2,5], Celine Gys[5], Maarten Roggeman[5], Fatima den Ouden[5], Ayaka Yasuda[6,1], Megasari Marsela[1], Pitsanu Khamnuan[1], Sachiko Itoh[2], Houman Goudarzi[4], Adrian Covaci[6], Reiko Kishi[2]

[1] Hokkaido University, Faculty of Health Sciences, Sapporo, Japan; [2] Hokkaido University, Center for Environmental and Health Sciences, Sapporo, Japan; [3] Hokkaido University, Creative Research Institution, Sapporo, Japan; [4] Hokkaido University, Faculty of Medicine, Sapporo, Japan; [5] University of Antwerp, Toxicological Center, Wilrijk, Belgium; [6] Hokkaido University of Health Sciences, Graduate School of Pharmacy, Tobetsu, Japan

Objectives: Phthalate esters (PE), organophosphate triesters (OPE), and bisphenols are widely used as plastic additives in daily products. Previous studies suggest associations between these chemicals with adverse health outcomes, such as immunotoxin effects, cardiovascular diseases, obesity and metabolic diseases. Disruption of cytokine levels maybe one possibility of underline mechanisms. This study aim to find associations between urinary chemical levels and cytokine levels.
Materials and Methods: The study is based on the birth cohort "Hokkaido Study on Environment and Children's Health". This is a cross-sectional analysis with 429 children who involved in 9-12 years old survey in Sapporo city, Japan. Urinary levels of ten PE metabolites, 13 OPE metabolites, seven bisphenols were measured in LC-MS/MS and GC-MS/MS, respectively. Seventeen cytokines in plasma were determined by Bio-Plex Pro Human Cytokine 17-plex panel. PE and OPE metabolites were molar sum to represent exposure level of parental compounds. Each chemical and cytokines were log-natural transformed and included into linear regression model.
Results: Metabolites of PEs were detected from almost all samples, BPA, BPF, and BPS were detected from 80% children with the highest median value with BPA (893 pg/mL). We found statistically significant positive associations between ΣDEHP and IL-7, BBzP and MIP-1b and TNF-α, BPA and IL-4, IL-6, and IL-7, whereas inverse associations between BBzP and IL-12, and PBF and IL-12 and GM-CSF. There were no clear associations found between OPE and cytokines.
Conclusion: IL-6 and INF-α are pro-inflammatory cytokines and essential for regulating cell-mediated immune responses. They play an important role in modulating not only the immune system, but also involved in the pathogenesis of many diseases, including Alzheimer's disease, Parkinson's disease, type 2 diabetes, and renal disorders. Increasing of these cytokines may be involved in the adverse outcome pathways of these diseases caused by environmental chemicals.
Fundings: JPMEERF20175053, JPMEERF20205002, JSPS JP21H04843

P43-16 | Identifying potential exposure sources among children with unusually high chemical concentrations in the Healthy Kids Minnesota biomonitoring program

Deanna Scher, Jessica Nelson, Sheila Amenumey

Minnesota Department of Health, Environmental Health Division, Saint Paul, USA

Objective

Healthy Kids Minnesota is an ongoing statewide program to measure chemicals of concern in preschool-aged children. If a child's urine sample has an unusually high level of a chemical, program staff contact the family to answer any questions they have and try to determine sources of exposure. Here, we provide examples in which the family interview provided clues on potential sources of exposure and subsequent actions taken.

Material and Methods

Recruitment for Healthy Kids Minnesota is conducted by local partners during state-required early childhood screenings. Families who agree to participate complete a questionnaire and help their child collect a urine sample. Nearly 1,000 children were enrolled during the 2021 and 2022 program cycles. Urine samples were analyzed for metals, pesticides, environmental phenols, phthalates, flame retardants, and air pollution chemicals. After all families were mailed their child's results along with information on ways to reduce exposures, program staff followed protocols to identify children with markedly elevated chemical levels and contacted these families by telephone.

Results

Follow-up with families identified likely sources and pathways of exposure including dietary habits (inorganic arsenic and frequent rice consumption); cultural practices (2-hydroxynapthalene and incense use); pesticides (2,5-dichlorophenol and moth repellants, pyrethroids and flea/tick pet products); behavioral factors (nickel and excessive sucking/chewing on outerwear and clothing); and medications (phthalates/parabens and topical treatments). Findings were used to make exposure reduction recommendations to families; inform discussions with subsequent families; revise the program questionnaire; plan statistical analyses; and guide public health actions.

Conclusions

One-on-one conversations with families of children with the highest chemical exposures helped identify likely sources and led to both individual and public health actions to reduce exposures. This type of effort can be a valuable complement to standard statistical analyses in biomonitoring studies to identify associations between biomarker levels and results from exposure surveys.

P43-18 | Dietary interventions to reduce heavy metal exposure in children: a systematic review

Su Ji Heo, Nalae Moon, Ju Hee Kim

Kyung Hee University, College of Nursing Science, Seoul, South Korea

Objective: Heavy metals accumulate in the environment, and ingestion is the main route of exposure. Children are more vulnerable to toxic metals due to their immature physiological development, which can significantly affect their normal growth and development. This study systematically reviews dietary interventions to reduce heavy metal exposure in children and proposes effective prevention strategies.
Materials and Methods: A systematic review was conducted using five databases (PubMed, Embase, Scopus, Web of Science, and Cochrane Library) and hand search (Google scholar) for articles published up to December 2024. The inclusion criteria focused on randomized controlled trials investigating dietary interventions to reduce heavy metal concentrations (lead, mercury, cadmium, arsenic) in children from infants to school-aged children.
Results: A total of 2,142 studies were identified through database searches, and after removing duplicates (n= 1,057) and studies without full text or non-English publications (n= 505), 580 studies were screened based on titles and abstracts. Following a full-text review, three studies were selected as eligible, and an additional three studies were identified through manual searches, resulting in a final selection of six studies. The study population ranged from 2.5 months to 10 years of age, with intervention durations varying from 4 to 9 months. The primary interventions were classified into nutritional supplementation (iron, zinc, calcium) aimed at inhibiting heavy metal absorption and educational interventions. Lead was identified as the primary heavy metal of concern. The effectiveness of the interventions was assessed through biomonitoring of heavy metal concentrations in blood and urine samples. Some studies demonstrated that iron and calcium supplementation reduced blood lead levels, while educational interventions had a positive impact on preventing heavy metal exposure.**Conclusion:** This study confirmed that dietary interventions effectively reduce heavy metal exposure in children. To support healthy development, dietary modifications and educational programs are essential for minimizing exposure.

P43-19 | Breastfeeding duration and serum organochlorine pesticides concentrations in residents from central-southern states of Mexico

Rodrigo Ugalde-Resano[1], Ángel Mérida-Ortega[1], Janet Flores Lujano[2], Juan C. Núñez-Enríquez[2], Mariano E. Cebrián[3], Lizbeth López-Carrillo[1]

[1] Instituto Nacional de Salud Pública, Centro de Investigación en Salud Poblacional, Cuernavaca, Mexico; [2] Instituto Mexicano del Seguro Social, Centro Médico Nacional Siglo XXI, Ciudad de México, Mexico; [3] Centro de Investigación y de Estudios Avanzados del Instituto Politécnico Naciona, Departamento de Toxicología, Ciudad de México, Mexico

It remains unclear how breastfeeding duration influences organochlorine pesticides (OCP) body burden concentrations in the offspring. The objective was to assess the association between breastfeeding duration and serum concentrations of 21 OCP in individuals under 20 years old from central-southern states of Mexico. We included 317 individuals that participated as controls in a population-based study in Puebla and Tlaxcala, Mexico (2021-2024). We collected data on breastfeeding history and sociodemographic characteristics through face-to-face

interviews. We determined serum concentrations of OCP using gas chromatography. We categorized breastfeeding duration into: never, 1–12 months, and >12 months. We used linear regression models to assess the associations between OCP serum concentrations and breastfeeding duration, adjusting by the minimum set of confounding variables according to a directed acyclic graph.
We found detectable concentrations in less than 10% of serum samples for most OCP, except: o,p'-DDE (11.36%), endosulfan sulfate (13.61%), hexachlorobenzene (17.35%), dieldrin (29.43%), oxychlordane (36.71%) and p,p'-DDE (95.58%). Serum concentrations of OCP did not vary across breastfeeding duration categories, except for p,p'-DDE which increased with increasing breastfeeding duration. Compared with those who were not breastfed, individuals who were breastfed for less than 1 year had positive, but not statistical significant, association with p,p'-DDE serum concentrations ($\beta_{\text{1-12 months vs. never breastfed}}$: 0.65; 95% CI: -0.08, 1.38), that reached significance among those who were breastfed for more than 12 months ($\beta_{\text{>12 months vs. never breastfed}}$: 0.76; 95% CI: 0.04, 1.49; p for trend = 0.064). Ever vs. never having been breastfed was positively associated with p,p'-DDE serum concentrations (β: 0.71; 95% CI: 0.04, 1.38).
Breastfeeding is widely recognized for its numerous benefits to health, however, in high-risk populations, might be also a source of persistent environmental organochlorine compounds. This work was supported by the Secretaría de Ciencia, Humanidades, Tecnología e Innovación (SECIHTI): FORDECYT-PRONACES 303019

P43-20 | Effects from prenatal and postnatal exposure to metals on the gut microbiota in children

Tsung-Lin Tsai[1], Chin-Chi Kuo[2], Chien-Wen Sun[3], Shih-Fen Tsai[3], Huei-Ju Liu[3], Hui-Ju Wen[3], Shu-Li Wang[3]

[1] China Medical University, Department of Public Health, Taichung, Taiwan; [2] China Medical University Hospital, Big Data Center, Taichung, Taiwan; [3] National Health Research Institutes, National Institute of Environmental Health Sciences, Miaoli, Taiwan

Objective
Some research supports the association between the composition or abundance of gut bacteria and human diseases (such as obesity and type 2 diabetes). Certain environmental toxicants, such as metals, may affect the diversity or balance of human gut microbiota. Reversely, gut microbiota might be associated with potential toxicokinetics, including metals' absorption and distribution. This study investigated the associations between prenatal and postnatal exposure to lead and cadmium and the abundance of gut microbiota in children.
Material and Methods
One hundred and eighteen children previously recruited in a birth cohort from central Taiwan were followed in 2015–2017. We collected maternal urinary samples during pregnancy and urinary samples and feces specimens in children aged 3–4 years. We quantified maternal and child urinary concentrations of lead and cadmium and extracted DNA from feces specimens. Shotgun Metagenomic Sequencing sequenced the composition (species) and abundance of gut microbiota.
Results
A total of 380 species were sequenced from 118 feces specimens of children. Increased maternal urinary lead concentration was significantly associated with elevated abundance of *Collinsella aerofaciens* (β = 2.12) and *Bacteroides caccae* (β = 1.70), and maternal urinary cadmium concentration showed positive associations with *Roseburia hominis*, *Bacteroides* sp. A1C1, *Collinsella aerofaciens*, *Bacteroides caccae*, *Bacteroides* sp. CACC 737,

and *Lachnoclostridium phocaeense* (β from 7.31 to 21.16). Child urinary lead concentration was significantly associated with an increased abundance of *Alistipes finegoldii*, *Collinsella aerofaciens*, and *Flintibacter* sp. KGMB00164 (β from 7.98 to 14.77). On the other hand, child urinary cadmium level showed a significantly negative association with *Coprococcus comes* (β = -46.40) and *Alistipes dispar* (β = -59.76).
ConclusionPrenatal or postnatal exposure to lead or cadmium may alter the abundance of gut microbiota potentially associated with human health. Further analysis will be performed to investigate the effects of lead and cadmium exposure on functional pathways.

P43-21 | Urinary Levels of Phthalate metabolites at 1.5- and 3-years with Early Adiposity Rebound and Overweight/Obesity in Japanese Children: Aichi Adjunct Study of the Japan Environment and Children’s Study

Yuki Ito[1], **Nayan C. Mohanto**[1], Sayaka Kato[1], Kayo Kaneko[1], Mayumi Sugiura-Ogasawara[2], Shinji Saitoh[3], Michihiro Kamijima[1]

[1] Nagoya City University Graduate School of Medical Sciences, Occupational and Environmental Health, Nagoya, Japan; [2] Nagoya City University Graduate School of Medical Sciences, Obstetrics and Gynecology, Nagoya, Japan; [3] Nagoya City University Graduate School of Medical Sciences, Pediatrics and Neonatology, Nagoya, Japan

Objective: Early adiposity rebound (EAR) is associated with late childhood and adult obesity. Phthalates are reported to affect the development of overweight and obesity. However, the relationship between early childhood phthalate exposure and EAR remains unclear. The objective of this study was to investigate the associations of 1,.5- and 3-year phthalate exposure with EAR and overweight/obesity in 7.5-year-old Japanese children.
Material and Methods: A total of 452 mother-child pairs were enrolled from the Aichi Regional Cohort of the Japan Environment and Children’s Study. The children were followed up at birth and at 1.5, 2, 3, 4, 5, 6, and 7.5 years of age for physical examinations. We measured 16 urinary metabolites of eight phthalates in urine from 1.5- and 3-year-old children. Latent class mixed model, binary logistic regression, and quantile g-computation were performed to identify body mass index (BMI) trajectories and investigate the relationships of single or mixed phthalate exposure with EAR and overweight/obesity.
Results: A one-unit increase in log_{10}-transformed 3-year-old Σdi(2-ethylhexyl) phthalate (ΣDEHP) exposure levels was significantly associated with 6-year-old BMI [β (99.9% CI) = 1.41(0.024, 2.79)] in girls. The urinary levels of mono-iso-butyl phthalate at 1.5 years and Σdi-isodecyl phthalate at 3 years were significantly associated with the repeated measures of longitudinal BMIs in girls [$β_{adjusted}$ (99.9% CI) =0.23 (0.0001, 0.47), and 0.20 (0.014, 0.39), respectively]. Each single phthalate exposure showed null associations with EAR or overweight/obesity in the 7.5-year-old children. Phthalate mixture exposure in 1.5- and 3-year-old children was not significantly associated with EAR.Conclusion: Early childhood phthalate exposure was not related to EAR or overweight/obesity in 7.5-year-old Japanese children. However, a few phthalates were positively associated with longitudinal BMIs in girls.

P43-22 | Quantifying the upper respiratory tract microbiome in children: nasal and oropharyngeal tropism and associations with the school air microbiome

Nicholas T. Minahan[1], Yue Leon Guo[1,2], Yu-Chen Chuang[2], Chi-Hsien Chen[2]

[1] National Health Research Institutes, National Institute of Environmental Health Sciences, Zhunan, Taiwan; [2] National Taiwan University (NTU) College of Medicine and NTU Hospital, Department of Environmental and Occupational Medicine, Taipei, Taiwan

Objective: The composition of the upper respiratory tract (URT) microbiome changes throughout life and in relation to respiratory allergy, infection, and systemic diseases. However, the extent to which airborne bacteria contribute to the URT microbiome remains unclear. This study aimed to quantify the nasal and oropharyngeal microbiomes of children to investigate site tropism and associations with the school air microbiome.
Material and Methods: Sampling was conducted during a cross-sectional survey on children's health in schools around Taiwan from December 2021 to April 2022 and repeated one year later. Airborne bacteria were passively sampled in classroom air for 24 hours before URT swab collection in each study wave. High-throughput sequencing and quantitative PCR targeting bacterial 16S rDNA were used to quantify bacterial genera, with URT bacterial abundance normalized to human DNA content. Generalized linear mixed models were used for analysis.
Results: Samples from 72 boys (144 nasal and 144 oropharyngeal samples) from 43 schools (86 air samples) were examined. A total of 20 genera were persistently detected in the nasal cavity of >25% of children and 46 genera in the oropharynx, with 13 overlapping. Among these, *Staphylococcus* and *Corynebacterium* were generally more abundant in the nasal cavity, while *Streptococcus*, *Prevotella*, *Neisseria*, *Gemella*, *Haemophilus*, *Granulicatella*, *Veillonella*, *Rothia*, *Porphyromonas*, *Lautropia*, and *Capnocytophaga* were more abundant in the oropharynx. The airborne abundance of *Streptococcus*, *Granulicatella*, and *Lautropia* significantly predicted their nasal abundance after adjusting for the indoor-to-outdoor temperature ratio but showed borderline significance when examining nasal-to-oropharynx abundance ratios.Conclusion: Most persistent bacterial genera in the nasal cavity exhibited stronger tropism for the oropharynx, and only a few were weakly associated with school air abundance. These findings indicate that personal factors explain most of the variation in URT microbiome abundance. This work received support from the National Science and Technology Council in Taiwan (NSTC 112-2314-B-002-224).

P43-23 | A characterization of infants' and children's time-activity patterns for use in soil and dust exposure estimation

Sara N. Lupolt[1,7], Lacey H. Etzkorn[2], Christina Huynh[1], Lesliam Quiros-Alcala[1], Erin DiMicco[3], Tracey J. Woodruff[3], Kristin Voegtline[5,6], Keeve E. Nachman[1,7]

[1] Johns Hopkins Bloomberg School of Public Health, Environmental Health & Engineering, Baltimore, USA; [2] Johns Hopkins Bloomberg School of Public Health, Biostatistics, Baltimore, USA; [3] University of California, San Franscisco, Program on Reproductive Health and the Environment, Obstetrics, Gynecology, and Reproductive Sciences, San Francisco, USA; [4] Johns Hopkins School of Medicine, Department of Pediatrics, Baltimore, USA; [5] Weill Cornell Medicine, Obstetrics and Gynecology, New York, USA; [6] Weill Cornell Medicine, Population Health Sciences, New York, USA; [7] Johns Hopkins Bloomberg School of Public Health, Risk Sciences and Public Policy Institute, Baltimore, USA

Background: Time-activity pattern data are key components of EPA's efforts to estimate soil and dust ingestion rates for use in risk assessments. However, the existing database of activity pattern data, which is mostly comprised of decades-old studies designed to estimate exposure to air pollution, limits its applicability to estimating soil and dust ingestion.
Objectives: We aimed to generate novel time-activity pattern data germane to soil and dust ingestion for children aged 6 months to 6 years. We explored potential differences in key exposure factors by child age, sex, fine and gross motor development, season, and day of the week, and compared estimates to the USEPA's Exposure Factors Handbook (EFH) recommendations.
Methods: Caregivers completed hourly time-activity surveys on one weekday and one weekend day in each season across one calendar year. We collected data on four microenvironments, seven macroactivities, and several play-related parameters specific to soil and dust exposure.
Results: We collected 1,010 participant-days of time-activity pattern data representing 242 children < 6 years. Children spent most of their day indoors, either sleeping (~734 minutes) or playing (~244 minutes); we observed notable differences in activity patterns across days and ages. On 97% of participant-days, children played with a mean of 45 hard toys/day, while on 83% of participant-days, children played with a mean 13 soft toys/day. Comfort object use was more prevalent than pacifier use across all age groups and highest (mean = 136 minutes/day) among 2 to < 3-year-olds. Pacifier use was rarely observed among children >3 years.
Conclusion/Significance: To advance soil and dust exposure estimation, we generated updated time activity data, including novel parameters specific to soil and dust ingestion for children under 6 years. These contribute to efforts to refine and advance EPA's current models of soil and dust ingestion in this vulnerable lifestage.

P43-24 | Associations of prenatal arsenic exposure withmyopia in primary school children: modifying effects of vitamin D levels

Juan Tong

Anhui Medical University, Hefei, China

The increasing role of environmental pollutants and nutrients in the development of myopia. Further longitudinal evidence is needed to elucidate the effects of early-life environmental pollutants on myopia and nutrients to protect against pollutant-induced myopia. We used mother-child dyads from the Ma'anshan Birth Cohort (n = 2028) to explore the modifying effects of maternal vitamin D status of prenatal and childhood arsenic exposure with refractive parameters and myopia. We measured serum or plasma arsenic concentrations during three trimesters, in cord blood, in childhood (5-year-old), and performed cycloplegic refraction in children aged 7 to 9 years (n = 1616). Primary outcomes were myopia and refractive parameters including axial length (AL), corneal radius of curvature, and spherical equivalent refraction error. Linear regression, logistic regression, and multiple informant models were performed for the association of prenatal and childhood arsenic exposure with vision health. Sex- and vitamin D status stratification analyses were also conducted. Cord serum arsenic was positively associated with AL (β = 0.15, 95%CI: 0.01, 0.29) and the risk of developing myopia (OR = 1.72, 95%CI: 1.07, 2.75). Among boys, a 1 ng/L increase in cord serum log_{10}-transformed arsenic resulted in larger AL and a higher risk of myopia. In the vitamin D deficient group (<20 ng/mL), cord serum arsenic showed positive associations with AL (0.26 [0.06, 0.46]) and the risk of myopia (1.99 [1.01, 3.90]). Late pregnancy arsenic exposure likely affects offspring development of myopia, especially in boys. Maternal sufficient vitamin D may confer protective effects against the development of myopia.

P43-25 | Environmental arsenic exposure and lung function in Yaqui children from southern Sonora, Mexico: Pilot study.

Ana G. Dévora-Figueroa[1], Jefferey L. Burgess[2], Paloma I. Beamer[2], Jose M. Guillen-Rodriguez[3], Anaid Vargas-Estrada[1], Iram Mondaca-Fernández[1], José J. Balderas-Cortés[1], **Maria M. Meza-Montenegro**[1]

[1] Instituto Tecnológico de Sonora, Natural Resources, Obregón, Mexico; [2] University of Arizona, College of Public Health, Tucson, USA; [3] University of Arizona, Arizona Cancer Center, Tucson, USA

Environmental arsenic exposure has been related with decreased lung function, respiratory symptoms, pneumonia, allergies, asthma, bronchitis and chronic obstructive pulmonary disease. In the Southwest US and Northern Mexico, drinking water exposure to arsenic is primarily found in the Hispanic population. Thus, this study was carried out in three Yaqui communities (Potam, Vicam and Cocorit) located in southern Sonora, Mexico, to explore the association between arsenic exposure by drinking water and serum pneumoproteins and lung function in children, who have been drinking well water for at least 5 years, were examined. Total arsenic was determined by inductively coupled plasma/optical emission spectrometry detecting values of 7.4 to 91 µg/l and 3.5 to 502.6 µg/l for drinking water and urine, respectively. Serum was analyzed for CC16 and MMP-9 using ELISA and lung function was evaluated by spirometry. A negative and significant association was found between urinary arsenic and the % predicted FEV1 and the FEV1/FVC ratio respectively. Increased levels of arsenic in drinking water declined significantly the values of the FEV1/FVC ratio and the same behavior was observed for this ratio when MMP-9 levels increased. Children with %

predicted FEV1 values less than 80 had the lowest levels of CC16 (Median 29.0 ng/ml, IQR 21.3, 37.4, *P* = 0.0148). Our study evidenced a damage in lung function in children exposed at low arsenic levels.

P43-26 | Predictors of PFAS exposure in Hispanic children with overweight/obesity from Northern Virginia

Rooshna Mohsin[1], Margaret T. Jones[2], Daniel Tomer[3], Jenna R. Krall[1], Allison McKay[4], Lameck Chiwaka[1], Roy R. Gerona[3], Michael S. Bloom[1]

[1] George Mason University, Department of Global and Community Health, College of Public Health, Fairfax, USA; [2] George Mason University, College of Education and Human Development, Sport, Recreation, and Tourism Management, Fairfax, USA; [3] University of California at San Francisco, Department of Obstetrics, Gynecology and Reproductive Sciences, San Francisco, USA; [4] George Mason University, Department of Nutrition and Food Studies, College of Public Health, Fairfax, USA

Objectives
To identify dietary, social, and acculturation predictors of perfluoroalkyl substances (PFAS) exposure in Hispanic children.
Materials and Methods
Hispanic children with overweight/obesity (n=126) were enrolled in a weight-loss intervention from 2017-2021. We measured children's anthropometry, and parents completed questionnaires about diet, socioeconomic factors (e.g., use of food assistance), and acculturation (e.g., English proficiency, child's place of birth). Children's blood samples were collected to measure 12 blood PFAS using liquid chromatography-tandem mass spectrometry (n=106). Generalized linear regression was used to estimate associations between dietary, socioeconomic, and acculturation factors with children's blood PFAS, adjusted for confounders.
Results
Children were 4-11 years old (mean=7.85); 90% had obesity (BMI > 95th age/sex percentile), and 50% had severe obesity (BMI ≥ 120% above 95th age/sex percentile). Greater consumption of burritos (mean difference (MD): 1.45 ng/mL, 95%CI: 0.56, 2.35) and cheeses (MD: 0.87 ng/mL, 95%CI: 0.01, 1.76) were associated with higher PFHpA. Greater consumption of fish (OR: 1.55, 95%CI: 1.17, 2.36) and beef (OR: 1.44, 95%CI: 1.01, 2.12) were associated with detectable PFBS. Worry about food was associated with higher PFOA (MD: 0.19 ng/mL, 95%CI: -0.04, 0.42) and PFNA (MD: 0.44 ng/mL, 95%CI: -0.05, 0.92). Using free/reduced school lunch was associated with lower PFHpA (MD: -0.61 ng/mL, 95%CI: -1.13, -0.10), PFOA (MD: -0.22 ng/mL, 95%CI: -0.44, -0.01), and PFHxS (MD: -0.64 ng/mL, 95%CI: -0.64, -0.06). Children born in the U.S. had lower PFHpA (MD: -0.54 ng/mL, 95%CI: -1.20, 0.09) and PFBS detection (OR: 0.25, 95%CI: 0.06, 0.88) than children born abroad. Longer maternal residence in the U.S. was associated with lower PFBS detection (OR: 0.92, 95%CI: 0.85, 0.99).
Conclusion
Dietary, social, and acculturation factors were associated with specific PFAS levels in Hispanic children, with varying associations across PFAS. Studies are needed to identify health implications of PFAS exposure in Hispanic children to inform future interventions.

P43-27 | Disparities in Environmental Chemical Exposure Among Young Children in Minnesota

Sheila Amenumey[1], Jessica Nelson[1], Jessie Carr[1], Carin Huset[2], Jason Peterson[2], Stefan Saravia[2]

[1] Minnesota Department of Health, Environmental Health, St Paul, USA; [2] Minnesota Department of Health, Public Health Laboratory, St Paul, USA

Objectives

Biomonitoring is a key component of the Minnesota Department of Health's (MDH) efforts to address public health concerns related to chemical exposures. Through the Healthy Kids Minnesota program, MDH collaborates with partners to measure six groups of chemical exposures in preschool-age children. This presentation explores disparities in exposure levels, potential sources—such as personal care products and incense—and their public health implications.

Materials and Methods

Recruitment for Healthy Kids Minnesota 2021 took place during state-mandated early childhood screenings from 8/17/2021 to 4/01/2021. Families who consented to participate completed an exposure survey and collected a urine sample from their child. A total of 454 children were enrolled in the 2021 program cycle. Urine samples were analyzed for metals, pesticides, environmental phenols, phthalates, flame retardants, and polycyclic aromatic hydrocarbons (PAHs). Statistical analyses were performed using SAS software version 9.2 on log-transformed biomonitoring results, demographic variables, and other exposure predictors from the survey.

Results

Preliminary analysis identified significant differences in chemical exposures based on race/ethnicity, household income, and exposure sources such as personal care products and incense use. Notable disparities were observed for environmental phenols, including 2,4-DCP, 2,5-DCP, BPS, BZP-3, MePB, PrPB, and TCS. Significant differences were also found for PAHs, including 1-PYR, 1-NAP, 2-NAP, 2-FLUO, and 3-FLUO, as well as for phthalate metabolites such as MEOHP, MBZP, MECPP, MECPTP, MEHHP, and MEP. Additionally, analysis of other chemicals indicated that certain groups of children may experience disproportionately higher exposures to these chemicals as well.

Conclusion

Statewide biomonitoring through Healthy Kids Minnesota provides essential data to identify vulnerable groups and support policy development aimed at reducing chemical exposures. Results indicate that certain groups of children experience significantly higher exposure to specific chemicals. These findings also underscore the need for further investigation into environmental and behavioral factors contributing to exposure disparities.

P43-28 | Secondhand Smoke and Polycyclic Aromatic Hydrocarbons Exposure: Associations with Epigenetic Aging among Children

Sneha Patil[1], Anne Bozack[2], Ajay Pillarisetti[3], Smita Patil[6], Kyle Steenland[5], Lance Waller[5], Jennifer Peel[4], Thomas Clasen[5], Dana B. Barr[5], Naveen Puttaswamy[1], Andres Cardenas[2]

[1] Sri Ramachandra Institute of Higher Education and Research, Chennai, India; [2] Stanford University, Stanford, USA; [3] University of California, Berkeley, USA; [4] Colorado State University, Fort Collins, USA; [5] Rollins School of Public Health, Emory University, Atlanta, USA; [6] Private Practitioner, Pune, India

Background: Smoking and polycyclic aromatic hydrocarbon (PAH) exposure have been associated with epigenetic age acceleration (EAA) in adults, but their impact on children remains less understood. We examined associations between secondhand smoke (SHS) and PAH exposure during prenatal, early postnatal, and current periods with EAA among preschool-aged children. **Methods:** DNA methylation was measured in buccal cells from 43 children (mean age: 4.1 years) in the Caries Risk from Exposure to environmental Tobacco Smoke - Household Air Pollution Intervention Network (CARES-HAPIN) cohort (2023-2025, Southern India). SHS exposure was assessed using urinary cotinine (COT) and 3-hydroxycotinine (3HC), while PAH exposure was measured through 2-naphthol (2NAP) and 1-hydroxypyrene (1PYR). Nicotine equivalents (NE2) were calculated as molar sum of COT + 3HC. EAA was estimated using the CpG and principal-component based Horvath's pan-tissue and skin&blood clocks, Pediatric Buccal Epigenetic clock (PedBE) and DNA methylation telomere length (DNAm TL). Associations between exposure and EAA were evaluated using OLS regression, adjusting for study site, mother's education, child's body mass index, child's age, and child's gender. **Results:** Chronological age correlated significantly with all epigenetic clocks ($r = 0.37–0.78$) and DNAm TL ($r = -0.38$). Current SHS exposure, as measured by urinary COT, was positively associated with Horvath pan-tissue EAA ($\beta = 0.385$ y, $p = 0.006$). 3HC and NE2 were linked to PedBE EAA ($\beta = 0.080$ y, $p = 0.04$; $\beta = 0.106$ y, $p = 0.03$, respectively). Among PAH metabolites, 2NAP was associated with PC skin&blood ($\beta = 0.495$ y, $p = 0.001$) and PedBE EAA ($\beta = 0.252$ y, $p = 0.026$), while 1PYR correlated with PC skin&blood EAA ($\beta = 0.222$ y, $p = 0.04$). **Conclusion:** These findings suggest that early-life exposure to SHS and PAHs may contribute to accelerated epigenetic aging in childhood, which could have implications for long-term health outcomes.

P43-29 | Exposure to arsenic associated with neurodevelopmental disorders in children from Arica

Claudia López Araya[1], Paola Rubilar Ramírez[2], María Pia Muñoz Quezada[1], Macarena Hirmas-Adauy[2], Verónica Iglesias Alamos[1]

[1] Universidad de Chile, Facultad de Medicina, Escuela de Salud Pública, Santiago, Chile; [2] Universidad del Desarrollo, Facultad de Medicina Clínica Alemana, Centro de Epidemiología y Políticas de Salud, Santiago, Chile

Objective: Arsenic exposure has been identified as a possible risk factor for the ocurrence of Neurodevelopmental Disorders (NDD). In the city of Arica, exposed to arsenic from both natural and antropogenic sources, research has been conducted to relate arsenic exposure to the prevalence of Attention Deficit Hyperactivity Disorders (ADHD). However, only a group with particular exposure characteristics has been studied, so it's necessary to consider a representative sample of the city, incorporate previously unmeasured variables, and explore other events, such as Autism Spectrum Disorder (ASD). The objective of this study was to evaluate the association between arsenic

exposure and the prevalence of neurodevelopmental disorders in children from the Arica city.

Material and Methods: Cross-sectional design. Analysis of secondary data from the FONIS study #SA22|0119. The sample consists of 450 children born between 2013 and 2016 in Arica. The outcome variable, diagnosis of NDD, was measured through parent self-reporting. The exposure variable corresponds to the current concentration of inorganic arsenic in urine, corrected by creatinine. A multiple logistic regression model adjusted for confounding variables was used to estimate the association.

Results: The prevalence of ADHD was 9.1%, ASD 5.3% and NDD 12%. The mean current inorganic arsenic concentration was 19.80 µg/g. A total of 7.56% presented values ≥ 35 µg/g. After adjusting for confounding variables (education of the mother or tutor, number of people living in the household, sex, and indigenous origin), the group with urinary arsenic ≥ 35 µg/g was more likely to present some NDD (OR: 2.93; 95% CI: 1.11 - 7.75). In the case of ADHD, the association was significant (OR: 3.85; 95% CI: 1.44 - 10.29).

Conclusion: The findings suggest an association between arsenic exposure and the prevalence of NDD. These results contribute to the evidence of arsenic's effect on the child population's neurodevelopment.

P43-30 | Associations of autism in children with prenatal exposure to extreme heat

David Luglio[1], Xin Yu[2], Jane Lin[3], Ting Chow[3], Mayra Martinez[3], Zhanghua Chen[4], Sandrah Eckel[4], Joel Schwartz[5], Frederick Lurmann[6], Nathan Pavlovic[6], Rob McConnell[4], Anny Xiang[3], Mostafijur Rahman[1]

[1] Tulane University, New Orleans, USA; [2] Icahn School of Medicine at Mount Sinai, New York, USA; [3] Kaiser Permanente Soutern California, Pasadena, USA; [4] University of Southern California, Los Angeles, USA; [5] Harvard University, Boston, USA; [6] Sonoma Technology, Petaluma, USA

Objective: To identify weekly associations of maximum and minimum temperature (T_{max} and T_{min}, respectively) during pregnancy on development of autism in children.

Methods: This retrospective birth cohort study included 294,937 singletons delivered at Kaiser Permanente Southern California hospitals from 2001 to 2014 and followed up to age 5. ASD diagnosis by age 5 were identified in electronic medical records with corresponding ICD codes. Weekly average T_{max} and T_{min} were estimated at the maternal residential addresses during pregnancy using the gridMET model. Cox proportional hazard model with nonlinear distributed lags was used to identify critical windows of exposure with hazard ratios (HR) comparing exposure to temperature at the 90th and 99th percentiles versus the 50th percentile. Models were mutually adjusted for T_{min} and T_{max} to assess their independent effects.

Results: A total of 4,076 children (80% male) had autism diagnosis by age 5. Exposure to extreme T_{min} during gestational weeks 1-13 and 26-37 were associated with increased risk of autism, with HR (95% CI) of 1.169 (1.040-1.299) for exposure during weeks 1-13, and 1.157 (1.030, 1.286) for weeks 26-37 comparing the 99th percentile to the 50th percentile. No association was observed for T_{max}.

Conclusions: Exposures to high T_{min} during early and late pregnancy, which serves as an indicator for hotter nights -- projected to become more frequent in a warming world – was associated with risk of autism in children.

Funding and acknowledgments: Funding was provided by NIEHS, EPA, and Kaiser Permanente Southern California Direct Community Benefit Funds.

P43-31 | General anesthesia in early childhood and neurodevelopment up to 4 years of age: The Japan Environment and Children's Study

Masayuki Shima[1,2], Takahisa Nagai[3,4], Narumi Tokuda[2], Yoshiko Yoda[3,5], Yasuhiro Takeshima[2,3], Munetaka Hirose[2,3], The Japan Environment and Children's Study (JECS) Group

[1] Hyogo Medical University, School of Nursing, Kobe, Japan; [2] Hyogo Medical University, Hyogo Regional Center for the Japan Environment and Children's study, Nishinomiya, Japan; [3] Hyogo Medical University, School of Medicine, Nishinomiya, Japan; [4] Taka Ladies Clinic, Osaka, Japan; [5] Kansai University of Welfare Sciences, Faculty of Health Sciences for Welfare, Kashiwara, Japan

Objective: The effects of general anesthesia on neurodevelopment in children remain controversial. We explored the relationship between general anesthesia and neurodevelopment in children participating in the Japan Environment and Children's Study (JECS).

Methods: This study included 69,653 children born between 37 and 41 weeks of pregnancy via single-vaginal delivery to mothers registered in the JECS between 2011 and 2014. Data were collected from mother-completed questionnaires and medical transcripts. Neurodevelopment was assessed every 6 months between 12–48 months of age, using the Japanese version of the Ages and Stages Questionnaires, Third Edition, comprising five developmental domains: communication, gross motor, fine motor, problem solving, and personal-social. Neurodevelopmental delay was defined as scores that were lower than the cut-off scores adjusted for each age in Japanese children. The associations between general anesthesia exposure during early childhood and neurodevelopmental delay were evaluated at each time-point. Adjusted odds ratio (OR) and 95% confidence interval (CI) were estimated after covariate adjustment using logistic regression models.

Results: Children who received general anesthesia before age 1 year had higher risks of neurodevelopmental delay in all five domains throughout the observational period. The largest risk was for gross motor delay at 18 months (adjusted OR: 3.51; 95% CI: 2.75–4.49). The effects on the incidence of neurodevelopmental delays after age 3 were not observed except for problem solving at 48 months. The risk of neurodevelopmental delay in children who first received general anesthesia after age 1 was considerably small in all domains.

Conclusions: This study suggests that general anesthesia administration before age 1 is associated with neurodevelopmental delay during 1–4 years of age. The risk of general anesthesia after age 1 may be small.

Acknowledgements: The authors thank all participants and cooperating health care providers. This study was funded by the Ministry of the Environment, Japan.

P43-32 | Association between exposure to organophosphorus and pyrethroid pesticides and attention-deficit/hyperactivity disorder in children in northern Taiwan

Mei-Lien Chen, Ya-Chun Yang

National Yang Ming Chiao Tung University, Institute of Environmental & Occupational Health Sciences, Beitou Dist., Taiwan

Objectives: Exposure to commonly used pesticides such as chlorpyrifos (CPF) and pyrethroids (PYRs) have been associated with attention deficit hyperactivity disorder (ADHD). This study aims to determine the correlation among urinary levels of CPF and PYRs metabolites, oxidative stress, and ADHD risk, assess the mediation effect of oxidative stress between exposure to CPF and PYRs and ADHD.

Methods: This study recruited 87 children diagnosed with ADHD and 129 control children from northern Taiwan. Urinary levels of 3,5,6-trichloro-2-pyridinol (TCPy), 3-(2,2-Dichlorovinyl)-2,2-dimethylcyclopropanecarboxylic acid (DCCA), and 3-Phenoxybenzoic acid (3-PBA), were measured using ultra-high performance liquid chromatography quadrupole time-of-flight mass spectrometry (UPLC-QTOF/MS). Four oxidative stress markers, including 8-hydroxy-2-deoxyguanosine (8-OHdG), 8-nitroguanine (8-NO_2-Gua), 8-iso-prostaglandin $F_{2\alpha}$ (8-iso-$PGF_{2\alpha}$), and 4-hydroxy-2-nonenoic acid-mercapturic acid (HNE-MA) were quantified using high-performance liquid chromatography-tandem mass spectrometry (HPLC-MS/MS). Statistical analyses were performed using SAS 9.4 and R 4.2.3.

Results: Urinary TCPy, DCCA, and 3-PBA levels were detected at 92.13%, 88.43%, and 89.81% of children respectively. ADHD children exhibited higher urinary DCCA (3.34 µg/g Cr vs. 2.13 µg/g Cr), 8-NO_2Gua (0.94 µg/g Cr vs. 0.64 µg/g Cr), and HNE-MA (39.31 µg/g Cr vs. 19.78 µg/g Cr). Children with higher DCCA levels showed 1.93 times higher risk of ADHD, whereas children in fourth quartile of 8-NO2Gua and HNE-MA had a 2.39-fold and 3.97-fold increased risk of ADHD, respectively. No mediation effect by oxidative stress was observed.

Conclusion: This study showed that higher levels of HNE-MA and exposure to DMP and DCCA were important risk factors for ADHD, warranting further research into the underlying mechanisms.

P43-33 | Association of Early-Life Exposure to Polycyclic Aromatic Hydrocarbons and Volatile Organic Compounds with Autistic Traits at Age 5: The Mothers and Children's Environmental Health (MOCEH) study

Soonsu Shin[1], Dajeong Ham[1], Sanghyuk Bae[1,2]

[1] The Catholic University of Korea, Department of Preventive Medicine, Seoul, South Korea; [2] The Catholic University of Korea, Environmental Health Center, Seoul, South Korea

Objective: Exposure to polycyclic aromatic hydrocarbons (PAHs) and volatile organic compounds (VOCs) has been suggested to affect neurodevelopment and neurocognitive function. This study aimed to examine the association between urinary biomarkers of PAHs and VOCs measured in children at ages 2–3 and their autistic traits at age 5.

Material and Methods: A total of 135 children from the Mothers and Children's Environmental Health (MOCEH) prospective cohort were included. Urinary concentrations of 1-hydroxypyrene (1-OHP), 2-naphthol (2-NAPH), and

hippuric acid (HA) were measured twice, at ages 2 and 3, with mean values used to represent cumulative exposure. All urinaty metablites were log-transformed. Autistic traits at age 5 were assessed using the Social Responsiveness Scale (SRS), considering both the continuous SRS T-score and a clinical cutoff (T-score ≥ 60) as outcomes. Multivariable linear and logistic regression models were applied to assess associations between each urinary metabolite and SRS T-scores, adjusting for potential confounders. Additionally, Bayesian Kernel Machine Regression (BKMR) models were used to estimate the overall effects of PAHs and VOCs.

Results: In the fully adjusted model, Urinary 2-NAPH concentrations were positively associated with both SRS T-scores at age 5 (β = 2.18, 95% confidence intervals: 0.55, 3.82) and increased odds of an SRS T-score ≥ 60 (odds ratio = 1.49, 95% confidence intervals: 1.02, 2.22). Consistent with the single-exposure results, the BKMR model demonstrated similar association patterns and identified 2-NAPH as the key contributor in the mixture model, but no significant overall mixture effect was observed.

Conclusions: Our findings suggest that early-life cumulative exposure to certain PAHs, particularly naphthalene, may be associated with increased autistic traits in children.

P43-34 | Effect of Essential and Toxic Metals on Children's Cognitive Function: Insights from a Mexico City Birth Cohort Using Multi-Media Biomarkers

Victor Florez-Garcia[1,2], Robert Wright[3], Alexander Keil[4], Martha Téllez-Rojo[5], Sandra Martínez-Medina[6], Guadalupe Estrada[7], Amy Kalkbrenner[2]

[1] University of Illinois Chicago, Division of Epidemiology and Biostatistics, School of Public Health, Chicago, USA; [2] University of Wisconsin Milwaukee, Joseph J. Zilber College of Public Health, Milwaukee, USA; [3] Icahn School of Medicine at Mount Sinai, Department of Environmental Medicine and Public Health, New York, USA; [4] National Institutes of Health, Epidemiology Branch, National Institute of Environmental Health Sciences, Department of Health and Human Services, Research Triangle Park, USA; [5] Instituto Nacional de Salud Pública, Centro de Investigación en Nutrición y Salud, Cuernavaca, Mexico; [6] National Institute of Perinatology, Department of Developmental Neurobiology, Ciudad de Mexico, Mexico; [7] National Institute of Perinatology, Department of Immunobiochemistry, Ciudad de Mexico, Mexico; [8] Department of Public Health, Universidad del Norte, Barranquilla, Colombia

Aim: This study examined how exposure to both non-essential and essential metals affect the intellectual function of Mexican children at 48 months of age. **Material and Methods**: We analyzed data from the Programming Research in Obesity, Growth, Environment, and Social Stressors (PROGRESS) birth cohort in Mexico City. Exposures comprised toxic metals (lead, cadmium, mercury, arsenic, strontium, barium, and cesium) and essential metals (manganese, copper, selenium, molybdenum, magnesium, and zinc) collected from blood, urine, hair, nails, and cord blood, during pregnancy and early childhood. Children's cognitive function was assessed using the McCarthy Scales of Children's Abilities, focusing on the General Cognitive Index(GCI). To capture the total body burden of metals during key developmental stages, we constructed a multi-media biomarker (MMB) index using the Weighted Quantile Sum Regression(WQS) approach. The MMB index incorporated all available biomarkers for each metal across different time points. We applied generalized linear models to estimate changes in GCI per interquartile increase in the MMB index (β) along with corresponding 95% confidence intervals (CIs) in both single- and multi-pollutant models. **Results**: We included 604 pairs of mother-child. In multi-pollutant models accounting for confounders, MMB-cadmium (β: -1.52, 95% CI: -3.03, -0.01) and MMB-arsenic (β: -1.53; 95% CI: -3.02, -0.03) were significantly associated with lower GCI. Conversely, MMB-manganese (β: 2.28; 95% CI: 0.77, 3.77), MMB-selenium (β: 2.07;

95% CI: 0.69, 3.43), and MMB-molybdenum (β: 1.76; 95% CI: 0.21, 3.31) exhibited the strongest positive associations with GCI. Discrepancies between single- and multipollutant models highlighted potential confounding effects for magnesium, molybdenum, barium, and zinc. **Conclusion**: Our findings contribute to the growing evidence on co-pollutant confounding in metal exposure studies and its implications for children's cognitive development. Additionally, this study underscores the need for further research on lesser-studied toxic metals, such as barium and cesium, which may have previously unrecognized effects on cognition.

P43-35 | Associations between urinary chemicals and estimated glomerular filtration rate (eGFR) in the Korean adolescents: Korean National Environmental Health Survey (KoNEHS) 2018–2020

Gowon Lee[1], Ah-Reum Jo[2], Inae Lee[3], Ba Reum Kwon[4], Yaerim Kim[5], Jeonghwan Lee[6], Jung Pyo Lee[6], Kyungho Choi[2]

[1] Korea National University of Transporation, Safety Engineering, Chungbuk, South Korea; [2] Seoul National University, Graduate School of Public Health, Seoul, South Korea; [3] Keimyung University, Department of Public Health, Daegu, South Korea; [4] Baylor University, Department of Environmental Science, Waco, USA; [5] Keimyung University, Division of Nephrology, Department of Internal Medicine, Daegu, South Korea; [6] Seoul National University Seoul Metropolitan Government Boramae Medical Center, Department of Internal Medicine, Seoul, South Korea

Research on chemical exposure and kidney function is ongoing, but studies on adolescents remain limited. The present study examined the association between urinary chemical exposure and estimated glomerular filtration rate (eGFR) in adolescents using data from the Korean National Environmental Health Survey (KoNEHS) 2018–2020. Among 828 adolescents aged 12–17 participants, 771 individuals were included, meeting the criteria of urinary chemical measurements, serum creatinine levels, and complete covariates (age, gender, BMI z-score, house income, smoking, alcohol drinking). Chemical exposure levels were adjusted using covariate-adjusted standardization (CAS), and kidney function was assessed using the eGFR formula from the Chronic Kidney Disease in Children Study under 25 (CKiDU25). Significant associations were observed between eGFR and MnBP, MBzP, MEOHP, MEHHP, MCPP, ethylparaben, tt-muconic acid, and BMA in the individual exposure models. Using the quantile g-computation model to assess mixed chemical exposure, overall exposure was significantly associated with a decrease in eGFR. The three chemicals with the highest weights were MBzP (0.21), ethylparaben (0.15), and BMA (0.12), in that order. Age-stratified analysis revealed a weaker association in the 12–14 age group. The reason for this was unclear; however, the characteristics of adolescents undergoing rapid growth may partly explain this finding. The 12–14 age group had greater variability and higher urinary creatinine levels. These factors need to be considered when evaluating the association between chemical exposure levels and health outcomes in adolescents. This study is significant as it investigates chemical exposure and its effects on kidney function in large-scale Korean adolescents.

P43-36 | Progress Status of Japan Environment and Children's Study

Michihiro Kamijima[1,2], Shin Yamazaki[2], Shoji F. Nakayama[2]

[1] Nagoya City University, Dept. of Occupational and Environmental Health, Nagoya, Japan; [2] National Institute of Environmental Studies, Tsukuba, Japan

Objective: Japan Environment and Children's Study (JECS) is a nationwide birth cohort study funded by the Ministry of the Environment, Japan (MOE) to clarify the impact of environmental factors on children's health and development in Japan. This presentation reports the progress status of JECS.

Methods: JECS initially registered 103,099 pregnancies in 15 Regional Centers across Japan between 2011 and 2014. Participating mothers have been requested to take part in the occasional questionnaire or face-to-face surveys about their children's health and environment since their pregnancies. Exposure to environmental chemicals is quantified primarily by biomonitoring methods.

Results: As of the end of 2024, more than 90% pairs of the mothers and children remained in the cohort, and biospecimens from children's fetal period through 12 years old were stored frozen. 502 original articles were published. JECS has been engaged in international collaborations by harmonizing methodologies and conducting facilitated data analyses. Based on the high follow-up ratio of the cohort, MOE revised the Basic Plan of JECS in 2023 to extend the follow-up period of children until they reach 18 years old, ideally around 40.

Conclusion: JECS has been contributing to the development of children's environmental health and its related field. The key issue at this stage is how to keep the cohort, especially by means of encouraging the children after they enter adolescence. Public awarenesses, especially communication among communities/schools about the significance of JECS is important as well as further promotion of the JECS research output/outcome.

Acknowledgements: The authors thank all the study participants and the collaborative researchers/staffs/organizations. Conclusions of this presentation are solely the responsibility of the authors and do not represent the official views of MOE.

P43-37 | How do the breathing efficiency and respiratory health of children from two regions of Poland differ: polluted Silesia and cleaner Pomerania?

Piotr Dąbrowiecki[1], Artur J. Badyda[2], Łukasz Adamkiewicz[3], Dominika Mucha[2], Andrzej Chciałowski[1]

[1] Military Institute of Medicine – National Research Institute, Department of Internal Medicine, Infectious Diseases, and Allergology, Warsaw, Poland; [2] Warsaw University of Technology, Department of Informatics and Environment Quality Research, Warsaw, Poland; [3] European Clean Air Center, Krakow, Poland

OBJECTIVE

Ambient air pollution negatively influences lung development in children, who may be more exposed to air pollution comparing to adults and have immature respiratory systems. The risk of decreased lung function is particularly important in children living in highly polluted areas. The air quality in Zabrze, located in the Upper Silesian Industrial Region, is among the worst in Europe, mainly due to households using solid fuels for heating purposes. Gdynia, located on the Baltic coast, is among the cleanest cities in Poland.

MATERIAL AND METHODS

Lung function and frequency of respiratory or allergic symptoms in children living in Zabrze and Gdynia, were measured. 258 children from Gdynia and 512 children from Zabrze aged 9-15 years, who were able to perform a correct spirometry, were examined. AioCare mobile spirometer, enabling data collection in cloud, was used. FVC, FEV_1, FEV_1/FVC index and PEF were collected. Participants' parents completed a survey about the frequencies of respiratory/allergic symptoms.

RESULTS

Children in Zabrze were exposed on higher concentrations of PM, NO_2 and SO_2. The mean values of FVC, FEV_1, and PEF were statistically significantly higher among children in Gdynia than in Zabrze (with p-values at the levels of <0.001, =0.032 and =0.018 respectively). However the frequencies of seasonal rhinorrhea (p=0.015) or cough episodes (p=0.022) were significantly higher in more polluted Zabrze than cleaner Gdynia. Other respiratory or allergic symptoms did not demonstrated statistical significance between the cities, thus they were considered as similar.

CONCLUSION

Lung function was significantly decreased in children living in Zabrze. Considering that both cohorts were similar in age, sex ratio, weight, and height, but greater percentage of children in Zabrze than in Gdynia lived near a major road or had a coal- or wood-burning stove in a living area, lower respiratory efficiency was likely because of poor air quality.

P43-38 | Burden of disease attributable to the exposome in children across 6 European cohorts

A. Rocabois[2], B. Wies[3], S. Boukari[4], M. Vrijheid[2], M. Nieuwenhuijsen[3], C. Philippat[2], S. Mathy[4], **R. Slama**[1]

[1] Inserm and ENS, PARSEC (Paris Research in Health Environment Climate), Paris, France; [2] Inserm, Team of Environmental epidemiology, IAB, Grenoble, France; [3] ISGlobal, Barcelona, Spain; [4] CNRS and University Grenoble-Alpes, GAEL (Grenoble Applied Laboratory in Economics), Grenoble, France

Background: Studies quantifying the exposome health burden in children considered a small number of chemicals, disregarding many exposures with likely effects.
Objectives: We assessed the impact of a large fraction of the exposome (including chemicals) on children's health.
Methods: We focused on 132 exposure-health outcome pairs corresponding to 'likely' or 'very likely' effects based on a previous assessment. Exposure-response functions were previously identified from meta-analyses or epidemiological studies. The health burden was assessed in children of HELIX cohorts from 6 European countries in whom exposures were measured with biomarkers, questionnaires or air pollution models. The health burden was expressed in disability-adjusted life years (DALYs) or as average variations for quantitative outcomes, and the corresponding intangible costs were estimated.
Results: The health impact could be estimated for 20 environmental risk factors belonging to 43 (33%) of the 132 considered exposure-outcome pairs, including in DALYs for 11 factors. About 5% (95% confidence interval, CI, 1, 10) of the childhood burden of disease, corresponding to 2.9 (95% CI, 0.6, 5.9) DALYs/1,000 children per year and to 3.7 times the Gross Domestic Product (GDP) per capita per 1,000 children, was attributable to these eleven risk factors. The largest contributors were tobacco smoke, particulate matter with a diameter under 2.5 μm, NO_2, pesticides, manganese, perfluoroalkyl substances and bisphenol A. The exposome entailed an estimated cumulated reduction in birth weight by 240 g (95% CI, 0, 560); and in intelligence quotient by 11 points (95% CI, 2, 30), which translated into total lifetime productivity costs of 5,539 times the GDP per capita per 1,000 children.
Discussion: Our study is one of the broadest environmental burden assessments in children in terms of number of chemicals considered. Missing data for a large proportion of factors, particularly for exposure-response functions, led to a probable underestimation of the impact.

P43-40 | Effects of co-exposure to multiple metals on children's anthropometric profile – PIPA Project – Rio de Janeiro, Brazil

N. D.D. Figueiredo[1, 2], C. I.R. Froes-Asmus[1]

[1] Federal University Of Rio de Janeiro, Public Health Institute, Rio de Janeiro, Brazil: [2] Souza Marques College, Public Health Department, Rio de Janeiro, Brazil

Aim: To investigate the relationship between environmental exposure to metals—lead, mercury, arsenic, and cadmium—during the first year of life and the anthropometric development profile in this population. Methods: Data were obtained from the birth cohort study of the PIPA Brazil Project. This study considered 463 children who returned for follow-ups at 3 months, 6 months, and 1 year and collected urine samples for metal analysis by inductively coupled plasma mass spectrometry. Anthropometric data were measured at each follow-up. Metal concentration descriptions were established for each follow-up. A generalized linear model analysis was performed to investigate the correlation between the metals studied and the outcomes of weight and length at 12 months. Adjustments were made for sex, income, and prematurity. Principal compounds analysis (PCA) was applied to evaluate mixture effects. Results: The study population is low to middle income with low maternal education levels. The investigated metals showed a positive correlation with each other. Throughout the follow-ups, an increase in arsenic concentrations ($p = 0.000$) was observed. The median increased from 0.3 (IQR 0.2 – 0.7) at 3 months of age to 2.7 (IQR 1.5 – 5.4) at 12 months. The concentrations of the other metals did not show significant variations. Mercury showed a negative correlation with weight ($r=-0.145$ $p= 0.001$) and length ($r = -0.117 p=0.01$), while arsenic showed a positive correlation with these parameters ($r= 0.160$ $p=0.02$ and $r = 0.11$ $p= 0.03$ for arsenic) and lead (r= 0.0.09 $p=0.04$ and $r = 0.10$ $p= 0.02$). Weight gain during the first year of life was positively correlated with arsenic ($r=0.16$, $p = 0.001$). Metal mixture was negatively correlated to weight gain ($r=-0.12$ $p=0.01$). The analysis suggests metal mixture effects on the outcomes investigated.

P44 | Climate and Health: Investiging Effects of Heatwaves and Hot Weather

P44-01 | Long-term Heatwave Exposure and Accelerated Aging: Evidence from a Longitudinal Cohort Study

Siyi Chen[1], Yuanyuan Yi[1], Cui Guo[1,2]

[1] The University of Hong Kong, Department of Urban Planning and Design, Faculty of Architecture, Hong Kong, Hong Kong; [2] The University of Hong Kong, Urban Systems Institute, Hong Kong, Hong Kong

Objective: Both climate change and the aging population represent pressing global challenges. This study aims to examine the association between long-term exposure to heatwaves and the acceleration of aging (AA).
Material and Methods: A total of 24,922 adults from a Taiwanese cohort, spanning the years 2008 to 2022, were included in this study. Heatwaves were defined as periods of at least two consecutive days during which the daily average temperature exceeded the 92.5th percentile over a two-year period. The long-term impacts of heatwaves were examined based on their characteristics, including frequency, duration, and intensity, as well as cumulative exposure. Linear mixed models, combined with inverse probability weights, were applied to estimate causal associations between heatwaves and AA. Concentration-response curves were plotted using the natural cubic spline function. Stratified analyses were conducted to identify vulnerable subgroups.
Results: Each interquartile-range increment in heatwave duration, intensity, and cumulative exposure was associated with an increase in AA, ranging from 0.026 to 0.030 years. A U-shaped relationship was identified between heatwave frequency and AA, with minimum effects observed at 10.6 occurrences. Manual workers, rural residents, and individuals living in communities with limited access to air conditioning demonstrated 1.5 to 4.5 times greater vulnerability compared to reference groups.
Conclusion: Long-term exposure to heatwaves is associated with accelerated aging. These findings advocate for further policy development to protect vulnerable populations and reduce climate-health inequities.
Funding: HKU Seed Fund for Collaborative Research, National Natural Science Foundation of China Young Scientists Fund.
Acknowledgement: Part of the data was authorized by the MJ Health Research Foundation.

P44-02 | Interactive effects of extreme heat and air pollution exposure on cardiovascular disease outcomes – A Systematic Review

Harshal R. Salve[1,2], Surbhi Kapoor[1,2], Sagnik Dey[5], Abhiyant Tiwari[3], Meenal M. Thakare[4]

[1] All India Institute of Medical Sciences, New Delhi, Centre for Community Medicine, New Delhi, India; [2] Collaborative for Air Pollution and Health Effects Research (CPAHER) India, Delhi, India; [3] NRDC India, Delhi, India; [4] All India Institute of Medical Sciences, Bilaspur, Department of Community and Family Medicine, Bilaspur, India; [5] Indian Insistute of Technology, Centre for Atmospheric Sciences, Delhi, India

Objectives: To assess the synergistic effect of extreme heat and air pollution on cardiovascular disease outcomes. **Material and Methods:** Literature review by two independent researchers through a systematic search of online scientific databases (PubMed, SCOPUS, EMBASE and Cochrane library) was carried out. Manual search of the reference lists from the identified papers was also done. Exposure variables considered in the study were heat events and all major air pollutants. Outcome variables were cardiovascular disease morbidity and mortality. Information regarding association (hazards ratio, odds ratio, relative risk etc.) between exposure and outcome

variable were retrieved. ROBINS E tool was used for methodological quality assessment of all the shortlisted articles. The protocol of the review was registered at PROSPERO (CRD420251002033). **Results:** Search using MESH, key words led to retrieval of 75 articles. After screening, applying inclusion and exclusion criteria 18 articles were included in the review. Heat exposure was measured as daily average temperature, extreme temperature with cut off range, range of maximum / minimum temperature using monitoring station or satellite based measurements. Exposure to air pollution was measured using monitoring stations, AI and satellite based assessment. Exposure assessment in all studies were ecological in nature. CVD related mortality due to air pollution exposure was reported higher during heat wave days/ higher daily average temperature. Males, elderly (> 65 years) reported higher risk. PM and O_3 exposure reported higher mortality during higher heat exposures with dose response relationship. Exposure to NO_2 reported decrease in CVD related mortality. Increase hospital admissions, IHD, heart failure, heart arrhythmias, conduction disorders, hypertensive disorders were reported with exposure to heat and air pollution. Exposure misclassification and confounding factors effects were identified as most common bias among included studies. **Conclusions:** Review suggested need of science driven policy action to mitigate synergistic effects of extreme heat and air pollution

P44-03 | A Decision-Support Tool for Assessing Heat-Mitigating Interventions to Improve Student Learning in Schools

Jinho Lee[1], M. Pilar Botana Martinez[1], Katherine H. Walsh[2], M. Patricia Fabian[1,3]

[1] Boston University School of Public Health, Department of Environmental Health, Boston, USA; [2] Boston Public Schools, Boston, USA; [3] Institute for Global Sustainability, Boston University, Boston, USA; [4] Boston University School of Public Health, Environmental Health, Boston, USA

Schools, the indoor environments where children spend 6-10 hours a day, are often in aging buildings equipped with insufficient cooling systems, exposing students to temperature extremes. Extreme heat exposure is rising worldwide and can hinder learning. Meanwhile, schools face competing demands as they balance indoor temperature with the need to meet energy goals. Given limited resources, it is important for school decision-makers to have tools to assess the effectiveness of cooling interventions such as installing or upgrading air conditioners (AC) and implementing high-reflectance roofs.

To address this gap, we developed a decision-support tool to estimate the impacts of cooling interventions on student test scores and school building energy consumption. This quantitative learning impact assessment tool is parameterized with sociodemographic data from Boston Public Schools (BPS), state-level standardized test scores and continuous indoor temperature data from ~4,000 BPS classroom sensors. Energy changes due to the interventions were calculated with EnergyPlus, a building energy simulation software.

We estimated the impact of cooling interventions under various scenarios. In the scenario of taking the test on the hottest day within the official testing period, installing high-reflectance roofs and central ACs led to a 4.86℉ temperature reduction (SD=5.97) and improved test scores with a greater impact among students who initially did not pass. For example, the math test score increased by 6.05 (SD=13.1) in the scenario. Additionally, it estimated that the test pass rates increased more in vulnerable groups (e.g., underserved racial groups, low-income households). The tool also estimated the energy consumption necessary to achieve indoor temperature goals.

This scalable framework can empower schools to prioritize sustainable interventions that mitigate excessive heat to promote learning, equity, and sustainability. Expanding the tool nationwide will help establish effective strategies for managing heat in U.S. schools, contributing to increased climate resilience and improved student learning.

P44-04 | Projected Urban Heat Island–Attributable Mortality in Tokyo Metropolitan Area, Japan, Across Age Groups and Geographies Under Future Climate and Demographic Scenarios

Jinyu He[1], Lina Madaniyazi[2], Paul L C Chua[4], Yoonhee Kim[3], Kazutaka Oka[1], Yasushi Honda[1,2], Masahiro Hashizume[4,2]

[1] National Institute for Environmental Studies, Center for Climate Change Adaptation, Ibaraki, Japan; [2] Department of Global Health, School of Tropical Medicine and Global Health, Nagasaki University, Nagasaki, Japan; [3] Department of Global Environmental Health, Graduate School of Medicine, The University of Tokyo, Tokyo, Japan; [4] Department of Global Health Policy, Graduate School of Medicine, The University of Tokyo, Tokyo, Japan

Objective

Urbanization and climate change interact to amplify heat exposure, exacerbating health risks through the Urban Heat Island (UHI) effect. This study projects UHI-attributable mortality in the Tokyo Metropolitan Area, Japan, considering future climate and demographic changes.

Materials and Methods

A space-time-stratified case-crossover design was applied to estimate temperature–mortality associations by age group. Temperature-attributable mortality was projected under climate and population scenarios. UHI-attributable mortality was defined as the difference in temperature-related deaths estimated under urban versus non-urban temperatures.

Results

Under RCP 8.5 and SSP5, UHI-attributable heat-related deaths are projected to reach 11,270 [95% empirical confidence interval (eCI): 6,251–16,088] by the 2090s. In contrast, reduction of cold-related deaths due to the UHI effect are expected to decline to 12,655 (95% eCI: 10,633–14,332). Population aging will exacerbate UHI-attributable heat-related mortality among older adults (≥65 years), rising to 11,933 (95% eCI: 5,555–17,827) by the 2090s Under RCP 8.5 and SSP5. While older adults bear the highest absolute burden, the relative mortality fraction remains notable in younger populations. Spatially, coastal areas will experience fewer reduction of cold-related deaths, whereas inland municipalities will see a rise in UHI-attributable heat-related mortality, exceeding reduction of cold-related deaths by the late 21st century.

Conclusion

The projected increase in UHI-attributable heat-related mortality highlights the need for targeted adaptation strategies, particularly for older populations and inland municipalities. Urban planning and climate adaptation policies should prioritize heat risk mitigation while considering reductions in cold-related mortality.

Funding: This study was supported by the Environmental Research and Technology Development Fund (JPMEERF20231007) from the Environmental Restoration and Conservation Agency, Ministry of the Environment, Japan.

P44-06 | Trends in Heat-related EMS Activations and Transports in the U.S.

Chris Lim[1], Jonathan Race[2], Parneet Kaur[2], Ladd Keith[6], Honghyok Kim[5], Ran Wei[4], Xiaojiang Li[3], Lynn Gerald[5], Clay Mann[2]

[1] The University of Arizona, Health and Urban Modeling Lab, Tucson, USA; [2] University of Utah, Salt Lake City, USA; [3] University of Pennsylvania, Philadelphia, USA; [4] University of California Riverside, Riverside, USA; [5] University of Illinois Chicago, Chicago, USA; [6] The University of Arizona, School of Architecture and Landscape Planning, Tucson, USA

Background: Heat-related illness (HRI) remains a critical public health issue in the United States, particularly as seasonal temperatures continue to rise. To characterize trends in HRI-related emergency medical services (EMS) calls, we analyzed multi-year data (2018–2023) from the National EMS Information System (NEMSIS) dataset spanning all participating states and encompassing both urban and non-urban service areas.
Methods: We constructed a cohort of EMS agencies reporting every year during the study period from the NEMSIS dataset to calculate total HRI-related calls and incidence rates by calendar year and state. Temporal patterns were examined by individual-level risk factors (age, sex, race/ethnicity), month, time of day, year, neighborhood-level median income, and urbanicity.
Results: Preliminary findings indicate an overall increase in the number and incidence rate of heat-related EMS activations over the study period, with the highest call volumes typically occurring during peak summer months (July–August). Highest number of calls were observed indoors and during late afternoons. Incidence rates were significantly elevated among the elderly (>75 years old), males, African American and Indigenous populations, urban areas, and those living in middle-income neighborhoods.
Discussion: These results indicate that both environmental conditions and population-level characteristics jointly contribute to heat-related illness risks. These findings could inform targeted interventions and resource planning to mitigate the burden of heat exposure. Future analyses will further explore the association between heat and heat-related EMS activations.

P44-07 | Heat Stress and Its Association with Severe Stunting and Underweight in Low Vegetation Areas: Insights from Ethiopia's National Food and Nutrition Strategy Survey.

Daniel A. Denssa[1], Desalew M. Moges[1], Bedasa Tessema[1], Feyissa Challa[1], Meseret Woldeyohannes[1], Alemnesh Petros[1], Yetayesh Maru[2], Meron Girma[1], Stanley Chitekwe[2], Ramadhani Noor[2], Shilpa Rao-Skirbekk[3], Eleni Papadopoulou[3], Masresha Tessema[1]

[1] Ethiopian Public Health Institute, Addis Ababa, Ethiopia; [2] UNICEF Ethiopia, Addis Ababa, Ethiopia; [3] Norwegian Institute of Public Health, Oslo, Norway

Climate change has emerged as the most significant global threat to human health in the 21st century, significantly impacting food and water security and contributing to rising rates of malnutrition, especially in developing countries. While climate-related shocks pose health risks to all population, children are particularly vulnerable due to their rapid metabolism and developing organ systems. According to a UNICEF report from 2021, nearly half of the world's

children are at extreme risk of worsening exposure to climate and environmental shocks. This study analyzes anthropometric data from 6,659 children in Ethiopia's recent food and nutrition baseline survey, alongside satellite-based Universal Thermal Climate Index (UTCI) and Normalized Difference Vegetation Index (NDVI) data, matched to GPS-specific locations of the measurements. All satellite data processing and analysis were conducted using Google Earth Engine, with statistical analyses and visualizations performed in STATA software and R programming. Descriptive statistics (mean ± SD) were used to present exposure duration. Multivariate logistic regression was applied to examine relationships between the two datasets. Regional analysis of heat stress using UTCI index showed that the Afar, Gambela, and Somali regions experience longer durations of heat stress (>26ºC), with exposure lasting 1,319, 1,129, and 899 hours, respectively, in the three months preceding anthropometric measurements. Similarly, the lowest NDVI (mean ± SD) was observed in the Somali region, at 0.26 (0.08), followed by Afar at 0.30 ± 0.14 during the year of child birth. In contrast, Gambela region exhibited the highest NDVI value of 0.65 ± 0.07, suggesting a better vegetation cover. The finding showed that children living in low vegetation areas (NDVI<0.44) have higher odds of experiencing severe stunting (OR=1.25, CI= 1.02- 1.54) and underweight (OR=1.76, CI=1.39-2.23) compared to children in moderate to high vegetation covers (NDVI≥0.44). Hence, any climate adaptation strategy should be tailored to local environmental conditions.

P44-08 | Heatwave characterization and human health effects in the Upper San Francisco Bay Estuary (Sacramento-San Joaquin Delta), 1999 to 2022

Brian Mahardja[1], Samuel Bashevkin[2], Catarina Pien[3], Dharshani Pearson[4], Brittany Davis[5], Rupa Basu[6], The summaries and conclusions made in this abstract and any future presentations are the sole opinions of the presenting author, D. Pearson, and do not reflect any statements or opinions from the respective agencies.

[1] US Bureau of Reclamation, Sacramento, USA; [2] California State Water Resources Control Board, Sacramento, USA; [3] US Bureau of Reclamation, Bay-Delta Office, Sacramento, USA; [4] 2448 Climate & Health Consulting, Walnut Creek, USA; [5] California Department of Water Resources, Division of Integrated Science & Engineering, Sacramento, USA; [6] California Office of Environmental Health Hazard Assessment, Oakland, USA

Introduction: A vital resource for California’s agricultural economy, the Sacramento-San Joaquin Delta (Delta) houses California’s central water supply, serving 27 million people and 7,000 square miles of farmland. This water supply is already over-resourced, but less predictable wet seasons coupled with higher outdoor temperatures further threatens its overall health. Here we describe Delta heatwave trends over from 1999 to 2022 using monitored climatic data and summarize health consequences to human populations from literature.

Methods: Using daily maximum temperature and average relative humidity data from GRIDMET, we created daily maximum apparent temperature (a combination of temperature and relative humidity) at the ZIP code population-weighted centroid-level. We then developed a heatwave definition based on the 90th percentile of maximum apparent temperature for each of five Delta cities (Stockton, Sacramento, West Sacramento, Pittsburg, and Isleton). In the literature synthesis, we considered such factors as residents’ potential lack of acclimatization and the Delta’s agricultural economy coupled with ecological, animal, and human interactions (“One Health”).

Results: Heatwave temperature cutoffs increased from west to east geographically. Overall, all five cities experienced an average of 97 heatwaves encompassing 395 days across the analysis period. Heatwaves with the longest duration occurred during winter months. Heatwaves occurred 0.4 days more frequently, on average, each

year (p = 0.0002). Reviewing previous studies showed increases in heat-related illnesses, renal failure, cardiovascular, and mental health events. Other outcomes include increases in coccidioidomycosis (fungal infection), gastroenteric and neurological diseases from harmful algal blooms, and potential complications from pesticide volatilization and pathogen aerosolization.**Discussion:** The “Delta Breeze” phenomenon helps keep temperatures cooler, but Delta residents may be less acclimated to extreme heat during stagnant periods. Extreme heat and drought could increase invasive vectors carrying viruses, such as chikungunya. Because of the area’s agricultural economy, outdoor workers and rural communities will likely face greater impacts.

P44-10 | Independent and joint effects of historic redlining, urban heat, and climate-related hazards on cardiometabolic disorders in children

Eun Kyung Lee[1], Brooks B. Gump[2], Megan B. Cole[3], Andrea R. Titus[4], Sadeer Al-Kindi[5,6]

[1] SUNY College of Environmental Science and Forestry, Department of Environmental Studies, Syracuse, USA; [2] Syracuse University, Department of Public Health, David B. Falk College of Sport & Human Dynamics, Syracuse, USA; [3] Boston University School of Public Health, Department of Health Law, Policy, and Management, Boston, USA; [4] New York University Grossman School of Medicine, Department of Population Health, New York, USA; [5] Houston Methodist Debakey Heart and Vascular Center, Houston, USA; [6] Weill Cornell Medicine, New York, USA

BACKGROUND AND AIM: Natural disasters--such as extreme temperatures, flooding, hurricanes, and wildfires--are increasing in frequency and severity, with lasting health impacts. These extreme weather events heighten the risk of cardiometabolic disorders (CMDs), particularly among vulnerable populations. Those most affected include residents of historically disinvested neighborhoods partly shaped by redlining, as well as children, racially and ethnically minoritized groups, and individuals with lower wealth. This study examines the effects of independent and co-exposure to redlining, urban heat, and climate hazards on CMDs in children.

MATERIALS AND METHODS: Using statewide hospital data from New York, the Climate Vulnerability Index, and satellite-imagery data, we applied Poisson regression to assess the independent and joint effects of historic redlining, urban heat, and climate hazards on CMD-related ED and outpatient visits (2020-2022), with stratified analyses by race/ethnicity. Exposure was defined by (1) disinvested neighborhoods based on historic redlining (A/B grades=non-redlined, C/D=redlined); and (2) climate-risk indicators, including (2a) vulnerability to multiple climate risks (e.g., flooding, hurricanes, wildfires) and (2b) independent climate risks (i.e., urban heat, measured by land surface temperatures).

RESULTS: Based on 230,150 healthcare visits, living in high-climate-risk areas was independently associated with a 22% higher risk of CMD. However, no significant independent risk was found for children residing in redlined or high-heat areas. Co-exposure to redlining and high-climate-risk increased CMD-related risk by 20%, with significant associations observed among children residing in redlined and high-urban-heat areas (RR=1.14; 95%CI: 1.10–1.17). Hispanic, Black, and Asian children faced greater CMD-related risks from redlining, climate risk, and urban heat than other racial groups (RRs: 1.23-2.02).

CONCLUSIONS: This study found that children in high-climate-risk areas--co-exposed to disinvested neighborhoods, urban heat, and racial/ethnic marginalization--had an elevated CMD risk. Targeted, season-specific interventions (e.g., heat refuges, winter CMD management, pre/post-disaster planning) and greater awareness for the most affected children could help mitigate these impacts

P44-11 | Effect of Heat Exposure on Electrolyte Loss, Dehydration, and Hypotension Among Agricultural Workers in Northwestern Bangladesh

Farjana Jahan, Mahbubur Rahman, Nazmul Hossain, Titly Sen, Ruhul Amin, Rezaul Hasan

icddr,b, Environmental Health and WASH, Dhaka, Bangladesh

Background: High humidity and heatwaves in Bangladesh contribute to farmers' heat exhaustion, reducing their productivity and increasing risk of developing chronic heat-related health conditions. There are lack of information on the effect of heat on the health of farmers in Bangladesh climate context. This study assesses the physiological and biochemical impacts of heat exposure during a typical work shift of agricultural workers in Bangladesh.

Method: We conducted a cross-sectional study among 100 farmers in Naogaon district in Bangladesh, evaluating key health parameters before and after a 6-hour work shift during summer (April 1- May 30, 2024). Blood pressure (BP), sweat electrolytes (Na, Cl), serum electrolytes (Na^+, K^+, $Cl^−$), urinary electrolytes, urine specific gravity, and body temperature were measured pre and post-shift. Statistical analyses were performed using independent-sample t-tests and Mann-Whitney U tests.

Results: Significant changes were observed post-shift. Systolic BP dropped from 116 mmHg (IQR: 106.33–121.67) to 105.5 mmHg (IQR: 99–113.83) ($p < 0.001$), and diastolic BP decreased from 66.5 mmHg (IQR: 60.67–73.33) to 60.5 mmHg (IQR: 54.5–65.33) ($p < 0.001$), indicating hypotensive effects post shift. Serum sodium levels showed a slight increase (153.8 to 157.35 mmol/L), while serum potassium declined (4.33 to 4.23 mmol/L), but was not statistically significant. Urinary potassium ($p = 0.01$) and chloride ($p = 0.02$) excretion increased, suggesting renal compensatory mechanisms for heat stress. Urine specific gravity increased significantly post-shift (>1.015) ($p < 0.001$), indicating fluid loss. Wet bulb globe temperature (WBGT) increased from 30.75°C to 32.40°C ($p < 0.001$), confirming substantial heat exposure during work hours and sweat electrolytes positively correlated with increasing WBGT.

Conclusion: Prolonged heat exposure in agricultural workers leads to dehydration, electrolyte losses, and hypotension, which may contribute to heat-related illnesses. Preventive strategies, including hydration interventions and occupational safety measures, are crucial for mitigating these risks.

P44-14 | Climate Resilience Index for Assessing Heat-Related Injury Risk: a National Survey in Taiwan

Yao-Yuan Wang[1], Hsiao-Yu Yang[1,2]

[1] National Taiwan University, Institute of Environmental and Occupational Health Sciences, Taipei, Taiwan; [2] National Taiwan University, Population Health Research Center, Taipei, Taiwan

Background:
With global warming, there is a need for effective methods to assess community climate resilience in responding to heat hazards. This study developed a Climate Resilience Index and examined its association with heat-related injuries.
Method:
A nationwide climate resilience survey was conducted in 2023. The Climate Resilience Index comprised three core dimensions: health resilience, community adaptation, and public health preparedness. Health resilience indicators included median household income, household expenditure, volunteer-to-service recipient ratio, education level, and the proportion of communities with development associations. Community adaptation was assessed through a nationwide online survey on heat adaptation behaviors. Public health preparedness included healthcare personnel and hospital beds per 10,000 population, green space ratio, medical institutions per 10,000 population, community development associations, and government-registered volunteers. Each indicator was standardized using Z-normalization and aggregated to generate a Climate Resilience Index at the county/city level.
Result:
The Climate Resilience Index was calculated for all counties and cities in Taiwan in 2023. Higher resilience scores were observed in Taipei City (2.57) and New Taipei City (2.52), where heat injury incidence rates were lower. Conversely, Hualien (-2.56) and Yunlin (-1.08) had higher heat injury rates (27.72 and 17.44, respectively). Regression analysis showed a significant negative correlation between the Climate Resilience Index and heat injury incidence ($\beta = -3.523$, $P < 0.01$), indicating higher resilience is associated with lower heat injury risk.
Conclusion:
Climate resilience is linked to heat injury risk. This Climate Resilience Index provides a valuable tool for community assessments, supporting targeted interventions to enhance resilience.

P44-15 | Associations between Temperature and Location of Injury or Condition among Heat-Related Emergency Department Visits

Chen Li[1], Hua Hao[2], Morgan Lane[2], Noah Scovronick[2], Rebecca Zhang[1], Stefanie Ebelt[2], Howard H. Chang[1]

[1] Emory University, Department of Biostatistics and Bioinformatics, Atlanta, USA; [2] Emory University, Gangarosa Department of Environmental Health, Atlanta, USA

High ambient temperature poses significant health risk globally. However, the relative importance of different exposure pathways leading to health risks remains unclear. For 9 US states during 2016-2018, patient-level ED visit records for heat exhaustion and heat stroke (HEAT), fluid and electrolyte imbalance (FEI), volume depletion (VD), and acute kidney injury (AKI) were identified via diagnosis codes. Co-diagnosed Y92 subcodes (Y codes) were used to categorize the patient's location at the time of injury or condition. Logistic regressions were used to estimate nonlinear associations between same-day temperature and Y codes for 11 non-residential versus residential locations among heat-related ED visits, including stratified analyses by patient age, race, and ethnicity. Odds ratios (OR) were calculated between the 95th versus 50th percentile of temperature. Overall, higher temperature was associated with increased risks of ED visits with Y codes for non-residential locations. HEAT ED visits were more likely to have Y codes for streets compared to residential locations (OR:1.68, 95% CI: 1.12-2.51). Similarly, VD visits were more likely to have Y codes for industrial area (OR: 2.68, 95% CI: 1.98-3.63), farms (OR:7.66, 95% CI: 4.05-14.50), recreation areas (OR:2.25, 95% CI: 1.78-2.84), and streets (OR:1.54, 95% CI: 1.39-1.70), but were less likely to have Y codes for public places (OR: 0.89, 95% CI: 0.84, 0.94). Similar associations were observed for FEI and AKI ED visits. Locations associated with higher heat risks may be due to exposure to outdoor temperature and activities, supporting the need to develop targeted strategies and interventions that minimize heat exposure in these areas.

P44-16 | The impact of extreme heat on the risk of car crashes, injuries, and fatalities in California

Jennifer Bailey[1,2], Rosana Aguilera[3], Katie Crist[5], Andrew Nakamura[6], Joan Casey[4,6], Tarik Benmarhnia[3,7]

[1] University of California San Diego, Herbert Wertheim School of Public Health and Human Longevity Science, San Diego, USA; [2] San Diego State University, School of Public Health, San Diego, USA; [3] University of California San Diego, Scripps Institution of Oceanography, La Jolla, USA; [4] University of Washington, Department of Environmental & Occupational Sciences, Seattle, USA; [5] San Diego State University, School of Exercise and Nutritional Sciences, San Diego, USA; [6] Columbia University, Mailman School of Public Health, New York, USA; [7] University of Rennes, Irset Institut de Recherche en Santé, Environnement et Travail (IRSET), Rennes, France

In the United States, car crashes are the leading cause of death for people between the ages of 1 and 54. California was the second deadliest state in 2022, experiencing an almost 30% increase in fatal crashes compared to 2019 and where, on average, a traffic crash resulting in a fatality or injury is reported every 3 minutes. As temperatures increase due to anthropogenic climate change, little is known about how heat impacts the likelihood of traffic collisions. In this study, we quantified the association between extreme heat and the risk of traffic collisions, injuries, and fatalities as

well as the impact of various effect modifiers for census tracts across California during the 2010-2020 summer months. We utilized high resolution gridded daily temperature data and calculated a variety of extreme heat event definitions incorporating threshold percentiles and days of duration to classify each day as a heatwave or non-heatwave day per census tract. A time stratified case-crossover design was used to assess the association between heatwaves and each traffic collision-related outcome of interest provided by the California Highway Patrol. Sociodemographic variables, collision-related factors, and neighborhood level built environment aspects were considered for their potential to modify the relationship between heat and collisions. As the analyses are ongoing, we anticipate that extreme heat events increase the odds of experiencing a traffic collision, injury, or fatality compared non-heatwave days, and that the odds estimates vary across individual and community-level effect modifiers such as age, sex, income, race/ethnicity, urbanicity, and walkability. This research is the first study that quantifies the association between heat and the risk of vehicle crashes, injuries, and fatalities in California. This investigation provides additional justification for policies that advocate for non-vehicular modes of transportation as a viable means to reduce emissions and improve safety in a warming world.

P44-18 | Too hot to move: Summer heat, sedentary behavior, and physical activity of children

Kevin Lanza[1], Baojiang Chen[1], Perry E. Sheffield[2], Deborah Salvo[3], David Gimeno Ruiz de Porras[4], Umberto Villa[3], Julia M. Gohlke[5]

[1] The University of Texas Health Science Center at Houston, Austin, USA; [2] Icahn School of Medicine at Mount Sinai, New York, USA; [3] University of Texas at Austin, Austin, USA; [4] The University of Texas Health Science Center at San Antonio, San Antonio, USA; [5] Virginia Tech, Blacksburg, USA

Objective: Most children in the United States do not meet health-based recommendations for sedentary behavior and physical activity, especially during summer; however, little is known about the role of extreme heat on these behaviors. We determined how outdoor temperatures relate to children's sedentary behavior and moderate-to-vigorous physical activity (MVPA) outdoors and indoors during summer in a hot and humid climate.

Material and Methods: This study, conducted from July 19–August 9, 2023, in Austin, Texas, United States, measured sedentary behavior and MVPA in 15sec intervals by accelerometers worn by children ages 8–10 (n = 25) from three schools for seven continuous days during waking hours. We used Evenson cut points to estimate physical activity intensity and 240lx and above from the accelerometer's ambient light sensor to signify outdoor time. Meteorological data originated from the Camp Mabry weather station. To test within-child associations between temperature and outcomes (sedentary behavior, MVPA) every 15 seconds, we specified multilevel mixed-effects negative binomial regression models for outdoor and indoor time separately. Final models adjusted for time of day; relative humidity; child's sex, race/ethnicity, and poverty level; and included random effects for child and date.

Results: On average during waking hours, temperatures were 36.3°C (SD = 3.3°C) and children spent over seven hours (443min, SD = 86min) being sedentary and 36min (SD = 30min) engaging in MVPA daily. In fully adjusted models, we found that higher temperatures are associated with increased sedentary behavior indoors (RR = 1.02, 95% CI 1.01–1.02, p<0.001) and decreased MVPA both outdoors (RR = 0.79, 95% CI 0.72–0.87, p<0.001) and indoors (RR = 0.92, 95% CI 0.89–0.95, p<0.001).

Conclusion: Extreme heat may cause children to engage in less MVPA and instead be sedentary indoors. Interventions should focus on promoting safe physical activity during summer, especially with projected warming from climate change.

P44-19 | Extreme heatwave and mortality risk among people with disabilities in Korea

Kyung-Hwa Choi[1,2], Jonghyuk Choi[1,2], Ho-Jang Kwon[1,2], Hyungryul Lim[3]

[1] Dankook University College of Medicine, Department of Preventive Medicine, Cheonan, South Korea; [2] Dankook University College of Medicine, Research Institute of Healthcare Bigdata, Cheonan, South Korea; [3] Ajou University School of Medicine, Department of Preventive Medicine and Public Health, Suwon, South Korea

Objective: This study evaluates whether extreme hot temperatures increase mortality risk among people with disabilities (PWD) compared to non-disabled (ND).
Methods: Data on daily death by age, sex, district were obtained from the Korean National Health Insurance Service (NHIS) for whole PWD (43.9 million person-years (PY) and 0.4 million death) and the Statistics Korea for ND (894 million PY and 1.5 million death) during the summer, 2002-2021. Disability type and severity were obtained via NHIS DB. Daily maximum temperatures (DMT) from May to September were obtained from the Korea Meteorological Administration. To assess mortality risk for 30℃ and 33℃ compared to 25℃ in DMT, we performed a case time series with a distributed lag non-linear model to DMT adjusting for relative humidity, population, and regional deprivation index. All analyses were stratified with disability, severity, type of disability, age (years, <65 and ≥65), and sex.
Results: Observations exceeding 33°C accounted for 8.0%. The mortality risk for 33℃ compared to 25℃ was higher in PWD (RR=1.07, 95% CI: 1.05–1.09) than ND (RR=1.04, 95% CI: 1.03–1.05). Severe disabilities showed greater risks (RR=1.08, 95% CI: 1.05–1.11) than mild disabilities. Risks at 33°C were significant for young PWD (RR=1.05, 95% CI: 1.01–1.10), old PWD (RR=1.07, 95% CI: 1.05–1.10), and old ND (RR=1.05, 95% CI: 1.04–1.06), while not in young ND. Intestinal/urinary fistula (RR=1.11 at 30°C, 95% CI: 1.04–1.17) and cardiac dysfunction (RR=1.26 at 33°C, 95% CI: 1.16–1.38), showed the highest risks among 15 disability types.
Conclusion: Extreme heat poses a higher mortality risk for PWD, particularly those with severe disabilities and certain health conditions. Further research is needed to explore underlying causes and various temperatures.
Funding: This work was supported by the National Research Foundation of Korea grant funded by the Korea government (No. 2023R1A2C1002801).

P44-20 | Lag associations of extreme heat and suicide mortality within race and sex groups in Michigan, USA, 2006-2024

Peter S. Larson[1,4], Jaemin Jeon[2], Abigail R. Nacht[2], Robert Melendez[3], Carina J. Gronlund[3], Douglas J. Wiebe[4,1]

[1] University of Michigan, Epidemiology, Ann Arbor, USA; [2] University of Michigan, Literature, Science and the Arts, Ann Arbor, USA; [3] University of Michigan, Institute for Social Research, Ann Arbor, USA; [4] University of Michigan, Injury Prevention Center, Ann Arbor, USA

Objective: Extreme temperature has been associated with risk for suicide in multiple studies but impacts might differ between demographic groups. This research uses vital record data from the United States to test associations of exposure to extreme temperature and suicide mortality for all suicide deaths and between sex and racial groups in a temperate Midwestern state in the United States

Materials and Methods: We obtained records for all deaths in Michigan, USA for years 2006 to 2024, using only those records which listed suicide related ICD10 codes as a cause of death. Data included age, sex and racial group. Latitude/longitude locations of residential addresses were added to the database. We extracted temperature for the date of death and all 21 days previous from raster datasets (PRISM Climate Group). We used a case-crossover design, where everyone served as their own control, staggering exposure days before and after the date of death. We tests lag associations of temperature/precipitation and mortality using stratified regression models within a directed non-linear modelling framework (DNLM), including the crossbasis for temperature.

Results: There were 31,128 suicides in Michigan between 2006 and 2024. 23,570 (75.7%) were male and 26,968 (86.6%) were white. Among all suicides, the cumulative effect of exposure to extreme temperatures (>90 degrees F) (21 days) was elevated (OR 1.26 95% CI [1.0, 1.58]). There were significant associations of temperature with suicide for Males (OR 1.38 95% CI [1.06, 1.79]) and for white people (OR 1.38 95% CI [1.08, 1.76]) but not for Females (OR .96 95% CI [0.6, 1.53]) or Black people (OR 0.9 95% CI[0.43, 1.90]).

Conclusions: Sustained exposure to extreme heat is associated with increased suicide risk in Michigan, USA particularly for Males and white decedents. Future work should explore demographic disparities in extreme weather and many other mental health and injury outcomes.

P44-21 | Mapping Community Health Vulnerability to Heat in a Mediterranean City of the Global South

Raquel B. Jimenez[1,2], Simon Inzunza[2], Nikole Guerrero[2], Magdalena Gil[3,2], Nicolas Bronfman[1,2]

[1] Universidad Andres Bello, School of Engineering, Santiago, Chile; [2] Research Center for Integrated Disaster Risk Management (CIGIDEN), Santiago, Chile; [3] Pontificia Universidad Católica, School of Government, Santiago, Chile

Aim: The global population increasingly experiences heatwaves and extreme heat events due to climate change, posing significant public health threats. The severity and distribution of heat-related health outcomes vary across geographic regions and demographic groups, with vulnerable communities often disproportionately impacted. Assessing heat vulnerability involves the complex interplay of demographic and ecological factors. This research developed a spatially explicit index to evaluate community health vulnerability to heat in Santiago, Chile—a Mediterranean city in the Global South.

Materials & Methods: Vulnerability was conceptualized as a multidimensional construct encompassing three dimensions: health susceptibility, exposure, and adaptive capacity, within the context of adverse public health outcomes. We estimated a sub-index for each dimension using principal component analysis, incorporating variables known to affect heat-related health outcomes informed by existing evidence, data availability, and local relevance. Each sub-index was normalized from 0 to 1 and combined multiplicatively to derive a heat vulnerability index (HVI) at the census tract level. We used descriptive statistics to analyze the distribution of sub-indices and the HVI, assessed the population's exposure across varying HVI levels, and explored associations between these measures and ambient temperature exposure.
Results: Districts with low adaptive capacity and high susceptibility often presented elevated exposure levels. Our analysis revealed that 1,279,409 individuals, or 20.3% of Santiago's population, reside in the highest vulnerability quintile. Additionally, 1.33 million (21.5%), 1.29 million (20.8%), and 1.23 million (19.7%) individuals belong to the lowest adaptive capacity decile, highest sensitivity decile, and highest exposure decile, respectively. Spatial distribution analyses showed significant differences in HVI, with the northern and western city sectors exhibiting the highest levels of vulnerability.
Conclusion: Understanding health vulnerability to heat in urban centers across diverse climatic contexts is crucial for developing effective preparedness and response strategies for extreme heat events.

P44-22 | Mortality risk attributable to extreme temperatures in 49 cities in Chile.

Salvador Ayala[1,2], Rodrigo Puentes[3], Alejandra Dünner[4], Natalia Santis[5], Alejandra Vaquero[4], Rayana Palharini[6], Mario Soto-Marchant[7], Janepsy Diaz[4]

[1] Universidad del Desarrollo, Santiago, Chile; [2] Universidad de Chile, Santiago, Chile; [3] Emory University, Gangarosa Department of Environmental Health, Rollins School of Public Health, Atlanta, USA; [4] Instituto de Salud Pública de Chile, Departamento Agencia Nacional de Dispositivos Médicos, Innovación y Desarrollo, Santiago, Chile; [5] Universidad de Chile, Departamento de Tecnología Médica, Facultad de Medicina, Santiago, Chile; [6] Universidad Tecnológica Metropolitana, Departamento de Prevención de Riesgos y Medio Ambiente, Santiago, Chile; [7] Universidad Diego Portales, Escuela de Tecnología Médica, Facultad de Salud y Odontología, Santiago, Chile

Objective: To estimate mortality attributable to non-optimal temperatures in 49 cities of Chile, from 2000 to 2019.
Material and Methods: We use all non-external causes of death and temperature data from ERA5-Land. We then fitted a time-series quasi-Poisson regression model controlling for time-varying confounders. We used a distributed lag non-linear framework to estimate the temperature-mortality associations in 49 cities in Chile and pooled the results in a multivariate meta-regression, including geographic zones and temperature as meta-predictors. Extreme temperature effects were evaluated for both cold and heat at each city's 2.5th and 97.5th temperature percentiles. We estimated relative risks (RR) and attributable deaths due to non-optimal temperatures for different percentile ranges. Associations were assessed by sex, age group, and causes (all, cardiovascular, and respiratory).
Results: We analyzed 1,276,642 deaths in the study period. At the country level, the all-cause attributable fraction (AF) for non-optimal temperatures was 5.90% (95% confidence interval (CI): 4.38%-7.13%). The AF for extreme cold was 0.67% (95% CI: 0.58%-0.73%), and 0.18% (95% CI: 0.11%-0.23%) for extreme heat. For cardiovascular diseases, the AF for non-optimal temperatures was 9.64% (95% CI: 6.52%-11.58%), 0.96% (95% CI: 0.80%-1.07%), and 0.18% (95% CI: 0.06%-0.26%) for total, extreme cold, and extreme heat, respectively. For respiratory diseases, the AF for non-optimal temperatures was 12.66% (95% CI: 6.60%-14.88%), 0.95% (95% CI: 0.61%-1.12%), and

0.32% (95% CI: 0.11%-0.40%) for total, extreme cold, and extreme heat, respectively. Geographic zones and temperature range meta-predictors did not explain substantial heterogeneity.
Conclusion: In Chile, the highest temperature-attributable deaths were caused by cold. However, heat-related mortality was acute in several cities. This study highlights city-specific population vulnerabilities to non-optimal temperatures; thus, climate assessments must consider local characteristics. Results from this study will contribute to evaluations of the effects of non-optimal temperatures on mortality and support the development of climate adaptation policies.

P44-24 | Forecast skill of the first Pan-European heat health early warning system during the deadliest summers of 2022-2024

Tomáš Janoš[1,2], Marcos Quijal-Zamorano[1], Natalia Shartova[1], Elisa Gallo[1], Raúl F. Méndez Turrubiates[1], Nadia D. Beltrán Barrón[1], Fabien Peyrusse[1], Joan Ballester[1]

[1] ISGlobal, Barcelona, Spain; [2] Masaryk University, RECETOX, Faculty of Science, Brno, Czech Republic

Continuous record-breaking summer temperatures and associated mortality burden raise the urgent question of how to better protect vulnerable populations. Here, we aimed to estimate heat-related mortality in Europe during the summers of 2022-2024 and to assess the forecast skill of Forecaster.health (*https://forecaster.health/*), a new generation of impact-based early warning systems, to anticipate health emergencies.
We used state-of-the-art epidemiological models fitted with the newly-created daily mortality database of the EARLY-ADAPT project, covering 654 contiguous regions across 32 European countries, and representing their entire population of 539 million people. These models were then combined with the daily timeseries of temperature observations or forecasts and the weekly timeseries of mortality counts to calculate the daily and summer heat-related mortality numbers over the years 2022-2024.
We estimated more than 180,000 heat related deaths in Europe during the last three summer seasons. The death toll in 2024 (62,669; 95% confidence interval = 36,288–85,025) largely exceeded the burden in 2023 (50,739; 28,945-69,024), but was somewhat smaller than that of 2022 (67,455; 37,449; 92,951). We showed that health emergency events can be reliably forecast at least one week in advance across the entire continent, even during these exceptionally warm summers (area under the ROC curve > 0.8). The forecast skill, as showed by AUC, was high even beyond this one-week horizon in Southern Europe, where heat-related risks and burden are stronger. Moreover, this skill was equal across all sex and age groups.
The findings of the study have important implications for public health agencies and other end-users across Europe. The adoption of the system would enable reliable heat-health emergency alerts at least one week ahead, which is the relevant time window in which stakeholders take effective actions to reduce preventable deaths. This offers invaluable potential, particularly in the most vulnerable countries and population groups.

P44-25 | Thresholds of heat warning system to reduce 50% of preventable heatstroke mortality in Japan

Vera Ling Hui Phung[1], Yasushi Honda[2], Kazutaka Oka[2], Lina Madaniyazi[3], Chris Fook Sheng Ng[1], Aurelio Tobias[4], Masahiro Hashizume[1]

[1] The University of Tokyo, Department of Global Health Policy, Tokyo, Japan; [2] National Institute for Environmental Studies, Center for Climate Change Adaptation, Tsukuba, Japan; [3] Nagasaki University, School of Tropical Medicine and Global Health, Nagasaki, Japan; [4] Spanish Council for Scientific Research, Institute of Environmental Assessment and Water Research, Barcelona, Spain

Objective: Heat warning system in Japan has been updated in 2020, with the implementation of heat alert issuance when the daily maximum wet bulb globe temperature (WBGT) exceeds 33°C, and community level mitigation measures when it exceeds 35°C. This study re-evaluated the system in consideration of heat-health relationship and identified the WBGT for a target of 50% reduction in preventable heat mortality.
Methods: With a time-stratified case-crossover design, we applied a conditional quasi-Poisson regression and distributed lag non-linear model to estimate attributable heatstroke mortality during summer months (May to September) from 2010 to 2019. In line with the national goal to reduce 50% preventable heatstroke mortality, we quantified the preventable numbers and identified key WBGT thresholds. Subgroup analyses were conducted for age groups, sex, and summer phases.
Results: There were 9,702 total heatstroke deaths during the study period. The preventable number was estimated approximately 2–3% under the current heat alert threshold (WBGT 33°C). In most prefectures, the key threshold was identified at approximately 31°C WBGT, while lower than this threshold may be necessary for prefectures with cooler climate conditions located in the north. Prefecture-specific key thresholds ranged approximately 24°C to 30°C in early summer and 28°C to 32°C in late summer.
Conclusion: WBGT at approximately 31°C was identified as the key threshold to reduce 50% heatstroke deaths in most of the prefectures in Japan. Early mitigation measures when these thresholds are reached could enhance the effectiveness of heat warning systems.

Acknowledgement: This research was performed by the Environment Research and Technology Development Fund (JPMEERF20231007) of the Environmental Restoration and Conservation Agency provided by Ministry of the Environment of Japan.

P44-26 | Comparing Heatwave Related Mortality Data from Distressed Counties to Affluent Counties in Central and Southern Central Appalachia

Ying Li, Miranda T. Pardue

East Tennessee State University, Environmental and Occupational Health and Safety Sciences, Johnson City, USA

The Appalachian Mountains are home to some of the most culturally rich places in the United States, but also some of the most impoverished communities as well. Several recent events support climate change across the globe. It is expected that Appalachian communities may suffer more dire consequences, as many communities lack strategies to help relieve some of the worst effects of climate change. Heatwaves are predicted to increase in duration and frequency over time, and communities that are not well prepared for the damaging effects of heatwaves can suffer unduly. This study aims to quantify the likelihood that people living in economically distressed counties in the Central and Southern Central regions of Appalachia will face heatwave related mortality more intensely than those who live in more affluent counties in the same regions. Twelve counties from each socioeconomic group have been selected based on the county economic status to analyze climate and mortality data over thirty-eight years starting in 1981 and ending in 2018. Data was collected during the warm season for each county, May 1st to September 30th, and compared to the mortality data from the same county during the same warm season. This study used all-cause mortality numbers from each of the twenty-four counties for the mortality data. The relative risk for each county in both the distressed and affluent categories was calculated. The average relative risk for each socioeconomic status were then compared. The results of this study did not show statistical significance in the likelihood that being in a socioeconomically distressed county increases one's chances of succumbing to heatwave related mortality in the Central and Southern Central regions of Appalachia.

P44-27 | Acute health effects of exposure to air pollution and heat in Florida

Xiuling Zhao, Bertram Melix, Christopher K. Uejio

Florida State Unversity, 1Department of Geography, Tallahassee, USA

Objective: Short-term exposure to air pollution and heat can individually or interactively trigger adverse health effects and further increase morbidity and mortality worldwide. However, health risk estimates from large scales like the national level have limited accuracy in local applications due to variations in pollution levels, climate, and population adaptation. This study focused on the state of Florida in the US, aiming to estimate the individual and interactive effects of O_3 and Heat on localized human health outcomes.
Methods: We applied time-series generalized additive models to estimate the individual and interactive effects of heat index and O_3 on emergency room visits (ER) for all causes (n=79378186), cardiovascular (n=1417836), respiratory (n=8453462), heat stress (n=581), immune diseases (n=4936), and mental health (n=1585520), using daily records from 2000 to 2016.Subgroup analysis by sex and age.

Results: For all causes, a 1 °C and μg/m^3 increase in heat index and O_3 were significantly associated with a 0.65% (95% confidence interval [CI]: 0.61%-0.68%) and 1.46% ([CI]: 1.29%-1.63%) increase in ER. The exposure-response relationship between heat and ER was inverted "U" shaped, while the relationship between O_3 and ER was nearly linear. The estimated risks of heat and O_3 were comparable within sex showing a little higher in males than in females, but much higher in younger people with age < 18 than older ones. The risks also varied among different types of diseases. Interaction effects between heat and O_3 were statistically significant for all types of diseases and subgroups, with stronger effects at higher levels of heat and O_3 concentrations.
Conclusion: This study suggests that increased exposure to heat and O_3 concentrations may increase morbidity for most of the diseases and all subgroups, and the risks could be intensified by the interaction between heat and O_3 in Florida.

P44-28 | Understanding The Relationship Between Drug Overdose Mortality Events and Heat Stress as a Function of Climate Change in West Virginia, 1999-2015

Uma M. Govindswamy[1], Yun Hang[1]

[1] University of Texas Health Science Center at Houston, Department of Environmental & Occupational Health Sciences, School of Public Health, Houston, USA; [2] University of Texas Health Science Center at Houston, Department of Environmental & Occupational Health Sciences, School of Public Health, Houston, USA

Climate change, and accompanying extreme weather, has a broad influence on human and environmental health. There are many studies that focus on the intersection of climate change induced heat stress and physical health. The body of literature detailing the relationship between climate and mental health is growing; however, there is a smaller focus on the impact on substance abuse. This study investigates how cumulative heat stress due to climate change influenced the drug crisis in West Virginia between 1999-2015. Using a data set that contained spatial, environmental, demographic, deprivation indices, and housing variables, we were able to conduct statistical and spatial analyses to investigate the correlation between heat stress and overdose mortality. The variety of additional variables gave further insight into the associated socio-economic stressors, including economic instability, limited access to healthcare, and challenges within the built environment. The findings of this study indicate that heat stress does play a small but significant role in the variability of overdose mortality deaths and reinforces the need for continued research on climate change and substance abuse. Based on the findings, we recommend that climate considerations are integrated into substance abuse policy and interventions to optimize resource allocation and programming sustainably and efficiently.

P44-29 | Cold Mortality Risk Vulnerability and Protection in China: The Role of Functional and Cognitive Impairments and the Trade-Offs Between Winter Heating and Air Pollution

J. S. Ji[1]

[1] Tsinghua University, Vanke School of Public Health, Beijing, China

Background: Cold-related mortality represents a significant health risk in China, particularly among older adults. The winter heating policy in northern China, intended to mitigate cold exposure, has inadvertently led to elevated levels of air pollution.
Methods: We assessed the Chinese Longitudinal Healthy Longevity Survey (CLHLS), comprising 13,527 participants (median age=89) and 4659 winter mortalities from 2008 to 2018. Daily cold spell exposure was assessed, and a time-varying Cox proportional hazard model was employed to account for the daily variation in cold spells and environmental exposure, adjusting for individual demographics, functional status, comorbidities, and socio-economic characteristics. Additionally, we examined the trade-offs between cold protection and air pollution-induced mortality in a cohort of 5,334 older adults living around the Huai River between 2000 and 2018.
Findings: Cold spell exposure significantly increased mortality risk, with hazard ratios (HRs) ranging from 1.87 to 2.06. The most vulnerable populations were those with functional impairments, particularly those dependent on activities of daily living (HRs ranging from 2.48 to 3.74), and those with cognitive impairments, including attention and memory deficits (HRs ranging from 2.23 to 2.41). Gender differences were observed, with older women facing a higher risk (HRs ranging from 2.17 to 2.27) compared to men (HRs ranging from 1.5 to 1.89). The winter heating policy was associated with a 22% reduction in mortality; however, the benefits were offset by a 27.8% increase in mortality due to elevated PM2.5 levels during the heating season.
Conclusion: The trade-off between cold protection and air pollution-induced mortality suggests that future policies should balance the protective effects of winter heating with efforts to reduce air pollution.

P45 | Environmental Exposures and Aging - PART II

P45-01 | Resilient Epigenomes: DNA methylation signatures of smoking in a high-longevity “Blue-Zone” region of Costa Rica

Andres Cardenas[1], Nicole Gladish[1], Dennis Khodasevich[1], Anne Bozack[1], Julie MacIsaac[2], Kristy Dever[2], Michael Kobor[2], William Dow[3], Luis Rosero-Bixby[4], David Rehkopf[1]

[1] Stanford University, Epidemiology and Population Health, Stanford, USA; [2] University of British Columbia, Department of Medical Genetics, Vancouver, Canada; [3] Division of Health Policy and Management, University of California, Berkeley, Berkeley, USA; [4] Centro Centroamericano de Población (CCP), Universidad de Costa Rica, San José, Costa Rica

Objective: Smoking remains highly prevalent despite extensive evidence demonstrating its contribution to morbidity and mortality. One of the most consistent and generalizable findings from epigenetic studies is the unique signature of smoking on the epigenome. We hypothesized that participants from a high-longevity "Blue-Zone" region would exhibit epigenomic resiliency to smoke exposure.

Material and methods: We evaluated smoking-associated DNA methylation signatures in N=1,021 Latino participants from the Costa Rican Study on Longevity and Healthy Aging (CRELES), including participants from the high-longevity region of the Nicoya Peninsula (n=190). We measured DNA methylation at 788,441 high-quality CpGs using the Illumina EPIC array. Linear models were used to test smoking associations adjusted for age, sex, BMI, education, wealth, cell-type composition, and region. Interaction models evaluated effect modification for smoking among participants in the high-longevity region compared to the rest.

Results: Participants were on average 79 years old, 54.3% female and 67 (6.6%) currently smoked. There were 8.4% current smokers in the high-longevity region and 6.1% in the rest of the sample. Overall, 256 CpGs were differentially methylated (FDR<0.05) among current smokers, recapitulating genes previously reported (*i.e. AHRR, GPR15*). There was evidence of effect modification ($FDR_{interaction}<0.05$) in the high-longevity region for 5 CpGs annotated to four genes (*DUS2, SERPINE2, FAM186B*, *XIRP2*). The top CpG mapped to *DUS2* was hypermethylated among smokers living in the high-longevity region (β=23.9%, P=4x10^{-32}) but not in the rest of the sample (P=0.27). Previous work has shown that *DUS2* is upregulated in pulmonary carcinogenesis.

Conclusion: In this Costa Rican cohort, we recapitulated previously reported DNA methylation associations among smokers. For smokers living in the high-longevity region there was evidence of differential response in genes associated with emphysema (*SERPINE2*), cardiovascular health (*XIRP2*), and lung cancer (*DUS2*). Our findings suggest that epigenomic resiliency may contribute to exceptional longevity in this region.

P45-02 | Prenatal and early-life arsenic exposure and epigenetic aging among adults in Northern Chile

Anne Bozack[1], Fenna Sillé[2], Catterina Ferreccio[4], Johanna Acevedo[3], Craig Steinmaus[5], Martyn Smith[5], Rosemarie de la Rosa[5], Andres Cardenas[1]

[1] Stanford University, Department of Epidemiology and Population Health, Stanford, USA; [2] Johns Hopkins University Bloomberg School of Public Health, Department of Environmental Health and Engineering, Baltimore, USA; [3] Universidad del Desarrollo, School of Medicine, Santiago, Chile; [4] Pontificia Universidad Católica de Chile, Advanced Center for Chronic Diseases (ACCDiS), School of Medicine, Santiago, Chile; [5] University of California, Berkeley, Division of Environmental Health Sciences, School of Public Health, Berkeley, USA

Objective: Arsenic exposure increases the risk of cancer and age-related diseases. Epigenetic clocks, biomarkers of biological aging, may be sensitive to arsenic exposure and help predict arsenic-related disease risk. Within a natural experiment, we investigated associations of prenatal and early-life arsenic exposure with epigenetic age deviation (EAD) in adulthood.

Materials and Methods: In Northern Chile, residents were exposed to high drinking water arsenic concentrations between 1958-1970, followed by a sharp decline after the installation of filtration plants. Water arsenic concentrations at birth were estimated using historical records. Among adults differentially exposed to arsenic (N=358, mean age=65.4 years), DNA methylation in blood clots was measured using the Illumina MethylationEPICv2.0 BeadChip. EAD was calculated for validated epigenetic clocks: Horvath1, Horvath2, Hannum, PCPhenoAge, PCGrimAge, DunedinPACE, and DNA methylation telomere length (DNAmTL). We tested associations for arsenic concentrations at birth with EAD using robust linear models adjusting for age, age^2, sex, cell-type proportions, smoking, race and ethnicity, BMI, and socioeconomic status (SES) in adulthood.

Results: Arsenic water concentrations at birth ranged from 0-860 ug/L. Each doubling of arsenic was associated with 0.19 years (95% confidence interval (CI)=0.08, 0.31) and 0.13 years (0.02, 0.24) greater Hannum and PCPhenoAge EAD, respectively, and -0.006 kilobase (-0.01, -0.001) lower DNAmTL. There was a significant interaction between arsenic and SES for associations with PCPhenoAge and DunedinPACE, (p_{int}<0.05). Among participants in the lowest two SES tertiles, arsenic was positively associated with PCPhenoAge (B=0.20; 95% CI=0.06, 0.35) and DunedinPACE (B=0.04 standard deviations; 95% CI=-0.001, 0.07); associations were null among participants in the highest SES tertile.

Conclusion: Arsenic exposure in early life was associated with increased biological aging in adulthood, with greater effects among participants with lower SES. Our results suggest that individuals with low SES might be at the highest risk for arsenic-associated health effects. Supported by NIEHS grant P42ES004705.

P45-03 | The effect of neighborhood environment on cognition among racially and ethnically diverse American older adults

Edna Patricia Mendoza[1], Kai Zhang[1], Yuexia Zhang[2], Sid O'Bryant[3,4], Raul Vintimilla[4], Leigh Johnson[3,6], Nicole Phillips[3,5], Malinee Neelamegam[1], Lubnaa Abdullah[4], Robert Barber[3,4], Zhengyang Zhou[1,3]

[1] University of North Texas Health Science Center, Department of Population and Community Health, Fort Worth, USA; [2] University of Texas at San Antonio, Department of Management Science and Statistics, San Antonio, USA; [3] University of North Texas Health Science Center, Institute for Translational Research, Fort Worth, USA; [4] University of North Texas Health Science Center, Family Medicine and Osteopathic Manipulative Medicine, Fort Worth, USA; [5] University of North Texas Health Science Center, Department of Microbiology, Immunology, and Genetics, Fort Worth, USA; [6] University of North Texas Health Science Center, Department of Pharmacology and Neuroscience, Fort Worth, USA

Objective: Residents of socioeconomically disadvantaged neighborhoods face heightened risks of adverse health outcomes, including cognitive impairment and Alzheimer's Disease (AD). Understanding the relationship between neighborhood socioeconomic conditions and cognitive aging is critical for addressing AD-related health disparities. This study investigates the association between neighborhood environment, measured by the Area Deprivation Index (ADI), and cognitive functions.

Material and Methods: The study analyzed data from 2,916 participants aged 50 and above from the Health and Aging Brain Study – Health Disparities cohort. Participants included African Americans (AA; n = 758), Hispanics (n = 1,077), and Non-Hispanic Whites (NHW; n = 1,081) from the Dallas/Fort Worth area in Texas. ADI was dichotomized into two categories to represent low (1-50 percentiles) and high (51-100 percentiles) neighborhood disadvantage. A doubly robust estimation model adjusted for propensity scores was used to estimate the total effect of low vs. high neighborhood disadvantage on four cognitive domains: episodic memory, executive function, processing speed, and language ability. Analyses were stratified by racial/ethnic groups.

Result: Statistically significant effects of ADI on all four cognitive domains were observed among Hispanic participants, indicating that residing in a disadvantaged neighborhood environment is associated with worse cognitive functioning. Specifically, Hispanics in areas with greater neighborhood disadvantage exhibited poorer performance in episodic memory ($\beta = -0.10$, 95% CI [-0.19, -0.01]), executive function ($\beta = -0.20$, 95% CI [-0.29, -0.10]), processing speed ($\beta = -0.25$, 95% CI [-0.26, -0.23]), and language ability ($\beta = -0.11$, 95% CI [-0.21, -0.01]). No statistically significant effects of ADI on cognition were observed among AA or NHW participants.

Conclusion: This study indicates that neighborhood environment differentially affects cognitive function among Hispanic, AA, and NHW populations and highlights the disproportionate cognitive burden experienced by Hispanic individuals living in socioeconomically disadvantaged neighborhoods. Future research should investigate the ecological mechanisms underlying these disparities.

P45-04 | Impact of Menopause and Cardiometabolic Risk Factors on the Incidence of Metabolic Dysfunction-associated Steatotic Liver Disease (MASLD) in Women

I-Jung Feng[1,2], Ming-Lun Yeh[2,3], Pei-Chien Tsai[3,4], Ming-Lung Yu[3]

[1] National Sun Yat-sen University, Institute of Precision Medicine, Kaohsiung, Taiwan; [2] National Sun Yat-sen University, School of Medicine and Doctoral Program of Clinical and Experimental Medicine, College of Medicine and Center of Excellence for Metabolic Associated Fatty Liver Disease, Kaohsiung, Taiwan; [3] Kaohsiung Medical University Hospital, Hepatobiliary Division, Department of Internal Medicine, Kaohsiung, Taiwan; [4] Kaohsiung Medical University Hospital, College of Medicine and Center for Liquid Biopsy and Cohort Research, Kaohsiung, Taiwan

Background and Aims: Recent studies have demonstrated a notable increase in the risk of metabolic dysfunction-associated steatotic liver disease (MASLD) among postmenopausal women. This heightened risk is potentially linked to changes in estrogen levels and a higher prevalence of cardiometabolic risk factors (CMRFs), such as obesity (BMI ≥ 23), hypertension, hypertriglyceridemia, reduced HDL, and diabetes mellitus (DM). This study aims to explore the impact of menopause on MASLD risk, with a particular focus on the distribution of these CMRFs.
Methods: We utilized a comprehensive dataset from the Taiwan MJ Health Database, encompassing records from 1996 to 2022. The cohort included women aged 45-65, stratified into postmenopausal and premenopausal groups. We assessed MASLD incidence in relation to five categorized CMRFs, with particular focus on the prevalence of DM and abnormal triglyceride levels, alongside the use of hormone replacement therapy (HRT) in postmenopausal women.
Results: Our findings reveal that postmenopausal women exhibit a higher incidence of MASLD compared to their premenopausal counterparts. Notably, the risk escalates significantly in postmenopausal women without overweight (BMI>=23). Furthermore, postmenopausal women using HRT displayed an elevated risk of MASLD when presenting with isolated triglyceride abnormalities, underscoring the nuanced interplay between menopausal hormonal changes and metabolic risk factors.
Conclusions: The study underscores the critical impact of menopause and specific CMRFs on MASLD risk among women. It highlights the need for targeted screening and management approaches for postmenopausal women, particularly those with DM and atypical lipid profiles. Our findings advocate for a nuanced understanding of how hormonal and metabolic factors converge to influence liver health in women post-menopause.

P45-05 | Alzheimer's Disease and Related Dementia Neuropathologies and Exposure to Air Pollution in the Adult Changes in Thought Study

Magali Blanco[1], Adam Szpiro[2], Caitlin Latimer[3], Dirk Keene[3], Jennifer Weuve[4], Paul Crane[5], Lianne Sheppard[1,2]

[1] University of Washington, Environmental and Occupational Health Sciences, Seattle, USA; [2] University of Washington, Biostatistics, Seattle, USA; [3] University of Washington, Laboratory Medicine and Pathology, Seattle, USA; [4] Boston University, Epidemiology, Boston, USA; [5] University of Washington, Medicine, Seattle, USA

Objective: Alzheimer's disease (AD) and related dementias (ADRD) have been linked to air pollution, a complex mixture composed of ultrafine particles (UFP, ≤100 nm), black carbon (BC), nitrogen oxides (NOx), fine particulate matter ($PM_{2.5}$), carbon dioxide (CO_2), and other pollutants. Still, few studies have investigated the underlying neuropathological changes. This study aimed to assess the role of air pollution mixtures in ADRD neuropathologic changes.
Material and Methods: Neuropathology data from the Adult Changes in Thought (ACT) autopsy cohort were used to examine associations between air pollution and neuropathologic markers. Metrics included AD neuropathologic change (Braak stage, CERAD score, Thal phase, overall AD change), vascular pathology (atherosclerosis, arteriosclerosis, cerebral amyloid angiopathy [CAA], infarcts), hippocampal sclerosis, and Lewy body disease. The sample included 356-797 donors, depending on the measure. We used Bayesian kernel machine regression (BKMR) to examine the adjusted association between air pollution mixtures and neuropathology severity.
Results: The cohort had a mean age at death of 89 years (SD: 7), with 57% female and 94% White participants. Most exhibited neuropathologic burden. Ten-year average exposures were 10,720 UFP pt/cm^3, 584 ng/m^3 of BC, 430 ppm of CO_2, 5.6 µg/m^3 of $PM_{2.5}$, and 16 ppb of NOx. BKMR identified NOx, BC, and $PM_{2.5}$ as having higher Posterior Inclusion Probabilities (PIPs) for CERAD score and gross infarcts in various regions, suggesting stronger associations with these outcomes than other pollutants. Higher NOx exposures (25th vs. 75th quantile) were associated with worse CERAD scores, while higher $PM_{2.5}$ exposures were associated with more chronic cerebral microinfarcts. Overall, air pollution mixtures were not consistently associated with neuropathologic burden.
Conclusion: Air pollution mixtures were not consistently linked to neuropathologic burden. However, elevated NOx and $PM_{2.5}$ levels were associated with worse CERAD scores and cerebral microinfarcts, suggesting that specific pollutants within air pollution may drive certain ADRD neuropathologic changes.

P45-06 | Association of Allostatic Load with Plasma Aβ 42/40 Ratio in Midlife: Evidence from the Child Health and Development Studies

Pam Factor-Litvak[1], Piera Cirillo[2], Isha Mhatre-Winters[3], Carolyn Accardi[4], Young-Mi Go[5], Dean Jones[6], Nickilou Krigbaum[7], Katrina Kezios[8], Barbara Cohn[9], Jason Richardson[10]

[1] Mailman School of Public Health Columbia University, Department of Epidemiology, New York, USA; [2] Child Health and Development Studies, Public Health Institute, Berkeley, USA; [3] University of Georgia, College of Veterinary Medicine, Athens, USA; [4] Emory University, Center for the Study of Human Health, Atlanta, USA; [5] Emory University School of Medicine, Department of Medicine, Atlanta, USA; [6] Emory University School of Medicine, Department of Medicine, Atlanta, USA; [7] Child Health and Development Studies, Public Health Institute, Berkeley, USA; [8] Mailman School of Public Health, Columbia University, Department of Epidemiology, New York, USA; [9] Child Health and Development Studies, Public Health Institute, Berkeley, USA; [10] University of Georgia, College of Veterinary Medicine, Athens, USA

BACKGROUND:

Allostatic load (AL) reflects the accumulation of stressors and results in dysregulation of metabolic, inflammatory, cardiovascular and neuroendocrine systems over the life course. Previous studies examining associations between AL and Alzheimer's Disease and Related Disorders (ADRD), use cognitive tests, self-reported status or white matter hyperintensity volumes as outcomes. Few use biomarkers, such as plasma Aβ 42/40 ratio. Here we examine associations between AL and plasma Aβ 42/40 ratio in a longitudinal birth cohort.

METHODS:

Offspring born into the Child Health and Development Studies pregnancy cohort from 1960 -1963 in Oakland, CA were recruited for a follow-up study of health disparities in 2010, when they were approximately 50. Participants (N = 367) completed cognitive function assessments, were interviewed and provided blood samples. Aβ 42/40 was measured from stored plasma taken in midlife. AL was assessed using 10 biomarkers reflecting metabolic, inflammatory, cardiovascular and neuroendocrine systems. Associations were estimated using binomial logistic regression, where the outcome, low Aβ 42/40 was defined as the lowest tertile compared to the higher tertiles. Models were adjusted for sex, race and educational attainment.

RESULTS:

Higher AL was associated with an increased risk of low Aβ 42/40 (estimated odds ratio = 1.20; 95% confidence interval: 1.08, 1.34; $p < .01$). High risk concentrations of biomarkers in the metabolic and inflammatory systems were individually associated with Aβ 42/40; one inflammatory and one neuroendocrine marker DHEA-S were borderline associated with Aβ 42/40; cardiovascular markers were not associated.

CONCLUSION:

In a sample of mid-life adults without known memory problems, allostatic load, reflecting chronic stressors, is associated with plasma Aβ 42/40, a predictor of ADRD. As many of the biomarkers used to define AL routinely are used in screening, early intervention may reduce the risk of ADRD later in life, but this requires confirmation in other studies.

P45-07 | Residential proximity to agricultural pesticide use and cognition in middle-age CHAMACOS Latinas

Robert Gunier[1], Lucia Calderon[2], Stephen Rauch[1], Katherine Kogut[1], Marcella Warner[1], Julianna Deardorff[1], Brenda Eskenazi[1], Jaqueline M. Torres[2]

[1] University of California, Berkeley, School of Public Health, Berkeley, USA; [2] University of California, San Francisco, Epidemiology & Biostatistics, San Francisco, USA

Objective: To examine the relationships between residential proximity to agricultural pesticide use and cognitive functioning in middle-age Latina women.
Methods: Women (N = 436) participating in the Center for the Health Assessment of Mothers and Children of Salinas (CHAMACOS) Maternal Cognition Study completed a neurocognitive battery (SOL-INCA2) that assessed verbal memory, verbal fluency, executive function, and a global composite score of cognitive performance. We estimated agricultural use of potentially neurotoxic pesticides within 1 km of residences averaged across the 20 years prior to the assessment using residential history and California's Pesticide Use Report data. We used linear regression models to assess associations between log10 transformed pesticide use and cognitive performance z-scores, adjusting for education, poverty level, age coming to the U.S., language of assessment and age at assessment.
Results: The mean age of the women was 48.7 (± 5.6) years old, and the vast majority (88.9%) were born in Mexico. A ten-fold increase in residential proximity to agricultural use of dimethoate and glyphosate were both associated with -0.16 standard deviations lower memory scores (95% CI = -0.34, 0.01). Residential proximity to greater use of organophosphates, carbamates, neonicotinoids and ethylene bisdithiocarbamates fungicides was associated with higher executive function z-scores (ranging from 0.10 to 0.13). Residential proximity to agricultural pesticide use was not associated with verbal fluency or global composite z-scores.
Conclusion: Long-term residential proximity to agricultural use of dimethoate and glyphosate may be related to poorer verbal memory at middle age. However, proximity to other agricultural pesticides appeared modestly protective of executive function possibly due to residual confounding. Follow-up assessments are needed to determine the relationship between residential proximity to pesticide use and cognitive decline.

P45-08 | The Effects of Air Pollution on Cognitive Impairment Among Adults and Elderly in Bangladesh: Findings from a Population-Based Study

Saira Tasmin[1], Habibul Ahsan[1], Syed Emdadul Haque[2], Benay K. Datta[2], Salma Akter[2], Golam Sarwar[2]

[1] The University of Chicago, Institute of Population and Precision Health, Chicago, USA; [2] UChicago Research Bangladesh (URB), Dhaka, Bangladesh

Background:
Emerging evidence suggests that air pollution—particularly fine particulate matter (PM2.5)—has been linked to cognitive decline in adults and the elderly. While ambient air pollution has been extensively studied, limited research has concurrently examined the role of household air pollution (HAP) in cognitive impairment.

Methods:
This study includes data from 185 adults aged 50 and over from the Health Effects of Arsenic Longitudinal Study (HEALS), a population-based cohort in a semi-urban region of Bangladesh. Annual ambient PM2.5 exposure at residential locations was estimated using satellite-based models. Household PM2.5 was measured using sensor-based air monitors in participants' homes for 7 days in two seasons. Cognitive function was assessed using the Bangla Mini-Mental State Examination (MMSE). Sociodemographic, environmental, and health-related factors were collected through structured questionnaires. Multivariate linear regression models were used to examine the associations between PM2.5 exposure and cognitive function, adjusting for potential confounders.
Results: Among 185 participants, 38% had cognitive impairment based on the MMSE score (cutoff >24 for mild cognitive impairment). PM2.5 exposure levels were high, with mean±SD concentrations of 67±0.13 μg/m³ for ambient PM2.5 and 125±65 μg/m³ for household PM2.5. Our results demonstrated that higher household PM2.5 levels were significantly associated with lower MMSE scores in fully adjusted models (Model 2), indicating a strong negative impact on cognitive function. However, no significant association was found between ambient PM2.5 and MMSE scores.
Conclusion:
Our findings provide evidence that household PM2.5 exposure is a significant risk factor for cognitive impairment in aging populations in Bangladesh. These results underscore the urgent need for targeted interventions and policy measures to mitigate household air pollution and protect cognitive health in highly polluted regions.

P45-09 | Identification of environmental chemicals targeting dementia genes and pathways using the Comparative Toxicogenomics Database

Scarlet Cockell[1], Sean Harris[1], Erin Ware[2], Kelly Bakulski[1]

[1] University of Michigan, School of Public Health, Ann Arbor, USA; [2] University of Michigan, Institute for Social Research, Ann Arbor, USA

Objective
Dementia is a prevalent public health issue. Though particular chemical exposures (lead, cadmium, air pollution) contribute to risk, a wide array of environmental chemical factors have not been evaluated for dementia risk. To identify novel chemical risk factors and the pathways that contribute to dementia, we screened thousands of chemicals for enrichment with dementia genes or dementia hallmark pathways.
Material and Methods
We used chemical and dementia human gene lists from the Comparative Toxicogenomics Database. We tested all chemicals with 15 or more gene annotations (n=2,270) and chemicals were categorized into 13 classes using medical subject headings. Dementia subtypes (Alzheimer's disease, frontotemporal dementia, Lewy body disease) and dementia pathways (endoplasmic reticulum stress, integrated stress response, proteostasis deficiencies, unfolded protein response, tauopathies) were included. Chi-squared enrichment tests and proportional reporting ratios (PRR) were calculated.
Results
The median number of genes annotated per chemical was 39.5. The number of chemicals per class ranged from 4 (macromolecular substances) to 1,231 (organic chemicals). Among dementia and pathway gene annotations, frontotemporal dementia had the smallest (n=14) and endoplasmic reticulum stress had the largest (n=316) number of genes. Of the 2,270 chemicals tested, 478 chemicals (21%) were enriched (FDR < 0.01) for at least one dementia

subtype or pathway, 44 (1.9%) were enriched for all eight. Endoplasmic reticulum stress had the most chemical associations (n=471): top hits included bisphenol a (PRR=6.8), benzo(a)pyrene (PRR=5.4), cadmium (PRR=4.7), and triphenyl phosphate(PRR=5.9).

Conclusion

We observed genes annotated to 478 chemicals were highly enriched for dementia subtypes or pathways. Since chemical exposures linked to dementia are potentially modifiable, our findings suggest broad classes of environmental chemical exposures may be connected to dementia.

Sources of funding/Acknowledgements This study is supported by the National Institute on Aging (R01 AG067592, R01 AG070897, R01 AG072396).

P45-10 | Boosting Antioxidant Defense Mitigates Oxidative Damage through Autophagy and Stress Response Modulation in Ultraviolet-B-Exposed Human Dermal Fibroblasts

Umar A. Sheikh[1,2], Aalim M. Bhat[1,2], Ragni Gupta[1,2], Mushtaq A. Malik[1,2], Tasduq A. Sheikh[1,2]

[1] CSIR-Indian Institute of Integrative Medicine, Pharmacology Division, Jammu, India; [2] Academy of Scientific & Innovative Research (AcSIR), Biological Sciences, Ghaziabad, India

Objective

Exposure to ultraviolet-B (UVB) radiation induces oxidative and endoplasmic reticulum (ER) stress, leading to DNA instability, cellular dysfunction, and skin photoaging. While previous studies have highlighted the role of reactive oxygen species (ROS) in exacerbating UVB-induced damage, the potential of enhancing antioxidant defenses to mitigate oxidative and ER stress while promoting cellular repair mechanisms such as autophagy remains underexplored. This study investigates the protective effects of antioxidant-mediated autophagy activation on UVB-exposed primary human dermal fibroblasts (HDFs).

Material and Methods

Primary HDFs were exposed to UVB radiation to induce oxidative and ER stress. The antioxidant Trolox, an analog of vitamin E (α-tocopherol), was used to enhance cellular defense mechanisms. ROS accumulation, ER stress markers, DNA damage, and cell viability were assessed. Additionally, autophagy activation and its role in mitigating oxidative damage were evaluated.

Results

UVB irradiation significantly increased ROS accumulation, triggering ER stress and impairing cellular viability by inducing DNA damage in HDFs. Treatment with Trolox effectively reduced ROS levels, alleviated oxidative and ER stress markers, and restored cellular homeostasis by mitigating DNA damage. Notably, Trolox treatment was associated with simultaneous activation of autophagy and antioxidant responses, facilitating cellular defense mechanisms. These findings suggest a mechanistic link between autophagy activation and the alleviation of oxidative stress-mediated ER stress, highlighting a synergistic interaction that mitigates oxidative DNA damage in HDFs.

Conclusion

Our study demonstrates that pharmacological enhancement of antioxidant defenses via Trolox reduces oxidative and ER stress, promotes autophagy activation, and restores cellular homeostasis in UVB-exposed HDFs. These findings provide valuable insights into therapeutic strategies aimed at enhancing skin resilience and mitigating the adverse effects of UVB exposure.

Funding Information

1. DBT India, (Project Number GAP-2166).
2. DST India, (INSPIRE Fellowship Award Number IF-160982).

P45-11 | Combined effects of air pollutant mixtures on Alzheimer's disease-related cortical atrophy

Heeseon Jang[1,2], Hyunji Park[1], Jaelim Cho[1,2], Changsoo Kim[1,2]

[1] Yonsei University College of Medicine, Department of Preventive Medicine, Seoul, South Korea; [2] Yonsei University College of Medicine, Institute of Human Complexity and Systems Science, Incheon, South Korea

Little is known about the combined effects of various air pollutants on Alzheimer's disease (AD)-specific brain structural pathologies. Hence, we aimed to examine the associations of air pollutant mixtures with subclinical brain imaging AD markers in dementia-free adults. This cross-sectional study included 649 dementia-free adults (≥50 years), dwelling in two metropolitan and two small cities in the Republic of Korea. All participants underwent brain magnetic resonance imaging (MRI) scans. The five-year average concentrations before enrollment of particulate matter with diameters ≤ 10 μm (PM10) and ≤ 2.5 μm (PM2.5), NO2, Mn in PM10 (Mn10), and volatile organic compounds (acetaldehyde, styrene, and toluene) were estimated at each participant's residential address. AD-related subclinical imaging markers, including the AD-specific cortical atrophy score and whole-brain cortical thickness, were derived from 3-T brain MRI scans. We performed Bayesian kernel machine regression (BKMR) to examine combined effects of seven air pollutant mixtures on AD-related subclinical imaging markers. The BKMR model showed that the overall effect of air pollutant mixtures on AD-specific cortical atrophy scores was significantly lower at the 25th percentile compared with that at the 50th percentile, whereas whole-brain cortical thickness was significantly greater. We also found that NO2 contributed the most to subclinical imaging markers within the mixture. The findings suggest that air pollutant mixtures may induce AD-specific cortical atrophy in dementia-free adults.
Acknowledgment: This study was funded by the Korea Environment Industry & Technology Institute (KEITI) through the Core Technology Development Project for Environmental Disease Prevention and Management, funded by the Korean Ministry of Environment (MOE) (Grant No. 2022003310011).

P45-12 | Preliminary findings from the Household Air Pollution Study: Indoor air pollutants and mild cognitive impairment in older adults

Katherine L. McKeon[1], Stephanie Utschig[2], Kelsey Fontenot[1], Derek Werthmann[1], Felicia Rabito[1]

[1] Tulane University, Department of Epidemiology/ Celia Scott Weatherhead School of Public Health and Tropical Medicine, New Orleans, USA; [2] Tulane University, School of Medicine, New Orleans, USA

Objective: Research on the impact of indoor air pollution (AP) on cognition is limited. This analysis examined the association between multiple indoor air pollutants and mild cognitive impairment (MCI) in older adults in New Orleans, Louisiana.

Methods: The Household Air Pollution Study (HAPS) is an ongoing (2021-2025) prospective cohort (n=100) study of adults age ≥60 years old in New Orleans, Louisiana, examining the impact of Particulate Matter ≤ 2.5 (PM2.5), Black Carbon (BC), and Nitrogen Dioxide (NO2) on various health indicators. AP concentration is measured inside the home at two time points, baseline and 6 months. Each sampling period is for 7 consecutive days. MCI is assessed at baseline using[FR1] [KM2] the Mini-Cog test, which is validated to detect early dementia. Scores range from 0-5, with ≤ 2 indicating MCI. MCI was modelled both as a continuous and categorical variable. Generalized linear models were run for the continuous score and logistic regression models were run with a threshold of 2 for presence of MCI. PM2.5, BC, and NO2 were modelled separately, and were treated as continuous variables.

Results: This pilot study includes 64 participants.The median age of participants was 72.53 years; sampling period median BC was 0.649 μg/m³, PM2.5 was 5.186 μg/m³, and NO2 was 6.9 ppb. The median MCI score was 4, and 11% scored ≤ 2 indicating MCI. Increasing PM2.5 was negatively associated with MCI treated as both a continuous variable (p = .0073) and a categorical variable (p = .033). Increasing NO2 was significantly associated with MCI ≤2 (p = .0372) but not when treated continuously. There was no association between BC and MCI.

Conclusion: These preliminary results suggest that exposure to PM2.5 and NO2 in the home may contribute to MCI in older adults. Additional studies are needed to further explore these findings.

P45-13 | Assessing causal exposure-response relationships between air pollutants and Alzheimer's disease and dementia incidence in the U.S. Medicare population

Tszshan Ma[1], Yaguang Wei[3,4], Joel Schwartz[3,5], Stefanie Ebelt[1,2], Thomas Wingo[6,7], Anke Hüls[1,2], Kyle Steenland[1,2]

[1] Emory University Rollins School of Public Health, Ganagarosa Department of Environmental Health, Atlanta, USA; [2] Emory University Rollins School of Public Health, Department of Epidemiology, Atlanta, USA; [3] Harvard T.H. Chan School of Public Health, Department of Environmental Health, Boston, USA; [4] Icahn School of Medicine at Mount Sinai, Department of Environmental Medicine, New York, USA; [5] Harvard T.H. Chan School of Public Health, Department of Epidemiology, Boston, USA; [6] University of California, Davis, Department of Neurology, Sacramento, USA; [7] University of California, Davis, Alzheimer's Disease Research Center, Sacramento, USA

Objective: Emerging evidence suggests that air pollution is a modifiable risk factor for Alzheimer's disease (AD) and related dementia (ADRD). However, most studies rely on association-based methods subject to residual confounding and cannot estimate the counterfactual impact of reducing air pollution exposure. This study examined both associative and causal exposure-response relationships between chronic exposure to PM2.5, NO2, and warm-season O3 and ADRD incidence.

Methods: We constructed a national, population-based cohort using Medicare data (2000–2016), with AD and dementia incidence ascertained from claims and high spatial resolution air pollution exposure estimates assigned by residential ZIP code. Exposure-response relationships were modeled using quasi-Poisson regression, regression adjustment, and inverse probability weighting, each coupled with decile-binned exposure, to flexibly assess exposure-response relationships.

Results: For $PM_{2.5}$, we observed a monotonic exposure-response relationship with both AD and dementia across all statistical methods. Causal models estimated stronger effects, with rate ratios (RRs) for AD increasing from 1.06 (95% CI: 1.04–1.08) at the 2nd decile (7.4 μg/m³) to 1.33 (95% CI: 1.31–1.36) at the 10th decile (14.5 μg/m³). Similar trends were observed for dementia, with RRs ranging from 1.03 (95% CI: 1.02–1.04) to 1.10 (95% CI: 1.09–1.11). Effects for NO_2 were less consistent. Conventional models suggested a nonlinear trend for AD, with an RR of 1.075 (95% CI: 1.064–1.085) at the highest decile (35.6 ppb), whereas causal models estimated attenuated effects. For dementia, both models showed small positive effects for NO_2. No clear associations were observed for O_3.

Conclusions: Chronic $PM_{2.5}$ and NO_2 exposures were significantly associated with ADRD incidence, with risks evident at levels below current regulatory thresholds. These findings underscore the need for stricter air quality standards to mitigate ADRD risk.

P45-14 | Source-specific $PM_{2.5}$ and Alzheimer's disease incidence: A national cohort study in the United States

Tszshan Ma[1], Qiao Zhu[1], Anke Hüls[1,2], Kyle Steenland[1,2]

[1] Emory University, Ganagarosa Department of Environmental Health, Rollins School of Public Health, Atlanta, USA; [2] Emory University, Department of Epidemiology, Rollins School of Public Health, Atlanta, USA

Introduction: Fine particulate matter ($PM_{2.5}$) air pollution is a known risk factor for Alzheimer's disease and related dementias (ADRD). While most studies focus on $PM_{2.5}$ mass exposure, fewer consider its diverse chemical composition and the relative importance of different emission sources.
Methods: We derived annual source-specific $PM_{2.5}$ exposure estimates at ZIP codes using two independent species datasets across the contiguous U.S. (2000–2018). (1) Monitoring-based $PM_{2.5}$ species data from 515 EPA stations were analyzed using Positive Matrix Factorization to resolve seven source factors. (2) Prediction-based $PM_{2.5}$ species data, modeled at high spatial resolution, were analyzed using Non-Negative Matrix Factorization to identify five source factors. We assessed associations between the two derived $PM_{2.5}$ source factor datasets and AD incidence in the nationwide Medicare cohort (2000–2018). Mixture analyses, including weighted quantile sum (WQS) regression and quantile g-computation (qgcomp), were used to evaluate joint effects and identify key contributors.
Results: Both source factor datasets consistently identified motor vehicle emissions, biomass and coal burning, and secondary sulfate as the most influential contributors to AD risk, characterized by high loadings of organic carbon, elemental carbon, sulfate, copper, and zinc. Each quintile increase in $PM_{2.5}$ source mixtures was associated with increased AD risk, with rate ratios ranging from 1.051 (95% CI: 1.048–1.054) to 1.109 (95% CI: 1.105–1.114) in national analyses across datasets and mixture methods. Regional analyses revealed geographic variations in source contributions.
Conclusion: In this cohort study, higher $PM_{2.5}$ levels, especially from motor vehicle emissions, solid fuel combustion, and secondary sulfate, were associated with increased AD risk. The consistency between the two source factor datasets strengthens evidence that specific $PM_{2.5}$ sources contribute to AD risk. These findings highlight the need for targeted air pollution reduction policies focusing on key emission sources to mitigate ADRD burden and promote healthy cognitive aging.

P45-15 | Effects of Long-Term Air Pollution Exposure on Brain Atrophy, Alzheimer's Disease Neurodegeneration, and Cerebrovascular Injury: Insights from the SOL-INCA-MRI Study

Xinmei Huang[1], Charles S. DeCarli[2], Hector M. Gonzalez[3], Robert Kaplan[4,5], Adam A. Szpiro[6], Marissa Childs[1], Paul K. Crane[7], Humberto Parada[8], Krista M. Perreira[9], Paola Filigrana[4], Christina M. Cordero[10], Haibo Zhou[11], Amber Pirzada[12], Martha L. Daviglus[12], Wassim Tarraf[13], Joel D. Kaufman[1,14]

[1] University of Washington, Department of Environmental and Occupational Health Sciences, Seattle, USA; [2] University of California, Davis, Department of Neurology, Davis, USA; [3] University of California, San Diego, Department of Neurosciences, San Diego, USA; [4] Albert Einstein College of Medicine, Department of Epidemiology and Population Health, New York, USA; [5] Fred Hutchinson Cancer Research Center, Division of Public Health Sciences, Seattle, USA; [6] University of Washington, Department of Biostatistics, Seattle, USA; [7] University of Washington, Department of Medicine, Seattle, USA; [8] San Diego State University, Division of Epidemiology and Biostatistics, San Diego, USA; [9] University of North Carolina School of Medicine, Department of Social Medicine, Chapel Hill, USA; [10] University of Miami, Department of Psychology and Behavioral Medicine Research Center, Miami, USA; [11] University of North Carolina School of Medicine, Department of Biostatistics, Chapel Hill, USA; [12] University of Illinois at Chicago, Institute for Minority Health Research, Chicago, USA; [13] Wayne State University, Institute of Gerontology and Department of Healthcare Sciences, Detroit, USA; [14] University of Washington, Department of Epidemiology, Seattle, USA

Objectives: Evidence suggests that long-term air pollution exposure increases dementia risk. Hispanic/Latino populations experience disproportionate exposure to air pollution, potentially contributing to brain health disparities. However, brain changes preceding dementia in this population remain understudied. We studied the association between ambient air pollution and magnetic resonance imaging (MRI)-based markers of brain health in the Study of Latinos – Investigation of Neurocognitive Aging MRI (SOL-INCA-MRI) study.

Materials and Methods: We included 2,258 SOL-INCA-MRI participants (aged 35–85 years) from the Bronx, Chicago, San Diego, and Miami SOL centers in this cross-sectional analysis, excluding those with a clinical stroke history or missing address information. MRI scans (2018–2022) assessed brain volume, cerebrovascular injury, and Alzheimer's disease (AD)-related neurodegeneration. Brain volume measures included total brain (TB) volume, lateral ventricle volume, and cortical gray matter (frontal, parietal, temporal, occipital). Cerebrovascular injury was assessed using white matter hyperintensities, cerebral microbleeds (presence/absence), and infarcts (presence/absence), while hippocampal volume served as an AD-related neurodegeneration marker. All brain volumes were residualized for total cranial volume and normalized to z-scores. Annual exposure to fine particulate matter ($PM_{2.5}$) and nitrogen dioxide in 2018 was estimated using validated spatiotemporal models at participants' residences. Linear and logistic regression models assessed associations between air pollution exposure and MRI markers, accounting for the sampling design and adjusting for demographic and socioeconomic variables, hypertension, mild cognitive impairment, diabetes, dyslipidemia, and Apolipoprotein E genotype.

Results: Preliminary findings revealed a significant association between $PM_{2.5}$ exposure and reduced TB volume, with a beta = -0.32 [95% CI: -0.63, -0.01] per 2 μg/m^3 increase in $PM_{2.5}$. No significant associations were observed with other MRI markers.

Conclusion: Our findings indicate a potential association between air pollution and reduced TB volume in a U.S. Hispanic/Latino population. Further longitudinal analyses are necessary to assess air pollution's role in neurodegenerative brain changes.

P45-16 | Impact of Phthalate Exposure on Epigenetic Aging and Renal Function: Insights from a Population-Based Study

Han-Bin Huang[1], Yu-Chen Chen[1], Wei-Yu Lin[2], Po-Chin Huang[3], Yuan-Ting C. Lo[1], None.

[1] National Defense Medical Center, School of Public Health, Taipei, Taiwan; [2] Cambridge, MRC Biostatistics Unit, Cambridge, UK; [3] National Health Research Institutes, National Institute of Environmental Health Sciences, Miaoli, Taiwan

Background

Emerging evidence suggests that exposure to phthalate esters (PAEs) may contribute to accelerated epigenetic aging and impaired renal function. However, epidemiological studies exploring the interrelationships among PAE exposure, epigenetic age acceleration (EAA), and renal function remain limited. This study aims to investigate whether EAA serves as a mediator in the relationship between PAE exposure and renal function and to assess potential interactions between PAE exposure and EAA on renal function.

Methods

This cross-sectional study utilized data from the Taiwan Biobank, including 818 participants aged 20 to 70 years. Urinary PAE metabolites were quantified, specifically ΣDBPm and ΣDEHPm. EAA was assessed using four epigenetic aging measures: AgeAccelResidual, AgeAccelHannum, AgeAccelPheno, and AgeAccelGrim. Renal function was evaluated using blood urea nitrogen (BUN), uric acid, and the albumin-to-creatinine ratio (ACR). Generalized linear models (GLMs) were employed to examine the associations and interactions among these variables. Additionally, Bayesian kernel machine regression was conducted to evaluate the overall impact of PAE exposure on EAA and renal function indicators. Mediation analysis was performed to assess potential mediating effects of EAA.

Results

We found a significant negative association between MCMHP and AgeAccelGrim (β = -0.199, 95% CI: -0.339, -0.060). Additionally, AgeAccelGrim was positively associated with uric acid (β = 0.007, 95% CI: 0.002, 0.013) and ACR (β = 0.028, 95% CI: 0.009, 0.046). Specifically, ΣDEHPm was positively associated with BUN (β = 0.031, 95% CI: 0.009, 0.054), while ΣDBPm and ΣDEHPm were positively associated with ACR (β = 0.098, 95% CI: 0.034, 0.162 for ΣDBPm; β = 0.088, 95% CI: 0.020, 0.156 for ΣDEHPm). Furthermore, significant interactions were observed between ΣDBPm, DEHP metabolites, AgeAccelGrim, and renal function indicators (ACR and uric acid).

Conclusion These findings suggest that both phthalate exposure and accelerated epigenetic aging are associated with an increased risk of renal impairment.

P45-17 | Heat-Related Healthcare Utilization and Costs Among Dementia Patients in the Ageing Population

Ejin Kim[1], Sera Kim[2], Jong-Tae Lee[2,3], Ho Kim[1,4]

[1] Seoul National University, Institute of Health and Environment, Graduate School of Public Health, Seoul, South Korea; [2] Korea University, Research and Management Center for Health Risk of Particulate Matter, Seoul, South Korea; [3] Korea University, School of Health Policy and Management, College of Health Sciences, Seoul, South Korea; [4] Seoul National University, Graduate School of Public Health, Seoul, South Korea

Objective

By 2024, 19.5% of South Koreans will be over 65, and 40% by 2025. 10% of 65-year-olds have dementia, and 3 million will by 2025. This study analyzes how heat exposure affects healthcare consumption and economic burden in dementia patients due to their susceptibility to environmental stressors, notably heat. This work will inform public health strategies and adaptive methods to reduce dementia-related heat-related health risks.

Material and Methods

We utilized national health insurance claims data from the National Health Insurance Service (NHIS) to define dementia patients aged 65 and older based on primary or secondary diagnosis codes (F00–F03). The association between daily hospital admissions and daily average temperature was analyzed using a Distributed Lag Nonlinear Model. Relative risks at the 95th percentile temperature were estimated compared to the Minimum Mortality Temperature (MMT) for each region. Attributable fractions were then calculated, which were used to estimate the proportion of total healthcare costs attributable to extreme heat among dementia patients.

Results

The pooled RR is 1.023 (95% CI: 1.018–1.029) for MMT vs. the 95th percentile and 1.038 (95% CI: 1.030–1.046) for MMT vs. the 97.5th percentile. The hospitalization cost was 262,834,108 KRW, accounting for approximately 3.9% of the medical expenses based on the 95th percentile of the hospitalization relative risk for dementia patients compared to MMT.

Conclusion

While population aging has made dementia a major public health issue with a great impact at both national and personal levels, global warming has resulted in a rising trend in average temperatures. This study evaluates the hazards experienced by dementia sufferers in high temperatures and approximates their contribution to healthcare expenses, therefore offering proof for treating these problems.

This research was supported by Basic Science Research Program through the National Research Foundation of Korea(NRF) funded by the Ministry of Education(RS-2024-00461921).

P45-18 | An analysis of toxic metals and cognitive function in nationally representative populations of older adults in the United States

Erika Walker[1], Sung Kyun Park[1], Tara Jenson[2], Xin Wang[1], Kelly Bakulski[1]

[1] University of Michigan, School of Public Health, Ann Arbor, USA; [2] Boston University, School of Public Health, Boston, USA

Objective: To evaluate associations between lead and cadmium exposure biomarkers and multiple cognitive function tests in two samples of older adults.
Material and Methods: Data from the National Health and Nutrition Examination Survey (NHANES)-III (1988-1994) and continuous NHANES (1999-2002, 2011-2014) were evaluated. Patella and tibia lead were estimated from blood lead and demographics. Cognitive function was measured in NHANES-III by the East Boston Memory Test and Mini Mental State Exam, and in continuous NHANES by the Digit Symbol Substitution (DSST), word recall, and animal fluency tests. Participants aged 60+ with at least one cognitive test, at least one lead (blood, patella, or tibia) or cadmium (urine) measure, and complete covariates (age, sex, race/ethnicity, education, smoking, BMI, alcohol, and creatinine) were included. Survey-weighted adjusted linear regression models evaluated associations between each combination of log_2-transformed metal exposure and z-score standardized cognitive outcome.
Results: Continuous NHANES participants (N=4,626) were on average 69.8 years old and 81.4% Non-Hispanic White. NHANES-III participants (N=4,974) were on average 70.2 years old and 85.3% Non-Hispanic White. Metal exposure levels were higher in NHANES-III than continuous NHANES (e.g. blood lead 4.4 vs. 2.2 μg/dL). In continuous NHANES, higher levels of all metal exposures were associated with lower cognitive function measured by the DSST (e.g. doubling of tibia lead, β=–0.47, 95% CI=–0.64, –0.31). Predicted tibia lead was associated with lower word recall scores (β=–0.41, 95% CI=–0.74, –0.08). Urinary cadmium was associated with lower animal fluency scores (β=–0.07, 95% CI=–0.13, –0.03). No associations between metal biomarkers and cognition were found in NHANES-III.
Conclusion: Cross-sectional associations of lead and cadmium with cognition varied across samples and cognitive test types. Higher levels of these metal biomarkers were consistently associated with lower DSST scores. Further investigations may consider more rigorous cognitive outcome assessment and longitudinal study design.

P45-19 | Lead Exposure and Alzheimer’s Disease Mortality Among US Adults: NHANES Analysis (1988-2008)

Aaron Grossman

Independent, Miami, USA

Background
Alzheimer's disease (AD) is a progressive neurodegenerative disorder with unclear etiology, although metal exposures, including lead (Pb), have been implicated. A previous study (Horton et al., 2019) using National Health and Nutrition Examination Survey (NHANES) data found a possible association between blood lead levels (BLL) and AD mortality; however, temporal biases may have influenced their findings.

Methods

Phase 1 replicated the prior study by Horton et al. using 5 cycles from Continuous NHANES (1999-2008) but with updated links to the 2019 National Death Index (NDI). This was then modified, incorporating calendar effect adjustments for temporal confounding and time-window exclusions for reverse causation. Phase 2 combined NHANES III (1988-1994) data with Continuous NHANES to enhance robustness.

Results

In Phase 1 (n= 8080), replication showed no overall association between BLL and AD mortality. Hazard ratios increased gradually across successive cycles from 1999-2008. Inclusion of a calendar effect adjustment for pooled cycles yielded an inverse, though nonsignificant, association (HR, 95% CI: 0.81 (0.64, 1.04). In Phase 2 (n = 21,308), the base analysis revealed inverse associations, strengthening with calendar adjustments (HR, 95% CI: 0.86 [0.70, 1.05], P = 0.13 vs. 0.57 [0.46, 0.70], P < 0.001, respectively).

Conclusions

Our findings suggest an inverse relationship between BLL and AD mortality, previously obscured by temporal confounding. This challenges prior findings and underscores the importance of accounting for temporal patterns in BLL data within public health research. Nonetheless, findings should be interpreted cautiously due to reliance on a single BLL measurement at baseline.

P45-20 | Identify local environmental factors for the prevalence of Alzheimer's disease in the United States

Jianyong Wu, Ria Martins, Yanni Cao

The Ohio State University, Environmental Health Sciences/College of Public Health, Columbus, USA

Alzheimer's disease (AD) is a neurodegenerative disorder that primarily affects the senior population and is one of the most common causes of dementia. Nearly one in ten adults over the age of 65 may suffer from this disease in the United States (US). Currently, there is no proven cure, posing significant challenges to public health.

The objective of this study is to identify influential environmental factors associated with AD prevalence in the US using explainable artificial intelligence (XAI). We obtained the prevalence of AD among adults aged 65 and above in all 3,142 counties of the US in a recent study and Environmental Quality Index dataset from the U.S. EPA, which includes 139 variables across five domains: air, water, land, built environment, and sociodemographic factors, representing ambient environmental quality in all counties.

We used two machine learning models, Random Forest and XGBoost, as base models to fit AD prevalence estimates. Additionally, we applied two explainable AI methods, Shapley Additive Explanations (SHAP) and Local Interpretable Model-Agnostic Explanations (LIME), to identify global and local factors influencing AD prevalence. The results indicate that the Environmental Quality Index (EQI) domains have varying impacts on AD prevalence, as revealed by SHAP values. Specifically, factors from the water domain exhibit a negative association with AD prevalence, whereas factors from the land, sociodemographic, and air domains show positive associations. At the individual factor level, we found that chloride (Cl) precipitation (water domain) is positively associated with AD prevalence, while the rate of recreation-related businesses (built environment domain) and median household value (sociodemographic domain) are negatively associated. Additionally, formaldehyde (air domain) has a positive relationship with AD prevalence. These findings can help identify high-risk areas, guide targeted prevention efforts, and support the development of precision public health strategies to mitigate AD risk at a localized level.

P45-21 | Analyzing Driving Behavior and Environmental Interactions to Assess Cognitive Decline in Preclinical Alzheimer's Disease

Kenan Li[1], Ganesh M. Babulal[2]

[1] Saint Louis University, Department of Epidemiology and Biostatistics, College for Public health and Social Justice, St. Louis, USA; [2] Washington University in St. Louis, Department of Neurology, School of Medicine, St. Louis, USA

Objective: Understanding the impact of environmental engagement on cognitive health is crucial for early detection and intervention in Alzheimer's disease (AD). This study examines the relationship between naturalistic driving behavior and cognitive decline in preclinical AD by integrating geospatial data on points of interest (POIs) via mobility patterns.

Material and Methods: Participants were drawn from The DRIVES Project at Washington University School of Medicine, a longitudinal study on driving and dementia. The cohort included community-dwelling residents (n=276) of the St. Louis metropolitan area who were ≥65 years old, cognitively normal (CDR=0), held a valid driver's license, and consented to biomarker testing. The present analysis focused on a subset of participants' driving data from 2023 (n>530k trips). We examined their trip characteristics, spatial mobility, and engagement with environmental settings to assess deviations in mobility patterns, route complexity, and visit frequency to key locations, including healthcare facilities, social venues, and recreational areas. These data were integrated with geospatial layers of POIs to explore behavioral and environmental interactions.

Results: Preliminary findings suggest that changes in driving behavior, such as reduced trip diversity, altered route efficiency, and decreased engagement with cognitively stimulating locations, may serve as early indicators of cognitive decline[GB1] . This study identifies potential digital biomarkers for early detection of AD-related neurocognitive changes by linking mobility patterns to environmental exposure.

Conclusion: This study advances exposure science and epidemiology by demonstrating how behavioral mobility patterns contribute to cognitive trajectories in preclinical AD. The findings provide novel insights into dementia risk assessment and inform intervention strategies to mitigate cognitive decline through lifestyle and environmental modifications.

Funding: This work was supported by NIH/NIA grants R01AG068183 and R01AG067428. We thank the participants of The DRIVES Project and the Knight Alzheimer's Disease Research Center staff and leadership for their contributions.

P45-22 | Change in Local Health and Social Infrastructure and Its Association with Social Isolation and Cognitive Function among Korean Older Adults: A Multilevel Analysis

Kyung-Shin Lee[1], Ji Eun Lee[2], Jeehye Lee[3], Hye Sook Min[1]

[1] National Medical Center, Public Health Research Institute, Seoul, South Korea; [2] Seoul National University, Department of Human Systems Medicine, College of Medicine, Seoul, South Korea; [3] Konkuk University, Department of Preventive Medicine, College of Medicine, Chungju-si, South Korea

OBJECTIVES:
This study aimed to investigate the influence of local health and social infrastructure on social isolation and cognitive function among older adults in Korea and to examine differences before and during the COVID-19 pandemic.
METHODS:
Data from the 2019 and 2021 Korea Community Health Surveys were analyzed. Social isolation was assessed based on five components: living alone or unmarried, unemployment, residential instability, lack of community trust, and absence of neighborhood support. Individuals were classified into two groups using the median score (cut-off: 3 points). Regional-level variables included the number of senior leisure facilities per 1,000 people, the financial independence rate, the number of culture facilities, the number of hospitals per 10,000 older adults, the number of long-term care facilities per 10,000 older adults, and greenness distribution. Multilevel logistic regression analysis was performed, with separate models for urban and rural areas to account for contextual differences.
RESULTS:
Among 142,614 older adults, 58.39% experienced social isolation, with a higher prevalence during COVID-19 (59.05%) compared to before the pandemic (57.64%) ($p < 0.0001$). In urban areas, community-level variance decreased slightly after adjusting for individual factors (Variance: 0.2392 → 0.2290), suggesting individual-level factors play a larger role. In contrast, community-level variance increased in rural areas after including community factors (Variance: 0.2396 → 0.2962), indicating the greater influence of regional characteristics. The interaction between social isolation and the COVID-19 period was not significant ($p > 0.05$), showing no notable difference in the effect of social isolation on cognitive function across periods.
CONCLUSIONS:
Social isolation's impact on cognitive function was shaped by community-level factors, especially in rural areas. Enhancing age-friendly environments and improving local infrastructure may help mitigate cognitive decline in socially isolated older adults. Rural contexts require particular attention when addressing cognitive health.

P45-23 | Associations between road, rail and aircraft traffic noise with cognitive function in the UK Biobank cohort

Rabiya Gangrekar[1], Xiangpu Gong[1], Calvin Jephcote[1,3], Sarah Johnson[1], Sana Suri[4], Wuxiang Xie[5], Charlotte Clark[6], Anna Hansell[1,2], Enock Havyarimana[1,2], Yutong Samuel Cai[1,2]

[1] University of Leicester, Centre for Environmental Health and Sustainability, Department of Population Health Sciences, Leicester, UK; [2] Leicester General Hospital, NIHR Leicester Biomedical Research Centre, Leicester, UK; [3] University of Leicester, School of Geography, Geology and Environment, Leicester, UK; [4] University of Oxford, Department of Psychiatry, Medical Sciences Division, Oxford, UK; [5] Peking University First Hospital, Peking University Clinical Research Institute, Beijing, China; [6] City St George's University of London, Population Health Research Institute, London, UK

Objective: Studies on the associations between noise pollution and cognitive function in adults remain limited. We herein examined the cross-sectional associations between road, rail and aircraft noise with cognitive performance in the UK Biobank cohort.
Methods: 499,893 mid-to-older aged adults recruited during 2006-2010 were included in the analyses (499,717, 442,921, 105,768 for road, rail and aircraft analyses, respectively.) Address-level average road noise for 2013 from minor and major roads were modelled using the CNOSSOS-EU framework; address-level average rail noise estimates for 2011 from major rail corridors were obtained from the government Department for Environment, Food and Rural Affairs; average aircraft noise estimates at postcode-level for 2011 were modelled using the ANCON model for participants residing in one of 44 districts partially or wholly encompassed with Civil Authority Aviation weighted 24-hour day-evening-night (L_{den}) aircraft noise contours. Reaction time, visuospatial memory, verbal-numerical reasoning, and prospective memory were self-administered visually via touchscreen at baseline. Multiple regression models were used to test associations between each traffic noise (L_{den} and L_{night}) and each cognitive domain, and a derived composite cognitive z-score. Analyses were adjusted for age, sex, socioeconomic status, AHA cardiovascular risk score, residential air pollution, and proximity to greenspace.
Results: Every 10dB higher in exposure to L_{den}road and L_{den}rail was associated with a higher error rate in visuospatial memory (RR:1.029(95%CI:0.998-1.061) and RR:1.017(95%CI:1.006-1.029), respectively). Higher exposure to rail noise (L_{night}rail <45dB vs. ≥45dB) was associated with lower performance on the visuospatial memory test (RR:1.207(95%CI:1.053-1.383) and worse cognitive function z-score (β-coef:0.050(95%CI:0.012-0.098). Exposure to the highest category of L_{night}aircraft (>50dB vs. <45dB) was significantly associated with slower reaction time (β-coef:18.48ms,95%CI:4.23-32.74) and a higher error rate in visuospatial memory (RR:1.452,95%CI:1.170-1.803).Conclusion: Higher traffic noise exposure, particularly L_{night}rail and L_{night}aircraft, was associated with lower global cognitive performance, suggesting that noise pollution may potentially contribute to cognitive impairment in adults.

P45-24 | Asian and Hispanic enclaves and hospitalizations with Parkinson's Disease in California Medicare beneficiaries

Veronica A. Wang[1], Scott Delaney[1], Daniel Mork[2], Michelle Audirac[2], Danielle Braun[2,3], Antonella Zanobetti[1]

[1] Harvard TH Chan School of Public Health, Environmental Health, Boston, USA; [2] Harvard TH Chan School of Public Health, Biostatistics, Boston, USA; [3] Dana Farber Cancer Institute, Data Science, Boston, USA

Objective: Living in ethnic enclaves may procure health benefits (e.g., social support, cultural continuity) despite disadvantages (e.g., cultural isolation, delayed assimilation). We investigated whether residing in Asian and Hispanic enclaves was associated with fewer hospitalizations with PD (first hospitalization with a Parkinson's Disease diagnosis code) among California Medicare Fee-For-Service beneficiaries (2010-2016).

Material and Methods: We considered three ethnic enclave binary exposure definitions using 2010 5-year American Community Survey variables: 1) cutoff (≥90th percentile % Asian/Hispanic residents), 2) spatial (local Moran's *I* to identify spatial clusters of Asian/Hispanic residents), and 3) dimension reduction (≥90th percentile composite score from principle component analysis on several acculturation variables). For each exposure definition and ethnic enclave, we fitted Poisson regressions adjusted for year, individual risk factors, atmospheric variables, with counts of hospitalizations with PD aggregated to the ZIP Code Tabulation Area level as the outcome. Subgroup analyses among Asian and Hispanic beneficiaries were also conducted.

Results: Among 3+ million beneficiaries aged ≥65 years in our study, ~1% experienced a hospitalization with PD. Residing in an Asian enclave regardless of beneficiary race/ethnicity was associated with higher rates of the outcome (IRRs of 1.13 [95% CI: 1.10, 1.16] for cutoff, 1.11 [95% CI: 1.08, 1.15] for spatial, and 1.10 [95% CI: 1.06, 1.14] for dimension reduction), while Hispanic enclaves appeared protective (IRRs of 0.94 [95% CI: 0.91, 0.96] for cutoff, 0.92 [95% CI 0.88, 0.97] for spatial, and 0.86 [95% CI: 0.82, 0.90] for dimension reduction). Asian beneficiaries residing in compared to outside of an Asian enclave experienced higher rates of hospitalization with PD; the converse was found for Hispanic beneficiaries.

Conclusion: A higher and lower rate of hospitalizations with PD was observed for residential Asian and Hispanic enclaves, respectively. Future work to clarify the multifaceted, context-dependent, and complex underlying mechanisms are needed to protect vulnerable populations.

P45-25 | Sleep disturbance and neighborhood disadvantage interact to affect cognitive health in older Mexican Americans

Yu Yu[1,2], Shiwen Li[3], Kimberly C. Paul[4], Yuyuan Lin[2], Beate Ritz[2,5]

[1] University of California Los Angeles, Center for Health Policy Research, Los Angeles, USA; [2] University of California Los Angeles, Department of Epidemiology, Los Angeles, USA; [3] University of Southern California, Department of Population and Public Health Sciences, Los Angeles, USA; [4] University of California Los Angeles, Department of Neurology, Los Angeles, USA; [5] University of California Los Angeles, Department of Environmental Health Sciences, Los Angeles, USA

Background: Both sleep disturbance and neighborhood disadvantage have been separately linked to cognitive impairment. Here, we aim to examine how sleep disturbance and neighborhood disadvantage interact to influence the development of incident dementia or cognitive impairment without dementia (CIND) among older Mexican Americans followed over almost a decade for cognitive decline.

Methods: We used 1,582 Mexican American participants from the Sacramento Area Latino Study on Aging conducted from 1998 to 2007. Sleep disturbance was defined according to five questions such as waking up to urinate during night and having trouble falling asleep at baseline and was converted into a standardized score (z-score). A neighborhood disadvantage index was created via principal component analysis based on percent of population with education below high school, linguistically isolated households, population with income below federal poverty line, population that were unemployed from 2010 American Community Survey. Using Cox proportional hazard models, we estimated the hazard of incident dementia or CIND according to the sleep disturbance score and further investigated whether neighborhood disadvantage modified the association between sleep disturbances and dementia or CIND.

Results: In total, 96 incident dementia and 150 incident dementia/CIND cases were identified during 10-years of follow-up. For each 1 standard error increase in the sleep disturbance score, the hazard of incident dementia increased 24% (HR= 1.24, 95%CI: 1.00, 1.55). The hazard ratio for sleep disturbance related dementia was stronger (HR=1.41, 95% CI: 1.04, 1.91) among those living in the disadvantaged neighborhood areas compared to those living in the advantaged areas.

Conclusion: Our study indicates that sleep disturbances adversely affect cognition in older Mexican Americans and that this effect may be modified by neighborhood disadvantages such that cognitive function in vulnerable populations is most affected.

P45-26 | Air toxics and and Late-life Dementia and Cognitive Impairment in Mexican Americans

Yu Yu[1,2], Jason Su[6], Sanjali Mitra[2], Karl O'Sharkey[2], Shiwen Li[4], Kimberly C. Paul[3], Beate Ritz[2,5]

[1] University of California Los Angeles, Center for Health Policy Research, Los Angeles, USA; [2] University of California Los Angeles, Department of Epidemiology, Los Angeles, USA; [3] University of California Los Angeles, Department of Neurology, Los Angeles, USA; [4] University of Southern California, Department of Population and Public Health Sciences, Los Angeles, USA; [5] University of California Los Angeles, Department of Environmental Health Sciences, Los Angeles, USA; [6] University of California Berkeley, Division of Environmental Health Sciences, School of Public Health, Berkeley, USA

Background Air pollution has been linked to cognitive impairment, yet studies on air toxics – specific components of particulate matter and/or volatile organic compounds – remain limited compared to criteria pollutants.

Objective We aim to examine whether long-term exposure to modeled traffic-related and industrial/urban-related air toxics including benzene, 1,3-butadiene, lead, nickel, chromium, zinc was associated with the development of incident dementia or cognitive impairment without dementia (CIND) among older Mexican Americans.

Methods We used 1,612 Mexican American participants from the Sacramento Area Latino Study on Aging conducted from 1998 to 2007. Land use regression models estimated concentrations of annual air toxics based on the participants' geocoded residential addresses. Using Cox proportional hazard models, we estimated the hazard of incident dementia or CIND from time-varying air toxics exposures calculated by averaging over 1-, 3-, 5- and 10-calendar years prior to the onset of an event for everyone in the risk set at the event time and each pollutant concentration was entered into models as a continuous variable scaled by its inter-quartile range (IQR).

Results Among the air toxics, higher 1,3-butadiene levels were positively associated with increased incident dementia/CIND and dementia only with a HR of 1.12 (95% CI: 1.00, 1.25) for incident dementia/CIND per IQR increase for 1,3-butadiene exposures. Benzene, zinc and nickel were also positively and more strongly associated with dementia/CIND or dementia alone, but the estimates 95% confidence intervals crossed the null. Associations remained robust when adjusting for other pollutants.**Conclusion** Our findings indicates that air toxics adversely affect cognition in older Mexican Americans, providing critical evidence that traffic- and industry-related air toxics contribute to cognitive impairment. Future studies exploring the mechanisms underlying the associations are needed.

P45-27 | DDE-Associated Serum Metabolome Perturbations in Midlife Adults: Relevance to Alzheimer's Disease

Carolyn Accardi[1], Mary Nellis[1], Bill Liang[1], ViLinh Tran[1], Young-Mi Go[1], Piera Cirillo[2], Nickilou Krigbaum[2], Pam Factor-Litvak[3], Isha Mhatre-Winters[4], Barbara Cohn[2], Jason Richardson[4], Dean Jones[1]

[1] Emory University, School of Medicine, Atlanta, USA; [2] Public Health Institute, Child Health and Development Studies, Oakland, USA; [3] Columbia University, Mailman School of Public Health, New York, USA; [4] University of Georgia, Isakson Center for Neurological Research, College of Veterinary Medicine, Athens, USA

Objective: Dichlorodiphenyltrichloroethane (DDT) and its primary metabolite dichlorodiphenyldichloroethylene (DDE) are associated with cognitive dysfunction and Alzheimer's Disease (AD). Prior research also shows specific metabolic associations with cognitive function and AD pathophysiology. This study used high resolution metabolomics (HRM) to test for DDE-associated metabolic perturbations in midlife linked to metabolic changes and mechanisms implicated in cognitive changes and development of AD.

Materials and Methods: This cross-sectional analysis of 362 subjects (172 females/190 males, ages 44.9-52.0 y, mean 46.8 y) is part of a prospective 60-y follow up study of the Child Health and Development Studies (CHDS) that investigates origins of health including environmental toxicant exposures. HRM analyses were performed using liquid chromatography-high-resolution mass spectrometry for serum metabolome and gas chromatography-mass spectrometry for *p,p'*-DDE measures. Metabolome-wide association study (MWAS) determined associations between midlife serum *p,p'*-DDE levels and the metabolome in untargeted and targeted HRM data. Significant metabolite and pathway changes were determined by regression analysis and *mummichog* pathway enrichment analysis.

Results: Midlife serum *p,p'*-DDE was strongly associated with four fatty acid metabolism pathways and to glycerophospholipid, carnitine, prostaglandin and alanine/aspartate metabolism. Serum *p,p'*-DDE significantly associated with 57 targeted analytes across metabolic processes. Lipid-related metabolites included 16 fatty acids in saturated, monosaturated, and polyunsaturated forms [notably, docosahexaenoic acid, eicosapentaenoic acid, arachidonic acid, docosapentanoic acid, linoleneate, linoleate, palmitate, laurate, myristate, oleate, stearate, heptanoate, palmitoleate], acylcarnitines [lauroylcarnitine, decanolycarnitine, octanolylcarnitine, propionylcarnitine], and cholesterol. Other significant metabolites included amino acids [serine, phenylalanine, histidine, valine, proline, leucine/isoleucine, aspartate], energy-related metabolites [citrate, b-hydroxybutyrate], serotonin-related metabolite [5-hydroxyindoleacetic acid], nucleotides [cytosine, adenosine monophosphate, ureidopriopionate, inosine], and choline.

Conclusions: Midlife metabolome associations with *p,p'*-DDE are suggestive of several metabolic alterations previously associated with AD, including fatty acid metabolism dysregulation, inflammation, mitochondrial dysfunction, oxidative stress and energy metabolism. The findings provide insight into mechanisms by which DDE may contribute to AD pathogenesis.

P45-28 | High-Resolution Metabolomics Reveals Potential Mechanisms of Gene-Environment Interactions Relevant to Alzheimer's Disease

Mary Nellis[1], Carolyn Accardi[1], Young-Mi Go[1], ViLinh Tran[1], Yongliang Liang[1], Piera Cirillo[2], Nickilou Krigbaum[2], Pam Factor-Litvak[3], Isha Mhatre-Winters[4], Barbara Cohn[2], Dean Jones[1], Jason Richardson[4]

[1] Emory University School of Medicine, Clinical Biomarkers Laboratory, Atlanta, USA; [2] Public Health Institute, Child Health and Development Studies, Oakland, USA; [3] Columbia University, Mailman School of Public Health, New York, USA; [4] University of Georgia College of Veterinary Medicine, Isakson Center for Neurological Disease Research, Athens, USA

Objective: Alzheimer's disease (AD) risk is associated with both genetic and environmental factors. The apolipoprotein E (APOE) E4 allele is the primary genetic risk factor, and pesticides, specifically dichlorodiphenyltrichloroethane (DDT) and its metabolite dichlorodiphenyldichloroethylene (DDE), are environmental risks for AD. Previously, we showed that serum DDE interacts with APOE4 genotype to result in worsened cognitive function in AD patients, suggesting interactive effects of these factors heighten AD risk. Here, we used high-resolution metabolomics (HRM) to evaluate metabolic responses associated with serum p,p'-DDE in individuals with and without APOE4.

Methods: Serum was collected from 362 subjects(mean age=46.8; 172 females) enrolled as a prospective follow-up of Child Health and Development Studies(CHDS). An APOE4 allele was present in 101 subjects(55 females). Serum p,p'-DDE was measured by gas chromatography/mass spectrometry(GCMS), and HRM was performed using established liquid chromatography/mass spectrometry(LCMS) methods. A targeted regression analysis of serum p,p'-DDE was performed in subjects with and without APOE4 using 193 confirmed serum metabolites and repeated within males and females.

Results: In subjects with an APOE4 allele, 15 metabolites were associated with p,p'-DDE levels, compared to 42 metabolites in subjects without APOE4. Multiple fatty acids were negatively associated with p,p'-DDE in all subjects. Notably, the ketone bodies acetoacetate($p=0.03$) and β-hydroxybutyrate($p=0.015$) were negatively associated with p,p'-DDE in subjects with APOE4. Sex-specific analysis showed that this association remained in females with APOE4, but not males, and was absent in males and females without APOE4.

Conclusion: The APOE4 genotype influences DDE-related effects on the metabolome and on ketone body metabolism in females. Acetoacetate and β-hydroxybutyrate have been found to be lower in AD, and ketogenic intervention has been reported to improve cognition in AD. The finding that ketone bodies decreased with DDE in APOE4 females suggests a mechanism for sex-specific gene-environment interactions in AD and a potential role for ketogenic interventions.

P46 | Environmental Exposures and Cancer - PART II

P46-01 | Long-term ambient ozone exposure and lung cancer mortality: a nested case-control study in Korea

Taiyue Jin[1], Seulbi Lee[2], Juhee Seo[3], Shinhee Ye[4], Soontae Kim[5], Jin-Kyoung Oh[1,6], Seyoung Kim[1], Byungmi Kim[1,6]

[1] National Cancer Center, Division of Cancer Prevention, National Cancer Control Institute, Goyang, South Korea; [2] National Health Insurance Service, Department of Big Data Strategy, Wonju, South Korea; [3] Statistics Korea, Vital Statistics Division, Daejeon, South Korea; [4] Korea Occupational Safety and Health Agency, Occupational Safety and Health Research Institute, Incheon, South Korea; [5] Ajou University, Department of Environmental and Safety Engineering, Suwon, South Korea; [6] National Cancer Center, Department of Cancer Control and Population Health, Graduate School of Cancer Science and Policy, Goyang, South Korea

Objective: The link between long-term exposure to tropospheric ozone (O_3) and risk of lung cancer mortality remains uncertain. We aimed to provide new insights into the association between long-term O_3 exposure and lung cancer mortality in Korea.
Material and Methods: A nested case-control study was conducted within a cancer-free cohort of 1,731,513 individuals who underwent health screenings provided by the National Health Insurance Service in 2006–2007. A total of 7,133 lung cancer deaths that occurred from January 1, 2012, to December 31, 2021, were matched to 28,532 controls at a 1:4 ratio based on propensity scores calculated based on age, sex, insurance fee, body mass index, smoking status, and alcohol consumption. Ambient O_3 concentrations were estimated using the Community Multiscale Air Quality (CMAQ) models and averaged both year-round and during the warm season (April to September) from 2006 to 2010. These estimates were then linked to participants' residential addresses to estimate O_3 exposure. Conditional logistic regression models were performed to evaluate the effects of 5-year O_3 exposure (with a 1-year lag) on the risk of lung cancer death, adjusted for ambient $PM_{2.5}$ and NO_2 concentrations.
Results: The averages of 5-year O_3 exposure were 24.1 ppb year-round and 29.0 ppb during the warm season. Elevated risk of lung cancer death was observed among participants in the 4th quintile of O_3 exposure compared with those in the 1st quintile (odds ratios [95% confidence intervals]: 1.12 [1.03-1.23] year-round and 1.12 [1.03-1.22] during the warm season). Although this effect was not significant among participants in the 5th quintile, a linear trend was found between them.**Conclusion:** Findings of this study suggest that long-term exposure to O_3 is associated with an excess risk of lung cancer mortality, underscoring the importance of addressing the O_3 burden for lung cancer prevention.

P46-02 | Lifetime cannabis smoking is associated with site-specific cancers: a two-sample Mendelian randomization study

Abram B. Kamiza[1], Elena Lukhere[2]

[1] Kamuzu University of Health Sciences, Blantyre, Malawi; [2] Malawi-Liverpool-Wellcome, Blantyre, Malawi

Background: The prevalence of cannabis smoking is increasing at an alarming rate due to its legalization and decriminalization in some countries. Epidemiological evidence regarding the association between cannabis smoking and cancer is inconsistent and conflicting. Herein, we performed a two-sample Mendelian randomization (MR) to investigate whether cannabis smoking is causally associated with site-specific cancers.

Methods: We identified 22 independent genetic variants that were strongly associated with cannabis smoking (pvalue < 5 x $10^{-8)}$ from a large meta-analysis of genome-wide association studies. Genome-wide association summary level data on site-specific cancers were obtained from FinnGen. MR were performed using the inverse-variance weighted (IVW) and multivariable method. Sensitivity analyses were performed using the simple median, weighted median, MR-Egger, and MR pleiotropy residual sum and outlier (MR-PRESSO) methods.
Results: Our multivariable IVW analyses adjusted for cigarette smoking found genetic liability to cannabis smoking to be causally associated with esophageal cancer (odds ratio [OR] =1.74, 95% confidence interval [CI] =1.29-2.15, pvalue =0.013) and lung cancer (OR=1.35, 95% CI = 1.35-1.58, pvalue=0.009). However, genetic liability to cannabis smoking exerted a protective effect against pancreatic cancer (OR=0.77, 95% CI =0.57-0.91, pvalue=0.032). Our sensitivity analyses found no evidence of horizontal pleiotropy between cannabis smoking and site-specific cancers.
Conclusion: Genetic liability to lifetime cannabis smoking was associated with esophageal, lung, and pancreatic cancers.

P46-03 | Residential Radon and Small Cell Lung Cancer in a radon-prone area. Are there differences by sex and onset age?

Alberto Ruano-Ravina[1], Lucía Martín-Gisbert[1], Eliana Torres-Cadavid[1], Virginia Calvo[2], Mariano Provencio[2], Luis Paz-Ares[2], Marcos Malumbres[4], Guadalupe Garcia[1], Monica Pérez-Ríos[1], Cristina Candal-Pedreira[1]

[1] University of Santiago de Compostela, Preventive Medicine and Public Health, Santiago de Compostela, Spain; [2] Hospital Puerta de Hierro, Oncology, Madrid, Spain; [3] Hospital 12 de Octubre, Oncology, Madrid, Spain; [4] Hospital Valle Hebrón, Research Institute, Barcelona, Spain

Objective: To analyze whether residential radon concentrations play any role on the onset age of Small Cell Lung Cancer (SCLC) and if there are differences by sex and smoking status.
Material and methods: Pooling study from different case-control studies in a radon-prone area. Cases recruited from 11 different hospitals had a confirmed pathological diagnosis and were older than 30 years. An alpha-track radon detector was placed in the participants' dwelling for at least three months. We performed a descriptive analysis to determine indoor radon concentrations by sex, age at diagnosis, and for different smoking status classified as never smokers, ex-smokers and current smokers at diagnosis. We performed a sub-analysis for participants exposed to more than 600 Bq/m^3.
Results: 510 SCLC cases included, 23.9% women. Median (IQR) age at diagnosis was 66 (59-72) years. Median (IQR) radon concentration was 123 Bq/m^3 (72-225). Medianduration of residence in the measured dwelling was 28 years (15-40). Men had a median radon concentration of 122 Bq/m^3, and women 137 Bq/m^3 (p = 0.98). There was a positive linear correlation between age at diagnosis and indoor radon concentration, overall and for both sexes. The higher the radon concentration, the older the age at diagnosis. Correlation is also present for each of the three smoking categories analyzed. When the analysis was restricted to radon concentrations of 600 Bq/m^3 or more we observed a downwards trend, with younger cases having a higher radon concentration.
Conclusions: There seems to be no difference in residential radon concentrations between men and women with SCLC. Residential radon concentrations seems to be positively correlated with age at diagnosis, overall, and for both sexes. People exposed to high radon concentration (≥600 Bq/m^3) might be diagnosed with SCLC at a younger age.
This study has been funded by the Spanish Association Against Cancer (SOSCLC project).

P46-04 | Exposure to household air pollution and its constituents in relation to stomach and esophageal cancer

Batel Blechter[1], Lützen Portengen[2], George Downward[2], Bryan Bassig[3], Wei Hu[1], Jason Wong[4], Mohammad Rahman[1], Yongliang Zhang[2], Bofu Ning[5], Jihua Li[6], Kaiyun Yang[7], H. Dean Hosgood[8], Debra Silverman[1], Nathaniel Rothman[1], Yunchao Huang[9], Roel Vermeulen[2], Qing Lan[1]

[1] National Cancer Institute, Division of Cancer Epidemiology and Genetics, Rockville, USA; [2] Utrecht University, Institute for Risk Assessment Sciences, Utrecht, Netherlands; [3] Inova Schar Cancer Institute, Inova Health System, Saville Cancer Screening and Prevention Center, Fairfax, USA; [4] National Heart Lung and Blood Institute, Epidemiology and Community Health Branch, Bethesda, USA; [5] Xuanwei Center for Disease Control and Prevention, Xuanwei, China; [6] Qujing Center for Diseases Control and Prevention, Quijing, China; [7] Third Affiliated Hospital of Kunming Medical University (Yunnan Tumor Hospital), Kunming, China; [8] Albert Einstein College of Medicine, Department of Epidemiology and Population Health, New York, USA; [9] Shanghai Cancer Institute, Shanghai Jiaotong University, Department of Epidemiology, Shanghai, China

Household air pollution (HAP) is a global health burden affecting about half of the world’s population, especially those in lower- and middle-income countries. In Xuanwei, China, individuals experience high concentrations of various HAP constituents, particularly polycyclic aromatic hydrocarbons (PAHs), from indoor cooking and heating with coal. Here, we investigated the association between HAP constituents and cause-specific mortality from stomach and esophageal cancer-two cancers with highest mortality rate after lung cancer.
The study was conducted in a cohort of 42,420 subjects from Xuanwei, China. Participants were followed from 1976 to 2011. Type of fuel used was collected through a questionnaire and categorized into lifetime smoky vs. other fuel use. Annual exposures were predicted for 43 HAP constituents and 5 clusters were identified using hierarchical clustering. Cox regression was used to estimate the association between lifetime smoky coal use and continuous pollutant clusters with stomach (n=143) and esophageal (n=39) cancer. All models were stratified by sex and birth cohort and adjusted for smoking status and education.
We found that lifetime smoky coal use was associated with an increased risk of stomach cancer mortality (hazard ratio [HR]=1.40, 95% confidence interval [CI]:1.00, 1.96) compared to other fuel use. Among specific combustion components, a large pollutant cluster, PAH37 (HR=1.59, 95% CI:1.19, 2.13 per standard deviation [SD]), was also associated with stomach cancer mortality. Similarly, lifetime smoky coal use (HR=2.84, 95% CI:1.38, 5.85) and PAH37 (HR=1.75, 95% CI:1.01, 3.04 per SD) were associated with esophageal cancer mortality. Stratified analyses found that the association between PAH37 and stomach cancer was higher among women compared to men (P-interaction=0.02).
We observed an association between HAP and its constituents and risk of mortality from stomach and esophageal cancer. Future work is needed to investigate potential underlying biological mechanisms especially as they pertain to observed differences by biological sex.

P46-05 | Geospatial Analysis of US County Glyphosate Usage and non-Hodgkin Lymphoma and Breast Cancer Rates

David Hollar, Raina Modi, Yudan Wei

Mercer University School of Medicine, Department of Community Medicine, Macon, USA

Objective: Glyphosate is one of the most commonly used herbicides in the United States, having been linked to an increased risk of non-Hodgkin's lymphoma (NHL) in multiple epidemiological studies. However, the evidence about its association as an endocrine disruptor with breast cancer is limited. This study analyzed possible geographic relationships between glyphosate use and these two cancers at the county level.
Material and Methods: Using data fromthe National Cancer Institute State Cancer Profiles, U.S. Geological Survey Annual Agricultural Pesticide Use, and the County Health Rankings, we geospatially mapped the independent variable glyphosate usage in kilograms (high estimate) on the dependent variable NHL or breast cancer rates per 100,000 population. Geospatial bivariate cluster mapping used GeoDa, including weighted Moran's I. Two weighted spatial regression models were tested on each cancer rate using glyphosate and demographic covariates for Model 1 plus percent smokers, obesity, physical inactivity, and insufficient sleep in Model 2.
Results: The glyphosate by lymphoma cluster map showed significant high-high counties located around the Great Lakes region, southeast Pennsylvania, central Florida and Louisiana. The Model 2 lymphoma regression showed R^2=0.45 with the four demographic variables and smoking being significant at a Bonferroni adjusted p<.005. The glyphosate by breast cancer cluster map showed significant high-high counties located primarily around the Great Lakes region and the North Carolina coastal plain, both notably agricultural areas. The breast cancer Model 2 showed R^2=0.45 with the four demographic variables and smoking, inactivity, and insufficient sleep being significant; glyphosate weakly correlated with p=.009.Conclusion: Counties with higher female, white, and smoking populations positively correlated with both cancers, with glyphosate showing a weak association with breast cancer county rates. Cluster mapping shows regional associations between glyphosate and both cancers in heavily agricultural areas, but not all such areas, indicating a need to investigate additional contributing factors.

P46-06 | A Prospective Exposome-Wide Association Study of Prostate Cancer

Dean Jones[1], Mary Nellis[1], Sami Teeny[1], Yongliang Liang[1], Zachery Jarrell[1], Nickilou Krigbaum[2], Piera Cirillo[2], Young-Mi Go[1], Barbara Cohn[2]

[1] Emory University, Medicine, Atlanta, USA; [2] Public Health Institute, Child Health Development Studies, Berkeley, USA

Objectives. Human cancers have multiple genetic and environmental risk factors, often developing over decades. In prior exposome research, we tested pregnancy serum for chemicals associated with subsequent diagnosis of breast cancer using untargeted mass spectral data and network analysis. The research demonstrated sensitivity to discover new suspected carcinogens. Here, we used untargeted mass spectral data for environmental chemicals in serum of young men to look for associations with subsequent diagnosis of prostate cancer (PCa).**Methods**: GC-Orbitrap was used to measure environmental chemicals in serum of 1104 men (average age=34.7y), who had subsequent PCa diagnosis (cases) or were not known to have PCa through linkage of fathers in the Child Health and

Development Studies to the California Cancer Registry and California Vital Statistics. Samples were analyzed with simultaneous analysis of pooled reference materials with >3500 environmental chemicals, and stable isotopic internal standards. Data were analyzed with PeakWalk, a software for automated identification and quantification of concurrently analyzed pooled reference materials. An Exposome-Wide Association Study (ExWAS) was used to test for environmental chemicals associated with PCa.

Results: Approximately 500 environmental chemicals were measured in at least one sample, and 371 chemicals with ³3 fragments for identification were present in 10% of samples. Of these, 103 passed additional criteria for rigorous identification. For high stringency identifications, pyrene was associated with PCa, consistent with previous research associating occupational PAH exposures with PCa. Other PCa risk candidates included insecticide, herbicide, commercial, and personal-use product chemicals. In blacks, who are at increased risk for early-onset and lethal PCa, a PFAS and a common commercial chemical were associated with PCa.
Conclusions: These results provide new suspected prostate carcinogens similar to chemicals previously linked to PCa. Additional validation is needed for candidate chemicals as well as to test for replication in independent cohorts and model systems.

P46-07 | Empowering Black Women Through Advocacy and Education on Endocrine Disrupting Chemicals (EDCs) and Breast Cancer Risk

Dede K. Teteh-Brooks[1], Ebony Andrews[2], Jazma Tapia[3], Pastor Rhonda Holbert[4], Tiah Tomlin-Harris[5], Elissia T. Franklin[6,7], Marissa Ericson[8]

[1] UT MD Anderson Cancer Center, Department of Health Disparities Research, Houston, USA; [2] The Ebony Experience, LLC, Upper Marlboro, USA; [3] City of Hope Comprehensive Cancer Center, Duarte, USA; [4] Celebrate Life Cancer Ministry, Los Angeles, USA; [5] My Style Matters, Inc., Atlanta, USA; [6] Silent Spring Institute, Boston, USA; [7] Research Her, LLC, South Holland, USA; [8] California Lutheran University, Thousand Oaks, USA

Objective: The Bench to Community Initiative (BCI) is a community-based program aimed at improving the quality of life for Black women, with a focus on Black Breast Cancer Survivors. The BCI symposium launched in 2020 to address education gaps on EDCs in hair and personal care products. This study assessed knowledge gained by participants of the 2024 symposium, *The Black Beauty Breakdown: A Black Woman's Guide to Uncovering the Connection Between Personal Care Products and Breast Cancer*.
Material and Methods: The three-day symposium, hosted via the BCI website and Zoom teleconferencing, covered EDC research and risks (Day 1), prevention through advocacy (Day 2), and conscious consumerism (Day 3). Recruitment strategies included partnerships with influencers through the Product Options in Women Engaged-Research (POWER) project, Claremont Graduate University for continuing education credits, BCI listserv, and partner organizations. The planning committee included public health experts, breast cancer survivors, and behavioral and biomedical specialists. Pre/post surveys assessed demographics and knowledge on research, advocacy, and consumerism. Open-ended questions captured testimonials and action commitments for the next two months.
Results: The symposium featured 10 sessions and 14 speakers, including researchers, advocates, nonprofits, and tech industry representatives. Twenty-one percent of participants completed both pre (n=501) and post (n=103) assessments. Participants were predominantly African American (46.3%), educated (66.7% with graduate degrees), and represented roles such as community members and health professionals from states including California

(24.1%), Texas, New York, Maryland, and Arizona (9.3% each), showed increased knowledge ($t(52)=6.097$, $p<0.001$). Forty-nine Certified Health Education Specialists earned continuing education credits, and 55% of attendees learned about the program through NCHEC. Post-survey respondents intend to share information with family, friends, colleagues, and healthcare providers.

Conclusion: The BCI symposium effectively increased community knowledge on environmental exposures, advocacy, and safer personal care product choices related to breast cancer risk.

P46-08 | Mapping the internal chemical exposome of breast cancer: regional differences, relationships with diet and socioeconomic factors

German Cano-Sancho[1], Eva Gorrochategui[2], Jade Chaker[2], Arthur David[2], Ke Zhou[3], Christine Bobin-Dubigeon[3], Caroline Goupille[4,5], Marie Potier-Cartereau[4], Martine Bellanger[3], Bruno Le Bizec[1], Jean-Philippe Antignac[1], Vincent Bessonneau[2]

[1] Oniris, INRAE, LABERCA, Nantes, France; [2] Univ Rennes, Inserm, EHESP, Irset (Institut de recherche en santé, environnement et travail) – UMR_S 1085, Rennes, France; [3] Institut de Cancérologie de l'Ouest (ICO, Nantes, France; [4] UMR INSERM U1069, Université de Tours, Tours, Tours, France; [5] CHRU de Tours, Tours, France

Breast cancer (BC) is the most common cancer in women worldwide and the second leading cause of cancer-related death. Evidence suggests that a growing list of environmental pollutants may play a role on the etiology or prognostic of BC. However, little is known about the complexity of chemical mixtures and their interplay with nonchemical factors, including individual factors, nutrition and the social environment. This interdisciplinary study aims to map the chemical, nutritional and social exposome and to characterize their associations and interactions with BC sub-types and hallmarks of aggressiveness.

Our study leverages three French BC cohorts (n=409) with extensive individual and clinical data as well as archived breast tissue and blood samples. The chemical exposome was characterized in blood and adipose tissue using both targeted and non-targeted approaches based on liquid and gas chromatography coupled to high-resolution mass spectrometric methods to ensure a large coverage of the chemical space. This comprehensive analytical framework allowed to quantify the levels of historical persistent organic pollutants and per/polyfluorinated substances, but also to identify emerging industrial compounds, pesticides, personal care products or food additives using advanced annotation methods. A subset of samples were further analyzed for nutritional and inflammatory biomarkers. Multivariate logistic and Cox regression were performed to assess the associations of individual factors with BC subtypes and outcomes. The mixture effects were explored using multipollutant models. Additionally, the composition and variability of chemical exposures were analyzed across regions, socioeconomic strata and dietary patterns. Preliminary results show that women with BC are exposed to complex mixtures of environmental pollutants, including both historical and emerging compounds specifically distributed across regions and cancer sub-types. Overall, this study underscores the large potential of implementing exposome-scale studies in breast cancer research, favoring a comprehensive characterization of chemical risk factors and their interactions with non-chemical dimensions.

P46-09 | Blood Sugar Level-Independent Impact of Metformin on Sarcoma Mortality: A Propensity Score-Matched Analysis Using a Nationwide Cancer Cohort

Hyewon Jo[1], Eunhee Ha[1,2], Jin-Hong Kim[3], Yi-Jun Kim[1,2]

[1] Ewha Womans University College of Medicine, 1Graduate Program in System Health Science and Engineering, Seoul, South Korea; [2] Ewha Womans University College of Medicine, Medicine, Seoul, South Korea; [3] Seoul National University, Department of Biological Sciences, College of Natural Sciences, Seoul, South Korea

There is a lack of evidence on whether metformin improves cancer survival independently of its diabetes treatment effects, particularly in sarcoma, where effective systemic therapy options are limited. This study aims to evaluate whether metformin improves survival in Chondrosarcoma patients, independent of its effects on fasting blood sugar levels, after adjusting for these levels.
This cohort study used a propensity score matching using data from the Korea-Clinical data Utilization network for Research Excellence (K-CURE) in Korea. Sarcoma patients diagnosed from January 2012 to December 2019. Follow up period was from January 2014 to December 2021. The primary outcome was overall survival(OS), assessed using hazard ratio(HR) after propensity score matching and adjusting for FBS, BMI, and organ function.
Of the 10,647 patients diagnosed with sarcoma between January 2012 and December 2019, 901 patients who were prescribed metformin matched 4,676 patients who were not prescribed. As a result of Cox proportional hazard model analysis, sarcoma patients in the metformin group experienced a 41.7% reduction in mortality [adjusted HR (aHR) 0.583 (95% confidence interval, 0.495-0.687)]. After propensity score matching, the risk of sarcoma patients prescribed metformin was reduced by 42.2% [0.578 (0.466-0.716)]. This result was the same even when performed for diabetes patients only [aHR 0.582 (0.491-0.691)], and with for cancer specific survival analysis [aHR 0.622 (0.511-0.758)].
Metformin significantly improved survival in sarcoma patients, even after adjusting for factors such as FBS, BMI which are controlled by metformin, and organ function which influence the decision to prescribe metformin. Metformin may be a viable therapeutic option for sarcoma, where systemic treatment options are limited.

P46-10 | Use of Hair Straighteners/Chemical Relaxers and Cancer Incidence

Jared T. Bailey[1], Che-Jung Chang[2], Symielle A. Gaston[2], Chandra L. Jackson[2], Dale P. Sandler[2], Katie M. O'Brien[2], Alexandra J. White[2]

[1] Emory University,Rollins School of Public Health, Gangarosa Department of Environmental Health, Atlanta, USA; [2] National Institute of Environmental Health Sciences, Epidemiology Branch, Research Triangle Park, USA

Objective. Hair straighteners and chemical relaxers contain endocrine disruptors and carcinogens. Frequent use of these products has been associated with increased incidence of breast, uterine, and ovarian cancers. We evaluated use of straighteners/relaxers in relation to the incidence of other cancer types.
Material and Methods. Participants in the Sister Study, a US-wide prospective study, who had no history of cancer at enrollment (2003-2009) were included in this analysis (N=46,287). Participants self-reported ever use and frequency of hair straightener/chemical relaxers in the 12 months before enrollment. Incident cancer diagnoses

(melanoma, non-Hodgkins's lymphoma, leukemia and thyroid, lung, pancreatic, colorectal, urinary, and kidney cancers) were reported and confirmed with pathology reports, when possible. We used multivariable Cox proportional hazards models to estimate hazard ratios (HRs) and 95% confidence intervals (CIs) for hair straightener/relaxer use in relation to incident cancers, adjusting for race and ethnicity, educational attainment, and smoking status.
Results. Approximately 10% of participants reported using a hair straightener/relaxer before enrollment and the median age at baseline was 55.5 years. During a median follow-up of 13.1 years, ever use of straightener/relaxers was associated with a higher incidence of pancreatic cancer (N=138 cases; HR=2.66, 95% CI: 1.25-5.66), thyroid cancer (N=225 cases; HR=1.71, 95% CI:1.01-2.89), and non-Hodgkin's lymphoma (N=313 cases; HR=1.62, 95% CI:0.94- 2.80). We saw negligible or imprecise associations for the remaining cancer types considered. There was little evidence of a dose-response association with increasing frequency of use in the past year, although confidence intervals were wide.
Conclusion. Use of hair straighteners/chemical relaxers may be associated with a higher incidence of pancreatic cancer, thyroid cancer, and non-Hodgkins Lymphoma. Further research is needed to confirm these findings.
Acknowledgements : This research was supported in part by the Intramural Research Program at the NIH, National Institute of Environmental Health Sciences (project Z1A ES103325 and ES103332).

P46-11 | Carcinogenic industrial air pollution and risk of non-Hodgkin lymphoma subtypes: a pooled analysis of 535,000 Americans

Jessica Madrigal[1], Danielle Duarte[1], Jared Fisher[1], Caroline Pruitt[1], Barry Graubard[2], Mary Ward[1], Debra Silverman[1], Sonja Berndt[1], Rena Jones[1]

[1] Occupational and Environmental Epidemiology Branch, Division of Cancer Epidemiology and Genetics, National Cancer Institute, National Institutes of Health, Department of Health and Human Services, Rockville, USA; [2] Biostatistics Branch, Division of Cancer Epidemiology and Genetics, National Cancer Institute, National Institutes of Health, Department of Health and Human Services, Rockville, USA

Objective: Industrial facilities emit numerous suspected and established lymphomagens (e.g., 1,3-butadiene, ethylene oxide, trichloroethylene), but the association of these agents with non-Hodgkin lymphoma (NHL) risk at environmental levels is unknown.
Methods: We used a nationwide emissions database to estimate airborne exposure to 15 human carcinogens from industrial sources for 534,939 participants pooled from the NIH-AARP Diet and Health Study (enrolled 1995-1996) and the Prostate, Lung, Colorectal, and Ovarian Cancer Screening Trial Cohort (enrolled 1993-2004). Over a median 18 years of follow-up, 11,473 participants developed NHL. For the 8 years pre-enrollment, we estimated inverse distance- wind direction-weighted average emissions within 3km, 5km, and 10km of the enrollment residence. Using Cox proportional hazards models adjusted for confounders, we evaluated risk overall and by lymphoma subtypes for tertiles of exposure compared to non-exposed.
Results: We observed no associations with risk of NHL overall. Increased marginal zone lymphoma risk was associated with the highest emissions of ethylene oxide and trichloroethylene at 3km (HR_{T3}=1.8, 95%CI=0.9-3.7, p-trend=0.07; HR_{T3}=1.6, 95%CI=1.1-2.3, p-trend=0.03, respectively) and remained elevated at 5km and 10km. Risk of lymphoplasmacytic lymphoma/Waldenström macroglobulinemia was 2-fold higher with monotonic trend ($p<0.05$) for increasing trichloroethylene (3km, 5km, and 10km) and 1,3-butadiene (10km) emissions. The highest emissions tertiles of beryllium, 1,2-dichloropropane, and pentachlorophenol at the 5km and 10km distances were associated with up to 50% increased risk of diffuse large B-cell lymphoma (DLBCL), with trend ($p<0.05$). Suggestive DLBCL

associations were seen for benzene at 3km (HR_{T3}=1.2, 95%CI=0.9-1.7, p-trend=0.3) but not at 5km or 10km. We observed no associations with chronic lymphocytic leukemia, follicular lymphoma, or multiple myeloma.

Conclusions: Our findings show that relatively higher levels of environmental emissions of suspected lymphomagens based on occupational studies, including 1,3-butadiene, ethylene oxide, and trichloroethylene, may contribute to the risk of specific lymphoid malignancies at levels found in the ambient environment.

P46-12 | Water arsenic levels and breast cancer riskin a United States-wide prospective cohort

Katherine Pullella[1], Maya Spaur[2], Jennifer L. Ish[1], Rena R. Jones[2], Alexandra J. White[1]

[1] National Institute of Enviromental Health Sciences, Epidemiology Branch, Research Triangle Park, USA; [2] National Cancer Institute, Occupational and Environmental Epidemiology Branch, Bethesda, USA

Objective: In the U.S., drinking water from unregulated private wells and federally regulated community water systems is an important source of exposure to inorganic arsenic, an established human carcinogen. Evidence links chronic exposure to low levels of inorganic arsenic (< 10.0 µg/L, the regulatory limit) with adverse health outcomes, including cardiovascular disease and cancer. However, the relationship between arsenic exposure and breast cancer risk remains inconclusive. We investigated the association between drinking water arsenic and breast cancer incidence in the U.S.

Materials and Methods: Sister Study participant's geocoded residential addresses at enrollment (2006 – 2008) were linked to county-level water arsenic concentrations using the EPA National Contaminant Occurrence database (2006–2011) for those who self-reported primarily using community water sources (n=29,530). Cox proportional hazards regression estimated hazard ratios (HR) and 95% confidence intervals (CI) for the association between water arsenic concentrations and breast cancer incidence, adjusting for age, ethnicity, household income and education. Subgroup analyses by tumor subtypes and menopausal status were conducted.

Results: Over 12.5 years (mean) of follow-up, 2,746 breast cancer cases (invasive and ductal carcinoma in situ) were diagnosed. The mean water arsenic level was 1.29 µg/L (20th– 80th percentile: 0.35–1.88 µg/L). We observed non-monotonic but elevated associations between increasing levels of water arsenic and invasive breast cancer risk (N= 2162; $HR_{Quintile(Q)5vs.Q1}$ = 1.11, 95%CI 0.97–1.24; P-trend = 0.4). Similar associations were observed for estrogen receptor positive tumors (N=1983; $HR_{Q2vs.Q1}$= 1.17, 95%CI 1.02–1.34; $HR_{Q5vs.Q1}$= 1.14, 95%CI 0.99–1.32; P–trend = 0.3). No differences were observed by menopausal status.

Conclusion: In a nationwide prospective cohort, we observed a weakly positive association between water arsenic levels and breast cancer incidence, particularly for invasive and ER+ breast cancers. These results suggest that exposure at levels under the regulatory limit may contribute to increased breast cancer risk in the U.S.

P46-13 | Toxic effects on the immune system of rural workers exposed to pesticides in Rio de Janeiro, Brazil.

Carolina P. Dias[1], Yngrid D.S. Cabral[1], Natalia Vellozo[2], Ubirani B. Otero[1], Landi Guilhermo[2], Marcia Sarpa[1,2]

[1] National Cancer Institute of Brazil, Cancer Prevention and Surveillance Coordination, Rio de Janeiro, Brazil; [2] Federal University of the State of Rio de Janeiro, Instituto Biomédico, Rio de Janeiro, Brazil

Objective: Analysis of the impact of pesticide use on the immune profile of rural workers in Casimiro de Abreu, Rio de Janeiro, Brazil.

Material and Methods: The study population consists of 80 rural workers occupationally exposed to pesticides, 25 rural residents, and 43 urban residents. For immune profile evaluation, the number of peripheral blood mononuclear cells (PBMCs) was quantified using PBMC culture from peripheral blood. From the generated supernatant, nitric oxide was measured using the Griess method. Additionally, plasma analysis was performed to quantify cytokines related to Th1, Th2, Th17, and Treg profiles.

Results: Rural workers (RW) and rural residents (RR) showed an increase in the PBMC number (RW: 18006.3; p<0,0001 / RR: 16447.9; p=0,0203) and lower production of nitric oxide (RW: 13.05uM; RR:7.32uM). Since workers and residents of rural areas showed similar quantifications in cytokine production, an intragroup analysis considering the length of residence was conducted and an increase in IFN-γ (4.89pg/mL; p=0,0444) was observed in those who lived over 40 years in the rural area. The inflammatory profile was also evidenced by the increase in IL-6 (12.37pg/mL; p=0,04) and IL-17A (178.09pg/mL; p=0,0457) in individuals over 60 years old.

Conclusion: There is a change in the immune response profile of both workers and residents in rural areas, highlighting not only the use of pesticides but also the duration of exposure. It’s important to emphasize the unrestricted use of pesticides in Brazil compared with other countries, such as those in Europe. This indicates the need for public policies, health surveillance, and effective legislation to protect public health.

P46-14 | Supporting Informed Decisions about Breast Cancer Screening in U.S. Communities with Known Environmental Contamination: A Pilot Pre-Post Study

Christine M. Gunn[2,3], Kayla Iuliano[1], Mia St. Angelo[1], **Megan E. Romano**[1,3]

[1] Dartmouth Geisel School of Medicine, Epidemiology, Lebanon, USA; [2] The Dartmouth Institute for Health Policy & Clinical Practice, Lebanon, USA; [3] Dartmouth Cancer Center, Cancer Population Sciences Program, Lebanon, USA

Objective: We tested the impact of a breast cancer screening decision aid (DA) that was adapted to include environmental risk information, on breast cancer risk perceptions, knowledge, and decisional conflict.

Materials and Methods: We conducted a pilot pre-post survey study in communities with known per- and polyfluoroalkyl substances (PFAS) contamination across four northeastern states in the United States. We used community-based recruitment and a regional survey panel to enroll 100 women ages 39-49, with no personal history of breast cancer, and no mammogram in the past 12 months. Participants completed a baseline survey, completed

the DA, and then a final survey. Baseline surveys measured demographics, information about residential drinking water, and PFAS-related risk perceptions. Paired pre-post survey measures included breast cancer risk perceptions, screening knowledge, and decisional conflict. We used paired t-tests for continuous and McNemar's tests for dichotomous outcomes to assess changes after DA use.

Results: Participants were majority non-Hispanic white (97%); 43% resided in micropolitan, small town, or rural areas, and commonly used private (unregulated) well water in their homes (35%). Only 19% had tested their water for PFAS, and 54% used a home water filter. While 62% did not experience PFAS-related stress, 25% often/constantly worried about PFAS health effects in the prior month. Before DA use, 25% felt it likely that they would develop breast cancer in the next 10 years, which reduced to 15% post-DA (p_=_0.06). Mean breast cancer screening knowledge scores increased from 8.1 pre-DA to 9.1 out of 10 post-DA use ($p<0.001$). Pre-DA, 66% were sure about the best screening schedule for them, which increased to 91% post-DA ($p<0.001$).

Conclusion: Use of a DA inclusive of environmental risk information reduced perceptions of individual breast cancer risk, improved knowledge, and reduced decisional conflict about breast cancer screening among women living in PFAS-contaminated communities.

P46-15 | Outdoor ultrafine particulate matter and risk of lung cancer in a prospective cohort of 400,000 Americans

Rena R. Jones[1], Jared A. Fisher[1], Joel D. Kaufman[2], Gerard Hoek[3], Debra T. Silverman[1]

[1] National Cancer Institute, Division of Cancer Epidemiology & Genetics, Occupational & Environmental Epidemiology Branch, Rockville, USA; [2] University of Washington, Department of Environmental and Occupational Health Sciences, Seattle, USA; [3] Institute for Risk Assessment Sciences, Utrecht University, Utrecht, Netherlands; [4] University Medical Center Utrecht, Utrecht, Netherlands

Objective: Fine particulate matter ($PM_{2.5}$, <2.5µm)is an established cause of lung cancer, but studies of ultrafine PM (<0.1µm) are extremely limited. We aimed to investigate the UFP-lung cancer association overall and by histologic subtype.

Materials and Methods: For 415,576 NIH-AARP Diet and Health Study participants aged 50+ years at enrollment (1995-1996), we estimated historical residential outdoor UFP number concentrations using a census block-level land use regression model and back-extrapolation via a spatiotemporal $PM_{2.5}$ model. We identified 19,429 lung cancers diagnosed during a mean of 23 years of prospective follow-up.We evaluated total and sex-specific associations between time-varying,10-year lagged UFP (per 10,000 particles/cm^3) and lung cancer overall and by major histologic subtype (adenocarcinoma, squamous cell carcinoma, small cell carcinoma) in Cox models adjusted for age (time scale) smoking, sex, race and ethnicity, state, and outdoor residential $PM_{2.5}$. We also evaluated relationships by smoking status.

Results: UFP concentrations were associated with increased risk of lung cancer overall (hazard ratio [HR] per 10,000 particles/cm^3=1.06, 95%CI=[1.03-1.10]) and for adenocarcinoma (HR=1.11 [1.06-1.16]). The adenocarcinoma association was stronger among men (HR=1.17 [1.10-1.25]) compared to women (HR=1.03 [0.96-1.11]; p-interaction=0.12). A positive association with squamous cell carcinoma was suggested among women (HR=1.13 [0.99-1.29] but not men (p-interaction=0.68). UFP was associated with increased risk of lung cancer overall in never smokers (HR=1.16 [1.03-1.30], and the association was weaker among former (HR=1.06 [1.02-1.11] and current

(HR=1.04 [0.99-1.09]) smokers. We found a similar pattern for adenocarcinoma: (HRs=1.18, 1.12, and 1.07, respectively). We observed no clear relationship with small cell carcinoma.
Conclusions: Outdoor UFP was associated with increased risk of lung cancer, particularly adenocarcinoma, in a large, geographically diverse U.S. cohort, contributing new evidence in support of this etiologic relationship. Further investigation of the heterogeneity in findings by sex is warranted.

P46-16 | Renal Cancer and Heavy Metals: A Systematic Review

Rokaya Elamrati, Narges Khanjani

Texas Tech University Health Sciences Center in El Paso, EL PASO, USA

The incidence of kidney tumors has steadily increased over recent decades, with environmental pollution, particularly heavy metal exposure, emerging as a potential contributing factor. This systematic review consolidates evidence on the associations between heavy metal exposures and renal cancer, particularly renal cell carcinoma (RCC). Following a comprehensive literature search and screening process, 18 studies were included, spanning diverse populations and methodologies. Meta-analysis was not possible due to the huge methodological heterogeneity of the articles. Significant positive associations with renal cancer were observed for cadmium, lead, copper, mercury, arsenic, and nickel, suggesting their role as potential risk factors. Mixed associations were reported for zinc, selenium, manganese, and iron, reflecting inconsistencies in findings across different studies and exposure assessments. In contrast, titanium, tungsten, chromium, and magnesium showed no significant association with renal cancer.
This review highlights the critical need for regulatory measures to limit cadmium and lead exposure, particularly in occupational and industrial settings, and underscores the possible role of selenium deficiency in causing RCC. Variability in study methodologies and exposure measurements points to the necessity of standardized approaches in future research. By identifying metals of significant public health concern and their associations with RCC, this review provides essential insights to guide preventive strategies and inform further investigations into the role of environmental contaminants in renal carcinogenesis.

P46-17 | A systematic approach to assessing evidence from mechanistic studies on the Key Characteristics of Carcinogens

Suril S. Mehta[1], Whitney D. Arroyave[2], William H. Bisson[2], Danila Cuomo[2], Stephen S. Ferguson[1], William M. Gwinn[1], Gloria D. Jahnke[1], Stephanie L. Smith-Roe[1], Amy Wang[1], Ruth M. Lunn[1]

[1] National Institute of Environmental Health Sciences, Research Triangle Park, USA; [2] Integrated Laboratory Systems – an Inotiv Company, Research Triangle Park, USA

Background: The Key Characteristics of Carcinogens (KCC) framework aids systematic reviewers in organizing and assessing an often large and unwieldy amount of mechanistic data for a given substance to reach a cancer hazard conclusion. However, wide variability exists within each KCC (e.g., KCC6 - Induces chronic inflammation) regarding the utility of various indicators or biomarkers (e.g., pro-inflammatory cytokines), the evidence types used (e.g., exposed humans), the numerous measurement methods used (e.g., serum concentrations of interleukins), and their informativeness in evaluating carcinogenicity. Transparent guidelines are needed to interpret the relative

informativeness of these KCC biomarkers and indicators in reaching scientific judgement regarding mechanisms of cancer.
Methods: We reviewed the published literature and consulted with experts on biomarkers, indicators, and assays for measuring KCCs. Using this information, a structured process was developed for evaluating and integrating mechanistic data, part of the upcoming Report on Carcinogens (RoC) Handbook for conducting cancer hazard evaluations.
Results: The updated RoC Handbook describes a fit-for-purpose structure to identify, evaluate, and integrate biological effects (e.g., KCCs) to reach conclusions on the level of evidence of carcinogenicity from mechanistic studies. For each KCC, guidance tables were developed on available assays, biomarkers, and indicators, and which biomarkers are most informative for evaluating mechanisms leading to cancer. These guidance tables are used for study quality evaluations of mechanistic studies, and for determining the confidence in KCC evidence. Importantly, we suggest what type of evidence is needed for high confidence for each KCC, and what type of evidence is needed to evaluate the entire body of mechanistic studies. Case examples of ongoing cancer hazard assessments are provided.
Conclusions: We offer a novel approach and resources to systematically evaluate the relative utility of each KCC biomarker to reach a judgement on the confidence of a body of evidence.

P46-18 | Primary Military Occupations and Risk for Papillary Thyroid Cancer among Officers and Enlisted Personnel

Susan Chadid[1,2], Cara Olsen[3], Matthew Horch[4], Christopher W. Edwards[5,6], Thanh D. Hoang[7], Marlene Sanchez[4], Yawei Zhang[8,9], Jordan McAdam[1,2], Jennifer A. Rusiecki[10]

[1] Henry M. Jackson Foundation for the Advancement of Military Medicine, Bethesda, USA; [2] Murtha Cancer Research Program, Bethesda, USA; [3] Uniformed Services University of the Health Sciences, Department of Preventive Medicine and Biostatistics, Bethesda, USA; [4] Uniformed Services University of the Health Sciences, Occupational & Environmental Medicine, Bethesda, USA; [5] Defense Center Public Health, Dayton, USA; [6] United States Air Force School of Aerospace Medicine, Dayton, USA; [7] Uniformed Services University of the Health Sciences, Department of Medicine/Division of Endocrinology, Bethesda, USA; [8] Yale School of Public Health, Department of Environmental Health Sciences, New Haven, USA; [9] Chinese Academy of Medical Sciences and Peking Union Medical College, Department of Cancer Prevention and Control, National Cancer Center, Beijing, China; [10] Uniformed Services University of the Health Sciences, Department of Medicine, Bethesda, USA

Introduction: Papillary thyroid cancer (PTC) incidence has increased in the U.S. since the 1980s, is even higher in military personnel, and is unexplained by better medical surveillance. We investigated risk from various military occupations on PTC incidence.
Methods: We carried out a nested case-control study ($N_{cases/controls}$ =736/736) within the U.S. military cohort matched on birth date, sex, race, and military component. PTC diagnosis was ascertained via the DoD Cancer Registry. Sourced from military records, military occupational coding and duration were grouped into 21 two-digit codes for enlisted personnel and officers. Using conditional logistic regression, we calculated odds ratios (ORs) and 95% confidence intervals for: (1) *Ever* vs *Never* in an occupation and (2) *Duration* in an occupation (5+ years vs Never).
Results: Among enlisted personnel, “Communications/Intelligence specialists” of younger age (<35 years) and male “Health Care Specialists” had increased risk (OR=1.43; 0.90-2.28 and OR=1.56; 0.83-2.93, respectively); “Craftworkers” had reduced risk (OR=0.44; 0.22-0.89). Among Officers, male “Health Care Officers” had increased risk (OR=2.33; 0.90-6.07), while those in “Tactical operations” had reduced risk (OR=0.50; 0.25-1.00). For enlisted

and officers combined, a greater risk was seen in younger "Health Care Officers" (OR=3.86;1.35-11.01) than in older. There were increased risks for working 5+ years in "Technical and Allied Specialists" (OR=2.50; 0.97-6.47), "Scientist/Professionals" (OR=3.25;1.06-9.97), "Intelligence Officer" (2.50;0.78-7.97), and "Health Care Officer" (1.60;1.01-2.53) occupations. Further categorizing by radiation exposure likelihood showed increased risk for men in "Health Care" occupations with Higher exposure likelihood (OR=1.80; 1.08-2.98) vs. Never.
Discussion: We observed inverse and positive associations for some military occupations with PTC. More consistent, increased risks were observed in health care occupations, notably in men, those diagnosed younger, and those with longer duration. Exposure to medical radiation may play a role in elevated risk for male health care professionals in our study.

P46-19 | Ambient air pollution and mortality in older breast cancer patients

Yaguang Wei[1,2], Edgar Castro[2], Kanhua Yin[3], Min Zhang[1], Hannah Thompson[1], Brent A. Coull[2], Joseph A. Sparano[4], Susan Teitelbaum[1], Robert O. Wright[1], Joel Schwartz[2,5]

[1] Icahn School of Medicine at Mount Sinai, Department of Environmental Medicine, New York, USA; [2] Harvard T.H. Chan School of Public Health, Department of Environmental Health, Boston, USA; [3] University of Missouri-Kansas City School of Medicine, Department of Surgery, Kansas City, USA; [4] Icahn School of Medicine at Mount Sinai, Department of Medicine, Division of Hematology and Medical Oncology, Tisch Cancer Institute, New York, USA; [5] Harvard T.H. Chan School of Public Health, Department of Epidemiology, Boston, USA

Objective: To evaluate associations between chronic exposures to fine particulate matter ($PM_{2.5}$), warm-season ozone, and nitrogen dioxide (NO_2) and mortality in a nationwide cohort of older breast cancer patients following diagnosis.

Methods: We constructed an open cohort from the linked SEER-Medicare database, which consisted of Medicare beneficiaries aged ≥65 years with primary diagnosis of breast cancer between 2000–2016.Patients were followed each year starting from 2000 or the first year of breast cancer diagnosis, whichever occurred later, until the earliest of death, loss to follow-up, or the study's end in 2016.Ambient concentrations of annual $PM_{2.5}$, warm-season ozone, and annual NO_2 were estimated using hybrid models at 1-km^2 grid resolution across the contiguous US. The estimates were aggregated and linked to patients' residential ZIP codes as proxy exposure measures.A three-pollutant Cox proportional hazard model was fitted to estimate the hazard rate of mortality per unit increase in exposures, adjusting for demographics, tumor characteristics, treatments, comorbidities, lifestyle factors, meteorological variables, and neighborhood-level characteristics.

Results: Among 593,333 patients included in the study, a 1-$\mu g/m^3$ increase in annual $PM_{2.5}$, 1-ppb increase in warm-season ozone, and 1-ppb increase in annual NO_2 were associated with hazard ratios for mortality of 1.0048 (95% confidence interval [CI]: 1.0026, 1.0070), 1.0021 (95% CI: 1.0013, 1.0029), and 1.0022 (95% CI: 1.0014, 1.0030), respectively. The effects were substantially larger at low exposure levels. Patients with breast cancer diagnosis at younger ages, those who received chemotherapy or radiation, and those diagnosed at later stages were more susceptible to the effects of $PM_{2.5}$ and NO_2.

Conclusions: Our findings identified air pollution as a risk factor for mortality for older breast cancer patients aged ≥65 years. Targeted strategies should be implemented to protect this at-risk population from the effect of air pollution to improve breast cancer survival.

P46-20 | A possible link between paraben exposure and skin cancer: a cross-sectional analysis of a national data

Yudan Wei[1], Jianmin Zhu[2], Carson C. Chambers[3]

[1] Mercer University School of Medicine, Department of Community Medicine, Macon, USA; [2] Fort Valley State University, Department of Mathematics and Computer Science, Fort Valley, USA; [3] Mercer University School of Medicine, MD Program, Macon, USA

Background & Objective: Parabens as antimicrobial preservatives are commonly present in a wide variety of cosmetic and personal care products. Long-term exposure through daily application of these endocrine-disrupting chemicals could be associated with increased risk of skin cancer. The objective of the study was to explore the potential relationship between paraben exposure and skin cancer in the general adult population.

Methods: A nationally representative subsample of 8068 adults, aged 20-85 years, in the 2005-2016 National Health and Nutrition Examination Survey was analyzed for the association. We examined prevalent skin cancer (including both melanoma and non-melanoma types) in association with urinary concentrations of parabens, including butylparaben (BP), ethylparaben (EP), methylparaben (MP), and propylparaben (PP), using multivariate logistic regression models, adjusting for potential confounders. To control for urine dilutions in spot urine samples, creatinine-adjusted urinary concentrations of parabens were determined for each individual.

Results: Of the study participants, 253 (weighted prevalence, 4.27%) were self-reported being diagnosed with skin cancer during 2005-2016. Individuals with skin cancer diagnosis showed a statistically significant increase in the geometric mean of urinary MP concentrations (69.58 µg/g creatinine) compared to non-skin cancer individuals (51.22 µg/g creatinine) ($p = 0.0094$). None of the other parabens showed statistically significant increase in urine concentrations among skin cancer individuals. After adjusting for potential confounders, we found that individuals in the highest quartile of urinary MP had significantly increased odds (OR=1.78; 95% CI: 1.03, 3.08) of prevalent skin cancer compared with those in the lowest quartile.

Conclusion: This study demonstrates a possible relationship between paraben exposure and skin canceramong US adults and provides a new insight into the potential role of long-term exposure through daily application of paraben-containing personal care products in skin cancer etiology. Prospective epidemiologic and mechanistic studies are warranted to further explore these interactions.

P46-21 | The Role of Zinc and IL-18 in the Pathogenesis of Gastric Cancer in High-Risk Areas of China and Its Potential Mechanisms

Jie Liu[1,2], Yongkang Li[1,2], Yanmei Gu[1,2], Yingying Wang[1,2], Yujie Wei[1,2], Lu Chen[1,2], Futian Tang[1,2], Yumin Li[1,2]

[1] Lanzhou University Second Hospital, Gansu Provincial Key Laboratory of Gastrointestinal Cancer, lanzhou, China; [2] Lanzhou University, Center for Digestive System Tumor Transformation and Innovation Engineering, Environmental Oncology Center, lanzhou, China

Objective: Gansu Province has the highest incidence and mortality rates of gastric cancer (GC) in China. This study aims to identify high-risk environmental factors and potential intervention targets.
Material and Methods: GC incidence data (2013–2021, n=76,411) from 87 counties and 262 hospitals in Gansu Province were collected to calculate ASIR. Environmental samples (water, soil, n=500 each) and biological samples (urine, serum) from 300 GC patients and 300 controls were analyzed via ICP-OES to assess element concentrations. CCK-8 and colony formation assays evaluated Zinc ($ZnCl_2$) effects on AGS and MKN45 cell proliferation. A mouse xenograft model (control, Zn-treated) was used for tumor sequencing, DEGs, GO, and KEGG analysis. Western Blot analyzed Zn regulation of IL-18 and AKT signaling. ELISA measured serum IL-18 levels in GC patients and controls. RNA-seq (10 paired GC and adjacent tissues) and IHC (241 GC patients) validated IL-18 expression. siRNA-mediated IL-18 knockdown and rIL-18 treatment assessed proliferation, migration, invasion, and AKT pathway activity, with six experimental groups (NC, rIL-18, si-IL18, NC+Zn, rIL-18+Zn, si-IL18+Zn). This study was ethically approved, and all participants provided written informed consent.
Results: High-risk GC areas were concentrated in Hexi, while low-risk areas were in Longnan. Zn levels were significantly lower in high-risk regions and GC patients ' urine and serum than in controls. Zn treatment inhibited GC cell proliferation, downregulated IL-18, and suppressed AKT phosphorylation. ELISA and RNA-seq confirmed IL-18 overexpression in GC serum and tissues, further validated by IHC. siRNA-mediated IL-18 knockdown reduced proliferation, invasion, and AKT signaling while altering the cell cycle. IL-18 promoted GC progression, whereas Zn intervention counteracted its effects. Combined Zn and IL-18 treatment further suppressed proliferation and invasion by inhibiting AKT phosphorylation.
Conclusion: IL-18 and Zn synergistically contribute to GC pathogenesis, providing potential targets for future GC diagnosis and treatment.

P46-22 | *Microplastic Exposure and Hepatocarcinogenesis: Multidimensional Molecular Crosstalk Networks and Emerging Precision Medicine Paradigms*

Libaihe Jing[1], Zihai Yang[2], Feifei Yang[3], Yumin Li[4]

[1] Lanzhou University, The Second Hospital & Clinical Medical School, Lanzhou, China; [2] Lanzhou University, The Second Hospital & Clinical Medical School, Lanzhou, China; [3] Lanzhou University, The Second Hospital & Clinical Medical School, Lanzhou, China; [4] Lanzhou University, The Second Hospital & Clinical Medical School, Lanzhou, China

This comprehensive review elucidates the multidimensional mechanisms by which microplastics (MPs), as emerging environmental contaminants, contribute to hepatocarcinogenesis and explores potential therapeutic countermeasures. Accumulating evidence indicates that MPs exert their oncogenic effects through physicochemical interactions, disrupting core signaling cascades in hepatocellular carcinoma (PI3K/AKT/mTOR, TGF-β/Smad, VEGF/VEGFR2, and Wnt/β-catenin pathways), inducing metabolic reprogramming and oxidative stress, while synergistically amplifying liver damage when combined with conventional risk factors like HBV/HCV infections. Mechanistically, MPs activate the aryl hydrocarbon receptor (AhR) to upregulate TGF-β1 expression, thereby driving hepatic fibrogenesis and subsequent malignant transformation. Their high surface adsorption capacity enhances carcinogenic potential through the concentrated delivery of hydrophobic pollutants (e.g., BPA, PAHs), while simultaneously triggering mitochondrial dysfunction and ferroptosis, alongside tumor microenvironment remodeling. Furthermore, MP-induced gut dysbiosis disrupts the gut-liver axis, accelerating NAFLD progression to hepatocellular carcinoma through chronic inflammatory pathways. In addition, microplastic exposure can also enhance tumor heterogeneity through epigenetic modification (such as the enrichment of H3K27me3, inhibiting the SOX9 gene) and immune escape (such as the upregulation of PD-L1). In response to the above mechanisms, we propose innovative treatment strategies based on existing research findings, including targeted nanodrug delivery systems, CRISPR-mediated epigenetic editing, immune combination therapies (anti-PD-L1/VEGF inhibitors), metabolic interventions (exogenous α-KG supplementation), and gut microbiota regulation. Future research needs to integrate multi-omics technologies to analyze the dynamic exposure effects of microplastics and develop precision medicine approaches to address the challenges of the environmental-host interaction in the prevention and treatment of liver cancer.

P46-23 | A narrative review of wood dust exposure and potential health risks in Vietnam

Hien T.T. Ngo[1], Aurora Le[2], Tran B. Huynh[3], Tuan N. Nguyen[4]

[1] Phenikaa University, Faculty of Public Health, Ha Noi, Vietnam; [2] Texas A&M University, Health Behavior, School of Public Health, College Station, TX 77843, USA; [3] University of Minnesota, Division of Environmental Health Sciences, School of Public Health, Minneapolis, MN 55455, USA; [4] Workplace Health Without Borders, US Branch, Ann Arbor, MI 48104, USA

Background: Wood dust, which is generated during various wood processing activities like grinding, sanding, cutting, milling, and debarking, remains a common occupational carcinogen today..
Objective: This review aims to examine the exposure to wood dust and the associated health risks in Vietnam.
Material and Methods: A narrative review was conducted using several databases, including online journals, reports, and Google Scholar, with keywords such as "wood dust," "wood dust-related legislation," "wood dust exposure," "wood dust-related health risk," and "Vietnam."

Results: In 1995, the International Agency for Research on Cancer classified wood dust as a Group 1 human carcinogen, as certain particle sizes (e.g., <4μm) from wood dust can deposit in the lungs. While hardwood dust (e.g., Oak, Teak, Mahogany) has been identified as a human carcinogen, the evidence linking softwood dust (e.g., Pine, Spruce, Fir) to carcinogenicity remains limited. Exposure to hardwood dust has been linked to various health issues, including sinonasal cancers, respiratory and digestive tract cancers, asthma, allergies, and an increased risk of lung cancer. Wood dust exposure significantly impacts workplace health, with 10% to 15% of workers being exposed. This exposure can lead to higher mortality rates among workers and may also affect the general population's mortality rate. In Vietnam, wood dust poses substantial environmental and health risks. A recent study at a wood processing company in southern Vietnam revealed that 11.8% of workers suffered from respiratory diseases. In a carpentry village, high percentages of workers reported suffering from eye diseases (67.5%), neurological disorders (55%), ear, nose, and throat diseases (36.3%), gastrointestinal issues (18.8%), and musculoskeletal diseases (25.0%).

Conclusion: Due to the significant health and social impacts of wood dust exposure, it is crucial to recognize it as a widespread occupational risk in Vietnam and to implement appropriate safety measures to protect workers in the workplace.

P46-24 | Environmental Exposure to Cadmium and Prostate Cancer Aggressiveness: A Population-Based Study

Sithembiso S. Msibi[1], Sarah Mayberry[1], Citlalli Lopez[1], Ping-Ching Hsu[2], MinJae Lee[1], Jeannette T. Bensen[3], Ebonee N. Butler[3], Laura Farnan[3], James L. Mohler[4], L. Joseph Su[1]

[1] Univesity of Texas Southwestern Medical Center, O'Donnell School of Public Health, Dallas, USA; [2] University of Arkansas for Medical Sciences, Fay W. Boozman College of Public Health, Little Rock, USA; [3] University of North Carolina, Gillings School of Public Health, Chapel Hill, USA; [4] Roswell Park Cancer Institute, Buffalo, USA

Background

Prostate cancer (PCa) is the second most common type of cancer among men worldwide. Exposure to cadmium contributes to PCa carcinogenesis and tumor progression through inflammation, endocrine disruption, epigenetic alterations, and oxidative stress. This study investigates the association between urinary cadmium levels and PCa aggressiveness.

Methods

We conducted an analysis of data from the population-based North Carolina-Louisiana Prostate Cancer Project (PCaP). A total of 1,498 participants were included with high (n = 396) or low (n = 1,102) aggressive PCa at diagnosis. Environmental exposure to cadmium was measured by analyzing urine samples from the participants using ultra-sensitive Inductively Coupled Plasma Mass Spectrometry (ICPMS). We assessed if cadmium exposure levels were associated with high aggressive PCa using multivariable logistic regression models after adjusting for demographic factors, including age, smoking status, waist-to-hip ratio (WHR), study site, race, and screening history.

Results

There was significantly higher cadmium exposure in Black individuals ($p < 0.01$) and Louisiana residents ($p = 0.03$) compared to White individuals and North Carolina residents. We found an overall positive association with elevated cadmium concentrations, with Quartile 3 showing significantly higher odds of aggressive PCa (OR: 1.99, 95% CI: 1.21–3.27, $p = 0.01$). Increased age (OR: 1.05, 95% CI: 1.03–1.07) and WHR (OR: 1.02, 95% CI: 1.01–1.03) also were linked to greater risk. Conversely, a history of PCa screening (OR: 0.39, 95% CI: 0.25–0.61) and White race (OR: 0.49, 95% CI: 0.35–0.68) were associated with lower odds of aggressive PCa.

Conclusions

Our findings highlight a concerning link between elevated cadmium exposure and increased likelihood of aggressive PCa, which suggests that cadmium may play a role in exacerbating PCa severity. Public health efforts should consider strategies to reduce cadmium exposure and mitigate its potential impact on PCa aggressiveness.

Funding: U.S. Department of Defense contract DAMD 17-03-2-0052/ award HT9425-23-1-0924

P46-25 | Mesothelioma cases and proximity to asbestos-related industries in the U.S.: A methodological approach for examining the neighborhood exposure pathway

Firoz Z. Abdoel Wahid[1], Christopher Garner[2], Yuhe Gao[3], Ye Ye[4], Michael Becich[5]

[1] University of Pittsburgh, School of Public Health/Dept. of Environmental and Occupational Health, Pittsburgh, USA; [2] University of Pittsburgh, School of Medicine/Department of Biomedical Informatics, Pittsburgh, USA; [3] University of Pittsburgh, School of Medicine/Department of Biomedical Informatics, Pittsburgh, USA; [4] University of Pittsburgh, School of Medicine/Department of Biomedical Informatics, Pittsburgh, USA; [5] University of Pittsburgh, School of Medicine/Department of Biomedical Informatics, Pittsburgh, USA

Objective

Mesothelioma, a rare and aggressive cancer primarily linked to asbestos exposure, has been extensively studied in occupational settings. However, non-occupational “neighborhood exposure” from nearby asbestos-related industries remains an overlooked yet important risk factor. This study presents a novel spatial-epidemiological framework to assess the relationship between mesothelioma cases and proximity to asbestos-handling industries in the U.S. Using data from the National Mesothelioma Virtual Bank (NMVB), this study aims to develop refined exposure metrics to improve risk assessment, guide prevention strategies, and inform environmental health policies.

Materials and Methods

This cross-sectional study integrates multiple data sources to systematically evaluate spatial exposure to asbestos. Mesothelioma case data from NMVB—including demographic, clinical information, and residential and occupational spanning the corresponding time periods —will be geocoded and mapped. Asbestos-related industrial sites from various time periods will be identified using EPA and OSHA records, with exposure categorized by distance to these sites (<1 km, 1–5 km, 5–10 km, >10 km). A composite exposure index will incorporate proximity and ambient asbestos levels, accounting for variations in historical site activity.

Advanced Geographic Information Systems (GIS) techniques, including spatial interpolation (e.g., Kriging) and clustering analysis (e.g., Getis-Ord Gi*), will visualize mesothelioma case distribution, industrial sites, and asbestos concentration estimates. Logistic regression models will assess associations between exposure levels and mesothelioma subtypes, controlling for demographic and occupational confounders. Sensitivity analyses will address potential selection bias and gaps in historical exposure data.

Conclusion

This study introduces a robust spatial-epidemiological approach to evaluating neighborhood asbestos exposure and mesothelioma risk. By integrating geospatial analysis with epidemiological data, findings will enhance exposure assessment and inform land-use planning, environmental health regulations, and risk mitigation policies. Future research should refine exposure classification and explore longitudinal health impacts of environmental asbestos exposure.

Funding & Acknowledgements

National Mesothelioma Virtual Bank for Translational Research

P47 | Advancing Exposomics: Comprehensive Assessment of Environmental and Lifestyle Exposures - PART II

P47-02 | Impact of environmental exposures on DNA methylation over the life-course in the EXPANSE project

Megi Vogli[1], Ayoung Jeong[2,3], Zhebin Yu[4], Judith Vonk[5,13], Dorina Ibi[6], Jaanika Kronberg[7], Petr Gregor[8], Lisa Maier[1,9], Miriam Leskien[1,9], Marta Cirach[10,11], Payam Dadvand[10,11], Ulrike Gehring[6], Kathrin Wolf[1], Melanie Waldenberger[1], Pavel Čupr[8], Kees de Hoogh[2,3], Gerard Koppelman[12,13], Erik Melén[14], Regina Pickford[1], Marie Standl[1], Jelle Vlaanderen[6], Roel Vermeulen[6], Nicole Probst-Hensch[2,3], Annette Peters[1]

[1] Helmholtz Zentrum München, Institute of Epidemiology, Munich, Germany; [2] Swiss Tropical and Public Health Institute, Allschwil, Switzerland; [3] University of Basel, Department of Public Health, Basel, Switzerland; [4] Karolinska Institute, Institute of Environmental Medicine, Stockholm, Sweden; [5] University of Groningen, University Medical Center Groningen, Department of Epidemiology, Groningen, Netherlands; [6] Utrecht University, Institute for Risk Assessment Sciences, Utrecht, Netherlands; [7] University of Tartu, Estonian Genome Centre, Institute of Genomics, Tartu, Estonia; [8] Masaryk University, RECETOX, Faculty of Science, Brno, Czech Republic; [9] Ludwig-Maximilians-Universität Munich, Institute for Medical Information Processing, Munich, Germany; [10] ISGlobal, Barcelona Institute for Global Health, Barcelona, Spain; [11] Universitat Pompeu Fabra, Barcelona, Spain; [12] University Medical Center Groningen, Department of Pediatric Pulmonology and Pediatric Allergology, Groningen, Netherlands; [13] University Medical Center Groningen, Groningen Research Institute for Asthma and COPD, Groningen, Netherlands; [14] Karolinska Institute and Sachs' Children and Youth Hospital, Department of Clinical Science and Education Södersjukhuset, Stockholm, Sweden

Objective: Living in an urban environment exposes the population to a mix of environmental and social factors, known as the Urban Exposome. These factors can have both protective and harmful effects on health. However, the underlying mechanisms are not yet fully understood. Environmental factors can influence DNA methylation. We therefore hypothesize that linking an urban exposome approach with epigenome-wide changes can contribute to understanding the effects of the urban exposome on human health across the lifespan.

Material and Methods: We conducted an inverse variance-weighted meta-analysis of epigenome-wide association studies of 7 European cohorts within the EXPANSE project. DNA methylation was assessed using Illumina Infinium Methylation 450K or MethylationEPIC arrays, covering 450/850K methylation sites, respectively. The urban exposures estimated at participants' home addresses included the annual exposures to PM2.5, NO_2, and O_3 (warm season), light at night (buffer 500m), Modified Soil Adjusted Vegetation Index (buffer 500m) and urbanicity**.**

Results: The total study population included 1778 (4-10 years), 878 adolescents (16 years), and 5975 adults (18-87 years). Children aged 4-6 showed 41.3% of significant differentially methylated regions (DMRs) associated with monthly NO_2, whereas children aged 8-10 years had 87.5% associated with urbanicity. In both age groups, the top five DMRs mapped to the same genes: *HOXA5*, *ZBTB22*, *CALCB/CALCA*, and *GNAS*. In adolescents, 41.9% of significant DMRs were associated with monthly PM2.5, while in adults, significant DMRs were generally associated with all exposures including greenness. Both life stages mapped to the *PEG10* and *MEST* genes.

Conclusion: Exposure to urban environmental factors can induce DNA methylation changes that may impact health across all life stages. Our findings suggest that both children and adults are particularly vulnerable to the urban exposome. Additionally, there is a distinct contrast in the methylation changes associated with greenness compared to other urban environmental factors.

P47-03 | Integrating Exposome Approach into a New National Human Biomonitoring Study in Greece: The HERACLES (Exposome and Health) study.

Catherine Gabriel[1,2], Maria-Nefeli Georgaki[1,2], Nafsika Papaioannou[1,2], Marianthi Kermenidou[1,2], Anthoula Chatzimpaloglou[1,2], Alkistis Kevrekidou[1,2], Ioanna Kantara[1,2], Elisavet Renieri[1,2], Spyros Karakitsios[1,2], Dimosthenis Sarigiannis[1,2]

[1] Aristotle University of Thessaloniki, Chemical Engineering, Thessaloniki, Greece; [2] Research Centre on the Exposome and Health (HERACLES), Center for Interdisciplinary Research and Innovation (KEDEK), Thessaloniki, Greece

Objectives:
The HERACLES study pioneers a holistic exposome framework in human biomonitoring, moving beyond single-chemical assessments to capture a broad spectrum of environmental exposures—including chemical, physical, and psychosocial stressors—throughout an individual's lifetime.
Materials and Methods:
A cross-sectional study is recruiting a representative Greek population sample across age groups: 300 children (6–11 years), 300 teenagers (12–17 years), and 500 adults (18+ years). Biological samples (blood, urine, and hair for children) are collected alongside detailed questionnaires on lifestyle, diet, occupational exposures, and environmental conditions. Environmental samples, including tap water, dust, soil, and air, are collected from participants' homes to assess potential household exposures. Additionally, all participants wear silicone wristbands as a passive sampler to measure personal exposure to environmental contaminants. High-resolution analytical techniques, including mass spectrometry, quantify a wide range of chemical exposures, alongside targeted chemical analyses. Advanced metabolomics assessment and statistical models assess cumulative exposures and stressor interactions, ensuring a comprehensive exposome assessment, and providing insights into environmental and personal exposure impacts on health. Ethical guidelines are strictly followed, with informed consent obtained.
Results:
Multi-level exposure assessments are ongoing, with preliminary data showing high participant engagement and compliance. Notably, Greek participants demonstrate exceptional willingness, reflecting a shift in public engagement with environmental initiatives aligned with the Green Deal. Initial findings reveal regional variations in exposure levels influenced by local environmental and socio-economic factors, with further analyses exploring their correlation with health outcomes.
Conclusion:
HERACLES marks a transformative step in environmental health research by integrating the exposome paradigm. This study provides a comprehensive assessment of environmental exposures and their health impacts, shaping evidence-based public health strategies and regulatory policies in Greece.
Acknowledgments: This project has received funding from the European Union's Horizon Europe research and innovation programme under Grant Agreement No 101057014.

P47-04 | Blood exposomics and breast cancer incidence: a prospective case-cohort analysis in the Sister Study

Che-Jung Chang[1], Katherine Pullella[1], Sami N. Teeny[2], Anna S. Young[3], Douglas Walker[3], Xin Hu[3], Dean Jones[2], Alexandra White[1]

[1] National Institute of Environmental Health Sciences, Epidemiology Branch, Research Triangle Park, USA; [2] Emory University, School of Medicine, Department of Medicine, Atlanta, USA; [3] Emory University, Rollins School of Public Health, Gangarosa Department of Environmental Health, Atlanta, USA

Objective: Breast cancer incidence is increasing yet there is little information about how environmental chemicals contribute to risk. This study leverages exposomics technology to examine a large number of chemicals in blood in relation to breast cancer incidence.
Methods: A case-cohort study of 1,559 invasive breast cancer cases and 2,623 non-cases was sampled from the Sister Study. Baseline blood samples (2003−2009) were analyzed in duplicate using gas chromatography-high-resolution mass spectrometry. Chemical features were extracted via XCMS and annotated using PeakWalk with in-house libraries (921 exogenous chemicals). Only chemicals with pairwise correlation >0.6 between fragments were retained. Chemical abundances were averaged across duplicates, log_2-transformed, and imputed with the minimal abundance/sqrt(2) if below detection. Self-reported breast cancer diagnosis was validated via medical records. Cox models were used to estimate hazard ratios (HRs) per interquartile range increase, adjusting for age, race, income, and multiple comparison with false discovery rate correction at 20%.
Results: Women had an average baseline age of 55.8 years, mostly of higher socioeconomic status and non-Hispanic White. Of 1,129 chemical fragments, 513 (≥40% detection frequency) were analyzed, yielding 230 unique chemicals. Over an average of 7.4 years follow-up, we found no significant association after multiple comparison correction. However, elevated HRs were observed for 3-hydroxycarbofuran (HR=1.16, 95%CI 1.03−1.30, p=0.01), ethyl myristate (HR=1.04, 95%CI 1.00−1.08, p=0.04), and PCB-28 (HR=1.07, 95%CI 1.01−1.14, p=0.02), while inverse associations were found for tributyl phosphate (HR=0.90, 95%CI 0.82−0.99, p=0.03) and 4-chloro-2-nitrotoluene (HR=0.90, 95%CI 0.81−1.00, p=0.04). Similar associations were observed across all fragments of these chemicals.
Conclusion: Although no significant association remained after multiple comparison correction, suggestive associations with pesticides, industrial and consumer product chemicals highlight the need for further research on modifiable environmental chemicals and breast cancer risk. This study also demonstrates exposomics as an efficient tool for comprehensively assessing chemical-breast cancer relationships or identifying novel associations.

P47-05 | Non-targeted Analysis of Plasma Samples Identifies Environmental Contaminants Linked to Euploid Early Pregnancy Loss

Dimitri Abrahamsson[1], Xiaowen Ji[3], Bahar Yilmaz[1], Amrita Nagasuri[4], Mona Aminbeidokhti[5], David Huang[1], Hakan Cakmak[1], Marcelle I. Cedars[1], Tomiko Oskotsky[6], Marina Sirota[2,6], Aleksandar Rajkovic[1]

[1] University of California, San Francisco, Department of Obstetrics, Gynecology and Reproductive Sciences, San Francisco, USA; [2] University of California, San Francisco, Department of Pediatrics, San Francisco, USA; [3] California Environmental Protection Agency, Department of Toxic Substances Control, Berkeley, USA; [4] University of California, San Francisco, Health Data Sciences, San Francisco, USA; [5] University of California, San Francisco, Pathology, San Francisco, USA; [6] University of California, San Francisco, Bakar Computational Health Sciences Institute, San Francisco, USA

Early pregnancy loss (EPL) affects approximately 15% of clinical pregnancies, with a subset of cases involving euploid losses that are not attributed to genetic abnormalities. This study aimed to investigate potential environmental exposures contributing to euploid EPL, utilizing a non-targeted analysis approach to compare affected cases with healthy pregnancies. Recent technological advances in high-resolution mass spectrometry (HRMS) have enabled us to study a wide spectrum of chemical exposures that would otherwise remain undetected with conventional targeted analysis approaches. Plasma samples were collected from 56 women with confirmed euploid EPL and 35 women with ongoing, healthy pregnancies. All EPL cases had normal uterine anatomy, and pregnancy tissue was verified as euploid by microarray. Plasma samples underwent cold methanol extraction and were analyzed using liquid chromatography-quadrupole HRMS in both positive and negative electrospray ionization (ESI) modes. Hierarchical clustering and multiple hypothesis correction (Benjamini-Hochberg) were applied for statistical analysis. Data analysis and visualization were performed using Python as the programming language, scikit-learn for statistics, and seaborn for visualizations. A total of 72,555 chemical features were detected, with 15,930 showing significant differential enrichment between EPL cases and controls ($p<0.01$). Hierarchical clustering revealed distinct metabolomic signatures between the cases and controls. Elevated levels of several exogenous compounds were found in EPL cases, including Deethylatrazine (metabolite of Atrazine, herbicide), N-Butylbenzenesulfonamide (plastic stabilizer), and Tebuthiuron (herbicide). The distinct exposure profiles observed in euploid EPL cases suggest that specific environmental contaminants may be associated with pregnancy loss. The identification of exogenous compounds highlights potential environmental risk factors, warranting further research into their role in euploid EPL pathophysiology.

P47-06 | BGS enzymatic hydrolysis tool suitable for EDCs monitoring in human and animal models

Elias Villalobos, Kura Biotech - Finden

Kura Biotech, Finden - Toxicology, Atlanta, USA

This literature review presents BGS™, a recombinant betaglucuronidase and arylsulftase enzyme used for various applications. When incorporated into liquid chromatography-tandem mass spectrometry quantification methods, it enables the detection of conjugated xenobiotics and steroid hormones in human and animal models.

Study	Incubation time	Incubation T°	Sample type	Application
(1)	30 min	52°C	Zebrafish	EDC; Steroid hormone profiling
(2)	30 (plasma) 60 (liver) min	52°C	Rat	Steroid hormone profiling
(3)	16 hours	45°C	Urine, plasma, breast milk	Human exposome biomonitoring
(4)	30 min	52°C	Urine, follicular, seminal fluid, plasma	Human exposome biomonitoring

The animal model studies incorporated BGS to measure steroid hormone fluctuations and gene expression changes in zebrafish(1), revealing endocrine-disrupting effects of environmental pollutants. Similarly, research in rat plasma and liver(2) presented an optimized method for steroid hormone profiling in tissue, broadening the available matrices to look into.

The human exposome studies explore using enzymatic hydrolysis to quantify total xenobiotic exposure, focusing on plasticizers, bisphenols, PFAS, and steroid hormones. These studies (3,4) demonstrated the effectiveness of enzymatic hydrolysis in different human matrices, accompanied by lower background noise due to its recombinant nature. Hence, incorporating an effective enzymatic tool ensures a more accurate measure of EDCs in human matrices.

As exposome monitoring advances, enzymatic hydrolysis can become a crucial step, facilitating cross-species comparisons, streamlining workflows, and enabling high-throughput analyses. Its integration with conjugated biomarker research can enhance method development in environmental health, toxicology, and clinical diagnostics, ensuring precise and consistent detection of chemical exposures and their effects.

References

1.

Maria Revenikioti, 2023, An Investigation of The Link Between Endocrine Disruption and Developmental Neurotoxicity Induced by Environmental Pollutants : In Zebrafish Embryos; Retrieved from https://urn.kb.se/resolve?urn=urn:nbn:se:uu:diva-505618

2.

Sara Evangelista et. al, Talanta, 2024, 266, 124981

3.

Yasmin Fareed et al, Exposome, 2022, 2(1), osac008

4.

Jaye Marchiandi et al, Environ. Sci. Technol. 2024, 58, 46, 20352–20365

P47-07 | A Multisystemic Exposomic Approach: Investigating the Combined Effects of Environmental Pollutants, Social Determinants, and Lifestyle Factors on Health Outcomes

Emmanuel Obeng-Gyasi

North Carolina Agricultural and Technical State University, Built Environment, Greensboro, USA

Despite growing recognition of the complex interplay between environmental exposures and health outcomes, relatively few studies have simultaneously examined the impact of multiple pollutants on multiple health outcomes within a single analytic framework. Most research to date has focused on isolated exposures or singular disease processes, failing to account for the reality that pollutants co-occur in complex mixtures and interact with biological systems through diverse mechanistic pathways. Likewise, disease processes rarely occur in isolation but emerge from the cumulative dysregulation of multiple physiological systems, often presenting as comorbid conditions such as cardiovascular dysfunction and stress-related mental health disorders. A more integrated approach is necessary to elucidate how environmental mixtures contribute to multisystemic health effects and to identify pathways through which combined exposures influence disease risk.

To address these limitations, this study employs an exposomic approach to investigate the simultaneous effects of multiple environmental pollutants, including heavy metals and per- and polyfluoroalkyl substances (PFAS), alongside social determinants and lifestyle factors, on both individual and cumulative health outcomes such as liver and kidney dysfunction, depression, and cardiovascular risk. Using data from NHANES, we applied structural equation modeling (SEM) to assess relationships between exposures and health outcomes, factor analysis to derive latent variables representing clusters of disease processes, and mixture models such as Bayesian Kernel Machine Regression (BKMR) to examine interactive effects between exposures and health outcomes.

Results indicate that exposure to chemical mixtures differentially influences individual organ system dysfunction and cumulative disease burden. Furthermore, social and lifestyle factors significantly modulate these relationships, providing additional insight into how disease processes manifest and progress.

By integrating multiple pollutants, social determinants, and diverse health outcomes, this study underscores the multisystemic consequences of environmental exposures and highlights the need for comprehensive public health strategies that address both environmental and psychosocial determinants of health disparities.

P47-08 | Temporal Patterns of Xenobiotics in Urine: A Non-Targeted LC-HRMS Analysis Pilot Study

Jinhyun Kwon[1], Sungkyoon Kim[1,2], Thomas McGrath[2]

[1] Seoul National University, Department of Environmental Health Sciences, Seoul, South Korea; [2] Seoul National University, Institute of Health and Environment, Seoul, South Korea

Introduction: Human exposome remains poorly understood due to the analytical challenges in measuring diverse chemical exposures and their temporal variations. This pilot study employs non-targeted LC-HRMS analysis to characterize the diurnal changes of xenobiotics in serially collected urine samples over 48 hours.

Materials and methods: Urine samples collected from healthy adult volunteers for 48 hours were pooled into a total of 7 samples per participant at 6-12hr intervals. Samples were prepared by solid phase extraction followed by filtration and analyzed in triplicate on a UPLC-QTOF-MS system in both ESI modes. QC measures included urine spiked with mixtures of 31 chemical standards. Data processing was conducted in R using patRoon version 2.3.3, applying the Open-MS peak picking algorithm, with normalization by the intensities of internal standards. Aligned features were screened against suspect lists from four nationwide biomonitoring surveys and annotated using MetFrag *in-silico* and online mass spectral databases following criteria described by Schymanski *et al*. (2014).

Results and discussion: In the preliminary results, 63,361 features were detected, and 832 features were matched with suspect lists. Phthalate metabolites, pyrethroid pesticide metabolites, and parabens (ethyl-, propyl-, and butyl-paraben) were tentatively identified across all time points with a maximum 16-fold intensity variation. Benzophenones were sporadically found along with metabolites of alternative plasticizers, fragrances, and UV filters, which is related to the use of personal care products.

Conclusion: This pilot study demonstrated a non-targeted workflow for analyzing xenobiotics and suggesting optimal sampling times for chemical exposure assessment. Further investigations are needed into how these temporal patterns vary within populations.

Acknowledgments: This research was supported by Brain Pool program funded by the Ministry of Science and ICT through the National Research Foundation of Korea (RS-2023-00281992) and the Ministry of Education of the Republic of Korea and the National Research Foundation of Korea (BK21 FOUR 5199990214126).

P47-09 | Community Wide Perspectives, Methodologies, and Applications in Exposomics: Results from the Network for Exposomics in the U.S. (NEXUS) Center for Exposome Research Coordination Survey

Krystal J. Godri Pollitt[1], Jeremy P. Koelmel[1], Randolph R. Singh[2], Thomas O. Metz[3]

[1] Yale University, Environmental Health Sciences, Yale School of Public Health, New Haven, USA; [2] Columbia University, Environmental Health Sciences, Columbia Mailman School of Public Health, New York, USA; [3] Biological Sciences Division, Pacific Northwest National Laboratory, Richland, USA

In 2005, Dr. Christopher Wild coined the term 'exposome' to describe an audacious concept-the suite of exposures (physical, chemical, social, lifestyle, ecosystems) a person experiences over a lifetime. Unfortunately, the scientific community was slow to embrace the concept. How could we measure the totality of exposures throughout the lifespan? Instead of measuring everything, the majority of which is likely inconsequential, why not focus on measuring those factors that have the greatest impact on health and that can actually be measured? NEXUS (Network of EXposomics in the United States), is a Center for Exposome Research Coordination (CERC), focused on operationalizing the concept by advancing measurement technologies and applying them to human health outcomes. One sub-goal of NEXUS is to aggregate data across a wide range of laboratories to facilitate knowledge exchange on the exposomic coverage of current and emerging technologies and identify streamlined, scalable, and harmonized analytical and computational infrastructures needed to systematically characterize the exposome. As a first step, a survey on methods and tools used to measure molecular features of the exposome was developed and sent out to over 1000 community members. The survey consisted of questions pertaining to the type of omics-studies employed by laboratories (e.g., lipidomics, genomics, proteomics, metabolomics, xenobiotics analysis, etc.), types of sample preparation and extraction methods employed for each omics-field, instrumentation, and data-processing methodologies. We will discuss survey results, and the design of a new exposome ring trial based on survey findings to solicit feedback from the community at ISES/ISEE. Ultimately, we hope that the results of this survey and the ring trial will inform the development of a harmonized method for measuring the exposome.

P47-10 | The Operationalization and Validation of Chronic Stress

Matthew Hill[3], Emmanuel Obeng-Gyasi[1], Sayed Mostafa[2]

[1] North Carolina A & T State University, Built Environment, Greensboro, USA; [2] North Carolina A & T State University, Mathematics, Greensboro, USA; [3] North Carolina A & T State University, Applied Science and Technology, Greensboro, USA

Objective: Environmental factors significantly influence physiological stress responses, increasing the risk of chronic diseases. Chronic stress, a silent epidemic, drives biological wear and tear known as allostatic load (AL), contributing to cardiovascular disease, diabetes, and mental health disorders. Despite its impact, accurately measuring chronic stress—particularly in the context of environmental exposures—remains a challenge, limiting efforts to mitigate its health effects.

This study examines multiple operationalizations of the Allostatic Load (AL) index and introduces the Chronic Stress Indicator (CSI), a novel measure that integrates socioeconomic and behavioral stressors alongside traditional biomarkers.

Materials and Methods: Using data from the MIDUS II Biomarker Project, a nationally representative sample of U.S. adults aged 34 to 84, we evaluated the utility of these models for stress-related health outcomes. Advanced statistical techniques, including the Boruta algorithm and factor analysis, refined biomarker selection and weighting for AL and CSI. Uncertainty analysis was conducted to further test the robustness of these constructs.

Results: Our findings show that chronic stress measures incorporating both physiological and socio-behavioral factors provided the most robust assessment of stress-related health risks. We also observed variability in how different stress models ranked, suggesting that different stress dimensions may be more relevant to specific health conditions.

Conclusion: The environment in which one lives plays a critical role in stress levels and long-term health. By improving how stress is measured, this study enhances the ability to assess the impact of environmental factors on stress and, subsequently, health outcomes. These advancements pave the way for more precise evaluations of environmental stressors and their role in shaping public health and health management strategies.

Funding for this work provided by Title III HBGI grant from the U.S. Department of Education

P47-11 | Exploring neighborhood hazard exposure and asthma risk in adults: interaction with social vulnerability

Yujing Chen[1], Farida S. Akhtari[4], Chirag J. Patel[5], Alison Motsinger-Reif[4], Ming Kei Chung[1,2]

[1] The Jockey Club School of Public Health and Primary Care, The Chinese University of Hong Kong, Hong Kong, Hong Kong; [2] Institute of Environment, Energy and Sustainability, The Chinese University of Hong Kong, Hong Kong, Hong Kong, Hong Kong; [3] Li Ka Shing Institute of Health Sciences, The Chinese University of Hong Kong, Hong Kong, Hong Kong; [4] Biostatistics and Computational Biology Branch, Division of Intramural Research, National Institute of Environmental Health Sciences, Durham, USA; [5] Department of Biomedical Informatics, Harvard Medical School, Harvard University, Boston, USA

Objective: We aim to investigate mixture effects of residential hazards on adult asthma risk across social vulnerability groups.

Materials and Methods: This cross-sectional study included 7092 adults from the Personalized Environment and Genes Study (PEGS), a North Carolina-based cohort (2002-current) with questionnaire-based health and exposure data, genomic data and geospatial linkage data. Adult asthma was defined as ever asthma diagnosis and recurrent attacks in adulthood. Exposure to 29 residential hazards was estimated using established spatiotemporal model and hazard maps, including multiple air pollutants and their chemical constituents, Toxics Release Inventory emissions, highway density, and proximity to waste or toxicant sites. The census tract-based social vulnerability index (SVI) composed of four demographic and socioeconomic themes was assigned to the residences according to the survey year. Exposome-wide associations for individual hazards with asthma were examined in high and low SVI groups (stratified by median percentile), and joint effects of mixture exposure were analyzed using quantile g-computation.

Results: Eight environmental hazards were positively associated with asthma in the high SVI group, four of which were also identified as asthma risk factors in the overall population [False discovery rate (FDR)< 0.05]. Eleven hazards showed stronger associations with asthma in the high SVI group compared to the low SVI group ($FDR_{interaction}$ < 0.10), particularly air pollutants. Combined exposure to these hazards was associated with a 21-23%

increase in asthma risk (per quintile increase), with organic matter in fine particulate matter and proximity to dry-cleaning solvent contamination being the primary contributors. Neighborhoods with vulnerabilities in household composition, housing, and transportation had higher hazard-related asthma risk ($FDR_{interaction} < 0.05$).
Conclusion: Individuals residing in high SVI neighborhoods experienced higher exposure to environmental hazards and an increased risk of asthma. Targeted interventions should prioritize communities with poor housing and transportation conditions to mitigate these risks.

P47-12 | Can Reducing the Use of Personal Care Products Lower Synthetic Phenol Exposure?

N. Jovanovic[1], S. Lyon-Caen[1], C. Thomsen[2], M. Casas[3], R. Slama[1], A. Rocabois[1], C. Chevrier[4], R. McEachan[5], T. Yang[5], A. Kaur Sakhi[2], M. Vrijheid[3], C. Philippat[1], A. Davias[6]

[1] Université Grenoble Alpes, Inserm, eam of Environmental Epidemiology Applied to Development and Respiratory Health, Grenoble, France; [2] Norwegian Institute of Public Health, Oslo, Norway; [3] IS-Global, Barcelona, Spain; [4] Univ Rennes, Inserm, EHESP, Institut de Recherche en Santé, Environnement et Travail - UMR_S 1085, Rennes, France; [5] Bradford Institute for Health Research, Bradford, UK; [6] Harvard T.H. Chan school of Public Health, Boston, Massachusetts, United States of America

Objectives: We conducted an intervention study aiming to reduce participants' use of Personal Care Products (PCPs) and cosmetics and evaluated the impacts of these behavioral changes on urinary phenol concentrations.
Materials and Methods: 103 women were recruited from Grenoble University’s campus (France). Participants recorded their baseline PCP usage over two days using a logbook-style app. Subsequently, during the intervention that lasted five days, participants were instructed to avoid their usual PCPs and only to use those provided by the research team. These did not contain the key chemicals of interest in their ingredients lists and were mostly not contained in plastic packaging. Participants recorded their PCP usage using a logbook-style app. They also collected all urine produced during each phase, that were, for each individual, combined into 24-hour pooled samples. Eleven phenols were assessed and median urinary concentrations for each stage were compared using a paired Wilcoxon rank test. Additionally, a Health Impact Assessment (HIA) was conducted to evaluate the intervention’s potential effects on asthma and IQ loss in children, within the hypothesis that the intervention will be applied to French pregnant women.
Results: Of the 11 phenols tested, 10 were detected in over 66% of samples, and 8 in more than 80%. Notably, median concentrations of several phenols had a statistically significant decrease during the intervention. That included Bisphenol A (median dropped by 40%), Methyl paraben (30%), Ethyl paraben (25%), and Propyl paraben (28%). The HIA suggested that the observed reduction in Bisphenol A concentrations, could prevent 6% (95% CI: 0.4%; 28.4%) of childhood asthma cases and prevent a loss of 0.45 (95% CI: -0.75; 2.48) IQ points.
Conclusion: The intervention effectively reduced urinary concentrations of bisphenol A and 3 parabens. and can have substantial impact for public health as highlighted by the HIA results.

P47-13 | Targeted Envirome and Microbiome Assay: A Comprehensive and High-Throughput LC-MS/MS Method for Multi-Class Serum Analysis.

Ravikumar Jagani[1], Hiraj Patel[1], Jasmin Chovatiya[1], Rosalind Wright[1,2], Robert O. Wright[1], Manish Arora[1], Mary S. Wolff[1], Jia Chen[1], **Syam S. Andra**[1]

[1] Icahn School of Medicine at Mount Sinai, Institute for Exposomic Research, Department of Environmental Medicine, New York City, USA; [2] Icahn School of Medicine at Mount Sinai, Department of Public Health, New York City, USA

Objective: To develop and validate a comprehensive liquid chromatography-tandem mass spectrometry (LC-MS/MS) assay for quantitative analysis of approximately 250 biomarkers in human serum, including environmental chemical exposures, their metabolites, and microbiota-derived endogenous compounds.

Material and Methods: The assay utilizes isotope dilution, solid-phase extraction, and reverse phase LC-MS/MS analyses in positive and negative electrospray ionization modes. Multiple reaction monitoring with isotopic standards and multi-point calibration curves enables biomarker detection and quantification. A single sample preparation method requiring 0.2 mL of serum followed by four tailored LC injections was optimized for a multi-class serum assay. Method performance was evaluated for linearity, sensitivity, precision, recovery, and matrix effects. The method was applied to quantify biomarkers of interest in NIST SRMs and pooled QC human serum. The method was adapted to a 96-well plate format for high-throughput analysis.

Results: The Targeted Envirome and Microbiome (TEAM) assay successfully detects and quantifies about 250 biomarkers across 25 chemical classes in serum. The assay includes 150 biomarkers from xenobiotic chemical classes belonging to environmental contaminants, diet-related compounds, industrial chemicals, personal care products, and pharmaceuticals. Additionally, it covers 100 biomarkers from microbiota-derived endogenous chemical classes, including amines, amino acids, arylsulfates, benzenediols, bile acids, carbohydrates, fatty acids, flavonoids, imidazoles, indoles, purines, pyrimidines, and quinolones. The TEAM assay detects both parent chemicals and their metabolite compounds in some cases, such as phthalates. The limits of detection range from 0.05 to 5.0 ng/mL, with most analytes ≤0.5 ng/mL. Extraction recovery rates range from 60% to 110%, with most analytes ≥80%. Good quantitative precision was observed, with inter-batch variation below 25% for most biomarkers.

Conclusion: The TEAM assay offers a comprehensive, high-throughput method for quantifying about 250 biomarkers in serum, including environmental chemicals, their metabolites, and microbiota-derived metabolites. This enhances our understanding of environmental and dietary influences on human health.

P47-14 | Improving the Characterisation of The perinatal chemical exposome: SS/NTS Based HRMS strategies for global internal exposure profiling

Tarek Moufawad[1], Solene Motteau[1], Ingrid Guiffard[1], Emmanuelle Bichon[1], Laurent Debrawer[2], Sylvia Sanquer[3], Marja Lamoree[4], Bruno Le Bizec[1], Jean-Philippe Antignac[1], German Cano-Sancho[1]

[1] Oniris, INRAE, LABERCA, 443000 Nantes, France; [2] Toxalim (Research Centre in Food Toxicology), INRAE UMR 1331, ENVT, INP-Purpan, Paul Sabatier University, Toulouse, France; [3] Service de Biochimie Métabolique, Hôpital Necker-Enfants Malades, GHU Paris Centre, AP-HP, F-75015 Paris, France; [4] Chemistry for Environment & Health, Amsterdam Institute for Life and Environment (A-LIFE), Vrije Universiteit Amsterdam, De Boelelaan 1085, 1081HV, Amesterdam, Netherlands; [5] French Institute for Agricultural and Environmental Research, Paris, France

The exposome encompasses all environmental and socio-economic factors encountered over a lifetime, complementing the genome in explaining their roles in health outcomes. Since the concept's introduction in 2005, significant progress has been made in advancing methods, tools, and knowledge to assess the exposome and its impact on human health. However, chemical exposure during the perinatal period remains understudied, largely due to the challenges of accessing appropriate and sufficient amount of biological samples required to conduct multiple analysis to detect a broad range of chemicals.

To overcome these limitations, the application of innovative suspect screening (SS) and non-targeted screening (NTS) approaches based on high-resolution mass spectrometry (HRMS) offers a more effective approach by one analysis to characterize and chemically profile a wider range of substances. These advanced analytical approaches allow, with a single analysis, for the detection of a wide array of compounds covering a broad spectrum of physicochemical properties. This strategy not only enhances global human biomonitoring but also improves the identification of chemicals of emerging concern in an early warning perspective.

We have developed streamlined workflows, from sample preparation to data analysis, to investigate various maternal (urine, serum, maternal milk) and foetal (cord blood, amniotic fluid) biological matrices from diverse mother-child cohorts. These methods were applied in a range of research projects conducted at national and EU level. The proposed presentation will describe these different proof-of-concepts enabling the determination of real-life chemical mixtures composing the internal exposure related to the perinatal period.

P47-15 | Characterizing and health risk assessment oforganophosphorus flame retardants (OPFRs):An exposome-wide approach and biomonitoring data

Wei-Hsiang Chang[1,2], Tzu-An Liu[1], Pei-Yu Wu[1], Yu-Hsuan Liu[2], Chia-Hui Chieh[2]

[1] National Cheng Kung University, Department of Food Safety/Hygiene and Risk Management, Tainan, Taiwan; [2] National Cheng Kung University, Department of Environmental and Occupational Health, Tainan, Taiwan

Objective: Organophosphate flame retardants (OPFRs) are substitutes for polybrominated diphenyl ethers and are widely used as flame retardants and plasticizers in commercial products. Human exposure to OPFRs has raised great concern due to associations with adverse health effects, especially in susceptible populations. This study investigates the total body burden and interaction of four exposure pathways of OPFRs in parent-child pairs, aiming to develop a multiple exposure-biomonitoring model (MEBM) to identify their contributions.
Material and Methods: Eleven OPFRs in residential environments (air, dust, dermal), selected foods, and corresponding urinary metabolites were measured in 89 parent-child pairs. Average daily doses from inhalation, dust ingestion, and dermal exposure were estimated. Total OPFR intake was back-calculated from urinary metabolites, and correlations between exposure pathways and internal doses were analyzed. The correlations between the OPFRs in four major pathways as well as the external and internal dose were explored.
Results: TBOEP was the most abundant OPFR, followed by TCIPP in house dust and skin wipe samples, while TCIPP dominated indoor/outdoor air samples. Significant correlations were found between TBP, TCIPP, TPHP, and TCP concentrations in skin wipe, indoor air, and house dust samples. TBOEP and TCIPP were the most abundant across all exposure pathways. DBP, DPHP, and BBOEP were the dominant OPFR metabolites in urine samples (detectability > 99%). Positive correlations between external exposure (air inhalation and dermal absorption) and internal intake were observed for TDCIPP and TBP.**Conclusion:** Dermal absorption is the main exposure pathway for OPFRs, though inhalation of air and ingestion of house dust are also significant. Our results show significant correlations between the internal dose of BDCIPP and the external dose of TDCIPP via inhalation and dermal absorption. The MEBM successfully explored the influence and contribution of multiple exposure pathways. Non-carcinogenic and carcinogenic risk assessments indicate no unacceptable risk for each pathway.

P47-17 | Importance of epidemiology and visualization to integrate non-chemical stressors into cumulative impact assessment for vulnerable communities

William Rish, Ann Verwiel

ToxStrategies LLC, Exposure Science Practice, Asheville, USA

Cumulative exposure to chemical and non-chemical stressors combine to influence susceptibility to health effects. Currently, the most common way to address exposure to non-chemical stressors in the context of cumulative impact assessment (CIA) is using screening tools. While screening tools can generally identify communities that have the potential for disproportionately higher exposure to chemical and non-chemical stressors, screening tools do not quantitatively assess the contribution of the interaction among chemical and non-chemical stressors to cumulative

health impacts. As part of a comprehensive review of CIA methods, a reoccurring recommendation was that multi-stakeholder collaboration using visualization tools in conjunction with epidemiological information could be used to account for and communicate causal relationships between non-chemical stressors, chemical stressors, and cumulative health impacts.

Our work involved a structured literature search that identified currently available information and methods for addressing the interaction of non-chemical and chemical stressors in CIA. A total of 74 articles were reviewed that used observational epidemiology to address potential associations between health effects and various other factors typically associated with vulnerable communities. The role of observational epidemiology encompassed in these studies is summarized along with opportunities, challenges, and limitations to using epidemiology for incorporating non-chemical and chemical stressor interactions into a CIA. In addition, available visualization tools are summarized in the context of their use in conjunction with epidemiological information for facilitating multi-stakeholder and multi-discipline planning and agreement on key associations among chemical and non-chemical stressors and cumulative health impacts, strength of associations, and quality of underlying evidence. This can improve the design of the CIA, the effectiveness of interventions, and acceptance of decisions based on the CIA findings.

P47-18 | Longitudinal Multi-Omics Profiling Of The Human Exposome In Crohn’s Disease

Xinyue Zhang[1,2], Xiangpin Lin[1], Meije B. Tiersma[1], Ji Hyun Kim[1], Jessi W. Li[1], Joseph C. Wu[1], Alka Goyal[3], Sarah E. Streett[4], Michael P. Snyder[1,5]

[1] Stanford Univresity, Department of Genetics, Stanford, USA; [2] Stanford University, Stanford Cardiovascular Institute, Stanford, USA; [3] Stanford University, Division of Pediatric Gastroenterology, Stanford, USA; [4] Stanford University, Division of Gastroenterology and Hepatology, Stanford, USA; [5] Stanford University, Stanford Center for Genomics and Personalized Medicine, Stanford, USA

Human health is determined by the genome, the environment and their interactions. Despite significant milestones on the genome, our knowledge of the environment remains remarkably limited. Increasing evidence suggested that the exposome, which is the collection of one’s lifetime environmental exposures, play an important role in the etiology of complex diseases, such as Crohn's disease (CD). CD is a type of inflammatory bowel disease, the exact cause of which is unknown. Traditional methods that focus on either one or one class of exposures or single time point design fail to provide a holistic view of the effects of environmental exposures on the disease trajectory. To this end, we adopted a precision environmental health approach to investigate individual human exposomes for potential triggers in the development and progression of CD using multi-omics tools. In this pilot study, we longitudinally monitored the airborne environmental, biological, and chemical exposures of CD adult and pediatric patients, along with a healthy control cohort, using a wearable device, termed exposometer for up to 12 months. The collected biological and chemical exposure samples were analyzed using non-targeted approaches, i.e. next generation sequencing and mass spectrometry, respectively. In addition, heart rate, daily steps count and sleep duration were measured by smartwatches. At-home blood microsampling and stool samples were collected for metabolome, immunome and gut microbiome profilings. By integrating environmental exposures, physiological measurements, metabolomics, immunome and microbiome profilings, our study not only improves our understanding of the environmental factors of CD etiology but also potentially opens new avenues for disease prevention and management.

P47-19 | Sensors and Metadata: Seeking input from the Exposure Health Community

Mollie Cummins[1,2], Sunho Im[1], Leela Jandhyala[3], Sukrut Shishupal[3], Rachel Nelson[3], Pavan Motagi[4], Katherine Sward[1,2], Julio Facelli[3,2], Ram Gouripeddi[3,2]

[1] University of Utah, College of Nursing, Salt Lake City, USA; [2] University of Utah, Center of Excellence for Exposure Health Informatics, Salt Lake City, USA; [3] University of Utah, Biomedical Informatics, Salt Lake City, USA; [4] University of Utah, Clinical and Translational Science Institute, Salt Lake City, USA

Sensors are commonly used to objectively measure the environment as well as the physiological state of human subjects to assess the effects of such exposures. Sensors measure a varied set of properties utilizing physical, chemical, or biological methods. In addition, sensors have diverse deployment characteristics and can be used indoors or outdoors, mobile or stationery. Finally, sensor measurement outputs have different semantics, which often need to be discovered and harmonized before such streaming data can be integrated and assimilated with other types of data across domains and varied temporal and spatial scales as needed for exposure health research.

Currently, there is a lack of informatics methods and tools that enable ease of discovering, harmonizing, and reusing sensor data and metadata. In addition, there is a lack of consensus on metadata standards and conceptual models representing the exposure domain. Investigators and curators of real-world data collections are often not incentivized or responsible for metadata discovery for data re-use. Moreover, there is a lack of accessible tools that enable scientists to discover, harmonize, store, and publish metadata.

In the SMARTER project (https://www.smarterexposurehealth.org/) project where we are developing logical models for representing diverse types of sensor metadata, and in doing so, discover and harmonize sensor and device metadata in a community-driven approach. Using a panel with expertise in sensor engineering, exposure health and informatics, we are also designing and evaluating a user-facing metadata repository that encompasses a library of data, sensors/ devices, and interfaces that support metadata submission and browsing. In this presenting, using exemplar sensors, we demonstrate the value of a community-driven in the development of FAIR shared and generalizable tools for metadata and data management, and seek to engage the ISES-ISEE community of experts in receiving further input on our metadata ecosystem and exposure health research use cases.

P47-20 | User stories, use cases and requirements for a generalizable informatics ecosystem for exposure health research

Ram Gouripeddi[1,2], Mollie Cummins[3,4], Pavan Motagi[2], Randy Madsen[2], Rob Smart[2], Katherine Sward[3,4], Julio Facelli[1,4]

[1] University of Utah, Biomedical Informatics, Salt Lake City, USA; [2] University of Utah, Clinical and Translational Science Institute, Salt Lake City, USA; [3] University of Utah, College of Nursing, Salt Lake City, USA; [4] University of Utah, Center of Excellence for Exposure Health Informatics, Salt Lake City, USA

Comprehensive quantification of effects of the modern environment and interventions on health requires the conduct of studies in the real-world using natural and quasi-experimental designs. Such studies need to consider data from ambient and study-specific environmental measurements, physiological effects of exposures, clinical, survey responses, as well contextual information reflecting the real-world and conduct of the study itself. In addition, the operations of such studies are a sociotechnical interplay between research teams consisting of exposure health scientists, research coordinators, sensor engineers, data aggregators, and informaticists, as well as participants, families, and clinicians. Performing exposure health studies are therefore complex logistical and resource-intensive endeavors that require scalable solutions.

Leveraging a user centered design approach, we are developing a generalizable informatics platform, Exposure Health Informatics Ecosystem (EHIE). By eliciting user stories and use cases, we developed a set of requirements that inform the development of this infrastructure. Key attributes of these requirements include: (1) Data: contributing concurrent, sequential, sub-threshold environmental exposures (exposome) which can span endogenous processes within the body, biological responses of adaptation to environment, physiological manifestations of these responses, and socio-behavioral factors; (2) Operations: ability to monitor current status of studies including sensor deployments and other data collection strategies; and (3) Assimilation: integrating data streams to support observational, active data collection and interventional study designs and analytics. To meet these needs, we are designing EHIE based on modern big data principles using a metadata-driven event-driven and hexagonal architecture patterns with the quality attributes of performance, reliability, and simplicity. We are adapting EHIE as a cloud based “Infrastructure as a Service” platform, increasing IoT sensor data reliability and security. In this presentation, we discuss and seek feedback on the user requirements and key functionalities of EHIE as a generalizable multi-scale, multi-omics ecosystem that provides robust pipelines for reproducible exposure research.

P47-21 | Untargeted Metabolomics and COPD Incidence: A Prospective Analysis from Three European Populations

Max J. Oosterwegel[1], Chrysovalantou Chatziioannou[2], Nivonirina Robinot[2], Pekka Keski-Rahkonen[2], Brooklynn R. McNeil[3], Randolph R. Singh[3], Gary W. Miller[3], Ayoung Jeong[4,5], Megi Vogli[6], Regina Pickford[6], Annette Peters[6], Peter Engelfriet[7], Monique Verschuren[7], Roel Vermeulen[1,8], Nicole Probst-Hensch[4,5], Jelle Vlaanderen[1], on behalf of the EXPANSE consortium

[1] Utrecht University, Institute for Risk Assessment Sciences, Utrecht, Netherlands; [2] International Agency for Research on Cancer, Nutrition and Metabolism Branch, Lyon, France; [3] Columbia University, Department of Environmental Health Sciences, Mailman School of Public Health, New York, USA; [4] Swiss Tropical and Public Health Institute, Allschwil, Switzerland; [5] University of Basel, Basel, Switzerland; [6] German Research Center for Environmental Health, Institute of Epidemiology, Helmholtz Zentrum München, Neuherberg, Germany; [7] National Institute for Public Health and the Environment, Centre for Nutrition, Prevention and Health Services, Bilthoven, Netherlands; [8] University Medical Center Utrecht, Julius Center for Health Sciences and Primary Care, Utrecht, Netherlands

Objective: Chronic obstructive pulmonary disease (COPD) is one of the leading causes of death in the industrialized world. Yet relatively little is known about risk factors other than smoking, air pollution and occupational exposures or through which metabolic pathways such factors operate.

Material and methods: We analyzed 1518 prospectively collected plasma samples from a case-control study nested within three European cohorts (Doetinchem Cohort Study, KORA, SAPALDIA). COPD was defined as the pre-bronchodilation FEV1/FVC ratio falling below 0.7 in a follow-up (3.5-16 years) after sample collection. Samples were analyzed using untargeted liquid and gas chromatography high resolution mass spectrometry (GC- and LC-HRMS). Controls were matched to cases on age, sex, follow-up time, and sample collection round. Case status was regressed on the intensity of each feature in separate conditional logistic regression models for each feature. Biological pathways linked to the top hits were explored using Mummichog. The false discovery rate (FDR) was controlled using the Benjamini-Hochberg procedure. Moreover, we used data on an additional 197 repeats to provide insight into how reflective a single measurement is of long-term averages (5 years).

Results: Hundreds of exogenous and endogenous markers were confidently identified. Nicotine and cotinine intensity levels were statistically significantly associated with COPD at an FDR of 10%. Considerable heterogeneity was observed across the different cohorts. Preliminary pathway analyses showed that metabolites involved in arachidonic acid metabolism were related to COPD in never-smokers. The intraclass correlation coefficient (ICC) of markers from the LC platform was 0.19 on average, while markers from the GC platform had an average ICC of 0.35.

Conclusion: We identified many exogenous and endogenous markers and assessed associations with COPD incidence.

P47-22 | Evaluating the Role of Exposome in the Progression of Chronic Obstructive Pulmonary Disease

Elizabeth Lin[1], John Huston[2], Emily Johnson[1], Gabrielee Valle[2], Jill Stewart[2], Jieqiong Zhou[1], Sheng Liu[1], Carolyn Rochester[2], Carrie Redlich[1,3], Charles Dela Cruz[2], Krystal Godri Pollitt[1]

[1] Yale School of Public Health, Environmental Health Sciences, New Haven, USA; [2] Yale School of Medicine, Yale Pulmonary, Critical Care & Sleep Medicine, New Haven, USA; [3] Yale School of Medicine, Yale Occupational and Environmental Medicine, New Haven, USA

Objective: Chronic Obstructive Pulmonary Disease (COPD) is a progressive lung disease that is characterized by chronic airflow obstruction. While individual environmental risk factors are linked to its onset and progression, the impact of the integrated ensemble of environmental influences to which patients are exposed has been underexplored. This study explored the relationship between chemical features of the exposome and COPD severity.
Material and Methods: Patients (n=37) from the Yale New Haven COPD Subspecialty Clinic wore wearable passive samplers (Fresh Air wristband) to assess chemical exposures. Stationary Fresh Air clips were placed outside participants' homes. A gas chromatography high resolution mass spectrometry was used to analyze passive samplers. Questionnaires on lifestyle, and built environment were additionally collected.
Results: Patients (mean 71 years, 68% female) spent 91% of the 5-day assessment period indoors. Over half (54%) had severe COPD symptoms, with a mean of 0.4 exacerbations and 1.4 hospitalizations due to acute COPD exacerbations in the prior year. Except for one, all patients have a history of smoking; among them, seven were current smokers. Suspect screening detected 309 chemicals, predominantly benzoids, which include polycyclic aromatic hydrocarbons (PAHs) from combustion sources. Exposures to 38 chemicals were quantified using targeted approaches, including nicotine (43% participants) and THC-9 (46%). Compared to outdoors, participants' indoor exposures were elevated for 27 (71%) of the assessed chemicals, most of which were PAHs, fragrances, flavoring agents, and hair dyes. After adjustments for covariates, di-n-octyl and di-n-butyl phthalate were found to be correlated with COPD severity. Di-n-octyl phthalate, galaxolide, benzothiazole, pyrene, and benzophenone, were associated with hospitalizations due to acute COPD exacerbations.
*Conclusion:*This pilot study demonstrated the feasibility of assessing the chemical exposure profile of COPD patients using a novel wearable passive sampler. Our results offer insights into potential environmental risk factors for COPD severity and exacerbations.

P47-23 | Enhancing drug screening in untargeted metabolomics with repository-scale analog search and controlled-vocabulary pharmacologic metadata

Haoqi Nina Zhao[1], Kine E. Kvitne[1,2], Shirley Tsunoda[1], Pieter Dorrestein[1]

[1] University of California San Diego, Skaggs School of Pharmacy and Pharmaceutical Sciences, La Jolla, USA; [2] University of Oslo, Department of Pharmacy, Oslo, Norway

Objective: Medication consumption represents a major human chemical exposure, yet studying their health impacts relies on costly and often incomplete medical records or self-reported data. Untargeted metabolomics offers an objective drug read-out, but it suffers from the limited reference spectra of drugs and even lower coverage for drug metabolites. Moreover, interpretation of the annotations requires extensive literature searches to understand the therapeutic indications of the drugs. There is currently no resource to provide objective, systematic, and efficient read-out of drugs in untargeted metabolomics.

Methods: We developed a MS/MS spectral library for drugs, metabolites, and analogs connected with drug metadata. Using GNPS Public Spectral Library, we extracted reference spectra through partial name searches against four drug knowledge bases and expanded coverage by identifying structurally similar analogs in 1.2 billion public MS/MS spectra.

Results: As of March 2025, the GNPS Drug Library contains 99,122 reference spectra of drugs (4,723 unique structures), 4,087 drug metabolite spectra (470 drugs), and 3,232 drug analog spectra (belonging to 577 drugs) derived from public data mining, and hand-curated drug metadata on the pharmacologic class, therapeutic area, therapeutic indication, and mechanism of action. In fecal samples from an HIV cohort, 169 drugs were detected, with HIV, cardiovascular, and antidepressant medications most prevalent. Notably, 33% of drugs were annotated alongside their metabolites or analogs, which strongly correlated with parent drug occurrence. We identified significant changes in *N*-acyl lipids linked to HIV status, which was attributed to HIV drug exposure based on metabolomics drug read-out. The changes were not observed using reported HIV drug consumption, highlighting the importance of performing metabolomics-based drug read-out.

Conclusion: A resource for systematic and efficient drug read-out was developed for untargeted metabolomics studies.

P48 | Breathing Easier, Living Longer: The Power of Greenspace - PART II

P48-01 | Mediating Effects of PM10 and PM2.5 on the Association Between Residential Greenness and Visual Impairment in 3-year-old children

Dajeong Ham[1], Sanghyuk Bae[1,2], Group Ko-CHENS Study group[3,4]

[1] The Catholic University of Korea, Department of Preventive Medicine, College of Medicine, Seoul, South Korea; [2] Environmental Health Center, The Catholic University of Korea, Department of Preventive Medicine, College of Medicine, Seoul, South Korea; [3] National Institute of Environmental Research, Ministry of Environment, Environmental Health Research Division, Department of Environmental Health Research, Incheon, South Korea; [4] Ministry of Environment, Environmental Health Policy Division, Office of Environmental Health, Sejong, South Korea

Background: A growing body of evidence shows the importance of nature, particularly green spaces, and their impact on a range of children's health outcomes. However, evidence of the association between green spaces and particulate matter with visual health for children remains limited.
Objectives: This study aims to examine the association between residential greenness and visual impairment in 3-year-old children at various buffer distances (200m, 300m, 500m, 1000m, 2000m) and to explore the mediating effects of PM10 and PM2.5.
Methods: We used the data from the Korean Children's Environmental Health Study. Among the cohort of 5,451, a total of 794 children were included in the analysis. NDVI was measured during pregnancy. Particulate matters were estimated using the kriging interpolation method for the first postnatal years. Visual impairment was defined as a score lower than 0.5 on Jin's vision chart results, corresponds to 20/40 on the Snellen chart. The association between green spaces, particulate matter, and visual impairment was analyzed using generalized additive model, logistic regression and causal mediation analysis.
Results: We found that an increase a 1% increase in NDVI is associated with a lower risk of visual impairment across all buffer distances (OR = 0.04–0.13, significant). A 1 μg/m³ increase in PM10 and PM2.5 is associated with 7% and a 14–15% higher risk of visual impairment. After categorizing indicators into quartiles, the adverse or protective effects on visual impairment were significant only in the second quartile. PM10 and PM2.5 mediation effects showed a significantly strong protective effect in the proximity buffer (200m: 22.2 and 22.6 (%), respectively).
Conclusion: Our findings suggest that particulate matter at closer distances plays a significant role in mitigating the risk of visual impairment in 3 years old children.

P48-02 | Comparing different metrics of residential proximity to greenspace and associations with stillbirth risk in Arkansas, USA

Kari Weber[1], Emine Bircan[1], Suman Maity[1], Wendy Nembhard[1], Sean Young[2]

[1] University of Arkansas for Medical Sciences, Epidemiology, Little Rock, USA; [2] UT Southwestern Medical Center, Dallas, USA

Objective: This study aimed to compare multiple estimates of proximity to greenspace and their associations with stillbirth (fetal death ≥ 20 weeks gestation).Materials and Methods: Women with pregnancies ending in stillbirth (cases, n=881) and livebirths (controls, n=491) between July 2016 – December 2021 were identified from the Arkansas Reproductive Health Monitoring System, an active surveillance system, in Arkansas, USA and invited to participate in the Birth Defects Study to Evaluate Pregnancy exposureS (BD-STEPS). Maternal residential addresses from were geocoded. Residential proximity to greenspace was estimated using average maximum normalized difference vegetation index (NDVI) within a 500m residential buffer (representing walking distance) and a 10-minute drive time buffer calculated in ArcGIS Pro, and distance in meters to the nearest park or recreation area was measured using data from USA Parks. Adjusted odds ratios and 95% confidence intervals comparing the top quartiles of NDVI to the lowest quartile and per 100m in distance to the nearest park were calculated using logistic regression.

Results: The majority of women lived in urban neighborhoods (66%) but cases were slightly more likely to live in rural areas than controls. On average, cases resided further from the nearest park than controls (4,750m vs. 4,400m) though the difference was not statistically significant. There were suggestive, non-significant inverse associations observed for those in the highest NDVI quartile (greenest areas) within the 10-minute drive time and 500m residential buffers compared to the lowest quartile.

Conclusion: In this initial analysis of greenspace and stillbirth, while no statistically significant differences were observed, suggestive associations for drive time buffer may support use of additional estimates of greenspace proximity to assess potential benefits. Arkansas is not rated highly for walkability in most areas so metrics that account for common modes of accessing greenspace (i.e. driving) may be more informative than “walking distance” buffers.

P48-05 | Green Space and Cognitive Health: A Longitudinal Study in Older Adults

Jaime Madrigano[1,2], Beth A. Griffin[2], Sarah E. Edgington[2], Carlos Calvo-Hernandez[2], Scott R. Stephenson[2], Charlie H. Nguyen[1], Regina A. Shih[3,2]

[1] Johns Hopkins Bloomberg School of Public Health, Department of Environmental Health and Engineering, Baltimore, USA; [2] RAND Corporation, Santa Monica, USA; [3] Emory Rollins School of Public Health, Department of Epidemiology, Atlanta, USA

Objective: The loss of cognitive function, a frequent antecedent of Alzheimer’s Disease and related dementias (ADRD), is an enormous public health burden and modifiable neighborhood factors may play a role in the risk for this health outcome. Exposure to green space is thought to influence cognitive health, though most evidence is based on

cross-sectional studies and few studies have assessed differences by demographic characteristics. We examined the role of residential exposure to green space on cognitive decline in a nationally representative sample of older adults.

Methods: We used data from the Health and Retirement Study, a nationally representative study of older adults born between 1931 and 1941. Data on green space were obtained from the US Geological Survey's National Land Cover Database. Cognitive outcomes were assessed in HRS participants every two years, beginning in 2000, through a modified version of the Telephone Interview for Cognitive Status (TICS-m). Sensitivity analyses to ascertain potential for bias in results are ongoing.

Results: After excluding residents who were institutionalized, missing cognitive outcomes, or missing geographic information, our sample included 7,272 participants. Unadjusted analyses showed participants living in areas with higher green space had lower baseline cognition scores. Results from mixed effects longitudinal regression models, adjusted for individual and neighborhood covariates, showed inconsistent results between green space exposure and cognitive decline, with an indication that the direction of the association was dependent upon living in an urban or non-urban area. Models stratified by race/ethnicity indicate that higher exposure to green space may slow cognitive decline in Hispanic participants.

Conclusion: The association between residential green space exposure and cognitive decline may vary based on sociodemographic and other neighborhood characteristics. Further, longitudinal findings may differ from those observed in cross-sectional studies. Further research assessing longitudinal associations between green space and cognitive health is warranted.

P48-06 | Exposure to different types of residential greenness during pregnancy and early childhood and attention-deficit/hyperactivity disorder diagnosis: A nested case-control study

Leire Luque García[1,2], Gonzalo Garcia-Baquero[3,2], Aitana Lertxundi[1,2], Wael K. Al-Delaimy[4], Tiffany C. Yang[5], Juana Maria Delgado-Saborit[6,7], Mònica Guxens[8,9], Rosemary R. McEachan[5], Martine Vrijheid[8,9], Marisa Estarlich[6,10], Mark Nieuwenhuijsen[8,9], Jesús Ibarluzea[2,11]

[1] University of the Basque Country (UPV/EHU), Department of Preventive Medicine and Public Health, Leioa, Spain; [2] Biogipuzkoa Health Research Institute, Environmental Epidemiology and Child Development Group, Donostia-San Sebastián, Spain; [3] University of Salamanca, Faculty of Biology, Salamanca, Spain; [4] University of California San Diego, Herbert Wertheim School of Public Health and Human Longevity Science, La Jolla, USA; [5] Bradford Institute for Health Research, Bradford Teaching Hospitals NHS Foundation Trust, Bradford, UK; [6] FISABIO–Universitat Jaume I–Universitat de València, Epidemiology and Environmental Health Joint Research Unit, Valencia, Spain; [7] Universitat Jaume I, Department of Medicine, School of Health Sciences, Castellón de la Plana, Spain; [8] ISGlobal, Barcelona, Spain; [9] Universitat Pompeu Fabra, Barcelona, Spain; [10] Valencia University, Nursing and Chiropody Faculty, Valencia, Spain; [11] University of the Basque Country (UPV/EHU), Faculty of Psychology, Donostia-San Sebastián, Spain

Background: Epidemiological studies suggest that exposure to greenness may protect children from attention-deficit/hyperactivity disorder (ADHD) diagnosis. However, evidence to date is limited while no previous research has independently investigated exposure to prenatal greenness.

Objective: We conducted a nested case-control study with data from Born in Bradford (BiB) and INfancia y Medio

Ambiente (INMA) birth cohorts to investigate the association between exposure to various types of residential greenness and ADHD diagnosis, considering both pregnancy and early childhood exposure periods independently. $PM_{2.5}$ was tested as a potential mediator of the association.
Methods: Children with ADHD were identified based on a confirmed medical diagnosis. Pregnancy and early childhood exposure to residential greenness were estimated through Normalized Difference Vegetation Index (NDVI) within 300-m, urban green space and natural green space percentages within 300-m, and the linear distance to the closest green space in meters. We performed a conditional logistic regression to analyze the association between the included greenness metrics and ADHD.
Results: We found no statistically significant associations between any of the pregnancy and early childhood greenness metrics and ADHD diagnosis in the BiB cohort. Further analysis on the INMA cohort found that higher urban green space percentage slightly increased the risk of ADHD diagnosis during both pregnancy (total effects: OR 1.04, 95 % CI 1.01 to 1.07, $p = 0.012$; direct effects: OR 1.06, 95 % CI 1.03 to 1.10, $p < 0.001$) and early childhood (total effects: OR 1.03, 95 % CI 1.00 to 1.07, $p = 0.042$; direct effects: OR 1.04, 95 % CI 1.00 to 1.07, $p = 0.033$). However, these associations were not supported by the sensitivity analyses.
Conclusions: We found both null and inconsistent associations between the included greenness metrics and ADHD. Further research is warranted to elucidate the potential role of different types of greenness in ADHD.

P48-07 | Comparison of the impact of dynamic and static virtual blue/green environments on stress reduction and physiological relaxation

Jingting Huang[1], **Linyan Li**[1,2]

[1] City University of Hong Kong, Department of Data Science, Hong Kong, Hong Kong; [2] City University of Hong Kong, Department of Infectious Disease and Public Health, Hong Kong, Hong Kong

Objective
Blue and green spaces are known to reduce stress, and their integration with virtual reality (VR) enables controlled testing of responses. This study compares dynamic and static elements to investigate whether motion within virtual environments enhances stress relief.
Materials and Methods
Forty young adults (52.5% female, aged 18–30) participated in a VR experiment assessing static and dynamic blue/green environments. Static environments featured stationary elements, while dynamic ones included moving components like flowing water or swaying leaves. A within-subjects crossover design was used. Participants watched a 3-minute urban video to induce stress, followed by self-reported stress, anxiety, and depression assessments. They then experienced alternating static and dynamic VR environments (3 minutes each) with 1-minute rest periods. Physiological responses (skin conductance level (SCL), heart rate (HR), respiratory rate, and heart rate variability (HRV) indices) were measured using a multimodal wristband. Post-exposure questionnaires assessed mood, stress, and immersion.
Results
All natural environments improved physiological responses compared to the urban setting. Dynamic blue spaces reduced SCL ($\Delta = -0.15$) and increased SDNN ($\Delta = +3.5$) and RMSSD ($\Delta = +5.8$) compared to static blue spaces, indicating enhanced autonomic regulation. Dynamic green spaces also decreased SCL ($\Delta = -0.01$) and increased SDNN ($\Delta = +5.65$) and RMSSD ($\Delta = +8.72$) compared to static green spaces. Dynamic environments were more

effective in reducing stress, with both blue and green settings providing greater physiological relaxation than the urban environment.

Conclusion

Dynamic environments were more effective in reducing stress than static ones, with both blue and green settings offering significant physiological relaxation. These findings highlight the importance of motion in natural spaces for stress relief.

Funding

This work was supported by a grant from the Research Grants Council of the Hong Kong Special Administrative Region, China (Project No. 21607223).

P48-08 | Urban Nature Profiles and Atherosclerotic Cardiovascular Disease Risk: A Study of Diverse Green Metrics.

Pablo Knobel[1], Aditi Singhvi[1], Kevin Lane[2], Rachel Litke[3], Charles Mobbs[3], Michael Hadley[4], Alex Federman[5], Itai Kloog[1], Maayan Yitshak Sade[1]

[1] Icahn School of Medicine at Mount Sinai, Department of Environmental Medicine, New York, USA; [2] Boston University School of Public Health, Department of Environmental Health, Boston, USA; [3] Icahn School of Medicine at Mount Sinai, Nash Family Department of Neuroscience, Friedman Brain Institute, New York, USA; [4] Icahn School of Medicine at Mount Sinai, Division of Cardiology, New York, USA; [5] Icahn School of Medicine at Mount Sinai, Division of General Internal Medicine, New York, USA

Objective: Urban exposure to nature has been linked to a reduced risk of cardiovascular disease. Yet, many studies simplify it to one-dimensional metrics, neglecting factors like accessibility, distribution, and diversity, which complicates practical applications. We classified urban nature neighborhoods into profiles and examined their association with atherosclerotic cardiovascular disease (ASCVD). We analyzed electronic health record data from the Mount Sinai Health System, identifying ASCVD cases, which include coronary heart disease, cerebrovascular disease, peripheral artery disease, and atherosclerotic disease.
Methods: Our study included 33,698 participants aged 55 and older, contributing 205,260 person-years of data and 9,612 events. We utilized k-means clustering to group participants' residences into five distinct exposure profiles based on eight urban nature metrics measured in the vicinity of participants' homes: Green View Index, number of trees, number of parks, access to large parks, tree biodiversity, tree canopy cover, grass and shrub cover, and water cover. Each metric reflects different aspects of urban nature, such as visual greenery, biodiversity, and accessibility. The profiles were designated as Low Overall Nature, Abundant Parks, Waterfront, Tree-Lined, and High Natural Coverage. We assessed the association of these clusters with ASCVD using Cox survival models adjusted for both individual and neighborhood-level sociodemographic factors and a spline for the year, censoring participants after they moved (14.9%), did not engage with the system for two years (30.1%), or died (4.5%).
Results: indicated a reduced risk of ASCVD as urban nature metrics increased: Waterfront with a hazard ratio of 0.88 (95% CI: 0.82–0.96), Park Access with 0.88 (95% CI: 0.83–0.93), Street Trees with 0.92 (95% CI: 0.87–0.97), and High Natural Coverage with 0.93 (95% CI: 0.86–1.00) compared to Low Nature.
Conclusion: Our findings underscore the benefits of urban nature in promoting cardiovascular health.

P48-09 | Assessment of the critical exposure windows for weekly greenspace exposure and term low birth weight risk

Seulkee Heo, Michelle Bell

Yale University, School of the Environment, New Haven, USA

The impact of greenspace on birth outcomes is often assessed using exposure averages during pregnancy or each trimester (1–13, 14–26, 27+ weeks). Evidence on the associations between weekly greenspace exposure and birthweight is limited, despite its significance in exploring mechanisms and improving care strategies for pregnant women. This case-control study investigated critical windows of gestational greenspace exposure and term low birth weight (LBW) estimates of the Enhanced Vegetation Index (EVI) and the birth certificate data from Connecticut, New Jersey, and Pennsylvania (2016–2020). Each included singleton term birth (n=788,275) was linked to weekly EVI estimates based on maternal residential ZIP codes and gestational age (1–40 weeks). Logistic regression with a distributed lag non-linear model was applied to estimate the exposure-response and lag-specific LBW risk for an interquartile range increase (0.200) in EVI, adjusting for season of conception, infant sex, smoking, maternal socioeconomic factors, average PM2.5 during pregnancy, and a cross-basis with spline functions of weekly temperature. The lag-specific odds ratios (ORs) showed an inverted U-shaped curve and significant associations during 1–9th and 30–40th gestation weeks. The OR for LBW in the 1st gestational week was 0.989 (95% CI: 0.977–0.999), and in the 9th week, it was 0.997 (95% CI: 0.992–1.000). The OR was 0.995 (95% CI: 0.991–1.000) and 0.984 (95% CI: 0.971–0.997) during the 30th and 40th weeks, respectively. Our regressions using trimester-specific EVI averages found significant impacts during the second and third trimesters. Results highlight the need for careful interpretation of the association between greenspace and birthweight as they may vary depending on the temporal scale of exposure (e.g., trimester vs. weekly). We underscore the importance of gestational greenspace exposure in reducing LBW risks, especially during early and late pregnancy, with implications for healthcare and public health policies.

P48-10 | Assessing Neighborhood Greenness Visibility Using AI Technology and Association with Pulmonary Function Changes in Older Adults

Weiju Zhou[1], Jiuxuan Gao[1], Xiaojie Guo[2], Yu'e Cha[2], Song Tang[2], Xiaoming Shi[2], John S. Ji[1]

[1] Tsinghua University, Vanke School of Public Health, Beijing, China; [2] Chinese Center for Disease Control and Prevention, National Institute of Environmental Health, Beijing, China

Background Limited studies have used artificial intelligence (AI) technology to assess neighborhood greenness exposure at eye level within micro-scale environments. Epidemiological evidence examining the impact of greenness visibility on pulmonaryfunction among older adults is scarce, and the underlying biological mechanisms remain largely unexplored.

Methods A longitudinal panel study was conducted in Jinan, Shandong Province, China, involving 76 healthy older adults monitored repeatedly over a five-month period. Pedestrian video data were collected using wearable digital cameras to capture micro-scale neighborhood greenness surrounding participants' residential buildings. Greenness

visibility was quantified using the panoramic segmentation function of the Detectron2 AI library. Pulmonary function indicators were assessed alongside multi-omics analyses of blood and urine samples. Causal inference test (CIT) and integrative pathway analysis (IPA) were performed to investigate the intricate associations between greenness visibility, biomarkers, and changes in pulmonary function, elucidating the underlying biological mechanisms.
Results Greenness visibility, extracted from pedestrian video data with analyzed using AI technology, demonstrated high accuracy in detecting neighborhood greenness and was significantly associated with improved forced vital capacity (FVC). An interquartile range (IQR) increase in greenness visibility corresponded to a 174.80mL (95%CI: 22.28mL to 327.30mL) increase in FVC. Multi-omics analyses identified mediating biomarkers, including β-Hydroxybutyric acid, N-amidino-L-aspartate(1-), PC(35:4), PC(42:5), and 69 blood transcriptomes (e.g., RUNX3, CD247, CAPN2). Additionally, 31 biological enrichment pathways, such as Th17 activation, nitric oxide metabolism, nucleotide excision repair, were implicated as potential mechanisms.
Conclusions This study highlights the role of neighborhood greenness visibility, assessed using AI-analyzed pedestrian video data, in improving pulmonary function among older adults. By identifying key biomarkers and enrichment pathways mediating these effects, the findings underscore the potential of targeted urban planning and public health strategies to enhance respiratory health through increased eye-level greenness exposure.

P48-11 | How tree canopy cover affects extreme heat: analyzing temperature effects of changing tree canopies within neighborhoods in California (2011-2021)

Andrew V. Nguyen, Neeta Thakur

University of California, San Francisco, Division of Pulmonary and Critical Care Medicine, School of Medicine, San Francisco, USA

Objective: In California, extreme heat (EH) events are increasing in frequency and severity and adversely affect the health of communities. Areas with highly urbanized development and less tree canopy cover (TCC) may be more susceptible to extreme heat events and the urban heat island effect. We investigate the association between change in TCC and change in EH events at the block group (BG) level and potential effect measure modification by socioeconomic/environmental characteristics in California from 2011 to 2021.

Material and Methods: We propensity score matched 23,196 BGs within counties by modeling the probability of increasing TCC over the study period as a function of population, housing characteristics, socioeconomic status, existing impervious surfaces, and demographic metrics. Every treated BGs was matched to four peer BGs within the same county with similar propensity of increasing TCC. We defined EH days as any BG-day with daily minimum or maximum temperatures exceeding the 95th percentile threshold for the specific BG and month. We estimated the change in EH metrics as a function of TCC change using generalized mixed models and a clustered sandwich estimator for robust standard errors. We additionally investigated effect measure modification by socioeconomic status via stratified models.

Results: A 10% increase in tree canopy cover was associated with a 0.4°C decrease in average daily maximum temperature (95% CI: 0.39, 0.41) and 0.78 fewer EH days annually (95% CI: 0.72, 0.84). These effects were more pronounced in low-income/resourced BGs and in BGs with higher initial canopy cover (>15%).

Conclusion: Our findings highlight the critical role of tree canopy cover in mitigating extreme heat, particularly with susceptible communities. Urban greening initiatives may consider prioritizing lower income neighborhoods and establishing a saturated tree canopy in public spaces to maximize temperature reduction benefits.

P48-12 | Impact of Green Spaces on Heat-Related Cardiorespiratory Hospitalizations in Brazil

Weeberb Requia[1], Leonardo Hoinaski[2], Paulo Saldiva[3], Petros Koutrakis[4]

[1] Fundação Getulio Vargas, Center for Environment and Public Health Studies, Brasilia, Brazil; [2] Federal University of Santa Catarina, Santa Catarina, Brazil; [3] University of Sao Paulo, School of Medicine, Sao Paulo, Brazil; [4] Harvard University, Harvard TH Chan School of Public Health, Boston, USA

Heat exposure poses a growing public health challenge, particularly in tropical regions such as Brazil. However, the extent to which green spaces mitigate heat-related health risks remains uncertain. This study examines the relationship between heat and cardiorespiratory hospital admissions in Brazil (2008–2018) and explores the potential mediating role of various green space metrics. Using a three-stage approach, we applied a time-stratified quasi-Poisson regression model to estimate state-level heat-health associations, followed by a meta-analysis to derive national effects and a meta-regression to evaluate the influence of green spaces. We analyzed four green space metrics: NDVI, urban green area, natural forest, and total green area.
Our findings indicate that extreme heat (99th percentile) significantly increased hospital admissions, with a 1% rise in circulatory admissions (RR: 1.01, 95% CI: 1.00–1.03) and a 26% increase in respiratory admissions (RR: 1.26, 95% CI: 1.22–1.30). Moderate heat (90th percentile) also elevated respiratory admissions (RR: 1.13, 95% CI: 1.11–1.15) but showed protective effects for certain subgroups in circulatory admissions. Regarding green spaces, our results suggest that NDVI, urban green areas, and total green coverage play a protective role against heat-related health risks, though their effectiveness varies by age and sex. Under extreme heat conditions, a one standard deviation (SD) increase in urban green area was linked to a 0.6% reduction in circulatory admissions among individuals aged 65 and older. For heat-related respiratory admissions, under moderate heat, a one SD increase in NDVI was associated with a 3.4% reduction in hospitalizations for the general population.
These findings highlight the importance of green spaces in mitigating heat-related hospitalizations, while also underscoring that their effectiveness depends on vegetation type, spatial distribution, and interactions with pollution. Our results reinforce the need for climate-adaptive urban planning and heat mitigation strategies tailored to local environmental and sociodemographic conditions.

P48-13 | Private residential gardens versus other greenspace types with obesity-related cancer incidence in the UK Biobank cohort.

Chinonso C. Odebeatu[1], Darsy Darssan[1], Charlotte Roscoe[2,3], Muktar Ahmed[4,5], Simon Reid[1], Nicholas J. Osborne[1,6]

[1] University of Queensland, School of Public Health, Faculty of Health, Medicine and Behavioural Science, Brisbane, Australia; [2] Oregon Health & Science University Portland State University, Environmental Systems and Human Health, School of Public Health, Portland, USA; [3] Harvard University, Department of Environmental Health, Harvard T.H Chan School of Public Health, Boston, USA; [4] University of South Australia, Cancer Epidemiology & Population Research Group, Adelaide, Australia; [5] Flinders University, Cancer Research, Flinders Health and Medical Research Institute, Adelaide, Australia; [6] University of New South Wales, School of Population Health, Sydney, Australia

Objective

We examined the associations between private residential gardens and other greenspace types with the incidence of overall and site-specific obesity-related cancer (ORC).

Material and Methods

The present longitudinal study used data from the UK Biobank participants recruited between 2006 and 2010, with follow-up censored until December 31, 2016. Private residential gardens and other greenspace types (e.g., public parks and allotments) within 100 m of participants' home locations were defined using Ordnance Survey MasterMap™ greenspace categories. The incidence of overall and 13 site-specific ORCs, including breast and uterine cancer, was identified via linkage to cancer registries. Cox proportional hazard models, adjusted for several covariates (including neighborhood deprivation), were used to estimate hazard ratios (HRs) and 95% confidence interval (CI). We conducted mediation analyses for physical activity, serum 25-hydroxyvitamin D [25(OHD)], particulate matter with diameter ≤2.5, and nitrogen dioxide (NO_2), along with subgroup and sensitivity analyses.

Results

Over a 7.82-year median follow-up, 9550 of 279,326 participants (mean age 55.15 ± 8.17 years) developed ORC. Higher exposure to private residential gardens was associated with lower risk of overall ORC (HR: 0.92; 95% CI: 0.88, 0.96), breast (HR: 0.91; 95%CI: 0.84, 0.98), and uterine cancer (HR: 0.80; 95%CI: 0.67, 0.96), but not other site-specific ORC. No association was found between other greenspace types and any ORC, except for uterine cancer. The inverse association between private gardens and overall ORC was partially mediated by NO_2 and stronger in less deprived neighborhoods, highly active individuals, and those with sufficient 25(OH)D (>50 nmol/L).

Conclusion

Our study suggests that people living in neighborhoods with more private gardens are at lower risk of developing obesity-related cancers, particularly breast and uterine cancer. This protective effect is more pronounced in less

deprived areas, highlighting the need for publicly accessible greenspaces, e.g., community gardens, to help reduce socioeconomic health disparities.

P48-14 | Residential greenspace and metabolic syndrome risk in the UK Biobank: mediation through multiple pathways

Chinonso C. Odebeatu[1], Darsy Darssan[1], Charlotte Roscoe[2,3], Simon Reid[1], Nicholas J. Osborne[1,4]

[1] The University of Queensland, School of Public Health, Faculty of Health, Medicine and Behavioural Science, Brisbane, Australia; [2] Oregon Health & Science University Portland State University, Environmental Systems and Human Health, School of Public Health, Portland, USA; [3] Harvard University, Department of Environmental Health, Harvard T.H Chan School of Public Health, Boston, USA; [4] University of New South Wales, School of Population Health, Sydney, Australia

Objective

We investigated the association and pathways between residential greenspace and the risk of metabolic syndrome (MetS) and its components.

Methods

This cross-sectional study included 221,028 UK Biobank participants aged between 38 to 73 years, recruited at baseline (2006-2010). Residential greenness was defined using Ordnance Survey MasterMap™ greenspace categories, including private gardens, and satellite-derived normalized difference vegetation index (NDVI). MetS was defined as having ≥3 of the five components – abdominal obesity, elevated triglycerides, elevated blood pressure, elevated fasting glucose, and reduced high-density lipoprotein-cholesterol. We used linear and logistic regression models, adjusting for several covariates, with stratified analyses by sex, and neighborhood deprivation. Where possible, we conducted independent and joint mediation effects of behavioral (physical activity, and sedentary time), environmental [particulate matter with a diameter of 10 micrometers (PM10)], nitrogen dioxide (NO2)], social (loneliness), and biomarker [serum 25-hydroxyvitamin D [25(OH)D] pathways in the greenspace-MetS association.

Results

Increased exposure to residential greenness (NDVI) was associated with a 29.3% lower risk of MetS (OR: 0.707; 95%CI: 0.633, 0.794), with stronger effects in males, and highly deprived individuals. For individual MetS components, higher NDVI was associated with reduced odds of abdominal obesity, elevated triglycerides, and high systolic blood pressure, but not with elevated diastolic blood pressure or high glucose levels. Notably, NO_2, PM_{10}, 25(OH)D, sedentary time, and loneliness each had individual mediation effects on MetS, with NO_2 exhibiting the strongest effect, followed by PM_{10} and 25(OH)D. In contrast, physical activity had a negative mediation effect. Collectively, these mediators accounted for 44.84% of the total protective effect of NDVI on MetS. Private gardens showed no beneficial effects for MetS, except in less deprived areas.

Conclusion

Our findings suggest that higher residential greenness may lower the risk of MetS through multiple pathways, but this depends on the greenspace indicator and neighborhood deprivation.

P48-15 | Change in green space exposure metrics caused by a large-scale greening intervention

Ray Yeager[1], Daniel Riggs[1], Rachel Keith[1], Daniel Fleisher[2], Ted Smith[1], Jay Turner[3], Aruni Bhatnagar[1]

[1] University of Louisville School of Medicine, Louisville, USA; [2] Hyphae Design Labs, Oakland, USA; [3] Washington University, Saint Louis, USA

Objective: We sought to evaluate changes in both traditional and detailed measures of urban green space that resulted from a large-scale neighborhood greening intervention.
Material and Methods: As part of the Green Heart Louisville Project, we planted 8,000 trees and shrubs in residential study area from 2019 to 2022. We collected coordinates of planted trees, 10m resolution satellite-based imagery, and Light Detection and Ranging data before and after planting. We calculated common green space metrics: Normalized Difference Vegetation Index (NDVI), total vegetation, and canopy cover. We also measured 2m resolution Leaf Area Index (LAI) and grass cover. We evaluated changes in planted areas compared with surrounding matched areas.
Results: We recorded the location of 4,983 trees that survived from initial planting through follow-up GPS logging in 2023, representing a 74.2% increase in number of trees. During and immediately after planting, we observed localized NDVI decreases due to mulching and grass disturbance. We observed mean annual NDVI increase of 0.025 in the planting area from 2019 through 2024, with localized changes between -0.018 and 0.089 mean within a 100m radius. There were LAI decreases in the planting area and nearby unplanted areas of -0.023 and 0.021, respectively, due to unrelated mortality of large canopy trees. However, we found localized increases in LAI among planted areas of up to 0.013 mean LAI within a 100m radius.
Conclusion: Studies evaluating the health effects of greening interventions may be unable to detect short-term changes in traditional green space metrics due to planting. Measurement of highly localized changes, characteristics, and numbers of trees planted may be more suitable for longitudinal analyses and yield actionable evidence to inform urban forestry practice.
Funding: This work was funded in-part by the U.S. National Institutes of Health, the Owsley Brown II Family Foundation, and The Nature Conservancy.

P48-16 | Association of residential greenness with autism spectrum disorder: a pilot study

Renata Rizzo[2], Tommaso Filippini[3], Eliana Pellegrino[1], Margherita Ferrante[1], Rita Barone[2], Maria Fiore[1]

[1] University of Catania, Department of Medical, Surgical and Advanced Technologies Sciences "G.F. Ingrassia", Catania, Italy; [2] University of Catania, Child Neuropsychiatry, Department of Clinical and Experimental Medicine University of Catania, Catania, Italy; [3] University of Modena and Reggio Emilia, Department of Biomedical, Metabolic and Neuroscience Sciences, Modena, Italy

Objective

This study aimed to investigate the association between exposure to greenness and risk of Autism spectrum disorder.

Material and Methods

Recruitment was carried out at the Child Neuropsychiatry Outpatient Clinics of the Policlinico "G. Rodolico- S. Marco" Hospital in Catania (Sicily). Green space exposure data were assessed using satellite data from the Copernicus Global Land Service, a component of the Land Monitoring Core Service. These data are used to calculate the Normalized Difference Vegetation Index (NDVI) as a proxy for green space exposure. Socio-demographic and clinical data were collected using a questionnaire built for this purpose. Data analysis was performed using restricted cubic spline and logistic regression models using the central quintile as the reference category, adjusting for age and sex. Sixty-one cases diagnosed from 2010 to 2016 and 93 controls were recruited.

Results

The study highlighted a non-linear trend in risk both when analyzing exposure relative to the year 2000 and in the following years up to 2016 (ORs 1.00; 1.46; 0.92; 0.94).

Conclusions

Exposure to green space can have beneficial effects due to lower air pollution, reduced stress and increased physical activity and social interaction. Furthermore, it should be noted that not only the availability of green space, but also the actual accessibility to such area should be considered as a relevant methodological issue to be considered when evaluating the relationship between greenness and nervous system disorders.

P48-17 | Exposure to green and blue areas and effects on cardiovascular disease in participants in the Longitudinal Study of Adult Health (ELSA-Brazil)

Nelzair A. Vianna[1], **Andre M. Fraga**[2], Maria da Conceição C. de Almeida[1], Thais Mauad[2], All other authors contributed to conceptualization in the first draft, technical inputs, case study material, review and editing of the final abstract.

[1] Fiocruz, Molecular Epidemiology and Biostatistics Laboratory, Salvador, Brazil; [2] University of Sao Paulo, School of Medicine, Sao Paulo, Brazil

Introduction: The correlation between green and blue urban areas, exposure and access, and health impact has been demonstrated in several studies. However there are very few studies investigating the association between exposure to green areas and correlates with human health outcomes in Brazilian cities. **Objective:** The aim was to evaluate exposure to green and blue areas and effects on cardiovascular health in participants in the Longitudinal Study of Adult Health (ELSA-Brazil) in Salvador, Bahia, Brazil. **Method:** This cross-sectional study used data from 1,929 participants from the Brazilian Longitudinal Study of Adult Health (ELSA-Brazil) living in Salvador, Bahia, to identify the correlation between the medical diagnosis of hypertension and diabetes and green and blue spaces. The city's orthophotos were classified using the random forest algorithm, radar images and the use of vectorized bases (shapefile). We used green and blue green areas in protection areas of 300m, 500m, and in neighborhood councils, in addition to the number of parks within a radius of 1 km as exposure indicators. Multivariate logistic regression models were used to explore the association between exposure indicators and health outcomes, adjusting for possible confounding factors. **Results:** The absence of parks was associated with higher prevalences and positive ORs for diabetes (1.27) and hypertension (1.46). Individuals participating in ELSA-Brasil with vegetation in protected areas 300 and 500 m from their homes did not present outcomes associated with such exposure, as did vegetation in neighborhood town halls. **Conclusion:** The results partially support the evidence that increasing green spaces contributes to better cardiovascular health and indicate the need to increase the number of qualified parks in cities.

P49 | Global Air Pollution: Sources, Trends, and Health Burdens

P49-01 | Has the National Clean Air Programme in India resulted in improvements in air quality?

Abinaya Sekar[1], Adithi Upadhya[2], Anumita Roychowdhury[3], Bhargav Krishna[4], Lucas R. Henneman[5], Ramya Sunder Raman[6], Pallavi Pant[1]

[1] Health Effects Institute, Boston, USA; [2] University of Liverpool, Liverpool, UK; [3] Centre for Science and Environment, Delhi, India; [4] Sustainable Futures Collaborative, Delhi, India; [5] George Mason University, Fairfax, USA; [6] Indian Institute of Science Education and Research Bhopal, Bhopal, India

Objective: Air pollution remains a major health risk in India. In 2019, the National Clean Air Programme (NCAP) was launched with the aim of reducing particulate matter levels. We assessed the impact of NCAP on availability and quality of air quality data and changes in levels of PM10 and PM2.5 between 2017-2023.

Methods: Ambient PM10 and PM2.5 data were obtained from regulatory stations. Data were tested for completeness: stations with data for at least 18 hours daily, 23 days monthly and 11 months annually were included. Changes were evaluated using multiple criteria: exceedance of annual Indian NAAQS and changes in annual averages between 2017-2023.

Results: Since the launch of NCAP, station coverage has increased by more than 120%, bolstering the availability of air quality data. Of the 314 PM10 and 318 PM2.5 stations, only 20 and 29 stations met the data completeness criteria for PM10 and PM2.5 respectively; in most cities, only one station met the criteria. Assessment of annual NAAQS exceedance provided inconsistent results across stations and was not reliable for capturing long-term trends. Citywide averages may not provide meaningful insights since the number of stations and data completeness vary significantly. Rolling 3-year averages better captured long-term trends; in some states, rolling means were higher than annual averages. Less than 10 stations showed a significant decrease in PM2.5, while none of the stations exhibited a strong decreasing trend for PM10.

Conclusion: Despite the increase in number of stations since 2019, for most cities, data are only available for the past two or three years. In cities where reductions were observed, trends often relied on data from a single station. Implementation of a variety of policies between 2017-2023 and limitations in data availability make it challenging to test the efficacy of NCAP on changes in air quality.

P49-02 | Landscape of on-road medium- and heavy-duty diesel vehicle fleets in the United States

Allison P. Patton[1], Kanok Boriboonsomsin[2], Jane Lin[3], Tara Ramani[4], Christopher Tessum[5], Deb Niemeier[6], Ellen K. Mantus[1], Marianne Hatzopoulou[7]

[1] Health Effects Institute, Boston, USA; [2] UC Riverside, CE-CERT, Riverside, USA; [3] University of Illinois Chicago, Civil Engineering, Chicago, USA; [4] Texas A&M Transportation Institute, Center for Advancing Research in Transportation Emissions, Energy and Health, College Station, USA; [5] University of Illinois at Urbana−Champaign, Department of Civil and Environmental Engineering, Urbana, USA; [6] University of Maryland, Department of Civil and Environmental Engineering, College Park, USA; [7] University of Toronto, Civil and Mineral Engineering, Toronto, Canada

Many freight vehicles continue to be operated without the most recent generation of technologies to control air pollutant emissions and also tend to be concentrated near human populations. Thus, air pollutant emissions from older medium- and heavy-duty diesel vehicles continue to affect health, and their transition to newer technologies likely carries substantial exposure and health benefits for communities residing in proximity to freight operation hotspots. Potential economic and societal opportunities *and* barriers also likely exist for owners or operators of older medium- and heavy-duty diesel vehicles. Therefore, HEI formed an expert Heavy-Duty Diesel Vehicle Fleet Turnover Panel to provide information on the current state of knowledge on the potential exposure and health benefits, opportunities, and barriers associated with accelerated fleet turnover. The Panel is summarizing key information on the current and anticipated fleet characteristics and turnover rates. They are comparing potential impacts from new medium- and heavy-duty vehicles (regardless of the specific technologies) to those from model years prior to the 2010 requirements for diesel particulate filters and selective catalytic conversion. Their work considers alternative fuels and powertrains and how past policies have affected the landscape. The summary of the literature is supplemented by a new case study of the potential opportunities and barriers associated with diesel fleet turnover in Chicago. The Panel's overview and findings will be published in an HEI Special Report on the landscape of on-road medium- and heavy-duty diesel vehicle fleets in the United States. The report is expected to inform government and industry, identify literature gaps, and guide HEI's continuing work on the health effects of the legacy diesel fleet.

P49-03 | Assessing $PM_{2.5}$ Air Pollution in Paramaribo, Suriname: Seasonal and Event-Related Trends

Ang Lê[1], Firoz Abdoel Wahid[1], Monique Pool[2], Maureen Lichtveld[1]

[1] University of Pittsburgh School of Public Health, Environmental and Occupational Health, Pittsburgh, USA; [2] Green Heritage Fund, Paramaribo, Suriname

Objective

This study examines $PM_{2.5}$ air pollution in the LMIC Suriname, with three objectives: (1) Compare $PM_{2.5}$ concentrations in 2022–2023 to WHO air quality standards, (2) Evaluate seasonal variations in $PM_{2.5}$ levels, particularly between the dry (February-May, September-December) and wet seasons (January-February, May-September), and (3) Analyze the impact of “Owru Yari”, Suriname’s New Year’s celebration, on $PM_{2.5}$ pollution. This is the first-ever analysis of air quality monitor data in Suriname, providing critical baseline data for air pollution research and policy development.

Materials and Methods

$PM_{2.5}$ data were collected using two – WHO-approved- IQR Air Quality Monitors in northern (low traffic) and southern (high traffic) Paramaribo, the capital of Suriname. Descriptive statistics and a Wilcoxon signed-rank test were used to compare seasonal and event-based differences. Statistical tests assessed whether: (1) $PM_{2.5}$ exceeded WHO standards (5.0 μg/m³), (2) $PM_{2.5}$ was higher in the dry season, and (3) $PM_{2.5}$ increased during Owru Yari (December 15 – January 6) compared to a control period (November 15 – December 6).

Results

$PM_{2.5}$ concentrations exceeded WHO standards in 2023 ($p < 2.2e-16$). Dry season levels were significantly higher than in the wet season ($p < 0.001$). During Owru Yari, both monitors recorded 2- to 3-fold increases in $PM_{2.5}$ compared to the control period ($p < 0.0006$).

Conclusion

As the first $PM_{2.5}$ study in Suriname, environmental causes need to be a part of a comprehensive analysis of why ischaemic heart disease and respiratory infections fall under the leading causes of morbidity, as $PM_{2.5}$ exposure is linked to respiratory, cardiovascular, and pregnancy-related health issues. Results underscore the need for air quality regulations and public health interventions.

Funding & Acknowledgements

Green Heritage Fund

P49-04 | Impact of the La Palma volcanic eruption on healthcare use in exposed and non-exposed adult populations

Cristina Candal-Pedreira[1,2], David Díaz-Pérez[2], Valle Velasco[2], Ciro Casanova[2], Esther Barreiro[2], Ana Cañas[2], Argelia Castaño[2], María Jesús Cruz Carmona[2], Xavier Muñoz[2], Francisco Javier Sánchez-Íñigo[2], Lucía Martín-Gisbert[2], Elena López-Pérez[1], Alberto Ruano-Ravina[1,2]

[1] University of Santiago de Compostela, Preventive Medicine, Santiago de Compostela, Spain; [2] Scientific committee of the ASHES project, Spanish Society of Neumology and Thoracic Surgery (SEPAR), Spain, Spain

Objective: To analyze the impact of the 2021 La Palma volcanic eruption on the use of health services in exposed and non-exposed adult populations during the eruptive and post-eruptive phases.
Methods: The ASHES study is a cohort study to assess the health impact of the La Palma volcanic eruption. Three exposure groups were considered: Group 1 (high exposure), Group 2 (moderate exposure) and Group 3 (unexposed). The analysis was restricted to a healthy adult population. A descriptive analysis of health service use and reasons for seeking care was performed, stratified by group and sex. The study protocol was approved.
Results: 472 subjects were included: 54 in Group 1, 333 in Group 2, 85 in Group 3. During the eruptive phase, 37.5% used healthcare services (no significant differences between groups, p=0.52), increasing to 75.1% after the eruption (significant differences, p=0.005). Primary care was the most used service across all groups in both phases. Healthcare visits increased by 30.7% (95%CI:12.0-38.2%) in the highly exposed, 42.5% (95%CI:35.7-49.3%) in the moderately exposed, and 29.6% (95%CI:14.9-44.3%) in the unexposed between the eruptive and post-eruptive phases. Women used healthcare services more than men. During the eruptive phase, respiratory symptoms were the main reason for consultation in highly exposed individuals(p=0.17), while mental health issues predominated in the moderately exposed(p=0.01). In the post-eruptive phase, respiratory visits increased from 5.1% to 10.8% overall and were the most common across all exposure groups. 7.4% of the highly exposed and 14.6% of the moderately exposed began taking anxiolytics after the eruption, compared to 2.4% of the unexposed(p<0.001).
Conclusion: The volcanic eruption increased healthcare utilization, particularly among exposed populations. Respiratory illnesses were the primary reason for consultation, while mental health issues were notable in the moderately exposed. The event led to increased anxiolytic use, underscoring its psychological impact.

P49-05 | Assessing global ozone exposure and health-relevant trends: Insights enabled by the Tropospheric Ozone Assessment Report

Gaige H. Kerr[1], Adeladza K. Amegah[2], Lulu Chen[1], James L. Crooks[3,4], Drew Shindell[5], James J. West[6]

[1] George Washington University, Milken Institute School of Public Health, Department of Environmental and Occupational Health, Washington, USA; [2] University of Cape Coast, Department of Biomedical Sciences, Accra, Ghana; [3] National Jewish Health, Division of Biostatistics and Bioinformatics, Denver, USA; [4] Colorado School of Public Health, Department of Epidemiology, Aurora, USA; [5] Duke University, Nicholas School of the Environment, Durham, USA; [6] University of North Carolina, Department of Environmental Sciences and Engineering, Gillings School of Global Public Health, Chapel Hill, USA

Objective: Long-term exposure to ambient ozone (O_3) is associated with impaired lung function and increased risk of cardiovascular and respiratory morbidity and mortality, and global health impact assessments consistently highlight

O_3 as a leading environmental risk factor for premature death. Underpinning our understanding of the health impacts of O_3 are measurements of O_3 concentrations, which are sparse in many low- and middle income nations worldwide.
Material and Methods: As part of the second Tropospheric Ozone Assessment Report (TOAR; https://igacproject.org/activities/TOAR/TOAR-II) Health Assessment, we leverage the observational TOAR-II database–a publicly-available, unified database of observed O_3 concentrations–and observationally constrained, high-resolution gridded estimates of surface-level O_3. We map the spatial variability and trends of surface-level O_3 globally using two health-relevant metrics that characterize chronic exposure. We also provide a scoping review of the literature on O_3-attributable health impacts and summarize state-of-the-science practices for conducting health impact assessments and characterizing associated uncertainty.
Results: While concentrations of other health-harming pollutants such as fine particulate matter and nitrogen dioxide have decreased in many world regions due to emissions reductions, we find that surface-level O_3 has significantly increased or exhibits non-significant changes across most world regions since 2000. Our literature review indicates that O_3-attributable premature deaths have also increased over this period, resulting in nearly 500,000 attributable deaths globally in 2020. Many countries with the largest positive trends in O_3-attributable mortality are countries that currently have low to moderate rates of attributable mortality.
Conclusion: We expect that our summary of the TOAR database will be beneficial for exposure scientists seeking to integrate global O_3 observations into their methodological frameworks and that our analysis of health-relevant O_3 trends derived from this database will spur additional research on exposure and health impacts in un- or under-monitored regions.

P49-06 | Associations of particulate matter and manganese exposures with plasma amyloid beta and phosphorylated tau concentrations

Jiyoon Yeom[1], Heeseon Jang[2], Hyunji Park[1], Yongjin Lee[4], Seunghan Hong[2], Kyungjun Jeong[4], Changsoo Kim[2,3], Jaelim Cho[2,3]

[1] Graduate School, Yonsei University, Department of Public Health, Seoul, South Korea; [2] Yonsei University College of Medicine, Department of Preventive Medicine, Seoul, South Korea; [3] Institute of Human Complexity and Systems Science, Yonsei University, Center for Systems and Translational Brain Science, Seoul, South Korea; [4] Institute of Environmental Research, College of Medicine, Yonsei University, Seoul, South Korea

Objective: Emerging evidence suggests that air pollution contributes to neurodegenerative diseases including Alzheimer's disease(AD). We aimed to investigate the associations of air pollution exposures including particulate matter(PM) and Manganese(Mn) with plasma amyloid beta(Aβ) and phosphorylated tau(p-tau) proteins, the key pathologies of AD.
Material and Methods: This study included 367 participants without history of neurological diseases(dementia, parkinson’s disorders, or stroke) from a community-based cohort in Korea. Ambient air pollution concentrations of PM with diameter ≤10㎛(PM_{10}), ≤2.5㎛($PM_{2.5}$), Mn with diameter ≤10㎛(Mn_{10}), and ≤2.5㎛($Mn_{2.5}$) were predicted based on residential addresses. Concentrations of plasma Aβ40, Aβ42 and p-tau proteins were collected and their ratios were calculated. Using a linear regression model adjusting for demographics, cardiovascular risk factors, and apolipoprotein E-ε4 allele carriership, we estimated the associations of ambient air pollution exposures with the blood-based markers of AD.
Results: The mean(standard deviation) age was 72.7(5.7) years. An increase in 10㎍/㎥ of $PM_{2.5}$ (β=-0.016; SE=0.001; *p*=0.012) and $Mn_{2.5}$ (β=-0.005; SE=0.003; *p*=0.039) was inversely associated with Aβ42/Aβ40 ratio. The

middle tertile of PM_{10} and Mn_{10} had a significantly lower Aβ42/Aβ40 ratio by -0.022 (SE=0.011; *p*=0.041) and -0.021 (SE=0.010; *p*=0.041) compared with the lowest tertile*(p for trend=0.010)*.
Conclusion: These findings suggest that exposure to ambient PM and Mn may induce Aβ accumulation and tau pathology, potentially accelerating AD progression in the elderly.
Acknowledgements: *This work was supported by Korea Environment Industry & Technology Institute(KEITI) through Core Technology Development Project for Environmental Diseases Prevention and Management, funded by Korea Ministry of Environment(MOE)(grant No.2022003310011), the National Research Foundation of Korea(NRF) grant funded by the Korea government (No. 2022R1F1A1069621), and (24192MFDS077) from Ministry of Food and Drug Safety in 2024.*

P49-07 | Exploring the Link Between Grandmaternal Air Pollution Exposure and Grandchild's ASD Risk: A Multigenerational Population-Based Study in California

Karl O'Sharkey[1], Ting Chow[1], Laura Thompson[2], Sanjali Mitra[1], Jason Su[3], Myles Cockburn[2], Zeyan Liew[4], Beate Ritz[1]

[1] University of California, Los Angeles, Epidemiology, Los Angeles, USA; [2] University of Southern California, Population and Public Health Sciences, Los Angeles, USA; [3] University of California, Berkeley, Environmental Health Sciences, Berkeley, USA; [4] Yale University, Environmental Health Sciences, Los Angeles, USA

Background: Autism Spectrum Disorder (ASD) is a neurodevelopmental disorder with increasing prevalence. While genetics play a strong causal role, among environmental factors, air pollution (AP) exposure in pregnancy and infancy has been strongly endorsed as a risk factor. However, potential multigenerational impacts through the exposure of the grandmother during her pregnancy remain unexplored.
Methods: Using a multigenerational, population-based cohort from California spanning three decades (1990-2019), we examined the association between grandmother's gestational AP exposure ($PM_{2.5}$, NO_2, O_3) and ASD risk in grandchildren using logistic regression, adjusting for maternal exposure in pregnancy or infant's exposure in the first year of life. We used continuous AP exposure as well as a categorical variable representing high and/or low (above or below median) exposure levels for both the grandmaternal and maternal pregnancies. Pregnancy and first year of life AP exposures were assigned using a land-use regression model with advanced machine-learning approaches.
Results: We observed associations between $PM_{2.5}$ (OR = 1.07, 95% CI: 1.05, 1.10)and NO_2 (OR = 1.09, 95% CI: 1.05, 1.13) exposure during the grandmaternal pregnancy and increased ASD risk in the grandchild. However, only for $PM_{2.5}$ did the increased effect estimates persist after adjusting for maternal pregnancy exposure (OR = 1.05; 95% CI: 1.02, 1.08). High compared to low exposures in both grandmaternal and maternal pregnancies to $PM_{2.5}$ (OR = 1.16, 95% CI: 1.11, 1.23) and NO_2 (OR = 1.12, 95% CI: 1.06, 1.17) showed the strongest joint effects for ASD in the grandchild.**Conclusions**: Multigenerational exposure to air pollution, particularly $PM_{2.5}$ exposure in grandmaternal pregnancy, may influence ASD risk. Our study also suggests that air pollution effects may compound by multigenerational exposures in ASD etiology.

P49-08 | Community exposures to ultrafine particles and benzene from traffic - preliminary evaluations

Leonid Turczynowicz[1], Felicia F. Maragkos[2], Lilliana Drapaniotis[3], Fawzia Kabir[4], Isaac A. Vann[5]

[1] University of Adelaide, Adelaide Exposure Science and Health, School of Public Health, Adelaide, Australia; [2] University of Adelaide, School of Public Health, Adelaide, Australia; [3] University of Adelaide, School of Public Health, Adelaide, Australia; [4] University of Adelaide, School of Public Health, Adelaide, Australia; [5] University of Adelaide, School of Public Health, Adelaide, Australia

Objective
Understanding community exposures to traffic emissions enables human health risk assessment supporting government transport policy initiatives through an evidenced-based process.
Material and Methods Ultrafine particle (UFP) exposures were evaluated at three bus shelters on different arterial roads in Adelaide, South Australia, during early morning peak traffic periods. Bus shelters were within 1 metre of the kerbside. Daily real-time UFP monitoring for two hours over one week at sedentary height, employed both a TSI P-Trak® 8525 and a Diffusion Size Classifier DiSCmini counter. Concurrent vehicle counts were recorded via an iPad application.
In Australia, cycling, a major pastime, generally occurs along major road lanes. Cyclists' personal exposures to total volatile organic compounds (VOCs) and benzene, toluene, ethyl benzene and xylenes (BTEX) were assessed over 2-3 hour early morning periods for 5 days along common road cycling lanes and an off-road cycling path. Distances ranged up to 40km and were tracked using a Garmin 800. Monitoring used Radiello™ (RAD145) passive samplers and a real-time VOC Ion Science Tiger ppb photo-ionisation detector.
Results
Bus shelter results reported vehicle counts of 1031-3537 with UFP averages of 2.9×10^3-17.4×10^3 pt/cc with peak counts exceeding 10^6pt/cc. Analyses generally reported significant associations between 2-h average particle counts and traffic frequency (multiple R^2 = 0.87, p=0.01).
Cycling results reported average concentrations of <1.5-5 μg m^{-3} (benzene); 2-20 μg m^{-3} (toluene); 0.5-2 μg m^{-3} (ethyl benzene); and 1.1-6 μg m^{-3} (xylenes). Peak exposures up to 350ppb VOCs were reported.
Conclusions
Elevated UFP exposures occur during bus wait times under peak traffic conditions in Adelaide with significant peak exposures.
Cycling along major roads within traffic dense areas contribute significantly to daily VOC and BTEX exposure. Benzene exposures increased cancer risk above target criteria, implying avoidance of regular cycling in high traffic areas was prudent.

P49-09 | County-level analysis of the association between ambient concentrations of trichloroethylene and Parkinson’s disease

Lynne P. Marshall, Lauren Lautermilch

Stantec, Health Sciences Advisory, Pittsburgh, USA

Objective:
Although some studies have reported a positive association between trichloroethylene (TCE) exposure and Parkinson’s disease (PD), most epidemiological analyses have focused only on occupational TCE exposures among populations such as military personnel. As of 2025, six U.S. states – California, Nebraska, South Carolina, Utah, Washington, and West Virginia – have state PD registries. We conducted an ecological analysis of the association between county-level, age-adjusted PD mortality rates and county-level ambient TCE concentrations in these states to explore the relationship between environmental TCE exposure and community PD risk.
Material and Methods:
Age-adjusted PD mortality rates from 2021 across six U.S. states were obtained from the Centers for Disease Control (CDC) and median county-level TCE air concentrations were obtained from the 1996 National Air Toxics Assessment (assumed a 25-year latency period). Exposure data was log (base 10) transformed due to right skewness. The exposure variable and covariates (county-level percentages of white individuals and males) were evaluated by tertile. Rate ratios (RRs) were calculated using Poisson regression. All analyses were conducted in SAS version 9.4.
Results:
Among 320 counties in the six states assessed, 12 were excluded due to PD mortality rate suppression. County-level, age-adjusted PD mortality rates (deaths per 100,000 population) ranged from 1.9 in Rich County, UT to 14.71 in Rock County, NE. We observed a statistically significant association between the highest tertile of county-level ambient TCE and county-level PD mortality (RR = 1.289; 95% CI: 1.147-1.450) compared to the lowest tertile of county-level ambient TCE, after adjustment for white race and sex.
Conclusion:
This analysis demonstrates a novel association between environmental TCE exposure and PD mortality. Additional research utilizing individual-level data from state PD registries and more granular exposure estimates may further explain this excess PD risk in association with low-level ambient TCE.

P49-10 | Impacts of Zero-Emission Truck Regulations on Tailpipe and Non-Tailpipe Air Pollutant Exposures and Health Risks in Southern California

Meredith Franklin[1], Jill Johnston[2], Harshit Gujral[1], Soroush Neyestani[2], Rima Habre[2], Jiachen Zhang[2]

[1] University of Toronto, Toronto, Canada; [2] University of Southern California, Los Angeles, USA

California has recently implemented two key regulations aimed at accelerating the transition to zero-emission trucks. These regulations are designed to meet phased targets for the percentage of zero-emission vehicles (ZEVs) in new truck sales, gradually replacing diesel-powered trucks with electric alternatives. The Advanced Clean Trucks (ACT) Rule and the Advanced Clean Fleets (ACF) Rule are central to this effort, mandating that manufacturers and fleet operators increase their adoption of zero-emission vehicles over time.
While these regulations are expected to significantly reduce emissions from the trucking sector, their implementation raises important questions about equity and environmental justice, particularly for the Los Angeles – Long Beach communities located along major freight corridors. Many of these communities experience higher levels of traffic-related air pollution due to their proximity to highways, ports, and distribution centers. Reducing emissions in these areas could yield substantial health benefits, but the spatial and temporal distribution of these benefits remains uncertain.
To assess the health equity implications of California's truck electrification policies, we adapted the Weather Research and Forecasting model coupled with Chemistry (WRF-Chem) to simulate diesel PM2.5, NOx, as well as tailpipe and non-tailpipe particulate matter (PM) emissions under different policy scenarios. Through these simulations, we analyzed changes in air pollution levels and coupled them with concentration response functions for asthma and mortality to assess health outcomes associated with projected exposures. By quantifying the health impacts of reduced PM2.5 concentrations, we provide a more comprehensive assessment of how these regulations influence public health, particularly in communities along freight corridors that have historically faced disproportionate pollution burdens.

P49-11 | Surgical Smoke in the Operating Room: Exploring the Role of Smoke Evacuators

Mohamed J Ruzmyn Vilcassim[1], Daniel Chu[2], Melanie Morris[2], Wendelyn Oslock[2,1]

[1] University of Alabama at Birmingham, Department of Environmental Health Sciences, Birmingham, USA; [2] University of Alabama at Birmingham, School of Medicine, Birmingham, USA

Background: Surgical smoke, primarily generated by electrocautery devices, contains harmful air pollutants such as particulate matter (PM) and volatile organic compounds (VOCs). However, the extent of exposure experienced by surgeons and operating room staff remains unclear. This study aimed to evaluate variations in air pollutant concentrations during frequent electrocautery use and assess the impact of smoke evacuators on exposure levels.
Methods: Air pollutant concentrations were measured during breast and amputation surgeries at an academic institution without mandatory smoke evacuator use. Operation details, room conditions, smoke evacuator presence, and 1-minute electrocautery observations were recorded. $PM_{2.5}$ concentrations were measured near the surgical field

and at the room's periphery using a PDR-1500 DataRAM air monitor. VOC levels were recorded near the smoke source and anesthetic gases. Cases were categorized based on smoke evacuator use, and peak and average concentrations were analyzed using t-tests and chi-square tests. PM collected on Teflon filters were analyzed using X-Ray Fluoresence (XRF) method to identify differences in elements due to surgical procedures.
Results: Ten surgeries were analyzed, with mastectomies being the most common (n=6, 60%). Smoke evacuators were used in 4 cases. Electrocautery was used for an average of 58.6 minutes. Without smoke evacuators, PM2.5 levels were significantly higher near the field (28.3 vs 4.3 μg/m³, p<0.0001) and at the periphery (3.8 vs 1.1 μg/m³, p<0.0001). Cases without evacuators experienced more frequent and severe $PM_{2.5}$ peaks, reaching up to 301 μg/m³, while those with evacuators peaked at 61.4 μg/m³. Conversely, VOC levels were higher with smoke evacuators (3052 vs 1191 ppb, p<0.0001).
Conclusion: Electrocautery use without smoke evacuators resulted in significantly higher $PM_{2.5}$ levels and frequent pollutant peaks. Further research is needed to assess exposure variations and particulate composition for improved risk evaluation.

This study was funded by the UAB Deep South Center for Occupational Health and Safety.

P49-12 | Global Assessment of Satellite Earth Observation Capabilities and Opportunities for Air Pollution and Health

Nathan Pavlovic[1], Carl Malings[2,3]

[1] Sonoma Technology, Petaluma, USA; [2] NASA GMAO, Greenbelt, USA; [3] Morgan State University, Baltimore, USA

Objective
Air pollution is a critical environmental exposure that contributes to a range of adverse health outcomes and millions of premature deaths annually across all regions of the world. In-situ air pollution observations are relatively sparse on a global scale due to the costs of establishing and maintaining such systems. Satellite Earth observation (EO) data can be used to assess regional and global air quality patterns and trends to complement in-situ monitoring, but data access and availability, interpretation, and analysis capacity often limit the use of EO in health-related applications. The Group on Earth Observations (GEO) Health Community of Practice's (CoP) Air Quality, Wildfires, and Respiratory Health Work Group (WG) assesses current capabilities, challenges, and opportunities for the use of EO in health decision-making.
Materials and Methods
Existing and proposed future use cases for health applications of EO were surveyed. Use cases highlighting the requirements, satellite data uses, capabilities and limitations, and regional considerations are reviewed. Based on use cases, decision-making processes were established to lower barriers to entry for the use of satellite data.
Results
The WG has compiled a list of current capabilities and future proposals for EO with potential health applications, including estimation of surface-level pollutants and emissions from polar orbiting, geostationary, and small-sat platforms. These capabilities are applicable to air pollution monitoring and management, emissions assessment and control, epidemiological analysis, public health; case studies of these applications are used to develop guidance for their use. Existing applications can be enhanced to encourage global applicability.
Conclusion

EO provides critical input to support health applications related to air pollution exposure, but significant gaps in current global applications exist. Challenges to uptake can be reduced through investment in additional EO applications, clear decision-making guidance, and open data and processes.

P49-13 | Health Effects of Air Pollution in East Africa: Current Evidence and Priorities for Future Action

Victor Nthusi[1], Egide Kalisa[2], Rebecca Nantanda[3], Alex Ndyabakira[4], Caradee Wright[5], **Pallavi Pant**[1]

[1] Health Effects Institute, Boston, USA; [2] University of Western Ontario, London, Canada; [3] Makerere Lung Institute, Kampala, Uganda; [4] Kampala Capital City Authority, Kampala, Uganda; [5] South African Medical Research Council, Pretoria, South Africa

Several cities and countries in East Africa are actively working to improve air quality, creating a growing demand for accessible and actionable air pollution data. This analysis is based on a scoping review on health effects of air pollution in eight East African countries and deliberations at an in-person workshop in February 2025. The review protocol, a detailed report and an interactive literature database have been published and are freely accessible.

A total of 34 studies on health effects on air pollution were identified for inclusion in the scoping review. The majority (76%) were published in or after 2019, yet 26.5% lacked any authors affiliated with institutions in East Africa. Thirteen out of 34 publications focused on respiratory health outcomes, either in children or adults. More than 25 of the 34 publications included in the review were cross-sectional studies. Furthermore, most publications included in this review were funded through international governments or philanthropies with limited investments from national or local governments. Several studies leveraged existing air quality and health data sources, including satellite-derived pollution estimates, demographic and health surveys, and established cohorts.
The current evidence on health effects of air pollution in East Africa is aligned with global evidence and provides a strong scientific basis for action on air pollution. While there are a growing number of cross-sectional studies, there is a need for longitudinal studies. Research is also needed on the relationship between exposure to air pollution and infectious diseases such as tuberculosis; this is especially relevant since tuberculosis continues to contribute to a substantial disease burden and. Finally, the focus must remain on support for local capacity and institutional strengthening, with an emphasis on policy-relevant research.

Acknowledgements: This work was supported by the Clean Air Fund.

P49-15 | Chemical speciation of daily ambient PM2.5 from seven Indian sites in GEOHealth HEALS

Prashant Rajput[1], Anubrati Dutta[1], Tripti Agarwal[1], Rani K. Lata[1], Sonal Singh[2], Mohan Deepa[3], Jyothi Menon[2], Suganthi Jaganathan[2], Siddhartha Mandal[2], Poornima Prabhakaran[2], Gagandeep K. Walia[2], Ruby Gupta[2], Sailesh Mohan[4], Chittaranjan Yajnik[5], Enakshi Ganguly[6], Vipin Gupta[7], Viswanathan Mohan[3], K. Srinath Reddy[8], Joel Schwartz[9], Dorairaj Prabhakaran[4], Tarun Gupta[10]

[1] Centre for Chronic Disease Control, New Delhi, India; [2] Centre for Chronic Disease Control; Ashoka University, Centre for Health Analytics Research and Trends (CHART)/Trivedi School of Biosciences, New Delhi; Sonipat, India; [3] Madras Diabetes Research Foundation, ICMR Collaborating Centre of Excellence, Chennai, India; [4] Centre for Chronic Disease Control; Public Health Foundation of India, New Delhi, India; [5] KEM Hospital Research Centre, Pune, India; [6] Mediciti Institute of Medical Sciences, Medchal, India; [7] Delhi University, Department of Anthropology, New Delhi, India; [8] Public Health Foundation of India, New Delhi, India; [9] Harvard University, Department of Environmental Health/Harvard T. H. Chan School of Public Health, Boston, USA; [10] Indian Institute of Technology Kanpur, Department of Civil Engineering, Kanpur, India

Background:
High-quality data of ambient PM2.5-bound chemical species are required for accurate exposure assessment. The lack of near continuously speciated PM2.5 data across India is a big challenge in assessing the geographical disparities in their health effects.

Objective:
One of the objectives of the “GEOHealth HEALS (Health Effects of Selected Environmental Exposomes Across the Life CourSe)-India” study is to assess chemical speciation of PM2.5 from seven Indian sites (Delhi, Sonipat, Bikaner, Pune, Hyderabad, Vizag, and Chennai).

Material and Methods:
Using low-volume impactor samplers, we are collecting daily ambient PM2.5 samples onto Teflon and Quartz filters from ~*10* locations per site both during the winter and summer since January 2024. So far, we have analyzed *346* samples along with *32* Teflon and *39* Quartz field blanks and carried out *16* sets of collocation experiments between the two types of monitors. PM2.5 was determined gravimetrically, whereas elemental and organic carbon (EC, OC) was analyzed on a DRI-EC-OC analyzer, and *32* metals were measured using X-ray fluorescence.

Results:
Average PM2.5 concentrations were 136.9 ± 73.2, 92.2 ± 46.7, 53.0 ± 23.7, 46.6 ± 11.6, and 23.7 ± 11.6 μg m^{-3} in Delhi, Sonipat, Bikaner, Chennai, and Pune, respectively. Large spatial and temporal variability in PM2.5 concentrations (*N=346*, Jan-early July 2024) was observed in Delhi and Sonipat followed by in Bikaner, whereas relatively less variability was observed in Chennai and Pune. On average, total carbonaceous aerosols (=1.6*OC+EC) and sum of metals contributed 24–46% and 24–41% to PM2.5, respectively.

Discussion:
Quantifying the chemical composition of ambient PM2.5 is crucial in understanding the disparities in their health effects. Data from this study and those from other ground monitoring stations like SPARTAN Network, and MISR aboard NASA’s Terra satellite will be used in conjunction with other predictors to develop exposure models for PM2.5 constituents.

P49-16 | Salivary Metabolomics and Air Pollution Exposure in Toddlers: Black Carbon and $PM_{2.5}$ are Associated with Pathways Related to Inflammatory Regulation

Roby Greenwald[1], YuJung G. Lin[1], Bisan Hammid[1], Nadia Alhadidi[1], Donghai Liang[2]

[1] Georgia State University, Population Health Sciences, Atlanta, USA; [2] Emory University, Environmental Health, Atlanta, USA

The Air Pollution Exposure in Child Care Settings (APECCS) study measures air pollution exposure in healthy children ages 3-4 years and explores biological mechanisms of response using salivary metabolomics. We coordinated exposure assessment activities at child care centers in Atlanta, Georgia, and we measured air pollutant levels indoors and outdoors at the centers, the homes of participating families, and in ambulatory settings using small backpacks accompanying the children in transport environments. Air pollutant measurements included $PM_{2.5}$, black carbon (BC), nitrogen dioxide (NO_2), ozone (O_3), particle number concentration (PNC), and elemental speciation of metals present if $PM_{2.5}$. Health outcome assessment included a metabolome-wide association study (MWAS) of saliva samples collected weekly. Untargeted metabolomics analysis measured the intensity of 5578 features using a C18 column for separation and 10274 features using a HILIC column. We assessed the association of exposure with metabolomic feature intensity using linear mixed-effects models and the lme4 package for R. We sequentially ran models for all ~16000 features in the database using the weekly exposure of each measured pollutant as the dependent variable and the change in feature intensity over the corresponding time period as the predictor while controlling for sex and race. We additionally included a random effect for subject. After adjusting resulting p-values for multiple comparisons, we performed pathway analysis using the mummichog network analysis tool for metabolomics. Numerous statistically-significant associations were observed for all pollutant metrics and various metabolomic pathways. Notably, exposure to $PM_{2.5}$ was strongly associated with multiple pathways related to inflammatory regulation. Exposure to BC was also strongly associated with inflammatory regulation and additionally to pathways identified as indicators of asthma pathogenesis. Analysis of this extensive dataset is ongoing, and it is likely that additional noteworthy observations will be reported at the time of presentation.

P49-17 | Long-Term Trends of Black Carbon in Pakistan's Metropolises: Exposure Assessment and Health Risks

Saadia Hina[1], Hamna Nisar[1], Salman Tariq[2], Eliani Ezani[3], Ammara Habib[4], Muhammad Ibrahim[1]

[1] Government College University Faisalabad, Department of Environmental Sciences, Faisalabad, Pakistan; [2] University of the Punjab, Centre for Remote Sensing, Lahore, Pakistan; [3] Universiti Putra Malaysia, Department of Environmental and Occupational Health, Serdang Selangor, Malaysia; [4] Urban Sector Policy and Management Services Unit (Pvt.) Limited, Lahore, Pakistan

Black carbon (BC), despite its relatively small contribution to total atmospheric aerosol loads, has gained increasing attention due to its significant impacts on air quality, human health, and climate change. This study investigates the long-term spatio-temporal trends of BC across major metropolitan cities in Pakistan using MERRA-2 reanalysis datasets spanning from 2001 to 2022. To identify BC hotspots, we employed Getis-Ord $G*i$, which revealed pronounced BC in Lahore and Karachi. Furthermore, the HYSPLIT model was utilized to provide insights into both

regional and transboundary contributions. Finally, health risk and exposure assessment were calculated based on average daily potential intake of BC. The results presented an increasing trend of BC in winter due to low planetary boundary layer and decreased trends in summers due to monsoon and the washout process. Among the metropolitans of Pakistan, the highest values of BC concentration were recorded in Karachi while lowest values have been observed in Islamabad. Our findings also revealed a consistent increase in BC levels, with Lahore exhibiting the highest population weighted exposure, followed by Karachi and Peshawar. Health risk assessments for adults and children, particularly in Lahore and Karachi. The lifetime cancer risk exceeded the acceptable threshold ($>10^{-6}$) in all cities, with Lahore showing the highest cancer risk values. Non-cancer risks (hazard quotient > 1) were also identified, highlighting potential adverse health effects. These results highlight the urgent need for targeted air quality management strategies and public health interventions to mitigate BC emissions and reduce associated health risks in Pakistan's urban centers.

P49-18 | The status of ambient PM2.5 concentrations in public schools' environment in Kampala City, Uganda: Preliminary findings from the Eastern Africa GEOHealth Hub Child Health Study

Samuel Etajak, Lynn Atuyambe, Eastern Africa GEOHealth Hub

Makerere University School Health, Kampala, Uganda

Introduction: Ambient air pollution remains a serious environmental risk factor for death in children. About 93% of children live in areas where air pollution exceeds WHO guidelines. In Africa, ambient air pollution is noted to have caused 394,000 deaths in children. The study aimed to assess the status of ambient PM2.5 concentrations in Public primary schools environment in Kampala City, Uganda
Methods: This was a cross-sectional study conducted in 2 public primary schools, Kitante Primary School in Central Division and St Martin Mulago in Kawempe Division. The status of ambient PM2.5 concentrations was measured using the QuantAQ Modulair PM equipment for a period of 3 months (September, October, and November 2024). The QuantAQ modulair PM was sited in the school compound away from obstructions from any obstructive objects. The equipment was mounted in a stand 4 meters above ground level. The QuantAQ modulair PM monitor measured PM1, PM2.5, and PM10.
Findings: The 24-hour PM2.5 concentrations was found to higher at 127.9 micrograms per cubic meter at St Martin Mulago Primary School in Kawempe division, which was 8 times above the World Health Organization (WHO) guidelines of 15 micrograms per cubic meter, compared to Kitante Primary School in Central division, which had 24-hour PM2.5 concentrations of 48.4 micrograms per cubic meter. $PM_{2.5}$ levels were notably higher in St Martin Primary School in Kawempe division mpala (127.9 $\mu g/m^3$) compared to Kitante Primary School (48.4 $\mu g/m^3$). The 24-hour average $PM_{2.5}$ exposures among school children was exceeding 15 $\mu g/m^3$ in line with the WHO Air Quality Guideline 2021.
Conclusion: The research revealed high levels of 24-hour average ambient PM2.5 concentrations school-children are exposed to in the school environment, thus underscoring the substantial health risks associated with high PM2.5 exposure.

P49-19 | Land Use Regression and Hotspot Analysis to Identify Ambient Sulphur Dioxide Monitoring Gaps in Saint John, Canada

Tsz Kin Siu[1], Christopher S. Greene[1], Kelvin C. Fong[1,2]

[1] Dalhousie University, Department of Earth and Environmental Sciences, Halifax, Canada; [2] George Washington University, Department of Environmental and Occupational Health, Milken Institute School of Public Health, Washington, D.C., USA

Objective:

Saint John, a medium-sized industrial city in New Brunswick, Canada, accounted for >70% of sulphur-related air pollution exceedances in the province. Due to limited spatial coverage, the ground-based monitoring network for sulphur dioxide (SO_2) does not measure air pollution in some communities. This study aimed to identify monitoring gaps by estimating high-resolution surface SO_2 concentrations then mapping hotspots from the model predictions.

Material and Methods:

We developed a two-step predictive model with gradient-boosted decision trees training on surface measurements from 13 monitoring stations during 2022–2023. Input variables included satellite remote sensing data and built environment features, comprising SO_2 column densities from the Tropospheric Monitoring Instrument (TROPOMI), meteorology, land cover and land use, road network, elevation, and population density. The predictions were generated by a classifier on SO_2-free and SO_2-polluted days at the first step, then a regressor on daily mean and maximum SO_2 pollution levels at the second step. Based on the estimated SO_2, we mapped hotspots by Getis-Ord Gi* and the 90th percentile. We determined the gap areas by population density, proximity to identified SO_2 hotspots and existing monitoring stations.

Results:

The classifier captured 96% of SO_2-polluted days and R^2 of the regressors for daily mean and maximum SO_2 were 0.46 and 0.44 respectively. Modelled hotspots during days with exceedances expanded from urban center to the northeastern, northwestern, and southeastern periphery, where SO_2 monitoring was absent; and near the paper and pulp mill, where the three nearby stations measured only hydrogen sulphide (H_2S). Wind speed and direction ranked high in variable importance, which might be associated with the spread of SO_2 hotspots from the emission sources to these suburban areas.

Conclusion:

SO_2 monitoring gaps existed in Saint John. Future efforts can apply our findings to fill in data gaps when examining population-level SO_2 exposure risk.

P49-20 | Evaluating Disparities in Fine Particulate Matter Monitoring in Eastern Canada

Tsz Kin Siu[1], Dainel G. Rainham[2], Rachel Y. Chang[3], Christopher S. Greene[1], Kelvin C. Fong[1,4]

[1] Dalhousie University, Department of Earth and Environmental Sciences, Halifax, Canada; [2] Dalhousie University, School of Health and Human Performance, Halifax, Canada; [3] Dalhousie University, Department of Physics and Atmospheric Science, Halifax, Canada; [4] George Washington University, Department of Environmental and Occupational Health, Milken Institute School of Public Health, Washington, D.C., USA

Objective:

Low-cost sensors for fine particulate matter ($PM_{2.5}$) have been increasingly used to complement regulatory monitoring. While disparities were found in $PM_{2.5}$ exposure, there is no investigation in Canada on monitoring disparities. This study aimed to evaluate the $PM_{2.5}$ monitoring coverage, gaps, and inequities in Ontario, Quebec, and the Atlantic provinces in Canada.

Material and Methods:

The locations and hourly measurements of regulatory monitoring stations and deployed low-cost sensors from January 2018 to September 2024 were obtained from the national air pollution surveillance network and PurpleAir, respectively. We applied kernel density estimation (KDE) to estimate the $PM_{2.5}$ monitor density. To account for spatiotemporal variability, we selected local bandwidths using model-derived surface $PM_{2.5}$, and weighted each monitor by the fraction of time being active. Based on the census dissemination area level $PM_{2.5}$ monitor densities, model-derived surface $PM_{2.5}$, and the Canadian Index of Multiple Deprivation (CIMD), we used quintile filtering methods and mixed-effects logistic regression to identify inequitably monitored communities and investigate the associations between low monitor density and each deprivation adjusted for population density.

Results:

Clusters of persistent monitoring gaps were found in coastal areas of Lake Huron and Georgian Bay, the south shore of St. Lawrence River, and the Gaspé Peninsula. Communities experiencing disproportionate $PM_{2.5}$ monitoring occurred in northwestern Ontario with Indigenous reserves, suburban Windsor in Ontario, and eastern suburbs of Hamilton in Ontario and Montreal in Quebec. Classification of monitoring inequities varied by whether $PM_{2.5}$ pollution and CIMD were compared regionally or across provinces. Ethno-cultural minorities and situationally vulnerable populations had higher odds (55%–92%) of low monitoring coverage in their communities. Notably, we found provincial variations.

Conclusion:

Current $PM_{2.5}$ monitoring in eastern Canada does not capture some materially deprived communities. KDE can be applied with approaches such as $PM_{2.5}$ standard attainment to improve monitoring coverage.

P49-21 | A study on the risk assessment of Metabolic Dysfunction-Associated Steatotic Liver Disease caused by the interaction between air pollution and genetics in Taiwan

Yu-Yan Fu[1], Chih-Da Wu[4], Chia C. Wang[3,5], Ming-Lung Yu[2,6], Fang-Yu Kuo[1], **Ya-Yun Cheng**[1,2], This work was supported in part by grants from the National Science and Technology Council [NSTC-113-2314-B-110-006-MY3]

[1] National Sun Yat-sen University, Department Of Medicine, College Of Medicine, Kaohsiung, Taiwan; [2] National Sun Yat-sen University, The Center of Excellence for Metabolic Associated Fatty Liver, Kaohsiung, Taiwan; [3] National Sun Yat-sen University, Aerosol Science Research Center (ASRC), Kaohsiung, Taiwan; [4] National Cheng Kung University, Department of Geomatics, Tainan, Taiwan; [5] National Sun Yat-sen University, Department of Chemistry, Kaohsiung, Taiwan; [6] Kaohsiung Medical University Hospital, Hepatobiliary Division, Department of Internal Medicine, Kaohsiung, Taiwan

Objective:

Asian populations tend to develop MASLD at a lower body mass index (BMI<23) compared to Western counterparts, highlighting the necessity for a deeper understanding of the influencing factors of MASLD in the Asian population. Growing evidence suggests that fine particulate matter and genetic factors contribute to a higher risk of MASLD. However, the gene-environmental interaction contributing to the lean MASLD is still unknown. Herein, we aimed to unveil the interaction between various genetic factors and air pollutants and their impact on lean MASLD.

Material and Methods

This retrospective cohort study analyzed 150,709 Taiwanese residents (2009–2016) from Taiwan Biobank, who were diagnosed with fatty liver disease with abdominal ultrasound. The MASLD was classified into obese (BMI ≥23) and lean subgroups (BMI<23). The levels of air pollution, $PM_{2.5}$, PM_{10}, NO_2, CO, SO_2, and O_3, were derived from 73 air pollution monitoring stations in Taiwan, and adjusted with the Land-use regression model. The associations between MASLD subpopulation with air pollutants, genetic factors, and gene-environment interactions were analyzed using logistic regression and chi-square tests.

Results

The GXE interaction between the C_T genotype of *TM6SF2* and exposure to air pollutants was associated with an increased risk of MASLD in the lean population, with 95% CIs for PM_{10} [1.036–2.841], $PM_{2.5}$ [1.230–3.353], and O_3 [1.121–2.928] across multiple models. The interaction between the C_T genotype of *APOE* and air pollutant exposure was associated with a reduced risk of MASLD in the lean population, with 95% CIs of [0.380-0.978] for PM_{10}, [0.362-0.965] for $PM_{2.5}$, [0.300-0.811] for SO_2 and O_3 for [0.371-0.969] across multiple models.

Conclusion

A synergistic effect was observed on the genotypes and air pollutants. Through a retrospective study, we found that genetic factors, air pollutants, and gene-environment interactions had a greater impact on lean MASLD than other subgroups.

P49-22 | Applying Positive Matrix Factorization to investigate source-specific coarse particulate matter in a rural environmentally burdened community

Yoshira O. Van Horne[1], WeiTing Liu[2], Elizabeth Kamai[2,3], Luis Olmedo[4], Esther Bejarano[4], Christian Torres[4], Rima Habre[2], Shohreh F. Farzan[2], Jill E. Johnston[2,3]

[1] University of California Los Angeles, Department of Environmental Health Sciences, Los Angeles, USA; [2] University of Southern California, Division of Environmental Health Sciences, Los Angeles, USA; [3] University of California Irvine, Department of Environmental and Occupational Health, Los Angeles, USA; [4] Comité Civico del Valle, Brawley, USA

Background:
In rural California, climate change and competing water demands are intensifying the desiccation of the Salton Sea, exposing more playa in this large land-locked “sea.” This study aimed to characterize source contributions to PM_{10} at five nearby schools.

Methods:
To characterize source composition, we collected two-week averaged PM_{10} samples from a rural environmentally burdened community in southeastern California near the Salton Sea. A total of 195 filters were collected from 2019-2022. Samples were analyzed for 30 elements using X-ray diffraction. Source factors were resolved via positive matrix factorization.

Results:
We identified five sources of PM_{10} pollution: 1) crustal (Al and Ca rich), 2) industrial (As, Cr, Rb, Se, Zr rich), 3) sea-spray (Cl and Na rich), 4) traffic (Black carbon, Cu, Pb, Zn rich), and 5) desert dust (Al, Ba, Co rich). We found seasonal variation in contributions. PM_{10} traffic contributions were highest in the winter season (31.4% PM_{10} contribution), consistent with when traffic counts are the highest. In comparison, PM_{10} sea-spray contributions were lowest in the winter season (5.8% PM_{10} contribution). During the spring and summer seasons, when arid conditions are prominent, PM_{10} sea-spray contributions were the highest (27.9% and 30.8% PM_{10} contribution, respectively), while industrial contributions were the lowest (13.6% and 7.9% PM_{10} contribution, respectively). In the fall season, PM_{10} industrial and traffic contributions were the highest, (32.6% and 20.1% PM_{10} contribution, respectively). Differences by site were also identified, with PM_{10} sea-spray contributions being the highest at sites closest to the Salton Sea (22.1% and 24.5% PM_{10} contribution), compared to other sites (19.4%, 17.9%, and 17.3% PM_{10} contribution).

Conclusions:
In our study, we identified crustal, industrial, sea-spray, traffic, and desert dust as sources contributing to PM_{10} composition. These findings raise concerns about the airborne exposures from a drying body of water on public health in vulnerable communities.

P49-23 | Household fuel type and urinary polycyclic aromatic hydrocarbon metabolites in a pregnancy cohort in Nairobi, Kenya

Anne M. Riederer[1], Christopher D. Simpson[1], Allison R. Sherris[1], Christopher Zuidema[1], Michael H. Paulsen[1], Abigail J. Gilbert[1], Judith Adhiambo[2], Lewis Olweywe[3], Barbra A. Richardson[4,5], Faridah H. Were[6], Priscilla W. Edemba[3], Elizabeth Maleche-Obimbo[2], Edmund Seto[1], Preston Omondi[3], Michael J. Gatari[7], Catherine J. Karr[1,8], Sarah Benki-Nugent[9]

[1] University of Washington, Department of Environmental & Occupational Health Sciences, Seattle, USA; [2] University of Nairobi, Department of Paediatrics and Child Health, Nairobi, Kenya; [3] Kenyatta National Hospital, Nairobi, Kenya; [4] University of Washington, Department of Global Health, Seattle, USA; [5] University of Washington, Department of Biostatistics, Seattle, USA; [6] University of Nairobi, Department of Chemistry, Faculty of Science and Technology, Nairobi, Kenya; [7] University of Nairobi, Institute of Nuclear Sciences, Nairobi, Kenya; [8] University of Washington, Department of Pediatrics, Seattle, USA; [9] University of Washington, Department of Medicine, Division of Allergy and Infectious Disease, Seattle, USA

Objective: Polycyclic aromatic hydrocarbon (PAH) exposures in pregnancy are a concern given their neurodevelopmental toxicity. Data from urban sub-Saharan African are scarce. We examined household fuel type as a source of PAH exposure in the Air Pollution and Brain Development prospective cohort study in Nairobi, Kenya.

Methods: Third trimester spot urine was collected from 374 participants and analyzed for 7 OH-PAHs using high performance liquid chromatography with fluorescence detection. Detection limits ranged from 0.002 ng/mL (2-hydroxyphenanthrene; 3- hydroxyphenanthrene) to 3.52 ng/mL (1-hydroxynaphthalene). 10 ng/mL and 200 ng/mL urine cotinine test strips were used to indicate secondhand smoke exposure and active smoking, respectively. Household characteristics and potential sources of air pollution exposure were collected via questionnaires at enrollment. Prior 24 h consumption of PAH-containing foods (chargrilled/fried/toasted) was reported at urine collection. We fit generalized linear models with robust standard errors of individual OH-PAHs versus household fuel type, controlling for specific gravity, urinary cotinine, other combustion in the home, prior 24 h PAH foods consumption, and education.

Results: Of 366 valid urine measurements, detection frequencies ranged from 95% (1-hydroxynaphthalene) to 100% (2-hydroxynaphthalene, 3-hydroxyphenanthrene). Median (inter-quartile range) concentrations ranged from 0.1 ng/mL (0.06-0.3 ng/mL) (3-hydroxynaphthalene) to 33 ng/mL (12-85 ng/mL) (1-hydroxynaphthalene). Participants reported daily/most/some days use of liquefied petroleum gas (LPG) only (n=117), mixed fuels (kerosene, LPG, and/or wood/charcoal) (n=176), kerosene only (n=61), and ethanol only (n=12). Some also used electricity (n=17), but none exclusively. 96% had <10 ng/mL cotinine. In adjusted models, urinary OH-PAHs were 81% (95% CI:38%-137%) (2-hydroxyphenanthrene) to 124% (58%-218%) (2/9-hydroxyfluorene) higher among kerosene only versus LPG only users; most (except 1-hydroxypyrene) were higher among mixed fuels vs. LPG only users.

Conclusion: These findings suggest household kerosene use is an important source PAH exposure for pregnant women in this setting, supporting efforts towards cleaner fuel usage. Funding: R01ES032153 (U.S. NIEHS)

P49-24 | Community monitoring of volatile organic compounds (VOCs) near the Bristol VA Landfill

Xiaoying Li[1], Marie J. Andales[1], Joshua Rudd[2], Tim Hilbert[3], Patrick Ryan[4], Joel Kellogg[5], Becky Evenden[5], Candace Brancato[3], Christine Kim[3], Erin Hayes[3], John Volckens[1]

[1] Colorado State University, Department of Mechanical Engineering, Fort Collins, USA; [2] King University, Bristol, USA; [3] University of Kentucky, Lexington, USA; [4] University of Cincinnati, Cincinnati, USA; [5] HOPE Bristol, Bristol, USA

Objective: Elevated temperature landfill events – the result of abnormal chemical reactions within the waste – can produce elevated odors and excess emissions of hazardous air pollutants. Despite the Bristol VA Landfill ceased operations in September 2022 following a series of elevated temperature events, residents continued to express concerns about noxious odors and potential health impacts from landfill emissions. To address these concerns, we co-developed a community-engaged study to monitor volatile organic compounds (VOCs) in ambient air, with a focus on benzene, toluene, ethylbenzene, and xylenes (BTEX). **Methods:** Seventeen residents living within two miles of the landfill were recruited to collect repeated, 8- or 12-hr VOC samples outside their homes from February 2024 through March 2025. Participants were trained to collect air samples onto Carbopack X sorbent tubes (Markes) using Ultrasonic Personal Air Samplers (Access Sensor Technologies). Samplers were pre-programmed for a set run time and actively logged key quality assurance measures, including flow-rate, battery voltage, and GPS location. Collected tubes were sent to Colorado State University for quantification of 39 VOCs using Thermal Desorption-Gas Chromatography-Mass Spectrometry (EPA Method TO-17). **Results:** The concentration ranges of benzene, toluene, ethylbenzene, and xylenes were 0.10-4.56, 0.02-2.72, 0.01-0.50, and 0.02-1.21 ppb, respectively, while they were 48.9%, 75.6%, 90.3%, and 96.0% higher on odorous days than non-odorous days, based on mixed-effects model analysis. We did not observe a clear decreasing trend in VOC levels with increasing distance from the landfill. **Conclusion:** This study successfully piloted a rigorous community-engaged approach to quantifying hazardous air pollution near an elevated-temperature landfill using portable, streamlined sampling technology with appropriate QA/QC controls. Although measured concentrations were below regulatory action levels, residents' perceptions of odor events were positively associated with increased BTEX concentrations, validating their suspicions of landfill emissions.

P49-25 | Unveiling Metabolic Signatures and Pathways Linking Fine Particulate Matter to ADRD: A Brain Metabolome-Wide Association Study

Donghai Liang[1,2], Youran Tan[1], Mengyuan Ren[1], Yasmith Farnan[1], Zhihao Jin[1], Marla Gearing[3,4], Allan I. Levey[4,5], James J. Lah[4,5], Aliza P. Wingo[6], Douglas I. Walker[1], Dean P. Jones[7], Thomas S. Wingo[8,9], Anke Huels[2,10]

[1] Emory University, Gangarosa Department of Environmental Health, Atlanta, USA; [2] Emory University, Department of Epidemiology, Atlanta, USA; [3] Emory University, Department of Pathology and Laboratory Medicine, Atlanta, USA; [4] Emory University, Department of Neurology, Atlanta, USA; [5] Emory University, Goizueta Alzheimer's Disease Research Center, Atlanta, USA; [6] University of California, Department of Psychiatry, Davis, Sacramento, USA; [7] Emory University, Department of Medicine, Atlanta, USA; [8] University of California, Department of Neurology, Davis, Sacramento, USA; [9] University of California, Alzheimer's Disease Research Center, Davis, Sacramento, USA; [10] Emory University, Department of Biostatistics and Bioinformatics, Atlanta, USA

Background: Air pollution, particularly fine particulate matter ($PM_{2.5}$), has emerged as a potential risk factor for Alzheimer's disease and related dementias (ADRD). However, the molecular signatures and pathways mediating this relationship remain poorly understood. Leveraging high-resolution metabolomics (HRM), we conducted a metabolome-wide association study (MWAS) to identify metabolic signatures linking $PM_{2.5}$ exposure to AD neuropathology.
Methods: We conducted untargeted HRM on 162 frontal cortex samples from the Emory Goizueta Alzheimer's Disease Research Center brain bank. $PM_{2.5}$ exposure was estimated using spatiotemporal models based on participants' residential histories. Linear regression models were applied to assess associations between $PM_{2.5}$ exposure, metabolic features, and AD neuropathology markers (Braak stage, CERAD, ABC scores), adjusting for potential confounders and correcting for multiple testing with FDR at 5%. Pathway enrichment analysis was conducted to reveal biological mechanisms, and key metabolites were confirmed with Level-1 annotation evidence.
Results: A total of 35,978 metabolic features were detected in brain tissue. Findings from the MWAS and pathway enrichment analysis inferred metabolic perturbations in 6 major pathways significantly associated with both $PM_{2.5}$ exposure in the year preceding death and AD neuropathology. Among these, disruptions in glutathione metabolism, mitochondrial function, and lipid peroxidation were notable, implicating oxidative stress and energy disruption as major contributors to $PM_{2.5}$-related neurotoxicity. Additionally, perturbations in key neurotransmitters, including dopamine and serotonin, were observed, suggesting impacts on neuronal signaling, independent of AD pathology. Notably, 18 metabolites confirmed with Level-1 evidence were implicated in amino acid, lipid, and nucleotide metabolism linking PM2.5 exposure to AD pathology. These included alterations in glucose, mannose, myo-inositol, and adenosine 5'-diphosphoribose, which have substantial roles in neuroinflammation and synaptic dysfunction.
Conclusion: This study highlights intermediate metabolites and biological pathways linking $PM_{2.5}$ exposure and AD neuropathology. Findings underscore the potential role of oxidative stress, neuroinflammation, and energy dysregulation in $PM_{2.5}$ neurotoxicity.

P49-26 | Association between long-term air pollution-induced hippocampal enlarged perivascular spaces

Hyunji Park[1], Young Noh[2], Joon-Kyung Seong[5], Heeseon Jang[3], Hyunah Son[1], Changsoo Kim[3,4], Jaelim Cho[3,4]

[1] Yonsei University Graduate School, Department of Public Health, Seoul, South Korea; [2] Gachon University, Neuroscience Research Institute, Incheon, South Korea; [3] Yonsei University College of Medicine, Department of Preventive Medicine, Seoul, South Korea; [4] Yonsei University, Institute of Human Complexity and Systems Science, Incheon, South Korea; [5] Korea University, Department of Bio-convergence Engineering, Seoul, South Korea

Objective: Recent evidence suggests that enlarged perivascular spaces in the hippocampus (H-ePVS) may serve as a potential neuroimaging marker for cognitive decline; however, the impact of environmental risk factors, such as air pollution, on H-ePVS remains unexplored. We aimed to investigate the association between long-term air pollution exposure and H-ePVS in cognitively healthy adults.

Material and Methods: This cross-sectional study included 422 adults aged 50 and older from the Environmental-Pollution-Induced Neurological Effects study, who underwent brain MRI scans between 2014 and 2018. The volume of H-ePVS was quantified using automated PVS segmentation methods. The fraction was calculated by dividing the H-ePVS volume by the intracranial volume. Concentrations of particulate matter with diameter≤10㎛ (PM10), ≤2.5㎛ (PM2.5), and manganese in PM10 (Mn10), PM2.5 (Mn2.5) were measured based on each participant's address. Linear regression models and beta regression models were used to evaluate the association between air pollution and H-ePVS volume and fraction. Generalized additive models were used to examine the non-linear relationship.

Results: The mean age was 66.7 (standard deviation 5.6) years. An increase of 10 ng/m³ in Mn10 was associated with an increased H-ePVS volume (β=42.41; standard error [SE] = 19.13; p=0.027) and H-ePVS fraction (β=0.220; SE=0.109; p=0.043), both of which exhibited non-linear associations (p for non-linearity<0.04). PM10 and PM2.5 were associated with increased H-ePVS volume and fraction, but these associations were not statistically significant.

Conclusion: These results suggest that long-term manganese exposure is associated with increased H-ePVS.

Acknowledgements: This work was supported by Korea Environment Industry & Technology Institute(KEITI) through Core Technology Development Project for Environmental Diseases Prevention and Management, funded by Korea Ministry of Environment(MOE)(grant No.2022003310011) and the Technology Innovation Program(or Industrial Strategic Technology Development Program-Program of Development of Biotechnology Industry Technologies) (RS-2024-00432128, (Part2) Development and Demonstration of Personalized Digital Healthcare and Care Services for Individuals with Severe Illness Experiences for Global Market Expansion) funded By the Ministry of Trade Industry & Energy (MOTIE, Korea).

P49-27 | Air Pollution and Survival in Patients with Amyotrophic Lateral Sclerosis in Michigan: A Survival Analysis

Meredith Pedde[1], Sara Adar[1], Dae Gyu Jang[2], Eva Feldman[2], Stephen Goutman[2]

[1] University of Michigan, Department of Epidemiology, Ann Arbor, USA; [2] University of Michigan, Department of Neurology, Ann Arbor, USA

OBJECTIVES: Amyotrophic lateral sclerosis (ALS) is a fatal neurodegenerative disease, and patients typically survive two to four years after diagnosis. While research has linked air pollution to disease incidence and aggravation, the role of air pollution on survival in patients with ALS remains unstudied largely because it is a rare disease, and most analyses have focused on administrative records. We examined the association between air pollution and survival among a prospective cohort of persons with ALS.

MATERIAL AND METHODS: We leveraged complete residential history and clinical data from 334 persons with ALS seen at the University of Michigan between 2009-2016. We estimated time-varying 1-year and 5-year average exposures of fine particulate matter ($PM_{2.5}$), black carbon (BC), nitrate, sulfate, sea salt, nitrogen dioxide (NO_2), and ozone (O_3) at their residential addresses. We used a Cox proportional hazards model to estimate the association between 1-year average pollutant exposures and survival after adjustment for covariates. Five-year average exposure periods were studied in sensitivity analyses.

RESULTS: The 334 study participants, 145 of whom died during the follow-up period, contributed an average of 3.0 ± 2.9 years of follow-up. One interquartile range (IQR) increases in 1-year average $PM_{2.5}$, BC, and O_3 were associated with 65%, 54%, and 18% increases in mortality, respectively ($PM_{2.5}$: HR 1.65, 95% CI: 0.98-2.76; BC: HR 1.54, 95% CI: 0.99-2.39; ozone: HR 1.18, 95% CI: 0.95-1.47), though results could not be distinguished from no association. One IQR increases in 5-year average BC, however, were strongly associated with increased mortality (HR 2.45, 95% CI: 1.36-4.42). One-year average nitrate, sulfate, and sea salt were not associated with mortality, while NO_2 exposure was associated with decreased mortality (HR 0.59, 95% CI: 0.36-0.97).

CONCLUSION: Higher levels of $PM_{2.5}$, BC, and O_3 were suggestively associated with increased mortality rates among persons with ALS.

P49-28 | Black Carbon Emissions in Jordan: Inventory, Projections, Health Implications, and Mitigation Potential (2022–2050)

Alham W. Al-Shurafat[1], Fayez A. Abdullah[2], Anas O. Ghawanmeh[2], Ayman S. Al Sharafat[1]

[1] Climate Action Network Jordan, Climate & Environment Department, Amman, Jordan; [2] Jordan University of Science and Technology, Civil Engineering Department, Irbid, Jordan

Jordan lacks a comprehensive national assessment of black carbon (BC) emissions, and mitigation potential. This gap hinders evidence-based policymaking to address BC's burden on public health and climate. This study aims to develop Jordan's first national BC inventory, project emission to 2050, assess health implications, and evaluate reduction potential.

Using the Low Emissions Analysis Platform–Integrated Benefits Calculator, we quantified 2022 BC emissions across emitting sectors and projected trends under a business-as-usual scenario to 2050 in collaboration with Jordan's Ministry of Environment. Health implications were estimated by linking BC-driven $PM_{2.5}$ exposure to premature deaths. Nationally planned Green House Gases mitigation measures were systematically reviewed in terms of BC reduction potential, prioritized through stakeholder consultations, and modeled across three implementation phases: short-(2020–2029), mid-(2030–2039), and long-term-(2040–2050).

In 2022, BC emissions totaled 2,739.6 $GgCO_2$-equivalent, dominated by transportation (59%) and energy (41%). Without intervention, emissions are projected to rise to 6,750 $GgCO_2$-equivalent by 2050, driven by population growth (2.2%) and GDP expansion (2.4%). BC contributed 1.7% to national $PM_{2.5}$ levels (20.76 µg/m³), linked to 30 annual premature deaths; with, natural sources (e.g., dust) accounted for 75% of $PM_{2.5}$ exposure. By 2050, BC's climate impact across countries could elevate global temperatures by 0.85°C, while BC-attributable premature deaths in Jordan may rise to 340 cases annually. Mitigation measures could reduce emissions by 40% by 2050.

Diesel consumption is a major emissions driver, requiring targeted actions such as stricter emission standards, and further transition to electric-vehicles. While natural resources contribute significantly to $PM_{2.5}$ levels, making desertification control crucial for public health, mitigating BC remains essential for air quality and climate. Prioritizing short-term measures, such as promoting cleaner fuels is key to effective BC reduction. Nationally planned measures can achieve 40% BC mitigation by 2050, underscoring the need for a dedicated BC-focused policy to fully address remaining potential.

P50 | Climate and Health: Health Outcomes Across the Lifespan

P50-02 | Short-term ambient temperature exposure and its influence on glucose metabolism in older women in Germany

Bo Sun[1,2], Nidhi Singh[1], Claudia Wigmann[1], Nitika Singh[3,4], Christian Herder[3,4], Tamara Schikowski[1,5]

[1] IUF-Leibniz Research Institute for Environmental Medicine, Düsseldorf, Germany; [2] Heinrich Heine University Düsseldorf, Medical Research School, Düsseldorf, Germany; [3] Leibniz Center for Diabetes Research at Heinrich Heine University Düsseldorf, Institute for Clinical Diabetology, German Diabetes Center, Düsseldorf, Germany; [4] German Center for Diabetes Research (DZD), Partner Düsseldorf, München-Neuherberg, Germany; [5] School of Public Health, University of Bielefeld, Bielefeld, Germany

Objective: Climatic factors, including temperature changes, affect glucose metabolism. However, the association between short-term temperature exposure and glucose metabolism in older populations remains understudied. This study investigated these associations in a cohort of older women.

Material and Methods: 379 German women aged 69-79 years from urban and rural areas were examined. Serum adiponectin, fasting glucose, insulin and glycated hemoglobin (HbA1c) were measured. Insulin resistance and beta-cell function were estimated using homeostasis model assessment (HOMA2 indices). Environmental exposures were obtained from COSMO-REA6 reanalysis datasets, including mean, minimum, maximum temperatures and diurnal temperature range (DTR). We used linear regression models up to 14 days lag of temperature, adjusted for age, BMI, smoking, socioeconomic status, and alcohol consumption. The best-fitting lags were selected using Akaike information criterion. Sensitivity analyses included additional adjustments and stratifications.

Results: Higher mean (range: 0.3-22.3°C), minimum, and maximum temperatures were associated with higher total adiponectin starting from lag 1, 1, 2, respectively. Increased DTR was associated with higher HbA1c and HOMA2%B from lag 1 and 5, respectively. The best-fitting lag indicated that a 5°C increase in maximum temperature was associated with an increase in total adiponectin (proportional change, PC=1.08, 95%CI: 1.03-1.12, lag 13). A 5°C increase in DTR was associated with higher HbA1c (PC=1.03, 95%CI: 1.02-1.05, lag 14) and HOMA2%B (PC=1.10, 95%CI: 1.04-1.15, lag 6). However, no significant association was found between DTR and HOMA2-IR (PC=0.94, 95%CI: 0.88-1.01). Associations remained robust after adjustment for physical activity, cardiovascular, respiratory disease, and air pollutants. Stronger associations were found in participants with higher BMI, impaired glucose metabolism, cardiovascular disease, and smoking.**Conclusion:** Short-term exposure to higher temperatures may have protective effects by increased circulating adiponectin levels, while DTR may affect beta-cell function and glucose control. The lack of association with insulin resistance underscores the complexity of these metabolic responses, warranting further research.

P50-03 | Changes in lipid mediators in response to short-term exposure to temperature

Daniel Riggs[1], Clara Sears[1], Rachel Keith[1], Amruta Nori-Sarma[2], Jay Turner[3], Aruni Bhatnagar[1], Brian Sansbury[1]

[1] University of Louisville, Christina Lee Brown Envirome Institute, Louisville, USA; [2] Harvard University, T.H. Chan School of Public Health, Boston, USA; [3] Washington University, Department of Chemical Engineering, St. Louis, USA

Objective: Extensive evidence shows that extreme-heat events increase cardiovascular mortality, and inflammation is likely to play a key role. The acute inflammatory response consists of an initiation phase followed by resolution phase, regulated in part by specialized pro-resolving mediators (SPMs). Interruption of SPM production can lead to defective resolution and result in chronic inflammation; however, the effects of ambient temperature exposure on resolution are unknown.

Methods: We used a subset of participants enrolled in the ongoing Longitudinal Evaluation of Environmental Cardiovascular Disease (LUCID) Study. Data were collected for an in-depth health examination via questionnaires, wearables devices, and blood and urine samples. The current analysis involves 17 participants, ages 30-50 years, across 4 visit dates between 2021 and 2022. Using targeted LC-MS/MS, we quantified 55 plasma lipid mediator species, derived from arachidonic, eicosapentaenoic, and docosahexaenoic acids. We calculated 24h temperature means and standard deviations from the visit date using ERA5-Land hourly data. We used adjusted linear mixed effects models to examine the association between daily temperatures and log-transformed lipid mediators.

Results: The mean age of participants was 39.4 years, with a majority being female (53%) and non-Hispanic White (83%). The median 24h temperature for the 4 study visits was 25.0, 8.2, 22.4, and 3.9 degrees Celsius. We found a positive association between mean daily temperature and arachidonic acid of 24.2% per 10 degrees Celsius (95% CI: 10.7, 39.4). We observed negative associations between prostaglandins (PGD2, PGJ2) and mean daily temperature. In analysis of SPM species, we observed positive associations with maresin-2 (4.4%; 95% CI:2.5, 6.3) and 17R-resolvin D3 (63.4%; 95% CI: 55.6, 71.7). Additionally, we found a negative association between daily temperature variability with resolvin D3 (-5.3; 95% CI: -8.6, -1.9)

Conclusion: Ambient temperature may increase inflammation by disrupting resolution. These findings could guide new targets for future prevention strategies.

P50-04 | Perceptions of Medical and Public Health Professionals on Climate Change and Emerging Health Challenges in Pakistan: A Multi-Scale Approach

Hira Tariq[1], Shahmeen Nazar[2], Umm-e-Rabab Rabab[3]

[1] The Aga Khan University Hospital, Community Health Sciences, Karachi, Pakistan; [2] university of Hamburg, Hamburg, Germany; [3] Liaquat National Medical College, Community Medicine, Karachi, Pakistan

Background and Objectives: Climate change is a significant global health threat, disproportionately impacting low- and middle-income countries. Urban centres like Karachi, Pakistan, face rising incidences of vector-borne and waterborne diseases because of changing climate (CC). This study aimed to explore the perceptions of healthcare professionals regarding climate change, its health impacts, and their role in addressing these challenges. Methods: A qualitative study was conducted using nine focus group discussions with 46 healthcare professionals, including doctors, nurses, and public health experts from three major hospitals and two postgraduate institutions in Karachi. Participants were recruited through snowball sampling, and data were collected using semi-structured interviews. Thematic content analysis was performed to identify manifest and latent themes. Results: The analysis revealed seven major themes: awareness of climate change, health impacts, economic and social consequences, environmental and natural disasters, the role of healthcare professionals, mitigation strategies, and policy challenges. Participants demonstrated varied levels of awareness, influenced by their educational background and professional specialization, with public health professionals exhibiting a broader understanding compared to other healthcare workers. Key concerns included the rise of infectious diseases, food insecurity, and urban resource strain. Participants identified barriers such as inadequate training, limited resources, and weak policy enforcement that hinder their ability to address climate impacts effectively. Conclusions: Healthcare professionals in Karachi play a critical role in mitigating climate-related health impacts. Enhanced education, integration of climate change into medical curricula, and interdisciplinary collaboration are essential. Strengthened policies and systemic investments are needed to empower healthcare workers as leaders in climate adaptation and mitigation efforts.

P50-05 | Temperatures extremes and mortality in federal prisons across the United States

Kristen Cowan[1], Jérémie Boudreault[2], Katherine LeMasters[4], Lauren Brinkley-Rubinstein[3]

[1] University of North Carolina at Chapel Hill, Chapel Hill, USA; [2] Institut national de la recherche scientifique, Centre Eau Terre Environnement, Quebec, Canada; [3] Duke University, Population Health Sciences, Durham, USA; [4] Colorado University Anschutz School of Medicine, General Internal Medicine, Aurora, USA

Objective: Temperature extremes in prisons are becoming more common due to climate change. Due to prisons' lack of air conditioning, aging population and high prevalence of chronic diseases, incarcerated people may be at higher risk of poor health outcomes during extreme weather. Our goal was to estimate associations between ambient daily temperature and all-cause mortality in federal prisons.

Material and Methods: Data on mortality in federal prisons including information on age, sex, race and type of death were obtained via Third City Project from 2009-2021. The locations of all prisons were linked with NASA Daymet

ambient daily mean temperature. A case cross-over design using a distributed lag non-linear mode was employed to estimate the temperature-mortality relationship for deaths occurring in summer (May-September) and winter months (November-March). Risk ratios of temperature exposure were compared to the median temperature in each location.
Results: 5,182 deaths were recorded in federal prisons from 2009-2021, 42% during summer months and 41% during winter months. Extreme temperatures (5th and 95th percentiles) were not associated with in an increase in mortality in federal prison when examining the cumulative effect across the US. However, an increase in mortality was noted at lag 2 days for extremely hot temperatures (95th percentile), and similarly for lag day 2 for extremely cold temperature (5th percentile), albeit not precise. Results were consistent across sensitivity and stratified analyses comparing other ways to quantify temperature exposure(such as temperature minimum and maximum), with some notable differences across race (white compared to nonwhite) and regions (south, northeast, west and Midwest).
Conclusions: Future studies are needed to help identify characteristics that may make individuals more or less vulnerable to the effects of extreme temperatures in federal prisons in the US. Furthermore, future work is needed to better document indoor temperatures through the use of indoor temperature sensors.

P50-06 | Comparison of extreme temperatures attributable mortality in two regions of the country and two zones of Santiago, Chile

Luis A. Cifuentes, Pablo L. Cea

Pontificia Universidad Católica de Chile, Centro de Cambio Global UC, santiago, Chile

Objective
To compare the mortality burden of extreme temperatures in two adjacent Chilean regions (Metropolitan Region (MR) and Valparaíso Region (VR)) and two zones of the City of Santiago. Most of the MR population is urban, residing in a valley between two mountain ranges. In the VR, approximately 60% of the population lives within 40 km of the coast with a Mediterranean climate.
Material and Methods
Death certificates were obtained from the Ministry of Health. Daily temperatures were obtained from the Chilean Meteorological Directorate. Population-weighted temperature was used to adjust DLNMs for each region and for each zone of Santiago. Excess cases and Attributable Fraction (AF) for cold days (AFC) and hot days (AFH) were computed based on the RR and the actual death count of each day, below and above the 10th and 90th percentile of temperatures.
Results
Results are reported as the average AF for the whole period. The effects of extreme weather were different between the two regions. In the MR, AFC was bigger than AFH until 2015 (1.7% vs 1.0% average for 1990-2015). In 2019 AFH was 2.6%, and AFC 1.5%. In the VR, AFH was always bigger than AFC (2.1% vs 1.5%) In 2019 AFH was 2.8%, and AFC 1.5%. For the east zone of Santiago, the impact of cold and hot days was the same (AF 1.4%). In the west zone, cold impacts are more than double the hot impacts (1.7% vs 0.7%)
Conclusions
Though exposure-response curves are similar, seasonal distribution of mortality and temperature influences the AF of extreme temperatures. While AFCs are similar across regions and within Santiago’s zones, AFHs vary without a clear pattern. The influence of income and other socioeconomic factors, such as housing quality, needs to be further explored if effective adaptation actions are to be taken.

P50-07 | Association Between Extreme Environmental Events and Hypertension: A Population-Based Study in Coastal Bangladesh

Md Maruf Haque Khan[1], Marium Salwa[1], Anika Tasnim[1], Md Redwanul Islam[1], Khandakar Fatema[1], Rakia Ishra[2], Saidur Rahman Mashreky[3], Md Atikul Islam[4], Jenni Koivisto[5], Syed Moniruzzaman[5], Johanna Gustavsson[5], Lars Nyberg[5], Md. Atiqul Haque[1]

[1] Bangabandhu Sheikh Mujib Medical University, Dhaka, Bangladesh; [2] American International University - Bangladesh, Dhaka, Bangladesh; [3] North South University, Dhaka, Bangladesh; [4] Khulna University, Khulna, Bangladesh; [5] Karlstad University, Karlstad, Sweden

Objective:
This study aimed to evaluate the association between lifetime exposure to extreme environmental events and the prevalence of hypertension in three coastal districts of Bangladesh.

Material and Methods:
In a cross-sectional study, 671 participants were recruited, and blood and urine samples were collected from 572 individuals. Hypertension was diagnosed based on three blood pressure measurements taken 10 minutes apart. Lifetime exposure to extreme environmental events was quantified by summing the number of events experienced. The nine events considered were earthquakes, river erosion, tsunamis/cyclones, tornadoes, drought, sea level rise, floods, extreme temperatures, and water salinity. Other covariates measured included lifestyle factors, body mass index (BMI, kg/m2), diabetes status, daily salt intake, and cholesterol level. Salt intake was measured from spot urine sample using the Kawasaki method.

Results:
The mean age of participants was 43.79 years (SD ±14.75). Nearly all participants reported exposure to approximately nine types of extreme events, with a mean exposure score of 8.85 (SD± 0.70). Men exhibited a higher exposure history than women. Significant gender differences were noted in both systolic ($p < 0.01$) and diastolic blood pressure ($p = 0.04$). Overall, 26% of participants were hypertensive. Women had a higher mean BMI (23.5 kg/m², SD± 4.2) compared to men (22.2 kg/m², SD± 3.4; $p < 0.01$), and the average daily salt intake was 13.3 grams (SD ±4.0). Participants with greater exposure to extreme disaster events had increased odds of hypertension, with an adjusted odds ratio of 5.25 (95% CI: 1.22–22.54). Additionally, age, BMI, and daily salt intake were significantly associated with hypertension.

Conclusion:
Lifetime exposure to extreme disaster events is significantly associated with hypertension. These findings highlight the increased risk among vulnerable populations in coastal Bangladesh in the context of climate change, providing insights for future public health strategies.

P50-08 | Association Between Extreme Temperature Events and Mortality in Older Adults in Costa Rica

Rocío D. Rodríguez-Báez[1,4], Zaray Miranda-Chacón[2], Carolina Santamaría-Ulloa[3]

[1] Universidad de Costa Rica, Programa de Doctorado en Ciencias, San Jose, Costa Rica; [2] Universidad de Costa Rica, Escuela de Medicina, San Jose, Costa Rica; [3] Universidad de Costa Rica, Instituto de Investigaciones en Salud, INISA, San Jose, Costa Rica; [4] Icahn School of Medicine at Mount Sinai, Environmental Medicine, New York, USA

Objectives

Climate change has intensified the frequency and severity of extreme temperature events, posing significant health risks. While extreme heat has been widely associated with increased mortality, recent studies suggest that cold temperatures also contribute to excess deaths, particularly among older adults. In tropical regions like Costa Rica, where temperature variability is relatively low, the health impacts of extreme temperature events remain underexplored. This study aims to assess the association between extreme temperature events (both heat and cold) and cause-specific mortality in the older adult population (≥65 years) in Costa Rica between 2000 and 2024.

Methods

We will conduct a time-series analysis using daily mortality data and meteorological records from 2000 to 2024. Extreme temperature events will be defined using relative thresholds based on temperature metrics, including daily maximum and minimum temperature, wet-bulb temperature, and the Universal Thermal Climate Index. We will employ a Distributed Lag Non-Linear Model within a Poisson regression framework to estimate the relative risk of mortality associated with extreme temperature events, adjusting for seasonality and long-term trends. Analyses will be stratified by cause of death to identify differential risks for cardiovascular and respiratory conditions.

Results

We hypothesize that extreme temperature events will be more frequent in recent years. We expect heat events to be associated with increased mortality, particularly for cardiovascular and respiratory diseases, and that cold events may also contribute to excess deaths in older adults. Additionally, we anticipate regional differences in the impact of extreme temperatures, with urban areas experiencing greater heat-related mortality due to the urban heat island effect, while rural and high-altitude regions may exhibit higher vulnerability to cold-related mortality.

Conclusions

This study will provide evidence on the health risks of temperature extremes in a tropical setting, informing climate adaptation and public health strategies in Costa Rica and similar regions.

P50-09 | The Effect of Drought on Childhood Nutritional Outcomes in Ethiopia: Evidence from the Young Lives Study

Shilpa Rao Skirbekk[2], **Amrutha G.s.**[1], Eleni Papadopoulou[2], Desalew Moges[3]

[1] Indian institute of Population Studies, Mumbai, India; [2] Norwegian Institute of Public Health (NIPH), Oslo, Norway; [3] Ethiopian Public Health Institute, Addis Ababa, Addis Ababa, Ethiopia

Early life exposure to extreme climate events, like drought can affect stunting in children. Using a robust longitudinal dataset on poverty and inequality: the Young Lives study (2002-2016) in Ethiopia, we investigate the association between drought shocks and childhood stunting. Using panel data probability models adjusted for potential confounders, we find that children exposed to drought shocks have an increased risk of being stunted than those not exposed to drought shocks (β=0.192, 95% CI= [0.062, 0.323]). Notably, crop-failure shocks and pests-on-crops shocks due to drought are the main pathways we examine. We highlight the need for targeted interventions in vulnerable settings.

P50-10 | The Compound Influence of Temperature Variability and Influenza on Seasonal Mortality Patterns in Europe

Ekaterina Borisova[1,2], Aleš Urban[1,2], Tomáš Janoš[3,4], Joan Ballester[3]

[1] Czech University of Life Sciences Prague, Faculty of Environmental Sciences, Prague, Czech Republic; [2] Institute of Atmospheric Physics of the Czech Academy of Sciences, Prague, Czech Republic; [3] ISGlobal, Barcelona, Spain; [4] Masaryk University, RECETOX, Faculty of Science, Brno, Czech Republic

Objective
Seasonal mortality patterns are influenced by a complex interaction between climatic factors, such as temperature variability, and epidemiological factors, such as the incidence of influenza-like illnesses. However, the extent to which year-to-year variations in mortality attributable to cold weather and seasonal influenza affect population vulnerability to extreme temperatures in subsequent warm seasons remains poorly understood.

Material and Methods
We analyzed daily temperature and mortality data from the EARLY-ADAPT dataset, integrated with weekly influenza-like illness counts from the ERVISS surveillance system, spanning multiple decades across the European regions. A two-stage mixed-effects meta-regression model was employed to quantify the associations between temperature variability, influenza activity, and mortality during the cold season, and their impact on heat-related mortality in the subsequent summer. Similarly, we examined how mortality in the summer season might influence population vulnerability to cold temperatures in the following winter.

Results
Our findings suggest that the interaction between influenza incidence and low temperatures significantly amplifies seasonal mortality risks. Additionally, high mortality rates in the winter season are associated with reduced heat-related mortality risk in the subsequent summer season. This indicates that high levels of cold-season mortality, driven by the combined effects of cold temperatures and influenza, may reduce population vulnerability to heat-related mortality in the following warm season. Known as the "mortality displacement", this phenomenon suggests

that the most vulnerable individuals are already affected during severe cold seasons, temporarily decreasing the pool of susceptible individuals during subsequent heatwaves.

Conclusion

This research highlights the complex relationship between climatic variability, respiratory infections, and seasonal mortality patterns, offering valuable insights for developing effective public health strategies aimed at mitigating temperature-related mortality risks. These findings underscore the importance of integrating epidemiological and climatic data to enhance public health adaptation strategies in the face of climate change.

P50-12 | Impact of Climate Anomalies and Air Pollutants on Spontaneous Miscarriage: Insights from the 2017 Ghana Maternal Health Survey

Prince M. Amegbor[1], Reginald Quansah[2], Lijuan Gu[3]

[1] New York University, Global and Environmental Health, New York, USA; [2] University of Ghana, School of Public Health, Accra, Ghana; [3] Chinese Academy of Sciences, Institute of Geographic Sciences and Natural Resources Research, Beijing, China

Background: Climate anomalies and ambient air pollutants (AAPs) are linked to adverse pregnancy outcomes, yet their impact on spontaneous miscarriage, particularly in sub-Saharan Africa, is underexplored. This study examines the effects of preconception exposure to climate anomalies, AAPs, and vegetation (measured by the Enhanced Vegetation Index, EVI) on spontaneous miscarriage risk among women in Ghana, with a focus on EVI's role as an effect modifier.

Methods: Data from the 2017 Ghana Maternal Health Survey were analyzed using Bayesian hierarchical linear and non-linear cloglog models to assess the relationship between environmental exposures and spontaneous miscarriage. Meteorological data from the Climatic Research Unit's gridded Time Series (1960–2017) and AAP and vegetation data from NASA's Goddard Earth Sciences Data were used. The analysis included interaction terms between EVI and environmental exposures.

Results: Preconception exposure to mean temperature anomalies and carbon monoxide (CO) was strongly associated with increased miscarriage risk (HR = 4.286 for temperature anomalies and HR = 1.013 for CO). Other AAPs, such as PM2.5 and ozone, were confounded by climate anomalies and maternal factors. EVI moderated the impact of environmental exposures, particularly reducing the effects of temperature anomalies and PM2.5 on miscarriage risk at higher EVI levels.

Conclusion: This study highlights the significant role of climate anomalies and AAPs in increasing spontaneous miscarriage risk in Ghana. Vegetation cover, as indicated by EVI, has the potential to mitigate the effects of environmental stressors. These findings emphasize the importance of policies addressing climate change and air pollution to protect maternal health, particularly in vulnerable regions.

P50-13 | Climate Change Anxiety in an Urban Context: Links to Mental Health and Environmental Stressors

Clara Zundel, Christine Lewis, Aidan Park, Gehad Badawy, Hilary Marusak

Wayne State University School of Medicine, Psychiatry and Behavioral Neurosciences, Detroit, USA

Objective: The indirect psychological impacts of climate change, such as existential threat and worry—known as climate change anxiety (CCA)—are understudied, particularly in U.S. urban settings. This study examines CCA in emerging adults in Detroit, MI, where socioenvironmental stressors may amplify its effects.
Material and Methods: This preliminary analysis includes 51 emerging adults (mean age 21.1 + 2.7 years; 37.3% female, 66.6% Black or African American, 15.7% Hispanic, 11.8% White, 7.8% Multiracial, 2.0% Middle Eastern or North African). CCA was measured using the Climate Change Anxiety Scale. Anxiety symptoms were assessed using the Generalized Anxiety Disorder-7 (GAD-7) and depression symptoms were measured using the 8-item Patient Health Questionnaire (PHQ-8). Two-sample t-tests were used to compare CCA, anxiety, and depression symptoms between the Detroit cohort and a national sample (N=284, mean age 23.2 + 3.9 years). Linear regression models were used to examine associations between CCA symptoms and anxiety and depression symptoms, controlling for age, sex, household income, and area deprivation index.
Results: Detroit emerging adults had significantly higher CCA scores (mean = 1.66 + 0.74) compared to the national sample (mean = 1.41 + 0.54; t =2.86, p =0.004). No differences were found in clinical anxiety or depression rates between cohorts. Greater CCA symptoms were associated with higher anxiety (β=2.88, p=0.021), even after adjusting for demographic and socioeconomic factors. No significant associations were observed for depressive symptoms. The number of climate-related events experienced in the past year—including severe storms, high-heat days, and high air pollution days—was positively correlated with CCA symptoms (r=0.30, p=0.035).
Conclusion: Detroit emerging adults experience heightened CCA, which is associated with anxiety symptoms. These results highlight CCA as an understudied contributor to anxiety in urban populations facing environmental stressors. As extreme weather events increase, addressing CCA may be important for mental health interventions in vulnerable communities.

P50-14 | Spatial Modelling Of Climate And Associated Risk Factors On Under-Five Mortality In Sub-Saharan Africa Using DHS Data

Aweke A. Mitku[1,2], Endeshaw Amuka[2], Yolandiu Ernst[1], Ngianga-Bakwin Kandala[4,3], Tobias Chirwa[4], Aweke Mitku is postdoc funded by SSCAB

[1] University of the Witwatersrand, Global Change Institute, johannesburg, South Africa; [2] Bahir Dar University, College of Science and Maritime, Bahir Dar, Ethiopia; [3] The University of Western Ontario, London, Canada; [4] University of the Witwatersrand, School of Public Health, johannesburg, South Africa

Abstract
Background: Sub-Saharan African countries(SSAs) exhibit significant spatial variation in under-five mortality rates, influenced by a range of geographic, socioeconomic, and environmental factors, often exacerbated by climate change. To lower the worldwide under-five mortality, it is crucial to investigate geographical distributions to pinpoint any climatic patterns and associated risk factors amongst children within and across SSAs countries.

Method: This study used the Demographic and Health Survey(DHS) data from SSAs collected using a two-stage sampling method. The spatial regression models were compared and employed to account for the spatial effect and associated risk factors on 34 Sub-Saharan African countries.
Results: The results showed that spatial dependence and Kenya, Burundi, Rwanda, and the Democratic Republic of the Congo were identified as high-risk countries of under-five child mortality. The general nesting spatial model with spatial lags component was found to be the most accurate model. According to the model results, higher temperature and travel time to the nearest city have been associated with increased under-five mortality rates. Similarly, the increase in the percentage of female children in the family and birth-order of child are associated with an increase in the under-five mortality rate in the countries studied. However, an increased percentage of rainfall has a protective effect on mortality among children under five in Sub-Saharan countries.
Conclusions: *In the 34 Sub-Saharan African countries, spatial disparities exist in under-five mortality. The study concluded that Kenya, Burundi, Rwanda, and the Democratic Republic of the Congo are hot-spot countries for under-five child mortality in the region.* There is regional variance in the death rate for children under five years of age, which is influenced by the neighborhoods. *Therefore, our research emphasizes how crucial it is to consider the geographical context of neighborhoods when developing strategies to minimize mortality in children under five.*

P50-15 | Mental Health Impacts of Climate Change in Pacific Island Countries: A Scoping Review

Haemin Park[1], Kristie L. Ebi[2], Jeremy J. Hess[2]

[1] University of Washington, Department of Environmental and Occupational Health Sciences, Seattle, USA; [2] University of Washington, Center for Health and the Global Environment, Seattle, USA

BACKGROUND AND AIM Pacific Island Countries (PICs) are among the most at-risk regions for climate change-related health risks, with mental health impacts, in particular, being significantly underreported. METHODS This scoping review synthesizes peer-reviewed literature and relevant international government reports to evaluate the mental health consequences of climate change across 22 PICs. Data were extracted from PubMed and Web of Science databases, using an artificial intelligence literature review tool, Elicit. The study investigates exposure to climate-related hazards, mental health outcomes, other consequences, cultural factors, coping strategies, and barriers and facilitators of mental health care. RESULTS Mental health in PICs is affected by direct and indirect impacts of climate change through exposure to multiple climate-sensitive hazards, sequelae of these exposures, such as loss of property, income, and social supports, and concomitant risks like interpersonal violence. Mental health impacts associated with climate change-related disasters and gradual climate changes include increases in depression, anxiety, and grief. Other indirect consequences, including food and water insecurity, socioeconomic disruption, migration, and violence further threaten mental health in the region. Cultural factors, including traditional knowledge, land connection, spiritual beliefs, and community resilience, are important mediators of mental health impacts. Coping strategies such as relocation, leadership-driven action, and community networks were identified. A key strategy, access to mental health services, remains challenging with limited numbers of mental health professionals. Barriers such as stigma, social customs, and preference for traditional healing are complicating factors in accessing mental health services. CONCLUSIONS A range of adverse mental health impacts are associated with exposure to climate-sensitive hazards in PICs, and mental health services are inadequate in the region. To address these challenges, it is essential to integrate mental health into national public health frameworks, enhance disaster

preparedness, increase access to mental health services, and conduct context-specific research to strengthen the region's adaptive capacity.

P50-16 | A Multi-Country Analysis of Vulnerability to Climate-Driven Pathogen Specific Moderate to Severe Diarrheal Disease

Megan Kowalcyk[1], Indira Bose[2], Kristopher Karnauskas[3], Elizabeth Carlton[4], Karen Kotloff[1], Andrea Buchwald[1]

[1] University of Maryland School of Medicine, Center for Vaccine Development and Global Health, Baltimore, USA; [2] London School of Hygiene & Tropical Medicine, Department of Population Health, London, UK; [3] University of Colorado Boulder, College of Arts and Sciences, Boulder, USA; [4] Colorado School of Public Health, Department of Environmental & Occupational Health, Aurora, USA

Objective

Vaccines are cost-effective public health interventions and should be considered as potential climate interventions. Using ten years of data (2008-2018) from two case-control studies pre-and-post rotavirus vaccine introduction (GEMS and VIDA, respectively) we examined the association between weather and pathogen-specific moderate-to-severe diarrhea (MSD).

Methods

We analyzed daily counts of rotavirus, *Shigella*, *Cryptosporidium*, and enterotoxigenic *E. coli* (ETEC) from MSD cases. Daily weather data from ERA5 and CHIRPS were used to measure days since the last rainfall >1 mm, cumulative precipitation in the one and two weeks prior, and lagged maximum temperature and total precipitation up to three weeks. Random forests identified influential weather variables for predicting the presence of each pathogen by site. We estimated individual vulnerability using factors that impact sensitivity and adaptive capacity to diarrheal disease. We used zero-inflated Poisson models, accounting for long term and seasonal trends, to model the association between weather variables and daily pathogen case counts stratified by rotavirus vaccine status and vulnerability.

Results

A total of 9378 MSD cases were recruited in GEMS and VIDA sites, 2707 in The Gambia, 3030 in Kenya, and 3641 in Mali. *Shigella* was the most prominent pathogen detected with increased incidence over time, while rotavirus incidence decreased after vaccine introduction. The associations between weather and pathogen-specific MSD are site and pathogen specific, with drought driving incidence in The Gambia, temperature in Kenya, and extreme precipitation in Mali. Few pathogen specific trends were seen apart from *Shigella,* which was driven by temperature across all sites. Additionally, these associations were modified by the introduction of rotavirus vaccination and vulnerability, with a decrease in risk of pathogen-specific diarrhea due to climate drivers after vaccine introduction.

Conclusion

Rotavirus vaccination introduction led to a decrease in the effect of weather on pathogen-specific MSD incidence, highlighting vaccination as a potential climate intervention.

P50-18 | Examining the Impact of Social and Environmental Conditions on COPD Mortality in the United States: A Systematic Review

C. Vo[1], D. T. Smith-Ireland[1], T. A. Wilson[1], I. K. Boyd[1], H. Gebrekristos[1]

[1] *Mercer University, Department of Public Health, Atlanta, Georgia, United States of America*

Objective: The objective of this systematic review is to determine the impact of structural, social, and environmental conditions on Chronic Obstructive Pulmonary Disease (COPD) mortality in the United States. By synthesizing existing studies, the review aims to identify key factors influencing COPD mortality and summarize evidence to reduce mortality risk among COPD patients.
Material and Methods: A systematic search was conducted in PubMed to identify relevant studies that examined structural, social, and environmental exposures and COPD mortality. We applied the WHO structural determinants of health framework to develop a comprehensive set of domains for the search strategy. All observational studies conducted in the US with participants older than 45 years old were included. All systematic review protocol management procedures were conducted in COVIDence.
Results: Of the 69 identified studies, ten studies fulfilled the eligibility criteria. One study found a significant association between the history of secondhand smoke exposure and COPD mortality among non-smokers. Five studies found significant associations between occupational exposures to dust, diesel exhaust, and vapors with COPD mortality. Mining and construction workers experienced significantly higher mortality risks than expected, as did those working in transportation particularly related to PM2.5 exposure, with a ≥1.46 per 10 μg/m³ increase. Limited green space and high social vulnerability index scores contribute to COPD mortality. Four studies found that exposure to extreme heat waves, cold waves, and aridity increases the risk of COPD mortality. These findings highlight the associations between social inequity, occupational exposure, extreme weather, air pollution, and COPD mortality.
Conclusion: This comprehensive systematic review underlines structural, social, and environmental factors in driving COPD mortality. The results call for additional research on the impact of structural policies and effects of climate conditions that may influence COPD mortality in the US to develop evidence-based strategies that protect populations at risk of COPD.

P51 | Environmental Exposures and Birth Outcomes - PART II

P51-01 | The Accra Birth Cohort: Investigating prenatal air pollution exposure, adverse birth outcomes, and infant respiratory infections in Accra, Ghana

Barbara E. Mottey[1], Carissa L. Lange[1], Abosede S. Alli[1], Kate Kyeremateng[2], Lilly Y. Adzrakor[3], Prince K. Anku[3], Selasie N. Clottey[3], Nancy Dery[3], Princess Fordjour[3], Geofrey K. Kotoka[3], Rhoda D. Kpabitey[3], Obed Kwao[3], Vivian Mensah[3], Philomina Oppong[3], John Quansah[3], Nana Ama Wilson[1], Zaida Yahuza[3], Maud Muosieyiri[2], Allison F. Hughes[3], Raphael E. Arku[1]

[1] University of Massachusetts, Amherst, Department of Environmental Health Sciences, Amherst, USA; [2] University of Massachusetts, Amherst, Department of Biostatistics and Epidemiology, Amherst, USA; [3] University of Ghana, Department of Physics, Accra, Ghana

Objective: Existing research demonstrates that children born to mothers who are chronically exposed to air pollution are at higher risk of being born too small or too early; decreasing their chance of survival and threatening future health of survivors. Yet, in sub-Saharan Africa (SSA), where urbanization is raising air pollution levels, prenatal and early-life exposures and their impacts on maternal and child health are less well characterized. Therefore, we established the Accra Birth Cohort (ABC) across 30 health facilities to quantify the effects of chronic maternal and early prenatal exposures to $PM_{2.5}$, black carbon (BC), and NO_2 on birth outcomes as well as lower respiratory infections in infants.

Materials and Methods: Between June 2023 and May 2024, ~3200 women (18-45years) were recruited. Maternal residential addresses were geocoded, and individual, household, and clinical data were collected at baseline and during follow-up in the 3rd trimester. Birth outcomes, including preterm birth, term birthweight and term low birthweight (LBW) were documented. The infants are being followed bi-weekly/monthly for up to 18 months to document episodes of respiratory tract infections. Exposures to $PM_{2.5}$, BC, and NO_2 are estimated using existing spatiotemporal land use regression models.

Results: The average maternal age was 28.8 years with 87% below age 35. About 16% primarily cooked with biomass and 43% were anemic at baseline. Three percent had miscarriage/still birth and 6% delivered preterm. Of 2,430 births, 10% were LBW and the mean birthweight was 3,058g (IQR: 2735-3400).

Conclusion: As SSA faces twin challenges of urbanization and environmental degradation, the ABC study aims to inform effective public health strategies and policy frameworks to enhance child health and survival in resource-limited settings. We anticipate the findings will offer vital contributions to the discourse on environmental health and child welfare, thereby guiding future research and interventions in similar urban settings.

P51-02 | Joint Effect of Air Pollution and Polyunsatruated Fatty Acid Intake in Early Pregnancy on Birth Weight in an African American Cohort

Benjamin R. Kessler[1], Anne L. Dunlop[1,2], Robert B. Hood[3], Stephanie M. Eick[1], Jiada J. Zhan[4], Susan S. Hoffman[5], Zhihao Jin[1], Youran Tan[1], Erin Ferranti[6], Jeremy Sarnat[1], Audrey J. Gaskins[5], Howard H. Chang[7], Christine Ekenga[1], Donghai Liang[1]

[1] Emory University, Gangarosa Department of Environmental Health, Atlanta, USA; [2] Emory University, Department of Gynecology & Obstetrics, Atlanta, USA; [3] Ohio State University, College of Public Health, Columbus, USA; [4] Emory University, Nutrition & Health Sciences Doctoral Program, Atlanta, USA; [5] Emory University, Department of Epidemiology, Atlanta, USA; [6] Emory University, Nell Hodgson Woodruff School of Nursing, Atlanta, USA; [7] Emory University, Department of Biostatistics & Bioinformatics, Atlanta, USA

Objective: Dietary polyunsaturated fatty acids (PUFAs) are crucial to healthy pregnancy outcomes because of their roles in fetal development and regulating inflammation. However, few studies have examined how dietary PUFAs and environmental exposures, both independently and jointly, influence birth outcomes. We therefore investigated the joint effect of prenatal exposure to traffic-related air pollutants (TRAPs) and dietary PUFAs on birth weight for gestational age z-scores (BWZ).

Materials and Methods: This study included participants from the Atlanta African American Maternal-Child Cohort recruited at prenatal clinic visits between 2014-2018 (n=297). Daily ambient carbon monoxide (CO), fine particulate matter ($PM_{2.5}$), and nitrogen oxide (NO_x) concentrations were estimated at participants' prenatal residential addresses at 250x250 m^2 spatial resolution from observations and numerical model outputs. Concentrations were averaged across the first trimester and full gestational periods. A food frequency questionnaire completed between 24-30 weeks gestation estimated dietary intake of PUFAs in early pregnancy. A sex-specific BWZ was determined using a US population-based reference for singleton births. Linear regression with multiplicative interactions terms was used to estimate the joint exposure effect on BWZ.

Results: When total omega-3 PUFA was held constant at its mean value, an IQR increase in $PM_{2.5}$ during the first trimester was associated with a 0.08 (0.02-0.18) unit decrease in BWZ. When the amount of PUFA was doubled, increased $PM_{2.5}$ was associated with a 1.12 (0.19-2.05) unit increase in BWZ (*p*-int=0.04). When total omega-6 PUFA was similarly held constant, increased $PM_{2.5}$ was associated with a 0.08 (0.02-0.17) unit decrease in BWZ. When omega-6 PUFA was doubled, increased $PM_{2.5}$ was associated with a 1.09 (0.16-2.02) unit increase in BWZ (*p*-int=0.09). These effects appear to be driven by the omega-3 PUFA linoleic acid (*p*-int=0.09) and the omega-6 PUFA a-linolenic (*p*-int=0.02).

Conclusion: Consumption of specific PUFAs may attenuate the negative effect of prenatal TRAP exposures on BWZ.

P51-03 | Associations between residential proximity to oil and gas development and adverse birth outcomes in a North American preconception cohort

Erin J. Campbell[1,2], Kaylin Vrkljan[1], Martha Koenig[1], Dmitrii Krivorotko[1], Jordan Kuiper[2], Nicole Deziel[4], Jonathan Buonocore[3], Amelia Wesselink[1], Lauren Wise[1], Mary Willis[1]

[1] Boston University School of Public Health, Department of Epidemiology, Boston, USA; [2] George Washington University Milken Institute School of Public Health, Department of Environmental and Occupational Health, Washington, USA; [3] Boston University School of Public Health, Department of Environmental Health, Boston, USA; [4] Yale School of Public Health, Department of Environmental Health Sciences, New Haven, USA

Objective: A growing body of literature has documented associations between living near oil and gas development and higher risk of adverse birth outcomes. However, no study has explored these associations using a prospective cohort study with primary data collection. Our objective is to estimate associations between living near oil and gas development and incidence of adverse birth outcomes.

Material and Methods: We used data from U.S. participants enrolled in Pregnancy Study Online (PRESTO), a prospective North American preconception cohort study of couples attempting conception without fertility treatment. Among participants whose pregnancy progressed beyond 20 weeks, we obtained birth outcome data from self-administered questionnaires. We measured small vulnerable newborn (SVN) status, a validated clinically relevant composite of low birthweight, preterm, and small-for gestational-age. Using participants' geocoded addresses, we calculated residential proximity measures to the nearest active oil and gas development site at conception. To evaluate the association between residential proximity to oil and gas development and risk of SVN, we used Cox proportional hazard models to estimate age-adjusted hazard ratios (HRs) and 95% CIs, with gestational weeks as the timescale.

Results: Among 5,373 participants in the analytic sample, 17.4% (n=932) gave birth to a newborn categorized as SVN, and 10.5% (n=562) resided within 5km of active oil and gas development. We found that residence within 5km of active oil and gas development was associated with an age-adjusted HR of 1.16 (95% CI: 0.94, 1.43), compared to residence greater than 10km from active oil and gas development. Associations were similar magnitude among components of SVN (e.g., low birthweight, preterm, and small-for gestational-age). All associations for >5km to 10km were null.

Conclusion: Although results were imprecise, our preliminary models indicate that closer residential proximity to oil and gas development is associated with slightly higher risk of SVN.

P51-04 | A Systematic Review and Meta-Analysis of Maternal Exposure to Unconventional Oil and Natural Gas Development Sites and Congenital Heart Defects in Newborns in the US

Sasan Faridi[1], Ata Rafiee[2], Fatemeh Yousefian[3], Omar Hahad[4,5], Hao Yin[6], Nasenien Nourkami-Tutdibi[7], Sanjay Rajagopalan[9,8], Sadeer Al-Kindi[10,11]

[1] Center for Air Pollution Research (CAPR), Institute for Environmental Research (IER), Tehran University of Medical Sciences, Tehran, Iran; [2] University of Alberta, Department of Medicine, Edmonton, Canada; [3] Emory University, Gangarosa Department of Environmental Health, Rollins School of Public Health, ATLANTA, USA; [4] Department of Cardiology, Cardiology I, University Medical Center of the Johannes Gutenberg-University Mainz, Mainz, Germany; [5] German Centre for Cardiovascular Research (DZHK), Partner Site Rhine-Main, Mainz, Germany; [6] Department of Economics, University of Southern California, Los Angeles, USA; [7] Hospital for General Pediatrics and Neonatology, Saarland University Medical Center, Homburg/Saa, Germany; [8] School of Medicine, Case Western Reserve University, Cleveland, USA; [9] Harrington Heart and Vascular Institute, University Hospitals, Cleveland, USA; [10] Division of Cardiovascular Prevention and Wellness, Houston Methodist, Houston, USA; [11] Center for Health and Nature, Houston, USA

An emerging environmental and public health concern in the US has been the rapid increase in fracking due to the Shale Oil boom and associated health issues as highlighted by numerous reviews and papers. Various studies indicated that pregnant women living near Unconventional Oil and Natural Gas (UONG) development sites may be more likely to give birth to infants with CHDs due to exposure to air, water, and soil pollution generated by these sites. We systematically searched three English-language databases—PubMed, Scopus, and the Web of Science Core Collection—from inception until September 21, 2024 to include all types of epidemiological studies that explored the associations between maternal residential proximity UONG sites and CHDs in the US.We screened a total of 282 unique studies. Six cohort and case-control studies, encompassing 4,499,494 births (including 88,638 cases of CHDs) conducted in Texas (two studies), Colorado (two), Ohio (one), and Oklahoma (one) between 1996 and 2017, were included. Four of these studies were used in the meta-analysis. All risk estimates were adjusted for socioeconomic status and behavioral factors (maternal alcohol use and maternal smoking). Living within 16 km of UONG sites during pregnancy was significantly associated with an increased risk of CHDs in newborns, with ORs of 1.09 (95% CI: 1.00–1.18), 1.15 (95% CI: 1.05–1.26), and 1.18 (95% CI: 1.06–1.31) for low, medium, and high exposure levels, respectively, compared to pregnant women with no recorded exposure within the same buffer. Our findings indicate that fetuses of mothers living in proximity to UONG sites are at an increased risk of CHDs. This highlights the urgent need for simple and practical interventions, such as enhancing access to greenspaces, to reduce associated exposures in these communities and the risk of total and certain types of CHDs in newborns.

P51-05 | Presence of gas stove, mold, water damage and birth outcomes in the Environmental influences on Child Health Outcomes (ECHO) Cohort

Jiwon Oh[1], Lesliam Quirós-Alcalá[2], Mohamad Burjak[3], Lisa A. Croen[4], Aisha S. Dickerson[3], Assiamira Ferrara[4], Rima Habre[5], Irva Hertz-Picciotto[1,9], Kundun Kamalakar[6], Margaret R. Karagas[7], Christine Loftus[8], Rebecca J. Schmidt[1,9], Julie B. Schweitzer[10,9], Annemarie Stroustrup[6], Yeyi Zhu[4], Deborah H. Bennett[1]

[1] University of California Davis, Department of Public Health Sciences, Davis, USA; [2] Johns Hopkins Bloomberg School of Public Health, Department of Environmental Health and Engineering, Baltimore, USA; [3] Johns Hopkins Bloomberg School of Public Health, Department of Epidemiology, Baltimore, USA; [4] Kaiser Permanente Northern California, Division of Research, Oakland, USA; [5] University of Southern California, Department of Population and Public Health Sciences, Los Angeles, USA; [6] Northwell Health Cohen Children's Medical Center, Division of Neonatology, New Hyde Park, USA; [7] Dartmouth Geisel School of Medicine, Department of Epidemiology, Lebanon, USA; [8] University of Washington, Department of Occupational and Environmental Health, Seattle, USA; [9] University of California Davis, MIND (Medical Investigations of Neurodevelopmental Disorders) Institute, Sacramento, USA; [10] University of California Davis, Department of Psychiatry and Behavioral Sciences, Sacramento, USA

Objective: Prenatal exposure to household environmental factors, including gas stoves, mold, and dampness, has been associated with adverse birth outcomes, though the evidence remains limited. This study examined associations between prenatal exposure to these household environmental factors with gestational duration and fetal growth.

Materials and Methods: This study included 9,648 mother-child dyads from 30 sites participating in the Environmental influences on Child Health Outcomes (ECHO) Cohort between 2005-2023. Participants retrospectively reported the presence of a gas stove, mold/mildew, and water leaks/damage in the home where they spent the majority of their pregnancy. Gestational age at birth and birth weight-for-gestational-age (BW-GA) z-scores were primarily obtained from maternal or child medical records and analyzed as continuous and categorical variables. Covariate-adjusted linear regression or multinomial logistic regression models with cohort as a random effect were used to examine associations between individual exposure variables and birth outcomes.

Results: Among participants, 53% reported having a gas stove, 14% reported mold/mildew, and 16% reported water damage in their home during pregnancy. Prenatal exposure to mold/mildew was associated with a reduction of 0.11 weeks in gestational duration (β = -0.11, 95% confidence interval [CI]: -0.21, 0.004) and a 26% higher odds of preterm birth (OR = 1.26, 95% CI: 1.00, 1.57) compared to those without exposure. Having a gas stove and water damage was not associated with gestational age or BW-GA z-scores.

Conclusions: In this geographically and sociodemographically diverse sample, prenatal exposure to mold/mildew was associated with a higher risk of preterm birth. Future analyses will explore potential effect modification by race/ethnicity, socioeconomic status, and exposure-related factors such as ventilation (hood and window use) and housing type, given that these exposures are often more prevalent in poorly maintained, low-income housing.

This research was supported by the ECHO Program, Office of the Director, National Institutes of Health.

P51-06 | Prenatal exposure to plastic-related chemicals is associated with increases in preterm birth

Junjie Yang[1], Dimitri Abrahamsson[1], Tracey Woodruff[1], June-Soo Park[3], Julian Edwards[3], Miaomiao Wang[3], Erin Demico[1], Rachel Morello-Frosch[2]

[1] University of California San Francisco, Department of Obstetrics, Gynecology & Reproductive Sciences, San Francisco, USA; [2] University of California Berkeley, Department of Environmental Science, Policy and Management, Berkeley, USA; [3] California Environmental Protection Agency, Department of Toxic Substances Control, Berkeley, USA

Plastic consumption has reached staggering volumes in recent years and is projected to reach 1,321 million tonnes (Mt) by 2060. This increase in production parallels a rise in human exposure to plastic-related chemicals. There is an urgent need to develop innovative analytical approaches that can address the wide range of chemical exposure to plastics, particularly during critical developmental periods. In our study, we performed suspect screening analysis for plastic exposures in 200 pregnant participants during second trimester, using liquid chromatography-quadrupole time-of-flight mass spectrometry (LC-QTOF-MS). We curated the in-house database of 5,278 plastic-related chemicals for suspect screening, based on the EPA PLASTICMAP chemical database of 10,547 chemicals associated with plastic production globally. We matched 2159 positive-mode, and 3609 negative-mode suspect chemical features against our database, of which 1127/1530 (positive/negative) features were prioritized for targeted fragmentation analysis based on detection frequency (DF >= 70%), peak area (>= 5000 counts) accurate mass matching (ppm <=5 ppm). We used an in-house developed algorithm to annotated suspect features by MS/MS spectrum and retention time matching. Among the 65 tentatively identified chemical features, 62 were not previously reported in existing exposome databases. We confirmed three candidates using reference standards: perfluorooctanesulfonic acid (PFAS compound), 8-hydroxyquinoline (antioxidant), and 1,3-diphenylguanidine (with multiple functions in rubber products). We tentatively identified eight plastic-related chemicals that are associated with increases in preterm birth, such as 12-Hydroxyoctadec-9-enoic acid and bis(2-ethylbutyl) phthalate as plasticizer in plastic products. Our findings indicated that exposures varied by educational level and nativity. Specifically, individuals with higher educational attainment had greater exposure to dimethyl terephthalate, while Native Americans experienced higher exposure to PFOS. In summary, our study highlights the potential of this suspect screening approach to identify and prioritize plastic chemical exposures during fetal development. This study was funded by NIH/NIEHS (grant numbers P30ES030284, R00ES032892, R01ES027051) and by UCSF.

P51-07 | Personal neonatal exposure to air toxics and low birthweight risk in an Indian megacity

Naveen Puttaswamy[1], Sudhakar R. Saidam[1], Divya Jayakumar[1], Sneha S. Patil[1], Sreekanth Vackacherla[1], Palaniyappan Narayanan[1], Krystal J. Godri Pollitt[2], Howard H. Chang[3], Dana B. Barr[3], Kalpana Balakrishnan[1]

[1] Sri Ramachandra Institute of Higher Education and Research, Faculty of Public Health, Chennai, India; [2] Yale University, School of Public Health, New Haven, USA; [3] Emory University, Rollins School of Public Health, Atlanta, USA

Objective: To assess personal neonatal exposure to air toxics and the association with birthweight in an urban mother–child cohort.

Materials and Methods: In the ongoing REACH (Reproductive effect from Exposures to Airborne Chemicals in urban Homes) cohort (n=300) study in Chennai, India, 24-hour personal exposures to fine particulate matter ($PM_{2.5}$), black carbon (BC) and polycyclic aromatic hydrocarbons (PAHs) were monitored each trimester (n=102) using a wearable PM sampler (UPAS v2.1). Monohydroxy metabolites of PAHs were determined in urine (n=102), and parent PAH compounds were measured in paired maternal and cord blood samples (n=30). Birth anthropometry (n=34) was measured within 24 hours of delivery.

Results: To date, 34 deliveries have occurred, with an average birthweight (±standard deviation) of 2837±329 grams. Personal exposures to $PM_{2.5}$ (44.2±19.1 μg/m^3), BC (8.3±3.8 μg/m^3), and total PAHs (570.4±309.6 ng/m^3) were 1.2 to 2 times higher in winter than summer. Correlations were observed between personal $PM_{2.5}$ mass concentration and BC (r=0.62, p=0.01) and personal PAHs and $PM_{2.5}$ mass concentration (r=0.72, p<0.001). Total PAHs in maternal and cord blood were highly correlated (r=0.75, p=0.001, n=30). Among the six metrics measured in the air and three exposure metrics evaluated in blood, negative relationships were found between average birthweight and maternal blood PAH levels (β=-0.0307, SE=0.017, p=0.042), personal total PAHs (β=-0.000462, SE=0.0002, p=0.055) and black carbon (β=-0.0311, SE=0.02, p=0.07),

Conclusion: Exposure to air toxics during pregnancy could decrease birthweight. For exposure variables examined, most showed negative associations with birthweight. With 100 more births in the REACH cohort by July 2025, we expect improved statistical power and precision.

P51-08 | Newborn metabolic perturbations associated with prenatal tobacco smoke exposure and early birth

Paula-Dene C. Nesbeth[1], Xiajie Lyu[1], Anne L. Dunlop[2], Youran Tan[1], Dana B. Barr[1], Volha Yakimavets[1], Stephanie Eick[1], Blake Rushing[3,4], Susan L. McRitchie[4], Susan Sumner[3,4], P. Barry Ryan[1], Elizabeth Corwin[5], Dean P. Jones[6], Donghai Liang[1]

[1] Emory University, Gangarosa Department of Environmental Health, Rollins School of Public Health, Atlanta, USA; [2] Emory University, Department of Gynecology and Obstetrics, School of Medicine, Atlanta, USA; [3] University of North Carolina at Chapel Hill, Department of Nutrition, Chapel Hill, USA; [4] University of North Carolina at Chapel Hill, Nutrition Research Institute, Kannapolis, USA; [5] Columbia University, School of Nursing, New York, USA; [6] Emory University, School of Medicine, Atlanta, USA

Objective: Tobacco exposure during pregnancy is an established risk factor for early birth including early term birth (ETB) and preterm birth (PTB). However, the underlying molecular mechanisms are not well understood. In this study, we determined the associations between metabolomic profiles of newborn dried blood spots and prenatal tobacco exposure and early birth.

Material and Methods: Our analytic samples consisted of 269 mother-child dyads in the Atlanta African American Maternal-Child Cohort (2016-2020). Established tobacco exposure biomarkers, cotinine and trans-3′-hydroxycotinine (3HC), were measured in maternal urine collected within 8-14 gestational weeks. Gestational age at birth was determined from medical records. Newborn dried blood spots were collected within 24-48 hours after birth and analyzed using untargeted high-resolution metabolomics. Metabolome-wide association studies adjusted for covariates along with metabolic pathway enrichment analyses and chemical annotations were used to determine newborn metabolite signals and pathways associated with prenatal tobacco exposure and early birth via the *meet-in-the-middle* approach.

Results: There were 84 (31.2%) ETB and 33 (12.3%) PTB in the cohort. The median creatinine-adjusted maternal urinary cotinine and 3HC concentrations were 5.03 µg/g and 9.45 µg/g, respectively. Maternal urinary cotinine and 3HC concentrations were associated ($p < 0.05$) with 648 and 503 metabolic signals, respectively. ETB and PTB were associated with ($p < 0.05$) 648 and 1,747 metabolic signals, respectively. Biopterin metabolism was associated with both tobacco exposure biomarkers and early birth (ETB and PTB). Early birth was also associated with lipid and fatty acid metabolic pathways. Riboflavin was positively associated and 5-hydroxytryptophan was negatively associated with both tobacco exposure biomarkers and early birth.

Conclusion: Taken together, these findings suggest that the newborn metabolome is perturbed by prenatal tobacco exposure and is associated with gestational age at birth. Perturbation in biopterin metabolism is a potential mechanism linking prenatal tobacco exposure to early birth.

P51-09 | In utero exposure to prolonged ambient heat and the negative impacts on adverse pregnancy outcomes in India: A cross-sectional study

Vineetha Vincent[1], Darsy Darssan[2], Sagnik Dey[3], Nicholas J Osborne[2]

[1] Indian Institute of Technology Delhi, UQIDAR, Hauz Khas, India; [2] The University of Queensland, School of Public Health, Brisbane, Australia; [3] Indian Institute of Technology Delhi, Centre for Atmospheric Sciences, Hauz Khas, India

Objective
Exposure to heat during pregnancy may increase the risk of maternal and child health. Studying these impacts in LMICs is crucial, as vulnerable populations face higher risks due to limited healthcare access and adaptive capacity. This research explores how ambient heat stress and prolonged heat conditions during the pregancy period impact adverse pregnancy outcomes like low birth weight, stillbirth, and the incidence of preterm births in India.
Materials and Methods
The study utilised a nationally representative population sample from the cross-sectional survey of India—the National Family Health Survey. This study employs a generalised logestic regression model as the primary model. The model further adjusted for potential confounding factors, including socioeconomic and demographic factors at individual and cluster levels. We estimated different heat exposures using participant's cluster geolocations.Further analysis was conducted on how different exposures, such as trimester exposure, in-utero exposure, and consecutive days above the 90th percentile during the pregnancy period, are associated with adverse birth outcomes.
Results
Among 209,266 participants, 29,873 low birth weight (LBW) cases, 2341 preterm birth cases, and 1740 stillbirth cases were identified from the survey. The exposure to heat conditions was estimated by calculating continuous days >90th percentile during the pregnancy period.In this period, there was an elevated risk of having LBW estimated by odds ratio 1.012 (95% CI: 1.006,1.018) and stillbirth 1.042 (95% CI: 1.020,1.065) in unadjusted models. In adjusted multivariable models, the adjusted odds ratios (aOR) are 1.020 (95% CI:1.018,1.022) for low birth weight and 1.038 (95% CI:1.016,1.061) for stillbirth.
Conclusion
We observed suggestive evidence for the association between exposure during pregnancy to ambient heat and adverse birth outcomes and the effect changes with different heat exposure ariables. The risk increases with the number of consecutive days above the 90th percentile during the pregnancy period.

P51-10 | Prenatal organophosphate esters exposure and neurobehavior, potential mediation by the gut microbiota in neonates: the Shanghai Maternal-Child Pairs Cohort

Yuhan Zhou[1], Yunhui Zhang[2]

[1] Shanghai University of Sport, Shanghai, China; [2] Fudan University, Shanghai, China

There is currently a dearth of epidemiological evidence regarding the impact of organophosphate esters (OPEs) on childhood neurobehavior and underlying role of gut microbiota. Cord blood samples and neonatal stools were collected. Strengths and Difficulties Questionnaire (SDQ) was applied to evaluate children's behavior. Generalized linear models, microbiome regression-based kernel association test, multivariate analysis by linear models and mediation analysis was used. Fetal exposure to tris (2-butoxy ethyl) phosphate (TBEP) was positively associated with conduct problems (β = 0.14, 95% CI: 0.04, 0.23) and general difficult behavior (β = 0.38, 95% CI: 0.09, 0.67). TBEP was also associated with altered neonatal gut microbiota, including increased diversity (β_{Chao1}= 0.11, 95% CI: (0.04, 0.19); β_{ACE}= 0.12, 95% CI: (0.05, 0.19)) and pathogens proportions, such as *Neisseria*, and *Corynebacterium*. Alpha diversity was significantly positively related to scores of the conduct problems subscale (β_{Chao1} = 0.39, 95% CI: (0.18, 0.60); β_{ACE} = 0.45, 95% CI: (0.22, 0.67)) and the general difficult behavior (β_{Chao1} = 1.07, 95% CI: (0.39, 1.74); β_{ACE} = 1.13, 95% CI: (0.42, 1.85)). Mediation analysis suggested that richness of neonatal gut microbiota accounted for over 30% of TBEP exposure-induced change in behavioral problems. Our results suggest neonatal gut microbiota may play a critical role in OPE neurotoxicity.
This study was supported by the National Key Research and Development Program of China (Grant No. 2022YFC2705004), the National Natural Science Foundation of China (Grant No. 82273585, 82304103), and the Shanghai 3-year Public Health Action Plan (GWVI-11.1–39).

P51-11 | Antenatal heat exposure and small airways dysfunction in early childhood: the Mother and Child in the Environment (MACE) birth cohort

Rajen N. Naidoo[1], Prakash Jeena[2], Lisa F. Ramsay[3], Sheena Muttoo[3], Jihada Cassim[3], on behalf of the Mother and Child in the Environment (MACE) Birth Cohort

[1] University of KwaZulu-Natal, Occupational and Environmental Health, Durban, South Africa; [2] University of KwaZulu-Natal, Paediatrics and Child Health, Durban, South Africa; [3] University of KwaZulu-Natal, Occupational and Environmental Health, Durban, South Africa; [4] University of KwaZulu-Natal, Occupational and Environmental Health, Durban, South Africa; [5] University of KwaZulu-Natal, Occupational and Environmental Health, Durban, South Africa

Background and Objective: Heat may have an impact on respiratory health across the life-cycle. Our aim was to determine whether antenatal exposure to heat is associated with small airways dysfunction as determined through oscillometry (OS) in early childhood.

Methods: Pregnant females, recruited at public antenatal clinics in their first trimester from low-socioeconomic communities in Durban, South Africa, were followed up until delivery. Newborns were followed up in infancy and annually, with early childhood oscillometry performed in 346 children. Daily maximum Land Surface Temperature

(LST) with 1km × 1km resolution was acquired from MODIS satellite data, providing estimates at each participant's residence. Daily maximum temperatures were averaged for the entire length of pregnancy, and for each trimester.
Results: Mean maximum temperatures were 27.8°C (sd=3.7), 28.2°C (3.7), 30.4°C (3.5) and 28.8°C (1.4) across trimesters 1-3 and the entire pregnancy respectively. The mean age of participating children was 7.3 years (sd=1.5), with 50.3% females. Maternal smoking during pregnancy was 16.7%, with regular environmental tobacco smoke reported by 38.5%. Low birth weight (LBW) was seen in 13.1%. Doctor diagnosed asthma was 6%, with 14.3% reporting an attack of wheezing in the past year. The median (interquartile range) oscillometry parameters of interest were: Rx_{5-20}: 2.4 (1.3-3.5); Ax: 40.9 (31.5-54.9); X5 z-score: 0.71 (-0.07-1.5) and R5 z-score: 0.28 (-2.6-0.84).
The relationship between heat metrics and small airways dysfunction were stronger when stratified by probable asthma and LBW respectively. Among probable asthmatics, third trimester effects were the strongest with inverted "U" relationships, peaking at approximately 30 °C. When stratified by LBW, evidence of reduced reactance was greater than increased resistance.
Conclusion: Antenatal heat exposure may influence small airways dysfunction among those with probable asthma or low birth weight, in a non-linear manner. Heat and LBW may increase the risk for reduced lung elasticity in early childhood.

P51-12 | Prenatal exposure to acrylamide, offspring's fetal size and size at birth: A Biomarker-based Cohort Study from Denmark

Damian Chandia-Poblete[1], Stéphane Tuffier[1], Efstathios Vryonidis[2], Thorhallur I. Halldorsson[3,4], Anne A. Bjerregaard[3,5], Dorte Rytter[6], Bodil H. Bech[6], Tine B. Henriksen[7], Margareta Törnqvist[2], Sjurdur F. Olsen[3,8], **Marie Pedersen**[1]

[1] University of Copenhagen, Department of Public health, Copenhagen, Denmark; [2] Stockholm University, Department of Environmental Science, Stockholm, Sweden; [3] Statens Serum Institut, Department of Epidemiology Research, Copenhagen, Denmark; [4] University of Iceland, Faculty of Food Science and Nutrition, Reykjavik, Iceland; [5] Copenhagen University Hospital - Bispebjerg and Frederiksberg Hospital, Centre for Clinical Research and Prevention, Copenhagen, Denmark; [6] Aarhus University, Department of Public Health, Aarhus, Denmark; [7] Aarhus University Hospital, Department of Paediatrics, Skejby, Denmark; [8] Harvard School of Public Health, Department of Nutrition, Boston, USA

Objective: Exposure to acrylamide (AA) from diet is common. We aimed to assess the associations between prenatal exposure to AA, early fetal size and size at birth.
Material and methods: Prenatal exposure to AA was quantified through hemoglobin adducts from AA (HbAA) measured with a high-throughput method in blood collected in gestational week 30 from women giving birth to liveborn singletons in 1988-89 in Aarhus, Denmark (N=996). Blood and information on maternal characteristics, clinical records of intrauterine growth restriction (IUGR), ultrasound scans of biparietal diameter (BPD) at the 16th gestational week, small-for-gestational-age (SGA), birth weight (BW), birth head circumference (BHC) and birth length (BL) of the offspring were also obtained from the Danish Fetal Origins 1988-89 cohort. We assessed adjusted associations in the whole cohort and after stratification by maternal smoking.
Results: The median (5th-95th percentile) was 92 (45-264) pmol/g Hb for HbAA, 35 (28-42) mm for BPD and 3.5 (2.6-4.3) kg for BW, respectively. Included were 33 infants classified as IUGR and 61 as SGA. AA was associated with a small non-significant reduction in BPD. Compared to the lowest quartile, the highest quartile of HbAA were associated with increased risk for IUGR (OR=4.1; 1.2, 16.0) and for SGA (OR=4.0; 1.4, 13.2) and a reduction in BW of -292 g (-423, -161). Each 10-pmol/g Hb increase in HbAA was associated with reduced size at birth: -11g (-18.3,

-4.5) for BW, -0.05 cm (-0.09, -0.01) for BHC, and –0.03 cm (-0.07, -0.01) for BL. The magnitude of most of these associations attenuated in non-smokers (N=366) and enlarged in smokers (N=269).

Conclusion: These findings add to the evidence that higher levels of prenatal exposure to AA is associated with reduced offspring's size at birth and calls for more research on exposure to AA early in prenatal development and on early fetal growth.

P51-13 | Association of organophosphate flame retardants exposure with ovarian reserve and in vitro fertilization-embryo transfer outcomes in women of reproductive age

Pengpeng Wang[1], Xiangkai Zhao[1], Baoxia Gu[2], Wei Wang[1]

[1] Zhengzhou University, Department of Occupational Health and Occupational Diseases, College of Public Health, Zhengzhou, China; [2] Zhengzhou University People's Hospital, Reproductive Medicine Center, Henan Provincial People's Hospital, Henan Joint International Research Laboratory of Reproductive Bioengineering, Zhengzhou, China

Background: Organophosphate flame retardants (OPFRs) are emerging environmental pollutants with reproductive and endocrine toxicity, whereas studies on the relationship between OPFRs exposure and reproductive health in women of childbearing age are limited.

Methods: A total of 555 females of reproductive age were recruited. The concentrations of 11 OPFRs in serum were measured using UPLC-MS/MS. Generalized Linear Regression, BKMR and Quantile g-Computation were utilized to assess the associations between single or mixed exposure of OPFRs and ovarian reserve function and in vitro fertilization-embryo transfer (IVF-ET) outcomes. IVF-ET outcomes were evaluated using indicators such as two-pronuclear count (2PNC), metaphase II oocyte count (MIIC), fertilization count (FC), and others. The logistic regression model to assess the effect of OPFR exposure on the risk of primary ovarian insufficiency (POI). The potential mediating role of ovarian function indicators in the association between serum OPFRs and IVF-ET outcomes were also examined.

Results: In the single-exposure model, the exposure level of blood 2-ethylhexyl diphenyl phosphate (EHDPP) was positively correlated with progesterone (β= 0.12, 95%*CI*: 0.02 to 0.23) and testosterone (β = 0.14, 95%*CI*: 0.04 to 0.24), EHDPP increased the risk of POI in women of childbearing age (*OR*= 3.14, 95%*CI*: 1.33 to 8.03). Mixed-exposure analyses showed that EHDPP is the major OPFR affecting ovarian reserve function and the risk of POI. In the single-exposure model, EHDPP was negatively associated with almost all IVF-ET outcome indicators. EHDPP consistently exhibits the largest negative weight on IVF-ET outcome indicators. Additionally, we observed significant mediating effects of testosterone in the associations between EHDPP and IVF-ET outcome indicators, including 2PNC, MIIC, FC, UBC, and UBR.

Conclusions: Exposure to OPFRs may be associated with diminished ovarian reserve and adverse IVF-ET outcomes. EHDPP is the major contributor to adverse effect. Testosterone plays a mediating role in the adverse outcomes of IVF-ET caused by EHDPP.

P51-14 | Stillbirth in São Paulo, Brazil – Insights from Health Registries for Surveillance and Research

Rafael Buralli[1,2], Lays J.P. Marques[3], Nelson Gouveia[2]

[1] Faculdade de Saúde Pública, Universidade de São Paulo, Departamento de Política, Gestão e Saúde, São Paulo, Brazil; [2] Faculdade de Medicina, Universidade de São Paulo, Departamento de Medicina Preventiva, São Paulo, Brazil; [3] Faculdade de Saúde Pública, Universidade de São Paulo, Departamento de Epidemiologia, São Paulo, Brazil

Despite its extreme burden for families and societies, stillbirth remains inadequately addressed in Brazil. This work aims to explore stillbirth registries in São Paulo, and implications for health surveillance and research. Methods: Descriptive study of stillbirths (fetal loss at ≥22 weeks of gestation or birth weight ≥500g) recorded in the Mortality Information System (SIM) for São Paulo Metropolitan Region (SPMR) in 2023. We analyzed frequencies of sociodemographic, epidemiological, etiological characteristics and data completeness. Results: 2,184 stillbirths were notified (monthly mean: 182; range: 143-215). Antepartum stillbirths comprised 86%, intrapartum 3%, while 11% lacked classification. Regarding gestational age, 74% were 22–36 weeks, and 16% were ≥36 weeks. Male stillbirths accounted for 52%, female for 45%. Missing data also affected maternal education (18%), age and pregnancy duration (10% each), and baby sex (3%). Most mothers were aged 20–34 years (59%) or 35–39 years (15%); and had ≥8 years of study (70%). The main underlying causes of deaths were intrauterine hypoxia (n=926; 42%); complications of placenta, cord, and membranes (n=295; 14%), unspecified causes (n=270; 12%); maternal conditions unrelated to present pregnancy (n=251; 12%); and maternal complications of pregnancy (n=109; 5%). Race/ethnicity was missing in all records. Nearly half (47%) of cases were not investigated. Conclusions: High proportion of late gestation and antepartum stillbirths indicate serious flaws in prenatal care, despite broad coverage. Non-specific causes accounted for a significant share of stillbirths. Lack of post-mortem investigation and incomplete records hinder the identification of stillbirth root causes and disparities, exacerbate structural inequities, and weaken health surveillance and research. Communication and data visualization strategies can enhance awareness, encouraging better reporting and prevention efforts. Enhancing health curriculum and professional training, improving data registration/analysis, and integrating health prevention and surveillance protocols are crucial steps to strengthening policies and reducing stillbirth.

P52 | Environmental Exposures and Cardiovascular Health - PART II

P52-01 | Impact of gaseous pollutants on the incidence of cardiovascular diseases: A case study from Karachi, Pakistan

Haider A. Khwaja[1], Omosehin Moyebi[1], Zafar Fatmi[2], Azhar Siddique[3], Mirza Hussain[1], David Carpenter[1]

[1] University at Albany, Environmental Health Sciences, Albany, USA; [2] The Aga Khan University, Community Health Sciences, Karachi, Pakistan; [3] Hammad Bin Khalifa University, Qatar Environment and Energy Institute, Doha, Qatar

No prior studies exist in Karachi, Pakistan evaluating the short-term effect of ambient air pollution on cardiovascular diseases (CVDs). We adopted a generalized linear model to quantitatively analyze the influences of gaseous pollutants (NO, NO_2, O_3, SO_2, and HNO_3) on morbidity due to CVDs. Increased risk of morbidity due to CVDs including CVD subtypes [hypertension (HTN), ischemic heart disease (IHD), myocardial infarction (MI), congestive heart failure (CHF), and arrhythmias] with gaseous pollutants were observed to be significantly correlated. NO, O_3 and SO_2 exhibited strong significant associations with both total CVD and all the subtypes. The effect estimates of pollutants varied by lag of pollutants' concentrations. Increase in NO caused a rise in total CVD at lag 1, 2, 3, and 5. O_3 and SO_2 showed a significant relationships to CVD hospital admissions (HAs) and emergency room visits (ER visits) at all the lag days (lag 0 – 5). Immediate effect was observed between NO and O_3 exposures with total CVD and all the subtypes at lag 1 and 2, while SO_2 has a delayed effect at lags 3 and 4. NO_2 was significantly associated with total CVD on the day of exposure (lag 0), while HNO_3 exhibited a statistically significant association with total CVD at lag 4. The largest significant effect for O_3 on arrhythmias was observed at the same day (RR= 1.20; 95%CI: 1.12 – 1.29). The gender, age, and seasonal significant association between pollutants and CVD did show differences in the magnitude. However, the risk of HAs and ER visits due to CVD was stronger among age-group 40 – 60. Exposure-response curve with NO and SO_2 was observed to have S-shaped relationship with CVD, while O_3 exhibited a J-shaped relationship with CVD.

P52-02 | Long-term exposure to fine particulate matter and the incidence of cardiovascular disease and accelerated aging in general adults in South Korea

Jongmin Oh[1,2], Jinwoo Cho[3], Yun-Chul Hong[2,4], Hyung-Jin Yoon[2,5], Eunhee Ha[1,6]

[1] Ewha Womans University College of Medicine, Department of Environmental Medicine, Seoul, South Korea; [2] Seoul National University College of Medicine, Department of Human Systems Medicine, Seoul, South Korea; [3] University of Pittsburgh, Department of Statistics, Pittsburgh, USA; [4] Seoul National University Medical Research Center, Institute of Environmental Medicine, Seoul, South Korea; [5] Seoul National University College of Medicine, Department of Biomedical Engineering, Seoul, South Korea; [6] Ewha Womans University, System Health & Engineering Major in Graduate School (BK21 Plus Program), Seoul, South Korea

Objectives

This study aim to evaluate the association between long-term exposure to fine particulate matter ($PM_{2.5}$) and incidence of cardiovascular disease (CVD) and accelerated aging (AA) in general adults in South Korea.

Methods

We utilized health check-up data from the National Health Insurance Service spanning from 2012 to 2014, and followed up the participants for 10 years. We defined the CVD event as first-time hospitalization for ischemic heart disease (IHD) or stroke during the study period, in individuals with no prior history of CVD. $PM_{2.5}$ was used as the primary exposure variable. A cardiovascular disease (CVD) prediction model was developed using the Cox proportional hazards model, adjusting for age, body mass index, systolic and diastolic blood pressure, waist circumference, fasting serum glucose, hemoglobin, GOT, GPT, GGT, total cholesterol, HDL, LDL, triglycerides, creatinine, eGFR, urine protein, smoking status, alcohol consumption, physical activity, and family history. Second, based on the CVD prediction model, we calculated biological age and AA, and evaluated the association between long-term exposure to $PM_{2.5}$ and AA.

Results

We analysed approximately 11 million general population (men: 5,631,377 and women: 6,089,404) from 2012 to 2014. For men, a 10 μg/m^3 increase in the 3-year moving average of $PM_{2.5}$ exposure was associated with a hazard ratio of 1.073 (95% CI: 1.056, 1.089). No association was observed for women. In the age-stratified analysis, for older women, a 10 μg/m^3 increase in $PM_{2.5}$ exposure was associated with a cardiovascular hospitalization risk of 1.050 (95% CI: 1.019, 1.082). Preliminary analyses indicate a potential positive association between the 3-year moving average of $PM_{2.5}$ exposure and AA.

Conclusions

Our findings suggest that long-term exposure to $PM_{2.5}$ may be linked to an increased risk of CVD and accelerated aging in the general population, though further analysis is needed to confirm this relationship.

P52-03 | Cardiovascular Emergency Hospitalization Risks of $PM_{2.5}$ Transition Metals: A Time-Stratified Case-Crossover Study

Lin Wang

Chinese Academy of Science, Institute of urban Environment, Xiamen, China

$PM_{2.5}$ pollution poses significant health risks in urban areas, yet the specific cardiovascular impacts of its hazardous components, especially transition metals, remain insufficiently understood. This study evaluated the associations of $PM_{2.5}$ components on acute myocardial infarction (AMI) and acute aortic dissections (AAD) emergency hospitalizations using a time-stratified case-crossover between 2017-2023 in Xiamen, China. We collected comprehensive data on daily air pollutants, $PM_{2.5}$ components (water-soluble ions, carbon components, metals, and other elements), and meteorological variables. Conditional logistic regressions were used to estimate odds ratios (OR) per interquartile range (IQR) of exposures. Our finding reveals significant short-term associations of exposures to air pollutants and $PM_{2.5}$ components with increased cardiovascular emergency hospitalizations. The strongest associations were observed between cumulative 3-day lagged (lag 0-3) $PM_{2.5}$ transition metals including Mn [odds ratio, OR = 1.106 (95% CI: 1.032-1.186)], Fe [OR = 1.078, (95% CI: 1.015-1.145)], V [OR = 1.117 (95% CI: 1.024-1.219)] and Zn [OR = 1.08, (95% CI: 1.005-1.161)] exposure with AMI. These associations were stronger among older (age >65 years), male patients and during colder seasons. Our study highlights the underexplored sub-acute cardiovascular risks of $PM_{2.5}$ transition metals, underscoring the need to integrate them into urban air quality management to promote environmental sustainability.

P52-04 | Air Pollution and Cardio-metabolic Disease Risk in the Multiethnic US Cohort (All of US)

Mohammad H. Shahriar, Andrew Craver, Rafique Islam, Jiajun Luo, Muhammad Ashique Haider Chowdhury, Saira Tasmin, Liz Connellan, Briseis Aschebrook-Kilfoy, Habibul Ahsan

The University of Chicago, Institute for Population and Precision Health, Chicago, USA

Introduction:
The NIH All of Us Research Program is going to recruit around 1 million diverse participants (around 633,000 already recruited) across the U.S. to investigate myriads of health outcomes. This study explores the association between PM2.5 exposure and cardio-metabolic disease risk.

Materials and Methods
This study utilized data from 393596 participants of the All of Us Research Program via the All of Us Researcher Workbench. Cardio-metabolic case identification was conducted using electronic health records (EHR) and self-reported Personal Medical History questionnaire responses. PM2.5 exposure data were obtained from NASA's Earth Observing System Data and Information Center and assigned based on participants' 3-digit zip code prefixes. Multivariate logistic regression models were applied to estimate the odds ratio (OR) and 95% confidence interval (CI).

Results
Participants were identified from EHR data and self-reported survey data (prevalent cases from 2016-2022), including Myocardial Infarction (MI) n=6536, Arrhythmia n=16293, Heart failure n=6536, Hypertension n=40649, and Diabetes Mellitus (DM) n=18797. Participants had a mean age of 55.92 years and an average BMI of 30.08; 57.44% were White, 17.82% Hispanic, 16.94% Black, and 2.64% Asian. The average PM2.5 level between 2014 and 2022 was 7.77 μg/m3 (range: 2.02-13.87). Analysis of EHR-based cardio-metabolic conditions indicated elevated odds of MI (OR 1.06, 95% CI 1.03-1.07), Type 2 DM (OR 1.09, 95% CI 1.07-1.09), Heart failure (OR 1.05, 95% CI 1.03-1.07), and Hypertension (OR 1.04, 95% CI 1.02–1.04) with 1 μg/m^3 increase in PM2.5. The association was stronger in the highest quartile of exposure compared to the lowest: MI (1.23), Hypertension (1.35), Type 2 DM (1.48), and Heart failure (1.23).

Conclusions
Our findings suggest a significant association between low PM2.5 exposure and increased risk of cardio-metabolic conditions. Further studies are necessary to validate and expand upon these findings.

P52-06 | Combined Effects of Arsenic, Cadmium, and Mercury with Cardiovascular Disease Risk: Insights from the All of Us Research Program

Oluwatobi L. Akinbode, Emmanuel Obeng-Gyasi

North Carolina A&T State University, Greensboro, USA

Background: Environmental exposure to heavy metals/metalloids like arsenic, cadmium, and mercury has been linked to adverse cardiovascular health outcomes. Using data from the *All of Us* research program, we examined associations between these exposures and six cardiovascular biomarkers: systolic blood pressure (SBP), HDL cholesterol, LDL cholesterol, C-reactive protein (CRP), total cholesterol, and triglycerides.

Methods: Among 136 participants (53.4% women), we assessed associations between cardiovascular biomarkers and metal/metalloid exposures through linear regression and Bayesian Kernel Machine Regression (BKMR). BKMR captured potential non-linear relationships and interactions, with posterior inclusion probabilities (PIPs) quantifying the contribution of each exposure. Univariate, bivariate, and overall exposure–response analyses in BKMR provided insights into individual and combined effects.

Results: The study population (mean age: 57.5 years) comprised 104 White, 10 Black (mean age: 63.2), 7 Hispanic, 3 Asian, and 12 of Other race. Men exhibited higher SBP, triglycerides, mercury, and arsenic levels, while women had higher CRP, LDL, HDL, total cholesterol, and cadmium levels. Black participants showed higher cardiovascular risk and inflammation markers (e.g., blood pressure, CRP), whereas Asians had the lowest levels across biomarkers. In linear regression, we identified significant positive associations between mercury and HDL cholesterol, arsenic and triglycerides, and arsenic and total cholesterol. BKMR results revealed that mercury had the highest predictive contribution for SBP, HDL cholesterol, and triglycerides; cadmium for CRP; and arsenic for LDL and total cholesterol. Univariate and bivariate exposure–response analyses demonstrated non-linear patterns, including U-shaped and inverted U-shaped relationships, for cadmium, particularly CRP or total cholesterol patterns undetectable with traditional linear regression.

Conclusion: Our study results highlight the influence of environmental metal/metalloid exposures on cardiovascular biomarkers, providing evidence of non-linear and interactive effects that warrant further investigation to understand their role in cardiovascular disease risk better.

P52-07 | Associations of serum per- and polyfluoroalkyl substances with prevalent diabetes and related blood biomarkers among adults in the Multi-Site Study

Anne P. Starling[1], Gayoung Lee[2], Jordan M. Bailey[3], Linda M. Brown[4], Judith M. Graber[5], Yerin Jung[6], Robert Laumbach[7], Tamarra James-Todd[8], Kayoko Kato[9], Celina N. Phillipson[10], Patrick Rago[11], Adam M. Schaefer[12], Carolina Villanueva[13], Yuting Wang[14], Marian Pavuk[15]

[1] University of North Carolina at Chapel Hill, Department of Epidemiology, Gillings School of Global Public Health, Chapel Hill, USA; [2] University of North Carolina at Chapel Hill, Department of Epidemiology, Gillings School of Global Public Health, Chapel Hill, USA; [3] Michigan Department of Health and Human Services, Environmental Health Bureau, Landing, USA; [4] RTI International, Research Triangle Park, USA; [5] Rutgers, The State University of New Jersey, Department of Biostatistics and Epidemiology, New Brunswick, USA; [6] University of California Irvine, Department of Environmental and Occupational Health, Joe C. Wen School of Population & Public Health, Irvine, USA; [7] Rutgers, The State University of New Jersey, Department of Environmental and Occupational Health and Justice, New Brunswick, USA; [8] Harvard T.H. Chan School of Public Health, Department of Environmental Health, Boston, USA; [9] Centers for Disease Control and Prevention, National Center for Environmental Health, Division of Laboratory Sciences, Atlanta, USA; [10] University of California Irvine, Department of Environmental and Occupational Health, Joe C. Wen School of Population & Public Health, Irvine, USA; [11] Centers for Disease Control and Prevention, Agency for Toxic Substances and Disease Registry, Office of Community Health Hazard Assessment, Atlanta, USA; [12] Abt Global, Cambridge, USA; [13] University of California Irvine, Environmental and Occupational Health, Joe C. Wen School of Population & Public Health, Irvine, USA; [14] Silent Spring Institute, Newton, USA; [15] Centers for Disease Control and Prevention, Agency for Toxic Substances and Disease Registry, Office of Community Health Hazard Assessment, Atlanta, USA

Objective: Previous studies suggest that exposure to certain per- and polyfluoroalkyl substances (PFAS) is linked to greater risk of diabetes; however, results are inconsistent. In this large, cross-sectional multi-site study of U.S. residents with historically elevated PFAS concentrations in drinking water, we estimated associations between serum PFAS and prevalent diabetes. We also examined the associations between PFAS and fasting glucose, insulin, and hemoglobin A1c (HbA1c).
Methods: Participants were adults recruited at eight sites (2019-2023) who completed questionnaires and blood sample collection. Serum was analyzed for seven PFAS; PFAS detected in >75% of participants were analyzed as continuous, while other PFAS were treated as binary at the limit of detection. Logistic regression models estimated associations between each PFAS and prevalent diabetes, based on self-report or medical record documentation. Among participants without diagnosed diabetes or current use of diabetes medication, linear regression models estimated associations between each PFAS and biomarker.
Results: Participants in this analysis (n=5,753) were 60% female with a mean age of 56 years. The prevalence of diabetes was 12.4%. Covariate-adjusted associations between individual PFAS and prevalent diabetes in adults were null or negative; for example, the odds ratio of diabetes for each log-unit increase in perfluorooctanoate (PFOA) was 0.83 (95% confidence interval 0.76, 0.91). Among participants eligible for biomarker analysis (n=4,582), associations with HbA1c were negative for PFOA and perfluorooctane sulfonate (PFOS) and positive for perfluorodecanoate (PFDA). The direction of association with glucose and insulin also varied by PFAS.
Conclusion: We did not observe increased odds of diabetes with higher blood PFAS concentrations. Causal interpretation is limited by the cross-sectional design that does not account for temporality; future studies will employ mixture models and historical PFAS levels. Funding for this study was provided by the Agency for Toxic Substances and Disease Registry, Centers for Disease Control and Prevention.

P52-08 | Is occupational particle exposure a risk factor for type II diabetes?

Karl Kilbo Edlund[1], Erik Sand[1], Martin Andersson[3], Leo Stockfelt[1,2]

[1] University of Gothenburg, School of Public Health and Community Medicine, Gothenburg, Sweden; [2] Sahlgrenska University Hospital, Occupational and Environmental Medicine, Gothenburg, Sweden; [3] Umeå University, Department of Epidemiology and Global Health, Umea, Sweden

Objective

Diabetes is a growing global health concern, with environmental and social dimensions. While ambient fine particles are recognised as a likely risk factor for diabetes, effects of occupational particle exposure are less studied – despite the typically much higher exposure levels. In this study, we investigate the association between occupational exposure to inorganic particles (dusts and fumes) and type 2 diabetes (T2DM) in a large Swedish cohort of construction workers.

Material and Methods

In this retrospective cohort study, we included approx. 300 000 male construction workers in Sweden, recruited through occupational health examinations in the 1970's to 1990's and followed through national healthcare registers. Exposure to nine forms of particles was determined using a job-exposure matrix and grouped as inorganic dusts and fumes, organic dusts and man-made fibres. T2DM cases were identified through validated national registers. Adjusted hazard ratios were determined using Cox models adjusted for age, year, and baseline smoking and body-mass index.

Preliminary results

Just above half of the included workers were exposed to inorganic dusts or fumes. High exposure to mineral dusts was associated with an increased risk of T2DM, but no association was seen for workers with low exposure or exposure to other particle types.

Conclusion

High occupational mineral dust exposure might increase the risk of T2DM. Further research is needed to expand evidence to other occupational groups and improve on and evaluate mitigation strategies.

This study is funded by the Swedish Research Council for Health, Working Life and Welfare and the Gothenburg Medical Society.

P52-09 | Associations between PFAS and their mixture and cardiovascular diseases among residents in Westhampton, NY: Comparison of three statistical models

Yichun Liu[1], Eric J. Rose[2], Wanhsiang Hsu[1,3], Elizabeth J. Mullin[4], Steven P. Forand[1]

[1] New York State Department of Health, Bureau of Environmental and Occupational Epidemiology, Albany, USA; [2] University at Albany, College of Integrated Health Sciences, Department of Epidemiology and Biostatistics, Rensselaer, USA; [3] University at Albany, College of Integrated Health Sciences, Department of Health Policy, Management and Behavior, Rensselaer, USA; [4] New York State Department of Health, Laboratory of Organic Analytical Chemistry, Wadsworth Center, Albany, USA

Background

Biomonitoring studies focusing on the associations between exposure to PFAS mixtures and the risk of adverse health outcomes are limited. Current studies primarily examine exposures to individual PFAS compounds. Because many communities may be exposed to multiple PFAS compounds at one time, studies that examine exposure to individual PFAS compounds as well as various PFAS mixtures are important when assessing the impact of PFAS exposures on human health. In this study, three statistical models were conducted to examine associations between individual PFAS compounds and PFAS mixtures and cardiovascular disease outcomes (CVDs).

Methods

Descriptive analyses were conducted to present PFAS serum levels among study participants in Westhampton, NY. PFAS levels are presented by health outcomes of interest. Multivariable models were fitted with logistic regressions with the generalized estimating equation (GEE) model, Bayesian kernel machine regression (BKMR) model, and weighted quantile sum (WQS) regression model to examine the association between PFAS exposures and CVDs.

Results

Among 161 participants, 57 participants reported CVD related health history (35.40%). The results of the GEE logistic regression model showed that the health outcome of CVDs was positively associated with serum levels of PFNA (odds ratio 8.47, 1.45-49.37). The results of the BKMR model suggested similar findings; PFNA and Me-PFOSA-AcOH were the most important exposure contributors. The WQS regression model identified PFNA as the most important exposure contributor based on the highest weight. The results of both BKMR model and WQS regression model suggested that PFAS mixtures were significantly and positively associated with increased risks of having CVDs.

Conclusion

The results of the comparison of three statistical models demonstrate the importance of applying multiple analysis approaches to ensure precision and stability of results when examining highly correlated data such as environmental exposures.

P52-10 | Influence of Heat Mitigation Strategies on Resting Heart Rate in Elderly Individuals During Summer: Insights from a Wearable Device Study

Chi-Hsien Chen, Yue Leon Guo

National Taiwan University Hospital, Department of Environmental and Occupational Medicine, Taipei, Taiwan

Background: The effect of elevated summer temperatures on resting heart rate (RHR) in older adults, and the role of heat adaptation strategies, are not well understood.

Objectives: This study aimed to evaluate the immediate impact of summer heat on RHR in elderly individuals and to determine how different heat adaptation behaviors may modify this response.

Methods: Eighty-three participants aged 65 and above in Taipei City were monitored from May to September 2021 using Garmin® smartwatches. RHR was defined as the lowest 30-minute heart rate recorded daily. Data on heat adaptation behaviors were collected in September. A mixed-effects model analyzed the effect of ambient temperature on RHR, with subgroup analyses and interaction terms assessing the modifying effects of these behaviors.

Results: Each 1°C increase in temperature over lag days 0 and 1 corresponded to a 0.11 beats per minute rise in RHR (95% CI 0.07-0.15, P<.0001). After adjusting for various heat adaptation behaviors, significant protective effects were observed for: reducing physical activity (B=-0.15, 95% CI -0.23 to -0.06, P=0.001), consuming iced water (B=-0.24, 95% CI -0.35 to -0.13, P<.0001), increasing naps or sleep duration (B=-0.28, 95% CI -0.46 to -0.1, P=.003), drinking additional water (≥500ml) (B=-0.10, 95% CI -0.19 to -0.01, P=.023), using air conditioning before and during sleep (B=-0.15, 95% CI -0.24 to -0.05, P=.002; B=-0.13, 95% CI -0.22 to -0.03, P=.007), and utilizing fans at night (B=-0.12, 95% CI -0.22 to -0.03, P=.012).

Conclusions: Elevated summer temperatures are associated with increased RHR in the elderly; however, specific heat adaptation behaviors can mitigate this effect. Combining smartwatch monitoring with personal behavior tracking offers a practical method for assessing individual responses to heat and the effectiveness of adaptive strategies.

P52-11 | Impacts of indoor high temperature on cardiovascular health among community-dwelling adults: A scoping review

Jianying Liao[1,2], Nibedita S Ray-Bennett[2,3], Joshua Vande Hey[1,4], Liu Yang[1,5], Yutong S. Cai[1,6]

[1] University of Leicester, Centre for Environmental Health and Sustainability, Leicester, UK; [2] University of Leicester, Institute for Environmental Futures, Leicester, UK; [3] University of Leicester, Avoidable Deaths Network, School of Business, Leicester, UK; [4] University of Leicester, Earth Observation Science Division, School of Physics and Astronomy, Leicester, UK; [5] Shanxi University, Institute of Environmental Science, Taiyuan, China; [6] Leicester General Hospital, NIHR Leicester Biomedical Research Centre, Leicester, UK

Objective

As global temperature rises, the frequency and intensity of heatwaves have increased, posing significant health risks, particularly for individuals with cardiovascular diseases (CVDs). Given that people spend the majority of their time indoors, high indoor temperature may exacerbate heat-related cardiovascular responses. This scoping review aimed to map the existing evidence on the impacts of high indoor temperature on CVD outcomes, including hypertension, symptom reporting, hospitalisation, and mortality.

Materials and Methods
A comprehensive literature search was conducted across PubMed, Medline, and Web of Science to identify studies published between January 2014 and December 2024. Following the Joanna Briggs Institute (JBI) scoping review framework, eligible studies were screened and data extracted based on study design, exposure assessment, population characteristics, and reported cardiovascular outcomes.
Results
Fifteen studies met the inclusion criteria, with most focusing on older adults (average age ranged from 36.3 to 79.6 years) and employing cross-sectional designs. Key findings indicated an inverse relationship between indoor temperature and blood pressure, particularly in colder periods. However, limited studies examined the effects of high indoor temperature, with mixed results regarding CVD outcomes such as emergency visits and blood pressure variability. No studies directly investigated the relationship between high indoor temperature and cardiovascular mortality. Methodological limitations, including heterogeneous exposure assessment and insufficient adjustment for confounders such as humidity and air pollution, were prevalent.
Conclusion
Current evidence remains insufficient to understand the adverse impacts of high indoor temperature on cardiovascular health. In the context of global warming, further research is warranted to better understand broader cardiovascular health risks of high indoor temperature, particularly among younger populations. Future studies should adopt robust study design, improve exposure assessment, and consider both environmental and behavioural factors to strengthen the evidence base for policy interventions.

P52-12 | Role of gut microbiota and metabolites between metal fume and the risk of cardiovascular diseases

Ruei-Jing Gao, Ching-Huang Lai

National Defense Medical Center, School of Public Health, Taipei, Taiwan

Objective
Long-term exposure to welding metal fumes increases inflammation and affects the intestinal barrier and gut microbiota, potentially triggering cardiovascular diseases (CVD). However, the interaction between welding fumes, gut microbiota, and CVD remains unclear. This study examines associations between metal exposure, CVD biomarkers, gut microbiota composition, and short-chain fatty acid (SCFA) metabolites.
Material and Methods
A longitudinal study was conducted on 56 shipyard workers in northern Taiwan (2022–2023). Personal air sampling was performed on Monday, and urine and stool samples were collected on Tuesday. CVD biomarkers included low-density lipoprotein (LDL), high-sensitivity C-reactive protein (hs-CRP), atherosclerosis index of plasma (AIP), and Framingham risk score (FCRS). Heavy metals were measured by inductively coupled plasma mass spectrometry (ICP-MS), and gut microbiota was analyzed via full-length 16S rDNA sequencing on the Illumina MiSeq platform. Statistical analysis was performed using SPSS 26.0 and R.
Results
Fecal iron and manganese levels categorized high- and low-dose groups, revealing significant beta diversity differences in gut microbiota. Adjusted generalized estimating equations (GEE) showed hs-CRP was positively associated with fecal Al, Cd, and Pb, while AIP correlated with Pb. SCFA concentrations of acetic, propionic, and

butyric acids were negatively associated with Al. At the genus level, Al and Hg increased Sutterella; As decreased Prevotella and Prevotellamassilia; Pb reduced Bacteroides and Lachnoclostridium; and AIP increased Megamonas.

Conclusion

Welding metal exposure increases CVD risk factors and alters gut microbiota composition. Al and Pb specifically enhance pathogenic gut microbiota. Future studies should explore the mediation effect between oxidative damage biomarkers and gut microbiota changes.

P52-14 | Childhood exposure to radioactive waste and cardiovascular disease: Evidence from the St. Louis Baby Teeth study

Michael Leung[1], Joyce J. Lin[1], Murray A. Mittleman[2], Justin G. Farmer[1], Ian W. Tang[1], Kaleigh McAlaine[1], Joseph J. Mangano[3], Marc G. Weisskopf[1,2]

[1] Harvard T.H. Chan School of Public Health, Environmental Health, Boston, USA; [2] Harvard T.H. Chan School of Public Health, Epidemiology, Boston, USA; [3] Radiation and Public Health Project, Ocean City, USA

Objective: Since the 1940s, Coldwater Creek—located in St. Louis, Missouri's northern suburbs—has been contaminated by radioactive waste produced by the Manhattan project's effort to refine uranium for the first atomic bomb. Here, in the St. Louis Baby Tooth—Later Life Health Study (SLBT), we evaluated whether residential proximity to Coldwater Creek during childhood was associated with increased cardiovascular disease (CVD) incidence, which we hypothesize are related based on prior mechanistic and epidemiologic evidence.

Material and Methods: We used data from 4,209 eligible SLBT participants. Self-reported CVD diagnoses were grouped into three categories: any CVD, atherosclerotic CVD, and hypertension. We used Cox models to estimate hazard ratios (HRs) and 95% CIs between our CVD outcomes and proximity of childhood residential addresses to Coldwater Creek (≤1km, >1-5km and >5km), adjusting for sociodemographics and neighborhood median income. We also stratified the analyses by sex.

Results: Among participants ≤1km from Coldwater Creek (~3% of our study population), the HR for any CVD was 1.35 (95% CI: 1.06-1.72) compared to those living >5km from the creek. Despite more imprecision in the estimates, there was suggestive evidence of an association when looking at atherosclerotic CVD (HR=1.72; 95% CI: 0.93-3.17) and hypertension (HR=1.23; 95% CI: 0.95-1.60). Our sex-stratified analyses also revealed that the associations were mainly driven by women. For example, among women living ≤1km from the Creek, the HR was 1.46 (95% CI: 1.03-2.05) for any CVD, 3.12 (95% CI: 1.33-7.35) for atherosclerotic CVD, and 1.42 (95% CI: 1.00-2.02) for hypertension; among men, the HR were 1.25 (95% CI: 0.88-1.76), 1.12 (95% CI: 0.46-2.76), and 1.05 (95% CI: 0.71-1.55), respectively.

Conclusion: Our study provides evidence that living near Coldwater Creek as a child is associated with a higher risk of developing CVD, where atherosclerotic subtypes and women were most impacted.

P52-15 | Potential Risks of Balneotherapy on Autonomic and Cardiovascular Physiological Effects: Focused on Seasonal Differences

Po-chun Wang[1,2], Ta-chen Su[3,4]

[1] National Taiwan University Hospital Hsin-Chu Branch, Department of Family Medicine, Hsinchu City, Taiwan; [2] Institute of Environmental and Occupational Health Science, National Taiwan University College of Public Health, Taipei City, Taiwan; [3] Tungs' Taichung MetroHarbor Hospital, Department of Medical Research, Taichung City, Taiwan; [4] National Taiwan University Hospital, Department of Internal Medicine, Taipei City, Taiwan

Objectives:
Bathing therapy such as sauna and hot spring has been shown to improve people's cardiovascular health in many studies. This study focused on the potential associated cardiovascular risks and seasonal autonomic effects of hot spring bathing, to provide suggestions on the seasonal difference for hot spring bathing. In addition, psychological state modulation which correlates with autonomic modulation would be demonstrated.

Material and Methods:
We recruited volunteers without cardiovascular disease for a cardiovascular health program in New Taipei City across four seasons. Hot spring temperature was maintained at 38–40°C, characterized as an acid-sulfate-chloride hot spring rich in iron. Cardiovascular function was assessed using an oscillometric BP device (DynaPulse 200M), while heart rate variability (HRV) was measured via HRV+ (BeneGear, Taipei, Taiwan) with 5-minute N–N interval segments analyzed. Participants' tympanic and environmental temperatures were also recorded. Each participant underwent assessments at five time points: baseline, two 20-minute bathing sessions, and at 20 and 40 minutes post-bathing. Psychological stress and environmental stress recovery were evaluated via self-administered questionnaires before and after bathing.

Results:
A total of 118 participants were recruited from March to December 2019. Hot spring bathing reduced cardiac workload and blood pressure (systolic, diastolic, and mean). HRV analysis revealed decreased standard deviation of NN intervals (SDNN), low-frequency power (LFP), and high-frequency power (HFP), with an increased LFP/HFP ratio during bathing. In summer, significant SDNN and LFP reduction persisted even after 40 minutes of rest, independent of ambient and body temperature effects. In psychological evaluation, positive response in relaxation, enjoying, pleasure and refreshing were found.

Conclusions: Hot spring bathing triggers sympathetic activation, especially in summer, which may implicate with increasing cardiac workload. However, it reduces cardiac stress, lowers blood pressure components, improves vascular compliance, and enhances psychological well-being and environmental stress recovery.

P52-16 | Tabacco consumption mediates the association between chronic psychosocial stress and the onset of metabolic syndrome in women.

Teresa C. García Aguilar[1], Ana C. Ariza-Gutiérrez[2], Belem Trejo Valdivia[2], Edgar Denova Gutiérrez[2], Martha María Téllez-Rojo[2], Carmen Hernández-Chavez[5], Robert Wright[4], Rosalind Wright[3], Marcela Tamayo-Ortiz[6]

[1] National Institute of Public Health, School of Public Health, Cuernavaca, Mexico; [2] National Institute of Public Health, Center for Research in Nutrition and Health, Cuernavaca, Mexico; [3] Icahn School of Medicine at Mount Sinai, Department of Environmental Medicine and Public Health, New York, USA; [4] Icahn School of Medicine at Mount Sinai, Co-Director, Mount Sinai Institute for Exposomic Research, New York, USA; [5] National Institute of Perinatology, PROGRESS cohort study, Mexico city, Mexico; [6] Columbia University Mailman School of Public Health, Department of Environmental Health Sciences, New York, USA

Objective: Lifestyle factors can modify the onset of metabolic syndrome (MetS), a cluster of simultaneous conditions including: high blood pressure, high blood sugar, excess body fat around the waist, and abnormal cholesterol or triglyceride levels. Cigarette smoking is highly related to micro and macrovascular problems and insulin resistance. Research is still needed on the joint association of chronic psychosocial stress (PS) and smoking with the incidence of MetS. We aimed to study the mediation role of smoking in the association between PS and MetS in women from the Programming Research in Obesity, Growth, Environment and Social Stressors (PROGRESS) cohort in Mexico City.

Methods: Chronic PS (low, medium and high) included measures from 1 to 4 years postpartum. MetS trajectories included (baseline: 4 years postpartum), 2- and 4-years follow-up. Smoking was assessed at baseline. We used multinomial logistic regression to model the association between chronic PS and smoking, and between chronic PS and MetS trajectories adjusted by smoking in 248 participants.

Results: At baseline, 158 women had experienced high chronic PS and 56 were current smokers; by the end of the follow-up 61 developed MetS. We saw an association between high chronic PS and smoking (OR 3.15 95% confidence interval (CI) 1.41, 7.03). The associations between chronic PS and MetS trajectories were mostly null; compared to women without MetS, smoking showed an increasing risk for women who developed MetS since the first follow-up (RR 2.64 95% CI: 0.75, 9.25) and by the end of follow-up (RR 1.60 95% CI: 0.71, 3.60) adjusted for age, parity, and marital status.

Conclusions: Our preliminary results suggest that modeling using structural equation models could advance our insight to the mediating role of smoking between chronic PS and MetS. Further longitudinal research should include other lifestyle factors such as diet and alcohol consumption.

P53 | Environmental Exposures During Pregnancy and Maternal/Infant Health Outcomes - PART II

P53-01 | Gestational $PM_{2.5}$ and NO_2 air pollution exposure and maternal immune activation and dysregulation in Rochester, New York and Pittsburgh, Pennsylvania

Catherine Yount[1], Kristin Scheible[2], Sally Thurston[3], Xing Qiu[3], Yihui Ge[4], Philip Hopke[1,5], Yan Lin[4], Richard Miller[6,7], Susan Murphy[16], Jessica Brunner[6], Pathik Wadhwa[13], Claudia Buss[14], Hyagriv Simhan[15], Jill Tarr[15], Emily Barrett[8,6], Thomas O'Connor[6,9], Junfeng Zhang[4], David Rich[1,7]

[1] University of Rochester Medical Center, Department of Public Health Sciences, Rochester, USA; [2] University of Rochester Medical Center, Department of Pediatrics, Rochester, USA; [3] University of Rochester Medical Center, Department of Biostatistics and Computational Biology, Rochester, USA; [4] Duke University, Nicholas School of the Environment & Duke Global Health Institute, Durham, USA; [5] Clarkson University, Potsdam, Institute for a Sustainable Environment, Rochester, USA; [6] University of Rochester Medical Center, Department of Obstetrics and Gynecology, Rochester, USA; [7] University of Rochester Medical Center, Department of Environmental Medicine, Rochester, USA; [8] Rutgers University School of Public Health, Department of Biostatistics and Epidemiology, Piscataway, USA; [9] University of Rochester, Department of Psychology, Rochester, USA; [10] University of Rochester School of Medicine and Dentistry, Department of Psychiatry, Rochester, USA; [11] University of Rochester School of Medicine and Dentistry, Department of Neuroscience, Rochester, USA; [12] University of Rochester School of Medicine and Dentistry, Department of Medicine, Rochester, USA; [13] University of California, Irvine School of Medicine, Department of Psychiatry and Human Behavior, Rochester, USA; [14] Charite University, Institute of Medical Psychology, Berlin, Germany; [15] University of Pittsburgh School of Medicine, Magee-Women's Research Institute, Pittsburgh, USA; [16] Duke University Medical Center, Department of Obstetrics and Gynecology, Durham, USA

Objective:
Air pollution has been associated with adverse pregnancy outcomes, but the underlying mechanisms remain unclear. We investigated whether short-term exposures to fine particulate matter ($PM_{2.5}$) and nitrogen dioxide (NO_2) are associated with maternal immune markers in pregnancy cohorts in Rochester, New York, and Pittsburgh, Pennsylvania.

Material and Methods:
Participants from the UPSIDE (Rochester, n= 273) and Magee ECHO (Pittsburgh, n= 106) cohorts provided blood samples in early-, mid-, and late-pregnancy. Immune markers, including those from the T-helper 1 (TH1), T-helper 2 (TH2), regulatory, and early activation pathways, were measured using Luminex assays. Daily $PM_{2.5}$ and NO_2 concentrations at residential addresses were estimated using spatiotemporal models. Generalized additive mixed-effects models were used to assess associations between interquartile range increases in pollutants (0–6-day lag) and immune marker levels, adjusting for gestational age, temperature, humidity, and relevant confounders.

Results:
Both cohorts had similar sociodemographic characteristics, and both showed NO_2-associated immune activation, with stronger effects in late-pregnancy. In Rochester, each 7.82 parts per billion (ppb) increase in NO_2 concentration was associated with a 0.24 pg/ml increase in interleukin-10 (IL-10) (95% confidence interval [CI]: 0.10, 0.38). In Pittsburgh, the same NO_2 increase was associated with a larger but less precise IL-10 increase (0.25 pg/ml, 95% CI: -0.05, 0.55). Unlike in Rochester, $PM_{2.5}$ in Pittsburgh was positively associated with IL-4 and IL-10. Across both cohorts, increased NO_2 and $PM_{2.5}$ were each associated with higher IL-4/interferon-gamma (IFN-γ) and IL-10/IFN-γ ratios.

Conclusion:
While both cohorts exhibited NO_2-related immune activation, associations with $PM_{2.5}$ were stronger in Pittsburgh, possibly due to differences in $PM_{2.5}$ composition. Findings suggest immune dysregulation associated with $PM_{2.5}$ and

NO_2 may influence pregnancy-related immune adaptations. Future research should examine associations between source-specific $PM_{2.5}$ and immune markers throughout pregnancy and assess the association of changes in immune markers relative to each other on pregnancy outcomes.

P53-02 | Prenatal $PM_{2.5}$exposure is associated with the development of the infant gut microbiome and fecal metabolome

Elizabeth A. Holzhausen[1], Haonan Li[1], Nathan Young[1], Devendra Paudel[1], Jeremy A. Sarnat[2], Fredrick Lurmann[3], Dean P. Jones[2], Michael I. Goran[4], Donghai Liang[2], Tanya L. Alderete[1]

[1] Johns Hopkins University, Baltimore, USA; [2] Emory University, Atlanta, USA; [3] Sonoma Technology Inc., Petaluma, USA; [4] Children's Hospital Los Angeles, Los Angeles, USA

Objective: Our previous work linked prenatal $PM_{2.5}$ exposure with the infant gut microbiome. Here, we used integrated, multi-omics analyses to assess the impact of prenatal $PM_{2.5}$ exposure on the infant gut microbiome and fecal metabolome, providing context to the biological consequences of $PM_{2.5}$ exposure.
Materials and Methods: Among 127 Latino mother-infant dyads from the Southern California Mother's Milk Study, we examined the associations between prenatal $PM_{2.5}$ exposure and the infant gut microbiome and fecal metabolome at 1-, 6-, 12-, 18-, and 24-months of age. The relationships between prenatal $PM_{2.5}$ exposure and gut microbial species were estimated using negative binomial mixed effects models adjusted for age, sex, socioeconomic status, season, maternal age, diet, mode of delivery, and maternal pre-pregnancy BMI. Among gut microbial species associated with prenatal $PM_{2.5}$ exposure, linear mixed effects models, adjusted for infant age, assessed their relationship with fecal metabolites. The Benjamini-Hochberg (BH) procedure was used to adjust for multiple testing ($P_{BH}<0.05$). Multi-omics analyses utilized mixOmics to identify key microbiota and metabolites linked to prenatal $PM_{2.5}$ exposure.
Results: Prenatal $PM_{2.5}$ was positively associated with 6 microbial species and negatively associated with 8 species. Higher prenatal $PM_{2.5}$ was associated a with higher abundance of 4 deleterious species and lower abundance of 5 beneficial species. Microbial abundance was linked with 147 metabolites with validated identification (i.e., Level-1). Multi-omics analysis identified metabolites acetyl-phenylalanine, guanidinosuccinate, indole, dethiobiotin, acetylglutamate, and isoleucine and microbial species *Selenomonas sputigena*, *[Clostridium] hylemonae*, and *Anaerocolumna chitinilytica* as most associated with prenatal $PM_{2.5}$ exposure.
Conclusion: Metabolites related to prenatal $PM_{2.5}$ are also linked with metabolic processes involving gut bacteria. These findings can provide insight into the possible pathophysiologic response to changes in the infant gut microbiome in the context of prenatal $PM_{2.5}$ exposure. Future work will incorporate untargeted fecal metabolites and will identify fecal metabolites of microbial origin.

P53-03 | Identifying critical windows and joint effects of prenatal air pollution and temperature exposure and lung function in Mexican schoolchildren: Findings from a prospective birth cohort study

Cheng-Yang Hu[1,2], Cecilia S. Alcala[1], Hector Lamadrid-Figueroa[3], Adriana Mercado Garcia[4], Marcela Tamayo-Ortiz[5], Ivan Gutierrez-Avila[1], Itai Kloog[1], Allan Just[6], Mike Z. He[1], Maayan Yitshak Sade[1], Nadya Rivera Rivera[1], Martha M. Tellez Rojo[4], Robert O. Wright[1,8], Rosalind J. Wright[1,7], Maria José Rosa[1,8]

[1] Icahn School of Medicine at Mount Sinai, Department of Environmental Medicine, New York, USA; [2] Anhui Medical University, Department of Epidemiology and Biostatistics, Hefei, China; [3] National Institute of Public Health, Department of Perinatal Health, Cuernavaca, Mexico; [4] National Institute of Public Health, Center for Nutrition and Health Research, Cuernavaca, Mexico; [5] Columbia University Mailman School of Public Health, Department of Environmental Health Sciences, New York, USA; [6] Brown University School of Public Health, Department of Epidemiology, Providence, USA; [7] Icahn School of Medicine at Mount Sinai, Department of Public Health, New York, USA; [8] Icahn School of Medicin at Mount Sinai, Institute for Exposomic Research, New York, USA

Objective: Exposure to air pollution and extreme temperatures during pregnancy has been associated with lung function in schoolchildren; however, windows of susceptibility have rarely been assessed and interactive effects are unknown. We aimed to identify critical windows of prenatal exposure to fine particulate matter ($PM_{2.5}$), nitrogen dioxide (NO_2), and extreme temperatures and their interactive effects on lung function in children and explore sex differences.

Materials and Methods: 429 mother-child dyads enrolled in the Programming Research in Obesity, Growth, Environment, and Social Stressors birth cohort study in Mexico City were included. Daily residential levels of pollutant/temperature exposures during pregnancy were generated from satellite-based models. Lung function was evaluated at ages 8–14 years-old, and modeled as age, height, and sex adjusted z-scores. Overall and sex-specific associations of exposures with lung function outcomes were evaluated using distributed lag non-linear models adjusted for covariates. Interactive effects were evaluated through the relative excess risk due to interaction and the attributable proportion.

Results: Prenatalhigher $PM_{2.5}$ exposure was associated with reduced lung function parameters: FEV_1 z-score (weeks 1–21, cumulative change: −0.23 [95% CI: −0.39, −0.07]), FVC z-score (weeks 13–19, cumulative change: −0.04 [95% CI: −0.08, −0.00]), $FEF_{25-75\%}$ z-score (weeks 1–20, cumulative change: −0.20 [95% CI: −0.36, −0.04]), and FEV_1/FVC ratio (weeks 6–16, cumulative change: −0.57 [95% CI: −0.11, −0.04]). Similarly, increased NO_2 exposure was associated with reduced FEV_1 z-score (weeks 1–16, cumulative change: −0.16 [95% CI: −0.31, −0.02]), $FEF_{25-75\%}$ z-score (weeks 13–16, −0.02 [95% CI: −0.04, −0.00]), and FEV_1/FVC ratio (weeks 6–15, −0.48 [95% CI: −0.96, −0.01]). In contrast, both warmer (weeks 1–8) and colder temperatures (weeks 9–18) showed positive associations with FVC z-score. Stronger associations were found in females. No interactive effects of air pollution and temperature were found.

Conclusions: Prenatal exposure to air pollution may impact long-term respiratory health in a sex-specific manner.

P53-04 | Hemoglobin levels, anemia, and solid fuel and mosquito coil use in pregnancy: Analysis of Accra Birth Cohort (ABC) baseline data

Nana Ama Wilson[1], Kate A. Kyeremateng[2], Barbara E. Mottey[1], Carissa Lange[1], Lilly Y. Adzrakor[3], Prince K. Anku[3], Selasie N. Clottey[3], Nancy Dery[3], Princess Fordjour[3], Geofrey K. Kotoka[3], Rhoda D. Kpabitey[3], Obed Kwao[3], Vivian Mensah[3], Philomina Oppong[3], John Quansah[3], Zaida Yahuza[3], Allison F. Hughes[3], Raphael E. Arku[1]

[1] University of Massachusetts, Amherst, Department of Environmental Health Sciences, Amherst, USA; [2] University of Massachusetts, Amherst, Department of Epidemiology, Amherst, USA; [3] University of Ghana, Legon, Department of Physics, Accra, Ghana

Background: Anemia in pregnancy is a well-recognized global health problem with health implications for mother and fetus. In sub-Saharan Africa (SSA), an estimated 45% of pregnant women are anemic. Besides the well-documented nutritional factors, identifying and addressing the modifiable environmental and behavioral risk factors will protect maternal and fetal health.

Objective: Using the ongoing Accra Birth Cohort (ABC) baseline data, we examined the associations of biomass fuel and mosquito coil use with hemoglobin levels and the risk of anemia in 2,326 pregnant women (mean age = 28.9 years), recruited across 30 health facilities in Accra, Ghana.

Method: We obtained data on hemoglobin levels at first antenatal visit along with self-reported primary cooking fuel, mosquito coil, and socio-demographic characteristics. We estimated associations of history of fuel [biomass versus clean fuel] and coil use with hemoglobin levels and anemia (hemoglobin < 11 g/dL) using linear and logistic regressions, adjusted for maternal age, BMI, gravida, marital status, educational level, infections, and income.

Results: The mean hemoglobin level was 11.1 g/dL (ranged: 5.3 -16.2), and 43.1% of the women were anemic at baseline. The prevalence of biomass and coil use were 15.7% and 43.8%, respectively. After adjustments, primary use of biomass fuel was associated with 0.24 g/dL lower hemoglobin (95% CI: -0.43, -0.04) and 31% greater odds of anemia [OR = 1.31 (95% CI: 0.97,1.77)] compared to clean fuel users. Similarly, mosquito coil use was associated with 0.12 g/dL lower hemoglobin (95% CI: -0.26, 0.01) and 24% greater odds of anemia [OR=1.24 (95% CI: 1.00,1.53)]) as compared with non-users.

Conclusion: Cooking with biomass fuels and the use of mosquito coil were independently associated with small decreases in hemoglobin levels during pregnancy. They also increased the odds of anemia. Quantitative exposure assessments are needed to confirm these associations.

P53-05 | Prenatal PM_{10} and $PM_{2.5}$ are associated with gut microbiome trajectories over the first two years of life

Nathan D. Young[1], Haonan Li[1], Elizabeth A. Holzhausen[1], Vishal Midya[2], Tanya L. Alderete[1]

[1] Johns Hopkins University, Department of Environmental Health and Engineering, Baltimore, USA; [2] Mount Sinai, Department of Environmental Medicine, New York, USA

Background: Research suggests prenatal exposure to particulate matter may alter the infant gut microbiome, which is critical for immune and metabolic development. However, most existing research only offers cross-sectional glimpses into these associations rather than longitudinal observations of how gut bacteria composition changes over time.

Methods: Participants included 93 mother-infant dyads from the Southern California Mother's Milk Study. Prenatal exposure to particulate matter (PM_{10}, $PM_{2.5}$) was modeled using residential address histories. We implemented a novel methodological approach called Recurrence Quantification Analysis (RQA), which quantifies patterns of stability in longitudinal time series. We applied RQA to estimate a measure of stability (recurrence rate) for relative microbe abundances across the 1-, 6-, 12-, 18-, and 24-month postpartum periods. Multivariable linear regression was then utilized to assess associations between $PM_{2.5}$ and PM_{10} exposure with microbe recurrence rates, controlling for mother age, prepregnancy body mass index, socioeconomic status, mode of delivery, season of birth, infant sex, cumulative proportion of breastfeedings, and age of solid food introduction.

Results: Higher $PM_{2.5}$ was associated with lower stability of beneficial microbes, such as Lactobacillus (a probiotic that prevents pathogenic diseases; β=-3.29, p=0.01), and was associated with higher stability of potentially harmful microbes, such as Kluyvera (which can cause opportunistic infections; β=3.68, p=0.02). Higher PM_{10} was also associated with lower stability of Lactobacillus (β=-1.19, p=0.01), as well as Pediococcus and Streptomyces (beneficial probiotics: β=-0.76, p=0.04 and β=-0.54, p=0.05 respectively). PM_{10} was also associated with higher stability of Kluyvera (β=1.24, p=0.01) and other potentially harmful microbes such as Desulfovibrio (an opportunistic pathobiont previously associated with Parkinson's; β=0.69, p=0.04).

Conclusion: Our study offers promising evidence that RQA may be a novel method for longitudinal gut microbiome analysis. Results suggest that exposure to $PM_{2.5}$ and PM_{10} may be associated with the biodynamic stability of several biologically relevant gut microbes in early life.

P53-06 | Biomarkers of placental and renal dysfunction in association with gestational phthalate exposure and preeclampsia

Erin McNell[1,2], Danielle Stevens[1], Suzanne Fenton[3], Elena Sinkovskaya[4], Ann Przybylska[4], George Saade[4], Alfred Abuhamad[4], **Kelly Ferguson**[1]

[1] National Institute of Environmental Health Sciences, Epidemiology Branch, Durham, USA; [2] University of North Carolina at Chapel Hill, Curriculum in Toxicology and Environmental Medicine, Chapel Hill, USA; [3] North Carolina State University, Center for Human Health and the Environment, Raleigh, USA; [4] Old Dominion University, Eastern Virginia Medical School, Norfolk, USA

Preeclampsia is characterized by maternal hypertension and acute organ dysfunction in pregnancy. Preeclampsia etiology remains unclear, but hypotheses involve placental and renal dysfunction. Exposure to endocrine-disrupting chemicals, including phthalates, may heighten risk. This study leverages urinary biomarkers to investigate these mechanisms in association with preeclampsia and phthalate exposure within a prospective pregnancy cohort (N=291). Repeated urine samples were collected; pregnancy-average phthalate metabolite concentrations were calculated, and urinary concentrations of placental and renal biomarkers were measured in early (median 13 weeks), middle (median 21 weeks), and late-pregnancy (median 33 weeks). Placental biomarkers included soluble Fms-like kinase-1 (sFlt-1), placental growth factor (PlGF), the sFlt-1:PlGF ratio, and soluble endoglin (sEng). Renal biomarkers included kidney injury molecule-1 (KIM-1), neutrophil gelatinase-associated lipocalin (NGAL), and nephrin. First, we examined associations of biomarkers in each pregnancy stage with hazard ratios (HRs) of preeclampsia using Cox proportional hazards models. Second, we examined associations of phthalate metabolites with biomarkers using linear mixed effects models. Covariates included maternal age, body mass index at enrollment, education, race and ethnicity, parity, and fetal sex. Late-pregnancy sFlt-1 was associated with increased HR of preeclampsia (HR: 3.48 [95% confidence interval: 1.41, 8.61]), whereas late-pregnancy PlGF was inversely associated with HR of preeclampsia (HR: 0.33 [0.15, 0.72]). sFlt-1:PlGF was increasingly associated with preeclampsia throughout gestation (early-pregnancy HR: 1.00 [0.49, 2.03], middle-pregnancy HR: 2.01 [0.81, 4.99], late-pregnancy HR: 9.00 [3.38, 23.98]). Middle-pregnancy NGAL was also associated with HR of preeclampsia (HR: 1.93 [1.16, 3.22]). Associations of KIM-1, sEng, and nephrin with preeclampsia, and of phthalate metabolites with placental and renal biomarkers, were null. In summary, sFlt-1, sFlt-1:PlGF, and NGAL were associated with elevated hazard of developing preeclampsia, though these biomarkers were not associated with phthalate metabolites. Our findings highlight the utility of urinary biomarkers and support that placental and renal dysfunction may be involved in preeclampsia.

P53-07 | Relationships between prenatal exposure to endocrine-disrupting chemicals and immune markers in neonatal blood spots

Jennifer Ames[1], Meredith Anderson[1], Paul Ashwood[2], Kimberly Berger[3], Kristen Lyall[4], Judy Van de Water[2,5], Lauren Weiss[6], Cathleen Yoshida[1], Lisa Croen[1]

[1] Kaiser Permanente Northern California, Division of Research, Pleasanton, USA; [2] University of California, Davis, MIND (Medical Investigations of Neurodevelopmental Disorders) Institute, Davis, USA; [3] Sequoia Foundation, Berkeley, USA; [4] Drexel University, A.J. Drexel Autism Institute, Philadelphia, USA; [5] University of California, Davis, Department of Public Health Sciences, Division of Rheumatology, Allergy and Clinical Immunology, Davis, USA; [6] University of California San Francisco, Weill Institute for Neurosciences, San Francisco, USA

Background and Aim: Prenatal exposure to endocrine-disrupting chemicals (EDCs) has been linked to adverse child neurodevelopment, possibly through early life immune activation. Some evidence suggests EDC exposures may influence the immune environment but whether EDCs influence neonatal immune physiology is unclear.
Methods: We analyzed data from 439 general population controls in the Early Markers for Autism (EMA) Study, a case-control study of children born in Southern California (2000-2003). Eleven polychlorinated biphenyls (PCBs), 6 brominated flame retardants (BFRs), 2 organochlorine pesticides (OCPs), 6 polyfluoroalkyl substances (PFAS), and 42 cytokines/chemokines were measured in neonatal blood spots. We assessed individual relationships between log-transformed EDCs and log-transformed immune markers with spearman correlations and linear regression models adjusted for child's sex, birth month and year, and maternal demographics.
Results: Spearman correlations showed 33 immune markers were weakly correlated with all 4 EDC classes (spearman rho ranging from -0.17 to 0.15). Several EDCs, including PBDE99, PBDE100, PBDE153, PCB28, and PFHxS, were positively associated with multiple immune markers spanning pathways of Th1 antiviral responses, Th2 allergic responses, chronic inflammation, and immune surveillance. Et-PFOSA was negatively associated with 8 immune markers implicated in chronic and acute inflammation and immune surveillance. Several immune markers, spanning Th1 (IP-10, I-TAC), Th2 (IL-13, Eotaxin-2), and immuno-developmental pathways (6CKINE, MPIF-1), were also correlated with multiple EDCs. After false-discovery rate correction, only four associations remained statistically significant in covariate-adjusted linear regression models: PCB194, PCB196/203, and PCB199 were positively associated with Eotaxin-2, a chemokine implicated in allergic inflammation and asthma while PCB28 was positively associated with MCP-2 (adj-β=0.07, 95% CI: 0.04, 0.10), a chemokine important to newborn immune defense.
Conclusions: These findings suggest that prenatal EDC exposure, particularly to PCBs, may influence diverse neonatal immune and inflammatory pathways. Future analyses will consider mixture approaches to account for correlations among EDCs and immune markers.

P53-08 | The role of melatonin in the effects of exposure to low-level artificial light at night during pregnancy on placental function and pregnancy outcome

Jiali Wang[1,2], Jian Xu[1,2]

[1] Shanghai Jiao Tong University, School of Medicine, The International Peace Maternity and Child Health Hospital, Shanghai, China; [2] Shanghai Key Laboratory of Embryo Original Diseases, Shanghai, China; [3] Shanghai Jiao Tong University School of Public Health, Department of Maternal and Child Health, Shanghai, China

Objective: Exposure to low-level artificial light at night (L-ALAN) during pregnancy is becoming increasingly common and is recognized as a potent factor inhibiting melatonin (MT) synthesis. MT regulates circadian rhythm synchronization and maintains placental immune homeostasis. This study aims to investigate the role of MT dysfunction in the effects of L-ALAN on placenta and pregnancy outcomes.
Materials and Methods: The mouse model of L-ALAN during pregnancy was established and three groups including the control (normal light-dark cycle), L-ALAN (exposed to light of 2 lux from 7pm-7am during pregnancy) and "L-ALAN + MT (gavage, 10 mg/kg/day)" groups were created. On gestational day 17.5, maternal serum MT and progesterone /estrogen levels were measured, spleen and liver indices were evaluated. Placental MT levels and placental function were assessed. RNA-seq analysis was performed to identify genes expressed differentially and related pathways. The survival rates of neonatal mice were evaluated.
Results: Compared to the control group, in the L-ALAN group, maternal serum and placenta MT levels decreased significantly, accompanied by disrupted progesterone and estrogen rhythms, spleen and liver indices decreased, and less weight gain and lower placental weight were observed. In the L-ALAN group, HE staining showed abnormal placental morphology and decreased cell proliferation. MT supplementation improved placental function. RNA-seq analysis revealed that L-ALAN upregulated inflammatory and oxidative stress pathways while inhibiting pathways related to vascular contraction, attachment, and migration in the placenta. Neonatal body weight and survival rate were lower in the L-ALAN group, whereas MT supplementation improved survival rate of neonatal mice.
Conclusion: L-ALAN during pregnancy reduced maternal and placenta MT levels, leading to placental dysfunction and maternal immune-hormonal imbalance, which may in turn resulted in decreased neonatal body weight and survival rate. MT supplementation mitigated these adverse pregnancy outcomes.This study was supported by the National Natural Science Foundation of China (82473657).

P53-09

This abstract has been withdrawn.

P53-10 | Association of phenol and paraben exposure with maternal blood pressure and hypertension during pregnancy in the PROTECT cohort

Julia R. Varshavsky[1,2], John D. Meeker[4], Emily Zimmerman[3], Megan L. Woodbury[2], Max T. Aung[5], Zaira Y. Rosario-Pabon[8], Amber L. Cathey[4], Carmen M. Vélez-Vega[8], José F. Cordero[6], Akram Alshawabkeh[2], Stephanie M. Eick[7]

[1] Northeastern University, Department of Public Health and Health Sciences, Boston, USA; [2] Northeastern University, Department of Civil and Environmental Engineering, Boston, USA; [3] Northeastern University, Department of Communication Sciences and Disorders, Boston, USA; [4] University of Michigan School of Public Health, Department of Environmental Health Sciences, Ann Arbor, USA; [5] University of Southern California, Department of Population and Public Health Sciences, Keck School of Medicine, Los Angeles, USA; [6] University of Georgia, Department of Epidemiology and Biostatistics, College of Public Health, Athens, USA; [7] Emory University, Gangarosa Department of Environmental Health, Rollins School of Public Health, Atlanta, USA; [8] University of Puerto Rico Medical Sciences Campus, Department of Social Sciences, Graduate School of Public Health, San Juan, Puerto Rico

Objective:
Phenols and parabens, chemicals used in consumer and personal care products, have been associated with pregnancy complications, but their role on maternal blood pressure and pregnancy-related hypertensive disorders, including gestational hypertension and preeclampsia, is not well understood.

Materials and Methods:
Using data from 1,433 PROTECT cohort study participants in Puerto Rico, we investigated associations between phenol and paraben exposures on maternal blood pressure. We included systolic and diastolic blood pressure (SBP and DBP) and hypertension during pregnancy, defined as stage 1 or 2 hypertension. Metabolites tested included two bisphenols, two dichlorophenols, four parabens, triclocarban, triclosan, and benzophenone-3. Individual metabolites and mixture effects on BP and hypertension were measured using generalized regression models and quantile g-computation cross-sectionally at two-time points during pregnancy (16–20 and 24–28 weeks' gestation).

Results:
Tested metabolites were associated with lower SBP and higher DBP at both time points in pregnancy, with evidence of non-linear exposure-response relationships for some metabolites. For example, the direction of association with SBP at 16–20 weeks' gestation was positive and inverse for medium and high methyl paraben (M-PB) exposure groups, respectively, compared to the low exposure group, while the DBP association at 24–28 weeks' gestation was inverse and positive for medium and high M-PB exposure groups, respectively. We also observed higher odds of hypertension during pregnancy at 24–28 weeks' gestation associated with phenols and parabens exposure at 16–20 weeks' gestation, with an adjusted mixture odds ratio (OR) = 1.48 (95% CI: 1.02, 2.15).

Conclusions:
Exposure to phenol and paraben metabolites may increase DBP and risk of hypertension later in pregnancy, which has important implications for fetal and maternal health, given that increased DBP is also a risk indicator for preeclampsia. Research on the role of the studied pollutants on gestational hypertension and its complications is warranted.

P53-11 | Prenatal PAH exposure and epigenetic gestational age acceleration in two New York City cohorts

Pei Wen Tung[1], Abigail Gaylord[1], Jia Guo[2], Frederica Perera[1], Shuang Wang[2], Julie Herbstman[1], Allison Kupsco[1]

[1] Columbia University, Department of Environmental Health Sciences, New York, USA; [2] Columbia University, Department of Biostatistics, New York, USA

Objective: Epigenetic gestational age acceleration (EGAA), measured via DNA methylation, is a biomarker for physiological development and may reflect the impacts of prenatal environmental exposure. Prenatal polycyclic aromatic hydrocarbons (PAHs) exposure has been linked to birth outcomes and neurodevelopmental disorders in human studies. Yet, impacts of PAH on cord blood EGAA remain underexplored. Thus, we hypothesized that prenatal PAH exposure would be associated with EGAA variations.
Material and Methods: Participants were enrolled in the Mothers and Newborns (MN; N=359; 1998-2006) and Fair Start (FS; N=129; 2015-onging) longitudinal birth cohorts in NYC. PAH levels were measured by personal backpack monitors worn by women for 48 hours during their third trimester. Cord blood DNA methylation was analyzed using 450K and EPIC arrays. EGAA was calculated using the Knight and Bohlin clocks. We examined the relationship between the sum of 8 airborne PAHs and EGAA, adjusted for relevant covariates, in each cohort individually using linear models, and combined in generalized estimating equations (GEE) with cohort as a cluster variable.
Results: The correlation between Knight and Bohlin clocks was 0.56. Though the effect direction was consistent across clocks and cohorts, we observed a significant association between total PAH and Knight EGAA in MN participants (β=0.90, 95% CI: [0.82, 0.98], p=0.02) and Bohlin EGAA (β=0.91, [0.84, 0.98], p=0.01) in FS participants. GEE model results showed every doubling of total PAH levels was associated with both Knight (β=0.87, [0.84, 0.91], p<0.001) and Bohlin EGAA (β=0.95, [0.92, 0.98], p=0.001).
Conclusion: The effect of prenatal PAH exposure on EGAA was consistent for both clocks and cohorts, highlighting their potential as biomarkers for environmental exposures. However, the implications of a positive association between PAH and EGAA remain to be elucidated. These findings underscore the need for further research into the long-term implications of PAH-related epigenetic changes for child health.

P53-12 | A time-series analysis for the impacts of gestational exposure to fine particulate matter on pregnancy loss in New Jersey, USA

Seulkee Heo[1], Michael Leung[2], Marianthi-Anna Kioumourtzoglou[3], Michelle Bell[1]

[1] Yale University, School of the Environment, New Haven, USA; [2] Harvard University, T.H. Chan School of Public Health, Boston, USA; [3] Columbia University, Mailman School of Public Health, New York, USA

Studies suggest impacts of air pollution on birth outcomes such as birthweight and preterm birth, but evidence is limited for pregnancy loss. This time-series analysis evaluated the impact of weekly fine particulate matter (PM2.5) exposure on pregnancy loss, assuming the weekly number of live birth-identified conceptions (LBIC) results from fetuses conceived in the same week, two weeks after the last menstrual period. The number of LBIC for each week for all 21 counties was calculated using birth certificate data in New Jersey, 1 January 2000 to 3 April 2016 (*n*=1,198,002). Population-weighted county-level weekly $PM_{2.5}$ levels were estimated from modeled data.

Multivariable Poisson regression models, adjusted for time trends and meteorologic factors, assessed the critical window of the pregnancy loss risk from the conception week to the 40th gestational week. We estimated relative risks (RRs) of LBIC counts for 10 μg/m³ increase in $PM_{2.5}$ weekly exposure. We found increasing risks of pregnancy loss (i.e., RR<1 for LBIC) for increases in exposures. The lag-specific results showed the highest associations for pregnancy loss during the 22nd gestational week (RR=0.993, 95% confidence intervals [CI]: 0.990, 0.995). Given the previous findings of 10–50% loss of all conceptions and our risk estimates, a 10 μg/m³ increase in exposure during 0–40 gestational weeks would contribute to 5.2 (95% CI: 3.9–6.6) to 9.3 (95% CI: 7.1–11.9) pregnancy losses per 1,000 conceptions per week. These findings have important implications for critical exposure windows of pregnancy loss for $PM_{2.5}$exposure through gestation, which can inform public health policies for the adverse health effects of air pollution on fetal health. This work was supported by the National Institute of Environmental Health Sciences and the National Institute on Aging.

P53-14 | Extreme Heat Exposure from Pregnancy to Postpartum and Cardiovascular Disease in Gestational Diabetes

Sohee Wang[1,2], Youngrin Kwag[1], Jieun Min[1,2], Minho Kim[3], Yoonkyung Chang[4], Tae-Jin Song[5], Eunhee Ha[1,6]

[1] Ewha Womans University, Department of Environmental Medicine, College of Medicine, Seoul, South Korea; [2] Ewha Womans University, Graduate Program in System Health Science and Engineering, College of Medicine, Seoul, South Korea; [3] Ewha Womans University Seoul Hospital, Information Department, Seoul, South Korea; [4] Ewha Womans University College of Medicine, Department of Neurology, Mokdong Hospital, Seoul, South Korea; [5] Ewha Womans University College of Medicine, Department of Neurology, Seoul Hospital, Seoul, South Korea; [6] Ewha Womans University, Institute of Ewha-SCL for Environmental Health (IESEH) College of Medicine, Seoul, South Korea

Objectives

Extreme heat exposure is an emerging public health concern during pregnancy and postpartum. However, its impact on postpartum cardiovascular disease (CVD) risk remains unclear. This study examines associations between extreme heat exposure and CVD risk up to two years postpartum, focusing on women with gestational diabetes mellitus (GDM).

Methods

We conducted a retrospective cohort study using National Health Insurance Service data of 453,367 women who gave birth between 2012 and 2018 in seven major metropolitan cities in South Korea. Gestational diabetes mellitus (GDM) was identified by ICD-10 code O24 (N=171,951). Cardiovascular disease (CVD) was defined by ICD-10 codes beginning with "I". Heat exposure was assessed by counting heatwave days (temperature ≥33°C) from pregnancy to two years postpartum, categorized in 10-day increments. Logistic regression was performed adjusting for GDM, gestational hypertension, maternal age, and income level.

Results

Higher heat exposure was associated with an increased risk of cardiovascular disease in the total population (OR [95% CI]: 1.01 [1.00, 1.01]), with a stronger effect in women with GDM (1.03 [1.01, 1.04]). Older mothers had a higher risk (1.05 [1.02, 1.08]) than younger ones (1.01 [1.00, 1.03]). Subgroup analyses (ICD-10 I70-79, I60-69, I20-25, I10-15) showed heat exposure increased cardiovascular risk in the total population (1.04 [1.03, 1.06]), women with GDM (1.06 [1.04, 1.09]) and was highest in older mothers (1.07 [1.03, 1.11]).

Conclusion

Extreme heat exposure from pregnancy to the postpartum is associated with an increased risk of cardiovascular disease, particularly in women with GDM, with a stronger effect observed in older mothers. These findings highlight the need for targeted interventions in high-risk populations.

Acknowledgement

This research was supported by the BK21 FOUR (Fostering Outstanding Universities for Research) funded by the Ministry of Education (MOE, Korea) and National Research Foundation of Korea (NRF-5199990614253, Education Research Center for 4IR-Based Health Care).

P53-15 | Evaluating the Mediating Role of Gestational Phthalate Exposure Biomarkers in the Associations Between Race and Ethnicity and Birthweight

Amir J. Lueth[1], Danielle R. Stevens[1], Elena Sinkovskaya[2], Ann Przybylska[2], George Saade[2], Alfred Abuhamad[2], Kelly K. Ferguson[2]

[1] National Institute of Environmental Health Sciences, Epidemiology Branch/ Perinatal Epidemiology, Durham, USA; [2] Eastern Virginia Medical School, Department of Obstetrics and Gynecology/ Division of Maternal-Fetal Medicine, Norfolk, USA

Background: Prenatal exposure to phthalates is higher among non-Hispanic Black (NHB) compared to non-Hispanic White (NHW) women and may contribute to racial/ethnic disparities in birth weight.
Objective: Investigate the mediating role of phthalate biomarkers in the association between race/ethnicity and birthweight z-scores.
Methods: We analyzed data from 271 pregnancies in the Human Placenta and Phthalates Study, a prospective cohort with recruitment from 2017-2020. We measured and averaged concentrations of phthalate metabolites in urine samples from up to eight-time points across pregnancy and created summary measures for metabolites of the same parent compound. Linear regression models were used to estimate the differences in phthalate exposure biomarkers by race/ethnicity, associations between race/ethnicity and birthweight z-scores, and associations between phthalate exposure biomarkers and birthweight z-scores. Mediation analysis examined whether phthalate exposure biomarkers contribute to the relationship between race/ethnicity and birthweight z-scores.
Results: NHB participants exhibited higher urinary concentrations of most phthalate metabolites than NHW participants. For example, monoethyl-phthalate (MEP) concentrations were 250% higher (95% CI: 173, 349) in NHB compared to NHW participants. Additionally, NHB participants had 0.43 lower birthweight z-scores (95% CI: -0.67, -

0.19) compared to NHW participants. Di-iso-butyl phthalate metabolites (ΣDiBP) and MEP were inversely associated with birthweight z-scores. For example, an interquartile range difference in ΣDiBP was associated with -0.16 (95% CI: 0.32, -0.002) lower birthweight z-scores. We performed mediation analyses for ΣDiBP, MEP, and Di-n-butyl phthalate. We observed a modest and non-significant mediation of the association between race/ethnicity and birthweight z-scores by these biomarkers.

Conclusions: In a diverse pregnancy cohort with well-characterized phthalate exposures, we observed differences in exposures by race/ethnicity, associations between race/ethnicity and birthweight z-scores, and associations between some exposures and birthweight z-scores. Yet, we observed only modest and non-significant mediation of the association between race/ethnicity and birthweight z-scores by phthalate exposure biomarkers.

P53-16 | Prenatal phthalate exposure is associated with higher fetoplacental ratio and lower placental weight

Ayushi Bommireddipalli, Linda Kahn, Whitney Cowell, Jacqueline Erler, Leonardo Trasande, Kurunthachalam Kannan, Shilpi Mehta-Lee

NYU Langone Health, Environmental Pediatrics, New York, USA

Objective:

Fetoplacental ratio (FPR), the ratio of birthweight (BW) to placental weight (PW), is an indicator of placental efficiency. Changes in FPR are linked to poor pregnancy outcomes and child health risks. Bisphenols and phthalates are endocrine disruptors found in plastics and personal care products that can cross the placenta and have been linked to pregnancy complications and adverse child health outcomes. We examined prenatal exposure to these chemicals in relation to FPR as a possible explanation for these risks.

Materials and Methods:

Our analysis included 392 participants in the NYU Children's Health and Environment Study with data on prenatal chemical exposure, BW, and PW from singleton live births. Chemical concentrations were creatinine-standardized and log-transformed. We calculated molar sums of bisphenols and metabolites of low and high molecular weight phthalates (LMWP, HMWP), diethylhexyl phthalate (DEHP), and antiandrogenic phthalates. Concentrations across trimesters were averaged. Linear regression models were adjusted for gestational age, fetal sex, parity, and pre-pregnancy body mass index. Analyses were stratified by fetal sex. A sensitivity analysis excluded preterm births.

Results:

HMWP were positively associated with FPR in the combined fetal sex sample (beta=0.26, [0.01, 0.50]) with a similar trend for DEHP and antiandrogenic phthalates (betas=0.21 [-0.04, 0.45] and 0.21 [-0.04, 0.45], respectively). Stratified analyses revealed that these results were driven by female fetuses, among which LMWP were also associated with higher FPR (beta=0.23 [0.003, 0.45]. While we observed no associations between chemicals and BW in combined or sex-stratified models, among female fetuses, HMWP, LMWP, DEHP, di-n-octylphthalate, and bisphenols had negative associations with PW. Results were consistent excluding preterm births.

Conclusion:

Among female fetuses, prenatal exposure to DEHP, HMWP, LMWP, and antiandrogenic phthalates are associated with higher FPR due to reduced PW. This may indicate increased placental efficiency, although further mechanistic research is necessary to confirm this theory.

P53-17 | Associations Between Persistent Organic Pollutant Exposure During Pregnancy and Post-delivery with Adiposity Trajectories in Women with Gestational Diabetes Mellitus

Chenyu Qiu[1], Wu Chen[1], Jiawen Liao[1], Zhenchun Yang[1], Jiayuan Hao[1], Frank D. Gilliland[1], Thomas A. Buchanan[2], Anny H. Xiang[3], Zhanghua Chen[1]

[1] Keck School of Medicine of the University of Southern California, Department of Population and Public Health Sciences, Los Angeles, USA; [2] Keck School of Medicine of the University of Southern California, Department of Medicine, Los Angeles, USA; [3] Kaiser Permanente Southern California, Department of Research & Evaluation, Pasadena, USA

Objective: This study aims to assess the longitudinal effects of persistent organic pollutants (POPs) during pregnancy and post-delivery on the trajectory of adiposity indicators over time among women with gestational diabetes mellitus (GDM).

Methods: 102 Hispanic women (mean age=30.7±5.7 years) were followed for up to 12 years after the index pregnancy with GDM. Plasma concentrations of 40 POPs including 21 polychlorinated biphenyls (PCBs), 7 organochlorine pesticides (OCPs), 5 polybrominated diphenyl ethers (PBDEs), 2,2',4,4',5,5'-hexabromobiphenyl, and 6 per- and polyfluoroalkyl substances (PFAS) were measured from archived samples collected at 3rd trimester of pregnancy and 15-60 months post-delivery. Adiposity indicators [body mass index (BMI) and body fat percentage] were obtained every 12-15 months until development of type 2 diabetes, lost-to-follow-up or 12-years post-delivery. Trajectories of adiposity indicators were analyzed for associations with averaged plasma concentrations of each POP during pregnancy and post-delivery using linear mixed effects models adjusted for age, breastfeeding and calorie intake.

Results:

Both BMI and body fat percentage significantly increased during the post-delivery period ($p=0.03$ and 0.05, respectively). Plasma concentrations of hexachlorobenzene, oxychlordane, and trans-nonachlor from pregnancy to post-delivery were associated with annual increases in BMI over 12 years ($p<0.05$, FDR<0.05), adjusting for covariates. Plasma perfluorononanoate (PFNA) and linear perfluorooctanoate (n-PFOA) were associated with annual increase in body fat percentage, while linear perfluorooctane sulfonate (n-PFOS), sum of perfluoromethylheptane sulfonates (Sm-PFOS), and their combined total were inversely associated with body fat percentage ($p<0.05$, FDR<0.05).

Conclusion:

Higher OCP, PFNA and n-PFOA levels during index pregnancy and postpartum were associated with faster increases in BMI and body fat percentage over 12-years after GDM-complicated pregnancy among Hispanic women, suggesting that POPs may have longitudinal effects on adiposity in high-risk group. PFNA and n-PFOA showed negative correlations with PFOS, indicating that further exposure mixture analysis is needed to identify detrimental PFAS affecting body fat.

P53-18 | Heavy Metal Exposure in Lebanese Infants: Cord Blood Analysis and Predictors of Prenatal Contamination

Emile Whaibeh[1,2], Jowy Abi Hanna[1,2], Norma Aouad[4], Alicia Abi Nader[1,2], Cyrille Khalil[2,3], Georges Abi Tayeh[4,5], Myriam Mrad[2,3]

[1] Saint-Joseph University of Beirut, Institut Supérieur de Santé Publique, Faculty of medicine, Beirut, Lebanon; [2] University of Balamand, Public Health Department, Faculty of Health Sciences, Beirut, Lebanon; [3] Health and Environment Response Agency (HERA), Beirut, Lebanon; [4] Hôtel-Dieu de France University Hospital, Beirut, Lebanon; [5] Saint Joseph Fertility Center, Beirut, Lebanon

Objective: The Environmental Exposures in Lebanese Infants (EELI) Study is a longitudinal birth cohort, examining environmental exposures' impact on infant neurocognitive and psychomotor development, focusing on air pollution (PM2.5), chemicals, and climate change's impacts. The current paper assesses toxic and essential heavy metal levels in Lebanese newborns' cord blood and their sociodemographic and environmental predictors.
Materials and Methods: Cord blood samples (N=75) were collected from newborns in Lebanon and analyzed for eight toxic metals—antimony, arsenic, chromium, nickel, cadmium, mercury, thallium, and lead—and six essential metals—molybdenum, manganese, cobalt, copper, zinc, and selenium—at RECETOX laboratories. Descriptive statistics, bivariate tests, and multivariate regression analyses identified significant exposure predictors.
Results: All toxic heavy metals were detected in cord blood samples, indicating prenatal exposure. Lead (median: 5.45 µg/L, 95th percentile: 22.4 µg/L) and mercury (median: 0.7 µg/L, 95th percentile: 2.01 µg/L) were among the highest detected. Essential metals such as zinc, copper, and manganese exhibited high variability. Proximity to highways, parking lots, and industrial areas was linked to increased antimony, arsenic, lead, and zinc levels, suggesting traffic and industrial emissions as exposure sources. Occupational factors, built environment features, and lifestyle elements—including home renovations, energy sources, and pesticide use—were also significant predictors.
Conclusion: This study provides an assessment of heavy metal toxicity in Lebanese infants. These findings emphasize the need for regulations, biomonitoring, and targeted interventions to mitigate maternal and fetal exposure. A comprehensive neurodevelopmental evaluation will be conducted to further investigate the impact of heavy metal exposure on early childhood development using the Ages and Stages Questionnaire.
Funding: This research is supported by an intramural fund from the University of Balamand and partially by an NIH Fogarty International Center grant (Reference: [1U01TW012237-01]). We thank RECETOX laboratories, the Ministry of Public Health, and the World Health Organization for their support.

P53-19 | Associations between brominated and organophosphate flame retardant levels and inflammatory biomarkers in maternal blood, cord blood, and placental tissue at mid-gestation

Vince Nguyen[1], Lin Li[2], Dana Goin[3], Neha Sehgal[4], Rachel Morello-Frosch[5,6], Tracey Woodruff[6], Julia Varshavsky[7], Stephanie Gaw[2], Joshua Robinson[2,6], **Stephaie Eick**[4]

[1] Emory University Rollins School of Public Health, Department of Epidemiology, Atlanta, USA; [2] University of California, San Francisco, Center for Reproductive Sciences, Department of Obstetrics, Gynecology and Reproductive Sciences, San Francisco, USA; [3] Columbia University Mailman School of Public Health, Department of Epidemiology, New York, USA; [4] Emory University Rollins School of Public Health, Gangarosa Department of Environmental Health, Atlanta, USA; [5] University of California, Berkeley, Department of Environmental Science, Policy and Management and School of Public Health, Berkeley, USA; [6] University of California, San Francisco, Program on Reproductive Health and the Environment, Department of Obstetrics, Gynecology and Reproductive Sciences, San Francisco, USA; [7] Northeastern University, Department of Public Health and Health Sciences, Boston, USA

Objective: Prenatal exposures to flame retardants (FRs), including legacy polybrominated diphenyl ethers (PBDEs) and alternative organophosphate FRs (OPFRs), are associated with adverse pregnancy outcomes.In animal models and cell models, inflammation has been proposed as a potential pathway of FR-toxicity; however, data in humans is limited.In this study, we leveraged mid-gestation maternal and fetal biospecimens to assess whether flame retardants are associated with diverse inflammatory mediators.

Material and Methods: Participants in this study included a demographically diverse group of pregnant women undergoing elective second trimester terminations in the San Francisco Bay Area (N=65). Four PBDEs (BDE-47, BDE-99, BDE-100, BDE-153) and four OPFRs (BCEP, BCIPP, BDCPP, DPHP) were quantified via mass spectrometry in mid-gestation maternal serum and urine samples, respectively. We measured 20 unique cytokines in matched maternal serum, cord blood, and placental tissue collected during mid-gestation by Luminex array. Linear regression was used to assess associations between individual FRs and cytokines, while quantile g-computation was used to assess mixture effects. All models were adjusted for gestational age and body mass index at time of sample collection, maternal age, education, insurance.

Results: A one-quartile increase in the mixture of PBDEs alone was significantly associated with increased maternal serum levels of CCL17 (Ψ=0.22, 95% CI=0.03, 0.42) and CCL2 (Ψ=0.21, 95% CI=0.06, 0.37). A one-quartile increase in the combined mixture of PBDEs and OPFRs was associated with significant increases in maternal serum levels of CCL2, CCL3, CC17, and CCL22. In contrast, BDE-47 and BDE-100 were associated with reductions in placental CCL2 in individual linear regression models (β=-0.57, 95% CI=-1.06, -0.0 and β=-0.44, 95% CI= -0.82, -0.06, respectively). No significant associations were observed in quantile g-computation models when cord blood cytokines were the outcome.**Conclusion:** Prenatal exposure to FRs, particularly PBDEs, was significantly associated with differences in maternal serum inflammatory profiles during mid-pregnancy.

P53-20 | Extreme Heat Exposure During Pregnancy and Birth Outcomes: A Review of Epidemiological Evidence and Policy Implications

Rebecca Ramos, Paulina L. Ramirez, Rietta S. Wagoner, Tuo Liu

University of Arizona, Public Health, Tucson, USA

Background/Objective:
Extreme heat events are increasing, raising concerns about their impact on maternal and infant health. Pregnant individuals are particularly vulnerable to heat exposure, which has been linked to adverse birth outcomes such as low birth weight, preterm birth, and stillbirth. This literature review synthesizes current epidemiological evidence on the relationship between maternal heat exposure and birth outcomes.

Material and Methods:
A comprehensive literature search was conducted using PubMed, Scopus, Web of Science, and Embase. Peer-reviewed epidemiological studies published in English that assessed maternal exposure to extreme heat and birth outcomes were included. Studies were evaluated for heat exposure characterization, birth outcome measures, statistical modeling approaches, and potential confounders such as gestational age and socioeconomic factors.

Results:
The review identified 20 studies from various geographical regions, with cohort and case-control study designs being the most common. Despite differences in heat exposure definitions (absolute temperature thresholds, percentiles, or heat waves), most studies reported a significant association between high temperatures and lower birth weights, mainly when exposure occurred during critical gestational windows. However, some studies found no association, potentially highlighting the need for standardized exposure assessment methods.

Conclusions:
The evidence suggests that maternal heat exposure increases the risk of adverse birth outcomes, emphasizing the need for targeted public health interventions. Policy recommendations include integrating heat risk education into prenatal care, strengthening workplace protections for pregnant individuals in high-heat environments, and expanding access to cooling resources in vulnerable communities. Further research is needed to standardize heat exposure metrics and explore the long-term health effects of prenatal heat exposure on offspring.

P53-21 | Widespread Pesticide Exposure Measured in Silicone Wristbands in a Diverse Cohort of Pregnant and Postpartum Women

Erin M. DeMicco[1], Aimee J. Lansdale[1], Jessica Chen[1], Joshua F. Robinson[1], Michelle L. Hladik[2], Tracey J. Woodruff[1]

[1] University of California, San Francisco, OB/Gyn & Reproductive Sciences, San Francisco, USA; [2] United States Geological Survey, California Water Science Center, Sacramento, USA

Objective:
To evaluate the effectiveness of silicone wristbands as a measure of pesticide exposure in a diverse cohort of pregnant women and to identify factors associated with differential exposure levels.

Material and Methods:
Participants from the UCSF Discovering cancer Risks from Environmental exposures And Maternal/child health (DREAM) Cohort were recruited from California's Central Valley (n=72) and the San Francisco Bay Area (n=21). Participants were assigned to wear silicone wristbands continuously for 7 or 14 days. The wristbands were analyzed by the United States Geological Survey for over 200 pesticides using gas chromatography-tandem mass spectrometry (GC-MS/MS) and liquid chromatography–tandem mass spectrometry (LC-MS/MS). Demographic information, residential location, occupational exposure, and lifestyle factors were collected through questionnaires to contextualize the exposure data.

Results:
79 different pesticides (ranging from 2 to 41 per sample) were detected across 93 wristbands (average wear 13 days). The most detected were insecticides (52%) and fungicides (33%), and the highest detected pesticide classes included azoles and benzothiazole derivatives (11), pyrethroids (10), neonicotinoids (7), and pyrazoles (7). We also detected the banned organochlorine dichlorodiphenyltrichloroethane (p,p'-DDT) and its major metabolites (p,p'-DDE, and p,p'-DDD), along with organophosphates (diazinon, malathion, and malathion oxon), all of which are known for their toxicity. Variations in pesticide levels were observed based on geographic location, with higher levels of insecticides (1.7-fold increase) and fungicides (1.5-fold increase) detected in the Central Valley compared to the San Francisco Bay Area.

Conclusion:
Silicone wristbands are effective tools for assessing pesticide exposure in pregnant and postpartum women. The study revealed widespread pesticide exposures, with geographic variations highlighting multiple chemicals that might contribute to stress pathways (inflammation, oxidative stress) and adverse health outcomes. These findings enhance understanding of pesticide exposures and their potential impacts on maternal health, informing targeted interventions to reduce exposure risks in vulnerable populations.

P53-22 | Identify Urinary Cotinine Cut-offs to Represent Active, Secondhand and Thirdhand Tobacco Smoke Exposure in Pregnancy

Yu Ni[1], Logan C. Dearborn[2], Catherine J. Karr[2], Adam A. Szpiro[2], Christine T. Loftus[2], Qi Zhao[3], Kaja LeWinn[4], Sheela Sathyanarayana[5], Marnie F. Hazlehurst[5], Penelope J.E. Quintana[1], Eunha Hoh[1], Georg Matt[1]

[1] San Diego State University, College of Health and Human Services, San Diego, USA; [2] University of Washington, School of Public Health, Seattle, USA; [3] The University of Tennessee Health Science Center, Memphis, USA; [4] University of California, San Francisco, School of Medicine, San Francisco, USA; [5] Seattle Children's Research Institute, Seattle, USA

Objective: Despite a significant decline in active smoking among U.S. pregnant women, secondhand smoke (SHS) remains a major public health concern. Toxic tobacco residue (thirdhand smoke, THS) is also widespread and persistent. An accurate determination of tobacco smoke exposure is essential for translational research purposes. We investigated urinary cotinine cut-offs for classifying tobacco smoke exposure in pregnant women, using self-reported data as reference.

Material: We analyzed data from 1,709 pregnant women in 11 cohorts from the Environmental Influences on Child Health Outcomes (ECHO) program. Data are publicly available from the NIH Data and Specimen Hub.

Methods: Urinary cotinine levels in the second trimester were corrected for dilution. We leveraged self-reported data on ECHO-wide and cohort-specific questionnaires to classify pregnant women into four tobacco exposure subgroups: active smokers, SHS exposure, THS exposure, and no exposure. Using these memberships as reference, we estimated the Receiver Operating Characteristic (ROC) curve with bootstrap resampling to determine optimal urinary cotinine cut-offs, maximizing the sum of sensitivity and specificity. We further employed a Gaussian mixture model fitted with an expectation-maximization (EM) algorithm to confirm our findings.

Results: The geometric means (geometric standard deviation [GSD]) in dilution-corrected cotinine were 530.67 (34.4), 1.82 (24.81), 0.20 (21.63), and 0.03 (11.03) ng/mL in subgroups of active smoker, SHS, THS, and no exposure, respectively. We determined optimal cut-offs of ≥6.24 ng/mL (Area Under the Curve [AUC]: 0.95) to classify active smokers and <1.18 ng/mL (AUC: 0.91) to classify no exposure. We distinguished THS from SHS at 1.24 ng/mL (AUC: 0.71), after excluding other exposure subgroups. The Gaussian mixture model identified four subgroups that supported primary findings.

Conclusion: We combined self-reported data and urinary cotinine to better classify pregnant women’s tobacco smoking exposure.This method may be applicable in future population studies or public health practice.

P53-23 | Effects of Exposure toLow-Level Artificial Light at Night During Pregnancy on Offspring Neurobehavioral Development

Li W. Wang[1,2], Jian Xu[1,2]

[1] Shanghai Jiao Tong University, The International Peace Maternity and Child Health Hospital, School of Medicine, Shanghai, China; [2] Shanghai Key Laboratory of Embryo Original Diseases, Shanghai, China; [3] Shanghai Jiao Tong University School of Public Health, Department of Maternal and Child Health, Shanghai, China

Objective

Exposure to low-level artificial light at night (ALAN) is a global environmental concern with recognized risks to human health. However, the effects and underlying mechanisms of low-level ALAN during pregnancy on offspring neurodevelopment remain unclear.

Materials and Methods

A mouse model of exposure to low-level (<2 lux) ALAN during pregnancy was established, with oral melatonin gavage administered as the therapeutic intervention. Offspring anxiety- and depression-like behaviors were assessed via open-field, elevated-plus-maze, sucrose-preference, tail-suspension, and forced-swim tests. Cognitive function was evaluated using novel object recognition, while circadian regulation of metabolism was monitored via metabolic cages. Based on preliminary findings, hippocampal transcriptome dynamics, metagenomic information of gut microbiota, and serum untargeted metabolomics using female offspring samples were analyzed.

Results

ALAN exposure significantly reduced recognition/discrimination indices, exploration time in the open-arms, sucrose preference in the novel-object-recognition/elevated-plus-maze, increased immobility time in tail-suspension and forced-swim tests, and induced nighttime metabolic disturbances in female offsprings, and such changes were mitigated by gestational melatonin intervention. However, male offsprings demonstrated slighter/less-significant changes except in the domain of depression-like behavior compared with female offsprings after prenatal ALAN. Enhanced hippocampal neuroinflammation and impaired hippocampal synaptic plasticity, increased gut microbiota α-diversity and abundance of depression-associated Oscillospiraceae members (*Oscillibacter sp.*, *Lawsonibacter sp.*, *Anaerotruncus*, *Pseudoflavonifractor*), *Prevotella sp. MGM2*, and *Parablautia*, while decreased abundance of probiotic members within the genus *Akkermansia* (*Akkermansia muciniphila*, *Akkermansia sp.*) and *Desulfovibrio* were all observed in ALAN female offsprings. Serum L-tryptophan, 5-HT, indole, indole-3-pyruvate, and lithocholic acid rhythms were disrupted, alongside increased daytime intake in ALAN female offsprings. These impairments were mitigated by prenatal melatonin intervention.

Conclusion

Gestational ALAN induces anxiety/depression-like behaviors and cognitive deficits mainly in ALAN female offsprings, potentially mediated by hippocampal synaptic dysfunction, gut microbiota dysbiosis, disrupted metabolic rhythms and tryptophan/bile acid metabolism, and these impairments were mitigated by prenatal melatonin administration.

P53-24 | Gestational PBDE Concentrations, MRI Functional Connectivity and Neurobehavioral Outcomes in Adolescents

Kim M. Cecil[1,3], Jonathan Dudley[2], Yingying Xu[2], Joseph Braun[4], Aimin Chen[5], Bruce Lanphear[6], Kimberly Yolton[2,3]

[1] Cincinnati Children's Hospital Medical Center, Radiology, Cincinnati, USA; [2] Cincinnati Children's Hospital Medical Center, Pediatrics, Cincinnati, USA; [3] University of Cincinnati College of Medicine, Environmental and Public Health Sciences, Cincinnati, USA; [4] Brown University School of Public Health, Epidemiology, Providence, USA; [5] University of Pennsylvania School of Medicine, Epidemiology, Philadelphia, USA; [6] Simon Fraser University, Health Sciences, Burnaby, Canada

Objective: Cognitive and behavioral deficits have been associated with polybrominated diphenyl ethers (PBDEs) concentrations in children, but the underlying mechanisms are unclear. We estimated the association of gestational PBDE serum concentrations and adolescent intrinsic functional connectivity in a prospective pregnancy and birth cohort longitudinally followed to adolescence. We determined whether functional connectivity patterns mediated the observed associations between gestational Σ_5BDE concentrations and outcomes at age 12.

Materials and Methods: We studied 143 children from the HOME Study (enrolled: 2003-2006). During pregnancy, we measured maternal serum concentrations of five PBDE congeners and created a summary variable (Σ_5BDE). At age 12, we acquired high resolution anatomical and resting state functional magnetic resonance neuroimaging to examine the relationship between gestational PBDE serum concentrations and global connectivity, adjusting for covariates. Image processing and analyses were performed using CONN and Statistical Parameter Mapping software. We assessed behaviors, executive function and mental health using the Behavioral Assessment System for Children (BASC), Behavior Rating Inventory of Executive Functioning (BRIEF), the Social Responsiveness Scale (SRS), the Spence Children’s Anxiety Scale (SCAS), and the Children’s Depression Inventory (CDI).

Results: Higher gestational PBDE serum concentrations (BDE-28, -47, -99, -100, and the Σ_5BDE) were associated with reduced global correlation in the primary visual cortex. Seed-to-voxel connectivity patterns showed significant mediation for associations between $\Sigma_5$5BDE and neurobehavioral outcomes (BASC Behavior Symptoms Index, SCAS total, SRS total, BRIEF-Cognitive Regulation Index, and CDI total T-scores, respectively) at age 12.

Conclusion: Gestational Σ_5BDE concentrations were associated with reduced global measures of functional connectivity within primary visual processing regions. Diminished connectivity between visual processing regions and regions within multiple intrinsic networks, including the default mode network, salience/ventral attention network, dorsal attention network, somatomotor network, and cerebellum, may partially explain the adverse associations of gestational PBDE exposure with neurobehavioral outcomes.

P53-25 | Outdoor temperature during pregnancy and its association with pre-and-postpartum demoralization in women from New York City

Eréndira Araujo Di Giuseppe[1], Yuqi Miao[2], Shuang Wang[2], Sarah Tom[3], Rebecca Kehm[3], Teresa Janevic[3], Haotian Wu[1], Parisa Tehranifar[3], Kendall Kruchten[1], Julie Herbstman[1], Marcela Tamayo-Ortiz[1]

[1] Columbia's Mailman School of Public Health, Environmental Health Sciences, NEW YORK, USA; [2] Columbia's Mailman School of Public Health, Biostatistics, NEW YORK, USA; [3] Columbia's Mailman School of Public Health, Epidemiology, NEW YORK, USA

Maternal mental health is key to improving maternal and child outcomes. However, little is known about environmental risk factors of demoralization, a measure of nonspecific psychological distress. We analyzed the association between outdoor temperature during pregnancy and demoralization during prenatal and- postpartum periods. The Mothers and Newborns Cohort of the Columbia Center for Children's Environmental Health includes718 women recruited in 1998 – 2006 from Northern Manhattan, Harlem, and South Bronx neighborhoods in New York City. Daily pregnancy outdoor air temperatures were derived from Daymet V4. Extreme hot days were considered as maximum temperatures >90°F and extreme cold days <25°F. For each season, we used the number of extreme hot and cold days and the average daily range (T_{max} - T_{min}). Demoralization was assessed during the third trimester and at 6 months, and 12 months postpartum using the PERI-D scale. Higher scores indicate more symptoms. Linear regression models were used, stratified by season, and adjusted for age, material hardship, BMI, education and home air conditioning (A/C) unit. Women were 25 ±4.9 years-old at delivery, 44% experienced material hardship, 51% had a healthy BMI, 65% had completed high-school, 67% had an A/C. During the winter, 10 additional extreme cold days was associated with a 0.03 increase in demoralization during pregnancy (p = 0.08) in crude models and with a 0.04 increase (p = 0.047) after adjustment. In the spring, an increase of 5°F in the average daily temperature difference was associated with a 0.17 increase in demoralization score at 12 months postpartum (p = 0.05) in crude models and with a 0.15 increase (p = 0.10) after adjustment. More extreme cold days and larger differences in average daily temperature were associated with increased demoralization scores during pregnancy and postpartum. Our results highlight the relevance of temperature on women's peripartum health and wellbeing.

P53-26 | Proximity to Active Polluted Sites during Gestation and Early Childhood Blood Pressure Trajectories

Sam J. Neally[1,2], Corinna Keeler[2], Celeste Butts-Jackson[3], Cathrine Hoyo[4], Chantel L. Martin[2], Kristen M. Rappazzo[5]

[1] US EPA, Oak Ridge Institute for Science and Education, Oak Ridge, USA; [2] UNC Gillings School of Public Health, Department of Epidemiology, Chapel Hill, USA; [3] North Carolina Agricultural and Technical State University, Department of Health Services Management, Greensboro, USA; [4] North Carolina State University, Department of Biological Sciences and Center for Human Health and the Environment, Raleigh, USA; [5] US EPA, Office of Research and Development, Research Triangle Park, USA

Objective: We examined associations between residential proximity to actively emitting polluted sites from the Toxics Release Inventory (TRI) during gestation and early childhood blood pressure (BP) trajectories in a North Carolina (NC) birth cohort.

Material and Methods: The Newborn Epigenetics Study (NEST) includes 2,681 participants recruited from prenatal visits at obstetrics care facilities in Durham County, NC (2005-2011). This analysis included participants with at least two clinical BP measurements from 3 to 9 years (n=1,040). The outcome was systolic BP percentile trajectories, using group-based trajectory modeling (low stable, low increasing, high stable, high decreasing).

The TRI program of the US Environmental Protection Agency requires annual chemical release reports from industrial facilities which manage, manufacture, release, or use toxic, carcinogenic, or otherwise harmful chemicals. Exposures were defined as having any TRI sites reported from 2005-2011 in NC within 1, 1.5, 2, and 3 mile buffers surrounding residence during gestation.

We used multinomial logistic regression to estimate odds ratios (OR (95%CI)) for relationships between having or not having TRI exposure and BP trajectory groups (ref=low stable) at each level of proximity, adjusting for age at delivery, race, marital status, and education.

Results: Associations ranged by TRI site proximity, with closer proximities having stronger point estimates but wider confidence intervals. After adjustment, children with gestational exposure to TRI sites had greater odds of having high stable compared to low stable BP trajectories at 1 mile (2.30 (1.21, 4.34)), 1.5 miles (1.78 (1.08, 2.91)), and 2 miles (1.89 (1.21, 2.95)); associations were attenuated but still positive at 3 miles (1.40 (0.92, 2.13)).

Conclusion: Closer residential proximities to actively emitting polluted sites during gestation may be associated with abnormal BP trajectories during early childhood. Analyses of different types of polluted sites and specific contaminants present at these sites may reveal additional associations.

P53-27 | Prenatal pesticide exposure and respiratory health outcomes in children residing in Mexico City

Cecilia S. Alcala[1], Hector Lamadrid-Figueroa[2], Marcela Tamayo-Ortiz[3], Adriana Mercado Garcia[4], Elena Colicino[1], Syam S. Andra[1], Jamil M. Lane[1], Martha M. Tellez-Rojo[4], Robert O. Wright[1,5], Rosalind J. Wright[1,5], Kecia N. Carroll[1,6], Maria José Rosa[1]

[1] Icahn School of Medicine at Mount Sinai, Department of Environmental Medicine, New York, USA; [2] National Institute of Public Health (INSP), Center for Population Health Research, Cuernavaca, Mexico; [3] Columbia University Mailman School of Public Health, Department of Environmental Health Sciences, New York, USA; [4] National Institute of Public Health, Center for Nutrition and Health Research, Cuernavaca, Mexico; [5] Icahn School of Medicine at Mount Sinai, USA., Kravis Children's Hospital, New York, USA; [6] Icahn School of Medicine at Mount Sinai, Institute for Exposomic Research, New York, USA

Objective: Prenatal exposure to pesticides may impact respiratory health in childhood. We examined associations between prenatal exposure to 12 organophosphate and pyrethroid pesticides and childhood asthma/wheeze in children and explored sex differences. Materials and Methods: We included 149 mother-child dyads from the Programming Research in Obesity, Growth, Environment, and Social Stressors birth cohort study in Mexico City. Pesticide metabolite concentrations were measured in 2nd trimester urine, (specific gravity-adjusted and log2-transformed). Reports of ever wheeze and wheeze in the past 12 months (current wheeze), and asthma were obtained using the International Study of Asthma and Allergies in Childhood questionnaire administered at 4-7 years of age. Associations were modeled for individual metabolites using modified Poisson regression and Bayesian Weighted Quantile Sum regression for mixture effects, adjusting for covariates, and stratified by sex. Results: A doubling of the prenatal concentration of cis-3-(2,2-dichlorovinyl)-2,2-dimethylcyclopropane carboxylic acid and 3,5,6-Trichloro-2-Pyridinol was associated with higher risk of current wheeze (RR: 1.14, 95% CI [1.03, 1.27]); and of asthma at 4-5 years (RR: 1.21, 95% CI [1.02, 1.45]) respectively. A doubling of dimethylthiophosphate and diethylphosphate was associated with ever wheeze at 6-7 years (RR: 1.11, 95% CI [1.00, 1.22]; RR: 1.15, 95% CI [1.02, 1.30]). In interaction analyses, associations were stronger in females compared to males at 6-7 years. We did not observe mixture associations between pesticide metabolites and any respiratory outcome. Conclusions: Higher prenatal pesticide metabolite concentrations were associated with an increased risk of wheezing and asthma in childhood. Future work examining outcomes including lung function trajectories will be important for understanding long-term impacts.

P54 | From Policy to Practice: Global Efforts to Eradicate Lead Exposure - PART II

P54-01 | Association between prenatal lead exposure and secondary sex ratio: Japan Environment and Children's Study

Nozomi Tatsuta[1], Miyuki Iwai-Shimada[1], Shoji F. Nakayama[1], Kunihiko Nakai[2]

[1] National Institute for Environmental Studies, Health and Environmental Risk Division, Tsukuba, Japan; [2] Tokai Gakuen University, School of Sport and Health Science, Miyoshi, Japan

Objective: The male birth rate (secondary sex ratio, SSR) has exhibited a decline in recent decades. Among the factors contributing to this decline is the influence of chemicals. Specifically, exposure to polychlorinated biphenyls and methylmercury has been reported to affect the SSR. Lead has been shown to cause adverse effects on pregnancy like miscarriage, premature birth, and low birth weight. However, there is limited research on this topic. This study investigated the relationship between blood lead levels (BLLs) and SSR.
Material and Methods: The analysis included 85,171 mothers and infants with complete data from the Japan Environment and Children's Study. The exposure biomarker was whole BLLs during pregnancy, and the outcome was infant SSR. The study population was divided into five groups based on the BLL, and multiple logistic regression analysis were used.
Results: The proportion of male infants born was 51.2%, and the median BLL of pregnant women was 5.85 ng/g. The results of the study indicated a positive correlation between the BLL of pregnant women and the proportion of male infants born, suggesting that the higher BLL, the higher the proportion of male births.
Conclusion: A positive association was identified between the BLL levels in pregnant women and the birth rate of male offspring. The relationship between environmental lead concentrations and human lead exposure is well-documented. As restrictions on leaded gasoline have been implemented, atmospheric lead concentrations have decreased, and monitoring data from other countries has shown a corresponding decrease in human BBL. This suggests a potential link between the decrease in atmospheric lead concentrations and changes in SSR. Given the documented findings indicating that paternal exposure to lead influences SRR, future investigations will focus on the impact of paternal blood lead concentrations on SSR.

Funding source: This study was funded by the Ministry of the Environment, Japan.

P54-02 | Lead and Cancer: A Narrative Review and Risk of Bias Assessment

Carol Cusack[1], Tara Serio[1], Noelle Henderson[1,4], Kathryn Egan[2], Zheng Li[3], Teresa Wang[1]

[1] Agency for Toxic Substances and Disease Registry (ATSDR), Office of Community Health Hazard Assessment, Atlanta, USA; [2] Agency for Toxic Substances and Disease Registry (ATSDR), Office of Science, Atlanta, USA; [3] Agency for Toxic Substances and Disease Registry (ATSDR), Office of Capacity Development and Applied Prevention Science, Atlanta, USA; [4] Oak Ridge Institute for Science and Education (ORISE) Fellowship Program, Atlanta, USA

Objective: The International Agency for Research on Cancer (IARC) classified inorganic lead as "probably carcinogenic to humans" (Group 2A) in 2006, based on limited human evidence and sufficient animal evidence from studies published prior to 2003. This narrative review synthesizes information from more recent epidemiologic studies using a risk-of-bias approach to overcome challenges in study variation.
Materials and Methods: A literature search was conducted for epidemiological studies published between 2003 and 2020 that evaluated lead exposure and site-specific cancers in humans. The National Toxicology Program's Office of Health Assessment and Translation tool was utilized to assess risk-of-bias. For each study, two reviewers independently abstracted data across 10 domains, including selection, confounding, attribution/exclusion, detection, reporting, and other sources of bias. Studies were categorized into Tiers reflecting overall risk-of-bias: Tier 1 (low), Tier 2 (moderate), or Tier 3 (high). Discrepancies were resolved by a third reviewer. Risk-of-bias results and a summary of findings were grouped by cancer site and body system.
Results: Of 591 search results, 43 studies met inclusion criteria. Thirteen studies were classified as Tier 1 and 30 studies were classified as Tier 2. Elevated lead exposure was most consistently and significantly associated with cancers of the respiratory, urinary, and digestive tracts. Additionally, breast, thyroid, and head and neck cancers, which were not previously examined in IARC's 2006 monograph, offered positive but more limited associations in terms of statistical significance. Common potential sources of bias included confounding and exposure classification bias.
Conclusions: This review highlights the value of standardized exposure assessments and robust confounder adjustments in additional epidemiologic studies to minimize potential for bias and further inform the association between lead and cancer in humans. Closing this research gap aligns with the Agency for Toxic Substances and Disease Registry's efforts to protect communities from the health risks of hazardous substances.

P54-03 | Biomarkers of Lead and Uranium Measurements from Postwar Fallujah, Iraq

Aaron J. Specht[1], Addison Vogt[3], Thomas R. Grier[1], Samira Alaani[5], Abdulqadar Alawi[5], Ian C. Lindsay[2], Ellen M. Wells[1,4], Kali Rubaii[2]

[1] Purdue University, School of Health Sciences, West Lafayette, USA; [2] Purdue University, Department of Anthropology, West Lafayette, USA; [3] Purdue University, Interdisciplinary Life Sciences, West Lafayette, USA; [4] Purdue University, Department of Public Health, West Lafayette, USA; [5] Fallujah Women and Children's Hospital, Fallujah, Iraq

This study investigated the accumulation of lead and uranium in bones of individuals from Fallujah, Iraq, an area with substantial historic military bombardment and extensive use of leaded gasoline. In 2022, 114 families were recruited from a women's hospital and completed in-depth interviews on reproductive history and environmental exposures. Additional biomarker sampling and follow-up interviews were conducted in fall 2024 on a subset of 68 participants (35 families). These biosamples were measured primarily for uranium and lead, which are both toxicants with both known neurological implications and no known safe level of exposure. Participant's average age was 34.7 years (95% confidence interval (CI): 32.9, 36.5). Bone lead and uranium were measured non-invasively onsite using a portable x-ray fluorescence device and blood and nail were collected and analyzed in the Trace Metals Lab at Purdue University. Results revealed average bone lead levels of 21.2 µg/g (95% CI: 18.5, 23.9) and bone uranium levels of 1.4 µg/g (95% CI: 1.2, 1.7) among participants. Average blood lead levels were 7.5 µg/dL (95% CI: 3.7, 11.3) and average nail lead levels were 1.6 µg/g (95% CI: 0, 5.4). Additionally, cumulative (bone) lead levels were between 300% and 2333% higher than contemporary populations in North America. This is the first study to quantify bone uranium levels in a living population and the first to identify cumulative uranium exposure in postwar areas likely arising from depleted uranium use during military action. This research emphasizes the environmental exposure to heavy metals, potentially linked to military activities, and its implications for public health in post-conflict regions.

P54-04 | Blood lead testing among children enrolled in Medicaid in the United States

Cheryl R. Cornwell, Audrey F. Pennington, Trina Williams, Perri Z. Ruckart, Paul B. Allwood, Joseph G. Courtney

Centers for Disease Control and Prevention, National Center for Environmental Health, Atlanta, USA

Objective: To assess the prevalence of blood lead testing among U.S. children enrolled in Medicaid, which requires enrollees to be tested at 12 and 24 months of age.
Material and Methods: We examined a cohort of children enrolled in Medicaid born between January 1, 2016, and December 31, 2019, from 42 U.S. states and Washington D.C. (n=6,294,037). We calculated the percentage of children who received a blood lead test at three ages: 12 months, 24 months, and at least one test by 3 years of age, overall and stratified by characteristics of interest. We assessed testing by U.S. region, state, and urbanicity given geographic variations in environmental sources of lead exposure. We estimated the associations between select characteristics and receiving a test using log-binomial regression. Models were adjusted for race/ethnicity, region, urbanicity, testing policy, well-child visit, and managed care enrollment.

Results: The percentages of children receiving a blood lead test at ages 12 months, 24 months, and by 3 years were 48%, 42%, and 63%, respectively. Testing by age 3 years was highest among children residing in the Northeast region (70%) and lowest in the West region (50%); it was lower among children living in rural counties (55%) than among children in large metropolitan counties (64%). In adjusted models, increased testing by age 3 years was associated with having ≥1 well-child visit (risk ratio (RR) [95% confidence interval (CI)] =16.6 [15.9, 16.5]) and living in a universal testing policy state (RR [95% CI] =1.06 [1.06, 1.07]).
Conclusion: Less than half of children in this analysis received a blood lead test at each prescribed age of 12 and 24 months. Understanding the patterns of blood lead testing among children enrolled in Medicaid could help guide public health programs to increase testing rates given continued risk of lead exposure in this population.

P54-05 | Distribution of urinary lead concentrations and exploration of socioeconomic predictors in a cohort of Ugandan adolescent girls and young women

Kristen Upson[1], Julio Landero[2], Juhee Lee[1,3], Renee Heffron[4], Andrew Mujugira[5], Flavia Matovu[6], Kenneth Mugwanya[7], Robert Wright[2], Michael Yin[8], Chenxi Li[1], Anne Marie Jukic[9], Quaker Harmon[9]

[1] Michigan State University, Department of Epidemiology and Biostatistics, College of Human Medicine, East Lansing, USA; [2] Icahn School of Medicine at Mount Sinai, Department of Environmental Medicine and Public Health, New York, USA; [3] Michigan State University, Department of Statistics and Probability, College of Natural Sciences, East Lansing, USA; [4] University of Alabama at Birmingham, Department of Medicine, Birmingham, USA; [5] Makerere University, Infectious Diseases Institute, College of Health Sciences, Kampala, Uganda; [6] Makerere University-Johns Hopkins University Research Collaboration, Kampala, Uganda; [7] University of Washington, Global Health, Seattle, USA; [8] Columbia University Irving Medical Center, Department of Medicine, Division of Infectious Diseases, New York, USA; [9] National Institute of Environmental Health Science, National Institutes of Health, Epidemiology Branch, Research Triangle Park, USA

Objective

Given the adverse health effects of lead, biomonitoring data are critical. Yet, these data are sparse in low-income and middle-income countries, particularly in Africa. We examined the distribution of urinary lead (uPb) concentrations and explored socioeconomic predictors in a cohort of Ugandan adolescent girls and young women.

Material and Methods

We conducted a cross-sectional analysis using baseline data (years 2018-2020) from the Kampala Women's Bone Study, which enrolled 499 HIV-negative women ages 16-25 years in Kampala, Uganda; none were pregnant, lactating, nor recent users of hormonal contraception. We measured uPb by inductively-coupled plasma-mass spectrometry (detection limit (DL) 0.188 µg/l; n=5 (1%) <DL). uPb concentrations were specific gravity-adjusted to account for urinary dilution. In non-smokers (n=494), we conducted multivariable linear regression to estimate the percent change in uPb concentrations and 95% CIs in relation to socioeconomic factors (collected by questionnaire), adjusting for age and education.

Results

In this Ugandan cohort, the median uPb concentration was 2.30 µg/l (interquartile range (IQR): 1.47, 3.53 µg/l). For comparison, this concentration is 14 times higher than the median uPb in US women ages 16-25 years (0.160 µg/l, IQR: 0.122, 0.252 µg/l, National Health and Nutrition Examination Survey 2017-2020). We observed higher uPb

concentrations with fewer years of educational attainment. Compared with 12-16 years of education, uPb concentrations were 25% higher (95% CI: 2%, 54%) and 20% higher (95% CI: -1, 45%) with 8-10 years and 11 years of education, respectively. uPb concentrations were 31% higher for participants receiving financial/material support from partner (95% CI: 2%, 70%) and 22% higher for participants reporting job loss worries in the past 3 months (95% CI: 2%, 46%).

Conclusion

This study contributes critical uPb data in a non-occupational, general Ugandan population and suggests higher uPb with indicators of lower socioeconomic status. Study funded by NICHD, NIH.

P54-06 | Development and validation of a questionnaire for screening sources of lead exposure in Mexican pregnant women

Larissa Betanzos-Robledo[1,2], Guadalupe Estrada-Gutiérrez[2], Alejandra Cantoral[3], Héctor Lamadrid-Figueroa[4], Julio Landero[5]

[1] National Autonomous University of Mexico, Doctoral Student, Mexico City, Mexico; [2] National Institute of Perinatology, Department of Immunobiochemistry, Mexico City, Mexico; [3] Iberoamericana University, Department of Health, Mexico City, Mexico; [4] National Institute of Public Health, Population Health Research Center, Cuernavaca, Mexico; [5] Icahn School of Medicine Mount Sinai, Department of Environmental Medicine and Public Health, New York, USA

Introduction: Pregnancy is the most critical window of time for human development, with a high sensitivity to exposure to lead (Pb), which has adverse toxic effects on maternal-infant health. However, the measurement of its levels is not part of the routine perinatal consultation due to its high cost and complexity.
Objective: To develop and validate a questionnaire as a screening tool at the first level of care to identify sources of Pb exposure in Mexican pregnant women.
Methodology: A literature review was carried out to identify existing validated questionnaires and the main sources of Pb exposure in the Mexican population, which were discussed with an expert committee and the questions of the questionnaire were designed. A content validity test was performed on a sample of 40 pregnant women participating in the Perinatal Cohort Biochemical and Epigenetic Origin of Overweight and Obesity (OBESO), from the National Institute of Perinatology “Dr. Isidro Espinosa de los Reyes” (INPer), Mexico City.
Results: The final questionnaire was composed of 21 questions and the application time is 10 minutes on average. The questions included the main sources of Pb exposure reported in the literature and discussed with the expert committee. Among them were the frequency of use of glazed ceramice; the frequency of consumption of foods with reported Pb content, such as rice and cocoa; condiments such as turmeric, paprika and pepper; and sausages such as sausage and ham; occupational exposure questions such as pottery; habits such as smoking and pica. As well as proximity of the home to factories, garbage dumps, polluted rivers, agricultural areas, construction sites or mines.
Conclusion: The content validity test allowed to ensure the understanding of the questions and their parsimoniousness. The questionnaire is currently in the stage of validation of concordance with Pb levels in maternal blood (gold standard).

P54-07 | Cord Blood Lead Screening in Lebanon: Findings from the Environmental Exposures in Lebanese Infants (EELI) Study

Emile Whaibeh[1,2], Bassam Eid[4], Jowy Abi Hanna[1,2], Norma Aouad[4], Joyce Haddad[5], **Georges Abi Tayeh**[4,6], Myriam Mrad[2,3]

[1] Saint-Joseph University of Beirut, Institut Supérieur de Santé Publique, Faculty of medicine, Beirut, Lebanon; [2] University of Balamand, Public Health Department, Faculty of Health Sciences, Beirut, Lebanon; [3] Health and Environment Response Agency (HERA), Beirut, Lebanon; [4] Hôtel-Dieu de France University Hospital, Beirut, Lebanon; [5] Ministry of Public Health, Directorate of Preventive Health Care, Beirut, Lebanon; [6] Saint Joseph Fertility Center, Beirut, Lebanon

Objective: Lead exposure is a significant public health concern for vulnerable populations, especially pregnant women and newborns. Despite the ban on leaded gasoline in Lebanon, other sources of lead exposure remain unregulated. This study aims to assess cord blood levels among pregnant women in Lebanon as part of the Environmental Exposures in Lebanese Infants (EELI) study.
Materials and Methods: Seventy-five umbilical cord blood samples were collected from 2021 to 2022. Samples were analyzed for lead content at RECETOX laboratories in the Czech Republic. Statistical analyses included bivariate tests to explore associations between lead levels and demographic or environmental factors, followed by multivariate analysis to identify independent predictors of elevated lead levels.
Results: The median lead concentration in cord blood was 0.545 µg/dL, with lead levels ranging from 0.238 to 5.44 µg/dL. Only one sample exceeded the CDC's threshold of 3.5 µg/dL. Higher maternal pre-pregnancy BMI was associated with elevated lead levels (OR=2.94, p=0.005). Active smoking during pregnancy significantly increased the odds of elevated lead levels (OR=14.94, p=0.035). Surprisingly, proximity to construction sites was associated with lower lead levels (OR=0.13, p=0.037).**Conclusion:** The findings highlight the presence of lead in all samples, indicating ongoing exposure risks. There is a need for continuous monitoring and public health interventions to reduce lead exposure in Lebanon. Recommendations include conducting larger screening campaigns, raising awareness, and establishing environmental monitoring programs.

Funding and Acknowledgments: The research is made possible by an intramural fund from the University of Balamand and partially by an NIH Fogarty International Center grant (Reference number: [1U01TW012237-01]). We thank the RECETOX laboratories, the Ministry of Public Health, and the World Health Organization for their invaluable assistance.

P54-08 | Strengths and Limitations of Performing Lead Exposure Assessment Using the All-Ages Lead Model

Tonia Burk

Agency for Toxic Substances & Disease Registry, Atlanta, USA

Lead exposure continues to pose significant health risks to the U.S. population, especially young children undergoing neurodevelopment. Physiologically based pharmacokinetic models are useful for estimating blood lead levels (BLLs) from environmental exposure in cases where reliable BLL data are not available. Modelled results are used to assess risk and to prioritize intervention activities to reduce exposure and minimize health risks.
The newest version of All-Ages Lead Model (AALM) was released in February 2024. The AALM models BLL from birth to 90 years of age for males and females from exposure for one or more days. The AALM estimates average BLL and the probability of exceeding a specified BLL during a specified age range. The AALM also presents transient BLL graphically and presents other organ system results throughout the time-period specified. Exposure factor inputs are used to account for intermittent exposure, eliminating the need to adjust the exposure point concentration.
Since its release, we explored the capability of the AALM in predicting BLLs in various exposure scenarios. In this assessment, we present the results and analyses of exposure scenarios that are simulated using the AALM. When appropriate, we compare the AALM results with those obtained from the two previously existing models (the Integrated Exposure Uptake Biokinetic model and the Adult Lead Methodology model). We discuss the strengths and limitations of the AALM. AALM uniquely simulates exposure less than 90 days and non-soil media for age 6+. The findings from this assessment will assist risk assessors across the globe in making appropriate decisions while using the AALM to conduct health risk assessment of lead exposure in various populations.
The findings and conclusions in this presentation have not been formally disseminated by the Agency for Toxic Substances and Disease Registry and should not be construed to represent any agency determination or policy.

P54-09 | Enhancing accessibility of the All Ages Lead Model: An R-based implementation

Maria E. Cardelino[1], Alexander Avramov[2], Eri Saikawa[1,2]

[1] Emory University, Gangarosa Department of Environmental Health, Rollins School of Public Health, Atlanta, USA; [2] Emory University, Department of Environmental Sciences, Atlanta, USA

Objective: The All Ages Lead Model (AALM) is a biokinetic model commonly used to predict blood lead levels from environmental exposures. Such models are important tools in lead exposure and risk assessment, especially for populations with high environmental lead burden like West Atlanta. However, the AALM has several limitations in its accessibility and usability. This project aims to modernize the AALM by transpiling it to R and integrating a user-friendly Shiny interface, allowing users to run it from any computer. Users may also enter blood lead levels to estimate critical intake parameters, a feature not yet available with the current AALM.
Methods: The original AALM Fortran code was sourced from GitHub and translated into several modules in R, maintaining complete fidelity to its biokinetic modeling framework. A Shiny-based interface was developed to allow fast, interactive scenario testing and additional visualization of lead biomarkers across the simulation time. The R-

based model was validated by comparing outputs to the existing model following simulations using the same inputs across many simulations. Bayesian inverse modeling functions were added to allow users to estimate intake parameters like soil ingestion rate given output (blood lead level) data.
Results: The R-based AALM provides an alternative to the existing model which is not compatible with many computers and whose source code is not accessible. The R-based AALM is compatible with any device that has R, and the Shiny interface gives the user more power to interact with the model through inverse modeling.
Conclusion: Translating the All Ages Lead Model to an R-based platform enhances code transparency and increases accessibility to the model. Additional functionalities allow for new ways to explore dose-response relationships and estimate intake parameters. By providing a universally compatible version of the model, this project supports broader engagement in lead exposure modeling and exposure assessment.

P54-10 | Drinking water lead exposures among private wells in New Jersey: A statewide stratified random sample

Jessie Gleason[1], Farzana Reza[1], Nicholas Procopio[3], Doug Haltmeier[2]

[1] New Jersey Department of Health, Environmental and Occupational Health Surveillance, Trenton, USA; [2] New Jersey Department of Health, Environmental and Chemical Laboratory Services, Ewing, USA; [3] New Jersey Department of Environmental Protection, Division of Science and Research, Trenton, USA

Little is known about lead in drinking water among homes served by private wells, which may be vulnerable because of geological characteristics of the groundwater (e.g. low pH), lack of corrosion control, housing age, and limited regulation. **Objective:** To evaluate potential lead in drinking water exposure in New Jersey (NJ) private wells. **Materials and Methods:** NJ has ~375,000 private wells, but residential addresses of these homes are unknown. We identified private well homes by overlaying tax parcel data with public water service boundaries. Parcels were classified into six strata based on estimated pH (very low, low, neutral/high) and housing age (pre-1986 and post-1986). Homes were randomly selected from each stratum mailed an invitation letter for free water testing for lead. Following online registration, well owners were mailed kits to self-collect a first draw sample (1L) and 3-minute flushed sample (250 mL). **Results:** A total of 8,838 letters were sent to randomly selected private well residential properties and 1,010 homeowners registered (11.4% registration rate), and 564 (55.8%) of these returned samples. First draw samples had a weighted mean lead concentration of 2.65 µg/L (95% CI 1.09, 4.20); concentrations ranged from <1 to 508 µg/L; and 7% had 5 µg/L or more. Among flush samples, the mean lead concentrations are meaningfully reduced relative to first draw (0.66 µg/L; 95% CI 0.45, 0.86); the maximum lead concentration among flush samples was 32 µg/L; and 1.5% had 5 µg/L or more. The highest average first draw concentrations were observed in the strata of homes with very low pH and older housing (4.56 µg/L; 95% CI 1.60, 7.52). **Conclusions:** This statewide citizen science initiative identified at-risk homes and finds generally low population-wide exposure to lead after flushing. **Funding:** This work is funded by the CDC-NJDOH Cooperative Agreement (Award #5 NUE1EH001413)

P54-11 | Recent Blood Lead Laboratory Test Results Ordered by Medical Facilities among Children Ages 1–17 Years in the United States, April 1, 2023–March 31, 2024

Tanya T. LeBlanc[1], Wenping Hu[1], Kibrten Hailu[1], Alan Woolf[2], Paul Allwood[1]

[1] Centers for Disease Control and Prevention, Lead Poisoning Prevention and Surveillance Branch, Atlanta, USA; [2] Harvard Medical School, Department of Pediatrics, Boston, USA

Objective: We sought to assess recent blood lead test results ordered by medical facilities among children ages 1-17 during a one-year period.
Material and Methods: Blood lead tests results among children ages 1-17 from commercial laboratory data reported to CDC's National Syndromic Surveillance Program (NSSP) from April 1, 2023–March 31, 2024, were assessed. Nine blood lead exposure groups were established (<1 µg/dL, 1 to <2.0 µg/dL, 2.0 to <3.5 µg/dL, 3.5 to <5.0 µg/dL, 5.0 to <10.0 µg/dL, 10.0 to <25.0 µg/dL, 25.0 to <45.0 µg/dL, 45.0 to <70.0 µg/dL and ≥70.0 µg/dL) and results compared by age, race, sex; and clinical reasons for test orders based on *International Classification of Disease* (ICD-10) medical coding and type of clinical care provider venues ordering the tests.
Results: Of 1,117,094 tests performed during the period, 5.3% (58,781) of lead positive tests had detectable blood lead levels at or above CDC's blood lead reference value (BLRV) of 3.5 µg/dL; 146,276 tests (13.1%) had blood lead levels 2.0 µg/dL or higher. Thirty-four tests performed on children under the age of 6 years had blood lead levels of 70.0 µg/dL or higher. Sixty percent (674,532, 60.4%) of tests were ordered by either pediatric or general practice care facilities leaving 39.6% (442,562) of tests ordered by other venues.
Conclusions: Protecting children from the adverse effects of lead exposure is important for promoting their long-term health and well-being. These findings highlight the value of incorporating blood lead testing into routine pediatric care to support improved health outcomes as children grow, learn, and age.

Disclaimer: The findings and conclusions in this abstract are those of the author(s) and do not necessarily represent the official position of the Centers for Disease Control and Prevention.

P54-12 | A decade of successful research and interventions by icddr,b in investigating and reducing lead exposure in Bangladesh

Mahbubur Rahman[1,4], Sanjida Adiba[1], Jesmin Sultana[1], Shaikh Sharif Hasan[1], Md. Musa Baker[1], Supta Sarker[1], Stephen P. Luby[2], Jenna E. Forsyth[2], Peter J. Winch[3]

[1] icddr,b, Environmental Health and WASH, Health Systems and Population Studies Division, Dhaka, Bangladesh; [2] Stanford University, Woods Institute for the Environment, Stanford, USA; [3] John Hopkins University, Bloomberg School of Public Health, Baltimore, USA; [4] Uppsala University, Global Health and Migration Unit, Department of Women's and Children's Health, Uppsala, Sweden

Background: Lead exposure poses a critical public health problem, particularly in developing countries like Bangladesh, affecting millions, including pregnant women and children. icddr,b's lead research journey started in 2012 with documentation through a household survey showing that 31% of pregnant women in rural Bangladesh had elevated blood lead levels (>5µg/dL), despite living in non-industrial areas. This finding led to investigations including

blood lead surveys, surveys of at-risk populations, and environment assessments to identify lead exposure sources. This has been followed by developing mitigation strategies and advocating for policy changes to reduce lead exposure.

Findings: Further investigations identified lead-soldered cans, adulterated turmeric and geophagia as the source of lead for the pregnant women. Isotope analysis confirmed adulterated turmeric as the most significant contributor of lead exposure amongst these women. On further exploration on how the turmeric gets contaminated with lead, we identified a yellow pigment consists of lead-chromate, called Puri, that gets added during polishing the dried turmeric to look shiny. These findings helped catalyze policy changes in Food Safety regulations, leading to a substantial reduction in lead levels in turmeric in the markets. Over the years, the studies expanded to a comprehensive approach addressing multiple sources, including lead-acid batteries, leaded paints and aluminium cookware. A landmark intervention at an abandoned lead-acid battery recycling site combined soil remediation with community education to help reduce lead exposure from the soil to the nearby residents. Currently, icddr,b research team is exploring the lead exposure from aluminium cookware and consumer products, generating evidence of being significant sources of lead exposure.

Conclusion: Over the past decade, icddr,b has made significant strides in identifying and mitigating lead exposure risks in Bangladesh. Through multidisciplinary research, targeted interventions and policy advocacy, our organization remains committed to reducing lead exposure and safeguarding the health of vulnerable populations in Bangladesh.

P54-13 | Sex-specific differences in epigenetic-based biomarkers of lead exposure and kidney function among a representative sample of older Americans

Shannon M. Sullivan[1], Charles Silkin[1], Trey Smith[2], Weihua Guan[3], Jessica D. Faul[2], Bharat Thyagarajan[1]

[1] University of Minnesota, Laboratory Medicine and Pathology, Minneapolis, USA; [2] University of Michigan, Survey Research Center, Institute for Social Research, Ann Arbor, USA; [3] University of Minnesota, Division of Biostatistics and Health Data Science, School of Public Health, Minneapolis, USA

Objective: We used previously developed epigenetic-based biomarkers of lead exposure investigate the role of lead exposure, a known nephrotoxin, in driving sex-based disparities in kidney disease, considering key risk factors such as age, diabetes, hypertension, and obesity.

Material and Methods: DNA methylation was measured in the Health and Retirement Study (HRS), a nationally representative sample of Americans aged 51 and older, using the Illumina MethylationEPIC BeadChip. Epigenetic-based lead biomarkers for blood, patella, tibia, and blood to bone ratios were standardized using one-standard deviation. Kidney disease was evaluated using the CKD-EPI Creatinine-Cystatin-C equation (2021) to estimate glomerular filtration rate (eGFR, mL/min/1.73m^2). Survey-weighted multinomial logistic and linear models were adjusted for demographics, comorbidities, smoking, and immune cell compositions.

Results: Among 3,804 participants (54% were female, mean age 68.5 years), adjusted survey-weighted mean epigenetic-based lead biomarkers were 11.5 µg/dL (blood), 5.27 µg/g (patella), 17.8 µg/g (tibia), 2.2 (blood/patella), and 0.65 (blood/tibia). Significant sex differences were observed in all lead biomarkers ($P<0.012$) except blood ($P=0.323$). In sex-stratified models, females had consistently higher odds of kidney decline with most lead biomarkers, except tibia lead. In contrast, males generally had lower odds of kidney decline, except for tibia lead and blood/patella lead. Notably, for both males and females, the largest differences are observed in the most severe

eGFR categories (<45 mL/min/1.73 m^2 as compared to Normal/high eGFR ≥90). Linear trends demonstrate a significant inverse association with reduced eGFR and tibia lead in males (=-1.35; 95%CI: -2.19, -0.51) and blood/tibia lead in females (=-0.93, 95% CI: -1.82, -0.04).
Conclusion: Sex-specific associations between epigenetic-biomarkers of lead exposure and eGFR highlight distinct effects of circulating versus stored lead on kidney disease. Findings suggest that sex differences in bone remodeling could contribute to lead mobilization, which in turn, may inversely impact kidney disease. Lead monitoring in aging populations is crucial.

P54-15 | Investigation of lead exposure following consumption of contaminated cinnamon-applesauce pouches — United States, November 2023–April 2024

Alyssa N. Troeschel[1], Melanie C. Buser[1], Perri Ruckart[1], Andrea Winquist[1], Natalie Cataldo[2], Katherine Arnold[2], Audrey F. Pennington[1], Jelonia T. Rumph[1], Madison Smith[1], Marisol Valenzuela Lara[1], Michael Yeh[1], Arthur Chang[1], Johnni Daniel[1]

[1] Centers for Disease Control and Prevention, Atlanta, USA; [2] Food and Drug Administration, College Park, USA

Objective: In November 2023, U.S. Centers for Disease Control and Prevention (CDC) initiated a national call for cases of lead poisoning following consumption of recalled cinnamon-applesauce pouches. Here, we estimate the extent of the outbreak and characterize reported cases.
Material and Methods: Cases were reported to CDC on a biweekly basis during November 21, 2023–April 12, 2024, using a standard template. CDC defined cases as blood lead levels (BLLs) ≥3.5 µg/dL within 3 months after consuming a recalled cinnamon-applesauce pouch. States further classified cases as suspect, probable, or confirmed depending on the type of blood lead test conducted (e.g., probable and confirmed status required results from a venous blood draw) and home investigation findings (e.g., confirmed status required a home investigation that ruled out other significant sources of lead exposure).
Results: Forty-four U.S. states, Washington D.C., and Puerto Rico reported 566 cases to CDC, including 130 confirmed, 401 probable, and 35 suspect cases. Most cases (96%) occurred in children aged <6 years, with 55% occurring in children aged <2 years (n=311/566). Among probable and confirmed cases (n=531), the median within-person maximum venous BLL was 7.2 µg/dL (range: 3.5–39.3 µg/dL); approximately 32% had BLLs ≥10 µg/dL. Symptoms were reported for 81 cases, including gastrointestinal (n=55) and developmental or behavioral symptoms (n=35).
Conclusions: Hundreds of children were reported as cases to CDC after consuming contaminated cinnamon-applesauce pouches. This incident highlights the importance of preventing lead contamination of food products, especially those marketed towards young children. Routine blood lead testing and follow-up to identify lead exposure sources through source testing remain a critical safety net for when primary prevention efforts fail. In cases of lead poisoning, clinicians and public health practitioners should maintain an index of suspicion for lesser-known sources of exposure, such as food.

P54-16 | The Decline of Perinatal Lead Exposure and the Rise of ASD: A Panel Analysis

Aaron Grossman

Independent, Miami, USA

Introduction: Prevalence of autism spectrum disorders (ASD) has been rising unabated for the last several decades. This study investigates whether the historical reduction in lead exposure may have unintentionally contributed to this surge, potentially by altering metal absorption dynamics.
Methods: We conducted a panel analysis of data from municipalities and counties across 10 US states from 2005 to 2020 to understand the association between elevated blood lead levels (EBLL, ≥5 μg/dL) and ASD prevalence in children aged six or in first grade. A six-year lag between lead measurement and ASD diagnosis was implemented. Single- and multi-state analyses were conducted.
Results: In individual state analyses, significant inverse associations between EBLL and ASD prevalence were observed. In fully adjusted aggregated models accounting for population density, income, race, and temporal trends, EBLL was inversely associated with ASD prevalence ($p<0.0001$). When stratified by population density quintiles, significant inverse associations were observed in the second and third quintiles, while the most rural and urban quintiles showed no significant association. Variance decomposition revealed that 20.0% of ASD prevalence variability was attributable to state-level differences ($p<0.05$), while 24.0% was due to county-level variations ($p<0.0001$).
Conclusions: This study provides the first evidence that reduced lead exposure may have unintentionally contributed to rising ASD prevalence. These findings suggest that the dramatic decline in environmental lead exposure over recent decades, while beneficial in many respects, may have had unintended and paradoxical consequences for neurodevelopmental disorders. Due to potential public health consequences, further research is needed to confirm the results.

P54-18 | Combating lead poisoning in Ethiopia: Insights From an Interministerial Collaboration

Abdulhalik W. Bushra[1,2], Meles Solomon[2]

[1] Fenot project, Evidence to Policy, Addis Ababa, Ethiopia; [2] Ministry of Health, Maternal CHild and Adolescent Health LEO, Addis Ababa, Ethiopia

Objective
According to WHO, lead poisoning in Ethiopia is a public health concern. In fact, by some estimates as many as 30 million children may have higher than 5μg/dl of blood lead level (BLL). Despite the evidence, there have not been coordinated lead poisoning mitigation efforts.
Methods
A team of experts from ten Minister offices in Ethiopia was established under the leadership of the Ministry of Health, Envirnmental Protection Authority (EPA), USAID, and GIZ. The team synthesized available evidence and developed

action plan that aims to: prioritize the specific area(s) that have the largest burden; raise awareness; secure government buy-in and engagement; and advance lead exposure mitigation policies and activities.

Results

The main sources of lead exposure in Ethiopia are contaminated drinking water, occupational exposures, and solid waste disposal. Informal laborers engaged in reconditioning lead-acid batteries in substandard settings are exposed to lead, posing a risk to themselves and their families. Significantly high blood lead levels were also found in women and children. EPA drafted a strict law to ban lead paint – which was finally adopted by the Ethiopia Council of Ministers in July 2018. This Law restricts lead limits to 90 ppm for manufacturing, import, export or sale of paint. A 'road map' detailing short, mid and long-term plans for recycling Used Lead Acid Batteries (ULABs) is under development. However, no regulations around cookware and waste generated from smelting or mining exists.

Conclusion

Given the lack of public awareness and limitations in regulation and enforcement, a need exists for effective legislation, ability to provide continuous support for advocacy, communication and control efforts. It is also important to build the capacity of institutions and their workforce with easy-to-use instruments and the knowledge and skill for testing, analyzing and preventing lead poisoning. We aknwledge USAID for coordinating and financial support.

P54-19 | Designing childhood lead exposure surveillance with governments in low- and middle-income countries

Yi Lu, Sumi Mehta, Yatin Pimple, Jesica Rodríguez-López, Maria Elena Arias Coronel, Edwin Siswono, Dan Kass

Vital Strategies, Environment, Climate, and Urban Health, New York, USA

Objective

Global estimates suggest lead exposure accounts for 71 million life years lost due to disability and death. Despite its harmful effects, surveillance systems that monitor lead exposure and sources are lacking in the countries most affected. We provide an overview of five pragmatic strategies considered when initiating surveillance in Asia and Latin America and discuss how resources and impacts of various options led to the choice of approaches taken under different scenarios.

Material and Methods

We initiated surveillance efforts in India, Indonesia, Colombia, and Peru in collaboration with local governments, international NGOs, and research organizations, In each geography, we summarized and compared key components of potential surveillance approaches, including design considerations and implementation challenges. Expected outputs and policy implications of options were also compared and contrasted.

Results

Government engagement and cross-sector collaboration varied significantly across the models and geographies. Working groups formed to lead initiatives were influenced by local policy context, institutional priorities and awareness, existing activities, and local stakeholders. The proposed surveillance designs also differed markedly in scope and implementation approach, and were influenced by the specific surveillance objectives, current surveillance and health system infrastructure, laboratory capacity, and the financial and human resources available. The probability of turning initial surveillance into routine programming, incorporation of results into data platforms, and

utilization of data to inform health resource allocation and policy development to reduce lead pollution, depends largely on the engagement and ownership by government partners.

Conclusion

Models and designs of blood lead surveillance should be developed based on existing in-country infrastructures and resources. While challenging and time-consuming on the front-end of the planning process, the buy-in of government leadership is key to ensuring sustainable and impactful surveillance programs.

P54-20 | Effect of pH on Biosorption Potential of *Micrococcus luteus* on Lead and Cadmium from Groundwater Sources in Shikira Community, Nigeria

Agatha A. Ikhumetse[1], Olabisi P. Abioye[2], Hyelamada A. Aminuwa[3], Sources of fundingThe study was not funded by any organization, the authors contributed their financial resources for the study.

[1] Federal University of Technology, Department of Microbiology, Minna, Nigeria; [2] Federal University of Technology, Department of Public Health, Minna, Nigeria; [3] Ahmadu Bello University, Department of Zoology, Zaria, Nigeria

Objective

The purpose of this study was to ascertain how pH affected the ability of *Micrococcus luteus* to biosorb lead and cadmium from groundwater sources in Shikira Community, Rafi Local Government Area, Niger State, Nigeria.

Material and Methods

Total viable counts and total coliform counts were done by the pour plate technique. Physicochemical properties and heavy metal contents of the water samples were determined using standard methods. Isolates were screened for heavy metal tolerance ability by culturing on nutrient broth supplemented with 5.50 mg/L lead [$(Pb(NO_3)_2$] and 1.50 mg/L cadmium ($CdSO_4$). *Micrococcus luteus* was chosen for biosorption studies because it demonstrated the highest resistance to the heavy metals. The effect of pH on the biosorption potential of the bacterial isolate was also determined.

Results

The results revealed that the total viable counts ranged from 0.00±0.00 cfu/mL to $165.67 \pm 3.75 \times 10^1$ cfu/mL while total coliforms ranged from 0.00±0.00 cfu/mL to$150.00 \pm 3.46 \times 10^1$ cfu/mL. The physicochemical parameters (conductivity, total hardness, total dissolved solids, phosphate, nitrate and chlorides) were within World Health Organization (WHO) permissible limits. Some samples had pH and fluoride levels within WHO permissible limit. The majority of the water samples' turbidity and biological oxygen demand (BOD_5) were higher than the WHO-recommended threshold. The findings of the heavy metal studies showed that zinc (Zn) was within the standard acceptable levels by WHO, but lead (Pb), cadmium (Cd), chromium (Cr), and mercury (Hg) had mean values over WHO allowed limits in all water samples. After 28 days, *Micrococcus luteus* removed lead and cadmium with an optimal removal efficiency of 98.21% and 88.56% at pH 4.20 and 4.80, respectively.

Conclusion

In light of the current study's findings that the bacterium effectively removed lead and cadmium from heavy metal-contaminated water, it can be used to support future investigations into the treatment of heavy metal-contaminated water.

P54-21 | Arsenic and Lead Levels in Drinking Water and Toenails: Association with Inflammation Biomarkers in Nova Scotia, Canada

Alexandra Del Favero-Campbell[1], Ellen Sweeney[2], David E. Fleming[3], Ian Weaver[4], Ethan Briand[3], W. Shane Journeay[5], Christopher S. Greene[1], Daniel Rainham[6], Kelvin C. Fong[7]

[1] Dalhousie University, Department of Earth and Environmental Sciences, Halifax, Canada; [2] Dalhousie University, Atlantic PATH, Faculty of Medicine, Halifax, Canada; [3] Mount Allison University, Department of Physics, Sackville, Canada; [4] Dalhousie University, Department of Psychology and Neuroscience, Halifax, Canada; [5] Dalhousie Medicine New Brunswick & Dalhousie University, Departments of Medicine and Community Health & Epidemiology, Saint John, Canada; [6] Dalhousie University, School of Health and Human Performance, Faculty of Health, Halifax, Canada; [7] George Washington University, Department of Environmental and Occupational Health, Washington DC, USA

Objective: Arsenic (As) and lead (Pb) contamination in drinking water is a public health concern in parts of Canada, including Nova Scotia. Chronic metals exposure may elevate inflammation, disrupt immune function, and affect oxidative stress regulation and associated metabolic processes. Given the high prevalence of metabolic disorders and chronic conditions in Nova Scotia, As and Pb exposure is suspected to increase chronic disease risk. Previous research on arsenic speciation profiles from toenail samples differed significantly between participants with chronic conditions, such as lung and kidney cancer, and healthy controls. However, the pathways and molecular mechanisms remain unclear. This study investigates whether these metals in Nova Scotia are associated with inflammation.
Methods: Using biological samples from two longitudinal cohort studies, the Atlantic Partnership for Tomorrow's Health (PATH) and the Canadian Longitudinal Study of Aging (CLSA), we assessed levels of two pro-inflammatory cytokines, Interleukin-6 (IL-6) and Tumor Necrosis Factor Alpha (TNF-α). Metal levels were estimated using well water data and participant toenail samples. Data from each cohort were analyzed separately and together to estimate the effect of metals on inflammation. Associations between metal exposure and cytokine levels were estimated using generalized additive models, adjusting for age, gender, and socioeconomic status.
Results: Well water arsenic concentrations ranged from 0.05μg/L to 3900μg/L and lead from 0.05μg/L to 1420μg/L, with the most polluted regions in the Central and Western Health Authority Regions of Nova Scotia. Initial analyses explored associations between elevated metal levels and inflammation biomarkers across cohorts. Further analyses identified susceptible groups for targeted interventions.
Conclusion: While heavy metal exposures have been linked to inflammation, the molecular pathways remain poorly understood. Our findings provide insights into a Canadian population with elevated metal exposure and a high chronic disease prevalence, informing the development of surveillance tools and public health strategies to mitigate environmental health risks.

P54-22 | Lead in Newark drinking water: A successful community-driven data collection and academic-community partnership

Sean Stratton[1,2], Adrienne Ettinger[1,3], Anthony Diaz[5], Bavisha Kalyan[6], Cathleen Doherty[4], Brian Buckley[2], Zorimar Rivera-Núñez[1,2]

[1] Rutgers University, School of Public Health, Piscataway, USA; [2] Rutgers University, Environmental and Occupational Health Sciences Institute, Piscataway, USA; [3] Rutgers University, Rutgers Health, Newark, USA; [4] Rutgers University, Deapratment of Arts and Sciences, Department of Earth and Planetary Science, Piscataway, USA; [5] Newark Water Coalition, Newark, USA; [6] University of British Columbia, Civil Engineering, Vancouver, Canada

From 2017-2019, Newark, NJ exceeded the U.S. Environmental Protection Agency action level for lead in drinking water of 15 mg/L (parts per billion, ppb). We established an academic-community partnership to address residents' concerns through the design and implementation of a water sampling project. We distributed 500 tap water sampling kits to residents in and around Newark with instructions on how to collect three sequential samples: first draw, and after 1 and 5 minutes of flushing the tap. Thirty-three percent (N=165) of kits were returned and 83% of those were from homes within the City of Newark for a total of 411 samples analyzed for lead. Lead was detected in all water samples with concentrations ranging from 0.01 to 685 ppb. Thirty-seven percent of homes had at least one sample ≥1 ppb; 17% had at least one sample ≥5 ppb. The 90th percentile of lead for first draw, and after 1- and 5-minute flush, were, respectively: 12.5,7.96, and 4.51 ppb. After a 5-minute flush, only 25% of homes with first draw samples. 1 ppb were reduced to <1 ppb, and 17% of homes with first draw samples above 5 ppb were reduced to below 5 ppb and even fewer of those (16%) were reduced to <1 ppb. Using a community science approach, we created a successful academic-community partnership that responded to residents' concerns about lead in drinking water. Additionally, we observed that common public health tap water flushing recommendations do not fully protect residents from lead in drinking water.

P54-23 | Urgent Need for Updating Blood Lead Reference Values in China: A National and International Perspective

X. Duan[1]

[1] University of Science and Technology Beijing, School of Energy and Environmental Engineering, Beijing, China

Despite significant reductions in blood lead levels (BLLs) among children in China over the past two decades, incidents of lead poisoning and elevated BLLs remain a persistent public health concern. This ongoing issue highlights the inadequacy of current hygienic regulations in effectively managing children's lead exposure. This study examines the revision processes of blood lead reference values (BLRVs) in various countries and evaluates the current BLLs and their changing trends in China. Additionally, it identifies potential sources of lead pollution and exposure pathways, emphasizing the importance of protecting children's health from lead-related hazards.

The study also reviews national measures and strategies for lead emission management and control, highlighting the necessity and urgency of updating China's BLRVs for children. Based on comprehensive analysis and considering China's specific environmental, health, and economic conditions, a proposed BLRV of 50 µg/L is suggested. This updated value is deemed more scientifically sound and protective of children's health compared to the current standards. Implementing this new BLRV could yield substantial economic benefits through improved cognitive development, as evidenced by enhanced IQ outcomes associated with lower lead exposure.

In conclusion, updating China's BLRVs is an urgent and necessary step to align with international best practices and to safeguard the health and development of Chinese children. This study underscores the importance of revising existing regulations and adopting a more stringent BLRV to mitigate the risks of lead exposure and its associated health impacts.

P55 | Occupational Exposures and Health - PART II

P55-01 | MRI signature of brain age underlying post-traumatic stress disorder in World Trade Center responders

Azzurra Invernizzi[1], Francesco La Rosa[2,3], Anna Sather[1], Elza Rechtman[1], Ismail Nabeel[1], R. Sean Morrison[4], Alison C. Pellecchia[5], Stephanie Santiago-Michels[6], Evelyn J. Bromet[7], Roberto Lucchini[8], Benjamin J. Luft[5,9], Sean A. Clouston[10], Erin Beck[2], Cheuk Y. Tang[1], Megan K. Horton[1]

[1] Icahn School of Medicine at Mount Sinai, Dept of Environmental Medicine, New York, USA; [2] Icahn School of Medicine at Mount Sinai, Department of Neurology, New York, USA; [3] Icahn School of Medicine at Mount Sinai, Department of Artificial Intelligence and Human Health, New York, USA; [4] Icahn School of Medicine at Mount Sinai, Department of Geriatrics and Palliative Medicine, New York, USA; [5] Renaissance School of Medicine at Stony Brook University, World Trade Center Health and Wellness Program, New York, USA; [6] City University of New York, Department of Psychology, The Graduate Center, New York, USA; [7] Renaissance School of Medicine at Stony Brook University, Department of Psychiatry, New York, USA; [8] Florida International University, Department of Environmental Health Sciences, Robert Stempel School of Public Health, Miami, USA; [9] Renaissance School of Medicine at Stony Brook University, Department of Medicine, New York, USA; [10] Renaissance School of Medicine at Stony Brook University, Program in Public Health and Department of Family, Population, and Preventive Medicine, New York, USA

Background. The men and women involved in rescue and recovery operations at the 9/11 World Trade Center (WTC) site have a greater prevalence (23%) of persistent, clinically significant post-traumatic stress disorder (PTSD). Recent structural and functional magnetic resonance imaging (MRI) studies demonstrate significant neural differences between WTC responders with and without PTSD. Here, we used brain age, a novel MRI-based data-driven biomarker optimized to detect accelerated structural aging and examined the impact of PTSD on this process.
Methods. Using BrainAgeNeXt, a novel convolutional neural network trained and validated on >11,000 magnetic resonance imaging (MRI) T1-weighted scans, we predicted brain age in WTC responders with PTSD (WTC-PTSD, n = 47) and age/sex matched responders without PTSD (non-PTSD, n = 52). Brain Age Difference (BAD) was then calculated for each WTC responder by subtracting chronological age from brain age. A positive BAD indicates that the responder's brain has aged more than expected for their chronological age.
Results. We found that BAD is significantly greater in WTC-PTSD compared to non-PTSD responders (BAD_{no_PTSD}=-0.43y; BAD_{WTC_PTSD}=3.07y; p < 0.001). Further, we found that WTC exposure duration (months working on site) moderates the association between PTSD and BAD (p=0.005).
Conclusion. Our results suggest that brain age is a relevant marker of structural damage in WTC responders with and without PTSD. In particular, PTSD may be a substantial risk factor for accelerated neurodegeneration in this vulnerable and aging population.

P55-02 | Occupational Risk Assessment for Elderly Workers in Thailand: Development of a Risk Assessment Tool

Kraiwuth Kallawicha[1], Pokkate Wongsasuluk[1], Ekarat Sombatsawat[2], Titaporn Luangwilai[3], Wattasit Siriwong[1], Narumol Bhummaphan[1]

[1] Chulalongkorn University, College of Public Health Sciences, Bangkok, Thailand; [2] Thammasart University, Faculty of Public Health, Pathumthani, Thailand; [3] Navamindradhiraj University, Department of Medical Research and Innovation, Faculty of Medicine Vajira Hospital, Bangkok, Germany

Objective: With an aging workforce, the decline in the working-age population presents economic and labor challenges. This study aims to identify occupational risk factors among elderly workers and develop a risk assessment tool to enhance workplace safety.
Material and Methods: A cross-sectional study was conducted among elderly workers (aged > 60 years) across Thailand's formal and informal sectors. A structured questionnaire on occupational hazards was developed to collect data on demographics, occupational status, health conditions, and workplace risks. The assessment tool was designed based on a literature review, expert consultation, and survey results.
Results: A total of 435 elderly workers participated, with most engaged in informal employment, particularly in agriculture (47.82%), trading and food vending (22.53%), and general labor (16.09%). The primary motivation for working was financial necessity. The most common health issues included high blood pressure (17.24%), musculoskeletal pain, vision impairment, and mobility difficulties. The risk assessment tool categorized risks into three sections: (A) age-related risks, (B) personal risk factors, and (C) occupational hazards. Risk levels were classified into four categories: low, moderate, relatively high, and high, with recommendations for risk mitigation. Pilot testing of the assessment tool indicated that most elderly workers faced minimal or no risks. However, factory workers exhibited moderate risks due to physical and ergonomic factors, while sales professionals faced moderate risks primarily due to their advanced age.
Conclusion: Occupational risks among elderly workers vary by job type, health status, and workplace conditions. The developed risk assessment tool provides a structured approach to identifying and mitigating these risks, promoting safer work environments and supporting sustainable employment for aging workers.

P55-05 | Health Effects of Occupational Exposure to Jet Fuels: A Fit-for-purpose Systematic Review of the Epidemiologic Literature

Alexander J. Lindahl[1], Elizabeth R. Heitz[2], Sorina E. Eftim[1], Kate Weinberger[1], Cary Haver[1], Justin G. Bergeron[2], Terra D. Vincent-Hall[2]

[1] ICF, Reston, USA; [2] U.S. Department of Veteran Affairs, Exposure Science Program, Health Outcomes Military Exposures, Washington, D.C., USA

Objective: Exposure to jet fuel is common in the military, but health impacts are incompletely characterized. The US Department of Veterans Affairs (VA) assessed potential health outcomes related to jet fuels used by the US military, including a review to determine the carcinogenic potential of jet fuels.
Methods: VA adapted the US EPA's Integrated Risk Information System (IRIS) methodology to systematically review cancer risks related to jet fuel exposure. Human and animal data were reviewed, and included studies underwent

study quality evaluations. Given the limited epidemiologic information, "*uninformative*" studies were considered during evidence synthesis. Mechanistic data were evaluated based on evidence related to key characteristics (KC) of human carcinogens.
Results: Ten epidemiologic studies met inclusion criteria for cancer outcomes, with nine considered *low* confidence and one considered *medium* confidence. Relevant studies reported positive associations between jet fuel exposure and incident kidney, bladder, and any cancer, as well as all cancer mortality in men and pancreatic and oropharyngeal cancer mortality in women. It was determined there was *slight* evidence of an association between jet fuel exposure and cancer outcomes from epidemiological studies. Findings were supported by evidence of renal cancer from animal toxicological studies and mechanistic data describing associations between jet fuel exposure and induction of inflammation, immunosuppression, and increased oxidative stress.
Conclusion: Based on the available human, animal, and mechanistic data, the weight-of-evidence was *suggestive of carcinogenicity*; a concern for potential carcinogenic effects in humans was raised, but data were not sufficient given the limited number of quality epidemiologic studies, inconsistent effects across jet fuels or study design in animal toxicological studies, and the complexity of associating cancer effects with a mixture. Future studies that better elucidate the relationship between jet fuel exposure and cancer risk are necessary to inform policies related to the health care needs of impacted Veterans.

P55-06 | The Long-term Impact of Fuel Exposure (LIFE) Study: Leveraging Administrative Data to Understand the Consequences of Military Exposures in Veterans

Terra D. Vincent-Hall[1], Elizabeth R. Heitz[1], Nicholas A. Tilton[1], Justin G. Bergeron[1], Jennifer A. Rusiecki[2], Aaron I. Schneiderman[1], Edward A. Sheriff[3], Warren S. Monks[4], Gregory Wolff[5]

[1] Department of Veterans Affairs, Health Outcomes Military Exposures, Washington, DC, USA; [2] Uniformed Services University of the Health Sciences, Department of Medicine, Bethesda, USA; [3] Defense Health Agency, Armed Forces Health Surveillance Division, Silver Spring, USA; [4] Defense Health Agency, Defense Centers for Public Health, Aberdeen, USA; [5] US Air Force School of Aerospace Medicine, Epidemiology Consult Service, Wright-Patterson AFB, USA

Objective: Previous studies indicate a probable relationship between jet fuel exposure and effects on various body systems, such as the nervous and respiratory systems. To better understand the risks for chronic outcomes associated with jet fuel exposure in in the military, the Department of Veterans Affairs (VA) is conducting the Long-term Impact of Fuel Exposure (LIFE) Study in collaboration with the Department of Defense (DoD).
Materials and Methods: This retrospective cohort study links administrative datasets from DoD and VA to identify Veterans who entered the service on or after January 1, 1995, with follow-up through December 31, 2023, to observe the occurrence of various health outcomes among the population. The Veterans selected for this study include those with potential occupational jet fuel exposure, as identified by military occupation codes, and a random sample of Veterans without jet-fuel related occupations. Where possible, DoD workspace monitoring data will be used to compare exposure potential across exposed career fields. Cox Proportional Hazards models will be used to estimate chronic disease risks related to jet fuel exposure. Effect modification due to demographic and military characteristics will also be assessed.
Results: The LIFE study is comprised of 1,351,407 Veterans, including 677,804 Veterans with potential occupational jet fuel exposure during their military service. Preliminary analyses suggest exposed Veterans exhibit different

patterns of risk related to certain outcomes (e.g., neurological, behavioral, respiratory) compared to Veterans that were not occupationally exposed, which is consistent with previous reports. Additionally, sex, service branch, and deployment also impact the risk of developing adverse outcomes in the exposed population.
Conclusion: The LIFE Study is the largest cohort study to investigate the consequences of jet fuel exposure in the military to date, and its findings may have implications for policies related to healthcare and disability benefits for impacted Service members and Veterans.

P55-07 | Initial Findings from an Investigation of Blood PFAS Concentrations Among Michigan Firefighters

Priyashi Manani, Carissa Hueter, **Jacob Carrick**, Matthew Geiger, Jennifer Gray, Elizabeth Wasilevich

Michigan Department of Health and Human Services, Lansing, USA

Objective: In 2021, MDHHS launched the PFAS in Firefighters of Michigan Surveillance (PFOMS) project to determine blood concentrations of per- and polyfluoroalkyl substances (PFAS) in Michigan firefighters. Firefighters have unique potential for exposure to PFAS through occupational sources such as Class B firefighting foam and protective gear.
Material and Methods: The PFOMS project 1) collected data on occupational and behavioral factors related to PFAS exposure through surveys, 2) measured concentrations of 39 PFAS in firefighters' blood and 3) analyzed drinking water samples collected from fire stations. Descriptive statistics were used to characterize firefighters' blood PFAS concentrations. Results were compared to U.S. population estimates from NHANES and to results from other studies. PFAS detection frequencies in fire station drinking water were also calculated.
Results: The PFOMS project enrolled 64 fire departments and 1,090 firefighters. Preliminary results indicate that all participants had detectable concentrations of PFAS. Prevalence of having detectable PFAS were higher or similar for 22 of 23 PFAS where U.S. population comparison data was available. Average blood PFAS concentrations were generally lower or similar compared to the U.S. population and other firefighter studies. Airport firefighters had higher average concentrations of eight PFAS compared to non-airport firefighters. PFAS was rarely detected in fire station drinking water.
Conclusion: The preliminary results from the PFOMS project do not indicate higher average concentrations of PFAS among Michigan firefighters compared to the U.S. population; however, some PFAS were detected more frequently. Differences in average PFAS concentrations were also identified between firefighter subgroups, indicating potential differences in exposure. MDHHS will continue to analyze this data to explore occupational factors that may contribute to differences in PFAS exposure.
PFOMS was funded by the Centers for Disease Control and Prevention (CDC) State Biomonitoring Cooperative Agreement and the State of Michigan (SOM).

P55-08 | Sources of Metal Exposures in Workers in MesoAmerican Nephropathy Occupational Study

Kathryn M. Rodgers[1], Ronald Glabonjat[2], Ana Navas-Acien[2], Patrick J. Parsons[3], Chistopher D. Palmer[3], Michael Light[1], Jocelyn Fimbres[1], Yorghos Tripodis[4], Emmanuel Jarquin[5], Ramon Garcia-Trabanino[6,7], Sinead A. Keogh[1], Zoe Petropoulos[1], Jessica H. Leibler[1], Daniel R. Brooks[8], Madeleine K. Scammell[1]

[1] Boston University School of Public Health, Department of Environmental Health, Boston, USA; [2] Columbia University Mailman School of Public Health, Department of Environmental Health Sciences, New York, USA; [3] New York State Department of Health, Wadsworth Center, Albany, USA; [4] Boston University School of Public Health, Department of Biostatistics, Boston, USA; [5] Agencia para el Desarrollo y la Salud Agropecuaria (AGDYSA), San Salvador, El Salvador; [6] Centro de Hemodialisis, San Salvador, El Salvador; [7] Emergency Social Fund for Health, Tierra Blanca, El Salvador; [8] Boston University School of Public Health, Department of Epidemiology, Boston, USA

Objective: Metal exposures are not well-characterized in Central American populations, which is a gap in understanding region-specific exposures that are relevant to region-specific diseases, notably chronic kidney disease of uncertain etiology (CKDu), also referred to as Mesoamerican Nephropathy.
Methods: We analyzed urine from 406 participants in January 2018 and repeated analyses in 194 participants in May 2021 for 15 metals/metalloids in a cohort of workers in the MesoAmerican Nephropathy Occupational Study (MANOS). We evaluated several predictors of urine metal concentrations, including smoking status, seafood and beverage consumption, agrochemical exposure, drinking water sources, type of industry, site of work, and home department (similar to a province).
Results: Eleven metals/metalloids were detected in at least 60% of participants. No participants exceeded health-based guidelines for cadmium or mercury. Four people’s total urinary arsenic concentrations exceeded >50 μg/L, though this study population had relatively high levels of dietary arsenic, making it is unclear what these exceedances meant for health. The most consistent trend among industries was that road construction workers had significantly higher urine cobalt concentrations compared to workers in all other industries. Workers at one sugarcane worksite had significantly higher urine concentrations of arsenic, cadmium, mercury, and lead, suggesting sources at that specific worksite location. Participants with private wells at home had significantly higher urine lead concentrations compared to those with municipal drinking water sources at home. While urine mercury was positively associated with working in corn, it was negatively associated with living in the same department as corn, suggesting a workplace exposure source. However, most elements were positively associated with living in three of the six departments, which suggests there are key sources associated with residential location apart from occupation.
Conclusion: The results from the study are important for understanding potential public health strategies to reduce metal exposures.

P55-09 | Pesticide use, hazard outlook, and farmers' health: challenges and opportunities for agri-health and food safety management in St. Vincent and the Grenadines

Lindonne Telesford, Odran Edwards, Sabrina Compton, Samuel Hale, Sandra Brianne Vandenborn, Masara Touza, Meryem Merve Ören, Leselle Pierre-Romain, Satesh Bidaisee, Prakash Ramdass, Yusuf Yakubu

St. George's University, St. George's, Grenada

Objectives:
Globally, low- and middle-income countries bear the highest burden of disease from indiscriminate use of agrochemicals. Few recent studies directly focus on pesticide use and health in the smaller Caribbean islands, leaving significant gaps to tailor policies and interventions in agri-health and food safety. This study aims to close this gap in one Caribbean country.

Methods:
A retrospective cross-sectional study was conducted with 212 farmers to assess pesticide use, hazard outlook and farmers' health in the three agricultural regions in St. Vincent and the Grenadines.

Results:
Over 90% of the farmers used commercial pesticides. There were no statistically significant associations between the use of pesticides and farmers' demographics. The percentage of farmers who reported using pesticides in the last 2 years were similar for those who completed primary and university education. A consistent pattern was not found in reported use of pesticides in the last 2 years across age groups. Over 40% of the farmers reported experiencing symptoms of rheumatoid arthritis and wheezing, respectively, and 15% reported having respiratory problems. Knowledge about natural pesticides was significantly associated with Region ($p < 0.05$). Personal protective equipment usage appears to be irregular. Training in pesticide handling, storage, and application was limited.

Conclusion:
Agri-health surveillance and food security systems are immediately necessary. Both men and women should be targeted in agri-health interventions. More investigations are needed from this baseline on relationships between pesticide use and demographics, health, and food safety practices.

P55-10 | Characterizing markers of acute kidney injury among hairdressers

Magdalena Fandino Del Rio[1], Grant Tore[1], Melissa DeSantiago[1], Wassim Obeid[2], Chirag Parikh[2], Katrina Randolph[3], Sarrah Davis[4], Walkiria Pool[5], Ana Rule[1], Lesliam Quirós-Alcalá[1]

[1] Johns Hopkins University, Department of Environmental Health and Engineering, Baltimore, USA; [2] Johns Hopkins Medicine, Department of Medicine, Baltimore, USA; [3] Tres Shadez Salon, Capitol Height, USA; [4] Taino, Inc., Waldorf, USA; [5] Center for Assisting Families, Hyatsville, USA

Objective: Hairdressers face chronic occupational exposures to many chemicals contained in hair care products and formed from heat application. In recent years, there have been several case reports of acute kidney injury associated with the use of hair products, including straighteners, but the impact of occupational exposures on the kidney health of hairdressers remains understudied. We characterized biomarkers of acute kidney injury among hairdressers and a comparison group of office workers.

Methods: We conducted a nested pilot study using data and biobanked urine samples collected from 13 hairdressers and 13 office workers, aged 21 to 56 years, matched by age and race/ethnicity. All participants provided urine samples at the end of one work shift. Using the MesoScale Discovery platform (MesoScale Diagnostics, LLC, Gaithersburg, MD USA), we measured five urinary biomarkers of kidney injury (NGAL, KIM-1), inflammation (IL-18, MCP-1), and repair (YKL-40), and assessed differences between occupational groups using non-parametric Wilcoxon rank tests.

Results: On average, hairdressers had 11.5 (SD:7.9) years of salon work experience. We observed strong positive correlations between creatinine-corrected kidney biomarkers in our study sample (Spearman correlations: 0.57-0.88, p-values<0.01). Median urinary levels of kidney injury biomarkers were consistently higher in hairdressers compared to office workers (in pg/mg-creatinine, NGAL: 45,216.32 vs. 32,673.49, p-value:0.54; KIM-1: 832.50 vs. 322.72, p-value:0.14; IL-18: 157.13 vs. 56.44 p-value:0.20; MCP-1: 690.71 vs. 158.09, p-value:0.09; and YKL-40: 1,202.24 vs. 355.66, p-value:0.20). Five participants (19.2%), all hairdressers, had ≥4 biomarker concentrations in the highest tertile, which we designated as subclinical kidney injury.

Conclusion: Preliminary findings from this pilot suggest that hairdressers may be at a higher risk of kidney damage due to increased occupational chemical exposures. Additionally, kidney injury itself could further elevate their susceptibility to the health effects of occupational chemical exposure. Future analyses will examine kidney injury biomarkers and chemical exposure levels.

P55-11 | Hazardous heat and wildfire $PM_{2.5}$ exposure among outdoor workers in the United States, 2010-2019

Abas Shkembi[1], Devon Payne-Sturges[1], Peter Song[2], Sung Kyun Park[1,3], Jon Zelner[3,4], Richard Neitzel[1]

[1] University of Michigan School of Public Health, Environmental Health Sciences, Ann Arbor, USA; [2] University of Michigan School of Public Health, Biostatistics, Ann Arbor, USA; [3] University of Michigan School of Public Health, Epidemiology, Ann Arbor, USA; [4] University of Michigan School of Public Health, Center for Social Epidemiology and Population Health, Ann Arbor, USA

Objective: Identifying populations most susceptible to climate change-related exposures is critical in order to develop effective interventions and policies to protect human health. The nearly 45 million Americans who regularly work outdoors are particularly susceptible. Not considering outdoor work as an important driver of heat and wildfire smoke exposure likely biases research on climate-related human health impacts. We sought to estimate the yearly, county-level prevalence of outdoor workers in the contiguous United States (CONUS) exposed to hazardous heat and wildfire $PM_{2.5}$ from 2010-2019.

Material and Methods: We simulated the daily number of outdoor workers exposed for every CONUS county by leveraging (i) large-scale, county-level data on daily-average web bulb globe temperature and wildfire $PM_{2.5}$ levels, (ii) work characteristics that influence exposure for all occupations from the Occupational Information Network (i.e., outdoor work, weekend work, metabolic rate, and work/rest allocation), and (iii) employment counts from the American Community Survey data. Overexposure for heat was determined according to 2023 ACGIH screening criteria for unacclimatized workers. Overexposure for wildfire smoke was determined according to a 2024 US NIOSH draft hazard review of wildfire smoke.

Results: Nationwide, there were 5.44 billion person-days of hazardous heat exposures from 2010-2019. Workers in Texas and Florida accounted for 46.9% of exposures annually (255 million person-days per year). For wildfire smoke exposure, we estimated around 497 million person-days of exposure to wildfire $PM_{2.5}$ ≥9 μg/m^3 between 2010-2019 for outdoor workers, with California, Texas, New York, and Washington accounting for a third of exposures. We observed substantial spatial heterogeneity in both exposures.

Conclusion: Characterizing work-related exposure to hazardous heat and wildfire smoke improves understanding of the true public health burden of these climate change-related hazards.

P55-12 | Occupational Heat Stress Exposure and Associated Factors among Workers at Omo Kuraz Sugar Estate, Ethiopia.

Dawit Siraw[1], Belay Simane[1], Abera Kumie[1], Kiros Birhane[2], Darby Jack[2]

[1] Addis Ababa University, Addis Ababa, Ethiopia; [2] Columbia University, New York, USA

Background: The sugar industry is a major employer in Ethiopia, with workers frequently exposed to high heat stress due to the hot climate and physically demanding harvesting tasks. This study assesses heat stress levels and workers' knowledge and perceptions at the Omo-Kuraz sugar estate.

Methods: Heat exposure was measured using the Wet Bulb Globe Temperature (WBGT) QUEST Temp 34 device during the peak harvesting season (March 16 – April 4, 2024), following ISO 7243 guidelines. Heat exposure data was collected every 15 minutes at a central site of the sugar estate farm and summarized hourly. Also, a total of 190 workers and 21 managers participated in assessing the heat exposure factors.

Results: Working hours were from 7:00–12:00 and 15:00–18:00. The average WBGT level was 28.18°C (SD = 2.29), ranging from 23.50°C to 30.75°C, often exceeding the 27.5°C threshold. Workers practiced extreme heat stress, with 77.89% who reported very hot conditions, and only 38.42% had access to drinking water. Heat-related symptoms were common (65.79%), with 50% undergoing illness. Heat exposure affected productivity, with 61.90% reporting absenteeism with an average of 18.62 days per year. Although 77.27% of managers believe hot weather affects work, 95.45% reported having no plan to handle heat waves, indicating inadequate preparedness for extreme heat events. Common coping mechanisms include working early in the morning (72.73%), hiring more workers to complete tasks before the heat intensifies (72.73%), and drinking water (63.64%).

Conclusion & Recommendation: Excessive heat exposure significantly affects sugarcane workers' health and productivity, which exceeds ISO safety standards. Interventions such as worker awareness and training, providing shaded rest areas, and improved drinking water access are useful to reduce heat stress risks.

Acknowledgement: This research was supported by the Fogarty International Center, NIH, under Award Number U01TW010094. The authors are responsible for the content, not the NIH's views.

P55-13 | Climate in context: Farmworker community experiences and public agency responses to compound extreme heat exposure in California agricultural occupational settings

Haley Ciborowski, Nicolas Lopez-Galvez, Penelope Quintana, Arely V. Ayala, Vladimir Quintana, Hurtado Stephanie, Trent Biggs, Corrie Monteverde, Ana Solorio, Briana Toji-Ruiz

San Diego State University, Public Health/Geography, San Diego, USA

Agricultural workers, essential to food systems and nutrition security, face growing risks of heat-related illness (HRI) due to rising extreme heat events. The Imperial Valley is one of the most productive agricultural regions in California, but the climate there results in the highest rates of HRI in the state. Despite the physically demanding nature of their work, farmworkers often lack adequate labor protections. As part of a large, mixed-methods study addressing extreme temperature exposure among farmworkers, we completed in-depth interviews with 25 farmworkers and 10 *mayordomos* (peer managers). We also held meetings with agencies at the county and state levels in public and

occupational health. Objectives were to contextualize the experience of and adaptation to compound exposure to extreme temperatures for farmworker communities; understand how they consume information about weather and HRI prevention; assess regulatory protections and resources at the county and state levels; and characterize HRI prevention in unregulated agricultural workplace settings. Findings suggest that most farmworkers have experienced symptoms of HRI and the mental stress of extreme heat exposure is burdensome. There is little to no training about HRI prevention for farmworkers or managers, but information is shared informally from experience. There is also no known enforcement of HRI prevention regulations. Peer managers are responsible for planning and adaptation for extreme heat events. Adaptation measures like nighttime shifts face challenges to safety and childcare logistics for workers. Information about extreme heat is consumed mostly via smartphone or the lived experience of how it "feels." County and state agencies feel pressure from the state to act on climate-related health issues but lack sufficient resources. Findings will improve HRI prevention resources, communication, and adaptation measures and foster collaboration between researchers, state agencies, and farmworker communities to develop evidence-based, community-driven solutions for extreme heat exposure. Funded by UCOP Climate Action Grants.

P55-14 | Influence of workload and work maneuver on thermoregulation and subjective perceptions of workers wearing safety helmets in outdoor workplaces

Tzu-Yu Liu, Hung-Pang Hsieh, Yi-Chun Lin, **Chen-Peng Chen**

China Medical University, Department of Occupational Safety and Health, Taichung, Taiwan

Objective:
Outdoor workers exposed to intensive heat may face thermal hazards when the burden of heat stress over-runs capacity of thermal strain. This study simulated heat exposure in outdoor environments of various heat loads with solar radiation to evaluate changes in thermoregulation and thermal sensations of participants wearing safety helmets.
Material and Methods:
This study was conducted in a microclimatic chamber pre-set at three temperatures (22°C, 26°C, and 30°C). The study simulated two work maneuvers, each lasting for an hour: the first maneuver consisted of 30 min of walking (235-360 W) followed by 30 min of stair-walking (360-465 W), while the second included the same activities but in the reversed order. Thermoregulatory indicators include core physiological metrics and skin-based thermo-physiological indicators.
Results:
The first maneuver (moderate-to-high intensity) resulted in a 9.86 % heart rate increase and a core temperature rise of less than 0.27 % during the initial phase, indicating effective thermoregulation. In contrast, the second sequence (high-to-moderate intensity) caused a 37.25 % heart rate increase, 1.72 times higher than the first maneuver, reflecting a greater cardiovascular load. High ambient temperatures (30 ^{o}C) and intense exertion significantly enhanced skin capillary blood flow, with significant statistical differences being observed between hot and cold seasons ($p < 0.05$). In cooler environments, skin moisture increased by only 6.88 %, one-tenth of the rise in hot seasons, suggesting reduced heat dissipation.
Conclusion:

The thermal adaptation and comfort under the same physical workload could be affected by the arrangement of work sequence. When working in warm environments for an extended duration, it is more appropriate to begin the work from a moderate metabolic rate and followed by work of a higher rate to minimize risk of thermal stress.
This study was financially supported by National Science and Technology Council, Taiwan (NSTC 113-2813-C-039-023-E and NSTC 112-2221-E-039-007-MY3).

P55-15 | Urinary Biomarkers of Kidney Inflammation and Oxidative Stress in Relation to Paddy Farming at High Ambient Temperature

Yan Lin[1], Qiumei Liu[2], Junfeng J. Zhang[1,2], Sameera Gunasekera[4], Mangala De Silva[3,2], Nishad Jayasundara[1,2]

[1] Duke University, Nicholas School of Environment, Durham, USA; [2] Duke University, Global Health Institute, Durham, USA; [3] University of Ruhuna, Department of Zoology, Matara, Sri Lanka; [4] Theme Institute Sri Lanka, Nugegoda, Sri Lanka

Objective: Increases in average temperature andheat extreme events can lead to acute kidney injury (AKI), especially among those involved in strenuous outdoor labor activities. This study aims to examine changes in markers of kidney inflammation and oxidative stress in tropical agricultural workers residing in a community at high risk of chronic kidney disease (CKD).
Material and Methods: We recruited healthy 39 male paddy farmers from Padaviya, Sri Lanka, a community with high prevalence of CKD of uncertain origins. We collected four urine samples from each farmer at the following time points: (1) early morning on farming day (baseline); (2) evening on the farming day; (3) early morning on the day following the farming day; and (4) early morning one month after the farming day. We used high-performance liquid chromatography with fluorescence detection to measure urinary levels of malondialdehyde and the ratio of kynurenine to tryptophan as biomarkers of oxidative stress and inflammation respectively.
Results: We did not observe significant difference in creatinine or tryptophan concentrations across four visits. Compared to the baseline, the creatinine-normalized malondialdehyde concentrations increased by 38.0% (95%CI: 18.6% to 57.4%) in the evening of the farming day and by 35.3% (95%CI: 15.9% to 54.7%) in the morning of day following farming. The ratio of kynurenine to tryptophan increased by 41.9% (95%CI: 6.7% to 88.7%) in the evening of the farming day and by 20.9% (95%CI: -9.1% to 60.8%) the morning of the day after farming. The increases in both malondialdehyde and kynurenine to tryptophan ratios were fully recovered to baseline levels one month later.
Conclusion: Strenuous farming in a hot environment led to reversible increases in urinary biomarkers of kidney inflammation and oxidative stress.

P55-17 | Occupational Human biomonitoring of flame retardants in WEEE sector in France: Impact of urinary temporal variability on the sampling design

Guillaume Antoine[1], Baninia Habchi[1], Nathalie Grova[1], François Zimmermann[2], Aurelie Remy[1], Sophie Ndaw[1]

[1] INRS (French National Research and Safety Institute for the Prevention of Occupational Accidents and Diseases), Department of Toxicology and Biomonitoring, Vandoeuvre-les-Nancy, France; [2] INRS (French National Research and Safety Institute for the Prevention of Occupational Accidents and Diseases), Department of Process Engineering, andoeuvre-les-Nancy, France

Objectives: Workers in E-waste sector are exposed to multi-chemicals including OPFRs (Organophosphate Flame Retardants). Assessing exposure of OPFRs by measuring metabolites in spot urine sample is difficult due to the limited knowledge of their half-lives and their temporal variability. This work aims to evaluate OPFRs temporal variability in E-waste workers' urine to identify the optimal sampling time for future occupational studies.
Materials and Methods: OPFR metabolites Bis(2-chloroethyl) phosphate (BCEP), Diphenylphosphate (DPP), Di(o-cresyl) phosphate (DoCP), Di(m-cresyl) phosphate (DmCP), Di(p-cresyl) phosphate (DpCP), Bis(1,3-dichloro-2-propyl) phosphate (BDCPP) and Bis-(1-chloro-2-propyl) phosphate (BCPP) were analyzed by mass spectrometry (LC-MS/MS) in urine specimens of workers collected during five working days in e-waste sector. Six exposed workers collected their urine from the first day's shift until the morning after the last day of work. Five control workers collected their first morning void, pre-shift urine and post-shift urine. Data were analyzed by various statistical approaches including the intraclass correlation coefficient (ICC).
Results: DoCP and BCPP were not detected in urine. DmCP and DpCP highlight a progressive weekly accumulation with a fair to good ICC-day (0.53) whereas, DPP, BDCPP, and BCEP show a rapid elimination profile (ICC-day <0.2). We applied an accurate grouping approach based on collection time and working day comparison. The results point out that the optimal urine sampling time is the “delayed post-shift” (2 to 5 hours after the shift) for BCEP and DPP and at the end of the workweek for DmCP &DpCP.
Conclusion: This study establishes the optimal urine sampling time for assessing occupational exposure to OPFRs. This approach can be extended to a broader range of FRs in the future, enhancing the efficiency and accuracy of occupational biomonitoring of these chemicals.

P55-18 | Life-time attributable risk of cancer compared to collective dose detriment in Sweden after the Chernobyl Nuclear Power Plant accident

Martin Tondel[1,2], Katja Gabrysch[3], Mats Isaksson[4], Christopher Rääf[5]

[1] Uppsala University, Department of Medical Sciences, Occupational and Environmental Medicine, Uppsala, Sweden; [2] Uppsala University Hospital, Department of Occupational and Environmental Medicine, Uppsala, Sweden; [3] Uppsala University, Uppsala Clinical Research Center, Uppsala, Sweden; [4] University of Gothenburg, Institute of Clinical Sciences, Department of Medical Radiation Sciences, Gothenburg, Sweden; [5] Lund University, Department of Translational Medicine, Medical Radiation Physics, Lund, Sweden

Objective: Life-time attributable risk (LAR) of cancer predicts the individual risk and can be translated into attributable number of incident cases (AC). AC was compared with the excess number of incident cancer cases based on the collective dose during the 50 years following the fallout in Sweden after the Chernobyl Nuclear Power Plant accident.
Material and methods: A dose calculation program included external dose from 134Cs and 137Cs and internal dose from 131I, 134Cs and 137Cs. Annual sex-specific population data in 1-year classes by municipality (n=290), counties (n=21) and future population projections was retrieved as open-source data from Statistics Sweden from 1986 to 2035. Estimated organ absorbed doses (mGy) was used to calculate the collective effective dose (manSv) with a method from the International Commission on Radiological Protection. Organ doses and risk coefficients for cancer provided by the United States Environmental Protection Agency was used to calculate LAR and AC.
Results: The municipality with the highest LAR was Härnösand municipality in Västernorrland county with 0.70 per thousand for males and 0.82 per thousand for females. AC in Sweden was estimated to 377 male and 448 female extra cancer cases over 50 years post-Chernobyl. The collective dose was calculated to 6,028 manSv whereas 2,148 manSv (36%) was due to internal exposure. Including skin cancer, the number of extra cases could be estimated to 665 from external and 368 from internal exposure. After excluding skin cancer, numbers were reduced to 277 from external and 154 from internal exposure in Sweden. Västernorrland county with only 3% of the Swedish population in 1986 got in total 18% of the collective dose.
Conclusion: LAR can be used in risk communication and collective dose is valuable for radiological protection. Our dose calculation manual and program code are freely available to be adopted to different fallout scenarios.

P55-19 | Relationship Between Metal Fume Particle Exposure, Gut Microbiota Health Index, and Lung Function

Chun-Chih Hsu, Dian-Ru Tsai, Ruei-Jing Gao, Ching-Huang Lai

National Defense Medical Center, School of Public Health, Taipei, Taiwan

Objective
Welding operations generate metal fume particles containing lead, manganese, nickel, zinc, cadmium, etc. These substances affect the respiratory system and may also impact the Gut Microbiotacomposition through inhalation and ingestion routes. With growing interest in the Gut-Lung Axis, this study aimed to investigate the Relationship Between Metal Fume Particle Exposure, Gut Microbiota Health Index, and lung function.

Material and Methods

This longitudinal study was conducted from 2022 to 2023, recruiting male workers from a shipyard in northern Taiwan. 80 and 74 participants were enrolled in 2022 and 2023, respectively. And 57 were repeated participants. Participants completed demographic surveys on Monday's workday. Urine and fecal samples were collected on Tuesday morning, along with lung function measurements. Urine samples were analyzed for heavy metals via ICP-MS. Lung function was assessed using spirometry. Gut microbiota was profiled with full-length 16S rRNA sequencing, and GMHI was calculated. Generalized estimating equations (GEE) in SPSS 27 examined the relationships between internal metal exposure, GMHI, and lung function.

Results

After adjusting for confounders, GEE analysis showed urinary aluminum ($\beta = -0.046$, 95% CI: -0.005 to -0.002, $p < 0.05$), iron ($\beta = -0.029$, 95% CI: -0.053 to -0.005, $p < 0.05$), and selenium ($\beta = -0.057$, 95% CI: -0.101 to -0.014, $p < 0.05$) were significant negative associations with FVC, respectively. GMHI was significantly positively associated with FEV1 ($\beta = 0.022$, 95% CI: -0.035 to -0.009, $p < 0.001$) and positively associated with FVC ($\beta = 0.026$, 95% CI: 0.014 to 0.038, $p < 0.001$).

Conclusion

Urinary metal levels are not significantly associated with GMHI. However, urinary selenium, aluminum, and iron were negatively associated with lung function, while GMHI was positively associated with lung function indices. These findings support the gut-lung axis and offer new insights into the health impacts of occupational metal exposure.

P55-20 | Association between metal fume particulate exposure and Dietary Inflammatory Index on Gut Microbiota composition.

Hsin-Hsu Shih, Shang-Yun Cheng, Ruei-Jing Gao, Ching-Huang Lai

National Defense Medical Center, School of Public Health, Taipei, Taiwan

Objective

Occupational heavy metal exposure poses a significant public health concern. Studies suggest that heavy metals can alter gut microbiota, increasing disease risk. However, the specific microbial responses to heavy metals and key microorganisms remain unclear. Diet significantly influences gut microbiota, and the Dietary Inflammatory Index (DII) serves as a tool to assess dietary effects on inflammation. This study investigates the combined impact of metal exposure and dietary inflammatory patterns on gut microbiota in an occupational setting.

Material and Methods

This longitudinal study was conducted at a shipyard in northern Taiwan (2022–2023), recruiting 80 and 74 male employees, with 56 having repeated measurements. A food frequency questionnaire (FFQ) was used to calculate DII and was categorized by tertiles. Urinary heavy metal levels were measured via Inductively Coupled Plasma Mass Spectrometry (ICP-MS). Gut microbiota composition was analyzed using third-generation full-length 16S rRNA gene sequencing. Statistical analyses were conducted using SPSS 27.0.

Results

Generalized estimating equation (GEE) analysis showed urinary cobalt (Co), zinc (Zn), and arsenic (As) concentrations were significantly positively associated with the Firmicutes-to-Bacteroidetes (F/B) ratio: Co (0.046, $p = 0.040$, 95% CI: 0.002, 0.09), Zn (0.054, $p = 0.003$, 95% CI: 0.018, 0.091), and As (0.056, $p = 0.011$, 95% CI: 0.013, 0.099). However, after adjusting for smoking and age, DII showed no significant association with the F/B ratio.

Conclusion

Occupational metal exposure significantly impacts gut microbiota composition, with Co, Zn, and As positively correlating with the F/B ratio, suggesting potential microbial dysbiosis. In contrast, dietary inflammatory patterns did not significantly influence gut microbiota, indicating that workplace metal exposure may have a more substantial effect than nutritional factors.

P55-21 | Comparison of shade materials for agricultural worker heat stress mitigation

John C. Flunker[2], Carolyn Reeb-Whitaker[1], **June T. Spector**[1,3]

[1] Washington State Department of Labor and Industries, Safety & Health Assessment & Research for Prevention (SHARP) Program, Tumwater, USA; [2] University of Iowa, Department of Epidemiology, Iowa City, USA; [3] University of Washington, Department of Environmental & Occupational Health Sciences, Seattle, USA

Objectives: Agricultural workers are at high risk of morbidity and mortality from workplace heat exposure. Shade contributes to reduced heat stress by blocking solar radiation. Though several US states have adopted occupational heat rules that require shade, implementation of specific shade approaches is not specified. We sought to compare different shade materials for agricultural worker heat stress mitigation to inform decision-making.
Materials & Methods: We conducted a quantitative field comparison study over three summer days in a real-world agricultural setting in Central Washington State. We examined the effect of five different shade materials on solar radiation and heat stress, measured every 10 minutes using Black Globe (BG) and Wet Bulb Globe Temperature (WBGT) sensors, respectively. Multivariable linear mixed effects regression models were used to examine the effect of different materials on BG and WBGT, while accounting for repeated measures and small cluster sizes.
Results: The mean (SD) dry air temperature across all study days was 28.3(1.3)°C [82.9(2.4)]°F. Shade materials reduced BG by 6.1-8.8°C and WBGT by 1.7-2.2°C, on average, depending on the material, compared to no shade. The largest reduction in BG (-8.81ºC) was seen for a hay tarp material (HAY; 95% Confidence Interval [CI]:-9.39,-8.23) followed by an 80% monofilament material (M80;-7.68ºC; 95%CI -8.27,-7.10). For WBGT, we also found the largest reductions among HAY (-2.22ºC; 95%CI -2.44,-2.00) and M80 (-2.07ºC; 95%CI -2.29,-1.84). We did not find substantial differences in BG and WBGTwhen comparing between different shade materials.
Conclusions: We found that all studied shade materials significantly reduced BG and WBGT compared to no shade, but we observed no substantial differences between materials in our study conditions. For similarly performing materials, practical factors that affect adoption and sustainability of shade approaches should be considered in workplace decision-making about which shade approach to pursue.

P55-22 | Beyond the Visible: Non-Targeted Detection of Occupational Airborne Exposures among Black Hairdressers

Lesliam Quirós-Alcalá[1], Krystal Pollitt[6], Magdalena Fandino Del Rio[1], Katrina Randolph[3], Sarrah Davis[4], Walkiria Pool[5], Melissa DeSantiago[1], Grant Tore[1], Monica McInerney[7], Julia Mandeville[2], Joanna Marroquin[2], Jean Zhou[6], Elizabeth Lin[6], Meredith McCormack[7], Ana M. Rule[1], Paloma Beamer[8], Kelly Palmer[8], Jodi Flaws[9], Meghan Rebuli[10], Anna Z. Pollack[2]

[1] Johns Hopkins University, Department of Environmental Health and Engineering, Bloomberg School of Public Health, Baltimore, USA; [2] George Mason University, Department of Global and Community Health, Fairfax, USA; [3] Tres Shadez Salon, Capitol Height, USA; [4] Taino, Inc., Waldorf, USA; [5] Center for Assisting Families, Hyatsville, USA; [6] Yale University, Department of Chemical & Environmental Engineering, New Haven, USA; [7] Johns Hopkins University, School of Medicine, Pulmonology and Critical Care, Baltimore, USA; [8] University of Arizona, Zuckerburg College of Public Health, Tucson, USA; [9] University of Illinois, Department of Nutritional Sciences, Urbana, USA; [10] University of North Carolina, Department of Pediatrics, Raleigh, USA

Background: There are over 700,000 US hairdressers, predominantly reproductive-aged women of low socioeconomic status; 30% are women of color. Hairdressers represent a “canary in a coal mine” due to occupational exposures to environmental contaminants potentially linked to endocrine disruption, respiratory, and reproductive effects. Occupational disparities to these contaminants may occur as hair products marketed to women of color may be more toxic than those marketed to White women. Many of these products exhibit hormonal activity, and are designed to alter hair structure and be used more frequently. Despite these concerns, exposure assessment studies in this workforce are lacking.

Methods:We assessed occupational exposures to airborne contaminants using non-targeted analysis among 7 Black hairdressers, aged 28-42 years, serving Black women. Hairdressers donned a wearable passive sampler (Fresh Air clip) in their breathing zone over 3 work shifts. We used thermal desorption gas chromatography high resolution mass spectrometry to quantify airborne contaminants.

Results: Non-targeted analysis revealed hairdressers were exposed to 216 chemicals in their workplace, including aromatic and oxygenated hydrocarbons, and oxygenated alkanes. The cheminformatics module of the EPA CompTox Dashboard was used to match potential health effects associated with detected chemicals. Information was available for 132 of the detected chemicals; 7 (5%) were identified as respiratory toxicants,102 (77%) as endocrine disruptors, and 23 (17%) as reproductive toxicants, including phthalates, musks, organometallics, and benzenoids. Based on EPA’s Chemical and Products Database, chemicals identified were predominantly fragrances, antimicrobials, preservatives, emollients, and adhesion promoters in personal care and hair products.

Conclusion: Non-targeted analysis of wearable passive samplers worn by hairdressers enabled comprehensive assessment of occupational exposures of concern and discovery of previously unidentified exposures. Exposures to these chemicals and other contaminants of concern will be examined further in an ongoing large-scale study to better understand and mitigate health risks in this vulnerable workforce.

P55-23 | Assessment of heat stress exposure of outdoor workers in Kuwait during the summer midday work ban.

Mariam Buqammaz[1,2], Perry Hystad[1], Barrak Alahmad[3], Laurel Kincl[1]

[1] Oregon State University, Environmental & Occupational Health Program, College of Health, Corvallis, USA; [2] Kuwait university, Department of Environmental & Occupational Health, Faculty of Public Health, Kuwait, Kuwait; [3] Harvard University, Department of Environmental Health, Harvard T.H. Chan School of Public Health, Boston, USA

Heat exposure in the workplace is a growing concern globally. There are important unanswered questions about the effectiveness of regulatory protective measures for outdoor workers; for example, in Kuwait's harsh desert climate, summer temperatures often reach extreme levels of 40 °C. Kuwait has a calendar-based midday work ban from June to August between 11:00 am and 4:00 pm. However, evidence of the effectiveness of this policy in reducing outdoor worker heat exposure remains limited. **Objective:** This study aimed to objectively measure heat stress risk among outdoor workers in Kuwait and evaluate how well the current midday summer ban reduces exposure to hazardous wet bulb globe temperature (WBGT) values. **Methods:** We evaluated heat exposure at three outdoor construction and roadwork worksites in Kuwait during the summer of 2024. We measured WBGT for 24 hours at two-time points in each location, one month apart. **Results:** The average WBGT ranged from 28°C to 30°C across the sites. Hourly WBGT values exceeded the American Conference of Governmental Industrial Hygienists Threshold Limit Values (TLVs) by 8 - 37% and 54 – 75% of 24 hours for the 3 sites for moderate and heavy work, respectively, when 21% (5 hours) is protected by the midday work ban. Exceeded TLVs suggest additional protective measures are needed. **Conclusion:** Our findings demonstrate that the current midday work ban does not fully protect workers from the full spectrum of hazardous heat exposure. Protective measures should extend beyond the current mid-day ban to include a comprehensive heat stress management program to protect its outdoor workers from heat stress.

P55-24 | Battling the Heat: Innovative Strategies for Municipal Workers in Kota Kinabalu, Sabah, Malaysia

Mei Ching Lim[1], Khamisah A. Lukman[2], Nelbon Giloi[2], Mohammad S. Jeffree[2], Sahipudin Saupin[2], Richard Avoi[2], Jac Fang Lim[3]

[1] South Seberang Perai District Health Office, NIBONG TEBAL, Malaysia; [2] Universiti Malaysia Sabah, Public Health Medicine Department, Kota Kinabalu, Malaysia; [3] Malaysian Industrial Hygiene Association (MIHA), Batu Caves, Malaysia

Introduction: The intensifying heatwaves resulting from climate change have highlighted the critical need to address heat-related health risks. This study focuses on evaluating the impact of the Heat-Awareness-Educational-and-Informative Tool (HAEIT) in reducing heat-related symptoms and mitigating heat strain among municipal workers.
Material and Methods: This mixed-method study involved 394 workers and incorporated both structured and open-ended questionnaires. Continuous heart rate monitoring was performed using smartwatches, while environmental parameters were assessed with iButton temperature loggers and WBGT (Wet Bulb Globe Temperature) heat-stress trackers. During the intervention phase, the Heat-Awareness Educational and Informative Tool (HAEIT) was implemented, complemented by the distribution of heat-reduction kits, which included a water bottle, handheld fan, cooling towel, and a heat-symptoms monitoring booklet. A follow-up evaluation was conducted three months post-

intervention. Data analysis utilized SPSS Version 28 for descriptive statistics, while RM-ANOVA, McNemar tests, and paired t-tests were applied to test the study's hypotheses.
Results: The average daily WBGT value (31.2±0.4°C) exceeded the threshold limit range (26.0°C–30.5°C), indicating a high vulnerability of workers to heat stress. Following the intervention, the prevalence of heat strain significantly dropped from 34.0% to 13.5% ($p<0.001$). Moreover, all heat-related symptoms—such as headaches, dizziness, fatigue, muscle pain, heat rash, and reduced concentration—showed marked reductions ($p<0.001$). Post-intervention, workers adopted healthier practices, including increased water consumption, taking more frequent breaks, and implementing additional heat-prevention strategies. Feedback from participants highlighted significant improvements in their awareness, knowledge, and behaviors. Additionally, the heat-reduction kits were widely regarded as practical and effective tools in managing heat stress.
Conclusion: This preliminary study, employing HAEIT and heat-reduction kits, offers valuable insights for optimizing these methods. The findings underscore their potential as sustainable, cost-effective solutions for mitigating heat-related risks in workplace settings.

P55-25 | Silica Exposure and Risk of Autoimmune Diseases: A Systematic Review

Glinda S. Cooper[3], Christine G. Parks[1], Emily C. Somers[2]

[1] National Institute of Environmental Health Sciences, Epidemiology Branch, Durham, USA; [2] University of Michigan, Environmental Health Sciences and Rheumatology, Ann Arbor, USA; [3] U.S. Environmental Protection Agency, Chemical & Pollutant Assessment Division (retired), Washington, USA

Objective: Numerous epidemiological studies have examined silica exposure in relation to risk of systemic sclerosis (SSc), rheumatoid arthritis (RA), systemic lupus erythematosus (SLE), and various forms of antineutrophil cytoplasmic antibody-associated vasculitis (ANCA-V). The objectives of our systematic review are to 1) facilitate the comparison across diseases and identify commonalities and differences with potential implications for mechanisms of action, 2) assess methodological strengths and weaknesses and recommend exposure measurement procedures for use in studies of different designs, and 3) examine available evidence pertaining to exposure intensity and other measures in relation to disease risk.
Methods: Our search of 4 databases yielded 30 case-control studies, 16 studies in occupational settings, and 12 studies of silicosis patients. We evaluated these studies across 7 domains relating to risk of bias.
Results: Moderate to strong associations (summary relative risks between 2.5 and 5.0) were observed across these studies between silica exposure for SSc, SLE, and ANCA-V, with a weaker association seen in RA. The most common deficiencies among studies set in cohorts were use of a relatively insensitive outcome assessment method (e.g., relying on cause of death data) and an inability to address potential confounding or effect modification, e.g., by smoking for RA. The most common deficiencies in case-control studies were the inclusion of prevalent, rather than incident, cases, and the use of exposure assessment methods with relatively low sensitivity or specificity (e.g., limited to occupational histories based on job titles).
Conclusions: Future epidemiological studies should focus on quantifying risk in relation to exposure levels to evaluate the utility of current exposure limits to address risk of autoimmune diseases. Occupational health professionals working in the silica industries should be aware of the potential autoimmune-related hazards, strategies to protect against exposures, and the need for active monitoring for early clinical markers (e.g., development of disease-specific autoantibodies).

P55-27 | The Association between Post-Acute Sequelae of SARS-CoV-2 Infection (PASC) and Working Environment: An Example of a Semiconductor Packaging Plant in Southern Taiwan

Ping-Chi Hsu[1], Fu-Jen Cheng[2], Yi-Ping Chuang[1]

[1] National Kaohsiung University of Science and Technology, Department of Safety, Health and Environmental Engineering, Kaohsiung City, Taiwan; [2] Kaohsiung Chang Gung Memorial Hospital, Chang Gung University, Department of Emergency Medicine, Kaohsiung City, Taiwan

The infection caused by the novel coronavirus (SARS-CoV-2) not only impacts individuals' short-term health but also leads to long-term health sequelae, collectively referred to as long COVID or Post-Acute Sequelae of SARS-CoV-2 Infection (PASC). The purpose of this study is to investigate the workers who were diagnosed for COVID-19 with the symptoms of subacute COVID-19 or PASC sequelae whether associated with their life styles, working environment, and health status at a semiconductor packaging plant in southern Taiwan. We used a questionnaire to survey employees who have been diagnosed with COVID-19 in the company. The questionnaire includes basic personal information, living habits, working environment, and health status, new coronavirus infection history and sequelae symptoms. A total of 350 questionnaires were distributed, 343 were recovered, and 333 were valid questionnaires. Simple and multiple logistic regression analyses were performed to identify factors associated with the appearance of subacute COVID-19 and PASC after the diagnosis; odds ratios (OR), adjusted odds ratios (aOR) and 95% confidence intervals (CI) were calculated to explore the relationship between variables and the interrelationship of PASC sequelae symptoms. There were 333 research subjects, with an average age of 37.57 years (SD=7.88), 55% were female and 45% were male. Univariate logistic regression analysis showed that female (OR=2.0, 95% CI 1.1–3.7), night shift (OR=4.8, 95% CI 2.4–9.8), exercise (OR=0.3, 95% CI 0.1–0.6), sleep quality (OR=0.4, 95% CI 0.2–0.8), and ongoing medical care (OR=5.6, 95% CI 2.9–11.0) were associated with an increase or decrease risk of the PASC. Multivariate logistic regression analysis demonstrated that night shift (aOR=4.3, 95% CI 1.8–10.5), exercise (aOR=0.3, 95% CI 0.1–0.8), and ongoing medical care (aOR=4.1, 95% CI 2.0–8.6) were significant independent predictors of PASC. In conclusion, this study confirmed the link between gender, shift, working environment and risk of persistent COVID-19 sequelae.

P55-28 | A qualitative study of employers' perspectives on COVID-19 impact and risk mitigation strategies

Trân B. Huỳnh[1], Kevin Moore[2], Grace N. Sembajwe[3], Ann M. Dale[4], Cynthia Ball[5], Claudia Parvanta[6]

[1] University of Minnesota, School of Public Health / Division of Environmental Health Sciences, Minneapolis, USA; [2] Oklahoma State University, Biosystems and Agricultural Engineering, Stillwater, USA; [3] Indiana University, School of Public Health-Bloomington / Environmental and Occupational Health, Bloomington, USA; [4] Washington University in St. Louis, School of Medicine / Institute for Public Health, St. Louis, USA; [5] University of Texas at Tyler, School of Medicine / Department of Public Health, Tyler, USA; [6] University of South Florida, College of Public Health, Tampa, USA

Objective: To document employers' perspectives on COVID-19 impact on their businesses, mitigation strategies implemented, and the utility of their emergency preparedness plans.
Material and Methods: Between October 2022 to January 2023, semi-structured interviews were conducted with 28 employers across the US. Transcripts were analyzed and summarized into themes.
Results: The pandemic impacted workplaces across different sectors, causing significant financial and operational challenges. Associated costs, such as revamping office buildings, procuring personal protective equipment, managing staff illness/absence, and implementing mitigation plans were burdensome. The shift to remote work was a common trend across industries to maintain productivity while ensuring safety. Healthcare organizations quickly pivoted to telehealth, which helped increase service utilization. Most employers used a combination of control strategies but thought that administrative controls, particularly policies around social distance/remote work, and PPE use, made the most difference to their companies, although a few emphasized vaccination. Engineering controls such as ventilation upgrades were often perceived as cost-prohibitive. Employers implemented a variety of administrative policies throughout the pandemic, and while these policies could boost morale for some, pushback from employees had to be managed to achieve success. Except for healthcare organizations, most large-sized firms had an emergency preparedness plan but did not have an infectious diseases component. Many small businesses did not have a plan.
Conclusion: Our study highlighted the need for employers to have a robust emergency preparedness plan that can respond to emerging infectious disease threats. Pushback around new policies in future epidemics may be mitigated by ongoing employee engagement and leadership support within a company's existing safety culture for developing the emergency/preparedness plan. Using a Total Worker Health® framework may enhance overall worker health and well-being.

P55-29 | Occupational Exposure to Antineoplastic Agents and Infertility Risk Among Nurses: A Systematic Review

Bridget E. Martin

George Washington University, Environmental and Occupational Health, Washington DC, USA

Background: Nurses face considerable risk of exposure to Antineoplastic Drugs (Ads) during drug preparation, administration, and patient care, potentially leading to adverse reproductive outcomes. Infertility, a growing concern, affects one in six people worldwide.

Objectives: To investigate the question: Does occupational exposure to antineoplastic agents significantly increase the risk of infertility among nurses? The study used the PECO framework and focused on nurses due to their high exposure risk.

Methods: A comprehensive search of Scopus, PubMed, Web of Science, CINAHL, and Google Scholar identified six studies assessing the association between AD exposure and nurse infertility. Using the Navigation Guide, studies were evaluated for risk of bias and quality of evidence, focusing on infertility and factors such as exposure duration and frequency.

Results: The six studies included in the review were conducted in Iran, the Netherlands, the United States, Canada, and Egypt. Infertility rates among exposed individuals ranged from 11.4% to 31%, compared to lower rates in control(unexposed) groups. A prolonged time to pregnancy (TTP) was also observed in the exposed groups, with significant findings reported regarding mean TTP and hazard ratios for pregnancy likelihood. The risk of bias was evaluated using the Navigation Guide systematic review methodology. Most studies received a high risk of bias rating for recruitment strategy, knowledge of exposure groups, and exposure assessment. The quality of evidence was categorized as moderate based on upgrading and downgrading methods.

Conclusions: The studies suggested an association between occupational exposure to ADs and infertility in nurses. The quality of the evidence was moderate, while the strength of the evidence was limited. There is an urgent need for improved safety measures and further research to guide policy changes that address regional disparities and investigate long-term effects. Healthcare institutions must acknowledge the potential dangers and implement comprehensive approaches to minimize the risks.

P55-31 | Healthcare Waste Management: Knowledge, Attitude, and Practices Among Healthcare Workers in Pinyudo Primary Hospital, Southwest Ethiopia

Wondwossen B. Eshete, **Opiew O. Ojoho**

Jimma University, Environmental Health Sciences & Technology, Jimma, Ethiopia

Background
Improper healthcare waste management (HCWM) poses significant risks to healthcare workers, patients, and the surrounding community. This study assessed the knowledge, attitude, and practices (KAP) of healthcare workers regarding HCWM and identified associated factors at Pinyudo Primary Hospital, Southwest Ethiopia.
Methods
A facility-based cross-sectional study was conducted from May to July 2023. A total of 157 healthcare workers were recruited using a systematic random sampling technique. Data were collected through a pretested, structured questionnaire and an observational checklist. Statistical analyses were performed using SPSS version 28. Descriptive statistics were computed, and logistic regression was conducted to identify factors associated with HCWM practices. A p-value of <0.05 was considered statistically significant.
Results
Among the 157 respondents, 50% demonstrated adequate knowledge, 54% had a positive attitude, and 50.3% exhibited adequate HCWM practices. Factors positively associated with improved HCWM practices included the presence of a waste management committee (AOR = 1.80, 95% CI: 1.03–3.15) and availability of HCWM guidelines (AOR = 2.43, 95% CI: 1.20–4.91). However, gaps remained in waste segregation, use of personal protective equipment (PPE), and waste treatment methods.
Conclusion
The study revealed gaps in HCWM knowledge and practices among healthcare workers. Establishing and strengthening HCWM committees, implementing regular training, and promoting behavioral change communication are recommended to enhance biomedical waste handling practices.

P56 | Per- and Poly-fluoroalkyl Substances: Sources, Measurements, Trends, and Health Effects - PART II

P56-01 | Prenatal Exposure to Per- and Polyfluoroalkyl Substances, Placental DNA Methylation, and Small Vulnerable Newborns: Insights from the Shanghai Maternal-Child Pairs Cohort

Yue Zhao, Liyi Zhang, **Yunhui Zhang**

Fudan University, Department of Environmental Health, Shanghai, China

Small vulnerable newborns (SVN), encompassing preterm birth, small for gestational age (SGA), and low birthweight (LBW), face heightened risks for lifelong health complications. Prenatal exposure to per- and polyfluoroalkyl substances (PFAS) has been associated with adverse birth outcomes, yet its role within the comprehensive SVN framework and underlying epigenetic mechanisms remains poorly understood. This study analyzed data from 1,021 mother-child pairs in the Shanghai Maternal-Child Pairs Cohort to explore associations between prenatal PFAS exposure, SVN, and placental DNA methylation . PFAS concentrations were measured in umbilical cord serum using high-performance liquid chromatography-triple quadrupole mass spectrometry, and placental DNA methylation was profiled with the Illumina Infinium HumanMethylationEPIC BeadChip. Higher exposure to 6:2 Cl-PFESA (OR: 1.383, 95% CI: 1.067–1.795) and ∑PFAS was significantly associated with increased SVN risk, while PFOA (OR: 1.824, 95% CI: 1.153–2.885), PFHxS (OR: 1.401, 95% CI: 1.074–1.829), 6:2 Cl-PFESA (OR: 1.372, 95% CI: 1.008–1.868), and ∑PFAS (OR: 1.673, 95% CI: 1.056–2.648) were specifically linked to the SGA but not preterm subtype. Differential methylation analysis identified 2,883 CpG sites enriched in pathways related to immune response, cell signaling, and placental function. High-dimensional mediation analysis identified 30 CpG sites mediating the PFAS-SVN relationship, with *FOXP1* emerging as a key gene implicated in placental and fetal development. This study identifies significant associations between prenatal PFAS exposure and the risk of SVN, with placental DNA methylation mediating key pathways in immune regulation, signal transduction, and placental function. The findings highlight sex-specific vulnerabilities, particularly in female neonates, and raise concerns about the safety of replacement PFAS compounds.

P56-02 | Understanding the relationship between particle size and per- and polyfluoroalkyl substance (PFAS) concentrations in house dust and its implications

Clara Eichler[1], Mahender Singh Rawat[2], Naomi Chang[1,3], Elizabeth Brown[2], Sujan Fernando[4], Thomas Holsen[2,4], Glenn Morrison[1], Andrea Ferro[2], Barbara Turpin[1]

[1] University of North Carolina at Chapel Hill, Department of Environmental Sciences and Engineering, Chapel Hill, USA; [2] Clarkson University, Department of Civil and Environmental Engineering, Potsdam, USA; [3] Colorado State University, Department of Systems Engineering, Fort Collins, USA; [4] Clarkson University, Center for Air and Aquatic Resources Engineering and Science, Potsdam, USA

Objective: Per- and polyfluoroalkyl substances (PFAS) are synthetic chemicals that are ubiquitously present in the environment, including in homes. Two major exposure pathways for PFAS indoors are inhalation and accidental ingestion of house dust. Previous studies investigating PFAS in dust usually report sieving the dust to one size fraction, ranging from <150 µm to >2,000 µm. The use of different size fractions makes it difficult to compare results

and provide reliable exposure estimates given the importance of particle size for exposure to PFAS in indoor dust. Thus, we are aiming to better understand the relationship between dust particle size and PFAS concentrations.
Materials and Methods: We collected dust from 10 homes in North Carolina and seven homes in New York, sieved the dust into multiple size fractions ranging from <63 µm to <2,000 µm, and used targeted liquid chromatography-tandem mass spectrometry (LC-MS/MS) and gas chromatography-mass spectrometry (GC-MS) methods to analyze the fractions for PFAS. Linear regression analysis was used to derive correlation coefficients and p-values.
Results: We found that several neutral PFAS are significantly and negatively correlated with dust particle size, i.e., higher concentrations were found in the smaller size fractions. This indicates that for neutral PFAS, partitioning to the dust particles from the gas phase is a dominant migration pathway. On the other hand, several perfluoroalkyl acids showed no clear correlation between particle size and concentration, suggesting that other migration pathways into the dust play a role, such as abrasion of fibers from upholstery, which would lead to higher concentrations in larger size fractions.
Conclusion: This work highlights the importance of the choice of size fraction when analyzing PFAS in dust and conducting exposure assessments.Funding: This work was, in part, funded by the Alfred P. Sloan Foundation, NSF GRFP, and CAARES at Clarkson University.

P56-03 | Per- and Polyfluoroalkyl Substances (PFAS) in Drinking Water and Breast Canceramong Women in the Military

Celia Byrne[1], Michelle Mellers[2], Stefanie Silsby[1], Caroline A. Hoffman[2], Lynsey L. Lewis[2], Barbara Fuhrman[1]

[1] Uniformed Services University of the Health Sciences, Preventive Medicine, Bethesda, USA; [2] Henry M Jackson Foundation for the Advancement of Military Medicine, Inc., Bethesda, USA

Objective: Previous studies indicated higher early-onset breast cancer rates among women in the U.S. Military than the U.S. population. The impact of the endocrine disrupting chemicals, Per- and Polyfluoroalkyl Substances (PFAS) on breast cancer in these younger women (< 50 years of age) remains uncertain. Many industrial and consumer products contain PFAS, but the long use of Aqueous Film Forming Foam (AFFF) for firefighting was a major source of contamination of drinking water on a number of US military installations.

Materials and Methods: In a nested case-control study among active duty military women we assessed the association of years assigned to 22 military installations with very high drinking water PFAS levels (> 70 ppt) and breast cancer. Between 2000 and 2022, there were 828 women diagnosed while on active duty with (708) invasive breast cancer (BC) or with (120) ductal carcinoma in situ (DCIS), who joined the military since 1990. We selected 1655 controls matched on age, race, and time in the military from active duty women without a reported breast cancer diagnosis at the time of the cases' diagnosis. Of the cases, 85% were under the age of 45 and 56% were non-White. Odds ratios estimated the rate ratio (RR) with 95% confidence intervals (CI) using conditional logistic regression.

Results: Compared to women never assigned to the 22 identified installations, those assigned three or more years had an RR=1.34 (95% CI:1.05-1.72) for invasive BC or DCIS. The RR increased for invasive BC only (RR=1.50; 95% CI:1.14-1.97), those diagnosed with invasive BC before 2016, (RR=1.57; 95% CI:1.05-2.32), and those diagnosed with invasive BC before the age of 45 (RR= 1.71; 95% CI:1.25-2.32).

Conclusion: These findings might in part provide an explanation for the reported higher rates of breast cancer among women serving in the U.S. Military.

Funding: CDMRP and DoD

P56-04 | Associations between PFAS perceptions and PFAS-related consumer spending across Michigan

Andrew Hoover, Sung Kyun Park

University of Michigan, School of Public Health, Ann Arbor, USA

Per- and polyfluoroalkyl substances (PFAS) have been linked to multiple negative health effects. Yet, few studies have investigated public opinions, which may affect consumer behaviors related to PFAS exposure. We examined how college students and residents throughout Michigan perceive PFAS and how these views impact PFAS-related consumer spending.

We conducted an online survey to assess PFAS perceptions, PFAS-related consumer spending, and sociodemographic factors among students at five public universities in Michigan and residents of Facebook groups in their corresponding cities. Perceptions were conceptualized as knowing what PFAS were and by a 13-point score to assess knowledge. Logistic regression models were used to examine associations between PFAS perceptions and whether people would pay more for $5 and $50 products labeled PFAS-free.

We included 3,489 responses, where 1,548 (44.4%) people knew what PFAS were, and the mean knowledge score was 7.11. The predominant age group was 18-22 (1,715 (49.2%)), gender was women (2,191 (62.8%)), and race/ethnicity was White/European (2,040 (58.5%)). Knowing what PFAS were was associated with a 1.61 (1.23-2.11) and 3.98 (2.52-6.31) odds ratio (OR) of paying more for a $5 product labeled PFAS-free for university and Facebook participants; it was associated with a 1.40 (1.10-1.79) and 3.65 (2.32-5.76) OR of paying more for a $50 product for university and Facebook participants. An interquartile range increase in PFAS knowledge score was associated with a 2.05 (1.72-2.44) and 2.30 (1.68-3.19) OR of paying more for a $5 product labeled PFAS-free for university and Facebook participants; it was associated with a 1.88 (1.60-2.21) and 2.19 (1.61-3.04) OR of paying more for a $50 product for university and Facebook participants.

These findings suggest that PFAS knowledge can influence consumer spending and could limit resulting PFAS exposure in the general population. Promoting public knowledge about PFAS may be a key strategy to lower PFAS exposure.

P56-07 | Prenatal concentrations of legacy and emerging per- and polyfluoroalkyl substances in a contemporary Canadian cohort: A descriptive analysis

Ashlyn Simpson[1], Michael Borghese[1], Susan MacPherson[1], Robin Shutt[1], Sara Packull-McCormick[1], Amy Metcalfe[2], Donna Slater[3], Lara Leijer[4], Janice Skiffington[2], Mandy Fisher[1], Tye Arbuckle[1], Jillian Ashley-Martin[1]

[1] Health Canada, Environmental Health Science and Research Bureau, Ottawa, Canada; [2] University of Calgary, Department of Obstetrics and Gynaecology, Calgary, Canada; [3] University of Calgary, Department of Physiology and Pharmacology, Calgary, Canada; [4] University of Calgary, Department of Pediatrics, Calgary, Canada

Objective: Despite the ubiquity of per- and polyfluoroalkyl substances (PFAS), disaggregated biomonitoring data remain scarce for emerging PFAS and during vulnerable life stages. These data are valuable for identifying subpopulations who may experience disproportionate PFAS exposure. We addressed these gaps by quantifying concentrations of legacy and emerging PFAS in a contemporary pregnancy cohort and investigating differences by social determinants and reproductive history.
Methods: We measured concentrations of 40 PFAS in 757 serum samples (mean 27 weeks gestation) from participants in the Prediction, Prevention and Interventions for Preterm Birth (P3) cohort study. Participants were recruited from Calgary, Alberta between 2021-2023 and provided sociodemographic and health history information by completing detailed online questionnaires during pregnancy. We modelled associations between maternal data (e.g., parity, household income, race, primary language, birth country) and geometric mean PFAS concentrations using ANOVA. We report significant differences (overall $p < 0.05$) for PFAS detected in the majority of participants.
Results: We detected 13 PFAS in ≥50% and of those, 8 in ≥98.5% of participants. Concentrations of perfluorooctanoic acid (PFOA), perfluorohexanesulphonic acid (PFHxS), and perfluorooctanesulfonic acid (PFOS) were 1.5 to 2.4-fold lower among participants with household incomes under the Canadian "low income cut-off". Compared to multipara, perfluorobutanoic acid (PFBA) , PFHxS, PFOA, PFOS and perfluorononanoic acid (PFNA) concentrations were 1.2 to 1.7-fold higher in nulliparous participants. PFHxS serum concentrations were higher among those who primarily spoke English at home (1.9-fold), and identified as White (1.6-fold). We observed null or non-monotonic results in models examining emerging PFAS.**Conclusions**: In this contemporary Canadian pregnancy cohort, concentrations of certain legacy – but not emerging - PFAS tended to be lower in participants who are nulliparous and those who may experience marginalization. Future research in the P3 cohort will examine PFAS in relation to maternal-infant health and differences by social and reproductive determinants.

P56-09 | Current Landscape of Risk Assessment Relevant Knowledge and Understanding of Per- and Polyfluoroalkyl Substances

Luke R. Calder, Jeong Weon Choi, Hyeong-Moo Shin

Baylor University, Environmental Science, Waco, USA

Background: Legacy per- and polyfluoroalkyl substances (PFAS) have been extensively studied for decades, yielding substantial exposure and toxicity data that enable risk assessment and the implementation of protective regulations. However, the U.S. Environmental Protection Agency (EPA) has identified over 14,000 PFAS, many of which lack sufficient data for comprehensive risk evaluation.

Methods: We compiled data from publicly available federal sources and performed literature searches to address data gaps. Beginning with over 14,000 identified PFAS from the EPA's PFAS Analytical Tools webpage, we investigated available toxicity and exposure information. Additionally, we reviewed environmental monitoring studies to identify potential research gaps. To further assess regulatory and health-based benchmarks, we searched for established biomonitoring data, as well as regulatory standards and guidance values, including drinking water standards, reference doses (RfDs), and tolerable daily intakes (TDIs) for each PFAS compound.

Results: The majority of identified PFAS lack the necessary data for human health risk assessments. Among the more than 14,000 PFAS, only 2,945 (19.9%) have available physiochemical property data, 354 (2.4%) have production data, and just 63 (0.4%) have biomonitoring data. Toxicity data are available for 511 PFAS (3.5%) from in vitro studies, 186 PFAS (1.3%) from in vivo studies, and 7,715 PFAS (52.4%) from in silico studies. Of the over 20,000 papers reviewed, water was the most frequently studied environmental medium (27%) followed by food (21%) and specialty products (13%). Only one PFAS (i.e., perfluorooctanoic acid) has an established cancer slope factor, while RfDs and TDIs have been determined for just four (<0.1%) and two PFAS (<0.1%), respectively.

Conclusions: This study provides a comprehensive summary of the current state of PFAS data essential for risk assessment. The identified data gaps can help guide research prioritization and inform future studies.

P56-10 | Associations between PFAS exposure and thyroid disease among adults in the Multi-Site Study

Michael S. Bloom[1], Lameck Chiwaka[1], Patrick Rago[2,3], John Adgate[4], Erin Bell[5], Linda Brown[6], Antonia Calafat[7], Judith M. Graber[8], Julianne Botelho[7], Shelby Kardel[4], Pamela O. Strickland[8], Anonna Saha[5], Adam Schaefer[9], Veronica M. Vieira[10], Teresa Wang[2], Marian Pavuk[2]

[1] George Mason University, Department of Global and Community Health, Fairfax, USA; [2] Centers for Disease Control and Prevention, Office of Community Health Hazard Assessment, Agency for Toxic Substances and Disease Registry, Atlanta, USA; [3] Oak Ridge Institute for Science and Education, Oak Ridge, USA; [4] University of Colorado Anschutz, Department of Environmental and Occupational Health, Aurora, USA; [5] University at Albany, State University of New York, Department of Environmental Health Sciences, Rensselaer, USA; [6] RTI International, Research Triangle Park, USA; [7] Centers for Disease Control and Prevention, National Center for Environmental Health, Atlanta, USA; [8] Rutgers University, Department of Biostatistics and Epidemiology, New Brunswick, USA; [9] Abt Global, Cambridge, USA; [10] University of California Irvine, Department of Environmental and Occupational Health, Irvine, USA

Objective: Exposure to per- and polyfluoroalkyl substances (PFAS) may affect thyroid function. Therefore, we investigated associations between PFAS exposure and thyroid disease in Multi-Site Study (MSS) participants aged $\geq$18 years.
Material and Methods: Participants (n=5771) completed questionnaires and provided a blood specimen. We quantified concentrations of PFHxS, PFOS, PFOA, PFNA, PFDA, PFUnDA, and MeFOSAA in blood serum using HPLC-MS/MS by the Centers for Disease Control and Prevention (CDC). We used logistic regression to estimate odds ratios (OR) and 95% confidence intervals for associations between serum PFAS concentrations as predictors of thyroid disease, defined by self-report, use of thyroid medications, or abstraction of medical records, adjusted for sex, age, education, smoking, and race/ethnicity. We tested for modification of the associations by sex. We used quantile-based g computation (qgcomp) to estimate the effect of the overall mixture of the seven measured PFAS on thyroid disease.
Results: Participants were 56 years old on average (range 18-100) and 60.2% were female. There were 867 (15.0%) cases of hypothyroid and 119 (2.1%) cases of hyperthyroid diseases. We found inverse associations between hypothyroid disease and PFOS (OR=0.92; 95%CI: 0.85-1.01), PFNA (OR=0.79; 95%CI: 0.72-0.88), PFDA (OR=0.76; 95%CI: 0.68-0.86), and PFUnDA (OR=0.79; 95%CI: 0.70-0.90), and null associations with hyperthyroid disease. The association between PFHxS and hypothyroid disease was modified by sex (p=0.08), with a positive association among females (OR=1.07; 95% CI: 1.00-1.15), and PFOS and hyperthyroid disease (p=0.08), with a positive association among males (OR=1.48; 95% CI: 0.95-2.30). The overall PFAS mixture was inversely associated with hypothyroid (ψ=-0.17; 95%CI -0.48, 0.14) and hyperthyroid (ψ=-0.17; 95%CI -0.92, 0.57) diseases using qgcomp, however these findings were not statistically significant.
Conclusion: Higher serum concentrations of certain PFAS were associated with lower odds of hypothyroid disease among adult residents of PFAS-contaminated areas enrolled in the MSS, although some associations varied in subgroups.

P56-11 | Association between Per- and polyfluoroalkyl substances (PFAS) concentrations in breast milk and the growth of infants in Korean mothers

Nalae Moon, Su Ji Heo, Ju Hee Kim

Kyung Hee University, College of Nursing Science, Seoul, South Korea

Objective: This study aims to elucidate the association between PFAS concentrations in the breast milk of Korean mothers and growth indicators in infants under 12 months according to the parity of mothers.
Material and methods: From May to August 2023, a study collected breast milk and sociodemographic data from 201 healthy mother-infant pairs in Korea, using stratified sampling by infant age, sex, and region. Nurses collected 20 mL of breast milk into polypropylene tubes, and stored at -70°C until analysis. The WHO standards were used to assess infant growth through weight-for-length z-scores (WFL). Five PFAS concentrations were measured using a UPLC-TSQ mass spectrometer. Multiple linear regression analyses explored the associations between PFAS concentrations in breast milk and infant WFL according to parity.
Results: Detection rates (LOD) of five PFAS were 92% (0.047 ng/mL) for perfluorooctanoic acid (PFOA), 46% (0.070 ng/mL) for perfluorooctane sulfonate (PFOS), 77% (0.013 ng/mL) for perfluorononanoic acid (PFNA), 43% (0.017 ng/mL) for perfluorodecanoic acid (PFDeA), and 4% (0.069 ng/mL) for perfluorohexane sulfonate (PFHxS). The mean PFOA concentration in primipara mothers' breast milk showed higher level than that of multipara mothers (0.127 vs 0.103 ng/mL, *p*-value 0.080). The results of the multiple linear regression analysis indicated that the concentrations of PFOA (B=-194, p=0.053) and PFOS (B=-490.4, p=0.030) in the breast milk of primiparous mothers were associated with a decrease in infants' WFL. Additionally, the concentration of PFOS (B=-399.9, p=0.094) was associated with a decrease in WFL in male, while for female, both PFOA (B=-207.1, p=0.087) and PFOS (B=-518.8, p=0.095) showed associations with a decrease in WFL at a p<0.1 level. In multiparous mothers, no significant association was found between PFAS concentrations in breast milk and infant WFL.
Conclusion: Primiparous mothers have higher breast milk PFAS levels, hindering infant growth. Reducing PFAS exposure in childbearing women is crucial.

P56-12 | Environmental Exposure to Emerging Per- and Polyfluoroalkyl Substances and Cardiovascular Health Outcomes: A Mixture Analysis

Rezaul Karim Ripon[1,3], Narayana G Prasad[2], Satish Govind[5], Sujata Saunik[4], Srikanth Sola[6], Mayra Volquez[7], Rajesh Melaram[8]

[1] Harvard T H Chan School of Public Health, Environmental Health, Boston, USA; [2] Public Health Literacy/BWH, Department of Cardiology, Miami, USA; [3] McHigher Centre of Health Research, Environmental Epidemiology, Dhaka, Bangladesh; [4] Harvard T H Chan School of Public Health, Takemi Fellow, Boston, USA; [5] Narayana Health, Department of Cardiology, Bengaluru, India; [6] Devic Earth, Founder, Bengaluru, India; [7] Public Health Literacy/UT Southwestern, Preventive Cardiology, Dallas, USA; [8] Texas A&M University, College of Nursing and Health sciences, Texas, USA

Objective: This study aims to investigate the impact of Per- and Polyfluoroalkyl Substances (PFAS) exposure on cardiovascular health outcomes, focusing on the relationships between specific PFAS compounds and conditions like heart attack, congestive heart failure, coronary heart disease, angina, and stroke.

Methodology: The study adopts a cross-sectional approach utilizing data from the National Health and Nutrition Examination Survey covering 2015-2020. Cardiovascular outcomes are assessed based on medical questionnaires, while PFAS exposure levels are measured using serum samples. Firth's logistic regression and generalized Weighted Quantile Sum (gWQS) regression assessed single and cumulative PFAS effects.

Results: Descriptive statistics indicate a sample comprising 48.0% male and 52.0% female participants. Firth logistic regression reveals significant associations between specific PFAS exposures and cardiovascular outcomes. 2-(n-methyl-pfosa) acetic acid is associated with an increased risk of heart attack (coefficient 0.278, p=0.031) and congestive heart failure (coefficient 0.330, p=0.026). N-perfluorooctanoic acid shows significant associations across all outcomes, including heart attack (coefficient 0.243, p<0.0001) and stroke (coefficient 0.138, p<0.0001). Mixture analysis using WQS regression indicates significant positive associations with heart attack (WQS coefficient 0.4728, p<0.001) and congestive heart failure (WQS coefficient 0.5806, p<0.001). N-perfluorooctanoic acid carries the highest mean weight (0.719) in the mixture analysis for heart attack.

Conclusion: The comprehensive analysis highlights significant associations between PFAS exposures and cardiovascular diseases. These findings emphasize the importance of public health interventions and policy actions to mitigate these environmental risks. Further research is recommended to explore these associations' underlying mechanisms and validate findings in broader populations.

P56-13 | Association between per- and polyfluoroalkyl substances and their substitutes, and thyroid capacity and homeostasis in Taiwanese

Po Chin Huang

National Health Research Institutes, National Institute of Environmental Health Sciences, Zhunan, Taiwan

Per- and polyfluoroalkyl substances (PFAS) are persistent environmental pollutants associated with endocrine disruption. This study investigates the associations between 20 PFAS exposure and their substitutes, and thyroid hormones, insulin-like growth factors, and related indices. A cross-sectional analysis was conducted on a cohort of adults (≥18 years, N=266) and minors (<18 years, N=73). Regression models were used to assess the associations between PFAS compounds and thyroid hormones (T_3, T_4, TSH, Free T_4, TBG), insulin-like growth factor-binding protein 3 (IGFBP-3), insulin-like growth factor 1 (IGF-1), and derived thyroid function indices, adjusting for potential confounders. Differences in PFAS detection rates, geometric means, and median levels between age groups were also analyzed. Significant differences in PFAS concentrations were observed between adults and minors. Minors exhibited significantly higher median concentrations of perfluorohexanoic acid (PFHxA, p=0.008), perfluorononanoic acid (PFNA, p=0.020), perfluorodecanoic acid (PFDA, p=0.002), perfluoroundecanoic acid (PFUnDA, p=0.001), and perfluorooctanoic acid (PFOA, p<0.001). Several PFAS compounds were significantly associated with thyroid function parameters. PFHpS was positively associated with T_3 (β = 0.042, p = 0.038), T4 (β = 0.044, p = 0.021), and TBG (β = 0.055, p = 0.004). PFNA was negatively associated with Free T_4 (β = -0.111, p = 0.037) but positively linked to SPINA-GD (β = 0.188, p = 0.023). PFOA showed a significant association with IGF-1 (β = 0.106, p = 0.008), whereas PFBS was negatively associated with T_3 (β = -0.028, p = 0.007). The total PFAS burden (ΣPFAS) was significantly elevated in minors, who also exhibited higher detection rates and concentrations of branched isomers of PFOA and perfluorooctane sulfonic acid (PFOS). These findings suggest that exposure to PFAS and their substitutes influence thyroid homeostasis, with significant age-related differences in PFAS accumulation.

P56-14 | Serum per- and polyfluoroalkyl substances (PFAS) and blood lipid levels among adults in the Multi-Site Study

Robert J. Laumbach[1], Judith M. Graber[2], Pamela Ohman-Strickland[2], John L. Adgate[3], Jordan M. Bailey[4], Scott Bartell[5], Linda M. Brown[6], Yerin Jung[5], Tamarra James-Todd[7], Kayoko Kato[8], Michael Lewin[9], Celina N. Phillipson[5], Patrick Rago[9], Anonna Saha[10], Adam Schaefer[11], Laurel Schaider[12], Anne P. Starling[13], Carolina Villanueva[5], Veronica Vieira[5], Yuting Wang[12], Meghan Weems[9], Alicia Legard[2], Alanna O'Neil[14], Adriana De Resende[2], Heather Butler[2], Kate Lubina[2], Marian Pavuk[9]

[1] Rutgers, The State University, Environmental and Occupational Health and Justice, New Brunswick, USA; [2] Rutgers, The State University of New Jersey, Department of Biostatistics and Epidemiology, New Brunswick, USA; [3] Colorado School of Public Health, Department of Environmental and Occupational Health, Aurora, USA; [4] Michigan Department of Health and Human Services, Environmental Health Bureau, Lansing, USA; [5] University of California, Irvine, Department of Environmental and Occupational Health, Joe C. Wen School of Population & Public Health, Irvine, USA; [6] RTI International, Research Triangle Park, USA; [7] Harvard T.H. Chan School of Public Health, Department of Environmental Health, Boston, USA; [8] Centers for Disease Control and Prevention, Divsion of Laboratory Sciences, National Center for Environmental Health, Atlanta, USA; [9] Centers for Disease Control and Prevention, Office of Community Health Hazards Assessment, Agency for Toxic Substances and Disease Registry, Atlanta, USA; [10] University of Albany, Department of Environmental Health, School of Public Health, Albany, USA; [11] Abt Global, Rockville, USA; [12] Silent Spring Institute, Newton, USA; [13] University of North Carolina at Chapel Hill, Department of Epidemiology, Gillings School of Public Health, Chapel Hill, USA; [14] Rutgers, The State University of New Jersey, Department of Environmental Sciences, New Brunswick, USA

Objective: Some previous studies suggest that exposure to certain per- and polyfluoroalkyl substances (PFAS) may disrupt blood lipid regulation. We studied associations between serum PFAS and blood lipid concentrations in adults in a large, cross-sectional, multi-site study of U.S. residents living in communities with a history of drinking water contaminated with PFAS.

Materials and Methods: Participants recruited from communities in eight U.S. states (2019-2023) completed standardized questionnaires and blood sample collection. Serum was analyzed for perfluorooctanate (PFOA), perfluorooctane sulfonic acid (PFOS), perfluorononanoate (PFNA), perfluorohexane sulfonic acid (PFHxS), perfluorodecanoate (PFDeA), perfluoroundecanoate (PFUnDA) and 2-(N-Methyl-perfluorooctane sulfonamido) acetic acid (MeFOSAA). PFAS detected in >75% of participants (PFOA, PFOS, PFNA, PFHxS) were analyzed as continuous variables. Among adult participants (aged ≥18 years) not using a lipid-lowering medication, associations between each PFAS and total cholesterol (TC), low-density lipoprotein cholesterol (LDL-C), high-density lipoprotein cholesterol (HDL-C), triglycerides (TG), and non-HDL cholesterol were modeled using linear regression, adjusted for age, sex, body mass index, race/ethnicity, income, education, cigarette and alcohol use.

Results: Participants in this analysis (n=4,180) were 64% female with a mean age of 52 years and a median TC of 196 mg/dL. In models with and without adjustment for covariates, PFOA, PFOS, PFNA, PFHxS, PFDeA, and PFUnDA were positively associated with TC, LDL-C, HDL-C, and non-HDL cholesterol; PFAS associations with TG were negative or null.

Conclusions: We observed positive associations between several serum PFAS and blood lipid concentrations. Although this cross-sectional design precludes causal inferences, these associations suggest potential implications for cardiovascular health. Future analyses will assess modeled PFAS mixtures and reconstructed serum PFAS back to year 2000. Funding for this study was provided by the Agency for Toxic Substances and Disease Registry, Centers for Disease Control and Prevention.

P56-15 | Blood profiles of per- and polyfluoroalkyl substance (PFAS) in communities across Canada: Data from the 2007–2019 Canadian Health Measures Survey (CHMS)

Tyler Pollock, Gabrielle Sauvageau, Subramanian Karthikeyan, Christine MacKinnon-Roy, Kavita Singh, Michael M. Borghese, Annie St-Amand

Health Canada, Environmental Health Science and Research Bureau, Ottawa, Canada

Background Health Canada's National Biomonitoring Program has generated twelve years of exposure data on environmental chemicals in people living in Canada through the Canadian Health Measures Survey (CHMS). In the past, these data were exclusively used to determine nationally representative chemical levels in the Canadian population. Here we sought to evaluate per- and polyfluoroalkyl substances (PFAS) exposures at the community-level using data from the 2007–2019 CHMS.

Materials and methods We selected five PFAS with detection rates over 60% in the Canadian population: PFOA, PFNA, PFDA, PFHxS, and PFOS. We calculated unweighted (non-representative) geometric mean PFAS levels for each of the 65 communities sampled across Canada. We compared profiles of the five PFAS among the total population and individual communities.

Results We found significant differences in PFAS levels among communities within each collection period. For each PFAS, levels were 1.5–3.5× higher in the community with the highest level compared to the community with the lowest level. In the most recent collection period, blood profiles of PFAS differed among certain communities. Levels of PFOA, PFNA, and PFDA were highest in select communities in Ontario, Quebec, and British Columbia. Levels of PFHxS were highest in communities in Ontario and British Columbia, and levels of PFOS were highest in communities in Ontario.

Conclusions Understanding chemical exposures at the community-level can provide insights into important variations that exist across the country. Exploring blood profiles of commonly detected PFAS can help identify exposure sources, including those of disproportionately impacted populations.

P56-16 | Exposure to Perfluoroalkyl Substances (PFAS) and Its Association with Liver Disease Progression from Hepatic Dysfunction to Hepatocellular Carcinoma

Bagavathy Shanmugam Karthikeyan[1], Naomy Kim[2], Catherine Mullins[1], Ju Dong Yang[2], Robert Haile[2], Vijay Pandyarajan[2], Douglas I. Walker[1]

[1] Emory University, Comprehensive Laboratory for Untargeted Exposome Science, Gangarosa Department of Environmental Health, Rollins School of Public Health, Atlanta, USA; [2] Cedars-Sinai Medical Center, Karsh Division of Gastroenterology and Hepatology, Los Angeles, USA

Objective
Growing evidence suggests that exposure to Perfluoroalkyl Substances (PFAS) is associated with metabolic dysfunction leading to Hepatocellular Carcinoma (HCC). However, the underlying mechanisms are poorly understood. We hypothesized that PFAS exposure is linked to metabolic changes that contribute to the progression of liver disease.
Materials and Methods
The cohort consists of plasma samples (N=300) from three stages of liver disease progression: Metabolic dysfunction-associated steatohepatitis (MASH), Cirrhosis, and HCC. Plasma levels of metabolites and PFAS were determined using untargeted liquid chromatography with high-resolution mass spectrometry (LC-HRMS).
Results
Statistical analysis indicates that PFAS exposure is associated with the progression of liver disease. Significant differences in PFAS concentration levels ($P < 0.05$) were observed across various stages, including MASH, Cirrhosis, and HCC. Specifically, seven PFAS (PFDA, PFHps, PFHxS, PFNA, PFOA, PFOS and PFPeAS) showed statistical differences in exposure levels across these stages. Additionally, we found significant variations in PFAS exposure among ethnic groups (Hispanic and Non-Hispanic) ($P < 0.05$). There were also statistical differences in exposure levels between patients who developed complications from Cirrhosis and HCC and those who did not. The analysis revealed a strong correlation between PFAS exposure and metabolite levels with advanced stages of liver disease progression, particularly HCC, compared MASH and Cirrhosis.
Conclusion
Overall, our results suggest that PFAS exposure interferes with metabolic function, potentially contributing to the progression of liver disease from hepatic dysfunction to hepatocellular carcinoma (HCC).
Funding sources and acknowledgements:
This project was supported with funding from NIEHS under award # R01ES032831.

P56-17 | Semi-automated sample preparation and high-throughput determination of PFAS in plasma

Tomohiko Isobe[1], Kenta Iwai[1], Hue T. Nguyen[1], Taiki Yamaji[2], Ayami Ono[2], Motoki Iwasaki[2], Manami Inoue[2], Shoichiro Tsugane[2], Norie Sawada[2], Shoji F. Nakayama[2]

[1] *National Institute for Environmental Studies, Tsukuba, Japan;* [2] *National Cancer Center, Tokyo, Japan*

Per- and polyfluoroalkyl substances (PFAS) have been used in a wide variety of industrial and household products such as aqueous film forming foam, surfactant, electrical/electronic appliance, nonstick cookware, food packaging and fabrics due to their unique properties including thermal and chemical stability and oil-, stain-, grease-, and water-repellency. Because of their persistent, bioaccumulative and long-range transport properties, exposure to and potential adverse health effects of PFAS have been of serious public concern worldwide. Despite historical and widespread use of PFAS, limited information is available on their blood levels in Japanese general population. For a large-scale biomonitoring of PFAS exposure, a high-throughput analytical method is required, but because many compounds are included as PFAS with a wide range of physico-chemical properties, the previous analytical methods accompany labourious preparation protocol and long analytical time. In this context, the present study developed an analytical method with semi-automated sample preparation and high-throughput determination of 35 PFAS compounds in plasma. Two hundred microliter of acetonitrile with 1% formic acid was added to 50 µl of plasma sample spiked with internal standards for deproteination. After centrifugation, supernatant was transferred to 96-well plate Captiva EMR-Lipid (Agilent Technologies, Inc.) for phospholipid removal. Eluate was collected in 96-well reservoir plate and injected to LC-MS/MS. All liquid handling was conducted using a Hamilton Microlab Star to improve reproducibility and avoid contamination. Chromatographic separation and quantification were achieved using an Agilent 1290 LC coupled to an Agilent 6495 MS/MS with a Poroshell 120 EC-C18 (2.1 x 50 mm, 2.7 µm) analytical column. A dual column system was employed to increase throughput, resulting in 6-min instrumental run time. After confirming quality control data, the developed method was applied to plasma samples to determine the levels and profiles of PFAS. Results of 90 samples from the preliminary survey will be presented.

P56-18 | Trends in Perfluoroalkyl and Polyfluoroalkyl Substances (PFASs) in Pregnant Californians from Measuring Analytes in Maternal Archives Samples (MAMAS) studies

Dina Dobraca[1], Kathleen Attfield[1], Songmei Gao[2], Sabrina Smith[2], June-Soo Park[2], Nerissa Wu[1]

[1] *California Dept. of Public Health, Richmond, USA;* [2] *California Dept. of Toxic Substances Control, Berkeley, USA*

Biomonitoring California routinely assesses California residents' exposures to environmental chemicals and designed the Measuring Analytes in Maternal Archives Samples (MAMAS) studies to determine serum PFAS trends in pregnant Californians and understand differences by demographic characteristics. Archived second trimester prenatal screening samples collected in 2012, 2015-2016, and 2016-2017 from regions across California were analyzed for PFASs. With race/ethnicity-stratified sampling, we disaggregated Asian ethnicity (i.e., Chinese, Filipina, Vietnamese). Multiple PFASs were detected in every participant. Highest exposed race/ethnic group varied by PFAS analyte; often Vietnamese participants displayed highest concentrations. Across MAMAS studies, we observed

decreasing concentrations of most legacy PFASs and indication of increasing perfluorobutanoic acid (PFBA) exposure.

P56-19 | Metabolic pathways connecting PFAS exposure to gestational age in the Health Outcomes and Measures of the Environment Study

Amber Hampton[1], Katherine Manz[2], Kurt Pennell[3], George Papandonatos[4], Aimin Chen[5], Bruce Lanphear[6], Kimberly Yolton[7], Joseph Braun[1], Elizabeth Costello[1]

[1] Brown University, Department of Epidemiology, Providence, USA; [2] University of Michigan, Department of Environmental Health Sciences, Ann Arbor, USA; [3] Brown University, School of Engineering, Providence, USA; [4] Brown University, Department of Biostatistics, Providence, USA; [5] Department of Biostatistics, Epidemiology and Informatics, University of Pennsylvania Perelman School of Medicine, Philadelphia, USA; [6] Simon Fraser University, Vancouver, Canada; [7] Department of Pediatrics, Cincinnati Children's Hospital Medical Center, Department of Pediatrics, Cincinnati Children's Hospital Medical Center, Cincinnati, USA

Objective

We investigated metabolome features and biological pathways associated with and underlying PFAS and gestational age (GA).

Background

Per- and polyfluoroalkyl substances (PFAS) have been associated with adverse gestational duration, but the underlying associated biological mechanisms remain largely unknown. Untargeted metabolomics offers an opportunity to agnostically evaluate thousands of metabolites and hundreds of biological pathways connecting PFAS to adverse birth outcomes, including GA.

Materials and Methods

In 264 pregnant people from the HOME Study, we quantified four PFAS (PFOA, PFOS, PFNA, PFHxS) in 1st trimester serum. We abstracted GA from medical charts, and measured 14,402 unique features in cord serum using untargeted metabolomics. We used covariate-adjusted quantile-based g-computation to evaluate associations between the PFAS mixture and GA. Next, we used covariate-adjusted quantile-based g-computation to assess the PFAS mixture's relationship with each metabolome feature, and covariate-adjusted linear regression for each feature's association with GA; these analyses were corrected for multiple testing using the *Benjamini–Hochberg* method. Next, we identified enriched metabolic pathways using the *mummichog* algorithm in MetaboAnalyst for both PFAS and GA. We then characterized overlapping metabolic pathways between PFAS, and GA using a *meet-in-the-middle* approach.

Results

Each quartile increase in the PFAS mixture was associated with three days shorter GA (95%CI: -5.2, -0.5). Accounting for multiple comparisons, we identified four metabolites, and 49 metabolic pathways associated with the PFAS mixture; and 71 metabolites and 15 metabolic pathways associated with GA. There were no overlapping individual metabolites that met statistical associations for both PFAS and GA. However, seven pathways were associated with both the PFAS mixture and GA; these were primarily amino acid and lipid metabolism pathways.

Conclusion

These findings suggest that PFAS may impact gestational duration through multiple biological pathways. Future studies are needed to replicate these findings with a larger sample size in other populations.

P56-20 | Per- and polyfluoroalkyl substances in breast milk in relation to cytokines and immune markers: findings from a pilot study

Joanna Marroquin[1], Virginia Espina[2], Jennifer Tender[3], Jasmin L. Flores[3], Erynn Richards[3], Sally Rucker[2], Kurunthachalam Kannan[4], Sahira Long[3], Amira Roess[1], **Anna Z. Pollack**[1]

[1] George Mason University, Global and Community Health Department, College of Public Health, Fairfax, USA; [2] George Mason University, Center for Applied Proteomics and Molecular Medicine, Fairfax, USA; [3] Children's National Hospital, Washington, DC, USA; [4] University at Albany, Department of Environmental Health Sciences, Albany, USA

Background: Per- and polyfluoroalkyl substances (PFAS) are used in a variety of consumer products. PFAS are widely detected in biomonitoring and may affect immune function. We sought to evaluate associations between PFAS in breast milk and cytokines in saliva among women in the first 3 months postpartum.
Methods: Women were recruited during pediatric infant appointments in Washington, DC. Participants provided a breast milk sample for measurement of per- and polyfluoroalkyl substances (n=47). Saliva samples from mothers were provided for measurement of immune markers and cytokines. Linear, odds, and mixed model regression was used to model associations between PFAS exposure and cytokines.
Results: Participants had a mean age of 31 years, 51% were non-Hispanic Black, 30% were White, and 14% were Latina. One quarter reported high school and 37% reported graduate school as their highest level of educational attainment. PFOS had the highest median levels (73.25 pg/ml), followed by PFNA (33.92), PFOA (24.07), PFHXA (20.14) and PFHXS (12.64). Cytokines were widely detected with the highest median values observed for IL8 (314.5), while IL6 (12.0 pg/ml), and TNF-alpha (10.0) were lower and IL10 was not detected. Immunoglobulin G median was 8.5 pg/ml (IQR 0, 24.7). PFOA and PFOS were not associated with cytokine levels. PFNA was associated with decreased IL6 (-2.2, 95% CI: -2.9, -1.4), IL8 (-1.8, 95% CI: -2.6, -1.0) and TNF-alpha (-1.1, 95% CI: -1.2, -0.6) after adjustment for maternal age and race/ethnicity. PFHxA was associated with increased IL6 (1.0, 95% CI: 0.1, 1.9), IL8 (1.0, 95% CI: 0.1, 1.9) and TNF-alpha (0.3, 95% CI: -0.3, 1.0). PFOS were associated inversely with IgG levels (-1.07, 95% CI: -18, -0.3).
Conclusion: Our findings in a diverse cohort of new mothers contribute to the literature that select PFAS were associated with maternal inflammatory and immune markers.

P56-21 | Association between prenatal exposure to per- and polyfluoroalkyl substances and pubertal development in boys and girls in the Spanish INMA cohort.

Sara Lopez de Calle[1,2], Aitana Lertxundi[1,2], Oihane Alvarez[3,4], Maria-Jose Lopez-Espinosa[3], Carmen Freire[5], Monica Guxens[6,7], Jean-Philippe Antignac[8], Martine Vrijheid[6,2], Amaia Irizar[1,2], German Cano-Sancho[8]

[1] Biodonostia, Unidad de Epidemiología Ambiental y Desarrollo Infantil, Facultad de Medicina, UPV-EHU, San Sebastian, Spain; [2] Spanish Consortium for Research on Epidemiology and Public Health (CIBERESP, Madrid, Spain; [3] Epidemiology and Environmental Health Joint Research Unit, Foundation for the Promotion of Health and Biomedical Research in the Valencian Region, FISABIO-Public Health, FISABIO, Universitat Jaume I/ Faculty of Nursing and Chiropody,Universitat de València, Valencia, Spain; [4] Applied Statistics and Operational Research, and Quality, Universitat Politècnica de València, Valencia, Spain; [5] Department of Legal Medicine, Toxicology, and Physical Anthropology, University of Granada, Granada, Spain; [6] ISGlobal, Barcelona, Spain; [7] ICREA, Barcelona, Spain; [8] Oniris, INRAE, LABERCA, Nantes, France

Per- and polyfluoroalkyl substances (PFAS) mayact as endocrine-disrupting chemicals due to their ability to interfere with hormone systems. PFAS can cross the placenta barrier and interfere with the child's development as this is a particularly vulnerable period for them. This prenatal exposure can cause latent effects on the child, which may emerge years later and potentially impact pubertal development. Nevertheless, studies assessing pubertal development are still scarce and results are inconclusive.

To evaluate the associations between prenatal exposure to PFAS and pubertal development in boys and girls.

PFAS concentrations including perfluorohexane sulfonic acid (PFHxS), perfluorooctanoic acid (PFOA), perfluorononanoic acid (PFNA), and perfluorooctane sulfonic acid (PFOS), were quantified in plasma collected during the first trimester of pregnancy from mothers participating in the Spanish INMA (Infancia y Medio Ambiente) cohort.

Generalized linear mixed models with Poisson distribution and logarithmic link function were used to evaluate the individual association between log-transformed PFAS concentrations and the risk of earlier puberty onset (PDS dichotomized; stage 1 vs. 2+). Models were adjusted for cohort, maternal age at delivery, maternal education level, and child’s age at pubertal assessment. Joint associations of PFAS as mixtures were assessed using Bayesian Kernel Machine Regression models.

Prenatal exposure to PFOA was statistically associated with an increased likelihood of earlier puberty among boys at 11 years (Relative Risk = 1.52 95% confidence interval =1.02, 2.27). No statistically significant associations were observed between prenatal PFAS exposure and risk of earlier puberty onset among girls. Multipollutant models are globally consistent with these findings.

These preliminary findings suggest that prenatal exposure to PFOA may be associated with earlier puberty in boys, highlighting the potential endocrine-disrupting effects of PFAS on pubertal timing. Further analyses are ongoing to further characterize the mixture effects and the associations with late onset.

P57 | Environmental Exposures and Sleep Health

P57-02 | The Association Between Temperature Anomalies, Air Pollutants, and Sleep-Disordered Breathing

I-Ping Lu[1], Ching-Huang Lai[2], Shih-Chun Candice Lung[3], Pei-Chih Wu[4], Tain-Junn Cheng[5]

[1] National Defense Medical Center, Department of Public Health, Taipei, Taiwan; [2] National Defense Medical Center, Department of Public Health, Taipei, Taiwan; [3] Academia Sinica, Research Center for Environmental Changes, Taipei, Taiwan; [4] Chang Jung Christian University, Environmental Research and Information Center, Tainan, Taiwan; [5] Chi Mei Medical Center, Departments of Neurology and Occupational Medicine, Tainan, Taiwan

Objective

Temperature variations due to climate change and air pollution may adversely affect respiratory and cardiovascular health, particularly during sleep when autonomic regulation is diminished. Research suggests that air pollutants [PM_{10}, $PM_{2.5}$, nitrogen dioxide (NO_2), and ozone (O_3)] can exacerbate airway inflammation and obstruction. At the same time, temperature anomalies may disrupt autonomic nervous regulation and vascular constriction, leading to abnormal breathing patterns and heart rate variability. However, the combined effects of temperature variations and air pollution on sleep-disordered breathing remain underexplored. This study investigates their associations with abnormal breathing indicators—Chest Effort Index (CEI) and Cyclic Variation of Heart Rate Index (CVHRI)—and evaluates potential interactions to inform public health and clinical strategies.

Material and Methods

Seven-day, 24-hour physiological monitoring data were collected from 11 stroke patients at a medical center using the medically certified RootiRx® sensor. Environmental data were sourced from Taiwan's Ministry of Environment and Central Weather Administration, including air pollutants (PM_{10}, $PM_{2.5}$, NO_2, O_3) and meteorological factors [temperature, relative humidity (RH)]. Spearman correlation and Generalized Additive Mixed Model (GAMM) analysis were used to assess the associations between air pollutants, meteorological factors, CEI, and CVHRI by R 4.4.1.

Results

A moderate positive correlation between $PM_{2.5}$ and PM_{10} ($\rho=0.5774$, $p<0.05$) and a weaker positive correlation with NO_2 ($\rho=0.2663$, $p<0.05$). O_3 correlated positively with temperature ($\rho=0.2805$, $p=0.0148$), while RH negatively correlated with PM_{10} ($\rho=-0.2883$, $p=0.0155$). Health risk analysis revealed significant correlations between $PM_{2.5}$ and CEI ($\rho=0.2857$, $p=0.0143$), and NO_2 and CVHRI ($\rho=0.2720$, $p=0.0199$). GAMM analysis showed significant effects on CEI ($p<0.01$), but no significant effects on CVHRI or their interactions.

Conclusion

Air pollutants ($PM_{2.5}$, NO_2) and temperature anomalies are significantly associated with sleep-disordered breathing, potentially increasing health risks. These findings emphasize the need for policies addressing environmental interactions and targeted interventions to mitigate sleep-related health effects.

P57-03 | Air pollution and sleep health in middle-aged and older adults: A systematic review and meta-analysis

Junxin Li[1], Man Wang[1,2], Youngmin Cho[1]

[1] Johns Hopkins University, Baltimore, USA; [2] Zhejiang University, Hangzhou, China

Exposure to air pollution may negatively impact sleep health. This systematic review and meta-analysis examined the association between air pollution and sleep health in middle-aged and older adults. PubMed, Web of Science, CINAHL and Embase were searched from inception to September 11, 2024. Included studies reported at least one air pollutants (e.g., PM_1 [particles < 1 μm], $PM_{2.5}$ [particles < 2.5 μm], PM_{10} [particles < 10 μm], CO [carbon monoxide], CO_2 [carbon dioxide], NO_2 [nitrogen dioxide], SO_2 [sulfur dioxide] and O_3 [ozone]) and sleep outcomes (e.g., poor sleep quality, prolonged sleep latency, low sleep efficiency, sleep deprivation, insomnia symptoms, excessive daytime sleepiness and obstructive sleep apnea). Twenty-five studies were included, with a final sample of at least 687,612 middle-aged and older adults. The pooled prevalence of poor sleep health is 45.6%. We conducted random-effects meta-analyses on nine studies using the DerSimonian-Laird estimator. Results showed that higher concentrations of PM_1 [OR (95% CI): 1.37 (1.07, 1.75)], $PM_{2.5}$ [OR (95% CI): 1.27 (1.12, 1.43)], PM_{10} [OR (95% CI): 1.10 (1.03, 1.19)] and NO_2 [OR (95% CI): 1.07 (1.01, 1.13)] were related to increased risks of poor sleep health. However, we did not find a significant relationship between O_3 concentrations and poor sleep health [OR (95% CI): 1.04 (0.88, 1.24)]. Indoor air pollution from the combustion of solid fuels was also associated with a higher risk of poor sleep health. Our findings highlight the critical roles of both indoor and outdoor air environments in promoting sleep health, underscoring the need for environmental interventions and public health policies to improve air quality and alleviate the burden of sleep disorders.

P57-04 | Evaluating the associations between heat exposure and sleep outcomes in rural Ghana

Kenneth A. Ae-Ngibise[1], Lewis White[2], Darby Jack[2], Solomon Nyame[1], Cascade Tuholske[3], Mohamed N. Mujtaba[1], Vaishnavi Kundel[4], Seyram Kaali[1], Steven Chillrud[5], Kwaku P. Asante[1], Alison Lee[4]

[1] Kintampo Health Research Centre, Ghana Health Service, Kintampo, Ghana; [2] Columbia University, Mailman School of Public Health, Department of Environmental Health Sciences, New York, USA; [3] Montana State University, Department of Earth Sciences, Bozeman, USA; [4] Icahn School of Medicine at Mount Sinai, Division of Pulmonary, Critical Care and Sleep Medicine, New York, USA; [5] Columbia University, Lamont Doherty Earth Observatory, New York, USA

Objective: Heat significantly disrupts thermoregulation, impairing sleep quality and duration, while also increasing risks of long-term health issues like fatigue and depression. This study examines the link between heat exposure and total sleep time (TST) among postpartum women in Ghana.

Material and Methods: Actigraphy data were collected from a total of 156 participants at four and eight years after birth, including single-night and multi-night recordings at both follow-ups. For the mult-inight study, the participants wore the wrist actigraphy device for a period of 5 to 7 days, while for the single-night study, they wore it over-night. The actigraphy data were paired with daily maximum wet-bulb globe temperature (WBGT) values from ERA5 reanalysis data specific to each participant's community. Mean WBGT was calculated for each participant over their

actigraphy study period and a linear regression model used to assess the relationship between average WBGT and TST.

Results: The results indicate that higher WBGT during the study period was significantly associated with reduced postpartum TST. Specifically, our model predicts that, on average, a 1°C increase in the average daily maximum WBGT over the sleep period is associated with a 16.5 minute reduction in the total sleep time ($\beta = -16.5$, $p < 0.0048$). This suggests warmer conditions challenge optimal sleep among postpartum women.

Conclusion: Heat exposure is linked to reduced postpartum sleep duration, highlighting the impact of elevated temperatures on sleep patterns. These findings emphasise the need to consider environmental factors, like temperature, in addressing sleep health during and after pregnancy.

P57-05 | Metals and children's sleep: a functional data analysis of longitudinal exposure data in the PROGRESS cohort

Sili Fan[1], Nia Mcrae[1], Inessa Lopez[1], Mahmoud Awawda[1], Libnit Torres-Olascoaga[2], María L. Pizano[3], Mauro Martinez[1], Mariana G. Figueiro[6], Lauren Petrick[1], Rosalind Wright[7], Shelley H. Liu[6], Jeff Goldsmith[5], Manish Arora[1], Martha M. Tellez-Rojo[2], Robert Wright[1], Allison Kupsco[4], Dinesh Barupal[1]

[1] Icahn School of Medicine at Mount Sinai, Environmental Medicine, New York, USA; [2] National Institute of Public Health, Center for Nutrition and Health Research, Cuernavaca, Mexico; [3] National Institute of Perinatology, Cuernavaca, Mexico; [4] Columbia University, Department of Environmental Health Sciences, New York, USA; [5] Columbia University, Department of Biostatistics, New York, USA; [6] Icahn School of Medicine at Mount Sinai, Department of Population Health Science and Policy, New York, USA; [7] Icahn School of Medicine at Mount Sinai, Department of Public Health, New York, USA

Children's sleep is an essential biological process for their overall health. Conversely, poor sleep has been associated with a range of social, medical and behavioral problems. Sleep is a highly coordinated biological process. We hypothesized that environmental exposures during the fetal and very early-life can influence sleep in the later stages of a child's life. Using 326 children from the deep-phenotyped Programming Research in Obesity, GRowth, Environment and Social Stress (PROGRESS) cohort's data repository, we compiled a dataset of pre/post-natal exposures to 14 metals (Li, Mg, Ca, Mn, Co, Ni, Cu, Zn, Sr, Mo, Sn, Ba, Pb, Bi) and sleep data. Metal exposures were measured using Laser Ablation Inductively Coupled Plasma Mass Spectrometry analysis of the children's naturally shed tooth samples. Weekly exposures were measured prenatally for the 11 weeks preceding birth and postnatally for 13 weeks following birth. Sleep outcomes were children's average sleep duration (hours) and sleep efficiency at age 48 months as measured and scored using Actigraphy for a period of seven days. We utilized scalar-on-function regression, a technique in the functional data analysis, in which exposures measured longitudinally treated as functions and sleep outcomes were scalar continuous variables to identify the time-varying associations between metal exposures and scaler sleep outcomes. Among the 14 metals, higher levels of Cobalt (Co) and Copper (Cu) at 6-12 weeks after birth showed significant negative associations with average sleep duration at age 48 months. Higher levels of Molybdenum (Mo) at 2-5 weeks after birth were negatively associated with sleep efficiency at age 48 months. These results suggest the importance of these metals exposures during the critical stage of children's development. Dysregulations in the essential metals, Cobalt, Copper and Molybdenum during the early-age may have disturbed the biological processes involved in determining children's sleep trajectories at the later stage of life.

P57-06 | Association between prenatal maternal phenol concentrations and psychosocial stress exposures, with early childhood sleep quality in the ECHO cohort

Sarah Geiger[1,2], Xiaoshuang Xun[4], Aruna Chandran[5], Cai Zhang[1,2], Zachary Sievers[2], Akram Alshawabkeh[6], Courtney Blackwell[14], Anne Dunlop[9], Dana Dabelea[8], Stephanie Eick[10], Dana Goin[11], Jonika Hash[12], Erica Jansen[13], Assiamira Ferrara[7], Maxwell Mansolf[14], Monique Hedderson[7], Louise O'Brien[15], Yeyi Zhu[7], Zhengmin Qian[16], Susan Schantz[1,3]

[1] University of Illinois at Urbana-Champaign, Beckman Institute for Advanced Science and Technology, Urbana, USA; [2] University of Illinois at Urbana-Champaign, Department of Health and Kinesiology, Champaign, USA; [3] University of Illinois at Urbana-Champaign, Department of Comparative Biosciences, Urbana, USA; [4] Johns Hopkins University, Bloomberg School of Public Health, Baltimore, USA; [5] Johns Hopkins University, Department of Epidemiology, Baltimore, USA; [6] Northeastern University, Boston, USA; [7] Kaiser Permanente Northern California, Division of Research, Oakland, USA; [8] University of Colorado, School of Medicine, Aurora, USA; [9] Emory University, Department of Gynecology & Obstetrics, Atlanta, USA; [10] Emory University, Gangarosa Department of Environmental Health, Atlanta, USA; [11] University of California San Francisco, Program on Reproductive Health and the Environment, San Francisco, USA; [12] University of Washington, Department of Child, Family, and Population Health Nursing, Seattle, USA; [13] University of Michigan Ann Arbor, Department of Nutritional Sciences, Ann Arbor, USA; [14] Northwestern University, Department of Medical Social Sciences, Chicago, USA; [15] University of Michigan Ann Arbor, Department of Obstetrics & Gynecology, Ann Arbor, USA; [16] Saint Louis University, College for Public Health & Social Justice, Saint Louis, USA

Objective: Early childhood sleep quality predicts sleep patterns and multiple physical and mental health outcomes through the life course. Previous work shows that maternal prenatal phenol concentrations and maternal prenatal stress are independently implicated in sleep quality in middle childhood. We examined associations of both phenol concentrations and maternal psychosocial stress with sleep outcomes among younger children, aged 1.5-5 years, and effect modification by child sex.

Material and Methods: Data were collected between 2008 and 2019 from the Environmental influences on Child Health Outcomes (ECHO) Cohort. Maternal urinary phenol concentrations were log-transformed; T-scores were used for Perceived Stress Scale (PSS), PROMIS Sleep Disturbance (PSD), and Child Behavior Checklist (CBCL) Sleep Problems measures. In separate linear mixed models assessed associations between 8 individual phenols, and PSS, with CBCL sleep problems (N=1,714) and PSD (N=1,119), controlling for maternal education, age, and alcohol intake. We also stratified by child sex.

Results: Prenatal PSS was associated with child CBCL Sleep Problems (ß=0.08, 95% CI=0.05,0.11) and PSD (ß=0.12, 95% CI=0.06,0.17), meaning for every 1-point increase in PSS T-score, there was a 0.08 (CBCL Sleep Problems) and 0.12 (PSD) point increase in child sleep quality T-scores, with higher scores indicating poorer quality sleep. There were no significant main effects of phenols on sleep outcomes. Bisphenol A (BPA) was inversely associated with sleep outcomes among male offspring only (CBCL Sleep Problems: ß=-0.30, 95% CI=-0.59,-0.01; PSD: ß=-0.70, 95% CI=-1.30,-0.09). Each one ng/mL increase in maternal methyl paraben (MEPB) was inversely associated with a 0.5-point PSD T-score decrease among males only (95% CI=-0.95,-0.06).

Conclusions: There were small, significant associations between prenatal PSS, BPA, and MEPB, and early childhood sleep outcomes. Efforts to reduce chemical and non-chemical stressors during the prenatal period may help to promote better child sleep health, one of the central tenets of child health and development.

P57-07 | Impact of Environmental Exposures on Sleep Quality: A Pilot Study with Real-time Monitoring

Jingting Huang[1], **Linyan Li**[1,2]

[1] City University of Hong Kong, Department of Data Science, Hong Kong, Hong Kong; [2] City University of Hong Kong, Department of Infectious Diseases and Public Health, Hong Kong, Hong Kong

Objective:
Emerging evidence suggests that indoor environmental quality influences sleep disturbances, yet real-time monitoring of these interactions remains limited. This pilot study examines the temporal relationships between multi-domain environmental exposures (air pollutants, humidity, temperature) and both objective and subjective sleep outcomes in urban young adults.

Materials and Methods:
Thirty healthy undergraduate students in Hong Kong participated in a 7-day ecological assessment. Real-time environmental monitoring was conducted using calibrated sensors placed in participants' bedrooms to measure temperature, humidity, particulate matter ($PM_{2.5}$, PM_{10}), CO_2, formaldehyde (HCHO), and volatile organic compounds (VOCs). Physiological sleep metrics, including total sleep time and wake-after-sleep-onset, were assessed via wristbands equipped with validated actigraphy algorithms. Subjective sleep quality, environmental comfort, and daytime fatigue were evaluated using a 15-item daily questionnaire with a 5-point Likert scale. Statistical analyses included Pearson correlations and mixed-effects models, adjusting for age, gender, and activity levels.

Results:
The average indoor temperature was 26°C, with a relative humidity of 62%. Notably, greater humidity fluctuations were significantly associated with shorter sleep duration ($\beta = -0.08$, $P < 0.05$). Air quality deteriorated during sleep, with increased levels of $PM_{2.5}$, PM_{10}, CO_2, and HCHO compared to pre-sleep conditions.

Conclusion:
This multimodal real-time monitoring approach uncovers previously undocumented nocturnal environmental exposures that may negatively impact sleep health in urban settings. While constrained by sample size and a single-city design, these findings offer valuable mechanistic insights for future intervention trials aimed at promoting sleep-friendly indoor environments.

P57-08 | Sleep Disordered Breathing: Critical Mediator in Mixed Toxicant Exposure Illness?

Brinton K. Berg[1], Jessica Y.F. Situ[1], Janhavi Kulkarni[1,2], Vincent Davidson[1], Bruce J. Miller[1], Beatrice A. Golomb[1]

[1] University of California, San Diego, Department of Medicine, La Jolla, USA; [2] Yale School of Public Health, Department of Environmental Health Sciences, New Haven, USA

Objective:To assess whether sleep-disordered-breathing (**SDB**) symptoms were present, new, and tied to other symptoms and markers in persons affected by the mixed toxicant exposure (**MTE**) following the East Palestine, Ohio (**EP**) train derailment. (Toxicants impair mitochondria, promoting SDB.)
Methods:55 adults (≤20-miles from the EP derailment) completed surveys, with optional blood tests. Presence of Gulf War illness (**GWI)**-like MTE-illness (Kansas criteria); MTE-illness severity (validated UCSD-20); and presumptive SDB (**PSDB**) defined by ≥1 of 4 SDB symptoms (prior 2-weeks) were assessed: daytime sleeping/napping, nocturia, night-sweats, nightmares. Labs (subset) included hsCRP, total protein and RBC membrane fatty acids.
Results:73% of participants met MTE-illness criteria of whom 71% had PSDB. In three-fourths of these, PSDB symptoms were new post-derailment. Mean UCSD-20 (possible 0-200) was 72.3. PSDB contributed powerfully: in age-sex adjusted regression (robust SEs): β(SE)[95% CI]p=46.6(11.3)[23.8-69.3]<0.001. (No PSDB symptom was part of the UCSD-20.) PSDB was unrelated to hsCRP (an inverse predictor of MTE-illness severity): p=0.77. Serum total protein (as an index of volume contraction) bore the expected relation to PSDB: logit β(SE)[95% CI]p= 2.35(1.16)[0.083-4.62]0.042. (SDB drives diuresis/natriuresis.) Eicosadienoic acid (RBC membrane), a marker of metabolism that strongly related to MTE-illness severity, was borderline-significantly predicted by PSDB: age-sex adjusted regression β(SE)[95% CI]p=0.025(0.012)[-0.0004-0.050]0.053. SDB also borderline-significantly related to (primarily dietary, from peanuts) behenic acid: β(SE)[95% CI]p=-0.059(0.029)[-0.119-0.001]0.055. (While not specific to MTE-illness, the possibility that peanut consumption is protective for respiratory disease – as is reported for tree nut consumption – may merit further study.)
Conclusion:PSDB is common/increased after EP MTE and linked to greater illness severity, mirroring Gulf War findings. SDB both results from and contributes to the mitochondrial/bioenergetic impairment mechanisms reported to underlie MTE-illness. SDB can contribute to multiple MTE comorbidities, and its assessment/treatment has prospects to significantly alleviate symptoms and mitigate comorbidities in EP and following other MTEs.

P58 | Wildland Fire, Smoke, and Health - PART II

P58-01 | Understanding the health impacts of co-occurring wildfire fine particulate matter and extreme temperatures

Carlos F. Gould[1], Sam Heft-Neal[2], Alexandra K. Heaney[1], Eran Bendavid[3], Marshall Burke[4]

[1] School of Public Health, University of California San Diego, La Jolla, USA; [2] Center of Food Security and Environment, Stanford University, Stanford, USA; [3] School of Medicine, Stanford University, Stanford, USA; [4] Doerr School of Sustainability, Stanford University, Stanford, USA

There is growing interest in understanding the impacts of compound climate hazards. Here, we quantify the effects of two such hazards -- extreme temperatures and wildfire-specific fine particulate matter (PM2.5) -- on healthcare utilization by linking 127 million all-cause ED visits from nonfederal hospitals in California (2006–2017) to daily ZIP code-level temperature and wildfire smoke data. We walk through the complexities of estimating the health impacts of temperatures and wildfire smoke: we first model temperature and wildfire smoke effects independently, then control for one exposure while estimating the other, and finally incorporate interactions ranging from simple linear forms to flexible nonlinear polynomials. We assessed same-day and cumulative lagged effects over 2, 7, 14, and 21 days. Consistent with existing evidence, extreme temperatures and wildfire smoke were both independently associated with increased ED visits. Controlling for each other only moderately attenuated associations. As compared to binary classifications (hot vs. not hot, smoky vs. not smoky, hot and smoky), nonlinear interaction models produced much larger estimates of increased ED visit rates, especially when a day was both hot and very smoky. Overall, days characterized by extreme heat combined with severe wildfire smoke produced disproportionately severe impacts, yet account for well below 1% of all observations. Future efforts to quantify the impacts of both extremes should account for nonlinear and lagged effects; otherwise, analyses may either emphasize only very rare events or obscure important but complex nonlinear relationships through averaging.

P58-02 | Association between wildfire $PM_{2.5}$ smoke exposure and respiratory and cardiovascular events among U.S. veterans

Caryn S. Yip[1], Michael P. Jones[2,3], Beiming Tang[4,5], Peter J. Kaboli[3,6], Margaret Carrel[7], Gregory R. Carmichael[8], Peter S. Thorne[1]

[1] University of Iowa, Department of Occupational & Environmental Health, Iowa City, USA; [2] University of Iowa, Department of Biostatistics, Iowa City, USA; [3] VA Healthcare System, Center for Comprehensive Access and Delivery Research and Evaluation (CADRE), Iowa City, USA; [4] National Oceanic and Atmospheric Administration (NOAA), Air Resources Laboratory (ARL), College Park, USA; [5] George Mason University, Center for Spatial Information Science and Systems (CSISS), Fairfax, USA; [6] University of Iowa, Department of Internal Medicine, Iowa City, USA; [7] University of Iowa, Department of Geographical and Sustainability Sciences, Iowa City, USA; [8] University of Iowa, College of Engineering, Iowa City, USA

Objective: Determine the extent to which exposure to wildfire $PM_{2.5}$ is associated with adverse respiratory and cardiovascular events in U.S. veterans.
Material and Methods: Retrospective cohort study including U.S. veterans enrolled in Veteran Health Administration primary care and whose home addresses were in Veterans Integrated Services Networks 19–22 (13 western states) during the November 2018 U.S. wildfires. Analyses included data from 1,000,314 veterans between October 2018 and December 2018. Wildfire $PM_{2.5}$ exposure was determined using a 1km^2 grid of $PM_{2.5}$ concentration from a

machine-learning downscaled air pollution model that included variables for meteorology, land cover, topography, population, and emissions. Outcomes of interest were respiratory events (asthma, chronic obstructive pulmonary disease (COPD), respiratory infections) and cardiovascular events (myocardial infarction, heart failure, and stroke) that required urgent care/emergency department visits or in-patient admission. Associations between $PM_{2.5}$ smoke and outcomes were estimated using Cox regression models.

Results: During the study period, wildfire-specific $PM_{2.5}$ smoke exposure ranged from 0.25 to 380 μg/m^3. There were a total of 15,146 respiratory events and 12,268 cardiovascular events over the study period. Increasing neighborhood disadvantage was consistently associated with respiratory and cardiovascular events. After adjusting for neighborhood disadvantage, each 10-μg/m^3 increase in wildfire-specific $PM_{2.5}$ exposure was associated with a hazard ratio of 1.05 (95% confidence interval, 1.03-1.07), 1.04 (1.01-1.07), 1.19 (1.14-1.24), and 1.09 (1.06-1.12) for respiratory, cardiovascular, asthma, and COPD events, respectively, among healthier veterans (83% of cohort). Among sicker veterans, each 10-μg/m^3 increase in $PM_{2.5}$ exposure was associated with a hazard ratio of 1.06 (1.02-1.11) for stroke.**Conclusion:**This study utilized a novel machine-learning downscaled $PM_{2.5}$ model to examine the association between $PM_{2.5}$ exposure and respiratory and cardiovascular events among U.S. veterans. After adjusting for neighborhood disadvantage, there was a significant association between $PM_{2.5}$ exposure and respiratory, asthma, COPD, and cardiovascular events among healthier veterans and stroke among sicker veterans.

P58-03 | Barriers and facilitators of early-life wildfire smoke protection in a rural, agricultural community: Implications for future interventions

Christine Loftus[1], Aileen Andrade-Torres[1], Javier Silva[1], Corbin Schuster[4], Jessica Black[2], Jari Tavira[2], Virginia Yelechin[2], Aaliyah Villa[2], Shelby Clark[2], Karolynn Tom[3], Catherine Karr[1]

[1] University of Washington, Seattle, USA; [2] Heritage University, Toppenish, USA; [3] Yakima Valley Community College, Yakima, USA; [4] Oregon State University, Corvallis, USA

Introduction: Millions of U.S. families are exposed to unhealthy levels of wildfire smoke (WFS) every year. A warming climate is accelerating this public health crisis, yet research on interventions to mitigate early-life WFS exposures lags. Little is known about adoption of protective behaviors, especially in vulnerable remote and marginalized communities. We aimed to address this critical knowledge gap in a rural, agricultural region of Washington State.

Methods: Community meetings informed research questions, a conceptual model for barriers and facilitators of self-protection, and a survey in English and Spanish for assessing past behaviors and potential determinants. Undergraduate interns at a local minority-serving university were trained in epidemiologic research methods and assisted with data collection. We used linear regression with robust standard errors to estimate bivariate associations between each predictor, standardized as a z-score, and total action score, a sum of the frequencies of six protective behaviors taken in past smoke events.

Results: Of N=199 respondents, all were parents, 84.8% were Hispanic, 55.3% were born in Mexico, and 58.8% had an annual income <40K. The most frequent protective action was staying indoors and the least was leaving the region, with 54.8% and 1.5% reporting doing so “often”, respectively. Impacts on child health in past WFS events and observations of community members taking action each had the strongest associations with action scores (beta=0.91 [95% CI: 0.50, 1.31] and 0.67 [95% CI: 0.16, 1.18] per standard deviation increase in predictor, respectively). Existing chronic health conditions of parent or child, general WFS knowledge, and practical knowledge about WFS mitigation were also associated with significantly more actions.

Discussion: These novel data identify facilitators of protective actions that could inform interventions, including the importance of community in motivating behaviors. Our multifaceted community-engaged methodology supported efficient data collection and provided valuable research experiences for local public health scientists-in-training.

P58-04 | A decision-support framework for efficient disaster research: rapid questionnaire design for perishable exposure and health data collection during wildfire events

Lilian Liu[1], Christine Loftus[1], Diana Rohlman[2], Edmund Seto[1], Elena Austin[1]

[1] University of Washington, Department of Environmental and Occupational Health Sciences, Seattle, USA; [2] Oregon State University, Department of Environmental and Molecular Toxicology, Corvallis, USA

Efficient disaster response research is critical to address wildfire (WF) impacts, especially in response to worsening events such as the January 2025 Los Angeles (LA) WFs. To our knowledge, there is no standardized, readily deployable framework for questionnaire development to facilitate rapid and reproducible exposure and health outcome data collection during WF events. Here we share updates on a new WF health questionnaire library and complementary decision-support framework to support rapid, efficient, and reproducible WF disaster response research.
We performed a systematic literature review on studies that assessed WF exposure and/or health impacts via questionnaires, plus a secondary search of questionnaires in REDCap for health outcomes identified in the review. Questionnaires were organized into a structured library. To create the full WF decision-support framework, we acquired additional WF impact assessment resources from literature review and the National Institutes of Environmental Health Sciences (NIEHS) Disaster Research Response (DR2) portal. We performed a thematic analysis of literature review studies and questionnaires to identify common elements/themes for the decision-support framework, which consisted of a flow diagram and a survey that produces a tailored list of questionnaires for unique WF study aims and needs.
N=30 peer-reviewed studies were identified for the literature review, yielding 100 questionnaires for the repository/library. N=21 eligible research resources were identified from DR2 portal. A framework flow diagram and REDCap survey with 3 themed sections were created. Feedback from professional research network members (e.g. DR2, University of Washington RAPID Facility, Public Health Extreme Event Research; total N = 40) helped improve the scope, content, and usability of the decision-support framework and the library, which is available and has been deployed to support LA Wildfire research. The final decision-support framework will be made available to the public as a research protocol on the DR2 portal for future WF research efforts.

P58-05 | Prenatal and infancy exposure to wildfire smoke and the development of atopic dermatitis in the ECHO Cohort

Logan C. Dearborn[1], Allison R. Sherris[1], Markus D. Boos[2], Adam A. Szpiro[3], James E. Gern[5], Amy Eapen[6], Jessica Wong[7], Judy L. Aschner[8], Carlos A. Camargo, Jr.[9,10], Yueh-Hsiu M. Chiu[11], Margaret R. Karagas[12], Christopher Miller[13], Sunni L. Mumford[7], Janet L. Peacock[12], Anne-Marie Singh[14], Zhaozhong Zhu[9], Catherine J. Karr[1,4], Christine T. Loftus[1]

[1] University of Washington, Department of Environmental and Occupational Health Sciences, Seattle, USA; [2] Seattle Children's Research Institute, Center for Clinical and Translational Research, Seattle, USA; [3] University of Washington, Department of Biostatistics, Seattle, USA; [4] University of Washington, Department of Pediatrics, Seattle, USA; [5] University of Wisconsin, Division of Allergy, Immunology & Rheumatology, Madison, USA; [6] Henry Ford Health System, Division of Allergy and Clinical Immunology, Detroit, USA; [7] University of Pennsylvania, Biostatistics and Epidemiology, Philadelphia, USA; [8] Hackensack Meridian School of Medicine, Pediatrics, Nutley, USA; [9] Harvard Medical School, Department of Emergency Medicine, Boston, USA; [10] Harvard T.H. Chan School of Public Health, Department of Epidemiology, Boston, USA; [11] Icahn School of Medicine at Mount Sinai, Environmental Medicine and Climate Science, New York, USA; [12] Geisel School of Medicine at Dartmouth, Department of Epidemiology, Hanover, USA; [13] Nationwide Children's Hospital, Primary Care Pediatrics, Columbus, USA; [14] University of Wisconsin, Department of Pediatrics, Madison, USA

Background

Growing evidence supports a biological link between wildfire smoke (WFS) and the development of atopic dermatitis (AD), but epidemiologic studies on this relationship are scarce. We assessed associations between prenatal and infancy exposures to WFS and development of AD in the U.S. Environmental Influences on Child Health Outcomes (ECHO) Cohort.

Methods

We included 4,145 mother-child dyads recruited between 2007-2020 at 11 ECHO cohort sites in the western United States. Estimates of daily census-tract exposure to WFS from a national spatiotemporal model were linked to residential address histories from conception through the first year of life. Prenatal and infancy exposure were evaluated as the number of days with wildfire-specific fine particulate matter ≥5 μg/m^3 during pregnancy and the first year of life. Three or more consecutive days ≥5 μg/m^3 were defined as WFS waves. Caregivers reported “ever AD” between child age 6 months-6 years. Associations were estimated using logistic regression mixed effect models with random intercepts for recruitment site, adjusted for sociodemographic, climatic, and neighborhood factors.

Results

Among eligible dyads, 30.3% reported AD. The median (interquartile range [IQR]) number of WFS days was 3 (6) during pregnancy and 6 (14) during infancy. WFS waves occurred in 34.6% of pregnancies and 51.0% of infancies. AD was not associated with prenatal exposure to WFS days (odds ratio [OR] per 10 WFS days: 0.98, 95% confidence interval [CI]: 0.89-1.09) or WFS waves (OR: 1.03, 95% CI: 0.88-1.20). However, we observed suggestive associations between AD and infancy WFS, though estimates were imprecise: OR: 1.03 per 10 days (95% CI: 0.96-1.11) and OR: 1.16 for ≥3-day WFS wave (95% CI: 0.96-1.29).

Conclusion

To our knowledge, this is the first epidemiological analysis suggesting that early-life WFS could influence AD risk. Understanding this relationship could inform public health interventions for reducing the burden of pediatric AD.

P58-06 | Investigating dependencies of inputs in assessing health impacts of wildfire smoke exposure in New Mexico during summer 2022

Olivia Sablan[1], Bonne Ford[2], Colin Hawkinson[3], Jihoon Jung[4], Chelsea Eastman Langer[3], Courtney Maichak[5], Kamal Jyoti Maji[6], Stephanie Moraga-McHaley[3], Armistead G. Russell[6], Chris K. Uejio[7], Melissa Vansickle[7], Emily V. Fischer[1], Jeffrey R. Pierce[1], Sheryl Magzamen[5]

[1] Colorado State University, Department of Atmospheric Science, Fort Collins, USA; [2] Colorado State University, Cooperative Institute for Research in the Atmosphere, Fort Collins, USA; [3] New Mexico Department of Health, Center of Health Protection, Environmental Health Epidemiology Bureau, Santa Fe, USA; [4] University of North Texas, Department of Geography and the Environment, Denton, USA; [5] Colorado State University, Department of Environmental and Radiological Health Sciences, Fort Collins, USA; [6] Georgia Institute of Technology, Department of Civil and Environmental Engineering, Atlanta, USA; [7] Florida State University, Department of Geography, Tallahassee, USA

As wildfires become more frequent and intense due to climate change, accurately assessing the health impacts of smoke exposure is critical. In 2022, New Mexico experienced the state's largest wildfire, Calf Canyon/Hermit's Peak. We investigated associations with a 10 μg m^{-3} increase in exposure to fine particulate matter ($PM_{2.5}$) from wildfire smoke and cardiorespiratory-related emergency department (ED) visits in New Mexico during 2022. We compared four smoke estimates in our analysis: 1) Regulatory-only 24-hour average product, which uses $PM_{2.5}$ measurements from the EPA regulatory-grade monitors, 2) Regulatory + PurpleAir product, which adds low-cost PurpleAir sensors to the Regulatory-only product, 3) modeled 24-hour average wildfire smoke $PM_{2.5}$ from the Community Multiscale Air Quality Modeling System (CMAQ), and 4) modeled 1-hour maximum wildfire smoke $PM_{2.5}$ from CMAQ. There were significant associations with all-respiratory ED visits, pneumonia, and heart failure with smoke exposure; however, the magnitude and significance of associations varied across the four smoke-exposure products. Modeled smoke estimates were less suitable for this epidemiological study, while the Regulatory + PA product provided useful exposure estimates, highlighting the importance of low-cost sensors in rural fire-impacted regions. We also tested two referent periods: April 6 - August 22, 2022 (during Calf Canyon/Hermit's Peak) and April 1 - September 30, 2022 (the wildfire season). Results were inconsistent across referent periods, likely due to limited smoke-free days in the shorter period, demonstrating the need for representative referent periods in smoke epidemiology. Additionally, we compared results for ED visits versus syndromic surveillance reports and associations varied more with all-cardiovascular related visits than the all-respiratory related and asthma ED visits/syndromic surveillance reports. Overall, we found the inputs to epidemiological studies must be thoroughly evaluated to produce meaningful results, as our results were very sensitive to different inputs of exposure estimates, referent period, and health data.

P58-07 | Medium-term exposure to wildfire smoke $PM_{2.5}$ and cardiorespiratory hospitalization risks

Yaguang Wei[1,2], Edgar Castro[2], Kanhua Yin[3], Alexandra Shtein[2], Bryan N. Vu[2], Mahdieh Danesh Yazdi[4,2], Longxiang Li[5], Yuxi Liu[6,7], Adjani A. Peralta[2], Joel D. Schwartz[2,7]

[1] Icahn School of Medicine at Mount Sinai, Department of Environmental Medicine and Climate Science, New York, USA; [2] Harvard T.H. Chan School of Public Health, Department of Environmental Health, Boston, USA; [3] University of Missouri-Kansas City School of Medicine, Department of Surgery, Kansas City, USA; [4] Renaissance School of Medicine at Stony Brook University, Program in Public Health, Department of Family, Population, and Preventive Medicine, Stony Brook, USA; [5] Emory University, Gangarosa Department of Environmental Health, Rollins School of Public Health, Atlanta, USA; [6] Brigham and Women's Hospital and Harvard Medical School, Department of Medicine, Boston, USA; [7] Harvard T.H. Chan School of Public Health, Department of Epidemiology, Boston, USA

Objective: Wildfire activity in the US has increased significantly in recent decades. Smoke $PM_{2.5}$, a primary wildfire emission, can spike for months after a wildfire begins, yet large-scale evidence of its health effects remains limited. We aimed to examine the associations of 3-month average exposure to smoke $PM_{2.5}$ with hospitalization risks for a comprehensive range of cardiorespiratory diseases.

Methods: Hospitalization records for the residents of 15 states between 2006–2016 were obtained from the State Inpatient Databases. Daily smoke $PM_{2.5}$ concentrations at 10 km^2 grid cells across the contiguous US were estimated using a machine learning model that incorporated ground, satellite, and reanalysis data, and were aggregated to ZIP codes to match the spatial resolution of hospitalization records. We extended traditional case-crossover design, a self-controlled design originally developed for studying acute effects, to examine associations between 3-month average exposure to smoke $PM_{2.5}$ and hospitalization risks for a comprehensive range of cardiovascular (ischemic heart disease, cerebrovascular disease, heart failure, arrhythmia, hypertension, other cardiovascular diseases) and respiratory diseases (acute respiratory infections, pneumonia, COPD, asthma, other respiratory diseases). We further assessed effects of single-month lagged exposures and explored subgroup differences based on neighborhood- and individual-level characteristics.

Results: We found that 3-month exposure to smoke $PM_{2.5}$ was associated or marginally associated with increased hospitalization risks for most cardiorespiratory diseases. Results for single-month lagged exposures suggested that these effects persisted up to 3 months after exposure. Subgroup analyses showed larger effects in neighborhoods with higher deprivation level or greater vegetation, as well as among ever-smokers.

Conclusion: Our findings provided unique insights into medium-term cardiorespiratory effects of smoke $PM_{2.5}$, which can persist for months, even after a wildfire has ended.

P58-08 | From Local Forecast Uncertainty to Seasonal Prediction: Improving Wildfire Smoke Forecasts through Climatological Analysis

Yunyao Li[1,2], Daniel Tong[2], Siqi Ma[2], Shinkuang Chang[2], Christine Pham[1], Bohua Huang[2], David Straus[2]

[1] The University of Texas at Arlington, Department of Earth and Environmental Health, Arlington, USA; [2] George Mason University, Department of Atmospheric, Oceanic & Earth Sciences, Fairfax, USA

Accurately predicting wildfire smoke transport and its air quality impacts is crucial for public health and emergency response. However, smoke forecasts remain uncertain due to variations in model resolution, emissions, and atmospheric transport. This study investigates forecast uncertainty using the 2025 Los Angeles wildfires as a case study, then extends the analysis to climatological wildfire smoke transport patterns to improve seasonal air quality predictions.

We analyzed wildfire smoke forecasts from multiple modeling systems, including those from the National Aeronautics and Space Administration (NASA), National Oceanic and Atmospheric Administration (NOAA), Environment and Climate Change Canada, and the Naval Research Laboratory. We assessed forecast uncertainties related to model resolution and fire emissions. To examine long-term variability, we conducted a 19-year (2003–2021) air quality simulation using the Weather Research and Forecasting model coupled with the Community Multiscale Air Quality model (WRF-CMAQ) with United States Environmental Protection Agency emissions. We applied Empirical Orthogonal Function (EOF) and Singular Value Decomposition (SVD) analyses to identify major climatological wildfire smoke transport patterns and their relationships with large-scale climate variability, including the North Atlantic Oscillation (NAO), Arctic Oscillation (AO), and El Niño-Southern Oscillation (ENSO).

The Los Angeles wildfire case study revealed significant discrepancies among forecasts, with model resolution and fire emissions playing key roles in transport uncertainty. Climatological analysis showed that positive AO and negative ENSO years amplify wildfire smoke impacts across the continental United States, while negative NAO years intensify smoke-related air quality deterioration in the eastern United States and reduce impacts in the western United States. Addressing short-term forecast uncertainty provides insights for improving seasonal wildfire smoke predictions. The link between wildfire air quality anomalies and climate variability can enhance long-term forecasting and risk assessment. This study is supported by NASA’s Health and Air Quality Program and NOAA’s Weather Program Office.

P58-10 | Laboratory Scale Wildland-Urban Interface (WUI) Fire:Multi-Route Human Exposure Risk Models

Cynthia Choo[1], David Kalafut[1], Mark Wilson[1], Timothy Nurkiewiez[2], Anand Ranpara[2], William T. Goldsmith[2], Thomas Batchelor[2], Marilyn Blank[1]

[1] UL Research Institutes, Chemical Insights Research Institute, Marietta, USA; [2] West Virginia University, Center for Inhalation Toxicology, Morgantown, USA

Objective: Residents in the wildland-urban interface (WUI) face exposure risks to a variety of wildfire hazards, including inhalation of combustion emissions and exposure to residual materials that infiltrate the indoors. WUI fire fuels contain synthetic materials leading to complex chemical profiles in emissions and residues that may contain volatile organic compounds (VOCs) and semi-volatile organic compounds (SVOCs). This research aims to (i) characterize and quantify VOCs and SVOCs compounds in emissions and residues and (ii) model exposure through multiple routes using concentrations measured from a laboratory-scale combustion system loaded with custom formulated pellets to simulate WUI fire fuels.
Material and Methods: Air samples from combustion emissions were collected onto 2,4-dinitrophenylhydrazine (DNPH) sorbent tubes for aldehyde measurements and Tenax® TA sorbent tubes for VOC analysis. A novel direct thermal desorption ash analysis method compliments the analysis of air samples providing VOC and SVOC levels in residual fire ashes. Average daily dose modeling for chemicals of concern was conducted with the USEPA ExpoFirst tool.
Results: Preliminary results show styrene was detected in emissions and ash of pellets containing WUI fire fuels while diethylhexyl-terephthalate (plasticizer) was only detected in ash. Total inhalation, ingestion and dermal modeled exposure to styrene from birth to age of 21 years old ranges from 1.42E-02 to 5.60E-02 mg/kg-day with the highest exposure potential at age 1 to 2 years old. The hazard quotient for non-cancer health risk for 21 years of exposure is 0.1 and cancer risk is 3 in 10,000 using conservative exposure assumptions.
Conclusions: Styrene, a probable human carcinogen, is also in a variety of common products. Chronic styrene exposure via multiple routes potentially increases the overall body burden. The laboratory scale combustion system allowed for controlled generation and chemical characterization of this complex combustion emission which is useful for exposure and health risk modeling.

P58-11 | The Effect of Wildfire Air Pollution on Local Emergency Department Admissions in Suburban New York

Mahdieh Danesh Yazdi[1], Nozomi Sasaki[1], Guanyu Huang[1], Minghao Qiu[1], Perry Sheffield[2]

[1] Stony Brook University, Program in Public Health, Stony Brook, USA; [2] Icahn School of Medicine at Mount Sinai, Department of Environmental Medicine, New York, USA

Objective: The objective of this study is to assess the effects of air pollution generated by wildfires on local emergency department (ED) visits in Long Island, New York.
Material and Methods: We used data from Stony Brook Hospital's emergency department, located in Suffolk County, New York to look at the health effects of the smog episode resulting from plumes generated by Canadian wildfires in June of 2023. To assess this effect, we calculated incidence rate ratios (IRRs) and confidence intervals (CIs) for ED

visits with cardiovascular and respiratory diseases. The event time period was June 6th – June 8th, 2023, during which pollution levels peaked in New York. For our reference group, we selected comparable time periods in other years, specifically: June 8th-June 10th, 2021; June 7th-June 9th, 2022; and June 4th- June 6th, 2024. These reference periods match the event by season, month, and day of week. We further examined changes in the rate of ED visits for specific diagnoses.
Results: We found that the smog event significantly increased the rate of ED visits for cardiovascular disease (IRR=3.44 (95% CI: 2.25-5.26)). We saw a similar increase for respiratory disease with an IRR of 3.79 (95% CI: 2.21-6.50). For asthma specifically, we found an IRR of 3.47 (95% CI: 1.63-7.39).
Conclusion: Exposure to wildfire-generated air pollution was associated with increases in ED visits for both cardiovascular diseases and respiratory diseases in eastern Long Island, New York.

P58-12 | Advancing new metrics for wildfire smoke exposure: Case study in Alaska to bridge public health, climate adaptation, and fire management

Micah Hahn[1], Nelsha Athauda[1], Zhiwei Dong[2], Melissa Bradley[1], Jingqiu Mao[2], Loretta Mickley[3]

[1] University of Alaska Anchorage, Institute for Circumpolar Health Studies, Anchorage, USA; [2] University of Alaska Fairbanks, Department of Chemistry and Biochemistry, Fairbanks, USA; [3] Harvard University, School of Engineering and Applied Sciences, Cambridge, USA

Objective: Exposure to wildfire smoke is a growing health concern in Alaska, but sparse air quality data in this largely rural region limits understanding of its impacts. Smoke metrics that capture episodic and cumulative exposure, along with disproportionate impacts on sensitive populations, can support community preparedness and wildfire response.
Materials and Methods: We used a hybrid approach integrating atmospheric transport model outputs with air quality monitoring data. Daily concentrations of fine particulate matter ($PM_{2.5}$) attributable to wildfire smoke were estimated at a 0.625° x 0.5° resolution across Alaska from 2003-2020. Estimates were aggregated to the census tract level to derive a series of wildfire smoke metrics characterizing population exposure. We calculated a Wildfire Smoke Social Vulnerability Index (WSSVI) that accounts for sensitive populations and adaptive capacity. We identified high-exposure census tracts and categorized them by the WSSVI.
Results: In years when over 2 million acres burned in Alaska during the study period, between 86-98% of census tracts experienced at least one day of medium density smoke, and 19-73% of tracts experienced heavy smoke. Census tracts in Northern Interior Alaska experienced over 300 days of poor air quality due to wildfire smoke (~10% of summer days), with the longest smoke waves lasting between 30-43 days in 2004, 2005, and 2009. Compared to census tracts with low smoke exposure, high exposure tracts had a higher proportion of women of childbearing age (22.6% vs. 21%), individuals living below the 150% poverty line (19.1% vs 17.3%), people who are housing cost burdened (24.8% vs 19.1%), and individuals in single-parent households (6.2% vs 5.4%).
Conclusion: Our results demonstrate the widespread and cumulative health burden of wildfire smoke in Alaska. Smoke metrics facilitate comparisons of health impact across years/regions/events, support fire management strategies aligned with public health goals, and inform preparedness and resilience planning.

P58-13 | Mental Health Outcomes in Children and Adolescents Following Wildfires: Insights from a Northern California Cohort

Mira G. Miles, Miriam Nuño, Kathryn C. Conlon, Irva Hertz-Picciotto

University of California Davis, Department of Public Health Sciences, Davis, USA

Objective: This study aims to examine the mental health impacts of wildfire-related experiences among children, with a focus on anxiety symptoms, depressed mood, and sleep disturbances.

Methods: In response to the October 2017 Northern California wildfires, which displaced approximately 100,000 individuals, an online survey was launched to recruit a cohort of wildfire survivors. The survey collected detailed information on household demographics, evacuation experiences, self-reported health outcomes, and unmet needs to assess the long-term effects of wildfire exposure. An adult household member reported information for all household members, including children. Most respondents accessed the survey through wildfire recovery Facebook groups and completed it within four to nine months after the fires. This study examines the experiences of 642 affected children.

Results: Although over 2,000 households participated in the survey, this study focuses on the experiences of 642 children and adolescents aged 5-17. Households with children were more likely to start but not complete the survey compared to those without children (66% vs. 42%). Anxiety and stress were the most commonly reported mental health symptoms, affecting nearly one-third of children. Other frequently reported symptoms included sleep disturbances and nightmares (21.8%), difficulty concentrating (16.0%), and depressed mood (12.5%). Children who evacuated two or more times, lost their home entirely, had a prior psychiatric diagnosis, or experienced the death of someone close to them were at higher risk of worsening mental health symptoms following the wildfire. These findings are consistent with open-ended survey responses describing household needs and impacts.

Conclusion: This study demonstrates the significant impact wildfires and evacuations can have on children's mental health. More research is needed to understand age-specific needs and find better ways to help children cope with the trauma of wildfire experiences that continue to devastate communities worldwide. Funding for this research comes from the National Institutes of Health.

P58-14 | Associations of autism with prenatal exposure to wildfire PM2.5

David Luglio[1], Xin Yu[2], Jane Lin[3], Ting Chow[3], Mayra Martinez[3], Zhanghua Chen[4], Sandrah Eckel[4], Joel Schwartz[5], Frederick Lurmann[6], Nathan Pavlovic[6], Rob McConnell[4], Anny Xiang[3], Mostafijur Rahman[1]

[1] Tulane University, New Orleans, USA; [2] Icahn School of Medicine at Mount Sinai, New York, USA; [3] Kaiser Permanente Southern California, Pasadena, USA; [4] University of Southern California, Los Angeles, USA; [5] Harvard University, Boston, USA; [6] Sonoma Technologies, Petaluna, USA

Objective: To assess risk of autism associated with exposure to prenatal wildfire PM2.5.

Methods: Effects of prenatal exposure to wildfire $PM_{2.5}$ on risk of autism was assessed using a retrospective pregnancy cohort (n = 204,374) based in southern California, using Cox proportional hazard models. Wildfire $PM_{2.5}$ exposure was estimated at maternal pregnancy addresses from 2006 – 2014 with a previously developed model. Three different metrics of wildfire $PM_{2.5}$ exposure were derived over pregnancy and trimesters: (1) mean

wildfire $PM_{2.5}$ concentration, (2) number of days of smoke exposure, (3) number of waves of smoke exposure (i.e., events of consecutive days of PM2.5 exposure). Additionally, analysis was conducted in relation to increased exposures days over specific $PM_{2.5}$ concentration thresholds (i.e., 3, 5, and 10 μg/m^3). Analysis was conducted on the full cohort, as well as the non-mover subset (75% of cohort).
Results: There were 3356 autism diagnoses by age 5. Autism risk was associated with increased number of wildfire exposure days, especially during the 3rd trimester, and were strongest when considering non-movers. Among non-movers, the hazard ratios (HRs) for autism associated with exposure to 1-5, 6-10, and >10 wildfire days, compared to no exposure in the 3rd trimester, were 1.108 (95% confidence interval, CI: 1.010, 1.215), 1.118 (0.957, 1.307), and 1.225 (1.043, 1.440), respectively. Increased frequency of waves of wildfire smoke (>3 μg/m^3 for 2 consecutive days) during 3rd trimester was also associated with autism risk in both the entire cohort [1.073 (1.009, 1.140)] and non-movers [1.267 (1.054, 1.205)]. No association was observed for increases of mean wildfire $PM_{2.5}$ concentration alone.
Conclusions: These findings underscore an importance of considering the frequency and duration of wildfire PM2.5 exposure, highlighting a potential late pregnancy window of susceptibility for autism risk.

Funding: Provided by NIEHS, EPA, and Kaiser Permanente Southern California Direct Community Benefit Funds.

P58-16 | Impact of the Wildfires on Physical Activity, Sleep, and Physiological Stress of Older Adults: A Cohort Study During 2025 Eaton Fire in Los Angeles

Jiawen Liao[1], Ruoxue Chen[2], Yan Lin[2], Jessilyn Dunn[2], Katherine Tian[2], Chenyu Qiu[1], Wu Chen[1], Zhenchun Yang[1], Jiayuan Hao[1], Yihui Ge[2], Mike Bergin[2], Frank Gilliland[1], Marilyn Black[3], Junfeng Zhang[2], Zhanghua Chen[1]

[1] University of Southern California, Population and Public Health Sciences, Los Angeles, USA; [2] Duke University, Durham, USA; [3] Underwriters Laboratories Inc, Chemical Insights Research Institute (CIRI), Marietta, USA

Objective: The Eaton Fire broke out in Los Angeles County on January 7th 2025, triggered widespread evacuations. We aim to evaluate changes in these metrics, objectively measured by digital wearables, during and after the 2025 Eaton Fire in the Los Angeles.

Material and Methods: This cohort study included 15 older adults [age mean (range): 73.2 (67 – 82) years, eight males and seven females] in Los Angeles County from December 2024 – Jan 2025. Participants wore the Oura Ring Gen 3 continuously at baseline (Dec 9th 2024 to Jan 6th 2025) before the fire, as well as during (Jan 7th – Jan 12th 2025) and after (Jan 13th – Jan 27th 2025) the fire. Daily measurements were remotely collected for physical activity (daily steps, activity, and sedentary time), sleep (sleep duration and sleep-wake transitions) and heart rate (HR) through the Oura Ring. Paired t-tests were conducted to assess changes in these biosignals during and after the fire vs baseline. Linear mixed-effects models were used to determine how evacuation alerts impacted these biosignals during and after fire.

Results: Among the fifteen participants, all living within 10 miles of the Eaton fire, and eight received evacuation alerts. During the fire, we found a significant increase in sedentary time (49.9 minutes/day; P=0.03), a reduction in high-intensity activity (-33 minutes/day; P=0.021), and a decrease in total sleep duration (-33 min/night; P=0.043). After the fire, physical activity reduction persisted, including step count (-731 steps/day; P=0.017) and low-intensity

activity (-22.4 minutes/day; P=0.028). Among participants who received evacuation alerts, sleep HR increased by 2.3 bpm (P=0.018) and 2.2 bpm (P=0.001) during and after the fire compared to baseline, and sleep-wake transitions increased by 0.8 events/nights (P=0.03) after the fire.

Conclusion: This study found that wildfires adversely impact physical activity, sleep, and physiological stress among older adults.

P58-17 | Evaluation of “Agricultural Pass” Program and Farmworkers’ Experiences Working during Wildfires in Sonoma County, California

Carly Hyland[1,2], Isabella Kaser[3], Moncerrat Hernandez[1], Dafne Cruz Rodriguez[1], Linda Gordon[4]

[1] University of California Berkeley School of Public Health, Environmental Health Sciences, Berkeley, USA; [2] University of California Agriculture and Natural Resources, Davis, USA; [3] Public Health Institute, Tracking California, Oakland, USA; [4] University of California School of Law, Human Rights Center, Berkeley, USA

Objective: Farmworkers are disproportionately exposed to and impacted by wildfire smoke, which will continue to worsen with climate change. Sonoma County is a winegrowing region in northern California that experienced record-breaking wildfires from 2017-2020; in 2022 the County passed an “Ag Pass” program that allows farm owners and full-time agricultural employees potential access to evacuated areas during disasters such as a wildfire. The goals of this study were to: 1) evaluate farmworkers’ experiences working during wildfires, 2) examine implications of the Ag Pass program on farmworker health and safety, and 3) develop policy recommendations to safeguard farmworkers’ health during wildfires.

Materials and Methods: We employed a local community engagement team to administer surveys and conduct open-ended interviews with farmworkers in Sonoma County to examine their experiences working during wildfires, including health impacts and barriers to accessing healthcare, and desired resources and training.

Results: Of the 1,011 survey participants, 763 (75.5%) had worked during a wildfire in Sonoma County since 2017; of these, 509 (66.7%) reported health impacts such as a headache (74.9%), sore throat (76.0%), and persistent cough (59.3%). Over 50% of participants who reported adverse health did not have health insurance, and many reported continuing to work while feeling ill to avoid losing income or their jobs. Surveys and interviews consistently revealed farmworkers felt it necessary to work in hazardous conditions (e.g., during active wildfires and poor air quality) to be able to pay for basic needs such as housing and groceries.

Conclusion: Our analysis identified gaps in farmworkers’ protection during wildfires and areas for improvement in the Ag Pass program, including consideration of air quality levels when the Ag Pass may be activated; health training requirements for program applicants; increased collaborations with trusted organizations to deliver health and safety information; and improved communication with employees at the workplace.

EP01 | E-posters

EP01-01 | Applying an air curtain to reduce welding fume under different crosswind conditions

Di-Zhong Lin[1], **Ming-Yeng Lin**[1], Chia-Ren Chu[2]

[1] National Cheng Kung University, Department of Environmental and Occupational Health, College of Medicine, Tainan, Taiwan; [2] National Central University, Department of Civil Engineering, College of Engineering, Taoyuan, Taiwan

Welders are exposed to harmful levels of metal fume during the welding process. Metal fumes contain high concentrations of particles, which can be hazardous to welders. Air curtains have previously been used to help reduce particle exposure. However, air curtains have not been applied to welding, and their effectiveness under different crosswinds remains unknown. In this study, we are the first to integrate the air curtain with a handheld welding device to help reduce ultrafine particle exposure. We further used the wind tunnel and Computational Fluid Dynamics (CFD) Models to investigate the air curtain's performance under different crosswind conditions (U_a = 0, 2, 4, 6 m/s). The Fast Mobility Particle Sizer (FMPS, Model 3091, TSI Inc., MN) was used to measure the particle number concentration and size distribution inside and outside the air curtain. The numerical simulations were verified by the wind tunnel experimental data. Preliminary results indicated that the air curtain is effective at reducing particle concentration with a sealing efficiency of about 80% when the crosswind is smaller than 2 m/s. Thus, the air curtain can significantly reduce the air pollution exposure level near the breathing zone, thereby improving the air quality and health of the welders.

EP01-02 | Mapping Air Quality Risk in Cape Town, South Africa: Implications for Environmental Justic

Meryl Jagarnath[1], Lerato Shikwambana[2]

[1] University of Cape Town, Division of Environmental Health, Cape Town, South Africa; [2] South African National Space Agency, Earth Observation, Pretoria, South Africa

Air pollution disproportionately impacts socially vulnerable communities, yet spatial analyzes that integrate social vulnerability indices (SVIs) and air quality indicators are limited in low- and middle-income countries (LMICs). This study provided the first comprehensive spatial assessment of air pollution risk in Cape Town, South Africa, combining air quality index (AQI) data derived from Sentinel-5P satellite measurements with a Principal Component Analysis (PCA)-generated SVI based on socioeconomic, demographic, and land use factors. Our analysis identified spatial hotspots where high levels of air pollution are associated with increased social vulnerability and showed that 40.3% of the population is at high to very high risk of air pollution. Areas of greatest concern include informal settlements and historically marginalized neighborhoods, highlighting the increased impact of environmental and social determinants of health. These findings highlight the utility of integrating SVI into air quality management frameworks to support equitable interventions. Policymakers, clinicians, and urban planners can leverage these insights to develop targeted strategies that address both air pollution exposure and underlying social inequities to promote environmental justice in urban contexts

EP01-03 | Prenatal exposure to Polycyclic Aromatic Hydrocarbons and infant growth trajectories in a prospective cohort study: The role of DNA methylation changes

Jinlang Lyu, Haijun Wang

Peking University, Maternal and Child Health, Beijing, China

Polycyclic aromatic hydrocarbons (PAHs), pervasive environmental pollutants, pose significant risks to maternal and fetal health, particularly during critical developmental stages. While prenatal PAH exposure has been linked to altered anthropometric measures of offsprings at birth, its impact on dynamic growth trajectories during infancy and underlying epigenetic mechanisms remain underexplored. Based on the Peking University Birth Cohort in Tongzhou (PKUBC-T) in China (N = 1803), internal exposure to PAHs were detected in maternal plasma during early pregnancy and genome-wide DNA methylation (DNAm) was measured in cord blood. The K-means for longitudinal data (KML) method was applied to obtain the growth trajectories of BMI Z-score from birth to three years old. Three trajectory groups were identified in this study. The trajectories were named based on their shapes and temporal patterns: rising high trajectory, stable medium trajectory, and stable low trajectory. When treated as tertile categories, compared to the lowest tertile group, risks of rising high trajectory significantly increased in the highest tertile groups of FLU, PHE and PYR. The third tertiles of ΣPAHs and ΣL-PAHs were also found to be positively associated with the risk of rising high trajectory. The weighted quantile sum index of the PAHs mixture showed a significant positive association with rising high trajectories, with PYR contributing the most to the associations. Our study also brings new insights by examining the underlying epigenetic mechanism. Four CpG cites (annotated to *TELO2*, *BHLHA9*, *CD226* and *CDC42*) were found to be mediate the association between PAHs exposure and infant growth trajectories. The study advances understanding of the epigenetic programming of infant growth influenced by environmental pollutants, emphasizing the need for targeted prenatal interventions and personalized risk assessments, particularly in populations with metabolic susceptibility.

EP01-04 | Reducing dental aerosol exposure through dental handpiece innovation

Ting-Yin Ji[1], Yung-Chun Chen[2], Chun-Juei Chou[3], Chih-Yung Wu[4], Sheng-He Wang[5], **Ming-Yeng Lin**[1]

[1] National Cheng Kung University, Department of Environmental and Occupational Health, College of Medicine, Tainan, Taiwan; [2] National Cheng Kung University, Department of Dentistry, College of Medicine, Tainan, Taiwan; [3] National Cheng Kung University, Department of Industrial Design, College of Planning and Design, Tainan, Taiwan; [4] National Cheng Kung University, Department of Aeronautics and Astronautics, College of Engineering, Tainan, Taiwan; [5] Southern Taiwan University of Science and Technology, Department of Mechanical Engineering, College of Engineering, Tainan, Taiwan

In dental clinics, a dental handpiece is commonly used in dental procedures. However, it can generate high concentrations of dental aerosols since water is constantly sprayed to cool the burr. Dental aerosols contain mist, droplets, ultrafine particles, and bioaerosols which can be hazardous to the people in the dental office. Currently, local exhaust ventilation is often used, but its aerosol removal efficiency varies and is generally large and bulky. In addition, hole towels are sometimes used to prevent the dental aerosol from soaking the patient's face. However, this can create anxiety among patients, especially children. To solve this problem, we are the first to integrate an air curtain outer shell that embeds the dental handpiece to reduce aerosol generation. Different test parameters, such as the opening width, opening angle, and flow rate of the air curtain, were investigated. We also used Particle Image Velocimetry (PIV) along with MATLAB image processing to evaluate the aerosol removal efficiency under different test parameters. Preliminary results indicate that the air curtain can help confine and reduce dental aerosol concentration by over 70%. This novel device will not only help reduce dental aerosol concentration but also make future dental clinics safer and more comfortable for both patients and health workers.

EP01-05 | Microplastic Exposure Disrupts Bile Acid Homeostasis via Gut Microbiota-Dependent Metabolic Reprogramming: A Multi-Omics Population Study

Zhijian Chen, Xiaofeng Wang, Pengcheng Tu, Xiaoming Lou

Zhejiang Provincial Center for Disease Control and Prevention, Department of Environmental Health, Hangzhou, China

Emerging evidence links microplastic exposure to gut microbial dysbiosis, but the interplay between human microplastic exposure and gut microbiome functionality remain poorly understood. In this study, we performed a cross-sectional multi-omics analysis that combined fecal microplastic quantification, metagenomic sequencing, and bile acid profiling to understand how microplastics disturb gut microbial ecosystems and their metabolic outputs. Among 227 participants, pyrolysis–gas chromatography/mass spectrometry (Py-GC/MS) detected the distribution and accumulation of 11 types of microplastics, with individuals experiencing high exposure showing significant shifts in gut microbiome β-diversity compared to those with low exposure. Metagenomic analysis pinpointed Blautia spp. and other taxa as key responders to the microplastic burden. Importantly, pathway analysis of the metagenomic data revealed significant alterations in microbial genes encoding bile acid-metabolizing enzymes in subjects with high exposure, corresponding to a distinct profile of secondary bile acids detected by LC-MS. These findings indicate that

microplastics disrupt bile acid homeostasis in humans by selectively modifying microbial bile acid transformation pathways. Our study establishes gut microbiota-dependent metabolic reprogramming of secondary bile acid biosynthesis as a novel mechanism through which environmental microplastics may exert toxicity and compromise human metabolic health.

EP01-06 | An exposure science certification framework to gain recognition and improve the professional competencies of exposure scientists

Gerald Bachler[1], Alison Connolly[2]

[1] EMSEA Training, Bad Ischl, Austria; [2] University College Dublin, UCD School of Public Health, Dublin, Ireland

Exposure science is essential for the in-depth understanding of human interaction with various stressors and evaluating associated health risks, which is particularly important for investigating exposure among vulnerable groups and identifying regions/populations or ‘hotspots’ of concern.
The European Chapter of the International Society of Exposure Science (ISES) was founded in 2017, and the 'European Exposure Science Strategy 2020-2030' was formulated shortly afterwards. One of the key thematic themes was the advancement of education, and the ISES Europe Education, Training and Communication (ETC) Working Group was established. A major strategic objective within this strategy was the development of a framework for certification and accreditation of exposure science courses and professionals. Exposure science certification will significantly advance the field by establishing a recognized professional identity and providing greater recognition for practitioners. It will help define and standardize the roles and profiles of exposure scientists while promoting the harmonization of educational and practical experiences. Additionally, certification will contribute to the development of clear career pathways for graduates and enhance the minimum competency standards for exposure scientists by encouraging continuous education and fostering scientific advancement.
To advance this objective, the European Registered Exposure Scientists (ERES) framework was created, adapted from current best practices in professional certifications from professional organisations in related fields, such as toxicology, industrial hygiene, and environmental science. The framework first developed the nine Domains of Expertise (DoE) for exposure science. Based on the DoE, two defined career pathways were proposed to obtain a qualification in exposure science: professional qualification in practising exposure science and accreditation of exposure science courses. Both qualification pathways will require continuous education and regular re-qualification. The ERES framework for exposure scientist certification will be presented, including an overview of how this framework could be extended worldwide.

EP01-07 | National HBM programmes: requirements for the establishment of national HBM programmes for evaluating exposures and their potential impacts on public health.

Alison Connolly[1], Richa Singh[1], Marike Kolossa-Gehring[2], André Conrad[2], Holger M. Koch[3]

[1] University College Dublin (UCD), UCD Centre for Safety & Health at Work, School of Public Health, Physiotherapy, and Sports Science, Dublin, Ireland; [2] German Environment Agency (Umweltbundesamt), Berlin, Germany; [3] Institute for Prevention and Occupational Medicine of the German Social Accident Insurance, Institute of the Ruhr-University Bochum (IPA), Bochum, Germany

The World Health Organization estimates a significant surge in global chemical production, doubling from 2017 to 2050, which can pose substantial health risks. Addressing this challenge requires timely and tailored measures, wherein human biomonitoring (HBM) can play a crucial role in the assessment of chemical exposures and their potential impacts on public health. HBM provides chemical exposure data that can measure exposures across a lifespan and contribute to early warning and action systems. Numerous countries have established robust national HBM programmes and have successfully showcased their potential to safeguard public health. However, many countries still lack such a programme.

Recognising this gap, the HBM4IRE (Human Biomonitoring for Ireland) feasibility study evaluated the foundational criteria for establishing a full-fledged national HBM programme by providing short-, medium-, and long-term policy goals. The study conducted an in-depth review of national HBM programmes available worldwide, a chemical awareness survey, and input from government agencies and departments. The project hosted a national stakeholder forum and included policymakers, national agencies, and departments. The forum involved a two-way knowledge transfer process, with international HBM experts presenting on topics, *inter alia*, national programmes, ethics, and analytics. A World Café was included, which is a structured discussion, to evaluate different aspects of national programmes such as policy impact, funding, communication and national synergies.

HBM4IRE developed a chemical prioritisation method and priority biomarker list, as well as a governing structure including roles and responsibilities, and identified opportunities for a national HBM programme. Furthermore, recommendations were provided for establishing a national programme while also aligning with harmonisation efforts of international projects (e.g. Human Biomonitoring for Europe, Partnership for the Assessment of Risks from Chemicals). These outputs can contribute to countries/regions attempting to initiate HBM programmes and will also augment the first cycle of the Human Biomonitoring for Ireland national programme.

EP01-08 | Urine arsenic and risk of CVD: A prospective case-cohort study in Danish never smokers

Meroona Gopang[1], Tesleem Babalola[1], Victoria Fruh[3], Clara Sears[5], James Harrington[2], Maria Tellez-Plaza[8], Tom Webster[3], Anne Tjonneland[6], Koren Mann[7], Gregory Wellenius[3], Brigit Claus Henn[3], Ole Raaschou-Nielsen[4], Jaymie Meliker[1]

[1] stonybrook university, Public Health, longisland, USA; [2] RTI, International, Public Health, north carolina, USA; [3] Boston University, Environmental Health, boston, USA; [4] Aarhus University, Environmental Science, Aarhus, Denmark; [5] University of Louisville, Public Health Sciences, Lousville, USA; [6] University of Copenhagen, Public Health, copenhagen, Denmark; [7] McGill University, Lady Davis Institute for Medical Research, Montreal, Canada; [8] Instituto de Salud Carlos III, Centro Nacional de Epidemiología, Madrid, Spain

Background: Chronic exposure to arsenic (*As*) is associated with cardiovascular disease (CVD), but risk is uncertain at lower levels of exposure.

Objective: To examine the associations of low-level urine arsenic (*As)* with incident stroke, acute myocardial infarction (AMI), and heart failure (HF) among never smokers.

Methods: Between 1993 and 1997, the Danish Diet, Cancer and Health (DCH) cohort enrolled 19,394 participants, ages 50-64 years, who reported never smoking. Among participants with baseline urine samples available for arsenic speciation, we identified incident cases of stroke (N = 457), AMI (N = 696), and HF (N = 803) using the Danish National Patient Registry. We also randomly selected a sub-cohort of 541 men and 548 women. Our primary indicator of exposure was the sum ∑ of arsenic species (iAs + MMA + DMA), corrected for arsenobetaine (AsB). We used modified Cox proportional hazards models to estimate hazard ratios (HR) for each incident CVD outcome, adjusted for creatinine, sex, BMI, education, and cotinine.

Results: Concentrations of ∑ of *As* species (iAs+MMA+DMA) were low, with median = 6.2 (IQR: 2.69 µg/l) after AsB correction, and median = 7.1 (IQR: 19.1 µg/l) without AsB correction. Associations with stroke (HR=1.08; 95% CI: 0.77-1.53), AMI (HR=0.96; 95% CI: 0.71-1.28), or HF (HR=0.86; 95% CI: 0.63-1.16) were null.

Conclusions: We do not find evidence of associations between urine *As* and risk of AMI, HF, or stroke among Danish never smokers.

Keywords: Acute Myocardial infarction, Case-cohort, Cardiovascular disease, Heart failure, Urine arsenic, Stroke.

EP01-09 | Quantifying the relationship between exposure to natural disasters and early-childhood immunization patterns in low- and middle-income countries (LMICs): findings from the Young Lives surveys

Brittany Shea[1], Jeffrey Shaman[2,1]

[1] Columbia University Mailman School of Public Health, Environmental Health Sciences, New York, USA; [2] Columbia Climate School, New York, USA

Objective:
Our aim is to quantify the relationship between exposure to natural disasters in Ethiopia, India, Peru, and Vietnam and early-childhood immunization patterns in majority low-income, marginalized communities using data from round 1 of the Young Lives longitudinal surveys conducted on cohorts of children and their communities.
Material and Methods:
We used data on the ~2,000 children surveyed per country aged 6-18 months in 2002, specifically leveraging household-level exposure data to natural disaster with individual-level vaccination status for multiple pre-natal and early-childhood vaccinations (Prenatal Tetanus, BCG (Bacillus Calmette-Guérin), Measles, Polio). We ran mixed effects binomial logistic regression models to assess the relationship between household-level exposure to a natural disaster and vaccination uptake for individual vaccinations while adjusting for individual and community-level variables. We also assessed effect modification by vaccination type.
Results:
Vaccination status was significantly associated with vaccination type, and the effect of household exposure to a natural disaster varied depending on the type of vaccination. For example, exposure to a natural disaster was significantly negatively associated with BCG uptake; and compared to BCG, exposure to a natural disaster had a stronger negative effect on measles uptake. In Ethiopia, we found a significant negative relationship between exposure to a natural disaster and vaccination uptake overall. A higher wealth index was significantly positively associated with vaccination uptake.
Conclusion:
We assessed whether exposure to a natural disaster is associated with missed early-childhood immunizations in four LMICs. We found that household exposure to natural disaster is negatively associated with vaccination uptake in Ethiopia and with uptake of specific immunizations across all countries. This study will help determine whether natural disasters reduce biological immunity against vaccine-preventable diseases and identify potential areas of intervention at the community-level to mitigate the effects of natural disasters and improve childhood vaccination uptake.

EP01-10 | Long-term Associations Between Mixtures of Industrial and Residential Air Pollutants ($PM_{2.5}$, SO_2, NO_2, O_3) and Neurological Disease-related Mortality in Canada

Sabit Cakmak[1], Kimberly Mitchell[1], Chris Hebbern[2], Toyip Olanian[3], Mathieu Rouleau[4], Ivana Popadic[5], Angelos Anastasopolos[6], Naizhuo Zhao[7], Michael Tjepkema[3]

[1] Health Canada, EHSRB, Ottawa, Canada; [2] Health Canada, Climate Change and Health Office, Ottawa, Canada; [3] Statistics Canada, Health Statistics, Ottawa, Canada; [4] Health Canada, WAQB, Ottawa, Canada; [5] ECCC, Meteorological Service of Canada, Montreal, Canada; [6] Health Canada, WAQB, Ottawa, Canada; [7] McGill University, Health Centre, Montreal, Canada; [8] Statistics Canada, Health Statistics, Ottawa, Canada

Objective: We investigated the long-term associations between exposure to total and industrially derived mixtures of fine particulate matter ($PM_{2.5}$), sulfur dioxide (SO_2), nitrogen dioxide (NO_2), and ozone (O_3) with all cause and cause-specific neurological mortality in Canada.

Material and Methods: The Canadian Census Health and Environment Cohort (CanCHEC) consists of approximately 3-million individuals aged 24 or older from the 2006 long-form census linked to mortality data. Industrial $PM_{2.5}$, SO_2, and NO_2 concentrations were estimated from sources including on-road, off-road, air-marine-rail, ore and mineral, electric power generation, manufacturing, oil and gas, and residential fuel combustion using the Global Environmental Multiscale-modelling Air Quality and Chemistry (GEM-MACH) model. Individuals were assigned an annual exposure value based on their postal code obtained from their annual tax file. Quantile g-computation models were used to calculate hazard ratios (HRs) for deaths from Alzheimer's, dementia and non-accidental causes per quartile increase in all air pollutants from each specific sector.

Results: We observed significant associations between the combined effects of air pollutants (NO_2, $PM_{2.5}$, O_3, and SO_2) and deaths from dementia for emissions from oil and gas industry [1.059 (1.030-1.088)]. Alzheimer's mortality was significantly associated with emissions from the on-road [1.215 (1.118-1.319)], air, marine and rail [1.116 (1.056-1.178)], ore and mineral [1.171 (1.104-1.243)], manufacturing [1.056 (1.005-1.108)], and residential fuel combustion [1.285 (1.158-1.425)]. No associations were found for off-road or electric power generation sectors. The contribution of each individual air pollutant to mortality risks varied by sector and neurological disease outcome.

Conclusion: Air pollution from specific industries increases the risk of neurological mortality. Oil and gas emissions were linked to higher dementia risk, while emissions from multiple industrial sources, including on-road transport, air-marine-rail, ore and mineral, manufacturing, and residential fuel combustion, were associated with Alzheimer's mortality.

EP01-11 | Anthropogenic Heat and Mortality in 186 countries and territories from 1990 to 2021: Socioeconomic Disparities in All-Cause and Cause-Specific Risks

Ting Xiao[1], Yiqian Zeng[1], Weiyi Chen[1], Dezhong Chen[1], Yiyue Yin[1], Hung Chak D. Ho[2], Xiang Qian Lao[1]

[1] City University of Hong Kong, Department of Biomedical Sciences, Hong Kong, Hong Kong; [2] City University of Hong Kong, Department of Public and International Affairs, Hong Kong, Hong Kong

Objective The long-term impact of anthropogenic heat emission (AHE) on human health remains poorly understood. This study aims to examine the association between AHE and all-cause and cause-specific mortality on a global scale, particularly focusing on disparities by the Socio-demographic Index (SDI).
Material and Methods This ecological study incorporates age-standardized mortality rates (ASMR) for all-cause mortality and cause-specific mortality and SDI data between 1990 and 2021 from the Global Burden of Disease (GBD) study. The Annual mean country-specific AHE was estimated using energy consumption information based on the second law of thermodynamics. Generalized additive mixed models were used to evaluate relationships between AHE and mortality, adjusting for climate variables, air quality, and socioeconomic data. Additionally, subgroup analyses were conducted using SDI categories to explore socioeconomic heterogeneity.
Results The global trend of AHE has shown an overall increase from 1990 to 2021. This upward trend is observed across all SDI groups other than the medium-high SDI group, which exhibits a fluctuating pattern. Globally, we found heterogeneous associations between AHE and mortality outcomes. Higher AHE levels were linked to reduced ASMR for all-cause mortality, with decreases of 3.86%, 7.85%, and 11.25% in the second, third, and fourth AHE quantiles, respectively. Subgroup analyses revealed similar patterns by SDI: in low, medium-high, and high SDI regions, AHE was associated with increased mortality, whereas in medium-low and medium groups, AHE was associated with reduced. Similar trends were observed for cause-specific outcomes, including mortality of neoplasms and cardiovascular and respiratory diseases.
Conclusion This study revealed the context-dependent relationship between AHE and mortality, shaped by socioeconomic development. These findings underscore the need for targeted climate adaptation policies that consider local socioeconomic contexts to mitigate the health impacts of AHE.

This study is supported by the New Research Initiatives/Infrastructure Support from the City University of Hong Kong (9610613).

EP01-12 | Extreme heat and pediatric emergency department visits in Santiago, Chile

Karla Yohannessen[1,3], Valentina Vergara[2], Isaias Vergara[2], Franco Alfaro[2]

[1] Universidad de Chile, Escuela de Salud Publica, Santiago, Chile; [2] Universidad de Chile, Escuela de Medicina, Santiago, Chile; [3] Universidad de Chile, Departamento de Pediatria y Cirugia Infantil, Santiago, Chile

Background and aim: Extreme heat poses significant health risks to children, who are more susceptible to its effects. It highlights the importance of understanding the diverse effects of extreme heat beyond mortality. This study assessed the association between extreme heat and daily emergency department visits among children (0 to 14 years) in Santiago, Chile.

Methods: We examined the average maximum temperature, relative humidity, and daily pediatric emergency department (ED) visits from 2011 to 2020 (warm season). We used ED visits from two pediatric hospitals. Daily meteorological data from the local weather station were obtained from the Meteorological Direction of Chile (DMC). We fit a conditional Poisson model with DNLM term for temperature, using three nodes and 7 days lag.
Results: There were 698 868 ED visits during the study period, an average of 426.6 per day. Children aged 5 to 14 years accounted for 47.6% of all visits (332 876 visits), followed by children aged 1 to 4 (35.6%; 248 379 visits), and children below 1 year of age (16.8%; 117 613 visits). Extreme heat, defined as the 95th percentile of the maximum temperature distribution, occurred at 32.3°C and was associated with an overall 34% increase in ED visits (RR: 1.34 95%CI 1.19-1.52) compared to the reference temperature of 18°C. This association was mainly found between the second and fourth days after the exposure. The most pronounced association was noted in children aged 5 to 14 (RR: 1.69 95%CI 1.40-2.03).

Conclusions: Extreme heat is associated with increased ED visits in children. Strategies to mitigate heat-related health risks among children should be developed as temperatures increase.

EP01-13 | Chronic exposure to ambient ozone, lung function, and risk of chronic obstructive pulmonary disease: a large prospective cohort study

Yiyue Yin, Dezhong Chen, Ting Xiao, Weiyi Chen, Yiqian Zeng, **Xiang Qian Lao**

City University Of Hong Kong, Biomedical Sciences, Hong Kong, Hong Kong

Objective:
The long-term effects of ozone on lung function remain limited and inconclusive, particularly among Asian adults. This study aims to investigate the impact of ambient ozone exposure on lung function and the incidence of COPD in Asian populations.
Materials and Methods:
We analyzed data from 338,765 participants aged 20 years or older from the MJ Health Management Institution cohort, recruited Taiwan and Hong Kong residents between 1998 and 2016, who underwent spirometric tests during medical examinations. Participants' addresses were matched with the annual 6-month ozone-season average of the daily maximum 8-hour concentration. We employed a linear mixed model to examine the associations between ozone concentrations and lung function, and a Cox proportional hazards regression model with time-dependent covariates to investigate the effects of ozone on COPD incidence.
Results:
A 1-ppb increase in ozone exposure was associated with a 0.14% decrease in forced expiratory volume in 1 second (FEV_1) (β -0.14%, 95% CI -0.16% to -0.13%), a 0.10% decrease in forced vital capacity (FVC) (β -0.10%, 95% CI -0.11% to -0.09%), a 0.14% decrease in maximum mid-expiratory flow (MMEF) (β -0.14%, 95% CI -0.16% to -0.12%), and a 0.05% decrease in the FEV_1:FVC ratio (β -0.05%, 95% CI -0.06% to -0.04%). Additionally, a 1-ppb increase in ambient ozone was associated with a 5% higher risk of COPD incidence (HR 1.05, 95% CI 1.04 to 1.06).
Conclusion:
Long-term exposure to ambient ozone is associated with reduced lung function and an increased risk of COPD incidence. These results highlight the critical need for worldwide efforts to reduce air pollution in order to enhance lung health and prevent COPD.

This study is supported by Health and Medical Research Fund (18190131), the Research Grants Council -General Research Fund (14109521) and the New Research Initiatives/Infrastructure Support from the City University of Hong Kong (9610613).

EP01-14 | Exploring the role of Indoor Air Quality, Housing Characteristics, and Greenspaces on the Psychological Wellbeing of Adolescents living with HIV in Durban, South Africa

Busisiwe Shezi[1,2], Hilbert Mendoza[3], Yusentha Balakrishna[7], Darshini Govindsasamy[4], Stanley Carries[4], Eugene Davids[5], Smanga Mkhwanazi[6], Nangipha Mnandi[6], Laura Corlin[8], Renee Street[9]

[1] South African Medical Research Council, Environment and Health Research Unit, Durban, South Africa; [2] University of Johannesburg, Department of Environmental Health, Johannesburg, South Africa; [3] University of Antwerp, Department of Family Medicine and Population Health, Antwerp, Belgium; [4] South African Medical Research Council, Health Systems Research Unit, Cape Town, South Africa; [5] University of Pretoria, Department of Psychology, Pretoria, South Africa; [6] South African Medical Research Council, Gender and Health Research Unit, Pretoria, South Africa; [7] South African Medical Research Council, Biostatistics Research Unit, Durban, South Africa; [8] Tufts University, Department of Public Health and Community Medicine, Boston, USA; [9] South African Medical Research Council, Environment and Health Research Unit, Cape Town, South Africa

Objective: This study aims to explore the role of indoor air quality, housing conditions, and greenspaces on the psychological well-being of adolescents living with HIV in Durban, South Africa.
Material and Methods: A cross-sectional, qualitative study design was used, nested within the Caregiver Wellbeing PLUS (CWeL+) study, evaluating a cash transfer and gender-transformative economic intervention. Twenty-three adolescents living with HIV were purposively selected to ensure gender and area representation. Data were collected through an arts-based approach, where adolescents drew their home environment and discussed them in semi-structured interviews with trained staff. Interviews were conducted in isiZulu, transcribed, and translated to English. Adolescents aged 18 and older provided consent, while those under 18 gave assent. Data were analyzed using thematic analysis.
Results: The data tell the story about the discomfort and stress that many adolescents experienced due to poor air quality, poor living conditions, and limited access to greenspaces. Participants highlighted concerns about poor air quality due to lack of ventilation, overcrowded living spaces, and poor waste management. Many adolescents described their homes as small, made of corrugated iron, poorly ventilated, with too many people living in them. Adolescents found peace in areas with trees and gardens, although they frequently faced barriers such as limited access to greenspaces and safety concerns in public parks. This was evident in both interviews and drawings, where parks were depicted as spaces of refuge but often linked with the presence of drug users. One adolescent expressed, ‘I would love to go to the park, but it is not safe; there are drug users, and I don’t feel comfortable.’
Conclusion: This study emphasizes the need to address environmental factors like housing quality, air pollution, and access to greenspaces to improve the psychological wellbeing of adolescents with HIV.

EP01-15 | Residential Green Environment and Psychological Wellbeing among Women Caregivers of Children and Adolescents living with HIV/AIDS in Durban, South Africa: A Mixed-Methods Study

Busisiwe Shezi[1,2], Hilbert Mendoza[3], Yusentha Balakrishna[7], Darshini Govindsasamy[4], Stanley Carries[4], Eugene Davids[5], Smanga Mkhwanazi[6], Nangipha Mnandi[6], Laura Corlin[8], Renee Street[9,2]

[1] South African Medical Research Council, Environment and Health Research Unit, Durban, South Africa; [2] University of Johannesburg, Department of Environmental Health, Johanneburg, South Africa; [3] University of Antwerp, Department of Family Medicine and Population Health, Antwerp, Belgium; [4] South African Medical Research Council, Health Systems Research Unit, Cape Town, South Africa; [5] University of Pretoria, Department of Psychology, Pretoria, South Africa; [6] South African Medical Research Council, Gender and Health Research Unit, Pretoria, South Africa; [7] South African Medical Research Council, Biostatistics Research Unit, Durban, South Africa; [8] Tufts University, Department of Public Health and Community Medicine, Boston, USA; [9] South African Medical Research Council, Environment and Health Research Unit, Cape Town, South Africa

Objective: This mixed-methods study investigates the effects of residential green spaces on the psychological wellbeing of women caregivers of children and adolescents living with HIV in Durban, South Africa.
Methods: Quantitative and qualitative studies assessed the impact of residential green spaces on the psychological well-being of women caregivers. A cross-sectional study design was used to explore the association between residential surrounding greenness and psychological wellbeing among 223 women caregivers. Residential greenness was measured using the Normalized Difference Vegetation Index in 100m and 300m buffers around their homes, categorized into tertiles. Psychological wellbeing was assessed using the Mental Health Continuum Short Form. Modified Poisson regression models were employed to calculate prevalence ratios with their 95% confidence intervals. Four focus group discussions, each with approximately six participants, explored caregivers' experiences with residential green spaces and their effects on psychological wellbeing. Data were analyzed using thematic analysis.
Results: Quantitative study findings revealed a potential inverse association between residential surrounding greenness and psychological well-being. For example, for NDVI 100m, we observed that those in the 'most green' tertile had a lower prevalence of poor psychological well-being (PR=0.89,95% CI:0.61-1.30) as compared to those in the ‘least green’ tertile. Similar results were observed for NDVI 300m. The qualitative data tell a story about the availability and quality of green spaces, use, positive impact on psychological wellbeing, and desirable changes. Many caregivers acknowledged the positive impact of parks on relaxation, socializing, and stress relief. However, they also highlighted safety concerns, particularly related to drug-related activities. One participant expressed, ‘Now they sell drugs there and when one person reported this nothing was done.’ Participants suggested improvements such as more recreational facilities, enhanced security, and better maintenance of parks.
Conclusion: To improve the mental health of caregivers, policymakers should prioritize the development and maintenance of safe, accessible green spaces.

EP01-16 | Long-term exposure of air pollution mixture and mortality: a prospective cohort study

Weiyi Chen[1], Shailesh Kharol[2,3], Jeffrey Brook[4], Xiang Qian Lao[1]

[1] City University of Hong Kong, Biomedical Sciences, Hong Kong, Hong Kong; [2] Environment and Climate Change Canada, Toronto, Canada; [3] AtmoAnalytics Inc., Brampton, Canada; [4] University of Toronto, Toronto, Canada

Objective
Epidemiological studies examining the health effects of air pollution have mainly focused on individual pollutants, such as $PM_{2.5}$. However, the reality of human exposure to mixtures of pollutants has been overlooked. Therefore, this study aims to explore the multi-pollutants and mortality in middle aged and older population.
Methods
Focusing on seven air pollutants ($PM_{2.5}$, CO, NO_2, SO_2, O_3, HCHO, and NH_3), we obtained the Chronic Air Pollution Index (CAPI) by using an approach of dimensionality reduction technique, which captures the largest variation in pollutant mixture from 2012 to 2014. Following 378,507 people in the UK Biobank cohort (2006-2019) for an average of 11 years, a total of 12,708 deaths were identified, among which 1,039 died from respiratory disease, 536 died from stroke, 536 died from cerebrovascular disease, 460 died from COPD, and 6,512 dies from cancer. A Cox regression model was used to analyze the association between CAPI and different-caused mortality. And we compare the CAPI model to single-pollutant model ($PM_{2.5}$) via the likelihood ratio test (LRT), the AIC, and the BIC.
Results
We found that people exposed to highest quartile of the CAPI generally had higher overall mortality (HR, 95%CI: 1.18, 1.09 to 1.28), particularly from respiratory disease (HR, 95%CI: 1.44, 1.08 to 1.93), COPD (HR, 95%CI: 1.68, 1.08 to 2.62), stroke (HR, 95%CI: 1.86, 1.26 to 2.72), cerebrovascular (HR, 95%CI: 1.86, 1.27 to 2.72), and cancer (HR, 95%CI: 1.13, 1.01 to 1.27), compared to those exposed to lowest quartile of the CAPI. And LRT suggested there was a significant difference between the CAPI model and the single-pollutant ($PM_{2.5}$) model ($p < 0.001$) when explaining overall mortality. And CAPI model had smaller AIC and BIC, which represents a better model.

This study is supported by the New Research Initiatives/Infrastructure Support from the City University of Hong Kong(9610613).

EP01-17 | Building cimate-resilient Health Systems in low-resource settings: insights from a Scoping Review

Eleni Papadopoulou, Katrine B. Frønsdal, Heather M. Ames, Sonja L. Myhre

Norwegian Institute Of Public Health, Oslo, Norway

We conducted a scoping review and synthesized evidence on interventions and strategies for strengthening climate-resilient health systems in low-resource settings. Using a structured search across multiple databases, 67 studies were identified for data extraction and data was synthesized according to the WHO health system pillars. Our findings highlight interventions across six health system pillars: strengthening infrastructure resilience, expanding community-based services, integrating climate-informed health programs, enhancing emergency preparedness, training health professionals, and developing robust health information systems. Strategies

emphasize the need for cross-sectoral collaboration, sustainable financing, and governance frameworks to support adaptation efforts. By synthesizing evidence from diverse contexts, this review provides a foundation for informed decision-making and policy development to enhance health system resilience in the face of climate change.

EP01-18 | Dust Loadings Collected from Children’s Hands and Surfaces Within Residential Settings.

Cristina Fayad Martinez[1], Annabella Cordovez[1], Maribeth Gidley[1], Helena Solo-Gabriele[1], Ali Pourmand[1], Arash Sharifi[1], Foluke Adelabu[2], Paloma Beamer[3], Olusola O. Ogunseye[3], Alesia Ferguson[2]

[1] University of Miami, Chemical, Environmental and Materials Engineering, Miami, USA; [2] North Carolina Agricultural and Technical State University, Built Environment, Greensboro, USA; [3] University of Arizona, Community, Environmental and Policy, Tucson, USA

Due to their play behaviors, which involve object and hand to mouth activities, children represent a vulnerable population for exposures to household dust. Dust in households can be found on surfaces and ultimately on children’s hands. The objective of this study was to use a new method to measure dust loading on children’s hands as well as collecting dust samples for surface dust loading estimates from inside homes. For hand dust loadings, the new method consists of using a Coulter Counter to determine the volume of particles washed off children’s hands followed by multiplying the volume by the density of dust estimated from vacuumed samples (average at 1.58 g/cm^3). A total of 101 children participated from across three different regions in the United States (North Carolina, Florida, Arizona). As for surface dust loadings, a total of 804 surface wipes and 99 vacuum samples were collected from 101 different households. Results showed that the overall median dust loading on children’s hands was of 11.1 ug/cm^2 (per total hand area), while surfaces measured at 37 ug/cm^2, and 168 ug/cm^2 for surface wipes and vacuum samples, respectively. When grouping loadings by region, there was a statistical difference between North Carolina and Florida in both children’s hands ($p = 0.039$) and surfaces ($p = 0.012$When comparing dust loadings between children’s hands and surfaces, no strong correlation was found ($p > 0.05$), suggesting that factors beyond total household dust likely influence dust accumulation on hands. These results will be used as part of the Dust Ingestion Children Study (DIRT; funded by the Environmental Protection Agency) which documents child play behavior through video translation for simulating soil/dust ingestion rates for the purpose of estimating soil/dust ingestion for young children within residential settings.

EP01-19 | Quantifying Children's Hands and Mouth Contact Patterns with Indoor Surfaces: A Micro-Activity Analysis Across Three States

Foluke Adelabu[1], Keziah Floyd[1], Daylan Dawkins[1], Isaiah Brathwaite[1], Jaden Holloway-Johnson[1], Helena Solo-Gabriele[2], Cristina Fayad Martinez[2], Maribeth Gidley[2], Paloma Beamer[3], Olusola Ogunseye[3], Alesia Ferguson[1]

[1] North Carolina Agricultural and Technical State University, Built Environment, Greensboro, North Carolina, United States of America; [2] University of Miami, Chemical, Environmental and Materials Engineering, Coral Gables, Florida, United States of America; [3] University of Arizona, Mel and Enid Zuckerman College of Public Health, Tucson, Arizona, United States of America

Young children's interactions with indoor surfaces through hands and mouth contacts represent critical pathways for potential exposure to environmental contaminants. This micro-activity study examined the frequency and duration of right hand and mouth contacts for 30 children aged 6 months to 6 years across three U.S. states (North

Carolina, Florida, and Arizona-10 children per state) using an advanced videotaping and video-translation methods. Analysis of 3-4 hours of indoor activity per child revealed distinct patterns for both mouth and right-hand contacts. Mouth contacts were predominantly characterized by non-contact periods (75.73% duration, 40.77 contacts/hour), "Not-In-View" (8.88% duration, 6.94 contacts/hour), "Food-Cont" (3.17% duration, 8.59 contacts/hour), and "Other_Food" (3.74% duration). Right hand contacts showed more diverse interactions, also dominated by "Nothing" (21.28% duration, 27.29 contacts/hour), followed by significant interactions with "Bedding/Towels" (14.75% duration, 6.24 contacts/hour), "Clothes" (9.97% duration, 9.12 contacts/hour), and "Not-In-View" (7.99% duration, 5.78 contacts/hour). Various surface types including "Wood_Wall/Furn" (4.38% duration), "Paper/Wrapper" (3.57% duration), and "Electronics" (4.71% duration) also showed notable contact patterns for hands. The higher frequency and diversity of hand contacts highlights the importance of hand-to-object and then hand-to mouth interactions in exposure assessment. These findings provide crucial quantitative data for understanding children's exposure patterns in indoor environments and can inform risk assessment strategies for chemical and biological exposures.

EP01-20 | Trends in Respiratory Disease-Related Hospitalizations and Mortality in Malaysia (2019-2023): A Post-Pandemic Epidemiological Analysis

Vanitha Thurairasu, Mohd Hasni Ja'afar

The National University of Malaysia, Department of Public Health Medicine, Cheras, Malaysia

Introduction: Respiratory diseases are a major global health concern, contributing significantly to hospitalizations and mortality worldwide. In Malaysia, respiratory illnesses have been among the leading causes of hospitalization and death, with notable fluctuations during the COVID-19 pandemic.

Objective: To analyze trends in respiratory disease-related hospitalizations and mortality from 2019 to 2023 in Malaysia, focusing on shifts before, during, and after the COVID-19 pandemic. It also examines temporal trends in respiratory disease-related hospitalizations and mortality in Malaysia from 2019 to 2023, comparing public and private healthcare settings to assess long-term health outcomes and policy implications.

Material and Methods: A retrospective study was conducted using Malaysia’s Ministry of Health hospitalization and mortality data from 2019 to 2023. The prevalence of respiratory diseases among hospitalized patients and respiratory-related deaths was analyzed. Temporal trends were assessed to identify shifts in disease burden pre-pandemic (2019), during the COVID-19 pandemic (2020-2021), and post-pandemic recovery (2022-2023). Hospitalization and mortality rates were compared between public and private hospitals.

Results: Respiratory diseases consistently ranked among the top three causes of hospitalization and mortality in public hospitals. A significant spike in hospitalizations was observed in 2021, with a corresponding increase in mortality rates. In private hospitals, respiratory diseases showed fluctuations, with a decline in hospitalizations in 2020 but a resurgence in 2022. The post-pandemic period (2022-2023) exhibited a slow decline in hospitalizations but persistent mortality. Differences in disease burden between public and private hospitals suggest variations in healthcare access and treatment availability.

Conclusion: The findings highlight the long-term impact of COVID-19 on respiratory disease burden in Malaysia. While hospitalization rates have declined post-pandemic, mortality trends indicate a need for enhanced respiratory health interventions. Strengthening air quality regulations, improving early diagnostic capabilities, and expanding public health initiatives for respiratory disease prevention are crucial for mitigating future health risks.

EP01-21 | Paradigm Shifts in Maternal Health and Pregnancy-Related Hospitalizations in Malaysia (2019-2023): An Epidemiological Perspective

Vanitha Thurairasu, Mohd Hasni Ja'afar

The National University of Malaysia, Department of Public Health Medicine, Cheras, Malaysia

Introduction: Maternal health remains a critical public health concern, with pregnancy, childbirth, and puerperium consistently ranking as the leading cause of hospitalization in Malaysia. The COVID-19 pandemic further disrupted healthcare services, necessitating an evaluation of its impact on maternal health trends. The long-term consequences of these disruptions on healthcare accessibility, maternal morbidity, and perinatal outcomes remain areas of concern.
Objective: The study analyzes the trends in maternal health and pregnancy-related hospitalizations in Malaysia from 2019 to 2023, assessing shifts before, during, and after the COVID-19 pandemic. It also assesses temporal trends in pregnancy-related hospitalizations from 2019 to 2023, comparing pre-pandemic, pandemic, and post-pandemic periods in both public and private healthcare settings.
Material and Methods: A retrospective study was conducted using hospitalization data from Malaysia's Ministry of Health (2019-2023). The prevalence of pregnancy-related hospitalizations was analyzed across different time periods: pre-pandemic (2019), pandemic (2020-2021), and post-pandemic recovery (2022-2023). Differences between public and private hospitals were examined to determine disparities in maternal healthcare system.
Results: Pregnancy, childbirth, and puerperium remained the top causes of hospitalization accounting for an average of 20.1% of total hospital admissions. A significant decline in hospitalizations was observed in 2020 ($p < 0.05$), coinciding with COVID-19-related disruptions, followed by a resurgence in 2022. Public hospitals managed 75.3% of all pregnancy-related hospitalizations, with a higher proportion of high-risk pregnancies ($p < 0.01$) compared to private hospitals. The average length of hospital stay for pregnancy-related complications increased by 12.5% in 2021, indicating potential delays in care access during the pandemic. Regional disparities were evident, with urban hospitals experiencing a 15.8% higher hospitalization rate than rural hospitals ($p < 0.05$).
Conclusion: The findings highlight the resilience of Malaysia's maternal healthcare system despite pandemic-related disruptions. Strengthening antenatal care and ensuring equitable healthcare access are crucial for improving maternal health outcomes.

EP01-22 | From Exposure to Epigenetics: Unraveling the Health Effects of Pesticides in Moroccan Agricultural Communities

Aziza Menouni[1], Radu C. Duca[3, 1], Imane Berni[2], Kaoutar Chbihi[2, 1], Samir El Jaafari[2], Lode Godderis[1, 4]

[1] Katholieke Universiteit of Leuven, Environment & Health Unit, Leuven, Belgium; [2] Moulay Ismail University of Meknes - Faculty of Sciences, Human Epidemiology and Environmental Health Team, Meknes, Morocco; [3] National Health Laboratory, Unit Environmental Hygiene and Human Biological Monitoring, Dudelange, Luxembourg; [4] IDEWE, External Service for Prevention and Protection at Work, Heverlee, Belgium

BACKGROUND AND AIM

In Morocco, pesticide exposure represents a critical occupational and environmental health issue, exacerbated by limited education, widespread misuse, and counterfeit and illegal products' presence. Increasing evidence suggests that pesticides can induce epigenetic modifications, particularly through alterations in DNA methylation. Our study aims to comprehensively investigate whether exposure to commonly used pesticides can lead to oxidative stress and DNA methylation among farmers and their families in Morocco.

METHODS

We initiated a cross-sectional study evaluating occupational exposure among 324 farmworkers and their families in Meknes (Morocco). Between 2021 and 2022, structured surveys assessed pesticide exposure and work practices. Blood samples were analyzed for acetylcholinesterase (AChE) and butyrylcholinesterase (BuChE) activity. Urinary pesticide metabolites were quantified using LC-MS and GC-MS, targeting 28 commonly used pesticides and some of their metabolites. Oxidative stress biomarkers, including glutathione (GSH) and 8-hydroxy-2'-deoxyguanosine (8-OHdG), were assessed. Global DNA methylation levels were measured in blood samples using LC-MS.

RESULTS

Educational status and prior training on pesticide use were significantly associated with increased knowledge, while awareness of health risks correlated with the use of personal protective equipment (PPE). Pesticide residues were detected in 77% of urine samples, with organophosphates and organochlorines being the most prevalent. Biomarkers of oxidative stress (GSH and 8-OHdG) showed significant associations with pesticide exposure. Notably, global DNA methylation levels were altered in the pesticide-exposed group compared to the less-exposed or unexposed population, suggesting potential epigenetic effects.

CONCLUSION

This study provides critical evidence supporting the need for structured human biomonitoring systems in Morocco to assess environmental and occupational exposures. Our findings highlight the potential long-term health risks associated with pesticide exposure and underscore the importance of preventive measures and policy interventions.

EP01-23 | Feeling the Heat: Community Perceptions of Extreme Heat Risks in Puerto Rico

Elizabeth M. Rojo[1], Jessica Alicea Vellón[2], Joselyn Wei-Chen[3], Ivonne Rivera Hernandez[2], José G. Perez Ramos[3]

[1] Duke University, Department of Population Health Sciences, Durham, USA; [2] HealthproMed, San Juan, Puerto Rico; [3] University of Rochester, School of Medicine and Dentistry, Rochester, USA

Background
Heat waves pose serious health risks, especially in communities with limited adaptive capacity. In the Caribbean, where climate change is intensifying extreme weather, understanding perceptions of heat-related health risks is crucial for public health preparedness. This study explores the relationship between geographic location and perceived health risks of extreme heat in the Puerto Rican island municipalities of Vieques and Culebra.
Methods
A cross-sectional survey (n=30) using a validated heat-health risk perception instrument was conducted in Spanish to assess perceived health risks, including probability, severity, and concern about heat hazards. Participants from Vieques (n=25) and Culebra (n=5) rated the likelihood of personal, family, and community members experiencing health harm from heat waves. Pearson correlation analysis examined associations between location and risk perception.
Results
Survey location was significantly negatively correlated with perceived probability of heat-related health harm for participants (p=0.0036) and their families (p=0.0010). Perceived severity of personal health harm was also negatively correlated with location (p=0.0046). These results suggest that location shifts from Vieques to Culebra, perceived health risks decline. Residents of Vieques reported significantly higher perceived risks than those in Culebra, indicating geographical disparities in risk awareness.
Discussion
Differences in risk perception may stem from variations in environmental exposure, community awareness, agency reduction, or healthcare access. While limited by sample size, these findings highlight the need for targeted interventions to enhance climate-related health awareness. Public health efforts should prioritize localized risk communication, community education, and sustainable adaptation programs in collaboration with local organizations and healthcare providers to mitigate long-term health impacts of extreme heat in these vulnerable island populations.

EP01-24 | BIONET: A Digital Sentinel Platform for Equitable Occupational Health Surveillance and Capacity Building in Africa

Aziza Menouni[1], Kaoutar Chbihi[1,2], Radu C. Duca[3,1], Emine A. Bajalan[1], Vivi Schlünssen[4], Salim Bounou[5], Samir El Jaafari[2], Lode Godderis[1,6], On Behalf of the Erasmus+ BIONET Consortium

[1] Katholieke Universiteit Leuven, Department of Primary Care & Public Health / Environment & Health Unit, Leuven, Belgium; [2] Moulay Ismail University of Meknes, Human Epidemiology and Environmental Health Team, Meknes, Morocco; [3] Laboratoire National de Santé, Unit Environmental Hygiene and Human Biological Monitoring, Dudelange, Luxembourg; [4] Aarhus University, Department of Public Health, Aarhus, Denmark; [5] Euro-Mediterranean University of Fez, Health Pole, Fez, Morocco; [6] IDEWE, External Service for Prevention and Protection at Work, Heverlee, Belgium

Introduction: In Africa, environmental and occupational health risks are often underreported. The BIONET Erasmus+ project aims to address this gap by understanding the occupational origins of diseases associated with emerging occupational settings while developing and implementing a digital sentinel surveillance system. This system includes training and guidance for occupational physicians (OCs) to report new work-related diseases in African countries.

Methods: A total of 30 OCs are recruited and trained in each partner country (Morocco, Benin, and Ethiopia), forming a sentinel network. Each OC was tasked with recruiting 50 workers during consultations over six months, leading to a target of 1,500 workers per country. Employees complete a paper-based questionnaire regarding their health status and work conditions, including their past-week exposure to 20 chemicals. Additionally, urine and blood samples are collected for human biomonitoring of common exposures.

Results: Preliminary findings of the pilot study conducted in Morocco indicate that 92% of surveyed employees experience physical constraints, 44% are exposed to at least one chemical, and 24% to biological agents. The most prevalent physical constraints include manual handling of loads, noise pollution, and thermal stress, while pesticide and fuel exposure represent the most common chemical risks.

Conclusion: The BIONET project advances the understanding of effective sentinel systems as a valuable approach for collecting high-quality, real-time data. This initiative not only aids in assessing occupational exposure but also facilitates the monitoring of workers' well-being, the prevention of emerging work-related diseases, and the development of targeted feedback mechanisms for preventive strategies in African countries.

EP01-25 | Oral Manganese Exposure and Neurotoxicity among Susceptible Populations: A Review of the Epidemiologic Literature

Ellen Hata, Francesca Branch, Deborah Segal, Ben Davis

U.S.Environmental Protection Agency, Center for Public Health and Environmental Assessment, District of Columbia, Washington, United States of America

Objective

Manganese (Mn) is an essential micronutrient, but excessive inhalation exposure has been associated with neurotoxicity in humans. However, the impact associated with oral Mn exposure remains unclear. Examining susceptibility factors in chemical health assessments can identify populations at highest risk for health effects. This review aimed to identify populations most susceptible to adverse neurological outcomes due to ingested Mn.

Materials and Methods

A population, exposure, comparator, and outcome (PECO) screening criteria were applied to identify studies exploring the association between oral Mn and any adverse health effect. PECO-relevant studies were further limited to those examining susceptibility factors as effect modifiers of neurological outcomes. An evidence map was developed, and a review was conducted to examine susceptibility factors modifying the association between ingested Mn and neurotoxicity in humans.

Results

Of the 288 studies that assessed susceptibility factors, 111 examined neurological outcomes. These studies highlighted factors including sex, lifestage, co-exposure to pollutants, body mass index, cognitive and nutritional factors, genetics, health conditions, socioeconomic status, education, race/ethnicity, location, and lifestyle. The three most investigated factors (sex, lifestage, and co-exposure to pollutants) were further evaluated to assess their impacts. Among studies reporting effects, females, young children aged <3, adults over 40, premenopausal women, and populations co-exposed to lead were found to be more susceptible to neurotoxicity following oral Mn exposure.

Conclusion

Our findings suggest that lifestage, sex, and co-exposure to pollutants are potential risk factors for detrimental neurological effects from oral Mn exposure.

Disclaimer: The views expressed are those of the authors and do not necessarily represent the views or policies of the U.S. EPA.

EP01-26 | Cadmium Exposure and Attention Deficient Hyperactivity Disorder Diagnoses in Children Living Near Coal-Burning Power Plants

Kristina M. Zierold[1], Lonnie Sears[2]

[1] *University of Alabama at Birmingham, Department of Environmental Health Sciences/Ryals School of Public Health, Birmingham, USA;* [2] *University of Louisville, Department of Pediatrics/School of Medicine, Louisville, USA*

OBJECTIVES: Cadmium is a neurotoxic metal ranked seventh by the US Agency for Toxic Substances and Disease Registry (ATSDR) for the greatest potential threat to human health. Limited and conflicting research exists that assesses the association between exposure to cadmium and Attention Deficient Hyperactivity Disorder (ADHD). Cadmium is emitted in large quantities from coal-fired power plants putting children at risk for mental health conditions. The aim of this study was to investigate the relationship between cadmium exposure and ADHD in children living near coal-fired power plants.

METHODS: Using a community-based research design, we enrolled 278 children aged 6-14 years old. Parents completed the Child Behavior Checklist which was reviewed for t-scores ≥ 60. Children with t-scores ≥ 60 were given a full clinical assessment by a pediatric psychologist. Cadmium exposure was determined from nail samples collected from children and analyzed by Inductively coupled plasma mass spectrometry (ICP-MS). Non-parametric statistical methods were used to evaluate the association between cadmium and ADHD.

RESULTS: The prevalence of ADHD was 18%, which is higher than the national prevalence. The median concentration of cadmium in nails of children with ADHD was 0.052 ug/g compared to those without ADHD (0.033 ug/g). Based on the Wilcoxon test, children with primary ADHD had significantly higher concentrations of cadmium in their nails (p=0.014). Generalized linear models controlled for sex, race, and age showed a significant increase in diagnoses of ADHD with increased cadmium concentrations (p=0.047).

CONCLUSIONS: Cadmium is emitted from coal-fired power plants and can be responsible for mental health conditions. Our study found that children living near coal-fired power plants are more likely to have ADHD compared to the US population. Additionally, as cadmium concentrations increased, the number of children diagnosed with ADHD increased. Regulations on cadmium emissions may not be protective enough to prevent harm to children.

EP01-27 | Heavy Metals from Coal-Fired Power Plants and Children's Neurobehavioral Performance

K. M. Zierold[1], C. Zhang[2], L. Sears[3]

[1] University of Alabama at Birmingham, Department of Environmental Health Sciences, Birmingham, Alabama, United States of America; [2] University of Louisville, Department of Geographic & Environmental Sciences, Louisville, Kentucky, United States of America; [3] University of Louisville, Department of Pediatrics, Louisville, Kentucky, United States of America

OBJECTIVES: Coal-fired power plants emit heavy metals in large quantities into the environment. Children from low-income and historically marginalized communities are more likely to be impacted from metal emissions from coal-fired power plants. Limited research has examined the harmful impact of heavy metal exposure from coal-fired power plants on children's neurobehavioral health. The objective of this study was to examine the associations between concentrations of heavy metals in children's nails, ambient metal exposures, and their linkages with neurobehavioral performance.

METHODS: Using a community-based research design, we enrolled 246 children aged 6-14 years residing in an urban community located near two coal-fired power plants. Children's neurobehavioral performance was assessed by the Behavior Assessment Research System using the Selective Attention Test (processing speed) and the Finger Tapping Test (fine motor speed). To determine concentrations of heavy metals (arsenic, cadmium, lead, manganese), nail samples from the children were collected and analyzed by Inductively coupled plasma mass spectrometry (ICP-MS). Geospatial analysis and regression models were conducted to evaluate associations between the BARS tests and metal pollutant concentrations in children's nails and ambient air.

RESULTS: Hot spots of children with significantly elevated levels of heavy metals in nails were observed in nearby neighborhoods of the two coal-fired power plants. While no direct associations between metal concentrations in children's nail samples and neurobehavioral performances were identified in the multiple linear regression models, a composite measure of exposure to airborne toxic pollutants showed statistically significant correlations with total tapping scores and average correct latency.

CONCLUSIONS: Children are a more vulnerable population that can be greatly impacted by exposure to heavy metals that are emitted into the environment from coal-fired power plants. Considering the abundant global distribution of coal-fired power plants, more research is needed to address children's heavy metal exposures in ambient air and the environment.

EP01-28 | Cooperative creation of a community environmental burden assessment in North Denver Colorado neighborhoods

Elizabeth K. Lunsford[1], Anna Kenyon[1], Nona Nyhart[3], Rocio Monroy-Tello[1], Sheryl Magzamen[1], Sheena Martenies[2]

[1] Colorado State University, Environmental and Radiological Health Sciences, Fort Collins, USA; [2] University of Illinois Urbana-Champaign, Health and Kinesiology, Urbana, USA; [3] Colorado State University, Anthropology, Fort Collins, USA

Environmental justice research has consistently highlighted relationships between harmful environmental exposures and adverse health outcomes, particularly among marginalized communities. In this study, we aimed to quantify the health impacts of increased air pollution exposures estimated in two communities in Denver, Colorado: Globeville and Elyria-Swansea (GES). We conducted a quantitative burden assessment using a Monte Carlo approach to estimate the health impacts attributable to air pollution in GES. Air pollution data from 2017-2021 for fine and coarse particulate matter (PM2.5, PM10), ozone (O3), nitrogen dioxide (NO2), sulfur dioxide (SO2), and carbon monoxide (CO) were obtained from EPA monitoring stations within the metropolitan area, and long-term concentrations in each neighborhood were estimated using inverse distance weighting. Health outcomes were selected based on community interest, and health data were obtained from the Global Burden of Disease. We used a health impact function to estimate deaths and incidence cases of disease attributable to the difference in air pollutant concentrations between GES and the least burdened neighborhood. NO2 and PM10 emerged as the two pollutants associated with the greatest number of attributable cases in both neighborhoods. In Globeville, 1.89 (95% CI: 0.73, 3.13) cases of cancer mortality, 10.02 (95% CI: 6.54, 13.71) cases of type two diabetes, and 7.12 (95% CI: 4.02, 11.01) cases of childhood asthma were attributable NO2 exposure compared to the least exposed neighborhood. In Elyria-Swansea, 2.77 (95% CI: 1.37, 4.27) cases of cancer mortality, 10.04 (95% CI: 1.42, 18.48) cases of type two diabetes, and 29.41 (95% CI: 12.20, 51.15) cases of childhood asthma were attributable to PM10 exposure. Our findings suggest that exposure to higher air pollutant concentrations in GES neighborhoods result in greater health burdens compared to other neighborhoods in Denver. Mitigating air pollution could significantly improve the health and well-being of community members. Funding: Central Interstate-70 expansion settlement

EP02 | E-posters (late breaking abstracts)

EP02-01 | Residential Ozone and Risk of Chronic Obstructive Pulmonary Disease in the United States: Demographic Differences in the All of Us Research Program

Shelton Lo[1], Gabriel Goodney[1], Hantao Wang[1], Jungeun Lim[1], Susan V. Czach[1], Jared Fisher[2], Joseph Shearer[1], Maryam Hashemian[1], Veronique Roger[1], Rena Jones[2], Jason Y. Wong[1]

[1] National Institutes of Health, National Cancer Institute, Bethesda, USA; [2] National Institutes of Health, National Heart Lung Blood Institute, Bethesda, USA

Background

Whether exposure to ozone concentrations consistently below United States (U.S.) regulatory limits (70 ppb, 8-hour average) is associated with incident chronic obstructive pulmonary disease (COPD) is unclear, particularly among demographic subgroups. Further, understanding of the complex interactions between ozone and atmospheric temperature remains limited. To address these knowledge gaps, we examined associations between residential ozone and incident COPD in a large prospective cohort study in the U.S., with focus on heterogeneity by demographic subgroups. Further, we examined whether the associations were modified by atmospheric temperature.

Methods

The All of Us research program followed >848,000 volunteers enrolled in 2017–2023. Annual average ozone concentrations from satellite data were linked to residential locations at enrollment for 376,535 individuals. We used multivariable Cox regression to estimate associations between ozone and incident COPD, adjusting for co-pollutants and potential confounders. Effect modification was assessed using cross-product terms and stratified analyses by annual average residential temperature, race, sex, age, income, and smoking status. Cell weighting was applied using the 2018–2022 American Community Survey to address non-probabilistic sampling.

Results

We identified 7,907 incident COPD cases over an average 4-year follow-up. Residential ozone concentrations (Median: 37.75 ppb, Min-Max: 29.79–51.39) were similar across demographic subgroups. Overall, we observed a positive, non-monotonic relationship between ozone and COPD risk, driven by the highest quartile of exposure ($HR_{Quartile\ 4vs1}$=1.09, 95%CI: 1.01-1.18). We found evidence of heterogeneity among different subgroups, with the most prominent, monotonic exposure-response relationship observed in male never-smokers ($P\text{-}trend_{continuous}=6.62\times10^{-6}$). Notably, we found a super-multiplicative interaction between ozone and temperature (β-interaction=0.01 (P-interaction=3.53×10^{-8})).

Conclusion

Even consistently below U.S. regulatory limits, the respiratory effects of ozone were apparent independent of smoking. These findings provide greater precision into which subgroups might be more susceptible. Furthermore, we found evidence suggesting that increased temperature strengthened the respiratory effects of ozone slightly.

EP02-02 | Spatial-temporal prediction of $PM_{2.5}$ components and its association with dementia and Alzheimer's disease in the Adult Changes in Thought study

Yayi Zhu[1], Adam Szpiro[2], Helen Suh[1]

[1] Tufts University, Department of Civil and Environmental Engineering, Medford, USA; [2] University of Washington, Department of Biostatistics, Seattle, USA

Objective: Exposures to airborne fine particles ($PM_{2.5}$) have been associated with adverse impacts on brain health. Dementia-associated neuropathology (DAN), such as Alzheimer's Disease (AD), microvascular brain injury (μVBI), and Lewy Body Pathology (LBP), is are key driver of morbidity, mortality, and escalating healthcare costs in the aging United States population. The objective of this research is to derive spatiotemporal predictions of $PM_{2.5}$ components to be used in an epidemiological analysis of the association between air pollution exposure and DAN in the Adult Changes in Thought (ACT) study, based in Seattle, WA.
Materials and Methods: We estimated $PM_{2.5}$ component exposure across the Western United States, specifically California, Oregon, and Washington, using data from 97 monitoring sites from 1999 to 2023, with daily average concentration records. We fit a generalized additive model (GAM) to predict $PM_{2.5}$ component concentrations, incorporating temporal factors (e.g., meteorological variables such as temperature, wind speed, pressure, and humidity) and spatial predictors (e.g., population density, traffic density, road proximity, elevation, and emissions data). We evaluated model performance using leave-one-site-out cross-validation (LOOCV).
Results: The model accurately predicted $PM_{2.5}$ component concentration across the region. For sulfur, the predicted mean was 0.27 μg/m³ (observed: 0.28 μg/m³), with an R^2 of 0.81 and mean squared error (MSE) of 0.007. For chlorine, the predicted mean was 0.047 μg/m³ (observed: 0.069 μg/m³), with R^2 = 0.79 and MSE = 0.0071. Arsenic, though present in lower concentrations, was predicted with a mean of 0.00029 μg/m³ (observed: 0.00032 μg/m³), R^2 = 0.61, and MSE = 1e-7.
Conclusion: This study provides accurate and validated spatiotemporal predictions of $PM_{2.5}$ components for use in epidemiologic analyses of DAN. Future work will assess the associations between long-term exposure to specific $PM_{2.5}$ components and DAN outcomes in the ACT cohort.

EP02-03 | Cadmium exposure, miR-30d-5p expression and Largeartery atherosclerosis stroke

Suli Huang[1], Ziquan Lv[2], Tian Wang[3], Dongju Zou[1], Jinling Liu[1]

[1] Shenzhen University, School of Public Health, Shenzhen, China; [2] Shenzhen Center for Disease Control and Prevention, Shenzhen, China; [3] Anhui University of Science and Technology, School of Public Health, Hefei, China

Background: Cadmium exposure has been implicated in the development of ischemic stroke (IS). MicroRNAs are potential mediators linking environmental exposure to health hazards. However, the underlying mechanisms by which cadmium exposure is associated with IS remain largely elusive.

Objectives: To uncover the role of miRNAs in the development of IS triggered by cadmium exposure.

Methods: Apolipoprotein E–deficient mice fed a Western diet were exposed to different dosages of $CdCl_2$ through drinking water. The in vitro experiments were employed to investigate the role of miRNAs in cadmium induced foam cell formation. Furthermore, plasma levels of candidate miRNAs were detected in a case-control population with 494 IS patients and 494 control subjects.

Results: In vivo experiments suggested that Cd could facilitate the progression of AS at a relatively low dosage (4mg/L). Alterations in the levels of miRNAs and genes involved in maintaining cholesterol homeostasis were identified. The induction of foam cell formation by Cd was further confirmed in vitro, accompanied by modified levels of miR-504-3p and miR-30d-5p. Genes involved in lipid uptake (*CD36*) or hydrolysis (*NCEH1*) was identified as novel target of these miRNAs, respectively. Plasma levels of miR-30d-5p might partially mediate the association between Cd exposure and stroke, accounting for a proportion of 16.4%. Moreover, miR-30d-5p significantly improved the ability to discriminate and reclassify patients with IS compared with the traditional risk factor model.

Conclusion: Cadmium might exert its atherogenic effect by modulating miR-30d-5p levels and interfering with foam cell formation within the vascular wall. The potential of plasma miR-30d-5p as biomarker for IS awaits validation in future perspective study.

EP02-05 | Scoping Literature Review on Effect Modifiers of Climate Change-Related Exposure and Low Birth Weight and Preterm Birth

Elizabeth F. Ludwig-Borycz, Adrianna Rojas, Ava Morisi, Ana Baylin, Sue Anne Bell, Cheryl Moyer, Manikandan Pandiyan, Arun Agrawal

University of Michigan, Ann Arbor, USA

Objective: We conducted a systematic review to assess the associations between climate change-related exposures and low birth weight (LBW) and preterm birth (PTB). We assessed results by income classification of the country, trimester of pregnancy, and effect modifiers.
Methods: Protocol was developed using PRISMA extension for scoping reviews. We conducted the search August 2023 across five (PubMed, EMBASE, Web of Science, Global Health, and Scopus) databases, 1,553 articles were initially identified and 78 were included in the study.
Results: The majority of literature, 69.6%, examined temperature as the climate change-related exposure and was conducted in high or upper-middle-income countries (80%) with very little being conducted in lower-middle 13.3% or low-income countries 6.7%. Studies in high-income countries consistently reported associations between heat exposure and increased PTB, Upper-middle-income countries showed stronger associations for PTB and LBW, and lower-middle- and low-income countries had even greater vulnerability. High-temperature exposure during all three trimesters significantly increased the risk of PTB, with greater effects in the third trimester. The impact of temperature on LBW also varies by trimester, with the first and second trimesters being particularly sensitive to temperature variability and the third being sensitive to high heat exposure. Urbanicity, air pollution, child's sex, maternal BMI, maternal age, maternal education, maternal race, and household income are all effect modifiers between temperature and PTB/LBW.
Conclusion: This information will aid public health practitioners, physicians, governments, and communities in making recommendations and tailoring interventions that are specific to the climate change-related exposures, trimesters, and sub-populations at most risk.

EP02-06 | Wildland-Urban Interface (WUI) Fires and the Spatial Distribution of Soil Heavy Metals in the Aftermath of the Lahaina Fire

David Kalafut[1], Mark Wilson[1], Patrick Chepaitis[1], Sumeet Saksena[3], Junfeng (Jim) Zhang[2]

[1] Chemical Insights Research Institute, Marietta, USA; [2] Duke University, Nicholas School of the Environment / Duke Global Health Institute, Durham, USA; [3] East West Cemter, Honolulu, USA

Wildland-Urban Interface (WUI) is the line, area, or zone where structures and other human development meet or intermingle with undeveloped wildland or vegetative fuels. WUI fires lead to the release of uncharacterized and complex mixtures of chemicals, particles, and heavy metals, which result from the combustion of the built environment and natural biomass. Little is known about the hazards associated with these WUI fires. Our research aims to investigate the spatial distribution of heavy metals in residual soil and how that relates to distances from burnt vehicles and buildings with differing construction dates within the fire footprint. In this study, we evaluated 63 homes/businesses across the Lahaina/Kula area. For each property, we obtained publicly available information related to construction and/or renovations. At each sample location, we gathered geospatial data with a Global

Navigation Satellite System (GNSS) receiver, conducted field soil screening using a handheld X-ray fluorescence (XRF) analyzer, and extracted a physical soil core of the top 3 inches. For homogeneity, we sieved the soil samples and tested them for metals via Inductively Coupled Plasma Mass Spectrometry (ICP-MS). We utilized historical imagery to identify vehicles that had been burned during the August 2023 Lahaina wildfires and spatially compared those locations to our sample locations. Our analysis revealed that areas within the burn footprint, particularly near burnt vehicles, showed 2.5-5 times higher target metal concentrations than unaffected or distant areas. These results support our hypothesis that elevated levels of heavy metals in soil correlate with proximity to locations of specific fire fuels. The findings from this project can help future first responders to screen sites more rapidly and to more clearly delineate “clean” sites from “dirty” sites.

EP02-07 | Prenatal fluoride exposure and autism spectrum disorder (ASD) symptoms among children in Los Angeles, California

Durdana Khan[1], Sandra P. Eckel[2], Howard Hu[2], Tingyu Yang[2], Shohreh F. Farzan[2], Carrie V. Breton[2], Theresa M. Bastain[2], Ashley J. Malin[1]

[1] University of Florida, Department of Epidemiology, College of Public Health and Health Professions and College of Medicine, Gainesville, USA; [2] University of Southern California, Department of Population and Public Health Sciences, Keck School of Medicine, Los Angeles, USA

Background: Early life fluoride exposure has been associated with adverse neurodevelopmental outcomes in children, even at relatively low levels. Two studies conducted in the United States (US) have found associations of prenatal or childhood fluoride exposure with symptoms or diagnoses of autism spectrum disorder (ASD). However, no study has examined associations between prenatal fluoride exposure and ASD-like traits. This study explored the association between maternal urinary fluoride levels during pregnancy and ASD-like traits in school-aged children in Los Angeles, California.
Methods: The study included 181 mother-child pairs from the Maternal and Developmental Risks from Environmental and Social Stressors (MADRES) prospective pregnancy and birth cohort. Maternal urinary fluoride adjusted for specific gravity (MUFsg) was measured in third trimester archived urine samples. ASD-like traits were assessed using the Social Responsiveness Scale Second Edition (SRS-2) at age 5 years. The SRS is a 65-item parent-report measure evaluating subdomains of social awareness, cognition, communication, motivation, and restricted interests and repetitive behavior (RRB), as well as total score. We related total score and each of the 5 subdomains to ASD-like traits, in 6 separate models. Higher SRS scores reflect more ASD-like traits. Linear regression analyses examined associations between MUFsg and SRS-2 T-scores, adjusting for maternal age, race, education, income, BMI, and child sex.
Results: Median (IQR) MUFsg and SRS-2 scores were 0.79 (0.63) mg/L and 51 (10), respectively. A 1-IQR (0.63mg/L) increase in MUFsg was associated with a nearly 2-point higher mean total SRS-2 score (β=1.87, 95% CI: 0.37, 3.38). Higher MUFsg levels were also associated with more difficulties in social communication (β=2.13, 95% CI: 0.59, 3.67), social motivation (β=1.81, 95% CI: 0.28, 3.35), and RRB (β=1.63, 95% CI: 0.05, 3.21).
Conclusions: Prenatal fluoride exposure may increase the risk of ASD-like traits in school-aged children. Additional prospective research is needed to further investigate these associations.

EP02-08 | Interpretable machine learning unveils key predictors and default values from database of human in vitro dermal absorption studies with pesticides

Fabiana C. Palma[1], **Danilo Sarti**[6], Daniele Lautenschalaeger[3], Marcela Giachini[5], Vanessa Lupianhez[3], Janaina Pires[2], Marina Sales[4], Patricia Faria[8], Jorg Wagner[9], Heloisa Kalvan[1], Marine Le Bras[10], Lisa Bertomeu[7]

[1] Instituto ProHuma de Estudos Científicos, Technical, Jundiai, Brazil; [2] Ouro Fino Quimica S.A, Ribeirão Preto, Brazil; [3] Bayer CropScience, Sao Paulo, Brazil; [4] Syngenta Proteção de Cultivos, São Paulo, Brazil; [5] Corteva Agriscience, Barueri, Brazil; [6] Consultant, Maynooth, Ireland; [7] Knoell France, Lyon, France; [8] Basf S.A, São Paulo, Brazil; [9] Knoell Germany GmbH, Mannheim, Germany; [10] Knoell Consult Schweiz GmbH, Basel, Switzerland

The skin is the primary route of exposure to pesticides for operators, workers, residents, and bystanders. Assessing dermal absorption is therefore essential for evaluating pesticide exposure. Initial risk assessment approaches often rely on default dermal absorption values (percent absorbed) or read-across data from similar formulations. This work compiled and evaluated the largest known database of human in vitro dermal absorption studies with pesticides, comprising 759 GLP-compliant studies covering 25 formulation types and 248 active substances at various in-use dilutions. Non-parametric statistical methods and interpretable machine learning techniques were applied. The objectives were to: i) identify relationships between dermal absorption and factors such as tested form (concentrate or dilution) and physical state (solid or liquid); ii) establish default dermal absorption values and percentiles for different formulation groups and types, using confidence intervals and bootstrap methods; iii) proposing scientifically supported read-across approaches. Bayesian Additive Regression Trees (BART) indicated that Log Pow and molecular weight are the most import predictors of dermal absorption. Proposed default values were derived using the upper bound of the 95% confidence interval for the 95th percentile and calculated via bootstrapping. Read-across possibilities are proposed, considering the statistical evaluation from the groupings and the dermal absorption differences within each group. When experimental data are unavailable for higher spray dilutions, a pro-rated correction is proposed.

EP02-10 | Is the Association between Serum PFAS Concentrations and Birth Weight Confounded by the Timing of Sample Collection: Results from Two Prospective Cohort Studies

Joseph Braun[1], Xiaoyan Wu[2,1], George Papandonatos[1], Juan Tong[2], Hong Gan[2], Shuman Tao[2], Meglong Geng[2], Chunmei Liang[2], Shuangqin Yan[3], Kun Huang[2], Aimin Chen[5], Kimberly Yolton[4], Fangbiao Tao[2]

[1] Brown University, Providence, USA; [2] Anhui Medical University, Anhui, China; [3] Ma'anshan Maternal and Child Healthcare Center, Child Healthcare, Anhui, China; [4] Cincinnati Children's Hospital Medical Center, Cincinnati, USA; [5] University of Pennsylvania, Philadelphia, USA

Background: Maternal blood concentrations of per- and polyfluoroalkyl substances (PFAS) during pregnancy have been consistently associated with reduced birthweight. However, these associations may be confounded by the timing of sample collection due to pregnancy-related changes in pharmacokinetics that impact both blood PFAS concentrations and birthweight.

Methods: We measured nine serum PFAS concentrations in each trimester among 728 women in the MABC Study, a prospective cohort study in Ma’anshan, China (enrolled 2013-2014). Using linear regression, quantile g-computation, and linear mixed models, we estimated covariate-adjusted differences in birthweight per doubling in individual PFAS, differences in birthweight with increased exposure to the PFAS mixture, and percent change in PFAS by infant birthweight, respectively. We repeated our analyses in 70 women-infant dyads from the HOME Study (Cincinnati, Ohio, enrolled 2003-2006) with repeated PFAS measures in the 2nd trimester and at delivery.

Results: The associations of individual PFAS and their mixture with birthweight became more strongly inverse for measurements taken later in pregnancy. For instance, each SD increase in $\log_2$-transformed PFOA in the 1st trimester was associated with a 6-gram decrease in birthweight (95% CI:-35, 22), while the 3rd trimester concentrations were associated with a 26-gram (95% CI:-54, 3) decrease. Concentrations of six PFAS declined at a greater rate among women who gave birth to babies at the 90th vs. 10th percentile of birthweight. PFOA declined by11.3% (95% CI:-12.7, -9.9) and 8.9% (95% CI:-10.3, -7.5) vs. per 13 weeks among women who gave birth to babies born at the 90th vs. 10th percentiles of birthweight, respectively. We observed a similar pattern of findings in the HOME Study.

Conclusions: These findings suggest that physiological changes in pregnancy confound associations between serum PFAS and birthweight. Studies with PFAS measures later in pregnancy may overestimate the true effect of PFAS in earlier or entire pregnancy on fetal growth.

EP02-11 | Polypropylene flock exposure and respiratory outcomes among manufacturing workers

Sarah Kleinschmidt[1], Karyn Leniek[1], Kara Andres[1], Paul Southall[2], Blair Marynicz[2], Oyebode Taiwo[1]

[1] 3M Company, Corporate Occupational Medicine Department, St. Paul, USA; [2] 3M Company, Environment Health and Safety, Newton Aycliffe, UK

Abstract

Occupational exposure to high concentrations of polypropylene flock may increase the risk of respiratory symptoms, illness, and disease. A cross-sectional study was conducted to evaluate the possible effects of exposure to polypropylene flock on respiratory health among workers at a UK manufacturing facility. Study participants completed a detailed health and work history questionnaire and spirometry testing; those with abnormal findings also received a physical examination. Exposure status was categorized as exposed (those who reported ever working in polypropylene production rooms) or unexposed (those who reported never working in polypropylene production rooms). A total of 55 workers (39 exposed and 16 unexposed) participated in the study. A significantly higher percentage of exposed workers (59%) reported the presence of phlegm in the past 12 months compared with unexposed workers (25%) (p=0.04). Other self-reported respiratory symptoms and illnesses (including nasal irritation, cough, wheeze, shortness of breath, chest tightness, pneumonia, bronchitis, COVID-19, hay fever, asthma and COPD) did not differ significantly by exposure status. There were no significant differences in FEV_1 % predicted, FVC % predicted and FEV_1/FVC% test results between the exposed and unexposed groups or when stratified by work status in the polypropylene production rooms. The most prevalent abnormal finding on the physical examination was nasal cavity erythema which was reported in 28% of exposed workers and none of the unexposed workers. No health conditions were determined to be work-related. Overall, this study observed limited evidence that exposure to polypropylene flock resulted in significant upper airway irritation and no evidence of increased risk of lower airway disease, such as asthma, chronic bronchitis, or interstitial lung disease (ILD). A longitudinal medical surveillance program to monitor workers with current exposure to polypropylene fiber above the working occupational exposure limit was recommended.

EP02-12 | Long-term effects of exposure to inorganic selenium through drinking water on the risk of type 2 diabetes in a Northern Italian community

Marco Vinceti[1,2], Marcella Malavolti[1], **Tommaso Filippini**[1,3]

[1] University of Modena and Reggio Emilia, Department of Biomedical, Metabolic and Neural Sciences, Modena, Italy; [2] Boston University School of Public Health, Department of Epidemiology, Boston, USA; [3] University of California Berkeley, School of Public Health, Berkeley, USA

Introduction: Selenium is a trace element with complex effects on human health due to its nutritional and toxicological properties, depending on its dose and chemical species and on other factors. Recent findings have shown that selenium exposure increases the risk of type-2 diabetes at unexpectedly low dietary intakes. This study aims at investigating diabetes risk following the detection of a unique “natural experiment” where a Northern Italian community during the 1970s–1980s was inadvertently exposed through drinking water to unusually high levels of selenium, close to the drinking water standard of 10 µg/L.
Methods: Using a retrospective cohort study design, we included all subjects who had been continually residing in this area and consuming tap water with 8-10 µg/L of inorganic hexavalent selenite cohorts from January 1st 1980 through December 31, 1985 (main exposed cohort), and from January 1, 1974 through December 31, 1985 (long-term exposure cohort). As unexposed cohorts we considered all the remaining municipal residents. We assessed diabetes prevalence at December 31, 2013, using disease registry data from the Local Health Authority.
Results: We included in the follow-up 3603 exposed and 67,463 unexposed individuals in the main cohorts, and 1310 exposed and 56,334 unexposed individuals in the long-term cohorts. Diabetes prevalence was rather similar in the exposed (8.7%) as compared with the unexposed (9.6%) main cohorts, while corresponding figures in the long-term cohorts were 9.8% and 10.4%, respectively.
Conclusions: Overall, these results do not provide evidence of an association between consumption of tap water with selenium content around the 10 µg/L standard and diabetes prevalence. Selective loss to the follow-up may have however occurred, limiting the reliability of these findings.

EP02-13 | Association of air pollution and pneumonia among children under 5 years in Ulaanbaatar, Mongolia

Oyu Tsogtbayar[1], Batzorig Bayartsogt[1], Yihui Ge[2], Ser-Od Khuyagaa[1], Gregory C. Gray[3], Damdindorj Boldbaatar[4], **Junfeng Zhang**[2,5], Davaalkham Dambadarjaa[1]

[1] Mongolian National University of Medical Sciences, School of Public Health, Ulaanbaatar, Mongolia; [2] Duke University, Nicholas School of the Environment, Durham, USA; [3] University of Texas Medical Branch, Department of Medicine, Division of Infectious Diseases, Galveston, USA; [4] Mongolian National University of Medical Sciences, Ulaanbaatar, Mongolia; [5] Duke University, Global Health Institute, Durham, USA

Childhood pneumonia is a major public health concern in Mongolia, exacerbated by air pollution and weather conditions. This study investigates the association between air pollution and pneumonia incidence among children under 5 in Ulaanbaatar.
We conducted a time-series analysis linking daily hospital visits for pneumonia disease (ICD10 code J18) for children under 5 years old with ambient concentrations of air pollutants ($PM_{2.5}$, PM_1, PM_{10}, SO_2, NO_2, O_3) in Ulaanbaatar from 2014-2019. We used over-dispersed, generalized additive models (GAM) to estimate associations, exploring lag effects up to 7 days. Covariates included time trend, weather conditions, day of the week, holidays and raw coal ban policy. We evaluated the effect of modification by age and sex.
A total of 48,488 cases of hospital visits for pneumonia were recorded for all children under 5 years old during the 6-year study period. For each interquartile range increase in daily average concentration of $PM_{2.5}$, PM_1, SO_2, NO_2, the risk of pneumonia hospital visits increased by 5.7% (RR = 1.057; CI: 1.015, 1.100), 6.5% (RR = 1.065; CI: 1.025, 1.106), 13.6% (RR = 1.136; CI: 1.023, 1.261) and 38.3% (RR = 1.383, CI: 1.108, 1.726) at lag day 2, respectively. We observed positive, but non-significant associations of PM_{10} and O_3. Infants under 1 year old responded sooner as a significant effect was seen at lag day 0 for NO_2 (RR=1.565;CI:1.161, 2.110) and PM_1 (RR=1.098; CI:1.046, 1.152). Boys showed a greater effect size for $PM_{2.5}$, PM_1 and NO_2. The estimated associations of PM_1 and NO_2 were relatively robust to the adjustments of co-pollutants.
These results provide evidence that short-term exposure to air pollution, especially NO_2 and PM_1, was associated with increased risk of daily hospital visits for pneumonia among children under 5 in Ulaanbaatar, Mongolia.

EP02-15 | Modeling Hourly Individual Temperature Exposure and Its Determinants Among Adults With Diabetes in Connecticut, USA

Xin Meng[1], Minjung Lee[2], Lingzhi Chu[1], Soohyun Nam[2], Kai Chen[1]

[1] Yale University, Department of Environmental Health Sciences, School of Public Health, New Haven, USA; [2] Yale University, School of Nursing, New Haven, USA

Background: Ambient temperature is commonly used as a proxy for individual exposure in epidemiological studies, yet substantial discrepancies exist between ambient and personal temperature, especially at finer temporal scales. These discrepancies may lead to exposure misclassification and biased health effect estimates. This study aimed to quantify and model the hourly difference between personal and ambient temperature exposure among adults with type 2 diabetes.

Methods:
We used the data from an ongoing panel study of 94 adults with type 2 diabetes in Connecticut, USA, who wore temperature sensors for two weeks. Personal dry-bulb temperature was recorded every 10 minutes and aggregated hourly. Ambient temperature at 1 km resolution was estimated using a machine learning–based spatiotemporal model. We examined the diurnal pattern of personal-ambient temperature difference using dynamic time warping (DTW) clustering. Linear mixed-effects models and extreme gradient boosting (XGBoost) with SHapley Additive Explanations (SHAP) explanations were used to identify key determinants of personal temperature and its discrepancy from ambient levels.
Results:
Personal temperature was on average 9.9°C higher than ambient temperature, with a consistent unimodal diurnal gap peaking at night and narrowing in the afternoon. The discrepancy was larger in cold months. Mixed-effects models identified ambient temperature, hour of day, month, solar radiation, albedo, and nightlight index as key predictors. The model explained 85.4% of the variance in temperature difference (marginal R^2). XGBoost models confirmed ambient temperature and hour of day as dominant predictors, with surface reflectivity (albedo) playing a consistent moderating role.**Conclusions:**
This study demonstrates that ambient temperature substantially underestimates personal thermal exposure, particularly during cold seasons and nighttime hours. Our results offer a practical framework to assess exposure misclassification, facilitating more accurate health assessments in climate epidemiology.

EP02-17 | Ambient Fungal Spores as Short-Term Predictors of Respiratory Virus Incidence and Mortality in Puerto Rico: Evidence from Machine Learning and Time-Series Modeling

Félix E. Rivera-Mariani[1,2], Armando Borrero-Aponte[3], Benjamín Bolaños-Rosero[4]

[1] Lynn University, College of Arts and Sciences, Boca Raton, USA; [2] RIPLRT Institute, Fort Lauderdale, USA; [3] University of Puerto Rico - Rio Piedras Campus, Natural Sciences Interdisciplinary Program, San Juan, Puerto Rico; [4] University of Puerto Rico - Medical Sciences Campus, Department of Microbiology, School of Medicine, San Juan, Puerto Rico

Objective: Fungal spores are known to exacerbate chronic respiratory conditions, yet their short-term influence on respiratory virus incidence and mortality remains underexplored. We investigated the potential of airborne fungal concentrations on predicting weekly COVID-19 and influenza cases and deaths in two health regions of Puerto Rico—San Juan (Metro) and Caguas—between 2022 and 2024.
Methods: Daily data on fungal spore and pollen concentrations were matched with epidemiological records of COVID-19 and influenza cases and deaths for the years 2022 to 2024. Data were aggregated weekly and analyzed using logistic regression, random forest classifiers, and Negative Binomial models. We evaluated both binary outcomes (weeks with 25% incidence of cases and death) and count outcomes, adjusting for season and health region. We also explored lagged relationships using Pearson correlation and distributed lag modeling strategies.
Results: Fungal spore concentrations—but not pollen—were strongly associated with weekly surges in flu and COVID-19 cases, particularly during fall in both regions. Random forest models predicted top-case weeks with high accuracy for flu and COVID-19 incidences (AUC = 0.95 for flu, 0.87–0.89 for COVID-19). In Caguas, fungal exposure was also a significant predictor of weekly flu mortality (IRR = 1.000016, p = 0.016). No mortality association was found in San Juan. Seasonal trends were pronounced, with lower incidence and mortality in spring and summer. Lag

analyses in Caguas showed that fungal exposure predicted COVID-19 mortality at 1 to 2-week lags ($r = 0.23$ and 0.21, $p < 0.01$).

Conclusions: Fungal spores were not only predictive of respiratory virus incidence, but also associated with short-term increases in influenza mortality, particularly in high-exposure settings like Caguas and fall season. These findings highlight the potential role of ambient fungi as an environmental risk factor and support its inclusion in seasonal surveillance for respiratory outbreak forecasting systems.

EP02-18 | Methods in Qualitative Data Analysis to Progress Community Health Equity

Rachel Righter[1], Kaitlyn Yale[2], Chloe Stewart[3], Lynne Marshall[4], Hannah Fairbanks[5]

[1] Stantec, Portland, USA; [2] Stantec, Arlington, USA; [3] Stantec, Denver, USA; [4] Valeo Sciences, Pittsburgh, USA; [5] Stantec, Pittsburgh, USA

There is increasing demand and requirements for local health departments (LHDs) to conduct community health assessments (CHAs) and develop community health improvement plans (CHIPs) to support Public Health Accreditation Board certification and achieve community health goals. A critical step for the CHA is collecting qualitative data, an invaluable resource in understanding concerns of priority populations and exploring root causes of community inequities. We sought to establish a qualitative analytical method that would facilitate 1) identifying themes within and across focus groups and 2) progressing the equity-centered initiatives of the CHA and CHIP. Transcriptions of ten population-specific focus groups from one urban region were provided. Each focus group followed the same interview guide consisting of nine health-related questions. Data was then stratified by question per speaker and analyzed through both deductive and inductive coding using a novel thematic topic guide. The guide included deductive parent topics and subtopics representing the five domains of the Social Determinants of Health (SDOH). Inductive coding was incorporated through the addition of common words or phrases as transcripts were reviewed. All codes were visually represented to support the determination of focused approaches to improve health equity. Deductive coding methods allowed for successful categorization of parent and subtopics by question within focus groups and allowed for clear identification of differences among focus groups. Inductive coding methods established the basis for revealing more detailed themes discussed within focus groups. Collectively, our results enabled discernment of unique concerns among priority populations. Results of our analysis provided an understanding of priority population's perspective to help identify CHA health priorities. Qualitative analysis methods harness the power of community voices and enable identification of population-specific needs to address unique health, safety, and equity concerns. Standardizing qualitative analysis methods can provide research transparency around equity initiatives and ensure resources are allocated more effectively.

EP02-19 | A case-control study of environmental chemical exposure and adenomyosis risk

Kristen Upson[1], Mandy Hall[1], Holly Harris[2,3], Sawsan As-Sanie[4], Victoria Holt[2,3]

[1] Michigan State University, Department of Epidemiology and Biostatistics, East Lansing, USA; [2] University of Washington, Department of Epidemiology, Seattle, USA; [3] Fred Hutchinson Cancer Center, Program in Epidemiology, Seattle, USA; [4] University of Michigan, Department of Obstetrics and Gynecology, Ann Arbor, USA

Objective

Adenomyosis, marked by endometrial glands and stroma within the myometrium, can confer debilitating symptoms. As estrogen is central to disease pathogenesis, environmental chemicals that are endocrine disruptive may alter adenomyosis risk. Yet, only one study has been published on this topic. We explored the association between self-reported exposure to a range of chemicals and adenomyosis.

Material and Methods

We conducted a case-control study among female enrollees ages 18-59 of an integrated healthcare system in Washington State. Cases had incident, pathology-confirmed adenomyosis diagnosed 2001-2006 (n=386). We employed two control groups: randomly selected age-matched enrollees with intact uteri ("population controls", n=323) and hysterectomy controls (n=233). Data on occupational and non-occupational exposure to chemical groups were collected by in-person interview. We conducted logistic regression to estimate adjusted ORs and 95% CIs, comparing cases to population and hysterectomy controls.

Results

Our data suggested increased adenomyosis risk with exposure to epoxy resins (cases vs. population controls: OR 1.8, 95%CI: 0.9-3.6; hysterectomy controls: OR 1.4, 95%CI: 0.7-2.9) and heavy metals (population controls: OR 1.5, 95%CI: 0.8-2.7; hysterectomy controls: OR 2.0, 95%CI: 1.0-4.1). Our data also suggested twice the risk of adenomyosis with lead exposure (population controls: OR 2.2, 95%CI: 0.8-6.3; hysterectomy controls: OR 2.0, 95%CI: 0.6-6.1); lead exposure mostly occurred from painting, home renovation, sanding, soldering, and stain glass work. The association with mercury exposure was inconsistent (cases vs. population controls: OR 1.1, 95%CI: 0.5-2.3; hysterectomy controls: OR 1.7, 95%CI: 0.7- 4.5). No association was observed with solvent exposure; results were inconclusive for exposure to herbicides, insecticides, fungicides, and rodenticides given few exposed participants.

Conclusion

Our results suggest that exposure to epoxy resins and heavy metals, particularly lead, are associated with increased adenomyosis risk. Given the exploratory nature of our study, further research is warranted. Study funded by NICHD, NIH.

EP02-20 | The association of per- and polyfluoroalkyl substances with inflammatorybiomarkers in US adolescents and the potential modification role of vitamin D

Chan Li

University At Buffalo, Buffalo, USA

Background: Per- and polyfluoroalkyl substances (PFAS) may disrupt immune function and contribute to inflammation. Vitamin D (VD), a key immunomodulator, may play a role in the association between PFAS and inflammation, but evidence is limited. **Objective:** To investigate the associations between PFAS exposure and inflammatory biomarkers in U.S. adolescents and to determine whether the associations are modified by VD. **Methods:** We analyzed data from 1,379 participants aged 12-20 years in the 2011-2018 National Health and Nutrition Examination Survey. PFAS measurements included perfluorooctane sulfonic acid (PFOS), perfluorooctanoic acid (PFOA), perfluorohexane sulfonic acid (PFHxS), perfluorononanoic acid (PFNA), and perfluorodecanoic acid (PFDeA). Covariate-adjusted linear regression models incorporating survey weights were used to examine associations between PFAS and biomarkers including high-sensitivity C-reactive protein (hCRP (mg/L)), alkaline phosphatase (ALP (IU/L)), neutrophils (10^3 cells/μL), and lymphocytes (10^3 cells/μL). Bayesian kernel machine regression (BKMR) was used to estimate the effect of PFAS as a mixture. Effect modification was assessed with stratified analysis by VD levels (insufficiency (< 50 nmol/L) versus sufficiency (≥ 50 nmol/L)). **Results:** We found negative associations between PFNA and hCRP (β: -0.40; 95% CI: -0.79, -0.01), and between PFOS (β: -0.20; 95% CI: -0.39, -0.02), PFDeA (β: -0.21; 95% CI: -0.41, -0.01) and neutrophils. No significant associations were observed for ALP or lymphocytes. BKMR showed robust results. Among adolescents with insufficient VD, PFOS was negatively associated with hCRP (β: -0.88; 95% CI: -1.71, -0.04), neutrophils (β: -0.28; 95% CI: -0.49, -0.06), and lymphocytes (β: -0.13; 95% CI: -0.22, -0.03), while PFNA was negatively associated with ALP (β: -9.04, 95% CI: -16.89, -1.19). Among adolescents with sufficient VD, no significant associations were found. **Conclusion:** Our findings suggest higher PFAS levels are associated with decreased levels of inflammatory markers indicating a potential immunosuppression effect in adolescents. The associations were much stronger among adolescents with insufficient VD.

EP02-21 | Long-Term Association Between Phthalate Exposure in Pregnancy and Midlife Bone Mineral Density among Women in Mexico City

Meizhen Yao[1], Marcela Tamayo-Ortiz[2], John D. Meeker[1], Juan Tamayo-Orozco[3], Belem Trejo-Valdivia[4], Martha M. Téllez-Rojo[4], Karen E. Peterson[1], Deborah J. Watkins[1]

[1] University of Michigan, School of Public Health, Ann Arbor, USA; [2] Columbia University, Mailman School of Public Health, New York, USA; [3] Mexican Committee for the Prevention of Osteoporosis, Mexico City, Mexico; [4] Instituto Nacional de Salud Pública, Cuernavaca, Mexico

Objective: Phthalates are widespread endocrine-disrupting chemicals (EDCs) that may affect bone metabolism, though human studies remain limited. This study investigated the long-term associations between phthalate exposure during pregnancy, a period of heightened bone activity, and bone mineral density (BMD) in midlife.

Material and Methods: Participants were women in the Early Life Exposure in Mexico to Environmental Toxicants (ELEMENT) cohort who had prenatal phthalate measurements and midlife BMD assessments (n=180), including 60 who received calcium supplementation during pregnancy as part of a clinical trial. Nine urinary phthalate metabolites were measured across up to three pregnancy trimesters, corrected for specific gravity, and a molar sum was calculated for di-2-ethylhexyl phthalate (ΣDEHP). BMD of the spine, hip, and forearm were assessed at a mean age of 50.1 years. We used linear models to estimate changes in BMD z-scores per doubling of geometric mean concentrations of individual phthalates across pregnancy, adjusting for age, BMI, and calcium supplementation. Effect modification by calcium supplementation was tested through interaction terms. Quantile-based g-computation was used to evaluate the overall effects of phthalate mixtures.

Results: Most phthalates were inversely associated with spine and hip BMD z-scores, whereas some showed significant positive associations with forearm BMD z-scores. Prenatal calcium supplementation significantly modified associations between phthalates and spine BMD, showing consistent negative trends in the placebo group and positive trends in the supplementation group (p-interaction=0.02). For example, a doubling in ΣDEHP was associated with 0.12 lower spine z-scores in the placebo group (95%CI= -0.28, 0.03) and 0.27 higher spine z-scores in the supplementation group (95%CI: -0.03,0.57). Similar patterns were seen for hip BMD. Mixture analysis results aligned with single-pollutant findings.

Conclusion: Phthalate exposure during pregnancy was associated with midlife BMD in a site-specific manner. Calcium supplementation in pregnancy modified these associations at the spine and hip, suggesting a potential protective role.

EP02-22 | Spatial Disparities in Long-Term $PM_{2.5}$ and O_3 Exposure Prior to Gastric Cancer Hospitalization in Shanxi, China

Mingyan Hao[1], Jianzhong Zheng[1]

[1] shanxi medical university, public health, taiyuan, China; [2] Shanxi Province Cancer Hospital/ Shanxi Hospital Affiliated to Cancer Hospital, Chinese Academy of Medical Sciences/Cancer Hospital Affiliated to Shanxi Medical University, hospital office, taiyuan, China

Background:
Gastric cancer remains a significant public health burden in northern China, particularly in regions with persistent air pollution. Long-term exposure to fine particulate matter ($PM_{2.5}$) and ozone (O_3) has been associated with gastrointestinal carcinogenesis. However, region-specific hospital-based exposure data remain scarce.
Objective:
To quantify five-year average ambient $PM_{2.5}$ and O_3 exposure among gastric cancer inpatients at Shanxi Cancer Hospital during late 2024, and to identify geographic disparities in exposure patterns.
Methods:
We included 462 patients diagnosed and hospitalized with gastric cancer between September and December 2024 at Shanxi Cancer Hospital. Residential addresses were extracted from hospital records, standardized, and geocoded using OpenStreetMap. Address data were linked to city-level annual average $PM_{2.5}$ and O_3 concentrations from OpenAQ (2019–2023). City-level exposure was assigned to each patient based on residence due to data availability constraints.
Results:
The five-year average $PM_{2.5}$ exposure was 41.7 μg/m³ (SD: 4.9), while O_3 exposure averaged 93.8 ppb (SD: 7.6). Substantial spatial variation was observed: higher exposure levels were recorded in Taiyuan, Linfen, and Datong, with lower concentrations in the southern municipalities. Patients in the highest exposure quartile were disproportionately from northern and industrialized regions.
Conclusions:
Hospitalized gastric cancer patients in late 2024 had experienced sustained exposure to elevated ambient air pollution in the preceding five years. These findings underscore the need for region-specific pollution control policies and highlight environmental risk disparities relevant to long-term gastric cancer prevention strategies in northern China.Future research is warranted to link long-term pollution exposure with individual-level cancer progression and survival outcomes

EP02-23 | Health Effects of Long-Term Exposure to $PM_{2.5}$ and Ozone in the Korean Genome and Epidemiology Study (KoGES): A Study Protocol

Soobin Jang

Daegu Haany University, Department of Preventive Medicine, College of Korean Medicine, Gyeongsan, South Korea

This study protocol outlines a planned investigation into the health effects of long-term air pollution exposure using data from the Korean Genome and Epidemiology Study (KoGES) community-based cohort. The study aims to examine associations between chronic exposure to fine particulate matter ($PM_{2.5}$) and ozone (O_3) and changes in blood metabolic biomarkers and pulmonary function, and to evaluate the modifying effects of meteorological conditions such as temperature and humidity.

The target population comprises adults aged 40–65 residing in Ansan and Anseong who participated in the KoGES baseline and six follow-up assessments. Health outcomes will be assessed through blood tests and spirometry measures. Ambient air pollution and meteorological data will be spatially and temporally linked to participants' residential addresses across the study period.

We hypothesize that long-term exposure to air pollutants adversely affects blood and lung function biomarkers and that meteorological conditions modify these associations. Statistical analyses will involve multivariable regression models to estimate the effects of air pollutants and interaction terms to evaluate effect modification by temperature and humidity.

This study addresses the urgent need to understand the compounding health risks posed by air pollution and climate variability. According to the World Health Organization, air pollution caused approximately 4.2 million premature deaths worldwide in 2019. South Korea has reported $PM_{2.5}$-related mortality rates higher than the OECD average. Evaluating intermediate biomarkers in a middle-aged population provides early insight into the progression toward severe health outcomes and mortality.

Findings from this study will support public health strategies aimed at mitigating air pollution-related risks in the context of climate change and provide evidence to inform national environmental health policies.

This protocol is currently under IRB review by the Korea Disease Control and Prevention Agency (KDCA).

EP02-24 | Assessment of Knowledge, Attitude, and Practice on the Effects of Pesticide Use among Households in Jimma Town, Southwest Ethiopia

Wondwossen B. Eshete[1], **Hiwot Tessema**[1]

[1] Jimma University, Environmental Health & Technology Department, Jimma, Ethiopia; [2] Jimma University, Environmental Health & Technology Department, Jimma, Ethiopia

Background
In Ethiopia, pesticides are widely used for both agricultural and domestic purposes. However, improper handling, inadequate knowledge, and unsafe practices contribute to environmental contamination, human poisoning, and chronic health effects. Urban populations, despite being exposed, are underrepresented in pesticide-related research. This study investigates the knowledge, attitudes, and practices (KAP) regarding pesticide use and its health and environmental impacts among households in Jimma Town, Southwest Ethiopia.
Methods
A cross-sectional study was conducted in January 2025 involving 419 randomly selected households from three purposely selected Kebeles (town administrations). Data were collected using structured, pre-tested questionnaires and analyzed using descriptive statistics.
Results
Pesticide use was reported by 90% of participants. While 73% could identify the pesticides used, mainly DDT (61.3%), only 60.1% were aware of associated health risks. Despite 76.1% supporting the use of personal protective equipment (PPE), only 24.8% reported its usage. Most participants did not receive any formal training (90.2%), and unsafe disposal of pesticide containers was common (53.7%).
ConclusionThere exists a considerable gap between knowledge and practice. Strengthened community education, strict regulation of pesticide sales, and improved accessibility to PPE are essential interventions.

EP02-26 | Associations Between Early-Life Chemical Exposures and ADHD Risk in Middle Childhood and Adolescence: A Multi-Cohort Study using ECHO data

Xiaolan Liao[1], Hongwan Li[2], Dan Wang[3], David Bard[1], Huajian Cai[4], Nyles Keenan[1]

[1] University of Oklahoma Health Sciences Center, Pediatrics, Oklahoma City, USA; [2] University of Oklahoma Health Sciences Center, Occupational & Environmental Health, Oklahoma City, USA; [3] University of Oklahoma, Sociology, Norman, USA; [4] University of Oklahoma, Psychology, Norman, USA

Objective:
To examine associations between chemical exposures during infancy and early childhood and the risk of attention-deficit/hyperactivity disorder (ADHD) in middle childhood and adolescence.
Material and Methods:
We analyzed data from the Environmental influences on Child Health Outcomes (ECHO) Program, which includes 10,284 participants aged 5–20 years across 41 U.S. cohorts with caregiver-reported ADHD status (Yes = 1,489; No = 8,795). Urinary biomarker data were available for 846 infants (46 weeks post–last menstrual period to 11 months) from 7 cohorts and 1,177 children (12 months to 5 years, 11 months) from 11 cohorts. We examined phthalates, phenols, and metals selected based on prior evidence and data availability. Wilcoxon rank-sum tests and logistic regression models adjusting for age, sex, and ethnicity were used to evaluate associations.
Results:
ADHD prevalence was 13% among infancy-exposed and 12% among early childhood–exposed participants. In infancy, mono-benzyl phthalate (MBZP; OR = 1.14, 95% CI: 1.04–1.24, false discovery rate [FDR] $p = .013$), mono-(2-ethyl-5-carboxypentyl) phthalate (MECPP; OR = 1.08, 95% CI: 1.05–1.12, FDR $p < .001$), mono-(2-ethyl-5-hydroxyhexyl) phthalate (MEHHP; OR = 1.24, 95% CI: 1.12–1.37, FDR $p < .001$), mono-(2-ethyl-5-oxohexyl) phthalate (MEOHP; OR = 1.22, 95% CI: 1.02–1.46, FDR $p < .001$), and mono-n-butyl phthalate (MNBP; OR = 1.22, 95% CI: 1.02–1.50, FDR $p = .056$) were associated with increased ADHD risk. In early childhood, molybdenum (OR = 1.12, 95% CI: 1.04–1.20, FDR $p = .032$) and manganese (OR = 2.67, 95% CI: 1.36–5.19, FDR $p = .043$) were significantly associated with ADHD. Thallium showed a positive association but did not remain significant after FDR correction.
Conclusion:
Exposures to specific phthalates and metals during early life may increase risk for ADHD. These findings support further investigation into modifiable environmental factors affecting neurodevelopment.

Funding: Supported by the OU DISC Faculty Seed Funding Program.

EP02-27 | Short-term nature exposures and the child skin microbiota — a randomized controlled trial

Yizhen Ding, Dongying Li, Terry Gentry, Chanam Lee, Galen Newman

Texas A&M University, Department of Landscape Architecture and Urban Planning, College Station, USA

Children are increasingly living in urban areas, and there is growing evidence that the environments they are exposed to play a critical role in their health. However, the effects of different environment types on human skin microbiota remain poorly understood. In this study, I experimentally tested the hypothesis that short-term exposure to various types of nature alter the skin microbiota of children, and that the effect varies depending on the type of environment. Specifically, I hypothesized that wooded areas and grass fields would have a greater effect on skin microbiota than paved areas and indoors. I conducted a randomized controlled trial involving 145 children aged 11 to 13, who were exposed to four different sites. Skin swabs were collected from two different fingers before and after a two-hour exposure. The results showed that grass field had an enriching and diversifying effect on skin microbiota. Moreover, specific microbial taxa shifted in response to different environmental exposures; notably, exposure to the grass field was associated with a reduced relative abundance of potentially pathogenic taxa. Overall, I found that nature exposure plays an important role in human microbiota alterations. My results suggest that incorporating short periods of green space exposure into school schedules may have beneficial effects for children's skin microbiota. Future investigations into the health impacts of permanent urban green space interventions are needed.

EP02-29 | Long-Term Ozone Exposure, Occupational Risk, and Aging-Related Health Outcomes: An Overview of Systematic Reviews

Soobin Jang

Daegu Haany University, Department of Prevent Medicine, College of Korean Medicine, Gyeongsan, South Korea

Ozone (O_3) is a secondary air pollutant formed from precursors such as nitrogen oxides (NO_x) and volatile organic compounds (VOCs). Although its atmospheric lifespan is short, long-term exposure to ambient ozone poses significant health risks, particularly for aging and occupationally exposed populations. This overview aims to summarize existing systematic reviews and meta-analyses addressing the occupational health impacts of long-term ozone exposure, with a focus on aging-related outcomes.
We reviewed 24 systematic reviews and meta-analyses published during last 10 years, identified through a structured PubMed search. The included studies examined long-term ozone exposure and outcomes such as mortality, respiratory and metabolic diseases, cognitive decline, and inflammatory biomarkers. Occupational groups with high exposure potential—such as construction workers, agricultural laborers, traffic-related occupations, and workers in industrial and energy sectors—were emphasized.
Findings indicate consistent associations between long-term ozone exposure and increased all-cause and cause-specific mortality, particularly due to respiratory and cardiovascular conditions. Elevated risks of type 2 diabetes, insulin resistance, and dyslipidemia were observed, especially in populations over 50. Multiple reviews linked long-term ozone exposure to increased incidence of neurodegenerative diseases, including dementia. Inflammatory pathways were frequently cited as mediating mechanisms. Importantly, modifying factors such as age, temperature,

co-pollutants (e.g., $PM_{2.5}$, NO_2), and behavioral risk factors (e.g., smoking, alcohol use) contributed to heterogeneity in observed effects, underscoring the vulnerability of older workers with cumulative exposure.
Our findings suggest the need to incorporate occupational and aging-related considerations into health risk assessments and public health policy. Better quantification of long-term ozone exposure in older worker populations is essential to inform targeted interventions and prevent chronic disease burdens associated with environmental exposures.

EP02-30 | Impact of Physical Environmental Features on Human Microbiota: Systematic Review and Meta-Analysis

Yizhen Ding[1], Dongying Li[1], Minxi Jiang[1], Yue Zhang[1], Chanam Lee[1]

[1] Texas A&M University, Department of Landscape Architecture and Urban Planning, College Station, USA; [2] University of California, Berkeley, Civil and Environmental Engineering, Berkeley, USA

Background
Physical environmental exposure can influence human microbiota. While some reviews have examined green spaces, evidence on built environments and urbanicity remains limited. We performed a systematic review and meta-analysis to comprehensively synthesize studies on physical environmental exposures across natural, built, and levels of urbanicity.
Methods
An initial literature scan was conducted in August 2022, followed by a comprehensive search completed on March 1st, 2025, using PubMed, Web of Science, and Environment Complete. Study quality and certainty of evidence were assessed. Study results were synthesized narratively, and a meta-analysis was conducted for environmental domains and microbiota alpha diversity.
Results
Twenty-eight studies were included. Environmental exposures were categorized into five domains: direct contact with natural materials, exposure to environment without direct contact, the home's surrounding natural environment, the home's surrounding built environment, and residential urbanicity level.
Strong certainty of evidence supports direct contact with natural materials increases microbiota richness, and moderate certainty supports changes in the Shannon index and microbiota composition changes. Home-surrounding natural environments were linked to higher microbiota alpha diversity (e.g., richness and Shannon index), and changes in composition, but evidence was rated as low due to inherent limitations of cross-sectional study designs. Urban living was associated with reduced alpha diversity, though meta-analysis was not significant, and evidence remained very low for both alpha diversity and beta-diversity. Other environmental domains lacked sufficient studies for meta-analysis.
Studies showed that direct contact with natural materials and environment exposure without direct contact altered gut and skin microbiota, which was linked to increased anti-inflammatory cytokines, decreased pro-inflammatory cytokines, changes in T regulatory cells, and reduced atopic conditions like allergies and asthma.

EP02-31 | Higher Maternal Urinary Fluoride is Associated with Gestational Diabetes Mellitus

Lucy F. Harvey[1], Tingyu Yang[2], Ashley J. Malin[3], Shohreh F. Farzan[2], Carrie V. Breton[2], Theresa M. Bastain[2]

[1] Keck School of Medicine of USC, University of Southern California, Los Angeles, USA; [2] Department of Population and Public Health Sciences, Keck School of Medicine of USC, University of Southern California, Los Angeles, USA; [3] Department of Epidemiology, College of Public Health and Health Professions, University of Florida, Gainesville, USA

Objective: To determine the association of first trimester specific gravity adjusted maternal urinary fluoride (MUF_{SG}) with gestational diabetes mellitus (GDM).
Material and Methods: We used data from the Maternal and Developmental Risks from Environmental and Social Stressors (MADRES) prospective pregnancy cohort, consisting mainly of Hispanic mothers in Los Angeles, California. MUF_{SG} was measured in urine collected during the first trimester from 12/2015 - 11/2019. Glucose intolerance (GI) was defined as a borderline (120 to <140 mg/dl) or positive (140 to <200 mg/dl) glucose challenge test (GCT) and an incomplete glucose tolerance test (GTT) or a GTT with one or more positive values. GDM was defined as a positive GCT and a GTT with two or more positive values, a diagnosis in the medical record, or a high-positive GCT result (>200) during the MADRES cohort index pregnancy. Sociodemographic characteristics were compared using the Wilcoxon-Mann Whitney or Kruskal-Wallis test. Logistic regression was used to examine associations between MUF_{SG} and GDM and GI per interquartile range (IQR) increase in MUF_{SG}. The models were adjusted for maternal age, race, BMI, education, income, cohabitation, parity, and season of conception.
Outcomes: There were 269 women included. The median (IQR) MUF_{SG} was 0.67μg/mL. The highest MUF_{SG} levels were observed in non-Hispanic white women (p=0.046) and those reporting four or more years of college (p=0.011). Lower MUF_{SG} levels were observed in women with annual incomes < $30,000 compared to incomes that were unknown or ≥ $30,000 ($p$=0.006). Each IQR increase in MUF_{SG} was associated with 72% increased odds of having GDM (OR = 1.72 (95% CI: 1.178 - 2.512)). MUF_{SG} exposure was not associated with GI (OR = 0.946 (95% CI: 0.701 - 1.277)).
Conclusions: In this prospective cohort study, higher first trimester fluoride exposure was associated with higher odds of having gestational diabetes mellitus.

EP02-32 | Restricted use pesticides in Hawaiʻi: community concerns and explorations of health associations

Rosana H. Weldon[1], Lisa Webster[5], Dylan Pilger[1], Kaiakahinaliʻi Kaʻōpua-Canonigo[4], Anne Frederick[4], Fern Holland[4]

[1] University of Hawaiʻi at Mānoa, Office of Public Health Studies, Honolulu, USA; [2] University of Hawaiʻi System, Office of Strategic Health Initiatives, Honolulu, USA; [3] University of Hawaiʻi at Mānoa, Honolulu, USA; [4] Hawaiʻi Alliance for Progressive Action, Kapaa, USA; [5] University of Hawaiʻi at Mānoa, National Disaster Preparedness Training Center, Honolulu, USA

Hawaiʻi has a long agricultural history and is an ideal location for growing crops due to its nearly year-round growing season. In 2018, Hawaiʻi passed legislation that required reporting of restricted use pesticide (RUP) applications. In 2019, some of the most highly used RUPs included two volatile fumigants that have been linked to asthma and cancer in the literature, 1,3-dichloropropene (1,3-D) and sodium methyldithiocarbamate (metam sodium). Over 200,000 pounds of 1,3-D and just about 100,000 lbs of metam sodium were used in the state with the use particularly concentrated in Central Oʻahu. Four focus groups were held with a total of 19 community members of Central Oʻahu to listen to their health and environmental concerns. In addition, associations between these RUP exposures and health issues were explored using GIS methods and publicly available data on asthma and cancer; thus, this pilot study was the first to use Hawaiʻi's RUP data in a public health context. Some of the themes that emerged from the focus groups included ways that participants could take further action to protect their families from pesticide exposure, the importance of voting for politicians that represent constituent interests, and awareness of pesticides and their health effects. GIS analyses suggest an association between RUP use and asthma, but not cancer. However, there were several challenges identified in attempting to use the RUP data. Data cleaning was time-consuming and required assumptions that likely introduced error. In addition, the location was represented as the tax map key which is not a standardized size and can vary widely in the range of acreage represented. The lack of precise location data can also introduce error in exposure estimates.

EP02-33 | Intrauterine PBDE Exposure is Associated with Body Fatness Among Children with Low Childhood Opportunity Index

Carmen Dickinson-Copeland[1], Muhammed Idris[2], Jabril Johnson[3], Stephanie Eick[4], Donghai Liang[4], Anne Dunlop[5]

[1] Morehouse School of Medicine, Microbiology, Biochemistry and Immunology/Department of Public Health Education, Atlanta, USA; [2] Morehouse School of Medicine, Department of Medicine, Altanta, USA; [3] Morehouse School of Medicine, Microbiology, Biochemistry and Immunology, Atlanta, USA; [4] Emory University, Gangarosa Department of Environmental Health/Rollins School of Public Health, Atlanta, USA; [5] Emory University, Department of Family & Preventive Medicine/School of Medicine, Atlanta, USA

Background: Polybrominated diphenyl ethers (PBDEs) are endocrine-disrupting chemicals with demonstrated persistence in human tissues and potential for long-term metabolic disruption. Emerging evidence suggests that in utero exposure to specific PBDE congeners may influence childhood body composition, but sex-specific effects and contextual influences remain underexplored. **Objective:** This study investigates whether prenatal exposure to polybrominated diphenyl ethers (PBDEs) influences childhood body mass index (BMI) trajectories in a longitudinal

cohort of African American mother-child dyads in Atlanta, Georgia, while accounting for maternal and neighborhood characteristics. **Methods:** We used longitudinal data from 465 children enrolled in the Atlanta African American Maternal-Child Cohort (2011–2022). PBDE concentrations were measured in maternal serum during pregnancy, and BMI z-scores were calculated using CDC standards and modeled using multilevel linear regression. Child Opportunity Index (COI) scores were incorporated in the models to evaluate the effect of area-level determinants. **Results:** PBDE99 was significantly associated with higher BMI z-scores in girls (β = 0.16, p = 0.044), and inversely, though insignificantly, associated in boys (β = -0.12, p = 0.077). PBDE85 and PBDE154 also showed positive associations in girls and boys, respectively. Maternal first-trimester BMI remained a strong and consistent predictor of child BMI. **Conclusions:** Findings suggest congener- and sex-specific effects of prenatal PBDE exposure on child adiposity. Including area-level metrics like the COI may improve early risk prediction strategies for pediatric metabolic outcomes

EP02-34 | Determinants of Urban Heat and Sociodemographics of Greenery Access: A Study of Mexico City Using Street View Imagery

Audrey Amezcua-Smith, Arianna Salazar Miranda, Michelle L. Bell

Yale University, School of the Environment, New Haven, USA

Objective: Latin America is a highly urbanized region with significant climate vulnerabilities. This includes Mexico City with its growing footprint, dense population, and inequitably distributed heat island. Mexico City’s streets play a critical role supporting active transportation and informal economies, which are vital to residents’ health and well-being. Temperature increases associated with planetary warming and local urbanization therefore necessitate cooling interventions to assure streets’ continued utility as a public space. Thus, we sought to understand street-level determinants of urban heat in Mexico City and geographic and sociodemographic correlates of heat and street greenness. *Materials & Methods:* We used Google Street View imagery and an image classification algorithm to characterize greenness and other potential correlates of temperature along the street network. We assessed streetscape features’ relationship with land surface temperature using 2018-2024 Landsat 30m data. Secondarily, we validated our findings using ambient temperature measurements from Mexico City’s meteorological monitoring network. Finally, we used 2020 Population and Housing Census data to analyze the sociodemographic correlates of street greenery and heat islands. *Results:* Preliminary results suggest an inverse relationship between street greenery and local temperatures, with the association’s strength depending on the type of greenness analyzed. Initial results suggest that associations are geographically heterogeneous, with temperatures increasing from west to east. Additionally, we identified sociodemographic differences in access to green streets and in neighborhood-level street view image availability, with less educated and more indigenous populations tending to have lower neighborhood street greenery and less coverage in our street view dataset. *Conclusion:* Our findings underscore the need for geographically targeted and socially equitable mitigation approaches to reduce urban heat islands and assure thermally comfortable, healthy streets. While our findings are most directly applicable to Mexico City, they can help inform scientific and urban planning efforts in climate adaptation in Latin American cities more broadly.

EP02-37 | Are You TILT-ed? A Study of Chemical Exposures, Autism, and ADHD

Aditi Mahesh, Rodolfo Rincon, Roger Perales, Claudia Miller

University of Texas Health Science Center at San Antonio, Environmental Health/ Family Medicine, San Antonio, USA

Objective: Toxicant-Induced Loss of Tolerance (TILT) is a two-stage disease process that can lead to the development of chemical intolerance (CI) with diverse multi-system symptoms. The TILT mechanism involves *sensitization* to foreign substances such as pesticides, cleaning agents, and fragrances, and later *triggering* of sensitized individuals' symptoms following tiny exposures to previously tolerated substances. In earlier studies, we found that maternal CI is associated with autism and attention deficit hyperactivity disorder (ADHD) in her children. This suggests the need for effective screening tools for CI.
Methods: In an online population-based study, we used the Quick Environmental Exposure and Sensitivity Survey (QEESI) to assess CI in respondents from India, Italy, Japan, Mexico, and the United States. In a separate study, U.S. adults completed the QEESI and answered questions regarding physician diagnosis of autism and ADHD in their children.
Results: Of the five countries, the prevalence of CI is greatest in India, with around 50% of the adult population meeting the QEESI criteria.Further, participants with QEESI scores in the top tenth versus bottom tenth percentile of U.S. adults had 5.7 times the risk of having a child with autism and 2.1 times of having one with ADHD.
Conclusion: These findings support our earlier study showing that maternal CI is associated with increased risk of autism and ADHD in children. Our results are consistent with studies by other groups showing increased rates of autism and ADHD in industrialized regions. The QEESI can identify prospective parents at high risk of CI and having a child with autism/ ADHD, affording opportunities to intervene with environmental counseling.

EP02-39 | Effect of health education program onparticulate matter for the elderly in South Korea

Jihee Choi[1], Hyeyun Kim[2], Sumi Chae[1]

[1] Korea Institute for Health and Social Affairs, Department of Healthcare Policy Research, Sejong, South Korea; [2] Korea Institute for Health and Social Affairs, Department of Universal Health Coverage Policy Research, Sejong, South Korea

Background and Aim: Particular matter poses significant health risks, particularly for the elderly who are a vulnerable population. This study aimed to design and provide health education programs with regard to preventing the effects of particular matter on the elderly, as well as to analyze whether there are differences in the improvements of knowledge, attitude, and practice between the educated and non-educated groups.
Methods: Conducted through "Developing a Visiting Health Education Program of Groups Sensitive to Particulate Pollution" project, the study involved 285 elderly individuals (142 educated, 143 non-educated). Educational content and evaluation tools were developed based on the Health Belief Model and Knowledge-Attitude-Practice Model. The educated group received two 10-minute in-person sessions over two weeks. Data were analyzed using various statistical tests, including multiple logistic regression.

Results: Unadjusted analyses of pre- and post-scores revealed statistically significant improvements in the educated group's awareness of particular matter, preventive behaviors, and attitude that such behaviors could protect their health. After adjusting for control variables in a multiple logistic regression analysis, the educated group demonstrated significantly greater improvement compared to the non-educated group. Improvements in the educated group were 3.21 times higher for knowledge, 2.71 times for practice, and 2.12 times for attitude toward particular matter exposure prevention.
Conclusion: Although older adults are generally considered less responsive to educational interventions due to age-related factors, this study suggests that continuous education can effectively enhance their knowledge and preventive behaviors regarding particulate matter exposure.

EP02-40 | Advancing Environmental Epidemiology Education: HHEAR's Role in Training the Next Generation Across the Educational Continuum

Sofia Bengoa[1,3], Jeanette Stingone[2], Susan Teitelbaum[1]

[1] Icahn School of Medicine at Mount Sinai, Department of Environmental Medicine, New York, USA; [2] Columbia University Mailman School of Public Health, Department of Epidemiology, New York, USA; [3] CUNY School of Public Health and Health Policy, Department of Epidemiology and Biostatistics, New York, USA

Objective: Environmental epidemiology is absent from formal education until graduate school, despite its growing importance in public health. The Human Health Exposure Analysis Resource (HHEAR) has supported environmental epidemiology education across the educational continuum, from high school to doctoral training, by engaging students in real-world data science and research experiences.
Materials and Methods: We adapted the epidemiology education continuum model by D'Agostino, Hlaing, and Stark, which outlines distinct learning goals at different academic levels. Through structured mentorship and applied learning opportunities, students supported core Data Center activities including semantic data mapping, metadata compilation, and analysis using publicly available environmental health data in the HHEAR repository. Projects were tailored to each student's academic level and background.
Results: High school students learned foundational epidemiologic and environmental health concepts and contributed to harmonization efforts through structured activities like creating semantic data dictionaries and compiling study metadata. Undergraduate students performed pooled analyses using repository data, addressing environmental exposures and birth outcomes, and presented posters demonstrating their ability to define exposures, conduct basic analyses, and communicate findings. Masters students participated in data harmonization and independently pursued projects such as analyzing autism-relevant ontologies and international categorizations of race/ethnicity. Doctoral students used the repository to apply advanced statistical methods (e.g., lagged kernel machine regression, multilevel modeling) to study complex exposure-outcome relationships and develop dissertation research questions.
Conclusion: HHEAR has successfully integrated environmental epidemiology into public health training at all academic levels. By aligning student engagement with stage-specific learning goals and providing access to real-world environmental health data, HHEAR helps build a scientifically literate public health workforce. This work demonstrates a model for embedding environmental health into epidemiology curricula through open data and applied mentorship.

EP02-41 | Global Health Benefits of Anthropogenic Emission Reductions on $PM_{2.5}$-, O_3- and NO_2-Associated Deaths by Sector, Season and City

Patrick Wiecko[1], Daven K. Henze[2,1]

[1] University of Colorado Boulder, Environmental Engineering Program, Boulder, USA; [2] University of Colorado Boulder, Mechanical Engineering Department, Boulder, USA

Long-term exposure to outdoor air pollution remains one of the largest risk factors for premature death, with the pollutants contributing the most to the global health burden being fine particulate matter ($PM_{2.5}$), ozone (O_3), and nitrogen dioxide (NO_2). Epidemiological studies have compiled hazard ratios that led to works quantifying the global mortality burdens of exposure to each of these respective pollutants, but these works lack insight into the sources of these pollutants: their emissions, direct or precursor. In the present work, we bridge the gap between the global deaths in 2019 associated with long-term exposure to $PM_{2.5}$, O_3, and NO_2 and the human-caused emissions responsible for the formation of those pollutants. We use the chemical transport model GEOS-Chem adjoint v35n to simulate hourly concentrations of $PM_{2.5}$, O_3 and NO_2 at a global 2°×2.5° resolution, with satellite data downscaling the final annual-averaged result to a neighborhood-scale 0.01°×0.01° resolution. The model uses a combination of anthropogenic and natural emissions and meteorology to calculate pollutant transport, production and removal processes. Using the results of this model, we quantify the global population-weighted, annual-averaged exposure to be 34.8 $\mu g/m^3$ $PM_{2.5}$, 47.7 ppb O_3 and 7.01 ppb NO_2, which translate to 4.36 (95% CI 3.38 – 5.25), 0.88 (95% CI 0.53 – 1.15), and 3.01 (95% CI 0.82 – 4.28) million deaths globally in 2019 associated with long-term $PM_{2.5}$, O_3, and NO_2 exposure, respectively. We then apply the model adjoint, which calculates the sensitivities of these exposure-associated global annual deaths to hourly emissions by model grid cell. We present this relationship through a hypothetical scenario where we incorporate uniform 10% anthropogenic emission reductions in each grid cell to quantify their effect on the global mortality burden by sector, season and city – a process that would otherwise take thousands of traditional model simulations to calculate.

EP02-42 | Effect of Greenness on Air Pollution and Mortality – An Ecological Study in Chennai

Alice Nikhita K J[1], Deep Chakraborty[2], Gokul Thanigaivasan[3], Santu Ghosh[4], Gurusamy Thangavel[5], Krishnendu Mukhopadhyay[6], Kalpana Balakrishnan[7], Sankar Sambandam[7]

[1] Sri Ramachandra Institute of Higher Education and Research, Department of Environmental Health Engineering, Chennai, India; [2] Sri Ramachandra Institute of Higher Education and Research, Department of Environmental Health Engineering, Chennai, India; [3] Sri Ramachandra Institute of Higher Education and Research, Department of Environmental Health Engineering, Chennai, India; [4] St John's Medical College, Departement of Biostatistics, Bangalore, India; [5] Sri Ramachandra Institute of Higher Education and Research, Department of Environmental Health Engineering, Chennai, India; [6] Sri Ramachandra Institute of Higher Education and Research, Department of Environmental Health Engineering, Chennai, India; [7] Sri Ramachandra Institute of Higher Education and Research, Department of Environmental Health Engineering, Chennai, India; [8] Sri Ramachandra Institute of Higher Education and Research, Department of Environmental Health Engineering, Chennai, India

Background: Air pollution was identified as the second leading risk factor for mortality in 2021 by the Global Burden of Disease study. Its impact is unavoidable in urban cities because of various sources of pollution. Nature, specifically, the presence of green spaces, has been identified by several studies to alleviate the air pollution levels. Therefore, we hypothesize that exploring the influence of green spaces on the relationship between air pollution and mortality is of significant public health interest, particularly in urban settings like Chennai, Tamil Nadu, India.

Objective: This study aimed to estimate the association between air pollution and all-cause mortality, and to determine the impact of green spaces on this association.

Material and Methods: We obtained air pollution data of PM10, PM2.5, NO2, SO2, and O3 from the Central and Tamil Nadu Pollution Control Boards (CPCB and TNPCB). Greenness exposure data were assessed using Normalised Difference Vegetation Index (NDVI) derived from Moderate Resolution Imaging Spectroradiometer (MODIS). Daily all-cause mortality data were obtained from the Greater Chennai Corporation (GCC). Using the Generalised Poisson Regression model, we have observed the impact of green space and the association of air pollution on all-cause mortality.

Results: We found 1.74% (95% CI: 0.35 – 3.13) increase in mortality per IQR increase of same day exposure to $PM_{2.5}$ and 1.58% (95% CI: 0.18– 2.98) increase in mortality per IQR increase of same day exposure to O_3. We have also observed that for one unit of increase in green space, there will be a 0.494% decrease in mortality rate.

Conclusion: Green spaces play a protective role in reducing the adverse effects of air pollution on mortality in urban Chennai. Strengthening urban greening initiatives may help mitigate pollution-related health risks.

EP02-43 | Residential lead service lines and infant blood lead levels

Joanna Balza[1], April Z. Dawson[1], David Nelson[1], Caitlin Kaeppler[2], Rachel Cusatis[1], Kathryn E. Flynn[1]

[1] Medical College of Wisconsin, Milwaukee, USA; [2] Children's Wisconsin, Milwaukee, USA

Introduction
Lead exposure has severe health impacts on children, including developmental delay, decreased intelligence quotient, and behavioral and learning problems. Water is one way children can be exposed, often through leaching of lead into the water supply through antiquated interior plumbing or lead service lines (LSL). Few studies have assessed the association between residing in a home with a LSL and infant blood lead level (BLL).
Methods
Data from the Wisconsin Department of Health Services was used to assess BLL of children ≤ 12 months of age in Milwaukee, WI. Data from the city of Milwaukee Department of Water Works was used to assess if there was a LSL serving the child's primary residence. Logistic regression for two binary outcomes, 1) BLL ≥ or <3.5μg/dL and 2) BLL ≥ or <5μg/dL was used to assess whether there is an association between BLL and LSL. Regression models were adjusted for age of child, sex of child, race/ethnicity, Medicaid status, poverty by zip code, season lead testing was performed, and age of the primary residence.
Results
After adjusting for all covariates, results showed that having a primary residence supplied by a LSL was associated with increased risk of BLL ≥3.5 μg/Dl (OR 1.60, 95% CI 1.04-2.46, p 0.031) and BLL ≥ 5μg/dL (OR 2.26, 95% CI 1.23-4.16, p 0.008) for children ≤ 12 months. Age of house was an independent correlate of BLL at both cut points.
Conclusion
Residing in a residence supplied by lead service lines was associated with increased odds of elevated blood lead levels in infants. Until complete removal of lead service lines -and other potential sources of water contamination- is achieved, interventions such as systematic educational efforts or distribution of water filters may be called for to mitigate the risk of lead exposure.

EP02-44 | Understanding the experiences of refugee resettlement workers related to lead hazards in residences of refugee families

Joanna Balza[1], Rachel Cusatis[1], Caitlin Kaeppler[2], Aprill Z. Dawson[1], David Nelson[1], Kathryn E. Flynn[1]

[1] Medical College of Wisconsin, Milwaukee, USA; [2] Children's Wisconsin, Milwaukee, USA

Background:
Lead poisoning can cause serious health issues, particularly developmental and behavioral problems in children. Refugee children may face increased risk of exposure to lead both before and after resettling in the United States.
Objectives:
To explore the factors that may increase the risk of lead exposure among refugee children following resettlement in the U.S., from the perspective of those involved in the resettlement process.
Methods:
Qualitative interviews were conducted with resettlement workers and other key stakeholders involved in refugee

resettlement. Thematic analysis was used to identify common barriers safe housing and challenges related to lead exposure post-resettlement.

Results:

Participants highlighted several risk factors for lead exposure, including inadequate housing conditions, widespread misperceptions about refugee populations, and a general lack of awareness about lead hazards. These barriers often limited refugee families' access to lead-safe housing.

Conclusions:

Improving refugee housing safety may require educational interventions for both families and landlords, expanded housing options, and enforcement of minimum safety standards in rental properties. These measures could reduce lead exposure risk and improve health outcomes for refugee children.

EP02-45 | Wildfire-derived $PM_{2.5}$ exposures and lung cancer risk in a US-based prospective cohort

Richard V. Remigio[1], Jared A. Fisher[1], Rosana Aguilera[2], Maria E. Kamenetsky[1], Neal Freedman[3], Jesse E. Bell[4,6], Tarik Benmarhnia[2], Rena R. Jones[1]

[1] National Cancer Institute, Occupational and Environmental Epidemiology Branch, Rockville, USA; [2] University of California San Diego, Scripps Institution of Oceanography, La Jolla, USA; [3] National Cancer Institute, Tobacco Research Branch, Rockville, USA; [4] Department of Environmental, Agricultural, and Occupational Health, University of Nebraska Medical Center, Omaha, USA; [5] University of Nebraska-Lincoln, School of Natural Resources, Lincoln, USA; [6] University of Nebraska-Lincoln, Daugherty Water for Food Global Institute, Lincoln, USA

Objective: Wildfire (WF)-derived particulate matter ($PM_{2.5}$) from wildfire smoke plumes is a mixture of modified biomass combustion by-products, including known and suspected carcinogens. We investigated the association between long-term WF exposure and lung cancer incidence in a large US-based prospective cohort.

Materials and Methods: We evaluated associations among participants in states exposed to WF-PM2.5 within the prospective NIH-AARP Diet and Health Study cohort, including 185,452 older adults residing in California, Florida, Louisiana, Pennsylvania, North Carolina, and New Jersey, enrolled in 1995-1996. We assessed exposures to WF-$PM_{2.5}$ from 2005 to 2018 at the ZIP code level using ensemble-based machine learning algorithms. We considered three exposure metrics and categorized them into quartiles: 1) three-year rolling WF-PM2.5 concentrations, 1) cumulative days of non-zero WF-$PM_{2.5}$ exposure, 3) and cumulative smoke-wave days. Referent groups included unexposed (Q0) and lowest exposures (Q1). We used discrete-time hazard models to estimate risk ratios (RR) and 95% confidence intervals (CI) for incident lung cancer, adjusting for total ambient $PM_{2.5}$, cigarette-smoking history, residence state, age, sex, and race-ethnicity. We also conducted analyses stratified by state.

Results: A total of 9,649 lung cancer cases were diagnosed throughout 2018. Participants were exposed to a median WF-$PM_{2.5}$ concentration of 0.24 μg/m^3 (interquartile range [IQR]=0.22), median of 25 (IQR=15) cumulative days of non-zero WF-$PM_{2.5}$ exposure days, and 2 (IQR=3) smoke-wave days. Overall, lung cancer risk was not positively associated with any exposure metric. Among New Jersey residents (N=1,487 cases), we observed increased risk at the highest exposure quartiles for WF-$PM_{2.5}$ concentrations (RR_{Q4vsQ1}=1.80, CI=1.50-2.16) and cumulative smoke wave days (RR_{Q4vsQ0}=2.40, CI=1.22-4.71). Associations within other states were inconsistent.

Conclusions: Our initial findings suggest that elevated WF-derived air pollution may be a lung cancer risk factor.

EP02-46 | Mapping actionable risk factors to address heat-related health disparities using a mixed methods matched case-control study

Stefanie Ebelt[1], Morgan Lane[1], Ayesha Saeed[1], Ivanna de Anda[1], Christine Ekenga[1], Ebony Coleman[1], Elizabeth Iledare[2], Adrienne Van Curen[3], Gretchen Bell[4], Saria Hassan[5], Noah Scovronick[1], Howard Chang[1]

[1] Emory University, Rollins School of Public Health, Atlanta, USA; [2] Grady Memorial Hospital, Atlanta, USA; [3] Emory University Hospital, Atlanta, USA; [4] Emory University Hospital Midtown, Atlanta, USA; [5] Emory University, School of Medicine, Atlanta, USA

Objective. A growing literature demonstrates impacts of heat on morbidity, including emergency department (ED) visits. However, gaps in understanding pathways leading to emergency healthcare utilization for heat-related illness (HRI) remain. Through Emory University's Climate and Health Actionable Research and Translation Center, we are examining risk factors for HRI ED visits within Atlanta's Emory and Grady healthcare systems.

Methods. We developed a survey and interview to assess demographic, physiological, social determinants of health, and knowledge, attitudes, and practice around factors hypothesized to impact susceptibility and exposure to heat. During summer 2024 and 2025, we are enrolling ED patients presenting with HRI and matched controls visiting the healthcare system for non-HRI reasons. Quantitative and qualitative approaches are employed to assess risk factors for HRI ED visits and characterize exposure pathway narratives on awareness, mitigation practices and constraints.

Results. During May-September 2024, we identified 90 ED patients with HRI. Twenty-five cases [average age=48 (range: 22-86) years; 44% female], and 19 matched controls were enrolled. While similarities exist among cases and controls, such as self-described general health and housing status, differences also emerge. For example, 20% of cases vs. 84% of controls recalled hearing about heat warnings in the previous summer. Among home characteristics, 76% of cases vs. 100% of controls had central air conditioning. Cases had more difficulty paying bills than controls. Among those working for pay in the previous 12 months, 53% of cases vs. 6% of controls worked partly or completely outdoors. Qualitative analyses are identifying themes (e.g., symptom-onset conditions, ED visit initiation, social and dietary determinants, and mitigating efforts) that are being used to translate and support the survey results.

Conclusions. Our study design allows engagement with patients and ascertainment of information unavailable in electronic medical records. Results are solidifying understanding of risk factors for emergency healthcare utilization for HRI.

EP02-47 | Understanding Skin and Air Exposure: A Paired Silicone Wristband Study

Steven G. O'Connell[1], Kelsie Younce[1], Kim Anderson[2], Marc Epstein[1]

[1] MyExposome, Inc., Corvallis, USA; [2] Oregon State University, EMT, Corvallis, USA

Objective: Silicone wristbands (SWBs) have emerged as promising personal environmental monitors due to their ease of use and broad chemical capture capabilities. However, the interpretation of SWB data remains challenging due to their non-selective absorption and potential for multiple routes of exposure being captured (atmospheric, dermal, or direct contact). This study aimed to quantify differences in chemical exposure and develop models to better distinguish exposure routes (contact versus atmospheric).
Material and Methods: In a paired-sample study, over 190 participants across the United States simultaneously wore silicone wristbands directly against the skin and inside metal housings around their necks for at least 8 hours per day over three days. Wristbands were extracted with ethyl acetate and analyzed quantitatively using GC-MS for over 1500 chemicals, including polycyclic aromatic hydrocarbons, flame retardants, pesticides, phthalates, fragrances, and other environmental contaminants. T-tests assessed differences in total detections and concentrations between configurations. Multivariate modeling (multinomial regression, random forest, and decision trees) incorporated physicochemical properties (boiling point, flash point, biodegradation half-life) to distinguish exposure sources.
Results: Wristbands worn directly against the skin showed significantly higher chemical detections and total concentrations after adjusting for lower housing uptake (Welch's t-tests, $p < 0.01$). Multivariate models leveraged physicochemical properties to classify exposure pathways, demonstrating the feasibility of predicting chemical source and route based on configuration-related quantitative differences.
Conclusion: These findings reinforce that SWBs directly capture multiple exposure routes, emphasizing the importance in exposure data interpretation for this technique. Utilizing physicochemical property-informed models improves differentiation between contact and atmospheric exposures, enhancing personalized exposure assessments. This work advances the practicality and accuracy of SWBs for broad environmental health applications.
Acknowledgments: Supported by SBIR funding from NIEHS under NIH #R44ES033586.

EP02-48 | Exploring freshwater cyanobacterial bloom exposure and Alzheimer’s incidence in the United States

Emma Lancaster[1,2], Jiyoung Lee[1,3], Jianyong Wu[1]

[1] The Ohio State University, Division of Environmental Health Sciences, College of Public Health, Columbus, USA; [2] The Ohio State University, Infectious Diseases Institute, Columbus, USA; [3] The Ohio State University, Department of Food Science and Technology, Columbus, USA

Objective

Freshwater harmful cyanobacterial blooms (HCBs) are increasing nationally, raising significant health risks from exposure to toxic metabolites (cyanotoxins). Exposure can illicit acute (gastrointestinal, respiratory, dermal) and chronic illnesses (liver cancer). Growing evidence suggests that cyanotoxins (Microcystin, β-N-methylamino-L-alanine) may also induce chronic neurodegenerative effects, including Alzheimer’s disease (AD), a leading cause of

death among seniors. Despite limited epidemiological studies, examining the complex relationship between chronic HCB exposure and AD in the U.S. is critically needed.

Material and Methods

Annual AD incidence rates were retrieved via the Centers for Medicare & Medicaid Services' claims data from 2017 to 2020. Cyanobacterial bloom extent and frequency data was acquired from the USEPA EnviroAtlas project, including > 2,000 freshwater lakes in the contiguous U.S. Data was aggregated at the ZIP-code level and hotspot analyses were used to identify and map regions in the U.S. with elevated AD incidence and HCB exposure risk. Multiple exposure metrics were used to evaluate whether populations with a high prevalence of AD are closer to freshwater lakes with a higher risk of HAB exposure.

Results

The spatial distribution of harmful cyanobacterial bloom risk and Alzheimer's incidence vary substantively in the U.S. Some states (New York, Florida, California) show evidence of significant global and local spatial clustering, with positive association between AD incidence and various HCB exposure metrics. For example, we found a significant increase of AD incidence rate in bloom area compared to non-bloom in Florida (64.45% increase, $p<0.05$). Hotspots within specific regions indicate potential target areas for future preventative measures.

Conclusion

We provide evidence that exposure to cyanotoxins from freshwater harmful cyanobacterial blooms may be a population-level risk factor for Alzheimer's disease. As HCBs distribution and AD prevalence are projected to rise in the U.S., this research will enhance our understanding of environmental influences on chronic health outcomes.

EP02-51 | Effect of Educational Intervention on older adults' Knowledge and Behaviors on Air pollution: A community-based pilot study

Myung-Sook Park[1], Hyuk Bang[1], Dong-Wook Lee[2], Hwan-Cheol Kim[2], Yun-Chul Hong[1]

[1] Seoul National University of Medical Research Center, Institute of Environmental Medicine, Seoul, South Korea; [2] Inha University School of Medicine, Department of Occupational and Environmental Medicine, Incheon, South Korea

Objective

Air pollution is a significant global issue that affects human health, with elderly being particularly vulnerable. This interventional study aimed to evaluate the effectiveness of a community- based environmental education program on improving knowledge and behavior related to particulate matter (PM) among older adults.

Material and Methods

This study was conducted from October to December 2024 at four senior community centers in Seoul, Korea. Two centers were assigned to the intervention group and two to the control group. Data was collected through pre- and post-tests. We used a quantitative questionnaire with three sections-demographics, knowledge, and behavior.

Only the intervention group received four education sessions on the health effects of PM, methods for checking air quality forecasts, and recommended actions on days with high PM levels. Video education was also provided twice a week during lunchtime. The education was delivered through group sessions and a digital platform using standardized materials.

Results

Our study found that older adults in the intervention group showed significant improvements in both their knowledge and behavioral changes related to PM. Compared to the control group, the intervention group showed a significantly increased understanding of PM, its health effects, and response and prevention behaviors related to PM. This enhanced knowledge directly led to behavioral changes, with significant improvements observed in preventive measures such as mask-wearing, indoor ventilation, and the use of air purifiers.

Conclusions

Our finding suggests that community-based education is effective in improving participants' knowledge and behavioral changes regarding PM. Improving health literacy and supporting tailored educational programs are essential for enhancing older adults' overall well-begin and fostering a healthier living environment amidst ongoing air pollution challenges.

EP02-52 | Association of heat exposure with workplace accidents in Germany

Katherine Ogurtsova, Clara Bracke, Barbara Hoffmann, Silvester Siegmann

Heinrich Heine University Düsseldorf, Institute of Occupational, Social and Environmental Medicine, Centre for Health and Society, Medical Faculty and University Hospital Düsseldorf, Düsseldorf, Germany

Objectives: Heat can significantly affect workplace safety and impose substantial economic burdens. We investigated the association between heat exposure and occupational accidents across several industrial workplaces in Germany.

Materials and Methods: We analysed retrospective workplace accident data collected by a company with four operating divisions, primary specialising in metal forging, in Germany 2018-2025. Daily mean (Tmean) and maximum (Tmax) air temperature was obtained from the nearest weather station operated by the German Weather Service. Heat exposure was defined as: (1) the number of consecutive days with Tmax at or above a given threshold (25-40°C), and (2) the Excessive Heat Factor (EHF), calculated as the product of the Excess Heat Acclimatisation Index, which compares current temperatures to the recent 30-day average, and the Excess Heat Absolute Index, which compares temperatures to long-term historical norms using the 95th-percentile of Tmean during the warm season over the previous five years. We conducted a case-crossover analysis with time-stratified referent selection to examine the association between heat exposure and accidents using conditional logistic regression adjusted to public and school holidays. We restricted the referents to the same day of the week, month and year as the index day.

Results: A total of 1,021 accidents were recorded over the 76-month study period. As the temperature threshold increased above 27°C, the odds of workplace accidents also rose. Each additional day with a Tmax≥32°C was significantly associated with a 39%-increase in the risk of an accident (OR = 1.39, 95%CI:1.08-1.78). Periods with excess heat (EHF>0) were associated with an 89%-increase in accident risk (OR = 1.89, 95%CI:1.03-3.48).

Conclusion: Heat exposure is associated with an increased risk of occupational accidents in the industrial sector in a region with temperate climate. Given the potential economic impact, further research is needed—covering larger populations and various groups.

Acknowledgements: no external funding.

EP02-53 | The Health Behaviors of Women in Health Access Resource Behavior Observation and Use Research (HARBOUR I) Project in the Eastern Caribbean

Steve D. Whittaker[1,2], Gabrielle Myers[2], Malika Benjamin[2], Kaylee Burroughs[2], Fiona Zhang[2]

[1] Clarence Fitzroy Bryant College, Arts, Science and General Studies, Basseterre, St. Kitts & Nevis; [2] Science Technology Research and Work Study (STRaWS), Research, Basseterre, St. Kitts & Nevis

The World Health Organization (WHO) frames the well-being of women as dependent on various factors. This study aimed to investigate the dynamics of health among female residents in the Eastern Caribbean nation of St. Kitts-Nevis (SKN). Surveys were administered to adult SKN residents between 2018 and 2020. Of the 222 female respondents, black (99%), single (82%), students (38%) and under 30 years old (62%), approximately 1 in 10 were unemployed. The most reported health issues were gastrointestinal problems (38%), depression (29%), ear, throat, and eye Infections (26%), exhaustion (26%), dermatological (21%), and musculoskeletal chronic pain (16%). About 1 in 2 women perceived inadequacy in the local healthcare system. Obstacles to seeking mental health counseling included concerns over cost (20%), privacy (53%), quality (41%) and efficacy (32%). In terms of perceived impediments to reproductive and sexual health, while 68% indicated having no issues procuring condoms, barriers were identified for oral or injectable birth control products (legal, 36%), and medical abortion (legal, 41%; safety, 53%). The probability of having (had) at least one child increased for women 30 and over (OR 2.36; 95%CI: 1.13 - 4.95) controlling for marital status, those in the medical field (OR 2.83; 95%CI: 1.20 - 7.21) and those living close to a ravine or ghaut (OR 2.54 95%CI: 1.49 - 4.40). Conversely being a student reduced the likelihood of motherhood (OR 0.32; 95%CI: 0.18 - 0.57). Mothers were more likely concerned about the cost of psychological counseling (OR 2.20; 95%CI: 1.13 - 4.39) and the cleanliness of doctor’s offices (OR 1.80; 95%CI: 1.04 - 3.18). Having more than one child complicates probabilities. Results suggest women in the Caribbean subregion face unique challenges in navigating local healthcare landscapes. Additional research, including incentivization, and interventions will be needed to better understand and improve outcomes for this vulnerable subpopulation.

EP02-54 | Nitrogen Dioxide and BMI are Associated with the Gut Microbiome in Young Adults Living in Denver, Colorado

Daisy Fry Brumit[1], William B. Patterson[2], Frederick Lurmann[3], Colleen Reid[4], Anthony Fodor[1], Tanya L. Alderete[5]

[1] University of North Carolina at Charlotte, Department Bioinformatics and Genomics, Charlotte, USA; [2] University of Colorado Anschutz, Aurora, USA; [3] Sonoma Technologies, Inc., Petaluma, USA; [4] University of Colorado Boulder, Department of Geography, Boulder, USA; [5] Johns Hopkins University, Department of Environmental Health and Engineering, Baltimore, USA

The human gut microbiome is a key player in chronic disease development and may be influenced by environmental factors such as air pollution. Previously, our research in Southern California linked ozone and traffic-related pollutants to decreased gut microbial diversity and changes in the relative abundance of certain taxa. This study aims to expand on these findings with a new cohort following young adults around Denver, Colorado by testing associations between human gut microbiota and ambient nitrogen dioxide (NO_2), ozone (O_3), and particulate matter (PM). Other features

such as BMI, dietary intake, and demographic factors are also considered. 78 young adults aged 18-25 years provided dietary recall, demographic, and residential data as well as stool samples for this study. Individual exposures to ambient air pollutants were modeled for up to one year prior to stool sample collection, and stool samples were processed with whole genome sequencing methods. We found significant associations between NO_2 and Shannon diversity, evenness, Bray-Curtis distance, and whole gut composition by way of PERMANOVA ($p < 0.05$). BMI was also significantly associated with whole gut composition as captured by PERMANOVA ($p < 0.05$). These findings are consistent with previous literature that links ambient air pollution to gut microbial composition and diversity, though these preliminary findings did not yield significant results for any one particular microbial taxon. This highlights a need for further, higher powered studies.

EP02-55 | Characterizing Variability in Personal Chemical Exposure to Improve Exposomics

Lisa Bramer[2], Holly Dixon[1], Alison Clark[1], Diana Rohlman[1], Katrina Waters[1,2], Kim Anderson[1]

[1] Oregon State University, Environmental and Molecular Toxicology, Corvallis, USA; [2] Pacific Northwest National Laboratory, Richland, USA

Understanding how chemical exposure varies within and between people over time is a critical component of characterizing the exposome—the totality of exposures over the lifespan. However, variability remains an understudied aspect of exposomic research. Our objective was to investigate trends in variability for chemical exposure data between and within participants. 35 people in Eugene, Oregon and 46 people in St. Helens, Oregon, wore silicone wristbands over multiple seasons, including a span of heavy wildfire smoke in Eugene. Each participant wore between four and 14 wristbands. We analyzed 586 wristbands for 94 (Eugene) and 58 (St. Helens) chemicals. While analytic tests differed between the studies, the same 43 polycyclic aromatic hydrocarbons (PAHs) were measured in both. We also evaluated three environmental variables for their impact on chemical concentrations. We fit generalized mixed effects models to each chemical, and used variance partitioning to understand and quantify sources of variability across environmental factors and inter- and intra-individual variables. We observed PAHs that were consistent within people across different days. For a subset of these PAHs, results did not agree well between studies, indicating the importance of measuring chemical data at different time points across studies. Environmental variables were not sufficient for explaining data variability for most chemicals. Only 21% and 30% of the modeled chemicals for Eugene and St. Helens, respectively, had a combined environmental variable R2 at or above 0.1. Yet, environmental factors still revealed valuable information; we observed higher combined R2 values for styrene, o-xylene, ethylbenzene, and phenanthrene in the Eugene detection model, which came from a combination of fine particulate matter and smoke density information. Funding was provided by the National Institute of Environmental Health Sciences with grant numbers T32ES007060, P42ES016465, and P30ES030287. This work has been submitted for publication to the Journal of Exposure Science and Environmental Epidemiology.

EP02-56 | Extraction and Analysis For Contaminants and Metabolomics Using Untargeted Mass Spectrometry Methods

Rachel Buckley, Christina Koleman, Ill Yang, Lauren Aleksunes, Brian Buckley

Rutgers University, Environmental Occupational Health Sciences Institute, Piscataway, USA

Metabolites are the most likely candidates for biomarker discovery of exposure because they are often a metabolite of the environmental contaminant. Screening for multiple/ unknown metabolites requires A *posteriori* identification of unknown mass spectrometry (MS) features, observed after liquid chromatography separation (LC-MS) and dissociation (MS/MS) scanning, followed by a data library search, commonly known as untargeted metabolomics. The results from a novel untargeted metabolomics LC-MS assay utilizing a high-resolution Q-Exactive orbitrap instrument in conjunction with a reverse phased C18 separation, are presented from a human embryonic kidney HEK293 cell culture exposed to media with or without 10% pooled human serum. The objective was to determine which xenobiotics can pass into the cell, as a potential predictor of biological uptake. The small molecule fraction was isolated from cell lysate utilizing solvent extraction, size exclusion filtration, and evaporation and reconstitution. The improved extraction method yielded a 2-fold increase in the recovery for a set of internal standards, compared to a previous method. New data filtering protocols and a split acquisition method were used to collect more data-dependent MS/MS spectra from analytes, increasing the number of features identified from ~150 to ~1000. The more efficient solvent reconstitution step created an approximate 10-fold increase in signal intensity and increased the number of identified features from ~1000 to ~1500 by reducing analyte loss (spike recovery 70-105%). Accurate mass and MS/MS fingerprint spectra from open-source libraries were used in tandem as a confirmatory criterion for feature identification. The high-resolution mass spectrum, greater signal, improved noise filtering, and iterative optimization to the analytical MS method, yielded an overall 10-fold increase in the number of molecules reliably identified by untargeted analysis. This method will be applied to exposure assessment studies for biomarker identification, with greater levels of confidence.
Supported by UC2HD113039 and P30ES005022.

EP02-57 | Prenatal Exposure to Essential Metal Mixtures and Leukocyte Telomere Length at Birth in the PROGRESS Cohort

Kshitij Sachdev[1], Shoshannah Eggers[1], María J. Rosa[2], Robert B. Wallace[1], Robert O. Wright[2], Martha M. Tellez-Rojo[3], Vishal Midya[2]

[1] The University of Iowa, College of Public Health, Department of Epidemiology, Iowa City, USA; [2] The Icahn School of Medicine at Mount Sinai, Department of Environmental Medicine and Climate Science, New York, USA; [3] National Institute of Public Health, Center for Nutrition and Health Research, Cuernavaca, Mexico

Objective:
Telomere length (TL) is a biomarker of biological aging that may be shaped by early-life environmental exposures. Prenatal exposure to toxic metals is associated with shorter TL in newborns, but effects of essential metals, and their combinations, remain poorly understood. This analysis investigates associations between a mixture of prenatal essential metal exposure and newborn TL.
Material and Methods:

We used data from the Programming Research in Obesity, Growth, Environment, and Social Stressors (PROGRESS) cohort in Mexico City. Exposures were maternal blood concentrations of essential metals, including cobalt (Co), chromium (Cr), copper (Cu), manganese (Mn), nickel (Ni), and zinc (Zn), measured at the second and third trimesters of pregnancy. Leukocyte TL was measured in cord blood. Covariates included child sex, white blood cell composition, maternal age, BMI, and socioeconomic status during pregnancy. We used covariate-adjusted weighted quantile sum (WQS) regression and repeated holdout WQS to estimate associations.
Results:
Analysis included 366 mother-child pairs. Average maternal age at delivery was 28.5 years, with 44% female infants. Higher prenatal exposure to essential metal mixtures was significantly associated with shorter telomere length at birth. In adjusted WQS, each one-decile increase in essential metal exposure was linked to 32.5% lower odds of telomere length in the top decile (OR=0.675; 95%CI: 0.472, 0.965; p=0.0312). Cu, Co, and Cr exposures from both trimesters were the most heavily weighted components in the mixture. Repeated holdout WQS showed a similar direction of effect, with borderline statistical significance (p=0.0787).
Conclusion:
Higher prenatal exposure to an essential metal mixture was associated with shorter leukocyte TL at birth. Consistent direction and borderline significance across repeated holdout models strengthen evidence of an inverse association. Findings suggest that essential metals, when considered together, may contribute to accelerated biological aging in early-life.
This project is supported by NIEHS R21ES035318 (Eggers & Midya).

EP02-58 | Human Chemical Exposure Through Flood Waters Following Extreme Precipitation Events: A Systematic Review

Karenna Roest, Jordan Kuiper

The George Washington University, Department of Environmental and Occupational Health, Washington, USA

Background: Flooding events often occur after precipitation events. With increased effects of climate change, precipitation events are becoming more frequent and more severe, thus leading to increased exposure of humans to flood waters. Flood waters can move toxic products, such as chemicals through a community thereby resulting in human exposure.
Objectives: We looked to conduct a systematic review to consider the effect of flood water exposure due to extreme precipitation events on chemical exposures experienced by humans.
Methods: We utilized the Navigation Guide framework to conduct a systematic review with a search spanning three databases in order to determine the quality and strength of the evidence of the current literature on floodwaters and chemical exposures.
Results: We found 15 relevant papers to include in the review, and four of which were deemed to be of high quality and high strength. The papers were mixed in their findings. 13 papers found some increased chemical exposures with flood water exposure, while six papers found no significant change among some chemicals, and one paper found an improvement in chemical concentration following flooding.*Discussion:* Future research is integral to be able to measure soil chemical concentrations prior and following floodwaters moving through an area following an extreme precipitation event. As the effects of climate change are continued to be felt, it is imperative that we are able to understand the types of chemical exposures humans are being exposed to through flood waters so that we can better prepare and mitigate future events.

EP02-59 | Heavy Metal(loid)s Exposure and Dietary Patterns among Women of Reproductive Age by Race/Ethnicity and Nativity, US NHANES 2011-2020

Kiran Y. Kui[1], Perrie O'Tierney-Ginn[3], MyDzung Chu[2,3]

[1] Tufts Clinical and Translational Science Institute, Boston, USA; [2] Tufts Medical Center, Institute for Clinical Research and Health Policy Studies, Boston, USA; [3] Tufts Medical Center, Woman, Mother + Baby Research Institute, Boston, USA

Background: Exposure to heavy metal(loids) such as inorganic arsenic (iAs), cadmium (Cd), and manganese (Mn) is linked to adverse maternal and fetal health outcomes. Persistent disparities exist across racial, ethnic, and nativity groups, but the underlying sources are not well understood.
Objective: We examined how major sources of heavy metal(loid) exposure—diet, smoking, and drinking water—are associated with biomarker concentrations of urinary iAs, blood Cd, and blood Mn among US women of reproductive age. We also assessed differences by race/ethnicity and acculturation (nativity and English language proficiency) using NHANES 2011–2020 data.
Methods: Major sources were identified from prior studies. Urinary iAs was calculated as the sum of As**III**, AsV, and their metabolites (MMA, DMA). Multivariable linear regression models estimated least squares geometric means (LSGM) for each biomarker by source, racial/ethnic group, and acculturation status, adjusting for covariates.
Results: Asian women had the highest LSGMs for urinary iAs (0.12 µg/g-creatinine, 95% CI: 0.11–0.15), blood Cd (0.39 µg/L, 95% CI: 0.37–0.42), and blood Mn (13.17 µg/L, 95% CI: 12.74–13.62) compared to other groups. Dietary factors—including white rice, brown rice, shellfish, and fish (p=0.001)—and public drinking water (p<0.001) were significant predictors of urinary iAs. Current smoking predicted higher blood Cd (p<0.001). Associations varied by race/ethnicity and acculturation (p<0.05). No measured sources were significantly associated with blood Mn. Among Asian women, an acculturation gradient emerged: foreign-born, non-English speakers had the highest biomarker levels and greater rice and shellfish intake, followed by foreign-born English speakers, then US-born women.**Conclusions:** Racial, ethnic, and nativity differences in heavy metal(loid) exposures are partly driven by diet, drinking water, and smoking. Addressing these sources may help reduce disparities in maternal health outcomes.

EP02-60 | Effect of reduced anthropogenic emissions on urban heat island dynamics in Prague

Tugba Dogan[1], Ales Urban[1,2], Martin Hanel[1]

[1] Czech University of Life Sciences Prague, Faculty of Environmental Sciences, Prague, Czech Republic; [2] Czech Academy of Sciences, Institute of Atmospheric Physics, Prague, Czech Republic

Objective
The urban heat island (UHI) effect is driven by land cover changes, reduced vegetation, anthropogenic heat emissions, dense urban morphology, and the thermal properties of construction materials, all of which alter energy balances and elevate temperatures in urban areas. UHI adversely affects thermal comfort, human health, and sustainability. The reduction in anthropogenic activity during the COVID-19 lockdown in 2020 provided a unique opportunity to examine how decreased anthropogenic emissions influence UHI dynamics. Previous studies suggest lockdown conditions led to declines in atmospheric UHI (AUHI) and surface UHI (SUHI), but the extent of these reductions remains uncertain due to confounding meteorological variables and urban-rural dynamics.
Material and Methods
This study investigates how the lockdown affected AUHI and SUHI in Prague by controlling for weather variability and urban-rural contrasts. To ensure robust comparisons, we selected meteorologically similar days across the lockdown period (March–April 2020) and reference period (March–April 2017–2019). SUHI intensity was assessed using MODIS satellite-derived land surface temperature, and AUHI variations were analyzed using near-surface air temperature records from Prague's meteorological stations.
Results
Our results reveal that urban SUHI intensity declined by 15% (0.1 °C) and AUHI in the city center decreased by 0.7 °C compared to the reference period. Satellite-based observations indicate a 12% reduction in aerosol optical depth and a 29% decline in nitrogen dioxide levels, supporting the hypothesis that reduced anthropogenic emissions contributed to weakened UHI effects. The highest decrease in mean SUHI was observed on Prague's outskirts, where rural land cover dominates, highlighting the importance of accounting for urban-rural dynamics when linking SUHI changes to anthropogenic heat flux.
Conclusion
Our findings advance the understanding of UHI dynamics by demonstrating the effects of reduced anthropogenic activities during the lockdown, providing policymakers with a comprehensive perspective on urban-rural microclimate interactions and their role in shaping the SUHI phenomenon.

EP02-61 | Effects of Repetitive Education-Based Intervention on Indoor Particulate Matter Exposure Among Older Adults: A Pilot Study in Smart Senior Centers in Seoul, Korea

Hyuk Bang[1], Myung-Sook Park[1], Dong-Wook Lee[2], Hwan-Cheol Kim[2], Yun-Chul Hong[1]

[1] Seoul National University of Medical Research Center, Institute of Environmental Medicine, Seoul, South Korea; [2] Inha University School of Medicine, Department of Occupational and Environmental Medicine, Incheon, South Korea

Background

Older adults are particularly vulnerable to the health effects of particulate matter (PM), and spend most of their time indoors. Improving indoor air quality (IAQ) through community-based behavioral interventions may be an effective strategy to reduce PM exposure in this population.

Methods

This pilot intervention study was conducted from October to December 2024 in four smart senior centers in Yangcheon-gu, Seoul. Two centers were assigned to the intervention group and two to the control group. The intervention group received four structured educational sessions covering the health effects of PM, how to check air quality forecasts, and recommended actions during high pollution days. Sessions were delivered through group education and digital platforms using standardized materials.

Indoor PM10 levels were continuously measured using IoT-based sensors installed in participants' homes for 10 weeks. One-minute data were aggregated into daily averages. Changes in PM10 levels were compared over time between the intervention and control groups.

Results

Analysis of PM10 measurements showed that indoor concentrations in the intervention group gradually decreased as the number of education sessions accumulated, while no such trend was observed in the control group. In the intervention group, the average PM10 concentration was 17.83 $\mu g/m^3$ before the intervention and 17.09 $\mu g/m^3$ during the intervention period, showing a reduction of 0.74 $\mu g/m^3$ (4.2%).

To assess this trend, we performed a simple linear regression using daily PM10 values from October 29 to December 16, which confirmed a statistically significant downward trend over time (slope = –0.159 $\mu g/m^3$/day, $p = 0.002$).

Conclusions

This pilot study suggests that repetitive education-based behavioral interventions implemented through smart senior centers may contribute to improvements in indoor air quality among older adults. Further studies with larger sample sizes and more rigorous statistical analyses are needed to validate these findings and assess long-term impacts.

EP02-62 | Applying Exposure Science and Personalized Interventions at the Household Level: VOC Reduction and Symptom Improvement in Chemically Susceptible Individuals

Elizabeth M.S. O'Nan[1], Aditi Mahesh[1], Roger Perales[1], Rodolfo Rincón[1], Carl Grimes[2], Claudia S. Miller[1]

[1] University of Texas Health Science Center at San Antonio, Department of Family and Community Medicine, San Antonio, USA; [2] Hayward Score, Carmel, USA

Objective:
Chemical intolerance (CI) is adaptive and usually initiated by one large or long term chemical exposure that evolves into multisystem symptoms triggered by low-level exposures to common indoor pollutants. CI is underrecognized and frequently misdiagnosed due to a lack of clinician training in environmental health. Patients often experience years of suffering and costly diagnostics without relief. Indoor exposures, including fragranced consumer products, combustion byproducts, and volatile organic compounds (VOCs), are significant but often overlooked contributors. Expanding on a published Environmental House Call (EHC) intervention, which demonstrated symptom and VOC reductions, we also describe a proposed remote EHC educational module designed to increase future accessibility.

Materials and Methods:
Thirty-seven individuals meeting validated Quick Environmental Exposure and Sensitivity Inventory (QEESI) criteria for CI participated in a structured five-visit EHC protocol over 12 months, as previously described by Perales et al. (2022). A physician-led team conducted walkthrough assessments, indoor air sampling (VOC analysis via GC-MS), and individualized education. Symptom changes were measured using the QEESI Symptom Star. Personalized action plans were developed and implemented with participant guidance.

Results:
The majority of participants demonstrated clinically significant symptom improvement, with average QEESI symptom severity scores decreasing from 61.7 to 28.3. Indoor VOC levels, especially from fragrances, solvents, and personal care products, decreased by up to 96%. To illustrate this approach in practice, we present a representative case highlighting key intervention steps and resulting outcomes, as outlined in Rincón et al. (2024).

Conclusion:
EHCs provide a feasible, effective strategy for reducing harmful indoor exposures and improving symptoms in chemically susceptible individuals. Broader adoption of this model could improve quality of life and reduce healthcare burden in this underserved population.

Funding and Acknowledgements: This work was supported by the TILT Research Foundation. We thank the participating individuals and TILT research team for their contributions.

EP02-64 | Temporal Trends and Regional Disparities in PFAS Detection and Exposure Through Infant Feeding: A Global Systematic Review

Mojirade M. Adetoba, Hans-Joachim Lehmler

University of Iowa, Occupational and Environmental Health, Iowa City, USA

Per- and polyfluoroalkyl substances (PFAS) in breastmilk and infant formula pose potential health risks to infants, leading to neurological disorders and weakened immune response during crucial developmental windows. Understanding temporal trends and regional variations in PFAS detection in infant diet is essential for addressing PFAS exposure across their lifespan. This systematic review examines temporal trends, regional variations, and methodological differences in PFAS detection in breast milk and infant formula worldwide.
A systematic search in PubMed, Scopus, and Embase was conducted for studies that reported PFAS levels in breast milk or infant formula. Data extraction included analytical techniques, detection limits, PFAS profiles, and PFAS levels. Infant intake estimates were calculated from the mean PFAS values reported in the included studies. Temporal trends and geographical variations in PFAS detection and concentrations were assessed using Kruskal-Wallis and ANOVA.
Thirty-eight studies were included, mostly from Asia and Europe compared to North America, with Africa and South America underrepresented. Most studies used LC-MS/MS methods to measure PFAS, with limits of detection as low as 0.0001 ng/mL. PFAS detection in breastmilk increased from 2020 - 2024 ($p = 0.0138$), shifting from legacy PFOS and PFOA to short-chain and emerging PFAS. PFOS and PFOA remained persistent (detected in > 90% studies), with no decrease over time ($p > 0.05$). Norway showed the highest PFAS concentration (603.1 ng/mL) while North America had the lowest. Daily PFAS intake was highest in breastfed infants, followed by formula-fed infants (up to 80,413 ng/kg/day). Mixed feeding infants showed moderate intake, with older infants (7–12 months) having lower intake due to diversified diets.
This review identified regional differences in the detection of PFAS in infants' diets and dietary exposure levels. These findings highlight the need for targeted actions and capacity building in underrepresented areas to reduce PFAS exposure in regions with elevated PFAS levels.

EP02-65 | Differential accumulation and toxicological effects of mercury and arsenic in african catfish: implication for food safety

Mojirade M. Adetoba[1], Oladapo T. Okareh[2]

[1] University of Iowa, Occupational and Environmental Health, Iowa City, USA; [2] University of Ibadan, Environmental health science, Ibadan, Nigeria

Mercury and arsenic pollution from industrial and agricultural activities poses serious health risks, especially to pregnant women and children in high-exposure areas. Chronic exposure to these metals through the consumption of contaminated fish, a vital protein source in low-income regions, has been linked to neurodevelopmental disorders, systemic toxicity, and cancers. This study aimed to determine the rates and patterns of mercury and arsenic accumulation in African catfish (*C. gariepinus*) and to evaluate the histopathological effects of these metals on fish organs.

Juvenile African catfish (n = 24, 100 – 150g) were exposed in controlled conditions to mercury chloride (0.2 mg/L) and arsenic (20 μg/L) over four weeks. Weekly mercury and arsenic concentrations were determined using titrimetric methods and atomic absorption spectrophotometry. Liver, muscle, and gill tissues were analyzed histologically to assess organ-specific toxic effects. Statistical analysis was conducted using ANOVA.

Mercury accumulates progressively over the four weeks (Week 4 > Week 3 > Week 2 > Week 1, $p<0.0001$) while arsenic decreases over time (Week 1 > Week 2 > Week 3 > Week 4, $p<0.0001$), reflecting distinct toxicokinetics. In mercury-exposed fish, lamellar fusion, epithelial lifting, and aneurysmal changes were observed in the gills, while the liver exhibited hepatocellular vacuolation, necrosis, and pigment buildup. Muscle tissues displayed fiber fragmentation and localized degeneration. Arsenic-exposed fish showed similar damage, including vacuolation in hepatocytes, hypertrophy and fusion of gill lamellae, and disrupted muscle fiber structure.

This study found differences in the accumulation of mercury and arsenic in African catfish, highlighting their morphological impacts and raising concerns about food safety and health risks for consumers. It highlights the need for continuous monitoring of heavy metal contamination in water and the establishment of safe consumption guidelines and water quality standards, particularly in low-income areas that rely on fish and lack strong regulations.

EP02-66 | Heat Exposure During Pregnancy and Its Association with Growth Outcomes in Bangladeshi Newborns

Ash Arian[1], Saira Tasmin[1], Lin Tong[1], Golam Sarwar[2], Alaya B. Jharna[2], Syed E. Haque[2], Habibul Ahsan[1,3]

[1] The University of Chicago, Institute for Population and Precision Health, Chicago, USA; [2] UChicago Research Bangladesh, Dhaka, Bangladesh; [3] The University of Chicago, Department of Public Health Sciences, Chicago, USA

Objective:
While maternal heat exposure during pregnancy has been associated with adverse birth outcomes in high-income settings, little is known about these effects in low- and middle-income countries (LMICs) such as Bangladesh, where climate extremes have intensified. This study investigates the association between gestational heat exposure and early-life growth outcomes in a rural Bangladeshi cohort.

Methods:
We used data from the Health Effects of Arsenic Longitudinal Study (HEALS), which followed pregnant women in Araihazar, Bangladesh between 2012 and 2023. Infant birth outcomes (weight, length, head circumference) and anthropometric measurements up to 31 days postpartum were recorded. Daily meteorological data were obtained from the Bangladesh Meteorological Department. Heat exposure was defined using year-specific percentiles of average daily temperature, with ≥95th percentile as “heat days” and ≥99th percentile as “extreme heat days.” Multivariable linear and logistic regression models were used to evaluate associations between trimester- and gestation-specific heat exposure and infant growth metrics, including continuous outcomes and binary indicators based on World Health Organization (WHO) growth standards.

Results:
Maternal exposure to higher ambient temperatures and extreme heat during the first trimester and across total gestation was significantly associated with reduced infant length and head circumference. Logistic models showed elevated odds of low height-for-age (LHA) and low birth weight (LBW) in relation to extreme heat days and higher average gestational temperature. Associations remained significant after adjusting for maternal sociodemographic and household factors.

Conclusion:
Gestational exposure to high ambient temperatures and extreme heat is associated with impaired early infant growth in a rural LMIC setting. As global temperatures rise, these findings underscore the need for public health strategies to mitigate the effects of heat stress on maternal and child health in vulnerable settings.

Funding:
This work was supported by the NIEHS Grant R24ES028532. We thank the participants and HEALS study team for their contributions.

EP02-67 | Elucidating the Effects of Household Air Pollution Exposure on Adverse Development in rural Bangladeshi communities: a Genome-Wide Association Study

Ash Arian[1], Saira Tasmin[1], Lin Tong[1], Golam Sarwar[2], Habibul Ahsan[1,3]

[1] The University of Chicago, Institute for Population and Precision Health, Chicago, USA; [2] UChicago Research Bangladesh, Dhaka, Bangladesh; [3] The University of Chicago, Department of Public Health Sciences, Chicago, USA

Objective

To investigate whether exposure to household air pollution (HAP), measured by PM2.5 and PM10 concentrations, is associated with adverse developmental outcomes, such as stunting, low birth weight, and reduced head circumference, and to explore whether individual genetic variation modifies susceptibility to these outcomes in a rural Bangladeshi population.

Materials and Methods

We analyzed maternal and infant phenotypes, including maternal height and weight, and infant height, weight, and head circumference. Exposure to HAP was quantified through direct measurement of PM2.5 and PM10 levels over a 7-day period. Multivariable linear and logistic regression models assessed associations between pollution levels and developmental outcomes: low height-for-age (LHA), low birth weight (LBW), and low head circumference (LHC), adjusting for sociodemographic and household factors. Genome-wide association studies (GWAS) were conducted using over 8 million single-nucleotide polymorphisms (SNPs) to identify potential genetic modifiers of pollution-related risk. Pollution levels were included as covariates in both linear and logistic GWAS models.

Results

Regression analyses found no statistically significant associations between HAP and developmental phenotypes. However, GWAS results identified several suggestive SNPs whose signals were magnified when PM2.5 and PM10 were included as covariates. Notable SNPs were located in or near genes involved in critical biological processes: TMEM131L (early development signaling), DGKB (energy metabolism), CHN2 (neurodevelopment), and EPN1 (cellular transport and nutrient uptake). No SNPs reached genome-wide significance, but findings indicate potential gene-environment interactions.

Conclusion

Although traditional models found no strong phenotypic associations, genomic analyses revealed variants potentially linked to heightened susceptibility to pollution-related developmental outcomes. These results highlight the need for further research into the biological mechanisms underlying environmental exposure and underscore the value of integrating genomic data into environmental health studies in low- and middle-income settings.

Funding and Acknowledgements

This work was supported by the NIEHS (R24ES028532). We thank the participants and HEALS field staff in Bangladesh.

EP02-68 | Effect of Coal Production on Chronic Respiratory Disease Mortality Rates in Kentucky Between 1980 and 2016

Aayush N. Gandhi, N/A

Dublin High School, Dublin, USA

Objective: Current public health literature has strongly associated coal mining with negative health and environmental impacts, ranging from chronic conditions to air pollution. However, existing research hasn't explored the effects of coal mining on specific health indicators over an extended period (>30 years) at the county level, data otherwise necessary to support policymakers when designing effective energy policies to transition away from coal.
Method: Our paper aimed to bridge this gap by creating a Panel Vector Autoregression model and subsequent impulse response functions in R to model the impact of increases in coal production on chronic respiratory mortality rates in Kentucky. This model was conducted on yearly county-level data from 1980 to 2016 which included information on total coal production, chronic respiratory mortality rates, total population, per-capita personal income, and high-school education rates. The latter three were included as exogenous variables.
Results: Our findings suggest that increased coal production may exacerbate chronic respiratory illnesses in the short term (within 2 years), after which respiratory mortality rates plateau, possibly due to other factors (e.g., genetics or inadequate health insurance).**Conclusion:** These findings underscore the need for policymakers to expedite the transition away from coal and refrain from permitting the construction of new mines or facilities that would increase coal production. Furthermore, our model's strong impulse response functions lay the foundation for modeling the short and long-term health effects of changes in coal production, thereby assisting legislators and researchers in designing similar models for energy policy proposals.

EP02-69 | INFLAMMATORY LIPID AND OXIDATIVE STRESS PATHWAYS IN PRE-DIAGNOSIS SERUM SAMPLES PROVIDE SIGNATURES FOR LETHAL PROSTATE CANCER

Nickilou Y. Krigbaum[1], Xin Hu[2], Piera M. Cirillo[1], Sami Teeny[3], Boris Minasenko[2], Young-Mi Go[3], Dean P. Jones[3], Barbara A. Cohn[1]

[1] Public Health Institute, Child Health and Development Studies, Berkeley, USA; [2] Emory University, Environmental Health, Atlanta, USA; [3] Emory University, School of Medicine, Atlanta, USA

Background: Prostate cancer is the second most common cancer and the second leading cause of cancer death among men in the United States. Black men are twice as likely to die from prostate cancer than other men.
Methods: We conducted a prospective Metabolome-Wide Association Study (MWAS) of lethal prostate cancer in men the CHDS cohort. We identified 111 cases of lethal prostate cancer in Black men (median age of 74 at death) and 258 cases in non-Black men (median age of 77 at death) via linkage to the California Cancer Registry. Controls were matched on birthyear (220 Black controls; 515 non-Black controls). We conducted High-Resolution Metabolomics (HRM) on pre-diagnostic, archived serum samples collected a median of 34.5 years before diagnosis.

Untargeted analyses was performed using Rodin (https://github.com/BM-Boris/rodin/tree/main). Feature intensities were log2 transformed and ANOVA was used to test for differential expression at raw $p < 0.05$. Features with $p < 0.05$ were used for pathway enrichment analysis with Rodin, using HILIC - ESI data.
Results: Metabolism of arachidonic acid, prostaglandin, and leukotriene were enriched for the non- Black men (adjusted p- value < 0.001). Metabolite putatively annotated as leukotriene A4 was increased in lethal prostate cases. In the Black men arachidonic acid was also enriched (p-value< .05) and metabolites putatively annotated as glutamate and leukotriene A4 were increased in lethal prostate cases. Tyrosine (p-value < .05), phosphatidylinositol phosphate (p-value < .05) and galactose (p-value <.05) metabolisms were associated with lethal prostate cancer only in Black men. These unique pathways suggest oxidative stress pathway signals unique to Black men years prior to cancer diagnosis.
Conclusion: Our results show there are overlapping (arachidonic acid pathway) and unique metabolic pathways tyrosine, phosphatidylinositol phosphate, and galactose) associated with lethal prostate cancer in two groups. These metabolic signatures can serve as indicators for prognosis and risk of lethality.

EP02-70 | Tracing the Ultrafine: Emission Profiles from Industrial Wood Combustion Units and Electrostatic Precipitators

Patricia Henegan, Richard Peltier

University of Massachusetts-Amherst, Environmental Health Sciences, Amherst, USA

Objective To investigate size-resolved particulate matter emissions from pellet and chip wood fuel appliances, and evaluate the effectiveness of emissions control strategies including electrostatic precipitators (ESP).
Materials & Methods Using a mobile laboratory equipped with a range of research instrumentation, we collected concurrent data with forty polytetrafluoroethylene filters from three industrial sized boilers. Diluted (~30x) aerosol samples were collected from these instruments and analyzed in situ for particle size distribution, as well as brown and black carbon fractions. Filters were returned to the laboratory for additional chemical composition analyses. Of the three sites, two had ESPs and one (Site 1) allowed us to sample with the ESP on/off.
Results During the winter of 2024-2025, we collected data from the stacks of 4 industrial boilers that use woodchip fuels in the northeastern United States. Average particulate matter with a diameter of 2.5 micrometers or less ($PM_{2.5}$) concentrations for each site varied greatly, which is hypothesized to be due to the differences in boiler models, wood fuel source, heating demand, or whether they have an operational ESP. Site 1 without the ESP recorded the highest average $PM_{2.5}$ concentration (2164 ± 442 μg/m³).The lowest average $PM_{2.5}$ concentration was observed at Site 1 when the ESP was operational (213 ± 231 μg/m³). Site 1 showed stronger for ultrafine particles with a diameter <100 ($t = 6.40$, 95% confidence interval 7025-13331[dN/log(dp)], $p<0.001$). Overall, the use of an ESP appears to increase the number of ultrafine particles (those particles with a diameter <100nm), as indicated by a statistically higher particulate count [dN/log(dp)] ($t=2.29$, 95% confidence interval 13942-11723 [dN/log(dp)], $p=0.023$).
Conclusion These findings spotlight the complex relationship between emission control technologies and $PM_{2.5}$ characteristics in industrial woodchip boilers. While ESPs effectively reduce overall emissions, they may inadvertently change the composition of ultrafine particles.

EP02-71 | Childhood Lymphoma subtypes and their Association with Unconventional Natural Gas Development (UNGD)

Evelyn Talbott[1], Jian Min Yuan[2], Vincent Arena[3], Fan Wu[4]

[1] University of Pittsburgh, Dept of Epidemiology, Pittsburgh, USA; [2] University of Pittsburgh, Dept of Epidemiology, Pittsburgh, USA; [3] University of Pittsburgh, Dept of Biostatistics, Pittsburgh, USA; [4] University of Pittsburgh, Dept of Occupational and Environmental Hlth, Pittsburgh, USA

(Background and Aim) The rapid growth of unconventional natural gas development (UNGD) raised concerns of potential exposures to hazardous chemicals. Few studies have examined the risk of childhood lymphoma subtypes with exposure to UNGD. We performed a secondary analysis for UNGD with risk of lymphoma subtypes.
Methods: A population-based case–control study of 498 children with leukemia, lymphoma, central nervous system neoplasms, and malignant bone tumors and 498 matched healthy children by date of birth, race, sex and county of residence in Southwestern Pennsylvania (PA) was conducted. Of the 105 lymphoma cases, 52 were Hodgkin's, 22 Non-Hodgkin's, and 5 Burkitt's lymphoma, 25 miscellaneous lymphoreticular neoplasm and 5 unspecified lymphoma. All cases were identified through PA Cancer Registry and control children from PA birth records. The matrix of exposure to UNGD was derived from official databases of PA Departments of Environmental Protection and Conservation and Natural Resources. Analysis was performed using conditional (on 105 lymphoma case-control pairs) and unconditional (105 lymphoma cases vs 498 controls) logistic regression with adjustment for multiple covariates.
Results: Exposure to UNGD within the 5-mile radius was associated with a statistically significant increased odds ratio of 2.53 for all lymphoma, 3.28 for Hodgkin's, and 4.78 for non-Hodgkin's lymphoma (all P's < 0.05) derived from unconditional logistic models. The odds ratios increased with increasing level of cumulative exposure to UNGD or with decreasing distance from residence to the closest UNGD site (all P's for trend < 0.05). No significant odds ratio was found for the other 3 subtypes of lymphoma.
Conclusion: Although immunodeficiency due to hereditary condition or exposure to human immunodeficiency virus (HIV) and Epstein-Barr Virus (EBV) are identified risk factors for both Hodgkin's and non-Hodgkin's lymphoma, the present analysis suggests that exposure to UNGD with mixed chemical compounds may promote the development of these lymphoma subtypes.

EP02-72 | Study results of PM1 and PM10 particulate matter concentrations in Ulaanbaatar city's household environments using low-cost sensors

Ulziimaa Daramragchaa[1], Davaalkham Dambadarjaa[1]

[1] Mongolian National University of Medical Sciences, School of Public Health, Ulaanbaatar, Mongolia; [2] Mongolian National University of Medical Sciences, Mongolian National University of Medical Sciences, Ulaanbaatar, Mongolia

Objective: To assess PM1 and PM10 concentrations in 120 selected households in Ulaanbaatar.
Materials and Methods: Indoor PM1 and PM10 concentrations were measured using PurpleAir real-time sensors in randomly selected Ulaanbaatar households between October 2023 and January 2024. Supplementary data on factors affecting the PM2.5 concentration were collected via questionnaires. Each measurement was taken in 10-minute intervals, yielding 51,309 data for analysis.
Results: PM1 concentrations were measured at 55.5 ± 53.2 μg/m³ in gers, 54.9 ± 46.7 μg/m³ in houses, and 31.6 ± 40.1 μg/m³ in apartments ($p<0.001$) and measuring PM10 concentrations were 110.6 ± 108.6 μg/m³ in gers, 110.6 ± 96.7 μg/m³ in houses, and 62.2 ± 83.0 μg/m³ in apartments ($p<0.001$) When considering the concentration of PM1, PM10 by heating type, PM1 was 55.3 ± 50.1 μg/m³ and PM10 was 110.6 ± 103.0 μg/m³ in households with stoves and furnaces, and PM1 was 31.6 ± 40.1 μg/m³ and PM10 was 62.2 ± 83.0 μg/m³ in households connected to the central heating system ($p<0.001$). Regarding the months of measurement, the highest concentration was observed in December 2023, at 77.1±94.1 μg/m³. The highest concentrations for both PM_1 and PM_{10} were recorded in January 2024, at PM_1: 64.8 ± 55.1 μg/m³, PM_{10}: 131.4 ± 116.0 μg/m³.
Conclusion:
1. Indoor PM10 concentrations in residential environments in Ulaanbaatar city were within the MNS4585:2016 Air Quality Standard, however, it was exceeded the WHO air quality guidelines, indicating an excessive risk of increasing morbidity and mortality among the population.
2.Indoor PM1 and PM10 concentrations in residential environments in Ulaanbaatar varies depending on location, type of housing, type of heating, and month of measurement.
Keywords: Particulate matter, direct monitoring device

EP02-73 | Lung function of adults living in highly polluted indoor areas in Ulaanbaatar, Mongolia

Davaalkham Dambadarjaa[1], Naidansusuren Tsendeekhuu[1], Serod Khuyagaa[1], Maralmaa Enkhbat[1], Anujin Munkhtulga[1], Bolor Munkhbayarlakh[1], Myagmarchuluun Sainnyambuu[1], Shatar Shaarii[1], Ulziimaa Daramragchaa[1], Damdindorj Boldbaatar[1], Robert Tighe[2], Junfeng Zhang[2]

[1] Mongolian National University of Medical Sciences, Ulaanbaatar, Mongolia; [2] Duke University, Durham, USA

Objective

Ulaanbaatar, the capital of Mongolia, is one of the most polluted cities in the world, especially during its long, harsh winters. Our objective was to study the impact of indoor air quality on lung function among adults living in the largest cities.

Material and Methods

A cross-sectional study was conducted enrolling non-smoking adults aged 18-60 years living in Darkhan and Ulaanbaatar, where raw and processed coals are used. Lung functions were assessed using a calibrated spirometer (Spirometry 100, Korea) measuring values of Forced Vital Capacity (FVC) and Forced Expiratory Volume in 1 second (FEV_1). PurpleAir sensors were used to measure indoor PM2.5 concentrations in each household. Data analyses were performed using STATA 18.0 software.

Results

A total of 241 subjects were enrolled during the winter of 2024, out of whom 46.5% were males. There was a significant decline in the FEV_1/FVC ratio with age, from 84.05 in participants under 29 to 78.74 in those over 50 ($p<0.05$). Normal weight individuals (82.23) had higher lung function compared to obese participants (80.83, $p<0.05$). Participants living in Darkhan city (81.99) had slightly better FEV_1/FVC ratios than those in Ulaanbaatar (81.03). Lower PM2.5 levels (<25 µg/m³) were associated with higher lung function (82.09), while exposure to higher PM2.5 reduced the mean (81.37). The highest values were observed among participants living in apartments (82.82) compared with those living in coal used houses (80.84) and gers (81.57). Participants exposed to PM2.5 levels greater than 25 µg/m³ had significantly lower values for FEV1/FVC predicted ($p<0.05$), PEF ($p=0.02$), FEF25–75 ($p=0.01$), VC ($p=0.02$), and MVV ($p=0.02$), Additionally, differences in FEV1/FVC predicted and MVV were also significant by type of residence ($p=0.03$).

Conclusion

Adults living in a coal used dwellings with higher PM2.5 exposure showed lower lung function that is associated with both large and small airway impairment.

EP02-74 | Comparison of ambient air pollution in cities with different types of coal use in Mongolia

Davaalkham Dambadarjaa, Serod Khuyagaa, Batzorig Bayartsogt, Ulziimaa Daramragchaa, Jargalsaikhan Galsuren, Damdindorj Boldbaatar

Mongolian National University of Medical Sciences, Ulaanbaatar, Mongolia

Objective: Since 2019, the capital city of Ulaanbaatar has introduced processed coal to reduce air pollution. This study aimed to examine the ambient air pollutants in cities that use processed and raw coal.
Materials and methods: A retrospective study was conducted using air pollutants (PM2.5, PM10, SO2, NO2, CO, O3) and climate data for 2013-2022 in Ulaanbaatar and Darkhan cities. Data from all environmental monitoring stations in these cities were included. Statistical analyses, including time series analysis, were performed using the R statistical software.
Results: The concentrations of ambient PM2.5 and PM10 have decreased significantly due to the use of processed coal in Ulaanbaatar city. There is an increase in PM2.5 and PM10 in Darkhan city, which uses raw coal. Comparing the average NO2 concentrations in Ulaanbaatar and Darkhan, they have started to decrease compared to the years before the use of improved fuel. However, no significant difference was observed in NO2 concentrations in Darkhan. The SO2 concentration in Ulaanbaatar has increased significantly since winter 2019, while in Darkhan it has decreased over the years. The daily average concentration of CO in Ulaanbaatar is 1.37 mg/m³, which is 0.14 times higher than the 2016 standard and 0.34 times higher than the WHO air quality guidelines (AQG:2021), with a slight increase.
Conclusion: The levels of PM2.5, PM10, and NO2 decreased significantly with the use of processed coal compared to raw coal in Ulaanbaatar city, while SO2 and CO levels increased. In Darkhan city, the concentrations of PM2.5 and PM10 are rising, whereas NO2 and SO2 levels are falling compared to many previous years.

EP02-75 | Determinants of plasma poly- and perfluoroalkyl substances during pregnancy: the Japan Environment and Children’s Study

Yonghang Lai[1], Shoji F. Nakayama[1], Yukiko Nishihama[2,1], Tomohiko Isobe[1]

[1] National Institute for Environmental Studies, Health and Environmental Risk Division, Tsukuba, Japan; [2] University of Tsukuba, Institute of Medicine, Tsukuba, Japan

Per- and polyfluoroalkyl substances (PFAS), associated with adverse health outcomes, are widely detected in human populations. This study investigated determinants of PFAS concentrations in plasma samples from pregnant women enrolled in the Japan Environment and Children’s Study between 2011 and 2014. Our models systematically evaluated over 200 determinants, identifying critical interactions and providing new insights into the effects of physical conditions, behaviors, and dietary intake on PFAS body burdens during pregnancy.
Multiple machine learning approaches were applied, with the XGBoost model exhibiting the highest predictive performance for seven PFAS quantified in over 50% of participants. Predictive accuracy was assessed using coefficient of determination (R^2) and root mean square error (*RMSE*).
The XGBoost model achieved R^2 values ranging from 0.34 (*RMSE*: 0.39 ng/mL, perfluorononanoic acid [PFNA]) to 0.85 (*RMSE*: 0.19 ng/mL, perfluoroundecanoic acid [PFUnDA]). Feature analysis, including Accumulated Local

Effects (ALE) and SHapley Additive exPlanations (SHAP) indicated regional variation as the most significant determinant, highlighting regional dietary patterns, particularly fish consumption, as potential exposure sources. Parity negatively correlated with most PFAS concentrations, whereas maternal age showed a positive association. A positive interaction between parity and age suggested that age-related PFAS accumulation could surpass parity-related reductions in PFAS levels. Additionally, numerous weaker interactions among determinants collectively influenced maternal plasma PFAS concentrations.

EP02-77 | A qualitative study on community engagement and implementation challenges in soil remediation for lead contamination in Mirzapur, Bangladesh

Mahbubur Rahman[1,2], Syeda Nurunnahar[2], Sanjida Adiba[2], Jesmin Sultana[2], Mitali Das[3], Gordon K. Binkhorst[4], Stephen P. Luby[5], Rubhana Raqib[6], Maria Kippler[7], Peter J. Winch[8], Syed Moshfiqur Rahman[1,2], This study was funded by Pure Earth and icddr,b. icddr,b is grateful to the Governments of Bangladesh and Canada for providing core/unrestricted support

[1] Uppsala University, Global Health and Migration Unit, Department of Women's and Children Health, Uppsala, Sweden; [2] International Centre for Diarrhoeal Disease Research Bangladesh (icddr,b), Environmental Health and WASH, Health System and Population Studies Division, Dhaka, Bangladesh; [3] Pure Earth, Bangladesh Country Office, Dhaka, Bangladesh; [4] Pure Earth, New York, New York, USA; [5] Stanford University, Woods Institute for the Environment, Stanford, USA; [6] International Center for Diarrhoeal Disease Research, Bangladesh (icddr,b), Nutrition Research Division, Dhaka, Bangladesh; [7] Karolinska Institutet, Institute of Environmental Medicine, Stockholm, Sweden; [8] John's Hopkins University, Johns Hopkins Bloomberg School of Public Health, Baltimore, USA

Objective :
Two abandoned informal lead-acid battery recycling operations in Mirzapur, Bangladesh, were identified to cause high levels of lead pollution in soil and impacts to the local population, especially children. This study evaluated community responses to a risk mitigation project implemented to mitigate community exposure by scraping and burying lead contaminated soil, house cleaning and community awareness efforts.
Methodology:
The study employed a qualitative design to explore the implementation process and community responses. The intervention activities were conducted from December 2021 through May 2022, and qualitative data collection was done in September 2023. In-depth interviews (n=30), key informant interviews (n=5) and two gender-segregated focus group discussions with 14 residents and stakeholders were conducted. Data were thematically analyzed using an inductive approach.
Results:
Following remediation, participants reported improved agricultural production and better livestock and children’s health. Their views of risk were greatly influenced by the economic concerns, giving livestock and agricultural effects precedence over the dangers of lead poisoning. Participants expressed concerns about the safety of soil excavation near homes and the burial of contaminated soil. The key informant acknowledged existing awareness efforts, also suggested that additional awareness activities and better dissemination of interventions could improve community participation further.
Conclusion:

The success of soil remediation and other risk mitigation activities depends not only on the technical process but also on effective community engagement. Participants noted positive impacts from the interventions but emphasized the need for ongoing awareness raising activities to improve understanding and acceptance. The findings highlight that for long term success and sustainability, technical solutions must be embedded within socially responsive and participatory frameworks. Based on the findings, relevant government and private sectors should take the lead in scaling up such soil remediation interventions in a similar high-risk context, ensuring that community engagement remains central to implementation.

EP02-78 | Exposure factor questionnaire development for personal care product usage among children

Mai Takagi, Tomohiko Isobe, Miyuki Iwai-Shimada, Yayoi Kobayashi, Shoji F. Nakayama

National Institute for Environmental Studies, The Health and Environmental Risk Division, Tsukuba, Japan

Personal care products (PCPs) contain functional additives such as parabens, phthalates and benzophenones, whose health effects have been of concern. The information about the exposure factor for a PCP usage, especially among children, is scarce. For a large-scale survey, it is important to develop a validated questionnaire that can be easily deployed. To develop an online version of the questionnaire, a study was conducted among a group of Japanese children at the age of 0–12 years and their caregivers living in Japan (103 pairs in winter and 110 pairs in summer). The study was approved by the internal review board and written consents were provided by all the participants. The caregivers were asked to record types of PCPs and frequency of their use by their children during a period of consecutive 7 days in February and September 2024. The quantity of each product used during the period was calculated by subtracting the weight of the product on the last day of the period from that on the first day. Median number of products used by children was 8.5 (95th percentile: 14.7) in winter and 8.0 (95th percentile: 14.0) in summer, resulting in no seasonal differences. The usage pattern of body/face cream, sunscreens, insect repellents and lip cream varied among seasons. The median mass of applied products among users (winter and summer) were 16 and 12 g/week for skincare products (sum of body cream, sunscreen and lip cream), 39 and 18 g/week for bath products (shampoo, conditioner and body/face soap) and 17 and 25 g/week for sanitary products (toothpaste, hand soap, sanitizer and insect repellents). The online questionnaire has been developed and validated by these data. Harmonization of the questionnaire among different countries and regions are anticipated.

EP02-79 | Estimating the causal effect of air pollution exposures on cardiovascular disease incidence using modified treatment policies

Salome Kakhaia[1,2], Lützen Portengen[2], Daniel Oberski[3], Marc Chadeau-Hyam[4], Roel Vermeulen[1,2]

[1] University Medical Center Utrecht, Department of Global Public Health and Bioethics, Julius Center for Health Sciences and Primary Care, Utrecht, Netherlands; [2] Utrecht University, Institute for Risk Assessment Sciences, Utrecht, Netherlands; [3] Utrecht University, Department of Methodology and Statistics, Faculty of Social and Behavioural Sciences, Utrecht, Netherlands; [4] Imperial College London, MRC Centre for Environment and Health & Department of Epidemiology and Biostatistics, Faculty of Medicine, School of Public Health, London, UK

One of the key goals of exposome research is to estimate the causal effects of modifiable environmental exposures on health outcomes to inform disease prevention policies. Most ambient air pollution is anthropogenic, affecting everyone and significantly contributing to the global disease burden, of which cardiovascular diseases (CVD) account for a major share.

We estimated the marginal effects of time-varying, continuous $PM_{2.5}$, and NO_2 exposures, as well as their joint (total) effect on the cumulative risk of cardiovascular diseases over 10 years of follow-up in the UK Biobank population. We defined air pollution interventions as modified treatment policies, modeling reduction in pollutant levels as a function of individually observed exposure levels and covariates. We estimated effects and accounted for (non-informative) censoring using a sequentially doubly-robust estimator, combining multiple statistical and machine learning methods in an ensemble learner to model exposure and outcome mechanisms. We also estimated the health impact of observed reductions in $PM_{2.5}$. We selected the covariate adjustment set for confounding based on expert knowledge, combined with results from a causal structure discovery approach.

The pollutants had a positive, additive joint effect on the outcome, which was less than the sum of the individual effects. A 25% reduction in both $PM_{2.5}$ and NO_2 levels was estimated to result in a 1.62% decrease in the cumulative incidence of CVD over the follow-up period. This amounts to 5,531 CVD-related events and deaths avoided in the population of 463,991 over 10 years. The observed reduction in $PM_{2.5}$ was estimated to have resulted in a 0.27% lower incidence of CVD (922 avoided events) compared to a counterfactual scenario in which exposures remained at baseline levels.

This framework of efficient non-parametric causal effect estimation based on flexible interventions can be a valuable tool for evaluating the impact of various environmental exposure interventions and informing policy.

EP02-80 | Impact of xAI Supercomputer Operations on Air Quality in Southwest Memphis, TN

Abu Mohammed Naser Titu, Namuun Batbaatar, Chunrong Jia

University of Memphis, Division of Epidemiology, Biostatistics, and Environmental Health, Please Select, USA

The xAI Supercomputer, located in Southwest Memphis, TN, began operation on September 1, 2024. Powered by 15 gas turbines, the launch raised concerns about potential air quality impacts. This study aimed to assess whether xAI operations significantly deteriorated the air quality in the nearby communities.

The community of most concern was Boxtown, a residential subdivision nearest to xAI (2.7 km). We used EPA's AERSCREEN dispersion model to estimate worst-case short-term and annual concentrations of criteria and annual air pollutants in Boxtown. As the modeling results indicated concerns for $PM_{2.5}$ pollution, daily 1 km-resolution aerosol optical depth (AOD) data collected between Jan 2023 and May 2025 from the MODIS MAIAC Level 2 product (MODIS/061/MCD19A2_GRANULES) were retrieved for Memphis. AOD was calibrated to $PM_{2.5}$ using air quality data measured at local EPA monitoring stations and applied to estimate $PM_{2.5}$ in Boxtown. A sharp regression discontinuity (RD) design centered on September 1, 2024, modeled $PM_{2.5}$ as a function of time, its square, a post-cut indicator, and their interactions, with month fixed effects and robust standard errors.

AERSCREEN estimates showed that all pollutants emitted from xAI turbines, except $PM_{2.5}$, remained below national standards in Boxtown. For $PM_{2.5}$, predicted daily and annual concentrations were 38 and 9.4 µg/m³, exceeding standards (35 and 9 µg/m³), primarily due to baseline levels (36 and 9.2 µg/m³). The estimated pre-cut slopes in $PM_{2.5}$ were –0.02 µg/m³ (95 % CI: –0.07 to 0.03). Immediate $PM_{2.5}$ shifts at the September 1, 2024, cutoff ranged from –3.92 to 2.78 µg/m³ with wide confidence intervals; no statistically significant post-cut changes in trend were observed.

xAI operations would not cause nonattainment of air pollution levels even in the worst-case scenario. The satellite data showed no significant changes in $PM_{2.5}$ levels after xAI launch. Efforts should target controlling $PM_{2.5}$ emissions at the regional level.

EP02-81 | Chronic Stress and Sex Modify Inflammatory Responses to Traffic-Related Air Pollution in rats

Jane E. Clougherty[1], Ayaka Suganuma[2], Keith Bein[3], Sihan Liu[2], Jaime Leon[2], Anthony Wexler[3,5], Chao-Yin Chen[2]

[1] Drexel University Dornsife School of Public Health, Environmental and Occupational Health, Philadelphia, USA; [2] University of California at Davis School of Medicine, Department of Pharmacology, Davis, USA; [3] University of California at Davis, Air Quality Research Center, Davis, USA; [4] University of California at Davis, Department of Mechanical and Aerospace Engineering, Davis, USA; [5] University of California at Davis, Department of Civil and Environmental Engineering, Davis, USA; [6] University of California at Davis, Department of Land, Air and Water Resources, Davis, USA

Background:
Traffic-related air pollution (TRAP) is associated with inflammation and cardiovascular disease, with evidence of greater susceptibility in individuals exposed to chronic stressors. This study aims to investigate pathways for this interaction.

Methods:
Thirteen-week-old Sprague Dawley rats (both sexes) were randomly assigned to no stress/ chronic mild unpredictable stress (CMUS), and to TRAP/ filtered air from Caldecott Tunnel in Oakland, CA. CMUS included twice daily randomized exposures to strobe light, loud noise, crowding, shaker, cage tilting, or no bedding, over three weeks. On weeks 2 and 3, rats were exposed to real-time TRAP. Plasma samples were collected weekly to assess inflammatory markers using Bio-Rad Rat Cytokine 23-Plex Assay. We used heatmaps and principal components analysis to identify response patterns in 23 cytokines from 95 animals, and derived factor scores to examine joint effects of stress, TRAP, and sex.

Results:
Suites of pro- and anti-inflammatory cytokines displayed common responses. One week of CMUS reduced IL-1β and MIP-1α levels only in females. Two weeks of TRAP reduced most cytokines - both pro- and anti-inflammatory cytokines – in males but not females, and not modified by stress. High loadings on Factor 1 (IFN-gamma, IL-1a, IL-2, IL-4, IL-6, IL-10, IL-12, IL-13, IL-17) were predicted by stress, sex, and batch. High loadings on Factor 2 (IL-1b, IL-7, MIP-1a, GM CSF) were predicted by TRAP.

Conclusion:
Preliminary results suggest sex-differing responses to stress and TRAP, and that TRAP evokes both pro- and anti-inflammatory responses. Future analyses will include longer exposures, larger sample, and heart rate variability measures.

EP02-82 | Ambient air PM2.5 pollution in two Mongolian cities with different types of coal

Buyantushig Boldbaatar, Enkhjargal Gombojav, Batzorig Bayartsogt, Davaalkham Dambadarjaa, On behalf of the "Mongolian Center for Environmental and Occupational Health" project funded by the NIH.

Mongolian National University of Medical Sciences, School of Public Health, Ulaanbaatar, Mongolia

Background: Approximately 99% of the global population lives in areas where PM2.5 levels surpass the World Health Organization's (WHO) air quality guideline of 15 μg/m³. In 2018, air pollution was linked to around 4.2 million deaths worldwide. In Mongolia, particularly in urban areas like Ulaanbaatar, air pollution becomes considerably more severe during winter, presenting a major public health issue. Therefore, comparing wintertime outdoor air quality between Ulaanbaatar, the capital, and Darkhan city is essential.

Objective: To evaluate and compare PM2.5 levels in the outdoor air of Ulaanbaatar and Darkhan cities during the winter.

Materials and Methods: The study was conducted from December 10, 2024, to February 19, 2025, in Ulaanbaatar and Darkhan. A total of 60 PurpleAir Classic+ sensors (30 in each city) were deployed, recording PM2.5 concentrations every 2 minutes. Data were analyzed using R software. The 24-hour average PM2.5 concentrations were compared against the Mongolian National Air Quality Standard (MNS4585:2016) and the 2021 WHO air quality guidelines.

Results: The average 24-hour PM2.5 concentration in Ulaanbaatar was 112.3±62.2 μg/m³, significantly higher than in Darkhan, which recorded 79.2±25.6 μg/m³ ($p<0.05$). Monthly averages in Ulaanbaatar were 119.9±67.7 μg/m³ in December, 113.5±60.8 μg/m³ in January, and 95.0±51.9 μg/m³ in February—all statistically significant ($p<0.05$). In contrast, Darkhan showed relatively consistent monthly averages: 79.1±22.2 μg/m³ in December, 78.7±28.6 μg/m³ in January, and 84.6±30.0 μg/m³ in February, with no significant differences ($p>0.05$). During the study period, Ulaanbaatar exceeded the MNS4585:2016 standard (50 μg/m³) on 69.8% of days, while Darkhan had 64.6%. Both cities exceeded the WHO guideline (15 μg/m³) on 93.4% of days.

Conclusion: While Darkhan experienced lower PM2.5 levels than Ulaanbaatar, both cities consistently surpassed the national and WHO air quality limits throughout the heating season.

EP02-83 | Co-methylation networks reveal nonlinear aging dynamics in mice

Jiachen Ai[1], Yihan Fan[2], Yinan Zheng[3], **Feng Gao**[4]

[1] University of California, Los Angeles, Department of Biostatistics, Los Angeles, USA; [2] University of California, Los Angeles, Department of Environmental Health Sciences, Los Angeles, USA; [3] Northwestern University, Department of Preventive Medicine, Chicago, USA; [4] University of California, Los Angeles, Department of Environmental Health Sciences, Los Angeles, USA

Objective
As a dynamic process that takes place through the whole lifespan, aging is closely related to human health and considered one of the most important risk factors of many diseases, such as Alzheimer's disease and cardiovascular diseases. In this study, we aim to explore the nonlinear dynamics of aging across multiple time points through co-methylation networks, which could reveal the temporal changes of interactions/correlations among differentially methylated regions (DMRs).

Material and Methods
We used a publicly available whole genome RRBS DNA methylation dataset of male mice that contains 82 mice with 5 different age groups (i.e., 3-month, 9-month, 15-month, 24-month, and 28 month). Co-methylation networks for each age group were constructed based on correlations between identified aging related DMRs. We then conducted network analysis to investigate the temporal changes of co-methylation networks based on network summary statistics. Gene ontology analysis was used to identify key biological processes from the top gene communities identified from the networks.

Results
Our results revealed the nonlinear changes of co-methylation networks in mice based on the network summary statistics. The temporal changes of correlations among DMRs corresponded to various biological functions at different stages of life, which is consistent with previous results using other omics data. These changes could be due to multiple mechanisms such as compensatory mechanisms. We further confirmed our results in a validation dataset.

Conclusion
Our study leveraged co-methylation networks to reveal the aging dynamics, which could provide valuable information on critical time windows of aging. Future work leveraging data from large population studies will provide more insights into human aging.

EP02-84 | Carcinogenic Industrial Air Pollution and Risk of Kidney Cancer: A Pooled Analysis of 535,000 Americans

Elizabeth Sharp[1,2], Jared Fisher[1], Caroline Pruitt[1], Jonathan Hofmann[1], Miranda Jones[2], Rena Jones[1], Jessica Madrigal[1]

[1] National Cancer Institute, Occupational and Environmental Epidemiology Branch, Division of Cancer Epidemiology and Genetics, Rockville, USA; [2] Johns Hopkins Blomberg School of Public Health, Department of Epidemiology, Baltimore, USA

Objective: Several industrial chemicals and metals are renal carcinogens based on evidence from occupational settings, but few studies have evaluated environmental exposure pathways. Our aim was to evaluate associations with kidney cancer risk for industrial air emissions of multiple *a priori* kidney carcinogens, and other agents classified as known or probable human carcinogens but with limited or no prior explicit links to kidney cancer.

Material and Methods: We estimated airborne exposure to 36 known and probable human carcinogens released from industrial facilities for 535,007 participants in the NIH-AARP Diet and Health Study (enrolled 1995–1996) and the Prostate, Lung, Colorectal, and Ovarian Cancer Screening Trial Cohort (enrolled 1993–2001). Incident kidney cancers (n=5,590) were identified over a median follow-up of 22 years through linkage with cancer registries. For the 8 years prior to enrollment, we estimated inverse distance- and wind direction-weighted average emission indices (AEI) for each chemical and metal within 5km and 10km of the residential address. Using Cox proportional hazards models adjusted for demographics and lifestyle factors, we assessed risk for AEI tertiles compared to non-exposed.

Results: Kidney cancer risk was elevated in the highest tertile of the beryllium AEI at 5km compared to non-exposed (HR_{T3}=1.52, CI: 0.97-2.39; p-trend=0.04) but not at 10km (HR_{T3}=1.08, CI: 0.83-1.40, p-trend=0.44). Risk was associated monotonically with the vinyl chloride AEI at 5km and 10km (5km HR_{T3}=1.40; CI: 1.01–1.96; p-trend=0.03; 10km HR_{T3}=1.36; CI: 1.12–1.65; p-trend=0.0007). Associations with the dichloromethane AEI were positive at both distances (5km HR_{T3}=1.09, CI: 0.99-1.19, p-trend=0.03 and 10km HR_{T3}=1.06, CI: 0.98-1.14, p-trend=0.12). We observed no associations with other chemicals and metals, including trichloroethylene or cadmium.

Conclusion: Our findings suggest that relatively higher levels of environmental emissions of specific chemicals and metals like vinyl chloride and beryllium at levels found in the ambient environment contribute to kidney cancer risk.

EP02-85 | Longitudinal association of volatile organic compound mixtures and hair cortisol

Cameron K. Stopforth[1,2], Daniel W. Riggs[1,3], Pawel Lorkiewicz[1,3], Shweta Srivastava[1,3], Tatiana Krivokhizhina[1,3], Kestutis Bendinskas[4], Rachel J. Keith[1,3], Sanjay Srivastava[1,3], Aruni Bhatnagar[1,3], Clara G. Sears[1,3]

[1] University of Louisville, Christina Lee Brown Envirome Institute, Louisville, USA; [2] University of Louisville, Department of Pharmocology and Toxicology, Louisville, USA; [3] University of Louisville, School of Medicine, Louisville, USA; [4] Stress Bioanalytics, LLC, Oswego, USA

Objective: Mixtures of volatile organic compounds (VOCs) are ubiquitous in the environment and exposure has been linked to cardiometabolic issues, but underlying mechanisms remain unclear. One potential mechanism may involve the hypothalamic-pituitary-adrenal (HPA) axis, which regulates stress responses, immune function, and energy metabolism. Thus, the objective was to determine the association between VOC metabolites and hair cortisol, an integrative marker of HPA axis activity. We hypothesized that higher levels of VOC metabolites would be associated with higher hair cortisol concentrations.

Methods: During three study visits (2019/2021/2022), we collected spot urine and hair samples from 127 adults (ages 26-72; 290 observations; Louisville, KY). Sixteen urinary metabolites from 13 parent VOCs were quantified using LC-MS/MS. Hair cortisol was quantified (pg/mg) using enzyme-linked immunosorbent assays. Using adjusted linear mixed models, we estimated the longitudinal associations between individual VOC metabolite and hair cortisol concentrations. We estimated the difference in hair cortisol per quartile increase in the VOC metabolite mixture using quantile-based g computation.

Results: At baseline, the mean age was 51±13 years. Most participants were women (61%) and reported their race as White (86%). A standard deviation increase in metabolites for styrene (MADA; 8.9% [-3.4, 22.7]), ethylene oxide (2HEMA; 7.7% [-6.9, 24.5]), and N,N-dimethylformamide (MCaMA; 5.8% [-6.6 , 19.9]) were associated with higher hair cortisol, but 95% confidence intervals were wide and included the null. We found opposite trends for metabolites of acrylonitrile (CyEMA; -6.7% [-17.5, 5.6]) and glycidol (23HPMA; -4.3% [-16.7, 9.9]). Furthermore, we did not find strong evidence of a positive association between the VOC metabolite mixture and hair cortisol (ψ = 1.0% [-16.8, 22.7]).

Conclusion: Our results did not show consistent positive or negative associations between VOC metabolites and hair cortisol. Analysis of the joint effect did not indicate potential additive or synergistic effects of the VOC mixture on HPA activity.

EP02-86 | Prenatal exposure to low-level PM2.5 and PM10 is associated with altered newborn anthropometric measures.

Rotem Kabir[1], Tal Michael[1], Ido Solt[2], Itay Kloog[3], Elkana Kohn[4], Ariela Hazan[4], Matitiahu Berkovitch[5], Anna Brik[4], Ori Hochwald[6], Liron Borenstein-Levin[6], Moshe Betser[7], Miki Moskovich[7], Ayelet Livne[8], Rimona Keidar[8], Zeev Weiner[2], Amalia Levy[1,9]

[1] Ben-Gurion University of the Negev, Department of Epidemiology, Biostatistics, and Community Health Sciences, School of Public Health, Faculty of Health Sciences, Beer Sheva, Israel; [2] Rambam Health Care Campus and Technion, Israel Institute of Technology, Department of Obstetrics and Gynecology and Bruce Rappaport Faculty of Medicine, Haifa, Israel; [3] Ben-Gurion University of the Negev, Department of Geography and Environmental Development, Beer Sheva, Israel; [4] Shamir (Assaf Harofeh) Medical Center and Tel-Aviv University, Clinical Pharmacology and Toxicology Unit, Pediatric Division and Sackler School of Medicine, Zerifin, Israel; [5] Shamir (Assaf Harofeh) Medical Center and Tel-Aviv University, Clinical Pharmacology and Toxicology and Unit The Andy Lebach Chair of Clinical Pharmacology and Toxicology, Sackler School of Medicine, Tel Aviv, Israel; [6] Rambam Health Care Campus and Technion, Israel Institute of Technology, Neonatal Intensive Care Unit and Bruce Rappaport Faculty of Medicine, Haifa, Israel; [7] Shamir (Assaf Harofeh) Medical Center, Delivery Rooms and Maternity Ward, Zerifin, Israel; [8] Shamir (Assaf Harofeh) Medical Center, Neonatal Intensive Care Unit, Zerifin, Israel; [9] Ben-Gurion University of the Negev, Environment and Health Epidemiology Research Center, School of Public Health, Faculty of Health Sciences, Beer Sheva, Israel

Objective

Accumulating evidence suggests that prenatal exposure to particulate matter (PM2.5 and PM10) is largely associated with adverse newborn's health outcomes. However, the relevant timing of exposures varies between studies and is yet unclear. Hence, we examined the association of several anthropometric measures with PM2.5 and PM10 exposure throughout weeks of gestation.

Material and Method:

We recruited 1060 mother-infant pairs from two major hospitals in Israel. Data regarding daily PM2.5 and PM10 predictions were estimated on a 1kmX1km grid from satellite-based models, geocoded and overlaid geographically per each mother using ArcGIS. We conducted Linear Mixed-Effects Models to examine the association between birth weight, length, and head circumference and trimestral as well as weekly exposures to PM2.5 and PM10 from the last menstrual period to the day of delivery.

Results:

Prenatal exposure to PM2.5 and PM10 throughout each trimester was significantly associated with decreased birthweight (PM2.5: b-trimester1= -0.021, CI: -0.046 to 0.002; b-trimester2= -0.035, CI: -0.059 to -0.012;b-trimester3= -0.033, CI:-0.057 to -0.0095 respectively; PM10:b-trimester1= -0.007, CI: -0.009 to 0.004; b-trimester2= -0.007, CI: -0.01 to -0.004;b-trimester3= -0.006, CI:-0.009 to -0.003 respectively). While head circumference was negatively associated with exposure to PM2.5 in the first trimester (b= -0.019, CI:-0.035 to -0.0036) birth length was positively associated with exposures during the second trimester (b=0.026, CI:0.006-0.046).

Conclusions:

Our findings suggest that PM2.5 and PM10 are significantly associated with reduction in birth weight. This critical period should be carefully assessed and could be the grounds for future clinical interventions. The trimestral associations of PM 2.5 with decreased head circumference and increased length could shed light on uncovered biological mechanisms that should be further studied.

EP02-87 | Developing Data-driven Wildfire Smoke Detection Using Wavelet-decomposed Surface Observations and Machine Learning

Meng Qi[1], Zhihao Jin[1], Zhuohan Chi[2], Danlu Zhang[2], Ke Xu[1], Alexander Xu[3], Dimple Pruthi[1], Wenhao Wang[1], Liu Yang[1]

[1] Emory University, Gangarosa Department of Environmental Health, Atlanta, USA; [2] Emory University, Department of Biostatistics and Bioinformatics, Atlanta, USA; [3] Johns Creek High School, Johns Creek, USA

Objective: Wildfire smoke poses an increasing threat to the public health in recent years. To assess its health impact, many efforts have estimated smoke contributed air pollutant concentrations. NOAA's Hazard Mapping System (HMS) is often used as a proxy to define smoke days in current studies, but its spatial accuracy is uncertain (e.g., a lofted smoke captured in HMS doesn't mean surface pollution), thus potentially leading to biased exposure and health assessment. This study aims to develop a smoke mask based on surface monitoring data only to improve smoke characterization.

Material and Methods: We leveraged two datasets for smoke mask development. First, we collected NOAA's Integrated Surface Database (ISD) which consists of airport-based surface meteorological observations. We extracted observations encoded as smoke or haze and label smoke days with smoke or haze percentage exceeding 5%. Second, we applied wavelet decomposition technique to extract smoke signals from hourly $PM_{2.5}$ measurements from Western Regional Climate Center (WRCC) database. Different levels of wavelets were aggregated to identify short/long smoke signals. XGBoost classifiers were trained using various predictors, including HMS, CMAQ simulations, fire counts and meteorological parameters.

Results: Using 10-fold cross-validation, the ISD-based model achieved an F1 score of 0.52, ROC AUC of 0.81, and average precision of 0.56 in our preliminary analysis. In contrast, the WRCC-based model underperformed (F1: 0.14; ROC: 0.64; AP: 0.09), likely due to its much smaller training size compared to ISD data. To improve model performance, future work will incorporate EPA and Purple Air measurements for wavelet decomposition to expand the training set. Final smoke mask will be applied to develop national smoke $PM_{2.5}$ models.

Conclusion: Surface monitoring data holds strong potential to identify wildfire smoke signals and enhance smoke exposure estimates thus strengthen the reliability of health effect assessment when combined with other smoke products such as HMS.

EP02-88 | Exposure to extreme weather and fall mortality involving traumatic brain injury: Results from vital records in Michigan, USA, 2006-2024

Peter S. Larson[1,2], Abigail Bretzin[1], Alice Buczkowski[2], Douglas Wiebe[1,2]

[1] University of Michigan, Injury Prevention Center, Ann Arbor, USA; [2] University of Michigan, Department of Epidemiology, School of Public Health, Ann Arbor, USA

Introduction: Falls are the number one cause of injury among people >65 in the United States. This research explores the epidemiology of adult fall mortality in Michigan and tests associations of temperature and precipitation with mortality risk.
Methods: We obtained records for all deaths in the State of Michigan from 2006-2024. We included all unintentional deaths which listed falls (ICD-10 W0X) and TBI as a contributing cause (S06). Deaths were linked to raster datasets of temperature and precipitation using listed residence. We tested for associations of fall mortality with daily maximum temperature and precipitation for up to 21 lag days preceding death using a case-crossover design in directed non-linear modeling framework.
Results: The re were 22,349 deaths listing falls as a contributing cause, 9,681 of which involved TBI. 58.9% were males. 8,791 (90.8%) and 612 (6.32%) were non-Hispanic white and Black, respectively. The average age was 79.9 (sd: 14.8) years. There were 518 TBI related deaths per year and a year-on-year increase of 20.9 (SE: 2.41). Deaths follow seasonal patterns, peaking in February and falling until mid-Summer. We found no association of cumulative exposure to extreme precipitation and deaths at any lag. However, we found evidence to suggest that cumulative exposure to extreme cold during the winter (95th percentile) was associated with fall mortality (OR 6.3 95\% CI (1.74, 22.75)). There was no association of extreme heat with fall related mortality during the summer months beyond lag day 0.
Conclusions: Results suggest short term exposure to extreme cold, but not heat, is associated with increased risk for TBI related fall deaths in Michigan. The lack of specificity of the timing/location of injury complicates accurate exposure assessment. Future work should assess influences of temperature on fatal and non-fatal TBI related falls in elderly populations using accurate exposure and location data.

EP02-89 | Do metals modify associations of sugar-sweetened beverages with dental caries in adolescence?

Karen Peterson[1], Michael Kaye[1], Erica Jansen[1], Yanxin Zhu[1], Guillermo Tamayo-Cabeza[2], Alejandra Cantoral[4], Juan S. Lara[2], Martha M. Tellez-Rojo[3], Jaclyn Goodrich[1], Angeles Martinez Mier[1]

[1] University of Michigan School of Public Health, Ann Arbor, USA; [2] Indiana University School of Dentistry, Indianapolis, USA; [3] Instituto Nacional de Salud Publica, Cuernavaca, Mexico; [4] Universidad Iberoamericana, Mexico City, Mexico

Objective:
Examine whether lead (Pb), cadmium (Cd) and arsenic (As) interact with intake of sugar-sweetened beverages (SSB) to increase risk of Decayed, Missing, Filled Teeth (DMFT) in adolescence.
Methods:
The analytic sample comprised 361 participants in the ELEMENT study aged 13.83 (SD +- 1.95) years with complete information on blood Pb, urinary Cd and As adjusted for urinary specific gravity, SSB intake; and DMFT assessed via an oral exam. Mean daily servings of 4 SSB categories (soda, fruit juice and flavored drinks, milk-based SSB (Yakult, chocolate milk, atole), tea or coffee with sugar) were estimated from a validated FFQ. As, Cd and Pb were IQR adjusted with Winsorization at 1.5x IQR. Beta Binomial regression was used to model associations of metals and SSB with the proportion of DMFT (out of a maximum DMFT of 16) in the study population overall and stratified by sex, adjusting for age. Interactions of metals with SSB were tested and explored in models stratified by exposure above/below the median.
Results:
Mean DMFT was 1.66 (range 0-16). Each milk-based SSB serving was associated with a statistically significant increased 5.12 odds of DMFT in males; sweetened tea/coffee was related to a 2.99 odds increase of females' DMFT. Cd was associated with a 1.07 odds increase in DMFT the overall sample and a 1.12 increase in males. Pb and As were not associated with DMFT in overall or stratified models. Stratified analysis showed that milk-based SSB increased the odds of DMFT (7.75, p=0.009) in the high Cd group only. In contrast, associations between tea/coffee with sugar and DMFT among females were stronger in the low compared to the high CD group (5.00, p=.01 vs 2.62, p=0.01).
Conclusions:
Cd exposure may modify the association of milk-based SSB and sweetened tea and coffee with DMFT score in a sex-specific manner.

EP02-90 | Blood Metal Levels Differ by Electronic Cigarette Use Behaviors and Device Characteristics: Findings From The EMIT Study

Anna Tillery[1], Angela Aherrera[2,1], Showry Peng[1], Rui Chen[1], Joyce Lin[3], Mina Tehrani[1], Donia Moustafa[1], Kathrin Schilling[4], Ronald Glabonjat[4], Jana Mihalic[1], Ana Navas-Acien[4], Ana M. Rule[1]

[1] Johns Hopkins School of Public Health, Environmental Health and Engineering, Baltimore, USA; [2] University of Pennsylvania, Perelman School of Medicine, Philadelphia, USA; [3] Harvard University TH Chan SPH, Environmental Health, Boston, USA; [4] Columbia University, Environmental Health, New York, USA

Introduction: Electronic cigarettes (e-cigs) rapidly evolved from “MOD” devices mostly used by smokers as a risk reduction strategy to “POD” devices with higher nicotine content also marketed to young non-smokers. E-cig aerosols are rich in metals. We hypothesized that differences between MOD and POD devices, and user behaviors, impact metal exposure.
Goal: Compare metal levels in blood of e-cig users and nonusers and by e-cig use behaviors and device type.
Methods: We collected from 63 participants (18 MOD, 26 POD, and 31 non-users): blood, e-cig aerosol, sociodemographics, use behaviors, and device characteristics by survey. Blood and aerosol metals were analyzed by inductively coupled plasma - mass spectrometry. Chi-squared, ANOVA, and regression analyses were used to assess relationships between variables and groups.
Results: Blood Cr was higher in POD and MOD users compared to non-users ($p=0.016$). Ni was higher in MOD users compared to other groups ($p=0.003$). Zn was higher in POD compared to MOD users ($p=0.007$). Nicotine percentage was positively associated with Pb ($p=0.011$), and Zn ($p=0.028$). The amount of e-liquid consumed per week was positively associated with Ni (0.007) and Sb ($p=0.002$). Using mint/menthol e-juice over fruit/candy flavors was positively associated with blood Zn ($p=0.028$) and Se ($p=0.038$). Earlier time to first vape in the morning was positively associated with Ni ($p=0.01$). A positive interaction between amount of e-juice per week and nicotine percentage was significant for Cd ($p=0.048$), Cr ($p=0.037$), and Sb ($p<0.001$).
Conclusions: E-cig heating components, differences in power between devices, and different chemistry of flavorings within the e-juice help explain differences in metal exposures. Our results show that device type and behaviors determine exposure to metals and could indicate differential health risks for e-cig users given what we know about chronic, low-dose metal inhalation. This information can help inform potential interventions and public health strategies.

EP02-92 | Exploring neighborhood environmental conditions among populations with autoimmune disease: a pilot-study within EXACT-PLAN

John L. Pearce[1], Jane Buckner[2], Cate Speake[2], Marc D. Natter, for the Carra Registry Investigators[3], Jill Norris[4], Wilson Liao[7], Judith James[6], Vaia L. Chatzi[5], Brigitte I. Frohnert[4]

[1] Medical University of South Carolina, Charleston, USA; [2] Benaroya Research Institute, Seattle, USA; [3] Boston Children's Hospital, Boston, USA; [4] University of Colorado - Anschutz, Aurora, USA; [5] University of Southern California, Los Angeles, USA; [6] Oklahoma Medical Research Foundation, Oklahoma City, USA; [7] University of California - San Francisco, San Francisco, USA

Objective. The goal of this pilot study was to establish a collective framework that allows EXACT-PLAN sites to begin the process of building exposome scale resources for populations with autoimmune disease. Material. We begin our pursuit of defining the external exposome by leveraging CDC’s Geospatial Research, Analysis, and Services Program (GRASP) 2024 environmental index data sets. These reflect a national, place-based tool designed to measure the cumulative impacts of environmental burden at the census tract scale. This included four indicators: Social Vulnerability; Environmental Pollution Burdens; Health Vulnerability; and Climate/Weather Burdens. Methods. We link our study populations using standardized geographic information systems (GIS) and spatial analytic methods in order to consistently assign proximal exposure to our study populations. Linked data were then categorized based on recommended percentile rankings and frequency distributions were calculated for each participating site. Results. Our framework established an EXACT-PLAN ‘Geographic Cohort’ consisting of a total population of N=43,578 across six EXACT-PLAN sites in our consortium. Findings reveal that our study populations experience a broad range of environmental burdens that vary by sites in the initiative. Census tracts with high burdens for social factors, air pollution, toxic releases, and climate burdens were identified among our populations. Conclusion. Examining links between the exposome and autoimmune disease is an important research area that can improve understanding of how environmental conditions influence autoimmune disease. This study provides a critical first step for more advanced approaches to the external exposome.

EP02-93 | Efficacy of in-home HEPA Filtration with Cognitive Function in a Randomized Cross-over Trial

Nicholas Pellegrino[1], Douglas Brugge[1], Misha Eliasziw[2], Richard Fortinsky[3]

[1] University of Connecticut, Department of Public Health Sciences, Farmington, USA; [2] Tufts University, Department of Public Health and Community Medicine, Boston, USA; [3] University of Connecticut, Center on Aging, Farmington, USA

Objective: Long-term exposure to particulate matter has been associated with adverse neurological outcomes, including dementia and depression. There are limited intervention studies on air filtration and its efficacy in reducing the cognitive effects of air pollution in humans. This preliminary study aimed to assess whether there was a benefit to cognitive function from HEPA air filtration compared to sham filtration for urban-dwelling, near-highway residents.

Materials and Methods: We analyzed data from 112 participants in the Home Air Filtration for Traffic-Related Air Pollution (HAFTRAP) study, which was a randomized cross-over trial in Somerville, MA, USA. Participants were randomized to receive either a high efficiency particulate arrestance (HEPA) air purifier for one month, a washout period of one month, and then a sham unit for one month or vice-versa. Time to complete the Trail Making Test was recorded at the beginning and end of each of the intervention periods. Results were stratified by age, education, and gender.
Results: There was no significant difference in overall completion time when comparing times before and after the HEPA intervention versus the sham intervention. When stratifying by age, however, a significant improvement was observed in the HEPA intervention for those older than 40 in time to completion of part B. There was no difference for those older than 40 in time to completion of Part A, and there was no different in either part of the Trail Making Test for those under 40. For education and gender, there were no significant differences between interventions in any group for either part of the Trail Making Test.
Conclusion: Our results suggest an improvement in cognitive performance based on the usage of HEPA to reduce particulate matter exposure in the household among healthy adults. Further research is needed on the short-term benefits of air filtration on cognition.

EP02-94 | The National Childhood Cancer Registry Data Platform: Linking Data from a Variety of Sources to Guide Future Childhood Cancer Cohort Research

Denise R. Lewis, Johanna L. Goderre

National Cancer Institute, Surveillance Research Program, Bethesda, USA

Objective: To promote childhood cancer cohort discovery. Materials and Methods: The National Cancer Institute's Childhood Cancer Data Initiative National Childhood Cancer Registry (NCCR) data platform is a source of relevant public health surveillance data to understand causes of incident cancers, treatment, recurrence, survival, comorbidities, subsequent primary cancers, and late effects of cancer among children and adolescents and young adults up to age 39 in the United States. Cancer case types are coded according to the International Classification of Childhood Cancer (ICCC) major category. Results: Currently, there are more than 1.5 million de-identified cases of cancer in the database, with most cases diagnosed between ages 20 and 39 years. The first incident cases in the cohort were recorded in 1995 through 2021. The three predominant case types include malignant epithelial neoplasms and malignant melanomas (more than 650,000 cases), central nervous system tumors (more than 165,000 cases), and lymphomas (more than 154,000 cases). Seven unique sources of data contribute additional information about tumor type from the SEER registries, non-clinical variables from area-based measures derived from the US Census Bureau, data from studies for enrollees in children's oncology group studies (COG), medical claims diagnoses, medical claims enrollment information from various insurers, medical claims procedures from various insurers, and pharmacy claims from various insurers. Researchers may submit a data request outlining their research goals to access detailed information about a specific cohort. The data request must be reviewed by the researcher's associated Institutional Review Board (IRB). Documentation of the IRB review including exempt from review or does not qualify as human research must be provided in the NCCR Data Platform. The NCCR Data Platform does not hold personally identifiable information (PII). Conclusion: the NCCR data platform will enhance our knowledge of childhood cancer. This work is funded by the US National Cancer Institute.

EP02-95 | Spatial Disparities of Industrial Pollution in Puerto Rico: A Mapping Analysis of On-Site Carcinogen Emissions and Cancer Incidence

Lenulisy Rosado-Estrada[1], Nancy R. Cardona-Cordero[2], Marievelisse Soto-Salgado[3], Pablo A. Méndez-Lázaro[4], Noreen Michael[5], Ana P. Ortiz[6]

[1] University of Puerto Rico Comprehensive Cancer Center, Division of Cancer Control and Population Sciences, San Juan, Puerto Rico; [2] University of Puerto Rico Comprehensive Cancer Center, Division of Cancer Control and Population Sciences, San Juan, Puerto Rico; [3] University of Puerto Rico Comprehensive Cancer Center, Division of Cancer Control and Population Sciences, San Juan, Puerto Rico; [4] University of Puerto Rico - Medical Sciences Campus, Department of Environmental Health, San Juan, Puerto Rico; [5] University of Virgin Islands, Caribbean Exploratory Research Center of Excellence, St. Thomas, U.S. Virgin Islands; [6] University of Puerto Rico Comprehensive Cancer Center, Division of Cancer Control and Population Sciences, San Juan, Puerto Rico

Industrial pollution is one of the main emerging contributors to environmental-related health risks. The EPA's Toxic Release Inventory (TRI) reports that between 2006 and 2020, 160 industries released 60.6 million pounds of toxic substances on-site in 53% of Puerto Rico's municipalities, with 100 industries releasing 33 different types of carcinogenic substances at least once. Pollution from these releases affects communities in the archipelago unevenly. This study describes the distribution of carcinogenic emissions and identifies municipalities most burdened by contamination.
We analyzed cancer incidence (2018-2022) and chemical releases (2006-2020) across municipalities using data from the Puerto Rico Central Cancer Registry and EPA's TRI database. We calculated the proportion of carcinogenic chemicals released and created bivariate choropleth maps to visualize the spatial intersection of cancer incidence and carcinogen burden. QGIS program was used for the analysis.
From 2018-2022, Jayuya (390.0), Bayamón (361.3), and Guayama (355.6) had the highest cancer rates per 100,000 in Puerto Rico. From 2006 to 2020, Carolina had the most industries releasing toxic substances. Guayama, Manatí, and Arecibo had the highest numbers of different chemicals released. Guayama (23%), Arecibo (31%), and Yabucoa (45%), had the highest percentages of carcinogenic chemicals, while the most pounds of on-site carcinogenic emissions were released in Ponce (11.5 million pounds). Bivariate spatial analysis showed that Guayama and Carolina faced high burdens of carcinogen emissions and high cancer incidence.
Findings suggest on-site carcinogenic emissions impact specific municipalities, emphasizing the need to understand how industrial pollution impacts environmental health equality in Puerto Rico. It highlights the urgency of targeted public health actions to address spatial disparities in exposure to carcinogenic pollutants.
Acknowledgements: This study was supported by the Caribbean Cancer Research Center on Environmental and Natural Hazards (CARIB-CARES) funded by the National Cancer Institute (NCI, award # 1P20CA294096-01).

EP02-96 | PFAS on Tap: From Municipal Drinking Water to Our Beer, A Lifespan of Exposure

Jennifer Hoponick Redmon, Nicole DeLuca, Andrew Kondash

RTI International, Research Triangle Park, USA

Background and Objective: Our study evaluated drinking water as a potential exposure medium through a widely consumed product for many adults: beer. While PFAS contamination in municipal systems is a known public health issue, this is the first study to identify the transfer of these persistent chemicals into beers purchased in U.S. stores. Our objective was to examine whether local drinking water PFAS contamination is reflected in beer, highlighting a pathway relevant to adults across geographic and socioeconomic lines.
Methods: We analyzed 23 commercially available beers from both national and craft breweries across the U.S., selected based on proximity to PFAS-impacted water systems, popularity, and geographical location. A modified version of EPA Method 533, optimized for beer matrices, was used to quantify PFAS analytes via LC-MS/MS. County-level water PFAS data were matched to each beer's brewery location to assess spatial correlation and exposure risk.
Results: PFAS were detected in 22 of 23 beers. PFOS, PFOA, and other legacy compounds were most prevalent, with several samples exceeding EPA's Maximum Contaminant Levels. Geographic clustering was observed in areas with known water contamination, particularly in North Carolina and Michigan.
Conclusions: Municipal drinking water is a primary source of PFAS in beer, representing a potential unmonitored route of exposure for adults. Our findings introduce additional concerns about cumulative PFAS exposure, especially for populations relying on public water sources. This work underscores the need for multi-sectoral data-driven decision making and risk mitigation to protect environmental health across the lifespan.

Keywords: PFAS, drinking water, beer, exposure pathways, environmental health equity

EP02-97 | Ambient Temperature and Urinary Total Protein: Direct and Blood Pressure Mediated Effects

Hoimonty Mazumder, Abu Mohd Naser

The University of Memphis, Division of Epidemiology, Biostatistics and Environmental Health, School of Public Health, Memphis, USA

Introduction: Extreme ambient temperatures have been associated with blood pressure changes and adverse kidney function. High blood pressure is also a risk factor for detrimental kidney function. We explored the direct and blood pressure mediating indirect relationship between lagged ambient temperature and urinary total protein excretion using mediation analyses.
Method: We used observational data from a study in southwest coastal Bangladesh. A total of 227 person-visit data of 24-hour urinary total protein (UTP) excretion is used as outcome. We downloaded satellite-derived meteorological gridded data (ambient temperature, dewpoint) of participant residences' during the study period using NASA Api. We estimated the direct and blood pressure–mediated effects of ambient temperature on UTP, a marker of kidney function, using Baron-Kenny's product method with linear mixed models that incorporated restricted cubic splines

and random intercepts at the participant, household, and community levels in both outcome and mediator models. We assessed the linearity between ambient temperature (lags 1 to 6) and UTP using Wald tests across varying degrees of freedom. For non-linearity, we used piecewise linear mixed-effects models to estimate associations between temperature and UTP.

Results: We found a linear association between UTP and lagged temperatures on days 1–4, and a non-linear association on days 5 and 6. For a 1ºC increase in lag 5 and lag 6 days ambient temperature was associated with 0.23 (95% CI: 0.08, 0.37; p = 0.003) and 0.38 mg/24 hours (95% CI: 0.14, 0.62; p = 0.002) change of UTP respectively (direct effects), when temperature was higher. The direct effects of ambient temperatures on UTP from lag 1 to 4 days and indirect BP-mediated effects in all lag days (1-6) were found to be insignificant.

Conclusion: Our finding suggests that high ambient temperature in lag day 5 and 6 demonstrated increased urinary total protein excretion.

EP02-99 | Creation of a Cumulative Exposure Index to Estimate Likelihood of Historic Pesticide Exposure in U.S. Veterans

Alison Doepker[1], Nicholas Rattray[2], Jason Cannon[1], Ellen Wells[1], In collaboration with the Richard L. Roudebush Veterans' Administration of Indianapolis, IN

[1] Purdue University, Human Health Sciences, West Lafayette, USA; [2] Richard L. Roudebush Veterans' Administration, Center for Health Information and Communication, Indianapolis, USA

U.S. veterans are a high-risk population for various environmental exposures, including pesticides. However, evaluating pesticide exposure among veterans has been challenging. Therefore, the objective of this analysis is to develop a cumulative exposure index (CEI) to estimate historic pesticide exposure in veterans. Exposure and demographic data were collected via survey from 110 U.S. veterans diagnosed with Parkinson's disease as part of a larger case-control study. Questions used to create the CEI include direct pesticide exposure, risk factors of exposure, and whether these were related to military responsibilities (classified as likely to be high/medium/low risk of exposure). Potential exposures were modified by frequency and duration of exposure and the total potential exposures were summed to create the final CEI. Of the 110 veterans, N=38 (34%) reported applying pesticides in their lifeline, N=20 (18%) reported applying or mixing pesticides while in the military, and N=9 (8%) reported wearing uniforms treated with pesticides. A significant correlation was found between CEI value and years spent in military service, which indicated a weak positive correlation (Spearman's ρ=0.29, p=0.002). Weak positive correlations were identified between CEI and both race and age at Parkinson's diagnoses, and weak negative correlations were identified between CEI and gender, ethnicity, and age, though none were statistically significant. Next steps will be to evaluate military versus non-military related pesticide exposure and to create a CEI for specific pesticides. It is our overarching goal to utilize the data collected to evaluate the relationship between pesticide exposure and Parkinson's disease.

EP02-100 | An expanded review and meta-analysis of associations between exposure to gasoline vapor and hematopoietic malignancies

Michael R. Hussey[1], Matthew Grespin[2], Allison Stock[3]

[1] J.S. Held LLC, Health Sciences, Redmond, USA; [2] J.S. Held LLC, Health Sciences, Denver, USA; [3] J.S. Held LLC, Health Sciences, New Orleans, USA

Objective:
Inhalation of gasoline vapor in occupational settings has been inconsistently linked to several hematopoietic malignancies yet inadequately explored in cancer subtypes. We sought to systematically review and analyze the relationship using recent scientific literature.

Material and Methods:
Following a PRISMA review framework, we searched four databases, PubMed, Embase, Medline, and Web of Science, for manuscripts relating gasoline exposure or employment in petroleum refining/distribution to acute myeloid leukemia (AML), acute lymphocytic leukemia (ALL), chronic myeloid leukemia (CML), chronic lymphocytic leukemia (CLL), Hodgkin's lymphoma (HL), non-Hodgkin's lymphoma (NHL), multiple myeloma (MM), and myelodysplastic syndrome (MDS) in human adults. Only the latest effect estimates for long-term cohorts were included to prevent overlap. Study quality was assessed using a modified Newcastle Ottawa Scale (NOS) and meta-analyses were performed on extracted data using the *meta* R package.

Results:
From 5,782 non-duplicate articles we identified 71 relevant studies for inclusion. Non-significant associations of AML mortality (mRR=1.11, 95%CI:0.99-1.25) and incidence were observed (mRR=1.64, 95%CI:0.88-3.04) in cohort studies of petroleum refining, distribution, and sales workers. NHL incidence was significantly associated with employment in occupational case-control studies (mOR=1.62, 95%CI: 1.20-2.20). Restriction to only studies involving likely gasoline vapor exposure made this effect non-significant, though AML remained positively associated with gasoline exposure. Analyses of duration of employment, period of hire, and job-type indicated the presence of population-specific differences in cancer risk independent of gasoline exposures. Further, uncontrolled confounding by smoking and other factors and incomplete assessment of mixture exposures also likely accounted for observed cancer trends.

Conclusion:
This study found petroleum refining, distribution, and other gasoline-linked professions not statistically significantly associated with AML incidence or mortality, consistent with prior reviews. However, these associations are likely accounted for by uncontrolled confounding, primarily by smoking. Future research should focus on the inclusion of gasoline-specific biomarkers and exposure assessments.

EP02-101 | Parental Occupational Exposure and Child Health Outcomes Scoping Review

Abigail P. Shirzadi, Tran Huynh

University of Minnesota Twin Cities, Division of Environmental Health Sciences, Minneapolis, USA

Objective: The objective of this scoping review is to understand the available evidence for parental occupational exposures and child health outcomes, as well as establish gaps in the literature for future investigation directions.
Introduction: There is evidence that parental exposures, both maternal and paternal, before and during pregnancy can affect the child’s health pre- and post-partum. However, there is less detail about the effects of parental occupational exposures on children’s health, especially because job exposures can be broad. This review aims to address this gap by compiling the available literature and making recommendations for future work to analyze the effects of occupational exposures on children’s health.
Inclusion criteria: The population of interest are parents who have a child and have reported their occupational history or exposures prior, during pregnancy, or postpartum. If there is follow-up postpartum, children up to 12 years old (pre-puberty) will be included in this review. The literature can be global and is not limited to U.S. studies. Studies must be focused on occupational exposures and the resultant health outcomes in offspring. Additionally, manuscripts with a job exposure component (job exposure matrices) will also be included as they permit for better detailing of job exposures..Methods: The initial search was done using OVID Medline. After this, additional literature extractions were replicated in Scopus. The date range was January 1st, 1970- June 6th, 2025. Only studies in English were considered. Studies were included based on the inclusion criteria and title-abstract screening will be done by two reviewers. Full texts were then reviewed based on the initial screening inclusion. Screening and review were done using Rayyan software.

Results: Thus far in this ongoing review, 696 unique manuscripts were imported to be screened. To date, 156 title/abstracts have been screened and 77 full-texts will be screened for inclusion.

EP02-103 | The National Human Biomonitoring Programme in Japan

Shoji F. Nakayama, Tomohiko Isobe, Miyuki Iwai-Shimada, Mai Takagi, Yayoi Kobayashi

National Institute for Environmental Studies, Health and Environmental Risk Division, Tsukuba, Japan

Since 2008, the Ministry of the Environment, Japan (MOEJ) has conducted a series of human biomonitoring (HBM) studies. However, the limited sample sizes of earlier studies restricted their ability to provide results representative of the national population. To address this, the MOEJ has launched a new, expanded HBM programme designed to produce nationally representative data.

The new programme will operate on a three-year cycle and employ stratified random sampling across the country. A total of 150 study locations will be selected nationwide, with approximately 20 households recruited from each location, yielding an estimated 3,000 to 5,000 samples per cycle.

To improve efficiency and participant engagement, the study will adopt digital tools, including electronic informed consent (e-consent) and web-based questionnaires. The questionnaire has been harmonized with those used in other international biomonitoring programmes, facilitating cross-country comparisons. Similarly, the target chemicals selected for analysis are aligned with global HBM initiatives, allowing Japan to contribute to and benefit from international data integration.

This presentation will provide an overview of the new national HBM programme, including its design, sampling strategy, digital infrastructure and alignment with global standards. The programme marks a significant advancement in Japan's capacity to assess environmental exposures and support evidence-based policymaking for public health protection.

EP02-104 | A country-level analysis of the impact of motor vehicle density on asthma prevalence

Akhilesh Anil Jakhete[1], Hina Narayan[1], Pranayaa Jeyaraman[2], Anand Gourishankar[1,3]

[1] George Washington University, Washington DC, USA; [2] Woodbridge Academy, Edison, USA; [3] Children's National Hospital, Washington DC, USA

Objective: To study the association between country-level motor vehicle density and asthma prevalence.
Materials and Methods: This study is an ecological analysis of publicly available data from 195 countries for the year 2019. Surrogate measures of traffic-related air pollution such as motor vehicle density and road length network, were used to assess their correlation with asthma prevalence. A zero-order correlation was also calculated between the country's gross domestic product (GDP) and its asthma prevalence. A multiple regression analysis was conducted to predict the prevalence of asthma in a country based on its motor vehicle density, GDP, and annual average temperatures.
Results: Asthma prevalence in a country was found to be moderately correlated with its motor vehicle density (Pearson correlation coefficient (PCC) = 0.53, $p < .0001$) and a weakly positive correlation with its GDP (PCC = 0.23, $p < .001$), but not with road length network (PCC = 0.12, $p = 0.13$). An iterative multivariate linear regression model was developed to predict asthma prevalence based on the country's motor vehicle density, GDP and annual average temperatures. The fitted regression model was: Asthma Prevalence = 1.8 + 5.3e-03*Motor Vehicle Density + 9.9e-08*GDP + 5.7e-02*Temperature (in °C) ± 1.6. Overall, the regression model was statistically significant (adjusted R2 = 0.35, $p < .0001$). Among the individual predictors examined, motor vehicle density ($t = 8.214$, $p < .0001$) and the annual average temperature ($t = 2.875$, $p = .005$) were significant predictors of asthma prevalence but GDP ($t = 1.593$, $p = .11$) was not.**Conclusion:** Asthma prevalence is significantly associated with motor vehicle density, even after accounting for annual average temperatures and GDP in this country-level analysis.

EP02-105 | Comparison of machine learning models for predicting suicidal ideation: Korean Community Health Survey (2017-2024)

Jieun Lee[1], Jeehye Lee[2], Hye Sook Min[3], Kyung-Shin Lee[4]

[1] Seoul National University, Department of Human Systems Medicine, Seoul, South Korea; [2] Konkuk University, Department of Preventive Medicine, Seoul, South Korea; [3] National Medical Center, Public Health Research Institute, Seoul, South Korea; [4] National Medical Center, Center for Public Healthcare Policy, Seoul, South Korea

Introduction

Older adult’s suicide remains a significant public health problem in South Korea, which has one of the highest suicide rates in this age group worldwide. Identifying risk factors for suicidal ideation is crucial for developing targeted prevention strategies. This study aims to identify risk factors associated with suicidal ideation in Korean adults aged 65 years or older using machine learning methods.***Methods***

We analyzed integrated data from the Korea Community Health Survey (2017-2024), which included 481,987 participants aged 65 years and older. Three prediction models were established by the logistic regression, least absolute shrinkage and selection operator (LASSO) regression, and random forest, respectively. We conducted stratified analyses to identify risk factors for suicidal ideation separately in groups with and without depressive symptoms. The data were split 80:20 for training and testing using appropriate questionnaire design weights.***Results***

The proportion of suicidal ideation among all participants was 6.7%. Results consistently highlighted depressive symptoms and perceived stress as the main risk domains for all three approaches, with slightly different top-ranking factors between the models: unmet medical needs in LASSO and logistic models, and survey year and age group in random forest model. Stratified analysis revealed that household income and employment status were high risk factors in the depressive symptoms group, and quality of life was high risk factors in the non-depressive symptoms group regarding suicidal ideation.***Conclusions***

Depressive symptoms, perceived stress have been shown to be major modifiable risk factors for suicidal thoughts in older Korean adults. Furthermore, the differential risk factors according to the presence of depressive symptoms suggest the need for tailored intervention approaches based on individual characteristics. These findings support the idea that integrated mental health screening and improved access to medical services are preventive strategies.

EP02-107 | Long-Term Mental Health Impacts of Wildfire Exposure

Lusine Gigoyan, Irva Hertz-Picciotto, Lusine Gigoyan

University of California Davis, Public Health Science, Davis, USA

Importance: Few studies have examined the persistence of mental health symptoms following wildfires beyond 1–2 years, particularly with attention to vulnerable subgroups, preexisting conditions and behavioral outcomes such as substance use, alcohol use, and smoking

Objectives: Investigate the risk factors associated with persistent mood/anxiety symptoms and behavioral symptoms following the wildfires over 3 years.

Exposure: 2018 Camp Fire

Outcomes: Outcomes included self-reported persistence of behavioral and mood-related symptoms at 12 months, 1–2 years, and 3 years after the wildfires.

Results: Among 508 adults, at 12 months post-wildfire 14% reported 1-3 symptoms, 34% reported 4 or more mood symptoms. At 1-2 years, 22% reported 1 to 3 symptoms, and 24% reported 4 or more symptoms mood symptoms. By 2-3 years post-wildfire 20% continued to report 1-3 symptoms and 22% reported 4 or more symptoms. Pre-existing mental or psychiatric conditions were significantly associated with the persistence of behavioral symptoms at all time points: RR = 2.52 (95% CI: 1.30–4.88) at 12 months, RR = 2.52 (95% CI: 1.28–4.96) at 1–2 years, and RR = 3.18 (95% CI: 1.36–7.45) at 2–3 years post-wildfire. Factors significantly associated with mood symptoms include living alone, significant home damage, loss of a family member or friend, loss of a pet during the wildfires, and a pre-existing mental or psychiatric condition.

Conclusion and relevance: Mental health symptoms may persist for up to three years following wildfire exposure, particularly among vulnerable populations with certain pre-existing conditions and high levels of fire-related exposure. While governmental agencies currently provide mental health support for up to nine months post-disaster, these findings underscore the need for sustained mental health services well beyond that period.Due to the reliance on self-reported data, additional research is needed to validate and build upon these findings.

EP02-110 | Rare-Event Weighted Wavelet Features Improve Random-Forest Performance in Air-Quality Prediction

Zhuohan Chi[2], Dimple Pruthi[1], Yang Liu[1]

[1] Emory University, Gangarosa Department of Environnment Health, Rollins School of Public Health, Decatur, USA; [2] Emory University, Department of Biostatistics and Bioinformatics, Rollins School of Public Health, Decatur, USA

Objective

To determine whether (i) discrete wavelet decomposition of meteorological and satellite variables and (ii) rare-event weighting jointly improve the out-of-bag (OOB) *r2* of a random-forest (RF) model that predicts daily PM2.5 concentrations during wildfire seasons in the California, United States (2019 – 2021).

Material and Methods

Daily PM2.5 observations from 143 EPA stations and 2130 Purple Air stations were merged with aerosol optical depth (AOD, MODIS), temperature, humidity, and wind fields, and etc.. Each predictor was decomposed with five mother wavelets (db4, db5, sym8, coif5, and haar); the first three detail levels were retained and appended to the raw series. Because extreme-smoke days (PM2.5 > 100 *µg/m3*) were under-represented, we applied inverse-prevalence weights during RF training. Hyper-parameters (trees = 500, mtry = √pp, min-node-size = 5) were fixed across scenarios to isolate the effect of feature engineering. Performance was evaluated with 10-fold spatial cross-validation and OOB R2.

Results

Adding wavelet features alone improved median OOB R2 from 0.62 to 0.69 (+11 %). Incorporating rare-event weighting further raised OOB R2 to 0.74 (+19 % relative, p<0.001). db4 produced the strongest incremental gain when applied to AOD (+0.05 R2), capturing sharp aerosol spikes. coif5 best enhanced relative humidity and temperature signals (+0.03 R2 combined), likely due to its smoother basis. Combining heterogeneous mother wavelets per variable outperformed any single-wavelet strategy.

Variable-importance analysis showed wavelet-transformed AOD (db4-D1, D2) and humidity (coif5-A1) entering the top 10 predictors; their partial-dependence plots revealed clearer non-linear thresholds around intense wildfire activity.

Conclusion

Wavelet decomposition tailored to each predictor, coupled with rare-event weighting, offers a simple yet powerful route to boost RF accuracy, especially under high-pollution conditions commonly missed by conventional models. These insights are transferable to other air-quality forecasting tasks where extreme events are rare but critical.

EP02-111 | The Ongoing Environmental Destruction and Degradation of Gaza: The Resulting Public Health Crisis

Ahlam Abuawad[1], Mark Griffiths[2], Graham H. Edwards[3], Adan Eftekhari[4], Mohammed Al-Ebweini[5], Husam Al-Najar[6], Abeer Butmeh[7], Rasha Abu Dayyeh[7], Mohamed El-Shewy[2], Amira Aker[8]

[1] Geisel School of Medicine at Dartmouth College, Epidemiology, Hanover, USA; [2] Newcastle University, School of Geography, Politics and Sociology, Newcastle, UK; [3] Trinity University, Earth and Environmental Geosciences, San Antonio, USA; [4] Harvard University, Cambridge, USA; [5] Coastal Municipalities Water Utility, Gaza Strip, Palestine; [6] Islamic University of Gaza, Gaza Strip, Palestine; [7] Palestinian Environmental NGOs Network, West Bank, Palestine; [8] Boston University, School of Public Health, Boston, USA

This essay examines the resulting environmental health conditions in Gaza since October 2023. Evidence is drawn from international agency reports and peer-reviewed literature to document destruction and public health impacts over 18 months. Key themes include water, sanitation, contamination, noise and air pollution, food insecurity, susceptible populations, health system collapse, and trauma.
Palestinians are beset with a public health crisis resulting from targeted environmental destruction. Water shortages and sewage contamination increase dehydration, famine, and disease. Air pollution from explosives and waste burning exposes residents to toxins. Waste management has collapsed, heightening disease outbreaks, including polio. Agricultural land is devastated, and food security is threatened with the aid blockade. Power grid destruction has crippled essential services. The health care system's collapse further exacerbates risks, especially for susceptible populations. In addition to mental and physical trauma, cultural and identity loss are immeasurable.
The crisis in Gaza has severe and lasting consequences for health, the environment, and regional stability. An end to the bombardment, along with urgent humanitarian aid, release of all hostages, infrastructure rebuilding, and environmental remediation, are essential to mitigate long-term impacts.
